# A novel strategy for dynamic modeling of genome-scale interaction networks

**DOI:** 10.1093/bioinformatics/btad079

**Published:** 2023-02-24

**Authors:** Pooya Borzou, Jafar Ghaisari, Iman Izadi, Yasin Eshraghi, Yousof Gheisari

**Affiliations:** Department of Electrical and Computer Engineering, Isfahan University of Technology, Isfahan 84156-83111, Iran; Department of Electrical and Computer Engineering, Isfahan University of Technology, Isfahan 84156-83111, Iran; Department of Electrical and Computer Engineering, Isfahan University of Technology, Isfahan 84156-83111, Iran; Regenerative Medicine Research Center, Isfahan University of Medical Sciences, Isfahan 81746-73476, Iran; Regenerative Medicine Research Center, Isfahan University of Medical Sciences, Isfahan 81746-73476, Iran

## Abstract

**Motivation:**

The recent availability of omics data allows the construction of holistic maps of interactions between numerous role-playing biomolecules. However, these networks are often static, ignoring the dynamic behavior of biological processes. On the other hand, dynamic models are commonly constructed on small scales. Hence, the construction of large-scale dynamic models that can quantitatively predict the time-course cellular behaviors remains a big challenge.

**Results:**

In this study, a pipeline is proposed for the automatic construction of large-scale dynamic models. The pipeline uses a list of biomolecules and their time-course trajectories in a given phenomenon as input. First, the interaction network of the biomolecules is constructed. To state the underlying molecular events of each interaction, it is translated into a map of biochemical reactions. Next, to define the kinetics of the reactions, an ordinary differential equation (ODE) is generated for each involved biomolecule. Finally, the parameters of the ODE system are estimated by a novel large-scale parameter approximation method. The high performance of the pipeline is demonstrated by modeling the response of a colorectal cancer cell line to different chemotherapy regimens. In conclusion, Systematic Protein Association Dynamic ANalyzer constructs genome-scale dynamic models, filling the gap between large-scale static and small-scale dynamic modeling strategies. This simulation approach allows for holistic quantitative predictions which are critical for the simulation of therapeutic interventions in precision medicine.

**Availability and implementation:**

Detailed information about the constructed large-scale model of colorectal cancer is available in supplementary data. The SPADAN toolbox source code is also available on GitHub (https://github.com/PooyaBorzou/SPADAN).

**Supplementary information:**

Supplementary data are available at *Bioinformatics* online.

## 1 Introduction

Although medicine has experienced great success in different areas such as surgical procedures, imaging techniques and diagnostic tests in the past decades, efficient management of complex disorders is yet an unmet goal. The recent availability of a huge amount of data has led to the understanding that most disorders are not caused by the malperformance of a few proteins but by the alteration of interactions between a large number of biomolecules (Agrawal *et al.*, 2018). Mapping the interactions between these role-playing elements, known as interactome networks, is essential to understand the physiological and pathological processes at a cell level (Yu *et al.*, 2008). However, these networks are static and represent the steady state of the system. Indeed, they provide only a snapshot of the system, ignoring the time-dependency nature of biomedical processes (Hecker *et al.*, 2009). On the other hand, dynamic modeling strategies allow for the generation of quantitative and predictive models which incorporate the dynamic behavior of such processes (Chung *et al.*, 2009; Khatibi *et al.*, 2017). Although such models are valuable tools to analyze and forecast the functions of biological systems, they are generally constructed on small scales. Therefore, the generation of predictive models of diseases that are both dynamic and holistic is yet a major challenge.

Constraint-based modeling is a common approach for the generation of genome-scale models of metabolism (Mahadevan and Schilling, 2003; O’Brien *et al.*, 2013; Ye *et al.*, 2022). Flux balance analysis is commonly employed to analyze these models which finds a proper flux distribution that satisfies the metabolic constraints and optimizes the objective functions (Gilbert *et al.*, 2019; Smallbone *et al.*, 2010). The applicability of this analysis approach has been extended by developing multi-scale Metabolic-Expression models that also include transcription and translation data to model cell metabolism (Bi *et al.*, 2022). Although classical constraint-based modeling is useful for phenotype prediction, biomarker discovery and drug target prediction, it only considers steady-state behaviors, missing the transient states and kinetics of biochemical reactions (Mahadevan and Schilling, 2003; Ye *et al.*, 2022). This limitation is addressed by developing the dynamic flux balance analysis which considers the rates of changes in flux constraints (Mahadevan *et al.*, 2002). Moreover, d-OptCom is an extension of conventional flux balance analysis proposed for the dynamic multi-species metabolic modeling of microbial communities. It incorporates substrate uptake kinetics, time-dependent biomass changes and extracellular concentrations of shared resources (Colarusso *et al.*, 2021; Zomorrodi *et al.*, 2014). Furthermore, model reduction has been proposed as an alternative strategy to add dynamic insight into constraint-based models (van Rosmalen *et al.*, 2021).

Ordinary differential equation (ODE) modeling permits a flexible modeling of non-stationary and non-linear biological systems (Liang and Kelemen, 2018). Hence, Smallbone *et al.* defined a pipeline for generating a genome-scale kinetic model from constraint-based models. It uses metabolite concentrations, fluxes, rate laws and available kinetic parameters. Parameters without available values are estimated using experimental data (Smallbone and Mendes, 2013). Furthermore, due to the difficulties of developing large-scale dynamic models, some algorithms were proposed for the automatization of modeling procedures; Smith *et al.* (2018) presented a python package, named DMPy, which uses metabolic networks containing the details of biochemical reactions as input and automatically converts it to a large set of differential equations. Then, the parameter values are collected from different databases to construct the dynamic model (Smith *et al.*, 2018).

Although genome-scale dynamic models of biochemical reactions have been constructed by a few investigators, to our knowledge, such models are not yet developed for protein–protein interaction (PPI) networks. This is partly due to the scarcity of experimental evidence on the rate of reactions. Furthermore, more details of chemical events in metabolic reactions are available than for the interactions between proteins. Indeed, PPI is a concise depiction of the interactions without going to the chemical details. Here, we introduce a novel strategy to automatically convert PPI networks to genome-wide dynamic models. In this pipeline, for a given list of proteins, the PPI network is constructed and then translated to the comprehensive language of biochemical reactions by considering the chemical details. Then, a series of ODEs is automatically generated to describe the kinetics of the reactions. In the next step, a large-scale and distributed parameter approximation algorithm is proposed to provide an estimation of system parameters based on high-throughput time-series experimental data. This strategy, which is named Systematic Protein Association Dynamic ANalyzer (SPADAN), allows a holistic insight into the dynamism of protein interactions and provides quantitative predictions of system behavior. The applicability of this approach was assessed by modeling the interactions of proteins in colorectal cancer (CRC) and predicting the response to specific chemotherapy agents.

## 2 Materials and methods

### 2.1 Data acquisition

A proteomics dataset (PXD007740) pertinent to the time-course exploration of proteomics and phosphoproteomics of CRC cells produced by Ressa *et al.* (2018) was retrieved from the ProteomeXchange database (Deutsch *et al.*, 2017). The analyzed RNAseq data of these cells were also obtained as a supplementary file of this report (Ressa *et al.*, 2018).

### 2.2 Proteomics and phosphoproteomics data analysis

Raw mass spectrometry data were analyzed with MaxQuant (version 1.6.8.0) integrated with Andromeda search engine against human-reviewed proteome from UniProt FASTA database. Trypsin was configured as a specific enzyme with a maximum of two missed cleavages. For proteomics data, cysteine carbamidomethylation was considered as fixed modification and methionine oxidation and N-terminal acetylation as variable modifications. For phosphopeptides, cysteine carbamidomethylation and phospho (STY) were selected. Proteins were quantified based on unique+razor peptides and two minimum ratio counts. A significance threshold of 0.1 was considered both for peptide spectrum match and protein false discovery rates. The ‘match between runs’ was enabled for all analyses. All quantified peptides and phosphopeptides were filtered for reverse, contaminant and only identified by sites. Also, phosphosites were retained if they were below a localization probability rate of 75%. To estimate the absolute abundance of proteins, ‘proteomic ruler’ plugin of Perseus was employed.

### 2.3 Hardware description

SPADAN is developed in MATLAB 2015b and its computation procedure was performed by a PC with 16 GB RAM and Intel^®^ Core™ i3-6100 CPU.

### 2.4 Calculation of function *S*

For the estimation of model parameters, the least square method is used in which the function *S* is calculated as the sum of squared errors between the model simulations and time-series experimental data. *S* is defined as below:

(1) $$S\;=\;\sum_{i=1}^{m}\sum_{j=1}^{n}{\left({\hat{y}}_{ij}-{\hat{e}}_{ij}\right)}^{2}\;,$$

where ${\hat{e}}_{ij}\;$is the normalized experimental data and ${\hat{y}}_{ij}$ is the normalized model simulation data. The variables i and j are the number of biological elements and time points, respectively. The total number of biomolecules and time points are also shown with m and n, respectively.

## 3 Results

**Fig. 1.**
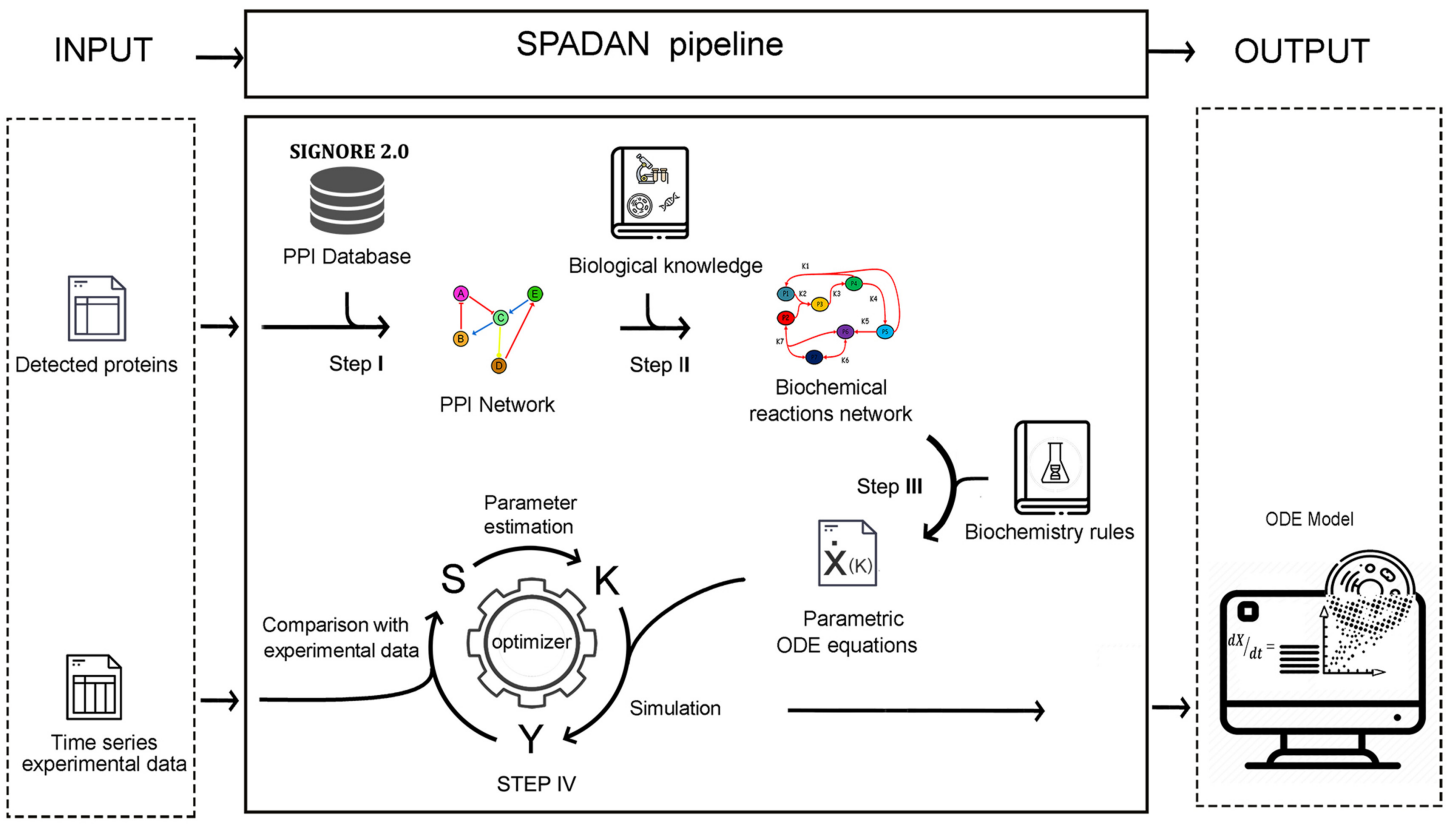
SPADAN modeling procedure pipeline. SPADAN toolbox starts from a list of biomolecules which are supposed to be involved in a given phenomenon. These biomolecules can be from different layers including proteins, phosphoproteins and RNA transcripts. In the pre-processing step, different isoforms of the same protein or different transcript variants are combined. In addition, proteins and transcripts expressed from the same gene are linked. Step I—Harvesting network interactions: SPADAN harvests experimentally verified interactions between input proteins from SIGNOR database. Each interaction in SIGNOR includes information about the direction of interaction, type of interaction (transcription, phosphorylation, complex formation, etc.), biochemical effect (activation, inhibition) and the reference reported in this interaction. In this step, the interactions are depicted as a PPI network. Step II—Converting the PPI network to a biochemical reaction network: Each edge in the PPI network is automatically expanded to a list of biochemical reactions according to the rules shown in Table 1. This results in the translation of the PPI network to a biochemical network which is more comprehensive. A simple example of this step is demonstrated in Figure 2. Step III—Converting the biochemical reaction network to a large-scale ODE system: Based on the biochemical kinetic law assigned to each type of interaction (mass action, Michaelis–Menten, Hill equation), SPADAN automatically generates a list of stochiometric ODEs which describe the kinetics of biochemical reactions as a function of reactants concentrations. In this step, SPADAN makes a parametric ODE system. Step IV—Parameter estimation: Unknown parameters in the ODE system are approximated using time-series experimental data by fitting the simulated concentration traces to their experimental equivalents. The proposed estimator performs numerous cycles of optimization to minimize the difference between simulated and experimental values. The output is a large-scale ODE model of the biological system, from which the experimental data were obtained. The ODE model is provided as an ‘m file’ which is simulatable in the MATLAB environment

This study aims at developing a strategy to generate dynamic insights of molecular interactions at the genome scale. Starting from time-series experimental data, the developed algorithm generates a PPI network and then translates the interactions into a biochemical language. Next, this biochemical network is converted to a series of ODEs. The parameters are estimated using a novel large-scale and distributed parameter estimation technique. The algorithm performs all of these steps automatically. This pipeline is schematically depicted in Figure 1 and the steps are described below.

### 3.1 Step I: Harvesting network interactions

After getting the list of proteins, SPADAN harvests interactions from the SIGNOR 2.0 database (Licata *et al.*, 2020) which includes experimentally validated protein interactions. For the sake of feasibility, we focused on common interaction types including phosphorylation, dephosphorylation, ubiquitination, binding and transcriptional regulation. The algorithm scans SIGNOR to find the interaction edges of which both nodes exist in the input list of proteins. The curated data from SIGNOR include the type of interactions (transcription, phosphorylation, complex formation, etc.), the direction of interactions, biochemical effect (activation or inhibition) and the evidence of the interaction. The harvested interactions are assembled to construct the PPI network. This graph provides a holistic static view of the interactions between the input proteins.

### 3.2 Step II: Converting the PPI network to a biochemical reaction network

The construction of PPI networks is a way to schematically illustrate the interactions between proteins. This depiction mode is concise and mainly focused on outcomes rather than processes. For instance, the phosphorylation of protein A by protein B is simply shown with a single edge, ignoring different underlying molecular processes. However, in terms of biochemical processes, this interaction is a set of reactions each with a different parameter. Similarly, other molecular interactions can be considered as a series of biochemical reactions (Table 1). In order to include transcriptional regulations, the participating genes are considered at DNA and RNA levels in addition to the protein level. For instance, in transcriptional upregulation, regulatory protein A binds the DNA of gene B and activates its transcription which then is translated to protein B. For genes without transcriptional regulation edges, a basal level of protein production is assumed. In addition, a degradation rate is assumed for all reactants and products.

**Table 1.** The ‘dictionary’ for converting interaction edges to their equivalent biochemical reactions

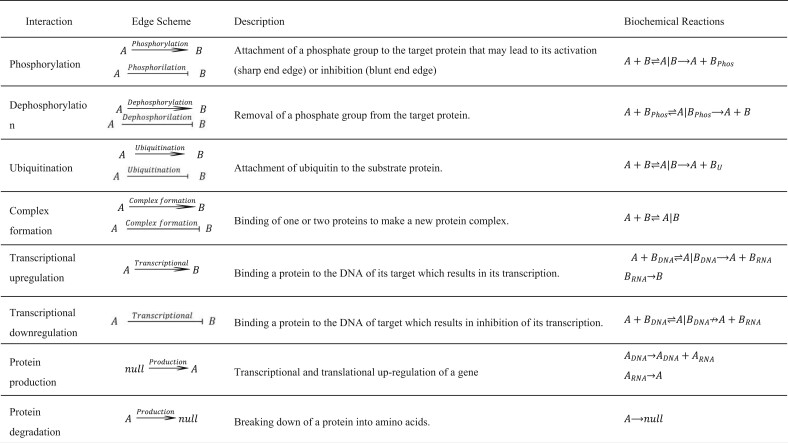

**Fig. 2.**
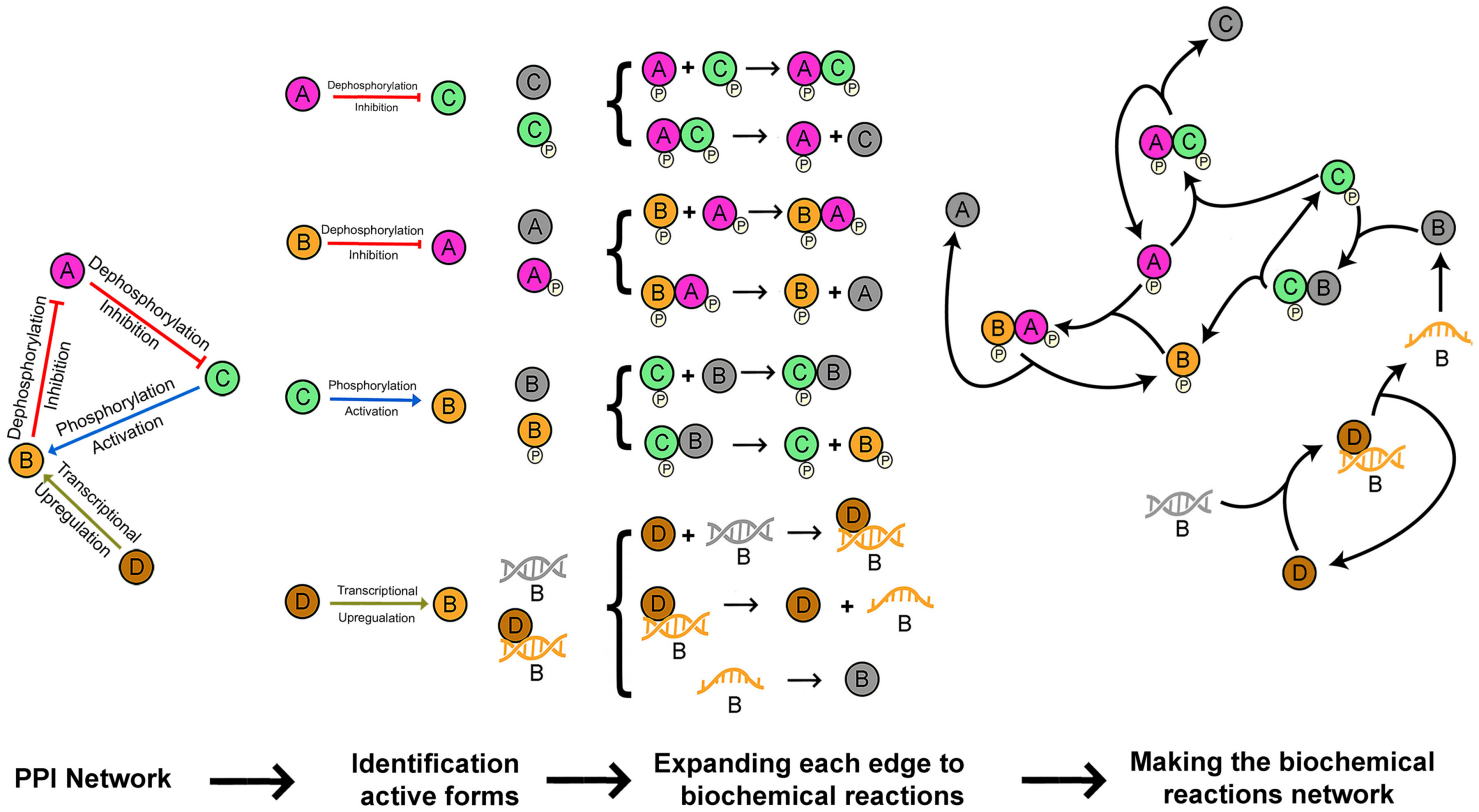
An example of converting a PPI network to its equivalent biochemical reaction network. (**A**) For the input list of proteins, the interaction types and activation or inhibition effects are harvested from SIGNOR. The complete list of interaction types is available in Table 1, not all of them are demonstrated in this simple example. The active form of each biomolecule is determined by scanning the interactions in which that biomolecule is involved. For example, considering the dephosphorylation inhibition edge from A to C, SPADAN detects that phosphorylated C is the active form. Inactive forms are shown by gray circles. (**B**) Considering the active forms of the molecules, each interaction is converted to a series of biochemical reactions. The rules for this conversion are shown in Table 1. (**C**) The biochemical reactions are assembled based on shared elements to construct the biochemical reaction network. All these steps are performed automatically

The translation from network language to biochemical reactions is an essential step in the construction of the model structure. A simple example of this process is schematically depicted in Figure 2 using the data harvested from SIGNOR. SPADAN determines the consequence of interactions in terms of activation or inhibition of the target protein. This information is stored as an ‘active mode table’ showing different forms of proteins in terms of Post-Translational Modification (PTM) and indicates which modification mode is the active state of each protein (Supplementary Table S1). We acknowledge that this is a simplification of the real world in which PTMs do not necessarily result in ON-and-OFF switch-like behaviors but may lead to partial augmentation or inhibition of basal activities. As well, the active state may not be restricted to one of the PTM modes.

An advantage of the developed algorithm is that it recognizes that each protein is present in different fractions at a given time point in terms of PTMs, bound to DNA or bound to other proteins. Indeed, the measured concentration of a given protein at a time is the sum of concentrations of these fractions. Each protein form is considered as an element and receives a unique ID in the algorithm. Furthermore, each element may participate in different reactions as there are overlaps between reactants and products of different reactions. Hence, the biochemical reactions are inter-connected and construct a biochemical network. In order to organize the data of metabolic reaction network for downstream computational processes, a ‘3D reactions matrix’ is generated with several 2D pages. Each page represents a biochemical reaction with two columns for reactants and products (Supplementary Fig. S1).

### 3.3 Step III: Converting the biochemical reaction network to a large-scale ODE system

The biochemical reaction network provides a comprehensive view on what is happening between interacting molecules. However, it does not explain the dynamic behavior of the system. In order to obtain dynamic insights, a large-scale ODE system is constructed by generating the kinetic equation for each biochemical reaction automatically. Although SPADAN is able to employ Michaels–Menten or Hill kinetics, all reactions in the CRC biochemical network are modeled based on mass action law in order to avoid pre-assumptions of those models. Consequently, the changing rate of concentrations in each reaction is written as a function of the concentrations of substrates and the kinetic parameter of the reaction. Since each molecule can take part in several reactions, its evolution with time is the algebraic sum of the concentration change rates in those reactions. PPI networks are generally constructed to provide a holistic description of the underlying events of complex biomedical phenomena, commonly composed of large numbers of interactions. The size of the model even expands when PPI networks are translated to biochemical networks. Thus, the ODE system constructed based on these networks has an unusually large scale. Therefore, applying novel strategies to decrease computational costs is of critical importance.

Since the ODE solver used in this article is based on numerical differentiation formulas, it calls the function F explained in Equation (2) in each time step of integration.

(2) $$\dot{X}=F(X,K),$$

where X and K are the matrices of state variables and model parameters, respectively. F is a non-linear parametric function of state variables.

On the other hand, completing the integration requires thousands of time steps. Thus, decreasing the computation cost of the function F has a considerable effect on the speed of model simulation and consequently on parameter estimation. In order to lower the computation cost of calculating F, the SPADAN calculates F by converting that to matrix multiplications as described further.

In order to make an ODE model from the biochemical reaction network, the concentration of each node in the network is considered as a state variable which is shown by x. Since reaction rates of the biochemical reactions in this article are calculated based on mass-action law, the rate of each reaction shown with v can be written as

(3) $$v_{i}=k_{F_{i}}x_{r1}x_{r2}x_{r3}\ldots -{\;k}_{B_{i}}x_{p1}x_{p2}x_{p3}\ldots ,$$

where i is the reaction number and $k_{F_{i}}$ and ${\;k}_{B_{i}}$ are the forward and backward reaction rate constants. In addition, $x_{r1\;}$, $x_{r2\;}$, $x_{r3}$, … represent the reactants and $x_{p1\;}$, $x_{p2\;}$, $x_{p3}$, … represent the products. Therefore, as explained in Equation (5), the derivative of each state variable is calculated by adding the reaction rates of which the equivalent node takes part in as product and subtracting the rates in which the equivalent node is a substrate where M is the total number of reactions and $v_{m}$ is the rate of the reaction *m*.

The non-linear structure of the equations in (2) and (3) shows that it is not possible to write the ODEs in the form of a linear ODE system as follows:

(4) $$\dot{X}=AX+B.$$

Thus, the ODE solver cannot calculate $\dot{X}$ by matrix multiplications which forces that to calculate each indice of $\dot{X}$ separately. This method of calculating increases computation cost exponentially by enlarging the size of the ODE system

(5) X˙k=∑m=1Mcmvm Cm=xk is a product in reaction m 1 xk is a substrate in reaction m-1  else 0 .

Considering the structure of the ODE system in this problem, the matrix of total reaction rates denoted by V can be calculated as

(6) $${V\left(X,K\right)}_{m\times 1}=K_{F}⊙X_{R1}⊙X_{R2}⊙X_{R3}⊙\ldots \;⊙X_{P1}-{\;K}_{B}⊙X_{P2}⊙X_{P3}⊙\ldots ,$$

where ⊙ is the element-wise product and $K_{F}$ and ${\;K}_{B}$ include the forward and backward reaction rate constants. $X_{Pj}$ and $X_{Rj}\;$include the list of $j_{\mathrm{th}}$ products and reactants for all reactions, respectively. The number of ${X_{R}}_{i}$s and ${X_{p}}_{i}$s depends on the maximum number of reactants and products of the reactions.

By analyzing the roles of each state variable in reactions, matrix H is given by

(7) hi,j=1, i is a product in reaction j-1, i is a substrate in reaction j0, i is not in reaction j.

Consequently, multiplying V by H results in the matrix $\dot{X}$ which is shown in Equation (8)

(8) $${\dot{X}}_{n\times 1}=\;F_{n\times 1}\left(X,K\right)=\;H_{n\times m}\times V_{m\times 1}.$$

In this method of calculating $\dot{X}$, in each time step of integration, the ODE solver calculates indices of $\dot{X}$ simultaneously by matrix multiplications which yields the same result with less computational cost. Testing this method on the non-linear ODE model of colon cancerous cells with 3347 state variables (which is explained further) shows that the proposed method could decrease mean calculation time from 2.5 s to 0.25 s for each run of ODE solving with similar initial values.

Taken together, using the SPADAN algorithm, the total biochemical reaction network is modeled by ODEs which have a set of unknown kinetic parameters denoted as K. In further steps, SPADAN attempts to find an acceptable approximation of the parameters using time-course experimental data.

### 3.4 Step IV: Parameter estimation

According to Equation (2), there is at least one kinetic parameter for each reaction of the biochemical reaction network. Therefore, ODE systems made from genome-wide networks have a large number of parameters. Except for metabolic reactions, there is not much experimental evidence available for the rate of protein reactions. Therefore, providing acceptable estimations of model parameters based on time-series measurements is of utmost importance here.

In order to estimate model parameters, SPADAN uses the least-squares method in which the gap between model simulations and experimental data is defined as an objective function called S which is explained in Equation (1). After that, model parameters are altered by an optimizer to minimize the objective function.

Due to the non-linear kinetics of biochemical reactions, the ODE systems that SPADAN operates on are mostly non-linear. Therefore, during development of this pipeline, the performances of non-linear optimization algorithms such as unconstrained quasi-newton, Nelder–Mead Simplex Method (Lagarias *et al.*, 1998) and Levenberg–Marquardt Algorithm (Moré, 1978) for large-scale parameter estimation problems were assessed. The results have shown that the progression toward the optimization solution becomes exponentially more complicated by increasing the number of unknown parameters and model equations. This occurs due to the numerical or analytical gradient calculations which are performed by the optimizers to find the traveling direction toward the optimum point. The gradient calculations need numerous times of model simulations which increase exponentially by increasing the number of unknown parameters. In addition, by enlarging the size of the ODE system, the time of each simulation run increases resulting in incrementing the total computation cost. Although the Nelder–Mead Simplex Method does not use derivatives and have a better performance for large-scale problems (Klein and Neira, 2014), the number of needed model simulations for each step progression is related to the number of unknown parameters making it difficult to find an answer in a feasible time. To overcome this challenge, we have proposed an approximation method that speeds up the optimizer progression by considering the interconnectivity between parameters and state variables in ODEs. Therefore, in this method, the total optimization problem is broken into several sub-optimal problems to find the best possible answer. This method is the upgraded version of the optimization method introduced in our previous study (Borzou *et al.*, 2019).

**Fig. 3.**
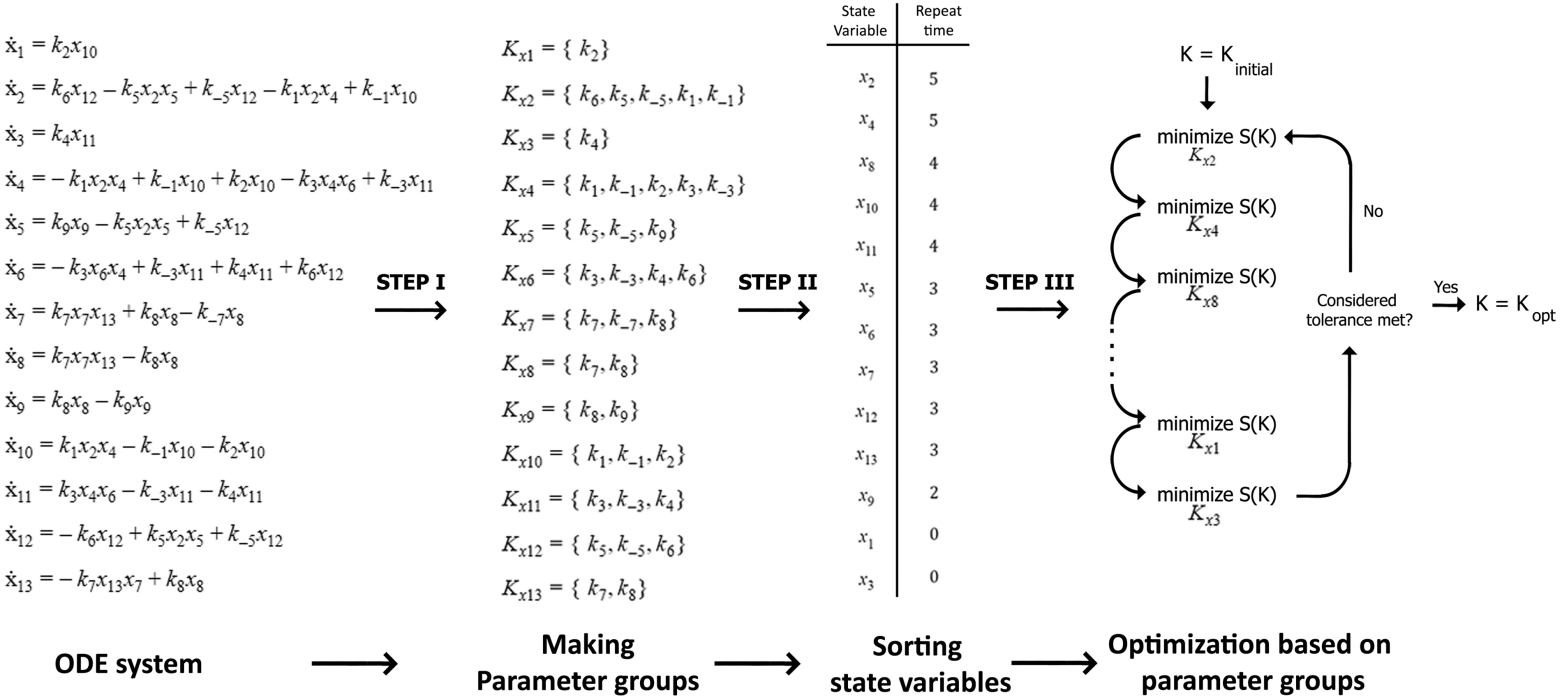
A simple example of the proposed parameter approximation algorithm. A supposed ODE system with 13 state variables is demonstrated in the left column. In the first step, a parameter group is made for each state variable. The group named as $K_{x_{i}}$ contains parameters existing in the equation of${\dot{\;x}}_{i}$. In Step II, the algorithm sorts the state variables based on the number of times repeated in the equations. In Step III, the optimizer solves a sub-optimal problem starting from the state variables with higher repetitions moving to less frequent variables. The sub-optimal problem for the state variable $x_{i}$ is to optimize the value of *S* in the space of parameters belonging to $K_{x_{i}}$. The algorithm is finished by reaching the considered tolerance of *S*

In the first step of the proposed parameter approximation method, the parameters existing in the derivative equation of each state variable are grouped together. As an example, in the ODE system shown in Figure 3, $K_{x2}$ includes the parameters that take part in the equation of ${\dot{x}}_{2}$. In the next step, state variables are sorted based on the times repeated in the equations as a measure of their influence on the entire system. In Step 3, the Nelder–Mead optimizer starts to find the optimum *S* by altering the parameters related to the first group only. In the further steps, the optimizer performs this procedure for the next parameter groups. After reaching the last group of parameters, the algorithm starts a new cycle from the first parameter group. This procedure continues until reaching the proposed termination tolerance. The details of this optimization algorithm are shown in Figure 3. In summary, the proposed approximation algorithm finds an estimation of the parameter values using time-course experimental data.

Taken together, SPADAN results in a large-scale ODE system, provided as an ‘m file’, describing the dynamics of interactions between all experimentally detected biomolecules in the considered biological situation. This pipeline can be exploited to model the dynamic behavior of a biological system as exemplified in the next section. It should be mentioned that the databases and the computational tools used in each step of the pipeline are shown in Supplementary Table S2.

## 4 Case study: applying SPADAN to develop a dynamic model of CRC

In order to assess the applicability of the developed algorithm to construct a dynamic model based on a time-course experimental data, we have here re-analyzed and exploited a time-course multi-layer expression profiling data originally generated by Ressa *et al.* (2018). These investigators assessed the response of WiDr CRC cells to vemurafenib and gefitinib as BRAF inhibitor (BRAFi) and EGFR inhibitor (EGFRi), respectively. The cells were harvested 0, 2, 6, 24 and 48 h after treatment and transcriptomics, proteomics and phosphoproteomics datasets were generated. They found the up-regulation of metabolic pathways and tyrosine kinases receptors under BRAF inhibition as a primary response. Also, the switching of energy sources in treated cells turns to a defensive state to compensate for MAPK signaling inhibition. Noteworthy, they extended the analyses to a PTPN11 knockout WiDr cell line, the data of which are not used in the current study. The expression profiles of four experimental groups, including no treatment control, cells treated with BRAFi, EGFRi or both are here explored.

**Fig. 4.**
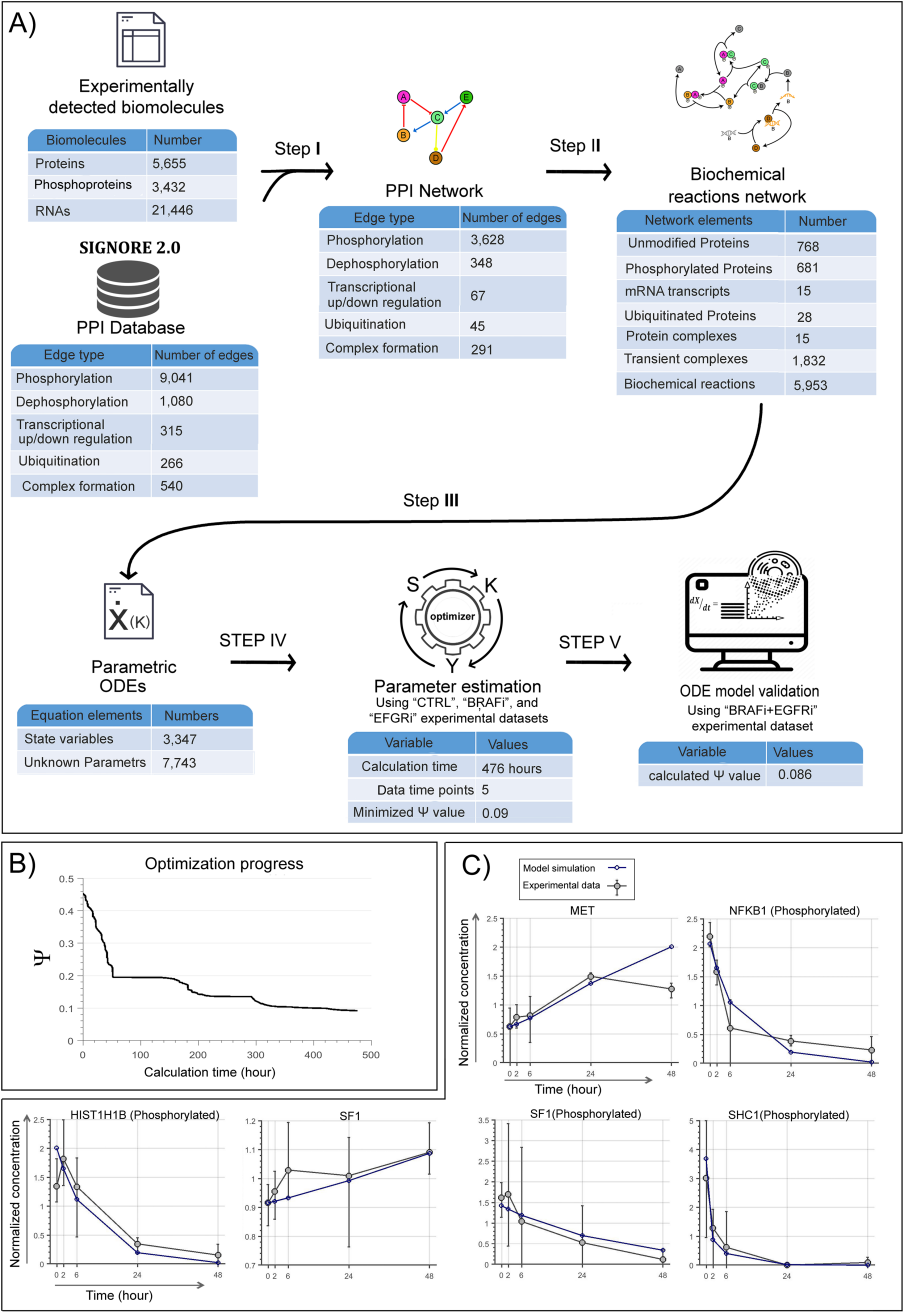
Case study modeling and validation. The validity of the pipeline was validated by the construction of a large-scale dynamic model of the response of a CRC cell line to chemotherapy agents. The experimental data include four experimental group including untreated control cells, treated by EGFRi, BRAFi or the combination of both drugs. The model is constructed with the first three groups and validated with the fourth one. (**A**) Statistics of different steps of modeling pipeline. (**B**) The progression of minimization of function *S* during parameter approximation process. After 476 h of calculation, the *S* value was decreased from $2.72\times 10^{7}$ to $1.13\times 10^{6}$ which equals to decreasing Ψ value from 0.45 to 0.09. (**C**) Model simulations versus experimental data for some representative genes. Gray circles show the mean value and error bars are the minimum and maximum of the three biological replicates at each time step

Using MaxQuant analysis and after filtration, 5655 proteins and 3432 phosphoproteins were identified. We were interested to consider the role and interactions of all identified genes, not only the differentially expressed ones. Hence, using SPADAN, the map of interactions between all identified proteins, phosphoproteins and transcripts in WiDr CRC cells was constructed (Fig. 4A). Next, the biochemical reaction network was constructed including 5953 reactions and 3347 nodes. This network was then converted to a large ODE system with 3347 equations and 7743 parameters.

### 4.1 Quantification of biomolecules

Proteomics data are generally expressed as relative quantifications with fold change values. However, the dynamic model simulation outputs are absolute concentrations. In order to make the comparison of experimental and simulation data possible, the absolute concentrations of proteins were estimated using the ‘proteome ruler’ plugin which uses histones as standards. Employing this technique is not feasible for phosphoproteins. Hence, in order to compare phosphoproteomics experimental and simulation data, mean normalization was performed for both datasets; intensities of each phosphoprotein were scaled to make the mean value of the five time points equal to one. In order to calculate RNA absolute concentrations, normalized RNA counts were divided by an estimated volume of mammalian cells which is about 8 µl (Sender *et al.*, 2016).

### 4.2 Approximation of basal concentrations

In ODE systems, initial values play an important role in the dynamic behavior of the system. Each protein concentration obtained from proteomics data is indeed the sum of concentrations of different states of that protein including phosphorylated form. In order to have an estimation of initial values of proteins and phosphoproteins, we relied on a previous study indicating that phosphoproteins constitute about 30% of total protein concentrations (Cohen, 2002). Hence, 30% and 70% of the measured concentration of each protein at the initial point was considered as the concentration of the phosphorylated and unmodified forms of that protein, respectively. We acknowledge that in real situations this 30/70 ratio is not exactly true for all proteins. However, in the lack of absolute quantitative data, especially for phosphoproteins, this approximation can be acceptable.

For transcriptomics data, absolute concentrations are available for both time 0 and other time steps as indicated above. For the genes with transcriptional regulation in the model, DNA level is also included which can be bound to transcription factors or in the unbound state. The total DNA concentration obviously remains constant and is equal to two copies divided by the estimated cell volume which is about 8 µl (Sender *et al.*, 2016).

### 4.3 Function *S*

In order to adjust model parameters, it is essential to compare model outputs with experimental data. In the exploited experimental data, measurements were performed with three biological replicates. The matrix of three biological replicates for each time point of experimental data is denoted by ${\underline{e}}_{i,j}$ as follows:

(9) $${\underline{e}}_{i,j}=\left[\begin{array}{c} e_{i,j,1}\\ e_{i,j,2}\\ e_{i,j,3} \end{array}\right],$$

where i is the gene number, j is the time point number and the third index represents the biological replicates.

As indicated above, each biomolecule can be present in different nodes of the biochemical network (Fig. 2). Hence, the simulated concentration of a given biomolecule is the summation of its concentration in the attributed nodes. Model simulation of the concentrations at each time point is calculated as matrix Y:

(10) $$Y_{2295\times 1}=\left[\begin{array}{c} {Y_{P}}_{765\times 1}\\ {Y_{\mathrm{Phos}}}_{765\times 1}\\ {Y_{\mathrm{RNA}}}_{765\times 1} \end{array}\right]=\left[\begin{array}{c} {C_{P}}_{765\times 3347}\\ {C_{\mathrm{Phos}}}_{765\times 3347}\\ {C_{\mathrm{RNA}}}_{765\times 3347} \end{array}\right]\times X_{3347\times 1},$$

$Y_{P}$, $Y_{\mathrm{Phospho}}$ and $Y_{\mathrm{RNA}}$ are three sets of model outputs which show the simulated concentrations of proteins, phosphoproteins and mRNAs at each time point, respectively. These values are the sum of concentrations of each biomolecule in different nodes of the biochemical reactions network and are calculated by multiplication of the *C* matrix to the *X*. *C* matrix consists of three binary matrices called $C_{P}$, $C_{\mathrm{Ph}}$ and $C_{\mathrm{RNA}}$ which are automatically made by SPADAN.

Considering the wide range of concentration scales, the sum of the squares of differences between model output and experimental data in different time points is calculated for each biomolecule and then divided by the average of experimental concentrations in the five time points.

As the concentration estimations are available for the proteomics and transcriptomics experimental data, the average of their concentrations in the five time points (${\tilde{e}}_{i}$) is calculated by

(11) $${\tilde{e}}_{i}=\frac{1}{5}\sum_{j=1}^{5}\mathrm{avg}\left(\;{\underline{e}}_{i,j}\right).$$

For each time point, if the simulation value ($y_{i,j})\;$is in the range of minimum and maximum of three experimental replicates shown with $\max({\underline{e}}_{i,j})$ and $\min({\underline{e}}_{i,j})$, $\alpha_{i,j}$ is considered zero and the difference between simulation and experimental data is ignored in calculating *S*. Otherwise, the residual between simulation data and mean value of the three replicates ($d_{i,j})\;$is used to calculate function *S* as the sum of squares of differences between simulation and experimental data. Variable $\alpha_{i,j}$ is calculated using

(12) $$\alpha_{i,j}=\frac{1}{2}s\mathrm{gn}\left(\;{(y}_{i,j}-\max({\underline{e}}_{i,j})){(y}_{i,j}-\min({\underline{e}}_{i,j}))\right)+\;\frac{1}{2}.$$

To calculate *S* relatively, it is calculated as follows:

(13) $$\begin{array}{c} d_{i,j}=\;y_{i,j}-\mathrm{avg}\left(\;{\underline{e}}_{i,j}\right)\\ {(S}_{p}\;\mathrm{or}\;S_{\mathrm{RNA}})=\sum_{i=1}^{768}\sum_{j=1}^{5}\alpha_{i,j}{\left(\frac{d_{i,j}}{{\tilde{e}}_{i}}\right)}^{2} \end{array},$$

where $S_{p}$ and $S_{\mathrm{RNA}}$ are the sum of squares for proteomics and transcriptomics levels, respectively. In addition, i is the genes number and j is the number of time points. An example of data normalization and *S* calculation is visualized in Supplementary Figure S2.

Since in this case study, absolute concentrations of phosphoproteomics experimental data were not available, both experimental data and model simulations were mean normalized prior to comparison of their trajectories. Mean normalization of experimental intensities was performed according to Equation (14)

(14) $${\bar{I}}_{i,r}=\frac{1}{5}\sum_{j=1}^{5}I_{i,j,r}\;\;\;\;\;e_{i,j,r}=\;\frac{I_{i,j,r}}{{\bar{I}}_{i,r}},$$

$I_{i,j,r}$ is the intensity of the $i_{\mathrm{th}}$ phosphoprotein at the $j_{\mathrm{th}}$ time point in the $r_{\mathrm{th}}$ biological replication.

After defining $e_{i,j,r}$ in Equation (14), the matrix ${\underline{e}}_{i,j}$ is constructed as Equation (9).

**Fig. 5.**
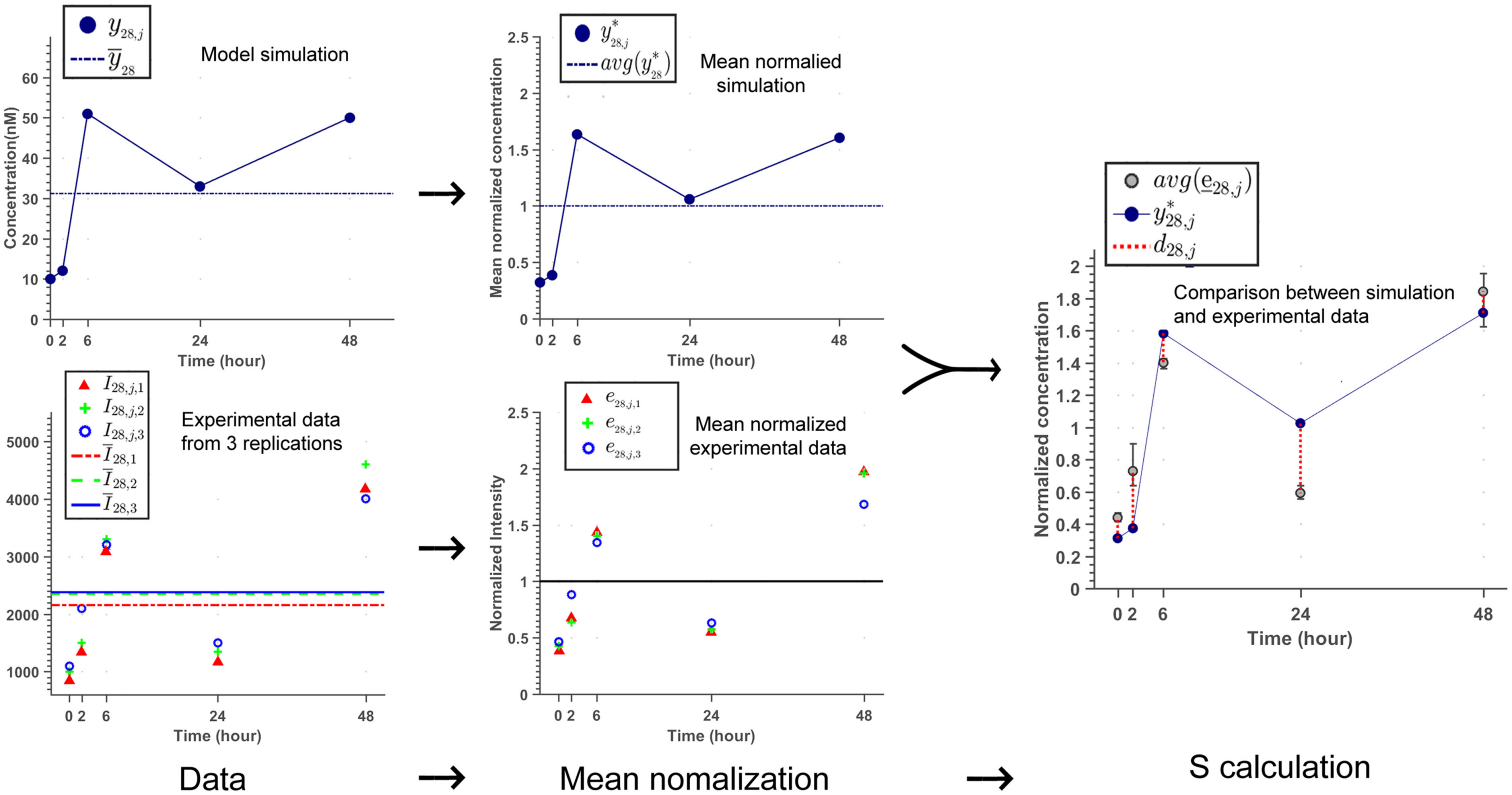
Representative example of data normalization in order to make model simulations and experimental measurements comparable. Since experimental data from phosphoproteins are intensities and model simulations are concentrations, they are both mean normalized to 1 for the calculation of *S* value

Similarly, model simulations are mean normalized by

(15) $${\bar{y}}_{i}=\frac{1}{5}\sum_{j=1}^{5}y_{i,j}\;{y^{*}}_{i,j}=\;\frac{y_{i,j}}{{\bar{y}}_{i}},$$

where ${y^{*}}_{i,j}$ is the mean normalized simulation value of the $i_{\mathrm{th}}$ phosphoprotein at the $j_{\mathrm{th}}$ time point. Next, the sum of squares for phosphoproteins, shown with $S_{\mathrm{ph}}$, is calculated by

(16) $$\begin{array}{c} \alpha_{i,j}=\frac{1}{2}s\mathrm{gn}\left({\;(y^{*}}_{i,j}-\max({\underline{e}}_{i,j})){(y}_{i,j}-\min({\underline{e}}_{i,j}))\right)+\;\frac{1}{2}\\ \begin{array}{c} d_{i,j}=\;{y^{*}}_{i,j}-\mathrm{avg}\left({\underline{e}}_{i,j}\right)\\ S_{\mathrm{ph}}=\sum_{i=1}^{768}\sum_{j=1}^{5}{{\alpha_{i,j}d}_{i,j}}^{2} \end{array}\; \end{array},$$

in which $d_{i,j}$ is ignored in the summation, if $\alpha_{i,j}$ is zero. A visual example of *S* calculation for the phosphoproteins is shown in Figure 5.

The normalized *S* values for all model sub-compartments are then summed to calculate${\;S}_{\mathrm{total}}$:

(17) $$S_{\mathrm{total}}=S_{p}+S_{\mathrm{ph}}+S_{\mathrm{RNA}}.$$

Using the SPADAN parameter approximation algorithm, $S_{\mathrm{total}}$ was decreased from $2.72\times 10^{7}$ to $1.13\times 10^{6}$ after 476 h of processing. In order to get a better insight about the mean value of *S* for each time point of the model, Ψ is declared in Equation (18) which is the mean value of root summed squared of the model residual for 768 biomolecules measured in triplicates at 5 time points.

(18) $$\Psi =\frac{\sqrt{S_{\mathrm{total}}}}{768\times 5\times 3}.$$

The progression of the proposed optimizer in minimizing Ψ from initial guess to approximated parameter values is shown in Figure 4B.

### 4.4 Model validation

After estimating model parameters to fit outputs to experimental data from control, EGFRi and BRAFi groups, the model was run in the situation of simultaneous treatment with EGFRi and BRAFi drugs. Simulation results were then compared with experimental data to assess the accuracy of the model prediction. The Ψ value, which shows the difference between model prediction and the experimental data for BRAFi + EGFRi dataset, was 0.083 indicating that the model could present an acceptable prediction. The comparison between model simulations and experimental data for some representative biomolecules is shown in Figure 4C.

## 5 Discussion

Despite invaluable insights provided by systems biology in the last few decades, a major unmet flaw is that the constructed models are either holistic or dynamic. In the top-down approach, big data are organized to generate holistic but static maps of interactions. On the other hand, in the bottom-up approach, mathematical predictive models can be constructed that incorporate the cell dynamism but focus on limited numbers of role players and ignore many critical elements. Pipelines for the construction of large-scale dynamic models are crucial for comprehensive investigations of complex disorders. The current study was aimed at the development of such a framework.

A bottleneck in constructing large dynamic networks is to identify the biochemical reactions involved in the interaction between two biomolecules. Indeed, every single edge between two proteins in a PPI network is a compact code that should be decoded to the more comprehensive language of biochemical reactions. We have generated a conversion list that could be assumed as a ‘dictionary’ for the translation of graph edges to reactions. Biochemical reactions are then converted to a set of ODEs. Hence, state-space equations can be extracted from large-scale networks using the above steps. Although in the presented case study, all equations are based on mass action, SPADAN allows to employ Michaelis–Menten or Hill kinetics as well.

In this study, we have employed time-course large-scale experimental data for colon cancer cells to assess the validity of the developed framework. An advantage of the constructed network is that it encompasses different layers of biomolecules including proteins, phosphoproteins and mRNA transcripts. Furthermore, the network is constructed with all identified proteins, not merely those differentially expressed, in order to consider the interactions of proteins that are not differential but can be involved in crucial interactions. Notably, in the large-scale model constructed by SPADAN, some complexities of gene–protein–reaction associations such as isozymes (multiple proteins–one function), promiscuous enzymes (one protein–multiple functions) and protein complexes (Machado *et al.*, 2016) are successfully included.

The constraint-based modeling approach has recently been improved by some investigators to consider the transient behaviors (Colarusso *et al.*, 2021; Zomorrodi *et al.*, 2014). In addition, the construction of large-scale kinetic models of metabolic networks has been attempted by a few investigators (Smallbone *et al.*, 2010; Smallbone and Mendes, 2013; Smith *et al.*, 2018). Despite the high merit of these works, they are limited by dependence on the availability of kinetic parameters (Smallbone *et al.*, 2010). Remarkably, the knowledge on the constant rates of the reactions is scarcer for PPI networks compared with metabolic systems. This indicates that parameter estimation is at the cornerstone of large-scale dynamic modeling of protein associations. The parameter estimation procedure is performed by minimizing the differences between trajectories of model outputs and time-course experimental measurements (Balsa-Canto *et al.*, 2010). Although a variety of optimizers are available for this purpose (Penas *et al.*, 2017), they could hardly be applied for large-scale ODE models. Therefore, in this study, a novel parameter approximation tool is developed and incorporated into SPADAN which masters the complexity of equations and the large dimensions of genome-scale models. Furthermore, to speed up the parameter estimation, this procedure has the potential to be parallelized on multi-core supercomputers. This enhanced computation approach would pave the way for more sophisticated analyses such as considering the stochasticity of biological reactions. However, it should be noted that the parameter estimation strategies, including the method employed in this study, may result in sets of parameters that are not unique. This is due to the complexity and non-linearity of biological systems and the inadequacy of time-course experimental data.

In conclusion, we have proposed a modeling and optimization method which can fill the gap between large-scale static and small-scale dynamic modeling strategies. This simulation scheme allows quantitative analysis of cell behavior and prediction of response to different therapeutic interventions, which is a major step toward precision medicine.

## Supplementary Material

btad079_Supplementary_Data
