# Supplementary material for: A novel strategy for dynamic modeling of genome-scale interaction networks
Source: Bioinformatics. 2023 Feb 24;39(2):btad079. doi: 10.1093/bioinformatics/btad079 (PMC9969830; doi:10.1093/bioinformatics/btad079)

A Novel Strategy for Dynamic Modelling of Genome-Scale Interaction Networks
Supplementary data

Table of contents

- PART1: supplementary tables and figures ………………………………………… 2
- PART2: List of biochemical reactions ………………………………………………… 5
- PART3: List of PPI edges ………………………………………………………………….143
- PART4: List of Genes informations (Ensemble and Uniprot codes) ……..191
- PART5: List of ODE state-space equations ……………………………………..346
- PART6: Estimated parameter values ………………………………………………..403
- PART7: Plots of model outputs vs 3 replications of experimental data ……………530

PART1: Supplementary tables and figures

*Supplementary Table 1. **Active mode table.** In the modeling procedure performed by SPADAN, for each protein, only one of the PTM modes is consumed as the active form. The table is automatically made by scanning the types and effects of PPI network edges.*

ID	protein	phosporilated protein	ubiquitinated protein
proteini 50	0	1	0
proteini 51	1	0	0
proteini 52	1	0	0
proteini 53	0	1	0
proteini 54	0	1	0
proteini 55	0	1	0
proteini 56	1	0	0
proteini 57	1	0	0
proteini 58	1	0	0
proteini 59	0	1	1
proteini 60	0	1	0
proteini 61	1	0	0
proteini 62	0	1	0
proteini 63	1	0	0
proteini 64	1	0	0
proteini 65	0	1	0

*Supplementary Table 2. **The list of databases and toolboxes used in each step of SPADAN**. The pipeline was developed in MATLAB 2015b. To perform SPADAN pipelines, most of the functions were written by the author, not by using any toolboxes.*

SPADAN step No.	Database(s) used	Toolbox(es) used
**STEPI**	SIGNOR 2.0, BioMart	________
**STEP II**	________	________
**STEP III**	________	________
**STEP IV**	________	ODE 15s, OptimTools

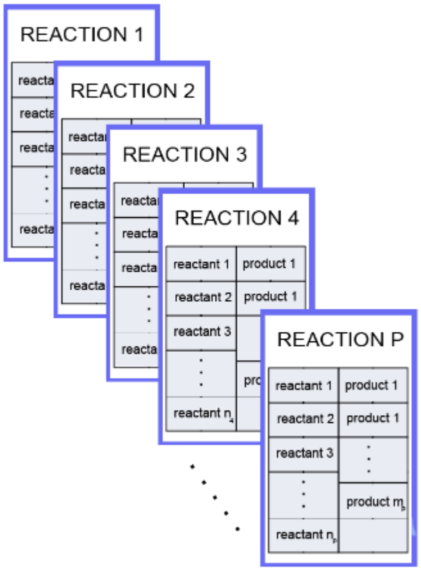

Supplementary Figure1. ***The 3D "reactions" matrix*** *is used by SPADAN to store the biochemical reactions network. Each page of the matrix represents one reaction in this network and includes IDs of reactants and products in the first and second columns, respectively. These two columns do not necessarily have the same size.*

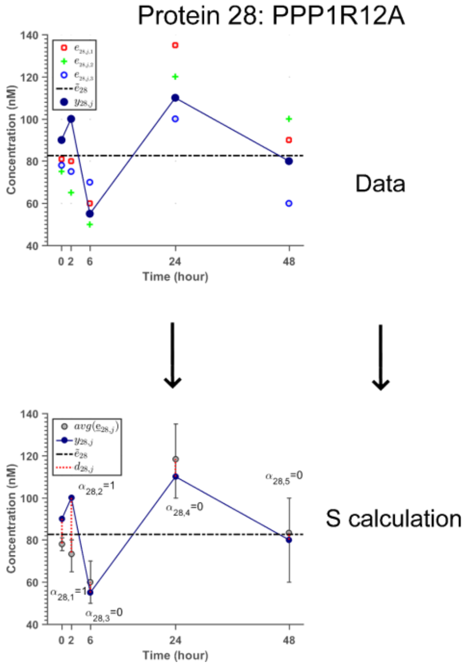

Supplementary Fig. 2. ***An example of comparing method between model simulation and experimental data*** *for RNAs and proteins. Three biological replications for each experimental data time point are shown with red, green, and blue markers. Since both experimental data and model simulation are absolute concentrations, there is no need for mean normalization. The method of calculating S is explained in (11) to (13).*

PART1: List of biochemical reactions in large-scale colon cancerous cell's model

*_p : Phosphorylated protein or protein complex
*_DNA : DNA of the Gene
*_u : Ubiquitinated protein
| : Complex between two elements
--> : One directional reaction
<--> : Two dorectional reaction

<table>
<tr><th>Reaction number</th><th>Reaction details</th></tr>
<tr><td>1</td><td>Q09472_p +Q92793_p <-->SIGNOR-C6 : K(1,1) for forward directionand K(1,2) for reverse direction</td></tr>
<tr><td>2</td><td>P19838_p +Q04206_p <-->SIGNOR-C13 : K(2,1) for forward directionand K(2,2) for reverse direction</td></tr>
<tr><td>3</td><td>P06493_p +P14635_p <-->SIGNOR-C17 : K(3,1) for forward directionand K(3,2) for reverse direction</td></tr>
<tr><td>4</td><td>P08047_p +P40763_p <-->SIGNOR-C74 : K(4,1) for forward directionand K(4,2) for reverse direction</td></tr>
<tr><td>5</td><td>P20248_p +P24941_p <-->SIGNOR-C83 : K(5,1) for forward directionand K(5,2) for reverse direction</td></tr>
<tr><td>6</td><td>P05412_p +P84022_p <-->SIGNOR-C86 : K(6,1) for forward directionand K(6,2) for reverse direction</td></tr>
<tr><td>7</td><td>P49815_p +Q92574 <-->SIGNOR-C101 : K(7,1) for forward directionand K(7,2) for reverse direction</td></tr>
<tr><td>8</td><td>P40763_p +P42224_p <-->SIGNOR-C118 : K(8,1) for forward directionand K(8,2) for reverse direction</td></tr>
<tr><td>9</td><td>Null <-->SIGNOR-C154 : K(9,1) for forward directionand K(9,2) for reverse direction</td></tr>
<tr><td>10</td><td>P31749_p +P55211 <-->P31749_p |P55211 : K(10,1) for forward directionand K(10,2) for reverse direction</td></tr>
<tr><td>11</td><td>P31749_p |P55211 -->P31749_p +P55211_p : K(11,1) for forward direction</td></tr>
<tr><td>12</td><td>P31749_p +P46527 <-->P31749_p |P46527 : K(12,1) for forward directionand K(12,2) for reverse direction</td></tr>
<tr><td>13</td><td>P31749_p |P46527 -->P31749_p +P46527_p : K(13,1) for forward direction</td></tr>
<tr><td>14</td><td>P31749_p +O14757 <-->P31749_p |O14757 : K(14,1) for forward directionand K(14,2) for reverse direction</td></tr>
<tr><td>15</td><td>P31749_p |O14757 -->P31749_p +O14757_p : K(15,1) for forward direction</td></tr>
<tr><td>16</td><td>P31749_p +Q12778 <-->P31749_p |Q12778 : K(16,1) for forward directionand K(16,2) for reverse direction</td></tr>
<tr><td>17</td><td>P31749_p |Q12778 -->P31749_p +Q12778_p : K(17,1) for forward direction</td></tr>
<tr><td>18</td><td>P31749_p +A8MYZ6 <-->P31749_p |A8MYZ6 : K(18,1) for forward directionand K(18,2) for reverse direction</td></tr>
<tr><td>19</td><td>P31749_p |A8MYZ6 -->P31749_p +A8MYZ6_p : K(19,1) for forward direction</td></tr>
<tr><td>20</td><td>P31749_p +P49840 <-->P31749_p |P49840 : K(20,1) for forward directionand K(20,2) for reverse direction</td></tr>
<tr><td>21</td><td>P31749_p |P49840 -->P31749_p +P49840_p : K(21,1) for forward direction</td></tr>
<tr><td>22</td><td>P31749_p +P09651 <-->P31749_p |P09651 : K(22,1) for forward directionand K(22,2) for reverse direction</td></tr>
<tr><td>23</td><td>P31749_p |P09651 -->P31749_p +P09651_p : K(23,1) for forward direction</td></tr>
<tr><td>24</td><td>P31749_p +P04792 <-->P31749_p |P04792 : K(24,1) for forward directionand K(24,2) for reverse direction</td></tr>
<tr><td>25</td><td>P31749_p |P04792 -->P31749_p +P04792_p : K(25,1) for forward direction</td></tr>
<tr><td>26</td><td>P31749_p +O43464 <-->P31749_p |O43464 : K(26,1) for forward directionand K(26,2) for reverse direction</td></tr>
<tr><td>27</td><td>P31749_p |O43464 -->P31749_p +O43464_p : K(27,1) for forward direction</td></tr>
<tr><td>28</td><td>P31749_p +P45985 <-->P31749_p |P45985 : K(28,1) for forward directionand K(28,2) for reverse direction</td></tr>
<tr><td>29</td><td>P31749_p |P45985 -->P31749_p +P45985_p : K(29,1) for forward direction</td></tr>
<tr><td>30</td><td>P31749_p +Q9UBP6 <-->P31749_p |Q9UBP6 : K(30,1) for forward directionand K(30,2) for reverse direction</td></tr>
<tr><td>31</td><td>P31749_p |Q9UBP6 -->P31749_p +Q9UBP6_p : K(31,1) for forward direction</td></tr>
<tr><td>32</td><td>P31749_p +O75376 <-->P31749_p |O75376 : K(32,1) for forward directionand K(32,2) for reverse direction</td></tr>
<tr><td>33</td><td>P31749_p |O75376 -->P31749_p +O75376_p : K(33,1) for forward direction</td></tr>
<tr><td>34</td><td>P31749_p +Q53EL6 <-->P31749_p |Q53EL6 : K(34,1) for forward directionand K(34,2) for reverse direction</td></tr>
<tr><td>35</td><td>P31749_p |Q53EL6 -->P31749_p +Q53EL6_p : K(35,1) for forward direction</td></tr>
<tr><td>36</td><td>P31749_p +P62136 <-->P31749_p |P62136 : K(36,1) for forward directionand K(36,2) for reverse direction</td></tr>
</table>

37	P31749_p |P62136 -->P31749_p +P62136_p : K(37,1) for forward direction
38	P31749_p +Q13188 <-->P31749_p |Q13188 : K(38,1) for forward directionand K(38,2) for reverse direction
39	P31749_p |Q13188 -->P31749_p +Q13188_p : K(39,1) for forward direction
40	P31749_p +Q13043 <-->P31749_p |Q13043 : K(40,1) for forward directionand K(40,2) for reverse direction
41	P31749_p |Q13043 -->P31749_p +Q13043_p : K(41,1) for forward direction
42	P31749_p +O60343 <-->P31749_p |O60343 : K(42,1) for forward directionand K(42,2) for reverse direction
43	P31749_p |O60343 -->P31749_p +O60343_p : K(43,1) for forward direction
44	P31749_p +P46937 <-->P31749_p |P46937 : K(44,1) for forward directionand K(44,2) for reverse direction
45	P31749_p |P46937 -->P31749_p +P46937_p : K(45,1) for forward direction
46	P31749_p +Q07352 <-->P31749_p |Q07352 : K(46,1) for forward directionand K(46,2) for reverse direction
47	P31749_p |Q07352 -->P31749_p +Q07352_p : K(47,1) for forward direction
48	P31749_p +Q15942 <-->P31749_p |Q15942 : K(48,1) for forward directionand K(48,2) for reverse direction
49	P31749_p |Q15942 -->P31749_p +Q15942_p : K(49,1) for forward direction
50	P31751_p +Q96B36 <-->P31751_p |Q96B36 : K(50,1) for forward directionand K(50,2) for reverse direction
51	P31751_p |Q96B36 -->P31751_p +Q96B36_p : K(51,1) for forward direction
52	P31751_p +Q92934 <-->P31751_p |Q92934 : K(52,1) for forward directionand K(52,2) for reverse direction
53	P31751_p |Q92934 -->P31751_p +Q92934_p : K(53,1) for forward direction
54	P31751_p +P15056 <-->P31751_p |P15056 : K(54,1) for forward directionand K(54,2) for reverse direction
55	P31751_p |P15056 -->P31751_p +P15056_p : K(55,1) for forward direction
56	P31751_p +P55211 <-->P31751_p |P55211 : K(56,1) for forward directionand K(56,2) for reverse direction
57	P31751_p |P55211 -->P31751_p +P55211_p : K(57,1) for forward direction
58	P31751_p +P46527 <-->P31751_p |P46527 : K(58,1) for forward directionand K(58,2) for reverse direction
59	P31751_p |P46527 -->P31751_p +P46527_p : K(59,1) for forward direction
60	P31751_p +P49840 <-->P31751_p |P49840 : K(60,1) for forward directionand K(60,2) for reverse direction
61	P31751_p |P49840 -->P31751_p +P49840_p : K(61,1) for forward direction
62	P31751_p +P04792 <-->P31751_p |P04792 : K(62,1) for forward directionand K(62,2) for reverse direction
63	P31751_p |P04792 -->P31751_p +P04792_p : K(63,1) for forward direction
64	P31751_p +O43464 <-->P31751_p |O43464 : K(64,1) for forward directionand K(64,2) for reverse direction
65	P31751_p |O43464 -->P31751_p +O43464_p : K(65,1) for forward direction
66	P31751_p +Q92945 <-->P31751_p |Q92945 : K(66,1) for forward directionand K(66,2) for reverse direction
67	P31751_p |Q92945 -->P31751_p +Q92945_p : K(67,1) for forward direction
68	P31751_p +Q13188 <-->P31751_p |Q13188 : K(68,1) for forward directionand K(68,2) for reverse direction
69	P31751_p |Q13188 -->P31751_p +Q13188_p : K(69,1) for forward direction
70	P31751_p +Q13043 <-->P31751_p |Q13043 : K(70,1) for forward directionand K(70,2) for reverse direction
71	P31751_p |Q13043 -->P31751_p +Q13043_p : K(71,1) for forward direction
72	P31751_p +P49815 <-->P31751_p |P49815 : K(72,1) for forward directionand K(72,2) for reverse direction
73	P31751_p |P49815 -->P31751_p +P49815_p : K(73,1) for forward direction
74	Q96GD4_p +Q9H410 <-->Q96GD4_p |Q9H410 : K(74,1) for forward directionand K(74,2) for reverse direction
75	Q96GD4_p |Q9H410 -->Q96GD4_p +Q9H410_p : K(75,1) for forward direction
76	Q96GD4_p +Q99661 <-->Q96GD4_p |Q99661 : K(76,1) for forward directionand K(76,2) for reverse direction
77	Q96GD4_p |Q99661 -->Q96GD4_p +Q99661_p : K(77,1) for forward direction
78	Q96GD4_p +O14777 <-->Q96GD4_p |O14777 : K(78,1) for forward directionand K(78,2) for reverse direction
79	Q96GD4_p |O14777 -->Q96GD4_p +O14777_p : K(79,1) for forward direction

80	Q96GD4_p +Q08J23 <-->Q96GD4_p |Q08J23 : K(80,1) for forward directionand K(80,2) for reverse direction
81	Q96GD4_p |Q08J23 -->Q96GD4_p +Q08J23_p : K(81,1) for forward direction
82	Q96GD4_p +P04637 <-->Q96GD4_p |P04637 : K(82,1) for forward directionand K(82,2) for reverse direction
83	Q96GD4_p |P04637 -->Q96GD4_p +P04637_p : K(83,1) for forward direction
84	Q96GD4_p +P08670 <-->Q96GD4_p |P08670 : K(84,1) for forward directionand K(84,2) for reverse direction
85	Q96GD4_p |P08670 -->Q96GD4_p +P08670_p : K(85,1) for forward direction
86	O14874 +P12694 <-->O14874 |P12694 : K(86,1) for forward directionand K(86,2) for reverse direction
87	O14874 |P12694 -->O14874 +P12694_p : K(87,1) for forward direction
88	Q9UQM7_p +Q9UQM7 <-->Q9UQM7_p |Q9UQM7 : K(88,1) for forward directionand K(88,2) for reverse direction
89	Q9UQM7_p |Q9UQM7 -->Q9UQM7_p +Q9UQM7_p : K(89,1) for forward direction
90	Q9UQM7_p +P16220 <-->Q9UQM7_p |P16220 : K(90,1) for forward directionand K(90,2) for reverse direction
91	Q9UQM7_p |P16220 -->Q9UQM7_p +P16220_p : K(91,1) for forward direction
92	Q9UQM7_p +Q05469 <-->Q9UQM7_p |Q05469 : K(92,1) for forward directionand K(92,2) for reverse direction
93	Q9UQM7_p |Q05469 -->Q9UQM7_p +Q05469_p : K(93,1) for forward direction
94	Q9UQM7_p +Q9Y618 <-->Q9UQM7_p |Q9Y618 : K(94,1) for forward directionand K(94,2) for reverse direction
95	Q9UQM7_p |Q9Y618 -->Q9UQM7_p +Q9Y618_p : K(95,1) for forward direction
96	Q9UQM7_p +Q96PM5 <-->Q9UQM7_p |Q96PM5 : K(96,1) for forward directionand K(96,2) for reverse direction
97	Q9UQM7_p |Q96PM5 -->Q9UQM7_p +Q96PM5_p : K(97,1) for forward direction
98	Q9UQM7_p +Q15796 <-->Q9UQM7_p |Q15796 : K(98,1) for forward directionand K(98,2) for reverse direction
99	Q9UQM7_p |Q15796 -->Q9UQM7_p +Q15796_p : K(99,1) for forward direction
100	Q9UQM7_p +P16949 <-->Q9UQM7_p |P16949 : K(100,1) for forward directionand K(100,2) for reverse direction
101	Q9UQM7_p |P16949 -->Q9UQM7_p +P16949_p : K(101,1) for forward direction
102	Q13554_p +P35222 <-->Q13554_p |P35222 : K(102,1) for forward directionand K(102,2) for reverse direction
103	Q13554_p |P35222 -->Q13554_p +P35222_p : K(103,1) for forward direction
104	Q13554_p +P49840 <-->Q13554_p |P49840 : K(104,1) for forward directionand K(104,2) for reverse direction
105	Q13554_p |P49840 -->Q13554_p +P49840_p : K(105,1) for forward direction
106	Q13554_p +P16949 <-->Q13554_p |P16949 : K(106,1) for forward directionand K(106,2) for reverse direction
107	Q13554_p |P16949 -->Q13554_p +P16949_p : K(107,1) for forward direction
108	Q13555 +Q9Y618 <-->Q13555 |Q9Y618 : K(108,1) for forward directionand K(108,2) for reverse direction
109	Q13555 |Q9Y618 -->Q13555 +Q9Y618_p : K(109,1) for forward direction
110	P06493_p +Q14790 <-->P06493_p |Q14790 : K(110,1) for forward directionand K(110,2) for reverse direction
111	P06493_p |Q14790 -->P06493_p +Q14790_p : K(111,1) for forward direction
112	P06493_p +P55211 <-->P06493_p |P55211 : K(112,1) for forward directionand K(112,2) for reverse direction
113	P06493_p |P55211 -->P06493_p +P55211_p : K(113,1) for forward direction
114	P06493_p +Q53EZ4 <-->P06493_p |Q53EZ4 : K(114,1) for forward directionand K(114,2) for reverse direction
115	P06493_p |Q53EZ4 -->P06493_p +Q53EZ4_p : K(115,1) for forward direction
116	P06493_p +O00571 <-->P06493_p |O00571 : K(116,1) for forward directionand K(116,2) for reverse direction
117	P06493_p |O00571 -->P06493_p +O00571_p : K(117,1) for forward direction
118	P06493_p +Q9H8V3 <-->P06493_p |Q9H8V3 : K(118,1) for forward directionand K(118,2) for reverse direction
119	P06493_p |Q9H8V3 -->P06493_p +Q9H8V3_p : K(119,1) for forward direction
120	P06493_p +O00418 <-->P06493_p |O00418 : K(120,1) for forward directionand K(120,2) for reverse direction
121	P06493_p |O00418 -->P06493_p +O00418_p : K(121,1) for forward direction
122	P06493_p +P00533 <-->P06493_p |P00533 : K(122,1) for forward directionand K(122,2) for reverse direction

123	P06493_p |P00533 -->P06493_p +P00533_p : K(123,1) for forward direction
124	P06493_p +P50548 <-->P06493_p |P50548 : K(124,1) for forward directionand K(124,2) for reverse direction
125	P06493_p |P50548 -->P06493_p +P50548_p : K(125,1) for forward direction
126	P06493_p +Q15910 <-->P06493_p |Q15910 : K(126,1) for forward directionand K(126,2) for reverse direction
127	P06493_p |Q15910 -->P06493_p +Q15910_p : K(127,1) for forward direction
128	P06493_p +Q12778 <-->P06493_p |Q12778 : K(128,1) for forward directionand K(128,2) for reverse direction
129	P06493_p |Q12778 -->P06493_p +Q12778_p : K(129,1) for forward direction
130	P06493_p +Q08379 <-->P06493_p |Q08379 : K(130,1) for forward directionand K(130,2) for reverse direction
131	P06493_p |Q08379 -->P06493_p +Q08379_p : K(131,1) for forward direction
132	P06493_p +P17096 <-->P06493_p |P17096 : K(132,1) for forward directionand K(132,2) for reverse direction
133	P06493_p |P17096 -->P06493_p +P17096_p : K(133,1) for forward direction
134	P06493_p +P52926 <-->P06493_p |P52926 : K(134,1) for forward directionand K(134,2) for reverse direction
135	P06493_p |P52926 -->P06493_p +P52926_p : K(135,1) for forward direction
136	P06493_p +P48200 <-->P06493_p |P48200 : K(136,1) for forward directionand K(136,2) for reverse direction
137	P06493_p |P48200 -->P06493_p +P48200_p : K(137,1) for forward direction
138	P06493_p +Q99661 <-->P06493_p |Q99661 : K(138,1) for forward directionand K(138,2) for reverse direction
139	P06493_p |Q99661 -->P06493_p +Q99661_p : K(139,1) for forward direction
140	P06493_p +Q14739 <-->P06493_p |Q14739 : K(140,1) for forward directionand K(140,2) for reverse direction
141	P06493_p |Q14739 -->P06493_p +Q14739_p : K(141,1) for forward direction
142	P06493_p +Q02750 <-->P06493_p |Q02750 : K(142,1) for forward directionand K(142,2) for reverse direction
143	P06493_p |Q02750 -->P06493_p +Q02750_p : K(143,1) for forward direction
144	P06493_p +P27816 <-->P06493_p |P27816 : K(144,1) for forward directionand K(144,2) for reverse direction
145	P06493_p |P27816 -->P06493_p +P27816_p : K(145,1) for forward direction
146	P06493_p +Q14980 <-->P06493_p |Q14980 : K(146,1) for forward directionand K(146,2) for reverse direction
147	P06493_p |Q14980 -->P06493_p +Q14980_p : K(147,1) for forward direction
148	P06493_p +Q9UKX7 <-->P06493_p |Q9UKX7 : K(148,1) for forward directionand K(148,2) for reverse direction
149	P06493_p |Q9UKX7 -->P06493_p +Q9UKX7_p : K(149,1) for forward direction
150	P06493_p +Q8NEB9 <-->P06493_p |Q8NEB9 : K(150,1) for forward directionand K(150,2) for reverse direction
151	P06493_p |Q8NEB9 -->P06493_p +Q8NEB9_p : K(151,1) for forward direction
152	P06493_p +Q15149 <-->P06493_p |Q15149 : K(152,1) for forward directionand K(152,2) for reverse direction
153	P06493_p |Q15149 -->P06493_p +Q15149_p : K(153,1) for forward direction
154	P06493_p +P29590 <-->P06493_p |P29590 : K(154,1) for forward directionand K(154,2) for reverse direction
155	P06493_p |P29590 -->P06493_p +P29590_p : K(155,1) for forward direction
156	P06493_p +P62136 <-->P06493_p |P62136 : K(156,1) for forward directionand K(156,2) for reverse direction
157	P06493_p |P62136 -->P06493_p +P62136_p : K(157,1) for forward direction
158	P06493_p +Q06830 <-->P06493_p |Q06830 : K(158,1) for forward directionand K(158,2) for reverse direction
159	P06493_p |Q06830 -->P06493_p +Q06830_p : K(159,1) for forward direction
160	P06493_p +P06400 <-->P06493_p |P06400 : K(160,1) for forward directionand K(160,2) for reverse direction
161	P06493_p |P06400 -->P06493_p +P06400_p : K(161,1) for forward direction
162	P06493_p +P31350 <-->P06493_p |P31350 : K(162,1) for forward directionand K(162,2) for reverse direction
163	P06493_p |P31350 -->P06493_p +P31350_p : K(163,1) for forward direction
164	P06493_p +Q9Y3Z3 <-->P06493_p |Q9Y3Z3 : K(164,1) for forward directionand K(164,2) for reverse direction
165	P06493_p |Q9Y3Z3 -->P06493_p +Q9Y3Z3_p : K(165,1) for forward direction

166	P06493_p +Q8IXJ6 <-->P06493_p |Q8IXJ6 : K(166,1) for forward directionand K(166,2) for reverse direction
167	P06493_p |Q8IXJ6 -->P06493_p +Q8IXJ6_p : K(167,1) for forward direction
168	P06493_p +Q13586 <-->P06493_p |Q13586 : K(168,1) for forward directionand K(168,2) for reverse direction
169	P06493_p |Q13586 -->P06493_p +Q13586_p : K(169,1) for forward direction
170	P06493_p +P04183 <-->P06493_p |P04183 : K(170,1) for forward directionand K(170,2) for reverse direction
171	P06493_p |P04183 -->P06493_p +P04183_p : K(171,1) for forward direction
172	P06493_p +Q92574 <-->P06493_p |Q92574 : K(172,1) for forward directionand K(172,2) for reverse direction
173	P06493_p |Q92574 -->P06493_p +Q92574_p : K(173,1) for forward direction
174	P06493_p +P08670 <-->P06493_p |P08670 : K(174,1) for forward directionand K(174,2) for reverse direction
175	P06493_p |P08670 -->P06493_p +P08670_p : K(175,1) for forward direction
176	P06493_p +P30291 <-->P06493_p |P30291 : K(176,1) for forward directionand K(176,2) for reverse direction
177	P06493_p |P30291 -->P06493_p +P30291_p : K(177,1) for forward direction
178	P06493_p +Q86WB0 <-->P06493_p |Q86WB0 : K(178,1) for forward directionand K(178,2) for reverse direction
179	P06493_p |Q86WB0 -->P06493_p +Q86WB0_p : K(179,1) for forward direction
180	P21127_p +O00303 <-->P21127_p |O00303 : K(180,1) for forward directionand K(180,2) for reverse direction
181	P21127_p |O00303 -->P21127_p +O00303_p : K(181,1) for forward direction
182	P24941_p +P46527 <-->P24941_p |P46527 : K(182,1) for forward directionand K(182,2) for reverse direction
183	P24941_p |P46527 -->P24941_p +P46527_p : K(183,1) for forward direction
184	P24941_p +Q12778 <-->P24941_p |Q12778 : K(184,1) for forward directionand K(184,2) for reverse direction
185	P24941_p |Q12778 -->P24941_p +Q12778_p : K(185,1) for forward direction
186	P24941_p +P25098 <-->P24941_p |P25098 : K(186,1) for forward directionand K(186,2) for reverse direction
187	P24941_p |P25098 -->P24941_p +P25098_p : K(187,1) for forward direction
188	P24941_p +P17096 <-->P24941_p |P17096 : K(188,1) for forward directionand K(188,2) for reverse direction
189	P24941_p |P17096 -->P24941_p +P17096_p : K(189,1) for forward direction
190	P24941_p +P49916 <-->P24941_p |P49916 : K(190,1) for forward directionand K(190,2) for reverse direction
191	P24941_p |P49916 -->P24941_p +P49916_p : K(191,1) for forward direction
192	P24941_p +P62136 <-->P24941_p |P62136 : K(192,1) for forward directionand K(192,2) for reverse direction
193	P24941_p |P62136 -->P24941_p +P62136_p : K(193,1) for forward direction
194	P24941_p +Q06830 <-->P24941_p |Q06830 : K(194,1) for forward directionand K(194,2) for reverse direction
195	P24941_p |Q06830 -->P24941_p +Q06830_p : K(195,1) for forward direction
196	P24941_p +P06400 <-->P24941_p |P06400 : K(196,1) for forward directionand K(196,2) for reverse direction
197	P24941_p |P06400 -->P24941_p +P06400_p : K(197,1) for forward direction
198	P24941_p +Q8IXJ6 <-->P24941_p |Q8IXJ6 : K(198,1) for forward directionand K(198,2) for reverse direction
199	P24941_p |Q8IXJ6 -->P24941_p +Q8IXJ6_p : K(199,1) for forward direction
200	P24941_p +P84022 <-->P24941_p |P84022 : K(200,1) for forward directionand K(200,2) for reverse direction
201	P24941_p |P84022 -->P24941_p +P84022_p : K(201,1) for forward direction
202	P24941_p +Q12800 <-->P24941_p |Q12800 : K(202,1) for forward directionand K(202,2) for reverse direction
203	P24941_p |Q12800 -->P24941_p +Q12800_p : K(203,1) for forward direction
204	P24941_p +P04183 <-->P24941_p |P04183 : K(204,1) for forward directionand K(204,2) for reverse direction
205	P24941_p |P04183 -->P24941_p +P04183_p : K(205,1) for forward direction
206	P24941_p +P30291 <-->P24941_p |P30291 : K(206,1) for forward directionand K(206,2) for reverse direction
207	P24941_p |P30291 -->P24941_p +P30291_p : K(207,1) for forward direction
208	P24941_p +Q86WB0 <-->P24941_p |Q86WB0 : K(208,1) for forward directionand K(208,2) for reverse direction

<table>
<tr><td>209</td><td>P24941_p |Q86WB0 -->P24941_p +Q86WB0_p : K(209,1) for forward direction</td></tr>
<tr><td>210</td><td>P11802_p +Q06830 <-->P11802_p |Q06830 : K(210,1) for forward directionand K(210,2) for reverse direction</td></tr>
<tr><td>211</td><td>P11802_p |Q06830 -->P11802_p +Q06830_p : K(211,1) for forward direction</td></tr>
<tr><td>212</td><td>P11802_p +P06400 <-->P11802_p |P06400 : K(212,1) for forward directionand K(212,2) for reverse direction</td></tr>
<tr><td>213</td><td>P11802_p |P06400 -->P11802_p +P06400_p : K(213,1) for forward direction</td></tr>
<tr><td>214</td><td>P11802_p +P84022 <-->P11802_p |P84022 : K(214,1) for forward directionand K(214,2) for reverse direction</td></tr>
<tr><td>215</td><td>P11802_p |P84022 -->P11802_p +P84022_p : K(215,1) for forward direction</td></tr>
<tr><td>216</td><td>Q00535_p +P07814 <-->Q00535_p |P07814 : K(216,1) for forward directionand K(216,2) for reverse direction</td></tr>
<tr><td>217</td><td>Q00535_p |P07814 -->Q00535_p +P07814_p : K(217,1) for forward direction</td></tr>
<tr><td>218</td><td>Q00535_p +Q8NEB9 <-->Q00535_p |Q8NEB9 : K(218,1) for forward directionand K(218,2) for reverse direction</td></tr>
<tr><td>219</td><td>Q00535_p |Q8NEB9 -->Q00535_p +Q8NEB9_p : K(219,1) for forward direction</td></tr>
<tr><td>220</td><td>Q00535_p +P62136 <-->Q00535_p |P62136 : K(220,1) for forward directionand K(220,2) for reverse direction</td></tr>
<tr><td>221</td><td>Q00535_p |P62136 -->Q00535_p +P62136_p : K(221,1) for forward direction</td></tr>
<tr><td>222</td><td>Q00535_p +P06400 <-->Q00535_p |P06400 : K(222,1) for forward directionand K(222,2) for reverse direction</td></tr>
<tr><td>223</td><td>Q00535_p |P06400 -->Q00535_p +P06400_p : K(223,1) for forward direction</td></tr>
<tr><td>224</td><td>Q00535_p +P16949 <-->Q00535_p |P16949 : K(224,1) for forward directionand K(224,2) for reverse direction</td></tr>
<tr><td>225</td><td>Q00535_p |P16949 -->Q00535_p +P16949_p : K(225,1) for forward direction</td></tr>
<tr><td>226</td><td>Q00535_p +Q15833 <-->Q00535_p |Q15833 : K(226,1) for forward directionand K(226,2) for reverse direction</td></tr>
<tr><td>227</td><td>Q00535_p |Q15833 -->Q00535_p +Q15833_p : K(227,1) for forward direction</td></tr>
<tr><td>228</td><td>Q00534 +P46527 <-->Q00534 |P46527 : K(228,1) for forward directionand K(228,2) for reverse direction</td></tr>
<tr><td>229</td><td>Q00534 |P46527 -->Q00534 +P46527_p : K(229,1) for forward direction</td></tr>
<tr><td>230</td><td>Q00534 +Q06830 <-->Q00534 |Q06830 : K(230,1) for forward directionand K(230,2) for reverse direction</td></tr>
<tr><td>231</td><td>Q00534 |Q06830 -->Q00534 +Q06830_p : K(231,1) for forward direction</td></tr>
<tr><td>232</td><td>Q00534 +P06400 <-->Q00534 |P06400 : K(232,1) for forward directionand K(232,2) for reverse direction</td></tr>
<tr><td>233</td><td>Q00534 |P06400 -->Q00534 +P06400_p : K(233,1) for forward direction</td></tr>
<tr><td>234</td><td>P50613_p +P24928 <-->P50613_p |P24928 : K(234,1) for forward directionand K(234,2) for reverse direction</td></tr>
<tr><td>235</td><td>P50613_p |P24928 -->P50613_p +P24928_p : K(235,1) for forward direction</td></tr>
<tr><td>236</td><td>P50750_p +Q96PM5 <-->P50750_p |Q96PM5 : K(236,1) for forward directionand K(236,2) for reverse direction</td></tr>
<tr><td>237</td><td>P50750_p |Q96PM5 -->P50750_p +Q96PM5_p : K(237,1) for forward direction</td></tr>
<tr><td>238</td><td>P50750_p +Q15797 <-->P50750_p |Q15797 : K(238,1) for forward directionand K(238,2) for reverse direction</td></tr>
<tr><td>239</td><td>P50750_p |Q15797 -->P50750_p +Q15797_p : K(239,1) for forward direction</td></tr>
<tr><td>240</td><td>P50750_p +P84022 <-->P50750_p |P84022 : K(240,1) for forward directionand K(240,2) for reverse direction</td></tr>
<tr><td>241</td><td>P50750_p |P84022 -->P50750_p +P84022_p : K(241,1) for forward direction</td></tr>
<tr><td>242</td><td>O14757_p +P19838 <-->O14757_p |P19838 : K(242,1) for forward directionand K(242,2) for reverse direction</td></tr>
<tr><td>243</td><td>O14757_p |P19838 -->O14757_p +P19838_p : K(243,1) for forward direction</td></tr>
<tr><td>244</td><td>O14757_p +P43405 <-->O14757_p |P43405 : K(244,1) for forward directionand K(244,2) for reverse direction</td></tr>
<tr><td>245</td><td>O14757_p |P43405 -->O14757_p +P43405_p : K(245,1) for forward direction</td></tr>
<tr><td>246</td><td>O14757_p +Q9UKI8 <-->O14757_p |Q9UKI8 : K(246,1) for forward directionand K(246,2) for reverse direction</td></tr>
<tr><td>247</td><td>O14757_p |Q9UKI8 -->O14757_p +Q9UKI8_p : K(247,1) for forward direction</td></tr>
<tr><td>248</td><td>O15111_p +O43524 <-->O15111_p |O43524 : K(248,1) for forward directionand K(248,2) for reverse direction</td></tr>
<tr><td>249</td><td>O15111_p |O43524 -->O15111_p +O43524_p : K(249,1) for forward direction</td></tr>
<tr><td>250</td><td>O15111_p +Q9Y618 <-->O15111_p |Q9Y618 : K(250,1) for forward directionand K(250,2) for reverse direction</td></tr>
<tr><td>251</td><td>O15111_p |Q9Y618 -->O15111_p +Q9Y618_p : K(251,1) for forward direction</td></tr>
</table>

<table>
<tr><td>252</td><td>O15111_p +P49815 <-->O15111_p |P49815 : K(252,1) for forward directionand K(252,2) for reverse direction</td></tr>
<tr><td>253</td><td>O15111_p |P49815 -->O15111_p +P49815_p : K(253,1) for forward direction</td></tr>
<tr><td>254</td><td>P41240_p +P12931 <-->P41240_p |P12931 : K(254,1) for forward directionand K(254,2) for reverse direction</td></tr>
<tr><td>255</td><td>P41240_p |P12931 -->P41240_p +P12931_p : K(255,1) for forward direction</td></tr>
<tr><td>256</td><td>P48729 +P35222 <-->P48729 |P35222 : K(256,1) for forward directionand K(256,2) for reverse direction</td></tr>
<tr><td>257</td><td>P48729 |P35222 -->P48729 +P35222_p : K(257,1) for forward direction</td></tr>
<tr><td>258</td><td>P48729 +Q9NRF8 <-->P48729 |Q9NRF8 : K(258,1) for forward directionand K(258,2) for reverse direction</td></tr>
<tr><td>259</td><td>P48729 |Q9NRF8 -->P48729 +Q9NRF8_p : K(259,1) for forward direction</td></tr>
<tr><td>260</td><td>P48729 +Q8TB45 <-->P48729 |Q8TB45 : K(260,1) for forward directionand K(260,2) for reverse direction</td></tr>
<tr><td>261</td><td>P48729 |Q8TB45 -->P48729 +Q8TB45_p : K(261,1) for forward direction</td></tr>
<tr><td>262</td><td>P48729 +Q13158 <-->P48729 |Q13158 : K(262,1) for forward directionand K(262,2) for reverse direction</td></tr>
<tr><td>263</td><td>P48729 |Q13158 -->P48729 +Q13158_p : K(263,1) for forward direction</td></tr>
<tr><td>264</td><td>P48729 +Q12778 <-->P48729 |Q12778 : K(264,1) for forward directionand K(264,2) for reverse direction</td></tr>
<tr><td>265</td><td>P48729 |Q12778 -->P48729 +Q12778_p : K(265,1) for forward direction</td></tr>
<tr><td>266</td><td>P48729 +O43524 <-->P48729 |O43524 : K(266,1) for forward directionand K(266,2) for reverse direction</td></tr>
<tr><td>267</td><td>P48729 |O43524 -->P48729 +O43524_p : K(267,1) for forward direction</td></tr>
<tr><td>268</td><td>P48729 +A8MYZ6 <-->P48729 |A8MYZ6 : K(268,1) for forward directionand K(268,2) for reverse direction</td></tr>
<tr><td>269</td><td>P48729 |A8MYZ6 -->P48729 +A8MYZ6_p : K(269,1) for forward direction</td></tr>
<tr><td>270</td><td>P48729 +P07910 <-->P48729 |P07910 : K(270,1) for forward directionand K(270,2) for reverse direction</td></tr>
<tr><td>271</td><td>P48729 |P07910 -->P48729 +P07910_p : K(271,1) for forward direction</td></tr>
<tr><td>272</td><td>P48729 +P62745 <-->P48729 |P62745 : K(272,1) for forward directionand K(272,2) for reverse direction</td></tr>
<tr><td>273</td><td>P48729 |P62745 -->P48729 +P62745_p : K(273,1) for forward direction</td></tr>
<tr><td>274</td><td>Q8N752_p +P35222 <-->Q8N752_p |P35222 : K(274,1) for forward directionand K(274,2) for reverse direction</td></tr>
<tr><td>275</td><td>Q8N752_p |P35222 -->Q8N752_p +P35222_p : K(275,1) for forward direction</td></tr>
<tr><td>276</td><td>P48730 +P35222 <-->P48730 |P35222 : K(276,1) for forward directionand K(276,2) for reverse direction</td></tr>
<tr><td>277</td><td>P48730 |P35222 -->P48730 +P35222_p : K(277,1) for forward direction</td></tr>
<tr><td>278</td><td>P48730 +P46937 <-->P48730 |P46937 : K(278,1) for forward directionand K(278,2) for reverse direction</td></tr>
<tr><td>279</td><td>P48730 |P46937 -->P48730 +P46937_p : K(279,1) for forward direction</td></tr>
<tr><td>280</td><td>P49674 +P35222 <-->P49674 |P35222 : K(280,1) for forward directionand K(280,2) for reverse direction</td></tr>
<tr><td>281</td><td>P49674 |P35222 -->P49674 +P35222_p : K(281,1) for forward direction</td></tr>
<tr><td>282</td><td>P49674 +O60716 <-->P49674 |O60716 : K(282,1) for forward directionand K(282,2) for reverse direction</td></tr>
<tr><td>283</td><td>P49674 |O60716 -->P49674 +O60716_p : K(283,1) for forward direction</td></tr>
<tr><td>284</td><td>P49674 +Q13541 <-->P49674 |Q13541 : K(284,1) for forward directionand K(284,2) for reverse direction</td></tr>
<tr><td>285</td><td>P49674 |Q13541 -->P49674 +Q13541_p : K(285,1) for forward direction</td></tr>
<tr><td>286</td><td>P49674 +P46937 <-->P49674 |P46937 : K(286,1) for forward directionand K(286,2) for reverse direction</td></tr>
<tr><td>287</td><td>P49674 |P46937 -->P49674 +P46937_p : K(287,1) for forward direction</td></tr>
<tr><td>288</td><td>P68400_p +Q15019 <-->P68400_p |Q15019 : K(288,1) for forward directionand K(288,2) for reverse direction</td></tr>
<tr><td>289</td><td>P68400_p |Q15019 -->P68400_p +Q15019_p : K(289,1) for forward direction</td></tr>
<tr><td>290</td><td>P68400_p +P18846 <-->P68400_p |P18846 : K(290,1) for forward directionand K(290,2) for reverse direction</td></tr>
<tr><td>291</td><td>P68400_p |P18846 -->P68400_p +P18846_p : K(291,1) for forward direction</td></tr>
<tr><td>292</td><td>P68400_p +Q9H257 <-->P68400_p |Q9H257 : K(292,1) for forward directionand K(292,2) for reverse direction</td></tr>
<tr><td>293</td><td>P68400_p |Q9H257 -->P68400_p +Q9H257_p : K(293,1) for forward direction</td></tr>
<tr><td>294</td><td>P68400_p +P42575 <-->P68400_p |P42575 : K(294,1) for forward directionand K(294,2) for reverse direction</td></tr>
</table>

<table>
<tr><td>295</td><td>P68400_p |P42575 -->P68400_p +P42575_p : K(295,1) for forward direction</td></tr>
<tr><td>296</td><td>P68400_p +P83916 <-->P68400_p |P83916 : K(296,1) for forward directionand K(296,2) for reverse direction</td></tr>
<tr><td>297</td><td>P68400_p |P83916 -->P68400_p +P83916_p : K(297,1) for forward direction</td></tr>
<tr><td>298</td><td>P68400_p +P35221 <-->P68400_p |P35221 : K(298,1) for forward directionand K(298,2) for reverse direction</td></tr>
<tr><td>299</td><td>P68400_p |P35221 -->P68400_p +P35221_p : K(299,1) for forward direction</td></tr>
<tr><td>300</td><td>P68400_p +O95786 <-->P68400_p |O95786 : K(300,1) for forward directionand K(300,2) for reverse direction</td></tr>
<tr><td>301</td><td>P68400_p |O95786 -->P68400_p +O95786_p : K(301,1) for forward direction</td></tr>
<tr><td>302</td><td>P68400_p +Q13541 <-->P68400_p |Q13541 : K(302,1) for forward directionand K(302,2) for reverse direction</td></tr>
<tr><td>303</td><td>P68400_p |Q13541 -->P68400_p +Q13541_p : K(303,1) for forward direction</td></tr>
<tr><td>304</td><td>P68400_p +P05114 <-->P68400_p |P05114 : K(304,1) for forward directionand K(304,2) for reverse direction</td></tr>
<tr><td>305</td><td>P68400_p |P05114 -->P68400_p +P05114_p : K(305,1) for forward direction</td></tr>
<tr><td>306</td><td>P68400_p +P08238 <-->P68400_p |P08238 : K(306,1) for forward directionand K(306,2) for reverse direction</td></tr>
<tr><td>307</td><td>P68400_p |P08238 -->P68400_p +P08238_p : K(307,1) for forward direction</td></tr>
<tr><td>308</td><td>P68400_p +P05412 <-->P68400_p |P05412 : K(308,1) for forward directionand K(308,2) for reverse direction</td></tr>
<tr><td>309</td><td>P68400_p |P05412 -->P68400_p +P05412_p : K(309,1) for forward direction</td></tr>
<tr><td>310</td><td>P68400_p +P29590 <-->P68400_p |P29590 : K(310,1) for forward directionand K(310,2) for reverse direction</td></tr>
<tr><td>311</td><td>P68400_p |P29590 -->P68400_p +P29590_p : K(311,1) for forward direction</td></tr>
<tr><td>312</td><td>P68400_p +Q9NYV6 <-->P68400_p |Q9NYV6 : K(312,1) for forward directionand K(312,2) for reverse direction</td></tr>
<tr><td>313</td><td>P68400_p |Q9NYV6 -->P68400_p +Q9NYV6_p : K(313,1) for forward direction</td></tr>
<tr><td>314</td><td>P68400_p +Q01105 <-->P68400_p |Q01105 : K(314,1) for forward directionand K(314,2) for reverse direction</td></tr>
<tr><td>315</td><td>P68400_p |Q01105 -->P68400_p +Q01105_p : K(315,1) for forward direction</td></tr>
<tr><td>316</td><td>P68400_p +Q9H2G2 <-->P68400_p |Q9H2G2 : K(316,1) for forward directionand K(316,2) for reverse direction</td></tr>
<tr><td>317</td><td>P68400_p |Q9H2G2 -->P68400_p +Q9H2G2_p : K(317,1) for forward direction</td></tr>
<tr><td>318</td><td>P68400_p +Q01082 <-->P68400_p |Q01082 : K(318,1) for forward directionand K(318,2) for reverse direction</td></tr>
<tr><td>319</td><td>P68400_p |Q01082 -->P68400_p +Q01082_p : K(319,1) for forward direction</td></tr>
<tr><td>320</td><td>P68400_p +Q9Y365 <-->P68400_p |Q9Y365 : K(320,1) for forward directionand K(320,2) for reverse direction</td></tr>
<tr><td>321</td><td>P68400_p |Q9Y365 -->P68400_p +Q9Y365_p : K(321,1) for forward direction</td></tr>
<tr><td>322</td><td>P68400_p +Q9Y4R8 <-->P68400_p |Q9Y4R8 : K(322,1) for forward directionand K(322,2) for reverse direction</td></tr>
<tr><td>323</td><td>P68400_p |Q9Y4R8 -->P68400_p +Q9Y4R8_p : K(323,1) for forward direction</td></tr>
<tr><td>324</td><td>P68400_p +O43156 <-->P68400_p |O43156 : K(324,1) for forward directionand K(324,2) for reverse direction</td></tr>
<tr><td>325</td><td>P68400_p |O43156 -->P68400_p +O43156_p : K(325,1) for forward direction</td></tr>
<tr><td>326</td><td>P68400_p +Q9Y6W5 <-->P68400_p |Q9Y6W5 : K(326,1) for forward directionand K(326,2) for reverse direction</td></tr>
<tr><td>327</td><td>P68400_p |Q9Y6W5 -->P68400_p +Q9Y6W5_p : K(327,1) for forward direction</td></tr>
<tr><td>328</td><td>P19784 +Q01105 <-->P19784 |Q01105 : K(328,1) for forward directionand K(328,2) for reverse direction</td></tr>
<tr><td>329</td><td>P19784 |Q01105 -->P19784 +Q01105_p : K(329,1) for forward direction</td></tr>
<tr><td>330</td><td>P67870_p +Q01105 <-->P67870_p |Q01105 : K(330,1) for forward directionand K(330,2) for reverse direction</td></tr>
<tr><td>331</td><td>P67870_p |Q01105 -->P67870_p +Q01105_p : K(331,1) for forward direction</td></tr>
<tr><td>332</td><td>Q13627_p +Q05193 <-->Q13627_p |Q05193 : K(332,1) for forward directionand K(332,2) for reverse direction</td></tr>
<tr><td>333</td><td>Q13627_p |Q05193 -->Q13627_p +Q05193_p : K(333,1) for forward direction</td></tr>
<tr><td>334</td><td>Q13627_p +Q12778 <-->Q13627_p |Q12778 : K(334,1) for forward directionand K(334,2) for reverse direction</td></tr>
<tr><td>335</td><td>Q13627_p |Q12778 -->Q13627_p +Q12778_p : K(335,1) for forward direction</td></tr>
<tr><td>336</td><td>Q13627_p +O43524 <-->Q13627_p |O43524 : K(336,1) for forward directionand K(336,2) for reverse direction</td></tr>
<tr><td>337</td><td>Q13627_p |O43524 -->Q13627_p +O43524_p : K(337,1) for forward direction</td></tr>
</table>

<table>
<tr><td>338</td><td>Q13627_p +A8MYZ6 <-->Q13627_p |A8MYZ6 : K(338,1) for forward directionand K(338,2) for reverse direction</td></tr>
<tr><td>339</td><td>Q13627_p |A8MYZ6 -->Q13627_p +A8MYZ6_p : K(339,1) for forward direction</td></tr>
<tr><td>340</td><td>O00418_p +P13639 <-->O00418_p |P13639 : K(340,1) for forward directionand K(340,2) for reverse direction</td></tr>
<tr><td>341</td><td>O00418_p |P13639 -->O00418_p +P13639_p : K(341,1) for forward direction</td></tr>
<tr><td>342</td><td>O00418_p +O00418 <-->O00418_p |O00418 : K(342,1) for forward directionand K(342,2) for reverse direction</td></tr>
<tr><td>343</td><td>O00418_p |O00418 -->O00418_p +O00418_p : K(343,1) for forward direction</td></tr>
<tr><td>344</td><td>P00533_p +P11171 <-->P00533_p |P11171 : K(344,1) for forward directionand K(344,2) for reverse direction</td></tr>
<tr><td>345</td><td>P00533_p |P11171 -->P00533_p +P11171_p : K(345,1) for forward direction</td></tr>
<tr><td>346</td><td>P00533_p +Q9UBN7 <-->P00533_p |Q9UBN7 : K(346,1) for forward directionand K(346,2) for reverse direction</td></tr>
<tr><td>347</td><td>P00533_p |Q9UBN7 -->P00533_p +Q9UBN7_p : K(347,1) for forward direction</td></tr>
<tr><td>348</td><td>P19525_p +P06493 <-->P19525_p |P06493 : K(348,1) for forward directionand K(348,2) for reverse direction</td></tr>
<tr><td>349</td><td>P19525_p |P06493 -->P19525_p +P06493_p : K(349,1) for forward direction</td></tr>
<tr><td>350</td><td>P19525_p +P05198 <-->P19525_p |P05198 : K(350,1) for forward directionand K(350,2) for reverse direction</td></tr>
<tr><td>351</td><td>P19525_p |P05198 -->P19525_p +P05198_p : K(351,1) for forward direction</td></tr>
<tr><td>352</td><td>P04626_p +P06493 <-->P04626_p |P06493 : K(352,1) for forward directionand K(352,2) for reverse direction</td></tr>
<tr><td>353</td><td>P04626_p |P06493 -->P04626_p +P06493_p : K(353,1) for forward direction</td></tr>
<tr><td>354</td><td>P04626_p +P04626 <-->P04626_p |P04626 : K(354,1) for forward directionand K(354,2) for reverse direction</td></tr>
<tr><td>355</td><td>P04626_p |P04626 -->P04626_p +P04626_p : K(355,1) for forward direction</td></tr>
<tr><td>356</td><td>P25098_p +Q16539 <-->P25098_p |Q16539 : K(356,1) for forward directionand K(356,2) for reverse direction</td></tr>
<tr><td>357</td><td>P25098_p |Q16539 -->P25098_p +Q16539_p : K(357,1) for forward direction</td></tr>
<tr><td>358</td><td>P49840_p +P13807 <-->P49840_p |P13807 : K(358,1) for forward directionand K(358,2) for reverse direction</td></tr>
<tr><td>359</td><td>P49840_p |P13807 -->P49840_p +P13807_p : K(359,1) for forward direction</td></tr>
<tr><td>360</td><td>P49840_p +P05412 <-->P49840_p |P05412 : K(360,1) for forward directionand K(360,2) for reverse direction</td></tr>
<tr><td>361</td><td>P49840_p |P05412 -->P49840_p +P05412_p : K(361,1) for forward direction</td></tr>
<tr><td>362</td><td>P49840_p +P01106 <-->P49840_p |P01106 : K(362,1) for forward directionand K(362,2) for reverse direction</td></tr>
<tr><td>363</td><td>P49840_p |P01106 -->P49840_p +P01106_p : K(363,1) for forward direction</td></tr>
<tr><td>364</td><td>P49840_p +P23246 <-->P49840_p |P23246 : K(364,1) for forward directionand K(364,2) for reverse direction</td></tr>
<tr><td>365</td><td>P49840_p |P23246 -->P49840_p +P23246_p : K(365,1) for forward direction</td></tr>
<tr><td>366</td><td>P49841_p +Q13144 <-->P49841_p |Q13144 : K(366,1) for forward directionand K(366,2) for reverse direction</td></tr>
<tr><td>367</td><td>P49841_p |Q13144 -->P49841_p +Q13144_p : K(367,1) for forward direction</td></tr>
<tr><td>368</td><td>P49841_p +Q9NQX3 <-->P49841_p |Q9NQX3 : K(368,1) for forward directionand K(368,2) for reverse direction</td></tr>
<tr><td>369</td><td>P49841_p |Q9NQX3 -->P49841_p +Q9NQX3_p : K(369,1) for forward direction</td></tr>
<tr><td>370</td><td>P49841_p +Q14103 <-->P49841_p |Q14103 : K(370,1) for forward directionand K(370,2) for reverse direction</td></tr>
<tr><td>371</td><td>P49841_p |Q14103 -->P49841_p +Q14103_p : K(371,1) for forward direction</td></tr>
<tr><td>372</td><td>P49841_p +Q00613 <-->P49841_p |Q00613 : K(372,1) for forward directionand K(372,2) for reverse direction</td></tr>
<tr><td>373</td><td>P49841_p |Q00613 -->P49841_p +Q00613_p : K(373,1) for forward direction</td></tr>
<tr><td>374</td><td>P49841_p +Q13887 <-->P49841_p |Q13887 : K(374,1) for forward directionand K(374,2) for reverse direction</td></tr>
<tr><td>375</td><td>P49841_p |Q13887 -->P49841_p +Q13887_p : K(375,1) for forward direction</td></tr>
<tr><td>376</td><td>P49841_p +Q07820 <-->P49841_p |Q07820 : K(376,1) for forward directionand K(376,2) for reverse direction</td></tr>
<tr><td>377</td><td>P49841_p |Q07820 -->P49841_p +Q07820_p : K(377,1) for forward direction</td></tr>
<tr><td>378</td><td>P49841_p +E9PAV3 <-->P49841_p |E9PAV3 : K(378,1) for forward directionand K(378,2) for reverse direction</td></tr>
<tr><td>379</td><td>P49841_p |E9PAV3 -->P49841_p +E9PAV3_p : K(379,1) for forward direction</td></tr>
<tr><td>380</td><td>P49841_p +Q9H0Z9 <-->P49841_p |Q9H0Z9 : K(380,1) for forward directionand K(380,2) for reverse direction</td></tr>
</table>

381	P49841_p |Q9H0Z9 -->P49841_p +Q9H0Z9_p : K(381,1) for forward direction
382	P49841_p +P23246 <-->P49841_p |P23246 : K(382,1) for forward directionand K(382,2) for reverse direction
383	P49841_p |P23246 -->P49841_p +P23246_p : K(383,1) for forward direction
384	P49841_p +Q15797 <-->P49841_p |Q15797 : K(384,1) for forward directionand K(384,2) for reverse direction
385	P49841_p |Q15797 -->P49841_p +Q15797_p : K(385,1) for forward direction
386	P35269 +P35269 <-->P35269 |P35269 : K(386,1) for forward directionand K(386,2) for reverse direction
387	P35269 |P35269 -->P35269 +P35269_p : K(387,1) for forward direction
388	Q13418_p +P23528 <-->Q13418_p |P23528 : K(388,1) for forward directionand K(388,2) for reverse direction
389	Q13418_p |P23528 -->Q13418_p +P23528_p : K(389,1) for forward direction
390	Q13418_p +O14974 <-->Q13418_p |O14974 : K(390,1) for forward directionand K(390,2) for reverse direction
391	Q13418_p |O14974 -->Q13418_p +O14974_p : K(391,1) for forward direction
392	Q02750_p +P35568 <-->Q02750_p |P35568 : K(392,1) for forward directionand K(392,2) for reverse direction
393	Q02750_p |P35568 -->Q02750_p +P35568_p : K(393,1) for forward direction
394	P45985_p +P19793 <-->P45985_p |P19793 : K(394,1) for forward directionand K(394,2) for reverse direction
395	P45985_p |P19793 -->P45985_p +P19793_p : K(395,1) for forward direction
396	O14733_p +Q13158 <-->O14733_p |Q13158 : K(396,1) for forward directionand K(396,2) for reverse direction
397	O14733_p |Q13158 -->O14733_p +Q13158_p : K(397,1) for forward direction
398	O43318_p +Q8WUI4 <-->O43318_p |Q8WUI4 : K(398,1) for forward directionand K(398,2) for reverse direction
399	O43318_p |Q8WUI4 -->O43318_p +Q8WUI4_p : K(399,1) for forward direction
400	O43318_p +Q15653 <-->O43318_p |Q15653 : K(400,1) for forward directionand K(400,2) for reverse direction
401	O43318_p |Q15653 -->O43318_p +Q15653_p : K(401,1) for forward direction
402	P28482_p +P49407 <-->P28482_p |P49407 : K(402,1) for forward directionand K(402,2) for reverse direction
403	P28482_p |P49407 -->P28482_p +P49407_p : K(403,1) for forward direction
404	P28482_p +Q14790 <-->P28482_p |Q14790 : K(404,1) for forward directionand K(404,2) for reverse direction
405	P28482_p |Q14790 -->P28482_p +Q14790_p : K(405,1) for forward direction
406	P28482_p +P46527 <-->P28482_p |P46527 : K(406,1) for forward directionand K(406,2) for reverse direction
407	P28482_p |P46527 -->P28482_p +P46527_p : K(407,1) for forward direction
408	P28482_p +Q53EZ4 <-->P28482_p |Q53EZ4 : K(408,1) for forward directionand K(408,2) for reverse direction
409	P28482_p |Q53EZ4 -->P28482_p +Q53EZ4_p : K(409,1) for forward direction
410	P28482_p +Q96RK0 <-->P28482_p |Q96RK0 : K(410,1) for forward directionand K(410,2) for reverse direction
411	P28482_p |Q96RK0 -->P28482_p +Q96RK0_p : K(411,1) for forward direction
412	P28482_p +Q13541 <-->P28482_p |Q13541 : K(412,1) for forward directionand K(412,2) for reverse direction
413	P28482_p |Q13541 -->P28482_p +Q13541_p : K(413,1) for forward direction
414	P28482_p +P50548 <-->P28482_p |P50548 : K(414,1) for forward directionand K(414,2) for reverse direction
415	P28482_p |P50548 -->P28482_p +P50548_p : K(415,1) for forward direction
416	P28482_p +P49841 <-->P28482_p |P49841 : K(416,1) for forward directionand K(416,2) for reverse direction
417	P28482_p |P49841 -->P28482_p +P49841_p : K(417,1) for forward direction
418	P28482_p +P35568 <-->P28482_p |P35568 : K(418,1) for forward directionand K(418,2) for reverse direction
419	P28482_p |P35568 -->P28482_p +P35568_p : K(419,1) for forward direction
420	P28482_p +Q07866 <-->P28482_p |Q07866 : K(420,1) for forward directionand K(420,2) for reverse direction
421	P28482_p |Q07866 -->P28482_p +Q07866_p : K(421,1) for forward direction
422	P28482_p +Q9UKX7 <-->P28482_p |Q9UKX7 : K(422,1) for forward directionand K(422,2) for reverse direction
423	P28482_p |Q9UKX7 -->P28482_p +Q9UKX7_p : K(423,1) for forward direction

<table>
<tr><td>424</td><td>P28482_p +P49585 <-->P28482_p |P49585 : K(424,1) for forward directionand K(424,2) for reverse direction</td></tr>
<tr><td>425</td><td>P28482_p |P49585 -->P28482_p +P49585_p : K(425,1) for forward direction</td></tr>
<tr><td>426</td><td>P28482_p +P24928 <-->P28482_p |P24928 : K(426,1) for forward directionand K(426,2) for reverse direction</td></tr>
<tr><td>427</td><td>P28482_p |P24928 -->P28482_p +P24928_p : K(427,1) for forward direction</td></tr>
<tr><td>428</td><td>P28482_p +Q13362 <-->P28482_p |Q13362 : K(428,1) for forward directionand K(428,2) for reverse direction</td></tr>
<tr><td>429</td><td>P28482_p |Q13362 -->P28482_p +Q13362_p : K(429,1) for forward direction</td></tr>
<tr><td>430</td><td>P28482_p +P19793 <-->P28482_p |P19793 : K(430,1) for forward directionand K(430,2) for reverse direction</td></tr>
<tr><td>431</td><td>P28482_p |P19793 -->P28482_p +P19793_p : K(431,1) for forward direction</td></tr>
<tr><td>432</td><td>P28482_p +Q15797 <-->P28482_p |Q15797 : K(432,1) for forward directionand K(432,2) for reverse direction</td></tr>
<tr><td>433</td><td>P28482_p |Q15797 -->P28482_p +Q15797_p : K(433,1) for forward direction</td></tr>
<tr><td>434</td><td>P28482_p +Q15796 <-->P28482_p |Q15796 : K(434,1) for forward directionand K(434,2) for reverse direction</td></tr>
<tr><td>435</td><td>P28482_p |Q15796 -->P28482_p +Q15796_p : K(435,1) for forward direction</td></tr>
<tr><td>436</td><td>P28482_p +P84022 <-->P28482_p |P84022 : K(436,1) for forward directionand K(436,2) for reverse direction</td></tr>
<tr><td>437</td><td>P28482_p |P84022 -->P28482_p +P84022_p : K(437,1) for forward direction</td></tr>
<tr><td>438</td><td>P28482_p +P16949 <-->P28482_p |P16949 : K(438,1) for forward directionand K(438,2) for reverse direction</td></tr>
<tr><td>439</td><td>P28482_p |P16949 -->P28482_p +P16949_p : K(439,1) for forward direction</td></tr>
<tr><td>440</td><td>P28482_p +Q12800 <-->P28482_p |Q12800 : K(440,1) for forward directionand K(440,2) for reverse direction</td></tr>
<tr><td>441</td><td>P28482_p |Q12800 -->P28482_p +Q12800_p : K(441,1) for forward direction</td></tr>
<tr><td>442</td><td>P28482_p +P50616 <-->P28482_p |P50616 : K(442,1) for forward directionand K(442,2) for reverse direction</td></tr>
<tr><td>443</td><td>P28482_p |P50616 -->P28482_p +P50616_p : K(443,1) for forward direction</td></tr>
<tr><td>444</td><td>P28482_p +Q92574 <-->P28482_p |Q92574 : K(444,1) for forward directionand K(444,2) for reverse direction</td></tr>
<tr><td>445</td><td>P28482_p |Q92574 -->P28482_p +Q92574_p : K(445,1) for forward direction</td></tr>
<tr><td>446</td><td>P28482_p +P49815 <-->P28482_p |P49815 : K(446,1) for forward directionand K(446,2) for reverse direction</td></tr>
<tr><td>447</td><td>P28482_p |P49815 -->P28482_p +P49815_p : K(447,1) for forward direction</td></tr>
<tr><td>448</td><td>P28482_p +P17480 <-->P28482_p |P17480 : K(448,1) for forward directionand K(448,2) for reverse direction</td></tr>
<tr><td>449</td><td>P28482_p |P17480 -->P28482_p +P17480_p : K(449,1) for forward direction</td></tr>
<tr><td>450</td><td>O15264_p +O00418 <-->O15264_p |O00418 : K(450,1) for forward directionand K(450,2) for reverse direction</td></tr>
<tr><td>451</td><td>O15264_p |O00418 -->O15264_p +O00418_p : K(451,1) for forward direction</td></tr>
<tr><td>452</td><td>O15264_p +P16949 <-->O15264_p |P16949 : K(452,1) for forward directionand K(452,2) for reverse direction</td></tr>
<tr><td>453</td><td>O15264_p |P16949 -->O15264_p +P16949_p : K(453,1) for forward direction</td></tr>
<tr><td>454</td><td>Q16539_p +P42574 <-->Q16539_p |P42574 : K(454,1) for forward directionand K(454,2) for reverse direction</td></tr>
<tr><td>455</td><td>Q16539_p |P42574 -->Q16539_p +P42574_p : K(455,1) for forward direction</td></tr>
<tr><td>456</td><td>Q16539_p +Q14790 <-->Q16539_p |Q14790 : K(456,1) for forward directionand K(456,2) for reverse direction</td></tr>
<tr><td>457</td><td>Q16539_p |Q14790 -->Q16539_p +Q14790_p : K(457,1) for forward direction</td></tr>
<tr><td>458</td><td>Q16539_p +P00533 <-->Q16539_p |P00533 : K(458,1) for forward directionand K(458,2) for reverse direction</td></tr>
<tr><td>459</td><td>Q16539_p |P00533 -->Q16539_p +P00533_p : K(459,1) for forward direction</td></tr>
<tr><td>460</td><td>Q16539_p +P49841 <-->Q16539_p |P49841 : K(460,1) for forward directionand K(460,2) for reverse direction</td></tr>
<tr><td>461</td><td>Q16539_p |P49841 -->Q16539_p +P49841_p : K(461,1) for forward direction</td></tr>
<tr><td>462</td><td>Q16539_p +P78356 <-->Q16539_p |P78356 : K(462,1) for forward directionand K(462,2) for reverse direction</td></tr>
<tr><td>463</td><td>Q16539_p |P78356 -->Q16539_p +P78356_p : K(463,1) for forward direction</td></tr>
<tr><td>464</td><td>Q16539_p +P06400 <-->Q16539_p |P06400 : K(464,1) for forward directionand K(464,2) for reverse direction</td></tr>
<tr><td>465</td><td>Q16539_p |P06400 -->Q16539_p +P06400_p : K(465,1) for forward direction</td></tr>
<tr><td>466</td><td>P27361_p +P49407 <-->P27361_p |P49407 : K(466,1) for forward directionand K(466,2) for reverse direction</td></tr>
</table>

467	P27361_p |P49407 -->P27361_p +P49407_p : K(467,1) for forward direction
468	P27361_p +Q92934 <-->P27361_p |Q92934 : K(468,1) for forward directionand K(468,2) for reverse direction
469	P27361_p |Q92934 -->P27361_p +Q92934_p : K(469,1) for forward direction
470	P27361_p +P15056 <-->P27361_p |P15056 : K(470,1) for forward directionand K(470,2) for reverse direction
471	P27361_p |P15056 -->P27361_p +P15056_p : K(471,1) for forward direction
472	P27361_p +Q14790 <-->P27361_p |Q14790 : K(472,1) for forward directionand K(472,2) for reverse direction
473	P27361_p |Q14790 -->P27361_p +Q14790_p : K(473,1) for forward direction
474	P27361_p +P46527 <-->P27361_p |P46527 : K(474,1) for forward directionand K(474,2) for reverse direction
475	P27361_p |P46527 -->P27361_p +P46527_p : K(475,1) for forward direction
476	P27361_p +Q96RK0 <-->P27361_p |Q96RK0 : K(476,1) for forward directionand K(476,2) for reverse direction
477	P27361_p |Q96RK0 -->P27361_p +Q96RK0_p : K(477,1) for forward direction
478	P27361_p +P00533 <-->P27361_p |P00533 : K(478,1) for forward directionand K(478,2) for reverse direction
479	P27361_p |P00533 -->P27361_p +P00533_p : K(479,1) for forward direction
480	P27361_p +P41212 <-->P27361_p |P41212 : K(480,1) for forward directionand K(480,2) for reverse direction
481	P27361_p |P41212 -->P27361_p +P41212_p : K(481,1) for forward direction
482	P27361_p +P25098 <-->P27361_p |P25098 : K(482,1) for forward directionand K(482,2) for reverse direction
483	P27361_p |P25098 -->P27361_p +P25098_p : K(483,1) for forward direction
484	P27361_p +P49841 <-->P27361_p |P49841 : K(484,1) for forward directionand K(484,2) for reverse direction
485	P27361_p |P49841 -->P27361_p +P49841_p : K(485,1) for forward direction
486	P27361_p +P61978 <-->P27361_p |P61978 : K(486,1) for forward directionand K(486,2) for reverse direction
487	P27361_p |P61978 -->P27361_p +P61978_p : K(487,1) for forward direction
488	P27361_p +Q00613 <-->P27361_p |Q00613 : K(488,1) for forward directionand K(488,2) for reverse direction
489	P27361_p |Q00613 -->P27361_p +Q00613_p : K(489,1) for forward direction
490	P27361_p +Q07866 <-->P27361_p |Q07866 : K(490,1) for forward directionand K(490,2) for reverse direction
491	P27361_p |Q07866 -->P27361_p +Q07866_p : K(491,1) for forward direction
492	P27361_p +Q9UKX7 <-->P27361_p |Q9UKX7 : K(492,1) for forward directionand K(492,2) for reverse direction
493	P27361_p |Q9UKX7 -->P27361_p +Q9UKX7_p : K(493,1) for forward direction
494	P27361_p +Q13153 <-->P27361_p |Q13153 : K(494,1) for forward directionand K(494,2) for reverse direction
495	P27361_p |Q13153 -->P27361_p +Q13153_p : K(495,1) for forward direction
496	P27361_p +P49585 <-->P27361_p |P49585 : K(496,1) for forward directionand K(496,2) for reverse direction
497	P27361_p |P49585 -->P27361_p +P49585_p : K(497,1) for forward direction
498	P27361_p +P24928 <-->P27361_p |P24928 : K(498,1) for forward directionand K(498,2) for reverse direction
499	P27361_p |P24928 -->P27361_p +P24928_p : K(499,1) for forward direction
500	P27361_p +Q13362 <-->P27361_p |Q13362 : K(500,1) for forward directionand K(500,2) for reverse direction
501	P27361_p |Q13362 -->P27361_p +Q13362_p : K(501,1) for forward direction
502	P27361_p +Q15797 <-->P27361_p |Q15797 : K(502,1) for forward directionand K(502,2) for reverse direction
503	P27361_p |Q15797 -->P27361_p +Q15797_p : K(503,1) for forward direction
504	P27361_p +Q15796 <-->P27361_p |Q15796 : K(504,1) for forward directionand K(504,2) for reverse direction
505	P27361_p |Q15796 -->P27361_p +Q15796_p : K(505,1) for forward direction
506	P27361_p +P84022 <-->P27361_p |P84022 : K(506,1) for forward directionand K(506,2) for reverse direction
507	P27361_p |P84022 -->P27361_p +P84022_p : K(507,1) for forward direction
508	P27361_p +Q07889 <-->P27361_p |Q07889 : K(508,1) for forward directionand K(508,2) for reverse direction
509	P27361_p |Q07889 -->P27361_p +Q07889_p : K(509,1) for forward direction

510	P27361_p +Q12800 <-->P27361_p |Q12800 : K(510,1) for forward directionand K(510,2) for reverse direction
511	P27361_p |Q12800 -->P27361_p +Q12800_p : K(511,1) for forward direction
512	P27361_p +P50616 <-->P27361_p |P50616 : K(512,1) for forward directionand K(512,2) for reverse direction
513	P27361_p |P50616 -->P27361_p +P50616_p : K(513,1) for forward direction
514	P27361_p +P17480 <-->P27361_p |P17480 : K(514,1) for forward directionand K(514,2) for reverse direction
515	P27361_p |P17480 -->P27361_p +P17480_p : K(515,1) for forward direction
516	P45983_p +Q12904 <-->P45983_p |Q12904 : K(516,1) for forward directionand K(516,2) for reverse direction
517	P45983_p |Q12904 -->P45983_p +Q12904_p : K(517,1) for forward direction
518	P45983_p +Q92934 <-->P45983_p |Q92934 : K(518,1) for forward directionand K(518,2) for reverse direction
519	P45983_p |Q92934 -->P45983_p +Q92934_p : K(519,1) for forward direction
520	P45983_p +Q07817 <-->P45983_p |Q07817 : K(520,1) for forward directionand K(520,2) for reverse direction
521	P45983_p |Q07817 -->P45983_p +Q07817_p : K(521,1) for forward direction
522	P45983_p +Q13363 <-->P45983_p |Q13363 : K(522,1) for forward directionand K(522,2) for reverse direction
523	P45983_p |Q13363 -->P45983_p +Q13363_p : K(523,1) for forward direction
524	P45983_p +P35568 <-->P45983_p |P35568 : K(524,1) for forward directionand K(524,2) for reverse direction
525	P45983_p |P35568 -->P45983_p +P35568_p : K(525,1) for forward direction
526	P45983_p +P19793 <-->P45983_p |P19793 : K(526,1) for forward directionand K(526,2) for reverse direction
527	P45983_p |P19793 -->P45983_p +P19793_p : K(527,1) for forward direction
528	P45983_p +P31947 <-->P45983_p |P31947 : K(528,1) for forward directionand K(528,2) for reverse direction
529	P45983_p |P31947 -->P45983_p +P31947_p : K(529,1) for forward direction
530	P45983_p +P42226 <-->P45983_p |P42226 : K(530,1) for forward directionand K(530,2) for reverse direction
531	P45983_p |P42226 -->P45983_p +P42226_p : K(531,1) for forward direction
532	P45983_p +P16949 <-->P45983_p |P16949 : K(532,1) for forward directionand K(532,2) for reverse direction
533	P45983_p |P16949 -->P45983_p +P16949_p : K(533,1) for forward direction
534	P45983_p +P31946 <-->P45983_p |P31946 : K(534,1) for forward directionand K(534,2) for reverse direction
535	P45983_p |P31946 -->P45983_p +P31946_p : K(535,1) for forward direction
536	P45983_p +P63104 <-->P45983_p |P63104 : K(536,1) for forward directionand K(536,2) for reverse direction
537	P45983_p |P63104 -->P45983_p +P63104_p : K(537,1) for forward direction
538	P45984_p +P35568 <-->P45984_p |P35568 : K(538,1) for forward directionand K(538,2) for reverse direction
539	P45984_p |P35568 -->P45984_p +P35568_p : K(539,1) for forward direction
540	P45984_p +Q9NYV6 <-->P45984_p |Q9NYV6 : K(540,1) for forward directionand K(540,2) for reverse direction
541	P45984_p |Q9NYV6 -->P45984_p +Q9NYV6_p : K(541,1) for forward direction
542	P45984_p +P19793 <-->P45984_p |P19793 : K(542,1) for forward directionand K(542,2) for reverse direction
543	P45984_p |P19793 -->P45984_p +P19793_p : K(543,1) for forward direction
544	P45984_p +P31947 <-->P45984_p |P31947 : K(544,1) for forward directionand K(544,2) for reverse direction
545	P45984_p |P31947 -->P45984_p +P31947_p : K(545,1) for forward direction
546	P45984_p +P16949 <-->P45984_p |P16949 : K(546,1) for forward directionand K(546,2) for reverse direction
547	P45984_p |P16949 -->P45984_p +P16949_p : K(547,1) for forward direction
548	P45984_p +P50616 <-->P45984_p |P50616 : K(548,1) for forward directionand K(548,2) for reverse direction
549	P45984_p |P50616 -->P45984_p +P50616_p : K(549,1) for forward direction
550	P45984_p +P63104 <-->P45984_p |P63104 : K(550,1) for forward directionand K(550,2) for reverse direction
551	P45984_p |P63104 -->P45984_p +P63104_p : K(551,1) for forward direction
552	P49137_p +P04792 <-->P49137_p |P04792 : K(552,1) for forward directionand K(552,2) for reverse direction

<table>
<tr><td>553</td><td>P49137_p |P04792 -->P49137_p +P04792_p : K(553,1) for forward direction</td></tr>
<tr><td>554</td><td>P49137_p +O95453 <-->P49137_p |O95453 : K(554,1) for forward directionand K(554,2) for reverse direction</td></tr>
<tr><td>555</td><td>P49137_p |O95453 -->P49137_p +O95453_p : K(555,1) for forward direction</td></tr>
<tr><td>556</td><td>P49137_p +Q07352 <-->P49137_p |Q07352 : K(556,1) for forward directionand K(556,2) for reverse direction</td></tr>
<tr><td>557</td><td>P49137_p |Q07352 -->P49137_p +Q07352_p : K(557,1) for forward direction</td></tr>
<tr><td>558</td><td>Q7KZI7 +Q92974 <-->Q7KZI7 |Q92974 : K(558,1) for forward directionand K(558,2) for reverse direction</td></tr>
<tr><td>559</td><td>Q7KZI7 |Q92974 -->Q7KZI7 +Q92974_p : K(559,1) for forward direction</td></tr>
<tr><td>560</td><td>Q7KZI7 +Q8WUI4 <-->Q7KZI7 |Q8WUI4 : K(560,1) for forward directionand K(560,2) for reverse direction</td></tr>
<tr><td>561</td><td>Q7KZI7 |Q8WUI4 -->Q7KZI7 +Q8WUI4_p : K(561,1) for forward direction</td></tr>
<tr><td>562</td><td>P42345_p +O75143 <-->P42345_p |O75143 : K(562,1) for forward directionand K(562,2) for reverse direction</td></tr>
<tr><td>563</td><td>P42345_p |O75143 -->P42345_p +O75143_p : K(563,1) for forward direction</td></tr>
<tr><td>564</td><td>P42345_p +Q8TB45 <-->P42345_p |Q8TB45 : K(564,1) for forward directionand K(564,2) for reverse direction</td></tr>
<tr><td>565</td><td>P42345_p |Q8TB45 -->P42345_p +Q8TB45_p : K(565,1) for forward direction</td></tr>
<tr><td>566</td><td>P42345_p +Q13542 <-->P42345_p |Q13542 : K(566,1) for forward directionand K(566,2) for reverse direction</td></tr>
<tr><td>567</td><td>P42345_p |Q13542 -->P42345_p +Q13542_p : K(567,1) for forward direction</td></tr>
<tr><td>568</td><td>Q13153_p +P52565 <-->Q13153_p |P52565 : K(568,1) for forward directionand K(568,2) for reverse direction</td></tr>
<tr><td>569</td><td>Q13153_p |P52565 -->Q13153_p +P52565_p : K(569,1) for forward direction</td></tr>
<tr><td>570</td><td>Q13153_p +Q92974 <-->Q13153_p |Q92974 : K(570,1) for forward directionand K(570,2) for reverse direction</td></tr>
<tr><td>571</td><td>Q13153_p |Q92974 -->Q13153_p +Q92974_p : K(571,1) for forward direction</td></tr>
<tr><td>572</td><td>Q13153_p +Q92934 <-->Q13153_p |Q92934 : K(572,1) for forward directionand K(572,2) for reverse direction</td></tr>
<tr><td>573</td><td>Q13153_p |Q92934 -->Q13153_p +Q92934_p : K(573,1) for forward direction</td></tr>
<tr><td>574</td><td>Q13153_p +P63167 <-->Q13153_p |P63167 : K(574,1) for forward directionand K(574,2) for reverse direction</td></tr>
<tr><td>575</td><td>Q13153_p |P63167 -->Q13153_p +P63167_p : K(575,1) for forward direction</td></tr>
<tr><td>576</td><td>Q13153_p +Q99661 <-->Q13153_p |Q99661 : K(576,1) for forward directionand K(576,2) for reverse direction</td></tr>
<tr><td>577</td><td>Q13153_p |Q99661 -->Q13153_p +Q99661_p : K(577,1) for forward direction</td></tr>
<tr><td>578</td><td>Q13153_p +P35240 <-->Q13153_p |P35240 : K(578,1) for forward directionand K(578,2) for reverse direction</td></tr>
<tr><td>579</td><td>Q13153_p |P35240 -->Q13153_p +P35240_p : K(579,1) for forward direction</td></tr>
<tr><td>580</td><td>Q13153_p +P18669 <-->Q13153_p |P18669 : K(580,1) for forward directionand K(580,2) for reverse direction</td></tr>
<tr><td>581</td><td>Q13153_p |P18669 -->Q13153_p +P18669_p : K(581,1) for forward direction</td></tr>
<tr><td>582</td><td>Q13153_p +P16949 <-->Q13153_p |P16949 : K(582,1) for forward directionand K(582,2) for reverse direction</td></tr>
<tr><td>583</td><td>Q13153_p |P16949 -->Q13153_p +P16949_p : K(583,1) for forward direction</td></tr>
<tr><td>584</td><td>Q13153_p +P30291 <-->Q13153_p |P30291 : K(584,1) for forward directionand K(584,2) for reverse direction</td></tr>
<tr><td>585</td><td>Q13153_p |P30291 -->Q13153_p +P30291_p : K(585,1) for forward direction</td></tr>
<tr><td>586</td><td>Q13177_p +P55210 <-->Q13177_p |P55210 : K(586,1) for forward directionand K(586,2) for reverse direction</td></tr>
<tr><td>587</td><td>Q13177_p |P55210 -->Q13177_p +P55210_p : K(587,1) for forward direction</td></tr>
<tr><td>588</td><td>Q13177_p +P35240 <-->Q13177_p |P35240 : K(588,1) for forward directionand K(588,2) for reverse direction</td></tr>
<tr><td>589</td><td>Q13177_p |P35240 -->Q13177_p +P35240_p : K(589,1) for forward direction</td></tr>
<tr><td>590</td><td>Q16512_p +Q92974 <-->Q16512_p |Q92974 : K(590,1) for forward directionand K(590,2) for reverse direction</td></tr>
<tr><td>591</td><td>Q16512_p |Q92974 -->Q16512_p +Q92974_p : K(591,1) for forward direction</td></tr>
<tr><td>592</td><td>Q16512_p +P00533 <-->Q16512_p |P00533 : K(592,1) for forward directionand K(592,2) for reverse direction</td></tr>
<tr><td>593</td><td>Q16512_p |P00533 -->Q16512_p +P00533_p : K(593,1) for forward direction</td></tr>
<tr><td>594</td><td>Q16512_p +Q12778 <-->Q16512_p |Q12778 : K(594,1) for forward directionand K(594,2) for reverse direction</td></tr>
<tr><td>595</td><td>Q16512_p |Q12778 -->Q16512_p +Q12778_p : K(595,1) for forward direction</td></tr>
</table>

596	Q16512_p +P18669 <-->Q16512_p |P18669 : K(596,1) for forward directionand K(596,2) for reverse direction
597	Q16512_p |P18669 -->Q16512_p +P18669_p : K(597,1) for forward direction
598	P53350_p +Q9UBB4 <-->P53350_p |Q9UBB4 : K(598,1) for forward directionand K(598,2) for reverse direction
599	P53350_p |Q9UBB4 -->P53350_p +Q9UBB4_p : K(599,1) for forward direction
600	P53350_p +Q13158 <-->P53350_p |Q13158 : K(600,1) for forward directionand K(600,2) for reverse direction
601	P53350_p |Q13158 -->P53350_p +Q13158_p : K(601,1) for forward direction
602	P53350_p +Q00613 <-->P53350_p |Q00613 : K(602,1) for forward directionand K(602,2) for reverse direction
603	P53350_p |Q00613 -->P53350_p +Q00613_p : K(603,1) for forward direction
604	P53350_p +P35568 <-->P53350_p |P35568 : K(604,1) for forward directionand K(604,2) for reverse direction
605	P53350_p |P35568 -->P53350_p +P35568_p : K(605,1) for forward direction
606	P53350_p +P47736 <-->P53350_p |P47736 : K(606,1) for forward directionand K(606,2) for reverse direction
607	P53350_p |P47736 -->P53350_p +P47736_p : K(607,1) for forward direction
608	P53350_p +P13693 <-->P53350_p |P13693 : K(608,1) for forward directionand K(608,2) for reverse direction
609	P53350_p |P13693 -->P53350_p +P13693_p : K(609,1) for forward direction
610	P53350_p +P30291 <-->P53350_p |P30291 : K(610,1) for forward directionand K(610,2) for reverse direction
611	P53350_p |P30291 -->P53350_p +P30291_p : K(611,1) for forward direction
612	Q13131_p +Q53ET0 <-->Q13131_p |Q53ET0 : K(612,1) for forward directionand K(612,2) for reverse direction
613	Q13131_p |Q53ET0 -->Q13131_p +Q53ET0_p : K(613,1) for forward direction
614	Q13131_p +Q13363 <-->Q13131_p |Q13363 : K(614,1) for forward directionand K(614,2) for reverse direction
615	Q13131_p |Q13363 -->Q13131_p +Q13363_p : K(615,1) for forward direction
616	Q13131_p +Q09472 <-->Q13131_p |Q09472 : K(616,1) for forward directionand K(616,2) for reverse direction
617	Q13131_p |Q09472 -->Q13131_p +Q09472_p : K(617,1) for forward direction
618	Q13131_p +Q92538 <-->Q13131_p |Q92538 : K(618,1) for forward directionand K(618,2) for reverse direction
619	Q13131_p |Q92538 -->Q13131_p +Q92538_p : K(619,1) for forward direction
620	Q13131_p +Q06210 <-->Q13131_p |Q06210 : K(620,1) for forward directionand K(620,2) for reverse direction
621	Q13131_p |Q06210 -->Q13131_p +Q06210_p : K(621,1) for forward direction
622	Q13131_p +Q8WUI4 <-->Q13131_p |Q8WUI4 : K(622,1) for forward directionand K(622,2) for reverse direction
623	Q13131_p |Q8WUI4 -->Q13131_p +Q8WUI4_p : K(623,1) for forward direction
624	Q13131_p +Q05469 <-->Q13131_p |Q05469 : K(624,1) for forward directionand K(624,2) for reverse direction
625	Q13131_p |Q05469 -->Q13131_p +Q05469_p : K(625,1) for forward direction
626	Q13131_p +P14859 <-->Q13131_p |P14859 : K(626,1) for forward directionand K(626,2) for reverse direction
627	Q13131_p |P14859 -->Q13131_p +P14859_p : K(627,1) for forward direction
628	Q13131_p +P06400 <-->Q13131_p |P06400 : K(628,1) for forward directionand K(628,2) for reverse direction
629	Q13131_p |P06400 -->Q13131_p +P06400_p : K(629,1) for forward direction
630	Q13131_p +Q9NYV6 <-->Q13131_p |Q9NYV6 : K(630,1) for forward directionand K(630,2) for reverse direction
631	Q13131_p |Q9NYV6 -->Q13131_p +Q9NYV6_p : K(631,1) for forward direction
632	Q13131_p +Q86TI0 <-->Q13131_p |Q86TI0 : K(632,1) for forward directionand K(632,2) for reverse direction
633	Q13131_p |Q86TI0 -->Q13131_p +Q86TI0_p : K(633,1) for forward direction
634	Q9Y478_p +Q8N122 <-->Q9Y478_p |Q8N122 : K(634,1) for forward directionand K(634,2) for reverse direction
635	Q9Y478_p |Q8N122 -->Q9Y478_p +Q8N122_p : K(635,1) for forward direction
636	P17612 +Q12802 <-->P17612 |Q12802 : K(636,1) for forward directionand K(636,2) for reverse direction
637	P17612 |Q12802 -->P17612 +Q12802_p : K(637,1) for forward direction
638	P17612 +P52565 <-->P17612 |P52565 : K(638,1) for forward directionand K(638,2) for reverse direction

639	P17612 |P52565 -->P17612 +P52565_p : K(639,1) for forward direction
640	P17612 +Q92934 <-->P17612 |Q92934 : K(640,1) for forward directionand K(640,2) for reverse direction
641	P17612 |Q92934 -->P17612 +Q92934_p : K(641,1) for forward direction
642	P17612 +P27708 <-->P17612 |P27708 : K(642,1) for forward directionand K(642,2) for reverse direction
643	P17612 |P27708 -->P17612 +P27708_p : K(643,1) for forward direction
644	P17612 +P17655 <-->P17612 |P17655 : K(644,1) for forward directionand K(644,2) for reverse direction
645	P17612 |P17655 -->P17612 +P17655_p : K(645,1) for forward direction
646	P17612 +P55211 <-->P17612 |P55211 : K(646,1) for forward directionand K(646,2) for reverse direction
647	P17612 |P55211 -->P17612 +P55211_p : K(647,1) for forward direction
648	P17612 +O60716 <-->P17612 |O60716 : K(648,1) for forward directionand K(648,2) for reverse direction
649	P17612 |O60716 -->P17612 +O60716_p : K(649,1) for forward direction
650	P17612 +P49840 <-->P17612 |P49840 : K(650,1) for forward directionand K(650,2) for reverse direction
651	P17612 |P49840 -->P17612 +P49840_p : K(651,1) for forward direction
652	P17612 +Q9BY41 <-->P17612 |Q9BY41 : K(652,1) for forward directionand K(652,2) for reverse direction
653	P17612 |Q9BY41 -->P17612 +Q9BY41_p : K(653,1) for forward direction
654	P17612 +P16144 <-->P17612 |P16144 : K(654,1) for forward directionand K(654,2) for reverse direction
655	P17612 |P16144 -->P17612 +P16144_p : K(655,1) for forward direction
656	P17612 +Q14847 <-->P17612 |Q14847 : K(656,1) for forward directionand K(656,2) for reverse direction
657	P17612 |Q14847 -->P17612 +Q14847_p : K(657,1) for forward direction
658	P17612 +Q05469 <-->P17612 |Q05469 : K(658,1) for forward directionand K(658,2) for reverse direction
659	P17612 |Q05469 -->P17612 +Q05469_p : K(659,1) for forward direction
660	P17612 +Q96PU5 <-->P17612 |Q96PU5 : K(660,1) for forward directionand K(660,2) for reverse direction
661	P17612 |Q96PU5 -->P17612 +Q96PU5_p : K(661,1) for forward direction
662	P17612 +P35240 <-->P17612 |P35240 : K(662,1) for forward directionand K(662,2) for reverse direction
663	P17612 |P35240 -->P17612 +P35240_p : K(663,1) for forward direction
664	P17612 +Q01970 <-->P17612 |Q01970 : K(664,1) for forward directionand K(664,2) for reverse direction
665	P17612 |Q01970 -->P17612 +Q01970_p : K(665,1) for forward direction
666	P17612 +Q15054 <-->P17612 |Q15054 : K(666,1) for forward directionand K(666,2) for reverse direction
667	P17612 |Q15054 -->P17612 +Q15054_p : K(667,1) for forward direction
668	P17612 +P19793 <-->P17612 |P19793 : K(668,1) for forward directionand K(668,2) for reverse direction
669	P17612 |P19793 -->P17612 +P19793_p : K(669,1) for forward direction
670	P17612 +P16949 <-->P17612 |P16949 : K(670,1) for forward directionand K(670,2) for reverse direction
671	P17612 |P16949 -->P17612 +P16949_p : K(671,1) for forward direction
672	P17252_p +P21399 <-->P17252_p |P21399 : K(672,1) for forward directionand K(672,2) for reverse direction
673	P17252_p |P21399 -->P17252_p +P21399_p : K(673,1) for forward direction
674	P17252_p +P52566 <-->P17252_p |P52566 : K(674,1) for forward directionand K(674,2) for reverse direction
675	P17252_p |P52566 -->P17252_p +P52566_p : K(675,1) for forward direction
676	P17252_p +P23528 <-->P17252_p |P23528 : K(676,1) for forward directionand K(676,2) for reverse direction
677	P17252_p |P23528 -->P17252_p +P23528_p : K(677,1) for forward direction
678	P17252_p +P30622 <-->P17252_p |P30622 : K(678,1) for forward directionand K(678,2) for reverse direction
679	P17252_p |P30622 -->P17252_p +P30622_p : K(679,1) for forward direction
680	P17252_p +Q9BR76 <-->P17252_p |Q9BR76 : K(680,1) for forward directionand K(680,2) for reverse direction
681	P17252_p |Q9BR76 -->P17252_p +Q9BR76_p : K(681,1) for forward direction

682	P17252_p +Q92793 <-->P17252_p |Q92793 : K(682,1) for forward directionand K(682,2) for reverse direction
683	P17252_p |Q92793 -->P17252_p +Q92793_p : K(683,1) for forward direction
684	P17252_p +P17812 <-->P17252_p |P17812 : K(684,1) for forward directionand K(684,2) for reverse direction
685	P17252_p |P17812 -->P17252_p +P17812_p : K(685,1) for forward direction
686	P17252_p +Q01844 <-->P17252_p |Q01844 : K(686,1) for forward directionand K(686,2) for reverse direction
687	P17252_p |Q01844 -->P17252_p +Q01844_p : K(687,1) for forward direction
688	P17252_p +P49840 <-->P17252_p |P49840 : K(688,1) for forward directionand K(688,2) for reverse direction
689	P17252_p |P49840 -->P17252_p +P49840_p : K(689,1) for forward direction
690	P17252_p +P49841 <-->P17252_p |P49841 : K(690,1) for forward directionand K(690,2) for reverse direction
691	P17252_p |P49841 -->P17252_p +P49841_p : K(691,1) for forward direction
692	P17252_p +P05114 <-->P17252_p |P05114 : K(692,1) for forward directionand K(692,2) for reverse direction
693	P17252_p |P05114 -->P17252_p +P05114_p : K(693,1) for forward direction
694	P17252_p +P05204 <-->P17252_p |P05204 : K(694,1) for forward directionand K(694,2) for reverse direction
695	P17252_p |P05204 -->P17252_p +P05204_p : K(695,1) for forward direction
696	P17252_p +P09651 <-->P17252_p |P09651 : K(696,1) for forward directionand K(696,2) for reverse direction
697	P17252_p |P09651 -->P17252_p +P09651_p : K(697,1) for forward direction
698	P17252_p +P16144 <-->P17252_p |P16144 : K(698,1) for forward directionand K(698,2) for reverse direction
699	P17252_p |P16144 -->P17252_p +P16144_p : K(699,1) for forward direction
700	P17252_p +P08581 <-->P17252_p |P08581 : K(700,1) for forward directionand K(700,2) for reverse direction
701	P17252_p |P08581 -->P17252_p +P08581_p : K(701,1) for forward direction
702	P17252_p +P24844 <-->P17252_p |P24844 : K(702,1) for forward directionand K(702,2) for reverse direction
703	P17252_p |P24844 -->P17252_p +P24844_p : K(703,1) for forward direction
704	P17252_p +Q15121 <-->P17252_p |Q15121 : K(704,1) for forward directionand K(704,2) for reverse direction
705	P17252_p |Q15121 -->P17252_p +Q15121_p : K(705,1) for forward direction
706	P17252_p +Q01970 <-->P17252_p |Q01970 : K(706,1) for forward directionand K(706,2) for reverse direction
707	P17252_p |Q01970 -->P17252_p +Q01970_p : K(707,1) for forward direction
708	P17252_p +Q05209 <-->P17252_p |Q05209 : K(708,1) for forward directionand K(708,2) for reverse direction
709	P17252_p |Q05209 -->P17252_p +Q05209_p : K(709,1) for forward direction
710	P17252_p +P29350 <-->P17252_p |P29350 : K(710,1) for forward directionand K(710,2) for reverse direction
711	P17252_p |P29350 -->P17252_p +P29350_p : K(711,1) for forward direction
712	Q05655_p +Q09472 <-->Q05655_p |Q09472 : K(712,1) for forward directionand K(712,2) for reverse direction
713	Q05655_p |Q09472 -->Q05655_p +Q09472_p : K(713,1) for forward direction
714	Q05655_p +P49840 <-->Q05655_p |P49840 : K(714,1) for forward directionand K(714,2) for reverse direction
715	Q05655_p |P49840 -->Q05655_p +P49840_p : K(715,1) for forward direction
716	Q05655_p +P17096 <-->Q05655_p |P17096 : K(716,1) for forward directionand K(716,2) for reverse direction
717	Q05655_p |P17096 -->Q05655_p +P17096_p : K(717,1) for forward direction
718	Q05655_p +P61978 <-->Q05655_p |P61978 : K(718,1) for forward directionand K(718,2) for reverse direction
719	Q05655_p |P61978 -->Q05655_p +P61978_p : K(719,1) for forward direction
720	Q05655_p +P35568 <-->Q05655_p |P35568 : K(720,1) for forward directionand K(720,2) for reverse direction
721	Q05655_p |P35568 -->Q05655_p +P35568_p : K(721,1) for forward direction
722	Q05655_p +P10588 <-->Q05655_p |P10588 : K(722,1) for forward directionand K(722,2) for reverse direction
723	Q05655_p |P10588 -->Q05655_p +P10588_p : K(723,1) for forward direction
724	Q05655_p +P31431 <-->Q05655_p |P31431 : K(724,1) for forward directionand K(724,2) for reverse direction

725	Q05655_p |P31431 -->Q05655_p +P31431_p : K(725,1) for forward direction
726	Q05655_p +Q13263 <-->Q05655_p |Q13263 : K(726,1) for forward directionand K(726,2) for reverse direction
727	Q05655_p |Q13263 -->Q05655_p +Q13263_p : K(727,1) for forward direction
728	Q05655_p +P31946 <-->Q05655_p |P31946 : K(728,1) for forward directionand K(728,2) for reverse direction
729	Q05655_p |P31946 -->Q05655_p +P31946_p : K(729,1) for forward direction
730	P41743_p +Q92934 <-->P41743_p |Q92934 : K(730,1) for forward directionand K(730,2) for reverse direction
731	P41743_p |Q92934 -->P41743_p +Q92934_p : K(731,1) for forward direction
732	P41743_p +P49757 <-->P41743_p |P49757 : K(732,1) for forward directionand K(732,2) for reverse direction
733	P41743_p |P49757 -->P41743_p +P49757_p : K(733,1) for forward direction
734	P78527_p +P49917 <-->P78527_p |P49917 : K(734,1) for forward directionand K(734,2) for reverse direction
735	P78527_p |P49917 -->P78527_p +P49917_p : K(735,1) for forward direction
736	P78527_p +P14859 <-->P78527_p |P14859 : K(736,1) for forward directionand K(736,2) for reverse direction
737	P78527_p |P14859 -->P78527_p +P14859_p : K(737,1) for forward direction
738	P78527_p +P78527 <-->P78527_p |P78527 : K(738,1) for forward directionand K(738,2) for reverse direction
739	P78527_p |P78527 -->P78527_p +P78527_p : K(739,1) for forward direction
740	Q13976_p +P04792 <-->Q13976_p |P04792 : K(740,1) for forward directionand K(740,2) for reverse direction
741	Q13976_p |P04792 -->Q13976_p +P04792_p : K(741,1) for forward direction
742	Q13976_p +Q14847 <-->Q13976_p |Q14847 : K(742,1) for forward directionand K(742,2) for reverse direction
743	Q13976_p |Q14847 -->Q13976_p +Q14847_p : K(743,1) for forward direction
744	Q13976_p +P50552 <-->Q13976_p |P50552 : K(744,1) for forward directionand K(744,2) for reverse direction
745	Q13976_p |P50552 -->Q13976_p +P50552_p : K(745,1) for forward direction
746	Q13523 +Q9Y2Y9 <-->Q13523 |Q9Y2Y9 : K(746,1) for forward directionand K(746,2) for reverse direction
747	Q13523 |Q9Y2Y9 -->Q13523 +Q9Y2Y9_p : K(747,1) for forward direction
748	Q05397_p +P12814 <-->Q05397_p |P12814 : K(748,1) for forward directionand K(748,2) for reverse direction
749	Q05397_p |P12814 -->Q05397_p +P12814_p : K(749,1) for forward direction
750	Q05397_p +O43707 <-->Q05397_p |O43707 : K(750,1) for forward directionand K(750,2) for reverse direction
751	Q05397_p |O43707 -->Q05397_p +O43707_p : K(751,1) for forward direction
752	Q13464 +Q9NRY4 <-->Q13464 |Q9NRY4 : K(752,1) for forward directionand K(752,2) for reverse direction
753	Q13464 |Q9NRY4 -->Q13464 +Q9NRY4_p : K(753,1) for forward direction
754	Q13464 +Q6WCQ1 <-->Q13464 |Q6WCQ1 : K(754,1) for forward directionand K(754,2) for reverse direction
755	Q13464 |Q6WCQ1 -->Q13464 +Q6WCQ1_p : K(755,1) for forward direction
756	Q13464 +P07737 <-->Q13464 |P07737 : K(756,1) for forward directionand K(756,2) for reverse direction
757	Q13464 |P07737 -->Q13464 +P07737_p : K(757,1) for forward direction
758	Q13464 +O14974 <-->Q13464 |O14974 : K(758,1) for forward directionand K(758,2) for reverse direction
759	Q13464 |O14974 -->Q13464 +O14974_p : K(759,1) for forward direction
760	Q15418_p +Q96RK0 <-->Q15418_p |Q96RK0 : K(760,1) for forward directionand K(760,2) for reverse direction
761	Q15418_p |Q96RK0 -->Q15418_p +Q96RK0_p : K(761,1) for forward direction
762	Q15418_p +Q8TB45 <-->Q15418_p |Q8TB45 : K(762,1) for forward directionand K(762,2) for reverse direction
763	Q15418_p |Q8TB45 -->Q15418_p +Q8TB45_p : K(763,1) for forward direction
764	Q15418_p +P49841 <-->Q15418_p |P49841 : K(764,1) for forward directionand K(764,2) for reverse direction
765	Q15418_p |P49841 -->Q15418_p +P49841_p : K(765,1) for forward direction
766	Q15418_p +Q9UBP6 <-->Q15418_p |Q9UBP6 : K(766,1) for forward directionand K(766,2) for reverse direction
767	Q15418_p |Q9UBP6 -->Q15418_p +Q9UBP6_p : K(767,1) for forward direction

<table>
<tr><td>768</td><td>Q15418_p +P49815 <-->Q15418_p |P49815 : K(768,1) for forward directionand K(768,2) for reverse direction</td></tr>
<tr><td>769</td><td>Q15418_p |P49815 -->Q15418_p +P49815_p : K(769,1) for forward direction</td></tr>
<tr><td>770</td><td>Q15418_p +P50552 <-->Q15418_p |P50552 : K(770,1) for forward directionand K(770,2) for reverse direction</td></tr>
<tr><td>771</td><td>Q15418_p |P50552 -->Q15418_p +P50552_p : K(771,1) for forward direction</td></tr>
<tr><td>772</td><td>P51812_p +Q92934 <-->P51812_p |Q92934 : K(772,1) for forward directionand K(772,2) for reverse direction</td></tr>
<tr><td>773</td><td>P51812_p |Q92934 -->P51812_p +Q92934_p : K(773,1) for forward direction</td></tr>
<tr><td>774</td><td>P51812_p +Q96RK0 <-->P51812_p |Q96RK0 : K(774,1) for forward directionand K(774,2) for reverse direction</td></tr>
<tr><td>775</td><td>P51812_p |Q96RK0 -->P51812_p +Q96RK0_p : K(775,1) for forward direction</td></tr>
<tr><td>776</td><td>P12931_p +P63010 <-->P12931_p |P63010 : K(776,1) for forward directionand K(776,2) for reverse direction</td></tr>
<tr><td>777</td><td>P12931_p |P63010 -->P12931_p +P63010_p : K(777,1) for forward direction</td></tr>
<tr><td>778</td><td>P12931_p +P52565 <-->P12931_p |P52565 : K(778,1) for forward directionand K(778,2) for reverse direction</td></tr>
<tr><td>779</td><td>P12931_p |P52565 -->P12931_p +P52565_p : K(779,1) for forward direction</td></tr>
<tr><td>780</td><td>P12931_p +P49407 <-->P12931_p |P49407 : K(780,1) for forward directionand K(780,2) for reverse direction</td></tr>
<tr><td>781</td><td>P12931_p |P49407 -->P12931_p +P49407_p : K(781,1) for forward direction</td></tr>
<tr><td>782</td><td>P12931_p +Q14790 <-->P12931_p |Q14790 : K(782,1) for forward directionand K(782,2) for reverse direction</td></tr>
<tr><td>783</td><td>P12931_p |Q14790 -->P12931_p +Q14790_p : K(783,1) for forward direction</td></tr>
<tr><td>784</td><td>P12931_p +P46527 <-->P12931_p |P46527 : K(784,1) for forward directionand K(784,2) for reverse direction</td></tr>
<tr><td>785</td><td>P12931_p |P46527 -->P12931_p +P46527_p : K(785,1) for forward direction</td></tr>
<tr><td>786</td><td>P12931_p +P23528 <-->P12931_p |P23528 : K(786,1) for forward directionand K(786,2) for reverse direction</td></tr>
<tr><td>787</td><td>P12931_p |P23528 -->P12931_p +P23528_p : K(787,1) for forward direction</td></tr>
<tr><td>788</td><td>P12931_p +Q14247 <-->P12931_p |Q14247 : K(788,1) for forward directionand K(788,2) for reverse direction</td></tr>
<tr><td>789</td><td>P12931_p |Q14247 -->P12931_p +Q14247_p : K(789,1) for forward direction</td></tr>
<tr><td>790</td><td>P12931_p +Q14118 <-->P12931_p |Q14118 : K(790,1) for forward directionand K(790,2) for reverse direction</td></tr>
<tr><td>791</td><td>P12931_p |Q14118 -->P12931_p +Q14118_p : K(791,1) for forward direction</td></tr>
<tr><td>792</td><td>P12931_p +P50402 <-->P12931_p |P50402 : K(792,1) for forward directionand K(792,2) for reverse direction</td></tr>
<tr><td>793</td><td>P12931_p |P50402 -->P12931_p +P50402_p : K(793,1) for forward direction</td></tr>
<tr><td>794</td><td>P12931_p +P61978 <-->P12931_p |P61978 : K(794,1) for forward directionand K(794,2) for reverse direction</td></tr>
<tr><td>795</td><td>P12931_p |P61978 -->P12931_p +P61978_p : K(795,1) for forward direction</td></tr>
<tr><td>796</td><td>P12931_p +P67775 <-->P12931_p |P67775 : K(796,1) for forward directionand K(796,2) for reverse direction</td></tr>
<tr><td>797</td><td>P12931_p |P67775 -->P12931_p +P67775_p : K(797,1) for forward direction</td></tr>
<tr><td>798</td><td>P12931_p +P60484 <-->P12931_p |P60484 : K(798,1) for forward directionand K(798,2) for reverse direction</td></tr>
<tr><td>799</td><td>P12931_p |P60484 -->P12931_p +P60484_p : K(799,1) for forward direction</td></tr>
<tr><td>800</td><td>P12931_p +P20936 <-->P12931_p |P20936 : K(800,1) for forward directionand K(800,2) for reverse direction</td></tr>
<tr><td>801</td><td>P12931_p |P20936 -->P12931_p +P20936_p : K(801,1) for forward direction</td></tr>
<tr><td>802</td><td>P12931_p +Q99961 <-->P12931_p |Q99961 : K(802,1) for forward directionand K(802,2) for reverse direction</td></tr>
<tr><td>803</td><td>P12931_p |Q99961 -->P12931_p +Q99961_p : K(803,1) for forward direction</td></tr>
<tr><td>804</td><td>Q96SB4_p +Q9Y5S9 <-->Q96SB4_p |Q9Y5S9 : K(804,1) for forward directionand K(804,2) for reverse direction</td></tr>
<tr><td>805</td><td>Q96SB4_p |Q9Y5S9 -->Q96SB4_p +Q9Y5S9_p : K(805,1) for forward direction</td></tr>
<tr><td>806</td><td>O94804 +P26038 <-->O94804 |P26038 : K(806,1) for forward directionand K(806,2) for reverse direction</td></tr>
<tr><td>807</td><td>O94804 |P26038 -->O94804 +P26038_p : K(807,1) for forward direction</td></tr>
<tr><td>808</td><td>P33981_p +P33981 <-->P33981_p |P33981 : K(808,1) for forward directionand K(808,2) for reverse direction</td></tr>
<tr><td>809</td><td>P33981_p |P33981 -->P33981_p +P33981_p : K(809,1) for forward direction</td></tr>
<tr><td>810</td><td>Q99986 +O75531 <-->Q99986 |O75531 : K(810,1) for forward directionand K(810,2) for reverse direction</td></tr>
</table>

<table>
<tr><td>811</td><td>Q99986 |O75531 -->Q99986 +O75531_p : K(811,1) for forward direction</td></tr>
<tr><td>812</td><td>P30291_p +P06493 <-->P30291_p |P06493 : K(812,1) for forward directionand K(812,2) for reverse direction</td></tr>
<tr><td>813</td><td>P30291_p |P06493 -->P30291_p +P06493_p : K(813,1) for forward direction</td></tr>
<tr><td>814</td><td>P30291_p +P24941 <-->P30291_p |P24941 : K(814,1) for forward directionand K(814,2) for reverse direction</td></tr>
<tr><td>815</td><td>P30291_p |P24941 -->P30291_p +P24941_p : K(815,1) for forward direction</td></tr>
<tr><td>816</td><td>P07947_p +P11802 <-->P07947_p |P11802 : K(816,1) for forward directionand K(816,2) for reverse direction</td></tr>
<tr><td>817</td><td>P07947_p |P11802 -->P07947_p +P11802_p : K(817,1) for forward direction</td></tr>
<tr><td>818</td><td>P31749_p +Q96B36 <-->P31749_p |Q96B36 : K(818,1) for forward directionand K(818,2) for reverse direction</td></tr>
<tr><td>819</td><td>P31749_p |Q96B36 -->P31749_p +Q96B36_p : K(819,1) for forward direction</td></tr>
<tr><td>820</td><td>P31749_p +Q92934 <-->P31749_p |Q92934 : K(820,1) for forward directionand K(820,2) for reverse direction</td></tr>
<tr><td>821</td><td>P31749_p |Q92934 -->P31749_p +Q92934_p : K(821,1) for forward direction</td></tr>
<tr><td>822</td><td>P31749_p +Q07812 <-->P31749_p |Q07812 : K(822,1) for forward directionand K(822,2) for reverse direction</td></tr>
<tr><td>823</td><td>P31749_p |Q07812 -->P31749_p +Q07812_p : K(823,1) for forward direction</td></tr>
<tr><td>824</td><td>P31749_p +P15056 <-->P31749_p |P15056 : K(824,1) for forward directionand K(824,2) for reverse direction</td></tr>
<tr><td>825</td><td>P31749_p |P15056 -->P31749_p +P15056_p : K(825,1) for forward direction</td></tr>
<tr><td>826</td><td>P31749_p +Q15910 <-->P31749_p |Q15910 : K(826,1) for forward directionand K(826,2) for reverse direction</td></tr>
<tr><td>827</td><td>P31749_p |Q15910 -->P31749_p +Q15910_p : K(827,1) for forward direction</td></tr>
<tr><td>828</td><td>P31749_p +O43524 <-->P31749_p |O43524 : K(828,1) for forward directionand K(828,2) for reverse direction</td></tr>
<tr><td>829</td><td>P31749_p |O43524 -->P31749_p +O43524_p : K(829,1) for forward direction</td></tr>
<tr><td>830</td><td>P31749_p +P49841 <-->P31749_p |P49841 : K(830,1) for forward directionand K(830,2) for reverse direction</td></tr>
<tr><td>831</td><td>P31749_p |P49841 -->P31749_p +P49841_p : K(831,1) for forward direction</td></tr>
<tr><td>832</td><td>P31749_p +P51617 <-->P31749_p |P51617 : K(832,1) for forward directionand K(832,2) for reverse direction</td></tr>
<tr><td>833</td><td>P31749_p |P51617 -->P31749_p +P51617_p : K(833,1) for forward direction</td></tr>
<tr><td>834</td><td>P31749_p +Q92945 <-->P31749_p |Q92945 : K(834,1) for forward directionand K(834,2) for reverse direction</td></tr>
<tr><td>835</td><td>P31749_p |Q92945 -->P31749_p +Q92945_p : K(835,1) for forward direction</td></tr>
<tr><td>836</td><td>P31749_p +P41279 <-->P31749_p |P41279 : K(836,1) for forward directionand K(836,2) for reverse direction</td></tr>
<tr><td>837</td><td>P31749_p |P41279 -->P31749_p +P41279_p : K(837,1) for forward direction</td></tr>
<tr><td>838</td><td>P31749_p +P42345 <-->P31749_p |P42345 : K(838,1) for forward directionand K(838,2) for reverse direction</td></tr>
<tr><td>839</td><td>P31749_p |P42345 -->P31749_p +P42345_p : K(839,1) for forward direction</td></tr>
<tr><td>840</td><td>P31749_p +Q13131 <-->P31749_p |Q13131 : K(840,1) for forward directionand K(840,2) for reverse direction</td></tr>
<tr><td>841</td><td>P31749_p |Q13131 -->P31749_p +Q13131_p : K(841,1) for forward direction</td></tr>
<tr><td>842</td><td>P31749_p +P18031 <-->P31749_p |P18031 : K(842,1) for forward directionand K(842,2) for reverse direction</td></tr>
<tr><td>843</td><td>P31749_p |P18031 -->P31749_p +P18031_p : K(843,1) for forward direction</td></tr>
<tr><td>844</td><td>P31749_p +P63000 <-->P31749_p |P63000 : K(844,1) for forward directionand K(844,2) for reverse direction</td></tr>
<tr><td>845</td><td>P31749_p |P63000 -->P31749_p +P63000_p : K(845,1) for forward direction</td></tr>
<tr><td>846</td><td>P31749_p +P49815 <-->P31749_p |P49815 : K(846,1) for forward directionand K(846,2) for reverse direction</td></tr>
<tr><td>847</td><td>P31749_p |P49815 -->P31749_p +P49815_p : K(847,1) for forward direction</td></tr>
<tr><td>848</td><td>P31751_p +Q12778 <-->P31751_p |Q12778 : K(848,1) for forward directionand K(848,2) for reverse direction</td></tr>
<tr><td>849</td><td>P31751_p |Q12778 -->P31751_p +Q12778_p : K(849,1) for forward direction</td></tr>
<tr><td>850</td><td>P31751_p +O43524 <-->P31751_p |O43524 : K(850,1) for forward directionand K(850,2) for reverse direction</td></tr>
<tr><td>851</td><td>P31751_p |O43524 -->P31751_p +O43524_p : K(851,1) for forward direction</td></tr>
<tr><td>852</td><td>P31751_p +P49841 <-->P31751_p |P49841 : K(852,1) for forward directionand K(852,2) for reverse direction</td></tr>
<tr><td>853</td><td>P31751_p |P49841 -->P31751_p +P49841_p : K(853,1) for forward direction</td></tr>
</table>

854	P31751_p +P18031 <-->P31751_p |P18031 : K(854,1) for forward directionand K(854,2) for reverse direction
855	P31751_p |P18031 -->P31751_p +P18031_p : K(855,1) for forward direction
856	Q9UQM7_p +Q12959 <-->Q9UQM7_p |Q12959 : K(856,1) for forward directionand K(856,2) for reverse direction
857	Q9UQM7_p |Q12959 -->Q9UQM7_p +Q12959_p : K(857,1) for forward direction
858	Q9UQM7_p +P00533 <-->Q9UQM7_p |P00533 : K(858,1) for forward directionand K(858,2) for reverse direction
859	Q9UQM7_p |P00533 -->Q9UQM7_p +P00533_p : K(859,1) for forward direction
860	Q13555 +P00533 <-->Q13555 |P00533 : K(860,1) for forward directionand K(860,2) for reverse direction
861	Q13555 |P00533 -->Q13555 +P00533_p : K(861,1) for forward direction
862	Q13555 +Q15746 <-->Q13555 |Q15746 : K(862,1) for forward directionand K(862,2) for reverse direction
863	Q13555 |Q15746 -->Q13555 +Q15746_p : K(863,1) for forward direction
864	Q5VT25 +Q9BZL4 <-->Q5VT25 |Q9BZL4 : K(864,1) for forward directionand K(864,2) for reverse direction
865	Q5VT25 |Q9BZL4 -->Q5VT25 +Q9BZL4_p : K(865,1) for forward direction
866	P06493_p +Q13541 <-->P06493_p |Q13541 : K(866,1) for forward directionand K(866,2) for reverse direction
867	P06493_p |Q13541 -->P06493_p +Q13541_p : K(867,1) for forward direction
868	P06493_p +P39748 <-->P06493_p |P39748 : K(868,1) for forward directionand K(868,2) for reverse direction
869	P06493_p |P39748 -->P06493_p +P39748_p : K(869,1) for forward direction
870	P06493_p +P33991 <-->P06493_p |P33991 : K(870,1) for forward directionand K(870,2) for reverse direction
871	P06493_p |P33991 -->P06493_p +P33991_p : K(871,1) for forward direction
872	P06493_p +P06748 <-->P06493_p |P06748 : K(872,1) for forward directionand K(872,2) for reverse direction
873	P06493_p |P06748 -->P06493_p +P06748_p : K(873,1) for forward direction
874	P06493_p +Q9UNZ2 <-->P06493_p |Q9UNZ2 : K(874,1) for forward directionand K(874,2) for reverse direction
875	P06493_p |Q9UNZ2 -->P06493_p +Q9UNZ2_p : K(875,1) for forward direction
876	P06493_p +O00443 <-->P06493_p |O00443 : K(876,1) for forward directionand K(876,2) for reverse direction
877	P06493_p |O00443 -->P06493_p +O00443_p : K(877,1) for forward direction
878	P24941_p +P33991 <-->P24941_p |P33991 : K(878,1) for forward directionand K(878,2) for reverse direction
879	P24941_p |P33991 -->P24941_p +P33991_p : K(879,1) for forward direction
880	P24941_p +P06748 <-->P24941_p |P06748 : K(880,1) for forward directionand K(880,2) for reverse direction
881	P24941_p |P06748 -->P24941_p +P06748_p : K(881,1) for forward direction
882	P24941_p +Q712K3 <-->P24941_p |Q712K3 : K(882,1) for forward directionand K(882,2) for reverse direction
883	P24941_p |Q712K3 -->P24941_p +Q712K3_p : K(883,1) for forward direction
884	Q00535_p +Q13153 <-->Q00535_p |Q13153 : K(884,1) for forward directionand K(884,2) for reverse direction
885	Q00535_p |Q13153 -->Q00535_p +Q13153_p : K(885,1) for forward direction
886	P41240_p +Q96J02 <-->P41240_p |Q96J02 : K(886,1) for forward directionand K(886,2) for reverse direction
887	P41240_p |Q96J02 -->P41240_p +Q96J02_p : K(887,1) for forward direction
888	P48729 +P27348 <-->P48729 |P27348 : K(888,1) for forward directionand K(888,2) for reverse direction
889	P48729 |P27348 -->P48729 +P27348_p : K(889,1) for forward direction
890	P48729 +P63104 <-->P48729 |P63104 : K(890,1) for forward directionand K(890,2) for reverse direction
891	P48729 |P63104 -->P48729 +P63104_p : K(891,1) for forward direction
892	P49674 +P49674 <-->P49674 |P49674 : K(892,1) for forward directionand K(892,2) for reverse direction
893	P49674 |P49674 -->P49674 +P49674_p : K(893,1) for forward direction
894	Q9HCP0 +Q12778 <-->Q9HCP0 |Q12778 : K(894,1) for forward directionand K(894,2) for reverse direction
895	Q9HCP0 |Q12778 -->Q9HCP0 +Q12778_p : K(895,1) for forward direction
896	P68400_p +Q9Y5B0 <-->P68400_p |Q9Y5B0 : K(896,1) for forward directionand K(896,2) for reverse direction

897	P68400_p |Q9Y5B0 -->P68400_p +Q9Y5B0_p : K(897,1) for forward direction
898	P68400_p +P12259 <-->P68400_p |P12259 : K(898,1) for forward directionand K(898,2) for reverse direction
899	P68400_p |P12259 -->P68400_p +P12259_p : K(899,1) for forward direction
900	P68400_p +Q02790 <-->P68400_p |Q02790 : K(900,1) for forward directionand K(900,2) for reverse direction
901	P68400_p |Q02790 -->P68400_p +Q02790_p : K(901,1) for forward direction
902	P68400_p +P06744 <-->P68400_p |P06744 : K(902,1) for forward directionand K(902,2) for reverse direction
903	P68400_p |P06744 -->P68400_p +P06744_p : K(903,1) for forward direction
904	P68400_p +P07910 <-->P68400_p |P07910 : K(904,1) for forward directionand K(904,2) for reverse direction
905	P68400_p |P07910 -->P68400_p +P07910_p : K(905,1) for forward direction
906	P68400_p +Q92598 <-->P68400_p |Q92598 : K(906,1) for forward directionand K(906,2) for reverse direction
907	P68400_p |Q92598 -->P68400_p +Q92598_p : K(907,1) for forward direction
908	P68400_p +P60484 <-->P68400_p |P60484 : K(908,1) for forward directionand K(908,2) for reverse direction
909	P68400_p |P60484 -->P68400_p +P60484_p : K(909,1) for forward direction
910	P68400_p +P08047 <-->P68400_p |P08047 : K(910,1) for forward directionand K(910,2) for reverse direction
911	P68400_p |P08047 -->P68400_p +P08047_p : K(911,1) for forward direction
912	P68400_p +Q08945 <-->P68400_p |Q08945 : K(912,1) for forward directionand K(912,2) for reverse direction
913	P68400_p |Q08945 -->P68400_p +Q08945_p : K(913,1) for forward direction
914	P68400_p +P04637 <-->P68400_p |P04637 : K(914,1) for forward directionand K(914,2) for reverse direction
915	P68400_p |P04637 -->P68400_p +P04637_p : K(915,1) for forward direction
916	P19784 +Q9Y5B0 <-->P19784 |Q9Y5B0 : K(916,1) for forward directionand K(916,2) for reverse direction
917	P19784 |Q9Y5B0 -->P19784 +Q9Y5B0_p : K(917,1) for forward direction
918	P19784 +Q13541 <-->P19784 |Q13541 : K(918,1) for forward directionand K(918,2) for reverse direction
919	P19784 |Q13541 -->P19784 +Q13541_p : K(919,1) for forward direction
920	P19784 +Q92598 <-->P19784 |Q92598 : K(920,1) for forward directionand K(920,2) for reverse direction
921	P19784 |Q92598 -->P19784 +Q92598_p : K(921,1) for forward direction
922	P19784 +P60484 <-->P19784 |P60484 : K(922,1) for forward directionand K(922,2) for reverse direction
923	P19784 |P60484 -->P19784 +P60484_p : K(923,1) for forward direction
924	P67870_p +Q9Y5B0 <-->P67870_p |Q9Y5B0 : K(924,1) for forward directionand K(924,2) for reverse direction
925	P67870_p |Q9Y5B0 -->P67870_p +Q9Y5B0_p : K(925,1) for forward direction
926	P53355_p +P53355 <-->P53355_p |P53355 : K(926,1) for forward directionand K(926,2) for reverse direction
927	P53355_p |P53355 -->P53355_p +P53355_p : K(927,1) for forward direction
928	P00533_p +P46108 <-->P00533_p |P46108 : K(928,1) for forward directionand K(928,2) for reverse direction
929	P00533_p |P46108 -->P00533_p +P46108_p : K(929,1) for forward direction
930	P25098_p +O76070 <-->P25098_p |O76070 : K(930,1) for forward directionand K(930,2) for reverse direction
931	P25098_p |O76070 -->P25098_p +O76070_p : K(931,1) for forward direction
932	P49840_p +P31749 <-->P49840_p |P31749 : K(932,1) for forward directionand K(932,2) for reverse direction
933	P49840_p |P31749 -->P49840_p +P31749_p : K(933,1) for forward direction
934	P49840_p +Q13144 <-->P49840_p |Q13144 : K(934,1) for forward directionand K(934,2) for reverse direction
935	P49840_p |Q13144 -->P49840_p +Q13144_p : K(935,1) for forward direction
936	P49841_p +P35222 <-->P49841_p |P35222 : K(936,1) for forward directionand K(936,2) for reverse direction
937	P49841_p |P35222 -->P49841_p +P35222_p : K(937,1) for forward direction
938	P49841_p +Q16555 <-->P49841_p |Q16555 : K(938,1) for forward directionand K(938,2) for reverse direction
939	P49841_p |Q16555 -->P49841_p +Q16555_p : K(939,1) for forward direction

940	P49841_p +Q13541 <-->P49841_p |Q13541 : K(940,1) for forward directionand K(940,2) for reverse direction
941	P49841_p |Q13541 -->P49841_p +Q13541_p : K(941,1) for forward direction
942	P49841_p +P13807 <-->P49841_p |P13807 : K(942,1) for forward directionand K(942,2) for reverse direction
943	P49841_p |P13807 -->P49841_p +P13807_p : K(943,1) for forward direction
944	P49841_p +P05412 <-->P49841_p |P05412 : K(944,1) for forward directionand K(944,2) for reverse direction
945	P49841_p |P05412 -->P49841_p +P05412_p : K(945,1) for forward direction
946	P49841_p +P60484 <-->P49841_p |P60484 : K(946,1) for forward directionand K(946,2) for reverse direction
947	P49841_p |P60484 -->P49841_p +P60484_p : K(947,1) for forward direction
948	P08069_p +P46108 <-->P08069_p |P46108 : K(948,1) for forward directionand K(948,2) for reverse direction
949	P08069_p |P46108 -->P08069_p +P46108_p : K(949,1) for forward direction
950	Q02750_p +P55211 <-->Q02750_p |P55211 : K(950,1) for forward directionand K(950,2) for reverse direction
951	Q02750_p |P55211 -->Q02750_p +P55211_p : K(951,1) for forward direction
952	P36507_p +P55211 <-->P36507_p |P55211 : K(952,1) for forward directionand K(952,2) for reverse direction
953	P36507_p |P55211 -->P36507_p +P55211_p : K(953,1) for forward direction
954	O14733_p +P35568 <-->O14733_p |P35568 : K(954,1) for forward directionand K(954,2) for reverse direction
955	O14733_p |P35568 -->O14733_p +P35568_p : K(955,1) for forward direction
956	P28482_p +Q92934 <-->P28482_p |Q92934 : K(956,1) for forward directionand K(956,2) for reverse direction
957	P28482_p |Q92934 -->P28482_p +Q92934_p : K(957,1) for forward direction
958	P28482_p +P15056 <-->P28482_p |P15056 : K(958,1) for forward directionand K(958,2) for reverse direction
959	P28482_p |P15056 -->P28482_p +P15056_p : K(959,1) for forward direction
960	P28482_p +P55211 <-->P28482_p |P55211 : K(960,1) for forward directionand K(960,2) for reverse direction
961	P28482_p |P55211 -->P28482_p +P55211_p : K(961,1) for forward direction
962	P28482_p +P00533 <-->P28482_p |P00533 : K(962,1) for forward directionand K(962,2) for reverse direction
963	P28482_p |P00533 -->P28482_p +P00533_p : K(963,1) for forward direction
964	P28482_p +Q02750 <-->P28482_p |Q02750 : K(964,1) for forward directionand K(964,2) for reverse direction
965	P28482_p |Q02750 -->P28482_p +Q02750_p : K(965,1) for forward direction
966	P28482_p +Q13153 <-->P28482_p |Q13153 : K(966,1) for forward directionand K(966,2) for reverse direction
967	P28482_p |Q13153 -->P28482_p +Q13153_p : K(967,1) for forward direction
968	P28482_p +Q07889 <-->P28482_p |Q07889 : K(968,1) for forward directionand K(968,2) for reverse direction
969	P28482_p |Q07889 -->P28482_p +Q07889_p : K(969,1) for forward direction
970	Q16539_p +O00418 <-->Q16539_p |O00418 : K(970,1) for forward directionand K(970,2) for reverse direction
971	Q16539_p |O00418 -->Q16539_p +O00418_p : K(971,1) for forward direction
972	Q16539_p +Q13541 <-->Q16539_p |Q13541 : K(972,1) for forward directionand K(972,2) for reverse direction
973	Q16539_p |Q13541 -->Q16539_p +Q13541_p : K(973,1) for forward direction
974	Q16539_p +Q92945 <-->Q16539_p |Q92945 : K(974,1) for forward directionand K(974,2) for reverse direction
975	Q16539_p |Q92945 -->Q16539_p +Q92945_p : K(975,1) for forward direction
976	Q16539_p +Q15750 <-->Q16539_p |Q15750 : K(976,1) for forward directionand K(976,2) for reverse direction
977	Q16539_p |Q15750 -->Q16539_p +Q15750_p : K(977,1) for forward direction
978	P27361_p +P55211 <-->P27361_p |P55211 : K(978,1) for forward directionand K(978,2) for reverse direction
979	P27361_p |P55211 -->P27361_p +P55211_p : K(979,1) for forward direction
980	P27361_p +P35568 <-->P27361_p |P35568 : K(980,1) for forward directionand K(980,2) for reverse direction
981	P27361_p |P35568 -->P27361_p +P35568_p : K(981,1) for forward direction
982	P27361_p +P16949 <-->P27361_p |P16949 : K(982,1) for forward directionand K(982,2) for reverse direction

983	P27361_p |P16949 -->P27361_p +P16949_p : K(983,1) for forward direction
984	P27361_p +Q93045 <-->P27361_p |Q93045 : K(984,1) for forward directionand K(984,2) for reverse direction
985	P27361_p |Q93045 -->P27361_p +Q93045_p : K(985,1) for forward direction
986	P27361_p +P49815 <-->P27361_p |P49815 : K(986,1) for forward directionand K(986,2) for reverse direction
987	P27361_p |P49815 -->P27361_p +P49815_p : K(987,1) for forward direction
988	P45983_p +Q00613 <-->P45983_p |Q00613 : K(988,1) for forward directionand K(988,2) for reverse direction
989	P45983_p |Q00613 -->P45983_p +Q00613_p : K(989,1) for forward direction
990	P49137_p +P63104 <-->P49137_p |P63104 : K(990,1) for forward directionand K(990,2) for reverse direction
991	P49137_p |P63104 -->P49137_p +P63104_p : K(991,1) for forward direction
992	P42345_p +Q96B36 <-->P42345_p |Q96B36 : K(992,1) for forward directionand K(992,2) for reverse direction
993	P42345_p |Q96B36 -->P42345_p +Q96B36_p : K(993,1) for forward direction
994	P42345_p +Q13541 <-->P42345_p |Q13541 : K(994,1) for forward directionand K(994,2) for reverse direction
995	P42345_p |Q13541 -->P42345_p +Q13541_p : K(995,1) for forward direction
996	P42345_p +P35568 <-->P42345_p |P35568 : K(996,1) for forward directionand K(996,2) for reverse direction
997	P42345_p |P35568 -->P42345_p +P35568_p : K(997,1) for forward direction
998	O95747_p +Q13153 <-->O95747_p |Q13153 : K(998,1) for forward directionand K(998,2) for reverse direction
999	O95747_p |Q13153 -->O95747_p +Q13153_p : K(999,1) for forward direction
1000	Q13153_p +Q13363 <-->Q13153_p |Q13363 : K(1000,1) for forward directionand K(1000,2) for reverse direction
1001	Q13153_p |Q13363 -->Q13153_p +Q13363_p : K(1001,1) for forward direction
1002	Q13153_p +Q15746 <-->Q13153_p |Q15746 : K(1002,1) for forward directionand K(1002,2) for reverse direction
1003	Q13153_p |Q15746 -->Q13153_p +Q15746_p : K(1003,1) for forward direction
1004	Q13177_p +Q15746 <-->Q13177_p |Q15746 : K(1004,1) for forward directionand K(1004,2) for reverse direction
1005	Q13177_p |Q15746 -->Q13177_p +Q15746_p : K(1005,1) for forward direction
1006	Q13177_p +P08670 <-->Q13177_p |P08670 : K(1006,1) for forward directionand K(1006,2) for reverse direction
1007	Q13177_p |P08670 -->Q13177_p +P08670_p : K(1007,1) for forward direction
1008	O15530_p +O43865 <-->O15530_p |O43865 : K(1008,1) for forward directionand K(1008,2) for reverse direction
1009	O15530_p |O43865 -->O15530_p +O43865_p : K(1009,1) for forward direction
1010	O15530_p +O15530 <-->O15530_p |O15530 : K(1010,1) for forward directionand K(1010,2) for reverse direction
1011	O15530_p |O15530 -->O15530_p +O15530_p : K(1011,1) for forward direction
1012	Q16512_p +P29966 <-->Q16512_p |P29966 : K(1012,1) for forward directionand K(1012,2) for reverse direction
1013	Q16512_p |P29966 -->Q16512_p +P29966_p : K(1013,1) for forward direction
1014	Q13131_p +Q13085 <-->Q13131_p |Q13085 : K(1014,1) for forward directionand K(1014,2) for reverse direction
1015	Q13131_p |Q13085 -->Q13131_p +Q13085_p : K(1015,1) for forward direction
1016	Q13131_p +O00418 <-->Q13131_p |O00418 : K(1016,1) for forward directionand K(1016,2) for reverse direction
1017	Q13131_p |O00418 -->Q13131_p +O00418_p : K(1017,1) for forward direction
1018	Q13131_p +Q13131 <-->Q13131_p |Q13131 : K(1018,1) for forward directionand K(1018,2) for reverse direction
1019	Q13131_p |Q13131 -->Q13131_p +Q13131_p : K(1019,1) for forward direction
1020	Q13131_p +Q8N122 <-->Q13131_p |Q8N122 : K(1020,1) for forward directionand K(1020,2) for reverse direction
1021	Q13131_p |Q8N122 -->Q13131_p +Q8N122_p : K(1021,1) for forward direction
1022	P17612 +P35611 <-->P17612 |P35611 : K(1022,1) for forward directionand K(1022,2) for reverse direction
1023	P17612 |P35611 -->P17612 +P35611_p : K(1023,1) for forward direction
1024	P17612 +P15056 <-->P17612 |P15056 : K(1024,1) for forward directionand K(1024,2) for reverse direction
1025	P17612 |P15056 -->P17612 +P15056_p : K(1025,1) for forward direction

1026	P17612 +P15924 <-->P17612 |P15924 : K(1026,1) for forward directionand K(1026,2) for reverse direction
1027	P17612 |P15924 -->P17612 +P15924_p : K(1027,1) for forward direction
1028	P17612 +Q14344 <-->P17612 |Q14344 : K(1028,1) for forward directionand K(1028,2) for reverse direction
1029	P17612 |Q14344 -->P17612 +Q14344_p : K(1029,1) for forward direction
1030	P17612 +P49841 <-->P17612 |P49841 : K(1030,1) for forward directionand K(1030,2) for reverse direction
1031	P17612 |P49841 -->P17612 +P49841_p : K(1031,1) for forward direction
1032	P17612 +P13807 <-->P17612 |P13807 : K(1032,1) for forward directionand K(1032,2) for reverse direction
1033	P17612 |P13807 -->P17612 +P13807_p : K(1033,1) for forward direction
1034	P17612 +P04035 <-->P17612 |P04035 : K(1034,1) for forward directionand K(1034,2) for reverse direction
1035	P17612 |P04035 -->P17612 +P04035_p : K(1035,1) for forward direction
1036	P17612 +P05114 <-->P17612 |P05114 : K(1036,1) for forward directionand K(1036,2) for reverse direction
1037	P17612 |P05114 -->P17612 +P05114_p : K(1037,1) for forward direction
1038	P17612 +Q12972 <-->P17612 |Q12972 : K(1038,1) for forward directionand K(1038,2) for reverse direction
1039	P17612 |Q12972 -->P17612 +Q12972_p : K(1039,1) for forward direction
1040	P17612 +Q96SB3 <-->P17612 |Q96SB3 : K(1040,1) for forward directionand K(1040,2) for reverse direction
1041	P17612 |Q96SB3 -->P17612 +Q96SB3_p : K(1041,1) for forward direction
1042	P17612 +Q13131 <-->P17612 |Q13131 : K(1042,1) for forward directionand K(1042,2) for reverse direction
1043	P17612 |Q13131 -->P17612 +Q13131_p : K(1043,1) for forward direction
1044	P17612 +P62834 <-->P17612 |P62834 : K(1044,1) for forward directionand K(1044,2) for reverse direction
1045	P17612 |P62834 -->P17612 +P62834_p : K(1045,1) for forward direction
1046	P17612 +O43665 <-->P17612 |O43665 : K(1046,1) for forward directionand K(1046,2) for reverse direction
1047	P17612 |O43665 -->P17612 +O43665_p : K(1047,1) for forward direction
1048	P17612 +P61586 <-->P17612 |P61586 : K(1048,1) for forward directionand K(1048,2) for reverse direction
1049	P17612 |P61586 -->P17612 +P61586_p : K(1049,1) for forward direction
1050	P17612 +Q01082 <-->P17612 |Q01082 : K(1050,1) for forward directionand K(1050,2) for reverse direction
1051	P17612 |Q01082 -->P17612 +Q01082_p : K(1051,1) for forward direction
1052	P17612 +Q93045 <-->P17612 |Q93045 : K(1052,1) for forward directionand K(1052,2) for reverse direction
1053	P17612 |Q93045 -->P17612 +Q93045_p : K(1053,1) for forward direction
1054	P17612 +Q05086 <-->P17612 |Q05086 : K(1054,1) for forward directionand K(1054,2) for reverse direction
1055	P17612 |Q05086 -->P17612 +Q05086_p : K(1055,1) for forward direction
1056	P17612 +P08670 <-->P17612 |P08670 : K(1056,1) for forward directionand K(1056,2) for reverse direction
1057	P17612 |P08670 -->P17612 +P08670_p : K(1057,1) for forward direction
1058	P17252_p +Q99418 <-->P17252_p |Q99418 : K(1058,1) for forward directionand K(1058,2) for reverse direction
1059	P17252_p |Q99418 -->P17252_p +Q99418_p : K(1059,1) for forward direction
1060	P17252_p +P17844 <-->P17252_p |P17844 : K(1060,1) for forward directionand K(1060,2) for reverse direction
1061	P17252_p |P17844 -->P17252_p +P17844_p : K(1061,1) for forward direction
1062	P17252_p +O60869 <-->P17252_p |O60869 : K(1062,1) for forward directionand K(1062,2) for reverse direction
1063	P17252_p |O60869 -->P17252_p +O60869_p : K(1063,1) for forward direction
1064	P17252_p +Q06210 <-->P17252_p |Q06210 : K(1064,1) for forward directionand K(1064,2) for reverse direction
1065	P17252_p |Q06210 -->P17252_p +Q06210_p : K(1065,1) for forward direction
1066	P17252_p +P29966 <-->P17252_p |P29966 : K(1066,1) for forward directionand K(1066,2) for reverse direction
1067	P17252_p |P29966 -->P17252_p +P29966_p : K(1067,1) for forward direction
1068	P17252_p +P29353 <-->P17252_p |P29353 : K(1068,1) for forward directionand K(1068,2) for reverse direction

1069	P17252_p |P29353 -->P17252_p +P29353_p : K(1069,1) for forward direction
1070	Q05655_p +P00533 <-->Q05655_p |P00533 : K(1070,1) for forward directionand K(1070,2) for reverse direction
1071	Q05655_p |P00533 -->Q05655_p +P00533_p : K(1071,1) for forward direction
1072	Q05655_p +P63104 <-->Q05655_p |P63104 : K(1072,1) for forward directionand K(1072,2) for reverse direction
1073	Q05655_p |P63104 -->Q05655_p +P63104_p : K(1073,1) for forward direction
1074	P78527_p +P15927 <-->P78527_p |P15927 : K(1074,1) for forward directionand K(1074,2) for reverse direction
1075	P78527_p |P15927 -->P78527_p +P15927_p : K(1075,1) for forward direction
1076	Q13976_p +Q01970 <-->Q13976_p |Q01970 : K(1076,1) for forward directionand K(1076,2) for reverse direction
1077	Q13976_p |Q01970 -->Q13976_p +Q01970_p : K(1077,1) for forward direction
1078	Q13976_p +Q15637 <-->Q13976_p |Q15637 : K(1078,1) for forward directionand K(1078,2) for reverse direction
1079	Q13976_p |Q15637 -->Q13976_p +Q15637_p : K(1079,1) for forward direction
1080	Q13464 +P08670 <-->Q13464 |P08670 : K(1080,1) for forward directionand K(1080,2) for reverse direction
1081	Q13464 |P08670 -->Q13464 +P08670_p : K(1081,1) for forward direction
1082	O75116 +O14974 <-->O75116 |O14974 : K(1082,1) for forward directionand K(1082,2) for reverse direction
1083	O75116 |O14974 -->O75116 +O14974_p : K(1083,1) for forward direction
1084	Q15418_p +Q92934 <-->Q15418_p |Q92934 : K(1084,1) for forward directionand K(1084,2) for reverse direction
1085	Q15418_p |Q92934 -->Q15418_p +Q92934_p : K(1085,1) for forward direction
1086	Q15418_p +O00418 <-->Q15418_p |O00418 : K(1086,1) for forward directionand K(1086,2) for reverse direction
1087	Q15418_p |O00418 -->Q15418_p +O00418_p : K(1087,1) for forward direction
1088	P51812_p +P49840 <-->P51812_p |P49840 : K(1088,1) for forward directionand K(1088,2) for reverse direction
1089	P51812_p |P49840 -->P51812_p +P49840_p : K(1089,1) for forward direction
1090	P51812_p +P05114 <-->P51812_p |P05114 : K(1090,1) for forward directionand K(1090,2) for reverse direction
1091	P51812_p |P05114 -->P51812_p +P05114_p : K(1091,1) for forward direction
1092	P51812_p +P05204 <-->P51812_p |P05204 : K(1092,1) for forward directionand K(1092,2) for reverse direction
1093	P51812_p |P05204 -->P51812_p +P05204_p : K(1093,1) for forward direction
1094	Q9HBY8_p +O43524 <-->Q9HBY8_p |O43524 : K(1094,1) for forward directionand K(1094,2) for reverse direction
1095	Q9HBY8_p |O43524 -->Q9HBY8_p +O43524_p : K(1095,1) for forward direction
1096	Q9Y2K2 +Q53ET0 <-->Q9Y2K2 |Q53ET0 : K(1096,1) for forward directionand K(1096,2) for reverse direction
1097	Q9Y2K2 |Q53ET0 -->Q9Y2K2 +Q53ET0_p : K(1097,1) for forward direction
1098	P12931_p +P56945 <-->P12931_p |P56945 : K(1098,1) for forward directionand K(1098,2) for reverse direction
1099	P12931_p |P56945 -->P12931_p +P56945_p : K(1099,1) for forward direction
1100	P12931_p +P35222 <-->P12931_p |P35222 : K(1100,1) for forward directionand K(1100,2) for reverse direction
1101	P12931_p |P35222 -->P12931_p +P35222_p : K(1101,1) for forward direction
1102	P12931_p +P01112 <-->P12931_p |P01112 : K(1102,1) for forward directionand K(1102,2) for reverse direction
1103	P12931_p |P01112 -->P12931_p +P01112_p : K(1103,1) for forward direction
1104	P12931_p +P10301 <-->P12931_p |P10301 : K(1104,1) for forward directionand K(1104,2) for reverse direction
1105	P12931_p |P10301 -->P12931_p +P10301_p : K(1105,1) for forward direction
1106	P12931_p +P12931 <-->P12931_p |P12931 : K(1106,1) for forward directionand K(1106,2) for reverse direction
1107	P12931_p |P12931 -->P12931_p +P12931_p : K(1107,1) for forward direction
1108	P12931_p +P18206 <-->P12931_p |P18206 : K(1108,1) for forward directionand K(1108,2) for reverse direction
1109	P12931_p |P18206 -->P12931_p +P18206_p : K(1109,1) for forward direction
1110	P37173_p +P37173 <-->P37173_p |P37173 : K(1110,1) for forward directionand K(1110,2) for reverse direction
1111	P37173_p |P37173 -->P37173_p +P37173_p : K(1111,1) for forward direction

<table>
<tr><td>1112</td><td>P17252_p +P22681 <-->P17252_p |P22681 : K(1112,1) for forward directionand K(1112,2) for reverse direction</td></tr>
<tr><td>1113</td><td>P17252_p |P22681 -->P17252_p +P22681_p : K(1113,1) for forward direction</td></tr>
<tr><td>1114</td><td>P12931_p +P29317 <-->P12931_p |P29317 : K(1114,1) for forward directionand K(1114,2) for reverse direction</td></tr>
<tr><td>1115</td><td>P12931_p |P29317 -->P12931_p +P29317_p : K(1115,1) for forward direction</td></tr>
<tr><td>1116</td><td>O15111_p +P19838 <-->O15111_p |P19838 : K(1116,1) for forward directionand K(1116,2) for reverse direction</td></tr>
<tr><td>1117</td><td>O15111_p |P19838 -->O15111_p +P19838_p : K(1117,1) for forward direction</td></tr>
<tr><td>1118</td><td>P00533_p +P00533 <-->P00533_p |P00533 : K(1118,1) for forward directionand K(1118,2) for reverse direction</td></tr>
<tr><td>1119</td><td>P00533_p |P00533 -->P00533_p +P00533_p : K(1119,1) for forward direction</td></tr>
<tr><td>1120</td><td>P49840_p +Q07820 <-->P49840_p |Q07820 : K(1120,1) for forward directionand K(1120,2) for reverse direction</td></tr>
<tr><td>1121</td><td>P49840_p |Q07820 -->P49840_p +Q07820_p : K(1121,1) for forward direction</td></tr>
<tr><td>1122</td><td>P49841_p +P01106 <-->P49841_p |P01106 : K(1122,1) for forward directionand K(1122,2) for reverse direction</td></tr>
<tr><td>1123</td><td>P49841_p |P01106 -->P49841_p +P01106_p : K(1123,1) for forward direction</td></tr>
<tr><td>1124</td><td>P49841_p +P84022 <-->P49841_p |P84022 : K(1124,1) for forward directionand K(1124,2) for reverse direction</td></tr>
<tr><td>1125</td><td>P49841_p |P84022 -->P49841_p +P84022_p : K(1125,1) for forward direction</td></tr>
<tr><td>1126</td><td>P28482_p +O43524 <-->P28482_p |O43524 : K(1126,1) for forward directionand K(1126,2) for reverse direction</td></tr>
<tr><td>1127</td><td>P28482_p |O43524 -->P28482_p +O43524_p : K(1127,1) for forward direction</td></tr>
<tr><td>1128</td><td>Q16539_p +Q15910 <-->Q16539_p |Q15910 : K(1128,1) for forward directionand K(1128,2) for reverse direction</td></tr>
<tr><td>1129</td><td>Q16539_p |Q15910 -->Q16539_p +Q15910_p : K(1129,1) for forward direction</td></tr>
<tr><td>1130</td><td>P27361_p +O43524 <-->P27361_p |O43524 : K(1130,1) for forward directionand K(1130,2) for reverse direction</td></tr>
<tr><td>1131</td><td>P27361_p |O43524 -->P27361_p +O43524_p : K(1131,1) for forward direction</td></tr>
<tr><td>1132</td><td>P17612 +P35568 <-->P17612 |P35568 : K(1132,1) for forward directionand K(1132,2) for reverse direction</td></tr>
<tr><td>1133</td><td>P17612 |P35568 -->P17612 +P35568_p : K(1133,1) for forward direction</td></tr>
<tr><td>1134</td><td>P17252_p +Q9UEY8 <-->P17252_p |Q9UEY8 : K(1134,1) for forward directionand K(1134,2) for reverse direction</td></tr>
<tr><td>1135</td><td>P17252_p |Q9UEY8 -->P17252_p +Q9UEY8_p : K(1135,1) for forward direction</td></tr>
<tr><td>1136</td><td>P17252_p +P08670 <-->P17252_p |P08670 : K(1136,1) for forward directionand K(1136,2) for reverse direction</td></tr>
<tr><td>1137</td><td>P17252_p |P08670 -->P17252_p +P08670_p : K(1137,1) for forward direction</td></tr>
<tr><td>1138</td><td>Q15418_p +P35568 <-->Q15418_p |P35568 : K(1138,1) for forward directionand K(1138,2) for reverse direction</td></tr>
<tr><td>1139</td><td>Q15418_p |P35568 -->Q15418_p +P35568_p : K(1139,1) for forward direction</td></tr>
<tr><td>1140</td><td>P17612 +P01100 <-->P17612 |P01100 : K(1140,1) for forward directionand K(1140,2) for reverse direction</td></tr>
<tr><td>1141</td><td>P17612 |P01100 -->P17612 +P01100_p : K(1141,1) for forward direction</td></tr>
<tr><td>1142</td><td>Q2M2I8 +P49757 <-->Q2M2I8 |P49757 : K(1142,1) for forward directionand K(1142,2) for reverse direction</td></tr>
<tr><td>1143</td><td>Q2M2I8 |P49757 -->Q2M2I8 +P49757_p : K(1143,1) for forward direction</td></tr>
<tr><td>1144</td><td>P31749_p +Q15027 <-->P31749_p |Q15027 : K(1144,1) for forward directionand K(1144,2) for reverse direction</td></tr>
<tr><td>1145</td><td>P31749_p |Q15027 -->P31749_p +Q15027_p : K(1145,1) for forward direction</td></tr>
<tr><td>1146</td><td>P31749_p +P53396 <-->P31749_p |P53396 : K(1146,1) for forward directionand K(1146,2) for reverse direction</td></tr>
<tr><td>1147</td><td>P31749_p |P53396 -->P31749_p +P53396_p : K(1147,1) for forward direction</td></tr>
<tr><td>1148</td><td>P31749_p +P53365 <-->P31749_p |P53365 : K(1148,1) for forward directionand K(1148,2) for reverse direction</td></tr>
<tr><td>1149</td><td>P31749_p |P53365 -->P31749_p +P53365_p : K(1149,1) for forward direction</td></tr>
<tr><td>1150</td><td>P31749_p +Q9Y2V2 <-->P31749_p |Q9Y2V2 : K(1150,1) for forward directionand K(1150,2) for reverse direction</td></tr>
<tr><td>1151</td><td>P31749_p |Q9Y2V2 -->P31749_p +Q9Y2V2_p : K(1151,1) for forward direction</td></tr>
<tr><td>1152</td><td>P31749_p +P78371 <-->P31749_p |P78371 : K(1152,1) for forward directionand K(1152,2) for reverse direction</td></tr>
<tr><td>1153</td><td>P31749_p |P78371 -->P31749_p +P78371_p : K(1153,1) for forward direction</td></tr>
<tr><td>1154</td><td>P31749_p +Q9BZQ8 <-->P31749_p |Q9BZQ8 : K(1154,1) for forward directionand K(1154,2) for reverse direction</td></tr>
</table>

<table>
<tr><td>1155</td><td>P31749_p |Q9BZQ8 -->P31749_p +Q9BZQ8_p : K(1155,1) for forward direction</td></tr>
<tr><td>1156</td><td>P31749_p +P42858 <-->P31749_p |P42858 : K(1156,1) for forward directionand K(1156,2) for reverse direction</td></tr>
<tr><td>1157</td><td>P31749_p |P42858 -->P31749_p +P42858_p : K(1157,1) for forward direction</td></tr>
<tr><td>1158</td><td>P31749_p +O60825 <-->P31749_p |O60825 : K(1158,1) for forward directionand K(1158,2) for reverse direction</td></tr>
<tr><td>1159</td><td>P31749_p |O60825 -->P31749_p +O60825_p : K(1159,1) for forward direction</td></tr>
<tr><td>1160</td><td>P31749_p +Q9H6Z4 <-->P31749_p |Q9H6Z4 : K(1160,1) for forward directionand K(1160,2) for reverse direction</td></tr>
<tr><td>1161</td><td>P31749_p |Q9H6Z4 -->P31749_p +Q9H6Z4_p : K(1161,1) for forward direction</td></tr>
<tr><td>1162</td><td>P31749_p +P63104 <-->P31749_p |P63104 : K(1162,1) for forward directionand K(1162,2) for reverse direction</td></tr>
<tr><td>1163</td><td>P31749_p |P63104 -->P31749_p +P63104_p : K(1163,1) for forward direction</td></tr>
<tr><td>1164</td><td>P31751_p +P53396 <-->P31751_p |P53396 : K(1164,1) for forward directionand K(1164,2) for reverse direction</td></tr>
<tr><td>1165</td><td>P31751_p |P53396 -->P31751_p +P53396_p : K(1165,1) for forward direction</td></tr>
<tr><td>1166</td><td>Q96GD4_p +Q9BQG0 <-->Q96GD4_p |Q9BQG0 : K(1166,1) for forward directionand K(1166,2) for reverse direction</td></tr>
<tr><td>1167</td><td>Q96GD4_p |Q9BQG0 -->Q96GD4_p +Q9BQG0_p : K(1167,1) for forward direction</td></tr>
<tr><td>1168</td><td>Q96GD4_p +Q8IX03 <-->Q96GD4_p |Q8IX03 : K(1168,1) for forward directionand K(1168,2) for reverse direction</td></tr>
<tr><td>1169</td><td>Q96GD4_p |Q8IX03 -->Q96GD4_p +Q8IX03_p : K(1169,1) for forward direction</td></tr>
<tr><td>1170</td><td>P11274_p +P27348 <-->P11274_p |P27348 : K(1170,1) for forward directionand K(1170,2) for reverse direction</td></tr>
<tr><td>1171</td><td>P11274_p |P27348 -->P11274_p +P27348_p : K(1171,1) for forward direction</td></tr>
<tr><td>1172</td><td>P11274_p +P63104 <-->P11274_p |P63104 : K(1172,1) for forward directionand K(1172,2) for reverse direction</td></tr>
<tr><td>1173</td><td>P11274_p |P63104 -->P11274_p +P63104_p : K(1173,1) for forward direction</td></tr>
<tr><td>1174</td><td>Q9UQM7_p +P05783 <-->Q9UQM7_p |P05783 : K(1174,1) for forward directionand K(1174,2) for reverse direction</td></tr>
<tr><td>1175</td><td>Q9UQM7_p |P05783 -->Q9UQM7_p +P05783_p : K(1175,1) for forward direction</td></tr>
<tr><td>1176</td><td>Q13554_p +Q01970 <-->Q13554_p |Q01970 : K(1176,1) for forward directionand K(1176,2) for reverse direction</td></tr>
<tr><td>1177</td><td>Q13554_p |Q01970 -->Q13554_p +Q01970_p : K(1177,1) for forward direction</td></tr>
<tr><td>1178</td><td>Q13557_p +P55327 <-->Q13557_p |P55327 : K(1178,1) for forward directionand K(1178,2) for reverse direction</td></tr>
<tr><td>1179</td><td>Q13557_p |P55327 -->Q13557_p +P55327_p : K(1179,1) for forward direction</td></tr>
<tr><td>1180</td><td>Q13557_p +P08670 <-->Q13557_p |P08670 : K(1180,1) for forward directionand K(1180,2) for reverse direction</td></tr>
<tr><td>1181</td><td>Q13557_p |P08670 -->Q13557_p +P08670_p : K(1181,1) for forward direction</td></tr>
<tr><td>1182</td><td>Q13555 +P21333 <-->Q13555 |P21333 : K(1182,1) for forward directionand K(1182,2) for reverse direction</td></tr>
<tr><td>1183</td><td>Q13555 |P21333 -->Q13555 +P21333_p : K(1183,1) for forward direction</td></tr>
<tr><td>1184</td><td>Q13555 +Q15121 <-->Q13555 |Q15121 : K(1184,1) for forward directionand K(1184,2) for reverse direction</td></tr>
<tr><td>1185</td><td>Q13555 |Q15121 -->Q13555 +Q15121_p : K(1185,1) for forward direction</td></tr>
<tr><td>1186</td><td>Q13555 +Q01970 <-->Q13555 |Q01970 : K(1186,1) for forward directionand K(1186,2) for reverse direction</td></tr>
<tr><td>1187</td><td>Q13555 |Q01970 -->Q13555 +Q01970_p : K(1187,1) for forward direction</td></tr>
<tr><td>1188</td><td>Q13555 +P35270 <-->Q13555 |P35270 : K(1188,1) for forward directionand K(1188,2) for reverse direction</td></tr>
<tr><td>1189</td><td>Q13555 |P35270 -->Q13555 +P35270_p : K(1189,1) for forward direction</td></tr>
<tr><td>1190</td><td>P06493_p +Q06481 <-->P06493_p |Q06481 : K(1190,1) for forward directionand K(1190,2) for reverse direction</td></tr>
<tr><td>1191</td><td>P06493_p |Q06481 -->P06493_p +Q06481_p : K(1191,1) for forward direction</td></tr>
<tr><td>1192</td><td>P06493_p +Q12959 <-->P06493_p |Q12959 : K(1192,1) for forward directionand K(1192,2) for reverse direction</td></tr>
<tr><td>1193</td><td>P06493_p |Q12959 -->P06493_p +Q12959_p : K(1193,1) for forward direction</td></tr>
<tr><td>1194</td><td>P06493_p +P29692 <-->P06493_p |P29692 : K(1194,1) for forward directionand K(1194,2) for reverse direction</td></tr>
<tr><td>1195</td><td>P06493_p |P29692 -->P06493_p +P29692_p : K(1195,1) for forward direction</td></tr>
<tr><td>1196</td><td>P06493_p +Q07666 <-->P06493_p |Q07666 : K(1196,1) for forward directionand K(1196,2) for reverse direction</td></tr>
<tr><td>1197</td><td>P06493_p |Q07666 -->P06493_p +Q07666_p : K(1197,1) for forward direction</td></tr>
</table>

<table>
<tr><td>1198</td><td>P06493_p +Q96KB5 <-->P06493_p |Q96KB5 : K(1198,1) for forward directionand K(1198,2) for reverse direction</td></tr>
<tr><td>1199</td><td>P06493_p |Q96KB5 -->P06493_p +Q96KB5_p : K(1199,1) for forward direction</td></tr>
<tr><td>1200</td><td>P06493_p +P18031 <-->P06493_p |P18031 : K(1200,1) for forward directionand K(1200,2) for reverse direction</td></tr>
<tr><td>1201</td><td>P06493_p |P18031 -->P06493_p +P18031_p : K(1201,1) for forward direction</td></tr>
<tr><td>1202</td><td>P06493_p +P17706 <-->P06493_p |P17706 : K(1202,1) for forward directionand K(1202,2) for reverse direction</td></tr>
<tr><td>1203</td><td>P06493_p |P17706 -->P06493_p +P17706_p : K(1203,1) for forward direction</td></tr>
<tr><td>1204</td><td>P06493_p +P61020 <-->P06493_p |P61020 : K(1204,1) for forward directionand K(1204,2) for reverse direction</td></tr>
<tr><td>1205</td><td>P06493_p |P61020 -->P06493_p +P61020_p : K(1205,1) for forward direction</td></tr>
<tr><td>1206</td><td>P06493_p +P47736 <-->P06493_p |P47736 : K(1206,1) for forward directionand K(1206,2) for reverse direction</td></tr>
<tr><td>1207</td><td>P06493_p |P47736 -->P06493_p +P47736_p : K(1207,1) for forward direction</td></tr>
<tr><td>1208</td><td>P06493_p +Q8N122 <-->P06493_p |Q8N122 : K(1208,1) for forward directionand K(1208,2) for reverse direction</td></tr>
<tr><td>1209</td><td>P06493_p |Q8N122 -->P06493_p +Q8N122_p : K(1209,1) for forward direction</td></tr>
<tr><td>1210</td><td>P06493_p +P11388 <-->P06493_p |P11388 : K(1210,1) for forward directionand K(1210,2) for reverse direction</td></tr>
<tr><td>1211</td><td>P06493_p |P11388 -->P06493_p +P11388_p : K(1211,1) for forward direction</td></tr>
<tr><td>1212</td><td>P24941_p +Q8WXE1 <-->P24941_p |Q8WXE1 : K(1212,1) for forward directionand K(1212,2) for reverse direction</td></tr>
<tr><td>1213</td><td>P24941_p |Q8WXE1 -->P24941_p +Q8WXE1_p : K(1213,1) for forward direction</td></tr>
<tr><td>1214</td><td>P24941_p +P50613 <-->P24941_p |P50613 : K(1214,1) for forward directionand K(1214,2) for reverse direction</td></tr>
<tr><td>1215</td><td>P24941_p |P50613 -->P24941_p +P50613_p : K(1215,1) for forward direction</td></tr>
<tr><td>1216</td><td>P24941_p +P17706 <-->P24941_p |P17706 : K(1216,1) for forward directionand K(1216,2) for reverse direction</td></tr>
<tr><td>1217</td><td>P24941_p |P17706 -->P24941_p +P17706_p : K(1217,1) for forward direction</td></tr>
<tr><td>1218</td><td>P24941_p +P30050 <-->P24941_p |P30050 : K(1218,1) for forward directionand K(1218,2) for reverse direction</td></tr>
<tr><td>1219</td><td>P24941_p |P30050 -->P24941_p +P30050_p : K(1219,1) for forward direction</td></tr>
<tr><td>1220</td><td>P24941_p +O75533 <-->P24941_p |O75533 : K(1220,1) for forward directionand K(1220,2) for reverse direction</td></tr>
<tr><td>1221</td><td>P24941_p |O75533 -->P24941_p +O75533_p : K(1221,1) for forward direction</td></tr>
<tr><td>1222</td><td>Q00535_p +P05067 <-->Q00535_p |P05067 : K(1222,1) for forward directionand K(1222,2) for reverse direction</td></tr>
<tr><td>1223</td><td>Q00535_p |P05067 -->Q00535_p +P05067_p : K(1223,1) for forward direction</td></tr>
<tr><td>1224</td><td>Q00535_p +P41236 <-->Q00535_p |P41236 : K(1224,1) for forward directionand K(1224,2) for reverse direction</td></tr>
<tr><td>1225</td><td>Q00535_p |P41236 -->Q00535_p +P41236_p : K(1225,1) for forward direction</td></tr>
<tr><td>1226</td><td>P50613_p +P04637 <-->P50613_p |P04637 : K(1226,1) for forward directionand K(1226,2) for reverse direction</td></tr>
<tr><td>1227</td><td>P50613_p |P04637 -->P50613_p +P04637_p : K(1227,1) for forward direction</td></tr>
<tr><td>1228</td><td>P50750_p +P04637 <-->P50750_p |P04637 : K(1228,1) for forward directionand K(1228,2) for reverse direction</td></tr>
<tr><td>1229</td><td>P50750_p |P04637 -->P50750_p +P04637_p : K(1229,1) for forward direction</td></tr>
<tr><td>1230</td><td>O96017_p +P29590 <-->O96017_p |P29590 : K(1230,1) for forward directionand K(1230,2) for reverse direction</td></tr>
<tr><td>1231</td><td>O96017_p |P29590 -->O96017_p +P29590_p : K(1231,1) for forward direction</td></tr>
<tr><td>1232</td><td>O96017_p +P33981 <-->O96017_p |P33981 : K(1232,1) for forward directionand K(1232,2) for reverse direction</td></tr>
<tr><td>1233</td><td>O96017_p |P33981 -->O96017_p +P33981_p : K(1233,1) for forward direction</td></tr>
<tr><td>1234</td><td>P41240_p +P06396 <-->P41240_p |P06396 : K(1234,1) for forward directionand K(1234,2) for reverse direction</td></tr>
<tr><td>1235</td><td>P41240_p |P06396 -->P41240_p +P06396_p : K(1235,1) for forward direction</td></tr>
<tr><td>1236</td><td>P48729 +O43865 <-->P48729 |O43865 : K(1236,1) for forward directionand K(1236,2) for reverse direction</td></tr>
<tr><td>1237</td><td>P48729 |O43865 -->P48729 +O43865_p : K(1237,1) for forward direction</td></tr>
<tr><td>1238</td><td>P48729 +Q13144 <-->P48729 |Q13144 : K(1238,1) for forward directionand K(1238,2) for reverse direction</td></tr>
<tr><td>1239</td><td>P48729 |Q13144 -->P48729 +Q13144_p : K(1239,1) for forward direction</td></tr>
<tr><td>1240</td><td>P48729 +P17931 <-->P48729 |P17931 : K(1240,1) for forward directionand K(1240,2) for reverse direction</td></tr>
</table>

1241	P48729 |P17931 -->P48729 +P17931_p : K(1241,1) for forward direction
1242	P48729 +P04637 <-->P48729 |P04637 : K(1242,1) for forward directionand K(1242,2) for reverse direction
1243	P48729 |P04637 -->P48729 +P04637_p : K(1243,1) for forward direction
1244	P68400_p +Q8NE71 <-->P68400_p |Q8NE71 : K(1244,1) for forward directionand K(1244,2) for reverse direction
1245	P68400_p |Q8NE71 -->P68400_p +Q8NE71_p : K(1245,1) for forward direction
1246	P68400_p +Q13085 <-->P68400_p |Q13085 : K(1246,1) for forward directionand K(1246,2) for reverse direction
1247	P68400_p |Q13085 -->P68400_p +Q13085_p : K(1247,1) for forward direction
1248	P68400_p +O00170 <-->P68400_p |O00170 : K(1248,1) for forward directionand K(1248,2) for reverse direction
1249	P68400_p |O00170 -->P68400_p +O00170_p : K(1249,1) for forward direction
1250	P68400_p +O14958 <-->P68400_p |O14958 : K(1250,1) for forward directionand K(1250,2) for reverse direction
1251	P68400_p |O14958 -->P68400_p +O14958_p : K(1251,1) for forward direction
1252	P68400_p +P09497 <-->P68400_p |P09497 : K(1252,1) for forward directionand K(1252,2) for reverse direction
1253	P68400_p |P09497 -->P68400_p +P09497_p : K(1253,1) for forward direction
1254	P68400_p +P24534 <-->P68400_p |P24534 : K(1254,1) for forward directionand K(1254,2) for reverse direction
1255	P68400_p |P24534 -->P68400_p +P24534_p : K(1255,1) for forward direction
1256	P68400_p +P29692 <-->P68400_p |P29692 : K(1256,1) for forward directionand K(1256,2) for reverse direction
1257	P68400_p |P29692 -->P68400_p +P29692_p : K(1257,1) for forward direction
1258	P68400_p +P60983 <-->P68400_p |P60983 : K(1258,1) for forward directionand K(1258,2) for reverse direction
1259	P68400_p |P60983 -->P68400_p +P60983_p : K(1259,1) for forward direction
1260	P68400_p +P13807 <-->P68400_p |P13807 : K(1260,1) for forward directionand K(1260,2) for reverse direction
1261	P68400_p |P13807 -->P68400_p +P13807_p : K(1261,1) for forward direction
1262	P68400_p +P17096 <-->P68400_p |P17096 : K(1262,1) for forward directionand K(1262,2) for reverse direction
1263	P68400_p |P17096 -->P68400_p +P17096_p : K(1263,1) for forward direction
1264	P68400_p +P07900 <-->P68400_p |P07900 : K(1264,1) for forward directionand K(1264,2) for reverse direction
1265	P68400_p |P07900 -->P68400_p +P07900_p : K(1265,1) for forward direction
1266	P68400_p +P11717 <-->P68400_p |P11717 : K(1266,1) for forward directionand K(1266,2) for reverse direction
1267	P68400_p |P11717 -->P68400_p +P11717_p : K(1267,1) for forward direction
1268	P68400_p +P35568 <-->P68400_p |P35568 : K(1268,1) for forward directionand K(1268,2) for reverse direction
1269	P68400_p |P35568 -->P68400_p +P35568_p : K(1269,1) for forward direction
1270	P68400_p +O43896 <-->P68400_p |O43896 : K(1270,1) for forward directionand K(1270,2) for reverse direction
1271	P68400_p |O43896 -->P68400_p +O43896_p : K(1271,1) for forward direction
1272	P68400_p +P45984 <-->P68400_p |P45984 : K(1272,1) for forward directionand K(1272,2) for reverse direction
1273	P68400_p |P45984 -->P68400_p +P45984_p : K(1273,1) for forward direction
1274	P68400_p +P25788 <-->P68400_p |P25788 : K(1274,1) for forward directionand K(1274,2) for reverse direction
1275	P68400_p |P25788 -->P68400_p +P25788_p : K(1275,1) for forward direction
1276	P68400_p +P18031 <-->P68400_p |P18031 : K(1276,1) for forward directionand K(1276,2) for reverse direction
1277	P68400_p |P18031 -->P68400_p +P18031_p : K(1277,1) for forward direction
1278	P68400_p +Q96EB6 <-->P68400_p |Q96EB6 : K(1278,1) for forward directionand K(1278,2) for reverse direction
1279	P68400_p |Q96EB6 -->P68400_p +Q96EB6_p : K(1279,1) for forward direction
1280	P68400_p +Q9UQE7 <-->P68400_p |Q9UQE7 : K(1280,1) for forward directionand K(1280,2) for reverse direction
1281	P68400_p |Q9UQE7 -->P68400_p +Q9UQE7_p : K(1281,1) for forward direction
1282	P68400_p +P11388 <-->P68400_p |P11388 : K(1282,1) for forward directionand K(1282,2) for reverse direction
1283	P68400_p |P11388 -->P68400_p +P11388_p : K(1283,1) for forward direction

1284	P19784 +Q13085 <-->P19784 |Q13085 : K(1284,1) for forward directionand K(1284,2) for reverse direction
1285	P19784 |Q13085 -->P19784 +Q13085_p : K(1285,1) for forward direction
1286	P19784 +O14958 <-->P19784 |O14958 : K(1286,1) for forward directionand K(1286,2) for reverse direction
1287	P19784 |O14958 -->P19784 +O14958_p : K(1287,1) for forward direction
1288	P19784 +P09497 <-->P19784 |P09497 : K(1288,1) for forward directionand K(1288,2) for reverse direction
1289	P19784 |P09497 -->P19784 +P09497_p : K(1289,1) for forward direction
1290	P19784 +P24534 <-->P19784 |P24534 : K(1290,1) for forward directionand K(1290,2) for reverse direction
1291	P19784 |P24534 -->P19784 +P24534_p : K(1291,1) for forward direction
1292	P19784 +P17096 <-->P19784 |P17096 : K(1292,1) for forward directionand K(1292,2) for reverse direction
1293	P19784 |P17096 -->P19784 +P17096_p : K(1293,1) for forward direction
1294	P19784 +P07910 <-->P19784 |P07910 : K(1294,1) for forward directionand K(1294,2) for reverse direction
1295	P19784 |P07910 -->P19784 +P07910_p : K(1295,1) for forward direction
1296	P19784 +O43896 <-->P19784 |O43896 : K(1296,1) for forward directionand K(1296,2) for reverse direction
1297	P19784 |O43896 -->P19784 +O43896_p : K(1297,1) for forward direction
1298	P19784 +P18031 <-->P19784 |P18031 : K(1298,1) for forward directionand K(1298,2) for reverse direction
1299	P19784 |P18031 -->P19784 +P18031_p : K(1299,1) for forward direction
1300	P19784 +Q9UQE7 <-->P19784 |Q9UQE7 : K(1300,1) for forward directionand K(1300,2) for reverse direction
1301	P19784 |Q9UQE7 -->P19784 +Q9UQE7_p : K(1301,1) for forward direction
1302	P67870_p +P67870 <-->P67870_p |P67870 : K(1302,1) for forward directionand K(1302,2) for reverse direction
1303	P67870_p |P67870 -->P67870_p +P67870_p : K(1303,1) for forward direction
1304	P67870_p +P35568 <-->P67870_p |P35568 : K(1304,1) for forward directionand K(1304,2) for reverse direction
1305	P67870_p |P35568 -->P67870_p +P35568_p : K(1305,1) for forward direction
1306	P67870_p +Q16625 <-->P67870_p |Q16625 : K(1306,1) for forward directionand K(1306,2) for reverse direction
1307	P67870_p |Q16625 -->P67870_p +Q16625_p : K(1307,1) for forward direction
1308	P53355_p +P25205 <-->P53355_p |P25205 : K(1308,1) for forward directionand K(1308,2) for reverse direction
1309	P53355_p |P25205 -->P53355_p +P25205_p : K(1309,1) for forward direction
1310	P53355_p +P46777 <-->P53355_p |P46777 : K(1310,1) for forward directionand K(1310,2) for reverse direction
1311	P53355_p |P46777 -->P53355_p +P46777_p : K(1311,1) for forward direction
1312	Q13627_p +O75533 <-->Q13627_p |O75533 : K(1312,1) for forward directionand K(1312,2) for reverse direction
1313	Q13627_p |O75533 -->Q13627_p +O75533_p : K(1313,1) for forward direction
1314	Q13627_p +Q07955 <-->Q13627_p |Q07955 : K(1314,1) for forward directionand K(1314,2) for reverse direction
1315	Q13627_p |Q07955 -->Q13627_p +Q07955_p : K(1315,1) for forward direction
1316	P00533_p +O60716 <-->P00533_p |O60716 : K(1316,1) for forward directionand K(1316,2) for reverse direction
1317	P00533_p |O60716 -->P00533_p +O60716_p : K(1317,1) for forward direction
1318	P00533_p +P15311 <-->P00533_p |P15311 : K(1318,1) for forward directionand K(1318,2) for reverse direction
1319	P00533_p |P15311 -->P00533_p +P15311_p : K(1319,1) for forward direction
1320	P00533_p +P62993 <-->P00533_p |P62993 : K(1320,1) for forward directionand K(1320,2) for reverse direction
1321	P00533_p |P62993 -->P00533_p +P62993_p : K(1321,1) for forward direction
1322	P00533_p +P20936 <-->P00533_p |P20936 : K(1322,1) for forward directionand K(1322,2) for reverse direction
1323	P00533_p |P20936 -->P00533_p +P20936_p : K(1323,1) for forward direction
1324	P00533_p +O14828 <-->P00533_p |O14828 : K(1324,1) for forward directionand K(1324,2) for reverse direction
1325	P00533_p |O14828 -->P00533_p +O14828_p : K(1325,1) for forward direction
1326	P00533_p +O75886 <-->P00533_p |O75886 : K(1326,1) for forward directionand K(1326,2) for reverse direction

<table>
<tr><td>1327</td><td>P00533_p |O75886 -->P00533_p +O75886_p : K(1327,1) for forward direction</td></tr>
<tr><td>1328</td><td>P21802 +P60484 <-->P21802 |P60484 : K(1328,1) for forward directionand K(1328,2) for reverse direction</td></tr>
<tr><td>1329</td><td>P21802 |P60484 -->P21802 +P60484_p : K(1329,1) for forward direction</td></tr>
<tr><td>1330</td><td>P25098_p +P09497 <-->P25098_p |P09497 : K(1330,1) for forward directionand K(1330,2) for reverse direction</td></tr>
<tr><td>1331</td><td>P25098_p |P09497 -->P25098_p +P09497_p : K(1331,1) for forward direction</td></tr>
<tr><td>1332</td><td>P49841_p +P05067 <-->P49841_p |P05067 : K(1332,1) for forward directionand K(1332,2) for reverse direction</td></tr>
<tr><td>1333</td><td>P49841_p |P05067 -->P49841_p +P05067_p : K(1333,1) for forward direction</td></tr>
<tr><td>1334</td><td>P49841_p +O60716 <-->P49841_p |O60716 : K(1334,1) for forward directionand K(1334,2) for reverse direction</td></tr>
<tr><td>1335</td><td>P49841_p |O60716 -->P49841_p +O60716_p : K(1335,1) for forward direction</td></tr>
<tr><td>1336</td><td>P28482_p +Q01844 <-->P28482_p |Q01844 : K(1336,1) for forward directionand K(1336,2) for reverse direction</td></tr>
<tr><td>1337</td><td>P28482_p |Q01844 -->P28482_p +Q01844_p : K(1337,1) for forward direction</td></tr>
<tr><td>1338</td><td>P28482_p +Q9H8Y8 <-->P28482_p |Q9H8Y8 : K(1338,1) for forward directionand K(1338,2) for reverse direction</td></tr>
<tr><td>1339</td><td>P28482_p |Q9H8Y8 -->P28482_p +Q9H8Y8_p : K(1339,1) for forward direction</td></tr>
<tr><td>1340</td><td>P28482_p +P49790 <-->P28482_p |P49790 : K(1340,1) for forward directionand K(1340,2) for reverse direction</td></tr>
<tr><td>1341</td><td>P28482_p |P49790 -->P28482_p +P49790_p : K(1341,1) for forward direction</td></tr>
<tr><td>1342</td><td>P28482_p +P47712 <-->P28482_p |P47712 : K(1342,1) for forward directionand K(1342,2) for reverse direction</td></tr>
<tr><td>1343</td><td>P28482_p |P47712 -->P28482_p +P47712_p : K(1343,1) for forward direction</td></tr>
<tr><td>1344</td><td>P28482_p +Q96SB3 <-->P28482_p |Q96SB3 : K(1344,1) for forward directionand K(1344,2) for reverse direction</td></tr>
<tr><td>1345</td><td>P28482_p |Q96SB3 -->P28482_p +Q96SB3_p : K(1345,1) for forward direction</td></tr>
<tr><td>1346</td><td>P28482_p +P23396 <-->P28482_p |P23396 : K(1346,1) for forward directionand K(1346,2) for reverse direction</td></tr>
<tr><td>1347</td><td>P28482_p |P23396 -->P28482_p +P23396_p : K(1347,1) for forward direction</td></tr>
<tr><td>1348</td><td>P28482_p +Q8N122 <-->P28482_p |Q8N122 : K(1348,1) for forward directionand K(1348,2) for reverse direction</td></tr>
<tr><td>1349</td><td>P28482_p |Q8N122 -->P28482_p +Q8N122_p : K(1349,1) for forward direction</td></tr>
<tr><td>1350</td><td>P28482_p +Q8IX03 <-->P28482_p |Q8IX03 : K(1350,1) for forward directionand K(1350,2) for reverse direction</td></tr>
<tr><td>1351</td><td>P28482_p |Q8IX03 -->P28482_p +Q8IX03_p : K(1351,1) for forward direction</td></tr>
<tr><td>1352</td><td>Q16539_p +Q01844 <-->Q16539_p |Q01844 : K(1352,1) for forward directionand K(1352,2) for reverse direction</td></tr>
<tr><td>1353</td><td>Q16539_p |Q01844 -->Q16539_p +Q01844_p : K(1353,1) for forward direction</td></tr>
<tr><td>1354</td><td>Q16539_p +P49023 <-->Q16539_p |P49023 : K(1354,1) for forward directionand K(1354,2) for reverse direction</td></tr>
<tr><td>1355</td><td>Q16539_p |P49023 -->Q16539_p +P49023_p : K(1355,1) for forward direction</td></tr>
<tr><td>1356</td><td>P27361_p +Q01844 <-->P27361_p |Q01844 : K(1356,1) for forward directionand K(1356,2) for reverse direction</td></tr>
<tr><td>1357</td><td>P27361_p |Q01844 -->P27361_p +Q01844_p : K(1357,1) for forward direction</td></tr>
<tr><td>1358</td><td>P27361_p +P49790 <-->P27361_p |P49790 : K(1358,1) for forward directionand K(1358,2) for reverse direction</td></tr>
<tr><td>1359</td><td>P27361_p |P49790 -->P27361_p +P49790_p : K(1359,1) for forward direction</td></tr>
<tr><td>1360</td><td>P27361_p +P23396 <-->P27361_p |P23396 : K(1360,1) for forward directionand K(1360,2) for reverse direction</td></tr>
<tr><td>1361</td><td>P27361_p |P23396 -->P27361_p +P23396_p : K(1361,1) for forward direction</td></tr>
<tr><td>1362</td><td>P27361_p +Q8IX03 <-->P27361_p |Q8IX03 : K(1362,1) for forward directionand K(1362,2) for reverse direction</td></tr>
<tr><td>1363</td><td>P27361_p |Q8IX03 -->P27361_p +Q8IX03_p : K(1363,1) for forward direction</td></tr>
<tr><td>1364</td><td>P49137_p +O15511 <-->P49137_p |O15511 : K(1364,1) for forward directionand K(1364,2) for reverse direction</td></tr>
<tr><td>1365</td><td>P49137_p |O15511 -->P49137_p +O15511_p : K(1365,1) for forward direction</td></tr>
<tr><td>1366</td><td>P49137_p +O00418 <-->P49137_p |O00418 : K(1366,1) for forward directionand K(1366,2) for reverse direction</td></tr>
<tr><td>1367</td><td>P49137_p |O00418 -->P49137_p +O00418_p : K(1367,1) for forward direction</td></tr>
<tr><td>1368</td><td>P49137_p +Q9NQC3 <-->P49137_p |Q9NQC3 : K(1368,1) for forward directionand K(1368,2) for reverse direction</td></tr>
<tr><td>1369</td><td>P49137_p |Q9NQC3 -->P49137_p +Q9NQC3_p : K(1369,1) for forward direction</td></tr>
</table>

1370	P49137_p +P49815 <-->P49137_p |P49815 : K(1370,1) for forward directionand K(1370,2) for reverse direction
1371	P49137_p |P49815 -->P49137_p +P49815_p : K(1371,1) for forward direction
1372	Q16644_p +O00418 <-->Q16644_p |O00418 : K(1372,1) for forward directionand K(1372,2) for reverse direction
1373	Q16644_p |O00418 -->Q16644_p +O00418_p : K(1373,1) for forward direction
1374	Q16644_p +P04792 <-->Q16644_p |P04792 : K(1374,1) for forward directionand K(1374,2) for reverse direction
1375	Q16644_p |P04792 -->Q16644_p +P04792_p : K(1375,1) for forward direction
1376	P15531_p +P15531 <-->P15531_p |P15531 : K(1376,1) for forward directionand K(1376,2) for reverse direction
1377	P15531_p |P15531 -->P15531_p +P15531_p : K(1377,1) for forward direction
1378	Q13153_p +P49023 <-->Q13153_p |P49023 : K(1378,1) for forward directionand K(1378,2) for reverse direction
1379	Q13153_p |P49023 -->Q13153_p +P49023_p : K(1379,1) for forward direction
1380	Q13153_p +P08670 <-->Q13153_p |P08670 : K(1380,1) for forward directionand K(1380,2) for reverse direction
1381	Q13153_p |P08670 -->Q13153_p +P08670_p : K(1381,1) for forward direction
1382	Q13177_p +P62753 <-->Q13177_p |P62753 : K(1382,1) for forward directionand K(1382,2) for reverse direction
1383	Q13177_p |P62753 -->Q13177_p +P62753_p : K(1383,1) for forward direction
1384	O96013_p +P49023 <-->O96013_p |P49023 : K(1384,1) for forward directionand K(1384,2) for reverse direction
1385	O96013_p |P49023 -->O96013_p +P49023_p : K(1385,1) for forward direction
1386	O96013_p +P62826 <-->O96013_p |P62826 : K(1386,1) for forward directionand K(1386,2) for reverse direction
1387	O96013_p |P62826 -->O96013_p +P62826_p : K(1387,1) for forward direction
1388	P53350_p +O96017 <-->P53350_p |O96017 : K(1388,1) for forward directionand K(1388,2) for reverse direction
1389	P53350_p |O96017 -->P53350_p +O96017_p : K(1389,1) for forward direction
1390	P53350_p +P35222 <-->P53350_p |P35222 : K(1390,1) for forward directionand K(1390,2) for reverse direction
1391	P53350_p |P35222 -->P53350_p +P35222_p : K(1391,1) for forward direction
1392	P53350_p +P30626 <-->P53350_p |P30626 : K(1392,1) for forward directionand K(1392,2) for reverse direction
1393	P53350_p |P30626 -->P53350_p +P30626_p : K(1393,1) for forward direction
1394	P17612 +O15551 <-->P17612 |O15551 : K(1394,1) for forward directionand K(1394,2) for reverse direction
1395	P17612 |O15551 -->P17612 +O15551_p : K(1395,1) for forward direction
1396	P17612 +Q9H3Z4 <-->P17612 |Q9H3Z4 : K(1396,1) for forward directionand K(1396,2) for reverse direction
1397	P17612 |Q9H3Z4 -->P17612 +Q9H3Z4_p : K(1397,1) for forward direction
1398	P17612 +P07900 <-->P17612 |P07900 : K(1398,1) for forward directionand K(1398,2) for reverse direction
1399	P17612 |P07900 -->P17612 +P07900_p : K(1399,1) for forward direction
1400	P17612 +P11717 <-->P17612 |P11717 : K(1400,1) for forward directionand K(1400,2) for reverse direction
1401	P17612 |P11717 -->P17612 +P11717_p : K(1401,1) for forward direction
1402	P17612 +P47736 <-->P17612 |P47736 : K(1402,1) for forward directionand K(1402,2) for reverse direction
1403	P17612 |P47736 -->P17612 +P47736_p : K(1403,1) for forward direction
1404	P17612 +Q9Y6E0 <-->P17612 |Q9Y6E0 : K(1404,1) for forward directionand K(1404,2) for reverse direction
1405	P17612 |Q9Y6E0 -->P17612 +Q9Y6E0_p : K(1405,1) for forward direction
1406	P17612 +P50552 <-->P17612 |P50552 : K(1406,1) for forward directionand K(1406,2) for reverse direction
1407	P17612 |P50552 -->P17612 +P50552_p : K(1407,1) for forward direction
1408	P17252_p +O75689 <-->P17252_p |O75689 : K(1408,1) for forward directionand K(1408,2) for reverse direction
1409	P17252_p |O75689 -->P17252_p +O75689_p : K(1409,1) for forward direction
1410	P17252_p +P07355 <-->P17252_p |P07355 : K(1410,1) for forward directionand K(1410,2) for reverse direction
1411	P17252_p |P07355 -->P17252_p +P07355_p : K(1411,1) for forward direction
1412	P17252_p +Q06481 <-->P17252_p |Q06481 : K(1412,1) for forward directionand K(1412,2) for reverse direction

<table>
<tr><td>1413</td><td>P17252_p |Q06481 -->P17252_p +Q06481_p : K(1413,1) for forward direction</td></tr>
<tr><td>1414</td><td>P17252_p +Q05193 <-->P17252_p |Q05193 : K(1414,1) for forward directionand K(1414,2) for reverse direction</td></tr>
<tr><td>1415</td><td>P17252_p |Q05193 -->P17252_p +Q05193_p : K(1415,1) for forward direction</td></tr>
<tr><td>1416</td><td>P17252_p +P06730 <-->P17252_p |P06730 : K(1416,1) for forward directionand K(1416,2) for reverse direction</td></tr>
<tr><td>1417</td><td>P17252_p |P06730 -->P17252_p +P06730_p : K(1417,1) for forward direction</td></tr>
<tr><td>1418</td><td>P17252_p +P56537 <-->P17252_p |P56537 : K(1418,1) for forward directionand K(1418,2) for reverse direction</td></tr>
<tr><td>1419</td><td>P17252_p |P56537 -->P17252_p +P56537_p : K(1419,1) for forward direction</td></tr>
<tr><td>1420</td><td>P17252_p +Q9Y624 <-->P17252_p |Q9Y624 : K(1420,1) for forward directionand K(1420,2) for reverse direction</td></tr>
<tr><td>1421</td><td>P17252_p |Q9Y624 -->P17252_p +Q9Y624_p : K(1421,1) for forward direction</td></tr>
<tr><td>1422</td><td>P17252_p +P60983 <-->P17252_p |P60983 : K(1422,1) for forward directionand K(1422,2) for reverse direction</td></tr>
<tr><td>1423</td><td>P17252_p |P60983 -->P17252_p +P60983_p : K(1423,1) for forward direction</td></tr>
<tr><td>1424</td><td>P17252_p +P05783 <-->P17252_p |P05783 : K(1424,1) for forward directionand K(1424,2) for reverse direction</td></tr>
<tr><td>1425</td><td>P17252_p |P05783 -->P17252_p +P05783_p : K(1425,1) for forward direction</td></tr>
<tr><td>1426</td><td>P17252_p +P02545 <-->P17252_p |P02545 : K(1426,1) for forward directionand K(1426,2) for reverse direction</td></tr>
<tr><td>1427</td><td>P17252_p |P02545 -->P17252_p +P02545_p : K(1427,1) for forward direction</td></tr>
<tr><td>1428</td><td>P17252_p +Q9UQ80 <-->P17252_p |Q9UQ80 : K(1428,1) for forward directionand K(1428,2) for reverse direction</td></tr>
<tr><td>1429</td><td>P17252_p |Q9UQ80 -->P17252_p +Q9UQ80_p : K(1429,1) for forward direction</td></tr>
<tr><td>1430</td><td>P17252_p +Q06124 <-->P17252_p |Q06124 : K(1430,1) for forward directionand K(1430,2) for reverse direction</td></tr>
<tr><td>1431</td><td>P17252_p |Q06124 -->P17252_p +Q06124_p : K(1431,1) for forward direction</td></tr>
<tr><td>1432</td><td>P17252_p +P11234 <-->P17252_p |P11234 : K(1432,1) for forward directionand K(1432,2) for reverse direction</td></tr>
<tr><td>1433</td><td>P17252_p |P11234 -->P17252_p +P11234_p : K(1433,1) for forward direction</td></tr>
<tr><td>1434</td><td>P17252_p +P27635 <-->P17252_p |P27635 : K(1434,1) for forward directionand K(1434,2) for reverse direction</td></tr>
<tr><td>1435</td><td>P17252_p |P27635 -->P17252_p +P27635_p : K(1435,1) for forward direction</td></tr>
<tr><td>1436</td><td>P17252_p +O00161 <-->P17252_p |O00161 : K(1436,1) for forward directionand K(1436,2) for reverse direction</td></tr>
<tr><td>1437</td><td>P17252_p |O00161 -->P17252_p +O00161_p : K(1437,1) for forward direction</td></tr>
<tr><td>1438</td><td>P17252_p +Q07617 <-->P17252_p |Q07617 : K(1438,1) for forward directionand K(1438,2) for reverse direction</td></tr>
<tr><td>1439</td><td>P17252_p |Q07617 -->P17252_p +Q07617_p : K(1439,1) for forward direction</td></tr>
<tr><td>1440</td><td>P17252_p +P12931 <-->P17252_p |P12931 : K(1440,1) for forward directionand K(1440,2) for reverse direction</td></tr>
<tr><td>1441</td><td>P17252_p |P12931 -->P17252_p +P12931_p : K(1441,1) for forward direction</td></tr>
<tr><td>1442</td><td>P17252_p +P61764 <-->P17252_p |P61764 : K(1442,1) for forward directionand K(1442,2) for reverse direction</td></tr>
<tr><td>1443</td><td>P17252_p |P61764 -->P17252_p +P61764_p : K(1443,1) for forward direction</td></tr>
<tr><td>1444</td><td>P17252_p +P18206 <-->P17252_p |P18206 : K(1444,1) for forward directionand K(1444,2) for reverse direction</td></tr>
<tr><td>1445</td><td>P17252_p |P18206 -->P17252_p +P18206_p : K(1445,1) for forward direction</td></tr>
<tr><td>1446</td><td>Q05655_p +Q92793 <-->Q05655_p |Q92793 : K(1446,1) for forward directionand K(1446,2) for reverse direction</td></tr>
<tr><td>1447</td><td>Q05655_p |Q92793 -->Q05655_p +Q92793_p : K(1447,1) for forward direction</td></tr>
<tr><td>1448</td><td>Q05655_p +P68104 <-->Q05655_p |P68104 : K(1448,1) for forward directionand K(1448,2) for reverse direction</td></tr>
<tr><td>1449</td><td>Q05655_p |P68104 -->Q05655_p +P68104_p : K(1449,1) for forward direction</td></tr>
<tr><td>1450</td><td>Q05655_p +P05198 <-->Q05655_p |P05198 : K(1450,1) for forward directionand K(1450,2) for reverse direction</td></tr>
<tr><td>1451</td><td>Q05655_p |P05198 -->Q05655_p +P05198_p : K(1451,1) for forward direction</td></tr>
<tr><td>1452</td><td>Q05655_p +P29966 <-->Q05655_p |P29966 : K(1452,1) for forward directionand K(1452,2) for reverse direction</td></tr>
<tr><td>1453</td><td>Q05655_p |P29966 -->Q05655_p +P29966_p : K(1453,1) for forward direction</td></tr>
<tr><td>1454</td><td>Q05655_p +Q05655 <-->Q05655_p |Q05655 : K(1454,1) for forward directionand K(1454,2) for reverse direction</td></tr>
<tr><td>1455</td><td>Q05655_p |Q05655 -->Q05655_p +Q05655_p : K(1455,1) for forward direction</td></tr>
</table>

1456	Q05655_p +P29353 <-->Q05655_p |P29353 : K(1456,1) for forward directionand K(1456,2) for reverse direction
1457	Q05655_p |P29353 -->Q05655_p +P29353_p : K(1457,1) for forward direction
1458	P78527_p +P07900 <-->P78527_p |P07900 : K(1458,1) for forward directionand K(1458,2) for reverse direction
1459	P78527_p |P07900 -->P78527_p +P07900_p : K(1459,1) for forward direction
1460	P78527_p +P05412 <-->P78527_p |P05412 : K(1460,1) for forward directionand K(1460,2) for reverse direction
1461	P78527_p |P05412 -->P78527_p +P05412_p : K(1461,1) for forward direction
1462	P78527_p +Q9H9Q4 <-->P78527_p |Q9H9Q4 : K(1462,1) for forward directionand K(1462,2) for reverse direction
1463	P78527_p |Q9H9Q4 -->P78527_p +Q9H9Q4_p : K(1463,1) for forward direction
1464	Q13976_p +P16220 <-->Q13976_p |P16220 : K(1464,1) for forward directionand K(1464,2) for reverse direction
1465	Q13976_p |P16220 -->Q13976_p +P16220_p : K(1465,1) for forward direction
1466	Q13976_p +P52943 <-->Q13976_p |P52943 : K(1466,1) for forward directionand K(1466,2) for reverse direction
1467	Q13976_p |P52943 -->Q13976_p +P52943_p : K(1467,1) for forward direction
1468	Q05397_p +P56945 <-->Q05397_p |P56945 : K(1468,1) for forward directionand K(1468,2) for reverse direction
1469	Q05397_p |P56945 -->Q05397_p +P56945_p : K(1469,1) for forward direction
1470	Q05397_p +P49023 <-->Q05397_p |P49023 : K(1470,1) for forward directionand K(1470,2) for reverse direction
1471	Q05397_p |P49023 -->Q05397_p +P49023_p : K(1471,1) for forward direction
1472	Q05397_p +Q99961 <-->Q05397_p |Q99961 : K(1472,1) for forward directionand K(1472,2) for reverse direction
1473	Q05397_p |Q99961 -->Q05397_p +Q99961_p : K(1473,1) for forward direction
1474	Q13464 +Q16555 <-->Q13464 |Q16555 : K(1474,1) for forward directionand K(1474,2) for reverse direction
1475	Q13464 |Q16555 -->Q13464 +Q16555_p : K(1475,1) for forward direction
1476	Q13464 +P35241 <-->Q13464 |P35241 : K(1476,1) for forward directionand K(1476,2) for reverse direction
1477	Q13464 |P35241 -->Q13464 +P35241_p : K(1477,1) for forward direction
1478	Q15418_p +Q9Y2V2 <-->Q15418_p |Q9Y2V2 : K(1478,1) for forward directionand K(1478,2) for reverse direction
1479	Q15418_p |Q9Y2V2 -->Q15418_p +Q9Y2V2_p : K(1479,1) for forward direction
1480	Q15418_p +Q9H6Z4 <-->Q15418_p |Q9H6Z4 : K(1480,1) for forward directionand K(1480,2) for reverse direction
1481	Q15418_p |Q9H6Z4 -->Q15418_p +Q9H6Z4_p : K(1481,1) for forward direction
1482	P51812_p +P05783 <-->P51812_p |P05783 : K(1482,1) for forward directionand K(1482,2) for reverse direction
1483	P51812_p |P05783 -->P51812_p +P05783_p : K(1483,1) for forward direction
1484	O75676_p +Q16695 <-->O75676_p |Q16695 : K(1484,1) for forward directionand K(1484,2) for reverse direction
1485	O75676_p |Q16695 -->O75676_p +Q16695_p : K(1485,1) for forward direction
1486	O75676_p +P05114 <-->O75676_p |P05114 : K(1486,1) for forward directionand K(1486,2) for reverse direction
1487	O75676_p |P05114 -->O75676_p +P05114_p : K(1487,1) for forward direction
1488	P12931_p +P04083 <-->P12931_p |P04083 : K(1488,1) for forward directionand K(1488,2) for reverse direction
1489	P12931_p |P04083 -->P12931_p +P04083_p : K(1489,1) for forward direction
1490	P12931_p +P52566 <-->P12931_p |P52566 : K(1490,1) for forward directionand K(1490,2) for reverse direction
1491	P12931_p |P52566 -->P12931_p +P52566_p : K(1491,1) for forward direction
1492	P12931_p +Q9H5V8 <-->P12931_p |Q9H5V8 : K(1492,1) for forward directionand K(1492,2) for reverse direction
1493	P12931_p |Q9H5V8 -->P12931_p +Q9H5V8_p : K(1493,1) for forward direction
1494	P12931_p +P62993 <-->P12931_p |P62993 : K(1494,1) for forward directionand K(1494,2) for reverse direction
1495	P12931_p |P62993 -->P12931_p +P62993_p : K(1495,1) for forward direction
1496	P12931_p +P08727 <-->P12931_p |P08727 : K(1496,1) for forward directionand K(1496,2) for reverse direction
1497	P12931_p |P08727 -->P12931_p +P08727_p : K(1497,1) for forward direction
1498	P12931_p +O43490 <-->P12931_p |O43490 : K(1498,1) for forward directionand K(1498,2) for reverse direction

1499	P12931_p |O43490 -->P12931_p +O43490_p : K(1499,1) for forward direction
1500	P12931_p +P51812 <-->P12931_p |P51812 : K(1500,1) for forward directionand K(1500,2) for reverse direction
1501	P12931_p |P51812 -->P12931_p +P51812_p : K(1501,1) for forward direction
1502	O00506 +Q9BUL8 <-->O00506 |Q9BUL8 : K(1502,1) for forward directionand K(1502,2) for reverse direction
1503	O00506 |Q9BUL8 -->O00506 +Q9BUL8_p : K(1503,1) for forward direction
1504	O43422 +P05198 <-->O43422 |P05198 : K(1504,1) for forward directionand K(1504,2) for reverse direction
1505	O43422 |P05198 -->O43422 +P05198_p : K(1505,1) for forward direction
1506	Q2M2I8 +Q9BXS5 <-->Q2M2I8 |Q9BXS5 : K(1506,1) for forward directionand K(1506,2) for reverse direction
1507	Q2M2I8 |Q9BXS5 -->Q2M2I8 +Q9BXS5_p : K(1507,1) for forward direction
1508	Q2M2I8 +Q96CW1 <-->Q2M2I8 |Q96CW1 : K(1508,1) for forward directionand K(1508,2) for reverse direction
1509	Q2M2I8 |Q96CW1 -->Q2M2I8 +Q96CW1_p : K(1509,1) for forward direction
1510	P31749_p +Q86V81 <-->P31749_p |Q86V81 : K(1510,1) for forward directionand K(1510,2) for reverse direction
1511	P31749_p |Q86V81 -->P31749_p +Q86V81_p : K(1511,1) for forward direction
1512	P31749_p +P24941 <-->P31749_p |P24941 : K(1512,1) for forward directionand K(1512,2) for reverse direction
1513	P31749_p |P24941 -->P31749_p +P24941_p : K(1513,1) for forward direction
1514	P31749_p +O15111 <-->P31749_p |O15111 : K(1514,1) for forward directionand K(1514,2) for reverse direction
1515	P31749_p |O15111 -->P31749_p +O15111_p : K(1515,1) for forward direction
1516	P31749_p +Q7L5N1 <-->P31749_p |Q7L5N1 : K(1516,1) for forward directionand K(1516,2) for reverse direction
1517	P31749_p |Q7L5N1 -->P31749_p +Q7L5N1_p : K(1517,1) for forward direction
1518	P31749_p +P35222 <-->P31749_p |P35222 : K(1518,1) for forward directionand K(1518,2) for reverse direction
1519	P31749_p |P35222 -->P31749_p +P35222_p : K(1519,1) for forward direction
1520	P31749_p +P26358 <-->P31749_p |P26358 : K(1520,1) for forward directionand K(1520,2) for reverse direction
1521	P31749_p |P26358 -->P31749_p +P26358_p : K(1521,1) for forward direction
1522	P31749_p +P23588 <-->P31749_p |P23588 : K(1522,1) for forward directionand K(1522,2) for reverse direction
1523	P31749_p |P23588 -->P31749_p +P23588_p : K(1523,1) for forward direction
1524	P31749_p +Q09472 <-->P31749_p |Q09472 : K(1524,1) for forward directionand K(1524,2) for reverse direction
1525	P31749_p |Q09472 -->P31749_p +Q09472_p : K(1525,1) for forward direction
1526	P31749_p +P84243 <-->P31749_p |P84243 : K(1526,1) for forward directionand K(1526,2) for reverse direction
1527	P31749_p |P84243 -->P31749_p +P84243_p : K(1527,1) for forward direction
1528	P31749_p +Q12906 <-->P31749_p |Q12906 : K(1528,1) for forward directionand K(1528,2) for reverse direction
1529	P31749_p |Q12906 -->P31749_p +Q12906_p : K(1529,1) for forward direction
1530	P31749_p +Q04912 <-->P31749_p |Q04912 : K(1530,1) for forward directionand K(1530,2) for reverse direction
1531	P31749_p |Q04912 -->P31749_p +Q04912_p : K(1531,1) for forward direction
1532	P31749_p +P78362 <-->P31749_p |P78362 : K(1532,1) for forward directionand K(1532,2) for reverse direction
1533	P31749_p |P78362 -->P31749_p +P78362_p : K(1533,1) for forward direction
1534	P31749_p +Q96S44 <-->P31749_p |Q96S44 : K(1534,1) for forward directionand K(1534,2) for reverse direction
1535	P31749_p |Q96S44 -->P31749_p +Q96S44_p : K(1535,1) for forward direction
1536	P31749_p +P55072 <-->P31749_p |P55072 : K(1536,1) for forward directionand K(1536,2) for reverse direction
1537	P31749_p |P55072 -->P31749_p +P55072_p : K(1537,1) for forward direction
1538	P31749_p +P08670 <-->P31749_p |P08670 : K(1538,1) for forward directionand K(1538,2) for reverse direction
1539	P31749_p |P08670 -->P31749_p +P08670_p : K(1539,1) for forward direction
1540	P31749_p +Q9H4A3 <-->P31749_p |Q9H4A3 : K(1540,1) for forward directionand K(1540,2) for reverse direction
1541	P31749_p |Q9H4A3 -->P31749_p +Q9H4A3_p : K(1541,1) for forward direction

1542	P31749_p +P67809 <-->P31749_p |P67809 : K(1542,1) for forward directionand K(1542,2) for reverse direction
1543	P31749_p |P67809 -->P31749_p +P67809_p : K(1543,1) for forward direction
1544	P31751_p +O15111 <-->P31751_p |O15111 : K(1544,1) for forward directionand K(1544,2) for reverse direction
1545	P31751_p |O15111 -->P31751_p +O15111_p : K(1545,1) for forward direction
1546	P31751_p +P16220 <-->P31751_p |P16220 : K(1546,1) for forward directionand K(1546,2) for reverse direction
1547	P31751_p |P16220 -->P31751_p +P16220_p : K(1547,1) for forward direction
1548	P31751_p +P35222 <-->P31751_p |P35222 : K(1548,1) for forward directionand K(1548,2) for reverse direction
1549	P31751_p |P35222 -->P31751_p +P35222_p : K(1549,1) for forward direction
1550	P31751_p +Q09472 <-->P31751_p |Q09472 : K(1550,1) for forward directionand K(1550,2) for reverse direction
1551	P31751_p |Q09472 -->P31751_p +Q09472_p : K(1551,1) for forward direction
1552	P31751_p +P15311 <-->P31751_p |P15311 : K(1552,1) for forward directionand K(1552,2) for reverse direction
1553	P31751_p |P15311 -->P31751_p +P15311_p : K(1553,1) for forward direction
1554	P31751_p +P98170 <-->P31751_p |P98170 : K(1554,1) for forward directionand K(1554,2) for reverse direction
1555	P31751_p |P98170 -->P31751_p +P98170_p : K(1555,1) for forward direction
1556	P10398_p +Q02750 <-->P10398_p |Q02750 : K(1556,1) for forward directionand K(1556,2) for reverse direction
1557	P10398_p |Q02750 -->P10398_p +Q02750_p : K(1557,1) for forward direction
1558	P10398_p +P36507 <-->P10398_p |P36507 : K(1558,1) for forward directionand K(1558,2) for reverse direction
1559	P10398_p |P36507 -->P10398_p +P36507_p : K(1559,1) for forward direction
1560	Q96GD4_p +Q96GD4 <-->Q96GD4_p |Q96GD4 : K(1560,1) for forward directionand K(1560,2) for reverse direction
1561	Q96GD4_p |Q96GD4 -->Q96GD4_p +Q96GD4_p : K(1561,1) for forward direction
1562	Q96GD4_p +Q96CF2 <-->Q96GD4_p |Q96CF2 : K(1562,1) for forward directionand K(1562,2) for reverse direction
1563	Q96GD4_p |Q96CF2 -->Q96GD4_p +Q96CF2_p : K(1563,1) for forward direction
1564	Q96GD4_p +O60879 <-->Q96GD4_p |O60879 : K(1564,1) for forward directionand K(1564,2) for reverse direction
1565	Q96GD4_p |O60879 -->Q96GD4_p +O60879_p : K(1565,1) for forward direction
1566	Q96GD4_p +P84243 <-->Q96GD4_p |P84243 : K(1566,1) for forward directionand K(1566,2) for reverse direction
1567	Q96GD4_p |P84243 -->Q96GD4_p +P84243_p : K(1567,1) for forward direction
1568	Q96GD4_p +P68431 <-->Q96GD4_p |P68431 : K(1568,1) for forward directionand K(1568,2) for reverse direction
1569	Q96GD4_p |P68431 -->Q96GD4_p +P68431_p : K(1569,1) for forward direction
1570	Q96GD4_p +Q9UPY8 <-->Q96GD4_p |Q9UPY8 : K(1570,1) for forward directionand K(1570,2) for reverse direction
1571	Q96GD4_p |Q9UPY8 -->Q96GD4_p +Q9UPY8_p : K(1571,1) for forward direction
1572	Q96GD4_p +P25490 <-->Q96GD4_p |P25490 : K(1572,1) for forward directionand K(1572,2) for reverse direction
1573	Q96GD4_p |P25490 -->Q96GD4_p +P25490_p : K(1573,1) for forward direction
1574	Q9UIG0_p +P16104 <-->Q9UIG0_p |P16104 : K(1574,1) for forward directionand K(1574,2) for reverse direction
1575	Q9UIG0_p |P16104 -->Q9UIG0_p +P16104_p : K(1575,1) for forward direction
1576	P15056 +P36507 <-->P15056 |P36507 : K(1576,1) for forward directionand K(1576,2) for reverse direction
1577	P15056 |P36507 -->P15056 +P36507_p : K(1577,1) for forward direction
1578	O60885 +P24928 <-->O60885 |P24928 : K(1578,1) for forward directionand K(1578,2) for reverse direction
1579	O60885 |P24928 -->O60885 +P24928_p : K(1579,1) for forward direction
1580	Q9UQM7_p +P16070 <-->Q9UQM7_p |P16070 : K(1580,1) for forward directionand K(1580,2) for reverse direction
1581	Q9UQM7_p |P16070 -->Q9UQM7_p +P16070_p : K(1581,1) for forward direction
1582	Q9UQM7_p +Q06210 <-->Q9UQM7_p |Q06210 : K(1582,1) for forward directionand K(1582,2) for reverse direction
1583	Q9UQM7_p |Q06210 -->Q9UQM7_p +Q06210_p : K(1583,1) for forward direction
1584	Q9UQM7_p +Q15121 <-->Q9UQM7_p |Q15121 : K(1584,1) for forward directionand K(1584,2) for reverse direction

1585	Q9UQM7_p |Q15121 -->Q9UQM7_p +Q15121_p : K(1585,1) for forward direction
1586	Q9UQM7_p +Q05397 <-->Q9UQM7_p |Q05397 : K(1586,1) for forward directionand K(1586,2) for reverse direction
1587	Q9UQM7_p |Q05397 -->Q9UQM7_p +Q05397_p : K(1587,1) for forward direction
1588	Q13554_p +Q13131 <-->Q13554_p |Q13131 : K(1588,1) for forward directionand K(1588,2) for reverse direction
1589	Q13554_p |Q13131 -->Q13554_p +Q13131_p : K(1589,1) for forward direction
1590	Q13554_p +P42224 <-->Q13554_p |P42224 : K(1590,1) for forward directionand K(1590,2) for reverse direction
1591	Q13554_p |P42224 -->Q13554_p +P42224_p : K(1591,1) for forward direction
1592	Q13557_p +Q13557 <-->Q13557_p |Q13557 : K(1592,1) for forward directionand K(1592,2) for reverse direction
1593	Q13557_p |Q13557 -->Q13557_p +Q13557_p : K(1593,1) for forward direction
1594	Q13557_p +P13688 <-->Q13557_p |P13688 : K(1594,1) for forward directionand K(1594,2) for reverse direction
1595	Q13557_p |P13688 -->Q13557_p +P13688_p : K(1595,1) for forward direction
1596	Q13555 +O43318 <-->Q13555 |O43318 : K(1596,1) for forward directionand K(1596,2) for reverse direction
1597	Q13555 |O43318 -->Q13555 +O43318_p : K(1597,1) for forward direction
1598	Q5VT25 +P24844 <-->Q5VT25 |P24844 : K(1598,1) for forward directionand K(1598,2) for reverse direction
1599	Q5VT25 |P24844 -->Q5VT25 +P24844_p : K(1599,1) for forward direction
1600	O00311_p +P49736 <-->O00311_p |P49736 : K(1600,1) for forward directionand K(1600,2) for reverse direction
1601	O00311_p |P49736 -->O00311_p +P49736_p : K(1601,1) for forward direction
1602	O00311_p +P33991 <-->O00311_p |P33991 : K(1602,1) for forward directionand K(1602,2) for reverse direction
1603	O00311_p |P33991 -->O00311_p +P33991_p : K(1603,1) for forward direction
1604	O00311_p +P33993 <-->O00311_p |P33993 : K(1604,1) for forward directionand K(1604,2) for reverse direction
1605	O00311_p |P33993 -->O00311_p +P33993_p : K(1605,1) for forward direction
1606	O00311_p +O75475 <-->O00311_p |O75475 : K(1606,1) for forward directionand K(1606,2) for reverse direction
1607	O00311_p |O75475 -->O00311_p +O75475_p : K(1607,1) for forward direction
1608	P06493_p +Q9H1A4 <-->P06493_p |Q9H1A4 : K(1608,1) for forward directionand K(1608,2) for reverse direction
1609	P06493_p |Q9H1A4 -->P06493_p +Q9H1A4_p : K(1609,1) for forward direction
1610	P06493_p +O60566 <-->P06493_p |O60566 : K(1610,1) for forward directionand K(1610,2) for reverse direction
1611	P06493_p |O60566 -->P06493_p +O60566_p : K(1611,1) for forward direction
1612	P06493_p +Q13042 <-->P06493_p |Q13042 : K(1612,1) for forward directionand K(1612,2) for reverse direction
1613	P06493_p |Q13042 -->P06493_p +Q13042_p : K(1613,1) for forward direction
1614	P06493_p +Q9UJX2 <-->P06493_p |Q9UJX2 : K(1614,1) for forward directionand K(1614,2) for reverse direction
1615	P06493_p |Q9UJX2 -->P06493_p +Q9UJX2_p : K(1615,1) for forward direction
1616	P06493_p +P30260 <-->P06493_p |P30260 : K(1616,1) for forward directionand K(1616,2) for reverse direction
1617	P06493_p |P30260 -->P06493_p +P30260_p : K(1617,1) for forward direction
1618	P06493_p +O00311 <-->P06493_p |O00311 : K(1618,1) for forward directionand K(1618,2) for reverse direction
1619	P06493_p |O00311 -->P06493_p +O00311_p : K(1619,1) for forward direction
1620	P06493_p +O14757 <-->P06493_p |O14757 : K(1620,1) for forward directionand K(1620,2) for reverse direction
1621	P06493_p |O14757 -->P06493_p +O14757_p : K(1621,1) for forward direction
1622	P06493_p +P68400 <-->P06493_p |P68400 : K(1622,1) for forward directionand K(1622,2) for reverse direction
1623	P06493_p |P68400 -->P06493_p +P68400_p : K(1623,1) for forward direction
1624	P06493_p +P67870 <-->P06493_p |P67870 : K(1624,1) for forward directionand K(1624,2) for reverse direction
1625	P06493_p |P67870 -->P06493_p +P67870_p : K(1625,1) for forward direction
1626	P06493_p +P26358 <-->P06493_p |P26358 : K(1626,1) for forward directionand K(1626,2) for reverse direction
1627	P06493_p |P26358 -->P06493_p +P26358_p : K(1627,1) for forward direction

1628	P06493_p +Q2NKX8 <-->P06493_p |Q2NKX8 : K(1628,1) for forward directionand K(1628,2) for reverse direction
1629	P06493_p |Q2NKX8 -->P06493_p +Q2NKX8_p : K(1629,1) for forward direction
1630	P06493_p +Q01167 <-->P06493_p |Q01167 : K(1630,1) for forward directionand K(1630,2) for reverse direction
1631	P06493_p |Q01167 -->P06493_p +Q01167_p : K(1631,1) for forward direction
1632	P06493_p +Q92993 <-->P06493_p |Q92993 : K(1632,1) for forward directionand K(1632,2) for reverse direction
1633	P06493_p |Q92993 -->P06493_p +Q92993_p : K(1633,1) for forward direction
1634	P06493_p +O95251 <-->P06493_p |O95251 : K(1634,1) for forward directionand K(1634,2) for reverse direction
1635	P06493_p |O95251 -->P06493_p +O95251_p : K(1635,1) for forward direction
1636	P06493_p +P52732 <-->P06493_p |P52732 : K(1636,1) for forward directionand K(1636,2) for reverse direction
1637	P06493_p |P52732 -->P06493_p +P52732_p : K(1637,1) for forward direction
1638	P06493_p +P05783 <-->P06493_p |P05783 : K(1638,1) for forward directionand K(1638,2) for reverse direction
1639	P06493_p |P05783 -->P06493_p +P05783_p : K(1639,1) for forward direction
1640	P06493_p +P02545 <-->P06493_p |P02545 : K(1640,1) for forward directionand K(1640,2) for reverse direction
1641	P06493_p |P02545 -->P06493_p +P02545_p : K(1641,1) for forward direction
1642	P06493_p +Q07820 <-->P06493_p |Q07820 : K(1642,1) for forward directionand K(1642,2) for reverse direction
1643	P06493_p |Q07820 -->P06493_p +Q07820_p : K(1643,1) for forward direction
1644	P06493_p +Q8TAP9 <-->P06493_p |Q8TAP9 : K(1644,1) for forward directionand K(1644,2) for reverse direction
1645	P06493_p |Q8TAP9 -->P06493_p +Q8TAP9_p : K(1645,1) for forward direction
1646	P06493_p +Q9NXR1 <-->P06493_p |Q9NXR1 : K(1646,1) for forward directionand K(1646,2) for reverse direction
1647	P06493_p |Q9NXR1 -->P06493_p +Q9NXR1_p : K(1647,1) for forward direction
1648	P06493_p +P15531 <-->P06493_p |P15531 : K(1648,1) for forward directionand K(1648,2) for reverse direction
1649	P06493_p |P15531 -->P06493_p +P15531_p : K(1649,1) for forward direction
1650	P06493_p +P52948 <-->P06493_p |P52948 : K(1650,1) for forward directionand K(1650,2) for reverse direction
1651	P06493_p |P52948 -->P06493_p +P52948_p : K(1651,1) for forward direction
1652	P06493_p +P46060 <-->P06493_p |P46060 : K(1652,1) for forward directionand K(1652,2) for reverse direction
1653	P06493_p |P46060 -->P06493_p +P46060_p : K(1653,1) for forward direction
1654	P06493_p +P15927 <-->P06493_p |P15927 : K(1654,1) for forward directionand K(1654,2) for reverse direction
1655	P06493_p |P15927 -->P06493_p +P15927_p : K(1655,1) for forward direction
1656	P06493_p +P23396 <-->P06493_p |P23396 : K(1656,1) for forward directionand K(1656,2) for reverse direction
1657	P06493_p |P23396 -->P06493_p +P23396_p : K(1657,1) for forward direction
1658	P06493_p +Q96EB6 <-->P06493_p |Q96EB6 : K(1658,1) for forward directionand K(1658,2) for reverse direction
1659	P06493_p |Q96EB6 -->P06493_p +Q96EB6_p : K(1659,1) for forward direction
1660	P06493_p +P08047 <-->P06493_p |P08047 : K(1660,1) for forward directionand K(1660,2) for reverse direction
1661	P06493_p |P08047 -->P06493_p +P08047_p : K(1661,1) for forward direction
1662	P06493_p +Q13501 <-->P06493_p |Q13501 : K(1662,1) for forward directionand K(1662,2) for reverse direction
1663	P06493_p |Q13501 -->P06493_p +Q13501_p : K(1663,1) for forward direction
1664	P06493_p +P04637 <-->P06493_p |P04637 : K(1664,1) for forward directionand K(1664,2) for reverse direction
1665	P06493_p |P04637 -->P06493_p +P04637_p : K(1665,1) for forward direction
1666	P06493_p +P22314 <-->P06493_p |P22314 : K(1666,1) for forward directionand K(1666,2) for reverse direction
1667	P06493_p |P22314 -->P06493_p +P22314_p : K(1667,1) for forward direction
1668	P06493_p +P49459 <-->P06493_p |P49459 : K(1668,1) for forward directionand K(1668,2) for reverse direction
1669	P06493_p |P49459 -->P06493_p +P49459_p : K(1669,1) for forward direction
1670	P06493_p +Q9Y5T5 <-->P06493_p |Q9Y5T5 : K(1670,1) for forward directionand K(1670,2) for reverse direction

1671	P06493_p |Q9Y5T5 -->P06493_p +Q9Y5T5_p : K(1671,1) for forward direction
1672	Q9UQ88 +P21127 <-->Q9UQ88 |P21127 : K(1672,1) for forward directionand K(1672,2) for reverse direction
1673	Q9UQ88 |P21127 -->Q9UQ88 +P21127_p : K(1673,1) for forward direction
1674	Q9NYV4 +P24928 <-->Q9NYV4 |P24928 : K(1674,1) for forward directionand K(1674,2) for reverse direction
1675	Q9NYV4 |P24928 -->Q9NYV4 +P24928_p : K(1675,1) for forward direction
1676	P24941_p +Q16204 <-->P24941_p |Q16204 : K(1676,1) for forward directionand K(1676,2) for reverse direction
1677	P24941_p |Q16204 -->P24941_p +Q16204_p : K(1677,1) for forward direction
1678	P24941_p +P20248 <-->P24941_p |P20248 : K(1678,1) for forward directionand K(1678,2) for reverse direction
1679	P24941_p |P20248 -->P24941_p +P20248_p : K(1679,1) for forward direction
1680	P24941_p +P24941 <-->P24941_p |P24941 : K(1680,1) for forward directionand K(1680,2) for reverse direction
1681	P24941_p |P24941 -->P24941_p +P24941_p : K(1681,1) for forward direction
1682	P24941_p +P17676 <-->P24941_p |P17676 : K(1682,1) for forward directionand K(1682,2) for reverse direction
1683	P24941_p |P17676 -->P24941_p +P17676_p : K(1683,1) for forward direction
1684	P24941_p +O14757 <-->P24941_p |O14757 : K(1684,1) for forward directionand K(1684,2) for reverse direction
1685	P24941_p |O14757 -->P24941_p +O14757_p : K(1685,1) for forward direction
1686	P24941_p +P38432 <-->P24941_p |P38432 : K(1686,1) for forward directionand K(1686,2) for reverse direction
1687	P24941_p |P38432 -->P24941_p +P38432_p : K(1687,1) for forward direction
1688	P24941_p +Q12959 <-->P24941_p |Q12959 : K(1688,1) for forward directionand K(1688,2) for reverse direction
1689	P24941_p |Q12959 -->P24941_p +Q12959_p : K(1689,1) for forward direction
1690	P24941_p +P26358 <-->P24941_p |P26358 : K(1690,1) for forward directionand K(1690,2) for reverse direction
1691	P24941_p |P26358 -->P24941_p +P26358_p : K(1691,1) for forward direction
1692	P24941_p +Q01167 <-->P24941_p |Q01167 : K(1692,1) for forward directionand K(1692,2) for reverse direction
1693	P24941_p |Q01167 -->P24941_p +Q01167_p : K(1693,1) for forward direction
1694	P24941_p +P49736 <-->P24941_p |P49736 : K(1694,1) for forward directionand K(1694,2) for reverse direction
1695	P24941_p |P49736 -->P24941_p +P49736_p : K(1695,1) for forward direction
1696	P24941_p +P25205 <-->P24941_p |P25205 : K(1696,1) for forward directionand K(1696,2) for reverse direction
1697	P24941_p |P25205 -->P24941_p +P25205_p : K(1697,1) for forward direction
1698	P24941_p +Q13416 <-->P24941_p |Q13416 : K(1698,1) for forward directionand K(1698,2) for reverse direction
1699	P24941_p |Q13416 -->P24941_p +Q13416_p : K(1699,1) for forward direction
1700	P24941_p +Q8IZL8 <-->P24941_p |Q8IZL8 : K(1700,1) for forward directionand K(1700,2) for reverse direction
1701	P24941_p |Q8IZL8 -->P24941_p +Q8IZL8_p : K(1701,1) for forward direction
1702	P24941_p +P10644 <-->P24941_p |P10644 : K(1702,1) for forward directionand K(1702,2) for reverse direction
1703	P24941_p |P10644 -->P24941_p +P10644_p : K(1703,1) for forward direction
1704	P24941_p +Q9NYV6 <-->P24941_p |Q9NYV6 : K(1704,1) for forward directionand K(1704,2) for reverse direction
1705	P24941_p |Q9NYV6 -->P24941_p +Q9NYV6_p : K(1705,1) for forward direction
1706	P24941_p +P04637 <-->P24941_p |P04637 : K(1706,1) for forward directionand K(1706,2) for reverse direction
1707	P24941_p |P04637 -->P24941_p +P04637_p : K(1707,1) for forward direction
1708	P24941_p +P49459 <-->P24941_p |P49459 : K(1708,1) for forward directionand K(1708,2) for reverse direction
1709	P24941_p |P49459 -->P24941_p +P49459_p : K(1709,1) for forward direction
1710	P11802_p +Q8IZL8 <-->P11802_p |Q8IZL8 : K(1710,1) for forward directionand K(1710,2) for reverse direction
1711	P11802_p |Q8IZL8 -->P11802_p +Q8IZL8_p : K(1711,1) for forward direction
1712	Q00535_p +P26358 <-->Q00535_p |P26358 : K(1712,1) for forward directionand K(1712,2) for reverse direction
1713	Q00535_p |P26358 -->Q00535_p +P26358_p : K(1713,1) for forward direction

1714	Q00535_p +O43464 <-->Q00535_p |O43464 : K(1714,1) for forward directionand K(1714,2) for reverse direction
1715	Q00535_p |O43464 -->Q00535_p +O43464_p : K(1715,1) for forward direction
1716	Q00535_p +P42858 <-->Q00535_p |P42858 : K(1716,1) for forward directionand K(1716,2) for reverse direction
1717	Q00535_p |P42858 -->Q00535_p +P42858_p : K(1717,1) for forward direction
1718	Q00535_p +Q05397 <-->Q00535_p |Q05397 : K(1718,1) for forward directionand K(1718,2) for reverse direction
1719	Q00535_p |Q05397 -->Q00535_p +Q05397_p : K(1719,1) for forward direction
1720	Q00535_p +P12931 <-->Q00535_p |P12931 : K(1720,1) for forward directionand K(1720,2) for reverse direction
1721	Q00535_p |P12931 -->Q00535_p +P12931_p : K(1721,1) for forward direction
1722	Q00535_p +P40763 <-->Q00535_p |P40763 : K(1722,1) for forward directionand K(1722,2) for reverse direction
1723	Q00535_p |P40763 -->Q00535_p +P40763_p : K(1723,1) for forward direction
1724	Q00535_p +Q9Y490 <-->Q00535_p |Q9Y490 : K(1724,1) for forward directionand K(1724,2) for reverse direction
1725	Q00535_p |Q9Y490 -->Q00535_p +Q9Y490_p : K(1725,1) for forward direction
1726	Q00535_p +P04637 <-->Q00535_p |P04637 : K(1726,1) for forward directionand K(1726,2) for reverse direction
1727	Q00535_p |P04637 -->Q00535_p +P04637_p : K(1727,1) for forward direction
1728	P50613_p +P06493 <-->P50613_p |P06493 : K(1728,1) for forward directionand K(1728,2) for reverse direction
1729	P50613_p |P06493 -->P50613_p +P06493_p : K(1729,1) for forward direction
1730	P50613_p +P11802 <-->P50613_p |P11802 : K(1730,1) for forward directionand K(1730,2) for reverse direction
1731	P50613_p |P11802 -->P50613_p +P11802_p : K(1731,1) for forward direction
1732	P50750_p +P24928 <-->P50750_p |P24928 : K(1732,1) for forward directionand K(1732,2) for reverse direction
1733	P50750_p |P24928 -->P50750_p +P24928_p : K(1733,1) for forward direction
1734	P50750_p +O00267 <-->P50750_p |O00267 : K(1734,1) for forward directionand K(1734,2) for reverse direction
1735	P50750_p |O00267 -->P50750_p +O00267_p : K(1735,1) for forward direction
1736	O14757_p +Q96GD4 <-->O14757_p |Q96GD4 : K(1736,1) for forward directionand K(1736,2) for reverse direction
1737	O14757_p |Q96GD4 -->O14757_p +Q96GD4_p : K(1737,1) for forward direction
1738	O14757_p +P54132 <-->O14757_p |P54132 : K(1738,1) for forward directionand K(1738,2) for reverse direction
1739	O14757_p |P54132 -->O14757_p +P54132_p : K(1739,1) for forward direction
1740	O14757_p +O00311 <-->O14757_p |O00311 : K(1740,1) for forward directionand K(1740,2) for reverse direction
1741	O14757_p |O00311 -->O14757_p +O00311_p : K(1741,1) for forward direction
1742	O14757_p +O00716 <-->O14757_p |O00716 : K(1742,1) for forward directionand K(1742,2) for reverse direction
1743	O14757_p |O00716 -->O14757_p +O00716_p : K(1743,1) for forward direction
1744	O14757_p +P84243 <-->O14757_p |P84243 : K(1744,1) for forward directionand K(1744,2) for reverse direction
1745	O14757_p |P84243 -->O14757_p +P84243_p : K(1745,1) for forward direction
1746	O14757_p +P06400 <-->O14757_p |P06400 : K(1746,1) for forward directionand K(1746,2) for reverse direction
1747	O14757_p |P06400 -->O14757_p +P06400_p : K(1747,1) for forward direction
1748	O14757_p +P30291 <-->O14757_p |P30291 : K(1748,1) for forward directionand K(1748,2) for reverse direction
1749	O14757_p |P30291 -->O14757_p +P30291_p : K(1749,1) for forward direction
1750	O96017_p +Q13362 <-->O96017_p |Q13362 : K(1750,1) for forward directionand K(1750,2) for reverse direction
1751	O96017_p |Q13362 -->O96017_p +Q13362_p : K(1751,1) for forward direction
1752	O96017_p +P18887 <-->O96017_p |P18887 : K(1752,1) for forward directionand K(1752,2) for reverse direction
1753	O96017_p |P18887 -->O96017_p +P18887_p : K(1753,1) for forward direction
1754	O15111_p +Q92793 <-->O15111_p |Q92793 : K(1754,1) for forward directionand K(1754,2) for reverse direction
1755	O15111_p |Q92793 -->O15111_p +Q92793_p : K(1755,1) for forward direction
1756	O15111_p +P84243 <-->O15111_p |P84243 : K(1756,1) for forward directionand K(1756,2) for reverse direction

1757	O15111_p |P84243 -->O15111_p +P84243_p : K(1757,1) for forward direction
1758	O15111_p +Q92985 <-->O15111_p |Q92985 : K(1758,1) for forward directionand K(1758,2) for reverse direction
1759	O15111_p |Q92985 -->O15111_p +Q92985_p : K(1759,1) for forward direction
1760	Q53ET0 +P31749 <-->Q53ET0 |P31749 : K(1760,1) for forward directionand K(1760,2) for reverse direction
1761	Q53ET0 |P31749 -->Q53ET0 +P31749_p : K(1761,1) for forward direction
1762	P48729 +P16220 <-->P48729 |P16220 : K(1762,1) for forward directionand K(1762,2) for reverse direction
1763	P48729 |P16220 -->P48729 +P16220_p : K(1763,1) for forward direction
1764	P48730 +O15169 <-->P48730 |O15169 : K(1764,1) for forward directionand K(1764,2) for reverse direction
1765	P48730 |O15169 -->P48730 +O15169_p : K(1765,1) for forward direction
1766	P48730 +O14641 <-->P48730 |O14641 : K(1766,1) for forward directionand K(1766,2) for reverse direction
1767	P48730 |O14641 -->P48730 +O14641_p : K(1767,1) for forward direction
1768	P48730 +P04637 <-->P48730 |P04637 : K(1768,1) for forward directionand K(1768,2) for reverse direction
1769	P48730 |P04637 -->P48730 +P04637_p : K(1769,1) for forward direction
1770	P48730 +Q96T88 <-->P48730 |Q96T88 : K(1770,1) for forward directionand K(1770,2) for reverse direction
1771	P48730 |Q96T88 -->P48730 +Q96T88_p : K(1771,1) for forward direction
1772	P49674 +O15169 <-->P49674 |O15169 : K(1772,1) for forward directionand K(1772,2) for reverse direction
1773	P49674 |O15169 -->P49674 +O15169_p : K(1773,1) for forward direction
1774	P49674 +O14641 <-->P49674 |O14641 : K(1774,1) for forward directionand K(1774,2) for reverse direction
1775	P49674 |O14641 -->P49674 +O14641_p : K(1775,1) for forward direction
1776	P68400_p +P33527 <-->P68400_p |P33527 : K(1776,1) for forward directionand K(1776,2) for reverse direction
1777	P68400_p |P33527 -->P68400_p +P33527_p : K(1777,1) for forward direction
1778	P68400_p +P31749 <-->P68400_p |P31749 : K(1778,1) for forward directionand K(1778,2) for reverse direction
1779	P68400_p |P31749 -->P68400_p +P31749_p : K(1779,1) for forward direction
1780	P68400_p +Q92688 <-->P68400_p |Q92688 : K(1780,1) for forward directionand K(1780,2) for reverse direction
1781	P68400_p |Q92688 -->P68400_p +Q92688_p : K(1781,1) for forward direction
1782	P68400_p +P52907 <-->P68400_p |P52907 : K(1782,1) for forward directionand K(1782,2) for reverse direction
1783	P68400_p |P52907 -->P68400_p +P52907_p : K(1783,1) for forward direction
1784	P68400_p +P45973 <-->P68400_p |P45973 : K(1784,1) for forward directionand K(1784,2) for reverse direction
1785	P68400_p |P45973 -->P68400_p +P45973_p : K(1785,1) for forward direction
1786	P68400_p +P30260 <-->P68400_p |P30260 : K(1786,1) for forward directionand K(1786,2) for reverse direction
1787	P68400_p |P30260 -->P68400_p +P30260_p : K(1787,1) for forward direction
1788	P68400_p +P06493 <-->P68400_p |P06493 : K(1788,1) for forward directionand K(1788,2) for reverse direction
1789	P68400_p |P06493 -->P68400_p +P06493_p : K(1789,1) for forward direction
1790	P68400_p +P30622 <-->P68400_p |P30622 : K(1790,1) for forward directionand K(1790,2) for reverse direction
1791	P68400_p |P30622 -->P68400_p +P30622_p : K(1791,1) for forward direction
1792	P68400_p +Q9ULV4 <-->P68400_p |Q9ULV4 : K(1792,1) for forward directionand K(1792,2) for reverse direction
1793	P68400_p |Q9ULV4 -->P68400_p +Q9ULV4_p : K(1793,1) for forward direction
1794	P68400_p +Q9UER7 <-->P68400_p |Q9UER7 : K(1794,1) for forward directionand K(1794,2) for reverse direction
1795	P68400_p |Q9UER7 -->P68400_p +Q9UER7_p : K(1795,1) for forward direction
1796	P68400_p +P35659 <-->P68400_p |P35659 : K(1796,1) for forward directionand K(1796,2) for reverse direction
1797	P68400_p |P35659 -->P68400_p +P35659_p : K(1797,1) for forward direction
1798	P68400_p +P20042 <-->P68400_p |P20042 : K(1798,1) for forward directionand K(1798,2) for reverse direction
1799	P68400_p |P20042 -->P68400_p +P20042_p : K(1799,1) for forward direction

<table>
<tr><td>1800</td><td>P68400_p +P55010 <-->P68400_p |P55010 : K(1800,1) for forward directionand K(1800,2) for reverse direction</td></tr>
<tr><td>1801</td><td>P68400_p |P55010 -->P68400_p +P55010_p : K(1801,1) for forward direction</td></tr>
<tr><td>1802</td><td>P68400_p +Q06265 <-->P68400_p |Q06265 : K(1802,1) for forward directionand K(1802,2) for reverse direction</td></tr>
<tr><td>1803</td><td>P68400_p |Q06265 -->P68400_p +Q06265_p : K(1803,1) for forward direction</td></tr>
<tr><td>1804</td><td>P68400_p +Q13547 <-->P68400_p |Q13547 : K(1804,1) for forward directionand K(1804,2) for reverse direction</td></tr>
<tr><td>1805</td><td>P68400_p |Q13547 -->P68400_p +Q13547_p : K(1805,1) for forward direction</td></tr>
<tr><td>1806</td><td>P68400_p +Q92769 <-->P68400_p |Q92769 : K(1806,1) for forward directionand K(1806,2) for reverse direction</td></tr>
<tr><td>1807</td><td>P68400_p |Q92769 -->P68400_p +Q92769_p : K(1807,1) for forward direction</td></tr>
<tr><td>1808</td><td>P68400_p +P49736 <-->P68400_p |P49736 : K(1808,1) for forward directionand K(1808,2) for reverse direction</td></tr>
<tr><td>1809</td><td>P68400_p |P49736 -->P68400_p +P49736_p : K(1809,1) for forward direction</td></tr>
<tr><td>1810</td><td>P68400_p +Q14676 <-->P68400_p |Q14676 : K(1810,1) for forward directionand K(1810,2) for reverse direction</td></tr>
<tr><td>1811</td><td>P68400_p |Q14676 -->P68400_p +Q14676_p : K(1811,1) for forward direction</td></tr>
<tr><td>1812</td><td>P68400_p +P35579 <-->P68400_p |P35579 : K(1812,1) for forward directionand K(1812,2) for reverse direction</td></tr>
<tr><td>1813</td><td>P68400_p |P35579 -->P68400_p +P35579_p : K(1813,1) for forward direction</td></tr>
<tr><td>1814</td><td>P68400_p +O14737 <-->P68400_p |O14737 : K(1814,1) for forward directionand K(1814,2) for reverse direction</td></tr>
<tr><td>1815</td><td>P68400_p |O14737 -->P68400_p +O14737_p : K(1815,1) for forward direction</td></tr>
<tr><td>1816</td><td>P68400_p +O75925 <-->P68400_p |O75925 : K(1816,1) for forward directionand K(1816,2) for reverse direction</td></tr>
<tr><td>1817</td><td>P68400_p |O75925 -->P68400_p +O75925_p : K(1817,1) for forward direction</td></tr>
<tr><td>1818</td><td>P68400_p +P48426 <-->P68400_p |P48426 : K(1818,1) for forward directionand K(1818,2) for reverse direction</td></tr>
<tr><td>1819</td><td>P68400_p |P48426 -->P68400_p +P48426_p : K(1819,1) for forward direction</td></tr>
<tr><td>1820</td><td>P68400_p +P41236 <-->P68400_p |P41236 : K(1820,1) for forward directionand K(1820,2) for reverse direction</td></tr>
<tr><td>1821</td><td>P68400_p |P41236 -->P68400_p +P41236_p : K(1821,1) for forward direction</td></tr>
<tr><td>1822</td><td>P68400_p +O43395 <-->P68400_p |O43395 : K(1822,1) for forward directionand K(1822,2) for reverse direction</td></tr>
<tr><td>1823</td><td>P68400_p |O43395 -->P68400_p +O43395_p : K(1823,1) for forward direction</td></tr>
<tr><td>1824</td><td>P68400_p +Q15185 <-->P68400_p |Q15185 : K(1824,1) for forward directionand K(1824,2) for reverse direction</td></tr>
<tr><td>1825</td><td>P68400_p |Q15185 -->P68400_p +Q15185_p : K(1825,1) for forward direction</td></tr>
<tr><td>1826</td><td>P68400_p +P46060 <-->P68400_p |P46060 : K(1826,1) for forward directionand K(1826,2) for reverse direction</td></tr>
<tr><td>1827</td><td>P68400_p |P46060 -->P68400_p +P46060_p : K(1827,1) for forward direction</td></tr>
<tr><td>1828</td><td>P68400_p +Q9UBF6 <-->P68400_p |Q9UBF6 : K(1828,1) for forward directionand K(1828,2) for reverse direction</td></tr>
<tr><td>1829</td><td>P68400_p |Q9UBF6 -->P68400_p +Q9UBF6_p : K(1829,1) for forward direction</td></tr>
<tr><td>1830</td><td>P68400_p +P05455 <-->P68400_p |P05455 : K(1830,1) for forward directionand K(1830,2) for reverse direction</td></tr>
<tr><td>1831</td><td>P68400_p |P05455 -->P68400_p +P05455_p : K(1831,1) for forward direction</td></tr>
<tr><td>1832</td><td>P68400_p +Q04724 <-->P68400_p |Q04724 : K(1832,1) for forward directionand K(1832,2) for reverse direction</td></tr>
<tr><td>1833</td><td>P68400_p |Q04724 -->P68400_p +Q04724_p : K(1833,1) for forward direction</td></tr>
<tr><td>1834</td><td>P68400_p +Q712K3 <-->P68400_p |Q712K3 : K(1834,1) for forward directionand K(1834,2) for reverse direction</td></tr>
<tr><td>1835</td><td>P68400_p |Q712K3 -->P68400_p +Q712K3_p : K(1835,1) for forward direction</td></tr>
<tr><td>1836</td><td>P68400_p +P18887 <-->P68400_p |P18887 : K(1836,1) for forward directionand K(1836,2) for reverse direction</td></tr>
<tr><td>1837</td><td>P68400_p |P18887 -->P68400_p +P18887_p : K(1837,1) for forward direction</td></tr>
<tr><td>1838</td><td>P68400_p +P25490 <-->P68400_p |P25490 : K(1838,1) for forward directionand K(1838,2) for reverse direction</td></tr>
<tr><td>1839</td><td>P68400_p |P25490 -->P68400_p +P25490_p : K(1839,1) for forward direction</td></tr>
<tr><td>1840</td><td>P19784 +Q00613 <-->P19784 |Q00613 : K(1840,1) for forward directionand K(1840,2) for reverse direction</td></tr>
<tr><td>1841</td><td>P19784 |Q00613 -->P19784 +Q00613_p : K(1841,1) for forward direction</td></tr>
<tr><td>1842</td><td>P53355_p +Q14457 <-->P53355_p |Q14457 : K(1842,1) for forward directionand K(1842,2) for reverse direction</td></tr>
</table>

1843	P53355_p |Q14457 -->P53355_p +Q14457_p : K(1843,1) for forward direction
1844	P53355_p +P04637 <-->P53355_p |P04637 : K(1844,1) for forward directionand K(1844,2) for reverse direction
1845	P53355_p |P04637 -->P53355_p +P04637_p : K(1845,1) for forward direction
1846	Q13627_p +Q13627 <-->Q13627_p |Q13627 : K(1846,1) for forward directionand K(1846,2) for reverse direction
1847	Q13627_p |Q13627 -->Q13627_p +Q13627_p : K(1847,1) for forward direction
1848	Q13627_p +P04637 <-->Q13627_p |P04637 : K(1848,1) for forward directionand K(1848,2) for reverse direction
1849	Q13627_p |P04637 -->Q13627_p +P04637_p : K(1849,1) for forward direction
1850	P00533_p +P04083 <-->P00533_p |P04083 : K(1850,1) for forward directionand K(1850,2) for reverse direction
1851	P00533_p |P04083 -->P00533_p +P04083_p : K(1851,1) for forward direction
1852	P00533_p +P42566 <-->P00533_p |P42566 : K(1852,1) for forward directionand K(1852,2) for reverse direction
1853	P00533_p |P42566 -->P00533_p +P42566_p : K(1853,1) for forward direction
1854	P00533_p +P21860 <-->P00533_p |P21860 : K(1854,1) for forward directionand K(1854,2) for reverse direction
1855	P00533_p |P21860 -->P00533_p +P21860_p : K(1855,1) for forward direction
1856	P00533_p +Q13480 <-->P00533_p |Q13480 : K(1856,1) for forward directionand K(1856,2) for reverse direction
1857	P00533_p |Q13480 -->P00533_p +Q13480_p : K(1857,1) for forward direction
1858	P00533_p +P09211 <-->P00533_p |P09211 : K(1858,1) for forward directionand K(1858,2) for reverse direction
1859	P00533_p |P09211 -->P00533_p +P09211_p : K(1859,1) for forward direction
1860	P00533_p +O14964 <-->P00533_p |O14964 : K(1860,1) for forward directionand K(1860,2) for reverse direction
1861	P00533_p |O14964 -->P00533_p +O14964_p : K(1861,1) for forward direction
1862	P00533_p +P12004 <-->P00533_p |P12004 : K(1862,1) for forward directionand K(1862,2) for reverse direction
1863	P00533_p |P12004 -->P00533_p +P12004_p : K(1863,1) for forward direction
1864	P00533_p +O00750 <-->P00533_p |O00750 : K(1864,1) for forward directionand K(1864,2) for reverse direction
1865	P00533_p |O00750 -->P00533_p +O00750_p : K(1865,1) for forward direction
1866	P00533_p +Q05397 <-->P00533_p |Q05397 : K(1866,1) for forward directionand K(1866,2) for reverse direction
1867	P00533_p |Q05397 -->P00533_p +Q05397_p : K(1867,1) for forward direction
1868	P00533_p +P18031 <-->P00533_p |P18031 : K(1868,1) for forward directionand K(1868,2) for reverse direction
1869	P00533_p |P18031 -->P00533_p +P18031_p : K(1869,1) for forward direction
1870	P00533_p +P42224 <-->P00533_p |P42224 : K(1870,1) for forward directionand K(1870,2) for reverse direction
1871	P00533_p |P42224 -->P00533_p +P42224_p : K(1871,1) for forward direction
1872	P00533_p +P51692 <-->P00533_p |P51692 : K(1872,1) for forward directionand K(1872,2) for reverse direction
1873	P00533_p |P51692 -->P00533_p +P51692_p : K(1873,1) for forward direction
1874	P00533_p +P52735 <-->P00533_p |P52735 : K(1874,1) for forward directionand K(1874,2) for reverse direction
1875	P00533_p |P52735 -->P00533_p +P52735_p : K(1875,1) for forward direction
1876	P19525_p +P19525 <-->P19525_p |P19525 : K(1876,1) for forward directionand K(1876,2) for reverse direction
1877	P19525_p |P19525 -->P19525_p +P19525_p : K(1877,1) for forward direction
1878	P19525_p +Q15172 <-->P19525_p |Q15172 : K(1878,1) for forward directionand K(1878,2) for reverse direction
1879	P19525_p |Q15172 -->P19525_p +Q15172_p : K(1879,1) for forward direction
1880	P19525_p +P04637 <-->P19525_p |P04637 : K(1880,1) for forward directionand K(1880,2) for reverse direction
1881	P19525_p |P04637 -->P19525_p +P04637_p : K(1881,1) for forward direction
1882	P29317_p +P29317 <-->P29317_p |P29317 : K(1882,1) for forward directionand K(1882,2) for reverse direction
1883	P29317_p |P29317 -->P29317_p +P29317_p : K(1883,1) for forward direction
1884	P04626_p +Q96N67 <-->P04626_p |Q96N67 : K(1884,1) for forward directionand K(1884,2) for reverse direction
1885	P04626_p |Q96N67 -->P04626_p +Q96N67_p : K(1885,1) for forward direction

1886	P21802 +P62993 <-->P21802 |P62993 : K(1886,1) for forward directionand K(1886,2) for reverse direction
1887	P21802 |P62993 -->P21802 +P62993_p : K(1887,1) for forward direction
1888	P25098_p +P15311 <-->P25098_p |P15311 : K(1888,1) for forward directionand K(1888,2) for reverse direction
1889	P25098_p |P15311 -->P25098_p +P15311_p : K(1889,1) for forward direction
1890	P25098_p +P05387 <-->P25098_p |P05387 : K(1890,1) for forward directionand K(1890,2) for reverse direction
1891	P25098_p |P05387 -->P25098_p +P05387_p : K(1891,1) for forward direction
1892	P49840_p +P49840 <-->P49840_p |P49840 : K(1892,1) for forward directionand K(1892,2) for reverse direction
1893	P49840_p |P49840 -->P49840_p +P49840_p : K(1893,1) for forward direction
1894	P49840_p +P49815 <-->P49840_p |P49815 : K(1894,1) for forward directionand K(1894,2) for reverse direction
1895	P49840_p |P49815 -->P49840_p +P49815_p : K(1895,1) for forward direction
1896	P49841_p +O15169 <-->P49841_p |O15169 : K(1896,1) for forward directionand K(1896,2) for reverse direction
1897	P49841_p |O15169 -->P49841_p +O15169_p : K(1897,1) for forward direction
1898	P49841_p +Q07812 <-->P49841_p |Q07812 : K(1898,1) for forward directionand K(1898,2) for reverse direction
1899	P49841_p |Q07812 -->P49841_p +Q07812_p : K(1899,1) for forward direction
1900	P49841_p +P17676 <-->P49841_p |P17676 : K(1900,1) for forward directionand K(1900,2) for reverse direction
1901	P49841_p |P17676 -->P49841_p +P17676_p : K(1901,1) for forward direction
1902	P49841_p +P16401 <-->P49841_p |P16401 : K(1902,1) for forward directionand K(1902,2) for reverse direction
1903	P49841_p |P16401 -->P49841_p +P16401_p : K(1903,1) for forward direction
1904	P49841_p +Q92993 <-->P49841_p |Q92993 : K(1904,1) for forward directionand K(1904,2) for reverse direction
1905	P49841_p |Q92993 -->P49841_p +Q92993_p : K(1905,1) for forward direction
1906	P49841_p +Q04206 <-->P49841_p |Q04206 : K(1906,1) for forward directionand K(1906,2) for reverse direction
1907	P49841_p |Q04206 -->P49841_p +Q04206_p : K(1907,1) for forward direction
1908	P49841_p +Q96R06 <-->P49841_p |Q96R06 : K(1908,1) for forward directionand K(1908,2) for reverse direction
1909	P49841_p |Q96R06 -->P49841_p +Q96R06_p : K(1909,1) for forward direction
1910	P08069_p +P08069 <-->P08069_p |P08069 : K(1910,1) for forward directionand K(1910,2) for reverse direction
1911	P08069_p |P08069 -->P08069_p +P08069_p : K(1911,1) for forward direction
1912	P08069_p +P35568 <-->P08069_p |P35568 : K(1912,1) for forward directionand K(1912,2) for reverse direction
1913	P08069_p |P35568 -->P08069_p +P35568_p : K(1913,1) for forward direction
1914	P08069_p +Q9Y4H2 <-->P08069_p |Q9Y4H2 : K(1914,1) for forward directionand K(1914,2) for reverse direction
1915	P08069_p |Q9Y4H2 -->P08069_p +Q9Y4H2_p : K(1915,1) for forward direction
1916	P08069_p +O15530 <-->P08069_p |O15530 : K(1916,1) for forward directionand K(1916,2) for reverse direction
1917	P08069_p |O15530 -->P08069_p +O15530_p : K(1917,1) for forward direction
1918	P08069_p +O00443 <-->P08069_p |O00443 : K(1918,1) for forward directionand K(1918,2) for reverse direction
1919	P08069_p |O00443 -->P08069_p +O00443_p : K(1919,1) for forward direction
1920	Q13418_p +P31749 <-->Q13418_p |P31749 : K(1920,1) for forward directionand K(1920,2) for reverse direction
1921	Q13418_p |P31749 -->Q13418_p +P31749_p : K(1921,1) for forward direction
1922	Q13418_p +Q13765 <-->Q13418_p |Q13765 : K(1922,1) for forward directionand K(1922,2) for reverse direction
1923	Q13418_p |Q13765 -->Q13418_p +Q13765_p : K(1923,1) for forward direction
1924	Q13418_p +E9PAV3 <-->Q13418_p |E9PAV3 : K(1924,1) for forward directionand K(1924,2) for reverse direction
1925	Q13418_p |E9PAV3 -->Q13418_p +E9PAV3_p : K(1925,1) for forward direction
1926	P51617_p +P40763 <-->P51617_p |P40763 : K(1926,1) for forward directionand K(1926,2) for reverse direction
1927	P51617_p |P40763 -->P51617_p +P40763_p : K(1927,1) for forward direction
1928	Q02750_p +P27361 <-->Q02750_p |P27361 : K(1928,1) for forward directionand K(1928,2) for reverse direction

1929	Q02750_p |P27361 -->Q02750_p +P27361_p : K(1929,1) for forward direction
1930	P36507_p +P28482 <-->P36507_p |P28482 : K(1930,1) for forward directionand K(1930,2) for reverse direction
1931	P36507_p |P28482 -->P36507_p +P28482_p : K(1931,1) for forward direction
1932	P36507_p +P27361 <-->P36507_p |P27361 : K(1932,1) for forward directionand K(1932,2) for reverse direction
1933	P36507_p |P27361 -->P36507_p +P27361_p : K(1933,1) for forward direction
1934	P45985_p +P45984 <-->P45985_p |P45984 : K(1934,1) for forward directionand K(1934,2) for reverse direction
1935	P45985_p |P45984 -->P45985_p +P45984_p : K(1935,1) for forward direction
1936	P52564_p +P46108 <-->P52564_p |P46108 : K(1936,1) for forward directionand K(1936,2) for reverse direction
1937	P52564_p |P46108 -->P52564_p +P46108_p : K(1937,1) for forward direction
1938	P52564_p +O15264 <-->P52564_p |O15264 : K(1938,1) for forward directionand K(1938,2) for reverse direction
1939	P52564_p |O15264 -->P52564_p +O15264_p : K(1939,1) for forward direction
1940	P52564_p +P45984 <-->P52564_p |P45984 : K(1940,1) for forward directionand K(1940,2) for reverse direction
1941	P52564_p |P45984 -->P52564_p +P45984_p : K(1941,1) for forward direction
1942	O14733_p +P45983 <-->O14733_p |P45983 : K(1942,1) for forward directionand K(1942,2) for reverse direction
1943	O14733_p |P45983 -->O14733_p +P45983_p : K(1943,1) for forward direction
1944	O14733_p +P45984 <-->O14733_p |P45984 : K(1944,1) for forward directionand K(1944,2) for reverse direction
1945	O14733_p |P45984 -->O14733_p +P45984_p : K(1945,1) for forward direction
1946	Q9Y2U5 +Q02750 <-->Q9Y2U5 |Q02750 : K(1946,1) for forward directionand K(1946,2) for reverse direction
1947	Q9Y2U5 |Q02750 -->Q9Y2U5 +Q02750_p : K(1947,1) for forward direction
1948	Q9NYL2_p +Q9NYL2 <-->Q9NYL2_p |Q9NYL2 : K(1948,1) for forward directionand K(1948,2) for reverse direction
1949	Q9NYL2_p |Q9NYL2 -->Q9NYL2_p +Q9NYL2_p : K(1949,1) for forward direction
1950	Q99759_p +Q99759 <-->Q99759_p |Q99759 : K(1950,1) for forward directionand K(1950,2) for reverse direction
1951	Q99759_p |Q99759 -->Q99759_p +Q99759_p : K(1951,1) for forward direction
1952	P41279_p +Q02750 <-->P41279_p |Q02750 : K(1952,1) for forward directionand K(1952,2) for reverse direction
1953	P41279_p |Q02750 -->P41279_p +Q02750_p : K(1953,1) for forward direction
1954	P41279_p +P36507 <-->P41279_p |P36507 : K(1954,1) for forward directionand K(1954,2) for reverse direction
1955	P41279_p |P36507 -->P41279_p +P36507_p : K(1955,1) for forward direction
1956	P41279_p +P45985 <-->P41279_p |P45985 : K(1956,1) for forward directionand K(1956,2) for reverse direction
1957	P41279_p |P45985 -->P41279_p +P45985_p : K(1957,1) for forward direction
1958	P41279_p +P53350 <-->P41279_p |P53350 : K(1958,1) for forward directionand K(1958,2) for reverse direction
1959	P41279_p |P53350 -->P41279_p +P53350_p : K(1959,1) for forward direction
1960	P28482_p +Q8IZP0 <-->P28482_p |Q8IZP0 : K(1960,1) for forward directionand K(1960,2) for reverse direction
1961	P28482_p |Q8IZP0 -->P28482_p +Q8IZP0_p : K(1961,1) for forward direction
1962	P28482_p +Q92974 <-->P28482_p |Q92974 : K(1962,1) for forward directionand K(1962,2) for reverse direction
1963	P28482_p |Q92974 -->P28482_p +Q92974_p : K(1963,1) for forward direction
1964	P28482_p +P15336 <-->P28482_p |P15336 : K(1964,1) for forward directionand K(1964,2) for reverse direction
1965	P28482_p |P15336 -->P28482_p +P15336_p : K(1965,1) for forward direction
1966	P28482_p +P27708 <-->P28482_p |P27708 : K(1966,1) for forward directionand K(1966,2) for reverse direction
1967	P28482_p |P27708 -->P28482_p +P27708_p : K(1967,1) for forward direction
1968	P28482_p +P17655 <-->P28482_p |P17655 : K(1968,1) for forward directionand K(1968,2) for reverse direction
1969	P28482_p |P17655 -->P28482_p +P17655_p : K(1969,1) for forward direction
1970	P28482_p +P24941 <-->P28482_p |P24941 : K(1970,1) for forward directionand K(1970,2) for reverse direction
1971	P28482_p |P24941 -->P28482_p +P24941_p : K(1971,1) for forward direction

1972	P28482_p +P17676 <-->P28482_p |P17676 : K(1972,1) for forward directionand K(1972,2) for reverse direction
1973	P28482_p |P17676 -->P28482_p +P17676_p : K(1973,1) for forward direction
1974	P28482_p +P68400 <-->P28482_p |P68400 : K(1974,1) for forward directionand K(1974,2) for reverse direction
1975	P28482_p |P68400 -->P28482_p +P68400_p : K(1975,1) for forward direction
1976	P28482_p +Q14247 <-->P28482_p |Q14247 : K(1976,1) for forward directionand K(1976,2) for reverse direction
1977	P28482_p |Q14247 -->P28482_p +Q14247_p : K(1977,1) for forward direction
1978	P28482_p +P53355 <-->P28482_p |P53355 : K(1978,1) for forward directionand K(1978,2) for reverse direction
1979	P28482_p |P53355 -->P28482_p +P53355_p : K(1979,1) for forward direction
1980	P28482_p +P19525 <-->P28482_p |P19525 : K(1980,1) for forward directionand K(1980,2) for reverse direction
1981	P28482_p |P19525 -->P28482_p +P19525_p : K(1981,1) for forward direction
1982	P28482_p +Q09472 <-->P28482_p |Q09472 : K(1982,1) for forward directionand K(1982,2) for reverse direction
1983	P28482_p |Q09472 -->P28482_p +Q09472_p : K(1983,1) for forward direction
1984	P28482_p +Q9UPT5 <-->P28482_p |Q9UPT5 : K(1984,1) for forward directionand K(1984,2) for reverse direction
1985	P28482_p |Q9UPT5 -->P28482_p +Q9UPT5_p : K(1985,1) for forward direction
1986	P28482_p +P78347 <-->P28482_p |P78347 : K(1986,1) for forward directionand K(1986,2) for reverse direction
1987	P28482_p |P78347 -->P28482_p +P78347_p : K(1987,1) for forward direction
1988	P28482_p +P84243 <-->P28482_p |P84243 : K(1988,1) for forward directionand K(1988,2) for reverse direction
1989	P28482_p |P84243 -->P28482_p +P84243_p : K(1989,1) for forward direction
1990	P28482_p +P17535 <-->P28482_p |P17535 : K(1990,1) for forward directionand K(1990,2) for reverse direction
1991	P28482_p |P17535 -->P28482_p +P17535_p : K(1991,1) for forward direction
1992	P28482_p +Q07666 <-->P28482_p |Q07666 : K(1992,1) for forward directionand K(1992,2) for reverse direction
1993	P28482_p |Q07666 -->P28482_p +Q07666_p : K(1993,1) for forward direction
1994	P28482_p +P49137 <-->P28482_p |P49137 : K(1994,1) for forward directionand K(1994,2) for reverse direction
1995	P28482_p |P49137 -->P28482_p +P49137_p : K(1995,1) for forward direction
1996	P28482_p +Q07820 <-->P28482_p |Q07820 : K(1996,1) for forward directionand K(1996,2) for reverse direction
1997	P28482_p |Q07820 -->P28482_p +Q07820_p : K(1997,1) for forward direction
1998	P28482_p +Q15648 <-->P28482_p |Q15648 : K(1998,1) for forward directionand K(1998,2) for reverse direction
1999	P28482_p |Q15648 -->P28482_p +Q15648_p : K(1999,1) for forward direction
2000	P28482_p +P01106 <-->P28482_p |P01106 : K(2000,1) for forward directionand K(2000,2) for reverse direction
2001	P28482_p |P01106 -->P28482_p +P01106_p : K(2001,1) for forward direction
2002	P28482_p +P09874 <-->P28482_p |P09874 : K(2002,1) for forward directionand K(2002,2) for reverse direction
2003	P28482_p |P09874 -->P28482_p +P09874_p : K(2003,1) for forward direction
2004	P28482_p +P29590 <-->P28482_p |P29590 : K(2004,1) for forward directionand K(2004,2) for reverse direction
2005	P28482_p |P29590 -->P28482_p +P29590_p : K(2005,1) for forward direction
2006	P28482_p +P30041 <-->P28482_p |P30041 : K(2006,1) for forward directionand K(2006,2) for reverse direction
2007	P28482_p |P30041 -->P28482_p +P30041_p : K(2007,1) for forward direction
2008	P28482_p +P51812 <-->P28482_p |P51812 : K(2008,1) for forward directionand K(2008,2) for reverse direction
2009	P28482_p |P51812 -->P28482_p +P51812_p : K(2009,1) for forward direction
2010	P28482_p +P19634 <-->P28482_p |P19634 : K(2010,1) for forward directionand K(2010,2) for reverse direction
2011	P28482_p |P19634 -->P28482_p +P19634_p : K(2011,1) for forward direction
2012	P28482_p +P08047 <-->P28482_p |P08047 : K(2012,1) for forward directionand K(2012,2) for reverse direction
2013	P28482_p |P08047 -->P28482_p +P08047_p : K(2013,1) for forward direction
2014	P28482_p +Q02447 <-->P28482_p |Q02447 : K(2014,1) for forward directionand K(2014,2) for reverse direction

2015	P28482_p |Q02447 -->P28482_p +Q02447_p : K(2015,1) for forward direction
2016	P28482_p +Q9NRA0 <-->P28482_p |Q9NRA0 : K(2016,1) for forward directionand K(2016,2) for reverse direction
2017	P28482_p |Q9NRA0 -->P28482_p +Q9NRA0_p : K(2017,1) for forward direction
2018	P28482_p +Q13586 <-->P28482_p |Q13586 : K(2018,1) for forward directionand K(2018,2) for reverse direction
2019	P28482_p |Q13586 -->P28482_p +Q13586_p : K(2019,1) for forward direction
2020	P28482_p +P12270 <-->P28482_p |P12270 : K(2020,1) for forward directionand K(2020,2) for reverse direction
2021	P28482_p |P12270 -->P28482_p +P12270_p : K(2021,1) for forward direction
2022	O15264_p +P04637 <-->O15264_p |P04637 : K(2022,1) for forward directionand K(2022,2) for reverse direction
2023	O15264_p |P04637 -->O15264_p +P04637_p : K(2023,1) for forward direction
2024	Q16539_p +P15336 <-->Q16539_p |P15336 : K(2024,1) for forward directionand K(2024,2) for reverse direction
2025	Q16539_p |P15336 -->Q16539_p +P15336_p : K(2025,1) for forward direction
2026	Q16539_p +Q9UIG0 <-->Q16539_p |Q9UIG0 : K(2026,1) for forward directionand K(2026,2) for reverse direction
2027	Q16539_p |Q9UIG0 -->Q16539_p +Q9UIG0_p : K(2027,1) for forward direction
2028	Q16539_p +Q15075 <-->Q16539_p |Q15075 : K(2028,1) for forward directionand K(2028,2) for reverse direction
2029	Q16539_p |Q15075 -->Q16539_p +Q15075_p : K(2029,1) for forward direction
2030	Q16539_p +Q15717 <-->Q16539_p |Q15717 : K(2030,1) for forward directionand K(2030,2) for reverse direction
2031	Q16539_p |Q15717 -->Q16539_p +Q15717_p : K(2031,1) for forward direction
2032	Q16539_p +P42566 <-->Q16539_p |P42566 : K(2032,1) for forward directionand K(2032,2) for reverse direction
2033	Q16539_p |P42566 -->Q16539_p +P42566_p : K(2033,1) for forward direction
2034	Q16539_p +O43524 <-->Q16539_p |O43524 : K(2034,1) for forward directionand K(2034,2) for reverse direction
2035	Q16539_p |O43524 -->Q16539_p +O43524_p : K(2035,1) for forward direction
2036	Q16539_p +P84243 <-->Q16539_p |P84243 : K(2036,1) for forward directionand K(2036,2) for reverse direction
2037	Q16539_p |P84243 -->Q16539_p +P84243_p : K(2037,1) for forward direction
2038	Q16539_p +P68431 <-->Q16539_p |P68431 : K(2038,1) for forward directionand K(2038,2) for reverse direction
2039	Q16539_p |P68431 -->Q16539_p +P68431_p : K(2039,1) for forward direction
2040	Q16539_p +O75676 <-->Q16539_p |O75676 : K(2040,1) for forward directionand K(2040,2) for reverse direction
2041	Q16539_p |O75676 -->Q16539_p +O75676_p : K(2041,1) for forward direction
2042	Q16539_p +P19634 <-->Q16539_p |P19634 : K(2042,1) for forward directionand K(2042,2) for reverse direction
2043	Q16539_p |P19634 -->Q16539_p +P19634_p : K(2043,1) for forward direction
2044	Q16539_p +P40763 <-->Q16539_p |P40763 : K(2044,1) for forward directionand K(2044,2) for reverse direction
2045	Q16539_p |P40763 -->Q16539_p +P40763_p : K(2045,1) for forward direction
2046	Q16539_p +P04637 <-->Q16539_p |P04637 : K(2046,1) for forward directionand K(2046,2) for reverse direction
2047	Q16539_p |P04637 -->Q16539_p +P04637_p : K(2047,1) for forward direction
2048	P27361_p +Q8IZP0 <-->P27361_p |Q8IZP0 : K(2048,1) for forward directionand K(2048,2) for reverse direction
2049	P27361_p |Q8IZP0 -->P27361_p +Q8IZP0_p : K(2049,1) for forward direction
2050	P27361_p +P78536 <-->P27361_p |P78536 : K(2050,1) for forward directionand K(2050,2) for reverse direction
2051	P27361_p |P78536 -->P27361_p +P78536_p : K(2051,1) for forward direction
2052	P27361_p +Q92974 <-->P27361_p |Q92974 : K(2052,1) for forward directionand K(2052,2) for reverse direction
2053	P27361_p |Q92974 -->P27361_p +Q92974_p : K(2053,1) for forward direction
2054	P27361_p +P15336 <-->P27361_p |P15336 : K(2054,1) for forward directionand K(2054,2) for reverse direction
2055	P27361_p |P15336 -->P27361_p +P15336_p : K(2055,1) for forward direction
2056	P27361_p +Q9UIG0 <-->P27361_p |Q9UIG0 : K(2056,1) for forward directionand K(2056,2) for reverse direction
2057	P27361_p |Q9UIG0 -->P27361_p +Q9UIG0_p : K(2057,1) for forward direction

2058	P27361_p +P27708 <-->P27361_p |P27708 : K(2058,1) for forward directionand K(2058,2) for reverse direction
2059	P27361_p |P27708 -->P27361_p +P27708_p : K(2059,1) for forward direction
2060	P27361_p +P17655 <-->P27361_p |P17655 : K(2060,1) for forward directionand K(2060,2) for reverse direction
2061	P27361_p |P17655 -->P27361_p +P17655_p : K(2061,1) for forward direction
2062	P27361_p +P17676 <-->P27361_p |P17676 : K(2062,1) for forward directionand K(2062,2) for reverse direction
2063	P27361_p |P17676 -->P27361_p +P17676_p : K(2063,1) for forward direction
2064	P27361_p +Q14247 <-->P27361_p |Q14247 : K(2064,1) for forward directionand K(2064,2) for reverse direction
2065	P27361_p |Q14247 -->P27361_p +Q14247_p : K(2065,1) for forward direction
2066	P27361_p +P01100 <-->P27361_p |P01100 : K(2066,1) for forward directionand K(2066,2) for reverse direction
2067	P27361_p |P01100 -->P27361_p +P01100_p : K(2067,1) for forward direction
2068	P27361_p +P78347 <-->P27361_p |P78347 : K(2068,1) for forward directionand K(2068,2) for reverse direction
2069	P27361_p |P78347 -->P27361_p +P78347_p : K(2069,1) for forward direction
2070	P27361_p +P84243 <-->P27361_p |P84243 : K(2070,1) for forward directionand K(2070,2) for reverse direction
2071	P27361_p |P84243 -->P27361_p +P84243_p : K(2071,1) for forward direction
2072	P27361_p +Q9UBN7 <-->P27361_p |Q9UBN7 : K(2072,1) for forward directionand K(2072,2) for reverse direction
2073	P27361_p |Q9UBN7 -->P27361_p +Q9UBN7_p : K(2073,1) for forward direction
2074	P27361_p +P05412 <-->P27361_p |P05412 : K(2074,1) for forward directionand K(2074,2) for reverse direction
2075	P27361_p |P05412 -->P27361_p +P05412_p : K(2075,1) for forward direction
2076	P27361_p +P17535 <-->P27361_p |P17535 : K(2076,1) for forward directionand K(2076,2) for reverse direction
2077	P27361_p |P17535 -->P27361_p +P17535_p : K(2077,1) for forward direction
2078	P27361_p +Q15046 <-->P27361_p |Q15046 : K(2078,1) for forward directionand K(2078,2) for reverse direction
2079	P27361_p |Q15046 -->P27361_p +Q15046_p : K(2079,1) for forward direction
2080	P27361_p +P49137 <-->P27361_p |P49137 : K(2080,1) for forward directionand K(2080,2) for reverse direction
2081	P27361_p |P49137 -->P27361_p +P49137_p : K(2081,1) for forward direction
2082	P27361_p +Q07820 <-->P27361_p |Q07820 : K(2082,1) for forward directionand K(2082,2) for reverse direction
2083	P27361_p |Q07820 -->P27361_p +Q07820_p : K(2083,1) for forward direction
2084	P27361_p +Q15648 <-->P27361_p |Q15648 : K(2084,1) for forward directionand K(2084,2) for reverse direction
2085	P27361_p |Q15648 -->P27361_p +Q15648_p : K(2085,1) for forward direction
2086	P27361_p +P05976 <-->P27361_p |P05976 : K(2086,1) for forward directionand K(2086,2) for reverse direction
2087	P27361_p |P05976 -->P27361_p +P05976_p : K(2087,1) for forward direction
2088	P27361_p +P47712 <-->P27361_p |P47712 : K(2088,1) for forward directionand K(2088,2) for reverse direction
2089	P27361_p |P47712 -->P27361_p +P47712_p : K(2089,1) for forward direction
2090	P27361_p +P29590 <-->P27361_p |P29590 : K(2090,1) for forward directionand K(2090,2) for reverse direction
2091	P27361_p |P29590 -->P27361_p +P29590_p : K(2091,1) for forward direction
2092	P27361_p +P51812 <-->P27361_p |P51812 : K(2092,1) for forward directionand K(2092,2) for reverse direction
2093	P27361_p |P51812 -->P27361_p +P51812_p : K(2093,1) for forward direction
2094	P27361_p +O75676 <-->P27361_p |O75676 : K(2094,1) for forward directionand K(2094,2) for reverse direction
2095	P27361_p |O75676 -->P27361_p +O75676_p : K(2095,1) for forward direction
2096	P27361_p +Q9NYV6 <-->P27361_p |Q9NYV6 : K(2096,1) for forward directionand K(2096,2) for reverse direction
2097	P27361_p |Q9NYV6 -->P27361_p +Q9NYV6_p : K(2097,1) for forward direction
2098	P27361_p +P19793 <-->P27361_p |P19793 : K(2098,1) for forward directionand K(2098,2) for reverse direction
2099	P27361_p |P19793 -->P27361_p +P19793_p : K(2099,1) for forward direction
2100	P27361_p +P08047 <-->P27361_p |P08047 : K(2100,1) for forward directionand K(2100,2) for reverse direction

2101	P27361_p |P08047 -->P27361_p +P08047_p : K(2101,1) for forward direction
2102	P27361_p +Q02447 <-->P27361_p |Q02447 : K(2102,1) for forward directionand K(2102,2) for reverse direction
2103	P27361_p |Q02447 -->P27361_p +Q02447_p : K(2103,1) for forward direction
2104	P27361_p +Q9NRA0 <-->P27361_p |Q9NRA0 : K(2104,1) for forward directionand K(2104,2) for reverse direction
2105	P27361_p |Q9NRA0 -->P27361_p +Q9NRA0_p : K(2105,1) for forward direction
2106	P27361_p +P40763 <-->P27361_p |P40763 : K(2106,1) for forward directionand K(2106,2) for reverse direction
2107	P27361_p |P40763 -->P27361_p +P40763_p : K(2107,1) for forward direction
2108	P27361_p +P04637 <-->P27361_p |P04637 : K(2108,1) for forward directionand K(2108,2) for reverse direction
2109	P27361_p |P04637 -->P27361_p +P04637_p : K(2109,1) for forward direction
2110	P45983_p +Q06481 <-->P45983_p |Q06481 : K(2110,1) for forward directionand K(2110,2) for reverse direction
2111	P45983_p |Q06481 -->P45983_p +Q06481_p : K(2111,1) for forward direction
2112	P45983_p +P05067 <-->P45983_p |P05067 : K(2112,1) for forward directionand K(2112,2) for reverse direction
2113	P45983_p |P05067 -->P45983_p +P05067_p : K(2113,1) for forward direction
2114	P45983_p +P15336 <-->P45983_p |P15336 : K(2114,1) for forward directionand K(2114,2) for reverse direction
2115	P45983_p |P15336 -->P45983_p +P15336_p : K(2115,1) for forward direction
2116	P45983_p +P16104 <-->P45983_p |P16104 : K(2116,1) for forward directionand K(2116,2) for reverse direction
2117	P45983_p |P16104 -->P45983_p +P16104_p : K(2117,1) for forward direction
2118	P45983_p +P84243 <-->P45983_p |P84243 : K(2118,1) for forward directionand K(2118,2) for reverse direction
2119	P45983_p |P84243 -->P45983_p +P84243_p : K(2119,1) for forward direction
2120	P45983_p +P61978 <-->P45983_p |P61978 : K(2120,1) for forward directionand K(2120,2) for reverse direction
2121	P45983_p |P61978 -->P45983_p +P61978_p : K(2121,1) for forward direction
2122	P45983_p +Q14653 <-->P45983_p |Q14653 : K(2122,1) for forward directionand K(2122,2) for reverse direction
2123	P45983_p |Q14653 -->P45983_p +Q14653_p : K(2123,1) for forward direction
2124	P45983_p +Q96J02 <-->P45983_p |Q96J02 : K(2124,1) for forward directionand K(2124,2) for reverse direction
2125	P45983_p |Q96J02 -->P45983_p +Q96J02_p : K(2125,1) for forward direction
2126	P45983_p +P17535 <-->P45983_p |P17535 : K(2126,1) for forward directionand K(2126,2) for reverse direction
2127	P45983_p |P17535 -->P45983_p +P17535_p : K(2127,1) for forward direction
2128	P45983_p +P45984 <-->P45983_p |P45984 : K(2128,1) for forward directionand K(2128,2) for reverse direction
2129	P45983_p |P45984 -->P45983_p +P45984_p : K(2129,1) for forward direction
2130	P45983_p +Q07820 <-->P45983_p |Q07820 : K(2130,1) for forward directionand K(2130,2) for reverse direction
2131	P45983_p |Q07820 -->P45983_p +Q07820_p : K(2131,1) for forward direction
2132	P45983_p +Q96EB6 <-->P45983_p |Q96EB6 : K(2132,1) for forward directionand K(2132,2) for reverse direction
2133	P45983_p |Q96EB6 -->P45983_p +Q96EB6_p : K(2133,1) for forward direction
2134	P45983_p +P08047 <-->P45983_p |P08047 : K(2134,1) for forward directionand K(2134,2) for reverse direction
2135	P45983_p |P08047 -->P45983_p +P08047_p : K(2135,1) for forward direction
2136	P45983_p +Q13043 <-->P45983_p |Q13043 : K(2136,1) for forward directionand K(2136,2) for reverse direction
2137	P45983_p |Q13043 -->P45983_p +Q13043_p : K(2137,1) for forward direction
2138	P45983_p +P04637 <-->P45983_p |P04637 : K(2138,1) for forward directionand K(2138,2) for reverse direction
2139	P45983_p |P04637 -->P45983_p +P04637_p : K(2139,1) for forward direction
2140	P45984_p +P15336 <-->P45984_p |P15336 : K(2140,1) for forward directionand K(2140,2) for reverse direction
2141	P45984_p |P15336 -->P45984_p +P15336_p : K(2141,1) for forward direction
2142	P45984_p +Q9UIG0 <-->P45984_p |Q9UIG0 : K(2142,1) for forward directionand K(2142,2) for reverse direction
2143	P45984_p |Q9UIG0 -->P45984_p +Q9UIG0_p : K(2143,1) for forward direction

2144	P45984_p +P35222 <-->P45984_p |P35222 : K(2144,1) for forward directionand K(2144,2) for reverse direction
2145	P45984_p |P35222 -->P45984_p +P35222_p : K(2145,1) for forward direction
2146	P45984_p +P16104 <-->P45984_p |P16104 : K(2146,1) for forward directionand K(2146,2) for reverse direction
2147	P45984_p |P16104 -->P45984_p +P16104_p : K(2147,1) for forward direction
2148	P45984_p +P84243 <-->P45984_p |P84243 : K(2148,1) for forward directionand K(2148,2) for reverse direction
2149	P45984_p |P84243 -->P45984_p +P84243_p : K(2149,1) for forward direction
2150	P45984_p +P05412 <-->P45984_p |P05412 : K(2150,1) for forward directionand K(2150,2) for reverse direction
2151	P45984_p |P05412 -->P45984_p +P05412_p : K(2151,1) for forward direction
2152	P45984_p +P01106 <-->P45984_p |P01106 : K(2152,1) for forward directionand K(2152,2) for reverse direction
2153	P45984_p |P01106 -->P45984_p +P01106_p : K(2153,1) for forward direction
2154	P45984_p +P40763 <-->P45984_p |P40763 : K(2154,1) for forward directionand K(2154,2) for reverse direction
2155	P45984_p |P40763 -->P45984_p +P40763_p : K(2155,1) for forward direction
2156	P45984_p +P04637 <-->P45984_p |P04637 : K(2156,1) for forward directionand K(2156,2) for reverse direction
2157	P45984_p |P04637 -->P45984_p +P04637_p : K(2157,1) for forward direction
2158	P49137_p +Q9NY61 <-->P49137_p |Q9NY61 : K(2158,1) for forward directionand K(2158,2) for reverse direction
2159	P49137_p |Q9NY61 -->P49137_p +Q9NY61_p : K(2159,1) for forward direction
2160	P49137_p +Q9UKV8 <-->P49137_p |Q9UKV8 : K(2160,1) for forward directionand K(2160,2) for reverse direction
2161	P49137_p |Q9UKV8 -->P49137_p +Q9UKV8_p : K(2161,1) for forward direction
2162	P49137_p +O95816 <-->P49137_p |O95816 : K(2162,1) for forward directionand K(2162,2) for reverse direction
2163	P49137_p |O95816 -->P49137_p +O95816_p : K(2163,1) for forward direction
2164	P49137_p +P16220 <-->P49137_p |P16220 : K(2164,1) for forward directionand K(2164,2) for reverse direction
2165	P49137_p |P16220 -->P49137_p +P16220_p : K(2165,1) for forward direction
2166	P49137_p +Q15717 <-->P49137_p |Q15717 : K(2166,1) for forward directionand K(2166,2) for reverse direction
2167	P49137_p |Q15717 -->P49137_p +Q15717_p : K(2167,1) for forward direction
2168	P49137_p +O75925 <-->P49137_p |O75925 : K(2168,1) for forward directionand K(2168,2) for reverse direction
2169	P49137_p |O75925 -->P49137_p +O75925_p : K(2169,1) for forward direction
2170	P49137_p +P53350 <-->P49137_p |P53350 : K(2170,1) for forward directionand K(2170,2) for reverse direction
2171	P49137_p |P53350 -->P49137_p +P53350_p : K(2171,1) for forward direction
2172	Q7KZI7 +Q8TEW0 <-->Q7KZI7 |Q8TEW0 : K(2172,1) for forward directionand K(2172,2) for reverse direction
2173	Q7KZI7 |Q8TEW0 -->Q7KZI7 +Q8TEW0_p : K(2173,1) for forward direction
2174	Q7KZI7 +P46939 <-->Q7KZI7 |P46939 : K(2174,1) for forward directionand K(2174,2) for reverse direction
2175	Q7KZI7 |P46939 -->Q7KZI7 +P46939_p : K(2175,1) for forward direction
2176	P08581_p +P08581 <-->P08581_p |P08581 : K(2176,1) for forward directionand K(2176,2) for reverse direction
2177	P08581_p |P08581 -->P08581_p +P08581_p : K(2177,1) for forward direction
2178	P08581_p +Q05397 <-->P08581_p |Q05397 : K(2178,1) for forward directionand K(2178,2) for reverse direction
2179	P08581_p |Q05397 -->P08581_p +Q05397_p : K(2179,1) for forward direction
2180	P42345_p +Q9H1K1 <-->P42345_p |Q9H1K1 : K(2180,1) for forward directionand K(2180,2) for reverse direction
2181	P42345_p |Q9H1K1 -->P42345_p +Q9H1K1_p : K(2181,1) for forward direction
2182	P42345_p +P40763 <-->P42345_p |P40763 : K(2182,1) for forward directionand K(2182,2) for reverse direction
2183	P42345_p |P40763 -->P42345_p +P40763_p : K(2183,1) for forward direction
2184	Q15746_p +P14649 <-->Q15746_p |P14649 : K(2184,1) for forward directionand K(2184,2) for reverse direction
2185	Q15746_p |P14649 -->Q15746_p +P14649_p : K(2185,1) for forward direction
2186	Q15746_p +P24844 <-->Q15746_p |P24844 : K(2186,1) for forward directionand K(2186,2) for reverse direction

<table>
<tr><td>2187</td><td>Q15746_p |P24844 -->Q15746_p +P24844_p : K(2187,1) for forward direction</td></tr>
<tr><td>2188</td><td>Q8TD19_p +Q8TD19 <-->Q8TD19_p |Q8TD19 : K(2188,1) for forward directionand K(2188,2) for reverse direction</td></tr>
<tr><td>2189</td><td>Q8TD19_p |Q8TD19 -->Q8TD19_p +Q8TD19_p : K(2189,1) for forward direction</td></tr>
<tr><td>2190</td><td>O95747_p +P55011 <-->O95747_p |P55011 : K(2190,1) for forward directionand K(2190,2) for reverse direction</td></tr>
<tr><td>2191</td><td>O95747_p |P55011 -->O95747_p +P55011_p : K(2191,1) for forward direction</td></tr>
<tr><td>2192</td><td>Q13153_p +P10398 <-->Q13153_p |P10398 : K(2192,1) for forward directionand K(2192,2) for reverse direction</td></tr>
<tr><td>2193</td><td>Q13153_p |P10398 -->Q13153_p +P10398_p : K(2193,1) for forward direction</td></tr>
<tr><td>2194</td><td>Q13153_p +O15143 <-->Q13153_p |O15143 : K(2194,1) for forward directionand K(2194,2) for reverse direction</td></tr>
<tr><td>2195</td><td>Q13153_p |O15143 -->Q13153_p +O15143_p : K(2195,1) for forward direction</td></tr>
<tr><td>2196</td><td>Q13153_p +P35222 <-->Q13153_p |P35222 : K(2196,1) for forward directionand K(2196,2) for reverse direction</td></tr>
<tr><td>2197</td><td>Q13153_p |P35222 -->Q13153_p +P35222_p : K(2197,1) for forward direction</td></tr>
<tr><td>2198</td><td>Q13153_p +Q14247 <-->Q13153_p |Q14247 : K(2198,1) for forward directionand K(2198,2) for reverse direction</td></tr>
<tr><td>2199</td><td>Q13153_p |Q14247 -->Q13153_p +Q14247_p : K(2199,1) for forward direction</td></tr>
<tr><td>2200</td><td>Q13153_p +P78545 <-->Q13153_p |P78545 : K(2200,1) for forward directionand K(2200,2) for reverse direction</td></tr>
<tr><td>2201</td><td>Q13153_p |P78545 -->Q13153_p +P78545_p : K(2201,1) for forward direction</td></tr>
<tr><td>2202</td><td>Q13153_p +P21333 <-->Q13153_p |P21333 : K(2202,1) for forward directionand K(2202,2) for reverse direction</td></tr>
<tr><td>2203</td><td>Q13153_p |P21333 -->Q13153_p +P21333_p : K(2203,1) for forward direction</td></tr>
<tr><td>2204</td><td>Q13153_p +P68431 <-->Q13153_p |P68431 : K(2204,1) for forward directionand K(2204,2) for reverse direction</td></tr>
<tr><td>2205</td><td>Q13153_p |P68431 -->Q13153_p +P68431_p : K(2205,1) for forward direction</td></tr>
<tr><td>2206</td><td>Q13153_p +Q13418 <-->Q13153_p |Q13418 : K(2206,1) for forward directionand K(2206,2) for reverse direction</td></tr>
<tr><td>2207</td><td>Q13153_p |Q13418 -->Q13153_p +Q13418_p : K(2207,1) for forward direction</td></tr>
<tr><td>2208</td><td>Q13153_p +Q9UQ80 <-->Q13153_p |Q9UQ80 : K(2208,1) for forward directionand K(2208,2) for reverse direction</td></tr>
<tr><td>2209</td><td>Q13153_p |Q9UQ80 -->Q13153_p +Q9UQ80_p : K(2209,1) for forward direction</td></tr>
<tr><td>2210</td><td>Q13153_p +P36871 <-->Q13153_p |P36871 : K(2210,1) for forward directionand K(2210,2) for reverse direction</td></tr>
<tr><td>2211</td><td>Q13153_p |P36871 -->Q13153_p +P36871_p : K(2211,1) for forward direction</td></tr>
<tr><td>2212</td><td>Q13153_p +P53350 <-->Q13153_p |P53350 : K(2212,1) for forward directionand K(2212,2) for reverse direction</td></tr>
<tr><td>2213</td><td>Q13153_p |P53350 -->Q13153_p +P53350_p : K(2213,1) for forward direction</td></tr>
<tr><td>2214</td><td>Q13153_p +Q99426 <-->Q13153_p |Q99426 : K(2214,1) for forward directionand K(2214,2) for reverse direction</td></tr>
<tr><td>2215</td><td>Q13153_p |Q99426 -->Q13153_p +Q99426_p : K(2215,1) for forward direction</td></tr>
<tr><td>2216</td><td>Q13177_p +P05412 <-->Q13177_p |P05412 : K(2216,1) for forward directionand K(2216,2) for reverse direction</td></tr>
<tr><td>2217</td><td>Q13177_p |P05412 -->Q13177_p +P05412_p : K(2217,1) for forward direction</td></tr>
<tr><td>2218</td><td>Q13177_p +Q13177 <-->Q13177_p |Q13177 : K(2218,1) for forward directionand K(2218,2) for reverse direction</td></tr>
<tr><td>2219</td><td>Q13177_p |Q13177 -->Q13177_p +Q13177_p : K(2219,1) for forward direction</td></tr>
<tr><td>2220</td><td>O96013_p +P35222 <-->O96013_p |P35222 : K(2220,1) for forward directionand K(2220,2) for reverse direction</td></tr>
<tr><td>2221</td><td>O96013_p |P35222 -->O96013_p +P35222_p : K(2221,1) for forward direction</td></tr>
<tr><td>2222</td><td>O96013_p +P18084 <-->O96013_p |P18084 : K(2222,1) for forward directionand K(2222,2) for reverse direction</td></tr>
<tr><td>2223</td><td>O96013_p |P18084 -->O96013_p +P18084_p : K(2223,1) for forward direction</td></tr>
<tr><td>2224</td><td>O96013_p +O96013 <-->O96013_p |O96013 : K(2224,1) for forward directionand K(2224,2) for reverse direction</td></tr>
<tr><td>2225</td><td>O96013_p |O96013 -->O96013_p +O96013_p : K(2225,1) for forward direction</td></tr>
<tr><td>2226</td><td>Q96KB5_p +P68431 <-->Q96KB5_p |P68431 : K(2226,1) for forward directionand K(2226,2) for reverse direction</td></tr>
<tr><td>2227</td><td>Q96KB5_p |P68431 -->Q96KB5_p +P68431_p : K(2227,1) for forward direction</td></tr>
<tr><td>2228</td><td>Q96KB5_p +Q06830 <-->Q96KB5_p |Q06830 : K(2228,1) for forward directionand K(2228,2) for reverse direction</td></tr>
<tr><td>2229</td><td>Q96KB5_p |Q06830 -->Q96KB5_p +Q06830_p : K(2229,1) for forward direction</td></tr>
</table>

2230	O15530_p +P31749 <-->O15530_p |P31749 : K(2230,1) for forward directionand K(2230,2) for reverse direction
2231	O15530_p |P31749 -->O15530_p +P31749_p : K(2231,1) for forward direction
2232	O15530_p +P36507 <-->O15530_p |P36507 : K(2232,1) for forward directionand K(2232,2) for reverse direction
2233	O15530_p |P36507 -->O15530_p +P36507_p : K(2233,1) for forward direction
2234	O15530_p +Q16512 <-->O15530_p |Q16512 : K(2234,1) for forward directionand K(2234,2) for reverse direction
2235	O15530_p |Q16512 -->O15530_p +Q16512_p : K(2235,1) for forward direction
2236	O15530_p +Q16513 <-->O15530_p |Q16513 : K(2236,1) for forward directionand K(2236,2) for reverse direction
2237	O15530_p |Q16513 -->O15530_p +Q16513_p : K(2237,1) for forward direction
2238	O15530_p +P17252 <-->O15530_p |P17252 : K(2238,1) for forward directionand K(2238,2) for reverse direction
2239	O15530_p |P17252 -->O15530_p +P17252_p : K(2239,1) for forward direction
2240	O15530_p +P51812 <-->O15530_p |P51812 : K(2240,1) for forward directionand K(2240,2) for reverse direction
2241	O15530_p |P51812 -->O15530_p +P51812_p : K(2241,1) for forward direction
2242	O15530_p +Q9HBY8 <-->O15530_p |Q9HBY8 : K(2242,1) for forward directionand K(2242,2) for reverse direction
2243	O15530_p |Q9HBY8 -->O15530_p +Q9HBY8_p : K(2243,1) for forward direction
2244	Q6A1A2 +P31749 <-->Q6A1A2 |P31749 : K(2244,1) for forward directionand K(2244,2) for reverse direction
2245	Q6A1A2 |P31749 -->Q6A1A2 +P31749_p : K(2245,1) for forward direction
2246	P14618 +P40763 <-->P14618 |P40763 : K(2246,1) for forward directionand K(2246,2) for reverse direction
2247	P14618 |P40763 -->P14618 +P40763_p : K(2247,1) for forward direction
2248	Q16512_p +Q9NYL2 <-->Q16512_p |Q9NYL2 : K(2248,1) for forward directionand K(2248,2) for reverse direction
2249	Q16512_p |Q9NYL2 -->Q16512_p +Q9NYL2_p : K(2249,1) for forward direction
2250	Q16512_p +Q16539 <-->Q16512_p |Q16539 : K(2250,1) for forward directionand K(2250,2) for reverse direction
2251	Q16512_p |Q16539 -->Q16512_p +Q16539_p : K(2251,1) for forward direction
2252	Q16512_p +P36871 <-->Q16512_p |P36871 : K(2252,1) for forward directionand K(2252,2) for reverse direction
2253	Q16512_p |P36871 -->Q16512_p +P36871_p : K(2253,1) for forward direction
2254	P53350_p +Q9H1A4 <-->P53350_p |Q9H1A4 : K(2254,1) for forward directionand K(2254,2) for reverse direction
2255	P53350_p |Q9H1A4 -->P53350_p +Q9H1A4_p : K(2255,1) for forward direction
2256	P53350_p +O60566 <-->P53350_p |O60566 : K(2256,1) for forward directionand K(2256,2) for reverse direction
2257	P53350_p |O60566 -->P53350_p +O60566_p : K(2257,1) for forward direction
2258	P53350_p +Q53EZ4 <-->P53350_p |Q53EZ4 : K(2258,1) for forward directionand K(2258,2) for reverse direction
2259	P53350_p |Q53EZ4 -->P53350_p +Q53EZ4_p : K(2259,1) for forward direction
2260	P53350_p +P30622 <-->P53350_p |P30622 : K(2260,1) for forward directionand K(2260,2) for reverse direction
2261	P53350_p |P30622 -->P53350_p +P30622_p : K(2261,1) for forward direction
2262	P53350_p +Q14203 <-->P53350_p |Q14203 : K(2262,1) for forward directionand K(2262,2) for reverse direction
2263	P53350_p |Q14203 -->P53350_p +Q14203_p : K(2263,1) for forward direction
2264	P53350_p +O14641 <-->P53350_p |O14641 : K(2264,1) for forward directionand K(2264,2) for reverse direction
2265	P53350_p |O14641 -->P53350_p +O14641_p : K(2265,1) for forward direction
2266	P53350_p +O95251 <-->P53350_p |O95251 : K(2266,1) for forward directionand K(2266,2) for reverse direction
2267	P53350_p |O95251 -->P53350_p +O95251_p : K(2267,1) for forward direction
2268	P53350_p +P06748 <-->P53350_p |P06748 : K(2268,1) for forward directionand K(2268,2) for reverse direction
2269	P53350_p |P06748 -->P53350_p +P06748_p : K(2269,1) for forward direction
2270	P53350_p +Q96CV9 <-->P53350_p |Q96CV9 : K(2270,1) for forward directionand K(2270,2) for reverse direction
2271	P53350_p |Q96CV9 -->P53350_p +Q96CV9_p : K(2271,1) for forward direction
2272	P53350_p +Q13526 <-->P53350_p |Q13526 : K(2272,1) for forward directionand K(2272,2) for reverse direction

2273	P53350_p |Q13526 -->P53350_p +Q13526_p : K(2273,1) for forward direction
2274	P53350_p +Q9H0H5 <-->P53350_p |Q9H0H5 : K(2274,1) for forward directionand K(2274,2) for reverse direction
2275	P53350_p |Q9H0H5 -->P53350_p +Q9H0H5_p : K(2275,1) for forward direction
2276	P53350_p +P62826 <-->P53350_p |P62826 : K(2276,1) for forward directionand K(2276,2) for reverse direction
2277	P53350_p |P62826 -->P53350_p +P62826_p : K(2277,1) for forward direction
2278	P53350_p +P11388 <-->P53350_p |P11388 : K(2278,1) for forward directionand K(2278,2) for reverse direction
2279	P53350_p |P11388 -->P53350_p +P11388_p : K(2279,1) for forward direction
2280	P53350_p +Q9H2D6 <-->P53350_p |Q9H2D6 : K(2280,1) for forward directionand K(2280,2) for reverse direction
2281	P53350_p |Q9H2D6 -->P53350_p +Q9H2D6_p : K(2281,1) for forward direction
2282	P53350_p +P08670 <-->P53350_p |P08670 : K(2282,1) for forward directionand K(2282,2) for reverse direction
2283	P53350_p |P08670 -->P53350_p +P08670_p : K(2283,1) for forward direction
2284	P53350_p +P25490 <-->P53350_p |P25490 : K(2284,1) for forward directionand K(2284,2) for reverse direction
2285	P53350_p |P25490 -->P53350_p +P25490_p : K(2285,1) for forward direction
2286	Q13131_p +Q12778 <-->Q13131_p |Q12778 : K(2286,1) for forward directionand K(2286,2) for reverse direction
2287	Q13131_p |Q12778 -->Q13131_p +Q12778_p : K(2287,1) for forward direction
2288	Q13131_p +Q9H0B6 <-->Q13131_p |Q9H0B6 : K(2288,1) for forward directionand K(2288,2) for reverse direction
2289	Q13131_p |Q9H0B6 -->Q13131_p +Q9H0B6_p : K(2289,1) for forward direction
2290	Q13131_p +P52292 <-->Q13131_p |P52292 : K(2290,1) for forward directionand K(2290,2) for reverse direction
2291	Q13131_p |P52292 -->Q13131_p +P52292_p : K(2291,1) for forward direction
2292	Q13131_p +Q9Y478 <-->Q13131_p |Q9Y478 : K(2292,1) for forward directionand K(2292,2) for reverse direction
2293	Q13131_p |Q9Y478 -->Q13131_p +Q9Y478_p : K(2293,1) for forward direction
2294	Q13131_p +P04637 <-->Q13131_p |P04637 : K(2294,1) for forward directionand K(2294,2) for reverse direction
2295	Q13131_p |P04637 -->Q13131_p +P04637_p : K(2295,1) for forward direction
2296	P17612 +Q16790 <-->P17612 |Q16790 : K(2296,1) for forward directionand K(2296,2) for reverse direction
2297	P17612 |Q16790 -->P17612 +Q16790_p : K(2297,1) for forward direction
2298	P17612 +Q13185 <-->P17612 |Q13185 : K(2298,1) for forward directionand K(2298,2) for reverse direction
2299	P17612 |Q13185 -->P17612 +Q13185_p : K(2299,1) for forward direction
2300	P17612 +P16070 <-->P17612 |P16070 : K(2300,1) for forward directionand K(2300,2) for reverse direction
2301	P17612 |P16070 -->P17612 +P16070_p : K(2301,1) for forward direction
2302	P17612 +Q8N752 <-->P17612 |Q8N752 : K(2302,1) for forward directionand K(2302,2) for reverse direction
2303	P17612 |Q8N752 -->P17612 +Q8N752_p : K(2303,1) for forward direction
2304	P17612 +Q9NP97 <-->P17612 |Q9NP97 : K(2304,1) for forward directionand K(2304,2) for reverse direction
2305	P17612 |Q9NP97 -->P17612 +Q9NP97_p : K(2305,1) for forward direction
2306	P17612 +P04626 <-->P17612 |P04626 : K(2306,1) for forward directionand K(2306,2) for reverse direction
2307	P17612 |P04626 -->P17612 +P04626_p : K(2307,1) for forward direction
2308	P17612 +P21333 <-->P17612 |P21333 : K(2308,1) for forward directionand K(2308,2) for reverse direction
2309	P17612 |P21333 -->P17612 +P21333_p : K(2309,1) for forward direction
2310	P17612 +P84243 <-->P17612 |P84243 : K(2310,1) for forward directionand K(2310,2) for reverse direction
2311	P17612 |P84243 -->P17612 +P84243_p : K(2311,1) for forward direction
2312	P17612 +Q14103 <-->P17612 |Q14103 : K(2312,1) for forward directionand K(2312,2) for reverse direction
2313	P17612 |Q14103 -->P17612 +Q14103_p : K(2313,1) for forward direction
2314	P17612 +Q00613 <-->P17612 |Q00613 : K(2314,1) for forward directionand K(2314,2) for reverse direction
2315	P17612 |Q00613 -->P17612 +Q00613_p : K(2315,1) for forward direction

2316	P17612 +Q9NXR1 <-->P17612 |Q9NXR1 : K(2316,1) for forward directionand K(2316,2) for reverse direction
2317	P17612 |Q9NXR1 -->P17612 +Q9NXR1_p : K(2317,1) for forward direction
2318	P17612 +P19838 <-->P17612 |P19838 : K(2318,1) for forward directionand K(2318,2) for reverse direction
2319	P17612 |P19838 -->P17612 +P19838_p : K(2319,1) for forward direction
2320	P17612 +Q14978 <-->P17612 |Q14978 : K(2320,1) for forward directionand K(2320,2) for reverse direction
2321	P17612 |Q14978 -->P17612 +Q14978_p : K(2321,1) for forward direction
2322	P17612 +Q13526 <-->P17612 |Q13526 : K(2322,1) for forward directionand K(2322,2) for reverse direction
2323	P17612 |Q13526 -->P17612 +Q13526_p : K(2323,1) for forward direction
2324	P17612 +Q14738 <-->P17612 |Q14738 : K(2324,1) for forward directionand K(2324,2) for reverse direction
2325	P17612 |Q14738 -->P17612 +Q14738_p : K(2325,1) for forward direction
2326	P17612 +P61224 <-->P17612 |P61224 : K(2326,1) for forward directionand K(2326,2) for reverse direction
2327	P17612 |P61224 -->P17612 +P61224_p : K(2327,1) for forward direction
2328	P17612 +Q04206 <-->P17612 |Q04206 : K(2328,1) for forward directionand K(2328,2) for reverse direction
2329	P17612 |Q04206 -->P17612 +Q04206_p : K(2329,1) for forward direction
2330	P17612 +P48436 <-->P17612 |P48436 : K(2330,1) for forward directionand K(2330,2) for reverse direction
2331	P17612 |P48436 -->P17612 +P48436_p : K(2331,1) for forward direction
2332	P17612 +P12931 <-->P17612 |P12931 : K(2332,1) for forward directionand K(2332,2) for reverse direction
2333	P17612 |P12931 -->P17612 +P12931_p : K(2333,1) for forward direction
2334	P17612 +Q07955 <-->P17612 |Q07955 : K(2334,1) for forward directionand K(2334,2) for reverse direction
2335	P17612 |Q07955 -->P17612 +Q07955_p : K(2335,1) for forward direction
2336	P17612 +Q00059 <-->P17612 |Q00059 : K(2336,1) for forward directionand K(2336,2) for reverse direction
2337	P17612 |Q00059 -->P17612 +Q00059_p : K(2337,1) for forward direction
2338	P17612 +P63104 <-->P17612 |P63104 : K(2338,1) for forward directionand K(2338,2) for reverse direction
2339	P17612 |P63104 -->P17612 +P63104_p : K(2339,1) for forward direction
2340	P17252_p +P35611 <-->P17252_p |P35611 : K(2340,1) for forward directionand K(2340,2) for reverse direction
2341	P17252_p |P35611 -->P17252_p +P35611_p : K(2341,1) for forward direction
2342	P17252_p +P04083 <-->P17252_p |P04083 : K(2342,1) for forward directionand K(2342,2) for reverse direction
2343	P17252_p |P04083 -->P17252_p +P04083_p : K(2343,1) for forward direction
2344	P17252_p +P00533 <-->P17252_p |P00533 : K(2344,1) for forward directionand K(2344,2) for reverse direction
2345	P17252_p |P00533 -->P17252_p +P00533_p : K(2345,1) for forward direction
2346	P17252_p +P15311 <-->P17252_p |P15311 : K(2346,1) for forward directionand K(2346,2) for reverse direction
2347	P17252_p |P15311 -->P17252_p +P15311_p : K(2347,1) for forward direction
2348	P17252_p +P46940 <-->P17252_p |P46940 : K(2348,1) for forward directionand K(2348,2) for reverse direction
2349	P17252_p |P46940 -->P17252_p +P46940_p : K(2349,1) for forward direction
2350	P17252_p +P35568 <-->P17252_p |P35568 : K(2350,1) for forward directionand K(2350,2) for reverse direction
2351	P17252_p |P35568 -->P17252_p +P35568_p : K(2351,1) for forward direction
2352	P17252_p +Q07954 <-->P17252_p |Q07954 : K(2352,1) for forward directionand K(2352,2) for reverse direction
2353	P17252_p |Q07954 -->P17252_p +Q07954_p : K(2353,1) for forward direction
2354	P17252_p +Q8NFA2 <-->P17252_p |Q8NFA2 : K(2354,1) for forward directionand K(2354,2) for reverse direction
2355	P17252_p |Q8NFA2 -->P17252_p +Q8NFA2_p : K(2355,1) for forward direction
2356	P17252_p +P47712 <-->P17252_p |P47712 : K(2356,1) for forward directionand K(2356,2) for reverse direction
2357	P17252_p |P47712 -->P17252_p +P47712_p : K(2357,1) for forward direction
2358	P17252_p +Q13393 <-->P17252_p |Q13393 : K(2358,1) for forward directionand K(2358,2) for reverse direction

2359	P17252_p |Q13393 -->P17252_p +Q13393_p : K(2359,1) for forward direction
2360	P17252_p +P17252 <-->P17252_p |P17252 : K(2360,1) for forward directionand K(2360,2) for reverse direction
2361	P17252_p |P17252 -->P17252_p +P17252_p : K(2361,1) for forward direction
2362	P17252_p +Q13976 <-->P17252_p |Q13976 : K(2362,1) for forward directionand K(2362,2) for reverse direction
2363	P17252_p |Q13976 -->P17252_p +Q13976_p : K(2363,1) for forward direction
2364	P17252_p +Q13769 <-->P17252_p |Q13769 : K(2364,1) for forward directionand K(2364,2) for reverse direction
2365	P17252_p |Q13769 -->P17252_p +Q13769_p : K(2365,1) for forward direction
2366	Q05655_p +O95817 <-->Q05655_p |O95817 : K(2366,1) for forward directionand K(2366,2) for reverse direction
2367	Q05655_p |O95817 -->Q05655_p +O95817_p : K(2367,1) for forward direction
2368	Q05655_p +P51398 <-->Q05655_p |P51398 : K(2368,1) for forward directionand K(2368,2) for reverse direction
2369	Q05655_p |P51398 -->Q05655_p +P51398_p : K(2369,1) for forward direction
2370	Q05655_p +P07108 <-->Q05655_p |P07108 : K(2370,1) for forward directionand K(2370,2) for reverse direction
2371	Q05655_p |P07108 -->Q05655_p +P07108_p : K(2371,1) for forward direction
2372	Q05655_p +O00429 <-->Q05655_p |O00429 : K(2372,1) for forward directionand K(2372,2) for reverse direction
2373	Q05655_p |O00429 -->Q05655_p +O00429_p : K(2373,1) for forward direction
2374	Q05655_p +Q15717 <-->Q05655_p |Q15717 : K(2374,1) for forward directionand K(2374,2) for reverse direction
2375	Q05655_p |Q15717 -->Q05655_p +Q15717_p : K(2375,1) for forward direction
2376	Q05655_p +P11413 <-->Q05655_p |P11413 : K(2376,1) for forward directionand K(2376,2) for reverse direction
2377	Q05655_p |P11413 -->Q05655_p +P11413_p : K(2377,1) for forward direction
2378	Q05655_p +P68431 <-->Q05655_p |P68431 : K(2378,1) for forward directionand K(2378,2) for reverse direction
2379	Q05655_p |P68431 -->Q05655_p +P68431_p : K(2379,1) for forward direction
2380	Q05655_p +P40189 <-->Q05655_p |P40189 : K(2380,1) for forward directionand K(2380,2) for reverse direction
2381	Q05655_p |P40189 -->Q05655_p +P40189_p : K(2381,1) for forward direction
2382	Q05655_p +P45983 <-->Q05655_p |P45983 : K(2382,1) for forward directionand K(2382,2) for reverse direction
2383	Q05655_p |P45983 -->Q05655_p +P45983_p : K(2383,1) for forward direction
2384	Q05655_p +Q9UQ80 <-->Q05655_p |Q9UQ80 : K(2384,1) for forward directionand K(2384,2) for reverse direction
2385	Q05655_p |Q9UQ80 -->Q05655_p +Q9UQ80_p : K(2385,1) for forward direction
2386	Q05655_p +P30086 <-->Q05655_p |P30086 : K(2386,1) for forward directionand K(2386,2) for reverse direction
2387	Q05655_p |P30086 -->Q05655_p +P30086_p : K(2387,1) for forward direction
2388	Q05655_p +O15162 <-->Q05655_p |O15162 : K(2388,1) for forward directionand K(2388,2) for reverse direction
2389	Q05655_p |O15162 -->Q05655_p +O15162_p : K(2389,1) for forward direction
2390	Q05655_p +P23396 <-->Q05655_p |P23396 : K(2390,1) for forward directionand K(2390,2) for reverse direction
2391	Q05655_p |P23396 -->Q05655_p +P23396_p : K(2391,1) for forward direction
2392	Q05655_p +P42224 <-->Q05655_p |P42224 : K(2392,1) for forward directionand K(2392,2) for reverse direction
2393	Q05655_p |P42224 -->Q05655_p +P42224_p : K(2393,1) for forward direction
2394	Q05655_p +P40763 <-->Q05655_p |P40763 : K(2394,1) for forward directionand K(2394,2) for reverse direction
2395	Q05655_p |P40763 -->Q05655_p +P40763_p : K(2395,1) for forward direction
2396	Q05655_p +P04637 <-->Q05655_p |P04637 : K(2396,1) for forward directionand K(2396,2) for reverse direction
2397	Q05655_p |P04637 -->Q05655_p +P04637_p : K(2397,1) for forward direction
2398	P41743_p +Q9H8V3 <-->P41743_p |Q9H8V3 : K(2398,1) for forward directionand K(2398,2) for reverse direction
2399	P41743_p |Q9H8V3 -->P41743_p +Q9H8V3_p : K(2399,1) for forward direction
2400	P41743_p +P15311 <-->P41743_p |P15311 : K(2400,1) for forward directionand K(2400,2) for reverse direction
2401	P41743_p |P15311 -->P41743_p +P15311_p : K(2401,1) for forward direction

2402	Q9BZL6_p +Q9UBF8 <-->Q9BZL6_p |Q9UBF8 : K(2402,1) for forward directionand K(2402,2) for reverse direction
2403	Q9BZL6_p |Q9UBF8 -->Q9BZL6_p +Q9UBF8_p : K(2403,1) for forward direction
2404	Q9BZL6_p +Q9BZL6 <-->Q9BZL6_p |Q9BZL6 : K(2404,1) for forward directionand K(2404,2) for reverse direction
2405	Q9BZL6_p |Q9BZL6 -->Q9BZL6_p +Q9BZL6_p : K(2405,1) for forward direction
2406	P78527_p +P42575 <-->P78527_p |P42575 : K(2406,1) for forward directionand K(2406,2) for reverse direction
2407	P78527_p |P42575 -->P78527_p +P42575_p : K(2407,1) for forward direction
2408	P78527_p +O96017 <-->P78527_p |O96017 : K(2408,1) for forward directionand K(2408,2) for reverse direction
2409	P78527_p |O96017 -->P78527_p +O96017_p : K(2409,1) for forward direction
2410	P78527_p +P16104 <-->P78527_p |P16104 : K(2410,1) for forward directionand K(2410,2) for reverse direction
2411	P78527_p |P16104 -->P78527_p +P16104_p : K(2411,1) for forward direction
2412	P78527_p +Q00839 <-->P78527_p |Q00839 : K(2412,1) for forward directionand K(2412,2) for reverse direction
2413	P78527_p |Q00839 -->P78527_p +Q00839_p : K(2413,1) for forward direction
2414	P78527_p +Q14653 <-->P78527_p |Q14653 : K(2414,1) for forward directionand K(2414,2) for reverse direction
2415	P78527_p |Q14653 -->P78527_p +Q14653_p : K(2415,1) for forward direction
2416	P78527_p +Q96T60 <-->P78527_p |Q96T60 : K(2416,1) for forward directionand K(2416,2) for reverse direction
2417	P78527_p |Q96T60 -->P78527_p +Q96T60_p : K(2417,1) for forward direction
2418	P78527_p +P04637 <-->P78527_p |P04637 : K(2418,1) for forward directionand K(2418,2) for reverse direction
2419	P78527_p |P04637 -->P78527_p +P04637_p : K(2419,1) for forward direction
2420	Q13976_p +Q9Y613 <-->Q13976_p |Q9Y613 : K(2420,1) for forward directionand K(2420,2) for reverse direction
2421	Q13976_p |Q9Y613 -->Q13976_p +Q9Y613_p : K(2421,1) for forward direction
2422	Q13976_p +P78347 <-->Q13976_p |P78347 : K(2422,1) for forward directionand K(2422,2) for reverse direction
2423	Q13976_p |P78347 -->Q13976_p +P78347_p : K(2423,1) for forward direction
2424	Q13976_p +Q13976 <-->Q13976_p |Q13976 : K(2424,1) for forward directionand K(2424,2) for reverse direction
2425	Q13976_p |Q13976 -->Q13976_p +Q13976_p : K(2425,1) for forward direction
2426	Q05397_p +Q05397 <-->Q05397_p |Q05397 : K(2426,1) for forward directionand K(2426,2) for reverse direction
2427	Q05397_p |Q05397 -->Q05397_p +Q05397_p : K(2427,1) for forward direction
2428	Q13464 +P35611 <-->Q13464 |P35611 : K(2428,1) for forward directionand K(2428,2) for reverse direction
2429	Q13464 |P35611 -->Q13464 +P35611_p : K(2429,1) for forward direction
2430	Q13464 +P15311 <-->Q13464 |P15311 : K(2430,1) for forward directionand K(2430,2) for reverse direction
2431	Q13464 |P15311 -->Q13464 +P15311_p : K(2431,1) for forward direction
2432	Q13464 +Q9Y613 <-->Q13464 |Q9Y613 : K(2432,1) for forward directionand K(2432,2) for reverse direction
2433	Q13464 |Q9Y613 -->Q13464 +Q9Y613_p : K(2433,1) for forward direction
2434	Q13464 +O14950 <-->Q13464 |O14950 : K(2434,1) for forward directionand K(2434,2) for reverse direction
2435	Q13464 |O14950 -->Q13464 +O14950_p : K(2435,1) for forward direction
2436	Q13464 +P24844 <-->Q13464 |P24844 : K(2436,1) for forward directionand K(2436,2) for reverse direction
2437	Q13464 |P24844 -->Q13464 +P24844_p : K(2437,1) for forward direction
2438	Q13464 +P60484 <-->Q13464 |P60484 : K(2438,1) for forward directionand K(2438,2) for reverse direction
2439	Q13464 |P60484 -->Q13464 +P60484_p : K(2439,1) for forward direction
2440	Q13464 +P61587 <-->Q13464 |P61587 : K(2440,1) for forward directionand K(2440,2) for reverse direction
2441	Q13464 |P61587 -->Q13464 +P61587_p : K(2441,1) for forward direction
2442	Q13464 +P48436 <-->Q13464 |P48436 : K(2442,1) for forward directionand K(2442,2) for reverse direction
2443	Q13464 |P48436 -->Q13464 +P48436_p : K(2443,1) for forward direction
2444	O75116 +Q16555 <-->O75116 |Q16555 : K(2444,1) for forward directionand K(2444,2) for reverse direction

2445	O75116 |Q16555 -->O75116 +Q16555_p : K(2445,1) for forward direction
2446	Q01973 +P12931 <-->Q01973 |P12931 : K(2446,1) for forward directionand K(2446,2) for reverse direction
2447	Q01973 |P12931 -->Q01973 +P12931_p : K(2447,1) for forward direction
2448	Q15418_p +P78371 <-->Q15418_p |P78371 : K(2448,1) for forward directionand K(2448,2) for reverse direction
2449	Q15418_p |P78371 -->Q15418_p +P78371_p : K(2449,1) for forward direction
2450	Q15418_p +P23588 <-->Q15418_p |P23588 : K(2450,1) for forward directionand K(2450,2) for reverse direction
2451	Q15418_p |P23588 -->Q15418_p +P23588_p : K(2451,1) for forward direction
2452	Q15418_p +P21333 <-->Q15418_p |P21333 : K(2452,1) for forward directionand K(2452,2) for reverse direction
2453	Q15418_p |P21333 -->Q15418_p +P21333_p : K(2453,1) for forward direction
2454	Q15418_p +P01100 <-->Q15418_p |P01100 : K(2454,1) for forward directionand K(2454,2) for reverse direction
2455	Q15418_p |P01100 -->Q15418_p +P01100_p : K(2455,1) for forward direction
2456	Q15418_p +P84243 <-->Q15418_p |P84243 : K(2456,1) for forward directionand K(2456,2) for reverse direction
2457	Q15418_p |P84243 -->Q15418_p +P84243_p : K(2457,1) for forward direction
2458	Q15418_p +P62753 <-->Q15418_p |P62753 : K(2458,1) for forward directionand K(2458,2) for reverse direction
2459	Q15418_p |P62753 -->Q15418_p +P62753_p : K(2459,1) for forward direction
2460	Q15418_p +Q8N122 <-->Q15418_p |Q8N122 : K(2460,1) for forward directionand K(2460,2) for reverse direction
2461	Q15418_p |Q8N122 -->Q15418_p +Q8N122_p : K(2461,1) for forward direction
2462	Q15418_p +P19634 <-->Q15418_p |P19634 : K(2462,1) for forward directionand K(2462,2) for reverse direction
2463	Q15418_p |P19634 -->Q15418_p +P19634_p : K(2463,1) for forward direction
2464	Q15418_p +Q8IX03 <-->Q15418_p |Q8IX03 : K(2464,1) for forward directionand K(2464,2) for reverse direction
2465	Q15418_p |Q8IX03 -->Q15418_p +Q8IX03_p : K(2465,1) for forward direction
2466	Q15418_p +P67809 <-->Q15418_p |P67809 : K(2466,1) for forward directionand K(2466,2) for reverse direction
2467	Q15418_p |P67809 -->Q15418_p +P67809_p : K(2467,1) for forward direction
2468	P51812_p +P19525 <-->P51812_p |P19525 : K(2468,1) for forward directionand K(2468,2) for reverse direction
2469	P51812_p |P19525 -->P51812_p +P19525_p : K(2469,1) for forward direction
2470	P51812_p +P84243 <-->P51812_p |P84243 : K(2470,1) for forward directionand K(2470,2) for reverse direction
2471	P51812_p |P84243 -->P51812_p +P84243_p : K(2471,1) for forward direction
2472	P51812_p +P33778 <-->P51812_p |P33778 : K(2472,1) for forward directionand K(2472,2) for reverse direction
2473	P51812_p |P33778 -->P51812_p +P33778_p : K(2473,1) for forward direction
2474	P51812_p +P68431 <-->P51812_p |P68431 : K(2474,1) for forward directionand K(2474,2) for reverse direction
2475	P51812_p |P68431 -->P51812_p +P68431_p : K(2475,1) for forward direction
2476	P51812_p +Q8IX03 <-->P51812_p |Q8IX03 : K(2476,1) for forward directionand K(2476,2) for reverse direction
2477	P51812_p |Q8IX03 -->P51812_p +Q8IX03_p : K(2477,1) for forward direction
2478	P51812_p +P67809 <-->P51812_p |P67809 : K(2478,1) for forward directionand K(2478,2) for reverse direction
2479	P51812_p |P67809 -->P51812_p +P67809_p : K(2479,1) for forward direction
2480	O75676_p +P18846 <-->O75676_p |P18846 : K(2480,1) for forward directionand K(2480,2) for reverse direction
2481	O75676_p |P18846 -->O75676_p +P18846_p : K(2481,1) for forward direction
2482	O75676_p +P16220 <-->O75676_p |P16220 : K(2482,1) for forward directionand K(2482,2) for reverse direction
2483	O75676_p |P16220 -->O75676_p +P16220_p : K(2483,1) for forward direction
2484	O75676_p +P01100 <-->O75676_p |P01100 : K(2484,1) for forward directionand K(2484,2) for reverse direction
2485	O75676_p |P01100 -->O75676_p +P01100_p : K(2485,1) for forward direction
2486	O75676_p +Q04206 <-->O75676_p |Q04206 : K(2486,1) for forward directionand K(2486,2) for reverse direction
2487	O75676_p |Q04206 -->O75676_p +Q04206_p : K(2487,1) for forward direction

2488	O75676_p +O75676 <-->O75676_p |O75676 : K(2488,1) for forward directionand K(2488,2) for reverse direction
2489	O75676_p |O75676 -->O75676_p +O75676_p : K(2489,1) for forward direction
2490	Q96Q15 +P04637 <-->Q96Q15 |P04637 : K(2490,1) for forward directionand K(2490,2) for reverse direction
2491	Q96Q15 |P04637 -->Q96Q15 +P04637_p : K(2491,1) for forward direction
2492	Q96Q15 +Q92900 <-->Q96Q15 |Q92900 : K(2492,1) for forward directionand K(2492,2) for reverse direction
2493	Q96Q15 |Q92900 -->Q96Q15 +Q92900_p : K(2493,1) for forward direction
2494	P12931_p +P07355 <-->P12931_p |P07355 : K(2494,1) for forward directionand K(2494,2) for reverse direction
2495	P12931_p |P07355 -->P12931_p +P07355_p : K(2495,1) for forward direction
2496	P12931_p +P10398 <-->P12931_p |P10398 : K(2496,1) for forward directionand K(2496,2) for reverse direction
2497	P12931_p |P10398 -->P12931_p +P10398_p : K(2497,1) for forward direction
2498	P12931_p +Q9NRY4 <-->P12931_p |Q9NRY4 : K(2498,1) for forward directionand K(2498,2) for reverse direction
2499	P12931_p |Q9NRY4 -->P12931_p +Q9NRY4_p : K(2499,1) for forward direction
2500	P12931_p +P60953 <-->P12931_p |P60953 : K(2500,1) for forward directionand K(2500,2) for reverse direction
2501	P12931_p |P60953 -->P12931_p +P60953_p : K(2501,1) for forward direction
2502	P12931_p +Q00610 <-->P12931_p |Q00610 : K(2502,1) for forward directionand K(2502,2) for reverse direction
2503	P12931_p |Q00610 -->P12931_p +Q00610_p : K(2503,1) for forward direction
2504	P12931_p +P00533 <-->P12931_p |P00533 : K(2504,1) for forward directionand K(2504,2) for reverse direction
2505	P12931_p |P00533 -->P12931_p +P00533_p : K(2505,1) for forward direction
2506	P12931_p +P06733 <-->P12931_p |P06733 : K(2506,1) for forward directionand K(2506,2) for reverse direction
2507	P12931_p |P06733 -->P12931_p +P06733_p : K(2507,1) for forward direction
2508	P12931_p +P15311 <-->P12931_p |P15311 : K(2508,1) for forward directionand K(2508,2) for reverse direction
2509	P12931_p |P15311 -->P12931_p +P15311_p : K(2509,1) for forward direction
2510	P12931_p +Q13480 <-->P12931_p |Q13480 : K(2510,1) for forward directionand K(2510,2) for reverse direction
2511	P12931_p |Q13480 -->P12931_p +Q13480_p : K(2511,1) for forward direction
2512	P12931_p +P06396 <-->P12931_p |P06396 : K(2512,1) for forward directionand K(2512,2) for reverse direction
2513	P12931_p |P06396 -->P12931_p +P06396_p : K(2513,1) for forward direction
2514	P12931_p +P08238 <-->P12931_p |P08238 : K(2514,1) for forward directionand K(2514,2) for reverse direction
2515	P12931_p |P08238 -->P12931_p +P08238_p : K(2515,1) for forward direction
2516	P12931_p +P08069 <-->P12931_p |P08069 : K(2516,1) for forward directionand K(2516,2) for reverse direction
2517	P12931_p |P08069 -->P12931_p +P08069_p : K(2517,1) for forward direction
2518	P12931_p +O15357 <-->P12931_p |O15357 : K(2518,1) for forward directionand K(2518,2) for reverse direction
2519	P12931_p |O15357 -->P12931_p +O15357_p : K(2519,1) for forward direction
2520	P12931_p +P01116 <-->P12931_p |P01116 : K(2520,1) for forward directionand K(2520,2) for reverse direction
2521	P12931_p |P01116 -->P12931_p +P01116_p : K(2521,1) for forward direction
2522	P12931_p +Q07954 <-->P12931_p |Q07954 : K(2522,1) for forward directionand K(2522,2) for reverse direction
2523	P12931_p |Q07954 -->P12931_p +Q07954_p : K(2523,1) for forward direction
2524	P12931_p +O95297 <-->P12931_p |O95297 : K(2524,1) for forward directionand K(2524,2) for reverse direction
2525	P12931_p |O95297 -->P12931_p +O95297_p : K(2525,1) for forward direction
2526	P12931_p +Q15746 <-->P12931_p |Q15746 : K(2526,1) for forward directionand K(2526,2) for reverse direction
2527	P12931_p |Q15746 -->P12931_p +Q15746_p : K(2527,1) for forward direction
2528	P12931_p +Q13177 <-->P12931_p |Q13177 : K(2528,1) for forward directionand K(2528,2) for reverse direction
2529	P12931_p |Q13177 -->P12931_p +Q13177_p : K(2529,1) for forward direction
2530	P12931_p +O15530 <-->P12931_p |O15530 : K(2530,1) for forward directionand K(2530,2) for reverse direction

2531	P12931_p |O15530 -->P12931_p +O15530_p : K(2531,1) for forward direction
2532	P12931_p +P41743 <-->P12931_p |P41743 : K(2532,1) for forward directionand K(2532,2) for reverse direction
2533	P12931_p |P41743 -->P12931_p +P41743_p : K(2533,1) for forward direction
2534	P12931_p +Q05397 <-->P12931_p |Q05397 : K(2534,1) for forward directionand K(2534,2) for reverse direction
2535	P12931_p |Q05397 -->P12931_p +Q05397_p : K(2535,1) for forward direction
2536	P12931_p +P29350 <-->P12931_p |P29350 : K(2536,1) for forward directionand K(2536,2) for reverse direction
2537	P12931_p |P29350 -->P12931_p +P29350_p : K(2537,1) for forward direction
2538	P12931_p +P63000 <-->P12931_p |P63000 : K(2538,1) for forward directionand K(2538,2) for reverse direction
2539	P12931_p |P63000 -->P12931_p +P63000_p : K(2539,1) for forward direction
2540	P12931_p +P63244 <-->P12931_p |P63244 : K(2540,1) for forward directionand K(2540,2) for reverse direction
2541	P12931_p |P63244 -->P12931_p +P63244_p : K(2541,1) for forward direction
2542	P12931_p +Q13813 <-->P12931_p |Q13813 : K(2542,1) for forward directionand K(2542,2) for reverse direction
2543	P12931_p |Q13813 -->P12931_p +Q13813_p : K(2543,1) for forward direction
2544	P12931_p +P51692 <-->P12931_p |P51692 : K(2544,1) for forward directionand K(2544,2) for reverse direction
2545	P12931_p |P51692 -->P12931_p +P51692_p : K(2545,1) for forward direction
2546	P12931_p +P37173 <-->P12931_p |P37173 : K(2546,1) for forward directionand K(2546,2) for reverse direction
2547	P12931_p |P37173 -->P12931_p +P37173_p : K(2547,1) for forward direction
2548	P12931_p +Q68CZ2 <-->P12931_p |Q68CZ2 : K(2548,1) for forward directionand K(2548,2) for reverse direction
2549	P12931_p |Q68CZ2 -->P12931_p +Q68CZ2_p : K(2549,1) for forward direction
2550	Q96SB4_p +Q96SB4 <-->Q96SB4_p |Q96SB4 : K(2550,1) for forward directionand K(2550,2) for reverse direction
2551	Q96SB4_p |Q96SB4 -->Q96SB4_p +Q96SB4_p : K(2551,1) for forward direction
2552	Q96SB4_p +Q07955 <-->Q96SB4_p |Q07955 : K(2552,1) for forward directionand K(2552,2) for reverse direction
2553	Q96SB4_p |Q07955 -->Q96SB4_p +Q07955_p : K(2553,1) for forward direction
2554	P78362_p +Q9UKV3 <-->P78362_p |Q9UKV3 : K(2554,1) for forward directionand K(2554,2) for reverse direction
2555	P78362_p |Q9UKV3 -->P78362_p +Q9UKV3_p : K(2555,1) for forward direction
2556	Q9Y6E0_p +Q9Y6E0 <-->Q9Y6E0_p |Q9Y6E0 : K(2556,1) for forward directionand K(2556,2) for reverse direction
2557	Q9Y6E0_p |Q9Y6E0 -->Q9Y6E0_p +Q9Y6E0_p : K(2557,1) for forward direction
2558	Q9Y6E0_p +Q15208 <-->Q9Y6E0_p |Q15208 : K(2558,1) for forward directionand K(2558,2) for reverse direction
2559	Q9Y6E0_p |Q15208 -->Q9Y6E0_p +Q15208_p : K(2559,1) for forward direction
2560	Q9Y6E0_p +Q9Y2H1 <-->Q9Y6E0_p |Q9Y2H1 : K(2560,1) for forward directionand K(2560,2) for reverse direction
2561	Q9Y6E0_p |Q9Y2H1 -->Q9Y6E0_p +Q9Y2H1_p : K(2561,1) for forward direction
2562	Q9P289 +P15311 <-->Q9P289 |P15311 : K(2562,1) for forward directionand K(2562,2) for reverse direction
2563	Q9P289 |P15311 -->Q9P289 +P15311_p : K(2563,1) for forward direction
2564	Q13188_p +Q9H8S9 <-->Q13188_p |Q9H8S9 : K(2564,1) for forward directionand K(2564,2) for reverse direction
2565	Q13188_p |Q9H8S9 -->Q13188_p +Q9H8S9_p : K(2565,1) for forward direction
2566	Q13188_p +Q13188 <-->Q13188_p |Q13188 : K(2566,1) for forward directionand K(2566,2) for reverse direction
2567	Q13188_p |Q13188 -->Q13188_p +Q13188_p : K(2567,1) for forward direction
2568	Q15208_p +Q15208 <-->Q15208_p |Q15208 : K(2568,1) for forward directionand K(2568,2) for reverse direction
2569	Q15208_p |Q15208 -->Q15208_p +Q15208_p : K(2569,1) for forward direction
2570	Q9Y2H1_p +Q9Y2H1 <-->Q9Y2H1_p |Q9Y2H1 : K(2570,1) for forward directionand K(2570,2) for reverse direction
2571	Q9Y2H1_p |Q9Y2H1 -->Q9Y2H1_p +Q9Y2H1_p : K(2571,1) for forward direction
2572	Q13043_p +Q12778 <-->Q13043_p |Q12778 : K(2572,1) for forward directionand K(2572,2) for reverse direction
2573	Q13043_p |Q12778 -->Q13043_p +Q12778_p : K(2573,1) for forward direction

2574	Q13043_p +O43524 <-->Q13043_p |O43524 : K(2574,1) for forward directionand K(2574,2) for reverse direction
2575	Q13043_p |O43524 -->Q13043_p +O43524_p : K(2575,1) for forward direction
2576	Q13043_p +P33778 <-->Q13043_p |P33778 : K(2576,1) for forward directionand K(2576,2) for reverse direction
2577	Q13043_p |P33778 -->Q13043_p +P33778_p : K(2577,1) for forward direction
2578	Q13043_p +Q9H8S9 <-->Q13043_p |Q9H8S9 : K(2578,1) for forward directionand K(2578,2) for reverse direction
2579	Q13043_p |Q9H8S9 -->Q13043_p +Q9H8S9_p : K(2579,1) for forward direction
2580	P43405_p +P29353 <-->P43405_p |P29353 : K(2580,1) for forward directionand K(2580,2) for reverse direction
2581	P43405_p |P29353 -->P43405_p +P29353_p : K(2581,1) for forward direction
2582	Q7L7X3 +Q13188 <-->Q7L7X3 |Q13188 : K(2582,1) for forward directionand K(2582,2) for reverse direction
2583	Q7L7X3 |Q13188 -->Q7L7X3 +Q13188_p : K(2583,1) for forward direction
2584	Q7L7X3 +Q13043 <-->Q7L7X3 |Q13043 : K(2584,1) for forward directionand K(2584,2) for reverse direction
2585	Q7L7X3 |Q13043 -->Q7L7X3 +Q13043_p : K(2585,1) for forward direction
2586	Q9UL54 +Q13188 <-->Q9UL54 |Q13188 : K(2586,1) for forward directionand K(2586,2) for reverse direction
2587	Q9UL54 |Q13188 -->Q9UL54 +Q13188_p : K(2587,1) for forward direction
2588	Q9UL54 +Q13043 <-->Q9UL54 |Q13043 : K(2588,1) for forward directionand K(2588,2) for reverse direction
2589	Q9UL54 |Q13043 -->Q9UL54 +Q13043_p : K(2589,1) for forward direction
2590	Q9H2K8_p +Q13188 <-->Q9H2K8_p |Q13188 : K(2590,1) for forward directionand K(2590,2) for reverse direction
2591	Q9H2K8_p |Q13188 -->Q9H2K8_p +Q13188_p : K(2591,1) for forward direction
2592	Q9H2K8_p +Q13043 <-->Q9H2K8_p |Q13043 : K(2592,1) for forward directionand K(2592,2) for reverse direction
2593	Q9H2K8_p |Q13043 -->Q9H2K8_p +Q13043_p : K(2593,1) for forward direction
2594	Q9UHD2 +P31749 <-->Q9UHD2 |P31749 : K(2594,1) for forward directionand K(2594,2) for reverse direction
2595	Q9UHD2 |P31749 -->Q9UHD2 +P31749_p : K(2595,1) for forward direction
2596	Q9UHD2 +Q04206 <-->Q9UHD2 |Q04206 : K(2596,1) for forward directionand K(2596,2) for reverse direction
2597	Q9UHD2 |Q04206 -->Q9UHD2 +Q04206_p : K(2597,1) for forward direction
2598	Q9UHD2 +Q13501 <-->Q9UHD2 |Q13501 : K(2598,1) for forward directionand K(2598,2) for reverse direction
2599	Q9UHD2 |Q13501 -->Q9UHD2 +Q13501_p : K(2599,1) for forward direction
2600	Q9UHD2 +P42226 <-->Q9UHD2 |P42226 : K(2600,1) for forward directionand K(2600,2) for reverse direction
2601	Q9UHD2 |P42226 -->Q9UHD2 +P42226_p : K(2601,1) for forward direction
2602	P21980 +P68431 <-->P21980 |P68431 : K(2602,1) for forward directionand K(2602,2) for reverse direction
2603	P21980 |P68431 -->P21980 +P68431_p : K(2603,1) for forward direction
2604	Q96S44_p +P04637 <-->Q96S44_p |P04637 : K(2604,1) for forward directionand K(2604,2) for reverse direction
2605	Q96S44_p |P04637 -->Q96S44_p +P04637_p : K(2605,1) for forward direction
2606	P33981_p +O96017 <-->P33981_p |O96017 : K(2606,1) for forward directionand K(2606,2) for reverse direction
2607	P33981_p |O96017 -->P33981_p +O96017_p : K(2607,1) for forward direction
2608	P33981_p +P38646 <-->P33981_p |P38646 : K(2608,1) for forward directionand K(2608,2) for reverse direction
2609	P33981_p |P38646 -->P33981_p +P38646_p : K(2609,1) for forward direction
2610	P33981_p +P04637 <-->P33981_p |P04637 : K(2610,1) for forward directionand K(2610,2) for reverse direction
2611	P33981_p |P04637 -->P33981_p +P04637_p : K(2611,1) for forward direction
2612	Q99986 +P15336 <-->Q99986 |P15336 : K(2612,1) for forward directionand K(2612,2) for reverse direction
2613	Q99986 |P15336 -->Q99986 +P15336_p : K(2613,1) for forward direction
2614	Q99986 +P68431 <-->Q99986 |P68431 : K(2614,1) for forward directionand K(2614,2) for reverse direction
2615	Q99986 |P68431 -->Q99986 +P68431_p : K(2615,1) for forward direction
2616	Q99986 +P05412 <-->Q99986 |P05412 : K(2616,1) for forward directionand K(2616,2) for reverse direction

2617	Q99986 |P05412 -->Q99986 +P05412_p : K(2617,1) for forward direction
2618	Q99986 +P04637 <-->Q99986 |P04637 : K(2618,1) for forward directionand K(2618,2) for reverse direction
2619	Q99986 |P04637 -->Q99986 +P04637_p : K(2619,1) for forward direction
2620	Q99986 +Q12888 <-->Q99986 |Q12888 : K(2620,1) for forward directionand K(2620,2) for reverse direction
2621	Q99986 |Q12888 -->Q99986 +Q12888_p : K(2621,1) for forward direction
2622	Q9H4A3_p +O95747 <-->Q9H4A3_p |O95747 : K(2622,1) for forward directionand K(2622,2) for reverse direction
2623	Q9H4A3_p |O95747 -->Q9H4A3_p +O95747_p : K(2623,1) for forward direction
2624	Q9H4A3_p +Q9UEW8 <-->Q9H4A3_p |Q9UEW8 : K(2624,1) for forward directionand K(2624,2) for reverse direction
2625	Q9H4A3_p |Q9UEW8 -->Q9H4A3_p +Q9UEW8_p : K(2625,1) for forward direction
2626	Q9H4A3_p +Q9H4A3 <-->Q9H4A3_p |Q9H4A3 : K(2626,1) for forward directionand K(2626,2) for reverse direction
2627	Q9H4A3_p |Q9H4A3 -->Q9H4A3_p +Q9H4A3_p : K(2627,1) for forward direction
2628	P31749_p +P35226 <-->P31749_p |P35226 : K(2628,1) for forward directionand K(2628,2) for reverse direction
2629	P31749_p |P35226 -->P31749_p +P35226_p : K(2629,1) for forward direction
2630	P31749_p +P16220 <-->P31749_p |P16220 : K(2630,1) for forward directionand K(2630,2) for reverse direction
2631	P31749_p |P16220 -->P31749_p +P16220_p : K(2631,1) for forward direction
2632	P31749_p +Q15121 <-->P31749_p |Q15121 : K(2632,1) for forward directionand K(2632,2) for reverse direction
2633	P31749_p |Q15121 -->P31749_p +Q15121_p : K(2633,1) for forward direction
2634	P31751_p +P35226 <-->P31751_p |P35226 : K(2634,1) for forward directionand K(2634,2) for reverse direction
2635	P31751_p |P35226 -->P31751_p +P35226_p : K(2635,1) for forward direction
2636	Q96GD4_p +Q9H0H5 <-->Q96GD4_p |Q9H0H5 : K(2636,1) for forward directionand K(2636,2) for reverse direction
2637	Q96GD4_p |Q9H0H5 -->Q96GD4_p +Q9H0H5_p : K(2637,1) for forward direction
2638	P15056 +Q02750 <-->P15056 |Q02750 : K(2638,1) for forward directionand K(2638,2) for reverse direction
2639	P15056 |Q02750 -->P15056 +Q02750_p : K(2639,1) for forward direction
2640	P27708_p +P27708 <-->P27708_p |P27708 : K(2640,1) for forward directionand K(2640,2) for reverse direction
2641	P27708_p |P27708 -->P27708_p +P27708_p : K(2641,1) for forward direction
2642	Q9UQM7_p +P16615 <-->Q9UQM7_p |P16615 : K(2642,1) for forward directionand K(2642,2) for reverse direction
2643	Q9UQM7_p |P16615 -->Q9UQM7_p +P16615_p : K(2643,1) for forward direction
2644	Q9UQM7_p +P17676 <-->Q9UQM7_p |P17676 : K(2644,1) for forward directionand K(2644,2) for reverse direction
2645	Q9UQM7_p |P17676 -->Q9UQM7_p +P17676_p : K(2645,1) for forward direction
2646	Q9UQM7_p +Q00613 <-->Q9UQM7_p |Q00613 : K(2646,1) for forward directionand K(2646,2) for reverse direction
2647	Q9UQM7_p |Q00613 -->Q9UQM7_p +Q00613_p : K(2647,1) for forward direction
2648	Q13554_p +Q13554 <-->Q13554_p |Q13554 : K(2648,1) for forward directionand K(2648,2) for reverse direction
2649	Q13554_p |Q13554 -->Q13554_p +Q13554_p : K(2649,1) for forward direction
2650	Q13555 +P18846 <-->Q13555 |P18846 : K(2650,1) for forward directionand K(2650,2) for reverse direction
2651	Q13555 |P18846 -->Q13555 +P18846_p : K(2651,1) for forward direction
2652	Q13555 +P42224 <-->Q13555 |P42224 : K(2652,1) for forward directionand K(2652,2) for reverse direction
2653	Q13555 |P42224 -->Q13555 +P42224_p : K(2653,1) for forward direction
2654	P06493_p +P18858 <-->P06493_p |P18858 : K(2654,1) for forward directionand K(2654,2) for reverse direction
2655	P06493_p |P18858 -->P06493_p +P18858_p : K(2655,1) for forward direction
2656	P06493_p +Q96GX5 <-->P06493_p |Q96GX5 : K(2656,1) for forward directionand K(2656,2) for reverse direction
2657	P06493_p |Q96GX5 -->P06493_p +Q96GX5_p : K(2657,1) for forward direction
2658	P24941_p +Q15910 <-->P24941_p |Q15910 : K(2658,1) for forward directionand K(2658,2) for reverse direction
2659	P24941_p |Q15910 -->P24941_p +Q15910_p : K(2659,1) for forward direction

2660	P24941_p +P54198 <-->P24941_p |P54198 : K(2660,1) for forward directionand K(2660,2) for reverse direction
2661	P24941_p |P54198 -->P24941_p +P54198_p : K(2661,1) for forward direction
2662	P24941_p +P18858 <-->P24941_p |P18858 : K(2662,1) for forward directionand K(2662,2) for reverse direction
2663	P24941_p |P18858 -->P24941_p +P18858_p : K(2663,1) for forward direction
2664	P24941_p +P23511 <-->P24941_p |P23511 : K(2664,1) for forward directionand K(2664,2) for reverse direction
2665	P24941_p |P23511 -->P24941_p +P23511_p : K(2665,1) for forward direction
2666	P24941_p +P08047 <-->P24941_p |P08047 : K(2666,1) for forward directionand K(2666,2) for reverse direction
2667	P24941_p |P08047 -->P24941_p +P08047_p : K(2667,1) for forward direction
2668	P24941_p +P17480 <-->P24941_p |P17480 : K(2668,1) for forward directionand K(2668,2) for reverse direction
2669	P24941_p |P17480 -->P24941_p +P17480_p : K(2669,1) for forward direction
2670	Q00535_p +P31146 <-->Q00535_p |P31146 : K(2670,1) for forward directionand K(2670,2) for reverse direction
2671	Q00535_p |P31146 -->Q00535_p +P31146_p : K(2671,1) for forward direction
2672	Q00535_p +Q05193 <-->Q00535_p |Q05193 : K(2672,1) for forward directionand K(2672,2) for reverse direction
2673	Q00535_p |Q05193 -->Q00535_p +Q05193_p : K(2673,1) for forward direction
2674	Q00535_p +P21860 <-->Q00535_p |P21860 : K(2674,1) for forward directionand K(2674,2) for reverse direction
2675	Q00535_p |P21860 -->Q00535_p +P21860_p : K(2675,1) for forward direction
2676	Q00535_p +P15311 <-->Q00535_p |P15311 : K(2676,1) for forward directionand K(2676,2) for reverse direction
2677	Q00535_p |P15311 -->Q00535_p +P15311_p : K(2677,1) for forward direction
2678	Q00535_p +Q9GZM8 <-->Q00535_p |Q9GZM8 : K(2678,1) for forward directionand K(2678,2) for reverse direction
2679	Q00535_p |Q9GZM8 -->Q00535_p +Q9GZM8_p : K(2679,1) for forward direction
2680	P50613_p +P21127 <-->P50613_p |P21127 : K(2680,1) for forward directionand K(2680,2) for reverse direction
2681	P50613_p |P21127 -->P50613_p +P21127_p : K(2681,1) for forward direction
2682	P50613_p +P24941 <-->P50613_p |P24941 : K(2682,1) for forward directionand K(2682,2) for reverse direction
2683	P50613_p |P24941 -->P50613_p +P24941_p : K(2683,1) for forward direction
2684	P50750_p +Q15596 <-->P50750_p |Q15596 : K(2684,1) for forward directionand K(2684,2) for reverse direction
2685	P50750_p |Q15596 -->P50750_p +Q15596_p : K(2685,1) for forward direction
2686	O14757_p +O14757 <-->O14757_p |O14757 : K(2686,1) for forward directionand K(2686,2) for reverse direction
2687	O14757_p |O14757 -->O14757_p +O14757_p : K(2687,1) for forward direction
2688	O14757_p +P04637 <-->O14757_p |P04637 : K(2688,1) for forward directionand K(2688,2) for reverse direction
2689	O14757_p |P04637 -->O14757_p +P04637_p : K(2689,1) for forward direction
2690	O96017_p +O96017 <-->O96017_p |O96017 : K(2690,1) for forward directionand K(2690,2) for reverse direction
2691	O96017_p |O96017 -->O96017_p +O96017_p : K(2691,1) for forward direction
2692	O96017_p +P06400 <-->O96017_p |P06400 : K(2692,1) for forward directionand K(2692,2) for reverse direction
2693	O96017_p |P06400 -->O96017_p +P06400_p : K(2693,1) for forward direction
2694	O15111_p +Q00653 <-->O15111_p |Q00653 : K(2694,1) for forward directionand K(2694,2) for reverse direction
2695	O15111_p |Q00653 -->O15111_p +Q00653_p : K(2695,1) for forward direction
2696	O15111_p +Q04206 <-->O15111_p |Q04206 : K(2696,1) for forward directionand K(2696,2) for reverse direction
2697	O15111_p |Q04206 -->O15111_p +Q04206_p : K(2697,1) for forward direction
2698	O15111_p +Q86VP1 <-->O15111_p |Q86VP1 : K(2698,1) for forward directionand K(2698,2) for reverse direction
2699	O15111_p |Q86VP1 -->O15111_p +Q86VP1_p : K(2699,1) for forward direction
2700	P46108_p +Q05397 <-->P46108_p |Q05397 : K(2700,1) for forward directionand K(2700,2) for reverse direction
2701	P46108_p |Q05397 -->P46108_p +Q05397_p : K(2701,1) for forward direction
2702	P41240_p +P41240 <-->P41240_p |P41240 : K(2702,1) for forward directionand K(2702,2) for reverse direction

2703	P41240_p |P41240 -->P41240_p +P41240_p : K(2703,1) for forward direction
2704	P48729 +O15169 <-->P48729 |O15169 : K(2704,1) for forward directionand K(2704,2) for reverse direction
2705	P48729 |O15169 -->P48729 +O15169_p : K(2705,1) for forward direction
2706	P48729 +P55957 <-->P48729 |P55957 : K(2706,1) for forward directionand K(2706,2) for reverse direction
2707	P48729 |P55957 -->P48729 +P55957_p : K(2707,1) for forward direction
2708	P48730 +Q00535 <-->P48730 |Q00535 : K(2708,1) for forward directionand K(2708,2) for reverse direction
2709	P48730 |Q00535 -->P48730 +Q00535_p : K(2709,1) for forward direction
2710	P49674 +P55957 <-->P49674 |P55957 : K(2710,1) for forward directionand K(2710,2) for reverse direction
2711	P49674 |P55957 -->P49674 +P55957_p : K(2711,1) for forward direction
2712	P68400_p +P27695 <-->P68400_p |P27695 : K(2712,1) for forward directionand K(2712,2) for reverse direction
2713	P68400_p |P27695 -->P68400_p +P27695_p : K(2713,1) for forward direction
2714	P68400_p +P55957 <-->P68400_p |P55957 : K(2714,1) for forward directionand K(2714,2) for reverse direction
2715	P68400_p |P55957 -->P68400_p +P55957_p : K(2715,1) for forward direction
2716	P68400_p +Q16543 <-->P68400_p |Q16543 : K(2716,1) for forward directionand K(2716,2) for reverse direction
2717	P68400_p |Q16543 -->P68400_p +Q16543_p : K(2717,1) for forward direction
2718	P68400_p +P12830 <-->P68400_p |P12830 : K(2718,1) for forward directionand K(2718,2) for reverse direction
2719	P68400_p |P12830 -->P68400_p +P12830_p : K(2719,1) for forward direction
2720	P68400_p +P35222 <-->P68400_p |P35222 : K(2720,1) for forward directionand K(2720,2) for reverse direction
2721	P68400_p |P35222 -->P68400_p +P35222_p : K(2721,1) for forward direction
2722	P68400_p +Q13144 <-->P68400_p |Q13144 : K(2722,1) for forward directionand K(2722,2) for reverse direction
2723	P68400_p |Q13144 -->P68400_p +Q13144_p : K(2723,1) for forward direction
2724	P68400_p +Q9UNN5 <-->P68400_p |Q9UNN5 : K(2724,1) for forward directionand K(2724,2) for reverse direction
2725	P68400_p |Q9UNN5 -->P68400_p +Q9UNN5_p : K(2725,1) for forward direction
2726	P68400_p +P52655 <-->P68400_p |P52655 : K(2726,1) for forward directionand K(2726,2) for reverse direction
2727	P68400_p |P52655 -->P68400_p +P52655_p : K(2727,1) for forward direction
2728	P68400_p +O15379 <-->P68400_p |O15379 : K(2728,1) for forward directionand K(2728,2) for reverse direction
2729	P68400_p |O15379 -->P68400_p +O15379_p : K(2729,1) for forward direction
2730	P68400_p +P30519 <-->P68400_p |P30519 : K(2730,1) for forward directionand K(2730,2) for reverse direction
2731	P68400_p |P30519 -->P68400_p +P30519_p : K(2731,1) for forward direction
2732	P68400_p +Q00613 <-->P68400_p |Q00613 : K(2732,1) for forward directionand K(2732,2) for reverse direction
2733	P68400_p |Q00613 -->P68400_p +Q00613_p : K(2733,1) for forward direction
2734	P68400_p +Q16666 <-->P68400_p |Q16666 : K(2734,1) for forward directionand K(2734,2) for reverse direction
2735	P68400_p |Q16666 -->P68400_p +Q16666_p : K(2735,1) for forward direction
2736	P68400_p +P18858 <-->P68400_p |P18858 : K(2736,1) for forward directionand K(2736,2) for reverse direction
2737	P68400_p |P18858 -->P68400_p +P18858_p : K(2737,1) for forward direction
2738	P68400_p +Q6VY07 <-->P68400_p |Q6VY07 : K(2738,1) for forward directionand K(2738,2) for reverse direction
2739	P68400_p |Q6VY07 -->P68400_p +Q6VY07_p : K(2739,1) for forward direction
2740	P68400_p +Q12972 <-->P68400_p |Q12972 : K(2740,1) for forward directionand K(2740,2) for reverse direction
2741	P68400_p |Q12972 -->P68400_p +Q12972_p : K(2741,1) for forward direction
2742	P68400_p +Q04206 <-->P68400_p |Q04206 : K(2742,1) for forward directionand K(2742,2) for reverse direction
2743	P68400_p |Q04206 -->P68400_p +Q04206_p : K(2743,1) for forward direction
2744	P19784 +P55957 <-->P19784 |P55957 : K(2744,1) for forward directionand K(2744,2) for reverse direction
2745	P19784 |P55957 -->P19784 +P55957_p : K(2745,1) for forward direction

<table>
<tr><td>2746</td><td>P19784 +Q16543 <-->P19784 |Q16543 : K(2746,1) for forward directionand K(2746,2) for reverse direction</td></tr>
<tr><td>2747</td><td>P19784 |Q16543 -->P19784 +Q16543_p : K(2747,1) for forward direction</td></tr>
<tr><td>2748</td><td>P19784 +Q13144 <-->P19784 |Q13144 : K(2748,1) for forward directionand K(2748,2) for reverse direction</td></tr>
<tr><td>2749</td><td>P19784 |Q13144 -->P19784 +Q13144_p : K(2749,1) for forward direction</td></tr>
<tr><td>2750</td><td>P19784 +P52655 <-->P19784 |P52655 : K(2750,1) for forward directionand K(2750,2) for reverse direction</td></tr>
<tr><td>2751</td><td>P19784 |P52655 -->P19784 +P52655_p : K(2751,1) for forward direction</td></tr>
<tr><td>2752</td><td>P19784 +Q13547 <-->P19784 |Q13547 : K(2752,1) for forward directionand K(2752,2) for reverse direction</td></tr>
<tr><td>2753</td><td>P19784 |Q13547 -->P19784 +Q13547_p : K(2753,1) for forward direction</td></tr>
<tr><td>2754</td><td>P19784 +Q92769 <-->P19784 |Q92769 : K(2754,1) for forward directionand K(2754,2) for reverse direction</td></tr>
<tr><td>2755</td><td>P19784 |Q92769 -->P19784 +Q92769_p : K(2755,1) for forward direction</td></tr>
<tr><td>2756</td><td>P19784 +P41236 <-->P19784 |P41236 : K(2756,1) for forward directionand K(2756,2) for reverse direction</td></tr>
<tr><td>2757</td><td>P19784 |P41236 -->P19784 +P41236_p : K(2757,1) for forward direction</td></tr>
<tr><td>2758</td><td>P19784 +Q12972 <-->P19784 |Q12972 : K(2758,1) for forward directionand K(2758,2) for reverse direction</td></tr>
<tr><td>2759</td><td>P19784 |Q12972 -->P19784 +Q12972_p : K(2759,1) for forward direction</td></tr>
<tr><td>2760</td><td>P19784 +Q712K3 <-->P19784 |Q712K3 : K(2760,1) for forward directionand K(2760,2) for reverse direction</td></tr>
<tr><td>2761</td><td>P19784 |Q712K3 -->P19784 +Q712K3_p : K(2761,1) for forward direction</td></tr>
<tr><td>2762</td><td>P19784 +P18887 <-->P19784 |P18887 : K(2762,1) for forward directionand K(2762,2) for reverse direction</td></tr>
<tr><td>2763</td><td>P19784 |P18887 -->P19784 +P18887_p : K(2763,1) for forward direction</td></tr>
<tr><td>2764</td><td>P67870_p +P55957 <-->P67870_p |P55957 : K(2764,1) for forward directionand K(2764,2) for reverse direction</td></tr>
<tr><td>2765</td><td>P67870_p |P55957 -->P67870_p +P55957_p : K(2765,1) for forward direction</td></tr>
<tr><td>2766</td><td>P67870_p +P35222 <-->P67870_p |P35222 : K(2766,1) for forward directionand K(2766,2) for reverse direction</td></tr>
<tr><td>2767</td><td>P67870_p |P35222 -->P67870_p +P35222_p : K(2767,1) for forward direction</td></tr>
<tr><td>2768</td><td>P67870_p +P55010 <-->P67870_p |P55010 : K(2768,1) for forward directionand K(2768,2) for reverse direction</td></tr>
<tr><td>2769</td><td>P67870_p |P55010 -->P67870_p +P55010_p : K(2769,1) for forward direction</td></tr>
<tr><td>2770</td><td>P67870_p +Q9UBF6 <-->P67870_p |Q9UBF6 : K(2770,1) for forward directionand K(2770,2) for reverse direction</td></tr>
<tr><td>2771</td><td>P67870_p |Q9UBF6 -->P67870_p +Q9UBF6_p : K(2771,1) for forward direction</td></tr>
<tr><td>2772</td><td>P67870_p +O60763 <-->P67870_p |O60763 : K(2772,1) for forward directionand K(2772,2) for reverse direction</td></tr>
<tr><td>2773</td><td>P67870_p |O60763 -->P67870_p +O60763_p : K(2773,1) for forward direction</td></tr>
<tr><td>2774</td><td>Q08345_p +Q08345 <-->Q08345_p |Q08345 : K(2774,1) for forward directionand K(2774,2) for reverse direction</td></tr>
<tr><td>2775</td><td>Q08345_p |Q08345 -->Q08345_p +Q08345_p : K(2775,1) for forward direction</td></tr>
<tr><td>2776</td><td>P00533_p +P04626 <-->P00533_p |P04626 : K(2776,1) for forward directionand K(2776,2) for reverse direction</td></tr>
<tr><td>2777</td><td>P00533_p |P04626 -->P00533_p +P04626_p : K(2777,1) for forward direction</td></tr>
<tr><td>2778</td><td>P00533_p +Q9UJM3 <-->P00533_p |Q9UJM3 : K(2778,1) for forward directionand K(2778,2) for reverse direction</td></tr>
<tr><td>2779</td><td>P00533_p |Q9UJM3 -->P00533_p +Q9UJM3_p : K(2779,1) for forward direction</td></tr>
<tr><td>2780</td><td>P00533_p +P40763 <-->P00533_p |P40763 : K(2780,1) for forward directionand K(2780,2) for reverse direction</td></tr>
<tr><td>2781</td><td>P00533_p |P40763 -->P00533_p +P40763_p : K(2781,1) for forward direction</td></tr>
<tr><td>2782</td><td>P49840_p +Q04206 <-->P49840_p |Q04206 : K(2782,1) for forward directionand K(2782,2) for reverse direction</td></tr>
<tr><td>2783</td><td>P49840_p |Q04206 -->P49840_p +Q04206_p : K(2783,1) for forward direction</td></tr>
<tr><td>2784</td><td>P49841_p +P53396 <-->P49841_p |P53396 : K(2784,1) for forward directionand K(2784,2) for reverse direction</td></tr>
<tr><td>2785</td><td>P49841_p |P53396 -->P49841_p +P53396_p : K(2785,1) for forward direction</td></tr>
<tr><td>2786</td><td>P49841_p +P12830 <-->P49841_p |P12830 : K(2786,1) for forward directionand K(2786,2) for reverse direction</td></tr>
<tr><td>2787</td><td>P49841_p |P12830 -->P49841_p +P12830_p : K(2787,1) for forward direction</td></tr>
<tr><td>2788</td><td>P49841_p +P16220 <-->P49841_p |P16220 : K(2788,1) for forward directionand K(2788,2) for reverse direction</td></tr>
</table>

2789	P49841_p |P16220 -->P49841_p +P16220_p : K(2789,1) for forward direction
2790	P49841_p +P49841 <-->P49841_p |P49841 : K(2790,1) for forward directionand K(2790,2) for reverse direction
2791	P49841_p |P49841 -->P49841_p +P49841_p : K(2791,1) for forward direction
2792	P49841_p +P41236 <-->P49841_p |P41236 : K(2792,1) for forward directionand K(2792,2) for reverse direction
2793	P49841_p |P41236 -->P49841_p +P41236_p : K(2793,1) for forward direction
2794	P49841_p +P04637 <-->P49841_p |P04637 : K(2794,1) for forward directionand K(2794,2) for reverse direction
2795	P49841_p |P04637 -->P49841_p +P04637_p : K(2795,1) for forward direction
2796	P49841_p +P49815 <-->P49841_p |P49815 : K(2796,1) for forward directionand K(2796,2) for reverse direction
2797	P49841_p |P49815 -->P49841_p +P49815_p : K(2797,1) for forward direction
2798	P01112 +P05412 <-->P01112 |P05412 : K(2798,1) for forward directionand K(2798,2) for reverse direction
2799	P01112 |P05412 -->P01112 +P05412_p : K(2799,1) for forward direction
2800	Q9Y6K9_u +Q04206 <-->Q9Y6K9_u |Q04206 : K(2800,1) for forward directionand K(2800,2) for reverse direction
2801	Q9Y6K9_u |Q04206 -->Q9Y6K9_u +Q04206_p : K(2801,1) for forward direction
2802	P40189_p +P40189 <-->P40189_p |P40189 : K(2802,1) for forward directionand K(2802,2) for reverse direction
2803	P40189_p |P40189 -->P40189_p +P40189_p : K(2803,1) for forward direction
2804	Q13418_p +Q13418 <-->Q13418_p |Q13418 : K(2804,1) for forward directionand K(2804,2) for reverse direction
2805	Q13418_p |Q13418 -->Q13418_p +Q13418_p : K(2805,1) for forward direction
2806	Q13418_p +O14950 <-->Q13418_p |O14950 : K(2806,1) for forward directionand K(2806,2) for reverse direction
2807	Q13418_p |O14950 -->Q13418_p +O14950_p : K(2807,1) for forward direction
2808	P51617_p +P51617 <-->P51617_p |P51617 : K(2808,1) for forward directionand K(2808,2) for reverse direction
2809	P51617_p |P51617 -->P51617_p +P51617_p : K(2809,1) for forward direction
2810	Q9NWZ3_p +P51617 <-->Q9NWZ3_p |P51617 : K(2810,1) for forward directionand K(2810,2) for reverse direction
2811	Q9NWZ3_p |P51617 -->Q9NWZ3_p +P51617_p : K(2811,1) for forward direction
2812	Q9NWZ3_p +Q9NWZ3 <-->Q9NWZ3_p |Q9NWZ3 : K(2812,1) for forward directionand K(2812,2) for reverse direction
2813	Q9NWZ3_p |Q9NWZ3 -->Q9NWZ3_p +Q9NWZ3_p : K(2813,1) for forward direction
2814	Q02750_p +P49841 <-->Q02750_p |P49841 : K(2814,1) for forward directionand K(2814,2) for reverse direction
2815	Q02750_p |P49841 -->Q02750_p +P49841_p : K(2815,1) for forward direction
2816	Q02750_p +Q02750 <-->Q02750_p |Q02750 : K(2816,1) for forward directionand K(2816,2) for reverse direction
2817	Q02750_p |Q02750 -->Q02750_p +Q02750_p : K(2817,1) for forward direction
2818	Q02750_p +P28482 <-->Q02750_p |P28482 : K(2818,1) for forward directionand K(2818,2) for reverse direction
2819	Q02750_p |P28482 -->Q02750_p +P28482_p : K(2819,1) for forward direction
2820	P46734_p +Q16539 <-->P46734_p |Q16539 : K(2820,1) for forward directionand K(2820,2) for reverse direction
2821	P46734_p |Q16539 -->P46734_p +Q16539_p : K(2821,1) for forward direction
2822	P45985_p +P45985 <-->P45985_p |P45985 : K(2822,1) for forward directionand K(2822,2) for reverse direction
2823	P45985_p |P45985 -->P45985_p +P45985_p : K(2823,1) for forward direction
2824	P45985_p +Q16539 <-->P45985_p |Q16539 : K(2824,1) for forward directionand K(2824,2) for reverse direction
2825	P45985_p |Q16539 -->P45985_p +Q16539_p : K(2825,1) for forward direction
2826	P45985_p +P45983 <-->P45985_p |P45983 : K(2826,1) for forward directionand K(2826,2) for reverse direction
2827	P45985_p |P45983 -->P45985_p +P45983_p : K(2827,1) for forward direction
2828	P52564_p +P52564 <-->P52564_p |P52564 : K(2828,1) for forward directionand K(2828,2) for reverse direction
2829	P52564_p |P52564 -->P52564_p +P52564_p : K(2829,1) for forward direction
2830	P52564_p +Q16539 <-->P52564_p |Q16539 : K(2830,1) for forward directionand K(2830,2) for reverse direction
2831	P52564_p |Q16539 -->P52564_p +Q16539_p : K(2831,1) for forward direction

<table>
<tr><td>2832</td><td>Q9Y2U5 +P45985 <-->Q9Y2U5 |P45985 : K(2832,1) for forward directionand K(2832,2) for reverse direction</td></tr>
<tr><td>2833</td><td>Q9Y2U5 |P45985 -->Q9Y2U5 +P45985_p : K(2833,1) for forward direction</td></tr>
<tr><td>2834</td><td>Q9NYL2_p +P45985 <-->Q9NYL2_p |P45985 : K(2834,1) for forward directionand K(2834,2) for reverse direction</td></tr>
<tr><td>2835</td><td>Q9NYL2_p |P45985 -->Q9NYL2_p +P45985_p : K(2835,1) for forward direction</td></tr>
<tr><td>2836</td><td>Q9NYL2_p +O14733 <-->Q9NYL2_p |O14733 : K(2836,1) for forward directionand K(2836,2) for reverse direction</td></tr>
<tr><td>2837</td><td>Q9NYL2_p |O14733 -->Q9NYL2_p +O14733_p : K(2837,1) for forward direction</td></tr>
<tr><td>2838</td><td>Q99759_p +P46734 <-->Q99759_p |P46734 : K(2838,1) for forward directionand K(2838,2) for reverse direction</td></tr>
<tr><td>2839</td><td>Q99759_p |P46734 -->Q99759_p +P46734_p : K(2839,1) for forward direction</td></tr>
<tr><td>2840</td><td>Q9Y6R4_p +P46734 <-->Q9Y6R4_p |P46734 : K(2840,1) for forward directionand K(2840,2) for reverse direction</td></tr>
<tr><td>2841</td><td>Q9Y6R4_p |P46734 -->Q9Y6R4_p +P46734_p : K(2841,1) for forward direction</td></tr>
<tr><td>2842</td><td>Q9Y6R4_p +P45985 <-->Q9Y6R4_p |P45985 : K(2842,1) for forward directionand K(2842,2) for reverse direction</td></tr>
<tr><td>2843</td><td>Q9Y6R4_p |P45985 -->Q9Y6R4_p +P45985_p : K(2843,1) for forward direction</td></tr>
<tr><td>2844</td><td>Q9Y6R4_p +P52564 <-->Q9Y6R4_p |P52564 : K(2844,1) for forward directionand K(2844,2) for reverse direction</td></tr>
<tr><td>2845</td><td>Q9Y6R4_p |P52564 -->Q9Y6R4_p +P52564_p : K(2845,1) for forward direction</td></tr>
<tr><td>2846</td><td>Q9Y6R4_p +Q9Y6R4 <-->Q9Y6R4_p |Q9Y6R4 : K(2846,1) for forward directionand K(2846,2) for reverse direction</td></tr>
<tr><td>2847</td><td>Q9Y6R4_p |Q9Y6R4 -->Q9Y6R4_p +Q9Y6R4_p : K(2847,1) for forward direction</td></tr>
<tr><td>2848</td><td>O43318_p +P46734 <-->O43318_p |P46734 : K(2848,1) for forward directionand K(2848,2) for reverse direction</td></tr>
<tr><td>2849</td><td>O43318_p |P46734 -->O43318_p +P46734_p : K(2849,1) for forward direction</td></tr>
<tr><td>2850</td><td>O43318_p +P45985 <-->O43318_p |P45985 : K(2850,1) for forward directionand K(2850,2) for reverse direction</td></tr>
<tr><td>2851</td><td>O43318_p |P45985 -->O43318_p +P45985_p : K(2851,1) for forward direction</td></tr>
<tr><td>2852</td><td>O43318_p +P52564 <-->O43318_p |P52564 : K(2852,1) for forward directionand K(2852,2) for reverse direction</td></tr>
<tr><td>2853</td><td>O43318_p |P52564 -->O43318_p +P52564_p : K(2853,1) for forward direction</td></tr>
<tr><td>2854</td><td>O43318_p +O43318 <-->O43318_p |O43318 : K(2854,1) for forward directionand K(2854,2) for reverse direction</td></tr>
<tr><td>2855</td><td>O43318_p |O43318 -->O43318_p +O43318_p : K(2855,1) for forward direction</td></tr>
<tr><td>2856</td><td>P28482_p +P01100 <-->P28482_p |P01100 : K(2856,1) for forward directionand K(2856,2) for reverse direction</td></tr>
<tr><td>2857</td><td>P28482_p |P01100 -->P28482_p +P01100_p : K(2857,1) for forward direction</td></tr>
<tr><td>2858</td><td>P28482_p +Q13480 <-->P28482_p |Q13480 : K(2858,1) for forward directionand K(2858,2) for reverse direction</td></tr>
<tr><td>2859</td><td>P28482_p |Q13480 -->P28482_p +Q13480_p : K(2859,1) for forward direction</td></tr>
<tr><td>2860</td><td>P28482_p +P05412 <-->P28482_p |P05412 : K(2860,1) for forward directionand K(2860,2) for reverse direction</td></tr>
<tr><td>2861</td><td>P28482_p |P05412 -->P28482_p +P05412_p : K(2861,1) for forward direction</td></tr>
<tr><td>2862</td><td>P28482_p +Q05469 <-->P28482_p |Q05469 : K(2862,1) for forward directionand K(2862,2) for reverse direction</td></tr>
<tr><td>2863</td><td>P28482_p |Q05469 -->P28482_p +Q05469_p : K(2863,1) for forward direction</td></tr>
<tr><td>2864</td><td>P28482_p +P28482 <-->P28482_p |P28482 : K(2864,1) for forward directionand K(2864,2) for reverse direction</td></tr>
<tr><td>2865</td><td>P28482_p |P28482 -->P28482_p +P28482_p : K(2865,1) for forward direction</td></tr>
<tr><td>2866</td><td>P28482_p +Q9NXR1 <-->P28482_p |Q9NXR1 : K(2866,1) for forward directionand K(2866,2) for reverse direction</td></tr>
<tr><td>2867</td><td>P28482_p |Q9NXR1 -->P28482_p +Q9NXR1_p : K(2867,1) for forward direction</td></tr>
<tr><td>2868</td><td>P28482_p +Q15418 <-->P28482_p |Q15418 : K(2868,1) for forward directionand K(2868,2) for reverse direction</td></tr>
<tr><td>2869</td><td>P28482_p |Q15418 -->P28482_p +Q15418_p : K(2869,1) for forward direction</td></tr>
<tr><td>2870</td><td>Q16539_p +P78536 <-->Q16539_p |P78536 : K(2870,1) for forward directionand K(2870,2) for reverse direction</td></tr>
<tr><td>2871</td><td>Q16539_p |P78536 -->Q16539_p +P78536_p : K(2871,1) for forward direction</td></tr>
<tr><td>2872</td><td>Q16539_p +P49137 <-->Q16539_p |P49137 : K(2872,1) for forward directionand K(2872,2) for reverse direction</td></tr>
<tr><td>2873</td><td>Q16539_p |P49137 -->Q16539_p +P49137_p : K(2873,1) for forward direction</td></tr>
<tr><td>2874</td><td>Q16539_p +Q16644 <-->Q16539_p |Q16644 : K(2874,1) for forward directionand K(2874,2) for reverse direction</td></tr>
</table>

<table>
<tr><td>2875</td><td>Q16539_p |Q16644 -->Q16539_p +Q16644_p : K(2875,1) for forward direction</td></tr>
<tr><td>2876</td><td>Q16539_p +P47712 <-->Q16539_p |P47712 : K(2876,1) for forward directionand K(2876,2) for reverse direction</td></tr>
<tr><td>2877</td><td>Q16539_p |P47712 -->Q16539_p +P47712_p : K(2877,1) for forward direction</td></tr>
<tr><td>2878</td><td>Q16539_p +P84022 <-->Q16539_p |P84022 : K(2878,1) for forward directionand K(2878,2) for reverse direction</td></tr>
<tr><td>2879</td><td>Q16539_p |P84022 -->Q16539_p +P84022_p : K(2879,1) for forward direction</td></tr>
<tr><td>2880</td><td>Q16539_p +P42224 <-->Q16539_p |P42224 : K(2880,1) for forward directionand K(2880,2) for reverse direction</td></tr>
<tr><td>2881</td><td>Q16539_p |P42224 -->Q16539_p +P42224_p : K(2881,1) for forward direction</td></tr>
<tr><td>2882</td><td>P27361_p +Q13480 <-->P27361_p |Q13480 : K(2882,1) for forward directionand K(2882,2) for reverse direction</td></tr>
<tr><td>2883</td><td>P27361_p |Q13480 -->P27361_p +Q13480_p : K(2883,1) for forward direction</td></tr>
<tr><td>2884</td><td>P27361_p +Q05469 <-->P27361_p |Q05469 : K(2884,1) for forward directionand K(2884,2) for reverse direction</td></tr>
<tr><td>2885</td><td>P27361_p |Q05469 -->P27361_p +Q05469_p : K(2885,1) for forward direction</td></tr>
<tr><td>2886</td><td>P27361_p +P27361 <-->P27361_p |P27361 : K(2886,1) for forward directionand K(2886,2) for reverse direction</td></tr>
<tr><td>2887</td><td>P27361_p |P27361 -->P27361_p +P27361_p : K(2887,1) for forward direction</td></tr>
<tr><td>2888</td><td>P27361_p +Q15418 <-->P27361_p |Q15418 : K(2888,1) for forward directionand K(2888,2) for reverse direction</td></tr>
<tr><td>2889</td><td>P27361_p |Q15418 -->P27361_p +Q15418_p : K(2889,1) for forward direction</td></tr>
<tr><td>2890</td><td>P27361_p +Q8N122 <-->P27361_p |Q8N122 : K(2890,1) for forward directionand K(2890,2) for reverse direction</td></tr>
<tr><td>2891</td><td>P27361_p |Q8N122 -->P27361_p +Q8N122_p : K(2891,1) for forward direction</td></tr>
<tr><td>2892</td><td>P45983_p +P31749 <-->P45983_p |P31749 : K(2892,1) for forward directionand K(2892,2) for reverse direction</td></tr>
<tr><td>2893</td><td>P45983_p |P31749 -->P45983_p +P31749_p : K(2893,1) for forward direction</td></tr>
<tr><td>2894</td><td>P45983_p +P05412 <-->P45983_p |P05412 : K(2894,1) for forward directionand K(2894,2) for reverse direction</td></tr>
<tr><td>2895</td><td>P45983_p |P05412 -->P45983_p +P05412_p : K(2895,1) for forward direction</td></tr>
<tr><td>2896</td><td>P45983_p +P01106 <-->P45983_p |P01106 : K(2896,1) for forward directionand K(2896,2) for reverse direction</td></tr>
<tr><td>2897</td><td>P45983_p |P01106 -->P45983_p +P01106_p : K(2897,1) for forward direction</td></tr>
<tr><td>2898</td><td>P45983_p +P49023 <-->P45983_p |P49023 : K(2898,1) for forward directionand K(2898,2) for reverse direction</td></tr>
<tr><td>2899</td><td>P45983_p |P49023 -->P45983_p +P49023_p : K(2899,1) for forward direction</td></tr>
<tr><td>2900</td><td>P45983_p +P42224 <-->P45983_p |P42224 : K(2900,1) for forward directionand K(2900,2) for reverse direction</td></tr>
<tr><td>2901</td><td>P45983_p |P42224 -->P45983_p +P42224_p : K(2901,1) for forward direction</td></tr>
<tr><td>2902</td><td>P45983_p +P40763 <-->P45983_p |P40763 : K(2902,1) for forward directionand K(2902,2) for reverse direction</td></tr>
<tr><td>2903</td><td>P45983_p |P40763 -->P45983_p +P40763_p : K(2903,1) for forward direction</td></tr>
<tr><td>2904</td><td>P49137_p +P09917 <-->P49137_p |P09917 : K(2904,1) for forward directionand K(2904,2) for reverse direction</td></tr>
<tr><td>2905</td><td>P49137_p |P09917 -->P49137_p +P09917_p : K(2905,1) for forward direction</td></tr>
<tr><td>2906</td><td>Q96GX5_p +P56211 <-->Q96GX5_p |P56211 : K(2906,1) for forward directionand K(2906,2) for reverse direction</td></tr>
<tr><td>2907</td><td>Q96GX5_p |P56211 -->Q96GX5_p +P56211_p : K(2907,1) for forward direction</td></tr>
<tr><td>2908</td><td>Q96GX5_p +O43768 <-->Q96GX5_p |O43768 : K(2908,1) for forward directionand K(2908,2) for reverse direction</td></tr>
<tr><td>2909</td><td>Q96GX5_p |O43768 -->Q96GX5_p +O43768_p : K(2909,1) for forward direction</td></tr>
<tr><td>2910</td><td>Q96GX5_p +Q96GX5 <-->Q96GX5_p |Q96GX5 : K(2910,1) for forward directionand K(2910,2) for reverse direction</td></tr>
<tr><td>2911</td><td>Q96GX5_p |Q96GX5 -->Q96GX5_p +Q96GX5_p : K(2911,1) for forward direction</td></tr>
<tr><td>2912</td><td>P08581_p +Q13480 <-->P08581_p |Q13480 : K(2912,1) for forward directionand K(2912,2) for reverse direction</td></tr>
<tr><td>2913</td><td>P08581_p |Q13480 -->P08581_p +Q13480_p : K(2913,1) for forward direction</td></tr>
<tr><td>2914</td><td>P42345_p +P31749 <-->P42345_p |P31749 : K(2914,1) for forward directionand K(2914,2) for reverse direction</td></tr>
<tr><td>2915</td><td>P42345_p |P31749 -->P42345_p +P31749_p : K(2915,1) for forward direction</td></tr>
<tr><td>2916</td><td>P42345_p +P42345 <-->P42345_p |P42345 : K(2916,1) for forward directionand K(2916,2) for reverse direction</td></tr>
<tr><td>2917</td><td>P42345_p |P42345 -->P42345_p +P42345_p : K(2917,1) for forward direction</td></tr>
</table>

<table>
<tr><td>2918</td><td>P42345_p +Q8N122 <-->P42345_p |Q8N122 : K(2918,1) for forward directionand K(2918,2) for reverse direction</td></tr>
<tr><td>2919</td><td>P42345_p |Q8N122 -->P42345_p +Q8N122_p : K(2919,1) for forward direction</td></tr>
<tr><td>2920</td><td>Q8TD19_p +Q8TDX7 <-->Q8TD19_p |Q8TDX7 : K(2920,1) for forward directionand K(2920,2) for reverse direction</td></tr>
<tr><td>2921</td><td>Q8TD19_p |Q8TDX7 -->Q8TD19_p +Q8TDX7_p : K(2921,1) for forward direction</td></tr>
<tr><td>2922</td><td>P22392_p +P22392 <-->P22392_p |P22392 : K(2922,1) for forward directionand K(2922,2) for reverse direction</td></tr>
<tr><td>2923</td><td>P22392_p |P22392 -->P22392_p +P22392_p : K(2923,1) for forward direction</td></tr>
<tr><td>2924</td><td>Q13153_p +Q02750 <-->Q13153_p |Q02750 : K(2924,1) for forward directionand K(2924,2) for reverse direction</td></tr>
<tr><td>2925</td><td>Q13153_p |Q02750 -->Q13153_p +Q02750_p : K(2925,1) for forward direction</td></tr>
<tr><td>2926</td><td>Q13153_p +Q13153 <-->Q13153_p |Q13153 : K(2926,1) for forward directionand K(2926,2) for reverse direction</td></tr>
<tr><td>2927</td><td>Q13153_p |Q13153 -->Q13153_p +Q13153_p : K(2927,1) for forward direction</td></tr>
<tr><td>2928</td><td>O15530_p +P31751 <-->O15530_p |P31751 : K(2928,1) for forward directionand K(2928,2) for reverse direction</td></tr>
<tr><td>2929</td><td>O15530_p |P31751 -->O15530_p +P31751_p : K(2929,1) for forward direction</td></tr>
<tr><td>2930</td><td>O15530_p +Q02750 <-->O15530_p |Q02750 : K(2930,1) for forward directionand K(2930,2) for reverse direction</td></tr>
<tr><td>2931</td><td>O15530_p |Q02750 -->O15530_p +Q02750_p : K(2931,1) for forward direction</td></tr>
<tr><td>2932</td><td>O15530_p +Q13153 <-->O15530_p |Q13153 : K(2932,1) for forward directionand K(2932,2) for reverse direction</td></tr>
<tr><td>2933</td><td>O15530_p |Q13153 -->O15530_p +Q13153_p : K(2933,1) for forward direction</td></tr>
<tr><td>2934</td><td>O15530_p +P53350 <-->O15530_p |P53350 : K(2934,1) for forward directionand K(2934,2) for reverse direction</td></tr>
<tr><td>2935</td><td>O15530_p |P53350 -->O15530_p +P53350_p : K(2935,1) for forward direction</td></tr>
<tr><td>2936</td><td>O15530_p +Q05655 <-->O15530_p |Q05655 : K(2936,1) for forward directionand K(2936,2) for reverse direction</td></tr>
<tr><td>2937</td><td>O15530_p |Q05655 -->O15530_p +Q05655_p : K(2937,1) for forward direction</td></tr>
<tr><td>2938</td><td>O15530_p +Q15418 <-->O15530_p |Q15418 : K(2938,1) for forward directionand K(2938,2) for reverse direction</td></tr>
<tr><td>2939</td><td>O15530_p |Q15418 -->O15530_p +Q15418_p : K(2939,1) for forward direction</td></tr>
<tr><td>2940</td><td>P46020_p +P46020 <-->P46020_p |P46020 : K(2940,1) for forward directionand K(2940,2) for reverse direction</td></tr>
<tr><td>2941</td><td>P46020_p |P46020 -->P46020_p +P46020_p : K(2941,1) for forward direction</td></tr>
<tr><td>2942</td><td>Q16512_p +Q16512 <-->Q16512_p |Q16512 : K(2942,1) for forward directionand K(2942,2) for reverse direction</td></tr>
<tr><td>2943</td><td>Q16512_p |Q16512 -->Q16512_p +Q16512_p : K(2943,1) for forward direction</td></tr>
<tr><td>2944</td><td>P53350_p +P14635 <-->P53350_p |P14635 : K(2944,1) for forward directionand K(2944,2) for reverse direction</td></tr>
<tr><td>2945</td><td>P53350_p |P14635 -->P53350_p +P14635_p : K(2945,1) for forward direction</td></tr>
<tr><td>2946</td><td>P53350_p +P01106 <-->P53350_p |P01106 : K(2946,1) for forward directionand K(2946,2) for reverse direction</td></tr>
<tr><td>2947</td><td>P53350_p |P01106 -->P53350_p +P01106_p : K(2947,1) for forward direction</td></tr>
<tr><td>2948</td><td>P53350_p +Q9Y266 <-->P53350_p |Q9Y266 : K(2948,1) for forward directionand K(2948,2) for reverse direction</td></tr>
<tr><td>2949</td><td>P53350_p |Q9Y266 -->P53350_p +Q9Y266_p : K(2949,1) for forward direction</td></tr>
<tr><td>2950</td><td>P67775 +O43524 <-->P67775 |O43524 : K(2950,1) for forward directionand K(2950,2) for reverse direction</td></tr>
<tr><td>2951</td><td>P67775 |O43524 -->P67775 +O43524_p : K(2951,1) for forward direction</td></tr>
<tr><td>2952</td><td>Q13131_p +O43524 <-->Q13131_p |O43524 : K(2952,1) for forward directionand K(2952,2) for reverse direction</td></tr>
<tr><td>2953</td><td>Q13131_p |O43524 -->Q13131_p +O43524_p : K(2953,1) for forward direction</td></tr>
<tr><td>2954</td><td>Q13131_p +Q9GZY8 <-->Q13131_p |Q9GZY8 : K(2954,1) for forward directionand K(2954,2) for reverse direction</td></tr>
<tr><td>2955</td><td>Q13131_p |Q9GZY8 -->Q13131_p +Q9GZY8_p : K(2955,1) for forward direction</td></tr>
<tr><td>2956</td><td>Q13131_p +O60825 <-->Q13131_p |O60825 : K(2956,1) for forward directionand K(2956,2) for reverse direction</td></tr>
<tr><td>2957</td><td>Q13131_p |O60825 -->Q13131_p +O60825_p : K(2957,1) for forward direction</td></tr>
<tr><td>2958</td><td>P17612 +P53396 <-->P17612 |P53396 : K(2958,1) for forward directionand K(2958,2) for reverse direction</td></tr>
<tr><td>2959</td><td>P17612 |P53396 -->P17612 +P53396_p : K(2959,1) for forward direction</td></tr>
<tr><td>2960</td><td>P17612 +P18846 <-->P17612 |P18846 : K(2960,1) for forward directionand K(2960,2) for reverse direction</td></tr>
</table>

<table>
<tr><td>2961</td><td>P17612 |P18846 -->P17612 +P18846_p : K(2961,1) for forward direction</td></tr>
<tr><td>2962</td><td>P17612 +P16220 <-->P17612 |P16220 : K(2962,1) for forward directionand K(2962,2) for reverse direction</td></tr>
<tr><td>2963</td><td>P17612 |P16220 -->P17612 +P16220_p : K(2963,1) for forward direction</td></tr>
<tr><td>2964</td><td>P17612 +P41240 <-->P17612 |P41240 : K(2964,1) for forward directionand K(2964,2) for reverse direction</td></tr>
<tr><td>2965</td><td>P17612 |P41240 -->P17612 +P41240_p : K(2965,1) for forward direction</td></tr>
<tr><td>2966</td><td>P17612 +P35222 <-->P17612 |P35222 : K(2966,1) for forward directionand K(2966,2) for reverse direction</td></tr>
<tr><td>2967</td><td>P17612 |P35222 -->P17612 +P35222_p : K(2967,1) for forward direction</td></tr>
<tr><td>2968</td><td>P17612 +Q93034 <-->P17612 |Q93034 : K(2968,1) for forward directionand K(2968,2) for reverse direction</td></tr>
<tr><td>2969</td><td>P17612 |Q93034 -->P17612 +Q93034_p : K(2969,1) for forward direction</td></tr>
<tr><td>2970</td><td>P17612 +O00418 <-->P17612 |O00418 : K(2970,1) for forward directionand K(2970,2) for reverse direction</td></tr>
<tr><td>2971</td><td>P17612 |O00418 -->P17612 +O00418_p : K(2971,1) for forward direction</td></tr>
<tr><td>2972</td><td>P17612 +P60983 <-->P17612 |P60983 : K(2972,1) for forward directionand K(2972,2) for reverse direction</td></tr>
<tr><td>2973</td><td>P17612 |P60983 -->P17612 +P60983_p : K(2973,1) for forward direction</td></tr>
<tr><td>2974</td><td>P17612 +P25098 <-->P17612 |P25098 : K(2974,1) for forward directionand K(2974,2) for reverse direction</td></tr>
<tr><td>2975</td><td>P17612 |P25098 -->P17612 +P25098_p : K(2975,1) for forward direction</td></tr>
<tr><td>2976</td><td>P17612 +Q07954 <-->P17612 |Q07954 : K(2976,1) for forward directionand K(2976,2) for reverse direction</td></tr>
<tr><td>2977</td><td>P17612 |Q07954 -->P17612 +Q07954_p : K(2977,1) for forward direction</td></tr>
<tr><td>2978</td><td>P17612 +O60825 <-->P17612 |O60825 : K(2978,1) for forward directionand K(2978,2) for reverse direction</td></tr>
<tr><td>2979</td><td>P17612 |O60825 -->P17612 +O60825_p : K(2979,1) for forward direction</td></tr>
<tr><td>2980</td><td>P17612 +P46020 <-->P17612 |P46020 : K(2980,1) for forward directionand K(2980,2) for reverse direction</td></tr>
<tr><td>2981</td><td>P17612 |P46020 -->P17612 +P46020_p : K(2981,1) for forward direction</td></tr>
<tr><td>2982</td><td>P17612 +O95295 <-->P17612 |O95295 : K(2982,1) for forward directionand K(2982,2) for reverse direction</td></tr>
<tr><td>2983</td><td>P17612 |O95295 -->P17612 +O95295_p : K(2983,1) for forward direction</td></tr>
<tr><td>2984</td><td>P17612 +P52888 <-->P17612 |P52888 : K(2984,1) for forward directionand K(2984,2) for reverse direction</td></tr>
<tr><td>2985</td><td>P17612 |P52888 -->P17612 +P52888_p : K(2985,1) for forward direction</td></tr>
<tr><td>2986</td><td>P13861_p +P13861 <-->P13861_p |P13861 : K(2986,1) for forward directionand K(2986,2) for reverse direction</td></tr>
<tr><td>2987</td><td>P13861_p |P13861 -->P13861_p +P13861_p : K(2987,1) for forward direction</td></tr>
<tr><td>2988</td><td>P17252_p +P25098 <-->P17252_p |P25098 : K(2988,1) for forward directionand K(2988,2) for reverse direction</td></tr>
<tr><td>2989</td><td>P17252_p |P25098 -->P17252_p +P25098_p : K(2989,1) for forward direction</td></tr>
<tr><td>2990</td><td>P17252_p +Q92686 <-->P17252_p |Q92686 : K(2990,1) for forward directionand K(2990,2) for reverse direction</td></tr>
<tr><td>2991</td><td>P17252_p |Q92686 -->P17252_p +Q92686_p : K(2991,1) for forward direction</td></tr>
<tr><td>2992</td><td>P17252_p +Q16625 <-->P17252_p |Q16625 : K(2992,1) for forward directionand K(2992,2) for reverse direction</td></tr>
<tr><td>2993</td><td>P17252_p |Q16625 -->P17252_p +Q16625_p : K(2993,1) for forward direction</td></tr>
<tr><td>2994</td><td>P17252_p +O60825 <-->P17252_p |O60825 : K(2994,1) for forward directionand K(2994,2) for reverse direction</td></tr>
<tr><td>2995</td><td>P17252_p |O60825 -->P17252_p +O60825_p : K(2995,1) for forward direction</td></tr>
<tr><td>2996</td><td>P17252_p +P31431 <-->P17252_p |P31431 : K(2996,1) for forward directionand K(2996,2) for reverse direction</td></tr>
<tr><td>2997</td><td>P17252_p |P31431 -->P17252_p +P31431_p : K(2997,1) for forward direction</td></tr>
<tr><td>2998</td><td>P17252_p +P04637 <-->P17252_p |P04637 : K(2998,1) for forward directionand K(2998,2) for reverse direction</td></tr>
<tr><td>2999</td><td>P17252_p |P04637 -->P17252_p +P04637_p : K(2999,1) for forward direction</td></tr>
<tr><td>3000</td><td>Q05655_p +P25098 <-->Q05655_p |P25098 : K(3000,1) for forward directionand K(3000,2) for reverse direction</td></tr>
<tr><td>3001</td><td>Q05655_p |P25098 -->Q05655_p +P25098_p : K(3001,1) for forward direction</td></tr>
<tr><td>3002</td><td>Q05655_p +Q13887 <-->Q05655_p |Q13887 : K(3002,1) for forward directionand K(3002,2) for reverse direction</td></tr>
<tr><td>3003</td><td>Q05655_p |Q13887 -->Q05655_p +Q13887_p : K(3003,1) for forward direction</td></tr>
</table>

3004	Q05655_p +P18433 <-->Q05655_p |P18433 : K(3004,1) for forward directionand K(3004,2) for reverse direction
3005	Q05655_p |P18433 -->Q05655_p +P18433_p : K(3005,1) for forward direction
3006	Q05655_p +P12931 <-->Q05655_p |P12931 : K(3006,1) for forward directionand K(3006,2) for reverse direction
3007	Q05655_p |P12931 -->Q05655_p +P12931_p : K(3007,1) for forward direction
3008	P78527_p +P31749 <-->P78527_p |P31749 : K(3008,1) for forward directionand K(3008,2) for reverse direction
3009	P78527_p |P31749 -->P78527_p +P31749_p : K(3009,1) for forward direction
3010	P78527_p +Q9H4A6 <-->P78527_p |Q9H4A6 : K(3010,1) for forward directionand K(3010,2) for reverse direction
3011	P78527_p |Q9H4A6 -->P78527_p +Q9H4A6_p : K(3011,1) for forward direction
3012	Q05397_p +O75962 <-->Q05397_p |O75962 : K(3012,1) for forward directionand K(3012,2) for reverse direction
3013	Q05397_p |O75962 -->Q05397_p +O75962_p : K(3013,1) for forward direction
3014	O43353_p +O43353 <-->O43353_p |O43353 : K(3014,1) for forward directionand K(3014,2) for reverse direction
3015	O43353_p |O43353 -->O43353_p +O43353_p : K(3015,1) for forward direction
3016	Q13464 +P45983 <-->Q13464 |P45983 : K(3016,1) for forward directionand K(3016,2) for reverse direction
3017	Q13464 |P45983 -->Q13464 +P45983_p : K(3017,1) for forward direction
3018	Q13464 +P26038 <-->Q13464 |P26038 : K(3018,1) for forward directionand K(3018,2) for reverse direction
3019	Q13464 |P26038 -->Q13464 +P26038_p : K(3019,1) for forward direction
3020	Q15418_p +P16220 <-->Q15418_p |P16220 : K(3020,1) for forward directionand K(3020,2) for reverse direction
3021	Q15418_p |P16220 -->Q15418_p +P16220_p : K(3021,1) for forward direction
3022	Q15418_p +Q15418 <-->Q15418_p |Q15418 : K(3022,1) for forward directionand K(3022,2) for reverse direction
3023	Q15418_p |Q15418 -->Q15418_p +Q15418_p : K(3023,1) for forward direction
3024	P51812_p +P16220 <-->P51812_p |P16220 : K(3024,1) for forward directionand K(3024,2) for reverse direction
3025	P51812_p |P16220 -->P51812_p +P16220_p : K(3025,1) for forward direction
3026	P51812_p +O60825 <-->P51812_p |O60825 : K(3026,1) for forward directionand K(3026,2) for reverse direction
3027	P51812_p |O60825 -->P51812_p +O60825_p : K(3027,1) for forward direction
3028	P12931_p +P31749 <-->P12931_p |P31749 : K(3028,1) for forward directionand K(3028,2) for reverse direction
3029	P12931_p |P31749 -->P12931_p +P31749_p : K(3029,1) for forward direction
3030	P12931_p +P13688 <-->P12931_p |P13688 : K(3030,1) for forward directionand K(3030,2) for reverse direction
3031	P12931_p |P13688 -->P12931_p +P13688_p : K(3031,1) for forward direction
3032	P12931_p +O60716 <-->P12931_p |O60716 : K(3032,1) for forward directionand K(3032,2) for reverse direction
3033	P12931_p |O60716 -->P12931_p +O60716_p : K(3033,1) for forward direction
3034	P12931_p +Q05193 <-->P12931_p |Q05193 : K(3034,1) for forward directionand K(3034,2) for reverse direction
3035	P12931_p |Q05193 -->P12931_p +Q05193_p : K(3035,1) for forward direction
3036	P12931_p +P78347 <-->P12931_p |P78347 : K(3036,1) for forward directionand K(3036,2) for reverse direction
3037	P12931_p |P78347 -->P12931_p +P78347_p : K(3037,1) for forward direction
3038	P12931_p +P14923 <-->P12931_p |P14923 : K(3038,1) for forward directionand K(3038,2) for reverse direction
3039	P12931_p |P14923 -->P12931_p +P14923_p : K(3039,1) for forward direction
3040	P12931_p +O15162 <-->P12931_p |O15162 : K(3040,1) for forward directionand K(3040,2) for reverse direction
3041	P12931_p |O15162 -->P12931_p +O15162_p : K(3041,1) for forward direction
3042	P12931_p +P18433 <-->P12931_p |P18433 : K(3042,1) for forward directionand K(3042,2) for reverse direction
3043	P12931_p |P18433 -->P12931_p +P18433_p : K(3043,1) for forward direction
3044	P12931_p +P29353 <-->P12931_p |P29353 : K(3044,1) for forward directionand K(3044,2) for reverse direction
3045	P12931_p |P29353 -->P12931_p +P29353_p : K(3045,1) for forward direction
3046	P12931_p +Q9UGK3 <-->P12931_p |Q9UGK3 : K(3046,1) for forward directionand K(3046,2) for reverse direction

3047	P12931_p |Q9UGK3 -->P12931_p +Q9UGK3_p : K(3047,1) for forward direction
3048	P12931_p +P42224 <-->P12931_p |P42224 : K(3048,1) for forward directionand K(3048,2) for reverse direction
3049	P12931_p |P42224 -->P12931_p +P42224_p : K(3049,1) for forward direction
3050	P12931_p +P40763 <-->P12931_p |P40763 : K(3050,1) for forward directionand K(3050,2) for reverse direction
3051	P12931_p |P40763 -->P12931_p +P40763_p : K(3051,1) for forward direction
3052	P12931_p +P09327 <-->P12931_p |P09327 : K(3052,1) for forward directionand K(3052,2) for reverse direction
3053	P12931_p |P09327 -->P12931_p +P09327_p : K(3053,1) for forward direction
3054	Q13043_p +Q13043 <-->Q13043_p |Q13043 : K(3054,1) for forward directionand K(3054,2) for reverse direction
3055	Q13043_p |Q13043 -->Q13043_p +Q13043_p : K(3055,1) for forward direction
3056	P43405_p +P17252 <-->P43405_p |P17252 : K(3056,1) for forward directionand K(3056,2) for reverse direction
3057	P43405_p |P17252 -->P43405_p +P17252_p : K(3057,1) for forward direction
3058	P43405_p +P78314 <-->P43405_p |P78314 : K(3058,1) for forward directionand K(3058,2) for reverse direction
3059	P43405_p |P78314 -->P43405_p +P78314_p : K(3059,1) for forward direction
3060	P43405_p +P43405 <-->P43405_p |P43405 : K(3060,1) for forward directionand K(3060,2) for reverse direction
3061	P43405_p |P43405 -->P43405_p +P43405_p : K(3061,1) for forward direction
3062	P43405_p +P68366 <-->P43405_p |P68366 : K(3062,1) for forward directionand K(3062,2) for reverse direction
3063	P43405_p |P68366 -->P43405_p +P68366_p : K(3063,1) for forward direction
3064	Q7L7X3 +P46734 <-->Q7L7X3 |P46734 : K(3064,1) for forward directionand K(3064,2) for reverse direction
3065	Q7L7X3 |P46734 -->Q7L7X3 +P46734_p : K(3065,1) for forward direction
3066	Q9UL54 +P46734 <-->Q9UL54 |P46734 : K(3066,1) for forward directionand K(3066,2) for reverse direction
3067	Q9UL54 |P46734 -->Q9UL54 +P46734_p : K(3067,1) for forward direction
3068	Q9UL54 +P52564 <-->Q9UL54 |P52564 : K(3068,1) for forward directionand K(3068,2) for reverse direction
3069	Q9UL54 |P52564 -->Q9UL54 +P52564_p : K(3069,1) for forward direction
3070	Q9H2K8_p +Q9H2K8 <-->Q9H2K8_p |Q9H2K8 : K(3070,1) for forward directionand K(3070,2) for reverse direction
3071	Q9H2K8_p |Q9H2K8 -->Q9H2K8_p +Q9H2K8_p : K(3071,1) for forward direction
3072	Q9UHD2 +Q14653 <-->Q9UHD2 |Q14653 : K(3072,1) for forward directionand K(3072,2) for reverse direction
3073	Q9UHD2 |Q14653 -->Q9UHD2 +Q14653_p : K(3073,1) for forward direction
3074	Q9UKI8 +P68431 <-->Q9UKI8 |P68431 : K(3074,1) for forward directionand K(3074,2) for reverse direction
3075	Q9UKI8 |P68431 -->Q9UKI8 +P68431_p : K(3075,1) for forward direction
3076	P33981_p +Q13257 <-->P33981_p |Q13257 : K(3076,1) for forward directionand K(3076,2) for reverse direction
3077	P33981_p |Q13257 -->P33981_p +Q13257_p : K(3077,1) for forward direction
3078	P07947_p +P07947 <-->P07947_p |P07947 : K(3078,1) for forward directionand K(3078,2) for reverse direction
3079	P07947_p |P07947 -->P07947_p +P07947_p : K(3079,1) for forward direction
3080	P31749_p +P54253 <-->P31749_p |P54253 : K(3080,1) for forward directionand K(3080,2) for reverse direction
3081	P31749_p |P54253 -->P31749_p +P54253_p : K(3081,1) for forward direction
3082	P31749_p +P98170 <-->P31749_p |P98170 : K(3082,1) for forward directionand K(3082,2) for reverse direction
3083	P31749_p |P98170 -->P31749_p +P98170_p : K(3083,1) for forward direction
3084	O96017_p +P04637 <-->O96017_p |P04637 : K(3084,1) for forward directionand K(3084,2) for reverse direction
3085	O96017_p |P04637 -->O96017_p +P04637_p : K(3085,1) for forward direction
3086	P49841_p +P19838 <-->P49841_p |P19838 : K(3086,1) for forward directionand K(3086,2) for reverse direction
3087	P49841_p |P19838 -->P49841_p +P19838_p : K(3087,1) for forward direction
3088	P06493_p +O43524 <-->P06493_p |O43524 : K(3088,1) for forward directionand K(3088,2) for reverse direction
3089	P06493_p |O43524 -->P06493_p +O43524_p : K(3089,1) for forward direction

3090	P11233_p +O43524 <-->P11233_p |O43524 : K(3090,1) for forward directionand K(3090,2) for reverse direction
3091	P11233_p |O43524 -->P11233_p +O43524_p : K(3091,1) for forward direction
3092	P24941_p +O43524 <-->P24941_p |O43524 : K(3092,1) for forward directionand K(3092,2) for reverse direction
3093	P24941_p |O43524 -->P24941_p +O43524_p : K(3093,1) for forward direction
3094	P40189_p +P27361 <-->P40189_p |P27361 : K(3094,1) for forward directionand K(3094,2) for reverse direction
3095	P40189_p |P27361 -->P40189_p +P27361_p : K(3095,1) for forward direction
3096	P42345_p +P51812 <-->P42345_p |P51812 : K(3096,1) for forward directionand K(3096,2) for reverse direction
3097	P42345_p |P51812 -->P42345_p +P51812_p : K(3097,1) for forward direction
3098	P45983_p +O43524 <-->P45983_p |O43524 : K(3098,1) for forward directionand K(3098,2) for reverse direction
3099	P45983_p |O43524 -->P45983_p +O43524_p : K(3099,1) for forward direction
3100	Q16512_p +O43524 <-->Q16512_p |O43524 : K(3100,1) for forward directionand K(3100,2) for reverse direction
3101	Q16512_p |O43524 -->Q16512_p +O43524_p : K(3101,1) for forward direction
3102	Q9HCP0 +O43524 <-->Q9HCP0 |O43524 : K(3102,1) for forward directionand K(3102,2) for reverse direction
3103	Q9HCP0 |O43524 -->Q9HCP0 +O43524_p : K(3103,1) for forward direction
3104	P27361_p +P01106 <-->P27361_p |P01106 : K(3104,1) for forward directionand K(3104,2) for reverse direction
3105	P27361_p |P01106 -->P27361_p +P01106_p : K(3105,1) for forward direction
3106	P27361_p +P09874 <-->P27361_p |P09874 : K(3106,1) for forward directionand K(3106,2) for reverse direction
3107	P27361_p |P09874 -->P27361_p +P09874_p : K(3107,1) for forward direction
3108	P27361_p +P24941 <-->P27361_p |P24941 : K(3108,1) for forward directionand K(3108,2) for reverse direction
3109	P27361_p |P24941 -->P27361_p +P24941_p : K(3109,1) for forward direction
3110	P27361_p +P68400 <-->P27361_p |P68400 : K(3110,1) for forward directionand K(3110,2) for reverse direction
3111	P27361_p |P68400 -->P27361_p +P68400_p : K(3111,1) for forward direction
3112	P27361_p +Q02750 <-->P27361_p |Q02750 : K(3112,1) for forward directionand K(3112,2) for reverse direction
3113	P27361_p |Q02750 -->P27361_p +Q02750_p : K(3113,1) for forward direction
3114	P27361_p +Q09472 <-->P27361_p |Q09472 : K(3114,1) for forward directionand K(3114,2) for reverse direction
3115	P27361_p |Q09472 -->P27361_p +Q09472_p : K(3115,1) for forward direction
3116	P27361_p +Q92574 <-->P27361_p |Q92574 : K(3116,1) for forward directionand K(3116,2) for reverse direction
3117	P27361_p |Q92574 -->P27361_p +Q92574_p : K(3117,1) for forward direction
3118	P27361_p +Q9NQX3 <-->P27361_p |Q9NQX3 : K(3118,1) for forward directionand K(3118,2) for reverse direction
3119	P27361_p |Q9NQX3 -->P27361_p +Q9NQX3_p : K(3119,1) for forward direction
3120	Q13177_p +Q02750 <-->Q13177_p |Q02750 : K(3120,1) for forward directionand K(3120,2) for reverse direction
3121	Q13177_p |Q02750 -->Q13177_p +Q02750_p : K(3121,1) for forward direction
3122	P45983_p +P35222 <-->P45983_p |P35222 : K(3122,1) for forward directionand K(3122,2) for reverse direction
3123	P45983_p |P35222 -->P45983_p +P35222_p : K(3123,1) for forward direction
3124	P45983_p +P84022 <-->P45983_p |P84022 : K(3124,1) for forward directionand K(3124,2) for reverse direction
3125	P45983_p |P84022 -->P45983_p +P84022_p : K(3125,1) for forward direction
3126	P17612 +Q16637 <-->P17612 |Q16637 : K(3126,1) for forward directionand K(3126,2) for reverse direction
3127	P17612 |Q16637 -->P17612 +Q16637_p : K(3127,1) for forward direction
3128	P31749_p +P15311 <-->P31749_p |P15311 : K(3128,1) for forward directionand K(3128,2) for reverse direction
3129	P31749_p |P15311 -->P31749_p +P15311_p : K(3129,1) for forward direction
3130	P51812_p +O00418 <-->P51812_p |O00418 : K(3130,1) for forward directionand K(3130,2) for reverse direction
3131	P51812_p |O00418 -->P51812_p +O00418_p : K(3131,1) for forward direction
3132	P51812_p +P01100 <-->P51812_p |P01100 : K(3132,1) for forward directionand K(3132,2) for reverse direction

3133	P51812_p |P01100 -->P51812_p +P01100_p : K(3133,1) for forward direction
3134	P51812_p +P19634 <-->P51812_p |P19634 : K(3134,1) for forward directionand K(3134,2) for reverse direction
3135	P51812_p |P19634 -->P51812_p +P19634_p : K(3135,1) for forward direction
3136	P51812_p +P21333 <-->P51812_p |P21333 : K(3136,1) for forward directionand K(3136,2) for reverse direction
3137	P51812_p |P21333 -->P51812_p +P21333_p : K(3137,1) for forward direction
3138	P51812_p +P23588 <-->P51812_p |P23588 : K(3138,1) for forward directionand K(3138,2) for reverse direction
3139	P51812_p |P23588 -->P51812_p +P23588_p : K(3139,1) for forward direction
3140	P51812_p +P35568 <-->P51812_p |P35568 : K(3140,1) for forward directionand K(3140,2) for reverse direction
3141	P51812_p |P35568 -->P51812_p +P35568_p : K(3141,1) for forward direction
3142	P51812_p +P49815 <-->P51812_p |P49815 : K(3142,1) for forward directionand K(3142,2) for reverse direction
3143	P51812_p |P49815 -->P51812_p +P49815_p : K(3143,1) for forward direction
3144	P51812_p +P49841 <-->P51812_p |P49841 : K(3144,1) for forward directionand K(3144,2) for reverse direction
3145	P51812_p |P49841 -->P51812_p +P49841_p : K(3145,1) for forward direction
3146	P51812_p +P50552 <-->P51812_p |P50552 : K(3146,1) for forward directionand K(3146,2) for reverse direction
3147	P51812_p |P50552 -->P51812_p +P50552_p : K(3147,1) for forward direction
3148	P51812_p +P62753 <-->P51812_p |P62753 : K(3148,1) for forward directionand K(3148,2) for reverse direction
3149	P51812_p |P62753 -->P51812_p +P62753_p : K(3149,1) for forward direction
3150	P51812_p +P78371 <-->P51812_p |P78371 : K(3150,1) for forward directionand K(3150,2) for reverse direction
3151	P51812_p |P78371 -->P51812_p +P78371_p : K(3151,1) for forward direction
3152	P51812_p +Q8N122 <-->P51812_p |Q8N122 : K(3152,1) for forward directionand K(3152,2) for reverse direction
3153	P51812_p |Q8N122 -->P51812_p +Q8N122_p : K(3153,1) for forward direction
3154	P51812_p +Q8TB45 <-->P51812_p |Q8TB45 : K(3154,1) for forward directionand K(3154,2) for reverse direction
3155	P51812_p |Q8TB45 -->P51812_p +Q8TB45_p : K(3155,1) for forward direction
3156	P51812_p +Q9H6Z4 <-->P51812_p |Q9H6Z4 : K(3156,1) for forward directionand K(3156,2) for reverse direction
3157	P51812_p |Q9H6Z4 -->P51812_p +Q9H6Z4_p : K(3157,1) for forward direction
3158	P51812_p +Q9UBP6 <-->P51812_p |Q9UBP6 : K(3158,1) for forward directionand K(3158,2) for reverse direction
3159	P51812_p |Q9UBP6 -->P51812_p +Q9UBP6_p : K(3159,1) for forward direction
3160	P51812_p +Q9Y2V2 <-->P51812_p |Q9Y2V2 : K(3160,1) for forward directionand K(3160,2) for reverse direction
3161	P51812_p |Q9Y2V2 -->P51812_p +Q9Y2V2_p : K(3161,1) for forward direction
3162	P51812_p +P51812 <-->P51812_p |P51812 : K(3162,1) for forward directionand K(3162,2) for reverse direction
3163	P51812_p |P51812 -->P51812_p +P51812_p : K(3163,1) for forward direction
3164	O15111_p +SIGNOR-C13 <-->O15111_p |SIGNOR-C13 : K(3164,1) for forward directionand K(3164,2) for reverse direction
3165	O15111_p |SIGNOR-C13 -->O15111_p +SIGNOR-C13_p : K(3165,1) for forward direction
3166	O75676_p +SIGNOR-C13 <-->O75676_p |SIGNOR-C13 : K(3166,1) for forward directionand K(3166,2) for reverse direction
3167	O75676_p |SIGNOR-C13 -->O75676_p +SIGNOR-C13_p : K(3167,1) for forward direction
3168	P17612 +SIGNOR-C13 <-->P17612 |SIGNOR-C13 : K(3168,1) for forward directionand K(3168,2) for reverse direction
3169	P17612 |SIGNOR-C13 -->P17612 +SIGNOR-C13_p : K(3169,1) for forward direction
3170	P31749_p +SIGNOR-C101 <-->P31749_p |SIGNOR-C101 : K(3170,1) for forward directionand K(3170,2) for reverse direction
3171	P31749_p |SIGNOR-C101 -->P31749_p +SIGNOR-C101_p : K(3171,1) for forward direction
3172	P31751_p +SIGNOR-C101 <-->P31751_p |SIGNOR-C101 : K(3172,1) for forward directionand K(3172,2) for reverse direction
3173	P31751_p |SIGNOR-C101 -->P31751_p +SIGNOR-C101_p : K(3173,1) for forward direction
3174	P45983_p +SIGNOR-C154 <-->P45983_p |SIGNOR-C154 : K(3174,1) for forward directionand K(3174,2) for reverse direction
3175	P45983_p |SIGNOR-C154 -->P45983_p +SIGNOR-C154_p : K(3175,1) for forward direction

3176	P49841_p +SIGNOR-C13 <-->P49841_p |SIGNOR-C13 : K(3176,1) for forward directionand K(3176,2) for reverse direction
3177	P49841_p |SIGNOR-C13 -->P49841_p +SIGNOR-C13_p : K(3177,1) for forward direction
3178	Q15418_p +SIGNOR-C101 <-->Q15418_p |SIGNOR-C101 : K(3178,1) for forward directionand K(3178,2) for reverse direction
3179	Q15418_p |SIGNOR-C101 -->Q15418_p +SIGNOR-C101_p : K(3179,1) for forward direction
3180	Q9Y6K9_u +SIGNOR-C13 <-->Q9Y6K9_u |SIGNOR-C13 : K(3180,1) for forward directionand K(3180,2) for reverse direction
3181	Q9Y6K9_u |SIGNOR-C13 -->Q9Y6K9_u +SIGNOR-C13_p : K(3181,1) for forward direction
3182	SIGNOR-C17 +O00311 <-->SIGNOR-C17 |O00311 : K(3182,1) for forward directionand K(3182,2) for reverse direction
3183	SIGNOR-C17 |O00311 -->SIGNOR-C17 +O00311_p : K(3183,1) for forward direction
3184	SIGNOR-C17 +O00571 <-->SIGNOR-C17 |O00571 : K(3184,1) for forward directionand K(3184,2) for reverse direction
3185	SIGNOR-C17 |O00571 -->SIGNOR-C17 +O00571_p : K(3185,1) for forward direction
3186	SIGNOR-C17 +P06748 <-->SIGNOR-C17 |P06748 : K(3186,1) for forward directionand K(3186,2) for reverse direction
3187	SIGNOR-C17 |P06748 -->SIGNOR-C17 +P06748_p : K(3187,1) for forward direction
3188	SIGNOR-C17 +P08047 <-->SIGNOR-C17 |P08047 : K(3188,1) for forward directionand K(3188,2) for reverse direction
3189	SIGNOR-C17 |P08047 -->SIGNOR-C17 +P08047_p : K(3189,1) for forward direction
3190	SIGNOR-C17 +P15531 <-->SIGNOR-C17 |P15531 : K(3190,1) for forward directionand K(3190,2) for reverse direction
3191	SIGNOR-C17 |P15531 -->SIGNOR-C17 +P15531_p : K(3191,1) for forward direction
3192	SIGNOR-C17 +P46060 <-->SIGNOR-C17 |P46060 : K(3192,1) for forward directionand K(3192,2) for reverse direction
3193	SIGNOR-C17 |P46060 -->SIGNOR-C17 +P46060_p : K(3193,1) for forward direction
3194	SIGNOR-C17 +P48200 <-->SIGNOR-C17 |P48200 : K(3194,1) for forward directionand K(3194,2) for reverse direction
3195	SIGNOR-C17 |P48200 -->SIGNOR-C17 +P48200_p : K(3195,1) for forward direction
3196	SIGNOR-C17 +P50548 <-->SIGNOR-C17 |P50548 : K(3196,1) for forward directionand K(3196,2) for reverse direction
3197	SIGNOR-C17 |P50548 -->SIGNOR-C17 +P50548_p : K(3197,1) for forward direction
3198	SIGNOR-C17 +P55211 <-->SIGNOR-C17 |P55211 : K(3198,1) for forward directionand K(3198,2) for reverse direction
3199	SIGNOR-C17 |P55211 -->SIGNOR-C17 +P55211_p : K(3199,1) for forward direction
3200	SIGNOR-C17 +Q07820 <-->SIGNOR-C17 |Q07820 : K(3200,1) for forward directionand K(3200,2) for reverse direction
3201	SIGNOR-C17 |Q07820 -->SIGNOR-C17 +Q07820_p : K(3201,1) for forward direction
3202	SIGNOR-C17 +Q13501 <-->SIGNOR-C17 |Q13501 : K(3202,1) for forward directionand K(3202,2) for reverse direction
3203	SIGNOR-C17 |Q13501 -->SIGNOR-C17 +Q13501_p : K(3203,1) for forward direction
3204	SIGNOR-C17 +Q14790 <-->SIGNOR-C17 |Q14790 : K(3204,1) for forward directionand K(3204,2) for reverse direction
3205	SIGNOR-C17 |Q14790 -->SIGNOR-C17 +Q14790_p : K(3205,1) for forward direction
3206	SIGNOR-C17 +Q15149 <-->SIGNOR-C17 |Q15149 : K(3206,1) for forward directionand K(3206,2) for reverse direction
3207	SIGNOR-C17 |Q15149 -->SIGNOR-C17 +Q15149_p : K(3207,1) for forward direction
3208	SIGNOR-C17 +Q86WB0 <-->SIGNOR-C17 |Q86WB0 : K(3208,1) for forward directionand K(3208,2) for reverse direction
3209	SIGNOR-C17 |Q86WB0 -->SIGNOR-C17 +Q86WB0_p : K(3209,1) for forward direction
3210	SIGNOR-C17 +Q92574 <-->SIGNOR-C17 |Q92574 : K(3210,1) for forward directionand K(3210,2) for reverse direction
3211	SIGNOR-C17 |Q92574 -->SIGNOR-C17 +Q92574_p : K(3211,1) for forward direction
3212	SIGNOR-C17 +Q92993 <-->SIGNOR-C17 |Q92993 : K(3212,1) for forward directionand K(3212,2) for reverse direction
3213	SIGNOR-C17 |Q92993 -->SIGNOR-C17 +Q92993_p : K(3213,1) for forward direction
3214	SIGNOR-C17 +Q96EB6 <-->SIGNOR-C17 |Q96EB6 : K(3214,1) for forward directionand K(3214,2) for reverse direction
3215	SIGNOR-C17 |Q96EB6 -->SIGNOR-C17 +Q96EB6_p : K(3215,1) for forward direction
3216	SIGNOR-C17 +Q96GX5 <-->SIGNOR-C17 |Q96GX5 : K(3216,1) for forward directionand K(3216,2) for reverse direction
3217	SIGNOR-C17 |Q96GX5 -->SIGNOR-C17 +Q96GX5_p : K(3217,1) for forward direction
3218	SIGNOR-C17 +Q96KB5 <-->SIGNOR-C17 |Q96KB5 : K(3218,1) for forward directionand K(3218,2) for reverse direction

3219	SIGNOR-C17 |Q96KB5 -->SIGNOR-C17 +Q96KB5_p : K(3219,1) for forward direction
3220	SIGNOR-C17 +Q9H8V3 <-->SIGNOR-C17 |Q9H8V3 : K(3220,1) for forward directionand K(3220,2) for reverse direction
3221	SIGNOR-C17 |Q9H8V3 -->SIGNOR-C17 +Q9H8V3_p : K(3221,1) for forward direction
3222	SIGNOR-C83 +O00311 <-->SIGNOR-C83 |O00311 : K(3222,1) for forward directionand K(3222,2) for reverse direction
3223	SIGNOR-C83 |O00311 -->SIGNOR-C83 +O00311_p : K(3223,1) for forward direction
3224	SIGNOR-C83 +P04637 <-->SIGNOR-C83 |P04637 : K(3224,1) for forward directionand K(3224,2) for reverse direction
3225	SIGNOR-C83 |P04637 -->SIGNOR-C83 +P04637_p : K(3225,1) for forward direction
3226	SIGNOR-C83 +P06400 <-->SIGNOR-C83 |P06400 : K(3226,1) for forward directionand K(3226,2) for reverse direction
3227	SIGNOR-C83 |P06400 -->SIGNOR-C83 +P06400_p : K(3227,1) for forward direction
3228	SIGNOR-C83 +P08047 <-->SIGNOR-C83 |P08047 : K(3228,1) for forward directionand K(3228,2) for reverse direction
3229	SIGNOR-C83 |P08047 -->SIGNOR-C83 +P08047_p : K(3229,1) for forward direction
3230	SIGNOR-C83 +P17480 <-->SIGNOR-C83 |P17480 : K(3230,1) for forward directionand K(3230,2) for reverse direction
3231	SIGNOR-C83 |P17480 -->SIGNOR-C83 +P17480_p : K(3231,1) for forward direction
3232	SIGNOR-C83 +P17676 <-->SIGNOR-C83 |P17676 : K(3232,1) for forward directionand K(3232,2) for reverse direction
3233	SIGNOR-C83 |P17676 -->SIGNOR-C83 +P17676_p : K(3233,1) for forward direction
3234	SIGNOR-C83 +P31749 <-->SIGNOR-C83 |P31749 : K(3234,1) for forward directionand K(3234,2) for reverse direction
3235	SIGNOR-C83 |P31749 -->SIGNOR-C83 +P31749_p : K(3235,1) for forward direction
3236	SIGNOR-C83 +P33991 <-->SIGNOR-C83 |P33991 : K(3236,1) for forward directionand K(3236,2) for reverse direction
3237	SIGNOR-C83 |P33991 -->SIGNOR-C83 +P33991_p : K(3237,1) for forward direction
3238	SIGNOR-C83 +Q13416 <-->SIGNOR-C83 |Q13416 : K(3238,1) for forward directionand K(3238,2) for reverse direction
3239	SIGNOR-C83 |Q13416 -->SIGNOR-C83 +Q13416_p : K(3239,1) for forward direction
3240	P27361_p +SIGNOR-C154 <-->P27361_p |SIGNOR-C154 : K(3240,1) for forward directionand K(3240,2) for reverse direction
3241	P27361_p |SIGNOR-C154 -->P27361_p +SIGNOR-C154_p : K(3241,1) for forward direction
3242	Q99504 +P16104_p <-->Q99504 |P16104_p : K(3242,1) for forward directionand K(3242,2) for reverse direction
3243	Q99504 |P16104_p -->Q99504 +P16104 : K(3243,1) for forward direction
3244	Q9P0J1 +Q15797_p <-->Q9P0J1 |Q15797_p : K(3244,1) for forward directionand K(3244,2) for reverse direction
3245	Q9P0J1 |Q15797_p -->Q9P0J1 +Q15797 : K(3245,1) for forward direction
3246	P35813 +O15169_p <-->P35813 |O15169_p : K(3246,1) for forward directionand K(3246,2) for reverse direction
3247	P35813 |O15169_p -->P35813 +O15169 : K(3247,1) for forward direction
3248	P35813 +Q15797_p <-->P35813 |Q15797_p : K(3248,1) for forward directionand K(3248,2) for reverse direction
3249	P35813 |Q15797_p -->P35813 +Q15797 : K(3249,1) for forward direction
3250	P35813 +Q15796_p <-->P35813 |Q15796_p : K(3250,1) for forward directionand K(3250,2) for reverse direction
3251	P35813 |Q15796_p -->P35813 +Q15796 : K(3251,1) for forward direction
3252	P35813 +P84022_p <-->P35813 |P84022_p : K(3252,1) for forward directionand K(3252,2) for reverse direction
3253	P35813 |P84022_p -->P35813 +P84022 : K(3253,1) for forward direction
3254	P62136 +P31749_p <-->P62136 |P31749_p : K(3254,1) for forward directionand K(3254,2) for reverse direction
3255	P62136 |P31749_p -->P62136 +P31749 : K(3255,1) for forward direction
3256	P62136 +P28482_p <-->P62136 |P28482_p : K(3256,1) for forward directionand K(3256,2) for reverse direction
3257	P62136 |P28482_p -->P62136 +P28482 : K(3257,1) for forward direction
3258	P62136 +P27361_p <-->P62136 |P27361_p : K(3258,1) for forward directionand K(3258,2) for reverse direction
3259	P62136 |P27361_p -->P62136 +P27361 : K(3259,1) for forward direction
3260	P62140 +P31749_p <-->P62140 |P31749_p : K(3260,1) for forward directionand K(3260,2) for reverse direction
3261	P62140 |P31749_p -->P62140 +P31749 : K(3261,1) for forward direction

3262	P67775 +P31749_p <-->P67775 |P31749_p : K(3262,1) for forward directionand K(3262,2) for reverse direction
3263	P67775 |P31749_p -->P67775 +P31749 : K(3263,1) for forward direction
3264	P67775 +P06730_p <-->P67775 |P06730_p : K(3264,1) for forward directionand K(3264,2) for reverse direction
3265	P67775 |P06730_p -->P67775 +P06730 : K(3265,1) for forward direction
3266	P67775 +Q02750_p <-->P67775 |Q02750_p : K(3266,1) for forward directionand K(3266,2) for reverse direction
3267	P67775 |Q02750_p -->P67775 +Q02750 : K(3267,1) for forward direction
3268	P67775 +P36507_p <-->P67775 |P36507_p : K(3268,1) for forward directionand K(3268,2) for reverse direction
3269	P67775 |P36507_p -->P67775 +P36507 : K(3269,1) for forward direction
3270	P67775 +Q99759_p <-->P67775 |Q99759_p : K(3270,1) for forward directionand K(3270,2) for reverse direction
3271	P67775 |Q99759_p -->P67775 +Q99759 : K(3271,1) for forward direction
3272	P67775 +O43318_p <-->P67775 |O43318_p : K(3272,1) for forward directionand K(3272,2) for reverse direction
3273	P67775 |O43318_p -->P67775 +O43318 : K(3273,1) for forward direction
3274	P67775 +P28482_p <-->P67775 |P28482_p : K(3274,1) for forward directionand K(3274,2) for reverse direction
3275	P67775 |P28482_p -->P67775 +P28482 : K(3275,1) for forward direction
3276	P67775 +P27361_p <-->P67775 |P27361_p : K(3276,1) for forward directionand K(3276,2) for reverse direction
3277	P67775 |P27361_p -->P67775 +P27361 : K(3277,1) for forward direction
3278	P67775 +Q9H0H5_p <-->P67775 |Q9H0H5_p : K(3278,1) for forward directionand K(3278,2) for reverse direction
3279	P67775 |Q9H0H5_p -->P67775 +Q9H0H5 : K(3279,1) for forward direction
3280	P67775 +Q04206_p <-->P67775 |Q04206_p : K(3280,1) for forward directionand K(3280,2) for reverse direction
3281	P67775 |Q04206_p -->P67775 +Q04206 : K(3281,1) for forward direction
3282	P67775 +P23396_p <-->P67775 |P23396_p : K(3282,1) for forward directionand K(3282,2) for reverse direction
3283	P67775 |P23396_p -->P67775 +P23396 : K(3283,1) for forward direction
3284	P67775 +P84022_p <-->P67775 |P84022_p : K(3284,1) for forward directionand K(3284,2) for reverse direction
3285	P67775 |P84022_p -->P67775 +P84022 : K(3285,1) for forward direction
3286	P67775 +Q13188_p <-->P67775 |Q13188_p : K(3286,1) for forward directionand K(3286,2) for reverse direction
3287	P67775 |Q13188_p -->P67775 +Q13188 : K(3287,1) for forward direction
3288	P67775 +Q13043_p <-->P67775 |Q13043_p : K(3288,1) for forward directionand K(3288,2) for reverse direction
3289	P67775 |Q13043_p -->P67775 +Q13043 : K(3289,1) for forward direction
3290	P62714 +P31749_p <-->P62714 |P31749_p : K(3290,1) for forward directionand K(3290,2) for reverse direction
3291	P62714 |P31749_p -->P62714 +P31749 : K(3291,1) for forward direction
3292	P62714 +P31751_p <-->P62714 |P31751_p : K(3292,1) for forward directionand K(3292,2) for reverse direction
3293	P62714 |P31751_p -->P62714 +P31751 : K(3293,1) for forward direction
3294	P62714 +P01106_p <-->P62714 |P01106_p : K(3294,1) for forward directionand K(3294,2) for reverse direction
3295	P62714 |P01106_p -->P62714 +P01106 : K(3295,1) for forward direction
3296	P62714 +P11233_p <-->P62714 |P11233_p : K(3296,1) for forward directionand K(3296,2) for reverse direction
3297	P62714 |P11233_p -->P62714 +P11233 : K(3297,1) for forward direction
3298	Q08209 +P21333_p <-->Q08209 |P21333_p : K(3298,1) for forward directionand K(3298,2) for reverse direction
3299	Q08209 |P21333_p -->Q08209 +P21333 : K(3299,1) for forward direction
3300	O00743 +O43318_p <-->O00743 |O43318_p : K(3300,1) for forward directionand K(3300,2) for reverse direction
3301	O00743 |O43318_p -->O00743 +O43318 : K(3301,1) for forward direction
3302	Q15257 +P31749_p <-->Q15257 |P31749_p : K(3302,1) for forward directionand K(3302,2) for reverse direction
3303	Q15257 |P31749_p -->Q15257 +P31749 : K(3303,1) for forward direction
3304	P18031_p +P31749_p <-->P18031_p |P31749_p : K(3304,1) for forward directionand K(3304,2) for reverse direction

3305	P18031_p |P31749_p -->P18031_p +P31749 : K(3305,1) for forward direction
3306	P18031_p +P11274_p <-->P18031_p |P11274_p : K(3306,1) for forward directionand K(3306,2) for reverse direction
3307	P18031_p |P11274_p -->P18031_p +P11274 : K(3307,1) for forward direction
3308	P18031_p +P35568_p <-->P18031_p |P35568_p : K(3308,1) for forward directionand K(3308,2) for reverse direction
3309	P18031_p |P35568_p -->P18031_p +P35568 : K(3309,1) for forward direction
3310	P18031_p +P08581_p <-->P18031_p |P08581_p : K(3310,1) for forward directionand K(3310,2) for reverse direction
3311	P18031_p |P08581_p -->P18031_p +P08581 : K(3311,1) for forward direction
3312	P18031_p +Q05655_p <-->P18031_p |Q05655_p : K(3312,1) for forward directionand K(3312,2) for reverse direction
3313	P18031_p |Q05655_p -->P18031_p +Q05655 : K(3313,1) for forward direction
3314	P18031_p +Q05397_p <-->P18031_p |Q05397_p : K(3314,1) for forward directionand K(3314,2) for reverse direction
3315	P18031_p |Q05397_p -->P18031_p +Q05397 : K(3315,1) for forward direction
3316	P18031_p +P40763_p <-->P18031_p |P40763_p : K(3316,1) for forward directionand K(3316,2) for reverse direction
3317	P18031_p |P40763_p -->P18031_p +P40763 : K(3317,1) for forward direction
3318	P18031_p +P51692_p <-->P18031_p |P51692_p : K(3318,1) for forward directionand K(3318,2) for reverse direction
3319	P18031_p |P51692_p -->P18031_p +P51692 : K(3319,1) for forward direction
3320	Q06124 +P35221_p <-->Q06124 |P35221_p : K(3320,1) for forward directionand K(3320,2) for reverse direction
3321	Q06124 |P35221_p -->Q06124 +P35221 : K(3321,1) for forward direction
3322	Q06124 +Q13480_p <-->Q06124 |Q13480_p : K(3322,1) for forward directionand K(3322,2) for reverse direction
3323	Q06124 |Q13480_p -->Q06124 +Q13480 : K(3323,1) for forward direction
3324	Q06124 +P35568_p <-->Q06124 |P35568_p : K(3324,1) for forward directionand K(3324,2) for reverse direction
3325	Q06124 |P35568_p -->Q06124 +P35568 : K(3325,1) for forward direction
3326	Q06124 +O95297_p <-->Q06124 |O95297_p : K(3326,1) for forward directionand K(3326,2) for reverse direction
3327	Q06124 |O95297_p -->Q06124 +O95297 : K(3327,1) for forward direction
3328	Q06124 +Q05397_p <-->Q06124 |Q05397_p : K(3328,1) for forward directionand K(3328,2) for reverse direction
3329	Q06124 |Q05397_p -->Q06124 +Q05397 : K(3329,1) for forward direction
3330	Q05209 +P56945_p <-->Q05209 |P56945_p : K(3330,1) for forward directionand K(3330,2) for reverse direction
3331	Q05209 |P56945_p -->Q05209 +P56945 : K(3331,1) for forward direction
3332	Q05209 +Q14161_p <-->Q05209 |Q14161_p : K(3332,1) for forward directionand K(3332,2) for reverse direction
3333	Q05209 |Q14161_p -->Q05209 +Q14161 : K(3333,1) for forward direction
3334	Q05209 +Q05397_p <-->Q05209 |Q05397_p : K(3334,1) for forward directionand K(3334,2) for reverse direction
3335	Q05209 |Q05397_p -->Q05209 +Q05397 : K(3335,1) for forward direction
3336	Q05209 +P29353_p <-->Q05209 |P29353_p : K(3336,1) for forward directionand K(3336,2) for reverse direction
3337	Q05209 |P29353_p -->Q05209 +P29353 : K(3337,1) for forward direction
3338	P17706 +P00533_p <-->P17706 |P00533_p : K(3338,1) for forward directionand K(3338,2) for reverse direction
3339	P17706 |P00533_p -->P17706 +P00533 : K(3339,1) for forward direction
3340	P17706 +Q02790_p <-->P17706 |Q02790_p : K(3340,1) for forward directionand K(3340,2) for reverse direction
3341	P17706 |Q02790_p -->P17706 +Q02790 : K(3341,1) for forward direction
3342	P17706 +P08581_p <-->P17706 |P08581_p : K(3342,1) for forward directionand K(3342,2) for reverse direction
3343	P17706 |P08581_p -->P17706 +P08581 : K(3343,1) for forward direction
3344	P17706 +P12931_p <-->P17706 |P12931_p : K(3344,1) for forward directionand K(3344,2) for reverse direction
3345	P17706 |P12931_p -->P17706 +P12931 : K(3345,1) for forward direction
3346	P17706 +P42224_p <-->P17706 |P42224_p : K(3346,1) for forward directionand K(3346,2) for reverse direction
3347	P17706 |P42224_p -->P17706 +P42224 : K(3347,1) for forward direction

3348	P17706 +P40763_p <-->P17706 |P40763_p : K(3348,1) for forward directionand K(3348,2) for reverse direction
3349	P17706 |P40763_p -->P17706 +P40763 : K(3349,1) for forward direction
3350	P17706 +O00401_p <-->P17706 |O00401_p : K(3350,1) for forward directionand K(3350,2) for reverse direction
3351	P17706 |O00401_p -->P17706 +O00401 : K(3351,1) for forward direction
3352	P29350_p +P63261_p <-->P29350_p |P63261_p : K(3352,1) for forward directionand K(3352,2) for reverse direction
3353	P29350_p |P63261_p -->P29350_p +P63261 : K(3353,1) for forward direction
3354	P29350_p +P00533_p <-->P29350_p |P00533_p : K(3354,1) for forward directionand K(3354,2) for reverse direction
3355	P29350_p |P00533_p -->P29350_p +P00533 : K(3355,1) for forward direction
3356	P29350_p +P78314_p <-->P29350_p |P78314_p : K(3356,1) for forward directionand K(3356,2) for reverse direction
3357	P29350_p |P78314_p -->P29350_p +P78314 : K(3357,1) for forward direction
3358	P29350_p +P40763_p <-->P29350_p |P40763_p : K(3358,1) for forward directionand K(3358,2) for reverse direction
3359	P29350_p |P40763_p -->P29350_p +P40763 : K(3359,1) for forward direction
3360	P29350_p +P43405_p <-->P29350_p |P43405_p : K(3360,1) for forward directionand K(3360,2) for reverse direction
3361	P29350_p |P43405_p -->P29350_p +P43405 : K(3361,1) for forward direction
3362	P43378 +P46459_p <-->P43378 |P46459_p : K(3362,1) for forward directionand K(3362,2) for reverse direction
3363	P43378 |P46459_p -->P43378 +P46459 : K(3363,1) for forward direction
3364	P10586 +P31749_p <-->P10586 |P31749_p : K(3364,1) for forward directionand K(3364,2) for reverse direction
3365	P10586 |P31749_p -->P10586 +P31749 : K(3365,1) for forward direction
3366	P24666 +P31749_p <-->P24666 |P31749_p : K(3366,1) for forward directionand K(3366,2) for reverse direction
3367	P24666 |P31749_p -->P24666 +P31749 : K(3367,1) for forward direction
3368	P24666 +P31751_p <-->P24666 |P31751_p : K(3368,1) for forward directionand K(3368,2) for reverse direction
3369	P24666 |P31751_p -->P24666 +P31751 : K(3369,1) for forward direction
3370	P24666 +P29317_p <-->P24666 |P29317_p : K(3370,1) for forward directionand K(3370,2) for reverse direction
3371	P24666 |P29317_p -->P24666 +P29317 : K(3371,1) for forward direction
3372	P24666 +P12931_p <-->P24666 |P12931_p : K(3372,1) for forward directionand K(3372,2) for reverse direction
3373	P24666 |P12931_p -->P24666 +P12931 : K(3373,1) for forward direction
3374	P51452 +P00533_p <-->P51452 |P00533_p : K(3374,1) for forward directionand K(3374,2) for reverse direction
3375	P51452 |P00533_p -->P51452 +P00533 : K(3375,1) for forward direction
3376	P51452 +P04626_p <-->P51452 |P04626_p : K(3376,1) for forward directionand K(3376,2) for reverse direction
3377	P51452 |P04626_p -->P51452 +P04626 : K(3377,1) for forward direction
3378	P51452 +P28482_p <-->P51452 |P28482_p : K(3378,1) for forward directionand K(3378,2) for reverse direction
3379	P51452 |P28482_p -->P51452 +P28482 : K(3379,1) for forward direction
3380	P51452 +P27361_p <-->P51452 |P27361_p : K(3380,1) for forward directionand K(3380,2) for reverse direction
3381	P51452 |P27361_p -->P51452 +P27361 : K(3381,1) for forward direction
3382	Q96GD0 +P23528_p <-->Q96GD0 |P23528_p : K(3382,1) for forward directionand K(3382,2) for reverse direction
3383	Q96GD0 |P23528_p -->Q96GD0 +P23528 : K(3383,1) for forward direction
3384	P35813 +P50750_p <-->P35813 |P50750_p : K(3384,1) for forward directionand K(3384,2) for reverse direction
3385	P35813 |P50750_p -->P35813 +P50750 : K(3385,1) for forward direction
3386	P35813 +Q16539_p <-->P35813 |Q16539_p : K(3386,1) for forward directionand K(3386,2) for reverse direction
3387	P35813 |Q16539_p -->P35813 +Q16539 : K(3387,1) for forward direction
3388	P35813 +Q13153_p <-->P35813 |Q13153_p : K(3388,1) for forward directionand K(3388,2) for reverse direction
3389	P35813 |Q13153_p -->P35813 +Q13153 : K(3389,1) for forward direction
3390	P62136 +O15169_p <-->P62136 |O15169_p : K(3390,1) for forward directionand K(3390,2) for reverse direction

3391	P62136 |O15169_p -->P62136 +O15169 : K(3391,1) for forward direction
3392	P62136 +P40763_p <-->P62136 |P40763_p : K(3392,1) for forward directionand K(3392,2) for reverse direction
3393	P62136 |P40763_p -->P62136 +P40763 : K(3393,1) for forward direction
3394	P62136 +P04637_p <-->P62136 |P04637_p : K(3394,1) for forward directionand K(3394,2) for reverse direction
3395	P62136 |P04637_p -->P62136 +P04637 : K(3395,1) for forward direction
3396	P62140 +O15169_p <-->P62140 |O15169_p : K(3396,1) for forward directionand K(3396,2) for reverse direction
3397	P62140 |O15169_p -->P62140 +O15169 : K(3397,1) for forward direction
3398	P62140 +P04637_p <-->P62140 |P04637_p : K(3398,1) for forward directionand K(3398,2) for reverse direction
3399	P62140 |P04637_p -->P62140 +P04637 : K(3399,1) for forward direction
3400	P36873 +P31749_p <-->P36873 |P31749_p : K(3400,1) for forward directionand K(3400,2) for reverse direction
3401	P36873 |P31749_p -->P36873 +P31749 : K(3401,1) for forward direction
3402	P36873 +O15169_p <-->P36873 |O15169_p : K(3402,1) for forward directionand K(3402,2) for reverse direction
3403	P36873 |O15169_p -->P36873 +O15169 : K(3403,1) for forward direction
3404	P36873 +Q9BY44_p <-->P36873 |Q9BY44_p : K(3404,1) for forward directionand K(3404,2) for reverse direction
3405	P36873 |Q9BY44_p -->P36873 +Q9BY44 : K(3405,1) for forward direction
3406	P36873 +P04637_p <-->P36873 |P04637_p : K(3406,1) for forward directionand K(3406,2) for reverse direction
3407	P36873 |P04637_p -->P36873 +P04637 : K(3407,1) for forward direction
3408	P67775 +P31751_p <-->P67775 |P31751_p : K(3408,1) for forward directionand K(3408,2) for reverse direction
3409	P67775 |P31751_p -->P67775 +P31751 : K(3409,1) for forward direction
3410	P67775 +O14757_p <-->P67775 |O14757_p : K(3410,1) for forward directionand K(3410,2) for reverse direction
3411	P67775 |O14757_p -->P67775 +O14757 : K(3411,1) for forward direction
3412	P67775 +P32519_p <-->P67775 |P32519_p : K(3412,1) for forward directionand K(3412,2) for reverse direction
3413	P67775 |P32519_p -->P67775 +P32519 : K(3413,1) for forward direction
3414	P67775 +Q13153_p <-->P67775 |Q13153_p : K(3414,1) for forward directionand K(3414,2) for reverse direction
3415	P67775 |Q13153_p -->P67775 +Q13153 : K(3415,1) for forward direction
3416	P67775 +Q05655_p <-->P67775 |Q05655_p : K(3416,1) for forward directionand K(3416,2) for reverse direction
3417	P67775 |Q05655_p -->P67775 +Q05655 : K(3417,1) for forward direction
3418	P67775 +P12931_p <-->P67775 |P12931_p : K(3418,1) for forward directionand K(3418,2) for reverse direction
3419	P67775 |P12931_p -->P67775 +P12931 : K(3419,1) for forward direction
3420	P67775 +P04637_p <-->P67775 |P04637_p : K(3420,1) for forward directionand K(3420,2) for reverse direction
3421	P67775 |P04637_p -->P67775 +P04637 : K(3421,1) for forward direction
3422	P67775 +Q12933_p <-->P67775 |Q12933_p : K(3422,1) for forward directionand K(3422,2) for reverse direction
3423	P67775 |Q12933_p -->P67775 +Q12933 : K(3423,1) for forward direction
3424	P62714 +O14757_p <-->P62714 |O14757_p : K(3424,1) for forward directionand K(3424,2) for reverse direction
3425	P62714 |O14757_p -->P62714 +O14757 : K(3425,1) for forward direction
3426	P62714 +P32519_p <-->P62714 |P32519_p : K(3426,1) for forward directionand K(3426,2) for reverse direction
3427	P62714 |P32519_p -->P62714 +P32519 : K(3427,1) for forward direction
3428	P62714 +P28482_p <-->P62714 |P28482_p : K(3428,1) for forward directionand K(3428,2) for reverse direction
3429	P62714 |P28482_p -->P62714 +P28482 : K(3429,1) for forward direction
3430	P62714 +Q13153_p <-->P62714 |Q13153_p : K(3430,1) for forward directionand K(3430,2) for reverse direction
3431	P62714 |Q13153_p -->P62714 +Q13153 : K(3431,1) for forward direction
3432	P62714 +Q05655_p <-->P62714 |Q05655_p : K(3432,1) for forward directionand K(3432,2) for reverse direction
3433	P62714 |Q05655_p -->P62714 +Q05655 : K(3433,1) for forward direction

3434	P62714 +P04637_p <-->P62714 |P04637_p : K(3434,1) for forward directionand K(3434,2) for reverse direction
3435	P62714 |P04637_p -->P62714 +P04637 : K(3435,1) for forward direction
3436	P62714 +Q12933_p <-->P62714 |Q12933_p : K(3436,1) for forward directionand K(3436,2) for reverse direction
3437	P62714 |Q12933_p -->P62714 +Q12933 : K(3437,1) for forward direction
3438	P60510 +O15379_p <-->P60510 |O15379_p : K(3438,1) for forward directionand K(3438,2) for reverse direction
3439	P60510 |O15379_p -->P60510 +O15379 : K(3439,1) for forward direction
3440	P60510 +Q9GZM8_p <-->P60510 |Q9GZM8_p : K(3440,1) for forward directionand K(3440,2) for reverse direction
3441	P60510 |Q9GZM8_p -->P60510 +Q9GZM8 : K(3441,1) for forward direction
3442	P53041 +Q16543_p <-->P53041 |Q16543_p : K(3442,1) for forward directionand K(3442,2) for reverse direction
3443	P53041 |Q16543_p -->P53041 +Q16543 : K(3443,1) for forward direction
3444	P60484_p +P16220_p <-->P60484_p |P16220_p : K(3444,1) for forward directionand K(3444,2) for reverse direction
3445	P60484_p |P16220_p -->P60484_p +P16220 : K(3445,1) for forward direction
3446	P60484_p +Q05397_p <-->P60484_p |Q05397_p : K(3446,1) for forward directionand K(3446,2) for reverse direction
3447	P60484_p |Q05397_p -->P60484_p +Q05397 : K(3447,1) for forward direction
3448	P18031_p +P00533_p <-->P18031_p |P00533_p : K(3448,1) for forward directionand K(3448,2) for reverse direction
3449	P18031_p |P00533_p -->P18031_p +P00533 : K(3449,1) for forward direction
3450	P18031_p +P08069_p <-->P18031_p |P08069_p : K(3450,1) for forward directionand K(3450,2) for reverse direction
3451	P18031_p |P08069_p -->P18031_p +P08069 : K(3451,1) for forward direction
3452	P18031_p +P18031_p <-->P18031_p |P18031_p : K(3452,1) for forward directionand K(3452,2) for reverse direction
3453	P18031_p |P18031_p -->P18031_p +P18031 : K(3453,1) for forward direction
3454	Q06124 +P00533_p <-->Q06124 |P00533_p : K(3454,1) for forward directionand K(3454,2) for reverse direction
3455	Q06124 |P00533_p -->Q06124 +P00533 : K(3455,1) for forward direction
3456	Q06124 +P12931_p <-->Q06124 |P12931_p : K(3456,1) for forward directionand K(3456,2) for reverse direction
3457	Q06124 |P12931_p -->Q06124 +P12931 : K(3457,1) for forward direction
3458	Q06124 +P42224_p <-->Q06124 |P42224_p : K(3458,1) for forward directionand K(3458,2) for reverse direction
3459	Q06124 |P42224_p -->Q06124 +P42224 : K(3459,1) for forward direction
3460	Q05209 +P12931_p <-->Q05209 |P12931_p : K(3460,1) for forward directionand K(3460,2) for reverse direction
3461	Q05209 |P12931_p -->Q05209 +P12931 : K(3461,1) for forward direction
3462	P17706 +P29353_p <-->P17706 |P29353_p : K(3462,1) for forward directionand K(3462,2) for reverse direction
3463	P17706 |P29353_p -->P17706 +P29353 : K(3463,1) for forward direction
3464	P17706 +P42226_p <-->P17706 |P42226_p : K(3464,1) for forward directionand K(3464,2) for reverse direction
3465	P17706 |P42226_p -->P17706 +P42226 : K(3465,1) for forward direction
3466	P29350_p +P12931_p <-->P29350_p |P12931_p : K(3466,1) for forward directionand K(3466,2) for reverse direction
3467	P29350_p |P12931_p -->P29350_p +P12931 : K(3467,1) for forward direction
3468	P18433_p +P18433_p <-->P18433_p |P18433_p : K(3468,1) for forward directionand K(3468,2) for reverse direction
3469	P18433_p |P18433_p -->P18433_p +P18433 : K(3469,1) for forward direction
3470	P16298 +P21333_p <-->P16298 |P21333_p : K(3470,1) for forward directionand K(3470,2) for reverse direction
3471	P16298 |P21333_p -->P16298 +P21333 : K(3471,1) for forward direction
3472	O75688 +P50750_p <-->O75688 |P50750_p : K(3472,1) for forward directionand K(3472,2) for reverse direction
3473	O75688 |P50750_p -->O75688 +P50750 : K(3473,1) for forward direction
3474	P62136 +O43865_p <-->P62136 |O43865_p : K(3474,1) for forward directionand K(3474,2) for reverse direction
3475	P62136 |O43865_p -->P62136 +O43865 : K(3475,1) for forward direction
3476	P62140 +O43865_p <-->P62140 |O43865_p : K(3476,1) for forward directionand K(3476,2) for reverse direction

3477	P62140 |O43865_p -->P62140 +O43865 : K(3477,1) for forward direction
3478	P36873 +O43865_p <-->P36873 |O43865_p : K(3478,1) for forward directionand K(3478,2) for reverse direction
3479	P36873 |O43865_p -->P36873 +O43865 : K(3479,1) for forward direction
3480	Q08209 +P50570_p <-->Q08209 |P50570_p : K(3480,1) for forward directionand K(3480,2) for reverse direction
3481	Q08209 |P50570_p -->Q08209 +P50570 : K(3481,1) for forward direction
3482	P16298 +P50570_p <-->P16298 |P50570_p : K(3482,1) for forward directionand K(3482,2) for reverse direction
3483	P16298 |P50570_p -->P16298 +P50570 : K(3483,1) for forward direction
3484	Q15262 +P00533_p <-->Q15262 |P00533_p : K(3484,1) for forward directionand K(3484,2) for reverse direction
3485	Q15262 |P00533_p -->Q15262 +P00533 : K(3485,1) for forward direction
3486	P62136 +P55211_p <-->P62136 |P55211_p : K(3486,1) for forward directionand K(3486,2) for reverse direction
3487	P62136 |P55211_p -->P62136 +P55211 : K(3487,1) for forward direction
3488	P62136 +P07737_p <-->P62136 |P07737_p : K(3488,1) for forward directionand K(3488,2) for reverse direction
3489	P62136 |P07737_p -->P62136 +P07737 : K(3489,1) for forward direction
3490	P62140 +P35240_p <-->P62140 |P35240_p : K(3490,1) for forward directionand K(3490,2) for reverse direction
3491	P62140 |P35240_p -->P62140 +P35240 : K(3491,1) for forward direction
3492	P36873 +P50750_p <-->P36873 |P50750_p : K(3492,1) for forward directionand K(3492,2) for reverse direction
3493	P36873 |P50750_p -->P36873 +P50750 : K(3493,1) for forward direction
3494	P67775 +P35222_p <-->P67775 |P35222_p : K(3494,1) for forward directionand K(3494,2) for reverse direction
3495	P67775 |P35222_p -->P67775 +P35222 : K(3495,1) for forward direction
3496	P67775 +P13639_p <-->P67775 |P13639_p : K(3496,1) for forward directionand K(3496,2) for reverse direction
3497	P67775 |P13639_p -->P67775 +P13639 : K(3497,1) for forward direction
3498	P67775 +O43524_p <-->P67775 |O43524_p : K(3498,1) for forward directionand K(3498,2) for reverse direction
3499	P67775 |O43524_p -->P67775 +O43524 : K(3499,1) for forward direction
3500	P67775 +P06400_p <-->P67775 |P06400_p : K(3500,1) for forward directionand K(3500,2) for reverse direction
3501	P67775 |P06400_p -->P67775 +P06400 : K(3501,1) for forward direction
3502	P67775 +Q12800_p <-->P67775 |Q12800_p : K(3502,1) for forward directionand K(3502,2) for reverse direction
3503	P67775 |Q12800_p -->P67775 +Q12800 : K(3503,1) for forward direction
3504	Q13362_p +P18846_p <-->Q13362_p |P18846_p : K(3504,1) for forward directionand K(3504,2) for reverse direction
3505	Q13362_p |P18846_p -->Q13362_p +P18846 : K(3505,1) for forward direction
3506	P60510 +O75531_p <-->P60510 |O75531_p : K(3506,1) for forward directionand K(3506,2) for reverse direction
3507	P60510 |O75531_p -->P60510 +O75531 : K(3507,1) for forward direction
3508	Q12974 +P24941_p <-->Q12974 |P24941_p : K(3508,1) for forward directionand K(3508,2) for reverse direction
3509	Q12974 |P24941_p -->Q12974 +P24941 : K(3509,1) for forward direction
3510	P18031_p +P12814_p <-->P18031_p |P12814_p : K(3510,1) for forward directionand K(3510,2) for reverse direction
3511	P18031_p |P12814_p -->P18031_p +P12814 : K(3511,1) for forward direction
3512	P18031_p +P12931_p <-->P18031_p |P12931_p : K(3512,1) for forward directionand K(3512,2) for reverse direction
3513	P18031_p |P12931_p -->P18031_p +P12931 : K(3513,1) for forward direction
3514	P18433_p +P12931_p <-->P18433_p |P12931_p : K(3514,1) for forward directionand K(3514,2) for reverse direction
3515	P18433_p |P12931_p -->P18433_p +P12931 : K(3515,1) for forward direction
3516	P10586 +P53355_p <-->P10586 |P53355_p : K(3516,1) for forward directionand K(3516,2) for reverse direction
3517	P10586 |P53355_p -->P10586 +P53355 : K(3517,1) for forward direction
3518	Q9P0J1 +P08559_p <-->Q9P0J1 |P08559_p : K(3518,1) for forward directionand K(3518,2) for reverse direction
3519	Q9P0J1 |P08559_p -->Q9P0J1 +P08559 : K(3519,1) for forward direction

3520	P35813 +P27986_p <-->P35813 |P27986_p : K(3520,1) for forward directionand K(3520,2) for reverse direction
3521	P35813 |P27986_p -->P35813 +P27986 : K(3521,1) for forward direction
3522	P62136 +P42575_p <-->P62136 |P42575_p : K(3522,1) for forward directionand K(3522,2) for reverse direction
3523	P62136 |P42575_p -->P62136 +P42575 : K(3523,1) for forward direction
3524	P62140 +P42575_p <-->P62140 |P42575_p : K(3524,1) for forward directionand K(3524,2) for reverse direction
3525	P62140 |P42575_p -->P62140 +P42575 : K(3525,1) for forward direction
3526	P36873 +P42575_p <-->P36873 |P42575_p : K(3526,1) for forward directionand K(3526,2) for reverse direction
3527	P36873 |P42575_p -->P36873 +P42575 : K(3527,1) for forward direction
3528	P67775 +O96017_p <-->P67775 |O96017_p : K(3528,1) for forward directionand K(3528,2) for reverse direction
3529	P67775 |O96017_p -->P67775 +O96017 : K(3529,1) for forward direction
3530	P67775 +Q8WUI4_p <-->P67775 |Q8WUI4_p : K(3530,1) for forward directionand K(3530,2) for reverse direction
3531	P67775 |Q8WUI4_p -->P67775 +Q8WUI4 : K(3531,1) for forward direction
3532	P67775 +P08047_p <-->P67775 |P08047_p : K(3532,1) for forward directionand K(3532,2) for reverse direction
3533	P67775 |P08047_p -->P67775 +P08047 : K(3533,1) for forward direction
3534	P62714 +O96017_p <-->P62714 |O96017_p : K(3534,1) for forward directionand K(3534,2) for reverse direction
3535	P62714 |O96017_p -->P62714 +O96017 : K(3535,1) for forward direction
3536	P62714 +Q8WUI4_p <-->P62714 |Q8WUI4_p : K(3536,1) for forward directionand K(3536,2) for reverse direction
3537	P62714 |Q8WUI4_p -->P62714 +Q8WUI4 : K(3537,1) for forward direction
3538	Q08209 +Q92934_p <-->Q08209 |Q92934_p : K(3538,1) for forward directionand K(3538,2) for reverse direction
3539	Q08209 |Q92934_p -->Q08209 +Q92934 : K(3539,1) for forward direction
3540	Q08209 +O00429_p <-->Q08209 |O00429_p : K(3540,1) for forward directionand K(3540,2) for reverse direction
3541	Q08209 |O00429_p -->Q08209 +O00429 : K(3541,1) for forward direction
3542	P16298 +Q92934_p <-->P16298 |Q92934_p : K(3542,1) for forward directionand K(3542,2) for reverse direction
3543	P16298 |Q92934_p -->P16298 +Q92934 : K(3543,1) for forward direction
3544	P16298 +O00429_p <-->P16298 |O00429_p : K(3544,1) for forward directionand K(3544,2) for reverse direction
3545	P16298 |O00429_p -->P16298 +O00429 : K(3545,1) for forward direction
3546	P60510 +Q04206_p <-->P60510 |Q04206_p : K(3546,1) for forward directionand K(3546,2) for reverse direction
3547	P60510 |Q04206_p -->P60510 +Q04206 : K(3547,1) for forward direction
3548	P60484_p +P60484_p <-->P60484_p |P60484_p : K(3548,1) for forward directionand K(3548,2) for reverse direction
3549	P60484_p |P60484_p -->P60484_p +P60484 : K(3549,1) for forward direction
3550	P18031_p +Q14247_p <-->P18031_p |Q14247_p : K(3550,1) for forward directionand K(3550,2) for reverse direction
3551	P18031_p |Q14247_p -->P18031_p +Q14247 : K(3551,1) for forward direction
3552	Q06124 +P01112_p <-->Q06124 |P01112_p : K(3552,1) for forward directionand K(3552,2) for reverse direction
3553	Q06124 |P01112_p -->Q06124 +P01112 : K(3553,1) for forward direction
3554	Q06124 +P01116_p <-->Q06124 |P01116_p : K(3554,1) for forward directionand K(3554,2) for reverse direction
3555	Q06124 |P01116_p -->Q06124 +P01116 : K(3555,1) for forward direction
3556	Q06124 +P01111_p <-->Q06124 |P01111_p : K(3556,1) for forward directionand K(3556,2) for reverse direction
3557	Q06124 |P01111_p -->Q06124 +P01111 : K(3557,1) for forward direction
3558	P29350_p +Q14790_p <-->P29350_p |Q14790_p : K(3558,1) for forward directionand K(3558,2) for reverse direction
3559	P29350_p |Q14790_p -->P29350_p +Q14790 : K(3559,1) for forward direction
3560	Q8IXW5 +P24928_p <-->Q8IXW5 |P24928_p : K(3560,1) for forward directionand K(3560,2) for reverse direction
3561	Q8IXW5 |P24928_p -->Q8IXW5 +P24928 : K(3561,1) for forward direction
3562	Q8TE77 +P23528_p <-->Q8TE77 |P23528_p : K(3562,1) for forward directionand K(3562,2) for reverse direction

3563	Q8TE77 |P23528_p -->Q8TE77 +P23528 : K(3563,1) for forward direction
3564	Q9NP77 +P24928_p <-->Q9NP77 |P24928_p : K(3564,1) for forward directionand K(3564,2) for reverse direction
3565	Q9NP77 |P24928_p -->Q9NP77 +P24928 : K(3565,1) for forward direction
3566	P18031_p +O75886_p <-->P18031_p |O75886_p : K(3566,1) for forward directionand K(3566,2) for reverse direction
3567	P18031_p |O75886_p -->P18031_p +O75886 : K(3567,1) for forward direction
3568	P62136 +P08047_p <-->P62136 |P08047_p : K(3568,1) for forward directionand K(3568,2) for reverse direction
3569	P62136 |P08047_p -->P62136 +P08047 : K(3569,1) for forward direction
3570	P62136 +Q02447_p <-->P62136 |Q02447_p : K(3570,1) for forward directionand K(3570,2) for reverse direction
3571	P62136 |Q02447_p -->P62136 +Q02447 : K(3571,1) for forward direction
3572	Q08209 +P05412_p <-->Q08209 |P05412_p : K(3572,1) for forward directionand K(3572,2) for reverse direction
3573	Q08209 |P05412_p -->Q08209 +P05412 : K(3573,1) for forward direction
3574	P67775 +SIGNOR-C13_p <-->P67775 |SIGNOR-C13_p : K(3574,1) for forward directionand K(3574,2) for reverse direction
3575	P67775 |SIGNOR-C13_p -->P67775 +SIGNOR-C13 : K(3575,1) for forward direction
3576	P22681 +P27986 <-->P22681 |P27986 : K(3576,1) for forward directionand K(3576,2) for reverse direction
3577	P22681 |P27986 -->P22681 +P27986_u : K(3577,1) for forward direction
3578	Q96PU5 +Q15796 <-->Q96PU5 |Q15796 : K(3578,1) for forward directionand K(3578,2) for reverse direction
3579	Q96PU5 |Q15796 -->Q96PU5 +Q15796_u : K(3579,1) for forward direction
3580	Q96PU5 +P84022 <-->Q96PU5 |P84022 : K(3580,1) for forward directionand K(3580,2) for reverse direction
3581	Q96PU5 |P84022 -->Q96PU5 +P84022_u : K(3581,1) for forward direction
3582	Q9HCE7 +Q15797 <-->Q9HCE7 |Q15797 : K(3582,1) for forward directionand K(3582,2) for reverse direction
3583	Q9HCE7 |Q15797 -->Q9HCE7 +Q15797_u : K(3583,1) for forward direction
3584	Q9HCE7 +Q99717 <-->Q9HCE7 |Q99717 : K(3584,1) for forward directionand K(3584,2) for reverse direction
3585	Q9HCE7 |Q99717 -->Q9HCE7 +Q99717_u : K(3585,1) for forward direction
3586	Q9UNE7 +Q15797 <-->Q9UNE7 |Q15797 : K(3586,1) for forward directionand K(3586,2) for reverse direction
3587	Q9UNE7 |Q15797 -->Q9UNE7 +Q15797_u : K(3587,1) for forward direction
3588	Q9UNE7 +Q99717 <-->Q9UNE7 |Q99717 : K(3588,1) for forward directionand K(3588,2) for reverse direction
3589	Q9UNE7 |Q99717 -->Q9UNE7 +Q99717_u : K(3589,1) for forward direction
3590	P19474 +P49915 <-->P19474 |P49915 : K(3590,1) for forward directionand K(3590,2) for reverse direction
3591	P19474 |P49915 -->P19474 +P49915_u : K(3591,1) for forward direction
3592	O15164 +P04637 <-->O15164 |P04637 : K(3592,1) for forward directionand K(3592,2) for reverse direction
3593	O15164 |P04637 -->O15164 +P04637_u : K(3593,1) for forward direction
3594	Q9HCE7 +P37173 <-->Q9HCE7 |P37173 : K(3594,1) for forward directionand K(3594,2) for reverse direction
3595	Q9HCE7 |P37173 -->Q9HCE7 +P37173_u : K(3595,1) for forward direction
3596	P22681 +P00533 <-->P22681 |P00533 : K(3596,1) for forward directionand K(3596,2) for reverse direction
3597	P22681 |P00533 -->P22681 +P00533_u : K(3597,1) for forward direction
3598	P22681 +P04626 <-->P22681 |P04626 : K(3598,1) for forward directionand K(3598,2) for reverse direction
3599	P22681 |P04626 -->P22681 +P04626_u : K(3599,1) for forward direction
3600	P22681 +P08581 <-->P22681 |P08581 : K(3600,1) for forward directionand K(3600,2) for reverse direction
3601	P22681 |P08581 -->P22681 +P08581_u : K(3601,1) for forward direction
3602	Q13616 +P46527 <-->Q13616 |P46527 : K(3602,1) for forward directionand K(3602,2) for reverse direction
3603	Q13616 |P46527 -->Q13616 +P46527_u : K(3603,1) for forward direction
3604	Q13616 +P35222 <-->Q13616 |P35222 : K(3604,1) for forward directionand K(3604,2) for reverse direction
3605	Q13616 |P35222 -->Q13616 +P35222_u : K(3605,1) for forward direction

3606	Q86YT6 +Q16637 <-->Q86YT6 |Q16637 : K(3606,1) for forward directionand K(3606,2) for reverse direction
3607	Q86YT6 |Q16637 -->Q86YT6 +Q16637_u : K(3607,1) for forward direction
3608	P62837 +P50542 <-->P62837 |P50542 : K(3608,1) for forward directionand K(3608,2) for reverse direction
3609	P62837 |P50542 -->P62837 +P50542_u : K(3609,1) for forward direction
3610	Q05086 +Q6IAA8 <-->Q05086 |Q6IAA8 : K(3610,1) for forward directionand K(3610,2) for reverse direction
3611	Q05086 |Q6IAA8 -->Q05086 +Q6IAA8_u : K(3611,1) for forward direction
3612	P98170_p +P42574 <-->P98170_p |P42574 : K(3612,1) for forward directionand K(3612,2) for reverse direction
3613	P98170_p |P42574 -->P98170_p +P42574_u : K(3613,1) for forward direction
3614	P98170_p +Q9NR28 <-->P98170_p |Q9NR28 : K(3614,1) for forward directionand K(3614,2) for reverse direction
3615	P98170_p |Q9NR28 -->P98170_p +Q9NR28_u : K(3615,1) for forward direction
3616	Q96J02_p +Q15796 <-->Q96J02_p |Q15796 : K(3616,1) for forward directionand K(3616,2) for reverse direction
3617	Q96J02_p |Q15796 -->Q96J02_p +Q15796_u : K(3617,1) for forward direction
3618	P62877 +P19838 <-->P62877 |P19838 : K(3618,1) for forward directionand K(3618,2) for reverse direction
3619	P62877 |P19838 -->P62877 +P19838_u : K(3619,1) for forward direction
3620	P62877 +Q00653 <-->P62877 |Q00653 : K(3620,1) for forward directionand K(3620,2) for reverse direction
3621	P62877 |Q00653 -->P62877 +Q00653_u : K(3621,1) for forward direction
3622	P49459_p +P12004 <-->P49459_p |P12004 : K(3622,1) for forward directionand K(3622,2) for reverse direction
3623	P49459_p |P12004 -->P49459_p +P12004_u : K(3623,1) for forward direction
3624	P61088 +P16104 <-->P61088 |P16104 : K(3624,1) for forward directionand K(3624,2) for reverse direction
3625	P61088 |P16104 -->P61088 +P16104_u : K(3625,1) for forward direction
3626	Q12933_p +O43318 <-->Q12933_p |O43318 : K(3626,1) for forward directionand K(3626,2) for reverse direction
3627	Q12933_p |O43318 -->Q12933_p +O43318_u : K(3627,1) for forward direction
3628	Q12933_p +Q13546 <-->Q12933_p |Q13546 : K(3628,1) for forward directionand K(3628,2) for reverse direction
3629	Q12933_p |Q13546 -->Q12933_p +Q13546_u : K(3629,1) for forward direction
3630	Q9Y4K3_u +P51617 <-->Q9Y4K3_u |P51617 : K(3630,1) for forward directionand K(3630,2) for reverse direction
3631	Q9Y4K3_u |P51617 -->Q9Y4K3_u +P51617_u : K(3631,1) for forward direction
3632	Q9Y4K3_u +O43318 <-->Q9Y4K3_u |O43318 : K(3632,1) for forward directionand K(3632,2) for reverse direction
3633	Q9Y4K3_u |O43318 -->Q9Y4K3_u +O43318_u : K(3633,1) for forward direction
3634	Q9Y4K3_u +Q9Y4K3 <-->Q9Y4K3_u |Q9Y4K3 : K(3634,1) for forward directionand K(3634,2) for reverse direction
3635	Q9Y4K3_u |Q9Y4K3 -->Q9Y4K3_u +Q9Y4K3_u : K(3635,1) for forward direction
3636	P61088 +Q12933 <-->P61088 |Q12933 : K(3636,1) for forward directionand K(3636,2) for reverse direction
3637	P61088 |Q12933 -->P61088 +Q12933_u : K(3637,1) for forward direction
3638	O43353_p +Q9Y6K9 <-->O43353_p |Q9Y6K9 : K(3638,1) for forward directionand K(3638,2) for reverse direction
3639	O43353_p |Q9Y6K9 -->O43353_p +Q9Y6K9_u : K(3639,1) for forward direction
3640	Q9HCE7 +Q15796 <-->Q9HCE7 |Q15796 : K(3640,1) for forward directionand K(3640,2) for reverse direction
3641	Q9HCE7 |Q15796 -->Q9HCE7 +Q15796_u : K(3641,1) for forward direction
3642	P17676_p +Q13887_dna <-->P17676_p |Q13887_dna : K(3642,1) for forward directionand K(3642,2) for reverse direction
3643	P17676_p |Q13887_dna -->Q13887_dna +Q13887_rna +P17676_p : K(3643,1) for forward direction
3644	Q13887_rna -->Q13887 +Q13887_rna : K(3644,1) for forward direction
3645	O43524_p +P46527_dna <-->O43524_p |P46527_dna : K(3645,1) for forward directionand K(3645,2) for reverse direction
3646	O43524_p |P46527_dna -->P46527_dna +P46527_rna +O43524_p : K(3646,1) for forward direction
3647	P46527_rna -->P46527 +P46527_rna : K(3647,1) for forward direction
3648	O43524_p +O43524_dna <-->O43524_p |O43524_dna : K(3648,1) for forward directionand K(3648,2) for reverse direction

3649	O43524_p |O43524_dna -->O43524_dna +O43524_rna +O43524_p : K(3649,1) for forward direction
3650	O43524_rna -->O43524 +O43524_rna : K(3650,1) for forward direction
3651	Q04206_p +P08047_p +P08581_dna <-->Q04206_p |P08047_p |P08581_dna : K(3651,1) for forward directionand K(3651,2) for reve
3652	Q04206_p |P08047_p |P08581_dna -->P08581_dna +P08581_rna +Q04206_p +P08047_p : K(3652,1) for forward direction
3653	P08581_rna -->P08581 +P08581_rna : K(3653,1) for forward direction
3654	P35226_p +P01106_p +P35226_dna <-->P35226_p |P01106_p |P35226_dna : K(3654,1) for forward directionand K(3654,2) for rever
3655	P35226_p |P01106_p |P35226_dna -->P35226_dna +P35226_rna +P35226_p +P01106_p : K(3655,1) for forward direction
3656	P35226_rna -->P35226 +P35226_rna : K(3656,1) for forward direction
3657	P17676_p +P21291_dna <-->P17676_p |P21291_dna : K(3657,1) for forward directionand K(3657,2) for reverse direction
3658	P17676_p |P21291_dna -->P21291_dna +P21291_rna +P17676_p : K(3658,1) for forward direction
3659	P21291_rna -->P21291 +P21291_rna : K(3659,1) for forward direction
3660	P17676_p +Q99988_dna <-->P17676_p |Q99988_dna : K(3660,1) for forward directionand K(3660,2) for reverse direction
3661	P17676_p |Q99988_dna -->Q99988_dna +Q99988_rna +P17676_p : K(3661,1) for forward direction
3662	Q99988_rna -->Q99988 +Q99988_rna : K(3662,1) for forward direction
3663	P17676_p +P05412_p +P17174_dna <-->P17676_p |P05412_p |P17174_dna : K(3663,1) for forward directionand K(3663,2) for rever
3664	P17676_p |P05412_p |P17174_dna -->P17174_dna +P17174_rna +P17676_p +P05412_p : K(3664,1) for forward direction
3665	P17174_rna -->P17174 +P17174_rna : K(3665,1) for forward direction
3666	P17676_p +P41440_dna <-->P17676_p |P41440_dna : K(3666,1) for forward directionand K(3666,2) for reverse direction
3667	P17676_p |P41440_dna -->P41440_dna +P41440_rna +P17676_p : K(3667,1) for forward direction
3668	P41440_rna -->P41440 +P41440_rna : K(3668,1) for forward direction
3669	P05412_p +P08047_p +P01137_dna <-->P05412_p |P08047_p |P01137_dna : K(3669,1) for forward directionand K(3669,2) for rever
3670	P05412_p |P08047_p |P01137_dna -->P01137_dna +P01137_rna +P05412_p +P08047_p : K(3670,1) for forward direction
3671	P01137_rna -->P01137 +P01137_rna : K(3671,1) for forward direction
3672	P14859 +P35580_dna <-->P14859 |P35580_dna : K(3672,1) for forward directionand K(3672,2) for reverse direction
3673	P14859 |P35580_dna -->P35580_dna +P35580_rna +P14859 : K(3673,1) for forward direction
3674	P35580_rna -->P35580 +P35580_rna : K(3674,1) for forward direction
3675	P08047_p +P11166_dna <-->P08047_p |P11166_dna : K(3675,1) for forward directionand K(3675,2) for reverse direction
3676	P08047_p |P11166_dna -->P11166_dna +P11166_rna +P08047_p : K(3676,1) for forward direction
3677	P11166_rna -->P11166 +P11166_rna : K(3677,1) for forward direction
3678	P04637_p +P25445_dna <-->P04637_p |P25445_dna : K(3678,1) for forward directionand K(3678,2) for reverse direction
3679	P04637_p |P25445_dna -->P25445_dna +P25445_rna +P04637_p : K(3679,1) for forward direction
3680	P25445_rna -->P25445 +P25445_rna : K(3680,1) for forward direction
3681	Q13363 +Q13547_p +O95471_dna <-->Q13363 |Q13547_p |O95471_dna : K(3681,1) for forward directionand K(3681,2) for reverse
3682	O95471_dna -->O95471_dna +O95471_rna : K(3682,1) for forward direction
3683	O95471_rna -->O95471 +O95471_rna : K(3683,1) for forward direction
3684	P84022_p +P01106_dna <-->P84022_p |P01106_dna : K(3684,1) for forward directionand K(3684,2) for reverse direction
3685	P01106_dna -->P01106_dna +P01106_rna : K(3685,1) for forward direction
3686	P01106_rna -->P01106 +P01106_rna : K(3686,1) for forward direction
3687	A8MYZ6 -->Null : K(3687,1) for forward direction
3688	E9PAV3 -->Null : K(3688,1) for forward direction
3689	O00161 -->Null : K(3689,1) for forward direction
3690	O00170 -->Null : K(3690,1) for forward direction
3691	O00267 -->Null : K(3691,1) for forward direction

3692	O00303 -->Null : K(3692,1) for forward direction
3693	O00311 -->Null : K(3693,1) for forward direction
3694	O00401 -->Null : K(3694,1) for forward direction
3695	O00418 -->Null : K(3695,1) for forward direction
3696	O00429 -->Null : K(3696,1) for forward direction
3697	O00443 -->Null : K(3697,1) for forward direction
3698	O00506 -->Null : K(3698,1) for forward direction
3699	O00571 -->Null : K(3699,1) for forward direction
3700	O00716 -->Null : K(3700,1) for forward direction
3701	O00743 -->Null : K(3701,1) for forward direction
3702	O00750 -->Null : K(3702,1) for forward direction
3703	O14641 -->Null : K(3703,1) for forward direction
3704	O14733 -->Null : K(3704,1) for forward direction
3705	O14737 -->Null : K(3705,1) for forward direction
3706	O14757 -->Null : K(3706,1) for forward direction
3707	O14777 -->Null : K(3707,1) for forward direction
3708	O14828 -->Null : K(3708,1) for forward direction
3709	O14874 -->Null : K(3709,1) for forward direction
3710	O14944 -->Null : K(3710,1) for forward direction
3711	O14950 -->Null : K(3711,1) for forward direction
3712	O14958 -->Null : K(3712,1) for forward direction
3713	O14964 -->Null : K(3713,1) for forward direction
3714	O14974 -->Null : K(3714,1) for forward direction
3715	O15111 -->Null : K(3715,1) for forward direction
3716	O15143 -->Null : K(3716,1) for forward direction
3717	O15162 -->Null : K(3717,1) for forward direction
3718	O15164 -->Null : K(3718,1) for forward direction
3719	O15169 -->Null : K(3719,1) for forward direction
3720	O15264 -->Null : K(3720,1) for forward direction
3721	O15357 -->Null : K(3721,1) for forward direction
3722	O15379 -->Null : K(3722,1) for forward direction
3723	O15511 -->Null : K(3723,1) for forward direction
3724	O15530 -->Null : K(3724,1) for forward direction
3725	O15551 -->Null : K(3725,1) for forward direction
3726	O43156 -->Null : K(3726,1) for forward direction
3727	O43318 -->Null : K(3727,1) for forward direction
3728	O43353 -->Null : K(3728,1) for forward direction
3729	O43395 -->Null : K(3729,1) for forward direction
3730	O43422 -->Null : K(3730,1) for forward direction
3731	O43464 -->Null : K(3731,1) for forward direction
3732	O43490 -->Null : K(3732,1) for forward direction
3733	O43524 -->Null : K(3733,1) for forward direction
3734	O43665 -->Null : K(3734,1) for forward direction

3735	O43707 -->Null : K(3735,1) for forward direction
3736	O43768 -->Null : K(3736,1) for forward direction
3737	O43865 -->Null : K(3737,1) for forward direction
3738	O43896 -->Null : K(3738,1) for forward direction
3739	O60343 -->Null : K(3739,1) for forward direction
3740	O60566 -->Null : K(3740,1) for forward direction
3741	O60716 -->Null : K(3741,1) for forward direction
3742	O60763 -->Null : K(3742,1) for forward direction
3743	O60825 -->Null : K(3743,1) for forward direction
3744	O60869 -->Null : K(3744,1) for forward direction
3745	O60879 -->Null : K(3745,1) for forward direction
3746	O60885 -->Null : K(3746,1) for forward direction
3747	O75116 -->Null : K(3747,1) for forward direction
3748	O75143 -->Null : K(3748,1) for forward direction
3749	O75376 -->Null : K(3749,1) for forward direction
3750	O75475 -->Null : K(3750,1) for forward direction
3751	O75531 -->Null : K(3751,1) for forward direction
3752	O75533 -->Null : K(3752,1) for forward direction
3753	O75676 -->Null : K(3753,1) for forward direction
3754	O75688 -->Null : K(3754,1) for forward direction
3755	O75689 -->Null : K(3755,1) for forward direction
3756	O75886 -->Null : K(3756,1) for forward direction
3757	O75925 -->Null : K(3757,1) for forward direction
3758	O75962 -->Null : K(3758,1) for forward direction
3759	O76070 -->Null : K(3759,1) for forward direction
3760	O94804 -->Null : K(3760,1) for forward direction
3761	O95251 -->Null : K(3761,1) for forward direction
3762	O95295 -->Null : K(3762,1) for forward direction
3763	O95297 -->Null : K(3763,1) for forward direction
3764	O95453 -->Null : K(3764,1) for forward direction
3765	O95471 -->Null : K(3765,1) for forward direction
3766	O95747 -->Null : K(3766,1) for forward direction
3767	O95786 -->Null : K(3767,1) for forward direction
3768	O95816 -->Null : K(3768,1) for forward direction
3769	O95817 -->Null : K(3769,1) for forward direction
3770	O96013 -->Null : K(3770,1) for forward direction
3771	O96017 -->Null : K(3771,1) for forward direction
3772	P00533 -->Null : K(3772,1) for forward direction
3773	P01100 -->Null : K(3773,1) for forward direction
3774	P01106 -->Null : K(3774,1) for forward direction
3775	P01111 -->Null : K(3775,1) for forward direction
3776	P01112 -->Null : K(3776,1) for forward direction
3777	P01116 -->Null : K(3777,1) for forward direction

3778	P01137 -->Null : K(3778,1) for forward direction
3779	P01344 -->Null : K(3779,1) for forward direction
3780	P02545 -->Null : K(3780,1) for forward direction
3781	P04035 -->Null : K(3781,1) for forward direction
3782	P04083 -->Null : K(3782,1) for forward direction
3783	P04183 -->Null : K(3783,1) for forward direction
3784	P04626 -->Null : K(3784,1) for forward direction
3785	P04637 -->Null : K(3785,1) for forward direction
3786	P04792 -->Null : K(3786,1) for forward direction
3787	P05067 -->Null : K(3787,1) for forward direction
3788	P05114 -->Null : K(3788,1) for forward direction
3789	P05198 -->Null : K(3789,1) for forward direction
3790	P05204 -->Null : K(3790,1) for forward direction
3791	P05387 -->Null : K(3791,1) for forward direction
3792	P05412 -->Null : K(3792,1) for forward direction
3793	P05455 -->Null : K(3793,1) for forward direction
3794	P05783 -->Null : K(3794,1) for forward direction
3795	P05976 -->Null : K(3795,1) for forward direction
3796	P06396 -->Null : K(3796,1) for forward direction
3797	P06400 -->Null : K(3797,1) for forward direction
3798	P06493 -->Null : K(3798,1) for forward direction
3799	P06730 -->Null : K(3799,1) for forward direction
3800	P06733 -->Null : K(3800,1) for forward direction
3801	P06744 -->Null : K(3801,1) for forward direction
3802	P06748 -->Null : K(3802,1) for forward direction
3803	P07108 -->Null : K(3803,1) for forward direction
3804	P07355 -->Null : K(3804,1) for forward direction
3805	P07737 -->Null : K(3805,1) for forward direction
3806	P07814 -->Null : K(3806,1) for forward direction
3807	P07900 -->Null : K(3807,1) for forward direction
3808	P07910 -->Null : K(3808,1) for forward direction
3809	P07947 -->Null : K(3809,1) for forward direction
3810	P08047 -->Null : K(3810,1) for forward direction
3811	P08069 -->Null : K(3811,1) for forward direction
3812	P08238 -->Null : K(3812,1) for forward direction
3813	P08559 -->Null : K(3813,1) for forward direction
3814	P08581 -->Null : K(3814,1) for forward direction
3815	P08670 -->Null : K(3815,1) for forward direction
3816	P08727 -->Null : K(3816,1) for forward direction
3817	P09211 -->Null : K(3817,1) for forward direction
3818	P09327 -->Null : K(3818,1) for forward direction
3819	P09497 -->Null : K(3819,1) for forward direction
3820	P09651 -->Null : K(3820,1) for forward direction

3821	P09874 -->Null : K(3821,1) for forward direction
3822	P09917 -->Null : K(3822,1) for forward direction
3823	P10301 -->Null : K(3823,1) for forward direction
3824	P10398 -->Null : K(3824,1) for forward direction
3825	P10586 -->Null : K(3825,1) for forward direction
3826	P10588 -->Null : K(3826,1) for forward direction
3827	P10644 -->Null : K(3827,1) for forward direction
3828	P11166 -->Null : K(3828,1) for forward direction
3829	P11171 -->Null : K(3829,1) for forward direction
3830	P11233 -->Null : K(3830,1) for forward direction
3831	P11234 -->Null : K(3831,1) for forward direction
3832	P11274 -->Null : K(3832,1) for forward direction
3833	P11388 -->Null : K(3833,1) for forward direction
3834	P11413 -->Null : K(3834,1) for forward direction
3835	P11717 -->Null : K(3835,1) for forward direction
3836	P11802 -->Null : K(3836,1) for forward direction
3837	P12004 -->Null : K(3837,1) for forward direction
3838	P12259 -->Null : K(3838,1) for forward direction
3839	P12270 -->Null : K(3839,1) for forward direction
3840	P12694 -->Null : K(3840,1) for forward direction
3841	P12814 -->Null : K(3841,1) for forward direction
3842	P12830 -->Null : K(3842,1) for forward direction
3843	P12931 -->Null : K(3843,1) for forward direction
3844	P13639 -->Null : K(3844,1) for forward direction
3845	P13688 -->Null : K(3845,1) for forward direction
3846	P13693 -->Null : K(3846,1) for forward direction
3847	P13807 -->Null : K(3847,1) for forward direction
3848	P13861 -->Null : K(3848,1) for forward direction
3849	P14618 -->Null : K(3849,1) for forward direction
3850	P14635 -->Null : K(3850,1) for forward direction
3851	P14649 -->Null : K(3851,1) for forward direction
3852	P14859 -->Null : K(3852,1) for forward direction
3853	P14923 -->Null : K(3853,1) for forward direction
3854	P15056 -->Null : K(3854,1) for forward direction
3855	P15311 -->Null : K(3855,1) for forward direction
3856	P15336 -->Null : K(3856,1) for forward direction
3857	P15531 -->Null : K(3857,1) for forward direction
3858	P15924 -->Null : K(3858,1) for forward direction
3859	P15927 -->Null : K(3859,1) for forward direction
3860	P16070 -->Null : K(3860,1) for forward direction
3861	P16104 -->Null : K(3861,1) for forward direction
3862	P16144 -->Null : K(3862,1) for forward direction
3863	P16220 -->Null : K(3863,1) for forward direction

3864	P16298 -->Null : K(3864,1) for forward direction
3865	P16401 -->Null : K(3865,1) for forward direction
3866	P16615 -->Null : K(3866,1) for forward direction
3867	P16949 -->Null : K(3867,1) for forward direction
3868	P17096 -->Null : K(3868,1) for forward direction
3869	P17174 -->Null : K(3869,1) for forward direction
3870	P17252 -->Null : K(3870,1) for forward direction
3871	P17480 -->Null : K(3871,1) for forward direction
3872	P17535 -->Null : K(3872,1) for forward direction
3873	P17612 -->Null : K(3873,1) for forward direction
3874	P17655 -->Null : K(3874,1) for forward direction
3875	P17676 -->Null : K(3875,1) for forward direction
3876	P17706 -->Null : K(3876,1) for forward direction
3877	P17812 -->Null : K(3877,1) for forward direction
3878	P17844 -->Null : K(3878,1) for forward direction
3879	P17931 -->Null : K(3879,1) for forward direction
3880	P18031 -->Null : K(3880,1) for forward direction
3881	P18084 -->Null : K(3881,1) for forward direction
3882	P18206 -->Null : K(3882,1) for forward direction
3883	P18433 -->Null : K(3883,1) for forward direction
3884	P18669 -->Null : K(3884,1) for forward direction
3885	P18846 -->Null : K(3885,1) for forward direction
3886	P18858 -->Null : K(3886,1) for forward direction
3887	P18887 -->Null : K(3887,1) for forward direction
3888	P19474 -->Null : K(3888,1) for forward direction
3889	P19525 -->Null : K(3889,1) for forward direction
3890	P19634 -->Null : K(3890,1) for forward direction
3891	P19784 -->Null : K(3891,1) for forward direction
3892	P19793 -->Null : K(3892,1) for forward direction
3893	P19838 -->Null : K(3893,1) for forward direction
3894	P20042 -->Null : K(3894,1) for forward direction
3895	P20248 -->Null : K(3895,1) for forward direction
3896	P20936 -->Null : K(3896,1) for forward direction
3897	P21127 -->Null : K(3897,1) for forward direction
3898	P21291 -->Null : K(3898,1) for forward direction
3899	P21333 -->Null : K(3899,1) for forward direction
3900	P21399 -->Null : K(3900,1) for forward direction
3901	P21802 -->Null : K(3901,1) for forward direction
3902	P21810 -->Null : K(3902,1) for forward direction
3903	P21860 -->Null : K(3903,1) for forward direction
3904	P21980 -->Null : K(3904,1) for forward direction
3905	P22314 -->Null : K(3905,1) for forward direction
3906	P22392 -->Null : K(3906,1) for forward direction

3907	P22681 -->Null : K(3907,1) for forward direction
3908	P23246 -->Null : K(3908,1) for forward direction
3909	P23396 -->Null : K(3909,1) for forward direction
3910	P23511 -->Null : K(3910,1) for forward direction
3911	P23528 -->Null : K(3911,1) for forward direction
3912	P23588 -->Null : K(3912,1) for forward direction
3913	P24534 -->Null : K(3913,1) for forward direction
3914	P24666 -->Null : K(3914,1) for forward direction
3915	P24844 -->Null : K(3915,1) for forward direction
3916	P24928 -->Null : K(3916,1) for forward direction
3917	P24941 -->Null : K(3917,1) for forward direction
3918	P25098 -->Null : K(3918,1) for forward direction
3919	P25205 -->Null : K(3919,1) for forward direction
3920	P25445 -->Null : K(3920,1) for forward direction
3921	P25490 -->Null : K(3921,1) for forward direction
3922	P25788 -->Null : K(3922,1) for forward direction
3923	P26038 -->Null : K(3923,1) for forward direction
3924	P26358 -->Null : K(3924,1) for forward direction
3925	P27348 -->Null : K(3925,1) for forward direction
3926	P27361 -->Null : K(3926,1) for forward direction
3927	P27635 -->Null : K(3927,1) for forward direction
3928	P27695 -->Null : K(3928,1) for forward direction
3929	P27708 -->Null : K(3929,1) for forward direction
3930	P27816 -->Null : K(3930,1) for forward direction
3931	P27986 -->Null : K(3931,1) for forward direction
3932	P28482 -->Null : K(3932,1) for forward direction
3933	P29317 -->Null : K(3933,1) for forward direction
3934	P29350 -->Null : K(3934,1) for forward direction
3935	P29353 -->Null : K(3935,1) for forward direction
3936	P29590 -->Null : K(3936,1) for forward direction
3937	P29692 -->Null : K(3937,1) for forward direction
3938	P29966 -->Null : K(3938,1) for forward direction
3939	P30041 -->Null : K(3939,1) for forward direction
3940	P30050 -->Null : K(3940,1) for forward direction
3941	P30086 -->Null : K(3941,1) for forward direction
3942	P30260 -->Null : K(3942,1) for forward direction
3943	P30291 -->Null : K(3943,1) for forward direction
3944	P30519 -->Null : K(3944,1) for forward direction
3945	P30622 -->Null : K(3945,1) for forward direction
3946	P30626 -->Null : K(3946,1) for forward direction
3947	P31146 -->Null : K(3947,1) for forward direction
3948	P31350 -->Null : K(3948,1) for forward direction
3949	P31431 -->Null : K(3949,1) for forward direction

3950	P31749 -->Null : K(3950,1) for forward direction
3951	P31751 -->Null : K(3951,1) for forward direction
3952	P31946 -->Null : K(3952,1) for forward direction
3953	P31947 -->Null : K(3953,1) for forward direction
3954	P32519 -->Null : K(3954,1) for forward direction
3955	P33527 -->Null : K(3955,1) for forward direction
3956	P33778 -->Null : K(3956,1) for forward direction
3957	P33981 -->Null : K(3957,1) for forward direction
3958	P33991 -->Null : K(3958,1) for forward direction
3959	P33993 -->Null : K(3959,1) for forward direction
3960	P35221 -->Null : K(3960,1) for forward direction
3961	P35222 -->Null : K(3961,1) for forward direction
3962	P35226 -->Null : K(3962,1) for forward direction
3963	P35240 -->Null : K(3963,1) for forward direction
3964	P35241 -->Null : K(3964,1) for forward direction
3965	P35269 -->Null : K(3965,1) for forward direction
3966	P35270 -->Null : K(3966,1) for forward direction
3967	P35568 -->Null : K(3967,1) for forward direction
3968	P35579 -->Null : K(3968,1) for forward direction
3969	P35580 -->Null : K(3969,1) for forward direction
3970	P35611 -->Null : K(3970,1) for forward direction
3971	P35659 -->Null : K(3971,1) for forward direction
3972	P35813 -->Null : K(3972,1) for forward direction
3973	P36507 -->Null : K(3973,1) for forward direction
3974	P36871 -->Null : K(3974,1) for forward direction
3975	P36873 -->Null : K(3975,1) for forward direction
3976	P37173 -->Null : K(3976,1) for forward direction
3977	P38432 -->Null : K(3977,1) for forward direction
3978	P38646 -->Null : K(3978,1) for forward direction
3979	P39748 -->Null : K(3979,1) for forward direction
3980	P40189 -->Null : K(3980,1) for forward direction
3981	P40763 -->Null : K(3981,1) for forward direction
3982	P41212 -->Null : K(3982,1) for forward direction
3983	P41236 -->Null : K(3983,1) for forward direction
3984	P41240 -->Null : K(3984,1) for forward direction
3985	P41279 -->Null : K(3985,1) for forward direction
3986	P41440 -->Null : K(3986,1) for forward direction
3987	P41743 -->Null : K(3987,1) for forward direction
3988	P42224 -->Null : K(3988,1) for forward direction
3989	P42226 -->Null : K(3989,1) for forward direction
3990	P42345 -->Null : K(3990,1) for forward direction
3991	P42566 -->Null : K(3991,1) for forward direction
3992	P42574 -->Null : K(3992,1) for forward direction

3993	P42575 -->Null : K(3993,1) for forward direction
3994	P42858 -->Null : K(3994,1) for forward direction
3995	P43378 -->Null : K(3995,1) for forward direction
3996	P43405 -->Null : K(3996,1) for forward direction
3997	P45973 -->Null : K(3997,1) for forward direction
3998	P45983 -->Null : K(3998,1) for forward direction
3999	P45984 -->Null : K(3999,1) for forward direction
4000	P45985 -->Null : K(4000,1) for forward direction
4001	P46020 -->Null : K(4001,1) for forward direction
4002	P46060 -->Null : K(4002,1) for forward direction
4003	P46108 -->Null : K(4003,1) for forward direction
4004	P46459 -->Null : K(4004,1) for forward direction
4005	P46527 -->Null : K(4005,1) for forward direction
4006	P46734 -->Null : K(4006,1) for forward direction
4007	P46777 -->Null : K(4007,1) for forward direction
4008	P46937 -->Null : K(4008,1) for forward direction
4009	P46939 -->Null : K(4009,1) for forward direction
4010	P46940 -->Null : K(4010,1) for forward direction
4011	P47712 -->Null : K(4011,1) for forward direction
4012	P47736 -->Null : K(4012,1) for forward direction
4013	P48200 -->Null : K(4013,1) for forward direction
4014	P48426 -->Null : K(4014,1) for forward direction
4015	P48436 -->Null : K(4015,1) for forward direction
4016	P48729 -->Null : K(4016,1) for forward direction
4017	P48730 -->Null : K(4017,1) for forward direction
4018	P49023 -->Null : K(4018,1) for forward direction
4019	P49137 -->Null : K(4019,1) for forward direction
4020	P49407 -->Null : K(4020,1) for forward direction
4021	P49459 -->Null : K(4021,1) for forward direction
4022	P49585 -->Null : K(4022,1) for forward direction
4023	P49674 -->Null : K(4023,1) for forward direction
4024	P49736 -->Null : K(4024,1) for forward direction
4025	P49757 -->Null : K(4025,1) for forward direction
4026	P49790 -->Null : K(4026,1) for forward direction
4027	P49815 -->Null : K(4027,1) for forward direction
4028	P49840 -->Null : K(4028,1) for forward direction
4029	P49841 -->Null : K(4029,1) for forward direction
4030	P49915 -->Null : K(4030,1) for forward direction
4031	P49916 -->Null : K(4031,1) for forward direction
4032	P49917 -->Null : K(4032,1) for forward direction
4033	P50402 -->Null : K(4033,1) for forward direction
4034	P50542 -->Null : K(4034,1) for forward direction
4035	P50548 -->Null : K(4035,1) for forward direction

4036	P50552 -->Null : K(4036,1) for forward direction
4037	P50570 -->Null : K(4037,1) for forward direction
4038	P50613 -->Null : K(4038,1) for forward direction
4039	P50616 -->Null : K(4039,1) for forward direction
4040	P50750 -->Null : K(4040,1) for forward direction
4041	P51398 -->Null : K(4041,1) for forward direction
4042	P51452 -->Null : K(4042,1) for forward direction
4043	P51617 -->Null : K(4043,1) for forward direction
4044	P51692 -->Null : K(4044,1) for forward direction
4045	P51812 -->Null : K(4045,1) for forward direction
4046	P52292 -->Null : K(4046,1) for forward direction
4047	P52564 -->Null : K(4047,1) for forward direction
4048	P52565 -->Null : K(4048,1) for forward direction
4049	P52566 -->Null : K(4049,1) for forward direction
4050	P52655 -->Null : K(4050,1) for forward direction
4051	P52732 -->Null : K(4051,1) for forward direction
4052	P52735 -->Null : K(4052,1) for forward direction
4053	P52888 -->Null : K(4053,1) for forward direction
4054	P52907 -->Null : K(4054,1) for forward direction
4055	P52926 -->Null : K(4055,1) for forward direction
4056	P52943 -->Null : K(4056,1) for forward direction
4057	P52948 -->Null : K(4057,1) for forward direction
4058	P53041 -->Null : K(4058,1) for forward direction
4059	P53350 -->Null : K(4059,1) for forward direction
4060	P53355 -->Null : K(4060,1) for forward direction
4061	P53365 -->Null : K(4061,1) for forward direction
4062	P53396 -->Null : K(4062,1) for forward direction
4063	P54132 -->Null : K(4063,1) for forward direction
4064	P54198 -->Null : K(4064,1) for forward direction
4065	P54253 -->Null : K(4065,1) for forward direction
4066	P55010 -->Null : K(4066,1) for forward direction
4067	P55011 -->Null : K(4067,1) for forward direction
4068	P55072 -->Null : K(4068,1) for forward direction
4069	P55210 -->Null : K(4069,1) for forward direction
4070	P55211 -->Null : K(4070,1) for forward direction
4071	P55327 -->Null : K(4071,1) for forward direction
4072	P55957 -->Null : K(4072,1) for forward direction
4073	P56211 -->Null : K(4073,1) for forward direction
4074	P56537 -->Null : K(4074,1) for forward direction
4075	P56945 -->Null : K(4075,1) for forward direction
4076	P60484 -->Null : K(4076,1) for forward direction
4077	P60510 -->Null : K(4077,1) for forward direction
4078	P60953 -->Null : K(4078,1) for forward direction

4079	P60983 -->Null : K(4079,1) for forward direction
4080	P61020 -->Null : K(4080,1) for forward direction
4081	P61088 -->Null : K(4081,1) for forward direction
4082	P61224 -->Null : K(4082,1) for forward direction
4083	P61586 -->Null : K(4083,1) for forward direction
4084	P61587 -->Null : K(4084,1) for forward direction
4085	P61764 -->Null : K(4085,1) for forward direction
4086	P61978 -->Null : K(4086,1) for forward direction
4087	P62136 -->Null : K(4087,1) for forward direction
4088	P62140 -->Null : K(4088,1) for forward direction
4089	P62258 -->Null : K(4089,1) for forward direction
4090	P62714 -->Null : K(4090,1) for forward direction
4091	P62745 -->Null : K(4091,1) for forward direction
4092	P62753 -->Null : K(4092,1) for forward direction
4093	P62826 -->Null : K(4093,1) for forward direction
4094	P62834 -->Null : K(4094,1) for forward direction
4095	P62837 -->Null : K(4095,1) for forward direction
4096	P62877 -->Null : K(4096,1) for forward direction
4097	P62993 -->Null : K(4097,1) for forward direction
4098	P63000 -->Null : K(4098,1) for forward direction
4099	P63010 -->Null : K(4099,1) for forward direction
4100	P63104 -->Null : K(4100,1) for forward direction
4101	P63167 -->Null : K(4101,1) for forward direction
4102	P63244 -->Null : K(4102,1) for forward direction
4103	P63261 -->Null : K(4103,1) for forward direction
4104	P67775 -->Null : K(4104,1) for forward direction
4105	P67809 -->Null : K(4105,1) for forward direction
4106	P67870 -->Null : K(4106,1) for forward direction
4107	P68104 -->Null : K(4107,1) for forward direction
4108	P68366 -->Null : K(4108,1) for forward direction
4109	P68400 -->Null : K(4109,1) for forward direction
4110	P68431 -->Null : K(4110,1) for forward direction
4111	P78314 -->Null : K(4111,1) for forward direction
4112	P78347 -->Null : K(4112,1) for forward direction
4113	P78356 -->Null : K(4113,1) for forward direction
4114	P78362 -->Null : K(4114,1) for forward direction
4115	P78371 -->Null : K(4115,1) for forward direction
4116	P78527 -->Null : K(4116,1) for forward direction
4117	P78536 -->Null : K(4117,1) for forward direction
4118	P78545 -->Null : K(4118,1) for forward direction
4119	P83916 -->Null : K(4119,1) for forward direction
4120	P84022 -->Null : K(4120,1) for forward direction
4121	P84243 -->Null : K(4121,1) for forward direction

4122	P98170 -->Null : K(4122,1) for forward direction
4123	Q00059 -->Null : K(4123,1) for forward direction
4124	Q00534 -->Null : K(4124,1) for forward direction
4125	Q00535 -->Null : K(4125,1) for forward direction
4126	Q00610 -->Null : K(4126,1) for forward direction
4127	Q00613 -->Null : K(4127,1) for forward direction
4128	Q00653 -->Null : K(4128,1) for forward direction
4129	Q00839 -->Null : K(4129,1) for forward direction
4130	Q01082 -->Null : K(4130,1) for forward direction
4131	Q01105 -->Null : K(4131,1) for forward direction
4132	Q01167 -->Null : K(4132,1) for forward direction
4133	Q01844 -->Null : K(4133,1) for forward direction
4134	Q01970 -->Null : K(4134,1) for forward direction
4135	Q01973 -->Null : K(4135,1) for forward direction
4136	Q02447 -->Null : K(4136,1) for forward direction
4137	Q02750 -->Null : K(4137,1) for forward direction
4138	Q02790 -->Null : K(4138,1) for forward direction
4139	Q04206 -->Null : K(4139,1) for forward direction
4140	Q04724 -->Null : K(4140,1) for forward direction
4141	Q04912 -->Null : K(4141,1) for forward direction
4142	Q04917 -->Null : K(4142,1) for forward direction
4143	Q05086 -->Null : K(4143,1) for forward direction
4144	Q05193 -->Null : K(4144,1) for forward direction
4145	Q05209 -->Null : K(4145,1) for forward direction
4146	Q05397 -->Null : K(4146,1) for forward direction
4147	Q05469 -->Null : K(4147,1) for forward direction
4148	Q05655 -->Null : K(4148,1) for forward direction
4149	Q06124 -->Null : K(4149,1) for forward direction
4150	Q06210 -->Null : K(4150,1) for forward direction
4151	Q06265 -->Null : K(4151,1) for forward direction
4152	Q06481 -->Null : K(4152,1) for forward direction
4153	Q06830 -->Null : K(4153,1) for forward direction
4154	Q07352 -->Null : K(4154,1) for forward direction
4155	Q07617 -->Null : K(4155,1) for forward direction
4156	Q07666 -->Null : K(4156,1) for forward direction
4157	Q07812 -->Null : K(4157,1) for forward direction
4158	Q07817 -->Null : K(4158,1) for forward direction
4159	Q07820 -->Null : K(4159,1) for forward direction
4160	Q07866 -->Null : K(4160,1) for forward direction
4161	Q07889 -->Null : K(4161,1) for forward direction
4162	Q07954 -->Null : K(4162,1) for forward direction
4163	Q07955 -->Null : K(4163,1) for forward direction
4164	Q08209 -->Null : K(4164,1) for forward direction

4165	Q08345 -->Null : K(4165,1) for forward direction
4166	Q08379 -->Null : K(4166,1) for forward direction
4167	Q08945 -->Null : K(4167,1) for forward direction
4168	Q08J23 -->Null : K(4168,1) for forward direction
4169	Q09472 -->Null : K(4169,1) for forward direction
4170	Q12778 -->Null : K(4170,1) for forward direction
4171	Q12800 -->Null : K(4171,1) for forward direction
4172	Q12802 -->Null : K(4172,1) for forward direction
4173	Q12888 -->Null : K(4173,1) for forward direction
4174	Q12904 -->Null : K(4174,1) for forward direction
4175	Q12906 -->Null : K(4175,1) for forward direction
4176	Q12933 -->Null : K(4176,1) for forward direction
4177	Q12959 -->Null : K(4177,1) for forward direction
4178	Q12972 -->Null : K(4178,1) for forward direction
4179	Q12974 -->Null : K(4179,1) for forward direction
4180	Q13042 -->Null : K(4180,1) for forward direction
4181	Q13043 -->Null : K(4181,1) for forward direction
4182	Q13085 -->Null : K(4182,1) for forward direction
4183	Q13131 -->Null : K(4183,1) for forward direction
4184	Q13144 -->Null : K(4184,1) for forward direction
4185	Q13153 -->Null : K(4185,1) for forward direction
4186	Q13158 -->Null : K(4186,1) for forward direction
4187	Q13177 -->Null : K(4187,1) for forward direction
4188	Q13185 -->Null : K(4188,1) for forward direction
4189	Q13188 -->Null : K(4189,1) for forward direction
4190	Q13257 -->Null : K(4190,1) for forward direction
4191	Q13263 -->Null : K(4191,1) for forward direction
4192	Q13308 -->Null : K(4192,1) for forward direction
4193	Q13362 -->Null : K(4193,1) for forward direction
4194	Q13363 -->Null : K(4194,1) for forward direction
4195	Q13393 -->Null : K(4195,1) for forward direction
4196	Q13416 -->Null : K(4196,1) for forward direction
4197	Q13418 -->Null : K(4197,1) for forward direction
4198	Q13464 -->Null : K(4198,1) for forward direction
4199	Q13480 -->Null : K(4199,1) for forward direction
4200	Q13501 -->Null : K(4200,1) for forward direction
4201	Q13523 -->Null : K(4201,1) for forward direction
4202	Q13526 -->Null : K(4202,1) for forward direction
4203	Q13541 -->Null : K(4203,1) for forward direction
4204	Q13542 -->Null : K(4204,1) for forward direction
4205	Q13546 -->Null : K(4205,1) for forward direction
4206	Q13547 -->Null : K(4206,1) for forward direction
4207	Q13554 -->Null : K(4207,1) for forward direction

4208	Q13555 -->Null : K(4208,1) for forward direction
4209	Q13557 -->Null : K(4209,1) for forward direction
4210	Q13586 -->Null : K(4210,1) for forward direction
4211	Q13616 -->Null : K(4211,1) for forward direction
4212	Q13627 -->Null : K(4212,1) for forward direction
4213	Q13765 -->Null : K(4213,1) for forward direction
4214	Q13769 -->Null : K(4214,1) for forward direction
4215	Q13813 -->Null : K(4215,1) for forward direction
4216	Q13887 -->Null : K(4216,1) for forward direction
4217	Q13976 -->Null : K(4217,1) for forward direction
4218	Q14103 -->Null : K(4218,1) for forward direction
4219	Q14118 -->Null : K(4219,1) for forward direction
4220	Q14161 -->Null : K(4220,1) for forward direction
4221	Q14203 -->Null : K(4221,1) for forward direction
4222	Q14247 -->Null : K(4222,1) for forward direction
4223	Q14344 -->Null : K(4223,1) for forward direction
4224	Q14457 -->Null : K(4224,1) for forward direction
4225	Q14653 -->Null : K(4225,1) for forward direction
4226	Q14676 -->Null : K(4226,1) for forward direction
4227	Q14738 -->Null : K(4227,1) for forward direction
4228	Q14739 -->Null : K(4228,1) for forward direction
4229	Q14790 -->Null : K(4229,1) for forward direction
4230	Q14847 -->Null : K(4230,1) for forward direction
4231	Q14978 -->Null : K(4231,1) for forward direction
4232	Q14980 -->Null : K(4232,1) for forward direction
4233	Q15019 -->Null : K(4233,1) for forward direction
4234	Q15027 -->Null : K(4234,1) for forward direction
4235	Q15046 -->Null : K(4235,1) for forward direction
4236	Q15054 -->Null : K(4236,1) for forward direction
4237	Q15075 -->Null : K(4237,1) for forward direction
4238	Q15121 -->Null : K(4238,1) for forward direction
4239	Q15149 -->Null : K(4239,1) for forward direction
4240	Q15172 -->Null : K(4240,1) for forward direction
4241	Q15185 -->Null : K(4241,1) for forward direction
4242	Q15208 -->Null : K(4242,1) for forward direction
4243	Q15257 -->Null : K(4243,1) for forward direction
4244	Q15262 -->Null : K(4244,1) for forward direction
4245	Q15418 -->Null : K(4245,1) for forward direction
4246	Q15596 -->Null : K(4246,1) for forward direction
4247	Q15637 -->Null : K(4247,1) for forward direction
4248	Q15648 -->Null : K(4248,1) for forward direction
4249	Q15653 -->Null : K(4249,1) for forward direction
4250	Q15717 -->Null : K(4250,1) for forward direction

4251	Q15746 -->Null : K(4251,1) for forward direction
4252	Q15750 -->Null : K(4252,1) for forward direction
4253	Q15796 -->Null : K(4253,1) for forward direction
4254	Q15797 -->Null : K(4254,1) for forward direction
4255	Q15833 -->Null : K(4255,1) for forward direction
4256	Q15910 -->Null : K(4256,1) for forward direction
4257	Q15942 -->Null : K(4257,1) for forward direction
4258	Q16204 -->Null : K(4258,1) for forward direction
4259	Q16512 -->Null : K(4259,1) for forward direction
4260	Q16513 -->Null : K(4260,1) for forward direction
4261	Q16539 -->Null : K(4261,1) for forward direction
4262	Q16543 -->Null : K(4262,1) for forward direction
4263	Q16555 -->Null : K(4263,1) for forward direction
4264	Q16625 -->Null : K(4264,1) for forward direction
4265	Q16637 -->Null : K(4265,1) for forward direction
4266	Q16644 -->Null : K(4266,1) for forward direction
4267	Q16666 -->Null : K(4267,1) for forward direction
4268	Q16695 -->Null : K(4268,1) for forward direction
4269	Q16790 -->Null : K(4269,1) for forward direction
4270	Q2M2I8 -->Null : K(4270,1) for forward direction
4271	Q2NKX8 -->Null : K(4271,1) for forward direction
4272	Q53EL6 -->Null : K(4272,1) for forward direction
4273	Q53ET0 -->Null : K(4273,1) for forward direction
4274	Q53EZ4 -->Null : K(4274,1) for forward direction
4275	Q5VT25 -->Null : K(4275,1) for forward direction
4276	Q68CZ2 -->Null : K(4276,1) for forward direction
4277	Q6A1A2 -->Null : K(4277,1) for forward direction
4278	Q6IAA8 -->Null : K(4278,1) for forward direction
4279	Q6VY07 -->Null : K(4279,1) for forward direction
4280	Q6WCQ1 -->Null : K(4280,1) for forward direction
4281	Q712K3 -->Null : K(4281,1) for forward direction
4282	Q7KZI7 -->Null : K(4282,1) for forward direction
4283	Q7L5N1 -->Null : K(4283,1) for forward direction
4284	Q7L7X3 -->Null : K(4284,1) for forward direction
4285	Q86TI0 -->Null : K(4285,1) for forward direction
4286	Q86V81 -->Null : K(4286,1) for forward direction
4287	Q86VP1 -->Null : K(4287,1) for forward direction
4288	Q86WB0 -->Null : K(4288,1) for forward direction
4289	Q86YT6 -->Null : K(4289,1) for forward direction
4290	Q8IX03 -->Null : K(4290,1) for forward direction
4291	Q8IXJ6 -->Null : K(4291,1) for forward direction
4292	Q8IXW5 -->Null : K(4292,1) for forward direction
4293	Q8IZL8 -->Null : K(4293,1) for forward direction

4294	Q8IZP0 -->Null : K(4294,1) for forward direction
4295	Q8N122 -->Null : K(4295,1) for forward direction
4296	Q8N752 -->Null : K(4296,1) for forward direction
4297	Q8NE71 -->Null : K(4297,1) for forward direction
4298	Q8NEB9 -->Null : K(4298,1) for forward direction
4299	Q8NFA2 -->Null : K(4299,1) for forward direction
4300	Q8TAP9 -->Null : K(4300,1) for forward direction
4301	Q8TB45 -->Null : K(4301,1) for forward direction
4302	Q8TD19 -->Null : K(4302,1) for forward direction
4303	Q8TDX7 -->Null : K(4303,1) for forward direction
4304	Q8TE77 -->Null : K(4304,1) for forward direction
4305	Q8TEW0 -->Null : K(4305,1) for forward direction
4306	Q8WUI4 -->Null : K(4306,1) for forward direction
4307	Q8WXE1 -->Null : K(4307,1) for forward direction
4308	Q92538 -->Null : K(4308,1) for forward direction
4309	Q92574 -->Null : K(4309,1) for forward direction
4310	Q92598 -->Null : K(4310,1) for forward direction
4311	Q92686 -->Null : K(4311,1) for forward direction
4312	Q92688 -->Null : K(4312,1) for forward direction
4313	Q92769 -->Null : K(4313,1) for forward direction
4314	Q92793 -->Null : K(4314,1) for forward direction
4315	Q92900 -->Null : K(4315,1) for forward direction
4316	Q92934 -->Null : K(4316,1) for forward direction
4317	Q92945 -->Null : K(4317,1) for forward direction
4318	Q92974 -->Null : K(4318,1) for forward direction
4319	Q92985 -->Null : K(4319,1) for forward direction
4320	Q92993 -->Null : K(4320,1) for forward direction
4321	Q93034 -->Null : K(4321,1) for forward direction
4322	Q93045 -->Null : K(4322,1) for forward direction
4323	Q96B36 -->Null : K(4323,1) for forward direction
4324	Q96CF2 -->Null : K(4324,1) for forward direction
4325	Q96CV9 -->Null : K(4325,1) for forward direction
4326	Q96CW1 -->Null : K(4326,1) for forward direction
4327	Q96EB6 -->Null : K(4327,1) for forward direction
4328	Q96GD0 -->Null : K(4328,1) for forward direction
4329	Q96GD4 -->Null : K(4329,1) for forward direction
4330	Q96GX5 -->Null : K(4330,1) for forward direction
4331	Q96J02 -->Null : K(4331,1) for forward direction
4332	Q96KB5 -->Null : K(4332,1) for forward direction
4333	Q96N67 -->Null : K(4333,1) for forward direction
4334	Q96PM5 -->Null : K(4334,1) for forward direction
4335	Q96PU5 -->Null : K(4335,1) for forward direction
4336	Q96Q15 -->Null : K(4336,1) for forward direction

4337	Q96R06 -->Null : K(4337,1) for forward direction
4338	Q96RK0 -->Null : K(4338,1) for forward direction
4339	Q96S44 -->Null : K(4339,1) for forward direction
4340	Q96SB3 -->Null : K(4340,1) for forward direction
4341	Q96SB4 -->Null : K(4341,1) for forward direction
4342	Q96T60 -->Null : K(4342,1) for forward direction
4343	Q96T88 -->Null : K(4343,1) for forward direction
4344	Q99418 -->Null : K(4344,1) for forward direction
4345	Q99426 -->Null : K(4345,1) for forward direction
4346	Q99504 -->Null : K(4346,1) for forward direction
4347	Q99594 -->Null : K(4347,1) for forward direction
4348	Q99661 -->Null : K(4348,1) for forward direction
4349	Q99717 -->Null : K(4349,1) for forward direction
4350	Q99759 -->Null : K(4350,1) for forward direction
4351	Q99961 -->Null : K(4351,1) for forward direction
4352	Q99986 -->Null : K(4352,1) for forward direction
4353	Q99988 -->Null : K(4353,1) for forward direction
4354	Q9BQG0 -->Null : K(4354,1) for forward direction
4355	Q9BR76 -->Null : K(4355,1) for forward direction
4356	Q9BUL8 -->Null : K(4356,1) for forward direction
4357	Q9BXS5 -->Null : K(4357,1) for forward direction
4358	Q9BY41 -->Null : K(4358,1) for forward direction
4359	Q9BY44 -->Null : K(4359,1) for forward direction
4360	Q9BZL4 -->Null : K(4360,1) for forward direction
4361	Q9BZL6 -->Null : K(4361,1) for forward direction
4362	Q9BZQ8 -->Null : K(4362,1) for forward direction
4363	Q9GZM8 -->Null : K(4363,1) for forward direction
4364	Q9GZY8 -->Null : K(4364,1) for forward direction
4365	Q9H0B6 -->Null : K(4365,1) for forward direction
4366	Q9H0H5 -->Null : K(4366,1) for forward direction
4367	Q9H0Z9 -->Null : K(4367,1) for forward direction
4368	Q9H1A4 -->Null : K(4368,1) for forward direction
4369	Q9H1K1 -->Null : K(4369,1) for forward direction
4370	Q9H257 -->Null : K(4370,1) for forward direction
4371	Q9H2D6 -->Null : K(4371,1) for forward direction
4372	Q9H2G2 -->Null : K(4372,1) for forward direction
4373	Q9H2K8 -->Null : K(4373,1) for forward direction
4374	Q9H3Z4 -->Null : K(4374,1) for forward direction
4375	Q9H410 -->Null : K(4375,1) for forward direction
4376	Q9H4A3 -->Null : K(4376,1) for forward direction
4377	Q9H4A6 -->Null : K(4377,1) for forward direction
4378	Q9H5V8 -->Null : K(4378,1) for forward direction
4379	Q9H6Z4 -->Null : K(4379,1) for forward direction

4380	Q9H8S9 -->Null : K(4380,1) for forward direction
4381	Q9H8V3 -->Null : K(4381,1) for forward direction
4382	Q9H8Y8 -->Null : K(4382,1) for forward direction
4383	Q9H9Q4 -->Null : K(4383,1) for forward direction
4384	Q9HBY8 -->Null : K(4384,1) for forward direction
4385	Q9HCE7 -->Null : K(4385,1) for forward direction
4386	Q9HCP0 -->Null : K(4386,1) for forward direction
4387	Q9NP77 -->Null : K(4387,1) for forward direction
4388	Q9NP97 -->Null : K(4388,1) for forward direction
4389	Q9NQC3 -->Null : K(4389,1) for forward direction
4390	Q9NQX3 -->Null : K(4390,1) for forward direction
4391	Q9NR28 -->Null : K(4391,1) for forward direction
4392	Q9NRA0 -->Null : K(4392,1) for forward direction
4393	Q9NRF8 -->Null : K(4393,1) for forward direction
4394	Q9NRY4 -->Null : K(4394,1) for forward direction
4395	Q9NWZ3 -->Null : K(4395,1) for forward direction
4396	Q9NXR1 -->Null : K(4396,1) for forward direction
4397	Q9NY61 -->Null : K(4397,1) for forward direction
4398	Q9NYL2 -->Null : K(4398,1) for forward direction
4399	Q9NYV4 -->Null : K(4399,1) for forward direction
4400	Q9NYV6 -->Null : K(4400,1) for forward direction
4401	Q9P0J1 -->Null : K(4401,1) for forward direction
4402	Q9P289 -->Null : K(4402,1) for forward direction
4403	Q9UBB4 -->Null : K(4403,1) for forward direction
4404	Q9UBF6 -->Null : K(4404,1) for forward direction
4405	Q9UBF8 -->Null : K(4405,1) for forward direction
4406	Q9UBI6 -->Null : K(4406,1) for forward direction
4407	Q9UBN7 -->Null : K(4407,1) for forward direction
4408	Q9UBP6 -->Null : K(4408,1) for forward direction
4409	Q9UER7 -->Null : K(4409,1) for forward direction
4410	Q9UEW8 -->Null : K(4410,1) for forward direction
4411	Q9UEY8 -->Null : K(4411,1) for forward direction
4412	Q9UGK3 -->Null : K(4412,1) for forward direction
4413	Q9UHD2 -->Null : K(4413,1) for forward direction
4414	Q9UIG0 -->Null : K(4414,1) for forward direction
4415	Q9UJM3 -->Null : K(4415,1) for forward direction
4416	Q9UJX2 -->Null : K(4416,1) for forward direction
4417	Q9UKI8 -->Null : K(4417,1) for forward direction
4418	Q9UKV3 -->Null : K(4418,1) for forward direction
4419	Q9UKV8 -->Null : K(4419,1) for forward direction
4420	Q9UKX7 -->Null : K(4420,1) for forward direction
4421	Q9UL54 -->Null : K(4421,1) for forward direction
4422	Q9ULV4 -->Null : K(4422,1) for forward direction

4423	Q9UNE7 -->Null : K(4423,1) for forward direction
4424	Q9UNN5 -->Null : K(4424,1) for forward direction
4425	Q9UNZ2 -->Null : K(4425,1) for forward direction
4426	Q9UPT5 -->Null : K(4426,1) for forward direction
4427	Q9UPY8 -->Null : K(4427,1) for forward direction
4428	Q9UQ80 -->Null : K(4428,1) for forward direction
4429	Q9UQ88 -->Null : K(4429,1) for forward direction
4430	Q9UQE7 -->Null : K(4430,1) for forward direction
4431	Q9UQM7 -->Null : K(4431,1) for forward direction
4432	Q9Y266 -->Null : K(4432,1) for forward direction
4433	Q9Y2H1 -->Null : K(4433,1) for forward direction
4434	Q9Y2K2 -->Null : K(4434,1) for forward direction
4435	Q9Y2U5 -->Null : K(4435,1) for forward direction
4436	Q9Y2V2 -->Null : K(4436,1) for forward direction
4437	Q9Y2Y9 -->Null : K(4437,1) for forward direction
4438	Q9Y365 -->Null : K(4438,1) for forward direction
4439	Q9Y3Z3 -->Null : K(4439,1) for forward direction
4440	Q9Y478 -->Null : K(4440,1) for forward direction
4441	Q9Y490 -->Null : K(4441,1) for forward direction
4442	Q9Y4H2 -->Null : K(4442,1) for forward direction
4443	Q9Y4K3 -->Null : K(4443,1) for forward direction
4444	Q9Y4R8 -->Null : K(4444,1) for forward direction
4445	Q9Y5B0 -->Null : K(4445,1) for forward direction
4446	Q9Y5S9 -->Null : K(4446,1) for forward direction
4447	Q9Y5T5 -->Null : K(4447,1) for forward direction
4448	Q9Y613 -->Null : K(4448,1) for forward direction
4449	Q9Y618 -->Null : K(4449,1) for forward direction
4450	Q9Y624 -->Null : K(4450,1) for forward direction
4451	Q9Y6E0 -->Null : K(4451,1) for forward direction
4452	Q9Y6K9 -->Null : K(4452,1) for forward direction
4453	Q9Y6R4 -->Null : K(4453,1) for forward direction
4454	Q9Y6W5 -->Null : K(4454,1) for forward direction
4455	O43524_rna -->Null : K(4455,1) for forward direction
4456	O95471_rna -->Null : K(4456,1) for forward direction
4457	P01106_rna -->Null : K(4457,1) for forward direction
4458	P01137_rna -->Null : K(4458,1) for forward direction
4459	P08581_rna -->Null : K(4459,1) for forward direction
4460	P11166_rna -->Null : K(4460,1) for forward direction
4461	P17174_rna -->Null : K(4461,1) for forward direction
4462	P21291_rna -->Null : K(4462,1) for forward direction
4463	P25445_rna -->Null : K(4463,1) for forward direction
4464	P35226_rna -->Null : K(4464,1) for forward direction
4465	P35580_rna -->Null : K(4465,1) for forward direction

4466	P41440_rna -->Null : K(4466,1) for forward direction
4467	P46527_rna -->Null : K(4467,1) for forward direction
4468	Q13887_rna -->Null : K(4468,1) for forward direction
4469	Q99988_rna -->Null : K(4469,1) for forward direction
4470	Null -->A8MYZ6 : K(4470,1) for forward direction
4471	Null -->E9PAV3 : K(4471,1) for forward direction
4472	Null -->O00161 : K(4472,1) for forward direction
4473	Null -->O00170 : K(4473,1) for forward direction
4474	Null -->O00267 : K(4474,1) for forward direction
4475	Null -->O00303 : K(4475,1) for forward direction
4476	Null -->O00311 : K(4476,1) for forward direction
4477	Null -->O00401 : K(4477,1) for forward direction
4478	Null -->O00418 : K(4478,1) for forward direction
4479	Null -->O00429 : K(4479,1) for forward direction
4480	Null -->O00443 : K(4480,1) for forward direction
4481	Null -->O00506 : K(4481,1) for forward direction
4482	Null -->O00571 : K(4482,1) for forward direction
4483	Null -->O00716 : K(4483,1) for forward direction
4484	Null -->O00743 : K(4484,1) for forward direction
4485	Null -->O00750 : K(4485,1) for forward direction
4486	Null -->O14641 : K(4486,1) for forward direction
4487	Null -->O14733 : K(4487,1) for forward direction
4488	Null -->O14737 : K(4488,1) for forward direction
4489	Null -->O14757 : K(4489,1) for forward direction
4490	Null -->O14777 : K(4490,1) for forward direction
4491	Null -->O14828 : K(4491,1) for forward direction
4492	Null -->O14874 : K(4492,1) for forward direction
4493	Null -->O14944 : K(4493,1) for forward direction
4494	Null -->O14950 : K(4494,1) for forward direction
4495	Null -->O14958 : K(4495,1) for forward direction
4496	Null -->O14964 : K(4496,1) for forward direction
4497	Null -->O14974 : K(4497,1) for forward direction
4498	Null -->O15111 : K(4498,1) for forward direction
4499	Null -->O15143 : K(4499,1) for forward direction
4500	Null -->O15162 : K(4500,1) for forward direction
4501	Null -->O15164 : K(4501,1) for forward direction
4502	Null -->O15169 : K(4502,1) for forward direction
4503	Null -->O15264 : K(4503,1) for forward direction
4504	Null -->O15357 : K(4504,1) for forward direction
4505	Null -->O15379 : K(4505,1) for forward direction
4506	Null -->O15511 : K(4506,1) for forward direction
4507	Null -->O15530 : K(4507,1) for forward direction
4508	Null -->O15551 : K(4508,1) for forward direction

4509	Null -->O43156 : K(4509,1) for forward direction
4510	Null -->O43318 : K(4510,1) for forward direction
4511	Null -->O43353 : K(4511,1) for forward direction
4512	Null -->O43395 : K(4512,1) for forward direction
4513	Null -->O43422 : K(4513,1) for forward direction
4514	Null -->O43464 : K(4514,1) for forward direction
4515	Null -->O43490 : K(4515,1) for forward direction
4516	Null -->O43665 : K(4516,1) for forward direction
4517	Null -->O43707 : K(4517,1) for forward direction
4518	Null -->O43768 : K(4518,1) for forward direction
4519	Null -->O43865 : K(4519,1) for forward direction
4520	Null -->O43896 : K(4520,1) for forward direction
4521	Null -->O60343 : K(4521,1) for forward direction
4522	Null -->O60566 : K(4522,1) for forward direction
4523	Null -->O60716 : K(4523,1) for forward direction
4524	Null -->O60763 : K(4524,1) for forward direction
4525	Null -->O60825 : K(4525,1) for forward direction
4526	Null -->O60869 : K(4526,1) for forward direction
4527	Null -->O60879 : K(4527,1) for forward direction
4528	Null -->O60885 : K(4528,1) for forward direction
4529	Null -->O75116 : K(4529,1) for forward direction
4530	Null -->O75143 : K(4530,1) for forward direction
4531	Null -->O75376 : K(4531,1) for forward direction
4532	Null -->O75475 : K(4532,1) for forward direction
4533	Null -->O75531 : K(4533,1) for forward direction
4534	Null -->O75533 : K(4534,1) for forward direction
4535	Null -->O75676 : K(4535,1) for forward direction
4536	Null -->O75688 : K(4536,1) for forward direction
4537	Null -->O75689 : K(4537,1) for forward direction
4538	Null -->O75886 : K(4538,1) for forward direction
4539	Null -->O75925 : K(4539,1) for forward direction
4540	Null -->O75962 : K(4540,1) for forward direction
4541	Null -->O76070 : K(4541,1) for forward direction
4542	Null -->O94804 : K(4542,1) for forward direction
4543	Null -->O95251 : K(4543,1) for forward direction
4544	Null -->O95295 : K(4544,1) for forward direction
4545	Null -->O95297 : K(4545,1) for forward direction
4546	Null -->O95453 : K(4546,1) for forward direction
4547	Null -->O95747 : K(4547,1) for forward direction
4548	Null -->O95786 : K(4548,1) for forward direction
4549	Null -->O95816 : K(4549,1) for forward direction
4550	Null -->O95817 : K(4550,1) for forward direction
4551	Null -->O96013 : K(4551,1) for forward direction

4552	Null -->O96017 : K(4552,1) for forward direction
4553	Null -->P00533 : K(4553,1) for forward direction
4554	Null -->P01100 : K(4554,1) for forward direction
4555	Null -->P01111 : K(4555,1) for forward direction
4556	Null -->P01112 : K(4556,1) for forward direction
4557	Null -->P01116 : K(4557,1) for forward direction
4558	Null -->P01344 : K(4558,1) for forward direction
4559	Null -->P02545 : K(4559,1) for forward direction
4560	Null -->P04035 : K(4560,1) for forward direction
4561	Null -->P04083 : K(4561,1) for forward direction
4562	Null -->P04183 : K(4562,1) for forward direction
4563	Null -->P04626 : K(4563,1) for forward direction
4564	Null -->P04637 : K(4564,1) for forward direction
4565	Null -->P04792 : K(4565,1) for forward direction
4566	Null -->P05067 : K(4566,1) for forward direction
4567	Null -->P05114 : K(4567,1) for forward direction
4568	Null -->P05198 : K(4568,1) for forward direction
4569	Null -->P05204 : K(4569,1) for forward direction
4570	Null -->P05387 : K(4570,1) for forward direction
4571	Null -->P05412 : K(4571,1) for forward direction
4572	Null -->P05455 : K(4572,1) for forward direction
4573	Null -->P05783 : K(4573,1) for forward direction
4574	Null -->P05976 : K(4574,1) for forward direction
4575	Null -->P06396 : K(4575,1) for forward direction
4576	Null -->P06400 : K(4576,1) for forward direction
4577	Null -->P06493 : K(4577,1) for forward direction
4578	Null -->P06730 : K(4578,1) for forward direction
4579	Null -->P06733 : K(4579,1) for forward direction
4580	Null -->P06744 : K(4580,1) for forward direction
4581	Null -->P06748 : K(4581,1) for forward direction
4582	Null -->P07108 : K(4582,1) for forward direction
4583	Null -->P07355 : K(4583,1) for forward direction
4584	Null -->P07737 : K(4584,1) for forward direction
4585	Null -->P07814 : K(4585,1) for forward direction
4586	Null -->P07900 : K(4586,1) for forward direction
4587	Null -->P07910 : K(4587,1) for forward direction
4588	Null -->P07947 : K(4588,1) for forward direction
4589	Null -->P08047 : K(4589,1) for forward direction
4590	Null -->P08069 : K(4590,1) for forward direction
4591	Null -->P08238 : K(4591,1) for forward direction
4592	Null -->P08559 : K(4592,1) for forward direction
4593	Null -->P08670 : K(4593,1) for forward direction
4594	Null -->P08727 : K(4594,1) for forward direction

4595	Null -->P09211 : K(4595,1) for forward direction
4596	Null -->P09327 : K(4596,1) for forward direction
4597	Null -->P09497 : K(4597,1) for forward direction
4598	Null -->P09651 : K(4598,1) for forward direction
4599	Null -->P09874 : K(4599,1) for forward direction
4600	Null -->P09917 : K(4600,1) for forward direction
4601	Null -->P10301 : K(4601,1) for forward direction
4602	Null -->P10398 : K(4602,1) for forward direction
4603	Null -->P10586 : K(4603,1) for forward direction
4604	Null -->P10588 : K(4604,1) for forward direction
4605	Null -->P10644 : K(4605,1) for forward direction
4606	Null -->P11171 : K(4606,1) for forward direction
4607	Null -->P11233 : K(4607,1) for forward direction
4608	Null -->P11234 : K(4608,1) for forward direction
4609	Null -->P11274 : K(4609,1) for forward direction
4610	Null -->P11388 : K(4610,1) for forward direction
4611	Null -->P11413 : K(4611,1) for forward direction
4612	Null -->P11717 : K(4612,1) for forward direction
4613	Null -->P11802 : K(4613,1) for forward direction
4614	Null -->P12004 : K(4614,1) for forward direction
4615	Null -->P12259 : K(4615,1) for forward direction
4616	Null -->P12270 : K(4616,1) for forward direction
4617	Null -->P12694 : K(4617,1) for forward direction
4618	Null -->P12814 : K(4618,1) for forward direction
4619	Null -->P12830 : K(4619,1) for forward direction
4620	Null -->P12931 : K(4620,1) for forward direction
4621	Null -->P13639 : K(4621,1) for forward direction
4622	Null -->P13688 : K(4622,1) for forward direction
4623	Null -->P13693 : K(4623,1) for forward direction
4624	Null -->P13807 : K(4624,1) for forward direction
4625	Null -->P13861 : K(4625,1) for forward direction
4626	Null -->P14618 : K(4626,1) for forward direction
4627	Null -->P14635 : K(4627,1) for forward direction
4628	Null -->P14649 : K(4628,1) for forward direction
4629	Null -->P14859 : K(4629,1) for forward direction
4630	Null -->P14923 : K(4630,1) for forward direction
4631	Null -->P15056 : K(4631,1) for forward direction
4632	Null -->P15311 : K(4632,1) for forward direction
4633	Null -->P15336 : K(4633,1) for forward direction
4634	Null -->P15531 : K(4634,1) for forward direction
4635	Null -->P15924 : K(4635,1) for forward direction
4636	Null -->P15927 : K(4636,1) for forward direction
4637	Null -->P16070 : K(4637,1) for forward direction

4638	Null -->P16104 : K(4638,1) for forward direction
4639	Null -->P16144 : K(4639,1) for forward direction
4640	Null -->P16220 : K(4640,1) for forward direction
4641	Null -->P16298 : K(4641,1) for forward direction
4642	Null -->P16401 : K(4642,1) for forward direction
4643	Null -->P16615 : K(4643,1) for forward direction
4644	Null -->P16949 : K(4644,1) for forward direction
4645	Null -->P17096 : K(4645,1) for forward direction
4646	Null -->P17252 : K(4646,1) for forward direction
4647	Null -->P17480 : K(4647,1) for forward direction
4648	Null -->P17535 : K(4648,1) for forward direction
4649	Null -->P17612 : K(4649,1) for forward direction
4650	Null -->P17655 : K(4650,1) for forward direction
4651	Null -->P17676 : K(4651,1) for forward direction
4652	Null -->P17706 : K(4652,1) for forward direction
4653	Null -->P17812 : K(4653,1) for forward direction
4654	Null -->P17844 : K(4654,1) for forward direction
4655	Null -->P17931 : K(4655,1) for forward direction
4656	Null -->P18031 : K(4656,1) for forward direction
4657	Null -->P18084 : K(4657,1) for forward direction
4658	Null -->P18206 : K(4658,1) for forward direction
4659	Null -->P18433 : K(4659,1) for forward direction
4660	Null -->P18669 : K(4660,1) for forward direction
4661	Null -->P18846 : K(4661,1) for forward direction
4662	Null -->P18858 : K(4662,1) for forward direction
4663	Null -->P18887 : K(4663,1) for forward direction
4664	Null -->P19474 : K(4664,1) for forward direction
4665	Null -->P19525 : K(4665,1) for forward direction
4666	Null -->P19634 : K(4666,1) for forward direction
4667	Null -->P19784 : K(4667,1) for forward direction
4668	Null -->P19793 : K(4668,1) for forward direction
4669	Null -->P19838 : K(4669,1) for forward direction
4670	Null -->P20042 : K(4670,1) for forward direction
4671	Null -->P20248 : K(4671,1) for forward direction
4672	Null -->P20936 : K(4672,1) for forward direction
4673	Null -->P21127 : K(4673,1) for forward direction
4674	Null -->P21333 : K(4674,1) for forward direction
4675	Null -->P21399 : K(4675,1) for forward direction
4676	Null -->P21802 : K(4676,1) for forward direction
4677	Null -->P21810 : K(4677,1) for forward direction
4678	Null -->P21860 : K(4678,1) for forward direction
4679	Null -->P21980 : K(4679,1) for forward direction
4680	Null -->P22314 : K(4680,1) for forward direction

4681	Null -->P22392 : K(4681,1) for forward direction
4682	Null -->P22681 : K(4682,1) for forward direction
4683	Null -->P23246 : K(4683,1) for forward direction
4684	Null -->P23396 : K(4684,1) for forward direction
4685	Null -->P23511 : K(4685,1) for forward direction
4686	Null -->P23528 : K(4686,1) for forward direction
4687	Null -->P23588 : K(4687,1) for forward direction
4688	Null -->P24534 : K(4688,1) for forward direction
4689	Null -->P24666 : K(4689,1) for forward direction
4690	Null -->P24844 : K(4690,1) for forward direction
4691	Null -->P24928 : K(4691,1) for forward direction
4692	Null -->P24941 : K(4692,1) for forward direction
4693	Null -->P25098 : K(4693,1) for forward direction
4694	Null -->P25205 : K(4694,1) for forward direction
4695	Null -->P25490 : K(4695,1) for forward direction
4696	Null -->P25788 : K(4696,1) for forward direction
4697	Null -->P26038 : K(4697,1) for forward direction
4698	Null -->P26358 : K(4698,1) for forward direction
4699	Null -->P27348 : K(4699,1) for forward direction
4700	Null -->P27361 : K(4700,1) for forward direction
4701	Null -->P27635 : K(4701,1) for forward direction
4702	Null -->P27695 : K(4702,1) for forward direction
4703	Null -->P27708 : K(4703,1) for forward direction
4704	Null -->P27816 : K(4704,1) for forward direction
4705	Null -->P27986 : K(4705,1) for forward direction
4706	Null -->P28482 : K(4706,1) for forward direction
4707	Null -->P29317 : K(4707,1) for forward direction
4708	Null -->P29350 : K(4708,1) for forward direction
4709	Null -->P29353 : K(4709,1) for forward direction
4710	Null -->P29590 : K(4710,1) for forward direction
4711	Null -->P29692 : K(4711,1) for forward direction
4712	Null -->P29966 : K(4712,1) for forward direction
4713	Null -->P30041 : K(4713,1) for forward direction
4714	Null -->P30050 : K(4714,1) for forward direction
4715	Null -->P30086 : K(4715,1) for forward direction
4716	Null -->P30260 : K(4716,1) for forward direction
4717	Null -->P30291 : K(4717,1) for forward direction
4718	Null -->P30519 : K(4718,1) for forward direction
4719	Null -->P30622 : K(4719,1) for forward direction
4720	Null -->P30626 : K(4720,1) for forward direction
4721	Null -->P31146 : K(4721,1) for forward direction
4722	Null -->P31350 : K(4722,1) for forward direction
4723	Null -->P31431 : K(4723,1) for forward direction

4724	Null -->P31749 : K(4724,1) for forward direction
4725	Null -->P31751 : K(4725,1) for forward direction
4726	Null -->P31946 : K(4726,1) for forward direction
4727	Null -->P31947 : K(4727,1) for forward direction
4728	Null -->P32519 : K(4728,1) for forward direction
4729	Null -->P33527 : K(4729,1) for forward direction
4730	Null -->P33778 : K(4730,1) for forward direction
4731	Null -->P33981 : K(4731,1) for forward direction
4732	Null -->P33991 : K(4732,1) for forward direction
4733	Null -->P33993 : K(4733,1) for forward direction
4734	Null -->P35221 : K(4734,1) for forward direction
4735	Null -->P35222 : K(4735,1) for forward direction
4736	Null -->P35240 : K(4736,1) for forward direction
4737	Null -->P35241 : K(4737,1) for forward direction
4738	Null -->P35269 : K(4738,1) for forward direction
4739	Null -->P35270 : K(4739,1) for forward direction
4740	Null -->P35568 : K(4740,1) for forward direction
4741	Null -->P35579 : K(4741,1) for forward direction
4742	Null -->P35611 : K(4742,1) for forward direction
4743	Null -->P35659 : K(4743,1) for forward direction
4744	Null -->P35813 : K(4744,1) for forward direction
4745	Null -->P36507 : K(4745,1) for forward direction
4746	Null -->P36871 : K(4746,1) for forward direction
4747	Null -->P36873 : K(4747,1) for forward direction
4748	Null -->P37173 : K(4748,1) for forward direction
4749	Null -->P38432 : K(4749,1) for forward direction
4750	Null -->P38646 : K(4750,1) for forward direction
4751	Null -->P39748 : K(4751,1) for forward direction
4752	Null -->P40189 : K(4752,1) for forward direction
4753	Null -->P40763 : K(4753,1) for forward direction
4754	Null -->P41212 : K(4754,1) for forward direction
4755	Null -->P41236 : K(4755,1) for forward direction
4756	Null -->P41240 : K(4756,1) for forward direction
4757	Null -->P41279 : K(4757,1) for forward direction
4758	Null -->P41743 : K(4758,1) for forward direction
4759	Null -->P42224 : K(4759,1) for forward direction
4760	Null -->P42226 : K(4760,1) for forward direction
4761	Null -->P42345 : K(4761,1) for forward direction
4762	Null -->P42566 : K(4762,1) for forward direction
4763	Null -->P42574 : K(4763,1) for forward direction
4764	Null -->P42575 : K(4764,1) for forward direction
4765	Null -->P42858 : K(4765,1) for forward direction
4766	Null -->P43378 : K(4766,1) for forward direction

4767	Null -->P43405 : K(4767,1) for forward direction
4768	Null -->P45973 : K(4768,1) for forward direction
4769	Null -->P45983 : K(4769,1) for forward direction
4770	Null -->P45984 : K(4770,1) for forward direction
4771	Null -->P45985 : K(4771,1) for forward direction
4772	Null -->P46020 : K(4772,1) for forward direction
4773	Null -->P46060 : K(4773,1) for forward direction
4774	Null -->P46108 : K(4774,1) for forward direction
4775	Null -->P46459 : K(4775,1) for forward direction
4776	Null -->P46734 : K(4776,1) for forward direction
4777	Null -->P46777 : K(4777,1) for forward direction
4778	Null -->P46937 : K(4778,1) for forward direction
4779	Null -->P46939 : K(4779,1) for forward direction
4780	Null -->P46940 : K(4780,1) for forward direction
4781	Null -->P47712 : K(4781,1) for forward direction
4782	Null -->P47736 : K(4782,1) for forward direction
4783	Null -->P48200 : K(4783,1) for forward direction
4784	Null -->P48426 : K(4784,1) for forward direction
4785	Null -->P48436 : K(4785,1) for forward direction
4786	Null -->P48729 : K(4786,1) for forward direction
4787	Null -->P48730 : K(4787,1) for forward direction
4788	Null -->P49023 : K(4788,1) for forward direction
4789	Null -->P49137 : K(4789,1) for forward direction
4790	Null -->P49407 : K(4790,1) for forward direction
4791	Null -->P49459 : K(4791,1) for forward direction
4792	Null -->P49585 : K(4792,1) for forward direction
4793	Null -->P49674 : K(4793,1) for forward direction
4794	Null -->P49736 : K(4794,1) for forward direction
4795	Null -->P49757 : K(4795,1) for forward direction
4796	Null -->P49790 : K(4796,1) for forward direction
4797	Null -->P49815 : K(4797,1) for forward direction
4798	Null -->P49840 : K(4798,1) for forward direction
4799	Null -->P49841 : K(4799,1) for forward direction
4800	Null -->P49915 : K(4800,1) for forward direction
4801	Null -->P49916 : K(4801,1) for forward direction
4802	Null -->P49917 : K(4802,1) for forward direction
4803	Null -->P50402 : K(4803,1) for forward direction
4804	Null -->P50542 : K(4804,1) for forward direction
4805	Null -->P50548 : K(4805,1) for forward direction
4806	Null -->P50552 : K(4806,1) for forward direction
4807	Null -->P50570 : K(4807,1) for forward direction
4808	Null -->P50613 : K(4808,1) for forward direction
4809	Null -->P50616 : K(4809,1) for forward direction

4810	Null -->P50750 : K(4810,1) for forward direction
4811	Null -->P51398 : K(4811,1) for forward direction
4812	Null -->P51452 : K(4812,1) for forward direction
4813	Null -->P51617 : K(4813,1) for forward direction
4814	Null -->P51692 : K(4814,1) for forward direction
4815	Null -->P51812 : K(4815,1) for forward direction
4816	Null -->P52292 : K(4816,1) for forward direction
4817	Null -->P52564 : K(4817,1) for forward direction
4818	Null -->P52565 : K(4818,1) for forward direction
4819	Null -->P52566 : K(4819,1) for forward direction
4820	Null -->P52655 : K(4820,1) for forward direction
4821	Null -->P52732 : K(4821,1) for forward direction
4822	Null -->P52735 : K(4822,1) for forward direction
4823	Null -->P52888 : K(4823,1) for forward direction
4824	Null -->P52907 : K(4824,1) for forward direction
4825	Null -->P52926 : K(4825,1) for forward direction
4826	Null -->P52943 : K(4826,1) for forward direction
4827	Null -->P52948 : K(4827,1) for forward direction
4828	Null -->P53041 : K(4828,1) for forward direction
4829	Null -->P53350 : K(4829,1) for forward direction
4830	Null -->P53355 : K(4830,1) for forward direction
4831	Null -->P53365 : K(4831,1) for forward direction
4832	Null -->P53396 : K(4832,1) for forward direction
4833	Null -->P54132 : K(4833,1) for forward direction
4834	Null -->P54198 : K(4834,1) for forward direction
4835	Null -->P54253 : K(4835,1) for forward direction
4836	Null -->P55010 : K(4836,1) for forward direction
4837	Null -->P55011 : K(4837,1) for forward direction
4838	Null -->P55072 : K(4838,1) for forward direction
4839	Null -->P55210 : K(4839,1) for forward direction
4840	Null -->P55211 : K(4840,1) for forward direction
4841	Null -->P55327 : K(4841,1) for forward direction
4842	Null -->P55957 : K(4842,1) for forward direction
4843	Null -->P56211 : K(4843,1) for forward direction
4844	Null -->P56537 : K(4844,1) for forward direction
4845	Null -->P56945 : K(4845,1) for forward direction
4846	Null -->P60484 : K(4846,1) for forward direction
4847	Null -->P60510 : K(4847,1) for forward direction
4848	Null -->P60953 : K(4848,1) for forward direction
4849	Null -->P60983 : K(4849,1) for forward direction
4850	Null -->P61020 : K(4850,1) for forward direction
4851	Null -->P61088 : K(4851,1) for forward direction
4852	Null -->P61224 : K(4852,1) for forward direction

4853	Null -->P61586 : K(4853,1) for forward direction
4854	Null -->P61587 : K(4854,1) for forward direction
4855	Null -->P61764 : K(4855,1) for forward direction
4856	Null -->P61978 : K(4856,1) for forward direction
4857	Null -->P62136 : K(4857,1) for forward direction
4858	Null -->P62140 : K(4858,1) for forward direction
4859	Null -->P62258 : K(4859,1) for forward direction
4860	Null -->P62714 : K(4860,1) for forward direction
4861	Null -->P62745 : K(4861,1) for forward direction
4862	Null -->P62753 : K(4862,1) for forward direction
4863	Null -->P62826 : K(4863,1) for forward direction
4864	Null -->P62834 : K(4864,1) for forward direction
4865	Null -->P62837 : K(4865,1) for forward direction
4866	Null -->P62877 : K(4866,1) for forward direction
4867	Null -->P62993 : K(4867,1) for forward direction
4868	Null -->P63000 : K(4868,1) for forward direction
4869	Null -->P63010 : K(4869,1) for forward direction
4870	Null -->P63104 : K(4870,1) for forward direction
4871	Null -->P63167 : K(4871,1) for forward direction
4872	Null -->P63244 : K(4872,1) for forward direction
4873	Null -->P63261 : K(4873,1) for forward direction
4874	Null -->P67775 : K(4874,1) for forward direction
4875	Null -->P67809 : K(4875,1) for forward direction
4876	Null -->P67870 : K(4876,1) for forward direction
4877	Null -->P68104 : K(4877,1) for forward direction
4878	Null -->P68366 : K(4878,1) for forward direction
4879	Null -->P68400 : K(4879,1) for forward direction
4880	Null -->P68431 : K(4880,1) for forward direction
4881	Null -->P78314 : K(4881,1) for forward direction
4882	Null -->P78347 : K(4882,1) for forward direction
4883	Null -->P78356 : K(4883,1) for forward direction
4884	Null -->P78362 : K(4884,1) for forward direction
4885	Null -->P78371 : K(4885,1) for forward direction
4886	Null -->P78527 : K(4886,1) for forward direction
4887	Null -->P78536 : K(4887,1) for forward direction
4888	Null -->P78545 : K(4888,1) for forward direction
4889	Null -->P83916 : K(4889,1) for forward direction
4890	Null -->P84022 : K(4890,1) for forward direction
4891	Null -->P84243 : K(4891,1) for forward direction
4892	Null -->P98170 : K(4892,1) for forward direction
4893	Null -->Q00059 : K(4893,1) for forward direction
4894	Null -->Q00534 : K(4894,1) for forward direction
4895	Null -->Q00535 : K(4895,1) for forward direction

4896	Null -->Q00610 : K(4896,1) for forward direction
4897	Null -->Q00613 : K(4897,1) for forward direction
4898	Null -->Q00653 : K(4898,1) for forward direction
4899	Null -->Q00839 : K(4899,1) for forward direction
4900	Null -->Q01082 : K(4900,1) for forward direction
4901	Null -->Q01105 : K(4901,1) for forward direction
4902	Null -->Q01167 : K(4902,1) for forward direction
4903	Null -->Q01844 : K(4903,1) for forward direction
4904	Null -->Q01970 : K(4904,1) for forward direction
4905	Null -->Q01973 : K(4905,1) for forward direction
4906	Null -->Q02447 : K(4906,1) for forward direction
4907	Null -->Q02750 : K(4907,1) for forward direction
4908	Null -->Q02790 : K(4908,1) for forward direction
4909	Null -->Q04206 : K(4909,1) for forward direction
4910	Null -->Q04724 : K(4910,1) for forward direction
4911	Null -->Q04912 : K(4911,1) for forward direction
4912	Null -->Q04917 : K(4912,1) for forward direction
4913	Null -->Q05086 : K(4913,1) for forward direction
4914	Null -->Q05193 : K(4914,1) for forward direction
4915	Null -->Q05209 : K(4915,1) for forward direction
4916	Null -->Q05397 : K(4916,1) for forward direction
4917	Null -->Q05469 : K(4917,1) for forward direction
4918	Null -->Q05655 : K(4918,1) for forward direction
4919	Null -->Q06124 : K(4919,1) for forward direction
4920	Null -->Q06210 : K(4920,1) for forward direction
4921	Null -->Q06265 : K(4921,1) for forward direction
4922	Null -->Q06481 : K(4922,1) for forward direction
4923	Null -->Q06830 : K(4923,1) for forward direction
4924	Null -->Q07352 : K(4924,1) for forward direction
4925	Null -->Q07617 : K(4925,1) for forward direction
4926	Null -->Q07666 : K(4926,1) for forward direction
4927	Null -->Q07812 : K(4927,1) for forward direction
4928	Null -->Q07817 : K(4928,1) for forward direction
4929	Null -->Q07820 : K(4929,1) for forward direction
4930	Null -->Q07866 : K(4930,1) for forward direction
4931	Null -->Q07889 : K(4931,1) for forward direction
4932	Null -->Q07954 : K(4932,1) for forward direction
4933	Null -->Q07955 : K(4933,1) for forward direction
4934	Null -->Q08209 : K(4934,1) for forward direction
4935	Null -->Q08345 : K(4935,1) for forward direction
4936	Null -->Q08379 : K(4936,1) for forward direction
4937	Null -->Q08945 : K(4937,1) for forward direction
4938	Null -->Q08J23 : K(4938,1) for forward direction

4939	Null -->Q09472 : K(4939,1) for forward direction
4940	Null -->Q12778 : K(4940,1) for forward direction
4941	Null -->Q12800 : K(4941,1) for forward direction
4942	Null -->Q12802 : K(4942,1) for forward direction
4943	Null -->Q12888 : K(4943,1) for forward direction
4944	Null -->Q12904 : K(4944,1) for forward direction
4945	Null -->Q12906 : K(4945,1) for forward direction
4946	Null -->Q12933 : K(4946,1) for forward direction
4947	Null -->Q12959 : K(4947,1) for forward direction
4948	Null -->Q12972 : K(4948,1) for forward direction
4949	Null -->Q12974 : K(4949,1) for forward direction
4950	Null -->Q13042 : K(4950,1) for forward direction
4951	Null -->Q13043 : K(4951,1) for forward direction
4952	Null -->Q13085 : K(4952,1) for forward direction
4953	Null -->Q13131 : K(4953,1) for forward direction
4954	Null -->Q13144 : K(4954,1) for forward direction
4955	Null -->Q13153 : K(4955,1) for forward direction
4956	Null -->Q13158 : K(4956,1) for forward direction
4957	Null -->Q13177 : K(4957,1) for forward direction
4958	Null -->Q13185 : K(4958,1) for forward direction
4959	Null -->Q13188 : K(4959,1) for forward direction
4960	Null -->Q13257 : K(4960,1) for forward direction
4961	Null -->Q13263 : K(4961,1) for forward direction
4962	Null -->Q13308 : K(4962,1) for forward direction
4963	Null -->Q13362 : K(4963,1) for forward direction
4964	Null -->Q13363 : K(4964,1) for forward direction
4965	Null -->Q13393 : K(4965,1) for forward direction
4966	Null -->Q13416 : K(4966,1) for forward direction
4967	Null -->Q13418 : K(4967,1) for forward direction
4968	Null -->Q13464 : K(4968,1) for forward direction
4969	Null -->Q13480 : K(4969,1) for forward direction
4970	Null -->Q13501 : K(4970,1) for forward direction
4971	Null -->Q13523 : K(4971,1) for forward direction
4972	Null -->Q13526 : K(4972,1) for forward direction
4973	Null -->Q13541 : K(4973,1) for forward direction
4974	Null -->Q13542 : K(4974,1) for forward direction
4975	Null -->Q13546 : K(4975,1) for forward direction
4976	Null -->Q13547 : K(4976,1) for forward direction
4977	Null -->Q13554 : K(4977,1) for forward direction
4978	Null -->Q13555 : K(4978,1) for forward direction
4979	Null -->Q13557 : K(4979,1) for forward direction
4980	Null -->Q13586 : K(4980,1) for forward direction
4981	Null -->Q13616 : K(4981,1) for forward direction

4982	Null -->Q13627 : K(4982,1) for forward direction
4983	Null -->Q13765 : K(4983,1) for forward direction
4984	Null -->Q13769 : K(4984,1) for forward direction
4985	Null -->Q13813 : K(4985,1) for forward direction
4986	Null -->Q13976 : K(4986,1) for forward direction
4987	Null -->Q14103 : K(4987,1) for forward direction
4988	Null -->Q14118 : K(4988,1) for forward direction
4989	Null -->Q14161 : K(4989,1) for forward direction
4990	Null -->Q14203 : K(4990,1) for forward direction
4991	Null -->Q14247 : K(4991,1) for forward direction
4992	Null -->Q14344 : K(4992,1) for forward direction
4993	Null -->Q14457 : K(4993,1) for forward direction
4994	Null -->Q14653 : K(4994,1) for forward direction
4995	Null -->Q14676 : K(4995,1) for forward direction
4996	Null -->Q14738 : K(4996,1) for forward direction
4997	Null -->Q14739 : K(4997,1) for forward direction
4998	Null -->Q14790 : K(4998,1) for forward direction
4999	Null -->Q14847 : K(4999,1) for forward direction
5000	Null -->Q14978 : K(5000,1) for forward direction
5001	Null -->Q14980 : K(5001,1) for forward direction
5002	Null -->Q15019 : K(5002,1) for forward direction
5003	Null -->Q15027 : K(5003,1) for forward direction
5004	Null -->Q15046 : K(5004,1) for forward direction
5005	Null -->Q15054 : K(5005,1) for forward direction
5006	Null -->Q15075 : K(5006,1) for forward direction
5007	Null -->Q15121 : K(5007,1) for forward direction
5008	Null -->Q15149 : K(5008,1) for forward direction
5009	Null -->Q15172 : K(5009,1) for forward direction
5010	Null -->Q15185 : K(5010,1) for forward direction
5011	Null -->Q15208 : K(5011,1) for forward direction
5012	Null -->Q15257 : K(5012,1) for forward direction
5013	Null -->Q15262 : K(5013,1) for forward direction
5014	Null -->Q15418 : K(5014,1) for forward direction
5015	Null -->Q15596 : K(5015,1) for forward direction
5016	Null -->Q15637 : K(5016,1) for forward direction
5017	Null -->Q15648 : K(5017,1) for forward direction
5018	Null -->Q15653 : K(5018,1) for forward direction
5019	Null -->Q15717 : K(5019,1) for forward direction
5020	Null -->Q15746 : K(5020,1) for forward direction
5021	Null -->Q15750 : K(5021,1) for forward direction
5022	Null -->Q15796 : K(5022,1) for forward direction
5023	Null -->Q15797 : K(5023,1) for forward direction
5024	Null -->Q15833 : K(5024,1) for forward direction

5025	Null -->Q15910 : K(5025,1) for forward direction
5026	Null -->Q15942 : K(5026,1) for forward direction
5027	Null -->Q16204 : K(5027,1) for forward direction
5028	Null -->Q16512 : K(5028,1) for forward direction
5029	Null -->Q16513 : K(5029,1) for forward direction
5030	Null -->Q16539 : K(5030,1) for forward direction
5031	Null -->Q16543 : K(5031,1) for forward direction
5032	Null -->Q16555 : K(5032,1) for forward direction
5033	Null -->Q16625 : K(5033,1) for forward direction
5034	Null -->Q16637 : K(5034,1) for forward direction
5035	Null -->Q16644 : K(5035,1) for forward direction
5036	Null -->Q16666 : K(5036,1) for forward direction
5037	Null -->Q16695 : K(5037,1) for forward direction
5038	Null -->Q16790 : K(5038,1) for forward direction
5039	Null -->Q2M2I8 : K(5039,1) for forward direction
5040	Null -->Q2NKX8 : K(5040,1) for forward direction
5041	Null -->Q53EL6 : K(5041,1) for forward direction
5042	Null -->Q53ET0 : K(5042,1) for forward direction
5043	Null -->Q53EZ4 : K(5043,1) for forward direction
5044	Null -->Q5VT25 : K(5044,1) for forward direction
5045	Null -->Q68CZ2 : K(5045,1) for forward direction
5046	Null -->Q6A1A2 : K(5046,1) for forward direction
5047	Null -->Q6IAA8 : K(5047,1) for forward direction
5048	Null -->Q6VY07 : K(5048,1) for forward direction
5049	Null -->Q6WCQ1 : K(5049,1) for forward direction
5050	Null -->Q712K3 : K(5050,1) for forward direction
5051	Null -->Q7KZI7 : K(5051,1) for forward direction
5052	Null -->Q7L5N1 : K(5052,1) for forward direction
5053	Null -->Q7L7X3 : K(5053,1) for forward direction
5054	Null -->Q86TI0 : K(5054,1) for forward direction
5055	Null -->Q86V81 : K(5055,1) for forward direction
5056	Null -->Q86VP1 : K(5056,1) for forward direction
5057	Null -->Q86WB0 : K(5057,1) for forward direction
5058	Null -->Q86YT6 : K(5058,1) for forward direction
5059	Null -->Q8IX03 : K(5059,1) for forward direction
5060	Null -->Q8IXJ6 : K(5060,1) for forward direction
5061	Null -->Q8IXW5 : K(5061,1) for forward direction
5062	Null -->Q8IZL8 : K(5062,1) for forward direction
5063	Null -->Q8IZP0 : K(5063,1) for forward direction
5064	Null -->Q8N122 : K(5064,1) for forward direction
5065	Null -->Q8N752 : K(5065,1) for forward direction
5066	Null -->Q8NE71 : K(5066,1) for forward direction
5067	Null -->Q8NEB9 : K(5067,1) for forward direction

5068	Null -->Q8NFA2 : K(5068,1) for forward direction
5069	Null -->Q8TAP9 : K(5069,1) for forward direction
5070	Null -->Q8TB45 : K(5070,1) for forward direction
5071	Null -->Q8TD19 : K(5071,1) for forward direction
5072	Null -->Q8TDX7 : K(5072,1) for forward direction
5073	Null -->Q8TE77 : K(5073,1) for forward direction
5074	Null -->Q8TEW0 : K(5074,1) for forward direction
5075	Null -->Q8WUI4 : K(5075,1) for forward direction
5076	Null -->Q8WXE1 : K(5076,1) for forward direction
5077	Null -->Q92538 : K(5077,1) for forward direction
5078	Null -->Q92574 : K(5078,1) for forward direction
5079	Null -->Q92598 : K(5079,1) for forward direction
5080	Null -->Q92686 : K(5080,1) for forward direction
5081	Null -->Q92688 : K(5081,1) for forward direction
5082	Null -->Q92769 : K(5082,1) for forward direction
5083	Null -->Q92793 : K(5083,1) for forward direction
5084	Null -->Q92900 : K(5084,1) for forward direction
5085	Null -->Q92934 : K(5085,1) for forward direction
5086	Null -->Q92945 : K(5086,1) for forward direction
5087	Null -->Q92974 : K(5087,1) for forward direction
5088	Null -->Q92985 : K(5088,1) for forward direction
5089	Null -->Q92993 : K(5089,1) for forward direction
5090	Null -->Q93034 : K(5090,1) for forward direction
5091	Null -->Q93045 : K(5091,1) for forward direction
5092	Null -->Q96B36 : K(5092,1) for forward direction
5093	Null -->Q96CF2 : K(5093,1) for forward direction
5094	Null -->Q96CV9 : K(5094,1) for forward direction
5095	Null -->Q96CW1 : K(5095,1) for forward direction
5096	Null -->Q96EB6 : K(5096,1) for forward direction
5097	Null -->Q96GD0 : K(5097,1) for forward direction
5098	Null -->Q96GD4 : K(5098,1) for forward direction
5099	Null -->Q96GX5 : K(5099,1) for forward direction
5100	Null -->Q96J02 : K(5100,1) for forward direction
5101	Null -->Q96KB5 : K(5101,1) for forward direction
5102	Null -->Q96N67 : K(5102,1) for forward direction
5103	Null -->Q96PM5 : K(5103,1) for forward direction
5104	Null -->Q96PU5 : K(5104,1) for forward direction
5105	Null -->Q96Q15 : K(5105,1) for forward direction
5106	Null -->Q96R06 : K(5106,1) for forward direction
5107	Null -->Q96RK0 : K(5107,1) for forward direction
5108	Null -->Q96S44 : K(5108,1) for forward direction
5109	Null -->Q96SB3 : K(5109,1) for forward direction
5110	Null -->Q96SB4 : K(5110,1) for forward direction

5111	Null -->Q96T60 : K(5111,1) for forward direction
5112	Null -->Q96T88 : K(5112,1) for forward direction
5113	Null -->Q99418 : K(5113,1) for forward direction
5114	Null -->Q99426 : K(5114,1) for forward direction
5115	Null -->Q99504 : K(5115,1) for forward direction
5116	Null -->Q99594 : K(5116,1) for forward direction
5117	Null -->Q99661 : K(5117,1) for forward direction
5118	Null -->Q99717 : K(5118,1) for forward direction
5119	Null -->Q99759 : K(5119,1) for forward direction
5120	Null -->Q99961 : K(5120,1) for forward direction
5121	Null -->Q99986 : K(5121,1) for forward direction
5122	Null -->Q9BQG0 : K(5122,1) for forward direction
5123	Null -->Q9BR76 : K(5123,1) for forward direction
5124	Null -->Q9BUL8 : K(5124,1) for forward direction
5125	Null -->Q9BXS5 : K(5125,1) for forward direction
5126	Null -->Q9BY41 : K(5126,1) for forward direction
5127	Null -->Q9BY44 : K(5127,1) for forward direction
5128	Null -->Q9BZL4 : K(5128,1) for forward direction
5129	Null -->Q9BZL6 : K(5129,1) for forward direction
5130	Null -->Q9BZQ8 : K(5130,1) for forward direction
5131	Null -->Q9GZM8 : K(5131,1) for forward direction
5132	Null -->Q9GZY8 : K(5132,1) for forward direction
5133	Null -->Q9H0B6 : K(5133,1) for forward direction
5134	Null -->Q9H0H5 : K(5134,1) for forward direction
5135	Null -->Q9H0Z9 : K(5135,1) for forward direction
5136	Null -->Q9H1A4 : K(5136,1) for forward direction
5137	Null -->Q9H1K1 : K(5137,1) for forward direction
5138	Null -->Q9H257 : K(5138,1) for forward direction
5139	Null -->Q9H2D6 : K(5139,1) for forward direction
5140	Null -->Q9H2G2 : K(5140,1) for forward direction
5141	Null -->Q9H2K8 : K(5141,1) for forward direction
5142	Null -->Q9H3Z4 : K(5142,1) for forward direction
5143	Null -->Q9H410 : K(5143,1) for forward direction
5144	Null -->Q9H4A3 : K(5144,1) for forward direction
5145	Null -->Q9H4A6 : K(5145,1) for forward direction
5146	Null -->Q9H5V8 : K(5146,1) for forward direction
5147	Null -->Q9H6Z4 : K(5147,1) for forward direction
5148	Null -->Q9H8S9 : K(5148,1) for forward direction
5149	Null -->Q9H8V3 : K(5149,1) for forward direction
5150	Null -->Q9H8Y8 : K(5150,1) for forward direction
5151	Null -->Q9H9Q4 : K(5151,1) for forward direction
5152	Null -->Q9HBY8 : K(5152,1) for forward direction
5153	Null -->Q9HCE7 : K(5153,1) for forward direction

5154	Null -->Q9HCP0 : K(5154,1) for forward direction
5155	Null -->Q9NP77 : K(5155,1) for forward direction
5156	Null -->Q9NP97 : K(5156,1) for forward direction
5157	Null -->Q9NQC3 : K(5157,1) for forward direction
5158	Null -->Q9NQX3 : K(5158,1) for forward direction
5159	Null -->Q9NR28 : K(5159,1) for forward direction
5160	Null -->Q9NRA0 : K(5160,1) for forward direction
5161	Null -->Q9NRF8 : K(5161,1) for forward direction
5162	Null -->Q9NRY4 : K(5162,1) for forward direction
5163	Null -->Q9NWZ3 : K(5163,1) for forward direction
5164	Null -->Q9NXR1 : K(5164,1) for forward direction
5165	Null -->Q9NY61 : K(5165,1) for forward direction
5166	Null -->Q9NYL2 : K(5166,1) for forward direction
5167	Null -->Q9NYV4 : K(5167,1) for forward direction
5168	Null -->Q9NYV6 : K(5168,1) for forward direction
5169	Null -->Q9P0J1 : K(5169,1) for forward direction
5170	Null -->Q9P289 : K(5170,1) for forward direction
5171	Null -->Q9UBB4 : K(5171,1) for forward direction
5172	Null -->Q9UBF6 : K(5172,1) for forward direction
5173	Null -->Q9UBF8 : K(5173,1) for forward direction
5174	Null -->Q9UBI6 : K(5174,1) for forward direction
5175	Null -->Q9UBN7 : K(5175,1) for forward direction
5176	Null -->Q9UBP6 : K(5176,1) for forward direction
5177	Null -->Q9UER7 : K(5177,1) for forward direction
5178	Null -->Q9UEW8 : K(5178,1) for forward direction
5179	Null -->Q9UEY8 : K(5179,1) for forward direction
5180	Null -->Q9UGK3 : K(5180,1) for forward direction
5181	Null -->Q9UHD2 : K(5181,1) for forward direction
5182	Null -->Q9UIG0 : K(5182,1) for forward direction
5183	Null -->Q9UJM3 : K(5183,1) for forward direction
5184	Null -->Q9UJX2 : K(5184,1) for forward direction
5185	Null -->Q9UKI8 : K(5185,1) for forward direction
5186	Null -->Q9UKV3 : K(5186,1) for forward direction
5187	Null -->Q9UKV8 : K(5187,1) for forward direction
5188	Null -->Q9UKX7 : K(5188,1) for forward direction
5189	Null -->Q9UL54 : K(5189,1) for forward direction
5190	Null -->Q9ULV4 : K(5190,1) for forward direction
5191	Null -->Q9UNE7 : K(5191,1) for forward direction
5192	Null -->Q9UNN5 : K(5192,1) for forward direction
5193	Null -->Q9UNZ2 : K(5193,1) for forward direction
5194	Null -->Q9UPT5 : K(5194,1) for forward direction
5195	Null -->Q9UPY8 : K(5195,1) for forward direction
5196	Null -->Q9UQ80 : K(5196,1) for forward direction

5197	Null -->Q9UQ88 : K(5197,1) for forward direction
5198	Null -->Q9UQE7 : K(5198,1) for forward direction
5199	Null -->Q9UQM7 : K(5199,1) for forward direction
5200	Null -->Q9Y266 : K(5200,1) for forward direction
5201	Null -->Q9Y2H1 : K(5201,1) for forward direction
5202	Null -->Q9Y2K2 : K(5202,1) for forward direction
5203	Null -->Q9Y2U5 : K(5203,1) for forward direction
5204	Null -->Q9Y2V2 : K(5204,1) for forward direction
5205	Null -->Q9Y2Y9 : K(5205,1) for forward direction
5206	Null -->Q9Y365 : K(5206,1) for forward direction
5207	Null -->Q9Y3Z3 : K(5207,1) for forward direction
5208	Null -->Q9Y478 : K(5208,1) for forward direction
5209	Null -->Q9Y490 : K(5209,1) for forward direction
5210	Null -->Q9Y4H2 : K(5210,1) for forward direction
5211	Null -->Q9Y4K3 : K(5211,1) for forward direction
5212	Null -->Q9Y4R8 : K(5212,1) for forward direction
5213	Null -->Q9Y5B0 : K(5213,1) for forward direction
5214	Null -->Q9Y5S9 : K(5214,1) for forward direction
5215	Null -->Q9Y5T5 : K(5215,1) for forward direction
5216	Null -->Q9Y613 : K(5216,1) for forward direction
5217	Null -->Q9Y618 : K(5217,1) for forward direction
5218	Null -->Q9Y624 : K(5218,1) for forward direction
5219	Null -->Q9Y6E0 : K(5219,1) for forward direction
5220	Null -->Q9Y6K9 : K(5220,1) for forward direction
5221	Null -->Q9Y6R4 : K(5221,1) for forward direction
5222	Null -->Q9Y6W5 : K(5222,1) for forward direction
5223	A8MYZ6_p -->A8MYZ6 : K(5223,1) for forward direction
5224	E9PAV3_p -->E9PAV3 : K(5224,1) for forward direction
5225	O00161_p -->O00161 : K(5225,1) for forward direction
5226	O00170_p -->O00170 : K(5226,1) for forward direction
5227	O00267_p -->O00267 : K(5227,1) for forward direction
5228	O00303_p -->O00303 : K(5228,1) for forward direction
5229	O00311_p -->O00311 : K(5229,1) for forward direction
5230	O00401_p -->O00401 : K(5230,1) for forward direction
5231	O00418_p -->O00418 : K(5231,1) for forward direction
5232	O00429_p -->O00429 : K(5232,1) for forward direction
5233	O00443_p -->O00443 : K(5233,1) for forward direction
5234	O00571_p -->O00571 : K(5234,1) for forward direction
5235	O00716_p -->O00716 : K(5235,1) for forward direction
5236	O00750_p -->O00750 : K(5236,1) for forward direction
5237	O14641_p -->O14641 : K(5237,1) for forward direction
5238	O14733_p -->O14733 : K(5238,1) for forward direction
5239	O14737_p -->O14737 : K(5239,1) for forward direction

5240	O14757_p -->O14757 : K(5240,1) for forward direction
5241	O14777_p -->O14777 : K(5241,1) for forward direction
5242	O14828_p -->O14828 : K(5242,1) for forward direction
5243	O14950_p -->O14950 : K(5243,1) for forward direction
5244	O14958_p -->O14958 : K(5244,1) for forward direction
5245	O14964_p -->O14964 : K(5245,1) for forward direction
5246	O14974_p -->O14974 : K(5246,1) for forward direction
5247	O15111_p -->O15111 : K(5247,1) for forward direction
5248	O15143_p -->O15143 : K(5248,1) for forward direction
5249	O15162_p -->O15162 : K(5249,1) for forward direction
5250	O15169_p -->O15169 : K(5250,1) for forward direction
5251	O15264_p -->O15264 : K(5251,1) for forward direction
5252	O15357_p -->O15357 : K(5252,1) for forward direction
5253	O15379_p -->O15379 : K(5253,1) for forward direction
5254	O15511_p -->O15511 : K(5254,1) for forward direction
5255	O15530_p -->O15530 : K(5255,1) for forward direction
5256	O15551_p -->O15551 : K(5256,1) for forward direction
5257	O43156_p -->O43156 : K(5257,1) for forward direction
5258	O43318_p -->O43318 : K(5258,1) for forward direction
5259	O43353_p -->O43353 : K(5259,1) for forward direction
5260	O43395_p -->O43395 : K(5260,1) for forward direction
5261	O43464_p -->O43464 : K(5261,1) for forward direction
5262	O43490_p -->O43490 : K(5262,1) for forward direction
5263	O43524_p -->O43524 : K(5263,1) for forward direction
5264	O43665_p -->O43665 : K(5264,1) for forward direction
5265	O43707_p -->O43707 : K(5265,1) for forward direction
5266	O43768_p -->O43768 : K(5266,1) for forward direction
5267	O43865_p -->O43865 : K(5267,1) for forward direction
5268	O43896_p -->O43896 : K(5268,1) for forward direction
5269	O60343_p -->O60343 : K(5269,1) for forward direction
5270	O60566_p -->O60566 : K(5270,1) for forward direction
5271	O60716_p -->O60716 : K(5271,1) for forward direction
5272	O60763_p -->O60763 : K(5272,1) for forward direction
5273	O60825_p -->O60825 : K(5273,1) for forward direction
5274	O60869_p -->O60869 : K(5274,1) for forward direction
5275	O60879_p -->O60879 : K(5275,1) for forward direction
5276	O75143_p -->O75143 : K(5276,1) for forward direction
5277	O75376_p -->O75376 : K(5277,1) for forward direction
5278	O75475_p -->O75475 : K(5278,1) for forward direction
5279	O75531_p -->O75531 : K(5279,1) for forward direction
5280	O75533_p -->O75533 : K(5280,1) for forward direction
5281	O75676_p -->O75676 : K(5281,1) for forward direction
5282	O75689_p -->O75689 : K(5282,1) for forward direction

5283	O75886_p -->O75886 : K(5283,1) for forward direction
5284	O75925_p -->O75925 : K(5284,1) for forward direction
5285	O75962_p -->O75962 : K(5285,1) for forward direction
5286	O76070_p -->O76070 : K(5286,1) for forward direction
5287	O95251_p -->O95251 : K(5287,1) for forward direction
5288	O95295_p -->O95295 : K(5288,1) for forward direction
5289	O95297_p -->O95297 : K(5289,1) for forward direction
5290	O95453_p -->O95453 : K(5290,1) for forward direction
5291	O95747_p -->O95747 : K(5291,1) for forward direction
5292	O95786_p -->O95786 : K(5292,1) for forward direction
5293	O95816_p -->O95816 : K(5293,1) for forward direction
5294	O95817_p -->O95817 : K(5294,1) for forward direction
5295	O96013_p -->O96013 : K(5295,1) for forward direction
5296	O96017_p -->O96017 : K(5296,1) for forward direction
5297	P00533_p -->P00533 : K(5297,1) for forward direction
5298	P01100_p -->P01100 : K(5298,1) for forward direction
5299	P01106_p -->P01106 : K(5299,1) for forward direction
5300	P01111_p -->P01111 : K(5300,1) for forward direction
5301	P01112_p -->P01112 : K(5301,1) for forward direction
5302	P01116_p -->P01116 : K(5302,1) for forward direction
5303	P02545_p -->P02545 : K(5303,1) for forward direction
5304	P04035_p -->P04035 : K(5304,1) for forward direction
5305	P04083_p -->P04083 : K(5305,1) for forward direction
5306	P04183_p -->P04183 : K(5306,1) for forward direction
5307	P04626_p -->P04626 : K(5307,1) for forward direction
5308	P04637_p -->P04637 : K(5308,1) for forward direction
5309	P04792_p -->P04792 : K(5309,1) for forward direction
5310	P05067_p -->P05067 : K(5310,1) for forward direction
5311	P05114_p -->P05114 : K(5311,1) for forward direction
5312	P05198_p -->P05198 : K(5312,1) for forward direction
5313	P05204_p -->P05204 : K(5313,1) for forward direction
5314	P05387_p -->P05387 : K(5314,1) for forward direction
5315	P05412_p -->P05412 : K(5315,1) for forward direction
5316	P05455_p -->P05455 : K(5316,1) for forward direction
5317	P05783_p -->P05783 : K(5317,1) for forward direction
5318	P05976_p -->P05976 : K(5318,1) for forward direction
5319	P06396_p -->P06396 : K(5319,1) for forward direction
5320	P06400_p -->P06400 : K(5320,1) for forward direction
5321	P06493_p -->P06493 : K(5321,1) for forward direction
5322	P06730_p -->P06730 : K(5322,1) for forward direction
5323	P06733_p -->P06733 : K(5323,1) for forward direction
5324	P06744_p -->P06744 : K(5324,1) for forward direction
5325	P06748_p -->P06748 : K(5325,1) for forward direction

5326	P07108_p -->P07108 : K(5326,1) for forward direction
5327	P07355_p -->P07355 : K(5327,1) for forward direction
5328	P07737_p -->P07737 : K(5328,1) for forward direction
5329	P07814_p -->P07814 : K(5329,1) for forward direction
5330	P07900_p -->P07900 : K(5330,1) for forward direction
5331	P07910_p -->P07910 : K(5331,1) for forward direction
5332	P07947_p -->P07947 : K(5332,1) for forward direction
5333	P08047_p -->P08047 : K(5333,1) for forward direction
5334	P08069_p -->P08069 : K(5334,1) for forward direction
5335	P08238_p -->P08238 : K(5335,1) for forward direction
5336	P08559_p -->P08559 : K(5336,1) for forward direction
5337	P08581_p -->P08581 : K(5337,1) for forward direction
5338	P08670_p -->P08670 : K(5338,1) for forward direction
5339	P08727_p -->P08727 : K(5339,1) for forward direction
5340	P09211_p -->P09211 : K(5340,1) for forward direction
5341	P09327_p -->P09327 : K(5341,1) for forward direction
5342	P09497_p -->P09497 : K(5342,1) for forward direction
5343	P09651_p -->P09651 : K(5343,1) for forward direction
5344	P09874_p -->P09874 : K(5344,1) for forward direction
5345	P09917_p -->P09917 : K(5345,1) for forward direction
5346	P10301_p -->P10301 : K(5346,1) for forward direction
5347	P10398_p -->P10398 : K(5347,1) for forward direction
5348	P10588_p -->P10588 : K(5348,1) for forward direction
5349	P10644_p -->P10644 : K(5349,1) for forward direction
5350	P11171_p -->P11171 : K(5350,1) for forward direction
5351	P11233_p -->P11233 : K(5351,1) for forward direction
5352	P11234_p -->P11234 : K(5352,1) for forward direction
5353	P11274_p -->P11274 : K(5353,1) for forward direction
5354	P11388_p -->P11388 : K(5354,1) for forward direction
5355	P11413_p -->P11413 : K(5355,1) for forward direction
5356	P11717_p -->P11717 : K(5356,1) for forward direction
5357	P11802_p -->P11802 : K(5357,1) for forward direction
5358	P12004_p -->P12004 : K(5358,1) for forward direction
5359	P12259_p -->P12259 : K(5359,1) for forward direction
5360	P12270_p -->P12270 : K(5360,1) for forward direction
5361	P12694_p -->P12694 : K(5361,1) for forward direction
5362	P12814_p -->P12814 : K(5362,1) for forward direction
5363	P12830_p -->P12830 : K(5363,1) for forward direction
5364	P12931_p -->P12931 : K(5364,1) for forward direction
5365	P13639_p -->P13639 : K(5365,1) for forward direction
5366	P13688_p -->P13688 : K(5366,1) for forward direction
5367	P13693_p -->P13693 : K(5367,1) for forward direction
5368	P13807_p -->P13807 : K(5368,1) for forward direction

5369	P13861_p -->P13861 : K(5369,1) for forward direction
5370	P14635_p -->P14635 : K(5370,1) for forward direction
5371	P14649_p -->P14649 : K(5371,1) for forward direction
5372	P14859_p -->P14859 : K(5372,1) for forward direction
5373	P14923_p -->P14923 : K(5373,1) for forward direction
5374	P15056_p -->P15056 : K(5374,1) for forward direction
5375	P15311_p -->P15311 : K(5375,1) for forward direction
5376	P15336_p -->P15336 : K(5376,1) for forward direction
5377	P15531_p -->P15531 : K(5377,1) for forward direction
5378	P15924_p -->P15924 : K(5378,1) for forward direction
5379	P15927_p -->P15927 : K(5379,1) for forward direction
5380	P16070_p -->P16070 : K(5380,1) for forward direction
5381	P16104_p -->P16104 : K(5381,1) for forward direction
5382	P16144_p -->P16144 : K(5382,1) for forward direction
5383	P16220_p -->P16220 : K(5383,1) for forward direction
5384	P16401_p -->P16401 : K(5384,1) for forward direction
5385	P16615_p -->P16615 : K(5385,1) for forward direction
5386	P16949_p -->P16949 : K(5386,1) for forward direction
5387	P17096_p -->P17096 : K(5387,1) for forward direction
5388	P17252_p -->P17252 : K(5388,1) for forward direction
5389	P17480_p -->P17480 : K(5389,1) for forward direction
5390	P17535_p -->P17535 : K(5390,1) for forward direction
5391	P17655_p -->P17655 : K(5391,1) for forward direction
5392	P17676_p -->P17676 : K(5392,1) for forward direction
5393	P17706_p -->P17706 : K(5393,1) for forward direction
5394	P17812_p -->P17812 : K(5394,1) for forward direction
5395	P17844_p -->P17844 : K(5395,1) for forward direction
5396	P17931_p -->P17931 : K(5396,1) for forward direction
5397	P18031_p -->P18031 : K(5397,1) for forward direction
5398	P18084_p -->P18084 : K(5398,1) for forward direction
5399	P18206_p -->P18206 : K(5399,1) for forward direction
5400	P18433_p -->P18433 : K(5400,1) for forward direction
5401	P18669_p -->P18669 : K(5401,1) for forward direction
5402	P18846_p -->P18846 : K(5402,1) for forward direction
5403	P18858_p -->P18858 : K(5403,1) for forward direction
5404	P18887_p -->P18887 : K(5404,1) for forward direction
5405	P19525_p -->P19525 : K(5405,1) for forward direction
5406	P19634_p -->P19634 : K(5406,1) for forward direction
5407	P19793_p -->P19793 : K(5407,1) for forward direction
5408	P19838_p -->P19838 : K(5408,1) for forward direction
5409	P20042_p -->P20042 : K(5409,1) for forward direction
5410	P20248_p -->P20248 : K(5410,1) for forward direction
5411	P20936_p -->P20936 : K(5411,1) for forward direction

5412	P21127_p -->P21127 : K(5412,1) for forward direction
5413	P21333_p -->P21333 : K(5413,1) for forward direction
5414	P21399_p -->P21399 : K(5414,1) for forward direction
5415	P21860_p -->P21860 : K(5415,1) for forward direction
5416	P22314_p -->P22314 : K(5416,1) for forward direction
5417	P22392_p -->P22392 : K(5417,1) for forward direction
5418	P22681_p -->P22681 : K(5418,1) for forward direction
5419	P23246_p -->P23246 : K(5419,1) for forward direction
5420	P23396_p -->P23396 : K(5420,1) for forward direction
5421	P23511_p -->P23511 : K(5421,1) for forward direction
5422	P23528_p -->P23528 : K(5422,1) for forward direction
5423	P23588_p -->P23588 : K(5423,1) for forward direction
5424	P24534_p -->P24534 : K(5424,1) for forward direction
5425	P24844_p -->P24844 : K(5425,1) for forward direction
5426	P24928_p -->P24928 : K(5426,1) for forward direction
5427	P24941_p -->P24941 : K(5427,1) for forward direction
5428	P25098_p -->P25098 : K(5428,1) for forward direction
5429	P25205_p -->P25205 : K(5429,1) for forward direction
5430	P25490_p -->P25490 : K(5430,1) for forward direction
5431	P25788_p -->P25788 : K(5431,1) for forward direction
5432	P26038_p -->P26038 : K(5432,1) for forward direction
5433	P26358_p -->P26358 : K(5433,1) for forward direction
5434	P27348_p -->P27348 : K(5434,1) for forward direction
5435	P27361_p -->P27361 : K(5435,1) for forward direction
5436	P27635_p -->P27635 : K(5436,1) for forward direction
5437	P27695_p -->P27695 : K(5437,1) for forward direction
5438	P27708_p -->P27708 : K(5438,1) for forward direction
5439	P27816_p -->P27816 : K(5439,1) for forward direction
5440	P27986_p -->P27986 : K(5440,1) for forward direction
5441	P28482_p -->P28482 : K(5441,1) for forward direction
5442	P29317_p -->P29317 : K(5442,1) for forward direction
5443	P29350_p -->P29350 : K(5443,1) for forward direction
5444	P29353_p -->P29353 : K(5444,1) for forward direction
5445	P29590_p -->P29590 : K(5445,1) for forward direction
5446	P29692_p -->P29692 : K(5446,1) for forward direction
5447	P29966_p -->P29966 : K(5447,1) for forward direction
5448	P30041_p -->P30041 : K(5448,1) for forward direction
5449	P30050_p -->P30050 : K(5449,1) for forward direction
5450	P30086_p -->P30086 : K(5450,1) for forward direction
5451	P30260_p -->P30260 : K(5451,1) for forward direction
5452	P30291_p -->P30291 : K(5452,1) for forward direction
5453	P30519_p -->P30519 : K(5453,1) for forward direction
5454	P30622_p -->P30622 : K(5454,1) for forward direction

5455	P30626_p -->P30626 : K(5455,1) for forward direction
5456	P31146_p -->P31146 : K(5456,1) for forward direction
5457	P31350_p -->P31350 : K(5457,1) for forward direction
5458	P31431_p -->P31431 : K(5458,1) for forward direction
5459	P31749_p -->P31749 : K(5459,1) for forward direction
5460	P31751_p -->P31751 : K(5460,1) for forward direction
5461	P31946_p -->P31946 : K(5461,1) for forward direction
5462	P31947_p -->P31947 : K(5462,1) for forward direction
5463	P32519_p -->P32519 : K(5463,1) for forward direction
5464	P33527_p -->P33527 : K(5464,1) for forward direction
5465	P33778_p -->P33778 : K(5465,1) for forward direction
5466	P33981_p -->P33981 : K(5466,1) for forward direction
5467	P33991_p -->P33991 : K(5467,1) for forward direction
5468	P33993_p -->P33993 : K(5468,1) for forward direction
5469	P35221_p -->P35221 : K(5469,1) for forward direction
5470	P35222_p -->P35222 : K(5470,1) for forward direction
5471	P35226_p -->P35226 : K(5471,1) for forward direction
5472	P35240_p -->P35240 : K(5472,1) for forward direction
5473	P35241_p -->P35241 : K(5473,1) for forward direction
5474	P35269_p -->P35269 : K(5474,1) for forward direction
5475	P35270_p -->P35270 : K(5475,1) for forward direction
5476	P35568_p -->P35568 : K(5476,1) for forward direction
5477	P35579_p -->P35579 : K(5477,1) for forward direction
5478	P35611_p -->P35611 : K(5478,1) for forward direction
5479	P35659_p -->P35659 : K(5479,1) for forward direction
5480	P36507_p -->P36507 : K(5480,1) for forward direction
5481	P36871_p -->P36871 : K(5481,1) for forward direction
5482	P37173_p -->P37173 : K(5482,1) for forward direction
5483	P38432_p -->P38432 : K(5483,1) for forward direction
5484	P38646_p -->P38646 : K(5484,1) for forward direction
5485	P39748_p -->P39748 : K(5485,1) for forward direction
5486	P40189_p -->P40189 : K(5486,1) for forward direction
5487	P40763_p -->P40763 : K(5487,1) for forward direction
5488	P41212_p -->P41212 : K(5488,1) for forward direction
5489	P41236_p -->P41236 : K(5489,1) for forward direction
5490	P41240_p -->P41240 : K(5490,1) for forward direction
5491	P41279_p -->P41279 : K(5491,1) for forward direction
5492	P41743_p -->P41743 : K(5492,1) for forward direction
5493	P42224_p -->P42224 : K(5493,1) for forward direction
5494	P42226_p -->P42226 : K(5494,1) for forward direction
5495	P42345_p -->P42345 : K(5495,1) for forward direction
5496	P42566_p -->P42566 : K(5496,1) for forward direction
5497	P42574_p -->P42574 : K(5497,1) for forward direction

5498	P42575_p -->P42575 : K(5498,1) for forward direction
5499	P42858_p -->P42858 : K(5499,1) for forward direction
5500	P43405_p -->P43405 : K(5500,1) for forward direction
5501	P45973_p -->P45973 : K(5501,1) for forward direction
5502	P45983_p -->P45983 : K(5502,1) for forward direction
5503	P45984_p -->P45984 : K(5503,1) for forward direction
5504	P45985_p -->P45985 : K(5504,1) for forward direction
5505	P46020_p -->P46020 : K(5505,1) for forward direction
5506	P46060_p -->P46060 : K(5506,1) for forward direction
5507	P46108_p -->P46108 : K(5507,1) for forward direction
5508	P46459_p -->P46459 : K(5508,1) for forward direction
5509	P46527_p -->P46527 : K(5509,1) for forward direction
5510	P46734_p -->P46734 : K(5510,1) for forward direction
5511	P46777_p -->P46777 : K(5511,1) for forward direction
5512	P46937_p -->P46937 : K(5512,1) for forward direction
5513	P46939_p -->P46939 : K(5513,1) for forward direction
5514	P46940_p -->P46940 : K(5514,1) for forward direction
5515	P47712_p -->P47712 : K(5515,1) for forward direction
5516	P47736_p -->P47736 : K(5516,1) for forward direction
5517	P48200_p -->P48200 : K(5517,1) for forward direction
5518	P48426_p -->P48426 : K(5518,1) for forward direction
5519	P48436_p -->P48436 : K(5519,1) for forward direction
5520	P49023_p -->P49023 : K(5520,1) for forward direction
5521	P49137_p -->P49137 : K(5521,1) for forward direction
5522	P49407_p -->P49407 : K(5522,1) for forward direction
5523	P49459_p -->P49459 : K(5523,1) for forward direction
5524	P49585_p -->P49585 : K(5524,1) for forward direction
5525	P49674_p -->P49674 : K(5525,1) for forward direction
5526	P49736_p -->P49736 : K(5526,1) for forward direction
5527	P49757_p -->P49757 : K(5527,1) for forward direction
5528	P49790_p -->P49790 : K(5528,1) for forward direction
5529	P49815_p -->P49815 : K(5529,1) for forward direction
5530	P49840_p -->P49840 : K(5530,1) for forward direction
5531	P49841_p -->P49841 : K(5531,1) for forward direction
5532	P49916_p -->P49916 : K(5532,1) for forward direction
5533	P49917_p -->P49917 : K(5533,1) for forward direction
5534	P50402_p -->P50402 : K(5534,1) for forward direction
5535	P50548_p -->P50548 : K(5535,1) for forward direction
5536	P50552_p -->P50552 : K(5536,1) for forward direction
5537	P50570_p -->P50570 : K(5537,1) for forward direction
5538	P50613_p -->P50613 : K(5538,1) for forward direction
5539	P50616_p -->P50616 : K(5539,1) for forward direction
5540	P50750_p -->P50750 : K(5540,1) for forward direction

5541	P51398_p -->P51398 : K(5541,1) for forward direction
5542	P51617_p -->P51617 : K(5542,1) for forward direction
5543	P51692_p -->P51692 : K(5543,1) for forward direction
5544	P51812_p -->P51812 : K(5544,1) for forward direction
5545	P52292_p -->P52292 : K(5545,1) for forward direction
5546	P52564_p -->P52564 : K(5546,1) for forward direction
5547	P52565_p -->P52565 : K(5547,1) for forward direction
5548	P52566_p -->P52566 : K(5548,1) for forward direction
5549	P52655_p -->P52655 : K(5549,1) for forward direction
5550	P52732_p -->P52732 : K(5550,1) for forward direction
5551	P52735_p -->P52735 : K(5551,1) for forward direction
5552	P52888_p -->P52888 : K(5552,1) for forward direction
5553	P52907_p -->P52907 : K(5553,1) for forward direction
5554	P52926_p -->P52926 : K(5554,1) for forward direction
5555	P52943_p -->P52943 : K(5555,1) for forward direction
5556	P52948_p -->P52948 : K(5556,1) for forward direction
5557	P53350_p -->P53350 : K(5557,1) for forward direction
5558	P53355_p -->P53355 : K(5558,1) for forward direction
5559	P53365_p -->P53365 : K(5559,1) for forward direction
5560	P53396_p -->P53396 : K(5560,1) for forward direction
5561	P54132_p -->P54132 : K(5561,1) for forward direction
5562	P54198_p -->P54198 : K(5562,1) for forward direction
5563	P54253_p -->P54253 : K(5563,1) for forward direction
5564	P55010_p -->P55010 : K(5564,1) for forward direction
5565	P55011_p -->P55011 : K(5565,1) for forward direction
5566	P55072_p -->P55072 : K(5566,1) for forward direction
5567	P55210_p -->P55210 : K(5567,1) for forward direction
5568	P55211_p -->P55211 : K(5568,1) for forward direction
5569	P55327_p -->P55327 : K(5569,1) for forward direction
5570	P55957_p -->P55957 : K(5570,1) for forward direction
5571	P56211_p -->P56211 : K(5571,1) for forward direction
5572	P56537_p -->P56537 : K(5572,1) for forward direction
5573	P56945_p -->P56945 : K(5573,1) for forward direction
5574	P60484_p -->P60484 : K(5574,1) for forward direction
5575	P60953_p -->P60953 : K(5575,1) for forward direction
5576	P60983_p -->P60983 : K(5576,1) for forward direction
5577	P61020_p -->P61020 : K(5577,1) for forward direction
5578	P61224_p -->P61224 : K(5578,1) for forward direction
5579	P61586_p -->P61586 : K(5579,1) for forward direction
5580	P61587_p -->P61587 : K(5580,1) for forward direction
5581	P61764_p -->P61764 : K(5581,1) for forward direction
5582	P61978_p -->P61978 : K(5582,1) for forward direction
5583	P62136_p -->P62136 : K(5583,1) for forward direction

5584	P62745_p -->P62745 : K(5584,1) for forward direction
5585	P62753_p -->P62753 : K(5585,1) for forward direction
5586	P62826_p -->P62826 : K(5586,1) for forward direction
5587	P62834_p -->P62834 : K(5587,1) for forward direction
5588	P62993_p -->P62993 : K(5588,1) for forward direction
5589	P63000_p -->P63000 : K(5589,1) for forward direction
5590	P63010_p -->P63010 : K(5590,1) for forward direction
5591	P63104_p -->P63104 : K(5591,1) for forward direction
5592	P63167_p -->P63167 : K(5592,1) for forward direction
5593	P63244_p -->P63244 : K(5593,1) for forward direction
5594	P63261_p -->P63261 : K(5594,1) for forward direction
5595	P67775_p -->P67775 : K(5595,1) for forward direction
5596	P67809_p -->P67809 : K(5596,1) for forward direction
5597	P67870_p -->P67870 : K(5597,1) for forward direction
5598	P68104_p -->P68104 : K(5598,1) for forward direction
5599	P68366_p -->P68366 : K(5599,1) for forward direction
5600	P68400_p -->P68400 : K(5600,1) for forward direction
5601	P68431_p -->P68431 : K(5601,1) for forward direction
5602	P78314_p -->P78314 : K(5602,1) for forward direction
5603	P78347_p -->P78347 : K(5603,1) for forward direction
5604	P78356_p -->P78356 : K(5604,1) for forward direction
5605	P78362_p -->P78362 : K(5605,1) for forward direction
5606	P78371_p -->P78371 : K(5606,1) for forward direction
5607	P78527_p -->P78527 : K(5607,1) for forward direction
5608	P78536_p -->P78536 : K(5608,1) for forward direction
5609	P78545_p -->P78545 : K(5609,1) for forward direction
5610	P83916_p -->P83916 : K(5610,1) for forward direction
5611	P84022_p -->P84022 : K(5611,1) for forward direction
5612	P84243_p -->P84243 : K(5612,1) for forward direction
5613	P98170_p -->P98170 : K(5613,1) for forward direction
5614	Q00059_p -->Q00059 : K(5614,1) for forward direction
5615	Q00535_p -->Q00535 : K(5615,1) for forward direction
5616	Q00610_p -->Q00610 : K(5616,1) for forward direction
5617	Q00613_p -->Q00613 : K(5617,1) for forward direction
5618	Q00653_p -->Q00653 : K(5618,1) for forward direction
5619	Q00839_p -->Q00839 : K(5619,1) for forward direction
5620	Q01082_p -->Q01082 : K(5620,1) for forward direction
5621	Q01105_p -->Q01105 : K(5621,1) for forward direction
5622	Q01167_p -->Q01167 : K(5622,1) for forward direction
5623	Q01844_p -->Q01844 : K(5623,1) for forward direction
5624	Q01970_p -->Q01970 : K(5624,1) for forward direction
5625	Q02447_p -->Q02447 : K(5625,1) for forward direction
5626	Q02750_p -->Q02750 : K(5626,1) for forward direction

5627	Q02790_p -->Q02790 : K(5627,1) for forward direction
5628	Q04206_p -->Q04206 : K(5628,1) for forward direction
5629	Q04724_p -->Q04724 : K(5629,1) for forward direction
5630	Q04912_p -->Q04912 : K(5630,1) for forward direction
5631	Q05086_p -->Q05086 : K(5631,1) for forward direction
5632	Q05193_p -->Q05193 : K(5632,1) for forward direction
5633	Q05209_p -->Q05209 : K(5633,1) for forward direction
5634	Q05397_p -->Q05397 : K(5634,1) for forward direction
5635	Q05469_p -->Q05469 : K(5635,1) for forward direction
5636	Q05655_p -->Q05655 : K(5636,1) for forward direction
5637	Q06124_p -->Q06124 : K(5637,1) for forward direction
5638	Q06210_p -->Q06210 : K(5638,1) for forward direction
5639	Q06265_p -->Q06265 : K(5639,1) for forward direction
5640	Q06481_p -->Q06481 : K(5640,1) for forward direction
5641	Q06830_p -->Q06830 : K(5641,1) for forward direction
5642	Q07352_p -->Q07352 : K(5642,1) for forward direction
5643	Q07617_p -->Q07617 : K(5643,1) for forward direction
5644	Q07666_p -->Q07666 : K(5644,1) for forward direction
5645	Q07812_p -->Q07812 : K(5645,1) for forward direction
5646	Q07817_p -->Q07817 : K(5646,1) for forward direction
5647	Q07820_p -->Q07820 : K(5647,1) for forward direction
5648	Q07866_p -->Q07866 : K(5648,1) for forward direction
5649	Q07889_p -->Q07889 : K(5649,1) for forward direction
5650	Q07954_p -->Q07954 : K(5650,1) for forward direction
5651	Q07955_p -->Q07955 : K(5651,1) for forward direction
5652	Q08345_p -->Q08345 : K(5652,1) for forward direction
5653	Q08379_p -->Q08379 : K(5653,1) for forward direction
5654	Q08945_p -->Q08945 : K(5654,1) for forward direction
5655	Q08J23_p -->Q08J23 : K(5655,1) for forward direction
5656	Q09472_p -->Q09472 : K(5656,1) for forward direction
5657	Q12778_p -->Q12778 : K(5657,1) for forward direction
5658	Q12800_p -->Q12800 : K(5658,1) for forward direction
5659	Q12802_p -->Q12802 : K(5659,1) for forward direction
5660	Q12888_p -->Q12888 : K(5660,1) for forward direction
5661	Q12904_p -->Q12904 : K(5661,1) for forward direction
5662	Q12906_p -->Q12906 : K(5662,1) for forward direction
5663	Q12933_p -->Q12933 : K(5663,1) for forward direction
5664	Q12959_p -->Q12959 : K(5664,1) for forward direction
5665	Q12972_p -->Q12972 : K(5665,1) for forward direction
5666	Q13042_p -->Q13042 : K(5666,1) for forward direction
5667	Q13043_p -->Q13043 : K(5667,1) for forward direction
5668	Q13085_p -->Q13085 : K(5668,1) for forward direction
5669	Q13131_p -->Q13131 : K(5669,1) for forward direction

5670	Q13144_p -->Q13144 : K(5670,1) for forward direction
5671	Q13153_p -->Q13153 : K(5671,1) for forward direction
5672	Q13158_p -->Q13158 : K(5672,1) for forward direction
5673	Q13177_p -->Q13177 : K(5673,1) for forward direction
5674	Q13185_p -->Q13185 : K(5674,1) for forward direction
5675	Q13188_p -->Q13188 : K(5675,1) for forward direction
5676	Q13257_p -->Q13257 : K(5676,1) for forward direction
5677	Q13263_p -->Q13263 : K(5677,1) for forward direction
5678	Q13362_p -->Q13362 : K(5678,1) for forward direction
5679	Q13363_p -->Q13363 : K(5679,1) for forward direction
5680	Q13393_p -->Q13393 : K(5680,1) for forward direction
5681	Q13416_p -->Q13416 : K(5681,1) for forward direction
5682	Q13418_p -->Q13418 : K(5682,1) for forward direction
5683	Q13480_p -->Q13480 : K(5683,1) for forward direction
5684	Q13501_p -->Q13501 : K(5684,1) for forward direction
5685	Q13526_p -->Q13526 : K(5685,1) for forward direction
5686	Q13541_p -->Q13541 : K(5686,1) for forward direction
5687	Q13542_p -->Q13542 : K(5687,1) for forward direction
5688	Q13547_p -->Q13547 : K(5688,1) for forward direction
5689	Q13554_p -->Q13554 : K(5689,1) for forward direction
5690	Q13557_p -->Q13557 : K(5690,1) for forward direction
5691	Q13586_p -->Q13586 : K(5691,1) for forward direction
5692	Q13627_p -->Q13627 : K(5692,1) for forward direction
5693	Q13765_p -->Q13765 : K(5693,1) for forward direction
5694	Q13769_p -->Q13769 : K(5694,1) for forward direction
5695	Q13813_p -->Q13813 : K(5695,1) for forward direction
5696	Q13887_p -->Q13887 : K(5696,1) for forward direction
5697	Q13976_p -->Q13976 : K(5697,1) for forward direction
5698	Q14103_p -->Q14103 : K(5698,1) for forward direction
5699	Q14118_p -->Q14118 : K(5699,1) for forward direction
5700	Q14161_p -->Q14161 : K(5700,1) for forward direction
5701	Q14203_p -->Q14203 : K(5701,1) for forward direction
5702	Q14247_p -->Q14247 : K(5702,1) for forward direction
5703	Q14344_p -->Q14344 : K(5703,1) for forward direction
5704	Q14457_p -->Q14457 : K(5704,1) for forward direction
5705	Q14653_p -->Q14653 : K(5705,1) for forward direction
5706	Q14676_p -->Q14676 : K(5706,1) for forward direction
5707	Q14738_p -->Q14738 : K(5707,1) for forward direction
5708	Q14739_p -->Q14739 : K(5708,1) for forward direction
5709	Q14790_p -->Q14790 : K(5709,1) for forward direction
5710	Q14847_p -->Q14847 : K(5710,1) for forward direction
5711	Q14978_p -->Q14978 : K(5711,1) for forward direction
5712	Q14980_p -->Q14980 : K(5712,1) for forward direction

5713	Q15019_p -->Q15019 : K(5713,1) for forward direction
5714	Q15027_p -->Q15027 : K(5714,1) for forward direction
5715	Q15046_p -->Q15046 : K(5715,1) for forward direction
5716	Q15054_p -->Q15054 : K(5716,1) for forward direction
5717	Q15075_p -->Q15075 : K(5717,1) for forward direction
5718	Q15121_p -->Q15121 : K(5718,1) for forward direction
5719	Q15149_p -->Q15149 : K(5719,1) for forward direction
5720	Q15172_p -->Q15172 : K(5720,1) for forward direction
5721	Q15185_p -->Q15185 : K(5721,1) for forward direction
5722	Q15208_p -->Q15208 : K(5722,1) for forward direction
5723	Q15418_p -->Q15418 : K(5723,1) for forward direction
5724	Q15596_p -->Q15596 : K(5724,1) for forward direction
5725	Q15637_p -->Q15637 : K(5725,1) for forward direction
5726	Q15648_p -->Q15648 : K(5726,1) for forward direction
5727	Q15653_p -->Q15653 : K(5727,1) for forward direction
5728	Q15717_p -->Q15717 : K(5728,1) for forward direction
5729	Q15746_p -->Q15746 : K(5729,1) for forward direction
5730	Q15750_p -->Q15750 : K(5730,1) for forward direction
5731	Q15796_p -->Q15796 : K(5731,1) for forward direction
5732	Q15797_p -->Q15797 : K(5732,1) for forward direction
5733	Q15833_p -->Q15833 : K(5733,1) for forward direction
5734	Q15910_p -->Q15910 : K(5734,1) for forward direction
5735	Q15942_p -->Q15942 : K(5735,1) for forward direction
5736	Q16204_p -->Q16204 : K(5736,1) for forward direction
5737	Q16512_p -->Q16512 : K(5737,1) for forward direction
5738	Q16513_p -->Q16513 : K(5738,1) for forward direction
5739	Q16539_p -->Q16539 : K(5739,1) for forward direction
5740	Q16543_p -->Q16543 : K(5740,1) for forward direction
5741	Q16555_p -->Q16555 : K(5741,1) for forward direction
5742	Q16625_p -->Q16625 : K(5742,1) for forward direction
5743	Q16637_p -->Q16637 : K(5743,1) for forward direction
5744	Q16644_p -->Q16644 : K(5744,1) for forward direction
5745	Q16666_p -->Q16666 : K(5745,1) for forward direction
5746	Q16695_p -->Q16695 : K(5746,1) for forward direction
5747	Q16790_p -->Q16790 : K(5747,1) for forward direction
5748	Q2NKX8_p -->Q2NKX8 : K(5748,1) for forward direction
5749	Q53EL6_p -->Q53EL6 : K(5749,1) for forward direction
5750	Q53ET0_p -->Q53ET0 : K(5750,1) for forward direction
5751	Q53EZ4_p -->Q53EZ4 : K(5751,1) for forward direction
5752	Q68CZ2_p -->Q68CZ2 : K(5752,1) for forward direction
5753	Q6VY07_p -->Q6VY07 : K(5753,1) for forward direction
5754	Q6WCQ1_p -->Q6WCQ1 : K(5754,1) for forward direction
5755	Q712K3_p -->Q712K3 : K(5755,1) for forward direction

5756	Q7L5N1_p -->Q7L5N1 : K(5756,1) for forward direction
5757	Q86TI0_p -->Q86TI0 : K(5757,1) for forward direction
5758	Q86V81_p -->Q86V81 : K(5758,1) for forward direction
5759	Q86VP1_p -->Q86VP1 : K(5759,1) for forward direction
5760	Q86WB0_p -->Q86WB0 : K(5760,1) for forward direction
5761	Q8IX03_p -->Q8IX03 : K(5761,1) for forward direction
5762	Q8IXJ6_p -->Q8IXJ6 : K(5762,1) for forward direction
5763	Q8IZL8_p -->Q8IZL8 : K(5763,1) for forward direction
5764	Q8IZP0_p -->Q8IZP0 : K(5764,1) for forward direction
5765	Q8N122_p -->Q8N122 : K(5765,1) for forward direction
5766	Q8N752_p -->Q8N752 : K(5766,1) for forward direction
5767	Q8NE71_p -->Q8NE71 : K(5767,1) for forward direction
5768	Q8NEB9_p -->Q8NEB9 : K(5768,1) for forward direction
5769	Q8NFA2_p -->Q8NFA2 : K(5769,1) for forward direction
5770	Q8TAP9_p -->Q8TAP9 : K(5770,1) for forward direction
5771	Q8TB45_p -->Q8TB45 : K(5771,1) for forward direction
5772	Q8TD19_p -->Q8TD19 : K(5772,1) for forward direction
5773	Q8TDX7_p -->Q8TDX7 : K(5773,1) for forward direction
5774	Q8TEW0_p -->Q8TEW0 : K(5774,1) for forward direction
5775	Q8WUI4_p -->Q8WUI4 : K(5775,1) for forward direction
5776	Q8WXE1_p -->Q8WXE1 : K(5776,1) for forward direction
5777	Q92538_p -->Q92538 : K(5777,1) for forward direction
5778	Q92574_p -->Q92574 : K(5778,1) for forward direction
5779	Q92598_p -->Q92598 : K(5779,1) for forward direction
5780	Q92686_p -->Q92686 : K(5780,1) for forward direction
5781	Q92688_p -->Q92688 : K(5781,1) for forward direction
5782	Q92769_p -->Q92769 : K(5782,1) for forward direction
5783	Q92793_p -->Q92793 : K(5783,1) for forward direction
5784	Q92900_p -->Q92900 : K(5784,1) for forward direction
5785	Q92934_p -->Q92934 : K(5785,1) for forward direction
5786	Q92945_p -->Q92945 : K(5786,1) for forward direction
5787	Q92974_p -->Q92974 : K(5787,1) for forward direction
5788	Q92985_p -->Q92985 : K(5788,1) for forward direction
5789	Q92993_p -->Q92993 : K(5789,1) for forward direction
5790	Q93034_p -->Q93034 : K(5790,1) for forward direction
5791	Q93045_p -->Q93045 : K(5791,1) for forward direction
5792	Q96B36_p -->Q96B36 : K(5792,1) for forward direction
5793	Q96CF2_p -->Q96CF2 : K(5793,1) for forward direction
5794	Q96CV9_p -->Q96CV9 : K(5794,1) for forward direction
5795	Q96CW1_p -->Q96CW1 : K(5795,1) for forward direction
5796	Q96EB6_p -->Q96EB6 : K(5796,1) for forward direction
5797	Q96GD4_p -->Q96GD4 : K(5797,1) for forward direction
5798	Q96GX5_p -->Q96GX5 : K(5798,1) for forward direction

5799	Q96J02_p -->Q96J02 : K(5799,1) for forward direction
5800	Q96KB5_p -->Q96KB5 : K(5800,1) for forward direction
5801	Q96N67_p -->Q96N67 : K(5801,1) for forward direction
5802	Q96PM5_p -->Q96PM5 : K(5802,1) for forward direction
5803	Q96PU5_p -->Q96PU5 : K(5803,1) for forward direction
5804	Q96R06_p -->Q96R06 : K(5804,1) for forward direction
5805	Q96RK0_p -->Q96RK0 : K(5805,1) for forward direction
5806	Q96S44_p -->Q96S44 : K(5806,1) for forward direction
5807	Q96SB3_p -->Q96SB3 : K(5807,1) for forward direction
5808	Q96SB4_p -->Q96SB4 : K(5808,1) for forward direction
5809	Q96T60_p -->Q96T60 : K(5809,1) for forward direction
5810	Q96T88_p -->Q96T88 : K(5810,1) for forward direction
5811	Q99418_p -->Q99418 : K(5811,1) for forward direction
5812	Q99426_p -->Q99426 : K(5812,1) for forward direction
5813	Q99661_p -->Q99661 : K(5813,1) for forward direction
5814	Q99759_p -->Q99759 : K(5814,1) for forward direction
5815	Q99961_p -->Q99961 : K(5815,1) for forward direction
5816	Q9BQG0_p -->Q9BQG0 : K(5816,1) for forward direction
5817	Q9BR76_p -->Q9BR76 : K(5817,1) for forward direction
5818	Q9BUL8_p -->Q9BUL8 : K(5818,1) for forward direction
5819	Q9BXS5_p -->Q9BXS5 : K(5819,1) for forward direction
5820	Q9BY41_p -->Q9BY41 : K(5820,1) for forward direction
5821	Q9BY44_p -->Q9BY44 : K(5821,1) for forward direction
5822	Q9BZL4_p -->Q9BZL4 : K(5822,1) for forward direction
5823	Q9BZL6_p -->Q9BZL6 : K(5823,1) for forward direction
5824	Q9BZQ8_p -->Q9BZQ8 : K(5824,1) for forward direction
5825	Q9GZM8_p -->Q9GZM8 : K(5825,1) for forward direction
5826	Q9GZY8_p -->Q9GZY8 : K(5826,1) for forward direction
5827	Q9H0B6_p -->Q9H0B6 : K(5827,1) for forward direction
5828	Q9H0H5_p -->Q9H0H5 : K(5828,1) for forward direction
5829	Q9H0Z9_p -->Q9H0Z9 : K(5829,1) for forward direction
5830	Q9H1A4_p -->Q9H1A4 : K(5830,1) for forward direction
5831	Q9H1K1_p -->Q9H1K1 : K(5831,1) for forward direction
5832	Q9H257_p -->Q9H257 : K(5832,1) for forward direction
5833	Q9H2D6_p -->Q9H2D6 : K(5833,1) for forward direction
5834	Q9H2G2_p -->Q9H2G2 : K(5834,1) for forward direction
5835	Q9H2K8_p -->Q9H2K8 : K(5835,1) for forward direction
5836	Q9H3Z4_p -->Q9H3Z4 : K(5836,1) for forward direction
5837	Q9H410_p -->Q9H410 : K(5837,1) for forward direction
5838	Q9H4A3_p -->Q9H4A3 : K(5838,1) for forward direction
5839	Q9H4A6_p -->Q9H4A6 : K(5839,1) for forward direction
5840	Q9H5V8_p -->Q9H5V8 : K(5840,1) for forward direction
5841	Q9H6Z4_p -->Q9H6Z4 : K(5841,1) for forward direction

5842	Q9H8S9_p -->Q9H8S9 : K(5842,1) for forward direction
5843	Q9H8V3_p -->Q9H8V3 : K(5843,1) for forward direction
5844	Q9H8Y8_p -->Q9H8Y8 : K(5844,1) for forward direction
5845	Q9H9Q4_p -->Q9H9Q4 : K(5845,1) for forward direction
5846	Q9HBY8_p -->Q9HBY8 : K(5846,1) for forward direction
5847	Q9NP97_p -->Q9NP97 : K(5847,1) for forward direction
5848	Q9NQC3_p -->Q9NQC3 : K(5848,1) for forward direction
5849	Q9NQX3_p -->Q9NQX3 : K(5849,1) for forward direction
5850	Q9NRA0_p -->Q9NRA0 : K(5850,1) for forward direction
5851	Q9NRF8_p -->Q9NRF8 : K(5851,1) for forward direction
5852	Q9NRY4_p -->Q9NRY4 : K(5852,1) for forward direction
5853	Q9NWZ3_p -->Q9NWZ3 : K(5853,1) for forward direction
5854	Q9NXR1_p -->Q9NXR1 : K(5854,1) for forward direction
5855	Q9NY61_p -->Q9NY61 : K(5855,1) for forward direction
5856	Q9NYL2_p -->Q9NYL2 : K(5856,1) for forward direction
5857	Q9NYV6_p -->Q9NYV6 : K(5857,1) for forward direction
5858	Q9UBB4_p -->Q9UBB4 : K(5858,1) for forward direction
5859	Q9UBF6_p -->Q9UBF6 : K(5859,1) for forward direction
5860	Q9UBF8_p -->Q9UBF8 : K(5860,1) for forward direction
5861	Q9UBN7_p -->Q9UBN7 : K(5861,1) for forward direction
5862	Q9UBP6_p -->Q9UBP6 : K(5862,1) for forward direction
5863	Q9UER7_p -->Q9UER7 : K(5863,1) for forward direction
5864	Q9UEW8_p -->Q9UEW8 : K(5864,1) for forward direction
5865	Q9UEY8_p -->Q9UEY8 : K(5865,1) for forward direction
5866	Q9UGK3_p -->Q9UGK3 : K(5866,1) for forward direction
5867	Q9UIG0_p -->Q9UIG0 : K(5867,1) for forward direction
5868	Q9UJM3_p -->Q9UJM3 : K(5868,1) for forward direction
5869	Q9UJX2_p -->Q9UJX2 : K(5869,1) for forward direction
5870	Q9UKI8_p -->Q9UKI8 : K(5870,1) for forward direction
5871	Q9UKV3_p -->Q9UKV3 : K(5871,1) for forward direction
5872	Q9UKV8_p -->Q9UKV8 : K(5872,1) for forward direction
5873	Q9UKX7_p -->Q9UKX7 : K(5873,1) for forward direction
5874	Q9ULV4_p -->Q9ULV4 : K(5874,1) for forward direction
5875	Q9UNN5_p -->Q9UNN5 : K(5875,1) for forward direction
5876	Q9UNZ2_p -->Q9UNZ2 : K(5876,1) for forward direction
5877	Q9UPT5_p -->Q9UPT5 : K(5877,1) for forward direction
5878	Q9UPY8_p -->Q9UPY8 : K(5878,1) for forward direction
5879	Q9UQ80_p -->Q9UQ80 : K(5879,1) for forward direction
5880	Q9UQE7_p -->Q9UQE7 : K(5880,1) for forward direction
5881	Q9UQM7_p -->Q9UQM7 : K(5881,1) for forward direction
5882	Q9Y266_p -->Q9Y266 : K(5882,1) for forward direction
5883	Q9Y2H1_p -->Q9Y2H1 : K(5883,1) for forward direction
5884	Q9Y2V2_p -->Q9Y2V2 : K(5884,1) for forward direction

5885	Q9Y2Y9_p -->Q9Y2Y9 : K(5885,1) for forward direction
5886	Q9Y365_p -->Q9Y365 : K(5886,1) for forward direction
5887	Q9Y3Z3_p -->Q9Y3Z3 : K(5887,1) for forward direction
5888	Q9Y478_p -->Q9Y478 : K(5888,1) for forward direction
5889	Q9Y490_p -->Q9Y490 : K(5889,1) for forward direction
5890	Q9Y4H2_p -->Q9Y4H2 : K(5890,1) for forward direction
5891	Q9Y4R8_p -->Q9Y4R8 : K(5891,1) for forward direction
5892	Q9Y5B0_p -->Q9Y5B0 : K(5892,1) for forward direction
5893	Q9Y5S9_p -->Q9Y5S9 : K(5893,1) for forward direction
5894	Q9Y5T5_p -->Q9Y5T5 : K(5894,1) for forward direction
5895	Q9Y613_p -->Q9Y613 : K(5895,1) for forward direction
5896	Q9Y618_p -->Q9Y618 : K(5896,1) for forward direction
5897	Q9Y624_p -->Q9Y624 : K(5897,1) for forward direction
5898	Q9Y6E0_p -->Q9Y6E0 : K(5898,1) for forward direction
5899	Q9Y6R4_p -->Q9Y6R4 : K(5899,1) for forward direction
5900	Q9Y6W5_p -->Q9Y6W5 : K(5900,1) for forward direction
5901	SIGNOR-C101_p -->SIGNOR-C101 : K(5901,1) for forward direction
5902	SIGNOR-C13_p -->SIGNOR-C13 : K(5902,1) for forward direction
5903	SIGNOR-C154_p -->SIGNOR-C154 : K(5903,1) for forward direction

PART2: List of PPI edges in large-scale colon cancerous cell's model

protein A				protein B				Directional	Effect	Edge type
ID	ID type	Name	type	ID	ID type	Name	type			
Q92793	UNIPROT	CREBBP	protein	Q04206	RELA	UNIPROT	protein	YES	up-regulates	acetylation
Q09472	UNIPROT	EP300	protein	P84243	H3F3A	UNIPROT	protein	YES	up-regulates	acetylation
Q09472	UNIPROT	EP300	protein	P52292	KPNA2	UNIPROT	protein	YES	up-regulates	acetylation
Q09472	UNIPROT	EP300	protein	Q04206	RELA	UNIPROT	protein	YES	up-regulates	acetylation
Q09472	UNIPROT	EP300	protein	P04637	TP53	UNIPROT	protein	YES	up-regulates	acetylation
Q09472	UNIPROT	EP300	protein	P04637	TP53	UNIPROT	protein	YES	up-regulates	acetylation
Q09472	UNIPROT	EP300	protein	Q04206	RELA	UNIPROT	protein	YES	up-regulates activity	acetylation
P31749	UNIPROT	AKT1	protein	P46527	CDKN1B	UNIPROT	protein	YES	down-regulates	binding
P31749	UNIPROT	AKT1	protein	P84022	SMAD3	UNIPROT	protein	YES	down-regulates	binding
P56945	UNIPROT	BCAR1	protein	P84022	SMAD3	UNIPROT	protein	YES	down-regulates	binding
Q07817	UNIPROT	BCL2L1	protein	Q07812	BAX	UNIPROT	protein	YES	down-regulates	binding
Q07817	UNIPROT	BCL2L1	protein	Q14457	BECN1	UNIPROT	protein	YES	down-regulates	binding
P60953	UNIPROT	CDC42	protein	P49841	GSK3B	UNIPROT	protein	YES	down-regulates	binding
P46527	UNIPROT	CDKN1B	protein	P06493	CDK1	UNIPROT	protein	YES	down-regulates	binding
P46527	UNIPROT	CDKN1B	protein	P24941	CDK2	UNIPROT	protein	YES	down-regulates	binding
P35221	UNIPROT	CTNNA1	protein	P46937	YAP1	UNIPROT	protein	YES	down-regulates	binding
P53355	UNIPROT	DAPK1	protein	P28482	MAPK1	UNIPROT	protein	YES	down-regulates	binding
Q9UER7	UNIPROT	DAXX	protein	P25445	FAS	UNIPROT	protein	YES	down-regulates	binding
Q9NR28	UNIPROT	DIABLO	protein	P98170	XIAP	UNIPROT	protein	YES	down-regulates	binding
Q9UJM3	UNIPROT	ERRFI1	protein	P00533	EGFR	UNIPROT	protein	YES	down-regulates	binding
Q9UBI6	UNIPROT	GNG12	protein	P17612	PRKACA	UNIPROT	protein	YES	down-regulates	binding
P49841	UNIPROT	GSK3B	protein	Q13144	EIF2B5	UNIPROT	protein	YES	down-regulates	binding
P49841	UNIPROT	GSK3B	protein	Q9Y6R4	MAP3K4	UNIPROT	protein	YES	down-regulates	binding
P49841	UNIPROT	GSK3B	protein	Q16539	MAPK14	UNIPROT	protein	YES	down-regulates	binding
Q13547	UNIPROT	HDAC1	protein	Q04206	RELA	UNIPROT	protein	YES	down-regulates	binding
O43464	UNIPROT	HTRA2	protein	Q15121	PEA15	UNIPROT	protein	YES	down-regulates	binding
O43464	UNIPROT	HTRA2	protein	P98170	XIAP	UNIPROT	protein	YES	down-regulates	binding
P45984	UNIPROT	MAPK9	protein	P05412	JUN	UNIPROT	protein	YES	down-regulates	binding
Q07820	UNIPROT	MCL1	protein	Q07812	BAX	UNIPROT	protein	YES	down-regulates	binding
P19838	UNIPROT	NFKB1	protein	P41279	MAP3K8	UNIPROT	protein	YES	down-regulates	binding
O75925	UNIPROT	PIAS1	protein	P42224	STAT1	UNIPROT	protein	YES	down-regulates	binding
O75925	UNIPROT	PIAS1	protein	P42224	STAT1	UNIPROT	protein	YES	down-regulates	binding
P41236	UNIPROT	PPP1R2	protein	P62136	PPP1CA	UNIPROT	protein	YES	down-regulates	binding
Q13362	UNIPROT	PPP2R5C	protein	P28482	MAPK1	UNIPROT	protein	YES	down-regulates	binding
Q13362	UNIPROT	PPP2R5C	protein	P27361	MAPK3	UNIPROT	protein	YES	down-regulates	binding
Q13308	UNIPROT	PTK7	protein	P35222	CTNNB1	UNIPROT	protein	YES	down-regulates	binding
P20936	UNIPROT	RASA1	protein	P01112	HRAS	UNIPROT	protein	YES	down-regulates	binding
P20936	UNIPROT	RASA1	protein	P01112	HRAS	UNIPROT	protein	YES	down-regulates	binding
P27635	UNIPROT	RPL10	protein	P05412	JUN	UNIPROT	protein	YES	down-regulates	binding
P27635	UNIPROT	RPL10	protein	P07947	YES1	UNIPROT	protein	YES	down-regulates	binding
P19793	UNIPROT	RXRA	protein	P35222	CTNNB1	UNIPROT	protein	YES	down-regulates	binding
Q01105	UNIPROT	SET	protein	P15531	NME1	UNIPROT	protein	YES	down-regulates	binding
Q01105	UNIPROT	SET	protein	P67775	PPP2CA	UNIPROT	protein	YES	down-regulates	binding
Q01105	UNIPROT	SET	protein	P62714	PPP2CB	UNIPROT	protein	YES	down-regulates	binding
Q15637	UNIPROT	SF1	protein	Q01844	EWSR1	UNIPROT	protein	YES	down-regulates	binding
P42224	UNIPROT	STAT1	protein	Q04206	RELA	UNIPROT	protein	YES	down-regulates	binding
P31946	UNIPROT	YWHAB	protein	P78362	SRPK2	UNIPROT	protein	YES	down-regulates	binding
P62258	UNIPROT	YWHAE	protein	P46527	CDKN1B	UNIPROT	protein	YES	down-regulates	binding
Q04917	UNIPROT	YWHAH	protein	P46527	CDKN1B	UNIPROT	protein	YES	down-regulates	binding
P27348	UNIPROT	YWHAQ	protein	P46527	CDKN1B	UNIPROT	protein	YES	down-regulates	binding
Q92934	UNIPROT	BAD	protein	Q07817	BCL2L1	UNIPROT	protein	YES	down-regulates activity	binding
Q92934	UNIPROT	BAD	protein	Q07817	BCL2L1	UNIPROT	protein	YES	down-regulates activity	binding
Q07817	UNIPROT	BCL2L1	protein	P55211	CASP9	UNIPROT	protein	YES	down-regulates activity	binding
P55957	UNIPROT	BID	protein	Q07817	BCL2L1	UNIPROT	protein	YES	down-regulates activity	binding
Q8TB45	UNIPROT	DEPTOR	protein	P42345	MTOR	UNIPROT	protein	YES	down-regulates activity	binding
Q9NR28	UNIPROT	DIABLO	protein	P98170	XIAP	UNIPROT	protein	YES	down-regulates activity	binding
Q9NR28	UNIPROT	DIABLO	protein	P98170	XIAP	UNIPROT	protein	YES	down-regulates activity	binding
Q13541	UNIPROT	EIF4EBP1	protein	P06730	EIF4E	UNIPROT	protein	YES	down-regulates activity	binding
P42566	UNIPROT	EPS15	protein	P00533	EGFR	UNIPROT	protein	YES	down-regulates activity	binding
Q9UJM3	UNIPROT	ERRFI1	protein	P00533	EGFR	UNIPROT	protein	YES	down-regulates activity	binding
Q9UJM3	UNIPROT	ERRFI1	protein	P04626	ERBB2	UNIPROT	protein	YES	down-regulates activity	binding
P01106	UNIPROT	MYC	protein	P84022	SMAD3	UNIPROT	protein	YES	down-regulates activity	binding
Q13153	UNIPROT	PAK1	protein	Q13153	PAK1	UNIPROT	protein	YES	down-regulates activity	binding
Q12972	UNIPROT	PPP1R8	protein	P62136	PPP1CA	UNIPROT	protein	YES	down-regulates activity	binding
P84022	UNIPROT	SMAD3	protein	P17676	CEBPB	UNIPROT	protein	YES	down-regulates activity	binding
P42226	UNIPROT	STAT6	protein	P42224	STAT1	UNIPROT	protein	YES	down-regulates activity	binding
P04637	UNIPROT	TP53	protein	Q07817	BCL2L1	UNIPROT	protein	YES	down-regulates activity	binding
P98170	UNIPROT	XIAP	protein	P42574	CASP3	UNIPROT	protein	YES	down-regulates activity	binding
P98170	UNIPROT	XIAP	protein	P42574	CASP3	UNIPROT	protein	YES	down-regulates activity	binding
Q9NR28	UNIPROT	DIABLO	protein	P98170	XIAP	UNIPROT	protein	YES	down-regulates quantity	binding
Q9UJM3	UNIPROT	ERRFI1	protein	P00533	EGFR	UNIPROT	protein	YES	down-regulates quantity by destabilization	binding
Q9UJM3	UNIPROT	ERRFI1	protein	P00533	EGFR	UNIPROT	protein	YES	down-regulates quantity by destabilization	binding
Q13526	UNIPROT	PIN1	protein	Q14653	IRF3	UNIPROT	protein	YES	down-regulates quantity by destabilization	binding
P98170	UNIPROT	XIAP	protein	P55210	CASP7	UNIPROT	protein	YES	down-regulates quantity by destabilization	binding
P98170	UNIPROT	XIAP	protein	P55210	CASP7	UNIPROT	protein	YES	down-regulates quantity by destabilization	binding
P98170	UNIPROT	XIAP	protein	P55211	CASP9	UNIPROT	protein	YES	down-regulates quantity by destabilization	binding
P98170	UNIPROT	XIAP	protein	P55211	CASP9	UNIPROT	protein	YES	down-regulates quantity by destabilization	binding
P98170	UNIPROT	XIAP	protein	P55211	CASP9	UNIPROT	protein	YES	down-regulates quantity by destabilization	binding
Q8IZP0	UNIPROT	ABI1	protein	P22681	CBL	UNIPROT	protein	YES	up-regulates	binding
O15169	UNIPROT	AXIN1	protein	P48730	CSNK1D	UNIPROT	protein	YES	up-regulates	binding
O15169	UNIPROT	AXIN1	protein	Q9Y6R4	MAP3K4	UNIPROT	protein	YES	up-regulates	binding
P55957	UNIPROT	BID	protein	Q07812	BAX	UNIPROT	protein	YES	up-regulates	binding
P55957	UNIPROT	BID	protein	Q07812	BAX	UNIPROT	protein	YES	up-regulates	binding
P55957	UNIPROT	BID	protein	Q07812	BAX	UNIPROT	protein	YES	up-regulates	binding
P55957	UNIPROT	BID	protein	Q07812	BAX	UNIPROT	protein	YES	up-regulates	binding
P12830	UNIPROT	CDH1	protein	P35221	CTNNA1	UNIPROT	protein	YES	up-regulates	binding
O15111	UNIPROT	CHUK	protein	Q92793	CREBBP	UNIPROT	protein	YES	up-regulates	binding
Q92793	UNIPROT	CREBBP	protein	P16220	CREB1	UNIPROT	protein	YES	up-regulates	binding
Q92793	UNIPROT	CREBBP	protein	P41240	CSK	UNIPROT	protein	YES	up-regulates	binding
P46108	UNIPROT	CRK	protein	P22681	CBL	UNIPROT	protein	YES	up-regulates	binding
P48730	UNIPROT	CSNK1D	protein	O15169	AXIN1	UNIPROT	protein	YES	up-regulates	binding
P35222	UNIPROT	CTNNB1	protein	Q09472	EP300	UNIPROT	protein	YES	up-regulates	binding

O60716	UNIPROT	CTNND1	protein	P63000	RAC1	UNIPROT	protein	YES	up-regulates	binding
O60716	UNIPROT	CTNND1	protein	P52735	VAV2	UNIPROT	protein	YES	up-regulates	binding
Q9UER7	UNIPROT	DAXX	protein	P37173	TGFBR2	UNIPROT	protein	YES	up-regulates	binding
P17844	UNIPROT	DDX5	protein	Q13547	HDAC1	UNIPROT	protein	YES	up-regulates	binding
P17844	UNIPROT	DDX5	protein	P04637	TP53	UNIPROT	protein	YES	up-regulates	binding
P00533	UNIPROT	EGFR	protein	P04626	ERBB2	UNIPROT	protein	YES	up-regulates	binding
P00533	UNIPROT	EGFR	protein	P27986	PIK3R1	UNIPROT	protein	YES	up-regulates	binding
P00533	UNIPROT	EGFR	protein	P27986	PIK3R1	UNIPROT	protein	YES	up-regulates	binding
P00533	UNIPROT	EGFR	protein	P29353	SHC1	UNIPROT	protein	YES	up-regulates	binding
Q09472	UNIPROT	EP300	protein	Q15797	SMAD1	UNIPROT	protein	YES	up-regulates	binding
Q09472	UNIPROT	EP300	protein	Q15797	SMAD1	UNIPROT	protein	YES	up-regulates	binding
P29317	UNIPROT	EPHA2	protein	P29353	SHC1	UNIPROT	protein	YES	up-regulates	binding
P04626	UNIPROT	ERBB2	protein	P00533	EGFR	UNIPROT	protein	YES	up-regulates	binding
P04626	UNIPROT	ERBB2	protein	P00533	EGFR	UNIPROT	protein	YES	up-regulates	binding
P04626	UNIPROT	ERBB2	protein	P21860	ERBB3	UNIPROT	protein	YES	up-regulates	binding
P04626	UNIPROT	ERBB2	protein	P21860	ERBB3	UNIPROT	protein	YES	up-regulates	binding
P04626	UNIPROT	ERBB2	protein	P29353	SHC1	UNIPROT	protein	YES	up-regulates	binding
P21860	UNIPROT	ERBB3	protein	P62993	GRB2	UNIPROT	protein	YES	up-regulates	binding
P21860	UNIPROT	ERBB3	protein	P62993	GRB2	UNIPROT	protein	YES	up-regulates	binding
O14944	UNIPROT	EREG	protein	P00533	EGFR	UNIPROT	protein	YES	up-regulates	binding
O14944	UNIPROT	EREG	protein	P00533	EGFR	UNIPROT	protein	YES	up-regulates	binding
O14944	UNIPROT	EREG	protein	P21860	ERBB3	UNIPROT	protein	YES	up-regulates	binding
O14944	UNIPROT	EREG	protein	P21860	ERBB3	UNIPROT	protein	YES	up-regulates	binding
P21333	UNIPROT	FLNA	protein	P45985	MAP2K4	UNIPROT	protein	YES	up-regulates	binding
P49915	UNIPROT	GMPS	protein	P04637	TP53	UNIPROT	protein	YES	up-regulates	binding
Q14344	UNIPROT	GNA13	protein	P61586	RHOA	UNIPROT	protein	YES	up-regulates	binding
Q9UBI6	UNIPROT	GNG12	protein	P31751	AKT2	UNIPROT	protein	YES	up-regulates	binding
P62993	UNIPROT	GRB2	protein	Q13480	GAB1	UNIPROT	protein	YES	up-regulates	binding
P49841	UNIPROT	GSK3B	protein	O15169	AXIN1	UNIPROT	protein	YES	up-regulates	binding
P49841	UNIPROT	GSK3B	protein	O15169	AXIN1	UNIPROT	protein	YES	up-regulates	binding
P16104	UNIPROT	H2AFX	protein	Q14676	MDC1	UNIPROT	protein	YES	up-regulates	binding
O15379	UNIPROT	HDAC3	protein	Q13547	HDAC1	UNIPROT	protein	YES	up-regulates	binding
P01112	UNIPROT	HRAS	protein	P10398	ARAF	UNIPROT	protein	YES	up-regulates	binding
P01112	UNIPROT	HRAS	protein	P15056	BRAF	UNIPROT	protein	YES	up-regulates	binding
P01112	UNIPROT	HRAS	protein	P15056	BRAF	UNIPROT	protein	YES	up-regulates	binding
P07900	UNIPROT	HSP90AA1	protein	P53041	PPP5C	UNIPROT	protein	YES	up-regulates	binding
P07900	UNIPROT	HSP90AA1	protein	P37173	TGFBR2	UNIPROT	protein	YES	up-regulates	binding
P08069	UNIPROT	IGF1R	protein	P27986	PIK3R1	UNIPROT	protein	YES	up-regulates	binding
P01344	UNIPROT	IGF2	protein	P11717	IGF2R	UNIPROT	protein	YES	up-regulates	binding
P40189	UNIPROT	IL6ST	protein	P29353	SHC1	UNIPROT	protein	YES	up-regulates	binding
Q15596	UNIPROT	NCOA2	protein	P19793	RXRA	UNIPROT	protein	YES	up-regulates	binding
P01111	UNIPROT	NRAS	protein	P10398	ARAF	UNIPROT	protein	YES	up-regulates	binding
Q14980	UNIPROT	NUMA1	protein	P68366	TUBA4A	UNIPROT	protein	YES	up-regulates	binding
P49757	UNIPROT	NUMB	protein	Q96J02	ITCH	UNIPROT	protein	YES	up-regulates	binding
P49757	UNIPROT	NUMB	protein	P04637	TP53	UNIPROT	protein	YES	up-regulates	binding
Q8NEB9	UNIPROT	PIK3C3	protein	P60484	PTEN	UNIPROT	protein	YES	up-regulates	binding
Q13526	UNIPROT	PIN1	protein	P01106	MYC	UNIPROT	protein	YES	up-regulates	binding
P29590	UNIPROT	PML	protein	Q15796	SMAD2	UNIPROT	protein	YES	up-regulates	binding
P29590	UNIPROT	PML	protein	P84022	SMAD3	UNIPROT	protein	YES	up-regulates	binding
P60484	UNIPROT	PTEN	protein	P35813	PPM1A	UNIPROT	protein	YES	up-regulates	binding
Q05397	UNIPROT	PTK2	protein	P27986	PIK3R1	UNIPROT	protein	YES	up-regulates	binding
P49023	UNIPROT	PXN	protein	Q13418	ILK	UNIPROT	protein	YES	up-regulates	binding
P63000	UNIPROT	RAC1	protein	P45983	MAPK8	UNIPROT	protein	YES	up-regulates	binding
P63000	UNIPROT	RAC1	protein	P45984	MAPK9	UNIPROT	protein	YES	up-regulates	binding
P63244	UNIPROT	RACK1	protein	Q13308	PTK7	UNIPROT	protein	YES	up-regulates	binding
Q04206	UNIPROT	RELA	protein	Q92793	CREBBP	UNIPROT	protein	YES	up-regulates	binding
Q04206	UNIPROT	RELA	protein	P05412	JUN	UNIPROT	protein	YES	up-regulates	binding
Q04206	UNIPROT	RELA	protein	P08047	SP1	UNIPROT	protein	YES	up-regulates	binding
P61586	UNIPROT	RHOA	protein	Q13393	PLD1	UNIPROT	protein	YES	up-regulates	binding
Q13464	UNIPROT	ROCK1	protein	Q15746	MYLK	UNIPROT	protein	YES	up-regulates	binding
P15927	UNIPROT	RPA2	protein	Q8WXE1	ATRIP	UNIPROT	protein	YES	up-regulates	binding
P31431	UNIPROT	SDC4	protein	O14641	DVL2	UNIPROT	protein	YES	up-regulates	binding
P29353	UNIPROT	SHC1	protein	P62993	GRB2	UNIPROT	protein	YES	up-regulates	binding
P29353	UNIPROT	SHC1	protein	P62993	GRB2	UNIPROT	protein	YES	up-regulates	binding
P29353	UNIPROT	SHC1	protein	P62993	GRB2	UNIPROT	protein	YES	up-regulates	binding
P29353	UNIPROT	SHC1	protein	P62993	GRB2	UNIPROT	protein	YES	up-regulates	binding
P29353	UNIPROT	SHC1	protein	Q07889	SOS1	UNIPROT	protein	YES	up-regulates	binding
Q15796	UNIPROT	SMAD2	protein	P16220	CREB1	UNIPROT	protein	YES	up-regulates	binding
Q9UEW8	UNIPROT	STK39	protein	Q16539	MAPK14	UNIPROT	protein	YES	up-regulates	binding
Q9UEW8	UNIPROT	STK39	protein	Q16539	MAPK14	UNIPROT	protein	YES	up-regulates	binding
Q9UL54	UNIPROT	TAOK2	protein	O14733	MAP2K7	UNIPROT	protein	YES	up-regulates	binding
P01137	UNIPROT	TGFB1	protein	P37173	TGFBR2	UNIPROT	protein	YES	up-regulates	binding
P37173	UNIPROT	TGFBR2	protein	P27986	PIK3R1	UNIPROT	protein	YES	up-regulates	binding
P04637	UNIPROT	TP53	protein	Q07812	BAX	UNIPROT	protein	YES	up-regulates	binding
P04637	UNIPROT	TP53	protein	Q07812	BAX	UNIPROT	protein	YES	up-regulates	binding
P04637	UNIPROT	TP53	protein	Q00653	NFKB2	UNIPROT	protein	YES	up-regulates	binding
P04637	UNIPROT	TP53	protein	Q15418	RPS6KA1	UNIPROT	protein	YES	up-regulates	binding
P12270	UNIPROT	TPR	protein	Q13257	MAD2L1	UNIPROT	protein	YES	up-regulates	binding
P52735	UNIPROT	VAV2	protein	P00533	EGFR	UNIPROT	protein	YES	up-regulates	binding
P46937	UNIPROT	YAP1	protein	P35222	CTNNB1	UNIPROT	protein	YES	up-regulates	binding
P46937	UNIPROT	YAP1	protein	Q15797	SMAD1	UNIPROT	protein	YES	up-regulates	binding
P46937	UNIPROT	YAP1	protein	Q99594	TEAD3	UNIPROT	protein	YES	up-regulates	binding
O15169	UNIPROT	AXIN1	protein	O15169	AXIN1	UNIPROT	protein	YES	up-regulates activity	binding
Q92934	UNIPROT	BAD	protein	P04637	TP53	UNIPROT	protein	YES	up-regulates activity	binding
Q07812	UNIPROT	BAX	protein	Q07812	BAX	UNIPROT	protein	YES	up-regulates activity	binding
Q07817	UNIPROT	BCL2L1	protein	Q92934	BAD	UNIPROT	protein	YES	up-regulates activity	binding
P60953	UNIPROT	CDC42	protein	Q13153	PAK1	UNIPROT	protein	YES	up-regulates activity	binding
P60953	UNIPROT	CDC42	protein	Q13177	PAK2	UNIPROT	protein	YES	up-regulates activity	binding
P30622	UNIPROT	CLIP1	protein	Q14203	DCTN1	UNIPROT	protein	YES	up-regulates activity	binding
Q53ET0	UNIPROT	CRTC2	protein	P16220	CREB1	UNIPROT	protein	YES	up-regulates activity	binding
P48729	UNIPROT	CSNK1A1	protein	P49841	GSK3B	UNIPROT	protein	YES	up-regulates activity	binding
P48729	UNIPROT	CSNK1A1	protein	P49841	GSK3B	UNIPROT	protein	YES	up-regulates activity	binding
O14641	UNIPROT	DVL2	protein	O15169	AXIN1	UNIPROT	protein	YES	up-regulates activity	binding
O14641	UNIPROT	DVL2	protein	O14641	DVL2	UNIPROT	protein	YES	up-regulates activity	binding
P00533	UNIPROT	EGFR	protein	P62993	GRB2	UNIPROT	protein	YES	up-regulates activity	binding
P00533	UNIPROT	EGFR	protein	P62993	GRB2	UNIPROT	protein	YES	up-regulates activity	binding
P00533	UNIPROT	EGFR	protein	P29353	SHC1	UNIPROT	protein	YES	up-regulates activity	binding
Q13158	UNIPROT	FADD	protein	Q14790	CASP8	UNIPROT	protein	YES	up-regulates activity	binding

Q13158	UNIPROT	FADD	protein	Q13546	RIPK1	UNIPROT	protein	YES	up-regulates activity	binding
Q13158	UNIPROT	FADD	protein	Q13546	RIPK1	UNIPROT	protein	YES	up-regulates activity	binding
P25445	UNIPROT	FAS	protein	Q13158	FADD	UNIPROT	protein	YES	up-regulates activity	binding
P25445	UNIPROT	FAS	protein	P25445	FAS	UNIPROT	protein	YES	up-regulates activity	binding
P25445	UNIPROT	FAS	protein	P25445	FAS	UNIPROT	protein	YES	up-regulates activity	binding
P25445	UNIPROT	FAS	protein	Q13546	RIPK1	UNIPROT	protein	YES	up-regulates activity	binding
P01100	UNIPROT	FOS	protein	P05412	JUN	UNIPROT	protein	YES	up-regulates activity	binding
P01112	UNIPROT	HRAS	protein	P15056	BRAF	UNIPROT	protein	YES	up-regulates activity	binding
P01344	UNIPROT	IGF2	protein	P08069	IGF1R	UNIPROT	protein	YES	up-regulates activity	binding
P51617	UNIPROT	IRAK1	protein	Q9Y4K3	TRAF6	UNIPROT	protein	YES	up-regulates activity	binding
P51617	UNIPROT	IRAK1	protein	Q9Y4K3	TRAF6	UNIPROT	protein	YES	up-regulates activity	binding
P35568	UNIPROT	IRS1	protein	P62993	GRB2	UNIPROT	protein	YES	up-regulates activity	binding
P35568	UNIPROT	IRS1	protein	P27986	PIK3R1	UNIPROT	protein	YES	up-regulates activity	binding
P01116	UNIPROT	KRAS	protein	P15056	BRAF	UNIPROT	protein	YES	up-regulates activity	binding
Q99759	UNIPROT	MAP3K3	protein	P45985	MAP2K4	UNIPROT	protein	YES	up-regulates activity	binding
O43318	UNIPROT	MAP3K7	protein	Q9Y6K9	IKBKG	UNIPROT	protein	YES	up-regulates activity	binding
P19838	UNIPROT	NFKB1	protein	Q04206	RELA	UNIPROT	protein	YES	up-regulates activity	binding
P23511	UNIPROT	NFYA	protein	P08047	SP1	UNIPROT	protein	YES	up-regulates activity	binding
P01111	UNIPROT	NRAS	protein	P15056	BRAF	UNIPROT	protein	YES	up-regulates activity	binding
Q8NEB9	UNIPROT	PIK3C3	protein	Q14457	BECN1	UNIPROT	protein	YES	up-regulates activity	binding
P29590	UNIPROT	PML	protein	Q15796	SMAD2	UNIPROT	protein	YES	up-regulates activity	binding
P29590	UNIPROT	PML	protein	P84022	SMAD3	UNIPROT	protein	YES	up-regulates activity	binding
Q12972	UNIPROT	PPP1R8	protein	Q15910	EZH2	UNIPROT	protein	YES	up-regulates activity	binding
P63000	UNIPROT	RAC1	protein	P45983	MAPK8	UNIPROT	protein	YES	up-regulates activity	binding
P63000	UNIPROT	RAC1	protein	Q13153	PAK1	UNIPROT	protein	YES	up-regulates activity	binding
P63000	UNIPROT	RAC1	protein	Q13177	PAK2	UNIPROT	protein	YES	up-regulates activity	binding
P61586	UNIPROT	RHOA	protein	Q13464	ROCK1	UNIPROT	protein	YES	up-regulates activity	binding
P61586	UNIPROT	RHOA	protein	Q13464	ROCK1	UNIPROT	protein	YES	up-regulates activity	binding
Q13546	UNIPROT	RIPK1	protein	Q14790	CASP8	UNIPROT	protein	YES	up-regulates activity	binding
Q13546	UNIPROT	RIPK1	protein	Q14790	CASP8	UNIPROT	protein	YES	up-regulates activity	binding
Q13546	UNIPROT	RIPK1	protein	P25445	FAS	UNIPROT	protein	YES	up-regulates activity	binding
Q13546	UNIPROT	RIPK1	protein	Q9Y6K9	IKBKG	UNIPROT	protein	YES	up-regulates activity	binding
Q13546	UNIPROT	RIPK1	protein	Q9Y4K3	TRAF6	UNIPROT	protein	YES	up-regulates activity	binding
P31431	UNIPROT	SDC4	protein	P63000	RAC1	UNIPROT	protein	YES	up-regulates activity	binding
Q15796	UNIPROT	SMAD2	protein	Q15796	SMAD2	UNIPROT	protein	YES	up-regulates activity	binding
P84022	UNIPROT	SMAD3	protein	P84022	SMAD3	UNIPROT	protein	YES	up-regulates activity	binding
P42224	UNIPROT	STAT1	protein	P42224	STAT1	UNIPROT	protein	YES	up-regulates activity	binding
Q15750	UNIPROT	TAB1	protein	O43318	MAP3K7	UNIPROT	protein	YES	up-regulates activity	binding
Q15750	UNIPROT	TAB1	protein	O43318	MAP3K7	UNIPROT	protein	YES	up-regulates activity	binding
Q15750	UNIPROT	TAB1	protein	Q16539	MAPK14	UNIPROT	protein	YES	up-regulates activity	binding
Q15750	UNIPROT	TAB1	protein	Q16539	MAPK14	UNIPROT	protein	YES	up-regulates activity	binding
Q9UL54	UNIPROT	TAOK2	protein	P46734	MAP2K3	UNIPROT	protein	YES	up-regulates activity	binding
Q9UL54	UNIPROT	TAOK2	protein	P45985	MAP2K4	UNIPROT	protein	YES	up-regulates activity	binding
Q9UL54	UNIPROT	TAOK2	protein	P52564	MAP2K6	UNIPROT	protein	YES	up-regulates activity	binding
P01137	UNIPROT	TGFB1	protein	P27986	PIK3R1	UNIPROT	protein	YES	up-regulates activity	binding
P01137	UNIPROT	TGFB1	protein	P01137	TGFB1	UNIPROT	protein	YES	up-regulates activity	binding
P01137	UNIPROT	TGFB1	protein	P37173	TGFBR2	UNIPROT	protein	YES	up-regulates activity	binding
P37173	UNIPROT	TGFBR2	protein	P27986	PIK3R1	UNIPROT	protein	YES	up-regulates activity	binding
P04637	UNIPROT	TP53	protein	Q07812	BAX	UNIPROT	protein	YES	up-regulates activity	binding
Q12933	UNIPROT	TRAF2	protein	Q13546	RIPK1	UNIPROT	protein	YES	up-regulates activity	binding
Q12933	UNIPROT	TRAF2	protein	P61088	UBE2N	UNIPROT	protein	YES	up-regulates activity	binding
Q9Y4K3	UNIPROT	TRAF6	protein	Q15750	TAB1	UNIPROT	protein	YES	up-regulates activity	binding
P49815	UNIPROT	TSC2	protein	Q92574	TSC1	UNIPROT	protein	YES	up-regulates activity	binding
P62258	UNIPROT	YWHAE	protein	Q9GZM8	NDEL1	UNIPROT	protein	YES	up-regulates activity	binding
O60716	UNIPROT	CTNND1	protein	P12830	CDH1	UNIPROT	protein	YES	up-regulates quantity by stabilization	binding
P42574	UNIPROT	CASP3	protein	P06396	GSN	UNIPROT	protein	YES	down-regulates	cleavage
P42574	UNIPROT	CASP3	protein	O75475	PSIP1	UNIPROT	protein	YES	down-regulates	cleavage
P42574	UNIPROT	CASP3	protein	Q13813	SPTAN1	UNIPROT	protein	YES	down-regulates	cleavage
P55210	UNIPROT	CASP7	protein	P09874	PARP1	UNIPROT	protein	YES	down-regulates	cleavage
P42574	UNIPROT	CASP3	protein	P31749	AKT1	UNIPROT	protein	YES	down-regulates activity	cleavage
P42574	UNIPROT	CASP3	protein	P09874	PARP1	UNIPROT	protein	YES	down-regulates activity	cleavage
Q14790	UNIPROT	CASP8	protein	Q13546	RIPK1	UNIPROT	protein	YES	down-regulates activity	cleavage
P42574	UNIPROT	CASP3	protein	Q9UKV3	ACIN1	UNIPROT	protein	YES	up-regulates	cleavage
P42574	UNIPROT	CASP3	protein	Q13464	ROCK1	UNIPROT	protein	YES	up-regulates	cleavage
Q14790	UNIPROT	CASP8	protein	P55210	CASP7	UNIPROT	protein	YES	up-regulates	cleavage
Q14790	UNIPROT	CASP8	protein	P55210	CASP7	UNIPROT	protein	YES	up-regulates	cleavage
P17655	UNIPROT	CAPN2	protein	P49840	GSK3A	UNIPROT	protein	YES	up-regulates activity	cleavage
P17655	UNIPROT	CAPN2	protein	P49841	GSK3B	UNIPROT	protein	YES	up-regulates activity	cleavage
P42574	UNIPROT	CASP3	protein	Q92934	BAD	UNIPROT	protein	YES	up-regulates activity	cleavage
P42574	UNIPROT	CASP3	protein	P55211	CASP9	UNIPROT	protein	YES	up-regulates activity	cleavage
P42574	UNIPROT	CASP3	protein	P55211	CASP9	UNIPROT	protein	YES	up-regulates activity	cleavage
P42574	UNIPROT	CASP3	protein	Q13043	STK4	UNIPROT	protein	YES	up-regulates activity	cleavage
P42574	UNIPROT	CASP3	protein	Q13043	STK4	UNIPROT	protein	YES	up-regulates activity	cleavage
Q14790	UNIPROT	CASP8	protein	P55957	BID	UNIPROT	protein	YES	up-regulates activity	cleavage
Q14790	UNIPROT	CASP8	protein	P42574	CASP3	UNIPROT	protein	YES	up-regulates activity	cleavage
Q14790	UNIPROT	CASP8	protein	P42574	CASP3	UNIPROT	protein	YES	up-regulates activity	cleavage
Q14790	UNIPROT	CASP8	protein	P42574	CASP3	UNIPROT	protein	YES	up-regulates activity	cleavage
P55211	UNIPROT	CASP9	protein	P42574	CASP3	UNIPROT	protein	YES	up-regulates activity	cleavage
P55211	UNIPROT	CASP9	protein	P42574	CASP3	UNIPROT	protein	YES	up-regulates activity	cleavage
Q96EB6	UNIPROT	SIRT1	protein	Q09472	EP300	UNIPROT	protein	YES	down-regulates	deacetylation
Q96EB6	UNIPROT	SIRT1	protein	Q09472	EP300	UNIPROT	protein	YES	down-regulates	deacetylation
Q96EB6	UNIPROT	SIRT1	protein	P04637	TP53	UNIPROT	protein	YES	down-regulates	deacetylation
Q9BY41	UNIPROT	HDAC8	protein	P04637	TP53	UNIPROT	protein	YES	down-regulates activity	deacetylation
Q96EB6	UNIPROT	SIRT1	protein	O43524	FOXO3	UNIPROT	protein	YES	down-regulates activity	deacetylation
Q96EB6	UNIPROT	SIRT1	protein	Q04206	RELA	UNIPROT	protein	YES	down-regulates activity	deacetylation
Q96EB6	UNIPROT	SIRT1	protein	Q12778	FOXO1	UNIPROT	protein	YES	down-regulates quantity by destabilization	deacetylation
Q96EB6	UNIPROT	SIRT1	protein	P31749	AKT1	UNIPROT	protein	YES	up-regulates activity	deacetylation
Q96EB6	UNIPROT	SIRT1	protein	Q12778	FOXO1	UNIPROT	protein	YES	up-regulates activity	deacetylation
Q96EB6	UNIPROT	SIRT1	protein	O43524	FOXO3	UNIPROT	protein	YES	up-regulates activity	deacetylation
Q96EB6	UNIPROT	SIRT1	protein	P35222	CTNNB1	UNIPROT	protein	YES	up-regulates quantity	deacetylation
Q99504	UNIPROT	EYA3	protein	P16104	H2AFX	UNIPROT	protein	YES	down-regulates	dephosphorylation
Q9P0J1	UNIPROT	PDP1	protein	Q15797	SMAD1	UNIPROT	protein	YES	down-regulates	dephosphorylation
P35813	UNIPROT	PPM1A	protein	O15169	AXIN1	UNIPROT	protein	YES	down-regulates	dephosphorylation
P35813	UNIPROT	PPM1A	protein	Q15797	SMAD1	UNIPROT	protein	YES	down-regulates	dephosphorylation
P35813	UNIPROT	PPM1A	protein	Q15796	SMAD2	UNIPROT	protein	YES	down-regulates	dephosphorylation
P35813	UNIPROT	PPM1A	protein	P84022	SMAD3	UNIPROT	protein	YES	down-regulates	dephosphorylation
P62136	UNIPROT	PPP1CA	protein	P31749	AKT1	UNIPROT	protein	YES	down-regulates	dephosphorylation
P62136	UNIPROT	PPP1CA	protein	P28482	MAPK1	UNIPROT	protein	YES	down-regulates	dephosphorylation

P62136	UNIPROT	PPP1CA	protein	P27361	MAPK3	UNIPROT	protein	YES	down-regulates	dephosphorylation
P62140	UNIPROT	PPP1CB	protein	P31749	AKT1	UNIPROT	protein	YES	down-regulates	dephosphorylation
P67775	UNIPROT	PPP2CA	protein	P31749	AKT1	UNIPROT	protein	YES	down-regulates	dephosphorylation
P67775	UNIPROT	PPP2CA	protein	P06730	EIF4E	UNIPROT	protein	YES	down-regulates	dephosphorylation
P67775	UNIPROT	PPP2CA	protein	Q02750	MAP2K1	UNIPROT	protein	YES	down-regulates	dephosphorylation
P67775	UNIPROT	PPP2CA	protein	P36507	MAP2K2	UNIPROT	protein	YES	down-regulates	dephosphorylation
P67775	UNIPROT	PPP2CA	protein	Q99759	MAP3K3	UNIPROT	protein	YES	down-regulates	dephosphorylation
P67775	UNIPROT	PPP2CA	protein	Q99759	MAP3K3	UNIPROT	protein	YES	down-regulates	dephosphorylation
P67775	UNIPROT	PPP2CA	protein	Q99759	MAP3K3	UNIPROT	protein	YES	down-regulates	dephosphorylation
P67775	UNIPROT	PPP2CA	protein	O43318	MAP3K7	UNIPROT	protein	YES	down-regulates	dephosphorylation
P67775	UNIPROT	PPP2CA	protein	P28482	MAPK1	UNIPROT	protein	YES	down-regulates	dephosphorylation
P67775	UNIPROT	PPP2CA	protein	P28482	MAPK1	UNIPROT	protein	YES	down-regulates	dephosphorylation
P67775	UNIPROT	PPP2CA	protein	P27361	MAPK3	UNIPROT	protein	YES	down-regulates	dephosphorylation
P67775	UNIPROT	PPP2CA	protein	P27361	MAPK3	UNIPROT	protein	YES	down-regulates	dephosphorylation
P67775	UNIPROT	PPP2CA	protein	Q9H0H5	RACGAP1	UNIPROT	protein	YES	down-regulates	dephosphorylation
P67775	UNIPROT	PPP2CA	protein	Q9H0H5	RACGAP1	UNIPROT	protein	YES	down-regulates	dephosphorylation
P67775	UNIPROT	PPP2CA	protein	Q04206	RELA	UNIPROT	protein	YES	down-regulates	dephosphorylation
P67775	UNIPROT	PPP2CA	protein	P23396	RPS3	UNIPROT	protein	YES	down-regulates	dephosphorylation
P67775	UNIPROT	PPP2CA	protein	P84022	SMAD3	UNIPROT	protein	YES	down-regulates	dephosphorylation
P67775	UNIPROT	PPP2CA	protein	P84022	SMAD3	UNIPROT	protein	YES	down-regulates	dephosphorylation
P67775	UNIPROT	PPP2CA	protein	Q13188	STK3	UNIPROT	protein	YES	down-regulates	dephosphorylation
P67775	UNIPROT	PPP2CA	protein	Q13043	STK4	UNIPROT	protein	YES	down-regulates	dephosphorylation
P62714	UNIPROT	PPP2CB	protein	P31749	AKT1	UNIPROT	protein	YES	down-regulates	dephosphorylation
P62714	UNIPROT	PPP2CB	protein	P31751	AKT2	UNIPROT	protein	YES	down-regulates	dephosphorylation
P62714	UNIPROT	PPP2CB	protein	P01106	MYC	UNIPROT	protein	YES	down-regulates	dephosphorylation
P62714	UNIPROT	PPP2CB	protein	P11233	RALA	UNIPROT	protein	YES	down-regulates	dephosphorylation
P62714	UNIPROT	PPP2CB	protein	P11233	RALA	UNIPROT	protein	YES	down-regulates	dephosphorylation
Q08209	UNIPROT	PPP3CA	protein	P21333	FLNA	UNIPROT	protein	YES	down-regulates	dephosphorylation
O00743	UNIPROT	PPP6C	protein	O43318	MAP3K7	UNIPROT	protein	YES	down-regulates	dephosphorylation
Q15257	UNIPROT	PTPA	protein	P31749	AKT1	UNIPROT	protein	YES	down-regulates	dephosphorylation
P18031	UNIPROT	PTPN1	protein	P31749	AKT1	UNIPROT	protein	YES	down-regulates	dephosphorylation
P18031	UNIPROT	PTPN1	protein	P11274	BCR	UNIPROT	protein	YES	down-regulates	dephosphorylation
P18031	UNIPROT	PTPN1	protein	P35568	IRS1	UNIPROT	protein	YES	down-regulates	dephosphorylation
P18031	UNIPROT	PTPN1	protein	P08581	MET	UNIPROT	protein	YES	down-regulates	dephosphorylation
P18031	UNIPROT	PTPN1	protein	P08581	MET	UNIPROT	protein	YES	down-regulates	dephosphorylation
P18031	UNIPROT	PTPN1	protein	P08581	MET	UNIPROT	protein	YES	down-regulates	dephosphorylation
P18031	UNIPROT	PTPN1	protein	P08581	MET	UNIPROT	protein	YES	down-regulates	dephosphorylation
P18031	UNIPROT	PTPN1	protein	Q05655	PRKCD	UNIPROT	protein	YES	down-regulates	dephosphorylation
P18031	UNIPROT	PTPN1	protein	Q05397	PTK2	UNIPROT	protein	YES	down-regulates	dephosphorylation
P18031	UNIPROT	PTPN1	protein	P40763	STAT3	UNIPROT	protein	YES	down-regulates	dephosphorylation
P18031	UNIPROT	PTPN1	protein	P51692	STAT5B	UNIPROT	protein	YES	down-regulates	dephosphorylation
Q06124	UNIPROT	PTPN11	protein	P35221	CTNNA1	UNIPROT	protein	YES	down-regulates	dephosphorylation
Q06124	UNIPROT	PTPN11	protein	Q13480	GAB1	UNIPROT	protein	YES	down-regulates	dephosphorylation
Q06124	UNIPROT	PTPN11	protein	Q13480	GAB1	UNIPROT	protein	YES	down-regulates	dephosphorylation
Q06124	UNIPROT	PTPN11	protein	P35568	IRS1	UNIPROT	protein	YES	down-regulates	dephosphorylation
Q06124	UNIPROT	PTPN11	protein	P35568	IRS1	UNIPROT	protein	YES	down-regulates	dephosphorylation
Q06124	UNIPROT	PTPN11	protein	P35568	IRS1	UNIPROT	protein	YES	down-regulates	dephosphorylation
Q06124	UNIPROT	PTPN11	protein	P35568	IRS1	UNIPROT	protein	YES	down-regulates	dephosphorylation
Q06124	UNIPROT	PTPN11	protein	O95297	MPZL1	UNIPROT	protein	YES	down-regulates	dephosphorylation
Q06124	UNIPROT	PTPN11	protein	Q05397	PTK2	UNIPROT	protein	YES	down-regulates	dephosphorylation
Q05209	UNIPROT	PTPN12	protein	P56945	BCAR1	UNIPROT	protein	YES	down-regulates	dephosphorylation
Q05209	UNIPROT	PTPN12	protein	Q14161	GIT2	UNIPROT	protein	YES	down-regulates	dephosphorylation
Q05209	UNIPROT	PTPN12	protein	Q14161	GIT2	UNIPROT	protein	YES	down-regulates	dephosphorylation
Q05209	UNIPROT	PTPN12	protein	Q14161	GIT2	UNIPROT	protein	YES	down-regulates	dephosphorylation
Q05209	UNIPROT	PTPN12	protein	Q05397	PTK2	UNIPROT	protein	YES	down-regulates	dephosphorylation
Q05209	UNIPROT	PTPN12	protein	P29353	SHC1	UNIPROT	protein	YES	down-regulates	dephosphorylation
Q05209	UNIPROT	PTPN12	protein	P29353	SHC1	UNIPROT	protein	YES	down-regulates	dephosphorylation
P17706	UNIPROT	PTPN2	protein	P00533	EGFR	UNIPROT	protein	YES	down-regulates	dephosphorylation
P17706	UNIPROT	PTPN2	protein	P00533	EGFR	UNIPROT	protein	YES	down-regulates	dephosphorylation
P17706	UNIPROT	PTPN2	protein	Q02790	FKBP4	UNIPROT	protein	YES	down-regulates	dephosphorylation
P17706	UNIPROT	PTPN2	protein	P08581	MET	UNIPROT	protein	YES	down-regulates	dephosphorylation
P17706	UNIPROT	PTPN2	protein	P08581	MET	UNIPROT	protein	YES	down-regulates	dephosphorylation
P17706	UNIPROT	PTPN2	protein	P12931	SRC	UNIPROT	protein	YES	down-regulates	dephosphorylation
P17706	UNIPROT	PTPN2	protein	P42224	STAT1	UNIPROT	protein	YES	down-regulates	dephosphorylation
P17706	UNIPROT	PTPN2	protein	P40763	STAT3	UNIPROT	protein	YES	down-regulates	dephosphorylation
P17706	UNIPROT	PTPN2	protein	O00401	WASL	UNIPROT	protein	YES	down-regulates	dephosphorylation
P29350	UNIPROT	PTPN6	protein	P63261	ACTG1	UNIPROT	protein	YES	down-regulates	dephosphorylation
P29350	UNIPROT	PTPN6	protein	P00533	EGFR	UNIPROT	protein	YES	down-regulates	dephosphorylation
P29350	UNIPROT	PTPN6	protein	P78314	SH3BP2	UNIPROT	protein	YES	down-regulates	dephosphorylation
P29350	UNIPROT	PTPN6	protein	P40763	STAT3	UNIPROT	protein	YES	down-regulates	dephosphorylation
P29350	UNIPROT	PTPN6	protein	P43405	SYK	UNIPROT	protein	YES	down-regulates	dephosphorylation
P43378	UNIPROT	PTPN9	protein	P46459	NSF	UNIPROT	protein	YES	down-regulates	dephosphorylation
P10586	UNIPROT	PTPRF	protein	P31749	AKT1	UNIPROT	protein	YES	down-regulates	dephosphorylation
P24666	UNIPROT	ACP1	protein	P31749	AKT1	UNIPROT	protein	YES	down-regulates activity	dephosphorylation
P24666	UNIPROT	ACP1	protein	P31751	AKT2	UNIPROT	protein	YES	down-regulates activity	dephosphorylation
P24666	UNIPROT	ACP1	protein	P29317	EPHA2	UNIPROT	protein	YES	down-regulates activity	dephosphorylation
P24666	UNIPROT	ACP1	protein	P29317	EPHA2	UNIPROT	protein	YES	down-regulates activity	dephosphorylation
P24666	UNIPROT	ACP1	protein	P29317	EPHA2	UNIPROT	protein	YES	down-regulates activity	dephosphorylation
P24666	UNIPROT	ACP1	protein	P29317	EPHA2	UNIPROT	protein	YES	down-regulates activity	dephosphorylation
P24666	UNIPROT	ACP1	protein	P29317	EPHA2	UNIPROT	protein	YES	down-regulates activity	dephosphorylation
P24666	UNIPROT	ACP1	protein	P12931	SRC	UNIPROT	protein	YES	down-regulates activity	dephosphorylation
P51452	UNIPROT	DUSP3	protein	P00533	EGFR	UNIPROT	protein	YES	down-regulates activity	dephosphorylation
P51452	UNIPROT	DUSP3	protein	P04626	ERBB2	UNIPROT	protein	YES	down-regulates activity	dephosphorylation
P51452	UNIPROT	DUSP3	protein	P04626	ERBB2	UNIPROT	protein	YES	down-regulates activity	dephosphorylation
P51452	UNIPROT	DUSP3	protein	P28482	MAPK1	UNIPROT	protein	YES	down-regulates activity	dephosphorylation
P51452	UNIPROT	DUSP3	protein	P27361	MAPK3	UNIPROT	protein	YES	down-regulates activity	dephosphorylation
P51452	UNIPROT	DUSP3	protein	P27361	MAPK3	UNIPROT	protein	YES	down-regulates activity	dephosphorylation
Q96GD0	UNIPROT	PDXP	protein	P23528	CFL1	UNIPROT	protein	YES	down-regulates activity	dephosphorylation
P35813	UNIPROT	PPM1A	protein	P50750	CDK9	UNIPROT	protein	YES	down-regulates activity	dephosphorylation
P35813	UNIPROT	PPM1A	protein	Q16539	MAPK14	UNIPROT	protein	YES	down-regulates activity	dephosphorylation
P35813	UNIPROT	PPM1A	protein	Q13153	PAK1	UNIPROT	protein	YES	down-regulates activity	dephosphorylation
P35813	UNIPROT	PPM1A	protein	Q13153	PAK1	UNIPROT	protein	YES	down-regulates activity	dephosphorylation
P35813	UNIPROT	PPM1A	protein	Q13153	PAK1	UNIPROT	protein	YES	down-regulates activity	dephosphorylation
P35813	UNIPROT	PPM1A	protein	Q15796	SMAD2	UNIPROT	protein	YES	down-regulates activity	dephosphorylation
P35813	UNIPROT	PPM1A	protein	P84022	SMAD3	UNIPROT	protein	YES	down-regulates activity	dephosphorylation
P62136	UNIPROT	PPP1CA	protein	P31749	AKT1	UNIPROT	protein	YES	down-regulates activity	dephosphorylation
P62136	UNIPROT	PPP1CA	protein	O15169	AXIN1	UNIPROT	protein	YES	down-regulates activity	dephosphorylation
P62136	UNIPROT	PPP1CA	protein	O15169	AXIN1	UNIPROT	protein	YES	down-regulates activity	dephosphorylation

P62136	UNIPROT	PPP1CA	protein	O15169	AXIN1	UNIPROT	protein	YES	down-regulates activity	dephosphorylation
P62136	UNIPROT	PPP1CA	protein	O15169	AXIN1	UNIPROT	protein	YES	down-regulates activity	dephosphorylation
P62136	UNIPROT	PPP1CA	protein	P40763	STAT3	UNIPROT	protein	YES	down-regulates activity	dephosphorylation
P62136	UNIPROT	PPP1CA	protein	P04637	TP53	UNIPROT	protein	YES	down-regulates activity	dephosphorylation
P62136	UNIPROT	PPP1CA	protein	P04637	TP53	UNIPROT	protein	YES	down-regulates activity	dephosphorylation
P62140	UNIPROT	PPP1CB	protein	P31749	AKT1	UNIPROT	protein	YES	down-regulates activity	dephosphorylation
P62140	UNIPROT	PPP1CB	protein	O15169	AXIN1	UNIPROT	protein	YES	down-regulates activity	dephosphorylation
P62140	UNIPROT	PPP1CB	protein	O15169	AXIN1	UNIPROT	protein	YES	down-regulates activity	dephosphorylation
P62140	UNIPROT	PPP1CB	protein	O15169	AXIN1	UNIPROT	protein	YES	down-regulates activity	dephosphorylation
P62140	UNIPROT	PPP1CB	protein	O15169	AXIN1	UNIPROT	protein	YES	down-regulates activity	dephosphorylation
P62140	UNIPROT	PPP1CB	protein	P04637	TP53	UNIPROT	protein	YES	down-regulates activity	dephosphorylation
P62140	UNIPROT	PPP1CB	protein	P04637	TP53	UNIPROT	protein	YES	down-regulates activity	dephosphorylation
P36873	UNIPROT	PPP1CC	protein	P31749	AKT1	UNIPROT	protein	YES	down-regulates activity	dephosphorylation
P36873	UNIPROT	PPP1CC	protein	O15169	AXIN1	UNIPROT	protein	YES	down-regulates activity	dephosphorylation
P36873	UNIPROT	PPP1CC	protein	O15169	AXIN1	UNIPROT	protein	YES	down-regulates activity	dephosphorylation
P36873	UNIPROT	PPP1CC	protein	O15169	AXIN1	UNIPROT	protein	YES	down-regulates activity	dephosphorylation
P36873	UNIPROT	PPP1CC	protein	O15169	AXIN1	UNIPROT	protein	YES	down-regulates activity	dephosphorylation
P36873	UNIPROT	PPP1CC	protein	Q9BY44	EIF2A	UNIPROT	protein	YES	down-regulates activity	dephosphorylation
P36873	UNIPROT	PPP1CC	protein	P04637	TP53	UNIPROT	protein	YES	down-regulates activity	dephosphorylation
P36873	UNIPROT	PPP1CC	protein	P04637	TP53	UNIPROT	protein	YES	down-regulates activity	dephosphorylation
P67775	UNIPROT	PPP2CA	protein	P31749	AKT1	UNIPROT	protein	YES	down-regulates activity	dephosphorylation
P67775	UNIPROT	PPP2CA	protein	P31749	AKT1	UNIPROT	protein	YES	down-regulates activity	dephosphorylation
P67775	UNIPROT	PPP2CA	protein	P31749	AKT1	UNIPROT	protein	YES	down-regulates activity	dephosphorylation
P67775	UNIPROT	PPP2CA	protein	P31749	AKT1	UNIPROT	protein	YES	down-regulates activity	dephosphorylation
P67775	UNIPROT	PPP2CA	protein	P31751	AKT2	UNIPROT	protein	YES	down-regulates activity	dephosphorylation
P67775	UNIPROT	PPP2CA	protein	P31751	AKT2	UNIPROT	protein	YES	down-regulates activity	dephosphorylation
P67775	UNIPROT	PPP2CA	protein	P31751	AKT2	UNIPROT	protein	YES	down-regulates activity	dephosphorylation
P67775	UNIPROT	PPP2CA	protein	P31751	AKT2	UNIPROT	protein	YES	down-regulates activity	dephosphorylation
P67775	UNIPROT	PPP2CA	protein	O14757	CHEK1	UNIPROT	protein	YES	down-regulates activity	dephosphorylation
P67775	UNIPROT	PPP2CA	protein	O14757	CHEK1	UNIPROT	protein	YES	down-regulates activity	dephosphorylation
P67775	UNIPROT	PPP2CA	protein	P32519	ELF1	UNIPROT	protein	YES	down-regulates activity	dephosphorylation
P67775	UNIPROT	PPP2CA	protein	P28482	MAPK1	UNIPROT	protein	YES	down-regulates activity	dephosphorylation
P67775	UNIPROT	PPP2CA	protein	P28482	MAPK1	UNIPROT	protein	YES	down-regulates activity	dephosphorylation
P67775	UNIPROT	PPP2CA	protein	Q13153	PAK1	UNIPROT	protein	YES	down-regulates activity	dephosphorylation
P67775	UNIPROT	PPP2CA	protein	Q13153	PAK1	UNIPROT	protein	YES	down-regulates activity	dephosphorylation
P67775	UNIPROT	PPP2CA	protein	Q13153	PAK1	UNIPROT	protein	YES	down-regulates activity	dephosphorylation
P67775	UNIPROT	PPP2CA	protein	Q05655	PRKCD	UNIPROT	protein	YES	down-regulates activity	dephosphorylation
P67775	UNIPROT	PPP2CA	protein	Q05655	PRKCD	UNIPROT	protein	YES	down-regulates activity	dephosphorylation
P67775	UNIPROT	PPP2CA	protein	Q05655	PRKCD	UNIPROT	protein	YES	down-regulates activity	dephosphorylation
P67775	UNIPROT	PPP2CA	protein	P12931	SRC	UNIPROT	protein	YES	down-regulates activity	dephosphorylation
P67775	UNIPROT	PPP2CA	protein	P04637	TP53	UNIPROT	protein	YES	down-regulates activity	dephosphorylation
P67775	UNIPROT	PPP2CA	protein	Q12933	TRAF2	UNIPROT	protein	YES	down-regulates activity	dephosphorylation
P62714	UNIPROT	PPP2CB	protein	O14757	CHEK1	UNIPROT	protein	YES	down-regulates activity	dephosphorylation
P62714	UNIPROT	PPP2CB	protein	O14757	CHEK1	UNIPROT	protein	YES	down-regulates activity	dephosphorylation
P62714	UNIPROT	PPP2CB	protein	P32519	ELF1	UNIPROT	protein	YES	down-regulates activity	dephosphorylation
P62714	UNIPROT	PPP2CB	protein	P28482	MAPK1	UNIPROT	protein	YES	down-regulates activity	dephosphorylation
P62714	UNIPROT	PPP2CB	protein	Q13153	PAK1	UNIPROT	protein	YES	down-regulates activity	dephosphorylation
P62714	UNIPROT	PPP2CB	protein	Q13153	PAK1	UNIPROT	protein	YES	down-regulates activity	dephosphorylation
P62714	UNIPROT	PPP2CB	protein	Q13153	PAK1	UNIPROT	protein	YES	down-regulates activity	dephosphorylation
P62714	UNIPROT	PPP2CB	protein	Q05655	PRKCD	UNIPROT	protein	YES	down-regulates activity	dephosphorylation
P62714	UNIPROT	PPP2CB	protein	Q05655	PRKCD	UNIPROT	protein	YES	down-regulates activity	dephosphorylation
P62714	UNIPROT	PPP2CB	protein	Q05655	PRKCD	UNIPROT	protein	YES	down-regulates activity	dephosphorylation
P62714	UNIPROT	PPP2CB	protein	P04637	TP53	UNIPROT	protein	YES	down-regulates activity	dephosphorylation
P62714	UNIPROT	PPP2CB	protein	Q12933	TRAF2	UNIPROT	protein	YES	down-regulates activity	dephosphorylation
P60510	UNIPROT	PPP4C	protein	O15379	HDAC3	UNIPROT	protein	YES	down-regulates activity	dephosphorylation
P60510	UNIPROT	PPP4C	protein	Q9GZM8	NDEL1	UNIPROT	protein	YES	down-regulates activity	dephosphorylation
P53041	UNIPROT	PPP5C	protein	Q16543	CDC37	UNIPROT	protein	YES	down-regulates activity	dephosphorylation
O00743	UNIPROT	PPP6C	protein	O43318	MAP3K7	UNIPROT	protein	YES	down-regulates activity	dephosphorylation
P60484	UNIPROT	PTEN	protein	P16220	CREB1	UNIPROT	protein	YES	down-regulates activity	dephosphorylation
P60484	UNIPROT	PTEN	protein	Q05397	PTK2	UNIPROT	protein	YES	down-regulates activity	dephosphorylation
P18031	UNIPROT	PTPN1	protein	P00533	EGFR	UNIPROT	protein	YES	down-regulates activity	dephosphorylation
P18031	UNIPROT	PTPN1	protein	P08069	IGF1R	UNIPROT	protein	YES	down-regulates activity	dephosphorylation
P18031	UNIPROT	PTPN1	protein	P08581	MET	UNIPROT	protein	YES	down-regulates activity	dephosphorylation
P18031	UNIPROT	PTPN1	protein	P08581	MET	UNIPROT	protein	YES	down-regulates activity	dephosphorylation
P18031	UNIPROT	PTPN1	protein	Q05397	PTK2	UNIPROT	protein	YES	down-regulates activity	dephosphorylation
P18031	UNIPROT	PTPN1	protein	P18031	PTPN1	UNIPROT	protein	YES	down-regulates activity	dephosphorylation
P18031	UNIPROT	PTPN1	protein	P18031	PTPN1	UNIPROT	protein	YES	down-regulates activity	dephosphorylation
P18031	UNIPROT	PTPN1	protein	P18031	PTPN1	UNIPROT	protein	YES	down-regulates activity	dephosphorylation
P18031	UNIPROT	PTPN1	protein	P18031	PTPN1	UNIPROT	protein	YES	down-regulates activity	dephosphorylation
P18031	UNIPROT	PTPN1	protein	P40763	STAT3	UNIPROT	protein	YES	down-regulates activity	dephosphorylation
P18031	UNIPROT	PTPN1	protein	P51692	STAT5B	UNIPROT	protein	YES	down-regulates activity	dephosphorylation
Q06124	UNIPROT	PTPN11	protein	P00533	EGFR	UNIPROT	protein	YES	down-regulates activity	dephosphorylation
Q06124	UNIPROT	PTPN11	protein	P00533	EGFR	UNIPROT	protein	YES	down-regulates activity	dephosphorylation
Q06124	UNIPROT	PTPN11	protein	Q13480	GAB1	UNIPROT	protein	YES	down-regulates activity	dephosphorylation
Q06124	UNIPROT	PTPN11	protein	Q13480	GAB1	UNIPROT	protein	YES	down-regulates activity	dephosphorylation
Q06124	UNIPROT	PTPN11	protein	Q13480	GAB1	UNIPROT	protein	YES	down-regulates activity	dephosphorylation
Q06124	UNIPROT	PTPN11	protein	P12931	SRC	UNIPROT	protein	YES	down-regulates activity	dephosphorylation
Q06124	UNIPROT	PTPN11	protein	P42224	STAT1	UNIPROT	protein	YES	down-regulates activity	dephosphorylation
Q06124	UNIPROT	PTPN11	protein	P42224	STAT1	UNIPROT	protein	YES	down-regulates activity	dephosphorylation
Q05209	UNIPROT	PTPN12	protein	Q05397	PTK2	UNIPROT	protein	YES	down-regulates activity	dephosphorylation
Q05209	UNIPROT	PTPN12	protein	P12931	SRC	UNIPROT	protein	YES	down-regulates activity	dephosphorylation
P17706	UNIPROT	PTPN2	protein	P08581	MET	UNIPROT	protein	YES	down-regulates activity	dephosphorylation
P17706	UNIPROT	PTPN2	protein	P08581	MET	UNIPROT	protein	YES	down-regulates activity	dephosphorylation
P17706	UNIPROT	PTPN2	protein	P29353	SHC1	UNIPROT	protein	YES	down-regulates activity	dephosphorylation
P17706	UNIPROT	PTPN2	protein	P42224	STAT1	UNIPROT	protein	YES	down-regulates activity	dephosphorylation
P17706	UNIPROT	PTPN2	protein	P42224	STAT1	UNIPROT	protein	YES	down-regulates activity	dephosphorylation
P17706	UNIPROT	PTPN2	protein	P40763	STAT3	UNIPROT	protein	YES	down-regulates activity	dephosphorylation
P17706	UNIPROT	PTPN2	protein	P42226	STAT6	UNIPROT	protein	YES	down-regulates activity	dephosphorylation
P29350	UNIPROT	PTPN6	protein	P12931	SRC	UNIPROT	protein	YES	down-regulates activity	dephosphorylation
P18433	UNIPROT	PTPRA	protein	P18433	PTPRA	UNIPROT	protein	YES	down-regulates activity	dephosphorylation
P35813	UNIPROT	PPM1A	protein	O15169	AXIN1	UNIPROT	protein	YES	down-regulates quantity by destabilization	dephosphorylation
P16298	UNIPROT	PPP3CB	protein	P21333	FLNA	UNIPROT	protein	YES	down-regulates quantity by destabilization	dephosphorylation
P35813	UNIPROT	PPM1A	protein	P50750	CDK9	UNIPROT	protein	YES	unknown	dephosphorylation
O75688	UNIPROT	PPM1B	protein	P50750	CDK9	UNIPROT	protein	YES	unknown	dephosphorylation
P62136	UNIPROT	PPP1CA	protein	O43865	AHCYL1	UNIPROT	protein	YES	unknown	dephosphorylation
P62140	UNIPROT	PPP1CB	protein	O43865	AHCYL1	UNIPROT	protein	YES	unknown	dephosphorylation
P36873	UNIPROT	PPP1CC	protein	O43865	AHCYL1	UNIPROT	protein	YES	unknown	dephosphorylation
Q08209	UNIPROT	PPP3CA	protein	P50570	DNM2	UNIPROT	protein	YES	unknown	dephosphorylation

P16298	UNIPROT	PPP3CB	protein	P50570	DNM2	UNIPROT	protein	YES	unknown	dephosphorylation
Q15262	UNIPROT	PTPRK	protein	P00533	EGFR	UNIPROT	protein	YES	unknown	dephosphorylation
Q15262	UNIPROT	PTPRK	protein	P00533	EGFR	UNIPROT	protein	YES	unknown	dephosphorylation
P62136	UNIPROT	PPP1CA	protein	P55211	CASP9	UNIPROT	protein	YES	up-regulates	dephosphorylation
P62136	UNIPROT	PPP1CA	protein	P07737	PFN1	UNIPROT	protein	YES	up-regulates	dephosphorylation
P62140	UNIPROT	PPP1CB	protein	P35240	NF2	UNIPROT	protein	YES	up-regulates	dephosphorylation
P36873	UNIPROT	PPP1CC	protein	P50750	CDK9	UNIPROT	protein	YES	up-regulates	dephosphorylation
P36873	UNIPROT	PPP1CC	protein	P50750	CDK9	UNIPROT	protein	YES	up-regulates	dephosphorylation
P67775	UNIPROT	PPP2CA	protein	P35222	CTNNB1	UNIPROT	protein	YES	up-regulates	dephosphorylation
P67775	UNIPROT	PPP2CA	protein	P13639	EEF2	UNIPROT	protein	YES	up-regulates	dephosphorylation
P67775	UNIPROT	PPP2CA	protein	P13639	EEF2	UNIPROT	protein	YES	up-regulates	dephosphorylation
P67775	UNIPROT	PPP2CA	protein	O43524	FOXO3	UNIPROT	protein	YES	up-regulates	dephosphorylation
P67775	UNIPROT	PPP2CA	protein	O43524	FOXO3	UNIPROT	protein	YES	up-regulates	dephosphorylation
P67775	UNIPROT	PPP2CA	protein	P06400	RB1	UNIPROT	protein	YES	up-regulates	dephosphorylation
P67775	UNIPROT	PPP2CA	protein	Q12800	TFCP2	UNIPROT	protein	YES	up-regulates	dephosphorylation
P67775	UNIPROT	PPP2CA	protein	Q12800	TFCP2	UNIPROT	protein	YES	up-regulates	dephosphorylation
Q13362	UNIPROT	PPP2R5C	protein	P18846	ATF1	UNIPROT	protein	YES	up-regulates	dephosphorylation
Q13362	UNIPROT	PPP2R5C	protein	P18846	ATF1	UNIPROT	protein	YES	up-regulates	dephosphorylation
Q13362	UNIPROT	PPP2R5C	protein	P18846	ATF1	UNIPROT	protein	YES	up-regulates	dephosphorylation
Q13362	UNIPROT	PPP2R5C	protein	P18846	ATF1	UNIPROT	protein	YES	up-regulates	dephosphorylation
P60510	UNIPROT	PPP4C	protein	O75531	BANF1	UNIPROT	protein	YES	up-regulates	dephosphorylation
P60510	UNIPROT	PPP4C	protein	O75531	BANF1	UNIPROT	protein	YES	up-regulates	dephosphorylation
Q12974	UNIPROT	PTP4A2	protein	P24941	CDK2	UNIPROT	protein	YES	up-regulates	dephosphorylation
P18031	UNIPROT	PTPN1	protein	P12814	ACTN1	UNIPROT	protein	YES	up-regulates	dephosphorylation
P18031	UNIPROT	PTPN1	protein	P12931	SRC	UNIPROT	protein	YES	up-regulates	dephosphorylation
P18433	UNIPROT	PTPRA	protein	P12931	SRC	UNIPROT	protein	YES	up-regulates	dephosphorylation
P10586	UNIPROT	PTPRF	protein	P53355	DAPK1	UNIPROT	protein	YES	up-regulates	dephosphorylation
P10586	UNIPROT	PTPRF	protein	P53355	DAPK1	UNIPROT	protein	YES	up-regulates	dephosphorylation
P24666	UNIPROT	ACP1	protein	P12931	SRC	UNIPROT	protein	YES	up-regulates activity	dephosphorylation
Q9P0J1	UNIPROT	PDP1	protein	P08559	PDHA1	UNIPROT	protein	YES	up-regulates activity	dephosphorylation
Q9P0J1	UNIPROT	PDP1	protein	P08559	PDHA1	UNIPROT	protein	YES	up-regulates activity	dephosphorylation
Q9P0J1	UNIPROT	PDP1	protein	P08559	PDHA1	UNIPROT	protein	YES	up-regulates activity	dephosphorylation
Q9P0J1	UNIPROT	PDP1	protein	P08559	PDHA1	UNIPROT	protein	YES	up-regulates activity	dephosphorylation
P35813	UNIPROT	PPM1A	protein	P27986	PIK3R1	UNIPROT	protein	YES	up-regulates activity	dephosphorylation
P62136	UNIPROT	PPP1CA	protein	P42575	CASP2	UNIPROT	protein	YES	up-regulates activity	dephosphorylation
P62136	UNIPROT	PPP1CA	protein	P55211	CASP9	UNIPROT	protein	YES	up-regulates activity	dephosphorylation
P62140	UNIPROT	PPP1CB	protein	P42575	CASP2	UNIPROT	protein	YES	up-regulates activity	dephosphorylation
P36873	UNIPROT	PPP1CC	protein	P42575	CASP2	UNIPROT	protein	YES	up-regulates activity	dephosphorylation
P67775	UNIPROT	PPP2CA	protein	O96017	CHEK2	UNIPROT	protein	YES	up-regulates activity	dephosphorylation
P67775	UNIPROT	PPP2CA	protein	Q8WUI4	HDAC7	UNIPROT	protein	YES	up-regulates activity	dephosphorylation
P67775	UNIPROT	PPP2CA	protein	Q8WUI4	HDAC7	UNIPROT	protein	YES	up-regulates activity	dephosphorylation
P67775	UNIPROT	PPP2CA	protein	Q8WUI4	HDAC7	UNIPROT	protein	YES	up-regulates activity	dephosphorylation
P67775	UNIPROT	PPP2CA	protein	Q8WUI4	HDAC7	UNIPROT	protein	YES	up-regulates activity	dephosphorylation
P67775	UNIPROT	PPP2CA	protein	P08047	SP1	UNIPROT	protein	YES	up-regulates activity	dephosphorylation
P62714	UNIPROT	PPP2CB	protein	O96017	CHEK2	UNIPROT	protein	YES	up-regulates activity	dephosphorylation
P62714	UNIPROT	PPP2CB	protein	Q8WUI4	HDAC7	UNIPROT	protein	YES	up-regulates activity	dephosphorylation
P62714	UNIPROT	PPP2CB	protein	Q8WUI4	HDAC7	UNIPROT	protein	YES	up-regulates activity	dephosphorylation
P62714	UNIPROT	PPP2CB	protein	Q8WUI4	HDAC7	UNIPROT	protein	YES	up-regulates activity	dephosphorylation
P62714	UNIPROT	PPP2CB	protein	Q8WUI4	HDAC7	UNIPROT	protein	YES	up-regulates activity	dephosphorylation
Q08209	UNIPROT	PPP3CA	protein	Q92934	BAD	UNIPROT	protein	YES	up-regulates activity	dephosphorylation
Q08209	UNIPROT	PPP3CA	protein	Q92934	BAD	UNIPROT	protein	YES	up-regulates activity	dephosphorylation
Q08209	UNIPROT	PPP3CA	protein	O00429	DNM1L	UNIPROT	protein	YES	up-regulates activity	dephosphorylation
P16298	UNIPROT	PPP3CB	protein	Q92934	BAD	UNIPROT	protein	YES	up-regulates activity	dephosphorylation
P16298	UNIPROT	PPP3CB	protein	Q92934	BAD	UNIPROT	protein	YES	up-regulates activity	dephosphorylation
P16298	UNIPROT	PPP3CB	protein	O00429	DNM1L	UNIPROT	protein	YES	up-regulates activity	dephosphorylation
P60510	UNIPROT	PPP4C	protein	Q04206	RELA	UNIPROT	protein	YES	up-regulates activity	dephosphorylation
P60484	UNIPROT	PTEN	protein	P60484	PTEN	UNIPROT	protein	YES	up-regulates activity	dephosphorylation
P60484	UNIPROT	PTEN	protein	P60484	PTEN	UNIPROT	protein	YES	up-regulates activity	dephosphorylation
P60484	UNIPROT	PTEN	protein	P60484	PTEN	UNIPROT	protein	YES	up-regulates activity	dephosphorylation
P18031	UNIPROT	PTPN1	protein	Q14247	CTTN	UNIPROT	protein	YES	up-regulates activity	dephosphorylation
P18031	UNIPROT	PTPN1	protein	P12931	SRC	UNIPROT	protein	YES	up-regulates activity	dephosphorylation
P18031	UNIPROT	PTPN1	protein	P12931	SRC	UNIPROT	protein	YES	up-regulates activity	dephosphorylation
Q06124	UNIPROT	PTPN11	protein	P01112	HRAS	UNIPROT	protein	YES	up-regulates activity	dephosphorylation
Q06124	UNIPROT	PTPN11	protein	P01116	KRAS	UNIPROT	protein	YES	up-regulates activity	dephosphorylation
Q06124	UNIPROT	PTPN11	protein	P01111	NRAS	UNIPROT	protein	YES	up-regulates activity	dephosphorylation
Q06124	UNIPROT	PTPN11	protein	P12931	SRC	UNIPROT	protein	YES	up-regulates activity	dephosphorylation
P29350	UNIPROT	PTPN6	protein	Q14790	CASP8	UNIPROT	protein	YES	up-regulates activity	dephosphorylation
P29350	UNIPROT	PTPN6	protein	Q14790	CASP8	UNIPROT	protein	YES	up-regulates activity	dephosphorylation
P29350	UNIPROT	PTPN6	protein	P12931	SRC	UNIPROT	protein	YES	up-regulates activity	dephosphorylation
P18433	UNIPROT	PTPRA	protein	P12931	SRC	UNIPROT	protein	YES	up-regulates activity	dephosphorylation
P18433	UNIPROT	PTPRA	protein	P12931	SRC	UNIPROT	protein	YES	up-regulates activity	dephosphorylation
Q8IXW5	UNIPROT	RPAP2	protein	P24928	POLR2A	UNIPROT	protein	YES	up-regulates activity	dephosphorylation
Q8IXW5	UNIPROT	RPAP2	protein	P24928	POLR2A	UNIPROT	protein	YES	up-regulates activity	dephosphorylation
Q8IXW5	UNIPROT	RPAP2	protein	P24928	POLR2A	UNIPROT	protein	YES	up-regulates activity	dephosphorylation
Q8IXW5	UNIPROT	RPAP2	protein	P24928	POLR2A	UNIPROT	protein	YES	up-regulates activity	dephosphorylation
Q8IXW5	UNIPROT	RPAP2	protein	P24928	POLR2A	UNIPROT	protein	YES	up-regulates activity	dephosphorylation
Q8IXW5	UNIPROT	RPAP2	protein	P24928	POLR2A	UNIPROT	protein	YES	up-regulates activity	dephosphorylation
Q8IXW5	UNIPROT	RPAP2	protein	P24928	POLR2A	UNIPROT	protein	YES	up-regulates activity	dephosphorylation
Q8IXW5	UNIPROT	RPAP2	protein	P24928	POLR2A	UNIPROT	protein	YES	up-regulates activity	dephosphorylation
Q8IXW5	UNIPROT	RPAP2	protein	P24928	POLR2A	UNIPROT	protein	YES	up-regulates activity	dephosphorylation
Q8IXW5	UNIPROT	RPAP2	protein	P24928	POLR2A	UNIPROT	protein	YES	up-regulates activity	dephosphorylation
Q8IXW5	UNIPROT	RPAP2	protein	P24928	POLR2A	UNIPROT	protein	YES	up-regulates activity	dephosphorylation
Q8IXW5	UNIPROT	RPAP2	protein	P24928	POLR2A	UNIPROT	protein	YES	up-regulates activity	dephosphorylation
Q8IXW5	UNIPROT	RPAP2	protein	P24928	POLR2A	UNIPROT	protein	YES	up-regulates activity	dephosphorylation
Q8IXW5	UNIPROT	RPAP2	protein	P24928	POLR2A	UNIPROT	protein	YES	up-regulates activity	dephosphorylation
Q8IXW5	UNIPROT	RPAP2	protein	P24928	POLR2A	UNIPROT	protein	YES	up-regulates activity	dephosphorylation
Q8IXW5	UNIPROT	RPAP2	protein	P24928	POLR2A	UNIPROT	protein	YES	up-regulates activity	dephosphorylation
Q8IXW5	UNIPROT	RPAP2	protein	P24928	POLR2A	UNIPROT	protein	YES	up-regulates activity	dephosphorylation
Q8IXW5	UNIPROT	RPAP2	protein	P24928	POLR2A	UNIPROT	protein	YES	up-regulates activity	dephosphorylation
Q8IXW5	UNIPROT	RPAP2	protein	P24928	POLR2A	UNIPROT	protein	YES	up-regulates activity	dephosphorylation
Q8IXW5	UNIPROT	RPAP2	protein	P24928	POLR2A	UNIPROT	protein	YES	up-regulates activity	dephosphorylation
Q8IXW5	UNIPROT	RPAP2	protein	P24928	POLR2A	UNIPROT	protein	YES	up-regulates activity	dephosphorylation
Q8IXW5	UNIPROT	RPAP2	protein	P24928	POLR2A	UNIPROT	protein	YES	up-regulates activity	dephosphorylation
Q8IXW5	UNIPROT	RPAP2	protein	P24928	POLR2A	UNIPROT	protein	YES	up-regulates activity	dephosphorylation
Q8IXW5	UNIPROT	RPAP2	protein	P24928	POLR2A	UNIPROT	protein	YES	up-regulates activity	dephosphorylation
Q8TE77	UNIPROT	SSH3	protein	P23528	CFL1	UNIPROT	protein	YES	up-regulates activity	dephosphorylation
Q9NP77	UNIPROT	SSU72	protein	P24928	POLR2A	UNIPROT	protein	YES	up-regulates activity	dephosphorylation
Q9NP77	UNIPROT	SSU72	protein	P24928	POLR2A	UNIPROT	protein	YES	up-regulates activity	dephosphorylation

Q9NP77	UNIPROT	SSU72	protein	P24928	POLR2A	UNIPROT	protein	YES	up-regulates activity	dephosphorylation
Q9NP77	UNIPROT	SSU72	protein	P24928	POLR2A	UNIPROT	protein	YES	up-regulates activity	dephosphorylation
Q9NP77	UNIPROT	SSU72	protein	P24928	POLR2A	UNIPROT	protein	YES	up-regulates activity	dephosphorylation
Q9NP77	UNIPROT	SSU72	protein	P24928	POLR2A	UNIPROT	protein	YES	up-regulates activity	dephosphorylation
Q9NP77	UNIPROT	SSU72	protein	P24928	POLR2A	UNIPROT	protein	YES	up-regulates activity	dephosphorylation
Q9NP77	UNIPROT	SSU72	protein	P24928	POLR2A	UNIPROT	protein	YES	up-regulates activity	dephosphorylation
Q9NP77	UNIPROT	SSU72	protein	P24928	POLR2A	UNIPROT	protein	YES	up-regulates activity	dephosphorylation
Q9NP77	UNIPROT	SSU72	protein	P24928	POLR2A	UNIPROT	protein	YES	up-regulates activity	dephosphorylation
Q9NP77	UNIPROT	SSU72	protein	P24928	POLR2A	UNIPROT	protein	YES	up-regulates activity	dephosphorylation
Q9NP77	UNIPROT	SSU72	protein	P24928	POLR2A	UNIPROT	protein	YES	up-regulates activity	dephosphorylation
Q9NP77	UNIPROT	SSU72	protein	P24928	POLR2A	UNIPROT	protein	YES	up-regulates activity	dephosphorylation
Q9NP77	UNIPROT	SSU72	protein	P24928	POLR2A	UNIPROT	protein	YES	up-regulates activity	dephosphorylation
Q9NP77	UNIPROT	SSU72	protein	P24928	POLR2A	UNIPROT	protein	YES	up-regulates activity	dephosphorylation
Q9NP77	UNIPROT	SSU72	protein	P24928	POLR2A	UNIPROT	protein	YES	up-regulates activity	dephosphorylation
Q9NP77	UNIPROT	SSU72	protein	P24928	POLR2A	UNIPROT	protein	YES	up-regulates activity	dephosphorylation
Q9NP77	UNIPROT	SSU72	protein	P24928	POLR2A	UNIPROT	protein	YES	up-regulates activity	dephosphorylation
Q9NP77	UNIPROT	SSU72	protein	P24928	POLR2A	UNIPROT	protein	YES	up-regulates activity	dephosphorylation
Q9NP77	UNIPROT	SSU72	protein	P24928	POLR2A	UNIPROT	protein	YES	up-regulates activity	dephosphorylation
Q9NP77	UNIPROT	SSU72	protein	P24928	POLR2A	UNIPROT	protein	YES	up-regulates activity	dephosphorylation
Q9NP77	UNIPROT	SSU72	protein	P24928	POLR2A	UNIPROT	protein	YES	up-regulates activity	dephosphorylation
Q9NP77	UNIPROT	SSU72	protein	P24928	POLR2A	UNIPROT	protein	YES	up-regulates activity	dephosphorylation
Q9NP77	UNIPROT	SSU72	protein	P24928	POLR2A	UNIPROT	protein	YES	up-regulates activity	dephosphorylation
P67775	UNIPROT	PPP2CA	protein	P04637	TP53	UNIPROT	protein	YES	up-regulates quantity by stabilization	dephosphorylation
P62714	UNIPROT	PPP2CB	protein	P04637	TP53	UNIPROT	protein	YES	up-regulates quantity by stabilization	dephosphorylation
P18031	UNIPROT	PTPN1	protein	O75886	STAM2	UNIPROT	protein	YES	up-regulates quantity by stabilization	dephosphorylation
Q07889	UNIPROT	SOS1	protein	P01116	KRAS	UNIPROT	protein	YES	up-regulates	guanine nucleotide exchange factor
Q07889	UNIPROT	SOS1	protein	P01116	KRAS	UNIPROT	protein	YES	up-regulates	guanine nucleotide exchange factor
Q07889	UNIPROT	SOS1	protein	P01116	KRAS	UNIPROT	protein	YES	up-regulates	guanine nucleotide exchange factor
Q07889	UNIPROT	SOS1	protein	P01116	KRAS	UNIPROT	protein	YES	up-regulates	guanine nucleotide exchange factor
P52735	UNIPROT	VAV2	protein	P63000	RAC1	UNIPROT	protein	YES	up-regulates	guanine nucleotide exchange factor
Q07889	UNIPROT	SOS1	protein	P01112	HRAS	UNIPROT	protein	YES	up-regulates activity	guanine nucleotide exchange factor
Q07889	UNIPROT	SOS1	protein	P01112	HRAS	UNIPROT	protein	YES	up-regulates activity	guanine nucleotide exchange factor
Q07889	UNIPROT	SOS1	protein	P01111	NRAS	UNIPROT	protein	YES	up-regulates activity	guanine nucleotide exchange factor
Q07889	UNIPROT	SOS1	protein	P01111	NRAS	UNIPROT	protein	YES	up-regulates activity	guanine nucleotide exchange factor
P31749	UNIPROT	AKT1	protein	P55211	CASP9	UNIPROT	protein	YES	down-regulates	phosphorylation
P31749	UNIPROT	AKT1	protein	P46527	CDKN1B	UNIPROT	protein	YES	down-regulates	phosphorylation
P31749	UNIPROT	AKT1	protein	P46527	CDKN1B	UNIPROT	protein	YES	down-regulates	phosphorylation
P31749	UNIPROT	AKT1	protein	P46527	CDKN1B	UNIPROT	protein	YES	down-regulates	phosphorylation
P31749	UNIPROT	AKT1	protein	P46527	CDKN1B	UNIPROT	protein	YES	down-regulates	phosphorylation
P31749	UNIPROT	AKT1	protein	O14757	CHEK1	UNIPROT	protein	YES	down-regulates	phosphorylation
P31749	UNIPROT	AKT1	protein	Q12778	FOXO1	UNIPROT	protein	YES	down-regulates	phosphorylation
P31749	UNIPROT	AKT1	protein	Q12778	FOXO1	UNIPROT	protein	YES	down-regulates	phosphorylation
P31749	UNIPROT	AKT1	protein	Q12778	FOXO1	UNIPROT	protein	YES	down-regulates	phosphorylation
P31749	UNIPROT	AKT1	protein	Q12778	FOXO1	UNIPROT	protein	YES	down-regulates	phosphorylation
P31749	UNIPROT	AKT1	protein	A8MYZ6	FOXO6	UNIPROT	protein	YES	down-regulates	phosphorylation
P31749	UNIPROT	AKT1	protein	A8MYZ6	FOXO6	UNIPROT	protein	YES	down-regulates	phosphorylation
P31749	UNIPROT	AKT1	protein	P49840	GSK3A	UNIPROT	protein	YES	down-regulates	phosphorylation
P31749	UNIPROT	AKT1	protein	P09651	HNRNPA1	UNIPROT	protein	YES	down-regulates	phosphorylation
P31749	UNIPROT	AKT1	protein	P04792	HSPB1	UNIPROT	protein	YES	down-regulates	phosphorylation
P31749	UNIPROT	AKT1	protein	O43464	HTRA2	UNIPROT	protein	YES	down-regulates	phosphorylation
P31749	UNIPROT	AKT1	protein	P45985	MAP2K4	UNIPROT	protein	YES	down-regulates	phosphorylation
P31749	UNIPROT	AKT1	protein	Q9UBP6	METTL1	UNIPROT	protein	YES	down-regulates	phosphorylation
P31749	UNIPROT	AKT1	protein	O75376	NCOR1	UNIPROT	protein	YES	down-regulates	phosphorylation
P31749	UNIPROT	AKT1	protein	Q53EL6	PDCD4	UNIPROT	protein	YES	down-regulates	phosphorylation
P31749	UNIPROT	AKT1	protein	Q53EL6	PDCD4	UNIPROT	protein	YES	down-regulates	phosphorylation
P31749	UNIPROT	AKT1	protein	Q53EL6	PDCD4	UNIPROT	protein	YES	down-regulates	phosphorylation
P31749	UNIPROT	AKT1	protein	P62136	PPP1CA	UNIPROT	protein	YES	down-regulates	phosphorylation
P31749	UNIPROT	AKT1	protein	P62136	PPP1CA	UNIPROT	protein	YES	down-regulates	phosphorylation
P31749	UNIPROT	AKT1	protein	Q13188	STK3	UNIPROT	protein	YES	down-regulates	phosphorylation
P31749	UNIPROT	AKT1	protein	Q13188	STK3	UNIPROT	protein	YES	down-regulates	phosphorylation
P31749	UNIPROT	AKT1	protein	Q13188	STK3	UNIPROT	protein	YES	down-regulates	phosphorylation
P31749	UNIPROT	AKT1	protein	Q13043	STK4	UNIPROT	protein	YES	down-regulates	phosphorylation
P31749	UNIPROT	AKT1	protein	Q13043	STK4	UNIPROT	protein	YES	down-regulates	phosphorylation
P31749	UNIPROT	AKT1	protein	O60343	TBC1D4	UNIPROT	protein	YES	down-regulates	phosphorylation
P31749	UNIPROT	AKT1	protein	P46937	YAP1	UNIPROT	protein	YES	down-regulates	phosphorylation
P31749	UNIPROT	AKT1	protein	Q07352	ZFP36L1	UNIPROT	protein	YES	down-regulates	phosphorylation
P31749	UNIPROT	AKT1	protein	Q15942	ZYX	UNIPROT	protein	YES	down-regulates	phosphorylation
P31751	UNIPROT	AKT2	protein	Q96B36	AKT1S1	UNIPROT	protein	YES	down-regulates	phosphorylation
P31751	UNIPROT	AKT2	protein	Q96B36	AKT1S1	UNIPROT	protein	YES	down-regulates	phosphorylation
P31751	UNIPROT	AKT2	protein	Q92934	BAD	UNIPROT	protein	YES	down-regulates	phosphorylation
P31751	UNIPROT	AKT2	protein	Q92934	BAD	UNIPROT	protein	YES	down-regulates	phosphorylation
P31751	UNIPROT	AKT2	protein	P15056	BRAF	UNIPROT	protein	YES	down-regulates	phosphorylation
P31751	UNIPROT	AKT2	protein	P15056	BRAF	UNIPROT	protein	YES	down-regulates	phosphorylation
P31751	UNIPROT	AKT2	protein	P15056	BRAF	UNIPROT	protein	YES	down-regulates	phosphorylation
P31751	UNIPROT	AKT2	protein	P55211	CASP9	UNIPROT	protein	YES	down-regulates	phosphorylation
P31751	UNIPROT	AKT2	protein	P55211	CASP9	UNIPROT	protein	YES	down-regulates	phosphorylation
P31751	UNIPROT	AKT2	protein	P46527	CDKN1B	UNIPROT	protein	YES	down-regulates	phosphorylation
P31751	UNIPROT	AKT2	protein	P49840	GSK3A	UNIPROT	protein	YES	down-regulates	phosphorylation
P31751	UNIPROT	AKT2	protein	P04792	HSPB1	UNIPROT	protein	YES	down-regulates	phosphorylation
P31751	UNIPROT	AKT2	protein	O43464	HTRA2	UNIPROT	protein	YES	down-regulates	phosphorylation
P31751	UNIPROT	AKT2	protein	Q92945	KHSRP	UNIPROT	protein	YES	down-regulates	phosphorylation
P31751	UNIPROT	AKT2	protein	Q13188	STK3	UNIPROT	protein	YES	down-regulates	phosphorylation
P31751	UNIPROT	AKT2	protein	Q13043	STK4	UNIPROT	protein	YES	down-regulates	phosphorylation
P31751	UNIPROT	AKT2	protein	P49815	TSC2	UNIPROT	protein	YES	down-regulates	phosphorylation
P31751	UNIPROT	AKT2	protein	P49815	TSC2	UNIPROT	protein	YES	down-regulates	phosphorylation
P31751	UNIPROT	AKT2	protein	P49815	TSC2	UNIPROT	protein	YES	down-regulates	phosphorylation
P31751	UNIPROT	AKT2	protein	P49815	TSC2	UNIPROT	protein	YES	down-regulates	phosphorylation
P31751	UNIPROT	AKT2	protein	P49815	TSC2	UNIPROT	protein	YES	down-regulates	phosphorylation
P31751	UNIPROT	AKT2	protein	P49815	TSC2	UNIPROT	protein	YES	down-regulates	phosphorylation
Q96GD4	UNIPROT	AURKB	protein	Q9H410	DSN1	UNIPROT	protein	YES	down-regulates	phosphorylation
Q96GD4	UNIPROT	AURKB	protein	Q9H410	DSN1	UNIPROT	protein	YES	down-regulates	phosphorylation
Q96GD4	UNIPROT	AURKB	protein	Q99661	KIF2C	UNIPROT	protein	YES	down-regulates	phosphorylation
Q96GD4	UNIPROT	AURKB	protein	Q99661	KIF2C	UNIPROT	protein	YES	down-regulates	phosphorylation
Q96GD4	UNIPROT	AURKB	protein	O14777	NDC80	UNIPROT	protein	YES	down-regulates	phosphorylation
Q96GD4	UNIPROT	AURKB	protein	O14777	NDC80	UNIPROT	protein	YES	down-regulates	phosphorylation
Q96GD4	UNIPROT	AURKB	protein	O14777	NDC80	UNIPROT	protein	YES	down-regulates	phosphorylation
Q96GD4	UNIPROT	AURKB	protein	Q08J23	NSUN2	UNIPROT	protein	YES	down-regulates	phosphorylation
Q96GD4	UNIPROT	AURKB	protein	P04637	TP53	UNIPROT	protein	YES	down-regulates	phosphorylation

Q96GD4	UNIPROT	AURKB	protein	P04637	TP53	UNIPROT	protein	YES	down-regulates	phosphorylation
Q96GD4	UNIPROT	AURKB	protein	P04637	TP53	UNIPROT	protein	YES	down-regulates	phosphorylation
Q96GD4	UNIPROT	AURKB	protein	P04637	TP53	UNIPROT	protein	YES	down-regulates	phosphorylation
Q96GD4	UNIPROT	AURKB	protein	P04637	TP53	UNIPROT	protein	YES	down-regulates	phosphorylation
Q96GD4	UNIPROT	AURKB	protein	P08670	VIM	UNIPROT	protein	YES	down-regulates	phosphorylation
O14874	UNIPROT	BCKDK	protein	P12694	BCKDHA	UNIPROT	protein	YES	down-regulates	phosphorylation
Q9UQM7	UNIPROT	CAMK2A	protein	Q9UQM7	CAMK2A	UNIPROT	protein	YES	down-regulates	phosphorylation
Q9UQM7	UNIPROT	CAMK2A	protein	Q9UQM7	CAMK2A	UNIPROT	protein	YES	down-regulates	phosphorylation
Q9UQM7	UNIPROT	CAMK2A	protein	Q9UQM7	CAMK2A	UNIPROT	protein	YES	down-regulates	phosphorylation
Q9UQM7	UNIPROT	CAMK2A	protein	P16220	CREB1	UNIPROT	protein	YES	down-regulates	phosphorylation
Q9UQM7	UNIPROT	CAMK2A	protein	P16220	CREB1	UNIPROT	protein	YES	down-regulates	phosphorylation
Q9UQM7	UNIPROT	CAMK2A	protein	P16220	CREB1	UNIPROT	protein	YES	down-regulates	phosphorylation
Q9UQM7	UNIPROT	CAMK2A	protein	Q05469	LIPE	UNIPROT	protein	YES	down-regulates	phosphorylation
Q9UQM7	UNIPROT	CAMK2A	protein	Q9Y618	NCOR2	UNIPROT	protein	YES	down-regulates	phosphorylation
Q9UQM7	UNIPROT	CAMK2A	protein	Q96PM5	RCHY1	UNIPROT	protein	YES	down-regulates	phosphorylation
Q9UQM7	UNIPROT	CAMK2A	protein	Q96PM5	RCHY1	UNIPROT	protein	YES	down-regulates	phosphorylation
Q9UQM7	UNIPROT	CAMK2A	protein	Q15796	SMAD2	UNIPROT	protein	YES	down-regulates	phosphorylation
Q9UQM7	UNIPROT	CAMK2A	protein	Q15796	SMAD2	UNIPROT	protein	YES	down-regulates	phosphorylation
Q9UQM7	UNIPROT	CAMK2A	protein	Q15796	SMAD2	UNIPROT	protein	YES	down-regulates	phosphorylation
Q9UQM7	UNIPROT	CAMK2A	protein	P16949	STMN1	UNIPROT	protein	YES	down-regulates	phosphorylation
Q9UQM7	UNIPROT	CAMK2A	protein	P16949	STMN1	UNIPROT	protein	YES	down-regulates	phosphorylation
Q13554	UNIPROT	CAMK2B	protein	P35222	CTNNB1	UNIPROT	protein	YES	down-regulates	phosphorylation
Q13554	UNIPROT	CAMK2B	protein	P35222	CTNNB1	UNIPROT	protein	YES	down-regulates	phosphorylation
Q13554	UNIPROT	CAMK2B	protein	P35222	CTNNB1	UNIPROT	protein	YES	down-regulates	phosphorylation
Q13554	UNIPROT	CAMK2B	protein	P49840	GSK3A	UNIPROT	protein	YES	down-regulates	phosphorylation
Q13554	UNIPROT	CAMK2B	protein	P16949	STMN1	UNIPROT	protein	YES	down-regulates	phosphorylation
Q13555	UNIPROT	CAMK2G	protein	Q9Y618	NCOR2	UNIPROT	protein	YES	down-regulates	phosphorylation
P06493	UNIPROT	CDK1	protein	Q14790	CASP8	UNIPROT	protein	YES	down-regulates	phosphorylation
P06493	UNIPROT	CDK1	protein	P55211	CASP9	UNIPROT	protein	YES	down-regulates	phosphorylation
P06493	UNIPROT	CDK1	protein	P55211	CASP9	UNIPROT	protein	YES	down-regulates	phosphorylation
P06493	UNIPROT	CDK1	protein	Q53EZ4	CEP55	UNIPROT	protein	YES	down-regulates	phosphorylation
P06493	UNIPROT	CDK1	protein	Q53EZ4	CEP55	UNIPROT	protein	YES	down-regulates	phosphorylation
P06493	UNIPROT	CDK1	protein	O00571	DDX3X	UNIPROT	protein	YES	down-regulates	phosphorylation
P06493	UNIPROT	CDK1	protein	O00571	DDX3X	UNIPROT	protein	YES	down-regulates	phosphorylation
P06493	UNIPROT	CDK1	protein	Q9H8V3	ECT2	UNIPROT	protein	YES	down-regulates	phosphorylation
P06493	UNIPROT	CDK1	protein	O00418	EEF2K	UNIPROT	protein	YES	down-regulates	phosphorylation
P06493	UNIPROT	CDK1	protein	P00533	EGFR	UNIPROT	protein	YES	down-regulates	phosphorylation
P06493	UNIPROT	CDK1	protein	P50548	ERF	UNIPROT	protein	YES	down-regulates	phosphorylation
P06493	UNIPROT	CDK1	protein	Q15910	EZH2	UNIPROT	protein	YES	down-regulates	phosphorylation
P06493	UNIPROT	CDK1	protein	Q15910	EZH2	UNIPROT	protein	YES	down-regulates	phosphorylation
P06493	UNIPROT	CDK1	protein	Q12778	FOXO1	UNIPROT	protein	YES	down-regulates	phosphorylation
P06493	UNIPROT	CDK1	protein	Q08379	GOLGA2	UNIPROT	protein	YES	down-regulates	phosphorylation
P06493	UNIPROT	CDK1	protein	P17096	HMGA1	UNIPROT	protein	YES	down-regulates	phosphorylation
P06493	UNIPROT	CDK1	protein	P17096	HMGA1	UNIPROT	protein	YES	down-regulates	phosphorylation
P06493	UNIPROT	CDK1	protein	P17096	HMGA1	UNIPROT	protein	YES	down-regulates	phosphorylation
P06493	UNIPROT	CDK1	protein	P52926	HMGA2	UNIPROT	protein	YES	down-regulates	phosphorylation
P06493	UNIPROT	CDK1	protein	P52926	HMGA2	UNIPROT	protein	YES	down-regulates	phosphorylation
P06493	UNIPROT	CDK1	protein	P48200	IREB2	UNIPROT	protein	YES	down-regulates	phosphorylation
P06493	UNIPROT	CDK1	protein	Q99661	KIF2C	UNIPROT	protein	YES	down-regulates	phosphorylation
P06493	UNIPROT	CDK1	protein	Q14739	LBR	UNIPROT	protein	YES	down-regulates	phosphorylation
P06493	UNIPROT	CDK1	protein	Q02750	MAP2K1	UNIPROT	protein	YES	down-regulates	phosphorylation
P06493	UNIPROT	CDK1	protein	Q02750	MAP2K1	UNIPROT	protein	YES	down-regulates	phosphorylation
P06493	UNIPROT	CDK1	protein	P27816	MAP4	UNIPROT	protein	YES	down-regulates	phosphorylation
P06493	UNIPROT	CDK1	protein	P27816	MAP4	UNIPROT	protein	YES	down-regulates	phosphorylation
P06493	UNIPROT	CDK1	protein	P27816	MAP4	UNIPROT	protein	YES	down-regulates	phosphorylation
P06493	UNIPROT	CDK1	protein	Q14980	NUMA1	UNIPROT	protein	YES	down-regulates	phosphorylation
P06493	UNIPROT	CDK1	protein	Q9UKX7	NUP50	UNIPROT	protein	YES	down-regulates	phosphorylation
P06493	UNIPROT	CDK1	protein	Q8NEB9	PIK3C3	UNIPROT	protein	YES	down-regulates	phosphorylation
P06493	UNIPROT	CDK1	protein	Q15149	PLEC	UNIPROT	protein	YES	down-regulates	phosphorylation
P06493	UNIPROT	CDK1	protein	Q15149	PLEC	UNIPROT	protein	YES	down-regulates	phosphorylation
P06493	UNIPROT	CDK1	protein	P29590	PML	UNIPROT	protein	YES	down-regulates	phosphorylation
P06493	UNIPROT	CDK1	protein	P62136	PPP1CA	UNIPROT	protein	YES	down-regulates	phosphorylation
P06493	UNIPROT	CDK1	protein	P62136	PPP1CA	UNIPROT	protein	YES	down-regulates	phosphorylation
P06493	UNIPROT	CDK1	protein	Q06830	PRDX1	UNIPROT	protein	YES	down-regulates	phosphorylation
P06493	UNIPROT	CDK1	protein	P06400	RB1	UNIPROT	protein	YES	down-regulates	phosphorylation
P06493	UNIPROT	CDK1	protein	P06400	RB1	UNIPROT	protein	YES	down-regulates	phosphorylation
P06493	UNIPROT	CDK1	protein	P06400	RB1	UNIPROT	protein	YES	down-regulates	phosphorylation
P06493	UNIPROT	CDK1	protein	P06400	RB1	UNIPROT	protein	YES	down-regulates	phosphorylation
P06493	UNIPROT	CDK1	protein	P06400	RB1	UNIPROT	protein	YES	down-regulates	phosphorylation
P06493	UNIPROT	CDK1	protein	P31350	RRM2	UNIPROT	protein	YES	down-regulates	phosphorylation
P06493	UNIPROT	CDK1	protein	Q9Y3Z3	SAMHD1	UNIPROT	protein	YES	down-regulates	phosphorylation
P06493	UNIPROT	CDK1	protein	Q8IXJ6	SIRT2	UNIPROT	protein	YES	down-regulates	phosphorylation
P06493	UNIPROT	CDK1	protein	Q13586	STIM1	UNIPROT	protein	YES	down-regulates	phosphorylation
P06493	UNIPROT	CDK1	protein	P04183	TK1	UNIPROT	protein	YES	down-regulates	phosphorylation
P06493	UNIPROT	CDK1	protein	P04183	TK1	UNIPROT	protein	YES	down-regulates	phosphorylation
P06493	UNIPROT	CDK1	protein	Q92574	TSC1	UNIPROT	protein	YES	down-regulates	phosphorylation
P06493	UNIPROT	CDK1	protein	Q92574	TSC1	UNIPROT	protein	YES	down-regulates	phosphorylation
P06493	UNIPROT	CDK1	protein	Q92574	TSC1	UNIPROT	protein	YES	down-regulates	phosphorylation
P06493	UNIPROT	CDK1	protein	P08670	VIM	UNIPROT	protein	YES	down-regulates	phosphorylation
P06493	UNIPROT	CDK1	protein	P30291	WEE1	UNIPROT	protein	YES	down-regulates	phosphorylation
P06493	UNIPROT	CDK1	protein	P30291	WEE1	UNIPROT	protein	YES	down-regulates	phosphorylation
P06493	UNIPROT	CDK1	protein	Q86WB0	ZC3HC1	UNIPROT	protein	YES	down-regulates	phosphorylation
P21127	UNIPROT	CDK11B	protein	O00303	EIF3F	UNIPROT	protein	YES	down-regulates	phosphorylation
P24941	UNIPROT	CDK2	protein	P46527	CDKN1B	UNIPROT	protein	YES	down-regulates	phosphorylation
P24941	UNIPROT	CDK2	protein	Q12778	FOXO1	UNIPROT	protein	YES	down-regulates	phosphorylation
P24941	UNIPROT	CDK2	protein	P25098	GRK2	UNIPROT	protein	YES	down-regulates	phosphorylation
P24941	UNIPROT	CDK2	protein	P17096	HMGA1	UNIPROT	protein	YES	down-regulates	phosphorylation
P24941	UNIPROT	CDK2	protein	P49916	LIG3	UNIPROT	protein	YES	down-regulates	phosphorylation
P24941	UNIPROT	CDK2	protein	P62136	PPP1CA	UNIPROT	protein	YES	down-regulates	phosphorylation
P24941	UNIPROT	CDK2	protein	Q06830	PRDX1	UNIPROT	protein	YES	down-regulates	phosphorylation
P24941	UNIPROT	CDK2	protein	P06400	RB1	UNIPROT	protein	YES	down-regulates	phosphorylation
P24941	UNIPROT	CDK2	protein	P06400	RB1	UNIPROT	protein	YES	down-regulates	phosphorylation
P24941	UNIPROT	CDK2	protein	Q8IXJ6	SIRT2	UNIPROT	protein	YES	down-regulates	phosphorylation
P24941	UNIPROT	CDK2	protein	P84022	SMAD3	UNIPROT	protein	YES	down-regulates	phosphorylation
P24941	UNIPROT	CDK2	protein	P84022	SMAD3	UNIPROT	protein	YES	down-regulates	phosphorylation
P24941	UNIPROT	CDK2	protein	P84022	SMAD3	UNIPROT	protein	YES	down-regulates	phosphorylation
P24941	UNIPROT	CDK2	protein	P84022	SMAD3	UNIPROT	protein	YES	down-regulates	phosphorylation
P24941	UNIPROT	CDK2	protein	P84022	SMAD3	UNIPROT	protein	YES	down-regulates	phosphorylation

P24941	UNIPROT	CDK2	protein	P84022	SMAD3	UNIPROT	protein	YES	down-regulates	phosphorylation
P24941	UNIPROT	CDK2	protein	Q12800	TFCP2	UNIPROT	protein	YES	down-regulates	phosphorylation
P24941	UNIPROT	CDK2	protein	P04183	TK1	UNIPROT	protein	YES	down-regulates	phosphorylation
P24941	UNIPROT	CDK2	protein	P04183	TK1	UNIPROT	protein	YES	down-regulates	phosphorylation
P24941	UNIPROT	CDK2	protein	P30291	WEE1	UNIPROT	protein	YES	down-regulates	phosphorylation
P24941	UNIPROT	CDK2	protein	Q86WB0	ZC3HC1	UNIPROT	protein	YES	down-regulates	phosphorylation
P11802	UNIPROT	CDK4	protein	Q06830	PRDX1	UNIPROT	protein	YES	down-regulates	phosphorylation
P11802	UNIPROT	CDK4	protein	P06400	RB1	UNIPROT	protein	YES	down-regulates	phosphorylation
P11802	UNIPROT	CDK4	protein	P06400	RB1	UNIPROT	protein	YES	down-regulates	phosphorylation
P11802	UNIPROT	CDK4	protein	P06400	RB1	UNIPROT	protein	YES	down-regulates	phosphorylation
P11802	UNIPROT	CDK4	protein	P06400	RB1	UNIPROT	protein	YES	down-regulates	phosphorylation
P11802	UNIPROT	CDK4	protein	P06400	RB1	UNIPROT	protein	YES	down-regulates	phosphorylation
P11802	UNIPROT	CDK4	protein	P84022	SMAD3	UNIPROT	protein	YES	down-regulates	phosphorylation
P11802	UNIPROT	CDK4	protein	P84022	SMAD3	UNIPROT	protein	YES	down-regulates	phosphorylation
P11802	UNIPROT	CDK4	protein	P84022	SMAD3	UNIPROT	protein	YES	down-regulates	phosphorylation
Q00535	UNIPROT	CDK5	protein	P07814	EPRS	UNIPROT	protein	YES	down-regulates	phosphorylation
Q00535	UNIPROT	CDK5	protein	P07814	EPRS	UNIPROT	protein	YES	down-regulates	phosphorylation
Q00535	UNIPROT	CDK5	protein	Q8NEB9	PIK3C3	UNIPROT	protein	YES	down-regulates	phosphorylation
Q00535	UNIPROT	CDK5	protein	P62136	PPP1CA	UNIPROT	protein	YES	down-regulates	phosphorylation
Q00535	UNIPROT	CDK5	protein	P62136	PPP1CA	UNIPROT	protein	YES	down-regulates	phosphorylation
Q00535	UNIPROT	CDK5	protein	P62136	PPP1CA	UNIPROT	protein	YES	down-regulates	phosphorylation
Q00535	UNIPROT	CDK5	protein	P06400	RB1	UNIPROT	protein	YES	down-regulates	phosphorylation
Q00535	UNIPROT	CDK5	protein	P16949	STMN1	UNIPROT	protein	YES	down-regulates	phosphorylation
Q00535	UNIPROT	CDK5	protein	Q15833	STXBP2	UNIPROT	protein	YES	down-regulates	phosphorylation
Q00534	UNIPROT	CDK6	protein	P46527	CDKN1B	UNIPROT	protein	YES	down-regulates	phosphorylation
Q00534	UNIPROT	CDK6	protein	Q06830	PRDX1	UNIPROT	protein	YES	down-regulates	phosphorylation
Q00534	UNIPROT	CDK6	protein	P06400	RB1	UNIPROT	protein	YES	down-regulates	phosphorylation
P50613	UNIPROT	CDK7	protein	P24928	POLR2A	UNIPROT	protein	YES	down-regulates	phosphorylation
P50613	UNIPROT	CDK7	protein	P24928	POLR2A	UNIPROT	protein	YES	down-regulates	phosphorylation
P50613	UNIPROT	CDK7	protein	P24928	POLR2A	UNIPROT	protein	YES	down-regulates	phosphorylation
P50613	UNIPROT	CDK7	protein	P24928	POLR2A	UNIPROT	protein	YES	down-regulates	phosphorylation
P50613	UNIPROT	CDK7	protein	P24928	POLR2A	UNIPROT	protein	YES	down-regulates	phosphorylation
P50613	UNIPROT	CDK7	protein	P24928	POLR2A	UNIPROT	protein	YES	down-regulates	phosphorylation
P50613	UNIPROT	CDK7	protein	P24928	POLR2A	UNIPROT	protein	YES	down-regulates	phosphorylation
P50613	UNIPROT	CDK7	protein	P24928	POLR2A	UNIPROT	protein	YES	down-regulates	phosphorylation
P50613	UNIPROT	CDK7	protein	P24928	POLR2A	UNIPROT	protein	YES	down-regulates	phosphorylation
P50613	UNIPROT	CDK7	protein	P24928	POLR2A	UNIPROT	protein	YES	down-regulates	phosphorylation
P50613	UNIPROT	CDK7	protein	P24928	POLR2A	UNIPROT	protein	YES	down-regulates	phosphorylation
P50613	UNIPROT	CDK7	protein	P24928	POLR2A	UNIPROT	protein	YES	down-regulates	phosphorylation
P50613	UNIPROT	CDK7	protein	P24928	POLR2A	UNIPROT	protein	YES	down-regulates	phosphorylation
P50613	UNIPROT	CDK7	protein	P24928	POLR2A	UNIPROT	protein	YES	down-regulates	phosphorylation
P50613	UNIPROT	CDK7	protein	P24928	POLR2A	UNIPROT	protein	YES	down-regulates	phosphorylation
P50613	UNIPROT	CDK7	protein	P24928	POLR2A	UNIPROT	protein	YES	down-regulates	phosphorylation
P50613	UNIPROT	CDK7	protein	P24928	POLR2A	UNIPROT	protein	YES	down-regulates	phosphorylation
P50613	UNIPROT	CDK7	protein	P24928	POLR2A	UNIPROT	protein	YES	down-regulates	phosphorylation
P50613	UNIPROT	CDK7	protein	P24928	POLR2A	UNIPROT	protein	YES	down-regulates	phosphorylation
P50613	UNIPROT	CDK7	protein	P24928	POLR2A	UNIPROT	protein	YES	down-regulates	phosphorylation
P50613	UNIPROT	CDK7	protein	P24928	POLR2A	UNIPROT	protein	YES	down-regulates	phosphorylation
P50613	UNIPROT	CDK7	protein	P24928	POLR2A	UNIPROT	protein	YES	down-regulates	phosphorylation
P50613	UNIPROT	CDK7	protein	P24928	POLR2A	UNIPROT	protein	YES	down-regulates	phosphorylation
P50750	UNIPROT	CDK9	protein	Q96PM5	RCHY1	UNIPROT	protein	YES	down-regulates	phosphorylation
P50750	UNIPROT	CDK9	protein	Q96PM5	RCHY1	UNIPROT	protein	YES	down-regulates	phosphorylation
P50750	UNIPROT	CDK9	protein	Q15797	SMAD1	UNIPROT	protein	YES	down-regulates	phosphorylation
P50750	UNIPROT	CDK9	protein	Q15797	SMAD1	UNIPROT	protein	YES	down-regulates	phosphorylation
P50750	UNIPROT	CDK9	protein	Q15797	SMAD1	UNIPROT	protein	YES	down-regulates	phosphorylation
P50750	UNIPROT	CDK9	protein	Q15797	SMAD1	UNIPROT	protein	YES	down-regulates	phosphorylation
P50750	UNIPROT	CDK9	protein	Q15797	SMAD1	UNIPROT	protein	YES	down-regulates	phosphorylation
P50750	UNIPROT	CDK9	protein	Q15797	SMAD1	UNIPROT	protein	YES	down-regulates	phosphorylation
P50750	UNIPROT	CDK9	protein	Q15797	SMAD1	UNIPROT	protein	YES	down-regulates	phosphorylation
P50750	UNIPROT	CDK9	protein	P84022	SMAD3	UNIPROT	protein	YES	down-regulates	phosphorylation
P50750	UNIPROT	CDK9	protein	P84022	SMAD3	UNIPROT	protein	YES	down-regulates	phosphorylation
P50750	UNIPROT	CDK9	protein	P84022	SMAD3	UNIPROT	protein	YES	down-regulates	phosphorylation
P50750	UNIPROT	CDK9	protein	P84022	SMAD3	UNIPROT	protein	YES	down-regulates	phosphorylation
P50750	UNIPROT	CDK9	protein	P84022	SMAD3	UNIPROT	protein	YES	down-regulates	phosphorylation
P50750	UNIPROT	CDK9	protein	P84022	SMAD3	UNIPROT	protein	YES	down-regulates	phosphorylation
O14757	UNIPROT	CHEK1	protein	P19838	NFKB1	UNIPROT	protein	YES	down-regulates	phosphorylation
O14757	UNIPROT	CHEK1	protein	P43405	SYK	UNIPROT	protein	YES	down-regulates	phosphorylation
O14757	UNIPROT	CHEK1	protein	Q9UKI8	TLK1	UNIPROT	protein	YES	down-regulates	phosphorylation
O15111	UNIPROT	CHUK	protein	O43524	FOXO3	UNIPROT	protein	YES	down-regulates	phosphorylation
O15111	UNIPROT	CHUK	protein	Q9Y618	NCOR2	UNIPROT	protein	YES	down-regulates	phosphorylation
O15111	UNIPROT	CHUK	protein	P49815	TSC2	UNIPROT	protein	YES	down-regulates	phosphorylation
P41240	UNIPROT	CSK	protein	P12931	SRC	UNIPROT	protein	YES	down-regulates	phosphorylation
P48729	UNIPROT	CSNK1A1	protein	P35222	CTNNB1	UNIPROT	protein	YES	down-regulates	phosphorylation
P48729	UNIPROT	CSNK1A1	protein	Q9NRF8	CTPS2	UNIPROT	protein	YES	down-regulates	phosphorylation
P48729	UNIPROT	CSNK1A1	protein	Q8TB45	DEPTOR	UNIPROT	protein	YES	down-regulates	phosphorylation
P48729	UNIPROT	CSNK1A1	protein	Q8TB45	DEPTOR	UNIPROT	protein	YES	down-regulates	phosphorylation
P48729	UNIPROT	CSNK1A1	protein	Q8TB45	DEPTOR	UNIPROT	protein	YES	down-regulates	phosphorylation
P48729	UNIPROT	CSNK1A1	protein	Q13158	FADD	UNIPROT	protein	YES	down-regulates	phosphorylation
P48729	UNIPROT	CSNK1A1	protein	Q12778	FOXO1	UNIPROT	protein	YES	down-regulates	phosphorylation
P48729	UNIPROT	CSNK1A1	protein	O43524	FOXO3	UNIPROT	protein	YES	down-regulates	phosphorylation
P48729	UNIPROT	CSNK1A1	protein	O43524	FOXO3	UNIPROT	protein	YES	down-regulates	phosphorylation
P48729	UNIPROT	CSNK1A1	protein	O43524	FOXO3	UNIPROT	protein	YES	down-regulates	phosphorylation
P48729	UNIPROT	CSNK1A1	protein	A8MYZ6	FOXO6	UNIPROT	protein	YES	down-regulates	phosphorylation
P48729	UNIPROT	CSNK1A1	protein	P07910	HNRNPC	UNIPROT	protein	YES	down-regulates	phosphorylation
P48729	UNIPROT	CSNK1A1	protein	P62745	RHOB	UNIPROT	protein	YES	down-regulates	phosphorylation
Q8N752	UNIPROT	CSNK1A1L	protein	P35222	CTNNB1	UNIPROT	protein	YES	down-regulates	phosphorylation
P48730	UNIPROT	CSNK1D	protein	P35222	CTNNB1	UNIPROT	protein	YES	down-regulates	phosphorylation
P48730	UNIPROT	CSNK1D	protein	P46937	YAP1	UNIPROT	protein	YES	down-regulates	phosphorylation
P48730	UNIPROT	CSNK1D	protein	P46937	YAP1	UNIPROT	protein	YES	down-regulates	phosphorylation
P48730	UNIPROT	CSNK1D	protein	P46937	YAP1	UNIPROT	protein	YES	down-regulates	phosphorylation
P48730	UNIPROT	CSNK1D	protein	P46937	YAP1	UNIPROT	protein	YES	down-regulates	phosphorylation
P49674	UNIPROT	CSNK1E	protein	P35222	CTNNB1	UNIPROT	protein	YES	down-regulates	phosphorylation
P49674	UNIPROT	CSNK1E	protein	O60716	CTNND1	UNIPROT	protein	YES	down-regulates	phosphorylation
P49674	UNIPROT	CSNK1E	protein	Q13541	EIF4EBP1	UNIPROT	protein	YES	down-regulates	phosphorylation
P49674	UNIPROT	CSNK1E	protein	Q13541	EIF4EBP1	UNIPROT	protein	YES	down-regulates	phosphorylation
P49674	UNIPROT	CSNK1E	protein	P46937	YAP1	UNIPROT	protein	YES	down-regulates	phosphorylation
P49674	UNIPROT	CSNK1E	protein	P46937	YAP1	UNIPROT	protein	YES	down-regulates	phosphorylation
P49674	UNIPROT	CSNK1E	protein	P46937	YAP1	UNIPROT	protein	YES	down-regulates	phosphorylation

P49674	UNIPROT	CSNK1E	protein	P46937	YAP1	UNIPROT	protein	YES	down-regulates	phosphorylation
P68400	UNIPROT	CSNK2A1	protein	Q15019		UNIPROT	protein	YES	down-regulates	phosphorylation
P68400	UNIPROT	CSNK2A1	protein	P18846	ATF1	UNIPROT	protein	YES	down-regulates	phosphorylation
P68400	UNIPROT	CSNK2A1	protein	P18846	ATF1	UNIPROT	protein	YES	down-regulates	phosphorylation
P68400	UNIPROT	CSNK2A1	protein	P18846	ATF1	UNIPROT	protein	YES	down-regulates	phosphorylation
P68400	UNIPROT	CSNK2A1	protein	P18846	ATF1	UNIPROT	protein	YES	down-regulates	phosphorylation
P68400	UNIPROT	CSNK2A1	protein	Q9H257	CARD9	UNIPROT	protein	YES	down-regulates	phosphorylation
P68400	UNIPROT	CSNK2A1	protein	Q9H257	CARD9	UNIPROT	protein	YES	down-regulates	phosphorylation
P68400	UNIPROT	CSNK2A1	protein	P42575	CASP2	UNIPROT	protein	YES	down-regulates	phosphorylation
P68400	UNIPROT	CSNK2A1	protein	P83916	CBX1	UNIPROT	protein	YES	down-regulates	phosphorylation
P68400	UNIPROT	CSNK2A1	protein	P35221	CTNNA1	UNIPROT	protein	YES	down-regulates	phosphorylation
P68400	UNIPROT	CSNK2A1	protein	O95786	DDX58	UNIPROT	protein	YES	down-regulates	phosphorylation
P68400	UNIPROT	CSNK2A1	protein	O95786	DDX58	UNIPROT	protein	YES	down-regulates	phosphorylation
P68400	UNIPROT	CSNK2A1	protein	O95786	DDX58	UNIPROT	protein	YES	down-regulates	phosphorylation
P68400	UNIPROT	CSNK2A1	protein	Q13541	EIF4EBP1	UNIPROT	protein	YES	down-regulates	phosphorylation
P68400	UNIPROT	CSNK2A1	protein	P05114	HMGN1	UNIPROT	protein	YES	down-regulates	phosphorylation
P68400	UNIPROT	CSNK2A1	protein	P05114	HMGN1	UNIPROT	protein	YES	down-regulates	phosphorylation
P68400	UNIPROT	CSNK2A1	protein	P05114	HMGN1	UNIPROT	protein	YES	down-regulates	phosphorylation
P68400	UNIPROT	CSNK2A1	protein	P05114	HMGN1	UNIPROT	protein	YES	down-regulates	phosphorylation
P68400	UNIPROT	CSNK2A1	protein	P08238	HSP90AB1	UNIPROT	protein	YES	down-regulates	phosphorylation
P68400	UNIPROT	CSNK2A1	protein	P08238	HSP90AB1	UNIPROT	protein	YES	down-regulates	phosphorylation
P68400	UNIPROT	CSNK2A1	protein	P05412	JUN	UNIPROT	protein	YES	down-regulates	phosphorylation
P68400	UNIPROT	CSNK2A1	protein	P05412	JUN	UNIPROT	protein	YES	down-regulates	phosphorylation
P68400	UNIPROT	CSNK2A1	protein	P29590	PML	UNIPROT	protein	YES	down-regulates	phosphorylation
P68400	UNIPROT	CSNK2A1	protein	P29590	PML	UNIPROT	protein	YES	down-regulates	phosphorylation
P68400	UNIPROT	CSNK2A1	protein	Q9NYV6	RRN3	UNIPROT	protein	YES	down-regulates	phosphorylation
P68400	UNIPROT	CSNK2A1	protein	Q9NYV6	RRN3	UNIPROT	protein	YES	down-regulates	phosphorylation
P68400	UNIPROT	CSNK2A1	protein	Q01105	SET	UNIPROT	protein	YES	down-regulates	phosphorylation
P68400	UNIPROT	CSNK2A1	protein	Q9H2G2	SLK	UNIPROT	protein	YES	down-regulates	phosphorylation
P68400	UNIPROT	CSNK2A1	protein	Q9H2G2	SLK	UNIPROT	protein	YES	down-regulates	phosphorylation
P68400	UNIPROT	CSNK2A1	protein	Q01082	SPTBN1	UNIPROT	protein	YES	down-regulates	phosphorylation
P68400	UNIPROT	CSNK2A1	protein	Q01082	SPTBN1	UNIPROT	protein	YES	down-regulates	phosphorylation
P68400	UNIPROT	CSNK2A1	protein	Q9Y365	STARD10	UNIPROT	protein	YES	down-regulates	phosphorylation
P68400	UNIPROT	CSNK2A1	protein	Q9Y4R8	TELO2	UNIPROT	protein	YES	down-regulates	phosphorylation
P68400	UNIPROT	CSNK2A1	protein	Q9Y4R8	TELO2	UNIPROT	protein	YES	down-regulates	phosphorylation
P68400	UNIPROT	CSNK2A1	protein	Q9Y4R8	TELO2	UNIPROT	protein	YES	down-regulates	phosphorylation
P68400	UNIPROT	CSNK2A1	protein	Q9Y4R8	TELO2	UNIPROT	protein	YES	down-regulates	phosphorylation
P68400	UNIPROT	CSNK2A1	protein	O43156	TTI1	UNIPROT	protein	YES	down-regulates	phosphorylation
P68400	UNIPROT	CSNK2A1	protein	Q9Y6W5	WASF2	UNIPROT	protein	YES	down-regulates	phosphorylation
P68400	UNIPROT	CSNK2A1	protein	Q9Y6W5	WASF2	UNIPROT	protein	YES	down-regulates	phosphorylation
P68400	UNIPROT	CSNK2A1	protein	Q9Y6W5	WASF2	UNIPROT	protein	YES	down-regulates	phosphorylation
P68400	UNIPROT	CSNK2A1	protein	Q9Y6W5	WASF2	UNIPROT	protein	YES	down-regulates	phosphorylation
P19784	UNIPROT	CSNK2A2	protein	Q01105	SET	UNIPROT	protein	YES	down-regulates	phosphorylation
P67870	UNIPROT	CSNK2B	protein	Q01105	SET	UNIPROT	protein	YES	down-regulates	phosphorylation
Q13627	UNIPROT	DYRK1A	protein	Q05193	DNM1	UNIPROT	protein	YES	down-regulates	phosphorylation
Q13627	UNIPROT	DYRK1A	protein	Q05193	DNM1	UNIPROT	protein	YES	down-regulates	phosphorylation
Q13627	UNIPROT	DYRK1A	protein	Q12778	FOXO1	UNIPROT	protein	YES	down-regulates	phosphorylation
Q13627	UNIPROT	DYRK1A	protein	O43524	FOXO3	UNIPROT	protein	YES	down-regulates	phosphorylation
Q13627	UNIPROT	DYRK1A	protein	A8MYZ6	FOXO6	UNIPROT	protein	YES	down-regulates	phosphorylation
O00418	UNIPROT	EEF2K	protein	P13639	EEF2	UNIPROT	protein	YES	down-regulates	phosphorylation
O00418	UNIPROT	EEF2K	protein	P13639	EEF2	UNIPROT	protein	YES	down-regulates	phosphorylation
O00418	UNIPROT	EEF2K	protein	P13639	EEF2	UNIPROT	protein	YES	down-regulates	phosphorylation
O00418	UNIPROT	EEF2K	protein	P13639	EEF2	UNIPROT	protein	YES	down-regulates	phosphorylation
O00418	UNIPROT	EEF2K	protein	O00418	EEF2K	UNIPROT	protein	YES	down-regulates	phosphorylation
P00533	UNIPROT	EGFR	protein	P11171	EPB41	UNIPROT	protein	YES	down-regulates	phosphorylation
P00533	UNIPROT	EGFR	protein	Q9UBN7	HDAC6	UNIPROT	protein	YES	down-regulates	phosphorylation
P19525	UNIPROT	EIF2AK2	protein	P06493	CDK1	UNIPROT	protein	YES	down-regulates	phosphorylation
P19525	UNIPROT	EIF2AK2	protein	P05198	EIF2S1	UNIPROT	protein	YES	down-regulates	phosphorylation
P04626	UNIPROT	ERBB2	protein	P06493	CDK1	UNIPROT	protein	YES	down-regulates	phosphorylation
P04626	UNIPROT	ERBB2	protein	P04626	ERBB2	UNIPROT	protein	YES	down-regulates	phosphorylation
P04626	UNIPROT	ERBB2	protein	P04626	ERBB2	UNIPROT	protein	YES	down-regulates	phosphorylation
P25098	UNIPROT	GRK2	protein	Q16539	MAPK14	UNIPROT	protein	YES	down-regulates	phosphorylation
P49840	UNIPROT	GSK3A	protein	P13807	GYS1	UNIPROT	protein	YES	down-regulates	phosphorylation
P49840	UNIPROT	GSK3A	protein	P05412	JUN	UNIPROT	protein	YES	down-regulates	phosphorylation
P49840	UNIPROT	GSK3A	protein	P05412	JUN	UNIPROT	protein	YES	down-regulates	phosphorylation
P49840	UNIPROT	GSK3A	protein	P05412	JUN	UNIPROT	protein	YES	down-regulates	phosphorylation
P49840	UNIPROT	GSK3A	protein	P05412	JUN	UNIPROT	protein	YES	down-regulates	phosphorylation
P49840	UNIPROT	GSK3A	protein	P01106	MYC	UNIPROT	protein	YES	down-regulates	phosphorylation
P49840	UNIPROT	GSK3A	protein	P23246	SFPQ	UNIPROT	protein	YES	down-regulates	phosphorylation
P49840	UNIPROT	GSK3A	protein	P23246	SFPQ	UNIPROT	protein	YES	down-regulates	phosphorylation
P49841	UNIPROT	GSK3B	protein	Q13144	EIF2B5	UNIPROT	protein	YES	down-regulates	phosphorylation
P49841	UNIPROT	GSK3B	protein	Q9NQX3	GPHN	UNIPROT	protein	YES	down-regulates	phosphorylation
P49841	UNIPROT	GSK3B	protein	Q14103	HNRNPD	UNIPROT	protein	YES	down-regulates	phosphorylation
P49841	UNIPROT	GSK3B	protein	Q00613	HSF1	UNIPROT	protein	YES	down-regulates	phosphorylation
P49841	UNIPROT	GSK3B	protein	Q13887	KLF5	UNIPROT	protein	YES	down-regulates	phosphorylation
P49841	UNIPROT	GSK3B	protein	Q07820	MCL1	UNIPROT	protein	YES	down-regulates	phosphorylation
P49841	UNIPROT	GSK3B	protein	E9PAV3	NACA	UNIPROT	protein	YES	down-regulates	phosphorylation
P49841	UNIPROT	GSK3B	protein	Q9H0Z9	RBM38	UNIPROT	protein	YES	down-regulates	phosphorylation
P49841	UNIPROT	GSK3B	protein	P23246	SFPQ	UNIPROT	protein	YES	down-regulates	phosphorylation
P49841	UNIPROT	GSK3B	protein	P23246	SFPQ	UNIPROT	protein	YES	down-regulates	phosphorylation
P49841	UNIPROT	GSK3B	protein	Q15797	SMAD1	UNIPROT	protein	YES	down-regulates	phosphorylation
P35269	UNIPROT	GTF2F1	protein	P35269	GTF2F1	UNIPROT	protein	YES	down-regulates	phosphorylation
P35269	UNIPROT	GTF2F1	protein	P35269	GTF2F1	UNIPROT	protein	YES	down-regulates	phosphorylation
Q13418	UNIPROT	ILK	protein	P23528	CFL1	UNIPROT	protein	YES	down-regulates	phosphorylation
Q13418	UNIPROT	ILK	protein	O14974	PPP1R12A	UNIPROT	protein	YES	down-regulates	phosphorylation
Q13418	UNIPROT	ILK	protein	O14974	PPP1R12A	UNIPROT	protein	YES	down-regulates	phosphorylation
Q02750	UNIPROT	MAP2K1	protein	P35568	IRS1	UNIPROT	protein	YES	down-regulates	phosphorylation
P45985	UNIPROT	MAP2K4	protein	P19793	RXRA	UNIPROT	protein	YES	down-regulates	phosphorylation
O14733	UNIPROT	MAP2K7	protein	Q13158	FADD	UNIPROT	protein	YES	down-regulates	phosphorylation
O43318	UNIPROT	MAP3K7	protein	Q8WUI4	HDAC7	UNIPROT	protein	YES	down-regulates	phosphorylation
O43318	UNIPROT	MAP3K7	protein	Q15653	NFKBIB	UNIPROT	protein	YES	down-regulates	phosphorylation
P28482	UNIPROT	MAPK1	protein	P49407	ARRB1	UNIPROT	protein	YES	down-regulates	phosphorylation
P28482	UNIPROT	MAPK1	protein	P49407	ARRB1	UNIPROT	protein	YES	down-regulates	phosphorylation
P28482	UNIPROT	MAPK1	protein	P49407	ARRB1	UNIPROT	protein	YES	down-regulates	phosphorylation
P28482	UNIPROT	MAPK1	protein	Q14790	CASP8	UNIPROT	protein	YES	down-regulates	phosphorylation
P28482	UNIPROT	MAPK1	protein	P46527	CDKN1B	UNIPROT	protein	YES	down-regulates	phosphorylation
P28482	UNIPROT	MAPK1	protein	Q53EZ4	CEP55	UNIPROT	protein	YES	down-regulates	phosphorylation
P28482	UNIPROT	MAPK1	protein	Q53EZ4	CEP55	UNIPROT	protein	YES	down-regulates	phosphorylation

P28482	UNIPROT	MAPK1	protein	Q96RK0	CIC	UNIPROT	protein	YES	down-regulates	phosphorylation
P28482	UNIPROT	MAPK1	protein	Q13541	EIF4EBP1	UNIPROT	protein	YES	down-regulates	phosphorylation
P28482	UNIPROT	MAPK1	protein	P50548	ERF	UNIPROT	protein	YES	down-regulates	phosphorylation
P28482	UNIPROT	MAPK1	protein	P49841	GSK3B	UNIPROT	protein	YES	down-regulates	phosphorylation
P28482	UNIPROT	MAPK1	protein	P35568	IRS1	UNIPROT	protein	YES	down-regulates	phosphorylation
P28482	UNIPROT	MAPK1	protein	P35568	IRS1	UNIPROT	protein	YES	down-regulates	phosphorylation
P28482	UNIPROT	MAPK1	protein	Q07866	KLC1	UNIPROT	protein	YES	down-regulates	phosphorylation
P28482	UNIPROT	MAPK1	protein	Q9UKX7	NUP50	UNIPROT	protein	YES	down-regulates	phosphorylation
P28482	UNIPROT	MAPK1	protein	P49585	PCYT1A	UNIPROT	protein	YES	down-regulates	phosphorylation
P28482	UNIPROT	MAPK1	protein	P24928	POLR2A	UNIPROT	protein	YES	down-regulates	phosphorylation
P28482	UNIPROT	MAPK1	protein	P24928	POLR2A	UNIPROT	protein	YES	down-regulates	phosphorylation
P28482	UNIPROT	MAPK1	protein	P24928	POLR2A	UNIPROT	protein	YES	down-regulates	phosphorylation
P28482	UNIPROT	MAPK1	protein	P24928	POLR2A	UNIPROT	protein	YES	down-regulates	phosphorylation
P28482	UNIPROT	MAPK1	protein	P24928	POLR2A	UNIPROT	protein	YES	down-regulates	phosphorylation
P28482	UNIPROT	MAPK1	protein	P24928	POLR2A	UNIPROT	protein	YES	down-regulates	phosphorylation
P28482	UNIPROT	MAPK1	protein	P24928	POLR2A	UNIPROT	protein	YES	down-regulates	phosphorylation
P28482	UNIPROT	MAPK1	protein	P24928	POLR2A	UNIPROT	protein	YES	down-regulates	phosphorylation
P28482	UNIPROT	MAPK1	protein	P24928	POLR2A	UNIPROT	protein	YES	down-regulates	phosphorylation
P28482	UNIPROT	MAPK1	protein	P24928	POLR2A	UNIPROT	protein	YES	down-regulates	phosphorylation
P28482	UNIPROT	MAPK1	protein	P24928	POLR2A	UNIPROT	protein	YES	down-regulates	phosphorylation
P28482	UNIPROT	MAPK1	protein	P24928	POLR2A	UNIPROT	protein	YES	down-regulates	phosphorylation
P28482	UNIPROT	MAPK1	protein	P24928	POLR2A	UNIPROT	protein	YES	down-regulates	phosphorylation
P28482	UNIPROT	MAPK1	protein	P24928	POLR2A	UNIPROT	protein	YES	down-regulates	phosphorylation
P28482	UNIPROT	MAPK1	protein	P24928	POLR2A	UNIPROT	protein	YES	down-regulates	phosphorylation
P28482	UNIPROT	MAPK1	protein	P24928	POLR2A	UNIPROT	protein	YES	down-regulates	phosphorylation
P28482	UNIPROT	MAPK1	protein	P24928	POLR2A	UNIPROT	protein	YES	down-regulates	phosphorylation
P28482	UNIPROT	MAPK1	protein	P24928	POLR2A	UNIPROT	protein	YES	down-regulates	phosphorylation
P28482	UNIPROT	MAPK1	protein	P24928	POLR2A	UNIPROT	protein	YES	down-regulates	phosphorylation
P28482	UNIPROT	MAPK1	protein	P24928	POLR2A	UNIPROT	protein	YES	down-regulates	phosphorylation
P28482	UNIPROT	MAPK1	protein	P24928	POLR2A	UNIPROT	protein	YES	down-regulates	phosphorylation
P28482	UNIPROT	MAPK1	protein	P24928	POLR2A	UNIPROT	protein	YES	down-regulates	phosphorylation
P28482	UNIPROT	MAPK1	protein	Q13362	PPP2R5C	UNIPROT	protein	YES	down-regulates	phosphorylation
P28482	UNIPROT	MAPK1	protein	P19793	RXRA	UNIPROT	protein	YES	down-regulates	phosphorylation
P28482	UNIPROT	MAPK1	protein	Q15797	SMAD1	UNIPROT	protein	YES	down-regulates	phosphorylation
P28482	UNIPROT	MAPK1	protein	Q15797	SMAD1	UNIPROT	protein	YES	down-regulates	phosphorylation
P28482	UNIPROT	MAPK1	protein	Q15797	SMAD1	UNIPROT	protein	YES	down-regulates	phosphorylation
P28482	UNIPROT	MAPK1	protein	Q15797	SMAD1	UNIPROT	protein	YES	down-regulates	phosphorylation
P28482	UNIPROT	MAPK1	protein	Q15797	SMAD1	UNIPROT	protein	YES	down-regulates	phosphorylation
P28482	UNIPROT	MAPK1	protein	Q15797	SMAD1	UNIPROT	protein	YES	down-regulates	phosphorylation
P28482	UNIPROT	MAPK1	protein	Q15797	SMAD1	UNIPROT	protein	YES	down-regulates	phosphorylation
P28482	UNIPROT	MAPK1	protein	Q15797	SMAD1	UNIPROT	protein	YES	down-regulates	phosphorylation
P28482	UNIPROT	MAPK1	protein	Q15797	SMAD1	UNIPROT	protein	YES	down-regulates	phosphorylation
P28482	UNIPROT	MAPK1	protein	Q15797	SMAD1	UNIPROT	protein	YES	down-regulates	phosphorylation
P28482	UNIPROT	MAPK1	protein	Q15797	SMAD1	UNIPROT	protein	YES	down-regulates	phosphorylation
P28482	UNIPROT	MAPK1	protein	Q15796	SMAD2	UNIPROT	protein	YES	down-regulates	phosphorylation
P28482	UNIPROT	MAPK1	protein	P84022	SMAD3	UNIPROT	protein	YES	down-regulates	phosphorylation
P28482	UNIPROT	MAPK1	protein	P84022	SMAD3	UNIPROT	protein	YES	down-regulates	phosphorylation
P28482	UNIPROT	MAPK1	protein	P84022	SMAD3	UNIPROT	protein	YES	down-regulates	phosphorylation
P28482	UNIPROT	MAPK1	protein	P84022	SMAD3	UNIPROT	protein	YES	down-regulates	phosphorylation
P28482	UNIPROT	MAPK1	protein	P84022	SMAD3	UNIPROT	protein	YES	down-regulates	phosphorylation
P28482	UNIPROT	MAPK1	protein	P84022	SMAD3	UNIPROT	protein	YES	down-regulates	phosphorylation
P28482	UNIPROT	MAPK1	protein	P84022	SMAD3	UNIPROT	protein	YES	down-regulates	phosphorylation
P28482	UNIPROT	MAPK1	protein	P84022	SMAD3	UNIPROT	protein	YES	down-regulates	phosphorylation
P28482	UNIPROT	MAPK1	protein	P84022	SMAD3	UNIPROT	protein	YES	down-regulates	phosphorylation
P28482	UNIPROT	MAPK1	protein	P16949	STMN1	UNIPROT	protein	YES	down-regulates	phosphorylation
P28482	UNIPROT	MAPK1	protein	Q12800	TFCP2	UNIPROT	protein	YES	down-regulates	phosphorylation
P28482	UNIPROT	MAPK1	protein	Q12800	TFCP2	UNIPROT	protein	YES	down-regulates	phosphorylation
P28482	UNIPROT	MAPK1	protein	P50616	TOB1	UNIPROT	protein	YES	down-regulates	phosphorylation
P28482	UNIPROT	MAPK1	protein	P50616	TOB1	UNIPROT	protein	YES	down-regulates	phosphorylation
P28482	UNIPROT	MAPK1	protein	P50616	TOB1	UNIPROT	protein	YES	down-regulates	phosphorylation
P28482	UNIPROT	MAPK1	protein	Q92574	TSC1	UNIPROT	protein	YES	down-regulates	phosphorylation
P28482	UNIPROT	MAPK1	protein	Q92574	TSC1	UNIPROT	protein	YES	down-regulates	phosphorylation
P28482	UNIPROT	MAPK1	protein	P49815	TSC2	UNIPROT	protein	YES	down-regulates	phosphorylation
P28482	UNIPROT	MAPK1	protein	P17480	UBTF	UNIPROT	protein	YES	down-regulates	phosphorylation
P28482	UNIPROT	MAPK1	protein	P17480	UBTF	UNIPROT	protein	YES	down-regulates	phosphorylation
O15264	UNIPROT	MAPK13	protein	O00418	EEF2K	UNIPROT	protein	YES	down-regulates	phosphorylation
O15264	UNIPROT	MAPK13	protein	P16949	STMN1	UNIPROT	protein	YES	down-regulates	phosphorylation
O15264	UNIPROT	MAPK13	protein	P16949	STMN1	UNIPROT	protein	YES	down-regulates	phosphorylation
O15264	UNIPROT	MAPK13	protein	P16949	STMN1	UNIPROT	protein	YES	down-regulates	phosphorylation
O15264	UNIPROT	MAPK13	protein	P16949	STMN1	UNIPROT	protein	YES	down-regulates	phosphorylation
Q16539	UNIPROT	MAPK14	protein	P42574	CASP3	UNIPROT	protein	YES	down-regulates	phosphorylation
Q16539	UNIPROT	MAPK14	protein	Q14790	CASP8	UNIPROT	protein	YES	down-regulates	phosphorylation
Q16539	UNIPROT	MAPK14	protein	P00533	EGFR	UNIPROT	protein	YES	down-regulates	phosphorylation
Q16539	UNIPROT	MAPK14	protein	P49841	GSK3B	UNIPROT	protein	YES	down-regulates	phosphorylation
Q16539	UNIPROT	MAPK14	protein	P49841	GSK3B	UNIPROT	protein	YES	down-regulates	phosphorylation
Q16539	UNIPROT	MAPK14	protein	P78356	PIP4K2B	UNIPROT	protein	YES	down-regulates	phosphorylation
Q16539	UNIPROT	MAPK14	protein	P06400	RB1	UNIPROT	protein	YES	down-regulates	phosphorylation
P27361	UNIPROT	MAPK3	protein	P49407	ARRB1	UNIPROT	protein	YES	down-regulates	phosphorylation
P27361	UNIPROT	MAPK3	protein	P49407	ARRB1	UNIPROT	protein	YES	down-regulates	phosphorylation
P27361	UNIPROT	MAPK3	protein	P49407	ARRB1	UNIPROT	protein	YES	down-regulates	phosphorylation
P27361	UNIPROT	MAPK3	protein	Q92934	BAD	UNIPROT	protein	YES	down-regulates	phosphorylation
P27361	UNIPROT	MAPK3	protein	P15056	BRAF	UNIPROT	protein	YES	down-regulates	phosphorylation
P27361	UNIPROT	MAPK3	protein	P15056	BRAF	UNIPROT	protein	YES	down-regulates	phosphorylation
P27361	UNIPROT	MAPK3	protein	Q14790	CASP8	UNIPROT	protein	YES	down-regulates	phosphorylation
P27361	UNIPROT	MAPK3	protein	P46527	CDKN1B	UNIPROT	protein	YES	down-regulates	phosphorylation
P27361	UNIPROT	MAPK3	protein	Q96RK0	CIC	UNIPROT	protein	YES	down-regulates	phosphorylation
P27361	UNIPROT	MAPK3	protein	P00533	EGFR	UNIPROT	protein	YES	down-regulates	phosphorylation
P27361	UNIPROT	MAPK3	protein	P41212	ETV6	UNIPROT	protein	YES	down-regulates	phosphorylation
P27361	UNIPROT	MAPK3	protein	P25098	GRK2	UNIPROT	protein	YES	down-regulates	phosphorylation
P27361	UNIPROT	MAPK3	protein	P49841	GSK3B	UNIPROT	protein	YES	down-regulates	phosphorylation
P27361	UNIPROT	MAPK3	protein	P61978	HNRNPK	UNIPROT	protein	YES	down-regulates	phosphorylation
P27361	UNIPROT	MAPK3	protein	Q00613	HSF1	UNIPROT	protein	YES	down-regulates	phosphorylation
P27361	UNIPROT	MAPK3	protein	Q07866	KLC1	UNIPROT	protein	YES	down-regulates	phosphorylation
P27361	UNIPROT	MAPK3	protein	Q9UKX7	NUP50	UNIPROT	protein	YES	down-regulates	phosphorylation
P27361	UNIPROT	MAPK3	protein	Q9UKX7	NUP50	UNIPROT	protein	YES	down-regulates	phosphorylation
P27361	UNIPROT	MAPK3	protein	Q13153	PAK1	UNIPROT	protein	YES	down-regulates	phosphorylation
P27361	UNIPROT	MAPK3	protein	P49585	PCYT1A	UNIPROT	protein	YES	down-regulates	phosphorylation

P27361	UNIPROT	MAPK3	protein	P24928	POLR2A	UNIPROT	protein	YES	down-regulates	phosphorylation
P27361	UNIPROT	MAPK3	protein	P24928	POLR2A	UNIPROT	protein	YES	down-regulates	phosphorylation
P27361	UNIPROT	MAPK3	protein	P24928	POLR2A	UNIPROT	protein	YES	down-regulates	phosphorylation
P27361	UNIPROT	MAPK3	protein	P24928	POLR2A	UNIPROT	protein	YES	down-regulates	phosphorylation
P27361	UNIPROT	MAPK3	protein	P24928	POLR2A	UNIPROT	protein	YES	down-regulates	phosphorylation
P27361	UNIPROT	MAPK3	protein	P24928	POLR2A	UNIPROT	protein	YES	down-regulates	phosphorylation
P27361	UNIPROT	MAPK3	protein	P24928	POLR2A	UNIPROT	protein	YES	down-regulates	phosphorylation
P27361	UNIPROT	MAPK3	protein	P24928	POLR2A	UNIPROT	protein	YES	down-regulates	phosphorylation
P27361	UNIPROT	MAPK3	protein	P24928	POLR2A	UNIPROT	protein	YES	down-regulates	phosphorylation
P27361	UNIPROT	MAPK3	protein	P24928	POLR2A	UNIPROT	protein	YES	down-regulates	phosphorylation
P27361	UNIPROT	MAPK3	protein	P24928	POLR2A	UNIPROT	protein	YES	down-regulates	phosphorylation
P27361	UNIPROT	MAPK3	protein	P24928	POLR2A	UNIPROT	protein	YES	down-regulates	phosphorylation
P27361	UNIPROT	MAPK3	protein	P24928	POLR2A	UNIPROT	protein	YES	down-regulates	phosphorylation
P27361	UNIPROT	MAPK3	protein	P24928	POLR2A	UNIPROT	protein	YES	down-regulates	phosphorylation
P27361	UNIPROT	MAPK3	protein	P24928	POLR2A	UNIPROT	protein	YES	down-regulates	phosphorylation
P27361	UNIPROT	MAPK3	protein	P24928	POLR2A	UNIPROT	protein	YES	down-regulates	phosphorylation
P27361	UNIPROT	MAPK3	protein	P24928	POLR2A	UNIPROT	protein	YES	down-regulates	phosphorylation
P27361	UNIPROT	MAPK3	protein	P24928	POLR2A	UNIPROT	protein	YES	down-regulates	phosphorylation
P27361	UNIPROT	MAPK3	protein	P24928	POLR2A	UNIPROT	protein	YES	down-regulates	phosphorylation
P27361	UNIPROT	MAPK3	protein	P24928	POLR2A	UNIPROT	protein	YES	down-regulates	phosphorylation
P27361	UNIPROT	MAPK3	protein	P24928	POLR2A	UNIPROT	protein	YES	down-regulates	phosphorylation
P27361	UNIPROT	MAPK3	protein	P24928	POLR2A	UNIPROT	protein	YES	down-regulates	phosphorylation
P27361	UNIPROT	MAPK3	protein	P24928	POLR2A	UNIPROT	protein	YES	down-regulates	phosphorylation
P27361	UNIPROT	MAPK3	protein	Q13362	PPP2R5C	UNIPROT	protein	YES	down-regulates	phosphorylation
P27361	UNIPROT	MAPK3	protein	Q15797	SMAD1	UNIPROT	protein	YES	down-regulates	phosphorylation
P27361	UNIPROT	MAPK3	protein	Q15796	SMAD2	UNIPROT	protein	YES	down-regulates	phosphorylation
P27361	UNIPROT	MAPK3	protein	P84022	SMAD3	UNIPROT	protein	YES	down-regulates	phosphorylation
P27361	UNIPROT	MAPK3	protein	Q07889	SOS1	UNIPROT	protein	YES	down-regulates	phosphorylation
P27361	UNIPROT	MAPK3	protein	Q07889	SOS1	UNIPROT	protein	YES	down-regulates	phosphorylation
P27361	UNIPROT	MAPK3	protein	Q07889	SOS1	UNIPROT	protein	YES	down-regulates	phosphorylation
P27361	UNIPROT	MAPK3	protein	Q07889	SOS1	UNIPROT	protein	YES	down-regulates	phosphorylation
P27361	UNIPROT	MAPK3	protein	Q07889	SOS1	UNIPROT	protein	YES	down-regulates	phosphorylation
P27361	UNIPROT	MAPK3	protein	Q12800	TFCP2	UNIPROT	protein	YES	down-regulates	phosphorylation
P27361	UNIPROT	MAPK3	protein	Q12800	TFCP2	UNIPROT	protein	YES	down-regulates	phosphorylation
P27361	UNIPROT	MAPK3	protein	P50616	TOB1	UNIPROT	protein	YES	down-regulates	phosphorylation
P27361	UNIPROT	MAPK3	protein	P50616	TOB1	UNIPROT	protein	YES	down-regulates	phosphorylation
P27361	UNIPROT	MAPK3	protein	P50616	TOB1	UNIPROT	protein	YES	down-regulates	phosphorylation
P27361	UNIPROT	MAPK3	protein	P50616	TOB1	UNIPROT	protein	YES	down-regulates	phosphorylation
P27361	UNIPROT	MAPK3	protein	P50616	TOB1	UNIPROT	protein	YES	down-regulates	phosphorylation
P27361	UNIPROT	MAPK3	protein	P50616	TOB1	UNIPROT	protein	YES	down-regulates	phosphorylation
P27361	UNIPROT	MAPK3	protein	P17480	UBTF	UNIPROT	protein	YES	down-regulates	phosphorylation
P27361	UNIPROT	MAPK3	protein	P17480	UBTF	UNIPROT	protein	YES	down-regulates	phosphorylation
P45983	UNIPROT	MAPK8	protein	Q12904	AIMP1	UNIPROT	protein	YES	down-regulates	phosphorylation
P45983	UNIPROT	MAPK8	protein	Q92934	BAD	UNIPROT	protein	YES	down-regulates	phosphorylation
P45983	UNIPROT	MAPK8	protein	Q07817	BCL2L1	UNIPROT	protein	YES	down-regulates	phosphorylation
P45983	UNIPROT	MAPK8	protein	Q13363	CTBP1	UNIPROT	protein	YES	down-regulates	phosphorylation
P45983	UNIPROT	MAPK8	protein	P35568	IRS1	UNIPROT	protein	YES	down-regulates	phosphorylation
P45983	UNIPROT	MAPK8	protein	P35568	IRS1	UNIPROT	protein	YES	down-regulates	phosphorylation
P45983	UNIPROT	MAPK8	protein	P35568	IRS1	UNIPROT	protein	YES	down-regulates	phosphorylation
P45983	UNIPROT	MAPK8	protein	P35568	IRS1	UNIPROT	protein	YES	down-regulates	phosphorylation
P45983	UNIPROT	MAPK8	protein	P35568	IRS1	UNIPROT	protein	YES	down-regulates	phosphorylation
P45983	UNIPROT	MAPK8	protein	P35568	IRS1	UNIPROT	protein	YES	down-regulates	phosphorylation
P45983	UNIPROT	MAPK8	protein	P35568	IRS1	UNIPROT	protein	YES	down-regulates	phosphorylation
P45983	UNIPROT	MAPK8	protein	P35568	IRS1	UNIPROT	protein	YES	down-regulates	phosphorylation
P45983	UNIPROT	MAPK8	protein	P35568	IRS1	UNIPROT	protein	YES	down-regulates	phosphorylation
P45983	UNIPROT	MAPK8	protein	P19793	RXRA	UNIPROT	protein	YES	down-regulates	phosphorylation
P45983	UNIPROT	MAPK8	protein	P31947	SFN	UNIPROT	protein	YES	down-regulates	phosphorylation
P45983	UNIPROT	MAPK8	protein	P42226	STAT6	UNIPROT	protein	YES	down-regulates	phosphorylation
P45983	UNIPROT	MAPK8	protein	P16949	STMN1	UNIPROT	protein	YES	down-regulates	phosphorylation
P45983	UNIPROT	MAPK8	protein	P31946	YWHAB	UNIPROT	protein	YES	down-regulates	phosphorylation
P45983	UNIPROT	MAPK8	protein	P63104	YWHAZ	UNIPROT	protein	YES	down-regulates	phosphorylation
P45984	UNIPROT	MAPK9	protein	P35568	IRS1	UNIPROT	protein	YES	down-regulates	phosphorylation
P45984	UNIPROT	MAPK9	protein	P35568	IRS1	UNIPROT	protein	YES	down-regulates	phosphorylation
P45984	UNIPROT	MAPK9	protein	Q9NYV6	RRN3	UNIPROT	protein	YES	down-regulates	phosphorylation
P45984	UNIPROT	MAPK9	protein	P19793	RXRA	UNIPROT	protein	YES	down-regulates	phosphorylation
P45984	UNIPROT	MAPK9	protein	P31947	SFN	UNIPROT	protein	YES	down-regulates	phosphorylation
P45984	UNIPROT	MAPK9	protein	P16949	STMN1	UNIPROT	protein	YES	down-regulates	phosphorylation
P45984	UNIPROT	MAPK9	protein	P50616	TOB1	UNIPROT	protein	YES	down-regulates	phosphorylation
P45984	UNIPROT	MAPK9	protein	P50616	TOB1	UNIPROT	protein	YES	down-regulates	phosphorylation
P45984	UNIPROT	MAPK9	protein	P50616	TOB1	UNIPROT	protein	YES	down-regulates	phosphorylation
P45984	UNIPROT	MAPK9	protein	P50616	TOB1	UNIPROT	protein	YES	down-regulates	phosphorylation
P45984	UNIPROT	MAPK9	protein	P50616	TOB1	UNIPROT	protein	YES	down-regulates	phosphorylation
P45984	UNIPROT	MAPK9	protein	P63104	YWHAZ	UNIPROT	protein	YES	down-regulates	phosphorylation
P49137	UNIPROT	MAPKAPK2	protein	P04792	HSPB1	UNIPROT	protein	YES	down-regulates	phosphorylation
P49137	UNIPROT	MAPKAPK2	protein	P04792	HSPB1	UNIPROT	protein	YES	down-regulates	phosphorylation
P49137	UNIPROT	MAPKAPK2	protein	P04792	HSPB1	UNIPROT	protein	YES	down-regulates	phosphorylation
P49137	UNIPROT	MAPKAPK2	protein	P04792	HSPB1	UNIPROT	protein	YES	down-regulates	phosphorylation
P49137	UNIPROT	MAPKAPK2	protein	P04792	HSPB1	UNIPROT	protein	YES	down-regulates	phosphorylation
P49137	UNIPROT	MAPKAPK2	protein	O95453	PARN	UNIPROT	protein	YES	down-regulates	phosphorylation
P49137	UNIPROT	MAPKAPK2	protein	Q07352	ZFP36L1	UNIPROT	protein	YES	down-regulates	phosphorylation
P49137	UNIPROT	MAPKAPK2	protein	Q07352	ZFP36L1	UNIPROT	protein	YES	down-regulates	phosphorylation
P49137	UNIPROT	MAPKAPK2	protein	Q07352	ZFP36L1	UNIPROT	protein	YES	down-regulates	phosphorylation
Q7KZI7	UNIPROT	MARK2	protein	Q92974	ARHGEF2	UNIPROT	protein	YES	down-regulates	phosphorylation
Q7KZI7	UNIPROT	MARK2	protein	Q92974	ARHGEF2	UNIPROT	protein	YES	down-regulates	phosphorylation
Q7KZI7	UNIPROT	MARK2	protein	Q8WUI4	HDAC7	UNIPROT	protein	YES	down-regulates	phosphorylation
P42345	UNIPROT	MTOR	protein	O75143	ATG13	UNIPROT	protein	YES	down-regulates	phosphorylation
P42345	UNIPROT	MTOR	protein	Q8TB45	DEPTOR	UNIPROT	protein	YES	down-regulates	phosphorylation
P42345	UNIPROT	MTOR	protein	Q8TB45	DEPTOR	UNIPROT	protein	YES	down-regulates	phosphorylation
P42345	UNIPROT	MTOR	protein	Q13542	EIF4EBP2	UNIPROT	protein	YES	down-regulates	phosphorylation
P42345	UNIPROT	MTOR	protein	Q13542	EIF4EBP2	UNIPROT	protein	YES	down-regulates	phosphorylation
Q13153	UNIPROT	PAK1	protein	P52565	ARHGDIA	UNIPROT	protein	YES	down-regulates	phosphorylation
Q13153	UNIPROT	PAK1	protein	P52565	ARHGDIA	UNIPROT	protein	YES	down-regulates	phosphorylation
Q13153	UNIPROT	PAK1	protein	Q92974	ARHGEF2	UNIPROT	protein	YES	down-regulates	phosphorylation
Q13153	UNIPROT	PAK1	protein	Q92934	BAD	UNIPROT	protein	YES	down-regulates	phosphorylation
Q13153	UNIPROT	PAK1	protein	Q92934	BAD	UNIPROT	protein	YES	down-regulates	phosphorylation
Q13153	UNIPROT	PAK1	protein	Q92934	BAD	UNIPROT	protein	YES	down-regulates	phosphorylation
Q13153	UNIPROT	PAK1	protein	P63167	DYNLL1	UNIPROT	protein	YES	down-regulates	phosphorylation
Q13153	UNIPROT	PAK1	protein	Q99661	KIF2C	UNIPROT	protein	YES	down-regulates	phosphorylation

Q13153	UNIPROT	PAK1	protein	Q99661	KIF2C	UNIPROT	protein	YES	down-regulates	phosphorylation
Q13153	UNIPROT	PAK1	protein	P35240	NF2	UNIPROT	protein	YES	down-regulates	phosphorylation
Q13153	UNIPROT	PAK1	protein	P18669	PGAM1	UNIPROT	protein	YES	down-regulates	phosphorylation
Q13153	UNIPROT	PAK1	protein	P18669	PGAM1	UNIPROT	protein	YES	down-regulates	phosphorylation
Q13153	UNIPROT	PAK1	protein	P16949	STMN1	UNIPROT	protein	YES	down-regulates	phosphorylation
Q13153	UNIPROT	PAK1	protein	P16949	STMN1	UNIPROT	protein	YES	down-regulates	phosphorylation
Q13153	UNIPROT	PAK1	protein	P16949	STMN1	UNIPROT	protein	YES	down-regulates	phosphorylation
Q13153	UNIPROT	PAK1	protein	P30291	WEE1	UNIPROT	protein	YES	down-regulates	phosphorylation
Q13177	UNIPROT	PAK2	protein	P55210	CASP7	UNIPROT	protein	YES	down-regulates	phosphorylation
Q13177	UNIPROT	PAK2	protein	P55210	CASP7	UNIPROT	protein	YES	down-regulates	phosphorylation
Q13177	UNIPROT	PAK2	protein	P55210	CASP7	UNIPROT	protein	YES	down-regulates	phosphorylation
Q13177	UNIPROT	PAK2	protein	P35240	NF2	UNIPROT	protein	YES	down-regulates	phosphorylation
Q16512	UNIPROT	PKN1	protein	Q92974	ARHGEF2	UNIPROT	protein	YES	down-regulates	phosphorylation
Q16512	UNIPROT	PKN1	protein	P00533	EGFR	UNIPROT	protein	YES	down-regulates	phosphorylation
Q16512	UNIPROT	PKN1	protein	Q12778	FOXO1	UNIPROT	protein	YES	down-regulates	phosphorylation
Q16512	UNIPROT	PKN1	protein	P18669	PGAM1	UNIPROT	protein	YES	down-regulates	phosphorylation
Q16512	UNIPROT	PKN1	protein	P18669	PGAM1	UNIPROT	protein	YES	down-regulates	phosphorylation
P53350	UNIPROT	PLK1	protein	Q9UBB4	ATXN10	UNIPROT	protein	YES	down-regulates	phosphorylation
P53350	UNIPROT	PLK1	protein	Q9UBB4	ATXN10	UNIPROT	protein	YES	down-regulates	phosphorylation
P53350	UNIPROT	PLK1	protein	Q13158	FADD	UNIPROT	protein	YES	down-regulates	phosphorylation
P53350	UNIPROT	PLK1	protein	Q00613	HSF1	UNIPROT	protein	YES	down-regulates	phosphorylation
P53350	UNIPROT	PLK1	protein	P35568	IRS1	UNIPROT	protein	YES	down-regulates	phosphorylation
P53350	UNIPROT	PLK1	protein	P47736	RAP1GAP	UNIPROT	protein	YES	down-regulates	phosphorylation
P53350	UNIPROT	PLK1	protein	P13693	TPT1	UNIPROT	protein	YES	down-regulates	phosphorylation
P53350	UNIPROT	PLK1	protein	P13693	TPT1	UNIPROT	protein	YES	down-regulates	phosphorylation
P53350	UNIPROT	PLK1	protein	P30291	WEE1	UNIPROT	protein	YES	down-regulates	phosphorylation
P53350	UNIPROT	PLK1	protein	P30291	WEE1	UNIPROT	protein	YES	down-regulates	phosphorylation
P53350	UNIPROT	PLK1	protein	P30291	WEE1	UNIPROT	protein	YES	down-regulates	phosphorylation
P53350	UNIPROT	PLK1	protein	P30291	WEE1	UNIPROT	protein	YES	down-regulates	phosphorylation
Q13131	UNIPROT	PRKAA1	protein	Q53ET0	CRTC2	UNIPROT	protein	YES	down-regulates	phosphorylation
Q13131	UNIPROT	PRKAA1	protein	Q13363	CTBP1	UNIPROT	protein	YES	down-regulates	phosphorylation
Q13131	UNIPROT	PRKAA1	protein	Q09472	EP300	UNIPROT	protein	YES	down-regulates	phosphorylation
Q13131	UNIPROT	PRKAA1	protein	Q92538	GBF1	UNIPROT	protein	YES	down-regulates	phosphorylation
Q13131	UNIPROT	PRKAA1	protein	Q06210	GFPT1	UNIPROT	protein	YES	down-regulates	phosphorylation
Q13131	UNIPROT	PRKAA1	protein	Q8WUI4	HDAC7	UNIPROT	protein	YES	down-regulates	phosphorylation
Q13131	UNIPROT	PRKAA1	protein	Q8WUI4	HDAC7	UNIPROT	protein	YES	down-regulates	phosphorylation
Q13131	UNIPROT	PRKAA1	protein	Q05469	LIPE	UNIPROT	protein	YES	down-regulates	phosphorylation
Q13131	UNIPROT	PRKAA1	protein	P14859	POU2F1	UNIPROT	protein	YES	down-regulates	phosphorylation
Q13131	UNIPROT	PRKAA1	protein	P14859	POU2F1	UNIPROT	protein	YES	down-regulates	phosphorylation
Q13131	UNIPROT	PRKAA1	protein	P06400	RB1	UNIPROT	protein	YES	down-regulates	phosphorylation
Q13131	UNIPROT	PRKAA1	protein	Q9NYV6	RRN3	UNIPROT	protein	YES	down-regulates	phosphorylation
Q13131	UNIPROT	PRKAA1	protein	Q86TI0	TBC1D1	UNIPROT	protein	YES	down-regulates	phosphorylation
Q9Y478	UNIPROT	PRKAB1	protein	Q8N122	RPTOR	UNIPROT	protein	YES	down-regulates	phosphorylation
P17612	UNIPROT	PRKACA	protein	Q12802	AKAP13	UNIPROT	protein	YES	down-regulates	phosphorylation
P17612	UNIPROT	PRKACA	protein	P52565	ARHGDIA	UNIPROT	protein	YES	down-regulates	phosphorylation
P17612	UNIPROT	PRKACA	protein	Q92934	BAD	UNIPROT	protein	YES	down-regulates	phosphorylation
P17612	UNIPROT	PRKACA	protein	Q92934	BAD	UNIPROT	protein	YES	down-regulates	phosphorylation
P17612	UNIPROT	PRKACA	protein	Q92934	BAD	UNIPROT	protein	YES	down-regulates	phosphorylation
P17612	UNIPROT	PRKACA	protein	Q92934	BAD	UNIPROT	protein	YES	down-regulates	phosphorylation
P17612	UNIPROT	PRKACA	protein	Q92934	BAD	UNIPROT	protein	YES	down-regulates	phosphorylation
P17612	UNIPROT	PRKACA	protein	Q92934	BAD	UNIPROT	protein	YES	down-regulates	phosphorylation
P17612	UNIPROT	PRKACA	protein	P27708	CAD	UNIPROT	protein	YES	down-regulates	phosphorylation
P17612	UNIPROT	PRKACA	protein	P17655	CAPN2	UNIPROT	protein	YES	down-regulates	phosphorylation
P17612	UNIPROT	PRKACA	protein	P55211	CASP9	UNIPROT	protein	YES	down-regulates	phosphorylation
P17612	UNIPROT	PRKACA	protein	P55211	CASP9	UNIPROT	protein	YES	down-regulates	phosphorylation
P17612	UNIPROT	PRKACA	protein	P55211	CASP9	UNIPROT	protein	YES	down-regulates	phosphorylation
P17612	UNIPROT	PRKACA	protein	O60716	CTNND1	UNIPROT	protein	YES	down-regulates	phosphorylation
P17612	UNIPROT	PRKACA	protein	P49840	GSK3A	UNIPROT	protein	YES	down-regulates	phosphorylation
P17612	UNIPROT	PRKACA	protein	Q9BY41	HDAC8	UNIPROT	protein	YES	down-regulates	phosphorylation
P17612	UNIPROT	PRKACA	protein	P16144	ITGB4	UNIPROT	protein	YES	down-regulates	phosphorylation
P17612	UNIPROT	PRKACA	protein	Q14847	LASP1	UNIPROT	protein	YES	down-regulates	phosphorylation
P17612	UNIPROT	PRKACA	protein	Q14847	LASP1	UNIPROT	protein	YES	down-regulates	phosphorylation
P17612	UNIPROT	PRKACA	protein	Q05469	LIPE	UNIPROT	protein	YES	down-regulates	phosphorylation
P17612	UNIPROT	PRKACA	protein	Q96PU5	NEDD4L	UNIPROT	protein	YES	down-regulates	phosphorylation
P17612	UNIPROT	PRKACA	protein	P35240	NF2	UNIPROT	protein	YES	down-regulates	phosphorylation
P17612	UNIPROT	PRKACA	protein	Q01970	PLCB3	UNIPROT	protein	YES	down-regulates	phosphorylation
P17612	UNIPROT	PRKACA	protein	Q15054	POLD3	UNIPROT	protein	YES	down-regulates	phosphorylation
P17612	UNIPROT	PRKACA	protein	P19793	RXRA	UNIPROT	protein	YES	down-regulates	phosphorylation
P17612	UNIPROT	PRKACA	protein	P16949	STMN1	UNIPROT	protein	YES	down-regulates	phosphorylation
P17612	UNIPROT	PRKACA	protein	P16949	STMN1	UNIPROT	protein	YES	down-regulates	phosphorylation
P17612	UNIPROT	PRKACA	protein	P16949	STMN1	UNIPROT	protein	YES	down-regulates	phosphorylation
P17612	UNIPROT	PRKACA	protein	P16949	STMN1	UNIPROT	protein	YES	down-regulates	phosphorylation
P17252	UNIPROT	PRKCA	protein	P21399	ACO1	UNIPROT	protein	YES	down-regulates	phosphorylation
P17252	UNIPROT	PRKCA	protein	P52566	ARHGDIB	UNIPROT	protein	YES	down-regulates	phosphorylation
P17252	UNIPROT	PRKCA	protein	P23528	CFL1	UNIPROT	protein	YES	down-regulates	phosphorylation
P17252	UNIPROT	PRKCA	protein	P23528	CFL1	UNIPROT	protein	YES	down-regulates	phosphorylation
P17252	UNIPROT	PRKCA	protein	P30622	CLIP1	UNIPROT	protein	YES	down-regulates	phosphorylation
P17252	UNIPROT	PRKCA	protein	Q9BR76	CORO1B	UNIPROT	protein	YES	down-regulates	phosphorylation
P17252	UNIPROT	PRKCA	protein	Q92793	CREBBP	UNIPROT	protein	YES	down-regulates	phosphorylation
P17252	UNIPROT	PRKCA	protein	P17812	CTPS1	UNIPROT	protein	YES	down-regulates	phosphorylation
P17252	UNIPROT	PRKCA	protein	Q01844	EWSR1	UNIPROT	protein	YES	down-regulates	phosphorylation
P17252	UNIPROT	PRKCA	protein	P49840	GSK3A	UNIPROT	protein	YES	down-regulates	phosphorylation
P17252	UNIPROT	PRKCA	protein	P49841	GSK3B	UNIPROT	protein	YES	down-regulates	phosphorylation
P17252	UNIPROT	PRKCA	protein	P05114	HMGN1	UNIPROT	protein	YES	down-regulates	phosphorylation
P17252	UNIPROT	PRKCA	protein	P05114	HMGN1	UNIPROT	protein	YES	down-regulates	phosphorylation
P17252	UNIPROT	PRKCA	protein	P05204	HMGN2	UNIPROT	protein	YES	down-regulates	phosphorylation
P17252	UNIPROT	PRKCA	protein	P05204	HMGN2	UNIPROT	protein	YES	down-regulates	phosphorylation
P17252	UNIPROT	PRKCA	protein	P09651	HNRNPA1	UNIPROT	protein	YES	down-regulates	phosphorylation
P17252	UNIPROT	PRKCA	protein	P16144	ITGB4	UNIPROT	protein	YES	down-regulates	phosphorylation
P17252	UNIPROT	PRKCA	protein	P16144	ITGB4	UNIPROT	protein	YES	down-regulates	phosphorylation
P17252	UNIPROT	PRKCA	protein	P08581	MET	UNIPROT	protein	YES	down-regulates	phosphorylation
P17252	UNIPROT	PRKCA	protein	P24844	MYL9	UNIPROT	protein	YES	down-regulates	phosphorylation
P17252	UNIPROT	PRKCA	protein	P24844	MYL9	UNIPROT	protein	YES	down-regulates	phosphorylation
P17252	UNIPROT	PRKCA	protein	P24844	MYL9	UNIPROT	protein	YES	down-regulates	phosphorylation
P17252	UNIPROT	PRKCA	protein	Q15121	PEA15	UNIPROT	protein	YES	down-regulates	phosphorylation
P17252	UNIPROT	PRKCA	protein	Q01970	PLCB3	UNIPROT	protein	YES	down-regulates	phosphorylation
P17252	UNIPROT	PRKCA	protein	Q05209	PTPN12	UNIPROT	protein	YES	down-regulates	phosphorylation
P17252	UNIPROT	PRKCA	protein	Q05209	PTPN12	UNIPROT	protein	YES	down-regulates	phosphorylation

P17252	UNIPROT	PRKCA	protein	P29350	PTPN6	UNIPROT	protein	YES	down-regulates	phosphorylation
Q05655	UNIPROT	PRKCD	protein	Q09472	EP300	UNIPROT	protein	YES	down-regulates	phosphorylation
Q05655	UNIPROT	PRKCD	protein	P49840	GSK3A	UNIPROT	protein	YES	down-regulates	phosphorylation
Q05655	UNIPROT	PRKCD	protein	P17096	HMGA1	UNIPROT	protein	YES	down-regulates	phosphorylation
Q05655	UNIPROT	PRKCD	protein	P17096	HMGA1	UNIPROT	protein	YES	down-regulates	phosphorylation
Q05655	UNIPROT	PRKCD	protein	P61978	HNRNPK	UNIPROT	protein	YES	down-regulates	phosphorylation
Q05655	UNIPROT	PRKCD	protein	P35568	IRS1	UNIPROT	protein	YES	down-regulates	phosphorylation
Q05655	UNIPROT	PRKCD	protein	P35568	IRS1	UNIPROT	protein	YES	down-regulates	phosphorylation
Q05655	UNIPROT	PRKCD	protein	P35568	IRS1	UNIPROT	protein	YES	down-regulates	phosphorylation
Q05655	UNIPROT	PRKCD	protein	P10588	NR2F6	UNIPROT	protein	YES	down-regulates	phosphorylation
Q05655	UNIPROT	PRKCD	protein	P31431	SDC4	UNIPROT	protein	YES	down-regulates	phosphorylation
Q05655	UNIPROT	PRKCD	protein	Q13263	TRIM28	UNIPROT	protein	YES	down-regulates	phosphorylation
Q05655	UNIPROT	PRKCD	protein	P31946	YWHAB	UNIPROT	protein	YES	down-regulates	phosphorylation
P41743	UNIPROT	PRKCI	protein	Q92934	BAD	UNIPROT	protein	YES	down-regulates	phosphorylation
P41743	UNIPROT	PRKCI	protein	Q92934	BAD	UNIPROT	protein	YES	down-regulates	phosphorylation
P41743	UNIPROT	PRKCI	protein	Q92934	BAD	UNIPROT	protein	YES	down-regulates	phosphorylation
P41743	UNIPROT	PRKCI	protein	P49757	NUMB	UNIPROT	protein	YES	down-regulates	phosphorylation
P78527	UNIPROT	PRKDC	protein	P49917	LIG4	UNIPROT	protein	YES	down-regulates	phosphorylation
P78527	UNIPROT	PRKDC	protein	P49917	LIG4	UNIPROT	protein	YES	down-regulates	phosphorylation
P78527	UNIPROT	PRKDC	protein	P49917	LIG4	UNIPROT	protein	YES	down-regulates	phosphorylation
P78527	UNIPROT	PRKDC	protein	P14859	POU2F1	UNIPROT	protein	YES	down-regulates	phosphorylation
P78527	UNIPROT	PRKDC	protein	P14859	POU2F1	UNIPROT	protein	YES	down-regulates	phosphorylation
P78527	UNIPROT	PRKDC	protein	P78527	PRKDC	UNIPROT	protein	YES	down-regulates	phosphorylation
Q13976	UNIPROT	PRKG1	protein	P04792	HSPB1	UNIPROT	protein	YES	down-regulates	phosphorylation
Q13976	UNIPROT	PRKG1	protein	P04792	HSPB1	UNIPROT	protein	YES	down-regulates	phosphorylation
Q13976	UNIPROT	PRKG1	protein	P04792	HSPB1	UNIPROT	protein	YES	down-regulates	phosphorylation
Q13976	UNIPROT	PRKG1	protein	Q14847	LASP1	UNIPROT	protein	YES	down-regulates	phosphorylation
Q13976	UNIPROT	PRKG1	protein	P50552	VASP	UNIPROT	protein	YES	down-regulates	phosphorylation
Q13976	UNIPROT	PRKG1	protein	P50552	VASP	UNIPROT	protein	YES	down-regulates	phosphorylation
Q13976	UNIPROT	PRKG1	protein	P50552	VASP	UNIPROT	protein	YES	down-regulates	phosphorylation
Q13523	UNIPROT	PRPF4B	protein	Q9Y2Y9	KLF13	UNIPROT	protein	YES	down-regulates	phosphorylation
Q05397	UNIPROT	PTK2	protein	P12814	ACTN1	UNIPROT	protein	YES	down-regulates	phosphorylation
Q05397	UNIPROT	PTK2	protein	O43707	ACTN4	UNIPROT	protein	YES	down-regulates	phosphorylation
Q05397	UNIPROT	PTK2	protein	O43707	ACTN4	UNIPROT	protein	YES	down-regulates	phosphorylation
Q13464	UNIPROT	ROCK1	protein	Q9NRY4	ARHGAP35	UNIPROT	protein	YES	down-regulates	phosphorylation
Q13464	UNIPROT	ROCK1	protein	Q9NRY4	ARHGAP35	UNIPROT	protein	YES	down-regulates	phosphorylation
Q13464	UNIPROT	ROCK1	protein	Q6WCQ1	MPRIP	UNIPROT	protein	YES	down-regulates	phosphorylation
Q13464	UNIPROT	ROCK1	protein	P07737	PFN1	UNIPROT	protein	YES	down-regulates	phosphorylation
Q13464	UNIPROT	ROCK1	protein	O14974	PPP1R12A	UNIPROT	protein	YES	down-regulates	phosphorylation
Q15418	UNIPROT	RPS6KA1	protein	Q96RK0	CIC	UNIPROT	protein	YES	down-regulates	phosphorylation
Q15418	UNIPROT	RPS6KA1	protein	Q8TB45	DEPTOR	UNIPROT	protein	YES	down-regulates	phosphorylation
Q15418	UNIPROT	RPS6KA1	protein	Q8TB45	DEPTOR	UNIPROT	protein	YES	down-regulates	phosphorylation
Q15418	UNIPROT	RPS6KA1	protein	Q8TB45	DEPTOR	UNIPROT	protein	YES	down-regulates	phosphorylation
Q15418	UNIPROT	RPS6KA1	protein	P49841	GSK3B	UNIPROT	protein	YES	down-regulates	phosphorylation
Q15418	UNIPROT	RPS6KA1	protein	Q9UBP6	METTL1	UNIPROT	protein	YES	down-regulates	phosphorylation
Q15418	UNIPROT	RPS6KA1	protein	P49815	TSC2	UNIPROT	protein	YES	down-regulates	phosphorylation
Q15418	UNIPROT	RPS6KA1	protein	P50552	VASP	UNIPROT	protein	YES	down-regulates	phosphorylation
P51812	UNIPROT	RPS6KA3	protein	Q92934	BAD	UNIPROT	protein	YES	down-regulates	phosphorylation
P51812	UNIPROT	RPS6KA3	protein	Q96RK0	CIC	UNIPROT	protein	YES	down-regulates	phosphorylation
P12931	UNIPROT	SRC	protein	P63010	AP2B1	UNIPROT	protein	YES	down-regulates	phosphorylation
P12931	UNIPROT	SRC	protein	P52565	ARHGDIA	UNIPROT	protein	YES	down-regulates	phosphorylation
P12931	UNIPROT	SRC	protein	P49407	ARRB1	UNIPROT	protein	YES	down-regulates	phosphorylation
P12931	UNIPROT	SRC	protein	Q14790	CASP8	UNIPROT	protein	YES	down-regulates	phosphorylation
P12931	UNIPROT	SRC	protein	P46527	CDKN1B	UNIPROT	protein	YES	down-regulates	phosphorylation
P12931	UNIPROT	SRC	protein	P46527	CDKN1B	UNIPROT	protein	YES	down-regulates	phosphorylation
P12931	UNIPROT	SRC	protein	P46527	CDKN1B	UNIPROT	protein	YES	down-regulates	phosphorylation
P12931	UNIPROT	SRC	protein	P23528	CFL1	UNIPROT	protein	YES	down-regulates	phosphorylation
P12931	UNIPROT	SRC	protein	Q14247	CTTN	UNIPROT	protein	YES	down-regulates	phosphorylation
P12931	UNIPROT	SRC	protein	Q14247	CTTN	UNIPROT	protein	YES	down-regulates	phosphorylation
P12931	UNIPROT	SRC	protein	Q14247	CTTN	UNIPROT	protein	YES	down-regulates	phosphorylation
P12931	UNIPROT	SRC	protein	Q14118	DAG1	UNIPROT	protein	YES	down-regulates	phosphorylation
P12931	UNIPROT	SRC	protein	P50402	EMD	UNIPROT	protein	YES	down-regulates	phosphorylation
P12931	UNIPROT	SRC	protein	P50402	EMD	UNIPROT	protein	YES	down-regulates	phosphorylation
P12931	UNIPROT	SRC	protein	P50402	EMD	UNIPROT	protein	YES	down-regulates	phosphorylation
P12931	UNIPROT	SRC	protein	P61978	HNRNPK	UNIPROT	protein	YES	down-regulates	phosphorylation
P12931	UNIPROT	SRC	protein	P61978	HNRNPK	UNIPROT	protein	YES	down-regulates	phosphorylation
P12931	UNIPROT	SRC	protein	P61978	HNRNPK	UNIPROT	protein	YES	down-regulates	phosphorylation
P12931	UNIPROT	SRC	protein	P61978	HNRNPK	UNIPROT	protein	YES	down-regulates	phosphorylation
P12931	UNIPROT	SRC	protein	P61978	HNRNPK	UNIPROT	protein	YES	down-regulates	phosphorylation
P12931	UNIPROT	SRC	protein	P61978	HNRNPK	UNIPROT	protein	YES	down-regulates	phosphorylation
P12931	UNIPROT	SRC	protein	P67775	PPP2CA	UNIPROT	protein	YES	down-regulates	phosphorylation
P12931	UNIPROT	SRC	protein	P60484	PTEN	UNIPROT	protein	YES	down-regulates	phosphorylation
P12931	UNIPROT	SRC	protein	P20936	RASA1	UNIPROT	protein	YES	down-regulates	phosphorylation
P12931	UNIPROT	SRC	protein	Q99961	SH3GL1	UNIPROT	protein	YES	down-regulates	phosphorylation
Q96SB4	UNIPROT	SRPK1	protein	Q9Y5S9	RBM8A	UNIPROT	protein	YES	down-regulates	phosphorylation
Q96SB4	UNIPROT	SRPK1	protein	Q9Y5S9	RBM8A	UNIPROT	protein	YES	down-regulates	phosphorylation
O94804	UNIPROT	STK10	protein	P26038	MSN	UNIPROT	protein	YES	down-regulates	phosphorylation
P33981	UNIPROT	TTK	protein	P33981	TTK	UNIPROT	protein	YES	down-regulates	phosphorylation
P33981	UNIPROT	TTK	protein	P33981	TTK	UNIPROT	protein	YES	down-regulates	phosphorylation
Q99986	UNIPROT	VRK1	protein	O75531	BANF1	UNIPROT	protein	YES	down-regulates	phosphorylation
Q99986	UNIPROT	VRK1	protein	O75531	BANF1	UNIPROT	protein	YES	down-regulates	phosphorylation
Q99986	UNIPROT	VRK1	protein	O75531	BANF1	UNIPROT	protein	YES	down-regulates	phosphorylation
Q99986	UNIPROT	VRK1	protein	O75531	BANF1	UNIPROT	protein	YES	down-regulates	phosphorylation
P30291	UNIPROT	WEE1	protein	P06493	CDK1	UNIPROT	protein	YES	down-regulates	phosphorylation
P30291	UNIPROT	WEE1	protein	P24941	CDK2	UNIPROT	protein	YES	down-regulates	phosphorylation
P07947	UNIPROT	YES1	protein	P11802	CDK4	UNIPROT	protein	YES	down-regulates	phosphorylation
P31749	UNIPROT	AKT1	protein	Q96B36	AKT1S1	UNIPROT	protein	YES	down-regulates activity	phosphorylation
P31749	UNIPROT	AKT1	protein	Q92934	BAD	UNIPROT	protein	YES	down-regulates activity	phosphorylation
P31749	UNIPROT	AKT1	protein	Q92934	BAD	UNIPROT	protein	YES	down-regulates activity	phosphorylation
P31749	UNIPROT	AKT1	protein	Q92934	BAD	UNIPROT	protein	YES	down-regulates activity	phosphorylation
P31749	UNIPROT	AKT1	protein	Q92934	BAD	UNIPROT	protein	YES	down-regulates activity	phosphorylation
P31749	UNIPROT	AKT1	protein	Q07812	BAX	UNIPROT	protein	YES	down-regulates activity	phosphorylation
P31749	UNIPROT	AKT1	protein	P15056	BRAF	UNIPROT	protein	YES	down-regulates activity	phosphorylation
P31749	UNIPROT	AKT1	protein	P15056	BRAF	UNIPROT	protein	YES	down-regulates activity	phosphorylation
P31749	UNIPROT	AKT1	protein	P55211	CASP9	UNIPROT	protein	YES	down-regulates activity	phosphorylation
P31749	UNIPROT	AKT1	protein	P55211	CASP9	UNIPROT	protein	YES	down-regulates activity	phosphorylation
P31749	UNIPROT	AKT1	protein	P55211	CASP9	UNIPROT	protein	YES	down-regulates activity	phosphorylation
P31749	UNIPROT	AKT1	protein	Q15910	EZH2	UNIPROT	protein	YES	down-regulates activity	phosphorylation

P31749	UNIPROT	AKT1	protein	Q12778	FOXO1	UNIPROT	protein	YES	down-regulates activity	phosphorylation
P31749	UNIPROT	AKT1	protein	O43524	FOXO3	UNIPROT	protein	YES	down-regulates activity	phosphorylation
P31749	UNIPROT	AKT1	protein	O43524	FOXO3	UNIPROT	protein	YES	down-regulates activity	phosphorylation
P31749	UNIPROT	AKT1	protein	O43524	FOXO3	UNIPROT	protein	YES	down-regulates activity	phosphorylation
P31749	UNIPROT	AKT1	protein	O43524	FOXO3	UNIPROT	protein	YES	down-regulates activity	phosphorylation
P31749	UNIPROT	AKT1	protein	P49841	GSK3B	UNIPROT	protein	YES	down-regulates activity	phosphorylation
P31749	UNIPROT	AKT1	protein	P49841	GSK3B	UNIPROT	protein	YES	down-regulates activity	phosphorylation
P31749	UNIPROT	AKT1	protein	P51617	IRAK1	UNIPROT	protein	YES	down-regulates activity	phosphorylation
P31749	UNIPROT	AKT1	protein	Q92945	KHSRP	UNIPROT	protein	YES	down-regulates activity	phosphorylation
P31749	UNIPROT	AKT1	protein	P41279	MAP3K8	UNIPROT	protein	YES	down-regulates activity	phosphorylation
P31749	UNIPROT	AKT1	protein	P41279	MAP3K8	UNIPROT	protein	YES	down-regulates activity	phosphorylation
P31749	UNIPROT	AKT1	protein	P42345	MTOR	UNIPROT	protein	YES	down-regulates activity	phosphorylation
P31749	UNIPROT	AKT1	protein	Q13131	PRKAA1	UNIPROT	protein	YES	down-regulates activity	phosphorylation
P31749	UNIPROT	AKT1	protein	P18031	PTPN1	UNIPROT	protein	YES	down-regulates activity	phosphorylation
P31749	UNIPROT	AKT1	protein	P63000	RAC1	UNIPROT	protein	YES	down-regulates activity	phosphorylation
P31749	UNIPROT	AKT1	protein	P63000	RAC1	UNIPROT	protein	YES	down-regulates activity	phosphorylation
P31749	UNIPROT	AKT1	protein	P49815	TSC2	UNIPROT	protein	YES	down-regulates activity	phosphorylation
P31749	UNIPROT	AKT1	protein	P49815	TSC2	UNIPROT	protein	YES	down-regulates activity	phosphorylation
P31751	UNIPROT	AKT2	protein	Q96B36	AKT1S1	UNIPROT	protein	YES	down-regulates activity	phosphorylation
P31751	UNIPROT	AKT2	protein	Q96B36	AKT1S1	UNIPROT	protein	YES	down-regulates activity	phosphorylation
P31751	UNIPROT	AKT2	protein	Q12778	FOXO1	UNIPROT	protein	YES	down-regulates activity	phosphorylation
P31751	UNIPROT	AKT2	protein	Q12778	FOXO1	UNIPROT	protein	YES	down-regulates activity	phosphorylation
P31751	UNIPROT	AKT2	protein	O43524	FOXO3	UNIPROT	protein	YES	down-regulates activity	phosphorylation
P31751	UNIPROT	AKT2	protein	O43524	FOXO3	UNIPROT	protein	YES	down-regulates activity	phosphorylation
P31751	UNIPROT	AKT2	protein	O43524	FOXO3	UNIPROT	protein	YES	down-regulates activity	phosphorylation
P31751	UNIPROT	AKT2	protein	P49841	GSK3B	UNIPROT	protein	YES	down-regulates activity	phosphorylation
P31751	UNIPROT	AKT2	protein	P49841	GSK3B	UNIPROT	protein	YES	down-regulates activity	phosphorylation
P31751	UNIPROT	AKT2	protein	P18031	PTPN1	UNIPROT	protein	YES	down-regulates activity	phosphorylation
Q9UQM7	UNIPROT	CAMK2A	protein	Q12959	DLG1	UNIPROT	protein	YES	down-regulates activity	phosphorylation
Q9UQM7	UNIPROT	CAMK2A	protein	P00533	EGFR	UNIPROT	protein	YES	down-regulates activity	phosphorylation
Q9UQM7	UNIPROT	CAMK2A	protein	P00533	EGFR	UNIPROT	protein	YES	down-regulates activity	phosphorylation
Q9UQM7	UNIPROT	CAMK2A	protein	P00533	EGFR	UNIPROT	protein	YES	down-regulates activity	phosphorylation
Q9UQM7	UNIPROT	CAMK2A	protein	P00533	EGFR	UNIPROT	protein	YES	down-regulates activity	phosphorylation
Q9UQM7	UNIPROT	CAMK2A	protein	P00533	EGFR	UNIPROT	protein	YES	down-regulates activity	phosphorylation
Q9UQM7	UNIPROT	CAMK2A	protein	P00533	EGFR	UNIPROT	protein	YES	down-regulates activity	phosphorylation
Q9UQM7	UNIPROT	CAMK2A	protein	P00533	EGFR	UNIPROT	protein	YES	down-regulates activity	phosphorylation
Q13555	UNIPROT	CAMK2G	protein	P00533	EGFR	UNIPROT	protein	YES	down-regulates activity	phosphorylation
Q13555	UNIPROT	CAMK2G	protein	P00533	EGFR	UNIPROT	protein	YES	down-regulates activity	phosphorylation
Q13555	UNIPROT	CAMK2G	protein	Q15746	MYLK	UNIPROT	protein	YES	down-regulates activity	phosphorylation
Q5VT25	UNIPROT	CDC42BPA	protein	Q9BZL4	PPP1R12C	UNIPROT	protein	YES	down-regulates activity	phosphorylation
P06493	UNIPROT	CDK1	protein	Q13541	EIF4EBP1	UNIPROT	protein	YES	down-regulates activity	phosphorylation
P06493	UNIPROT	CDK1	protein	P39748	FEN1	UNIPROT	protein	YES	down-regulates activity	phosphorylation
P06493	UNIPROT	CDK1	protein	P33991	MCM4	UNIPROT	protein	YES	down-regulates activity	phosphorylation
P06493	UNIPROT	CDK1	protein	P06748	NPM1	UNIPROT	protein	YES	down-regulates activity	phosphorylation
P06493	UNIPROT	CDK1	protein	P06748	NPM1	UNIPROT	protein	YES	down-regulates activity	phosphorylation
P06493	UNIPROT	CDK1	protein	P06748	NPM1	UNIPROT	protein	YES	down-regulates activity	phosphorylation
P06493	UNIPROT	CDK1	protein	P06748	NPM1	UNIPROT	protein	YES	down-regulates activity	phosphorylation
P06493	UNIPROT	CDK1	protein	P06748	NPM1	UNIPROT	protein	YES	down-regulates activity	phosphorylation
P06493	UNIPROT	CDK1	protein	P06748	NPM1	UNIPROT	protein	YES	down-regulates activity	phosphorylation
P06493	UNIPROT	CDK1	protein	Q9UNZ2	NSFL1C	UNIPROT	protein	YES	down-regulates activity	phosphorylation
P06493	UNIPROT	CDK1	protein	O00443	PIK3C2A	UNIPROT	protein	YES	down-regulates activity	phosphorylation
P24941	UNIPROT	CDK2	protein	P33991	MCM4	UNIPROT	protein	YES	down-regulates activity	phosphorylation
P24941	UNIPROT	CDK2	protein	P33991	MCM4	UNIPROT	protein	YES	down-regulates activity	phosphorylation
P24941	UNIPROT	CDK2	protein	P33991	MCM4	UNIPROT	protein	YES	down-regulates activity	phosphorylation
P24941	UNIPROT	CDK2	protein	P33991	MCM4	UNIPROT	protein	YES	down-regulates activity	phosphorylation
P24941	UNIPROT	CDK2	protein	P06748	NPM1	UNIPROT	protein	YES	down-regulates activity	phosphorylation
P24941	UNIPROT	CDK2	protein	P06748	NPM1	UNIPROT	protein	YES	down-regulates activity	phosphorylation
P24941	UNIPROT	CDK2	protein	P84022	SMAD3	UNIPROT	protein	YES	down-regulates activity	phosphorylation
P24941	UNIPROT	CDK2	protein	P84022	SMAD3	UNIPROT	protein	YES	down-regulates activity	phosphorylation
P24941	UNIPROT	CDK2	protein	P84022	SMAD3	UNIPROT	protein	YES	down-regulates activity	phosphorylation
P24941	UNIPROT	CDK2	protein	Q712K3	UBE2R2	UNIPROT	protein	YES	down-regulates activity	phosphorylation
P11802	UNIPROT	CDK4	protein	P84022	SMAD3	UNIPROT	protein	YES	down-regulates activity	phosphorylation
P11802	UNIPROT	CDK4	protein	P84022	SMAD3	UNIPROT	protein	YES	down-regulates activity	phosphorylation
P11802	UNIPROT	CDK4	protein	P84022	SMAD3	UNIPROT	protein	YES	down-regulates activity	phosphorylation
Q00535	UNIPROT	CDK5	protein	Q13153	PAK1	UNIPROT	protein	YES	down-regulates activity	phosphorylation
P41240	UNIPROT	CSK	protein	Q96J02	ITCH	UNIPROT	protein	YES	down-regulates activity	phosphorylation
P48729	UNIPROT	CSNK1A1	protein	P07910	HNRNPC	UNIPROT	protein	YES	down-regulates activity	phosphorylation
P48729	UNIPROT	CSNK1A1	protein	P07910	HNRNPC	UNIPROT	protein	YES	down-regulates activity	phosphorylation
P48729	UNIPROT	CSNK1A1	protein	P27348	YWHAQ	UNIPROT	protein	YES	down-regulates activity	phosphorylation
P48729	UNIPROT	CSNK1A1	protein	P63104	YWHAZ	UNIPROT	protein	YES	down-regulates activity	phosphorylation
P49674	UNIPROT	CSNK1E	protein	P49674	CSNK1E	UNIPROT	protein	YES	down-regulates activity	phosphorylation
P49674	UNIPROT	CSNK1E	protein	P49674	CSNK1E	UNIPROT	protein	YES	down-regulates activity	phosphorylation
P49674	UNIPROT	CSNK1E	protein	P49674	CSNK1E	UNIPROT	protein	YES	down-regulates activity	phosphorylation
P49674	UNIPROT	CSNK1E	protein	P49674	CSNK1E	UNIPROT	protein	YES	down-regulates activity	phosphorylation
P49674	UNIPROT	CSNK1E	protein	P49674	CSNK1E	UNIPROT	protein	YES	down-regulates activity	phosphorylation
P49674	UNIPROT	CSNK1E	protein	P49674	CSNK1E	UNIPROT	protein	YES	down-regulates activity	phosphorylation
P49674	UNIPROT	CSNK1E	protein	P49674	CSNK1E	UNIPROT	protein	YES	down-regulates activity	phosphorylation
P49674	UNIPROT	CSNK1E	protein	P49674	CSNK1E	UNIPROT	protein	YES	down-regulates activity	phosphorylation
P49674	UNIPROT	CSNK1E	protein	P35222	CTNNB1	UNIPROT	protein	YES	down-regulates activity	phosphorylation
Q9HCP0	UNIPROT	CSNK1G1	protein	Q12778	FOXO1	UNIPROT	protein	YES	down-regulates activity	phosphorylation
Q9HCP0	UNIPROT	CSNK1G1	protein	Q12778	FOXO1	UNIPROT	protein	YES	down-regulates activity	phosphorylation
P68400	UNIPROT	CSNK2A1	protein	Q9Y5B0	CTDP1	UNIPROT	protein	YES	down-regulates activity	phosphorylation
P68400	UNIPROT	CSNK2A1	protein	Q9Y5B0	CTDP1	UNIPROT	protein	YES	down-regulates activity	phosphorylation
P68400	UNIPROT	CSNK2A1	protein	P12259	F5	UNIPROT	protein	YES	down-regulates activity	phosphorylation
P68400	UNIPROT	CSNK2A1	protein	Q02790	FKBP4	UNIPROT	protein	YES	down-regulates activity	phosphorylation
P68400	UNIPROT	CSNK2A1	protein	P06744	GPI	UNIPROT	protein	YES	down-regulates activity	phosphorylation
P68400	UNIPROT	CSNK2A1	protein	P07910	HNRNPC	UNIPROT	protein	YES	down-regulates activity	phosphorylation
P68400	UNIPROT	CSNK2A1	protein	Q92598	HSPH1	UNIPROT	protein	YES	down-regulates activity	phosphorylation
P68400	UNIPROT	CSNK2A1	protein	P60484	PTEN	UNIPROT	protein	YES	down-regulates activity	phosphorylation
P68400	UNIPROT	CSNK2A1	protein	P60484	PTEN	UNIPROT	protein	YES	down-regulates activity	phosphorylation
P68400	UNIPROT	CSNK2A1	protein	P60484	PTEN	UNIPROT	protein	YES	down-regulates activity	phosphorylation
P68400	UNIPROT	CSNK2A1	protein	P60484	PTEN	UNIPROT	protein	YES	down-regulates activity	phosphorylation
P68400	UNIPROT	CSNK2A1	protein	P60484	PTEN	UNIPROT	protein	YES	down-regulates activity	phosphorylation
P68400	UNIPROT	CSNK2A1	protein	P60484	PTEN	UNIPROT	protein	YES	down-regulates activity	phosphorylation
P68400	UNIPROT	CSNK2A1	protein	P08047	SP1	UNIPROT	protein	YES	down-regulates activity	phosphorylation
P68400	UNIPROT	CSNK2A1	protein	Q08945	SSRP1	UNIPROT	protein	YES	down-regulates activity	phosphorylation
P68400	UNIPROT	CSNK2A1	protein	Q08945	SSRP1	UNIPROT	protein	YES	down-regulates activity	phosphorylation
P68400	UNIPROT	CSNK2A1	protein	Q08945	SSRP1	UNIPROT	protein	YES	down-regulates activity	phosphorylation

P68400	UNIPROT	CSNK2A1	protein	P04637	TP53	UNIPROT	protein	YES	down-regulates activity	phosphorylation
P19784	UNIPROT	CSNK2A2	protein	Q9Y5B0	CTDP1	UNIPROT	protein	YES	down-regulates activity	phosphorylation
P19784	UNIPROT	CSNK2A2	protein	Q9Y5B0	CTDP1	UNIPROT	protein	YES	down-regulates activity	phosphorylation
P19784	UNIPROT	CSNK2A2	protein	Q13541	EIF4EBP1	UNIPROT	protein	YES	down-regulates activity	phosphorylation
P19784	UNIPROT	CSNK2A2	protein	Q92598	HSPH1	UNIPROT	protein	YES	down-regulates activity	phosphorylation
P19784	UNIPROT	CSNK2A2	protein	P60484	PTEN	UNIPROT	protein	YES	down-regulates activity	phosphorylation
P19784	UNIPROT	CSNK2A2	protein	P60484	PTEN	UNIPROT	protein	YES	down-regulates activity	phosphorylation
P19784	UNIPROT	CSNK2A2	protein	P60484	PTEN	UNIPROT	protein	YES	down-regulates activity	phosphorylation
P67870	UNIPROT	CSNK2B	protein	Q9Y5B0	CTDP1	UNIPROT	protein	YES	down-regulates activity	phosphorylation
P67870	UNIPROT	CSNK2B	protein	Q9Y5B0	CTDP1	UNIPROT	protein	YES	down-regulates activity	phosphorylation
P53355	UNIPROT	DAPK1	protein	P53355	DAPK1	UNIPROT	protein	YES	down-regulates activity	phosphorylation
Q13627	UNIPROT	DYRK1A	protein	Q12778	FOXO1	UNIPROT	protein	YES	down-regulates activity	phosphorylation
Q13627	UNIPROT	DYRK1A	protein	O43524	FOXO3	UNIPROT	protein	YES	down-regulates activity	phosphorylation
P00533	UNIPROT	EGFR	protein	P46108	CRK	UNIPROT	protein	YES	down-regulates activity	phosphorylation
P25098	UNIPROT	GRK2	protein	O76070	SNCG	UNIPROT	protein	YES	down-regulates activity	phosphorylation
P49840	UNIPROT	GSK3A	protein	P31749	AKT1	UNIPROT	protein	YES	down-regulates activity	phosphorylation
P49840	UNIPROT	GSK3A	protein	Q13144	EIF2B5	UNIPROT	protein	YES	down-regulates activity	phosphorylation
P49841	UNIPROT	GSK3B	protein	P35222	CTNNB1	UNIPROT	protein	YES	down-regulates activity	phosphorylation
P49841	UNIPROT	GSK3B	protein	P35222	CTNNB1	UNIPROT	protein	YES	down-regulates activity	phosphorylation
P49841	UNIPROT	GSK3B	protein	P35222	CTNNB1	UNIPROT	protein	YES	down-regulates activity	phosphorylation
P49841	UNIPROT	GSK3B	protein	P35222	CTNNB1	UNIPROT	protein	YES	down-regulates activity	phosphorylation
P49841	UNIPROT	GSK3B	protein	P35222	CTNNB1	UNIPROT	protein	YES	down-regulates activity	phosphorylation
P49841	UNIPROT	GSK3B	protein	P35222	CTNNB1	UNIPROT	protein	YES	down-regulates activity	phosphorylation
P49841	UNIPROT	GSK3B	protein	P35222	CTNNB1	UNIPROT	protein	YES	down-regulates activity	phosphorylation
P49841	UNIPROT	GSK3B	protein	P35222	CTNNB1	UNIPROT	protein	YES	down-regulates activity	phosphorylation
P49841	UNIPROT	GSK3B	protein	P35222	CTNNB1	UNIPROT	protein	YES	down-regulates activity	phosphorylation
P49841	UNIPROT	GSK3B	protein	P35222	CTNNB1	UNIPROT	protein	YES	down-regulates activity	phosphorylation
P49841	UNIPROT	GSK3B	protein	P35222	CTNNB1	UNIPROT	protein	YES	down-regulates activity	phosphorylation
P49841	UNIPROT	GSK3B	protein	Q16555	DPYSL2	UNIPROT	protein	YES	down-regulates activity	phosphorylation
P49841	UNIPROT	GSK3B	protein	Q16555	DPYSL2	UNIPROT	protein	YES	down-regulates activity	phosphorylation
P49841	UNIPROT	GSK3B	protein	Q13144	EIF2B5	UNIPROT	protein	YES	down-regulates activity	phosphorylation
P49841	UNIPROT	GSK3B	protein	Q13144	EIF2B5	UNIPROT	protein	YES	down-regulates activity	phosphorylation
P49841	UNIPROT	GSK3B	protein	Q13541	EIF4EBP1	UNIPROT	protein	YES	down-regulates activity	phosphorylation
P49841	UNIPROT	GSK3B	protein	Q13541	EIF4EBP1	UNIPROT	protein	YES	down-regulates activity	phosphorylation
P49841	UNIPROT	GSK3B	protein	Q13541	EIF4EBP1	UNIPROT	protein	YES	down-regulates activity	phosphorylation
P49841	UNIPROT	GSK3B	protein	P13807	GYS1	UNIPROT	protein	YES	down-regulates activity	phosphorylation
P49841	UNIPROT	GSK3B	protein	P13807	GYS1	UNIPROT	protein	YES	down-regulates activity	phosphorylation
P49841	UNIPROT	GSK3B	protein	P13807	GYS1	UNIPROT	protein	YES	down-regulates activity	phosphorylation
P49841	UNIPROT	GSK3B	protein	P13807	GYS1	UNIPROT	protein	YES	down-regulates activity	phosphorylation
P49841	UNIPROT	GSK3B	protein	P13807	GYS1	UNIPROT	protein	YES	down-regulates activity	phosphorylation
P49841	UNIPROT	GSK3B	protein	P13807	GYS1	UNIPROT	protein	YES	down-regulates activity	phosphorylation
P49841	UNIPROT	GSK3B	protein	P13807	GYS1	UNIPROT	protein	YES	down-regulates activity	phosphorylation
P49841	UNIPROT	GSK3B	protein	P13807	GYS1	UNIPROT	protein	YES	down-regulates activity	phosphorylation
P49841	UNIPROT	GSK3B	protein	Q14103	HNRNPD	UNIPROT	protein	YES	down-regulates activity	phosphorylation
P49841	UNIPROT	GSK3B	protein	Q00613	HSF1	UNIPROT	protein	YES	down-regulates activity	phosphorylation
P49841	UNIPROT	GSK3B	protein	P05412	JUN	UNIPROT	protein	YES	down-regulates activity	phosphorylation
P49841	UNIPROT	GSK3B	protein	P05412	JUN	UNIPROT	protein	YES	down-regulates activity	phosphorylation
P49841	UNIPROT	GSK3B	protein	P05412	JUN	UNIPROT	protein	YES	down-regulates activity	phosphorylation
P49841	UNIPROT	GSK3B	protein	P60484	PTEN	UNIPROT	protein	YES	down-regulates activity	phosphorylation
P08069	UNIPROT	IGF1R	protein	P46108	CRK	UNIPROT	protein	YES	down-regulates activity	phosphorylation
Q02750	UNIPROT	MAP2K1	protein	P55211	CASP9	UNIPROT	protein	YES	down-regulates activity	phosphorylation
P36507	UNIPROT	MAP2K2	protein	P55211	CASP9	UNIPROT	protein	YES	down-regulates activity	phosphorylation
O14733	UNIPROT	MAP2K7	protein	P35568	IRS1	UNIPROT	protein	YES	down-regulates activity	phosphorylation
P28482	UNIPROT	MAPK1	protein	Q92934	BAD	UNIPROT	protein	YES	down-regulates activity	phosphorylation
P28482	UNIPROT	MAPK1	protein	P15056	BRAF	UNIPROT	protein	YES	down-regulates activity	phosphorylation
P28482	UNIPROT	MAPK1	protein	P55211	CASP9	UNIPROT	protein	YES	down-regulates activity	phosphorylation
P28482	UNIPROT	MAPK1	protein	P55211	CASP9	UNIPROT	protein	YES	down-regulates activity	phosphorylation
P28482	UNIPROT	MAPK1	protein	P00533	EGFR	UNIPROT	protein	YES	down-regulates activity	phosphorylation
P28482	UNIPROT	MAPK1	protein	Q13541	EIF4EBP1	UNIPROT	protein	YES	down-regulates activity	phosphorylation
P28482	UNIPROT	MAPK1	protein	Q13541	EIF4EBP1	UNIPROT	protein	YES	down-regulates activity	phosphorylation
P28482	UNIPROT	MAPK1	protein	Q13541	EIF4EBP1	UNIPROT	protein	YES	down-regulates activity	phosphorylation
P28482	UNIPROT	MAPK1	protein	Q13541	EIF4EBP1	UNIPROT	protein	YES	down-regulates activity	phosphorylation
P28482	UNIPROT	MAPK1	protein	Q13541	EIF4EBP1	UNIPROT	protein	YES	down-regulates activity	phosphorylation
P28482	UNIPROT	MAPK1	protein	Q02750	MAP2K1	UNIPROT	protein	YES	down-regulates activity	phosphorylation
P28482	UNIPROT	MAPK1	protein	Q02750	MAP2K1	UNIPROT	protein	YES	down-regulates activity	phosphorylation
P28482	UNIPROT	MAPK1	protein	Q13153	PAK1	UNIPROT	protein	YES	down-regulates activity	phosphorylation
P28482	UNIPROT	MAPK1	protein	Q07889	SOS1	UNIPROT	protein	YES	down-regulates activity	phosphorylation
P28482	UNIPROT	MAPK1	protein	Q07889	SOS1	UNIPROT	protein	YES	down-regulates activity	phosphorylation
P28482	UNIPROT	MAPK1	protein	Q07889	SOS1	UNIPROT	protein	YES	down-regulates activity	phosphorylation
P28482	UNIPROT	MAPK1	protein	Q07889	SOS1	UNIPROT	protein	YES	down-regulates activity	phosphorylation
P28482	UNIPROT	MAPK1	protein	Q07889	SOS1	UNIPROT	protein	YES	down-regulates activity	phosphorylation
P28482	UNIPROT	MAPK1	protein	Q07889	SOS1	UNIPROT	protein	YES	down-regulates activity	phosphorylation
P28482	UNIPROT	MAPK1	protein	P49815	TSC2	UNIPROT	protein	YES	down-regulates activity	phosphorylation
O15264	UNIPROT	MAPK13	protein	O00418	EEF2K	UNIPROT	protein	YES	down-regulates activity	phosphorylation
O15264	UNIPROT	MAPK13	protein	O00418	EEF2K	UNIPROT	protein	YES	down-regulates activity	phosphorylation
Q16539	UNIPROT	MAPK14	protein	O00418	EEF2K	UNIPROT	protein	YES	down-regulates activity	phosphorylation
Q16539	UNIPROT	MAPK14	protein	Q13541	EIF4EBP1	UNIPROT	protein	YES	down-regulates activity	phosphorylation
Q16539	UNIPROT	MAPK14	protein	Q13541	EIF4EBP1	UNIPROT	protein	YES	down-regulates activity	phosphorylation
Q16539	UNIPROT	MAPK14	protein	Q13541	EIF4EBP1	UNIPROT	protein	YES	down-regulates activity	phosphorylation
Q16539	UNIPROT	MAPK14	protein	Q92945	KHSRP	UNIPROT	protein	YES	down-regulates activity	phosphorylation
Q16539	UNIPROT	MAPK14	protein	Q15750	TAB1	UNIPROT	protein	YES	down-regulates activity	phosphorylation
Q16539	UNIPROT	MAPK14	protein	Q15750	TAB1	UNIPROT	protein	YES	down-regulates activity	phosphorylation
Q16539	UNIPROT	MAPK14	protein	Q15750	TAB1	UNIPROT	protein	YES	down-regulates activity	phosphorylation
P27361	UNIPROT	MAPK3	protein	P55211	CASP9	UNIPROT	protein	YES	down-regulates activity	phosphorylation
P27361	UNIPROT	MAPK3	protein	P61978	HNRNPK	UNIPROT	protein	YES	down-regulates activity	phosphorylation
P27361	UNIPROT	MAPK3	protein	P35568	IRS1	UNIPROT	protein	YES	down-regulates activity	phosphorylation
P27361	UNIPROT	MAPK3	protein	P16949	STMN1	UNIPROT	protein	YES	down-regulates activity	phosphorylation
P27361	UNIPROT	MAPK3	protein	P16949	STMN1	UNIPROT	protein	YES	down-regulates activity	phosphorylation
P27361	UNIPROT	MAPK3	protein	Q93045	STMN2	UNIPROT	protein	YES	down-regulates activity	phosphorylation
P27361	UNIPROT	MAPK3	protein	Q93045	STMN2	UNIPROT	protein	YES	down-regulates activity	phosphorylation
P27361	UNIPROT	MAPK3	protein	P49815	TSC2	UNIPROT	protein	YES	down-regulates activity	phosphorylation
P27361	UNIPROT	MAPK3	protein	P49815	TSC2	UNIPROT	protein	YES	down-regulates activity	phosphorylation
P27361	UNIPROT	MAPK3	protein	P49815	TSC2	UNIPROT	protein	YES	down-regulates activity	phosphorylation
P45983	UNIPROT	MAPK8	protein	Q00613	HSF1	UNIPROT	protein	YES	down-regulates activity	phosphorylation
P49137	UNIPROT	MAPKAPK2	protein	P63104	YWHAZ	UNIPROT	protein	YES	down-regulates activity	phosphorylation
P42345	UNIPROT	MTOR	protein	Q96B36	AKT1S1	UNIPROT	protein	YES	down-regulates activity	phosphorylation
P42345	UNIPROT	MTOR	protein	Q96B36	AKT1S1	UNIPROT	protein	YES	down-regulates activity	phosphorylation
P42345	UNIPROT	MTOR	protein	Q96B36	AKT1S1	UNIPROT	protein	YES	down-regulates activity	phosphorylation

P42345	UNIPROT	MTOR	protein	Q96B36	AKT1S1	UNIPROT	protein	YES	down-regulates activity	phosphorylation
P42345	UNIPROT	MTOR	protein	Q13541	EIF4EBP1	UNIPROT	protein	YES	down-regulates activity	phosphorylation
P42345	UNIPROT	MTOR	protein	Q13541	EIF4EBP1	UNIPROT	protein	YES	down-regulates activity	phosphorylation
P42345	UNIPROT	MTOR	protein	Q13541	EIF4EBP1	UNIPROT	protein	YES	down-regulates activity	phosphorylation
P42345	UNIPROT	MTOR	protein	Q13541	EIF4EBP1	UNIPROT	protein	YES	down-regulates activity	phosphorylation
P42345	UNIPROT	MTOR	protein	Q13541	EIF4EBP1	UNIPROT	protein	YES	down-regulates activity	phosphorylation
P42345	UNIPROT	MTOR	protein	Q13541	EIF4EBP1	UNIPROT	protein	YES	down-regulates activity	phosphorylation
P42345	UNIPROT	MTOR	protein	Q13541	EIF4EBP1	UNIPROT	protein	YES	down-regulates activity	phosphorylation
P42345	UNIPROT	MTOR	protein	Q13541	EIF4EBP1	UNIPROT	protein	YES	down-regulates activity	phosphorylation
P42345	UNIPROT	MTOR	protein	Q13541	EIF4EBP1	UNIPROT	protein	YES	down-regulates activity	phosphorylation
P42345	UNIPROT	MTOR	protein	Q13541	EIF4EBP1	UNIPROT	protein	YES	down-regulates activity	phosphorylation
P42345	UNIPROT	MTOR	protein	Q13541	EIF4EBP1	UNIPROT	protein	YES	down-regulates activity	phosphorylation
P42345	UNIPROT	MTOR	protein	Q13541	EIF4EBP1	UNIPROT	protein	YES	down-regulates activity	phosphorylation
P42345	UNIPROT	MTOR	protein	Q13541	EIF4EBP1	UNIPROT	protein	YES	down-regulates activity	phosphorylation
P42345	UNIPROT	MTOR	protein	Q13541	EIF4EBP1	UNIPROT	protein	YES	down-regulates activity	phosphorylation
P42345	UNIPROT	MTOR	protein	Q13541	EIF4EBP1	UNIPROT	protein	YES	down-regulates activity	phosphorylation
P42345	UNIPROT	MTOR	protein	Q13541	EIF4EBP1	UNIPROT	protein	YES	down-regulates activity	phosphorylation
P42345	UNIPROT	MTOR	protein	Q13541	EIF4EBP1	UNIPROT	protein	YES	down-regulates activity	phosphorylation
P42345	UNIPROT	MTOR	protein	Q13541	EIF4EBP1	UNIPROT	protein	YES	down-regulates activity	phosphorylation
P42345	UNIPROT	MTOR	protein	P35568	IRS1	UNIPROT	protein	YES	down-regulates activity	phosphorylation
P42345	UNIPROT	MTOR	protein	P35568	IRS1	UNIPROT	protein	YES	down-regulates activity	phosphorylation
P42345	UNIPROT	MTOR	protein	P35568	IRS1	UNIPROT	protein	YES	down-regulates activity	phosphorylation
P42345	UNIPROT	MTOR	protein	P35568	IRS1	UNIPROT	protein	YES	down-regulates activity	phosphorylation
P42345	UNIPROT	MTOR	protein	P35568	IRS1	UNIPROT	protein	YES	down-regulates activity	phosphorylation
P42345	UNIPROT	MTOR	protein	P35568	IRS1	UNIPROT	protein	YES	down-regulates activity	phosphorylation
P42345	UNIPROT	MTOR	protein	P35568	IRS1	UNIPROT	protein	YES	down-regulates activity	phosphorylation
O95747	UNIPROT	OXSR1	protein	Q13153	PAK1	UNIPROT	protein	YES	down-regulates activity	phosphorylation
Q13153	UNIPROT	PAK1	protein	Q13363	CTBP1	UNIPROT	protein	YES	down-regulates activity	phosphorylation
Q13153	UNIPROT	PAK1	protein	Q15746	MYLK	UNIPROT	protein	YES	down-regulates activity	phosphorylation
Q13177	UNIPROT	PAK2	protein	Q15746	MYLK	UNIPROT	protein	YES	down-regulates activity	phosphorylation
Q13177	UNIPROT	PAK2	protein	Q15746	MYLK	UNIPROT	protein	YES	down-regulates activity	phosphorylation
Q13177	UNIPROT	PAK2	protein	P08670	VIM	UNIPROT	protein	YES	down-regulates activity	phosphorylation
Q13177	UNIPROT	PAK2	protein	P08670	VIM	UNIPROT	protein	YES	down-regulates activity	phosphorylation
Q13177	UNIPROT	PAK2	protein	P08670	VIM	UNIPROT	protein	YES	down-regulates activity	phosphorylation
Q13177	UNIPROT	PAK2	protein	P08670	VIM	UNIPROT	protein	YES	down-regulates activity	phosphorylation
O15530	UNIPROT	PDPK1	protein	O43865	AHCYL1	UNIPROT	protein	YES	down-regulates activity	phosphorylation
O15530	UNIPROT	PDPK1	protein	O15530	PDPK1	UNIPROT	protein	YES	down-regulates activity	phosphorylation
Q16512	UNIPROT	PKN1	protein	P29966	MARCKS	UNIPROT	protein	YES	down-regulates activity	phosphorylation
Q16512	UNIPROT	PKN1	protein	P29966	MARCKS	UNIPROT	protein	YES	down-regulates activity	phosphorylation
Q16512	UNIPROT	PKN1	protein	P29966	MARCKS	UNIPROT	protein	YES	down-regulates activity	phosphorylation
Q13131	UNIPROT	PRKAA1	protein	Q13085	ACACA	UNIPROT	protein	YES	down-regulates activity	phosphorylation
Q13131	UNIPROT	PRKAA1	protein	O00418	EEF2K	UNIPROT	protein	YES	down-regulates activity	phosphorylation
Q13131	UNIPROT	PRKAA1	protein	O00418	EEF2K	UNIPROT	protein	YES	down-regulates activity	phosphorylation
Q13131	UNIPROT	PRKAA1	protein	Q13131	PRKAA1	UNIPROT	protein	YES	down-regulates activity	phosphorylation
Q13131	UNIPROT	PRKAA1	protein	Q8N122	RPTOR	UNIPROT	protein	YES	down-regulates activity	phosphorylation
Q13131	UNIPROT	PRKAA1	protein	Q8N122	RPTOR	UNIPROT	protein	YES	down-regulates activity	phosphorylation
P17612	UNIPROT	PRKACA	protein	P35611	ADD1	UNIPROT	protein	YES	down-regulates activity	phosphorylation
P17612	UNIPROT	PRKACA	protein	P35611	ADD1	UNIPROT	protein	YES	down-regulates activity	phosphorylation
P17612	UNIPROT	PRKACA	protein	P35611	ADD1	UNIPROT	protein	YES	down-regulates activity	phosphorylation
P17612	UNIPROT	PRKACA	protein	Q92934	BAD	UNIPROT	protein	YES	down-regulates activity	phosphorylation
P17612	UNIPROT	PRKACA	protein	Q92934	BAD	UNIPROT	protein	YES	down-regulates activity	phosphorylation
P17612	UNIPROT	PRKACA	protein	Q92934	BAD	UNIPROT	protein	YES	down-regulates activity	phosphorylation
P17612	UNIPROT	PRKACA	protein	P15056	BRAF	UNIPROT	protein	YES	down-regulates activity	phosphorylation
P17612	UNIPROT	PRKACA	protein	P27708	CAD	UNIPROT	protein	YES	down-regulates activity	phosphorylation
P17612	UNIPROT	PRKACA	protein	P15924	DSP	UNIPROT	protein	YES	down-regulates activity	phosphorylation
P17612	UNIPROT	PRKACA	protein	Q14344	GNA13	UNIPROT	protein	YES	down-regulates activity	phosphorylation
P17612	UNIPROT	PRKACA	protein	P49841	GSK3B	UNIPROT	protein	YES	down-regulates activity	phosphorylation
P17612	UNIPROT	PRKACA	protein	P13807	GYS1	UNIPROT	protein	YES	down-regulates activity	phosphorylation
P17612	UNIPROT	PRKACA	protein	P13807	GYS1	UNIPROT	protein	YES	down-regulates activity	phosphorylation
P17612	UNIPROT	PRKACA	protein	P04035	HMGCR	UNIPROT	protein	YES	down-regulates activity	phosphorylation
P17612	UNIPROT	PRKACA	protein	P05114	HMGN1	UNIPROT	protein	YES	down-regulates activity	phosphorylation
P17612	UNIPROT	PRKACA	protein	Q12972	PPP1R8	UNIPROT	protein	YES	down-regulates activity	phosphorylation
P17612	UNIPROT	PRKACA	protein	Q12972	PPP1R8	UNIPROT	protein	YES	down-regulates activity	phosphorylation
P17612	UNIPROT	PRKACA	protein	Q96SB3	PPP1R9B	UNIPROT	protein	YES	down-regulates activity	phosphorylation
P17612	UNIPROT	PRKACA	protein	Q13131	PRKAA1	UNIPROT	protein	YES	down-regulates activity	phosphorylation
P17612	UNIPROT	PRKACA	protein	Q13131	PRKAA1	UNIPROT	protein	YES	down-regulates activity	phosphorylation
P17612	UNIPROT	PRKACA	protein	P62834	RAP1A	UNIPROT	protein	YES	down-regulates activity	phosphorylation
P17612	UNIPROT	PRKACA	protein	O43665	RGS10	UNIPROT	protein	YES	down-regulates activity	phosphorylation
P17612	UNIPROT	PRKACA	protein	P61586	RHOA	UNIPROT	protein	YES	down-regulates activity	phosphorylation
P17612	UNIPROT	PRKACA	protein	Q01082	SPTBN1	UNIPROT	protein	YES	down-regulates activity	phosphorylation
P17612	UNIPROT	PRKACA	protein	Q93045	STMN2	UNIPROT	protein	YES	down-regulates activity	phosphorylation
P17612	UNIPROT	PRKACA	protein	Q93045	STMN2	UNIPROT	protein	YES	down-regulates activity	phosphorylation
P17612	UNIPROT	PRKACA	protein	Q05086	UBE3A	UNIPROT	protein	YES	down-regulates activity	phosphorylation
P17612	UNIPROT	PRKACA	protein	P08670	VIM	UNIPROT	protein	YES	down-regulates activity	phosphorylation
P17612	UNIPROT	PRKACA	protein	P08670	VIM	UNIPROT	protein	YES	down-regulates activity	phosphorylation
P17612	UNIPROT	PRKACA	protein	P08670	VIM	UNIPROT	protein	YES	down-regulates activity	phosphorylation
P17612	UNIPROT	PRKACA	protein	P08670	VIM	UNIPROT	protein	YES	down-regulates activity	phosphorylation
P17612	UNIPROT	PRKACA	protein	P08670	VIM	UNIPROT	protein	YES	down-regulates activity	phosphorylation
P17612	UNIPROT	PRKACA	protein	P08670	VIM	UNIPROT	protein	YES	down-regulates activity	phosphorylation
P17252	UNIPROT	PRKCA	protein	Q99418	CYTH2	UNIPROT	protein	YES	down-regulates activity	phosphorylation
P17252	UNIPROT	PRKCA	protein	P17844	DDX5	UNIPROT	protein	YES	down-regulates activity	phosphorylation
P17252	UNIPROT	PRKCA	protein	O60869	EDF1	UNIPROT	protein	YES	down-regulates activity	phosphorylation
P17252	UNIPROT	PRKCA	protein	Q06210	GFPT1	UNIPROT	protein	YES	down-regulates activity	phosphorylation
P17252	UNIPROT	PRKCA	protein	P29966	MARCKS	UNIPROT	protein	YES	down-regulates activity	phosphorylation
P17252	UNIPROT	PRKCA	protein	P29966	MARCKS	UNIPROT	protein	YES	down-regulates activity	phosphorylation
P17252	UNIPROT	PRKCA	protein	P29966	MARCKS	UNIPROT	protein	YES	down-regulates activity	phosphorylation
P17252	UNIPROT	PRKCA	protein	P29353	SHC1	UNIPROT	protein	YES	down-regulates activity	phosphorylation
Q05655	UNIPROT	PRKCD	protein	P00533	EGFR	UNIPROT	protein	YES	down-regulates activity	phosphorylation
Q05655	UNIPROT	PRKCD	protein	P35568	IRS1	UNIPROT	protein	YES	down-regulates activity	phosphorylation
Q05655	UNIPROT	PRKCD	protein	P63104	YWHAZ	UNIPROT	protein	YES	down-regulates activity	phosphorylation
P78527	UNIPROT	PRKDC	protein	P15927	RPA2	UNIPROT	protein	YES	down-regulates activity	phosphorylation
P78527	UNIPROT	PRKDC	protein	P15927	RPA2	UNIPROT	protein	YES	down-regulates activity	phosphorylation
P78527	UNIPROT	PRKDC	protein	P15927	RPA2	UNIPROT	protein	YES	down-regulates activity	phosphorylation
P78527	UNIPROT	PRKDC	protein	P15927	RPA2	UNIPROT	protein	YES	down-regulates activity	phosphorylation
P78527	UNIPROT	PRKDC	protein	P15927	RPA2	UNIPROT	protein	YES	down-regulates activity	phosphorylation
Q13976	UNIPROT	PRKG1	protein	Q01970	PLCB3	UNIPROT	protein	YES	down-regulates activity	phosphorylation
Q13976	UNIPROT	PRKG1	protein	Q01970	PLCB3	UNIPROT	protein	YES	down-regulates activity	phosphorylation
Q13976	UNIPROT	PRKG1	protein	Q15637	SF1	UNIPROT	protein	YES	down-regulates activity	phosphorylation

Q05397	UNIPROT	PTK2	protein	P12814	ACTN1	UNIPROT	protein	YES	down-regulates activity	phosphorylation
Q13464	UNIPROT	ROCK1	protein	O14974	PPP1R12A	UNIPROT	protein	YES	down-regulates activity	phosphorylation
Q13464	UNIPROT	ROCK1	protein	P08670	VIM	UNIPROT	protein	YES	down-regulates activity	phosphorylation
O75116	UNIPROT	ROCK2	protein	O14974	PPP1R12A	UNIPROT	protein	YES	down-regulates activity	phosphorylation
Q15418	UNIPROT	RPS6KA1	protein	Q92934	BAD	UNIPROT	protein	YES	down-regulates activity	phosphorylation
Q15418	UNIPROT	RPS6KA1	protein	Q92934	BAD	UNIPROT	protein	YES	down-regulates activity	phosphorylation
Q15418	UNIPROT	RPS6KA1	protein	Q92934	BAD	UNIPROT	protein	YES	down-regulates activity	phosphorylation
Q15418	UNIPROT	RPS6KA1	protein	Q92934	BAD	UNIPROT	protein	YES	down-regulates activity	phosphorylation
Q15418	UNIPROT	RPS6KA1	protein	O00418	EEF2K	UNIPROT	protein	YES	down-regulates activity	phosphorylation
P51812	UNIPROT	RPS6KA3	protein	P49840	GSK3A	UNIPROT	protein	YES	down-regulates activity	phosphorylation
P51812	UNIPROT	RPS6KA3	protein	P05114	HMGN1	UNIPROT	protein	YES	down-regulates activity	phosphorylation
P51812	UNIPROT	RPS6KA3	protein	P05114	HMGN1	UNIPROT	protein	YES	down-regulates activity	phosphorylation
P51812	UNIPROT	RPS6KA3	protein	P05204	HMGN2	UNIPROT	protein	YES	down-regulates activity	phosphorylation
P51812	UNIPROT	RPS6KA3	protein	P05204	HMGN2	UNIPROT	protein	YES	down-regulates activity	phosphorylation
Q9HBY8	UNIPROT	SGK2	protein	O43524	FOXO3	UNIPROT	protein	YES	down-regulates activity	phosphorylation
Q9HBY8	UNIPROT	SGK2	protein	O43524	FOXO3	UNIPROT	protein	YES	down-regulates activity	phosphorylation
Q9Y2K2	UNIPROT	SIK3	protein	Q53ET0	CRTC2	UNIPROT	protein	YES	down-regulates activity	phosphorylation
Q9Y2K2	UNIPROT	SIK3	protein	Q53ET0	CRTC2	UNIPROT	protein	YES	down-regulates activity	phosphorylation
Q9Y2K2	UNIPROT	SIK3	protein	Q53ET0	CRTC2	UNIPROT	protein	YES	down-regulates activity	phosphorylation
P12931	UNIPROT	SRC	protein	P56945	BCAR1	UNIPROT	protein	YES	down-regulates activity	phosphorylation
P12931	UNIPROT	SRC	protein	P56945	BCAR1	UNIPROT	protein	YES	down-regulates activity	phosphorylation
P12931	UNIPROT	SRC	protein	P56945	BCAR1	UNIPROT	protein	YES	down-regulates activity	phosphorylation
P12931	UNIPROT	SRC	protein	P56945	BCAR1	UNIPROT	protein	YES	down-regulates activity	phosphorylation
P12931	UNIPROT	SRC	protein	P56945	BCAR1	UNIPROT	protein	YES	down-regulates activity	phosphorylation
P12931	UNIPROT	SRC	protein	P56945	BCAR1	UNIPROT	protein	YES	down-regulates activity	phosphorylation
P12931	UNIPROT	SRC	protein	P56945	BCAR1	UNIPROT	protein	YES	down-regulates activity	phosphorylation
P12931	UNIPROT	SRC	protein	P56945	BCAR1	UNIPROT	protein	YES	down-regulates activity	phosphorylation
P12931	UNIPROT	SRC	protein	P56945	BCAR1	UNIPROT	protein	YES	down-regulates activity	phosphorylation
P12931	UNIPROT	SRC	protein	P56945	BCAR1	UNIPROT	protein	YES	down-regulates activity	phosphorylation
P12931	UNIPROT	SRC	protein	P35222	CTNNB1	UNIPROT	protein	YES	down-regulates activity	phosphorylation
P12931	UNIPROT	SRC	protein	Q14247	CTTN	UNIPROT	protein	YES	down-regulates activity	phosphorylation
P12931	UNIPROT	SRC	protein	P01112	HRAS	UNIPROT	protein	YES	down-regulates activity	phosphorylation
P12931	UNIPROT	SRC	protein	P10301	RRAS	UNIPROT	protein	YES	down-regulates activity	phosphorylation
P12931	UNIPROT	SRC	protein	P12931	SRC	UNIPROT	protein	YES	down-regulates activity	phosphorylation
P12931	UNIPROT	SRC	protein	P18206	VCL	UNIPROT	protein	YES	down-regulates activity	phosphorylation
P12931	UNIPROT	SRC	protein	P18206	VCL	UNIPROT	protein	YES	down-regulates activity	phosphorylation
P37173	UNIPROT	TGFBR2	protein	P37173	TGFBR2	UNIPROT	protein	YES	down-regulates activity	phosphorylation
P37173	UNIPROT	TGFBR2	protein	P37173	TGFBR2	UNIPROT	protein	YES	down-regulates activity	phosphorylation
P17252	UNIPROT	PRKCA	protein	P22681	CBL	UNIPROT	protein	YES	down-regulates quantity	phosphorylation
P17252	UNIPROT	PRKCA	protein	P22681	CBL	UNIPROT	protein	YES	down-regulates quantity	phosphorylation
P17252	UNIPROT	PRKCA	protein	P22681	CBL	UNIPROT	protein	YES	down-regulates quantity	phosphorylation
P17252	UNIPROT	PRKCA	protein	P22681	CBL	UNIPROT	protein	YES	down-regulates quantity	phosphorylation
P12931	UNIPROT	SRC	protein	P29317	EPHA2	UNIPROT	protein	YES	down-regulates quantity	phosphorylation
P31749	UNIPROT	AKT1	protein	Q12778	FOXO1	UNIPROT	protein	YES	down-regulates quantity by destabilization	phosphorylation
P31749	UNIPROT	AKT1	protein	O43524	FOXO3	UNIPROT	protein	YES	down-regulates quantity by destabilization	phosphorylation
P31749	UNIPROT	AKT1	protein	O43524	FOXO3	UNIPROT	protein	YES	down-regulates quantity by destabilization	phosphorylation
P31749	UNIPROT	AKT1	protein	O43524	FOXO3	UNIPROT	protein	YES	down-regulates quantity by destabilization	phosphorylation
P31751	UNIPROT	AKT2	protein	O43524	FOXO3	UNIPROT	protein	YES	down-regulates quantity by destabilization	phosphorylation
P31751	UNIPROT	AKT2	protein	O43524	FOXO3	UNIPROT	protein	YES	down-regulates quantity by destabilization	phosphorylation
P31751	UNIPROT	AKT2	protein	O43524	FOXO3	UNIPROT	protein	YES	down-regulates quantity by destabilization	phosphorylation
O15111	UNIPROT	CHUK	protein	P19838	NFKB1	UNIPROT	protein	YES	down-regulates quantity by destabilization	phosphorylation
O15111	UNIPROT	CHUK	protein	P19838	NFKB1	UNIPROT	protein	YES	down-regulates quantity by destabilization	phosphorylation
O15111	UNIPROT	CHUK	protein	P19838	NFKB1	UNIPROT	protein	YES	down-regulates quantity by destabilization	phosphorylation
O15111	UNIPROT	CHUK	protein	P19838	NFKB1	UNIPROT	protein	YES	down-regulates quantity by destabilization	phosphorylation
O15111	UNIPROT	CHUK	protein	P19838	NFKB1	UNIPROT	protein	YES	down-regulates quantity by destabilization	phosphorylation
O15111	UNIPROT	CHUK	protein	P19838	NFKB1	UNIPROT	protein	YES	down-regulates quantity by destabilization	phosphorylation
O15111	UNIPROT	CHUK	protein	P19838	NFKB1	UNIPROT	protein	YES	down-regulates quantity by destabilization	phosphorylation
P00533	UNIPROT	EGFR	protein	P00533	EGFR	UNIPROT	protein	YES	down-regulates quantity by destabilization	phosphorylation
P49840	UNIPROT	GSK3A	protein	Q07820	MCL1	UNIPROT	protein	YES	down-regulates quantity by destabilization	phosphorylation
P49841	UNIPROT	GSK3B	protein	Q07820	MCL1	UNIPROT	protein	YES	down-regulates quantity by destabilization	phosphorylation
P49841	UNIPROT	GSK3B	protein	P01106	MYC	UNIPROT	protein	YES	down-regulates quantity by destabilization	phosphorylation
P49841	UNIPROT	GSK3B	protein	P01106	MYC	UNIPROT	protein	YES	down-regulates quantity by destabilization	phosphorylation
P49841	UNIPROT	GSK3B	protein	P01106	MYC	UNIPROT	protein	YES	down-regulates quantity by destabilization	phosphorylation
P49841	UNIPROT	GSK3B	protein	P84022	SMAD3	UNIPROT	protein	YES	down-regulates quantity by destabilization	phosphorylation
P28482	UNIPROT	MAPK1	protein	O43524	FOXO3	UNIPROT	protein	YES	down-regulates quantity by destabilization	phosphorylation
P28482	UNIPROT	MAPK1	protein	O43524	FOXO3	UNIPROT	protein	YES	down-regulates quantity by destabilization	phosphorylation
P28482	UNIPROT	MAPK1	protein	O43524	FOXO3	UNIPROT	protein	YES	down-regulates quantity by destabilization	phosphorylation
Q16539	UNIPROT	MAPK14	protein	Q15910	EZH2	UNIPROT	protein	YES	down-regulates quantity by destabilization	phosphorylation
P27361	UNIPROT	MAPK3	protein	O43524	FOXO3	UNIPROT	protein	YES	down-regulates quantity by destabilization	phosphorylation
P27361	UNIPROT	MAPK3	protein	O43524	FOXO3	UNIPROT	protein	YES	down-regulates quantity by destabilization	phosphorylation
P27361	UNIPROT	MAPK3	protein	O43524	FOXO3	UNIPROT	protein	YES	down-regulates quantity by destabilization	phosphorylation
P45983	UNIPROT	MAPK8	protein	P35568	IRS1	UNIPROT	protein	YES	down-regulates quantity by destabilization	phosphorylation
P17612	UNIPROT	PRKACA	protein	P35568	IRS1	UNIPROT	protein	YES	down-regulates quantity by destabilization	phosphorylation
P17612	UNIPROT	PRKACA	protein	P35568	IRS1	UNIPROT	protein	YES	down-regulates quantity by destabilization	phosphorylation
P17612	UNIPROT	PRKACA	protein	P35568	IRS1	UNIPROT	protein	YES	down-regulates quantity by destabilization	phosphorylation
P17252	UNIPROT	PRKCA	protein	Q9UEY8	ADD3	UNIPROT	protein	YES	down-regulates quantity by destabilization	phosphorylation
P17252	UNIPROT	PRKCA	protein	P08670	VIM	UNIPROT	protein	YES	down-regulates quantity by destabilization	phosphorylation
P17252	UNIPROT	PRKCA	protein	P08670	VIM	UNIPROT	protein	YES	down-regulates quantity by destabilization	phosphorylation
P17252	UNIPROT	PRKCA	protein	P08670	VIM	UNIPROT	protein	YES	down-regulates quantity by destabilization	phosphorylation
P17252	UNIPROT	PRKCA	protein	P08670	VIM	UNIPROT	protein	YES	down-regulates quantity by destabilization	phosphorylation
P17252	UNIPROT	PRKCA	protein	P08670	VIM	UNIPROT	protein	YES	down-regulates quantity by destabilization	phosphorylation
P17252	UNIPROT	PRKCA	protein	P08670	VIM	UNIPROT	protein	YES	down-regulates quantity by destabilization	phosphorylation
P17252	UNIPROT	PRKCA	protein	P08670	VIM	UNIPROT	protein	YES	down-regulates quantity by destabilization	phosphorylation
P17252	UNIPROT	PRKCA	protein	P08670	VIM	UNIPROT	protein	YES	down-regulates quantity by destabilization	phosphorylation
P17252	UNIPROT	PRKCA	protein	P08670	VIM	UNIPROT	protein	YES	down-regulates quantity by destabilization	phosphorylation
P17252	UNIPROT	PRKCA	protein	P08670	VIM	UNIPROT	protein	YES	down-regulates quantity by destabilization	phosphorylation
P17252	UNIPROT	PRKCA	protein	P08670	VIM	UNIPROT	protein	YES	down-regulates quantity by destabilization	phosphorylation
Q15418	UNIPROT	RPS6KA1	protein	P35568	IRS1	UNIPROT	protein	YES	down-regulates quantity by destabilization	phosphorylation
P17612	UNIPROT	PRKACA	protein	P01100	FOS	UNIPROT	protein	YES	down-regulates quantity by repression	phosphorylation
Q2M2I8	UNIPROT	AAK1	protein	P49757	NUMB	UNIPROT	protein	YES	unknown	phosphorylation
P31749	UNIPROT	AKT1	protein	Q15027	ACAP1	UNIPROT	protein	YES	unknown	phosphorylation
P31749	UNIPROT	AKT1	protein	P53396	ACLY	UNIPROT	protein	YES	unknown	phosphorylation
P31749	UNIPROT	AKT1	protein	P53365	ARFIP2	UNIPROT	protein	YES	unknown	phosphorylation
P31749	UNIPROT	AKT1	protein	Q9Y2V2	CARHSP1	UNIPROT	protein	YES	unknown	phosphorylation
P31749	UNIPROT	AKT1	protein	P78371	CCT2	UNIPROT	protein	YES	unknown	phosphorylation
P31749	UNIPROT	AKT1	protein	Q9BZQ8	FAM129A	UNIPROT	protein	YES	unknown	phosphorylation
P31749	UNIPROT	AKT1	protein	P42858	HTT	UNIPROT	protein	YES	unknown	phosphorylation
P31749	UNIPROT	AKT1	protein	P42345	MTOR	UNIPROT	protein	YES	unknown	phosphorylation

P31749	UNIPROT	AKT1	protein	O60825	PFKFB2	UNIPROT	protein	YES	unknown	phosphorylation
P31749	UNIPROT	AKT1	protein	O60825	PFKFB2	UNIPROT	protein	YES	unknown	phosphorylation
P31749	UNIPROT	AKT1	protein	Q9H6Z4	RANBP3	UNIPROT	protein	YES	unknown	phosphorylation
P31749	UNIPROT	AKT1	protein	O60343	TBC1D4	UNIPROT	protein	YES	unknown	phosphorylation
P31749	UNIPROT	AKT1	protein	P63104	YWHAZ	UNIPROT	protein	YES	unknown	phosphorylation
P31749	UNIPROT	AKT1	protein	P63104	YWHAZ	UNIPROT	protein	YES	unknown	phosphorylation
P31751	UNIPROT	AKT2	protein	P53396	ACLY	UNIPROT	protein	YES	unknown	phosphorylation
Q96GD4	UNIPROT	AURKB	protein	Q9BQG0	MYBBP1A	UNIPROT	protein	YES	unknown	phosphorylation
Q96GD4	UNIPROT	AURKB	protein	Q8IX03	WWC1	UNIPROT	protein	YES	unknown	phosphorylation
P11274	UNIPROT	BCR	protein	P27348	YWHAQ	UNIPROT	protein	YES	unknown	phosphorylation
P11274	UNIPROT	BCR	protein	P63104	YWHAZ	UNIPROT	protein	YES	unknown	phosphorylation
Q9UQM7	UNIPROT	CAMK2A	protein	P05783	KRT18	UNIPROT	protein	YES	unknown	phosphorylation
Q13554	UNIPROT	CAMK2B	protein	Q01970	PLCB3	UNIPROT	protein	YES	unknown	phosphorylation
Q13557	UNIPROT	CAMK2D	protein	P55327	TPD52	UNIPROT	protein	YES	unknown	phosphorylation
Q13557	UNIPROT	CAMK2D	protein	P55327	TPD52	UNIPROT	protein	YES	unknown	phosphorylation
Q13557	UNIPROT	CAMK2D	protein	P08670	VIM	UNIPROT	protein	YES	unknown	phosphorylation
Q13555	UNIPROT	CAMK2G	protein	P21333	FLNA	UNIPROT	protein	YES	unknown	phosphorylation
Q13555	UNIPROT	CAMK2G	protein	Q15121	PEA15	UNIPROT	protein	YES	unknown	phosphorylation
Q13555	UNIPROT	CAMK2G	protein	Q01970	PLCB3	UNIPROT	protein	YES	unknown	phosphorylation
Q13555	UNIPROT	CAMK2G	protein	P35270	SPR	UNIPROT	protein	YES	unknown	phosphorylation
P06493	UNIPROT	CDK1	protein	Q06481	APLP2	UNIPROT	protein	YES	unknown	phosphorylation
P06493	UNIPROT	CDK1	protein	Q12959	DLG1	UNIPROT	protein	YES	unknown	phosphorylation
P06493	UNIPROT	CDK1	protein	Q12959	DLG1	UNIPROT	protein	YES	unknown	phosphorylation
P06493	UNIPROT	CDK1	protein	P29692	EEF1D	UNIPROT	protein	YES	unknown	phosphorylation
P06493	UNIPROT	CDK1	protein	Q07666	KHDRBS1	UNIPROT	protein	YES	unknown	phosphorylation
P06493	UNIPROT	CDK1	protein	P06748	NPM1	UNIPROT	protein	YES	unknown	phosphorylation
P06493	UNIPROT	CDK1	protein	Q96KB5	PBK	UNIPROT	protein	YES	unknown	phosphorylation
P06493	UNIPROT	CDK1	protein	P18031	PTPN1	UNIPROT	protein	YES	unknown	phosphorylation
P06493	UNIPROT	CDK1	protein	P17706	PTPN2	UNIPROT	protein	YES	unknown	phosphorylation
P06493	UNIPROT	CDK1	protein	P61020	RAB5B	UNIPROT	protein	YES	unknown	phosphorylation
P06493	UNIPROT	CDK1	protein	P47736	RAP1GAP	UNIPROT	protein	YES	unknown	phosphorylation
P06493	UNIPROT	CDK1	protein	Q8N122	RPTOR	UNIPROT	protein	YES	unknown	phosphorylation
P06493	UNIPROT	CDK1	protein	Q8N122	RPTOR	UNIPROT	protein	YES	unknown	phosphorylation
P06493	UNIPROT	CDK1	protein	P31350	RRM2	UNIPROT	protein	YES	unknown	phosphorylation
P06493	UNIPROT	CDK1	protein	P11388	TOP2A	UNIPROT	protein	YES	unknown	phosphorylation
P06493	UNIPROT	CDK1	protein	P11388	TOP2A	UNIPROT	protein	YES	unknown	phosphorylation
P06493	UNIPROT	CDK1	protein	P11388	TOP2A	UNIPROT	protein	YES	unknown	phosphorylation
P06493	UNIPROT	CDK1	protein	P11388	TOP2A	UNIPROT	protein	YES	unknown	phosphorylation
P06493	UNIPROT	CDK1	protein	Q92574	TSC1	UNIPROT	protein	YES	unknown	phosphorylation
P06493	UNIPROT	CDK1	protein	Q92574	TSC1	UNIPROT	protein	YES	unknown	phosphorylation
P06493	UNIPROT	CDK1	protein	Q92574	TSC1	UNIPROT	protein	YES	unknown	phosphorylation
P21127	UNIPROT	CDK11B	protein	O00303	EIF3F	UNIPROT	protein	YES	unknown	phosphorylation
P24941	UNIPROT	CDK2	protein	Q8WXE1	ATRIP	UNIPROT	protein	YES	unknown	phosphorylation
P24941	UNIPROT	CDK2	protein	P50613	CDK7	UNIPROT	protein	YES	unknown	phosphorylation
P24941	UNIPROT	CDK2	protein	P06748	NPM1	UNIPROT	protein	YES	unknown	phosphorylation
P24941	UNIPROT	CDK2	protein	P17706	PTPN2	UNIPROT	protein	YES	unknown	phosphorylation
P24941	UNIPROT	CDK2	protein	P30050	RPL12	UNIPROT	protein	YES	unknown	phosphorylation
P24941	UNIPROT	CDK2	protein	O75533	SF3B1	UNIPROT	protein	YES	unknown	phosphorylation
P24941	UNIPROT	CDK2	protein	P84022	SMAD3	UNIPROT	protein	YES	unknown	phosphorylation
P24941	UNIPROT	CDK2	protein	P84022	SMAD3	UNIPROT	protein	YES	unknown	phosphorylation
P11802	UNIPROT	CDK4	protein	P84022	SMAD3	UNIPROT	protein	YES	unknown	phosphorylation
P11802	UNIPROT	CDK4	protein	P84022	SMAD3	UNIPROT	protein	YES	unknown	phosphorylation
Q00535	UNIPROT	CDK5	protein	P05067	APP	UNIPROT	protein	YES	unknown	phosphorylation
Q00535	UNIPROT	CDK5	protein	P41236	PPP1R2	UNIPROT	protein	YES	unknown	phosphorylation
P50613	UNIPROT	CDK7	protein	P04637	TP53	UNIPROT	protein	YES	unknown	phosphorylation
P50750	UNIPROT	CDK9	protein	P04637	TP53	UNIPROT	protein	YES	unknown	phosphorylation
O96017	UNIPROT	CHEK2	protein	P29590	PML	UNIPROT	protein	YES	unknown	phosphorylation
O96017	UNIPROT	CHEK2	protein	P33981	TTK	UNIPROT	protein	YES	unknown	phosphorylation
P41240	UNIPROT	CSK	protein	P06396	GSN	UNIPROT	protein	YES	unknown	phosphorylation
P41240	UNIPROT	CSK	protein	P06396	GSN	UNIPROT	protein	YES	unknown	phosphorylation
P41240	UNIPROT	CSK	protein	P06396	GSN	UNIPROT	protein	YES	unknown	phosphorylation
P41240	UNIPROT	CSK	protein	P06396	GSN	UNIPROT	protein	YES	unknown	phosphorylation
P41240	UNIPROT	CSK	protein	P06396	GSN	UNIPROT	protein	YES	unknown	phosphorylation
P48729	UNIPROT	CSNK1A1	protein	O43865	AHCYL1	UNIPROT	protein	YES	unknown	phosphorylation
P48729	UNIPROT	CSNK1A1	protein	Q13144	EIF2B5	UNIPROT	protein	YES	unknown	phosphorylation
P48729	UNIPROT	CSNK1A1	protein	Q13144	EIF2B5	UNIPROT	protein	YES	unknown	phosphorylation
P48729	UNIPROT	CSNK1A1	protein	P17931	LGALS3	UNIPROT	protein	YES	unknown	phosphorylation
P48729	UNIPROT	CSNK1A1	protein	P04637	TP53	UNIPROT	protein	YES	unknown	phosphorylation
P68400	UNIPROT	CSNK2A1	protein	Q8NE71	ABCF1	UNIPROT	protein	YES	unknown	phosphorylation
P68400	UNIPROT	CSNK2A1	protein	Q8NE71	ABCF1	UNIPROT	protein	YES	unknown	phosphorylation
P68400	UNIPROT	CSNK2A1	protein	Q13085	ACACA	UNIPROT	protein	YES	unknown	phosphorylation
P68400	UNIPROT	CSNK2A1	protein	O00170	AIP	UNIPROT	protein	YES	unknown	phosphorylation
P68400	UNIPROT	CSNK2A1	protein	O14958	CASQ2	UNIPROT	protein	YES	unknown	phosphorylation
P68400	UNIPROT	CSNK2A1	protein	P09497	CLTB	UNIPROT	protein	YES	unknown	phosphorylation
P68400	UNIPROT	CSNK2A1	protein	P09497	CLTB	UNIPROT	protein	YES	unknown	phosphorylation
P68400	UNIPROT	CSNK2A1	protein	P24534	EEF1B2	UNIPROT	protein	YES	unknown	phosphorylation
P68400	UNIPROT	CSNK2A1	protein	P24534	EEF1B2	UNIPROT	protein	YES	unknown	phosphorylation
P68400	UNIPROT	CSNK2A1	protein	P29692	EEF1D	UNIPROT	protein	YES	unknown	phosphorylation
P68400	UNIPROT	CSNK2A1	protein	P60983	GMFB	UNIPROT	protein	YES	unknown	phosphorylation
P68400	UNIPROT	CSNK2A1	protein	P13807	GYS1	UNIPROT	protein	YES	unknown	phosphorylation
P68400	UNIPROT	CSNK2A1	protein	P13807	GYS1	UNIPROT	protein	YES	unknown	phosphorylation
P68400	UNIPROT	CSNK2A1	protein	P13807	GYS1	UNIPROT	protein	YES	unknown	phosphorylation
P68400	UNIPROT	CSNK2A1	protein	P13807	GYS1	UNIPROT	protein	YES	unknown	phosphorylation
P68400	UNIPROT	CSNK2A1	protein	P13807	GYS1	UNIPROT	protein	YES	unknown	phosphorylation
P68400	UNIPROT	CSNK2A1	protein	P13807	GYS1	UNIPROT	protein	YES	unknown	phosphorylation
P68400	UNIPROT	CSNK2A1	protein	P17096	HMGA1	UNIPROT	protein	YES	unknown	phosphorylation
P68400	UNIPROT	CSNK2A1	protein	P17096	HMGA1	UNIPROT	protein	YES	unknown	phosphorylation
P68400	UNIPROT	CSNK2A1	protein	P17096	HMGA1	UNIPROT	protein	YES	unknown	phosphorylation
P68400	UNIPROT	CSNK2A1	protein	P07900	HSP90AA1	UNIPROT	protein	YES	unknown	phosphorylation
P68400	UNIPROT	CSNK2A1	protein	P07900	HSP90AA1	UNIPROT	protein	YES	unknown	phosphorylation
P68400	UNIPROT	CSNK2A1	protein	P11717	IGF2R	UNIPROT	protein	YES	unknown	phosphorylation
P68400	UNIPROT	CSNK2A1	protein	P11717	IGF2R	UNIPROT	protein	YES	unknown	phosphorylation
P68400	UNIPROT	CSNK2A1	protein	P35568	IRS1	UNIPROT	protein	YES	unknown	phosphorylation
P68400	UNIPROT	CSNK2A1	protein	P35568	IRS1	UNIPROT	protein	YES	unknown	phosphorylation
P68400	UNIPROT	CSNK2A1	protein	P35568	IRS1	UNIPROT	protein	YES	unknown	phosphorylation
P68400	UNIPROT	CSNK2A1	protein	P35568	IRS1	UNIPROT	protein	YES	unknown	phosphorylation
P68400	UNIPROT	CSNK2A1	protein	P35568	IRS1	UNIPROT	protein	YES	unknown	phosphorylation
P68400	UNIPROT	CSNK2A1	protein	P35568	IRS1	UNIPROT	protein	YES	unknown	phosphorylation

P68400	UNIPROT	CSNK2A1	protein	O43896	KIF1C	UNIPROT	protein	YES	unknown	phosphorylation
P68400	UNIPROT	CSNK2A1	protein	P45984	MAPK9	UNIPROT	protein	YES	unknown	phosphorylation
P68400	UNIPROT	CSNK2A1	protein	P45984	MAPK9	UNIPROT	protein	YES	unknown	phosphorylation
P68400	UNIPROT	CSNK2A1	protein	P25788	PSMA3	UNIPROT	protein	YES	unknown	phosphorylation
P68400	UNIPROT	CSNK2A1	protein	P25788	PSMA3	UNIPROT	protein	YES	unknown	phosphorylation
P68400	UNIPROT	CSNK2A1	protein	P18031	PTPN1	UNIPROT	protein	YES	unknown	phosphorylation
P68400	UNIPROT	CSNK2A1	protein	Q96EB6	SIRT1	UNIPROT	protein	YES	unknown	phosphorylation
P68400	UNIPROT	CSNK2A1	protein	Q96EB6	SIRT1	UNIPROT	protein	YES	unknown	phosphorylation
P68400	UNIPROT	CSNK2A1	protein	Q9UQE7	SMC3	UNIPROT	protein	YES	unknown	phosphorylation
P68400	UNIPROT	CSNK2A1	protein	P11388	TOP2A	UNIPROT	protein	YES	unknown	phosphorylation
P68400	UNIPROT	CSNK2A1	protein	P11388	TOP2A	UNIPROT	protein	YES	unknown	phosphorylation
P19784	UNIPROT	CSNK2A2	protein	Q13085	ACACA	UNIPROT	protein	YES	unknown	phosphorylation
P19784	UNIPROT	CSNK2A2	protein	O14958	CASQ2	UNIPROT	protein	YES	unknown	phosphorylation
P19784	UNIPROT	CSNK2A2	protein	P09497	CLTB	UNIPROT	protein	YES	unknown	phosphorylation
P19784	UNIPROT	CSNK2A2	protein	P09497	CLTB	UNIPROT	protein	YES	unknown	phosphorylation
P19784	UNIPROT	CSNK2A2	protein	P24534	EEF1B2	UNIPROT	protein	YES	unknown	phosphorylation
P19784	UNIPROT	CSNK2A2	protein	P24534	EEF1B2	UNIPROT	protein	YES	unknown	phosphorylation
P19784	UNIPROT	CSNK2A2	protein	P17096	HMGA1	UNIPROT	protein	YES	unknown	phosphorylation
P19784	UNIPROT	CSNK2A2	protein	P17096	HMGA1	UNIPROT	protein	YES	unknown	phosphorylation
P19784	UNIPROT	CSNK2A2	protein	P17096	HMGA1	UNIPROT	protein	YES	unknown	phosphorylation
P19784	UNIPROT	CSNK2A2	protein	P07910	HNRNPC	UNIPROT	protein	YES	unknown	phosphorylation
P19784	UNIPROT	CSNK2A2	protein	O43896	KIF1C	UNIPROT	protein	YES	unknown	phosphorylation
P19784	UNIPROT	CSNK2A2	protein	P18031	PTPN1	UNIPROT	protein	YES	unknown	phosphorylation
P19784	UNIPROT	CSNK2A2	protein	Q9UQE7	SMC3	UNIPROT	protein	YES	unknown	phosphorylation
P67870	UNIPROT	CSNK2B	protein	P67870	CSNK2B	UNIPROT	protein	YES	unknown	phosphorylation
P67870	UNIPROT	CSNK2B	protein	P35568	IRS1	UNIPROT	protein	YES	unknown	phosphorylation
P67870	UNIPROT	CSNK2B	protein	P35568	IRS1	UNIPROT	protein	YES	unknown	phosphorylation
P67870	UNIPROT	CSNK2B	protein	P35568	IRS1	UNIPROT	protein	YES	unknown	phosphorylation
P67870	UNIPROT	CSNK2B	protein	P35568	IRS1	UNIPROT	protein	YES	unknown	phosphorylation
P67870	UNIPROT	CSNK2B	protein	P35568	IRS1	UNIPROT	protein	YES	unknown	phosphorylation
P67870	UNIPROT	CSNK2B	protein	P35568	IRS1	UNIPROT	protein	YES	unknown	phosphorylation
P67870	UNIPROT	CSNK2B	protein	Q16625	OCLN	UNIPROT	protein	YES	unknown	phosphorylation
P67870	UNIPROT	CSNK2B	protein	Q16625	OCLN	UNIPROT	protein	YES	unknown	phosphorylation
P53355	UNIPROT	DAPK1	protein	P25205	MCM3	UNIPROT	protein	YES	unknown	phosphorylation
P53355	UNIPROT	DAPK1	protein	P46777	RPL5	UNIPROT	protein	YES	unknown	phosphorylation
Q13627	UNIPROT	DYRK1A	protein	O75533	SF3B1	UNIPROT	protein	YES	unknown	phosphorylation
Q13627	UNIPROT	DYRK1A	protein	Q07955	SRSF1	UNIPROT	protein	YES	unknown	phosphorylation
P00533	UNIPROT	EGFR	protein	O60716	CTNND1	UNIPROT	protein	YES	unknown	phosphorylation
P00533	UNIPROT	EGFR	protein	P15311	EZR	UNIPROT	protein	YES	unknown	phosphorylation
P00533	UNIPROT	EGFR	protein	P62993	GRB2	UNIPROT	protein	YES	unknown	phosphorylation
P00533	UNIPROT	EGFR	protein	P20936	RASA1	UNIPROT	protein	YES	unknown	phosphorylation
P00533	UNIPROT	EGFR	protein	O14828	SCAMP3	UNIPROT	protein	YES	unknown	phosphorylation
P00533	UNIPROT	EGFR	protein	O75886	STAM2	UNIPROT	protein	YES	unknown	phosphorylation
P21802	UNIPROT	FGFR2	protein	P60484	PTEN	UNIPROT	protein	YES	unknown	phosphorylation
P25098	UNIPROT	GRK2	protein	P09497	CLTB	UNIPROT	protein	YES	unknown	phosphorylation
P49841	UNIPROT	GSK3B	protein	P05067	APP	UNIPROT	protein	YES	unknown	phosphorylation
P49841	UNIPROT	GSK3B	protein	O60716	CTNND1	UNIPROT	protein	YES	unknown	phosphorylation
P49841	UNIPROT	GSK3B	protein	O60716	CTNND1	UNIPROT	protein	YES	unknown	phosphorylation
P28482	UNIPROT	MAPK1	protein	Q01844	EWSR1	UNIPROT	protein	YES	unknown	phosphorylation
P28482	UNIPROT	MAPK1	protein	Q9H8Y8	GORASP2	UNIPROT	protein	YES	unknown	phosphorylation
P28482	UNIPROT	MAPK1	protein	P49790	NUP153	UNIPROT	protein	YES	unknown	phosphorylation
P28482	UNIPROT	MAPK1	protein	P49790	NUP153	UNIPROT	protein	YES	unknown	phosphorylation
P28482	UNIPROT	MAPK1	protein	P47712	PLA2G4A	UNIPROT	protein	YES	unknown	phosphorylation
P28482	UNIPROT	MAPK1	protein	Q96SB3	PPP1R9B	UNIPROT	protein	YES	unknown	phosphorylation
P28482	UNIPROT	MAPK1	protein	P23396	RPS3	UNIPROT	protein	YES	unknown	phosphorylation
P28482	UNIPROT	MAPK1	protein	Q8N122	RPTOR	UNIPROT	protein	YES	unknown	phosphorylation
P28482	UNIPROT	MAPK1	protein	Q8N122	RPTOR	UNIPROT	protein	YES	unknown	phosphorylation
P28482	UNIPROT	MAPK1	protein	Q8N122	RPTOR	UNIPROT	protein	YES	unknown	phosphorylation
P28482	UNIPROT	MAPK1	protein	P84022	SMAD3	UNIPROT	protein	YES	unknown	phosphorylation
P28482	UNIPROT	MAPK1	protein	P84022	SMAD3	UNIPROT	protein	YES	unknown	phosphorylation
P28482	UNIPROT	MAPK1	protein	Q8IX03	WWC1	UNIPROT	protein	YES	unknown	phosphorylation
Q16539	UNIPROT	MAPK14	protein	Q01844	EWSR1	UNIPROT	protein	YES	unknown	phosphorylation
Q16539	UNIPROT	MAPK14	protein	P49023	PXN	UNIPROT	protein	YES	unknown	phosphorylation
P27361	UNIPROT	MAPK3	protein	Q01844	EWSR1	UNIPROT	protein	YES	unknown	phosphorylation
P27361	UNIPROT	MAPK3	protein	P49790	NUP153	UNIPROT	protein	YES	unknown	phosphorylation
P27361	UNIPROT	MAPK3	protein	P49790	NUP153	UNIPROT	protein	YES	unknown	phosphorylation
P27361	UNIPROT	MAPK3	protein	P23396	RPS3	UNIPROT	protein	YES	unknown	phosphorylation
P27361	UNIPROT	MAPK3	protein	Q8IX03	WWC1	UNIPROT	protein	YES	unknown	phosphorylation
P49137	UNIPROT	MAPKAPK2	protein	O15511	ARPC5	UNIPROT	protein	YES	unknown	phosphorylation
P49137	UNIPROT	MAPKAPK2	protein	O00418	EEF2K	UNIPROT	protein	YES	unknown	phosphorylation
P49137	UNIPROT	MAPKAPK2	protein	Q9NQC3	RTN4	UNIPROT	protein	YES	unknown	phosphorylation
P49137	UNIPROT	MAPKAPK2	protein	P49815	TSC2	UNIPROT	protein	YES	unknown	phosphorylation
Q16644	UNIPROT	MAPKAPK3	protein	O00418	EEF2K	UNIPROT	protein	YES	unknown	phosphorylation
Q16644	UNIPROT	MAPKAPK3	protein	P04792	HSPB1	UNIPROT	protein	YES	unknown	phosphorylation
Q16644	UNIPROT	MAPKAPK3	protein	P04792	HSPB1	UNIPROT	protein	YES	unknown	phosphorylation
Q16644	UNIPROT	MAPKAPK3	protein	P04792	HSPB1	UNIPROT	protein	YES	unknown	phosphorylation
P15531	UNIPROT	NME1	protein	P15531	NME1	UNIPROT	protein	YES	unknown	phosphorylation
P15531	UNIPROT	NME1	protein	P15531	NME1	UNIPROT	protein	YES	unknown	phosphorylation
P15531	UNIPROT	NME1	protein	P15531	NME1	UNIPROT	protein	YES	unknown	phosphorylation
Q13153	UNIPROT	PAK1	protein	P49023	PXN	UNIPROT	protein	YES	unknown	phosphorylation
Q13153	UNIPROT	PAK1	protein	P08670	VIM	UNIPROT	protein	YES	unknown	phosphorylation
Q13177	UNIPROT	PAK2	protein	P62753	RPS6	UNIPROT	protein	YES	unknown	phosphorylation
Q13177	UNIPROT	PAK2	protein	P62753	RPS6	UNIPROT	protein	YES	unknown	phosphorylation
Q13177	UNIPROT	PAK2	protein	P62753	RPS6	UNIPROT	protein	YES	unknown	phosphorylation
Q13177	UNIPROT	PAK2	protein	P62753	RPS6	UNIPROT	protein	YES	unknown	phosphorylation
O96013	UNIPROT	PAK4	protein	P49023	PXN	UNIPROT	protein	YES	unknown	phosphorylation
O96013	UNIPROT	PAK4	protein	P62826	RAN	UNIPROT	protein	YES	unknown	phosphorylation
O15530	UNIPROT	PDPK1	protein	O15530	PDPK1	UNIPROT	protein	YES	unknown	phosphorylation
O15530	UNIPROT	PDPK1	protein	O15530	PDPK1	UNIPROT	protein	YES	unknown	phosphorylation
O15530	UNIPROT	PDPK1	protein	O15530	PDPK1	UNIPROT	protein	YES	unknown	phosphorylation
O15530	UNIPROT	PDPK1	protein	O15530	PDPK1	UNIPROT	protein	YES	unknown	phosphorylation
Q16512	UNIPROT	PKN1	protein	P29966	MARCKS	UNIPROT	protein	YES	unknown	phosphorylation
Q16512	UNIPROT	PKN1	protein	P29966	MARCKS	UNIPROT	protein	YES	unknown	phosphorylation
Q16512	UNIPROT	PKN1	protein	P29966	MARCKS	UNIPROT	protein	YES	unknown	phosphorylation
P53350	UNIPROT	PLK1	protein	O96017	CHEK2	UNIPROT	protein	YES	unknown	phosphorylation
P53350	UNIPROT	PLK1	protein	P35222	CTNNB1	UNIPROT	protein	YES	unknown	phosphorylation
P53350	UNIPROT	PLK1	protein	P30626	SRI	UNIPROT	protein	YES	unknown	phosphorylation
P17612	UNIPROT	PRKACA	protein	P27708	CAD	UNIPROT	protein	YES	unknown	phosphorylation

P17612	UNIPROT	PRKACA	protein	O15551	CLDN3	UNIPROT	protein	YES	unknown	phosphorylation
P17612	UNIPROT	PRKACA	protein	Q9H3Z4	DNAJC5	UNIPROT	protein	YES	unknown	phosphorylation
P17612	UNIPROT	PRKACA	protein	P13807	GYS1	UNIPROT	protein	YES	unknown	phosphorylation
P17612	UNIPROT	PRKACA	protein	P07900	HSP90AA1	UNIPROT	protein	YES	unknown	phosphorylation
P17612	UNIPROT	PRKACA	protein	P11717	IGF2R	UNIPROT	protein	YES	unknown	phosphorylation
P17612	UNIPROT	PRKACA	protein	P47736	RAP1GAP	UNIPROT	protein	YES	unknown	phosphorylation
P17612	UNIPROT	PRKACA	protein	P47736	RAP1GAP	UNIPROT	protein	YES	unknown	phosphorylation
P17612	UNIPROT	PRKACA	protein	Q9Y6E0	STK24	UNIPROT	protein	YES	unknown	phosphorylation
P17612	UNIPROT	PRKACA	protein	P50552	VASP	UNIPROT	protein	YES	unknown	phosphorylation
P17612	UNIPROT	PRKACA	protein	P50552	VASP	UNIPROT	protein	YES	unknown	phosphorylation
P17252	UNIPROT	PRKCA	protein	O75689	ADAP1	UNIPROT	protein	YES	unknown	phosphorylation
P17252	UNIPROT	PRKCA	protein	O75689	ADAP1	UNIPROT	protein	YES	unknown	phosphorylation
P17252	UNIPROT	PRKCA	protein	P07355	ANXA2	UNIPROT	protein	YES	unknown	phosphorylation
P17252	UNIPROT	PRKCA	protein	Q06481	APLP2	UNIPROT	protein	YES	unknown	phosphorylation
P17252	UNIPROT	PRKCA	protein	Q05193	DNM1	UNIPROT	protein	YES	unknown	phosphorylation
P17252	UNIPROT	PRKCA	protein	P06730	EIF4E	UNIPROT	protein	YES	unknown	phosphorylation
P17252	UNIPROT	PRKCA	protein	P56537	EIF6	UNIPROT	protein	YES	unknown	phosphorylation
P17252	UNIPROT	PRKCA	protein	Q9Y624	F11R	UNIPROT	protein	YES	unknown	phosphorylation
P17252	UNIPROT	PRKCA	protein	Q9Y624	F11R	UNIPROT	protein	YES	unknown	phosphorylation
P17252	UNIPROT	PRKCA	protein	P60983	GMFB	UNIPROT	protein	YES	unknown	phosphorylation
P17252	UNIPROT	PRKCA	protein	P60983	GMFB	UNIPROT	protein	YES	unknown	phosphorylation
P17252	UNIPROT	PRKCA	protein	P60983	GMFB	UNIPROT	protein	YES	unknown	phosphorylation
P17252	UNIPROT	PRKCA	protein	P05783	KRT18	UNIPROT	protein	YES	unknown	phosphorylation
P17252	UNIPROT	PRKCA	protein	P02545	LMNA	UNIPROT	protein	YES	unknown	phosphorylation
P17252	UNIPROT	PRKCA	protein	P29966	MARCKS	UNIPROT	protein	YES	unknown	phosphorylation
P17252	UNIPROT	PRKCA	protein	P29966	MARCKS	UNIPROT	protein	YES	unknown	phosphorylation
P17252	UNIPROT	PRKCA	protein	P29966	MARCKS	UNIPROT	protein	YES	unknown	phosphorylation
P17252	UNIPROT	PRKCA	protein	P29966	MARCKS	UNIPROT	protein	YES	unknown	phosphorylation
P17252	UNIPROT	PRKCA	protein	P29966	MARCKS	UNIPROT	protein	YES	unknown	phosphorylation
P17252	UNIPROT	PRKCA	protein	P29966	MARCKS	UNIPROT	protein	YES	unknown	phosphorylation
P17252	UNIPROT	PRKCA	protein	P29966	MARCKS	UNIPROT	protein	YES	unknown	phosphorylation
P17252	UNIPROT	PRKCA	protein	P29966	MARCKS	UNIPROT	protein	YES	unknown	phosphorylation
P17252	UNIPROT	PRKCA	protein	P29966	MARCKS	UNIPROT	protein	YES	unknown	phosphorylation
P17252	UNIPROT	PRKCA	protein	Q9UQ80	PA2G4	UNIPROT	protein	YES	unknown	phosphorylation
P17252	UNIPROT	PRKCA	protein	Q9UQ80	PA2G4	UNIPROT	protein	YES	unknown	phosphorylation
P17252	UNIPROT	PRKCA	protein	Q06124	PTPN11	UNIPROT	protein	YES	unknown	phosphorylation
P17252	UNIPROT	PRKCA	protein	P11234	RALB	UNIPROT	protein	YES	unknown	phosphorylation
P17252	UNIPROT	PRKCA	protein	P27635	RPL10	UNIPROT	protein	YES	unknown	phosphorylation
P17252	UNIPROT	PRKCA	protein	O00161	SNAP23	UNIPROT	protein	YES	unknown	phosphorylation
P17252	UNIPROT	PRKCA	protein	O00161	SNAP23	UNIPROT	protein	YES	unknown	phosphorylation
P17252	UNIPROT	PRKCA	protein	O00161	SNAP23	UNIPROT	protein	YES	unknown	phosphorylation
P17252	UNIPROT	PRKCA	protein	Q07617	SPAG1	UNIPROT	protein	YES	unknown	phosphorylation
P17252	UNIPROT	PRKCA	protein	P12931	SRC	UNIPROT	protein	YES	unknown	phosphorylation
P17252	UNIPROT	PRKCA	protein	P61764	STXBP1	UNIPROT	protein	YES	unknown	phosphorylation
P17252	UNIPROT	PRKCA	protein	P18206	VCL	UNIPROT	protein	YES	unknown	phosphorylation
P17252	UNIPROT	PRKCA	protein	P18206	VCL	UNIPROT	protein	YES	unknown	phosphorylation
Q05655	UNIPROT	PRKCD	protein	Q92793	CREBBP	UNIPROT	protein	YES	unknown	phosphorylation
Q05655	UNIPROT	PRKCD	protein	P68104	EEF1A1	UNIPROT	protein	YES	unknown	phosphorylation
Q05655	UNIPROT	PRKCD	protein	P05198	EIF2S1	UNIPROT	protein	YES	unknown	phosphorylation
Q05655	UNIPROT	PRKCD	protein	P29966	MARCKS	UNIPROT	protein	YES	unknown	phosphorylation
Q05655	UNIPROT	PRKCD	protein	P29966	MARCKS	UNIPROT	protein	YES	unknown	phosphorylation
Q05655	UNIPROT	PRKCD	protein	P29966	MARCKS	UNIPROT	protein	YES	unknown	phosphorylation
Q05655	UNIPROT	PRKCD	protein	Q05655	PRKCD	UNIPROT	protein	YES	unknown	phosphorylation
Q05655	UNIPROT	PRKCD	protein	Q05655	PRKCD	UNIPROT	protein	YES	unknown	phosphorylation
Q05655	UNIPROT	PRKCD	protein	Q05655	PRKCD	UNIPROT	protein	YES	unknown	phosphorylation
Q05655	UNIPROT	PRKCD	protein	Q05655	PRKCD	UNIPROT	protein	YES	unknown	phosphorylation
Q05655	UNIPROT	PRKCD	protein	P29353	SHC1	UNIPROT	protein	YES	unknown	phosphorylation
P78527	UNIPROT	PRKDC	protein	P07900	HSP90AA1	UNIPROT	protein	YES	unknown	phosphorylation
P78527	UNIPROT	PRKDC	protein	P07900	HSP90AA1	UNIPROT	protein	YES	unknown	phosphorylation
P78527	UNIPROT	PRKDC	protein	P05412	JUN	UNIPROT	protein	YES	unknown	phosphorylation
P78527	UNIPROT	PRKDC	protein	Q9H9Q4	NHEJ1	UNIPROT	protein	YES	unknown	phosphorylation
P78527	UNIPROT	PRKDC	protein	P15927	RPA2	UNIPROT	protein	YES	unknown	phosphorylation
P78527	UNIPROT	PRKDC	protein	P15927	RPA2	UNIPROT	protein	YES	unknown	phosphorylation
P78527	UNIPROT	PRKDC	protein	P15927	RPA2	UNIPROT	protein	YES	unknown	phosphorylation
Q13976	UNIPROT	PRKG1	protein	P16220	CREB1	UNIPROT	protein	YES	unknown	phosphorylation
Q13976	UNIPROT	PRKG1	protein	P52943	CRIP2	UNIPROT	protein	YES	unknown	phosphorylation
Q05397	UNIPROT	PTK2	protein	P56945	BCAR1	UNIPROT	protein	YES	unknown	phosphorylation
Q05397	UNIPROT	PTK2	protein	P56945	BCAR1	UNIPROT	protein	YES	unknown	phosphorylation
Q05397	UNIPROT	PTK2	protein	P49023	PXN	UNIPROT	protein	YES	unknown	phosphorylation
Q05397	UNIPROT	PTK2	protein	P49023	PXN	UNIPROT	protein	YES	unknown	phosphorylation
Q05397	UNIPROT	PTK2	protein	Q99961	SH3GL1	UNIPROT	protein	YES	unknown	phosphorylation
Q13464	UNIPROT	ROCK1	protein	Q16555	DPYSL2	UNIPROT	protein	YES	unknown	phosphorylation
Q13464	UNIPROT	ROCK1	protein	P35241	RDX	UNIPROT	protein	YES	unknown	phosphorylation
Q15418	UNIPROT	RPS6KA1	protein	Q9Y2V2	CARHSP1	UNIPROT	protein	YES	unknown	phosphorylation
Q15418	UNIPROT	RPS6KA1	protein	Q9H6Z4	RANBP3	UNIPROT	protein	YES	unknown	phosphorylation
P51812	UNIPROT	RPS6KA3	protein	P05114	HMGN1	UNIPROT	protein	YES	unknown	phosphorylation
P51812	UNIPROT	RPS6KA3	protein	P05783	KRT18	UNIPROT	protein	YES	unknown	phosphorylation
O75676	UNIPROT	RPS6KA4	protein	Q16695	HIST3H3	UNIPROT	protein	YES	unknown	phosphorylation
O75676	UNIPROT	RPS6KA4	protein	Q16695	HIST3H3	UNIPROT	protein	YES	unknown	phosphorylation
O75676	UNIPROT	RPS6KA4	protein	P05114	HMGN1	UNIPROT	protein	YES	unknown	phosphorylation
P12931	UNIPROT	SRC	protein	P04083	ANXA1	UNIPROT	protein	YES	unknown	phosphorylation
P12931	UNIPROT	SRC	protein	P52566	ARHGDIB	UNIPROT	protein	YES	unknown	phosphorylation
P12931	UNIPROT	SRC	protein	P52566	ARHGDIB	UNIPROT	protein	YES	unknown	phosphorylation
P12931	UNIPROT	SRC	protein	Q9H5V8	CDCP1	UNIPROT	protein	YES	unknown	phosphorylation
P12931	UNIPROT	SRC	protein	P62993	GRB2	UNIPROT	protein	YES	unknown	phosphorylation
P12931	UNIPROT	SRC	protein	P08727	KRT19	UNIPROT	protein	YES	unknown	phosphorylation
P12931	UNIPROT	SRC	protein	O43490	PROM1	UNIPROT	protein	YES	unknown	phosphorylation
P12931	UNIPROT	SRC	protein	O43490	PROM1	UNIPROT	protein	YES	unknown	phosphorylation
P12931	UNIPROT	SRC	protein	P51812	RPS6KA3	UNIPROT	protein	YES	unknown	phosphorylation
P12931	UNIPROT	SRC	protein	Q99961	SH3GL1	UNIPROT	protein	YES	unknown	phosphorylation
O00506	UNIPROT	STK25	protein	Q9BUL8	PDCD10	UNIPROT	protein	YES	unknown	phosphorylation
O00506	UNIPROT	STK25	protein	Q9BUL8	PDCD10	UNIPROT	protein	YES	unknown	phosphorylation
O43422	UNIPROT	THAP12	protein	P05198	EIF2S1	UNIPROT	protein	YES	unknown	phosphorylation
Q2M2I8	UNIPROT	AAK1	protein	Q9BXS5	AP1M1	UNIPROT	protein	YES	up-regulates	phosphorylation
Q2M2I8	UNIPROT	AAK1	protein	Q96CW1	AP2M1	UNIPROT	protein	YES	up-regulates	phosphorylation
Q2M2I8	UNIPROT	AAK1	protein	P49757	NUMB	UNIPROT	protein	YES	up-regulates	phosphorylation
P31749	UNIPROT	AKT1	protein	Q86V81	ALYREF	UNIPROT	protein	YES	up-regulates	phosphorylation
P31749	UNIPROT	AKT1	protein	P24941	CDK2	UNIPROT	protein	YES	up-regulates	phosphorylation

P31749	UNIPROT	AKT1	protein	O15111	CHUK	UNIPROT	protein	YES	up-regulates	phosphorylation
P31749	UNIPROT	AKT1	protein	Q7L5N1	COPS6	UNIPROT	protein	YES	up-regulates	phosphorylation
P31749	UNIPROT	AKT1	protein	P35222	CTNNB1	UNIPROT	protein	YES	up-regulates	phosphorylation
P31749	UNIPROT	AKT1	protein	P26358	DNMT1	UNIPROT	protein	YES	up-regulates	phosphorylation
P31749	UNIPROT	AKT1	protein	P23588	EIF4B	UNIPROT	protein	YES	up-regulates	phosphorylation
P31749	UNIPROT	AKT1	protein	Q09472	EP300	UNIPROT	protein	YES	up-regulates	phosphorylation
P31749	UNIPROT	AKT1	protein	Q09472	EP300	UNIPROT	protein	YES	up-regulates	phosphorylation
P31749	UNIPROT	AKT1	protein	P84243	H3F3A	UNIPROT	protein	YES	up-regulates	phosphorylation
P31749	UNIPROT	AKT1	protein	Q12906	ILF3	UNIPROT	protein	YES	up-regulates	phosphorylation
P31749	UNIPROT	AKT1	protein	Q04912	MST1R	UNIPROT	protein	YES	up-regulates	phosphorylation
P31749	UNIPROT	AKT1	protein	P42345	MTOR	UNIPROT	protein	YES	up-regulates	phosphorylation
P31749	UNIPROT	AKT1	protein	P78362	SRPK2	UNIPROT	protein	YES	up-regulates	phosphorylation
P31749	UNIPROT	AKT1	protein	Q96S44	TP53RK	UNIPROT	protein	YES	up-regulates	phosphorylation
P31749	UNIPROT	AKT1	protein	P55072	VCP	UNIPROT	protein	YES	up-regulates	phosphorylation
P31749	UNIPROT	AKT1	protein	P55072	VCP	UNIPROT	protein	YES	up-regulates	phosphorylation
P31749	UNIPROT	AKT1	protein	P55072	VCP	UNIPROT	protein	YES	up-regulates	phosphorylation
P31749	UNIPROT	AKT1	protein	P08670	VIM	UNIPROT	protein	YES	up-regulates	phosphorylation
P31749	UNIPROT	AKT1	protein	Q9H4A3	WNK1	UNIPROT	protein	YES	up-regulates	phosphorylation
P31749	UNIPROT	AKT1	protein	P67809	YBX1	UNIPROT	protein	YES	up-regulates	phosphorylation
P31749	UNIPROT	AKT1	protein	P67809	YBX1	UNIPROT	protein	YES	up-regulates	phosphorylation
P31751	UNIPROT	AKT2	protein	O15111	CHUK	UNIPROT	protein	YES	up-regulates	phosphorylation
P31751	UNIPROT	AKT2	protein	P16220	CREB1	UNIPROT	protein	YES	up-regulates	phosphorylation
P31751	UNIPROT	AKT2	protein	P35222	CTNNB1	UNIPROT	protein	YES	up-regulates	phosphorylation
P31751	UNIPROT	AKT2	protein	Q09472	EP300	UNIPROT	protein	YES	up-regulates	phosphorylation
P31751	UNIPROT	AKT2	protein	Q09472	EP300	UNIPROT	protein	YES	up-regulates	phosphorylation
P31751	UNIPROT	AKT2	protein	P15311	EZR	UNIPROT	protein	YES	up-regulates	phosphorylation
P31751	UNIPROT	AKT2	protein	P98170	XIAP	UNIPROT	protein	YES	up-regulates	phosphorylation
P10398	UNIPROT	ARAF	protein	Q02750	MAP2K1	UNIPROT	protein	YES	up-regulates	phosphorylation
P10398	UNIPROT	ARAF	protein	Q02750	MAP2K1	UNIPROT	protein	YES	up-regulates	phosphorylation
P10398	UNIPROT	ARAF	protein	P36507	MAP2K2	UNIPROT	protein	YES	up-regulates	phosphorylation
Q96GD4	UNIPROT	AURKB	protein	Q96GD4	AURKB	UNIPROT	protein	YES	up-regulates	phosphorylation
Q96GD4	UNIPROT	AURKB	protein	Q96CF2	CHMP4C	UNIPROT	protein	YES	up-regulates	phosphorylation
Q96GD4	UNIPROT	AURKB	protein	Q96CF2	CHMP4C	UNIPROT	protein	YES	up-regulates	phosphorylation
Q96GD4	UNIPROT	AURKB	protein	Q96CF2	CHMP4C	UNIPROT	protein	YES	up-regulates	phosphorylation
Q96GD4	UNIPROT	AURKB	protein	O60879	DIAPH2	UNIPROT	protein	YES	up-regulates	phosphorylation
Q96GD4	UNIPROT	AURKB	protein	P84243	H3F3A	UNIPROT	protein	YES	up-regulates	phosphorylation
Q96GD4	UNIPROT	AURKB	protein	P84243	H3F3A	UNIPROT	protein	YES	up-regulates	phosphorylation
Q96GD4	UNIPROT	AURKB	protein	P84243	H3F3A	UNIPROT	protein	YES	up-regulates	phosphorylation
Q96GD4	UNIPROT	AURKB	protein	P68431	HIST1H3A	UNIPROT	protein	YES	up-regulates	phosphorylation
Q96GD4	UNIPROT	AURKB	protein	P68431	HIST1H3A	UNIPROT	protein	YES	up-regulates	phosphorylation
Q96GD4	UNIPROT	AURKB	protein	P68431	HIST1H3A	UNIPROT	protein	YES	up-regulates	phosphorylation
Q96GD4	UNIPROT	AURKB	protein	P68431	HIST1H3A	UNIPROT	protein	YES	up-regulates	phosphorylation
Q96GD4	UNIPROT	AURKB	protein	Q99661	KIF2C	UNIPROT	protein	YES	up-regulates	phosphorylation
Q96GD4	UNIPROT	AURKB	protein	Q9UPY8	MAPRE3	UNIPROT	protein	YES	up-regulates	phosphorylation
Q96GD4	UNIPROT	AURKB	protein	P25490	YY1	UNIPROT	protein	YES	up-regulates	phosphorylation
Q96GD4	UNIPROT	AURKB	protein	P25490	YY1	UNIPROT	protein	YES	up-regulates	phosphorylation
Q9UIG0	UNIPROT	BAZ1B	protein	P16104	H2AFX	UNIPROT	protein	YES	up-regulates	phosphorylation
P15056	UNIPROT	BRAF	protein	P36507	MAP2K2	UNIPROT	protein	YES	up-regulates	phosphorylation
P15056	UNIPROT	BRAF	protein	P36507	MAP2K2	UNIPROT	protein	YES	up-regulates	phosphorylation
O60885	UNIPROT	BRD4	protein	P24928	POLR2A	UNIPROT	protein	YES	up-regulates	phosphorylation
Q9UQM7	UNIPROT	CAMK2A	protein	Q9UQM7	CAMK2A	UNIPROT	protein	YES	up-regulates	phosphorylation
Q9UQM7	UNIPROT	CAMK2A	protein	P16070	CD44	UNIPROT	protein	YES	up-regulates	phosphorylation
Q9UQM7	UNIPROT	CAMK2A	protein	Q06210	GFPT1	UNIPROT	protein	YES	up-regulates	phosphorylation
Q9UQM7	UNIPROT	CAMK2A	protein	Q15121	PEA15	UNIPROT	protein	YES	up-regulates	phosphorylation
Q9UQM7	UNIPROT	CAMK2A	protein	Q05397	PTK2	UNIPROT	protein	YES	up-regulates	phosphorylation
Q13554	UNIPROT	CAMK2B	protein	Q13131	PRKAA1	UNIPROT	protein	YES	up-regulates	phosphorylation
Q13554	UNIPROT	CAMK2B	protein	P42224	STAT1	UNIPROT	protein	YES	up-regulates	phosphorylation
Q13557	UNIPROT	CAMK2D	protein	Q13557	CAMK2D	UNIPROT	protein	YES	up-regulates	phosphorylation
Q13557	UNIPROT	CAMK2D	protein	P13688	CEACAM1	UNIPROT	protein	YES	up-regulates	phosphorylation
Q13555	UNIPROT	CAMK2G	protein	O43318	MAP3K7	UNIPROT	protein	YES	up-regulates	phosphorylation
Q5VT25	UNIPROT	CDC42BPA	protein	P24844	MYL9	UNIPROT	protein	YES	up-regulates	phosphorylation
Q5VT25	UNIPROT	CDC42BPA	protein	P24844	MYL9	UNIPROT	protein	YES	up-regulates	phosphorylation
O00311	UNIPROT	CDC7	protein	P49736	MCM2	UNIPROT	protein	YES	up-regulates	phosphorylation
O00311	UNIPROT	CDC7	protein	P49736	MCM2	UNIPROT	protein	YES	up-regulates	phosphorylation
O00311	UNIPROT	CDC7	protein	P49736	MCM2	UNIPROT	protein	YES	up-regulates	phosphorylation
O00311	UNIPROT	CDC7	protein	P49736	MCM2	UNIPROT	protein	YES	up-regulates	phosphorylation
O00311	UNIPROT	CDC7	protein	P49736	MCM2	UNIPROT	protein	YES	up-regulates	phosphorylation
O00311	UNIPROT	CDC7	protein	P49736	MCM2	UNIPROT	protein	YES	up-regulates	phosphorylation
O00311	UNIPROT	CDC7	protein	P49736	MCM2	UNIPROT	protein	YES	up-regulates	phosphorylation
O00311	UNIPROT	CDC7	protein	P49736	MCM2	UNIPROT	protein	YES	up-regulates	phosphorylation
O00311	UNIPROT	CDC7	protein	P49736	MCM2	UNIPROT	protein	YES	up-regulates	phosphorylation
O00311	UNIPROT	CDC7	protein	P49736	MCM2	UNIPROT	protein	YES	up-regulates	phosphorylation
O00311	UNIPROT	CDC7	protein	P49736	MCM2	UNIPROT	protein	YES	up-regulates	phosphorylation
O00311	UNIPROT	CDC7	protein	P33991	MCM4	UNIPROT	protein	YES	up-regulates	phosphorylation
O00311	UNIPROT	CDC7	protein	P33993	MCM7	UNIPROT	protein	YES	up-regulates	phosphorylation
O00311	UNIPROT	CDC7	protein	O75475	PSIP1	UNIPROT	protein	YES	up-regulates	phosphorylation
P06493	UNIPROT	CDK1	protein	Q9H1A4	ANAPC1	UNIPROT	protein	YES	up-regulates	phosphorylation
P06493	UNIPROT	CDK1	protein	O60566	BUB1B	UNIPROT	protein	YES	up-regulates	phosphorylation
P06493	UNIPROT	CDK1	protein	Q13042	CDC16	UNIPROT	protein	YES	up-regulates	phosphorylation
P06493	UNIPROT	CDK1	protein	Q9UJX2	CDC23	UNIPROT	protein	YES	up-regulates	phosphorylation
P06493	UNIPROT	CDK1	protein	P30260	CDC27	UNIPROT	protein	YES	up-regulates	phosphorylation
P06493	UNIPROT	CDK1	protein	P30260	CDC27	UNIPROT	protein	YES	up-regulates	phosphorylation
P06493	UNIPROT	CDK1	protein	O00311	CDC7	UNIPROT	protein	YES	up-regulates	phosphorylation
P06493	UNIPROT	CDK1	protein	O14757	CHEK1	UNIPROT	protein	YES	up-regulates	phosphorylation
P06493	UNIPROT	CDK1	protein	O14757	CHEK1	UNIPROT	protein	YES	up-regulates	phosphorylation
P06493	UNIPROT	CDK1	protein	P68400	CSNK2A1	UNIPROT	protein	YES	up-regulates	phosphorylation
P06493	UNIPROT	CDK1	protein	P68400	CSNK2A1	UNIPROT	protein	YES	up-regulates	phosphorylation
P06493	UNIPROT	CDK1	protein	P68400	CSNK2A1	UNIPROT	protein	YES	up-regulates	phosphorylation
P06493	UNIPROT	CDK1	protein	P68400	CSNK2A1	UNIPROT	protein	YES	up-regulates	phosphorylation
P06493	UNIPROT	CDK1	protein	P67870	CSNK2B	UNIPROT	protein	YES	up-regulates	phosphorylation
P06493	UNIPROT	CDK1	protein	P26358	DNMT1	UNIPROT	protein	YES	up-regulates	phosphorylation
P06493	UNIPROT	CDK1	protein	Q9H8V3	ECT2	UNIPROT	protein	YES	up-regulates	phosphorylation
P06493	UNIPROT	CDK1	protein	Q9H8V3	ECT2	UNIPROT	protein	YES	up-regulates	phosphorylation
P06493	UNIPROT	CDK1	protein	Q2NKX8	ERCC6L	UNIPROT	protein	YES	up-regulates	phosphorylation
P06493	UNIPROT	CDK1	protein	Q01167	FOXK2	UNIPROT	protein	YES	up-regulates	phosphorylation
P06493	UNIPROT	CDK1	protein	Q01167	FOXK2	UNIPROT	protein	YES	up-regulates	phosphorylation
P06493	UNIPROT	CDK1	protein	Q92993	KAT5	UNIPROT	protein	YES	up-regulates	phosphorylation
P06493	UNIPROT	CDK1	protein	Q92993	KAT5	UNIPROT	protein	YES	up-regulates	phosphorylation

P06493	UNIPROT	CDK1	protein	O95251	KAT7	UNIPROT	protein	YES	up-regulates	phosphorylation
P06493	UNIPROT	CDK1	protein	O95251	KAT7	UNIPROT	protein	YES	up-regulates	phosphorylation
P06493	UNIPROT	CDK1	protein	P52732	KIF11	UNIPROT	protein	YES	up-regulates	phosphorylation
P06493	UNIPROT	CDK1	protein	P05783	KRT18	UNIPROT	protein	YES	up-regulates	phosphorylation
P06493	UNIPROT	CDK1	protein	P02545	LMNA	UNIPROT	protein	YES	up-regulates	phosphorylation
P06493	UNIPROT	CDK1	protein	P02545	LMNA	UNIPROT	protein	YES	up-regulates	phosphorylation
P06493	UNIPROT	CDK1	protein	P02545	LMNA	UNIPROT	protein	YES	up-regulates	phosphorylation
P06493	UNIPROT	CDK1	protein	Q07820	MCL1	UNIPROT	protein	YES	up-regulates	phosphorylation
P06493	UNIPROT	CDK1	protein	Q07820	MCL1	UNIPROT	protein	YES	up-regulates	phosphorylation
P06493	UNIPROT	CDK1	protein	Q8TAP9	MPLKIP	UNIPROT	protein	YES	up-regulates	phosphorylation
P06493	UNIPROT	CDK1	protein	Q8TAP9	MPLKIP	UNIPROT	protein	YES	up-regulates	phosphorylation
P06493	UNIPROT	CDK1	protein	Q8TAP9	MPLKIP	UNIPROT	protein	YES	up-regulates	phosphorylation
P06493	UNIPROT	CDK1	protein	Q9NXR1	NDE1	UNIPROT	protein	YES	up-regulates	phosphorylation
P06493	UNIPROT	CDK1	protein	P15531	NME1	UNIPROT	protein	YES	up-regulates	phosphorylation
P06493	UNIPROT	CDK1	protein	P52948	NUP98	UNIPROT	protein	YES	up-regulates	phosphorylation
P06493	UNIPROT	CDK1	protein	P52948	NUP98	UNIPROT	protein	YES	up-regulates	phosphorylation
P06493	UNIPROT	CDK1	protein	P52948	NUP98	UNIPROT	protein	YES	up-regulates	phosphorylation
P06493	UNIPROT	CDK1	protein	P46060	RANGAP1	UNIPROT	protein	YES	up-regulates	phosphorylation
P06493	UNIPROT	CDK1	protein	P46060	RANGAP1	UNIPROT	protein	YES	up-regulates	phosphorylation
P06493	UNIPROT	CDK1	protein	P46060	RANGAP1	UNIPROT	protein	YES	up-regulates	phosphorylation
P06493	UNIPROT	CDK1	protein	P15927	RPA2	UNIPROT	protein	YES	up-regulates	phosphorylation
P06493	UNIPROT	CDK1	protein	P15927	RPA2	UNIPROT	protein	YES	up-regulates	phosphorylation
P06493	UNIPROT	CDK1	protein	P23396	RPS3	UNIPROT	protein	YES	up-regulates	phosphorylation
P06493	UNIPROT	CDK1	protein	Q96EB6	SIRT1	UNIPROT	protein	YES	up-regulates	phosphorylation
P06493	UNIPROT	CDK1	protein	P08047	SP1	UNIPROT	protein	YES	up-regulates	phosphorylation
P06493	UNIPROT	CDK1	protein	Q13501	SQSTM1	UNIPROT	protein	YES	up-regulates	phosphorylation
P06493	UNIPROT	CDK1	protein	Q13501	SQSTM1	UNIPROT	protein	YES	up-regulates	phosphorylation
P06493	UNIPROT	CDK1	protein	P04637	TP53	UNIPROT	protein	YES	up-regulates	phosphorylation
P06493	UNIPROT	CDK1	protein	P04637	TP53	UNIPROT	protein	YES	up-regulates	phosphorylation
P06493	UNIPROT	CDK1	protein	P22314	UBA1	UNIPROT	protein	YES	up-regulates	phosphorylation
P06493	UNIPROT	CDK1	protein	P22314	UBA1	UNIPROT	protein	YES	up-regulates	phosphorylation
P06493	UNIPROT	CDK1	protein	P22314	UBA1	UNIPROT	protein	YES	up-regulates	phosphorylation
P06493	UNIPROT	CDK1	protein	P49459	UBE2A	UNIPROT	protein	YES	up-regulates	phosphorylation
P06493	UNIPROT	CDK1	protein	Q9Y5T5	USP16	UNIPROT	protein	YES	up-regulates	phosphorylation
Q9UQ88	UNIPROT	CDK11A	protein	P21127	CDK11B	UNIPROT	protein	YES	up-regulates	phosphorylation
Q9NYV4	UNIPROT	CDK12	protein	P24928	POLR2A	UNIPROT	protein	YES	up-regulates	phosphorylation
Q9NYV4	UNIPROT	CDK12	protein	P24928	POLR2A	UNIPROT	protein	YES	up-regulates	phosphorylation
Q9NYV4	UNIPROT	CDK12	protein	P24928	POLR2A	UNIPROT	protein	YES	up-regulates	phosphorylation
Q9NYV4	UNIPROT	CDK12	protein	P24928	POLR2A	UNIPROT	protein	YES	up-regulates	phosphorylation
Q9NYV4	UNIPROT	CDK12	protein	P24928	POLR2A	UNIPROT	protein	YES	up-regulates	phosphorylation
Q9NYV4	UNIPROT	CDK12	protein	P24928	POLR2A	UNIPROT	protein	YES	up-regulates	phosphorylation
Q9NYV4	UNIPROT	CDK12	protein	P24928	POLR2A	UNIPROT	protein	YES	up-regulates	phosphorylation
Q9NYV4	UNIPROT	CDK12	protein	P24928	POLR2A	UNIPROT	protein	YES	up-regulates	phosphorylation
Q9NYV4	UNIPROT	CDK12	protein	P24928	POLR2A	UNIPROT	protein	YES	up-regulates	phosphorylation
Q9NYV4	UNIPROT	CDK12	protein	P24928	POLR2A	UNIPROT	protein	YES	up-regulates	phosphorylation
Q9NYV4	UNIPROT	CDK12	protein	P24928	POLR2A	UNIPROT	protein	YES	up-regulates	phosphorylation
Q9NYV4	UNIPROT	CDK12	protein	P24928	POLR2A	UNIPROT	protein	YES	up-regulates	phosphorylation
Q9NYV4	UNIPROT	CDK12	protein	P24928	POLR2A	UNIPROT	protein	YES	up-regulates	phosphorylation
P24941	UNIPROT	CDK2	protein	Q8WXE1	ATRIP	UNIPROT	protein	YES	up-regulates	phosphorylation
P24941	UNIPROT	CDK2	protein	Q16204	CCDC6	UNIPROT	protein	YES	up-regulates	phosphorylation
P24941	UNIPROT	CDK2	protein	P20248	CCNA2	UNIPROT	protein	YES	up-regulates	phosphorylation
P24941	UNIPROT	CDK2	protein	P24941	CDK2	UNIPROT	protein	YES	up-regulates	phosphorylation
P24941	UNIPROT	CDK2	protein	P50613	CDK7	UNIPROT	protein	YES	up-regulates	phosphorylation
P24941	UNIPROT	CDK2	protein	P17676	CEBPB	UNIPROT	protein	YES	up-regulates	phosphorylation
P24941	UNIPROT	CDK2	protein	P17676	CEBPB	UNIPROT	protein	YES	up-regulates	phosphorylation
P24941	UNIPROT	CDK2	protein	O14757	CHEK1	UNIPROT	protein	YES	up-regulates	phosphorylation
P24941	UNIPROT	CDK2	protein	O14757	CHEK1	UNIPROT	protein	YES	up-regulates	phosphorylation
P24941	UNIPROT	CDK2	protein	P38432	COIL	UNIPROT	protein	YES	up-regulates	phosphorylation
P24941	UNIPROT	CDK2	protein	Q12959	DLG1	UNIPROT	protein	YES	up-regulates	phosphorylation
P24941	UNIPROT	CDK2	protein	Q12959	DLG1	UNIPROT	protein	YES	up-regulates	phosphorylation
P24941	UNIPROT	CDK2	protein	P26358	DNMT1	UNIPROT	protein	YES	up-regulates	phosphorylation
P24941	UNIPROT	CDK2	protein	Q01167	FOXK2	UNIPROT	protein	YES	up-regulates	phosphorylation
P24941	UNIPROT	CDK2	protein	Q01167	FOXK2	UNIPROT	protein	YES	up-regulates	phosphorylation
P24941	UNIPROT	CDK2	protein	P49736	MCM2	UNIPROT	protein	YES	up-regulates	phosphorylation
P24941	UNIPROT	CDK2	protein	P25205	MCM3	UNIPROT	protein	YES	up-regulates	phosphorylation
P24941	UNIPROT	CDK2	protein	Q13416	ORC2	UNIPROT	protein	YES	up-regulates	phosphorylation
P24941	UNIPROT	CDK2	protein	Q13416	ORC2	UNIPROT	protein	YES	up-regulates	phosphorylation
P24941	UNIPROT	CDK2	protein	Q13416	ORC2	UNIPROT	protein	YES	up-regulates	phosphorylation
P24941	UNIPROT	CDK2	protein	Q8IZL8	PELP1	UNIPROT	protein	YES	up-regulates	phosphorylation
P24941	UNIPROT	CDK2	protein	P10644	PRKAR1A	UNIPROT	protein	YES	up-regulates	phosphorylation
P24941	UNIPROT	CDK2	protein	Q9NYV6	RRN3	UNIPROT	protein	YES	up-regulates	phosphorylation
P24941	UNIPROT	CDK2	protein	O75533	SF3B1	UNIPROT	protein	YES	up-regulates	phosphorylation
P24941	UNIPROT	CDK2	protein	O75533	SF3B1	UNIPROT	protein	YES	up-regulates	phosphorylation
P24941	UNIPROT	CDK2	protein	P04637	TP53	UNIPROT	protein	YES	up-regulates	phosphorylation
P24941	UNIPROT	CDK2	protein	P49459	UBE2A	UNIPROT	protein	YES	up-regulates	phosphorylation
P11802	UNIPROT	CDK4	protein	Q8IZL8	PELP1	UNIPROT	protein	YES	up-regulates	phosphorylation
P11802	UNIPROT	CDK4	protein	Q8IZL8	PELP1	UNIPROT	protein	YES	up-regulates	phosphorylation
Q00535	UNIPROT	CDK5	protein	P26358	DNMT1	UNIPROT	protein	YES	up-regulates	phosphorylation
Q00535	UNIPROT	CDK5	protein	O43464	HTRA2	UNIPROT	protein	YES	up-regulates	phosphorylation
Q00535	UNIPROT	CDK5	protein	P42858	HTT	UNIPROT	protein	YES	up-regulates	phosphorylation
Q00535	UNIPROT	CDK5	protein	P42858	HTT	UNIPROT	protein	YES	up-regulates	phosphorylation
Q00535	UNIPROT	CDK5	protein	Q05397	PTK2	UNIPROT	protein	YES	up-regulates	phosphorylation
Q00535	UNIPROT	CDK5	protein	P12931	SRC	UNIPROT	protein	YES	up-regulates	phosphorylation
Q00535	UNIPROT	CDK5	protein	P40763	STAT3	UNIPROT	protein	YES	up-regulates	phosphorylation
Q00535	UNIPROT	CDK5	protein	Q9Y490	TLN1	UNIPROT	protein	YES	up-regulates	phosphorylation
Q00535	UNIPROT	CDK5	protein	P04637	TP53	UNIPROT	protein	YES	up-regulates	phosphorylation
Q00535	UNIPROT	CDK5	protein	P04637	TP53	UNIPROT	protein	YES	up-regulates	phosphorylation
Q00535	UNIPROT	CDK5	protein	P04637	TP53	UNIPROT	protein	YES	up-regulates	phosphorylation
Q00535	UNIPROT	CDK5	protein	P04637	TP53	UNIPROT	protein	YES	up-regulates	phosphorylation
Q00534	UNIPROT	CDK6	protein	P46527	CDKN1B	UNIPROT	protein	YES	up-regulates	phosphorylation
P50613	UNIPROT	CDK7	protein	P06493	CDK1	UNIPROT	protein	YES	up-regulates	phosphorylation
P50613	UNIPROT	CDK7	protein	P11802	CDK4	UNIPROT	protein	YES	up-regulates	phosphorylation
P50613	UNIPROT	CDK7	protein	P04637	TP53	UNIPROT	protein	YES	up-regulates	phosphorylation
P50613	UNIPROT	CDK7	protein	P04637	TP53	UNIPROT	protein	YES	up-regulates	phosphorylation
P50613	UNIPROT	CDK7	protein	P04637	TP53	UNIPROT	protein	YES	up-regulates	phosphorylation
P50613	UNIPROT	CDK7	protein	P04637	TP53	UNIPROT	protein	YES	up-regulates	phosphorylation
P50750	UNIPROT	CDK9	protein	P24928	POLR2A	UNIPROT	protein	YES	up-regulates	phosphorylation
P50750	UNIPROT	CDK9	protein	P24928	POLR2A	UNIPROT	protein	YES	up-regulates	phosphorylation

P50750	UNIPROT	CDK9	protein	P24928	POLR2A	UNIPROT	protein	YES	up-regulates	phosphorylation
P50750	UNIPROT	CDK9	protein	P24928	POLR2A	UNIPROT	protein	YES	up-regulates	phosphorylation
P50750	UNIPROT	CDK9	protein	P24928	POLR2A	UNIPROT	protein	YES	up-regulates	phosphorylation
P50750	UNIPROT	CDK9	protein	P24928	POLR2A	UNIPROT	protein	YES	up-regulates	phosphorylation
P50750	UNIPROT	CDK9	protein	P24928	POLR2A	UNIPROT	protein	YES	up-regulates	phosphorylation
P50750	UNIPROT	CDK9	protein	P24928	POLR2A	UNIPROT	protein	YES	up-regulates	phosphorylation
P50750	UNIPROT	CDK9	protein	P24928	POLR2A	UNIPROT	protein	YES	up-regulates	phosphorylation
P50750	UNIPROT	CDK9	protein	P24928	POLR2A	UNIPROT	protein	YES	up-regulates	phosphorylation
P50750	UNIPROT	CDK9	protein	P24928	POLR2A	UNIPROT	protein	YES	up-regulates	phosphorylation
P50750	UNIPROT	CDK9	protein	P24928	POLR2A	UNIPROT	protein	YES	up-regulates	phosphorylation
P50750	UNIPROT	CDK9	protein	P24928	POLR2A	UNIPROT	protein	YES	up-regulates	phosphorylation
P50750	UNIPROT	CDK9	protein	P24928	POLR2A	UNIPROT	protein	YES	up-regulates	phosphorylation
P50750	UNIPROT	CDK9	protein	P24928	POLR2A	UNIPROT	protein	YES	up-regulates	phosphorylation
P50750	UNIPROT	CDK9	protein	P24928	POLR2A	UNIPROT	protein	YES	up-regulates	phosphorylation
P50750	UNIPROT	CDK9	protein	P24928	POLR2A	UNIPROT	protein	YES	up-regulates	phosphorylation
P50750	UNIPROT	CDK9	protein	P24928	POLR2A	UNIPROT	protein	YES	up-regulates	phosphorylation
P50750	UNIPROT	CDK9	protein	P24928	POLR2A	UNIPROT	protein	YES	up-regulates	phosphorylation
P50750	UNIPROT	CDK9	protein	P24928	POLR2A	UNIPROT	protein	YES	up-regulates	phosphorylation
P50750	UNIPROT	CDK9	protein	P24928	POLR2A	UNIPROT	protein	YES	up-regulates	phosphorylation
P50750	UNIPROT	CDK9	protein	P24928	POLR2A	UNIPROT	protein	YES	up-regulates	phosphorylation
P50750	UNIPROT	CDK9	protein	P24928	POLR2A	UNIPROT	protein	YES	up-regulates	phosphorylation
P50750	UNIPROT	CDK9	protein	P24928	POLR2A	UNIPROT	protein	YES	up-regulates	phosphorylation
P50750	UNIPROT	CDK9	protein	P24928	POLR2A	UNIPROT	protein	YES	up-regulates	phosphorylation
P50750	UNIPROT	CDK9	protein	Q15797	SMAD1	UNIPROT	protein	YES	up-regulates	phosphorylation
P50750	UNIPROT	CDK9	protein	P84022	SMAD3	UNIPROT	protein	YES	up-regulates	phosphorylation
P50750	UNIPROT	CDK9	protein	O00267	SUPT5H	UNIPROT	protein	YES	up-regulates	phosphorylation
P50750	UNIPROT	CDK9	protein	O00267	SUPT5H	UNIPROT	protein	YES	up-regulates	phosphorylation
P50750	UNIPROT	CDK9	protein	O00267	SUPT5H	UNIPROT	protein	YES	up-regulates	phosphorylation
P50750	UNIPROT	CDK9	protein	O00267	SUPT5H	UNIPROT	protein	YES	up-regulates	phosphorylation
P50750	UNIPROT	CDK9	protein	O00267	SUPT5H	UNIPROT	protein	YES	up-regulates	phosphorylation
P50750	UNIPROT	CDK9	protein	O00267	SUPT5H	UNIPROT	protein	YES	up-regulates	phosphorylation
P50750	UNIPROT	CDK9	protein	O00267	SUPT5H	UNIPROT	protein	YES	up-regulates	phosphorylation
P50750	UNIPROT	CDK9	protein	P04637	TP53	UNIPROT	protein	YES	up-regulates	phosphorylation
P50750	UNIPROT	CDK9	protein	P04637	TP53	UNIPROT	protein	YES	up-regulates	phosphorylation
P50750	UNIPROT	CDK9	protein	P04637	TP53	UNIPROT	protein	YES	up-regulates	phosphorylation
O14757	UNIPROT	CHEK1	protein	Q96GD4	AURKB	UNIPROT	protein	YES	up-regulates	phosphorylation
O14757	UNIPROT	CHEK1	protein	P54132	BLM	UNIPROT	protein	YES	up-regulates	phosphorylation
O14757	UNIPROT	CHEK1	protein	O00311	CDC7	UNIPROT	protein	YES	up-regulates	phosphorylation
O14757	UNIPROT	CHEK1	protein	O00716	E2F3	UNIPROT	protein	YES	up-regulates	phosphorylation
O14757	UNIPROT	CHEK1	protein	P84243	H3F3A	UNIPROT	protein	YES	up-regulates	phosphorylation
O14757	UNIPROT	CHEK1	protein	P06400	RB1	UNIPROT	protein	YES	up-regulates	phosphorylation
O14757	UNIPROT	CHEK1	protein	P30291	WEE1	UNIPROT	protein	YES	up-regulates	phosphorylation
O96017	UNIPROT	CHEK2	protein	Q13362	PPP2R5C	UNIPROT	protein	YES	up-regulates	phosphorylation
O96017	UNIPROT	CHEK2	protein	P33981	TTK	UNIPROT	protein	YES	up-regulates	phosphorylation
O96017	UNIPROT	CHEK2	protein	P18887	XRCC1	UNIPROT	protein	YES	up-regulates	phosphorylation
O15111	UNIPROT	CHUK	protein	Q92793	CREBBP	UNIPROT	protein	YES	up-regulates	phosphorylation
O15111	UNIPROT	CHUK	protein	Q92793	CREBBP	UNIPROT	protein	YES	up-regulates	phosphorylation
O15111	UNIPROT	CHUK	protein	P84243	H3F3A	UNIPROT	protein	YES	up-regulates	phosphorylation
O15111	UNIPROT	CHUK	protein	Q92985	IRF7	UNIPROT	protein	YES	up-regulates	phosphorylation
Q53ET0	UNIPROT	CRTC2	protein	P31749	AKT1	UNIPROT	protein	YES	up-regulates	phosphorylation
P48729	UNIPROT	CSNK1A1	protein	P16220	CREB1	UNIPROT	protein	YES	up-regulates	phosphorylation
P48729	UNIPROT	CSNK1A1	protein	P16220	CREB1	UNIPROT	protein	YES	up-regulates	phosphorylation
P48729	UNIPROT	CSNK1A1	protein	P16220	CREB1	UNIPROT	protein	YES	up-regulates	phosphorylation
P48729	UNIPROT	CSNK1A1	protein	P17931	LGALS3	UNIPROT	protein	YES	up-regulates	phosphorylation
P48730	UNIPROT	CSNK1D	protein	O15169	AXIN1	UNIPROT	protein	YES	up-regulates	phosphorylation
P48730	UNIPROT	CSNK1D	protein	O14641	DVL2	UNIPROT	protein	YES	up-regulates	phosphorylation
P48730	UNIPROT	CSNK1D	protein	O14641	DVL2	UNIPROT	protein	YES	up-regulates	phosphorylation
P48730	UNIPROT	CSNK1D	protein	P04637	TP53	UNIPROT	protein	YES	up-regulates	phosphorylation
P48730	UNIPROT	CSNK1D	protein	P04637	TP53	UNIPROT	protein	YES	up-regulates	phosphorylation
P48730	UNIPROT	CSNK1D	protein	P04637	TP53	UNIPROT	protein	YES	up-regulates	phosphorylation
P48730	UNIPROT	CSNK1D	protein	Q96T88	UHRF1	UNIPROT	protein	YES	up-regulates	phosphorylation
P49674	UNIPROT	CSNK1E	protein	O15169	AXIN1	UNIPROT	protein	YES	up-regulates	phosphorylation
P49674	UNIPROT	CSNK1E	protein	O14641	DVL2	UNIPROT	protein	YES	up-regulates	phosphorylation
P49674	UNIPROT	CSNK1E	protein	O14641	DVL2	UNIPROT	protein	YES	up-regulates	phosphorylation
P68400	UNIPROT	CSNK2A1	protein	P33527	ABCC1	UNIPROT	protein	YES	up-regulates	phosphorylation
P68400	UNIPROT	CSNK2A1	protein	P31749	AKT1	UNIPROT	protein	YES	up-regulates	phosphorylation
P68400	UNIPROT	CSNK2A1	protein	P31749	AKT1	UNIPROT	protein	YES	up-regulates	phosphorylation
P68400	UNIPROT	CSNK2A1	protein	Q92688	ANP32B	UNIPROT	protein	YES	up-regulates	phosphorylation
P68400	UNIPROT	CSNK2A1	protein	Q92688	ANP32B	UNIPROT	protein	YES	up-regulates	phosphorylation
P68400	UNIPROT	CSNK2A1	protein	P18846	ATF1	UNIPROT	protein	YES	up-regulates	phosphorylation
P68400	UNIPROT	CSNK2A1	protein	P52907	CAPZA1	UNIPROT	protein	YES	up-regulates	phosphorylation
P68400	UNIPROT	CSNK2A1	protein	P45973	CBX5	UNIPROT	protein	YES	up-regulates	phosphorylation
P68400	UNIPROT	CSNK2A1	protein	P45973	CBX5	UNIPROT	protein	YES	up-regulates	phosphorylation
P68400	UNIPROT	CSNK2A1	protein	P45973	CBX5	UNIPROT	protein	YES	up-regulates	phosphorylation
P68400	UNIPROT	CSNK2A1	protein	P45973	CBX5	UNIPROT	protein	YES	up-regulates	phosphorylation
P68400	UNIPROT	CSNK2A1	protein	P30260	CDC27	UNIPROT	protein	YES	up-regulates	phosphorylation
P68400	UNIPROT	CSNK2A1	protein	P06493	CDK1	UNIPROT	protein	YES	up-regulates	phosphorylation
P68400	UNIPROT	CSNK2A1	protein	P30622	CLIP1	UNIPROT	protein	YES	up-regulates	phosphorylation
P68400	UNIPROT	CSNK2A1	protein	Q9ULV4	CORO1C	UNIPROT	protein	YES	up-regulates	phosphorylation
P68400	UNIPROT	CSNK2A1	protein	Q9UER7	DAXX	UNIPROT	protein	YES	up-regulates	phosphorylation
P68400	UNIPROT	CSNK2A1	protein	Q9UER7	DAXX	UNIPROT	protein	YES	up-regulates	phosphorylation
P68400	UNIPROT	CSNK2A1	protein	P35659	DEK	UNIPROT	protein	YES	up-regulates	phosphorylation
P68400	UNIPROT	CSNK2A1	protein	P35659	DEK	UNIPROT	protein	YES	up-regulates	phosphorylation
P68400	UNIPROT	CSNK2A1	protein	P20042	EIF2S2	UNIPROT	protein	YES	up-regulates	phosphorylation
P68400	UNIPROT	CSNK2A1	protein	P20042	EIF2S2	UNIPROT	protein	YES	up-regulates	phosphorylation
P68400	UNIPROT	CSNK2A1	protein	P55010	EIF5	UNIPROT	protein	YES	up-regulates	phosphorylation
P68400	UNIPROT	CSNK2A1	protein	P55010	EIF5	UNIPROT	protein	YES	up-regulates	phosphorylation
P68400	UNIPROT	CSNK2A1	protein	P55010	EIF5	UNIPROT	protein	YES	up-regulates	phosphorylation
P68400	UNIPROT	CSNK2A1	protein	Q06265	EXOSC9	UNIPROT	protein	YES	up-regulates	phosphorylation
P68400	UNIPROT	CSNK2A1	protein	Q06265	EXOSC9	UNIPROT	protein	YES	up-regulates	phosphorylation
P68400	UNIPROT	CSNK2A1	protein	Q13547	HDAC1	UNIPROT	protein	YES	up-regulates	phosphorylation
P68400	UNIPROT	CSNK2A1	protein	Q13547	HDAC1	UNIPROT	protein	YES	up-regulates	phosphorylation
P68400	UNIPROT	CSNK2A1	protein	Q92769	HDAC2	UNIPROT	protein	YES	up-regulates	phosphorylation
P68400	UNIPROT	CSNK2A1	protein	Q92769	HDAC2	UNIPROT	protein	YES	up-regulates	phosphorylation
P68400	UNIPROT	CSNK2A1	protein	P49736	MCM2	UNIPROT	protein	YES	up-regulates	phosphorylation
P68400	UNIPROT	CSNK2A1	protein	Q14676	MDC1	UNIPROT	protein	YES	up-regulates	phosphorylation
P68400	UNIPROT	CSNK2A1	protein	Q14676	MDC1	UNIPROT	protein	YES	up-regulates	phosphorylation
P68400	UNIPROT	CSNK2A1	protein	Q14676	MDC1	UNIPROT	protein	YES	up-regulates	phosphorylation

P68400	UNIPROT	CSNK2A1	protein	Q14676	MDC1	UNIPROT	protein	YES	up-regulates	phosphorylation
P68400	UNIPROT	CSNK2A1	protein	Q14676	MDC1	UNIPROT	protein	YES	up-regulates	phosphorylation
P68400	UNIPROT	CSNK2A1	protein	Q14676	MDC1	UNIPROT	protein	YES	up-regulates	phosphorylation
P68400	UNIPROT	CSNK2A1	protein	P35579	MYH9	UNIPROT	protein	YES	up-regulates	phosphorylation
P68400	UNIPROT	CSNK2A1	protein	P35579	MYH9	UNIPROT	protein	YES	up-regulates	phosphorylation
P68400	UNIPROT	CSNK2A1	protein	P35579	MYH9	UNIPROT	protein	YES	up-regulates	phosphorylation
P68400	UNIPROT	CSNK2A1	protein	P35579	MYH9	UNIPROT	protein	YES	up-regulates	phosphorylation
P68400	UNIPROT	CSNK2A1	protein	O14737	PDCD5	UNIPROT	protein	YES	up-regulates	phosphorylation
P68400	UNIPROT	CSNK2A1	protein	O75925	PIAS1	UNIPROT	protein	YES	up-regulates	phosphorylation
P68400	UNIPROT	CSNK2A1	protein	O75925	PIAS1	UNIPROT	protein	YES	up-regulates	phosphorylation
P68400	UNIPROT	CSNK2A1	protein	O75925	PIAS1	UNIPROT	protein	YES	up-regulates	phosphorylation
P68400	UNIPROT	CSNK2A1	protein	P48426	PIP4K2A	UNIPROT	protein	YES	up-regulates	phosphorylation
P68400	UNIPROT	CSNK2A1	protein	P41236	PPP1R2	UNIPROT	protein	YES	up-regulates	phosphorylation
P68400	UNIPROT	CSNK2A1	protein	P41236	PPP1R2	UNIPROT	protein	YES	up-regulates	phosphorylation
P68400	UNIPROT	CSNK2A1	protein	O43395	PRPF3	UNIPROT	protein	YES	up-regulates	phosphorylation
P68400	UNIPROT	CSNK2A1	protein	Q15185	PTGES3	UNIPROT	protein	YES	up-regulates	phosphorylation
P68400	UNIPROT	CSNK2A1	protein	Q15185	PTGES3	UNIPROT	protein	YES	up-regulates	phosphorylation
P68400	UNIPROT	CSNK2A1	protein	P46060	RANGAP1	UNIPROT	protein	YES	up-regulates	phosphorylation
P68400	UNIPROT	CSNK2A1	protein	Q9UBF6	RNF7	UNIPROT	protein	YES	up-regulates	phosphorylation
P68400	UNIPROT	CSNK2A1	protein	P05455	SSB	UNIPROT	protein	YES	up-regulates	phosphorylation
P68400	UNIPROT	CSNK2A1	protein	Q04724	TLE1	UNIPROT	protein	YES	up-regulates	phosphorylation
P68400	UNIPROT	CSNK2A1	protein	Q04724	TLE1	UNIPROT	protein	YES	up-regulates	phosphorylation
P68400	UNIPROT	CSNK2A1	protein	P11388	TOP2A	UNIPROT	protein	YES	up-regulates	phosphorylation
P68400	UNIPROT	CSNK2A1	protein	Q712K3	UBE2R2	UNIPROT	protein	YES	up-regulates	phosphorylation
P68400	UNIPROT	CSNK2A1	protein	P18887	XRCC1	UNIPROT	protein	YES	up-regulates	phosphorylation
P68400	UNIPROT	CSNK2A1	protein	P18887	XRCC1	UNIPROT	protein	YES	up-regulates	phosphorylation
P68400	UNIPROT	CSNK2A1	protein	P18887	XRCC1	UNIPROT	protein	YES	up-regulates	phosphorylation
P68400	UNIPROT	CSNK2A1	protein	P18887	XRCC1	UNIPROT	protein	YES	up-regulates	phosphorylation
P68400	UNIPROT	CSNK2A1	protein	P18887	XRCC1	UNIPROT	protein	YES	up-regulates	phosphorylation
P68400	UNIPROT	CSNK2A1	protein	P18887	XRCC1	UNIPROT	protein	YES	up-regulates	phosphorylation
P68400	UNIPROT	CSNK2A1	protein	P18887	XRCC1	UNIPROT	protein	YES	up-regulates	phosphorylation
P68400	UNIPROT	CSNK2A1	protein	P25490	YY1	UNIPROT	protein	YES	up-regulates	phosphorylation
P19784	UNIPROT	CSNK2A2	protein	Q00613	HSF1	UNIPROT	protein	YES	up-regulates	phosphorylation
P53355	UNIPROT	DAPK1	protein	Q14457	BECN1	UNIPROT	protein	YES	up-regulates	phosphorylation
P53355	UNIPROT	DAPK1	protein	Q14457	BECN1	UNIPROT	protein	YES	up-regulates	phosphorylation
P53355	UNIPROT	DAPK1	protein	P04637	TP53	UNIPROT	protein	YES	up-regulates	phosphorylation
P53355	UNIPROT	DAPK1	protein	P04637	TP53	UNIPROT	protein	YES	up-regulates	phosphorylation
Q13627	UNIPROT	DYRK1A	protein	Q13627	DYRK1A	UNIPROT	protein	YES	up-regulates	phosphorylation
Q13627	UNIPROT	DYRK1A	protein	Q13627	DYRK1A	UNIPROT	protein	YES	up-regulates	phosphorylation
Q13627	UNIPROT	DYRK1A	protein	P04637	TP53	UNIPROT	protein	YES	up-regulates	phosphorylation
P00533	UNIPROT	EGFR	protein	P04083	ANXA1	UNIPROT	protein	YES	up-regulates	phosphorylation
P00533	UNIPROT	EGFR	protein	P42566	EPS15	UNIPROT	protein	YES	up-regulates	phosphorylation
P00533	UNIPROT	EGFR	protein	P21860	ERBB3	UNIPROT	protein	YES	up-regulates	phosphorylation
P00533	UNIPROT	EGFR	protein	P21860	ERBB3	UNIPROT	protein	YES	up-regulates	phosphorylation
P00533	UNIPROT	EGFR	protein	P15311	EZR	UNIPROT	protein	YES	up-regulates	phosphorylation
P00533	UNIPROT	EGFR	protein	Q13480	GAB1	UNIPROT	protein	YES	up-regulates	phosphorylation
P00533	UNIPROT	EGFR	protein	Q13480	GAB1	UNIPROT	protein	YES	up-regulates	phosphorylation
P00533	UNIPROT	EGFR	protein	Q13480	GAB1	UNIPROT	protein	YES	up-regulates	phosphorylation
P00533	UNIPROT	EGFR	protein	Q13480	GAB1	UNIPROT	protein	YES	up-regulates	phosphorylation
P00533	UNIPROT	EGFR	protein	Q13480	GAB1	UNIPROT	protein	YES	up-regulates	phosphorylation
P00533	UNIPROT	EGFR	protein	P09211	GSTP1	UNIPROT	protein	YES	up-regulates	phosphorylation
P00533	UNIPROT	EGFR	protein	P09211	GSTP1	UNIPROT	protein	YES	up-regulates	phosphorylation
P00533	UNIPROT	EGFR	protein	P09211	GSTP1	UNIPROT	protein	YES	up-regulates	phosphorylation
P00533	UNIPROT	EGFR	protein	O14964	HGS	UNIPROT	protein	YES	up-regulates	phosphorylation
P00533	UNIPROT	EGFR	protein	P12004	PCNA	UNIPROT	protein	YES	up-regulates	phosphorylation
P00533	UNIPROT	EGFR	protein	O00750	PIK3C2B	UNIPROT	protein	YES	up-regulates	phosphorylation
P00533	UNIPROT	EGFR	protein	Q05397	PTK2	UNIPROT	protein	YES	up-regulates	phosphorylation
P00533	UNIPROT	EGFR	protein	Q05397	PTK2	UNIPROT	protein	YES	up-regulates	phosphorylation
P00533	UNIPROT	EGFR	protein	P18031	PTPN1	UNIPROT	protein	YES	up-regulates	phosphorylation
P00533	UNIPROT	EGFR	protein	P42224	STAT1	UNIPROT	protein	YES	up-regulates	phosphorylation
P00533	UNIPROT	EGFR	protein	P42224	STAT1	UNIPROT	protein	YES	up-regulates	phosphorylation
P00533	UNIPROT	EGFR	protein	P51692	STAT5B	UNIPROT	protein	YES	up-regulates	phosphorylation
P00533	UNIPROT	EGFR	protein	P51692	STAT5B	UNIPROT	protein	YES	up-regulates	phosphorylation
P00533	UNIPROT	EGFR	protein	P51692	STAT5B	UNIPROT	protein	YES	up-regulates	phosphorylation
P00533	UNIPROT	EGFR	protein	P52735	VAV2	UNIPROT	protein	YES	up-regulates	phosphorylation
P00533	UNIPROT	EGFR	protein	P52735	VAV2	UNIPROT	protein	YES	up-regulates	phosphorylation
P00533	UNIPROT	EGFR	protein	P52735	VAV2	UNIPROT	protein	YES	up-regulates	phosphorylation
P19525	UNIPROT	EIF2AK2	protein	P19525	EIF2AK2	UNIPROT	protein	YES	up-regulates	phosphorylation
P19525	UNIPROT	EIF2AK2	protein	P19525	EIF2AK2	UNIPROT	protein	YES	up-regulates	phosphorylation
P19525	UNIPROT	EIF2AK2	protein	P19525	EIF2AK2	UNIPROT	protein	YES	up-regulates	phosphorylation
P19525	UNIPROT	EIF2AK2	protein	P19525	EIF2AK2	UNIPROT	protein	YES	up-regulates	phosphorylation
P19525	UNIPROT	EIF2AK2	protein	P19525	EIF2AK2	UNIPROT	protein	YES	up-regulates	phosphorylation
P19525	UNIPROT	EIF2AK2	protein	P19525	EIF2AK2	UNIPROT	protein	YES	up-regulates	phosphorylation
P19525	UNIPROT	EIF2AK2	protein	P19525	EIF2AK2	UNIPROT	protein	YES	up-regulates	phosphorylation
P19525	UNIPROT	EIF2AK2	protein	Q15172	PPP2R5A	UNIPROT	protein	YES	up-regulates	phosphorylation
P19525	UNIPROT	EIF2AK2	protein	P04637	TP53	UNIPROT	protein	YES	up-regulates	phosphorylation
P29317	UNIPROT	EPHA2	protein	P29317	EPHA2	UNIPROT	protein	YES	up-regulates	phosphorylation
P29317	UNIPROT	EPHA2	protein	P29317	EPHA2	UNIPROT	protein	YES	up-regulates	phosphorylation
P29317	UNIPROT	EPHA2	protein	P29317	EPHA2	UNIPROT	protein	YES	up-regulates	phosphorylation
P29317	UNIPROT	EPHA2	protein	P29317	EPHA2	UNIPROT	protein	YES	up-regulates	phosphorylation
P04626	UNIPROT	ERBB2	protein	Q96N67	DOCK7	UNIPROT	protein	YES	up-regulates	phosphorylation
P04626	UNIPROT	ERBB2	protein	P04626	ERBB2	UNIPROT	protein	YES	up-regulates	phosphorylation
P04626	UNIPROT	ERBB2	protein	P04626	ERBB2	UNIPROT	protein	YES	up-regulates	phosphorylation
P04626	UNIPROT	ERBB2	protein	P04626	ERBB2	UNIPROT	protein	YES	up-regulates	phosphorylation
P04626	UNIPROT	ERBB2	protein	P04626	ERBB2	UNIPROT	protein	YES	up-regulates	phosphorylation
P21802	UNIPROT	FGFR2	protein	P62993	GRB2	UNIPROT	protein	YES	up-regulates	phosphorylation
P25098	UNIPROT	GRK2	protein	P15311	EZR	UNIPROT	protein	YES	up-regulates	phosphorylation
P25098	UNIPROT	GRK2	protein	P05387	RPLP2	UNIPROT	protein	YES	up-regulates	phosphorylation
P25098	UNIPROT	GRK2	protein	P05387	RPLP2	UNIPROT	protein	YES	up-regulates	phosphorylation
P49840	UNIPROT	GSK3A	protein	P49840	GSK3A	UNIPROT	protein	YES	up-regulates	phosphorylation
P49840	UNIPROT	GSK3A	protein	P49815	TSC2	UNIPROT	protein	YES	up-regulates	phosphorylation
P49840	UNIPROT	GSK3A	protein	P49815	TSC2	UNIPROT	protein	YES	up-regulates	phosphorylation
P49841	UNIPROT	GSK3B	protein	O15169	AXIN1	UNIPROT	protein	YES	up-regulates	phosphorylation
P49841	UNIPROT	GSK3B	protein	Q07812	BAX	UNIPROT	protein	YES	up-regulates	phosphorylation
P49841	UNIPROT	GSK3B	protein	P17676	CEBPB	UNIPROT	protein	YES	up-regulates	phosphorylation
P49841	UNIPROT	GSK3B	protein	P17676	CEBPB	UNIPROT	protein	YES	up-regulates	phosphorylation
P49841	UNIPROT	GSK3B	protein	P17676	CEBPB	UNIPROT	protein	YES	up-regulates	phosphorylation
P49841	UNIPROT	GSK3B	protein	P16401	HIST1H1B	UNIPROT	protein	YES	up-regulates	phosphorylation

P49841	UNIPROT	GSK3B	protein	Q92993	KAT5	UNIPROT	protein	YES	up-regulates	phosphorylation
P49841	UNIPROT	GSK3B	protein	Q04206	RELA	UNIPROT	protein	YES	up-regulates	phosphorylation
P49841	UNIPROT	GSK3B	protein	Q04206	RELA	UNIPROT	protein	YES	up-regulates	phosphorylation
P49841	UNIPROT	GSK3B	protein	Q96R06	SPAG5	UNIPROT	protein	YES	up-regulates	phosphorylation
P49841	UNIPROT	GSK3B	protein	Q96R06	SPAG5	UNIPROT	protein	YES	up-regulates	phosphorylation
P49841	UNIPROT	GSK3B	protein	Q96R06	SPAG5	UNIPROT	protein	YES	up-regulates	phosphorylation
P49841	UNIPROT	GSK3B	protein	Q96R06	SPAG5	UNIPROT	protein	YES	up-regulates	phosphorylation
P08069	UNIPROT	IGF1R	protein	P08069	IGF1R	UNIPROT	protein	YES	up-regulates	phosphorylation
P08069	UNIPROT	IGF1R	protein	P08069	IGF1R	UNIPROT	protein	YES	up-regulates	phosphorylation
P08069	UNIPROT	IGF1R	protein	P35568	IRS1	UNIPROT	protein	YES	up-regulates	phosphorylation
P08069	UNIPROT	IGF1R	protein	P35568	IRS1	UNIPROT	protein	YES	up-regulates	phosphorylation
P08069	UNIPROT	IGF1R	protein	P35568	IRS1	UNIPROT	protein	YES	up-regulates	phosphorylation
P08069	UNIPROT	IGF1R	protein	P35568	IRS1	UNIPROT	protein	YES	up-regulates	phosphorylation
P08069	UNIPROT	IGF1R	protein	P35568	IRS1	UNIPROT	protein	YES	up-regulates	phosphorylation
P08069	UNIPROT	IGF1R	protein	P35568	IRS1	UNIPROT	protein	YES	up-regulates	phosphorylation
P08069	UNIPROT	IGF1R	protein	P35568	IRS1	UNIPROT	protein	YES	up-regulates	phosphorylation
P08069	UNIPROT	IGF1R	protein	P35568	IRS1	UNIPROT	protein	YES	up-regulates	phosphorylation
P08069	UNIPROT	IGF1R	protein	P35568	IRS1	UNIPROT	protein	YES	up-regulates	phosphorylation
P08069	UNIPROT	IGF1R	protein	P35568	IRS1	UNIPROT	protein	YES	up-regulates	phosphorylation
P08069	UNIPROT	IGF1R	protein	Q9Y4H2	IRS2	UNIPROT	protein	YES	up-regulates	phosphorylation
P08069	UNIPROT	IGF1R	protein	O15530	PDPK1	UNIPROT	protein	YES	up-regulates	phosphorylation
P08069	UNIPROT	IGF1R	protein	O15530	PDPK1	UNIPROT	protein	YES	up-regulates	phosphorylation
P08069	UNIPROT	IGF1R	protein	O15530	PDPK1	UNIPROT	protein	YES	up-regulates	phosphorylation
P08069	UNIPROT	IGF1R	protein	O15530	PDPK1	UNIPROT	protein	YES	up-regulates	phosphorylation
P08069	UNIPROT	IGF1R	protein	O00443	PIK3C2A	UNIPROT	protein	YES	up-regulates	phosphorylation
Q13418	UNIPROT	ILK	protein	P31749	AKT1	UNIPROT	protein	YES	up-regulates	phosphorylation
Q13418	UNIPROT	ILK	protein	Q13765	NACA	UNIPROT	protein	YES	up-regulates	phosphorylation
Q13418	UNIPROT	ILK	protein	E9PAV3	NACA	UNIPROT	protein	YES	up-regulates	phosphorylation
P51617	UNIPROT	IRAK1	protein	P40763	STAT3	UNIPROT	protein	YES	up-regulates	phosphorylation
Q02750	UNIPROT	MAP2K1	protein	P27361	MAPK3	UNIPROT	protein	YES	up-regulates	phosphorylation
Q02750	UNIPROT	MAP2K1	protein	P27361	MAPK3	UNIPROT	protein	YES	up-regulates	phosphorylation
Q02750	UNIPROT	MAP2K1	protein	P27361	MAPK3	UNIPROT	protein	YES	up-regulates	phosphorylation
P36507	UNIPROT	MAP2K2	protein	P28482	MAPK1	UNIPROT	protein	YES	up-regulates	phosphorylation
P36507	UNIPROT	MAP2K2	protein	P28482	MAPK1	UNIPROT	protein	YES	up-regulates	phosphorylation
P36507	UNIPROT	MAP2K2	protein	P27361	MAPK3	UNIPROT	protein	YES	up-regulates	phosphorylation
P36507	UNIPROT	MAP2K2	protein	P27361	MAPK3	UNIPROT	protein	YES	up-regulates	phosphorylation
P45985	UNIPROT	MAP2K4	protein	P45984	MAPK9	UNIPROT	protein	YES	up-regulates	phosphorylation
P52564	UNIPROT	MAP2K6	protein	P46108	CRK	UNIPROT	protein	YES	up-regulates	phosphorylation
P52564	UNIPROT	MAP2K6	protein	O15264	MAPK13	UNIPROT	protein	YES	up-regulates	phosphorylation
P52564	UNIPROT	MAP2K6	protein	O15264	MAPK13	UNIPROT	protein	YES	up-regulates	phosphorylation
P52564	UNIPROT	MAP2K6	protein	P45984	MAPK9	UNIPROT	protein	YES	up-regulates	phosphorylation
O14733	UNIPROT	MAP2K7	protein	P45983	MAPK8	UNIPROT	protein	YES	up-regulates	phosphorylation
O14733	UNIPROT	MAP2K7	protein	P45983	MAPK8	UNIPROT	protein	YES	up-regulates	phosphorylation
O14733	UNIPROT	MAP2K7	protein	P45983	MAPK8	UNIPROT	protein	YES	up-regulates	phosphorylation
O14733	UNIPROT	MAP2K7	protein	P45984	MAPK9	UNIPROT	protein	YES	up-regulates	phosphorylation
O14733	UNIPROT	MAP2K7	protein	P45984	MAPK9	UNIPROT	protein	YES	up-regulates	phosphorylation
O14733	UNIPROT	MAP2K7	protein	P45984	MAPK9	UNIPROT	protein	YES	up-regulates	phosphorylation
Q9Y2U5	UNIPROT	MAP3K2	protein	Q02750	MAP2K1	UNIPROT	protein	YES	up-regulates	phosphorylation
Q9NYL2	UNIPROT	MAP3K20	protein	Q9NYL2	MAP3K20	UNIPROT	protein	YES	up-regulates	phosphorylation
Q9NYL2	UNIPROT	MAP3K20	protein	Q9NYL2	MAP3K20	UNIPROT	protein	YES	up-regulates	phosphorylation
Q9NYL2	UNIPROT	MAP3K20	protein	Q9NYL2	MAP3K20	UNIPROT	protein	YES	up-regulates	phosphorylation
Q99759	UNIPROT	MAP3K3	protein	Q99759	MAP3K3	UNIPROT	protein	YES	up-regulates	phosphorylation
P41279	UNIPROT	MAP3K8	protein	Q02750	MAP2K1	UNIPROT	protein	YES	up-regulates	phosphorylation
P41279	UNIPROT	MAP3K8	protein	Q02750	MAP2K1	UNIPROT	protein	YES	up-regulates	phosphorylation
P41279	UNIPROT	MAP3K8	protein	Q02750	MAP2K1	UNIPROT	protein	YES	up-regulates	phosphorylation
P41279	UNIPROT	MAP3K8	protein	Q02750	MAP2K1	UNIPROT	protein	YES	up-regulates	phosphorylation
P41279	UNIPROT	MAP3K8	protein	P36507	MAP2K2	UNIPROT	protein	YES	up-regulates	phosphorylation
P41279	UNIPROT	MAP3K8	protein	P36507	MAP2K2	UNIPROT	protein	YES	up-regulates	phosphorylation
P41279	UNIPROT	MAP3K8	protein	P45985	MAP2K4	UNIPROT	protein	YES	up-regulates	phosphorylation
P41279	UNIPROT	MAP3K8	protein	P53350	PLK1	UNIPROT	protein	YES	up-regulates	phosphorylation
P28482	UNIPROT	MAPK1	protein	Q8IZP0	ABI1	UNIPROT	protein	YES	up-regulates	phosphorylation
P28482	UNIPROT	MAPK1	protein	Q8IZP0	ABI1	UNIPROT	protein	YES	up-regulates	phosphorylation
P28482	UNIPROT	MAPK1	protein	Q8IZP0	ABI1	UNIPROT	protein	YES	up-regulates	phosphorylation
P28482	UNIPROT	MAPK1	protein	Q92974	ARHGEF2	UNIPROT	protein	YES	up-regulates	phosphorylation
P28482	UNIPROT	MAPK1	protein	P15336	ATF2	UNIPROT	protein	YES	up-regulates	phosphorylation
P28482	UNIPROT	MAPK1	protein	P15336	ATF2	UNIPROT	protein	YES	up-regulates	phosphorylation
P28482	UNIPROT	MAPK1	protein	P27708	CAD	UNIPROT	protein	YES	up-regulates	phosphorylation
P28482	UNIPROT	MAPK1	protein	P17655	CAPN2	UNIPROT	protein	YES	up-regulates	phosphorylation
P28482	UNIPROT	MAPK1	protein	P24941	CDK2	UNIPROT	protein	YES	up-regulates	phosphorylation
P28482	UNIPROT	MAPK1	protein	P46527	CDKN1B	UNIPROT	protein	YES	up-regulates	phosphorylation
P28482	UNIPROT	MAPK1	protein	P46527	CDKN1B	UNIPROT	protein	YES	up-regulates	phosphorylation
P28482	UNIPROT	MAPK1	protein	P17676	CEBPB	UNIPROT	protein	YES	up-regulates	phosphorylation
P28482	UNIPROT	MAPK1	protein	P68400	CSNK2A1	UNIPROT	protein	YES	up-regulates	phosphorylation
P28482	UNIPROT	MAPK1	protein	P68400	CSNK2A1	UNIPROT	protein	YES	up-regulates	phosphorylation
P28482	UNIPROT	MAPK1	protein	Q14247	CTTN	UNIPROT	protein	YES	up-regulates	phosphorylation
P28482	UNIPROT	MAPK1	protein	Q14247	CTTN	UNIPROT	protein	YES	up-regulates	phosphorylation
P28482	UNIPROT	MAPK1	protein	Q14247	CTTN	UNIPROT	protein	YES	up-regulates	phosphorylation
P28482	UNIPROT	MAPK1	protein	Q14247	CTTN	UNIPROT	protein	YES	up-regulates	phosphorylation
P28482	UNIPROT	MAPK1	protein	P53355	DAPK1	UNIPROT	protein	YES	up-regulates	phosphorylation
P28482	UNIPROT	MAPK1	protein	P19525	EIF2AK2	UNIPROT	protein	YES	up-regulates	phosphorylation
P28482	UNIPROT	MAPK1	protein	Q09472	EP300	UNIPROT	protein	YES	up-regulates	phosphorylation
P28482	UNIPROT	MAPK1	protein	Q09472	EP300	UNIPROT	protein	YES	up-regulates	phosphorylation
P28482	UNIPROT	MAPK1	protein	Q09472	EP300	UNIPROT	protein	YES	up-regulates	phosphorylation
P28482	UNIPROT	MAPK1	protein	P50548	ERF	UNIPROT	protein	YES	up-regulates	phosphorylation
P28482	UNIPROT	MAPK1	protein	P50548	ERF	UNIPROT	protein	YES	up-regulates	phosphorylation
P28482	UNIPROT	MAPK1	protein	P50548	ERF	UNIPROT	protein	YES	up-regulates	phosphorylation
P28482	UNIPROT	MAPK1	protein	Q9UPT5	EXOC7	UNIPROT	protein	YES	up-regulates	phosphorylation
P28482	UNIPROT	MAPK1	protein	P49841	GSK3B	UNIPROT	protein	YES	up-regulates	phosphorylation
P28482	UNIPROT	MAPK1	protein	P78347	GTF2I	UNIPROT	protein	YES	up-regulates	phosphorylation
P28482	UNIPROT	MAPK1	protein	P78347	GTF2I	UNIPROT	protein	YES	up-regulates	phosphorylation
P28482	UNIPROT	MAPK1	protein	P84243	H3F3A	UNIPROT	protein	YES	up-regulates	phosphorylation
P28482	UNIPROT	MAPK1	protein	P17535	JUND	UNIPROT	protein	YES	up-regulates	phosphorylation
P28482	UNIPROT	MAPK1	protein	Q07666	KHDRBS1	UNIPROT	protein	YES	up-regulates	phosphorylation
P28482	UNIPROT	MAPK1	protein	Q07666	KHDRBS1	UNIPROT	protein	YES	up-regulates	phosphorylation
P28482	UNIPROT	MAPK1	protein	Q07666	KHDRBS1	UNIPROT	protein	YES	up-regulates	phosphorylation
P28482	UNIPROT	MAPK1	protein	P49137	MAPKAPK2	UNIPROT	protein	YES	up-regulates	phosphorylation
P28482	UNIPROT	MAPK1	protein	P49137	MAPKAPK2	UNIPROT	protein	YES	up-regulates	phosphorylation
P28482	UNIPROT	MAPK1	protein	P49137	MAPKAPK2	UNIPROT	protein	YES	up-regulates	phosphorylation

P28482	UNIPROT	MAPK1	protein	Q07820	MCL1	UNIPROT	protein	YES	up-regulates	phosphorylation
P28482	UNIPROT	MAPK1	protein	Q07820	MCL1	UNIPROT	protein	YES	up-regulates	phosphorylation
P28482	UNIPROT	MAPK1	protein	Q15648	MED1	UNIPROT	protein	YES	up-regulates	phosphorylation
P28482	UNIPROT	MAPK1	protein	Q15648	MED1	UNIPROT	protein	YES	up-regulates	phosphorylation
P28482	UNIPROT	MAPK1	protein	P01106	MYC	UNIPROT	protein	YES	up-regulates	phosphorylation
P28482	UNIPROT	MAPK1	protein	Q9UKX7	NUP50	UNIPROT	protein	YES	up-regulates	phosphorylation
P28482	UNIPROT	MAPK1	protein	Q9UKX7	NUP50	UNIPROT	protein	YES	up-regulates	phosphorylation
P28482	UNIPROT	MAPK1	protein	P09874	PARP1	UNIPROT	protein	YES	up-regulates	phosphorylation
P28482	UNIPROT	MAPK1	protein	P09874	PARP1	UNIPROT	protein	YES	up-regulates	phosphorylation
P28482	UNIPROT	MAPK1	protein	P29590	PML	UNIPROT	protein	YES	up-regulates	phosphorylation
P28482	UNIPROT	MAPK1	protein	P29590	PML	UNIPROT	protein	YES	up-regulates	phosphorylation
P28482	UNIPROT	MAPK1	protein	P29590	PML	UNIPROT	protein	YES	up-regulates	phosphorylation
P28482	UNIPROT	MAPK1	protein	P29590	PML	UNIPROT	protein	YES	up-regulates	phosphorylation
P28482	UNIPROT	MAPK1	protein	P29590	PML	UNIPROT	protein	YES	up-regulates	phosphorylation
P28482	UNIPROT	MAPK1	protein	P29590	PML	UNIPROT	protein	YES	up-regulates	phosphorylation
P28482	UNIPROT	MAPK1	protein	P30041	PRDX6	UNIPROT	protein	YES	up-regulates	phosphorylation
P28482	UNIPROT	MAPK1	protein	P23396	RPS3	UNIPROT	protein	YES	up-regulates	phosphorylation
P28482	UNIPROT	MAPK1	protein	P51812	RPS6KA3	UNIPROT	protein	YES	up-regulates	phosphorylation
P28482	UNIPROT	MAPK1	protein	P19634	SLC9A1	UNIPROT	protein	YES	up-regulates	phosphorylation
P28482	UNIPROT	MAPK1	protein	Q15796	SMAD2	UNIPROT	protein	YES	up-regulates	phosphorylation
P28482	UNIPROT	MAPK1	protein	Q15796	SMAD2	UNIPROT	protein	YES	up-regulates	phosphorylation
P28482	UNIPROT	MAPK1	protein	Q15796	SMAD2	UNIPROT	protein	YES	up-regulates	phosphorylation
P28482	UNIPROT	MAPK1	protein	Q15796	SMAD2	UNIPROT	protein	YES	up-regulates	phosphorylation
P28482	UNIPROT	MAPK1	protein	P08047	SP1	UNIPROT	protein	YES	up-regulates	phosphorylation
P28482	UNIPROT	MAPK1	protein	P08047	SP1	UNIPROT	protein	YES	up-regulates	phosphorylation
P28482	UNIPROT	MAPK1	protein	P08047	SP1	UNIPROT	protein	YES	up-regulates	phosphorylation
P28482	UNIPROT	MAPK1	protein	P08047	SP1	UNIPROT	protein	YES	up-regulates	phosphorylation
P28482	UNIPROT	MAPK1	protein	Q02447	SP3	UNIPROT	protein	YES	up-regulates	phosphorylation
P28482	UNIPROT	MAPK1	protein	Q9NRA0	SPHK2	UNIPROT	protein	YES	up-regulates	phosphorylation
P28482	UNIPROT	MAPK1	protein	Q9NRA0	SPHK2	UNIPROT	protein	YES	up-regulates	phosphorylation
P28482	UNIPROT	MAPK1	protein	Q13586	STIM1	UNIPROT	protein	YES	up-regulates	phosphorylation
P28482	UNIPROT	MAPK1	protein	P12270	TPR	UNIPROT	protein	YES	up-regulates	phosphorylation
P28482	UNIPROT	MAPK1	protein	P12270	TPR	UNIPROT	protein	YES	up-regulates	phosphorylation
P28482	UNIPROT	MAPK1	protein	P12270	TPR	UNIPROT	protein	YES	up-regulates	phosphorylation
P28482	UNIPROT	MAPK1	protein	P12270	TPR	UNIPROT	protein	YES	up-regulates	phosphorylation
O15264	UNIPROT	MAPK13	protein	P04637	TP53	UNIPROT	protein	YES	up-regulates	phosphorylation
O15264	UNIPROT	MAPK13	protein	P04637	TP53	UNIPROT	protein	YES	up-regulates	phosphorylation
Q16539	UNIPROT	MAPK14	protein	P15336	ATF2	UNIPROT	protein	YES	up-regulates	phosphorylation
Q16539	UNIPROT	MAPK14	protein	P15336	ATF2	UNIPROT	protein	YES	up-regulates	phosphorylation
Q16539	UNIPROT	MAPK14	protein	P15336	ATF2	UNIPROT	protein	YES	up-regulates	phosphorylation
Q16539	UNIPROT	MAPK14	protein	P15336	ATF2	UNIPROT	protein	YES	up-regulates	phosphorylation
Q16539	UNIPROT	MAPK14	protein	P15336	ATF2	UNIPROT	protein	YES	up-regulates	phosphorylation
Q16539	UNIPROT	MAPK14	protein	P15336	ATF2	UNIPROT	protein	YES	up-regulates	phosphorylation
Q16539	UNIPROT	MAPK14	protein	Q9UIG0	BAZ1B	UNIPROT	protein	YES	up-regulates	phosphorylation
Q16539	UNIPROT	MAPK14	protein	Q15075	EEA1	UNIPROT	protein	YES	up-regulates	phosphorylation
Q16539	UNIPROT	MAPK14	protein	Q15717	ELAVL1	UNIPROT	protein	YES	up-regulates	phosphorylation
Q16539	UNIPROT	MAPK14	protein	Q15717	ELAVL1	UNIPROT	protein	YES	up-regulates	phosphorylation
Q16539	UNIPROT	MAPK14	protein	P42566	EPS15	UNIPROT	protein	YES	up-regulates	phosphorylation
Q16539	UNIPROT	MAPK14	protein	O43524	FOXO3	UNIPROT	protein	YES	up-regulates	phosphorylation
Q16539	UNIPROT	MAPK14	protein	P84243	H3F3A	UNIPROT	protein	YES	up-regulates	phosphorylation
Q16539	UNIPROT	MAPK14	protein	P68431	HIST1H3A	UNIPROT	protein	YES	up-regulates	phosphorylation
Q16539	UNIPROT	MAPK14	protein	O75676	RPS6KA4	UNIPROT	protein	YES	up-regulates	phosphorylation
Q16539	UNIPROT	MAPK14	protein	O75676	RPS6KA4	UNIPROT	protein	YES	up-regulates	phosphorylation
Q16539	UNIPROT	MAPK14	protein	O75676	RPS6KA4	UNIPROT	protein	YES	up-regulates	phosphorylation
Q16539	UNIPROT	MAPK14	protein	O75676	RPS6KA4	UNIPROT	protein	YES	up-regulates	phosphorylation
Q16539	UNIPROT	MAPK14	protein	O75676	RPS6KA4	UNIPROT	protein	YES	up-regulates	phosphorylation
Q16539	UNIPROT	MAPK14	protein	O75676	RPS6KA4	UNIPROT	protein	YES	up-regulates	phosphorylation
Q16539	UNIPROT	MAPK14	protein	P19634	SLC9A1	UNIPROT	protein	YES	up-regulates	phosphorylation
Q16539	UNIPROT	MAPK14	protein	P19634	SLC9A1	UNIPROT	protein	YES	up-regulates	phosphorylation
Q16539	UNIPROT	MAPK14	protein	P19634	SLC9A1	UNIPROT	protein	YES	up-regulates	phosphorylation
Q16539	UNIPROT	MAPK14	protein	P19634	SLC9A1	UNIPROT	protein	YES	up-regulates	phosphorylation
Q16539	UNIPROT	MAPK14	protein	P19634	SLC9A1	UNIPROT	protein	YES	up-regulates	phosphorylation
Q16539	UNIPROT	MAPK14	protein	P19634	SLC9A1	UNIPROT	protein	YES	up-regulates	phosphorylation
Q16539	UNIPROT	MAPK14	protein	P40763	STAT3	UNIPROT	protein	YES	up-regulates	phosphorylation
Q16539	UNIPROT	MAPK14	protein	P40763	STAT3	UNIPROT	protein	YES	up-regulates	phosphorylation
Q16539	UNIPROT	MAPK14	protein	P04637	TP53	UNIPROT	protein	YES	up-regulates	phosphorylation
Q16539	UNIPROT	MAPK14	protein	P04637	TP53	UNIPROT	protein	YES	up-regulates	phosphorylation
Q16539	UNIPROT	MAPK14	protein	P04637	TP53	UNIPROT	protein	YES	up-regulates	phosphorylation
Q16539	UNIPROT	MAPK14	protein	P04637	TP53	UNIPROT	protein	YES	up-regulates	phosphorylation
P27361	UNIPROT	MAPK3	protein	Q8IZP0	ABI1	UNIPROT	protein	YES	up-regulates	phosphorylation
P27361	UNIPROT	MAPK3	protein	P78536	ADAM17	UNIPROT	protein	YES	up-regulates	phosphorylation
P27361	UNIPROT	MAPK3	protein	Q92974	ARHGEF2	UNIPROT	protein	YES	up-regulates	phosphorylation
P27361	UNIPROT	MAPK3	protein	P15336	ATF2	UNIPROT	protein	YES	up-regulates	phosphorylation
P27361	UNIPROT	MAPK3	protein	P15336	ATF2	UNIPROT	protein	YES	up-regulates	phosphorylation
P27361	UNIPROT	MAPK3	protein	P15336	ATF2	UNIPROT	protein	YES	up-regulates	phosphorylation
P27361	UNIPROT	MAPK3	protein	P15336	ATF2	UNIPROT	protein	YES	up-regulates	phosphorylation
P27361	UNIPROT	MAPK3	protein	Q9UIG0	BAZ1B	UNIPROT	protein	YES	up-regulates	phosphorylation
P27361	UNIPROT	MAPK3	protein	P27708	CAD	UNIPROT	protein	YES	up-regulates	phosphorylation
P27361	UNIPROT	MAPK3	protein	P17655	CAPN2	UNIPROT	protein	YES	up-regulates	phosphorylation
P27361	UNIPROT	MAPK3	protein	P17676	CEBPB	UNIPROT	protein	YES	up-regulates	phosphorylation
P27361	UNIPROT	MAPK3	protein	Q14247	CTTN	UNIPROT	protein	YES	up-regulates	phosphorylation
P27361	UNIPROT	MAPK3	protein	Q14247	CTTN	UNIPROT	protein	YES	up-regulates	phosphorylation
P27361	UNIPROT	MAPK3	protein	Q14247	CTTN	UNIPROT	protein	YES	up-regulates	phosphorylation
P27361	UNIPROT	MAPK3	protein	Q14247	CTTN	UNIPROT	protein	YES	up-regulates	phosphorylation
P27361	UNIPROT	MAPK3	protein	P01100	FOS	UNIPROT	protein	YES	up-regulates	phosphorylation
P27361	UNIPROT	MAPK3	protein	P01100	FOS	UNIPROT	protein	YES	up-regulates	phosphorylation
P27361	UNIPROT	MAPK3	protein	P01100	FOS	UNIPROT	protein	YES	up-regulates	phosphorylation
P27361	UNIPROT	MAPK3	protein	P01100	FOS	UNIPROT	protein	YES	up-regulates	phosphorylation
P27361	UNIPROT	MAPK3	protein	P78347	GTF2I	UNIPROT	protein	YES	up-regulates	phosphorylation
P27361	UNIPROT	MAPK3	protein	P78347	GTF2I	UNIPROT	protein	YES	up-regulates	phosphorylation
P27361	UNIPROT	MAPK3	protein	P84243	H3F3A	UNIPROT	protein	YES	up-regulates	phosphorylation
P27361	UNIPROT	MAPK3	protein	Q9UBN7	HDAC6	UNIPROT	protein	YES	up-regulates	phosphorylation
P27361	UNIPROT	MAPK3	protein	Q9UBN7	HDAC6	UNIPROT	protein	YES	up-regulates	phosphorylation
P27361	UNIPROT	MAPK3	protein	P05412	JUN	UNIPROT	protein	YES	up-regulates	phosphorylation
P27361	UNIPROT	MAPK3	protein	P05412	JUN	UNIPROT	protein	YES	up-regulates	phosphorylation
P27361	UNIPROT	MAPK3	protein	P17535	JUND	UNIPROT	protein	YES	up-regulates	phosphorylation
P27361	UNIPROT	MAPK3	protein	Q15046	KARS	UNIPROT	protein	YES	up-regulates	phosphorylation
P27361	UNIPROT	MAPK3	protein	P49137	MAPKAPK2	UNIPROT	protein	YES	up-regulates	phosphorylation

P27361	UNIPROT	MAPK3	protein	P49137	MAPKAPK2	UNIPROT	protein	YES	up-regulates	phosphorylation
P27361	UNIPROT	MAPK3	protein	Q07820	MCL1	UNIPROT	protein	YES	up-regulates	phosphorylation
P27361	UNIPROT	MAPK3	protein	Q15648	MED1	UNIPROT	protein	YES	up-regulates	phosphorylation
P27361	UNIPROT	MAPK3	protein	Q15648	MED1	UNIPROT	protein	YES	up-regulates	phosphorylation
P27361	UNIPROT	MAPK3	protein	P05976	MYL1	UNIPROT	protein	YES	up-regulates	phosphorylation
P27361	UNIPROT	MAPK3	protein	P47712	PLA2G4A	UNIPROT	protein	YES	up-regulates	phosphorylation
P27361	UNIPROT	MAPK3	protein	P29590	PML	UNIPROT	protein	YES	up-regulates	phosphorylation
P27361	UNIPROT	MAPK3	protein	P23396	RPS3	UNIPROT	protein	YES	up-regulates	phosphorylation
P27361	UNIPROT	MAPK3	protein	P51812	RPS6KA3	UNIPROT	protein	YES	up-regulates	phosphorylation
P27361	UNIPROT	MAPK3	protein	P51812	RPS6KA3	UNIPROT	protein	YES	up-regulates	phosphorylation
P27361	UNIPROT	MAPK3	protein	P51812	RPS6KA3	UNIPROT	protein	YES	up-regulates	phosphorylation
P27361	UNIPROT	MAPK3	protein	O75676	RPS6KA4	UNIPROT	protein	YES	up-regulates	phosphorylation
P27361	UNIPROT	MAPK3	protein	Q9NYV6	RRN3	UNIPROT	protein	YES	up-regulates	phosphorylation
P27361	UNIPROT	MAPK3	protein	Q9NYV6	RRN3	UNIPROT	protein	YES	up-regulates	phosphorylation
P27361	UNIPROT	MAPK3	protein	P19793	RXRA	UNIPROT	protein	YES	up-regulates	phosphorylation
P27361	UNIPROT	MAPK3	protein	Q15796	SMAD2	UNIPROT	protein	YES	up-regulates	phosphorylation
P27361	UNIPROT	MAPK3	protein	Q15796	SMAD2	UNIPROT	protein	YES	up-regulates	phosphorylation
P27361	UNIPROT	MAPK3	protein	Q15796	SMAD2	UNIPROT	protein	YES	up-regulates	phosphorylation
P27361	UNIPROT	MAPK3	protein	Q15796	SMAD2	UNIPROT	protein	YES	up-regulates	phosphorylation
P27361	UNIPROT	MAPK3	protein	Q15796	SMAD2	UNIPROT	protein	YES	up-regulates	phosphorylation
P27361	UNIPROT	MAPK3	protein	Q15796	SMAD2	UNIPROT	protein	YES	up-regulates	phosphorylation
P27361	UNIPROT	MAPK3	protein	Q15796	SMAD2	UNIPROT	protein	YES	up-regulates	phosphorylation
P27361	UNIPROT	MAPK3	protein	Q15796	SMAD2	UNIPROT	protein	YES	up-regulates	phosphorylation
P27361	UNIPROT	MAPK3	protein	Q15796	SMAD2	UNIPROT	protein	YES	up-regulates	phosphorylation
P27361	UNIPROT	MAPK3	protein	Q15796	SMAD2	UNIPROT	protein	YES	up-regulates	phosphorylation
P27361	UNIPROT	MAPK3	protein	Q15796	SMAD2	UNIPROT	protein	YES	up-regulates	phosphorylation
P27361	UNIPROT	MAPK3	protein	Q15796	SMAD2	UNIPROT	protein	YES	up-regulates	phosphorylation
P27361	UNIPROT	MAPK3	protein	Q15796	SMAD2	UNIPROT	protein	YES	up-regulates	phosphorylation
P27361	UNIPROT	MAPK3	protein	Q15796	SMAD2	UNIPROT	protein	YES	up-regulates	phosphorylation
P27361	UNIPROT	MAPK3	protein	Q15796	SMAD2	UNIPROT	protein	YES	up-regulates	phosphorylation
P27361	UNIPROT	MAPK3	protein	P08047	SP1	UNIPROT	protein	YES	up-regulates	phosphorylation
P27361	UNIPROT	MAPK3	protein	P08047	SP1	UNIPROT	protein	YES	up-regulates	phosphorylation
P27361	UNIPROT	MAPK3	protein	P08047	SP1	UNIPROT	protein	YES	up-regulates	phosphorylation
P27361	UNIPROT	MAPK3	protein	P08047	SP1	UNIPROT	protein	YES	up-regulates	phosphorylation
P27361	UNIPROT	MAPK3	protein	P08047	SP1	UNIPROT	protein	YES	up-regulates	phosphorylation
P27361	UNIPROT	MAPK3	protein	P08047	SP1	UNIPROT	protein	YES	up-regulates	phosphorylation
P27361	UNIPROT	MAPK3	protein	Q02447	SP3	UNIPROT	protein	YES	up-regulates	phosphorylation
P27361	UNIPROT	MAPK3	protein	Q9NRA0	SPHK2	UNIPROT	protein	YES	up-regulates	phosphorylation
P27361	UNIPROT	MAPK3	protein	Q9NRA0	SPHK2	UNIPROT	protein	YES	up-regulates	phosphorylation
P27361	UNIPROT	MAPK3	protein	P40763	STAT3	UNIPROT	protein	YES	up-regulates	phosphorylation
P27361	UNIPROT	MAPK3	protein	P40763	STAT3	UNIPROT	protein	YES	up-regulates	phosphorylation
P27361	UNIPROT	MAPK3	protein	P40763	STAT3	UNIPROT	protein	YES	up-regulates	phosphorylation
P27361	UNIPROT	MAPK3	protein	P40763	STAT3	UNIPROT	protein	YES	up-regulates	phosphorylation
P27361	UNIPROT	MAPK3	protein	P04637	TP53	UNIPROT	protein	YES	up-regulates	phosphorylation
P45983	UNIPROT	MAPK8	protein	Q06481	APLP2	UNIPROT	protein	YES	up-regulates	phosphorylation
P45983	UNIPROT	MAPK8	protein	P05067	APP	UNIPROT	protein	YES	up-regulates	phosphorylation
P45983	UNIPROT	MAPK8	protein	P05067	APP	UNIPROT	protein	YES	up-regulates	phosphorylation
P45983	UNIPROT	MAPK8	protein	P15336	ATF2	UNIPROT	protein	YES	up-regulates	phosphorylation
P45983	UNIPROT	MAPK8	protein	P15336	ATF2	UNIPROT	protein	YES	up-regulates	phosphorylation
P45983	UNIPROT	MAPK8	protein	P15336	ATF2	UNIPROT	protein	YES	up-regulates	phosphorylation
P45983	UNIPROT	MAPK8	protein	P15336	ATF2	UNIPROT	protein	YES	up-regulates	phosphorylation
P45983	UNIPROT	MAPK8	protein	P16104	H2AFX	UNIPROT	protein	YES	up-regulates	phosphorylation
P45983	UNIPROT	MAPK8	protein	P84243	H3F3A	UNIPROT	protein	YES	up-regulates	phosphorylation
P45983	UNIPROT	MAPK8	protein	P61978	HNRNPK	UNIPROT	protein	YES	up-regulates	phosphorylation
P45983	UNIPROT	MAPK8	protein	P61978	HNRNPK	UNIPROT	protein	YES	up-regulates	phosphorylation
P45983	UNIPROT	MAPK8	protein	Q14653	IRF3	UNIPROT	protein	YES	up-regulates	phosphorylation
P45983	UNIPROT	MAPK8	protein	Q96J02	ITCH	UNIPROT	protein	YES	up-regulates	phosphorylation
P45983	UNIPROT	MAPK8	protein	P17535	JUND	UNIPROT	protein	YES	up-regulates	phosphorylation
P45983	UNIPROT	MAPK8	protein	P45984	MAPK9	UNIPROT	protein	YES	up-regulates	phosphorylation
P45983	UNIPROT	MAPK8	protein	Q07820	MCL1	UNIPROT	protein	YES	up-regulates	phosphorylation
P45983	UNIPROT	MAPK8	protein	Q07820	MCL1	UNIPROT	protein	YES	up-regulates	phosphorylation
P45983	UNIPROT	MAPK8	protein	Q96EB6	SIRT1	UNIPROT	protein	YES	up-regulates	phosphorylation
P45983	UNIPROT	MAPK8	protein	Q96EB6	SIRT1	UNIPROT	protein	YES	up-regulates	phosphorylation
P45983	UNIPROT	MAPK8	protein	Q96EB6	SIRT1	UNIPROT	protein	YES	up-regulates	phosphorylation
P45983	UNIPROT	MAPK8	protein	P08047	SP1	UNIPROT	protein	YES	up-regulates	phosphorylation
P45983	UNIPROT	MAPK8	protein	P08047	SP1	UNIPROT	protein	YES	up-regulates	phosphorylation
P45983	UNIPROT	MAPK8	protein	Q13043	STK4	UNIPROT	protein	YES	up-regulates	phosphorylation
P45983	UNIPROT	MAPK8	protein	P04637	TP53	UNIPROT	protein	YES	up-regulates	phosphorylation
P45983	UNIPROT	MAPK8	protein	P04637	TP53	UNIPROT	protein	YES	up-regulates	phosphorylation
P45983	UNIPROT	MAPK8	protein	P04637	TP53	UNIPROT	protein	YES	up-regulates	phosphorylation
P45984	UNIPROT	MAPK9	protein	P15336	ATF2	UNIPROT	protein	YES	up-regulates	phosphorylation
P45984	UNIPROT	MAPK9	protein	P15336	ATF2	UNIPROT	protein	YES	up-regulates	phosphorylation
P45984	UNIPROT	MAPK9	protein	P15336	ATF2	UNIPROT	protein	YES	up-regulates	phosphorylation
P45984	UNIPROT	MAPK9	protein	P15336	ATF2	UNIPROT	protein	YES	up-regulates	phosphorylation
P45984	UNIPROT	MAPK9	protein	Q9UIG0	BAZ1B	UNIPROT	protein	YES	up-regulates	phosphorylation
P45984	UNIPROT	MAPK9	protein	P35222	CTNNB1	UNIPROT	protein	YES	up-regulates	phosphorylation
P45984	UNIPROT	MAPK9	protein	P35222	CTNNB1	UNIPROT	protein	YES	up-regulates	phosphorylation
P45984	UNIPROT	MAPK9	protein	P16104	H2AFX	UNIPROT	protein	YES	up-regulates	phosphorylation
P45984	UNIPROT	MAPK9	protein	P84243	H3F3A	UNIPROT	protein	YES	up-regulates	phosphorylation
P45984	UNIPROT	MAPK9	protein	P35568	IRS1	UNIPROT	protein	YES	up-regulates	phosphorylation
P45984	UNIPROT	MAPK9	protein	P35568	IRS1	UNIPROT	protein	YES	up-regulates	phosphorylation
P45984	UNIPROT	MAPK9	protein	P05412	JUN	UNIPROT	protein	YES	up-regulates	phosphorylation
P45984	UNIPROT	MAPK9	protein	P05412	JUN	UNIPROT	protein	YES	up-regulates	phosphorylation
P45984	UNIPROT	MAPK9	protein	P05412	JUN	UNIPROT	protein	YES	up-regulates	phosphorylation
P45984	UNIPROT	MAPK9	protein	P01106	MYC	UNIPROT	protein	YES	up-regulates	phosphorylation
P45984	UNIPROT	MAPK9	protein	P01106	MYC	UNIPROT	protein	YES	up-regulates	phosphorylation
P45984	UNIPROT	MAPK9	protein	P40763	STAT3	UNIPROT	protein	YES	up-regulates	phosphorylation
P45984	UNIPROT	MAPK9	protein	P04637	TP53	UNIPROT	protein	YES	up-regulates	phosphorylation
P45984	UNIPROT	MAPK9	protein	P04637	TP53	UNIPROT	protein	YES	up-regulates	phosphorylation
P49137	UNIPROT	MAPKAPK2	protein	Q9NY61	AATF	UNIPROT	protein	YES	up-regulates	phosphorylation
P49137	UNIPROT	MAPKAPK2	protein	Q9UKV8	AGO2	UNIPROT	protein	YES	up-regulates	phosphorylation
P49137	UNIPROT	MAPKAPK2	protein	O95816	BAG2	UNIPROT	protein	YES	up-regulates	phosphorylation
P49137	UNIPROT	MAPKAPK2	protein	P16220	CREB1	UNIPROT	protein	YES	up-regulates	phosphorylation
P49137	UNIPROT	MAPKAPK2	protein	Q15717	ELAVL1	UNIPROT	protein	YES	up-regulates	phosphorylation
P49137	UNIPROT	MAPKAPK2	protein	O75925	PIAS1	UNIPROT	protein	YES	up-regulates	phosphorylation
P49137	UNIPROT	MAPKAPK2	protein	P53350	PLK1	UNIPROT	protein	YES	up-regulates	phosphorylation
Q7KZI7	UNIPROT	MARK2	protein	Q8TEW0	PARD3	UNIPROT	protein	YES	up-regulates	phosphorylation
Q7KZI7	UNIPROT	MARK2	protein	P46939	UTRN	UNIPROT	protein	YES	up-regulates	phosphorylation

P08581	UNIPROT	MET	protein	P08581	MET	UNIPROT	protein	YES	up-regulates	phosphorylation
P08581	UNIPROT	MET	protein	P08581	MET	UNIPROT	protein	YES	up-regulates	phosphorylation
P08581	UNIPROT	MET	protein	Q05397	PTK2	UNIPROT	protein	YES	up-regulates	phosphorylation
P08581	UNIPROT	MET	protein	Q05397	PTK2	UNIPROT	protein	YES	up-regulates	phosphorylation
P08581	UNIPROT	MET	protein	Q05397	PTK2	UNIPROT	protein	YES	up-regulates	phosphorylation
P08581	UNIPROT	MET	protein	Q05397	PTK2	UNIPROT	protein	YES	up-regulates	phosphorylation
P08581	UNIPROT	MET	protein	Q05397	PTK2	UNIPROT	protein	YES	up-regulates	phosphorylation
P08581	UNIPROT	MET	protein	Q05397	PTK2	UNIPROT	protein	YES	up-regulates	phosphorylation
P08581	UNIPROT	MET	protein	Q05397	PTK2	UNIPROT	protein	YES	up-regulates	phosphorylation
P08581	UNIPROT	MET	protein	Q05397	PTK2	UNIPROT	protein	YES	up-regulates	phosphorylation
P42345	UNIPROT	MTOR	protein	Q9H1K1	ISCU	UNIPROT	protein	YES	up-regulates	phosphorylation
P42345	UNIPROT	MTOR	protein	P40763	STAT3	UNIPROT	protein	YES	up-regulates	phosphorylation
P42345	UNIPROT	MTOR	protein	P40763	STAT3	UNIPROT	protein	YES	up-regulates	phosphorylation
Q15746	UNIPROT	MYLK	protein	P14649	MYL6B	UNIPROT	protein	YES	up-regulates	phosphorylation
Q15746	UNIPROT	MYLK	protein	P24844	MYL9	UNIPROT	protein	YES	up-regulates	phosphorylation
Q15746	UNIPROT	MYLK	protein	P24844	MYL9	UNIPROT	protein	YES	up-regulates	phosphorylation
Q8TD19	UNIPROT	NEK9	protein	Q8TD19	NEK9	UNIPROT	protein	YES	up-regulates	phosphorylation
Q8TD19	UNIPROT	NEK9	protein	Q8TD19	NEK9	UNIPROT	protein	YES	up-regulates	phosphorylation
P15531	UNIPROT	NME1	protein	P15531	NME1	UNIPROT	protein	YES	up-regulates	phosphorylation
O95747	UNIPROT	OXSR1	protein	P55011	SLC12A2	UNIPROT	protein	YES	up-regulates	phosphorylation
O95747	UNIPROT	OXSR1	protein	P55011	SLC12A2	UNIPROT	protein	YES	up-regulates	phosphorylation
O95747	UNIPROT	OXSR1	protein	P55011	SLC12A2	UNIPROT	protein	YES	up-regulates	phosphorylation
O95747	UNIPROT	OXSR1	protein	P55011	SLC12A2	UNIPROT	protein	YES	up-regulates	phosphorylation
O95747	UNIPROT	OXSR1	protein	P55011	SLC12A2	UNIPROT	protein	YES	up-regulates	phosphorylation
Q13153	UNIPROT	PAK1	protein	P10398	ARAF	UNIPROT	protein	YES	up-regulates	phosphorylation
Q13153	UNIPROT	PAK1	protein	O15143	ARPC1B	UNIPROT	protein	YES	up-regulates	phosphorylation
Q13153	UNIPROT	PAK1	protein	P35222	CTNNB1	UNIPROT	protein	YES	up-regulates	phosphorylation
Q13153	UNIPROT	PAK1	protein	Q14247	CTTN	UNIPROT	protein	YES	up-regulates	phosphorylation
Q13153	UNIPROT	PAK1	protein	Q14247	CTTN	UNIPROT	protein	YES	up-regulates	phosphorylation
Q13153	UNIPROT	PAK1	protein	Q14247	CTTN	UNIPROT	protein	YES	up-regulates	phosphorylation
Q13153	UNIPROT	PAK1	protein	Q14247	CTTN	UNIPROT	protein	YES	up-regulates	phosphorylation
Q13153	UNIPROT	PAK1	protein	P78545	ELF3	UNIPROT	protein	YES	up-regulates	phosphorylation
Q13153	UNIPROT	PAK1	protein	P21333	FLNA	UNIPROT	protein	YES	up-regulates	phosphorylation
Q13153	UNIPROT	PAK1	protein	P68431	HIST1H3A	UNIPROT	protein	YES	up-regulates	phosphorylation
Q13153	UNIPROT	PAK1	protein	Q13418	ILK	UNIPROT	protein	YES	up-regulates	phosphorylation
Q13153	UNIPROT	PAK1	protein	Q13418	ILK	UNIPROT	protein	YES	up-regulates	phosphorylation
Q13153	UNIPROT	PAK1	protein	Q9UQ80	PA2G4	UNIPROT	protein	YES	up-regulates	phosphorylation
Q13153	UNIPROT	PAK1	protein	P36871	PGM1	UNIPROT	protein	YES	up-regulates	phosphorylation
Q13153	UNIPROT	PAK1	protein	P53350	PLK1	UNIPROT	protein	YES	up-regulates	phosphorylation
Q13153	UNIPROT	PAK1	protein	Q99426	TBCB	UNIPROT	protein	YES	up-regulates	phosphorylation
Q13153	UNIPROT	PAK1	protein	Q99426	TBCB	UNIPROT	protein	YES	up-regulates	phosphorylation
Q13177	UNIPROT	PAK2	protein	P05412	JUN	UNIPROT	protein	YES	up-regulates	phosphorylation
Q13177	UNIPROT	PAK2	protein	P05412	JUN	UNIPROT	protein	YES	up-regulates	phosphorylation
Q13177	UNIPROT	PAK2	protein	P05412	JUN	UNIPROT	protein	YES	up-regulates	phosphorylation
Q13177	UNIPROT	PAK2	protein	P05412	JUN	UNIPROT	protein	YES	up-regulates	phosphorylation
Q13177	UNIPROT	PAK2	protein	P05412	JUN	UNIPROT	protein	YES	up-regulates	phosphorylation
Q13177	UNIPROT	PAK2	protein	Q13177	PAK2	UNIPROT	protein	YES	up-regulates	phosphorylation
Q13177	UNIPROT	PAK2	protein	Q13177	PAK2	UNIPROT	protein	YES	up-regulates	phosphorylation
O96013	UNIPROT	PAK4	protein	P35222	CTNNB1	UNIPROT	protein	YES	up-regulates	phosphorylation
O96013	UNIPROT	PAK4	protein	P18084	ITGB5	UNIPROT	protein	YES	up-regulates	phosphorylation
O96013	UNIPROT	PAK4	protein	P18084	ITGB5	UNIPROT	protein	YES	up-regulates	phosphorylation
O96013	UNIPROT	PAK4	protein	O96013	PAK4	UNIPROT	protein	YES	up-regulates	phosphorylation
O96013	UNIPROT	PAK4	protein	P62826	RAN	UNIPROT	protein	YES	up-regulates	phosphorylation
Q96KB5	UNIPROT	PBK	protein	P68431	HIST1H3A	UNIPROT	protein	YES	up-regulates	phosphorylation
Q96KB5	UNIPROT	PBK	protein	Q06830	PRDX1	UNIPROT	protein	YES	up-regulates	phosphorylation
O15530	UNIPROT	PDPK1	protein	P31749	AKT1	UNIPROT	protein	YES	up-regulates	phosphorylation
O15530	UNIPROT	PDPK1	protein	P31749	AKT1	UNIPROT	protein	YES	up-regulates	phosphorylation
O15530	UNIPROT	PDPK1	protein	P31749	AKT1	UNIPROT	protein	YES	up-regulates	phosphorylation
O15530	UNIPROT	PDPK1	protein	P31749	AKT1	UNIPROT	protein	YES	up-regulates	phosphorylation
O15530	UNIPROT	PDPK1	protein	P31749	AKT1	UNIPROT	protein	YES	up-regulates	phosphorylation
O15530	UNIPROT	PDPK1	protein	P36507	MAP2K2	UNIPROT	protein	YES	up-regulates	phosphorylation
O15530	UNIPROT	PDPK1	protein	Q16512	PKN1	UNIPROT	protein	YES	up-regulates	phosphorylation
O15530	UNIPROT	PDPK1	protein	Q16513	PKN2	UNIPROT	protein	YES	up-regulates	phosphorylation
O15530	UNIPROT	PDPK1	protein	P17252	PRKCA	UNIPROT	protein	YES	up-regulates	phosphorylation
O15530	UNIPROT	PDPK1	protein	P51812	RPS6KA3	UNIPROT	protein	YES	up-regulates	phosphorylation
O15530	UNIPROT	PDPK1	protein	P51812	RPS6KA3	UNIPROT	protein	YES	up-regulates	phosphorylation
O15530	UNIPROT	PDPK1	protein	Q9HBY8	SGK2	UNIPROT	protein	YES	up-regulates	phosphorylation
Q6A1A2	UNIPROT	PDPK2P	protein	P31749	AKT1	UNIPROT	protein	YES	up-regulates	phosphorylation
P14618	UNIPROT	PKM	protein	P40763	STAT3	UNIPROT	protein	YES	up-regulates	phosphorylation
Q16512	UNIPROT	PKN1	protein	Q9NYL2	MAP3K20	UNIPROT	protein	YES	up-regulates	phosphorylation
Q16512	UNIPROT	PKN1	protein	Q16539	MAPK14	UNIPROT	protein	YES	up-regulates	phosphorylation
Q16512	UNIPROT	PKN1	protein	P36871	PGM1	UNIPROT	protein	YES	up-regulates	phosphorylation
P53350	UNIPROT	PLK1	protein	Q9H1A4	ANAPC1	UNIPROT	protein	YES	up-regulates	phosphorylation
P53350	UNIPROT	PLK1	protein	O60566	BUB1B	UNIPROT	protein	YES	up-regulates	phosphorylation
P53350	UNIPROT	PLK1	protein	O60566	BUB1B	UNIPROT	protein	YES	up-regulates	phosphorylation
P53350	UNIPROT	PLK1	protein	O60566	BUB1B	UNIPROT	protein	YES	up-regulates	phosphorylation
P53350	UNIPROT	PLK1	protein	O60566	BUB1B	UNIPROT	protein	YES	up-regulates	phosphorylation
P53350	UNIPROT	PLK1	protein	Q53EZ4	CEP55	UNIPROT	protein	YES	up-regulates	phosphorylation
P53350	UNIPROT	PLK1	protein	O96017	CHEK2	UNIPROT	protein	YES	up-regulates	phosphorylation
P53350	UNIPROT	PLK1	protein	P30622	CLIP1	UNIPROT	protein	YES	up-regulates	phosphorylation
P53350	UNIPROT	PLK1	protein	Q14203	DCTN1	UNIPROT	protein	YES	up-regulates	phosphorylation
P53350	UNIPROT	PLK1	protein	O14641	DVL2	UNIPROT	protein	YES	up-regulates	phosphorylation
P53350	UNIPROT	PLK1	protein	O95251	KAT7	UNIPROT	protein	YES	up-regulates	phosphorylation
P53350	UNIPROT	PLK1	protein	P06748	NPM1	UNIPROT	protein	YES	up-regulates	phosphorylation
P53350	UNIPROT	PLK1	protein	Q96CV9	OPTN	UNIPROT	protein	YES	up-regulates	phosphorylation
P53350	UNIPROT	PLK1	protein	Q13526	PIN1	UNIPROT	protein	YES	up-regulates	phosphorylation
P53350	UNIPROT	PLK1	protein	Q9H0H5	RACGAP1	UNIPROT	protein	YES	up-regulates	phosphorylation
P53350	UNIPROT	PLK1	protein	Q9H0H5	RACGAP1	UNIPROT	protein	YES	up-regulates	phosphorylation
P53350	UNIPROT	PLK1	protein	Q9H0H5	RACGAP1	UNIPROT	protein	YES	up-regulates	phosphorylation
P53350	UNIPROT	PLK1	protein	Q9H0H5	RACGAP1	UNIPROT	protein	YES	up-regulates	phosphorylation
P53350	UNIPROT	PLK1	protein	P62826	RAN	UNIPROT	protein	YES	up-regulates	phosphorylation
P53350	UNIPROT	PLK1	protein	P11388	TOP2A	UNIPROT	protein	YES	up-regulates	phosphorylation
P53350	UNIPROT	PLK1	protein	P11388	TOP2A	UNIPROT	protein	YES	up-regulates	phosphorylation
P53350	UNIPROT	PLK1	protein	P11388	TOP2A	UNIPROT	protein	YES	up-regulates	phosphorylation
P53350	UNIPROT	PLK1	protein	Q9H2D6	TRIOBP	UNIPROT	protein	YES	up-regulates	phosphorylation
P53350	UNIPROT	PLK1	protein	Q9H2D6	TRIOBP	UNIPROT	protein	YES	up-regulates	phosphorylation
P53350	UNIPROT	PLK1	protein	P08670	VIM	UNIPROT	protein	YES	up-regulates	phosphorylation
P53350	UNIPROT	PLK1	protein	P25490	YY1	UNIPROT	protein	YES	up-regulates	phosphorylation

Q13131	UNIPROT	PRKAA1	protein	O00418	EEF2K	UNIPROT	protein	YES	up-regulates	phosphorylation
Q13131	UNIPROT	PRKAA1	protein	Q12778	FOXO1	UNIPROT	protein	YES	up-regulates	phosphorylation
Q13131	UNIPROT	PRKAA1	protein	Q06210	GFPT1	UNIPROT	protein	YES	up-regulates	phosphorylation
Q13131	UNIPROT	PRKAA1	protein	Q9H0B6	KLC2	UNIPROT	protein	YES	up-regulates	phosphorylation
Q13131	UNIPROT	PRKAA1	protein	P52292	KPNA2	UNIPROT	protein	YES	up-regulates	phosphorylation
Q13131	UNIPROT	PRKAA1	protein	Q9Y478	PRKAB1	UNIPROT	protein	YES	up-regulates	phosphorylation
Q13131	UNIPROT	PRKAA1	protein	P04637	TP53	UNIPROT	protein	YES	up-regulates	phosphorylation
P17612	UNIPROT	PRKACA	protein	Q12802	AKAP13	UNIPROT	protein	YES	up-regulates	phosphorylation
P17612	UNIPROT	PRKACA	protein	Q16790	CA9	UNIPROT	protein	YES	up-regulates	phosphorylation
P17612	UNIPROT	PRKACA	protein	Q13185	CBX3	UNIPROT	protein	YES	up-regulates	phosphorylation
P17612	UNIPROT	PRKACA	protein	P16070	CD44	UNIPROT	protein	YES	up-regulates	phosphorylation
P17612	UNIPROT	PRKACA	protein	Q8N752	CSNK1A1L	UNIPROT	protein	YES	up-regulates	phosphorylation
P17612	UNIPROT	PRKACA	protein	Q9NP97	DYNLRB1	UNIPROT	protein	YES	up-regulates	phosphorylation
P17612	UNIPROT	PRKACA	protein	P04626	ERBB2	UNIPROT	protein	YES	up-regulates	phosphorylation
P17612	UNIPROT	PRKACA	protein	P21333	FLNA	UNIPROT	protein	YES	up-regulates	phosphorylation
P17612	UNIPROT	PRKACA	protein	P84243	H3F3A	UNIPROT	protein	YES	up-regulates	phosphorylation
P17612	UNIPROT	PRKACA	protein	Q14103	HNRNPD	UNIPROT	protein	YES	up-regulates	phosphorylation
P17612	UNIPROT	PRKACA	protein	Q00613	HSF1	UNIPROT	protein	YES	up-regulates	phosphorylation
P17612	UNIPROT	PRKACA	protein	Q9NXR1	NDE1	UNIPROT	protein	YES	up-regulates	phosphorylation
P17612	UNIPROT	PRKACA	protein	P35240	NF2	UNIPROT	protein	YES	up-regulates	phosphorylation
P17612	UNIPROT	PRKACA	protein	P19838	NFKB1	UNIPROT	protein	YES	up-regulates	phosphorylation
P17612	UNIPROT	PRKACA	protein	Q14978	NOLC1	UNIPROT	protein	YES	up-regulates	phosphorylation
P17612	UNIPROT	PRKACA	protein	Q13526	PIN1	UNIPROT	protein	YES	up-regulates	phosphorylation
P17612	UNIPROT	PRKACA	protein	Q14738	PPP2R5D	UNIPROT	protein	YES	up-regulates	phosphorylation
P17612	UNIPROT	PRKACA	protein	P61224	RAP1B	UNIPROT	protein	YES	up-regulates	phosphorylation
P17612	UNIPROT	PRKACA	protein	Q04206	RELA	UNIPROT	protein	YES	up-regulates	phosphorylation
P17612	UNIPROT	PRKACA	protein	P48436	SOX9	UNIPROT	protein	YES	up-regulates	phosphorylation
P17612	UNIPROT	PRKACA	protein	P48436	SOX9	UNIPROT	protein	YES	up-regulates	phosphorylation
P17612	UNIPROT	PRKACA	protein	P12931	SRC	UNIPROT	protein	YES	up-regulates	phosphorylation
P17612	UNIPROT	PRKACA	protein	Q07955	SRSF1	UNIPROT	protein	YES	up-regulates	phosphorylation
P17612	UNIPROT	PRKACA	protein	Q00059	TFAM	UNIPROT	protein	YES	up-regulates	phosphorylation
P17612	UNIPROT	PRKACA	protein	P63104	YWHAZ	UNIPROT	protein	YES	up-regulates	phosphorylation
P17252	UNIPROT	PRKCA	protein	P35611	ADD1	UNIPROT	protein	YES	up-regulates	phosphorylation
P17252	UNIPROT	PRKCA	protein	P35611	ADD1	UNIPROT	protein	YES	up-regulates	phosphorylation
P17252	UNIPROT	PRKCA	protein	P04083	ANXA1	UNIPROT	protein	YES	up-regulates	phosphorylation
P17252	UNIPROT	PRKCA	protein	P17812	CTPS1	UNIPROT	protein	YES	up-regulates	phosphorylation
P17252	UNIPROT	PRKCA	protein	P00533	EGFR	UNIPROT	protein	YES	up-regulates	phosphorylation
P17252	UNIPROT	PRKCA	protein	P15311	EZR	UNIPROT	protein	YES	up-regulates	phosphorylation
P17252	UNIPROT	PRKCA	protein	P46940	IQGAP1	UNIPROT	protein	YES	up-regulates	phosphorylation
P17252	UNIPROT	PRKCA	protein	P46940	IQGAP1	UNIPROT	protein	YES	up-regulates	phosphorylation
P17252	UNIPROT	PRKCA	protein	P46940	IQGAP1	UNIPROT	protein	YES	up-regulates	phosphorylation
P17252	UNIPROT	PRKCA	protein	P35568	IRS1	UNIPROT	protein	YES	up-regulates	phosphorylation
P17252	UNIPROT	PRKCA	protein	Q07954	LRP1	UNIPROT	protein	YES	up-regulates	phosphorylation
P17252	UNIPROT	PRKCA	protein	Q07954	LRP1	UNIPROT	protein	YES	up-regulates	phosphorylation
P17252	UNIPROT	PRKCA	protein	Q07954	LRP1	UNIPROT	protein	YES	up-regulates	phosphorylation
P17252	UNIPROT	PRKCA	protein	Q07954	LRP1	UNIPROT	protein	YES	up-regulates	phosphorylation
P17252	UNIPROT	PRKCA	protein	Q8NFA2	NOXO1	UNIPROT	protein	YES	up-regulates	phosphorylation
P17252	UNIPROT	PRKCA	protein	P47712	PLA2G4A	UNIPROT	protein	YES	up-regulates	phosphorylation
P17252	UNIPROT	PRKCA	protein	Q13393	PLD1	UNIPROT	protein	YES	up-regulates	phosphorylation
P17252	UNIPROT	PRKCA	protein	Q13393	PLD1	UNIPROT	protein	YES	up-regulates	phosphorylation
P17252	UNIPROT	PRKCA	protein	Q13393	PLD1	UNIPROT	protein	YES	up-regulates	phosphorylation
P17252	UNIPROT	PRKCA	protein	P17252	PRKCA	UNIPROT	protein	YES	up-regulates	phosphorylation
P17252	UNIPROT	PRKCA	protein	P17252	PRKCA	UNIPROT	protein	YES	up-regulates	phosphorylation
P17252	UNIPROT	PRKCA	protein	Q13976	PRKG1	UNIPROT	protein	YES	up-regulates	phosphorylation
P17252	UNIPROT	PRKCA	protein	Q13769	THOC5	UNIPROT	protein	YES	up-regulates	phosphorylation
P17252	UNIPROT	PRKCA	protein	Q13769	THOC5	UNIPROT	protein	YES	up-regulates	phosphorylation
Q05655	UNIPROT	PRKCD	protein	O95817	BAG3	UNIPROT	protein	YES	up-regulates	phosphorylation
Q05655	UNIPROT	PRKCD	protein	P51398	DAP3	UNIPROT	protein	YES	up-regulates	phosphorylation
Q05655	UNIPROT	PRKCD	protein	P07108	DBI	UNIPROT	protein	YES	up-regulates	phosphorylation
Q05655	UNIPROT	PRKCD	protein	O00429	DNM1L	UNIPROT	protein	YES	up-regulates	phosphorylation
Q05655	UNIPROT	PRKCD	protein	Q15717	ELAVL1	UNIPROT	protein	YES	up-regulates	phosphorylation
Q05655	UNIPROT	PRKCD	protein	Q15717	ELAVL1	UNIPROT	protein	YES	up-regulates	phosphorylation
Q05655	UNIPROT	PRKCD	protein	P11413	G6PD	UNIPROT	protein	YES	up-regulates	phosphorylation
Q05655	UNIPROT	PRKCD	protein	P11413	G6PD	UNIPROT	protein	YES	up-regulates	phosphorylation
Q05655	UNIPROT	PRKCD	protein	P68431	HIST1H3A	UNIPROT	protein	YES	up-regulates	phosphorylation
Q05655	UNIPROT	PRKCD	protein	P40189	IL6ST	UNIPROT	protein	YES	up-regulates	phosphorylation
Q05655	UNIPROT	PRKCD	protein	P45983	MAPK8	UNIPROT	protein	YES	up-regulates	phosphorylation
Q05655	UNIPROT	PRKCD	protein	Q9UQ80	PA2G4	UNIPROT	protein	YES	up-regulates	phosphorylation
Q05655	UNIPROT	PRKCD	protein	P30086	PEBP1	UNIPROT	protein	YES	up-regulates	phosphorylation
Q05655	UNIPROT	PRKCD	protein	O15162	PLSCR1	UNIPROT	protein	YES	up-regulates	phosphorylation
Q05655	UNIPROT	PRKCD	protein	Q05655	PRKCD	UNIPROT	protein	YES	up-regulates	phosphorylation
Q05655	UNIPROT	PRKCD	protein	Q05655	PRKCD	UNIPROT	protein	YES	up-regulates	phosphorylation
Q05655	UNIPROT	PRKCD	protein	Q05655	PRKCD	UNIPROT	protein	YES	up-regulates	phosphorylation
Q05655	UNIPROT	PRKCD	protein	Q05655	PRKCD	UNIPROT	protein	YES	up-regulates	phosphorylation
Q05655	UNIPROT	PRKCD	protein	Q05655	PRKCD	UNIPROT	protein	YES	up-regulates	phosphorylation
Q05655	UNIPROT	PRKCD	protein	Q05655	PRKCD	UNIPROT	protein	YES	up-regulates	phosphorylation
Q05655	UNIPROT	PRKCD	protein	P23396	RPS3	UNIPROT	protein	YES	up-regulates	phosphorylation
Q05655	UNIPROT	PRKCD	protein	P23396	RPS3	UNIPROT	protein	YES	up-regulates	phosphorylation
Q05655	UNIPROT	PRKCD	protein	P23396	RPS3	UNIPROT	protein	YES	up-regulates	phosphorylation
Q05655	UNIPROT	PRKCD	protein	P29353	SHC1	UNIPROT	protein	YES	up-regulates	phosphorylation
Q05655	UNIPROT	PRKCD	protein	P42224	STAT1	UNIPROT	protein	YES	up-regulates	phosphorylation
Q05655	UNIPROT	PRKCD	protein	P40763	STAT3	UNIPROT	protein	YES	up-regulates	phosphorylation
Q05655	UNIPROT	PRKCD	protein	P04637	TP53	UNIPROT	protein	YES	up-regulates	phosphorylation
P41743	UNIPROT	PRKCI	protein	Q9H8V3	ECT2	UNIPROT	protein	YES	up-regulates	phosphorylation
P41743	UNIPROT	PRKCI	protein	P15311	EZR	UNIPROT	protein	YES	up-regulates	phosphorylation
Q9BZL6	UNIPROT	PRKD2	protein	Q9UBF8	PI4KB	UNIPROT	protein	YES	up-regulates	phosphorylation
Q9BZL6	UNIPROT	PRKD2	protein	Q9BZL6	PRKD2	UNIPROT	protein	YES	up-regulates	phosphorylation
P78527	UNIPROT	PRKDC	protein	P42575	CASP2	UNIPROT	protein	YES	up-regulates	phosphorylation
P78527	UNIPROT	PRKDC	protein	O96017	CHEK2	UNIPROT	protein	YES	up-regulates	phosphorylation
P78527	UNIPROT	PRKDC	protein	P16104	H2AFX	UNIPROT	protein	YES	up-regulates	phosphorylation
P78527	UNIPROT	PRKDC	protein	Q00839	HNRNPU	UNIPROT	protein	YES	up-regulates	phosphorylation
P78527	UNIPROT	PRKDC	protein	Q14653	IRF3	UNIPROT	protein	YES	up-regulates	phosphorylation
P78527	UNIPROT	PRKDC	protein	Q96T60	PNKP	UNIPROT	protein	YES	up-regulates	phosphorylation
P78527	UNIPROT	PRKDC	protein	Q96T60	PNKP	UNIPROT	protein	YES	up-regulates	phosphorylation
P78527	UNIPROT	PRKDC	protein	P78527	PRKDC	UNIPROT	protein	YES	up-regulates	phosphorylation
P78527	UNIPROT	PRKDC	protein	P78527	PRKDC	UNIPROT	protein	YES	up-regulates	phosphorylation
P78527	UNIPROT	PRKDC	protein	P15927	RPA2	UNIPROT	protein	YES	up-regulates	phosphorylation
P78527	UNIPROT	PRKDC	protein	P04637	TP53	UNIPROT	protein	YES	up-regulates	phosphorylation

Q13976	UNIPROT	PRKG1	protein	Q9Y613	FHOD1	UNIPROT	protein	YES	up-regulates	phosphorylation
Q13976	UNIPROT	PRKG1	protein	Q9Y613	FHOD1	UNIPROT	protein	YES	up-regulates	phosphorylation
Q13976	UNIPROT	PRKG1	protein	P78347	GTF2I	UNIPROT	protein	YES	up-regulates	phosphorylation
Q13976	UNIPROT	PRKG1	protein	P78347	GTF2I	UNIPROT	protein	YES	up-regulates	phosphorylation
Q13976	UNIPROT	PRKG1	protein	Q13976	PRKG1	UNIPROT	protein	YES	up-regulates	phosphorylation
Q05397	UNIPROT	PTK2	protein	O43707	ACTN4	UNIPROT	protein	YES	up-regulates	phosphorylation
Q05397	UNIPROT	PTK2	protein	Q05397	PTK2	UNIPROT	protein	YES	up-regulates	phosphorylation
Q05397	UNIPROT	PTK2	protein	Q05397	PTK2	UNIPROT	protein	YES	up-regulates	phosphorylation
Q05397	UNIPROT	PTK2	protein	Q05397	PTK2	UNIPROT	protein	YES	up-regulates	phosphorylation
Q05397	UNIPROT	PTK2	protein	Q05397	PTK2	UNIPROT	protein	YES	up-regulates	phosphorylation
Q05397	UNIPROT	PTK2	protein	Q05397	PTK2	UNIPROT	protein	YES	up-regulates	phosphorylation
Q05397	UNIPROT	PTK2	protein	Q05397	PTK2	UNIPROT	protein	YES	up-regulates	phosphorylation
Q05397	UNIPROT	PTK2	protein	Q05397	PTK2	UNIPROT	protein	YES	up-regulates	phosphorylation
Q05397	UNIPROT	PTK2	protein	Q05397	PTK2	UNIPROT	protein	YES	up-regulates	phosphorylation
Q13464	UNIPROT	ROCK1	protein	P35611	ADD1	UNIPROT	protein	YES	up-regulates	phosphorylation
Q13464	UNIPROT	ROCK1	protein	P35611	ADD1	UNIPROT	protein	YES	up-regulates	phosphorylation
Q13464	UNIPROT	ROCK1	protein	P15311	EZR	UNIPROT	protein	YES	up-regulates	phosphorylation
Q13464	UNIPROT	ROCK1	protein	Q9Y613	FHOD1	UNIPROT	protein	YES	up-regulates	phosphorylation
Q13464	UNIPROT	ROCK1	protein	Q9Y613	FHOD1	UNIPROT	protein	YES	up-regulates	phosphorylation
Q13464	UNIPROT	ROCK1	protein	Q9Y613	FHOD1	UNIPROT	protein	YES	up-regulates	phosphorylation
Q13464	UNIPROT	ROCK1	protein	O14950	MYL12B	UNIPROT	protein	YES	up-regulates	phosphorylation
Q13464	UNIPROT	ROCK1	protein	O14950	MYL12B	UNIPROT	protein	YES	up-regulates	phosphorylation
Q13464	UNIPROT	ROCK1	protein	P24844	MYL9	UNIPROT	protein	YES	up-regulates	phosphorylation
Q13464	UNIPROT	ROCK1	protein	P60484	PTEN	UNIPROT	protein	YES	up-regulates	phosphorylation
Q13464	UNIPROT	ROCK1	protein	P60484	PTEN	UNIPROT	protein	YES	up-regulates	phosphorylation
Q13464	UNIPROT	ROCK1	protein	P61587	RND3	UNIPROT	protein	YES	up-regulates	phosphorylation
Q13464	UNIPROT	ROCK1	protein	P48436	SOX9	UNIPROT	protein	YES	up-regulates	phosphorylation
O75116	UNIPROT	ROCK2	protein	Q16555	DPYSL2	UNIPROT	protein	YES	up-regulates	phosphorylation
Q01973	UNIPROT	ROR1	protein	P12931	SRC	UNIPROT	protein	YES	up-regulates	phosphorylation
Q15418	UNIPROT	RPS6KA1	protein	P78371	CCT2	UNIPROT	protein	YES	up-regulates	phosphorylation
Q15418	UNIPROT	RPS6KA1	protein	P23588	EIF4B	UNIPROT	protein	YES	up-regulates	phosphorylation
Q15418	UNIPROT	RPS6KA1	protein	P21333	FLNA	UNIPROT	protein	YES	up-regulates	phosphorylation
Q15418	UNIPROT	RPS6KA1	protein	P01100	FOS	UNIPROT	protein	YES	up-regulates	phosphorylation
Q15418	UNIPROT	RPS6KA1	protein	P84243	H3F3A	UNIPROT	protein	YES	up-regulates	phosphorylation
Q15418	UNIPROT	RPS6KA1	protein	P84243	H3F3A	UNIPROT	protein	YES	up-regulates	phosphorylation
Q15418	UNIPROT	RPS6KA1	protein	P84243	H3F3A	UNIPROT	protein	YES	up-regulates	phosphorylation
Q15418	UNIPROT	RPS6KA1	protein	P62753	RPS6	UNIPROT	protein	YES	up-regulates	phosphorylation
Q15418	UNIPROT	RPS6KA1	protein	P62753	RPS6	UNIPROT	protein	YES	up-regulates	phosphorylation
Q15418	UNIPROT	RPS6KA1	protein	P62753	RPS6	UNIPROT	protein	YES	up-regulates	phosphorylation
Q15418	UNIPROT	RPS6KA1	protein	P62753	RPS6	UNIPROT	protein	YES	up-regulates	phosphorylation
Q15418	UNIPROT	RPS6KA1	protein	Q8N122	RPTOR	UNIPROT	protein	YES	up-regulates	phosphorylation
Q15418	UNIPROT	RPS6KA1	protein	Q8N122	RPTOR	UNIPROT	protein	YES	up-regulates	phosphorylation
Q15418	UNIPROT	RPS6KA1	protein	Q8N122	RPTOR	UNIPROT	protein	YES	up-regulates	phosphorylation
Q15418	UNIPROT	RPS6KA1	protein	P19634	SLC9A1	UNIPROT	protein	YES	up-regulates	phosphorylation
Q15418	UNIPROT	RPS6KA1	protein	Q8IX03	WWC1	UNIPROT	protein	YES	up-regulates	phosphorylation
Q15418	UNIPROT	RPS6KA1	protein	Q8IX03	WWC1	UNIPROT	protein	YES	up-regulates	phosphorylation
Q15418	UNIPROT	RPS6KA1	protein	P67809	YBX1	UNIPROT	protein	YES	up-regulates	phosphorylation
P51812	UNIPROT	RPS6KA3	protein	P19525	EIF2AK2	UNIPROT	protein	YES	up-regulates	phosphorylation
P51812	UNIPROT	RPS6KA3	protein	P84243	H3F3A	UNIPROT	protein	YES	up-regulates	phosphorylation
P51812	UNIPROT	RPS6KA3	protein	P84243	H3F3A	UNIPROT	protein	YES	up-regulates	phosphorylation
P51812	UNIPROT	RPS6KA3	protein	P84243	H3F3A	UNIPROT	protein	YES	up-regulates	phosphorylation
P51812	UNIPROT	RPS6KA3	protein	P33778	HIST1H2BB	UNIPROT	protein	YES	up-regulates	phosphorylation
P51812	UNIPROT	RPS6KA3	protein	P68431	HIST1H3A	UNIPROT	protein	YES	up-regulates	phosphorylation
P51812	UNIPROT	RPS6KA3	protein	P68431	HIST1H3A	UNIPROT	protein	YES	up-regulates	phosphorylation
P51812	UNIPROT	RPS6KA3	protein	P68431	HIST1H3A	UNIPROT	protein	YES	up-regulates	phosphorylation
P51812	UNIPROT	RPS6KA3	protein	Q8IX03	WWC1	UNIPROT	protein	YES	up-regulates	phosphorylation
P51812	UNIPROT	RPS6KA3	protein	Q8IX03	WWC1	UNIPROT	protein	YES	up-regulates	phosphorylation
P51812	UNIPROT	RPS6KA3	protein	P67809	YBX1	UNIPROT	protein	YES	up-regulates	phosphorylation
O75676	UNIPROT	RPS6KA4	protein	P18846	ATF1	UNIPROT	protein	YES	up-regulates	phosphorylation
O75676	UNIPROT	RPS6KA4	protein	P18846	ATF1	UNIPROT	protein	YES	up-regulates	phosphorylation
O75676	UNIPROT	RPS6KA4	protein	P16220	CREB1	UNIPROT	protein	YES	up-regulates	phosphorylation
O75676	UNIPROT	RPS6KA4	protein	P01100	FOS	UNIPROT	protein	YES	up-regulates	phosphorylation
O75676	UNIPROT	RPS6KA4	protein	P01100	FOS	UNIPROT	protein	YES	up-regulates	phosphorylation
O75676	UNIPROT	RPS6KA4	protein	Q04206	RELA	UNIPROT	protein	YES	up-regulates	phosphorylation
O75676	UNIPROT	RPS6KA4	protein	O75676	RPS6KA4	UNIPROT	protein	YES	up-regulates	phosphorylation
Q96Q15	UNIPROT	SMG1	protein	P04637	TP53	UNIPROT	protein	YES	up-regulates	phosphorylation
Q96Q15	UNIPROT	SMG1	protein	Q92900	UPF1	UNIPROT	protein	YES	up-regulates	phosphorylation
Q96Q15	UNIPROT	SMG1	protein	Q92900	UPF1	UNIPROT	protein	YES	up-regulates	phosphorylation
Q96Q15	UNIPROT	SMG1	protein	Q92900	UPF1	UNIPROT	protein	YES	up-regulates	phosphorylation
P12931	UNIPROT	SRC	protein	P04083	ANXA1	UNIPROT	protein	YES	up-regulates	phosphorylation
P12931	UNIPROT	SRC	protein	P07355	ANXA2	UNIPROT	protein	YES	up-regulates	phosphorylation
P12931	UNIPROT	SRC	protein	P10398	ARAF	UNIPROT	protein	YES	up-regulates	phosphorylation
P12931	UNIPROT	SRC	protein	Q9NRY4	ARHGAP35	UNIPROT	protein	YES	up-regulates	phosphorylation
P12931	UNIPROT	SRC	protein	P56945	BCAR1	UNIPROT	protein	YES	up-regulates	phosphorylation
P12931	UNIPROT	SRC	protein	P56945	BCAR1	UNIPROT	protein	YES	up-regulates	phosphorylation
P12931	UNIPROT	SRC	protein	P56945	BCAR1	UNIPROT	protein	YES	up-regulates	phosphorylation
P12931	UNIPROT	SRC	protein	P60953	CDC42	UNIPROT	protein	YES	up-regulates	phosphorylation
P12931	UNIPROT	SRC	protein	Q00610	CLTC	UNIPROT	protein	YES	up-regulates	phosphorylation
P12931	UNIPROT	SRC	protein	P35222	CTNNB1	UNIPROT	protein	YES	up-regulates	phosphorylation
P12931	UNIPROT	SRC	protein	P00533	EGFR	UNIPROT	protein	YES	up-regulates	phosphorylation
P12931	UNIPROT	SRC	protein	P06733	ENO1	UNIPROT	protein	YES	up-regulates	phosphorylation
P12931	UNIPROT	SRC	protein	P06733	ENO1	UNIPROT	protein	YES	up-regulates	phosphorylation
P12931	UNIPROT	SRC	protein	P15311	EZR	UNIPROT	protein	YES	up-regulates	phosphorylation
P12931	UNIPROT	SRC	protein	P15311	EZR	UNIPROT	protein	YES	up-regulates	phosphorylation
P12931	UNIPROT	SRC	protein	P15311	EZR	UNIPROT	protein	YES	up-regulates	phosphorylation
P12931	UNIPROT	SRC	protein	Q13480	GAB1	UNIPROT	protein	YES	up-regulates	phosphorylation
P12931	UNIPROT	SRC	protein	P06396	GSN	UNIPROT	protein	YES	up-regulates	phosphorylation
P12931	UNIPROT	SRC	protein	P08238	HSP90AB1	UNIPROT	protein	YES	up-regulates	phosphorylation
P12931	UNIPROT	SRC	protein	P08069	IGF1R	UNIPROT	protein	YES	up-regulates	phosphorylation
P12931	UNIPROT	SRC	protein	P08069	IGF1R	UNIPROT	protein	YES	up-regulates	phosphorylation
P12931	UNIPROT	SRC	protein	P08069	IGF1R	UNIPROT	protein	YES	up-regulates	phosphorylation
P12931	UNIPROT	SRC	protein	P08069	IGF1R	UNIPROT	protein	YES	up-regulates	phosphorylation
P12931	UNIPROT	SRC	protein	O15357	INPPL1	UNIPROT	protein	YES	up-regulates	phosphorylation
P12931	UNIPROT	SRC	protein	O15357	INPPL1	UNIPROT	protein	YES	up-regulates	phosphorylation
P12931	UNIPROT	SRC	protein	P01116	KRAS	UNIPROT	protein	YES	up-regulates	phosphorylation
P12931	UNIPROT	SRC	protein	Q07954	LRP1	UNIPROT	protein	YES	up-regulates	phosphorylation
P12931	UNIPROT	SRC	protein	O95297	MPZL1	UNIPROT	protein	YES	up-regulates	phosphorylation
P12931	UNIPROT	SRC	protein	O95297	MPZL1	UNIPROT	protein	YES	up-regulates	phosphorylation

P12931	UNIPROT	SRC	protein	Q15746	MYLK	UNIPROT	protein	YES	up-regulates	phosphorylation
P12931	UNIPROT	SRC	protein	Q15746	MYLK	UNIPROT	protein	YES	up-regulates	phosphorylation
P12931	UNIPROT	SRC	protein	Q15746	MYLK	UNIPROT	protein	YES	up-regulates	phosphorylation
P12931	UNIPROT	SRC	protein	Q15746	MYLK	UNIPROT	protein	YES	up-regulates	phosphorylation
P12931	UNIPROT	SRC	protein	Q13177	PAK2	UNIPROT	protein	YES	up-regulates	phosphorylation
P12931	UNIPROT	SRC	protein	O15530	PDPK1	UNIPROT	protein	YES	up-regulates	phosphorylation
P12931	UNIPROT	SRC	protein	O15530	PDPK1	UNIPROT	protein	YES	up-regulates	phosphorylation
P12931	UNIPROT	SRC	protein	P41743	PRKCI	UNIPROT	protein	YES	up-regulates	phosphorylation
P12931	UNIPROT	SRC	protein	P41743	PRKCI	UNIPROT	protein	YES	up-regulates	phosphorylation
P12931	UNIPROT	SRC	protein	P41743	PRKCI	UNIPROT	protein	YES	up-regulates	phosphorylation
P12931	UNIPROT	SRC	protein	Q05397	PTK2	UNIPROT	protein	YES	up-regulates	phosphorylation
P12931	UNIPROT	SRC	protein	Q05397	PTK2	UNIPROT	protein	YES	up-regulates	phosphorylation
P12931	UNIPROT	SRC	protein	Q05397	PTK2	UNIPROT	protein	YES	up-regulates	phosphorylation
P12931	UNIPROT	SRC	protein	Q05397	PTK2	UNIPROT	protein	YES	up-regulates	phosphorylation
P12931	UNIPROT	SRC	protein	Q05397	PTK2	UNIPROT	protein	YES	up-regulates	phosphorylation
P12931	UNIPROT	SRC	protein	Q05397	PTK2	UNIPROT	protein	YES	up-regulates	phosphorylation
P12931	UNIPROT	SRC	protein	Q05397	PTK2	UNIPROT	protein	YES	up-regulates	phosphorylation
P12931	UNIPROT	SRC	protein	Q05397	PTK2	UNIPROT	protein	YES	up-regulates	phosphorylation
P12931	UNIPROT	SRC	protein	P29350	PTPN6	UNIPROT	protein	YES	up-regulates	phosphorylation
P12931	UNIPROT	SRC	protein	P29350	PTPN6	UNIPROT	protein	YES	up-regulates	phosphorylation
P12931	UNIPROT	SRC	protein	P63000	RAC1	UNIPROT	protein	YES	up-regulates	phosphorylation
P12931	UNIPROT	SRC	protein	P63244	RACK1	UNIPROT	protein	YES	up-regulates	phosphorylation
P12931	UNIPROT	SRC	protein	P63244	RACK1	UNIPROT	protein	YES	up-regulates	phosphorylation
P12931	UNIPROT	SRC	protein	P51812	RPS6KA3	UNIPROT	protein	YES	up-regulates	phosphorylation
P12931	UNIPROT	SRC	protein	Q13813	SPTAN1	UNIPROT	protein	YES	up-regulates	phosphorylation
P12931	UNIPROT	SRC	protein	P12931	SRC	UNIPROT	protein	YES	up-regulates	phosphorylation
P12931	UNIPROT	SRC	protein	P12931	SRC	UNIPROT	protein	YES	up-regulates	phosphorylation
P12931	UNIPROT	SRC	protein	P51692	STAT5B	UNIPROT	protein	YES	up-regulates	phosphorylation
P12931	UNIPROT	SRC	protein	P37173	TGFBR2	UNIPROT	protein	YES	up-regulates	phosphorylation
P12931	UNIPROT	SRC	protein	Q68CZ2	TNS3	UNIPROT	protein	YES	up-regulates	phosphorylation
P12931	UNIPROT	SRC	protein	Q68CZ2	TNS3	UNIPROT	protein	YES	up-regulates	phosphorylation
P12931	UNIPROT	SRC	protein	Q68CZ2	TNS3	UNIPROT	protein	YES	up-regulates	phosphorylation
P12931	UNIPROT	SRC	protein	Q68CZ2	TNS3	UNIPROT	protein	YES	up-regulates	phosphorylation
Q96SB4	UNIPROT	SRPK1	protein	Q96SB4	SRPK1	UNIPROT	protein	YES	up-regulates	phosphorylation
Q96SB4	UNIPROT	SRPK1	protein	Q96SB4	SRPK1	UNIPROT	protein	YES	up-regulates	phosphorylation
Q96SB4	UNIPROT	SRPK1	protein	Q96SB4	SRPK1	UNIPROT	protein	YES	up-regulates	phosphorylation
Q96SB4	UNIPROT	SRPK1	protein	Q07955	SRSF1	UNIPROT	protein	YES	up-regulates	phosphorylation
P78362	UNIPROT	SRPK2	protein	Q9UKV3	ACIN1	UNIPROT	protein	YES	up-regulates	phosphorylation
Q9Y6E0	UNIPROT	STK24	protein	Q9Y6E0	STK24	UNIPROT	protein	YES	up-regulates	phosphorylation
Q9Y6E0	UNIPROT	STK24	protein	Q15208	STK38	UNIPROT	protein	YES	up-regulates	phosphorylation
Q9Y6E0	UNIPROT	STK24	protein	Q9Y2H1	STK38L	UNIPROT	protein	YES	up-regulates	phosphorylation
Q9P289	UNIPROT	STK26	protein	P15311	EZR	UNIPROT	protein	YES	up-regulates	phosphorylation
Q13188	UNIPROT	STK3	protein	Q9H8S9	MOB1A	UNIPROT	protein	YES	up-regulates	phosphorylation
Q13188	UNIPROT	STK3	protein	Q9H8S9	MOB1A	UNIPROT	protein	YES	up-regulates	phosphorylation
Q13188	UNIPROT	STK3	protein	Q9H8S9	MOB1A	UNIPROT	protein	YES	up-regulates	phosphorylation
Q13188	UNIPROT	STK3	protein	Q9H8S9	MOB1A	UNIPROT	protein	YES	up-regulates	phosphorylation
Q13188	UNIPROT	STK3	protein	Q9H8S9	MOB1A	UNIPROT	protein	YES	up-regulates	phosphorylation
Q13188	UNIPROT	STK3	protein	Q13188	STK3	UNIPROT	protein	YES	up-regulates	phosphorylation
Q15208	UNIPROT	STK38	protein	Q15208	STK38	UNIPROT	protein	YES	up-regulates	phosphorylation
Q15208	UNIPROT	STK38	protein	Q15208	STK38	UNIPROT	protein	YES	up-regulates	phosphorylation
Q15208	UNIPROT	STK38	protein	Q15208	STK38	UNIPROT	protein	YES	up-regulates	phosphorylation
Q15208	UNIPROT	STK38	protein	Q15208	STK38	UNIPROT	protein	YES	up-regulates	phosphorylation
Q9Y2H1	UNIPROT	STK38L	protein	Q9Y2H1	STK38L	UNIPROT	protein	YES	up-regulates	phosphorylation
Q13043	UNIPROT	STK4	protein	Q12778	FOXO1	UNIPROT	protein	YES	up-regulates	phosphorylation
Q13043	UNIPROT	STK4	protein	Q12778	FOXO1	UNIPROT	protein	YES	up-regulates	phosphorylation
Q13043	UNIPROT	STK4	protein	O43524	FOXO3	UNIPROT	protein	YES	up-regulates	phosphorylation
Q13043	UNIPROT	STK4	protein	O43524	FOXO3	UNIPROT	protein	YES	up-regulates	phosphorylation
Q13043	UNIPROT	STK4	protein	P33778	HIST1H2BB	UNIPROT	protein	YES	up-regulates	phosphorylation
Q13043	UNIPROT	STK4	protein	Q9H8S9	MOB1A	UNIPROT	protein	YES	up-regulates	phosphorylation
Q13043	UNIPROT	STK4	protein	Q9H8S9	MOB1A	UNIPROT	protein	YES	up-regulates	phosphorylation
Q13043	UNIPROT	STK4	protein	Q9H8S9	MOB1A	UNIPROT	protein	YES	up-regulates	phosphorylation
Q13043	UNIPROT	STK4	protein	Q9H8S9	MOB1A	UNIPROT	protein	YES	up-regulates	phosphorylation
P43405	UNIPROT	SYK	protein	P29353	SHC1	UNIPROT	protein	YES	up-regulates	phosphorylation
P43405	UNIPROT	SYK	protein	P29353	SHC1	UNIPROT	protein	YES	up-regulates	phosphorylation
P43405	UNIPROT	SYK	protein	P29353	SHC1	UNIPROT	protein	YES	up-regulates	phosphorylation
Q7L7X3	UNIPROT	TAOK1	protein	Q13188	STK3	UNIPROT	protein	YES	up-regulates	phosphorylation
Q7L7X3	UNIPROT	TAOK1	protein	Q13043	STK4	UNIPROT	protein	YES	up-regulates	phosphorylation
Q9UL54	UNIPROT	TAOK2	protein	Q13188	STK3	UNIPROT	protein	YES	up-regulates	phosphorylation
Q9UL54	UNIPROT	TAOK2	protein	Q13043	STK4	UNIPROT	protein	YES	up-regulates	phosphorylation
Q9H2K8	UNIPROT	TAOK3	protein	Q13188	STK3	UNIPROT	protein	YES	up-regulates	phosphorylation
Q9H2K8	UNIPROT	TAOK3	protein	Q13043	STK4	UNIPROT	protein	YES	up-regulates	phosphorylation
Q9UHD2	UNIPROT	TBK1	protein	P31749	AKT1	UNIPROT	protein	YES	up-regulates	phosphorylation
Q9UHD2	UNIPROT	TBK1	protein	Q04206	RELA	UNIPROT	protein	YES	up-regulates	phosphorylation
Q9UHD2	UNIPROT	TBK1	protein	Q13501	SQSTM1	UNIPROT	protein	YES	up-regulates	phosphorylation
Q9UHD2	UNIPROT	TBK1	protein	P42226	STAT6	UNIPROT	protein	YES	up-regulates	phosphorylation
Q9UHD2	UNIPROT	TBK1	protein	P42226	STAT6	UNIPROT	protein	YES	up-regulates	phosphorylation
P21980	UNIPROT	TGM2	protein	P68431	HIST1H3A	UNIPROT	protein	YES	up-regulates	phosphorylation
Q96S44	UNIPROT	TP53RK	protein	P04637	TP53	UNIPROT	protein	YES	up-regulates	phosphorylation
P33981	UNIPROT	TTK	protein	O96017	CHEK2	UNIPROT	protein	YES	up-regulates	phosphorylation
P33981	UNIPROT	TTK	protein	P38646	HSPA9	UNIPROT	protein	YES	up-regulates	phosphorylation
P33981	UNIPROT	TTK	protein	P38646	HSPA9	UNIPROT	protein	YES	up-regulates	phosphorylation
P33981	UNIPROT	TTK	protein	P04637	TP53	UNIPROT	protein	YES	up-regulates	phosphorylation
P33981	UNIPROT	TTK	protein	P33981	TTK	UNIPROT	protein	YES	up-regulates	phosphorylation
P33981	UNIPROT	TTK	protein	P33981	TTK	UNIPROT	protein	YES	up-regulates	phosphorylation
P33981	UNIPROT	TTK	protein	P33981	TTK	UNIPROT	protein	YES	up-regulates	phosphorylation
P33981	UNIPROT	TTK	protein	P33981	TTK	UNIPROT	protein	YES	up-regulates	phosphorylation
P33981	UNIPROT	TTK	protein	P33981	TTK	UNIPROT	protein	YES	up-regulates	phosphorylation
Q99986	UNIPROT	VRK1	protein	P15336	ATF2	UNIPROT	protein	YES	up-regulates	phosphorylation
Q99986	UNIPROT	VRK1	protein	P15336	ATF2	UNIPROT	protein	YES	up-regulates	phosphorylation
Q99986	UNIPROT	VRK1	protein	P68431	HIST1H3A	UNIPROT	protein	YES	up-regulates	phosphorylation
Q99986	UNIPROT	VRK1	protein	P05412	JUN	UNIPROT	protein	YES	up-regulates	phosphorylation
Q99986	UNIPROT	VRK1	protein	P05412	JUN	UNIPROT	protein	YES	up-regulates	phosphorylation
Q99986	UNIPROT	VRK1	protein	P04637	TP53	UNIPROT	protein	YES	up-regulates	phosphorylation
Q99986	UNIPROT	VRK1	protein	Q12888	TP53BP1	UNIPROT	protein	YES	up-regulates	phosphorylation
Q9H4A3	UNIPROT	WNK1	protein	O95747	OXSR1	UNIPROT	protein	YES	up-regulates	phosphorylation
Q9H4A3	UNIPROT	WNK1	protein	O95747	OXSR1	UNIPROT	protein	YES	up-regulates	phosphorylation
Q9H4A3	UNIPROT	WNK1	protein	Q9UEW8	STK39	UNIPROT	protein	YES	up-regulates	phosphorylation
Q9H4A3	UNIPROT	WNK1	protein	Q9UEW8	STK39	UNIPROT	protein	YES	up-regulates	phosphorylation

Q9H4A3	UNIPROT	WNK1	protein	Q9UEW8	STK39	UNIPROT	protein	YES	up-regulates	phosphorylation
Q9H4A3	UNIPROT	WNK1	protein	Q9UEW8	STK39	UNIPROT	protein	YES	up-regulates	phosphorylation
Q9H4A3	UNIPROT	WNK1	protein	Q9UEW8	STK39	UNIPROT	protein	YES	up-regulates	phosphorylation
Q9H4A3	UNIPROT	WNK1	protein	Q9H4A3	WNK1	UNIPROT	protein	YES	up-regulates	phosphorylation
Q9H4A3	UNIPROT	WNK1	protein	Q9H4A3	WNK1	UNIPROT	protein	YES	up-regulates	phosphorylation
P31749	UNIPROT	AKT1	protein	Q07812	BAX	UNIPROT	protein	YES	up-regulates activity	phosphorylation
P31749	UNIPROT	AKT1	protein	P35226	BMI1	UNIPROT	protein	YES	up-regulates activity	phosphorylation
P31749	UNIPROT	AKT1	protein	P16220	CREB1	UNIPROT	protein	YES	up-regulates activity	phosphorylation
P31749	UNIPROT	AKT1	protein	P41279	MAP3K8	UNIPROT	protein	YES	up-regulates activity	phosphorylation
P31749	UNIPROT	AKT1	protein	P41279	MAP3K8	UNIPROT	protein	YES	up-regulates activity	phosphorylation
P31749	UNIPROT	AKT1	protein	Q15121	PEA15	UNIPROT	protein	YES	up-regulates activity	phosphorylation
P31749	UNIPROT	AKT1	protein	O60825	PFKFB2	UNIPROT	protein	YES	up-regulates activity	phosphorylation
P31749	UNIPROT	AKT1	protein	O60825	PFKFB2	UNIPROT	protein	YES	up-regulates activity	phosphorylation
P31749	UNIPROT	AKT1	protein	O60825	PFKFB2	UNIPROT	protein	YES	up-regulates activity	phosphorylation
P31749	UNIPROT	AKT1	protein	O60825	PFKFB2	UNIPROT	protein	YES	up-regulates activity	phosphorylation
P31751	UNIPROT	AKT2	protein	P35226	BMI1	UNIPROT	protein	YES	up-regulates activity	phosphorylation
Q96GD4	UNIPROT	AURKB	protein	Q9H0H5	RACGAP1	UNIPROT	protein	YES	up-regulates activity	phosphorylation
Q96GD4	UNIPROT	AURKB	protein	Q9H0H5	RACGAP1	UNIPROT	protein	YES	up-regulates activity	phosphorylation
Q96GD4	UNIPROT	AURKB	protein	Q9H0H5	RACGAP1	UNIPROT	protein	YES	up-regulates activity	phosphorylation
Q96GD4	UNIPROT	AURKB	protein	Q9H0H5	RACGAP1	UNIPROT	protein	YES	up-regulates activity	phosphorylation
Q96GD4	UNIPROT	AURKB	protein	Q9H0H5	RACGAP1	UNIPROT	protein	YES	up-regulates activity	phosphorylation
Q96GD4	UNIPROT	AURKB	protein	Q9H0H5	RACGAP1	UNIPROT	protein	YES	up-regulates activity	phosphorylation
P15056	UNIPROT	BRAF	protein	Q02750	MAP2K1	UNIPROT	protein	YES	up-regulates activity	phosphorylation
P15056	UNIPROT	BRAF	protein	Q02750	MAP2K1	UNIPROT	protein	YES	up-regulates activity	phosphorylation
P15056	UNIPROT	BRAF	protein	Q02750	MAP2K1	UNIPROT	protein	YES	up-regulates activity	phosphorylation
P15056	UNIPROT	BRAF	protein	Q02750	MAP2K1	UNIPROT	protein	YES	up-regulates activity	phosphorylation
P27708	UNIPROT	CAD	protein	P27708	CAD	UNIPROT	protein	YES	up-regulates activity	phosphorylation
Q9UQM7	UNIPROT	CAMK2A	protein	P16615	ATP2A2	UNIPROT	protein	YES	up-regulates activity	phosphorylation
Q9UQM7	UNIPROT	CAMK2A	protein	P16070	CD44	UNIPROT	protein	YES	up-regulates activity	phosphorylation
Q9UQM7	UNIPROT	CAMK2A	protein	P17676	CEBPB	UNIPROT	protein	YES	up-regulates activity	phosphorylation
Q9UQM7	UNIPROT	CAMK2A	protein	Q00613	HSF1	UNIPROT	protein	YES	up-regulates activity	phosphorylation
Q13554	UNIPROT	CAMK2B	protein	Q13554	CAMK2B	UNIPROT	protein	YES	up-regulates activity	phosphorylation
Q13555	UNIPROT	CAMK2G	protein	P18846	ATF1	UNIPROT	protein	YES	up-regulates activity	phosphorylation
Q13555	UNIPROT	CAMK2G	protein	P42224	STAT1	UNIPROT	protein	YES	up-regulates activity	phosphorylation
P06493	UNIPROT	CDK1	protein	P18858	LIG1	UNIPROT	protein	YES	up-regulates activity	phosphorylation
P06493	UNIPROT	CDK1	protein	Q96GX5	MASTL	UNIPROT	protein	YES	up-regulates activity	phosphorylation
P06493	UNIPROT	CDK1	protein	Q96GX5	MASTL	UNIPROT	protein	YES	up-regulates activity	phosphorylation
P24941	UNIPROT	CDK2	protein	Q15910	EZH2	UNIPROT	protein	YES	up-regulates activity	phosphorylation
P24941	UNIPROT	CDK2	protein	P54198	HIRA	UNIPROT	protein	YES	up-regulates activity	phosphorylation
P24941	UNIPROT	CDK2	protein	P18858	LIG1	UNIPROT	protein	YES	up-regulates activity	phosphorylation
P24941	UNIPROT	CDK2	protein	P18858	LIG1	UNIPROT	protein	YES	up-regulates activity	phosphorylation
P24941	UNIPROT	CDK2	protein	P18858	LIG1	UNIPROT	protein	YES	up-regulates activity	phosphorylation
P24941	UNIPROT	CDK2	protein	P23511	NFYA	UNIPROT	protein	YES	up-regulates activity	phosphorylation
P24941	UNIPROT	CDK2	protein	P23511	NFYA	UNIPROT	protein	YES	up-regulates activity	phosphorylation
P24941	UNIPROT	CDK2	protein	P08047	SP1	UNIPROT	protein	YES	up-regulates activity	phosphorylation
P24941	UNIPROT	CDK2	protein	P17480	UBTF	UNIPROT	protein	YES	up-regulates activity	phosphorylation
Q00535	UNIPROT	CDK5	protein	P31146	CORO1A	UNIPROT	protein	YES	up-regulates activity	phosphorylation
Q00535	UNIPROT	CDK5	protein	Q05193	DNM1	UNIPROT	protein	YES	up-regulates activity	phosphorylation
Q00535	UNIPROT	CDK5	protein	Q05193	DNM1	UNIPROT	protein	YES	up-regulates activity	phosphorylation
Q00535	UNIPROT	CDK5	protein	P21860	ERBB3	UNIPROT	protein	YES	up-regulates activity	phosphorylation
Q00535	UNIPROT	CDK5	protein	P21860	ERBB3	UNIPROT	protein	YES	up-regulates activity	phosphorylation
Q00535	UNIPROT	CDK5	protein	P15311	EZR	UNIPROT	protein	YES	up-regulates activity	phosphorylation
Q00535	UNIPROT	CDK5	protein	Q9GZM8	NDEL1	UNIPROT	protein	YES	up-regulates activity	phosphorylation
Q00535	UNIPROT	CDK5	protein	Q9GZM8	NDEL1	UNIPROT	protein	YES	up-regulates activity	phosphorylation
Q00535	UNIPROT	CDK5	protein	Q9GZM8	NDEL1	UNIPROT	protein	YES	up-regulates activity	phosphorylation
P50613	UNIPROT	CDK7	protein	P21127	CDK11B	UNIPROT	protein	YES	up-regulates activity	phosphorylation
P50613	UNIPROT	CDK7	protein	P24941	CDK2	UNIPROT	protein	YES	up-regulates activity	phosphorylation
P50750	UNIPROT	CDK9	protein	Q15596	NCOA2	UNIPROT	protein	YES	up-regulates activity	phosphorylation
P50750	UNIPROT	CDK9	protein	Q15596	NCOA2	UNIPROT	protein	YES	up-regulates activity	phosphorylation
P50750	UNIPROT	CDK9	protein	Q15596	NCOA2	UNIPROT	protein	YES	up-regulates activity	phosphorylation
P50750	UNIPROT	CDK9	protein	Q15596	NCOA2	UNIPROT	protein	YES	up-regulates activity	phosphorylation
O14757	UNIPROT	CHEK1	protein	O14757	CHEK1	UNIPROT	protein	YES	up-regulates activity	phosphorylation
O14757	UNIPROT	CHEK1	protein	O14757	CHEK1	UNIPROT	protein	YES	up-regulates activity	phosphorylation
O14757	UNIPROT	CHEK1	protein	P04637	TP53	UNIPROT	protein	YES	up-regulates activity	phosphorylation
O14757	UNIPROT	CHEK1	protein	P04637	TP53	UNIPROT	protein	YES	up-regulates activity	phosphorylation
O14757	UNIPROT	CHEK1	protein	P04637	TP53	UNIPROT	protein	YES	up-regulates activity	phosphorylation
O14757	UNIPROT	CHEK1	protein	P04637	TP53	UNIPROT	protein	YES	up-regulates activity	phosphorylation
O14757	UNIPROT	CHEK1	protein	P04637	TP53	UNIPROT	protein	YES	up-regulates activity	phosphorylation
O14757	UNIPROT	CHEK1	protein	P04637	TP53	UNIPROT	protein	YES	up-regulates activity	phosphorylation
O14757	UNIPROT	CHEK1	protein	P04637	TP53	UNIPROT	protein	YES	up-regulates activity	phosphorylation
O96017	UNIPROT	CHEK2	protein	O96017	CHEK2	UNIPROT	protein	YES	up-regulates activity	phosphorylation
O96017	UNIPROT	CHEK2	protein	O96017	CHEK2	UNIPROT	protein	YES	up-regulates activity	phosphorylation
O96017	UNIPROT	CHEK2	protein	O96017	CHEK2	UNIPROT	protein	YES	up-regulates activity	phosphorylation
O96017	UNIPROT	CHEK2	protein	O96017	CHEK2	UNIPROT	protein	YES	up-regulates activity	phosphorylation
O96017	UNIPROT	CHEK2	protein	O96017	CHEK2	UNIPROT	protein	YES	up-regulates activity	phosphorylation
O96017	UNIPROT	CHEK2	protein	P06400	RB1	UNIPROT	protein	YES	up-regulates activity	phosphorylation
O15111	UNIPROT	CHUK	protein	Q00653	NFKB2	UNIPROT	protein	YES	up-regulates activity	phosphorylation
O15111	UNIPROT	CHUK	protein	Q00653	NFKB2	UNIPROT	protein	YES	up-regulates activity	phosphorylation
O15111	UNIPROT	CHUK	protein	Q04206	RELA	UNIPROT	protein	YES	up-regulates activity	phosphorylation
O15111	UNIPROT	CHUK	protein	Q04206	RELA	UNIPROT	protein	YES	up-regulates activity	phosphorylation
O15111	UNIPROT	CHUK	protein	Q04206	RELA	UNIPROT	protein	YES	up-regulates activity	phosphorylation
O15111	UNIPROT	CHUK	protein	Q86VP1	TAX1BP1	UNIPROT	protein	YES	up-regulates activity	phosphorylation
O15111	UNIPROT	CHUK	protein	Q86VP1	TAX1BP1	UNIPROT	protein	YES	up-regulates activity	phosphorylation
P46108	UNIPROT	CRK	protein	Q05397	PTK2	UNIPROT	protein	YES	up-regulates activity	phosphorylation
P41240	UNIPROT	CSK	protein	P41240	CSK	UNIPROT	protein	YES	up-regulates activity	phosphorylation
P48729	UNIPROT	CSNK1A1	protein	O15169	AXIN1	UNIPROT	protein	YES	up-regulates activity	phosphorylation
P48729	UNIPROT	CSNK1A1	protein	O15169	AXIN1	UNIPROT	protein	YES	up-regulates activity	phosphorylation
P48729	UNIPROT	CSNK1A1	protein	O15169	AXIN1	UNIPROT	protein	YES	up-regulates activity	phosphorylation
P48729	UNIPROT	CSNK1A1	protein	P55957	BID	UNIPROT	protein	YES	up-regulates activity	phosphorylation
P48729	UNIPROT	CSNK1A1	protein	P55957	BID	UNIPROT	protein	YES	up-regulates activity	phosphorylation
P48730	UNIPROT	CSNK1D	protein	Q00535	CDK5	UNIPROT	protein	YES	up-regulates activity	phosphorylation
P49674	UNIPROT	CSNK1E	protein	P55957	BID	UNIPROT	protein	YES	up-regulates activity	phosphorylation
P49674	UNIPROT	CSNK1E	protein	P55957	BID	UNIPROT	protein	YES	up-regulates activity	phosphorylation
P49674	UNIPROT	CSNK1E	protein	O14641	DVL2	UNIPROT	protein	YES	up-regulates activity	phosphorylation
P68400	UNIPROT	CSNK2A1	protein	P27695	APEX1	UNIPROT	protein	YES	up-regulates activity	phosphorylation
P68400	UNIPROT	CSNK2A1	protein	P55957	BID	UNIPROT	protein	YES	up-regulates activity	phosphorylation
P68400	UNIPROT	CSNK2A1	protein	P55957	BID	UNIPROT	protein	YES	up-regulates activity	phosphorylation
P68400	UNIPROT	CSNK2A1	protein	Q16543	CDC37	UNIPROT	protein	YES	up-regulates activity	phosphorylation

P68400	UNIPROT	CSNK2A1	protein	P12830	CDH1	UNIPROT	protein	YES	up-regulates activity	phosphorylation
P68400	UNIPROT	CSNK2A1	protein	P12830	CDH1	UNIPROT	protein	YES	up-regulates activity	phosphorylation
P68400	UNIPROT	CSNK2A1	protein	P12830	CDH1	UNIPROT	protein	YES	up-regulates activity	phosphorylation
P68400	UNIPROT	CSNK2A1	protein	P35222	CTNNB1	UNIPROT	protein	YES	up-regulates activity	phosphorylation
P68400	UNIPROT	CSNK2A1	protein	P35222	CTNNB1	UNIPROT	protein	YES	up-regulates activity	phosphorylation
P68400	UNIPROT	CSNK2A1	protein	P35222	CTNNB1	UNIPROT	protein	YES	up-regulates activity	phosphorylation
P68400	UNIPROT	CSNK2A1	protein	P35222	CTNNB1	UNIPROT	protein	YES	up-regulates activity	phosphorylation
P68400	UNIPROT	CSNK2A1	protein	Q13144	EIF2B5	UNIPROT	protein	YES	up-regulates activity	phosphorylation
P68400	UNIPROT	CSNK2A1	protein	Q13144	EIF2B5	UNIPROT	protein	YES	up-regulates activity	phosphorylation
P68400	UNIPROT	CSNK2A1	protein	P55010	EIF5	UNIPROT	protein	YES	up-regulates activity	phosphorylation
P68400	UNIPROT	CSNK2A1	protein	Q9UNN5	FAF1	UNIPROT	protein	YES	up-regulates activity	phosphorylation
P68400	UNIPROT	CSNK2A1	protein	Q9UNN5	FAF1	UNIPROT	protein	YES	up-regulates activity	phosphorylation
P68400	UNIPROT	CSNK2A1	protein	P52655	GTF2A1	UNIPROT	protein	YES	up-regulates activity	phosphorylation
P68400	UNIPROT	CSNK2A1	protein	P52655	GTF2A1	UNIPROT	protein	YES	up-regulates activity	phosphorylation
P68400	UNIPROT	CSNK2A1	protein	P52655	GTF2A1	UNIPROT	protein	YES	up-regulates activity	phosphorylation
P68400	UNIPROT	CSNK2A1	protein	P52655	GTF2A1	UNIPROT	protein	YES	up-regulates activity	phosphorylation
P68400	UNIPROT	CSNK2A1	protein	Q92769	HDAC2	UNIPROT	protein	YES	up-regulates activity	phosphorylation
P68400	UNIPROT	CSNK2A1	protein	Q92769	HDAC2	UNIPROT	protein	YES	up-regulates activity	phosphorylation
P68400	UNIPROT	CSNK2A1	protein	O15379	HDAC3	UNIPROT	protein	YES	up-regulates activity	phosphorylation
P68400	UNIPROT	CSNK2A1	protein	P30519	HMOX2	UNIPROT	protein	YES	up-regulates activity	phosphorylation
P68400	UNIPROT	CSNK2A1	protein	Q00613	HSF1	UNIPROT	protein	YES	up-regulates activity	phosphorylation
P68400	UNIPROT	CSNK2A1	protein	Q16666	IFI16	UNIPROT	protein	YES	up-regulates activity	phosphorylation
P68400	UNIPROT	CSNK2A1	protein	P18858	LIG1	UNIPROT	protein	YES	up-regulates activity	phosphorylation
P68400	UNIPROT	CSNK2A1	protein	Q6VY07	PACS1	UNIPROT	protein	YES	up-regulates activity	phosphorylation
P68400	UNIPROT	CSNK2A1	protein	P41236	PPP1R2	UNIPROT	protein	YES	up-regulates activity	phosphorylation
P68400	UNIPROT	CSNK2A1	protein	Q12972	PPP1R8	UNIPROT	protein	YES	up-regulates activity	phosphorylation
P68400	UNIPROT	CSNK2A1	protein	Q12972	PPP1R8	UNIPROT	protein	YES	up-regulates activity	phosphorylation
P68400	UNIPROT	CSNK2A1	protein	Q04206	RELA	UNIPROT	protein	YES	up-regulates activity	phosphorylation
P68400	UNIPROT	CSNK2A1	protein	Q04206	RELA	UNIPROT	protein	YES	up-regulates activity	phosphorylation
P68400	UNIPROT	CSNK2A1	protein	P04637	TP53	UNIPROT	protein	YES	up-regulates activity	phosphorylation
P68400	UNIPROT	CSNK2A1	protein	P18887	XRCC1	UNIPROT	protein	YES	up-regulates activity	phosphorylation
P19784	UNIPROT	CSNK2A2	protein	P55957	BID	UNIPROT	protein	YES	up-regulates activity	phosphorylation
P19784	UNIPROT	CSNK2A2	protein	P55957	BID	UNIPROT	protein	YES	up-regulates activity	phosphorylation
P19784	UNIPROT	CSNK2A2	protein	Q16543	CDC37	UNIPROT	protein	YES	up-regulates activity	phosphorylation
P19784	UNIPROT	CSNK2A2	protein	Q13144	EIF2B5	UNIPROT	protein	YES	up-regulates activity	phosphorylation
P19784	UNIPROT	CSNK2A2	protein	Q13144	EIF2B5	UNIPROT	protein	YES	up-regulates activity	phosphorylation
P19784	UNIPROT	CSNK2A2	protein	P52655	GTF2A1	UNIPROT	protein	YES	up-regulates activity	phosphorylation
P19784	UNIPROT	CSNK2A2	protein	P52655	GTF2A1	UNIPROT	protein	YES	up-regulates activity	phosphorylation
P19784	UNIPROT	CSNK2A2	protein	P52655	GTF2A1	UNIPROT	protein	YES	up-regulates activity	phosphorylation
P19784	UNIPROT	CSNK2A2	protein	P52655	GTF2A1	UNIPROT	protein	YES	up-regulates activity	phosphorylation
P19784	UNIPROT	CSNK2A2	protein	Q13547	HDAC1	UNIPROT	protein	YES	up-regulates activity	phosphorylation
P19784	UNIPROT	CSNK2A2	protein	Q13547	HDAC1	UNIPROT	protein	YES	up-regulates activity	phosphorylation
P19784	UNIPROT	CSNK2A2	protein	Q92769	HDAC2	UNIPROT	protein	YES	up-regulates activity	phosphorylation
P19784	UNIPROT	CSNK2A2	protein	Q92769	HDAC2	UNIPROT	protein	YES	up-regulates activity	phosphorylation
P19784	UNIPROT	CSNK2A2	protein	Q92769	HDAC2	UNIPROT	protein	YES	up-regulates activity	phosphorylation
P19784	UNIPROT	CSNK2A2	protein	P41236	PPP1R2	UNIPROT	protein	YES	up-regulates activity	phosphorylation
P19784	UNIPROT	CSNK2A2	protein	P41236	PPP1R2	UNIPROT	protein	YES	up-regulates activity	phosphorylation
P19784	UNIPROT	CSNK2A2	protein	P41236	PPP1R2	UNIPROT	protein	YES	up-regulates activity	phosphorylation
P19784	UNIPROT	CSNK2A2	protein	Q12972	PPP1R8	UNIPROT	protein	YES	up-regulates activity	phosphorylation
P19784	UNIPROT	CSNK2A2	protein	Q12972	PPP1R8	UNIPROT	protein	YES	up-regulates activity	phosphorylation
P19784	UNIPROT	CSNK2A2	protein	Q712K3	UBE2R2	UNIPROT	protein	YES	up-regulates activity	phosphorylation
P19784	UNIPROT	CSNK2A2	protein	P18887	XRCC1	UNIPROT	protein	YES	up-regulates activity	phosphorylation
P19784	UNIPROT	CSNK2A2	protein	P18887	XRCC1	UNIPROT	protein	YES	up-regulates activity	phosphorylation
P19784	UNIPROT	CSNK2A2	protein	P18887	XRCC1	UNIPROT	protein	YES	up-regulates activity	phosphorylation
P67870	UNIPROT	CSNK2B	protein	P55957	BID	UNIPROT	protein	YES	up-regulates activity	phosphorylation
P67870	UNIPROT	CSNK2B	protein	P55957	BID	UNIPROT	protein	YES	up-regulates activity	phosphorylation
P67870	UNIPROT	CSNK2B	protein	P35222	CTNNB1	UNIPROT	protein	YES	up-regulates activity	phosphorylation
P67870	UNIPROT	CSNK2B	protein	P35222	CTNNB1	UNIPROT	protein	YES	up-regulates activity	phosphorylation
P67870	UNIPROT	CSNK2B	protein	P35222	CTNNB1	UNIPROT	protein	YES	up-regulates activity	phosphorylation
P67870	UNIPROT	CSNK2B	protein	P55010	EIF5	UNIPROT	protein	YES	up-regulates activity	phosphorylation
P67870	UNIPROT	CSNK2B	protein	P55010	EIF5	UNIPROT	protein	YES	up-regulates activity	phosphorylation
P67870	UNIPROT	CSNK2B	protein	P55010	EIF5	UNIPROT	protein	YES	up-regulates activity	phosphorylation
P67870	UNIPROT	CSNK2B	protein	Q9UBF6	RNF7	UNIPROT	protein	YES	up-regulates activity	phosphorylation
P67870	UNIPROT	CSNK2B	protein	O60763	USO1	UNIPROT	protein	YES	up-regulates activity	phosphorylation
Q08345	UNIPROT	DDR1	protein	Q08345	DDR1	UNIPROT	protein	YES	up-regulates activity	phosphorylation
Q13627	UNIPROT	DYRK1A	protein	Q13627	DYRK1A	UNIPROT	protein	YES	up-regulates activity	phosphorylation
Q13627	UNIPROT	DYRK1A	protein	Q13627	DYRK1A	UNIPROT	protein	YES	up-regulates activity	phosphorylation
P00533	UNIPROT	EGFR	protein	P00533	EGFR	UNIPROT	protein	YES	up-regulates activity	phosphorylation
P00533	UNIPROT	EGFR	protein	P00533	EGFR	UNIPROT	protein	YES	up-regulates activity	phosphorylation
P00533	UNIPROT	EGFR	protein	P00533	EGFR	UNIPROT	protein	YES	up-regulates activity	phosphorylation
P00533	UNIPROT	EGFR	protein	P00533	EGFR	UNIPROT	protein	YES	up-regulates activity	phosphorylation
P00533	UNIPROT	EGFR	protein	P00533	EGFR	UNIPROT	protein	YES	up-regulates activity	phosphorylation
P00533	UNIPROT	EGFR	protein	P00533	EGFR	UNIPROT	protein	YES	up-regulates activity	phosphorylation
P00533	UNIPROT	EGFR	protein	P00533	EGFR	UNIPROT	protein	YES	up-regulates activity	phosphorylation
P00533	UNIPROT	EGFR	protein	P00533	EGFR	UNIPROT	protein	YES	up-regulates activity	phosphorylation
P00533	UNIPROT	EGFR	protein	P00533	EGFR	UNIPROT	protein	YES	up-regulates activity	phosphorylation
P00533	UNIPROT	EGFR	protein	P00533	EGFR	UNIPROT	protein	YES	up-regulates activity	phosphorylation
P00533	UNIPROT	EGFR	protein	P00533	EGFR	UNIPROT	protein	YES	up-regulates activity	phosphorylation
P00533	UNIPROT	EGFR	protein	P00533	EGFR	UNIPROT	protein	YES	up-regulates activity	phosphorylation
P00533	UNIPROT	EGFR	protein	P04626	ERBB2	UNIPROT	protein	YES	up-regulates activity	phosphorylation
P00533	UNIPROT	EGFR	protein	Q9UJM3	ERRFI1	UNIPROT	protein	YES	up-regulates activity	phosphorylation
P00533	UNIPROT	EGFR	protein	Q13480	GAB1	UNIPROT	protein	YES	up-regulates activity	phosphorylation
P00533	UNIPROT	EGFR	protein	Q13480	GAB1	UNIPROT	protein	YES	up-regulates activity	phosphorylation
P00533	UNIPROT	EGFR	protein	Q13480	GAB1	UNIPROT	protein	YES	up-regulates activity	phosphorylation
P00533	UNIPROT	EGFR	protein	Q13480	GAB1	UNIPROT	protein	YES	up-regulates activity	phosphorylation
P00533	UNIPROT	EGFR	protein	O14964	HGS	UNIPROT	protein	YES	up-regulates activity	phosphorylation
P00533	UNIPROT	EGFR	protein	P40763	STAT3	UNIPROT	protein	YES	up-regulates activity	phosphorylation
P00533	UNIPROT	EGFR	protein	P40763	STAT3	UNIPROT	protein	YES	up-regulates activity	phosphorylation
P00533	UNIPROT	EGFR	protein	P51692	STAT5B	UNIPROT	protein	YES	up-regulates activity	phosphorylation
P19525	UNIPROT	EIF2AK2	protein	P19525	EIF2AK2	UNIPROT	protein	YES	up-regulates activity	phosphorylation
P19525	UNIPROT	EIF2AK2	protein	P19525	EIF2AK2	UNIPROT	protein	YES	up-regulates activity	phosphorylation
P19525	UNIPROT	EIF2AK2	protein	P19525	EIF2AK2	UNIPROT	protein	YES	up-regulates activity	phosphorylation
P19525	UNIPROT	EIF2AK2	protein	P19525	EIF2AK2	UNIPROT	protein	YES	up-regulates activity	phosphorylation
P19525	UNIPROT	EIF2AK2	protein	P19525	EIF2AK2	UNIPROT	protein	YES	up-regulates activity	phosphorylation
P19525	UNIPROT	EIF2AK2	protein	P19525	EIF2AK2	UNIPROT	protein	YES	up-regulates activity	phosphorylation
P04626	UNIPROT	ERBB2	protein	P04626	ERBB2	UNIPROT	protein	YES	up-regulates activity	phosphorylation
P04626	UNIPROT	ERBB2	protein	P04626	ERBB2	UNIPROT	protein	YES	up-regulates activity	phosphorylation
P04626	UNIPROT	ERBB2	protein	P04626	ERBB2	UNIPROT	protein	YES	up-regulates activity	phosphorylation

P04626	UNIPROT	ERBB2	protein	P04626	ERBB2	UNIPROT	protein	YES	up-regulates activity	phosphorylation
P49840	UNIPROT	GSK3A	protein	Q04206	RELA	UNIPROT	protein	YES	up-regulates activity	phosphorylation
P49841	UNIPROT	GSK3B	protein	P53396	ACLY	UNIPROT	protein	YES	up-regulates activity	phosphorylation
P49841	UNIPROT	GSK3B	protein	P53396	ACLY	UNIPROT	protein	YES	up-regulates activity	phosphorylation
P49841	UNIPROT	GSK3B	protein	O15169	AXIN1	UNIPROT	protein	YES	up-regulates activity	phosphorylation
P49841	UNIPROT	GSK3B	protein	O15169	AXIN1	UNIPROT	protein	YES	up-regulates activity	phosphorylation
P49841	UNIPROT	GSK3B	protein	P12830	CDH1	UNIPROT	protein	YES	up-regulates activity	phosphorylation
P49841	UNIPROT	GSK3B	protein	P17676	CEBPB	UNIPROT	protein	YES	up-regulates activity	phosphorylation
P49841	UNIPROT	GSK3B	protein	P16220	CREB1	UNIPROT	protein	YES	up-regulates activity	phosphorylation
P49841	UNIPROT	GSK3B	protein	P49841	GSK3B	UNIPROT	protein	YES	up-regulates activity	phosphorylation
P49841	UNIPROT	GSK3B	protein	P49841	GSK3B	UNIPROT	protein	YES	up-regulates activity	phosphorylation
P49841	UNIPROT	GSK3B	protein	P41236	PPP1R2	UNIPROT	protein	YES	up-regulates activity	phosphorylation
P49841	UNIPROT	GSK3B	protein	Q04206	RELA	UNIPROT	protein	YES	up-regulates activity	phosphorylation
P49841	UNIPROT	GSK3B	protein	P04637	TP53	UNIPROT	protein	YES	up-regulates activity	phosphorylation
P49841	UNIPROT	GSK3B	protein	P49815	TSC2	UNIPROT	protein	YES	up-regulates activity	phosphorylation
P01112	UNIPROT	HRAS	protein	P05412	JUN	UNIPROT	protein	YES	up-regulates activity	phosphorylation
P01112	UNIPROT	HRAS	protein	P05412	JUN	UNIPROT	protein	YES	up-regulates activity	phosphorylation
P01112	UNIPROT	HRAS	protein	P05412	JUN	UNIPROT	protein	YES	up-regulates activity	phosphorylation
P01112	UNIPROT	HRAS	protein	P05412	JUN	UNIPROT	protein	YES	up-regulates activity	phosphorylation
P08069	UNIPROT	IGF1R	protein	P08069	IGF1R	UNIPROT	protein	YES	up-regulates activity	phosphorylation
P08069	UNIPROT	IGF1R	protein	P08069	IGF1R	UNIPROT	protein	YES	up-regulates activity	phosphorylation
P08069	UNIPROT	IGF1R	protein	P08069	IGF1R	UNIPROT	protein	YES	up-regulates activity	phosphorylation
P08069	UNIPROT	IGF1R	protein	P08069	IGF1R	UNIPROT	protein	YES	up-regulates activity	phosphorylation
P08069	UNIPROT	IGF1R	protein	P08069	IGF1R	UNIPROT	protein	YES	up-regulates activity	phosphorylation
P08069	UNIPROT	IGF1R	protein	P08069	IGF1R	UNIPROT	protein	YES	up-regulates activity	phosphorylation
Q9Y6K9	UNIPROT	IKBKG	protein	Q04206	RELA	UNIPROT	protein	YES	up-regulates activity	phosphorylation
P40189	UNIPROT	IL6ST	protein	P40189	IL6ST	UNIPROT	protein	YES	up-regulates activity	phosphorylation
P40189	UNIPROT	IL6ST	protein	P40189	IL6ST	UNIPROT	protein	YES	up-regulates activity	phosphorylation
Q13418	UNIPROT	ILK	protein	P31749	AKT1	UNIPROT	protein	YES	up-regulates activity	phosphorylation
Q13418	UNIPROT	ILK	protein	Q13418	ILK	UNIPROT	protein	YES	up-regulates activity	phosphorylation
Q13418	UNIPROT	ILK	protein	O14950	MYL12B	UNIPROT	protein	YES	up-regulates activity	phosphorylation
Q13418	UNIPROT	ILK	protein	O14950	MYL12B	UNIPROT	protein	YES	up-regulates activity	phosphorylation
P51617	UNIPROT	IRAK1	protein	P51617	IRAK1	UNIPROT	protein	YES	up-regulates activity	phosphorylation
P51617	UNIPROT	IRAK1	protein	P51617	IRAK1	UNIPROT	protein	YES	up-regulates activity	phosphorylation
P51617	UNIPROT	IRAK1	protein	P51617	IRAK1	UNIPROT	protein	YES	up-regulates activity	phosphorylation
P51617	UNIPROT	IRAK1	protein	P51617	IRAK1	UNIPROT	protein	YES	up-regulates activity	phosphorylation
P51617	UNIPROT	IRAK1	protein	P51617	IRAK1	UNIPROT	protein	YES	up-regulates activity	phosphorylation
Q9NWZ3	UNIPROT	IRAK4	protein	P51617	IRAK1	UNIPROT	protein	YES	up-regulates activity	phosphorylation
Q9NWZ3	UNIPROT	IRAK4	protein	P51617	IRAK1	UNIPROT	protein	YES	up-regulates activity	phosphorylation
Q9NWZ3	UNIPROT	IRAK4	protein	P51617	IRAK1	UNIPROT	protein	YES	up-regulates activity	phosphorylation
Q9NWZ3	UNIPROT	IRAK4	protein	P51617	IRAK1	UNIPROT	protein	YES	up-regulates activity	phosphorylation
Q9NWZ3	UNIPROT	IRAK4	protein	Q9NWZ3	IRAK4	UNIPROT	protein	YES	up-regulates activity	phosphorylation
Q9NWZ3	UNIPROT	IRAK4	protein	Q9NWZ3	IRAK4	UNIPROT	protein	YES	up-regulates activity	phosphorylation
Q9NWZ3	UNIPROT	IRAK4	protein	Q9NWZ3	IRAK4	UNIPROT	protein	YES	up-regulates activity	phosphorylation
Q9NWZ3	UNIPROT	IRAK4	protein	Q9NWZ3	IRAK4	UNIPROT	protein	YES	up-regulates activity	phosphorylation
Q02750	UNIPROT	MAP2K1	protein	P49841	GSK3B	UNIPROT	protein	YES	up-regulates activity	phosphorylation
Q02750	UNIPROT	MAP2K1	protein	Q02750	MAP2K1	UNIPROT	protein	YES	up-regulates activity	phosphorylation
Q02750	UNIPROT	MAP2K1	protein	P28482	MAPK1	UNIPROT	protein	YES	up-regulates activity	phosphorylation
Q02750	UNIPROT	MAP2K1	protein	P28482	MAPK1	UNIPROT	protein	YES	up-regulates activity	phosphorylation
Q02750	UNIPROT	MAP2K1	protein	P28482	MAPK1	UNIPROT	protein	YES	up-regulates activity	phosphorylation
P46734	UNIPROT	MAP2K3	protein	Q16539	MAPK14	UNIPROT	protein	YES	up-regulates activity	phosphorylation
P46734	UNIPROT	MAP2K3	protein	Q16539	MAPK14	UNIPROT	protein	YES	up-regulates activity	phosphorylation
P46734	UNIPROT	MAP2K3	protein	Q16539	MAPK14	UNIPROT	protein	YES	up-regulates activity	phosphorylation
P46734	UNIPROT	MAP2K3	protein	Q16539	MAPK14	UNIPROT	protein	YES	up-regulates activity	phosphorylation
P46734	UNIPROT	MAP2K3	protein	Q16539	MAPK14	UNIPROT	protein	YES	up-regulates activity	phosphorylation
P45985	UNIPROT	MAP2K4	protein	P45985	MAP2K4	UNIPROT	protein	YES	up-regulates activity	phosphorylation
P45985	UNIPROT	MAP2K4	protein	P45985	MAP2K4	UNIPROT	protein	YES	up-regulates activity	phosphorylation
P45985	UNIPROT	MAP2K4	protein	Q16539	MAPK14	UNIPROT	protein	YES	up-regulates activity	phosphorylation
P45985	UNIPROT	MAP2K4	protein	Q16539	MAPK14	UNIPROT	protein	YES	up-regulates activity	phosphorylation
P45985	UNIPROT	MAP2K4	protein	P45983	MAPK8	UNIPROT	protein	YES	up-regulates activity	phosphorylation
P45985	UNIPROT	MAP2K4	protein	P45983	MAPK8	UNIPROT	protein	YES	up-regulates activity	phosphorylation
P45985	UNIPROT	MAP2K4	protein	P45983	MAPK8	UNIPROT	protein	YES	up-regulates activity	phosphorylation
P52564	UNIPROT	MAP2K6	protein	P52564	MAP2K6	UNIPROT	protein	YES	up-regulates activity	phosphorylation
P52564	UNIPROT	MAP2K6	protein	O15264	MAPK13	UNIPROT	protein	YES	up-regulates activity	phosphorylation
P52564	UNIPROT	MAP2K6	protein	Q16539	MAPK14	UNIPROT	protein	YES	up-regulates activity	phosphorylation
P52564	UNIPROT	MAP2K6	protein	Q16539	MAPK14	UNIPROT	protein	YES	up-regulates activity	phosphorylation
P52564	UNIPROT	MAP2K6	protein	Q16539	MAPK14	UNIPROT	protein	YES	up-regulates activity	phosphorylation
P52564	UNIPROT	MAP2K6	protein	Q16539	MAPK14	UNIPROT	protein	YES	up-regulates activity	phosphorylation
P52564	UNIPROT	MAP2K6	protein	Q16539	MAPK14	UNIPROT	protein	YES	up-regulates activity	phosphorylation
O14733	UNIPROT	MAP2K7	protein	P45984	MAPK9	UNIPROT	protein	YES	up-regulates activity	phosphorylation
O14733	UNIPROT	MAP2K7	protein	P45984	MAPK9	UNIPROT	protein	YES	up-regulates activity	phosphorylation
O14733	UNIPROT	MAP2K7	protein	P45984	MAPK9	UNIPROT	protein	YES	up-regulates activity	phosphorylation
Q9Y2U5	UNIPROT	MAP3K2	protein	P45985	MAP2K4	UNIPROT	protein	YES	up-regulates activity	phosphorylation
Q9NYL2	UNIPROT	MAP3K20	protein	P45985	MAP2K4	UNIPROT	protein	YES	up-regulates activity	phosphorylation
Q9NYL2	UNIPROT	MAP3K20	protein	O14733	MAP2K7	UNIPROT	protein	YES	up-regulates activity	phosphorylation
Q9NYL2	UNIPROT	MAP3K20	protein	O14733	MAP2K7	UNIPROT	protein	YES	up-regulates activity	phosphorylation
Q99759	UNIPROT	MAP3K3	protein	P46734	MAP2K3	UNIPROT	protein	YES	up-regulates activity	phosphorylation
Q9Y6R4	UNIPROT	MAP3K4	protein	P46734	MAP2K3	UNIPROT	protein	YES	up-regulates activity	phosphorylation
Q9Y6R4	UNIPROT	MAP3K4	protein	P45985	MAP2K4	UNIPROT	protein	YES	up-regulates activity	phosphorylation
Q9Y6R4	UNIPROT	MAP3K4	protein	P52564	MAP2K6	UNIPROT	protein	YES	up-regulates activity	phosphorylation
Q9Y6R4	UNIPROT	MAP3K4	protein	P52564	MAP2K6	UNIPROT	protein	YES	up-regulates activity	phosphorylation
Q9Y6R4	UNIPROT	MAP3K4	protein	Q9Y6R4	MAP3K4	UNIPROT	protein	YES	up-regulates activity	phosphorylation
O43318	UNIPROT	MAP3K7	protein	P46734	MAP2K3	UNIPROT	protein	YES	up-regulates activity	phosphorylation
O43318	UNIPROT	MAP3K7	protein	P46734	MAP2K3	UNIPROT	protein	YES	up-regulates activity	phosphorylation
O43318	UNIPROT	MAP3K7	protein	P45985	MAP2K4	UNIPROT	protein	YES	up-regulates activity	phosphorylation
O43318	UNIPROT	MAP3K7	protein	P52564	MAP2K6	UNIPROT	protein	YES	up-regulates activity	phosphorylation
O43318	UNIPROT	MAP3K7	protein	P52564	MAP2K6	UNIPROT	protein	YES	up-regulates activity	phosphorylation
O43318	UNIPROT	MAP3K7	protein	O43318	MAP3K7	UNIPROT	protein	YES	up-regulates activity	phosphorylation
O43318	UNIPROT	MAP3K7	protein	O43318	MAP3K7	UNIPROT	protein	YES	up-regulates activity	phosphorylation
O43318	UNIPROT	MAP3K7	protein	O43318	MAP3K7	UNIPROT	protein	YES	up-regulates activity	phosphorylation
O43318	UNIPROT	MAP3K7	protein	O43318	MAP3K7	UNIPROT	protein	YES	up-regulates activity	phosphorylation
O43318	UNIPROT	MAP3K7	protein	O43318	MAP3K7	UNIPROT	protein	YES	up-regulates activity	phosphorylation
P28482	UNIPROT	MAPK1	protein	P01100	FOS	UNIPROT	protein	YES	up-regulates activity	phosphorylation
P28482	UNIPROT	MAPK1	protein	P01100	FOS	UNIPROT	protein	YES	up-regulates activity	phosphorylation
P28482	UNIPROT	MAPK1	protein	P01100	FOS	UNIPROT	protein	YES	up-regulates activity	phosphorylation
P28482	UNIPROT	MAPK1	protein	P01100	FOS	UNIPROT	protein	YES	up-regulates activity	phosphorylation
P28482	UNIPROT	MAPK1	protein	Q13480	GAB1	UNIPROT	protein	YES	up-regulates activity	phosphorylation
P28482	UNIPROT	MAPK1	protein	Q13480	GAB1	UNIPROT	protein	YES	up-regulates activity	phosphorylation

P28482	UNIPROT	MAPK1	protein	Q13480	GAB1	UNIPROT	protein	YES	up-regulates activity	phosphorylation
P28482	UNIPROT	MAPK1	protein	Q13480	GAB1	UNIPROT	protein	YES	up-regulates activity	phosphorylation
P28482	UNIPROT	MAPK1	protein	Q13480	GAB1	UNIPROT	protein	YES	up-regulates activity	phosphorylation
P28482	UNIPROT	MAPK1	protein	Q13480	GAB1	UNIPROT	protein	YES	up-regulates activity	phosphorylation
P28482	UNIPROT	MAPK1	protein	P35568	IRS1	UNIPROT	protein	YES	up-regulates activity	phosphorylation
P28482	UNIPROT	MAPK1	protein	P35568	IRS1	UNIPROT	protein	YES	up-regulates activity	phosphorylation
P28482	UNIPROT	MAPK1	protein	P05412	JUN	UNIPROT	protein	YES	up-regulates activity	phosphorylation
P28482	UNIPROT	MAPK1	protein	Q05469	LIPE	UNIPROT	protein	YES	up-regulates activity	phosphorylation
P28482	UNIPROT	MAPK1	protein	P28482	MAPK1	UNIPROT	protein	YES	up-regulates activity	phosphorylation
P28482	UNIPROT	MAPK1	protein	P28482	MAPK1	UNIPROT	protein	YES	up-regulates activity	phosphorylation
P28482	UNIPROT	MAPK1	protein	P01106	MYC	UNIPROT	protein	YES	up-regulates activity	phosphorylation
P28482	UNIPROT	MAPK1	protein	Q9NXR1	NDE1	UNIPROT	protein	YES	up-regulates activity	phosphorylation
P28482	UNIPROT	MAPK1	protein	Q9NXR1	NDE1	UNIPROT	protein	YES	up-regulates activity	phosphorylation
P28482	UNIPROT	MAPK1	protein	Q15418	RPS6KA1	UNIPROT	protein	YES	up-regulates activity	phosphorylation
P28482	UNIPROT	MAPK1	protein	Q15418	RPS6KA1	UNIPROT	protein	YES	up-regulates activity	phosphorylation
P28482	UNIPROT	MAPK1	protein	Q15418	RPS6KA1	UNIPROT	protein	YES	up-regulates activity	phosphorylation
P28482	UNIPROT	MAPK1	protein	Q15418	RPS6KA1	UNIPROT	protein	YES	up-regulates activity	phosphorylation
P28482	UNIPROT	MAPK1	protein	Q15418	RPS6KA1	UNIPROT	protein	YES	up-regulates activity	phosphorylation
P28482	UNIPROT	MAPK1	protein	Q15418	RPS6KA1	UNIPROT	protein	YES	up-regulates activity	phosphorylation
P28482	UNIPROT	MAPK1	protein	Q15418	RPS6KA1	UNIPROT	protein	YES	up-regulates activity	phosphorylation
P28482	UNIPROT	MAPK1	protein	Q15418	RPS6KA1	UNIPROT	protein	YES	up-regulates activity	phosphorylation
Q16539	UNIPROT	MAPK14	protein	P78536	ADAM17	UNIPROT	protein	YES	up-regulates activity	phosphorylation
Q16539	UNIPROT	MAPK14	protein	P15336	ATF2	UNIPROT	protein	YES	up-regulates activity	phosphorylation
Q16539	UNIPROT	MAPK14	protein	P15336	ATF2	UNIPROT	protein	YES	up-regulates activity	phosphorylation
Q16539	UNIPROT	MAPK14	protein	Q15075	EEA1	UNIPROT	protein	YES	up-regulates activity	phosphorylation
Q16539	UNIPROT	MAPK14	protein	Q15910	EZH2	UNIPROT	protein	YES	up-regulates activity	phosphorylation
Q16539	UNIPROT	MAPK14	protein	P49137	MAPKAPK2	UNIPROT	protein	YES	up-regulates activity	phosphorylation
Q16539	UNIPROT	MAPK14	protein	P49137	MAPKAPK2	UNIPROT	protein	YES	up-regulates activity	phosphorylation
Q16539	UNIPROT	MAPK14	protein	P49137	MAPKAPK2	UNIPROT	protein	YES	up-regulates activity	phosphorylation
Q16539	UNIPROT	MAPK14	protein	P49137	MAPKAPK2	UNIPROT	protein	YES	up-regulates activity	phosphorylation
Q16539	UNIPROT	MAPK14	protein	P49137	MAPKAPK2	UNIPROT	protein	YES	up-regulates activity	phosphorylation
Q16539	UNIPROT	MAPK14	protein	P49137	MAPKAPK2	UNIPROT	protein	YES	up-regulates activity	phosphorylation
Q16539	UNIPROT	MAPK14	protein	Q16644	MAPKAPK3	UNIPROT	protein	YES	up-regulates activity	phosphorylation
Q16539	UNIPROT	MAPK14	protein	P47712	PLA2G4A	UNIPROT	protein	YES	up-regulates activity	phosphorylation
Q16539	UNIPROT	MAPK14	protein	P84022	SMAD3	UNIPROT	protein	YES	up-regulates activity	phosphorylation
Q16539	UNIPROT	MAPK14	protein	P84022	SMAD3	UNIPROT	protein	YES	up-regulates activity	phosphorylation
Q16539	UNIPROT	MAPK14	protein	P84022	SMAD3	UNIPROT	protein	YES	up-regulates activity	phosphorylation
Q16539	UNIPROT	MAPK14	protein	P42224	STAT1	UNIPROT	protein	YES	up-regulates activity	phosphorylation
Q16539	UNIPROT	MAPK14	protein	P04637	TP53	UNIPROT	protein	YES	up-regulates activity	phosphorylation
Q16539	UNIPROT	MAPK14	protein	P04637	TP53	UNIPROT	protein	YES	up-regulates activity	phosphorylation
Q16539	UNIPROT	MAPK14	protein	P04637	TP53	UNIPROT	protein	YES	up-regulates activity	phosphorylation
Q16539	UNIPROT	MAPK14	protein	P04637	TP53	UNIPROT	protein	YES	up-regulates activity	phosphorylation
Q16539	UNIPROT	MAPK14	protein	P04637	TP53	UNIPROT	protein	YES	up-regulates activity	phosphorylation
Q16539	UNIPROT	MAPK14	protein	P04637	TP53	UNIPROT	protein	YES	up-regulates activity	phosphorylation
P27361	UNIPROT	MAPK3	protein	Q13480	GAB1	UNIPROT	protein	YES	up-regulates activity	phosphorylation
P27361	UNIPROT	MAPK3	protein	Q13480	GAB1	UNIPROT	protein	YES	up-regulates activity	phosphorylation
P27361	UNIPROT	MAPK3	protein	Q13480	GAB1	UNIPROT	protein	YES	up-regulates activity	phosphorylation
P27361	UNIPROT	MAPK3	protein	Q13480	GAB1	UNIPROT	protein	YES	up-regulates activity	phosphorylation
P27361	UNIPROT	MAPK3	protein	Q13480	GAB1	UNIPROT	protein	YES	up-regulates activity	phosphorylation
P27361	UNIPROT	MAPK3	protein	Q13480	GAB1	UNIPROT	protein	YES	up-regulates activity	phosphorylation
P27361	UNIPROT	MAPK3	protein	P35568	IRS1	UNIPROT	protein	YES	up-regulates activity	phosphorylation
P27361	UNIPROT	MAPK3	protein	P35568	IRS1	UNIPROT	protein	YES	up-regulates activity	phosphorylation
P27361	UNIPROT	MAPK3	protein	Q05469	LIPE	UNIPROT	protein	YES	up-regulates activity	phosphorylation
P27361	UNIPROT	MAPK3	protein	P27361	MAPK3	UNIPROT	protein	YES	up-regulates activity	phosphorylation
P27361	UNIPROT	MAPK3	protein	P27361	MAPK3	UNIPROT	protein	YES	up-regulates activity	phosphorylation
P27361	UNIPROT	MAPK3	protein	P27361	MAPK3	UNIPROT	protein	YES	up-regulates activity	phosphorylation
P27361	UNIPROT	MAPK3	protein	Q15418	RPS6KA1	UNIPROT	protein	YES	up-regulates activity	phosphorylation
P27361	UNIPROT	MAPK3	protein	Q15418	RPS6KA1	UNIPROT	protein	YES	up-regulates activity	phosphorylation
P27361	UNIPROT	MAPK3	protein	Q15418	RPS6KA1	UNIPROT	protein	YES	up-regulates activity	phosphorylation
P27361	UNIPROT	MAPK3	protein	Q15418	RPS6KA1	UNIPROT	protein	YES	up-regulates activity	phosphorylation
P27361	UNIPROT	MAPK3	protein	Q15418	RPS6KA1	UNIPROT	protein	YES	up-regulates activity	phosphorylation
P27361	UNIPROT	MAPK3	protein	Q15418	RPS6KA1	UNIPROT	protein	YES	up-regulates activity	phosphorylation
P27361	UNIPROT	MAPK3	protein	Q15418	RPS6KA1	UNIPROT	protein	YES	up-regulates activity	phosphorylation
P27361	UNIPROT	MAPK3	protein	Q15418	RPS6KA1	UNIPROT	protein	YES	up-regulates activity	phosphorylation
P27361	UNIPROT	MAPK3	protein	Q8N122	RPTOR	UNIPROT	protein	YES	up-regulates activity	phosphorylation
P27361	UNIPROT	MAPK3	protein	Q8N122	RPTOR	UNIPROT	protein	YES	up-regulates activity	phosphorylation
P27361	UNIPROT	MAPK3	protein	Q8N122	RPTOR	UNIPROT	protein	YES	up-regulates activity	phosphorylation
P27361	UNIPROT	MAPK3	protein	Q8N122	RPTOR	UNIPROT	protein	YES	up-regulates activity	phosphorylation
P27361	UNIPROT	MAPK3	protein	Q8N122	RPTOR	UNIPROT	protein	YES	up-regulates activity	phosphorylation
P27361	UNIPROT	MAPK3	protein	Q15796	SMAD2	UNIPROT	protein	YES	up-regulates activity	phosphorylation
P27361	UNIPROT	MAPK3	protein	Q15796	SMAD2	UNIPROT	protein	YES	up-regulates activity	phosphorylation
P27361	UNIPROT	MAPK3	protein	Q15796	SMAD2	UNIPROT	protein	YES	up-regulates activity	phosphorylation
P27361	UNIPROT	MAPK3	protein	P08047	SP1	UNIPROT	protein	YES	up-regulates activity	phosphorylation
P27361	UNIPROT	MAPK3	protein	P08047	SP1	UNIPROT	protein	YES	up-regulates activity	phosphorylation
P27361	UNIPROT	MAPK3	protein	P40763	STAT3	UNIPROT	protein	YES	up-regulates activity	phosphorylation
P45983	UNIPROT	MAPK8	protein	P31749	AKT1	UNIPROT	protein	YES	up-regulates activity	phosphorylation
P45983	UNIPROT	MAPK8	protein	P61978	HNRNPK	UNIPROT	protein	YES	up-regulates activity	phosphorylation
P45983	UNIPROT	MAPK8	protein	P61978	HNRNPK	UNIPROT	protein	YES	up-regulates activity	phosphorylation
P45983	UNIPROT	MAPK8	protein	Q96J02	ITCH	UNIPROT	protein	YES	up-regulates activity	phosphorylation
P45983	UNIPROT	MAPK8	protein	Q96J02	ITCH	UNIPROT	protein	YES	up-regulates activity	phosphorylation
P45983	UNIPROT	MAPK8	protein	Q96J02	ITCH	UNIPROT	protein	YES	up-regulates activity	phosphorylation
P45983	UNIPROT	MAPK8	protein	Q96J02	ITCH	UNIPROT	protein	YES	up-regulates activity	phosphorylation
P45983	UNIPROT	MAPK8	protein	Q96J02	ITCH	UNIPROT	protein	YES	up-regulates activity	phosphorylation
P45983	UNIPROT	MAPK8	protein	P05412	JUN	UNIPROT	protein	YES	up-regulates activity	phosphorylation
P45983	UNIPROT	MAPK8	protein	P05412	JUN	UNIPROT	protein	YES	up-regulates activity	phosphorylation
P45983	UNIPROT	MAPK8	protein	P01106	MYC	UNIPROT	protein	YES	up-regulates activity	phosphorylation
P45983	UNIPROT	MAPK8	protein	P01106	MYC	UNIPROT	protein	YES	up-regulates activity	phosphorylation
P45983	UNIPROT	MAPK8	protein	P49023	PXN	UNIPROT	protein	YES	up-regulates activity	phosphorylation
P45983	UNIPROT	MAPK8	protein	P42224	STAT1	UNIPROT	protein	YES	up-regulates activity	phosphorylation
P45983	UNIPROT	MAPK8	protein	P40763	STAT3	UNIPROT	protein	YES	up-regulates activity	phosphorylation
P45983	UNIPROT	MAPK8	protein	P04637	TP53	UNIPROT	protein	YES	up-regulates activity	phosphorylation
P49137	UNIPROT	MAPKAPK2	protein	P09917	ALOX5	UNIPROT	protein	YES	up-regulates activity	phosphorylation
P49137	UNIPROT	MAPKAPK2	protein	P16220	CREB1	UNIPROT	protein	YES	up-regulates activity	phosphorylation
P49137	UNIPROT	MAPKAPK2	protein	P16220	CREB1	UNIPROT	protein	YES	up-regulates activity	phosphorylation
Q96GX5	UNIPROT	MASTL	protein	P56211	ARPP19	UNIPROT	protein	YES	up-regulates activity	phosphorylation
Q96GX5	UNIPROT	MASTL	protein	O43768	ENSA	UNIPROT	protein	YES	up-regulates activity	phosphorylation
Q96GX5	UNIPROT	MASTL	protein	Q96GX5	MASTL	UNIPROT	protein	YES	up-regulates activity	phosphorylation
P08581	UNIPROT	MET	protein	Q13480	GAB1	UNIPROT	protein	YES	up-regulates activity	phosphorylation

P08581	UNIPROT	MET	protein	Q13480	GAB1	UNIPROT	protein	YES	up-regulates activity	phosphorylation
P08581	UNIPROT	MET	protein	Q13480	GAB1	UNIPROT	protein	YES	up-regulates activity	phosphorylation
P08581	UNIPROT	MET	protein	Q13480	GAB1	UNIPROT	protein	YES	up-regulates activity	phosphorylation
P42345	UNIPROT	MTOR	protein	P31749	AKT1	UNIPROT	protein	YES	up-regulates activity	phosphorylation
P42345	UNIPROT	MTOR	protein	P31749	AKT1	UNIPROT	protein	YES	up-regulates activity	phosphorylation
P42345	UNIPROT	MTOR	protein	P31749	AKT1	UNIPROT	protein	YES	up-regulates activity	phosphorylation
P42345	UNIPROT	MTOR	protein	P42345	MTOR	UNIPROT	protein	YES	up-regulates activity	phosphorylation
P42345	UNIPROT	MTOR	protein	P42345	MTOR	UNIPROT	protein	YES	up-regulates activity	phosphorylation
P42345	UNIPROT	MTOR	protein	Q8N122	RPTOR	UNIPROT	protein	YES	up-regulates activity	phosphorylation
P42345	UNIPROT	MTOR	protein	Q8N122	RPTOR	UNIPROT	protein	YES	up-regulates activity	phosphorylation
P42345	UNIPROT	MTOR	protein	Q8N122	RPTOR	UNIPROT	protein	YES	up-regulates activity	phosphorylation
P42345	UNIPROT	MTOR	protein	Q8N122	RPTOR	UNIPROT	protein	YES	up-regulates activity	phosphorylation
Q8TD19	UNIPROT	NEK9	protein	Q8TDX7	NEK7	UNIPROT	protein	YES	up-regulates activity	phosphorylation
P15531	UNIPROT	NME1	protein	P15531	NME1	UNIPROT	protein	YES	up-regulates activity	phosphorylation
P22392	UNIPROT	NME2	protein	P22392	NME2	UNIPROT	protein	YES	up-regulates activity	phosphorylation
P22392	UNIPROT	NME2	protein	P22392	NME2	UNIPROT	protein	YES	up-regulates activity	phosphorylation
Q13153	UNIPROT	PAK1	protein	Q02750	MAP2K1	UNIPROT	protein	YES	up-regulates activity	phosphorylation
Q13153	UNIPROT	PAK1	protein	Q13153	PAK1	UNIPROT	protein	YES	up-regulates activity	phosphorylation
Q13153	UNIPROT	PAK1	protein	Q13153	PAK1	UNIPROT	protein	YES	up-regulates activity	phosphorylation
Q13177	UNIPROT	PAK2	protein	Q13177	PAK2	UNIPROT	protein	YES	up-regulates activity	phosphorylation
Q13177	UNIPROT	PAK2	protein	Q13177	PAK2	UNIPROT	protein	YES	up-regulates activity	phosphorylation
Q13177	UNIPROT	PAK2	protein	Q13177	PAK2	UNIPROT	protein	YES	up-regulates activity	phosphorylation
Q13177	UNIPROT	PAK2	protein	Q13177	PAK2	UNIPROT	protein	YES	up-regulates activity	phosphorylation
Q13177	UNIPROT	PAK2	protein	Q13177	PAK2	UNIPROT	protein	YES	up-regulates activity	phosphorylation
Q13177	UNIPROT	PAK2	protein	Q13177	PAK2	UNIPROT	protein	YES	up-regulates activity	phosphorylation
Q13177	UNIPROT	PAK2	protein	Q13177	PAK2	UNIPROT	protein	YES	up-regulates activity	phosphorylation
O96013	UNIPROT	PAK4	protein	O96013	PAK4	UNIPROT	protein	YES	up-regulates activity	phosphorylation
O15530	UNIPROT	PDPK1	protein	P31749	AKT1	UNIPROT	protein	YES	up-regulates activity	phosphorylation
O15530	UNIPROT	PDPK1	protein	P31749	AKT1	UNIPROT	protein	YES	up-regulates activity	phosphorylation
O15530	UNIPROT	PDPK1	protein	P31751	AKT2	UNIPROT	protein	YES	up-regulates activity	phosphorylation
O15530	UNIPROT	PDPK1	protein	P31751	AKT2	UNIPROT	protein	YES	up-regulates activity	phosphorylation
O15530	UNIPROT	PDPK1	protein	P31751	AKT2	UNIPROT	protein	YES	up-regulates activity	phosphorylation
O15530	UNIPROT	PDPK1	protein	Q02750	MAP2K1	UNIPROT	protein	YES	up-regulates activity	phosphorylation
O15530	UNIPROT	PDPK1	protein	Q13153	PAK1	UNIPROT	protein	YES	up-regulates activity	phosphorylation
O15530	UNIPROT	PDPK1	protein	O15530	PDPK1	UNIPROT	protein	YES	up-regulates activity	phosphorylation
O15530	UNIPROT	PDPK1	protein	O15530	PDPK1	UNIPROT	protein	YES	up-regulates activity	phosphorylation
O15530	UNIPROT	PDPK1	protein	P53350	PLK1	UNIPROT	protein	YES	up-regulates activity	phosphorylation
O15530	UNIPROT	PDPK1	protein	Q05655	PRKCD	UNIPROT	protein	YES	up-regulates activity	phosphorylation
O15530	UNIPROT	PDPK1	protein	Q15418	RPS6KA1	UNIPROT	protein	YES	up-regulates activity	phosphorylation
O15530	UNIPROT	PDPK1	protein	Q9HBY8	SGK2	UNIPROT	protein	YES	up-regulates activity	phosphorylation
O15530	UNIPROT	PDPK1	protein	Q9HBY8	SGK2	UNIPROT	protein	YES	up-regulates activity	phosphorylation
P46020	UNIPROT	PHKA1	protein	P46020	PHKA1	UNIPROT	protein	YES	up-regulates activity	phosphorylation
P46020	UNIPROT	PHKA1	protein	P46020	PHKA1	UNIPROT	protein	YES	up-regulates activity	phosphorylation
P46020	UNIPROT	PHKA1	protein	P46020	PHKA1	UNIPROT	protein	YES	up-regulates activity	phosphorylation
Q16512	UNIPROT	PKN1	protein	Q16512	PKN1	UNIPROT	protein	YES	up-regulates activity	phosphorylation
Q16512	UNIPROT	PKN1	protein	Q16512	PKN1	UNIPROT	protein	YES	up-regulates activity	phosphorylation
Q16512	UNIPROT	PKN1	protein	Q16512	PKN1	UNIPROT	protein	YES	up-regulates activity	phosphorylation
P53350	UNIPROT	PLK1	protein	P14635	CCNB1	UNIPROT	protein	YES	up-regulates activity	phosphorylation
P53350	UNIPROT	PLK1	protein	P14635	CCNB1	UNIPROT	protein	YES	up-regulates activity	phosphorylation
P53350	UNIPROT	PLK1	protein	P14635	CCNB1	UNIPROT	protein	YES	up-regulates activity	phosphorylation
P53350	UNIPROT	PLK1	protein	P14635	CCNB1	UNIPROT	protein	YES	up-regulates activity	phosphorylation
P53350	UNIPROT	PLK1	protein	P01106	MYC	UNIPROT	protein	YES	up-regulates activity	phosphorylation
P53350	UNIPROT	PLK1	protein	Q9Y266	NUDC	UNIPROT	protein	YES	up-regulates activity	phosphorylation
P53350	UNIPROT	PLK1	protein	Q9Y266	NUDC	UNIPROT	protein	YES	up-regulates activity	phosphorylation
P67775	UNIPROT	PPP2CA	protein	O43524	FOXO3	UNIPROT	protein	YES	up-regulates activity	phosphorylation
Q13131	UNIPROT	PRKAA1	protein	O00418	EEF2K	UNIPROT	protein	YES	up-regulates activity	phosphorylation
Q13131	UNIPROT	PRKAA1	protein	O00418	EEF2K	UNIPROT	protein	YES	up-regulates activity	phosphorylation
Q13131	UNIPROT	PRKAA1	protein	O43524	FOXO3	UNIPROT	protein	YES	up-regulates activity	phosphorylation
Q13131	UNIPROT	PRKAA1	protein	O43524	FOXO3	UNIPROT	protein	YES	up-regulates activity	phosphorylation
Q13131	UNIPROT	PRKAA1	protein	O43524	FOXO3	UNIPROT	protein	YES	up-regulates activity	phosphorylation
Q13131	UNIPROT	PRKAA1	protein	O43524	FOXO3	UNIPROT	protein	YES	up-regulates activity	phosphorylation
Q13131	UNIPROT	PRKAA1	protein	O43524	FOXO3	UNIPROT	protein	YES	up-regulates activity	phosphorylation
Q13131	UNIPROT	PRKAA1	protein	O43524	FOXO3	UNIPROT	protein	YES	up-regulates activity	phosphorylation
Q13131	UNIPROT	PRKAA1	protein	O43524	FOXO3	UNIPROT	protein	YES	up-regulates activity	phosphorylation
Q13131	UNIPROT	PRKAA1	protein	O43524	FOXO3	UNIPROT	protein	YES	up-regulates activity	phosphorylation
Q13131	UNIPROT	PRKAA1	protein	Q9GZY8	MFF	UNIPROT	protein	YES	up-regulates activity	phosphorylation
Q13131	UNIPROT	PRKAA1	protein	Q9GZY8	MFF	UNIPROT	protein	YES	up-regulates activity	phosphorylation
Q13131	UNIPROT	PRKAA1	protein	O60825	PFKFB2	UNIPROT	protein	YES	up-regulates activity	phosphorylation
P17612	UNIPROT	PRKACA	protein	P53396	ACLY	UNIPROT	protein	YES	up-regulates activity	phosphorylation
P17612	UNIPROT	PRKACA	protein	P18846	ATF1	UNIPROT	protein	YES	up-regulates activity	phosphorylation
P17612	UNIPROT	PRKACA	protein	P16220	CREB1	UNIPROT	protein	YES	up-regulates activity	phosphorylation
P17612	UNIPROT	PRKACA	protein	P16220	CREB1	UNIPROT	protein	YES	up-regulates activity	phosphorylation
P17612	UNIPROT	PRKACA	protein	P16220	CREB1	UNIPROT	protein	YES	up-regulates activity	phosphorylation
P17612	UNIPROT	PRKACA	protein	P16220	CREB1	UNIPROT	protein	YES	up-regulates activity	phosphorylation
P17612	UNIPROT	PRKACA	protein	P41240	CSK	UNIPROT	protein	YES	up-regulates activity	phosphorylation
P17612	UNIPROT	PRKACA	protein	P35222	CTNNB1	UNIPROT	protein	YES	up-regulates activity	phosphorylation
P17612	UNIPROT	PRKACA	protein	P35222	CTNNB1	UNIPROT	protein	YES	up-regulates activity	phosphorylation
P17612	UNIPROT	PRKACA	protein	P35222	CTNNB1	UNIPROT	protein	YES	up-regulates activity	phosphorylation
P17612	UNIPROT	PRKACA	protein	Q93034	CUL5	UNIPROT	protein	YES	up-regulates activity	phosphorylation
P17612	UNIPROT	PRKACA	protein	O00418	EEF2K	UNIPROT	protein	YES	up-regulates activity	phosphorylation
P17612	UNIPROT	PRKACA	protein	O00418	EEF2K	UNIPROT	protein	YES	up-regulates activity	phosphorylation
P17612	UNIPROT	PRKACA	protein	P60983	GMFB	UNIPROT	protein	YES	up-regulates activity	phosphorylation
P17612	UNIPROT	PRKACA	protein	P60983	GMFB	UNIPROT	protein	YES	up-regulates activity	phosphorylation
P17612	UNIPROT	PRKACA	protein	P25098	GRK2	UNIPROT	protein	YES	up-regulates activity	phosphorylation
P17612	UNIPROT	PRKACA	protein	Q14847	LASP1	UNIPROT	protein	YES	up-regulates activity	phosphorylation
P17612	UNIPROT	PRKACA	protein	Q14847	LASP1	UNIPROT	protein	YES	up-regulates activity	phosphorylation
P17612	UNIPROT	PRKACA	protein	Q05469	LIPE	UNIPROT	protein	YES	up-regulates activity	phosphorylation
P17612	UNIPROT	PRKACA	protein	Q05469	LIPE	UNIPROT	protein	YES	up-regulates activity	phosphorylation
P17612	UNIPROT	PRKACA	protein	Q05469	LIPE	UNIPROT	protein	YES	up-regulates activity	phosphorylation
P17612	UNIPROT	PRKACA	protein	Q07954	LRP1	UNIPROT	protein	YES	up-regulates activity	phosphorylation
P17612	UNIPROT	PRKACA	protein	Q14978	NOLC1	UNIPROT	protein	YES	up-regulates activity	phosphorylation
P17612	UNIPROT	PRKACA	protein	Q14978	NOLC1	UNIPROT	protein	YES	up-regulates activity	phosphorylation
P17612	UNIPROT	PRKACA	protein	O60825	PFKFB2	UNIPROT	protein	YES	up-regulates activity	phosphorylation
P17612	UNIPROT	PRKACA	protein	P46020	PHKA1	UNIPROT	protein	YES	up-regulates activity	phosphorylation
P17612	UNIPROT	PRKACA	protein	O95295	SNAPIN	UNIPROT	protein	YES	up-regulates activity	phosphorylation
P17612	UNIPROT	PRKACA	protein	P12931	SRC	UNIPROT	protein	YES	up-regulates activity	phosphorylation
P17612	UNIPROT	PRKACA	protein	P52888	THOP1	UNIPROT	protein	YES	up-regulates activity	phosphorylation
P17612	UNIPROT	PRKACA	protein	P50552	VASP	UNIPROT	protein	YES	up-regulates activity	phosphorylation

P17612	UNIPROT	PRKACA	protein	P50552	VASP	UNIPROT	protein	YES	up-regulates activity	phosphorylation
P13861	UNIPROT	PRKAR2A	protein	P13861	PRKAR2A	UNIPROT	protein	YES	up-regulates activity	phosphorylation
P17252	UNIPROT	PRKCA	protein	P25098	GRK2	UNIPROT	protein	YES	up-regulates activity	phosphorylation
P17252	UNIPROT	PRKCA	protein	P02545	LMNA	UNIPROT	protein	YES	up-regulates activity	phosphorylation
P17252	UNIPROT	PRKCA	protein	P02545	LMNA	UNIPROT	protein	YES	up-regulates activity	phosphorylation
P17252	UNIPROT	PRKCA	protein	Q92686	NRGN	UNIPROT	protein	YES	up-regulates activity	phosphorylation
P17252	UNIPROT	PRKCA	protein	Q16625	OCLN	UNIPROT	protein	YES	up-regulates activity	phosphorylation
P17252	UNIPROT	PRKCA	protein	O60825	PFKFB2	UNIPROT	protein	YES	up-regulates activity	phosphorylation
P17252	UNIPROT	PRKCA	protein	P31431	SDC4	UNIPROT	protein	YES	up-regulates activity	phosphorylation
P17252	UNIPROT	PRKCA	protein	P04637	TP53	UNIPROT	protein	YES	up-regulates activity	phosphorylation
Q05655	UNIPROT	PRKCD	protein	P25098	GRK2	UNIPROT	protein	YES	up-regulates activity	phosphorylation
Q05655	UNIPROT	PRKCD	protein	P40189	IL6ST	UNIPROT	protein	YES	up-regulates activity	phosphorylation
Q05655	UNIPROT	PRKCD	protein	Q13887	KLF5	UNIPROT	protein	YES	up-regulates activity	phosphorylation
Q05655	UNIPROT	PRKCD	protein	P18433	PTPRA	UNIPROT	protein	YES	up-regulates activity	phosphorylation
Q05655	UNIPROT	PRKCD	protein	P18433	PTPRA	UNIPROT	protein	YES	up-regulates activity	phosphorylation
Q05655	UNIPROT	PRKCD	protein	P12931	SRC	UNIPROT	protein	YES	up-regulates activity	phosphorylation
P78527	UNIPROT	PRKDC	protein	P31749	AKT1	UNIPROT	protein	YES	up-regulates activity	phosphorylation
P78527	UNIPROT	PRKDC	protein	Q9H4A6	GOLPH3	UNIPROT	protein	YES	up-regulates activity	phosphorylation
P78527	UNIPROT	PRKDC	protein	Q9H4A6	GOLPH3	UNIPROT	protein	YES	up-regulates activity	phosphorylation
P78527	UNIPROT	PRKDC	protein	P78527	PRKDC	UNIPROT	protein	YES	up-regulates activity	phosphorylation
P78527	UNIPROT	PRKDC	protein	P78527	PRKDC	UNIPROT	protein	YES	up-regulates activity	phosphorylation
P78527	UNIPROT	PRKDC	protein	P78527	PRKDC	UNIPROT	protein	YES	up-regulates activity	phosphorylation
P78527	UNIPROT	PRKDC	protein	P78527	PRKDC	UNIPROT	protein	YES	up-regulates activity	phosphorylation
P78527	UNIPROT	PRKDC	protein	P78527	PRKDC	UNIPROT	protein	YES	up-regulates activity	phosphorylation
P78527	UNIPROT	PRKDC	protein	P78527	PRKDC	UNIPROT	protein	YES	up-regulates activity	phosphorylation
P78527	UNIPROT	PRKDC	protein	P78527	PRKDC	UNIPROT	protein	YES	up-regulates activity	phosphorylation
Q05397	UNIPROT	PTK2	protein	O75962	TRIO	UNIPROT	protein	YES	up-regulates activity	phosphorylation
O43353	UNIPROT	RIPK2	protein	O43353	RIPK2	UNIPROT	protein	YES	up-regulates activity	phosphorylation
Q13464	UNIPROT	ROCK1	protein	P45983	MAPK8	UNIPROT	protein	YES	up-regulates activity	phosphorylation
Q13464	UNIPROT	ROCK1	protein	P26038	MSN	UNIPROT	protein	YES	up-regulates activity	phosphorylation
Q13464	UNIPROT	ROCK1	protein	P35241	RDX	UNIPROT	protein	YES	up-regulates activity	phosphorylation
Q15418	UNIPROT	RPS6KA1	protein	P16220	CREB1	UNIPROT	protein	YES	up-regulates activity	phosphorylation
Q15418	UNIPROT	RPS6KA1	protein	Q15418	RPS6KA1	UNIPROT	protein	YES	up-regulates activity	phosphorylation
P51812	UNIPROT	RPS6KA3	protein	P16220	CREB1	UNIPROT	protein	YES	up-regulates activity	phosphorylation
P51812	UNIPROT	RPS6KA3	protein	O60825	PFKFB2	UNIPROT	protein	YES	up-regulates activity	phosphorylation
P51812	UNIPROT	RPS6KA3	protein	O60825	PFKFB2	UNIPROT	protein	YES	up-regulates activity	phosphorylation
O75676	UNIPROT	RPS6KA4	protein	P18846	ATF1	UNIPROT	protein	YES	up-regulates activity	phosphorylation
P12931	UNIPROT	SRC	protein	P31749	AKT1	UNIPROT	protein	YES	up-regulates activity	phosphorylation
P12931	UNIPROT	SRC	protein	P31749	AKT1	UNIPROT	protein	YES	up-regulates activity	phosphorylation
P12931	UNIPROT	SRC	protein	P31749	AKT1	UNIPROT	protein	YES	up-regulates activity	phosphorylation
P12931	UNIPROT	SRC	protein	P10398	ARAF	UNIPROT	protein	YES	up-regulates activity	phosphorylation
P12931	UNIPROT	SRC	protein	P56945	BCAR1	UNIPROT	protein	YES	up-regulates activity	phosphorylation
P12931	UNIPROT	SRC	protein	P13688	CEACAM1	UNIPROT	protein	YES	up-regulates activity	phosphorylation
P12931	UNIPROT	SRC	protein	P13688	CEACAM1	UNIPROT	protein	YES	up-regulates activity	phosphorylation
P12931	UNIPROT	SRC	protein	P35222	CTNNB1	UNIPROT	protein	YES	up-regulates activity	phosphorylation
P12931	UNIPROT	SRC	protein	O60716	CTNND1	UNIPROT	protein	YES	up-regulates activity	phosphorylation
P12931	UNIPROT	SRC	protein	O60716	CTNND1	UNIPROT	protein	YES	up-regulates activity	phosphorylation
P12931	UNIPROT	SRC	protein	O60716	CTNND1	UNIPROT	protein	YES	up-regulates activity	phosphorylation
P12931	UNIPROT	SRC	protein	O60716	CTNND1	UNIPROT	protein	YES	up-regulates activity	phosphorylation
P12931	UNIPROT	SRC	protein	O60716	CTNND1	UNIPROT	protein	YES	up-regulates activity	phosphorylation
P12931	UNIPROT	SRC	protein	O60716	CTNND1	UNIPROT	protein	YES	up-regulates activity	phosphorylation
P12931	UNIPROT	SRC	protein	O60716	CTNND1	UNIPROT	protein	YES	up-regulates activity	phosphorylation
P12931	UNIPROT	SRC	protein	O60716	CTNND1	UNIPROT	protein	YES	up-regulates activity	phosphorylation
P12931	UNIPROT	SRC	protein	Q05193	DNM1	UNIPROT	protein	YES	up-regulates activity	phosphorylation
P12931	UNIPROT	SRC	protein	Q05193	DNM1	UNIPROT	protein	YES	up-regulates activity	phosphorylation
P12931	UNIPROT	SRC	protein	P00533	EGFR	UNIPROT	protein	YES	up-regulates activity	phosphorylation
P12931	UNIPROT	SRC	protein	P00533	EGFR	UNIPROT	protein	YES	up-regulates activity	phosphorylation
P12931	UNIPROT	SRC	protein	P00533	EGFR	UNIPROT	protein	YES	up-regulates activity	phosphorylation
P12931	UNIPROT	SRC	protein	P00533	EGFR	UNIPROT	protein	YES	up-regulates activity	phosphorylation
P12931	UNIPROT	SRC	protein	Q13480	GAB1	UNIPROT	protein	YES	up-regulates activity	phosphorylation
P12931	UNIPROT	SRC	protein	Q13480	GAB1	UNIPROT	protein	YES	up-regulates activity	phosphorylation
P12931	UNIPROT	SRC	protein	Q13480	GAB1	UNIPROT	protein	YES	up-regulates activity	phosphorylation
P12931	UNIPROT	SRC	protein	Q13480	GAB1	UNIPROT	protein	YES	up-regulates activity	phosphorylation
P12931	UNIPROT	SRC	protein	P78347	GTF2I	UNIPROT	protein	YES	up-regulates activity	phosphorylation
P12931	UNIPROT	SRC	protein	P78347	GTF2I	UNIPROT	protein	YES	up-regulates activity	phosphorylation
P12931	UNIPROT	SRC	protein	P08069	IGF1R	UNIPROT	protein	YES	up-regulates activity	phosphorylation
P12931	UNIPROT	SRC	protein	P08069	IGF1R	UNIPROT	protein	YES	up-regulates activity	phosphorylation
P12931	UNIPROT	SRC	protein	P08069	IGF1R	UNIPROT	protein	YES	up-regulates activity	phosphorylation
P12931	UNIPROT	SRC	protein	P08069	IGF1R	UNIPROT	protein	YES	up-regulates activity	phosphorylation
P12931	UNIPROT	SRC	protein	P08069	IGF1R	UNIPROT	protein	YES	up-regulates activity	phosphorylation
P12931	UNIPROT	SRC	protein	P14923	JUP	UNIPROT	protein	YES	up-regulates activity	phosphorylation
P12931	UNIPROT	SRC	protein	Q07954	LRP1	UNIPROT	protein	YES	up-regulates activity	phosphorylation
P12931	UNIPROT	SRC	protein	O15530	PDPK1	UNIPROT	protein	YES	up-regulates activity	phosphorylation
P12931	UNIPROT	SRC	protein	O15162	PLSCR1	UNIPROT	protein	YES	up-regulates activity	phosphorylation
P12931	UNIPROT	SRC	protein	O15162	PLSCR1	UNIPROT	protein	YES	up-regulates activity	phosphorylation
P12931	UNIPROT	SRC	protein	P18433	PTPRA	UNIPROT	protein	YES	up-regulates activity	phosphorylation
P12931	UNIPROT	SRC	protein	P29353	SHC1	UNIPROT	protein	YES	up-regulates activity	phosphorylation
P12931	UNIPROT	SRC	protein	P29353	SHC1	UNIPROT	protein	YES	up-regulates activity	phosphorylation
P12931	UNIPROT	SRC	protein	Q9UGK3	STAP2	UNIPROT	protein	YES	up-regulates activity	phosphorylation
P12931	UNIPROT	SRC	protein	Q9UGK3	STAP2	UNIPROT	protein	YES	up-regulates activity	phosphorylation
P12931	UNIPROT	SRC	protein	P42224	STAT1	UNIPROT	protein	YES	up-regulates activity	phosphorylation
P12931	UNIPROT	SRC	protein	P40763	STAT3	UNIPROT	protein	YES	up-regulates activity	phosphorylation
P12931	UNIPROT	SRC	protein	P40763	STAT3	UNIPROT	protein	YES	up-regulates activity	phosphorylation
P12931	UNIPROT	SRC	protein	P09327	VIL1	UNIPROT	protein	YES	up-regulates activity	phosphorylation
P12931	UNIPROT	SRC	protein	P09327	VIL1	UNIPROT	protein	YES	up-regulates activity	phosphorylation
P12931	UNIPROT	SRC	protein	P09327	VIL1	UNIPROT	protein	YES	up-regulates activity	phosphorylation
Q13043	UNIPROT	STK4	protein	Q13043	STK4	UNIPROT	protein	YES	up-regulates activity	phosphorylation
Q13043	UNIPROT	STK4	protein	Q13043	STK4	UNIPROT	protein	YES	up-regulates activity	phosphorylation
Q13043	UNIPROT	STK4	protein	Q13043	STK4	UNIPROT	protein	YES	up-regulates activity	phosphorylation
Q13043	UNIPROT	STK4	protein	Q13043	STK4	UNIPROT	protein	YES	up-regulates activity	phosphorylation
P43405	UNIPROT	SYK	protein	P17252	PRKCA	UNIPROT	protein	YES	up-regulates activity	phosphorylation
P43405	UNIPROT	SYK	protein	P78314	SH3BP2	UNIPROT	protein	YES	up-regulates activity	phosphorylation
P43405	UNIPROT	SYK	protein	P78314	SH3BP2	UNIPROT	protein	YES	up-regulates activity	phosphorylation
P43405	UNIPROT	SYK	protein	P78314	SH3BP2	UNIPROT	protein	YES	up-regulates activity	phosphorylation
P43405	UNIPROT	SYK	protein	P43405	SYK	UNIPROT	protein	YES	up-regulates activity	phosphorylation
P43405	UNIPROT	SYK	protein	P43405	SYK	UNIPROT	protein	YES	up-regulates activity	phosphorylation
P43405	UNIPROT	SYK	protein	P43405	SYK	UNIPROT	protein	YES	up-regulates activity	phosphorylation
P43405	UNIPROT	SYK	protein	P43405	SYK	UNIPROT	protein	YES	up-regulates activity	phosphorylation

P43405	UNIPROT	SYK	protein	P43405	SYK	UNIPROT	protein	YES	up-regulates activity	phosphorylation
P43405	UNIPROT	SYK	protein	P43405	SYK	UNIPROT	protein	YES	up-regulates activity	phosphorylation
P43405	UNIPROT	SYK	protein	P68366	TUBA4A	UNIPROT	protein	YES	up-regulates activity	phosphorylation
Q7L7X3	UNIPROT	TAOK1	protein	P46734	MAP2K3	UNIPROT	protein	YES	up-regulates activity	phosphorylation
Q9UL54	UNIPROT	TAOK2	protein	P46734	MAP2K3	UNIPROT	protein	YES	up-regulates activity	phosphorylation
Q9UL54	UNIPROT	TAOK2	protein	P52564	MAP2K6	UNIPROT	protein	YES	up-regulates activity	phosphorylation
Q9H2K8	UNIPROT	TAOK3	protein	Q9H2K8	TAOK3	UNIPROT	protein	YES	up-regulates activity	phosphorylation
Q9H2K8	UNIPROT	TAOK3	protein	Q9H2K8	TAOK3	UNIPROT	protein	YES	up-regulates activity	phosphorylation
Q9UHD2	UNIPROT	TBK1	protein	Q14653	IRF3	UNIPROT	protein	YES	up-regulates activity	phosphorylation
Q9UHD2	UNIPROT	TBK1	protein	Q14653	IRF3	UNIPROT	protein	YES	up-regulates activity	phosphorylation
Q9UHD2	UNIPROT	TBK1	protein	Q14653	IRF3	UNIPROT	protein	YES	up-regulates activity	phosphorylation
Q9UHD2	UNIPROT	TBK1	protein	Q14653	IRF3	UNIPROT	protein	YES	up-regulates activity	phosphorylation
Q9UHD2	UNIPROT	TBK1	protein	Q14653	IRF3	UNIPROT	protein	YES	up-regulates activity	phosphorylation
Q9UHD2	UNIPROT	TBK1	protein	Q14653	IRF3	UNIPROT	protein	YES	up-regulates activity	phosphorylation
Q9UHD2	UNIPROT	TBK1	protein	Q14653	IRF3	UNIPROT	protein	YES	up-regulates activity	phosphorylation
Q9UHD2	UNIPROT	TBK1	protein	Q14653	IRF3	UNIPROT	protein	YES	up-regulates activity	phosphorylation
P37173	UNIPROT	TGFBR2	protein	P37173	TGFBR2	UNIPROT	protein	YES	up-regulates activity	phosphorylation
P37173	UNIPROT	TGFBR2	protein	P37173	TGFBR2	UNIPROT	protein	YES	up-regulates activity	phosphorylation
P37173	UNIPROT	TGFBR2	protein	P37173	TGFBR2	UNIPROT	protein	YES	up-regulates activity	phosphorylation
P37173	UNIPROT	TGFBR2	protein	P37173	TGFBR2	UNIPROT	protein	YES	up-regulates activity	phosphorylation
P37173	UNIPROT	TGFBR2	protein	P37173	TGFBR2	UNIPROT	protein	YES	up-regulates activity	phosphorylation
Q9UKI8	UNIPROT	TLK1	protein	P68431	HIST1H3A	UNIPROT	protein	YES	up-regulates activity	phosphorylation
P33981	UNIPROT	TTK	protein	Q13257	MAD2L1	UNIPROT	protein	YES	up-regulates activity	phosphorylation
P07947	UNIPROT	YES1	protein	P07947	YES1	UNIPROT	protein	YES	up-regulates activity	phosphorylation
P31749	UNIPROT	AKT1	protein	P54253	ATXN1	UNIPROT	protein	YES	up-regulates quantity by stabilization	phosphorylation
P31749	UNIPROT	AKT1	protein	P98170	XIAP	UNIPROT	protein	YES	up-regulates quantity by stabilization	phosphorylation
O96017	UNIPROT	CHEK2	protein	P04637	TP53	UNIPROT	protein	YES	up-regulates quantity by stabilization	phosphorylation
O96017	UNIPROT	CHEK2	protein	P04637	TP53	UNIPROT	protein	YES	up-regulates quantity by stabilization	phosphorylation
O96017	UNIPROT	CHEK2	protein	P04637	TP53	UNIPROT	protein	YES	up-regulates quantity by stabilization	phosphorylation
O96017	UNIPROT	CHEK2	protein	P04637	TP53	UNIPROT	protein	YES	up-regulates quantity by stabilization	phosphorylation
O96017	UNIPROT	CHEK2	protein	P04637	TP53	UNIPROT	protein	YES	up-regulates quantity by stabilization	phosphorylation
O96017	UNIPROT	CHEK2	protein	P04637	TP53	UNIPROT	protein	YES	up-regulates quantity by stabilization	phosphorylation
O96017	UNIPROT	CHEK2	protein	P04637	TP53	UNIPROT	protein	YES	up-regulates quantity by stabilization	phosphorylation
O96017	UNIPROT	CHEK2	protein	P04637	TP53	UNIPROT	protein	YES	up-regulates quantity by stabilization	phosphorylation
O96017	UNIPROT	CHEK2	protein	P04637	TP53	UNIPROT	protein	YES	up-regulates quantity by stabilization	phosphorylation
O96017	UNIPROT	CHEK2	protein	P04637	TP53	UNIPROT	protein	YES	up-regulates quantity by stabilization	phosphorylation
O96017	UNIPROT	CHEK2	protein	P04637	TP53	UNIPROT	protein	YES	up-regulates quantity by stabilization	phosphorylation
O96017	UNIPROT	CHEK2	protein	P04637	TP53	UNIPROT	protein	YES	up-regulates quantity by stabilization	phosphorylation
O96017	UNIPROT	CHEK2	protein	P04637	TP53	UNIPROT	protein	YES	up-regulates quantity by stabilization	phosphorylation
O96017	UNIPROT	CHEK2	protein	P04637	TP53	UNIPROT	protein	YES	up-regulates quantity by stabilization	phosphorylation
O96017	UNIPROT	CHEK2	protein	P04637	TP53	UNIPROT	protein	YES	up-regulates quantity by stabilization	phosphorylation
O96017	UNIPROT	CHEK2	protein	P04637	TP53	UNIPROT	protein	YES	up-regulates quantity by stabilization	phosphorylation
O96017	UNIPROT	CHEK2	protein	P04637	TP53	UNIPROT	protein	YES	up-regulates quantity by stabilization	phosphorylation
O96017	UNIPROT	CHEK2	protein	P04637	TP53	UNIPROT	protein	YES	up-regulates quantity by stabilization	phosphorylation
P49841	UNIPROT	GSK3B	protein	P19838	NFKB1	UNIPROT	protein	YES	up-regulates quantity by stabilization	phosphorylation
P49841	UNIPROT	GSK3B	protein	P19838	NFKB1	UNIPROT	protein	YES	up-regulates quantity by stabilization	phosphorylation
Q16539	UNIPROT	MAPK14	protein	P04637	TP53	UNIPROT	protein	YES	up-regulates quantity by stabilization	phosphorylation
P45983	UNIPROT	MAPK8	protein	P04637	TP53	UNIPROT	protein	YES	up-regulates quantity by stabilization	phosphorylation
P31749	UNIPROT	AKT1	protein	Q12778	FOXO1	UNIPROT	protein	YES	down-regulates	relocalization
Q96CW1	UNIPROT	AP2M1	protein	P00533	EGFR	UNIPROT	protein	YES	down-regulates	relocalization
Q9H6Z4	UNIPROT	RANBP3	protein	P84022	SMAD3	UNIPROT	protein	YES	down-regulates	relocalization
Q9H6Z4	UNIPROT	RANBP3	protein	Q15796	SMAD2	UNIPROT	protein	YES	down-regulates activity	relocalization
Q9H6Z4	UNIPROT	RANBP3	protein	Q15796	SMAD2	UNIPROT	protein	YES	down-regulates activity	relocalization
Q9H6Z4	UNIPROT	RANBP3	protein	P84022	SMAD3	UNIPROT	protein	YES	down-regulates activity	relocalization
Q9H6Z4	UNIPROT	RANBP3	protein	P84022	SMAD3	UNIPROT	protein	YES	down-regulates activity	relocalization
Q07812	UNIPROT	BAX	protein	Q9NR28	DIABLO	UNIPROT	protein	YES	up-regulates	relocalization
Q07812	UNIPROT	BAX	protein	O43464	HTRA2	UNIPROT	protein	YES	up-regulates	relocalization
P00533	UNIPROT	EGFR	protein	P22681	CBL	UNIPROT	protein	YES	up-regulates	relocalization
P00533	UNIPROT	EGFR	protein	P22681	CBL	UNIPROT	protein	YES	up-regulates	relocalization
P04626	UNIPROT	ERBB2	protein	P62993	GRB2	UNIPROT	protein	YES	up-regulates	relocalization
P62993	UNIPROT	GRB2	protein	P22681	CBL	UNIPROT	protein	YES	up-regulates	relocalization
Q8NEB9	UNIPROT	PIK3C3	protein	P31749	AKT1	UNIPROT	protein	YES	up-regulates	relocalization
P53350	UNIPROT	PLK1	protein	Q2NKX8	ERCC6L	UNIPROT	protein	YES	up-regulates	relocalization
P62993	UNIPROT	GRB2	protein	Q07889	SOS1	UNIPROT	protein	YES	up-regulates activity	relocalization
P62993	UNIPROT	GRB2	protein	Q07889	SOS1	UNIPROT	protein	YES	up-regulates activity	relocalization
P62993	UNIPROT	GRB2	protein	Q07889	SOS1	UNIPROT	protein	YES	up-regulates activity	relocalization
Q9GZY8	UNIPROT	MFF	protein	O00429	DNM1L	UNIPROT	protein	YES	up-regulates activity	relocalization
P15056	UNIPROT	BRAF	protein	P01137	TGFB1	UNIPROT	protein	YES	up-regulates quantity	relocalization
O75925	UNIPROT	PIAS1	protein	P17844	DDX5	UNIPROT	protein	YES	down-regulates	sumoylation
O75925	UNIPROT	PIAS1	protein	P17844	DDX5	UNIPROT	protein	YES	up-regulates	sumoylation
O75925	UNIPROT	PIAS1	protein	P15927	RPA2	UNIPROT	protein	YES	up-regulates	sumoylation
O75925	UNIPROT	PIAS1	protein	Q12888	TP53BP1	UNIPROT	protein	YES	up-regulates	sumoylation
O75925	UNIPROT	PIAS1	protein	P31749	AKT1	UNIPROT	protein	YES	up-regulates activity	sumoylation
Q13363	UNIPROT	CTBP1	protein	O95471	CLDN7	UNIPROT	protein	YES	down-regulates quantity by repression	transcriptional regulation
Q13547	UNIPROT	HDAC1	protein	O95471	CLDN7	UNIPROT	protein	YES	down-regulates quantity by repression	transcriptional regulation
P84022	UNIPROT	SMAD3	protein	P01106	MYC	UNIPROT	protein	YES	down-regulates quantity by repression	transcriptional regulation
P17676	UNIPROT	CEBPB	protein	Q13887	KLF5	UNIPROT	protein	YES	up-regulates	transcriptional regulation
O43524	UNIPROT	FOXO3	protein	P46527	CDKN1B	UNIPROT	protein	YES	up-regulates quantity	transcriptional regulation
O43524	UNIPROT	FOXO3	protein	O43524	FOXO3	UNIPROT	protein	YES	up-regulates quantity	transcriptional regulation
Q04206	UNIPROT	RELA	protein	P08581	MET	UNIPROT	protein	YES	up-regulates quantity	transcriptional regulation
P35226	UNIPROT	BMI1	protein	P35226	BMI1	UNIPROT	protein	YES	up-regulates quantity by expression	transcriptional regulation
P17676	UNIPROT	CEBPB	protein	P21291	CSRP1	UNIPROT	protein	YES	up-regulates quantity by expression	transcriptional regulation
P17676	UNIPROT	CEBPB	protein	Q99988	GDF15	UNIPROT	protein	YES	up-regulates quantity by expression	transcriptional regulation
P17676	UNIPROT	CEBPB	protein	P17174	GOT1	UNIPROT	protein	YES	up-regulates quantity by expression	transcriptional regulation
P17676	UNIPROT	CEBPB	protein	P41440	SLC19A1	UNIPROT	protein	YES	up-regulates quantity by expression	transcriptional regulation
P05412	UNIPROT	JUN	protein	P17174	GOT1	UNIPROT	protein	YES	up-regulates quantity by expression	transcriptional regulation
P05412	UNIPROT	JUN	protein	P01137	TGFB1	UNIPROT	protein	YES	up-regulates quantity by expression	transcriptional regulation
P01106	UNIPROT	MYC	protein	P35226	BMI1	UNIPROT	protein	YES	up-regulates quantity by expression	transcriptional regulation
P14859	UNIPROT	POU2F1	protein	P35580	MYH10	UNIPROT	protein	YES	up-regulates quantity by expression	transcriptional regulation
P08047	UNIPROT	SP1	protein	P08581	MET	UNIPROT	protein	YES	up-regulates quantity by expression	transcriptional regulation
P08047	UNIPROT	SP1	protein	P11166	SLC2A1	UNIPROT	protein	YES	up-regulates quantity by expression	transcriptional regulation
P08047	UNIPROT	SP1	protein	P01137	TGFB1	UNIPROT	protein	YES	up-regulates quantity by expression	transcriptional regulation
P04637	UNIPROT	TP53	protein	P25445	FAS	UNIPROT	protein	YES	up-regulates quantity by expression	transcriptional regulation
P35222	UNIPROT	CTNNB1	protein	P68431	HIST1H3A	UNIPROT	protein	YES	up-regulates	trimethylation
P22681	UNIPROT	CBL	protein	P27986	PIK3R1	UNIPROT	protein	YES	down-regulates	ubiquitination
Q96PU5	UNIPROT	NEDD4L	protein	Q15796	SMAD2	UNIPROT	protein	YES	down-regulates	ubiquitination
Q96PU5	UNIPROT	NEDD4L	protein	P84022	SMAD3	UNIPROT	protein	YES	down-regulates	ubiquitination
Q9HCE7	UNIPROT	SMURF1	protein	Q15797	SMAD1	UNIPROT	protein	YES	down-regulates	ubiquitination

Q9HCE7	UNIPROT	SMURF1	protein	Q99717	SMAD5	UNIPROT	protein	YES	down-regulates	ubiquitination
Q9HCE7	UNIPROT	SMURF1	protein	Q99717	SMAD5	UNIPROT	protein	YES	down-regulates	ubiquitination
Q9UNE7	UNIPROT	STUB1	protein	Q15797	SMAD1	UNIPROT	protein	YES	down-regulates	ubiquitination
Q9UNE7	UNIPROT	STUB1	protein	Q15797	SMAD1	UNIPROT	protein	YES	down-regulates	ubiquitination
Q9UNE7	UNIPROT	STUB1	protein	Q15797	SMAD1	UNIPROT	protein	YES	down-regulates	ubiquitination
Q9UNE7	UNIPROT	STUB1	protein	Q99717	SMAD5	UNIPROT	protein	YES	down-regulates	ubiquitination
Q9UNE7	UNIPROT	STUB1	protein	Q99717	SMAD5	UNIPROT	protein	YES	down-regulates	ubiquitination
P19474	UNIPROT	TRIM21	protein	P49915	GMPS	UNIPROT	protein	YES	down-regulates	ubiquitination
O15164	UNIPROT	TRIM24	protein	P04637	TP53	UNIPROT	protein	YES	down-regulates	ubiquitination
Q96PU5	UNIPROT	NEDD4L	protein	Q15796	SMAD2	UNIPROT	protein	YES	down-regulates activity	ubiquitination
Q96PU5	UNIPROT	NEDD4L	protein	P84022	SMAD3	UNIPROT	protein	YES	down-regulates activity	ubiquitination
Q9HCE7	UNIPROT	SMURF1	protein	P37173	TGFBR2	UNIPROT	protein	YES	down-regulates activity	ubiquitination
P22681	UNIPROT	CBL	protein	P00533	EGFR	UNIPROT	protein	YES	down-regulates quantity by destabilization	ubiquitination
P22681	UNIPROT	CBL	protein	P04626	ERBB2	UNIPROT	protein	YES	down-regulates quantity by destabilization	ubiquitination
P22681	UNIPROT	CBL	protein	P08581	MET	UNIPROT	protein	YES	down-regulates quantity by destabilization	ubiquitination
Q13616	UNIPROT	CUL1	protein	P46527	CDKN1B	UNIPROT	protein	YES	down-regulates quantity by destabilization	ubiquitination
Q13616	UNIPROT	CUL1	protein	P35222	CTNNB1	UNIPROT	protein	YES	down-regulates quantity by destabilization	ubiquitination
Q86YT6	UNIPROT	MIB1	protein	Q16637	SMN1	UNIPROT	protein	YES	down-regulates quantity by destabilization	ubiquitination
P62837	UNIPROT	UBE2D2	protein	P50542	PEX5	UNIPROT	protein	YES	down-regulates quantity by destabilization	ubiquitination
Q05086	UNIPROT	UBE3A	protein	Q6IAA8	LAMTOR1	UNIPROT	protein	YES	down-regulates quantity by destabilization	ubiquitination
P98170	UNIPROT	XIAP	protein	P42574	CASP3	UNIPROT	protein	YES	down-regulates quantity by destabilization	ubiquitination
P98170	UNIPROT	XIAP	protein	Q9NR28	DIABLO	UNIPROT	protein	YES	down-regulates quantity by destabilization	ubiquitination
Q96J02	UNIPROT	ITCH	protein	Q15796	SMAD2	UNIPROT	protein	YES	up-regulates	ubiquitination
P62877	UNIPROT	RBX1	protein	P19838	NFKB1	UNIPROT	protein	YES	up-regulates	ubiquitination
P62877	UNIPROT	RBX1	protein	Q00653	NFKB2	UNIPROT	protein	YES	up-regulates	ubiquitination
P49459	UNIPROT	UBE2A	protein	P12004	PCNA	UNIPROT	protein	YES	up-regulates	ubiquitination
P49459	UNIPROT	UBE2A	protein	P12004	PCNA	UNIPROT	protein	YES	up-regulates	ubiquitination
P61088	UNIPROT	UBE2N	protein	P16104	H2AFX	UNIPROT	protein	YES	up-regulates	ubiquitination
Q12933	UNIPROT	TRAF2	protein	O43318	MAP3K7	UNIPROT	protein	YES	up-regulates activity	ubiquitination
Q12933	UNIPROT	TRAF2	protein	Q13546	RIPK1	UNIPROT	protein	YES	up-regulates activity	ubiquitination
Q12933	UNIPROT	TRAF2	protein	Q13546	RIPK1	UNIPROT	protein	YES	up-regulates activity	ubiquitination
Q12933	UNIPROT	TRAF2	protein	Q13546	RIPK1	UNIPROT	protein	YES	up-regulates activity	ubiquitination
Q12933	UNIPROT	TRAF2	protein	Q13546	RIPK1	UNIPROT	protein	YES	up-regulates activity	ubiquitination
Q9Y4K3	UNIPROT	TRAF6	protein	P51617	IRAK1	UNIPROT	protein	YES	up-regulates activity	ubiquitination
Q9Y4K3	UNIPROT	TRAF6	protein	O43318	MAP3K7	UNIPROT	protein	YES	up-regulates activity	ubiquitination
Q9Y4K3	UNIPROT	TRAF6	protein	Q9Y4K3	TRAF6	UNIPROT	protein	YES	up-regulates activity	ubiquitination
Q9Y4K3	UNIPROT	TRAF6	protein	Q9Y4K3	TRAF6	UNIPROT	protein	YES	up-regulates activity	ubiquitination
P61088	UNIPROT	UBE2N	protein	Q12933	TRAF2	UNIPROT	protein	YES	up-regulates activity	ubiquitination
P31749	UNIPROT	AKT1	protein	P49841	GSK3B	UNIPROT	protein	YES	down-regulates activity	
P17252	UNIPROT	PRKCA	protein	P27635	RPL10	UNIPROT	protein	YES	unknown	
P16220	UNIPROT	CREB1	protein	P17676	CEBPB	UNIPROT	protein	YES	up-regulates	
Q96EB6	UNIPROT	SIRT1	protein	O75376	NCOR1	UNIPROT	protein	YES	up-regulates	
Q96EB6	UNIPROT	SIRT1	protein	Q9Y618	NCOR2	UNIPROT	protein	YES	up-regulates	
P40763	UNIPROT	STAT3	protein	P42574	CASP3	UNIPROT	protein	YES	up-regulates	
P61586	UNIPROT	RHOA	protein	P07737	PFN1	UNIPROT	protein	YES	up-regulates activity	
Q13546	UNIPROT	RIPK1	protein	O43318	MAP3K7	UNIPROT	protein	YES	up-regulates activity	
Q13546	UNIPROT	RIPK1	protein	O43318	MAP3K7	UNIPROT	protein	YES	up-regulates activity	
Q96EB6	UNIPROT	SIRT1	protein	O43524	FOXO3	UNIPROT	protein	NO	down-regulates activity	deacetylation
P62136	UNIPROT	PPP1CA	protein	P08047	SP1	UNIPROT	protein	NO	down-regulates activity	dephosphorylation
P62136	UNIPROT	PPP1CA	protein	Q02447	SP3	UNIPROT	protein	NO	down-regulates activity	dephosphorylation
Q8IXJ6	UNIPROT	SIRT2	protein	P01106	MYC	UNIPROT	protein	NO	up-regulates quantity by stabilization	stabilization
P31749	UNIPROT	AKT1	protein	P46527	CDKN1B	UNIPROT	protein	NO	down-regulates	transcriptional regulation
P48436	UNIPROT	SOX9	protein	P17676	CEBPB	UNIPROT	protein	NO	down-regulates	transcriptional regulation
P31749	UNIPROT	AKT1	protein	P46527	CDKN1B	UNIPROT	protein	NO	down-regulates quantity by repression	transcriptional regulation
P35226	UNIPROT	BMI1	protein	P60484	PTEN	UNIPROT	protein	NO	down-regulates quantity by repression	transcriptional regulation
P16220	UNIPROT	CREB1	protein	P10588	NR2F6	UNIPROT	protein	NO	down-regulates quantity by repression	transcriptional regulation
Q13547	UNIPROT	HDAC1	protein	P26358	DNMT1	UNIPROT	protein	NO	down-regulates quantity by repression	transcriptional regulation
P01106	UNIPROT	MYC	protein	P46527	CDKN1B	UNIPROT	protein	NO	down-regulates quantity by repression	transcriptional regulation
P15531	UNIPROT	NME1	protein	P08581	MET	UNIPROT	protein	NO	down-regulates quantity by repression	transcriptional regulation
P84022	UNIPROT	SMAD3	protein	P01106	MYC	UNIPROT	protein	NO	down-regulates quantity by repression	transcriptional regulation
P48436	UNIPROT	SOX9	protein	P17676	CEBPB	UNIPROT	protein	NO	down-regulates quantity by repression	transcriptional regulation
P01137	UNIPROT	TGFB1	protein	P20248	CCNA2	UNIPROT	protein	NO	down-regulates quantity by repression	transcriptional regulation
P01137	UNIPROT	TGFB1	protein	P11802	CDK4	UNIPROT	protein	NO	down-regulates quantity by repression	transcriptional regulation
P27361	UNIPROT	MAPK3	protein	P48436	SOX9	UNIPROT	protein	NO	up-regulates	transcriptional regulation
P40763	UNIPROT	STAT3	protein	P01137	TGFB1	UNIPROT	protein	NO	up-regulates	transcriptional regulation
P31749	UNIPROT	AKT1	protein	P11166	SLC2A1	UNIPROT	protein	NO	up-regulates quantity by expression	transcriptional regulation
P31751	UNIPROT	AKT2	protein	P35222	CTNNB1	UNIPROT	protein	NO	up-regulates quantity by expression	transcriptional regulation
P16220	UNIPROT	CREB1	protein	Q07817	BCL2L1	UNIPROT	protein	NO	up-regulates quantity by expression	transcriptional regulation
P16220	UNIPROT	CREB1	protein	P35222	CTNNB1	UNIPROT	protein	NO	up-regulates quantity by expression	transcriptional regulation
P16220	UNIPROT	CREB1	protein	P01100	FOS	UNIPROT	protein	NO	up-regulates quantity by expression	transcriptional regulation
P16220	UNIPROT	CREB1	protein	P14618	PKM	UNIPROT	protein	NO	up-regulates quantity by expression	transcriptional regulation
P16220	UNIPROT	CREB1	protein	P14618	PKM	UNIPROT	protein	NO	up-regulates quantity by expression	transcriptional regulation
P35222	UNIPROT	CTNNB1	protein	P01106	MYC	UNIPROT	protein	NO	up-regulates quantity by expression	transcriptional regulation
P35222	UNIPROT	CTNNB1	protein	P01106	MYC	UNIPROT	protein	NO	up-regulates quantity by expression	transcriptional regulation
P26358	UNIPROT	DNMT1	protein	O95817	BAG3	UNIPROT	protein	NO	up-regulates quantity by expression	transcriptional regulation
P52926	UNIPROT	HMGA2	protein	P20248	CCNA2	UNIPROT	protein	NO	up-regulates quantity by expression	transcriptional regulation
O43318	UNIPROT	MAP3K7	protein	P19838	NFKB1	UNIPROT	protein	NO	up-regulates quantity by expression	transcriptional regulation
P27361	UNIPROT	MAPK3	protein	P48436	SOX9	UNIPROT	protein	NO	up-regulates quantity by expression	transcriptional regulation
P01106	UNIPROT	MYC	protein	P20248	CCNA2	UNIPROT	protein	NO	up-regulates quantity by expression	transcriptional regulation
P01106	UNIPROT	MYC	protein	P11802	CDK4	UNIPROT	protein	NO	up-regulates quantity by expression	transcriptional regulation
P01106	UNIPROT	MYC	protein	Q00534	CDK6	UNIPROT	protein	NO	up-regulates quantity by expression	transcriptional regulation
P01106	UNIPROT	MYC	protein	Q13616	CUL1	UNIPROT	protein	NO	up-regulates quantity by expression	transcriptional regulation
P01106	UNIPROT	MYC	protein	Q8IXJ6	SIRT2	UNIPROT	protein	NO	up-regulates quantity by expression	transcriptional regulation
Q04206	UNIPROT	RELA	protein	Q12933	TRAF2	UNIPROT	protein	NO	up-regulates quantity by expression	transcriptional regulation
P08047	UNIPROT	SP1	protein	P09917	ALOX5	UNIPROT	protein	NO	up-regulates quantity by expression	transcriptional regulation
P08047	UNIPROT	SP1	protein	P41440	SLC19A1	UNIPROT	protein	NO	up-regulates quantity by expression	transcriptional regulation
P42224	UNIPROT	STAT1	protein	Q92985	IRF7	UNIPROT	protein	NO	up-regulates quantity by expression	transcriptional regulation
P04637	UNIPROT	TP53	protein	Q07812	BAX	UNIPROT	protein	NO	up-regulates quantity by expression	transcriptional regulation
P04637	UNIPROT	TP53	protein	Q07812	BAX	UNIPROT	protein	NO	up-regulates quantity by expression	transcriptional regulation
P04637	UNIPROT	TP53	protein	P55957	BID	UNIPROT	protein	NO	up-regulates quantity by expression	transcriptional regulation
P04637	UNIPROT	TP53	protein	P00533	EGFR	UNIPROT	protein	NO	up-regulates quantity by expression	transcriptional regulation
P67809	UNIPROT	YBX1	protein	P12830	CDH1	UNIPROT	protein	NO	up-regulates quantity by expression	transcriptional regulation
O43353	UNIPROT	RIPK2	protein	Q9Y6K9	IKBKG	UNIPROT	protein	NO	up-regulates activity	ubiquitination
P31749	UNIPROT	AKT1	protein	P25445	FAS	UNIPROT	protein	NO	down-regulates	
Q9UIG0	UNIPROT	BAZ1B	protein	Q14676	MDC1	UNIPROT	protein	NO	down-regulates	
P04792	UNIPROT	HSPB1	protein	P42574	CASP3	UNIPROT	protein	NO	down-regulates	
P04792	UNIPROT	HSPB1	protein	Q9NR28	DIABLO	UNIPROT	protein	NO	down-regulates	
O15357	UNIPROT	INPPL1	protein	P31749	AKT1	UNIPROT	protein	NO	down-regulates	

Q02750	UNIPROT	MAP2K1	protein	Q16539	MAPK14	UNIPROT	protein	NO	down-regulates	
P28482	UNIPROT	MAPK1	protein	Q16539	MAPK14	UNIPROT	protein	NO	down-regulates	
P28482	UNIPROT	MAPK1	protein	Q16539	MAPK14	UNIPROT	protein	NO	down-regulates	
Q16539	UNIPROT	MAPK14	protein	Q13541	EIF4EBP1	UNIPROT	protein	NO	down-regulates	
P01106	UNIPROT	MYC	protein	Q15796	SMAD2	UNIPROT	protein	NO	down-regulates	
Q15121	UNIPROT	PEA15	protein	P28482	MAPK1	UNIPROT	protein	NO	down-regulates	
Q15121	UNIPROT	PEA15	protein	P27361	MAPK3	UNIPROT	protein	NO	down-regulates	
P53350	UNIPROT	PLK1	protein	P04637	TP53	UNIPROT	protein	NO	down-regulates	
Q13131	UNIPROT	PRKAA1	protein	P01137	TGFB1	UNIPROT	protein	NO	down-regulates	
Q05655	UNIPROT	PRKCD	protein	P35568	IRS1	UNIPROT	protein	NO	down-regulates	
P17706	UNIPROT	PTPN2	protein	P31749	AKT1	UNIPROT	protein	NO	down-regulates	
P29350	UNIPROT	PTPN6	protein	P42226	STAT6	UNIPROT	protein	NO	down-regulates	
O75676	UNIPROT	RPS6KA4	protein	P04637	TP53	UNIPROT	protein	NO	down-regulates	
P01137	UNIPROT	TGFB1	protein	P24941	CDK2	UNIPROT	protein	NO	down-regulates	
P31751	UNIPROT	AKT2	protein	Q16539	MAPK14	UNIPROT	protein	NO	down-regulates activity	
Q16539	UNIPROT	MAPK14	protein	P31751	AKT2	UNIPROT	protein	NO	down-regulates activity	
P60484	UNIPROT	PTEN	protein	P31749	AKT1	UNIPROT	protein	NO	down-regulates activity	
P60484	UNIPROT	PTEN	protein	P31749	AKT1	UNIPROT	protein	NO	down-regulates activity	
Q92574	UNIPROT	TSC1	protein	P42345	MTOR	UNIPROT	protein	NO	down-regulates activity	
P49815	UNIPROT	TSC2	protein	P42345	MTOR	UNIPROT	protein	NO	down-regulates activity	
P78536	UNIPROT	ADAM17	protein	P00533	EGFR	UNIPROT	protein	NO	up-regulates	
P31749	UNIPROT	AKT1	protein	P17612	PRKACA	UNIPROT	protein	NO	up-regulates	
P31751	UNIPROT	AKT2	protein	P42345	MTOR	UNIPROT	protein	NO	up-regulates	
P31751	UNIPROT	AKT2	protein	P19838	NFKB1	UNIPROT	protein	NO	up-regulates	
P05067	UNIPROT	APP	protein	P49840	GSK3A	UNIPROT	protein	NO	up-regulates	
O15169	UNIPROT	AXIN1	protein	P84022	SMAD3	UNIPROT	protein	NO	up-regulates	
Q07812	UNIPROT	BAX	protein	Q9NR28	DIABLO	UNIPROT	protein	NO	up-regulates	
Q9NR28	UNIPROT	DIABLO	protein	P55211	CASP9	UNIPROT	protein	NO	up-regulates	
P25445	UNIPROT	FAS	protein	Q9UER7	DAXX	UNIPROT	protein	NO	up-regulates	
P21802	UNIPROT	FGFR2	protein	Q02750	MAP2K1	UNIPROT	protein	NO	up-regulates	
O14964	UNIPROT	HGS	protein	Q15796	SMAD2	UNIPROT	protein	NO	up-regulates	
P08069	UNIPROT	IGF1R	protein	P41240	CSK	UNIPROT	protein	NO	up-regulates	
Q99759	UNIPROT	MAP3K3	protein	P52564	MAP2K6	UNIPROT	protein	NO	up-regulates	
P45983	UNIPROT	MAPK8	protein	Q07812	BAX	UNIPROT	protein	NO	up-regulates	
P45984	UNIPROT	MAPK9	protein	Q07812	BAX	UNIPROT	protein	NO	up-regulates	
P49757	UNIPROT	NUMB	protein	P04637	TP53	UNIPROT	protein	NO	up-regulates	
Q8NEB9	UNIPROT	PIK3C3	protein	P31749	AKT1	UNIPROT	protein	NO	up-regulates	
Q13131	UNIPROT	PRKAA1	protein	Q96EB6	SIRT1	UNIPROT	protein	NO	up-regulates	
P17612	UNIPROT	PRKACA	protein	P31751	AKT2	UNIPROT	protein	NO	up-regulates	
P63000	UNIPROT	RAC1	protein	Q16539	MAPK14	UNIPROT	protein	NO	up-regulates	
P06400	UNIPROT	RB1	protein	O15379	HDAC3	UNIPROT	protein	NO	up-regulates	
Q14790	UNIPROT	CASP8	protein	P55211	CASP9	UNIPROT	protein	NO	up-regulates activity	
P60953	UNIPROT	CDC42	protein	Q16539	MAPK14	UNIPROT	protein	NO	up-regulates activity	
P00533	UNIPROT	EGFR	protein	P01106	MYC	UNIPROT	protein	NO	up-regulates activity	
Q9H4A6	UNIPROT	GOLPH3	protein	P42345	MTOR	UNIPROT	protein	NO	up-regulates activity	
P08069	UNIPROT	IGF1R	protein	P31749	AKT1	UNIPROT	protein	NO	up-regulates activity	
Q9Y6R4	UNIPROT	MAP3K4	protein	Q16539	MAPK14	UNIPROT	protein	NO	up-regulates activity	
O43318	UNIPROT	MAP3K7	protein	Q9Y6R4	MAP3K4	UNIPROT	protein	NO	up-regulates activity	
Q16539	UNIPROT	MAPK14	protein	P31751	AKT2	UNIPROT	protein	NO	up-regulates activity	
P63000	UNIPROT	RAC1	protein	P45983	MAPK8	UNIPROT	protein	NO	up-regulates activity	
P51812	UNIPROT	RPS6KA3	protein	Q13257	MAD2L1	UNIPROT	protein	NO	up-regulates activity	
P29353	UNIPROT	SHC1	protein	P28482	MAPK1	UNIPROT	protein	NO	up-regulates activity	
P29353	UNIPROT	SHC1	protein	P27361	MAPK3	UNIPROT	protein	NO	up-regulates activity	
P01137	UNIPROT	TGFB1	protein	P29353	SHC1	UNIPROT	protein	NO	up-regulates activity	
Q9Y4K3	UNIPROT	TRAF6	protein	P41279	MAP3K8	UNIPROT	protein	NO	up-regulates activity	
P40763	UNIPROT	STAT3	protein	P01106	MYC	UNIPROT	protein	NO	up-regulates quantity by expression	
P01137	UNIPROT	TGFB1	protein	P46527	CDKN1B	UNIPROT	protein	NO	up-regulates quantity by expression	
O14733	UNIPROT	MAP2K7	protein	P45983	JNK	SIGNOR	proteinfamily	YES	up-regulates activity	phosphorylation
O15111	UNIPROT	CHUK	protein	O43524	FOXO	SIGNOR	proteinfamily	YES	down-regulates	phosphorylation
O15357	UNIPROT	INPPL1	protein	P31749	AKT	SIGNOR	proteinfamily	NO	down-regulates	
O15530	UNIPROT	PDPK1	protein	P31749	AKT	SIGNOR	proteinfamily	YES	up-regulates	phosphorylation
O15530	UNIPROT	PDPK1	protein	P31749	AKT	SIGNOR	proteinfamily	YES	up-regulates	phosphorylation
O15530	UNIPROT	PDPK1	protein	P31749	AKT	SIGNOR	proteinfamily	YES	up-regulates	phosphorylation
O15530	UNIPROT	PDPK1	protein	P31749	AKT	SIGNOR	proteinfamily	YES	up-regulates activity	phosphorylation
O15530	UNIPROT	PDPK1	protein	P31749	AKT	SIGNOR	proteinfamily	YES	up-regulates activity	phosphorylation
O15530	UNIPROT	PDPK1	protein	Q02750	MEK1/2	SIGNOR	proteinfamily	YES	up-regulates	phosphorylation
O15530	UNIPROT	PDPK1	protein	Q02750	MEK1/2	SIGNOR	proteinfamily	YES	up-regulates activity	phosphorylation
O15530	UNIPROT	PDPK1	protein	P51812	RPS6K	SIGNOR	proteinfamily	YES	up-regulates activity	phosphorylation
O75925	UNIPROT	PIAS1	protein	P31749	AKT	SIGNOR	proteinfamily	YES	up-regulates activity	sumoylation
P04637	UNIPROT	TP53	protein	P51812	RPS6K	SIGNOR	proteinfamily	YES	up-regulates	binding
P06493	UNIPROT	CDK1	protein	Q02750	MEK1/2	SIGNOR	proteinfamily	YES	down-regulates	phosphorylation
P06493	UNIPROT	CDK1	protein	O43524	FOXO	SIGNOR	proteinfamily	YES	down-regulates	phosphorylation
P08069	UNIPROT	IGF1R	protein	P31749	AKT	SIGNOR	proteinfamily	NO	up-regulates activity	
P10398	UNIPROT	ARAF	protein	Q02750	MEK1/2	SIGNOR	proteinfamily	YES	up-regulates	phosphorylation
P10398	UNIPROT	ARAF	protein	Q02750	MEK1/2	SIGNOR	proteinfamily	YES	up-regulates	phosphorylation
P11233	UNIPROT	RALA	protein	O43524	FOXO	SIGNOR	proteinfamily	YES	up-regulates activity	phosphorylation
P11233	UNIPROT	RALA	protein	O43524	FOXO	SIGNOR	proteinfamily	YES	up-regulates activity	phosphorylation
P12931	UNIPROT	SRC	protein	P31749	AKT	SIGNOR	proteinfamily	YES	up-regulates activity	phosphorylation
P12931	UNIPROT	SRC	protein	P31749	AKT	SIGNOR	proteinfamily	YES	up-regulates activity	phosphorylation
P12931	UNIPROT	SRC	protein	P31749	AKT	SIGNOR	proteinfamily	YES	up-regulates activity	phosphorylation
P15056	UNIPROT	BRAF	protein	Q02750	MEK1/2	SIGNOR	proteinfamily	YES	up-regulates	phosphorylation
P15056	UNIPROT	BRAF	protein	Q02750	MEK1/2	SIGNOR	proteinfamily	YES	up-regulates activity	phosphorylation
P15056	UNIPROT	BRAF	protein	Q02750	MEK1/2	SIGNOR	proteinfamily	YES	up-regulates activity	phosphorylation
P15056	UNIPROT	BRAF	protein	Q02750	MEK1/2	SIGNOR	proteinfamily	YES	up-regulates activity	phosphorylation
P16220	UNIPROT	CREB1	protein	O43524	FOXO	SIGNOR	proteinfamily	YES	down-regulates activity	binding
P17706	UNIPROT	PTPN2	protein	P31749	AKT	SIGNOR	proteinfamily	NO	down-regulates	
P18031	UNIPROT	PTPN1	protein	P31749	AKT	SIGNOR	proteinfamily	YES	down-regulates	dephosphorylation
P21802	UNIPROT	FGFR2	protein	Q02750	MEK1/2	SIGNOR	proteinfamily	NO	up-regulates	
P24666	UNIPROT	ACP1	protein	P31749	AKT	SIGNOR	proteinfamily	YES	down-regulates activity	dephosphorylation
P24941	UNIPROT	CDK2	protein	O43524	FOXO	SIGNOR	proteinfamily	YES	down-regulates	phosphorylation
P24941	UNIPROT	CDK2	protein	O43524	FOXO	SIGNOR	proteinfamily	YES	down-regulates	phosphorylation
P27361	UNIPROT	MAPK3	protein	P51812	RPS6K	SIGNOR	proteinfamily	YES	up-regulates activity	phosphorylation
P27361	UNIPROT	MAPK3	protein	P51812	RPS6K	SIGNOR	proteinfamily	YES	up-regulates activity	phosphorylation
P27361	UNIPROT	MAPK3	protein	P51812	RPS6K	SIGNOR	proteinfamily	YES	up-regulates activity	phosphorylation
P27361	UNIPROT	MAPK3	protein	P51812	RPS6K	SIGNOR	proteinfamily	YES	up-regulates activity	phosphorylation
P27361	UNIPROT	MAPK3	protein	P51812	RPS6K	SIGNOR	proteinfamily	YES	up-regulates activity	phosphorylation
P27361	UNIPROT	MAPK3	protein	P51812	RPS6K	SIGNOR	proteinfamily	YES	up-regulates activity	phosphorylation
P27361	UNIPROT	MAPK3	protein	P51812	RPS6K	SIGNOR	proteinfamily	YES	up-regulates activity	phosphorylation

P27361	UNIPROT	MAPK3	protein	P51812	RPS6K	SIGNOR	proteinfamily	YES	up-regulates activity	phosphorylation
P27361	UNIPROT	MAPK3	protein	O43524	FOXO	SIGNOR	proteinfamily	YES	down-regulates quantity by destabilization	phosphorylation
P27361	UNIPROT	MAPK3	protein	O43524	FOXO	SIGNOR	proteinfamily	YES	down-regulates quantity by destabilization	phosphorylation
P27361	UNIPROT	MAPK3	protein	O43524	FOXO	SIGNOR	proteinfamily	YES	down-regulates quantity by destabilization	phosphorylation
P28482	UNIPROT	MAPK1	protein	Q02750	MEK1/2	SIGNOR	proteinfamily	YES	down-regulates activity	phosphorylation
P28482	UNIPROT	MAPK1	protein	Q02750	MEK1/2	SIGNOR	proteinfamily	YES	down-regulates activity	phosphorylation
P28482	UNIPROT	MAPK1	protein	P51812	RPS6K	SIGNOR	proteinfamily	YES	up-regulates activity	phosphorylation
P28482	UNIPROT	MAPK1	protein	P51812	RPS6K	SIGNOR	proteinfamily	YES	up-regulates activity	phosphorylation
P28482	UNIPROT	MAPK1	protein	P51812	RPS6K	SIGNOR	proteinfamily	YES	up-regulates activity	phosphorylation
P28482	UNIPROT	MAPK1	protein	P51812	RPS6K	SIGNOR	proteinfamily	YES	up-regulates activity	phosphorylation
P28482	UNIPROT	MAPK1	protein	P51812	RPS6K	SIGNOR	proteinfamily	YES	up-regulates activity	phosphorylation
P28482	UNIPROT	MAPK1	protein	P51812	RPS6K	SIGNOR	proteinfamily	YES	up-regulates activity	phosphorylation
P28482	UNIPROT	MAPK1	protein	P51812	RPS6K	SIGNOR	proteinfamily	YES	up-regulates activity	phosphorylation
P28482	UNIPROT	MAPK1	protein	P51812	RPS6K	SIGNOR	proteinfamily	YES	up-regulates activity	phosphorylation
P28482	UNIPROT	MAPK1	protein	O43524	FOXO	SIGNOR	proteinfamily	YES	down-regulates quantity by destabilization	phosphorylation
P28482	UNIPROT	MAPK1	protein	O43524	FOXO	SIGNOR	proteinfamily	YES	down-regulates quantity by destabilization	phosphorylation
P28482	UNIPROT	MAPK1	protein	O43524	FOXO	SIGNOR	proteinfamily	YES	down-regulates quantity by destabilization	phosphorylation
P29353	UNIPROT	SHC1	protein	P27361	ERK1/2	SIGNOR	proteinfamily	NO	up-regulates activity	
P31749	UNIPROT	AKT1	protein	O43524	FOXO	SIGNOR	proteinfamily	YES	down-regulates	phosphorylation
P31749	UNIPROT	AKT1	protein	O43524	FOXO	SIGNOR	proteinfamily	YES	down-regulates	phosphorylation
P31749	UNIPROT	AKT1	protein	O43524	FOXO	SIGNOR	proteinfamily	YES	down-regulates	phosphorylation
P31749	UNIPROT	AKT1	protein	O43524	FOXO	SIGNOR	proteinfamily	YES	down-regulates	phosphorylation
P31749	UNIPROT	AKT1	protein	O43524	FOXO	SIGNOR	proteinfamily	YES	down-regulates	phosphorylation
P31749	UNIPROT	AKT1	protein	O43524	FOXO	SIGNOR	proteinfamily	YES	down-regulates	phosphorylation
P31749	UNIPROT	AKT1	protein	O43524	FOXO	SIGNOR	proteinfamily	YES	down-regulates	phosphorylation
P31749	UNIPROT	AKT1	protein	O43524	FOXO	SIGNOR	proteinfamily	YES	down-regulates	phosphorylation
P31749	UNIPROT	AKT1	protein	O43524	FOXO	SIGNOR	proteinfamily	YES	down-regulates	phosphorylation
P31749	UNIPROT	AKT1	protein	O43524	FOXO	SIGNOR	proteinfamily	YES	down-regulates activity	phosphorylation
P31749	UNIPROT	AKT1	protein	O43524	FOXO	SIGNOR	proteinfamily	YES	down-regulates activity	phosphorylation
P31749	UNIPROT	AKT1	protein	O43524	FOXO	SIGNOR	proteinfamily	YES	down-regulates activity	phosphorylation
P31749	UNIPROT	AKT1	protein	O43524	FOXO	SIGNOR	proteinfamily	YES	down-regulates activity	phosphorylation
P31749	UNIPROT	AKT1	protein	O43524	FOXO	SIGNOR	proteinfamily	YES	down-regulates activity	phosphorylation
P31749	UNIPROT	AKT1	protein	O43524	FOXO	SIGNOR	proteinfamily	YES	down-regulates activity	phosphorylation
P31749	UNIPROT	AKT1	protein	O43524	FOXO	SIGNOR	proteinfamily	YES	down-regulates activity	phosphorylation
P31749	UNIPROT	AKT1	protein	O43524	FOXO	SIGNOR	proteinfamily	YES	down-regulates quantity by destabilization	phosphorylation
P31749	UNIPROT	AKT1	protein	O43524	FOXO	SIGNOR	proteinfamily	YES	down-regulates quantity by destabilization	phosphorylation
P31749	UNIPROT	AKT1	protein	O43524	FOXO	SIGNOR	proteinfamily	YES	down-regulates quantity by destabilization	phosphorylation
P31749	UNIPROT	AKT1	protein	O43524	FOXO	SIGNOR	proteinfamily	YES	down-regulates quantity by destabilization	phosphorylation
P31749	UNIPROT	AKT1	protein	O43524	FOXO	SIGNOR	proteinfamily	YES	down-regulates	relocalization
P31751	UNIPROT	AKT2	protein	O43524	FOXO	SIGNOR	proteinfamily	YES	down-regulates activity	phosphorylation
P31751	UNIPROT	AKT2	protein	O43524	FOXO	SIGNOR	proteinfamily	YES	down-regulates activity	phosphorylation
P31751	UNIPROT	AKT2	protein	O43524	FOXO	SIGNOR	proteinfamily	YES	down-regulates activity	phosphorylation
P31751	UNIPROT	AKT2	protein	O43524	FOXO	SIGNOR	proteinfamily	YES	down-regulates activity	phosphorylation
P31751	UNIPROT	AKT2	protein	O43524	FOXO	SIGNOR	proteinfamily	YES	down-regulates activity	phosphorylation
P31751	UNIPROT	AKT2	protein	O43524	FOXO	SIGNOR	proteinfamily	YES	down-regulates activity	phosphorylation
P31751	UNIPROT	AKT2	protein	O43524	FOXO	SIGNOR	proteinfamily	YES	down-regulates activity	phosphorylation
P31751	UNIPROT	AKT2	protein	O43524	FOXO	SIGNOR	proteinfamily	YES	down-regulates activity	phosphorylation
P31751	UNIPROT	AKT2	protein	O43524	FOXO	SIGNOR	proteinfamily	YES	down-regulates quantity by destabilization	phosphorylation
P31751	UNIPROT	AKT2	protein	O43524	FOXO	SIGNOR	proteinfamily	YES	down-regulates quantity by destabilization	phosphorylation
P31751	UNIPROT	AKT2	protein	O43524	FOXO	SIGNOR	proteinfamily	YES	down-regulates quantity by destabilization	phosphorylation
P36507	UNIPROT	MAP2K2	protein	P27361	ERK1/2	SIGNOR	proteinfamily	YES	up-regulates	phosphorylation
P36873	UNIPROT	PPP1CC	protein	P31749	AKT	SIGNOR	proteinfamily	YES	down-regulates activity	dephosphorylation
P40189	UNIPROT	IL6ST	protein	P27361	ERK1/2	SIGNOR	proteinfamily	NO	up-regulates	phosphorylation
P41279	UNIPROT	MAP3K8	protein	P27361	ERK1/2	SIGNOR	proteinfamily	NO	up-regulates activity	
P41279	UNIPROT	MAP3K8	protein	Q02750	MEK1/2	SIGNOR	proteinfamily	YES	up-regulates	phosphorylation
P41279	UNIPROT	MAP3K8	protein	Q02750	MEK1/2	SIGNOR	proteinfamily	YES	up-regulates	phosphorylation
P42345	UNIPROT	MTOR	protein	P31749	AKT	SIGNOR	proteinfamily	YES	up-regulates activity	phosphorylation
P42345	UNIPROT	MTOR	protein	P31749	AKT	SIGNOR	proteinfamily	YES	up-regulates activity	phosphorylation
P42345	UNIPROT	MTOR	protein	P51812	RPS6K	SIGNOR	proteinfamily	YES	up-regulates activity	phosphorylation
P42574	UNIPROT	CASP3	protein	P31749	AKT	SIGNOR	proteinfamily	YES	down-regulates activity	cleavage
P45983	UNIPROT	MAPK8	protein	O43524	FOXO	SIGNOR	proteinfamily	YES	up-regulates	phosphorylation
P45983	UNIPROT	MAPK8	protein	O43524	FOXO	SIGNOR	proteinfamily	YES	up-regulates	phosphorylation
P45985	UNIPROT	MAP2K4	protein	P45983	JNK	SIGNOR	proteinfamily	YES	up-regulates activity	phosphorylation
P45985	UNIPROT	MAP2K4	protein	P45983	JNK	SIGNOR	proteinfamily	YES	up-regulates activity	phosphorylation
P48729	UNIPROT	CSNK1A1	protein	O43524	FOXO	SIGNOR	proteinfamily	YES	down-regulates	phosphorylation
P48729	UNIPROT	CSNK1A1	protein	O43524	FOXO	SIGNOR	proteinfamily	YES	down-regulates	phosphorylation
P48729	UNIPROT	CSNK1A1	protein	O43524	FOXO	SIGNOR	proteinfamily	YES	down-regulates	phosphorylation
P48729	UNIPROT	CSNK1A1	protein	O43524	FOXO	SIGNOR	proteinfamily	YES	down-regulates	phosphorylation
P49840	UNIPROT	GSK3A	protein	P31749	AKT	SIGNOR	proteinfamily	YES	down-regulates activity	phosphorylation
P60484	UNIPROT	PTEN	protein	P31749	AKT	SIGNOR	proteinfamily	NO	down-regulates activity	
P60484	UNIPROT	PTEN	protein	P31749	AKT	SIGNOR	proteinfamily	NO	down-regulates activity	
P60953	UNIPROT	CDC42	protein	Q13177	PAK	SIGNOR	proteinfamily	YES	up-regulates activity	binding
P62136	UNIPROT	PPP1CA	protein	P31749	AKT	SIGNOR	proteinfamily	YES	down-regulates	dephosphorylation
P62136	UNIPROT	PPP1CA	protein	P31749	AKT	SIGNOR	proteinfamily	YES	down-regulates activity	dephosphorylation
P62140	UNIPROT	PPP1CB	protein	P31749	AKT	SIGNOR	proteinfamily	YES	down-regulates activity	dephosphorylation
P62714	UNIPROT	PPP2CB	protein	P31749	AKT	SIGNOR	proteinfamily	YES	down-regulates	dephosphorylation
P62714	UNIPROT	PPP2CB	protein	P31749	AKT	SIGNOR	proteinfamily	YES	down-regulates activity	dephosphorylation
P63000	UNIPROT	RAC1	protein	Q13177	PAK	SIGNOR	proteinfamily	YES	up-regulates activity	binding
P67775	UNIPROT	PPP2CA	protein	P31749	AKT	SIGNOR	proteinfamily	YES	down-regulates	dephosphorylation
P67775	UNIPROT	PPP2CA	protein	P31749	AKT	SIGNOR	proteinfamily	YES	down-regulates activity	dephosphorylation
P67775	UNIPROT	PPP2CA	protein	P31749	AKT	SIGNOR	proteinfamily	YES	down-regulates activity	dephosphorylation
P67775	UNIPROT	PPP2CA	protein	P31749	AKT	SIGNOR	proteinfamily	YES	down-regulates activity	dephosphorylation
P67775	UNIPROT	PPP2CA	protein	Q02750	MEK1/2	SIGNOR	proteinfamily	YES	down-regulates	dephosphorylation
P67775	UNIPROT	PPP2CA	protein	O43524	FOXO	SIGNOR	proteinfamily	YES	up-regulates	dephosphorylation
P67775	UNIPROT	PPP2CA	protein	O43524	FOXO	SIGNOR	proteinfamily	YES	up-regulates	dephosphorylation
P67775	UNIPROT	PPP2CA	protein	O43524	FOXO	SIGNOR	proteinfamily	YES	up-regulates	dephosphorylation
P68400	UNIPROT	CSNK2A1	protein	P31749	AKT	SIGNOR	proteinfamily	YES	up-regulates	phosphorylation
P68400	UNIPROT	CSNK2A1	protein	P31749	AKT	SIGNOR	proteinfamily	YES	up-regulates	phosphorylation
P78527	UNIPROT	PRKDC	protein	P31749	AKT	SIGNOR	proteinfamily	YES	up-regulates activity	phosphorylation
Q02750	UNIPROT	MAP2K1	protein	P27361	ERK1/2	SIGNOR	proteinfamily	YES	up-regulates activity	phosphorylation
Q13043	UNIPROT	STK4	protein	O43524	FOXO	SIGNOR	proteinfamily	YES	up-regulates	phosphorylation
Q13043	UNIPROT	STK4	protein	O43524	FOXO	SIGNOR	proteinfamily	YES	up-regulates	phosphorylation
Q13043	UNIPROT	STK4	protein	O43524	FOXO	SIGNOR	proteinfamily	YES	up-regulates	phosphorylation
Q13043	UNIPROT	STK4	protein	O43524	FOXO	SIGNOR	proteinfamily	YES	up-regulates	phosphorylation
Q13043	UNIPROT	STK4	protein	O43524	FOXO	SIGNOR	proteinfamily	YES	up-regulates	phosphorylation
Q13131	UNIPROT	PRKAA1	protein	O43524	FOXO	SIGNOR	proteinfamily	YES	up-regulates	phosphorylation
Q13131	UNIPROT	PRKAA1	protein	O43524	FOXO	SIGNOR	proteinfamily	YES	up-regulates	phosphorylation
Q13131	UNIPROT	PRKAA1	protein	O43524	FOXO	SIGNOR	proteinfamily	YES	up-regulates activity	phosphorylation

Q13131	UNIPROT	PRKAA1	protein	O43524	FOXO	SIGNOR	proteinfamily	YES	up-regulates activity	phosphorylation
Q13131	UNIPROT	PRKAA1	protein	O43524	FOXO	SIGNOR	proteinfamily	YES	up-regulates activity	phosphorylation
Q13131	UNIPROT	PRKAA1	protein	O43524	FOXO	SIGNOR	proteinfamily	YES	up-regulates activity	phosphorylation
Q13131	UNIPROT	PRKAA1	protein	O43524	FOXO	SIGNOR	proteinfamily	YES	up-regulates activity	phosphorylation
Q13131	UNIPROT	PRKAA1	protein	O43524	FOXO	SIGNOR	proteinfamily	YES	up-regulates activity	phosphorylation
Q13131	UNIPROT	PRKAA1	protein	O43524	FOXO	SIGNOR	proteinfamily	YES	up-regulates activity	phosphorylation
Q13153	UNIPROT	PAK1	protein	Q02750	MEK1/2	SIGNOR	proteinfamily	YES	up-regulates activity	phosphorylation
Q13418	UNIPROT	ILK	protein	P31749	AKT	SIGNOR	proteinfamily	YES	up-regulates	phosphorylation
Q13418	UNIPROT	ILK	protein	P31749	AKT	SIGNOR	proteinfamily	YES	up-regulates activity	phosphorylation
Q13627	UNIPROT	DYRK1A	protein	O43524	FOXO	SIGNOR	proteinfamily	YES	down-regulates	phosphorylation
Q13627	UNIPROT	DYRK1A	protein	O43524	FOXO	SIGNOR	proteinfamily	YES	down-regulates	phosphorylation
Q13627	UNIPROT	DYRK1A	protein	O43524	FOXO	SIGNOR	proteinfamily	YES	down-regulates	phosphorylation
Q13627	UNIPROT	DYRK1A	protein	O43524	FOXO	SIGNOR	proteinfamily	YES	down-regulates activity	phosphorylation
Q13627	UNIPROT	DYRK1A	protein	O43524	FOXO	SIGNOR	proteinfamily	YES	down-regulates activity	phosphorylation
Q15257	UNIPROT	PTPA	protein	P31749	AKT	SIGNOR	proteinfamily	YES	down-regulates	dephosphorylation
Q15750	UNIPROT	TAB1	protein	P45983	JNK	SIGNOR	proteinfamily	YES	up-regulates	binding
Q15750	UNIPROT	TAB1	protein	Q16539	p38	SIGNOR	proteinfamily	YES	up-regulates	binding
Q16512	UNIPROT	PKN1	protein	O43524	FOXO	SIGNOR	proteinfamily	YES	down-regulates	phosphorylation
Q16539	UNIPROT	MAPK14	protein	P31749	AKT	SIGNOR	proteinfamily	NO	down-regulates activity	
Q16539	UNIPROT	MAPK14	protein	P31749	AKT	SIGNOR	proteinfamily	NO	up-regulates activity	
Q16539	UNIPROT	MAPK14	protein	O43524	FOXO	SIGNOR	proteinfamily	YES	up-regulates	phosphorylation
Q53ET0	UNIPROT	CRTC2	protein	P31749	AKT	SIGNOR	proteinfamily	YES	up-regulates	phosphorylation
Q6A1A2	UNIPROT	PDPK2P	protein	P31749	AKT	SIGNOR	proteinfamily	YES	up-regulates	phosphorylation
Q8NEB9	UNIPROT	PIK3C3	protein	P31749	AKT	SIGNOR	proteinfamily	YES	up-regulates	relocalization
Q8NEB9	UNIPROT	PIK3C3	protein	P31749	AKT	SIGNOR	proteinfamily	NO	up-regulates	
Q96EB6	UNIPROT	SIRT1	protein	P31749	AKT	SIGNOR	proteinfamily	YES	up-regulates activity	deacetylation
Q96EB6	UNIPROT	SIRT1	protein	O43524	FOXO	SIGNOR	proteinfamily	YES	down-regulates quantity by destabilization	binding
Q96EB6	UNIPROT	SIRT1	protein	O43524	FOXO	SIGNOR	proteinfamily	YES	up-regulates	deacetylation
Q96EB6	UNIPROT	SIRT1	protein	O43524	FOXO	SIGNOR	proteinfamily	YES	up-regulates activity	deacetylation
Q96EB6	UNIPROT	SIRT1	protein	O43524	FOXO	SIGNOR	proteinfamily	NO	down-regulates	
Q96EB6	UNIPROT	SIRT1	protein	O43524	FOXO	SIGNOR	proteinfamily	NO	down-regulates activity	
Q9HBY8	UNIPROT	SGK2	protein	O43524	FOXO	SIGNOR	proteinfamily	YES	down-regulates activity	phosphorylation
Q9HBY8	UNIPROT	SGK2	protein	O43524	FOXO	SIGNOR	proteinfamily	YES	down-regulates activity	phosphorylation
Q9HCP0	UNIPROT	CSNK1G1	protein	O43524	FOXO	SIGNOR	proteinfamily	YES	down-regulates activity	phosphorylation
Q9HCP0	UNIPROT	CSNK1G1	protein	O43524	FOXO	SIGNOR	proteinfamily	YES	down-regulates activity	phosphorylation
Q9UHD2	UNIPROT	TBK1	protein	P31749	AKT	SIGNOR	proteinfamily	YES	up-regulates	phosphorylation
Q9Y2U5	UNIPROT	MAP3K2	protein	Q02750	MEK1/2	SIGNOR	proteinfamily	YES	up-regulates	phosphorylation
P27361	SIGNOR	ERK1/2	proteinfamily	P00533	EGFR	UNIPROT	protein	YES	down-regulates	phosphorylation
P27361	SIGNOR	ERK1/2	proteinfamily	P01100	FOS	UNIPROT	protein	YES	up-regulates	phosphorylation
P27361	SIGNOR	ERK1/2	proteinfamily	P01100	FOS	UNIPROT	protein	YES	up-regulates	phosphorylation
P27361	SIGNOR	ERK1/2	proteinfamily	P01100	FOS	UNIPROT	protein	YES	up-regulates	phosphorylation
P27361	SIGNOR	ERK1/2	proteinfamily	P01100	FOS	UNIPROT	protein	YES	up-regulates	phosphorylation
P27361	SIGNOR	ERK1/2	proteinfamily	P01106	MYC	UNIPROT	protein	YES	up-regulates quantity by stabilization	phosphorylation
P27361	SIGNOR	ERK1/2	proteinfamily	P05412	JUN	UNIPROT	protein	YES	up-regulates activity	phosphorylation
P27361	SIGNOR	ERK1/2	proteinfamily	P05412	JUN	UNIPROT	protein	UNK	up-regulates activity	phosphorylation
P27361	SIGNOR	ERK1/2	proteinfamily	P08047	SP1	UNIPROT	protein	YES	up-regulates activity	phosphorylation
P27361	SIGNOR	ERK1/2	proteinfamily	P09874	PARP1	UNIPROT	protein	YES	up-regulates	phosphorylation
P27361	SIGNOR	ERK1/2	proteinfamily	P09874	PARP1	UNIPROT	protein	YES	up-regulates	phosphorylation
P27361	SIGNOR	ERK1/2	proteinfamily	P15056	BRAF	UNIPROT	protein	YES	down-regulates	phosphorylation
P27361	SIGNOR	ERK1/2	proteinfamily	P17676	CEBPB	UNIPROT	protein	YES	up-regulates activity	phosphorylation
P27361	SIGNOR	ERK1/2	proteinfamily	P19793	RXRA	UNIPROT	protein	YES	up-regulates	phosphorylation
P27361	SIGNOR	ERK1/2	proteinfamily	P24941	CDK2	UNIPROT	protein	YES	up-regulates	phosphorylation
P27361	SIGNOR	ERK1/2	proteinfamily	P27361	MAPK3	UNIPROT	protein	YES	up-regulates activity	phosphorylation
P27361	SIGNOR	ERK1/2	proteinfamily	P46527	CDKN1B	UNIPROT	protein	YES	up-regulates	phosphorylation
P27361	SIGNOR	ERK1/2	proteinfamily	P46527	CDKN1B	UNIPROT	protein	YES	up-regulates	phosphorylation
P27361	SIGNOR	ERK1/2	proteinfamily	P48436	SOX9	UNIPROT	protein	NO	up-regulates	transcriptional regulation
P27361	SIGNOR	ERK1/2	proteinfamily	P49815	TSC2	UNIPROT	protein	YES	down-regulates	phosphorylation
P27361	SIGNOR	ERK1/2	proteinfamily	P49815	TSC2	UNIPROT	protein	YES	down-regulates activity	phosphorylation
P27361	SIGNOR	ERK1/2	proteinfamily	P49815	TSC2	UNIPROT	protein	YES	down-regulates activity	phosphorylation
P27361	SIGNOR	ERK1/2	proteinfamily	P51812	RPS6KA3	UNIPROT	protein	YES	up-regulates	phosphorylation
P27361	SIGNOR	ERK1/2	proteinfamily	P51812	RPS6KA3	UNIPROT	protein	YES	up-regulates	phosphorylation
P27361	SIGNOR	ERK1/2	proteinfamily	P51812	RPS6KA3	UNIPROT	protein	YES	up-regulates	phosphorylation
P27361	SIGNOR	ERK1/2	proteinfamily	P61978	HNRNPK	UNIPROT	protein	YES	up-regulates	phosphorylation
P27361	SIGNOR	ERK1/2	proteinfamily	P68400	CSNK2A1	UNIPROT	protein	YES	up-regulates	phosphorylation
P27361	SIGNOR	ERK1/2	proteinfamily	P68400	CSNK2A1	UNIPROT	protein	YES	up-regulates	phosphorylation
P27361	SIGNOR	ERK1/2	proteinfamily	P78536	ADAM17	UNIPROT	protein	YES	up-regulates	phosphorylation
P27361	SIGNOR	ERK1/2	proteinfamily	P84243	H3F3A	UNIPROT	protein	YES	up-regulates	phosphorylation
P27361	SIGNOR	ERK1/2	proteinfamily	Q02750	MAP2K1	UNIPROT	protein	YES	down-regulates activity	phosphorylation
P27361	SIGNOR	ERK1/2	proteinfamily	Q02750	MAP2K1	UNIPROT	protein	YES	down-regulates activity	phosphorylation
P27361	SIGNOR	ERK1/2	proteinfamily	Q07889	SOS1	UNIPROT	protein	YES	down-regulates	phosphorylation
P27361	SIGNOR	ERK1/2	proteinfamily	Q07889	SOS1	UNIPROT	protein	YES	down-regulates	phosphorylation
P27361	SIGNOR	ERK1/2	proteinfamily	Q07889	SOS1	UNIPROT	protein	YES	down-regulates activity	phosphorylation
P27361	SIGNOR	ERK1/2	proteinfamily	Q07889	SOS1	UNIPROT	protein	YES	down-regulates activity	phosphorylation
P27361	SIGNOR	ERK1/2	proteinfamily	Q07889	SOS1	UNIPROT	protein	YES	down-regulates activity	phosphorylation
P27361	SIGNOR	ERK1/2	proteinfamily	Q07889	SOS1	UNIPROT	protein	YES	down-regulates activity	phosphorylation
P27361	SIGNOR	ERK1/2	proteinfamily	Q09472	EP300	UNIPROT	protein	YES	up-regulates	phosphorylation
P27361	SIGNOR	ERK1/2	proteinfamily	Q09472	EP300	UNIPROT	protein	YES	up-regulates	phosphorylation
P27361	SIGNOR	ERK1/2	proteinfamily	Q09472	EP300	UNIPROT	protein	YES	up-regulates	phosphorylation
P27361	SIGNOR	ERK1/2	proteinfamily	Q13480	GAB1	UNIPROT	protein	YES	down-regulates	phosphorylation
P27361	SIGNOR	ERK1/2	proteinfamily	Q13480	GAB1	UNIPROT	protein	YES	down-regulates	phosphorylation
P27361	SIGNOR	ERK1/2	proteinfamily	Q13480	GAB1	UNIPROT	protein	YES	down-regulates	phosphorylation
P27361	SIGNOR	ERK1/2	proteinfamily	Q13480	GAB1	UNIPROT	protein	YES	down-regulates	phosphorylation
P27361	SIGNOR	ERK1/2	proteinfamily	Q14790	CASP8	UNIPROT	protein	YES	down-regulates	phosphorylation
P27361	SIGNOR	ERK1/2	proteinfamily	Q15418	RPS6KA1	UNIPROT	protein	YES	up-regulates activity	phosphorylation
P27361	SIGNOR	ERK1/2	proteinfamily	Q15418	RPS6KA1	UNIPROT	protein	YES	up-regulates activity	phosphorylation
P27361	SIGNOR	ERK1/2	proteinfamily	Q15418	RPS6KA1	UNIPROT	protein	YES	up-regulates activity	phosphorylation
P27361	SIGNOR	ERK1/2	proteinfamily	Q15418	RPS6KA1	UNIPROT	protein	YES	up-regulates activity	phosphorylation
P27361	SIGNOR	ERK1/2	proteinfamily	Q15418	RPS6KA1	UNIPROT	protein	YES	up-regulates activity	phosphorylation
P27361	SIGNOR	ERK1/2	proteinfamily	Q15418	RPS6KA1	UNIPROT	protein	YES	up-regulates activity	phosphorylation
P27361	SIGNOR	ERK1/2	proteinfamily	Q15796	SMAD2	UNIPROT	protein	YES	down-regulates	phosphorylation
P27361	SIGNOR	ERK1/2	proteinfamily	Q15796	SMAD2	UNIPROT	protein	YES	up-regulates	phosphorylation
P27361	SIGNOR	ERK1/2	proteinfamily	Q15796	SMAD2	UNIPROT	protein	YES	up-regulates	phosphorylation
P27361	SIGNOR	ERK1/2	proteinfamily	Q15796	SMAD2	UNIPROT	protein	YES	up-regulates	phosphorylation
P27361	SIGNOR	ERK1/2	proteinfamily	Q15796	SMAD2	UNIPROT	protein	YES	up-regulates	phosphorylation
P27361	SIGNOR	ERK1/2	proteinfamily	Q15796	SMAD2	UNIPROT	protein	YES	up-regulates	phosphorylation
P27361	SIGNOR	ERK1/2	proteinfamily	Q15796	SMAD2	UNIPROT	protein	YES	up-regulates	phosphorylation
P27361	SIGNOR	ERK1/2	proteinfamily	Q16539	MAPK14	UNIPROT	protein	NO	down-regulates	
P27361	SIGNOR	ERK1/2	proteinfamily	Q92574	TSC1	UNIPROT	protein	YES	down-regulates	phosphorylation

P27361	SIGNOR	ERK1/2	proteinfamily	Q92934	BAD	UNIPROT	protein	YES	down-regulates activity	phosphorylation
P27361	SIGNOR	ERK1/2	proteinfamily	Q96RK0	CIC	UNIPROT	protein	YES	down-regulates	phosphorylation
P27361	SIGNOR	ERK1/2	proteinfamily	Q9NQX3	GPHN	UNIPROT	protein	YES	down-regulates	phosphorylation
P27361	SIGNOR	ERK1/2	proteinfamily	Q9UBN7	HDAC6	UNIPROT	protein	YES	up-regulates	phosphorylation
P27361	SIGNOR	ERK1/2	proteinfamily	Q9UBN7	HDAC6	UNIPROT	protein	YES	up-regulates	phosphorylation
P27361	SIGNOR	ERK1/2	proteinfamily	Q02750	MEK1/2	SIGNOR	proteinfamily	YES	down-regulates activity	phosphorylation
P27361	SIGNOR	ERK1/2	proteinfamily	Q02750	MEK1/2	SIGNOR	proteinfamily	YES	down-regulates activity	phosphorylation
P27361	SIGNOR	ERK1/2	proteinfamily	P51812	RPS6K	SIGNOR	proteinfamily	YES	up-regulates activity	phosphorylation
P27361	SIGNOR	ERK1/2	proteinfamily	P51812	RPS6K	SIGNOR	proteinfamily	YES	up-regulates activity	phosphorylation
P27361	SIGNOR	ERK1/2	proteinfamily	P51812	RPS6K	SIGNOR	proteinfamily	YES	up-regulates activity	phosphorylation
P27361	SIGNOR	ERK1/2	proteinfamily	P51812	RPS6K	SIGNOR	proteinfamily	YES	up-regulates activity	phosphorylation
P27361	SIGNOR	ERK1/2	proteinfamily	P51812	RPS6K	SIGNOR	proteinfamily	YES	up-regulates activity	phosphorylation
P27361	SIGNOR	ERK1/2	proteinfamily	P51812	RPS6K	SIGNOR	proteinfamily	YES	up-regulates activity	phosphorylation
Q13177	SIGNOR	PAK	proteinfamily	Q02750	MAP2K1	UNIPROT	protein	YES	up-regulates	phosphorylation
Q13177	SIGNOR	PAK	proteinfamily	Q02750	MEK1/2	SIGNOR	proteinfamily	YES	up-regulates	phosphorylation
P45983	SIGNOR	JNK	proteinfamily	P05412	JUN	UNIPROT	protein	YES	up-regulates activity	phosphorylation
P45983	SIGNOR	JNK	proteinfamily	P05412	JUN	UNIPROT	protein	YES	up-regulates activity	phosphorylation
P45983	SIGNOR	JNK	proteinfamily	P05412	JUN	UNIPROT	protein	YES	up-regulates activity	phosphorylation
P45983	SIGNOR	JNK	proteinfamily	P05412	JUN	UNIPROT	protein	YES	up-regulates activity	phosphorylation
P45983	SIGNOR	JNK	proteinfamily	P05412	JUN	UNIPROT	protein	YES	up-regulates activity	phosphorylation
P45983	SIGNOR	JNK	proteinfamily	P15336	ATF2	UNIPROT	protein	YES	up-regulates	phosphorylation
P45983	SIGNOR	JNK	proteinfamily	P15336	ATF2	UNIPROT	protein	YES	up-regulates	phosphorylation
P45983	SIGNOR	JNK	proteinfamily	P35222	CTNNB1	UNIPROT	protein	YES	down-regulates	phosphorylation
P45983	SIGNOR	JNK	proteinfamily	P35222	CTNNB1	UNIPROT	protein	YES	down-regulates	phosphorylation
P45983	SIGNOR	JNK	proteinfamily	P35222	CTNNB1	UNIPROT	protein	YES	down-regulates	phosphorylation
P45983	SIGNOR	JNK	proteinfamily	P35568	IRS1	UNIPROT	protein	YES	down-regulates quantity by destabilization	phosphorylation
P45983	SIGNOR	JNK	proteinfamily	P61978	HNRNPK	UNIPROT	protein	YES	up-regulates	phosphorylation
P45983	SIGNOR	JNK	proteinfamily	P61978	HNRNPK	UNIPROT	protein	YES	up-regulates	phosphorylation
P45983	SIGNOR	JNK	proteinfamily	P84022	SMAD3	UNIPROT	protein	YES	up-regulates activity	phosphorylation
P45983	SIGNOR	JNK	proteinfamily	Q07817	BCL2L1	UNIPROT	protein	YES	down-regulates	phosphorylation
P45983	SIGNOR	JNK	proteinfamily	Q07817	BCL2L1	UNIPROT	protein	YES	down-regulates	phosphorylation
P45983	SIGNOR	JNK	proteinfamily	Q07817	BCL2L1	UNIPROT	protein	YES	down-regulates	phosphorylation
P45983	SIGNOR	JNK	proteinfamily	O43524	FOXO	SIGNOR	proteinfamily	YES	up-regulates	phosphorylation
P45983	SIGNOR	JNK	proteinfamily	O43524	FOXO	SIGNOR	proteinfamily	YES	up-regulates	phosphorylation
P45983	SIGNOR	JNK	proteinfamily	O43524	FOXO	SIGNOR	proteinfamily	YES	up-regulates	phosphorylation
P45983	SIGNOR	JNK	proteinfamily	O43524	FOXO	SIGNOR	proteinfamily	YES	up-regulates	phosphorylation
P17612	SIGNOR	PKA	proteinfamily	Q16637	SMN1	UNIPROT	protein	YES	up-regulates quantity by stabilization	phosphorylation
Q08209	SIGNOR	PP2B	proteinfamily	P05412	JUN	UNIPROT	protein	YES	up-regulates	dephosphorylation
Q7L7X3	SIGNOR	TAOK	proteinfamily	Q13043	STK4	UNIPROT	protein	YES	up-regulates	phosphorylation
Q7L7X3	SIGNOR	TAOK	proteinfamily	Q13188	STK3	UNIPROT	protein	YES	up-regulates	phosphorylation
Q7L7X3	SIGNOR	TAOK	proteinfamily	Q13043	STK3/4	SIGNOR	proteinfamily	YES	up-regulates activity	phosphorylation
Q99594	SIGNOR	TEAD	proteinfamily	P46937	YAP1	UNIPROT	protein	YES	up-regulates activity	binding
P31749	SIGNOR	AKT	proteinfamily	A8MYZ6	FOXO6	UNIPROT	protein	YES	down-regulates	phosphorylation
P31749	SIGNOR	AKT	proteinfamily	O14757	CHEK1	UNIPROT	protein	YES	down-regulates	phosphorylation
P31749	SIGNOR	AKT	proteinfamily	O15111	CHUK	UNIPROT	protein	YES	up-regulates	phosphorylation
P31749	SIGNOR	AKT	proteinfamily	O43464	HTRA2	UNIPROT	protein	YES	down-regulates	phosphorylation
P31749	SIGNOR	AKT	proteinfamily	O43524	FOXO3	UNIPROT	protein	YES	down-regulates activity	phosphorylation
P31749	SIGNOR	AKT	proteinfamily	O43524	FOXO3	UNIPROT	protein	YES	down-regulates activity	phosphorylation
P31749	SIGNOR	AKT	proteinfamily	O43524	FOXO3	UNIPROT	protein	YES	down-regulates activity	phosphorylation
P31749	SIGNOR	AKT	proteinfamily	O43524	FOXO3	UNIPROT	protein	YES	down-regulates quantity by destabilization	phosphorylation
P31749	SIGNOR	AKT	proteinfamily	O43524	FOXO3	UNIPROT	protein	YES	down-regulates quantity by destabilization	phosphorylation
P31749	SIGNOR	AKT	proteinfamily	O43524	FOXO3	UNIPROT	protein	YES	down-regulates quantity by destabilization	phosphorylation
P31749	SIGNOR	AKT	proteinfamily	O60343	TBC1D4	UNIPROT	protein	YES	down-regulates	phosphorylation
P31749	SIGNOR	AKT	proteinfamily	O60343	TBC1D4	UNIPROT	protein	YES	unknown	phosphorylation
P31749	SIGNOR	AKT	proteinfamily	O60343	TBC1D4	UNIPROT	protein	YES	unknown	phosphorylation
P31749	SIGNOR	AKT	proteinfamily	O60825	PFKFB2	UNIPROT	protein	YES	unknown	phosphorylation
P31749	SIGNOR	AKT	proteinfamily	O60825	PFKFB2	UNIPROT	protein	YES	unknown	phosphorylation
P31749	SIGNOR	AKT	proteinfamily	O60825	PFKFB2	UNIPROT	protein	YES	up-regulates activity	phosphorylation
P31749	SIGNOR	AKT	proteinfamily	O60825	PFKFB2	UNIPROT	protein	YES	up-regulates activity	phosphorylation
P31749	SIGNOR	AKT	proteinfamily	O60825	PFKFB2	UNIPROT	protein	YES	up-regulates activity	phosphorylation
P31749	SIGNOR	AKT	proteinfamily	O60825	PFKFB2	UNIPROT	protein	YES	up-regulates activity	phosphorylation
P31749	SIGNOR	AKT	proteinfamily	O75376	NCOR1	UNIPROT	protein	YES	down-regulates	phosphorylation
P31749	SIGNOR	AKT	proteinfamily	P04792	HSPB1	UNIPROT	protein	YES	down-regulates	phosphorylation
P31749	SIGNOR	AKT	proteinfamily	P08670	VIM	UNIPROT	protein	YES	up-regulates	phosphorylation
P31749	SIGNOR	AKT	proteinfamily	P09651	HNRNPA1	UNIPROT	protein	YES	down-regulates	phosphorylation
P31749	SIGNOR	AKT	proteinfamily	P11166	SLC2A1	UNIPROT	protein	NO	up-regulates quantity by expression	transcriptional regulation
P31749	SIGNOR	AKT	proteinfamily	P15056	BRAF	UNIPROT	protein	YES	down-regulates	phosphorylation
P31749	SIGNOR	AKT	proteinfamily	P15056	BRAF	UNIPROT	protein	YES	down-regulates	phosphorylation
P31749	SIGNOR	AKT	proteinfamily	P15056	BRAF	UNIPROT	protein	YES	down-regulates	phosphorylation
P31749	SIGNOR	AKT	proteinfamily	P15311	EZR	UNIPROT	protein	YES	up-regulates	phosphorylation
P31749	SIGNOR	AKT	proteinfamily	P16220	CREB1	UNIPROT	protein	YES	up-regulates activity	phosphorylation
P31749	SIGNOR	AKT	proteinfamily	P16220	CREB1	UNIPROT	protein	NO	up-regulates activity	phosphorylation
P31749	SIGNOR	AKT	proteinfamily	P17612	PRKACA	UNIPROT	protein	NO	up-regulates	
P31749	SIGNOR	AKT	proteinfamily	P18031	PTPN1	UNIPROT	protein	YES	down-regulates activity	phosphorylation
P31749	SIGNOR	AKT	proteinfamily	P23588	EIF4B	UNIPROT	protein	YES	up-regulates	phosphorylation
P31749	SIGNOR	AKT	proteinfamily	P24941	CDK2	UNIPROT	protein	YES	up-regulates	phosphorylation
P31749	SIGNOR	AKT	proteinfamily	P25445	FAS	UNIPROT	protein	NO	down-regulates	
P31749	SIGNOR	AKT	proteinfamily	P26358	DNMT1	UNIPROT	protein	YES	up-regulates	phosphorylation
P31749	SIGNOR	AKT	proteinfamily	P35222	CTNNB1	UNIPROT	protein	YES	up-regulates	phosphorylation
P31749	SIGNOR	AKT	proteinfamily	P35222	CTNNB1	UNIPROT	protein	YES	up-regulates	phosphorylation
P31749	SIGNOR	AKT	proteinfamily	P35222	CTNNB1	UNIPROT	protein	NO	up-regulates quantity by expression	transcriptional regulation
P31749	SIGNOR	AKT	proteinfamily	P35226	BMI1	UNIPROT	protein	YES	up-regulates activity	phosphorylation
P31749	SIGNOR	AKT	proteinfamily	P41279	MAP3K8	UNIPROT	protein	YES	down-regulates activity	phosphorylation
P31749	SIGNOR	AKT	proteinfamily	P41279	MAP3K8	UNIPROT	protein	YES	down-regulates activity	phosphorylation
P31749	SIGNOR	AKT	proteinfamily	P41279	MAP3K8	UNIPROT	protein	YES	up-regulates activity	phosphorylation
P31749	SIGNOR	AKT	proteinfamily	P41279	MAP3K8	UNIPROT	protein	YES	up-regulates activity	phosphorylation
P31749	SIGNOR	AKT	proteinfamily	P42345	MTOR	UNIPROT	protein	YES	down-regulates activity	phosphorylation
P31749	SIGNOR	AKT	proteinfamily	P42345	MTOR	UNIPROT	protein	NO	up-regulates	
P31749	SIGNOR	AKT	proteinfamily	P42858	HTT	UNIPROT	protein	YES	unknown	phosphorylation
P31749	SIGNOR	AKT	proteinfamily	P45985	MAP2K4	UNIPROT	protein	YES	down-regulates	phosphorylation
P31749	SIGNOR	AKT	proteinfamily	P46527	CDKN1B	UNIPROT	protein	YES	down-regulates	binding
P31749	SIGNOR	AKT	proteinfamily	P46527	CDKN1B	UNIPROT	protein	YES	down-regulates activity	phosphorylation
P31749	SIGNOR	AKT	proteinfamily	P46527	CDKN1B	UNIPROT	protein	YES	down-regulates activity	phosphorylation
P31749	SIGNOR	AKT	proteinfamily	P46527	CDKN1B	UNIPROT	protein	YES	down-regulates activity	phosphorylation
P31749	SIGNOR	AKT	proteinfamily	P46937	YAP1	UNIPROT	protein	YES	down-regulates	phosphorylation
P31749	SIGNOR	AKT	proteinfamily	P49815	TSC2	UNIPROT	protein	YES	down-regulates activity	phosphorylation
P31749	SIGNOR	AKT	proteinfamily	P49815	TSC2	UNIPROT	protein	YES	down-regulates activity	phosphorylation
P31749	SIGNOR	AKT	proteinfamily	P49840	GSK3A	UNIPROT	protein	YES	down-regulates	phosphorylation

P31749	SIGNOR	AKT	proteinfamily	P49841	GSK3B	UNIPROT	protein	YES	down-regulates	phosphorylation
P31749	SIGNOR	AKT	proteinfamily	P49841	GSK3B	UNIPROT	protein	YES	down-regulates activity	phosphorylation
P31749	SIGNOR	AKT	proteinfamily	P49841	GSK3B	UNIPROT	protein	YES	down-regulates activity	phosphorylation
P31749	SIGNOR	AKT	proteinfamily	P51617	IRAK1	UNIPROT	protein	YES	down-regulates activity	phosphorylation
P31749	SIGNOR	AKT	proteinfamily	P53365	ARFIP2	UNIPROT	protein	YES	unknown	phosphorylation
P31749	SIGNOR	AKT	proteinfamily	P53396	ACLY	UNIPROT	protein	YES	unknown	phosphorylation
P31749	SIGNOR	AKT	proteinfamily	P54253	ATXN1	UNIPROT	protein	YES	up-regulates quantity by stabilization	phosphorylation
P31749	SIGNOR	AKT	proteinfamily	P55072	VCP	UNIPROT	protein	YES	up-regulates	phosphorylation
P31749	SIGNOR	AKT	proteinfamily	P55072	VCP	UNIPROT	protein	YES	up-regulates	phosphorylation
P31749	SIGNOR	AKT	proteinfamily	P55072	VCP	UNIPROT	protein	YES	up-regulates	phosphorylation
P31749	SIGNOR	AKT	proteinfamily	P55211	CASP9	UNIPROT	protein	YES	down-regulates activity	phosphorylation
P31749	SIGNOR	AKT	proteinfamily	P55211	CASP9	UNIPROT	protein	YES	down-regulates activity	phosphorylation
P31749	SIGNOR	AKT	proteinfamily	P55211	CASP9	UNIPROT	protein	YES	down-regulates activity	phosphorylation
P31749	SIGNOR	AKT	proteinfamily	P62136	PPP1CA	UNIPROT	protein	YES	down-regulates	phosphorylation
P31749	SIGNOR	AKT	proteinfamily	P62136	PPP1CA	UNIPROT	protein	YES	down-regulates	phosphorylation
P31749	SIGNOR	AKT	proteinfamily	P63000	RAC1	UNIPROT	protein	YES	down-regulates activity	phosphorylation
P31749	SIGNOR	AKT	proteinfamily	P63000	RAC1	UNIPROT	protein	YES	down-regulates activity	phosphorylation
P31749	SIGNOR	AKT	proteinfamily	P63104	YWHAZ	UNIPROT	protein	YES	unknown	phosphorylation
P31749	SIGNOR	AKT	proteinfamily	P67809	YBX1	UNIPROT	protein	YES	up-regulates	phosphorylation
P31749	SIGNOR	AKT	proteinfamily	P78362	SRPK2	UNIPROT	protein	YES	up-regulates	phosphorylation
P31749	SIGNOR	AKT	proteinfamily	P78371	CCT2	UNIPROT	protein	YES	unknown	phosphorylation
P31749	SIGNOR	AKT	proteinfamily	P84022	SMAD3	UNIPROT	protein	YES	down-regulates	binding
P31749	SIGNOR	AKT	proteinfamily	P84243	H3F3A	UNIPROT	protein	YES	up-regulates	phosphorylation
P31749	SIGNOR	AKT	proteinfamily	P98170	XIAP	UNIPROT	protein	YES	up-regulates quantity by stabilization	phosphorylation
P31749	SIGNOR	AKT	proteinfamily	Q04912	MST1R	UNIPROT	protein	YES	up-regulates	phosphorylation
P31749	SIGNOR	AKT	proteinfamily	Q07352	ZFP36L1	UNIPROT	protein	YES	down-regulates	phosphorylation
P31749	SIGNOR	AKT	proteinfamily	Q07812	BAX	UNIPROT	protein	YES	down-regulates activity	phosphorylation
P31749	SIGNOR	AKT	proteinfamily	Q07812	BAX	UNIPROT	protein	YES	up-regulates activity	phosphorylation
P31749	SIGNOR	AKT	proteinfamily	Q09472	EP300	UNIPROT	protein	YES	up-regulates	phosphorylation
P31749	SIGNOR	AKT	proteinfamily	Q09472	EP300	UNIPROT	protein	YES	up-regulates	phosphorylation
P31749	SIGNOR	AKT	proteinfamily	Q12778	FOXO1	UNIPROT	protein	YES	down-regulates	phosphorylation
P31749	SIGNOR	AKT	proteinfamily	Q12778	FOXO1	UNIPROT	protein	YES	down-regulates	phosphorylation
P31749	SIGNOR	AKT	proteinfamily	Q12778	FOXO1	UNIPROT	protein	YES	down-regulates	phosphorylation
P31749	SIGNOR	AKT	proteinfamily	Q12778	FOXO1	UNIPROT	protein	YES	down-regulates	phosphorylation
P31749	SIGNOR	AKT	proteinfamily	Q12778	FOXO1	UNIPROT	protein	YES	down-regulates activity	phosphorylation
P31749	SIGNOR	AKT	proteinfamily	Q12778	FOXO1	UNIPROT	protein	YES	down-regulates quantity by destabilization	phosphorylation
P31749	SIGNOR	AKT	proteinfamily	Q12778	FOXO1	UNIPROT	protein	YES	down-regulates	relocalization
P31749	SIGNOR	AKT	proteinfamily	Q12906	ILF3	UNIPROT	protein	YES	up-regulates	phosphorylation
P31749	SIGNOR	AKT	proteinfamily	Q13043	STK4	UNIPROT	protein	YES	down-regulates	phosphorylation
P31749	SIGNOR	AKT	proteinfamily	Q13043	STK4	UNIPROT	protein	YES	down-regulates	phosphorylation
P31749	SIGNOR	AKT	proteinfamily	Q13131	PRKAA1	UNIPROT	protein	YES	down-regulates activity	phosphorylation
P31749	SIGNOR	AKT	proteinfamily	Q13188	STK3	UNIPROT	protein	YES	down-regulates	phosphorylation
P31749	SIGNOR	AKT	proteinfamily	Q13188	STK3	UNIPROT	protein	YES	down-regulates	phosphorylation
P31749	SIGNOR	AKT	proteinfamily	Q13188	STK3	UNIPROT	protein	YES	down-regulates	phosphorylation
P31749	SIGNOR	AKT	proteinfamily	Q15027	ACAP1	UNIPROT	protein	YES	unknown	phosphorylation
P31749	SIGNOR	AKT	proteinfamily	Q15121	PEA15	UNIPROT	protein	YES	up-regulates activity	phosphorylation
P31749	SIGNOR	AKT	proteinfamily	Q15910	EZH2	UNIPROT	protein	YES	down-regulates activity	phosphorylation
P31749	SIGNOR	AKT	proteinfamily	Q15942	ZYX	UNIPROT	protein	YES	down-regulates	phosphorylation
P31749	SIGNOR	AKT	proteinfamily	Q16539	MAPK14	UNIPROT	protein	NO	down-regulates activity	
P31749	SIGNOR	AKT	proteinfamily	Q53EL6	PDCD4	UNIPROT	protein	YES	down-regulates	phosphorylation
P31749	SIGNOR	AKT	proteinfamily	Q53EL6	PDCD4	UNIPROT	protein	YES	down-regulates	phosphorylation
P31749	SIGNOR	AKT	proteinfamily	Q53EL6	PDCD4	UNIPROT	protein	YES	down-regulates	phosphorylation
P31749	SIGNOR	AKT	proteinfamily	Q7L5N1	COPS6	UNIPROT	protein	YES	up-regulates	phosphorylation
P31749	SIGNOR	AKT	proteinfamily	Q86V81	ALYREF	UNIPROT	protein	YES	up-regulates	phosphorylation
P31749	SIGNOR	AKT	proteinfamily	Q92934	BAD	UNIPROT	protein	YES	down-regulates activity	phosphorylation
P31749	SIGNOR	AKT	proteinfamily	Q92934	BAD	UNIPROT	protein	YES	down-regulates activity	phosphorylation
P31749	SIGNOR	AKT	proteinfamily	Q92934	BAD	UNIPROT	protein	YES	down-regulates activity	phosphorylation
P31749	SIGNOR	AKT	proteinfamily	Q92934	BAD	UNIPROT	protein	YES	down-regulates activity	phosphorylation
P31749	SIGNOR	AKT	proteinfamily	Q92945	KHSRP	UNIPROT	protein	YES	down-regulates activity	phosphorylation
P31749	SIGNOR	AKT	proteinfamily	Q96B36	AKT1S1	UNIPROT	protein	YES	down-regulates activity	phosphorylation
P31749	SIGNOR	AKT	proteinfamily	Q96S44	TP53RK	UNIPROT	protein	YES	up-regulates	phosphorylation
P31749	SIGNOR	AKT	proteinfamily	Q9BZQ8	FAM129A	UNIPROT	protein	YES	unknown	phosphorylation
P31749	SIGNOR	AKT	proteinfamily	Q9H4A3	WNK1	UNIPROT	protein	YES	up-regulates	phosphorylation
P31749	SIGNOR	AKT	proteinfamily	Q9H6Z4	RANBP3	UNIPROT	protein	YES	unknown	phosphorylation
P31749	SIGNOR	AKT	proteinfamily	Q9UBP6	METTL1	UNIPROT	protein	YES	down-regulates	phosphorylation
P31749	SIGNOR	AKT	proteinfamily	Q9Y2V2	CARHSP1	UNIPROT	protein	YES	unknown	phosphorylation
P31749	SIGNOR	AKT	proteinfamily	O43524	FOXO	SIGNOR	proteinfamily	YES	down-regulates	phosphorylation
P31749	SIGNOR	AKT	proteinfamily	O43524	FOXO	SIGNOR	proteinfamily	YES	down-regulates	phosphorylation
P31749	SIGNOR	AKT	proteinfamily	O43524	FOXO	SIGNOR	proteinfamily	YES	down-regulates	phosphorylation
P31749	SIGNOR	AKT	proteinfamily	O43524	FOXO	SIGNOR	proteinfamily	YES	down-regulates	phosphorylation
P31749	SIGNOR	AKT	proteinfamily	O43524	FOXO	SIGNOR	proteinfamily	YES	down-regulates	phosphorylation
P31749	SIGNOR	AKT	proteinfamily	O43524	FOXO	SIGNOR	proteinfamily	YES	down-regulates	phosphorylation
P31749	SIGNOR	AKT	proteinfamily	O43524	FOXO	SIGNOR	proteinfamily	YES	down-regulates	phosphorylation
P31749	SIGNOR	AKT	proteinfamily	O43524	FOXO	SIGNOR	proteinfamily	YES	down-regulates	phosphorylation
P31749	SIGNOR	AKT	proteinfamily	O43524	FOXO	SIGNOR	proteinfamily	YES	down-regulates	phosphorylation
P31749	SIGNOR	AKT	proteinfamily	O43524	FOXO	SIGNOR	proteinfamily	YES	down-regulates activity	phosphorylation
P31749	SIGNOR	AKT	proteinfamily	O43524	FOXO	SIGNOR	proteinfamily	YES	down-regulates activity	phosphorylation
P31749	SIGNOR	AKT	proteinfamily	O43524	FOXO	SIGNOR	proteinfamily	YES	down-regulates activity	phosphorylation
P31749	SIGNOR	AKT	proteinfamily	O43524	FOXO	SIGNOR	proteinfamily	YES	down-regulates activity	phosphorylation
P31749	SIGNOR	AKT	proteinfamily	O43524	FOXO	SIGNOR	proteinfamily	YES	down-regulates activity	phosphorylation
P31749	SIGNOR	AKT	proteinfamily	O43524	FOXO	SIGNOR	proteinfamily	YES	down-regulates activity	phosphorylation
P31749	SIGNOR	AKT	proteinfamily	O43524	FOXO	SIGNOR	proteinfamily	YES	down-regulates quantity by destabilization	phosphorylation
P31749	SIGNOR	AKT	proteinfamily	O43524	FOXO	SIGNOR	proteinfamily	YES	down-regulates quantity by destabilization	phosphorylation
P31749	SIGNOR	AKT	proteinfamily	O43524	FOXO	SIGNOR	proteinfamily	YES	down-regulates quantity by destabilization	phosphorylation
P31749	SIGNOR	AKT	proteinfamily	O43524	FOXO	SIGNOR	proteinfamily	YES	down-regulates quantity by destabilization	phosphorylation
P31749	SIGNOR	AKT	proteinfamily	O43524	FOXO	SIGNOR	proteinfamily	YES	down-regulates	relocalization
Q02750	SIGNOR	MEK1/2	proteinfamily	P27361	MAPK3	UNIPROT	protein	YES	up-regulates	phosphorylation
Q02750	SIGNOR	MEK1/2	proteinfamily	P27361	MAPK3	UNIPROT	protein	YES	up-regulates	phosphorylation
Q02750	SIGNOR	MEK1/2	proteinfamily	P27361	MAPK3	UNIPROT	protein	YES	up-regulates	phosphorylation
Q02750	SIGNOR	MEK1/2	proteinfamily	P28482	MAPK1	UNIPROT	protein	YES	up-regulates activity	phosphorylation
Q02750	SIGNOR	MEK1/2	proteinfamily	P28482	MAPK1	UNIPROT	protein	YES	up-regulates activity	phosphorylation
Q02750	SIGNOR	MEK1/2	proteinfamily	P28482	MAPK1	UNIPROT	protein	YES	up-regulates activity	phosphorylation
Q02750	SIGNOR	MEK1/2	proteinfamily	P35568	IRS1	UNIPROT	protein	YES	down-regulates	phosphorylation
Q02750	SIGNOR	MEK1/2	proteinfamily	P49841	GSK3B	UNIPROT	protein	YES	up-regulates activity	phosphorylation
Q02750	SIGNOR	MEK1/2	proteinfamily	P27361	ERK1/2	SIGNOR	proteinfamily	YES	up-regulates	phosphorylation
Q02750	SIGNOR	MEK1/2	proteinfamily	Q02750	MEK1/2	SIGNOR	proteinfamily	NO	down-regulates	
P51812	SIGNOR	RPS6K	proteinfamily	O00418	EEF2K	UNIPROT	protein	YES	down-regulates activity	phosphorylation
P51812	SIGNOR	RPS6K	proteinfamily	P01100	FOS	UNIPROT	protein	YES	up-regulates	phosphorylation

P51812	SIGNOR	RPS6K	proteinfamily	P16220	CREB1	UNIPROT	protein	YES	up-regulates activity	phosphorylation
P51812	SIGNOR	RPS6K	proteinfamily	P19634	SLC9A1	UNIPROT	protein	YES	up-regulates	phosphorylation
P51812	SIGNOR	RPS6K	proteinfamily	P21333	FLNA	UNIPROT	protein	YES	up-regulates	phosphorylation
P51812	SIGNOR	RPS6K	proteinfamily	P23588	EIF4B	UNIPROT	protein	YES	up-regulates	phosphorylation
P51812	SIGNOR	RPS6K	proteinfamily	P35568	IRS1	UNIPROT	protein	YES	down-regulates quantity by destabilization	phosphorylation
P51812	SIGNOR	RPS6K	proteinfamily	P49815	TSC2	UNIPROT	protein	YES	down-regulates	phosphorylation
P51812	SIGNOR	RPS6K	proteinfamily	P49841	GSK3B	UNIPROT	protein	YES	down-regulates	phosphorylation
P51812	SIGNOR	RPS6K	proteinfamily	P50552	VASP	UNIPROT	protein	YES	down-regulates	phosphorylation
P51812	SIGNOR	RPS6K	proteinfamily	P62753	RPS6	UNIPROT	protein	YES	up-regulates	phosphorylation
P51812	SIGNOR	RPS6K	proteinfamily	P62753	RPS6	UNIPROT	protein	YES	up-regulates	phosphorylation
P51812	SIGNOR	RPS6K	proteinfamily	P62753	RPS6	UNIPROT	protein	YES	up-regulates	phosphorylation
P51812	SIGNOR	RPS6K	proteinfamily	P62753	RPS6	UNIPROT	protein	YES	up-regulates	phosphorylation
P51812	SIGNOR	RPS6K	proteinfamily	P67809	YBX1	UNIPROT	protein	YES	up-regulates	phosphorylation
P51812	SIGNOR	RPS6K	proteinfamily	P78371	CCT2	UNIPROT	protein	YES	up-regulates	phosphorylation
P51812	SIGNOR	RPS6K	proteinfamily	P84243	H3F3A	UNIPROT	protein	YES	up-regulates	phosphorylation
P51812	SIGNOR	RPS6K	proteinfamily	P84243	H3F3A	UNIPROT	protein	YES	up-regulates	phosphorylation
P51812	SIGNOR	RPS6K	proteinfamily	P84243	H3F3A	UNIPROT	protein	YES	up-regulates	phosphorylation
P51812	SIGNOR	RPS6K	proteinfamily	Q8IX03	WWC1	UNIPROT	protein	YES	up-regulates	phosphorylation
P51812	SIGNOR	RPS6K	proteinfamily	Q8IX03	WWC1	UNIPROT	protein	YES	up-regulates	phosphorylation
P51812	SIGNOR	RPS6K	proteinfamily	Q8N122	RPTOR	UNIPROT	protein	YES	up-regulates	phosphorylation
P51812	SIGNOR	RPS6K	proteinfamily	Q8N122	RPTOR	UNIPROT	protein	YES	up-regulates	phosphorylation
P51812	SIGNOR	RPS6K	proteinfamily	Q8N122	RPTOR	UNIPROT	protein	YES	up-regulates	phosphorylation
P51812	SIGNOR	RPS6K	proteinfamily	Q8TB45	DEPTOR	UNIPROT	protein	YES	down-regulates	phosphorylation
P51812	SIGNOR	RPS6K	proteinfamily	Q8TB45	DEPTOR	UNIPROT	protein	YES	down-regulates	phosphorylation
P51812	SIGNOR	RPS6K	proteinfamily	Q8TB45	DEPTOR	UNIPROT	protein	YES	down-regulates	phosphorylation
P51812	SIGNOR	RPS6K	proteinfamily	Q92934	BAD	UNIPROT	protein	YES	down-regulates activity	phosphorylation
P51812	SIGNOR	RPS6K	proteinfamily	Q92934	BAD	UNIPROT	protein	YES	down-regulates activity	phosphorylation
P51812	SIGNOR	RPS6K	proteinfamily	Q92934	BAD	UNIPROT	protein	YES	down-regulates activity	phosphorylation
P51812	SIGNOR	RPS6K	proteinfamily	Q96RK0	CIC	UNIPROT	protein	YES	down-regulates	phosphorylation
P51812	SIGNOR	RPS6K	proteinfamily	Q9H6Z4	RANBP3	UNIPROT	protein	YES	unknown	phosphorylation
P51812	SIGNOR	RPS6K	proteinfamily	Q9UBP6	METTL1	UNIPROT	protein	YES	down-regulates	phosphorylation
P51812	SIGNOR	RPS6K	proteinfamily	Q9Y2V2	CARHSP1	UNIPROT	protein	YES	unknown	phosphorylation
P51812	SIGNOR	RPS6K	proteinfamily	P51812	RPS6K	SIGNOR	proteinfamily	YES	up-regulates activity	phosphorylation
O43524	SIGNOR	FOXO	proteinfamily	P46527	CDKN1B	UNIPROT	protein	YES	up-regulates quantity	transcriptional regulation
Q9HCE7	SIGNOR	SMURF	proteinfamily	P37173	TGFBR2	UNIPROT	protein	YES	down-regulates activity	ubiquitination
Q9HCE7	SIGNOR	SMURF	proteinfamily	Q15796	SMAD2	UNIPROT	protein	YES	down-regulates activity	ubiquitination
P00533	SIGNOR	ErbB receptor family	proteinfamily	P62993	GRB2	UNIPROT	protein	YES	up-regulates activity	binding
P21802	SIGNOR	RTKs	proteinfamily	P62993	GRB2	UNIPROT	protein	YES	up-regulates activity	binding
P01344	SIGNOR	GF	proteinfamily	P21802	RTKs	SIGNOR	proteinfamily	YES	up-regulates activity	binding
Q13043	SIGNOR	STK3/4	proteinfamily	Q9H8S9	Mob1	SIGNOR	proteinfamily	YES	up-regulates activity	phosphorylation
P01137	SIGNOR	TGFb	proteinfamily	P37173	TGFBR2	UNIPROT	protein	YES	up-regulates activity	binding
P01137	SIGNOR	TGFb	proteinfamily	P37173	TGFBR2	UNIPROT	protein	YES	up-regulates activity	binding
P01137	SIGNOR	TGFb	proteinfamily	P84022	SMAD3	UNIPROT	protein	NO	up-regulates	
Q04917	SIGNOR	14-3-3	proteinfamily	O43524	FOXO3	UNIPROT	protein	YES	down-regulates	binding
Q04917	SIGNOR	14-3-3	proteinfamily	O43524	FOXO3	UNIPROT	protein	YES	down-regulates	binding
Q04917	SIGNOR	14-3-3	proteinfamily	P46937	YAP1	UNIPROT	protein	YES	down-regulates	binding
Q04917	SIGNOR	14-3-3	proteinfamily	P46937	YAP1	UNIPROT	protein	YES	down-regulates	binding
Q04917	SIGNOR	14-3-3	proteinfamily	Q92934	BAD	UNIPROT	protein	YES	down-regulates	binding
Q04917	SIGNOR	14-3-3	proteinfamily	Q92934	BAD	UNIPROT	protein	YES	down-regulates	binding
Q04917	SIGNOR	14-3-3	proteinfamily	Q92945	KHSRP	UNIPROT	protein	YES	down-regulates	binding
Q04917	SIGNOR	14-3-3	proteinfamily	Q96B36	AKT1S1	UNIPROT	protein	YES	down-regulates	binding
Q04917	SIGNOR	14-3-3	proteinfamily	O43524	FOXO	SIGNOR	proteinfamily	YES	down-regulates	binding
Q04917	SIGNOR	14-3-3	proteinfamily	O43524	FOXO	SIGNOR	proteinfamily	YES	down-regulates	binding
O15111	UNIPROT	CHUK	protein	SIGNOR-C13	NfKb-p65/p50	SIGNOR	complex	YES	down-regulates	phosphorylation
O43318	UNIPROT	MAP3K7	protein	SIGNOR-C13	NfKb-p65/p50	SIGNOR	complex	NO	up-regulates activity	
O75676	UNIPROT	RPS6KA4	protein	SIGNOR-C13	NfKb-p65/p50	SIGNOR	complex	YES	up-regulates	phosphorylation
P01137	UNIPROT	TGFB1	protein	SIGNOR-C13	NfKb-p65/p50	SIGNOR	complex	NO	up-regulates	
P04637	UNIPROT	TP53	protein	SIGNOR-C6	CBP/p300	SIGNOR	complex	YES	up-regulates	binding
P05412	UNIPROT	JUN	protein	SIGNOR-C86	SMAD3/JUN	SIGNOR	complex	YES	form complex	binding
P06493	UNIPROT	CDK1	protein	SIGNOR-C17	CyclinB/CDK1	SIGNOR	complex	YES	form complex	binding
P08047	UNIPROT	SP1	protein	SIGNOR-C74	SP1/STAT3	SIGNOR	complex	YES	form complex	binding
P14635	UNIPROT	CCNB1	protein	SIGNOR-C17	CyclinB/CDK1	SIGNOR	complex	YES	form complex	binding
P17612	UNIPROT	PRKACA	protein	SIGNOR-C13	NfKb-p65/p50	SIGNOR	complex	YES	up-regulates	phosphorylation
P17612	UNIPROT	PRKACA	protein	SIGNOR-C13	NfKb-p65/p50	SIGNOR	complex	YES	up-regulates	phosphorylation
P19838	UNIPROT	NFKB1	protein	SIGNOR-C13	NfKb-p65/p50	SIGNOR	complex	YES	form complex	binding
P20248	UNIPROT	CCNA2	protein	SIGNOR-C83	CyclinA2/CDK2	SIGNOR	complex	YES	form complex	binding
P24941	UNIPROT	CDK2	protein	SIGNOR-C83	CyclinA2/CDK2	SIGNOR	complex	YES	form complex	binding
P31749	UNIPROT	AKT1	protein	SIGNOR-C101	TSC1/TSC2	SIGNOR	complex	YES	down-regulates activity	phosphorylation
P31749	UNIPROT	AKT1	protein	SIGNOR-C101	TSC1/TSC2	SIGNOR	complex	YES	down-regulates activity	phosphorylation
P31751	UNIPROT	AKT2	protein	SIGNOR-C101	TSC1/TSC2	SIGNOR	complex	YES	down-regulates activity	phosphorylation
P40763	UNIPROT	STAT3	protein	SIGNOR-C118	STAT1/STAT3	SIGNOR	complex	YES	form complex	binding
P40763	UNIPROT	STAT3	protein	SIGNOR-C74	SP1/STAT3	SIGNOR	complex	YES	form complex	binding
P42224	UNIPROT	STAT1	protein	SIGNOR-C118	STAT1/STAT3	SIGNOR	complex	YES	form complex	binding
P42224	UNIPROT	STAT1	protein	SIGNOR-C13	NfKb-p65/p50	SIGNOR	complex	YES	down-regulates	binding
P42226	UNIPROT	STAT6	protein	SIGNOR-C13	NfKb-p65/p50	SIGNOR	complex	YES	down-regulates activity	binding
P45983	UNIPROT	MAPK8	protein	SIGNOR-C154	AP1	SIGNOR	complex	YES	up-regulates activity	phosphorylation
P45983	UNIPROT	MAPK8	protein	SIGNOR-C154	AP1	SIGNOR	complex	YES	up-regulates activity	phosphorylation
P49815	UNIPROT	TSC2	protein	SIGNOR-C101	TSC1/TSC2	SIGNOR	complex	YES	form complex	binding
P49841	UNIPROT	GSK3B	protein	SIGNOR-C13	NfKb-p65/p50	SIGNOR	complex	YES	up-regulates	phosphorylation
P49841	UNIPROT	GSK3B	protein	SIGNOR-C13	NfKb-p65/p50	SIGNOR	complex	YES	up-regulates	phosphorylation
P49841	UNIPROT	GSK3B	protein	SIGNOR-C13	NfKb-p65/p50	SIGNOR	complex	NO	up-regulates activity	
P67775	UNIPROT	PPP2CA	protein	SIGNOR-C13	NfKb-p65/p50	SIGNOR	complex	YES	down-regulates	dephosphorylation
P84022	UNIPROT	SMAD3	protein	SIGNOR-C86	SMAD3/JUN	SIGNOR	complex	YES	form complex	binding
Q04206	UNIPROT	RELA	protein	SIGNOR-C13	NfKb-p65/p50	SIGNOR	complex	YES	form complex	binding
Q04206	UNIPROT	RELA	protein	SIGNOR-C6	CBP/p300	SIGNOR	complex	YES	up-regulates	binding
Q09472	UNIPROT	EP300	protein	SIGNOR-C6	CBP/p300	SIGNOR	complex	YES	form complex	binding
Q13547	UNIPROT	HDAC1	protein	SIGNOR-C13	NfKb-p65/p50	SIGNOR	complex	YES	down-regulates	binding
Q15418	UNIPROT	RPS6KA1	protein	SIGNOR-C101	TSC1/TSC2	SIGNOR	complex	YES	down-regulates activity	phosphorylation
Q92574	UNIPROT	TSC1	protein	SIGNOR-C101	TSC1/TSC2	SIGNOR	complex	YES	form complex	binding
Q92793	UNIPROT	CREBBP	protein	SIGNOR-C6	CBP/p300	SIGNOR	complex	YES	form complex	binding
Q9Y6K9	UNIPROT	IKBKG	protein	SIGNOR-C13	NfKb-p65/p50	SIGNOR	complex	YES	up-regulates	phosphorylation
SIGNOR-C101	SIGNOR	TSC1/TSC2	complex	P42345	MTOR	UNIPROT	protein	NO	down-regulates activity	
SIGNOR-C13	SIGNOR	NfKb-p65/p50	complex	P04637	TP53	UNIPROT	protein	NO	up-regulates quantity by expression	transcriptional regulation
SIGNOR-C13	SIGNOR	NfKb-p65/p50	complex	P05412	JUN	UNIPROT	protein	YES	up-regulates	binding
SIGNOR-C13	SIGNOR	NfKb-p65/p50	complex	P08047	SP1	UNIPROT	protein	YES	up-regulates	binding
SIGNOR-C13	SIGNOR	NfKb-p65/p50	complex	P21810	BGN	UNIPROT	protein	NO	up-regulates quantity by expression	transcriptional regulation
SIGNOR-C13	SIGNOR	NfKb-p65/p50	complex	P41279	MAP3K8	UNIPROT	protein	YES	down-regulates	binding
SIGNOR-C17	SIGNOR	CyclinB/CDK1	complex	O00311	CDC7	UNIPROT	protein	YES	up-regulates activity	phosphorylation

SIGNOR-C17	SIGNOR	CyclinB/CDK1	complex	O00571	DDX3X	UNIPROT	protein	YES	down-regulates	phosphorylation
SIGNOR-C17	SIGNOR	CyclinB/CDK1	complex	O00571	DDX3X	UNIPROT	protein	YES	down-regulates	phosphorylation
SIGNOR-C17	SIGNOR	CyclinB/CDK1	complex	P06748	NPM1	UNIPROT	protein	YES	down-regulates activity	phosphorylation
SIGNOR-C17	SIGNOR	CyclinB/CDK1	complex	P06748	NPM1	UNIPROT	protein	YES	down-regulates activity	phosphorylation
SIGNOR-C17	SIGNOR	CyclinB/CDK1	complex	P06748	NPM1	UNIPROT	protein	YES	down-regulates activity	phosphorylation
SIGNOR-C17	SIGNOR	CyclinB/CDK1	complex	P08047	SP1	UNIPROT	protein	YES	up-regulates	phosphorylation
SIGNOR-C17	SIGNOR	CyclinB/CDK1	complex	P15531	NME1	UNIPROT	protein	YES	up-regulates	phosphorylation
SIGNOR-C17	SIGNOR	CyclinB/CDK1	complex	P46060	RANGAP1	UNIPROT	protein	YES	up-regulates	phosphorylation
SIGNOR-C17	SIGNOR	CyclinB/CDK1	complex	P46060	RANGAP1	UNIPROT	protein	YES	up-regulates	phosphorylation
SIGNOR-C17	SIGNOR	CyclinB/CDK1	complex	P46060	RANGAP1	UNIPROT	protein	YES	up-regulates	phosphorylation
SIGNOR-C17	SIGNOR	CyclinB/CDK1	complex	P48200	IREB2	UNIPROT	protein	YES	down-regulates	phosphorylation
SIGNOR-C17	SIGNOR	CyclinB/CDK1	complex	P50548	ERF	UNIPROT	protein	YES	down-regulates	phosphorylation
SIGNOR-C17	SIGNOR	CyclinB/CDK1	complex	P55211	CASP9	UNIPROT	protein	YES	down-regulates	phosphorylation
SIGNOR-C17	SIGNOR	CyclinB/CDK1	complex	P55211	CASP9	UNIPROT	protein	YES	down-regulates	phosphorylation
SIGNOR-C17	SIGNOR	CyclinB/CDK1	complex	Q07820	MCL1	UNIPROT	protein	YES	up-regulates	phosphorylation
SIGNOR-C17	SIGNOR	CyclinB/CDK1	complex	Q07820	MCL1	UNIPROT	protein	YES	up-regulates	phosphorylation
SIGNOR-C17	SIGNOR	CyclinB/CDK1	complex	Q13501	SQSTM1	UNIPROT	protein	YES	up-regulates	phosphorylation
SIGNOR-C17	SIGNOR	CyclinB/CDK1	complex	Q13501	SQSTM1	UNIPROT	protein	YES	up-regulates	phosphorylation
SIGNOR-C17	SIGNOR	CyclinB/CDK1	complex	Q14790	CASP8	UNIPROT	protein	YES	down-regulates	phosphorylation
SIGNOR-C17	SIGNOR	CyclinB/CDK1	complex	Q15149	PLEC	UNIPROT	protein	YES	down-regulates	phosphorylation
SIGNOR-C17	SIGNOR	CyclinB/CDK1	complex	Q15149	PLEC	UNIPROT	protein	YES	down-regulates	phosphorylation
SIGNOR-C17	SIGNOR	CyclinB/CDK1	complex	Q86WB0	ZC3HC1	UNIPROT	protein	YES	down-regulates	phosphorylation
SIGNOR-C17	SIGNOR	CyclinB/CDK1	complex	Q92574	TSC1	UNIPROT	protein	YES	down-regulates	phosphorylation
SIGNOR-C17	SIGNOR	CyclinB/CDK1	complex	Q92574	TSC1	UNIPROT	protein	YES	down-regulates	phosphorylation
SIGNOR-C17	SIGNOR	CyclinB/CDK1	complex	Q92574	TSC1	UNIPROT	protein	YES	down-regulates	phosphorylation
SIGNOR-C17	SIGNOR	CyclinB/CDK1	complex	Q92993	KAT5	UNIPROT	protein	YES	up-regulates activity	phosphorylation
SIGNOR-C17	SIGNOR	CyclinB/CDK1	complex	Q92993	KAT5	UNIPROT	protein	YES	up-regulates activity	phosphorylation
SIGNOR-C17	SIGNOR	CyclinB/CDK1	complex	Q96EB6	SIRT1	UNIPROT	protein	YES	up-regulates	phosphorylation
SIGNOR-C17	SIGNOR	CyclinB/CDK1	complex	Q96GX5	MASTL	UNIPROT	protein	YES	up-regulates activity	phosphorylation
SIGNOR-C17	SIGNOR	CyclinB/CDK1	complex	Q96GX5	MASTL	UNIPROT	protein	YES	up-regulates activity	phosphorylation
SIGNOR-C17	SIGNOR	CyclinB/CDK1	complex	Q96KB5	PBK	UNIPROT	protein	YES	unknown	phosphorylation
SIGNOR-C17	SIGNOR	CyclinB/CDK1	complex	Q96KB5	PBK	UNIPROT	protein	YES	up-regulates activity	phosphorylation
SIGNOR-C17	SIGNOR	CyclinB/CDK1	complex	Q9H8V3	ECT2	UNIPROT	protein	YES	down-regulates	phosphorylation
SIGNOR-C17	SIGNOR	CyclinB/CDK1	complex	Q9H8V3	ECT2	UNIPROT	protein	YES	up-regulates	phosphorylation
SIGNOR-C6	SIGNOR	CBP/p300	complex	P17844	DDX5	UNIPROT	protein	YES	up-regulates	binding
SIGNOR-C6	SIGNOR	CBP/p300	complex	P84022	SMAD3	UNIPROT	protein	YES	up-regulates activity	acetylation
SIGNOR-C6	SIGNOR	CBP/p300	complex	P84022	SMAD3	UNIPROT	protein	YES	up-regulates activity	acetylation
SIGNOR-C6	SIGNOR	CBP/p300	complex	P84243	H3F3A	UNIPROT	protein	YES	up-regulates	acetylation
SIGNOR-C6	SIGNOR	CBP/p300	complex	Q04206	RELA	UNIPROT	protein	YES	up-regulates	acetylation
SIGNOR-C6	SIGNOR	CBP/p300	complex	Q15796	SMAD2	UNIPROT	protein	YES	up-regulates activity	acetylation
SIGNOR-C6	SIGNOR	CBP/p300	complex	Q15797	SMAD1	UNIPROT	protein	YES	up-regulates	binding
SIGNOR-C6	SIGNOR	CBP/p300	complex	Q15797	SMAD1	UNIPROT	protein	YES	up-regulates	binding
SIGNOR-C83	SIGNOR	CyclinA2/CDK2	complex	O00311	CDC7	UNIPROT	protein	YES	up-regulates activity	phosphorylation
SIGNOR-C83	SIGNOR	CyclinA2/CDK2	complex	P04637	TP53	UNIPROT	protein	YES	unknown	phosphorylation
SIGNOR-C83	SIGNOR	CyclinA2/CDK2	complex	P04637	TP53	UNIPROT	protein	YES	up-regulates	phosphorylation
SIGNOR-C83	SIGNOR	CyclinA2/CDK2	complex	P06400	RB1	UNIPROT	protein	YES	down-regulates	phosphorylation
SIGNOR-C83	SIGNOR	CyclinA2/CDK2	complex	P08047	SP1	UNIPROT	protein	YES	up-regulates activity	phosphorylation
SIGNOR-C83	SIGNOR	CyclinA2/CDK2	complex	P17480	UBTF	UNIPROT	protein	YES	up-regulates	phosphorylation
SIGNOR-C83	SIGNOR	CyclinA2/CDK2	complex	P17676	CEBPB	UNIPROT	protein	YES	up-regulates	phosphorylation
SIGNOR-C83	SIGNOR	CyclinA2/CDK2	complex	P17676	CEBPB	UNIPROT	protein	YES	up-regulates	phosphorylation
SIGNOR-C83	SIGNOR	CyclinA2/CDK2	complex	P31749	AKT1	UNIPROT	protein	YES	up-regulates activity	phosphorylation
SIGNOR-C83	SIGNOR	CyclinA2/CDK2	complex	P31749	AKT1	UNIPROT	protein	YES	up-regulates activity	phosphorylation
SIGNOR-C83	SIGNOR	CyclinA2/CDK2	complex	P33991	MCM4	UNIPROT	protein	YES	down-regulates	phosphorylation
SIGNOR-C83	SIGNOR	CyclinA2/CDK2	complex	P33991	MCM4	UNIPROT	protein	YES	down-regulates	phosphorylation
SIGNOR-C83	SIGNOR	CyclinA2/CDK2	complex	P33991	MCM4	UNIPROT	protein	YES	down-regulates	phosphorylation
SIGNOR-C83	SIGNOR	CyclinA2/CDK2	complex	Q13416	ORC2	UNIPROT	protein	YES	up-regulates	phosphorylation
SIGNOR-C83	SIGNOR	CyclinA2/CDK2	complex	Q13416	ORC2	UNIPROT	protein	YES	up-regulates	phosphorylation
SIGNOR-C83	SIGNOR	CyclinA2/CDK2	complex	P31749	AKT	SIGNOR	proteinfamily	YES	up-regulates activity	phosphorylation
SIGNOR-C83	SIGNOR	CyclinA2/CDK2	complex	P31749	AKT	SIGNOR	proteinfamily	YES	up-regulates activity	phosphorylation
P27361	SIGNOR	ERK1/2	proteinfamily	SIGNOR-C154	AP1	SIGNOR	complex	YES	up-regulates	phosphorylation
P27361	SIGNOR	ERK1/2	proteinfamily	SIGNOR-C154	AP1	SIGNOR	complex	YES	up-regulates	phosphorylation
P27361	SIGNOR	ERK1/2	proteinfamily	SIGNOR-C154	AP1	SIGNOR	complex	YES	up-regulates	phosphorylation
P27361	SIGNOR	ERK1/2	proteinfamily	SIGNOR-C154	AP1	SIGNOR	complex	YES	up-regulates	phosphorylation
P45983	SIGNOR	JNK	proteinfamily	SIGNOR-C154	AP1	SIGNOR	complex	YES	up-regulates activity	binding
P31749	SIGNOR	AKT	proteinfamily	SIGNOR-C101	TSC1/TSC2	SIGNOR	complex	YES	down-regulates activity	phosphorylation

PART3: List of Genes informations
in large-scale colon cancerous cell's model

Gene number	1	2	3
UNIPROT ID	A8MYZ6	E9PAV3	O00161
Name	FOXO6	NACA	SNAP23
Ensemble Gene ID	ENSG00000204060	ENSG00000196531	ENSG00000092531
Ensemble Protein IDs	ENSP00000493184	ENSP00000349212	ENSP00000249647
	ENSP00000368880	ENSP00000447821	ENSP00000380327
		ENSP00000377469	ENSP00000207062
		ENSP00000446801	ENSP00000454214
		ENSP00000448039	ENSP00000454799
		ENSP00000447705	ENSP00000457535
		ENSP00000446745	ENSP00000455339
		ENSP00000447133	ENSP00000455472
		ENSP00000450383	ENSP00000455652
		ENSP00000446933	ENSP00000458012
		ENSP00000447764	ENSP00000454842
		ENSP00000447207	ENSP00000456035
		ENSP00000447666	ENSP00000456144
		ENSP00000448035	ENSP00000455102
		ENSP00000403817	ENSP00000456000
			ENSP00000485888
			ENSP00000486335

4	5	6	7	8
O00170	O00267	O00303	O00311	O00401
AIP	SUPT5H	EIF3F	CDC7	WASL
ENSG00000110711	ENSG00000196235	ENSG00000175390	ENSG00000097046	ENSG00000106299
ENSP00000279146	ENSP00000470252	ENSP00000431800	ENSP00000234626	ENSP00000223023
ENSP00000434982	ENSP00000352117	ENSP00000310040	ENSP00000393139	
ENSP00000476993	ENSP00000469090	ENSP00000432489	ENSP00000398077	
	ENSP00000404029	ENSP00000434286		
	ENSP00000384505	ENSP00000491198		
	ENSP00000471561	ENSP00000485772		
	ENSP00000472675	ENSP00000486798		
	ENSP00000470595	ENSP00000487000		
	ENSP00000469915	ENSP00000492080		
		ENSP00000487359		

9	10	11	12	13
O00418	O00429	O00443	O00506	O00571
EEF2K	DNM1L	PIK3C2A	STK25	DDX3X
ENSG00000103319	ENSG00000087470	ENSG00000011405	ENSG00000115694	ENSG00000215301
ENSP00000263026	ENSP00000415131	ENSP00000265970	ENSP00000325748	ENSP00000487224
ENSP00000456243	ENSP00000449089	ENSP00000436244	ENSP00000384162	ENSP00000486516
	ENSP00000450399	ENSP00000436969	ENSP00000385687	ENSP00000486720
	ENSP00000266481		ENSP00000384444	ENSP00000478443
	ENSP00000448610		ENSP00000385541	ENSP00000392494
	ENSP00000370388		ENSP00000446008	ENSP00000382840
	ENSP00000404160		ENSP00000444886	ENSP00000494040
	ENSP00000350948		ENSP00000404469	ENSP00000486306
	ENSP00000450401		ENSP00000399212	ENSP00000486514
	ENSP00000447845		ENSP00000417020	ENSP00000487062
	ENSP00000447013		ENSP00000403607	ENSP00000486443
	ENSP00000390090		ENSP00000395104	ENSP00000493997
	ENSP00000396030		ENSP00000404960	ENSP00000480647
	ENSP00000448936		ENSP00000412617	ENSP00000414281
	ENSP00000447788		ENSP00000403866	ENSP00000494588
	ENSP00000448105		ENSP00000401114	ENSP00000493819
	ENSP00000448802		ENSP00000406208	ENSP00000494952
	ENSP00000451288		ENSP00000414191	ENSP00000496222
			ENSP00000404552	ENSP00000494767
				ENSP00000493976
				ENSP00000496663
				ENSP00000495264
				ENSP00000496043
				ENSP00000495377
				ENSP00000494905
				ENSP00000493687
				ENSP00000493475
				ENSP00000496257
				ENSP00000496524
				ENSP00000496052
				ENSP00000494518
				ENSP00000493795
				ENSP00000496356
				ENSP00000494887
				ENSP00000496076
				ENSP00000487626
				ENSP00000479790
				ENSP00000353284
				ENSP00000336725

14	15	16	17	18
O00716	O00743	O00750	O14641	O14733
E2F3	PPP6C	PIK3C2B	DVL2	MAP2K7
ENSG00000112242	ENSG00000119414	ENSG00000133056	ENSG00000004975	ENSG00000076984
ENSP00000262904	ENSP00000362648	ENSP00000356155	ENSP00000005340	ENSP00000381068
ENSP00000443418	ENSP00000392147	ENSP00000356152	ENSP00000459797	ENSP00000381070
ENSP00000483249	ENSP00000411744	ENSP00000401795	ENSP00000458465	ENSP00000381066
	ENSP00000416287	ENSP00000409554	ENSP00000459991	
		ENSP00000400561	ENSP00000458209	

19	20	21	22	23
O14737	O14757	O14777	O14828	O14874
PDCD5	CHEK1	NDC80	SCAMP3	BCKDK
ENSG00000105185	ENSG00000149554	ENSG00000080986	ENSG00000116521	ENSG00000103507
ENSP00000466214	ENSP00000412504	ENSP00000261597	ENSP00000307275	ENSP00000378405
ENSP00000476525	ENSP00000442317	ENSP00000461227	ENSP00000347540	ENSP00000378404
ENSP00000221784	ENSP00000435371	ENSP00000476919	ENSP00000461521	ENSP00000287507
ENSP00000468741	ENSP00000432890	ENSP00000458236	ENSP00000458542	ENSP00000219794
ENSP00000368620	ENSP00000481616			ENSP00000456479
ENSP00000467737	ENSP00000388648			ENSP00000455866
	ENSP00000434141			ENSP00000454947
	ENSP00000391090			ENSP00000457226
	ENSP00000434646			ENSP00000457821
	ENSP00000431525			
	ENSP00000431815			
	ENSP00000432470			
	ENSP00000433103			
	ENSP00000477588			
	ENSP00000278916			
	ENSP00000481614			

24	25	26	27	28
O14944	O14950	O14958	O14964	O14974
EREG	MYL12B	CASQ2	HGS	PPP1R12A
ENSG00000124882	ENSG00000118680	ENSG00000118729	ENSG00000185359	ENSG00000058272
ENSP00000244869	ENSP00000462171	ENSP00000261448	ENSP00000331201	ENSP00000389168
	ENSP00000217652		ENSP00000459685	ENSP00000416769
	ENSP00000463614		ENSP00000458987	ENSP00000449514
	ENSP00000441231		ENSP00000458603	ENSP00000446855
	ENSP00000462865		ENSP00000458775	ENSP00000261207
	ENSP00000463009		ENSP00000460130	ENSP00000450059
	ENSP00000464359		ENSP00000458789	ENSP00000489242
	ENSP00000237500		ENSP00000460165	ENSP00000449647
	ENSP00000463559			ENSP00000449666
	ENSP00000383037			ENSP00000447144
	ENSP00000464464			ENSP00000446816
	ENSP00000279022			ENSP00000450061
	ENSP00000217313			ENSP00000447338
				ENSP00000449843

29	30	31	32	33
O15111	O15143	O15162	O15164	O15169
CHUK	ARPC1B	PLSCR1	TRIM24	AXIN1
ENSG00000213341	ENSG00000130429	ENSG00000188313	ENSG00000122779	ENSG00000103126
ENSP00000359424	ENSP00000252725	ENSP00000345494	ENSP00000340507	ENSP00000262320
	ENSP00000389631	ENSP00000411675	ENSP00000390829	ENSP00000346935
	ENSP00000413173	ENSP00000419680		ENSP00000416835
	ENSP00000398620	ENSP00000419228		
	ENSP00000494033	ENSP00000417792		
	ENSP00000410238	ENSP00000417469		
	ENSP00000413067	ENSP00000418654		
	ENSP00000408758	ENSP00000417733		
	ENSP00000403324	ENSP00000420006		
	ENSP00000398110	ENSP00000417588		
	ENSP00000388802	ENSP00000418103		
	ENSP00000403211	ENSP00000418550		
	ENSP00000496599	ENSP00000420523		
	ENSP00000408508			
	ENSP00000399955			

34	35	36	37	38
O15264	O15357	O15379	O15511	O15530
MAPK13	INPPL1	HDAC3	ARPC5	PDPK1
ENSG00000156711	ENSG00000165458	ENSG00000171720	ENSG00000162704	ENSG00000140992
ENSP00000211287	ENSP00000298229	ENSP00000302967	ENSP00000352918	ENSP00000344220
ENSP00000362871	ENSP00000444619	ENSP00000429099	ENSP00000294742	ENSP00000395357
ENSP00000362864	ENSP00000440904	ENSP00000430782	ENSP00000356504	ENSP00000268673
	ENSP00000440512			ENSP00000455492
	ENSP00000440018			ENSP00000455025
	ENSP00000444630			ENSP00000455684
	ENSP00000437852			ENSP00000373876
	ENSP00000441094			ENSP00000455551
	ENSP00000321775			ENSP00000455438
	ENSP00000446360			

39	40	41	42	43
O15551	O43156	O43318	O43353	O43395
CLDN3	TTI1	MAP3K7	RIPK2	PRPF3
ENSG00000165215	ENSG00000101407	ENSG00000135341	ENSG00000104312	ENSG00000117360
ENSP00000378577	ENSP00000362547	ENSP00000358331	ENSP00000220751	ENSP00000315379
	ENSP00000362546	ENSP00000358333	ENSP00000429271	
	ENSP00000407270	ENSP00000358338	ENSP00000427736	
	ENSP00000477545	ENSP00000358335		
		ENSP00000358326		

44	45	46	47	48
O43422	O43464	O43490	O43524	O43665
THAP12	HTRA2	PROM1	FOXO3	RGS10
ENSG00000137492	ENSG00000115317	ENSG00000007062	ENSG00000118689	ENSG00000148908
ENSP00000260045	ENSP00000258080	ENSP00000426090	ENSP00000339527	ENSP00000358097
ENSP00000433966	ENSP00000312893	ENSP00000427346	ENSP00000385824	ENSP00000358099
	ENSP00000399166	ENSP00000426809	ENSP00000446316	ENSP00000376605
		ENSP00000415481		
		ENSP00000438045		
		ENSP00000443620		
		ENSP00000423860		
		ENSP00000424738		
		ENSP00000424569		
		ENSP00000425927		
		ENSP00000422704		
		ENSP00000423793		

49	50	51	52	53
O43707	O43768	O43865	O43896	O60343
ACTN4	ENSA	AHCYL1	KIF1C	TBC1D4
ENSG00000130402	ENSG00000143420	ENSG00000168710	ENSG00000129250	ENSG00000136111
ENSP00000252699	ENSP00000358010	ENSP00000358814	ENSP00000320821	ENSP00000366863
ENSP00000439497	ENSP00000354835	ENSP00000352092	ENSP00000458697	ENSP00000366852
ENSP00000467190	ENSP00000355239	ENSP00000377238		ENSP00000395986
ENSP00000465176	ENSP00000426110			ENSP00000396932
ENSP00000398393	ENSP00000341743			
ENSP00000411187	ENSP00000271690			
ENSP00000489220	ENSP00000422343			
ENSP00000488962	ENSP00000421458			
ENSP00000489202	ENSP00000424242			
ENSP00000489474	ENSP00000355220			
ENSP00000489516	ENSP00000358012			
ENSP00000489448	ENSP00000492428			
	ENSP00000348921			

54	55	56	57	58
O60566	O60716	O60763	O60825	O60869
BUB1B	CTNND1	USO1	PFKFB2	EDF1
ENSG00000156970	ENSG00000198561	ENSG00000138768	ENSG00000123836	ENSG00000107223
ENSP00000287598	ENSP00000436543	ENSP00000444850	ENSP00000356047	ENSP00000360712
ENSP00000398470	ENSP00000354907	ENSP00000264904	ENSP00000356046	ENSP00000224073
ENSP00000453861	ENSP00000436323		ENSP00000439420	ENSP00000360711
ENSP00000453643	ENSP00000409930		ENSP00000484988	
	ENSP00000382004		ENSP00000408717	
	ENSP00000354785		ENSP00000484769	
	ENSP00000354823			
	ENSP00000432075			
	ENSP00000437156			
	ENSP00000351527			
	ENSP00000434949			
	ENSP00000437051			
	ENSP00000435379			
	ENSP00000432243			
	ENSP00000436744			
	ENSP00000413586			
	ENSP00000434900			
	ENSP00000435266			
	ENSP00000432623			
	ENSP00000433158			
	ENSP00000435494			
	ENSP00000434672			
	ENSP00000433276			
	ENSP00000433334			
	ENSP00000437327			
	ENSP00000403518			
	ENSP00000434017			
	ENSP00000435789			
	ENSP00000432041			
	ENSP00000434808			
	ENSP00000435242			
	ENSP00000432299			
	ENSP00000431600			
	ENSP00000434202			
	ENSP00000431208			

59	60	61	62	63
O60879	O60885	O75116	O75143	O75376
DIAPH2	BRD4	ROCK2	ATG13	NCOR1
ENSG00000147202	ENSG00000141867	ENSG00000134318	ENSG00000175224	ENSG00000141027
ENSP00000362140	ENSP00000263377	ENSP00000317985	ENSP00000433756	ENSP00000268712
ENSP00000321348	ENSP00000360901	ENSP00000385509	ENSP00000434390	ENSP00000379192
ENSP00000362152	ENSP00000353112	ENSP00000261535	ENSP00000431974	ENSP00000379189
ENSP00000348082	ENSP00000470481	ENSP00000395957	ENSP00000433543	ENSP00000379190
ENSP00000362145	ENSP00000470108	ENSP00000481789	ENSP00000352500	ENSP00000461959
	ENSP00000471240		ENSP00000432412	ENSP00000474008
	ENSP00000486621		ENSP00000463808	ENSP00000463169
			ENSP00000464274	ENSP00000462281
			ENSP00000436931	ENSP00000389839
			ENSP00000463550	ENSP00000407998
			ENSP00000463998	ENSP00000462308
			ENSP00000462644	ENSP00000387727
			ENSP00000462167	ENSP00000462794
			ENSP00000464091	ENSP00000410784
			ENSP00000378919	ENSP00000379198
			ENSP00000434125	
			ENSP00000436174	
			ENSP00000431877	
			ENSP00000437191	

64	65	66	67	68
O75475	O75531	O75533	O75676	O75688
PSIP1	BANF1	SF3B1	RPS6KA4	PPM1B
ENSG00000164985	ENSG00000175334	ENSG00000115524	ENSG00000162302	ENSG00000138032
ENSP00000370114	ENSP00000310275	ENSP00000335321	ENSP00000333896	ENSP00000387287
ENSP00000370109	ENSP00000416128	ENSP00000428820	ENSP00000435580	ENSP00000282412
ENSP00000370091	ENSP00000433760	ENSP00000402997	ENSP00000435314	ENSP00000367813
ENSP00000370092	ENSP00000432867	ENSP00000409435	ENSP00000432945	ENSP00000326089
ENSP00000380653	ENSP00000431785	ENSP00000419896		ENSP00000390087
ENSP00000477077		ENSP00000392117		ENSP00000386982

69	70	71	72	73
O75689	O75886	O75925	O75962	O76070
ADAP1	STAM2	PIAS1	TRIO	SNCG
ENSG00000105963	ENSG00000115145	ENSG00000033800	ENSG00000038382	ENSG00000173267
ENSP00000265846	ENSP00000263904	ENSP00000465676	ENSP00000339299	ENSP00000361087
ENSP00000407267		ENSP00000317163	ENSP00000339291	ENSP00000344658
ENSP00000442682		ENSP00000381648	ENSP00000421555	
ENSP00000481154		ENSP00000443238	ENSP00000445592	
ENSP00000414436		ENSP00000479025	ENSP00000426342	
ENSP00000409129		ENSP00000465312	ENSP00000492184	
ENSP00000404217		ENSP00000468166	ENSP00000483869	
ENSP00000391315		ENSP00000466596		
ENSP00000416545		ENSP00000468017		
ENSP00000394973		ENSP00000249636		
ENSP00000478011		ENSP00000438574		
		ENSP00000456721		
		ENSP00000457698		
		ENSP00000456558		

74	75	76	77	78
O94804	O95251	O95295	O95297	O95453
STK10	KAT7	SNAPIN	MPZL1	PARN
ENSG00000072786	ENSG00000136504	ENSG00000143553	ENSG00000197965	ENSG00000140694
ENSP00000176763	ENSP00000259021	ENSP00000357674	ENSP00000352513	ENSP00000387911
ENSP00000428806	ENSP00000413415		ENSP00000375968	ENSP00000410525
	ENSP00000424577		ENSP00000420455	ENSP00000345456
	ENSP00000423385		ENSP00000399490	ENSP00000444381
	ENSP00000398961		ENSP00000356827	ENSP00000456279
	ENSP00000421368			ENSP00000458103
	ENSP00000409477			ENSP00000445659
				ENSP00000456982
				ENSP00000478668
				ENSP00000484279
				ENSP00000487634
				ENSP00000488554
				ENSP00000488415
				ENSP00000488466
				ENSP00000487870
				ENSP00000488491

79	80	81	82	83
O95471	O95747	O95786	O95816	O95817
CLDN7	OXSR1	DDX58	BAG2	BAG3
ENSG00000181885	ENSG00000172939	ENSG00000107201	ENSG00000112208	ENSG00000151929
ENSP00000353475	ENSP00000311713	ENSP00000369213	ENSP00000359727	ENSP00000358081
ENSP00000396638	ENSP00000415851	ENSP00000369197		ENSP00000410036
ENSP00000460550	ENSP00000398356			
ENSP00000460796				
ENSP00000465963				
ENSP00000467220				
ENSP00000445131				

84	85	86	87	88
O96013	O96017	P00533	P01100	P01106
PAK4	CHEK2	EGFR	FOS	MYC
ENSG00000130669	ENSG00000183765	ENSG00000146648	ENSG00000170345	ENSG00000136997
ENSP00000470284	ENSP00000385747	ENSP00000342376	ENSP00000306245	ENSP00000367207
ENSP00000326864	ENSP00000329178	ENSP00000345973	ENSP00000442268	ENSP00000479618
ENSP00000351049	ENSP00000386087	ENSP00000413843	ENSP00000452590	ENSP00000259523
ENSP00000469413	ENSP00000372023	ENSP00000275493	ENSP00000450519	ENSP00000493139
ENSP00000471157	ENSP00000404567	ENSP00000415559	ENSP00000452443	ENSP00000429441
ENSP00000353625	ENSP00000412901	ENSP00000413354	ENSP00000452386	ENSP00000493145
ENSP00000471318	ENSP00000404400	ENSP00000492462	ENSP00000452440	ENSP00000478887
ENSP00000472862	ENSP00000384919	ENSP00000395243	ENSP00000451786	ENSP00000430235
ENSP00000473215	ENSP00000384835		ENSP00000450886	ENSP00000430226
ENSP00000472061	ENSP00000329012			
	ENSP00000416721			
	ENSP00000394430			
	ENSP00000402225			
	ENSP00000397478			
	ENSP00000403659			
	ENSP00000483777			
	ENSP00000390244			
	ENSP00000396903			
	ENSP00000408065			
	ENSP00000387451			
	ENSP00000381099			
	ENSP00000372006			

89	90	91	92	93
P01111	P01112	P01116	P01137	P01344
NRAS	HRAS	KRAS	TGFB1	IGF2
ENSG00000213281	ENSG00000174775	ENSG00000133703	ENSG00000105329	ENSG00000167244
ENSP00000358548	ENSP00000380722	ENSP00000308495	ENSP00000221930	ENSP00000370802
ENSP00000457423	ENSP00000380723	ENSP00000256078	ENSP00000472767	ENSP00000370813
ENSP00000457791	ENSP00000434023	ENSP00000452512		ENSP00000414497
	ENSP00000309845	ENSP00000451856		ENSP00000391826
	ENSP00000388246			ENSP00000370799
	ENSP00000407586			ENSP00000370796
	ENSP00000488225			ENSP00000402047
	ENSP00000488757			ENSP00000493122
	ENSP00000488296			
	ENSP00000482366			
	ENSP00000480317			
	ENSP00000480686			
	ENSP00000488397			

94	95	96	97	98
P02545	P04035	P04083	P04183	P04626
LMNA	HMGCR	ANXA1	TK1	ERBB2
ENSG00000160789	ENSG00000113161	ENSG00000135046	ENSG00000167900	ENSG00000141736
ENSP00000357284	ENSP00000426745	ENSP00000257497	ENSP00000301634	ENSP00000462438
ENSP00000357283	ENSP00000287936	ENSP00000366109	ENSP00000468425	ENSP00000269571
ENSP00000357282	ENSP00000340816	ENSP00000412489	ENSP00000468556	ENSP00000385185
ENSP00000395597	ENSP00000427340	ENSP00000414013	ENSP00000467121	ENSP00000446466
ENSP00000421821	ENSP00000409100		ENSP00000468278	ENSP00000462808
ENSP00000292304	ENSP00000421378			ENSP00000404047
ENSP00000424518	ENSP00000420871			ENSP00000463714
ENSP00000357280				ENSP00000462024
ENSP00000426535				ENSP00000463427
ENSP00000424977				ENSP00000463719
ENSP00000355292				ENSP00000464420
				ENSP00000464252
				ENSP00000463002

99	100	101	102	103
P04637	P04792	P05067	P05114	P05198
TP53	HSPB1	APP	HMGN1	EIF2S1
ENSG00000141510	ENSG00000106211	ENSG00000142192	ENSG00000205581	ENSG00000134001
ENSP00000484409	ENSP00000248553	ENSP00000284981	ENSP00000370125	ENSP00000256383
ENSP00000478499	ENSP00000414357	ENSP00000346129	ENSP00000288344	ENSP00000425299
ENSP00000481179	ENSP00000405285	ENSP00000345463	ENSP00000395291	ENSP00000451975
ENSP00000478219		ENSP00000350578	ENSP00000414421	ENSP00000452034
ENSP00000269305		ENSP00000387483	ENSP00000370124	
ENSP00000481638		ENSP00000351796	ENSP00000396693	
ENSP00000398846		ENSP00000398879	ENSP00000370123	
ENSP00000391127		ENSP00000396923	ENSP00000395787	
ENSP00000482222		ENSP00000406539	ENSP00000495398	
ENSP00000480868		ENSP00000352760		
ENSP00000391478				
ENSP00000482537				
ENSP00000482258				
ENSP00000410739				
ENSP00000488924				
ENSP00000352610				
ENSP00000484375				
ENSP00000481401				
ENSP00000477531				
ENSP00000458393				
ENSP00000425104				
ENSP00000423862				
ENSP00000424104				
ENSP00000473895				
ENSP00000426252				
ENSP00000482903				

104	105	106	107	108
P05204	P05387	P05412	P05455	P05783
HMGN2	RPLP2	JUN	SSB	KRT18
ENSG00000198830	ENSG00000177600	ENSG00000177606	ENSG00000138385	ENSG00000111057
ENSP00000355228	ENSP00000322419	ENSP00000360266	ENSP00000260956	ENSP00000373487
ENSP00000481160	ENSP00000431240		ENSP00000386636	ENSP00000373489
	ENSP00000433443		ENSP00000397029	ENSP00000447278
			ENSP00000395758	
			ENSP00000396890	

109	110	111	112	113
P05976	P06396	P06400	P06493	P06730
MYL1	GSN	RB1	CDK1	EIF4E
ENSG00000168530	ENSG00000148180	ENSG00000139687	ENSG00000170312	ENSG00000151247
ENSP00000343321	ENSP00000362929	ENSP00000267163	ENSP00000378699	ENSP00000389624
ENSP00000307280	ENSP00000362914	ENSP00000496556	ENSP00000362915	ENSP00000280892
ENSP00000379210	ENSP00000445823	ENSP00000434702	ENSP00000325970	ENSP00000425561
ENSP00000292327	ENSP00000362924	ENSP00000496005	ENSP00000430665	ENSP00000426602
ENSP00000393455	ENSP00000404226		ENSP00000397973	ENSP00000421287
	ENSP00000409358		ENSP00000482996	ENSP00000423977
	ENSP00000489067			
	ENSP00000377882			
	ENSP00000362912			

114	115	116	117	118
P06733	P06744	P06748	P07108	P07355
ENO1	GPI	NPM1	DBI	ANXA2
ENSG00000074800	ENSG00000105220	ENSG00000181163	ENSG00000155368	ENSG00000182718
ENSP00000234590	ENSP00000405573	ENSP00000428755	ENSP00000348116	ENSP00000379342
ENSP00000493526	ENSP00000348877	ENSP00000296930	ENSP00000486281	ENSP00000387545
ENSP00000495530	ENSP00000464797	ENSP00000341168	ENSP00000386486	ENSP00000346032
ENSP00000466406	ENSP00000468298	ENSP00000377408	ENSP00000311117	ENSP00000411352
ENSP00000494470	ENSP00000466191	ENSP00000429485	ENSP00000486361	ENSP00000453754
ENSP00000495946	ENSP00000496041	ENSP00000428647	ENSP00000376815	ENSP00000453464
ENSP00000495568	ENSP00000465858		ENSP00000439012	ENSP00000453039
ENSP00000496562	ENSP00000464798		ENSP00000442917	ENSP00000453841
ENSP00000495581	ENSP00000467221		ENSP00000440698	ENSP00000453859
ENSP00000495805	ENSP00000493517			ENSP00000454096
ENSP00000468392	ENSP00000467590			ENSP00000452876
	ENSP00000466851			ENSP00000453556
	ENSP00000468201			ENSP00000453356
	ENSP00000495129			ENSP00000453925
	ENSP00000467670			ENSP00000453579
				ENSP00000452921
				ENSP00000453869
				ENSP00000452895
				ENSP00000453174
				ENSP00000453663
				ENSP00000452981
				ENSP00000453739
				ENSP00000453000
				ENSP00000453694
				ENSP00000453770
				ENSP00000452858
				ENSP00000452958
				ENSP00000453566
				ENSP00000453945
				ENSP00000453742

119	120	121	122	123
P07737	P07814	P07900	P07910	P07947
PFN1	EPRS	HSP90AA1	HNRNPC	YES1
ENSG00000108518	ENSG00000136628	ENSG00000080824	ENSG00000092199	ENSG00000176105
ENSP00000225655	ENSP00000355890	ENSP00000216281	ENSP00000450725	ENSP00000462468
ENSP00000465019	ENSP00000477245	ENSP00000335153	ENSP00000451291	ENSP00000324740
ENSP00000460363	ENSP00000477493	ENSP00000451400	ENSP00000442816	ENSP00000464380
		ENSP00000450712	ENSP00000451652	
		ENSP00000452241	ENSP00000450629	
		ENSP00000489370	ENSP00000452276	
			ENSP00000450544	
			ENSP00000451176	
			ENSP00000404848	
			ENSP00000338095	
			ENSP00000451187	
			ENSP00000450548	
			ENSP00000451708	
			ENSP00000450790	
			ENSP00000452599	
			ENSP00000452214	
			ENSP00000450601	
			ENSP00000452545	
			ENSP00000452021	
			ENSP00000452213	
			ENSP00000452185	
			ENSP00000450467	
			ENSP00000451292	
			ENSP00000452573	
			ENSP00000451892	
			ENSP00000450669	
			ENSP00000404559	

124	125	126	127	128
P08047	P08069	P08238	P08559	P08581
SP1	IGF1R	HSP90AB1	PDHA1	MET
ENSG00000185591	ENSG00000140443	ENSG00000096384	ENSG00000131828	ENSG00000105976
ENSP00000329357	ENSP00000268035	ENSP00000360709	ENSP00000394382	ENSP00000380860
ENSP00000404263	ENSP00000453007	ENSP00000325875	ENSP00000440761	ENSP00000317272
ENSP00000457804	ENSP00000453630	ENSP00000360609	ENSP00000438550	ENSP00000410980
ENSP00000458133	ENSP00000454115	ENSP00000481908	ENSP00000369134	ENSP00000413857
	ENSP00000456950		ENSP00000406473	ENSP00000398776
	ENSP00000453180		ENSP00000404616	ENSP00000398140
			ENSP00000348062	
			ENSP00000369133	
			ENSP00000369132	

129	130	131	132	133
P08670	P08727	P09211	P09327	P09497
RTEL1-TNFRSF6B	KRT19	GSTP1	VIL1	CLTB
ENSG00000026036	ENSG00000171345	ENSG00000084207	ENSG00000127831	ENSG00000175416
ENSP00000457428	ENSP00000355124	ENSP00000381607	ENSP00000248444	ENSP00000309415
ENSP00000446007	ENSP00000466625	ENSP00000381604	ENSP00000409270	ENSP00000310812
ENSP00000224237	ENSP00000408759	ENSP00000493538	ENSP00000412657	ENSP00000424739
ENSP00000489830		ENSP00000494477	ENSP00000375962	ENSP00000422978
ENSP00000490509		ENSP00000484929	ENSP00000394030	ENSP00000427229
ENSP00000435613		ENSP00000484686		
ENSP00000431702				

134	135	136	137	138
P09651	P09874	P09917	P10301	P10398
HNRNPA1	PARP1	ALOX5	RRAS	ARAF
ENSG00000135486	ENSG00000143799	ENSG00000012779	ENSG00000126458	ENSG00000078061
ENSP00000448617	ENSP00000355759	ENSP00000363512	ENSP00000246792	ENSP00000366244
ENSP00000449913	ENSP00000355757	ENSP00000437634		ENSP00000366238
ENSP00000341826	ENSP00000355755	ENSP00000483803		ENSP00000290277
ENSP00000447260	ENSP00000485852	ENSP00000484468		
ENSP00000448229		ENSP00000479958		
ENSP00000448117		ENSP00000479452		
ENSP00000333504				
ENSP00000447782				
ENSP00000448917				
ENSP00000446486				
ENSP00000350090				

139	140	141	142	143
P10586	P10588	P10644	P11166	P11171
PTPRF	NR2F6	PRKAR1A	SLC2A1	EPB41
ENSG00000142949	ENSG00000160113	ENSG00000108946	ENSG00000117394	ENSG00000159023
ENSP00000353030	ENSP00000291442	ENSP00000351410	ENSP00000416293	ENSP00000362906
ENSP00000398822	ENSP00000471686	ENSP00000464977	ENSP00000486694	ENSP00000290100
ENSP00000413306		ENSP00000445625	ENSP00000487213	ENSP00000362904
ENSP00000391764		ENSP00000466459	ENSP00000361578	ENSP00000362903
ENSP00000408952		ENSP00000468106	ENSP00000491942	ENSP00000317597
ENSP00000416343		ENSP00000376475		ENSP00000345259
ENSP00000361484		ENSP00000465445		ENSP00000496326
ENSP00000483557		ENSP00000464715		ENSP00000496138
		ENSP00000466722		ENSP00000495138
		ENSP00000376474		ENSP00000493575
		ENSP00000464701		ENSP00000494212
		ENSP00000467500		ENSP00000496751
		ENSP00000467311		ENSP00000496675
		ENSP00000466649		ENSP00000494945
		ENSP00000467867		ENSP00000494244
		ENSP00000465013		ENSP00000494042
		ENSP00000466314		ENSP00000494435
		ENSP00000465740		ENSP00000495300
				ENSP00000495763
				ENSP00000490911
				ENSP00000493958
				ENSP00000494357
				ENSP00000496077
				ENSP00000495565
				ENSP00000348397

144	145	146	147	148
P11233	P11234	P11274	P11388	P11413
RALA	RALB	BCR	TOP2A	G6PD
ENSG00000006451	ENSG00000144118	ENSG00000186716	ENSG00000131747	ENSG00000160211
ENSP00000005257	ENSP00000272519	ENSP00000303507	ENSP00000411532	ENSP00000483686
ENSP00000388975	ENSP00000414224	ENSP00000352535	ENSP00000464055	ENSP00000377194
ENSP00000413227	ENSP00000402866	ENSP00000396531	ENSP00000462908	ENSP00000358633
	ENSP00000393782			ENSP00000377192
	ENSP00000398162			ENSP00000395599
	ENSP00000407062			ENSP00000400648
	ENSP00000487037			ENSP00000394690

149	150	151	152	153
P11717	P11802	P12004	P12259	P12270
IGF2R	CDK4	PCNA	F5	TPR
ENSG00000197081	ENSG00000135446	ENSG00000132646	ENSG00000198734	ENSG00000047410
ENSP00000349437	ENSP00000257904	ENSP00000368438	ENSP00000356771	ENSP00000356448
ENSP00000473880	ENSP00000447005	ENSP00000368458	ENSP00000356770	ENSP00000483425
	ENSP00000448885			ENSP00000408103
	ENSP00000447779			
	ENSP00000447274			
	ENSP00000448098			
	ENSP00000449391			
	ENSP00000449179			
	ENSP00000448963			
	ENSP00000446763			
	ENSP00000449725			
	ENSP00000316889			

154	155	156	157	158
P12694	P12814	P12830	P12931	P13639
BCKDHA	ACTN1	CDH1	SRC	EEF2
ENSG00000248098	ENSG00000072110	ENSG00000039068	ENSG00000197122	ENSG00000167658
ENSP00000269980	ENSP00000193403	ENSP00000261769	ENSP00000362680	ENSP00000307940
ENSP00000416000	ENSP00000377941	ENSP00000414946	ENSP00000362668	ENSP00000471265
ENSP00000440345	ENSP00000414272	ENSP00000454782	ENSP00000362659	
ENSP00000445809	ENSP00000439828	ENSP00000458139	ENSP00000350941	
ENSP00000445727	ENSP00000366035	ENSP00000463170		
	ENSP00000452254	ENSP00000478360		
	ENSP00000444422	ENSP00000480664		
	ENSP00000450625	ENSP00000481063		
	ENSP00000450903			
	ENSP00000450764			
	ENSP00000450925			
	ENSP00000450618			
	ENSP00000452423			
	ENSP00000451086			

159	160	161	162	163
P13688	P13693	P13807	P13861	P14618
CEACAM1	TPT1	GYS1	PRKAR2A	PKM
ENSG00000079385	ENSG00000133112	ENSG00000104812	ENSG00000114302	ENSG00000067225
ENSP00000244291	ENSP00000431872	ENSP00000317904	ENSP00000265563	ENSP00000455736
ENSP00000161559	ENSP00000368345	ENSP00000263276	ENSP00000394041	ENSP00000334983
ENSP00000384709	ENSP00000368344	ENSP00000470072	ENSP00000296446	ENSP00000455901
ENSP00000384083	ENSP00000433091		ENSP00000409239	ENSP00000456970
ENSP00000351165	ENSP00000432457		ENSP00000400840	ENSP00000403365
ENSP00000471918	ENSP00000368350		ENSP00000416438	ENSP00000320171
ENSP00000471566	ENSP00000339051		ENSP00000411432	ENSP00000456004
ENSP00000472633	ENSP00000433738			ENSP00000457253
	ENSP00000477781			ENSP00000455584
				ENSP00000457198
				ENSP00000457830
				ENSP00000456984
				ENSP00000457420
				ENSP00000454668
				ENSP00000373745
				ENSP00000456100

164	165	166	167	168
P14635	P14649	P14859	P14923	P15056
CCNB1	MYL6B	POU2F1	JUP	BRAF
ENSG00000134057	ENSG00000196465	ENSG00000143190	ENSG00000173801	ENSG00000157764
ENSP00000256442	ENSP00000450385	ENSP00000356840	ENSP00000377507	ENSP00000288602
ENSP00000424588	ENSP00000446643	ENSP00000401217	ENSP00000311113	ENSP00000419060
ENSP00000423387	ENSP00000448198	ENSP00000441285	ENSP00000377508	ENSP00000496776
ENSP00000426092	ENSP00000446965	ENSP00000356836	ENSP00000389886	ENSP00000493543
ENSP00000425103		ENSP00000271411	ENSP00000394146	ENSP00000493678
ENSP00000426230		ENSP00000404571	ENSP00000411449	ENSP00000494784
		ENSP00000453094	ENSP00000401034	ENSP00000418033
		ENSP00000414660	ENSP00000468347	ENSP00000495132
		ENSP00000415993	ENSP00000409948	ENSP00000493783
			ENSP00000467065	ENSP00000420119
				ENSP00000495858

169	170	171	172	173
P15311	P15336	P15531	P15924	P15927
EZR	ATF2	NME1	DSP	RPA2
ENSG00000092820	ENSG00000115966	ENSG00000239672	ENSG00000096696	ENSG00000117748
ENSP00000338934	ENSP00000264110	ENSP00000376892	ENSP00000369129	ENSP00000363021
ENSP00000356042	ENSP00000340576	ENSP00000337060	ENSP00000396591	ENSP00000363017
ENSP00000376016	ENSP00000387093	ENSP00000013034		ENSP00000363015
	ENSP00000376327	ENSP00000429318		ENSP00000413541
	ENSP00000386282	ENSP00000428553		ENSP00000387649
	ENSP00000386526	ENSP00000435498		
	ENSP00000407911	ENSP00000387682		
	ENSP00000386326			
	ENSP00000406995			
	ENSP00000412293			
	ENSP00000389504			
	ENSP00000402190			
	ENSP00000410224			
	ENSP00000402229			
	ENSP00000410086			
	ENSP00000414171			
	ENSP00000416854			
	ENSP00000437952			

174	175	176	177	178
P16070	P16104	P16144	P16220	P16298
CD44	H2AFX	ITGB4	CREB1	PPP3CB
ENSG00000026508	ENSG00000188486	ENSG00000132470	ENSG00000118260	ENSG00000107758
ENSP00000263398	ENSP00000434024	ENSP00000463651	ENSP00000405539	ENSP00000353881
ENSP00000398632	ENSP00000364310	ENSP00000200181	ENSP00000387699	ENSP00000378305
ENSP00000389830	ENSP00000297012	ENSP00000405536	ENSP00000236995	ENSP00000378306
ENSP00000392331		ENSP00000400217	ENSP00000407227	ENSP00000398022
ENSP00000278386		ENSP00000463788	ENSP00000403678	ENSP00000343147
ENSP00000404447		ENSP00000464259	ENSP00000392428	
ENSP00000309732		ENSP00000462494	ENSP00000404890	
ENSP00000435377			ENSP00000405711	
ENSP00000432704			ENSP00000391125	
ENSP00000395953			ENSP00000412016	
ENSP00000432405			ENSP00000401803	
ENSP00000398099				
ENSP00000434465				
ENSP00000279452				
ENSP00000434530				
ENSP00000432718				
ENSP00000433189				
ENSP00000436549				
ENSP00000436623				
ENSP00000436980				
ENSP00000278385				
ENSP00000435321				
ENSP00000436451				
ENSP00000434418				
ENSP00000431860				
ENSP00000434920				
ENSP00000492449				

179	180	181	182	183
P16401	P16615	P16949	P17096	P17174
HIST1H1B	ATP2A2	STMN1	HMGA1	GOT1
ENSG00000184357	ENSG00000174437	ENSG00000117632	ENSG00000137309	ENSG00000120053
ENSP00000330074	ENSP00000311186	ENSP00000410452	ENSP00000288245	ENSP00000359539
	ENSP00000440045	ENSP00000387858	ENSP00000385693	
	ENSP00000447406	ENSP00000350531	ENSP00000308227	
	ENSP00000366913	ENSP00000363409	ENSP00000363230	
	ENSP00000449454	ENSP00000382633	ENSP00000399888	
	ENSP00000450407	ENSP00000407567		

184	185	186	187	188
P17252	P17480	P17535	P17612	P17655
PRKCA	UBTF	JUND	PRKACA	CAPN2
ENSG00000154229	ENSG00000108312	ENSG00000130522	ENSG00000072062	ENSG00000162909
ENSP00000408695	ENSP00000345297	ENSP00000252818	ENSP00000309591	ENSP00000295006
ENSP00000462087	ENSP00000302640	ENSP00000475153	ENSP00000466651	ENSP00000413158
ENSP00000284384	ENSP00000437180		ENSP00000466976	ENSP00000399949
	ENSP00000390669		ENSP00000468352	
	ENSP00000377231		ENSP00000466289	
	ENSP00000432925		ENSP00000466660	
	ENSP00000435708			
	ENSP00000431539			
	ENSP00000433625			
	ENSP00000431295			
	ENSP00000433046			

189	190	191	192	193
P17676	P17706	P17812	P17844	P17931
CEBPB	PTPN2	CTPS1	DDX5	LGALS3
ENSG00000172216	ENSG00000175354	ENSG00000171793	ENSG00000108654	ENSG00000131981
ENSP00000305422	ENSP00000320298	ENSP00000361704	ENSP00000225792	ENSP00000254301
	ENSP00000466936	ENSP00000361699	ENSP00000403085	ENSP00000451526
	ENSP00000320546		ENSP00000440276	ENSP00000451381
	ENSP00000311857		ENSP00000464566	
	ENSP00000464937		ENSP00000462885	
	ENSP00000464719		ENSP00000464659	
	ENSP00000467043		ENSP00000462222	
	ENSP00000467823		ENSP00000463975	
	ENSP00000496181		ENSP00000464337	
	ENSP00000493693		ENSP00000464304	
	ENSP00000494336		ENSP00000464064	
	ENSP00000468012		ENSP00000463541	
	ENSP00000468155		ENSP00000463101	
	ENSP00000468408		ENSP00000464587	
	ENSP00000466206		ENSP00000465791	
			ENSP00000462206	
			ENSP00000463903	
			ENSP00000463168	
			ENSP00000485764	

194	195	196	197	198
P18031	P18084	P18206	P18433	P18669
PTPN1	ITGB5	VCL	PTPRA	PGAM1
ENSG00000196396	ENSG00000082781	ENSG00000035403	ENSG00000132670	ENSG00000171314
ENSP00000360683	ENSP00000296181	ENSP00000211998	ENSP00000369756	ENSP00000359991
ENSP00000437732	ENSP00000420814	ENSP00000361841	ENSP00000216877	
	ENSP00000477446	ENSP00000485551	ENSP00000314568	
	ENSP00000418367		ENSP00000348468	
	ENSP00000418079		ENSP00000382787	
	ENSP00000477230		ENSP00000414089	
	ENSP00000419198		ENSP00000390775	
	ENSP00000476655		ENSP00000394132	

199	200	201	202	203
P18846	P18858	P18887	P19474	P19525
ATF1	LIG1	XRCC1	TRIM21	EIF2AK2
ENSG00000123268	ENSG00000105486	ENSG00000073050	ENSG00000132109	ENSG00000055332
ENSP00000262053	ENSP00000263274	ENSP00000262887	ENSP00000254436	ENSP00000233057
ENSP00000448592	ENSP00000442841	ENSP00000443671	ENSP00000434053	ENSP00000385014
ENSP00000448421	ENSP00000471836	ENSP00000470391		ENSP00000378559
ENSP00000448987	ENSP00000483027	ENSP00000470045		ENSP00000393921
ENSP00000448921	ENSP00000471380	ENSP00000472128		ENSP00000374663
	ENSP00000472331	ENSP00000471159		
	ENSP00000445928			
	ENSP00000471861			
	ENSP00000469638			
	ENSP00000472085			
	ENSP00000441531			

204	205	206	207	208
P19634	P19784	P19793	P19838	P20042
SLC9A1	CSNK2A2	RXRA	NFKB1	EIF2S2
ENSG00000090020	ENSG00000070770	ENSG00000186350	ENSG00000109320	ENSG00000125977
ENSP00000263980	ENSP00000262506	ENSP00000419692	ENSP00000226574	ENSP00000364119
ENSP00000363199	ENSP00000456606	ENSP00000352900	ENSP00000378297	
ENSP00000363197	ENSP00000457911	ENSP00000482458	ENSP00000424790	
	ENSP00000454874		ENSP00000469340	
			ENSP00000420904	
			ENSP00000426147	
			ENSP00000423877	
			ENSP00000424815	

209	210	211	212	213
P20248	P20936	P21127	P21291	P21333
CCNA2	RASA1	CDK11B	CSRP1	FLNA
ENSG00000145386	ENSG00000145715	ENSG00000248333	ENSG00000159176	ENSG00000196924
ENSP00000274026	ENSP00000274376	ENSP00000463048	ENSP00000356275	ENSP00000358866
ENSP00000481380	ENSP00000423395	ENSP00000464036	ENSP00000434147	ENSP00000353467
	ENSP00000411221	ENSP00000464016	ENSP00000436792	ENSP00000416926
	ENSP00000422008	ENSP00000481846	ENSP00000345079	ENSP00000408921
	ENSP00000420905	ENSP00000484222	ENSP00000432110	ENSP00000358872
		ENSP00000485937	ENSP00000483864	ENSP00000397824
		ENSP00000485705	ENSP00000436491	ENSP00000405458
		ENSP00000485849	ENSP00000437218	ENSP00000398215
		ENSP00000384442		ENSP00000480593
		ENSP00000351629		
		ENSP00000367900		
		ENSP00000348529		
		ENSP00000367905		
		ENSP00000422149		
		ENSP00000462289		
		ENSP00000350403		
		ENSP00000423900		
		ENSP00000383874		

214	215	216	217	218
P21399	P21802	P21810	P21860	P21980
ACO1	FGFR2	BGN	ERBB3	TGM2
ENSG00000122729	ENSG00000066468	ENSG00000182492	ENSG00000065361	ENSG00000198959
ENSP00000369255	ENSP00000351276	ENSP00000327336	ENSP00000267101	ENSP00000355330
ENSP00000309477	ENSP00000348559	ENSP00000402525	ENSP00000415753	ENSP00000362502
ENSP00000438733	ENSP00000358056		ENSP00000447510	ENSP00000387642
	ENSP00000263451		ENSP00000408340	
	ENSP00000410294		ENSP00000495453	
	ENSP00000353262		ENSP00000448636	
	ENSP00000358052		ENSP00000449138	
	ENSP00000358054		ENSP00000448671	
	ENSP00000352309		ENSP00000448483	
	ENSP00000350166		ENSP00000449713	
	ENSP00000358057		ENSP00000482073	
	ENSP00000474011		ENSP00000448946	
	ENSP00000491912		ENSP00000449129	
	ENSP00000474109		ENSP00000448729	
	ENSP00000358055			
	ENSP00000404219			
	ENSP00000337665			
	ENSP00000481464			
	ENSP00000484892			
	ENSP00000490905			
	ENSP00000484154			
	ENSP00000309878			

219	220	221	222	223
P22314	P22392	P22681	P23246	P23396
UBA1	AC006254.1	CBL	SFPQ	RPS3
ENSG00000130985	ENSG00000243696	ENSG00000110395	ENSG00000116560	ENSG00000149273
ENSP00000366568	ENSP00000422253	ENSP00000264033	ENSP00000349748	ENSP00000434643
ENSP00000338413	ENSP00000426976	ENSP00000489218	ENSP00000425071	ENSP00000278572
ENSP00000415033	ENSP00000425581	ENSP00000489324	ENSP00000424440	ENSP00000436971
ENSP00000397816	ENSP00000421064	ENSP00000490763		ENSP00000433821
ENSP00000389583	ENSP00000426901	ENSP00000489556		ENSP00000432542
ENSP00000404796	ENSP00000376886			ENSP00000433155
ENSP00000401101	ENSP00000376880			ENSP00000432458
ENSP00000366481				ENSP00000416745
				ENSP00000431834
				ENSP00000436335
				ENSP00000433421
				ENSP00000435457
				ENSP00000431737
				ENSP00000435566
				ENSP00000436556
				ENSP00000436476
				ENSP00000437105

224	225	226	227	228
P23511	P23528	P23588	P24534	P24666
NFYA	CFL1	EIF4B	EEF1B2	ACP1
ENSG00000001167	ENSG00000172757	ENSG00000063046	ENSG00000114942	ENSG00000143727
ENSP00000345702	ENSP00000432660	ENSP00000262056	ENSP00000236957	ENSP00000272067
ENSP00000229418	ENSP00000309629	ENSP00000412530	ENSP00000376056	ENSP00000272065
	ENSP00000432155	ENSP00000448399	ENSP00000376055	ENSP00000385404
	ENSP00000433910	ENSP00000447192	ENSP00000407730	ENSP00000410331
	ENSP00000432226	ENSP00000388806	ENSP00000397793	ENSP00000389681
	ENSP00000431696	ENSP00000448365	ENSP00000393501	ENSP00000408596
	ENSP00000433131	ENSP00000449746	ENSP00000399917	ENSP00000384184
	ENSP00000436431	ENSP00000450324	ENSP00000387407	ENSP00000384307
	ENSP00000436899	ENSP00000448980	ENSP00000489993	ENSP00000411121
	ENSP00000433308		ENSP00000490214	
	ENSP00000432153		ENSP00000490326	
			ENSP00000490885	
			ENSP00000490893	
			ENSP00000490192	
			ENSP00000490022	
			ENSP00000490503	

229	230	231	232	233
P24844	P24928	P24941	P25098	P25205
MYL9	POLR2A	CDK2	GRK2	MCM3
ENSG00000101335	ENSG00000181222	ENSG00000123374	ENSG00000173020	ENSG00000112118
ENSP00000462171	ENSP00000461879	ENSP00000266970	ENSP00000312262	ENSP00000229854
ENSP00000217652		ENSP00000243067	ENSP00000434126	ENSP00000472940
ENSP00000463614		ENSP00000450983		ENSP00000480987
ENSP00000441231		ENSP00000452514		ENSP00000388647
ENSP00000462865		ENSP00000393605		ENSP00000407651
ENSP00000463009		ENSP00000452138		ENSP00000470057
ENSP00000464359				
ENSP00000237500				
ENSP00000463559				
ENSP00000383037				
ENSP00000464464				
ENSP00000279022				
ENSP00000217313				

234	235	236	237	238
P25445	P25490	P25788	P26038	P26358
FAS	YY1	PSMA3	MSN	DNMT1
ENSG00000026103	ENSG00000100811	ENSG00000100567	ENSG00000147065	ENSG00000130816
ENSP00000347979	ENSP00000262238	ENSP00000216455	ENSP00000353408	ENSP00000345739
ENSP00000420975	ENSP00000451329	ENSP00000390491	ENSP00000477441	ENSP00000352516
ENSP00000349896	ENSP00000452140	ENSP00000451461		ENSP00000466657
ENSP00000425159	ENSP00000452225	ENSP00000452437		ENSP00000467125
ENSP00000423755		ENSP00000452056		ENSP00000467260
ENSP00000424113		ENSP00000450573		ENSP00000464958
ENSP00000422453				ENSP00000467050
ENSP00000347426				ENSP00000465223
ENSP00000477997				ENSP00000465993
ENSP00000345601				ENSP00000468359
				ENSP00000468062
				ENSP00000465555

239	240	241	242	243
P27348	P27361	P27635	P27695	P27708
YWHAQ	MAPK3	RPL10	APEX1	CAD
ENSG00000134308	ENSG00000102882	ENSG00000147403	ENSG00000100823	ENSG00000084774
ENSP00000371267	ENSP00000263025	ENSP00000358832	ENSP00000451979	ENSP00000264705
ENSP00000238081	ENSP00000378628	ENSP00000341730	ENSP00000216714	ENSP00000384510
ENSP00000398990	ENSP00000378625	ENSP00000413436	ENSP00000381111	ENSP00000414742
	ENSP00000327293	ENSP00000388600	ENSP00000451327	ENSP00000388740
	ENSP00000433746	ENSP00000395025	ENSP00000452137	ENSP00000405416
	ENSP00000432742	ENSP00000385621	ENSP00000450604	
	ENSP00000432479	ENSP00000406125	ENSP00000452304	
	ENSP00000436772	ENSP00000407754	ENSP00000452460	
	ENSP00000378626	ENSP00000405064	ENSP00000451491	
	ENSP00000432292	ENSP00000398047	ENSP00000451170	
	ENSP00000433639	ENSP00000479103	ENSP00000452212	
			ENSP00000451060	
			ENSP00000452418	
			ENSP00000416414	

244	245	246	247	248
P27816	P27986	P28482	P29317	P29350
MAP4	PIK3R1	MAPK1	EPHA2	PTPN6
ENSG00000047849	ENSG00000145675	ENSG00000100030	ENSG00000142627	ENSG00000111679
ENSP00000379083	ENSP00000428056	ENSP00000215832	ENSP00000351209	ENSP00000382376
ENSP00000353375	ENSP00000429277	ENSP00000381803		ENSP00000326010
ENSP00000402767	ENSP00000323512	ENSP00000440842		ENSP00000391592
ENSP00000397414	ENSP00000338554			ENSP00000443393
ENSP00000416743	ENSP00000430098			ENSP00000444805
ENSP00000407602	ENSP00000428566			ENSP00000438740
ENSP00000409731	ENSP00000430126			ENSP00000476175
ENSP00000334770	ENSP00000429766			ENSP00000441044
ENSP00000373242	ENSP00000431058			ENSP00000445646
ENSP00000415689	ENSP00000430424			ENSP00000440114
ENSP00000487797	ENSP00000429156			ENSP00000446345
ENSP00000487698				

249	250	251	252	253
P29353	P29590	P29692	P29966	P30041
SHC1	PML	EEF1D	MARCKS	PRDX6
ENSG00000160691	ENSG00000140464	ENSG00000104529	ENSG00000277443	ENSG00000117592
ENSP00000357430	ENSP00000378567	ENSP00000436507	ENSP00000478061	ENSP00000342026
ENSP00000401303	ENSP00000378564	ENSP00000391944		
ENSP00000357438	ENSP00000268059	ENSP00000431763		
ENSP00000357435	ENSP00000315434	ENSP00000378551		
ENSP00000357434	ENSP00000268058	ENSP00000434872		
ENSP00000396333	ENSP00000455838	ENSP00000435697		
ENSP00000404908	ENSP00000455612	ENSP00000410059		
ENSP00000398864	ENSP00000456486	ENSP00000388261		
ENSP00000398441	ENSP00000456277	ENSP00000317399		
ENSP00000396162	ENSP00000394642	ENSP00000433784		
	ENSP00000395576	ENSP00000435015		
	ENSP00000457023	ENSP00000435117		
	ENSP00000353004	ENSP00000434070		
	ENSP00000457032	ENSP00000432881		
	ENSP00000456989	ENSP00000436045		
	ENSP00000457669	ENSP00000432429		
	ENSP00000458061	ENSP00000437276		
	ENSP00000456329	ENSP00000433611		
		ENSP00000436880		
		ENSP00000431460		
		ENSP00000434691		
		ENSP00000436542		
		ENSP00000431833		
		ENSP00000434448		
		ENSP00000433412		
		ENSP00000436933		
		ENSP00000431742		
		ENSP00000431933		
		ENSP00000435477		
		ENSP00000431613		
		ENSP00000434123		
		ENSP00000437117		
		ENSP00000434389		
		ENSP00000432221		
		ENSP00000432919		
		ENSP00000432941		
		ENSP00000433698		
		ENSP00000436251		
		ENSP00000437223		

254	255	256	257	258
P30050	P30086	P30260	P30291	P30519
RPL12	PEBP1	CDC27	WEE1	HMOX2
ENSG00000197958	ENSG00000089220	ENSG00000004897	ENSG00000166483	ENSG00000103415
ENSP00000354739	ENSP00000261313	ENSP00000066544	ENSP00000402084	ENSP00000459214
ENSP00000441179		ENSP00000434614	ENSP00000299613	ENSP00000394103
		ENSP00000460830	ENSP00000434446	ENSP00000219700
		ENSP00000432211	ENSP00000434148	ENSP00000460926
		ENSP00000460279	ENSP00000435945	ENSP00000381595
		ENSP00000437339	ENSP00000432284	ENSP00000484423
		ENSP00000432105		ENSP00000391637
		ENSP00000460132		ENSP00000385100
		ENSP00000458904		ENSP00000484467
		ENSP00000459307		ENSP00000458797
				ENSP00000459445
				ENSP00000461428
				ENSP00000458824
				ENSP00000458592
				ENSP00000460992
				ENSP00000459203
				ENSP00000477572
				ENSP00000488880
				ENSP00000487673
				ENSP00000483319
				ENSP00000488769
				ENSP00000488579
				ENSP00000481811
				ENSP00000481295
				ENSP00000484422
				ENSP00000478785
				ENSP00000488170
				ENSP00000488577
				ENSP00000487635
				ENSP00000488477
				ENSP00000487781
				ENSP00000488598
				ENSP00000488904
				ENSP00000480515

259	260	261	262	263
P30622	P30626	P31146	P31350	P31431
CLIP1	SRI	CORO1A	RRM2	SDC4
ENSG00000130779	ENSG00000075142	ENSG00000102879	ENSG00000171848	ENSG00000124145
ENSP00000303585	ENSP00000265729	ENSP00000219150	ENSP00000493399	ENSP00000361818
ENSP00000445531	ENSP00000378137	ENSP00000455552	ENSP00000353770	
ENSP00000439093	ENSP00000391148	ENSP00000456266	ENSP00000302955	
ENSP00000351665	ENSP00000397609	ENSP00000456756	ENSP00000484183	
ENSP00000479322	ENSP00000418512	ENSP00000456457	ENSP00000419177	
ENSP00000355314	ENSP00000415271	ENSP00000457332		
ENSP00000438743		ENSP00000454752		
ENSP00000437617		ENSP00000457509		
ENSP00000437786				
ENSP00000441409				
ENSP00000446379				
ENSP00000439472				
ENSP00000445387				

264	265	266	267	268
P31749	P31751	P31946	P31947	P32519
AKT1	AKT2	YWHAB	SFN	ELF1
ENSG00000142208	ENSG00000105221	ENSG00000166913	ENSG00000175793	ENSG00000120690
ENSP00000451828	ENSP00000375892	ENSP00000300161	ENSP00000340989	ENSP00000239882
ENSP00000384293	ENSP00000309428	ENSP00000361930		ENSP00000486912
ENSP00000270202	ENSP00000399532	ENSP00000487924		ENSP00000487240
ENSP00000385326	ENSP00000470822	ENSP00000394729		ENSP00000489586
ENSP00000450688	ENSP00000375719	ENSP00000394558		ENSP00000384135
ENSP00000451166	ENSP00000463368	ENSP00000488178		
ENSP00000451470	ENSP00000462469			
ENSP00000450681	ENSP00000472382			
ENSP00000481526	ENSP00000471369			
ENSP00000451824	ENSP00000463262			
	ENSP00000462919			
	ENSP00000472371			
	ENSP00000462776			
	ENSP00000463086			
	ENSP00000396968			
	ENSP00000463686			
	ENSP00000470604			
	ENSP00000404083			
	ENSP00000375891			
	ENSP00000407999			
	ENSP00000462022			
	ENSP00000403842			
	ENSP00000351095			
	ENSP00000396532			
	ENSP00000462715			
	ENSP00000463806			
	ENSP00000392458			
	ENSP00000403890			

269	270	271	272	273
P33527	P33778	P33981	P33991	P33993
ABCC1	HIST1H2BB	TTK	MCM4	CLEC3A
ENSG00000103222	ENSG00000276410	ENSG00000112742	ENSG00000104738	ENSG00000166509
ENSP00000382342	ENSP00000358151	ENSP00000422936	ENSP00000430194	ENSP00000299642
ENSP00000461615	ENSP00000482674	ENSP00000230510	ENSP00000262105	ENSP00000457211
ENSP00000459693	ENSP00000477527	ENSP00000358813	ENSP00000430329	ENSP00000344006
ENSP00000382340	ENSP00000476136	ENSP00000421406	ENSP00000427829	ENSP00000307288
ENSP00000483316	ENSP00000342886	ENSP00000424851	ENSP00000428833	ENSP00000483795
ENSP00000481080	ENSP00000475213	ENSP00000421636	ENSP00000427875	ENSP00000346171
ENSP00000478647		ENSP00000427483	ENSP00000430864	ENSP00000445904
		ENSP00000487202		ENSP00000411295

274	275	276	277	278
P35221	P35222	P35226	P35240	P35241
CTNNA1	CTNNB1	BMI1	NF2	RDX
ENSG00000044115	ENSG00000168036	ENSG00000168283	ENSG00000186575	ENSG00000137710
ENSP00000304669	ENSP00000385604	ENSP00000365851	ENSP00000344666	ENSP00000437301
ENSP00000438476	ENSP00000344456	ENSP00000397912	ENSP00000384029	ENSP00000432112
ENSP00000428439	ENSP00000379488	ENSP00000399220	ENSP00000354897	ENSP00000384136
ENSP00000429636	ENSP00000379486	ENSP00000390768	ENSP00000384797	ENSP00000433580
ENSP00000428049	ENSP00000494411		ENSP00000335652	ENSP00000436277
ENSP00000430304	ENSP00000400508		ENSP00000340626	ENSP00000342830
ENSP00000428202	ENSP00000496180		ENSP00000395885	ENSP00000445826
ENSP00000431033	ENSP00000496067		ENSP00000354529	ENSP00000496121
ENSP00000430626	ENSP00000495244		ENSP00000355183	ENSP00000494060
ENSP00000430671	ENSP00000387455		ENSP00000380891	ENSP00000496414
ENSP00000430078	ENSP00000496385		ENSP00000409921	ENSP00000494693
ENSP00000429457	ENSP00000412219			ENSP00000496503
ENSP00000427821	ENSP00000496295			ENSP00000432829
ENSP00000429142	ENSP00000494422			ENSP00000431560
ENSP00000429738	ENSP00000495794			ENSP00000494706
ENSP00000430379	ENSP00000495450			ENSP00000437140
ENSP00000429569	ENSP00000494845			ENSP00000434881
ENSP00000428044	ENSP00000411226			ENSP00000432572
ENSP00000430841	ENSP00000494849			ENSP00000435930
ENSP00000428088	ENSP00000495286			
ENSP00000431118	ENSP00000495161			
ENSP00000430240	ENSP00000493610			
ENSP00000429103	ENSP00000493533			
ENSP00000430177	ENSP00000496020			
ENSP00000430981	ENSP00000496649			
ENSP00000430623	ENSP00000494654			
ENSP00000428894	ENSP00000494263			
ENSP00000482738	ENSP00000495719			
ENSP00000428710	ENSP00000495426			
ENSP00000428686	ENSP00000496511			
ENSP00000430076	ENSP00000495992			
ENSP00000486200	ENSP00000496021			
	ENSP00000494467			
	ENSP00000494914			
	ENSP00000493583			
	ENSP00000494053			
	ENSP00000494780			
	ENSP00000494677			
	ENSP00000409302			

279	280	281	282	283
P35269	P35270	P35568	P35579	P35580
GTF2F1	SPR	IRS1	MYH9	MYH10
ENSG00000125651	ENSG00000116096	ENSG00000169047	ENSG00000100345	ENSG00000133026
ENSP00000377969	ENSP00000234454	ENSP00000304895	ENSP00000216181	ENSP00000353590
ENSP00000469091			ENSP00000384631	ENSP00000269243
ENSP00000471403			ENSP00000414852	ENSP00000369315
ENSP00000471542				ENSP00000408220

284	285	286	287	288
P35611	P35659	P35813	P36507	P36871
ADD1	DEK	PPM1A	MAP2K2	PGM1
ENSG00000087274	ENSG00000124795	ENSG00000100614	ENSG00000126934	ENSG00000079739
ENSP00000348100	ENSP00000380414	ENSP00000327255	ENSP00000262948	ENSP00000360125
ENSP00000381191	ENSP00000244776	ENSP00000378514	ENSP00000471763	ENSP00000360124
ENSP00000381197	ENSP00000421165	ENSP00000314850	ENSP00000378336	ENSP00000443449
ENSP00000381193	ENSP00000423427	ENSP00000437200		
ENSP00000264758	ENSP00000422380	ENSP00000436445		
ENSP00000425703	ENSP00000425399	ENSP00000431453		
ENSP00000421918	ENSP00000423553	ENSP00000435398		
ENSP00000421907		ENSP00000435575		
ENSP00000426700				
ENSP00000423024				
ENSP00000424143				
ENSP00000438069				
ENSP00000441388				
ENSP00000399828				

289	290	291	292	293
P36873	P37173	P38432	P38646	P39748
PPP1CC	TGFBR2	COIL	HSPA9	FEN1
ENSG00000186298	ENSG00000163513	ENSG00000121058	ENSG00000113013	ENSG00000168496
ENSP00000335084	ENSP00000295754	ENSP00000240316	ENSP00000297185	ENSP00000305480
ENSP00000341779	ENSP00000351905	ENSP00000460217	ENSP00000421892	ENSP00000445692
ENSP00000447528			ENSP00000429475	ENSP00000460402
ENSP00000447090			ENSP00000421311	
ENSP00000447122			ENSP00000423759	
ENSP00000448981			ENSP00000420944	
ENSP00000448437			ENSP00000425598	
ENSP00000482210			ENSP00000423098	

294	295	296	297	298
P40189	P40763	P41212	P41236	P41240
IL6ST	STAT3	ETV6	PPP1R2	CSK
ENSG00000134352	ENSG00000168610	ENSG00000139083	ENSG00000184203	ENSG00000103653
ENSP00000370698	ENSP00000264657	ENSP00000379658	ENSP00000484580	ENSP00000220003
ENSP00000462158	ENSP00000467000	ENSP00000441463	ENSP00000415175	ENSP00000414764
ENSP00000370694	ENSP00000467985	ENSP00000266427	ENSP00000400238	ENSP00000454906
ENSP00000435399	ENSP00000384943		ENSP00000406725	ENSP00000457497
ENSP00000370687	ENSP00000373923		ENSP00000486328	ENSP00000457714
ENSP00000338799	ENSP00000467173		ENSP00000490297	ENSP00000454640
ENSP00000426224				
ENSP00000370693				
ENSP00000370686				
ENSP00000463896				

299	300	301	302	303
P41279	P41440	P41743	P42224	P42226
MAP3K8	SLC19A1	PRKCI	STAT1	STAT6
ENSG00000107968	ENSG00000173638	ENSG00000163558	ENSG00000115415	ENSG00000166888
ENSP00000263056	ENSP00000308895	ENSP00000295797	ENSP00000354394	ENSP00000300134
ENSP00000364470	ENSP00000369347		ENSP00000386244	ENSP00000445409
ENSP00000443610	ENSP00000441772		ENSP00000376136	ENSP00000438451
ENSP00000409653	ENSP00000393988		ENSP00000388240	ENSP00000451742
ENSP00000364471	ENSP00000457278		ENSP00000388772	ENSP00000444530
ENSP00000391275	ENSP00000401850		ENSP00000394512	ENSP00000401486
	ENSP00000411345		ENSP00000376137	ENSP00000451909
	ENSP00000435780		ENSP00000402548	ENSP00000450428
			ENSP00000411398	ENSP00000450921
			ENSP00000416019	ENSP00000451546
			ENSP00000438703	ENSP00000452394
				ENSP00000452373
				ENSP00000451074
				ENSP00000452203
				ENSP00000450510
				ENSP00000450665
				ENSP00000451209
				ENSP00000450747
				ENSP00000450732
				ENSP00000491116

304	305	306	307	308
P42345	P42566	P42574	P42575	P42858
MTOR	EPS15	CASP3	CASP2	HTT
ENSG00000198793	ENSG00000085832	ENSG00000164305	ENSG00000106144	ENSG00000197386
ENSP00000354558	ENSP00000360798	ENSP00000311032	ENSP00000312664	ENSP00000347184
ENSP00000366034	ENSP00000360795	ENSP00000428929	ENSP00000376656	ENSP00000425743
ENSP00000398745	ENSP00000474739	ENSP00000377210	ENSP00000340030	
		ENSP00000428372	ENSP00000481929	
		ENSP00000377213		
		ENSP00000407142		

309	310	311	312	313
P43378	P43405	P45973	P45983	P45984
PTPN9	SYK	CBX5	MAPK8	MAPK9
ENSG00000169410	ENSG00000165025	ENSG00000094916	ENSG00000107643	ENSG00000050748
ENSP00000482732	ENSP00000364907	ENSP00000209875	ENSP00000363304	ENSP00000394560
ENSP00000455934	ENSP00000364904	ENSP00000401009	ENSP00000363297	ENSP00000377028
ENSP00000303554	ENSP00000364899	ENSP00000449207	ENSP00000363294	ENSP00000389338
	ENSP00000364898	ENSP00000450190	ENSP00000363291	ENSP00000345524
			ENSP00000378974	ENSP00000397422
			ENSP00000353483	ENSP00000321410
			ENSP00000387936	ENSP00000377029
			ENSP00000393223	ENSP00000430608
			ENSP00000397729	ENSP00000443149
			ENSP00000363289	ENSP00000380262

314	315	316	317	318
P45985	P46020	P46060	P46108	P46459
MAP2K4	PHKA1	RANGAP1	CRK	AC171558.5
ENSG00000065559	ENSG00000067177	ENSG00000100401	ENSG00000167193	ENSG00000278178
ENSP00000262445	ENSP00000362643	ENSP00000385866	ENSP00000381942	ENSP00000481382
ENSP00000410402	ENSP00000342469	ENSP00000348577	ENSP00000300574	ENSP00000485766
ENSP00000463507	ENSP00000441251	ENSP00000401470	ENSP00000459505	ENSP00000381293
ENSP00000473582	ENSP00000362646	ENSP00000393407		ENSP00000461448
ENSP00000473472	ENSP00000362640	ENSP00000394787		ENSP00000460152
ENSP00000473606		ENSP00000408743		ENSP00000458184
ENSP00000473458		ENSP00000404842		ENSP00000458221
ENSP00000473511				ENSP00000459646
ENSP00000467239				ENSP00000467779
				ENSP00000481714
				ENSP00000488517
				ENSP00000487668
				ENSP00000488185
				ENSP00000488015
				ENSP00000488480
				ENSP00000478614
				ENSP00000487474
				ENSP00000487064
				ENSP00000486903
				ENSP00000486150
				ENSP00000486400
				ENSP00000487479

319	320	321	322	323
P46527	P46734	P46777	P46937	P46939
CDKN1B	MAP2K3	RPL5	YAP1	UTRN
ENSG00000111276	ENSG00000034152	ENSG00000122406	ENSG00000137693	ENSG00000152818
ENSP00000228872	ENSP00000345083	ENSP00000359345	ENSP00000434134	ENSP00000356515
ENSP00000379629	ENSP00000319139	ENSP00000495549	ENSP00000282441	ENSP00000390879
ENSP00000407597	ENSP00000478619	ENSP00000495589	ENSP00000487519	ENSP00000487153
	ENSP00000355081	ENSP00000493811	ENSP00000431574	ENSP00000396276
	ENSP00000378869	ENSP00000473675	ENSP00000435602	ENSP00000356496
	ENSP00000431203	ENSP00000359338	ENSP00000445635	ENSP00000356495
	ENSP00000463202		ENSP00000331023	ENSP00000356494
	ENSP00000463417		ENSP00000478927	ENSP00000398923
	ENSP00000464043		ENSP00000431626	ENSP00000404205
	ENSP00000434068			ENSP00000387927
	ENSP00000462178			
	ENSP00000486923			

324	325	326	327	328
P46940	P47712	P47736	P48200	P48426
IQGAP1	PLA2G4A	RAP1GAP	IREB2	PIP4K2A
ENSG00000140575	ENSG00000116711	ENSG00000076864	ENSG00000136381	ENSG00000150867
ENSP00000268182	ENSP00000356436	ENSP00000363897	ENSP00000452938	ENSP00000365757
ENSP00000488618		ENSP00000441661	ENSP00000453172	ENSP00000442098
ENSP00000453181		ENSP00000434033	ENSP00000453532	ENSP00000326294
ENSP00000452723		ENSP00000363895	ENSP00000258886	ENSP00000473858
ENSP00000484647		ENSP00000363893	ENSP00000453058	
ENSP00000480393		ENSP00000431364	ENSP00000454063	
		ENSP00000315420	ENSP00000453863	
		ENSP00000392717		
		ENSP00000290101		

329	330	331	332	333
P48436	P48729	P48730	P49023	P49137
SOX9	CSNK1A1	CSNK1D	PXN	MAPKAPK2
ENSG00000125398	ENSG00000113712	ENSG00000141551	ENSG00000089159	ENSG00000162889
ENSP00000245479	ENSP00000261798	ENSP00000324464	ENSP00000395536	ENSP00000294981
ENSP00000495527	ENSP00000421689	ENSP00000376146	ENSP00000228307	ENSP00000356070
	ENSP00000367074	ENSP00000462079	ENSP00000391283	
	ENSP00000426747	ENSP00000381531	ENSP00000267257	
	ENSP00000427031	ENSP00000463906	ENSP00000490896	
	ENSP00000475319	ENSP00000269361	ENSP00000443887	
	ENSP00000475969	ENSP00000385769	ENSP00000489977	
	ENSP00000425366	ENSP00000463757	ENSP00000489840	
	ENSP00000475633	ENSP00000464551	ENSP00000490352	
	ENSP00000430252	ENSP00000462144	ENSP00000446893	
	ENSP00000421268	ENSP00000352929	ENSP00000443745	
	ENSP00000369126	ENSP00000384074	ENSP00000447180	
		ENSP00000380044	ENSP00000446607	
		ENSP00000400494	ENSP00000446650	
		ENSP00000403610		
		ENSP00000412194		
		ENSP00000407235		
		ENSP00000384426		
		ENSP00000397460		
		ENSP00000403057		
		ENSP00000412335		

334	335	336	337	338
P49407	P49459	P49585	P49674	P49736
ARRB1	UBE2A	PCYT1A	CSNK1E	MCM2
ENSG00000137486	ENSG00000077721	ENSG00000161217	ENSG00000213923	ENSG00000073111
ENSP00000409581	ENSP00000360613	ENSP00000292823	ENSP00000324464	ENSP00000265056
ENSP00000353124	ENSP00000486599	ENSP00000394617	ENSP00000376146	ENSP00000420007
ENSP00000436530	ENSP00000486550	ENSP00000392397	ENSP00000462079	ENSP00000419802
ENSP00000433171	ENSP00000335027	ENSP00000390968	ENSP00000381531	ENSP00000418930
ENSP00000434196	ENSP00000486153	ENSP00000400430	ENSP00000463906	ENSP00000417800
ENSP00000436352	ENSP00000486347	ENSP00000390458	ENSP00000269361	ENSP00000420528
ENSP00000433137	ENSP00000487203	ENSP00000402283	ENSP00000385769	
		ENSP00000397888	ENSP00000463757	
		ENSP00000402015	ENSP00000464551	
		ENSP00000391405	ENSP00000462144	
		ENSP00000393341	ENSP00000352929	
			ENSP00000384074	
			ENSP00000380044	
			ENSP00000400494	
			ENSP00000403610	
			ENSP00000412194	
			ENSP00000407235	
			ENSP00000384426	
			ENSP00000397460	
			ENSP00000403057	
			ENSP00000412335	

339	340	341	342	343
P49757	P49790	P49815	P49840	P49841
NUMB	NUP153	TSC2	GSK3A	GSK3B
ENSG00000133961	ENSG00000124789	ENSG00000103197	ENSG00000105723	ENSG00000082701
ENSP00000452416	ENSP00000262077	ENSP00000219476	ENSP00000222330	ENSP00000264235
ENSP00000348644	ENSP00000478627	ENSP00000371978	ENSP00000412663	ENSP00000324806
ENSP00000451117	ENSP00000444029	ENSP00000384468	ENSP00000381301	
ENSP00000451300		ENSP00000399232		
ENSP00000451625		ENSP00000344383		
ENSP00000452888		ENSP00000494747		
ENSP00000453209		ENSP00000495927		
ENSP00000452069		ENSP00000495644		
ENSP00000450817		ENSP00000495948		
ENSP00000441258		ENSP00000494514		
ENSP00000352563		ENSP00000496317		
ENSP00000347169		ENSP00000495921		
ENSP00000446001		ENSP00000494393		
ENSP00000451513		ENSP00000495146		
ENSP00000451326		ENSP00000496611		
ENSP00000452357		ENSP00000406980		
ENSP00000451374		ENSP00000496262		
ENSP00000451559		ENSP00000493846		
ENSP00000451959		ENSP00000495099		
		ENSP00000454487		
		ENSP00000493990		
		ENSP00000493912		
		ENSP00000387937		
		ENSP00000455997		
		ENSP00000495459		
		ENSP00000496610		
		ENSP00000460310		
		ENSP00000458541		
		ENSP00000455817		
		ENSP00000495110		

344	345	346	347	348
P49915	P49916	P49917	P50402	P50542
GMPS	LIG3	LIG4	EMD	PEX5
ENSG00000163655	ENSG00000005156	ENSG00000174405	ENSG00000102119	ENSG00000139197
ENSP00000419851	ENSP00000367787	ENSP00000402030	ENSP00000358857	ENSP00000400647
ENSP00000295920	ENSP00000262327	ENSP00000484288	ENSP00000358850	ENSP00000266563
	ENSP00000468479	ENSP00000385955	ENSP00000401081	ENSP00000407401
	ENSP00000467063	ENSP00000349393		ENSP00000410159
	ENSP00000467757	ENSP00000480814		ENSP00000266564
	ENSP00000466278			ENSP00000441553
	ENSP00000465288			ENSP00000439025
				ENSP00000438494
				ENSP00000438772
				ENSP00000440833
				ENSP00000443500
				ENSP00000391601
				ENSP00000379877
				ENSP00000445630
				ENSP00000437938
				ENSP00000440711
				ENSP00000444787

349	350	351	352	353
P50548	P50552	P50570	P50613	P50616
ERF	VASP	DNM2	CDK7	TOB1
ENSG00000105722	ENSG00000125753	ENSG00000079805	ENSG00000134058	ENSG00000141232
ENSP00000222329	ENSP00000245932	ENSP00000468734	ENSP00000256443	ENSP00000427695
ENSP00000388173	ENSP00000467061	ENSP00000352721	ENSP00000425043	ENSP00000268957
ENSP00000469274	ENSP00000464742	ENSP00000373905	ENSP00000425893	
ENSP00000468962	ENSP00000467773	ENSP00000347890	ENSP00000426372	
	ENSP00000467005	ENSP00000386192	ENSP00000425983	
	ENSP00000466319	ENSP00000467430	ENSP00000426561	
		ENSP00000466603	ENSP00000422737	
		ENSP00000466914	ENSP00000422027	
		ENSP00000466621	ENSP00000421559	
			ENSP00000422121	
			ENSP00000486415	
			ENSP00000486479	
			ENSP00000479116	
			ENSP00000477911	
			ENSP00000477833	
			ENSP00000478968	
			ENSP00000477752	
			ENSP00000479440	
			ENSP00000483754	
			ENSP00000483013	
			ENSP00000478296	
			ENSP00000478687	
			ENSP00000485943	
			ENSP00000486424	

354	355	356	357	358
P50750	P51398	P51452	P51617	P51692
CDK9	DAP3	DUSP3	IRAK1	STAT5B
ENSG00000136807	ENSG00000132676	ENSG00000108861	ENSG00000184216	ENSG00000173757
ENSP00000362361	ENSP00000476592	ENSP00000226004	ENSP00000358997	ENSP00000293328
ENSP00000395872	ENSP00000357320	ENSP00000466069	ENSP00000358991	ENSP00000398379
	ENSP00000341692	ENSP00000468604	ENSP00000377291	
	ENSP00000412605	ENSP00000467424	ENSP00000399974	
	ENSP00000445003		ENSP00000411809	
	ENSP00000476899		ENSP00000358990	
	ENSP00000476889		ENSP00000410953	
	ENSP00000476816		ENSP00000406249	
	ENSP00000476291		ENSP00000392662	
	ENSP00000476840		ENSP00000403730	
	ENSP00000477025			
	ENSP00000477265			
	ENSP00000476558			
	ENSP00000476624			
	ENSP00000477139			
	ENSP00000477453			
	ENSP00000476706			

359	360	361	362	363
P51812	P52292	P52564	P52565	P52566
RPS6KA3	KPNA2	MAP2K6	ARHGDIA	ARHGDIB
ENSG00000177189	ENSG00000182481	ENSG00000108984	ENSG00000141522	ENSG00000111348
ENSP00000368884	ENSP00000332455	ENSP00000468348	ENSP00000269321	ENSP00000228945
ENSP00000494131	ENSP00000438483	ENSP00000467213	ENSP00000383556	ENSP00000444860
ENSP00000496271	ENSP00000463320	ENSP00000477701	ENSP00000441348	ENSP00000440560
ENSP00000494967	ENSP00000463602	ENSP00000351997	ENSP00000464205	ENSP00000445185
ENSP00000493862	ENSP00000462331	ENSP00000466143	ENSP00000463939	ENSP00000445263
ENSP00000495776		ENSP00000464916	ENSP00000462960	ENSP00000437454
ENSP00000494220			ENSP00000461956	ENSP00000445450
ENSP00000496226			ENSP00000462209	
ENSP00000407655			ENSP00000463530	
ENSP00000495839			ENSP00000462323	
ENSP00000495462				
ENSP00000493487				
ENSP00000494769				
ENSP00000495974				
ENSP00000494866				
ENSP00000388512				

364	365	366	367	368
P52655	P52732	P52735	P52888	P52907
GTF2A1	KIF11	VAV2	THOP1	CAPZA1
ENSG00000165417	ENSG00000138160	ENSG00000160293	ENSG00000172009	ENSG00000116489
ENSP00000452454	ENSP00000260731	ENSP00000385362	ENSP00000304467	ENSP00000263168
ENSP00000409492		ENSP00000360916	ENSP00000378638	
ENSP00000298173		ENSP00000360917	ENSP00000465545	
			ENSP00000467226	
			ENSP00000466653	
			ENSP00000465855	
			ENSP00000465895	
			ENSP00000464786	

369	370	371	372	373
P52926	P52943	P52948	P53041	P53350
HMGA2	CRIP2	NUP98	PPP5C	PLK1
ENSG00000149948	ENSG00000182809	ENSG00000110713	ENSG00000011485	ENSG00000166851
ENSP00000384026	ENSP00000426119	ENSP00000316032	ENSP00000012443	ENSP00000300093
ENSP00000437747	ENSP00000328521	ENSP00000347404	ENSP00000434329	ENSP00000460266
ENSP00000377206	ENSP00000446875	ENSP00000380199	ENSP00000375786	ENSP00000459688
ENSP00000443372	ENSP00000437723	ENSP00000380202		ENSP00000460084
ENSP00000407306		ENSP00000352091		
ENSP00000346658		ENSP00000432275		
ENSP00000437621		ENSP00000413146		
ENSP00000439317		ENSP00000433515		
ENSP00000377205		ENSP00000433226		
ENSP00000440919		ENSP00000436226		
		ENSP00000432142		
		ENSP00000380208		
		ENSP00000434698		

374	375	376	377	378
P53355	P53365	P53396	P54132	P54198
DAPK1	ARFIP2	ACLY	BLM	HIRA
ENSG00000196730	ENSG00000132254	ENSG00000131473	ENSG00000197299	ENSG00000100084
ENSP00000350785	ENSP00000254584	ENSP00000253792	ENSP00000347232	ENSP00000345350
ENSP00000417076	ENSP00000379998	ENSP00000466259	ENSP00000454158	ENSP00000263208
ENSP00000386135	ENSP00000391427	ENSP00000445349	ENSP00000453359	ENSP00000404792
ENSP00000419026	ENSP00000398375	ENSP00000377474		
ENSP00000484267	ENSP00000434564	ENSP00000345398		
ENSP00000418885	ENSP00000434124	ENSP00000464717		
ENSP00000417746	ENSP00000484121	ENSP00000468705		

379	380	381	382	383
P54253	P55010	P55011	P55072	P55210
ATXN1	EIF5	SLC12A2	VCP	CASP7
ENSG00000124788	ENSG00000100664	ENSG00000064651	ENSG00000165280	ENSG00000165806
ENSP00000244769	ENSP00000216554	ENSP00000262461	ENSP00000351777	ENSP00000358337
ENSP00000416360	ENSP00000376477	ENSP00000340878	ENSP00000392088	ENSP00000298701
ENSP00000493530	ENSP00000453743	ENSP00000427109	ENSP00000399456	ENSP00000358324
ENSP00000450642	ENSP00000453633	ENSP00000486323		ENSP00000358321
	ENSP00000452636			ENSP00000398107
	ENSP00000453396			ENSP00000478999
	ENSP00000453856			ENSP00000478285
	ENSP00000452883			ENSP00000480584
	ENSP00000452615			ENSP00000400094
	ENSP00000453468			ENSP00000358327

384	385	386	387	388
P55211	P55327	P55957	P56211	P56537
CASP9	TPD52	BID	ARPP19	EIF6
ENSG00000132906	ENSG00000076554	ENSG00000015475	ENSG00000128989	ENSG00000242372
ENSP00000330237	ENSP00000368390	ENSP00000318822	ENSP00000455625	ENSP00000363559
ENSP00000255256	ENSP00000429915	ENSP00000382674	ENSP00000249822	ENSP00000363566
ENSP00000365051	ENSP00000429309	ENSP00000382667	ENSP00000454234	ENSP00000363574
ENSP00000449584	ENSP00000429351	ENSP00000344594	ENSP00000457805	ENSP00000395907
ENSP00000383588	ENSP00000429708	ENSP00000449236	ENSP00000455986	ENSP00000411450
ENSP00000408691	ENSP00000368391	ENSP00000480414	ENSP00000455151	ENSP00000415044
ENSP00000449216	ENSP00000410222	ENSP00000483709	ENSP00000454297	ENSP00000390897
ENSP00000396540	ENSP00000428951	ENSP00000483534	ENSP00000457631	
ENSP00000411304	ENSP00000430646	ENSP00000477773	ENSP00000455610	
ENSP00000480785	ENSP00000430421	ENSP00000382669	ENSP00000454612	
	ENSP00000430323	ENSP00000481991	ENSP00000454341	
	ENSP00000430140		ENSP00000457123	
	ENSP00000430865			

389	390	391	392	393
P56945	P60484	P60510	P60953	P60983
BCAR1	PTEN	PPP4C	CDC42	GMFB
ENSG00000050820	ENSG00000171862	ENSG00000149923	ENSG00000070831	ENSG00000197045
ENSP00000377074	ENSP00000361021	ENSP00000279387	ENSP00000341072	ENSP00000350757
ENSP00000162330	ENSP00000483066	ENSP00000455995	ENSP00000314458	ENSP00000479543
ENSP00000440370		ENSP00000455394	ENSP00000383118	ENSP00000452410
ENSP00000443841		ENSP00000458079	ENSP00000398327	ENSP00000451473
ENSP00000391669		ENSP00000461609		ENSP00000451920
ENSP00000440415		ENSP00000457089		ENSP00000487065
ENSP00000392708		ENSP00000457915		
ENSP00000377072		ENSP00000486140		
ENSP00000455166				
ENSP00000454702				
ENSP00000456625				
ENSP00000455810				
ENSP00000456929				
ENSP00000458088				
ENSP00000457457				
ENSP00000457097				
ENSP00000457231				
ENSP00000442161				

394	395	396	397	398
P61020	P61088	P61224	P61586	P61587
RAB5B	UBE2N	RAP1B	RHOA	RND3
ENSG00000111540	ENSG00000177889	ENSG00000127314	ENSG00000067560	ENSG00000115963
ENSP00000450168	ENSP00000316176	ENSP00000250559	ENSP00000400175	ENSP00000364886
ENSP00000353444	ENSP00000449352	ENSP00000377085	ENSP00000394483	ENSP00000263895
ENSP00000391319	ENSP00000450260	ENSP00000441275	ENSP00000413587	ENSP00000386576
ENSP00000450285	ENSP00000447009	ENSP00000439966	ENSP00000408402	ENSP00000411950
ENSP00000447895	ENSP00000448352	ENSP00000399986		ENSP00000395997
ENSP00000449762		ENSP00000446318		
ENSP00000449554		ENSP00000444830		
ENSP00000487368		ENSP00000440014		
		ENSP00000401095		
		ENSP00000446027		
		ENSP00000390972		
		ENSP00000445138		
		ENSP00000444786		
		ENSP00000437415		
		ENSP00000444467		
		ENSP00000438088		
		ENSP00000445090		
		ENSP00000441952		
		ENSP00000444060		
		ENSP00000368270		
		ENSP00000440466		
		ENSP00000443775		
		ENSP00000444924		
		ENSP00000445977		
		ENSP00000440635		
		ENSP00000440708		
		ENSP00000438311		
		ENSP00000443851		

399	400	401	402	403
P61764	P61978	P62136	P62140	P62258
STXBP1	HNRNPK	PPP1CA	PPP1CB	KIR3DL3
ENSG00000136854	ENSG00000165119	ENSG00000172531	ENSG00000213639	ENSG00000274480
ENSP00000362399	ENSP00000365458	ENSP00000326031	ENSP00000296122	ENSP00000481489
ENSP00000362396	ENSP00000317788	ENSP00000365936	ENSP00000378769	ENSP00000264335
ENSP00000489791	ENSP00000353552	ENSP00000350974	ENSP00000351298	ENSP00000461762
ENSP00000486944	ENSP00000365439	ENSP00000431146	ENSP00000390715	ENSP00000464883
ENSP00000490810	ENSP00000473957	ENSP00000439568	ENSP00000398839	ENSP00000458386
ENSP00000487211	ENSP00000409456	ENSP00000438409	ENSP00000414918	ENSP00000466324
ENSP00000490613	ENSP00000475098		ENSP00000394589	ENSP00000461766
ENSP00000490903			ENSP00000388056	ENSP00000460712
ENSP00000489762				ENSP00000487356
ENSP00000489655				ENSP00000481059
ENSP00000490674				ENSP00000487228
ENSP00000490519				ENSP00000486697
ENSP00000485680				ENSP00000487432
ENSP00000485895				ENSP00000486352
ENSP00000490199				ENSP00000487508
ENSP00000486814				
ENSP00000494727				
ENSP00000485361				
ENSP00000485397				

404	405	406	407	408
P62714	P62745	P62753	P62826	P62834
PPP2CB	RHOB	RPS6	RAN	RAP1A
ENSG00000104695	ENSG00000143878	ENSG00000137154	ENSG00000132341	ENSG00000116473
ENSP00000221138	ENSP00000272233	ENSP00000369757	ENSP00000446215	ENSP00000348786
ENSP00000429927		ENSP00000369745	ENSP00000376176	ENSP00000358723
ENSP00000428142		ENSP00000369743	ENSP00000396127	ENSP00000396741
ENSP00000430758		ENSP00000369741	ENSP00000437950	
ENSP00000428618			ENSP00000441210	
ENSP00000428866			ENSP00000444042	
			ENSP00000483687	
			ENSP00000376174	

409	410	411	412	413
P62837	P62877	P62993	P63000	P63010
UBE2D2	RBX1	GRB2	RAC1	AP2B1
ENSG00000131508	ENSG00000100387	ENSG00000177885	ENSG00000136238	ENSG00000006125
ENSP00000429613	ENSP00000216225	ENSP00000376346	ENSP00000258737	ENSP00000482315
ENSP00000381717		ENSP00000376347	ENSP00000348461	ENSP00000483185
ENSP00000381718		ENSP00000376345	ENSP00000304283	ENSP00000482835
ENSP00000424941		ENSP00000339007	ENSP00000463590	
ENSP00000426523		ENSP00000317360	ENSP00000462421	
ENSP00000253815		ENSP00000463525		
ENSP00000396901		ENSP00000464279		
ENSP00000378280		ENSP00000462785		
ENSP00000378282				
ENSP00000345285				
ENSP00000318494				
ENSP00000337208				
ENSP00000344069				
ENSP00000349722				
ENSP00000378283				
ENSP00000337262				
ENSP00000426620				
ENSP00000426586				
ENSP00000424359				
ENSP00000421904				
ENSP00000427270				
ENSP00000422939				
ENSP00000422134				
ENSP00000424710				
ENSP00000421794				
ENSP00000423487				
ENSP00000423692				
ENSP00000423324				
ENSP00000425762				
ENSP00000423869				
ENSP00000421310				
ENSP00000481600				

414	415	416	417	418
P63104	P63167	P63244	P63261	P67775
YWHAZ	DYNLL1	RACK1	ACTG1	PPP2CA
ENSG00000164924	ENSG00000088986	ENSG00000204628	ENSG00000184009	ENSG00000113575
ENSP00000379287	ENSP00000376297	ENSP00000426909	ENSP00000458435	ENSP00000418447
ENSP00000379286	ENSP00000447907	ENSP00000420936	ENSP00000331514	ENSP00000430869
ENSP00000309503	ENSP00000242577	ENSP00000422198	ENSP00000458162	ENSP00000428816
ENSP00000430072	ENSP00000376296	ENSP00000426708	ENSP00000459124	
ENSP00000379283	ENSP00000446614	ENSP00000424654	ENSP00000461672	
ENSP00000379281	ENSP00000448834	ENSP00000426960	ENSP00000477968	
ENSP00000395114	ENSP00000448608	ENSP00000422316	ENSP00000493648	
ENSP00000398599	ENSP00000446596	ENSP00000426101	ENSP00000462823	
ENSP00000379288	ENSP00000447494	ENSP00000421838	ENSP00000461407	
ENSP00000428860	ENSP00000449088	ENSP00000427677	ENSP00000460464	
ENSP00000429623		ENSP00000424760	ENSP00000459119	
ENSP00000428775		ENSP00000366013	ENSP00000460660	
ENSP00000379278		ENSP00000426046	ENSP00000466346	
ENSP00000430058		ENSP00000422768		
ENSP00000429041		ENSP00000425008		
ENSP00000416551		ENSP00000423569		
ENSP00000394880		ENSP00000422029		
ENSP00000428381		ENSP00000421416		
ENSP00000430219		ENSP00000426111		
		ENSP00000426270		
		ENSP00000424237		
		ENSP00000421356		
		ENSP00000486170		

419	420	421	422	423
P67809	P67870	P68104	P68366	P68400
YBX1	CSNK2B	EEF1A1	TUBA4A	CSNK2A1
ENSG00000065978	ENSG00000204435	ENSG00000156508	ENSG00000127824	ENSG00000101266
ENSP00000361626	ENSP00000365042	ENSP00000339063	ENSP00000248437	ENSP00000339247
ENSP00000405937	ENSP00000365025	ENSP00000339053	ENSP00000375938	ENSP00000217244
ENSP00000389639	ENSP00000365026	ENSP00000330054	ENSP00000396212	ENSP00000383076
	ENSP00000365046	ENSP00000348651	ENSP00000408194	ENSP00000496569
	ENSP00000372925	ENSP00000392366	ENSP00000416992	ENSP00000495248
	ENSP00000372919	ENSP00000479055	ENSP00000396061	ENSP00000495998
	ENSP00000382980	ENSP00000479609	ENSP00000404740	ENSP00000481572
	ENSP00000382984			ENSP00000383086
	ENSP00000372923			ENSP00000493697
	ENSP00000477593			ENSP00000494445
	ENSP00000483317			ENSP00000495902
	ENSP00000478364			ENSP00000493931
	ENSP00000407018			ENSP00000495414
	ENSP00000394855			ENSP00000494288
	ENSP00000412520			ENSP00000495422
	ENSP00000413092			ENSP00000496146
	ENSP00000407864			ENSP00000495439
	ENSP00000477727			ENSP00000493791
	ENSP00000480237			ENSP00000495387
	ENSP00000484115			ENSP00000496152
	ENSP00000415303			ENSP00000494370
	ENSP00000413469			ENSP00000495912
	ENSP00000395275			ENSP00000494038
	ENSP00000415615			ENSP00000493704
	ENSP00000389767			ENSP00000477147
	ENSP00000484705			ENSP00000495139
	ENSP00000478586			ENSP00000495079
	ENSP00000478717			ENSP00000476547
	ENSP00000415237			ENSP00000495942
	ENSP00000411322			ENSP00000476486
	ENSP00000410802			ENSP00000493643
	ENSP00000410942			ENSP00000496232
	ENSP00000407844			ENSP00000496115
	ENSP00000483938			ENSP00000493474
	ENSP00000480787			ENSP00000473553
	ENSP00000478919			
	ENSP00000400188			
	ENSP00000409510			
	ENSP00000407379			

424	425	426	427	428
P68431	P78314	P78347	P78356	P78362
ATP9B	SH3BP2	GTF2I	PIP4K2B	SRPK2
ENSG00000274273	ENSG00000087266	ENSG00000263001	ENSG00000276293	ENSG00000135250
ENSP00000385479	ENSP00000409746	ENSP00000460070	ENSP00000482548	ENSP00000377262
ENSP00000358154	ENSP00000424072	ENSP00000484526	ENSP00000477549	ENSP00000349863
ENSP00000333277	ENSP00000403231	ENSP00000482476	ENSP00000480776	ENSP00000419791
ENSP00000355781	ENSP00000424846	ENSP00000477837	ENSP00000487739	ENSP00000417946
ENSP00000355780	ENSP00000422605	ENSP00000404240		ENSP00000420208
ENSP00000355778	ENSP00000348685	ENSP00000405022		ENSP00000418786
ENSP00000355779	ENSP00000388152			ENSP00000419240
ENSP00000254810	ENSP00000422168			ENSP00000417357
ENSP00000468714	ENSP00000422796			ENSP00000417446
ENSP00000465813	ENSP00000423275			
ENSP00000466020	ENSP00000424917			
ENSP00000467165	ENSP00000424105			
ENSP00000466663	ENSP00000423823			
ENSP00000465403	ENSP00000425537			
ENSP00000468484	ENSP00000426818			
ENSP00000482146				
ENSP00000349768				
ENSP00000345062				
ENSP00000479886				
ENSP00000480826				
ENSP00000484841				
ENSP00000484658				
ENSP00000366999				
ENSP00000489282				
ENSP00000482271				
ENSP00000484095				
ENSP00000484638				
ENSP00000358160				
ENSP00000483283				
ENSP00000352252				
ENSP00000478759				
ENSP00000481266				
ENSP00000483200				
ENSP00000483308				
ENSP00000478077				
ENSP00000478727				
ENSP00000478584				
ENSP00000484286				
ENSP00000479497				

429	430	431	432	433
P78371	P78527	P78536	P78545	P83916
CCT2	PRKDC	ADAM17	ELF3	CBX1
ENSG00000166226	ENSG00000253729	ENSG00000151694	ENSG00000163435	ENSG00000108468
ENSP00000299300	ENSP00000313420	ENSP00000309968	ENSP00000352673	ENSP00000377060
ENSP00000445471	ENSP00000345182	ENSP00000418728	ENSP00000356253	ENSP00000225603
ENSP00000441847	ENSP00000442177	ENSP00000480552	ENSP00000356252	ENSP00000462242
ENSP00000450153	ENSP00000441157		ENSP00000405162	ENSP00000385413
				ENSP00000465985
				ENSP00000393179

434	435	436	437	438
P84022	P84243	P98170	Q00059	Q00534
SMAD3	H3F3A	XIAP	TFAM	CDK6
ENSG00000166949	ENSG00000163041	ENSG00000101966	ENSG00000108064	ENSG00000105810
ENSP00000332973	ENSP00000385479	ENSP00000360242	ENSP00000420588	ENSP00000265734
ENSP00000437757	ENSP00000358154	ENSP00000347858	ENSP00000363002	ENSP00000397087
ENSP00000401133	ENSP00000333277	ENSP00000395230	ENSP00000378776	
ENSP00000445348	ENSP00000355781	ENSP00000405529		
ENSP00000453082	ENSP00000355780	ENSP00000400637		
ENSP00000453788	ENSP00000355778			
ENSP00000455095	ENSP00000355779			
ENSP00000458060	ENSP00000254810			
ENSP00000453684	ENSP00000468714			
ENSP00000454165	ENSP00000465813			
ENSP00000452767	ENSP00000466020			
ENSP00000455540	ENSP00000467165			
	ENSP00000466663			
	ENSP00000465403			
	ENSP00000468484			
	ENSP00000482146			
	ENSP00000349768			
	ENSP00000345062			
	ENSP00000479886			
	ENSP00000480826			
	ENSP00000484841			
	ENSP00000484658			
	ENSP00000366999			
	ENSP00000489282			
	ENSP00000482271			
	ENSP00000484095			
	ENSP00000484638			
	ENSP00000358160			
	ENSP00000483283			
	ENSP00000352252			
	ENSP00000478759			
	ENSP00000481266			
	ENSP00000483200			
	ENSP00000483308			
	ENSP00000478077			
	ENSP00000478727			
	ENSP00000478584			
	ENSP00000484286			
	ENSP00000479497			

439	440	441	442	443
Q00535	Q00610	Q00613	Q00653	Q00839
CDK5	CLTC	HSF1	NFKB2	HNRNPU
ENSG00000164885	ENSG00000141367	ENSG00000185122	ENSG00000077150	ENSG00000153187
ENSP00000419782	ENSP00000376763	ENSP00000431512	ENSP00000358983	ENSP00000393151
ENSP00000297518	ENSP00000269122	ENSP00000383590	ENSP00000189444	ENSP00000491215
	ENSP00000462592	ENSP00000481007	ENSP00000410256	ENSP00000482621
	ENSP00000462252	ENSP00000436616	ENSP00000470826	ENSP00000492010
	ENSP00000463336	ENSP00000484174	ENSP00000471586	ENSP00000491685
	ENSP00000463068	ENSP00000480423		ENSP00000479468
	ENSP00000465200			ENSP00000410728
	ENSP00000461977			ENSP00000491340
	ENSP00000479606			ENSP00000491601
				ENSP00000490988
				ENSP00000283179
				ENSP00000491305
				ENSP00000491903
				ENSP00000491294
				ENSP00000492620
				ENSP00000491807
				ENSP00000491491
				ENSP00000492573
				ENSP00000491263

444	445	446	447	448
Q01082	Q01105	Q01167	Q01844	Q01970
SPTBN1	SET	FOXK2	EWSR1	PLCB3
ENSG00000115306	ENSG00000119335	ENSG00000141568	ENSG00000182944	ENSG00000149782
ENSP00000349259	ENSP00000361777	ENSP00000436108	ENSP00000381031	ENSP00000279230
ENSP00000334156	ENSP00000387321	ENSP00000335677	ENSP00000385726	ENSP00000443631
ENSP00000374630	ENSP00000318012	ENSP00000433167	ENSP00000400142	ENSP00000324660
ENSP00000479037	ENSP00000361771	ENSP00000432663	ENSP00000327456	
	ENSP00000410806	ENSP00000461540	ENSP00000331699	
	ENSP00000361773		ENSP00000416171	
	ENSP00000480946		ENSP00000406824	
	ENSP00000483053		ENSP00000405947	
			ENSP00000412670	
			ENSP00000395200	
			ENSP00000330516	
			ENSP00000393637	
			ENSP00000353204	
			ENSP00000330896	
			ENSP00000486709	

449	450	451	452	453
Q01973	Q02447	Q02750	Q02790	Q04206
ROR1	SP3	MAP2K1	FKBP4	RELA
ENSG00000185483	ENSG00000172845	ENSG00000169032	ENSG00000004478	ENSG00000173039
ENSP00000360121	ENSP00000310301	ENSP00000302486	ENSP00000001008	ENSP00000384273
ENSP00000360120	ENSP00000492253	ENSP00000456438	ENSP00000439703	ENSP00000311508
ENSP00000441637	ENSP00000406140		ENSP00000446368	ENSP00000432537
	ENSP00000413665		ENSP00000438491	ENSP00000435290
			ENSP00000442193	ENSP00000433526
			ENSP00000486800	ENSP00000431304
				ENSP00000437137
				ENSP00000434008
				ENSP00000433144
				ENSP00000432922
				ENSP00000434372
				ENSP00000436545
				ENSP00000431153
				ENSP00000434745
				ENSP00000434098
				ENSP00000435531
				ENSP00000433616
				ENSP00000483705
				ENSP00000479572

454	455	456	457	458
Q04724	Q04912	Q04917	Q05086	Q05193
TLE1	MST1R	YWHAH	UBE3A	DNM1
ENSG00000196781	ENSG00000164078	ENSG00000128245	ENSG00000114062	ENSG00000106976
ENSP00000365682	ENSP00000296474	ENSP00000248975	ENSP00000490111	ENSP00000362014
ENSP00000365667	ENSP00000341325	ENSP00000408468	ENSP00000490258	ENSP00000419225
ENSP00000391347	ENSP00000482642	ENSP00000380629	ENSP00000490557	ENSP00000345680
ENSP00000365646	ENSP00000407926	ENSP00000406747	ENSP00000411258	ENSP00000377219
ENSP00000365735	ENSP00000393294		ENSP00000481796	ENSP00000420045
ENSP00000365720	ENSP00000414792		ENSP00000486349	ENSP00000487310
ENSP00000265284	ENSP00000481084		ENSP00000232165	ENSP00000486525
ENSP00000365727	ENSP00000482827		ENSP00000401265	ENSP00000490285
ENSP00000417906			ENSP00000457771	ENSP00000486437
ENSP00000417245			ENSP00000381045	ENSP00000489096
ENSP00000415423			ENSP00000489625	ENSP00000489985
ENSP00000397822			ENSP00000490563	ENSP00000420808
ENSP00000417715			ENSP00000487178	ENSP00000490555
ENSP00000412567			ENSP00000473822	ENSP00000486770
ENSP00000409313			ENSP00000487217	
ENSP00000417844			ENSP00000487534	
ENSP00000418409			ENSP00000486388	
ENSP00000417102			ENSP00000485958	
ENSP00000400334			ENSP00000487550	
			ENSP00000487148	
			ENSP00000487552	
			ENSP00000486900	
			ENSP00000485732	
			ENSP00000486601	
			ENSP00000486066	

459	460	461	462	463
Q05209	Q05397	Q05469	Q05655	Q06124
PTPN12	PTK2	LIPE	PRKCD	PTPN11
ENSG00000127947	ENSG00000169398	ENSG00000079435	ENSG00000163932	ENSG00000179295
ENSP00000248594	ENSP00000429911	ENSP00000244289	ENSP00000378217	ENSP00000376376
ENSP00000392429	ENSP00000429474	ENSP00000469545	ENSP00000331602	ENSP00000340944
ENSP00000397991	ENSP00000341189	ENSP00000472218	ENSP00000419726	ENSP00000489597
ENSP00000428735	ENSP00000378644	ENSP00000469990	ENSP00000418106	ENSP00000437013
ENSP00000404050	ENSP00000429929	ENSP00000472531	ENSP00000419629	ENSP00000489541
ENSP00000392526	ENSP00000429170	ENSP00000469268		ENSP00000491593
ENSP00000430726	ENSP00000429082	ENSP00000469030		
ENSP00000413449	ENSP00000430916			
ENSP00000397364	ENSP00000428492			
ENSP00000385079	ENSP00000428570			
	ENSP00000429129			
	ENSP00000429023			
	ENSP00000403416			
	ENSP00000430603			
	ENSP00000430828			
	ENSP00000429437			
	ENSP00000427774			
	ENSP00000431106			
	ENSP00000427972			
	ENSP00000429702			
	ENSP00000428593			
	ENSP00000429958			
	ENSP00000428482			
	ENSP00000428380			
	ENSP00000428232			
	ENSP00000429001			
	ENSP00000427825			
	ENSP00000429895			
	ENSP00000427762			
	ENSP00000428792			
	ENSP00000430277			
	ENSP00000427904			
	ENSP00000429530			
	ENSP00000427795			
	ENSP00000430951			
	ENSP00000430044			
	ENSP00000429907			
	ENSP00000429409			
	ENSP00000430037			

464	465	466	467	468
Q06210	Q06265	Q06481	Q06830	Q07352
GFPT1	EXOSC9	APLP2	PRDX1	ZFP36L1
ENSG00000198380	ENSG00000123737	ENSG00000084234	ENSG00000117450	ENSG00000185650
ENSP00000354347	ENSP00000243498	ENSP00000435914	ENSP00000361152	ENSP00000388402
ENSP00000349860	ENSP00000368984	ENSP00000263574	ENSP00000262746	ENSP00000337386
	ENSP00000423476	ENSP00000263575	ENSP00000361150	ENSP00000451093
	ENSP00000421845	ENSP00000345444	ENSP00000407034	ENSP00000450784
	ENSP00000422205	ENSP00000278756	ENSP00000389047	ENSP00000450600
	ENSP00000425782	ENSP00000434592		ENSP00000452119
	ENSP00000424504	ENSP00000433582		
		ENSP00000434059		
		ENSP00000434379		
		ENSP00000435470		

469	470	471	472	473
Q07617	Q07666	Q07812	Q07817	Q07820
SPAG1	KHDRBS1	BAX	BCL2L1	MCL1
ENSG00000104450	ENSG00000121774	ENSG00000087088	ENSG00000171552	ENSG00000143384
ENSP00000427716	ENSP00000313829	ENSP00000263262	ENSP00000365230	ENSP00000358022
ENSP00000428070	ENSP00000417731	ENSP00000426328	ENSP00000365223	ENSP00000309973
ENSP00000373450		ENSP00000348871	ENSP00000302564	ENSP00000477624
ENSP00000251809		ENSP00000389971	ENSP00000406203	
		ENSP00000346461	ENSP00000411252	
		ENSP00000293288	ENSP00000395545	
		ENSP00000426184	ENSP00000390760	
		ENSP00000441413	ENSP00000389688	
		ENSP00000375744	ENSP00000401173	

474	475	476	477	478
Q07866	Q07889	Q07954	Q07955	Q08209
KLC1	SOS1	LRP1	SRSF1	PPP3CA
ENSG00000126214	ENSG00000115904	ENSG00000123384	ENSG00000136450	ENSG00000138814
ENSP00000341154	ENSP00000387784	ENSP00000243077	ENSP00000462215	ENSP00000422781
ENSP00000374394	ENSP00000384675	ENSP00000341264	ENSP00000463223	ENSP00000378323
ENSP00000452487	ENSP00000378479	ENSP00000451449	ENSP00000258962	ENSP00000320580
ENSP00000334618	ENSP00000393899	ENSP00000451737	ENSP00000462616	ENSP00000378322
ENSP00000334523		ENSP00000451622	ENSP00000463042	ENSP00000422990
ENSP00000246489		ENSP00000451012	ENSP00000462687	ENSP00000426565
ENSP00000488938			ENSP00000463917	ENSP00000431619
ENSP00000450616				ENSP00000434599
ENSP00000450648				
ENSP00000414982				
ENSP00000450786				
ENSP00000369377				
ENSP00000450617				
ENSP00000452481				
ENSP00000451242				
ENSP00000412693				
ENSP00000441347				
ENSP00000452167				
ENSP00000452095				
ENSP00000451732				
ENSP00000442554				
ENSP00000451691				

479	480	481	482	483
Q08345	Q08379	Q08945	Q08J23	Q09472
DDR1	GOLGA2	SSRP1	NSUN2	EP300
ENSG00000137332	ENSG00000167110	ENSG00000149136	ENSG00000037474	ENSG00000100393
ENSP00000383262	ENSP00000416097	ENSP00000278412	ENSP00000264670	ENSP00000263253
ENSP00000383261	ENSP00000478799	ENSP00000489564	ENSP00000420957	ENSP00000489397
ENSP00000372868	ENSP00000476265	ENSP00000431154	ENSP00000421783	ENSP00000488981
ENSP00000259875	ENSP00000409271	ENSP00000434546	ENSP00000426459	
ENSP00000383265	ENSP00000411598			
ENSP00000449307	ENSP00000473603			
ENSP00000448377	ENSP00000491980			
ENSP00000484588	ENSP00000481685			
ENSP00000394161				
ENSP00000408790				
ENSP00000383258				
ENSP00000392422				
ENSP00000406089				
ENSP00000404001				
ENSP00000408328				
ENSP00000482421				
ENSP00000318217				
ENSP00000407699				
ENSP00000406091				
ENSP00000365753				
ENSP00000365752				
ENSP00000421978				
ENSP00000422442				
ENSP00000365751				
ENSP00000427552				
ENSP00000365754				
ENSP00000405039				
ENSP00000365759				
ENSP00000424346				
ENSP00000421189				
ENSP00000426420				
ENSP00000420833				
ENSP00000421753				
ENSP00000424468				
ENSP00000427369				
ENSP00000422331				
ENSP00000392413				
ENSP00000424703				
ENSP00000425713				

484	485	486	487	488
Q12778	Q12800	Q12802	Q12888	Q12904
FOXO1	TFCP2	AKAP13	TP53BP1	AIMP1
ENSG00000150907	ENSG00000135457	ENSG00000170776	ENSG00000067369	ENSG00000164022
ENSP00000493184	ENSP00000257915	ENSP00000453634	ENSP00000371475	ENSP00000350699
ENSP00000368880	ENSP00000447991	ENSP00000378026	ENSP00000263801	ENSP00000378191
	ENSP00000449742	ENSP00000354718	ENSP00000393497	ENSP00000405248
	ENSP00000449280	ENSP00000453768	ENSP00000413010	ENSP00000423681
		ENSP00000453620	ENSP00000371470	
		ENSP00000452622	ENSP00000416212	
		ENSP00000452698	ENSP00000392048	
		ENSP00000483091	ENSP00000398508	
		ENSP00000481485	ENSP00000471624	
		ENSP00000477780	ENSP00000388028	
		ENSP00000453205		
		ENSP00000482058		
		ENSP00000480789		
		ENSP00000378018		

489	490	491	492	493
Q12906	Q12933	Q12959	Q12972	Q12974
ILF3	TRAF2	DLG1	PPP1R8	PTP4A2
ENSG00000129351	ENSG00000127191	ENSG00000075711	ENSG00000117751	ENSG00000184007
ENSP00000384660	ENSP00000247668	ENSP00000345731	ENSP00000311677	ENSP00000473259
ENSP00000465219	ENSP00000405860	ENSP00000407531	ENSP00000236412	ENSP00000409260
ENSP00000250241	ENSP00000406524	ENSP00000398939	ENSP00000363042	ENSP00000473490
ENSP00000465515	ENSP00000397653	ENSP00000413238	ENSP00000410206	ENSP00000493688
ENSP00000468181		ENSP00000391732		ENSP00000435941
ENSP00000468156		ENSP00000396658		ENSP00000433050
ENSP00000404121		ENSP00000411278		ENSP00000433800
ENSP00000468134		ENSP00000376187		ENSP00000437251
ENSP00000466396		ENSP00000396768		ENSP00000433375
ENSP00000465654		ENSP00000376186		ENSP00000486151
ENSP00000468323		ENSP00000398702		ENSP00000486246
ENSP00000466991		ENSP00000412579		
ENSP00000468092		ENSP00000396474		
ENSP00000464974		ENSP00000376185		
ENSP00000467704		ENSP00000414189		
ENSP00000465831		ENSP00000393771		
ENSP00000467950		ENSP00000390403		
ENSP00000467372		ENSP00000400169		
ENSP00000466724		ENSP00000350303		
ENSP00000466235				

494	495	496	497	498
Q13042	Q13043	Q13085	Q13131	Q13144
CDC16	STK4	ACACA	PRKAA1	EIF2B5
ENSG00000130177	ENSG00000101109	ENSG00000275176	ENSG00000132356	ENSG00000145191
ENSP00000348554	ENSP00000361892	ENSP00000478577	ENSP00000380317	ENSP00000273783
ENSP00000364459	ENSP00000361887	ENSP00000479901	ENSP00000346148	ENSP00000414775
ENSP00000252457	ENSP00000482076	ENSP00000483674	ENSP00000296800	ENSP00000408198
ENSP00000364457	ENSP00000479564	ENSP00000480031		
ENSP00000353549	ENSP00000443514	ENSP00000488846		
ENSP00000252458		ENSP00000488724		
ENSP00000486027		ENSP00000488139		
		ENSP00000487997		
		ENSP00000488112		
		ENSP00000488223		
		ENSP00000487874		
		ENSP00000478588		
		ENSP00000488774		
		ENSP00000488145		
		ENSP00000483300		
		ENSP00000482269		
		ENSP00000478547		
		ENSP00000482368		
		ENSP00000483969		
		ENSP00000480371		
		ENSP00000482064		
		ENSP00000482498		
		ENSP00000483696		
		ENSP00000479621		
		ENSP00000481900		
		ENSP00000477912		
		ENSP00000483852		
		ENSP00000484948		

499	500	501	502	503
Q13153	Q13158	Q13177	Q13185	Q13188
PAK1	FADD	PAK2	CBX3	STK3
ENSG00000149269	ENSG00000168040	ENSG00000180370	ENSG00000122565	ENSG00000104375
ENSP00000348696	ENSP00000301838	ENSP00000314067	ENSP00000336687	ENSP00000390500
ENSP00000278568		ENSP00000402927	ENSP00000379670	ENSP00000429744
ENSP00000433423			ENSP00000408672	ENSP00000428014
ENSP00000433211			ENSP00000387348	ENSP00000429369
ENSP00000431906			ENSP00000473748	ENSP00000428167
ENSP00000431541				ENSP00000428295
ENSP00000437261				ENSP00000482260
ENSP00000433518				
ENSP00000432477				
ENSP00000433004				
ENSP00000435983				
ENSP00000433737				

504	505	506	507	508
Q13257	Q13263	Q13308	Q13362	Q13363
MAD2L1	TRIM28	PTK7	PPP2R5C	CTBP1
ENSG00000164109	ENSG00000130726	ENSG00000112655	ENSG00000078304	ENSG00000159692
ENSP00000296509	ENSP00000253024	ENSP00000230419	ENSP00000329009	ENSP00000372411
ENSP00000332295	ENSP00000342232	ENSP00000325462	ENSP00000262239	ENSP00000290921
ENSP00000425702	ENSP00000472586	ENSP00000326029	ENSP00000408389	ENSP00000422148
	ENSP00000473126	ENSP00000325992	ENSP00000333905	ENSP00000422364
	ENSP00000470503	ENSP00000230418	ENSP00000412324	ENSP00000424575
	ENSP00000471303	ENSP00000418754	ENSP00000450525	ENSP00000424684
		ENSP00000417607	ENSP00000454035	ENSP00000422730
		ENSP00000420322	ENSP00000450931	ENSP00000426470
		ENSP00000419037	ENSP00000452632	ENSP00000425053
		ENSP00000420165	ENSP00000453875	ENSP00000420983
		ENSP00000420765	ENSP00000450867	ENSP00000423872
		ENSP00000418545	ENSP00000454124	ENSP00000487417
		ENSP00000419096	ENSP00000452500	
		ENSP00000418462	ENSP00000452630	
		ENSP00000420186	ENSP00000451179	
		ENSP00000418386	ENSP00000450743	
			ENSP00000452533	
			ENSP00000452124	
			ENSP00000453906	
			ENSP00000450816	
			ENSP00000450626	

509	510	511	512	513
Q13393	Q13416	Q13418	Q13464	Q13480
PLD1	ORC2	ILK	ROCK1	GAB1
ENSG00000075651	ENSG00000115942	ENSG00000166333	ENSG00000067900	ENSG00000109458
ENSP00000348681	ENSP00000234296	ENSP00000299421	ENSP00000382697	ENSP00000262995
ENSP00000342793	ENSP00000386390	ENSP00000403487	ENSP00000489185	ENSP00000262994
ENSP00000395556	ENSP00000396641	ENSP00000435323	ENSP00000462004	ENSP00000427435
ENSP00000391164		ENSP00000379975		ENSP00000426668
ENSP00000400639		ENSP00000434492		ENSP00000424554
ENSP00000486967		ENSP00000479932		ENSP00000425921
ENSP00000328422		ENSP00000439606		ENSP00000420867
		ENSP00000432617		ENSP00000426297
		ENSP00000436062		
		ENSP00000480597		
		ENSP00000487498		

514	515	516	517	518
Q13501	Q13523	Q13526	Q13541	Q13542
SQSTM1	PRPF4B	PIN1	EIF4EBP1	EIF4EBP2
ENSG00000161011	ENSG00000112739	ENSG00000127445	ENSG00000187840	ENSG00000148730
ENSP00000374455	ENSP00000433547	ENSP00000247970	ENSP00000340691	ENSP00000362314
ENSP00000353944	ENSP00000337194	ENSP00000466962		
ENSP00000427308	ENSP00000433714	ENSP00000466656		
ENSP00000394534		ENSP00000467197		
ENSP00000424195		ENSP00000466780		
ENSP00000405061				
ENSP00000425957				
ENSP00000424477				
ENSP00000487071				

519	520	521	522	523
Q13546	Q13547	Q13554	Q13555	Q13557
RIPK1	HDAC1	CAMK2B	CAMK2G	CAMK2D
ENSG00000137275	ENSG00000116478	ENSG00000058404	ENSG00000148660	ENSG00000145349
ENSP00000259808	ENSP00000362649	ENSP00000326375	ENSP00000277853	ENSP00000378032
ENSP00000369773	ENSP00000407859	ENSP00000379098	ENSP00000315599	ENSP00000339740
		ENSP00000397937	ENSP00000307082	ENSP00000423482
		ENSP00000351542	ENSP00000378243	ENSP00000378030
		ENSP00000326427	ENSP00000410298	ENSP00000369098
		ENSP00000326544	ENSP00000393784	ENSP00000422566
		ENSP00000326518	ENSP00000361851	ENSP00000296402
		ENSP00000258682	ENSP00000405561	ENSP00000424247
		ENSP00000379096	ENSP00000319060	ENSP00000425824
		ENSP00000390292		ENSP00000423677
		ENSP00000428912		ENSP00000424245
		ENSP00000410445		ENSP00000423753
		ENSP00000326600		
		ENSP00000388951		
		ENSP00000390419		
		ENSP00000400387		
		ENSP00000388445		
		ENSP00000261793		
		ENSP00000381412		
		ENSP00000427580		
		ENSP00000426607		

524	525	526	527	528
Q13586	Q13616	Q13627	Q13765	Q13769
STIM1	CUL1	DYRK1A	NACA	THOC5
ENSG00000167323	ENSG00000055130	ENSG00000157540	ENSG00000196531	ENSG00000100296
ENSP00000300737	ENSP00000387160	ENSP00000342690	ENSP00000349212	ENSP00000420306
ENSP00000436208	ENSP00000473318	ENSP00000381932	ENSP00000447821	ENSP00000380971
ENSP00000432210	ENSP00000326804	ENSP00000381929	ENSP00000377469	ENSP00000380969
ENSP00000434848	ENSP00000482123	ENSP00000340373	ENSP00000446801	ENSP00000380970
ENSP00000433949		ENSP00000495632	ENSP00000448039	ENSP00000416500
ENSP00000431878		ENSP00000496748	ENSP00000447705	ENSP00000444493
ENSP00000434200		ENSP00000495571	ENSP00000446745	ENSP00000393247
ENSP00000431191		ENSP00000494544	ENSP00000447133	ENSP00000413505
ENSP00000432378		ENSP00000494572	ENSP00000450383	ENSP00000415425
ENSP00000432383		ENSP00000493627	ENSP00000446933	ENSP00000416804
ENSP00000434767		ENSP00000494698	ENSP00000447764	ENSP00000393420
ENSP00000433266		ENSP00000412269	ENSP00000447207	ENSP00000388039
ENSP00000478059		ENSP00000495908	ENSP00000447666	
		ENSP00000398483	ENSP00000448035	
		ENSP00000493653	ENSP00000403817	
		ENSP00000494897		
		ENSP00000495596		
		ENSP00000494536		

529	530	531	532	533
Q13813	Q13887	Q13976	Q14103	Q14118
SPTAN1	KLF5	PRKG1	HNRNPD	DAG1
ENSG00000197694	ENSG00000102554	ENSG00000185532	ENSG00000138668	ENSG00000173402
ENSP00000361816	ENSP00000366915	ENSP00000363097	ENSP00000313199	ENSP00000312435
ENSP00000361824	ENSP00000440407	ENSP00000363092	ENSP00000313327	ENSP00000439334
ENSP00000350882		ENSP00000384200	ENSP00000305860	ENSP00000442600
ENSP00000486308		ENSP00000494124	ENSP00000426446	ENSP00000440590
ENSP00000486547		ENSP00000493966	ENSP00000424002	ENSP00000438421
ENSP00000490229		ENSP00000496551	ENSP00000420926	ENSP00000440705
ENSP00000490740		ENSP00000495279	ENSP00000421952	ENSP00000412067
ENSP00000490890		ENSP00000363087	ENSP00000426666	ENSP00000405859
ENSP00000489629			ENSP00000422615	ENSP00000388833
ENSP00000487444			ENSP00000425439	ENSP00000387859
ENSP00000489647				ENSP00000415321
ENSP00000490179				ENSP00000410145
ENSP00000490332				ENSP00000401382
ENSP00000490109				ENSP00000402443
ENSP00000486605				ENSP00000401805

534	535	536	537	538
Q14161	Q14203	Q14247	Q14344	Q14457
GIT2	DCTN1	CTTN	GNA13	BECN1
ENSG00000139436	ENSG00000204843	ENSG00000085733	ENSG00000120063	ENSG00000126581
ENSP00000347464	ENSP00000354791	ENSP00000317189	ENSP00000400717	ENSP00000355231
ENSP00000391813	ENSP00000487724	ENSP00000301843	ENSP00000439647	ENSP00000465364
ENSP00000354282	ENSP00000387270	ENSP00000365745		ENSP00000468597
ENSP00000447465	ENSP00000386406	ENSP00000431421		ENSP00000416173
ENSP00000450348	ENSP00000386843	ENSP00000436613		ENSP00000466419
ENSP00000448832	ENSP00000377571			ENSP00000468104
ENSP00000473637	ENSP00000416711			ENSP00000465964
ENSP00000323833	ENSP00000387327			ENSP00000466281
ENSP00000346585	ENSP00000414315			ENSP00000468461
	ENSP00000404038			ENSP00000466766
	ENSP00000402509			ENSP00000465658
	ENSP00000395312			ENSP00000467937
	ENSP00000413268			ENSP00000479791
	ENSP00000409363			
	ENSP00000400059			
	ENSP00000407484			
	ENSP00000487279			
	ENSP00000366875			

539	540	541	542	543
Q14653	Q14676	Q14738	Q14739	Q14790
IRF3	MDC1	PPP2R5D	LBR	CASP8
ENSG00000126456	ENSG00000137337	ENSG00000112640	ENSG00000143815	ENSG00000064012
ENSP00000310127	ENSP00000365588	ENSP00000417963	ENSP00000272163	ENSP00000376091
ENSP00000471896	ENSP00000408962	ENSP00000377669	ENSP00000339883	ENSP00000412523
ENSP00000366344	ENSP00000400979	ENSP00000420674	ENSP00000388059	ENSP00000264275
ENSP00000469113	ENSP00000396989	ENSP00000420550	ENSP00000416554	ENSP00000376087
ENSP00000471358	ENSP00000411310	ENSP00000230402		ENSP00000351273
ENSP00000472700	ENSP00000483601	ENSP00000419756		ENSP00000325722
ENSP00000470512	ENSP00000373060	ENSP00000420529		ENSP00000264274
ENSP00000400378	ENSP00000396121			ENSP00000391709
ENSP00000470431	ENSP00000392784			ENSP00000396869
ENSP00000468911	ENSP00000405991			ENSP00000388306
ENSP00000470436	ENSP00000390040			ENSP00000407378
ENSP00000472601	ENSP00000448066			ENSP00000397528
ENSP00000470069	ENSP00000396484			ENSP00000390641
ENSP00000472582	ENSP00000396124			ENSP00000390346
ENSP00000473128	ENSP00000390489			ENSP00000394434
ENSP00000472223	ENSP00000398151			ENSP00000376094
ENSP00000469067	ENSP00000408831			
ENSP00000471517	ENSP00000447771			
ENSP00000471402	ENSP00000416511			
ENSP00000473176	ENSP00000397642			
ENSP00000470140	ENSP00000396389			
ENSP00000473232	ENSP00000393886			
ENSP00000470905	ENSP00000387743			
ENSP00000366339	ENSP00000447851			
	ENSP00000415212			
	ENSP00000400305			
	ENSP00000397126			
	ENSP00000394074			
	ENSP00000398474			
	ENSP00000450002			
	ENSP00000404318			
	ENSP00000410383			
	ENSP00000392833			
	ENSP00000414163			
	ENSP00000405806			
	ENSP00000448232			
	ENSP00000387429			
	ENSP00000413610			
	ENSP00000388355			

544	545	546	547	548
Q14847	Q14978	Q14980	Q15019	Q15027
LASP1	NOLC1	NUMA1		ACAP1
ENSG00000002834	ENSG00000166197	ENSG00000137497	ENSG00000168385	ENSG00000072818
ENSP00000325240	ENSP00000474710	ENSP00000260051	ENSP00000375834	ENSP00000158762
ENSP00000401048	ENSP00000475080	ENSP00000351851	ENSP00000353157	ENSP00000458173
ENSP00000392853	ENSP00000385410	ENSP00000377298	ENSP00000375832	ENSP00000459422
ENSP00000468664	ENSP00000473910	ENSP00000478624	ENSP00000385109	
ENSP00000414803	ENSP00000474924	ENSP00000480172	ENSP00000385172	
ENSP00000391897	ENSP00000473927	ENSP00000440954	ENSP00000479861	
ENSP00000462564	ENSP00000474140	ENSP00000446396	ENSP00000397195	
	ENSP00000359024	ENSP00000444880	ENSP00000389174	
		ENSP00000442936	ENSP00000404484	
		ENSP00000442761	ENSP00000386001	
		ENSP00000439759	ENSP00000384525	
		ENSP00000438821	ENSP00000397290	
		ENSP00000438589	ENSP00000406181	
		ENSP00000439092	ENSP00000394666	
		ENSP00000444175	ENSP00000399767	
		ENSP00000439576	ENSP00000413031	
		ENSP00000357955	ENSP00000403287	
		ENSP00000438331	ENSP00000399195	
		ENSP00000438318	ENSP00000412434	
		ENSP00000441598	ENSP00000404417	
		ENSP00000441674	ENSP00000409596	
		ENSP00000444831	ENSP00000391717	
		ENSP00000442331	ENSP00000408902	
		ENSP00000481541	ENSP00000408296	
			ENSP00000402256	

549	550	551	552	553
Q15046	Q15054	Q15075	Q15121	Q15149
KARS	POLD3	EEA1	PEA15	PLEC
ENSG00000065427	ENSG00000077514	ENSG00000102189	ENSG00000162734	ENSG00000178209
ENSP00000325448	ENSP00000263681	ENSP00000317955	ENSP00000353660	ENSP00000344848
ENSP00000303043	ENSP00000432951	ENSP00000394312	ENSP00000357055	ENSP00000350277
ENSP00000454512	ENSP00000436018	ENSP00000446785	ENSP00000357056	ENSP00000346602
ENSP00000455818	ENSP00000431239	ENSP00000449682		ENSP00000381756
ENSP00000456185	ENSP00000432233			ENSP00000323856
ENSP00000456786	ENSP00000437231			ENSP00000347044
ENSP00000455488	ENSP00000432969			ENSP00000348702
ENSP00000462057	ENSP00000436781			ENSP00000388180
ENSP00000458028				ENSP00000434583
				ENSP00000433982
				ENSP00000437303
				ENSP00000433557
				ENSP00000434490
				ENSP00000436702

554	555	556	557	558
Q15172	Q15185	Q15208	Q15257	Q15262
PPP2R5A	PTGES3	STK38	PTPA	PTPRK
ENSG00000066027	ENSG00000110958	ENSG00000112079	ENSG00000119383	ENSG00000152894
ENSP00000261461	ENSP00000262033	ENSP00000229812	ENSP00000351885	ENSP00000357209
ENSP00000442866	ENSP00000405299		ENSP00000377036	ENSP00000432973
	ENSP00000402385		ENSP00000347109	ENSP00000357196
	ENSP00000414892		ENSP00000349726	ENSP00000357198
	ENSP00000389090		ENSP00000337448	ENSP00000357193
	ENSP00000482075		ENSP00000395499	ENSP00000357190
			ENSP00000335200	ENSP00000406825
			ENSP00000337412	ENSP00000408180
			ENSP00000406997	ENSP00000432960
			ENSP00000399069	ENSP00000434058
			ENSP00000403542	ENSP00000434116
			ENSP00000393092	ENSP00000376243
			ENSP00000400314	ENSP00000437299
			ENSP00000393796	ENSP00000483766
			ENSP00000396660	ENSP00000477791
			ENSP00000405100	ENSP00000484742
			ENSP00000408316	ENSP00000486442
			ENSP00000389312	ENSP00000487066
			ENSP00000408726	ENSP00000487005
			ENSP00000416522	ENSP00000485812
			ENSP00000393196	ENSP00000487578
			ENSP00000394001	ENSP00000486036
			ENSP00000416661	ENSP00000487445
			ENSP00000387726	ENSP00000486448
			ENSP00000411604	ENSP00000482183
			ENSP00000394338	ENSP00000485893

559	560	561	562	563
Q15418	Q15596	Q15637	Q15648	Q15653
ABO	NCOA2	SF1	MED1	NFKBIB
ENSG00000281879	ENSG00000140396	ENSG00000168066	ENSG00000125686	ENSG00000104825
ENSP00000496236	ENSP00000399968	ENSP00000366604	ENSP00000377828	ENSP00000459728
ENSP00000494079	ENSP00000430148	ENSP00000366607	ENSP00000300651	ENSP00000312988
ENSP00000487108	ENSP00000429132	ENSP00000227503	ENSP00000463307	ENSP00000461617
ENSP00000494984	ENSP00000430348	ENSP00000366611	ENSP00000462441	ENSP00000458685
ENSP00000486083	ENSP00000430850	ENSP00000334414		ENSP00000438598
ENSP00000363283		ENSP00000396793		ENSP00000375929
ENSP00000431651		ENSP00000407962		ENSP00000489145
ENSP00000432281		ENSP00000391198		ENSP00000489138
ENSP00000435412		ENSP00000395927		ENSP00000489318
ENSP00000434616		ENSP00000419062		ENSP00000489191
ENSP00000436990		ENSP00000388470		ENSP00000489042
ENSP00000363281		ENSP00000406354		ENSP00000489085
ENSP00000433039		ENSP00000486693		
ENSP00000355831				
ENSP00000363278				
ENSP00000383967				
ENSP00000403548				
ENSP00000486881				
ENSP00000487166				
ENSP00000487553				
ENSP00000487349				
ENSP00000485797				
ENSP00000486119				
ENSP00000486031				
ENSP00000487239				
ENSP00000487154				
ENSP00000486849				
ENSP00000486179				
ENSP00000487207				

564	565	566	567	568
Q15717	Q15746	Q15750	Q15796	Q15797
ELAVL1	MYLK	TAB1	SMAD2	SMAD1
ENSG00000066044	ENSG00000065534	ENSG00000100324	ENSG00000175387	ENSG00000170365
ENSP00000385269	ENSP00000354004	ENSP00000216160	ENSP00000262160	ENSP00000441954
ENSP00000472197	ENSP00000428967	ENSP00000333049	ENSP00000384449	ENSP00000446474
ENSP00000471011	ENSP00000463691		ENSP00000466193	ENSP00000425749
ENSP00000470727	ENSP00000320622		ENSP00000349282	ENSP00000424279
	ENSP00000462118		ENSP00000467075	ENSP00000426696
	ENSP00000418335		ENSP00000466254	ENSP00000427330
	ENSP00000346846		ENSP00000465355	ENSP00000422954
	ENSP00000352088		ENSP00000468202	ENSP00000427474
	ENSP00000353452		ENSP00000465971	ENSP00000427650
	ENSP00000417798		ENSP00000468732	ENSP00000305769
	ENSP00000422984			ENSP00000377652
				ENSP00000426568
				ENSP00000424959
				ENSP00000424649
				ENSP00000425270
				ENSP00000427002
				ENSP00000422706
				ENSP00000421601

569	570	571	572	573
Q15833	Q15910	Q15942	Q16204	Q16512
STXBP2	EZH2	ZYX	CCDC6	PKN1
ENSG00000076944	ENSG00000106462	ENSG00000159840	ENSG00000108091	ENSG00000123143
ENSP00000413606	ENSP00000417062	ENSP00000324422	ENSP00000263102	ENSP00000242783
ENSP00000221283	ENSP00000419711	ENSP00000376642		ENSP00000343325
ENSP00000409471	ENSP00000223193	ENSP00000413467		ENSP00000467422
ENSP00000470313	ENSP00000320147	ENSP00000400537		ENSP00000465842
ENSP00000471161	ENSP00000419050	ENSP00000346417		ENSP00000466367
ENSP00000471327	ENSP00000419856	ENSP00000411230		ENSP00000466819
ENSP00000471585	ENSP00000417377	ENSP00000403714		ENSP00000465867
ENSP00000471737	ENSP00000417704			ENSP00000468497
ENSP00000473040				
ENSP00000473406				
ENSP00000478797				

574	575	576	577	578
Q16513	Q16539	Q16543	Q16555	Q16625
PKN2	MAPK14	CDC37	DPYSL2	OCLN
ENSG00000065243	ENSG00000112062	ENSG00000105401	ENSG00000092964	ENSG00000197822
ENSP00000359552	ENSP00000229795	ENSP00000222005	ENSP00000309539	ENSP00000347379
ENSP00000359544	ENSP00000229794	ENSP00000465941	ENSP00000431117	ENSP00000379719
ENSP00000317851	ENSP00000308669	ENSP00000464889	ENSP00000427985	ENSP00000445940
ENSP00000401125	ENSP00000419837	ENSP00000467747	ENSP00000427954	
ENSP00000389076	ENSP00000417531	ENSP00000465724		
	ENSP00000417065	ENSP00000464971		
	ENSP00000419141			
	ENSP00000478079			

579	580	581	582	583
Q16637	Q16644	Q16666	Q16695	Q16790
KIR2DS4	MAPKAPK3	IFI16	HIST3H3	CA9
ENSG00000275351	ENSG00000114738	ENSG00000163565	ENSG00000168148	ENSG00000107159
ENSP00000479356	ENSP00000396467	ENSP00000357113	ENSP00000385479	ENSP00000367608
ENSP00000479177	ENSP00000350639	ENSP00000357114	ENSP00000358154	ENSP00000482050
ENSP00000484234	ENSP00000478922	ENSP00000295809	ENSP00000333277	
ENSP00000428128	ENSP00000410970	ENSP00000352740	ENSP00000355781	
ENSP00000370083	ENSP00000402045	ENSP00000404325	ENSP00000355780	
ENSP00000424926	ENSP00000394894	ENSP00000458084	ENSP00000355778	
ENSP00000305857		ENSP00000406406	ENSP00000355779	
ENSP00000423298		ENSP00000407052	ENSP00000254810	
ENSP00000422679		ENSP00000456094	ENSP00000468714	
ENSP00000430657		ENSP00000456129	ENSP00000465813	
ENSP00000486539		ENSP00000342741	ENSP00000466020	
ENSP00000487206			ENSP00000467165	
ENSP00000487029			ENSP00000466663	
ENSP00000480906			ENSP00000465403	
ENSP00000482966			ENSP00000468484	
ENSP00000487289			ENSP00000482146	
ENSP00000487186			ENSP00000349768	
ENSP00000487162			ENSP00000345062	
ENSP00000486022			ENSP00000479886	
ENSP00000487193			ENSP00000480826	
ENSP00000477738			ENSP00000484841	
ENSP00000370119			ENSP00000484658	
ENSP00000486152			ENSP00000366999	
ENSP00000370118			ENSP00000489282	
ENSP00000479279			ENSP00000482271	
ENSP00000370117			ENSP00000484095	
ENSP00000424282			ENSP00000484638	
ENSP00000424799			ENSP00000358160	
ENSP00000475824			ENSP00000483283	
ENSP00000475331			ENSP00000352252	
ENSP00000486268			ENSP00000478759	
ENSP00000492675			ENSP00000481266	
ENSP00000481427			ENSP00000483200	
ENSP00000487421			ENSP00000483308	
ENSP00000487015			ENSP00000478077	
ENSP00000479920			ENSP00000478727	
ENSP00000486922			ENSP00000478584	
ENSP00000486030			ENSP00000484286	
ENSP00000485777			ENSP00000479497	

584	585	586	587	588
Q2M2I8	Q2NKX8	Q53EL6	Q53ET0	Q53EZ4
AAK1	ERCC6L	PDCD4	CRTC2	CEP55
ENSG00000115977	ENSG00000186871	ENSG00000150593	ENSG00000160741	ENSG00000138180
ENSP00000386456	ENSP00000334675	ENSP00000280154	ENSP00000357622	ENSP00000360540
ENSP00000385181	ENSP00000362761	ENSP00000376816	ENSP00000305873	ENSP00000389150
ENSP00000485350		ENSP00000394668	ENSP00000434115	
ENSP00000386342			ENSP00000357619	
ENSP00000485296				
ENSP00000485409				

589	590	591	592	593
Q5VT25	Q68CZ2	Q6A1A2	Q6IAA8	Q6VY07
CDC42BPA	TNS3		LAMTOR1	PACS1
ENSG00000143776	ENSG00000136205		ENSG00000149357	ENSG00000175115
ENSP00000355729	ENSP00000312143	ENSP00000344220	ENSP00000278671	ENSP00000316454
ENSP00000335341	ENSP00000389285	ENSP00000395357	ENSP00000440738	ENSP00000437150
ENSP00000355731	ENSP00000388318	ENSP00000268673	ENSP00000445170	ENSP00000432639
ENSP00000415388	ENSP00000387990	ENSP00000455492	ENSP00000438717	ENSP00000437274
ENSP00000401051	ENSP00000414358	ENSP00000455025	ENSP00000439011	ENSP00000435037
ENSP00000407120	ENSP00000396914	ENSP00000455684	ENSP00000439482	ENSP00000432858
ENSP00000408165	ENSP00000409415	ENSP00000373876		ENSP00000437052
ENSP00000355728	ENSP00000388142	ENSP00000455551		ENSP00000433991
ENSP00000355726	ENSP00000407464	ENSP00000455438		ENSP00000434012
				ENSP00000432365

594	595	596	597	598
Q6WCQ1	Q712K3	Q7KZI7	Q7L5N1	Q7L7X3
MPRIP	UBE2R2	MARK2	COPS6	TAOK1
ENSG00000133030	ENSG00000107341	ENSG00000072518	ENSG00000168090	ENSG00000160551
ENSP00000379156	ENSP00000263228	ENSP00000425765	ENSP00000304102	ENSP00000261716
ENSP00000342379		ENSP00000355091	ENSP00000388865	ENSP00000438819
ENSP00000462532		ENSP00000294247	ENSP00000400617	ENSP00000464562
ENSP00000379151		ENSP00000423974	ENSP00000413702	
ENSP00000462688		ENSP00000421075		
ENSP00000491358		ENSP00000386128		
ENSP00000464983		ENSP00000385751		
ENSP00000317786		ENSP00000423452		
ENSP00000412147		ENSP00000444956		
ENSP00000411723		ENSP00000444859		
ENSP00000464276		ENSP00000437509		
ENSP00000465902		ENSP00000423844		
ENSP00000463367		ENSP00000415494		
		ENSP00000367041		

599	600	601	602	603
Q86TI0	Q86V81	Q86VP1	Q86WB0	Q86YT6
TBC1D1	ALYREF	TAX1BP1	ZC3HC1	MIB1
ENSG00000065882	ENSG00000183684	ENSG00000106052	ENSG00000091732	ENSG00000101752
ENSP00000423651	ENSP00000331817	ENSP00000265393	ENSP00000351052	ENSP00000261537
ENSP00000261439		ENSP00000379612	ENSP00000353933	
ENSP00000383994		ENSP00000391907	ENSP00000309301	
ENSP00000396877		ENSP00000444811	ENSP00000419509	
ENSP00000421641		ENSP00000411490	ENSP00000418533	
ENSP00000425655		ENSP00000395290	ENSP00000417682	
ENSP00000397640		ENSP00000386515	ENSP00000420068	
ENSP00000410167		ENSP00000391215	ENSP00000417217	
ENSP00000478039		ENSP00000412575		
		ENSP00000477282		

604	605	606	607	608
Q8IX03	Q8IXJ6	Q8IXW5	Q8IZL8	Q8IZP0
WWC1	SIRT2	RPAP2	PELP1	ABI1
ENSG00000113645	ENSG00000068903	ENSG00000122484	ENSG00000141456	ENSG00000136754
ENSP00000265293	ENSP00000249396	ENSP00000476948	ENSP00000461625	ENSP00000365340
ENSP00000427772	ENSP00000375931		ENSP00000460300	ENSP00000365312
ENSP00000377473	ENSP00000404309		ENSP00000461380	ENSP00000352114
ENSP00000429339	ENSP00000400338		ENSP00000460959	ENSP00000365309
ENSP00000428084	ENSP00000411393		ENSP00000461419	ENSP00000365336
	ENSP00000385146		ENSP00000458985	ENSP00000365308
	ENSP00000401203		ENSP00000301396	ENSP00000279599
	ENSP00000407272		ENSP00000416231	ENSP00000365310
	ENSP00000409690			ENSP00000440101
	ENSP00000397022			ENSP00000365307
	ENSP00000408023			ENSP00000365330
	ENSP00000351809			ENSP00000439646
	ENSP00000481932			
	ENSP00000489602			
	ENSP00000488940			
	ENSP00000488998			
	ENSP00000489020			
	ENSP00000489426			
	ENSP00000489036			
	ENSP00000489495			
	ENSP00000489481			
	ENSP00000489066			
	ENSP00000489332			
	ENSP00000489238			
	ENSP00000489360			
	ENSP00000489070			

609	610	611	612	613
Q8N122	Q8N752	Q8NE71	Q8NEB9	Q8NFA2
RPTOR	CSNK1A1L	ABCF1	PIK3C3	NOXO1
ENSG00000141564	ENSG00000180138	ENSG00000204574	ENSG00000078142	ENSG00000196408
ENSP00000460136	ENSP00000261798	ENSP00000313603	ENSP00000262039	ENSP00000348435
ENSP00000307272	ENSP00000421689	ENSP00000365728	ENSP00000381845	ENSP00000346195
ENSP00000442479	ENSP00000367074	ENSP00000405512	ENSP00000465621	ENSP00000380450
ENSP00000459701	ENSP00000426747	ENSP00000440893	ENSP00000464909	ENSP00000456800
ENSP00000460940	ENSP00000427031	ENSP00000445100	ENSP00000466946	ENSP00000454644
	ENSP00000475319	ENSP00000373081	ENSP00000472926	
	ENSP00000475969	ENSP00000373082	ENSP00000471957	
	ENSP00000425366	ENSP00000406926	ENSP00000473061	
	ENSP00000475633	ENSP00000383452	ENSP00000492528	
	ENSP00000430252	ENSP00000411327		
	ENSP00000421268	ENSP00000391102		
	ENSP00000369126	ENSP00000406756		
		ENSP00000397199		
		ENSP00000393143		
		ENSP00000414373		
		ENSP00000394994		
		ENSP00000390550		
		ENSP00000403526		
		ENSP00000389065		
		ENSP00000406948		
		ENSP00000397875		
		ENSP00000413319		
		ENSP00000389472		
		ENSP00000416890		
		ENSP00000394785		
		ENSP00000404726		
		ENSP00000412553		
		ENSP00000401969		
		ENSP00000395308		

614	615	616	617	618
Q8TAP9	Q8TB45	Q8TD19	Q8TDX7	Q8TE77
MPLKIP	DEPTOR	NEK9	NEK7	SSH3
ENSG00000168303	ENSG00000155792	ENSG00000119638	ENSG00000151414	ENSG00000172830
ENSP00000304553	ENSP00000430457	ENSP00000238616	ENSP00000356355	ENSP00000312081
ENSP00000495808	ENSP00000286234	ENSP00000451604	ENSP00000444621	ENSP00000365948
		ENSP00000450943	ENSP00000356353	ENSP00000431788
		ENSP00000452537	ENSP00000392745	ENSP00000310055
		ENSP00000452544	ENSP00000439095	ENSP00000432974
			ENSP00000375835	ENSP00000480198
				ENSP00000433902

619	620	621	622	623
Q8TEW0	Q8WUI4	Q8WXE1	Q92538	Q92574
PARD3	HDAC7	ATRIP	GBF1	TSC1
ENSG00000148498	ENSG00000061273	ENSG00000164053	ENSG00000107862	ENSG00000165699
ENSP00000363921	ENSP00000080059	ENSP00000302338	ENSP00000359000	ENSP00000298552
ENSP00000363920	ENSP00000351326	ENSP00000323099		ENSP00000444017
ENSP00000340591	ENSP00000448532	ENSP00000400930		ENSP00000394524
ENSP00000363926	ENSP00000404394	ENSP00000349620		ENSP00000495546
ENSP00000311986	ENSP00000448305	ENSP00000489608		ENSP00000495428
ENSP00000363908	ENSP00000446538	ENSP00000489136		ENSP00000496772
ENSP00000341844	ENSP00000449171	ENSP00000491983		ENSP00000496263
ENSP00000440857	ENSP00000369984	ENSP00000489199		ENSP00000494682
ENSP00000443147	ENSP00000447191	ENSP00000489041		ENSP00000495158
ENSP00000363922	ENSP00000482003			ENSP00000495554
ENSP00000363905	ENSP00000448448			ENSP00000494502
ENSP00000363900	ENSP00000401872			ENSP00000493773
ENSP00000444429	ENSP00000396159			ENSP00000494685
	ENSP00000410068			ENSP00000493608
	ENSP00000411058			ENSP00000494104
	ENSP00000389501			ENSP00000496691
	ENSP00000388561			ENSP00000494743
	ENSP00000412155			ENSP00000495598
	ENSP00000397236			ENSP00000496398
	ENSP00000400811			ENSP00000494847
	ENSP00000403149			ENSP00000493963
	ENSP00000387792			ENSP00000496292
	ENSP00000390415			ENSP00000494365
				ENSP00000496066
				ENSP00000495821
				ENSP00000495654
				ENSP00000493639
				ENSP00000386093
				ENSP00000494639
				ENSP00000494916
				ENSP00000496126
				ENSP00000495533
				ENSP00000495830

624	625	626	627	628
Q92598	Q92686	Q92688	Q92769	Q92793
HSPH1	NRGN	ANP32B	HDAC2	CREBBP
ENSG00000120694	ENSG00000154146	ENSG00000136938	ENSG00000196591	ENSG00000005339
ENSP00000318687	ENSP00000284292	ENSP00000345848	ENSP00000430432	ENSP00000262367
ENSP00000487365	ENSP00000399591		ENSP00000430008	ENSP00000371502
ENSP00000369768			ENSP00000357621	ENSP00000490244
ENSP00000408991			ENSP00000417026	ENSP00000461002
ENSP00000473512			ENSP00000427861	ENSP00000460474
ENSP00000396090			ENSP00000429901	ENSP00000459490
			ENSP00000428599	ENSP00000458254
			ENSP00000428024	
			ENSP00000428653	
			ENSP00000429236	
			ENSP00000428861	
			ENSP00000428989	

629	630	631	632	633
Q92900	Q92934	Q92945	Q92974	Q92985
UPF1	BAD	KHSRP	ARHGEF2	AP006285.3
ENSG00000005007	ENSG00000002330	ENSG00000088247	ENSG00000116584	ENSG00000276565
ENSP00000262803	ENSP00000378040	ENSP00000381216	ENSP00000315325	ENSP00000483175
ENSP00000470142	ENSP00000309103	ENSP00000469804	ENSP00000354837	ENSP00000331803
	ENSP00000440575	ENSP00000472328	ENSP00000314787	ENSP00000380704
	ENSP00000378039	ENSP00000473254	ENSP00000476916	ENSP00000433903
	ENSP00000438975	ENSP00000472347	ENSP00000476689	ENSP00000434607
	ENSP00000439202	ENSP00000471146	ENSP00000477448	ENSP00000329411
		ENSP00000471262	ENSP00000477299	ENSP00000380697
		ENSP00000469830	ENSP00000476724	ENSP00000434009
		ENSP00000469431	ENSP00000476699	ENSP00000436696
		ENSP00000478033	ENSP00000476532	ENSP00000432403
			ENSP00000476518	ENSP00000434524
			ENSP00000357298	ENSP00000380700
				ENSP00000488666
				ENSP00000480358
				ENSP00000488591
				ENSP00000488581
				ENSP00000488039
				ENSP00000479615
				ENSP00000488372
				ENSP00000487875
				ENSP00000479277

634	635	636	637	638
Q92993	Q93034	Q93045	Q96B36	Q96CF2
KAT5	CUL5	STMN2	AKT1S1	CHMP4C
ENSG00000172977	ENSG00000166266	ENSG00000104435	ENSG00000204673	ENSG00000164695
ENSP00000366245	ENSP00000376808	ENSP00000220876	ENSP00000375709	ENSP00000297265
ENSP00000344955	ENSP00000435376	ENSP00000429243	ENSP00000375708	
ENSP00000340330	ENSP00000436494	ENSP00000430102	ENSP00000341698	
ENSP00000434765	ENSP00000431221		ENSP00000375707	
ENSP00000435939			ENSP00000375711	
ENSP00000436247			ENSP00000375710	
ENSP00000436000			ENSP00000375706	
ENSP00000436012			ENSP00000472839	
ENSP00000431819				
ENSP00000434993				
ENSP00000435766				

639	640	641	642	643
Q96CV9	Q96CW1	Q96EB6	Q96GD0	Q96GD4
ITIH5	AP2M1	SIRT1	PDXP	AURKB
ENSG00000123243	ENSG00000161203	ENSG00000096717	ENSG00000241360	ENSG00000178999
ENSP00000380333	ENSP00000371894	ENSP00000212015	ENSP00000215904	ENSP00000313950
ENSP00000485414	ENSP00000292807	ENSP00000384508	ENSP00000385336	ENSP00000443869
ENSP00000419386	ENSP00000409081	ENSP00000384063		ENSP00000463999
ENSP00000380332	ENSP00000402051	ENSP00000409208		ENSP00000462207
ENSP00000263036	ENSP00000403362			ENSP00000462858
ENSP00000368040	ENSP00000402592			ENSP00000461981
ENSP00000368032	ENSP00000404031			ENSP00000463636
ENSP00000368027	ENSP00000395438			ENSP00000462930
ENSP00000368022	ENSP00000404326			ENSP00000464031
ENSP00000368021	ENSP00000416688			ENSP00000462027
ENSP00000484961	ENSP00000481563			
ENSP00000414747				
ENSP00000481473				
ENSP00000400356				

644	645	646	647	648
Q96GX5	Q96J02	Q96KB5	Q96N67	Q96PM5
MASTL	ITCH	PBK	DOCK7	RCHY1
ENSG00000120539	ENSG00000078747	ENSG00000168078	ENSG00000116641	ENSG00000163743
ENSP00000365113	ENSP00000363998	ENSP00000301905	ENSP00000251157	ENSP00000321239
ENSP00000365107	ENSP00000445608	ENSP00000428489	ENSP00000413583	ENSP00000370220
ENSP00000343446	ENSP00000262650	ENSP00000428438	ENSP00000340742	ENSP00000424631
ENSP00000478938	ENSP00000492520	ENSP00000427892	ENSP00000489284	ENSP00000423976
	ENSP00000491176		ENSP00000489499	ENSP00000421084
			ENSP00000489124	ENSP00000424472
			ENSP00000384446	ENSP00000421958
			ENSP00000490888	ENSP00000427205
			ENSP00000489412	
			ENSP00000490079	
			ENSP00000489037	
			ENSP00000489450	
			ENSP00000488988	
			ENSP00000483062	
			ENSP00000489164	
			ENSP00000490677	

649	650	651	652	653
Q96PU5	Q96Q15	Q96R06	Q96RK0	Q96S44
NEDD4L	SMG1	SPAG5	CIC	TP53RK
ENSG00000049759	ENSG00000157106	ENSG00000076382	ENSG00000079432	ENSG00000172315
ENSP00000348847	ENSP00000402515	ENSP00000323300	ENSP00000458663	ENSP00000361186
ENSP00000383199	ENSP00000456259	ENSP00000466061	ENSP00000459719	ENSP00000361174
ENSP00000372301	ENSP00000455861	ENSP00000466021	ENSP00000459704	
ENSP00000256830	ENSP00000484278	ENSP00000464280	ENSP00000160740	
ENSP00000393395	ENSP00000458558	ENSP00000464474	ENSP00000458543	
ENSP00000350569	ENSP00000460854	ENSP00000463095	ENSP00000459011	
ENSP00000468546	ENSP00000458380	ENSP00000464400	ENSP00000460230	
ENSP00000405440	ENSP00000432825	ENSP00000462663		
ENSP00000389406	ENSP00000455453	ENSP00000444223		
ENSP00000411947	ENSP00000456025			
ENSP00000465669	ENSP00000481150			
ENSP00000479462				
ENSP00000466776				
ENSP00000466789				
ENSP00000467072				
ENSP00000467768				
ENSP00000490696				
ENSP00000468332				

654	655	656	657	658
Q96SB3	Q96SB4	Q96T60	Q96T88	Q99418
PPP1R9B	SRPK1	PNKP	UHRF1	CYTH2
ENSG00000108819	ENSG00000096063	ENSG00000039650	ENSG00000276043	ENSG00000105443
ENSP00000475417	ENSP00000362931	ENSP00000323511	ENSP00000484739	ENSP00000408236
	ENSP00000391069	ENSP00000472300	ENSP00000478601	ENSP00000493357
	ENSP00000354674	ENSP00000490737	ENSP00000478348	ENSP00000493227
	ENSP00000422886	ENSP00000486037	ENSP00000485604	ENSP00000375753
	ENSP00000424170	ENSP00000468896	ENSP00000478171	ENSP00000314566
	ENSP00000425172	ENSP00000473137	ENSP00000480214	ENSP00000472602
	ENSP00000424068	ENSP00000486707	ENSP00000479617	ENSP00000486578
	ENSP00000424323	ENSP00000469826		ENSP00000482082
		ENSP00000471097		
		ENSP00000491347		
		ENSP00000486898		
		ENSP00000486500		
		ENSP00000489983		
		ENSP00000469848		
		ENSP00000470887		
		ENSP00000470346		
		ENSP00000490539		
		ENSP00000490791		
		ENSP00000489771		

659	660	661	662	663
Q99426	Q99504	Q99594	Q99661	Q99717
TBCB	EYA3	TEAD3	KIF2C	SMAD5
ENSG00000105254	ENSG00000158161	ENSG00000007866	ENSG00000142945	ENSG00000113658
ENSP00000221855	ENSP00000362978	ENSP00000492431	ENSP00000361298	ENSP00000441954
ENSP00000467487	ENSP00000362970	ENSP00000384577	ENSP00000361291	ENSP00000446474
ENSP00000465447	ENSP00000442558	ENSP00000345772	ENSP00000410346	ENSP00000425749
ENSP00000465979	ENSP00000405587	ENSP00000416400	ENSP00000395050	ENSP00000424279
ENSP00000467172	ENSP00000362971		ENSP00000402172	ENSP00000426696
ENSP00000467825				ENSP00000427330
ENSP00000468055				ENSP00000422954
ENSP00000485894				ENSP00000427474
				ENSP00000427650
				ENSP00000305769
				ENSP00000377652
				ENSP00000426568
				ENSP00000424959
				ENSP00000424649
				ENSP00000425270
				ENSP00000427002
				ENSP00000422706
				ENSP00000421601

664	665	666	667	668
Q99759	Q99961	Q99986	Q99988	Q9BQG0
MAP3K3	SH3GL1	VRK1	GDF15	MYBBP1A
ENSG00000198909	ENSG00000141985	ENSG00000100749	ENSG00000130513	ENSG00000132382
ENSP00000387246	ENSP00000269886	ENSP00000216639	ENSP00000252809	ENSP00000254718
ENSP00000343463	ENSP00000470792	ENSP00000450820	ENSP00000469819	ENSP00000370968
ENSP00000386313	ENSP00000404568	ENSP00000451682	ENSP00000470531	ENSP00000458919
ENSP00000461988	ENSP00000471260	ENSP00000451412		ENSP00000459692
ENSP00000354485	ENSP00000469766			ENSP00000460087
ENSP00000354927	ENSP00000472640			
ENSP00000464130				
ENSP00000462086				
ENSP00000462917				
ENSP00000464532				
ENSP00000464482				

669	670	671	672	673
Q9BR76	Q9BUL8	Q9BXS5	Q9BY41	Q9BY44
CORO1B	PDCD10	AP1M1	HDAC8	EIF2A
ENSG00000172725	ENSG00000114209	ENSG00000072958	ENSG00000147099	ENSG00000144895
ENSP00000340211	ENSP00000376506	ENSP00000291439	ENSP00000362674	ENSP00000420537
ENSP00000377471	ENSP00000418317	ENSP00000388996	ENSP00000414486	ENSP00000417229
ENSP00000479949	ENSP00000420553	ENSP00000466858	ENSP00000362660	ENSP00000385292
ENSP00000445330	ENSP00000420021	ENSP00000465224	ENSP00000362657	ENSP00000419456
ENSP00000438056	ENSP00000417202	ENSP00000411498	ENSP00000362655	ENSP00000418698
ENSP00000486585	ENSP00000417309	ENSP00000468140	ENSP00000362691	ENSP00000273435
	ENSP00000417118	ENSP00000467721	ENSP00000362685	ENSP00000417575
	ENSP00000420266	ENSP00000467417	ENSP00000362669	ENSP00000420495
	ENSP00000417876	ENSP00000482949	ENSP00000416489	ENSP00000420493
	ENSP00000420308	ENSP00000468015	ENSP00000396424	ENSP00000420611
	ENSP00000420424		ENSP00000400180	
	ENSP00000420014		ENSP00000362672	
	ENSP00000420450		ENSP00000362661	
	ENSP00000418160		ENSP00000398997	
			ENSP00000409778	

674	675	676	677	678
Q9BZL4	Q9BZL6	Q9BZQ8	Q9GZM8	Q9GZY8
PPP1R12C	PRKD2	FAM129A	NDEL1	MFF
ENSG00000125503	ENSG00000105287	ENSG00000135842	ENSG00000166579	ENSG00000168958
ENSP00000263433	ENSP00000469106	ENSP00000356481	ENSP00000379643	ENSP00000304898
ENSP00000387833	ENSP00000291281	ENSP00000414039	ENSP00000379642	ENSP00000302037
ENSP00000468148	ENSP00000472744		ENSP00000459889	ENSP00000346498
ENSP00000465957	ENSP00000470804		ENSP00000460436	ENSP00000386964
	ENSP00000393978		ENSP00000460996	ENSP00000386641
	ENSP00000472913		ENSP00000461729	ENSP00000338412
	ENSP00000470442		ENSP00000459875	ENSP00000304134
	ENSP00000469064		ENSP00000459863	ENSP00000390165
	ENSP00000470363		ENSP00000461748	ENSP00000431725
	ENSP00000470919		ENSP00000459918	ENSP00000432447
	ENSP00000472987		ENSP00000488050	ENSP00000415996
			ENSP00000488199	ENSP00000436920
			ENSP00000487642	ENSP00000435354
			ENSP00000488828	ENSP00000436289
			ENSP00000488895	ENSP00000411386
			ENSP00000488637	ENSP00000391829
			ENSP00000488454	ENSP00000407547
			ENSP00000488694	ENSP00000415313
			ENSP00000487817	ENSP00000375912
			ENSP00000487625	
			ENSP00000333982	
			ENSP00000384963	
			ENSP00000462052	
			ENSP00000464154	
			ENSP00000463430	
			ENSP00000463492	
			ENSP00000369364	
			ENSP00000462458	
			ENSP00000462759	
			ENSP00000463351	
			ENSP00000464441	
			ENSP00000464634	

679	680	681	682	683
Q9H0B6	Q9H0H5	Q9H0Z9	Q9H1A4	Q9H1K1
KLC2	RACGAP1	RBM38	ANAPC1	ISCU
ENSG00000174996	ENSG00000161800	ENSG00000132819	ENSG00000153107	ENSG00000136003
ENSP00000377631	ENSP00000404190	ENSP00000348538	ENSP00000339109	ENSP00000310623
ENSP00000314837	ENSP00000309871	ENSP00000407848	ENSP00000494863	ENSP00000376554
ENSP00000377630	ENSP00000404808	ENSP00000343718	ENSP00000396695	ENSP00000445598
ENSP00000408484	ENSP00000449374	ENSP00000345248	ENSP00000405375	ENSP00000411108
ENSP00000399403	ENSP00000449370	ENSP00000360263	ENSP00000486322	ENSP00000446606
ENSP00000436577	ENSP00000446889	ENSP00000368341		ENSP00000437854
ENSP00000437026	ENSP00000449565	ENSP00000396898		ENSP00000443272
ENSP00000396952	ENSP00000448507	ENSP00000319551		
ENSP00000377641	ENSP00000449574	ENSP00000426222		
ENSP00000434538	ENSP00000448697	ENSP00000421971		
ENSP00000431253	ENSP00000446642			
ENSP00000377629	ENSP00000447429			
	ENSP00000449963			
	ENSP00000450064			
	ENSP00000449170			
	ENSP00000448860			
	ENSP00000449620			
	ENSP00000449669			
	ENSP00000447393			
	ENSP00000448968			
	ENSP00000450074			
	ENSP00000447177			
	ENSP00000449186			
	ENSP00000448707			
	ENSP00000449959			
	ENSP00000448136			

684	685	686	687	688
Q9H257	Q9H2D6	Q9H2G2	Q9H2K8	Q9H3Z4
CARD9	TRIOBP	SLK	TAOK3	DNAJC5
ENSG00000187796	ENSG00000100106	ENSG00000065613	ENSG00000135090	ENSG00000101152
ENSP00000360799	ENSP00000384312	ENSP00000336824	ENSP00000416374	ENSP00000354111
ENSP00000360797	ENSP00000383913	ENSP00000358770	ENSP00000376317	ENSP00000434744
ENSP00000451368	ENSP00000386026		ENSP00000441932	ENSP00000473290
ENSP00000493113	ENSP00000496394		ENSP00000443834	ENSP00000436783
	ENSP00000340312		ENSP00000443487	
	ENSP00000495322		ENSP00000439620	
	ENSP00000399006		ENSP00000437389	
	ENSP00000400680		ENSP00000443465	
	ENSP00000396946		ENSP00000438820	
	ENSP00000407542		ENSP00000444057	
	ENSP00000387881		ENSP00000440315	
			ENSP00000441071	
			ENSP00000442541	
			ENSP00000438766	

689	690	691	692	693
Q9H410	Q9H4A3	Q9H4A6	Q9H5V8	Q9H6Z4
DSN1	WNK1	GOLPH3	CDCP1	RANBP3
ENSG00000149636	ENSG00000060237	ENSG00000113384	ENSG00000163814	ENSG00000031823
ENSP00000404463	ENSP00000313059	ENSP00000265070	ENSP00000296129	ENSP00000404837
ENSP00000362855	ENSP00000341292	ENSP00000426752	ENSP00000399342	ENSP00000341483
ENSP00000362839	ENSP00000444465			ENSP00000034275
ENSP00000389810	ENSP00000433548			ENSP00000466879
ENSP00000362845	ENSP00000441972			ENSP00000465085
ENSP00000395760	ENSP00000392542			ENSP00000464774
ENSP00000399654	ENSP00000438313			ENSP00000468815
	ENSP00000446462			ENSP00000466510
	ENSP00000439552			ENSP00000466761
	ENSP00000460651			ENSP00000468211
	ENSP00000459380			ENSP00000466970
	ENSP00000446253			ENSP00000464705
	ENSP00000437503			ENSP00000467760
	ENSP00000438985			ENSP00000464986
				ENSP00000467560
				ENSP00000466638

694	695	696	697	698
Q9H8S9	Q9H8V3	Q9H8Y8	Q9H9Q4	Q9HBY8
MOB1A	ECT2	GORASP2	NHEJ1	SGK2
ENSG00000114978	ENSG00000114346	ENSG00000115806	ENSG00000187736	ENSG00000101049
ENSP00000379364	ENSP00000232458	ENSP00000234160	ENSP00000387290	ENSP00000362192
	ENSP00000376457	ENSP00000413945	ENSP00000349313	ENSP00000362184
	ENSP00000415876	ENSP00000401808	ENSP00000408966	ENSP00000340608
	ENSP00000412259	ENSP00000398191	ENSP00000394896	ENSP00000412214
	ENSP00000443160	ENSP00000399889	ENSP00000407201	ENSP00000392795
	ENSP00000403501		ENSP00000408421	ENSP00000362168
	ENSP00000403446			ENSP00000436841
	ENSP00000412331			ENSP00000481432
	ENSP00000391695			ENSP00000396222
	ENSP00000412028			
	ENSP00000389108			
	ENSP00000411674			
	ENSP00000397788			

699	700	701	702	703
Q9HCE7	Q9HCP0	Q9NP77	Q9NP97	Q9NQC3
KIR2DL3	CSNK1G1	SSU72	DYNLRB1	RTN4
ENSG00000284132	ENSG00000169118	ENSG00000160075	ENSG00000125971	ENSG00000115310
ENSP00000492143	ENSP00000305777	ENSP00000291386	ENSP00000349679	ENSP00000378107
ENSP00000491888	ENSP00000475325	ENSP00000351955	ENSP00000300469	ENSP00000384471
ENSP00000355326	ENSP00000475724		ENSP00000363979	ENSP00000349944
ENSP00000354621	ENSP00000476088			ENSP00000337838
ENSP00000491036	ENSP00000489165			ENSP00000322147
ENSP00000492201	ENSP00000489233			ENSP00000350365
	ENSP00000489116			ENSP00000378109
	ENSP00000489050			ENSP00000385650
	ENSP00000489310			ENSP00000489133
	ENSP00000488990			ENSP00000397808
	ENSP00000475686			ENSP00000411628
	ENSP00000489172			ENSP00000384825
	ENSP00000489090			
	ENSP00000489008			
	ENSP00000489277			
	ENSP00000489534			
	ENSP00000489046			

704	705	706	707	708
Q9NQX3	Q9NR28	Q9NRA0	Q9NRF8	Q9NRY4
GPHN	AC068831.7	SPHK2	CTPS2	ARHGAP35
ENSG00000171723	ENSG00000284946	ENSG00000063176	ENSG00000047230	ENSG00000160007
ENSP00000312771	ENSP00000494429	ENSP00000245222	ENSP00000352222	ENSP00000385720
ENSP00000417901	ENSP00000493953	ENSP00000470092	ENSP00000369590	ENSP00000483730
ENSP00000452220	ENSP00000496165	ENSP00000469158	ENSP00000401264	
ENSP00000438404	ENSP00000495878	ENSP00000471205	ENSP00000400431	
ENSP00000451935	ENSP00000496662	ENSP00000471180		
ENSP00000451056	ENSP00000442360	ENSP00000410044		
ENSP00000451790	ENSP00000320343	ENSP00000471111		
ENSP00000451725	ENSP00000267169	ENSP00000469448		
ENSP00000450706	ENSP00000494535	ENSP00000470679		
ENSP00000452009	ENSP00000339963	ENSP00000472983		
ENSP00000450442	ENSP00000441139	ENSP00000341091		
	ENSP00000390818			
	ENSP00000440971			
	ENSP00000440653			
	ENSP00000390103			
	ENSP00000411638			
	ENSP00000398495			
	ENSP00000493911			
	ENSP00000495792			
	ENSP00000496127			
	ENSP00000494184			
	ENSP00000494048			
	ENSP00000494805			
	ENSP00000496557			
	ENSP00000495127			
	ENSP00000495742			

709	710	711	712	713
Q9NWZ3	Q9NXR1	Q9NY61	Q9NYL2	Q9NYV4
IRAK4	NDE1	AATF	MAP3K20	CDK12
ENSG00000198001	ENSG00000072864	ENSG00000275700	ENSG00000091436	ENSG00000167258
ENSP00000408734	ENSP00000379643	ENSP00000477848	ENSP00000387259	ENSP00000407720
ENSP00000479889	ENSP00000379642	ENSP00000479926	ENSP00000340257	ENSP00000398880
ENSP00000446490	ENSP00000459889	ENSP00000480472	ENSP00000364361	ENSP00000464641
ENSP00000390327	ENSP00000460436	ENSP00000477881	ENSP00000439414	ENSP00000453329
ENSP00000446571	ENSP00000460996	ENSP00000481894	ENSP00000411923	
ENSP00000449553	ENSP00000461729	ENSP00000488862		
ENSP00000449693	ENSP00000459875	ENSP00000488221		
ENSP00000449848	ENSP00000459863	ENSP00000487925		
ENSP00000449317	ENSP00000461748	ENSP00000482816		
ENSP00000448197	ENSP00000459918	ENSP00000485653		
ENSP00000449859	ENSP00000488050	ENSP00000485302		
ENSP00000390651	ENSP00000488199			
	ENSP00000487642			
	ENSP00000488828			
	ENSP00000488895			
	ENSP00000488637			
	ENSP00000488454			
	ENSP00000488694			
	ENSP00000487817			
	ENSP00000487625			
	ENSP00000333982			
	ENSP00000384963			
	ENSP00000462052			
	ENSP00000464154			
	ENSP00000463430			
	ENSP00000463492			
	ENSP00000369364			
	ENSP00000462458			
	ENSP00000462759			
	ENSP00000463351			
	ENSP00000464441			
	ENSP00000464634			

714	715	716	717	718
Q9NYV6	Q9P0J1	Q9P289	Q9UBB4	Q9UBF6
RRN3	PDP1	STK26	ATXN10	RNF7
ENSG00000085721	ENSG00000164951	ENSG00000134602	ENSG00000130638	ENSG00000114125
ENSP00000198767	ENSP00000297598	ENSP00000377867	ENSP00000252934	ENSP00000273480
ENSP00000318484	ENSP00000428317	ENSP00000377868	ENSP00000370449	ENSP00000419084
ENSP00000454238	ENSP00000430380	ENSP00000419702	ENSP00000391117	ENSP00000376725
ENSP00000454530	ENSP00000379503	ENSP00000418753	ENSP00000385624	ENSP00000419339
ENSP00000402027	ENSP00000430931	ENSP00000346755	ENSP00000491659	ENSP00000419979
ENSP00000480743	ENSP00000430366			
ENSP00000487821	ENSP00000428433			
ENSP00000488651	ENSP00000430655			
ENSP00000487746	ENSP00000429492			
ENSP00000487970				

719	720	721	722	723
Q9UBF8	Q9UBI6	Q9UBN7	Q9UBP6	Q9UER7
PI4KB	GNG12	HDAC6	METTL1	DAXX
ENSG00000143393	ENSG00000172380	ENSG00000094631	ENSG00000037897	ENSG00000204209
ENSP00000357868	ENSP00000360021	ENSP00000334061	ENSP00000314441	ENSP00000266000
ENSP00000357867		ENSP00000365804	ENSP00000257848	ENSP00000363668
ENSP00000433149		ENSP00000392815	ENSP00000447838	ENSP00000396876
ENSP00000357866		ENSP00000403370	ENSP00000449397	ENSP00000482399
ENSP00000357869		ENSP00000365831	ENSP00000447988	ENSP00000406008
ENSP00000410974		ENSP00000365795	ENSP00000448342	ENSP00000390008
ENSP00000391546		ENSP00000496727		ENSP00000382014
ENSP00000432426		ENSP00000402189		ENSP00000372539
ENSP00000413599		ENSP00000494447		ENSP00000477633
ENSP00000431501		ENSP00000495151		ENSP00000483394
ENSP00000394719		ENSP00000493921		ENSP00000408215
		ENSP00000496046		ENSP00000409756
		ENSP00000496013		ENSP00000411119
		ENSP00000393916		ENSP00000382281
		ENSP00000494503		ENSP00000372681
		ENSP00000496207		ENSP00000480448
		ENSP00000402751		ENSP00000478810
		ENSP00000394377		ENSP00000415898
		ENSP00000494230		ENSP00000411700
		ENSP00000496032		ENSP00000407623
		ENSP00000494293		ENSP00000410772
		ENSP00000494007		ENSP00000404376
		ENSP00000495872		ENSP00000479172
		ENSP00000495932		ENSP00000483517
		ENSP00000397697		ENSP00000403986
				ENSP00000397612
				ENSP00000387575
				ENSP00000394108
				ENSP00000404623
				ENSP00000484830
				ENSP00000412433
				ENSP00000412177
				ENSP00000406649

724	725	726	727	728
Q9UEW8	Q9UEY8	Q9UGK3	Q9UHD2	Q9UIG0
STK39	ADD3	STAP2	TBK1	BAZ1B
ENSG00000198648	ENSG00000148700	ENSG00000178078	ENSG00000183735	ENSG00000009954
ENSP00000348278	ENSP00000353286	ENSP00000468927	ENSP00000329967	ENSP00000342434
	ENSP00000348381	ENSP00000471052	ENSP00000445834	ENSP00000385442
	ENSP00000277900	ENSP00000469657	ENSP00000445628	
		ENSP00000471281	ENSP00000444428	
		ENSP00000470321		
		ENSP00000472851		

729	730	731	732	733
Q9UJM3	Q9UJX2	Q9UKI8	Q9UKV3	Q9UKV8
ERRFI1	CDC23	TLK1	ACIN1	AGO2
ENSG00000116285	ENSG00000094880	ENSG00000198586	ENSG00000100813	ENSG00000123908
ENSP00000366702	ENSP00000378350	ENSP00000411099	ENSP00000345541	ENSP00000220592
ENSP00000466958	ENSP00000378348	ENSP00000354089	ENSP00000350073	ENSP00000430176
ENSP00000465100	ENSP00000422505	ENSP00000428113	ENSP00000262710	ENSP00000430164
ENSP00000466454	ENSP00000422902	ENSP00000409222	ENSP00000380502	ENSP00000431056
ENSP00000467030	ENSP00000423704	ENSP00000352810	ENSP00000451328	
		ENSP00000387313	ENSP00000451138	
		ENSP00000393165	ENSP00000483298	
			ENSP00000474349	
			ENSP00000451410	
			ENSP00000481955	
			ENSP00000405677	

734	735	736	737	738
Q9UKX7	Q9UL54	Q9ULV4	Q9UNE7	Q9UNN5
NUP50	TAOK2	CORO1C	STUB1	FAF1
ENSG00000093000	ENSG00000149930	ENSG00000110880	ENSG00000103266	ENSG00000185104
ENSP00000345895	ENSP00000310094	ENSP00000261401	ENSP00000457228	ENSP00000379457
ENSP00000385555	ENSP00000440336	ENSP00000438341	ENSP00000219548	ENSP00000434929
ENSP00000379403	ENSP00000279394	ENSP00000447534	ENSP00000456875	ENSP00000360843
ENSP00000415062	ENSP00000393048	ENSP00000394496	ENSP00000456591	
ENSP00000394450		ENSP00000447337	ENSP00000457583	
ENSP00000388426		ENSP00000415554	ENSP00000457090	
ENSP00000397960		ENSP00000448130	ENSP00000476751	
ENSP00000398297		ENSP00000449658		
ENSP00000400465		ENSP00000449330		
ENSP00000391987		ENSP00000447989		
ENSP00000416264		ENSP00000448527		
		ENSP00000448195		
		ENSP00000447049		

739	740	741	742	743
Q9UNZ2	Q9UPT5	Q9UPY8	Q9UQ80	Q9UQ88
NSFL1C	EXOC7	MAPRE3	PA2G4	CDK11A
ENSG00000088833	ENSG00000182473	ENSG00000084764	ENSG00000170515	ENSG00000008128
ENSP00000338643	ENSP00000333806	ENSP00000233121	ENSP00000302886	ENSP00000463048
ENSP00000418529	ENSP00000468404	ENSP00000383915	ENSP00000448557	ENSP00000464036
ENSP00000216879	ENSP00000334100	ENSP00000385715	ENSP00000447615	ENSP00000464016
ENSP00000452019	ENSP00000489266	ENSP00000391705	ENSP00000447389	ENSP00000481846
ENSP00000434067	ENSP00000404322			ENSP00000484222
ENSP00000451675	ENSP00000466682			ENSP00000485937
ENSP00000473400	ENSP00000384368			ENSP00000485705
ENSP00000473616	ENSP00000349767			ENSP00000485849
	ENSP00000467042			ENSP00000384442
	ENSP00000389950			ENSP00000351629
	ENSP00000389723			ENSP00000367900
	ENSP00000468364			ENSP00000348529
	ENSP00000385066			ENSP00000367905
	ENSP00000475431			ENSP00000422149
				ENSP00000462289
				ENSP00000350403
				ENSP00000423900
				ENSP00000383874

744	745	746	747	748
Q9UQE7	Q9UQM7	Q9Y266	Q9Y2H1	Q9Y2K2
SMC3	CAMK2A	NUDC	STK38L	SIK3
ENSG00000108055	ENSG00000070808	ENSG00000090273	ENSG00000211455	ENSG00000160584
ENSP00000354720	ENSP00000326375	ENSP00000319664	ENSP00000373684	ENSP00000364449
	ENSP00000379098	ENSP00000404020	ENSP00000437856	ENSP00000390442
	ENSP00000397937	ENSP00000400981	ENSP00000439457	ENSP00000391295
	ENSP00000351542		ENSP00000445898	ENSP00000392849
	ENSP00000326427		ENSP00000443838	ENSP00000392761
	ENSP00000326544		ENSP00000443456	ENSP00000414347
	ENSP00000326518		ENSP00000442253	
	ENSP00000258682		ENSP00000440279	
	ENSP00000379096		ENSP00000385174	
	ENSP00000390292		ENSP00000443961	
	ENSP00000428912			
	ENSP00000410445			
	ENSP00000326600			
	ENSP00000388951			
	ENSP00000390419			
	ENSP00000400387			
	ENSP00000388445			
	ENSP00000261793			
	ENSP00000381412			
	ENSP00000427580			
	ENSP00000426607			

749	750	751	752	753
Q9Y2U5	Q9Y2V2	Q9Y2Y9	Q9Y365	Q9Y3Z3
MAP3K2	CARHSP1	KLF13	STARD10	SAMHD1
ENSG00000169967	ENSG00000153048	ENSG00000169926	ENSG00000214530	ENSG00000101347
ENSP00000387246	ENSP00000379838	ENSP00000302456	ENSP00000438792	ENSP00000262878
ENSP00000343463	ENSP00000311847	ENSP00000452609	ENSP00000335247	ENSP00000493536
ENSP00000386313	ENSP00000455284	ENSP00000452867	ENSP00000445708	ENSP00000494520
ENSP00000461988	ENSP00000455855	ENSP00000484904	ENSP00000440016	ENSP00000494823
ENSP00000354485	ENSP00000481550	ENSP00000481494	ENSP00000443548	ENSP00000493810
ENSP00000354927	ENSP00000483591		ENSP00000438357	ENSP00000494979
ENSP00000464130	ENSP00000480144		ENSP00000445657	ENSP00000494313
ENSP00000462086	ENSP00000478055		ENSP00000442414	ENSP00000495667
ENSP00000462917	ENSP00000480542		ENSP00000444027	ENSP00000494436
ENSP00000464532	ENSP00000455016		ENSP00000443597	ENSP00000493928
ENSP00000464482	ENSP00000455522		ENSP00000445886	ENSP00000494354
	ENSP00000457864		ENSP00000441589	ENSP00000495432
	ENSP00000456909		ENSP00000440924	ENSP00000496075
	ENSP00000455988		ENSP00000443523	ENSP00000495448
	ENSP00000460812		ENSP00000438924	ENSP00000495381
	ENSP00000457213		ENSP00000440302	ENSP00000494271
				ENSP00000496474
				ENSP00000495737
				ENSP00000495645

754	755	756	757	758
Q9Y478	Q9Y490	Q9Y4H2	Q9Y4K3	Q9Y4R8
PRKAB1	TLN1	IRS2	TRAF6	TELO2
ENSG00000111725	ENSG00000137076	ENSG00000185950	ENSG00000175104	ENSG00000100726
ENSP00000229328	ENSP00000316029	ENSP00000365016	ENSP00000433623	ENSP00000262319
ENSP00000441369			ENSP00000337853	ENSP00000456383
ENSP00000444816				ENSP00000457461
ENSP00000439235				
ENSP00000437993				
ENSP00000486936				

759	760	761	762	763
Q9Y5B0	Q9Y5S9	Q9Y5T5	Q9Y613	Q9Y618
CTDP1	AC099850.2	USP16	FHOD1	NCOR2
ENSG00000060069	ENSG00000265303	ENSG00000156256	ENSG00000135723	ENSG00000196498
ENSP00000484525	ENSP00000464167	ENSP00000382857	ENSP00000258201	ENSP00000348551
ENSP00000075430	ENSP00000463058	ENSP00000382858	ENSP00000462807	ENSP00000384018
ENSP00000465119	ENSP00000488265	ENSP00000334808	ENSP00000462988	ENSP00000384202
ENSP00000467563	ENSP00000358313	ENSP00000382855	ENSP00000458085	ENSP00000400281
ENSP00000299543	ENSP00000488887		ENSP00000464011	ENSP00000391389
	ENSP00000487764			ENSP00000407357
				ENSP00000398963
				ENSP00000396044
				ENSP00000400687
				ENSP00000402808
				ENSP00000408247
				ENSP00000405367
				ENSP00000443689
				ENSP00000403034
				ENSP00000385618

764	765	766	767	768
Q9Y624	Q9Y6E0	Q9Y6K9	Q9Y6R4	Q9Y6W5
F11R	STK24	IKBKG	MAP3K4	WASF2
ENSG00000158769	ENSG00000102572	ENSG00000269335	ENSG00000085511	ENSG00000158195
ENSP00000357005	ENSP00000442539	ENSP00000483825	ENSP00000355886	ENSP00000483313
ENSP00000440812	ENSP00000365730	ENSP00000471166	ENSP00000375986	ENSP00000439883
ENSP00000481591	ENSP00000365737	ENSP00000478616	ENSP00000439377	
	ENSP00000402810	ENSP00000479662	ENSP00000355887	
	ENSP00000402764	ENSP00000394934	ENSP00000297332	
	ENSP00000380651	ENSP00000390368	ENSP00000445018	
		ENSP00000480431	ENSP00000446303	
		ENSP00000395205	ENSP00000390508	
		ENSP00000398579		
		ENSP00000478979		
		ENSP00000483381		
		ENSP00000484023		
		ENSP00000479144		
		ENSP00000484275		

768	768	768	768	768	768	768
SIGNOR-C101	SIGNOR-C118	SIGNOR-C13	SIGNOR-C154	SIGNOR-C17	SIGNOR-C6	SIGNOR-C74

768	768
SIGNOR-C83	SIGNOR-C86

PART4: List of ODE state-space equations in large-scale colon cancerous cell's model

num	Ordinary Differential Equation
1	X_dot(1) =K4470_1 + K18_2*X1491 + K268_2*X1616 + K338_2*X1651 - K3687_1*X1 + K5223_1*X782 - K18_1*X1*X1031 - K268_1*X1*X330 - K338_1*X1*X1250
2	X_dot(2) =K4471_1 + K378_2*X1671 + K1924_2*X2444 - K3688_1*X2 + K5224_1*X904 - K378_1*X2*X915 - K1924_1*X2*X1310
3	X_dot(3) =K4472_1 + K1436_2*X2200 - K3689_1*X3 + K5225_1*X1141 - K1436_1*X3*X1318
4	X_dot(4) =K4473_1 + K1248_2*X2106 - K3690_1*X4 + K5226_1*X1095 - K1248_1*X4*X1195
5	X_dot(5) =K4474_1 + K1734_2*X2349 - K3691_1*X5 + K5227_1*X1219 - K1734_1*X5*X1451
6	X_dot(6) =K4475_1 + K180_2*X1572 - K3692_1*X6 + K5228_1*X848 - K180_1*X6*X1209
7	X_dot(7) =K4476_1 + K1618_2*X2291 + K1740_2*X2352 + K3182_2*X3073 + K3222_2*X3093 - K3693_1*X7 + K5229_1*X1194 - K1618_1*X7*X891 - K1740_1*X7*X780 - K3182_1*X7*X773 - K3222_1*X7*X776
8	X_dot(8) =K4477_1 + K3351_1*X3157 - K3694_1*X8 + K5230_1*X1448
9	X_dot(9) =K4478_1 + K120_2*X1542 + K342_2*X1653 + K450_2*X1707 + K970_2*X1967 + K1016_2*X1990 + K1086_2*X2025 + K1366_2*X2165 + K1372_2*X2168 + K2970_2*X2967 + K3130_2*X3047 - K3695_1*X9 + K5231_1*X822 - K120_1*X9*X891 - K342_1*X9*X822 - K450_1*X9*X1272 - K970_1*X9*X894 - K1016_1*X9*X1006 - K1086_1*X9*X1411 - K1366_1*X9*X1282 - K1372_1*X9*X1412 - K2970_1*X9*X187 - K3130_1*X9*X1154
10	X_dot(10) =K4479_1 + K2372_2*X2668 - K3696_1*X10 + K3541_1*X3252 + K3545_1*X3254 + K5232_1*X1346 - K2372_1*X10*X1145
11	X_dot(11) =K4480_1 + K876_2*X1920 + K1918_2*X2441 - K3697_1*X11 + K5233_1*X1016 - K876_1*X11*X891 - K1918_1*X11*X1267
12	X_dot(12) =K4481_1 + K1502_2*X2233 + K1503_1*X2233 - K3698_1*X12 - K1502_1*X12*X670
13	X_dot(13) =K4482_1 + K116_2*X1540 + K3184_2*X3074 - K3699_1*X13 + K5234_1*X820 - K116_1*X13*X891 - K3184_1*X13*X773
14	X_dot(14) =K4483_1 + K1742_2*X2353 - K3700_1*X14 + K5235_1*X1221 - K1742_1*X14*X780
15	X_dot(15) =K4484_1 + K3300_2*X3132 + K3301_1*X3132 - K3701_1*X15 - K3300_1*X15*X1185
16	X_dot(16) =K4485_1 + K1864_2*X2414 - K3702_1*X16 + K5236_1*X1257 - K1864_1*X16*X823
17	X_dot(17) =K4486_1 + K1766_2*X2365 + K1774_2*X2369 + K2264_2*X2614 - K3703_1*X17 + K5237_1*X1225 - K1766_1*X17*X331 - K1774_1*X17*X337 - K2264_1*X17*X1276
18	X_dot(18) =K4487_1 + K2836_2*X2900 - K3704_1*X18 + K5238_1*X1408 - K2836_1*X18*X1274
19	X_dot(19) =K4488_1 + K1814_2*X2389 - K3705_1*X19 + K5239_1*X1241 - K1814_1*X19*X1195
20	X_dot(20) =K4489_1 + K14_2*X1489 + K1620_2*X2292 + K1684_2*X2324 + K2686_2*X2825 - K3706_1*X20 + K3411_1*X3187 + K3425_1*X3194 + K5240_1*X780 - K14_1*X20*X1031 - K1620_1*X20*X891 - K1684_1*X20*X1000 - K2686_1*X20*X780
21	X_dot(21) =K4490_1 + K78_2*X1521 - K3707_1*X21 + K5241_1*X805 - K78_1*X21*X1173
22	X_dot(22) =K4491_1 + K1324_2*X2144 - K3708_1*X22 + K5242_1*X1114 - K1324_1*X22*X823
23	X_dot(23) =K4492_1 + K86_2*X1525 + K87_1*X1525 - K3709_1*X23 - K86_1*X23*X154
24	X_dot(24) =K4493_1 - K3710_1*X24
25	X_dot(25) =K4494_1 + K2434_2*X2699 + K2806_2*X2885 - K3711_1*X25 + K5243_1*X1356 - K2434_1*X25*X512 - K2806_1*X25*X1310
26	X_dot(26) =K4495_1 + K1250_2*X2107 + K1286_2*X2125 - K3712_1*X26 + K5244_1*X1096 - K1286_1*X26*X205 - K1250_1*X26*X1195
27	X_dot(27) =K4496_1 + K1860_2*X2412 - K3713_1*X27 + K5245_1*X1255 - K1860_1*X27*X823
28	X_dot(28) =K4497_1 + K390_2*X1677 + K758_2*X1861 + K1082_2*X2023 - K3714_1*X28 + K5246_1*X908 - K390_1*X28*X1310 - K758_1*X28*X512 - K1082_1*X28*X61
29	X_dot(29) =K4498_1 + K1514_2*X2239 + K1544_2*X2254 - K3715_1*X29 + K5247_1*X1159 - K1514_1*X29*X1031 - K1544_1*X29*X1418
30	X_dot(30) =K4499_1 + K2194_2*X2579 - K3716_1*X30 + K5248_1*X1308 - K2194_1*X30*X925
31	X_dot(31) =K4500_1 + K2388_2*X2676 + K3040_2*X3002 - K3717_1*X31 + K5249_1*X1350 - K2388_1*X31*X1145 - K3040_1*X31*X861
32	X_dot(32) =K4501_1 - K3718_1*X32 + K3592_2*X3278 + K3593_1*X3278 - K3592_1*X32*X99
33	X_dot(33) =K4502_1 + K1764_2*X2364 + K1772_2*X2368 + K1896_2*X2430 + K2704_2*X2834 + K3247_1*X3105 + K3391_1*X3177 + K3397_1*X3180 + K3403_1*X3183 - K3719_1*X33 + K5250_1*X1224 - K1764_1*X33*X331 - K1772_1*X33*X337 - K1896_1*X33*X915 - K2704_1*X33*X330
34	X_dot(34) =K4503_1 + K1938_2*X2451 - K3720_1*X34 + K5251_1*X1272 - K1938_1*X34*X1407
35	X_dot(35) =K4504_1 + K2518_2*X2741 - K3721_1*X35 + K5252_1*X1363 - K2518_1*X35*X861
36	X_dot(36) =K4505_1 + K2728_2*X2846 - K3722_1*X36 + K3439_1*X3201 + K5253_1*X1399 - K2728_1*X36*X1195
37	X_dot(37) =K4506_1 + K1364_2*X2164 - K3723_1*X37 + K5254_1*X1121 - K1364_1*X37*X1282
38	X_dot(38) =K4507_1 + K1010_2*X1987 + K1916_2*X2440 + K2530_2*X2747 - K3724_1*X38 + K5255_1*X1036 - K1010_1*X38*X1036 - K1916_1*X38*X1267 - K2530_1*X38*X861
39	X_dot(39) =K4508_1 + K1394_2*X2179 - K3725_1*X39 + K5256_1*X1128 - K1394_1*X39*X187
40	X_dot(40) =K4509_1 + K324_2*X1644 - K3726_1*X40 + K5257_1*X885 - K324_1*X40*X1195
41	X_dot(41) =K4510_1 + K1596_2*X2280 + K2854_2*X2909 + K3273_1*X3118 + K3301_1*X3132 - K3727_1*X41 + K3626_2*X3295 + K3632_2*X3298 + K5258_1*X1185 - K1596_1*X41*X522 - K2854_1*X41*X1185 - K3626_1*X41*X1454 - K3632_1*X41*X1484
42	X_dot(42) =K4511_1 + K3014_2*X2989 - K3728_1*X42 + K5259_1*X1431 - K3014_1*X42*X1431
43	X_dot(43) =K4512_1 + K1822_2*X2393 - K3729_1*X43 + K5260_1*X1244 - K1822_1*X43*X1195
44	X_dot(44) =K4513_1 + K1504_2*X2234 + K1505_1*X2234 - K3730_1*X44 - K1504_1*X44*X103
45	X_dot(45) =K4514_1 + K26_2*X1495 + K64_2*X1514 + K1714_2*X2339 - K3731_1*X45 + K5261_1*X786 - K26_1*X45*X1031 - K64_1*X45*X1418 - K1714_1*X45*X1393
46	X_dot(46) =K4515_1 + K1498_2*X2231 - K3732_1*X46 + K5262_1*X1153 - K1498_1*X46*X861
47	X_dot(47) =K248_2*X1606 + K266_2*X1615 + K336_2*X1650 + K828_2*X1896 + K850_2*X1907 + K1094_2*X2029 + K1126_2*X2045 + K1130_2*X2047 + K2034_2*X2499 + K2574_2*X2769 + K2950_2*X2957 + K2952_2*X2958 + K3088_2*X3026 + K3090_2*X3027 + K3092_2*X3028 + K3098_2*X3031 + K3100_2*X3032 + K3102_2*X3033 - K3733_1*X47 + K3499_1*X3231 + K3650_1*X3311 + K5263_1*X860 - K266_1*X47*X330 - K248_1*X47*X1159 - K336_1*X47*X1250 - K828_1*X47*X1031 - K850_1*X47*X1418 - K1094_1*X47*X1319 - K1126_1*X47*X1271 - K1130_1*X47*X1270 - K2034_1*X47*X894 - K2574_1*X47*X793 - K2950_1*X47*X418 - K2952_1*X47*X1006 - K3102_1*X47*X700 - K3088_1*X47*X891 - K3092_1*X47*X1000 - K3098_1*X47*X1273 - K3100_1*X47*X1316 - K3090_1*X47*X1445
48	X_dot(48) =K4516_1 + K1046_2*X2005 - K3734_1*X48 + K5264_1*X1046 - K1046_1*X48*X187
49	X_dot(49) =K4517_1 + K750_2*X1857 - K3735_1*X49 + K5265_1*X984 - K750_1*X49*X1181
50	X_dot(50) =K4518_1 + K2908_2*X2936 - K3736_1*X50 + K5266_1*X1415 - K2908_1*X50*X1383
51	X_dot(51) =K4519_1 + K1008_2*X1986 + K1236_2*X2100 - K3737_1*X51 + K3475_1*X3219 + K3477_1*X3220 + K3479_1*X3221 + K5267_1*X1035 - K1008_1*X51*X1036 - K1236_1*X51*X330
52	X_dot(52) =K4520_1 + K1270_2*X2117 + K1296_2*X2130 - K3738_1*X52 + K5268_1*X1102 - K1296_1*X52*X205 - K1270_1*X52*X1195
53	X_dot(53) =K4521_1 + K42_2*X1503 - K3739_1*X53 + K5269_1*X794 - K42_1*X53*X1031
54	X_dot(54) =K4522_1 + K1610_2*X2287 + K2256_2*X2610 - K3740_1*X54 + K5270_1*X1190 - K1610_1*X54*X891 - K2256_1*X54*X1276
55	X_dot(55) =K4523_1 + K282_2*X1623 + K648_2*X1806 + K1316_2*X2140 + K1334_2*X2149 + K3032_2*X2998 - K3741_1*X55 + K5271_1*X867 - K282_1*X55*X337 - K648_1*X55*X187 - K1316_1*X55*X823 - K1334_1*X55*X915 - K3032_1*X55*X861
56	X_dot(56) =K4524_1 + K2772_2*X2868 - K3742_1*X56 + K5272_1*X1403 - K2772_1*X56*X1107

57	X_dot(57) =K4525_1 + K1158_2*X2061 + K2956_2*X2960 + K2978_2*X2971 + K2994_2*X2979 + K3026_2*X2995 - K3743_1*X57 + K5273_1*X1071 - K1158_1*X57*X1031 - K2978_1*X57*X187 - K2956_1*X57*X1006 - K2994_1*X57*X1318 - K3026_1*X57*X1154
58	X_dot(58) =K4526_1 + K1062_2*X2013 - K3744_1*X58 + K5274_1*X1051 - K1062_1*X58*X1318
59	X_dot(59) =K4527_1 + K1564_2*X2264 - K3745_1*X59 + K5275_1*X1175 - K1564_1*X59*X1173
60	X_dot(60) =K4528_1 + K1578_2*X2271 + K1579_1*X2271 - K3746_1*X60 - K1578_1*X60*X230
61	X_dot(61) =K4529_1 + K1082_2*X2023 + K1083_1*X2023 + K2444_2*X2704 + K2445_1*X2704 - K3747_1*X61 - K1082_1*X28*X61 - K2444_1*X61*X577
62	X_dot(62) =K4530_1 + K562_2*X1763 - K3748_1*X62 + K5276_1*X936 - K562_1*X62*X1005
63	X_dot(63) =K4531_1 + K32_2*X1498 - K3749_1*X63 + K5277_1*X789 - K32_1*X63*X1031
64	X_dot(64) =K4532_1 + K1606_2*X2285 - K3750_1*X64 + K5278_1*X1188 - K1606_1*X64*X1194
65	X_dot(65) =K4533_1 + K810_2*X1887 - K3751_1*X65 + K3507_1*X3235 + K5279_1*X999 - K810_1*X65*X666
66	X_dot(66) =K4534_1 + K1220_2*X2092 + K1312_2*X2138 - K3752_1*X66 + K5280_1*X1089 - K1220_1*X66*X1000 - K1312_1*X66*X1250
67	X_dot(67) =K4535_1 + K2040_2*X2502 + K2094_2*X2529 + K2488_2*X2726 - K3753_1*X67 + K5281_1*X1293 - K2040_1*X67*X894 - K2094_1*X67*X1270 - K2488_1*X67*X1293
68	X_dot(68) =K4536_1 - K3754_1*X68 + K3472_2*X3218 + K3473_1*X3218 - K3472_1*X68*X1451
69	X_dot(69) =K4537_1 + K1408_2*X2186 - K3755_1*X69 + K5282_1*X1131 - K1408_1*X69*X1318
70	X_dot(70) =K4538_1 + K1326_2*X2145 - K3756_1*X70 + K3567_1*X3265 + K5283_1*X1115 - K1326_1*X70*X823
71	X_dot(71) =K4539_1 + K1816_2*X2390 + K2168_2*X2566 - K3757_1*X71 + K5284_1*X1242 - K1816_1*X71*X1195 - K2168_1*X71*X1282
72	X_dot(72) =K4540_1 + K3012_2*X2988 - K3758_1*X72 + K5285_1*X1430 - K3012_1*X72*X1181
73	X_dot(73) =K4541_1 + K930_2*X1947 - K3759_1*X73 + K5286_1*X1030 - K930_1*X73*X849
74	X_dot(74) =K4542_1 + K806_2*X1885 + K807_1*X1885 - K3760_1*X74 - K806_1*X74*X237
75	X_dot(75) =K4543_1 + K1634_2*X2299 + K2266_2*X2615 - K3761_1*X75 + K5287_1*X1199 - K1634_1*X75*X891 - K2266_1*X75*X1276
76	X_dot(76) =K4544_1 + K2982_2*X2973 - K3762_1*X76 + K5288_1*X1424 - K2982_1*X76*X187
77	X_dot(77) =K4545_1 + K2524_2*X2744 + K3327_1*X3145 - K3763_1*X77 + K5289_1*X1365 - K2524_1*X77*X861
78	X_dot(78) =K4546_1 + K554_2*X1759 - K3764_1*X78 + K5290_1*X934 - K554_1*X78*X1282
79	X_dot(79) =K3683_1*X3344 - K3765_1*X79
80	X_dot(80) =K4547_1 + K2622_2*X2793 - K3766_1*X80 + K5291_1*X1377 - K2622_1*X80*X1169
81	X_dot(81) =K4548_1 + K300_2*X1632 - K3767_1*X81 + K5292_1*X875 - K300_1*X81*X1195
82	X_dot(82) =K4549_1 + K2162_2*X2563 - K3768_1*X82 + K5293_1*X1300 - K2162_1*X82*X1282
83	X_dot(83) =K4550_1 + K2366_2*X2665 - K3769_1*X83 + K5294_1*X1343 - K2366_1*X83*X1145
84	X_dot(84) =K4551_1 + K2224_2*X2594 - K3770_1*X84 + K5295_1*X1315 - K2224_1*X84*X1315
85	X_dot(85) =K4552_1 + K1388_2*X2176 + K2408_2*X2686 + K2606_2*X2785 + K2690_2*X2827 - K3771_1*X85 + K3529_1*X3246 + K3535_1*X3249 + K5296_1*X1126 - K1388_1*X85*X1276 - K2408_1*X85*X980 - K2606_1*X85*X998 - K2690_1*X85*X1126
86	X_dot(86) =K4553_1 + K122_2*X1543 + K458_2*X1711 + K478_2*X1721 + K592_2*X1778 + K858_2*X1911 + K860_2*X1912 + K962_2*X1963 + K1070_2*X2017 + K1118_2*X2041 + K2344_2*X2654 + K2504_2*X2734 + K3339_1*X3151 + K3355_1*X3159 + K3375_1*X3169 + K3449_1*X3206 + K3455_1*X3209 - K3772_1*X86 + K3485_1*X3224 + K3596_2*X3280 + K5297_1*X823 - K122_1*X86*X891 - K458_1*X86*X894 - K478_1*X86*X1270 - K592_1*X86*X1316 - K860_1*X86*X522 - K858_1*X86*X810 - K962_1*X86*X1271 - K1070_1*X86*X1145 - K1118_1*X86*X823 - K2344_1*X86*X1318 - K2504_1*X86*X861 - K3596_1*X86*X221
87	X_dot(87) =K4554_1 + K1140_2*X2052 + K2066_2*X2515 + K2454_2*X2709 + K2484_2*X2724 + K2856_2*X2910 + K3132_2*X3048 - K3773_1*X87 + K5298_1*X1063 - K1140_1*X87*X187 - K2066_1*X87*X1270 - K2454_1*X87*X1411 - K2484_1*X87*X1293 - K2856_1*X87*X1271 - K3132_1*X87*X1154
88	X_dot(88) =K362_2*X1663 + K1122_2*X2043 + K2000_2*X2482 + K2152_2*X2558 + K2896_2*X2930 + K2946_2*X2955 + K3104_2*X3034 + K3295_1*X3129 - K3774_1*X88 + K3686_1*X3347 + K5299_1*X896 - K362_1*X88*X783 - K1122_1*X88*X915 - K2000_1*X88*X1271 - K2152_1*X88*X1103 - K2896_1*X88*X1273 - K2946_1*X88*X1276 - K3104_1*X88*X1270
89	X_dot(89) =K4555_1 - K3775_1*X89 + K3557_1*X3260 + K5300_1*X1458
90	X_dot(90) =K4556_1 + K1102_2*X2033 + K2798_2*X2881 + K2799_1*X2881 - K3776_1*X90 + K3553_1*X3258 + K5301_1*X1056 - K1102_1*X90*X861 - K2798_1*X90*X106
91	X_dot(91) =K4557_1 + K2520_2*X2742 - K3777_1*X91 + K3555_1*X3259 + K5302_1*X1364 - K2520_1*X91*X861
92	X_dot(92) =K3671_1*X3332 - K3778_1*X92
93	X_dot(93) =K4558_1 - K3779_1*X93
94	X_dot(94) =K4559_1 + K1426_2*X2195 + K1640_2*X2302 - K3780_1*X94 + K5303_1*X1136 - K1426_1*X94*X1318 - K1640_1*X94*X891
95	X_dot(95) =K4560_1 + K1034_2*X1999 - K3781_1*X95 + K5304_1*X1042 - K1034_1*X95*X187
96	X_dot(96) =K4561_1 + K1488_2*X2226 + K1850_2*X2407 + K2342_2*X2653 - K3782_1*X96 + K5305_1*X1150 - K1488_1*X96*X861 - K1850_1*X96*X823 - K2342_1*X96*X1318
97	X_dot(97) =K4562_1 + K170_2*X1567 + K204_2*X1584 - K3783_1*X97 + K5306_1*X844 - K170_1*X97*X891 - K204_1*X97*X1000
98	X_dot(98) =K4563_1 + K354_2*X1659 + K2306_2*X2635 + K2776_2*X2870 + K3377_1*X3170 - K3784_1*X98 + K3598_2*X3281 + K5307_1*X893 - K354_1*X98*X893 - K2306_1*X98*X187 - K2776_1*X98*X823 - K3598_1*X98*X221
99	X_dot(99) =K4564_1 + K82_2*X1523 + K914_2*X1939 + K1226_2*X2095 + K1228_2*X2096 + K1242_2*X2103 + K1664_2*X2314 + K1706_2*X2335 + K1726_2*X2345 + K1768_2*X2366 + K1844_2*X2404 + K1848_2*X2406 + K1880_2*X2422 + K2022_2*X2493 + K2046_2*X2505 + K2108_2*X2536 + K2138_2*X2551 + K2156_2*X2560 + K2294_2*X2629 + K2396_2*X2680 + K2418_2*X2691 + K2490_2*X2727 + K2604_2*X2784 + K2610_2*X2787 + K2618_2*X2791 + K2688_2*X2826 + K2794_2*X2879 + K2998_2*X2981 + K3084_2*X3024 + K3224_2*X3094 + K3395_1*X3179 + K3399_1*X3181 + K3407_1*X3185 + K3421_1*X3192 + K3435_1*X3199 - K3785_1*X99 + K3592_2*X3278 + K5308_1*X807 - K82_1*X99*X1173 - K914_1*X99*X1195 - K1242_1*X99*X330 - K1226_1*X99*X1087 - K1228_1*X99*X1451 - K1664_1*X99*X891 - K1768_1*X99*X331 - K1706_1*X99*X1000 - K1726_1*X99*X1393 - K1844_1*X99*X1028 - K1848_1*X99*X1250 - K1880_1*X99*X1260 - K2046_1*X99*X894 - K2022_1*X99*X1272 - K2108_1*X99*X1270 - K2138_1*X99*X1273 - K2156_1*X99*X1103 - K2294_1*X99*X1006 - K2396_1*X99*X1145 - K2418_1*X99*X980 - K2490_1*X99*X650 - K2618_1*X99*X666 - K2610_1*X99*X998 - K2604_1*X99*X1167 - K2688_1*X99*X780 - K2794_1*X99*X915 - K2998_1*X99*X1318 - K3084_1*X99*X1126 - K3224_1*X99*X776 - K3592_1*X32*X99
100	X_dot(100) =K4565_1 + K24_2*X1494 + K62_2*X1513 + K552_2*X1758 + K740_2*X1852 + K1374_2*X2169 - K3786_1*X100 + K5309_1*X785 - K24_1*X100*X1031 - K62_1*X100*X1418 - K552_1*X100*X1282 - K740_1*X100*X1341 - K1374_1*X100*X1412
101	X_dot(101) =K4566_1 + K1222_2*X2093 + K1332_2*X2148 + K2112_2*X2538 - K3787_1*X101 + K5310_1*X1090 - K1222_1*X101*X1393 - K1332_1*X101*X915 - K2112_1*X101*X1273
102	X_dot(102) =K4567_1 + K304_2*X1634 + K692_2*X1828 + K1036_2*X2000 + K1090_2*X2027 + K1486_2*X2225 - K3788_1*X102 + K5311_1*X876 - K304_1*X102*X1195 - K692_1*X102*X1318 - K1036_1*X102*X187 - K1090_1*X102*X1154 - K1486_1*X102*X1293
103	X_dot(103) =K4568_1 + K350_2*X1657 + K1450_2*X2207 + K1504_2*X2234 - K3789_1*X103 + K5312_1*X892 - K350_1*X103*X1260 - K1504_1*X44*X103 - K1450_1*X103*X1145
104	X_dot(104) =K4569_1 + K694_2*X1829 + K1092_2*X2028 - K3790_1*X104 + K5313_1*X969 - K694_1*X104*X1318 - K1092_1*X104*X1154
105	X_dot(105) =K4570_1 + K1890_2*X2427 - K3791_1*X105 + K5314_1*X1263 - K1890_1*X105*X849

106	X_dot(106) =K4571_1 + K308_2*X1636 + K360_2*X1662 + K944_2*X1954 + K1460_2*X2212 + K2074_2*X2519 + K2150_2*X2557 + K2216_2*X2590 + K2616_2*X2790 + K2798_2*X2881 + K2860_2*X2912 + K2894_2*X2929 - K3792_1*X106 + K3573_1*X3268 + K5315_1*X878 - K308_1*X106*X1195 - K360_1*X106*X783 - K944_1*X106*X915 - K1460_1*X106*X980 - K2074_1*X106*X1270 - K2150_1*X106*X1103 - K2216_1*X106*X1313 - K2616_1*X106*X666 - K2798_1*X90*X106 - K2860_1*X106*X1271 - K2894_1*X106*X1273
107	X_dot(107) =K4572_1 + K1830_2*X2397 - K3793_1*X107 + K5316_1*X1247 - K1830_1*X107*X1195
108	X_dot(108) =K4573_1 + K1174_2*X2069 + K1424_2*X2194 + K1482_2*X2223 + K1638_2*X2301 - K3794_1*X108 + K5317_1*X1075 - K1174_1*X108*X810 - K1424_1*X108*X1318 - K1482_1*X108*X1154 - K1638_1*X108*X891
109	X_dot(109) =K4574_1 + K2086_2*X2525 - K3795_1*X109 + K5318_1*X1296 - K2086_1*X109*X1270
110	X_dot(110) =K4575_1 + K1234_2*X2099 + K2512_2*X2738 - K3796_1*X110 + K5319_1*X1092 - K1234_1*X110*X1391 - K2512_1*X110*X861
111	X_dot(111) =K4576_1 + K160_2*X1562 + K196_2*X1580 + K212_2*X1588 + K222_2*X1593 + K232_2*X1598 + K464_2*X1714 + K628_2*X1796 + K1746_2*X2355 + K2692_2*X2828 + K3226_2*X3095 - K3797_1*X111 + K3501_1*X3232 + K5320_1*X839 - K160_1*X111*X891 - K232_1*X111*X438 - K196_1*X111*X1000 - K212_1*X111*X1001 - K222_1*X111*X1393 - K464_1*X111*X894 - K628_1*X111*X1006 - K1746_1*X111*X780 - K2692_1*X111*X1126 - K3226_1*X111*X776
112	X_dot(112) =K4577_1 + K348_2*X1656 + K352_2*X1658 + K812_2*X1888 + K1728_2*X2346 + K1788_2*X2376 - K3798_1*X112 + K5321_1*X891 - K352_1*X112*X893 - K348_1*X112*X1260 - K812_1*X112*X846 - K1728_1*X112*X1087 - K1788_1*X112*X1195
113	X_dot(113) =K4578_1 + K1416_2*X2190 + K3265_1*X3114 - K3799_1*X113 + K5322_1*X1133 - K1416_1*X113*X1318
114	X_dot(114) =K4579_1 + K2506_2*X2735 - K3800_1*X114 + K5323_1*X1362 - K2506_1*X114*X861
115	X_dot(115) =K4580_1 + K902_2*X1933 - K3801_1*X115 + K5324_1*X1024 - K902_1*X115*X1195
116	X_dot(116) =K4581_1 + K872_2*X1918 + K880_2*X1922 + K2268_2*X2616 + K3186_2*X3075 - K3802_1*X116 + K5325_1*X1014 - K872_1*X116*X891 - K880_1*X116*X1000 - K2268_1*X116*X1276 - K3186_1*X116*X773
117	X_dot(117) =K4582_1 + K2370_2*X2667 - K3803_1*X117 + K5326_1*X1345 - K2370_1*X117*X1145
118	X_dot(118) =K4583_1 + K1410_2*X2187 + K2494_2*X2729 - K3804_1*X118 + K5327_1*X1132 - K1410_1*X118*X1318 - K2494_1*X118*X861
119	X_dot(119) =K4584_1 + K756_2*X1860 + K3489_1*X3226 - K3805_1*X119 + K5328_1*X987 - K756_1*X119*X512
120	X_dot(120) =K4585_1 + K216_2*X1590 - K3806_1*X120 + K5329_1*X853 - K216_1*X120*X1393
121	X_dot(121) =K4586_1 + K1264_2*X2114 + K1398_2*X2181 + K1458_2*X2211 - K3807_1*X121 + K5330_1*X1100 - K1264_1*X121*X1195 - K1398_1*X121*X187 - K1458_1*X121*X980
122	X_dot(122) =K4587_1 + K270_2*X1617 + K904_2*X1934 + K1294_2*X2129 - K3808_1*X122 + K5331_1*X865 - K270_1*X122*X330 - K904_1*X122*X1195 - K1294_1*X122*X205
123	X_dot(123) =K4588_1 + K3078_2*X3021 - K3809_1*X123 + K5332_1*X1439 - K3078_1*X123*X1439
124	X_dot(124) =K4589_1 + K910_2*X1937 + K1660_2*X2312 + K2012_2*X2488 + K2100_2*X2532 + K2134_2*X2549 + K2666_2*X2815 + K3188_2*X3076 + K3228_2*X3096 - K3810_1*X124 + K3533_1*X3248 + K3569_1*X3266 + K5333_1*X1026 - K910_1*X124*X1195 - K1660_1*X124*X891 - K2012_1*X124*X1271 - K2100_1*X124*X1270 - K2134_1*X124*X1273 - K2666_1*X124*X1000 - K3188_1*X124*X773 - K3228_1*X124*X776
125	X_dot(125) =K4590_1 + K1910_2*X2437 + K2516_2*X2740 + K3451_1*X3207 - K3811_1*X125 + K5334_1*X1267 - K1910_1*X125*X1267 - K2516_1*X125*X861
126	X_dot(126) =K4591_1 + K306_2*X1635 + K2514_2*X2739 - K3812_1*X126 + K5335_1*X877 - K306_1*X126*X1195 - K2514_1*X126*X861
127	X_dot(127) =K4592_1 - K3813_1*X127 + K3519_1*X3241 + K5336_1*X1456
128	X_dot(128) =K700_2*X1832 + K2176_2*X2570 + K3311_1*X3137 + K3343_1*X3153 - K3814_1*X128 + K3600_2*X3282 + K3653_1*X3314 + K5337_1*X970 - K700_1*X128*X1318 - K2176_1*X128*X970 - K3600_1*X128*X221
129	X_dot(129) =K4593_1 + K84_2*X1524 + K174_2*X1569 + K1006_2*X1985 + K1056_2*X2010 + K1080_2*X2022 + K1136_2*X2050 + K1180_2*X2072 + K1380_2*X2172 + K1538_2*X2251 + K2282_2*X2623 - K3815_1*X129 + K5338_1*X808 - K84_1*X129*X1173 - K174_1*X129*X891 - K1056_1*X129*X187 - K1080_1*X129*X512 - K1006_1*X129*X1313 - K1136_1*X129*X1318 - K1180_1*X129*X1183 - K1380_1*X129*X925 - K1538_1*X129*X1031 - K2282_1*X129*X1276
130	X_dot(130) =K4594_1 + K1496_2*X2230 - K3816_1*X130 + K5339_1*X1152 - K1496_1*X130*X861
131	X_dot(131) =K4595_1 + K1858_2*X2411 - K3817_1*X131 + K5340_1*X1254 - K1858_1*X131*X823
132	X_dot(132) =K4596_1 + K3052_2*X3008 - K3818_1*X132 + K5341_1*X1434 - K3052_1*X132*X861
133	X_dot(133) =K4597_1 + K1252_2*X2108 + K1288_2*X2126 + K1330_2*X2147 - K3819_1*X133 + K5342_1*X1097 - K1288_1*X133*X205 - K1252_1*X133*X1195 - K1330_1*X133*X849
134	X_dot(134) =K4598_1 + K22_2*X1493 + K696_2*X1830 - K3820_1*X134 + K5343_1*X784 - K22_1*X134*X1031 - K696_1*X134*X1318
135	X_dot(135) =K4599_1 + K2002_2*X2483 + K3106_2*X3035 - K3821_1*X135 + K5344_1*X1284 - K2002_1*X135*X1271 - K3106_1*X135*X1270
136	X_dot(136) =K4600_1 + K2904_2*X2934 - K3822_1*X136 + K5345_1*X1413 - K2904_1*X136*X1282
137	X_dot(137) =K4601_1 + K1104_2*X2034 - K3823_1*X137 + K5346_1*X1057 - K1104_1*X137*X861
138	X_dot(138) =K4602_1 + K2192_2*X2578 + K2496_2*X2730 - K3824_1*X138 + K5347_1*X1307 - K2192_1*X138*X925 - K2496_1*X138*X861
139	X_dot(139) =K4603_1 + K3364_2*X3164 + K3365_1*X3164 - K3825_1*X139 + K3516_2*X3240 + K3517_1*X3240 - K3364_1*X139*X1031 - K3516_1*X139*X1028
140	X_dot(140) =K4604_1 + K722_2*X1843 - K3826_1*X140 + K5348_1*X975 - K722_1*X140*X1145
141	X_dot(141) =K4605_1 + K1702_2*X2333 - K3827_1*X141 + K5349_1*X1216 - K1702_1*X141*X1000
142	X_dot(142) =K3677_1*X3338 - K3828_1*X142
143	X_dot(143) =K4606_1 + K344_2*X1654 - K3829_1*X143 + K5350_1*X889 - K344_1*X143*X823
144	X_dot(144) =K4607_1 + K3297_1*X3130 - K3830_1*X144 + K5351_1*X1445
145	X_dot(145) =K4608_1 + K1432_2*X2198 - K3831_1*X145 + K5352_1*X1139 - K1432_1*X145*X1318
146	X_dot(146) =K4609_1 + K3307_1*X3135 - K3832_1*X146 + K5353_1*X1446
147	X_dot(147) =K4610_1 + K1210_2*X2087 + K1282_2*X2123 + K2278_2*X2621 - K3833_1*X147 + K5354_1*X1085 - K1210_1*X147*X891 - K1282_1*X147*X1195 - K2278_1*X147*X1276
148	X_dot(148) =K4611_1 + K2376_2*X2670 - K3834_1*X148 + K5355_1*X1347 - K2376_1*X148*X1145
149	X_dot(149) =K4612_1 + K1266_2*X2115 + K1400_2*X2182 - K3835_1*X149 + K5356_1*X1101 - K1266_1*X149*X1195 - K1400_1*X149*X187
150	X_dot(150) =K4613_1 + K816_2*X1890 + K1730_2*X2347 - K3836_1*X150 + K5357_1*X1001 - K816_1*X150*X1439 - K1730_1*X150*X1087
151	X_dot(151) =K4614_1 + K1862_2*X2413 - K3837_1*X151 + K3622_2*X3293 + K5358_1*X1256 - K1862_1*X151*X823 - K3622_1*X151*X1207
152	X_dot(152) =K4615_1 + K898_2*X1931 - K3838_1*X152 + K5359_1*X1022 - K898_1*X152*X1195
153	X_dot(153) =K4616_1 + K2020_2*X2492 - K3839_1*X153 + K5360_1*X1289 - K2020_1*X153*X1271
154	X_dot(154) =K4617_1 + K86_2*X1525 - K3840_1*X154 + K5361_1*X809 - K86_1*X23*X154
155	X_dot(155) =K4618_1 + K748_2*X1856 + K3511_1*X3237 - K3841_1*X155 + K5362_1*X983 - K748_1*X155*X1181
156	X_dot(156) =K4619_1 + K2718_2*X2841 + K2786_2*X2875 - K3842_1*X156 + K5363_1*X1396 - K2718_1*X156*X1195 - K2786_1*X156*X915
157	X_dot(157) =K4620_1 + K254_2*X1609 + K1106_2*X2035 + K1440_2*X2202 + K1720_2*X2342 + K2332_2*X2648 + K2446_2*X2705 + K3006_2*X2985 + K3345_1*X3154 + K3373_1*X3168 + K3419_1*X3191 + K3457_1*X3210 + K3461_1*X3212 + K3467_1*X3215 + K3513_1*X3238 + K3515_1*X3239 - K3843_1*X157 + K5364_1*X861 - K254_1*X157*X1391 - K1106_1*X157*X861 - K1440_1*X157*X1318 - K1720_1*X157*X1393 - K2332_1*X157*X187 - K2446_1*X157*X449 - K3006_1*X157*X1145
158	X_dot(158) =K4621_1 + K340_2*X1652 + K3497_1*X3230 - K3844_1*X158 + K5365_1*X888 - K340_1*X158*X822
159	X_dot(159) =K4622_1 + K1594_2*X2279 + K3030_2*X2997 - K3845_1*X159 + K5366_1*X1184 - K1594_1*X159*X1183 - K3030_1*X159*X861
160	X_dot(160) =K4623_1 + K608_2*X1786 - K3846_1*X160 + K5367_1*X945 - K608_1*X160*X1276

161	X_dot(161) =K4624_1 + K358_2*X1661 + K942_2*X1953 + K1032_2*X1998 + K1260_2*X2112 - K3847_1*X161 + K5368_1*X895 - K358_1*X161*X783 - K942_1*X161*X915 - K1032_1*X161*X187 - K1260_1*X161*X1195
162	X_dot(162) =K4625_1 + K2986_2*X2975 - K3848_1*X162 + K5369_1*X1426 - K2986_1*X162*X1426
163	X_dot(163) =K4626_1 + K2246_2*X2605 + K2247_1*X2605 - K3849_1*X163 - K2246_1*X163*X295
164	X_dot(164) =K4627_1 + K2944_2*X2954 - K3850_1*X164 + K5370_1*X1420 - K2944_1*X164*X1276
165	X_dot(165) =K4628_1 + K2184_2*X2574 - K3851_1*X165 + K5371_1*X1304 - K2184_1*X165*X1010
166	X_dot(166) =K4629_1 + K626_2*X1795 + K736_2*X1850 - K3852_1*X166 + K3672_2*X3333 + K3673_1*X3333 + K5372_1*X950 - K626_1*X166*X1006 - K736_1*X166*X980 - K3672_1*X166*X3334
167	X_dot(167) =K4630_1 + K3038_2*X3001 - K3853_1*X167 + K5373_1*X1432 - K3038_1*X167*X861
168	X_dot(168) =K4631_1 + K54_2*X1509 + K470_2*X1717 + K824_2*X1894 + K958_2*X1961 + K1024_2*X1994 + K1576_2*X2270 + K1577_1*X2270 + K2638_2*X2801 + K2639_1*X2801 - K3854_1*X168 + K5374_1*X800 - K54_1*X168*X1418 - K470_1*X168*X1270 - K824_1*X168*X1031 - K1024_1*X168*X187 - K958_1*X168*X1271 - K1576_1*X168*X287 - K2638_1*X168*X451
169	X_dot(169) =K4632_1 + K1318_2*X2141 + K1552_2*X2258 + K1888_2*X2426 + K2346_2*X2655 + K2400_2*X2682 + K2430_2*X2697 + K2508_2*X2736 + K2562_2*X2763 + K2676_2*X2820 + K3128_2*X3046 - K3855_1*X169 + K5375_1*X1112 - K1318_1*X169*X823 - K1552_1*X169*X1418 - K1888_1*X169*X849 - K2346_1*X169*X1318 - K2430_1*X169*X512 - K2400_1*X169*X1366 - K2508_1*X169*X861 - K2562_1*X169*X716 - K2676_1*X169*X1393 - K3128_1*X169*X1031
170	X_dot(170) =K4633_1 + K1964_2*X2464 + K2024_2*X2494 + K2054_2*X2509 + K2114_2*X2539 + K2140_2*X2552 + K2612_2*X2788 - K3856_1*X170 + K5376_1*X1278 - K1964_1*X170*X1271 - K2024_1*X170*X894 - K2054_1*X170*X1270 - K2114_1*X170*X1273 - K2140_1*X170*X1103 - K2612_1*X170*X666
171	X_dot(171) =K4634_1 + K1376_2*X2170 + K1648_2*X2306 + K3190_2*X3077 - K3857_1*X171 + K5377_1*X1123 - K1376_1*X171*X1123 - K1648_1*X171*X891 - K3190_1*X171*X773
172	X_dot(172) =K4635_1 + K1026_2*X1995 - K3858_1*X172 + K5378_1*X1040 - K1026_1*X172*X187
173	X_dot(173) =K4636_1 + K1074_2*X2019 + K1654_2*X2309 - K3859_1*X173 + K5379_1*X1053 - K1074_1*X173*X980 - K1654_1*X173*X891
174	X_dot(174) =K4637_1 + K1580_2*X2272 + K2300_2*X2632 - K3860_1*X174 + K5380_1*X1180 - K1580_1*X174*X810 - K2300_1*X174*X187
175	X_dot(175) =K4638_1 + K1574_2*X2269 + K2116_2*X2540 + K2146_2*X2555 + K2410_2*X2687 + K3243_1*X3103 - K3861_1*X175 + K3624_2*X3294 + K5381_1*X1179 - K1574_1*X175*X1290 - K2116_1*X175*X1273 - K2146_1*X175*X1103 - K2410_1*X175*X980 - K3624_1*X175*X395
176	X_dot(176) =K4639_1 + K654_2*X1809 + K698_2*X1831 - K3862_1*X176 + K5382_1*X957 - K654_1*X176*X187 - K698_1*X176*X1318
177	X_dot(177) =K4640_1 + K90_2*X1527 + K1464_2*X2214 + K1546_2*X2255 + K1762_2*X2363 + K2164_2*X2564 + K2482_2*X2723 + K2630_2*X2797 + K2788_2*X2876 + K2962_2*X2963 + K3020_2*X2992 + K3024_2*X2994 + K3445_1*X3204 - K3863_1*X177 + K5383_1*X811 - K90_1*X177*X810 - K1464_1*X177*X1341 - K1546_1*X177*X1418 - K1762_1*X177*X330 - K2164_1*X177*X1282 - K2482_1*X177*X1293 - K2630_1*X177*X1031 - K2788_1*X177*X915 - K2962_1*X177*X187 - K3024_1*X177*X1154 - K3020_1*X177*X1411
178	X_dot(178) =K4641_1 + K3470_2*X3217 + K3471_1*X3217 + K3482_2*X3223 + K3483_1*X3223 + K3542_2*X3253 + K3543_1*X3253 + K3544_2*X3254 + K3545_1*X3254 - K3864_1*X178 - K3470_1*X178*X1077 - K3542_1*X178*X799 - K3482_1*X178*X1455 - K3544_1*X178*X1346
179	X_dot(179) =K4642_1 + K1902_2*X2433 - K3865_1*X179 + K5384_1*X1264 - K1902_1*X179*X915
180	X_dot(180) =K4643_1 + K2642_2*X2803 - K3866_1*X180 + K5385_1*X1380 - K2642_1*X180*X810
181	X_dot(181) =K4644_1 + K100_2*X1532 + K106_2*X1535 + K224_2*X1594 + K438_2*X1701 + K452_2*X1708 + K532_2*X1748 + K546_2*X1755 + K582_2*X1773 + K670_2*X1817 + K982_2*X1973 - K3867_1*X181 + K5386_1*X816 - K100_1*X181*X810 - K106_1*X181*X1381 - K224_1*X181*X1393 - K438_1*X181*X1271 - K452_1*X181*X1272 - K546_1*X181*X1103 - K532_1*X181*X1273 - K582_1*X181*X925 - K670_1*X181*X187 - K982_1*X181*X1270
182	X_dot(182) =K4645_1 + K132_2*X1548 + K188_2*X1576 + K716_2*X1840 + K1262_2*X2113 + K1292_2*X2128 - K3868_1*X182 + K5387_1*X827 - K132_1*X182*X891 - K188_1*X182*X1000 - K716_1*X182*X1145 - K1292_1*X182*X205 - K1262_1*X182*X1195
183	X_dot(183) =K3665_1*X3326 - K3869_1*X183
184	X_dot(184) =K4646_1 + K2238_2*X2601 + K2360_2*X2662 + K3056_2*X3010 - K3870_1*X184 + K5388_1*X1318 - K2238_1*X184*X1036 - K2360_1*X184*X1318 - K3056_1*X184*X858
185	X_dot(185) =K4647_1 + K448_2*X1706 + K514_2*X1739 + K2668_2*X2816 + K3230_2*X3097 - K3871_1*X185 + K5389_1*X920 - K448_1*X185*X1271 - K514_1*X185*X1270 - K2668_1*X185*X1000 - K3230_1*X185*X776
186	X_dot(186) =K4648_1 + K1990_2*X2477 + K2076_2*X2520 + K2126_2*X2545 - K3872_1*X186 + K5390_1*X1281 - K1990_1*X186*X1271 - K2076_1*X186*X1270 - K2126_1*X186*X1273
187	X_dot(187) =K4649_1 + K636_2*X1800 + K637_1*X1800 + K638_2*X1801 + K639_1*X1801 + K640_2*X1802 + K641_1*X1802 + K642_2*X1803 + K643_1*X1803 + K644_2*X1804 + K645_1*X1804 + K646_2*X1805 + K647_1*X1805 + K648_2*X1806 + K649_1*X1806 + K650_2*X1807 + K651_1*X1807 + K652_2*X1808 + K653_1*X1808 + K654_2*X1809 + K655_1*X1809 + K656_2*X1810 + K657_1*X1810 + K658_2*X1811 + K659_1*X1811 + K660_2*X1812 + K661_1*X1812 + K662_2*X1813 + K663_1*X1813 + K664_2*X1814 + K665_1*X1814 + K666_2*X1815 + K667_1*X1815 + K668_2*X1816 + K669_1*X1816 + K670_2*X1817 + K671_1*X1817 + K1022_2*X1993 + K1023_1*X1993 + K1024_2*X1994 + K1025_1*X1994 + K1026_2*X1995 + K1027_1*X1995 + K1028_2*X1996 + K1029_1*X1996 + K1030_2*X1997 + K1031_1*X1997 + K1032_2*X1998 + K1033_1*X1998 + K1034_2*X1999 + K1035_1*X1999 + K1036_2*X2000 + K1037_1*X2000 + K1038_2*X2001 + K1039_1*X2001 + K1040_2*X2002 + K1041_1*X2002 + K1042_2*X2003 + K1043_1*X2003 + K1044_2*X2004 + K1045_1*X2004 + K1046_2*X2005 + K1047_1*X2005 + K1048_2*X2006 + K1049_1*X2006 + K1050_2*X2007 + K1051_1*X2007 + K1052_2*X2008 + K1053_1*X2008 + K1054_2*X2009 + K1055_1*X2009 + K1056_2*X2010 + K1057_1*X2010 + K1132_2*X2048 + K1133_1*X2048 + K1140_2*X2052 + K1141_1*X2052 + K1394_2*X2179 + K1395_1*X2179 + K1396_2*X2180 + K1397_1*X2180 + K1398_2*X2181 + K1399_1*X2181 + K1400_2*X2182 + K1401_1*X2182 + K1402_2*X2183 + K1403_1*X2183 + K1404_2*X2184 + K1405_1*X2184 + K1406_2*X2185 + K1407_1*X2185 + K2296_2*X2630 + K2297_1*X2630 + K2298_2*X2631 + K2299_1*X2631 + K2300_2*X2632 + K2301_1*X2632 + K2302_2*X2633 + K2303_1*X2633 + K2304_2*X2634 + K2305_1*X2634 + K2306_2*X2635 + K2307_1*X2635 + K2308_2*X2636 + K2309_1*X2636 + K2310_2*X2637 + K2311_1*X2637 + K2312_2*X2638 + K2313_1*X2638 + K2314_2*X2639 + K2315_1*X2639 + K2316_2*X2640 + K2317_1*X2640 + K2318_2*X2641 + K2319_1*X2641 + K2320_2*X2642 + K2321_1*X2642 + K2322_2*X2643 + K2323_1*X2643 + K2324_2*X2644 + K2325_1*X2644 + K2326_2*X2645 + K2327_1*X2645 + K2328_2*X2646 + K2329_1*X2646 + K2330_2*X2647 + K2331_1*X2647 + K2332_2*X2648 + K2333_1*X2648 + K2334_2*X2649 + K2335_1*X2649 + K2336_2*X2650 + K2337_1*X2650 + K2338_2*X2651 + K2339_1*X2651 + K2958_2*X2961 + K2959_1*X2961 + K2960_2*X2962 + K2961_1*X2962 + K2962_2*X2963 + K2963_1*X2963 + K2964_2*X2964 + K2965_1*X2964 + K2966_2*X2965 + K2967_1*X2965 + K2968_2*X2966 + K2969_1*X2966 + K2970_2*X2967 + K2971_1*X2967 + K2972_2*X2968 + K2973_1*X2968 + K2974_2*X2969 + K2975_1*X2969 + K2976_2*X2970 + K2977_1*X2970 + K2978_2*X2971 + K2979_1*X2971 + K2980_2*X2972 + K2981_1*X2972 + K2982_2*X2973 + K2983_1*X2973 + K2984_2*X2974 + K2985_1*X2974 + K3126_2*X3045 + K3127_1*X3045 + K3168_2*X3066 + K3169_1*X3066 - K3873_1*X187 - K648_1*X55*X187 - K644_1*X187*X188 - K642_1*X187*X243 - K654_1*X176*X187 - K638_1*X187*X362 - K650_1*X187*X342 - K646_1*X187*X384 - K636_1*X187*X486 - K670_1*X181*X187 - K668_1*X187*X206 - K662_1*X187*X277 - K640_1*X187*X630 - K658_1*X187*X461 - K664_1*X187*X448 - K656_1*X187*X544 - K652_1*X187*X672 - K666_1*X187*X550 - K660_1*X187*X649 - K1024_1*X168*X187 - K1026_1*X172*X187 - K1034_1*X95*X187 - K1036_1*X102*X187 - K1032_1*X161*X187 - K1022_1*X187*X284 - K1046_1*X48*X187 - K1030_1*X187*X343 - K1056_1*X129*X187 - K1028_1*X187*X537 - K1044_1*X187*X408 - K1038_1*X187*X492 - K1048_1*X187*X397 - K1042_1*X187*X497 - K1050_1*X187*X444 - K1054_1*X187*X457 - K1040_1*X187*X654 - K1052_1*X187*X636 - K1140_1*X87*X187 - K1132_1*X187*X281 - K1394_1*X39*X187 - K1398_1*X121*X187 - K1400_1*X149*X187 - K1402_1*X187*X326 - K1406_1*X187*X350 - K1396_1*X187*X688 - K1404_1*X187*X765 - K2306_1*X98*X187 - K2300_1*X174*X187 - K2308_1*X187*X213 - K2318_1*X187*X207 - K2332_1*X157*X187 - K2298_1*X187*X502 - K2310_1*X187*X435 - K2296_1*X187*X583 - K2314_1*X187*X441 - K2330_1*X187*X329 - K2302_1*X187*X610 - K2312_1*X187*X532 - K2326_1*X187*X396 - K2328_1*X187*X453 - K2322_1*X187*X516 - K2304_1*X187*X702 - K2320_1*X187*X545 - K2324_1*X187*X541 - K2338_1*X187*X414 - K2336_1*X187*X437 - K2334_1*X187*X477 - K2316_1*X187*X710 - K2970_1*X9*X187 - K2962_1*X177*X187 - K2960_1*X187*X199 - K2978_1*X57*X187 - K2982_1*X76*X187 - K2966_1*X187*X275 - K2964_1*X187*X298 - K2958_1*X187*X376 - K2974_1*X187*X232 - K2972_1*X187*X393 - K2980_1*X187*X315 - K2984_1*X187*X367 - K2976_1*X187*X476 - K2968_1*X187*X635 - K3126_1*X187*X579 - K3168_1*X187*X771
188	X_dot(188) =K4650_1 + K644_2*X1804 + K1968_2*X2466 + K2060_2*X2512 - K3874_1*X188 + K5391_1*X955 - K644_1*X187*X188 - K1968_1*X188*X1271 - K2060_1*X188*X1270

189	X_dot(189) =K4651_1 + K1682_2*X2323 + K1900_2*X2432 + K1972_2*X2468 + K2062_2*X2513 + K2644_2*X2804 + K3232_2*X3098 - K3875_1*X189 + K5392_1*X1212 - K1682_1*X189*X1000 - K1900_1*X189*X915 - K1972_1*X189*X1271 - K2062_1*X189*X1270 - K2644_1*X189*X810 - K3232_1*X189*X776
190	X_dot(190) =K4652_1 + K1202_2*X2083 + K1216_2*X2090 + K3338_2*X3151 + K3339_1*X3151 + K3340_2*X3152 + K3341_1*X3152 + K3342_2*X3153 + K3343_1*X3153 + K3344_2*X3154 + K3345_1*X3154 + K3346_2*X3155 + K3347_1*X3155 + K3348_2*X3156 + K3349_1*X3156 + K3350_2*X3157 + K3351_1*X3157 + K3462_2*X3213 + K3463_1*X3213 + K3464_2*X3214 + K3465_1*X3214 - K3876_1*X190 + K5393_1*X1083 - K1202_1*X190*X891 - K1216_1*X190*X1000 - K3338_1*X190*X823 - K3344_1*X190*X861 - K3342_1*X190*X970 - K3340_1*X190*X1023 - K3346_1*X190*X1182 - K3348_1*X190*X1217 - K3350_1*X190*X1448 - K3464_1*X190*X931 - K3462_1*X190*X1052
191	X_dot(191) =K4653_1 + K684_2*X1824 - K3877_1*X191 + K5394_1*X967 - K684_1*X191*X1318
192	X_dot(192) =K4654_1 + K1060_2*X2012 - K3878_1*X192 + K5395_1*X1050 - K1060_1*X192*X1318
193	X_dot(193) =K4655_1 + K1240_2*X2102 - K3879_1*X193 + K5396_1*X1093 - K1240_1*X193*X330
194	X_dot(194) =K4656_1 + K842_2*X1903 + K854_2*X1909 + K1200_2*X2082 + K1276_2*X2120 + K1298_2*X2131 + K1868_2*X2416 + K3453_1*X3208 - K3880_1*X194 + K5397_1*X1007 - K842_1*X194*X1031 - K854_1*X194*X1418 - K1200_1*X194*X891 - K1298_1*X194*X205 - K1276_1*X194*X1195 - K1868_1*X194*X823
195	X_dot(195) =K4657_1 + K2222_2*X2593 - K3881_1*X195 + K5398_1*X1314 - K2222_1*X195*X1315
196	X_dot(196) =K4658_1 + K1108_2*X2036 + K1444_2*X2204 - K3882_1*X196 + K5399_1*X1058 - K1108_1*X196*X861 - K1444_1*X196*X1318
197	X_dot(197) =K4659_1 + K3004_2*X2984 + K3042_2*X3003 + K3469_1*X3216 - K3883_1*X197 + K5400_1*X1428 - K3004_1*X197*X1145 - K3042_1*X197*X861
198	X_dot(198) =K4660_1 + K580_2*X1772 + K596_2*X1780 - K3884_1*X198 + K5401_1*X941 - K580_1*X198*X925 - K596_1*X198*X1316
199	X_dot(199) =K4661_1 + K290_2*X1627 + K2480_2*X2722 + K2650_2*X2807 + K2960_2*X2962 + K3505_1*X3234 - K3885_1*X199 + K5402_1*X870 - K290_1*X199*X1195 - K2480_1*X199*X1293 - K2650_1*X199*X522 - K2960_1*X187*X199
200	X_dot(200) =K4662_1 + K2654_2*X2809 + K2662_2*X2813 + K2736_2*X2850 - K3886_1*X200 + K5403_1*X1382 - K2654_1*X200*X891 - K2662_1*X200*X1000 - K2736_1*X200*X1195
201	X_dot(201) =K4663_1 + K1752_2*X2358 + K1836_2*X2400 + K2762_2*X2863 - K3887_1*X201 + K5404_1*X1222 - K1752_1*X201*X1126 - K1836_1*X201*X1195 - K2762_1*X201*X205
202	X_dot(202) =K4664_1 - K3888_1*X202 + K3590_2*X3277 + K3591_1*X3277 - K3590_1*X202*X344
203	X_dot(203) =K4665_1 + K1876_2*X2420 + K1980_2*X2472 + K2468_2*X2716 - K3889_1*X203 + K5405_1*X1260 - K1876_1*X203*X1260 - K1980_1*X203*X1271 - K2468_1*X203*X1154
204	X_dot(204) =K4666_1 + K2010_2*X2487 + K2042_2*X2503 + K2462_2*X2713 + K3134_2*X3049 - K3890_1*X204 + K5406_1*X1286 - K2042_1*X204*X894 - K2010_1*X204*X1271 - K2462_1*X204*X1411 - K3134_1*X204*X1154
205	X_dot(205) =K4667_1 + K328_2*X1646 + K329_1*X1646 + K916_2*X1940 + K917_1*X1940 + K918_2*X1941 + K919_1*X1941 + K920_2*X1942 + K921_1*X1942 + K922_2*X1943 + K923_1*X1943 + K1284_2*X2124 + K1285_1*X2124 + K1286_2*X2125 + K1287_1*X2125 + K1288_2*X2126 + K1289_1*X2126 + K1290_2*X2127 + K1291_1*X2127 + K1292_2*X2128 + K1293_1*X2128 + K1294_2*X2129 + K1295_1*X2129 + K1296_2*X2130 + K1297_1*X2130 + K1298_2*X2131 + K1299_1*X2131 + K1300_2*X2132 + K1301_1*X2132 + K1840_2*X2402 + K1841_1*X2402 + K2744_2*X2854 + K2745_1*X2854 + K2746_2*X2855 + K2747_1*X2855 + K2748_2*X2856 + K2749_1*X2856 + K2750_2*X2857 + K2751_1*X2857 + K2752_2*X2858 + K2753_1*X2858 + K2754_2*X2859 + K2755_1*X2859 + K2756_2*X2860 + K2757_1*X2860 + K2758_2*X2861 + K2759_1*X2861 + K2760_2*X2862 + K2761_1*X2862 + K2762_2*X2863 + K2763_1*X2863 - K3891_1*X205 - K328_1*X205*X445 - K922_1*X205*X390 - K918_1*X205*X517 - K920_1*X205*X624 - K916_1*X205*X759 - K1286_1*X26*X205 - K1296_1*X52*X205 - K1288_1*X133*X205 - K1294_1*X122*X205 - K1292_1*X182*X205 - K1290_1*X205*X227 - K1298_1*X194*X205 - K1284_1*X205*X496 - K1300_1*X205*X744 - K1840_1*X205*X441 - K2762_1*X201*X205 - K2744_1*X205*X386 - K2756_1*X205*X297 - K2750_1*X205*X364 - K2748_1*X205*X498 - K2746_1*X205*X576 - K2752_1*X205*X520 - K2758_1*X205*X492 - K2754_1*X205*X627 - K2760_1*X205*X595
206	X_dot(206) =K4668_1 + K394_2*X1679 + K430_2*X1697 + K526_2*X1745 + K542_2*X1753 + K668_2*X1816 + K2098_2*X2531 - K3892_1*X206 + K5407_1*X910 - K394_1*X206*X787 - K430_1*X206*X1271 - K542_1*X206*X1103 - K526_1*X206*X1273 - K668_1*X187*X206 - K2098_1*X206*X1270
207	X_dot(207) =K4669_1 + K242_2*X1603 + K1116_2*X2040 + K2318_2*X2641 + K3086_2*X3025 - K3893_1*X207 + K3618_2*X3291 + K5408_1*X857 - K242_1*X207*X780 - K1116_1*X207*X1159 - K2318_1*X187*X207 - K3086_1*X207*X915 - K3618_1*X207*X410
208	X_dot(208) =K4670_1 + K1798_2*X2381 - K3894_1*X208 + K5409_1*X1234 - K1798_1*X208*X1195
209	X_dot(209) =K4671_1 + K1678_2*X2321 - K3895_1*X209 + K5410_1*X1211 - K1678_1*X209*X1000
210	X_dot(210) =K4672_1 + K800_2*X1882 + K1322_2*X2143 - K3896_1*X210 + K5411_1*X994 - K800_1*X210*X861 - K1322_1*X210*X823
211	X_dot(211) =K4673_1 + K1672_2*X2318 + K2680_2*X2822 - K3897_1*X211 + K5412_1*X1209 - K1672_1*X211*X743 - K2680_1*X211*X1087
212	X_dot(212) =K3659_1*X3320 - K3898_1*X212
213	X_dot(213) =K4674_1 + K1182_2*X2073 + K2202_2*X2583 + K2308_2*X2636 + K2452_2*X2708 + K3136_2*X3050 + K3299_1*X3131 + K3471_1*X3217 - K3899_1*X213 + K5413_1*X1077 - K1182_1*X213*X522 - K2202_1*X213*X925 - K2308_1*X187*X213 - K2452_1*X213*X1411 - K3136_1*X213*X1154
214	X_dot(214) =K4675_1 + K672_2*X1818 - K3900_1*X214 + K5414_1*X962 - K672_1*X214*X1318
215	X_dot(215) =K4676_1 + K1328_2*X2146 + K1329_1*X2146 + K1886_2*X2425 + K1887_1*X2425 - K3901_1*X215 - K1328_1*X215*X390 - K1886_1*X215*X411
216	X_dot(216) =K4677_1 - K3902_1*X216
217	X_dot(217) =K4678_1 + K1854_2*X2409 + K2674_2*X2819 - K3903_1*X217 + K5415_1*X1252 - K1854_1*X217*X823 - K2674_1*X217*X1393
218	X_dot(218) =K4679_1 + K2602_2*X2783 + K2603_1*X2783 - K3904_1*X218 - K2602_1*X218*X424
219	X_dot(219) =K4680_1 + K1666_2*X2315 - K3905_1*X219 + K5416_1*X1206 - K1666_1*X219*X891
220	X_dot(220) =K4681_1 + K2922_2*X2943 - K3906_1*X220 + K5417_1*X1417 - K2922_1*X220*X1417
221	X_dot(221) =K4682_1 + K1112_2*X2038 + K3576_2*X3270 + K3577_1*X3270 + K3596_2*X3280 + K3597_1*X3280 + K3598_2*X3281 + K3599_1*X3281 + K3600_2*X3282 - K3907_1*X221 + K3601_1*X3282 + K5418_1*X1060 - K1112_1*X221*X1318 - K3576_1*X221*X245 - K3596_1*X86*X221 - K3598_1*X98*X221 - K3600_1*X128*X221
222	X_dot(222) =K4683_1 + K364_2*X1664 + K382_2*X1673 - K3908_1*X222 + K5419_1*X897 - K364_1*X222*X783 - K382_1*X222*X915
223	X_dot(223) =K4684_1 + K1346_2*X2155 + K1360_2*X2162 + K1656_2*X2310 + K2390_2*X2677 + K3283_1*X3123 - K3909_1*X223 + K5420_1*X1119 - K1346_1*X223*X1271 - K1360_1*X223*X1270 - K1656_1*X223*X891 - K2390_1*X223*X1145
224	X_dot(224) =K4685_1 + K2664_2*X2814 - K3910_1*X224 + K5421_1*X1385 - K2664_1*X224*X1000
225	X_dot(225) =K4686_1 + K388_2*X1676 + K676_2*X1820 + K786_2*X1875 + K3383_1*X3173 + K3563_1*X3263 - K3911_1*X225 + K5422_1*X907 - K388_1*X225*X1310 - K676_1*X225*X1318 - K786_1*X225*X861
226	X_dot(226) =K4687_1 + K1522_2*X2243 + K2450_2*X2707 + K3138_2*X3051 - K3912_1*X226 + K5423_1*X1162 - K1522_1*X226*X1031 - K2450_1*X226*X1411 - K3138_1*X226*X1154
227	X_dot(227) =K4688_1 + K1254_2*X2109 + K1290_2*X2127 - K3913_1*X227 + K5424_1*X1098 - K1290_1*X205*X227 - K1254_1*X227*X1195
228	X_dot(228) =K4689_1 + K3366_2*X3165 + K3367_1*X3165 + K3368_2*X3166 + K3369_1*X3166 + K3370_2*X3167 + K3371_1*X3167 + K3372_2*X3168 + K3373_1*X3168 - K3914_1*X228 - K3372_1*X228*X861 - K3366_1*X228*X1031 - K3370_1*X228*X1061 - K3368_1*X228*X1418
229	X_dot(229) =K4690_1 + K702_2*X1833 + K1598_2*X2281 + K2186_2*X2575 + K2436_2*X2700 - K3915_1*X229 + K5425_1*X971 - K702_1*X229*X1318 - K1598_1*X229*X589 - K2186_1*X229*X1010 - K2436_1*X229*X512

230	X_dot(230) =K4691_1 + K234_2*X1599 + K426_2*X1695 + K498_2*X1731 + K1578_2*X2271 + K1674_2*X2319 + K1732_2*X2348 + K3561_1*X3262 + K3565_1*X3264 - K3916_1*X230 + K5426_1*X855 - K234_1*X230*X1087 - K426_1*X230*X1271 - K498_1*X230*X1270 - K1578_1*X60*X230 - K1674_1*X230*X713 - K1732_1*X230*X1451
231	X_dot(231) =K4692_1 + K814_2*X1889 + K1512_2*X2238 + K1680_2*X2322 + K1970_2*X2467 + K2682_2*X2823 + K3108_2*X3036 + K3509_1*X3236 - K3917_1*X231 + K5427_1*X1000 - K814_1*X231*X846 - K1512_1*X231*X1031 - K1680_1*X231*X1000 - K1970_1*X231*X1271 - K2682_1*X231*X1087 - K3108_1*X231*X1270
232	X_dot(232) =K4693_1 + K186_2*X1575 + K482_2*X1723 + K2974_2*X2969 + K2988_2*X2976 + K3000_2*X2982 - K3918_1*X232 + K5428_1*X849 - K186_1*X232*X1000 - K482_1*X232*X1270 - K2974_1*X187*X232 - K3000_1*X232*X1145 - K2988_1*X232*X1318
233	X_dot(233) =K4694_1 + K1308_2*X2136 + K1696_2*X2330 - K3919_1*X233 + K5429_1*X1109 - K1308_1*X233*X1028 - K1696_1*X233*X1000
234	X_dot(234) =K3680_1*X3341 - K3920_1*X234
235	X_dot(235) =K4695_1 + K1572_2*X2268 + K1838_2*X2401 + K2284_2*X2624 - K3921_1*X235 + K5430_1*X1178 - K1572_1*X235*X1173 - K1838_1*X235*X1195 - K2284_1*X235*X1276
236	X_dot(236) =K4696_1 + K1274_2*X2119 - K3922_1*X236 + K5431_1*X1104 - K1274_1*X236*X1195
237	X_dot(237) =K4697_1 + K806_2*X1885 + K3018_2*X2991 - K3923_1*X237 + K5432_1*X997 - K806_1*X74*X237 - K3018_1*X237*X512
238	X_dot(238) =K4698_1 + K1520_2*X2242 + K1626_2*X2295 + K1690_2*X2327 + K1712_2*X2338 - K3924_1*X238 + K5433_1*X1161 - K1520_1*X238*X1031 - K1626_1*X238*X891 - K1690_1*X238*X1000 - K1712_1*X238*X1393
239	X_dot(239) =K4699_1 + K888_2*X1926 + K1170_2*X2067 - K3925_1*X239 + K5434_1*X1019 - K888_1*X239*X330 - K1170_1*X239*X1446
240	X_dot(240) =K4700_1 + K1928_2*X2446 + K1932_2*X2448 + K2886_2*X2925 + K3094_2*X3029 + K3259_1*X3111 + K3277_1*X3120 + K3381_1*X3172 - K3926_1*X240 + K5435_1*X1270 - K1928_1*X240*X831 - K1932_1*X240*X1172 - K2886_1*X240*X1270 - K3094_1*X240*X1348
241	X_dot(241) =K4701_1 + K1434_2*X2199 - K3927_1*X241 + K5436_1*X1140 - K1434_1*X241*X1318
242	X_dot(242) =K4702_1 + K2712_2*X2838 - K3928_1*X242 + K5437_1*X1394 - K2712_1*X242*X1195
243	X_dot(243) =K4703_1 + K642_2*X1803 + K1966_2*X2465 + K2058_2*X2511 + K2640_2*X2802 - K3929_1*X243 + K5438_1*X954 - K642_1*X187*X243 - K1966_1*X243*X1271 - K2058_1*X243*X1270 - K2640_1*X243*X954
244	X_dot(244) =K4704_1 + K144_2*X1554 - K3930_1*X244 + K5439_1*X832 - K144_1*X244*X891
245	X_dot(245) =K4705_1 + K3521_1*X3242 + K3576_2*X3270 - K3931_1*X245 + K5440_1*X1457 - K3576_1*X221*X245
246	X_dot(246) =K4706_1 + K1930_2*X2447 + K2818_2*X2891 + K2864_2*X2914 + K3257_1*X3110 + K3275_1*X3119 + K3379_1*X3171 + K3429_1*X3196 - K3932_1*X246 + K5441_1*X1271 - K1930_1*X246*X1172 - K2818_1*X246*X831 - K2864_1*X246*X1271
247	X_dot(247) =K4707_1 + K1114_2*X2039 + K1882_2*X2423 + K3371_1*X3167 - K3933_1*X247 + K5442_1*X1061 - K1114_1*X247*X861 - K1882_1*X247*X1061
248	X_dot(248) =K4708_1 + K710_2*X1837 + K2536_2*X2750 - K3934_1*X248 + K5443_1*X974 - K710_1*X248*X1318 - K2536_1*X248*X861
249	X_dot(249) =K4709_1 + K1068_2*X2016 + K1456_2*X2210 + K2580_2*X2772 + K3044_2*X3004 + K3337_1*X3150 + K3463_1*X3213 - K3935_1*X249 + K5444_1*X1052 - K1068_1*X249*X1318 - K1456_1*X249*X1145 - K2580_1*X249*X858 - K3044_1*X249*X861
250	X_dot(250) =K4710_1 + K154_2*X1559 + K310_2*X1637 + K1230_2*X2097 + K2004_2*X2484 + K2090_2*X2527 - K3936_1*X250 + K5445_1*X837 - K154_1*X250*X891 - K310_1*X250*X1195 - K1230_1*X250*X1126 - K2004_1*X250*X1271 - K2090_1*X250*X1270
251	X_dot(251) =K4711_1 + K1194_2*X2079 + K1256_2*X2110 - K3937_1*X251 + K5446_1*X1080 - K1194_1*X251*X891 - K1256_1*X251*X1195
252	X_dot(252) =K4712_1 + K1012_2*X1988 + K1066_2*X2015 + K1452_2*X2208 - K3938_1*X252 + K5447_1*X1037 - K1012_1*X252*X1316 - K1066_1*X252*X1318 - K1452_1*X252*X1145
253	X_dot(253) =K4713_1 + K2006_2*X2485 - K3939_1*X253 + K5448_1*X1285 - K2006_1*X253*X1271
254	X_dot(254) =K4714_1 + K1218_2*X2091 - K3940_1*X254 + K5449_1*X1088 - K1218_1*X254*X1000
255	X_dot(255) =K4715_1 + K2386_2*X2675 - K3941_1*X255 + K5450_1*X1349 - K2386_1*X255*X1145
256	X_dot(256) =K4716_1 + K1616_2*X2290 + K1786_2*X2375 - K3942_1*X256 + K5451_1*X1193 - K1616_1*X256*X891 - K1786_1*X256*X1195
257	X_dot(257) =K4717_1 + K176_2*X1570 + K206_2*X1585 + K584_2*X1774 + K610_2*X1787 + K1748_2*X2356 - K3943_1*X257 + K5452_1*X846 - K176_1*X257*X891 - K206_1*X257*X1000 - K584_1*X257*X925 - K610_1*X257*X1276 - K1748_1*X257*X780
258	X_dot(258) =K4718_1 + K2730_2*X2847 - K3944_1*X258 + K5453_1*X1400 - K2730_1*X258*X1195
259	X_dot(259) =K4719_1 + K678_2*X1821 + K1790_2*X2377 + K2260_2*X2612 - K3945_1*X259 + K5454_1*X964 - K678_1*X259*X1318 - K1790_1*X259*X1195 - K2260_1*X259*X1276
260	X_dot(260) =K4720_1 + K1392_2*X2178 - K3946_1*X260 + K5455_1*X1127 - K1392_1*X260*X1276
261	X_dot(261) =K4721_1 + K2670_2*X2817 - K3947_1*X261 + K5456_1*X1386 - K2670_1*X261*X1393
262	X_dot(262) =K4722_1 + K162_2*X1563 - K3948_1*X262 + K5457_1*X840 - K162_1*X262*X891
263	X_dot(263) =K4723_1 + K724_2*X1844 + K2996_2*X2980 - K3949_1*X263 + K5458_1*X976 - K724_1*X263*X1145 - K2996_1*X263*X1318
264	X_dot(264) =K4724_1 + K932_2*X1948 + K1760_2*X2362 + K1778_2*X2371 + K1920_2*X2442 + K2230_2*X2597 + K2244_2*X2604 + K2594_2*X2779 + K2892_2*X2928 + K2914_2*X2939 + K3008_2*X2986 + K3028_2*X2996 + K3234_2*X3099 + K3255_1*X3109 + K3261_1*X3112 + K3263_1*X3113 + K3291_1*X3127 + K3303_1*X3133 + K3305_1*X3134 + K3365_1*X3164 + K3367_1*X3165 + K3401_1*X3182 - K3950_1*X264 + K5459_1*X1031 - K932_1*X264*X783 - K1760_1*X264*X587 - K1778_1*X264*X1195 - K1920_1*X264*X1310 - K2244_1*X264*X591 - K2230_1*X264*X1036 - K2594_1*X264*X727 - K2914_1*X264*X1005 - K2892_1*X264*X1273 - K3008_1*X264*X980 - K3028_1*X264*X861 - K3234_1*X264*X776
265	X_dot(265) =K4725_1 + K2928_2*X2946 + K3293_1*X3128 + K3369_1*X3166 + K3409_1*X3186 - K3951_1*X265 + K5460_1*X1418 - K2928_1*X265*X1036
266	X_dot(266) =K4726_1 + K534_2*X1749 + K728_2*X1846 - K3952_1*X266 + K5461_1*X932 - K534_1*X266*X1273 - K728_1*X266*X1145
267	X_dot(267) =K4727_1 + K528_2*X1746 + K544_2*X1754 - K3953_1*X267 + K5462_1*X930 - K544_1*X267*X1103 - K528_1*X267*X1273
268	X_dot(268) =K4728_1 + K3413_1*X3188 + K3427_1*X3195 - K3954_1*X268 + K5463_1*X1453
269	X_dot(269) =K4729_1 + K1776_2*X2370 - K3955_1*X269 + K5464_1*X1227 - K1776_1*X269*X1195
270	X_dot(270) =K4730_1 + K2472_2*X2718 + K2576_2*X2770 - K3956_1*X270 + K5465_1*X1358 - K2472_1*X270*X1154 - K2576_1*X270*X793
271	X_dot(271) =K4731_1 + K808_2*X1886 + K1232_2*X2098 - K3957_1*X271 + K5466_1*X998 - K808_1*X271*X998 - K1232_1*X271*X1126
272	X_dot(272) =K4732_1 + K870_2*X1917 + K878_2*X1921 + K1602_2*X2283 + K3236_2*X3100 - K3958_1*X272 + K5467_1*X1013 - K870_1*X272*X891 - K878_1*X272*X1000 - K1602_1*X272*X1194 - K3236_1*X272*X776
273	X_dot(273) =K4733_1 + K1604_2*X2284 - K3959_1*X273 + K5468_1*X1187 - K1604_1*X273*X1194
274	X_dot(274) =K4734_1 + K298_2*X1631 + K3321_1*X3142 - K3960_1*X274 + K5469_1*X874 - K298_1*X274*X1195
275	X_dot(275) =K4735_1 + K102_2*X1533 + K256_2*X1610 + K274_2*X1619 + K276_2*X1620 + K280_2*X1622 + K936_2*X1950 + K1100_2*X2032 + K1390_2*X2177 + K1518_2*X2241 + K1548_2*X2256 + K2144_2*X2554 + K2196_2*X2580 + K2220_2*X2592 + K2720_2*X2842 + K2766_2*X2865 + K2966_2*X2965 + K3122_2*X3043 + K3495_1*X3229 + K3604_2*X3284 - K3961_1*X275 + K5470_1*X817 - K102_1*X275*X1381 - K256_1*X275*X330 - K276_1*X275*X331 - K280_1*X275*X337 - K274_1*X275*X1330 - K936_1*X275*X915 - K1100_1*X275*X861 - K1390_1*X275*X1276 - K1518_1*X275*X1031 - K1548_1*X275*X1418 - K2144_1*X275*X1103 - K2196_1*X275*X925 - K2220_1*X275*X1315 - K2720_1*X275*X1195 - K2766_1*X275*X1107 - K2966_1*X187*X275 - K3122_1*X275*X1273 - K3604_1*X275*X525
276	X_dot(276) =K2628_2*X2796 + K2634_2*X2799 + K3656_1*X3317 - K3962_1*X276 + K5471_1*X1379 - K2628_1*X276*X1031 - K2634_1*X276*X1418
277	X_dot(277) =K4736_1 + K578_2*X1771 + K588_2*X1776 + K662_2*X1813 + K3491_1*X3227 - K3963_1*X277 + K5472_1*X940 - K578_1*X277*X925 - K662_1*X187*X277 - K588_1*X277*X1313
278	X_dot(278) =K4737_1 + K1476_2*X2220 - K3964_1*X278 + K5473_1*X1148 - K1476_1*X278*X512
279	X_dot(279) =K4738_1 + K386_2*X1675 + K387_1*X1675 - K3965_1*X279 + K5474_1*X906 - K386_1*X279^2

280	X_dot(280) =K4739_1 + K1188_2*X2076 - K3966_1*X280 + K5475_1*X1078 - K1188_1*X280*X522
281	X_dot(281) =K4740_1 + K392_2*X1678 + K418_2*X1691 + K524_2*X1744 + K538_2*X1751 + K604_2*X1784 + K720_2*X1842 + K954_2*X1959 + K980_2*X1972 + K996_2*X1980 + K1132_2*X2048 + K1138_2*X2051 + K1268_2*X2116 + K1304_2*X2134 + K1912_2*X2438 + K2350_2*X2657 + K3140_2*X3052 + K3309_1*X3136 + K3325_1*X3144 - K3967_1*X281 + K5476_1*X909 - K392_1*X281*X831 - K418_1*X281*X1271 - K538_1*X281*X1103 - K524_1*X281*X1273 - K604_1*X281*X1276 - K720_1*X281*X1145 - K954_1*X281*X1408 - K996_1*X281*X1005 - K980_1*X281*X1270 - K1132_1*X187*X281 - K1138_1*X281*X1411 - K1268_1*X281*X1195 - K1304_1*X281*X1107 - K1912_1*X281*X1267 - K2350_1*X281*X1318 - K3140_1*X281*X1154
282	X_dot(282) =K4741_1 + K1812_2*X2388 - K3968_1*X282 + K5477_1*X1240 - K1812_1*X282*X1195
283	X_dot(283) =K3674_1*X3335 - K3969_1*X283
284	X_dot(284) =K4742_1 + K1022_2*X1993 + K2340_2*X2652 + K2428_2*X2696 - K3970_1*X284 + K5478_1*X1039 - K1022_1*X187*X284 - K2340_1*X284*X1318 - K2428_1*X284*X512
285	X_dot(285) =K4743_1 + K1796_2*X2380 - K3971_1*X285 + K5479_1*X1233 - K1796_1*X285*X1195
286	X_dot(286) =K4744_1 + K3246_2*X3105 + K3247_1*X3105 + K3248_2*X3106 + K3249_1*X3106 + K3250_2*X3107 + K3251_1*X3107 + K3252_2*X3108 + K3253_1*X3108 + K3384_2*X3174 + K3385_1*X3174 + K3386_2*X3175 + K3387_1*X3175 + K3388_2*X3176 + K3389_1*X3176 + K3520_2*X3242 + K3521_1*X3242 - K3972_1*X286 - K3250_1*X286*X815 - K3248_1*X286*X856 - K3252_1*X286*X851 - K3246_1*X286*X1224 - K3386_1*X286*X894 - K3388_1*X286*X925 - K3384_1*X286*X1451 - K3520_1*X286*X1457
287	X_dot(287) =K4745_1 + K1558_2*X2261 + K1576_2*X2270 + K1954_2*X2459 + K2232_2*X2598 + K3269_1*X3116 - K3973_1*X287 + K5480_1*X1172 - K1576_1*X168*X287 - K1558_1*X287*X1307 - K1954_1*X287*X1004 - K2232_1*X287*X1036
288	X_dot(288) =K4746_1 + K2210_2*X2587 + K2252_2*X2608 - K3974_1*X288 + K5481_1*X1311 - K2210_1*X288*X925 - K2252_1*X288*X1316
289	X_dot(289) =K4747_1 + K3400_2*X3182 + K3401_1*X3182 + K3402_2*X3183 + K3403_1*X3183 + K3404_2*X3184 + K3405_1*X3184 + K3406_2*X3185 + K3407_1*X3185 + K3478_2*X3221 + K3479_1*X3221 + K3492_2*X3228 + K3493_1*X3228 + K3526_2*X3245 + K3527_1*X3245 - K3975_1*X289 - K3406_1*X289*X807 - K3400_1*X289*X1031 - K3402_1*X289*X1224 - K3404_1*X289*X1452 - K3478_1*X289*X1035 - K3526_1*X289*X872 - K3492_1*X289*X1451
290	X_dot(290) =K4748_1 + K1110_2*X2037 + K2546_2*X2755 + K3594_2*X3279 - K3976_1*X290 + K5482_1*X1059 - K1110_1*X290*X1059 - K2546_1*X290*X861 - K3594_1*X290*X699
291	X_dot(291) =K4749_1 + K1686_2*X2325 - K3977_1*X291 + K5483_1*X1213 - K1686_1*X291*X1000
292	X_dot(292) =K4750_1 + K2608_2*X2786 - K3978_1*X292 + K5484_1*X1375 - K2608_1*X292*X998
293	X_dot(293) =K4751_1 + K868_2*X1916 - K3979_1*X293 + K5485_1*X1012 - K868_1*X293*X891
294	X_dot(294) =K4752_1 + K2380_2*X2672 + K2802_2*X2883 - K3980_1*X294 + K5486_1*X1348 - K2380_1*X294*X1145 - K2802_1*X294*X1348
295	X_dot(295) =K4753_1 + K1722_2*X2343 + K1926_2*X2445 + K2044_2*X2504 + K2106_2*X2535 + K2154_2*X2559 + K2182_2*X2573 + K2246_2*X2605 + K2394_2*X2679 + K2780_2*X2872 + K2902_2*X2933 + K3050_2*X3007 + K3317_1*X3140 + K3349_1*X3156 + K3359_1*X3161 + K3393_1*X3178 - K3981_1*X295 + K5487_1*X1217 - K1722_1*X295*X1393 - K1926_1*X295*X1003 - K2044_1*X295*X894 - K2106_1*X295*X1270 - K2246_1*X163*X295 - K2154_1*X295*X1103 - K2182_1*X295*X1005 - K2394_1*X295*X1145 - K2780_1*X295*X823 - K2902_1*X295*X1273 - K3050_1*X295*X861
296	X_dot(296) =K4754_1 + K480_2*X1722 - K3982_1*X296 + K5488_1*X923 - K480_1*X296*X1270
297	X_dot(297) =K4755_1 + K1224_2*X2094 + K1820_2*X2392 + K2756_2*X2860 + K2792_2*X2878 - K3983_1*X297 + K5489_1*X1091 - K1224_1*X297*X1393 - K1820_1*X297*X1195 - K2756_1*X205*X297 - K2792_1*X297*X915
298	X_dot(298) =K4756_1 + K2702_2*X2833 + K2964_2*X2964 - K3984_1*X298 + K5490_1*X1391 - K2702_1*X298*X1391 - K2964_1*X187*X298
299	X_dot(299) =K4757_1 + K836_2*X1900 - K3985_1*X299 + K5491_1*X1004 - K836_1*X299*X1031
300	X_dot(300) =K3668_1*X3329 - K3986_1*X300
301	X_dot(301) =K4758_1 + K2532_2*X2748 - K3987_1*X301 + K5492_1*X1366 - K2532_1*X301*X861
302	X_dot(302) =K4759_1 + K1590_2*X2277 + K1870_2*X2417 + K2392_2*X2678 + K2652_2*X2808 + K2880_2*X2922 + K2900_2*X2932 + K3048_2*X3006 + K3347_1*X3155 + K3459_1*X3211 - K3988_1*X302 + K5493_1*X1182 - K1590_1*X302*X1381 - K1870_1*X302*X823 - K2392_1*X302*X1145 - K2652_1*X302*X522 - K2880_1*X302*X894 - K2900_1*X302*X1273 - K3048_1*X302*X861
303	X_dot(303) =K4760_1 + K530_2*X1747 + K2600_2*X2782 + K3465_1*X3214 - K3989_1*X303 + K5494_1*X931 - K530_1*X303*X1273 - K2600_1*X303*X727
304	X_dot(304) =K4761_1 + K838_2*X1901 + K2916_2*X2940 - K3990_1*X304 + K5495_1*X1005 - K838_1*X304*X1031 - K2916_1*X304*X1005
305	X_dot(305) =K4762_1 + K1852_2*X2408 + K2032_2*X2498 - K3991_1*X305 + K5496_1*X1251 - K1852_1*X305*X823 - K2032_1*X305*X894
306	X_dot(306) =K4763_1 + K454_2*X1709 + K3612_2*X3288 - K3992_1*X306 + K5497_1*X921 - K454_1*X306*X894 - K3612_1*X306*X1171
307	X_dot(307) =K4764_1 + K294_2*X1629 + K2406_2*X2685 + K3523_1*X3243 + K3525_1*X3244 + K3527_1*X3245 - K3993_1*X307 + K5498_1*X872 - K294_1*X307*X1195 - K2406_1*X307*X980
308	X_dot(308) =K4765_1 + K1156_2*X2060 + K1716_2*X2340 - K3994_1*X308 + K5499_1*X1070 - K1156_1*X308*X1031 - K1716_1*X308*X1393
309	X_dot(309) =K4766_1 + K3362_2*X3163 + K3363_1*X3163 - K3995_1*X309 - K3362_1*X309*X1450
310	X_dot(310) =K4767_1 + K244_2*X1604 + K3060_2*X3012 + K3361_1*X3162 - K3996_1*X310 + K5500_1*X858 - K244_1*X310*X780 - K3060_1*X310*X858
311	X_dot(311) =K4768_1 + K1784_2*X2374 - K3997_1*X311 + K5501_1*X1230 - K1784_1*X311*X1195
312	X_dot(312) =K4769_1 + K1942_2*X2453 + K2382_2*X2673 + K2826_2*X2895 + K3016_2*X2990 - K3998_1*X312 + K5502_1*X1273 - K1942_1*X312*X1408 - K2382_1*X312*X1145 - K2826_1*X312*X787 - K3016_1*X312*X512
313	X_dot(313) =K4770_1 + K1272_2*X2118 + K1934_2*X2449 + K1940_2*X2452 + K1944_2*X2454 + K2128_2*X2546 - K3999_1*X313 + K5503_1*X1103 - K1272_1*X313*X1195 - K1934_1*X313*X787 - K1940_1*X313*X1407 - K1944_1*X313*X1408 - K2128_1*X313*X1273
314	X_dot(314) =K4771_1 + K28_2*X1496 + K1956_2*X2460 + K2822_2*X2893 + K2832_2*X2898 + K2834_2*X2899 + K2842_2*X2903 + K2850_2*X2907 - K4000_1*X314 + K5504_1*X787 - K28_1*X314*X1031 - K1956_1*X314*X1004 - K2822_1*X314*X787 - K2832_1*X314*X749 - K2834_1*X314*X1274 - K2850_1*X314*X1185 - K2842_1*X314*X1410
315	X_dot(315) =K4772_1 + K2940_2*X2952 + K2980_2*X2972 - K4001_1*X315 + K5505_1*X1419 - K2980_1*X187*X315 - K2940_1*X315*X1419
316	X_dot(316) =K4773_1 + K1652_2*X2308 + K1826_2*X2395 + K3192_2*X3078 - K4002_1*X316 + K5506_1*X1204 - K1652_1*X316*X891 - K1826_1*X316*X1195 - K3192_1*X316*X773
317	X_dot(317) =K4774_1 + K928_2*X1946 + K948_2*X1956 + K1936_2*X2450 - K4003_1*X317 + K5507_1*X1029 - K928_1*X317*X823 - K948_1*X317*X1267 - K1936_1*X317*X1407
318	X_dot(318) =K4775_1 + K3363_1*X3163 - K4004_1*X318 + K5508_1*X1450
319	X_dot(319) =K12_2*X1488 + K58_2*X1511 + K182_2*X1573 + K228_2*X1596 + K406_2*X1685 + K474_2*X1719 + K784_2*X1874 + K3602_2*X3283 + K3647_1*X3308 - K4005_1*X319 + K5509_1*X779 - K12_1*X319*X1031 - K58_1*X319*X1418 - K228_1*X319*X438 - K182_1*X319*X1000 - K406_1*X319*X1271 - K474_1*X319*X1270 - K784_1*X319*X861 - K3602_1*X319*X525
320	X_dot(320) =K4776_1 + K2838_2*X2901 + K2840_2*X2902 + K2848_2*X2906 + K3064_2*X3014 + K3066_2*X3015 - K4006_1*X320 + K5510_1*X1409 - K2838_1*X320*X1275 - K2848_1*X320*X1185 - K2840_1*X320*X1410 - K3064_1*X320*X598 - K3066_1*X320*X735
321	X_dot(321) =K4777_1 + K1310_2*X2137 - K4007_1*X321 + K5511_1*X1110 - K1310_1*X321*X1028
322	X_dot(322) =K4778_1 + K44_2*X1504 + K278_2*X1621 + K286_2*X1625 - K4008_1*X322 + K5512_1*X795 - K44_1*X322*X1031 - K278_1*X322*X331 - K286_1*X322*X337
323	X_dot(323) =K4779_1 + K2174_2*X2569 - K4009_1*X323 + K5513_1*X1302 - K2174_1*X323*X596
324	X_dot(324) =K4780_1 + K2348_2*X2656 - K4010_1*X324 + K5514_1*X1337 - K2348_1*X324*X1318

325	X_dot(325) =K4781_1 + K1342_2*X2153 + K2088_2*X2526 + K2356_2*X2660 + K2876_2*X2920 - K4011_1*X325 + K5515_1*X1118 - K1342_1*X325*X1271 - K2088_1*X325*X1270 - K2356_1*X325*X1318 - K2876_1*X325*X894
326	X_dot(326) =K4782_1 + K606_2*X1785 + K1206_2*X2085 + K1402_2*X2183 - K4012_1*X326 + K5516_1*X944 - K606_1*X326*X1276 - K1206_1*X326*X891 - K1402_1*X187*X326
327	X_dot(327) =K4783_1 + K136_2*X1550 + K3194_2*X3079 - K4013_1*X327 + K5517_1*X829 - K136_1*X327*X891 - K3194_1*X327*X773
328	X_dot(328) =K4784_1 + K1818_2*X2391 - K4014_1*X328 + K5518_1*X1243 - K1818_1*X328*X1195
329	X_dot(329) =K4785_1 + K2330_2*X2647 + K2442_2*X2703 - K4015_1*X329 + K5519_1*X1335 - K2330_1*X187*X329 - K2442_1*X329*X512
330	X_dot(330) =K4786_1 + K256_2*X1610 + K257_1*X1610 + K258_2*X1611 + K259_1*X1611 + K260_2*X1612 + K261_1*X1612 + K262_2*X1613 + K263_1*X1613 + K264_2*X1614 + K265_1*X1614 + K266_2*X1615 + K267_1*X1615 + K268_2*X1616 + K269_1*X1616 + K270_2*X1617 + K271_1*X1617 + K272_2*X1618 + K273_1*X1618 + K888_2*X1926 + K889_1*X1926 + K890_2*X1927 + K891_1*X1927 + K1236_2*X2100 + K1237_1*X2100 + K1238_2*X2101 + K1239_1*X2101 + K1240_2*X2102 + K1241_1*X2102 + K1242_2*X2103 + K1243_1*X2103 + K1762_2*X2363 + K1763_1*X2363 + K2704_2*X2834 + K2705_1*X2834 + K2706_2*X2835 + K2707_1*X2835 - K4016_1*X330 - K268_1*X1*X330 - K266_1*X47*X330 - K270_1*X122*X330 - K256_1*X275*X330 - K262_1*X330*X500 - K264_1*X330*X484 - K272_1*X330*X405 - K260_1*X330*X615 - K258_1*X330*X707 - K888_1*X239*X330 - K890_1*X330*X414 - K1236_1*X51*X330 - K1242_1*X99*X330 - K1240_1*X193*X330 - K1238_1*X330*X498 - K1762_1*X177*X330 - K2704_1*X33*X330 - K2706_1*X330*X386
331	X_dot(331) =K4787_1 + K276_2*X1620 + K277_1*X1620 + K278_2*X1621 + K279_1*X1621 + K1764_2*X2364 + K1765_1*X2364 + K1766_2*X2365 + K1767_1*X2365 + K1768_2*X2366 + K1769_1*X2366 + K1770_2*X2367 + K1771_1*X2367 + K2708_2*X2836 + K2709_1*X2836 - K4017_1*X331 - K276_1*X275*X331 - K278_1*X322*X331 - K1764_1*X33*X331 - K1766_1*X17*X331 - K1768_1*X99*X331 - K1770_1*X331*X657 - K2708_1*X331*X439
332	X_dot(332) =K4788_1 + K1354_2*X2159 + K1378_2*X2171 + K1384_2*X2174 + K1470_2*X2217 + K2898_2*X2931 - K4018_1*X332 + K5520_1*X1120 - K1354_1*X332*X894 - K1378_1*X332*X925 - K1384_1*X332*X1315 - K1470_1*X332*X1181 - K2898_1*X332*X1273
333	X_dot(333) =K4789_1 + K1994_2*X2479 + K2080_2*X2522 + K2872_2*X2918 - K4019_1*X333 + K5521_1*X1282 - K1994_1*X333*X1271 - K2080_1*X333*X1270 - K2872_1*X333*X894
334	X_dot(334) =K4790_1 + K402_2*X1683 + K466_2*X1715 + K780_2*X1872 - K4020_1*X334 + K5522_1*X913 - K402_1*X334*X1271 - K466_1*X334*X1270 - K780_1*X334*X861
335	X_dot(335) =K4791_1 + K1668_2*X2316 + K1708_2*X2336 - K4021_1*X335 + K5523_1*X1207 - K1668_1*X335*X891 - K1708_1*X335*X1000
336	X_dot(336) =K4792_1 + K424_2*X1694 + K496_2*X1730 - K4022_1*X336 + K5524_1*X917 - K424_1*X336*X1271 - K496_1*X336*X1270
337	X_dot(337) =K4793_1 + K280_2*X1622 + K281_1*X1622 + K282_2*X1623 + K283_1*X1623 + K284_2*X1624 + K285_1*X1624 + K286_2*X1625 + K287_1*X1625 + K892_2*X1928 + K893_1*X1928 + K1772_2*X2368 + K1773_1*X2368 + K1774_2*X2369 + K1775_1*X2369 + K2710_2*X2837 + K2711_1*X2837 - K4023_1*X337 + K5525_1*X1020 - K892_1*X337^2 - K282_1*X55*X337 - K280_1*X275*X337 - K286_1*X322*X337 - K284_1*X337*X517 - K1772_1*X33*X337 - K1774_1*X17*X337 - K2710_1*X337*X386
338	X_dot(338) =K4794_1 + K1600_2*X2282 + K1694_2*X2329 + K1808_2*X2386 - K4024_1*X338 + K5526_1*X1186 - K1600_1*X338*X1194 - K1694_1*X338*X1000 - K1808_1*X338*X1195
339	X_dot(339) =K4795_1 + K732_2*X1848 + K1142_2*X2053 - K4025_1*X339 + K5527_1*X978 - K732_1*X339*X1366 - K1142_1*X339*X584
340	X_dot(340) =K4796_1 + K1340_2*X2152 + K1358_2*X2161 - K4026_1*X340 + K5528_1*X1117 - K1340_1*X340*X1271 - K1358_1*X340*X1270
341	X_dot(341) =K4797_1 + K72_2*X1518 + K252_2*X1608 + K446_2*X1705 + K768_2*X1866 + K846_2*X1905 + K986_2*X1975 + K1370_2*X2167 + K1894_2*X2429 + K2796_2*X2880 + K3142_2*X3053 - K4027_1*X341 + K5529_1*X802 - K72_1*X341*X1418 - K252_1*X341*X1159 - K446_1*X341*X1271 - K768_1*X341*X1411 - K846_1*X341*X1031 - K986_1*X341*X1270 - K1370_1*X341*X1282 - K1894_1*X341*X783 - K2796_1*X341*X915 - K3142_1*X341*X1154
342	X_dot(342) =K4798_1 + K20_2*X1492 + K60_2*X1512 + K104_2*X1534 + K650_2*X1807 + K688_2*X1826 + K714_2*X1839 + K1088_2*X2026 + K1892_2*X2428 - K4028_1*X342 + K5530_1*X783 - K20_1*X342*X1031 - K60_1*X342*X1418 - K104_1*X342*X1381 - K650_1*X187*X342 - K688_1*X342*X1318 - K714_1*X342*X1145 - K1088_1*X342*X1154 - K1892_1*X342*X783
343	X_dot(343) =K4799_1 + K416_2*X1690 + K460_2*X1712 + K484_2*X1724 + K690_2*X1827 + K764_2*X1864 + K830_2*X1897 + K852_2*X1908 + K1030_2*X1997 + K2790_2*X2877 + K2814_2*X2889 + K3144_2*X3054 - K4029_1*X343 + K5531_1*X915 - K416_1*X343*X1271 - K460_1*X343*X894 - K484_1*X343*X1270 - K690_1*X343*X1318 - K764_1*X343*X1411 - K830_1*X343*X1031 - K852_1*X343*X1418 - K1030_1*X187*X343 - K2790_1*X343*X915 - K2814_1*X343*X831 - K3144_1*X343*X1154
344	X_dot(344) =K4800_1 + K3590_2*X3277 - K4030_1*X344 - K3590_1*X202*X344
345	X_dot(345) =K4801_1 + K190_2*X1577 - K4031_1*X345 + K5532_1*X850 - K190_1*X345*X1000
346	X_dot(346) =K4802_1 + K734_2*X1849 - K4032_1*X346 + K5533_1*X979 - K734_1*X346*X980
347	X_dot(347) =K4803_1 + K792_2*X1878 - K4033_1*X347 + K5534_1*X991 - K792_1*X347*X861
348	X_dot(348) =K4804_1 + K3608_2*X3286 - K4034_1*X348 - K3608_1*X348*X409
349	X_dot(349) =K4805_1 + K124_2*X1544 + K414_2*X1689 + K3196_2*X3080 - K4035_1*X349 + K5535_1*X824 - K124_1*X349*X891 - K414_1*X349*X1271 - K3196_1*X349*X773
350	X_dot(350) =K4806_1 + K744_2*X1854 + K770_2*X1867 + K1406_2*X2185 + K3146_2*X3055 - K4036_1*X350 + K5536_1*X981 - K744_1*X350*X1341 - K770_1*X350*X1411 - K1406_1*X187*X350 - K3146_1*X350*X1154
351	X_dot(351) =K4807_1 + K3481_1*X3222 + K3483_1*X3223 - K4037_1*X351 + K5537_1*X1455
352	X_dot(352) =K4808_1 + K1214_2*X2089 - K4038_1*X352 + K5538_1*X1087 - K1214_1*X352*X1000
353	X_dot(353) =K4809_1 + K442_2*X1703 + K512_2*X1738 + K548_2*X1756 - K4039_1*X353 + K5539_1*X919 - K442_1*X353*X1271 - K512_1*X353*X1270 - K548_1*X353*X1103
354	X_dot(354) =K4810_1 + K3385_1*X3174 + K3473_1*X3218 + K3493_1*X3228 - K4040_1*X354 + K5540_1*X1451
355	X_dot(355) =K4811_1 + K2368_2*X2666 - K4041_1*X355 + K5541_1*X1344 - K2368_1*X355*X1145
356	X_dot(356) =K4812_1 + K3374_2*X3169 + K3375_1*X3169 + K3376_2*X3170 + K3377_1*X3170 + K3378_2*X3171 + K3379_1*X3171 + K3380_2*X3172 + K3381_1*X3172 - K4042_1*X356 - K3374_1*X356*X823 - K3376_1*X356*X893 - K3378_1*X356*X1271 - K3380_1*X356*X1270
357	X_dot(357) =K4813_1 + K832_2*X1898 + K2808_2*X2886 + K2810_2*X2887 + K3630_2*X3297 - K4043_1*X357 + K5542_1*X1003 - K832_1*X357*X1031 - K2808_1*X357*X1003 - K2810_1*X357*X1406 - K3630_1*X357*X1484
358	X_dot(358) =K4814_1 + K1872_2*X2418 + K2544_2*X2754 + K3319_1*X3141 - K4044_1*X358 + K5543_1*X1258 - K1872_1*X358*X823 - K2544_1*X358*X861
359	X_dot(359) =K4815_1 + K1500_2*X2232 + K2008_2*X2486 + K2092_2*X2528 + K2240_2*X2602 + K3096_2*X3030 + K3162_2*X3063 - K4045_1*X359 + K5544_1*X1154 - K1500_1*X359*X861 - K2008_1*X359*X1271 - K2092_1*X359*X1270 - K2240_1*X359*X1036 - K3096_1*X359*X1005 - K3162_1*X359*X1154
360	X_dot(360) =K4816_1 + K2290_2*X2627 - K4046_1*X360 + K5545_1*X1326 - K2290_1*X360*X1006
361	X_dot(361) =K4817_1 + K2828_2*X2896 + K2844_2*X2904 + K2852_2*X2908 + K3068_2*X3016 - K4047_1*X361 + K5546_1*X1407 - K2828_1*X361*X1407 - K2852_1*X361*X1185 - K2844_1*X361*X1410 - K3068_1*X361*X735
362	X_dot(362) =K4818_1 + K568_2*X1766 + K638_2*X1801 + K778_2*X1871 - K4048_1*X362 + K5547_1*X938 - K638_1*X187*X362 - K568_1*X362*X925 - K778_1*X362*X861
363	X_dot(363) =K4819_1 + K674_2*X1819 + K1490_2*X2227 - K4049_1*X363 + K5548_1*X963 - K674_1*X363*X1318 - K1490_1*X363*X861
364	X_dot(364) =K4820_1 + K2726_2*X2845 + K2750_2*X2857 - K4050_1*X364 + K5549_1*X1398 - K2750_1*X205*X364 - K2726_1*X364*X1195
365	X_dot(365) =K4821_1 + K1636_2*X2300 - K4051_1*X365 + K5550_1*X1200 - K1636_1*X365*X891
366	X_dot(366) =K4822_1 + K1874_2*X2419 - K4052_1*X366 + K5551_1*X1259 - K1874_1*X366*X823

367	X_dot(367) =K4823_1 + K2984_2*X2974 - K4053_1*X367 + K5552_1*X1425 - K2984_1*X187*X367
368	X_dot(368) =K4824_1 + K1782_2*X2373 - K4054_1*X368 + K5553_1*X1229 - K1782_1*X368*X1195
369	X_dot(369) =K4825_1 + K134_2*X1549 - K4055_1*X369 + K5554_1*X828 - K134_1*X369*X891
370	X_dot(370) =K4826_1 + K1466_2*X2215 - K4056_1*X370 + K5555_1*X1147 - K1466_1*X370*X1341
371	X_dot(371) =K4827_1 + K1650_2*X2307 - K4057_1*X371 + K5556_1*X1203 - K1650_1*X371*X891
372	X_dot(372) =K4828_1 + K3442_2*X3203 + K3443_1*X3203 - K4058_1*X372 - K3442_1*X372*X1395
373	X_dot(373) =K4829_1 + K1958_2*X2461 + K2170_2*X2567 + K2212_2*X2588 + K2934_2*X2949 - K4059_1*X373 + K5557_1*X1276 - K1958_1*X373*X1004 - K2170_1*X373*X1282 - K2212_1*X373*X925 - K2934_1*X373*X1036
374	X_dot(374) =K4830_1 + K926_2*X1945 + K1978_2*X2471 + K3517_1*X3240 - K4060_1*X374 + K5558_1*X1028 - K926_1*X374*X1028 - K1978_1*X374*X1271
375	X_dot(375) =K4831_1 + K1148_2*X2056 - K4061_1*X375 + K5559_1*X1066 - K1148_1*X375*X1031
376	X_dot(376) =K4832_1 + K1146_2*X2055 + K1164_2*X2064 + K2784_2*X2874 + K2958_2*X2961 - K4062_1*X376 + K5560_1*X1065 - K1146_1*X376*X1031 - K1164_1*X376*X1418 - K2784_1*X376*X915 - K2958_1*X187*X376
377	X_dot(377) =K4833_1 + K1738_2*X2351 - K4063_1*X377 + K5561_1*X1220 - K1738_1*X377*X780
378	X_dot(378) =K4834_1 + K2660_2*X2812 - K4064_1*X378 + K5562_1*X1384 - K2660_1*X378*X1000
379	X_dot(379) =K4835_1 + K3080_2*X3022 - K4065_1*X379 + K5563_1*X1440 - K3080_1*X379*X1031
380	X_dot(380) =K4836_1 + K1800_2*X2382 + K2768_2*X2866 - K4066_1*X380 + K5564_1*X1235 - K1800_1*X380*X1195 - K2768_1*X380*X1107
381	X_dot(381) =K4837_1 + K2190_2*X2577 - K4067_1*X381 + K5565_1*X1306 - K2190_1*X381*X1377
382	X_dot(382) =K4838_1 + K1536_2*X2250 - K4068_1*X382 + K5566_1*X1168 - K1536_1*X382*X1031
383	X_dot(383) =K4839_1 + K586_2*X1775 - K4069_1*X383 + K5567_1*X942 - K586_1*X383*X1313
384	X_dot(384) =K4840_1 + K10_2*X1487 + K56_2*X1510 + K112_2*X1538 + K646_2*X1805 + K950_2*X1957 + K952_2*X1958 + K960_2*X1962 + K978_2*X1971 + K3198_2*X3081 + K3487_1*X3225 - K4070_1*X384 + K5568_1*X778 - K10_1*X384*X1031 - K56_1*X384*X1418 - K112_1*X384*X891 - K646_1*X187*X384 - K950_1*X384*X831 - K952_1*X384*X1172 - K960_1*X384*X1271 - K978_1*X384*X1270 - K3198_1*X384*X773
385	X_dot(385) =K4841_1 + K1178_2*X2071 - K4071_1*X385 + K5569_1*X1076 - K1178_1*X385*X1183
386	X_dot(386) =K4842_1 + K2706_2*X2835 + K2710_2*X2837 + K2714_2*X2839 + K2744_2*X2854 + K2764_2*X2864 - K4072_1*X386 + K5570_1*X1392 - K2706_1*X330*X386 - K2710_1*X337*X386 - K2744_1*X205*X386 - K2714_1*X386*X1195 - K2764_1*X386*X1107
387	X_dot(387) =K4843_1 + K2906_2*X2935 - K4073_1*X387 + K5571_1*X1414 - K2906_1*X387*X1383
388	X_dot(388) =K4844_1 + K1418_2*X2191 - K4074_1*X388 + K5572_1*X1134 - K1418_1*X388*X1318
389	X_dot(389) =K4845_1 + K1098_2*X2031 + K1468_2*X2216 + K3331_1*X3147 - K4075_1*X389 + K5573_1*X1055 - K1098_1*X389*X861 - K1468_1*X389*X1181
390	X_dot(390) =K4846_1 + K798_2*X1881 + K908_2*X1936 + K922_2*X1943 + K946_2*X1955 + K1328_2*X2146 + K2438_2*X2701 + K3549_1*X3256 - K4076_1*X390 + K5574_1*X993 - K798_1*X390*X861 - K922_1*X205*X390 - K908_1*X390*X1195 - K946_1*X390*X915 - K1328_1*X215*X390 - K2438_1*X390*X512
391	X_dot(391) =K4847_1 + K3438_2*X3201 + K3439_1*X3201 + K3440_2*X3202 + K3441_1*X3202 + K3506_2*X3235 + K3507_1*X3235 + K3546_2*X3255 + K3547_1*X3255 - K4077_1*X391 - K3438_1*X391*X1399 - K3440_1*X391*X1387 - K3506_1*X391*X999 - K3546_1*X391*X1265
392	X_dot(392) =K4848_1 + K2500_2*X2732 - K4078_1*X392 + K5575_1*X1360 - K2500_1*X392*X861
393	X_dot(393) =K4849_1 + K1258_2*X2111 + K1422_2*X2193 + K2972_2*X2968 - K4079_1*X393 + K5576_1*X1099 - K1258_1*X393*X1195 - K1422_1*X393*X1318 - K2972_1*X187*X393
394	X_dot(394) =K4850_1 + K1204_2*X2084 - K4080_1*X394 + K5577_1*X1084 - K1204_1*X394*X891
395	X_dot(395) =K4851_1 + K3624_2*X3294 + K3625_1*X3294 + K3636_2*X3300 + K3637_1*X3300 - K4081_1*X395 - K3624_1*X175*X395 - K3636_1*X395*X490
396	X_dot(396) =K4852_1 + K2326_2*X2645 - K4082_1*X396 + K5578_1*X1334 - K2326_1*X187*X396
397	X_dot(397) =K4853_1 + K1048_2*X2006 - K4083_1*X397 + K5579_1*X1047 - K1048_1*X187*X397
398	X_dot(398) =K4854_1 + K2440_2*X2702 - K4084_1*X398 + K5580_1*X1357 - K2440_1*X398*X512
399	X_dot(399) =K4855_1 + K1442_2*X2203 - K4085_1*X399 + K5581_1*X1143 - K1442_1*X399*X1318
400	X_dot(400) =K4856_1 + K486_2*X1725 + K718_2*X1841 + K794_2*X1879 + K2120_2*X2542 - K4086_1*X400 + K5582_1*X924 - K486_1*X400*X1270 - K718_1*X400*X1145 - K794_1*X400*X861 - K2120_1*X400*X1273
401	X_dot(401) =K4857_1 + K36_2*X1500 + K156_2*X1560 + K192_2*X1578 + K220_2*X1592 + K3254_2*X3109 + K3255_1*X3109 + K3256_2*X3110 + K3257_1*X3110 + K3258_2*X3111 + K3259_1*X3111 + K3390_2*X3177 + K3391_1*X3177 + K3392_2*X3178 + K3393_1*X3178 + K3394_2*X3179 + K3395_1*X3179 + K3474_2*X3219 + K3475_1*X3219 + K3486_2*X3225 + K3487_1*X3225 + K3488_2*X3226 + K3489_1*X3226 + K3522_2*X3243 + K3523_1*X3243 + K3568_2*X3266 + K3569_1*X3266 + K3570_2*X3267 + K3571_1*X3267 - K4087_1*X401 + K5583_1*X791 - K36_1*X401*X1031 - K156_1*X401*X891 - K192_1*X401*X1000 - K220_1*X401*X1393 - K3254_1*X401*X1031 - K3256_1*X401*X1271 - K3258_1*X401*X1270 - K3394_1*X401*X807 - K3390_1*X401*X1224 - K3392_1*X401*X1217 - K3486_1*X401*X778 - K3474_1*X401*X1035 - K3488_1*X401*X987 - K3522_1*X401*X872 - K3568_1*X401*X1026 - K3570_1*X401*X1287
402	X_dot(402) =K4858_1 + K3260_2*X3112 + K3261_1*X3112 + K3396_2*X3180 + K3397_1*X3180 + K3398_2*X3181 + K3399_1*X3181 + K3476_2*X3220 + K3477_1*X3220 + K3490_2*X3227 + K3491_1*X3227 + K3524_2*X3244 + K3525_1*X3244 - K4088_1*X402 - K3260_1*X402*X1031 - K3398_1*X402*X807 - K3396_1*X402*X1224 - K3476_1*X402*X1035 - K3490_1*X402*X940 - K3524_1*X402*X872
403	X_dot(403) =K4859_1 - K4089_1*X403
404	X_dot(404) =K4860_1 + K3290_2*X3127 + K3291_1*X3127 + K3292_2*X3128 + K3293_1*X3128 + K3294_2*X3129 + K3295_1*X3129 + K3296_2*X3130 + K3297_1*X3130 + K3424_2*X3194 + K3425_1*X3194 + K3426_2*X3195 + K3427_1*X3195 + K3428_2*X3196 + K3429_1*X3196 + K3430_2*X3197 + K3431_1*X3197 + K3432_2*X3198 + K3433_1*X3198 + K3434_2*X3199 + K3435_1*X3199 + K3436_2*X3200 + K3437_1*X3200 + K3534_2*X3249 + K3535_1*X3249 + K3536_2*X3250 + K3537_1*X3250 - K4090_1*X404 - K3294_1*X404*X896 - K3290_1*X404*X1031 - K3292_1*X404*X1418 - K3296_1*X404*X1445 - K3424_1*X404*X780 - K3434_1*X404*X807 - K3430_1*X404*X925 - K3432_1*X404*X1145 - K3428_1*X404*X1271 - K3426_1*X404*X1453 - K3436_1*X404*X1454 - K3536_1*X404*X911 - K3534_1*X404*X1126
405	X_dot(405) =K4861_1 + K272_2*X1618 - K4091_1*X405 + K5584_1*X866 - K272_1*X330*X405
406	X_dot(406) =K4862_1 + K1382_2*X2173 + K2458_2*X2711 + K3148_2*X3056 - K4092_1*X406 + K5585_1*X1124 - K1382_1*X406*X1313 - K2458_1*X406*X1411 - K3148_1*X406*X1154
407	X_dot(407) =K4863_1 + K1386_2*X2175 + K2276_2*X2620 - K4093_1*X407 + K5586_1*X1125 - K1386_1*X407*X1315 - K2276_1*X407*X1276
408	X_dot(408) =K4864_1 + K1044_2*X2004 - K4094_1*X408 + K5587_1*X1045 - K1044_1*X187*X408
409	X_dot(409) =K4865_1 + K3608_2*X3286 + K3609_1*X3286 - K4095_1*X409 - K3608_1*X348*X409
410	X_dot(410) =K4866_1 + K3618_2*X3291 + K3619_1*X3291 + K3620_2*X3292 + K3621_1*X3292 - K4096_1*X410 - K3618_1*X207*X410 - K3620_1*X410*X442
411	X_dot(411) =K4867_1 + K1320_2*X2142 + K1494_2*X2229 + K1886_2*X2425 - K4097_1*X411 + K5588_1*X1113 - K1320_1*X411*X823 - K1494_1*X411*X861 - K1886_1*X215*X411
412	X_dot(412) =K4868_1 + K844_2*X1904 + K2538_2*X2751 - K4098_1*X412 + K5589_1*X1008 - K844_1*X412*X1031 - K2538_1*X412*X861
413	X_dot(413) =K4869_1 + K776_2*X1870 - K4099_1*X413 + K5590_1*X988 - K776_1*X413*X861

414	X_dot(414) =K4870_1 + K536_2*X1750 + K550_2*X1757 + K890_2*X1927 + K990_2*X1977 + K1072_2*X2018 + K1162_2*X2063 + K1172_2*X2068 + K2338_2*X2651 - K4100_1*X414 + K5591_1*X933 - K550_1*X414*X1103 - K536_1*X414*X1273 - K890_1*X330*X414 - K990_1*X414*X1282 - K1072_1*X414*X1145 - K1162_1*X414*X1031 - K1172_1*X414*X1446 - K2338_1*X187*X414
415	X_dot(415) =K4871_1 + K574_2*X1769 - K4101_1*X415 + K5592_1*X939 - K574_1*X415*X925
416	X_dot(416) =K4872_1 + K2540_2*X2752 - K4102_1*X416 + K5593_1*X1367 - K2540_1*X416*X861
417	X_dot(417) =K4873_1 + K3353_1*X3158 - K4103_1*X417 + K5594_1*X1449
418	X_dot(418) =K4874_1 + K796_2*X1880 + K2950_2*X2957 + K2951_1*X2957 + K3262_2*X3113 + K3263_1*X3113 + K3264_2*X3114 + K3265_1*X3114 + K3266_2*X3115 + K3267_1*X3115 + K3268_2*X3116 + K3269_1*X3116 + K3270_2*X3117 + K3271_1*X3117 + K3272_2*X3118 + K3273_1*X3118 + K3274_2*X3119 + K3275_1*X3119 + K3276_2*X3120 + K3277_1*X3120 + K3278_2*X3121 + K3279_1*X3121 + K3280_2*X3122 + K3281_1*X3122 + K3282_2*X3123 + K3283_1*X3123 + K3284_2*X3124 + K3285_1*X3124 + K3286_2*X3125 + K3287_1*X3125 + K3288_2*X3126 + K3289_1*X3126 + K3408_2*X3186 + K3409_1*X3186 + K3410_2*X3187 + K3411_1*X3187 + K3412_2*X3188 + K3413_1*X3188 + K3414_2*X3189 + K3415_1*X3189 + K3416_2*X3190 + K3417_1*X3190 + K3418_2*X3191 + K3419_1*X3191 + K3420_2*X3192 + K3421_1*X3192 + K3422_2*X3193 + K3423_1*X3193 + K3494_2*X3229 + K3495_1*X3229 + K3496_2*X3230 + K3497_1*X3230 + K3498_2*X3231 + K3499_1*X3231 + K3500_2*X3232 + K3501_1*X3232 + K3502_2*X3233 + K3503_1*X3233 + K3528_2*X3246 + K3529_1*X3246 + K3530_2*X3247 + K3531_1*X3247 + K3532_2*X3248 + K3533_1*X3248 + K3574_2*X3269 + K3575_1*X3269 - K4104_1*X418 + K5595_1*X992 - K796_1*X418*X861 - K2950_1*X47*X418 - K3266_1*X418*X831 - K3262_1*X418*X1031 - K3286_1*X418*X792 - K3288_1*X418*X793 - K3284_1*X418*X851 - K3264_1*X418*X1133 - K3268_1*X418*X1172 - K3272_1*X418*X1185 - K3282_1*X418*X1119 - K3270_1*X418*X1275 - K3274_1*X418*X1271 - K3276_1*X418*X1270 - K3280_1*X418*X1265 - K3278_1*X418*X1323 - K3410_1*X418*X780 - K3420_1*X418*X807 - K3418_1*X418*X861 - K3414_1*X418*X925 - K3416_1*X418*X1145 - K3408_1*X418*X1418 - K3412_1*X418*X1453 - K3422_1*X418*X1454 - K3494_1*X418*X817 - K3500_1*X418*X839 - K3498_1*X418*X860 - K3496_1*X418*X888 - K3502_1*X418*X852 - K3530_1*X418*X911 - K3532_1*X418*X1026 - K3528_1*X418*X1126 - K3574_1*X418*X1442
419	X_dot(419) =K4875_1 + K1542_2*X2253 + K2466_2*X2715 + K2478_2*X2721 - K4105_1*X419 + K5596_1*X1170 - K1542_1*X419*X1031 - K2478_1*X419*X1154 - K2466_1*X419*X1411
420	X_dot(420) =K4876_1 + K1302_2*X2133 + K1624_2*X2294 - K4106_1*X420 + K5597_1*X1107 - K1302_1*X420*X1107 - K1624_1*X420*X891
421	X_dot(421) =K4877_1 + K1448_2*X2206 - K4107_1*X421 + K5598_1*X1144 - K1448_1*X421*X1145
422	X_dot(422) =K4878_1 + K3062_2*X3013 - K4108_1*X422 + K5599_1*X1436 - K3062_1*X422*X858
423	X_dot(423) =K4879_1 + K1622_2*X2293 + K1974_2*X2469 + K3110_2*X3037 - K4109_1*X423 + K5600_1*X1195 - K1622_1*X423*X891 - K1974_1*X423*X1271 - K3110_1*X423*X1270
424	X_dot(424) =K4880_1 + K1568_2*X2266 + K2038_2*X2501 + K2204_2*X2584 + K2226_2*X2595 + K2378_2*X2671 + K2474_2*X2719 + K2602_2*X2783 + K2614_2*X2789 + K3074_2*X3019 - K4110_1*X424 + K5601_1*X1176 - K1568_1*X424*X1173 - K2038_1*X424*X894 - K2204_1*X424*X925 - K2226_1*X424*X1082 - K2378_1*X424*X1145 - K2474_1*X424*X1154 - K2602_1*X218*X424 - K2614_1*X424*X666 - K3074_1*X424*X731
425	X_dot(425) =K4881_1 + K3058_2*X3011 + K3357_1*X3160 - K4111_1*X425 + K5602_1*X1435 - K3058_1*X425*X858
426	X_dot(426) =K4882_1 + K1986_2*X2475 + K2068_2*X2516 + K2422_2*X2693 + K3036_2*X3000 - K4112_1*X426 + K5603_1*X1280 - K1986_1*X426*X1271 - K2068_1*X426*X1270 - K2422_1*X426*X1341 - K3036_1*X426*X861
427	X_dot(427) =K4883_1 + K462_2*X1713 - K4113_1*X427 + K5604_1*X922 - K462_1*X427*X894
428	X_dot(428) =K4884_1 + K1532_2*X2248 - K4114_1*X428 + K5605_1*X1166 - K1532_1*X428*X1031
429	X_dot(429) =K4885_1 + K1152_2*X2058 + K2448_2*X2706 + K3150_2*X3057 - K4115_1*X429 + K5606_1*X1068 - K1152_1*X429*X1031 - K2448_1*X429*X1411 - K3150_1*X429*X1154
430	X_dot(430) =K4886_1 + K738_2*X1851 - K4116_1*X430 + K5607_1*X980 - K738_1*X430*X980
431	X_dot(431) =K4887_1 + K2050_2*X2507 + K2870_2*X2917 - K4117_1*X431 + K5608_1*X1294 - K2050_1*X431*X1270 - K2870_1*X431*X894
432	X_dot(432) =K4888_1 + K2200_2*X2582 - K4118_1*X432 + K5609_1*X1309 - K2200_1*X432*X925
433	X_dot(433) =K4889_1 + K296_2*X1630 - K4119_1*X433 + K5610_1*X873 - K296_1*X433*X1195
434	X_dot(434) =K4890_1 + K200_2*X1582 + K214_2*X1589 + K240_2*X1602 + K436_2*X1700 + K506_2*X1735 + K1124_2*X2044 + K2878_2*X2921 + K3124_2*X3044 + K3253_1*X3108 + K3285_1*X3124 + K3580_2*X3272 - K4120_1*X434 + K5611_1*X851 - K200_1*X434*X1000 - K214_1*X434*X1001 - K240_1*X434*X1451 - K436_1*X434*X1271 - K506_1*X434*X1270 - K1124_1*X434*X915 - K2878_1*X434*X894 - K3124_1*X434*X1273 - K3580_1*X434*X649
435	X_dot(435) =K4891_1 + K1526_2*X2245 + K1566_2*X2265 + K1744_2*X2354 + K1756_2*X2360 + K1988_2*X2476 + K2036_2*X2500 + K2070_2*X2517 + K2118_2*X2541 + K2148_2*X2556 + K2310_2*X2637 + K2456_2*X2710 + K2470_2*X2717 - K4121_1*X435 + K5612_1*X1163 - K1526_1*X435*X1031 - K1566_1*X435*X1173 - K1744_1*X435*X780 - K1756_1*X435*X1159 - K1988_1*X435*X1271 - K2036_1*X435*X894 - K2070_1*X435*X1270 - K2118_1*X435*X1273 - K2148_1*X435*X1103 - K2310_1*X187*X435 - K2470_1*X435*X1154 - K2456_1*X435*X1411
436	X_dot(436) =K4892_1 + K1554_2*X2259 + K3082_2*X3023 - K4122_1*X436 + K5613_1*X1171 - K1554_1*X436*X1418 - K3082_1*X436*X1031
437	X_dot(437) =K4893_1 + K2336_2*X2650 - K4123_1*X437 + K5614_1*X1336 - K2336_1*X187*X437
438	X_dot(438) =K4894_1 + K228_2*X1596 + K229_1*X1596 + K230_2*X1597 + K231_1*X1597 + K232_2*X1598 + K233_1*X1598 - K4124_1*X438 - K232_1*X111*X438 - K228_1*X319*X438 - K230_1*X438*X467
439	X_dot(439) =K4895_1 + K2708_2*X2836 - K4125_1*X439 + K5615_1*X1393 - K2708_1*X331*X439
440	X_dot(440) =K4896_1 + K2502_2*X2733 - K4126_1*X440 + K5616_1*X1361 - K2502_1*X440*X861
441	X_dot(441) =K4897_1 + K372_2*X1668 + K488_2*X1726 + K602_2*X1783 + K988_2*X1976 + K1840_2*X2402 + K2314_2*X2639 + K2646_2*X2805 + K2732_2*X2848 - K4127_1*X441 + K5617_1*X901 - K372_1*X441*X915 - K488_1*X441*X1270 - K602_1*X441*X1276 - K988_1*X441*X1273 - K1840_1*X205*X441 - K2314_1*X187*X441 - K2646_1*X441*X810 - K2732_1*X441*X1195
442	X_dot(442) =K4898_1 + K2694_2*X2829 + K3620_2*X3292 - K4128_1*X442 + K5618_1*X1389 - K2694_1*X442*X1159 - K3620_1*X410*X442
443	X_dot(443) =K4899_1 + K2412_2*X2688 - K4129_1*X443 + K5619_1*X1353 - K2412_1*X443*X980
444	X_dot(444) =K4900_1 + K318_2*X1641 + K1050_2*X2007 - K4130_1*X444 + K5620_1*X882 - K318_1*X444*X1195 - K1050_1*X187*X444
445	X_dot(445) =K4901_1 + K314_2*X1639 + K328_2*X1646 + K330_2*X1647 - K4131_1*X445 + K5621_1*X880 - K328_1*X205*X445 - K314_1*X445*X1195 - K330_1*X445*X1107
446	X_dot(446) =K4902_1 + K1630_2*X2297 + K1692_2*X2328 - K4132_1*X446 + K5622_1*X1197 - K1630_1*X446*X891 - K1692_1*X446*X1000
447	X_dot(447) =K4903_1 + K686_2*X1825 + K1336_2*X2150 + K1352_2*X2158 + K1356_2*X2160 - K4133_1*X447 + K5623_1*X968 - K686_1*X447*X1318 - K1352_1*X447*X894 - K1336_1*X447*X1271 - K1356_1*X447*X1270
448	X_dot(448) =K4904_1 + K664_2*X1814 + K706_2*X1835 + K1076_2*X2020 + K1176_2*X2070 + K1186_2*X2075 - K4134_1*X448 + K5624_1*X960 - K664_1*X187*X448 - K706_1*X448*X1318 - K1076_1*X448*X1341 - K1186_1*X448*X522 - K1176_1*X448*X1381
449	X_dot(449) =K4905_1 + K2446_2*X2705 + K2447_1*X2705 - K4135_1*X449 - K2446_1*X157*X449
450	X_dot(450) =K4906_1 + K2014_2*X2489 + K2102_2*X2533 + K3571_1*X3267 - K4136_1*X450 + K5625_1*X1287 - K2014_1*X450*X1271 - K2102_1*X450*X1270
451	X_dot(451) =K4907_1 + K142_2*X1553 + K964_2*X1964 + K1556_2*X2260 + K1946_2*X2455 + K1952_2*X2458 + K2638_2*X2801 + K2816_2*X2890 + K2924_2*X2944 + K2930_2*X2947 + K3112_2*X3038 + K3120_2*X3042 + K3267_1*X3115 - K4137_1*X451 + K5626_1*X831 - K142_1*X451*X891 - K964_1*X451*X1271 - K1556_1*X451*X1307 - K1946_1*X451*X749 - K1952_1*X451*X1004 - K2638_1*X168*X451 - K2816_1*X451*X831 - K2924_1*X451*X925 - K2930_1*X451*X1036 - K3112_1*X451*X1270 - K3120_1*X451*X1313
452	X_dot(452) =K4908_1 + K900_2*X1932 + K3341_1*X3152 - K4138_1*X452 + K5627_1*X1023 - K900_1*X452*X1195

453	X_dot(453) =K4909_1 + K1906_2*X2435 + K2328_2*X2646 + K2486_2*X2725 + K2596_2*X2780 + K2696_2*X2830 + K2742_2*X2853 + K2782_2*X2873 + K2800_2*X2882 + K3281_1*X3122 + K3547_1*X3255 - K4139_1*X453 + K5628_1*X1265 - K1906_1*X453*X915 - K2328_1*X187*X453 - K2486_1*X453*X1293 - K2596_1*X453*X727 - K2696_1*X453*X1159 - K2782_1*X453*X783 - K2742_1*X453*X1195 - K2800_1*X453*X1486
454	X_dot(454) =K4910_1 + K1832_2*X2398 - K4140_1*X454 + K5629_1*X1248 - K1832_1*X454*X1195
455	X_dot(455) =K4911_1 + K1530_2*X2247 - K4141_1*X455 + K5630_1*X1165 - K1530_1*X455*X1031
456	X_dot(456) =K4912_1 - K4142_1*X456
457	X_dot(457) =K4913_1 + K1054_2*X2009 + K3610_2*X3287 + K3611_1*X3287 - K4143_1*X457 + K5631_1*X1048 - K1054_1*X187*X457 - K3610_1*X457*X592
458	X_dot(458) =K4914_1 + K332_2*X1648 + K1414_2*X2189 + K2672_2*X2818 + K3034_2*X2999 - K4144_1*X458 + K5632_1*X887 - K332_1*X458*X1250 - K1414_1*X458*X1318 - K2672_1*X458*X1393 - K3034_1*X458*X861
459	X_dot(459) =K4915_1 + K708_2*X1836 + K3330_2*X3147 + K3331_1*X3147 + K3332_2*X3148 + K3333_1*X3148 + K3334_2*X3149 + K3335_1*X3149 + K3336_2*X3150 + K3337_1*X3150 + K3460_2*X3212 + K3461_1*X3212 - K4145_1*X459 + K5633_1*X973 - K708_1*X459*X1318 - K3330_1*X459*X1055 - K3336_1*X459*X1052 - K3334_1*X459*X1181 - K3332_1*X459*X1447 - K3460_1*X459*X861
460	X_dot(460) =K4916_1 + K1586_2*X2275 + K1718_2*X2341 + K1866_2*X2415 + K2178_2*X2571 + K2426_2*X2695 + K2534_2*X2749 + K2700_2*X2832 + K3315_1*X3139 + K3329_1*X3146 + K3335_1*X3149 + K3447_1*X3205 - K4146_1*X460 + K5634_1*X1181 - K1586_1*X460*X810 - K1718_1*X460*X1393 - K1866_1*X460*X823 - K2178_1*X460*X970 - K2426_1*X460*X1181 - K2534_1*X460*X861 - K2700_1*X460*X1029
461	X_dot(461) =K4917_1 + K92_2*X1528 + K624_2*X1794 + K658_2*X1811 + K2862_2*X2913 + K2884_2*X2924 - K4147_1*X461 + K5635_1*X812 - K92_1*X461*X810 - K658_1*X187*X461 - K624_1*X461*X1006 - K2862_1*X461*X1271 - K2884_1*X461*X1270
462	X_dot(462) =K4918_1 + K1454_2*X2209 + K2936_2*X2950 + K3313_1*X3138 + K3417_1*X3190 + K3433_1*X3198 - K4148_1*X462 + K5636_1*X1145 - K1454_1*X462*X1145 - K2936_1*X462*X1036
463	X_dot(463) =K4919_1 + K1430_2*X2197 + K3320_2*X3142 + K3321_1*X3142 + K3322_2*X3143 + K3323_1*X3143 + K3324_2*X3144 + K3325_1*X3144 + K3326_2*X3145 + K3327_1*X3145 + K3328_2*X3146 + K3329_1*X3146 + K3454_2*X3209 + K3455_1*X3209 + K3456_2*X3210 + K3457_1*X3210 + K3458_2*X3211 + K3459_1*X3211 + K3552_2*X3258 + K3553_1*X3258 + K3554_2*X3259 + K3555_1*X3259 + K3556_2*X3260 + K3557_1*X3260 - K4149_1*X463 + K5637_1*X1138 - K1430_1*X463*X1318 - K3320_1*X463*X874 - K3324_1*X463*X909 - K3328_1*X463*X1181 - K3322_1*X463*X1253 - K3326_1*X463*X1365 - K3454_1*X463*X823 - K3456_1*X463*X861 - K3458_1*X463*X1182 - K3552_1*X463*X1056 - K3554_1*X463*X1364 - K3556_1*X463*X1458
464	X_dot(464) =K4920_1 + K620_2*X1792 + K1064_2*X2014 + K1582_2*X2273 - K4150_1*X464 + K5638_1*X949 - K620_1*X464*X1006 - K1064_1*X464*X1318 - K1582_1*X464*X810
465	X_dot(465) =K4921_1 + K1802_2*X2383 - K4151_1*X465 + K5639_1*X1236 - K1802_1*X465*X1195
466	X_dot(466) =K4922_1 + K1190_2*X2077 + K1412_2*X2188 + K2110_2*X2537 - K4152_1*X466 + K5640_1*X1079 - K1190_1*X466*X891 - K1412_1*X466*X1318 - K2110_1*X466*X1273
467	X_dot(467) =K4923_1 + K158_2*X1561 + K194_2*X1579 + K210_2*X1587 + K230_2*X1597 + K2228_2*X2596 - K4153_1*X467 + K5641_1*X838 - K158_1*X467*X891 - K230_1*X438*X467 - K194_1*X467*X1000 - K210_1*X467*X1001 - K2228_1*X467*X1082
468	X_dot(468) =K4924_1 + K46_2*X1505 + K556_2*X1760 - K4154_1*X468 + K5642_1*X796 - K46_1*X468*X1031 - K556_1*X468*X1282
469	X_dot(469) =K4925_1 + K1438_2*X2201 - K4155_1*X469 + K5643_1*X1142 - K1438_1*X469*X1318
470	X_dot(470) =K4926_1 + K1196_2*X2080 + K1992_2*X2478 - K4156_1*X470 + K5644_1*X1081 - K1196_1*X470*X891 - K1992_1*X470*X1271
471	X_dot(471) =K4927_1 + K822_2*X1893 + K1898_2*X2431 - K4157_1*X471 + K5645_1*X1002 - K822_1*X471*X1031 - K1898_1*X471*X915
472	X_dot(472) =K4928_1 + K520_2*X1742 - K4158_1*X472 + K5646_1*X928 - K520_1*X472*X1273
473	X_dot(473) =K4929_1 + K376_2*X1670 + K1120_2*X2042 + K1642_2*X2303 + K1996_2*X2480 + K2082_2*X2523 + K2130_2*X2547 + K3200_2*X3082 - K4159_1*X473 + K5647_1*X903 - K376_1*X473*X915 - K1120_1*X473*X783 - K1642_1*X473*X891 - K1996_1*X473*X1271 - K2082_1*X473*X1270 - K2130_1*X473*X1273 - K3200_1*X473*X773
474	X_dot(474) =K4930_1 + K420_2*X1692 + K490_2*X1727 - K4160_1*X474 + K5648_1*X916 - K420_1*X474*X1271 - K490_1*X474*X1270
475	X_dot(475) =K4931_1 + K508_2*X1736 + K968_2*X1966 - K4161_1*X475 + K5649_1*X926 - K508_1*X475*X1270 - K968_1*X475*X1271
476	X_dot(476) =K4932_1 + K2352_2*X2658 + K2522_2*X2743 + K2976_2*X2970 - K4162_1*X476 + K5650_1*X1338 - K2352_1*X476*X1318 - K2522_1*X476*X861 - K2976_1*X187*X476
477	X_dot(477) =K4933_1 + K1314_2*X2139 + K2334_2*X2649 + K2552_2*X2758 - K4163_1*X477 + K5651_1*X1111 - K1314_1*X477*X1250 - K2334_1*X187*X477 - K2552_1*X477*X1370
478	X_dot(478) =K4934_1 + K3298_2*X3131 + K3299_1*X3131 + K3480_2*X3222 + K3481_1*X3222 + K3538_2*X3251 + K3539_1*X3251 + K3540_2*X3252 + K3541_1*X3252 + K3572_2*X3268 + K3573_1*X3268 - K4164_1*X478 - K3298_1*X478*X1077 - K3538_1*X478*X799 - K3480_1*X478*X1455 - K3572_1*X478*X878 - K3540_1*X478*X1346
479	X_dot(479) =K4935_1 + K2774_2*X2869 - K4165_1*X479 + K5652_1*X1404 - K2774_1*X479*X1404
480	X_dot(480) =K4936_1 + K130_2*X1547 - K4166_1*X480 + K5653_1*X826 - K130_1*X480*X891
481	X_dot(481) =K4937_1 + K912_2*X1938 - K4167_1*X481 + K5654_1*X1027 - K912_1*X481*X1195
482	X_dot(482) =K4938_1 + K80_2*X1522 - K4168_1*X482 + K5655_1*X806 - K80_1*X482*X1173
483	X_dot(483) =K4939_1 + K616_2*X1790 + K712_2*X1838 + K1524_2*X2244 + K1550_2*X2257 + K1982_2*X2473 + K3114_2*X3039 - K4169_1*X483 + K5656_1*X947 - K616_1*X483*X1006 - K712_1*X483*X1145 - K1524_1*X483*X1031 - K1550_1*X483*X1418 - K1982_1*X483*X1271 - K3114_1*X483*X1270
484	X_dot(484) =K4940_1 + K16_2*X1490 + K128_2*X1546 + K184_2*X1574 + K264_2*X1614 + K334_2*X1649 + K594_2*X1779 + K848_2*X1906 + K894_2*X1929 + K2286_2*X2625 + K2572_2*X2768 - K4170_1*X484 + K5657_1*X781 - K16_1*X484*X1031 - K128_1*X484*X891 - K184_1*X484*X1000 - K264_1*X330*X484 - K334_1*X484*X1250 - K594_1*X484*X1316 - K894_1*X484*X700 - K848_1*X484*X1418 - K2286_1*X484*X1006 - K2572_1*X484*X793
485	X_dot(485) =K4941_1 + K202_2*X1583 + K440_2*X1702 + K510_2*X1737 + K3503_1*X3233 - K4171_1*X485 + K5658_1*X852 - K202_1*X485*X1000 - K440_1*X485*X1271 - K510_1*X485*X1270
486	X_dot(486) =K4942_1 + K636_2*X1800 - K4172_1*X486 + K5659_1*X953 - K636_1*X187*X486
487	X_dot(487) =K4943_1 + K2620_2*X2792 - K4173_1*X487 + K5660_1*X1376 - K2620_1*X487*X666
488	X_dot(488) =K4944_1 + K516_2*X1740 - K4174_1*X488 + K5661_1*X927 - K516_1*X488*X1273
489	X_dot(489) =K4945_1 + K1528_2*X2246 - K4175_1*X489 + K5662_1*X1164 - K1528_1*X489*X1031
490	X_dot(490) =K4946_1 + K3423_1*X3193 + K3437_1*X3200 + K3636_2*X3300 - K4176_1*X490 + K5663_1*X1454 - K3636_1*X395*X490
491	X_dot(491) =K4947_1 + K856_2*X1910 + K1192_2*X2078 + K1688_2*X2326 - K4177_1*X491 + K5664_1*X1009 - K856_1*X491*X810 - K1192_1*X491*X891 - K1688_1*X491*X1000
492	X_dot(492) =K4948_1 + K1038_2*X2001 + K2740_2*X2852 + K2758_2*X2861 - K4178_1*X492 + K5665_1*X1043 - K1038_1*X187*X492 - K2758_1*X205*X492 - K2740_1*X492*X1195
493	X_dot(493) =K4949_1 + K3508_2*X3236 + K3509_1*X3236 - K4179_1*X493 - K3508_1*X493*X1000
494	X_dot(494) =K4950_1 + K1612_2*X2288 - K4180_1*X494 + K5666_1*X1191 - K1612_1*X494*X891
495	X_dot(495) =K4951_1 + K40_2*X1502 + K70_2*X1517 + K2136_2*X2550 + K2584_2*X2774 + K2588_2*X2776 + K2592_2*X2778 + K3054_2*X3009 + K3289_1*X3126 - K4181_1*X495 + K5667_1*X793 - K40_1*X495*X1031 - K70_1*X495*X1418 - K2136_1*X495*X1273 - K2584_1*X495*X598 - K2588_1*X495*X735 - K2592_1*X495*X1437 - K3054_1*X495*X793

496	X_dot(496) =K4952_1 + K1014_2*X1989 + K1246_2*X2105 + K1284_2*X2124 - K4182_1*X496 + K5668_1*X1038 - K1014_1*X496*X1006 - K1284_1*X205*X496 - K1246_1*X496*X1195
497	X_dot(497) =K4953_1 + K840_2*X1902 + K1018_2*X1991 + K1042_2*X2003 + K1588_2*X2276 - K4183_1*X497 + K5669_1*X1006 - K840_1*X497*X1031 - K1042_1*X187*X497 - K1018_1*X497*X1006 - K1588_1*X497*X1381
498	X_dot(498) =K4954_1 + K366_2*X1665 + K934_2*X1949 + K1238_2*X2101 + K2722_2*X2843 + K2748_2*X2856 - K4184_1*X498 + K5670_1*X898 - K366_1*X498*X915 - K934_1*X498*X783 - K1238_1*X330*X498 - K2748_1*X205*X498 - K2722_1*X498*X1195
499	X_dot(499) =K4955_1 + K494_2*X1729 + K884_2*X1924 + K966_2*X1965 + K998_2*X1981 + K2926_2*X2945 + K2932_2*X2948 + K3389_1*X3176 + K3415_1*X3189 + K3431_1*X3197 - K4185_1*X499 + K5671_1*X925 - K494_1*X499*X1270 - K884_1*X499*X1393 - K966_1*X499*X1271 - K998_1*X499*X1377 - K2926_1*X499*X925 - K2932_1*X499*X1036
500	X_dot(500) =K4956_1 + K262_2*X1613 + K396_2*X1680 + K600_2*X1782 - K4186_1*X500 + K5672_1*X864 - K262_1*X330*X500 - K396_1*X500*X1408 - K600_1*X500*X1276
501	X_dot(501) =K4957_1 + K2218_2*X2591 + K2528_2*X2746 - K4187_1*X501 + K5673_1*X1313 - K2218_1*X501*X1313 - K2528_1*X501*X861
502	X_dot(502) =K4958_1 + K2298_2*X2631 - K4188_1*X502 + K5674_1*X1329 - K2298_1*X187*X502
503	X_dot(503) =K4959_1 + K38_2*X1501 + K68_2*X1516 + K2566_2*X2765 + K2582_2*X2773 + K2586_2*X2775 + K2590_2*X2777 + K3287_1*X3125 - K4189_1*X503 + K5675_1*X792 - K38_1*X503*X1031 - K68_1*X503*X1418 - K2582_1*X503*X598 - K2566_1*X503*X792 - K2586_1*X503*X735 - K2590_1*X503*X1437
504	X_dot(504) =K4960_1 + K3076_2*X3020 - K4190_1*X504 + K5676_1*X1438 - K3076_1*X504*X998
505	X_dot(505) =K4961_1 + K726_2*X1845 - K4191_1*X505 + K5677_1*X977 - K726_1*X505*X1145
506	X_dot(506) =K4962_1 - K4192_1*X506
507	X_dot(507) =K4963_1 + K428_2*X1696 + K500_2*X1732 + K1750_2*X2357 - K4193_1*X507 + K5678_1*X918 - K428_1*X507*X1271 - K500_1*X507*X1270 - K1750_1*X507*X1126
508	X_dot(508) =K4964_1 + K522_2*X1743 + K614_2*X1789 + K1000_2*X1982 + K3681_2*X3342 - K4194_1*X508 + K5679_1*X929 - K522_1*X508*X1273 - K614_1*X508*X1006 - K1000_1*X508*X925 - K3681_1*X508*X1237*X3343
509	X_dot(509) =K4965_1 + K2358_2*X2661 - K4195_1*X509 + K5680_1*X1340 - K2358_1*X509*X1318
510	X_dot(510) =K4966_1 + K1698_2*X2331 + K3238_2*X3101 - K4196_1*X510 + K5681_1*X1214 - K1698_1*X510*X1000 - K3238_1*X510*X776
511	X_dot(511) =K4967_1 + K2206_2*X2585 + K2804_2*X2884 - K4197_1*X511 + K5682_1*X1310 - K2206_1*X511*X925 - K2804_1*X511*X1310
512	X_dot(512) =K4968_1 + K752_2*X1858 + K753_1*X1858 + K754_2*X1859 + K755_1*X1859 + K756_2*X1860 + K757_1*X1860 + K758_2*X1861 + K759_1*X1861 + K1080_2*X2022 + K1081_1*X2022 + K1474_2*X2219 + K1475_1*X2219 + K1476_2*X2220 + K1477_1*X2220 + K2428_2*X2696 + K2429_1*X2696 + K2430_2*X2697 + K2431_1*X2697 + K2432_2*X2698 + K2433_1*X2698 + K2434_2*X2699 + K2435_1*X2699 + K2436_2*X2700 + K2437_1*X2700 + K2438_2*X2701 + K2439_1*X2701 + K2440_2*X2702 + K2441_1*X2702 + K2442_2*X2703 + K2443_1*X2703 + K3016_2*X2990 + K3017_1*X2990 + K3018_2*X2991 + K3019_1*X2991 - K4198_1*X512 - K758_1*X28*X512 - K756_1*X119*X512 - K754_1*X512*X594 - K752_1*X512*X708 - K1080_1*X129*X512 - K1476_1*X278*X512 - K1474_1*X512*X577 - K2434_1*X25*X512 - K2430_1*X169*X512 - K2428_1*X284*X512 - K2436_1*X229*X512 - K2442_1*X329*X512 - K2438_1*X390*X512 - K2440_1*X398*X512 - K2432_1*X512*X762 - K3018_1*X237*X512 - K3016_1*X312*X512
513	X_dot(513) =K4969_1 + K1856_2*X2410 + K2510_2*X2737 + K2858_2*X2911 + K2882_2*X2923 + K2912_2*X2938 + K3323_1*X3143 - K4199_1*X513 + K5683_1*X1253 - K1856_1*X513*X823 - K2510_1*X513*X861 - K2858_1*X513*X1271 - K2882_1*X513*X1270 - K2912_1*X513*X970
514	X_dot(514) =K4970_1 + K1662_2*X2313 + K2598_2*X2781 + K3202_2*X3083 - K4200_1*X514 + K5684_1*X1205 - K1662_1*X514*X891 - K2598_1*X514*X727 - K3202_1*X514*X773
515	X_dot(515) =K4971_1 + K746_2*X1855 + K747_1*X1855 - K4201_1*X515 - K746_1*X515*X751
516	X_dot(516) =K4972_1 + K2272_2*X2618 + K2322_2*X2643 - K4202_1*X516 + K5685_1*X1322 - K2322_1*X187*X516 - K2272_1*X516*X1276
517	X_dot(517) =K4973_1 + K284_2*X1624 + K302_2*X1633 + K412_2*X1688 + K866_2*X1915 + K918_2*X1941 + K940_2*X1952 + K972_2*X1968 + K994_2*X1979 - K4203_1*X517 + K5686_1*X868 - K284_1*X337*X517 - K302_1*X517*X1195 - K412_1*X517*X1271 - K918_1*X205*X517 - K866_1*X517*X891 - K940_1*X517*X915 - K972_1*X517*X894 - K994_1*X517*X1005
518	X_dot(518) =K4974_1 + K566_2*X1765 - K4204_1*X518 + K5687_1*X937 - K566_1*X518*X1005
519	X_dot(519) =K4975_1 + K3628_2*X3296 - K4205_1*X519 - K3628_1*X519*X1454
520	X_dot(520) =K4976_1 + K1804_2*X2384 + K2752_2*X2858 - K4206_1*X520 + K5688_1*X1237 - K1804_1*X520*X1195 - K2752_1*X205*X520
521	X_dot(521) =K4977_1 + K2648_2*X2806 - K4207_1*X521 + K5689_1*X1381 - K2648_1*X521*X1381
522	X_dot(522) =K4978_1 + K108_2*X1536 + K109_1*X1536 + K860_2*X1912 + K861_1*X1912 + K862_2*X1913 + K863_1*X1913 + K1182_2*X2073 + K1183_1*X2073 + K1184_2*X2074 + K1185_1*X2074 + K1186_2*X2075 + K1187_1*X2075 + K1188_2*X2076 + K1189_1*X2076 + K1596_2*X2280 + K1597_1*X2280 + K2650_2*X2807 + K2651_1*X2807 + K2652_2*X2808 + K2653_1*X2808 - K4208_1*X522 - K108_1*X522*X763 - K860_1*X86*X522 - K862_1*X522*X565 - K1182_1*X213*X522 - K1188_1*X280*X522 - K1186_1*X448*X522 - K1184_1*X522*X552 - K1596_1*X41*X522 - K2650_1*X199*X522 - K2652_1*X302*X522
523	X_dot(523) =K4979_1 + K1592_2*X2278 - K4209_1*X523 + K5690_1*X1183 - K1592_1*X523*X1183
524	X_dot(524) =K4980_1 + K168_2*X1566 + K2018_2*X2491 - K4210_1*X524 + K5691_1*X843 - K168_1*X524*X891 - K2018_1*X524*X1271
525	X_dot(525) =K4981_1 + K3602_2*X3283 + K3603_1*X3283 + K3604_2*X3284 + K3605_1*X3284 - K4211_1*X525 - K3604_1*X275*X525 - K3602_1*X319*X525
526	X_dot(526) =K4982_1 + K1846_2*X2405 - K4212_1*X526 + K5692_1*X1250 - K1846_1*X526*X1250
527	X_dot(527) =K4983_1 + K1922_2*X2443 - K4213_1*X527 + K5693_1*X1269 - K1922_1*X527*X1310
528	X_dot(528) =K4984_1 + K2364_2*X2664 - K4214_1*X528 + K5694_1*X1342 - K2364_1*X528*X1318
529	X_dot(529) =K4985_1 + K2542_2*X2753 - K4215_1*X529 + K5695_1*X1368 - K2542_1*X529*X861
530	X_dot(530) =K374_2*X1669 + K3002_2*X2983 + K3644_1*X3305 - K4216_1*X530 + K5696_1*X902 - K374_1*X530*X915 - K3002_1*X530*X1145
531	X_dot(531) =K4986_1 + K2362_2*X2663 + K2424_2*X2694 - K4217_1*X531 + K5697_1*X1341 - K2362_1*X531*X1318 - K2424_1*X531*X1341
532	X_dot(532) =K4987_1 + K370_2*X1667 + K2312_2*X2638 - K4218_1*X532 + K5698_1*X900 - K370_1*X532*X915 - K2312_1*X187*X532
533	X_dot(533) =K4988_1 + K790_2*X1877 - K4219_1*X533 + K5699_1*X990 - K790_1*X533*X861
534	X_dot(534) =K4989_1 + K3333_1*X3148 - K4220_1*X534 + K5700_1*X1447
535	X_dot(535) =K4990_1 + K2262_2*X2613 - K4221_1*X535 + K5701_1*X1320 - K2262_1*X535*X1276
536	X_dot(536) =K4991_1 + K788_2*X1876 + K1976_2*X2470 + K2064_2*X2514 + K2198_2*X2581 + K3551_1*X3257 - K4222_1*X536 + K5702_1*X989 - K788_1*X536*X861 - K1976_1*X536*X1271 - K2064_1*X536*X1270 - K2198_1*X536*X925
537	X_dot(537) =K4992_1 + K1028_2*X1996 - K4223_1*X537 + K5703_1*X1041 - K1028_1*X187*X537
538	X_dot(538) =K4993_1 + K1842_2*X2403 - K4224_1*X538 + K5704_1*X1249 - K1842_1*X538*X1028
539	X_dot(539) =K4994_1 + K2122_2*X2543 + K2414_2*X2689 + K3072_2*X3018 - K4225_1*X539 + K5705_1*X1297 - K2122_1*X539*X1273 - K2414_1*X539*X980 - K3072_1*X539*X727
540	X_dot(540) =K4995_1 + K1810_2*X2387 - K4226_1*X540 + K5706_1*X1239 - K1810_1*X540*X1195
541	X_dot(541) =K4996_1 + K2324_2*X2644 - K4227_1*X541 + K5707_1*X1333 - K2324_1*X187*X541
542	X_dot(542) =K4997_1 + K140_2*X1552 - K4228_1*X542 + K5708_1*X830 - K140_1*X542*X891
543	X_dot(543) =K4998_1 + K110_2*X1537 + K404_2*X1684 + K456_2*X1710 + K472_2*X1718 + K782_2*X1873 + K3204_2*X3084 + K3559_1*X3261 - K4229_1*X543 + K5709_1*X818 - K110_1*X543*X891 - K404_1*X543*X1271 - K456_1*X543*X894 - K472_1*X543*X1270 - K782_1*X543*X861 - K3204_1*X543*X773
544	X_dot(544) =K4999_1 + K656_2*X1810 + K742_2*X1853 - K4230_1*X544 + K5710_1*X958 - K656_1*X187*X544 - K742_1*X544*X1341

545	X_dot(545) =K5000_1 + K2320_2*X2642 - K4231_1*X545 + K5711_1*X1332 - K2320_1*X187*X545
546	X_dot(546) =K5001_1 + K146_2*X1555 - K4232_1*X546 + K5712_1*X833 - K146_1*X546*X891
547	X_dot(547) =K5002_1 + K288_2*X1626 - K4233_1*X547 + K5713_1*X869 - K288_1*X547*X1195
548	X_dot(548) =K5003_1 + K1144_2*X2054 - K4234_1*X548 + K5714_1*X1064 - K1144_1*X548*X1031
549	X_dot(549) =K5004_1 + K2078_2*X2521 - K4235_1*X549 + K5715_1*X1295 - K2078_1*X549*X1270
550	X_dot(550) =K5005_1 + K666_2*X1815 - K4236_1*X550 + K5716_1*X961 - K666_1*X187*X550
551	X_dot(551) =K5006_1 + K2028_2*X2496 - K4237_1*X551 + K5717_1*X1291 - K2028_1*X551*X894
552	X_dot(552) =K5007_1 + K704_2*X1834 + K1184_2*X2074 + K1584_2*X2274 + K2632_2*X2798 - K4238_1*X552 + K5718_1*X972 - K704_1*X552*X1318 - K1184_1*X522*X552 - K1584_1*X552*X810 - K2632_1*X552*X1031
553	X_dot(553) =K5008_1 + K152_2*X1558 + K3206_2*X3085 - K4239_1*X553 + K5719_1*X836 - K152_1*X553*X891 - K3206_1*X553*X773
554	X_dot(554) =K5009_1 + K1878_2*X2421 - K4240_1*X554 + K5720_1*X1261 - K1878_1*X554*X1260
555	X_dot(555) =K5010_1 + K1824_2*X2394 - K4241_1*X555 + K5721_1*X1245 - K1824_1*X555*X1195
556	X_dot(556) =K5011_1 + K2558_2*X2761 + K2568_2*X2766 - K4242_1*X556 + K5722_1*X1372 - K2558_1*X556*X1130 - K2568_1*X556*X1372
557	X_dot(557) =K5012_1 + K3302_2*X3133 + K3303_1*X3133 - K4243_1*X557 - K3302_1*X557*X1031
558	X_dot(558) =K5013_1 + K3484_2*X3224 + K3485_1*X3224 - K4244_1*X558 - K3484_1*X558*X823
559	X_dot(559) =K5014_1 + K2868_2*X2916 + K2888_2*X2926 + K2938_2*X2951 + K3022_2*X2993 - K4245_1*X559 + K5723_1*X1411 - K2868_1*X559*X1271 - K2888_1*X559*X1270 - K2938_1*X559*X1036 - K3022_1*X559*X1411
560	X_dot(560) =K5015_1 + K2684_2*X2824 - K4246_1*X560 + K5724_1*X1388 - K2684_1*X560*X1451
561	X_dot(561) =K5016_1 + K1078_2*X2021 - K4247_1*X561 + K5725_1*X1054 - K1078_1*X561*X1341
562	X_dot(562) =K5017_1 + K1998_2*X2481 + K2084_2*X2524 - K4248_1*X562 + K5726_1*X1283 - K1998_1*X562*X1271 - K2084_1*X562*X1270
563	X_dot(563) =K5018_1 + K400_2*X1682 - K4249_1*X563 + K5727_1*X912 - K400_1*X563*X1185
564	X_dot(564) =K5019_1 + K2030_2*X2497 + K2166_2*X2565 + K2374_2*X2669 - K4250_1*X564 + K5728_1*X1292 - K2030_1*X564*X894 - K2166_1*X564*X1282 - K2374_1*X564*X1145
565	X_dot(565) =K5020_1 + K862_2*X1913 + K1002_2*X1983 + K1004_2*X1984 + K2526_2*X2745 - K4251_1*X565 + K5729_1*X1010 - K862_1*X522*X565 - K1002_1*X565*X925 - K1004_1*X565*X1313 - K2526_1*X565*X861
566	X_dot(566) =K5021_1 + K976_2*X1970 - K4252_1*X566 + K5730_1*X1033 - K976_1*X566*X894
567	X_dot(567) =K5022_1 + K98_2*X1531 + K434_2*X1699 + K504_2*X1734 + K3251_1*X3107 + K3578_2*X3271 + K3616_2*X3290 + K3640_2*X3302 - K4253_1*X567 + K5731_1*X815 - K98_1*X567*X810 - K434_1*X567*X1271 - K504_1*X567*X1270 - K3578_1*X567*X649 - K3640_1*X567*X699 - K3616_1*X567*X1018
568	X_dot(568) =K5023_1 + K238_2*X1601 + K384_2*X1674 + K432_2*X1698 + K502_2*X1733 + K3245_1*X3104 + K3249_1*X3106 + K3582_2*X3273 + K3586_2*X3275 - K4254_1*X568 + K5732_1*X856 - K238_1*X568*X1451 - K384_1*X568*X915 - K432_1*X568*X1271 - K502_1*X568*X1270 - K3582_1*X568*X699 - K3586_1*X568*X737
569	X_dot(569) =K5024_1 + K226_2*X1595 - K4255_1*X569 + K5733_1*X854 - K226_1*X569*X1393
570	X_dot(570) =K5025_1 + K126_2*X1545 + K826_2*X1895 + K1128_2*X2046 + K2658_2*X2811 - K4256_1*X570 + K5734_1*X825 - K126_1*X570*X891 - K826_1*X570*X1031 - K1128_1*X570*X894 - K2658_1*X570*X1000
571	X_dot(571) =K5026_1 + K48_2*X1506 - K4257_1*X571 + K5735_1*X797 - K48_1*X571*X1031
572	X_dot(572) =K5027_1 + K1676_2*X2320 - K4258_1*X572 + K5736_1*X1210 - K1676_1*X572*X1000
573	X_dot(573) =K5028_1 + K2234_2*X2599 + K2942_2*X2953 - K4259_1*X573 + K5737_1*X1316 - K2234_1*X573*X1036 - K2942_1*X573*X1316
574	X_dot(574) =K5029_1 + K2236_2*X2600 - K4260_1*X574 + K5738_1*X1317 - K2236_1*X574*X1036
575	X_dot(575) =K5030_1 + K356_2*X1660 + K2250_2*X2607 + K2820_2*X2892 + K2824_2*X2894 + K2830_2*X2897 + K3387_1*X3175 - K4261_1*X575 + K5739_1*X894 - K356_1*X575*X849 - K2250_1*X575*X1316 - K2824_1*X575*X787 - K2820_1*X575*X1409 - K2830_1*X575*X1407
576	X_dot(576) =K5031_1 + K2716_2*X2840 + K2746_2*X2855 + K3443_1*X3203 - K4262_1*X576 + K5740_1*X1395 - K2746_1*X205*X576 - K2716_1*X576*X1195
577	X_dot(577) =K5032_1 + K938_2*X1951 + K1474_2*X2219 + K2444_2*X2704 - K4263_1*X577 + K5741_1*X1032 - K938_1*X577*X915 - K1474_1*X512*X577 - K2444_1*X61*X577
578	X_dot(578) =K5033_1 + K1306_2*X2135 + K2992_2*X2978 - K4264_1*X578 + K5742_1*X1108 - K1306_1*X578*X1107 - K2992_1*X578*X1318
579	X_dot(579) =K5034_1 + K3126_2*X3045 + K3606_2*X3285 - K4265_1*X579 + K5743_1*X1441 - K3126_1*X187*X579 - K3606_1*X579*X603
580	X_dot(580) =K5035_1 + K2874_2*X2919 - K4266_1*X580 + K5744_1*X1412 - K2874_1*X580*X894
581	X_dot(581) =K5036_1 + K2734_2*X2849 - K4267_1*X581 + K5745_1*X1401 - K2734_1*X581*X1195
582	X_dot(582) =K5037_1 + K1484_2*X2224 - K4268_1*X582 + K5746_1*X1149 - K1484_1*X582*X1293
583	X_dot(583) =K5038_1 + K2296_2*X2630 - K4269_1*X583 + K5747_1*X1328 - K2296_1*X187*X583
584	X_dot(584) =K5039_1 + K1142_2*X2053 + K1143_1*X2053 + K1506_2*X2235 + K1507_1*X2235 + K1508_2*X2236 + K1509_1*X2236 - K4270_1*X584 - K1142_1*X339*X584 - K1508_1*X584*X640 - K1506_1*X584*X671
585	X_dot(585) =K5040_1 + K1628_2*X2296 - K4271_1*X585 + K5748_1*X1196 - K1628_1*X585*X891
586	X_dot(586) =K5041_1 + K34_2*X1499 - K4272_1*X586 + K5749_1*X790 - K34_1*X586*X1031
587	X_dot(587) =K5042_1 + K612_2*X1788 + K1096_2*X2030 + K1760_2*X2362 + K1761_1*X2362 - K4273_1*X587 + K5750_1*X946 - K612_1*X587*X1006 - K1096_1*X587*X748 - K1760_1*X264*X587
588	X_dot(588) =K5043_1 + K114_2*X1539 + K408_2*X1686 + K2258_2*X2611 - K4274_1*X588 + K5751_1*X819 - K114_1*X588*X891 - K408_1*X588*X1271 - K2258_1*X588*X1276
589	X_dot(589) =K5044_1 + K864_2*X1914 + K865_1*X1914 + K1598_2*X2281 + K1599_1*X2281 - K4275_1*X589 - K864_1*X589*X674 - K1598_1*X229*X589
590	X_dot(590) =K5045_1 + K2548_2*X2756 - K4276_1*X590 + K5752_1*X1369 - K2548_1*X590*X861
591	X_dot(591) =K5046_1 + K2244_2*X2604 + K2245_1*X2604 - K4277_1*X591 - K2244_1*X264*X591
592	X_dot(592) =K5047_1 + K3610_2*X3287 - K4278_1*X592 - K3610_1*X457*X592
593	X_dot(593) =K5048_1 + K2738_2*X2851 - K4279_1*X593 + K5753_1*X1402 - K2738_1*X593*X1195
594	X_dot(594) =K5049_1 + K754_2*X1859 - K4280_1*X594 + K5754_1*X986 - K754_1*X512*X594
595	X_dot(595) =K5050_1 + K882_2*X1923 + K1834_2*X2399 + K2760_2*X2862 - K4281_1*X595 + K5755_1*X1017 - K882_1*X595*X1000 - K1834_1*X595*X1195 - K2760_1*X205*X595
596	X_dot(596) =K5051_1 + K558_2*X1761 + K559_1*X1761 + K560_2*X1762 + K561_1*X1762 + K2172_2*X2568 + K2173_1*X2568 + K2174_2*X2569 + K2175_1*X2569 - K4282_1*X596 - K558_1*X596*X632 - K560_1*X596*X620 - K2174_1*X323*X596 - K2172_1*X596*X619
597	X_dot(597) =K5052_1 + K1516_2*X2240 - K4283_1*X597 + K5756_1*X1160 - K1516_1*X597*X1031
598	X_dot(598) =K5053_1 + K2582_2*X2773 + K2583_1*X2773 + K2584_2*X2774 + K2585_1*X2774 + K3064_2*X3014 + K3065_1*X3014 - K4284_1*X598 - K2582_1*X503*X598 - K2584_1*X495*X598 - K3064_1*X320*X598
599	X_dot(599) =K5054_1 + K632_2*X1798 - K4285_1*X599 + K5757_1*X951 - K632_1*X599*X1006
600	X_dot(600) =K5055_1 + K1510_2*X2237 - K4286_1*X600 + K5758_1*X1158 - K1510_1*X600*X1031
601	X_dot(601) =K5056_1 + K2698_2*X2831 - K4287_1*X601 + K5759_1*X1390 - K2698_1*X601*X1159
602	X_dot(602) =K5057_1 + K178_2*X1571 + K208_2*X1586 + K3208_2*X3086 - K4288_1*X602 + K5760_1*X847 - K178_1*X602*X891 - K208_1*X602*X1000 - K3208_1*X602*X773
603	X_dot(603) =K5058_1 + K3606_2*X3285 + K3607_1*X3285 - K4289_1*X603 - K3606_1*X579*X603

604	X_dot(604) =K5059_1 + K1168_2*X2066 + K1350_2*X2157 + K1362_2*X2163 + K2464_2*X2714 + K2476_2*X2720 - K4290_1*X604 + K5761_1*X1074 - K1168_1*X604*X1173 - K1350_1*X604*X1271 - K1362_1*X604*X1270 - K2476_1*X604*X1154 - K2464_1*X604*X1411
605	X_dot(605) =K5060_1 + K166_2*X1565 + K198_2*X1581 - K4291_1*X605 + K5762_1*X842 - K166_1*X605*X891 - K198_1*X605*X1000
606	X_dot(606) =K5061_1 + K3560_2*X3262 + K3561_1*X3262 - K4292_1*X606 - K3560_1*X606*X855
607	X_dot(607) =K5062_1 + K1700_2*X2332 + K1710_2*X2337 - K4293_1*X607 + K5763_1*X1215 - K1700_1*X607*X1000 - K1710_1*X607*X1001
608	X_dot(608) =K5063_1 + K1960_2*X2462 + K2048_2*X2506 - K4294_1*X608 + K5764_1*X1277 - K1960_1*X608*X1271 - K2048_1*X608*X1270
609	X_dot(609) =K5064_1 + K634_2*X1799 + K1020_2*X1992 + K1208_2*X2086 + K1348_2*X2156 + K2460_2*X2712 + K2890_2*X2927 + K2918_2*X2941 + K3152_2*X3058 - K4295_1*X609 + K5765_1*X952 - K634_1*X609*X1327 - K1020_1*X609*X1006 - K1208_1*X609*X891 - K1348_1*X609*X1271 - K2460_1*X609*X1411 - K2890_1*X609*X1270 - K2918_1*X609*X1005 - K3152_1*X609*X1154
610	X_dot(610) =K5065_1 + K2302_2*X2633 - K4296_1*X610 + K5766_1*X1330 - K2302_1*X187*X610
611	X_dot(611) =K5066_1 + K1244_2*X2104 - K4297_1*X611 + K5767_1*X1094 - K1244_1*X611*X1195
612	X_dot(612) =K5067_1 + K150_2*X1557 + K218_2*X1591 - K4298_1*X612 + K5768_1*X835 - K150_1*X612*X891 - K218_1*X612*X1393
613	X_dot(613) =K5068_1 + K2354_2*X2659 - K4299_1*X613 + K5769_1*X1339 - K2354_1*X613*X1318
614	X_dot(614) =K5069_1 + K1644_2*X2304 - K4300_1*X614 + K5770_1*X1201 - K1644_1*X614*X891
615	X_dot(615) =K5070_1 + K260_2*X1612 + K564_2*X1764 + K762_2*X1863 + K3154_2*X3059 - K4301_1*X615 + K5771_1*X863 - K260_1*X330*X615 - K564_1*X615*X1005 - K762_1*X615*X1411 - K3154_1*X615*X1154
616	X_dot(616) =K5071_1 + K2188_2*X2576 - K4302_1*X616 + K5772_1*X1305 - K2188_1*X616*X1305
617	X_dot(617) =K5072_1 + K2920_2*X2942 - K4303_1*X617 + K5773_1*X1416 - K2920_1*X617*X1305
618	X_dot(618) =K5073_1 + K3562_2*X3263 + K3563_1*X3263 - K4304_1*X618 - K3562_1*X618*X907
619	X_dot(619) =K5074_1 + K2172_2*X2568 - K4305_1*X619 + K5774_1*X1301 - K2172_1*X596*X619
620	X_dot(620) =K5075_1 + K398_2*X1681 + K560_2*X1762 + K622_2*X1793 + K3531_1*X3247 + K3537_1*X3250 - K4306_1*X620 + K5775_1*X911 - K398_1*X620*X1185 - K560_1*X596*X620 - K622_1*X620*X1006
621	X_dot(621) =K5076_1 + K1212_2*X2088 - K4307_1*X621 + K5776_1*X1086 - K1212_1*X621*X1000
622	X_dot(622) =K5077_1 + K618_2*X1791 - K4308_1*X622 + K5777_1*X948 - K618_1*X622*X1006
623	X_dot(623) =K5078_1 + K172_2*X1568 + K444_2*X1704 + K7_2*X769 + K3116_2*X3040 + K3210_2*X3087 - K4309_1*X623 + K5778_1*X845 - K172_1*X623*X891 - K444_1*X623*X1271 - K7_1*X623*X802 - K3116_1*X623*X1270 - K3210_1*X623*X773
624	X_dot(624) =K5079_1 + K906_2*X1935 + K920_2*X1942 - K4310_1*X624 + K5779_1*X1025 - K920_1*X205*X624 - K906_1*X624*X1195
625	X_dot(625) =K5080_1 + K2990_2*X2977 - K4311_1*X625 + K5780_1*X1427 - K2990_1*X625*X1318
626	X_dot(626) =K5081_1 + K1780_2*X2372 - K4312_1*X626 + K5781_1*X1228 - K1780_1*X626*X1195
627	X_dot(627) =K5082_1 + K1806_2*X2385 + K2754_2*X2859 - K4313_1*X627 + K5782_1*X1238 - K1806_1*X627*X1195 - K2754_1*X205*X627
628	X_dot(628) =K5083_1 + K682_2*X1823 + K1446_2*X2205 + K1754_2*X2359 - K4314_1*X628 + K5783_1*X966 - K682_1*X628*X1318 - K1446_1*X628*X1145 - K1754_1*X628*X1159
629	X_dot(629) =K5084_1 + K2492_2*X2728 - K4315_1*X629 + K5784_1*X1359 - K2492_1*X629*X650
630	X_dot(630) =K5085_1 + K52_2*X1508 + K468_2*X1716 + K518_2*X1741 + K572_2*X1768 + K640_2*X1802 + K730_2*X1847 + K772_2*X1868 + K820_2*X1892 + K956_2*X1960 + K1084_2*X2024 + K3539_1*X3251 + K3543_1*X3253 - K4316_1*X630 + K5785_1*X799 - K52_1*X630*X1418 - K468_1*X630*X1270 - K518_1*X630*X1273 - K640_1*X187*X630 - K572_1*X630*X925 - K730_1*X630*X1366 - K772_1*X630*X1154 - K820_1*X630*X1031 - K956_1*X630*X1271 - K1084_1*X630*X1411
631	X_dot(631) =K5086_1 + K66_2*X1515 + K834_2*X1899 + K974_2*X1969 - K4317_1*X631 + K5786_1*X801 - K66_1*X631*X1418 - K834_1*X631*X1031 - K974_1*X631*X894
632	X_dot(632) =K5087_1 + K558_2*X1761 + K570_2*X1767 + K590_2*X1777 + K1962_2*X2463 + K2052_2*X2508 - K4318_1*X632 + K5787_1*X935 - K558_1*X596*X632 - K570_1*X632*X925 - K590_1*X632*X1316 - K1962_1*X632*X1271 - K2052_1*X632*X1270
633	X_dot(633) =K5088_1 + K1758_2*X2361 - K4319_1*X633 + K5788_1*X1223 - K1758_1*X633*X1159
634	X_dot(634) =K5089_1 + K1632_2*X2298 + K1904_2*X2434 + K3212_2*X3088 - K4320_1*X634 + K5789_1*X1198 - K1632_1*X634*X891 - K1904_1*X634*X915 - K3212_1*X634*X773
635	X_dot(635) =K5090_1 + K2968_2*X2966 - K4321_1*X635 + K5790_1*X1423 - K2968_1*X187*X635
636	X_dot(636) =K5091_1 + K984_2*X1974 + K1052_2*X2008 - K4322_1*X636 + K5791_1*X1034 - K1052_1*X187*X636 - K984_1*X636*X1270
637	X_dot(637) =K5092_1 + K50_2*X1507 + K818_2*X1891 + K992_2*X1978 - K4323_1*X637 + K5792_1*X798 - K50_1*X637*X1418 - K818_1*X637*X1031 - K992_1*X637*X1005
638	X_dot(638) =K5093_1 + K1562_2*X2263 - K4324_1*X638 + K5793_1*X1174 - K1562_1*X638*X1173
639	X_dot(639) =K5094_1 + K2270_2*X2617 - K4325_1*X639 + K5794_1*X1321 - K2270_1*X639*X1276
640	X_dot(640) =K5095_1 + K1508_2*X2236 - K4326_1*X640 + K5795_1*X1157 - K1508_1*X584*X640
641	X_dot(641) =K5096_1 + K1278_2*X2121 + K1658_2*X2311 + K2132_2*X2548 + K3214_2*X3089 - K4327_1*X641 + K5796_1*X1105 - K1278_1*X641*X1195 - K1658_1*X641*X891 - K2132_1*X641*X1273 - K3214_1*X641*X773
642	X_dot(642) =K5097_1 + K3382_2*X3173 + K3383_1*X3173 - K4328_1*X642 - K3382_1*X642*X907
643	X_dot(643) =K5098_1 + K1560_2*X2262 + K1736_2*X2350 - K4329_1*X643 + K5797_1*X1173 - K1560_1*X643*X1173 - K1736_1*X643*X780
644	X_dot(644) =K5099_1 + K2656_2*X2810 + K2910_2*X2937 + K3216_2*X3090 - K4330_1*X644 + K5798_1*X1383 - K2656_1*X644*X891 - K2910_1*X644*X1383 - K3216_1*X644*X773
645	X_dot(645) =K5100_1 + K886_2*X1925 + K2124_2*X2544 - K4331_1*X645 + K5799_1*X1018 - K886_1*X645*X1391 - K2124_1*X645*X1273
646	X_dot(646) =K5101_1 + K1198_2*X2081 + K3218_2*X3091 - K4332_1*X646 + K5800_1*X1082 - K1198_1*X646*X891 - K3218_1*X646*X773
647	X_dot(647) =K5102_1 + K1884_2*X2424 - K4333_1*X647 + K5801_1*X1262 - K1884_1*X647*X893
648	X_dot(648) =K5103_1 + K96_2*X1530 + K236_2*X1600 - K4334_1*X648 + K5802_1*X814 - K96_1*X648*X810 - K236_1*X648*X1451
649	X_dot(649) =K5104_1 + K660_2*X1812 + K3578_2*X3271 + K3579_1*X3271 + K3580_2*X3272 + K3581_1*X3272 - K4335_1*X649 + K5803_1*X959 - K660_1*X187*X649 - K3580_1*X434*X649 - K3578_1*X567*X649
650	X_dot(650) =K5105_1 + K2490_2*X2727 + K2491_1*X2727 + K2492_2*X2728 + K2493_1*X2728 - K4336_1*X650 - K2490_1*X99*X650 - K2492_1*X629*X650
651	X_dot(651) =K5106_1 + K1908_2*X2436 - K4337_1*X651 + K5804_1*X1266 - K1908_1*X651*X915
652	X_dot(652) =K5107_1 + K410_2*X1687 + K476_2*X1720 + K760_2*X1862 + K774_2*X1869 - K4338_1*X652 + K5805_1*X914 - K410_1*X652*X1271 - K476_1*X652*X1270 - K774_1*X652*X1154 - K760_1*X652*X1411
653	X_dot(653) =K5108_1 + K1534_2*X2249 - K4339_1*X653 + K5806_1*X1167 - K1534_1*X653*X1031
654	X_dot(654) =K5109_1 + K1040_2*X2002 + K1344_2*X2154 - K4340_1*X654 + K5807_1*X1044 - K1040_1*X187*X654 - K1344_1*X654*X1271
655	X_dot(655) =K5110_1 + K2550_2*X2757 - K4341_1*X655 + K5808_1*X1370 - K2550_1*X655*X1370
656	X_dot(656) =K5111_1 + K2416_2*X2690 - K4342_1*X656 + K5809_1*X1354 - K2416_1*X656*X980
657	X_dot(657) =K5112_1 + K1770_2*X2367 - K4343_1*X657 + K5810_1*X1226 - K1770_1*X331*X657
658	X_dot(658) =K5113_1 + K1058_2*X2011 - K4344_1*X658 + K5811_1*X1049 - K1058_1*X658*X1318
659	X_dot(659) =K5114_1 + K2214_2*X2589 - K4345_1*X659 + K5812_1*X1312 - K2214_1*X659*X925
660	X_dot(660) =K5115_1 + K3242_2*X3103 + K3243_1*X3103 - K4346_1*X660 - K3242_1*X660*X1179
661	X_dot(661) =K5116_1 - K4347_1*X661
662	X_dot(662) =K5117_1 + K76_2*X1520 + K138_2*X1551 + K576_2*X1770 - K4348_1*X662 + K5813_1*X804 - K76_1*X662*X1173 - K138_1*X662*X891 - K576_1*X662*X925

663	X_dot(663) =K5118_1 + K3584_2*X3274 + K3588_2*X3276 - K4349_1*X663 - K3584_1*X663*X699 - K3588_1*X663*X737
664	X_dot(664) =K5119_1 + K1950_2*X2457 + K3271_1*X3117 - K4350_1*X664 + K5814_1*X1275 - K1950_1*X664*X1275
665	X_dot(665) =K5120_1 + K802_2*X1883 + K1472_2*X2218 - K4351_1*X665 + K5815_1*X995 - K802_1*X665*X861 - K1472_1*X665*X1181
666	X_dot(666) =K5121_1 + K810_2*X1887 + K811_1*X1887 + K2612_2*X2788 + K2613_1*X2788 + K2614_2*X2789 + K2615_1*X2789 + K2616_2*X2790 + K2617_1*X2790 + K2618_2*X2791 + K2619_1*X2791 + K2620_2*X2792 + K2621_1*X2792 - K4352_1*X666 - K810_1*X65*X666 - K2616_1*X106*X666 - K2618_1*X99*X666 - K2612_1*X170*X666 - K2614_1*X424*X666 - K2620_1*X487*X666
667	X_dot(667) =K3662_1*X3323 - K4353_1*X667
668	X_dot(668) =K5122_1 + K1166_2*X2065 - K4354_1*X668 + K5816_1*X1073 - K1166_1*X668*X1173
669	X_dot(669) =K5123_1 + K680_2*X1822 - K4355_1*X669 + K5817_1*X965 - K680_1*X669*X1318
670	X_dot(670) =K5124_1 + K1502_2*X2233 - K4356_1*X670 + K5818_1*X1155 - K1502_1*X12*X670
671	X_dot(671) =K5125_1 + K1506_2*X2235 - K4357_1*X671 + K5819_1*X1156 - K1506_1*X584*X671
672	X_dot(672) =K5126_1 + K652_2*X1808 - K4358_1*X672 + K5820_1*X956 - K652_1*X187*X672
673	X_dot(673) =K5127_1 + K3405_1*X3184 - K4359_1*X673 + K5821_1*X1452
674	X_dot(674) =K5128_1 + K864_2*X1914 - K4360_1*X674 + K5822_1*X1011 - K864_1*X589*X674
675	X_dot(675) =K5129_1 + K2404_2*X2684 - K4361_1*X675 + K5823_1*X1352 - K2404_1*X675*X1352
676	X_dot(676) =K5130_1 + K1154_2*X2059 - K4362_1*X676 + K5824_1*X1069 - K1154_1*X676*X1031
677	X_dot(677) =K5131_1 + K2678_2*X2821 + K3441_1*X3202 - K4363_1*X677 + K5825_1*X1387 - K2678_1*X677*X1393
678	X_dot(678) =K5132_1 + K2954_2*X2959 - K4364_1*X678 + K5826_1*X1422 - K2954_1*X678*X1006
679	X_dot(679) =K5133_1 + K2288_2*X2626 - K4365_1*X679 + K5827_1*X1325 - K2288_1*X679*X1006
680	X_dot(680) =K5134_1 + K2274_2*X2619 + K2636_2*X2800 + K3279_1*X3121 - K4366_1*X680 + K5828_1*X1323 - K2274_1*X680*X1276 - K2636_1*X680*X1173
681	X_dot(681) =K5135_1 + K380_2*X1672 - K4367_1*X681 + K5829_1*X905 - K380_1*X681*X915
682	X_dot(682) =K5136_1 + K1608_2*X2286 + K2254_2*X2609 - K4368_1*X682 + K5830_1*X1189 - K1608_1*X682*X891 - K2254_1*X682*X1276
683	X_dot(683) =K5137_1 + K2180_2*X2572 - K4369_1*X683 + K5831_1*X1303 - K2180_1*X683*X1005
684	X_dot(684) =K5138_1 + K292_2*X1628 - K4370_1*X684 + K5832_1*X871 - K292_1*X684*X1195
685	X_dot(685) =K5139_1 + K2280_2*X2622 - K4371_1*X685 + K5833_1*X1324 - K2280_1*X685*X1276
686	X_dot(686) =K5140_1 + K316_2*X1640 - K4372_1*X686 + K5834_1*X881 - K316_1*X686*X1195
687	X_dot(687) =K5141_1 + K3070_2*X3017 - K4373_1*X687 + K5835_1*X1437 - K3070_1*X687*X1437
688	X_dot(688) =K5142_1 + K1396_2*X2180 - K4374_1*X688 + K5836_1*X1129 - K1396_1*X187*X688
689	X_dot(689) =K5143_1 + K74_2*X1519 - K4375_1*X689 + K5837_1*X803 - K74_1*X689*X1173
690	X_dot(690) =K5144_1 + K1540_2*X2252 + K2626_2*X2795 - K4376_1*X690 + K5838_1*X1169 - K1540_1*X690*X1031 - K2626_1*X690*X1169
691	X_dot(691) =K5145_1 + K3010_2*X2987 - K4377_1*X691 + K5839_1*X1429 - K3010_1*X691*X980
692	X_dot(692) =K5146_1 + K1492_2*X2228 - K4378_1*X692 + K5840_1*X1151 - K1492_1*X692*X861
693	X_dot(693) =K5147_1 + K1160_2*X2062 + K1480_2*X2222 + K3156_2*X3060 - K4379_1*X693 + K5841_1*X1072 - K1160_1*X693*X1031 - K1480_1*X693*X1411 - K3156_1*X693*X1154
694	X_dot(694) =K5148_1 + K2564_2*X2764 + K2578_2*X2771 - K4380_1*X694 + K5842_1*X1374 - K2564_1*X694*X792 - K2578_1*X694*X793
695	X_dot(695) =K5149_1 + K118_2*X1541 + K2398_2*X2681 + K3220_2*X3092 - K4381_1*X695 + K5843_1*X821 - K118_1*X695*X891 - K2398_1*X695*X1366 - K3220_1*X695*X773
696	X_dot(696) =K5150_1 + K1338_2*X2151 - K4382_1*X696 + K5844_1*X1116 - K1338_1*X696*X1271
697	X_dot(697) =K5151_1 + K1462_2*X2213 - K4383_1*X697 + K5845_1*X1146 - K1462_1*X697*X980
698	X_dot(698) =K5152_1 + K2242_2*X2603 - K4384_1*X698 + K5846_1*X1319 - K2242_1*X698*X1036
699	X_dot(699) =K5153_1 + K3582_2*X3273 + K3583_1*X3273 + K3584_2*X3274 + K3585_1*X3274 + K3594_2*X3279 + K3595_1*X3279 + K3640_2*X3302 + K3641_1*X3302 - K4385_1*X699 - K3594_1*X290*X699 - K3582_1*X568*X699 - K3584_1*X663*X699 - K3640_1*X567*X699
700	X_dot(700) =K5154_1 + K894_2*X1929 + K895_1*X1929 + K3102_2*X3033 + K3103_1*X3033 - K4386_1*X700 - K894_1*X484*X700 - K3102_1*X47*X700
701	X_dot(701) =K5155_1 + K3564_2*X3264 + K3565_1*X3264 - K4387_1*X701 - K3564_1*X701*X855
702	X_dot(702) =K5156_1 + K2304_2*X2634 - K4388_1*X702 + K5847_1*X1331 - K2304_1*X187*X702
703	X_dot(703) =K5157_1 + K1368_2*X2166 - K4389_1*X703 + K5848_1*X1122 - K1368_1*X703*X1282
704	X_dot(704) =K5158_1 + K368_2*X1666 + K3118_2*X3041 - K4390_1*X704 + K5849_1*X899 - K368_1*X704*X915 - K3118_1*X704*X1270
705	X_dot(705) =K5159_1 + K3614_2*X3289 - K4391_1*X705 - K3614_1*X705*X1171
706	X_dot(706) =K5160_1 + K2016_2*X2490 + K2104_2*X2534 - K4392_1*X706 + K5850_1*X1288 - K2016_1*X706*X1271 - K2104_1*X706*X1270
707	X_dot(707) =K5161_1 + K258_2*X1611 - K4393_1*X707 + K5851_1*X862 - K258_1*X330*X707
708	X_dot(708) =K5162_1 + K752_2*X1858 + K2498_2*X2731 - K4394_1*X708 + K5852_1*X985 - K752_1*X512*X708 - K2498_1*X708*X861
709	X_dot(709) =K5163_1 + K2812_2*X2888 - K4395_1*X709 + K5853_1*X1406 - K2812_1*X709*X1406
710	X_dot(710) =K5164_1 + K1646_2*X2305 + K2316_2*X2640 + K2866_2*X2915 - K4396_1*X710 + K5854_1*X1202 - K1646_1*X710*X891 - K2316_1*X187*X710 - K2866_1*X710*X1271
711	X_dot(711) =K5165_1 + K2158_2*X2561 - K4397_1*X711 + K5855_1*X1298 - K2158_1*X711*X1282
712	X_dot(712) =K5166_1 + K1948_2*X2456 + K2248_2*X2606 - K4398_1*X712 + K5856_1*X1274 - K1948_1*X712*X1274 - K2248_1*X712*X1316
713	X_dot(713) =K5167_1 + K1674_2*X2319 + K1675_1*X2319 - K4399_1*X713 - K1674_1*X230*X713
714	X_dot(714) =K5168_1 + K312_2*X1638 + K540_2*X1752 + K630_2*X1797 + K1704_2*X2334 + K2096_2*X2530 - K4400_1*X714 + K5857_1*X879 - K312_1*X714*X1195 - K540_1*X714*X1103 - K630_1*X714*X1006 - K1704_1*X714*X1000 - K2096_1*X714*X1270
715	X_dot(715) =K5169_1 + K3244_2*X3104 + K3245_1*X3104 + K3518_2*X3241 + K3519_1*X3241 - K4401_1*X715 - K3244_1*X715*X856 - K3518_1*X715*X1456
716	X_dot(716) =K5170_1 + K2562_2*X2763 + K2563_1*X2763 - K4402_1*X716 - K2562_1*X169*X716
717	X_dot(717) =K5171_1 + K598_2*X1781 - K4403_1*X717 + K5858_1*X943 - K598_1*X717*X1276
718	X_dot(718) =K5172_1 + K1828_2*X2396 + K2770_2*X2867 - K4404_1*X718 + K5859_1*X1246 - K1828_1*X718*X1195 - K2770_1*X718*X1107
719	X_dot(719) =K5173_1 + K2402_2*X2683 - K4405_1*X719 + K5860_1*X1351 - K2402_1*X719*X1352
720	X_dot(720) =K5174_1 - K4406_1*X720
721	X_dot(721) =K5175_1 + K346_2*X1655 + K2072_2*X2518 - K4407_1*X721 + K5861_1*X890 - K346_1*X721*X823 - K2072_1*X721*X1270
722	X_dot(722) =K5176_1 + K30_2*X1497 + K766_2*X1865 + K3158_2*X3061 - K4408_1*X722 + K5862_1*X788 - K30_1*X722*X1031 - K766_1*X722*X1411 - K3158_1*X722*X1154
723	X_dot(723) =K5177_1 + K1794_2*X2379 - K4409_1*X723 + K5863_1*X1232 - K1794_1*X723*X1195
724	X_dot(724) =K5178_1 + K2624_2*X2794 - K4410_1*X724 + K5864_1*X1378 - K2624_1*X724*X1169
725	X_dot(725) =K5179_1 + K1134_2*X2049 - K4411_1*X725 + K5865_1*X1062 - K1134_1*X725*X1318
726	X_dot(726) =K5180_1 + K3046_2*X3005 - K4412_1*X726 + K5866_1*X1433 - K3046_1*X726*X861
727	X_dot(727) =K5181_1 + K2594_2*X2779 + K2595_1*X2779 + K2596_2*X2780 + K2597_1*X2780 + K2598_2*X2781 + K2599_1*X2781 + K2600_2*X2782 + K2601_1*X2782 + K3072_2*X3018 + K3073_1*X3018 - K4413_1*X727 - K2594_1*X264*X727 - K2600_1*X303*X727 - K2596_1*X453*X727 - K2598_1*X514*X727 - K3072_1*X539*X727
728	X_dot(728) =K5182_1 + K2026_2*X2495 + K2056_2*X2510 + K2142_2*X2553 - K4414_1*X728 + K5867_1*X1290 - K2026_1*X728*X894 - K2056_1*X728*X1270 - K2142_1*X728*X1103

729	X_dot(729) =K5183_1 + K2778_2*X2871 - K4415_1*X729 + K5868_1*X1405 - K2778_1*X729*X823
730	X_dot(730) =K5184_1 + K1614_2*X2289 - K4416_1*X730 + K5869_1*X1192 - K1614_1*X730*X891
731	X_dot(731) =K5185_1 + K246_2*X1605 + K3074_2*X3019 + K3075_1*X3019 - K4417_1*X731 + K5870_1*X859 - K246_1*X731*X780 - K3074_1*X424*X731
732	X_dot(732) =K5186_1 + K2554_2*X2759 - K4418_1*X732 + K5871_1*X1371 - K2554_1*X732*X1166
733	X_dot(733) =K5187_1 + K2160_2*X2562 - K4419_1*X733 + K5872_1*X1299 - K2160_1*X733*X1282
734	X_dot(734) =K5188_1 + K148_2*X1556 + K422_2*X1693 + K492_2*X1728 - K4420_1*X734 + K5873_1*X834 - K148_1*X734*X891 - K422_1*X734*X1271 - K492_1*X734*X1270
735	X_dot(735) =K5189_1 + K2586_2*X2775 + K2587_1*X2775 + K2588_2*X2776 + K2589_1*X2776 + K3066_2*X3015 + K3067_1*X3015 + K3068_2*X3016 + K3069_1*X3016 - K4421_1*X735 - K2586_1*X503*X735 - K2588_1*X495*X735 - K3066_1*X320*X735 - K3068_1*X361*X735
736	X_dot(736) =K5190_1 + K1792_2*X2378 - K4422_1*X736 + K5874_1*X1231 - K1792_1*X736*X1195
737	X_dot(737) =K5191_1 + K3586_2*X3275 + K3587_1*X3275 + K3588_2*X3276 + K3589_1*X3276 - K4423_1*X737 - K3586_1*X568*X737 - K3588_1*X663*X737
738	X_dot(738) =K5192_1 + K2724_2*X2844 - K4424_1*X738 + K5875_1*X1397 - K2724_1*X738*X1195
739	X_dot(739) =K5193_1 + K874_2*X1919 - K4425_1*X739 + K5876_1*X1015 - K874_1*X739*X891
740	X_dot(740) =K5194_1 + K1984_2*X2474 - K4426_1*X740 + K5877_1*X1279 - K1984_1*X740*X1271
741	X_dot(741) =K5195_1 + K1570_2*X2267 - K4427_1*X741 + K5878_1*X1177 - K1570_1*X741*X1173
742	X_dot(742) =K5196_1 + K1428_2*X2196 + K2208_2*X2586 + K2384_2*X2674 - K4428_1*X742 + K5879_1*X1137 - K1428_1*X742*X1318 - K2208_1*X742*X925 - K2384_1*X742*X1145
743	X_dot(743) =K5197_1 + K1672_2*X2318 + K1673_1*X2318 - K4429_1*X743 - K1672_1*X211*X743
744	X_dot(744) =K5198_1 + K1280_2*X2122 + K1300_2*X2132 - K4430_1*X744 + K5880_1*X1106 - K1300_1*X205*X744 - K1280_1*X744*X1195
745	X_dot(745) =K5199_1 + K88_2*X1526 - K4431_1*X745 + K5881_1*X810 - K88_1*X745*X810
746	X_dot(746) =K5200_1 + K2948_2*X2956 - K4432_1*X746 + K5882_1*X1421 - K2948_1*X746*X1276
747	X_dot(747) =K5201_1 + K2560_2*X2762 + K2570_2*X2767 - K4433_1*X747 + K5883_1*X1373 - K2560_1*X747*X1130 - K2570_1*X747*X1373
748	X_dot(748) =K5202_1 + K1096_2*X2030 + K1097_1*X2030 - K4434_1*X748 - K1096_1*X587*X748
749	X_dot(749) =K5203_1 + K1946_2*X2455 + K1947_1*X2455 + K2832_2*X2898 + K2833_1*X2898 - K4435_1*X749 - K1946_1*X451*X749 - K2832_1*X314*X749
750	X_dot(750) =K5204_1 + K1150_2*X2057 + K1478_2*X2221 + K3160_2*X3062 - K4436_1*X750 + K5884_1*X1067 - K1150_1*X750*X1031 - K1478_1*X750*X1411 - K3160_1*X750*X1154
751	X_dot(751) =K5205_1 + K746_2*X1855 - K4437_1*X751 + K5885_1*X982 - K746_1*X515*X751
752	X_dot(752) =K5206_1 + K320_2*X1642 - K4438_1*X752 + K5886_1*X883 - K320_1*X752*X1195
753	X_dot(753) =K5207_1 + K164_2*X1564 - K4439_1*X753 + K5887_1*X841 - K164_1*X753*X891
754	X_dot(754) =K5208_1 + K2292_2*X2628 - K4440_1*X754 + K5888_1*X1327 - K2292_1*X754*X1006
755	X_dot(755) =K5209_1 + K1724_2*X2344 - K4441_1*X755 + K5889_1*X1218 - K1724_1*X755*X1393
756	X_dot(756) =K5210_1 + K1914_2*X2439 - K4442_1*X756 + K5890_1*X1268 - K1914_1*X756*X1267
757	X_dot(757) =K5211_1 + K3634_2*X3299 - K4443_1*X757 - K3634_1*X757*X1484
758	X_dot(758) =K5212_1 + K322_2*X1643 - K4444_1*X758 + K5891_1*X884 - K322_1*X758*X1195
759	X_dot(759) =K5213_1 + K896_2*X1930 + K916_2*X1940 + K924_2*X1944 - K4445_1*X759 + K5892_1*X1021 - K916_1*X205*X759 - K896_1*X759*X1195 - K924_1*X759*X1107
760	X_dot(760) =K5214_1 + K804_2*X1884 - K4446_1*X760 + K5893_1*X996 - K804_1*X760*X1370
761	X_dot(761) =K5215_1 + K1670_2*X2317 - K4447_1*X761 + K5894_1*X1208 - K1670_1*X761*X891
762	X_dot(762) =K5216_1 + K2420_2*X2692 + K2432_2*X2698 - K4448_1*X762 + K5895_1*X1355 - K2432_1*X512*X762 - K2420_1*X762*X1341
763	X_dot(763) =K5217_1 + K94_2*X1529 + K108_2*X1536 + K250_2*X1607 - K4449_1*X763 + K5896_1*X813 - K108_1*X522*X763 - K94_1*X763*X810 - K250_1*X763*X1159
764	X_dot(764) =K5218_1 + K1420_2*X2192 - K4450_1*X764 + K5897_1*X1135 - K1420_1*X764*X1318
765	X_dot(765) =K5219_1 + K1404_2*X2184 + K2556_2*X2760 - K4451_1*X765 + K5898_1*X1130 - K1404_1*X187*X765 - K2556_1*X765*X1130
766	X_dot(766) =K5220_1 + K3638_2*X3301 - K4452_1*X766 - K3638_1*X766*X1431
767	X_dot(767) =K5221_1 + K2846_2*X2905 - K4453_1*X767 + K5899_1*X1410 - K2846_1*X767*X1410
768	X_dot(768) =K5222_1 + K326_2*X1645 - K4454_1*X768 + K5900_1*X886 - K326_1*X768*X1195
769	X_dot(769) =K3170_2*X3067 - K7_2*X769 + K3172_2*X3068 + K3178_2*X3071 + K5901_1*X1443 + K7_1*X623*X802 - K3170_1*X769*X1031 - K3172_1*X769*X1418 - K3178_1*X769*X1411
770	X_dot(770) =K8_1*X1182*X1217 - K8_2*X770
771	X_dot(771) =K3164_2*X3064 - K2_2*X771 + K3166_2*X3065 + K3168_2*X3066 + K3176_2*X3070 + K3180_2*X3072 + K3575_1*X3269 + K5902_1*X1442 + K2_1*X857*X1265 - K3168_1*X187*X771 - K3176_1*X771*X915 - K3164_1*X771*X1159 - K3166_1*X771*X1293 - K3180_1*X771*X1486
772	X_dot(772) =K9_1 - K9_2*X772 + K3174_2*X3069 + K3240_2*X3102 + K5903_1*X1444 - K3174_1*X772*X1273 - K3240_1*X772*X1270
773	X_dot(773) =K3182_2*X3073 - K3_2*X773 + K3183_1*X3073 + K3184_2*X3074 + K3185_1*X3074 + K3186_2*X3075 + K3187_1*X3075 + K3188_2*X3076 + K3189_1*X3076 + K3190_2*X3077 + K3191_1*X3077 + K3192_2*X3078 + K3193_1*X3078 + K3194_2*X3079 + K3195_1*X3079 + K3196_2*X3080 + K3197_1*X3080 + K3198_2*X3081 + K3199_1*X3081 + K3200_2*X3082 + K3201_1*X3082 + K3202_2*X3083 + K3203_1*X3083 + K3204_2*X3084 + K3205_1*X3084 + K3206_2*X3085 + K3207_1*X3085 + K3208_2*X3086 + K3209_1*X3086 + K3210_2*X3087 + K3211_1*X3087 + K3212_2*X3088 + K3213_1*X3088 + K3214_2*X3089 + K3215_1*X3089 + K3216_2*X3090 + K3217_1*X3090 + K3218_2*X3091 + K3219_1*X3091 + K3220_2*X3092 + K3221_1*X3092 + K3_1*X891*X1420 - K3182_1*X7*X773 - K3184_1*X13*X773 - K3186_1*X116*X773 - K3188_1*X124*X773 - K3190_1*X171*X773 - K3192_1*X316*X773 - K3194_1*X327*X773 - K3196_1*X349*X773 - K3198_1*X384*X773 - K3200_1*X473*X773 - K3202_1*X514*X773 - K3204_1*X543*X773 - K3206_1*X553*X773 - K3208_1*X602*X773 - K3210_1*X623*X773 - K3212_1*X634*X773 - K3214_1*X641*X773 - K3216_1*X644*X773 - K3218_1*X646*X773 - K3220_1*X695*X773
774	X_dot(774) =K1_1*X947*X966 - K1_2*X774
775	X_dot(775) =K4_1*X1026*X1217 - K4_2*X775
776	X_dot(776) =K3222_2*X3093 - K5_2*X776 + K3223_1*X3093 + K3224_2*X3094 + K3225_1*X3094 + K3226_2*X3095 + K3227_1*X3095 + K3228_2*X3096 + K3229_1*X3096 + K3230_2*X3097 + K3231_1*X3097 + K3232_2*X3098 + K3233_1*X3098 + K3234_2*X3099 + K3235_1*X3099 + K3236_2*X3100 + K3237_1*X3100 + K3238_2*X3101 + K3239_1*X3101 + K5_1*X1000*X1211 - K3222_1*X7*X776 - K3224_1*X99*X776 - K3226_1*X111*X776 - K3228_1*X124*X776 - K3230_1*X185*X776 - K3232_1*X189*X776 - K3234_1*X264*X776 - K3236_1*X272*X776 - K3238_1*X510*X776
777	X_dot(777) =K6_1*X851*X878 - K6_2*X777
778	X_dot(778) =K11_1*X1487 + K57_1*X1510 + K113_1*X1538 + K647_1*X1805 + K951_1*X1957 + K953_1*X1958 + K961_1*X1962 + K979_1*X1971 + K3199_1*X3081 + K3486_2*X3225 - K5568_1*X778 - K3486_1*X401*X778
779	X_dot(779) =K13_1*X1488 + K59_1*X1511 + K183_1*X1573 + K229_1*X1596 + K407_1*X1685 + K475_1*X1719 + K785_1*X1874 - K5509_1*X779

780	X_dot(780) =K15_1*X1489 + K242_2*X1603 + K243_1*X1603 + K244_2*X1604 + K245_1*X1604 + K246_2*X1605 + K247_1*X1605 + K1621_1*X2292 + K1685_1*X2324 + K1736_2*X2350 + K1737_1*X2350 + K1738_2*X2351 + K1739_1*X2351 + K1740_2*X2352 + K1741_1*X2352 + K1742_2*X2353 + K1743_1*X2353 + K1744_2*X2354 + K1745_1*X2354 + K1746_2*X2355 + K1747_1*X2355 + K1748_2*X2356 + K1749_1*X2356 + K2686_2*X2825 + K2687_1*X2825 + K2688_2*X2826 + K2689_1*X2826 + K3410_2*X3187 + K3424_2*X3194 - K5240_1*X780 - K242_1*X207*X780 - K244_1*X310*X780 - K246_1*X731*X780 - K1740_1*X7*X780 - K1742_1*X14*X780 - K1746_1*X111*X780 - K1748_1*X257*X780 - K1738_1*X377*X780 - K1744_1*X435*X780 - K1736_1*X643*X780 - K2686_1*X20*X780 - K2688_1*X99*X780 - K3410_1*X418*X780 - K3424_1*X404*X780
781	X_dot(781) =K17_1*X1490 + K129_1*X1546 + K185_1*X1574 + K265_1*X1614 + K335_1*X1649 + K595_1*X1779 + K849_1*X1906 + K895_1*X1929 + K2287_1*X2625 + K2573_1*X2768 - K5657_1*X781
782	X_dot(782) =K19_1*X1491 + K269_1*X1616 + K339_1*X1651 - K5223_1*X782
783	X_dot(783) =K21_1*X1492 + K61_1*X1512 + K105_1*X1534 + K358_2*X1661 + K359_1*X1661 + K360_2*X1662 + K361_1*X1662 + K362_2*X1663 + K363_1*X1663 + K364_2*X1664 + K365_1*X1664 + K651_1*X1807 + K689_1*X1826 + K715_1*X1839 + K932_2*X1948 + K933_1*X1948 + K934_2*X1949 + K935_1*X1949 + K1089_1*X2026 + K1120_2*X2042 + K1121_1*X2042 + K1892_2*X2428 + K1893_1*X2428 + K1894_2*X2429 + K1895_1*X2429 + K2782_2*X2873 + K2783_1*X2873 - K5530_1*X783 - K360_1*X106*X783 - K362_1*X88*X783 - K358_1*X161*X783 - K364_1*X222*X783 - K932_1*X264*X783 - K934_1*X498*X783 - K1120_1*X473*X783 - K1892_1*X342*X783 - K1894_1*X341*X783 - K2782_1*X453*X783
784	X_dot(784) =K23_1*X1493 + K697_1*X1830 - K5343_1*X784
785	X_dot(785) =K25_1*X1494 + K63_1*X1513 + K553_1*X1758 + K741_1*X1852 + K1375_1*X2169 - K5309_1*X785
786	X_dot(786) =K27_1*X1495 + K65_1*X1514 + K1715_1*X2339 - K5261_1*X786
787	X_dot(787) =K29_1*X1496 + K394_2*X1679 + K395_1*X1679 + K1934_2*X2449 + K1935_1*X2449 + K1957_1*X2460 + K2822_2*X2893 + K2823_1*X2893 + K2824_2*X2894 + K2825_1*X2894 + K2826_2*X2895 + K2827_1*X2895 + K2833_1*X2898 + K2835_1*X2899 + K2843_1*X2903 + K2851_1*X2907 - K5504_1*X787 - K394_1*X206*X787 - K1934_1*X313*X787 - K2822_1*X314*X787 - K2826_1*X312*X787 - K2824_1*X575*X787
788	X_dot(788) =K31_1*X1497 + K767_1*X1865 + K3159_1*X3061 - K5862_1*X788
789	X_dot(789) =K33_1*X1498 - K5277_1*X789
790	X_dot(790) =K35_1*X1499 - K5749_1*X790
791	X_dot(791) =K37_1*X1500 + K157_1*X1560 + K193_1*X1578 + K221_1*X1592 - K5583_1*X791
792	X_dot(792) =K39_1*X1501 + K69_1*X1516 + K2564_2*X2764 + K2565_1*X2764 + K2566_2*X2765 + K2567_1*X2765 + K2583_1*X2773 + K2587_1*X2775 + K2591_1*X2777 + K3286_2*X3125 - K5675_1*X792 - K2566_1*X503*X792 - K2564_1*X694*X792 - K3286_1*X418*X792
793	X_dot(793) =K41_1*X1502 + K71_1*X1517 + K2137_1*X2550 + K2572_2*X2768 + K2573_1*X2768 + K2574_2*X2769 + K2575_1*X2769 + K2576_2*X2770 + K2577_1*X2770 + K2578_2*X2771 + K2579_1*X2771 + K2585_1*X2774 + K2589_1*X2776 + K2593_1*X2778 + K3054_2*X3009 + K3055_1*X3009 + K3288_2*X3126 - K5667_1*X793 - K2574_1*X47*X793 - K2576_1*X270*X793 - K2572_1*X484*X793 - K2578_1*X694*X793 - K3054_1*X495*X793 - K3288_1*X418*X793
794	X_dot(794) =K43_1*X1503 - K5269_1*X794
795	X_dot(795) =K45_1*X1504 + K279_1*X1621 + K287_1*X1625 - K5512_1*X795
796	X_dot(796) =K47_1*X1505 + K557_1*X1760 - K5642_1*X796
797	X_dot(797) =K49_1*X1506 - K5735_1*X797
798	X_dot(798) =K51_1*X1507 + K819_1*X1891 + K993_1*X1978 - K5792_1*X798
799	X_dot(799) =K53_1*X1508 + K469_1*X1716 + K519_1*X1741 + K573_1*X1768 + K641_1*X1802 + K731_1*X1847 + K773_1*X1868 + K821_1*X1892 + K957_1*X1960 + K1085_1*X2024 + K3538_2*X3251 + K3542_2*X3253 - K5785_1*X799 - K3542_1*X178*X799 - K3538_1*X478*X799
800	X_dot(800) =K55_1*X1509 + K471_1*X1717 + K825_1*X1894 + K959_1*X1961 + K1025_1*X1994 - K5374_1*X800
801	X_dot(801) =K67_1*X1515 + K835_1*X1899 + K975_1*X1969 - K5786_1*X801
802	X_dot(802) =K73_1*X1518 + K253_1*X1608 + K447_1*X1705 + K769_1*X1866 + K847_1*X1905 + K987_1*X1975 + K1371_1*X2167 + K1895_1*X2429 + K7_2*X769 + K2797_1*X2880 + K3143_1*X3053 - K5529_1*X802 - K7_1*X623*X802
803	X_dot(803) =K75_1*X1519 - K5837_1*X803
804	X_dot(804) =K77_1*X1520 + K139_1*X1551 + K577_1*X1770 - K5813_1*X804
805	X_dot(805) =K79_1*X1521 - K5241_1*X805
806	X_dot(806) =K81_1*X1522 - K5655_1*X806
807	X_dot(807) =K83_1*X1523 + K915_1*X1939 + K1227_1*X2095 + K1229_1*X2096 + K1243_1*X2103 + K1665_1*X2314 + K1707_1*X2335 + K1727_1*X2345 + K1769_1*X2366 + K1845_1*X2404 + K1849_1*X2406 + K1881_1*X2422 + K2023_1*X2493 + K2047_1*X2505 + K2109_1*X2536 + K2139_1*X2551 + K2157_1*X2560 + K2295_1*X2629 + K2397_1*X2680 + K2419_1*X2691 + K2491_1*X2727 + K2605_1*X2784 + K2611_1*X2787 + K2619_1*X2791 + K2689_1*X2826 + K2795_1*X2879 + K2999_1*X2981 + K3085_1*X3024 + K3225_1*X3094 + K3394_2*X3179 + K3398_2*X3181 + K3406_2*X3185 + K3420_2*X3192 + K3434_2*X3199 + K3678_2*X3339 + K3679_1*X3339 - K5308_1*X807 - K3394_1*X401*X807 - K3406_1*X289*X807 - K3398_1*X402*X807 - K3420_1*X418*X807 - K3434_1*X404*X807 - K3678_1*X807*X3340
808	X_dot(808) =K85_1*X1524 + K175_1*X1569 + K1007_1*X1985 + K1057_1*X2010 + K1081_1*X2022 + K1137_1*X2050 + K1181_1*X2072 + K1381_1*X2172 + K1539_1*X2251 + K2283_1*X2623 - K5338_1*X808
809	X_dot(809) =K87_1*X1525 - K5361_1*X809
810	X_dot(810) =K88_2*X1526 + K89_1*X1526 + K90_2*X1527 + K91_1*X1527 + K92_2*X1528 + K93_1*X1528 + K94_2*X1529 + K95_1*X1529 + K96_2*X1530 + K97_1*X1530 + K98_2*X1531 + K99_1*X1531 + K100_2*X1532 + K101_1*X1532 + K856_2*X1910 + K857_1*X1910 + K858_2*X1911 + K859_1*X1911 + K1174_2*X2069 + K1175_1*X2069 + K1580_2*X2272 + K1581_1*X2272 + K1582_2*X2273 + K1583_1*X2273 + K1584_2*X2274 + K1585_1*X2274 + K1586_2*X2275 + K1587_1*X2275 + K2642_2*X2803 + K2643_1*X2803 + K2644_2*X2804 + K2645_1*X2804 + K2646_2*X2805 + K2647_1*X2805 - K5881_1*X810 - K90_1*X177*X810 - K100_1*X181*X810 - K92_1*X461*X810 - K98_1*X567*X810 - K96_1*X648*X810 - K88_1*X745*X810 - K94_1*X763*X810 - K858_1*X86*X810 - K856_1*X491*X810 - K1174_1*X108*X810 - K1580_1*X174*X810 - K1582_1*X464*X810 - K1586_1*X460*X810 - K1584_1*X552*X810 - K2642_1*X180*X810 - K2644_1*X189*X810 - K2646_1*X441*X810
811	X_dot(811) =K91_1*X1527 + K1465_1*X2214 + K1547_1*X2255 + K1763_1*X2363 + K2165_1*X2564 + K2483_1*X2723 + K2631_1*X2797 + K2789_1*X2876 + K2963_1*X2963 + K3021_1*X2992 + K3025_1*X2994 + K3444_2*X3204 - K5383_1*X811 - K3444_1*X811*X993
812	X_dot(812) =K93_1*X1528 + K625_1*X1794 + K659_1*X1811 + K2863_1*X2913 + K2885_1*X2924 - K5635_1*X812
813	X_dot(813) =K95_1*X1529 + K109_1*X1536 + K251_1*X1607 - K5896_1*X813
814	X_dot(814) =K97_1*X1530 + K237_1*X1600 - K5802_1*X814
815	X_dot(815) =K99_1*X1531 + K435_1*X1699 + K505_1*X1734 + K3250_2*X3107 - K5731_1*X815 - K3250_1*X286*X815
816	X_dot(816) =K101_1*X1532 + K107_1*X1535 + K225_1*X1594 + K439_1*X1701 + K453_1*X1708 + K533_1*X1748 + K547_1*X1755 + K583_1*X1773 + K671_1*X1817 + K983_1*X1973 - K5386_1*X816
817	X_dot(817) =K103_1*X1533 + K257_1*X1610 + K275_1*X1619 + K277_1*X1620 + K281_1*X1622 + K937_1*X1950 + K1101_1*X2032 + K1391_1*X2177 + K1519_1*X2241 + K1549_1*X2256 + K2145_1*X2554 + K2197_1*X2580 + K2221_1*X2592 + K2721_1*X2842 + K2767_1*X2865 + K2967_1*X2965 + K3123_1*X3043 + K3494_2*X3229 - K5470_1*X817 - K3494_1*X418*X817
818	X_dot(818) =K111_1*X1537 + K405_1*X1684 + K457_1*X1710 + K473_1*X1718 + K783_1*X1873 + K3205_1*X3084 + K3558_2*X3261 - K5709_1*X818 - K3558_1*X818*X974
819	X_dot(819) =K115_1*X1539 + K409_1*X1686 + K2259_1*X2611 - K5751_1*X819
820	X_dot(820) =K117_1*X1540 + K3185_1*X3074 - K5234_1*X820
821	X_dot(821) =K119_1*X1541 + K2399_1*X2681 + K3221_1*X3092 - K5843_1*X821

822	X_dot(822) =K121_1*X1542 + K340_2*X1652 + K341_1*X1652 + K342_2*X1653 + K343_1*X1653 + K451_1*X1707 + K971_1*X1967 + K1017_1*X1990 + K1087_1*X2025 + K1367_1*X2165 + K1373_1*X2168 + K2971_1*X2967 + K3131_1*X3047 - K5231_1*X822 - K342_1*X9*X822 - K340_1*X158*X822
823	X_dot(823) =K123_1*X1543 + K344_2*X1654 + K345_1*X1654 + K346_2*X1655 + K347_1*X1655 + K459_1*X1711 + K479_1*X1721 + K593_1*X1778 + K859_1*X1911 + K861_1*X1912 + K928_2*X1946 + K929_1*X1946 + K963_1*X1963 + K1071_1*X2017 + K1118_2*X2041 + K1119_1*X2041 + K1316_2*X2140 + K1317_1*X2140 + K1318_2*X2141 + K1319_1*X2141 + K1320_2*X2142 + K1321_1*X2142 + K1322_2*X2143 + K1323_1*X2143 + K1324_2*X2144 + K1325_1*X2144 + K1326_2*X2145 + K1327_1*X2145 + K1850_2*X2407 + K1851_1*X2407 + K1852_2*X2408 + K1853_1*X2408 + K1854_2*X2409 + K1855_1*X2409 + K1856_2*X2410 + K1857_1*X2410 + K1858_2*X2411 + K1859_1*X2411 + K1860_2*X2412 + K1861_1*X2412 + K1862_2*X2413 + K1863_1*X2413 + K1864_2*X2414 + K1865_1*X2414 + K1866_2*X2415 + K1867_1*X2415 + K1868_2*X2416 + K1869_1*X2416 + K1870_2*X2417 + K1871_1*X2417 + K1872_2*X2418 + K1873_1*X2418 + K1874_2*X2419 + K1875_1*X2419 + K2345_1*X2654 + K2505_1*X2734 + K2776_2*X2870 + K2777_1*X2870 + K2778_2*X2871 + K2779_1*X2871 + K2780_2*X2872 + K2781_1*X2872 + K3338_2*X3151 + K3354_2*X3159 + K3374_2*X3169 + K3448_2*X3206 + K3454_2*X3209 + K3484_2*X3224 - K5297_1*X823 - K344_1*X143*X823 - K346_1*X721*X823 - K928_1*X317*X823 - K1118_1*X86*X823 - K1316_1*X55*X823 - K1324_1*X22*X823 - K1326_1*X70*X823 - K1318_1*X169*X823 - K1322_1*X210*X823 - K1320_1*X411*X823 - K1850_1*X96*X823 - K1860_1*X27*X823 - K1864_1*X16*X823 - K1858_1*X131*X823 - K1854_1*X217*X823 - K1862_1*X151*X823 - K1852_1*X305*X823 - K1868_1*X194*X823 - K1870_1*X302*X823 - K1856_1*X513*X823 - K1872_1*X358*X823 - K1874_1*X366*X823 - K1866_1*X460*X823 - K2776_1*X98*X823 - K2780_1*X295*X823 - K2778_1*X729*X823 - K3338_1*X190*X823 - K3374_1*X356*X823 - K3354_1*X823*X974 - K3454_1*X463*X823 - K3484_1*X558*X823 - K3448_1*X823*X1007
824	X_dot(824) =K125_1*X1544 + K415_1*X1689 + K3197_1*X3080 - K5535_1*X824
825	X_dot(825) =K127_1*X1545 + K827_1*X1895 + K1129_1*X2046 + K2659_1*X2811 - K5734_1*X825
826	X_dot(826) =K131_1*X1547 - K5653_1*X826
827	X_dot(827) =K133_1*X1548 + K189_1*X1576 + K717_1*X1840 + K1263_1*X2113 + K1293_1*X2128 - K5387_1*X827
828	X_dot(828) =K135_1*X1549 - K5554_1*X828
829	X_dot(829) =K137_1*X1550 + K3195_1*X3079 - K5517_1*X829
830	X_dot(830) =K141_1*X1552 - K5708_1*X830
831	X_dot(831) =K143_1*X1553 + K392_2*X1678 + K393_1*X1678 + K950_2*X1957 + K951_1*X1957 + K965_1*X1964 + K1557_1*X2260 + K1928_2*X2446 + K1929_1*X2446 + K1947_1*X2455 + K1953_1*X2458 + K2639_1*X2801 + K2814_2*X2889 + K2815_1*X2889 + K2816_2*X2890 + K2817_1*X2890 + K2818_2*X2891 + K2819_1*X2891 + K2925_1*X2944 + K2931_1*X2947 + K3113_1*X3038 + K3121_1*X3042 + K3266_2*X3115 - K5626_1*X831 - K392_1*X281*X831 - K950_1*X384*X831 - K1928_1*X240*X831 - K2818_1*X246*X831 - K2814_1*X343*X831 - K2816_1*X451*X831 - K3266_1*X418*X831
832	X_dot(832) =K145_1*X1554 - K5439_1*X832
833	X_dot(833) =K147_1*X1555 - K5712_1*X833
834	X_dot(834) =K149_1*X1556 + K423_1*X1693 + K493_1*X1728 - K5873_1*X834
835	X_dot(835) =K151_1*X1557 + K219_1*X1591 - K5768_1*X835
836	X_dot(836) =K153_1*X1558 + K3207_1*X3085 - K5719_1*X836
837	X_dot(837) =K155_1*X1559 + K311_1*X1637 + K1231_1*X2097 + K2005_1*X2484 + K2091_1*X2527 - K5445_1*X837
838	X_dot(838) =K159_1*X1561 + K195_1*X1579 + K211_1*X1587 + K231_1*X1597 + K2229_1*X2596 - K5641_1*X838
839	X_dot(839) =K161_1*X1562 + K197_1*X1580 + K213_1*X1588 + K223_1*X1593 + K233_1*X1598 + K465_1*X1714 + K629_1*X1796 + K1747_1*X2355 + K2693_1*X2828 + K3227_1*X3095 + K3500_2*X3232 - K5320_1*X839 - K3500_1*X418*X839
840	X_dot(840) =K163_1*X1563 - K5457_1*X840
841	X_dot(841) =K165_1*X1564 - K5887_1*X841
842	X_dot(842) =K167_1*X1565 + K199_1*X1581 - K5762_1*X842
843	X_dot(843) =K169_1*X1566 + K2019_1*X2491 - K5691_1*X843
844	X_dot(844) =K171_1*X1567 + K205_1*X1584 - K5306_1*X844
845	X_dot(845) =K173_1*X1568 + K445_1*X1704 + K3117_1*X3040 + K3211_1*X3087 - K5778_1*X845
846	X_dot(846) =K177_1*X1570 + K207_1*X1585 + K585_1*X1774 + K611_1*X1787 + K812_2*X1888 + K813_1*X1888 + K814_2*X1889 + K815_1*X1889 + K1749_1*X2356 - K5452_1*X846 - K812_1*X112*X846 - K814_1*X231*X846
847	X_dot(847) =K179_1*X1571 + K209_1*X1586 + K3209_1*X3086 - K5760_1*X847
848	X_dot(848) =K181_1*X1572 - K5228_1*X848
849	X_dot(849) =K187_1*X1575 + K356_2*X1660 + K357_1*X1660 + K483_1*X1723 + K930_2*X1947 + K931_1*X1947 + K1330_2*X2147 + K1331_1*X2147 + K1888_2*X2426 + K1889_1*X2426 + K1890_2*X2427 + K1891_1*X2427 + K2975_1*X2969 + K2989_1*X2976 + K3001_1*X2982 - K5428_1*X849 - K356_1*X575*X849 - K930_1*X73*X849 - K1330_1*X133*X849 - K1890_1*X105*X849 - K1888_1*X169*X849
850	X_dot(850) =K191_1*X1577 - K5532_1*X850
851	X_dot(851) =K201_1*X1582 + K215_1*X1589 + K241_1*X1602 + K437_1*X1700 + K507_1*X1735 + K1125_1*X2044 + K6_2*X777 + K2879_1*X2921 + K3125_1*X3044 + K3252_2*X3108 + K3284_2*X3124 + K3684_2*X3345 - K5611_1*X851 - K6_1*X851*X878 - K3252_1*X286*X851 - K3284_1*X418*X851 - K3684_1*X851*X3346
852	X_dot(852) =K203_1*X1583 + K441_1*X1702 + K511_1*X1737 + K3502_2*X3233 - K5658_1*X852 - K3502_1*X418*X852
853	X_dot(853) =K217_1*X1590 - K5329_1*X853
854	X_dot(854) =K227_1*X1595 - K5733_1*X854
855	X_dot(855) =K235_1*X1599 + K427_1*X1695 + K499_1*X1731 + K1579_1*X2271 + K1675_1*X2319 + K1733_1*X2348 + K3560_2*X3262 + K3564_2*X3264 - K5426_1*X855 - K3560_1*X606*X855 - K3564_1*X701*X855
856	X_dot(856) =K239_1*X1601 + K385_1*X1674 + K433_1*X1698 + K503_1*X1733 + K3244_2*X3104 + K3248_2*X3106 - K5732_1*X856 - K3248_1*X286*X856 - K3244_1*X715*X856
857	X_dot(857) =K243_1*X1603 + K1117_1*X2040 + K2_2*X771 + K2319_1*X2641 + K3087_1*X3025 - K5408_1*X857 - K2_1*X857*X1265
858	X_dot(858) =K245_1*X1604 + K2580_2*X2772 + K2581_1*X2772 + K3056_2*X3010 + K3057_1*X3010 + K3058_2*X3011 + K3059_1*X3011 + K3060_2*X3012 + K3061_1*X3012 + K3062_2*X3013 + K3063_1*X3013 + K3360_2*X3162 - K5500_1*X858 - K2580_1*X249*X858 - K3056_1*X184*X858 - K3060_1*X310*X858 - K3058_1*X425*X858 - K3062_1*X422*X858 - K3360_1*X858*X974
859	X_dot(859) =K247_1*X1605 - K5870_1*X859
860	X_dot(860) =K249_1*X1606 + K267_1*X1615 + K337_1*X1650 + K829_1*X1896 + K851_1*X1907 + K1095_1*X2029 + K1127_1*X2045 + K1131_1*X2047 + K2035_1*X2499 + K2575_1*X2769 + K2951_1*X2957 + K2953_1*X2958 + K3089_1*X3026 + K3091_1*X3027 + K3093_1*X3028 + K3099_1*X3031 + K3101_1*X3032 + K3103_1*X3033 + K3498_2*X3231 + K3645_2*X3306 + K3646_1*X3306 + K3648_2*X3309 + K3649_1*X3309 - K5263_1*X860 - K3498_1*X418*X860 - K3645_1*X860*X3307 - K3648_1*X860*X3310

861	X_dot(861) =K255_1*X1609 + K776_2*X1870 + K777_1*X1870 + K778_2*X1871 + K779_1*X1871 + K780_2*X1872 + K781_1*X1872 + K782_2*X1873 + K783_1*X1873 + K784_2*X1874 + K785_1*X1874 + K786_2*X1875 + K787_1*X1875 + K788_2*X1876 + K789_1*X1876 + K790_2*X1877 + K791_1*X1877 + K792_2*X1878 + K793_1*X1878 + K794_2*X1879 + K795_1*X1879 + K796_2*X1880 + K797_1*X1880 + K798_2*X1881 + K799_1*X1881 + K800_2*X1882 + K801_1*X1882 + K802_2*X1883 + K803_1*X1883 + K1098_2*X2031 + K1099_1*X2031 + K1100_2*X2032 + K1101_1*X2032 + K1102_2*X2033 + K1103_1*X2033 + K1104_2*X2034 + K1105_1*X2034 + K1106_2*X2035 + K1107_1*X2035 + K1108_2*X2036 + K1109_1*X2036 + K1114_2*X2039 + K1115_1*X2039 + K1441_1*X2202 + K1488_2*X2226 + K1489_1*X2226 + K1490_2*X2227 + K1491_1*X2227 + K1492_2*X2228 + K1493_1*X2228 + K1494_2*X2229 + K1495_1*X2229 + K1496_2*X2230 + K1497_1*X2230 + K1498_2*X2231 + K1499_1*X2231 + K1500_2*X2232 + K1501_1*X2232 + K1721_1*X2342 + K2333_1*X2648 + K2447_1*X2705 + K2494_2*X2729 + K2495_1*X2729 + K2496_2*X2730 + K2497_1*X2730 + K2498_2*X2731 + K2499_1*X2731 + K2500_2*X2732 + K2501_1*X2732 + K2502_2*X2733 + K2503_1*X2733 + K2504_2*X2734 + K2505_1*X2734 + K2506_2*X2735 + K2507_1*X2735 + K2508_2*X2736 + K2509_1*X2736 + K2510_2*X2737 + K2511_1*X2737 + K2512_2*X2738 + K2513_1*X2738 + K2514_2*X2739 + K2515_1*X2739 + K2516_2*X2740 + K2517_1*X2740 + K2518_2*X2741 + K2519_1*X2741 + K2520_2*X2742 + K2521_1*X2742 + K2522_2*X2743 + K2523_1*X2743 + K2524_2*X2744 + K2525_1*X2744 + K2526_2*X2745 + K2527_1*X2745 + K2528_2*X2746 + K2529_1*X2746 + K2530_2*X2747 + K2531_1*X2747 + K2532_2*X2748 + K2533_1*X2748 + K2534_2*X2749 + K2535_1*X2749 + K2536_2*X2750 + K2537_1*X2750 + K2538_2*X2751 + K2539_1*X2751 + K2540_2*X2752 + K2541_1*X2752 + K2542_2*X2753 + K2543_1*X2753 + K2544_2*X2754 + K2545_1*X2754 + K2546_2*X2755 + K2547_1*X2755 + K2548_2*X2756 + K2549_1*X2756 + K3007_1*X2985 + K3028_2*X2996 + K3029_1*X2996 + K3030_2*X2997 + K3031_1*X2997 + K3032_2*X2998 + K3033_1*X2998 + K3034_2*X2999 + K3035_1*X2999 + K3036_2*X3000 + K3037_1*X3000 + K3038_2*X3001 + K3039_1*X3001 + K3040_2*X3002 + K3041_1*X3002 + K3042_2*X3003 + K3043_1*X3003 + K3044_2*X3004 + K3045_1*X3004 + K3046_2*X3005 + K3047_1*X3005 + K3048_2*X3006 + K3049_1*X3006 + K3050_2*X3007 + K3051_1*X3007 + K3052_2*X3008 + K3053_1*X3008 + K3344_2*X3154 + K3372_2*X3168 + K3418_2*X3191 + K3456_2*X3210 + K3460_2*X3212 + K3466_2*X3215 + K3512_2*X3238 + K3514_2*X3239 - K5364_1*X861 - K786_1*X225*X861 - K780_1*X334*X861 - K778_1*X362*X861 - K784_1*X319*X861 - K776_1*X413*X861 - K800_1*X210*X861 - K792_1*X347*X861 - K794_1*X400*X861 - K782_1*X543*X861 - K798_1*X390*X861 - K796_1*X418*X861 - K788_1*X536*X861 - K790_1*X533*X861 - K802_1*X665*X861 - K1102_1*X90*X861 - K1104_1*X137*X861 - K1106_1*X157*X861 - K1100_1*X275*X861 - K1108_1*X196*X861 - K1098_1*X389*X861 - K1114_1*X247*X861 - K1488_1*X96*X861 - K1498_1*X46*X861 - K1496_1*X130*X861 - K1490_1*X363*X861 - K1494_1*X411*X861 - K1500_1*X359*X861 - K1492_1*X692*X861 - K2494_1*X118*X861 - K2496_1*X138*X861 - K2504_1*X86*X861 - K2506_1*X114*X861 - K2518_1*X35*X861 - K2512_1*X110*X861 - K2508_1*X169*X861 - K2514_1*X126*X861 - K2516_1*X125*X861 - K2520_1*X91*X861 - K2524_1*X77*X861 - K2530_1*X38*X861 - K2500_1*X392*X861 - K2502_1*X440*X861 - K2536_1*X248*X861 - K2510_1*X513*X861 - K2532_1*X301*X861 - K2498_1*X708*X861 - K2522_1*X476*X861 - K2546_1*X290*X861 - K2528_1*X501*X861 - K2538_1*X412*X861 - K2544_1*X358*X861 - K2534_1*X460*X861 - K2540_1*X416*X861 - K2526_1*X565*X861 - K2542_1*X529*X861 - K2548_1*X590*X861 - K3032_1*X55*X861 - K3040_1*X31*X861 - K3030_1*X159*X861 - K3028_1*X264*X861 - K3038_1*X167*X861 - K3042_1*X197*X861 - K3052_1*X132*X861 - K3044_1*X249*X861 - K3048_1*X302*X861 - K3036_1*X426*X861 - K3050_1*X295*X861 - K3034_1*X458*X861 - K3046_1*X726*X861 - K3344_1*X190*X861 - K3372_1*X228*X861 - K3418_1*X418*X861 - K3456_1*X463*X861 - K3460_1*X459*X861 - K3466_1*X861*X974 - K3512_1*X861*X1007 - K3514_1*X861*X1428
862	X_dot(862) =K259_1*X1611 - K5851_1*X862
863	X_dot(863) =K261_1*X1612 + K565_1*X1764 + K763_1*X1863 + K3155_1*X3059 - K5771_1*X863
864	X_dot(864) =K263_1*X1613 + K397_1*X1680 + K601_1*X1782 - K5672_1*X864
865	X_dot(865) =K271_1*X1617 + K905_1*X1934 + K1295_1*X2129 - K5331_1*X865
866	X_dot(866) =K273_1*X1618 - K5584_1*X866
867	X_dot(867) =K283_1*X1623 + K649_1*X1806 + K1317_1*X2140 + K1335_1*X2149 + K3033_1*X2998 - K5271_1*X867
868	X_dot(868) =K285_1*X1624 + K303_1*X1633 + K413_1*X1688 + K867_1*X1915 + K919_1*X1941 + K941_1*X1952 + K973_1*X1968 + K995_1*X1979 - K5686_1*X868
869	X_dot(869) =K289_1*X1626 - K5713_1*X869
870	X_dot(870) =K291_1*X1627 + K2481_1*X2722 + K2651_1*X2807 + K2961_1*X2962 + K3504_2*X3234 - K5402_1*X870 - K3504_1*X870*X918
871	X_dot(871) =K293_1*X1628 - K5832_1*X871
872	X_dot(872) =K295_1*X1629 + K2407_1*X2685 + K3522_2*X3243 + K3524_2*X3244 + K3526_2*X3245 - K5498_1*X872 - K3526_1*X289*X872 - K3522_1*X401*X872 - K3524_1*X402*X872
873	X_dot(873) =K297_1*X1630 - K5610_1*X873
874	X_dot(874) =K299_1*X1631 + K3320_2*X3142 - K5469_1*X874 - K3320_1*X463*X874
875	X_dot(875) =K301_1*X1632 - K5292_1*X875
876	X_dot(876) =K305_1*X1634 + K693_1*X1828 + K1037_1*X2000 + K1091_1*X2027 + K1487_1*X2225 - K5311_1*X876
877	X_dot(877) =K307_1*X1635 + K2515_1*X2739 - K5335_1*X877
878	X_dot(878) =K309_1*X1636 + K361_1*X1662 + K945_1*X1954 + K1461_1*X2212 + K2075_1*X2519 + K2151_1*X2557 + K2217_1*X2590 + K6_2*X777 + K2617_1*X2790 + K2799_1*X2881 + K2861_1*X2912 + K2895_1*X2929 + K3572_2*X3268 + K3663_2*X3324 + K3664_1*X3324 + K3669_2*X3330 + K3670_1*X3330 - K5315_1*X878 - K6_1*X851*X878 - K3572_1*X478*X878 - K3669_1*X878*X1026*X3331 - K3663_1*X878*X1212*X3325
879	X_dot(879) =K313_1*X1638 + K541_1*X1752 + K631_1*X1797 + K1705_1*X2334 + K2097_1*X2530 - K5857_1*X879
880	X_dot(880) =K315_1*X1639 + K329_1*X1646 + K331_1*X1647 - K5621_1*X880
881	X_dot(881) =K317_1*X1640 - K5834_1*X881
882	X_dot(882) =K319_1*X1641 + K1051_1*X2007 - K5620_1*X882
883	X_dot(883) =K321_1*X1642 - K5886_1*X883
884	X_dot(884) =K323_1*X1643 - K5891_1*X884
885	X_dot(885) =K325_1*X1644 - K5257_1*X885
886	X_dot(886) =K327_1*X1645 - K5900_1*X886
887	X_dot(887) =K333_1*X1648 + K1415_1*X2189 + K2673_1*X2818 + K3035_1*X2999 - K5632_1*X887
888	X_dot(888) =K341_1*X1652 + K3496_2*X3230 - K5365_1*X888 - K3496_1*X418*X888
889	X_dot(889) =K345_1*X1654 - K5350_1*X889
890	X_dot(890) =K347_1*X1655 + K2073_1*X2518 - K5861_1*X890

891	X_dot(891) =K110_2*X1537 + K111_1*X1537 + K112_2*X1538 + K113_1*X1538 + K114_2*X1539 + K115_1*X1539 + K116_2*X1540 + K117_1*X1540 + K118_2*X1541 + K119_1*X1541 + K120_2*X1542 + K121_1*X1542 + K122_2*X1543 + K123_1*X1543 + K124_2*X1544 + K125_1*X1544 + K126_2*X1545 + K127_1*X1545 + K128_2*X1546 + K129_1*X1546 + K130_2*X1547 + K131_1*X1547 + K132_2*X1548 + K133_1*X1548 + K134_2*X1549 + K135_1*X1549 + K136_2*X1550 + K137_1*X1550 + K138_2*X1551 + K139_1*X1551 + K140_2*X1552 + K141_1*X1552 + K142_2*X1553 + K143_1*X1553 + K144_2*X1554 + K145_1*X1554 + K146_2*X1555 + K147_1*X1555 + K148_2*X1556 + K149_1*X1556 + K150_2*X1557 + K151_1*X1557 + K152_2*X1558 + K153_1*X1558 + K154_2*X1559 + K155_1*X1559 + K156_2*X1560 + K157_1*X1560 + K158_2*X1561 + K159_1*X1561 + K160_2*X1562 + K161_1*X1562 + K162_2*X1563 + K163_1*X1563 + K164_2*X1564 + K165_1*X1564 + K166_2*X1565 + K167_1*X1565 + K168_2*X1566 + K169_1*X1566 + K170_2*X1567 + K171_1*X1567 + K172_2*X1568 + K173_1*X1568 + K174_2*X1569 + K175_1*X1569 + K176_2*X1570 + K177_1*X1570 + K178_2*X1571 + K179_1*X1571 + K349_1*X1656 + K353_1*X1658 + K813_1*X1888 + K866_2*X1915 + K867_1*X1915 + K868_2*X1916 + K869_1*X1916 + K870_2*X1917 + K871_1*X1917 + K872_2*X1918 + K873_1*X1918 + K874_2*X1919 + K875_1*X1919 + K876_2*X1920 + K877_1*X1920 + K1190_2*X2077 + K1191_1*X2077 + K1192_2*X2078 + K1193_1*X2078 + K1194_2*X2079 + K1195_1*X2079 + K1196_2*X2080 + K1197_1*X2080 + K1198_2*X2081 + K1199_1*X2081 + K1200_2*X2082 + K1201_1*X2082 + K1202_2*X2083 + K1203_1*X2083 + K1204_2*X2084 + K1205_1*X2084 + K1206_2*X2085 + K1207_1*X2085 + K1208_2*X2086 + K1209_1*X2086 + K1210_2*X2087 + K1211_1*X2087 + K1608_2*X2286 + K1609_1*X2286 + K1610_2*X2287 + K1611_1*X2287 + K1612_2*X2288 + K1613_1*X2288 + K1614_2*X2289 + K1615_1*X2289 + K1616_2*X2290 + K1617_1*X2290 + K1618_2*X2291 + K1619_1*X2291 + K1620_2*X2292 + K1621_1*X2292 + K1622_2*X2293 + K1623_1*X2293 + K1624_2*X2294 + K1625_1*X2294 + K1626_2*X2295 + K1627_1*X2295 + K1628_2*X2296 + K1629_1*X2296 + K1630_2*X2297 + K1631_1*X2297 + K1632_2*X2298 + K1633_1*X2298 + K1634_2*X2299 + K1635_1*X2299 + K1636_2*X2300 + K1637_1*X2300 + K1638_2*X2301 + K1639_1*X2301 + K1640_2*X2302 + K1641_1*X2302 + K1642_2*X2303 + K1643_1*X2303 + K1644_2*X2304 + K1645_1*X2304 + K1646_2*X2305 + K1647_1*X2305 + K1648_2*X2306 + K1649_1*X2306 + K1650_2*X2307 + K1651_1*X2307 + K1652_2*X2308 + K1653_1*X2308 + K1654_2*X2309 + K1655_1*X2309 + K1656_2*X2310 + K1657_1*X2310 + K1658_2*X2311 + K1659_1*X2311 + K1660_2*X2312 + K1661_1*X2312 + K1662_2*X2313 + K1663_1*X2313 + K1664_2*X2314 + K1665_1*X2314 + K1666_2*X2315 + K1667_1*X2315 + K1668_2*X2316 + K1669_1*X2316 + K1670_2*X2317 + K1671_1*X2317 + K1729_1*X2346 + K1789_1*X2376 + K3_2*X773 + K2654_2*X2809 + K2655_1*X2809 + K2656_2*X2810 + K2657_1*X2810 + K3088_2*X3026 + K3089_1*X3026 - K5321_1*X891 - K116_1*X13*X891 - K120_1*X9*X891 - K122_1*X86*X891 - K132_1*X182*X891 - K112_1*X384*X891 - K124_1*X349*X891 - K110_1*X543*X891 - K144_1*X244*X891 - K136_1*X327*X891 - K134_1*X369*X891 - K160_1*X111*X891 - K114_1*X588*X891 - K128_1*X484*X891 - K130_1*X480*X891 - K154_1*X250*X891 - K170_1*X97*X891 - K126_1*X570*X891 - K174_1*X129*X891 - K142_1*X451*X891 - K118_1*X695*X891 - K162_1*X262*X891 - K140_1*X542*X891 - K156_1*X401*X891 - K146_1*X546*X891 - K176_1*X257*X891 - K138_1*X662*X891 - K158_1*X467*X891 - K152_1*X553*X891 - K150_1*X612*X891 - K168_1*X524*X891 - K148_1*X734*X891 - K166_1*X605*X891 - K172_1*X623*X891 - K178_1*X602*X891 - K164_1*X753*X891 - K876_1*X11*X891 - K872_1*X116*X891 - K870_1*X272*X891 - K868_1*X293*X891 - K866_1*X517*X891 - K874_1*X739*X891 - K1194_1*X251*X891 - K1200_1*X194*X891 - K1202_1*X190*X891 - K1210_1*X147*X891 - K1190_1*X466*X891 - K1206_1*X326*X891 - K1192_1*X491*X891 - K1196_1*X470*X891 - K1204_1*X394*X891 - K1198_1*X646*X891 - K1208_1*X609*X891 - K1610_1*X54*X891 - K1618_1*X7*X891 - K1620_1*X20*X891 - K1634_1*X75*X891 - K1616_1*X256*X891 - K1638_1*X108*X891 - K1640_1*X94*X891 - K1626_1*X238*X891 - K1612_1*X494*X891 - K1622_1*X423*X891 - K1648_1*X171*X891 - K1624_1*X420*X891 - K1654_1*X173*X891 - K1660_1*X124*X891 - K1636_1*X365*X891 - K1664_1*X99*X891 - K1630_1*X446*X891 - K1608_1*X682*X891 - K1656_1*X223*X891 - K1652_1*X316*X891 - K1628_1*X585*X891 - K1614_1*X730*X891 - K1650_1*X371*X891 - K1666_1*X219*X891 - K1642_1*X473*X891 - K1632_1*X634*X891 - K1668_1*X335*X891 - K1644_1*X614*X891 -
892	X_dot(892) =K351_1*X1657 + K1451_1*X2207 + K1505_1*X2234 - K5312_1*X892
893	X_dot(893) =K352_2*X1658 + K353_1*X1658 + K354_2*X1659 + K355_1*X1659 + K1884_2*X2424 + K1885_1*X2424 + K2307_1*X2635 + K2777_1*X2870 + K3376_2*X3170 - K5307_1*X893 - K352_1*X112*X893 - K354_1*X98*X893 - K1884_1*X647*X893 - K3376_1*X356*X893
894	X_dot(894) =K357_1*X1660 + K454_2*X1709 + K455_1*X1709 + K456_2*X1710 + K457_1*X1710 + K458_2*X1711 + K459_1*X1711 + K460_2*X1712 + K461_1*X1712 + K462_2*X1713 + K463_1*X1713 + K464_2*X1714 + K465_1*X1714 + K970_2*X1967 + K971_1*X1967 + K972_2*X1968 + K973_1*X1968 + K974_2*X1969 + K975_1*X1969 + K976_2*X1970 + K977_1*X1970 + K1128_2*X2046 + K1129_1*X2046 + K1352_2*X2158 + K1353_1*X2158 + K1354_2*X2159 + K1355_1*X2159 + K2024_2*X2494 + K2025_1*X2494 + K2026_2*X2495 + K2027_1*X2495 + K2028_2*X2496 + K2029_1*X2496 + K2030_2*X2497 + K2031_1*X2497 + K2032_2*X2498 + K2033_1*X2498 + K2034_2*X2499 + K2035_1*X2499 + K2036_2*X2500 + K2037_1*X2500 + K2038_2*X2501 + K2039_1*X2501 + K2040_2*X2502 + K2041_1*X2502 + K2042_2*X2503 + K2043_1*X2503 + K2044_2*X2504 + K2045_1*X2504 + K2046_2*X2505 + K2047_1*X2505 + K2251_1*X2607 + K2821_1*X2892 + K2825_1*X2894 + K2831_1*X2897 + K2870_2*X2917 + K2871_1*X2917 + K2872_2*X2918 + K2873_1*X2918 + K2874_2*X2919 + K2875_1*X2919 + K2876_2*X2920 + K2877_1*X2920 + K2878_2*X2921 + K2879_1*X2921 + K2880_2*X2922 + K2881_1*X2922 + K3386_2*X3175 - K5739_1*X894 - K458_1*X86*X894 - K464_1*X111*X894 - K454_1*X306*X894 - K460_1*X343*X894 - K462_1*X427*X894 - K456_1*X543*X894 - K970_1*X9*X894 - K972_1*X517*X894 - K976_1*X566*X894 - K974_1*X631*X894 - K1128_1*X570*X894 - K1354_1*X332*X894 - K1352_1*X447*X894 - K2034_1*X47*X894 - K2024_1*X170*X894 - K2040_1*X67*X894 - K2046_1*X99*X894 - K2042_1*X204*X894 - K2032_1*X305*X894 - K2044_1*X295*X894 - K2036_1*X435*X894 - K2038_1*X424*X894 - K2028_1*X551*X894 - K2030_1*X564*X894 - K2026_1*X728*X894 - K2872_1*X333*X894 - K2876_1*X325*X894 - K2880_1*X302*X894 - K2870_1*X431*X894 - K2878_1*X434*X894 - K2874_1*X580*X894 - K3386_1*X286*X894
895	X_dot(895) =K359_1*X1661 + K943_1*X1953 + K1033_1*X1998 + K1261_1*X2112 - K5368_1*X895
896	X_dot(896) =K363_1*X1663 + K1123_1*X2043 + K2001_1*X2482 + K2153_1*X2558 + K2897_1*X2930 + K2947_1*X2955 + K3105_1*X3034 + K3294_2*X3129 + K3654_2*X3315 + K3655_1*X3315 - K5299_1*X896 - K3294_1*X404*X896 - K3654_1*X896*X1379*X3316
897	X_dot(897) =K365_1*X1664 + K383_1*X1673 - K5419_1*X897
898	X_dot(898) =K367_1*X1665 + K935_1*X1949 + K1239_1*X2101 + K2723_1*X2843 + K2749_1*X2856 - K5670_1*X898
899	X_dot(899) =K369_1*X1666 + K3119_1*X3041 - K5849_1*X899
900	X_dot(900) =K371_1*X1667 + K2313_1*X2638 - K5698_1*X900
901	X_dot(901) =K373_1*X1668 + K489_1*X1726 + K603_1*X1783 + K989_1*X1976 + K1841_1*X2402 + K2315_1*X2639 + K2647_1*X2805 + K2733_1*X2848 - K5617_1*X901
902	X_dot(902) =K375_1*X1669 + K3003_1*X2983 - K5696_1*X902
903	X_dot(903) =K377_1*X1670 + K1121_1*X2042 + K1643_1*X2303 + K1997_1*X2480 + K2083_1*X2523 + K2131_1*X2547 + K3201_1*X3082 - K5647_1*X903
904	X_dot(904) =K379_1*X1671 + K1925_1*X2444 - K5224_1*X904
905	X_dot(905) =K381_1*X1672 - K5829_1*X905
906	X_dot(906) =K387_1*X1675 - K5474_1*X906
907	X_dot(907) =K389_1*X1676 + K677_1*X1820 + K787_1*X1875 + K3382_2*X3173 + K3562_2*X3263 - K5422_1*X907 - K3382_1*X642*X907 - K3562_1*X618*X907
908	X_dot(908) =K391_1*X1677 + K759_1*X1861 + K1083_1*X2023 - K5246_1*X908
909	X_dot(909) =K393_1*X1678 + K419_1*X1691 + K525_1*X1744 + K539_1*X1751 + K605_1*X1784 + K721_1*X1842 + K955_1*X1959 + K981_1*X1972 + K997_1*X1980 + K1133_1*X2048 + K1139_1*X2051 + K1269_1*X2116 + K1305_1*X2134 + K1913_1*X2438 + K2351_1*X2657 + K3141_1*X3052 + K3308_2*X3136 + K3324_2*X3144 - K5476_1*X909 - K3324_1*X463*X909 - K3308_1*X909*X1007
910	X_dot(910) =K395_1*X1679 + K431_1*X1697 + K527_1*X1745 + K543_1*X1753 + K669_1*X1816 + K2099_1*X2531 - K5407_1*X910
911	X_dot(911) =K399_1*X1681 + K561_1*X1762 + K623_1*X1793 + K3530_2*X3247 + K3536_2*X3250 - K5775_1*X911 - K3530_1*X418*X911 - K3536_1*X404*X911
912	X_dot(912) =K401_1*X1682 - K5727_1*X912
913	X_dot(913) =K403_1*X1683 + K467_1*X1715 + K781_1*X1872 - K5522_1*X913
914	X_dot(914) =K411_1*X1687 + K477_1*X1720 + K761_1*X1862 + K775_1*X1869 - K5805_1*X914

915	X_dot(915) =K366_2*X1665 + K367_1*X1665 + K368_2*X1666 + K369_1*X1666 + K370_2*X1667 + K371_1*X1667 + K372_2*X1668 + K373_1*X1668 + K374_2*X1669 + K375_1*X1669 + K376_2*X1670 + K377_1*X1670 + K378_2*X1671 + K379_1*X1671 + K380_2*X1672 + K381_1*X1672 + K382_2*X1673 + K383_1*X1673 + K384_2*X1674 + K385_1*X1674 + K417_1*X1690 + K461_1*X1712 + K485_1*X1724 + K691_1*X1827 + K765_1*X1864 + K831_1*X1897 + K853_1*X1908 + K936_2*X1950 + K937_1*X1950 + K938_2*X1951 + K939_1*X1951 + K940_2*X1952 + K941_1*X1952 + K942_2*X1953 + K943_1*X1953 + K944_2*X1954 + K945_1*X1954 + K946_2*X1955 + K947_1*X1955 + K1031_1*X1997 + K1122_2*X2043 + K1123_1*X2043 + K1124_2*X2044 + K1125_1*X2044 + K1332_2*X2148 + K1333_1*X2148 + K1334_2*X2149 + K1335_1*X2149 + K1896_2*X2430 + K1897_1*X2430 + K1898_2*X2431 + K1899_1*X2431 + K1900_2*X2432 + K1901_1*X2432 + K1902_2*X2433 + K1903_1*X2433 + K1904_2*X2434 + K1905_1*X2434 + K1906_2*X2435 + K1907_1*X2435 + K1908_2*X2436 + K1909_1*X2436 + K2784_2*X2874 + K2785_1*X2874 + K2786_2*X2875 + K2787_1*X2875 + K2788_2*X2876 + K2789_1*X2876 + K2790_2*X2877 + K2791_1*X2877 + K2792_2*X2878 + K2793_1*X2878 + K2794_2*X2879 + K2795_1*X2879 + K2796_2*X2880 + K2797_1*X2880 + K2815_1*X2889 + K3086_2*X3025 + K3087_1*X3025 + K3145_1*X3054 + K3176_2*X3070 + K3177_1*X3070 - K5531_1*X915 - K378_1*X2*X915 - K382_1*X222*X915 - K366_1*X498*X915 - K372_1*X441*X915 - K370_1*X532*X915 - K376_1*X473*X915 - K374_1*X530*X915 - K368_1*X704*X915 - K384_1*X568*X915 - K380_1*X681*X915 - K944_1*X106*X915 - K942_1*X161*X915 - K936_1*X275*X915 - K946_1*X390*X915 - K940_1*X517*X915 - K938_1*X577*X915 - K1122_1*X88*X915 - K1124_1*X434*X915 - K1334_1*X55*X915 - K1332_1*X101*X915 - K1896_1*X33*X915 - K1900_1*X189*X915 - K1902_1*X179*X915 - K1898_1*X471*X915 - K1906_1*X453*X915 - K1904_1*X634*X915 - K1908_1*X651*X915 - K2786_1*X156*X915 - K2794_1*X99*X915 - K2788_1*X177*X915 - K2784_1*X376*X915 - K2792_1*X297*X915 - K2790_1*X343*X915 - K2796_1*X341*X915 - K3086_1*X207*X915 - K3176_1*X771*X915
916	X_dot(916) =K421_1*X1692 + K491_1*X1727 - K5648_1*X916
917	X_dot(917) =K425_1*X1694 + K497_1*X1730 - K5524_1*X917
918	X_dot(918) =K429_1*X1696 + K501_1*X1732 + K1751_1*X2357 + K3504_2*X3234 + K3505_1*X3234 - K5678_1*X918 - K3504_1*X870*X918
919	X_dot(919) =K443_1*X1703 + K513_1*X1738 + K549_1*X1756 - K5539_1*X919
920	X_dot(920) =K449_1*X1706 + K515_1*X1739 + K2669_1*X2816 + K3231_1*X3097 - K5389_1*X920
921	X_dot(921) =K455_1*X1709 - K5497_1*X921
922	X_dot(922) =K463_1*X1713 - K5604_1*X922
923	X_dot(923) =K481_1*X1722 - K5488_1*X923
924	X_dot(924) =K487_1*X1725 + K719_1*X1841 + K795_1*X1879 + K2121_1*X2542 - K5582_1*X924
925	X_dot(925) =K495_1*X1729 + K568_2*X1766 + K569_1*X1766 + K570_2*X1767 + K571_1*X1767 + K572_2*X1768 + K573_1*X1768 + K574_2*X1769 + K575_1*X1769 + K576_2*X1770 + K577_1*X1770 + K578_2*X1771 + K579_1*X1771 + K580_2*X1772 + K581_1*X1772 + K582_2*X1773 + K583_1*X1773 + K584_2*X1774 + K585_1*X1774 + K885_1*X1924 + K967_1*X1965 + K999_1*X1981 + K1000_2*X1982 + K1001_1*X1982 + K1002_2*X1983 + K1003_1*X1983 + K1378_2*X2171 + K1379_1*X2171 + K1380_2*X2172 + K1381_1*X2172 + K2192_2*X2578 + K2193_1*X2578 + K2194_2*X2579 + K2195_1*X2579 + K2196_2*X2580 + K2197_1*X2580 + K2198_2*X2581 + K2199_1*X2581 + K2200_2*X2582 + K2201_1*X2582 + K2202_2*X2583 + K2203_1*X2583 + K2204_2*X2584 + K2205_1*X2584 + K2206_2*X2585 + K2207_1*X2585 + K2208_2*X2586 + K2209_1*X2586 + K2210_2*X2587 + K2211_1*X2587 + K2212_2*X2588 + K2213_1*X2588 + K2214_2*X2589 + K2215_1*X2589 + K2924_2*X2944 + K2925_1*X2944 + K2926_2*X2945 + K2927_1*X2945 + K2933_1*X2948 + K3388_2*X3176 + K3414_2*X3189 + K3430_2*X3197 - K5671_1*X925 - K580_1*X198*X925 - K582_1*X181*X925 - K568_1*X362*X925 - K578_1*X277*X925 - K584_1*X257*X925 - K574_1*X415*X925 - K570_1*X632*X925 - K572_1*X630*X925 - K576_1*X662*X925 - K1000_1*X508*X925 - K1002_1*X565*X925 - K1380_1*X129*X925 - K1378_1*X332*X925 - K2194_1*X30*X925 - K2192_1*X138*X925 - K2202_1*X213*X925 - K2196_1*X275*X925 - K2210_1*X288*X925 - K2200_1*X432*X925 - K2204_1*X424*X925 - K2212_1*X373*X925 - K2198_1*X536*X925 - K2206_1*X511*X925 - K2214_1*X659*X925 - K2208_1*X742*X925 - K2924_1*X451*X925 - K2926_1*X499*X925 - K3388_1*X286*X925 - K3414_1*X418*X925 - K3430_1*X404*X925
926	X_dot(926) =K509_1*X1736 + K969_1*X1966 - K5649_1*X926
927	X_dot(927) =K517_1*X1740 - K5661_1*X927
928	X_dot(928) =K521_1*X1742 - K5646_1*X928
929	X_dot(929) =K523_1*X1743 + K615_1*X1789 + K1001_1*X1982 - K5679_1*X929
930	X_dot(930) =K529_1*X1746 + K545_1*X1754 - K5462_1*X930
931	X_dot(931) =K531_1*X1747 + K2601_1*X2782 + K3464_2*X3214 - K5494_1*X931 - K3464_1*X190*X931
932	X_dot(932) =K535_1*X1749 + K729_1*X1846 - K5461_1*X932
933	X_dot(933) =K537_1*X1750 + K551_1*X1757 + K891_1*X1927 + K991_1*X1977 + K1073_1*X2018 + K1163_1*X2063 + K1173_1*X2068 + K2339_1*X2651 - K5591_1*X933
934	X_dot(934) =K555_1*X1759 - K5290_1*X934
935	X_dot(935) =K559_1*X1761 + K571_1*X1767 + K591_1*X1777 + K1963_1*X2463 + K2053_1*X2508 - K5787_1*X935
936	X_dot(936) =K563_1*X1763 - K5276_1*X936
937	X_dot(937) =K567_1*X1765 - K5687_1*X937
938	X_dot(938) =K569_1*X1766 + K639_1*X1801 + K779_1*X1871 - K5547_1*X938
939	X_dot(939) =K575_1*X1769 - K5592_1*X939
940	X_dot(940) =K579_1*X1771 + K589_1*X1776 + K663_1*X1813 + K3490_2*X3227 - K5472_1*X940 - K3490_1*X402*X940
941	X_dot(941) =K581_1*X1772 + K597_1*X1780 - K5401_1*X941
942	X_dot(942) =K587_1*X1775 - K5567_1*X942
943	X_dot(943) =K599_1*X1781 - K5858_1*X943
944	X_dot(944) =K607_1*X1785 + K1207_1*X2085 + K1403_1*X2183 - K5516_1*X944
945	X_dot(945) =K609_1*X1786 - K5367_1*X945
946	X_dot(946) =K613_1*X1788 + K1097_1*X2030 - K5750_1*X946
947	X_dot(947) =K617_1*X1790 + K713_1*X1838 + K1525_1*X2244 + K1551_1*X2257 + K1983_1*X2473 + K1_2*X774 + K3115_1*X3039 - K5656_1*X947 - K1_1*X947*X966
948	X_dot(948) =K619_1*X1791 - K5777_1*X948
949	X_dot(949) =K621_1*X1792 + K1065_1*X2014 + K1583_1*X2273 - K5638_1*X949
950	X_dot(950) =K627_1*X1795 + K737_1*X1850 - K5372_1*X950
951	X_dot(951) =K633_1*X1798 - K5757_1*X951
952	X_dot(952) =K635_1*X1799 + K1021_1*X1992 + K1209_1*X2086 + K1349_1*X2156 + K2461_1*X2712 + K2891_1*X2927 + K2919_1*X2941 + K3153_1*X3058 - K5765_1*X952
953	X_dot(953) =K637_1*X1800 - K5659_1*X953
954	X_dot(954) =K643_1*X1803 + K1967_1*X2465 + K2059_1*X2511 + K2640_2*X2802 + K2641_1*X2802 - K5438_1*X954 - K2640_1*X243*X954
955	X_dot(955) =K645_1*X1804 + K1969_1*X2466 + K2061_1*X2512 - K5391_1*X955
956	X_dot(956) =K653_1*X1808 - K5820_1*X956
957	X_dot(957) =K655_1*X1809 + K699_1*X1831 - K5382_1*X957
958	X_dot(958) =K657_1*X1810 + K743_1*X1853 - K5710_1*X958
959	X_dot(959) =K661_1*X1812 - K5803_1*X959
960	X_dot(960) =K665_1*X1814 + K707_1*X1835 + K1077_1*X2020 + K1177_1*X2070 + K1187_1*X2075 - K5624_1*X960
961	X_dot(961) =K667_1*X1815 - K5716_1*X961
962	X_dot(962) =K673_1*X1818 - K5414_1*X962

963	X_dot(963) =K675_1*X1819 + K1491_1*X2227 - K5548_1*X963
964	X_dot(964) =K679_1*X1821 + K1791_1*X2377 + K2261_1*X2612 - K5454_1*X964
965	X_dot(965) =K681_1*X1822 - K5817_1*X965
966	X_dot(966) =K683_1*X1823 + K1447_1*X2205 + K1755_1*X2359 + K1_2*X774 - K5783_1*X966 - K1_1*X947*X966
967	X_dot(967) =K685_1*X1824 - K5394_1*X967
968	X_dot(968) =K687_1*X1825 + K1337_1*X2150 + K1353_1*X2158 + K1357_1*X2160 - K5623_1*X968
969	X_dot(969) =K695_1*X1829 + K1093_1*X2028 - K5313_1*X969
970	X_dot(970) =K701_1*X1832 + K2176_2*X2570 + K2177_1*X2570 + K2178_2*X2571 + K2179_1*X2571 + K2912_2*X2938 + K2913_1*X2938 + K3310_2*X3137 + K3342_2*X3153 - K5337_1*X970 - K2176_1*X128*X970 - K2178_1*X460*X970 - K2912_1*X513*X970 - K3342_1*X190*X970 - K3310_1*X970*X1007
971	X_dot(971) =K703_1*X1833 + K1599_1*X2281 + K2187_1*X2575 + K2437_1*X2700 - K5425_1*X971
972	X_dot(972) =K705_1*X1834 + K1185_1*X2074 + K1585_1*X2274 + K2633_1*X2798 - K5718_1*X972
973	X_dot(973) =K709_1*X1836 - K5633_1*X973
974	X_dot(974) =K711_1*X1837 + K2537_1*X2750 + K3352_2*X3158 + K3353_1*X3158 + K3354_2*X3159 + K3355_1*X3159 + K3356_2*X3160 + K3357_1*X3160 + K3358_2*X3161 + K3359_1*X3161 + K3360_2*X3162 + K3361_1*X3162 + K3466_2*X3215 + K3467_1*X3215 + K3558_2*X3261 + K3559_1*X3261 - K5443_1*X974 - K3354_1*X823*X974 - K3360_1*X858*X974 - K3358_1*X974*X1217 - K3352_1*X974*X1449 - K3356_1*X974*X1435 - K3466_1*X861*X974 - K3558_1*X818*X974
975	X_dot(975) =K723_1*X1843 - K5348_1*X975
976	X_dot(976) =K725_1*X1844 + K2997_1*X2980 - K5458_1*X976
977	X_dot(977) =K727_1*X1845 - K5677_1*X977
978	X_dot(978) =K733_1*X1848 + K1143_1*X2053 - K5527_1*X978
979	X_dot(979) =K735_1*X1849 - K5533_1*X979
980	X_dot(980) =K734_2*X1849 + K735_1*X1849 + K736_2*X1850 + K737_1*X1850 + K738_2*X1851 + K739_1*X1851 + K1074_2*X2019 + K1075_1*X2019 + K1458_2*X2211 + K1459_1*X2211 + K1460_2*X2212 + K1461_1*X2212 + K1462_2*X2213 + K1463_1*X2213 + K2406_2*X2685 + K2407_1*X2685 + K2408_2*X2686 + K2409_1*X2686 + K2410_2*X2687 + K2411_1*X2687 + K2412_2*X2688 + K2413_1*X2688 + K2414_2*X2689 + K2415_1*X2689 + K2416_2*X2690 + K2417_1*X2690 + K2418_2*X2691 + K2419_1*X2691 + K3008_2*X2986 + K3009_1*X2986 + K3010_2*X2987 + K3011_1*X2987 - K5607_1*X980 - K736_1*X166*X980 - K734_1*X346*X980 - K738_1*X430*X980 - K1074_1*X173*X980 - K1458_1*X121*X980 - K1460_1*X106*X980 - K1462_1*X697*X980 - K2408_1*X85*X980 - K2410_1*X175*X980 - K2418_1*X99*X980 - K2406_1*X307*X980 - K2412_1*X443*X980 - K2414_1*X539*X980 - K2416_1*X656*X980 - K3008_1*X264*X980 - K3010_1*X691*X980
981	X_dot(981) =K745_1*X1854 + K771_1*X1867 + K1407_1*X2185 + K3147_1*X3055 - K5536_1*X981
982	X_dot(982) =K747_1*X1855 - K5885_1*X982
983	X_dot(983) =K749_1*X1856 + K3510_2*X3237 - K5362_1*X983 - K3510_1*X983*X1007
984	X_dot(984) =K751_1*X1857 - K5265_1*X984
985	X_dot(985) =K753_1*X1858 + K2499_1*X2731 - K5852_1*X985
986	X_dot(986) =K755_1*X1859 - K5754_1*X986
987	X_dot(987) =K757_1*X1860 + K3488_2*X3226 - K5328_1*X987 - K3488_1*X401*X987
988	X_dot(988) =K777_1*X1870 - K5590_1*X988
989	X_dot(989) =K789_1*X1876 + K1977_1*X2470 + K2065_1*X2514 + K2199_1*X2581 + K3550_2*X3257 - K5702_1*X989 - K3550_1*X989*X1007
990	X_dot(990) =K791_1*X1877 - K5699_1*X990
991	X_dot(991) =K793_1*X1878 - K5534_1*X991
992	X_dot(992) =K797_1*X1880 - K5595_1*X992
993	X_dot(993) =K799_1*X1881 + K909_1*X1936 + K923_1*X1943 + K947_1*X1955 + K1329_1*X2146 + K2439_1*X2701 + K3444_2*X3204 + K3445_1*X3204 + K3446_2*X3205 + K3447_1*X3205 + K3548_2*X3256 + K3549_1*X3256 - K5574_1*X993 - K3548_1*X993^2 - K3444_1*X811*X993 - K3446_1*X993*X1181
994	X_dot(994) =K801_1*X1882 + K1323_1*X2143 - K5411_1*X994
995	X_dot(995) =K803_1*X1883 + K1473_1*X2218 - K5815_1*X995
996	X_dot(996) =K805_1*X1884 - K5893_1*X996
997	X_dot(997) =K807_1*X1885 + K3019_1*X2991 - K5432_1*X997
998	X_dot(998) =K808_2*X1886 + K809_1*X1886 + K1233_1*X2098 + K2606_2*X2785 + K2607_1*X2785 + K2608_2*X2786 + K2609_1*X2786 + K2610_2*X2787 + K2611_1*X2787 + K3076_2*X3020 + K3077_1*X3020 - K5466_1*X998 - K808_1*X271*X998 - K2606_1*X85*X998 - K2610_1*X99*X998 - K2608_1*X292*X998 - K3076_1*X504*X998
999	X_dot(999) =K811_1*X1887 + K3506_2*X3235 - K5279_1*X999 - K3506_1*X391*X999
1000	X_dot(1000) =K182_2*X1573 + K183_1*X1573 + K184_2*X1574 + K185_1*X1574 + K186_2*X1575 + K187_1*X1575 + K188_2*X1576 + K189_1*X1576 + K190_2*X1577 + K191_1*X1577 + K192_2*X1578 + K193_1*X1578 + K194_2*X1579 + K195_1*X1579 + K196_2*X1580 + K197_1*X1580 + K198_2*X1581 + K199_1*X1581 + K200_2*X1582 + K201_1*X1582 + K202_2*X1583 + K203_1*X1583 + K204_2*X1584 + K205_1*X1584 + K206_2*X1585 + K207_1*X1585 + K208_2*X1586 + K209_1*X1586 + K815_1*X1889 + K878_2*X1921 + K879_1*X1921 + K880_2*X1922 + K881_1*X1922 + K882_2*X1923 + K883_1*X1923 + K1212_2*X2088 + K1213_1*X2088 + K1214_2*X2089 + K1215_1*X2089 + K1216_2*X2090 + K1217_1*X2090 + K1218_2*X2091 + K1219_1*X2091 + K1220_2*X2092 + K1221_1*X2092 + K1513_1*X2238 + K1676_2*X2320 + K1677_1*X2320 + K1678_2*X2321 + K1679_1*X2321 + K1680_2*X2322 + K1681_1*X2322 + K1682_2*X2323 + K1683_1*X2323 + K1684_2*X2324 + K1685_1*X2324 + K1686_2*X2325 + K1687_1*X2325 + K1688_2*X2326 + K1689_1*X2326 + K1690_2*X2327 + K1691_1*X2327 + K1692_2*X2328 + K1693_1*X2328 + K1694_2*X2329 + K1695_1*X2329 + K1696_2*X2330 + K1697_1*X2330 + K1698_2*X2331 + K1699_1*X2331 + K1700_2*X2332 + K1701_1*X2332 + K1702_2*X2333 + K1703_1*X2333 + K1704_2*X2334 + K1705_1*X2334 + K1706_2*X2335 + K1707_1*X2335 + K1708_2*X2336 + K1709_1*X2336 + K1971_1*X2467 + K5_2*X776 + K2658_2*X2811 + K2659_1*X2811 + K2660_2*X2812 + K2661_1*X2812 + K2662_2*X2813 + K2663_1*X2813 + K2664_2*X2814 + K2665_1*X2814 + K2666_2*X2815 + K2667_1*X2815 + K2668_2*X2816 + K2669_1*X2816 + K2683_1*X2823 + K3092_2*X3028 + K3093_1*X3028 + K3109_1*X3036 + K3508_2*X3236 - K5427_1*X1000 - K188_1*X182*X1000 - K196_1*X111*X1000 - K186_1*X232*X1000 - K204_1*X97*X1000 - K182_1*X319*X1000 - K190_1*X345*X1000 - K206_1*X257*X1000 - K192_1*X401*X1000 - K184_1*X484*X1000 - K194_1*X467*X1000 - K200_1*X434*X1000 - K202_1*X485*X1000 - K198_1*X605*X1000 - K208_1*X602*X1000 - K880_1*X116*X1000 - K878_1*X272*X1000 - K882_1*X595*X1000 - K1220_1*X66*X1000 - K1216_1*X190*X1000 - K1218_1*X254*X1000 - K1214_1*X352*X1000 - K1212_1*X621*X1000 - K1684_1*X20*X1000 - K1678_1*X209*X1000 - K1682_1*X189*X1000 - K1680_1*X231*X1000 - K1690_1*X238*X1000 - K1686_1*X291*X1000 - K1706_1*X99*X1000 - K1702_1*X141*X1000 - K1696_1*X233*X1000 - K1694_1*X338*X1000 - K1676_1*X572*X1000 - K1692_1*X446*X1000 - K1688_1*X491*X1000 - K1708_1*X335*X1000 - K1698_1*X510*X1000 - K1700_1*X607*X1000 - K1704_1*X714*X1000 - K5_1*X1000*X1211 - K2666_1*X124*X1000 - K2662_1*X200*X1000 - K2664_1*X224*X1000 - K2668_1*X185*X1000 - K2660_1*X378*X1000 - K2658_1*X570*X1000 - K3092_1*X47*X1000 - K3508_1*X493*X1000
1001	X_dot(1001) =K210_2*X1587 + K211_1*X1587 + K212_2*X1588 + K213_1*X1588 + K214_2*X1589 + K215_1*X1589 + K817_1*X1890 + K1710_2*X2337 + K1711_1*X2337 + K1731_1*X2347 - K5357_1*X1001 - K212_1*X111*X1001 - K210_1*X467*X1001 - K214_1*X434*X1001 - K1710_1*X607*X1001
1002	X_dot(1002) =K823_1*X1893 + K1899_1*X2431 - K5645_1*X1002
1003	X_dot(1003) =K833_1*X1898 + K1926_2*X2445 + K1927_1*X2445 + K2808_2*X2886 + K2809_1*X2886 + K2811_1*X2887 - K5542_1*X1003 - K1926_1*X295*X1003 - K2808_1*X357*X1003

1004	X_dot(1004) =K837_1*X1900 + K1952_2*X2458 + K1953_1*X2458 + K1954_2*X2459 + K1955_1*X2459 + K1956_2*X2460 + K1957_1*X2460 + K1958_2*X2461 + K1959_1*X2461 - K5491_1*X1004 - K1954_1*X287*X1004 - K1956_1*X314*X1004 - K1958_1*X373*X1004 - K1952_1*X451*X1004
1005	X_dot(1005) =K562_2*X1763 + K563_1*X1763 + K564_2*X1764 + K565_1*X1764 + K566_2*X1765 + K567_1*X1765 + K839_1*X1901 + K992_2*X1978 + K993_1*X1978 + K994_2*X1979 + K995_1*X1979 + K996_2*X1980 + K997_1*X1980 + K2180_2*X2572 + K2181_1*X2572 + K2182_2*X2573 + K2183_1*X2573 + K2914_2*X2939 + K2915_1*X2939 + K2916_2*X2940 + K2917_1*X2940 + K2918_2*X2941 + K2919_1*X2941 + K3096_2*X3030 + K3097_1*X3030 - K5495_1*X1005 - K562_1*X62*X1005 - K566_1*X518*X1005 - K564_1*X615*X1005 - K996_1*X281*X1005 - K994_1*X517*X1005 - K992_1*X637*X1005 - K2182_1*X295*X1005 - K2180_1*X683*X1005 - K2914_1*X264*X1005 - K2916_1*X304*X1005 - K2918_1*X609*X1005 - K3096_1*X359*X1005
1006	X_dot(1006) =K612_2*X1788 + K613_1*X1788 + K614_2*X1789 + K615_1*X1789 + K616_2*X1790 + K617_1*X1790 + K618_2*X1791 + K619_1*X1791 + K620_2*X1792 + K621_1*X1792 + K622_2*X1793 + K623_1*X1793 + K624_2*X1794 + K625_1*X1794 + K626_2*X1795 + K627_1*X1795 + K628_2*X1796 + K629_1*X1796 + K630_2*X1797 + K631_1*X1797 + K632_2*X1798 + K633_1*X1798 + K841_1*X1902 + K1014_2*X1989 + K1015_1*X1989 + K1016_2*X1990 + K1017_1*X1990 + K1018_2*X1991 + K1019_1*X1991 + K1020_2*X1992 + K1021_1*X1992 + K1043_1*X2003 + K1589_1*X2276 + K2286_2*X2625 + K2287_1*X2625 + K2288_2*X2626 + K2289_1*X2626 + K2290_2*X2627 + K2291_1*X2627 + K2292_2*X2628 + K2293_1*X2628 + K2294_2*X2629 + K2295_1*X2629 + K2952_2*X2958 + K2953_1*X2958 + K2954_2*X2959 + K2955_1*X2959 + K2956_2*X2960 + K2957_1*X2960 - K5669_1*X1006 - K628_1*X111*X1006 - K626_1*X166*X1006 - K616_1*X483*X1006 - K614_1*X508*X1006 - K620_1*X464*X1006 - K624_1*X461*X1006 - K612_1*X587*X1006 - K618_1*X622*X1006 - K622_1*X620*X1006 - K632_1*X599*X1006 - K630_1*X714*X1006 - K1016_1*X9*X1006 - K1014_1*X496*X1006 - K1018_1*X497*X1006 - K1020_1*X609*X1006 - K2294_1*X99*X1006 - K2290_1*X360*X1006 - K2286_1*X484*X1006 - K2288_1*X679*X1006 - K2292_1*X754*X1006 - K2952_1*X47*X1006 - K2956_1*X57*X1006 - K2954_1*X678*X1006
1007	X_dot(1007) =K843_1*X1903 + K855_1*X1909 + K1201_1*X2082 + K1277_1*X2120 + K1299_1*X2131 + K1869_1*X2416 + K3304_2*X3134 + K3305_1*X3134 + K3306_2*X3135 + K3307_1*X3135 + K3308_2*X3136 + K3309_1*X3136 + K3310_2*X3137 + K3311_1*X3137 + K3312_2*X3138 + K3313_1*X3138 + K3314_2*X3139 + K3315_1*X3139 + K3316_2*X3140 + K3317_1*X3140 + K3318_2*X3141 + K3319_1*X3141 + K3448_2*X3206 + K3449_1*X3206 + K3450_2*X3207 + K3451_1*X3207 + K3452_2*X3208 + K3453_1*X3208 + K3510_2*X3237 + K3511_1*X3237 + K3512_2*X3238 + K3513_1*X3238 + K3550_2*X3257 + K3551_1*X3257 + K3566_2*X3265 + K3567_1*X3265 - K5397_1*X1007 - K3452_1*X1007^2 - K3308_1*X909*X1007 - K3310_1*X970*X1007 - K3304_1*X1007*X1031 - K3312_1*X1007*X1145 - K3314_1*X1007*X1181 - K3316_1*X1007*X1217 - K3318_1*X1007*X1258 - K3306_1*X1007*X1446 - K3448_1*X823*X1007 - K3450_1*X1007*X1267 - K3512_1*X861*X1007 - K3510_1*X983*X1007 - K3550_1*X989*X1007 - K3566_1*X1007*X1115
1008	X_dot(1008) =K845_1*X1904 + K2539_1*X2751 - K5589_1*X1008
1009	X_dot(1009) =K857_1*X1910 + K1193_1*X2078 + K1689_1*X2326 - K5664_1*X1009
1010	X_dot(1010) =K863_1*X1913 + K1003_1*X1983 + K1005_1*X1984 + K2184_2*X2574 + K2185_1*X2574 + K2186_2*X2575 + K2187_1*X2575 + K2527_1*X2745 - K5729_1*X1010 - K2184_1*X165*X1010 - K2186_1*X229*X1010
1011	X_dot(1011) =K865_1*X1914 - K5822_1*X1011
1012	X_dot(1012) =K869_1*X1916 - K5485_1*X1012
1013	X_dot(1013) =K871_1*X1917 + K879_1*X1921 + K1603_1*X2283 + K3237_1*X3100 - K5467_1*X1013
1014	X_dot(1014) =K873_1*X1918 + K881_1*X1922 + K2269_1*X2616 + K3187_1*X3075 - K5325_1*X1014
1015	X_dot(1015) =K875_1*X1919 - K5876_1*X1015
1016	X_dot(1016) =K877_1*X1920 + K1919_1*X2441 - K5233_1*X1016
1017	X_dot(1017) =K883_1*X1923 + K1835_1*X2399 + K2761_1*X2862 - K5755_1*X1017
1018	X_dot(1018) =K887_1*X1925 + K2125_1*X2544 + K3616_2*X3290 + K3617_1*X3290 - K5799_1*X1018 - K3616_1*X567*X1018
1019	X_dot(1019) =K889_1*X1926 + K1171_1*X2067 - K5434_1*X1019
1020	X_dot(1020) =K893_1*X1928 - K5525_1*X1020
1021	X_dot(1021) =K897_1*X1930 + K917_1*X1940 + K925_1*X1944 - K5892_1*X1021
1022	X_dot(1022) =K899_1*X1931 - K5359_1*X1022
1023	X_dot(1023) =K901_1*X1932 + K3340_2*X3152 - K5627_1*X1023 - K3340_1*X190*X1023
1024	X_dot(1024) =K903_1*X1933 - K5324_1*X1024
1025	X_dot(1025) =K907_1*X1935 + K921_1*X1942 - K5779_1*X1025
1026	X_dot(1026) =K911_1*X1937 + K1661_1*X2312 + K2013_1*X2488 + K2101_1*X2532 + K2135_1*X2549 + K4_2*X775 + K2667_1*X2815 + K3189_1*X3076 + K3229_1*X3096 + K3532_2*X3248 + K3568_2*X3266 + K3651_2*X3312 + K3652_1*X3312 + K3669_2*X3330 + K3670_1*X3330 + K3675_2*X3336 + K3676_1*X3336 - K5333_1*X1026 - K4_1*X1026*X1217 - K3532_1*X418*X1026 - K3568_1*X401*X1026 - K3675_1*X1026*X3337 - K3669_1*X878*X1026*X3331 - K3651_1*X1026*X1265*X3313
1027	X_dot(1027) =K913_1*X1938 - K5654_1*X1027
1028	X_dot(1028) =K926_2*X1945 + K927_1*X1945 + K1308_2*X2136 + K1309_1*X2136 + K1310_2*X2137 + K1311_1*X2137 + K1842_2*X2403 + K1843_1*X2403 + K1844_2*X2404 + K1845_1*X2404 + K1979_1*X2471 + K3516_2*X3240 - K5558_1*X1028 - K926_1*X374*X1028 - K1308_1*X233*X1028 - K1310_1*X321*X1028 - K1844_1*X99*X1028 - K1842_1*X538*X1028 - K3516_1*X139*X1028
1029	X_dot(1029) =K929_1*X1946 + K949_1*X1956 + K1937_1*X2450 + K2700_2*X2832 + K2701_1*X2832 - K5507_1*X1029 - K2700_1*X460*X1029
1030	X_dot(1030) =K931_1*X1947 - K5286_1*X1030

1031	X_dot(1031) =K10_2*X1487 + K11_1*X1487 + K12_2*X1488 + K13_1*X1488 + K14_2*X1489 + K15_1*X1489 + K16_2*X1490 + K17_1*X1490 + K18_2*X1491 + K19_1*X1491 + K20_2*X1492 + K21_1*X1492 + K22_2*X1493 + K23_1*X1493 + K24_2*X1494 + K25_1*X1494 + K26_2*X1495 + K27_1*X1495 + K28_2*X1496 + K29_1*X1496 + K30_2*X1497 + K31_1*X1497 + K32_2*X1498 + K33_1*X1498 + K34_2*X1499 + K35_1*X1499 + K36_2*X1500 + K37_1*X1500 + K38_2*X1501 + K39_1*X1501 + K40_2*X1502 + K41_1*X1502 + K42_2*X1503 + K43_1*X1503 + K44_2*X1504 + K45_1*X1504 + K46_2*X1505 + K47_1*X1505 + K48_2*X1506 + K49_1*X1506 + K818_2*X1891 + K819_1*X1891 + K820_2*X1892 + K821_1*X1892 + K822_2*X1893 + K823_1*X1893 + K824_2*X1894 + K825_1*X1894 + K826_2*X1895 + K827_1*X1895 + K828_2*X1896 + K829_1*X1896 + K830_2*X1897 + K831_1*X1897 + K832_2*X1898 + K833_1*X1898 + K834_2*X1899 + K835_1*X1899 + K836_2*X1900 + K837_1*X1900 + K838_2*X1901 + K839_1*X1901 + K840_2*X1902 + K841_1*X1902 + K842_2*X1903 + K843_1*X1903 + K844_2*X1904 + K845_1*X1904 + K846_2*X1905 + K847_1*X1905 + K933_1*X1948 + K1144_2*X2054 + K1145_1*X2054 + K1146_2*X2055 + K1147_1*X2055 + K1148_2*X2056 + K1149_1*X2056 + K1150_2*X2057 + K1151_1*X2057 + K1152_2*X2058 + K1153_1*X2058 + K1154_2*X2059 + K1155_1*X2059 + K1156_2*X2060 + K1157_1*X2060 + K1158_2*X2061 + K1159_1*X2061 + K1160_2*X2062 + K1161_1*X2062 + K1162_2*X2063 + K1163_1*X2063 + K1510_2*X2237 + K1511_1*X2237 + K1512_2*X2238 + K1513_1*X2238 + K1514_2*X2239 + K1515_1*X2239 + K1516_2*X2240 + K1517_1*X2240 + K1518_2*X2241 + K1519_1*X2241 + K1520_2*X2242 + K1521_1*X2242 + K1522_2*X2243 + K1523_1*X2243 + K1524_2*X2244 + K1525_1*X2244 + K1526_2*X2245 + K1527_1*X2245 + K1528_2*X2246 + K1529_1*X2246 + K1530_2*X2247 + K1531_1*X2247 + K1532_2*X2248 + K1533_1*X2248 + K1534_2*X2249 + K1535_1*X2249 + K1536_2*X2250 + K1537_1*X2250 + K1538_2*X2251 + K1539_1*X2251 + K1540_2*X2252 + K1541_1*X2252 + K1542_2*X2253 + K1543_1*X2253 + K1761_1*X2362 + K1779_1*X2371 + K1921_1*X2442 + K2231_1*X2597 + K2245_1*X2604 + K2595_1*X2779 + K2628_2*X2796 + K2629_1*X2796 + K2630_2*X2797 + K2631_1*X2797 + K2632_2*X2798 + K2633_1*X2798 + K2893_1*X2928 + K2915_1*X2939 + K3009_1*X2986 + K3029_1*X2996 + K3080_2*X3022 + K3081_1*X3022 + K3082_2*X3023 + K3083_1*X3023 + K3128_2*X3046 + K3129_1*X3046 + K3170_2*X3067 + K3171_1*X3067 + K3235_1*X3099 + K3254_2*X3109 + K3260_2*X3112 + K3262_2*X3113 + K3290_2*X3127 + K3302_2*X3133 + K3304_2*X3134 + K3364_2*X3164 + K3366_2*X3165 + K3400_2*X3182 - K5459_1*X1031 - K14_1*X20*X1031 - K18_1*X1*X1031 - K26_1*X45*X1031 - K24_1*X100*X1031 - K22_1*X134*X1031 - K32_1*X63*X1031 - K12_1*X319*X1031 - K42_1*X53*X1031 - K10_1*X384*X1031 - K20_1*X342*X1031 - K28_1*X314*X1031 - K16_1*X484*X1031 - K36_1*X401*X1031 - K44_1*X322*X1031 - K38_1*X503*X1031 - K40_1*X495*X1031 - K34_1*X586*X1031 - K46_1*X468*X1031 - K30_1*X722*X1031 - K48_1*X571*X1031 - K828_1*X47*X1031 - K824_1*X168*X1031 - K842_1*X194*X1031 - K830_1*X343*X1031 - K836_1*X299*X1031 - K832_1*X357*X1031 - K838_1*X304*X1031 - K822_1*X471*X1031 - K846_1*X341*X1031 - K818_1*X637*X1031 - K820_1*X630*X1031 - K826_1*X570*X1031 - K844_1*X412*X1031 - K840_1*X497*X1031 - K834_1*X631*X1031 - K1158_1*X57*X1031 - K1146_1*X376*X1031 - K1148_1*X375*X1031 - K1156_1*X308*X1031 - K1152_1*X429*X1031 - K1144_1*X548*X1031 - K1162_1*X414*X1031 - K1154_1*X676*X1031 - K1150_1*X750*X1031 - K1160_1*X693*X1031 - K1514_1*X29*X1031 - K1512_1*X231*X1031 - K1520_1*X238*X1031 - K1522_1*X226*X1031 - K1518_1*X275*X1031 - K1538_1*X129*X1031 - K1526_1*X435*X1031 - K1510_1*X600*X1031 - K1524_1*X483*X1031 - K1536_1*X382*X1031 - K1532_1*X428*X1031 - K1530_1*X455*X1031 - K1516_1*X597*X1031 - K1528_1*X489*X1031 - K1542_1*X419*X1031 - K1534_1*X653*X1031 - K1540_1*X690*X1031 - K2630_1*X177*X1031 - K2628_1*X276*X1031 - K2632_1*X552*X1031 - K3080_1*X379*X1031 - K3082_1*X436*X1031 - K3128_1*X169*X1031 - K3170_1*X769*X1031 - K3254_1*X401*X1031 - K3260_1*X402*X1031 - K3262_1*X418*X1031 - K3290_1*X404*X1031 - K3302_1*X557*X1031 - K3364_1*X139*X1031 - K3366_1*X228*X1031 - K3304_1*X1007*X1031 - K3400_1*X289*X1031
1032	X_dot(1032) =K939_1*X1951 + K1475_1*X2219 + K2445_1*X2704 - K5741_1*X1032
1033	X_dot(1033) =K977_1*X1970 - K5730_1*X1033
1034	X_dot(1034) =K985_1*X1974 + K1053_1*X2008 - K5791_1*X1034
1035	X_dot(1035) =K1009_1*X1986 + K1237_1*X2100 + K3474_2*X3219 + K3476_2*X3220 + K3478_2*X3221 - K5267_1*X1035 - K3478_1*X289*X1035 - K3474_1*X401*X1035 - K3476_1*X402*X1035
1036	X_dot(1036) =K1008_2*X1986 + K1009_1*X1986 + K1010_2*X1987 + K1011_1*X1987 + K1917_1*X2440 + K2230_2*X2597 + K2231_1*X2597 + K2232_2*X2598 + K2233_1*X2598 + K2234_2*X2599 + K2235_1*X2599 + K2236_2*X2600 + K2237_1*X2600 + K2238_2*X2601 + K2239_1*X2601 + K2240_2*X2602 + K2241_1*X2602 + K2242_2*X2603 + K2243_1*X2603 + K2531_1*X2747 + K2928_2*X2946 + K2929_1*X2946 + K2930_2*X2947 + K2931_1*X2947 + K2932_2*X2948 + K2933_1*X2948 + K2934_2*X2949 + K2935_1*X2949 + K2936_2*X2950 + K2937_1*X2950 + K2938_2*X2951 + K2939_1*X2951 - K5255_1*X1036 - K1008_1*X51*X1036 - K1010_1*X38*X1036 - K2230_1*X264*X1036 - K2238_1*X184*X1036 - K2232_1*X287*X1036 - K2240_1*X359*X1036 - K2234_1*X573*X1036 - K2236_1*X574*X1036 - K2242_1*X698*X1036 - K2928_1*X265*X1036 - K2934_1*X373*X1036 - K2930_1*X451*X1036 - K2932_1*X499*X1036 - K2936_1*X462*X1036 - K2938_1*X559*X1036
1037	X_dot(1037) =K1013_1*X1988 + K1067_1*X2015 + K1453_1*X2208 - K5447_1*X1037
1038	X_dot(1038) =K1015_1*X1989 + K1247_1*X2105 + K1285_1*X2124 - K5668_1*X1038
1039	X_dot(1039) =K1023_1*X1993 + K2341_1*X2652 + K2429_1*X2696 - K5478_1*X1039
1040	X_dot(1040) =K1027_1*X1995 - K5378_1*X1040
1041	X_dot(1041) =K1029_1*X1996 - K5703_1*X1041
1042	X_dot(1042) =K1035_1*X1999 - K5304_1*X1042
1043	X_dot(1043) =K1039_1*X2001 + K2741_1*X2852 + K2759_1*X2861 - K5665_1*X1043
1044	X_dot(1044) =K1041_1*X2002 + K1345_1*X2154 - K5807_1*X1044
1045	X_dot(1045) =K1045_1*X2004 - K5587_1*X1045
1046	X_dot(1046) =K1047_1*X2005 - K5264_1*X1046
1047	X_dot(1047) =K1049_1*X2006 - K5579_1*X1047
1048	X_dot(1048) =K1055_1*X2009 - K5631_1*X1048
1049	X_dot(1049) =K1059_1*X2011 - K5811_1*X1049
1050	X_dot(1050) =K1061_1*X2012 - K5395_1*X1050
1051	X_dot(1051) =K1063_1*X2013 - K5274_1*X1051
1052	X_dot(1052) =K1069_1*X2016 + K1457_1*X2210 + K2581_1*X2772 + K3045_1*X3004 + K3336_2*X3150 + K3462_2*X3213 - K5444_1*X1052 - K3336_1*X459*X1052 - K3462_1*X190*X1052
1053	X_dot(1053) =K1075_1*X2019 + K1655_1*X2309 - K5379_1*X1053
1054	X_dot(1054) =K1079_1*X2021 - K5725_1*X1054
1055	X_dot(1055) =K1099_1*X2031 + K1469_1*X2216 + K3330_2*X3147 - K5573_1*X1055 - K3330_1*X459*X1055
1056	X_dot(1056) =K1103_1*X2033 + K3552_2*X3258 - K5301_1*X1056 - K3552_1*X463*X1056
1057	X_dot(1057) =K1105_1*X2034 - K5346_1*X1057
1058	X_dot(1058) =K1109_1*X2036 + K1445_1*X2204 - K5399_1*X1058
1059	X_dot(1059) =K1110_2*X2037 + K1111_1*X2037 + K2547_1*X2755 - K5482_1*X1059 - K1110_1*X290*X1059
1060	X_dot(1060) =K1113_1*X2038 - K5418_1*X1060
1061	X_dot(1061) =K1115_1*X2039 + K1882_2*X2423 + K1883_1*X2423 + K3370_2*X3167 - K5442_1*X1061 - K1882_1*X247*X1061 - K3370_1*X228*X1061
1062	X_dot(1062) =K1135_1*X2049 - K5865_1*X1062
1063	X_dot(1063) =K1141_1*X2052 + K2067_1*X2515 + K2455_1*X2709 + K2485_1*X2724 + K2857_1*X2910 + K3133_1*X3048 - K5298_1*X1063
1064	X_dot(1064) =K1145_1*X2054 - K5714_1*X1064
1065	X_dot(1065) =K1147_1*X2055 + K1165_1*X2064 + K2785_1*X2874 + K2959_1*X2961 - K5560_1*X1065
1066	X_dot(1066) =K1149_1*X2056 - K5559_1*X1066
1067	X_dot(1067) =K1151_1*X2057 + K1479_1*X2221 + K3161_1*X3062 - K5884_1*X1067
1068	X_dot(1068) =K1153_1*X2058 + K2449_1*X2706 + K3151_1*X3057 - K5606_1*X1068
1069	X_dot(1069) =K1155_1*X2059 - K5824_1*X1069
1070	X_dot(1070) =K1157_1*X2060 + K1717_1*X2340 - K5499_1*X1070
1071	X_dot(1071) =K1159_1*X2061 + K2957_1*X2960 + K2979_1*X2971 + K2995_1*X2979 + K3027_1*X2995 - K5273_1*X1071
1072	X_dot(1072) =K1161_1*X2062 + K1481_1*X2222 + K3157_1*X3060 - K5841_1*X1072
1073	X_dot(1073) =K1167_1*X2065 - K5816_1*X1073
1074	X_dot(1074) =K1169_1*X2066 + K1351_1*X2157 + K1363_1*X2163 + K2465_1*X2714 + K2477_1*X2720 - K5761_1*X1074

1075	X_dot(1075) =K1175_1*X2069 + K1425_1*X2194 + K1483_1*X2223 + K1639_1*X2301 - K5317_1*X1075
1076	X_dot(1076) =K1179_1*X2071 - K5569_1*X1076
1077	X_dot(1077) =K1183_1*X2073 + K2203_1*X2583 + K2309_1*X2636 + K2453_1*X2708 + K3137_1*X3050 + K3298_2*X3131 + K3470_2*X3217 - K5413_1*X1077 - K3298_1*X478*X1077 - K3470_1*X178*X1077
1078	X_dot(1078) =K1189_1*X2076 - K5475_1*X1078
1079	X_dot(1079) =K1191_1*X2077 + K1413_1*X2188 + K2111_1*X2537 - K5640_1*X1079
1080	X_dot(1080) =K1195_1*X2079 + K1257_1*X2110 - K5446_1*X1080
1081	X_dot(1081) =K1197_1*X2080 + K1993_1*X2478 - K5644_1*X1081
1082	X_dot(1082) =K1199_1*X2081 + K2226_2*X2595 + K2227_1*X2595 + K2228_2*X2596 + K2229_1*X2596 + K3219_1*X3091 - K5800_1*X1082 - K2226_1*X424*X1082 - K2228_1*X467*X1082
1083	X_dot(1083) =K1203_1*X2083 + K1217_1*X2090 - K5393_1*X1083
1084	X_dot(1084) =K1205_1*X2084 - K5577_1*X1084
1085	X_dot(1085) =K1211_1*X2087 + K1283_1*X2123 + K2279_1*X2621 - K5354_1*X1085
1086	X_dot(1086) =K1213_1*X2088 - K5776_1*X1086
1087	X_dot(1087) =K234_2*X1599 + K235_1*X1599 + K1215_1*X2089 + K1226_2*X2095 + K1227_1*X2095 + K1728_2*X2346 + K1729_1*X2346 + K1730_2*X2347 + K1731_1*X2347 + K2680_2*X2822 + K2681_1*X2822 + K2682_2*X2823 + K2683_1*X2823 - K5538_1*X1087 - K234_1*X230*X1087 - K1226_1*X99*X1087 - K1728_1*X112*X1087 - K1730_1*X150*X1087 - K2680_1*X211*X1087 - K2682_1*X231*X1087
1088	X_dot(1088) =K1219_1*X2091 - K5449_1*X1088
1089	X_dot(1089) =K1221_1*X2092 + K1313_1*X2138 - K5280_1*X1089
1090	X_dot(1090) =K1223_1*X2093 + K1333_1*X2148 + K2113_1*X2538 - K5310_1*X1090
1091	X_dot(1091) =K1225_1*X2094 + K1821_1*X2392 + K2757_1*X2860 + K2793_1*X2878 - K5489_1*X1091
1092	X_dot(1092) =K1235_1*X2099 + K2513_1*X2738 - K5319_1*X1092
1093	X_dot(1093) =K1241_1*X2102 - K5396_1*X1093
1094	X_dot(1094) =K1245_1*X2104 - K5767_1*X1094
1095	X_dot(1095) =K1249_1*X2106 - K5226_1*X1095
1096	X_dot(1096) =K1251_1*X2107 + K1287_1*X2125 - K5244_1*X1096
1097	X_dot(1097) =K1253_1*X2108 + K1289_1*X2126 + K1331_1*X2147 - K5342_1*X1097
1098	X_dot(1098) =K1255_1*X2109 + K1291_1*X2127 - K5424_1*X1098
1099	X_dot(1099) =K1259_1*X2111 + K1423_1*X2193 + K2973_1*X2968 - K5576_1*X1099
1100	X_dot(1100) =K1265_1*X2114 + K1399_1*X2181 + K1459_1*X2211 - K5330_1*X1100
1101	X_dot(1101) =K1267_1*X2115 + K1401_1*X2182 - K5356_1*X1101
1102	X_dot(1102) =K1271_1*X2117 + K1297_1*X2130 - K5268_1*X1102
1103	X_dot(1103) =K538_2*X1751 + K539_1*X1751 + K540_2*X1752 + K541_1*X1752 + K542_2*X1753 + K543_1*X1753 + K544_2*X1754 + K545_1*X1754 + K546_2*X1755 + K547_1*X1755 + K548_2*X1756 + K549_1*X1756 + K550_2*X1757 + K551_1*X1757 + K1273_1*X2118 + K1935_1*X2449 + K1941_1*X2452 + K1945_1*X2454 + K2129_1*X2546 + K2140_2*X2552 + K2141_1*X2552 + K2142_2*X2553 + K2143_1*X2553 + K2144_2*X2554 + K2145_1*X2554 + K2146_2*X2555 + K2147_1*X2555 + K2148_2*X2556 + K2149_1*X2556 + K2150_2*X2557 + K2151_1*X2557 + K2152_2*X2558 + K2153_1*X2558 + K2154_2*X2559 + K2155_1*X2559 + K2156_2*X2560 + K2157_1*X2560 - K5503_1*X1103 - K542_1*X206*X1103 - K546_1*X181*X1103 - K538_1*X281*X1103 - K544_1*X267*X1103 - K548_1*X353*X1103 - K550_1*X414*X1103 - K540_1*X714*X1103 - K2140_1*X170*X1103 - K2150_1*X106*X1103 - K2152_1*X88*X1103 - K2146_1*X175*X1103 - K2156_1*X99*X1103 - K2144_1*X275*X1103 - K2154_1*X295*X1103 - K2148_1*X435*X1103 - K2142_1*X728*X1103
1104	X_dot(1104) =K1275_1*X2119 - K5431_1*X1104
1105	X_dot(1105) =K1279_1*X2121 + K1659_1*X2311 + K2133_1*X2548 + K3215_1*X3089 - K5796_1*X1105
1106	X_dot(1106) =K1281_1*X2122 + K1301_1*X2132 - K5880_1*X1106
1107	X_dot(1107) =K330_2*X1647 + K331_1*X1647 + K924_2*X1944 + K925_1*X1944 + K1302_2*X2133 + K1303_1*X2133 + K1304_2*X2134 + K1305_1*X2134 + K1306_2*X2135 + K1307_1*X2135 + K1625_1*X2294 + K2764_2*X2864 + K2765_1*X2864 + K2766_2*X2865 + K2767_1*X2865 + K2768_2*X2866 + K2769_1*X2866 + K2770_2*X2867 + K2771_1*X2867 + K2772_2*X2868 + K2773_1*X2868 - K5597_1*X1107 - K330_1*X445*X1107 - K924_1*X759*X1107 - K1304_1*X281*X1107 - K1302_1*X420*X1107 - K1306_1*X578*X1107 - K2772_1*X56*X1107 - K2766_1*X275*X1107 - K2764_1*X386*X1107 - K2768_1*X380*X1107 - K2770_1*X718*X1107
1108	X_dot(1108) =K1307_1*X2135 + K2993_1*X2978 - K5742_1*X1108
1109	X_dot(1109) =K1309_1*X2136 + K1697_1*X2330 - K5429_1*X1109
1110	X_dot(1110) =K1311_1*X2137 - K5511_1*X1110
1111	X_dot(1111) =K1315_1*X2139 + K2335_1*X2649 + K2553_1*X2758 - K5651_1*X1111
1112	X_dot(1112) =K1319_1*X2141 + K1553_1*X2258 + K1889_1*X2426 + K2347_1*X2655 + K2401_1*X2682 + K2431_1*X2697 + K2509_1*X2736 + K2563_1*X2763 + K2677_1*X2820 + K3129_1*X3046 - K5375_1*X1112
1113	X_dot(1113) =K1321_1*X2142 + K1495_1*X2229 + K1887_1*X2425 - K5588_1*X1113
1114	X_dot(1114) =K1325_1*X2144 - K5242_1*X1114
1115	X_dot(1115) =K1327_1*X2145 + K3566_2*X3265 - K5283_1*X1115 - K3566_1*X1007*X1115
1116	X_dot(1116) =K1339_1*X2151 - K5844_1*X1116
1117	X_dot(1117) =K1341_1*X2152 + K1359_1*X2161 - K5528_1*X1117
1118	X_dot(1118) =K1343_1*X2153 + K2089_1*X2526 + K2357_1*X2660 + K2877_1*X2920 - K5515_1*X1118
1119	X_dot(1119) =K1347_1*X2155 + K1361_1*X2162 + K1657_1*X2310 + K2391_1*X2677 + K3282_2*X3123 - K5420_1*X1119 - K3282_1*X418*X1119
1120	X_dot(1120) =K1355_1*X2159 + K1379_1*X2171 + K1385_1*X2174 + K1471_1*X2217 + K2899_1*X2931 - K5520_1*X1120
1121	X_dot(1121) =K1365_1*X2164 - K5254_1*X1121
1122	X_dot(1122) =K1369_1*X2166 - K5848_1*X1122
1123	X_dot(1123) =K1376_2*X2170 + K1377_1*X2170 + K1649_1*X2306 + K3191_1*X3077 - K5377_1*X1123 - K1376_1*X171*X1123
1124	X_dot(1124) =K1383_1*X2173 + K2459_1*X2711 + K3149_1*X3056 - K5585_1*X1124
1125	X_dot(1125) =K1387_1*X2175 + K2277_1*X2620 - K5586_1*X1125
1126	X_dot(1126) =K1230_2*X2097 + K1231_1*X2097 + K1232_2*X2098 + K1233_1*X2098 + K1389_1*X2176 + K1750_2*X2357 + K1751_1*X2357 + K1752_2*X2358 + K1753_1*X2358 + K2409_1*X2686 + K2607_1*X2785 + K2690_2*X2827 + K2691_1*X2827 + K2692_2*X2828 + K2693_1*X2828 + K3084_2*X3024 + K3085_1*X3024 + K3528_2*X3246 + K3534_2*X3249 - K5296_1*X1126 - K1230_1*X250*X1126 - K1232_1*X271*X1126 - K1752_1*X201*X1126 - K1750_1*X507*X1126 - K2690_1*X85*X1126 - K2692_1*X111*X1126 - K3084_1*X99*X1126 - K3528_1*X418*X1126 - K3534_1*X404*X1126
1127	X_dot(1127) =K1393_1*X2178 - K5455_1*X1127
1128	X_dot(1128) =K1395_1*X2179 - K5256_1*X1128
1129	X_dot(1129) =K1397_1*X2180 - K5836_1*X1129
1130	X_dot(1130) =K1405_1*X2184 + K2556_2*X2760 + K2557_1*X2760 + K2558_2*X2761 + K2559_1*X2761 + K2560_2*X2762 + K2561_1*X2762 - K5898_1*X1130 - K2558_1*X556*X1130 - K2556_1*X765*X1130 - K2560_1*X747*X1130
1131	X_dot(1131) =K1409_1*X2186 - K5282_1*X1131
1132	X_dot(1132) =K1411_1*X2187 + K2495_1*X2729 - K5327_1*X1132
1133	X_dot(1133) =K1417_1*X2190 + K3264_2*X3114 - K5322_1*X1133 - K3264_1*X418*X1133

1134	X_dot(1134) =K1419_1*X2191 - K5572_1*X1134
1135	X_dot(1135) =K1421_1*X2192 - K5897_1*X1135
1136	X_dot(1136) =K1427_1*X2195 + K1641_1*X2302 - K5303_1*X1136
1137	X_dot(1137) =K1429_1*X2196 + K2209_1*X2586 + K2385_1*X2674 - K5879_1*X1137
1138	X_dot(1138) =K1431_1*X2197 - K5637_1*X1138
1139	X_dot(1139) =K1433_1*X2198 - K5352_1*X1139
1140	X_dot(1140) =K1435_1*X2199 - K5436_1*X1140
1141	X_dot(1141) =K1437_1*X2200 - K5225_1*X1141
1142	X_dot(1142) =K1439_1*X2201 - K5643_1*X1142
1143	X_dot(1143) =K1443_1*X2203 - K5581_1*X1143
1144	X_dot(1144) =K1449_1*X2206 - K5598_1*X1144
1145	X_dot(1145) =K712_2*X1838 + K713_1*X1838 + K714_2*X1839 + K715_1*X1839 + K716_2*X1840 + K717_1*X1840 + K718_2*X1841 + K719_1*X1841 + K720_2*X1842 + K721_1*X1842 + K722_2*X1843 + K723_1*X1843 + K724_2*X1844 + K725_1*X1844 + K726_2*X1845 + K727_1*X1845 + K728_2*X1846 + K729_1*X1846 + K1070_2*X2017 + K1071_1*X2017 + K1072_2*X2018 + K1073_1*X2018 + K1446_2*X2205 + K1447_1*X2205 + K1448_2*X2206 + K1449_1*X2206 + K1450_2*X2207 + K1451_1*X2207 + K1452_2*X2208 + K1453_1*X2208 + K1454_2*X2209 + K1455_1*X2209 + K1456_2*X2210 + K1457_1*X2210 + K2366_2*X2665 + K2367_1*X2665 + K2368_2*X2666 + K2369_1*X2666 + K2370_2*X2667 + K2371_1*X2667 + K2372_2*X2668 + K2373_1*X2668 + K2374_2*X2669 + K2375_1*X2669 + K2376_2*X2670 + K2377_1*X2670 + K2378_2*X2671 + K2379_1*X2671 + K2380_2*X2672 + K2381_1*X2672 + K2382_2*X2673 + K2383_1*X2673 + K2384_2*X2674 + K2385_1*X2674 + K2386_2*X2675 + K2387_1*X2675 + K2388_2*X2676 + K2389_1*X2676 + K2390_2*X2677 + K2391_1*X2677 + K2392_2*X2678 + K2393_1*X2678 + K2394_2*X2679 + K2395_1*X2679 + K2396_2*X2680 + K2397_1*X2680 + K2937_1*X2950 + K3000_2*X2982 + K3001_1*X2982 + K3002_2*X2983 + K3003_1*X2983 + K3004_2*X2984 + K3005_1*X2984 + K3006_2*X2985 + K3007_1*X2985 + K3312_2*X3138 + K3416_2*X3190 + K3432_2*X3198 - K5636_1*X1145 - K716_1*X182*X1145 - K722_1*X140*X1145 - K720_1*X281*X1145 - K714_1*X342*X1145 - K724_1*X263*X1145 - K728_1*X266*X1145 - K718_1*X400*X1145 - K712_1*X483*X1145 - K726_1*X505*X1145 - K1070_1*X86*X1145 - K1072_1*X414*X1145 - K1450_1*X103*X1145 - K1452_1*X252*X1145 - K1456_1*X249*X1145 - K1448_1*X421*X1145 - K1454_1*X462*X1145 - K1446_1*X628*X1145 - K2372_1*X10*X1145 - K2366_1*X83*X1145 - K2370_1*X117*X1145 - K2376_1*X148*X1145 - K2388_1*X31*X1145 - K2368_1*X355*X1145 - K2396_1*X99*X1145 - K2380_1*X294*X1145 - K2386_1*X255*X1145 - K2390_1*X223*X1145 - K2382_1*X312*X1145 - K2378_1*X424*X1145 - K2392_1*X302*X1145 - K2394_1*X295*X1145 - K2374_1*X564*X1145 - K2384_1*X742*X1145 - K3006_1*X157*X1145 - K3000_1*X232*X1145 - K3004_1*X197*X1145 - K3002_1*X530*X1145 - K3312_1*X1007*X1145 - K3416_1*X418*X1145 - K3432_1*X404*X1145
1146	X_dot(1146) =K1463_1*X2213 - K5845_1*X1146
1147	X_dot(1147) =K1467_1*X2215 - K5555_1*X1147
1148	X_dot(1148) =K1477_1*X2220 - K5473_1*X1148
1149	X_dot(1149) =K1485_1*X2224 - K5746_1*X1149
1150	X_dot(1150) =K1489_1*X2226 + K1851_1*X2407 + K2343_1*X2653 - K5305_1*X1150
1151	X_dot(1151) =K1493_1*X2228 - K5840_1*X1151
1152	X_dot(1152) =K1497_1*X2230 - K5339_1*X1152
1153	X_dot(1153) =K1499_1*X2231 - K5262_1*X1153
1154	X_dot(1154) =K772_2*X1868 + K773_1*X1868 + K774_2*X1869 + K775_1*X1869 + K1088_2*X2026 + K1089_1*X2026 + K1090_2*X2027 + K1091_1*X2027 + K1092_2*X2028 + K1093_1*X2028 + K1482_2*X2223 + K1483_1*X2223 + K1501_1*X2232 + K2009_1*X2486 + K2093_1*X2528 + K2241_1*X2602 + K2468_2*X2716 + K2469_1*X2716 + K2470_2*X2717 + K2471_1*X2717 + K2472_2*X2718 + K2473_1*X2718 + K2474_2*X2719 + K2475_1*X2719 + K2476_2*X2720 + K2477_1*X2720 + K2478_2*X2721 + K2479_1*X2721 + K3024_2*X2994 + K3025_1*X2994 + K3026_2*X2995 + K3027_1*X2995 + K3097_1*X3030 + K3130_2*X3047 + K3131_1*X3047 + K3132_2*X3048 + K3133_1*X3048 + K3134_2*X3049 + K3135_1*X3049 + K3136_2*X3050 + K3137_1*X3050 + K3138_2*X3051 + K3139_1*X3051 + K3140_2*X3052 + K3141_1*X3052 + K3142_2*X3053 + K3143_1*X3053 + K3144_2*X3054 + K3145_1*X3054 + K3146_2*X3055 + K3147_1*X3055 + K3148_2*X3056 + K3149_1*X3056 + K3150_2*X3057 + K3151_1*X3057 + K3152_2*X3058 + K3153_1*X3058 + K3154_2*X3059 + K3155_1*X3059 + K3156_2*X3060 + K3157_1*X3060 + K3158_2*X3061 + K3159_1*X3061 + K3160_2*X3062 + K3161_1*X3062 + K3162_2*X3063 + K3163_1*X3063 - K5544_1*X1154 - K772_1*X630*X1154 - K774_1*X652*X1154 - K1090_1*X102*X1154 - K1092_1*X104*X1154 - K1088_1*X342*X1154 - K1482_1*X108*X1154 - K2468_1*X203*X1154 - K2472_1*X270*X1154 - K2470_1*X435*X1154 - K2474_1*X424*X1154 - K2478_1*X419*X1154 - K2476_1*X604*X1154 - K3026_1*X57*X1154 - K3024_1*X177*X1154 - K3130_1*X9*X1154 - K3132_1*X87*X1154 - K3134_1*X204*X1154 - K3136_1*X213*X1154 - K3138_1*X226*X1154 - K3140_1*X281*X1154 - K3142_1*X341*X1154 - K3144_1*X343*X1154 - K3146_1*X350*X1154 - K3148_1*X406*X1154 - K3150_1*X429*X1154 - K3162_1*X359*X1154 - K3152_1*X609*X1154 - K3154_1*X615*X1154 - K3156_1*X693*X1154 - K3158_1*X722*X1154 - K3160_1*X750*X1154
1155	X_dot(1155) =K1503_1*X2233 - K5818_1*X1155
1156	X_dot(1156) =K1507_1*X2235 - K5819_1*X1156
1157	X_dot(1157) =K1509_1*X2236 - K5795_1*X1157
1158	X_dot(1158) =K1511_1*X2237 - K5758_1*X1158
1159	X_dot(1159) =K248_2*X1606 + K249_1*X1606 + K250_2*X1607 + K251_1*X1607 + K252_2*X1608 + K253_1*X1608 + K1116_2*X2040 + K1117_1*X2040 + K1515_1*X2239 + K1545_1*X2254 + K1754_2*X2359 + K1755_1*X2359 + K1756_2*X2360 + K1757_1*X2360 + K1758_2*X2361 + K1759_1*X2361 + K2694_2*X2829 + K2695_1*X2829 + K2696_2*X2830 + K2697_1*X2830 + K2698_2*X2831 + K2699_1*X2831 + K3164_2*X3064 + K3165_1*X3064 - K5247_1*X1159 - K248_1*X47*X1159 - K252_1*X341*X1159 - K250_1*X763*X1159 - K1116_1*X207*X1159 - K1756_1*X435*X1159 - K1754_1*X628*X1159 - K1758_1*X633*X1159 - K2694_1*X442*X1159 - K2696_1*X453*X1159 - K2698_1*X601*X1159 - K3164_1*X771*X1159
1160	X_dot(1160) =K1517_1*X2240 - K5756_1*X1160
1161	X_dot(1161) =K1521_1*X2242 + K1627_1*X2295 + K1691_1*X2327 + K1713_1*X2338 - K5433_1*X1161
1162	X_dot(1162) =K1523_1*X2243 + K2451_1*X2707 + K3139_1*X3051 - K5423_1*X1162
1163	X_dot(1163) =K1527_1*X2245 + K1567_1*X2265 + K1745_1*X2354 + K1757_1*X2360 + K1989_1*X2476 + K2037_1*X2500 + K2071_1*X2517 + K2119_1*X2541 + K2149_1*X2556 + K2311_1*X2637 + K2457_1*X2710 + K2471_1*X2717 - K5612_1*X1163
1164	X_dot(1164) =K1529_1*X2246 - K5662_1*X1164
1165	X_dot(1165) =K1531_1*X2247 - K5630_1*X1165
1166	X_dot(1166) =K1533_1*X2248 + K2554_2*X2759 + K2555_1*X2759 - K5605_1*X1166 - K2554_1*X732*X1166
1167	X_dot(1167) =K1535_1*X2249 + K2604_2*X2784 + K2605_1*X2784 - K5806_1*X1167 - K2604_1*X99*X1167
1168	X_dot(1168) =K1537_1*X2250 - K5566_1*X1168
1169	X_dot(1169) =K1541_1*X2252 + K2622_2*X2793 + K2623_1*X2793 + K2624_2*X2794 + K2625_1*X2794 + K2626_2*X2795 + K2627_1*X2795 - K5838_1*X1169 - K2622_1*X80*X1169 - K2626_1*X690*X1169 - K2624_1*X724*X1169
1170	X_dot(1170) =K1543_1*X2253 + K2467_1*X2715 + K2479_1*X2721 - K5596_1*X1170
1171	X_dot(1171) =K1555_1*X2259 + K3083_1*X3023 + K3612_2*X3288 + K3613_1*X3288 + K3614_2*X3289 + K3615_1*X3289 - K5613_1*X1171 - K3612_1*X306*X1171 - K3614_1*X705*X1171
1172	X_dot(1172) =K952_2*X1958 + K953_1*X1958 + K1559_1*X2261 + K1577_1*X2270 + K1930_2*X2447 + K1931_1*X2447 + K1932_2*X2448 + K1933_1*X2448 + K1955_1*X2459 + K2233_1*X2598 + K3268_2*X3116 - K5480_1*X1172 - K952_1*X384*X1172 - K1930_1*X246*X1172 - K1932_1*X240*X1172 - K3268_1*X418*X1172

1173	X_dot(1173) =K74_2*X1519 + K75_1*X1519 + K76_2*X1520 + K77_1*X1520 + K78_2*X1521 + K79_1*X1521 + K80_2*X1522 + K81_1*X1522 + K82_2*X1523 + K83_1*X1523 + K84_2*X1524 + K85_1*X1524 + K1166_2*X2065 + K1167_1*X2065 + K1168_2*X2066 + K1169_1*X2066 + K1560_2*X2262 + K1561_1*X2262 + K1562_2*X2263 + K1563_1*X2263 + K1564_2*X2264 + K1565_1*X2264 + K1566_2*X2265 + K1567_1*X2265 + K1568_2*X2266 + K1569_1*X2266 + K1570_2*X2267 + K1571_1*X2267 + K1572_2*X2268 + K1573_1*X2268 + K1737_1*X2350 + K2636_2*X2800 + K2637_1*X2800 - K5797_1*X1173 - K78_1*X21*X1173 - K82_1*X99*X1173 - K84_1*X129*X1173 - K80_1*X482*X1173 - K76_1*X662*X1173 - K74_1*X689*X1173 - K1168_1*X604*X1173 - K1166_1*X668*X1173 - K1564_1*X59*X1173 - K1572_1*X235*X1173 - K1566_1*X435*X1173 - K1568_1*X424*X1173 - K1560_1*X643*X1173 - K1562_1*X638*X1173 - K1570_1*X741*X1173 - K2636_1*X680*X1173
1174	X_dot(1174) =K1563_1*X2263 - K5793_1*X1174
1175	X_dot(1175) =K1565_1*X2264 - K5275_1*X1175
1176	X_dot(1176) =K1569_1*X2266 + K2039_1*X2501 + K2205_1*X2584 + K2227_1*X2595 + K2379_1*X2671 + K2475_1*X2719 + K2603_1*X2783 + K2615_1*X2789 + K3075_1*X3019 - K5601_1*X1176
1177	X_dot(1177) =K1571_1*X2267 - K5878_1*X1177
1178	X_dot(1178) =K1573_1*X2268 + K1839_1*X2401 + K2285_1*X2624 - K5430_1*X1178
1179	X_dot(1179) =K1575_1*X2269 + K2117_1*X2540 + K2147_1*X2555 + K2411_1*X2687 + K3242_2*X3103 - K5381_1*X1179 - K3242_1*X660*X1179
1180	X_dot(1180) =K1581_1*X2272 + K2301_1*X2632 - K5380_1*X1180
1181	X_dot(1181) =K748_2*X1856 + K749_1*X1856 + K750_2*X1857 + K751_1*X1857 + K1468_2*X2216 + K1469_1*X2216 + K1470_2*X2217 + K1471_1*X2217 + K1472_2*X2218 + K1473_1*X2218 + K1587_1*X2275 + K1719_1*X2341 + K1867_1*X2415 + K2179_1*X2571 + K2426_2*X2695 + K2427_1*X2695 + K2535_1*X2749 + K2701_1*X2832 + K3012_2*X2988 + K3013_1*X2988 + K3314_2*X3139 + K3328_2*X3146 + K3334_2*X3149 + K3446_2*X3205 - K5634_1*X1181 - K750_1*X49*X1181 - K748_1*X155*X1181 - K1470_1*X332*X1181 - K1468_1*X389*X1181 - K1472_1*X665*X1181 - K2426_1*X460*X1181 - K3012_1*X72*X1181 - K3328_1*X463*X1181 - K3334_1*X459*X1181 - K3314_1*X1007*X1181 - K3446_1*X993*X1181
1182	X_dot(1182) =K1591_1*X2277 + K1871_1*X2417 + K8_2*X770 + K2393_1*X2678 + K2653_1*X2808 + K2881_1*X2922 + K2901_1*X2932 + K3049_1*X3006 + K3346_2*X3155 + K3458_2*X3211 - K5493_1*X1182 - K8_1*X1182*X1217 - K3346_1*X190*X1182 - K3458_1*X463*X1182
1183	X_dot(1183) =K1178_2*X2071 + K1179_1*X2071 + K1180_2*X2072 + K1181_1*X2072 + K1592_2*X2278 + K1593_1*X2278 + K1594_2*X2279 + K1595_1*X2279 - K5690_1*X1183 - K1180_1*X129*X1183 - K1178_1*X385*X1183 - K1594_1*X159*X1183 - K1592_1*X523*X1183
1184	X_dot(1184) =K1595_1*X2279 + K3031_1*X2997 - K5366_1*X1184
1185	X_dot(1185) =K398_2*X1681 + K399_1*X1681 + K400_2*X1682 + K401_1*X1682 + K1597_1*X2280 + K2848_2*X2906 + K2849_1*X2906 + K2850_2*X2907 + K2851_1*X2907 + K2852_2*X2908 + K2853_1*X2908 + K2854_2*X2909 + K2855_1*X2909 + K3272_2*X3118 + K3300_2*X3132 - K5258_1*X1185 - K400_1*X563*X1185 - K398_1*X620*X1185 - K2854_1*X41*X1185 - K2848_1*X320*X1185 - K2850_1*X314*X1185 - K2852_1*X361*X1185 - K3300_1*X15*X1185 - K3272_1*X418*X1185
1186	X_dot(1186) =K1601_1*X2282 + K1695_1*X2329 + K1809_1*X2386 - K5526_1*X1186
1187	X_dot(1187) =K1605_1*X2284 - K5468_1*X1187
1188	X_dot(1188) =K1607_1*X2285 - K5278_1*X1188
1189	X_dot(1189) =K1609_1*X2286 + K2255_1*X2609 - K5830_1*X1189
1190	X_dot(1190) =K1611_1*X2287 + K2257_1*X2610 - K5270_1*X1190
1191	X_dot(1191) =K1613_1*X2288 - K5666_1*X1191
1192	X_dot(1192) =K1615_1*X2289 - K5869_1*X1192
1193	X_dot(1193) =K1617_1*X2290 + K1787_1*X2375 - K5451_1*X1193
1194	X_dot(1194) =K1600_2*X2282 + K1601_1*X2282 + K1602_2*X2283 + K1603_1*X2283 + K1604_2*X2284 + K1605_1*X2284 + K1606_2*X2285 + K1607_1*X2285 + K1619_1*X2291 + K1741_1*X2352 + K3183_1*X3073 + K3223_1*X3093 - K5229_1*X1194 - K1606_1*X64*X1194 - K1602_1*X272*X1194 - K1604_1*X273*X1194 - K1600_1*X338*X1194
1195	X_dot(1195) =K288_2*X1626 + K289_1*X1626 + K290_2*X1627 + K291_1*X1627 + K292_2*X1628 + K293_1*X1628 + K294_2*X1629 + K295_1*X1629 + K296_2*X1630 + K297_1*X1630 + K298_2*X1631 + K299_1*X1631 + K300_2*X1632 + K301_1*X1632 + K302_2*X1633 + K303_1*X1633 + K304_2*X1634 + K305_1*X1634 + K306_2*X1635 + K307_1*X1635 + K308_2*X1636 + K309_1*X1636 + K310_2*X1637 + K311_1*X1637 + K312_2*X1638 + K313_1*X1638 + K314_2*X1639 + K315_1*X1639 + K316_2*X1640 + K317_1*X1640 + K318_2*X1641 + K319_1*X1641 + K320_2*X1642 + K321_1*X1642 + K322_2*X1643 + K323_1*X1643 + K324_2*X1644 + K325_1*X1644 + K326_2*X1645 + K327_1*X1645 + K896_2*X1930 + K897_1*X1930 + K898_2*X1931 + K899_1*X1931 + K900_2*X1932 + K901_1*X1932 + K902_2*X1933 + K903_1*X1933 + K904_2*X1934 + K905_1*X1934 + K906_2*X1935 + K907_1*X1935 + K908_2*X1936 + K909_1*X1936 + K910_2*X1937 + K911_1*X1937 + K912_2*X1938 + K913_1*X1938 + K914_2*X1939 + K915_1*X1939 + K1244_2*X2104 + K1245_1*X2104 + K1246_2*X2105 + K1247_1*X2105 + K1248_2*X2106 + K1249_1*X2106 + K1250_2*X2107 + K1251_1*X2107 + K1252_2*X2108 + K1253_1*X2108 + K1254_2*X2109 + K1255_1*X2109 + K1256_2*X2110 + K1257_1*X2110 + K1258_2*X2111 + K1259_1*X2111 + K1260_2*X2112 + K1261_1*X2112 + K1262_2*X2113 + K1263_1*X2113 + K1264_2*X2114 + K1265_1*X2114 + K1266_2*X2115 + K1267_1*X2115 + K1268_2*X2116 + K1269_1*X2116 + K1270_2*X2117 + K1271_1*X2117 + K1272_2*X2118 + K1273_1*X2118 + K1274_2*X2119 + K1275_1*X2119 + K1276_2*X2120 + K1277_1*X2120 + K1278_2*X2121 + K1279_1*X2121 + K1280_2*X2122 + K1281_1*X2122 + K1282_2*X2123 + K1283_1*X2123 + K1623_1*X2293 + K1776_2*X2370 + K1777_1*X2370 + K1778_2*X2371 + K1779_1*X2371 + K1780_2*X2372 + K1781_1*X2372 + K1782_2*X2373 + K1783_1*X2373 + K1784_2*X2374 + K1785_1*X2374 + K1786_2*X2375 + K1787_1*X2375 + K1788_2*X2376 + K1789_1*X2376 + K1790_2*X2377 + K1791_1*X2377 + K1792_2*X2378 + K1793_1*X2378 + K1794_2*X2379 + K1795_1*X2379 + K1796_2*X2380 + K1797_1*X2380 + K1798_2*X2381 + K1799_1*X2381 + K1800_2*X2382 + K1801_1*X2382 + K1802_2*X2383 + K1803_1*X2383 + K1804_2*X2384 + K1805_1*X2384 + K1806_2*X2385 + K1807_1*X2385 + K1808_2*X2386 + K1809_1*X2386 + K1810_2*X2387 + K1811_1*X2387 + K1812_2*X2388 + K1813_1*X2388 + K1814_2*X2389 + K1815_1*X2389 + K1816_2*X2390 + K1817_1*X2390 + K1818_2*X2391 + K1819_1*X2391 + K1820_2*X2392 + K1821_1*X2392 + K1822_2*X2393 + K1823_1*X2393 + K1824_2*X2394 + K1825_1*X2394 + K1826_2*X2395 + K1827_1*X2395 + K1828_2*X2396 + K1829_1*X2396 + K1830_2*X2397 + K1831_1*X2397 + K1832_2*X2398 + K1833_1*X2398 + K1834_2*X2399 + K1835_1*X2399 + K1836_2*X2400 + K1837_1*X2400 + K1838_2*X2401 + K1839_1*X2401 + K1975_1*X2469 + K2712_2*X2838 + K2713_1*X2838 + K2714_2*X2839 + K2715_1*X2839 + K2716_2*X2840 + K2717_1*X2840 + K2718_2*X2841 + K2719_1*X2841 + K2720_2*X2842 + K2721_1*X2842 + K2722_2*X2843 + K2723_1*X2843 + K2724_2*X2844 + K2725_1*X2844 + K2726_2*X2845 + K2727_1*X2845 + K2728_2*X2846 + K2729_1*X2846 + K2730_2*X2847 + K2731_1*X2847 + K2732_2*X2848 + K2733_1*X2848 + K2734_2*X2849 + K2735_1*X2849 + K2736_2*X2850 + K2737_1*X2850 + K2738_2*X2851 + K2739_1*X2851 + K2740_2*X2852 + K2741_1*X2852 + K2742_2*X2853 + K2743_1*X2853 + K3111_1*X3037 - K5600_1*X1195 - K300_1*X81*X1195 - K290_1*X199*X1195 - K304_1*X102*X1195 - K306_1*X126*X1195 - K308_1*X106*X1195 - K294_1*X307*X1195 - K298_1*X274*X1195 - K324_1*X40*X1195 - K310_1*X250*X1195 - K296_1*X433*X1195 - K288_1*X547*X1195 - K302_1*X517*X1195 - K314_1*X445*X1195 - K292_1*X684*X1195 - K318_1*X444*X1195 - K312_1*X714*X1195 - K316_1*X686*X1195 - K320_1*X752*X1195 - K322_1*X758*X1195 - K326_1*X768*X1195 - K898_1*X152*X1195 - K902_1*X115*X1195 - K904_1*X122*X1195 - K910_1*X124*X1195 - K914_1*X99*X1195 - K900_1*X452*X1195 - K908_1*X390*X1195 - K912_1*X481*X1195 - K906_1*X624*X1195 - K896_1*X759*X1195 - K1248_1*X4*X1195 - K1250_1*X26*X1195 - K1252_1*X133*X1195 - K1270_1*X52*X1195 - K1260_1*X161*X1195 - K1264_1*X121*X1195 - K1254_1*X227*X1195 - K1262_1*X182*X1195 - K1266_1*X149*X1195 - K1256_1*X251*X1195 - K1276_1*X194*X1195 - K1246_1*X496*X1195 - K1268_1*X281*X1195 - K1282_1*X147*X1195 - K1258_1*X393*X1195 - K1274_1*X236*X1195 - K1272_1*X313*X1195 - K1244_1*X611*X1195 - K1278_1*X641*X1195 - K1280_1*X744*X1195 - K1788_1*X112*X1195 - K1776_1*X269*X1195 - K1778_1*X264*X1195 - K1786_1*X256*X1195 - K1784_1*X311*X1195 - K1790_1*X259*X1195 - K1814_1*X19*X1195 - K1782_1*X368*X1195 - K1798_1*X208*X1195 - K1816_1*X71*X1195 -
1196	X_dot(1196) =K1629_1*X2296 - K5748_1*X1196
1197	X_dot(1197) =K1631_1*X2297 + K1693_1*X2328 - K5622_1*X1197
1198	X_dot(1198) =K1633_1*X2298 + K1905_1*X2434 + K3213_1*X3088 - K5789_1*X1198
1199	X_dot(1199) =K1635_1*X2299 + K2267_1*X2615 - K5287_1*X1199
1200	X_dot(1200) =K1637_1*X2300 - K5550_1*X1200
1201	X_dot(1201) =K1645_1*X2304 - K5770_1*X1201
1202	X_dot(1202) =K1647_1*X2305 + K2317_1*X2640 + K2867_1*X2915 - K5854_1*X1202
1203	X_dot(1203) =K1651_1*X2307 - K5556_1*X1203
1204	X_dot(1204) =K1653_1*X2308 + K1827_1*X2395 + K3193_1*X3078 - K5506_1*X1204
1205	X_dot(1205) =K1663_1*X2313 + K2599_1*X2781 + K3203_1*X3083 - K5684_1*X1205
1206	X_dot(1206) =K1667_1*X2315 - K5416_1*X1206

1207	X_dot(1207) =K1669_1*X2316 + K1709_1*X2336 + K3622_2*X3293 + K3623_1*X3293 - K5523_1*X1207 - K3622_1*X151*X1207
1208	X_dot(1208) =K1671_1*X2317 - K5894_1*X1208
1209	X_dot(1209) =K180_2*X1572 + K181_1*X1572 + K1673_1*X2318 + K2681_1*X2822 - K5412_1*X1209 - K180_1*X6*X1209
1210	X_dot(1210) =K1677_1*X2320 - K5736_1*X1210
1211	X_dot(1211) =K1679_1*X2321 + K5_2*X776 - K5410_1*X1211 - K5_1*X1000*X1211
1212	X_dot(1212) =K1683_1*X2323 + K1901_1*X2432 + K1973_1*X2468 + K2063_1*X2513 + K2645_1*X2804 + K3233_1*X3098 + K3642_2*X3303 + K3643_1*X3303 + K3657_2*X3318 + K3658_1*X3318 + K3660_2*X3321 + K3661_1*X3321 + K3663_2*X3324 + K3664_1*X3324 + K3666_2*X3327 + K3667_1*X3327 - K5392_1*X1212 - K3642_1*X1212*X3304 - K3657_1*X1212*X3319 - K3660_1*X1212*X3322 - K3666_1*X1212*X3328 - K3663_1*X878*X1212*X3325
1213	X_dot(1213) =K1687_1*X2325 - K5483_1*X1213
1214	X_dot(1214) =K1699_1*X2331 + K3239_1*X3101 - K5681_1*X1214
1215	X_dot(1215) =K1701_1*X2332 + K1711_1*X2337 - K5763_1*X1215
1216	X_dot(1216) =K1703_1*X2333 - K5349_1*X1216
1217	X_dot(1217) =K1723_1*X2343 + K1927_1*X2445 + K2045_1*X2504 + K2107_1*X2535 + K2155_1*X2559 + K2183_1*X2573 + K4_2*X775 + K2247_1*X2605 + K8_2*X770 + K2395_1*X2679 + K2781_1*X2872 + K2903_1*X2933 + K3051_1*X3007 + K3316_2*X3140 + K3348_2*X3156 + K3358_2*X3161 + K3392_2*X3178 - K5487_1*X1217 - K4_1*X1026*X1217 - K8_1*X1182*X1217 - K3348_1*X190*X1217 - K3316_1*X1007*X1217 - K3392_1*X401*X1217 - K3358_1*X974*X1217
1218	X_dot(1218) =K1725_1*X2344 - K5889_1*X1218
1219	X_dot(1219) =K1735_1*X2349 - K5227_1*X1219
1220	X_dot(1220) =K1739_1*X2351 - K5561_1*X1220
1221	X_dot(1221) =K1743_1*X2353 - K5235_1*X1221
1222	X_dot(1222) =K1753_1*X2358 + K1837_1*X2400 + K2763_1*X2863 - K5404_1*X1222
1223	X_dot(1223) =K1759_1*X2361 - K5788_1*X1223
1224	X_dot(1224) =K1765_1*X2364 + K1773_1*X2368 + K1897_1*X2430 + K2705_1*X2834 + K3246_2*X3105 + K3390_2*X3177 + K3396_2*X3180 + K3402_2*X3183 - K5250_1*X1224 - K3246_1*X286*X1224 - K3390_1*X401*X1224 - K3402_1*X289*X1224 - K3396_1*X402*X1224
1225	X_dot(1225) =K1767_1*X2365 + K1775_1*X2369 + K2265_1*X2614 - K5237_1*X1225
1226	X_dot(1226) =K1771_1*X2367 - K5810_1*X1226
1227	X_dot(1227) =K1777_1*X2370 - K5464_1*X1227
1228	X_dot(1228) =K1781_1*X2372 - K5781_1*X1228
1229	X_dot(1229) =K1783_1*X2373 - K5553_1*X1229
1230	X_dot(1230) =K1785_1*X2374 - K5501_1*X1230
1231	X_dot(1231) =K1793_1*X2378 - K5874_1*X1231
1232	X_dot(1232) =K1795_1*X2379 - K5863_1*X1232
1233	X_dot(1233) =K1797_1*X2380 - K5479_1*X1233
1234	X_dot(1234) =K1799_1*X2381 - K5409_1*X1234
1235	X_dot(1235) =K1801_1*X2382 + K2769_1*X2866 - K5564_1*X1235
1236	X_dot(1236) =K1803_1*X2383 - K5639_1*X1236
1237	X_dot(1237) =K1805_1*X2384 + K2753_1*X2858 + K3681_2*X3342 - K5688_1*X1237 - K3681_1*X508*X1237*X3343
1238	X_dot(1238) =K1807_1*X2385 + K2755_1*X2859 - K5782_1*X1238
1239	X_dot(1239) =K1811_1*X2387 - K5706_1*X1239
1240	X_dot(1240) =K1813_1*X2388 - K5477_1*X1240
1241	X_dot(1241) =K1815_1*X2389 - K5239_1*X1241
1242	X_dot(1242) =K1817_1*X2390 + K2169_1*X2566 - K5284_1*X1242
1243	X_dot(1243) =K1819_1*X2391 - K5518_1*X1243
1244	X_dot(1244) =K1823_1*X2393 - K5260_1*X1244
1245	X_dot(1245) =K1825_1*X2394 - K5721_1*X1245
1246	X_dot(1246) =K1829_1*X2396 + K2771_1*X2867 - K5859_1*X1246
1247	X_dot(1247) =K1831_1*X2397 - K5316_1*X1247
1248	X_dot(1248) =K1833_1*X2398 - K5629_1*X1248
1249	X_dot(1249) =K1843_1*X2403 - K5704_1*X1249
1250	X_dot(1250) =K332_2*X1648 + K333_1*X1648 + K334_2*X1649 + K335_1*X1649 + K336_2*X1650 + K337_1*X1650 + K338_2*X1651 + K339_1*X1651 + K1312_2*X2138 + K1313_1*X2138 + K1314_2*X2139 + K1315_1*X2139 + K1846_2*X2405 + K1847_1*X2405 + K1848_2*X2406 + K1849_1*X2406 - K5692_1*X1250 - K338_1*X1*X1250 - K336_1*X47*X1250 - K332_1*X458*X1250 - K334_1*X484*X1250 - K1312_1*X66*X1250 - K1314_1*X477*X1250 - K1848_1*X99*X1250 - K1846_1*X526*X1250
1251	X_dot(1251) =K1853_1*X2408 + K2033_1*X2498 - K5496_1*X1251
1252	X_dot(1252) =K1855_1*X2409 + K2675_1*X2819 - K5415_1*X1252
1253	X_dot(1253) =K1857_1*X2410 + K2511_1*X2737 + K2859_1*X2911 + K2883_1*X2923 + K2913_1*X2938 + K3322_2*X3143 - K5683_1*X1253 - K3322_1*X463*X1253
1254	X_dot(1254) =K1859_1*X2411 - K5340_1*X1254
1255	X_dot(1255) =K1861_1*X2412 - K5245_1*X1255
1256	X_dot(1256) =K1863_1*X2413 - K5358_1*X1256
1257	X_dot(1257) =K1865_1*X2414 - K5236_1*X1257
1258	X_dot(1258) =K1873_1*X2418 + K2545_1*X2754 + K3318_2*X3141 - K5543_1*X1258 - K3318_1*X1007*X1258
1259	X_dot(1259) =K1875_1*X2419 - K5551_1*X1259
1260	X_dot(1260) =K348_2*X1656 + K349_1*X1656 + K350_2*X1657 + K351_1*X1657 + K1876_2*X2420 + K1877_1*X2420 + K1878_2*X2421 + K1879_1*X2421 + K1880_2*X2422 + K1881_1*X2422 + K1981_1*X2472 + K2469_1*X2716 - K5405_1*X1260 - K348_1*X112*X1260 - K350_1*X103*X1260 - K1880_1*X99*X1260 - K1876_1*X203*X1260 - K1878_1*X554*X1260
1261	X_dot(1261) =K1879_1*X2421 - K5720_1*X1261
1262	X_dot(1262) =K1885_1*X2424 - K5801_1*X1262
1263	X_dot(1263) =K1891_1*X2427 - K5314_1*X1263
1264	X_dot(1264) =K1903_1*X2433 - K5384_1*X1264
1265	X_dot(1265) =K1907_1*X2435 + K2_2*X771 + K2329_1*X2646 + K2487_1*X2725 + K2597_1*X2780 + K2697_1*X2830 + K2743_1*X2853 + K2783_1*X2873 + K2801_1*X2882 + K3280_2*X3122 + K3546_2*X3255 + K3651_2*X3312 + K3652_1*X3312 - K5628_1*X1265 - K2_1*X857*X1265 - K3280_1*X418*X1265 - K3546_1*X391*X1265 - K3651_1*X1026*X1265*X3313
1266	X_dot(1266) =K1909_1*X2436 - K5804_1*X1266
1267	X_dot(1267) =K948_2*X1956 + K949_1*X1956 + K1910_2*X2437 + K1911_1*X2437 + K1912_2*X2438 + K1913_1*X2438 + K1914_2*X2439 + K1915_1*X2439 + K1916_2*X2440 + K1917_1*X2440 + K1918_2*X2441 + K1919_1*X2441 + K2517_1*X2740 + K3450_2*X3207 - K5334_1*X1267 - K948_1*X317*X1267 - K1918_1*X11*X1267 - K1916_1*X38*X1267 - K1910_1*X125*X1267 - K1912_1*X281*X1267 - K1914_1*X756*X1267 - K3450_1*X1007*X1267
1268	X_dot(1268) =K1915_1*X2439 - K5890_1*X1268

1269	X_dot(1269) =K1923_1*X2443 - K5693_1*X1269
1270	X_dot(1270) =K466_2*X1715 + K467_1*X1715 + K468_2*X1716 + K469_1*X1716 + K470_2*X1717 + K471_1*X1717 + K472_2*X1718 + K473_1*X1718 + K474_2*X1719 + K475_1*X1719 + K476_2*X1720 + K477_1*X1720 + K478_2*X1721 + K479_1*X1721 + K480_2*X1722 + K481_1*X1722 + K482_2*X1723 + K483_1*X1723 + K484_2*X1724 + K485_1*X1724 + K486_2*X1725 + K487_1*X1725 + K488_2*X1726 + K489_1*X1726 + K490_2*X1727 + K491_1*X1727 + K492_2*X1728 + K493_1*X1728 + K494_2*X1729 + K495_1*X1729 + K496_2*X1730 + K497_1*X1730 + K498_2*X1731 + K499_1*X1731 + K500_2*X1732 + K501_1*X1732 + K502_2*X1733 + K503_1*X1733 + K504_2*X1734 + K505_1*X1734 + K506_2*X1735 + K507_1*X1735 + K508_2*X1736 + K509_1*X1736 + K510_2*X1737 + K511_1*X1737 + K512_2*X1738 + K513_1*X1738 + K514_2*X1739 + K515_1*X1739 + K978_2*X1971 + K979_1*X1971 + K980_2*X1972 + K981_1*X1972 + K982_2*X1973 + K983_1*X1973 + K984_2*X1974 + K985_1*X1974 + K986_2*X1975 + K987_1*X1975 + K1130_2*X2047 + K1131_1*X2047 + K1356_2*X2160 + K1357_1*X2160 + K1358_2*X2161 + K1359_1*X2161 + K1360_2*X2162 + K1361_1*X2162 + K1362_2*X2163 + K1363_1*X2163 + K1929_1*X2446 + K1933_1*X2448 + K2048_2*X2506 + K2049_1*X2506 + K2050_2*X2507 + K2051_1*X2507 + K2052_2*X2508 + K2053_1*X2508 + K2054_2*X2509 + K2055_1*X2509 + K2056_2*X2510 + K2057_1*X2510 + K2058_2*X2511 + K2059_1*X2511 + K2060_2*X2512 + K2061_1*X2512 + K2062_2*X2513 + K2063_1*X2513 + K2064_2*X2514 + K2065_1*X2514 + K2066_2*X2515 + K2067_1*X2515 + K2068_2*X2516 + K2069_1*X2516 + K2070_2*X2517 + K2071_1*X2517 + K2072_2*X2518 + K2073_1*X2518 + K2074_2*X2519 + K2075_1*X2519 + K2076_2*X2520 + K2077_1*X2520 + K2078_2*X2521 + K2079_1*X2521 + K2080_2*X2522 + K2081_1*X2522 + K2082_2*X2523 + K2083_1*X2523 + K2084_2*X2524 + K2085_1*X2524 + K2086_2*X2525 + K2087_1*X2525 + K2088_2*X2526 + K2089_1*X2526 + K2090_2*X2527 + K2091_1*X2527 + K2092_2*X2528 + K2093_1*X2528 + K2094_2*X2529 + K2095_1*X2529 + K2096_2*X2530 + K2097_1*X2530 + K2098_2*X2531 + K2099_1*X2531 + K2100_2*X2532 + K2101_1*X2532 + K2102_2*X2533 + K2103_1*X2533 + K2104_2*X2534 + K2105_1*X2534 + K2106_2*X2535 + K2107_1*X2535 + K2108_2*X2536 + K2109_1*X2536 + K2882_2*X2923 + K2883_1*X2923 + K2884_2*X2924 + K2885_1*X2924 + K2886_2*X2925 + K2887_1*X2925 + K2888_2*X2926 + K2889_1*X2926 + K2890_2*X2927 + K2891_1*X2927 + K3095_1*X3029 + K3104_2*X3034 + K3105_1*X3034 + K3106_2*X3035 + K3107_1*X3035 + K3108_2*X3036 + K3109_1*X3036 + K3110_2*X3037 + K3111_1*X3037 + K3112_2*X3038 + K3113_1*X3038 + K3114_2*X3039 + K3115_1*X3039 + K3116_2*X3040 + K3117_1*X3040 + K3118_2*X3041 + K3119_1*X3041 + K3240_2*X3102 + K3241_1*X3102 + K3258_2*X3111 + K3276_2*X3120 + K3380_2*X3172 - K5435_1*X1270 - K478_1*X86*X1270 - K470_1*X168*X1270 - K466_1*X334*X1270 - K482_1*X232*X1270 - K474_1*X319*X1270 - K480_1*X296*X1270 - K484_1*X343*X1270 - K498_1*X230*X1270 - K486_1*X400*X1270 - K472_1*X543*X1270 - K496_1*X336*X1270 - K468_1*X630*X1270 - K488_1*X441*X1270 - K514_1*X185*X1270 - K490_1*X474*X1270 - K476_1*X652*X1270 - K494_1*X499*X1270 - K512_1*X353*X1270 - K506_1*X434*X1270 - K500_1*X507*X1270 - K508_1*X475*X1270 - K510_1*X485*X1270 - K502_1*X568*X1270 - K504_1*X567*X1270 - K492_1*X734*X1270 - K982_1*X181*X1270 - K980_1*X281*X1270 - K978_1*X384*X1270 - K986_1*X341*X1270 - K984_1*X636*X1270 - K1130_1*X47*X1270 - K1360_1*X223*X1270 - K1358_1*X340*X1270 - K1356_1*X447*X1270 - K1362_1*X604*X1270 - K2054_1*X170*X1270 - K2066_1*X87*X1270 - K2060_1*X188*X1270 - K2062_1*X189*X1270 - K2058_1*X243*X1270 - K2074_1*X106*X1270 - K2050_1*X431*X1270 - K2076_1*X186*X1270 - K2086_1*X109*X1270 - K2094_1*X67*X1270 - K2048_1*X608*X1270 - K2068_1*X426*X1270 - K2100_1*X124*X1270 - K2080_1*X333*X1270 - K2070_1*X435*X1270 - K2090_1*X250*X1270 - K2052_1*X632*X1270 - K2064_1*X536*X1270 - K2108_1*X99*X1270 - K2098_1*X206*X1270 - K2088_1*X325*X1270 - K2092_1*X359*X1270 - K2056_1*X728*X1270 - K2082_1*X473*X1270 - K2078_1*X549*X1270 - K2106_1*X295*X1270 - K2084_1*X562*X1270 - K2072_1*X721*X1270 - K2102_1*X450*X1270 - K2096_1*X714*X1270 - K2104_1*X706*X1270 - K2886_1*X240*X1270 - K2884_1*X461*X1270 - K2882_1*X513*X1270 - K2888_1*X559*X1270 - K2890_1*X609*X1270 - K3104_1*X88*X1270 - K3106_1*X135*X1270 - K3108_1*X231*X1270 - K3110_1*X423*X1270 - K3112_1*X451*X1270 - K3114_1*X483*X1270 - K3116_1*X623*X1270 - K3118_1*X704*X1270 - K3258_1*X401*X1270 - K3240_1*X772*X1270 - K3276_1*X418*X1270 -
1271	X_dot(1271) =K402_2*X1683 + K403_1*X1683 + K404_2*X1684 + K405_1*X1684 + K406_2*X1685 + K407_1*X1685 + K408_2*X1686 + K409_1*X1686 + K410_2*X1687 + K411_1*X1687 + K412_2*X1688 + K413_1*X1688 + K414_2*X1689 + K415_1*X1689 + K416_2*X1690 + K417_1*X1690 + K418_2*X1691 + K419_1*X1691 + K420_2*X1692 + K421_1*X1692 + K422_2*X1693 + K423_1*X1693 + K424_2*X1694 + K425_1*X1694 + K426_2*X1695 + K427_1*X1695 + K428_2*X1696 + K429_1*X1696 + K430_2*X1697 + K431_1*X1697 + K432_2*X1698 + K433_1*X1698 + K434_2*X1699 + K435_1*X1699 + K436_2*X1700 + K437_1*X1700 + K438_2*X1701 + K439_1*X1701 + K440_2*X1702 + K441_1*X1702 + K442_2*X1703 + K443_1*X1703 + K444_2*X1704 + K445_1*X1704 + K446_2*X1705 + K447_1*X1705 + K448_2*X1706 + K449_1*X1706 + K956_2*X1960 + K957_1*X1960 + K958_2*X1961 + K959_1*X1961 + K960_2*X1962 + K961_1*X1962 + K962_2*X1963 + K963_1*X1963 + K964_2*X1964 + K965_1*X1964 + K966_2*X1965 + K967_1*X1965 + K968_2*X1966 + K969_1*X1966 + K1126_2*X2045 + K1127_1*X2045 + K1336_2*X2150 + K1337_1*X2150 + K1338_2*X2151 + K1339_1*X2151 + K1340_2*X2152 + K1341_1*X2152 + K1342_2*X2153 + K1343_1*X2153 + K1344_2*X2154 + K1345_1*X2154 + K1346_2*X2155 + K1347_1*X2155 + K1348_2*X2156 + K1349_1*X2156 + K1350_2*X2157 + K1351_1*X2157 + K1931_1*X2447 + K1960_2*X2462 + K1961_1*X2462 + K1962_2*X2463 + K1963_1*X2463 + K1964_2*X2464 + K1965_1*X2464 + K1966_2*X2465 + K1967_1*X2465 + K1968_2*X2466 + K1969_1*X2466 + K1970_2*X2467 + K1971_1*X2467 + K1972_2*X2468 + K1973_1*X2468 + K1974_2*X2469 + K1975_1*X2469 + K1976_2*X2470 + K1977_1*X2470 + K1978_2*X2471 + K1979_1*X2471 + K1980_2*X2472 + K1981_1*X2472 + K1982_2*X2473 + K1983_1*X2473 + K1984_2*X2474 + K1985_1*X2474 + K1986_2*X2475 + K1987_1*X2475 + K1988_2*X2476 + K1989_1*X2476 + K1990_2*X2477 + K1991_1*X2477 + K1992_2*X2478 + K1993_1*X2478 + K1994_2*X2479 + K1995_1*X2479 + K1996_2*X2480 + K1997_1*X2480 + K1998_2*X2481 + K1999_1*X2481 + K2000_2*X2482 + K2001_1*X2482 + K2002_2*X2483 + K2003_1*X2483 + K2004_2*X2484 + K2005_1*X2484 + K2006_2*X2485 + K2007_1*X2485 + K2008_2*X2486 + K2009_1*X2486 + K2010_2*X2487 + K2011_1*X2487 + K2012_2*X2488 + K2013_1*X2488 + K2014_2*X2489 + K2015_1*X2489 + K2016_2*X2490 + K2017_1*X2490 + K2018_2*X2491 + K2019_1*X2491 + K2020_2*X2492 + K2021_1*X2492 + K2819_1*X2891 + K2856_2*X2910 + K2857_1*X2910 + K2858_2*X2911 + K2859_1*X2911 + K2860_2*X2912 + K2861_1*X2912 + K2862_2*X2913 + K2863_1*X2913 + K2864_2*X2914 + K2865_1*X2914 + K2866_2*X2915 + K2867_1*X2915 + K2868_2*X2916 + K2869_1*X2916 + K3256_2*X3110 + K3274_2*X3119 + K3378_2*X3171 + K3428_2*X3196 - K5441_1*X1271 - K402_1*X334*X1271 - K406_1*X319*X1271 - K418_1*X281*X1271 - K414_1*X349*X1271 - K426_1*X230*X1271 - K416_1*X343*X1271 - K430_1*X206*X1271 - K438_1*X181*X1271 - K424_1*X336*X1271 - K404_1*X543*X1271 - K412_1*X517*X1271 - K448_1*X185*X1271 - K408_1*X588*X1271 - K420_1*X474*X1271 - K410_1*X652*X1271 - K442_1*X353*X1271 - K428_1*X507*X1271 - K436_1*X434*X1271 - K446_1*X341*X1271 - K440_1*X485*X1271 - K432_1*X568*X1271 - K434_1*X567*X1271 - K422_1*X734*X1271 - K444_1*X623*X1271 - K962_1*X86*X1271 - K958_1*X168*X1271 - K960_1*X384*X1271 - K964_1*X451*X1271 - K968_1*X475*X1271 - K966_1*X499*X1271 - K956_1*X630*X1271 - K1126_1*X47*X1271 - K1346_1*X223*X1271 - K1340_1*X340*X1271 - K1342_1*X325*X1271 - K1336_1*X447*X1271 - K1338_1*X696*X1271 - K1348_1*X609*X1271 - K1344_1*X654*X1271 - K1350_1*X604*X1271 - K1964_1*X170*X1271 - K1968_1*X188*X1271 - K1966_1*X243*X1271 - K1972_1*X189*X1271 - K1970_1*X231*X1271 - K1980_1*X203*X1271 - K1990_1*X186*X1271 - K2000_1*X88*X1271 - K1978_1*X374*X1271 - K2002_1*X135*X1271 - K1974_1*X423*X1271 - K1960_1*X608*X1271 - K2012_1*X124*X1271 - K1962_1*X632*X1271 - K1994_1*X333*X1271 - K1986_1*X426*X1271 - K2004_1*X250*X1271 - K1976_1*X536*X1271 - K1982_1*X483*X1271 - K2010_1*X204*X1271 - K2006_1*X253*X1271 - K1988_1*X435*X1271 - K2020_1*X153*X1271 - K1992_1*X470*X1271 - K1996_1*X473*X1271 - K2008_1*X359*X1271 - K1998_1*X562*X1271 - K1984_1*X740*X1271 - K2014_1*X450*X1271 - K2018_1*X524*X1271 - K2016_1*X706*X1271 - K2856_1*X87*X1271 - K2860_1*X106*X1271 - K2864_1*X246*X1271 - K2862_1*X461*X1271 - K2858_1*X513*X1271 - K2868_1*X559*X1271 - K2866_1*X710*X1271 - K3256_1*X401*X1271 - K3274_1*X418*X1271 - K3378_1*X356*X1271 - K3428_1*X404*X1271
1272	X_dot(1272) =K450_2*X1707 + K451_1*X1707 + K452_2*X1708 + K453_1*X1708 + K1939_1*X2451 + K2022_2*X2493 + K2023_1*X2493 - K5251_1*X1272 - K450_1*X9*X1272 - K452_1*X181*X1272 - K2022_1*X99*X1272
1273	X_dot(1273) =K516_2*X1740 + K517_1*X1740 + K518_2*X1741 + K519_1*X1741 + K520_2*X1742 + K521_1*X1742 + K522_2*X1743 + K523_1*X1743 + K524_2*X1744 + K525_1*X1744 + K526_2*X1745 + K527_1*X1745 + K528_2*X1746 + K529_1*X1746 + K530_2*X1747 + K531_1*X1747 + K532_2*X1748 + K533_1*X1748 + K534_2*X1749 + K535_1*X1749 + K536_2*X1750 + K537_1*X1750 + K988_2*X1976 + K989_1*X1976 + K1943_1*X2453 + K2110_2*X2537 + K2111_1*X2537 + K2112_2*X2538 + K2113_1*X2538 + K2114_2*X2539 + K2115_1*X2539 + K2116_2*X2540 + K2117_1*X2540 + K2118_2*X2541 + K2119_1*X2541 + K2120_2*X2542 + K2121_1*X2542 + K2122_2*X2543 + K2123_1*X2543 + K2124_2*X2544 + K2125_1*X2544 + K2126_2*X2545 + K2127_1*X2545 + K2128_2*X2546 + K2129_1*X2546 + K2130_2*X2547 + K2131_1*X2547 + K2132_2*X2548 + K2133_1*X2548 + K2134_2*X2549 + K2135_1*X2549 + K2136_2*X2550 + K2137_1*X2550 + K2138_2*X2551 + K2139_1*X2551 + K2383_1*X2673 + K2827_1*X2895 + K2892_2*X2928 + K2893_1*X2928 + K2894_2*X2929 + K2895_1*X2929 + K2896_2*X2930 + K2897_1*X2930 + K2898_2*X2931 + K2899_1*X2931 + K2900_2*X2932 + K2901_1*X2932 + K2902_2*X2933 + K2903_1*X2933 + K3017_1*X2990 + K3098_2*X3031 + K3099_1*X3031 + K3122_2*X3043 + K3123_1*X3043 + K3124_2*X3044 + K3125_1*X3044 + K3174_2*X3069 + K3175_1*X3069 - K5502_1*X1273 - K526_1*X206*X1273 - K532_1*X181*X1273 - K524_1*X281*X1273 - K528_1*X267*X1273 - K530_1*X303*X1273 - K534_1*X266*X1273 - K516_1*X488*X1273 - K520_1*X472*X1273 - K522_1*X508*X1273 - K536_1*X414*X1273 - K518_1*X630*X1273 - K988_1*X441*X1273 - K2112_1*X101*X1273 - K2114_1*X170*X1273 - K2116_1*X175*X1273 - K2126_1*X186*X1273 - K2134_1*X124*X1273 - K2138_1*X99*X1273 - K2110_1*X466*X1273 - K2128_1*X313*X1273 - K2120_1*X400*X1273 - K2118_1*X435*X1273 - K2122_1*X539*X1273 - K2130_1*X473*X1273 - K2136_1*X495*X1273 - K2124_1*X645*X1273 - K2132_1*X641*X1273 - K2894_1*X106*X1273 - K2896_1*X88*X1273 - K2892_1*X264*X1273 - K2900_1*X302*X1273 - K2898_1*X332*X1273 - K2902_1*X295*X1273 - K3098_1*X47*X1273 - K3122_1*X275*X1273 - K3124_1*X434*X1273 - K3174_1*X772*X1273
1274	X_dot(1274) =K1948_2*X2456 + K1949_1*X2456 + K2249_1*X2606 + K2834_2*X2899 + K2835_1*X2899 + K2836_2*X2900 + K2837_1*X2900 - K5856_1*X1274 - K1948_1*X712*X1274 - K2836_1*X18*X1274 - K2834_1*X314*X1274
1275	X_dot(1275) =K1950_2*X2457 + K1951_1*X2457 + K2838_2*X2901 + K2839_1*X2901 + K3270_2*X3117 - K5814_1*X1275 - K1950_1*X664*X1275 - K2838_1*X320*X1275 - K3270_1*X418*X1275

1276	X_dot(1276) =K598_2*X1781 + K599_1*X1781 + K600_2*X1782 + K601_1*X1782 + K602_2*X1783 + K603_1*X1783 + K604_2*X1784 + K605_1*X1784 + K606_2*X1785 + K607_1*X1785 + K608_2*X1786 + K609_1*X1786 + K610_2*X1787 + K611_1*X1787 + K1388_2*X2176 + K1389_1*X2176 + K1390_2*X2177 + K1391_1*X2177 + K1392_2*X2178 + K1393_1*X2178 + K1959_1*X2461 + K2171_1*X2567 + K2213_1*X2588 + K2254_2*X2609 + K2255_1*X2609 + K2256_2*X2610 + K2257_1*X2610 + K2258_2*X2611 + K2259_1*X2611 + K2260_2*X2612 + K2261_1*X2612 + K2262_2*X2613 + K2263_1*X2613 + K2264_2*X2614 + K2265_1*X2614 + K2266_2*X2615 + K2267_1*X2615 + K2268_2*X2616 + K2269_1*X2616 + K2270_2*X2617 + K2271_1*X2617 + K2272_2*X2618 + K2273_1*X2618 + K2274_2*X2619 + K2275_1*X2619 + K2276_2*X2620 + K2277_1*X2620 + K2278_2*X2621 + K2279_1*X2621 + K2280_2*X2622 + K2281_1*X2622 + K2282_2*X2623 + K2283_1*X2623 + K2284_2*X2624 + K2285_1*X2624 + K2935_1*X2949 + K2944_2*X2954 + K2945_1*X2954 + K2946_2*X2955 + K2947_1*X2955 + K2948_2*X2956 + K2949_1*X2956 - K5557_1*X1276 - K608_1*X160*X1276 - K604_1*X281*X1276 - K610_1*X257*X1276 - K606_1*X326*X1276 - K602_1*X441*X1276 - K600_1*X500*X1276 - K598_1*X717*X1276 - K1388_1*X85*X1276 - K1390_1*X275*X1276 - K1392_1*X260*X1276 - K2256_1*X54*X1276 - K2264_1*X17*X1276 - K2266_1*X75*X1276 - K2268_1*X116*X1276 - K2260_1*X259*X1276 - K2278_1*X147*X1276 - K2282_1*X129*X1276 - K2284_1*X235*X1276 - K2262_1*X535*X1276 - K2276_1*X407*X1276 - K2258_1*X588*X1276 - K2254_1*X682*X1276 - K2272_1*X516*X1276 - K2270_1*X639*X1276 - K2274_1*X680*X1276 - K2280_1*X685*X1276 - K2946_1*X88*X1276 - K2944_1*X164*X1276 - K2948_1*X746*X1276
1277	X_dot(1277) =K1961_1*X2462 + K2049_1*X2506 - K5764_1*X1277
1278	X_dot(1278) =K1965_1*X2464 + K2025_1*X2494 + K2055_1*X2509 + K2115_1*X2539 + K2141_1*X2552 + K2613_1*X2788 - K5376_1*X1278
1279	X_dot(1279) =K1985_1*X2474 - K5877_1*X1279
1280	X_dot(1280) =K1987_1*X2475 + K2069_1*X2516 + K2423_1*X2693 + K3037_1*X3000 - K5603_1*X1280
1281	X_dot(1281) =K1991_1*X2477 + K2077_1*X2520 + K2127_1*X2545 - K5390_1*X1281
1282	X_dot(1282) =K552_2*X1758 + K553_1*X1758 + K554_2*X1759 + K555_1*X1759 + K556_2*X1760 + K557_1*X1760 + K990_2*X1977 + K991_1*X1977 + K1364_2*X2164 + K1365_1*X2164 + K1366_2*X2165 + K1367_1*X2165 + K1368_2*X2166 + K1369_1*X2166 + K1370_2*X2167 + K1371_1*X2167 + K1995_1*X2479 + K2081_1*X2522 + K2158_2*X2561 + K2159_1*X2561 + K2160_2*X2562 + K2161_1*X2562 + K2162_2*X2563 + K2163_1*X2563 + K2164_2*X2564 + K2165_1*X2564 + K2166_2*X2565 + K2167_1*X2565 + K2168_2*X2566 + K2169_1*X2566 + K2170_2*X2567 + K2171_1*X2567 + K2873_1*X2918 + K2904_2*X2934 + K2905_1*X2934 - K5521_1*X1282 - K554_1*X78*X1282 - K552_1*X100*X1282 - K556_1*X468*X1282 - K990_1*X414*X1282 - K1366_1*X9*X1282 - K1364_1*X37*X1282 - K1370_1*X341*X1282 - K1368_1*X703*X1282 - K2162_1*X82*X1282 - K2168_1*X71*X1282 - K2164_1*X177*X1282 - K2170_1*X373*X1282 - K2166_1*X564*X1282 - K2158_1*X711*X1282 - K2160_1*X733*X1282 - K2904_1*X136*X1282
1283	X_dot(1283) =K1999_1*X2481 + K2085_1*X2524 - K5726_1*X1283
1284	X_dot(1284) =K2003_1*X2483 + K3107_1*X3035 - K5344_1*X1284
1285	X_dot(1285) =K2007_1*X2485 - K5448_1*X1285
1286	X_dot(1286) =K2011_1*X2487 + K2043_1*X2503 + K2463_1*X2713 + K3135_1*X3049 - K5406_1*X1286
1287	X_dot(1287) =K2015_1*X2489 + K2103_1*X2533 + K3570_2*X3267 - K5625_1*X1287 - K3570_1*X401*X1287
1288	X_dot(1288) =K2017_1*X2490 + K2105_1*X2534 - K5850_1*X1288
1289	X_dot(1289) =K2021_1*X2492 - K5360_1*X1289
1290	X_dot(1290) =K1574_2*X2269 + K1575_1*X2269 + K2027_1*X2495 + K2057_1*X2510 + K2143_1*X2553 - K5867_1*X1290 - K1574_1*X175*X1290
1291	X_dot(1291) =K2029_1*X2496 - K5717_1*X1291
1292	X_dot(1292) =K2031_1*X2497 + K2167_1*X2565 + K2375_1*X2669 - K5728_1*X1292
1293	X_dot(1293) =K1484_2*X2224 + K1485_1*X2224 + K1486_2*X2225 + K1487_1*X2225 + K2041_1*X2502 + K2095_1*X2529 + K2480_2*X2722 + K2481_1*X2722 + K2482_2*X2723 + K2483_1*X2723 + K2484_2*X2724 + K2485_1*X2724 + K2486_2*X2725 + K2487_1*X2725 + K2488_2*X2726 + K2489_1*X2726 + K3166_2*X3065 + K3167_1*X3065 - K5281_1*X1293 - K1486_1*X102*X1293 - K1484_1*X582*X1293 - K2484_1*X87*X1293 - K2488_1*X67*X1293 - K2482_1*X177*X1293 - K2480_1*X199*X1293 - K2486_1*X453*X1293 - K3166_1*X771*X1293
1294	X_dot(1294) =K2051_1*X2507 + K2871_1*X2917 - K5608_1*X1294
1295	X_dot(1295) =K2079_1*X2521 - K5715_1*X1295
1296	X_dot(1296) =K2087_1*X2525 - K5318_1*X1296
1297	X_dot(1297) =K2123_1*X2543 + K2415_1*X2689 + K3073_1*X3018 - K5705_1*X1297
1298	X_dot(1298) =K2159_1*X2561 - K5855_1*X1298
1299	X_dot(1299) =K2161_1*X2562 - K5872_1*X1299
1300	X_dot(1300) =K2163_1*X2563 - K5293_1*X1300
1301	X_dot(1301) =K2173_1*X2568 - K5774_1*X1301
1302	X_dot(1302) =K2175_1*X2569 - K5513_1*X1302
1303	X_dot(1303) =K2181_1*X2572 - K5831_1*X1303
1304	X_dot(1304) =K2185_1*X2574 - K5371_1*X1304
1305	X_dot(1305) =K2188_2*X2576 + K2189_1*X2576 + K2920_2*X2942 + K2921_1*X2942 - K5772_1*X1305 - K2188_1*X616*X1305 - K2920_1*X617*X1305
1306	X_dot(1306) =K2191_1*X2577 - K5565_1*X1306
1307	X_dot(1307) =K1556_2*X2260 + K1557_1*X2260 + K1558_2*X2261 + K1559_1*X2261 + K2193_1*X2578 + K2497_1*X2730 - K5347_1*X1307 - K1558_1*X287*X1307 - K1556_1*X451*X1307
1308	X_dot(1308) =K2195_1*X2579 - K5248_1*X1308
1309	X_dot(1309) =K2201_1*X2582 - K5609_1*X1309
1310	X_dot(1310) =K388_2*X1676 + K389_1*X1676 + K390_2*X1677 + K391_1*X1677 + K1920_2*X2442 + K1921_1*X2442 + K1922_2*X2443 + K1923_1*X2443 + K1924_2*X2444 + K1925_1*X2444 + K2207_1*X2585 + K2804_2*X2884 + K2805_1*X2884 + K2806_2*X2885 + K2807_1*X2885 - K5682_1*X1310 - K390_1*X28*X1310 - K388_1*X225*X1310 - K1924_1*X2*X1310 - K1920_1*X264*X1310 - K1922_1*X527*X1310 - K2806_1*X25*X1310 - K2804_1*X511*X1310
1311	X_dot(1311) =K2211_1*X2587 + K2253_1*X2608 - K5481_1*X1311
1312	X_dot(1312) =K2215_1*X2589 - K5812_1*X1312
1313	X_dot(1313) =K586_2*X1775 + K587_1*X1775 + K588_2*X1776 + K589_1*X1776 + K1004_2*X1984 + K1005_1*X1984 + K1006_2*X1985 + K1007_1*X1985 + K1382_2*X2173 + K1383_1*X2173 + K2216_2*X2590 + K2217_1*X2590 + K2218_2*X2591 + K2219_1*X2591 + K2529_1*X2746 + K3120_2*X3042 + K3121_1*X3042 - K5673_1*X1313 - K588_1*X277*X1313 - K586_1*X383*X1313 - K1006_1*X129*X1313 - K1004_1*X565*X1313 - K1382_1*X406*X1313 - K2216_1*X106*X1313 - K2218_1*X501*X1313 - K3120_1*X451*X1313
1314	X_dot(1314) =K2223_1*X2593 - K5398_1*X1314
1315	X_dot(1315) =K1384_2*X2174 + K1385_1*X2174 + K1386_2*X2175 + K1387_1*X2175 + K2220_2*X2592 + K2221_1*X2592 + K2222_2*X2593 + K2223_1*X2593 + K2224_2*X2594 + K2225_1*X2594 - K5295_1*X1315 - K1384_1*X332*X1315 - K1386_1*X407*X1315 - K2224_1*X84*X1315 - K2222_1*X195*X1315 - K2220_1*X275*X1315
1316	X_dot(1316) =K590_2*X1777 + K591_1*X1777 + K592_2*X1778 + K593_1*X1778 + K594_2*X1779 + K595_1*X1779 + K596_2*X1780 + K597_1*X1780 + K1012_2*X1988 + K1013_1*X1988 + K2235_1*X2599 + K2248_2*X2606 + K2249_1*X2606 + K2250_2*X2607 + K2251_1*X2607 + K2252_2*X2608 + K2253_1*X2608 + K2942_2*X2953 + K2943_1*X2953 + K3100_2*X3032 + K3101_1*X3032 - K5737_1*X1316 - K592_1*X86*X1316 - K596_1*X198*X1316 - K594_1*X484*X1316 - K590_1*X632*X1316 - K1012_1*X252*X1316 - K2252_1*X288*X1316 - K2250_1*X575*X1316 - K2248_1*X712*X1316 - K2942_1*X573*X1316 - K3100_1*X47*X1316
1317	X_dot(1317) =K2237_1*X2600 - K5738_1*X1317

1318	X_dot(1318) =K672_2*X1818 + K673_1*X1818 + K674_2*X1819 + K675_1*X1819 + K676_2*X1820 + K677_1*X1820 + K678_2*X1821 + K679_1*X1821 + K680_2*X1822 + K681_1*X1822 + K682_2*X1823 + K683_1*X1823 + K684_2*X1824 + K685_1*X1824 + K686_2*X1825 + K687_1*X1825 + K688_2*X1826 + K689_1*X1826 + K690_2*X1827 + K691_1*X1827 + K692_2*X1828 + K693_1*X1828 + K694_2*X1829 + K695_1*X1829 + K696_2*X1830 + K697_1*X1830 + K698_2*X1831 + K699_1*X1831 + K700_2*X1832 + K701_1*X1832 + K702_2*X1833 + K703_1*X1833 + K704_2*X1834 + K705_1*X1834 + K706_2*X1835 + K707_1*X1835 + K708_2*X1836 + K709_1*X1836 + K710_2*X1837 + K711_1*X1837 + K1058_2*X2011 + K1059_1*X2011 + K1060_2*X2012 + K1061_1*X2012 + K1062_2*X2013 + K1063_1*X2013 + K1064_2*X2014 + K1065_1*X2014 + K1066_2*X2015 + K1067_1*X2015 + K1068_2*X2016 + K1069_1*X2016 + K1112_2*X2038 + K1113_1*X2038 + K1134_2*X2049 + K1135_1*X2049 + K1136_2*X2050 + K1137_1*X2050 + K1408_2*X2186 + K1409_1*X2186 + K1410_2*X2187 + K1411_1*X2187 + K1412_2*X2188 + K1413_1*X2188 + K1414_2*X2189 + K1415_1*X2189 + K1416_2*X2190 + K1417_1*X2190 + K1418_2*X2191 + K1419_1*X2191 + K1420_2*X2192 + K1421_1*X2192 + K1422_2*X2193 + K1423_1*X2193 + K1424_2*X2194 + K1425_1*X2194 + K1426_2*X2195 + K1427_1*X2195 + K1428_2*X2196 + K1429_1*X2196 + K1430_2*X2197 + K1431_1*X2197 + K1432_2*X2198 + K1433_1*X2198 + K1434_2*X2199 + K1435_1*X2199 + K1436_2*X2200 + K1437_1*X2200 + K1438_2*X2201 + K1439_1*X2201 + K1440_2*X2202 + K1441_1*X2202 + K1442_2*X2203 + K1443_1*X2203 + K1444_2*X2204 + K1445_1*X2204 + K2239_1*X2601 + K2340_2*X2652 + K2341_1*X2652 + K2342_2*X2653 + K2343_1*X2653 + K2344_2*X2654 + K2345_1*X2654 + K2346_2*X2655 + K2347_1*X2655 + K2348_2*X2656 + K2349_1*X2656 + K2350_2*X2657 + K2351_1*X2657 + K2352_2*X2658 + K2353_1*X2658 + K2354_2*X2659 + K2355_1*X2659 + K2356_2*X2660 + K2357_1*X2660 + K2358_2*X2661 + K2359_1*X2661 + K2360_2*X2662 + K2361_1*X2662 + K2362_2*X2663 + K2363_1*X2663 + K2364_2*X2664 + K2365_1*X2664 + K2988_2*X2976 + K2989_1*X2976 + K2990_2*X2977 + K2991_1*X2977 + K2992_2*X2978 + K2993_1*X2978 + K2994_2*X2979 + K2995_1*X2979 + K2996_2*X2980 + K2997_1*X2980 + K2998_2*X2981 + K2999_1*X2981 + K3057_1*X3010 - K5388_1*X1318 - K672_1*X214*X1318 - K676_1*X225*X1318 - K692_1*X102*X1318 - K684_1*X191*X1318 - K678_1*X259*X1318 - K694_1*X104*X1318 - K696_1*X134*X1318 - K674_1*X363*X1318 - K700_1*X128*X1318 - K698_1*X176*X1318 - K688_1*X342*X1318 - K690_1*X343*X1318 - K702_1*X229*X1318 - K686_1*X447*X1318 - K710_1*X248*X1318 - K682_1*X628*X1318 - K680_1*X669*X1318 - K706_1*X448*X1318 - K708_1*X459*X1318 - K704_1*X552*X1318 - K1062_1*X58*X1318 - K1060_1*X192*X1318 - K1066_1*X252*X1318 - K1068_1*X249*X1318 - K1064_1*X464*X1318 - K1058_1*X658*X1318 - K1112_1*X221*X1318 - K1136_1*X129*X1318 - K1134_1*X725*X1318 - K1408_1*X69*X1318 - K1410_1*X118*X1318 - K1416_1*X113*X1318 - K1424_1*X108*X1318 - K1426_1*X94*X1318 - K1436_1*X3*X1318 - K1432_1*X145*X1318 - K1440_1*X157*X1318 - K1418_1*X388*X1318 - K1434_1*X241*X1318 - K1412_1*X466*X1318 - K1414_1*X458*X1318 - K1422_1*X393*X1318 - K1444_1*X196*X1318 - K1430_1*X463*X1318 - K1442_1*X399*X1318 - K1438_1*X469*X1318 - K1420_1*X764*X1318 - K1428_1*X742*X1318 - K2342_1*X96*X1318 - K2344_1*X86*X1318 - K2346_1*X169*X1318 - K2340_1*X284*X1318 - K2350_1*X281*X1318 - K2360_1*X184*X1318 - K2348_1*X324*X1318 - K2356_1*X325*X1318 - K2352_1*X476*X1318 - K2358_1*X509*X1318 - K2362_1*X531*X1318 - K2354_1*X613*X1318 - K2364_1*X528*X1318 - K2994_1*X57*X1318 - K2998_1*X99*X1318 - K2988_1*X232*X1318 - K2996_1*X263*X1318 - K2992_1*X578*X1318 - K2990_1*X625*X1318
1319	X_dot(1319) =K1094_2*X2029 + K1095_1*X2029 + K2243_1*X2603 - K5846_1*X1319 - K1094_1*X47*X1319
1320	X_dot(1320) =K2263_1*X2613 - K5701_1*X1320
1321	X_dot(1321) =K2271_1*X2617 - K5794_1*X1321
1322	X_dot(1322) =K2273_1*X2618 + K2323_1*X2643 - K5685_1*X1322
1323	X_dot(1323) =K2275_1*X2619 + K2637_1*X2800 + K3278_2*X3121 - K5828_1*X1323 - K3278_1*X418*X1323
1324	X_dot(1324) =K2281_1*X2622 - K5833_1*X1324
1325	X_dot(1325) =K2289_1*X2626 - K5827_1*X1325
1326	X_dot(1326) =K2291_1*X2627 - K5545_1*X1326
1327	X_dot(1327) =K634_2*X1799 + K635_1*X1799 + K2293_1*X2628 - K5888_1*X1327 - K634_1*X609*X1327
1328	X_dot(1328) =K2297_1*X2630 - K5747_1*X1328
1329	X_dot(1329) =K2299_1*X2631 - K5674_1*X1329
1330	X_dot(1330) =K274_2*X1619 + K275_1*X1619 + K2303_1*X2633 - K5766_1*X1330 - K274_1*X275*X1330
1331	X_dot(1331) =K2305_1*X2634 - K5847_1*X1331
1332	X_dot(1332) =K2321_1*X2642 - K5711_1*X1332
1333	X_dot(1333) =K2325_1*X2644 - K5707_1*X1333
1334	X_dot(1334) =K2327_1*X2645 - K5578_1*X1334
1335	X_dot(1335) =K2331_1*X2647 + K2443_1*X2703 - K5519_1*X1335
1336	X_dot(1336) =K2337_1*X2650 - K5614_1*X1336
1337	X_dot(1337) =K2349_1*X2656 - K5514_1*X1337
1338	X_dot(1338) =K2353_1*X2658 + K2523_1*X2743 + K2977_1*X2970 - K5650_1*X1338
1339	X_dot(1339) =K2355_1*X2659 - K5769_1*X1339
1340	X_dot(1340) =K2359_1*X2661 - K5680_1*X1340
1341	X_dot(1341) =K740_2*X1852 + K741_1*X1852 + K742_2*X1853 + K743_1*X1853 + K744_2*X1854 + K745_1*X1854 + K1076_2*X2020 + K1077_1*X2020 + K1078_2*X2021 + K1079_1*X2021 + K1464_2*X2214 + K1465_1*X2214 + K1466_2*X2215 + K1467_1*X2215 + K2363_1*X2663 + K2420_2*X2692 + K2421_1*X2692 + K2422_2*X2693 + K2423_1*X2693 + K2424_2*X2694 + K2425_1*X2694 - K5697_1*X1341 - K740_1*X100*X1341 - K744_1*X350*X1341 - K742_1*X544*X1341 - K1076_1*X448*X1341 - K1078_1*X561*X1341 - K1464_1*X177*X1341 - K1466_1*X370*X1341 - K2422_1*X426*X1341 - K2424_1*X531*X1341 - K2420_1*X762*X1341
1342	X_dot(1342) =K2365_1*X2664 - K5694_1*X1342
1343	X_dot(1343) =K2367_1*X2665 - K5294_1*X1343
1344	X_dot(1344) =K2369_1*X2666 - K5541_1*X1344
1345	X_dot(1345) =K2371_1*X2667 - K5326_1*X1345
1346	X_dot(1346) =K2373_1*X2668 + K3540_2*X3252 + K3544_2*X3254 - K5232_1*X1346 - K3544_1*X178*X1346 - K3540_1*X478*X1346
1347	X_dot(1347) =K2377_1*X2670 - K5355_1*X1347
1348	X_dot(1348) =K2381_1*X2672 + K2802_2*X2883 + K2803_1*X2883 + K3094_2*X3029 + K3095_1*X3029 - K5486_1*X1348 - K2802_1*X294*X1348 - K3094_1*X240*X1348
1349	X_dot(1349) =K2387_1*X2675 - K5450_1*X1349
1350	X_dot(1350) =K2389_1*X2676 + K3041_1*X3002 - K5249_1*X1350
1351	X_dot(1351) =K2403_1*X2683 - K5860_1*X1351
1352	X_dot(1352) =K2402_2*X2683 + K2403_1*X2683 + K2404_2*X2684 + K2405_1*X2684 - K5823_1*X1352 - K2404_1*X675*X1352 - K2402_1*X719*X1352
1353	X_dot(1353) =K2413_1*X2688 - K5619_1*X1353
1354	X_dot(1354) =K2417_1*X2690 - K5809_1*X1354
1355	X_dot(1355) =K2421_1*X2692 + K2433_1*X2698 - K5895_1*X1355
1356	X_dot(1356) =K2435_1*X2699 + K2807_1*X2885 - K5243_1*X1356
1357	X_dot(1357) =K2441_1*X2702 - K5580_1*X1357
1358	X_dot(1358) =K2473_1*X2718 + K2577_1*X2770 - K5465_1*X1358
1359	X_dot(1359) =K2493_1*X2728 - K5784_1*X1359
1360	X_dot(1360) =K2501_1*X2732 - K5575_1*X1360
1361	X_dot(1361) =K2503_1*X2733 - K5616_1*X1361
1362	X_dot(1362) =K2507_1*X2735 - K5323_1*X1362
1363	X_dot(1363) =K2519_1*X2741 - K5252_1*X1363
1364	X_dot(1364) =K2521_1*X2742 + K3554_2*X3259 - K5302_1*X1364 - K3554_1*X463*X1364

1365	X_dot(1365) =K2525_1*X2744 + K3326_2*X3145 - K5289_1*X1365 - K3326_1*X463*X1365
1366	X_dot(1366) =K730_2*X1847 + K731_1*X1847 + K732_2*X1848 + K733_1*X1848 + K2398_2*X2681 + K2399_1*X2681 + K2400_2*X2682 + K2401_1*X2682 + K2533_1*X2748 - K5492_1*X1366 - K732_1*X339*X1366 - K730_1*X630*X1366 - K2400_1*X169*X1366 - K2398_1*X695*X1366
1367	X_dot(1367) =K2541_1*X2752 - K5593_1*X1367
1368	X_dot(1368) =K2543_1*X2753 - K5695_1*X1368
1369	X_dot(1369) =K2549_1*X2756 - K5752_1*X1369
1370	X_dot(1370) =K804_2*X1884 + K805_1*X1884 + K2550_2*X2757 + K2551_1*X2757 + K2552_2*X2758 + K2553_1*X2758 - K5808_1*X1370 - K804_1*X760*X1370 - K2552_1*X477*X1370 - K2550_1*X655*X1370
1371	X_dot(1371) =K2555_1*X2759 - K5871_1*X1371
1372	X_dot(1372) =K2559_1*X2761 + K2568_2*X2766 + K2569_1*X2766 - K5722_1*X1372 - K2568_1*X556*X1372
1373	X_dot(1373) =K2561_1*X2762 + K2570_2*X2767 + K2571_1*X2767 - K5883_1*X1373 - K2570_1*X747*X1373
1374	X_dot(1374) =K2565_1*X2764 + K2579_1*X2771 - K5842_1*X1374
1375	X_dot(1375) =K2609_1*X2786 - K5484_1*X1375
1376	X_dot(1376) =K2621_1*X2792 - K5660_1*X1376
1377	X_dot(1377) =K998_2*X1981 + K999_1*X1981 + K2190_2*X2577 + K2191_1*X2577 + K2623_1*X2793 - K5291_1*X1377 - K998_1*X499*X1377 - K2190_1*X381*X1377
1378	X_dot(1378) =K2625_1*X2794 - K5864_1*X1378
1379	X_dot(1379) =K2629_1*X2796 + K2635_1*X2799 + K3654_2*X3315 + K3655_1*X3315 - K5471_1*X1379 - K3654_1*X896*X1379*X3316
1380	X_dot(1380) =K2643_1*X2803 - K5385_1*X1380
1381	X_dot(1381) =K102_2*X1533 + K103_1*X1533 + K104_2*X1534 + K105_1*X1534 + K106_2*X1535 + K107_1*X1535 + K1176_2*X2070 + K1177_1*X2070 + K1588_2*X2276 + K1589_1*X2276 + K1590_2*X2277 + K1591_1*X2277 + K2648_2*X2806 + K2649_1*X2806 - K5689_1*X1381 - K106_1*X181*X1381 - K102_1*X275*X1381 - K104_1*X342*X1381 - K1176_1*X448*X1381 - K1590_1*X302*X1381 - K1588_1*X497*X1381 - K2648_1*X521*X1381
1382	X_dot(1382) =K2655_1*X2809 + K2663_1*X2813 + K2737_1*X2850 - K5403_1*X1382
1383	X_dot(1383) =K2657_1*X2810 + K2906_2*X2935 + K2907_1*X2935 + K2908_2*X2936 + K2909_1*X2936 + K2910_2*X2937 + K2911_1*X2937 + K3217_1*X3090 - K5798_1*X1383 - K2908_1*X50*X1383 - K2906_1*X387*X1383 - K2910_1*X644*X1383
1384	X_dot(1384) =K2661_1*X2812 - K5562_1*X1384
1385	X_dot(1385) =K2665_1*X2814 - K5421_1*X1385
1386	X_dot(1386) =K2671_1*X2817 - K5456_1*X1386
1387	X_dot(1387) =K2679_1*X2821 + K3440_2*X3202 - K5825_1*X1387 - K3440_1*X391*X1387
1388	X_dot(1388) =K2685_1*X2824 - K5724_1*X1388
1389	X_dot(1389) =K2695_1*X2829 - K5618_1*X1389
1390	X_dot(1390) =K2699_1*X2831 - K5759_1*X1390
1391	X_dot(1391) =K254_2*X1609 + K255_1*X1609 + K886_2*X1925 + K887_1*X1925 + K1234_2*X2099 + K1235_1*X2099 + K2702_2*X2833 + K2703_1*X2833 + K2965_1*X2964 - K5490_1*X1391 - K254_1*X157*X1391 - K886_1*X645*X1391 - K1234_1*X110*X1391 - K2702_1*X298*X1391
1392	X_dot(1392) =K2707_1*X2835 + K2711_1*X2837 + K2715_1*X2839 + K2745_1*X2854 + K2765_1*X2864 - K5570_1*X1392
1393	X_dot(1393) =K216_2*X1590 + K217_1*X1590 + K218_2*X1591 + K219_1*X1591 + K220_2*X1592 + K221_1*X1592 + K222_2*X1593 + K223_1*X1593 + K224_2*X1594 + K225_1*X1594 + K226_2*X1595 + K227_1*X1595 + K884_2*X1924 + K885_1*X1924 + K1222_2*X2093 + K1223_1*X2093 + K1224_2*X2094 + K1225_1*X2094 + K1712_2*X2338 + K1713_1*X2338 + K1714_2*X2339 + K1715_1*X2339 + K1716_2*X2340 + K1717_1*X2340 + K1718_2*X2341 + K1719_1*X2341 + K1720_2*X2342 + K1721_1*X2342 + K1722_2*X2343 + K1723_1*X2343 + K1724_2*X2344 + K1725_1*X2344 + K1726_2*X2345 + K1727_1*X2345 + K2670_2*X2817 + K2671_1*X2817 + K2672_2*X2818 + K2673_1*X2818 + K2674_2*X2819 + K2675_1*X2819 + K2676_2*X2820 + K2677_1*X2820 + K2678_2*X2821 + K2679_1*X2821 + K2709_1*X2836 - K5615_1*X1393 - K216_1*X120*X1393 - K222_1*X111*X1393 - K224_1*X181*X1393 - K220_1*X401*X1393 - K218_1*X612*X1393 - K226_1*X569*X1393 - K884_1*X499*X1393 - K1222_1*X101*X1393 - K1224_1*X297*X1393 - K1714_1*X45*X1393 - K1720_1*X157*X1393 - K1712_1*X238*X1393 - K1726_1*X99*X1393 - K1716_1*X308*X1393 - K1722_1*X295*X1393 - K1718_1*X460*X1393 - K1724_1*X755*X1393 - K2676_1*X169*X1393 - K2674_1*X217*X1393 - K2670_1*X261*X1393 - K2672_1*X458*X1393 - K2678_1*X677*X1393
1394	X_dot(1394) =K2713_1*X2838 - K5437_1*X1394
1395	X_dot(1395) =K2717_1*X2840 + K2747_1*X2855 + K3442_2*X3203 - K5740_1*X1395 - K3442_1*X372*X1395
1396	X_dot(1396) =K2719_1*X2841 + K2787_1*X2875 - K5363_1*X1396
1397	X_dot(1397) =K2725_1*X2844 - K5875_1*X1397
1398	X_dot(1398) =K2727_1*X2845 + K2751_1*X2857 - K5549_1*X1398
1399	X_dot(1399) =K2729_1*X2846 + K3438_2*X3201 - K5253_1*X1399 - K3438_1*X391*X1399
1400	X_dot(1400) =K2731_1*X2847 - K5453_1*X1400
1401	X_dot(1401) =K2735_1*X2849 - K5745_1*X1401
1402	X_dot(1402) =K2739_1*X2851 - K5753_1*X1402
1403	X_dot(1403) =K2773_1*X2868 - K5272_1*X1403
1404	X_dot(1404) =K2774_2*X2869 + K2775_1*X2869 - K5652_1*X1404 - K2774_1*X479*X1404
1405	X_dot(1405) =K2779_1*X2871 - K5868_1*X1405
1406	X_dot(1406) =K2810_2*X2887 + K2811_1*X2887 + K2812_2*X2888 + K2813_1*X2888 - K5853_1*X1406 - K2810_1*X357*X1406 - K2812_1*X709*X1406
1407	X_dot(1407) =K1936_2*X2450 + K1937_1*X2450 + K1938_2*X2451 + K1939_1*X2451 + K1940_2*X2452 + K1941_1*X2452 + K2828_2*X2896 + K2829_1*X2896 + K2830_2*X2897 + K2831_1*X2897 + K2845_1*X2904 + K2853_1*X2908 + K3069_1*X3016 - K5546_1*X1407 - K1938_1*X34*X1407 - K1936_1*X317*X1407 - K1940_1*X313*X1407 - K2828_1*X361*X1407 - K2830_1*X575*X1407
1408	X_dot(1408) =K396_2*X1680 + K397_1*X1680 + K954_2*X1959 + K955_1*X1959 + K1942_2*X2453 + K1943_1*X2453 + K1944_2*X2454 + K1945_1*X2454 + K2837_1*X2900 - K5238_1*X1408 - K396_1*X500*X1408 - K954_1*X281*X1408 - K1942_1*X312*X1408 - K1944_1*X313*X1408
1409	X_dot(1409) =K2820_2*X2892 + K2821_1*X2892 + K2839_1*X2901 + K2841_1*X2902 + K2849_1*X2906 + K3065_1*X3014 + K3067_1*X3015 - K5510_1*X1409 - K2820_1*X575*X1409
1410	X_dot(1410) =K2840_2*X2902 + K2841_1*X2902 + K2842_2*X2903 + K2843_1*X2903 + K2844_2*X2904 + K2845_1*X2904 + K2846_2*X2905 + K2847_1*X2905 - K5899_1*X1410 - K2840_1*X320*X1410 - K2842_1*X314*X1410 - K2844_1*X361*X1410 - K2846_1*X767*X1410

1411	X_dot(1411) =K760_2*X1862 + K761_1*X1862 + K762_2*X1863 + K763_1*X1863 + K764_2*X1864 + K765_1*X1864 + K766_2*X1865 + K767_1*X1865 + K768_2*X1866 + K769_1*X1866 + K770_2*X1867 + K771_1*X1867 + K1084_2*X2024 + K1085_1*X2024 + K1086_2*X2025 + K1087_1*X2025 + K1138_2*X2051 + K1139_1*X2051 + K1478_2*X2221 + K1479_1*X2221 + K1480_2*X2222 + K1481_1*X2222 + K2448_2*X2706 + K2449_1*X2706 + K2450_2*X2707 + K2451_1*X2707 + K2452_2*X2708 + K2453_1*X2708 + K2454_2*X2709 + K2455_1*X2709 + K2456_2*X2710 + K2457_1*X2710 + K2458_2*X2711 + K2459_1*X2711 + K2460_2*X2712 + K2461_1*X2712 + K2462_2*X2713 + K2463_1*X2713 + K2464_2*X2714 + K2465_1*X2714 + K2466_2*X2715 + K2467_1*X2715 + K2869_1*X2916 + K2889_1*X2926 + K2939_1*X2951 + K3020_2*X2992 + K3021_1*X2992 + K3022_2*X2993 + K3023_1*X2993 + K3178_2*X3071 + K3179_1*X3071 - K5723_1*X1411 - K764_1*X343*X1411 - K768_1*X341*X1411 - K770_1*X350*X1411 - K762_1*X615*X1411 - K760_1*X652*X1411 - K766_1*X722*X1411 - K1086_1*X9*X1411 - K1084_1*X630*X1411 - K1138_1*X281*X1411 - K1480_1*X693*X1411 - K1478_1*X750*X1411 - K2454_1*X87*X1411 - K2450_1*X226*X1411 - K2452_1*X213*X1411 - K2462_1*X204*X1411 - K2448_1*X429*X1411 - K2458_1*X406*X1411 - K2456_1*X435*X1411 - K2466_1*X419*X1411 - K2460_1*X609*X1411 - K2464_1*X604*X1411 - K3020_1*X177*X1411 - K3022_1*X559*X1411 - K3178_1*X769*X1411
1412	X_dot(1412) =K1372_2*X2168 + K1373_1*X2168 + K1374_2*X2169 + K1375_1*X2169 + K2875_1*X2919 - K5744_1*X1412 - K1372_1*X9*X1412 - K1374_1*X100*X1412
1413	X_dot(1413) =K2905_1*X2934 - K5345_1*X1413
1414	X_dot(1414) =K2907_1*X2935 - K5571_1*X1414
1415	X_dot(1415) =K2909_1*X2936 - K5266_1*X1415
1416	X_dot(1416) =K2921_1*X2942 - K5773_1*X1416
1417	X_dot(1417) =K2922_2*X2943 + K2923_1*X2943 - K5417_1*X1417 - K2922_1*X220*X1417
1418	X_dot(1418) =K50_2*X1507 + K51_1*X1507 + K52_2*X1508 + K53_1*X1508 + K54_2*X1509 + K55_1*X1509 + K56_2*X1510 + K57_1*X1510 + K58_2*X1511 + K59_1*X1511 + K60_2*X1512 + K61_1*X1512 + K62_2*X1513 + K63_1*X1513 + K64_2*X1514 + K65_1*X1514 + K66_2*X1515 + K67_1*X1515 + K68_2*X1516 + K69_1*X1516 + K70_2*X1517 + K71_1*X1517 + K72_2*X1518 + K73_1*X1518 + K848_2*X1906 + K849_1*X1906 + K850_2*X1907 + K851_1*X1907 + K852_2*X1908 + K853_1*X1908 + K854_2*X1909 + K855_1*X1909 + K1164_2*X2064 + K1165_1*X2064 + K1544_2*X2254 + K1545_1*X2254 + K1546_2*X2255 + K1547_1*X2255 + K1548_2*X2256 + K1549_1*X2256 + K1550_2*X2257 + K1551_1*X2257 + K1552_2*X2258 + K1553_1*X2258 + K1554_2*X2259 + K1555_1*X2259 + K2634_2*X2799 + K2635_1*X2799 + K2929_1*X2946 + K3172_2*X3068 + K3173_1*X3068 + K3292_2*X3128 + K3368_2*X3166 + K3408_2*X3186 - K5460_1*X1418 - K64_1*X45*X1418 - K54_1*X168*X1418 - K62_1*X100*X1418 - K58_1*X319*X1418 - K60_1*X342*X1418 - K56_1*X384*X1418 - K72_1*X341*X1418 - K50_1*X637*X1418 - K52_1*X630*X1418 - K68_1*X503*X1418 - K70_1*X495*X1418 - K66_1*X631*X1418 - K850_1*X47*X1418 - K854_1*X194*X1418 - K852_1*X343*X1418 - K848_1*X484*X1418 - K1164_1*X376*X1418 - K1544_1*X29*X1418 - K1546_1*X177*X1418 - K1552_1*X169*X1418 - K1548_1*X275*X1418 - K1554_1*X436*X1418 - K1550_1*X483*X1418 - K2634_1*X276*X1418 - K3172_1*X769*X1418 - K3292_1*X404*X1418 - K3368_1*X228*X1418 - K3408_1*X418*X1418
1419	X_dot(1419) =K2940_2*X2952 + K2941_1*X2952 + K2981_1*X2972 - K5505_1*X1419 - K2940_1*X315*X1419
1420	X_dot(1420) =K3_2*X773 + K2945_1*X2954 - K5370_1*X1420 - K3_1*X891*X1420
1421	X_dot(1421) =K2949_1*X2956 - K5882_1*X1421
1422	X_dot(1422) =K2955_1*X2959 - K5826_1*X1422
1423	X_dot(1423) =K2969_1*X2966 - K5790_1*X1423
1424	X_dot(1424) =K2983_1*X2973 - K5288_1*X1424
1425	X_dot(1425) =K2985_1*X2974 - K5552_1*X1425
1426	X_dot(1426) =K2986_2*X2975 + K2987_1*X2975 - K5369_1*X1426 - K2986_1*X162*X1426
1427	X_dot(1427) =K2991_1*X2977 - K5780_1*X1427
1428	X_dot(1428) =K3005_1*X2984 + K3043_1*X3003 + K3468_2*X3216 + K3469_1*X3216 + K3514_2*X3239 + K3515_1*X3239 - K5400_1*X1428 - K3468_1*X1428^2 - K3514_1*X861*X1428
1429	X_dot(1429) =K3011_1*X2987 - K5839_1*X1429
1430	X_dot(1430) =K3013_1*X2988 - K5285_1*X1430
1431	X_dot(1431) =K3014_2*X2989 + K3015_1*X2989 + K3638_2*X3301 + K3639_1*X3301 - K5259_1*X1431 - K3014_1*X42*X1431 - K3638_1*X766*X1431
1432	X_dot(1432) =K3039_1*X3001 - K5373_1*X1432
1433	X_dot(1433) =K3047_1*X3005 - K5866_1*X1433
1434	X_dot(1434) =K3053_1*X3008 - K5341_1*X1434
1435	X_dot(1435) =K3059_1*X3011 + K3356_2*X3160 - K5602_1*X1435 - K3356_1*X974*X1435
1436	X_dot(1436) =K3063_1*X3013 - K5599_1*X1436
1437	X_dot(1437) =K2590_2*X2777 + K2591_1*X2777 + K2592_2*X2778 + K2593_1*X2778 + K3070_2*X3017 + K3071_1*X3017 - K5835_1*X1437 - K2590_1*X503*X1437 - K2592_1*X495*X1437 - K3070_1*X687*X1437
1438	X_dot(1438) =K3077_1*X3020 - K5676_1*X1438
1439	X_dot(1439) =K816_2*X1890 + K817_1*X1890 + K3078_2*X3021 + K3079_1*X3021 - K5332_1*X1439 - K816_1*X150*X1439 - K3078_1*X123*X1439
1440	X_dot(1440) =K3081_1*X3022 - K5563_1*X1440
1441	X_dot(1441) =K3127_1*X3045 - K5743_1*X1441
1442	X_dot(1442) =K3165_1*X3064 + K3167_1*X3065 + K3169_1*X3066 + K3177_1*X3070 + K3181_1*X3072 + K3574_2*X3269 - K5902_1*X1442 - K3574_1*X418*X1442
1443	X_dot(1443) =K3171_1*X3067 + K3173_1*X3068 + K3179_1*X3071 - K5901_1*X1443
1444	X_dot(1444) =K3175_1*X3069 + K3241_1*X3102 - K5903_1*X1444
1445	X_dot(1445) =K3090_2*X3027 + K3091_1*X3027 + K3296_2*X3130 - K5351_1*X1445 - K3090_1*X47*X1445 - K3296_1*X404*X1445
1446	X_dot(1446) =K1170_2*X2067 + K1171_1*X2067 + K1172_2*X2068 + K1173_1*X2068 + K3306_2*X3135 - K5353_1*X1446 - K1170_1*X239*X1446 - K1172_1*X414*X1446 - K3306_1*X1007*X1446
1447	X_dot(1447) =K3332_2*X3148 - K5700_1*X1447 - K3332_1*X459*X1447
1448	X_dot(1448) =K3350_2*X3157 - K5230_1*X1448 - K3350_1*X190*X1448
1449	X_dot(1449) =K3352_2*X3158 - K5594_1*X1449 - K3352_1*X974*X1449
1450	X_dot(1450) =K3362_2*X3163 - K5508_1*X1450 - K3362_1*X309*X1450
1451	X_dot(1451) =K236_2*X1600 + K237_1*X1600 + K238_2*X1601 + K239_1*X1601 + K240_2*X1602 + K241_1*X1602 + K1228_2*X2096 + K1229_1*X2096 + K1732_2*X2348 + K1733_1*X2348 + K1734_2*X2349 + K1735_1*X2349 + K2684_2*X2824 + K2685_1*X2824 + K3384_2*X3174 + K3472_2*X3218 + K3492_2*X3228 - K5540_1*X1451 - K240_1*X434*X1451 - K238_1*X568*X1451 - K236_1*X648*X1451 - K1228_1*X99*X1451 - K1734_1*X5*X1451 - K1732_1*X230*X1451 - K2684_1*X560*X1451 - K3384_1*X286*X1451 - K3472_1*X68*X1451 - K3492_1*X289*X1451
1452	X_dot(1452) =K3404_2*X3184 - K5821_1*X1452 - K3404_1*X289*X1452
1453	X_dot(1453) =K3412_2*X3188 + K3426_2*X3195 - K5463_1*X1453 - K3412_1*X418*X1453 - K3426_1*X404*X1453

1454	X_dot(1454) =K3422_2*X3193 + K3436_2*X3200 + K3626_2*X3295 + K3627_1*X3295 + K3628_2*X3296 + K3629_1*X3296 - K5663_1*X1454 - K3422_1*X418*X1454 - K3436_1*X404*X1454 - K3626_1*X41*X1454 - K3628_1*X519*X1454
1455	X_dot(1455) =K3480_2*X3222 + K3482_2*X3223 - K5537_1*X1455 - K3482_1*X178*X1455 - K3480_1*X478*X1455
1456	X_dot(1456) =K3518_2*X3241 - K5336_1*X1456 - K3518_1*X715*X1456
1457	X_dot(1457) =K3520_2*X3242 - K5440_1*X1457 - K3520_1*X286*X1457
1458	X_dot(1458) =K3556_2*X3260 - K5300_1*X1458 - K3556_1*X463*X1458
1459	X_dot(1459) =K3577_1*X3270
1460	X_dot(1460) =K3579_1*X3271 + K3617_1*X3290 + K3641_1*X3302
1461	X_dot(1461) =K3581_1*X3272
1462	X_dot(1462) =K3583_1*X3273 + K3587_1*X3275
1463	X_dot(1463) =K3585_1*X3274 + K3589_1*X3276
1464	X_dot(1464) =K3591_1*X3277
1465	X_dot(1465) =K3593_1*X3278
1466	X_dot(1466) =K3595_1*X3279
1467	X_dot(1467) =K3597_1*X3280
1468	X_dot(1468) =K3599_1*X3281
1469	X_dot(1469) =K3601_1*X3282
1470	X_dot(1470) =K3603_1*X3283
1471	X_dot(1471) =K3605_1*X3284
1472	X_dot(1472) =K3607_1*X3285
1473	X_dot(1473) =K3609_1*X3286
1474	X_dot(1474) =K3611_1*X3287
1475	X_dot(1475) =K3613_1*X3288
1476	X_dot(1476) =K3615_1*X3289
1477	X_dot(1477) =K3619_1*X3291
1478	X_dot(1478) =K3621_1*X3292
1479	X_dot(1479) =K3623_1*X3293
1480	X_dot(1480) =K3625_1*X3294
1481	X_dot(1481) =K3627_1*X3295 + K3633_1*X3298
1482	X_dot(1482) =K3629_1*X3296
1483	X_dot(1483) =K3631_1*X3297
1484	X_dot(1484) =K3630_2*X3297 + K3631_1*X3297 + K3632_2*X3298 + K3633_1*X3298 + K3634_2*X3299 + K3635_1*X3299 - K3632_1*X41*X1484 - K3630_1*X357*X1484 - K3634_1*X757*X1484
1485	X_dot(1485) =K3637_1*X3300
1486	X_dot(1486) =K2800_2*X2882 + K2801_1*X2882 + K3180_2*X3072 + K3181_1*X3072 + K3639_1*X3301 - K2800_1*X453*X1486 - K3180_1*X771*X1486
1487	X_dot(1487) =K10_1*X384*X1031 - K11_1*X1487 - K10_2*X1487
1488	X_dot(1488) =K12_1*X319*X1031 - K13_1*X1488 - K12_2*X1488
1489	X_dot(1489) =K14_1*X20*X1031 - K15_1*X1489 - K14_2*X1489
1490	X_dot(1490) =K16_1*X484*X1031 - K17_1*X1490 - K16_2*X1490
1491	X_dot(1491) =K18_1*X1*X1031 - K19_1*X1491 - K18_2*X1491
1492	X_dot(1492) =K20_1*X342*X1031 - K21_1*X1492 - K20_2*X1492
1493	X_dot(1493) =K22_1*X134*X1031 - K23_1*X1493 - K22_2*X1493
1494	X_dot(1494) =K24_1*X100*X1031 - K25_1*X1494 - K24_2*X1494
1495	X_dot(1495) =K26_1*X45*X1031 - K27_1*X1495 - K26_2*X1495
1496	X_dot(1496) =K28_1*X314*X1031 - K29_1*X1496 - K28_2*X1496
1497	X_dot(1497) =K30_1*X722*X1031 - K31_1*X1497 - K30_2*X1497
1498	X_dot(1498) =K32_1*X63*X1031 - K33_1*X1498 - K32_2*X1498
1499	X_dot(1499) =K34_1*X586*X1031 - K35_1*X1499 - K34_2*X1499
1500	X_dot(1500) =K36_1*X401*X1031 - K37_1*X1500 - K36_2*X1500
1501	X_dot(1501) =K38_1*X503*X1031 - K39_1*X1501 - K38_2*X1501
1502	X_dot(1502) =K40_1*X495*X1031 - K41_1*X1502 - K40_2*X1502
1503	X_dot(1503) =K42_1*X53*X1031 - K43_1*X1503 - K42_2*X1503
1504	X_dot(1504) =K44_1*X322*X1031 - K45_1*X1504 - K44_2*X1504
1505	X_dot(1505) =K46_1*X468*X1031 - K47_1*X1505 - K46_2*X1505
1506	X_dot(1506) =K48_1*X571*X1031 - K49_1*X1506 - K48_2*X1506
1507	X_dot(1507) =K50_1*X637*X1418 - K51_1*X1507 - K50_2*X1507
1508	X_dot(1508) =K52_1*X630*X1418 - K53_1*X1508 - K52_2*X1508
1509	X_dot(1509) =K54_1*X168*X1418 - K55_1*X1509 - K54_2*X1509
1510	X_dot(1510) =K56_1*X384*X1418 - K57_1*X1510 - K56_2*X1510
1511	X_dot(1511) =K58_1*X319*X1418 - K59_1*X1511 - K58_2*X1511
1512	X_dot(1512) =K60_1*X342*X1418 - K61_1*X1512 - K60_2*X1512
1513	X_dot(1513) =K62_1*X100*X1418 - K63_1*X1513 - K62_2*X1513
1514	X_dot(1514) =K64_1*X45*X1418 - K65_1*X1514 - K64_2*X1514
1515	X_dot(1515) =K66_1*X631*X1418 - K67_1*X1515 - K66_2*X1515
1516	X_dot(1516) =K68_1*X503*X1418 - K69_1*X1516 - K68_2*X1516
1517	X_dot(1517) =K70_1*X495*X1418 - K71_1*X1517 - K70_2*X1517
1518	X_dot(1518) =K72_1*X341*X1418 - K73_1*X1518 - K72_2*X1518
1519	X_dot(1519) =K74_1*X689*X1173 - K75_1*X1519 - K74_2*X1519
1520	X_dot(1520) =K76_1*X662*X1173 - K77_1*X1520 - K76_2*X1520
1521	X_dot(1521) =K78_1*X21*X1173 - K79_1*X1521 - K78_2*X1521
1522	X_dot(1522) =K80_1*X482*X1173 - K81_1*X1522 - K80_2*X1522
1523	X_dot(1523) =K82_1*X99*X1173 - K83_1*X1523 - K82_2*X1523
1524	X_dot(1524) =K84_1*X129*X1173 - K85_1*X1524 - K84_2*X1524
1525	X_dot(1525) =K86_1*X23*X154 - K87_1*X1525 - K86_2*X1525
1526	X_dot(1526) =K88_1*X745*X810 - K89_1*X1526 - K88_2*X1526
1527	X_dot(1527) =K90_1*X177*X810 - K91_1*X1527 - K90_2*X1527
1528	X_dot(1528) =K92_1*X461*X810 - K93_1*X1528 - K92_2*X1528
1529	X_dot(1529) =K94_1*X763*X810 - K95_1*X1529 - K94_2*X1529
1530	X_dot(1530) =K96_1*X648*X810 - K97_1*X1530 - K96_2*X1530

1531	X_dot(1531) =K98_1*X567*X810 - K99_1*X1531 - K98_2*X1531
1532	X_dot(1532) =K100_1*X181*X810 - K101_1*X1532 - K100_2*X1532
1533	X_dot(1533) =K102_1*X275*X1381 - K103_1*X1533 - K102_2*X1533
1534	X_dot(1534) =K104_1*X342*X1381 - K105_1*X1534 - K104_2*X1534
1535	X_dot(1535) =K106_1*X181*X1381 - K107_1*X1535 - K106_2*X1535
1536	X_dot(1536) =K108_1*X522*X763 - K109_1*X1536 - K108_2*X1536
1537	X_dot(1537) =K110_1*X543*X891 - K111_1*X1537 - K110_2*X1537
1538	X_dot(1538) =K112_1*X384*X891 - K113_1*X1538 - K112_2*X1538
1539	X_dot(1539) =K114_1*X588*X891 - K115_1*X1539 - K114_2*X1539
1540	X_dot(1540) =K116_1*X13*X891 - K117_1*X1540 - K116_2*X1540
1541	X_dot(1541) =K118_1*X695*X891 - K119_1*X1541 - K118_2*X1541
1542	X_dot(1542) =K120_1*X9*X891 - K121_1*X1542 - K120_2*X1542
1543	X_dot(1543) =K122_1*X86*X891 - K123_1*X1543 - K122_2*X1543
1544	X_dot(1544) =K124_1*X349*X891 - K125_1*X1544 - K124_2*X1544
1545	X_dot(1545) =K126_1*X570*X891 - K127_1*X1545 - K126_2*X1545
1546	X_dot(1546) =K128_1*X484*X891 - K129_1*X1546 - K128_2*X1546
1547	X_dot(1547) =K130_1*X480*X891 - K131_1*X1547 - K130_2*X1547
1548	X_dot(1548) =K132_1*X182*X891 - K133_1*X1548 - K132_2*X1548
1549	X_dot(1549) =K134_1*X369*X891 - K135_1*X1549 - K134_2*X1549
1550	X_dot(1550) =K136_1*X327*X891 - K137_1*X1550 - K136_2*X1550
1551	X_dot(1551) =K138_1*X662*X891 - K139_1*X1551 - K138_2*X1551
1552	X_dot(1552) =K140_1*X542*X891 - K141_1*X1552 - K140_2*X1552
1553	X_dot(1553) =K142_1*X451*X891 - K143_1*X1553 - K142_2*X1553
1554	X_dot(1554) =K144_1*X244*X891 - K145_1*X1554 - K144_2*X1554
1555	X_dot(1555) =K146_1*X546*X891 - K147_1*X1555 - K146_2*X1555
1556	X_dot(1556) =K148_1*X734*X891 - K149_1*X1556 - K148_2*X1556
1557	X_dot(1557) =K150_1*X612*X891 - K151_1*X1557 - K150_2*X1557
1558	X_dot(1558) =K152_1*X553*X891 - K153_1*X1558 - K152_2*X1558
1559	X_dot(1559) =K154_1*X250*X891 - K155_1*X1559 - K154_2*X1559
1560	X_dot(1560) =K156_1*X401*X891 - K157_1*X1560 - K156_2*X1560
1561	X_dot(1561) =K158_1*X467*X891 - K159_1*X1561 - K158_2*X1561
1562	X_dot(1562) =K160_1*X111*X891 - K161_1*X1562 - K160_2*X1562
1563	X_dot(1563) =K162_1*X262*X891 - K163_1*X1563 - K162_2*X1563
1564	X_dot(1564) =K164_1*X753*X891 - K165_1*X1564 - K164_2*X1564
1565	X_dot(1565) =K166_1*X605*X891 - K167_1*X1565 - K166_2*X1565
1566	X_dot(1566) =K168_1*X524*X891 - K169_1*X1566 - K168_2*X1566
1567	X_dot(1567) =K170_1*X97*X891 - K171_1*X1567 - K170_2*X1567
1568	X_dot(1568) =K172_1*X623*X891 - K173_1*X1568 - K172_2*X1568
1569	X_dot(1569) =K174_1*X129*X891 - K175_1*X1569 - K174_2*X1569
1570	X_dot(1570) =K176_1*X257*X891 - K177_1*X1570 - K176_2*X1570
1571	X_dot(1571) =K178_1*X602*X891 - K179_1*X1571 - K178_2*X1571
1572	X_dot(1572) =K180_1*X6*X1209 - K181_1*X1572 - K180_2*X1572
1573	X_dot(1573) =K182_1*X319*X1000 - K183_1*X1573 - K182_2*X1573
1574	X_dot(1574) =K184_1*X484*X1000 - K185_1*X1574 - K184_2*X1574
1575	X_dot(1575) =K186_1*X232*X1000 - K187_1*X1575 - K186_2*X1575
1576	X_dot(1576) =K188_1*X182*X1000 - K189_1*X1576 - K188_2*X1576
1577	X_dot(1577) =K190_1*X345*X1000 - K191_1*X1577 - K190_2*X1577
1578	X_dot(1578) =K192_1*X401*X1000 - K193_1*X1578 - K192_2*X1578
1579	X_dot(1579) =K194_1*X467*X1000 - K195_1*X1579 - K194_2*X1579
1580	X_dot(1580) =K196_1*X111*X1000 - K197_1*X1580 - K196_2*X1580
1581	X_dot(1581) =K198_1*X605*X1000 - K199_1*X1581 - K198_2*X1581
1582	X_dot(1582) =K200_1*X434*X1000 - K201_1*X1582 - K200_2*X1582
1583	X_dot(1583) =K202_1*X485*X1000 - K203_1*X1583 - K202_2*X1583
1584	X_dot(1584) =K204_1*X97*X1000 - K205_1*X1584 - K204_2*X1584
1585	X_dot(1585) =K206_1*X257*X1000 - K207_1*X1585 - K206_2*X1585
1586	X_dot(1586) =K208_1*X602*X1000 - K209_1*X1586 - K208_2*X1586
1587	X_dot(1587) =K210_1*X467*X1001 - K211_1*X1587 - K210_2*X1587
1588	X_dot(1588) =K212_1*X111*X1001 - K213_1*X1588 - K212_2*X1588
1589	X_dot(1589) =K214_1*X434*X1001 - K215_1*X1589 - K214_2*X1589
1590	X_dot(1590) =K216_1*X120*X1393 - K217_1*X1590 - K216_2*X1590
1591	X_dot(1591) =K218_1*X612*X1393 - K219_1*X1591 - K218_2*X1591
1592	X_dot(1592) =K220_1*X401*X1393 - K221_1*X1592 - K220_2*X1592
1593	X_dot(1593) =K222_1*X111*X1393 - K223_1*X1593 - K222_2*X1593
1594	X_dot(1594) =K224_1*X181*X1393 - K225_1*X1594 - K224_2*X1594
1595	X_dot(1595) =K226_1*X569*X1393 - K227_1*X1595 - K226_2*X1595
1596	X_dot(1596) =K228_1*X319*X438 - K229_1*X1596 - K228_2*X1596
1597	X_dot(1597) =K230_1*X438*X467 - K231_1*X1597 - K230_2*X1597
1598	X_dot(1598) =K232_1*X111*X438 - K233_1*X1598 - K232_2*X1598
1599	X_dot(1599) =K234_1*X230*X1087 - K235_1*X1599 - K234_2*X1599
1600	X_dot(1600) =K236_1*X648*X1451 - K237_1*X1600 - K236_2*X1600
1601	X_dot(1601) =K238_1*X568*X1451 - K239_1*X1601 - K238_2*X1601
1602	X_dot(1602) =K240_1*X434*X1451 - K241_1*X1602 - K240_2*X1602
1603	X_dot(1603) =K242_1*X207*X780 - K243_1*X1603 - K242_2*X1603
1604	X_dot(1604) =K244_1*X310*X780 - K245_1*X1604 - K244_2*X1604
1605	X_dot(1605) =K246_1*X731*X780 - K247_1*X1605 - K246_2*X1605
1606	X_dot(1606) =K248_1*X47*X1159 - K249_1*X1606 - K248_2*X1606
1607	X_dot(1607) =K250_1*X763*X1159 - K251_1*X1607 - K250_2*X1607
1608	X_dot(1608) =K252_1*X341*X1159 - K253_1*X1608 - K252_2*X1608
1609	X_dot(1609) =K254_1*X157*X1391 - K255_1*X1609 - K254_2*X1609
1610	X_dot(1610) =K256_1*X275*X330 - K257_1*X1610 - K256_2*X1610

1611	X_dot(1611) =K258_1*X330*X707 - K259_1*X1611 - K258_2*X1611
1612	X_dot(1612) =K260_1*X330*X615 - K261_1*X1612 - K260_2*X1612
1613	X_dot(1613) =K262_1*X330*X500 - K263_1*X1613 - K262_2*X1613
1614	X_dot(1614) =K264_1*X330*X484 - K265_1*X1614 - K264_2*X1614
1615	X_dot(1615) =K266_1*X47*X330 - K267_1*X1615 - K266_2*X1615
1616	X_dot(1616) =K268_1*X1*X330 - K269_1*X1616 - K268_2*X1616
1617	X_dot(1617) =K270_1*X122*X330 - K271_1*X1617 - K270_2*X1617
1618	X_dot(1618) =K272_1*X330*X405 - K273_1*X1618 - K272_2*X1618
1619	X_dot(1619) =K274_1*X275*X1330 - K275_1*X1619 - K274_2*X1619
1620	X_dot(1620) =K276_1*X275*X331 - K277_1*X1620 - K276_2*X1620
1621	X_dot(1621) =K278_1*X322*X331 - K279_1*X1621 - K278_2*X1621
1622	X_dot(1622) =K280_1*X275*X337 - K281_1*X1622 - K280_2*X1622
1623	X_dot(1623) =K282_1*X55*X337 - K283_1*X1623 - K282_2*X1623
1624	X_dot(1624) =K284_1*X337*X517 - K285_1*X1624 - K284_2*X1624
1625	X_dot(1625) =K286_1*X322*X337 - K287_1*X1625 - K286_2*X1625
1626	X_dot(1626) =K288_1*X547*X1195 - K289_1*X1626 - K288_2*X1626
1627	X_dot(1627) =K290_1*X199*X1195 - K291_1*X1627 - K290_2*X1627
1628	X_dot(1628) =K292_1*X684*X1195 - K293_1*X1628 - K292_2*X1628
1629	X_dot(1629) =K294_1*X307*X1195 - K295_1*X1629 - K294_2*X1629
1630	X_dot(1630) =K296_1*X433*X1195 - K297_1*X1630 - K296_2*X1630
1631	X_dot(1631) =K298_1*X274*X1195 - K299_1*X1631 - K298_2*X1631
1632	X_dot(1632) =K300_1*X81*X1195 - K301_1*X1632 - K300_2*X1632
1633	X_dot(1633) =K302_1*X517*X1195 - K303_1*X1633 - K302_2*X1633
1634	X_dot(1634) =K304_1*X102*X1195 - K305_1*X1634 - K304_2*X1634
1635	X_dot(1635) =K306_1*X126*X1195 - K307_1*X1635 - K306_2*X1635
1636	X_dot(1636) =K308_1*X106*X1195 - K309_1*X1636 - K308_2*X1636
1637	X_dot(1637) =K310_1*X250*X1195 - K311_1*X1637 - K310_2*X1637
1638	X_dot(1638) =K312_1*X714*X1195 - K313_1*X1638 - K312_2*X1638
1639	X_dot(1639) =K314_1*X445*X1195 - K315_1*X1639 - K314_2*X1639
1640	X_dot(1640) =K316_1*X686*X1195 - K317_1*X1640 - K316_2*X1640
1641	X_dot(1641) =K318_1*X444*X1195 - K319_1*X1641 - K318_2*X1641
1642	X_dot(1642) =K320_1*X752*X1195 - K321_1*X1642 - K320_2*X1642
1643	X_dot(1643) =K322_1*X758*X1195 - K323_1*X1643 - K322_2*X1643
1644	X_dot(1644) =K324_1*X40*X1195 - K325_1*X1644 - K324_2*X1644
1645	X_dot(1645) =K326_1*X768*X1195 - K327_1*X1645 - K326_2*X1645
1646	X_dot(1646) =K328_1*X205*X445 - K329_1*X1646 - K328_2*X1646
1647	X_dot(1647) =K330_1*X445*X1107 - K331_1*X1647 - K330_2*X1647
1648	X_dot(1648) =K332_1*X458*X1250 - K333_1*X1648 - K332_2*X1648
1649	X_dot(1649) =K334_1*X484*X1250 - K335_1*X1649 - K334_2*X1649
1650	X_dot(1650) =K336_1*X47*X1250 - K337_1*X1650 - K336_2*X1650
1651	X_dot(1651) =K338_1*X1*X1250 - K339_1*X1651 - K338_2*X1651
1652	X_dot(1652) =K340_1*X158*X822 - K341_1*X1652 - K340_2*X1652
1653	X_dot(1653) =K342_1*X9*X822 - K343_1*X1653 - K342_2*X1653
1654	X_dot(1654) =K344_1*X143*X823 - K345_1*X1654 - K344_2*X1654
1655	X_dot(1655) =K346_1*X721*X823 - K347_1*X1655 - K346_2*X1655
1656	X_dot(1656) =K348_1*X112*X1260 - K349_1*X1656 - K348_2*X1656
1657	X_dot(1657) =K350_1*X103*X1260 - K351_1*X1657 - K350_2*X1657
1658	X_dot(1658) =K352_1*X112*X893 - K353_1*X1658 - K352_2*X1658
1659	X_dot(1659) =K354_1*X98*X893 - K355_1*X1659 - K354_2*X1659
1660	X_dot(1660) =K356_1*X575*X849 - K357_1*X1660 - K356_2*X1660
1661	X_dot(1661) =K358_1*X161*X783 - K359_1*X1661 - K358_2*X1661
1662	X_dot(1662) =K360_1*X106*X783 - K361_1*X1662 - K360_2*X1662
1663	X_dot(1663) =K362_1*X88*X783 - K363_1*X1663 - K362_2*X1663
1664	X_dot(1664) =K364_1*X222*X783 - K365_1*X1664 - K364_2*X1664
1665	X_dot(1665) =K366_1*X498*X915 - K367_1*X1665 - K366_2*X1665
1666	X_dot(1666) =K368_1*X704*X915 - K369_1*X1666 - K368_2*X1666
1667	X_dot(1667) =K370_1*X532*X915 - K371_1*X1667 - K370_2*X1667
1668	X_dot(1668) =K372_1*X441*X915 - K373_1*X1668 - K372_2*X1668
1669	X_dot(1669) =K374_1*X530*X915 - K375_1*X1669 - K374_2*X1669
1670	X_dot(1670) =K376_1*X473*X915 - K377_1*X1670 - K376_2*X1670
1671	X_dot(1671) =K378_1*X2*X915 - K379_1*X1671 - K378_2*X1671
1672	X_dot(1672) =K380_1*X681*X915 - K381_1*X1672 - K380_2*X1672
1673	X_dot(1673) =K382_1*X222*X915 - K383_1*X1673 - K382_2*X1673
1674	X_dot(1674) =K384_1*X568*X915 - K385_1*X1674 - K384_2*X1674
1675	X_dot(1675) =K386_1*X279^2 - K387_1*X1675 - K386_2*X1675
1676	X_dot(1676) =K388_1*X225*X1310 - K389_1*X1676 - K388_2*X1676
1677	X_dot(1677) =K390_1*X28*X1310 - K391_1*X1677 - K390_2*X1677
1678	X_dot(1678) =K392_1*X281*X831 - K393_1*X1678 - K392_2*X1678
1679	X_dot(1679) =K394_1*X206*X787 - K395_1*X1679 - K394_2*X1679
1680	X_dot(1680) =K396_1*X500*X1408 - K397_1*X1680 - K396_2*X1680
1681	X_dot(1681) =K398_1*X620*X1185 - K399_1*X1681 - K398_2*X1681
1682	X_dot(1682) =K400_1*X563*X1185 - K401_1*X1682 - K400_2*X1682
1683	X_dot(1683) =K402_1*X334*X1271 - K403_1*X1683 - K402_2*X1683
1684	X_dot(1684) =K404_1*X543*X1271 - K405_1*X1684 - K404_2*X1684
1685	X_dot(1685) =K406_1*X319*X1271 - K407_1*X1685 - K406_2*X1685
1686	X_dot(1686) =K408_1*X588*X1271 - K409_1*X1686 - K408_2*X1686
1687	X_dot(1687) =K410_1*X652*X1271 - K411_1*X1687 - K410_2*X1687
1688	X_dot(1688) =K412_1*X517*X1271 - K413_1*X1688 - K412_2*X1688
1689	X_dot(1689) =K414_1*X349*X1271 - K415_1*X1689 - K414_2*X1689
1690	X_dot(1690) =K416_1*X343*X1271 - K417_1*X1690 - K416_2*X1690

1691	X_dot(1691) =K418_1*X281*X1271 - K419_1*X1691 - K418_2*X1691
1692	X_dot(1692) =K420_1*X474*X1271 - K421_1*X1692 - K420_2*X1692
1693	X_dot(1693) =K422_1*X734*X1271 - K423_1*X1693 - K422_2*X1693
1694	X_dot(1694) =K424_1*X336*X1271 - K425_1*X1694 - K424_2*X1694
1695	X_dot(1695) =K426_1*X230*X1271 - K427_1*X1695 - K426_2*X1695
1696	X_dot(1696) =K428_1*X507*X1271 - K429_1*X1696 - K428_2*X1696
1697	X_dot(1697) =K430_1*X206*X1271 - K431_1*X1697 - K430_2*X1697
1698	X_dot(1698) =K432_1*X568*X1271 - K433_1*X1698 - K432_2*X1698
1699	X_dot(1699) =K434_1*X567*X1271 - K435_1*X1699 - K434_2*X1699
1700	X_dot(1700) =K436_1*X434*X1271 - K437_1*X1700 - K436_2*X1700
1701	X_dot(1701) =K438_1*X181*X1271 - K439_1*X1701 - K438_2*X1701
1702	X_dot(1702) =K440_1*X485*X1271 - K441_1*X1702 - K440_2*X1702
1703	X_dot(1703) =K442_1*X353*X1271 - K443_1*X1703 - K442_2*X1703
1704	X_dot(1704) =K444_1*X623*X1271 - K445_1*X1704 - K444_2*X1704
1705	X_dot(1705) =K446_1*X341*X1271 - K447_1*X1705 - K446_2*X1705
1706	X_dot(1706) =K448_1*X185*X1271 - K449_1*X1706 - K448_2*X1706
1707	X_dot(1707) =K450_1*X9*X1272 - K451_1*X1707 - K450_2*X1707
1708	X_dot(1708) =K452_1*X181*X1272 - K453_1*X1708 - K452_2*X1708
1709	X_dot(1709) =K454_1*X306*X894 - K455_1*X1709 - K454_2*X1709
1710	X_dot(1710) =K456_1*X543*X894 - K457_1*X1710 - K456_2*X1710
1711	X_dot(1711) =K458_1*X86*X894 - K459_1*X1711 - K458_2*X1711
1712	X_dot(1712) =K460_1*X343*X894 - K461_1*X1712 - K460_2*X1712
1713	X_dot(1713) =K462_1*X427*X894 - K463_1*X1713 - K462_2*X1713
1714	X_dot(1714) =K464_1*X111*X894 - K465_1*X1714 - K464_2*X1714
1715	X_dot(1715) =K466_1*X334*X1270 - K467_1*X1715 - K466_2*X1715
1716	X_dot(1716) =K468_1*X630*X1270 - K469_1*X1716 - K468_2*X1716
1717	X_dot(1717) =K470_1*X168*X1270 - K471_1*X1717 - K470_2*X1717
1718	X_dot(1718) =K472_1*X543*X1270 - K473_1*X1718 - K472_2*X1718
1719	X_dot(1719) =K474_1*X319*X1270 - K475_1*X1719 - K474_2*X1719
1720	X_dot(1720) =K476_1*X652*X1270 - K477_1*X1720 - K476_2*X1720
1721	X_dot(1721) =K478_1*X86*X1270 - K479_1*X1721 - K478_2*X1721
1722	X_dot(1722) =K480_1*X296*X1270 - K481_1*X1722 - K480_2*X1722
1723	X_dot(1723) =K482_1*X232*X1270 - K483_1*X1723 - K482_2*X1723
1724	X_dot(1724) =K484_1*X343*X1270 - K485_1*X1724 - K484_2*X1724
1725	X_dot(1725) =K486_1*X400*X1270 - K487_1*X1725 - K486_2*X1725
1726	X_dot(1726) =K488_1*X441*X1270 - K489_1*X1726 - K488_2*X1726
1727	X_dot(1727) =K490_1*X474*X1270 - K491_1*X1727 - K490_2*X1727
1728	X_dot(1728) =K492_1*X734*X1270 - K493_1*X1728 - K492_2*X1728
1729	X_dot(1729) =K494_1*X499*X1270 - K495_1*X1729 - K494_2*X1729
1730	X_dot(1730) =K496_1*X336*X1270 - K497_1*X1730 - K496_2*X1730
1731	X_dot(1731) =K498_1*X230*X1270 - K499_1*X1731 - K498_2*X1731
1732	X_dot(1732) =K500_1*X507*X1270 - K501_1*X1732 - K500_2*X1732
1733	X_dot(1733) =K502_1*X568*X1270 - K503_1*X1733 - K502_2*X1733
1734	X_dot(1734) =K504_1*X567*X1270 - K505_1*X1734 - K504_2*X1734
1735	X_dot(1735) =K506_1*X434*X1270 - K507_1*X1735 - K506_2*X1735
1736	X_dot(1736) =K508_1*X475*X1270 - K509_1*X1736 - K508_2*X1736
1737	X_dot(1737) =K510_1*X485*X1270 - K511_1*X1737 - K510_2*X1737
1738	X_dot(1738) =K512_1*X353*X1270 - K513_1*X1738 - K512_2*X1738
1739	X_dot(1739) =K514_1*X185*X1270 - K515_1*X1739 - K514_2*X1739
1740	X_dot(1740) =K516_1*X488*X1273 - K517_1*X1740 - K516_2*X1740
1741	X_dot(1741) =K518_1*X630*X1273 - K519_1*X1741 - K518_2*X1741
1742	X_dot(1742) =K520_1*X472*X1273 - K521_1*X1742 - K520_2*X1742
1743	X_dot(1743) =K522_1*X508*X1273 - K523_1*X1743 - K522_2*X1743
1744	X_dot(1744) =K524_1*X281*X1273 - K525_1*X1744 - K524_2*X1744
1745	X_dot(1745) =K526_1*X206*X1273 - K527_1*X1745 - K526_2*X1745
1746	X_dot(1746) =K528_1*X267*X1273 - K529_1*X1746 - K528_2*X1746
1747	X_dot(1747) =K530_1*X303*X1273 - K531_1*X1747 - K530_2*X1747
1748	X_dot(1748) =K532_1*X181*X1273 - K533_1*X1748 - K532_2*X1748
1749	X_dot(1749) =K534_1*X266*X1273 - K535_1*X1749 - K534_2*X1749
1750	X_dot(1750) =K536_1*X414*X1273 - K537_1*X1750 - K536_2*X1750
1751	X_dot(1751) =K538_1*X281*X1103 - K539_1*X1751 - K538_2*X1751
1752	X_dot(1752) =K540_1*X714*X1103 - K541_1*X1752 - K540_2*X1752
1753	X_dot(1753) =K542_1*X206*X1103 - K543_1*X1753 - K542_2*X1753
1754	X_dot(1754) =K544_1*X267*X1103 - K545_1*X1754 - K544_2*X1754
1755	X_dot(1755) =K546_1*X181*X1103 - K547_1*X1755 - K546_2*X1755
1756	X_dot(1756) =K548_1*X353*X1103 - K549_1*X1756 - K548_2*X1756
1757	X_dot(1757) =K550_1*X414*X1103 - K551_1*X1757 - K550_2*X1757
1758	X_dot(1758) =K552_1*X100*X1282 - K553_1*X1758 - K552_2*X1758
1759	X_dot(1759) =K554_1*X78*X1282 - K555_1*X1759 - K554_2*X1759
1760	X_dot(1760) =K556_1*X468*X1282 - K557_1*X1760 - K556_2*X1760
1761	X_dot(1761) =K558_1*X596*X632 - K559_1*X1761 - K558_2*X1761
1762	X_dot(1762) =K560_1*X596*X620 - K561_1*X1762 - K560_2*X1762
1763	X_dot(1763) =K562_1*X62*X1005 - K563_1*X1763 - K562_2*X1763
1764	X_dot(1764) =K564_1*X615*X1005 - K565_1*X1764 - K564_2*X1764
1765	X_dot(1765) =K566_1*X518*X1005 - K567_1*X1765 - K566_2*X1765
1766	X_dot(1766) =K568_1*X362*X925 - K569_1*X1766 - K568_2*X1766
1767	X_dot(1767) =K570_1*X632*X925 - K571_1*X1767 - K570_2*X1767
1768	X_dot(1768) =K572_1*X630*X925 - K573_1*X1768 - K572_2*X1768
1769	X_dot(1769) =K574_1*X415*X925 - K575_1*X1769 - K574_2*X1769
1770	X_dot(1770) =K576_1*X662*X925 - K577_1*X1770 - K576_2*X1770

1771	X_dot(1771) =K578_1*X277*X925 - K579_1*X1771 - K578_2*X1771
1772	X_dot(1772) =K580_1*X198*X925 - K581_1*X1772 - K580_2*X1772
1773	X_dot(1773) =K582_1*X181*X925 - K583_1*X1773 - K582_2*X1773
1774	X_dot(1774) =K584_1*X257*X925 - K585_1*X1774 - K584_2*X1774
1775	X_dot(1775) =K586_1*X383*X1313 - K587_1*X1775 - K586_2*X1775
1776	X_dot(1776) =K588_1*X277*X1313 - K589_1*X1776 - K588_2*X1776
1777	X_dot(1777) =K590_1*X632*X1316 - K591_1*X1777 - K590_2*X1777
1778	X_dot(1778) =K592_1*X86*X1316 - K593_1*X1778 - K592_2*X1778
1779	X_dot(1779) =K594_1*X484*X1316 - K595_1*X1779 - K594_2*X1779
1780	X_dot(1780) =K596_1*X198*X1316 - K597_1*X1780 - K596_2*X1780
1781	X_dot(1781) =K598_1*X717*X1276 - K599_1*X1781 - K598_2*X1781
1782	X_dot(1782) =K600_1*X500*X1276 - K601_1*X1782 - K600_2*X1782
1783	X_dot(1783) =K602_1*X441*X1276 - K603_1*X1783 - K602_2*X1783
1784	X_dot(1784) =K604_1*X281*X1276 - K605_1*X1784 - K604_2*X1784
1785	X_dot(1785) =K606_1*X326*X1276 - K607_1*X1785 - K606_2*X1785
1786	X_dot(1786) =K608_1*X160*X1276 - K609_1*X1786 - K608_2*X1786
1787	X_dot(1787) =K610_1*X257*X1276 - K611_1*X1787 - K610_2*X1787
1788	X_dot(1788) =K612_1*X587*X1006 - K613_1*X1788 - K612_2*X1788
1789	X_dot(1789) =K614_1*X508*X1006 - K615_1*X1789 - K614_2*X1789
1790	X_dot(1790) =K616_1*X483*X1006 - K617_1*X1790 - K616_2*X1790
1791	X_dot(1791) =K618_1*X622*X1006 - K619_1*X1791 - K618_2*X1791
1792	X_dot(1792) =K620_1*X464*X1006 - K621_1*X1792 - K620_2*X1792
1793	X_dot(1793) =K622_1*X620*X1006 - K623_1*X1793 - K622_2*X1793
1794	X_dot(1794) =K624_1*X461*X1006 - K625_1*X1794 - K624_2*X1794
1795	X_dot(1795) =K626_1*X166*X1006 - K627_1*X1795 - K626_2*X1795
1796	X_dot(1796) =K628_1*X111*X1006 - K629_1*X1796 - K628_2*X1796
1797	X_dot(1797) =K630_1*X714*X1006 - K631_1*X1797 - K630_2*X1797
1798	X_dot(1798) =K632_1*X599*X1006 - K633_1*X1798 - K632_2*X1798
1799	X_dot(1799) =K634_1*X609*X1327 - K635_1*X1799 - K634_2*X1799
1800	X_dot(1800) =K636_1*X187*X486 - K637_1*X1800 - K636_2*X1800
1801	X_dot(1801) =K638_1*X187*X362 - K639_1*X1801 - K638_2*X1801
1802	X_dot(1802) =K640_1*X187*X630 - K641_1*X1802 - K640_2*X1802
1803	X_dot(1803) =K642_1*X187*X243 - K643_1*X1803 - K642_2*X1803
1804	X_dot(1804) =K644_1*X187*X188 - K645_1*X1804 - K644_2*X1804
1805	X_dot(1805) =K646_1*X187*X384 - K647_1*X1805 - K646_2*X1805
1806	X_dot(1806) =K648_1*X55*X187 - K649_1*X1806 - K648_2*X1806
1807	X_dot(1807) =K650_1*X187*X342 - K651_1*X1807 - K650_2*X1807
1808	X_dot(1808) =K652_1*X187*X672 - K653_1*X1808 - K652_2*X1808
1809	X_dot(1809) =K654_1*X176*X187 - K655_1*X1809 - K654_2*X1809
1810	X_dot(1810) =K656_1*X187*X544 - K657_1*X1810 - K656_2*X1810
1811	X_dot(1811) =K658_1*X187*X461 - K659_1*X1811 - K658_2*X1811
1812	X_dot(1812) =K660_1*X187*X649 - K661_1*X1812 - K660_2*X1812
1813	X_dot(1813) =K662_1*X187*X277 - K663_1*X1813 - K662_2*X1813
1814	X_dot(1814) =K664_1*X187*X448 - K665_1*X1814 - K664_2*X1814
1815	X_dot(1815) =K666_1*X187*X550 - K667_1*X1815 - K666_2*X1815
1816	X_dot(1816) =K668_1*X187*X206 - K669_1*X1816 - K668_2*X1816
1817	X_dot(1817) =K670_1*X181*X187 - K671_1*X1817 - K670_2*X1817
1818	X_dot(1818) =K672_1*X214*X1318 - K673_1*X1818 - K672_2*X1818
1819	X_dot(1819) =K674_1*X363*X1318 - K675_1*X1819 - K674_2*X1819
1820	X_dot(1820) =K676_1*X225*X1318 - K677_1*X1820 - K676_2*X1820
1821	X_dot(1821) =K678_1*X259*X1318 - K679_1*X1821 - K678_2*X1821
1822	X_dot(1822) =K680_1*X669*X1318 - K681_1*X1822 - K680_2*X1822
1823	X_dot(1823) =K682_1*X628*X1318 - K683_1*X1823 - K682_2*X1823
1824	X_dot(1824) =K684_1*X191*X1318 - K685_1*X1824 - K684_2*X1824
1825	X_dot(1825) =K686_1*X447*X1318 - K687_1*X1825 - K686_2*X1825
1826	X_dot(1826) =K688_1*X342*X1318 - K689_1*X1826 - K688_2*X1826
1827	X_dot(1827) =K690_1*X343*X1318 - K691_1*X1827 - K690_2*X1827
1828	X_dot(1828) =K692_1*X102*X1318 - K693_1*X1828 - K692_2*X1828
1829	X_dot(1829) =K694_1*X104*X1318 - K695_1*X1829 - K694_2*X1829
1830	X_dot(1830) =K696_1*X134*X1318 - K697_1*X1830 - K696_2*X1830
1831	X_dot(1831) =K698_1*X176*X1318 - K699_1*X1831 - K698_2*X1831
1832	X_dot(1832) =K700_1*X128*X1318 - K701_1*X1832 - K700_2*X1832
1833	X_dot(1833) =K702_1*X229*X1318 - K703_1*X1833 - K702_2*X1833
1834	X_dot(1834) =K704_1*X552*X1318 - K705_1*X1834 - K704_2*X1834
1835	X_dot(1835) =K706_1*X448*X1318 - K707_1*X1835 - K706_2*X1835
1836	X_dot(1836) =K708_1*X459*X1318 - K709_1*X1836 - K708_2*X1836
1837	X_dot(1837) =K710_1*X248*X1318 - K711_1*X1837 - K710_2*X1837
1838	X_dot(1838) =K712_1*X483*X1145 - K713_1*X1838 - K712_2*X1838
1839	X_dot(1839) =K714_1*X342*X1145 - K715_1*X1839 - K714_2*X1839
1840	X_dot(1840) =K716_1*X182*X1145 - K717_1*X1840 - K716_2*X1840
1841	X_dot(1841) =K718_1*X400*X1145 - K719_1*X1841 - K718_2*X1841
1842	X_dot(1842) =K720_1*X281*X1145 - K721_1*X1842 - K720_2*X1842
1843	X_dot(1843) =K722_1*X140*X1145 - K723_1*X1843 - K722_2*X1843
1844	X_dot(1844) =K724_1*X263*X1145 - K725_1*X1844 - K724_2*X1844
1845	X_dot(1845) =K726_1*X505*X1145 - K727_1*X1845 - K726_2*X1845
1846	X_dot(1846) =K728_1*X266*X1145 - K729_1*X1846 - K728_2*X1846
1847	X_dot(1847) =K730_1*X630*X1366 - K731_1*X1847 - K730_2*X1847
1848	X_dot(1848) =K732_1*X339*X1366 - K733_1*X1848 - K732_2*X1848
1849	X_dot(1849) =K734_1*X346*X980 - K735_1*X1849 - K734_2*X1849
1850	X_dot(1850) =K736_1*X166*X980 - K737_1*X1850 - K736_2*X1850

1851	X_dot(1851) =K738_1*X430*X980 - K739_1*X1851 - K738_2*X1851
1852	X_dot(1852) =K740_1*X100*X1341 - K741_1*X1852 - K740_2*X1852
1853	X_dot(1853) =K742_1*X544*X1341 - K743_1*X1853 - K742_2*X1853
1854	X_dot(1854) =K744_1*X350*X1341 - K745_1*X1854 - K744_2*X1854
1855	X_dot(1855) =K746_1*X515*X751 - K747_1*X1855 - K746_2*X1855
1856	X_dot(1856) =K748_1*X155*X1181 - K749_1*X1856 - K748_2*X1856
1857	X_dot(1857) =K750_1*X49*X1181 - K751_1*X1857 - K750_2*X1857
1858	X_dot(1858) =K752_1*X512*X708 - K753_1*X1858 - K752_2*X1858
1859	X_dot(1859) =K754_1*X512*X594 - K755_1*X1859 - K754_2*X1859
1860	X_dot(1860) =K756_1*X119*X512 - K757_1*X1860 - K756_2*X1860
1861	X_dot(1861) =K758_1*X28*X512 - K759_1*X1861 - K758_2*X1861
1862	X_dot(1862) =K760_1*X652*X1411 - K761_1*X1862 - K760_2*X1862
1863	X_dot(1863) =K762_1*X615*X1411 - K763_1*X1863 - K762_2*X1863
1864	X_dot(1864) =K764_1*X343*X1411 - K765_1*X1864 - K764_2*X1864
1865	X_dot(1865) =K766_1*X722*X1411 - K767_1*X1865 - K766_2*X1865
1866	X_dot(1866) =K768_1*X341*X1411 - K769_1*X1866 - K768_2*X1866
1867	X_dot(1867) =K770_1*X350*X1411 - K771_1*X1867 - K770_2*X1867
1868	X_dot(1868) =K772_1*X630*X1154 - K773_1*X1868 - K772_2*X1868
1869	X_dot(1869) =K774_1*X652*X1154 - K775_1*X1869 - K774_2*X1869
1870	X_dot(1870) =K776_1*X413*X861 - K777_1*X1870 - K776_2*X1870
1871	X_dot(1871) =K778_1*X362*X861 - K779_1*X1871 - K778_2*X1871
1872	X_dot(1872) =K780_1*X334*X861 - K781_1*X1872 - K780_2*X1872
1873	X_dot(1873) =K782_1*X543*X861 - K783_1*X1873 - K782_2*X1873
1874	X_dot(1874) =K784_1*X319*X861 - K785_1*X1874 - K784_2*X1874
1875	X_dot(1875) =K786_1*X225*X861 - K787_1*X1875 - K786_2*X1875
1876	X_dot(1876) =K788_1*X536*X861 - K789_1*X1876 - K788_2*X1876
1877	X_dot(1877) =K790_1*X533*X861 - K791_1*X1877 - K790_2*X1877
1878	X_dot(1878) =K792_1*X347*X861 - K793_1*X1878 - K792_2*X1878
1879	X_dot(1879) =K794_1*X400*X861 - K795_1*X1879 - K794_2*X1879
1880	X_dot(1880) =K796_1*X418*X861 - K797_1*X1880 - K796_2*X1880
1881	X_dot(1881) =K798_1*X390*X861 - K799_1*X1881 - K798_2*X1881
1882	X_dot(1882) =K800_1*X210*X861 - K801_1*X1882 - K800_2*X1882
1883	X_dot(1883) =K802_1*X665*X861 - K803_1*X1883 - K802_2*X1883
1884	X_dot(1884) =K804_1*X760*X1370 - K805_1*X1884 - K804_2*X1884
1885	X_dot(1885) =K806_1*X74*X237 - K807_1*X1885 - K806_2*X1885
1886	X_dot(1886) =K808_1*X271*X998 - K809_1*X1886 - K808_2*X1886
1887	X_dot(1887) =K810_1*X65*X666 - K811_1*X1887 - K810_2*X1887
1888	X_dot(1888) =K812_1*X112*X846 - K813_1*X1888 - K812_2*X1888
1889	X_dot(1889) =K814_1*X231*X846 - K815_1*X1889 - K814_2*X1889
1890	X_dot(1890) =K816_1*X150*X1439 - K817_1*X1890 - K816_2*X1890
1891	X_dot(1891) =K818_1*X637*X1031 - K819_1*X1891 - K818_2*X1891
1892	X_dot(1892) =K820_1*X630*X1031 - K821_1*X1892 - K820_2*X1892
1893	X_dot(1893) =K822_1*X471*X1031 - K823_1*X1893 - K822_2*X1893
1894	X_dot(1894) =K824_1*X168*X1031 - K825_1*X1894 - K824_2*X1894
1895	X_dot(1895) =K826_1*X570*X1031 - K827_1*X1895 - K826_2*X1895
1896	X_dot(1896) =K828_1*X47*X1031 - K829_1*X1896 - K828_2*X1896
1897	X_dot(1897) =K830_1*X343*X1031 - K831_1*X1897 - K830_2*X1897
1898	X_dot(1898) =K832_1*X357*X1031 - K833_1*X1898 - K832_2*X1898
1899	X_dot(1899) =K834_1*X631*X1031 - K835_1*X1899 - K834_2*X1899
1900	X_dot(1900) =K836_1*X299*X1031 - K837_1*X1900 - K836_2*X1900
1901	X_dot(1901) =K838_1*X304*X1031 - K839_1*X1901 - K838_2*X1901
1902	X_dot(1902) =K840_1*X497*X1031 - K841_1*X1902 - K840_2*X1902
1903	X_dot(1903) =K842_1*X194*X1031 - K843_1*X1903 - K842_2*X1903
1904	X_dot(1904) =K844_1*X412*X1031 - K845_1*X1904 - K844_2*X1904
1905	X_dot(1905) =K846_1*X341*X1031 - K847_1*X1905 - K846_2*X1905
1906	X_dot(1906) =K848_1*X484*X1418 - K849_1*X1906 - K848_2*X1906
1907	X_dot(1907) =K850_1*X47*X1418 - K851_1*X1907 - K850_2*X1907
1908	X_dot(1908) =K852_1*X343*X1418 - K853_1*X1908 - K852_2*X1908
1909	X_dot(1909) =K854_1*X194*X1418 - K855_1*X1909 - K854_2*X1909
1910	X_dot(1910) =K856_1*X491*X810 - K857_1*X1910 - K856_2*X1910
1911	X_dot(1911) =K858_1*X86*X810 - K859_1*X1911 - K858_2*X1911
1912	X_dot(1912) =K860_1*X86*X522 - K861_1*X1912 - K860_2*X1912
1913	X_dot(1913) =K862_1*X522*X565 - K863_1*X1913 - K862_2*X1913
1914	X_dot(1914) =K864_1*X589*X674 - K865_1*X1914 - K864_2*X1914
1915	X_dot(1915) =K866_1*X517*X891 - K867_1*X1915 - K866_2*X1915
1916	X_dot(1916) =K868_1*X293*X891 - K869_1*X1916 - K868_2*X1916
1917	X_dot(1917) =K870_1*X272*X891 - K871_1*X1917 - K870_2*X1917
1918	X_dot(1918) =K872_1*X116*X891 - K873_1*X1918 - K872_2*X1918
1919	X_dot(1919) =K874_1*X739*X891 - K875_1*X1919 - K874_2*X1919
1920	X_dot(1920) =K876_1*X11*X891 - K877_1*X1920 - K876_2*X1920
1921	X_dot(1921) =K878_1*X272*X1000 - K879_1*X1921 - K878_2*X1921
1922	X_dot(1922) =K880_1*X116*X1000 - K881_1*X1922 - K880_2*X1922
1923	X_dot(1923) =K882_1*X595*X1000 - K883_1*X1923 - K882_2*X1923
1924	X_dot(1924) =K884_1*X499*X1393 - K885_1*X1924 - K884_2*X1924
1925	X_dot(1925) =K886_1*X645*X1391 - K887_1*X1925 - K886_2*X1925
1926	X_dot(1926) =K888_1*X239*X330 - K889_1*X1926 - K888_2*X1926
1927	X_dot(1927) =K890_1*X330*X414 - K891_1*X1927 - K890_2*X1927
1928	X_dot(1928) =K892_1*X337^2 - K893_1*X1928 - K892_2*X1928
1929	X_dot(1929) =K894_1*X484*X700 - K895_1*X1929 - K894_2*X1929
1930	X_dot(1930) =K896_1*X759*X1195 - K897_1*X1930 - K896_2*X1930

1931	X_dot(1931) =K898_1*X152*X1195 - K899_1*X1931 - K898_2*X1931
1932	X_dot(1932) =K900_1*X452*X1195 - K901_1*X1932 - K900_2*X1932
1933	X_dot(1933) =K902_1*X115*X1195 - K903_1*X1933 - K902_2*X1933
1934	X_dot(1934) =K904_1*X122*X1195 - K905_1*X1934 - K904_2*X1934
1935	X_dot(1935) =K906_1*X624*X1195 - K907_1*X1935 - K906_2*X1935
1936	X_dot(1936) =K908_1*X390*X1195 - K909_1*X1936 - K908_2*X1936
1937	X_dot(1937) =K910_1*X124*X1195 - K911_1*X1937 - K910_2*X1937
1938	X_dot(1938) =K912_1*X481*X1195 - K913_1*X1938 - K912_2*X1938
1939	X_dot(1939) =K914_1*X99*X1195 - K915_1*X1939 - K914_2*X1939
1940	X_dot(1940) =K916_1*X205*X759 - K917_1*X1940 - K916_2*X1940
1941	X_dot(1941) =K918_1*X205*X517 - K919_1*X1941 - K918_2*X1941
1942	X_dot(1942) =K920_1*X205*X624 - K921_1*X1942 - K920_2*X1942
1943	X_dot(1943) =K922_1*X205*X390 - K923_1*X1943 - K922_2*X1943
1944	X_dot(1944) =K924_1*X759*X1107 - K925_1*X1944 - K924_2*X1944
1945	X_dot(1945) =K926_1*X374*X1028 - K927_1*X1945 - K926_2*X1945
1946	X_dot(1946) =K928_1*X317*X823 - K929_1*X1946 - K928_2*X1946
1947	X_dot(1947) =K930_1*X73*X849 - K931_1*X1947 - K930_2*X1947
1948	X_dot(1948) =K932_1*X264*X783 - K933_1*X1948 - K932_2*X1948
1949	X_dot(1949) =K934_1*X498*X783 - K935_1*X1949 - K934_2*X1949
1950	X_dot(1950) =K936_1*X275*X915 - K937_1*X1950 - K936_2*X1950
1951	X_dot(1951) =K938_1*X577*X915 - K939_1*X1951 - K938_2*X1951
1952	X_dot(1952) =K940_1*X517*X915 - K941_1*X1952 - K940_2*X1952
1953	X_dot(1953) =K942_1*X161*X915 - K943_1*X1953 - K942_2*X1953
1954	X_dot(1954) =K944_1*X106*X915 - K945_1*X1954 - K944_2*X1954
1955	X_dot(1955) =K946_1*X390*X915 - K947_1*X1955 - K946_2*X1955
1956	X_dot(1956) =K948_1*X317*X1267 - K949_1*X1956 - K948_2*X1956
1957	X_dot(1957) =K950_1*X384*X831 - K951_1*X1957 - K950_2*X1957
1958	X_dot(1958) =K952_1*X384*X1172 - K953_1*X1958 - K952_2*X1958
1959	X_dot(1959) =K954_1*X281*X1408 - K955_1*X1959 - K954_2*X1959
1960	X_dot(1960) =K956_1*X630*X1271 - K957_1*X1960 - K956_2*X1960
1961	X_dot(1961) =K958_1*X168*X1271 - K959_1*X1961 - K958_2*X1961
1962	X_dot(1962) =K960_1*X384*X1271 - K961_1*X1962 - K960_2*X1962
1963	X_dot(1963) =K962_1*X86*X1271 - K963_1*X1963 - K962_2*X1963
1964	X_dot(1964) =K964_1*X451*X1271 - K965_1*X1964 - K964_2*X1964
1965	X_dot(1965) =K966_1*X499*X1271 - K967_1*X1965 - K966_2*X1965
1966	X_dot(1966) =K968_1*X475*X1271 - K969_1*X1966 - K968_2*X1966
1967	X_dot(1967) =K970_1*X9*X894 - K971_1*X1967 - K970_2*X1967
1968	X_dot(1968) =K972_1*X517*X894 - K973_1*X1968 - K972_2*X1968
1969	X_dot(1969) =K974_1*X631*X894 - K975_1*X1969 - K974_2*X1969
1970	X_dot(1970) =K976_1*X566*X894 - K977_1*X1970 - K976_2*X1970
1971	X_dot(1971) =K978_1*X384*X1270 - K979_1*X1971 - K978_2*X1971
1972	X_dot(1972) =K980_1*X281*X1270 - K981_1*X1972 - K980_2*X1972
1973	X_dot(1973) =K982_1*X181*X1270 - K983_1*X1973 - K982_2*X1973
1974	X_dot(1974) =K984_1*X636*X1270 - K985_1*X1974 - K984_2*X1974
1975	X_dot(1975) =K986_1*X341*X1270 - K987_1*X1975 - K986_2*X1975
1976	X_dot(1976) =K988_1*X441*X1273 - K989_1*X1976 - K988_2*X1976
1977	X_dot(1977) =K990_1*X414*X1282 - K991_1*X1977 - K990_2*X1977
1978	X_dot(1978) =K992_1*X637*X1005 - K993_1*X1978 - K992_2*X1978
1979	X_dot(1979) =K994_1*X517*X1005 - K995_1*X1979 - K994_2*X1979
1980	X_dot(1980) =K996_1*X281*X1005 - K997_1*X1980 - K996_2*X1980
1981	X_dot(1981) =K998_1*X499*X1377 - K999_1*X1981 - K998_2*X1981
1982	X_dot(1982) =K1000_1*X508*X925 - K1001_1*X1982 - K1000_2*X1982
1983	X_dot(1983) =K1002_1*X565*X925 - K1003_1*X1983 - K1002_2*X1983
1984	X_dot(1984) =K1004_1*X565*X1313 - K1005_1*X1984 - K1004_2*X1984
1985	X_dot(1985) =K1006_1*X129*X1313 - K1007_1*X1985 - K1006_2*X1985
1986	X_dot(1986) =K1008_1*X51*X1036 - K1009_1*X1986 - K1008_2*X1986
1987	X_dot(1987) =K1010_1*X38*X1036 - K1011_1*X1987 - K1010_2*X1987
1988	X_dot(1988) =K1012_1*X252*X1316 - K1013_1*X1988 - K1012_2*X1988
1989	X_dot(1989) =K1014_1*X496*X1006 - K1015_1*X1989 - K1014_2*X1989
1990	X_dot(1990) =K1016_1*X9*X1006 - K1017_1*X1990 - K1016_2*X1990
1991	X_dot(1991) =K1018_1*X497*X1006 - K1019_1*X1991 - K1018_2*X1991
1992	X_dot(1992) =K1020_1*X609*X1006 - K1021_1*X1992 - K1020_2*X1992
1993	X_dot(1993) =K1022_1*X187*X284 - K1023_1*X1993 - K1022_2*X1993
1994	X_dot(1994) =K1024_1*X168*X187 - K1025_1*X1994 - K1024_2*X1994
1995	X_dot(1995) =K1026_1*X172*X187 - K1027_1*X1995 - K1026_2*X1995
1996	X_dot(1996) =K1028_1*X187*X537 - K1029_1*X1996 - K1028_2*X1996
1997	X_dot(1997) =K1030_1*X187*X343 - K1031_1*X1997 - K1030_2*X1997
1998	X_dot(1998) =K1032_1*X161*X187 - K1033_1*X1998 - K1032_2*X1998
1999	X_dot(1999) =K1034_1*X95*X187 - K1035_1*X1999 - K1034_2*X1999
2000	X_dot(2000) =K1036_1*X102*X187 - K1037_1*X2000 - K1036_2*X2000
2001	X_dot(2001) =K1038_1*X187*X492 - K1039_1*X2001 - K1038_2*X2001
2002	X_dot(2002) =K1040_1*X187*X654 - K1041_1*X2002 - K1040_2*X2002
2003	X_dot(2003) =K1042_1*X187*X497 - K1043_1*X2003 - K1042_2*X2003
2004	X_dot(2004) =K1044_1*X187*X408 - K1045_1*X2004 - K1044_2*X2004
2005	X_dot(2005) =K1046_1*X48*X187 - K1047_1*X2005 - K1046_2*X2005
2006	X_dot(2006) =K1048_1*X187*X397 - K1049_1*X2006 - K1048_2*X2006
2007	X_dot(2007) =K1050_1*X187*X444 - K1051_1*X2007 - K1050_2*X2007
2008	X_dot(2008) =K1052_1*X187*X636 - K1053_1*X2008 - K1052_2*X2008
2009	X_dot(2009) =K1054_1*X187*X457 - K1055_1*X2009 - K1054_2*X2009
2010	X_dot(2010) =K1056_1*X129*X187 - K1057_1*X2010 - K1056_2*X2010

2011	X_dot(2011) =K1058_1*X658*X1318 - K1059_1*X2011 - K1058_2*X2011
2012	X_dot(2012) =K1060_1*X192*X1318 - K1061_1*X2012 - K1060_2*X2012
2013	X_dot(2013) =K1062_1*X58*X1318 - K1063_1*X2013 - K1062_2*X2013
2014	X_dot(2014) =K1064_1*X464*X1318 - K1065_1*X2014 - K1064_2*X2014
2015	X_dot(2015) =K1066_1*X252*X1318 - K1067_1*X2015 - K1066_2*X2015
2016	X_dot(2016) =K1068_1*X249*X1318 - K1069_1*X2016 - K1068_2*X2016
2017	X_dot(2017) =K1070_1*X86*X1145 - K1071_1*X2017 - K1070_2*X2017
2018	X_dot(2018) =K1072_1*X414*X1145 - K1073_1*X2018 - K1072_2*X2018
2019	X_dot(2019) =K1074_1*X173*X980 - K1075_1*X2019 - K1074_2*X2019
2020	X_dot(2020) =K1076_1*X448*X1341 - K1077_1*X2020 - K1076_2*X2020
2021	X_dot(2021) =K1078_1*X561*X1341 - K1079_1*X2021 - K1078_2*X2021
2022	X_dot(2022) =K1080_1*X129*X512 - K1081_1*X2022 - K1080_2*X2022
2023	X_dot(2023) =K1082_1*X28*X61 - K1083_1*X2023 - K1082_2*X2023
2024	X_dot(2024) =K1084_1*X630*X1411 - K1085_1*X2024 - K1084_2*X2024
2025	X_dot(2025) =K1086_1*X9*X1411 - K1087_1*X2025 - K1086_2*X2025
2026	X_dot(2026) =K1088_1*X342*X1154 - K1089_1*X2026 - K1088_2*X2026
2027	X_dot(2027) =K1090_1*X102*X1154 - K1091_1*X2027 - K1090_2*X2027
2028	X_dot(2028) =K1092_1*X104*X1154 - K1093_1*X2028 - K1092_2*X2028
2029	X_dot(2029) =K1094_1*X47*X1319 - K1095_1*X2029 - K1094_2*X2029
2030	X_dot(2030) =K1096_1*X587*X748 - K1097_1*X2030 - K1096_2*X2030
2031	X_dot(2031) =K1098_1*X389*X861 - K1099_1*X2031 - K1098_2*X2031
2032	X_dot(2032) =K1100_1*X275*X861 - K1101_1*X2032 - K1100_2*X2032
2033	X_dot(2033) =K1102_1*X90*X861 - K1103_1*X2033 - K1102_2*X2033
2034	X_dot(2034) =K1104_1*X137*X861 - K1105_1*X2034 - K1104_2*X2034
2035	X_dot(2035) =K1106_1*X157*X861 - K1107_1*X2035 - K1106_2*X2035
2036	X_dot(2036) =K1108_1*X196*X861 - K1109_1*X2036 - K1108_2*X2036
2037	X_dot(2037) =K1110_1*X290*X1059 - K1111_1*X2037 - K1110_2*X2037
2038	X_dot(2038) =K1112_1*X221*X1318 - K1113_1*X2038 - K1112_2*X2038
2039	X_dot(2039) =K1114_1*X247*X861 - K1115_1*X2039 - K1114_2*X2039
2040	X_dot(2040) =K1116_1*X207*X1159 - K1117_1*X2040 - K1116_2*X2040
2041	X_dot(2041) =K1118_1*X86*X823 - K1119_1*X2041 - K1118_2*X2041
2042	X_dot(2042) =K1120_1*X473*X783 - K1121_1*X2042 - K1120_2*X2042
2043	X_dot(2043) =K1122_1*X88*X915 - K1123_1*X2043 - K1122_2*X2043
2044	X_dot(2044) =K1124_1*X434*X915 - K1125_1*X2044 - K1124_2*X2044
2045	X_dot(2045) =K1126_1*X47*X1271 - K1127_1*X2045 - K1126_2*X2045
2046	X_dot(2046) =K1128_1*X570*X894 - K1129_1*X2046 - K1128_2*X2046
2047	X_dot(2047) =K1130_1*X47*X1270 - K1131_1*X2047 - K1130_2*X2047
2048	X_dot(2048) =K1132_1*X187*X281 - K1133_1*X2048 - K1132_2*X2048
2049	X_dot(2049) =K1134_1*X725*X1318 - K1135_1*X2049 - K1134_2*X2049
2050	X_dot(2050) =K1136_1*X129*X1318 - K1137_1*X2050 - K1136_2*X2050
2051	X_dot(2051) =K1138_1*X281*X1411 - K1139_1*X2051 - K1138_2*X2051
2052	X_dot(2052) =K1140_1*X87*X187 - K1141_1*X2052 - K1140_2*X2052
2053	X_dot(2053) =K1142_1*X339*X584 - K1143_1*X2053 - K1142_2*X2053
2054	X_dot(2054) =K1144_1*X548*X1031 - K1145_1*X2054 - K1144_2*X2054
2055	X_dot(2055) =K1146_1*X376*X1031 - K1147_1*X2055 - K1146_2*X2055
2056	X_dot(2056) =K1148_1*X375*X1031 - K1149_1*X2056 - K1148_2*X2056
2057	X_dot(2057) =K1150_1*X750*X1031 - K1151_1*X2057 - K1150_2*X2057
2058	X_dot(2058) =K1152_1*X429*X1031 - K1153_1*X2058 - K1152_2*X2058
2059	X_dot(2059) =K1154_1*X676*X1031 - K1155_1*X2059 - K1154_2*X2059
2060	X_dot(2060) =K1156_1*X308*X1031 - K1157_1*X2060 - K1156_2*X2060
2061	X_dot(2061) =K1158_1*X57*X1031 - K1159_1*X2061 - K1158_2*X2061
2062	X_dot(2062) =K1160_1*X693*X1031 - K1161_1*X2062 - K1160_2*X2062
2063	X_dot(2063) =K1162_1*X414*X1031 - K1163_1*X2063 - K1162_2*X2063
2064	X_dot(2064) =K1164_1*X376*X1418 - K1165_1*X2064 - K1164_2*X2064
2065	X_dot(2065) =K1166_1*X668*X1173 - K1167_1*X2065 - K1166_2*X2065
2066	X_dot(2066) =K1168_1*X604*X1173 - K1169_1*X2066 - K1168_2*X2066
2067	X_dot(2067) =K1170_1*X239*X1446 - K1171_1*X2067 - K1170_2*X2067
2068	X_dot(2068) =K1172_1*X414*X1446 - K1173_1*X2068 - K1172_2*X2068
2069	X_dot(2069) =K1174_1*X108*X810 - K1175_1*X2069 - K1174_2*X2069
2070	X_dot(2070) =K1176_1*X448*X1381 - K1177_1*X2070 - K1176_2*X2070
2071	X_dot(2071) =K1178_1*X385*X1183 - K1179_1*X2071 - K1178_2*X2071
2072	X_dot(2072) =K1180_1*X129*X1183 - K1181_1*X2072 - K1180_2*X2072
2073	X_dot(2073) =K1182_1*X213*X522 - K1183_1*X2073 - K1182_2*X2073
2074	X_dot(2074) =K1184_1*X522*X552 - K1185_1*X2074 - K1184_2*X2074
2075	X_dot(2075) =K1186_1*X448*X522 - K1187_1*X2075 - K1186_2*X2075
2076	X_dot(2076) =K1188_1*X280*X522 - K1189_1*X2076 - K1188_2*X2076
2077	X_dot(2077) =K1190_1*X466*X891 - K1191_1*X2077 - K1190_2*X2077
2078	X_dot(2078) =K1192_1*X491*X891 - K1193_1*X2078 - K1192_2*X2078
2079	X_dot(2079) =K1194_1*X251*X891 - K1195_1*X2079 - K1194_2*X2079
2080	X_dot(2080) =K1196_1*X470*X891 - K1197_1*X2080 - K1196_2*X2080
2081	X_dot(2081) =K1198_1*X646*X891 - K1199_1*X2081 - K1198_2*X2081
2082	X_dot(2082) =K1200_1*X194*X891 - K1201_1*X2082 - K1200_2*X2082
2083	X_dot(2083) =K1202_1*X190*X891 - K1203_1*X2083 - K1202_2*X2083
2084	X_dot(2084) =K1204_1*X394*X891 - K1205_1*X2084 - K1204_2*X2084
2085	X_dot(2085) =K1206_1*X326*X891 - K1207_1*X2085 - K1206_2*X2085
2086	X_dot(2086) =K1208_1*X609*X891 - K1209_1*X2086 - K1208_2*X2086
2087	X_dot(2087) =K1210_1*X147*X891 - K1211_1*X2087 - K1210_2*X2087
2088	X_dot(2088) =K1212_1*X621*X1000 - K1213_1*X2088 - K1212_2*X2088
2089	X_dot(2089) =K1214_1*X352*X1000 - K1215_1*X2089 - K1214_2*X2089
2090	X_dot(2090) =K1216_1*X190*X1000 - K1217_1*X2090 - K1216_2*X2090

2091	X_dot(2091) =K1218_1*X254*X1000 - K1219_1*X2091 - K1218_2*X2091
2092	X_dot(2092) =K1220_1*X66*X1000 - K1221_1*X2092 - K1220_2*X2092
2093	X_dot(2093) =K1222_1*X101*X1393 - K1223_1*X2093 - K1222_2*X2093
2094	X_dot(2094) =K1224_1*X297*X1393 - K1225_1*X2094 - K1224_2*X2094
2095	X_dot(2095) =K1226_1*X99*X1087 - K1227_1*X2095 - K1226_2*X2095
2096	X_dot(2096) =K1228_1*X99*X1451 - K1229_1*X2096 - K1228_2*X2096
2097	X_dot(2097) =K1230_1*X250*X1126 - K1231_1*X2097 - K1230_2*X2097
2098	X_dot(2098) =K1232_1*X271*X1126 - K1233_1*X2098 - K1232_2*X2098
2099	X_dot(2099) =K1234_1*X110*X1391 - K1235_1*X2099 - K1234_2*X2099
2100	X_dot(2100) =K1236_1*X51*X330 - K1237_1*X2100 - K1236_2*X2100
2101	X_dot(2101) =K1238_1*X330*X498 - K1239_1*X2101 - K1238_2*X2101
2102	X_dot(2102) =K1240_1*X193*X330 - K1241_1*X2102 - K1240_2*X2102
2103	X_dot(2103) =K1242_1*X99*X330 - K1243_1*X2103 - K1242_2*X2103
2104	X_dot(2104) =K1244_1*X611*X1195 - K1245_1*X2104 - K1244_2*X2104
2105	X_dot(2105) =K1246_1*X496*X1195 - K1247_1*X2105 - K1246_2*X2105
2106	X_dot(2106) =K1248_1*X4*X1195 - K1249_1*X2106 - K1248_2*X2106
2107	X_dot(2107) =K1250_1*X26*X1195 - K1251_1*X2107 - K1250_2*X2107
2108	X_dot(2108) =K1252_1*X133*X1195 - K1253_1*X2108 - K1252_2*X2108
2109	X_dot(2109) =K1254_1*X227*X1195 - K1255_1*X2109 - K1254_2*X2109
2110	X_dot(2110) =K1256_1*X251*X1195 - K1257_1*X2110 - K1256_2*X2110
2111	X_dot(2111) =K1258_1*X393*X1195 - K1259_1*X2111 - K1258_2*X2111
2112	X_dot(2112) =K1260_1*X161*X1195 - K1261_1*X2112 - K1260_2*X2112
2113	X_dot(2113) =K1262_1*X182*X1195 - K1263_1*X2113 - K1262_2*X2113
2114	X_dot(2114) =K1264_1*X121*X1195 - K1265_1*X2114 - K1264_2*X2114
2115	X_dot(2115) =K1266_1*X149*X1195 - K1267_1*X2115 - K1266_2*X2115
2116	X_dot(2116) =K1268_1*X281*X1195 - K1269_1*X2116 - K1268_2*X2116
2117	X_dot(2117) =K1270_1*X52*X1195 - K1271_1*X2117 - K1270_2*X2117
2118	X_dot(2118) =K1272_1*X313*X1195 - K1273_1*X2118 - K1272_2*X2118
2119	X_dot(2119) =K1274_1*X236*X1195 - K1275_1*X2119 - K1274_2*X2119
2120	X_dot(2120) =K1276_1*X194*X1195 - K1277_1*X2120 - K1276_2*X2120
2121	X_dot(2121) =K1278_1*X641*X1195 - K1279_1*X2121 - K1278_2*X2121
2122	X_dot(2122) =K1280_1*X744*X1195 - K1281_1*X2122 - K1280_2*X2122
2123	X_dot(2123) =K1282_1*X147*X1195 - K1283_1*X2123 - K1282_2*X2123
2124	X_dot(2124) =K1284_1*X205*X496 - K1285_1*X2124 - K1284_2*X2124
2125	X_dot(2125) =K1286_1*X26*X205 - K1287_1*X2125 - K1286_2*X2125
2126	X_dot(2126) =K1288_1*X133*X205 - K1289_1*X2126 - K1288_2*X2126
2127	X_dot(2127) =K1290_1*X205*X227 - K1291_1*X2127 - K1290_2*X2127
2128	X_dot(2128) =K1292_1*X182*X205 - K1293_1*X2128 - K1292_2*X2128
2129	X_dot(2129) =K1294_1*X122*X205 - K1295_1*X2129 - K1294_2*X2129
2130	X_dot(2130) =K1296_1*X52*X205 - K1297_1*X2130 - K1296_2*X2130
2131	X_dot(2131) =K1298_1*X194*X205 - K1299_1*X2131 - K1298_2*X2131
2132	X_dot(2132) =K1300_1*X205*X744 - K1301_1*X2132 - K1300_2*X2132
2133	X_dot(2133) =K1302_1*X420*X1107 - K1303_1*X2133 - K1302_2*X2133
2134	X_dot(2134) =K1304_1*X281*X1107 - K1305_1*X2134 - K1304_2*X2134
2135	X_dot(2135) =K1306_1*X578*X1107 - K1307_1*X2135 - K1306_2*X2135
2136	X_dot(2136) =K1308_1*X233*X1028 - K1309_1*X2136 - K1308_2*X2136
2137	X_dot(2137) =K1310_1*X321*X1028 - K1311_1*X2137 - K1310_2*X2137
2138	X_dot(2138) =K1312_1*X66*X1250 - K1313_1*X2138 - K1312_2*X2138
2139	X_dot(2139) =K1314_1*X477*X1250 - K1315_1*X2139 - K1314_2*X2139
2140	X_dot(2140) =K1316_1*X55*X823 - K1317_1*X2140 - K1316_2*X2140
2141	X_dot(2141) =K1318_1*X169*X823 - K1319_1*X2141 - K1318_2*X2141
2142	X_dot(2142) =K1320_1*X411*X823 - K1321_1*X2142 - K1320_2*X2142
2143	X_dot(2143) =K1322_1*X210*X823 - K1323_1*X2143 - K1322_2*X2143
2144	X_dot(2144) =K1324_1*X22*X823 - K1325_1*X2144 - K1324_2*X2144
2145	X_dot(2145) =K1326_1*X70*X823 - K1327_1*X2145 - K1326_2*X2145
2146	X_dot(2146) =K1328_1*X215*X390 - K1329_1*X2146 - K1328_2*X2146
2147	X_dot(2147) =K1330_1*X133*X849 - K1331_1*X2147 - K1330_2*X2147
2148	X_dot(2148) =K1332_1*X101*X915 - K1333_1*X2148 - K1332_2*X2148
2149	X_dot(2149) =K1334_1*X55*X915 - K1335_1*X2149 - K1334_2*X2149
2150	X_dot(2150) =K1336_1*X447*X1271 - K1337_1*X2150 - K1336_2*X2150
2151	X_dot(2151) =K1338_1*X696*X1271 - K1339_1*X2151 - K1338_2*X2151
2152	X_dot(2152) =K1340_1*X340*X1271 - K1341_1*X2152 - K1340_2*X2152
2153	X_dot(2153) =K1342_1*X325*X1271 - K1343_1*X2153 - K1342_2*X2153
2154	X_dot(2154) =K1344_1*X654*X1271 - K1345_1*X2154 - K1344_2*X2154
2155	X_dot(2155) =K1346_1*X223*X1271 - K1347_1*X2155 - K1346_2*X2155
2156	X_dot(2156) =K1348_1*X609*X1271 - K1349_1*X2156 - K1348_2*X2156
2157	X_dot(2157) =K1350_1*X604*X1271 - K1351_1*X2157 - K1350_2*X2157
2158	X_dot(2158) =K1352_1*X447*X894 - K1353_1*X2158 - K1352_2*X2158
2159	X_dot(2159) =K1354_1*X332*X894 - K1355_1*X2159 - K1354_2*X2159
2160	X_dot(2160) =K1356_1*X447*X1270 - K1357_1*X2160 - K1356_2*X2160
2161	X_dot(2161) =K1358_1*X340*X1270 - K1359_1*X2161 - K1358_2*X2161
2162	X_dot(2162) =K1360_1*X223*X1270 - K1361_1*X2162 - K1360_2*X2162
2163	X_dot(2163) =K1362_1*X604*X1270 - K1363_1*X2163 - K1362_2*X2163
2164	X_dot(2164) =K1364_1*X37*X1282 - K1365_1*X2164 - K1364_2*X2164
2165	X_dot(2165) =K1366_1*X9*X1282 - K1367_1*X2165 - K1366_2*X2165
2166	X_dot(2166) =K1368_1*X703*X1282 - K1369_1*X2166 - K1368_2*X2166
2167	X_dot(2167) =K1370_1*X341*X1282 - K1371_1*X2167 - K1370_2*X2167
2168	X_dot(2168) =K1372_1*X9*X1412 - K1373_1*X2168 - K1372_2*X2168
2169	X_dot(2169) =K1374_1*X100*X1412 - K1375_1*X2169 - K1374_2*X2169
2170	X_dot(2170) =K1376_1*X171*X1123 - K1377_1*X2170 - K1376_2*X2170

2171	X_dot(2171) =K1378_1*X332*X925 - K1379_1*X2171 - K1378_2*X2171
2172	X_dot(2172) =K1380_1*X129*X925 - K1381_1*X2172 - K1380_2*X2172
2173	X_dot(2173) =K1382_1*X406*X1313 - K1383_1*X2173 - K1382_2*X2173
2174	X_dot(2174) =K1384_1*X332*X1315 - K1385_1*X2174 - K1384_2*X2174
2175	X_dot(2175) =K1386_1*X407*X1315 - K1387_1*X2175 - K1386_2*X2175
2176	X_dot(2176) =K1388_1*X85*X1276 - K1389_1*X2176 - K1388_2*X2176
2177	X_dot(2177) =K1390_1*X275*X1276 - K1391_1*X2177 - K1390_2*X2177
2178	X_dot(2178) =K1392_1*X260*X1276 - K1393_1*X2178 - K1392_2*X2178
2179	X_dot(2179) =K1394_1*X39*X187 - K1395_1*X2179 - K1394_2*X2179
2180	X_dot(2180) =K1396_1*X187*X688 - K1397_1*X2180 - K1396_2*X2180
2181	X_dot(2181) =K1398_1*X121*X187 - K1399_1*X2181 - K1398_2*X2181
2182	X_dot(2182) =K1400_1*X149*X187 - K1401_1*X2182 - K1400_2*X2182
2183	X_dot(2183) =K1402_1*X187*X326 - K1403_1*X2183 - K1402_2*X2183
2184	X_dot(2184) =K1404_1*X187*X765 - K1405_1*X2184 - K1404_2*X2184
2185	X_dot(2185) =K1406_1*X187*X350 - K1407_1*X2185 - K1406_2*X2185
2186	X_dot(2186) =K1408_1*X69*X1318 - K1409_1*X2186 - K1408_2*X2186
2187	X_dot(2187) =K1410_1*X118*X1318 - K1411_1*X2187 - K1410_2*X2187
2188	X_dot(2188) =K1412_1*X466*X1318 - K1413_1*X2188 - K1412_2*X2188
2189	X_dot(2189) =K1414_1*X458*X1318 - K1415_1*X2189 - K1414_2*X2189
2190	X_dot(2190) =K1416_1*X113*X1318 - K1417_1*X2190 - K1416_2*X2190
2191	X_dot(2191) =K1418_1*X388*X1318 - K1419_1*X2191 - K1418_2*X2191
2192	X_dot(2192) =K1420_1*X764*X1318 - K1421_1*X2192 - K1420_2*X2192
2193	X_dot(2193) =K1422_1*X393*X1318 - K1423_1*X2193 - K1422_2*X2193
2194	X_dot(2194) =K1424_1*X108*X1318 - K1425_1*X2194 - K1424_2*X2194
2195	X_dot(2195) =K1426_1*X94*X1318 - K1427_1*X2195 - K1426_2*X2195
2196	X_dot(2196) =K1428_1*X742*X1318 - K1429_1*X2196 - K1428_2*X2196
2197	X_dot(2197) =K1430_1*X463*X1318 - K1431_1*X2197 - K1430_2*X2197
2198	X_dot(2198) =K1432_1*X145*X1318 - K1433_1*X2198 - K1432_2*X2198
2199	X_dot(2199) =K1434_1*X241*X1318 - K1435_1*X2199 - K1434_2*X2199
2200	X_dot(2200) =K1436_1*X3*X1318 - K1437_1*X2200 - K1436_2*X2200
2201	X_dot(2201) =K1438_1*X469*X1318 - K1439_1*X2201 - K1438_2*X2201
2202	X_dot(2202) =K1440_1*X157*X1318 - K1441_1*X2202 - K1440_2*X2202
2203	X_dot(2203) =K1442_1*X399*X1318 - K1443_1*X2203 - K1442_2*X2203
2204	X_dot(2204) =K1444_1*X196*X1318 - K1445_1*X2204 - K1444_2*X2204
2205	X_dot(2205) =K1446_1*X628*X1145 - K1447_1*X2205 - K1446_2*X2205
2206	X_dot(2206) =K1448_1*X421*X1145 - K1449_1*X2206 - K1448_2*X2206
2207	X_dot(2207) =K1450_1*X103*X1145 - K1451_1*X2207 - K1450_2*X2207
2208	X_dot(2208) =K1452_1*X252*X1145 - K1453_1*X2208 - K1452_2*X2208
2209	X_dot(2209) =K1454_1*X462*X1145 - K1455_1*X2209 - K1454_2*X2209
2210	X_dot(2210) =K1456_1*X249*X1145 - K1457_1*X2210 - K1456_2*X2210
2211	X_dot(2211) =K1458_1*X121*X980 - K1459_1*X2211 - K1458_2*X2211
2212	X_dot(2212) =K1460_1*X106*X980 - K1461_1*X2212 - K1460_2*X2212
2213	X_dot(2213) =K1462_1*X697*X980 - K1463_1*X2213 - K1462_2*X2213
2214	X_dot(2214) =K1464_1*X177*X1341 - K1465_1*X2214 - K1464_2*X2214
2215	X_dot(2215) =K1466_1*X370*X1341 - K1467_1*X2215 - K1466_2*X2215
2216	X_dot(2216) =K1468_1*X389*X1181 - K1469_1*X2216 - K1468_2*X2216
2217	X_dot(2217) =K1470_1*X332*X1181 - K1471_1*X2217 - K1470_2*X2217
2218	X_dot(2218) =K1472_1*X665*X1181 - K1473_1*X2218 - K1472_2*X2218
2219	X_dot(2219) =K1474_1*X512*X577 - K1475_1*X2219 - K1474_2*X2219
2220	X_dot(2220) =K1476_1*X278*X512 - K1477_1*X2220 - K1476_2*X2220
2221	X_dot(2221) =K1478_1*X750*X1411 - K1479_1*X2221 - K1478_2*X2221
2222	X_dot(2222) =K1480_1*X693*X1411 - K1481_1*X2222 - K1480_2*X2222
2223	X_dot(2223) =K1482_1*X108*X1154 - K1483_1*X2223 - K1482_2*X2223
2224	X_dot(2224) =K1484_1*X582*X1293 - K1485_1*X2224 - K1484_2*X2224
2225	X_dot(2225) =K1486_1*X102*X1293 - K1487_1*X2225 - K1486_2*X2225
2226	X_dot(2226) =K1488_1*X96*X861 - K1489_1*X2226 - K1488_2*X2226
2227	X_dot(2227) =K1490_1*X363*X861 - K1491_1*X2227 - K1490_2*X2227
2228	X_dot(2228) =K1492_1*X692*X861 - K1493_1*X2228 - K1492_2*X2228
2229	X_dot(2229) =K1494_1*X411*X861 - K1495_1*X2229 - K1494_2*X2229
2230	X_dot(2230) =K1496_1*X130*X861 - K1497_1*X2230 - K1496_2*X2230
2231	X_dot(2231) =K1498_1*X46*X861 - K1499_1*X2231 - K1498_2*X2231
2232	X_dot(2232) =K1500_1*X359*X861 - K1501_1*X2232 - K1500_2*X2232
2233	X_dot(2233) =K1502_1*X12*X670 - K1503_1*X2233 - K1502_2*X2233
2234	X_dot(2234) =K1504_1*X44*X103 - K1505_1*X2234 - K1504_2*X2234
2235	X_dot(2235) =K1506_1*X584*X671 - K1507_1*X2235 - K1506_2*X2235
2236	X_dot(2236) =K1508_1*X584*X640 - K1509_1*X2236 - K1508_2*X2236
2237	X_dot(2237) =K1510_1*X600*X1031 - K1511_1*X2237 - K1510_2*X2237
2238	X_dot(2238) =K1512_1*X231*X1031 - K1513_1*X2238 - K1512_2*X2238
2239	X_dot(2239) =K1514_1*X29*X1031 - K1515_1*X2239 - K1514_2*X2239
2240	X_dot(2240) =K1516_1*X597*X1031 - K1517_1*X2240 - K1516_2*X2240
2241	X_dot(2241) =K1518_1*X275*X1031 - K1519_1*X2241 - K1518_2*X2241
2242	X_dot(2242) =K1520_1*X238*X1031 - K1521_1*X2242 - K1520_2*X2242
2243	X_dot(2243) =K1522_1*X226*X1031 - K1523_1*X2243 - K1522_2*X2243
2244	X_dot(2244) =K1524_1*X483*X1031 - K1525_1*X2244 - K1524_2*X2244
2245	X_dot(2245) =K1526_1*X435*X1031 - K1527_1*X2245 - K1526_2*X2245
2246	X_dot(2246) =K1528_1*X489*X1031 - K1529_1*X2246 - K1528_2*X2246
2247	X_dot(2247) =K1530_1*X455*X1031 - K1531_1*X2247 - K1530_2*X2247
2248	X_dot(2248) =K1532_1*X428*X1031 - K1533_1*X2248 - K1532_2*X2248
2249	X_dot(2249) =K1534_1*X653*X1031 - K1535_1*X2249 - K1534_2*X2249
2250	X_dot(2250) =K1536_1*X382*X1031 - K1537_1*X2250 - K1536_2*X2250

2251	X_dot(2251) =K1538_1*X129*X1031 - K1539_1*X2251 - K1538_2*X2251
2252	X_dot(2252) =K1540_1*X690*X1031 - K1541_1*X2252 - K1540_2*X2252
2253	X_dot(2253) =K1542_1*X419*X1031 - K1543_1*X2253 - K1542_2*X2253
2254	X_dot(2254) =K1544_1*X29*X1418 - K1545_1*X2254 - K1544_2*X2254
2255	X_dot(2255) =K1546_1*X177*X1418 - K1547_1*X2255 - K1546_2*X2255
2256	X_dot(2256) =K1548_1*X275*X1418 - K1549_1*X2256 - K1548_2*X2256
2257	X_dot(2257) =K1550_1*X483*X1418 - K1551_1*X2257 - K1550_2*X2257
2258	X_dot(2258) =K1552_1*X169*X1418 - K1553_1*X2258 - K1552_2*X2258
2259	X_dot(2259) =K1554_1*X436*X1418 - K1555_1*X2259 - K1554_2*X2259
2260	X_dot(2260) =K1556_1*X451*X1307 - K1557_1*X2260 - K1556_2*X2260
2261	X_dot(2261) =K1558_1*X287*X1307 - K1559_1*X2261 - K1558_2*X2261
2262	X_dot(2262) =K1560_1*X643*X1173 - K1561_1*X2262 - K1560_2*X2262
2263	X_dot(2263) =K1562_1*X638*X1173 - K1563_1*X2263 - K1562_2*X2263
2264	X_dot(2264) =K1564_1*X59*X1173 - K1565_1*X2264 - K1564_2*X2264
2265	X_dot(2265) =K1566_1*X435*X1173 - K1567_1*X2265 - K1566_2*X2265
2266	X_dot(2266) =K1568_1*X424*X1173 - K1569_1*X2266 - K1568_2*X2266
2267	X_dot(2267) =K1570_1*X741*X1173 - K1571_1*X2267 - K1570_2*X2267
2268	X_dot(2268) =K1572_1*X235*X1173 - K1573_1*X2268 - K1572_2*X2268
2269	X_dot(2269) =K1574_1*X175*X1290 - K1575_1*X2269 - K1574_2*X2269
2270	X_dot(2270) =K1576_1*X168*X287 - K1577_1*X2270 - K1576_2*X2270
2271	X_dot(2271) =K1578_1*X60*X230 - K1579_1*X2271 - K1578_2*X2271
2272	X_dot(2272) =K1580_1*X174*X810 - K1581_1*X2272 - K1580_2*X2272
2273	X_dot(2273) =K1582_1*X464*X810 - K1583_1*X2273 - K1582_2*X2273
2274	X_dot(2274) =K1584_1*X552*X810 - K1585_1*X2274 - K1584_2*X2274
2275	X_dot(2275) =K1586_1*X460*X810 - K1587_1*X2275 - K1586_2*X2275
2276	X_dot(2276) =K1588_1*X497*X1381 - K1589_1*X2276 - K1588_2*X2276
2277	X_dot(2277) =K1590_1*X302*X1381 - K1591_1*X2277 - K1590_2*X2277
2278	X_dot(2278) =K1592_1*X523*X1183 - K1593_1*X2278 - K1592_2*X2278
2279	X_dot(2279) =K1594_1*X159*X1183 - K1595_1*X2279 - K1594_2*X2279
2280	X_dot(2280) =K1596_1*X41*X522 - K1597_1*X2280 - K1596_2*X2280
2281	X_dot(2281) =K1598_1*X229*X589 - K1599_1*X2281 - K1598_2*X2281
2282	X_dot(2282) =K1600_1*X338*X1194 - K1601_1*X2282 - K1600_2*X2282
2283	X_dot(2283) =K1602_1*X272*X1194 - K1603_1*X2283 - K1602_2*X2283
2284	X_dot(2284) =K1604_1*X273*X1194 - K1605_1*X2284 - K1604_2*X2284
2285	X_dot(2285) =K1606_1*X64*X1194 - K1607_1*X2285 - K1606_2*X2285
2286	X_dot(2286) =K1608_1*X682*X891 - K1609_1*X2286 - K1608_2*X2286
2287	X_dot(2287) =K1610_1*X54*X891 - K1611_1*X2287 - K1610_2*X2287
2288	X_dot(2288) =K1612_1*X494*X891 - K1613_1*X2288 - K1612_2*X2288
2289	X_dot(2289) =K1614_1*X730*X891 - K1615_1*X2289 - K1614_2*X2289
2290	X_dot(2290) =K1616_1*X256*X891 - K1617_1*X2290 - K1616_2*X2290
2291	X_dot(2291) =K1618_1*X7*X891 - K1619_1*X2291 - K1618_2*X2291
2292	X_dot(2292) =K1620_1*X20*X891 - K1621_1*X2292 - K1620_2*X2292
2293	X_dot(2293) =K1622_1*X423*X891 - K1623_1*X2293 - K1622_2*X2293
2294	X_dot(2294) =K1624_1*X420*X891 - K1625_1*X2294 - K1624_2*X2294
2295	X_dot(2295) =K1626_1*X238*X891 - K1627_1*X2295 - K1626_2*X2295
2296	X_dot(2296) =K1628_1*X585*X891 - K1629_1*X2296 - K1628_2*X2296
2297	X_dot(2297) =K1630_1*X446*X891 - K1631_1*X2297 - K1630_2*X2297
2298	X_dot(2298) =K1632_1*X634*X891 - K1633_1*X2298 - K1632_2*X2298
2299	X_dot(2299) =K1634_1*X75*X891 - K1635_1*X2299 - K1634_2*X2299
2300	X_dot(2300) =K1636_1*X365*X891 - K1637_1*X2300 - K1636_2*X2300
2301	X_dot(2301) =K1638_1*X108*X891 - K1639_1*X2301 - K1638_2*X2301
2302	X_dot(2302) =K1640_1*X94*X891 - K1641_1*X2302 - K1640_2*X2302
2303	X_dot(2303) =K1642_1*X473*X891 - K1643_1*X2303 - K1642_2*X2303
2304	X_dot(2304) =K1644_1*X614*X891 - K1645_1*X2304 - K1644_2*X2304
2305	X_dot(2305) =K1646_1*X710*X891 - K1647_1*X2305 - K1646_2*X2305
2306	X_dot(2306) =K1648_1*X171*X891 - K1649_1*X2306 - K1648_2*X2306
2307	X_dot(2307) =K1650_1*X371*X891 - K1651_1*X2307 - K1650_2*X2307
2308	X_dot(2308) =K1652_1*X316*X891 - K1653_1*X2308 - K1652_2*X2308
2309	X_dot(2309) =K1654_1*X173*X891 - K1655_1*X2309 - K1654_2*X2309
2310	X_dot(2310) =K1656_1*X223*X891 - K1657_1*X2310 - K1656_2*X2310
2311	X_dot(2311) =K1658_1*X641*X891 - K1659_1*X2311 - K1658_2*X2311
2312	X_dot(2312) =K1660_1*X124*X891 - K1661_1*X2312 - K1660_2*X2312
2313	X_dot(2313) =K1662_1*X514*X891 - K1663_1*X2313 - K1662_2*X2313
2314	X_dot(2314) =K1664_1*X99*X891 - K1665_1*X2314 - K1664_2*X2314
2315	X_dot(2315) =K1666_1*X219*X891 - K1667_1*X2315 - K1666_2*X2315
2316	X_dot(2316) =K1668_1*X335*X891 - K1669_1*X2316 - K1668_2*X2316
2317	X_dot(2317) =K1670_1*X761*X891 - K1671_1*X2317 - K1670_2*X2317
2318	X_dot(2318) =K1672_1*X211*X743 - K1673_1*X2318 - K1672_2*X2318
2319	X_dot(2319) =K1674_1*X230*X713 - K1675_1*X2319 - K1674_2*X2319
2320	X_dot(2320) =K1676_1*X572*X1000 - K1677_1*X2320 - K1676_2*X2320
2321	X_dot(2321) =K1678_1*X209*X1000 - K1679_1*X2321 - K1678_2*X2321
2322	X_dot(2322) =K1680_1*X231*X1000 - K1681_1*X2322 - K1680_2*X2322
2323	X_dot(2323) =K1682_1*X189*X1000 - K1683_1*X2323 - K1682_2*X2323
2324	X_dot(2324) =K1684_1*X20*X1000 - K1685_1*X2324 - K1684_2*X2324
2325	X_dot(2325) =K1686_1*X291*X1000 - K1687_1*X2325 - K1686_2*X2325
2326	X_dot(2326) =K1688_1*X491*X1000 - K1689_1*X2326 - K1688_2*X2326
2327	X_dot(2327) =K1690_1*X238*X1000 - K1691_1*X2327 - K1690_2*X2327
2328	X_dot(2328) =K1692_1*X446*X1000 - K1693_1*X2328 - K1692_2*X2328
2329	X_dot(2329) =K1694_1*X338*X1000 - K1695_1*X2329 - K1694_2*X2329
2330	X_dot(2330) =K1696_1*X233*X1000 - K1697_1*X2330 - K1696_2*X2330

2331	X_dot(2331) =K1698_1*X510*X1000 - K1699_1*X2331 - K1698_2*X2331
2332	X_dot(2332) =K1700_1*X607*X1000 - K1701_1*X2332 - K1700_2*X2332
2333	X_dot(2333) =K1702_1*X141*X1000 - K1703_1*X2333 - K1702_2*X2333
2334	X_dot(2334) =K1704_1*X714*X1000 - K1705_1*X2334 - K1704_2*X2334
2335	X_dot(2335) =K1706_1*X99*X1000 - K1707_1*X2335 - K1706_2*X2335
2336	X_dot(2336) =K1708_1*X335*X1000 - K1709_1*X2336 - K1708_2*X2336
2337	X_dot(2337) =K1710_1*X607*X1001 - K1711_1*X2337 - K1710_2*X2337
2338	X_dot(2338) =K1712_1*X238*X1393 - K1713_1*X2338 - K1712_2*X2338
2339	X_dot(2339) =K1714_1*X45*X1393 - K1715_1*X2339 - K1714_2*X2339
2340	X_dot(2340) =K1716_1*X308*X1393 - K1717_1*X2340 - K1716_2*X2340
2341	X_dot(2341) =K1718_1*X460*X1393 - K1719_1*X2341 - K1718_2*X2341
2342	X_dot(2342) =K1720_1*X157*X1393 - K1721_1*X2342 - K1720_2*X2342
2343	X_dot(2343) =K1722_1*X295*X1393 - K1723_1*X2343 - K1722_2*X2343
2344	X_dot(2344) =K1724_1*X755*X1393 - K1725_1*X2344 - K1724_2*X2344
2345	X_dot(2345) =K1726_1*X99*X1393 - K1727_1*X2345 - K1726_2*X2345
2346	X_dot(2346) =K1728_1*X112*X1087 - K1729_1*X2346 - K1728_2*X2346
2347	X_dot(2347) =K1730_1*X150*X1087 - K1731_1*X2347 - K1730_2*X2347
2348	X_dot(2348) =K1732_1*X230*X1451 - K1733_1*X2348 - K1732_2*X2348
2349	X_dot(2349) =K1734_1*X5*X1451 - K1735_1*X2349 - K1734_2*X2349
2350	X_dot(2350) =K1736_1*X643*X780 - K1737_1*X2350 - K1736_2*X2350
2351	X_dot(2351) =K1738_1*X377*X780 - K1739_1*X2351 - K1738_2*X2351
2352	X_dot(2352) =K1740_1*X7*X780 - K1741_1*X2352 - K1740_2*X2352
2353	X_dot(2353) =K1742_1*X14*X780 - K1743_1*X2353 - K1742_2*X2353
2354	X_dot(2354) =K1744_1*X435*X780 - K1745_1*X2354 - K1744_2*X2354
2355	X_dot(2355) =K1746_1*X111*X780 - K1747_1*X2355 - K1746_2*X2355
2356	X_dot(2356) =K1748_1*X257*X780 - K1749_1*X2356 - K1748_2*X2356
2357	X_dot(2357) =K1750_1*X507*X1126 - K1751_1*X2357 - K1750_2*X2357
2358	X_dot(2358) =K1752_1*X201*X1126 - K1753_1*X2358 - K1752_2*X2358
2359	X_dot(2359) =K1754_1*X628*X1159 - K1755_1*X2359 - K1754_2*X2359
2360	X_dot(2360) =K1756_1*X435*X1159 - K1757_1*X2360 - K1756_2*X2360
2361	X_dot(2361) =K1758_1*X633*X1159 - K1759_1*X2361 - K1758_2*X2361
2362	X_dot(2362) =K1760_1*X264*X587 - K1761_1*X2362 - K1760_2*X2362
2363	X_dot(2363) =K1762_1*X177*X330 - K1763_1*X2363 - K1762_2*X2363
2364	X_dot(2364) =K1764_1*X33*X331 - K1765_1*X2364 - K1764_2*X2364
2365	X_dot(2365) =K1766_1*X17*X331 - K1767_1*X2365 - K1766_2*X2365
2366	X_dot(2366) =K1768_1*X99*X331 - K1769_1*X2366 - K1768_2*X2366
2367	X_dot(2367) =K1770_1*X331*X657 - K1771_1*X2367 - K1770_2*X2367
2368	X_dot(2368) =K1772_1*X33*X337 - K1773_1*X2368 - K1772_2*X2368
2369	X_dot(2369) =K1774_1*X17*X337 - K1775_1*X2369 - K1774_2*X2369
2370	X_dot(2370) =K1776_1*X269*X1195 - K1777_1*X2370 - K1776_2*X2370
2371	X_dot(2371) =K1778_1*X264*X1195 - K1779_1*X2371 - K1778_2*X2371
2372	X_dot(2372) =K1780_1*X626*X1195 - K1781_1*X2372 - K1780_2*X2372
2373	X_dot(2373) =K1782_1*X368*X1195 - K1783_1*X2373 - K1782_2*X2373
2374	X_dot(2374) =K1784_1*X311*X1195 - K1785_1*X2374 - K1784_2*X2374
2375	X_dot(2375) =K1786_1*X256*X1195 - K1787_1*X2375 - K1786_2*X2375
2376	X_dot(2376) =K1788_1*X112*X1195 - K1789_1*X2376 - K1788_2*X2376
2377	X_dot(2377) =K1790_1*X259*X1195 - K1791_1*X2377 - K1790_2*X2377
2378	X_dot(2378) =K1792_1*X736*X1195 - K1793_1*X2378 - K1792_2*X2378
2379	X_dot(2379) =K1794_1*X723*X1195 - K1795_1*X2379 - K1794_2*X2379
2380	X_dot(2380) =K1796_1*X285*X1195 - K1797_1*X2380 - K1796_2*X2380
2381	X_dot(2381) =K1798_1*X208*X1195 - K1799_1*X2381 - K1798_2*X2381
2382	X_dot(2382) =K1800_1*X380*X1195 - K1801_1*X2382 - K1800_2*X2382
2383	X_dot(2383) =K1802_1*X465*X1195 - K1803_1*X2383 - K1802_2*X2383
2384	X_dot(2384) =K1804_1*X520*X1195 - K1805_1*X2384 - K1804_2*X2384
2385	X_dot(2385) =K1806_1*X627*X1195 - K1807_1*X2385 - K1806_2*X2385
2386	X_dot(2386) =K1808_1*X338*X1195 - K1809_1*X2386 - K1808_2*X2386
2387	X_dot(2387) =K1810_1*X540*X1195 - K1811_1*X2387 - K1810_2*X2387
2388	X_dot(2388) =K1812_1*X282*X1195 - K1813_1*X2388 - K1812_2*X2388
2389	X_dot(2389) =K1814_1*X19*X1195 - K1815_1*X2389 - K1814_2*X2389
2390	X_dot(2390) =K1816_1*X71*X1195 - K1817_1*X2390 - K1816_2*X2390
2391	X_dot(2391) =K1818_1*X328*X1195 - K1819_1*X2391 - K1818_2*X2391
2392	X_dot(2392) =K1820_1*X297*X1195 - K1821_1*X2392 - K1820_2*X2392
2393	X_dot(2393) =K1822_1*X43*X1195 - K1823_1*X2393 - K1822_2*X2393
2394	X_dot(2394) =K1824_1*X555*X1195 - K1825_1*X2394 - K1824_2*X2394
2395	X_dot(2395) =K1826_1*X316*X1195 - K1827_1*X2395 - K1826_2*X2395
2396	X_dot(2396) =K1828_1*X718*X1195 - K1829_1*X2396 - K1828_2*X2396
2397	X_dot(2397) =K1830_1*X107*X1195 - K1831_1*X2397 - K1830_2*X2397
2398	X_dot(2398) =K1832_1*X454*X1195 - K1833_1*X2398 - K1832_2*X2398
2399	X_dot(2399) =K1834_1*X595*X1195 - K1835_1*X2399 - K1834_2*X2399
2400	X_dot(2400) =K1836_1*X201*X1195 - K1837_1*X2400 - K1836_2*X2400
2401	X_dot(2401) =K1838_1*X235*X1195 - K1839_1*X2401 - K1838_2*X2401
2402	X_dot(2402) =K1840_1*X205*X441 - K1841_1*X2402 - K1840_2*X2402
2403	X_dot(2403) =K1842_1*X538*X1028 - K1843_1*X2403 - K1842_2*X2403
2404	X_dot(2404) =K1844_1*X99*X1028 - K1845_1*X2404 - K1844_2*X2404
2405	X_dot(2405) =K1846_1*X526*X1250 - K1847_1*X2405 - K1846_2*X2405
2406	X_dot(2406) =K1848_1*X99*X1250 - K1849_1*X2406 - K1848_2*X2406
2407	X_dot(2407) =K1850_1*X96*X823 - K1851_1*X2407 - K1850_2*X2407
2408	X_dot(2408) =K1852_1*X305*X823 - K1853_1*X2408 - K1852_2*X2408
2409	X_dot(2409) =K1854_1*X217*X823 - K1855_1*X2409 - K1854_2*X2409
2410	X_dot(2410) =K1856_1*X513*X823 - K1857_1*X2410 - K1856_2*X2410

2411	X_dot(2411) =K1858_1*X131*X823 - K1859_1*X2411 - K1858_2*X2411
2412	X_dot(2412) =K1860_1*X27*X823 - K1861_1*X2412 - K1860_2*X2412
2413	X_dot(2413) =K1862_1*X151*X823 - K1863_1*X2413 - K1862_2*X2413
2414	X_dot(2414) =K1864_1*X16*X823 - K1865_1*X2414 - K1864_2*X2414
2415	X_dot(2415) =K1866_1*X460*X823 - K1867_1*X2415 - K1866_2*X2415
2416	X_dot(2416) =K1868_1*X194*X823 - K1869_1*X2416 - K1868_2*X2416
2417	X_dot(2417) =K1870_1*X302*X823 - K1871_1*X2417 - K1870_2*X2417
2418	X_dot(2418) =K1872_1*X358*X823 - K1873_1*X2418 - K1872_2*X2418
2419	X_dot(2419) =K1874_1*X366*X823 - K1875_1*X2419 - K1874_2*X2419
2420	X_dot(2420) =K1876_1*X203*X1260 - K1877_1*X2420 - K1876_2*X2420
2421	X_dot(2421) =K1878_1*X554*X1260 - K1879_1*X2421 - K1878_2*X2421
2422	X_dot(2422) =K1880_1*X99*X1260 - K1881_1*X2422 - K1880_2*X2422
2423	X_dot(2423) =K1882_1*X247*X1061 - K1883_1*X2423 - K1882_2*X2423
2424	X_dot(2424) =K1884_1*X647*X893 - K1885_1*X2424 - K1884_2*X2424
2425	X_dot(2425) =K1886_1*X215*X411 - K1887_1*X2425 - K1886_2*X2425
2426	X_dot(2426) =K1888_1*X169*X849 - K1889_1*X2426 - K1888_2*X2426
2427	X_dot(2427) =K1890_1*X105*X849 - K1891_1*X2427 - K1890_2*X2427
2428	X_dot(2428) =K1892_1*X342*X783 - K1893_1*X2428 - K1892_2*X2428
2429	X_dot(2429) =K1894_1*X341*X783 - K1895_1*X2429 - K1894_2*X2429
2430	X_dot(2430) =K1896_1*X33*X915 - K1897_1*X2430 - K1896_2*X2430
2431	X_dot(2431) =K1898_1*X471*X915 - K1899_1*X2431 - K1898_2*X2431
2432	X_dot(2432) =K1900_1*X189*X915 - K1901_1*X2432 - K1900_2*X2432
2433	X_dot(2433) =K1902_1*X179*X915 - K1903_1*X2433 - K1902_2*X2433
2434	X_dot(2434) =K1904_1*X634*X915 - K1905_1*X2434 - K1904_2*X2434
2435	X_dot(2435) =K1906_1*X453*X915 - K1907_1*X2435 - K1906_2*X2435
2436	X_dot(2436) =K1908_1*X651*X915 - K1909_1*X2436 - K1908_2*X2436
2437	X_dot(2437) =K1910_1*X125*X1267 - K1911_1*X2437 - K1910_2*X2437
2438	X_dot(2438) =K1912_1*X281*X1267 - K1913_1*X2438 - K1912_2*X2438
2439	X_dot(2439) =K1914_1*X756*X1267 - K1915_1*X2439 - K1914_2*X2439
2440	X_dot(2440) =K1916_1*X38*X1267 - K1917_1*X2440 - K1916_2*X2440
2441	X_dot(2441) =K1918_1*X11*X1267 - K1919_1*X2441 - K1918_2*X2441
2442	X_dot(2442) =K1920_1*X264*X1310 - K1921_1*X2442 - K1920_2*X2442
2443	X_dot(2443) =K1922_1*X527*X1310 - K1923_1*X2443 - K1922_2*X2443
2444	X_dot(2444) =K1924_1*X2*X1310 - K1925_1*X2444 - K1924_2*X2444
2445	X_dot(2445) =K1926_1*X295*X1003 - K1927_1*X2445 - K1926_2*X2445
2446	X_dot(2446) =K1928_1*X240*X831 - K1929_1*X2446 - K1928_2*X2446
2447	X_dot(2447) =K1930_1*X246*X1172 - K1931_1*X2447 - K1930_2*X2447
2448	X_dot(2448) =K1932_1*X240*X1172 - K1933_1*X2448 - K1932_2*X2448
2449	X_dot(2449) =K1934_1*X313*X787 - K1935_1*X2449 - K1934_2*X2449
2450	X_dot(2450) =K1936_1*X317*X1407 - K1937_1*X2450 - K1936_2*X2450
2451	X_dot(2451) =K1938_1*X34*X1407 - K1939_1*X2451 - K1938_2*X2451
2452	X_dot(2452) =K1940_1*X313*X1407 - K1941_1*X2452 - K1940_2*X2452
2453	X_dot(2453) =K1942_1*X312*X1408 - K1943_1*X2453 - K1942_2*X2453
2454	X_dot(2454) =K1944_1*X313*X1408 - K1945_1*X2454 - K1944_2*X2454
2455	X_dot(2455) =K1946_1*X451*X749 - K1947_1*X2455 - K1946_2*X2455
2456	X_dot(2456) =K1948_1*X712*X1274 - K1949_1*X2456 - K1948_2*X2456
2457	X_dot(2457) =K1950_1*X664*X1275 - K1951_1*X2457 - K1950_2*X2457
2458	X_dot(2458) =K1952_1*X451*X1004 - K1953_1*X2458 - K1952_2*X2458
2459	X_dot(2459) =K1954_1*X287*X1004 - K1955_1*X2459 - K1954_2*X2459
2460	X_dot(2460) =K1956_1*X314*X1004 - K1957_1*X2460 - K1956_2*X2460
2461	X_dot(2461) =K1958_1*X373*X1004 - K1959_1*X2461 - K1958_2*X2461
2462	X_dot(2462) =K1960_1*X608*X1271 - K1961_1*X2462 - K1960_2*X2462
2463	X_dot(2463) =K1962_1*X632*X1271 - K1963_1*X2463 - K1962_2*X2463
2464	X_dot(2464) =K1964_1*X170*X1271 - K1965_1*X2464 - K1964_2*X2464
2465	X_dot(2465) =K1966_1*X243*X1271 - K1967_1*X2465 - K1966_2*X2465
2466	X_dot(2466) =K1968_1*X188*X1271 - K1969_1*X2466 - K1968_2*X2466
2467	X_dot(2467) =K1970_1*X231*X1271 - K1971_1*X2467 - K1970_2*X2467
2468	X_dot(2468) =K1972_1*X189*X1271 - K1973_1*X2468 - K1972_2*X2468
2469	X_dot(2469) =K1974_1*X423*X1271 - K1975_1*X2469 - K1974_2*X2469
2470	X_dot(2470) =K1976_1*X536*X1271 - K1977_1*X2470 - K1976_2*X2470
2471	X_dot(2471) =K1978_1*X374*X1271 - K1979_1*X2471 - K1978_2*X2471
2472	X_dot(2472) =K1980_1*X203*X1271 - K1981_1*X2472 - K1980_2*X2472
2473	X_dot(2473) =K1982_1*X483*X1271 - K1983_1*X2473 - K1982_2*X2473
2474	X_dot(2474) =K1984_1*X740*X1271 - K1985_1*X2474 - K1984_2*X2474
2475	X_dot(2475) =K1986_1*X426*X1271 - K1987_1*X2475 - K1986_2*X2475
2476	X_dot(2476) =K1988_1*X435*X1271 - K1989_1*X2476 - K1988_2*X2476
2477	X_dot(2477) =K1990_1*X186*X1271 - K1991_1*X2477 - K1990_2*X2477
2478	X_dot(2478) =K1992_1*X470*X1271 - K1993_1*X2478 - K1992_2*X2478
2479	X_dot(2479) =K1994_1*X333*X1271 - K1995_1*X2479 - K1994_2*X2479
2480	X_dot(2480) =K1996_1*X473*X1271 - K1997_1*X2480 - K1996_2*X2480
2481	X_dot(2481) =K1998_1*X562*X1271 - K1999_1*X2481 - K1998_2*X2481
2482	X_dot(2482) =K2000_1*X88*X1271 - K2001_1*X2482 - K2000_2*X2482
2483	X_dot(2483) =K2002_1*X135*X1271 - K2003_1*X2483 - K2002_2*X2483
2484	X_dot(2484) =K2004_1*X250*X1271 - K2005_1*X2484 - K2004_2*X2484
2485	X_dot(2485) =K2006_1*X253*X1271 - K2007_1*X2485 - K2006_2*X2485
2486	X_dot(2486) =K2008_1*X359*X1271 - K2009_1*X2486 - K2008_2*X2486
2487	X_dot(2487) =K2010_1*X204*X1271 - K2011_1*X2487 - K2010_2*X2487
2488	X_dot(2488) =K2012_1*X124*X1271 - K2013_1*X2488 - K2012_2*X2488
2489	X_dot(2489) =K2014_1*X450*X1271 - K2015_1*X2489 - K2014_2*X2489
2490	X_dot(2490) =K2016_1*X706*X1271 - K2017_1*X2490 - K2016_2*X2490

2491	X_dot(2491) =K2018_1*X524*X1271 - K2019_1*X2491 - K2018_2*X2491
2492	X_dot(2492) =K2020_1*X153*X1271 - K2021_1*X2492 - K2020_2*X2492
2493	X_dot(2493) =K2022_1*X99*X1272 - K2023_1*X2493 - K2022_2*X2493
2494	X_dot(2494) =K2024_1*X170*X894 - K2025_1*X2494 - K2024_2*X2494
2495	X_dot(2495) =K2026_1*X728*X894 - K2027_1*X2495 - K2026_2*X2495
2496	X_dot(2496) =K2028_1*X551*X894 - K2029_1*X2496 - K2028_2*X2496
2497	X_dot(2497) =K2030_1*X564*X894 - K2031_1*X2497 - K2030_2*X2497
2498	X_dot(2498) =K2032_1*X305*X894 - K2033_1*X2498 - K2032_2*X2498
2499	X_dot(2499) =K2034_1*X47*X894 - K2035_1*X2499 - K2034_2*X2499
2500	X_dot(2500) =K2036_1*X435*X894 - K2037_1*X2500 - K2036_2*X2500
2501	X_dot(2501) =K2038_1*X424*X894 - K2039_1*X2501 - K2038_2*X2501
2502	X_dot(2502) =K2040_1*X67*X894 - K2041_1*X2502 - K2040_2*X2502
2503	X_dot(2503) =K2042_1*X204*X894 - K2043_1*X2503 - K2042_2*X2503
2504	X_dot(2504) =K2044_1*X295*X894 - K2045_1*X2504 - K2044_2*X2504
2505	X_dot(2505) =K2046_1*X99*X894 - K2047_1*X2505 - K2046_2*X2505
2506	X_dot(2506) =K2048_1*X608*X1270 - K2049_1*X2506 - K2048_2*X2506
2507	X_dot(2507) =K2050_1*X431*X1270 - K2051_1*X2507 - K2050_2*X2507
2508	X_dot(2508) =K2052_1*X632*X1270 - K2053_1*X2508 - K2052_2*X2508
2509	X_dot(2509) =K2054_1*X170*X1270 - K2055_1*X2509 - K2054_2*X2509
2510	X_dot(2510) =K2056_1*X728*X1270 - K2057_1*X2510 - K2056_2*X2510
2511	X_dot(2511) =K2058_1*X243*X1270 - K2059_1*X2511 - K2058_2*X2511
2512	X_dot(2512) =K2060_1*X188*X1270 - K2061_1*X2512 - K2060_2*X2512
2513	X_dot(2513) =K2062_1*X189*X1270 - K2063_1*X2513 - K2062_2*X2513
2514	X_dot(2514) =K2064_1*X536*X1270 - K2065_1*X2514 - K2064_2*X2514
2515	X_dot(2515) =K2066_1*X87*X1270 - K2067_1*X2515 - K2066_2*X2515
2516	X_dot(2516) =K2068_1*X426*X1270 - K2069_1*X2516 - K2068_2*X2516
2517	X_dot(2517) =K2070_1*X435*X1270 - K2071_1*X2517 - K2070_2*X2517
2518	X_dot(2518) =K2072_1*X721*X1270 - K2073_1*X2518 - K2072_2*X2518
2519	X_dot(2519) =K2074_1*X106*X1270 - K2075_1*X2519 - K2074_2*X2519
2520	X_dot(2520) =K2076_1*X186*X1270 - K2077_1*X2520 - K2076_2*X2520
2521	X_dot(2521) =K2078_1*X549*X1270 - K2079_1*X2521 - K2078_2*X2521
2522	X_dot(2522) =K2080_1*X333*X1270 - K2081_1*X2522 - K2080_2*X2522
2523	X_dot(2523) =K2082_1*X473*X1270 - K2083_1*X2523 - K2082_2*X2523
2524	X_dot(2524) =K2084_1*X562*X1270 - K2085_1*X2524 - K2084_2*X2524
2525	X_dot(2525) =K2086_1*X109*X1270 - K2087_1*X2525 - K2086_2*X2525
2526	X_dot(2526) =K2088_1*X325*X1270 - K2089_1*X2526 - K2088_2*X2526
2527	X_dot(2527) =K2090_1*X250*X1270 - K2091_1*X2527 - K2090_2*X2527
2528	X_dot(2528) =K2092_1*X359*X1270 - K2093_1*X2528 - K2092_2*X2528
2529	X_dot(2529) =K2094_1*X67*X1270 - K2095_1*X2529 - K2094_2*X2529
2530	X_dot(2530) =K2096_1*X714*X1270 - K2097_1*X2530 - K2096_2*X2530
2531	X_dot(2531) =K2098_1*X206*X1270 - K2099_1*X2531 - K2098_2*X2531
2532	X_dot(2532) =K2100_1*X124*X1270 - K2101_1*X2532 - K2100_2*X2532
2533	X_dot(2533) =K2102_1*X450*X1270 - K2103_1*X2533 - K2102_2*X2533
2534	X_dot(2534) =K2104_1*X706*X1270 - K2105_1*X2534 - K2104_2*X2534
2535	X_dot(2535) =K2106_1*X295*X1270 - K2107_1*X2535 - K2106_2*X2535
2536	X_dot(2536) =K2108_1*X99*X1270 - K2109_1*X2536 - K2108_2*X2536
2537	X_dot(2537) =K2110_1*X466*X1273 - K2111_1*X2537 - K2110_2*X2537
2538	X_dot(2538) =K2112_1*X101*X1273 - K2113_1*X2538 - K2112_2*X2538
2539	X_dot(2539) =K2114_1*X170*X1273 - K2115_1*X2539 - K2114_2*X2539
2540	X_dot(2540) =K2116_1*X175*X1273 - K2117_1*X2540 - K2116_2*X2540
2541	X_dot(2541) =K2118_1*X435*X1273 - K2119_1*X2541 - K2118_2*X2541
2542	X_dot(2542) =K2120_1*X400*X1273 - K2121_1*X2542 - K2120_2*X2542
2543	X_dot(2543) =K2122_1*X539*X1273 - K2123_1*X2543 - K2122_2*X2543
2544	X_dot(2544) =K2124_1*X645*X1273 - K2125_1*X2544 - K2124_2*X2544
2545	X_dot(2545) =K2126_1*X186*X1273 - K2127_1*X2545 - K2126_2*X2545
2546	X_dot(2546) =K2128_1*X313*X1273 - K2129_1*X2546 - K2128_2*X2546
2547	X_dot(2547) =K2130_1*X473*X1273 - K2131_1*X2547 - K2130_2*X2547
2548	X_dot(2548) =K2132_1*X641*X1273 - K2133_1*X2548 - K2132_2*X2548
2549	X_dot(2549) =K2134_1*X124*X1273 - K2135_1*X2549 - K2134_2*X2549
2550	X_dot(2550) =K2136_1*X495*X1273 - K2137_1*X2550 - K2136_2*X2550
2551	X_dot(2551) =K2138_1*X99*X1273 - K2139_1*X2551 - K2138_2*X2551
2552	X_dot(2552) =K2140_1*X170*X1103 - K2141_1*X2552 - K2140_2*X2552
2553	X_dot(2553) =K2142_1*X728*X1103 - K2143_1*X2553 - K2142_2*X2553
2554	X_dot(2554) =K2144_1*X275*X1103 - K2145_1*X2554 - K2144_2*X2554
2555	X_dot(2555) =K2146_1*X175*X1103 - K2147_1*X2555 - K2146_2*X2555
2556	X_dot(2556) =K2148_1*X435*X1103 - K2149_1*X2556 - K2148_2*X2556
2557	X_dot(2557) =K2150_1*X106*X1103 - K2151_1*X2557 - K2150_2*X2557
2558	X_dot(2558) =K2152_1*X88*X1103 - K2153_1*X2558 - K2152_2*X2558
2559	X_dot(2559) =K2154_1*X295*X1103 - K2155_1*X2559 - K2154_2*X2559
2560	X_dot(2560) =K2156_1*X99*X1103 - K2157_1*X2560 - K2156_2*X2560
2561	X_dot(2561) =K2158_1*X711*X1282 - K2159_1*X2561 - K2158_2*X2561
2562	X_dot(2562) =K2160_1*X733*X1282 - K2161_1*X2562 - K2160_2*X2562
2563	X_dot(2563) =K2162_1*X82*X1282 - K2163_1*X2563 - K2162_2*X2563
2564	X_dot(2564) =K2164_1*X177*X1282 - K2165_1*X2564 - K2164_2*X2564
2565	X_dot(2565) =K2166_1*X564*X1282 - K2167_1*X2565 - K2166_2*X2565
2566	X_dot(2566) =K2168_1*X71*X1282 - K2169_1*X2566 - K2168_2*X2566
2567	X_dot(2567) =K2170_1*X373*X1282 - K2171_1*X2567 - K2170_2*X2567
2568	X_dot(2568) =K2172_1*X596*X619 - K2173_1*X2568 - K2172_2*X2568
2569	X_dot(2569) =K2174_1*X323*X596 - K2175_1*X2569 - K2174_2*X2569
2570	X_dot(2570) =K2176_1*X128*X970 - K2177_1*X2570 - K2176_2*X2570

2571	X_dot(2571) =K2178_1*X460*X970 - K2179_1*X2571 - K2178_2*X2571
2572	X_dot(2572) =K2180_1*X683*X1005 - K2181_1*X2572 - K2180_2*X2572
2573	X_dot(2573) =K2182_1*X295*X1005 - K2183_1*X2573 - K2182_2*X2573
2574	X_dot(2574) =K2184_1*X165*X1010 - K2185_1*X2574 - K2184_2*X2574
2575	X_dot(2575) =K2186_1*X229*X1010 - K2187_1*X2575 - K2186_2*X2575
2576	X_dot(2576) =K2188_1*X616*X1305 - K2189_1*X2576 - K2188_2*X2576
2577	X_dot(2577) =K2190_1*X381*X1377 - K2191_1*X2577 - K2190_2*X2577
2578	X_dot(2578) =K2192_1*X138*X925 - K2193_1*X2578 - K2192_2*X2578
2579	X_dot(2579) =K2194_1*X30*X925 - K2195_1*X2579 - K2194_2*X2579
2580	X_dot(2580) =K2196_1*X275*X925 - K2197_1*X2580 - K2196_2*X2580
2581	X_dot(2581) =K2198_1*X536*X925 - K2199_1*X2581 - K2198_2*X2581
2582	X_dot(2582) =K2200_1*X432*X925 - K2201_1*X2582 - K2200_2*X2582
2583	X_dot(2583) =K2202_1*X213*X925 - K2203_1*X2583 - K2202_2*X2583
2584	X_dot(2584) =K2204_1*X424*X925 - K2205_1*X2584 - K2204_2*X2584
2585	X_dot(2585) =K2206_1*X511*X925 - K2207_1*X2585 - K2206_2*X2585
2586	X_dot(2586) =K2208_1*X742*X925 - K2209_1*X2586 - K2208_2*X2586
2587	X_dot(2587) =K2210_1*X288*X925 - K2211_1*X2587 - K2210_2*X2587
2588	X_dot(2588) =K2212_1*X373*X925 - K2213_1*X2588 - K2212_2*X2588
2589	X_dot(2589) =K2214_1*X659*X925 - K2215_1*X2589 - K2214_2*X2589
2590	X_dot(2590) =K2216_1*X106*X1313 - K2217_1*X2590 - K2216_2*X2590
2591	X_dot(2591) =K2218_1*X501*X1313 - K2219_1*X2591 - K2218_2*X2591
2592	X_dot(2592) =K2220_1*X275*X1315 - K2221_1*X2592 - K2220_2*X2592
2593	X_dot(2593) =K2222_1*X195*X1315 - K2223_1*X2593 - K2222_2*X2593
2594	X_dot(2594) =K2224_1*X84*X1315 - K2225_1*X2594 - K2224_2*X2594
2595	X_dot(2595) =K2226_1*X424*X1082 - K2227_1*X2595 - K2226_2*X2595
2596	X_dot(2596) =K2228_1*X467*X1082 - K2229_1*X2596 - K2228_2*X2596
2597	X_dot(2597) =K2230_1*X264*X1036 - K2231_1*X2597 - K2230_2*X2597
2598	X_dot(2598) =K2232_1*X287*X1036 - K2233_1*X2598 - K2232_2*X2598
2599	X_dot(2599) =K2234_1*X573*X1036 - K2235_1*X2599 - K2234_2*X2599
2600	X_dot(2600) =K2236_1*X574*X1036 - K2237_1*X2600 - K2236_2*X2600
2601	X_dot(2601) =K2238_1*X184*X1036 - K2239_1*X2601 - K2238_2*X2601
2602	X_dot(2602) =K2240_1*X359*X1036 - K2241_1*X2602 - K2240_2*X2602
2603	X_dot(2603) =K2242_1*X698*X1036 - K2243_1*X2603 - K2242_2*X2603
2604	X_dot(2604) =K2244_1*X264*X591 - K2245_1*X2604 - K2244_2*X2604
2605	X_dot(2605) =K2246_1*X163*X295 - K2247_1*X2605 - K2246_2*X2605
2606	X_dot(2606) =K2248_1*X712*X1316 - K2249_1*X2606 - K2248_2*X2606
2607	X_dot(2607) =K2250_1*X575*X1316 - K2251_1*X2607 - K2250_2*X2607
2608	X_dot(2608) =K2252_1*X288*X1316 - K2253_1*X2608 - K2252_2*X2608
2609	X_dot(2609) =K2254_1*X682*X1276 - K2255_1*X2609 - K2254_2*X2609
2610	X_dot(2610) =K2256_1*X54*X1276 - K2257_1*X2610 - K2256_2*X2610
2611	X_dot(2611) =K2258_1*X588*X1276 - K2259_1*X2611 - K2258_2*X2611
2612	X_dot(2612) =K2260_1*X259*X1276 - K2261_1*X2612 - K2260_2*X2612
2613	X_dot(2613) =K2262_1*X535*X1276 - K2263_1*X2613 - K2262_2*X2613
2614	X_dot(2614) =K2264_1*X17*X1276 - K2265_1*X2614 - K2264_2*X2614
2615	X_dot(2615) =K2266_1*X75*X1276 - K2267_1*X2615 - K2266_2*X2615
2616	X_dot(2616) =K2268_1*X116*X1276 - K2269_1*X2616 - K2268_2*X2616
2617	X_dot(2617) =K2270_1*X639*X1276 - K2271_1*X2617 - K2270_2*X2617
2618	X_dot(2618) =K2272_1*X516*X1276 - K2273_1*X2618 - K2272_2*X2618
2619	X_dot(2619) =K2274_1*X680*X1276 - K2275_1*X2619 - K2274_2*X2619
2620	X_dot(2620) =K2276_1*X407*X1276 - K2277_1*X2620 - K2276_2*X2620
2621	X_dot(2621) =K2278_1*X147*X1276 - K2279_1*X2621 - K2278_2*X2621
2622	X_dot(2622) =K2280_1*X685*X1276 - K2281_1*X2622 - K2280_2*X2622
2623	X_dot(2623) =K2282_1*X129*X1276 - K2283_1*X2623 - K2282_2*X2623
2624	X_dot(2624) =K2284_1*X235*X1276 - K2285_1*X2624 - K2284_2*X2624
2625	X_dot(2625) =K2286_1*X484*X1006 - K2287_1*X2625 - K2286_2*X2625
2626	X_dot(2626) =K2288_1*X679*X1006 - K2289_1*X2626 - K2288_2*X2626
2627	X_dot(2627) =K2290_1*X360*X1006 - K2291_1*X2627 - K2290_2*X2627
2628	X_dot(2628) =K2292_1*X754*X1006 - K2293_1*X2628 - K2292_2*X2628
2629	X_dot(2629) =K2294_1*X99*X1006 - K2295_1*X2629 - K2294_2*X2629
2630	X_dot(2630) =K2296_1*X187*X583 - K2297_1*X2630 - K2296_2*X2630
2631	X_dot(2631) =K2298_1*X187*X502 - K2299_1*X2631 - K2298_2*X2631
2632	X_dot(2632) =K2300_1*X174*X187 - K2301_1*X2632 - K2300_2*X2632
2633	X_dot(2633) =K2302_1*X187*X610 - K2303_1*X2633 - K2302_2*X2633
2634	X_dot(2634) =K2304_1*X187*X702 - K2305_1*X2634 - K2304_2*X2634
2635	X_dot(2635) =K2306_1*X98*X187 - K2307_1*X2635 - K2306_2*X2635
2636	X_dot(2636) =K2308_1*X187*X213 - K2309_1*X2636 - K2308_2*X2636
2637	X_dot(2637) =K2310_1*X187*X435 - K2311_1*X2637 - K2310_2*X2637
2638	X_dot(2638) =K2312_1*X187*X532 - K2313_1*X2638 - K2312_2*X2638
2639	X_dot(2639) =K2314_1*X187*X441 - K2315_1*X2639 - K2314_2*X2639
2640	X_dot(2640) =K2316_1*X187*X710 - K2317_1*X2640 - K2316_2*X2640
2641	X_dot(2641) =K2318_1*X187*X207 - K2319_1*X2641 - K2318_2*X2641
2642	X_dot(2642) =K2320_1*X187*X545 - K2321_1*X2642 - K2320_2*X2642
2643	X_dot(2643) =K2322_1*X187*X516 - K2323_1*X2643 - K2322_2*X2643
2644	X_dot(2644) =K2324_1*X187*X541 - K2325_1*X2644 - K2324_2*X2644
2645	X_dot(2645) =K2326_1*X187*X396 - K2327_1*X2645 - K2326_2*X2645
2646	X_dot(2646) =K2328_1*X187*X453 - K2329_1*X2646 - K2328_2*X2646
2647	X_dot(2647) =K2330_1*X187*X329 - K2331_1*X2647 - K2330_2*X2647
2648	X_dot(2648) =K2332_1*X157*X187 - K2333_1*X2648 - K2332_2*X2648
2649	X_dot(2649) =K2334_1*X187*X477 - K2335_1*X2649 - K2334_2*X2649
2650	X_dot(2650) =K2336_1*X187*X437 - K2337_1*X2650 - K2336_2*X2650

2651	X_dot(2651) =K2338_1*X187*X414 - K2339_1*X2651 - K2338_2*X2651
2652	X_dot(2652) =K2340_1*X284*X1318 - K2341_1*X2652 - K2340_2*X2652
2653	X_dot(2653) =K2342_1*X96*X1318 - K2343_1*X2653 - K2342_2*X2653
2654	X_dot(2654) =K2344_1*X86*X1318 - K2345_1*X2654 - K2344_2*X2654
2655	X_dot(2655) =K2346_1*X169*X1318 - K2347_1*X2655 - K2346_2*X2655
2656	X_dot(2656) =K2348_1*X324*X1318 - K2349_1*X2656 - K2348_2*X2656
2657	X_dot(2657) =K2350_1*X281*X1318 - K2351_1*X2657 - K2350_2*X2657
2658	X_dot(2658) =K2352_1*X476*X1318 - K2353_1*X2658 - K2352_2*X2658
2659	X_dot(2659) =K2354_1*X613*X1318 - K2355_1*X2659 - K2354_2*X2659
2660	X_dot(2660) =K2356_1*X325*X1318 - K2357_1*X2660 - K2356_2*X2660
2661	X_dot(2661) =K2358_1*X509*X1318 - K2359_1*X2661 - K2358_2*X2661
2662	X_dot(2662) =K2360_1*X184*X1318 - K2361_1*X2662 - K2360_2*X2662
2663	X_dot(2663) =K2362_1*X531*X1318 - K2363_1*X2663 - K2362_2*X2663
2664	X_dot(2664) =K2364_1*X528*X1318 - K2365_1*X2664 - K2364_2*X2664
2665	X_dot(2665) =K2366_1*X83*X1145 - K2367_1*X2665 - K2366_2*X2665
2666	X_dot(2666) =K2368_1*X355*X1145 - K2369_1*X2666 - K2368_2*X2666
2667	X_dot(2667) =K2370_1*X117*X1145 - K2371_1*X2667 - K2370_2*X2667
2668	X_dot(2668) =K2372_1*X10*X1145 - K2373_1*X2668 - K2372_2*X2668
2669	X_dot(2669) =K2374_1*X564*X1145 - K2375_1*X2669 - K2374_2*X2669
2670	X_dot(2670) =K2376_1*X148*X1145 - K2377_1*X2670 - K2376_2*X2670
2671	X_dot(2671) =K2378_1*X424*X1145 - K2379_1*X2671 - K2378_2*X2671
2672	X_dot(2672) =K2380_1*X294*X1145 - K2381_1*X2672 - K2380_2*X2672
2673	X_dot(2673) =K2382_1*X312*X1145 - K2383_1*X2673 - K2382_2*X2673
2674	X_dot(2674) =K2384_1*X742*X1145 - K2385_1*X2674 - K2384_2*X2674
2675	X_dot(2675) =K2386_1*X255*X1145 - K2387_1*X2675 - K2386_2*X2675
2676	X_dot(2676) =K2388_1*X31*X1145 - K2389_1*X2676 - K2388_2*X2676
2677	X_dot(2677) =K2390_1*X223*X1145 - K2391_1*X2677 - K2390_2*X2677
2678	X_dot(2678) =K2392_1*X302*X1145 - K2393_1*X2678 - K2392_2*X2678
2679	X_dot(2679) =K2394_1*X295*X1145 - K2395_1*X2679 - K2394_2*X2679
2680	X_dot(2680) =K2396_1*X99*X1145 - K2397_1*X2680 - K2396_2*X2680
2681	X_dot(2681) =K2398_1*X695*X1366 - K2399_1*X2681 - K2398_2*X2681
2682	X_dot(2682) =K2400_1*X169*X1366 - K2401_1*X2682 - K2400_2*X2682
2683	X_dot(2683) =K2402_1*X719*X1352 - K2403_1*X2683 - K2402_2*X2683
2684	X_dot(2684) =K2404_1*X675*X1352 - K2405_1*X2684 - K2404_2*X2684
2685	X_dot(2685) =K2406_1*X307*X980 - K2407_1*X2685 - K2406_2*X2685
2686	X_dot(2686) =K2408_1*X85*X980 - K2409_1*X2686 - K2408_2*X2686
2687	X_dot(2687) =K2410_1*X175*X980 - K2411_1*X2687 - K2410_2*X2687
2688	X_dot(2688) =K2412_1*X443*X980 - K2413_1*X2688 - K2412_2*X2688
2689	X_dot(2689) =K2414_1*X539*X980 - K2415_1*X2689 - K2414_2*X2689
2690	X_dot(2690) =K2416_1*X656*X980 - K2417_1*X2690 - K2416_2*X2690
2691	X_dot(2691) =K2418_1*X99*X980 - K2419_1*X2691 - K2418_2*X2691
2692	X_dot(2692) =K2420_1*X762*X1341 - K2421_1*X2692 - K2420_2*X2692
2693	X_dot(2693) =K2422_1*X426*X1341 - K2423_1*X2693 - K2422_2*X2693
2694	X_dot(2694) =K2424_1*X531*X1341 - K2425_1*X2694 - K2424_2*X2694
2695	X_dot(2695) =K2426_1*X460*X1181 - K2427_1*X2695 - K2426_2*X2695
2696	X_dot(2696) =K2428_1*X284*X512 - K2429_1*X2696 - K2428_2*X2696
2697	X_dot(2697) =K2430_1*X169*X512 - K2431_1*X2697 - K2430_2*X2697
2698	X_dot(2698) =K2432_1*X512*X762 - K2433_1*X2698 - K2432_2*X2698
2699	X_dot(2699) =K2434_1*X25*X512 - K2435_1*X2699 - K2434_2*X2699
2700	X_dot(2700) =K2436_1*X229*X512 - K2437_1*X2700 - K2436_2*X2700
2701	X_dot(2701) =K2438_1*X390*X512 - K2439_1*X2701 - K2438_2*X2701
2702	X_dot(2702) =K2440_1*X398*X512 - K2441_1*X2702 - K2440_2*X2702
2703	X_dot(2703) =K2442_1*X329*X512 - K2443_1*X2703 - K2442_2*X2703
2704	X_dot(2704) =K2444_1*X61*X577 - K2445_1*X2704 - K2444_2*X2704
2705	X_dot(2705) =K2446_1*X157*X449 - K2447_1*X2705 - K2446_2*X2705
2706	X_dot(2706) =K2448_1*X429*X1411 - K2449_1*X2706 - K2448_2*X2706
2707	X_dot(2707) =K2450_1*X226*X1411 - K2451_1*X2707 - K2450_2*X2707
2708	X_dot(2708) =K2452_1*X213*X1411 - K2453_1*X2708 - K2452_2*X2708
2709	X_dot(2709) =K2454_1*X87*X1411 - K2455_1*X2709 - K2454_2*X2709
2710	X_dot(2710) =K2456_1*X435*X1411 - K2457_1*X2710 - K2456_2*X2710
2711	X_dot(2711) =K2458_1*X406*X1411 - K2459_1*X2711 - K2458_2*X2711
2712	X_dot(2712) =K2460_1*X609*X1411 - K2461_1*X2712 - K2460_2*X2712
2713	X_dot(2713) =K2462_1*X204*X1411 - K2463_1*X2713 - K2462_2*X2713
2714	X_dot(2714) =K2464_1*X604*X1411 - K2465_1*X2714 - K2464_2*X2714
2715	X_dot(2715) =K2466_1*X419*X1411 - K2467_1*X2715 - K2466_2*X2715
2716	X_dot(2716) =K2468_1*X203*X1154 - K2469_1*X2716 - K2468_2*X2716
2717	X_dot(2717) =K2470_1*X435*X1154 - K2471_1*X2717 - K2470_2*X2717
2718	X_dot(2718) =K2472_1*X270*X1154 - K2473_1*X2718 - K2472_2*X2718
2719	X_dot(2719) =K2474_1*X424*X1154 - K2475_1*X2719 - K2474_2*X2719
2720	X_dot(2720) =K2476_1*X604*X1154 - K2477_1*X2720 - K2476_2*X2720
2721	X_dot(2721) =K2478_1*X419*X1154 - K2479_1*X2721 - K2478_2*X2721
2722	X_dot(2722) =K2480_1*X199*X1293 - K2481_1*X2722 - K2480_2*X2722
2723	X_dot(2723) =K2482_1*X177*X1293 - K2483_1*X2723 - K2482_2*X2723
2724	X_dot(2724) =K2484_1*X87*X1293 - K2485_1*X2724 - K2484_2*X2724
2725	X_dot(2725) =K2486_1*X453*X1293 - K2487_1*X2725 - K2486_2*X2725
2726	X_dot(2726) =K2488_1*X67*X1293 - K2489_1*X2726 - K2488_2*X2726
2727	X_dot(2727) =K2490_1*X99*X650 - K2491_1*X2727 - K2490_2*X2727
2728	X_dot(2728) =K2492_1*X629*X650 - K2493_1*X2728 - K2492_2*X2728
2729	X_dot(2729) =K2494_1*X118*X861 - K2495_1*X2729 - K2494_2*X2729
2730	X_dot(2730) =K2496_1*X138*X861 - K2497_1*X2730 - K2496_2*X2730

2731	X_dot(2731) =K2498_1*X708*X861 - K2499_1*X2731 - K2498_2*X2731
2732	X_dot(2732) =K2500_1*X392*X861 - K2501_1*X2732 - K2500_2*X2732
2733	X_dot(2733) =K2502_1*X440*X861 - K2503_1*X2733 - K2502_2*X2733
2734	X_dot(2734) =K2504_1*X86*X861 - K2505_1*X2734 - K2504_2*X2734
2735	X_dot(2735) =K2506_1*X114*X861 - K2507_1*X2735 - K2506_2*X2735
2736	X_dot(2736) =K2508_1*X169*X861 - K2509_1*X2736 - K2508_2*X2736
2737	X_dot(2737) =K2510_1*X513*X861 - K2511_1*X2737 - K2510_2*X2737
2738	X_dot(2738) =K2512_1*X110*X861 - K2513_1*X2738 - K2512_2*X2738
2739	X_dot(2739) =K2514_1*X126*X861 - K2515_1*X2739 - K2514_2*X2739
2740	X_dot(2740) =K2516_1*X125*X861 - K2517_1*X2740 - K2516_2*X2740
2741	X_dot(2741) =K2518_1*X35*X861 - K2519_1*X2741 - K2518_2*X2741
2742	X_dot(2742) =K2520_1*X91*X861 - K2521_1*X2742 - K2520_2*X2742
2743	X_dot(2743) =K2522_1*X476*X861 - K2523_1*X2743 - K2522_2*X2743
2744	X_dot(2744) =K2524_1*X77*X861 - K2525_1*X2744 - K2524_2*X2744
2745	X_dot(2745) =K2526_1*X565*X861 - K2527_1*X2745 - K2526_2*X2745
2746	X_dot(2746) =K2528_1*X501*X861 - K2529_1*X2746 - K2528_2*X2746
2747	X_dot(2747) =K2530_1*X38*X861 - K2531_1*X2747 - K2530_2*X2747
2748	X_dot(2748) =K2532_1*X301*X861 - K2533_1*X2748 - K2532_2*X2748
2749	X_dot(2749) =K2534_1*X460*X861 - K2535_1*X2749 - K2534_2*X2749
2750	X_dot(2750) =K2536_1*X248*X861 - K2537_1*X2750 - K2536_2*X2750
2751	X_dot(2751) =K2538_1*X412*X861 - K2539_1*X2751 - K2538_2*X2751
2752	X_dot(2752) =K2540_1*X416*X861 - K2541_1*X2752 - K2540_2*X2752
2753	X_dot(2753) =K2542_1*X529*X861 - K2543_1*X2753 - K2542_2*X2753
2754	X_dot(2754) =K2544_1*X358*X861 - K2545_1*X2754 - K2544_2*X2754
2755	X_dot(2755) =K2546_1*X290*X861 - K2547_1*X2755 - K2546_2*X2755
2756	X_dot(2756) =K2548_1*X590*X861 - K2549_1*X2756 - K2548_2*X2756
2757	X_dot(2757) =K2550_1*X655*X1370 - K2551_1*X2757 - K2550_2*X2757
2758	X_dot(2758) =K2552_1*X477*X1370 - K2553_1*X2758 - K2552_2*X2758
2759	X_dot(2759) =K2554_1*X732*X1166 - K2555_1*X2759 - K2554_2*X2759
2760	X_dot(2760) =K2556_1*X765*X1130 - K2557_1*X2760 - K2556_2*X2760
2761	X_dot(2761) =K2558_1*X556*X1130 - K2559_1*X2761 - K2558_2*X2761
2762	X_dot(2762) =K2560_1*X747*X1130 - K2561_1*X2762 - K2560_2*X2762
2763	X_dot(2763) =K2562_1*X169*X716 - K2563_1*X2763 - K2562_2*X2763
2764	X_dot(2764) =K2564_1*X694*X792 - K2565_1*X2764 - K2564_2*X2764
2765	X_dot(2765) =K2566_1*X503*X792 - K2567_1*X2765 - K2566_2*X2765
2766	X_dot(2766) =K2568_1*X556*X1372 - K2569_1*X2766 - K2568_2*X2766
2767	X_dot(2767) =K2570_1*X747*X1373 - K2571_1*X2767 - K2570_2*X2767
2768	X_dot(2768) =K2572_1*X484*X793 - K2573_1*X2768 - K2572_2*X2768
2769	X_dot(2769) =K2574_1*X47*X793 - K2575_1*X2769 - K2574_2*X2769
2770	X_dot(2770) =K2576_1*X270*X793 - K2577_1*X2770 - K2576_2*X2770
2771	X_dot(2771) =K2578_1*X694*X793 - K2579_1*X2771 - K2578_2*X2771
2772	X_dot(2772) =K2580_1*X249*X858 - K2581_1*X2772 - K2580_2*X2772
2773	X_dot(2773) =K2582_1*X503*X598 - K2583_1*X2773 - K2582_2*X2773
2774	X_dot(2774) =K2584_1*X495*X598 - K2585_1*X2774 - K2584_2*X2774
2775	X_dot(2775) =K2586_1*X503*X735 - K2587_1*X2775 - K2586_2*X2775
2776	X_dot(2776) =K2588_1*X495*X735 - K2589_1*X2776 - K2588_2*X2776
2777	X_dot(2777) =K2590_1*X503*X1437 - K2591_1*X2777 - K2590_2*X2777
2778	X_dot(2778) =K2592_1*X495*X1437 - K2593_1*X2778 - K2592_2*X2778
2779	X_dot(2779) =K2594_1*X264*X727 - K2595_1*X2779 - K2594_2*X2779
2780	X_dot(2780) =K2596_1*X453*X727 - K2597_1*X2780 - K2596_2*X2780
2781	X_dot(2781) =K2598_1*X514*X727 - K2599_1*X2781 - K2598_2*X2781
2782	X_dot(2782) =K2600_1*X303*X727 - K2601_1*X2782 - K2600_2*X2782
2783	X_dot(2783) =K2602_1*X218*X424 - K2603_1*X2783 - K2602_2*X2783
2784	X_dot(2784) =K2604_1*X99*X1167 - K2605_1*X2784 - K2604_2*X2784
2785	X_dot(2785) =K2606_1*X85*X998 - K2607_1*X2785 - K2606_2*X2785
2786	X_dot(2786) =K2608_1*X292*X998 - K2609_1*X2786 - K2608_2*X2786
2787	X_dot(2787) =K2610_1*X99*X998 - K2611_1*X2787 - K2610_2*X2787
2788	X_dot(2788) =K2612_1*X170*X666 - K2613_1*X2788 - K2612_2*X2788
2789	X_dot(2789) =K2614_1*X424*X666 - K2615_1*X2789 - K2614_2*X2789
2790	X_dot(2790) =K2616_1*X106*X666 - K2617_1*X2790 - K2616_2*X2790
2791	X_dot(2791) =K2618_1*X99*X666 - K2619_1*X2791 - K2618_2*X2791
2792	X_dot(2792) =K2620_1*X487*X666 - K2621_1*X2792 - K2620_2*X2792
2793	X_dot(2793) =K2622_1*X80*X1169 - K2623_1*X2793 - K2622_2*X2793
2794	X_dot(2794) =K2624_1*X724*X1169 - K2625_1*X2794 - K2624_2*X2794
2795	X_dot(2795) =K2626_1*X690*X1169 - K2627_1*X2795 - K2626_2*X2795
2796	X_dot(2796) =K2628_1*X276*X1031 - K2629_1*X2796 - K2628_2*X2796
2797	X_dot(2797) =K2630_1*X177*X1031 - K2631_1*X2797 - K2630_2*X2797
2798	X_dot(2798) =K2632_1*X552*X1031 - K2633_1*X2798 - K2632_2*X2798
2799	X_dot(2799) =K2634_1*X276*X1418 - K2635_1*X2799 - K2634_2*X2799
2800	X_dot(2800) =K2636_1*X680*X1173 - K2637_1*X2800 - K2636_2*X2800
2801	X_dot(2801) =K2638_1*X168*X451 - K2639_1*X2801 - K2638_2*X2801
2802	X_dot(2802) =K2640_1*X243*X954 - K2641_1*X2802 - K2640_2*X2802
2803	X_dot(2803) =K2642_1*X180*X810 - K2643_1*X2803 - K2642_2*X2803
2804	X_dot(2804) =K2644_1*X189*X810 - K2645_1*X2804 - K2644_2*X2804
2805	X_dot(2805) =K2646_1*X441*X810 - K2647_1*X2805 - K2646_2*X2805
2806	X_dot(2806) =K2648_1*X521*X1381 - K2649_1*X2806 - K2648_2*X2806
2807	X_dot(2807) =K2650_1*X199*X522 - K2651_1*X2807 - K2650_2*X2807
2808	X_dot(2808) =K2652_1*X302*X522 - K2653_1*X2808 - K2652_2*X2808
2809	X_dot(2809) =K2654_1*X200*X891 - K2655_1*X2809 - K2654_2*X2809
2810	X_dot(2810) =K2656_1*X644*X891 - K2657_1*X2810 - K2656_2*X2810

2811	X_dot(2811) =K2658_1*X570*X1000 - K2659_1*X2811 - K2658_2*X2811
2812	X_dot(2812) =K2660_1*X378*X1000 - K2661_1*X2812 - K2660_2*X2812
2813	X_dot(2813) =K2662_1*X200*X1000 - K2663_1*X2813 - K2662_2*X2813
2814	X_dot(2814) =K2664_1*X224*X1000 - K2665_1*X2814 - K2664_2*X2814
2815	X_dot(2815) =K2666_1*X124*X1000 - K2667_1*X2815 - K2666_2*X2815
2816	X_dot(2816) =K2668_1*X185*X1000 - K2669_1*X2816 - K2668_2*X2816
2817	X_dot(2817) =K2670_1*X261*X1393 - K2671_1*X2817 - K2670_2*X2817
2818	X_dot(2818) =K2672_1*X458*X1393 - K2673_1*X2818 - K2672_2*X2818
2819	X_dot(2819) =K2674_1*X217*X1393 - K2675_1*X2819 - K2674_2*X2819
2820	X_dot(2820) =K2676_1*X169*X1393 - K2677_1*X2820 - K2676_2*X2820
2821	X_dot(2821) =K2678_1*X677*X1393 - K2679_1*X2821 - K2678_2*X2821
2822	X_dot(2822) =K2680_1*X211*X1087 - K2681_1*X2822 - K2680_2*X2822
2823	X_dot(2823) =K2682_1*X231*X1087 - K2683_1*X2823 - K2682_2*X2823
2824	X_dot(2824) =K2684_1*X560*X1451 - K2685_1*X2824 - K2684_2*X2824
2825	X_dot(2825) =K2686_1*X20*X780 - K2687_1*X2825 - K2686_2*X2825
2826	X_dot(2826) =K2688_1*X99*X780 - K2689_1*X2826 - K2688_2*X2826
2827	X_dot(2827) =K2690_1*X85*X1126 - K2691_1*X2827 - K2690_2*X2827
2828	X_dot(2828) =K2692_1*X111*X1126 - K2693_1*X2828 - K2692_2*X2828
2829	X_dot(2829) =K2694_1*X442*X1159 - K2695_1*X2829 - K2694_2*X2829
2830	X_dot(2830) =K2696_1*X453*X1159 - K2697_1*X2830 - K2696_2*X2830
2831	X_dot(2831) =K2698_1*X601*X1159 - K2699_1*X2831 - K2698_2*X2831
2832	X_dot(2832) =K2700_1*X460*X1029 - K2701_1*X2832 - K2700_2*X2832
2833	X_dot(2833) =K2702_1*X298*X1391 - K2703_1*X2833 - K2702_2*X2833
2834	X_dot(2834) =K2704_1*X33*X330 - K2705_1*X2834 - K2704_2*X2834
2835	X_dot(2835) =K2706_1*X330*X386 - K2707_1*X2835 - K2706_2*X2835
2836	X_dot(2836) =K2708_1*X331*X439 - K2709_1*X2836 - K2708_2*X2836
2837	X_dot(2837) =K2710_1*X337*X386 - K2711_1*X2837 - K2710_2*X2837
2838	X_dot(2838) =K2712_1*X242*X1195 - K2713_1*X2838 - K2712_2*X2838
2839	X_dot(2839) =K2714_1*X386*X1195 - K2715_1*X2839 - K2714_2*X2839
2840	X_dot(2840) =K2716_1*X576*X1195 - K2717_1*X2840 - K2716_2*X2840
2841	X_dot(2841) =K2718_1*X156*X1195 - K2719_1*X2841 - K2718_2*X2841
2842	X_dot(2842) =K2720_1*X275*X1195 - K2721_1*X2842 - K2720_2*X2842
2843	X_dot(2843) =K2722_1*X498*X1195 - K2723_1*X2843 - K2722_2*X2843
2844	X_dot(2844) =K2724_1*X738*X1195 - K2725_1*X2844 - K2724_2*X2844
2845	X_dot(2845) =K2726_1*X364*X1195 - K2727_1*X2845 - K2726_2*X2845
2846	X_dot(2846) =K2728_1*X36*X1195 - K2729_1*X2846 - K2728_2*X2846
2847	X_dot(2847) =K2730_1*X258*X1195 - K2731_1*X2847 - K2730_2*X2847
2848	X_dot(2848) =K2732_1*X441*X1195 - K2733_1*X2848 - K2732_2*X2848
2849	X_dot(2849) =K2734_1*X581*X1195 - K2735_1*X2849 - K2734_2*X2849
2850	X_dot(2850) =K2736_1*X200*X1195 - K2737_1*X2850 - K2736_2*X2850
2851	X_dot(2851) =K2738_1*X593*X1195 - K2739_1*X2851 - K2738_2*X2851
2852	X_dot(2852) =K2740_1*X492*X1195 - K2741_1*X2852 - K2740_2*X2852
2853	X_dot(2853) =K2742_1*X453*X1195 - K2743_1*X2853 - K2742_2*X2853
2854	X_dot(2854) =K2744_1*X205*X386 - K2745_1*X2854 - K2744_2*X2854
2855	X_dot(2855) =K2746_1*X205*X576 - K2747_1*X2855 - K2746_2*X2855
2856	X_dot(2856) =K2748_1*X205*X498 - K2749_1*X2856 - K2748_2*X2856
2857	X_dot(2857) =K2750_1*X205*X364 - K2751_1*X2857 - K2750_2*X2857
2858	X_dot(2858) =K2752_1*X205*X520 - K2753_1*X2858 - K2752_2*X2858
2859	X_dot(2859) =K2754_1*X205*X627 - K2755_1*X2859 - K2754_2*X2859
2860	X_dot(2860) =K2756_1*X205*X297 - K2757_1*X2860 - K2756_2*X2860
2861	X_dot(2861) =K2758_1*X205*X492 - K2759_1*X2861 - K2758_2*X2861
2862	X_dot(2862) =K2760_1*X205*X595 - K2761_1*X2862 - K2760_2*X2862
2863	X_dot(2863) =K2762_1*X201*X205 - K2763_1*X2863 - K2762_2*X2863
2864	X_dot(2864) =K2764_1*X386*X1107 - K2765_1*X2864 - K2764_2*X2864
2865	X_dot(2865) =K2766_1*X275*X1107 - K2767_1*X2865 - K2766_2*X2865
2866	X_dot(2866) =K2768_1*X380*X1107 - K2769_1*X2866 - K2768_2*X2866
2867	X_dot(2867) =K2770_1*X718*X1107 - K2771_1*X2867 - K2770_2*X2867
2868	X_dot(2868) =K2772_1*X56*X1107 - K2773_1*X2868 - K2772_2*X2868
2869	X_dot(2869) =K2774_1*X479*X1404 - K2775_1*X2869 - K2774_2*X2869
2870	X_dot(2870) =K2776_1*X98*X823 - K2777_1*X2870 - K2776_2*X2870
2871	X_dot(2871) =K2778_1*X729*X823 - K2779_1*X2871 - K2778_2*X2871
2872	X_dot(2872) =K2780_1*X295*X823 - K2781_1*X2872 - K2780_2*X2872
2873	X_dot(2873) =K2782_1*X453*X783 - K2783_1*X2873 - K2782_2*X2873
2874	X_dot(2874) =K2784_1*X376*X915 - K2785_1*X2874 - K2784_2*X2874
2875	X_dot(2875) =K2786_1*X156*X915 - K2787_1*X2875 - K2786_2*X2875
2876	X_dot(2876) =K2788_1*X177*X915 - K2789_1*X2876 - K2788_2*X2876
2877	X_dot(2877) =K2790_1*X343*X915 - K2791_1*X2877 - K2790_2*X2877
2878	X_dot(2878) =K2792_1*X297*X915 - K2793_1*X2878 - K2792_2*X2878
2879	X_dot(2879) =K2794_1*X99*X915 - K2795_1*X2879 - K2794_2*X2879
2880	X_dot(2880) =K2796_1*X341*X915 - K2797_1*X2880 - K2796_2*X2880
2881	X_dot(2881) =K2798_1*X90*X106 - K2799_1*X2881 - K2798_2*X2881
2882	X_dot(2882) =K2800_1*X453*X1486 - K2801_1*X2882 - K2800_2*X2882
2883	X_dot(2883) =K2802_1*X294*X1348 - K2803_1*X2883 - K2802_2*X2883
2884	X_dot(2884) =K2804_1*X511*X1310 - K2805_1*X2884 - K2804_2*X2884
2885	X_dot(2885) =K2806_1*X25*X1310 - K2807_1*X2885 - K2806_2*X2885
2886	X_dot(2886) =K2808_1*X357*X1003 - K2809_1*X2886 - K2808_2*X2886
2887	X_dot(2887) =K2810_1*X357*X1406 - K2811_1*X2887 - K2810_2*X2887
2888	X_dot(2888) =K2812_1*X709*X1406 - K2813_1*X2888 - K2812_2*X2888
2889	X_dot(2889) =K2814_1*X343*X831 - K2815_1*X2889 - K2814_2*X2889
2890	X_dot(2890) =K2816_1*X451*X831 - K2817_1*X2890 - K2816_2*X2890

2891	X_dot(2891) =K2818_1*X246*X831 - K2819_1*X2891 - K2818_2*X2891
2892	X_dot(2892) =K2820_1*X575*X1409 - K2821_1*X2892 - K2820_2*X2892
2893	X_dot(2893) =K2822_1*X314*X787 - K2823_1*X2893 - K2822_2*X2893
2894	X_dot(2894) =K2824_1*X575*X787 - K2825_1*X2894 - K2824_2*X2894
2895	X_dot(2895) =K2826_1*X312*X787 - K2827_1*X2895 - K2826_2*X2895
2896	X_dot(2896) =K2828_1*X361*X1407 - K2829_1*X2896 - K2828_2*X2896
2897	X_dot(2897) =K2830_1*X575*X1407 - K2831_1*X2897 - K2830_2*X2897
2898	X_dot(2898) =K2832_1*X314*X749 - K2833_1*X2898 - K2832_2*X2898
2899	X_dot(2899) =K2834_1*X314*X1274 - K2835_1*X2899 - K2834_2*X2899
2900	X_dot(2900) =K2836_1*X18*X1274 - K2837_1*X2900 - K2836_2*X2900
2901	X_dot(2901) =K2838_1*X320*X1275 - K2839_1*X2901 - K2838_2*X2901
2902	X_dot(2902) =K2840_1*X320*X1410 - K2841_1*X2902 - K2840_2*X2902
2903	X_dot(2903) =K2842_1*X314*X1410 - K2843_1*X2903 - K2842_2*X2903
2904	X_dot(2904) =K2844_1*X361*X1410 - K2845_1*X2904 - K2844_2*X2904
2905	X_dot(2905) =K2846_1*X767*X1410 - K2847_1*X2905 - K2846_2*X2905
2906	X_dot(2906) =K2848_1*X320*X1185 - K2849_1*X2906 - K2848_2*X2906
2907	X_dot(2907) =K2850_1*X314*X1185 - K2851_1*X2907 - K2850_2*X2907
2908	X_dot(2908) =K2852_1*X361*X1185 - K2853_1*X2908 - K2852_2*X2908
2909	X_dot(2909) =K2854_1*X41*X1185 - K2855_1*X2909 - K2854_2*X2909
2910	X_dot(2910) =K2856_1*X87*X1271 - K2857_1*X2910 - K2856_2*X2910
2911	X_dot(2911) =K2858_1*X513*X1271 - K2859_1*X2911 - K2858_2*X2911
2912	X_dot(2912) =K2860_1*X106*X1271 - K2861_1*X2912 - K2860_2*X2912
2913	X_dot(2913) =K2862_1*X461*X1271 - K2863_1*X2913 - K2862_2*X2913
2914	X_dot(2914) =K2864_1*X246*X1271 - K2865_1*X2914 - K2864_2*X2914
2915	X_dot(2915) =K2866_1*X710*X1271 - K2867_1*X2915 - K2866_2*X2915
2916	X_dot(2916) =K2868_1*X559*X1271 - K2869_1*X2916 - K2868_2*X2916
2917	X_dot(2917) =K2870_1*X431*X894 - K2871_1*X2917 - K2870_2*X2917
2918	X_dot(2918) =K2872_1*X333*X894 - K2873_1*X2918 - K2872_2*X2918
2919	X_dot(2919) =K2874_1*X580*X894 - K2875_1*X2919 - K2874_2*X2919
2920	X_dot(2920) =K2876_1*X325*X894 - K2877_1*X2920 - K2876_2*X2920
2921	X_dot(2921) =K2878_1*X434*X894 - K2879_1*X2921 - K2878_2*X2921
2922	X_dot(2922) =K2880_1*X302*X894 - K2881_1*X2922 - K2880_2*X2922
2923	X_dot(2923) =K2882_1*X513*X1270 - K2883_1*X2923 - K2882_2*X2923
2924	X_dot(2924) =K2884_1*X461*X1270 - K2885_1*X2924 - K2884_2*X2924
2925	X_dot(2925) =K2886_1*X240*X1270 - K2887_1*X2925 - K2886_2*X2925
2926	X_dot(2926) =K2888_1*X559*X1270 - K2889_1*X2926 - K2888_2*X2926
2927	X_dot(2927) =K2890_1*X609*X1270 - K2891_1*X2927 - K2890_2*X2927
2928	X_dot(2928) =K2892_1*X264*X1273 - K2893_1*X2928 - K2892_2*X2928
2929	X_dot(2929) =K2894_1*X106*X1273 - K2895_1*X2929 - K2894_2*X2929
2930	X_dot(2930) =K2896_1*X88*X1273 - K2897_1*X2930 - K2896_2*X2930
2931	X_dot(2931) =K2898_1*X332*X1273 - K2899_1*X2931 - K2898_2*X2931
2932	X_dot(2932) =K2900_1*X302*X1273 - K2901_1*X2932 - K2900_2*X2932
2933	X_dot(2933) =K2902_1*X295*X1273 - K2903_1*X2933 - K2902_2*X2933
2934	X_dot(2934) =K2904_1*X136*X1282 - K2905_1*X2934 - K2904_2*X2934
2935	X_dot(2935) =K2906_1*X387*X1383 - K2907_1*X2935 - K2906_2*X2935
2936	X_dot(2936) =K2908_1*X50*X1383 - K2909_1*X2936 - K2908_2*X2936
2937	X_dot(2937) =K2910_1*X644*X1383 - K2911_1*X2937 - K2910_2*X2937
2938	X_dot(2938) =K2912_1*X513*X970 - K2913_1*X2938 - K2912_2*X2938
2939	X_dot(2939) =K2914_1*X264*X1005 - K2915_1*X2939 - K2914_2*X2939
2940	X_dot(2940) =K2916_1*X304*X1005 - K2917_1*X2940 - K2916_2*X2940
2941	X_dot(2941) =K2918_1*X609*X1005 - K2919_1*X2941 - K2918_2*X2941
2942	X_dot(2942) =K2920_1*X617*X1305 - K2921_1*X2942 - K2920_2*X2942
2943	X_dot(2943) =K2922_1*X220*X1417 - K2923_1*X2943 - K2922_2*X2943
2944	X_dot(2944) =K2924_1*X451*X925 - K2925_1*X2944 - K2924_2*X2944
2945	X_dot(2945) =K2926_1*X499*X925 - K2927_1*X2945 - K2926_2*X2945
2946	X_dot(2946) =K2928_1*X265*X1036 - K2929_1*X2946 - K2928_2*X2946
2947	X_dot(2947) =K2930_1*X451*X1036 - K2931_1*X2947 - K2930_2*X2947
2948	X_dot(2948) =K2932_1*X499*X1036 - K2933_1*X2948 - K2932_2*X2948
2949	X_dot(2949) =K2934_1*X373*X1036 - K2935_1*X2949 - K2934_2*X2949
2950	X_dot(2950) =K2936_1*X462*X1036 - K2937_1*X2950 - K2936_2*X2950
2951	X_dot(2951) =K2938_1*X559*X1036 - K2939_1*X2951 - K2938_2*X2951
2952	X_dot(2952) =K2940_1*X315*X1419 - K2941_1*X2952 - K2940_2*X2952
2953	X_dot(2953) =K2942_1*X573*X1316 - K2943_1*X2953 - K2942_2*X2953
2954	X_dot(2954) =K2944_1*X164*X1276 - K2945_1*X2954 - K2944_2*X2954
2955	X_dot(2955) =K2946_1*X88*X1276 - K2947_1*X2955 - K2946_2*X2955
2956	X_dot(2956) =K2948_1*X746*X1276 - K2949_1*X2956 - K2948_2*X2956
2957	X_dot(2957) =K2950_1*X47*X418 - K2951_1*X2957 - K2950_2*X2957
2958	X_dot(2958) =K2952_1*X47*X1006 - K2953_1*X2958 - K2952_2*X2958
2959	X_dot(2959) =K2954_1*X678*X1006 - K2955_1*X2959 - K2954_2*X2959
2960	X_dot(2960) =K2956_1*X57*X1006 - K2957_1*X2960 - K2956_2*X2960
2961	X_dot(2961) =K2958_1*X187*X376 - K2959_1*X2961 - K2958_2*X2961
2962	X_dot(2962) =K2960_1*X187*X199 - K2961_1*X2962 - K2960_2*X2962
2963	X_dot(2963) =K2962_1*X177*X187 - K2963_1*X2963 - K2962_2*X2963
2964	X_dot(2964) =K2964_1*X187*X298 - K2965_1*X2964 - K2964_2*X2964
2965	X_dot(2965) =K2966_1*X187*X275 - K2967_1*X2965 - K2966_2*X2965
2966	X_dot(2966) =K2968_1*X187*X635 - K2969_1*X2966 - K2968_2*X2966
2967	X_dot(2967) =K2970_1*X9*X187 - K2971_1*X2967 - K2970_2*X2967
2968	X_dot(2968) =K2972_1*X187*X393 - K2973_1*X2968 - K2972_2*X2968
2969	X_dot(2969) =K2974_1*X187*X232 - K2975_1*X2969 - K2974_2*X2969
2970	X_dot(2970) =K2976_1*X187*X476 - K2977_1*X2970 - K2976_2*X2970

2971	X_dot(2971) =K2978_1*X57*X187 - K2979_1*X2971 - K2978_2*X2971
2972	X_dot(2972) =K2980_1*X187*X315 - K2981_1*X2972 - K2980_2*X2972
2973	X_dot(2973) =K2982_1*X76*X187 - K2983_1*X2973 - K2982_2*X2973
2974	X_dot(2974) =K2984_1*X187*X367 - K2985_1*X2974 - K2984_2*X2974
2975	X_dot(2975) =K2986_1*X162*X1426 - K2987_1*X2975 - K2986_2*X2975
2976	X_dot(2976) =K2988_1*X232*X1318 - K2989_1*X2976 - K2988_2*X2976
2977	X_dot(2977) =K2990_1*X625*X1318 - K2991_1*X2977 - K2990_2*X2977
2978	X_dot(2978) =K2992_1*X578*X1318 - K2993_1*X2978 - K2992_2*X2978
2979	X_dot(2979) =K2994_1*X57*X1318 - K2995_1*X2979 - K2994_2*X2979
2980	X_dot(2980) =K2996_1*X263*X1318 - K2997_1*X2980 - K2996_2*X2980
2981	X_dot(2981) =K2998_1*X99*X1318 - K2999_1*X2981 - K2998_2*X2981
2982	X_dot(2982) =K3000_1*X232*X1145 - K3001_1*X2982 - K3000_2*X2982
2983	X_dot(2983) =K3002_1*X530*X1145 - K3003_1*X2983 - K3002_2*X2983
2984	X_dot(2984) =K3004_1*X197*X1145 - K3005_1*X2984 - K3004_2*X2984
2985	X_dot(2985) =K3006_1*X157*X1145 - K3007_1*X2985 - K3006_2*X2985
2986	X_dot(2986) =K3008_1*X264*X980 - K3009_1*X2986 - K3008_2*X2986
2987	X_dot(2987) =K3010_1*X691*X980 - K3011_1*X2987 - K3010_2*X2987
2988	X_dot(2988) =K3012_1*X72*X1181 - K3013_1*X2988 - K3012_2*X2988
2989	X_dot(2989) =K3014_1*X42*X1431 - K3015_1*X2989 - K3014_2*X2989
2990	X_dot(2990) =K3016_1*X312*X512 - K3017_1*X2990 - K3016_2*X2990
2991	X_dot(2991) =K3018_1*X237*X512 - K3019_1*X2991 - K3018_2*X2991
2992	X_dot(2992) =K3020_1*X177*X1411 - K3021_1*X2992 - K3020_2*X2992
2993	X_dot(2993) =K3022_1*X559*X1411 - K3023_1*X2993 - K3022_2*X2993
2994	X_dot(2994) =K3024_1*X177*X1154 - K3025_1*X2994 - K3024_2*X2994
2995	X_dot(2995) =K3026_1*X57*X1154 - K3027_1*X2995 - K3026_2*X2995
2996	X_dot(2996) =K3028_1*X264*X861 - K3029_1*X2996 - K3028_2*X2996
2997	X_dot(2997) =K3030_1*X159*X861 - K3031_1*X2997 - K3030_2*X2997
2998	X_dot(2998) =K3032_1*X55*X861 - K3033_1*X2998 - K3032_2*X2998
2999	X_dot(2999) =K3034_1*X458*X861 - K3035_1*X2999 - K3034_2*X2999
3000	X_dot(3000) =K3036_1*X426*X861 - K3037_1*X3000 - K3036_2*X3000
3001	X_dot(3001) =K3038_1*X167*X861 - K3039_1*X3001 - K3038_2*X3001
3002	X_dot(3002) =K3040_1*X31*X861 - K3041_1*X3002 - K3040_2*X3002
3003	X_dot(3003) =K3042_1*X197*X861 - K3043_1*X3003 - K3042_2*X3003
3004	X_dot(3004) =K3044_1*X249*X861 - K3045_1*X3004 - K3044_2*X3004
3005	X_dot(3005) =K3046_1*X726*X861 - K3047_1*X3005 - K3046_2*X3005
3006	X_dot(3006) =K3048_1*X302*X861 - K3049_1*X3006 - K3048_2*X3006
3007	X_dot(3007) =K3050_1*X295*X861 - K3051_1*X3007 - K3050_2*X3007
3008	X_dot(3008) =K3052_1*X132*X861 - K3053_1*X3008 - K3052_2*X3008
3009	X_dot(3009) =K3054_1*X495*X793 - K3055_1*X3009 - K3054_2*X3009
3010	X_dot(3010) =K3056_1*X184*X858 - K3057_1*X3010 - K3056_2*X3010
3011	X_dot(3011) =K3058_1*X425*X858 - K3059_1*X3011 - K3058_2*X3011
3012	X_dot(3012) =K3060_1*X310*X858 - K3061_1*X3012 - K3060_2*X3012
3013	X_dot(3013) =K3062_1*X422*X858 - K3063_1*X3013 - K3062_2*X3013
3014	X_dot(3014) =K3064_1*X320*X598 - K3065_1*X3014 - K3064_2*X3014
3015	X_dot(3015) =K3066_1*X320*X735 - K3067_1*X3015 - K3066_2*X3015
3016	X_dot(3016) =K3068_1*X361*X735 - K3069_1*X3016 - K3068_2*X3016
3017	X_dot(3017) =K3070_1*X687*X1437 - K3071_1*X3017 - K3070_2*X3017
3018	X_dot(3018) =K3072_1*X539*X727 - K3073_1*X3018 - K3072_2*X3018
3019	X_dot(3019) =K3074_1*X424*X731 - K3075_1*X3019 - K3074_2*X3019
3020	X_dot(3020) =K3076_1*X504*X998 - K3077_1*X3020 - K3076_2*X3020
3021	X_dot(3021) =K3078_1*X123*X1439 - K3079_1*X3021 - K3078_2*X3021
3022	X_dot(3022) =K3080_1*X379*X1031 - K3081_1*X3022 - K3080_2*X3022
3023	X_dot(3023) =K3082_1*X436*X1031 - K3083_1*X3023 - K3082_2*X3023
3024	X_dot(3024) =K3084_1*X99*X1126 - K3085_1*X3024 - K3084_2*X3024
3025	X_dot(3025) =K3086_1*X207*X915 - K3087_1*X3025 - K3086_2*X3025
3026	X_dot(3026) =K3088_1*X47*X891 - K3089_1*X3026 - K3088_2*X3026
3027	X_dot(3027) =K3090_1*X47*X1445 - K3091_1*X3027 - K3090_2*X3027
3028	X_dot(3028) =K3092_1*X47*X1000 - K3093_1*X3028 - K3092_2*X3028
3029	X_dot(3029) =K3094_1*X240*X1348 - K3095_1*X3029 - K3094_2*X3029
3030	X_dot(3030) =K3096_1*X359*X1005 - K3097_1*X3030 - K3096_2*X3030
3031	X_dot(3031) =K3098_1*X47*X1273 - K3099_1*X3031 - K3098_2*X3031
3032	X_dot(3032) =K3100_1*X47*X1316 - K3101_1*X3032 - K3100_2*X3032
3033	X_dot(3033) =K3102_1*X47*X700 - K3103_1*X3033 - K3102_2*X3033
3034	X_dot(3034) =K3104_1*X88*X1270 - K3105_1*X3034 - K3104_2*X3034
3035	X_dot(3035) =K3106_1*X135*X1270 - K3107_1*X3035 - K3106_2*X3035
3036	X_dot(3036) =K3108_1*X231*X1270 - K3109_1*X3036 - K3108_2*X3036
3037	X_dot(3037) =K3110_1*X423*X1270 - K3111_1*X3037 - K3110_2*X3037
3038	X_dot(3038) =K3112_1*X451*X1270 - K3113_1*X3038 - K3112_2*X3038
3039	X_dot(3039) =K3114_1*X483*X1270 - K3115_1*X3039 - K3114_2*X3039
3040	X_dot(3040) =K3116_1*X623*X1270 - K3117_1*X3040 - K3116_2*X3040
3041	X_dot(3041) =K3118_1*X704*X1270 - K3119_1*X3041 - K3118_2*X3041
3042	X_dot(3042) =K3120_1*X451*X1313 - K3121_1*X3042 - K3120_2*X3042
3043	X_dot(3043) =K3122_1*X275*X1273 - K3123_1*X3043 - K3122_2*X3043
3044	X_dot(3044) =K3124_1*X434*X1273 - K3125_1*X3044 - K3124_2*X3044
3045	X_dot(3045) =K3126_1*X187*X579 - K3127_1*X3045 - K3126_2*X3045
3046	X_dot(3046) =K3128_1*X169*X1031 - K3129_1*X3046 - K3128_2*X3046
3047	X_dot(3047) =K3130_1*X9*X1154 - K3131_1*X3047 - K3130_2*X3047
3048	X_dot(3048) =K3132_1*X87*X1154 - K3133_1*X3048 - K3132_2*X3048
3049	X_dot(3049) =K3134_1*X204*X1154 - K3135_1*X3049 - K3134_2*X3049
3050	X_dot(3050) =K3136_1*X213*X1154 - K3137_1*X3050 - K3136_2*X3050

3051	X_dot(3051) =K3138_1*X226*X1154 - K3139_1*X3051 - K3138_2*X3051
3052	X_dot(3052) =K3140_1*X281*X1154 - K3141_1*X3052 - K3140_2*X3052
3053	X_dot(3053) =K3142_1*X341*X1154 - K3143_1*X3053 - K3142_2*X3053
3054	X_dot(3054) =K3144_1*X343*X1154 - K3145_1*X3054 - K3144_2*X3054
3055	X_dot(3055) =K3146_1*X350*X1154 - K3147_1*X3055 - K3146_2*X3055
3056	X_dot(3056) =K3148_1*X406*X1154 - K3149_1*X3056 - K3148_2*X3056
3057	X_dot(3057) =K3150_1*X429*X1154 - K3151_1*X3057 - K3150_2*X3057
3058	X_dot(3058) =K3152_1*X609*X1154 - K3153_1*X3058 - K3152_2*X3058
3059	X_dot(3059) =K3154_1*X615*X1154 - K3155_1*X3059 - K3154_2*X3059
3060	X_dot(3060) =K3156_1*X693*X1154 - K3157_1*X3060 - K3156_2*X3060
3061	X_dot(3061) =K3158_1*X722*X1154 - K3159_1*X3061 - K3158_2*X3061
3062	X_dot(3062) =K3160_1*X750*X1154 - K3161_1*X3062 - K3160_2*X3062
3063	X_dot(3063) =K3162_1*X359*X1154 - K3163_1*X3063 - K3162_2*X3063
3064	X_dot(3064) =K3164_1*X771*X1159 - K3165_1*X3064 - K3164_2*X3064
3065	X_dot(3065) =K3166_1*X771*X1293 - K3167_1*X3065 - K3166_2*X3065
3066	X_dot(3066) =K3168_1*X187*X771 - K3169_1*X3066 - K3168_2*X3066
3067	X_dot(3067) =K3170_1*X769*X1031 - K3171_1*X3067 - K3170_2*X3067
3068	X_dot(3068) =K3172_1*X769*X1418 - K3173_1*X3068 - K3172_2*X3068
3069	X_dot(3069) =K3174_1*X772*X1273 - K3175_1*X3069 - K3174_2*X3069
3070	X_dot(3070) =K3176_1*X771*X915 - K3177_1*X3070 - K3176_2*X3070
3071	X_dot(3071) =K3178_1*X769*X1411 - K3179_1*X3071 - K3178_2*X3071
3072	X_dot(3072) =K3180_1*X771*X1486 - K3181_1*X3072 - K3180_2*X3072
3073	X_dot(3073) =K3182_1*X7*X773 - K3183_1*X3073 - K3182_2*X3073
3074	X_dot(3074) =K3184_1*X13*X773 - K3185_1*X3074 - K3184_2*X3074
3075	X_dot(3075) =K3186_1*X116*X773 - K3187_1*X3075 - K3186_2*X3075
3076	X_dot(3076) =K3188_1*X124*X773 - K3189_1*X3076 - K3188_2*X3076
3077	X_dot(3077) =K3190_1*X171*X773 - K3191_1*X3077 - K3190_2*X3077
3078	X_dot(3078) =K3192_1*X316*X773 - K3193_1*X3078 - K3192_2*X3078
3079	X_dot(3079) =K3194_1*X327*X773 - K3195_1*X3079 - K3194_2*X3079
3080	X_dot(3080) =K3196_1*X349*X773 - K3197_1*X3080 - K3196_2*X3080
3081	X_dot(3081) =K3198_1*X384*X773 - K3199_1*X3081 - K3198_2*X3081
3082	X_dot(3082) =K3200_1*X473*X773 - K3201_1*X3082 - K3200_2*X3082
3083	X_dot(3083) =K3202_1*X514*X773 - K3203_1*X3083 - K3202_2*X3083
3084	X_dot(3084) =K3204_1*X543*X773 - K3205_1*X3084 - K3204_2*X3084
3085	X_dot(3085) =K3206_1*X553*X773 - K3207_1*X3085 - K3206_2*X3085
3086	X_dot(3086) =K3208_1*X602*X773 - K3209_1*X3086 - K3208_2*X3086
3087	X_dot(3087) =K3210_1*X623*X773 - K3211_1*X3087 - K3210_2*X3087
3088	X_dot(3088) =K3212_1*X634*X773 - K3213_1*X3088 - K3212_2*X3088
3089	X_dot(3089) =K3214_1*X641*X773 - K3215_1*X3089 - K3214_2*X3089
3090	X_dot(3090) =K3216_1*X644*X773 - K3217_1*X3090 - K3216_2*X3090
3091	X_dot(3091) =K3218_1*X646*X773 - K3219_1*X3091 - K3218_2*X3091
3092	X_dot(3092) =K3220_1*X695*X773 - K3221_1*X3092 - K3220_2*X3092
3093	X_dot(3093) =K3222_1*X7*X776 - K3223_1*X3093 - K3222_2*X3093
3094	X_dot(3094) =K3224_1*X99*X776 - K3225_1*X3094 - K3224_2*X3094
3095	X_dot(3095) =K3226_1*X111*X776 - K3227_1*X3095 - K3226_2*X3095
3096	X_dot(3096) =K3228_1*X124*X776 - K3229_1*X3096 - K3228_2*X3096
3097	X_dot(3097) =K3230_1*X185*X776 - K3231_1*X3097 - K3230_2*X3097
3098	X_dot(3098) =K3232_1*X189*X776 - K3233_1*X3098 - K3232_2*X3098
3099	X_dot(3099) =K3234_1*X264*X776 - K3235_1*X3099 - K3234_2*X3099
3100	X_dot(3100) =K3236_1*X272*X776 - K3237_1*X3100 - K3236_2*X3100
3101	X_dot(3101) =K3238_1*X510*X776 - K3239_1*X3101 - K3238_2*X3101
3102	X_dot(3102) =K3240_1*X772*X1270 - K3241_1*X3102 - K3240_2*X3102
3103	X_dot(3103) =K3242_1*X660*X1179 - K3243_1*X3103 - K3242_2*X3103
3104	X_dot(3104) =K3244_1*X715*X856 - K3245_1*X3104 - K3244_2*X3104
3105	X_dot(3105) =K3246_1*X286*X1224 - K3247_1*X3105 - K3246_2*X3105
3106	X_dot(3106) =K3248_1*X286*X856 - K3249_1*X3106 - K3248_2*X3106
3107	X_dot(3107) =K3250_1*X286*X815 - K3251_1*X3107 - K3250_2*X3107
3108	X_dot(3108) =K3252_1*X286*X851 - K3253_1*X3108 - K3252_2*X3108
3109	X_dot(3109) =K3254_1*X401*X1031 - K3255_1*X3109 - K3254_2*X3109
3110	X_dot(3110) =K3256_1*X401*X1271 - K3257_1*X3110 - K3256_2*X3110
3111	X_dot(3111) =K3258_1*X401*X1270 - K3259_1*X3111 - K3258_2*X3111
3112	X_dot(3112) =K3260_1*X402*X1031 - K3261_1*X3112 - K3260_2*X3112
3113	X_dot(3113) =K3262_1*X418*X1031 - K3263_1*X3113 - K3262_2*X3113
3114	X_dot(3114) =K3264_1*X418*X1133 - K3265_1*X3114 - K3264_2*X3114
3115	X_dot(3115) =K3266_1*X418*X831 - K3267_1*X3115 - K3266_2*X3115
3116	X_dot(3116) =K3268_1*X418*X1172 - K3269_1*X3116 - K3268_2*X3116
3117	X_dot(3117) =K3270_1*X418*X1275 - K3271_1*X3117 - K3270_2*X3117
3118	X_dot(3118) =K3272_1*X418*X1185 - K3273_1*X3118 - K3272_2*X3118
3119	X_dot(3119) =K3274_1*X418*X1271 - K3275_1*X3119 - K3274_2*X3119
3120	X_dot(3120) =K3276_1*X418*X1270 - K3277_1*X3120 - K3276_2*X3120
3121	X_dot(3121) =K3278_1*X418*X1323 - K3279_1*X3121 - K3278_2*X3121
3122	X_dot(3122) =K3280_1*X418*X1265 - K3281_1*X3122 - K3280_2*X3122
3123	X_dot(3123) =K3282_1*X418*X1119 - K3283_1*X3123 - K3282_2*X3123
3124	X_dot(3124) =K3284_1*X418*X851 - K3285_1*X3124 - K3284_2*X3124
3125	X_dot(3125) =K3286_1*X418*X792 - K3287_1*X3125 - K3286_2*X3125
3126	X_dot(3126) =K3288_1*X418*X793 - K3289_1*X3126 - K3288_2*X3126
3127	X_dot(3127) =K3290_1*X404*X1031 - K3291_1*X3127 - K3290_2*X3127
3128	X_dot(3128) =K3292_1*X404*X1418 - K3293_1*X3128 - K3292_2*X3128
3129	X_dot(3129) =K3294_1*X404*X896 - K3295_1*X3129 - K3294_2*X3129
3130	X_dot(3130) =K3296_1*X404*X1445 - K3297_1*X3130 - K3296_2*X3130

3131	X_dot(3131) =K3298_1*X478*X1077 - K3299_1*X3131 - K3298_2*X3131
3132	X_dot(3132) =K3300_1*X15*X1185 - K3301_1*X3132 - K3300_2*X3132
3133	X_dot(3133) =K3302_1*X557*X1031 - K3303_1*X3133 - K3302_2*X3133
3134	X_dot(3134) =K3304_1*X1007*X1031 - K3305_1*X3134 - K3304_2*X3134
3135	X_dot(3135) =K3306_1*X1007*X1446 - K3307_1*X3135 - K3306_2*X3135
3136	X_dot(3136) =K3308_1*X909*X1007 - K3309_1*X3136 - K3308_2*X3136
3137	X_dot(3137) =K3310_1*X970*X1007 - K3311_1*X3137 - K3310_2*X3137
3138	X_dot(3138) =K3312_1*X1007*X1145 - K3313_1*X3138 - K3312_2*X3138
3139	X_dot(3139) =K3314_1*X1007*X1181 - K3315_1*X3139 - K3314_2*X3139
3140	X_dot(3140) =K3316_1*X1007*X1217 - K3317_1*X3140 - K3316_2*X3140
3141	X_dot(3141) =K3318_1*X1007*X1258 - K3319_1*X3141 - K3318_2*X3141
3142	X_dot(3142) =K3320_1*X463*X874 - K3321_1*X3142 - K3320_2*X3142
3143	X_dot(3143) =K3322_1*X463*X1253 - K3323_1*X3143 - K3322_2*X3143
3144	X_dot(3144) =K3324_1*X463*X909 - K3325_1*X3144 - K3324_2*X3144
3145	X_dot(3145) =K3326_1*X463*X1365 - K3327_1*X3145 - K3326_2*X3145
3146	X_dot(3146) =K3328_1*X463*X1181 - K3329_1*X3146 - K3328_2*X3146
3147	X_dot(3147) =K3330_1*X459*X1055 - K3331_1*X3147 - K3330_2*X3147
3148	X_dot(3148) =K3332_1*X459*X1447 - K3333_1*X3148 - K3332_2*X3148
3149	X_dot(3149) =K3334_1*X459*X1181 - K3335_1*X3149 - K3334_2*X3149
3150	X_dot(3150) =K3336_1*X459*X1052 - K3337_1*X3150 - K3336_2*X3150
3151	X_dot(3151) =K3338_1*X190*X823 - K3339_1*X3151 - K3338_2*X3151
3152	X_dot(3152) =K3340_1*X190*X1023 - K3341_1*X3152 - K3340_2*X3152
3153	X_dot(3153) =K3342_1*X190*X970 - K3343_1*X3153 - K3342_2*X3153
3154	X_dot(3154) =K3344_1*X190*X861 - K3345_1*X3154 - K3344_2*X3154
3155	X_dot(3155) =K3346_1*X190*X1182 - K3347_1*X3155 - K3346_2*X3155
3156	X_dot(3156) =K3348_1*X190*X1217 - K3349_1*X3156 - K3348_2*X3156
3157	X_dot(3157) =K3350_1*X190*X1448 - K3351_1*X3157 - K3350_2*X3157
3158	X_dot(3158) =K3352_1*X974*X1449 - K3353_1*X3158 - K3352_2*X3158
3159	X_dot(3159) =K3354_1*X823*X974 - K3355_1*X3159 - K3354_2*X3159
3160	X_dot(3160) =K3356_1*X974*X1435 - K3357_1*X3160 - K3356_2*X3160
3161	X_dot(3161) =K3358_1*X974*X1217 - K3359_1*X3161 - K3358_2*X3161
3162	X_dot(3162) =K3360_1*X858*X974 - K3361_1*X3162 - K3360_2*X3162
3163	X_dot(3163) =K3362_1*X309*X1450 - K3363_1*X3163 - K3362_2*X3163
3164	X_dot(3164) =K3364_1*X139*X1031 - K3365_1*X3164 - K3364_2*X3164
3165	X_dot(3165) =K3366_1*X228*X1031 - K3367_1*X3165 - K3366_2*X3165
3166	X_dot(3166) =K3368_1*X228*X1418 - K3369_1*X3166 - K3368_2*X3166
3167	X_dot(3167) =K3370_1*X228*X1061 - K3371_1*X3167 - K3370_2*X3167
3168	X_dot(3168) =K3372_1*X228*X861 - K3373_1*X3168 - K3372_2*X3168
3169	X_dot(3169) =K3374_1*X356*X823 - K3375_1*X3169 - K3374_2*X3169
3170	X_dot(3170) =K3376_1*X356*X893 - K3377_1*X3170 - K3376_2*X3170
3171	X_dot(3171) =K3378_1*X356*X1271 - K3379_1*X3171 - K3378_2*X3171
3172	X_dot(3172) =K3380_1*X356*X1270 - K3381_1*X3172 - K3380_2*X3172
3173	X_dot(3173) =K3382_1*X642*X907 - K3383_1*X3173 - K3382_2*X3173
3174	X_dot(3174) =K3384_1*X286*X1451 - K3385_1*X3174 - K3384_2*X3174
3175	X_dot(3175) =K3386_1*X286*X894 - K3387_1*X3175 - K3386_2*X3175
3176	X_dot(3176) =K3388_1*X286*X925 - K3389_1*X3176 - K3388_2*X3176
3177	X_dot(3177) =K3390_1*X401*X1224 - K3391_1*X3177 - K3390_2*X3177
3178	X_dot(3178) =K3392_1*X401*X1217 - K3393_1*X3178 - K3392_2*X3178
3179	X_dot(3179) =K3394_1*X401*X807 - K3395_1*X3179 - K3394_2*X3179
3180	X_dot(3180) =K3396_1*X402*X1224 - K3397_1*X3180 - K3396_2*X3180
3181	X_dot(3181) =K3398_1*X402*X807 - K3399_1*X3181 - K3398_2*X3181
3182	X_dot(3182) =K3400_1*X289*X1031 - K3401_1*X3182 - K3400_2*X3182
3183	X_dot(3183) =K3402_1*X289*X1224 - K3403_1*X3183 - K3402_2*X3183
3184	X_dot(3184) =K3404_1*X289*X1452 - K3405_1*X3184 - K3404_2*X3184
3185	X_dot(3185) =K3406_1*X289*X807 - K3407_1*X3185 - K3406_2*X3185
3186	X_dot(3186) =K3408_1*X418*X1418 - K3409_1*X3186 - K3408_2*X3186
3187	X_dot(3187) =K3410_1*X418*X780 - K3411_1*X3187 - K3410_2*X3187
3188	X_dot(3188) =K3412_1*X418*X1453 - K3413_1*X3188 - K3412_2*X3188
3189	X_dot(3189) =K3414_1*X418*X925 - K3415_1*X3189 - K3414_2*X3189
3190	X_dot(3190) =K3416_1*X418*X1145 - K3417_1*X3190 - K3416_2*X3190
3191	X_dot(3191) =K3418_1*X418*X861 - K3419_1*X3191 - K3418_2*X3191
3192	X_dot(3192) =K3420_1*X418*X807 - K3421_1*X3192 - K3420_2*X3192
3193	X_dot(3193) =K3422_1*X418*X1454 - K3423_1*X3193 - K3422_2*X3193
3194	X_dot(3194) =K3424_1*X404*X780 - K3425_1*X3194 - K3424_2*X3194
3195	X_dot(3195) =K3426_1*X404*X1453 - K3427_1*X3195 - K3426_2*X3195
3196	X_dot(3196) =K3428_1*X404*X1271 - K3429_1*X3196 - K3428_2*X3196
3197	X_dot(3197) =K3430_1*X404*X925 - K3431_1*X3197 - K3430_2*X3197
3198	X_dot(3198) =K3432_1*X404*X1145 - K3433_1*X3198 - K3432_2*X3198
3199	X_dot(3199) =K3434_1*X404*X807 - K3435_1*X3199 - K3434_2*X3199
3200	X_dot(3200) =K3436_1*X404*X1454 - K3437_1*X3200 - K3436_2*X3200
3201	X_dot(3201) =K3438_1*X391*X1399 - K3439_1*X3201 - K3438_2*X3201
3202	X_dot(3202) =K3440_1*X391*X1387 - K3441_1*X3202 - K3440_2*X3202
3203	X_dot(3203) =K3442_1*X372*X1395 - K3443_1*X3203 - K3442_2*X3203
3204	X_dot(3204) =K3444_1*X811*X993 - K3445_1*X3204 - K3444_2*X3204
3205	X_dot(3205) =K3446_1*X993*X1181 - K3447_1*X3205 - K3446_2*X3205
3206	X_dot(3206) =K3448_1*X823*X1007 - K3449_1*X3206 - K3448_2*X3206
3207	X_dot(3207) =K3450_1*X1007*X1267 - K3451_1*X3207 - K3450_2*X3207
3208	X_dot(3208) =K3452_1*X1007^2 - K3453_1*X3208 - K3452_2*X3208
3209	X_dot(3209) =K3454_1*X463*X823 - K3455_1*X3209 - K3454_2*X3209
3210	X_dot(3210) =K3456_1*X463*X861 - K3457_1*X3210 - K3456_2*X3210

3211	X_dot(3211) =K3458_1*X463*X1182 - K3459_1*X3211 - K3458_2*X3211
3212	X_dot(3212) =K3460_1*X459*X861 - K3461_1*X3212 - K3460_2*X3212
3213	X_dot(3213) =K3462_1*X190*X1052 - K3463_1*X3213 - K3462_2*X3213
3214	X_dot(3214) =K3464_1*X190*X931 - K3465_1*X3214 - K3464_2*X3214
3215	X_dot(3215) =K3466_1*X861*X974 - K3467_1*X3215 - K3466_2*X3215
3216	X_dot(3216) =K3468_1*X1428^2 - K3469_1*X3216 - K3468_2*X3216
3217	X_dot(3217) =K3470_1*X178*X1077 - K3471_1*X3217 - K3470_2*X3217
3218	X_dot(3218) =K3472_1*X68*X1451 - K3473_1*X3218 - K3472_2*X3218
3219	X_dot(3219) =K3474_1*X401*X1035 - K3475_1*X3219 - K3474_2*X3219
3220	X_dot(3220) =K3476_1*X402*X1035 - K3477_1*X3220 - K3476_2*X3220
3221	X_dot(3221) =K3478_1*X289*X1035 - K3479_1*X3221 - K3478_2*X3221
3222	X_dot(3222) =K3480_1*X478*X1455 - K3481_1*X3222 - K3480_2*X3222
3223	X_dot(3223) =K3482_1*X178*X1455 - K3483_1*X3223 - K3482_2*X3223
3224	X_dot(3224) =K3484_1*X558*X823 - K3485_1*X3224 - K3484_2*X3224
3225	X_dot(3225) =K3486_1*X401*X778 - K3487_1*X3225 - K3486_2*X3225
3226	X_dot(3226) =K3488_1*X401*X987 - K3489_1*X3226 - K3488_2*X3226
3227	X_dot(3227) =K3490_1*X402*X940 - K3491_1*X3227 - K3490_2*X3227
3228	X_dot(3228) =K3492_1*X289*X1451 - K3493_1*X3228 - K3492_2*X3228
3229	X_dot(3229) =K3494_1*X418*X817 - K3495_1*X3229 - K3494_2*X3229
3230	X_dot(3230) =K3496_1*X418*X888 - K3497_1*X3230 - K3496_2*X3230
3231	X_dot(3231) =K3498_1*X418*X860 - K3499_1*X3231 - K3498_2*X3231
3232	X_dot(3232) =K3500_1*X418*X839 - K3501_1*X3232 - K3500_2*X3232
3233	X_dot(3233) =K3502_1*X418*X852 - K3503_1*X3233 - K3502_2*X3233
3234	X_dot(3234) =K3504_1*X870*X918 - K3505_1*X3234 - K3504_2*X3234
3235	X_dot(3235) =K3506_1*X391*X999 - K3507_1*X3235 - K3506_2*X3235
3236	X_dot(3236) =K3508_1*X493*X1000 - K3509_1*X3236 - K3508_2*X3236
3237	X_dot(3237) =K3510_1*X983*X1007 - K3511_1*X3237 - K3510_2*X3237
3238	X_dot(3238) =K3512_1*X861*X1007 - K3513_1*X3238 - K3512_2*X3238
3239	X_dot(3239) =K3514_1*X861*X1428 - K3515_1*X3239 - K3514_2*X3239
3240	X_dot(3240) =K3516_1*X139*X1028 - K3517_1*X3240 - K3516_2*X3240
3241	X_dot(3241) =K3518_1*X715*X1456 - K3519_1*X3241 - K3518_2*X3241
3242	X_dot(3242) =K3520_1*X286*X1457 - K3521_1*X3242 - K3520_2*X3242
3243	X_dot(3243) =K3522_1*X401*X872 - K3523_1*X3243 - K3522_2*X3243
3244	X_dot(3244) =K3524_1*X402*X872 - K3525_1*X3244 - K3524_2*X3244
3245	X_dot(3245) =K3526_1*X289*X872 - K3527_1*X3245 - K3526_2*X3245
3246	X_dot(3246) =K3528_1*X418*X1126 - K3529_1*X3246 - K3528_2*X3246
3247	X_dot(3247) =K3530_1*X418*X911 - K3531_1*X3247 - K3530_2*X3247
3248	X_dot(3248) =K3532_1*X418*X1026 - K3533_1*X3248 - K3532_2*X3248
3249	X_dot(3249) =K3534_1*X404*X1126 - K3535_1*X3249 - K3534_2*X3249
3250	X_dot(3250) =K3536_1*X404*X911 - K3537_1*X3250 - K3536_2*X3250
3251	X_dot(3251) =K3538_1*X478*X799 - K3539_1*X3251 - K3538_2*X3251
3252	X_dot(3252) =K3540_1*X478*X1346 - K3541_1*X3252 - K3540_2*X3252
3253	X_dot(3253) =K3542_1*X178*X799 - K3543_1*X3253 - K3542_2*X3253
3254	X_dot(3254) =K3544_1*X178*X1346 - K3545_1*X3254 - K3544_2*X3254
3255	X_dot(3255) =K3546_1*X391*X1265 - K3547_1*X3255 - K3546_2*X3255
3256	X_dot(3256) =K3548_1*X993^2 - K3549_1*X3256 - K3548_2*X3256
3257	X_dot(3257) =K3550_1*X989*X1007 - K3551_1*X3257 - K3550_2*X3257
3258	X_dot(3258) =K3552_1*X463*X1056 - K3553_1*X3258 - K3552_2*X3258
3259	X_dot(3259) =K3554_1*X463*X1364 - K3555_1*X3259 - K3554_2*X3259
3260	X_dot(3260) =K3556_1*X463*X1458 - K3557_1*X3260 - K3556_2*X3260
3261	X_dot(3261) =K3558_1*X818*X974 - K3559_1*X3261 - K3558_2*X3261
3262	X_dot(3262) =K3560_1*X606*X855 - K3561_1*X3262 - K3560_2*X3262
3263	X_dot(3263) =K3562_1*X618*X907 - K3563_1*X3263 - K3562_2*X3263
3264	X_dot(3264) =K3564_1*X701*X855 - K3565_1*X3264 - K3564_2*X3264
3265	X_dot(3265) =K3566_1*X1007*X1115 - K3567_1*X3265 - K3566_2*X3265
3266	X_dot(3266) =K3568_1*X401*X1026 - K3569_1*X3266 - K3568_2*X3266
3267	X_dot(3267) =K3570_1*X401*X1287 - K3571_1*X3267 - K3570_2*X3267
3268	X_dot(3268) =K3572_1*X478*X878 - K3573_1*X3268 - K3572_2*X3268
3269	X_dot(3269) =K3574_1*X418*X1442 - K3575_1*X3269 - K3574_2*X3269
3270	X_dot(3270) =K3576_1*X221*X245 - K3577_1*X3270 - K3576_2*X3270
3271	X_dot(3271) =K3578_1*X567*X649 - K3579_1*X3271 - K3578_2*X3271
3272	X_dot(3272) =K3580_1*X434*X649 - K3581_1*X3272 - K3580_2*X3272
3273	X_dot(3273) =K3582_1*X568*X699 - K3583_1*X3273 - K3582_2*X3273
3274	X_dot(3274) =K3584_1*X663*X699 - K3585_1*X3274 - K3584_2*X3274
3275	X_dot(3275) =K3586_1*X568*X737 - K3587_1*X3275 - K3586_2*X3275
3276	X_dot(3276) =K3588_1*X663*X737 - K3589_1*X3276 - K3588_2*X3276
3277	X_dot(3277) =K3590_1*X202*X344 - K3591_1*X3277 - K3590_2*X3277
3278	X_dot(3278) =K3592_1*X32*X99 - K3593_1*X3278 - K3592_2*X3278
3279	X_dot(3279) =K3594_1*X290*X699 - K3595_1*X3279 - K3594_2*X3279
3280	X_dot(3280) =K3596_1*X86*X221 - K3597_1*X3280 - K3596_2*X3280
3281	X_dot(3281) =K3598_1*X98*X221 - K3599_1*X3281 - K3598_2*X3281
3282	X_dot(3282) =K3600_1*X128*X221 - K3601_1*X3282 - K3600_2*X3282
3283	X_dot(3283) =K3602_1*X319*X525 - K3603_1*X3283 - K3602_2*X3283
3284	X_dot(3284) =K3604_1*X275*X525 - K3605_1*X3284 - K3604_2*X3284
3285	X_dot(3285) =K3606_1*X579*X603 - K3607_1*X3285 - K3606_2*X3285
3286	X_dot(3286) =K3608_1*X348*X409 - K3609_1*X3286 - K3608_2*X3286
3287	X_dot(3287) =K3610_1*X457*X592 - K3611_1*X3287 - K3610_2*X3287
3288	X_dot(3288) =K3612_1*X306*X1171 - K3613_1*X3288 - K3612_2*X3288
3289	X_dot(3289) =K3614_1*X705*X1171 - K3615_1*X3289 - K3614_2*X3289
3290	X_dot(3290) =K3616_1*X567*X1018 - K3617_1*X3290 - K3616_2*X3290

3291	X_dot(3291) =K3618_1*X207*X410 - K3619_1*X3291 - K3618_2*X3291
3292	X_dot(3292) =K3620_1*X410*X442 - K3621_1*X3292 - K3620_2*X3292
3293	X_dot(3293) =K3622_1*X151*X1207 - K3623_1*X3293 - K3622_2*X3293
3294	X_dot(3294) =K3624_1*X175*X395 - K3625_1*X3294 - K3624_2*X3294
3295	X_dot(3295) =K3626_1*X41*X1454 - K3627_1*X3295 - K3626_2*X3295
3296	X_dot(3296) =K3628_1*X519*X1454 - K3629_1*X3296 - K3628_2*X3296
3297	X_dot(3297) =K3630_1*X357*X1484 - K3631_1*X3297 - K3630_2*X3297
3298	X_dot(3298) =K3632_1*X41*X1484 - K3633_1*X3298 - K3632_2*X3298
3299	X_dot(3299) =K3634_1*X757*X1484 - K3635_1*X3299 - K3634_2*X3299
3300	X_dot(3300) =K3636_1*X395*X490 - K3637_1*X3300 - K3636_2*X3300
3301	X_dot(3301) =K3638_1*X766*X1431 - K3639_1*X3301 - K3638_2*X3301
3302	X_dot(3302) =K3640_1*X567*X699 - K3641_1*X3302 - K3640_2*X3302
3303	X_dot(3303) =K3642_1*X1212*X3304 - K3643_1*X3303 - K3642_2*X3303
3304	X_dot(3304) =K3642_2*X3303 + K3643_1*X3303 - K3642_1*X1212*X3304
3305	X_dot(3305) =K3643_1*X3303 - K4468_1*X3305
3306	X_dot(3306) =K3645_1*X860*X3307 - K3646_1*X3306 - K3645_2*X3306
3307	X_dot(3307) =K3645_2*X3306 + K3646_1*X3306 - K3645_1*X860*X3307
3308	X_dot(3308) =K3646_1*X3306 - K4467_1*X3308
3309	X_dot(3309) =K3648_1*X860*X3310 - K3649_1*X3309 - K3648_2*X3309
3310	X_dot(3310) =K3648_2*X3309 + K3649_1*X3309 - K3648_1*X860*X3310
3311	X_dot(3311) =K3649_1*X3309 - K4455_1*X3311
3312	X_dot(3312) =K3651_1*X1026*X1265*X3313 - K3652_1*X3312 - K3651_2*X3312
3313	X_dot(3313) =K3651_2*X3312 + K3652_1*X3312 - K3651_1*X1026*X1265*X3313
3314	X_dot(3314) =K3652_1*X3312 - K4459_1*X3314
3315	X_dot(3315) =K3654_1*X896*X1379*X3316 - K3655_1*X3315 - K3654_2*X3315
3316	X_dot(3316) =K3654_2*X3315 + K3655_1*X3315 - K3654_1*X896*X1379*X3316
3317	X_dot(3317) =K3655_1*X3315 - K4464_1*X3317
3318	X_dot(3318) =K3657_1*X1212*X3319 - K3658_1*X3318 - K3657_2*X3318
3319	X_dot(3319) =K3657_2*X3318 + K3658_1*X3318 - K3657_1*X1212*X3319
3320	X_dot(3320) =K3658_1*X3318 - K4462_1*X3320
3321	X_dot(3321) =K3660_1*X1212*X3322 - K3661_1*X3321 - K3660_2*X3321
3322	X_dot(3322) =K3660_2*X3321 + K3661_1*X3321 - K3660_1*X1212*X3322
3323	X_dot(3323) =K3661_1*X3321 - K4469_1*X3323
3324	X_dot(3324) =K3663_1*X878*X1212*X3325 - K3664_1*X3324 - K3663_2*X3324
3325	X_dot(3325) =K3663_2*X3324 + K3664_1*X3324 - K3663_1*X878*X1212*X3325
3326	X_dot(3326) =K3664_1*X3324 - K4461_1*X3326
3327	X_dot(3327) =K3666_1*X1212*X3328 - K3667_1*X3327 - K3666_2*X3327
3328	X_dot(3328) =K3666_2*X3327 + K3667_1*X3327 - K3666_1*X1212*X3328
3329	X_dot(3329) =K3667_1*X3327 - K4466_1*X3329
3330	X_dot(3330) =K3669_1*X878*X1026*X3331 - K3670_1*X3330 - K3669_2*X3330
3331	X_dot(3331) =K3669_2*X3330 + K3670_1*X3330 - K3669_1*X878*X1026*X3331
3332	X_dot(3332) =K3670_1*X3330 - K4458_1*X3332
3333	X_dot(3333) =K3672_1*X166*X3334 - K3673_1*X3333 - K3672_2*X3333
3334	X_dot(3334) =K3672_2*X3333 + K3673_1*X3333 - K3672_1*X166*X3334
3335	X_dot(3335) =K3673_1*X3333 - K4465_1*X3335
3336	X_dot(3336) =K3675_1*X1026*X3337 - K3676_1*X3336 - K3675_2*X3336
3337	X_dot(3337) =K3675_2*X3336 + K3676_1*X3336 - K3675_1*X1026*X3337
3338	X_dot(3338) =K3676_1*X3336 - K4460_1*X3338
3339	X_dot(3339) =K3678_1*X807*X3340 - K3679_1*X3339 - K3678_2*X3339
3340	X_dot(3340) =K3678_2*X3339 + K3679_1*X3339 - K3678_1*X807*X3340
3341	X_dot(3341) =K3679_1*X3339 - K4463_1*X3341
3342	X_dot(3342) =K3681_1*X508*X1237*X3343 - K3681_2*X3342
3343	X_dot(3343) =K3681_2*X3342 - K3681_1*X508*X1237*X3343
3344	X_dot(3344) =K3682_1*X3343 - K4456_1*X3344
3345	X_dot(3345) =K3684_1*X851*X3346 - K3684_2*X3345
3346	X_dot(3346) =K3684_2*X3345 - K3684_1*X851*X3346
3347	X_dot(3347) =K3685_1*X3346 - K4457_1*X3347

PART5: Estimated parameter values in large-scale colon cancerous cell's model		
parameter names	Forward reaction	Reverse reaction
K 1	0.1	0.1
K 2	0.1	0.1
K 3	0.1	0.1
K 4	0.1	0.1
K 5	0.1	0.1
K 6	0.1	0.1
K 7	0.193901662	0.877657589
K 8	0.1	0.1
K 9	0.1	0.1
K 10	0.104420179	0.115799184
K 11	0.1	
K 12	0.100600241	0.112367215
K 13	0.1	
K 14	0.099547393	0.119333659
K 15	0.1	
K 16	0.098526875	0.123772632
K 17	0.1	
K 18	0.105957037	0.1872662
K 19	0.1	
K 20	0.103087885	0.111623293
K 21	0.1	
K 22	0.103847656	0.127198672
K 23	0.1	
K 24	0.100976947	0.138394947
K 25	0.1	
K 26	0.112128727	0.189217968
K 27	0.1	
K 28	0.09577491	0.193224277
K 29	0.1	
K 30	0.100650571	0.12161425
K 31	0.1	
K 32	0.122705826	0.119336983
K 33	0.1	
K 34	0.118432817	0.124481596
K 35	0.1	
K 36	0.100960506	0.196557454
K 37	0.1	
K 38	0.101883789	0.118837896
K 39	0.1	
K 40	0.103623682	0.128997564
K 41	0.1	
K 42	0.115072092	0.124728747
K 43	0.1	

K 44	0.105911093	0.185351873
K 45	0.1	
K 46	0.125948065	0.114466147
K 47	0.1	
K 48	0.129970734	0.117774569
K 49	0.1	
K 50	0.107645341	0.117274528
K 51	0.1	
K 52	0.094705029	0.156737796
K 53	0.1	
K 54	0.099661971	0.99214334
K 55	0.1	
K 56	0.100977611	0.159116182
K 57	0.1	
K 58	0.1060273	0.121626842
K 59	0.1	
K 60	0.095947024	0.134698949
K 61	0.1	
K 62	0.110501548	0.11321628
K 63	0.1	
K 64	0.110922899	0.112325526
K 65	0.1	
K 66	0.121340885	0.173335523
K 67	0.1	
K 68	0.101883789	0.118837896
K 69	0.1	
K 70	0.099313776	0.12595557
K 71	0.1	
K 72	0.103857019	0.132984547
K 73	0.1	
K 74	0.134612166	0.118925477
K 75	0.1	
K 76	0.170459151	0.158416876
K 77	0.1	
K 78	0.125267228	0.115548614
K 79	0.1	
K 80	0.133874461	0.136256169
K 81	0.1	
K 82	0.100663226	0.166322555
K 83	0.1	
K 84	0.101848143	0.155714418
K 85	0.1	
K 86	0.444255431	0.279747367
K 87	0.118074386	
K 88	0.134384933	0.153831735
K 89	0.1	
K 90	0.101523207	0.126637588

K 91	0.1	
K 92	0.114705053	0.135766473
K 93	0.1	
K 94	0.103014514	0.117272
K 95	0.1	
K 96	0.108553325	0.121743747
K 97	0.1	
K 98	0.100812531	0.121896869
K 99	0.1	
K 100	0.101308601	0.113861134
K 101	0.1	
K 102	0.100910655	0.113699123
K 103	0.1	
K 104	0.100932543	0.134753575
K 105	0.1	
K 106	0.101308601	0.113861134
K 107	0.1	
K 108	0.116495316	0.111271986
K 109	0.100939697	
K 110	0.101883789	0.118837896
K 111	0.1	
K 112	0.102244235	0.141434668
K 113	0.1	
K 114	0.110462772	0.121619283
K 115	0.1	
K 116	0.135979296	0.125895396
K 117	0.1	
K 118	0.124240513	0.138575845
K 119	0.1	
K 120	0.107279773	0.988313252
K 121	0.1	
K 122	0.100676007	0.113239291
K 123	0.1	
K 124	0.104863081	0.164888144
K 125	0.1	
K 126	0.112496294	0.969693446
K 127	0.1	
K 128	0.102079109	0.171513565
K 129	0.1	
K 130	0.124164585	0.198627965
K 131	0.1	
K 132	0.089564695	0.173153976
K 133	0.1	
K 134	0.124891322	0.124735916
K 135	0.1	
K 136	0.132247225	0.115428899
K 137	0.1	

K 138	0.130457352	0.155573422
K 139	0.1	
K 140	0.100629926	0.138983989
K 141	0.1	
K 142	0.102326719	0.135714184
K 143	0.1	
K 144	0.117565568	0.119113462
K 145	0.1	
K 146	0.141636214	0.131175157
K 147	0.1	
K 148	0.103876143	0.123812141
K 149	0.1	
K 150	0.129516482	0.113394989
K 151	0.1	
K 152	0.107694966	0.997192287
K 153	0.1	
K 154	0.106245844	0.117662374
K 155	0.1	
K 156	0.100960506	0.196557454
K 157	0.1	
K 158	0.102676855	0.128992933
K 159	0.1	
K 160	0.101253906	0.112539625
K 161	0.1	
K 162	0.120106703	0.138889814
K 163	0.1	
K 164	0.114936826	0.127277665
K 165	0.1	
K 166	0.104051231	0.197764389
K 167	0.1	
K 168	0.100144428	0.119233559
K 169	0.1	
K 170	0.095583319	0.117787112
K 171	0.1	
K 172	0.181002667	0.185936481
K 173	0.1	
K 174	0.104467552	0.136413326
K 175	0.1	
K 176	0.10098504	0.136715384
K 177	0.1	
K 178	0.105193009	0.175158412
K 179	0.1	
K 180	0.122563886	0.12279625
K 181	0.1	
K 182	0.10072981	0.177979792
K 183	0.1	
K 184	0.102523319	0.111248454

K 185	0.1	
K 186	0.116833615	0.127444495
K 187	0.1	
K 188	0.102749906	0.925518417
K 189	0.1	
K 190	0.116660699	0.118926467
K 191	0.1	
K 192	0.100960506	0.196557454
K 193	0.1	
K 194	0.094300789	0.114433722
K 195	0.1	
K 196	0.101253906	0.112539625
K 197	0.1	
K 198	0.114863556	0.11324293
K 199	0.1	
K 200	0.112309605	0.141928537
K 201	0.1	
K 202	0.104100063	0.115199982
K 203	0.1	
K 204	0.129099233	0.178414722
K 205	0.1	
K 206	0.102168782	0.118448653
K 207	0.1	
K 208	0.119889396	0.119889396
K 209	0.1	
K 210	0.129815571	0.983131553
K 211	0.1	
K 212	0.101253906	0.112539625
K 213	0.1	
K 214	0.101162254	0.125251156
K 215	0.1	
K 216	0.113356829	0.117632726
K 217	0.1	
K 218	0.113227748	0.11413347
K 219	0.1	
K 220	0.100960506	0.196557454
K 221	0.1	
K 222	0.101253906	0.112539625
K 223	0.1	
K 224	0.101308601	0.113861134
K 225	0.1	
K 226	0.12810008	0.118981893
K 227	0.1	
K 228	0.100821236	0.119172981
K 229	0.102812726	
K 230	0.094620366	0.136332899
K 231	0.100784227	

K 232	0.102340942	0.123494215
K 233	0.103319575	
K 234	0.09973054	0.119267518
K 235	0.1	
K 236	0.095466768	0.167372645
K 237	0.1	
K 238	0.100022962	0.122962395
K 239	0.1	
K 240	0.105729281	0.142972513
K 241	0.1	
K 242	0.100832291	0.183229793
K 243	0.1	
K 244	0.130757472	0.145722577
K 245	0.1	
K 246	0.1049501	0.989424791
K 247	0.1	
K 248	0.100082029	0.192238975
K 249	0.1	
K 250	0.109921385	0.177428288
K 251	0.1	
K 252	0.091198607	0.116358131
K 253	0.1	
K 254	0.099751521	0.179961858
K 255	0.1	
K 256	0.106668599	0.111452275
K 257	0.1	
K 258	0.139448566	0.124826333
K 259	0.1	
K 260	0.112725386	0.112285829
K 261	0.1	
K 262	0.107745504	0.192867315
K 263	0.1	
K 264	0.103385673	0.163277561
K 265	0.1	
K 266	0.095676779	0.118252449
K 267	0.1	
K 268	0.11216034	0.123161212
K 269	0.1	
K 270	0.102400824	0.126845123
K 271	0.1	
K 272	0.123516314	0.125443895
K 273	0.1	
K 274	0.101418378	0.111233179
K 275	0.1	
K 276	0.099196881	0.126781558
K 277	0.10116031	
K 278	0.108182233	0.117627918

K 279	0.102050702	
K 280	0.102421354	0.122398165
K 281	0.101152157	
K 282	0.126651687	0.143231816
K 283	0.100629591	
K 284	0.111460272	0.979179117
K 285	0.100590882	
K 286	0.115824454	0.195797972
K 287	0.10040517	
K 288	0.123832781	0.129862919
K 289	0.1	
K 290	0.110954316	0.121354448
K 291	0.1	
K 292	0.118728713	0.11837617
K 293	0.1	
K 294	0.100533843	0.143127292
K 295	0.1	
K 296	0.107184784	0.131614868
K 297	0.1	
K 298	0.096387213	0.119789267
K 299	0.1	
K 300	0.102476894	0.131349123
K 301	0.1	
K 302	0.103866492	0.134473578
K 303	0.1	
K 304	0.102746072	0.113554828
K 305	0.1	
K 306	0.10737747	0.184215734
K 307	0.1	
K 308	0.100993514	0.199351388
K 309	0.1	
K 310	0.109976942	0.115137614
K 311	0.1	
K 312	0.125458797	0.113125117
K 313	0.1	
K 314	0.113468356	0.116742238
K 315	0.1	
K 316	0.125912868	0.117342198
K 317	0.1	
K 318	0.114815534	0.972349822
K 319	0.1	
K 320	0.157852185	0.122656424
K 321	0.1	
K 322	0.123911717	0.114565199
K 323	0.1	
K 324	0.137779178	0.123379377
K 325	0.1	

K 326	0.142446933	0.194364988
K 327	0.1	
K 328	0.106787184	0.116527312
K 329	0.1	
K 330	0.090509267	0.115142561
K 331	0.1	
K 332	0.106182136	0.169677469
K 333	0.1	
K 334	0.100792501	0.125815438
K 335	0.1	
K 336	0.10023204	0.193674146
K 337	0.1	
K 338	0.114282094	0.122362393
K 339	0.1	
K 340	0.134541631	0.133834969
K 341	0.1	
K 342	0.107248134	0.997144488
K 343	0.1	
K 344	0.12343081	0.123947944
K 345	0.1	
K 346	0.125336344	0.166814453
K 347	0.1	
K 348	0.10490274	0.138621527
K 349	0.1	
K 350	0.096643149	0.125964811
K 351	0.1	
K 352	0.113834093	0.113994592
K 353	0.1	
K 354	0.131049128	0.166538869
K 355	0.1	
K 356	0.105591281	0.128662987
K 357	0.1	
K 358	0.098164911	0.129879188
K 359	0.1	
K 360	0.100993514	0.199351388
K 361	0.1	
K 362	0.104149159	0.988569234
K 363	0.1	
K 364	0.112084401	0.111289438
K 365	0.1	
K 366	0.093301138	0.136376837
K 367	0.1	
K 368	0.105204484	0.126145129
K 369	0.1	
K 370	0.111013299	0.117324478
K 371	0.1	
K 372	0.136306128	0.168655518

K 373	0.1	
K 374	0.104299896	0.177345293
K 375	0.1	
K 376	0.101772491	0.117724913
K 377	0.1	
K 378	0.094116129	0.989798668
K 379	0.1	
K 380	0.133282704	0.124496591
K 381	0.1	
K 382	0.104585192	0.197194367
K 383	0.1	
K 384	0.100022962	0.122962395
K 385	0.1	
K 386	0.171120054	0.143948467
K 387	0.010363162	
K 388	0.114815156	0.119179776
K 389	0.1	
K 390	0.115440397	0.146265836
K 391	0.1	
K 392	0.100812454	0.181245435
K 393	0.1	
K 394	0.113168062	0.13322326
K 395	0.1	
K 396	0.103965519	0.182463729
K 397	0.1	
K 398	0.151240718	0.128696179
K 399	0.1	
K 400	0.156192847	0.118661776
K 401	0.1	
K 402	0.108616135	0.198656392
K 403	0.1	
K 404	0.101883789	0.118837896
K 405	0.1	
K 406	0.100998434	0.178527735
K 407	0.1	
K 408	0.100000348	0.112488216
K 409	0.1	
K 410	0.120524172	0.157652196
K 411	0.1	
K 412	0.103531874	0.135739418
K 413	0.1	
K 414	0.112626771	0.11183366
K 415	0.1	
K 416	0.1012036	0.11236
K 417	0.1	
K 418	0.100812454	0.181245435
K 419	0.1	

K 420	0.1125241	0.183259734
K 421	0.1	
K 422	0.106514056	0.111449585
K 423	0.1	
K 424	0.110132583	0.173168153
K 425	0.1	
K 426	0.103437286	0.116816568
K 427	0.1	
K 428	0.103965079	0.114291847
K 429	0.1	
K 430	0.110470415	0.197226175
K 431	0.1	
K 432	0.100022962	0.122962395
K 433	0.1	
K 434	0.100472815	0.122211487
K 435	0.1	
K 436	0.104209723	0.118251722
K 437	0.1	
K 438	0.101308601	0.113861134
K 439	0.1	
K 440	0.115109998	0.141634545
K 441	0.1	
K 442	0.10310299	0.119224927
K 443	0.1	
K 444	0.105796349	0.113187499
K 445	0.1	
K 446	0.109626225	0.182525566
K 447	0.1	
K 448	0.105278162	0.111465624
K 449	0.1	
K 450	0.103778809	0.998273575
K 451	0.1	
K 452	0.101308601	0.113861134
K 453	0.1	
K 454	0.091878904	0.124978669
K 455	0.1	
K 456	0.101883789	0.118837896
K 457	0.1	
K 458	0.099668334	0.114892229
K 459	0.1	
K 460	0.1012036	0.11236
K 461	0.1	
K 462	0.11083692	0.126527928
K 463	0.1	
K 464	0.101253906	0.112539625
K 465	0.1	
K 466	0.102986891	0.189883278

K 467	0.1	
K 468	0.116459454	0.142772238
K 469	0.1	
K 470	0.101681951	0.989545889
K 471	0.1	
K 472	0.101883789	0.118837896
K 473	0.1	
K 474	0.100910487	0.184429385
K 475	0.1	
K 476	0.137731767	0.114677629
K 477	0.1	
K 478	0.101670121	0.157789444
K 479	0.1	
K 480	0.130690873	0.123213971
K 481	0.1	
K 482	0.111775009	0.111918987
K 483	0.1	
K 484	0.1012036	0.11236
K 485	0.1	
K 486	0.107966703	0.111446531
K 487	0.1	
K 488	0.098634089	0.152168945
K 489	0.1	
K 490	0.122184131	0.112385158
K 491	0.1	
K 492	0.107702242	0.196429563
K 493	0.1	
K 494	0.10227881	0.122788136
K 495	0.1	
K 496	0.11554826	0.168955785
K 497	0.1	
K 498	0.093132736	0.114647435
K 499	0.1	
K 500	0.101608597	0.153229299
K 501	0.1	
K 502	0.100022962	0.122962395
K 503	0.1	
K 504	0.103530263	0.118335963
K 505	0.1	
K 506	0.101849214	0.999937465
K 507	0.1	
K 508	0.123760846	0.113139358
K 509	0.1	
K 510	0.100293539	0.132336133
K 511	0.1	
K 512	0.108938474	0.112635626
K 513	0.1	

K 514	0.106168161	0.118936415
K 515	0.1	
K 516	0.101078706	0.119393245
K 517	0.1	
K 518	0.116426945	0.177448188
K 519	0.1	
K 520	0.125183938	0.179553189
K 521	0.1	
K 522	0.111248528	0.117189912
K 523	0.1	
K 524	0.100812454	0.181245435
K 525	0.1	
K 526	0.114176436	0.939913344
K 527	0.1	
K 528	0.124565804	0.126897177
K 529	0.1	
K 530	0.109172273	0.117247729
K 531	0.1	
K 532	0.101308601	0.113861134
K 533	0.1	
K 534	0.113381034	0.11348117
K 535	0.1	
K 536	0.10158518	0.115851855
K 537	0.1	
K 538	0.100812454	0.181245435
K 539	0.1	
K 540	0.104639923	0.192565799
K 541	0.1	
K 542	0.108921722	0.983715562
K 543	0.1	
K 544	0.104554157	0.128434768
K 545	0.1	
K 546	0.101308601	0.113861134
K 547	0.1	
K 548	0.104632986	0.111531325
K 549	0.1	
K 550	0.10158518	0.115851855
K 551	0.1	
K 552	0.119119889	0.197261269
K 553	0.1	
K 554	0.137150923	0.118673225
K 555	0.1	
K 556	0.110325346	0.144397646
K 557	0.1	
K 558	0.074828665	0.123954178
K 559	0.107377863	
K 560	0.157092834	0.119179498

K 561	0.104913869	
K 562	0.122165605	0.128163115
K 563	0.1	
K 564	0.101692537	0.116917739
K 565	0.1	
K 566	0.099219305	0.129348156
K 567	0.1	
K 568	0.103698376	0.113388563
K 569	0.1	
K 570	0.108543654	0.999542951
K 571	0.1	
K 572	0.101989551	0.163358965
K 573	0.1	
K 574	0.115668702	0.194139956
K 575	0.1	
K 576	0.135036564	0.159216615
K 577	0.1	
K 578	0.112967533	0.783263922
K 579	0.1	
K 580	0.103510747	0.129747186
K 581	0.1	
K 582	0.101308601	0.113861134
K 583	0.1	
K 584	0.104179774	0.997546998
K 585	0.1	
K 586	0.11334038	0.127967173
K 587	0.1	
K 588	0.145312675	0.122123518
K 589	0.1	
K 590	0.109343012	0.973795274
K 591	0.1	
K 592	0.102391011	0.115795838
K 593	0.1	
K 594	0.101473997	0.113338217
K 595	0.1	
K 596	0.120927252	0.123189597
K 597	0.1	
K 598	0.109078169	0.123538697
K 599	0.1	
K 600	0.107623894	0.168195965
K 601	0.1	
K 602	0.119083433	0.133337713
K 603	0.1	
K 604	0.100812454	0.181245435
K 605	0.1	
K 606	0.130939479	0.121934818
K 607	0.1	

K 608	0.120249109	0.118639137
K 609	0.1	
K 610	0.101176361	0.114661127
K 611	0.1	
K 612	0.107645833	0.111222689
K 613	0.1	
K 614	0.102510234	0.121536651
K 615	0.1	
K 616	0.10201	0.121
K 617	0.1	
K 618	0.149293102	0.125957892
K 619	0.1	
K 620	0.10235655	0.117916979
K 621	0.1	
K 622	0.08768828	0.111566762
K 623	0.1	
K 624	0.103542041	0.999486294
K 625	0.1	
K 626	0.111235856	0.132464371
K 627	0.1	
K 628	0.101253906	0.112539625
K 629	0.1	
K 630	0.15108441	0.176768111
K 631	0.1	
K 632	0.132057598	0.12373444
K 633	0.1	
K 634	0.10158518	0.115851855
K 635	0.1	
K 636	0.128295132	0.125161513
K 637	0.1	
K 638	0.10441712	0.131793896
K 639	0.1	
K 640	0.076113748	0.151569445
K 641	0.1	
K 642	0.10323387	0.166592369
K 643	0.1	
K 644	0.097992868	0.117515792
K 645	0.1	
K 646	0.118015417	0.162249913
K 647	0.1	
K 648	0.122344774	0.123439478
K 649	0.1	
K 650	0.105381608	0.133886736
K 651	0.1	
K 652	0.131190421	0.121999143
K 653	0.1	
K 654	0.120852925	0.997491853

K 655	0.1	
K 656	0.126060599	0.112684369
K 657	0.1	
K 658	0.115730893	0.162595269
K 659	0.1	
K 660	0.104030894	0.98259777
K 661	0.1	
K 662	0.133560549	0.125882755
K 663	0.1	
K 664	0.11215372	0.11226617
K 665	0.1	
K 666	0.127534546	0.116833618
K 667	0.1	
K 668	0.112619727	0.964632442
K 669	0.1	
K 670	0.101308601	0.113861134
K 671	0.1	
K 672	0.133410709	0.114331657
K 673	0.1	
K 674	0.120155179	0.114321361
K 675	0.1	
K 676	0.114021002	0.129668918
K 677	0.1	
K 678	0.106555604	0.127744164
K 679	0.1	
K 680	0.13102387	0.114615483
K 681	0.1	
K 682	0.100563285	0.152918153
K 683	0.1	
K 684	0.146689498	0.125925352
K 685	0.1	
K 686	0.111127648	0.185111673
K 687	0.1	
K 688	0.103837671	0.153991755
K 689	0.1	
K 690	0.1012036	0.11236
K 691	0.1	
K 692	0.092436205	0.152598566
K 693	0.1	
K 694	0.117659828	0.114519758
K 695	0.1	
K 696	0.095211265	0.112262516
K 697	0.1	
K 698	0.098093421	0.118445439
K 699	0.1	
K 700	0.104939536	0.113319333
K 701	0.1	

K 702	0.105944356	0.186136297
K 703	0.1	
K 704	0.106495833	0.173636687
K 705	0.1	
K 706	0.107619016	0.182362474
K 707	0.1	
K 708	0.101505625	0.1155625
K 709	0.1	
K 710	0.1184331	0.114338555
K 711	0.1	
K 712	0.10201	0.121
K 713	0.1	
K 714	0.095023929	0.995911536
K 715	0.1	
K 716	0.111152453	0.121441526
K 717	0.1	
K 718	0.132156301	0.113725798
K 719	0.1	
K 720	0.100812454	0.181245435
K 721	0.1	
K 722	0.136897215	0.126473889
K 723	0.1	
K 724	0.096581811	0.181598272
K 725	0.1	
K 726	0.138516277	0.131318539
K 727	0.1	
K 728	0.107798581	0.123936314
K 729	0.1	
K 730	0.117286958	0.113256137
K 731	0.1	
K 732	0.112609795	0.119667516
K 733	0.1	
K 734	0.134384933	0.153831735
K 735	0.1	
K 736	0.106141027	0.113154724
K 737	0.1	
K 738	0.167241647	0.156594794
K 739	0.1	
K 740	0.116322202	0.113981875
K 741	0.1	
K 742	0.108784558	0.13621882
K 743	0.1	
K 744	0.118747264	0.192249933
K 745	0.1	
K 746	0.225286627	0.985937376
K 747	0.184009189	
K 748	0.104495077	0.118574624

K 749	0.1	
K 750	0.114313933	0.112323135
K 751	0.1	
K 752	0.115093851	0.145157698
K 753	0.100237861	
K 754	0.162313412	0.167272887
K 755	0.099838874	
K 756	0.128477896	0.129379292
K 757	0.099770789	
K 758	0.105307661	0.164271793
K 759	0.099823818	
K 760	0.128418576	0.136322457
K 761	0.1	
K 762	0.110107822	0.984445691
K 763	0.1	
K 764	0.1012036	0.11236
K 765	0.1	
K 766	0.120804232	0.183513172
K 767	0.1	
K 768	0.114463385	0.123588987
K 769	0.1	
K 770	0.101835449	0.161821364
K 771	0.1	
K 772	0.114626374	0.165921365
K 773	0.1	
K 774	0.116339978	0.998694263
K 775	0.1	
K 776	0.106482247	0.114222627
K 777	0.1	
K 778	0.109635589	0.111289174
K 779	0.1	
K 780	0.107242504	0.113398347
K 781	0.1	
K 782	0.101883789	0.118837896
K 783	0.1	
K 784	0.101074135	0.182446895
K 785	0.1	
K 786	0.103356431	0.116538517
K 787	0.1	
K 788	0.108771663	0.121985874
K 789	0.1	
K 790	0.12351095	0.995363389
K 791	0.1	
K 792	0.130438721	0.148784445
K 793	0.1	
K 794	0.113546091	0.962169789
K 795	0.1	

K 796	0.100247672	0.12476718
K 797	0.1	
K 798	0.116746832	0.113483923
K 799	0.1	
K 800	0.107818767	0.112769982
K 801	0.1	
K 802	0.124261692	0.1257216
K 803	0.1	
K 804	0.117962027	0.187668916
K 805	0.1	
K 806	0.108507104	0.116351849
K 807	0.097229384	
K 808	0.116914203	0.175986337
K 809	0.1	
K 810	0.115998182	0.173626585
K 811	0.10075	
K 812	0.112708381	0.115673458
K 813	0.1	
K 814	0.101883789	0.118837896
K 815	0.1	
K 816	0.126460651	0.113136535
K 817	0.1	
K 818	0.111166663	0.118761294
K 819	0.1	
K 820	0.097650272	0.172791682
K 821	0.1	
K 822	0.10836042	0.119683579
K 823	0.1	
K 824	0.09985435	0.971625862
K 825	0.1	
K 826	0.106820042	0.119427732
K 827	0.1	
K 828	0.100624331	0.198478789
K 829	0.1	
K 830	0.1012036	0.11236
K 831	0.1	
K 832	0.130953499	0.125967356
K 833	0.1	
K 834	0.113588537	0.151241996
K 835	0.1	
K 836	0.128986312	0.126828159
K 837	0.1	
K 838	0.13171857	0.11278618
K 839	0.1	
K 840	0.100399614	0.137195459
K 841	0.1	
K 842	0.095215466	0.188241762

K 843	0.1	
K 844	0.113855949	0.181362996
K 845	0.1	
K 846	0.118150351	0.134941166
K 847	0.1	
K 848	0.098861808	0.111826523
K 849	0.1	
K 850	0.09976568	0.196485522
K 851	0.1	
K 852	0.1012036	0.11236
K 853	0.1	
K 854	0.10752978	0.133257729
K 855	0.1	
K 856	0.105967686	0.111539339
K 857	0.1	
K 858	0.102078999	0.111697283
K 859	0.1	
K 860	0.101841992	0.126758892
K 861	0.100939697	
K 862	0.133313141	0.133254862
K 863	0.100939697	
K 864	0.140359918	0.126378256
K 865	0.09832717	
K 866	0.101540753	0.128516224
K 867	0.1	
K 868	0.121958297	0.11832724
K 869	0.1	
K 870	0.10687707	0.111771357
K 871	0.1	
K 872	0.101782343	0.145515263
K 873	0.1	
K 874	0.115131262	0.112963694
K 875	0.1	
K 876	0.126431992	0.113627563
K 877	0.1	
K 878	0.101019205	0.119792794
K 879	0.1	
K 880	0.099874829	0.147865147
K 881	0.1	
K 882	0.117316996	0.198825429
K 883	0.1	
K 884	0.10139185	0.122788136
K 885	0.1	
K 886	0.114121134	0.118776773
K 887	0.1	
K 888	0.13907225	0.117256951
K 889	0.1	

K 890	0.10158518	0.115851855
K 891	0.1	
K 892	0.100290407	0.984572714
K 893	0.100349838	
K 894	0.159452072	0.143376364
K 895	0.104549013	
K 896	0.119655704	0.197323385
K 897	0.1	
K 898	0.145020648	0.133346998
K 899	0.1	
K 900	0.120570501	0.142263329
K 901	0.1	
K 902	0.117111082	0.129938384
K 903	0.1	
K 904	0.105805849	0.119942716
K 905	0.1	
K 906	0.095135888	0.1131425
K 907	0.1	
K 908	0.082447413	0.113494362
K 909	0.1	
K 910	0.114884906	0.111598519
K 911	0.1	
K 912	0.122409521	0.115471825
K 913	0.1	
K 914	0.100663226	0.166322555
K 915	0.1	
K 916	0.101478997	0.195824683
K 917	0.1	
K 918	0.10268466	0.124198993
K 919	0.1	
K 920	0.132008346	0.121412152
K 921	0.1	
K 922	0.124834712	0.174314868
K 923	0.1	
K 924	0.120336339	0.161432447
K 925	0.1	
K 926	0.111070653	0.195872436
K 927	0.1	
K 928	0.101786222	0.116227755
K 929	0.1	
K 930	0.126538137	0.13123633
K 931	0.1	
K 932	0.060535233	0.526258186
K 933	0.1	
K 934	0.109904591	0.116215444
K 935	0.1	
K 936	0.10123802	0.112249176

K 937	0.1	
K 938	0.097608038	0.155659254
K 939	0.1	
K 940	0.106938343	0.124984469
K 941	0.1	
K 942	0.137399385	0.119688214
K 943	0.1	
K 944	0.100993514	0.199351388
K 945	0.1	
K 946	0.130516274	0.987856355
K 947	0.1	
K 948	0.10511963	0.118347958
K 949	0.1	
K 950	0.110157855	0.863513524
K 951	0.1	
K 952	0.106629209	0.171635459
K 953	0.1	
K 954	0.100812454	0.181245435
K 955	0.1	
K 956	0.103642101	0.185185429
K 957	0.1	
K 958	0.100185888	0.981915217
K 959	0.1	
K 960	0.104936605	0.983381564
K 961	0.1	
K 962	0.102485288	0.111268633
K 963	0.1	
K 964	0.102612738	0.131261329
K 965	0.1	
K 966	0.10106213	0.122788136
K 967	0.1	
K 968	0.105262035	0.115624353
K 969	0.1	
K 970	0.11600726	0.981596868
K 971	0.1	
K 972	0.10014018	0.139125111
K 973	0.1	
K 974	0.111858821	0.139134828
K 975	0.1	
K 976	0.143261722	0.125575242
K 977	0.1	
K 978	0.110355765	0.967158968
K 979	0.1	
K 980	0.100812454	0.181245435
K 981	0.1	
K 982	0.101308601	0.113861134
K 983	0.1	

K 984	0.105823241	0.111274743
K 985	0.1	
K 986	0.111818225	0.112922149
K 987	0.1	
K 988	0.097180585	0.125733696
K 989	0.1	
K 990	0.10158518	0.115851855
K 991	0.1	
K 992	0.105757665	0.176122581
K 993	0.1	
K 994	0.101408627	0.124746726
K 995	0.1	
K 996	0.100812454	0.181245435
K 997	0.1	
K 998	0.10227881	0.122788136
K 999	0.1	
K 1000	0.118794478	0.117629263
K 1001	0.1	
K 1002	0.108577846	0.144143685
K 1003	0.1	
K 1004	0.116491438	0.139485413
K 1005	0.1	
K 1006	0.102491263	0.136428949
K 1007	0.1	
K 1008	0.104819936	0.118937348
K 1009	0.1	
K 1010	0.110465729	0.174894575
K 1011	0.1	
K 1012	0.104796744	0.173766873
K 1013	0.1	
K 1014	0.101145286	0.116572229
K 1015	0.1	
K 1016	0.099028093	0.987153748
K 1017	0.1	
K 1018	0.106350031	0.119362948
K 1019	0.1	
K 1020	0.10158518	0.115851855
K 1021	0.1	
K 1022	0.107120024	0.189299319
K 1023	0.1	
K 1024	0.101681951	0.139321414
K 1025	0.1	
K 1026	0.137871242	0.12761732
K 1027	0.1	
K 1028	0.126378042	0.118815278
K 1029	0.1	
K 1030	0.1012036	0.11236

K 1031	0.1	
K 1032	0.115864632	0.132281696
K 1033	0.1	
K 1034	0.114132532	0.157428246
K 1035	0.1	
K 1036	0.092786731	0.117366772
K 1037	0.1	
K 1038	0.114265554	0.113295275
K 1039	0.1	
K 1040	0.103474928	0.11534865
K 1041	0.1	
K 1042	0.122739913	0.116362541
K 1043	0.1	
K 1044	0.122443588	0.113884197
K 1045	0.1	
K 1046	0.130618191	0.123698982
K 1047	0.1	
K 1048	0.09350091	0.111278745
K 1049	0.1	
K 1050	0.12413097	0.116871363
K 1051	0.1	
K 1052	0.115762246	0.11281978
K 1053	0.1	
K 1054	0.103939337	0.126846463
K 1055	0.1	
K 1056	0.109501209	0.142172665
K 1057	0.1	
K 1058	0.1255417	0.118721593
K 1059	0.1	
K 1060	0.096507258	0.111291993
K 1061	0.1	
K 1062	0.115419843	0.126412947
K 1063	0.1	
K 1064	0.101390435	0.119416415
K 1065	0.1	
K 1066	0.121591002	0.163423969
K 1067	0.1	
K 1068	0.098191823	0.171477615
K 1069	0.1	
K 1070	0.101722355	0.118429893
K 1071	0.1	
K 1072	0.10158518	0.115851855
K 1073	0.1	
K 1074	0.102662733	0.188487722
K 1075	0.1	
K 1076	0.103440575	0.116182385
K 1077	0.1	

K 1078	0.128671588	0.137558353
K 1079	0.1	
K 1080	0.098675072	0.112897973
K 1081	0.100237861	
K 1082	0.102722012	0.152694586
K 1083	0.108780175	
K 1084	0.120677839	0.14121779
K 1085	0.1	
K 1086	0.135924602	0.977814532
K 1087	0.1	
K 1088	0.106403527	0.141722148
K 1089	0.1	
K 1090	0.141053822	0.957537823
K 1091	0.1	
K 1092	0.102198385	0.111767575
K 1093	0.1	
K 1094	0.100070939	0.197539744
K 1095	0.1	
K 1096	0.183647277	0.147791473
K 1097	0.12707348	
K 1098	0.11056484	0.117183919
K 1099	0.1	
K 1100	0.100918998	0.111815875
K 1101	0.1	
K 1102	0.103943481	0.157752887
K 1103	0.1	
K 1104	0.133107198	0.123791131
K 1105	0.1	
K 1106	0.100799602	0.179961858
K 1107	0.1	
K 1108	0.116812107	0.115549947
K 1109	0.1	
K 1110	0.107345282	0.147448493
K 1111	0.1	
K 1112	0.0999826	0.992276963
K 1113	0.1	
K 1114	0.113809532	0.112327417
K 1115	0.1	
K 1116	0.103264555	0.183229793
K 1117	0.1	
K 1118	0.101457823	0.111388834
K 1119	0.1	
K 1120	0.101772491	0.117724913
K 1121	0.1	
K 1122	0.100305725	0.114389944
K 1123	0.1	
K 1124	0.104549726	0.982958772

K 1125	0.1	
K 1126	0.100994957	0.197397891
K 1127	0.1	
K 1128	0.109906406	0.115673484
K 1129	0.1	
K 1130	0.099961783	0.194487997
K 1131	0.1	
K 1132	0.100812454	0.181245435
K 1133	0.1	
K 1134	0.140562866	0.117132856
K 1135	0.1	
K 1136	0.10283093	0.179119477
K 1137	0.1	
K 1138	0.100812454	0.181245435
K 1139	0.1	
K 1140	0.136255849	0.122819622
K 1141	0.1	
K 1142	0.159013351	0.152415667
K 1143	0.104980567	
K 1144	0.124494596	0.122935192
K 1145	0.1	
K 1146	0.099838599	0.165738751
K 1147	0.1	
K 1148	0.122954203	0.11478837
K 1149	0.1	
K 1150	0.10636683	0.183954973
K 1151	0.1	
K 1152	0.114192353	0.161452536
K 1153	0.1	
K 1154	0.1273066	0.11995653
K 1155	0.1	
K 1156	0.109742607	0.114314625
K 1157	0.1	
K 1158	0.100288494	0.134349119
K 1159	0.1	
K 1160	0.106060829	0.128229568
K 1161	0.1	
K 1162	0.10158518	0.115851855
K 1163	0.1	
K 1164	0.106625007	0.129113211
K 1165	0.1	
K 1166	0.124646751	0.116978354
K 1167	0.1	
K 1168	0.11869957	0.111616824
K 1169	0.1	
K 1170	0.110073121	0.127915233
K 1171	0.1	

K 1172	0.10158518	0.115851855
K 1173	0.1	
K 1174	0.134565479	0.112731274
K 1175	0.1	
K 1176	0.102269892	0.115445683
K 1177	0.1	
K 1178	0.095175561	0.114262169
K 1179	0.1	
K 1180	0.100717704	0.121495528
K 1181	0.1	
K 1182	0.105926803	0.176923464
K 1183	0.100939697	
K 1184	0.112341165	0.146618299
K 1185	0.100939697	
K 1186	0.108485664	0.124985833
K 1187	0.100939697	
K 1188	0.104356717	0.132112933
K 1189	0.100939697	
K 1190	0.105334115	0.118394117
K 1191	0.1	
K 1192	0.118370628	0.172668768
K 1193	0.1	
K 1194	0.128406956	0.117739671
K 1195	0.1	
K 1196	0.103096067	0.169319963
K 1197	0.1	
K 1198	0.116728891	0.114124662
K 1199	0.1	
K 1200	0.108117427	0.123651366
K 1201	0.1	
K 1202	0.100476801	0.147688328
K 1203	0.1	
K 1204	0.109332562	0.113445799
K 1205	0.1	
K 1206	0.104423393	0.137433674
K 1207	0.1	
K 1208	0.10158518	0.115851855
K 1209	0.1	
K 1210	0.112057505	0.128625845
K 1211	0.1	
K 1212	0.138504228	0.124743726
K 1213	0.1	
K 1214	0.143178726	0.131588792
K 1215	0.1	
K 1216	0.100476801	0.147688328
K 1217	0.1	
K 1218	0.135888654	0.131519984

K 1219	0.1	
K 1220	0.117785604	0.198949345
K 1221	0.1	
K 1222	0.10546091	0.111123587
K 1223	0.1	
K 1224	0.104362679	0.119762233
K 1225	0.1	
K 1226	0.100663226	0.166322555
K 1227	0.1	
K 1228	0.100663226	0.166322555
K 1229	0.1	
K 1230	0.116271303	0.111711845
K 1231	0.1	
K 1232	0.105328279	0.113928964
K 1233	0.1	
K 1234	0.120447444	0.165782789
K 1235	0.1	
K 1236	0.103500514	0.169168755
K 1237	0.1	
K 1238	0.112260511	0.111114814
K 1239	0.1	
K 1240	0.095602306	0.122823395
K 1241	0.1	
K 1242	0.100663226	0.166322555
K 1243	0.1	
K 1244	0.119935793	0.1252346
K 1245	0.1	
K 1246	0.117658462	0.152835532
K 1247	0.1	
K 1248	0.132322411	0.117754574
K 1249	0.1	
K 1250	0.111323498	0.137591688
K 1251	0.1	
K 1252	0.116467682	0.198422798
K 1253	0.1	
K 1254	0.12754764	0.137579616
K 1255	0.1	
K 1256	0.112464518	0.127367865
K 1257	0.1	
K 1258	0.105978079	0.193843314
K 1259	0.1	
K 1260	0.129673831	0.187581825
K 1261	0.1	
K 1262	0.100202875	0.163823577
K 1263	0.1	
K 1264	0.112428621	0.122242489
K 1265	0.1	

K 1266	0.103819693	0.111877288
K 1267	0.1	
K 1268	0.100812454	0.181245435
K 1269	0.1	
K 1270	0.123525175	0.135198364
K 1271	0.1	
K 1272	0.107023453	0.996874119
K 1273	0.1	
K 1274	0.134568693	0.124127716
K 1275	0.1	
K 1276	0.11048386	0.121965311
K 1277	0.1	
K 1278	0.105953726	0.174515629
K 1279	0.1	
K 1280	0.102857529	0.119286614
K 1281	0.1	
K 1282	0.102892032	0.198563525
K 1283	0.1	
K 1284	0.111691852	0.142963692
K 1285	0.1	
K 1286	0.146700876	0.112653996
K 1287	0.1	
K 1288	0.110658846	0.166233884
K 1289	0.1	
K 1290	0.112538584	0.117755433
K 1291	0.1	
K 1292	0.115359238	0.126882929
K 1293	0.1	
K 1294	0.089980497	0.117186918
K 1295	0.1	
K 1296	0.147364711	0.132834799
K 1297	0.1	
K 1298	0.108740709	0.159496167
K 1299	0.1	
K 1300	0.125791673	0.112731818
K 1301	0.1	
K 1302	0.119547127	0.187838595
K 1303	0.1	
K 1304	0.100812454	0.181245435
K 1305	0.1	
K 1306	0.138165218	0.113273289
K 1307	0.1	
K 1308	0.123232203	0.118961145
K 1309	0.1	
K 1310	0.080088164	0.117328216
K 1311	0.1	
K 1312	0.115769601	0.111352898

K 1313	0.1	
K 1314	0.108693458	0.118665933
K 1315	0.1	
K 1316	0.126966089	0.112642866
K 1317	0.1	
K 1318	0.102182591	0.136288797
K 1319	0.1	
K 1320	0.108890051	0.189823897
K 1321	0.1	
K 1322	0.108158486	0.136163482
K 1323	0.1	
K 1324	0.123934868	0.113425786
K 1325	0.1	
K 1326	0.147992567	0.11848147
K 1327	0.1	
K 1328	0.109298586	0.147387115
K 1329	0.102680718	
K 1330	0.103622885	0.143375797
K 1331	0.1	
K 1332	0.116840482	0.169656963
K 1333	0.1	
K 1334	0.108454252	0.172288859
K 1335	0.1	
K 1336	0.097028693	0.152295259
K 1337	0.1	
K 1338	0.12211378	0.116552663
K 1339	0.1	
K 1340	0.098879495	0.992315592
K 1341	0.1	
K 1342	0.115877789	0.121852511
K 1343	0.1	
K 1344	0.104886058	0.116684731
K 1345	0.1	
K 1346	0.101041653	0.196711733
K 1347	0.1	
K 1348	0.10158518	0.115851855
K 1349	0.1	
K 1350	0.115750856	0.14168879
K 1351	0.1	
K 1352	0.110141206	0.11416184
K 1353	0.1	
K 1354	0.110252803	0.115866444
K 1355	0.1	
K 1356	0.100567688	0.97238222
K 1357	0.1	
K 1358	0.120956295	0.117225279
K 1359	0.1	

K 1360	0.111533947	0.169198832
K 1361	0.1	
K 1362	0.142834472	0.132615527
K 1363	0.1	
K 1364	0.129002273	0.118411538
K 1365	0.1	
K 1366	0.110014054	0.128284552
K 1367	0.1	
K 1368	0.125697096	0.111179185
K 1369	0.1	
K 1370	0.105092792	0.189943994
K 1371	0.1	
K 1372	0.124384242	0.146532893
K 1373	0.1	
K 1374	0.109312407	0.117632684
K 1375	0.1	
K 1376	0.104489316	0.185218146
K 1377	0.1	
K 1378	0.112611875	0.113612455
K 1379	0.1	
K 1380	0.097966449	0.116348684
K 1381	0.1	
K 1382	0.086773573	0.113611166
K 1383	0.1	
K 1384	0.118684341	0.11549563
K 1385	0.1	
K 1386	0.11735309	0.114536474
K 1387	0.1	
K 1388	0.108074726	0.133364699
K 1389	0.1	
K 1390	0.101220464	0.112514466
K 1391	0.1	
K 1392	0.132492121	0.136373132
K 1393	0.1	
K 1394	0.113776626	0.115496694
K 1395	0.1	
K 1396	0.129696218	0.118326587
K 1397	0.1	
K 1398	0.110507406	0.118647554
K 1399	0.1	
K 1400	0.113007412	0.111898573
K 1401	0.1	
K 1402	0.137503899	0.126118523
K 1403	0.1	
K 1404	0.113987331	0.196445259
K 1405	0.105	
K 1406	0.098901166	0.126411987

K 1407	0.1	
K 1408	0.124828787	0.132169119
K 1409	0.1	
K 1410	0.10286977	0.119522289
K 1411	0.1	
K 1412	0.10467414	0.114282728
K 1413	0.1	
K 1414	0.104234247	0.194824984
K 1415	0.1	
K 1416	0.158806052	0.121862728
K 1417	0.1	
K 1418	0.119838335	0.162925355
K 1419	0.1	
K 1420	0.10682187	0.127631288
K 1421	0.1	
K 1422	0.105416445	0.111133918
K 1423	0.1	
K 1424	0.104054724	0.113599115
K 1425	0.1	
K 1426	0.093809698	0.114643775
K 1427	0.1	
K 1428	0.122584261	0.137646385
K 1429	0.1	
K 1430	0.100811889	0.181188949
K 1431	0.1	
K 1432	0.121023362	0.118169213
K 1433	0.1	
K 1434	0.097110591	0.128585835
K 1435	0.1	
K 1436	0.140592087	0.129913186
K 1437	0.1	
K 1438	0.125808632	0.132692919
K 1439	0.1	
K 1440	0.100799602	0.179961858
K 1441	0.1	
K 1442	0.150598791	0.125689856
K 1443	0.1	
K 1444	0.128105587	0.116412154
K 1445	0.1	
K 1446	0.10940254	0.152186837
K 1447	0.1	
K 1448	0.121989741	0.119471267
K 1449	0.1	
K 1450	0.101559091	0.113542716
K 1451	0.1	
K 1452	0.102199449	0.174118444
K 1453	0.1	

K 1454	0.10826716	0.113692228
K 1455	0.1	
K 1456	0.122297186	0.954871742
K 1457	0.1	
K 1458	0.108531045	0.182592615
K 1459	0.1	
K 1460	0.100993514	0.199351388
K 1461	0.1	
K 1462	0.142521332	0.165738769
K 1463	0.1	
K 1464	0.102479364	0.127965178
K 1465	0.1	
K 1466	0.13091991	0.112638984
K 1467	0.1	
K 1468	0.100385258	0.114571319
K 1469	0.1	
K 1470	0.091196516	0.172477227
K 1471	0.1	
K 1472	0.108417945	0.117664215
K 1473	0.1	
K 1474	0.120623823	0.171531834
K 1475	0.099853382	
K 1476	0.13868787	0.134937485
K 1477	0.100237861	
K 1478	0.114940288	0.152931969
K 1479	0.1	
K 1480	0.103728543	0.198161698
K 1481	0.1	
K 1482	0.109669286	0.111269749
K 1483	0.1	
K 1484	0.097821485	0.117133615
K 1485	0.1	
K 1486	0.120891294	0.127864996
K 1487	0.1	
K 1488	0.105296219	0.149189913
K 1489	0.1	
K 1490	0.106134427	0.116116713
K 1491	0.1	
K 1492	0.122843134	0.119699624
K 1493	0.1	
K 1494	0.118292798	0.121132127
K 1495	0.1	
K 1496	0.099474787	0.114814246
K 1497	0.1	
K 1498	0.104155948	0.119618276
K 1499	0.1	
K 1500	0.105997803	0.157293189

K 1501	0.1	
K 1502	0.071045365	0.134549418
K 1503	0.187193666	
K 1504	0.115058571	0.113317853
K 1505	0.105102366	
K 1506	0.108502356	0.111398751
K 1507	0.09839776	
K 1508	0.097747819	0.121743962
K 1509	0.098830779	
K 1510	0.132942339	0.122433894
K 1511	0.1	
K 1512	0.101883789	0.118837896
K 1513	0.1	
K 1514	0.127781956	0.165666545
K 1515	0.1	
K 1516	0.11995855	0.122964899
K 1517	0.1	
K 1518	0.100899615	0.111514295
K 1519	0.1	
K 1520	0.114818268	0.123459922
K 1521	0.1	
K 1522	0.102207165	0.115344212
K 1523	0.1	
K 1524	0.10201	0.121
K 1525	0.1	
K 1526	0.103784817	0.122783188
K 1527	0.1	
K 1528	0.106662345	0.163247396
K 1529	0.1	
K 1530	0.131037055	0.132436418
K 1531	0.1	
K 1532	0.141105625	0.124358155
K 1533	0.1	
K 1534	0.131949899	0.124471221
K 1535	0.1	
K 1536	0.11858881	0.168754119
K 1537	0.1	
K 1538	0.101294146	0.176526673
K 1539	0.1	
K 1540	0.110005628	0.119424292
K 1541	0.1	
K 1542	0.108199284	0.114361355
K 1543	0.1	
K 1544	0.112688189	0.171579579
K 1545	0.1	
K 1546	0.102612329	0.136128199
K 1547	0.1	

K 1548	0.100950755	0.19863792
K 1549	0.1	
K 1550	0.10201	0.121
K 1551	0.1	
K 1552	0.102569603	0.129126614
K 1553	0.1	
K 1554	0.105542635	0.198215369
K 1555	0.1	
K 1556	0.100516522	0.134747226
K 1557	0.1	
K 1558	0.106321119	0.111239629
K 1559	0.1	
K 1560	0.112008067	0.116272464
K 1561	0.1	
K 1562	0.122494587	0.123329891
K 1563	0.1	
K 1564	0.148584674	0.134339696
K 1565	0.1	
K 1566	0.102335479	0.123543594
K 1567	0.1	
K 1568	0.100407458	0.143696623
K 1569	0.1	
K 1570	0.118653004	0.137653385
K 1571	0.1	
K 1572	0.108564686	0.116481575
K 1573	0.1	
K 1574	0.097309306	0.148645265
K 1575	0.1	
K 1576	0.134155716	0.158469486
K 1577	0.101681951	
K 1578	0.038858319	0.755527428
K 1579	0.271333638	
K 1580	0.108512494	0.113547462
K 1581	0.1	
K 1582	0.094681768	0.111668446
K 1583	0.1	
K 1584	0.098307109	0.113156548
K 1585	0.1	
K 1586	0.097871297	0.12965545
K 1587	0.1	
K 1588	0.131603888	0.165831273
K 1589	0.1	
K 1590	0.107208048	0.154257193
K 1591	0.1	
K 1592	0.097255168	0.125429947
K 1593	0.1	
K 1594	0.11754285	0.112459512

K 1595	0.1	
K 1596	0.108424925	0.116915715
K 1597	0.100939697	
K 1598	0.103950352	0.115611197
K 1599	0.102314864	
K 1600	0.11920032	0.115987158
K 1601	0.1	
K 1602	0.112530092	0.992869835
K 1603	0.1	
K 1604	0.14848744	0.131777755
K 1605	0.1	
K 1606	0.12038919	0.139414565
K 1607	0.1	
K 1608	0.146705627	0.151719113
K 1609	0.1	
K 1610	0.140454751	0.122496893
K 1611	0.1	
K 1612	0.122569781	0.112861742
K 1613	0.1	
K 1614	0.132782178	0.123423329
K 1615	0.1	
K 1616	0.123048044	0.132436724
K 1617	0.1	
K 1618	0.119758259	0.136555782
K 1619	0.1	
K 1620	0.092261913	0.97833377
K 1621	0.1	
K 1622	0.10427641	0.119542544
K 1623	0.1	
K 1624	0.115917325	0.999635838
K 1625	0.1	
K 1626	0.1392038	0.147665935
K 1627	0.1	
K 1628	0.143966697	0.123424867
K 1629	0.1	
K 1630	0.107380341	0.118817196
K 1631	0.1	
K 1632	0.105600812	0.12432167
K 1633	0.1	
K 1634	0.129525344	0.116522874
K 1635	0.1	
K 1636	0.139494324	0.114253839
K 1637	0.1	
K 1638	0.101394568	0.128995994
K 1639	0.1	
K 1640	0.107483133	0.115599887
K 1641	0.1	

K 1642	0.101772491	0.117724913
K 1643	0.1	
K 1644	0.119174507	0.121986932
K 1645	0.1	
K 1646	0.120945261	0.158625329
K 1647	0.1	
K 1648	0.096078486	0.149481952
K 1649	0.1	
K 1650	0.128286195	0.128382479
K 1651	0.1	
K 1652	0.10518261	0.173588119
K 1653	0.1	
K 1654	0.107125019	0.111454665
K 1655	0.1	
K 1656	0.107369844	0.137539823
K 1657	0.1	
K 1658	0.117239024	0.135318126
K 1659	0.1	
K 1660	0.098115617	0.112283813
K 1661	0.1	
K 1662	0.097910223	0.113265163
K 1663	0.1	
K 1664	0.100663226	0.166322555
K 1665	0.1	
K 1666	0.115103989	0.116337119
K 1667	0.1	
K 1668	0.114359678	0.175892664
K 1669	0.1	
K 1670	0.14785682	0.144621353
K 1671	0.1	
K 1672	0.131879833	0.115663517
K 1673	0.108959391	
K 1674	0.116754232	0.11294948
K 1675	0.102337569	
K 1676	0.13987924	0.126431557
K 1677	0.1	
K 1678	0.136927202	0.132783433
K 1679	0.1	
K 1680	0.101883789	0.118837896
K 1681	0.1	
K 1682	0.10201	0.121
K 1683	0.1	
K 1684	0.142087683	0.176455853
K 1685	0.1	
K 1686	0.127311639	0.116435586
K 1687	0.1	
K 1688	0.103261909	0.149643739

K 1689	0.1	
K 1690	0.12299524	0.172584836
K 1691	0.1	
K 1692	0.105421372	0.112412876
K 1693	0.1	
K 1694	0.105157233	0.113966232
K 1695	0.1	
K 1696	0.106744616	0.137812349
K 1697	0.1	
K 1698	0.110588011	0.114857867
K 1699	0.1	
K 1700	0.118949428	0.123537476
K 1701	0.1	
K 1702	0.130563797	0.124991245
K 1703	0.1	
K 1704	0.100851798	0.155884992
K 1705	0.1	
K 1706	0.100663226	0.166322555
K 1707	0.1	
K 1708	0.125271444	0.137473841
K 1709	0.1	
K 1710	0.109344243	0.168226765
K 1711	0.1	
K 1712	0.102373052	0.152621485
K 1713	0.1	
K 1714	0.116717139	0.147865738
K 1715	0.1	
K 1716	0.126907587	0.154463699
K 1717	0.1	
K 1718	0.104761145	0.132954588
K 1719	0.1	
K 1720	0.099259628	0.179961858
K 1721	0.1	
K 1722	0.10038908	0.144682856
K 1723	0.1	
K 1724	0.098058514	0.131212843
K 1725	0.1	
K 1726	0.100663226	0.166322555
K 1727	0.1	
K 1728	0.104092385	0.996368496
K 1729	0.1	
K 1730	0.104913295	0.114496937
K 1731	0.1	
K 1732	0.102305097	0.118154543
K 1733	0.1	
K 1734	0.111633436	0.123116525
K 1735	0.1	

K 1736	0.139004307	0.128139862
K 1737	0.1	
K 1738	0.104117979	0.131451677
K 1739	0.1	
K 1740	0.104518441	0.117722884
K 1741	0.1	
K 1742	0.178737967	0.145957432
K 1743	0.1	
K 1744	0.09879896	0.126684944
K 1745	0.1	
K 1746	0.101253906	0.112539625
K 1747	0.1	
K 1748	0.112050249	0.112631266
K 1749	0.1	
K 1750	0.110916516	0.134516269
K 1751	0.1	
K 1752	0.106903397	0.181121982
K 1753	0.1	
K 1754	0.107310235	0.116721377
K 1755	0.1	
K 1756	0.102356647	0.121139926
K 1757	0.1	
K 1758	0.128466344	0.139833235
K 1759	0.1	
K 1760	0.125246666	0.13422186
K 1761	0.097132896	
K 1762	0.103163009	0.11897753
K 1763	0.1	
K 1764	0.132645631	0.113537248
K 1765	0.102050702	
K 1766	0.109215384	0.113877562
K 1767	0.100998726	
K 1768	0.100161254	0.112829132
K 1769	0.102050702	
K 1770	0.151327089	0.127272685
K 1771	0.10080782	
K 1772	0.112782632	0.161359761
K 1773	0.101152157	
K 1774	0.124711335	0.112888332
K 1775	0.100456687	
K 1776	0.120027677	0.133276598
K 1777	0.1	
K 1778	0.148015154	0.656837292
K 1779	0.1	
K 1780	0.129409327	0.119772544
K 1781	0.1	
K 1782	0.108717807	0.11778825

K 1783	0.1	
K 1784	0.120358273	0.121866663
K 1785	0.1	
K 1786	0.105239616	0.114327377
K 1787	0.1	
K 1788	0.112711156	0.167528652
K 1789	0.1	
K 1790	0.102551526	0.121443277
K 1791	0.1	
K 1792	0.113214232	0.115831171
K 1793	0.1	
K 1794	0.123685781	0.118785356
K 1795	0.1	
K 1796	0.131100099	0.126633827
K 1797	0.1	
K 1798	0.127214705	0.122592214
K 1799	0.1	
K 1800	0.106181706	0.113374198
K 1801	0.1	
K 1802	0.137182748	0.135999939
K 1803	0.1	
K 1804	0.107423045	0.114522712
K 1805	0.1	
K 1806	0.110031791	0.985618442
K 1807	0.1	
K 1808	0.107916663	0.167962341
K 1809	0.1	
K 1810	0.140672255	0.119326531
K 1811	0.1	
K 1812	0.097183189	0.123668618
K 1813	0.1	
K 1814	0.148401532	0.126553543
K 1815	0.1	
K 1816	0.126649024	0.114931893
K 1817	0.1	
K 1818	0.132817859	0.124714492
K 1819	0.1	
K 1820	0.104930408	0.111749617
K 1821	0.1	
K 1822	0.125485673	0.123485149
K 1823	0.1	
K 1824	0.122120937	0.112327849
K 1825	0.1	
K 1826	0.108961181	0.191823589
K 1827	0.1	
K 1828	0.106020229	0.196919572
K 1829	0.1	

K 1830	0.130908868	0.131134152
K 1831	0.1	
K 1832	0.133843572	0.11181798
K 1833	0.1	
K 1834	0.101862976	0.195696612
K 1835	0.1	
K 1836	0.111714379	0.151391519
K 1837	0.1	
K 1838	0.101831824	0.115129648
K 1839	0.1	
K 1840	0.107433476	0.133899419
K 1841	0.1	
K 1842	0.120721781	0.114926294
K 1843	0.1	
K 1844	0.100663226	0.166322555
K 1845	0.1	
K 1846	0.142521332	0.165738769
K 1847	0.1	
K 1848	0.100663226	0.166322555
K 1849	0.1	
K 1850	0.107876744	0.113163893
K 1851	0.1	
K 1852	0.124903016	0.129765367
K 1853	0.1	
K 1854	0.137613844	0.118717212
K 1855	0.1	
K 1856	0.106623668	0.987494547
K 1857	0.1	
K 1858	0.098370513	0.125552899
K 1859	0.1	
K 1860	0.134931113	0.119192815
K 1861	0.1	
K 1862	0.098380123	0.119874178
K 1863	0.1	
K 1864	0.161577679	0.161726954
K 1865	0.1	
K 1866	0.103065509	0.124821649
K 1867	0.1	
K 1868	0.110740233	0.163122844
K 1869	0.1	
K 1870	0.133846071	0.112177931
K 1871	0.1	
K 1872	0.130414123	0.147992867
K 1873	0.1	
K 1874	0.130382022	0.122824887
K 1875	0.1	
K 1876	0.102735634	0.162674823

K 1877	0.1	
K 1878	0.14495849	0.129665344
K 1879	0.1	
K 1880	0.100663226	0.166322555
K 1881	0.1	
K 1882	0.100681977	0.198686274
K 1883	0.1	
K 1884	0.12485064	0.128435295
K 1885	0.1	
K 1886	0.119799147	0.142335249
K 1887	0.103158687	
K 1888	0.103060576	0.129632879
K 1889	0.1	
K 1890	0.120398618	0.112149192
K 1891	0.1	
K 1892	0.108368409	0.116798387
K 1893	0.1	
K 1894	0.111264861	0.813462494
K 1895	0.1	
K 1896	0.113123136	0.1317298
K 1897	0.1	
K 1898	0.099555315	0.166469796
K 1899	0.1	
K 1900	0.10201	0.121
K 1901	0.1	
K 1902	0.10271738	0.129713169
K 1903	0.1	
K 1904	0.118209479	0.192366295
K 1905	0.1	
K 1906	0.101313976	0.162161281
K 1907	0.1	
K 1908	0.120426567	0.124285384
K 1909	0.1	
K 1910	0.113421059	0.118665715
K 1911	0.1	
K 1912	0.100812454	0.181245435
K 1913	0.1	
K 1914	0.134449854	0.124727667
K 1915	0.1	
K 1916	0.119747248	0.122799335
K 1917	0.1	
K 1918	0.108907105	0.134925785
K 1919	0.1	
K 1920	0.096906448	0.859849924
K 1921	0.1	
K 1922	0.102832341	0.127452884
K 1923	0.1	

K 1924	0.119955994	0.113279196
K 1925	0.1	
K 1926	0.100446829	0.144682856
K 1927	0.1	
K 1928	0.103384922	0.137564827
K 1929	0.1	
K 1930	0.091994746	0.111149132
K 1931	0.1	
K 1932	0.09995918	0.128871387
K 1933	0.1	
K 1934	0.107205033	0.97331429
K 1935	0.1	
K 1936	0.109355861	0.168933218
K 1937	0.1	
K 1938	0.119388222	0.122144376
K 1939	0.1	
K 1940	0.107205033	0.945878419
K 1941	0.1	
K 1942	0.119104908	0.119939432
K 1943	0.1	
K 1944	0.107023453	0.117786838
K 1945	0.1	
K 1946	0.099346372	0.161483293
K 1947	0.103431701	
K 1948	0.136347398	0.119597765
K 1949	0.1	
K 1950	0.130166808	0.111826397
K 1951	0.1	
K 1952	0.102526857	0.13919695
K 1953	0.1	
K 1954	0.1092077	0.993819377
K 1955	0.1	
K 1956	0.107357141	0.143219167
K 1957	0.1	
K 1958	0.14871757	0.138218169
K 1959	0.1	
K 1960	0.123417632	0.114926922
K 1961	0.1	
K 1962	0.10772778	0.944544855
K 1963	0.1	
K 1964	0.10201	0.121
K 1965	0.1	
K 1966	0.09955469	0.111138772
K 1967	0.1	
K 1968	0.098368502	0.116774237
K 1969	0.1	
K 1970	0.101883789	0.118837896

K 1971	0.1	
K 1972	0.10201	0.121
K 1973	0.1	
K 1974	0.109108403	0.123289699
K 1975	0.1	
K 1976	0.084219598	0.114345112
K 1977	0.1	
K 1978	0.117556644	0.116222638
K 1979	0.1	
K 1980	0.106080547	0.167237632
K 1981	0.1	
K 1982	0.10201	0.121
K 1983	0.1	
K 1984	0.127949199	0.121697833
K 1985	0.1	
K 1986	0.101657075	0.135498659
K 1987	0.1	
K 1988	0.095926249	0.124612671
K 1989	0.1	
K 1990	0.103344516	0.118141393
K 1991	0.1	
K 1992	0.117068312	0.115298358
K 1993	0.1	
K 1994	0.112926699	0.145764655
K 1995	0.1	
K 1996	0.101772491	0.117724913
K 1997	0.1	
K 1998	0.144352883	0.116599179
K 1999	0.1	
K 2000	0.101139136	0.113773196
K 2001	0.1	
K 2002	0.133831654	0.114363177
K 2003	0.1	
K 2004	0.132031688	0.167984599
K 2005	0.1	
K 2006	0.105042718	0.129788942
K 2007	0.1	
K 2008	0.101903456	0.111635145
K 2009	0.1	
K 2010	0.138962331	0.13899137
K 2011	0.1	
K 2012	0.100628276	0.923184684
K 2013	0.1	
K 2014	0.111473289	0.114637529
K 2015	0.1	
K 2016	0.137449611	0.132879328
K 2017	0.1	

K 2018	0.129598242	0.167948997
K 2019	0.1	
K 2020	0.124657568	0.12858464
K 2021	0.1	
K 2022	0.100663226	0.166322555
K 2023	0.1	
K 2024	0.10201	0.121
K 2025	0.1	
K 2026	0.103658885	0.179493531
K 2027	0.1	
K 2028	0.119083458	0.114659895
K 2029	0.1	
K 2030	0.118119399	0.114746248
K 2031	0.1	
K 2032	0.103820765	0.129461589
K 2033	0.1	
K 2034	0.100733256	0.193498618
K 2035	0.1	
K 2036	0.102524513	0.122961454
K 2037	0.1	
K 2038	0.099688325	0.143696623
K 2039	0.1	
K 2040	0.105004257	0.111191986
K 2041	0.1	
K 2042	0.104460154	0.132192146
K 2043	0.1	
K 2044	0.099664092	0.144682856
K 2045	0.1	
K 2046	0.100663226	0.166322555
K 2047	0.1	
K 2048	0.100411036	0.116795756
K 2049	0.1	
K 2050	0.105780345	0.114458753
K 2051	0.1	
K 2052	0.108118122	0.943193672
K 2053	0.1	
K 2054	0.10201	0.121
K 2055	0.1	
K 2056	0.119360822	0.116227389
K 2057	0.1	
K 2058	0.099009973	0.121272562
K 2059	0.1	
K 2060	0.106908947	0.112698623
K 2061	0.1	
K 2062	0.10201	0.121
K 2063	0.1	
K 2064	0.10698874	0.166316721

K 2065	0.1	
K 2066	0.112868443	0.173814336
K 2067	0.1	
K 2068	0.105483721	0.112751688
K 2069	0.1	
K 2070	0.102450786	0.151484297
K 2071	0.1	
K 2072	0.112563988	0.17876894
K 2073	0.1	
K 2074	0.100993514	0.199351388
K 2075	0.1	
K 2076	0.1164322	0.115975131
K 2077	0.1	
K 2078	0.115167736	0.133895389
K 2079	0.1	
K 2080	0.107991133	0.163113444
K 2081	0.1	
K 2082	0.101772491	0.117724913
K 2083	0.1	
K 2084	0.11286707	0.14165355
K 2085	0.1	
K 2086	0.124495013	0.128727843
K 2087	0.1	
K 2088	0.105830993	0.112198167
K 2089	0.1	
K 2090	0.103447641	0.175635199
K 2091	0.1	
K 2092	0.111101484	0.117737699
K 2093	0.1	
K 2094	0.101640123	0.112757243
K 2095	0.1	
K 2096	0.0989512	0.125452649
K 2097	0.1	
K 2098	0.113607514	0.126723754
K 2099	0.1	
K 2100	0.111257131	0.984756563
K 2101	0.1	
K 2102	0.119046552	0.191274568
K 2103	0.1	
K 2104	0.135888825	0.128177624
K 2105	0.1	
K 2106	0.100446829	0.144682856
K 2107	0.1	
K 2108	0.100663226	0.166322555
K 2109	0.1	
K 2110	0.104010782	0.184722972
K 2111	0.1	

K 2112	0.098725731	0.181937557
K 2113	0.1	
K 2114	0.10201	0.121
K 2115	0.1	
K 2116	0.099646894	0.935259882
K 2117	0.1	
K 2118	0.103650107	0.117228137
K 2119	0.1	
K 2120	0.111896076	0.946233586
K 2121	0.1	
K 2122	0.106443018	0.124865129
K 2123	0.1	
K 2124	0.135974902	0.129631331
K 2125	0.1	
K 2126	0.10910663	0.155254817
K 2127	0.1	
K 2128	0.101867525	0.175169438
K 2129	0.1	
K 2130	0.101772491	0.117724913
K 2131	0.1	
K 2132	0.107264534	0.112674635
K 2133	0.1	
K 2134	0.112686414	0.986856313
K 2135	0.1	
K 2136	0.10340468	0.122567614
K 2137	0.1	
K 2138	0.100663226	0.166322555
K 2139	0.1	
K 2140	0.10201	0.121
K 2141	0.1	
K 2142	0.102708124	0.167362528
K 2143	0.1	
K 2144	0.101182674	0.195616623
K 2145	0.1	
K 2146	0.100791331	0.994112658
K 2147	0.1	
K 2148	0.104480432	0.112927847
K 2149	0.1	
K 2150	0.100190089	0.199351388
K 2151	0.1	
K 2152	0.10161725	0.11248316
K 2153	0.1	
K 2154	0.100446829	0.144682856
K 2155	0.1	
K 2156	0.100663226	0.166322555
K 2157	0.1	
K 2158	0.099798325	0.113914224

K 2159	0.1	
K 2160	0.134436567	0.116748971
K 2161	0.1	
K 2162	0.139663683	0.135862299
K 2163	0.1	
K 2164	0.100817994	0.123437977
K 2165	0.1	
K 2166	0.102005338	0.116799139
K 2167	0.1	
K 2168	0.106207874	0.151163529
K 2169	0.1	
K 2170	0.110466261	0.128189939
K 2171	0.1	
K 2172	0.133992087	0.121845359
K 2173	0.10511405	
K 2174	0.003003848	0.321248487
K 2175	0.098518166	
K 2176	0.096251923	0.197985453
K 2177	0.1	
K 2178	0.10416309	0.121345291
K 2179	0.1	
K 2180	0.124461133	0.125691678
K 2181	0.1	
K 2182	0.100446829	0.144682856
K 2183	0.1	
K 2184	0.13108398	0.124616917
K 2185	0.1	
K 2186	0.102086609	0.115291731
K 2187	0.1	
K 2188	0.142521332	0.165738769
K 2189	0.1	
K 2190	0.001414328	0.149772457
K 2191	0.1	
K 2192	0.104548661	0.111475688
K 2193	0.1	
K 2194	0.11104807	0.117651158
K 2195	0.1	
K 2196	0.101270664	0.192451745
K 2197	0.1	
K 2198	0.126447827	0.174144285
K 2199	0.1	
K 2200	0.128600022	0.122544356
K 2201	0.1	
K 2202	0.094039642	0.111354656
K 2203	0.1	
K 2204	0.10034933	0.143696623
K 2205	0.1	

K 2206	0.107072995	0.193192287
K 2207	0.1	
K 2208	0.094237705	0.114335118
K 2209	0.1	
K 2210	0.118675461	0.117693856
K 2211	0.1	
K 2212	0.126411271	0.167224994
K 2213	0.1	
K 2214	0.128384836	0.126481694
K 2215	0.1	
K 2216	0.100993514	0.199351388
K 2217	0.1	
K 2218	0.025493695	0.116185538
K 2219	0.1	
K 2220	0.101460054	0.117973712
K 2221	0.1	
K 2222	0.199656923	0.256899414
K 2223	0.1	
K 2224	0.135907	0.155651689
K 2225	0.1	
K 2226	0.099846893	0.143696623
K 2227	0.1	
K 2228	0.123635893	0.162997369
K 2229	0.1	
K 2230	0.0935895	0.992987917
K 2231	0.1	
K 2232	0.125451436	0.111712113
K 2233	0.1	
K 2234	0.126075027	0.114351563
K 2235	0.1	
K 2236	0.13362824	0.116537448
K 2237	0.1	
K 2238	0.106797393	0.973644582
K 2239	0.1	
K 2240	0.112324764	0.959778363
K 2241	0.1	
K 2242	0.135290177	0.123738397
K 2243	0.1	
K 2244	0.06297522	0.535435274
K 2245	0.390810913	
K 2246	0.108399705	0.132819134
K 2247	0.11088276	
K 2248	0.102656016	0.126986847
K 2249	0.1	
K 2250	0.10117863	0.111171175
K 2251	0.1	
K 2252	0.113138479	0.158664392

K 2253	0.1	
K 2254	0.134216787	0.152875571
K 2255	0.1	
K 2256	0.107040481	0.124925249
K 2257	0.1	
K 2258	0.104122342	0.164185895
K 2259	0.1	
K 2260	0.107727385	0.118636798
K 2261	0.1	
K 2262	0.118325447	0.123114966
K 2263	0.1	
K 2264	0.107028769	0.198322285
K 2265	0.1	
K 2266	0.111838901	0.177271427
K 2267	0.1	
K 2268	0.112391092	0.188137267
K 2269	0.1	
K 2270	0.132594895	0.113124223
K 2271	0.1	
K 2272	0.123972306	0.11869966
K 2273	0.1	
K 2274	0.113937248	0.115914748
K 2275	0.1	
K 2276	0.125048067	0.138916136
K 2277	0.1	
K 2278	0.105753842	0.15712566
K 2279	0.1	
K 2280	0.130609233	0.121843894
K 2281	0.1	
K 2282	0.101306757	0.174467362
K 2283	0.1	
K 2284	0.10451187	0.198929632
K 2285	0.1	
K 2286	0.09785476	0.172419347
K 2287	0.1	
K 2288	0.140359441	0.127792571
K 2289	0.1	
K 2290	0.127623143	0.124741455
K 2291	0.1	
K 2292	0.115498873	0.138668487
K 2293	0.1	
K 2294	0.100663226	0.166322555
K 2295	0.1	
K 2296	0.119363009	0.123731594
K 2297	0.1	
K 2298	0.11062593	0.118714268
K 2299	0.1	

K 2300	0.130092055	0.129458684
K 2301	0.1	
K 2302	0.114649703	0.122344628
K 2303	0.1	
K 2304	0.105112314	0.112314734
K 2305	0.1	
K 2306	0.101876065	0.126163455
K 2307	0.1	
K 2308	0.133062595	0.146561685
K 2309	0.1	
K 2310	0.103284357	0.11631945
K 2311	0.1	
K 2312	0.101872336	0.111429374
K 2313	0.1	
K 2314	0.112044743	0.188466742
K 2315	0.1	
K 2316	0.114267706	0.114323599
K 2317	0.1	
K 2318	0.100832291	0.183229793
K 2319	0.1	
K 2320	0.118952037	0.117547357
K 2321	0.1	
K 2322	0.107163434	0.121764257
K 2323	0.1	
K 2324	0.111315658	0.125199993
K 2325	0.1	
K 2326	0.120876585	0.112532295
K 2327	0.1	
K 2328	0.10408225	0.149829886
K 2329	0.1	
K 2330	0.128771132	0.113177183
K 2331	0.1	
K 2332	0.099577121	0.179961858
K 2333	0.1	
K 2334	0.101825902	0.122845639
K 2335	0.1	
K 2336	0.120586029	0.122768197
K 2337	0.1	
K 2338	0.10158518	0.115851855
K 2339	0.1	
K 2340	0.120881355	0.163345413
K 2341	0.1	
K 2342	0.115346606	0.193265442
K 2343	0.1	
K 2344	0.105034407	0.176251499
K 2345	0.1	
K 2346	0.102961313	0.123259167

K 2347	0.1	
K 2348	0.121017582	0.129459216
K 2349	0.1	
K 2350	0.100812454	0.181245435
K 2351	0.1	
K 2352	0.114901585	0.112188648
K 2353	0.1	
K 2354	0.128862398	0.122466315
K 2355	0.1	
K 2356	0.112882128	0.18834465
K 2357	0.1	
K 2358	0.1587058	0.171839461
K 2359	0.1	
K 2360	0.1073381	0.116635929
K 2361	0.1	
K 2362	0.10500858	0.11347254
K 2363	0.1	
K 2364	0.109750163	0.124648462
K 2365	0.1	
K 2366	0.135766642	0.133989445
K 2367	0.1	
K 2368	0.13036917	0.121795721
K 2369	0.1	
K 2370	0.122240763	0.124526982
K 2371	0.1	
K 2372	0.102236396	0.116425681
K 2373	0.1	
K 2374	0.121475303	0.155985855
K 2375	0.1	
K 2376	0.137295127	0.128133457
K 2377	0.1	
K 2378	0.100216385	0.143696623
K 2379	0.1	
K 2380	0.104814372	0.113299916
K 2381	0.1	
K 2382	0.116744881	0.119796642
K 2383	0.1	
K 2384	0.104558363	0.111276965
K 2385	0.1	
K 2386	0.121788836	0.119996829
K 2387	0.1	
K 2388	0.109099565	0.114954968
K 2389	0.1	
K 2390	0.11152327	0.112135584
K 2391	0.1	
K 2392	0.106902703	0.198127397
K 2393	0.1	

K 2394	0.100396291	0.144682856
K 2395	0.1	
K 2396	0.100663226	0.166322555
K 2397	0.1	
K 2398	0.098730993	0.127637921
K 2399	0.1	
K 2400	0.103847147	0.122397878
K 2401	0.1	
K 2402	0.189392848	0.135274543
K 2403	0.1	
K 2404	0.011081668	0.175171929
K 2405	0.1	
K 2406	0.149914827	0.118836817
K 2407	0.1	
K 2408	0.103440739	0.11228588
K 2409	0.1	
K 2410	0.099661519	0.115962184
K 2411	0.1	
K 2412	0.142521332	0.142734531
K 2413	0.1	
K 2414	0.102148326	0.191318112
K 2415	0.1	
K 2416	0.163000677	0.227718584
K 2417	0.1	
K 2418	0.100663226	0.166322555
K 2419	0.1	
K 2420	0.13613711	0.116258145
K 2421	0.1	
K 2422	0.103719027	0.115567916
K 2423	0.1	
K 2424	0.126791381	0.115225275
K 2425	0.1	
K 2426	0.103771863	0.111294342
K 2427	0.1	
K 2428	0.107342478	0.123294552
K 2429	0.100237861	
K 2430	0.109712957	0.121297368
K 2431	0.099808193	
K 2432	0.117213889	0.128392748
K 2433	0.100237861	
K 2434	0.111298837	0.168123261
K 2435	0.100237861	
K 2436	0.111188932	0.997414345
K 2437	0.100237861	
K 2438	0.115974138	0.112138467
K 2439	0.100237861	
K 2440	0.122370601	0.118986598

K 2441	0.100237861	
K 2442	0.101406961	0.114276993
K 2443	0.100237861	
K 2444	0.100444721	0.985589769
K 2445	0.110967998	
K 2446	0.123075192	0.116556678
K 2447	0.11018087	
K 2448	0.108320475	0.193262724
K 2449	0.1	
K 2450	0.098380126	0.17352744
K 2451	0.1	
K 2452	0.127105102	0.954936423
K 2453	0.1	
K 2454	0.1004037	0.137414623
K 2455	0.1	
K 2456	0.109347418	0.112453153
K 2457	0.1	
K 2458	0.113754411	0.11319979
K 2459	0.1	
K 2460	0.10158518	0.115851855
K 2461	0.1	
K 2462	0.112343248	0.117884913
K 2463	0.1	
K 2464	0.112982785	0.158126229
K 2465	0.1	
K 2466	0.102924453	0.11129244
K 2467	0.1	
K 2468	0.101699688	0.113259835
K 2469	0.1	
K 2470	0.102572144	0.112263865
K 2471	0.1	
K 2472	0.100239767	0.14185538
K 2473	0.1	
K 2474	0.099939132	0.143696623
K 2475	0.1	
K 2476	0.09695553	0.113283385
K 2477	0.1	
K 2478	0.102988635	0.178512199
K 2479	0.1	
K 2480	0.102962894	0.111778971
K 2481	0.1	
K 2482	0.101202133	0.136475912
K 2483	0.1	
K 2484	0.109876358	0.134426285
K 2485	0.1	
K 2486	0.103289797	0.972261145
K 2487	0.1	

K 2488	0.121931198	0.118298914
K 2489	0.1	
K 2490	0.098995225	0.117552882
K 2491	0.102035295	
K 2492	0.132894529	0.115159937
K 2493	0.103710058	
K 2494	0.103181076	0.112371385
K 2495	0.1	
K 2496	0.131524726	0.114494445
K 2497	0.1	
K 2498	0.101478858	0.114962371
K 2499	0.1	
K 2500	0.10207484	0.121187382
K 2501	0.1	
K 2502	0.101346246	0.115896249
K 2503	0.1	
K 2504	0.10073055	0.116978454
K 2505	0.1	
K 2506	0.106078613	0.134326666
K 2507	0.1	
K 2508	0.101992689	0.114953196
K 2509	0.1	
K 2510	0.103402608	0.963951993
K 2511	0.1	
K 2512	0.10183412	0.191896623
K 2513	0.1	
K 2514	0.111409834	0.119312432
K 2515	0.1	
K 2516	0.115309646	0.124426692
K 2517	0.1	
K 2518	0.124234743	0.124644627
K 2519	0.1	
K 2520	0.127390282	0.151367814
K 2521	0.1	
K 2522	0.116834481	0.178723467
K 2523	0.1	
K 2524	0.137935445	0.12942678
K 2525	0.1	
K 2526	0.1156248	0.173384356
K 2527	0.1	
K 2528	0.108536086	0.127437155
K 2529	0.1	
K 2530	0.111180556	0.127637774
K 2531	0.1	
K 2532	0.130558977	0.127534385
K 2533	0.1	
K 2534	0.100491584	0.121847413

K 2535	0.1	
K 2536	0.127575952	0.143165147
K 2537	0.1	
K 2538	0.102968367	0.127312894
K 2539	0.1	
K 2540	0.096081563	0.15888482
K 2541	0.1	
K 2542	0.11796163	0.114621916
K 2543	0.1	
K 2544	0.138956126	0.137996112
K 2545	0.1	
K 2546	0.094056954	0.132752127
K 2547	0.1	
K 2548	0.121046295	0.126183458
K 2549	0.1	
K 2550	0.121141888	0.133185389
K 2551	0.1	
K 2552	0.105637236	0.191911524
K 2553	0.1	
K 2554	0.124262144	0.135853518
K 2555	0.1	
K 2556	0.10446198	0.112842363
K 2557	0.1	
K 2558	0.125603072	0.114621615
K 2559	0.1	
K 2560	0.125159214	0.112456398
K 2561	0.1	
K 2562	0.101216456	0.127565322
K 2563	0.109206634	
K 2564	0.091097095	0.141551293
K 2565	0.1	
K 2566	0.101883789	0.118837896
K 2567	0.1	
K 2568	0.107548725	0.111999768
K 2569	0.1	
K 2570	0.109355036	0.126622741
K 2571	0.1	
K 2572	0.104452341	0.137733638
K 2573	0.1	
K 2574	0.097273296	0.19658644
K 2575	0.1	
K 2576	0.107014878	0.128988544
K 2577	0.1	
K 2578	0.102477728	0.161622969
K 2579	0.1	
K 2580	0.122175528	0.924163658
K 2581	0.1	

K 2582	0.101654153	0.117712386
K 2583	0.099658511	
K 2584	0.09894629	0.122211584
K 2585	0.100337511	
K 2586	0.099438817	0.137778497
K 2587	0.09872522	
K 2588	0.109721295	0.128651287
K 2589	0.101042968	
K 2590	0.101883789	0.118837896
K 2591	0.1	
K 2592	0.099941869	0.111597426
K 2593	0.1	
K 2594	0.156103516	0.149332236
K 2595	0.099920038	
K 2596	0.105948301	0.15698328
K 2597	0.10487019	
K 2598	0.122300729	0.114447927
K 2599	0.100597426	
K 2600	0.115730665	0.112168544
K 2601	0.101321572	
K 2602	0.102606904	0.125675418
K 2603	0.110070931	
K 2604	0.100663226	0.166322555
K 2605	0.1	
K 2606	0.107773444	0.124323279
K 2607	0.1	
K 2608	0.106866453	0.126212561
K 2609	0.1	
K 2610	0.100663226	0.166322555
K 2611	0.1	
K 2612	0.105344452	0.1277575
K 2613	0.10075	
K 2614	0.101190243	0.111924348
K 2615	0.10075	
K 2616	0.101750965	0.117596524
K 2617	0.10075	
K 2618	0.103953655	0.114181997
K 2619	0.10075	
K 2620	0.135524144	0.118313915
K 2621	0.10075	
K 2622	0.133217315	0.135191559
K 2623	0.1	
K 2624	0.134720378	0.128481137
K 2625	0.1	
K 2626	0.129545614	0.116854296
K 2627	0.1	
K 2628	0.094743779	0.986634689

K 2629	0.1	
K 2630	0.092714842	0.121128982
K 2631	0.1	
K 2632	0.099397181	0.125463142
K 2633	0.1	
K 2634	0.108397382	0.12118275
K 2635	0.1	
K 2636	0.096412031	0.112647598
K 2637	0.1	
K 2638	0.099037969	0.945954736
K 2639	0.106766048	
K 2640	0.111905698	0.126347833
K 2641	0.1	
K 2642	0.036605464	0.159678412
K 2643	0.1	
K 2644	0.10201	0.121
K 2645	0.1	
K 2646	0.097491533	0.131181553
K 2647	0.1	
K 2648	0.134384933	0.153831735
K 2649	0.1	
K 2650	0.105797788	0.123649611
K 2651	0.100939697	
K 2652	0.08532825	0.141875459
K 2653	0.100939697	
K 2654	0.119242758	0.113632271
K 2655	0.1	
K 2656	0.107148238	0.188375625
K 2657	0.1	
K 2658	0.106829944	0.127294696
K 2659	0.1	
K 2660	0.1115101	0.117159811
K 2661	0.1	
K 2662	0.109335102	0.175274445
K 2663	0.1	
K 2664	0.128829836	0.122478754
K 2665	0.1	
K 2666	0.099662757	0.967433144
K 2667	0.1	
K 2668	0.109519718	0.997926624
K 2669	0.1	
K 2670	0.143762058	0.126352183
K 2671	0.1	
K 2672	0.111833019	0.999156689
K 2673	0.1	
K 2674	0.109023639	0.133866775
K 2675	0.1	

K 2676	0.102761929	0.988414218
K 2677	0.1	
K 2678	0.130709837	0.137497274
K 2679	0.1	
K 2680	0.096743107	0.955865174
K 2681	0.1	
K 2682	0.101883789	0.118837896
K 2683	0.1	
K 2684	0.118953959	0.117958163
K 2685	0.1	
K 2686	0.135815022	0.122514248
K 2687	0.1	
K 2688	0.100663226	0.166322555
K 2689	0.1	
K 2690	0.100520535	0.991957664
K 2691	0.1	
K 2692	0.101253906	0.112539625
K 2693	0.1	
K 2694	0.102889492	0.127123438
K 2695	0.1	
K 2696	0.103838254	0.144685237
K 2697	0.1	
K 2698	0.134566288	0.129523115
K 2699	0.1	
K 2700	0.100224586	0.124397882
K 2701	0.1	
K 2702	0.11577401	0.111154149
K 2703	0.1	
K 2704	0.110448561	0.135815927
K 2705	0.1	
K 2706	0.137870321	0.167699396
K 2707	0.1	
K 2708	0.107157548	0.112973575
K 2709	0.10090725	
K 2710	0.11342921	0.113228796
K 2711	0.100549306	
K 2712	0.127285961	0.119735339
K 2713	0.1	
K 2714	0.124438205	0.995155733
K 2715	0.1	
K 2716	0.100217622	0.11941162
K 2717	0.1	
K 2718	0.128545609	0.113536824
K 2719	0.1	
K 2720	0.10090897	0.117266328
K 2721	0.1	
K 2722	0.109841627	0.147724338

K 2723	0.1	
K 2724	0.129864164	0.115674264
K 2725	0.1	
K 2726	0.105850717	0.113993988
K 2727	0.1	
K 2728	0.132760416	0.136741851
K 2729	0.1	
K 2730	0.122468568	0.121386573
K 2731	0.1	
K 2732	0.082453756	0.113551727
K 2733	0.1	
K 2734	0.107198809	0.158953888
K 2735	0.1	
K 2736	0.107118564	0.127182523
K 2737	0.1	
K 2738	0.134150213	0.119627327
K 2739	0.1	
K 2740	0.106476412	0.977597272
K 2741	0.1	
K 2742	0.104985459	0.14528815
K 2743	0.1	
K 2744	0.100071776	0.114349875
K 2745	0.1	
K 2746	0.100229521	0.112777338
K 2747	0.1	
K 2748	0.098645177	0.983144197
K 2749	0.1	
K 2750	0.127577138	0.115516784
K 2751	0.1	
K 2752	0.120790644	0.114584977
K 2753	0.1	
K 2754	0.100138598	0.113813244
K 2755	0.1	
K 2756	0.110899632	0.121879793
K 2757	0.1	
K 2758	0.117399018	0.983195274
K 2759	0.1	
K 2760	0.103624559	0.1797139
K 2761	0.1	
K 2762	0.101776627	0.151676364
K 2763	0.1	
K 2764	0.114120969	0.138495269
K 2765	0.1	
K 2766	0.101358917	0.116615557
K 2767	0.1	
K 2768	0.128307754	0.119514298
K 2769	0.1	

K 2770	0.115779385	0.116165619
K 2771	0.1	
K 2772	0.086222143	0.123362389
K 2773	0.1	
K 2774	0.142521332	0.165738769
K 2775	0.1	
K 2776	0.139127482	0.128272175
K 2777	0.1	
K 2778	0.143651669	0.112849438
K 2779	0.1	
K 2780	0.100446829	0.144682856
K 2781	0.1	
K 2782	0.102596268	0.143873862
K 2783	0.1	
K 2784	0.110480519	0.112391469
K 2785	0.1	
K 2786	0.100388205	0.118194128
K 2787	0.1	
K 2788	0.103597621	0.121376358
K 2789	0.1	
K 2790	0.1012036	0.11236
K 2791	0.1	
K 2792	0.107306009	0.112892654
K 2793	0.1	
K 2794	0.100663226	0.166322555
K 2795	0.1	
K 2796	0.123017041	0.128254844
K 2797	0.1	
K 2798	0.109231571	0.172995112
K 2799	0.104683999	
K 2800	0.104307104	0.145532765
K 2801	0.1	
K 2802	0.122814858	0.115216184
K 2803	0.1	
K 2804	0.119836788	0.1966727
K 2805	0.1	
K 2806	0.100135165	0.116972888
K 2807	0.1	
K 2808	0.111500712	0.131694862
K 2809	0.1	
K 2810	0.102614674	0.186948385
K 2811	0.1	
K 2812	0.142521332	0.165738769
K 2813	0.1	
K 2814	0.1012036	0.11236
K 2815	0.1	
K 2816	0.09877734	0.115267912

K 2817	0.1	
K 2818	0.095088214	0.113362215
K 2819	0.1	
K 2820	0.105340726	0.1144534
K 2821	0.1	
K 2822	0.115409182	0.127379843
K 2823	0.1	
K 2824	0.103620754	0.119976175
K 2825	0.1	
K 2826	0.096019795	0.134567434
K 2827	0.1	
K 2828	0.085516859	0.113246558
K 2829	0.1	
K 2830	0.118636246	0.112525266
K 2831	0.1	
K 2832	0.135925805	0.136599823
K 2833	0.101271752	
K 2834	0.113399089	0.158872544
K 2835	0.1	
K 2836	0.143793583	0.12176727
K 2837	0.1	
K 2838	0.111761546	0.175241434
K 2839	0.1	
K 2840	0.113414714	0.138798992
K 2841	0.1	
K 2842	0.105564758	0.139236688
K 2843	0.1	
K 2844	0.114806578	0.111884882
K 2845	0.1	
K 2846	0.155092975	0.127557426
K 2847	0.1	
K 2848	0.123633067	0.111485286
K 2849	0.1	
K 2850	0.107034287	0.112577424
K 2851	0.1	
K 2852	0.123287009	0.119722145
K 2853	0.1	
K 2854	0.13271378	0.113414637
K 2855	0.1	
K 2856	0.130996269	0.151587893
K 2857	0.1	
K 2858	0.106623668	0.161338127
K 2859	0.1	
K 2860	0.100993514	0.199351388
K 2861	0.1	
K 2862	0.1096934	0.135279749
K 2863	0.1	

K 2864	0.107737693	0.194113674
K 2865	0.1	
K 2866	0.103883499	0.11553223
K 2867	0.1	
K 2868	0.105677958	0.132555375
K 2869	0.1	
K 2870	0.126224062	0.115543914
K 2871	0.1	
K 2872	0.117678281	0.145723479
K 2873	0.1	
K 2874	0.140276657	0.123173649
K 2875	0.1	
K 2876	0.094564642	0.12372174
K 2877	0.1	
K 2878	0.094926343	0.115828144
K 2879	0.1	
K 2880	0.108479076	0.122782869
K 2881	0.1	
K 2882	0.100830498	0.193535521
K 2883	0.1	
K 2884	0.118459625	0.125256613
K 2885	0.1	
K 2886	0.101822503	0.126716993
K 2887	0.1	
K 2888	0.101038499	0.113157232
K 2889	0.1	
K 2890	0.10158518	0.115851855
K 2891	0.1	
K 2892	0.087538405	0.112473572
K 2893	0.1	
K 2894	0.100993514	0.199351388
K 2895	0.1	
K 2896	0.100979152	0.114313863
K 2897	0.1	
K 2898	0.092046276	0.143838932
K 2899	0.1	
K 2900	0.106352742	0.119976112
K 2901	0.1	
K 2902	0.100387358	0.124217824
K 2903	0.1	
K 2904	0.142873033	0.123779898
K 2905	0.1	
K 2906	0.134217117	0.148831863
K 2907	0.1	
K 2908	0.138575669	0.118247198
K 2909	0.1	
K 2910	0.115201465	0.162455342

K 2911	0.1	
K 2912	0.106784834	0.174118956
K 2913	0.1	
K 2914	0.041889267	0.875416949
K 2915	0.1	
K 2916	0.100389888	0.113226973
K 2917	0.1	
K 2918	0.10158518	0.115851855
K 2919	0.1	
K 2920	0.137687076	0.144773612
K 2921	0.1	
K 2922	0.091868245	0.158683242
K 2923	0.1	
K 2924	0.102214097	0.126672558
K 2925	0.1	
K 2926	0.096817005	0.122788136
K 2927	0.1	
K 2928	0.099930681	0.111272128
K 2929	0.1	
K 2930	0.100922599	0.122279592
K 2931	0.1	
K 2932	0.10067265	0.122788136
K 2933	0.1	
K 2934	0.119737108	0.14966648
K 2935	0.1	
K 2936	0.105221252	0.115632766
K 2937	0.1	
K 2938	0.101835449	0.127339164
K 2939	0.1	
K 2940	0.10660594	0.116368943
K 2941	0.1	
K 2942	0.104956434	0.153268938
K 2943	0.1	
K 2944	0.117456202	0.139616455
K 2945	0.1	
K 2946	0.100782356	0.111997122
K 2947	0.1	
K 2948	0.13280502	0.117854729
K 2949	0.1	
K 2950	0.10242419	0.112492794
K 2951	0.100247672	
K 2952	0.100620792	0.199891754
K 2953	0.1	
K 2954	0.138507176	0.125738532
K 2955	0.1	
K 2956	0.103593151	0.113822555
K 2957	0.1	

K 2958	0.102857491	0.117954763
K 2959	0.1	
K 2960	0.108363742	0.991745246
K 2961	0.1	
K 2962	0.104548689	0.131132687
K 2963	0.1	
K 2964	0.111746592	0.112782768
K 2965	0.1	
K 2966	0.10120203	0.116288287
K 2967	0.1	
K 2968	0.121525912	0.119629738
K 2969	0.1	
K 2970	0.106232138	0.167195432
K 2971	0.1	
K 2972	0.117000811	0.162261832
K 2973	0.1	
K 2974	0.122022177	0.145824176
K 2975	0.1	
K 2976	0.102912946	0.183681487
K 2977	0.1	
K 2978	0.100756894	0.115378185
K 2979	0.1	
K 2980	0.126571335	0.115387656
K 2981	0.1	
K 2982	0.121640369	0.127532688
K 2983	0.1	
K 2984	0.12456055	0.123493988
K 2985	0.1	
K 2986	0.135808945	0.135427426
K 2987	0.1	
K 2988	0.118710959	0.927266618
K 2989	0.1	
K 2990	0.129031254	0.119926628
K 2991	0.1	
K 2992	0.111487974	0.122452837
K 2993	0.1	
K 2994	0.135483331	0.131541395
K 2995	0.1	
K 2996	0.112849271	0.176119367
K 2997	0.1	
K 2998	0.100663226	0.166322555
K 2999	0.1	
K 3000	0.127578019	0.891822713
K 3001	0.1	
K 3002	0.100478211	0.165682341
K 3003	0.1	
K 3004	0.111984471	0.169791853

K 3005	0.1	
K 3006	0.099901042	0.953996186
K 3007	0.1	
K 3008	0.094299774	0.554294293
K 3009	0.1	
K 3010	0.167241647	0.156594794
K 3011	0.1	
K 3012	0.128152832	0.13131579
K 3013	0.1	
K 3014	0.142521332	0.165738769
K 3015	0.1	
K 3016	0.126660959	0.123123523
K 3017	0.100237861	
K 3018	0.110322125	0.117258824
K 3019	0.09979198	
K 3020	0.103246378	0.145734567
K 3021	0.1	
K 3022	0.103269296	0.998253163
K 3023	0.1	
K 3024	0.101889517	0.126165765
K 3025	0.1	
K 3026	0.108596889	0.11199657
K 3027	0.1	
K 3028	0.094805386	0.138765323
K 3029	0.1	
K 3030	0.113899361	0.159892442
K 3031	0.1	
K 3032	0.106636944	0.952153782
K 3033	0.1	
K 3034	0.108717506	0.135758398
K 3035	0.1	
K 3036	0.10807331	0.111747295
K 3037	0.1	
K 3038	0.092587014	0.119293127
K 3039	0.1	
K 3040	0.12705856	0.147322259
K 3041	0.1	
K 3042	0.123236265	0.197835343
K 3043	0.1	
K 3044	0.125129237	0.122164132
K 3045	0.1	
K 3046	0.123859561	0.139414648
K 3047	0.1	
K 3048	0.100868934	0.117548146
K 3049	0.1	
K 3050	0.099584268	0.139213752
K 3051	0.1	

K 3052	0.103645586	0.113898825
K 3053	0.1	
K 3054	0.105594704	0.157152292
K 3055	0.1	
K 3056	0.107138298	0.118343625
K 3057	0.1	
K 3058	0.13535013	0.1396835
K 3059	0.1	
K 3060	0.167511825	0.142219224
K 3061	0.1	
K 3062	0.122110266	0.115241654
K 3063	0.1	
K 3064	0.100138088	0.147424275
K 3065	0.106526692	
K 3066	0.117812728	0.896557774
K 3067	0.10530359	
K 3068	0.151811798	0.142541584
K 3069	0.090016995	
K 3070	0.142521332	0.165738769
K 3071	0.1	
K 3072	0.101363683	0.165933849
K 3073	0.100914961	
K 3074	0.105461635	0.982392715
K 3075	0.104626257	
K 3076	0.106445839	0.123913652
K 3077	0.1	
K 3078	0.112236869	0.184536693
K 3079	0.1	
K 3080	0.150439348	0.164461477
K 3081	0.1	
K 3082	0.112842856	0.99462383
K 3083	0.1	
K 3084	0.100663226	0.166322555
K 3085	0.1	
K 3086	0.095128353	0.183229793
K 3087	0.1	
K 3088	0.097526567	0.196642615
K 3089	0.1	
K 3090	0.10021727	0.193211621
K 3091	0.1	
K 3092	0.100186228	0.198219656
K 3093	0.1	
K 3094	0.102144092	0.124254828
K 3095	0.1	
K 3096	0.111161648	0.159434296
K 3097	0.1	
K 3098	0.099610067	0.197245115

K 3099	0.1	
K 3100	0.099806433	0.196324452
K 3101	0.1	
K 3102	0.114411458	0.114427268
K 3103	0.103972178	
K 3104	0.099999898	0.118198349
K 3105	0.1	
K 3106	0.116421093	0.165871364
K 3107	0.1	
K 3108	0.101883789	0.118837896
K 3109	0.1	
K 3110	0.104281016	0.113283737
K 3111	0.1	
K 3112	0.103732366	0.11937824
K 3113	0.1	
K 3114	0.10201	0.121
K 3115	0.1	
K 3116	0.126271387	0.488552418
K 3117	0.1	
K 3118	0.101989586	0.133813794
K 3119	0.1	
K 3120	0.105659045	0.116774588
K 3121	0.1	
K 3122	0.100891024	0.182678548
K 3123	0.1	
K 3124	0.105283932	0.129369847
K 3125	0.1	
K 3126	0.089183946	0.886346535
K 3127	0.1	
K 3128	0.103145336	0.949867487
K 3129	0.1	
K 3130	0.108281459	0.843756317
K 3131	0.1	
K 3132	0.110170914	0.831912626
K 3133	0.1	
K 3134	0.110649853	0.141927968
K 3135	0.1	
K 3136	0.105124994	0.121787186
K 3137	0.1	
K 3138	0.124801959	0.175681622
K 3139	0.1	
K 3140	0.100812454	0.181245435
K 3141	0.1	
K 3142	0.087996917	0.116468295
K 3143	0.1	
K 3144	0.1012036	0.11236
K 3145	0.1	

K 3146	0.106819717	0.985327936
K 3147	0.1	
K 3148	0.114017571	0.193266119
K 3149	0.1	
K 3150	0.107660614	0.996919189
K 3151	0.1	
K 3152	0.10158518	0.115851855
K 3153	0.1	
K 3154	0.102525489	0.118813377
K 3155	0.1	
K 3156	0.104297585	0.179591181
K 3157	0.1	
K 3158	0.117273786	0.114314475
K 3159	0.1	
K 3160	0.106093342	0.156924764
K 3161	0.1	
K 3162	0.108080117	0.171474939
K 3163	0.1	
K 3164	0.1	0.1
K 3165	0.1	
K 3166	0.1	0.1
K 3167	0.1	
K 3168	0.1	0.1
K 3169	0.1	
K 3170	0.1	0.1
K 3171	0.1	
K 3172	0.1	0.1
K 3173	0.1	
K 3174	0.1	0.1
K 3175	0.1	
K 3176	0.1	0.1
K 3177	0.1	
K 3178	0.1	0.1
K 3179	0.1	
K 3180	0.1	0.1
K 3181	0.1	
K 3182	0.119342214	0.111297942
K 3183	0.1	
K 3184	0.101887195	0.97264223
K 3185	0.1	
K 3186	0.106569736	0.11151822
K 3187	0.1	
K 3188	0.11194052	0.129879648
K 3189	0.1	
K 3190	0.112031448	0.197192384
K 3191	0.1	
K 3192	0.119772443	0.112851896

K 3193	0.1	
K 3194	0.10465561	0.121343544
K 3195	0.1	
K 3196	0.104144777	0.151991668
K 3197	0.1	
K 3198	0.109716764	0.116838315
K 3199	0.1	
K 3200	0.101772491	0.117724913
K 3201	0.1	
K 3202	0.100714237	0.171358335
K 3203	0.1	
K 3204	0.101883789	0.118837896
K 3205	0.1	
K 3206	0.123790053	0.11361173
K 3207	0.1	
K 3208	0.105960371	0.174826272
K 3209	0.1	
K 3210	0.137845155	0.725828154
K 3211	0.1	
K 3212	0.104526285	0.133633264
K 3213	0.1	
K 3214	0.102091117	0.117486989
K 3215	0.1	
K 3216	0.106819613	0.192152185
K 3217	0.1	
K 3218	0.139948857	0.111249734
K 3219	0.1	
K 3220	0.137418487	0.122392849
K 3221	0.1	
K 3222	0.124511316	0.134987244
K 3223	0.1	
K 3224	0.100663226	0.166322555
K 3225	0.1	
K 3226	0.101253906	0.112539625
K 3227	0.1	
K 3228	0.108602685	0.116527216
K 3229	0.1	
K 3230	0.108133733	0.177742696
K 3231	0.1	
K 3232	0.10201	0.121
K 3233	0.1	
K 3234	0.068796957	0.759176427
K 3235	0.1	
K 3236	0.104252182	0.192287724
K 3237	0.1	
K 3238	0.129168004	0.148642974
K 3239	0.1	

K 3240	0.1	0.1
K 3241	0.1	
K 3242	0.127079662	0.12499244
K 3243	0.144276316	
K 3244	0.105771915	0.117597662
K 3245	0.105520985	
K 3246	0.103712248	0.173548332
K 3247	0.112404055	
K 3248	0.101256508	0.136352228
K 3249	0.101052607	
K 3250	0.103061401	0.147994332
K 3251	0.099891002	
K 3252	0.101654319	0.129198234
K 3253	0.105885574	
K 3254	0.100960506	0.196557454
K 3255	0.084125275	
K 3256	0.100960506	0.196557454
K 3257	0.098750074	
K 3258	0.100960506	0.196557454
K 3259	0.102260059	
K 3260	0.101505625	0.1155625
K 3261	0.062502619	
K 3262	0.100247672	0.12476718
K 3263	0.11360821	
K 3264	0.100247672	0.12476718
K 3265	0.099774073	
K 3266	0.100247672	0.12476718
K 3267	0.103299761	
K 3268	0.100247672	0.12476718
K 3269	0.107472995	
K 3270	0.100247672	0.12476718
K 3271	0.131981612	
K 3272	0.100247672	0.12476718
K 3273	0.111851513	
K 3274	0.100247672	0.12476718
K 3275	0.100449247	
K 3276	0.100247672	0.12476718
K 3277	0.10217137	
K 3278	0.100247672	0.12476718
K 3279	0.099983055	
K 3280	0.100247672	0.12476718
K 3281	0.102938988	
K 3282	0.100247672	0.12476718
K 3283	0.082954711	
K 3284	0.100247672	0.12476718
K 3285	0.098359756	
K 3286	0.100247672	0.12476718

K 3287	0.102136126	
K 3288	0.100247672	0.12476718
K 3289	0.098082795	
K 3290	0.101101581	0.111158134
K 3291	0.090827732	
K 3292	0.101101581	0.111158134
K 3293	0.116567343	
K 3294	0.101101581	0.111158134
K 3295	0.10321873	
K 3296	0.101101581	0.111158134
K 3297	0.107568959	
K 3298	0.101772491	0.117724913
K 3299	0.104341694	
K 3300	0.006458297	0.174295888
K 3301	0.077106457	
K 3302	0.130307136	0.128592459
K 3303	0.094781232	
K 3304	0.1	0.1
K 3305	0.119694139	
K 3306	0.1	0.1
K 3307	0.119010833	
K 3308	0.1	0.1
K 3309	0.100812454	
K 3310	0.1	0.1
K 3311	0.103615928	
K 3312	0.1	0.1
K 3313	0.105464251	
K 3314	0.1	0.1
K 3315	0.098055911	
K 3316	0.1	0.1
K 3317	0.094889508	
K 3318	0.1	0.1
K 3319	0.150244287	
K 3320	0.100811889	0.181188949
K 3321	0.120947356	
K 3322	0.100811889	0.181188949
K 3323	0.107651809	
K 3324	0.100811889	0.181188949
K 3325	0.10163094	
K 3326	0.100811889	0.181188949
K 3327	0.1378893	
K 3328	0.100811889	0.181188949
K 3329	0.104187736	
K 3330	0.101505625	0.1155625
K 3331	0.097364872	
K 3332	0.101505625	0.1155625
K 3333	0.121195069	

K 3334	0.101505625	0.1155625
K 3335	0.106558286	
K 3336	0.101505625	0.1155625
K 3337	0.091946473	
K 3338	0.100476801	0.147688328
K 3339	0.102664877	
K 3340	0.100476801	0.147688328
K 3341	0.111015291	
K 3342	0.100476801	0.147688328
K 3343	0.099573684	
K 3344	0.100476801	0.147688328
K 3345	0.101280215	
K 3346	0.102984586	0.147688328
K 3347	0.096275347	
K 3348	0.100476801	0.147688328
K 3349	0.102132626	
K 3350	0.100476801	0.147688328
K 3351	0.140409874	
K 3352	0.1	0.1
K 3353	0.118352211	
K 3354	0.1	0.1
K 3355	0.10127177	
K 3356	0.1	0.1
K 3357	0.119537822	
K 3358	0.1	0.1
K 3359	0.100446829	
K 3360	0.1	0.1
K 3361	0.145034296	
K 3362	0.142521332	0.165738769
K 3363	0.200529499	
K 3364	0.111374391	0.111884573
K 3365	0.091633023	
K 3366	0.103895068	0.11842864
K 3367	0.242970535	
K 3368	0.10399154	0.156623851
K 3369	0.143113059	
K 3370	0.100132369	0.114177168
K 3371	0.09751684	
K 3372	0.098852223	0.153565362
K 3373	0.106308115	
K 3374	0.102154337	0.121543367
K 3375	0.104494713	
K 3376	0.102154337	0.121543367
K 3377	0.091141685	
K 3378	0.102154337	0.121543367
K 3379	0.106372751	
K 3380	0.102154337	0.121543367

K 3381	0.102963079	
K 3382	0.104041687	0.192399618
K 3383	0.128338238	
K 3384	0.095002921	0.139655485
K 3385	0.122112355	
K 3386	0.102601853	0.139346243
K 3387	0.112231531	
K 3388	0.104338956	0.132861289
K 3389	0.10387438	
K 3390	0.100960506	0.196557454
K 3391	0.094424263	
K 3392	0.100960506	0.196557454
K 3393	0.10121834	
K 3394	0.100960506	0.196557454
K 3395	0.101630102	
K 3396	0.101505625	0.1155625
K 3397	0.110922478	
K 3398	0.101505625	0.1155625
K 3399	0.102178836	
K 3400	0.101308601	0.113861134
K 3401	0.1011323	
K 3402	0.101308601	0.113861134
K 3403	0.08445982	
K 3404	0.101308601	0.113861134
K 3405	0.094439264	
K 3406	0.101308601	0.113861134
K 3407	0.101980506	
K 3408	0.100247672	0.12476718
K 3409	0.111562449	
K 3410	0.100247672	0.12476718
K 3411	0.098909522	
K 3412	0.100247672	0.12476718
K 3413	0.118243567	
K 3414	0.100247672	0.12476718
K 3415	0.101348717	
K 3416	0.100247672	0.12476718
K 3417	0.1006558	
K 3418	0.100247672	0.12476718
K 3419	0.099914524	
K 3420	0.100247672	0.12476718
K 3421	0.10091254	
K 3422	0.100247672	0.12476718
K 3423	0.102917097	
K 3424	0.101101581	0.111158134
K 3425	0.100739829	
K 3426	0.101101581	0.111158134
K 3427	0.134070907	

K 3428	0.101101581	0.111158134
K 3429	0.108924511	
K 3430	0.101101581	0.111158134
K 3431	0.102021012	
K 3432	0.101101581	0.111158134
K 3433	0.111331689	
K 3434	0.101101581	0.111158134
K 3435	0.101772113	
K 3436	0.101101581	0.111158134
K 3437	0.100473586	
K 3438	0.098963252	0.122141784
K 3439	0.108570952	
K 3440	0.100868283	0.119945573
K 3441	0.112917346	
K 3442	0.135621167	0.128279374
K 3443	0.109436712	
K 3444	0.1	0.1
K 3445	0.100889767	
K 3446	0.1	0.1
K 3447	0.103289006	
K 3448	0.1	0.1
K 3449	0.100325365	
K 3450	0.1	0.1
K 3451	0.103113574	
K 3452	0.1	0.1
K 3453	0.107565058	
K 3454	0.100811889	0.181188949
K 3455	0.100441712	
K 3456	0.100811889	0.181188949
K 3457	0.100639659	
K 3458	0.100811889	0.181188949
K 3459	0.074873399	
K 3460	0.101505625	0.1155625
K 3461	0.100977108	
K 3462	0.100476801	0.147688328
K 3463	0.104620302	
K 3464	0.100476801	0.147688328
K 3465	0.095025371	
K 3466	0.1	0.1
K 3467	0.100799602	
K 3468	0.1	0.1
K 3469	0.102225675	
K 3470	0.124636567	0.113553145
K 3471	0.13551301	
K 3472	0.108848266	0.112673629
K 3473	0.104574972	
K 3474	0.100960506	0.196557454

K 3475	0.11932086	
K 3476	0.101505625	0.1155625
K 3477	0.108158885	
K 3478	0.101308601	0.113861134
K 3479	0.101634003	
K 3480	0.101772491	0.117724913
K 3481	0.120016352	
K 3482	0.112342387	0.13237365
K 3483	0.106928899	
K 3484	0.127496055	0.126625822
K 3485	0.12208526	
K 3486	0.100960506	0.196557454
K 3487	0.124360921	
K 3488	0.100960506	0.196557454
K 3489	0.098177362	
K 3490	0.101505625	0.1155625
K 3491	0.119184164	
K 3492	0.101308601	0.113861134
K 3493	0.112191632	
K 3494	0.100247672	0.12476718
K 3495	0.101113164	
K 3496	0.100247672	0.12476718
K 3497	0.094738897	
K 3498	0.100247672	0.12476718
K 3499	0.101650602	
K 3500	0.100247672	0.12476718
K 3501	0.101504684	
K 3502	0.100247672	0.12476718
K 3503	0.103518871	
K 3504	0.1	0.1
K 3505	0.109517045	
K 3506	0.106887327	0.117435617
K 3507	0.094201475	
K 3508	0.13083963	0.116866794
K 3509	0.127105351	
K 3510	0.1	0.1
K 3511	0.09883285	
K 3512	0.1	0.1
K 3513	0.100799602	
K 3514	0.1	0.1
K 3515	0.099967602	
K 3516	0.101186958	0.119365695
K 3517	0.141592382	
K 3518	0.115138702	0.135182298
K 3519	0.130870054	
K 3520	0.099872826	0.129519653
K 3521	0.129178003	

K 3522	0.100960506	0.196557454
K 3523	0.086322426	
K 3524	0.101505625	0.1155625
K 3525	0.099753205	
K 3526	0.101308601	0.113861134
K 3527	0.113764415	
K 3528	0.100247672	0.12476718
K 3529	0.105261872	
K 3530	0.100247672	0.12476718
K 3531	0.097530388	
K 3532	0.100247672	0.12476718
K 3533	0.097976136	
K 3534	0.101101581	0.111158134
K 3535	0.105020491	
K 3536	0.101101581	0.111158134
K 3537	0.1066006	
K 3538	0.101772491	0.117724913
K 3539	0.096871429	
K 3540	0.101772491	0.117724913
K 3541	0.102981844	
K 3542	0.120071795	0.157616163
K 3543	0.093424468	
K 3544	0.127792348	0.939934999
K 3545	0.118547556	
K 3546	0.09846202	0.114567959
K 3547	0.105058911	
K 3548	0.1	0.1
K 3549	0.097387381	
K 3550	0.1	0.1
K 3551	0.097514133	
K 3552	0.100811889	0.181188949
K 3553	0.119561589	
K 3554	0.099998442	0.181188949
K 3555	0.121759812	
K 3556	0.100811889	0.181188949
K 3557	0.147525058	
K 3558	0.1	0.1
K 3559	0.101883789	
K 3560	0.171900068	0.153195696
K 3561	0.173709265	
K 3562	0.098722663	0.149956313
K 3563	0.100697113	
K 3564	0.090583079	0.136484817
K 3565	0.092975139	
K 3566	0.1	0.1
K 3567	0.128697481	
K 3568	0.100960506	0.196557454

K 3569	0.104985024	
K 3570	0.100960506	0.196557454
K 3571	0.103379864	
K 3572	0.101772491	0.117724913
K 3573	0.102783615	
K 3574	0.100247672	0.12476718
K 3575	0.100247672	
K 3576	0.114465998	0.121373366
K 3577	0.100064495	
K 3578	0.097539645	0.987867637
K 3579	0.104030894	
K 3580	0.100685144	0.189562233
K 3581	0.109232438	
K 3582	0.105965089	0.141864532
K 3583	0.10043926	
K 3584	0.101690722	0.167331492
K 3585	0.100795425	
K 3586	0.100883202	0.117517521
K 3587	0.110688555	
K 3588	0.110220631	0.116298537
K 3589	0.114205211	
K 3590	0.065480693	0.567153697
K 3591	0.29620643	
K 3592	0.103805273	0.974652219
K 3593	0.111144472	
K 3594	0.118741426	0.147754488
K 3595	0.099520786	
K 3596	0.104221976	0.155925447
K 3597	0.105798638	
K 3598	0.122817386	0.137293826
K 3599	0.100760608	
K 3600	0.112119493	0.155971765
K 3601	0.10013777	
K 3602	0.092879316	0.129483364
K 3603	0.100706757	
K 3604	0.100101293	0.119953759
K 3605	0.108457771	
K 3606	0.124135641	0.872999933
K 3607	0.686251908	
K 3608	0.302406663	0.211282795
K 3609	0.153383662	
K 3610	0.141104896	0.117629657
K 3611	0.103051655	
K 3612	0.120432	0.122153255
K 3613	0.1	
K 3614	0.147611708	0.124728288
K 3615	0.1	

K 3616	0.100090633	0.113829192
K 3617	0.1	
K 3618	0.095858034	0.112176824
K 3619	0.100415737	
K 3620	0.106409596	0.112522292
K 3621	0.116852306	
K 3622	0.118230274	0.112394495
K 3623	0.1	
K 3624	0.109163017	0.112692776
K 3625	0.097734877	
K 3626	0.118865177	0.117278977
K 3627	0.1	
K 3628	0.002991954	0.165167779
K 3629	0.1	
K 3630	0.104969494	0.129728437
K 3631	0.1	
K 3632	0.118694708	0.935646127
K 3633	0.1	
K 3634	0.135183018	0.181864296
K 3635	0.1	
K 3636	0.103836351	0.115151668
K 3637	0.097827319	
K 3638	0.135183018	0.181864296
K 3639	0.1	
K 3640	0.106292912	0.141485911
K 3641	0.105611598	
K 3642	0.1	0.1
K 3643	0.1	
K 3644	0.101180119	
K 3645	0.1	0.1
K 3646	0.1	
K 3647	0.101044735	
K 3648	0.1	0.1
K 3649	0.1	
K 3650	0.10062312	
K 3651	0.1	0.1
K 3652	0.1	
K 3653	0.102088346	
K 3654	0.1	0.1
K 3655	0.1	
K 3656	0.111204553	
K 3657	0.1	0.1
K 3658	0.1	
K 3659	0.124441941	
K 3660	0.1	0.1
K 3661	0.1	
K 3662	4.11528E-05	

K 3663	0.1	0.1
K 3664	0.1	
K 3665	1.574352691	
K 3666	0.1	0.1
K 3667	0.1	
K 3668	0	
K 3669	0.1	0.1
K 3670	0.1	
K 3671	2.525188717	
K 3672	0.108757009	0.149275952
K 3673	0.097795353	
K 3674	1.90925E-05	
K 3675	0.1	0.1
K 3676	0.1	
K 3677	4.11528E-05	
K 3678	0.1	0.1
K 3679	0.1	
K 3680	3.016267154	
K 3681	0.103551539	0.174991946
K 3682	0.1	
K 3683	0.000699523	
K 3684	0.1	0.1
K 3685	0.1	
K 3686	0.101527231	
K 3687	0.024410017	
K 3688	0.021812679	
K 3689	0.002133859	
K 3690	0.03465785	
K 3691	0.000508374	
K 3692	0.006405382	
K 3693	0.011373256	
K 3694	0.006454136	
K 3695	1.96288E-10	
K 3696	0.03219109	
K 3697	0.026725467	
K 3698	0.001764009	
K 3699	0.018369938	
K 3700	0.000523338	
K 3701	0.03905768	
K 3702	0.001043085	
K 3703	0.042417161	
K 3704	0.00049493	
K 3705	0.003886366	
K 3706	4.44577E-10	
K 3707	0.013849506	
K 3708	0.024286075	
K 3709	0.12707348	

K 3710	0.000548354	
K 3711	0.036215512	
K 3712	0.024866022	
K 3713	0.007614311	
K 3714	0.063847273	
K 3715	0.02357753	
K 3716	0.014981251	
K 3717	0.026601528	
K 3718	0.099137904	
K 3719	1.47753E-11	
K 3720	0.013669267	
K 3721	0.001469323	
K 3722	0.002119624	
K 3723	0.00734589	
K 3724	0.046926314	
K 3725	0.083222377	
K 3726	0.00547978	
K 3727	0.012257447	
K 3728	0.000492407	
K 3729	0.008920576	
K 3730	0.098264604	
K 3731	0.040632287	
K 3732	0.006139249	
K 3733	0.097119697	
K 3734	0.017026289	
K 3735	0.011566245	
K 3736	0.013488438	
K 3737	0.049032993	
K 3738	0.007504828	
K 3739	0.008194011	
K 3740	0.007648004	
K 3741	0.020338163	
K 3742	0.008789354	
K 3743	1.33929E-08	
K 3744	0.027333961	
K 3745	0.006029372	
K 3746	0.120366981	
K 3747	0.105989833	
K 3748	0.001082921	
K 3749	0.004351904	
K 3750	0.000439968	
K 3751	0.039568187	
K 3752	0.026945029	
K 3753	0.044378448	
K 3754	0.098847846	
K 3755	0.005453855	
K 3756	0.005720044	

K 3757	0.025659276	
K 3758	0.000495646	
K 3759	0.010825067	
K 3760	0.102546297	
K 3761	0.026221668	
K 3762	0.010966612	
K 3763	0.007622738	
K 3764	0.001050518	
K 3765	5.51883E-09	
K 3766	0.008886772	
K 3767	0.003419499	
K 3768	0.017901877	
K 3769	0.005924059	
K 3770	0.000489917	
K 3771	0.084686691	
K 3772	0.07268185	
K 3773	7.7685E-11	
K 3774	0.092119328	
K 3775	0.002530857	
K 3776	0.046407807	
K 3777	0.019720692	
K 3778	8.53091E-07	
K 3779	0.000127542	
K 3780	0.026981696	
K 3781	0.007142615	
K 3782	0.042444228	
K 3783	0.033153935	
K 3784	0.002764971	
K 3785	0.0729	
K 3786	0.022997979	
K 3787	0.05700612	
K 3788	0.009580293	
K 3789	0.040441501	
K 3790	0.026445269	
K 3791	0.00295174	
K 3792	0.100993514	
K 3793	0.013906249	
K 3794	0.030889882	
K 3795	0.000808028	
K 3796	0.024224185	
K 3797	0.081	
K 3798	0.060127391	
K 3799	0.019422216	
K 3800	0.035767297	
K 3801	0.00617065	
K 3802	0.062037478	
K 3803	0.011608599	

K 3804	0.039166968	
K 3805	0.031471655	
K 3806	0.009344638	
K 3807	0.041974109	
K 3808	0.040774763	
K 3809	0.033447156	
K 3810	1.83789E-10	
K 3811	0.035371239	
K 3812	0.044537991	
K 3813	0.048836993	
K 3814	0.058809231	
K 3815	0.056216322	
K 3816	0.026655335	
K 3817	0.013362778	
K 3818	0.01740791	
K 3819	0.047148744	
K 3820	0.022781965	
K 3821	0.019775477	
K 3822	0.000497639	
K 3823	0.035378691	
K 3824	0.02584468	
K 3825	0.04411951	
K 3826	0.000516532	
K 3827	0.006057925	
K 3828	1.69113E-13	
K 3829	0.001350895	
K 3830	0.011173753	
K 3831	0.003094639	
K 3832	0.004744787	
K 3833	0.042082935	
K 3834	0.002010596	
K 3835	0.035542103	
K 3836	0.025464415	
K 3837	0.027294823	
K 3838	0.000601534	
K 3839	0.000697774	
K 3840	0.00784633	
K 3841	0.005979156	
K 3842	0.026599765	
K 3843	0.105839582	
K 3844	0.044366951	
K 3845	0.029432218	
K 3846	0.015866739	
K 3847	0.008547417	
K 3848	0.000666463	
K 3849	0.043221292	
K 3850	0.000495068	

K 3851	0.00050976	
K 3852	0.053479877	
K 3853	0.020838933	
K 3854	0.100028407	
K 3855	0.057903759	
K 3856	0.081	
K 3857	0.071056217	
K 3858	0.022456695	
K 3859	0.029706534	
K 3860	0.029813606	
K 3861	0.105655188	
K 3862	0.018452432	
K 3863	0.06711821	
K 3864	0.000468596	
K 3865	0.004007086	
K 3866	0.001645578	
K 3867	0.081	
K 3868	9.98107E-05	
K 3869	0.002720523	
K 3870	0.046978043	
K 3871	0.062037597	
K 3872	0.04516869	
K 3873	0.1	
K 3874	0.045591553	
K 3875	0.081	
K 3876	0.099550631	
K 3877	0.000497389	
K 3878	0.000196592	
K 3879	0.006913728	
K 3880	0.02948036	
K 3881	2.5014E-06	
K 3882	0.023624752	
K 3883	0.0353242	
K 3884	0.017727994	
K 3885	0.064946951	
K 3886	0.046411073	
K 3887	0.048986516	
K 3888	0.130662852	
K 3889	0.055383383	
K 3890	0.005331163	
K 3891	0.1	
K 3892	4.20752E-07	
K 3893	0.100814526	
K 3894	0.009535159	
K 3895	0.000496904	
K 3896	0.028574667	
K 3897	0.108932364	

K 3898	0.000327548	
K 3899	0.005918273	
K 3900	0.006002613	
K 3901	0.103549753	
K 3902	0.000966745	
K 3903	0.007465879	
K 3904	0.102290128	
K 3905	0.016756709	
K 3906	0.074158774	
K 3907	0.100760608	
K 3908	0.029705072	
K 3909	0.063608514	
K 3910	0.009270881	
K 3911	0.014183739	
K 3912	0.053283354	
K 3913	0.018708388	
K 3914	0.008897804	
K 3915	0.062392816	
K 3916	0.098155229	
K 3917	0.081	
K 3918	0.024124889	
K 3919	0.030076945	
K 3920	6.99273E-07	
K 3921	0.047041587	
K 3922	0.006477352	
K 3923	0.039685842	
K 3924	2.69915E-05	
K 3925	0.017974648	
K 3926	0.081419323	
K 3927	0.029727404	
K 3928	0.010287024	
K 3929	0.074547351	
K 3930	0.004063555	
K 3931	0.005681755	
K 3932	0.075948664	
K 3933	0.047038566	
K 3934	0.024947186	
K 3935	0.017937429	
K 3936	0.025604559	
K 3937	0.017895064	
K 3938	0.053511743	
K 3939	0.005021571	
K 3940	0.028365794	
K 3941	0.003929967	
K 3942	0.007579657	
K 3943	0.076678045	
K 3944	0.004264693	

K 3945	0.041211916	
K 3946	0.011199147	
K 3947	0.000523133	
K 3948	0.003101194	
K 3949	0.036980787	
K 3950	0.090071658	
K 3951	0.046671047	
K 3952	0.029699858	
K 3953	0.025620576	
K 3954	0.018715476	
K 3955	0.001532583	
K 3956	0.092236542	
K 3957	0.026503423	
K 3958	0.063566203	
K 3959	0.009301303	
K 3960	0.033211449	
K 3961	0.074095805	
K 3962	0.088423235	
K 3963	1.00001E-05	
K 3964	0.000294173	
K 3965	0.022919766	
K 3966	0.000523847	
K 3967	0.081	
K 3968	0.002651966	
K 3969	0.015884881	
K 3970	0.043719637	
K 3971	0.010875675	
K 3972	0.062960732	
K 3973	0.023225555	
K 3974	0.022113259	
K 3975	0.081	
K 3976	0.085925247	
K 3977	0.004004745	
K 3978	0.009134675	
K 3979	0.002929396	
K 3980	0.025998932	
K 3981	0.104212315	
K 3982	0.000509878	
K 3983	0.062392816	
K 3984	0.026459227	
K 3985	0.008763324	
K 3986	0.0038125	
K 3987	0.003789849	
K 3988	0.032945792	
K 3989	0.044507481	
K 3990	0.025715008	
K 3991	0.037485694	

K 3992	0.018048836	
K 3993	1.70026E-06	
K 3994	0.026227306	
K 3995	0.000492407	
K 3996	0.013027568	
K 3997	0.009158573	
K 3998	7.86659E-05	
K 3999	0.076678045	
K 4000	1.24265E-08	
K 4001	0.025739563	
K 4002	0.045636358	
K 4003	0.047288304	
K 4004	0.001996843	
K 4005	0.08967349	
K 4006	8.7312E-05	
K 4007	0.020471544	
K 4008	0.045717296	
K 4009	0.020710279	
K 4010	0.000202946	
K 4011	0.022036646	
K 4012	4.33682E-09	
K 4013	0.028974447	
K 4014	0.009291627	
K 4015	0.024752373	
K 4016	0.1	
K 4017	0.099313666	
K 4018	0.022529656	
K 4019	0.041640929	
K 4020	0.046729996	
K 4021	0.025997033	
K 4022	0.038633802	
K 4023	0.101152157	
K 4024	0.03957231	
K 4025	0.007754936	
K 4026	0.018081617	
K 4027	7.26784E-09	
K 4028	0.047478881	
K 4029	0.081	
K 4030	0.02666898	
K 4031	0.011709024	
K 4032	0.000493324	
K 4033	0.016658494	
K 4034	0.112453863	
K 4035	0.050530845	
K 4036	0.064392952	
K 4037	0.005300595	
K 4038	0.00049804	

K 4039	0.045946799	
K 4040	0.035985368	
K 4041	0.003707082	
K 4042	0.081	
K 4043	0.008065298	
K 4044	0.018949037	
K 4045	0.011392698	
K 4046	0.012093116	
K 4047	2.91557E-05	
K 4048	0.040711185	
K 4049	0.022228229	
K 4050	0.024987793	
K 4051	0.005671591	
K 4052	0.005945203	
K 4053	0.008642146	
K 4054	0.006008685	
K 4055	0.003729463	
K 4056	0.00389756	
K 4057	0.006189857	
K 4058	0.014424574	
K 4059	0.000114571	
K 4060	0.036599694	
K 4061	0.003862315	
K 4062	0.063204303	
K 4063	0.092322485	
K 4064	0.015148638	
K 4065	0.000581326	
K 4066	0.030111223	
K 4067	0.009386183	
K 4068	0.026249572	
K 4069	0.004566776	
K 4070	1.16304E-08	
K 4071	0.011642103	
K 4072	0.000181797	
K 4073	0.000642198	
K 4074	0.003919056	
K 4075	0.047411301	
K 4076	8.35309E-10	
K 4077	0.097665811	
K 4078	0.016770913	
K 4079	0.050936753	
K 4080	0.008685713	
K 4081	0.046748586	
K 4082	0.001427925	
K 4083	0.025789932	
K 4084	0.00050642	
K 4085	0.009208548	

K 4086	0.034611707	
K 4087	0.0729	
K 4088	0.081	
K 4089	0.001607772	
K 4090	0.0729	
K 4091	0.00221052	
K 4092	0.046436788	
K 4093	0.026928459	
K 4094	0.017277297	
K 4095	2.47866E-06	
K 4096	0.047617957	
K 4097	0.042023655	
K 4098	0.038290246	
K 4099	0.04930827	
K 4100	0.081	
K 4101	0.044895689	
K 4102	0.034261138	
K 4103	0.009839555	
K 4104	0.100247672	
K 4105	0.048344764	
K 4106	0.03564231	
K 4107	0.004004989	
K 4108	0.030900051	
K 4109	0.042307223	
K 4110	0.100436966	
K 4111	0.010919305	
K 4112	0.059312609	
K 4113	0.031812027	
K 4114	0.008918844	
K 4115	0.051029974	
K 4116	1.32351E-05	
K 4117	0.026246871	
K 4118	0.004444688	
K 4119	0.001076984	
K 4120	0.05304097	
K 4121	0.064245499	
K 4122	0.04760123	
K 4123	0.004393937	
K 4124	0.100487549	
K 4125	0.034284962	
K 4126	0.029744292	
K 4127	2.4418E-08	
K 4128	0.102626638	
K 4129	0.000492407	
K 4130	0.01340301	
K 4131	0.038014697	
K 4132	0.025955916	

K 4133	0.063566203	
K 4134	0.001993508	
K 4135	0.003432008	
K 4136	0.035841481	
K 4137	0.062232256	
K 4138	0.021309905	
K 4139	0.05301432	
K 4140	0.009156561	
K 4141	0.007060181	
K 4142	0.001969791	
K 4143	0.09864823	
K 4144	0.064392952	
K 4145	0.081	
K 4146	0.052065962	
K 4147	0.005139097	
K 4148	0.053635191	
K 4149	0.100811889	
K 4150	0.045252155	
K 4151	0.006716841	
K 4152	0.05141013	
K 4153	0.010031648	
K 4154	0.026074353	
K 4155	0.008691448	
K 4156	0.032251332	
K 4157	0.030241238	
K 4158	0.004091735	
K 4159	0.081	
K 4160	0.026320876	
K 4161	0.026558136	
K 4162	0.041179837	
K 4163	0.045135441	
K 4164	0.081	
K 4165	0.000492407	
K 4166	0.004376874	
K 4167	0.0079958	
K 4168	0.00085263	
K 4169	0.081	
K 4170	0.069721581	
K 4171	0.059190019	
K 4172	0.012631678	
K 4173	0.013827877	
K 4174	0.001328863	
K 4175	0.039396576	
K 4176	0.104268074	
K 4177	0.051067971	
K 4178	0.055048446	
K 4179	0.008205624	

K 4180	0.003731157	
K 4181	0.082714596	
K 4182	0.047533685	
K 4183	0.011366845	
K 4184	0.025008411	
K 4185	0.083931124	
K 4186	0.046815861	
K 4187	0.045161325	
K 4188	0.010862068	
K 4189	0.081	
K 4190	0.02525938	
K 4191	0.009361649	
K 4192	1.57328E-05	
K 4193	0.066029532	
K 4194	0.024781543	
K 4195	0.000509938	
K 4196	0.024789118	
K 4197	0.026519703	
K 4198	0.100237861	
K 4199	0.076723008	
K 4200	0.058907544	
K 4201	6.91804E-05	
K 4202	0.030265654	
K 4203	0.046339804	
K 4204	0.000510361	
K 4205	0.005569179	
K 4206	0.029841099	
K 4207	0.000493324	
K 4208	0.100939697	
K 4209	0.000499568	
K 4210	0.030039331	
K 4211	0.102211368	
K 4212	0.000492407	
K 4213	0.005841579	
K 4214	0.008985479	
K 4215	0.049040708	
K 4216	0.081384398	
K 4217	0.025866803	
K 4218	0.027283599	
K 4219	0.01562963	
K 4220	0.004357642	
K 4221	0.002950092	
K 4222	0.014593456	
K 4223	0.001759683	
K 4224	0.005453689	
K 4225	0.05358126	
K 4226	0.000500506	

K 4227	0.0037532	
K 4228	0.003355202	
K 4229	0.081	
K 4230	0.027227751	
K 4231	0.004778452	
K 4232	0.006427016	
K 4233	0.010256877	
K 4234	0.000318117	
K 4235	0.003450688	
K 4236	0.006810453	
K 4237	0.039662224	
K 4238	0.062261423	
K 4239	0.030176691	
K 4240	0.007042513	
K 4241	0.000603285	
K 4242	0.027381392	
K 4243	0.011101756	
K 4244	0.025976143	
K 4245	0.064392952	
K 4246	0.000395305	
K 4247	0.003979614	
K 4248	0.027743821	
K 4249	0.012155297	
K 4250	0.033020523	
K 4251	0.015144903	
K 4252	0.007086185	
K 4253	0.087945055	
K 4254	0.100022962	
K 4255	0.006740985	
K 4256	0.063606288	
K 4257	0.004777121	
K 4258	0.00049886	
K 4259	0.025939563	
K 4260	0.011630005	
K 4261	0.032418856	
K 4262	0.046773104	
K 4263	0.064252802	
K 4264	0.025579219	
K 4265	0.089331201	
K 4266	0.009255806	
K 4267	0.090495845	
K 4268	0.018383836	
K 4269	0.001318634	
K 4270	0.098261974	
K 4271	0.013794875	
K 4272	0.001081665	
K 4273	0.101445503	

K 4274	0.043958994	
K 4275	0.103635708	
K 4276	0.000498371	
K 4277	9.26133E-06	
K 4278	0.00016971	
K 4279	0.013970362	
K 4280	0.000521793	
K 4281	0.044294253	
K 4282	0.107952188	
K 4283	0.006046877	
K 4284	0.097300883	
K 4285	0.000484489	
K 4286	0.00930967	
K 4287	0.000479744	
K 4288	0.04516869	
K 4289	0.043286682	
K 4290	5.9874E-06	
K 4291	0.026085243	
K 4292	0.000544958	
K 4293	0.035926479	
K 4294	0.026782477	
K 4295	0.081	
K 4296	0.033005701	
K 4297	0.025799698	
K 4298	0.026570161	
K 4299	0.000496393	
K 4300	0.000499739	
K 4301	0.063334579	
K 4302	0.000492407	
K 4303	0.000516458	
K 4304	0.098007656	
K 4305	0.000527085	
K 4306	0.000391357	
K 4307	0.000499329	
K 4308	0.026746755	
K 4309	0.000665948	
K 4310	0.022719745	
K 4311	0.006985773	
K 4312	0.004380401	
K 4313	0.041233618	
K 4314	0.044357938	
K 4315	0.01215822	
K 4316	6.44085E-10	
K 4317	0.048379579	
K 4318	0.074604758	
K 4319	0.00039057	
K 4320	0.044149807	

K 4321	0.008331351	
K 4322	0.026753156	
K 4323	0.046482617	
K 4324	0.000500311	
K 4325	0.008415888	
K 4326	0.00050995	
K 4327	0.060512782	
K 4328	0.091848965	
K 4329	0.007640724	
K 4330	0.045816157	
K 4331	0.007697847	
K 4332	0.007573551	
K 4333	0.01028379	
K 4334	0.048265782	
K 4335	0.095232485	
K 4336	0.099742095	
K 4337	0.000492038	
K 4338	3.63979E-10	
K 4339	0.0083912	
K 4340	0.025740631	
K 4341	0.003423817	
K 4342	0.015292413	
K 4343	0.013291035	
K 4344	0.009267086	
K 4345	0.003812168	
K 4346	0.000499885	
K 4347	0.000219024	
K 4348	5.72221E-05	
K 4349	0.101128352	
K 4350	0.004732475	
K 4351	0.030110289	
K 4352	0.10075	
K 4353	1.69113E-13	
K 4354	0.013565037	
K 4355	0.009660306	
K 4356	6.40954E-05	
K 4357	0.091289799	
K 4358	0.000476901	
K 4359	0.039075075	
K 4360	0.000507042	
K 4361	0.000813626	
K 4362	0.010343259	
K 4363	0.002140594	
K 4364	0.006252286	
K 4365	0.001354335	
K 4366	0.047907655	
K 4367	0.023434528	

K 4368	0.004572466	
K 4369	0.004867754	
K 4370	0.017898807	
K 4371	0.010119634	
K 4372	0.003804454	
K 4373	0.000492407	
K 4374	0.008415941	
K 4375	0.010377735	
K 4376	0.027571828	
K 4377	1.32351E-05	
K 4378	0.008647843	
K 4379	0.046246677	
K 4380	0.093751396	
K 4381	0.001361448	
K 4382	0.015624114	
K 4383	0.000492407	
K 4384	0.009114073	
K 4385	0.082878975	
K 4386	0.049244636	
K 4387	0.018581907	
K 4388	0.018241234	
K 4389	0.030678315	
K 4390	0.082128689	
K 4391	0.012292229	
K 4392	0.024604512	
K 4393	0.000502915	
K 4394	0.034355235	
K 4395	0.000492407	
K 4396	0.0453681	
K 4397	0.020691884	
K 4398	0.016504298	
K 4399	0.095227189	
K 4400	0.000331187	
K 4401	0.059753484	
K 4402	0.09697666	
K 4403	0.004049168	
K 4404	0.028496114	
K 4405	0.028867218	
K 4406	0.008066867	
K 4407	0.02546832	
K 4408	0.038910892	
K 4409	0.01221238	
K 4410	0.024727894	
K 4411	0.002810932	
K 4412	0.011912578	
K 4413	0.097200869	
K 4414	0.050512465	

K 4415	0.009982813	
K 4416	0.001523734	
K 4417	0.101730163	
K 4418	0.014551799	
K 4419	0.011102801	
K 4420	0.042593616	
K 4421	0.099380012	
K 4422	1.47675E-06	
K 4423	0.047056413	
K 4424	0.002718196	
K 4425	0.021005504	
K 4426	0.006239635	
K 4427	0.003482169	
K 4428	0.037725784	
K 4429	0.021983835	
K 4430	0.027026118	
K 4431	0.000493324	
K 4432	0.019142394	
K 4433	0.027403396	
K 4434	0.000594802	
K 4435	0.098805706	
K 4436	0.048487435	
K 4437	0.012717576	
K 4438	0.019902419	
K 4439	0.016819836	
K 4440	0.007976546	
K 4441	0.043271382	
K 4442	0.014958195	
K 4443	0.00367061	
K 4444	0.016383331	
K 4445	0.0453681	
K 4446	0.001047584	
K 4447	0.00054346	
K 4448	0.007637492	
K 4449	0.045817798	
K 4450	0.013452803	
K 4451	0.027124416	
K 4452	0.00367061	
K 4453	0.009223797	
K 4454	0.022088861	
K 4455	0.1	
K 4456	0.1	
K 4457	0.1	
K 4458	0.1	
K 4459	0.1	
K 4460	0.1	
K 4461	0.1	

K 4462	0.1	
K 4463	0.1	
K 4464	0.1	
K 4465	0.1	
K 4466	0.1	
K 4467	0.1	
K 4468	0.1	
K 4469	0.1	
K 4470	0.094038906	
K 4471	0.12399281	
K 4472	0.117160675	
K 4473	0.111925755	
K 4474	0.129642365	
K 4475	0.13268673	
K 4476	0.079865222	
K 4477	0.116475712	
K 4478	0.099008674	
K 4479	0.125110706	
K 4480	0.116307402	
K 4481	0.010524619	
K 4482	0.119562129	
K 4483	0.004500888	
K 4484	0.163295244	
K 4485	0.071701201	
K 4486	0.099668688	
K 4487	0.092641015	
K 4488	0.097198552	
K 4489	0.095092209	
K 4490	0.096463706	
K 4491	0.122996628	
K 4492	2.47866E-06	
K 4493	0.00528708	
K 4494	0.100071296	
K 4495	0.030433745	
K 4496	0.110615023	
K 4497	0.102329057	
K 4498	0.111882108	
K 4499	0.129710893	
K 4500	0.104705598	
K 4501	0.099832214	
K 4502	0.096300621	
K 4503	0.100175215	
K 4504	0.094119735	
K 4505	0.08791081	
K 4506	0.115171535	
K 4507	0.090354567	
K 4508	0.082730322	

K 4509	0.088358922	
K 4510	0.10005168	
K 4511	0.000492407	
K 4512	0.120075321	
K 4513	0.096167271	
K 4514	0.0928811	
K 4515	0.131980275	
K 4516	0.096444561	
K 4517	0.099574381	
K 4518	0.122971269	
K 4519	0.102166621	
K 4520	0.084479337	
K 4521	0.126940979	
K 4522	0.092711858	
K 4523	0.097068137	
K 4524	0.133458374	
K 4525	0.085383147	
K 4526	0.115236265	
K 4527	0.092679293	
K 4528	0.083600017	
K 4529	0.09910922	
K 4530	0.093505263	
K 4531	0.084215033	
K 4532	0.111408967	
K 4533	0.112037459	
K 4534	0.103182953	
K 4535	0.09028425	
K 4536	0.099621343	
K 4537	0.104659079	
K 4538	0.108101926	
K 4539	0.109129763	
K 4540	0.091362495	
K 4541	0.120621674	
K 4542	0.101238098	
K 4543	0.097352294	
K 4544	0.095121365	
K 4545	0.089754916	
K 4546	0.097511623	
K 4547	0.124959343	
K 4548	0.126967097	
K 4549	0.113286595	
K 4550	0.130335467	
K 4551	0.013705661	
K 4552	0.09379542	
K 4553	0.102179091	
K 4554	0.095149163	
K 4555	0.007840759	

K 4556	0.101077273	
K 4557	0.102593707	
K 4558	0.000605605	
K 4559	0.121139937	
K 4560	0.089041143	
K 4561	0.10616635	
K 4562	0.110048068	
K 4563	0.07744006	
K 4564	0.100663226	
K 4565	0.087710152	
K 4566	0.097093634	
K 4567	0.120280574	
K 4568	0.09925118	
K 4569	0.103010126	
K 4570	0.130883339	
K 4571	0.085526627	
K 4572	0.140645895	
K 4573	0.105691505	
K 4574	0.082424007	
K 4575	0.105842641	
K 4576	0.101253906	
K 4577	0.101104407	
K 4578	0.11890387	
K 4579	0.118285587	
K 4580	0.142014727	
K 4581	0.099940733	
K 4582	0.10018033	
K 4583	0.110404186	
K 4584	0.110136454	
K 4585	0.12854378	
K 4586	0.098373627	
K 4587	0.109356133	
K 4588	0.106518813	
K 4589	0.098018356	
K 4590	0.104300541	
K 4591	0.105738111	
K 4592	0.149797108	
K 4593	0.094346277	
K 4594	0.132891735	
K 4595	0.138241728	
K 4596	0.120341889	
K 4597	0.098226649	
K 4598	0.134455728	
K 4599	0.099204078	
K 4600	0.091086378	
K 4601	0.108807544	
K 4602	0.107633436	

K 4603	0.10055466	
K 4604	0.089107774	
K 4605	0.126276664	
K 4606	0.125608299	
K 4607	0.131290412	
K 4608	0.113408135	
K 4609	0.104564068	
K 4610	0.10585097	
K 4611	0.128438411	
K 4612	0.099527675	
K 4613	0.108254578	
K 4614	0.114135051	
K 4615	0.008902295	
K 4616	0.133679877	
K 4617	0.166982534	
K 4618	0.117830486	
K 4619	0.120722988	
K 4620	0.100799602	
K 4621	0.107710334	
K 4622	0.122527805	
K 4623	0.123541072	
K 4624	0.092727625	
K 4625	0.152858134	
K 4626	0.10692569	
K 4627	0.097378752	
K 4628	0.087684812	
K 4629	0.095258749	
K 4630	0.150496365	
K 4631	0.101681951	
K 4632	0.110964023	
K 4633	0.10201	
K 4634	0.097052569	
K 4635	0.140636463	
K 4636	0.117509058	
K 4637	0.106076241	
K 4638	0.105655188	
K 4639	0.126002175	
K 4640	0.100153924	
K 4641	0.084839182	
K 4642	0.156728714	
K 4643	0.37396953	
K 4644	0.101308601	
K 4645	0.093653915	
K 4646	0.096845321	
K 4647	0.102724288	
K 4648	0.102144539	
K 4649	0.1	

K 4650	0.100624278	
K 4651	0.10201	
K 4652	0.092658791	
K 4653	0.131669624	
K 4654	0.128901453	
K 4655	0.135807913	
K 4656	0.104360985	
K 4657	0.002600472	
K 4658	0.102735494	
K 4659	0.107444031	
K 4660	0.10680678	
K 4661	0.093360882	
K 4662	0.117327711	
K 4663	0.11850961	
K 4664	0.050977428	
K 4665	0.117732707	
K 4666	0.090374769	
K 4667	0.1	
K 4668	0.090611272	
K 4669	0.100901881	
K 4670	0.118162988	
K 4671	0.09537799	
K 4672	0.117948101	
K 4673	0.100428798	
K 4674	0.107823689	
K 4675	0.102106444	
K 4676	0.085815937	
K 4677	0.007580963	
K 4678	0.092846295	
K 4679	0.100048497	
K 4680	0.125148888	
K 4681	0.112128661	
K 4682	0.100760608	
K 4683	0.107507322	
K 4684	0.100232907	
K 4685	0.096810832	
K 4686	0.110097352	
K 4687	0.099111505	
K 4688	0.094462789	
K 4689	0.107438309	
K 4690	0.100422926	
K 4691	0.097133117	
K 4692	0.101883789	
K 4693	0.097713392	
K 4694	0.116656611	
K 4695	0.090394313	
K 4696	0.129789023	

K 4697	0.115557448	
K 4698	0.090580755	
K 4699	0.105583864	
K 4700	0.102625752	
K 4701	0.145125482	
K 4702	0.128321648	
K 4703	0.101473179	
K 4704	0.129369946	
K 4705	0.10414206	
K 4706	0.10229617	
K 4707	0.098784216	
K 4708	0.105600602	
K 4709	0.130655638	
K 4710	0.088488222	
K 4711	0.106044291	
K 4712	0.104209314	
K 4713	0.155139189	
K 4714	0.143724099	
K 4715	0.133131156	
K 4716	0.093780198	
K 4717	0.092249544	
K 4718	0.10484456	
K 4719	0.10946059	
K 4720	0.105832097	
K 4721	0.089530693	
K 4722	0.155219414	
K 4723	0.10185096	
K 4724	0.06630733	
K 4725	0.10346037	
K 4726	0.105230146	
K 4727	0.104116508	
K 4728	0.106370789	
K 4729	0.094773131	
K 4730	0.105980095	
K 4731	0.106275921	
K 4732	0.1019682	
K 4733	0.103982959	
K 4734	0.102474874	
K 4735	0.100700681	
K 4736	0.070663453	
K 4737	0.115798258	
K 4738	0.120498771	
K 4739	0.106106972	
K 4740	0.100812454	
K 4741	0.135686005	
K 4742	0.109449351	
K 4743	0.130789922	

K 4744	0.097471343	
K 4745	0.114071538	
K 4746	0.107089907	
K 4747	0.101308601	
K 4748	0.093382801	
K 4749	0.103293048	
K 4750	0.115831564	
K 4751	0.126018297	
K 4752	0.108468352	
K 4753	0.100446829	
K 4754	0.092332168	
K 4755	0.098519682	
K 4756	0.106114777	
K 4757	0.091554038	
K 4758	0.120777255	
K 4759	0.107970962	
K 4760	0.100350286	
K 4761	0.109653477	
K 4762	0.101696944	
K 4763	0.098032533	
K 4764	0.095956686	
K 4765	0.099498095	
K 4766	0.000492407	
K 4767	0.00339384	
K 4768	0.120574188	
K 4769	0.085994044	
K 4770	0.092249544	
K 4771	0.086148576	
K 4772	0.099602627	
K 4773	0.100680255	
K 4774	0.10498826	
K 4775	0.215455092	
K 4776	0.115063188	
K 4777	0.12914509	
K 4778	0.106080769	
K 4779	0.128987157	
K 4780	0.144449568	
K 4781	0.12701401	
K 4782	0.099944756	
K 4783	0.100209529	
K 4784	0.101522137	
K 4785	0.105755903	
K 4786	0.1	
K 4787	0.102050702	
K 4788	0.116451173	
K 4789	0.089340364	
K 4790	0.105102024	

K 4791	0.105669402	
K 4792	0.120964668	
K 4793	0.101152157	
K 4794	0.09839572	
K 4795	0.083033019	
K 4796	0.1270313	
K 4797	0.104444122	
K 4798	0.099596832	
K 4799	0.1012036	
K 4800	0.12508732	
K 4801	0.138624341	
K 4802	0.031151513	
K 4803	0.103344952	
K 4804	0.009915419	
K 4805	0.096582534	
K 4806	0.098621991	
K 4807	0.120898118	
K 4808	0.09632029	
K 4809	0.089864381	
K 4810	0.097183356	
K 4811	0.106121264	
K 4812	0.102154337	
K 4813	0.111698082	
K 4814	0.007464492	
K 4815	0.114633739	
K 4816	0.112490215	
K 4817	0.077716702	
K 4818	0.108705524	
K 4819	0.107079137	
K 4820	0.107373632	
K 4821	0.10330942	
K 4822	0.101492655	
K 4823	0.106980169	
K 4824	0.131366327	
K 4825	0.103958926	
K 4826	0.129161552	
K 4827	0.122836998	
K 4828	0.12316991	
K 4829	0.082069577	
K 4830	0.10694836	
K 4831	0.097881986	
K 4832	0.100308759	
K 4833	0.104074453	
K 4834	0.09649818	
K 4835	0.010117181	
K 4836	0.108624892	
K 4837	0.15768837	

K 4838	0.121319642	
K 4839	0.127402138	
K 4840	0.079405755	
K 4841	0.142822146	
K 4842	0.108425066	
K 4843	0.098662042	
K 4844	0.109461282	
K 4845	0.098582487	
K 4846	0.072708373	
K 4847	0.098471089	
K 4848	0.112970875	
K 4849	0.101209855	
K 4850	0.11744443	
K 4851	0.113344522	
K 4852	0.109003613	
K 4853	0.122055586	
K 4854	0.096224108	
K 4855	0.099106605	
K 4856	0.106756041	
K 4857	0.100960506	
K 4858	0.101505625	
K 4859	0.004100885	
K 4860	0.101101581	
K 4861	0.104640668	
K 4862	0.104925226	
K 4863	0.10437326	
K 4864	0.097361501	
K 4865	0.097820869	
K 4866	0.111952627	
K 4867	0.114325926	
K 4868	0.135215122	
K 4869	0.102995396	
K 4870	0.10158518	
K 4871	0.100690292	
K 4872	0.104536616	
K 4873	0.107788504	
K 4874	0.100247672	
K 4875	0.109756532	
K 4876	0.117933821	
K 4877	0.123820156	
K 4878	0.098240257	
K 4879	0.106338977	
K 4880	0.100436966	
K 4881	0.086739635	
K 4882	0.10690457	
K 4883	0.124599106	
K 4884	0.093971694	

K 4885	0.112164784	
K 4886	0.099812003	
K 4887	0.103463925	
K 4888	0.096346486	
K 4889	0.099970633	
K 4890	0.107745895	
K 4891	0.100366637	
K 4892	0.100665063	
K 4893	0.107958105	
K 4894	0.09779579	
K 4895	0.099275769	
K 4896	0.119539237	
K 4897	0.107335165	
K 4898	0.091298071	
K 4899	0.165073871	
K 4900	0.116673321	
K 4901	0.105904253	
K 4902	0.108934081	
K 4903	0.108005392	
K 4904	0.115880275	
K 4905	0.141029363	
K 4906	0.105102618	
K 4907	0.102619828	
K 4908	0.117038236	
K 4909	0.098927401	
K 4910	0.105734941	
K 4911	0.097043323	
K 4912	0.03424742	
K 4913	0.100783784	
K 4914	0.106145359	
K 4915	0.101505625	
K 4916	0.109494592	
K 4917	0.083664875	
K 4918	0.099293736	
K 4919	0.100811889	
K 4920	0.106132655	
K 4921	0.098901841	
K 4922	0.101382428	
K 4923	0.102261378	
K 4924	0.10788619	
K 4925	0.092122828	
K 4926	0.108075951	
K 4927	0.128892183	
K 4928	0.126304453	
K 4929	0.101772491	
K 4930	0.104225804	
K 4931	0.104393495	

K 4932	0.089181382	
K 4933	0.105611335	
K 4934	0.101772491	
K 4935	0.000492407	
K 4936	0.129888163	
K 4937	0.125371674	
K 4938	0.118582798	
K 4939	0.10201	
K 4940	0.099904229	
K 4941	0.103263117	
K 4942	0.092204307	
K 4943	0.136212918	
K 4944	0.115177897	
K 4945	0.119413824	
K 4946	0.103625688	
K 4947	0.094685628	
K 4948	0.105294613	
K 4949	0.098032392	
K 4950	0.096218899	
K 4951	0.105594704	
K 4952	0.103276854	
K 4953	0.093014861	
K 4954	0.097746041	
K 4955	0.101903292	
K 4956	0.103833737	
K 4957	0.137965298	
K 4958	0.130390969	
K 4959	0.101883789	
K 4960	0.129933494	
K 4961	0.125332902	
K 4962	0.069588251	
K 4963	0.097503656	
K 4964	0.094737747	
K 4965	0.013082124	
K 4966	0.103634303	
K 4967	0.111296078	
K 4968	0.100237861	
K 4969	0.091969672	
K 4970	0.103683485	
K 4971	0.095713089	
K 4972	0.106604526	
K 4973	0.10624331	
K 4974	0.135006376	
K 4975	0.189773288	
K 4976	0.108189594	
K 4977	0.031151513	
K 4978	0.100939697	

K 4979	0.139022692	
K 4980	0.108652809	
K 4981	0.102039665	
K 4982	0.000492407	
K 4983	0.121341356	
K 4984	0.128730239	
K 4985	0.103647412	
K 4986	0.107809111	
K 4987	0.117509281	
K 4988	0.110653572	
K 4989	0.012934988	
K 4990	0.10164111	
K 4991	0.11782475	
K 4992	0.098315906	
K 4993	0.092520682	
K 4994	0.102757459	
K 4995	0.096687295	
K 4996	0.13221956	
K 4997	0.120370386	
K 4998	0.101883789	
K 4999	0.114141075	
K 5000	0.133407421	
K 5001	0.124169347	
K 5002	0.125038545	
K 5003	0.099226184	
K 5004	0.098499734	
K 5005	0.098741079	
K 5006	0.112912684	
K 5007	0.101613952	
K 5008	0.106889597	
K 5009	0.096491815	
K 5010	0.131118661	
K 5011	0.103806964	
K 5012	0.10691606	
K 5013	0.100193626	
K 5014	0.11585915	
K 5015	0.105590947	
K 5016	0.138741378	
K 5017	0.003092258	
K 5018	0.09762881	
K 5019	0.101045313	
K 5020	0.094064106	
K 5021	0.095274376	
K 5022	0.103530263	
K 5023	0.104840746	
K 5024	0.112675728	
K 5025	0.097042845	

K 5026	0.125484907	
K 5027	0.129162861	
K 5028	0.106885775	
K 5029	0.133503682	
K 5030	0.094505151	
K 5031	0.113935401	
K 5032	0.102100585	
K 5033	0.116168906	
K 5034	0.057252208	
K 5035	0.097049618	
K 5036	0.091503261	
K 5037	0.119547033	
K 5038	0.098918964	
K 5039	0.102704265	
K 5040	0.096157407	
K 5041	0.124394792	
K 5042	0.064681247	
K 5043	0.10387013	
K 5044	0.100210506	
K 5045	0.128137935	
K 5046	0.554899131	
K 5047	0.113379571	
K 5048	0.090774413	
K 5049	0.000877942	
K 5050	0.10316064	
K 5051	0.102687439	
K 5052	0.100742732	
K 5053	0.103138816	
K 5054	0.09556685	
K 5055	0.126013098	
K 5056	0.096539037	
K 5057	0.102423633	
K 5058	0.257179666	
K 5059	0.08236494	
K 5060	0.105715575	
K 5061	0.00397561	
K 5062	0.091705533	
K 5063	0.115879574	
K 5064	0.10158518	
K 5065	0.100684207	
K 5066	0.131945523	
K 5067	0.101276597	
K 5068	0.097268164	
K 5069	0.096689279	
K 5070	0.098066751	
K 5071	0.000492407	
K 5072	0.039888579	

K 5073	0.107632401	
K 5074	0.094202481	
K 5075	0.089906311	
K 5076	0.096146353	
K 5077	0.129594814	
K 5078	0.003681103	
K 5079	0.118922846	
K 5080	0.093777775	
K 5081	0.105848206	
K 5082	0.124842888	
K 5083	0.09594192	
K 5084	0.130499806	
K 5085	0.082470212	
K 5086	0.10095437	
K 5087	0.110967232	
K 5088	0.105384003	
K 5089	0.09141768	
K 5090	0.120025558	
K 5091	0.10316318	
K 5092	0.101889164	
K 5093	0.129161713	
K 5094	0.093938812	
K 5095	0.119521646	
K 5096	0.093852419	
K 5097	0.089000656	
K 5098	0.097827274	
K 5099	0.097767179	
K 5100	0.096107659	
K 5101	0.096637113	
K 5102	0.108967214	
K 5103	0.09974181	
K 5104	0.100085537	
K 5105	0.090648555	
K 5106	0.095350279	
K 5107	0.079206685	
K 5108	0.096369435	
K 5109	0.105654903	
K 5110	0.106720399	
K 5111	0.345781613	
K 5112	0.131715414	
K 5113	0.10297953	
K 5114	0.129358543	
K 5115	0.092316632	
K 5116	0.00012967	
K 5117	0.016657686	
K 5118	0.096790238	
K 5119	0.091039639	

K 5120	0.107369318	
K 5121	0.09	
K 5122	0.124599046	
K 5123	0.12027958	
K 5124	0.133263711	
K 5125	0.081496231	
K 5126	0.097414685	
K 5127	0.132752245	
K 5128	0.093937942	
K 5129	0.230420945	
K 5130	0.097870875	
K 5131	0.089657474	
K 5132	0.0990115	
K 5133	0.096928778	
K 5134	0.100557124	
K 5135	0.097163978	
K 5136	0.096947977	
K 5137	0.129618141	
K 5138	0.105546925	
K 5139	0.095785113	
K 5140	0.123593102	
K 5141	0.000492407	
K 5142	0.12405912	
K 5143	0.096943988	
K 5144	0.10138629	
K 5145	0.099812003	
K 5146	0.094927616	
K 5147	0.101917111	
K 5148	0.103378994	
K 5149	0.074679112	
K 5150	0.121499346	
K 5151	0.000492407	
K 5152	0.091594997	
K 5153	0.096332948	
K 5154	0.104934074	
K 5155	0.150918213	
K 5156	0.122073019	
K 5157	0.10303068	
K 5158	0.106586446	
K 5159	0.105808283	
K 5160	0.024171464	
K 5161	0.093405888	
K 5162	0.117387065	
K 5163	0.000492407	
K 5164	0.086878478	
K 5165	0.119496756	
K 5166	0.106160956	

K 5167	0.094416605	
K 5168	0.067228769	
K 5169	0.09320779	
K 5170	0.102223158	
K 5171	0.124709178	
K 5172	0.111766881	
K 5173	0.010096827	
K 5174	2.659849701	
K 5175	0.103546588	
K 5176	0.092240933	
K 5177	0.097474116	
K 5178	0.103812927	
K 5179	0.116162271	
K 5180	0.092637585	
K 5181	0.100910936	
K 5182	0.095100713	
K 5183	0.097007035	
K 5184	0.099584997	
K 5185	0.096243839	
K 5186	0.099851085	
K 5187	0.121966098	
K 5188	0.102360093	
K 5189	0.10530359	
K 5190	0.126307532	
K 5191	0.09742954	
K 5192	0.100723737	
K 5193	0.128560488	
K 5194	0.121826057	
K 5195	0.102465044	
K 5196	0.105789581	
K 5197	0.133831736	
K 5198	0.116776693	
K 5199	0.031151513	
K 5200	0.135449682	
K 5201	0.103219696	
K 5202	0.000492407	
K 5203	0.110081643	
K 5204	0.105132418	
K 5205	0.024336965	
K 5206	0.125479286	
K 5207	0.126907512	
K 5208	0.107371884	
K 5209	0.121991632	
K 5210	0.094670319	
K 5211	0.00367061	
K 5212	0.09745868	
K 5213	0.086878478	

K 5214	0.137444557	
K 5215	0.010703886	
K 5216	0.097247654	
K 5217	0.09936881	
K 5218	0.143739474	
K 5219	0.104945083	
K 5220	0.00367061	
K 5221	0.099000437	
K 5222	0.138095976	
K 5223	0.117586135	
K 5224	0.10758669	
K 5225	0.093276675	
K 5226	0.103335285	
K 5227	0.098989814	
K 5228	0.093027326	
K 5229	0.098628609	
K 5230	0.106538727	
K 5231	0.097738714	
K 5232	0.100890237	
K 5233	0.103667278	
K 5234	0.096532774	
K 5235	0.177504564	
K 5236	0.116794296	
K 5237	0.095148207	
K 5238	0.132577839	
K 5239	0.115611085	
K 5240	0.106269498	
K 5241	0.12411371	
K 5242	0.103068837	
K 5243	0.11611625	
K 5244	0.113856186	
K 5245	0.133896476	
K 5246	0.102189544	
K 5247	0.113993455	
K 5248	0.104192235	
K 5249	0.114538183	
K 5250	0.133699513	
K 5251	0.119222373	
K 5252	0.116724475	
K 5253	0.121988786	
K 5254	0.108725824	
K 5255	0.112742128	
K 5256	0.111547506	
K 5257	0.129343947	
K 5258	0.103790892	
K 5259	0.142734531	
K 5260	0.089371364	

K 5261	0.118457003	
K 5262	0.122809421	
K 5263	0.100952999	
K 5264	0.121831275	
K 5265	0.141824669	
K 5266	0.096438385	
K 5267	0.105776613	
K 5268	0.135174642	
K 5269	0.102444157	
K 5270	0.116632044	
K 5271	0.088912988	
K 5272	0.113906862	
K 5273	0.126929335	
K 5274	0.11337294	
K 5275	0.125489369	
K 5276	0.125870296	
K 5277	0.129935941	
K 5278	0.107034937	
K 5279	0.110541319	
K 5280	0.103248337	
K 5281	0.105150988	
K 5282	0.153374643	
K 5283	0.111554516	
K 5284	0.111689081	
K 5285	0.12347633	
K 5286	0.096225745	
K 5287	0.108178007	
K 5288	0.124398803	
K 5289	0.112532839	
K 5290	0.116070807	
K 5291	0.099564127	
K 5292	0.130176455	
K 5293	0.129538289	
K 5294	0.090010179	
K 5295	0.144900012	
K 5296	0.097335071	
K 5297	0.100166573	
K 5298	0.110869303	
K 5299	0.10210687	
K 5300	0.174011305	
K 5301	0.102504078	
K 5302	0.108575352	
K 5303	0.102768917	
K 5304	0.144399454	
K 5305	0.116104266	
K 5306	0.101702875	
K 5307	0.103469491	

K 5308	0.100663226	
K 5309	0.09891931	
K 5310	0.094075637	
K 5311	0.104794346	
K 5312	0.103991954	
K 5313	0.113584363	
K 5314	0.09713475	
K 5315	0.100993514	
K 5316	0.080301154	
K 5317	0.095383852	
K 5318	0.130949313	
K 5319	0.11194216	
K 5320	0.101253906	
K 5321	0.100477391	
K 5322	0.111785376	
K 5323	0.128494674	
K 5324	0.097882438	
K 5325	0.099133272	
K 5326	0.13654362	
K 5327	0.102895925	
K 5328	0.111354755	
K 5329	0.095678957	
K 5330	0.096766847	
K 5331	0.094042446	
K 5332	0.11699262	
K 5333	0.093126232	
K 5334	0.121176583	
K 5335	0.100042711	
K 5336	0.058445525	
K 5337	0.099595901	
K 5338	0.100677912	
K 5339	0.117687019	
K 5340	0.122165791	
K 5341	0.129778829	
K 5342	0.106232142	
K 5343	0.101612779	
K 5344	0.095349114	
K 5345	0.117507032	
K 5346	0.155417539	
K 5347	0.106413847	
K 5348	0.134936751	
K 5349	0.096265094	
K 5350	0.097085302	
K 5351	0.116272494	
K 5352	0.138962618	
K 5353	0.140794126	
K 5354	0.100040588	

K 5355	0.09352383	
K 5356	0.109003995	
K 5357	0.105248781	
K 5358	0.103758205	
K 5359	0.162576674	
K 5360	0.097127231	
K 5361	0.007019563	
K 5362	0.157931197	
K 5363	0.094089456	
K 5364	0.100799602	
K 5365	0.00459968	
K 5366	0.100929461	
K 5367	0.105357897	
K 5368	0.112682689	
K 5369	0.007113682	
K 5370	0.145850378	
K 5371	0.121121648	
K 5372	0.118978432	
K 5373	0.125960897	
K 5374	0.100008019	
K 5375	0.104219689	
K 5376	0.10201	
K 5377	0.10285606	
K 5378	0.089344219	
K 5379	0.124794674	
K 5380	0.100499831	
K 5381	0.102292937	
K 5382	0.093068304	
K 5383	0.100954976	
K 5384	0.097797143	
K 5385	0.023695801	
K 5386	0.101308601	
K 5387	0.110733793	
K 5388	0.106768522	
K 5389	0.098311323	
K 5390	0.102423633	
K 5391	0.104639133	
K 5392	0.10201	
K 5393	0.100476801	
K 5394	0.09145624	
K 5395	0.146104541	
K 5396	0.132794665	
K 5397	0.098772395	
K 5398	0.144257043	
K 5399	0.104789683	
K 5400	0.103950685	
K 5401	0.101935574	

K 5402	0.099796133	
K 5403	0.096543798	
K 5404	0.106573821	
K 5405	0.111537448	
K 5406	0.11436168	
K 5407	0.111257708	
K 5408	0.103138863	
K 5409	0.095559153	
K 5410	0.123600172	
K 5411	0.126168113	
K 5412	0.102000253	
K 5413	0.105106548	
K 5414	0.133595264	
K 5415	0.112075991	
K 5416	0.107994838	
K 5417	0.102003656	
K 5418	0.099471156	
K 5419	0.107559452	
K 5420	0.09574103	
K 5421	0.138759178	
K 5422	0.094806012	
K 5423	0.101823018	
K 5424	0.100002153	
K 5425	0.102893822	
K 5426	0.09925565	
K 5427	0.101883789	
K 5428	0.119382942	
K 5429	0.106550067	
K 5430	0.108475585	
K 5431	0.084941968	
K 5432	0.104804493	
K 5433	0.090912176	
K 5434	0.099895237	
K 5435	0.101454973	
K 5436	0.150842385	
K 5437	0.10107737	
K 5438	0.101178337	
K 5439	0.09396047	
K 5440	0.146875251	
K 5441	0.105731466	
K 5442	0.117110382	
K 5443	0.111177801	
K 5444	0.097146286	
K 5445	0.088258354	
K 5446	0.1018606	
K 5447	0.100642812	
K 5448	0.116467717	

K 5449	0.097050377	
K 5450	0.095115858	
K 5451	0.110526594	
K 5452	0.100158413	
K 5453	0.154186865	
K 5454	0.101530472	
K 5455	0.14370801	
K 5456	0.130062362	
K 5457	0.09041322	
K 5458	0.132562875	
K 5459	0.103379799	
K 5460	0.112627767	
K 5461	0.128743279	
K 5462	0.101750636	
K 5463	0.118938774	
K 5464	0.143332955	
K 5465	0.098806833	
K 5466	0.108923111	
K 5467	0.099922593	
K 5468	0.141522461	
K 5469	0.115583767	
K 5470	0.100548637	
K 5471	0.108288891	
K 5472	0.122254091	
K 5473	0.11261072	
K 5474	0.129452285	
K 5475	0.128973439	
K 5476	0.100812454	
K 5477	0.118174089	
K 5478	0.099338176	
K 5479	0.095134312	
K 5480	0.084500808	
K 5481	0.107956607	
K 5482	0.103182229	
K 5483	0.129543564	
K 5484	0.161210936	
K 5485	0.113673192	
K 5486	0.106328508	
K 5487	0.100326659	
K 5488	0.142759566	
K 5489	0.097410632	
K 5490	0.102579364	
K 5491	0.129728953	
K 5492	0.089063068	
K 5493	0.096480481	
K 5494	0.112368567	
K 5495	0.11573755	

K 5496	0.103244357	
K 5497	0.127504331	
K 5498	0.125726564	
K 5499	0.12046441	
K 5500	0.129095917	
K 5501	0.096437908	
K 5502	0.101132995	
K 5503	0.101867525	
K 5504	0.104629604	
K 5505	0.106348083	
K 5506	0.100658229	
K 5507	0.102476385	
K 5508	0.057077402	
K 5509	0.10350453	
K 5510	0.123909532	
K 5511	0.165535806	
K 5512	0.102344537	
K 5513	0.132616667	
K 5514	0.089329833	
K 5515	0.09364013	
K 5516	0.105796045	
K 5517	0.110860872	
K 5518	0.144996511	
K 5519	0.12022955	
K 5520	0.101280444	
K 5521	0.128039969	
K 5522	0.099193021	
K 5523	0.112792903	
K 5524	0.110164671	
K 5525	0.100364341	
K 5526	0.096194519	
K 5527	0.111429807	
K 5528	0.094725739	
K 5529	0.101127792	
K 5530	0.10654259	
K 5531	0.1012036	
K 5532	0.111928576	
K 5533	0.140656207	
K 5534	0.126452215	
K 5535	0.112632707	
K 5536	0.097636426	
K 5537	0.128637882	
K 5538	0.118162158	
K 5539	0.118489687	
K 5540	0.11798011	
K 5541	0.113271934	
K 5542	0.128613257	

K 5543	0.110532648	
K 5544	0.107542066	
K 5545	0.095858064	
K 5546	0.091148451	
K 5547	0.103024883	
K 5548	0.107143143	
K 5549	0.106498508	
K 5550	0.123166123	
K 5551	0.125143066	
K 5552	0.117312724	
K 5553	0.118686733	
K 5554	0.132358115	
K 5555	0.091823217	
K 5556	0.099441907	
K 5557	0.096396555	
K 5558	0.101103227	
K 5559	0.130238521	
K 5560	0.098020757	
K 5561	0.103197414	
K 5562	0.138709777	
K 5563	0.148105119	
K 5564	0.100477033	
K 5565	0.16904602	
K 5566	0.123306286	
K 5567	0.10345608	
K 5568	0.110376632	
K 5569	0.123236974	
K 5570	0.093936109	
K 5571	0.128947518	
K 5572	0.140594942	
K 5573	0.117603917	
K 5574	0.104255624	
K 5575	0.128514128	
K 5576	0.105455308	
K 5577	0.135824899	
K 5578	0.124176882	
K 5579	0.122879597	
K 5580	0.134827683	
K 5581	0.12793823	
K 5582	0.097037218	
K 5583	0.100960506	
K 5584	0.134101593	
K 5585	0.101070697	
K 5586	0.107452054	
K 5587	0.121820457	
K 5588	0.107358398	
K 5589	0.097125586	

K 5590	0.1228095	
K 5591	0.10158518	
K 5592	0.115987229	
K 5593	0.145284233	
K 5594	0.148769419	
K 5595	0.100247672	
K 5596	0.108972766	
K 5597	0.108810083	
K 5598	0.100666559	
K 5599	0.116975549	
K 5600	0.105671392	
K 5601	0.100436966	
K 5602	0.111477095	
K 5603	0.09759696	
K 5604	0.095206918	
K 5605	0.130967906	
K 5606	0.099743933	
K 5607	0.120745929	
K 5608	0.106429335	
K 5609	0.138262691	
K 5610	0.135527051	
K 5611	0.100214348	
K 5612	0.100649462	
K 5613	0.118392732	
K 5614	0.123385103	
K 5615	0.118219967	
K 5616	0.122517183	
K 5617	0.097062312	
K 5618	0.102046129	
K 5619	0.000492407	
K 5620	0.09431566	
K 5621	0.10396559	
K 5622	0.115255386	
K 5623	0.101019205	
K 5624	0.109375268	
K 5625	0.099474258	
K 5626	0.103274707	
K 5627	0.152384255	
K 5628	0.104674306	
K 5629	0.12099646	
K 5630	0.135842658	
K 5631	0.093641687	
K 5632	0.097636426	
K 5633	0.101505625	
K 5634	0.099918778	
K 5635	0.114527666	
K 5636	0.106222004	

K 5637	0.100811889	
K 5638	0.102363252	
K 5639	0.128129645	
K 5640	0.104901227	
K 5641	0.105955456	
K 5642	0.109405397	
K 5643	0.121256694	
K 5644	0.104887026	
K 5645	0.100356795	
K 5646	0.11468737	
K 5647	0.101772491	
K 5648	0.108453588	
K 5649	0.108977816	
K 5650	0.115787063	
K 5651	0.102132418	
K 5652	0.142734531	
K 5653	0.099869263	
K 5654	0.095615944	
K 5655	0.094254066	
K 5656	0.10201	
K 5657	0.104137456	
K 5658	0.105662283	
K 5659	0.131421469	
K 5660	0.104637821	
K 5661	0.130367762	
K 5662	0.110023224	
K 5663	0.091399426	
K 5664	0.110054602	
K 5665	0.108692417	
K 5666	0.145315984	
K 5667	0.098615895	
K 5668	0.106423866	
K 5669	0.103396792	
K 5670	0.098206448	
K 5671	0.101293299	
K 5672	0.09973266	
K 5673	0.117245047	
K 5674	0.100365461	
K 5675	0.101883789	
K 5676	0.159228866	
K 5677	0.102439399	
K 5678	0.105003447	
K 5679	0.103768796	
K 5680	0.167335042	
K 5681	0.118163246	
K 5682	0.107317237	
K 5683	0.100199127	

K 5684	0.094658547	
K 5685	0.11734506	
K 5686	0.1048787	
K 5687	0.127470163	
K 5688	0.098797015	
K 5689	0.140656207	
K 5690	0.128555002	
K 5691	0.099819229	
K 5692	0.142734531	
K 5693	0.103153431	
K 5694	0.155756	
K 5695	0.11023909	
K 5696	0.105606463	
K 5697	0.103365619	
K 5698	0.104044489	
K 5699	0.121327506	
K 5700	0.166261087	
K 5701	0.127575622	
K 5702	0.108417	
K 5703	0.128294439	
K 5704	0.136550871	
K 5705	0.107344214	
K 5706	0.128981926	
K 5707	0.099965705	
K 5708	0.139389516	
K 5709	0.101883789	
K 5710	0.103947668	
K 5711	0.094427177	
K 5712	0.09536712	
K 5713	0.107327965	
K 5714	0.1276987	
K 5715	0.151916249	
K 5716	0.119465002	
K 5717	0.104546535	
K 5718	0.098638035	
K 5719	0.100382917	
K 5720	0.132020541	
K 5721	0.105904547	
K 5722	0.115893859	
K 5723	0.096994433	
K 5724	0.136891469	
K 5725	0.029577784	
K 5726	0.119350145	
K 5727	0.134002704	
K 5728	0.092210584	
K 5729	0.103313169	
K 5730	0.126237324	

K 5731	0.103530263	
K 5732	0.099979455	
K 5733	0.115021216	
K 5734	0.099480548	
K 5735	0.096872476	
K 5736	0.098273077	
K 5737	0.112408205	
K 5738	0.097370094	
K 5739	0.111270613	
K 5740	0.107181719	
K 5741	0.100119132	
K 5742	0.117196508	
K 5743	0.108816439	
K 5744	0.127378584	
K 5745	0.109640012	
K 5746	0.128688128	
K 5747	0.128473853	
K 5748	0.124188009	
K 5749	0.09307812	
K 5750	0.10534354	
K 5751	0.1029823	
K 5752	0.097442164	
K 5753	0.132799829	
K 5754	0.163200338	
K 5755	0.10408606	
K 5756	0.150010365	
K 5757	0.122480465	
K 5758	0.104264543	
K 5759	0.122410194	
K 5760	0.102144539	
K 5761	0.099259778	
K 5762	0.116646019	
K 5763	0.106741944	
K 5764	0.101599696	
K 5765	0.10158518	
K 5766	0.132587403	
K 5767	0.08767493	
K 5768	0.104287947	
K 5769	0.147140389	
K 5770	0.129899074	
K 5771	0.100560819	
K 5772	0.142734531	
K 5773	0.136330656	
K 5774	0.12674365	
K 5775	0.08953047	
K 5776	0.1310662	
K 5777	0.113545987	

K 5778	0.063571241	
K 5779	0.104125697	
K 5780	0.120140499	
K 5781	0.133814423	
K 5782	0.102507865	
K 5783	0.113592506	
K 5784	0.106074924	
K 5785	0.089560096	
K 5786	0.099794387	
K 5787	0.105141485	
K 5788	0.120733914	
K 5789	0.108402848	
K 5790	0.097855158	
K 5791	0.106302567	
K 5792	0.100966739	
K 5793	0.0935081	
K 5794	0.130832022	
K 5795	0.100316961	
K 5796	0.101219015	
K 5797	0.111689925	
K 5798	0.108796839	
K 5799	0.105577575	
K 5800	0.095710548	
K 5801	0.128024184	
K 5802	0.107306708	
K 5803	0.104030894	
K 5804	0.126216391	
K 5805	0.128611036	
K 5806	0.132257378	
K 5807	0.109687848	
K 5808	0.133112623	
K 5809	0.018660954	
K 5810	0.103519285	
K 5811	0.12545709	
K 5812	0.097321973	
K 5813	0.117768462	
K 5814	0.127475467	
K 5815	0.106118446	
K 5816	0.094347117	
K 5817	0.112375823	
K 5818	0.279290293	
K 5819	0.107156928	
K 5820	0.14189048	
K 5821	0.11126275	
K 5822	0.131033716	
K 5823	0.098977831	
K 5824	0.128854295	

K 5825	0.119906128	
K 5826	0.124684284	
K 5827	0.130845931	
K 5828	0.116076954	
K 5829	0.133443985	
K 5830	0.116700284	
K 5831	0.097266249	
K 5832	0.12556695	
K 5833	0.131611489	
K 5834	0.097690849	
K 5835	0.142734531	
K 5836	0.095023729	
K 5837	0.128899929	
K 5838	0.111572705	
K 5839	0.120745929	
K 5840	0.127950204	
K 5841	0.105122479	
K 5842	0.104256653	
K 5843	0.129926462	
K 5844	0.101822998	
K 5845	0.142734531	
K 5846	0.136879333	
K 5847	0.117736971	
K 5848	0.114429321	
K 5849	0.105049927	
K 5850	0.13249852	
K 5851	0.130558191	
K 5852	0.102331203	
K 5853	0.142734531	
K 5854	0.103832473	
K 5855	0.1247791	
K 5856	0.098266172	
K 5857	0.106693032	
K 5858	0.136291604	
K 5859	0.105406074	
K 5860	0.138729539	
K 5861	0.114162847	
K 5862	0.108098771	
K 5863	0.12330359	
K 5864	0.11171	
K 5865	0.091436249	
K 5866	0.12664243	
K 5867	0.107116512	
K 5868	0.12433718	
K 5869	0.126277634	
K 5870	0.100987698	
K 5871	0.117790721	

K 5872	0.090480189	
K 5873	0.10022676	
K 5874	0.132402237	
K 5875	0.123386243	
K 5876	0.098406541	
K 5877	0.098576993	
K 5878	0.109073068	
K 5879	0.112265131	
K 5880	0.10412367	
K 5881	0.140656207	
K 5882	0.100721777	
K 5883	0.11794074	
K 5884	0.101076308	
K 5885	0.216222986	
K 5886	0.115717321	
K 5887	0.09753175	
K 5888	0.140876408	
K 5889	0.094447929	
K 5890	0.114253102	
K 5891	0.14536695	
K 5892	0.104399094	
K 5893	0.106815928	
K 5894	0.129987952	
K 5895	0.109101324	
K 5896	0.104503229	
K 5897	0.101193415	
K 5898	0.112678854	
K 5899	0.120179936	
K 5900	0.109187375	
K 5901	0.1	
K 5902	0.1	
K 5903	0.1	

PART6: Plots of model outputs vs 3 replications of experimental data in large-scale colon cancerous cell's model

- Errorbars shows the maximum and minimum values in 3 biological replications of experimental data in each time point.
- The blue circles show the mean value in 3 biological replications of experimental data in each time point.
- The first 3 rows of plots (Ctrl, GEF and PLX) were used for parameter approximation and the 4th row shows estimation of the model comparing with experimental data in GEF+PLX dataset

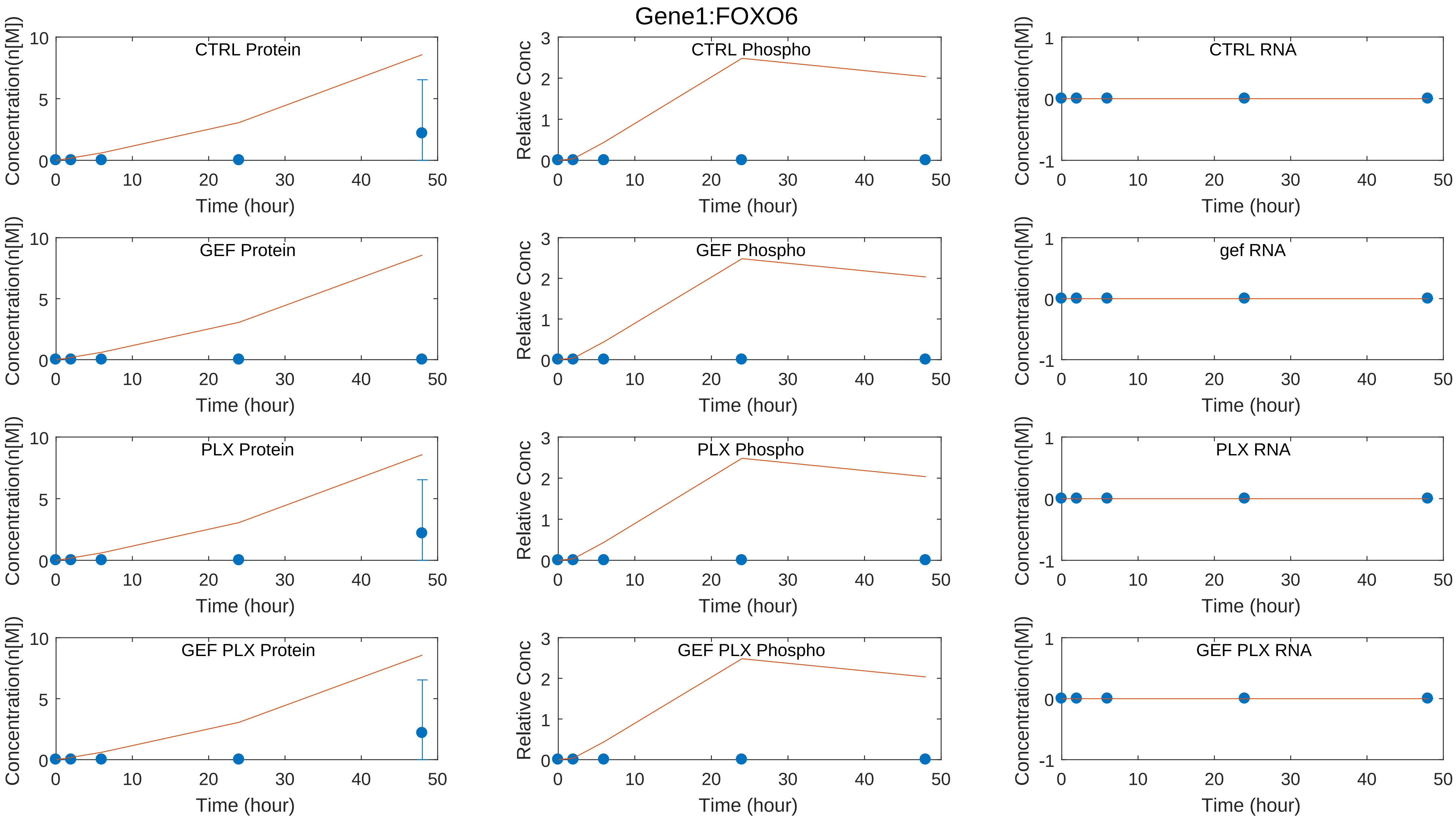

Gene1:FOXO6
CTRL Protein
CTRL Phospho
CTRL RNA
GEF Protein
GEF Phospho
gef RNA
PLX Protein
PLX Phospho
PLX RNA
GEF PLX Protein
GEF PLX Phospho
GEF PLX RNA
Concentration(n[M])
Relative Conc
Time (hour)

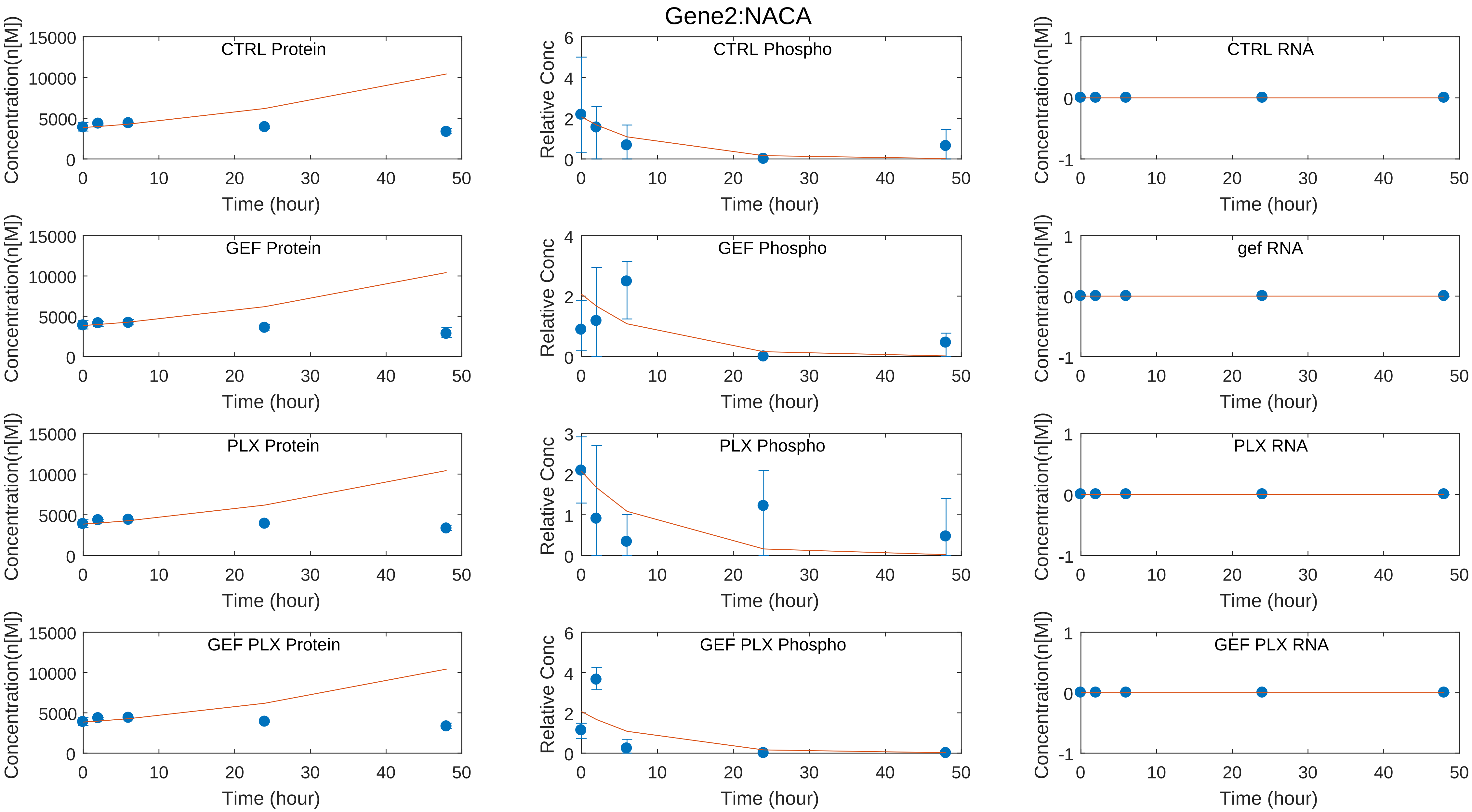
Gene2:NACA
CTRL Protein
CTRL Phospho
CTRL RNA
GEF Protein
GEF Phospho
gef RNA
PLX Protein
PLX Phospho
PLX RNA
GEF PLX Protein
GEF PLX Phospho
GEF PLX RNA
Concentration(n[M])
Relative Conc
Time (hour)

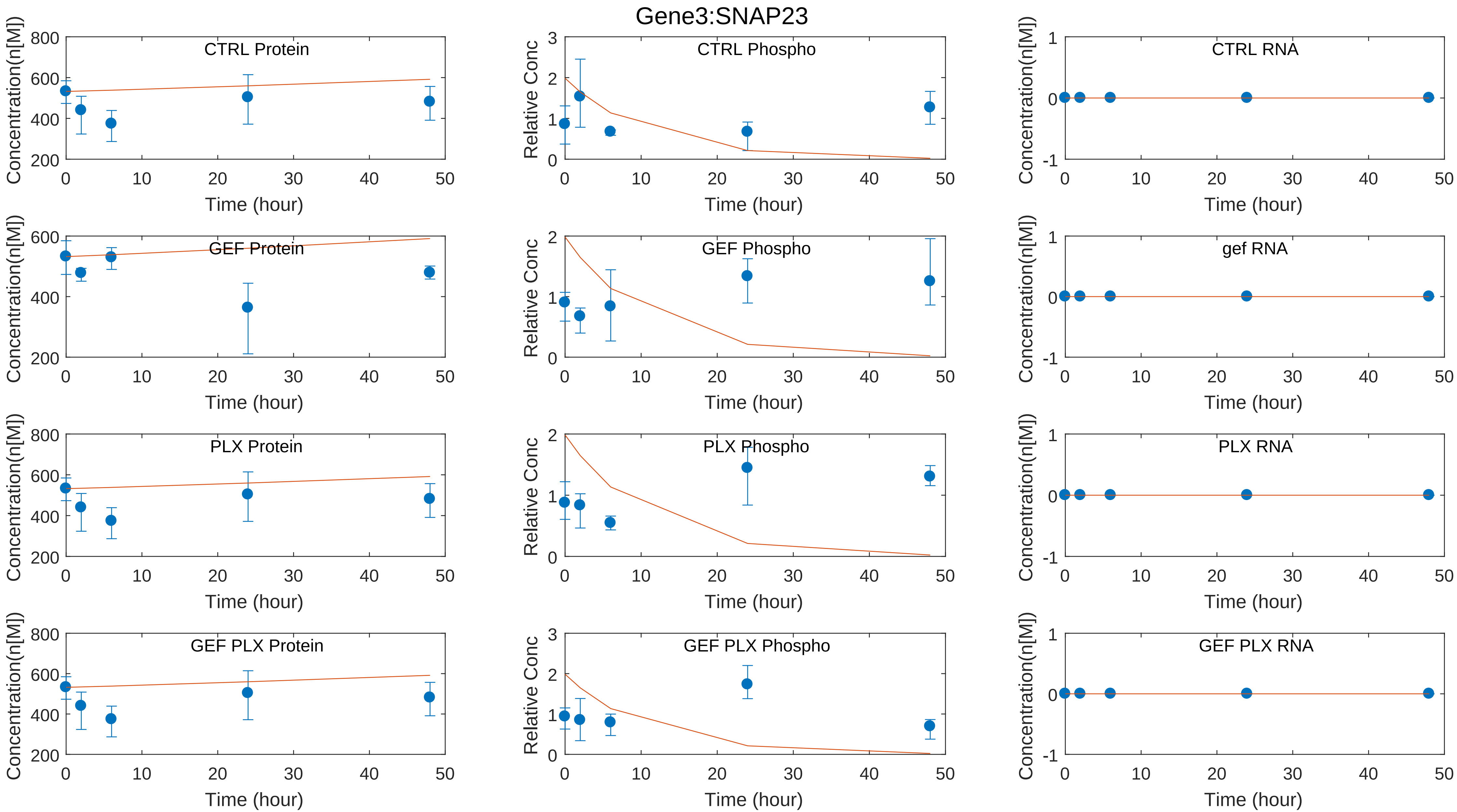

Gene3:SNAP23
CTRL Protein
CTRL Phospho
CTRL RNA
GEF Protein
GEF Phospho
gef RNA
PLX Protein
PLX Phospho
PLX RNA
GEF PLX Protein
GEF PLX Phospho
GEF PLX RNA
Concentration(n[M])
Relative Conc
Time (hour)

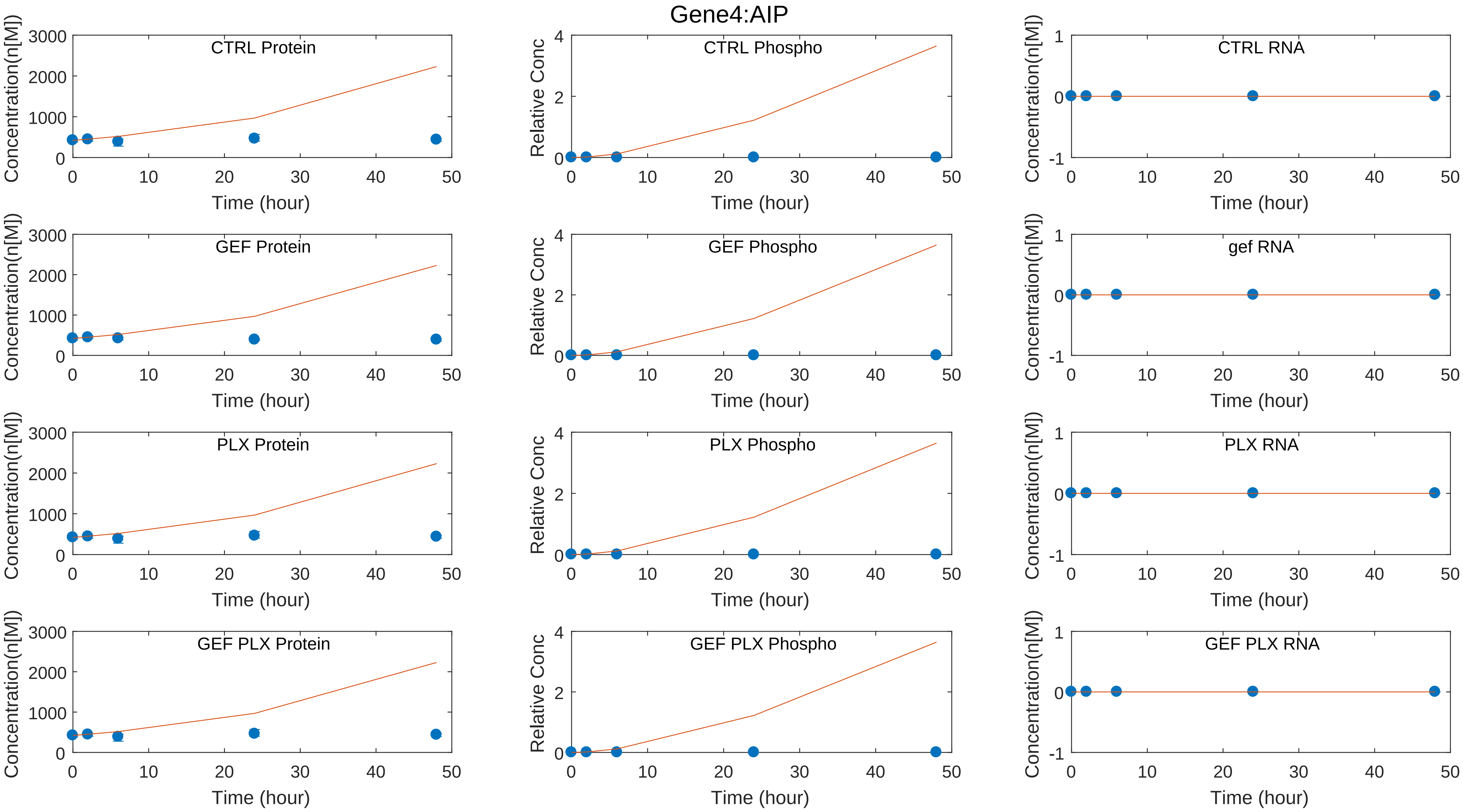
Gene4:AIP
CTRL Protein
CTRL Phospho
CTRL RNA
GEF Protein
GEF Phospho
gef RNA
PLX Protein
PLX Phospho
PLX RNA
GEF PLX Protein
GEF PLX Phospho
GEF PLX RNA
Concentration(n[M])
Relative Conc
Time (hour)

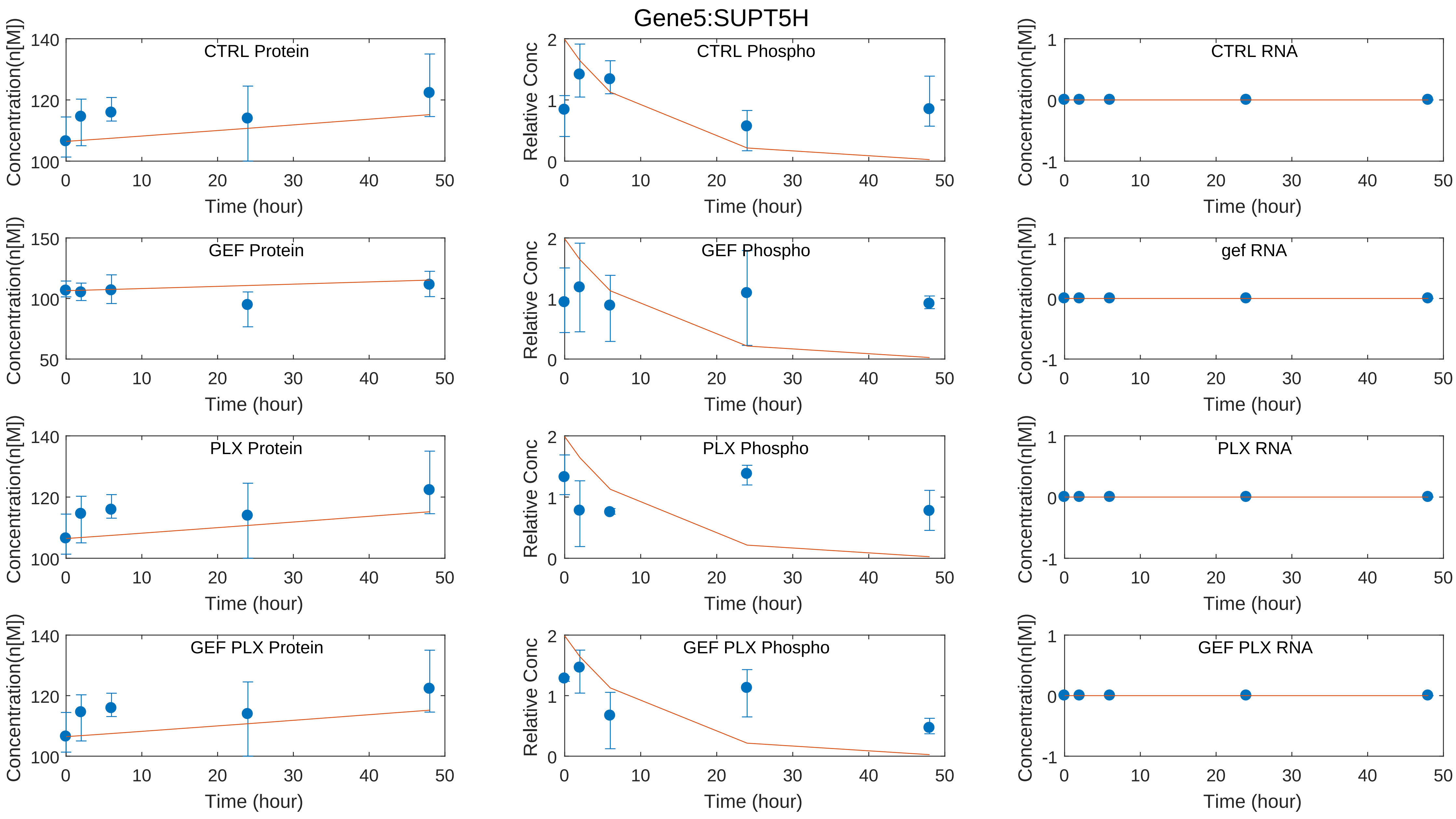

Gene5:SUPT5H
CTRL Protein
CTRL Phospho
CTRL RNA
GEF Protein
GEF Phospho
gef RNA
PLX Protein
PLX Phospho
PLX RNA
GEF PLX Protein
GEF PLX Phospho
GEF PLX RNA
Concentration(n[M])
Relative Conc
Time (hour)

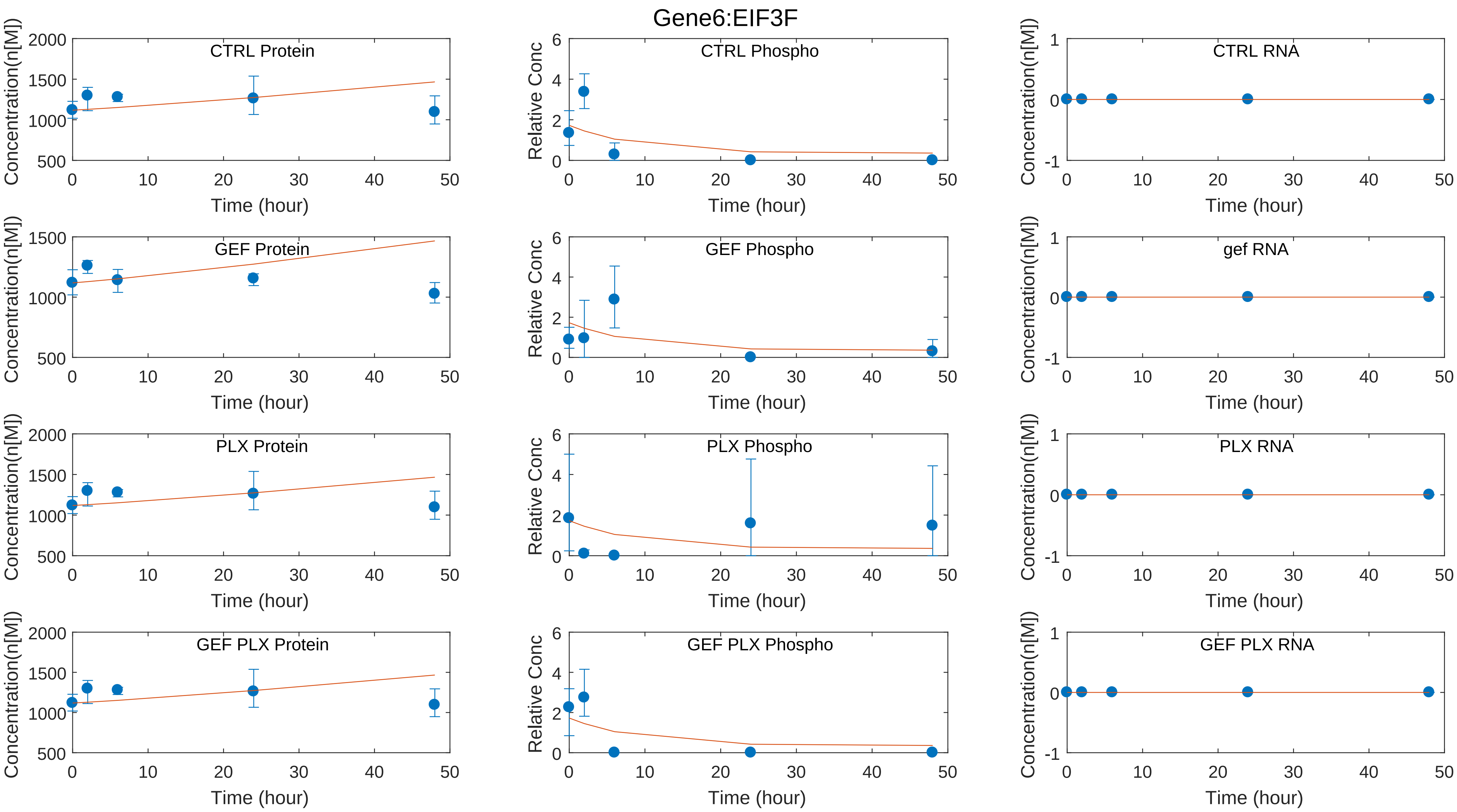

Gene6:EIF3F
CTRL Protein
CTRL Phospho
CTRL RNA
GEF Protein
GEF Phospho
gef RNA
PLX Protein
PLX Phospho
PLX RNA
GEF PLX Protein
GEF PLX Phospho
GEF PLX RNA
Concentration(n[M])
Relative Conc
Time (hour)

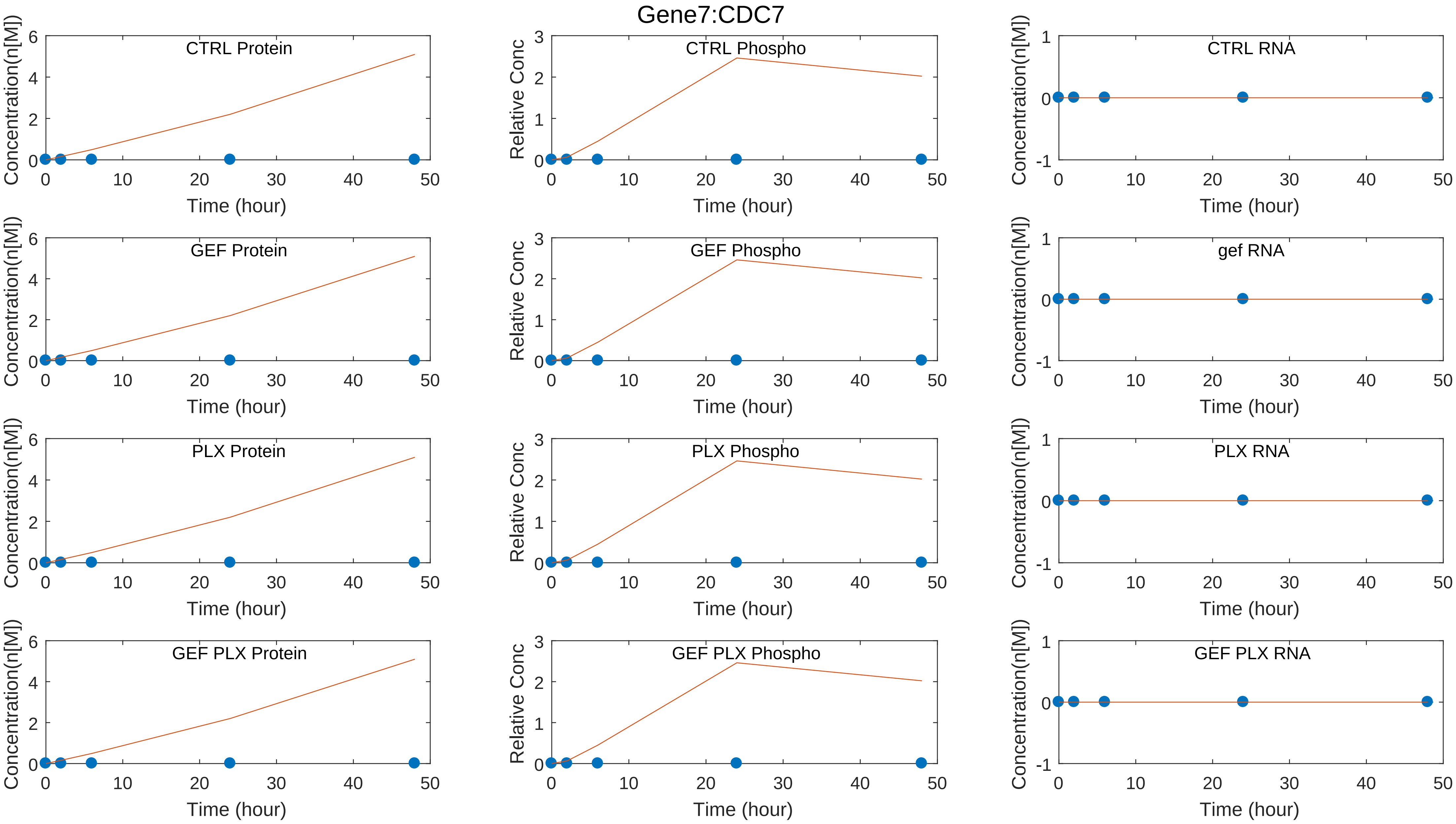
Gene7:CDC7
CTRL Protein
CTRL Phospho
CTRL RNA
GEF Protein
GEF Phospho
gef RNA
PLX Protein
PLX Phospho
PLX RNA
GEF PLX Protein
GEF PLX Phospho
GEF PLX RNA
Concentration(n[M])
Relative Conc
Time (hour)

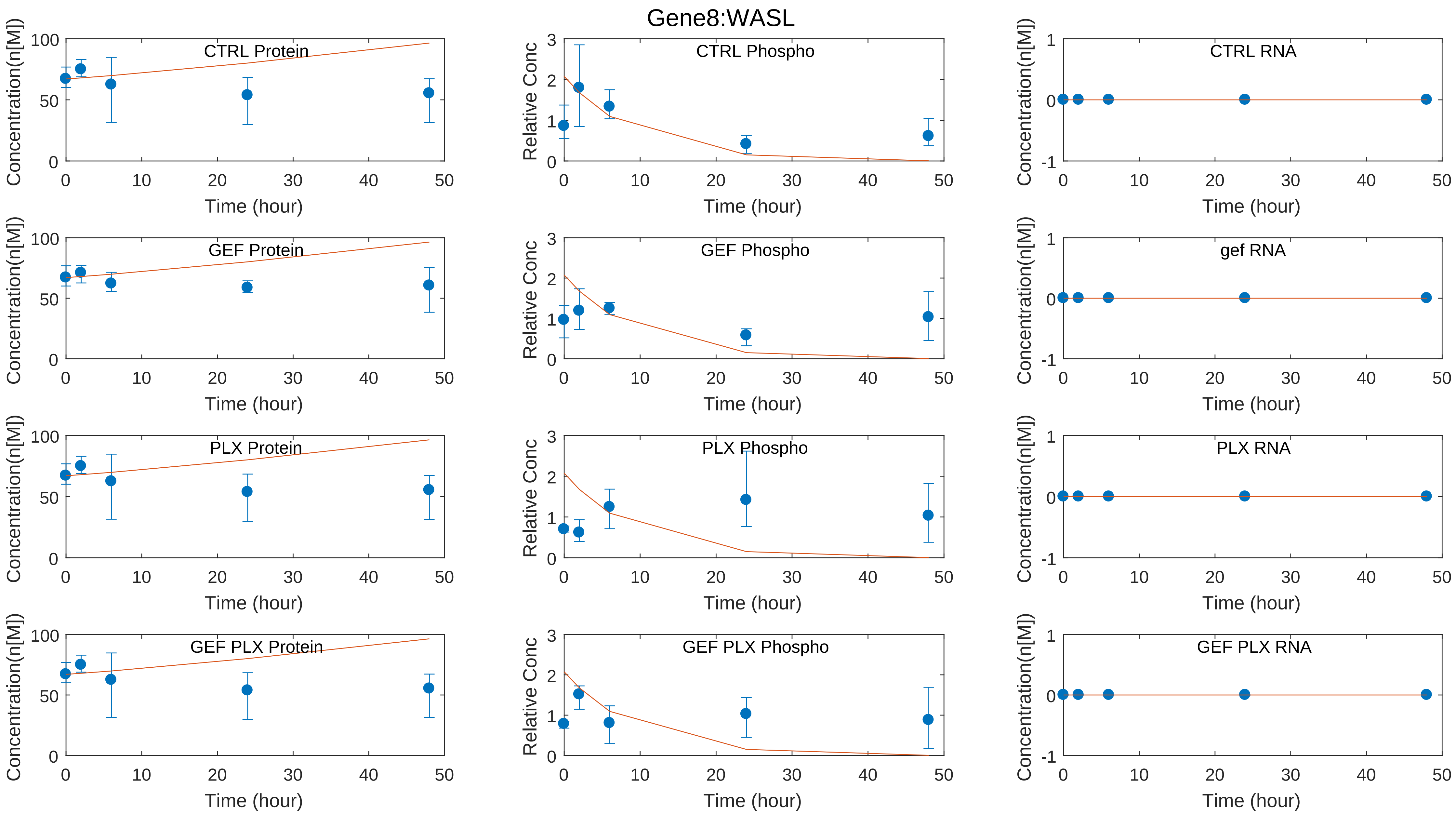

Gene8:WASL
CTRL Protein
CTRL Phospho
CTRL RNA
GEF Protein
GEF Phospho
gef RNA
PLX Protein
PLX Phospho
PLX RNA
GEF PLX Protein
GEF PLX Phospho
GEF PLX RNA
Concentration(n[M])
Relative Conc
Time (hour)

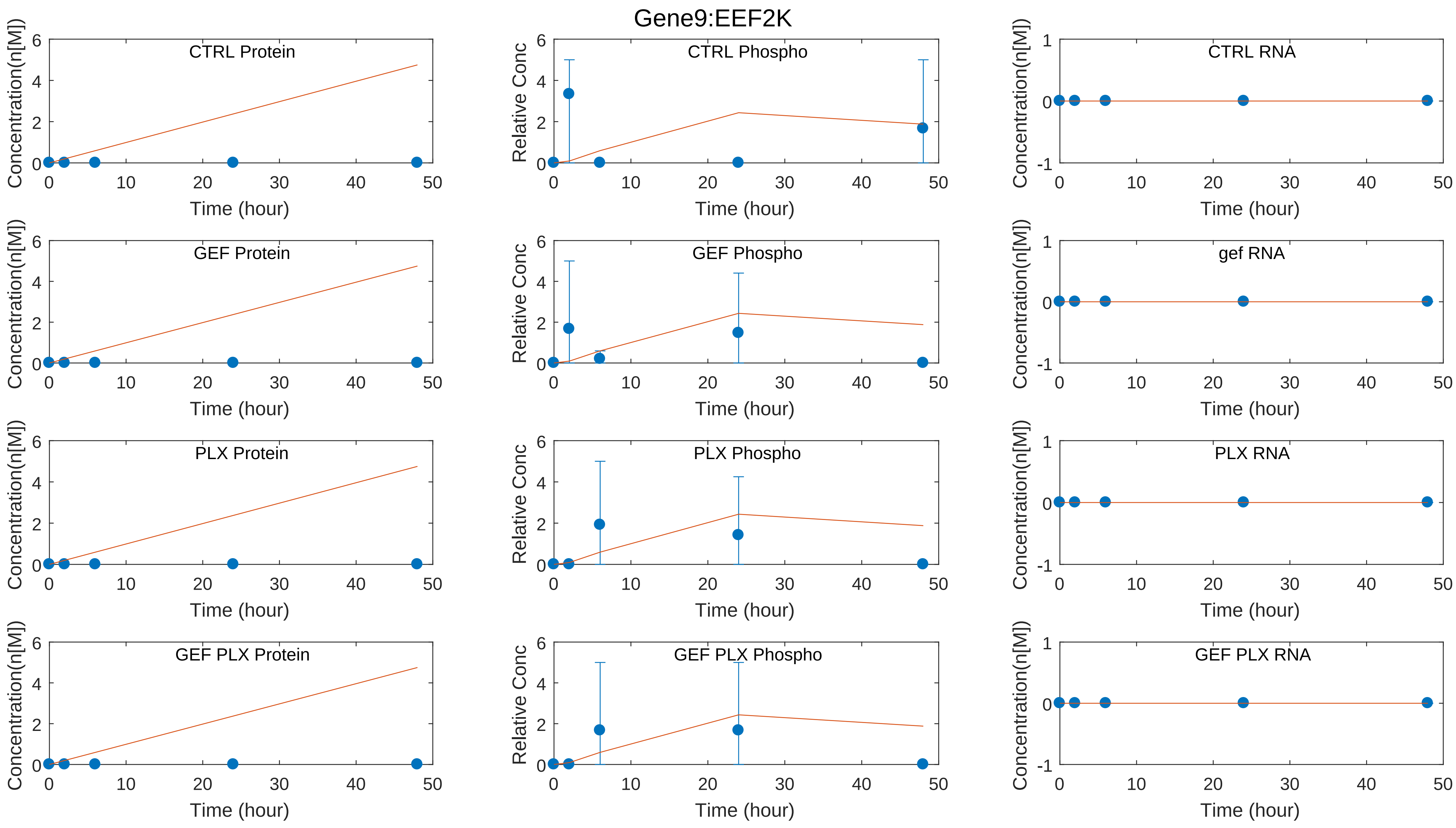

Gene9:EEF2K
CTRL Protein
CTRL Phospho
CTRL RNA
GEF Protein
GEF Phospho
gef RNA
PLX Protein
PLX Phospho
PLX RNA
GEF PLX Protein
GEF PLX Phospho
GEF PLX RNA
Concentration(n[M])
Relative Conc
Time (hour)

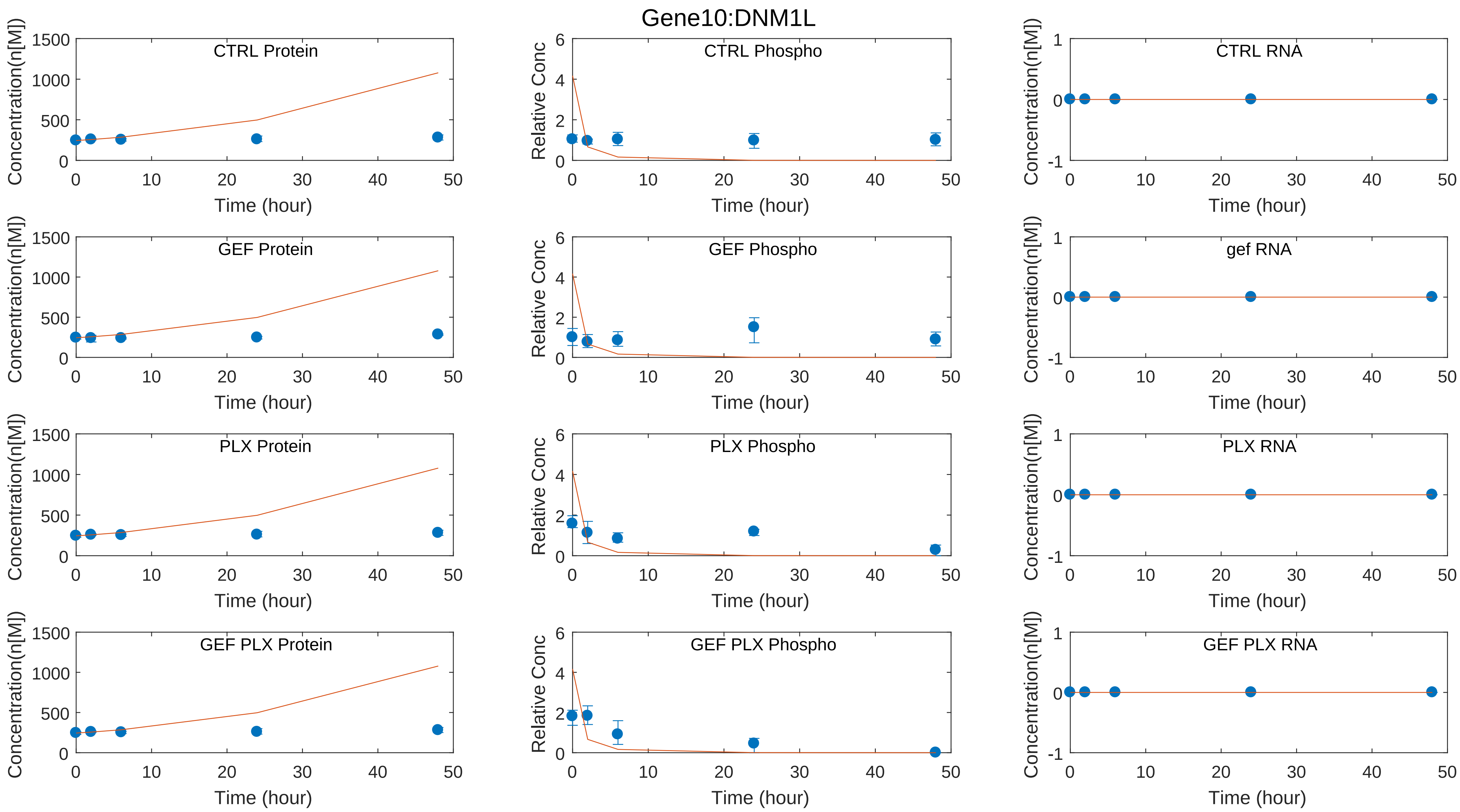
Gene10:DNM1L
CTRL Protein
GEF Protein
PLX Protein
GEF PLX Protein
CTRL Phospho
GEF Phospho
PLX Phospho
GEF PLX Phospho
CTRL RNA
gef RNA
PLX RNA
GEF PLX RNA
Concentration(n[M])
Relative Conc
Time (hour)

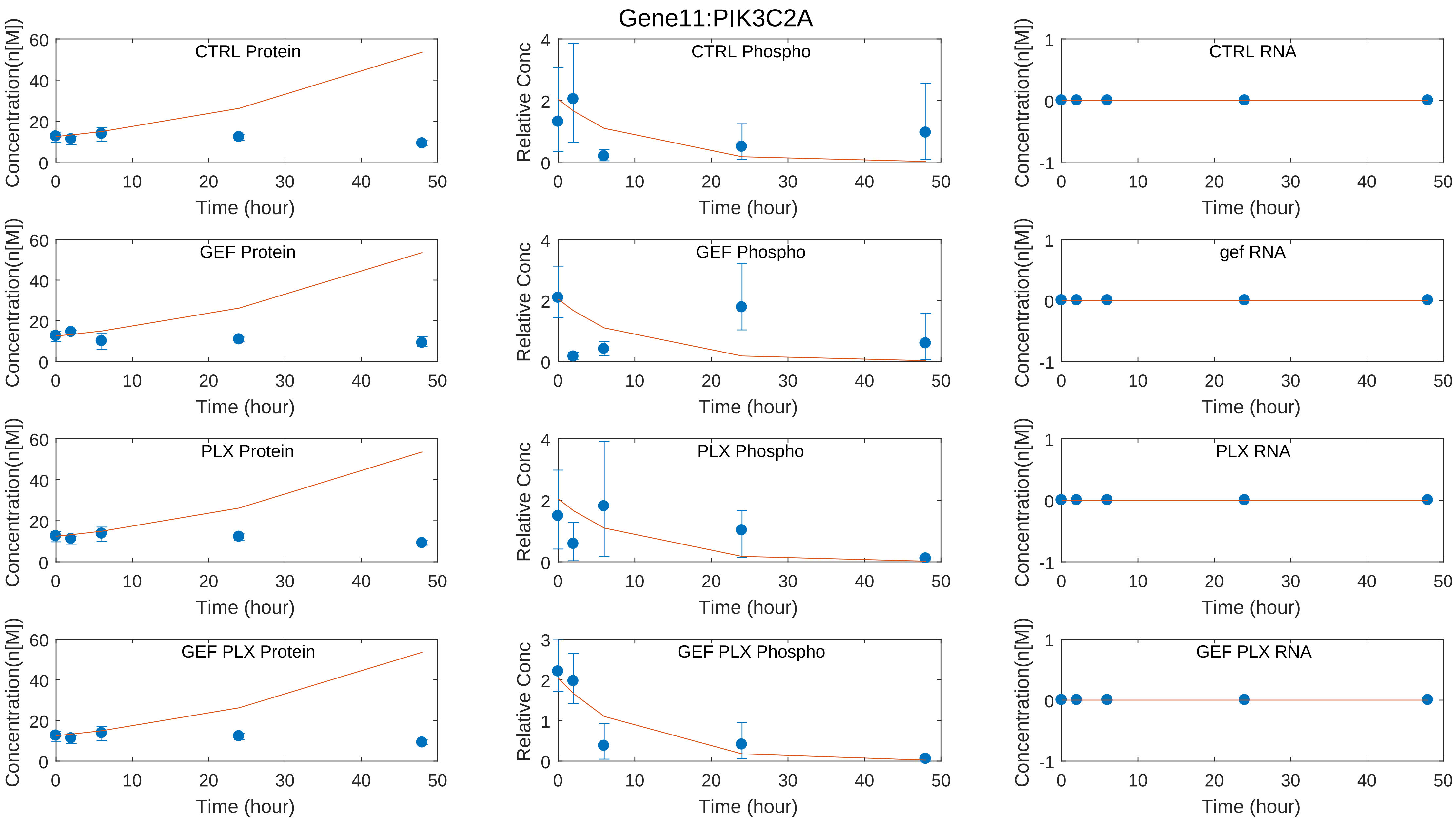
Gene11:PIK3C2A
CTRL Protein
GEF Protein
PLX Protein
GEF PLX Protein
CTRL Phospho
GEF Phospho
PLX Phospho
GEF PLX Phospho
CTRL RNA
gef RNA
PLX RNA
GEF PLX RNA
Concentration(n[M])
Relative Conc
Time (hour)

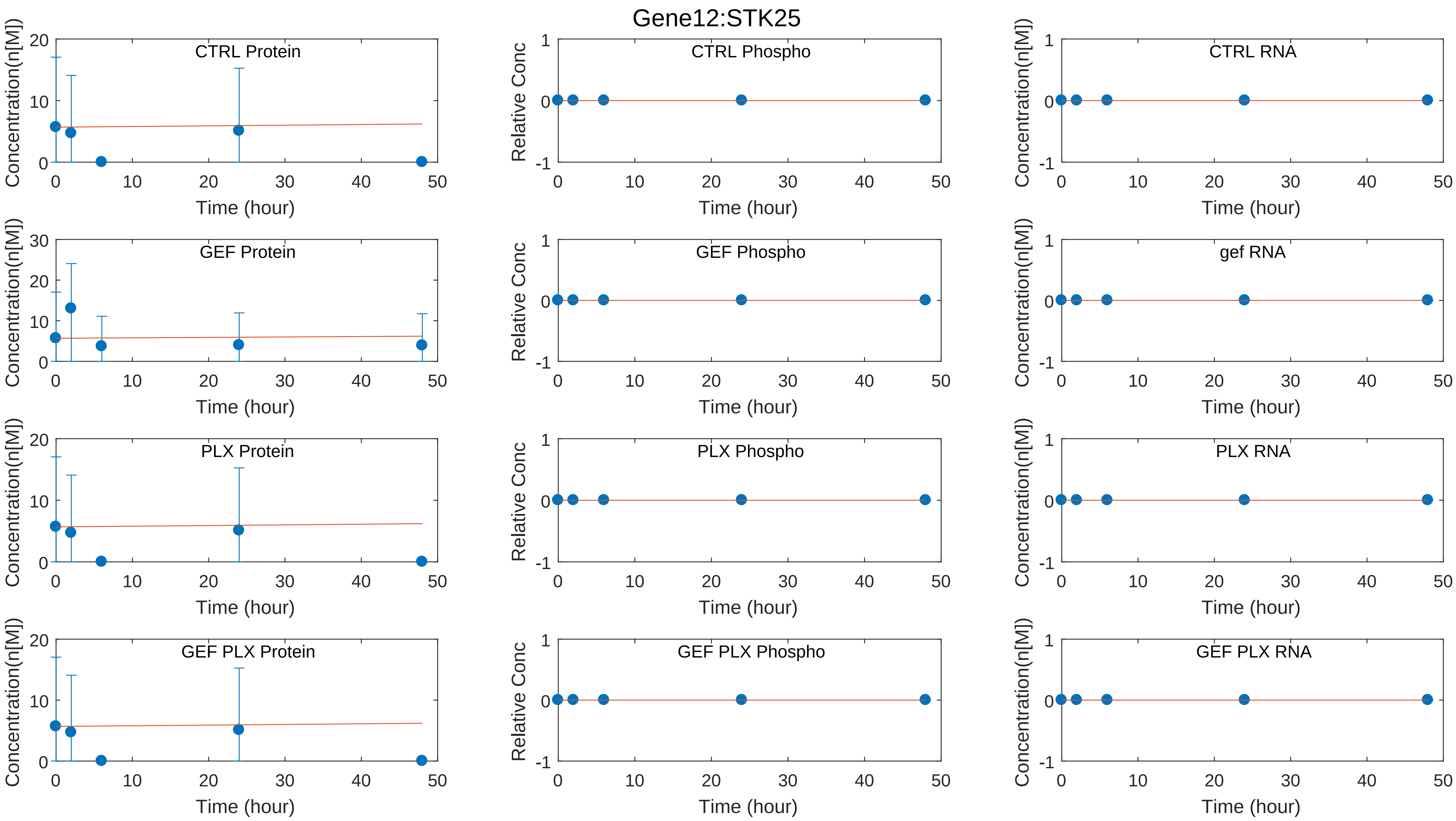
Gene12:STK25
CTRL Protein
CTRL Phospho
CTRL RNA
GEF Protein
GEF Phospho
gef RNA
PLX Protein
PLX Phospho
PLX RNA
GEF PLX Protein
GEF PLX Phospho
GEF PLX RNA
Concentration(n[M])
Relative Conc
Time (hour)

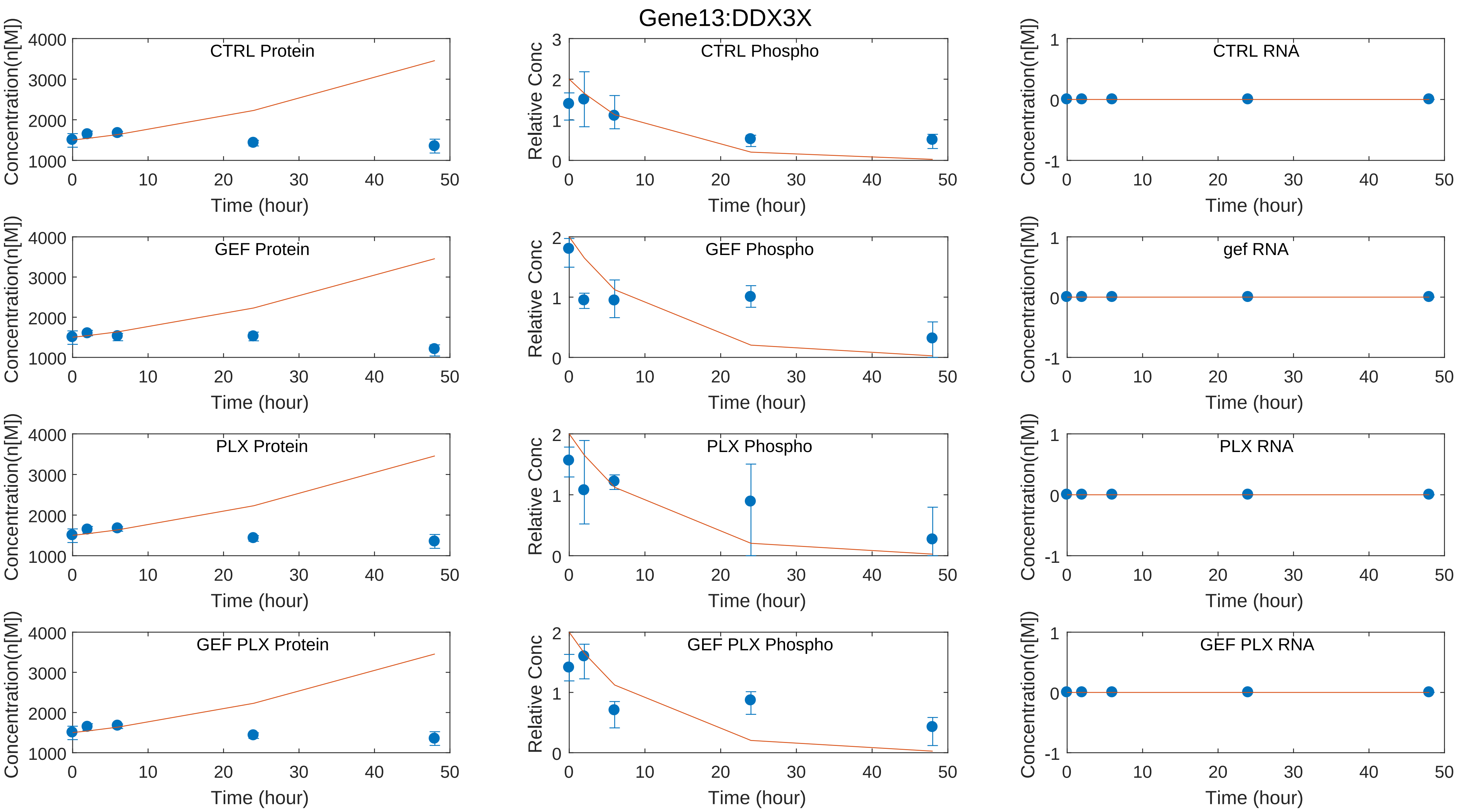
Gene13:DDX3X
CTRL Protein
CTRL Phospho
CTRL RNA
GEF Protein
GEF Phospho
gef RNA
PLX Protein
PLX Phospho
PLX RNA
GEF PLX Protein
GEF PLX Phospho
GEF PLX RNA
Concentration(n[M])
Relative Conc
Time (hour)

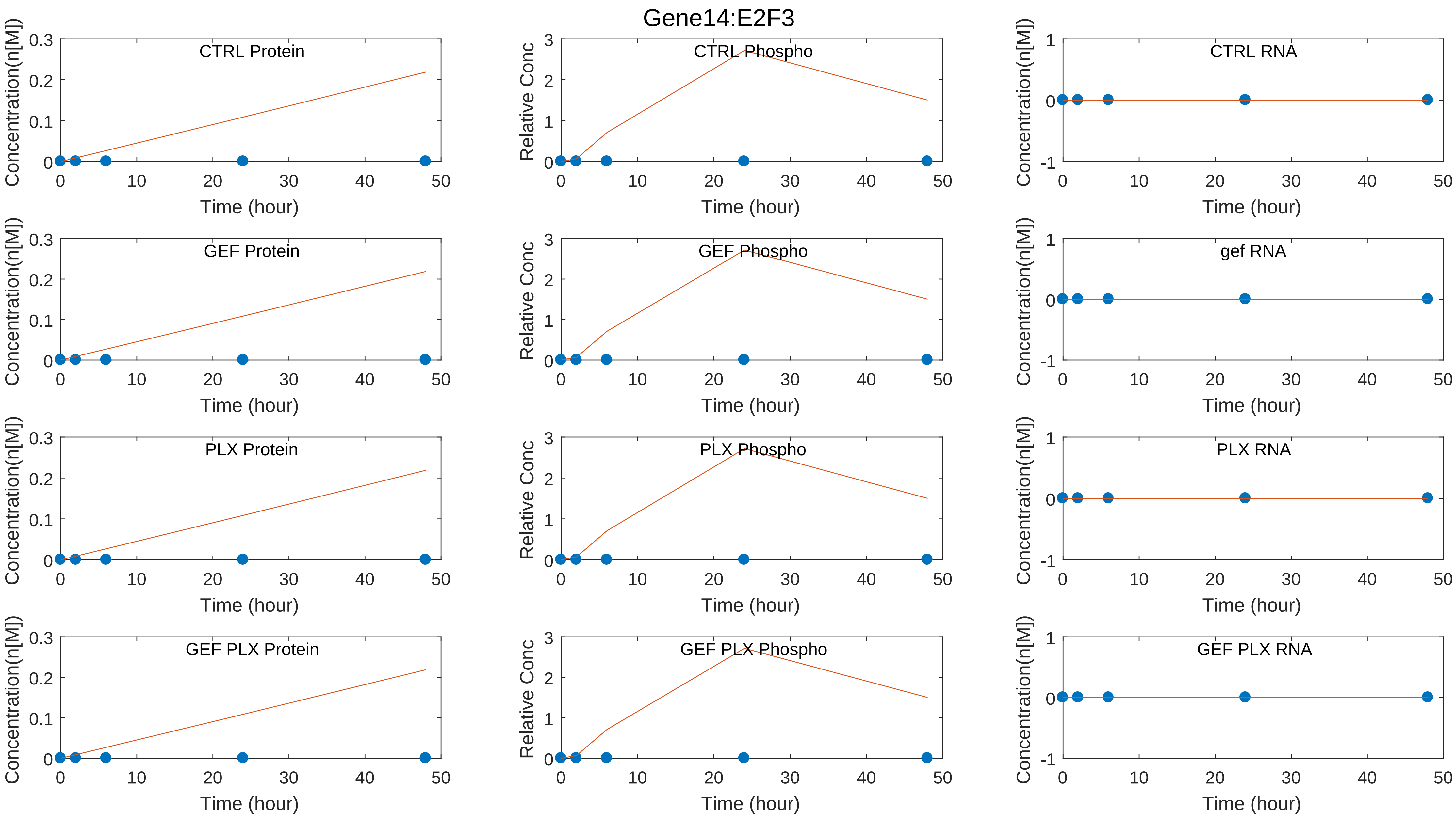
Gene14:E2F3
CTRL Protein
CTRL Phospho
CTRL RNA
GEF Protein
GEF Phospho
gef RNA
PLX Protein
PLX Phospho
PLX RNA
GEF PLX Protein
GEF PLX Phospho
GEF PLX RNA
Concentration(n[M])
Relative Conc
Time (hour)

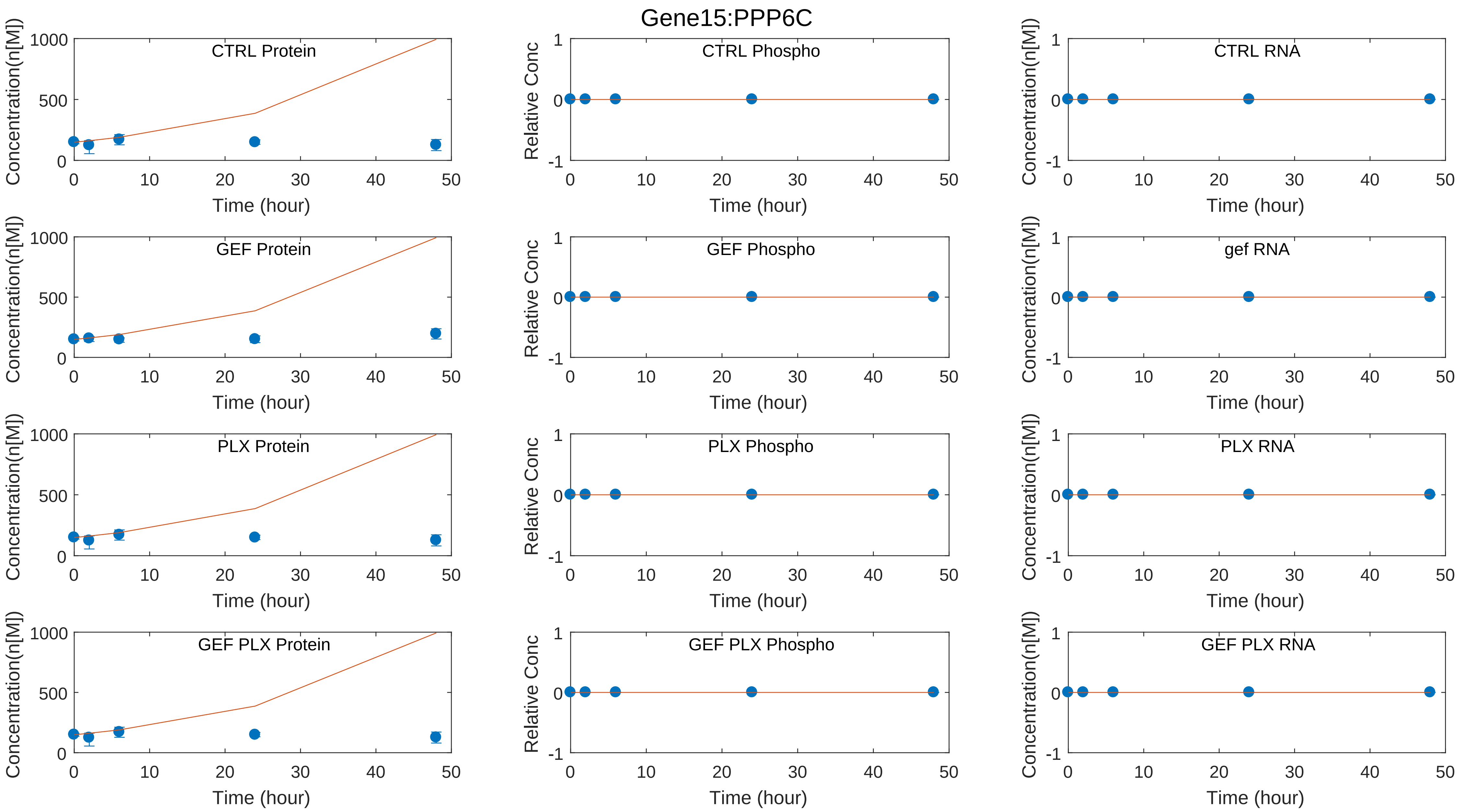
Gene15:PPP6C
CTRL Protein
CTRL Phospho
CTRL RNA
GEF Protein
GEF Phospho
gef RNA
PLX Protein
PLX Phospho
PLX RNA
GEF PLX Protein
GEF PLX Phospho
GEF PLX RNA
Concentration(n[M])
Relative Conc
Time (hour)
0
10
20
30
40
50
1000
500
1
-1

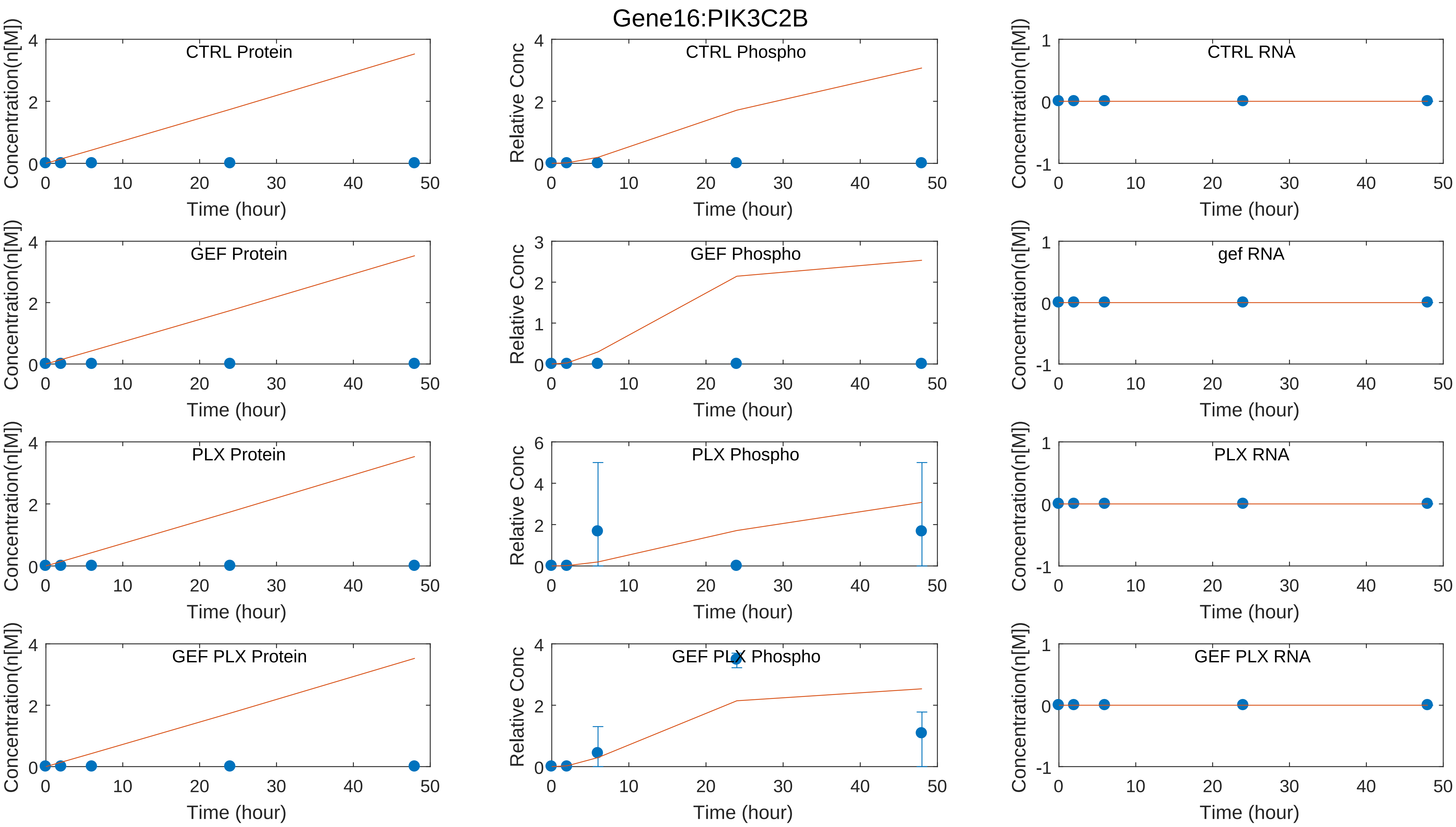

Gene16:PIK3C2B
CTRL Protein
CTRL Phospho
CTRL RNA
GEF Protein
GEF Phospho
gef RNA
PLX Protein
PLX Phospho
PLX RNA
GEF PLX Protein
GEF PLX Phospho
GEF PLX RNA
Concentration(n[M])
Relative Conc
Time (hour)

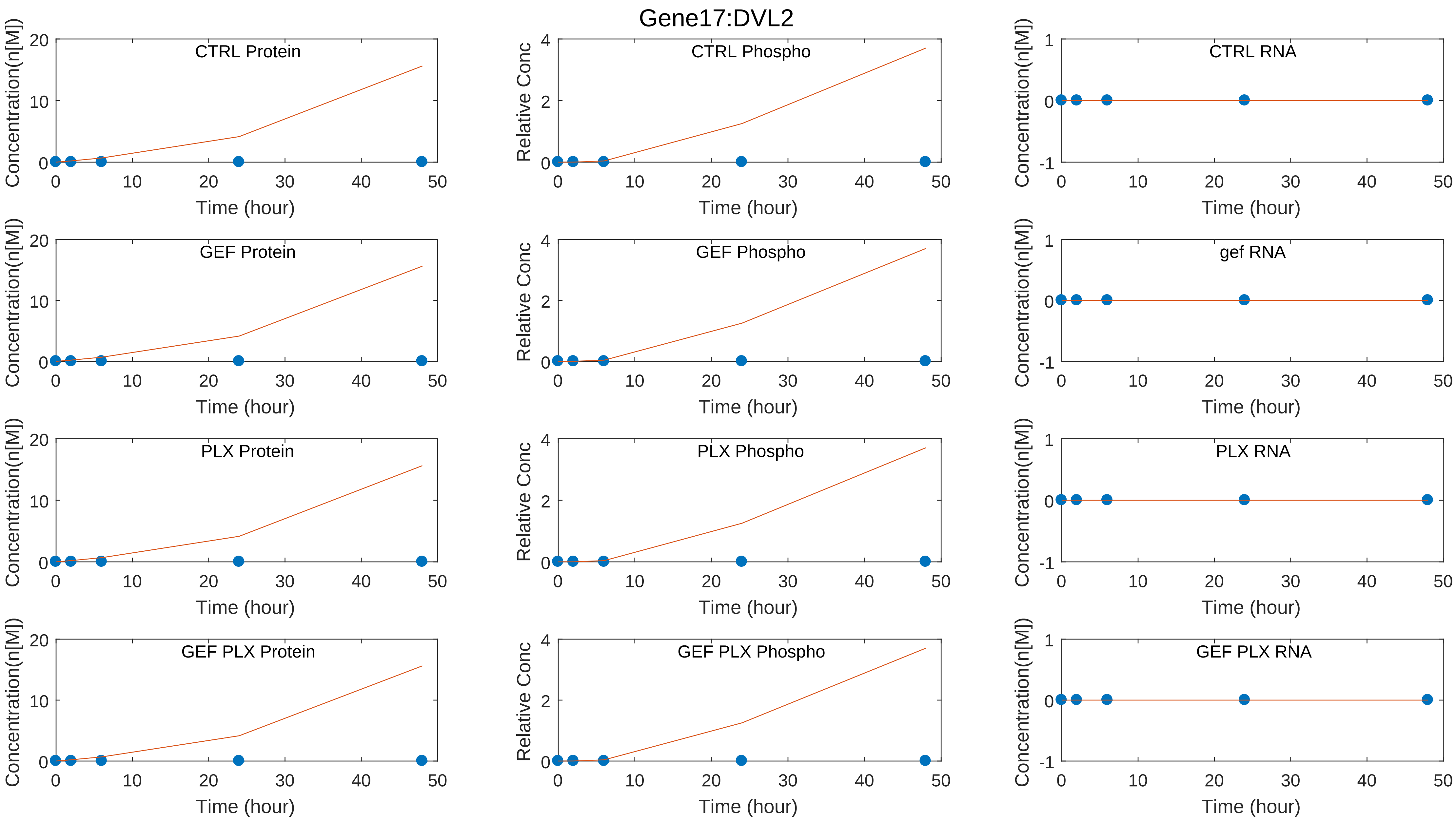

Gene17:DVL2
CTRL Protein
CTRL Phospho
CTRL RNA
GEF Protein
GEF Phospho
gef RNA
PLX Protein
PLX Phospho
PLX RNA
GEF PLX Protein
GEF PLX Phospho
GEF PLX RNA
Concentration(n[M])
Relative Conc
Time (hour)

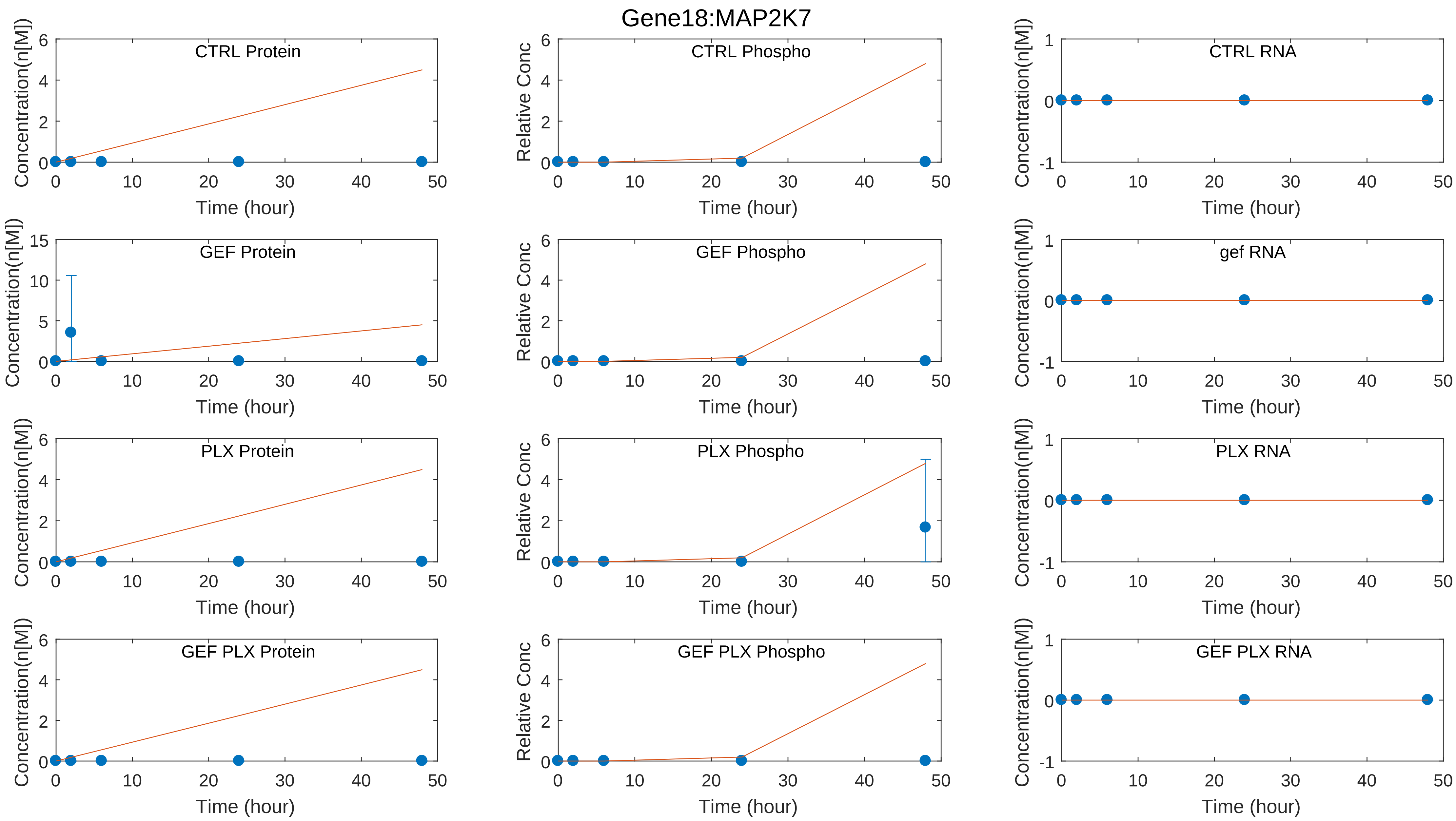
Gene18:MAP2K7
CTRL Protein
CTRL Phospho
CTRL RNA
GEF Protein
GEF Phospho
gef RNA
PLX Protein
PLX Phospho
PLX RNA
GEF PLX Protein
GEF PLX Phospho
GEF PLX RNA
Concentration(n[M])
Relative Conc
Time (hour)

Gene19:PDCD5

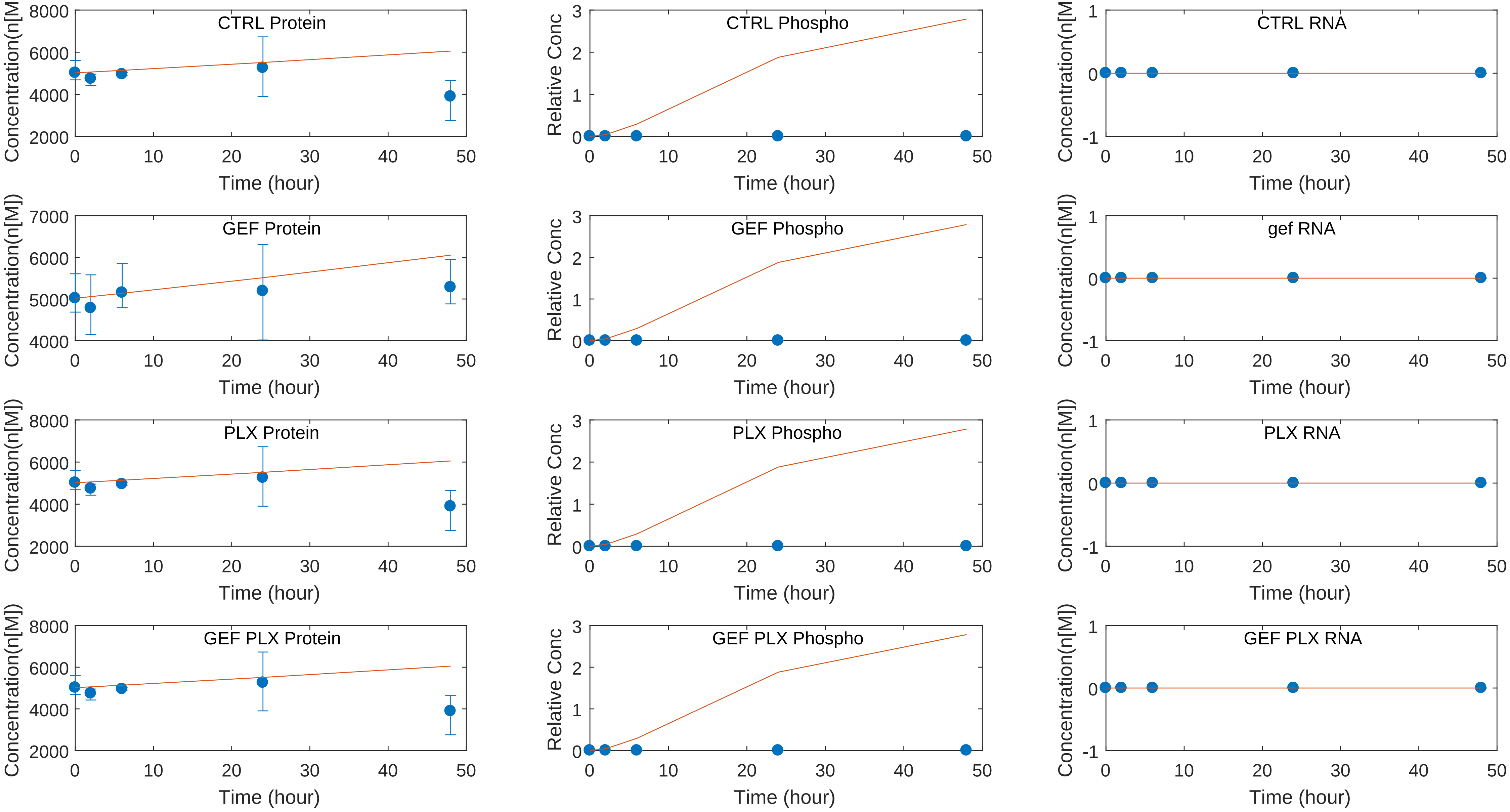

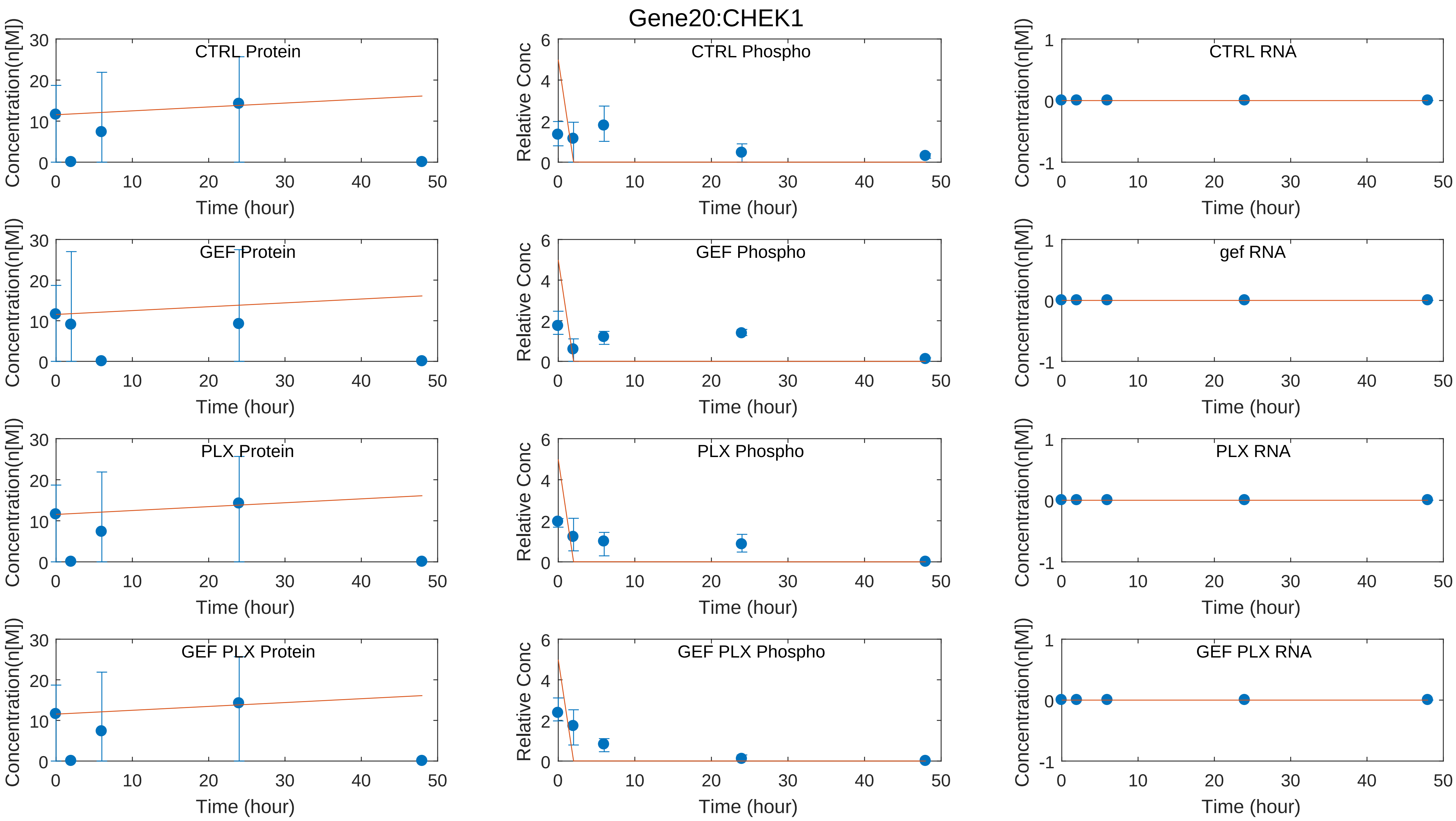
Gene20:CHEK1
CTRL Protein
CTRL Phospho
CTRL RNA
GEF Protein
GEF Phospho
gef RNA
PLX Protein
PLX Phospho
PLX RNA
GEF PLX Protein
GEF PLX Phospho
GEF PLX RNA
Concentration(n[M])
Relative Conc
Time (hour)

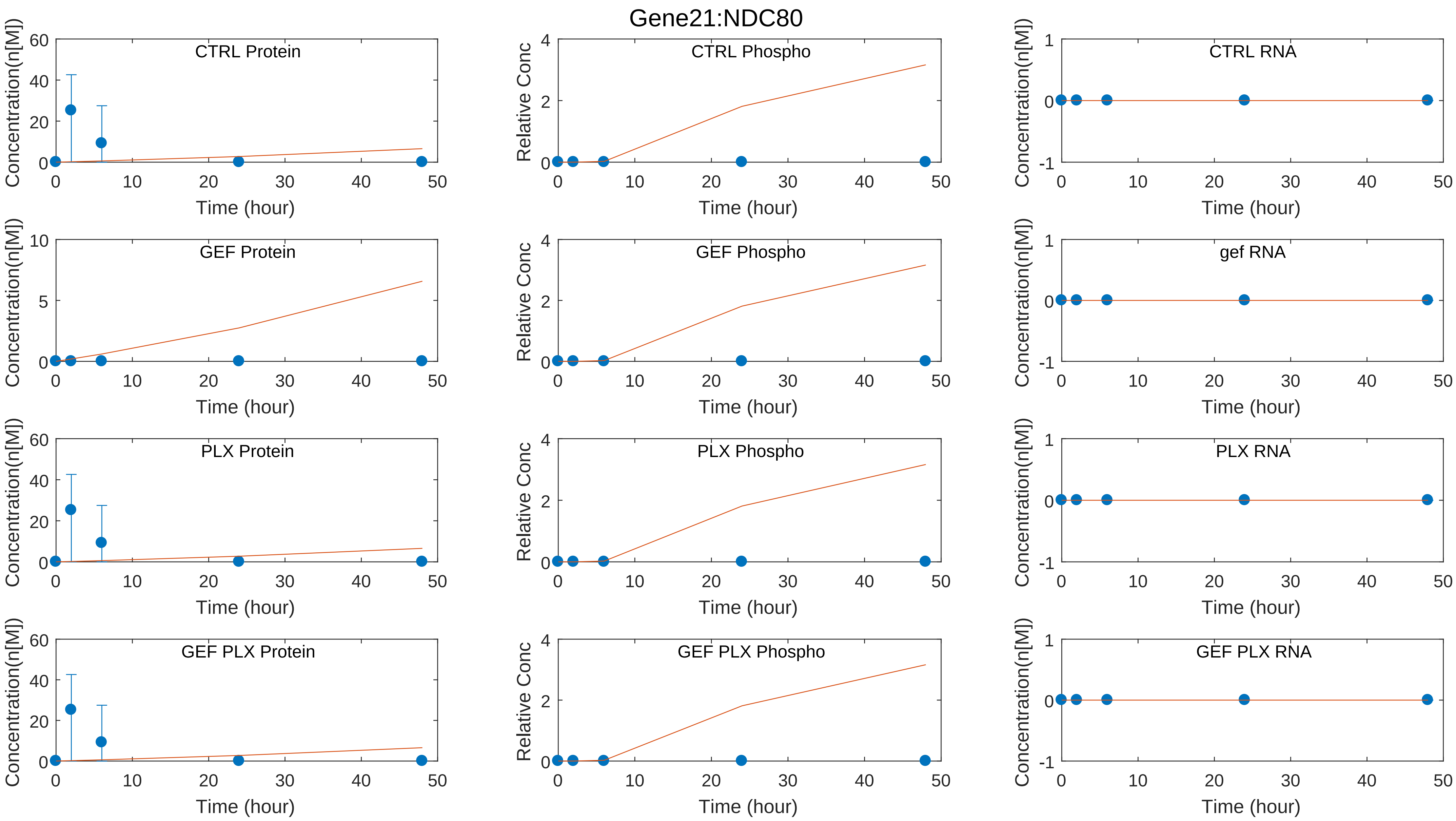

Gene21:NDC80
CTRL Protein
CTRL Phospho
CTRL RNA
GEF Protein
GEF Phospho
gef RNA
PLX Protein
PLX Phospho
PLX RNA
GEF PLX Protein
GEF PLX Phospho
GEF PLX RNA
Concentration(n[M])
Relative Conc
Time (hour)

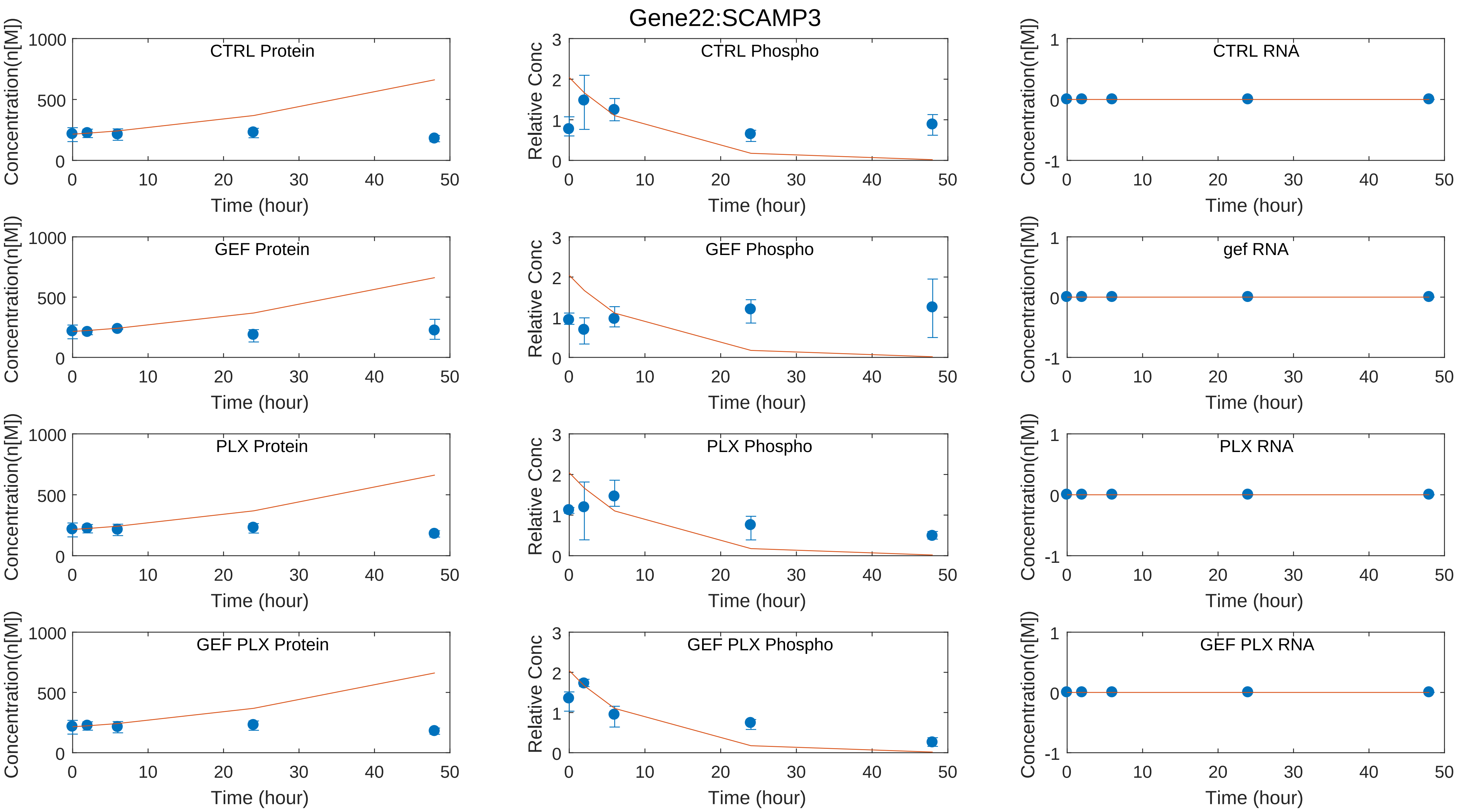

Gene22:SCAMP3
CTRL Protein
CTRL Phospho
CTRL RNA
GEF Protein
GEF Phospho
gef RNA
PLX Protein
PLX Phospho
PLX RNA
GEF PLX Protein
GEF PLX Phospho
GEF PLX RNA
Concentration(n[M])
Relative Conc
Time (hour)

Gene23:BCKDK

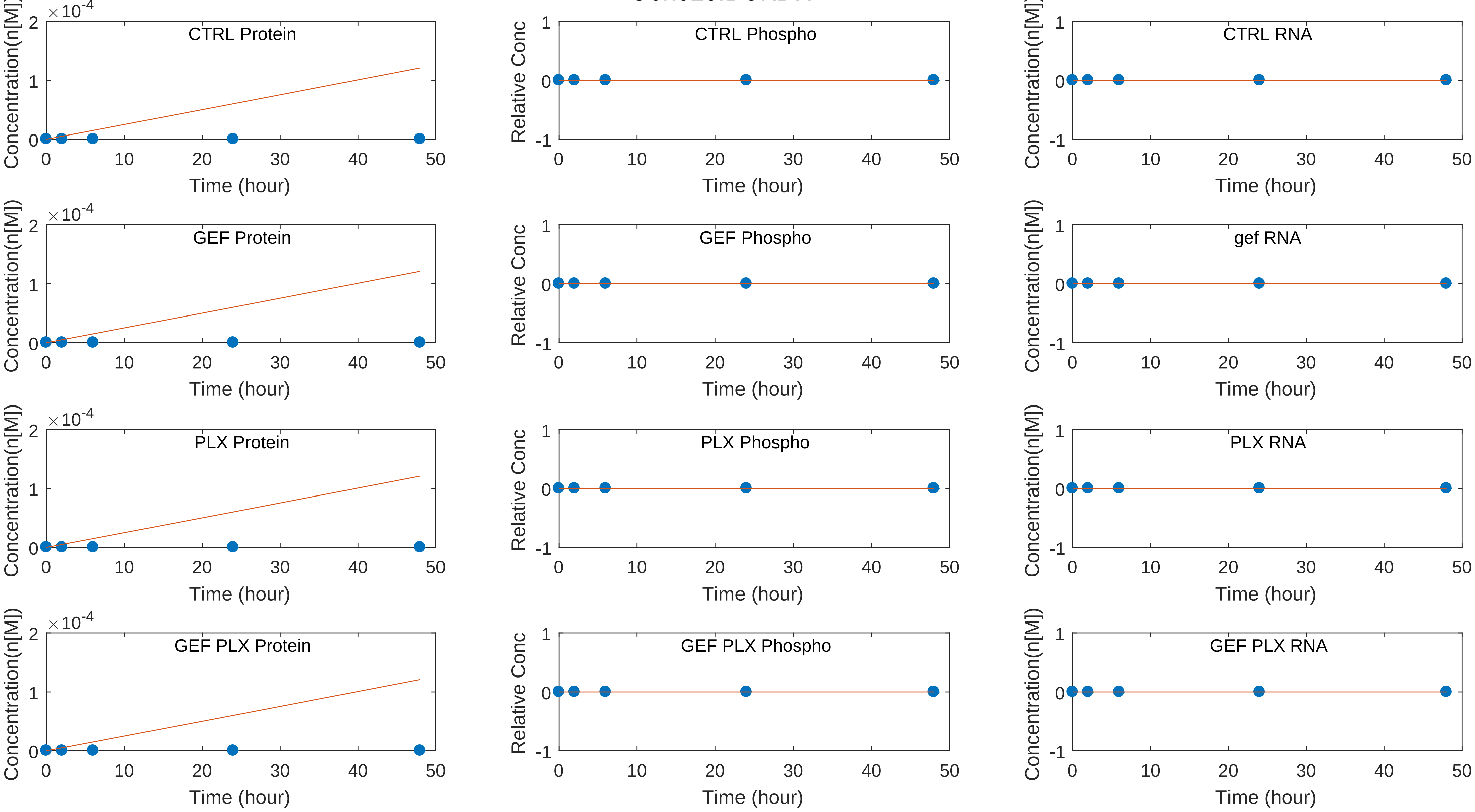

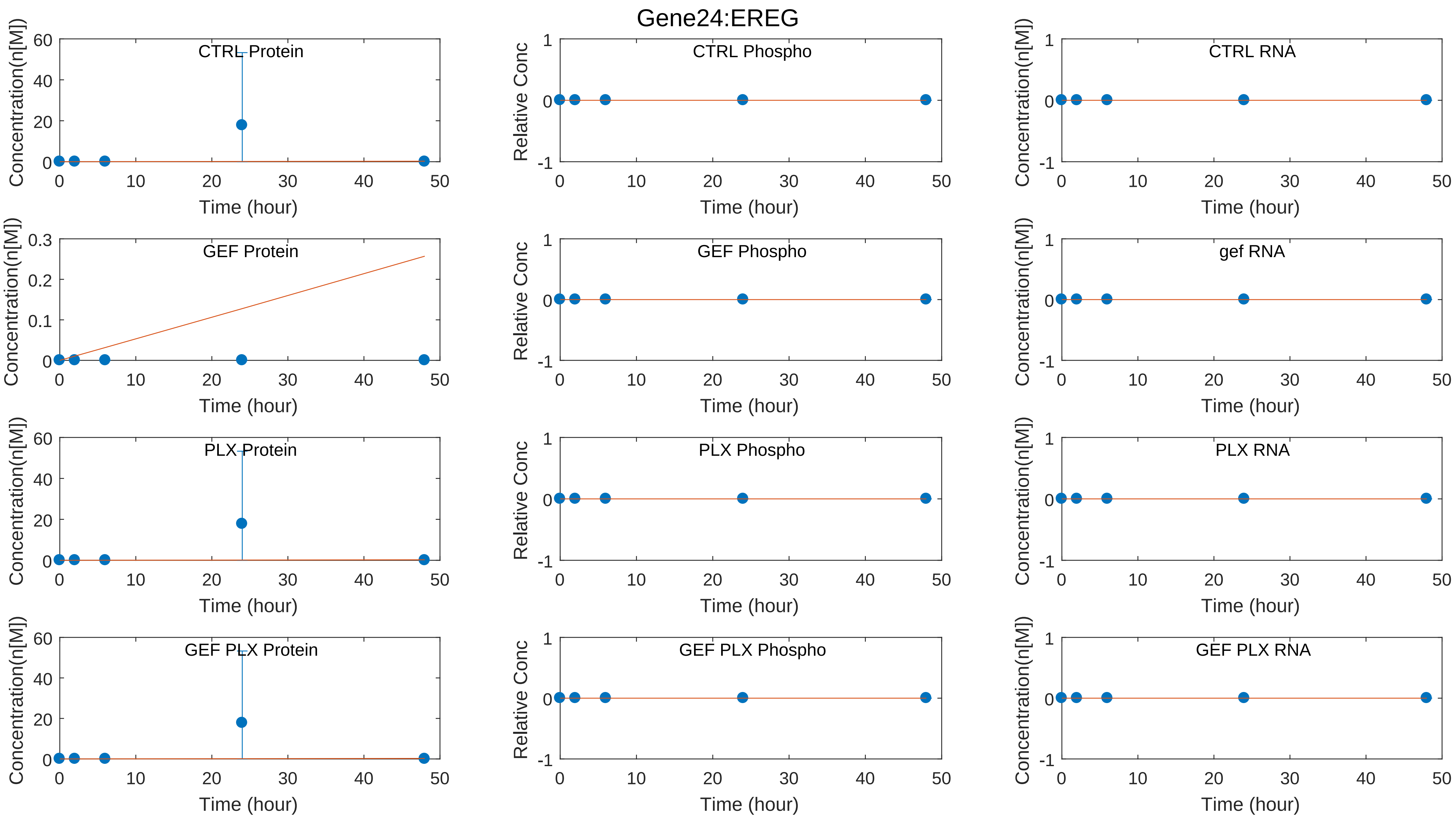
Gene24:EREG
CTRL Protein
CTRL Phospho
CTRL RNA
GEF Protein
GEF Phospho
gef RNA
PLX Protein
PLX Phospho
PLX RNA
GEF PLX Protein
GEF PLX Phospho
GEF PLX RNA
Concentration(n[M])
Relative Conc
Time (hour)

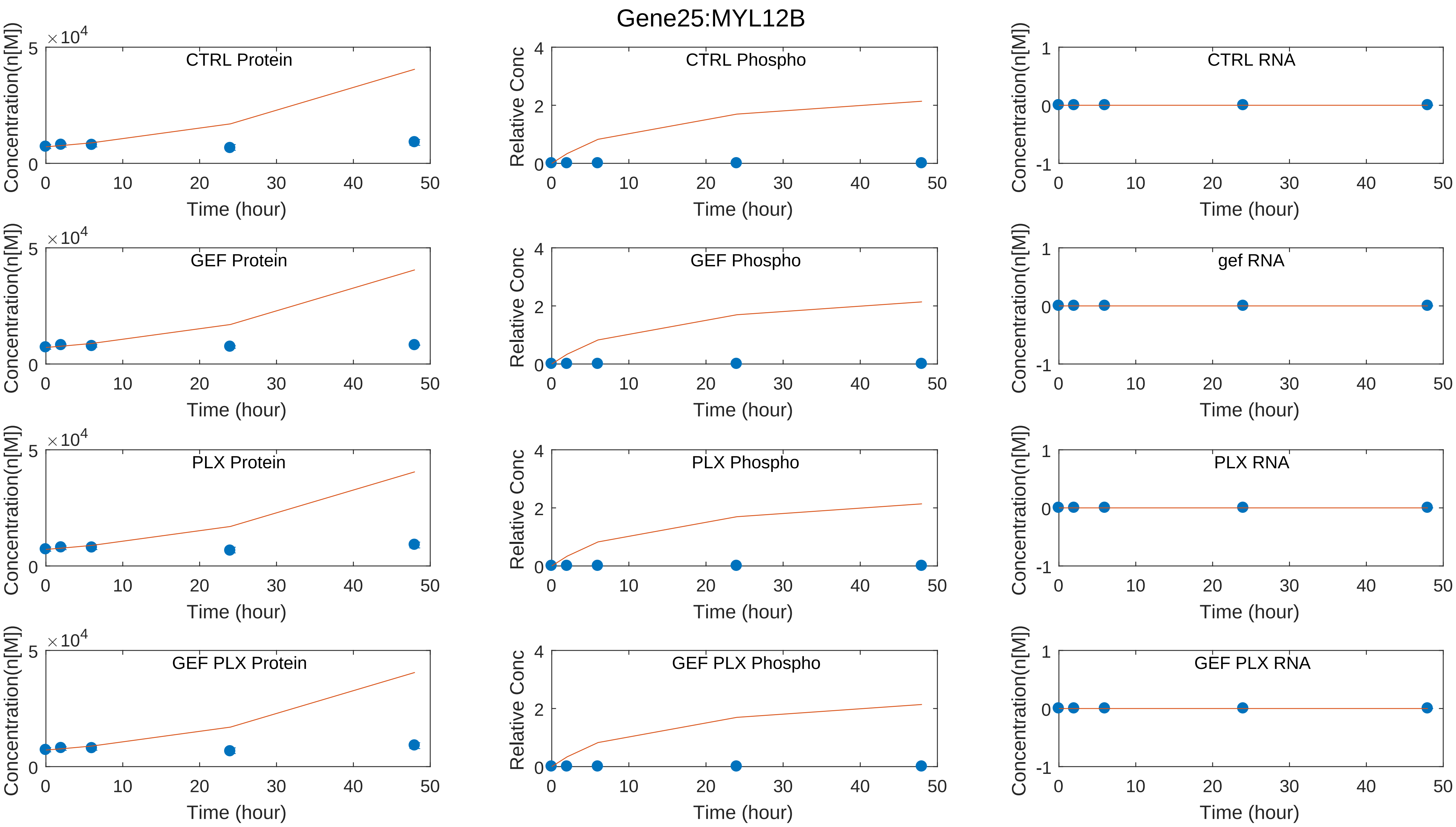

Gene25:MYL12B
CTRL Protein
CTRL Phospho
CTRL RNA
GEF Protein
GEF Phospho
gef RNA
PLX Protein
PLX Phospho
PLX RNA
GEF PLX Protein
GEF PLX Phospho
GEF PLX RNA
Concentration(n[M])
Relative Conc
Time (hour)
×10^4

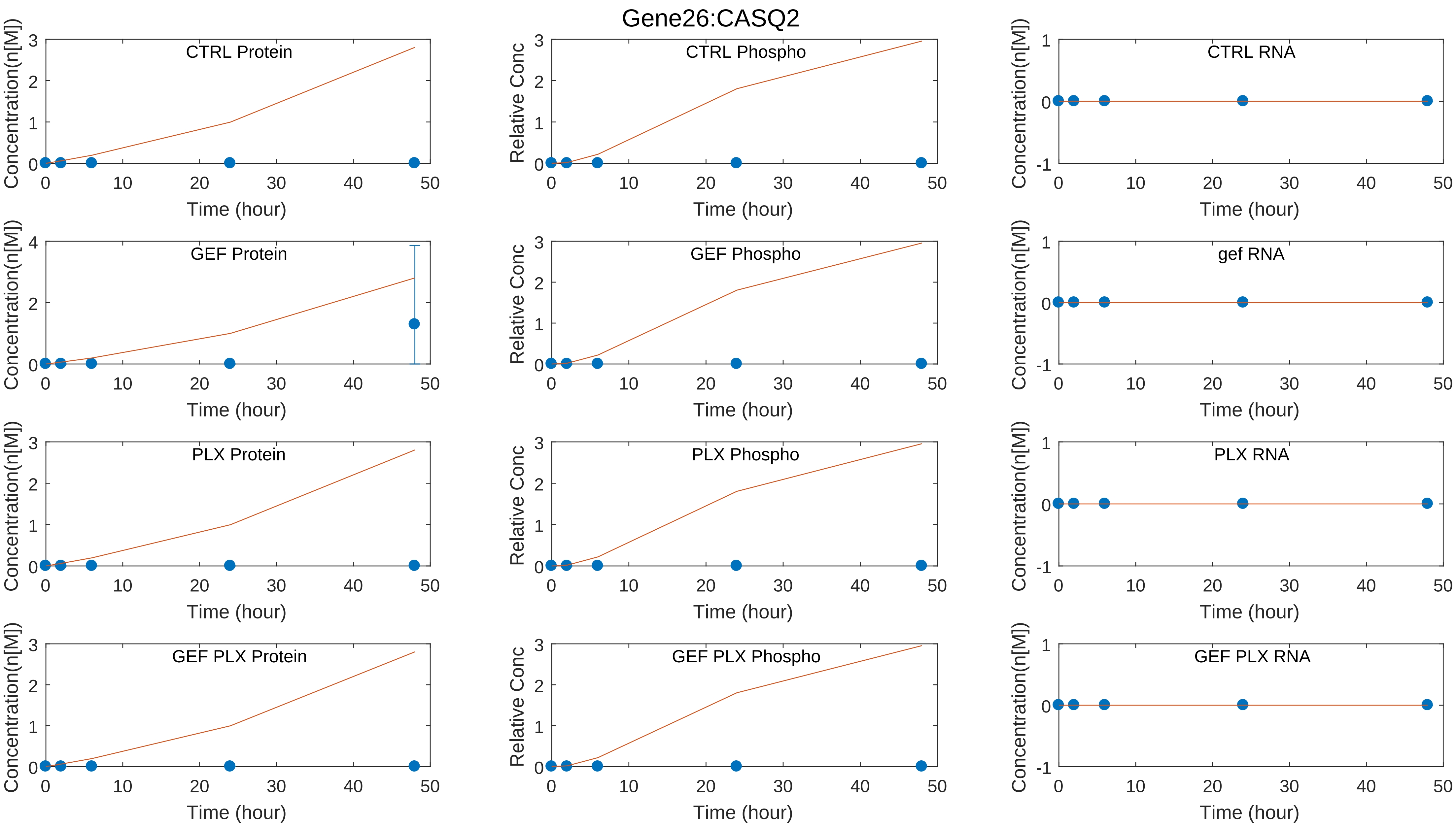

Gene26:CASQ2
CTRL Protein
CTRL Phospho
CTRL RNA
GEF Protein
GEF Phospho
gef RNA
PLX Protein
PLX Phospho
PLX RNA
GEF PLX Protein
GEF PLX Phospho
GEF PLX RNA
Concentration(n[M])
Relative Conc
Time (hour)

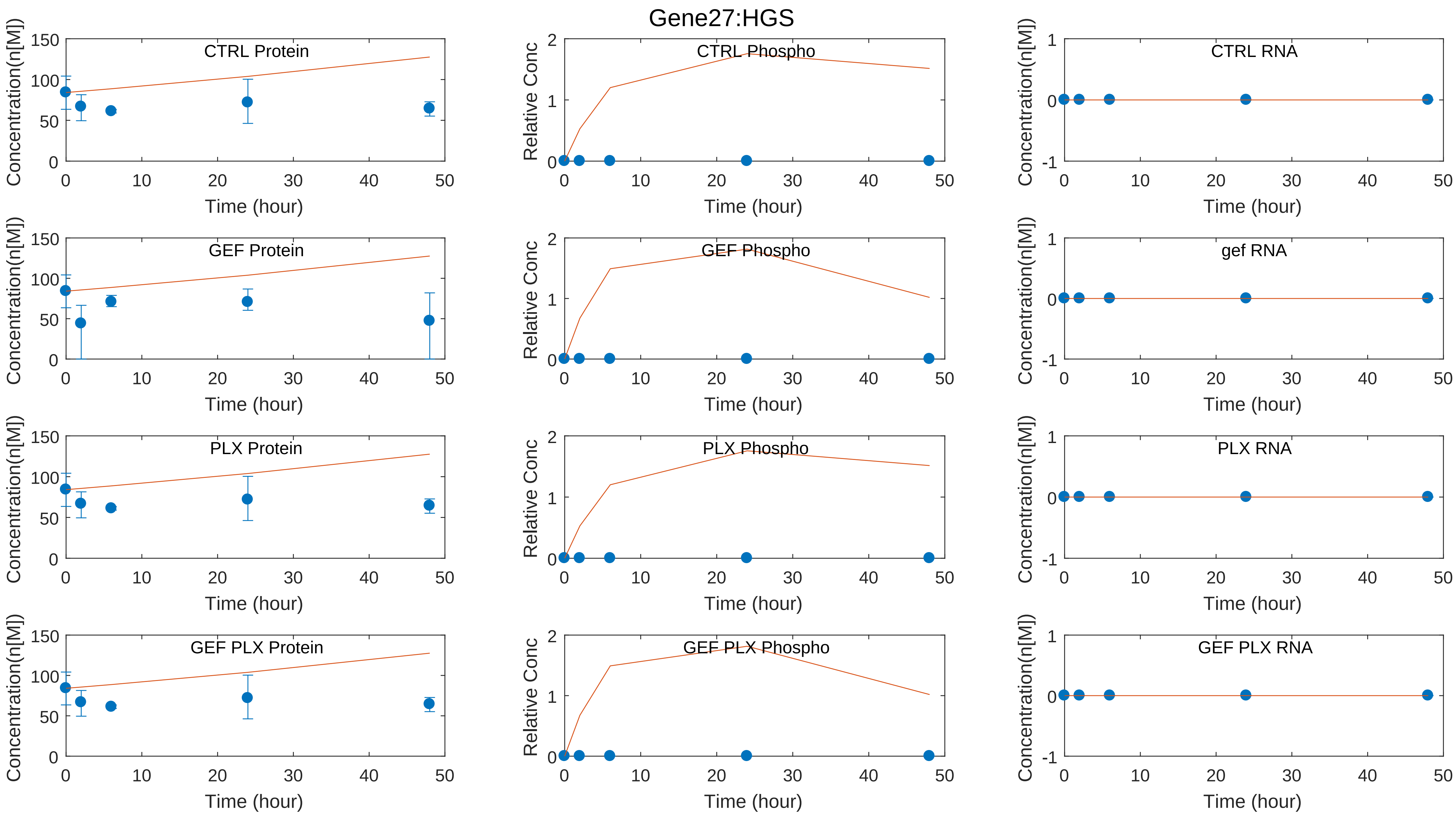

Gene27:HGS
CTRL Protein
CTRL Phospho
CTRL RNA
GEF Protein
GEF Phospho
gef RNA
PLX Protein
PLX Phospho
PLX RNA
GEF PLX Protein
GEF PLX Phospho
GEF PLX RNA
Concentration(n[M])
Relative Conc
Time (hour)

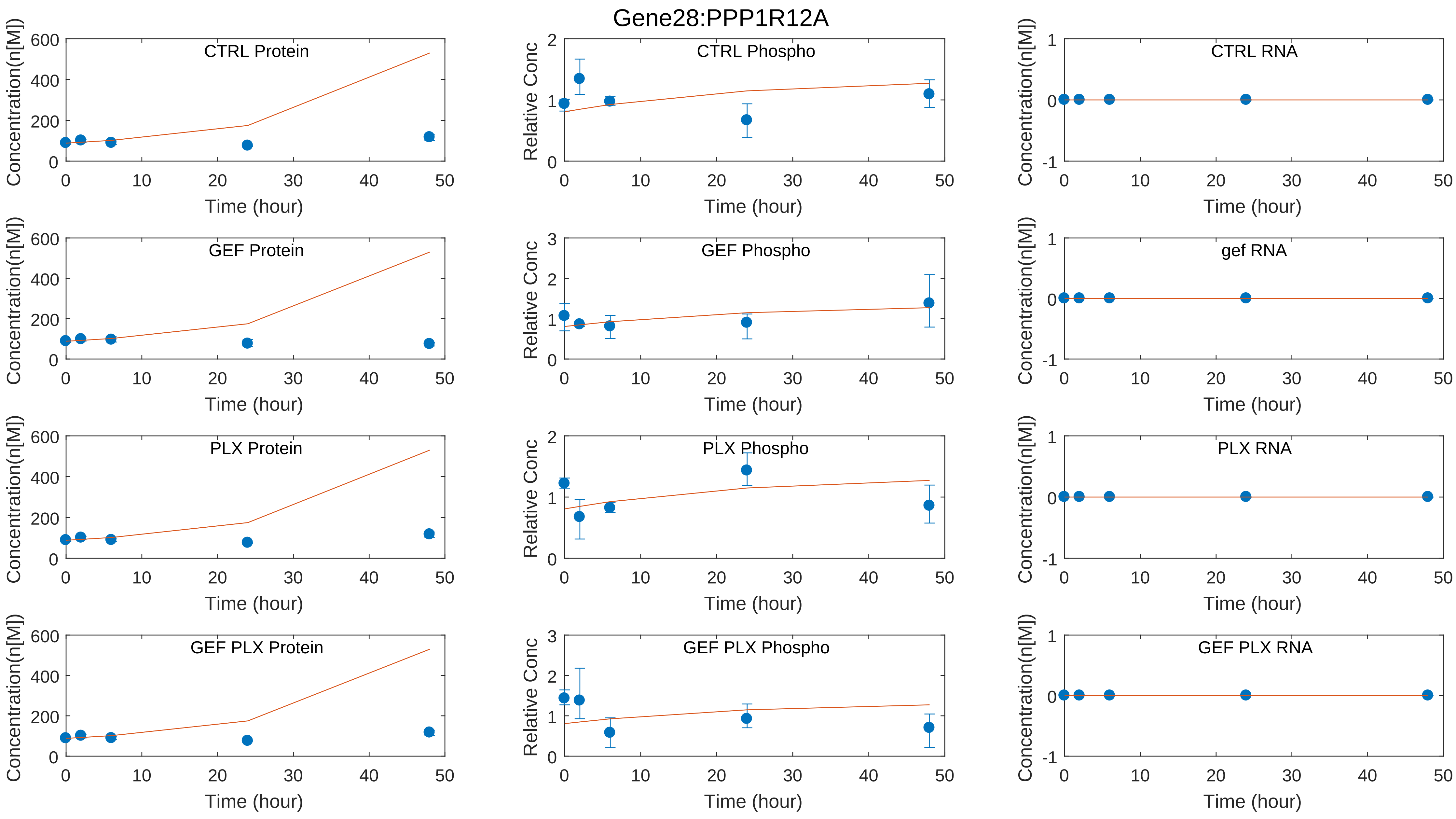
Gene28:PPP1R12A
CTRL Protein
CTRL Phospho
CTRL RNA
GEF Protein
GEF Phospho
gef RNA
PLX Protein
PLX Phospho
PLX RNA
GEF PLX Protein
GEF PLX Phospho
GEF PLX RNA
Concentration(n[M])
Relative Conc
Time (hour)

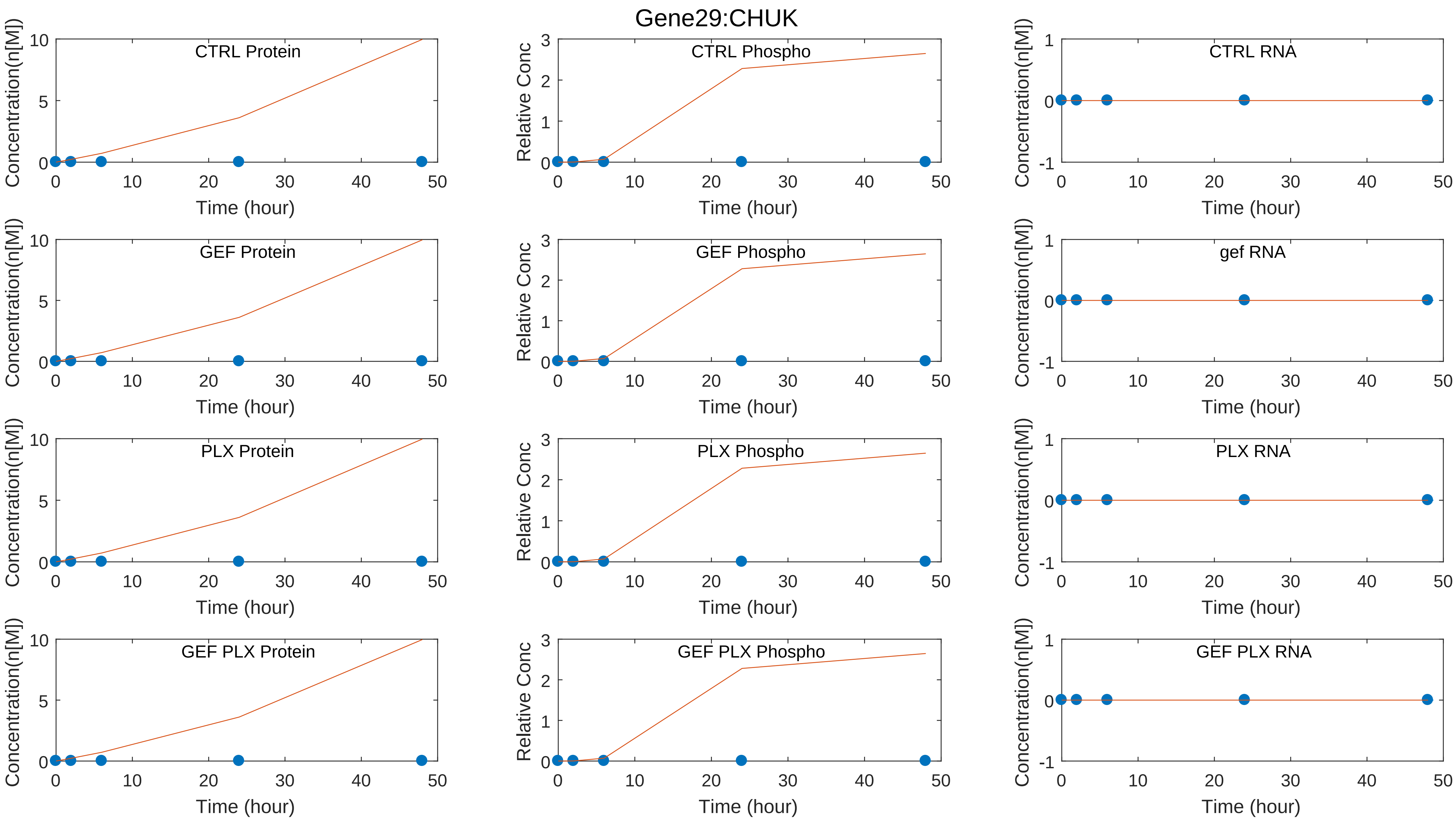
Gene29:CHUK
CTRL Protein
CTRL Phospho
CTRL RNA
GEF Protein
GEF Phospho
gef RNA
PLX Protein
PLX Phospho
PLX RNA
GEF PLX Protein
GEF PLX Phospho
GEF PLX RNA
Concentration(n[M])
Relative Conc
Time (hour)

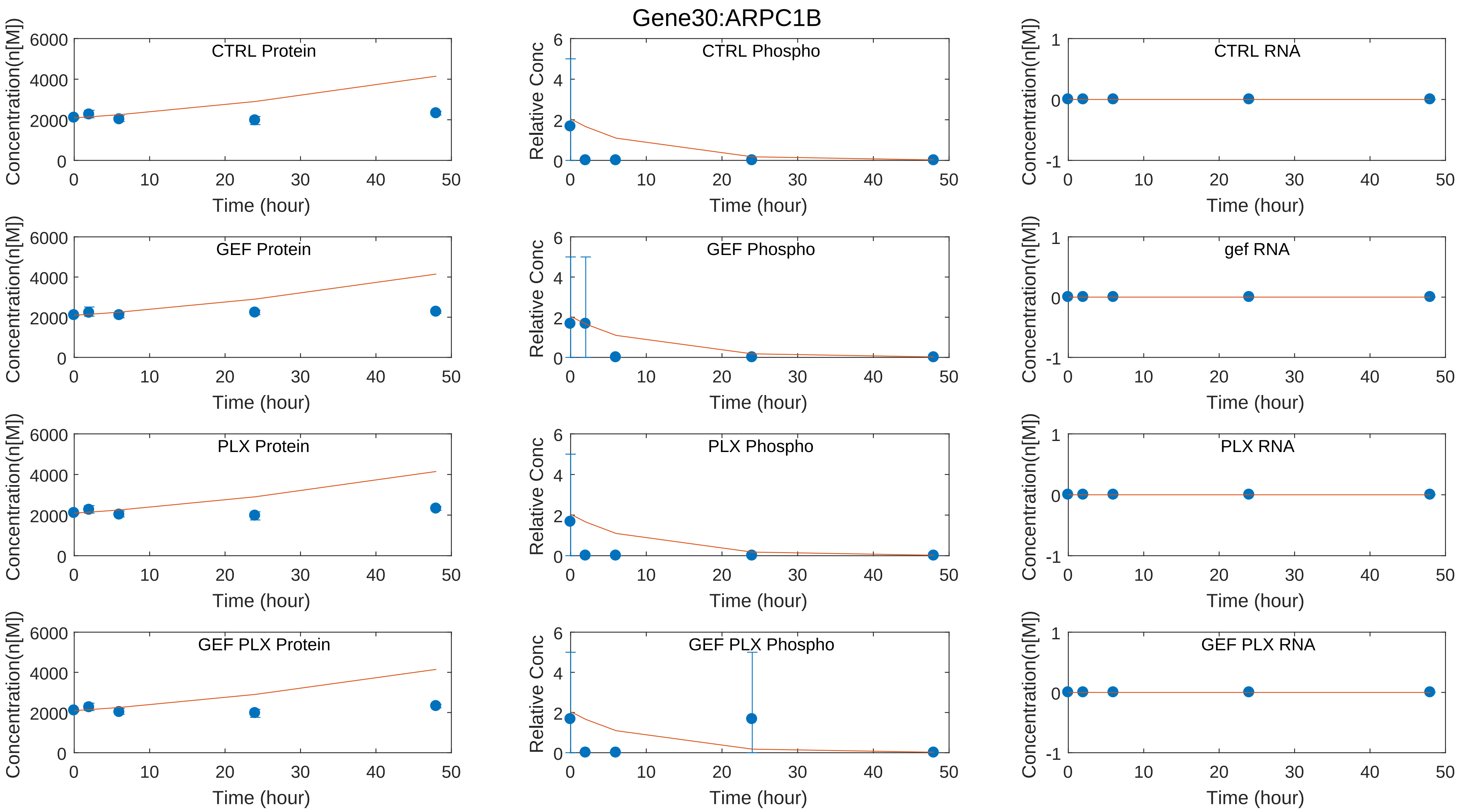

Gene30:ARPC1B
CTRL Protein
CTRL Phospho
CTRL RNA
GEF Protein
GEF Phospho
gef RNA
PLX Protein
PLX Phospho
PLX RNA
GEF PLX Protein
GEF PLX Phospho
GEF PLX RNA
Concentration(n[M])
Relative Conc
Time (hour)

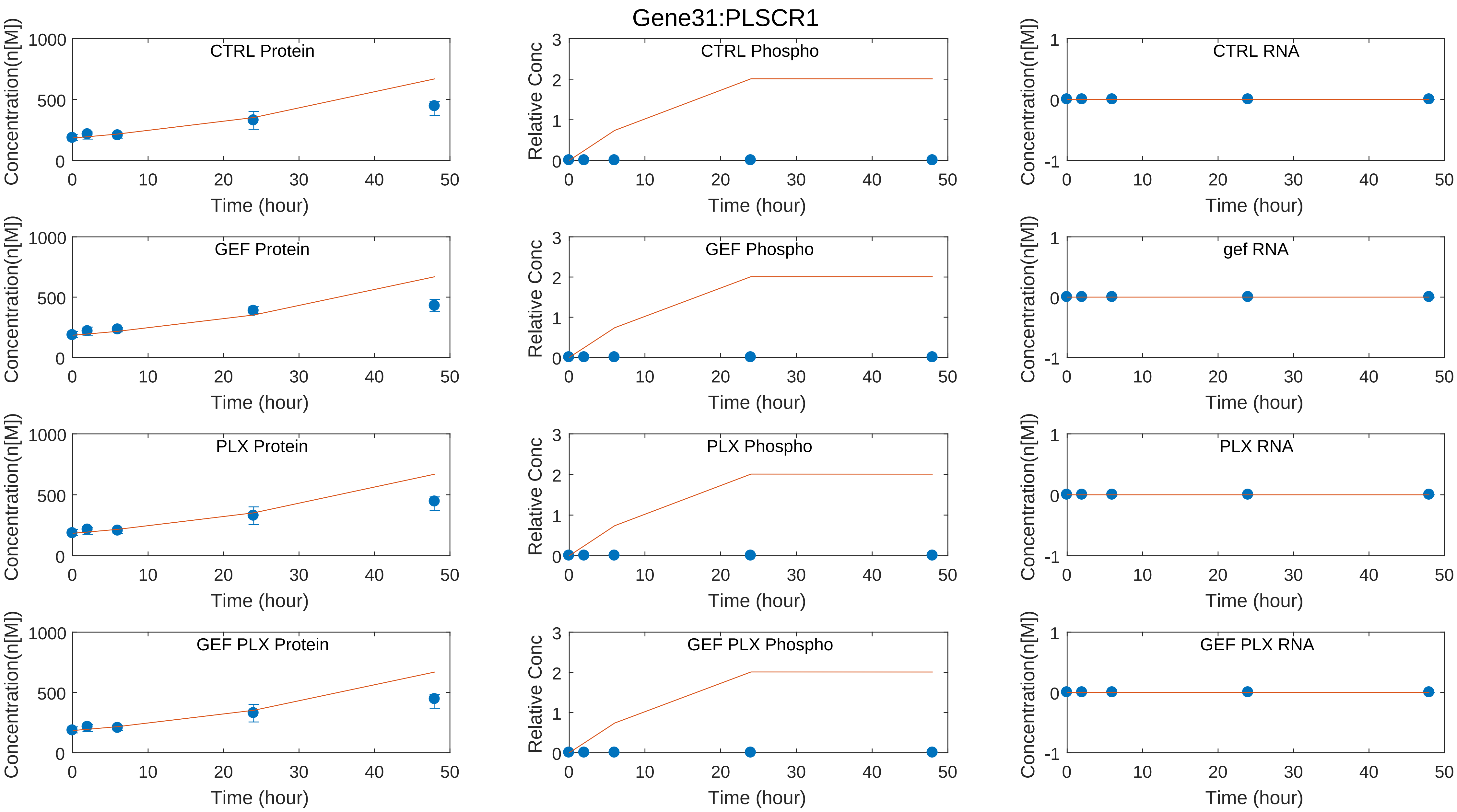

Gene31:PLSCR1
CTRL Protein
GEF Protein
PLX Protein
GEF PLX Protein
CTRL Phospho
GEF Phospho
PLX Phospho
GEF PLX Phospho
CTRL RNA
gef RNA
PLX RNA
GEF PLX RNA
Concentration(n[M])
Relative Conc
Time (hour)

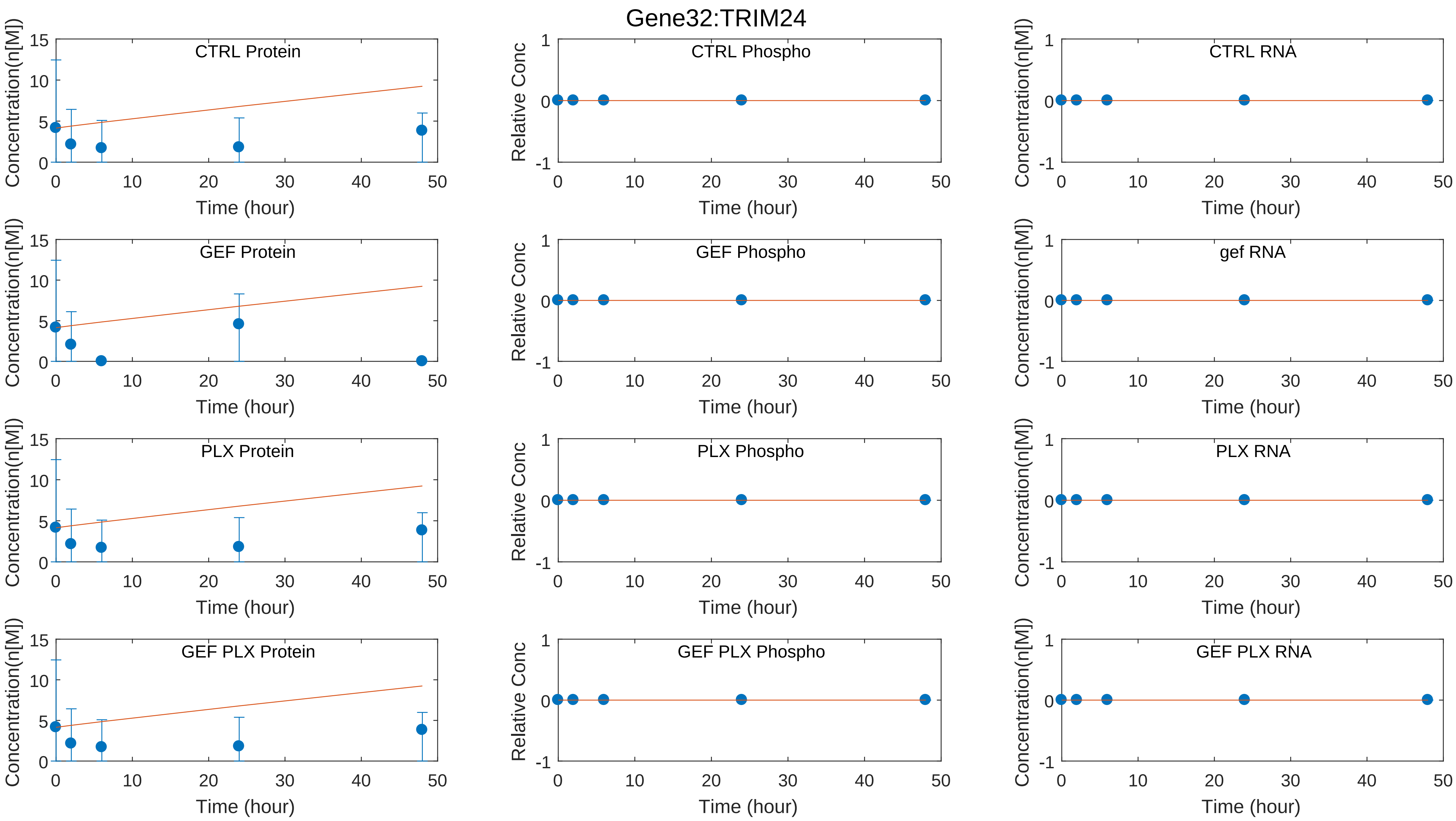
Gene32:TRIM24
CTRL Protein
CTRL Phospho
CTRL RNA
GEF Protein
GEF Phospho
gef RNA
PLX Protein
PLX Phospho
PLX RNA
GEF PLX Protein
GEF PLX Phospho
GEF PLX RNA
Concentration(n[M])
Relative Conc
Time (hour)

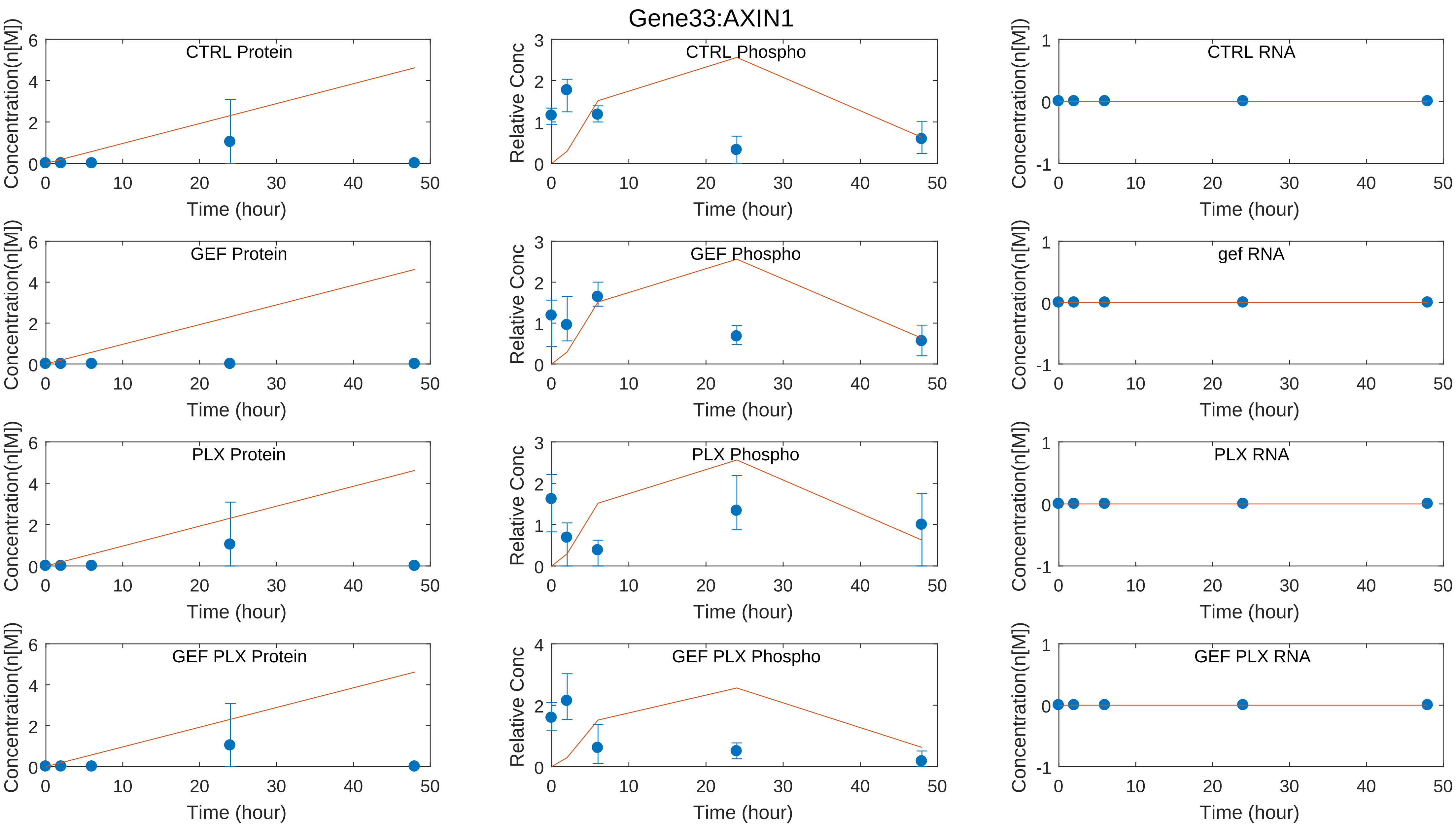

Gene33:AXIN1
CTRL Protein
CTRL Phospho
CTRL RNA
GEF Protein
GEF Phospho
gef RNA
PLX Protein
PLX Phospho
PLX RNA
GEF PLX Protein
GEF PLX Phospho
GEF PLX RNA
Concentration(n[M])
Relative Conc
Time (hour)
0
10
20
30
40
50

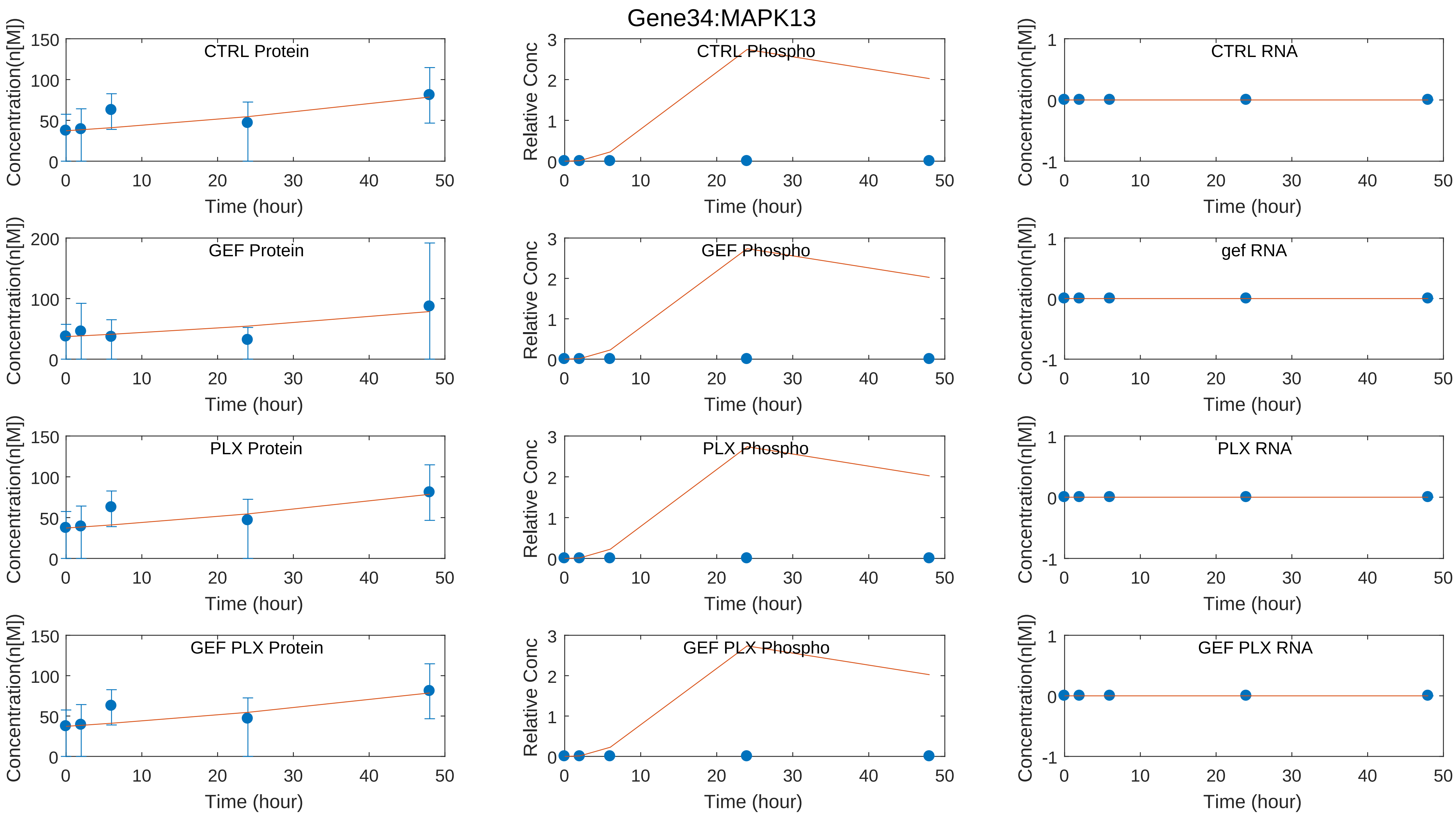

Gene34:MAPK13
CTRL Protein
CTRL Phospho
CTRL RNA
GEF Protein
GEF Phospho
gef RNA
PLX Protein
PLX Phospho
PLX RNA
GEF PLX Protein
GEF PLX Phospho
GEF PLX RNA
Concentration(n[M])
Relative Conc
Time (hour)

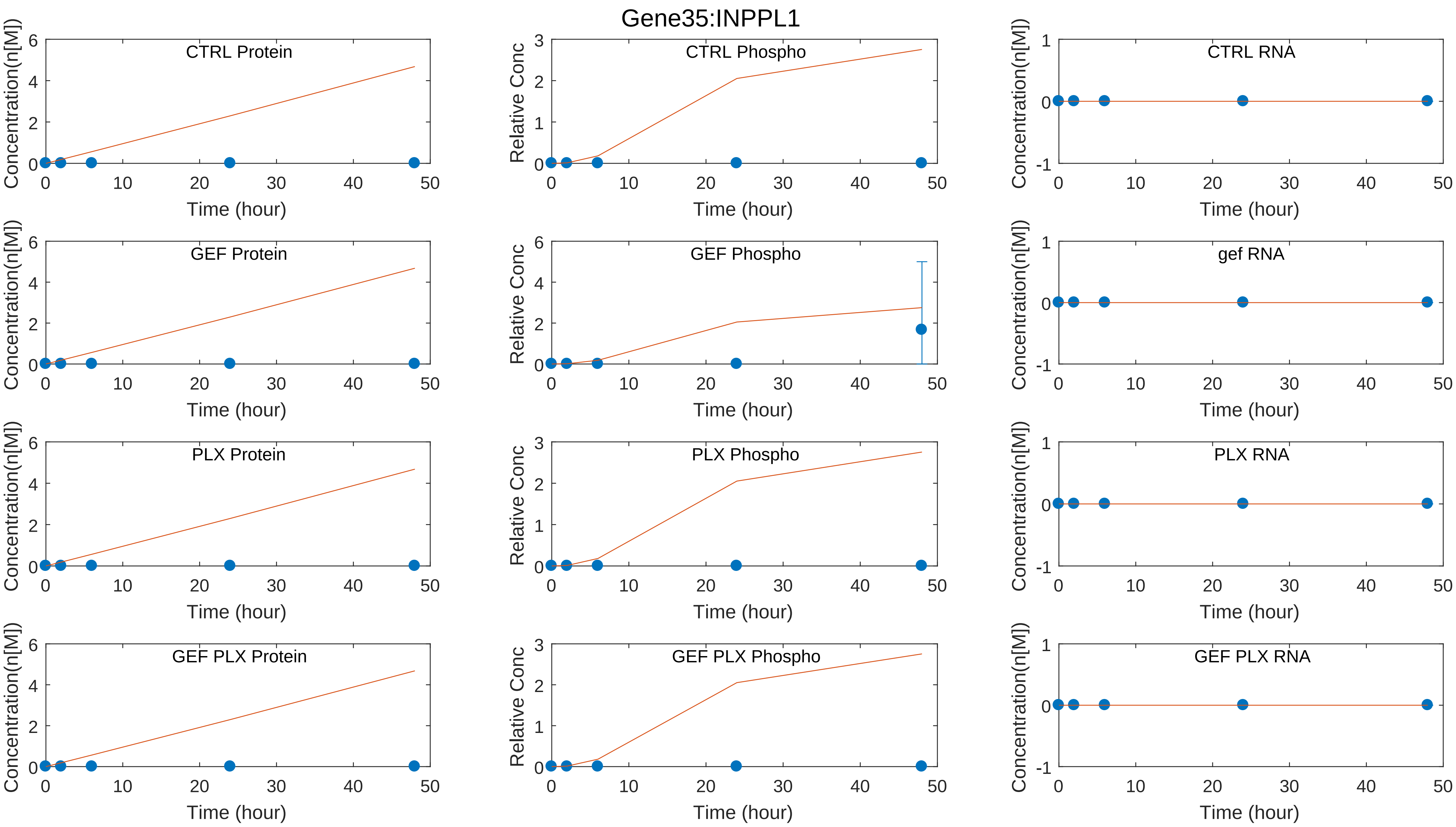

Gene35:INPPL1
CTRL Protein
CTRL Phospho
CTRL RNA
GEF Protein
GEF Phospho
gef RNA
PLX Protein
PLX Phospho
PLX RNA
GEF PLX Protein
GEF PLX Phospho
GEF PLX RNA
Concentration(n[M])
Relative Conc
Time (hour)

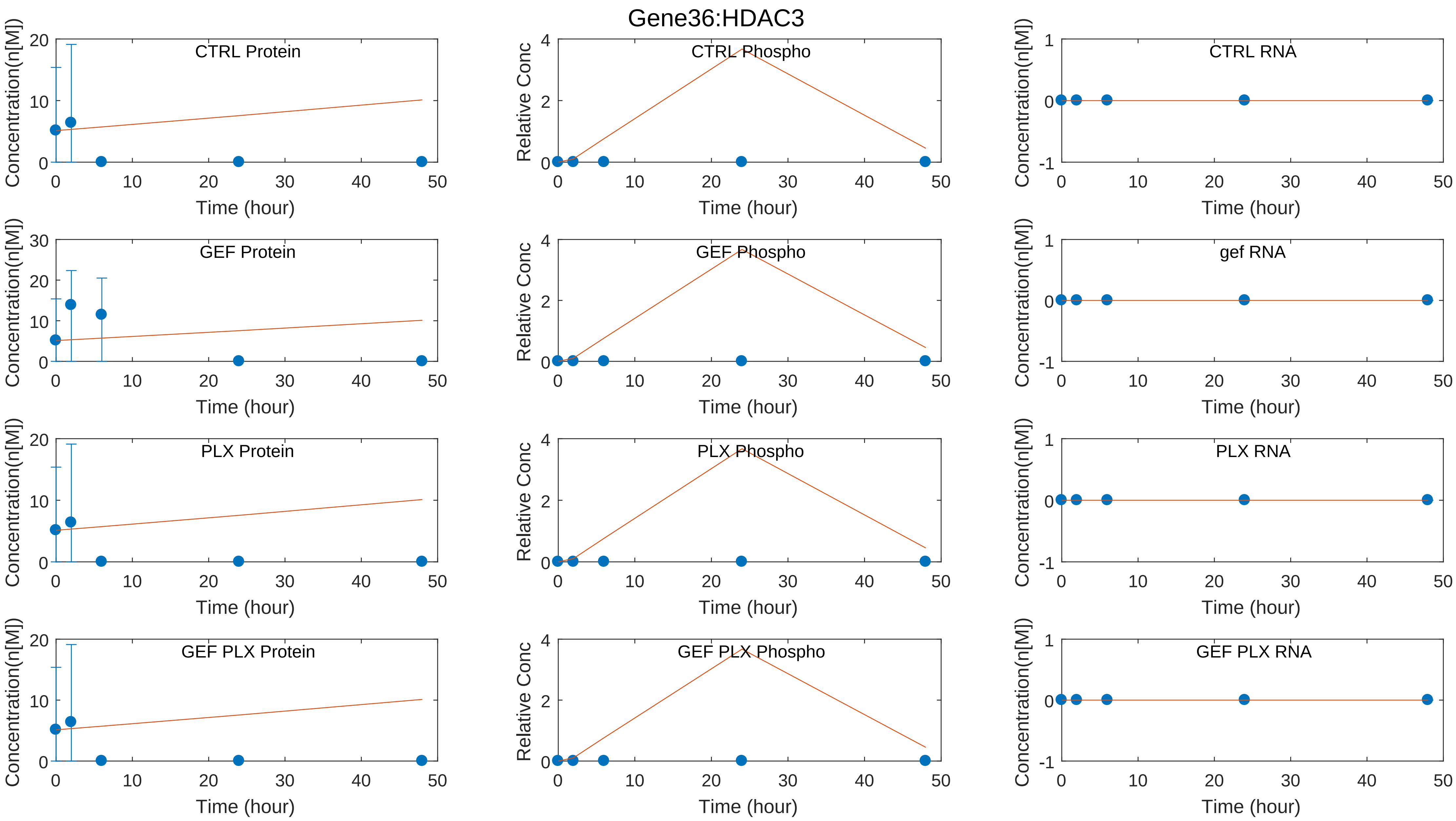
Gene36:HDAC3
CTRL Protein
CTRL Phospho
CTRL RNA
GEF Protein
GEF Phospho
gef RNA
PLX Protein
PLX Phospho
PLX RNA
GEF PLX Protein
GEF PLX Phospho
GEF PLX RNA
Concentration(n[M])
Relative Conc
Time (hour)

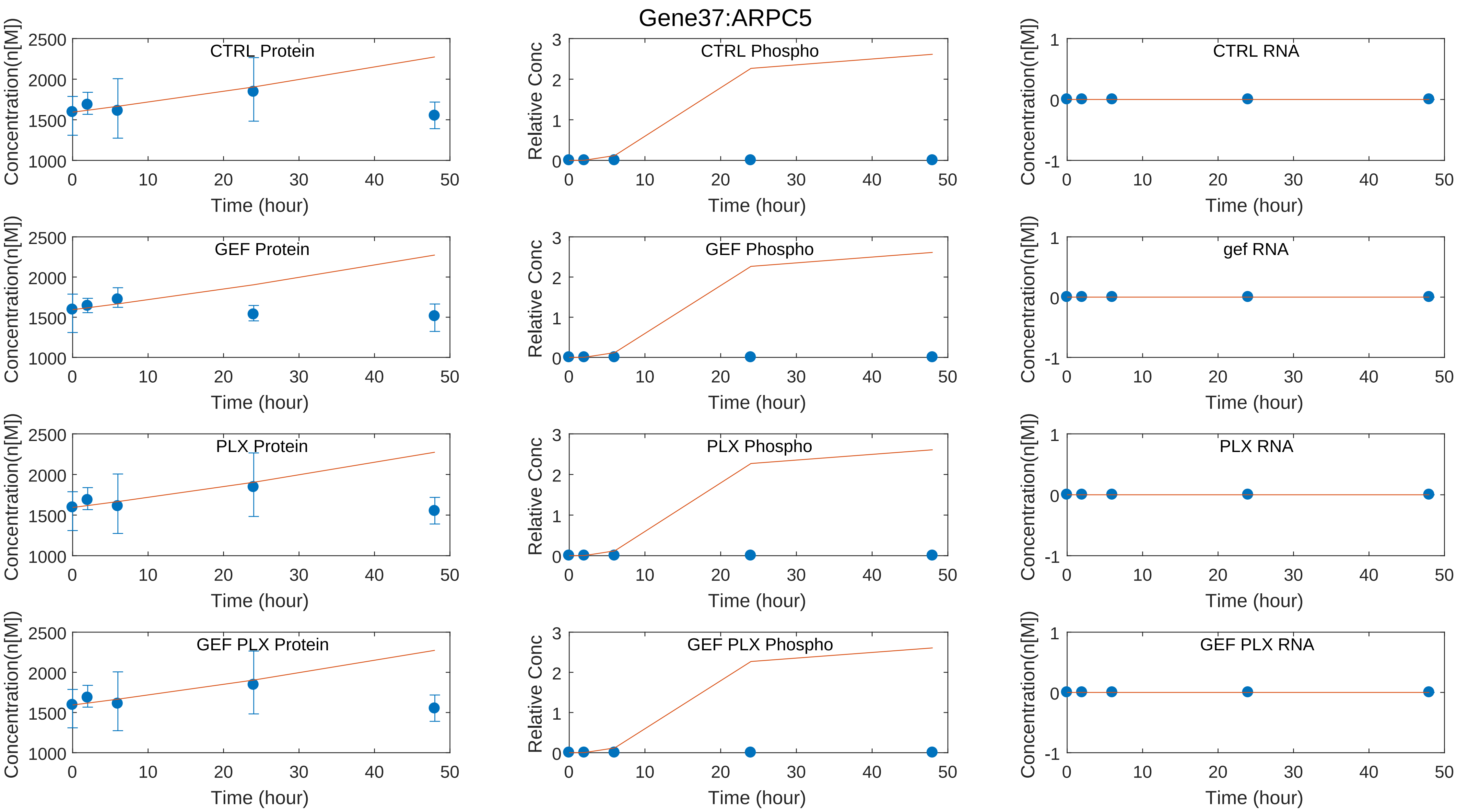

Gene37:ARPC5
CTRL Protein
GEF Protein
PLX Protein
GEF PLX Protein
CTRL Phospho
GEF Phospho
PLX Phospho
GEF PLX Phospho
CTRL RNA
gef RNA
PLX RNA
GEF PLX RNA
Concentration(n[M])
Relative Conc
Time (hour)

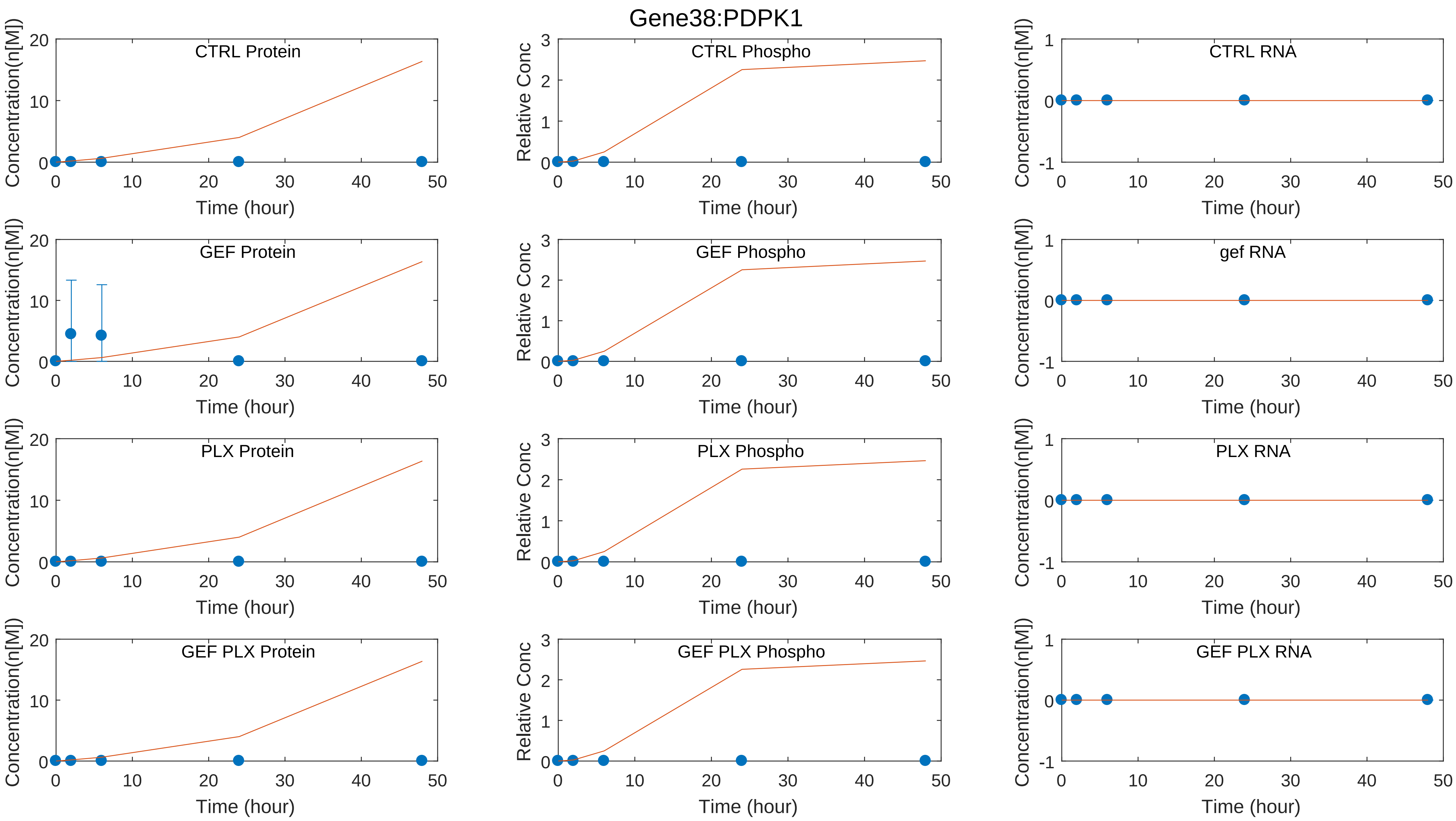

Gene38:PDPK1
CTRL Protein
CTRL Phospho
CTRL RNA
GEF Protein
GEF Phospho
gef RNA
PLX Protein
PLX Phospho
PLX RNA
GEF PLX Protein
GEF PLX Phospho
GEF PLX RNA
Concentration(n[M])
Relative Conc
Time (hour)

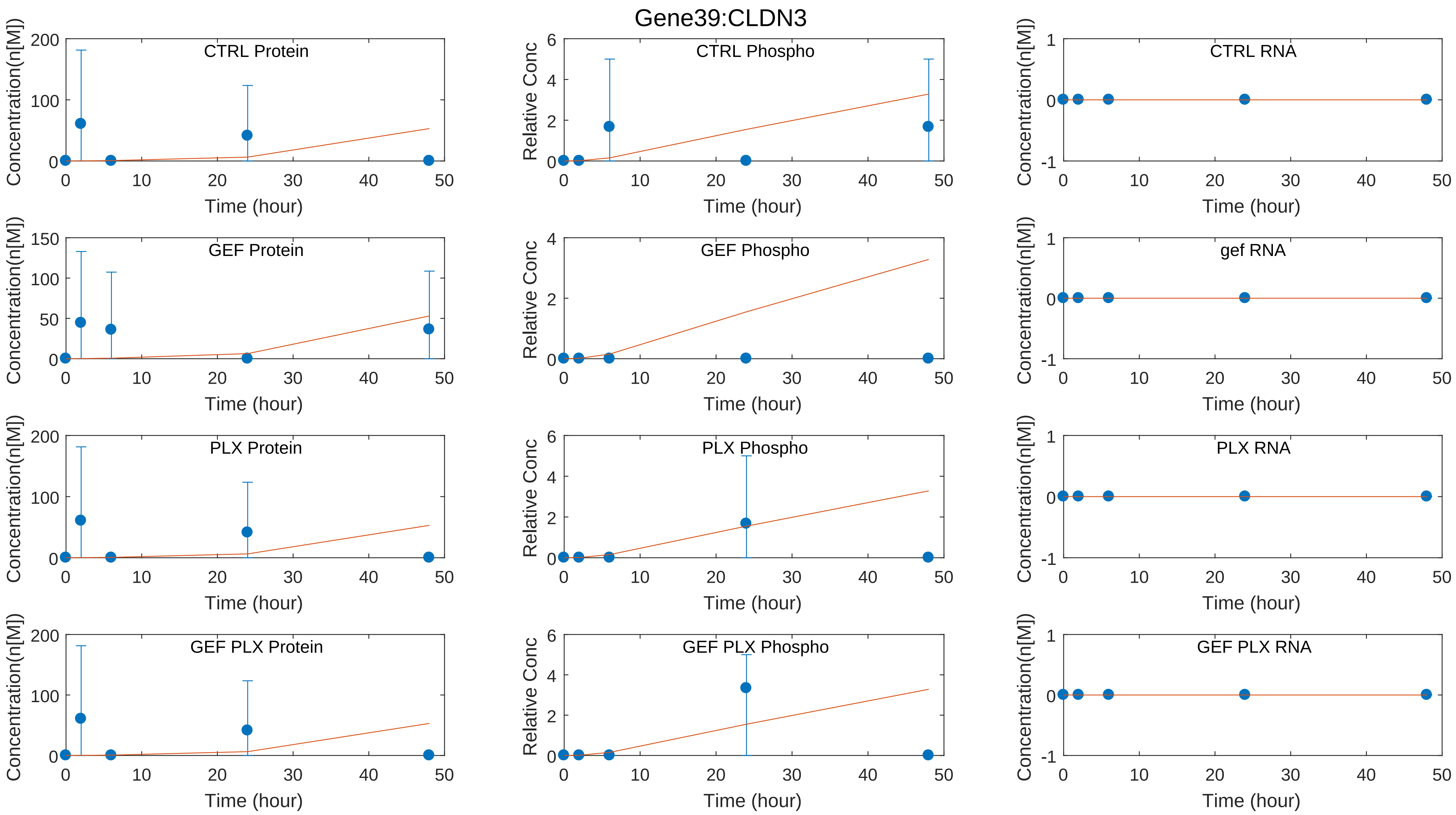

Gene39:CLDN3
CTRL Protein
CTRL Phospho
CTRL RNA
GEF Protein
GEF Phospho
gef RNA
PLX Protein
PLX Phospho
PLX RNA
GEF PLX Protein
GEF PLX Phospho
GEF PLX RNA
Concentration(n[M])
Relative Conc
Time (hour)

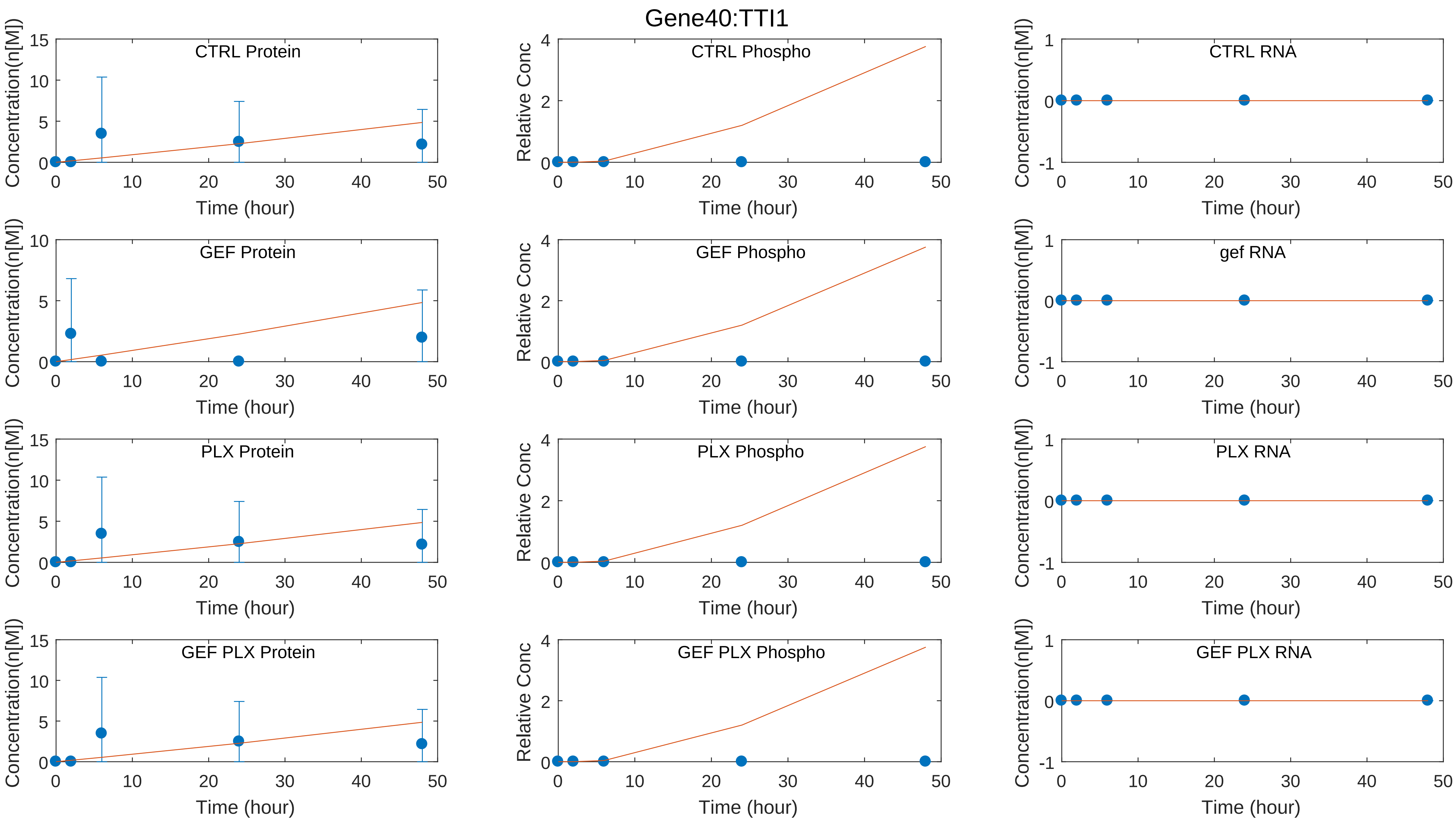
Gene40:TTI1
CTRL Protein
CTRL Phospho
CTRL RNA
GEF Protein
GEF Phospho
gef RNA
PLX Protein
PLX Phospho
PLX RNA
GEF PLX Protein
GEF PLX Phospho
GEF PLX RNA
Concentration(n[M])
Relative Conc
Time (hour)

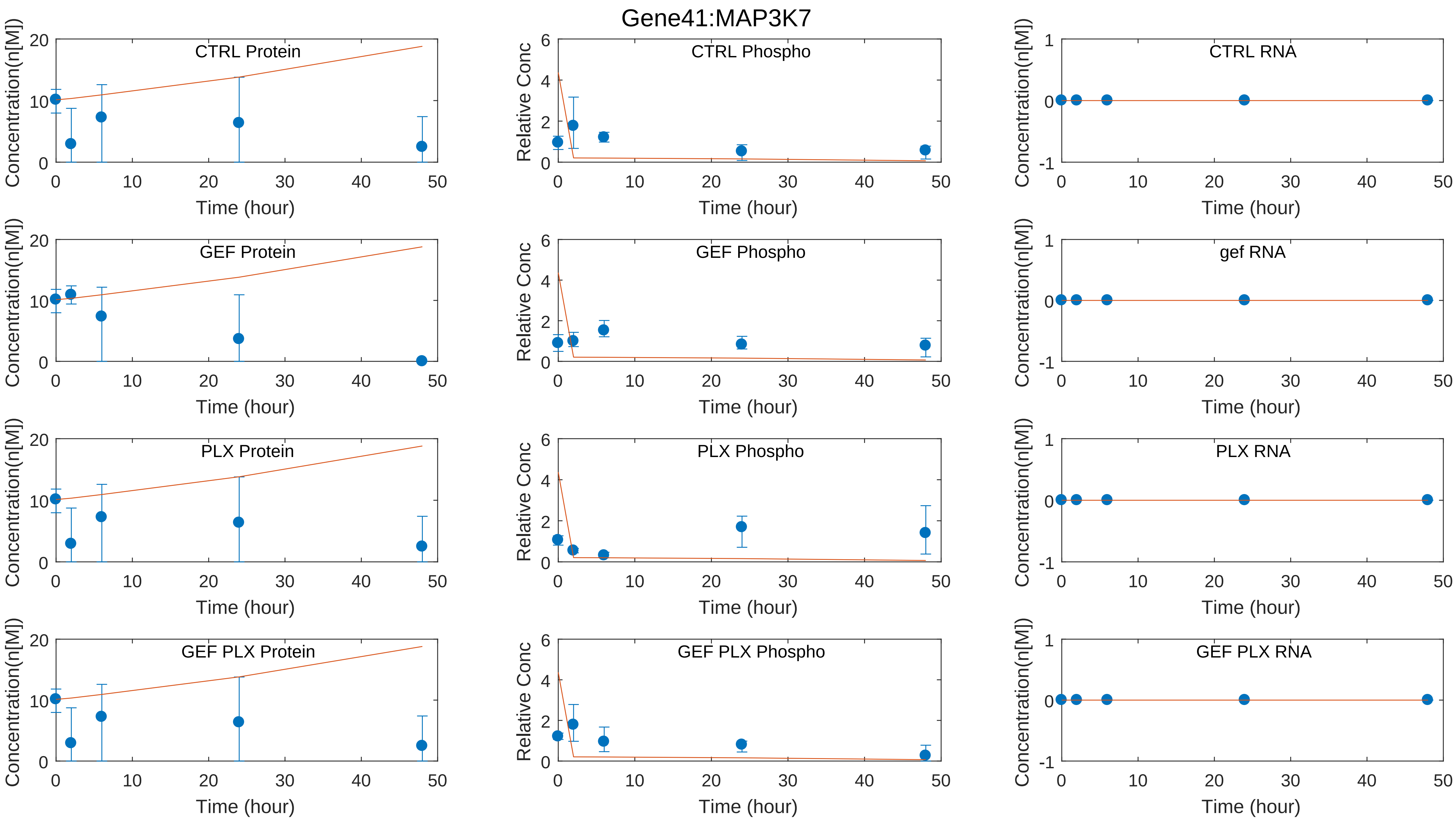

Gene41:MAP3K7
CTRL Protein
CTRL Phospho
CTRL RNA
GEF Protein
GEF Phospho
gef RNA
PLX Protein
PLX Phospho
PLX RNA
GEF PLX Protein
GEF PLX Phospho
GEF PLX RNA
Concentration(n[M])
Relative Conc
Time (hour)

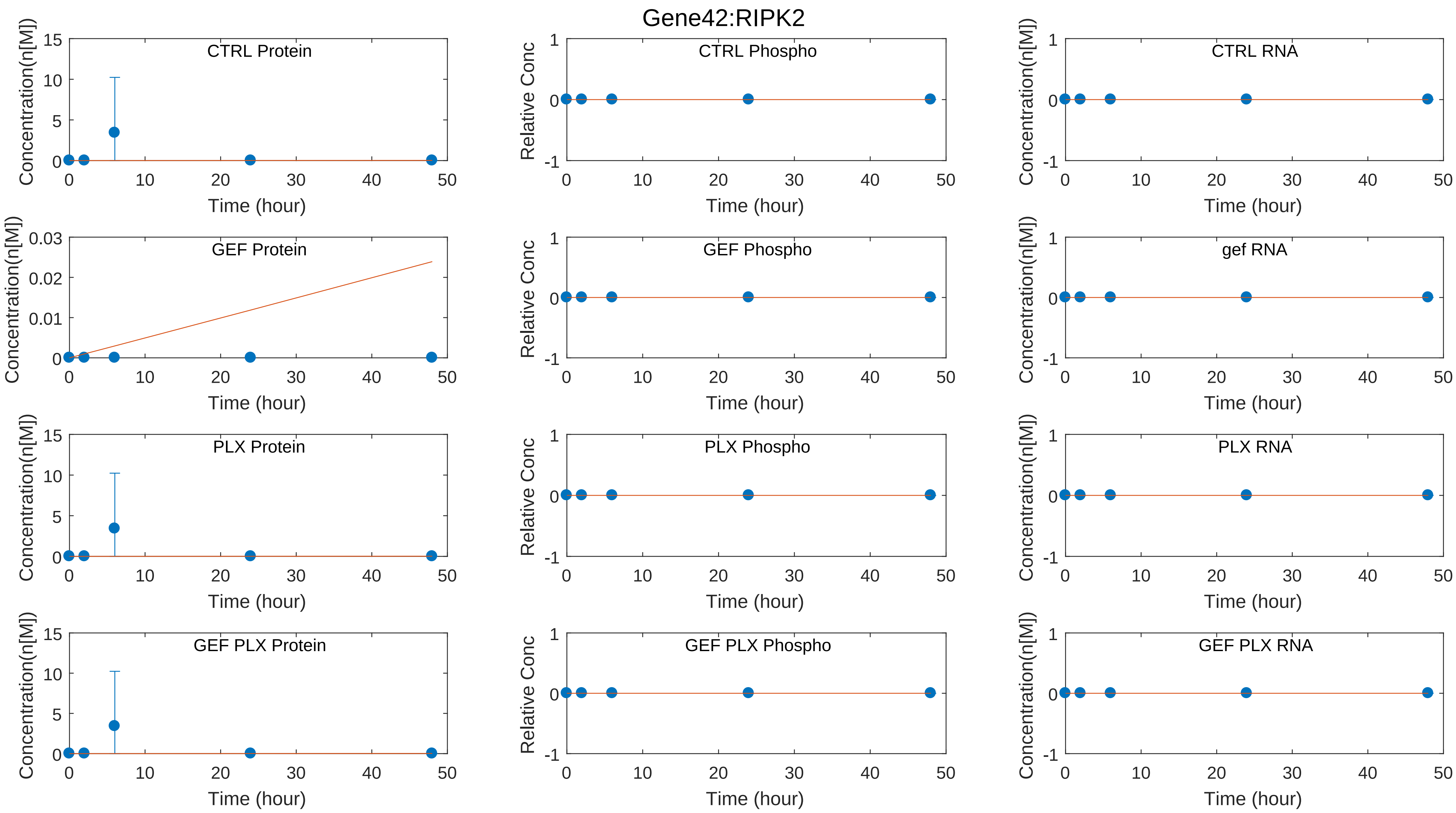

Gene42:RIPK2
CTRL Protein
CTRL Phospho
CTRL RNA
GEF Protein
GEF Phospho
gef RNA
PLX Protein
PLX Phospho
PLX RNA
GEF PLX Protein
GEF PLX Phospho
GEF PLX RNA
Concentration(n[M])
Relative Conc
Time (hour)

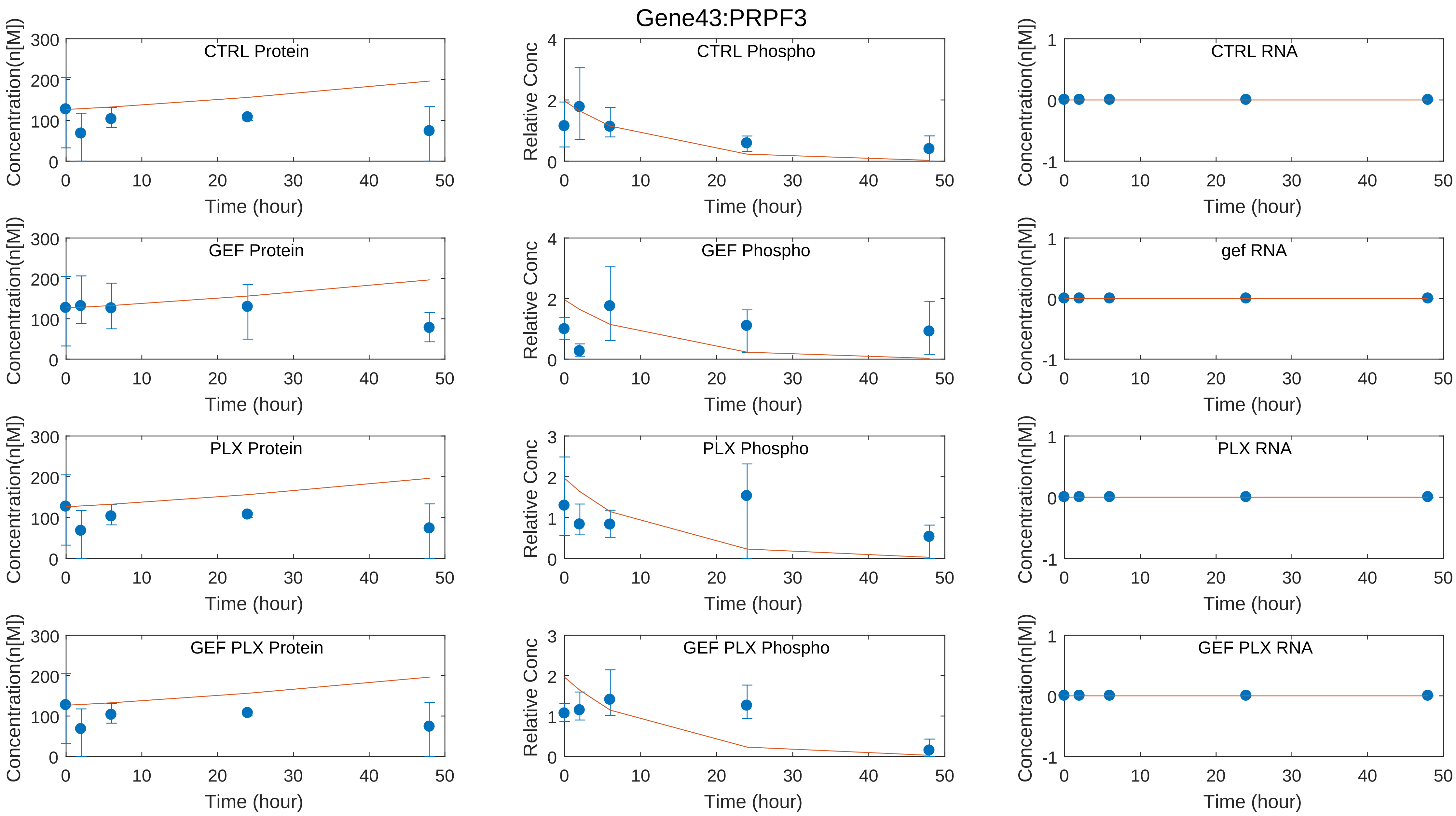
Gene43:PRPF3
CTRL Protein
CTRL Phospho
CTRL RNA
GEF Protein
GEF Phospho
gef RNA
PLX Protein
PLX Phospho
PLX RNA
GEF PLX Protein
GEF PLX Phospho
GEF PLX RNA
Concentration(n[M])
Relative Conc
Time (hour)

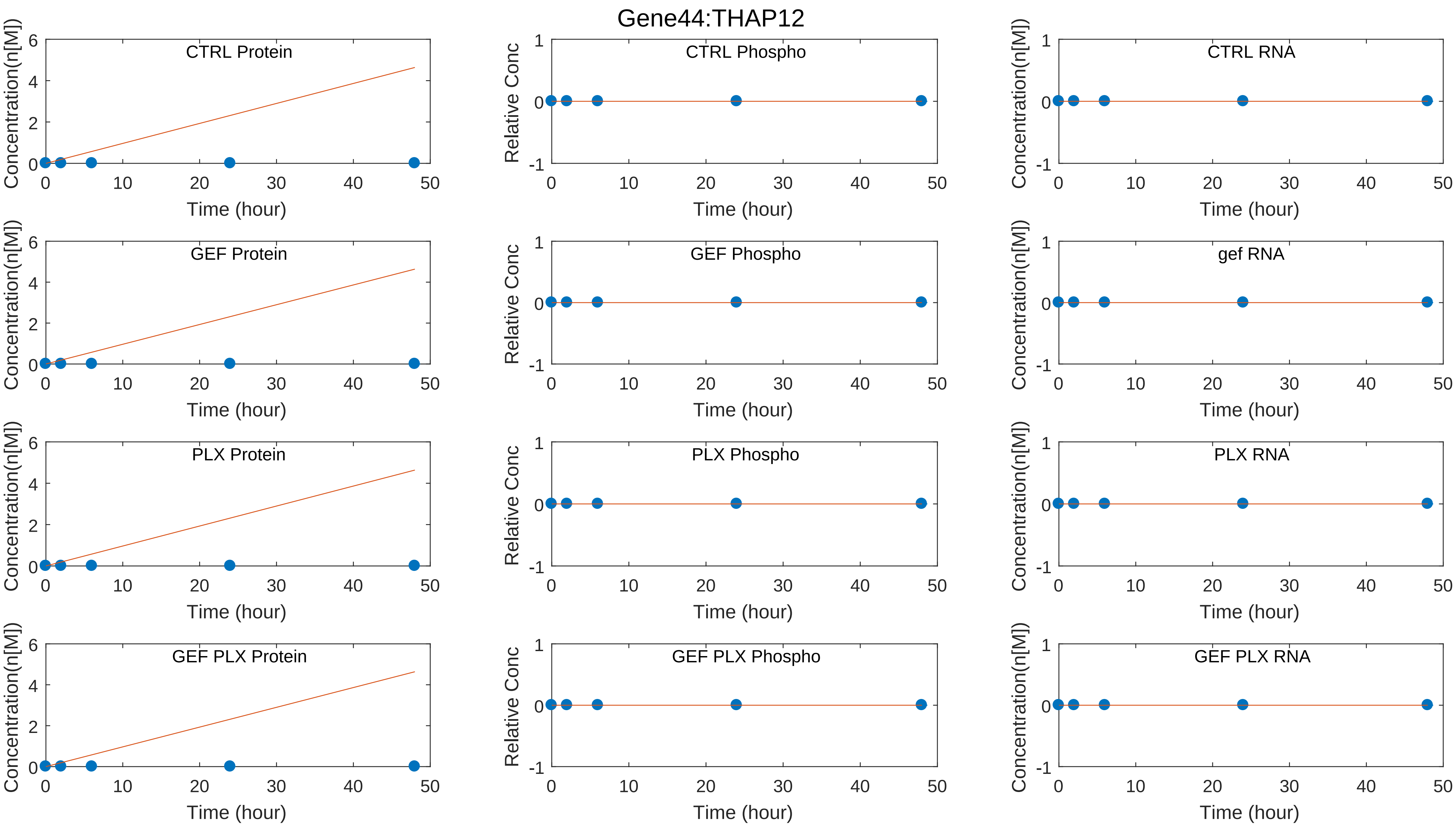
Gene44:THAP12
CTRL Protein
CTRL Phospho
CTRL RNA
GEF Protein
GEF Phospho
gef RNA
PLX Protein
PLX Phospho
PLX RNA
GEF PLX Protein
GEF PLX Phospho
GEF PLX RNA
Concentration(n[M])
Relative Conc
Time (hour)

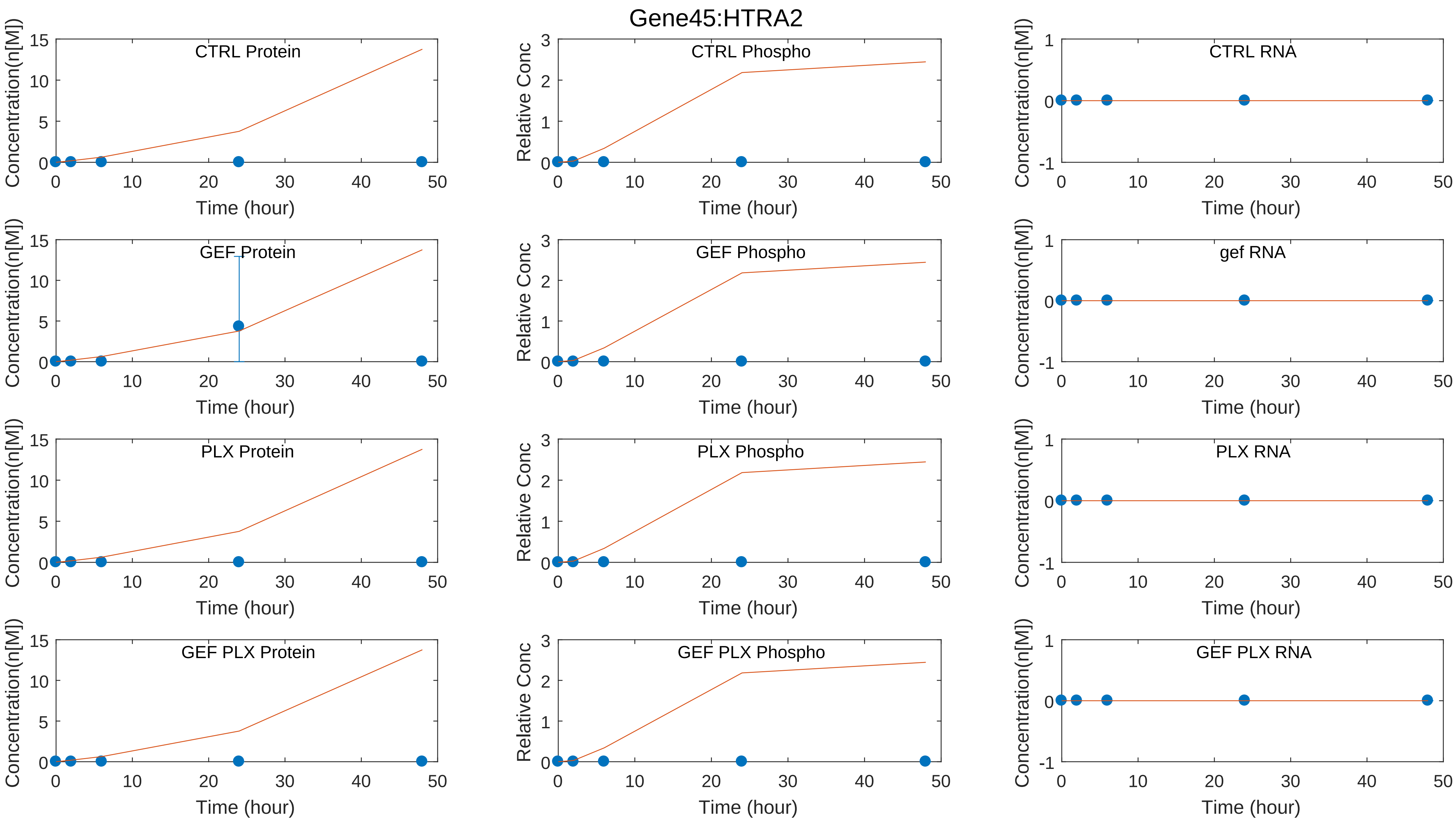

Gene45:HTRA2
CTRL Protein
CTRL Phospho
CTRL RNA
GEF Protein
GEF Phospho
gef RNA
PLX Protein
PLX Phospho
PLX RNA
GEF PLX Protein
GEF PLX Phospho
GEF PLX RNA
Concentration(n[M])
Relative Conc
Time (hour)

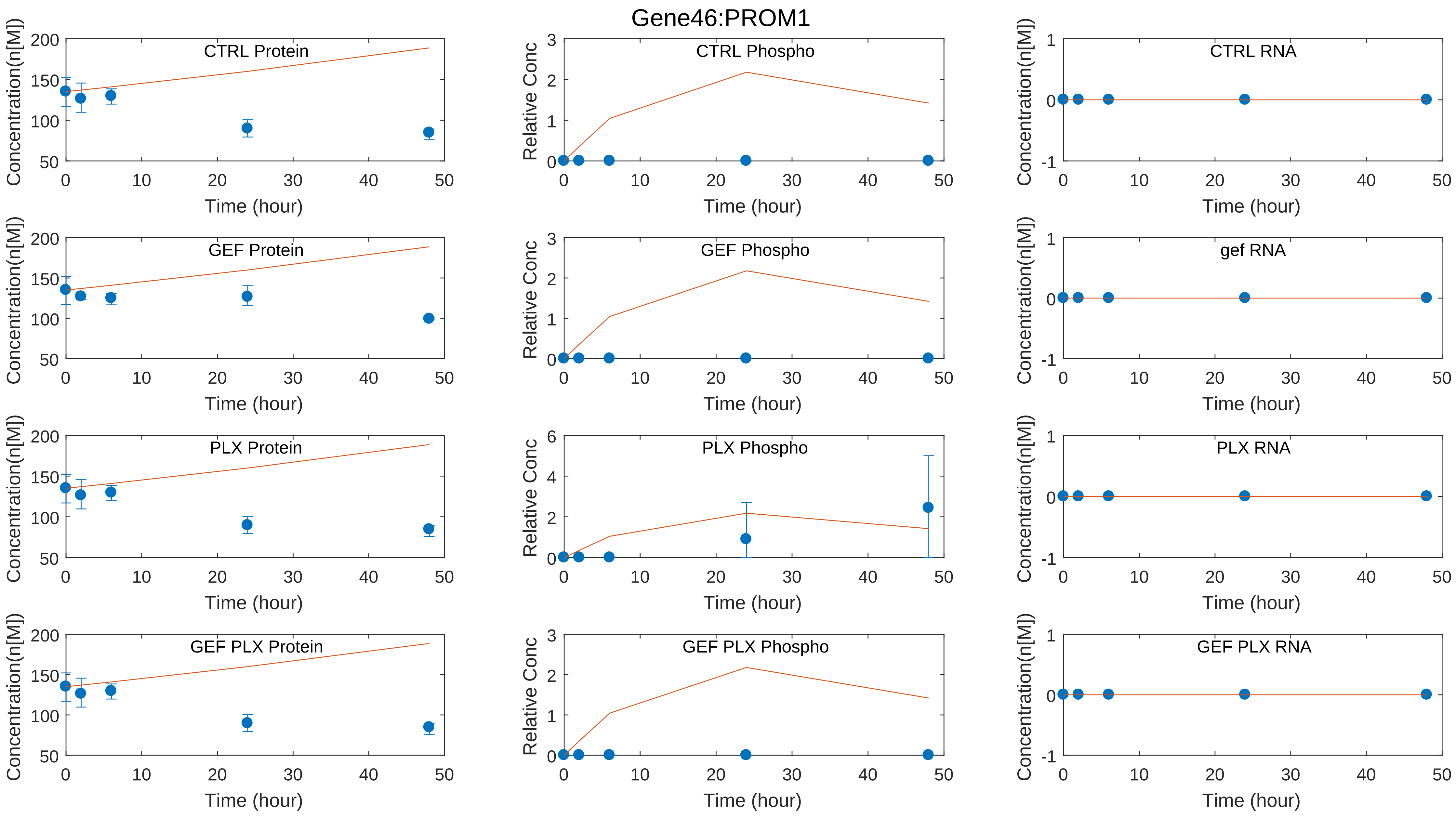

Gene46:PROM1
CTRL Protein
CTRL Phospho
CTRL RNA
GEF Protein
GEF Phospho
gef RNA
PLX Protein
PLX Phospho
PLX RNA
GEF PLX Protein
GEF PLX Phospho
GEF PLX RNA
Concentration(n[M])
Relative Conc
Time (hour)

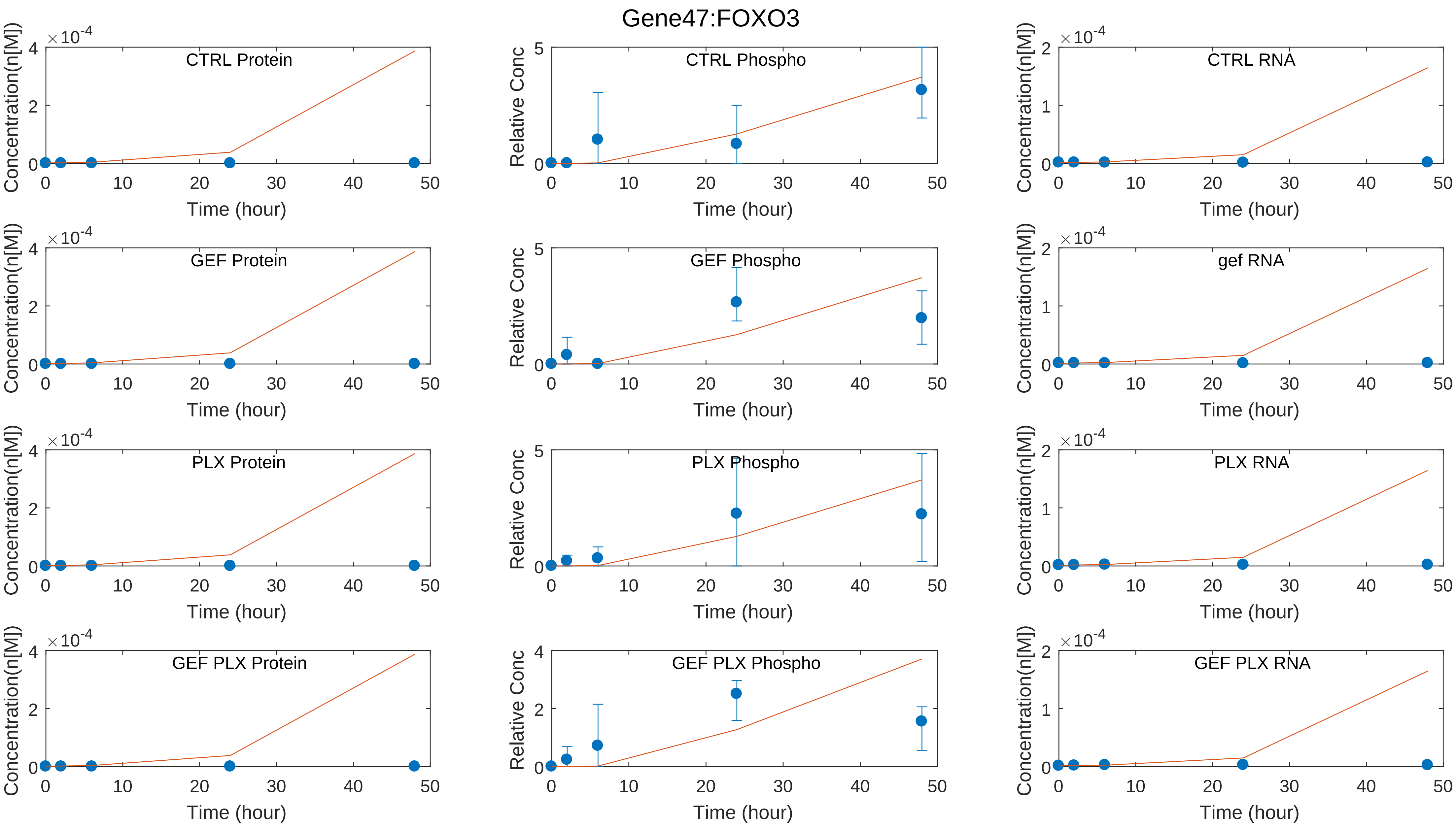

Gene47:FOXO3
CTRL Protein
CTRL Phospho
CTRL RNA
GEF Protein
GEF Phospho
gef RNA
PLX Protein
PLX Phospho
PLX RNA
GEF PLX Protein
GEF PLX Phospho
GEF PLX RNA
Concentration(n[M])
Relative Conc
Time (hour)
×10^-4

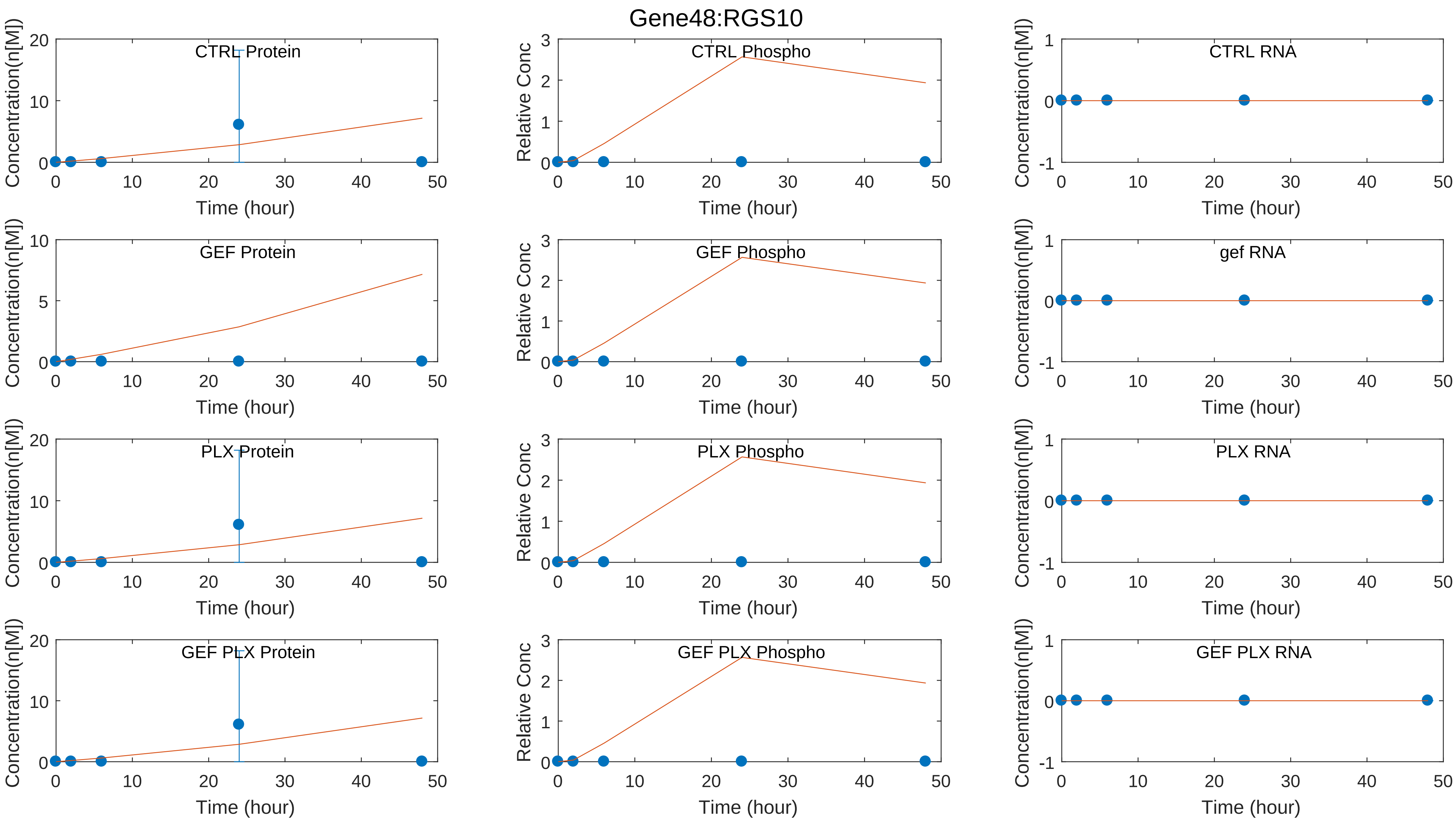
Gene48:RGS10
CTRL Protein
CTRL Phospho
CTRL RNA
GEF Protein
GEF Phospho
gef RNA
PLX Protein
PLX Phospho
PLX RNA
GEF PLX Protein
GEF PLX Phospho
GEF PLX RNA
Concentration(n[M])
Relative Conc
Time (hour)

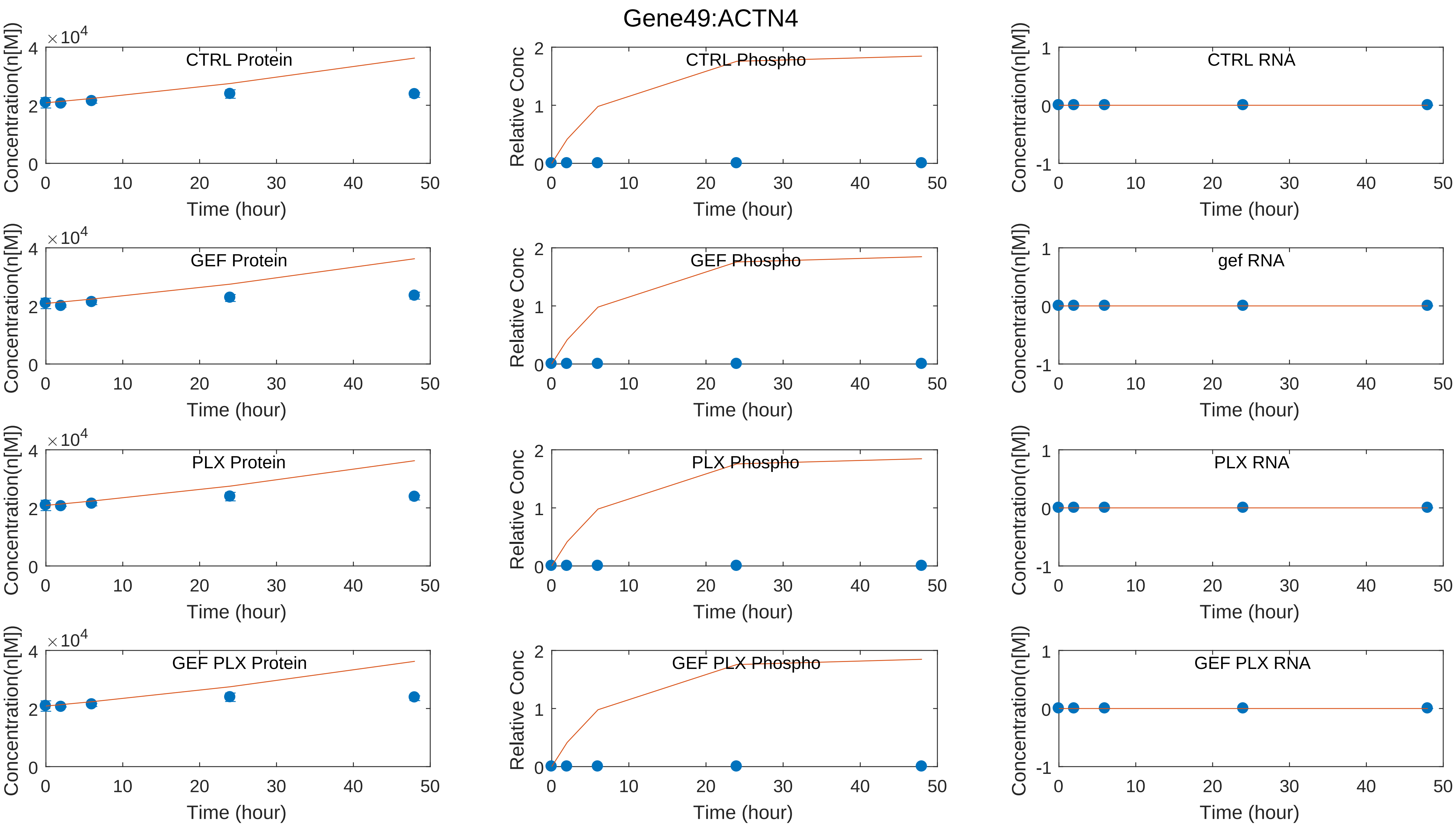
Gene49:ACTN4
CTRL Protein
CTRL Phospho
CTRL RNA
GEF Protein
GEF Phospho
gef RNA
PLX Protein
PLX Phospho
PLX RNA
GEF PLX Protein
GEF PLX Phospho
GEF PLX RNA
Concentration(n[M])
Relative Conc
Time (hour)
×10^4

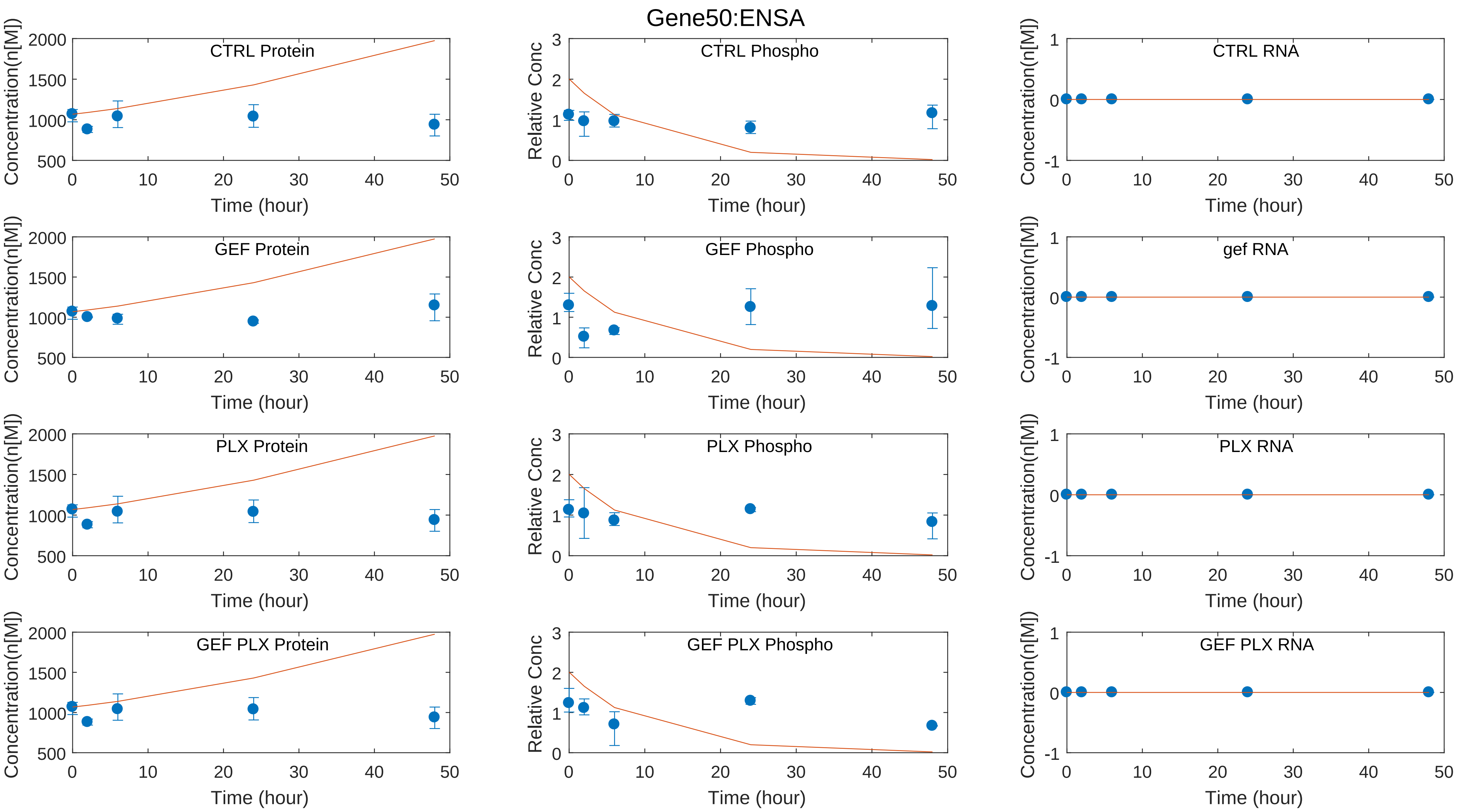

Gene50:ENSA
CTRL Protein
GEF Protein
PLX Protein
GEF PLX Protein
CTRL Phospho
GEF Phospho
PLX Phospho
GEF PLX Phospho
CTRL RNA
gef RNA
PLX RNA
GEF PLX RNA
Concentration(n[M])
Relative Conc
Time (hour)

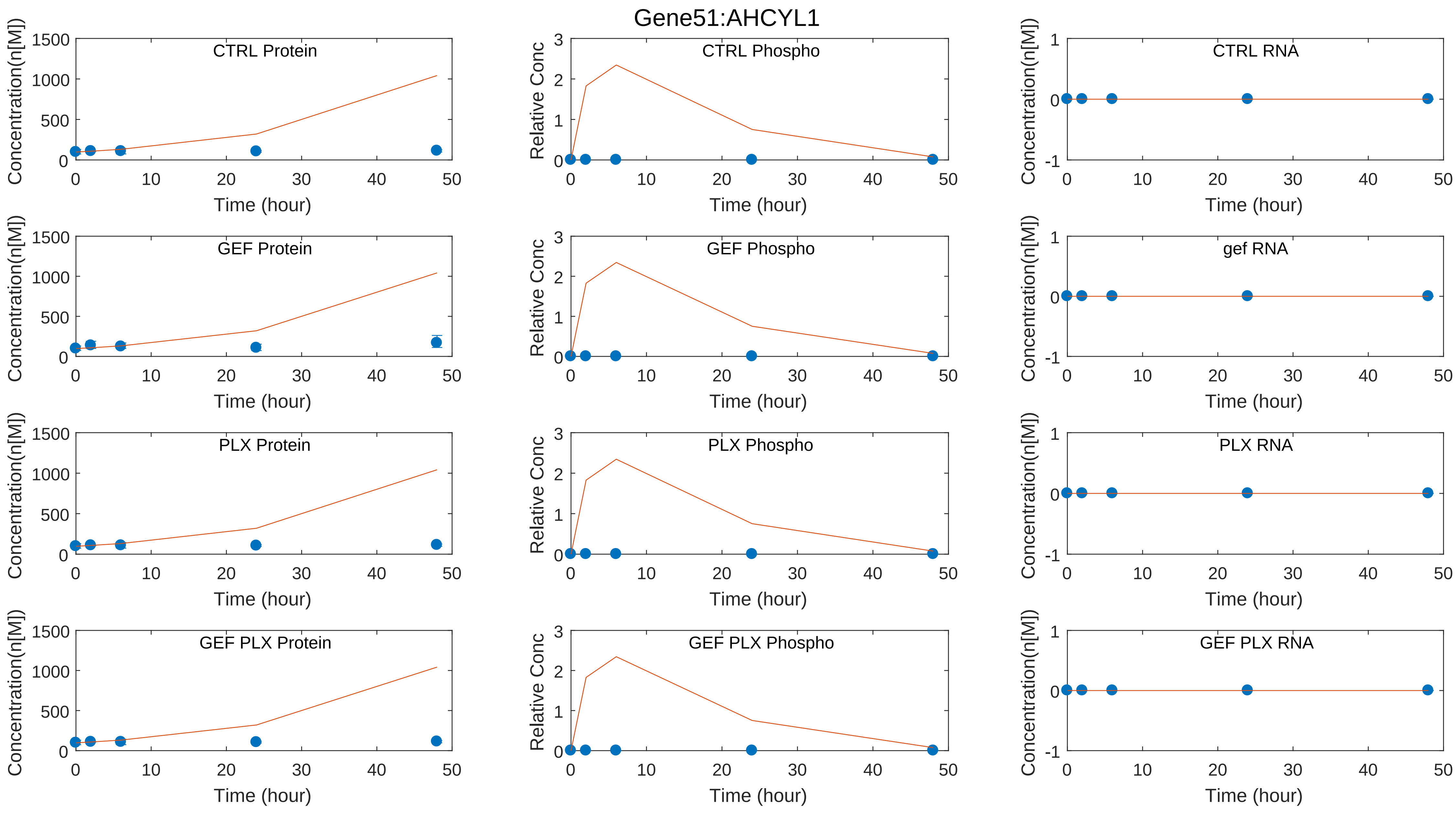
Gene51:AHCYL1
CTRL Protein
CTRL Phospho
CTRL RNA
GEF Protein
GEF Phospho
gef RNA
PLX Protein
PLX Phospho
PLX RNA
GEF PLX Protein
GEF PLX Phospho
GEF PLX RNA
Concentration(n[M])
Relative Conc
Time (hour)

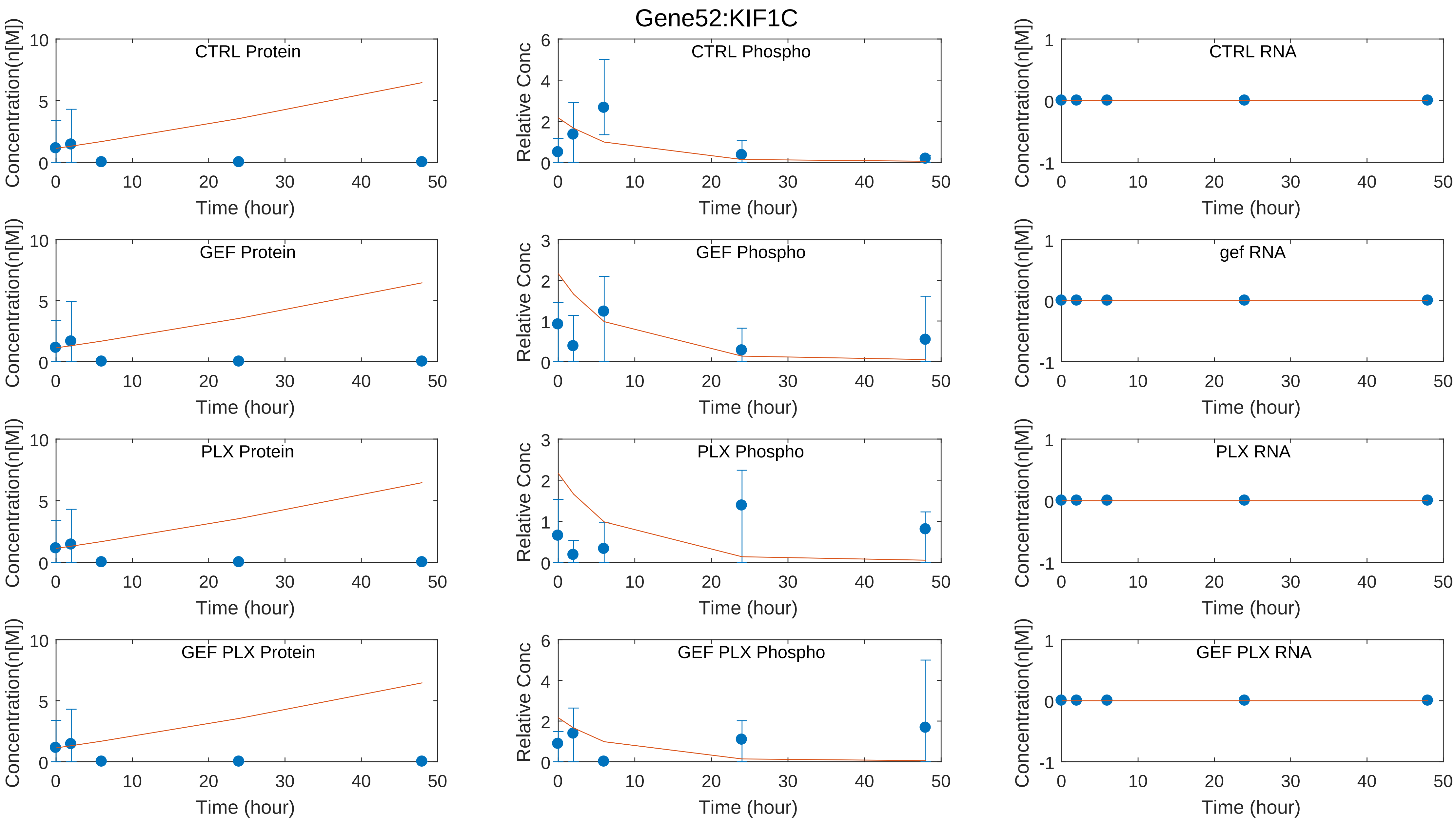
Gene52:KIF1C
CTRL Protein
GEF Protein
PLX Protein
GEF PLX Protein
CTRL Phospho
GEF Phospho
PLX Phospho
GEF PLX Phospho
CTRL RNA
gef RNA
PLX RNA
GEF PLX RNA
Concentration(n[M])
Relative Conc
Time (hour)

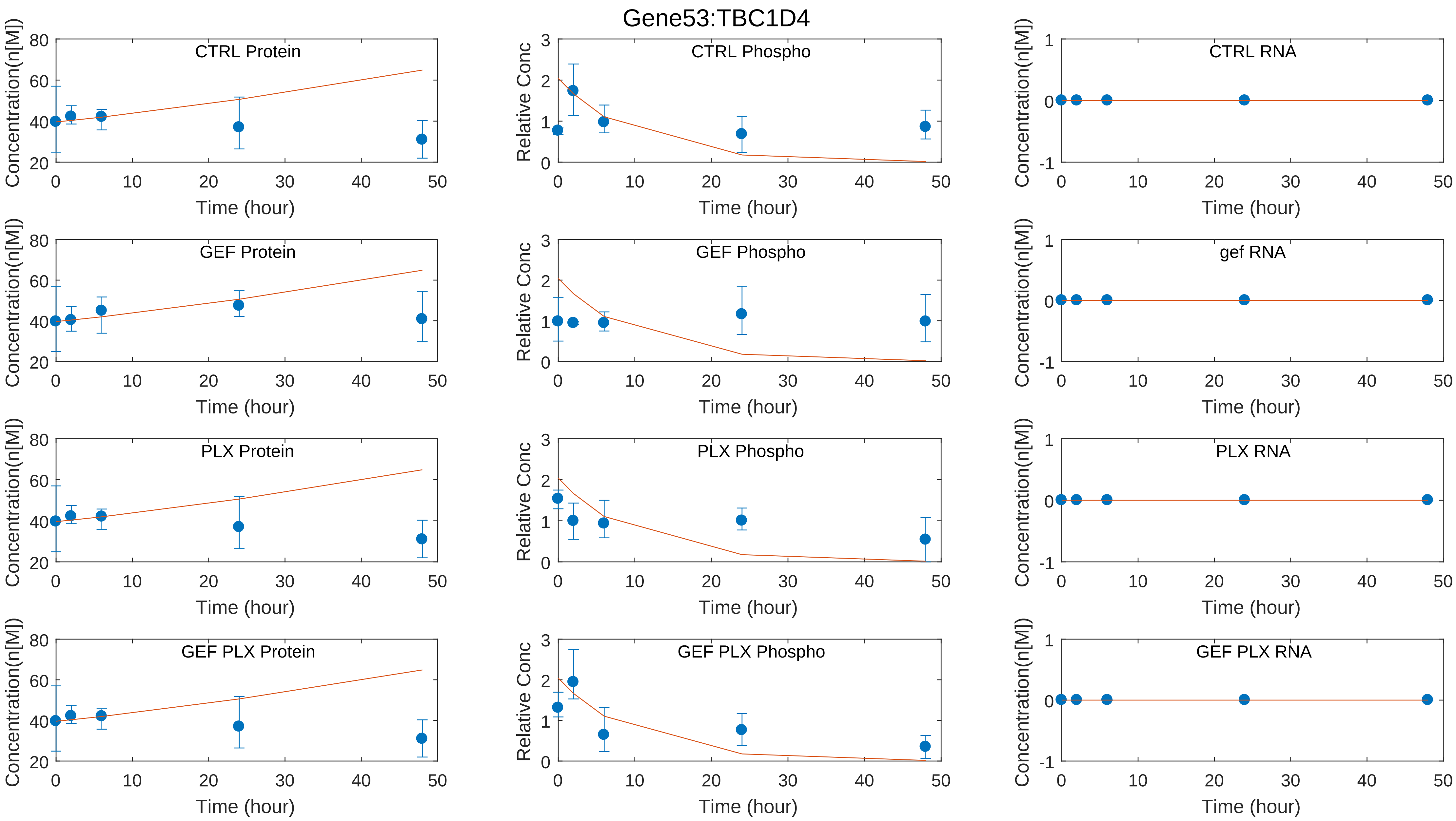

Gene53:TBC1D4
CTRL Protein
CTRL Phospho
CTRL RNA
GEF Protein
GEF Phospho
gef RNA
PLX Protein
PLX Phospho
PLX RNA
GEF PLX Protein
GEF PLX Phospho
GEF PLX RNA
Concentration(n[M])
Relative Conc
Time (hour)

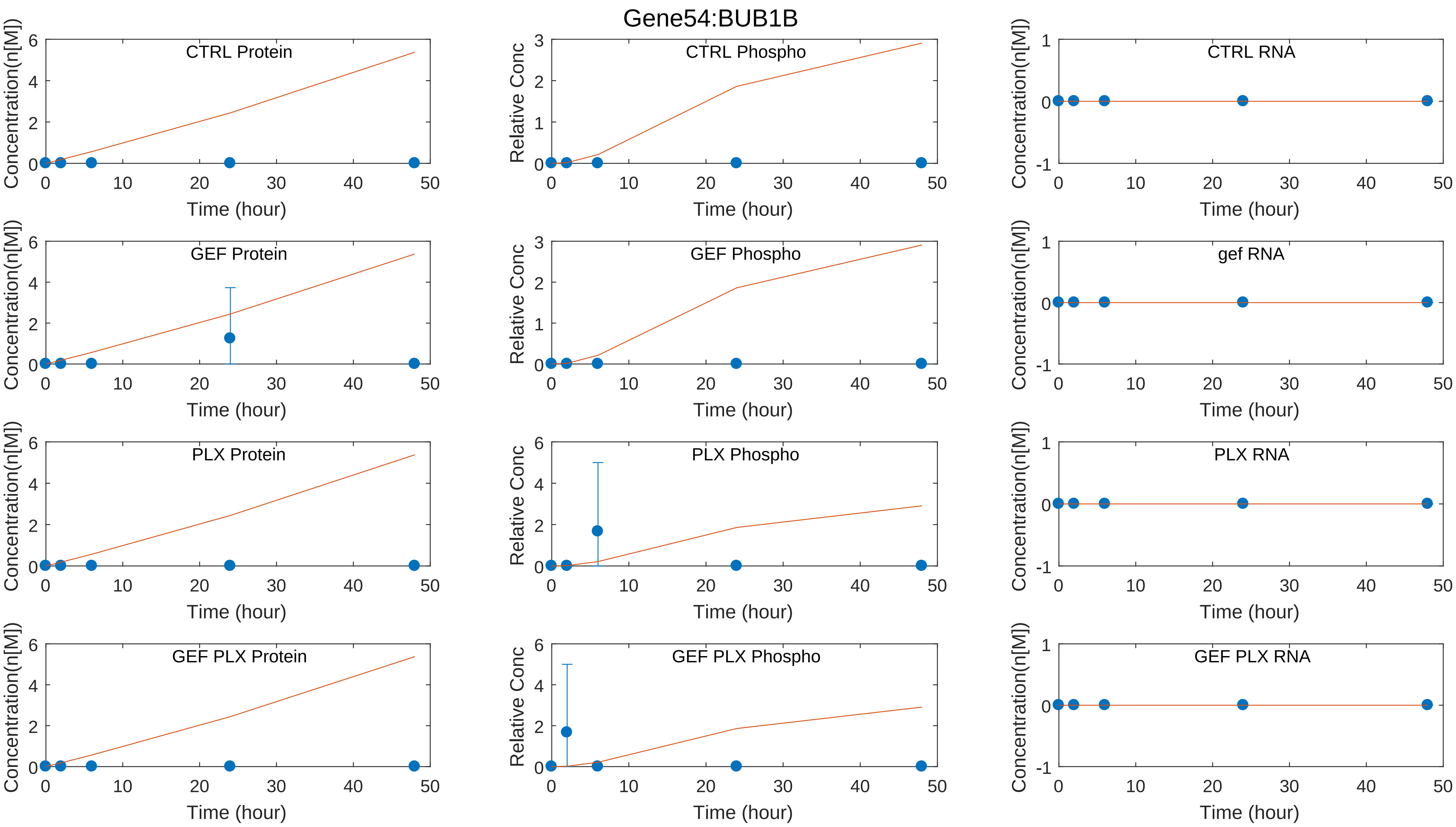

Gene54:BUB1B
CTRL Protein
CTRL Phospho
CTRL RNA
GEF Protein
GEF Phospho
gef RNA
PLX Protein
PLX Phospho
PLX RNA
GEF PLX Protein
GEF PLX Phospho
GEF PLX RNA
Concentration(n[M])
Relative Conc
Time (hour)

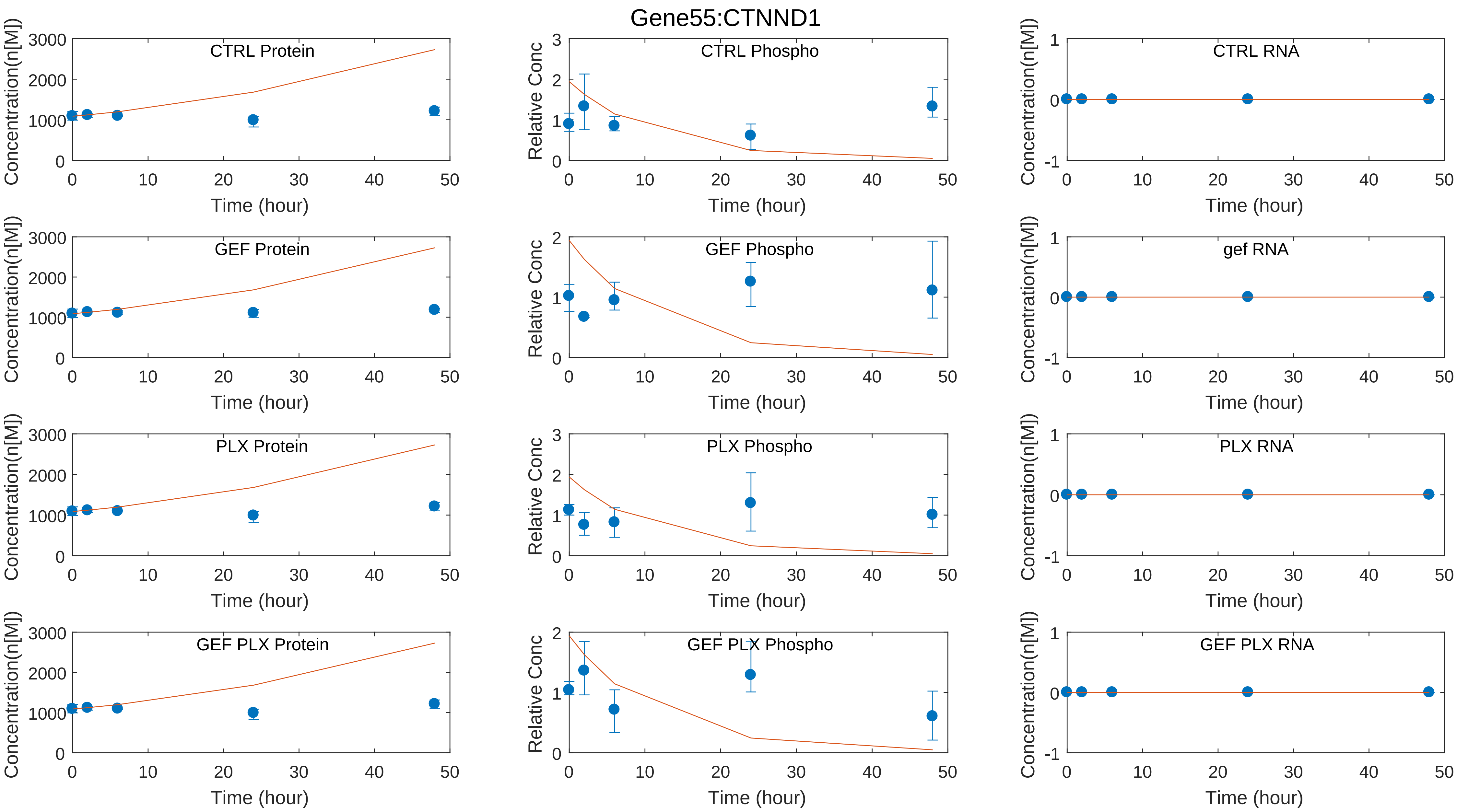
Gene55:CTNND1
CTRL Protein
CTRL Phospho
CTRL RNA
GEF Protein
GEF Phospho
gef RNA
PLX Protein
PLX Phospho
PLX RNA
GEF PLX Protein
GEF PLX Phospho
GEF PLX RNA
Concentration(n[M])
Relative Conc
Time (hour)

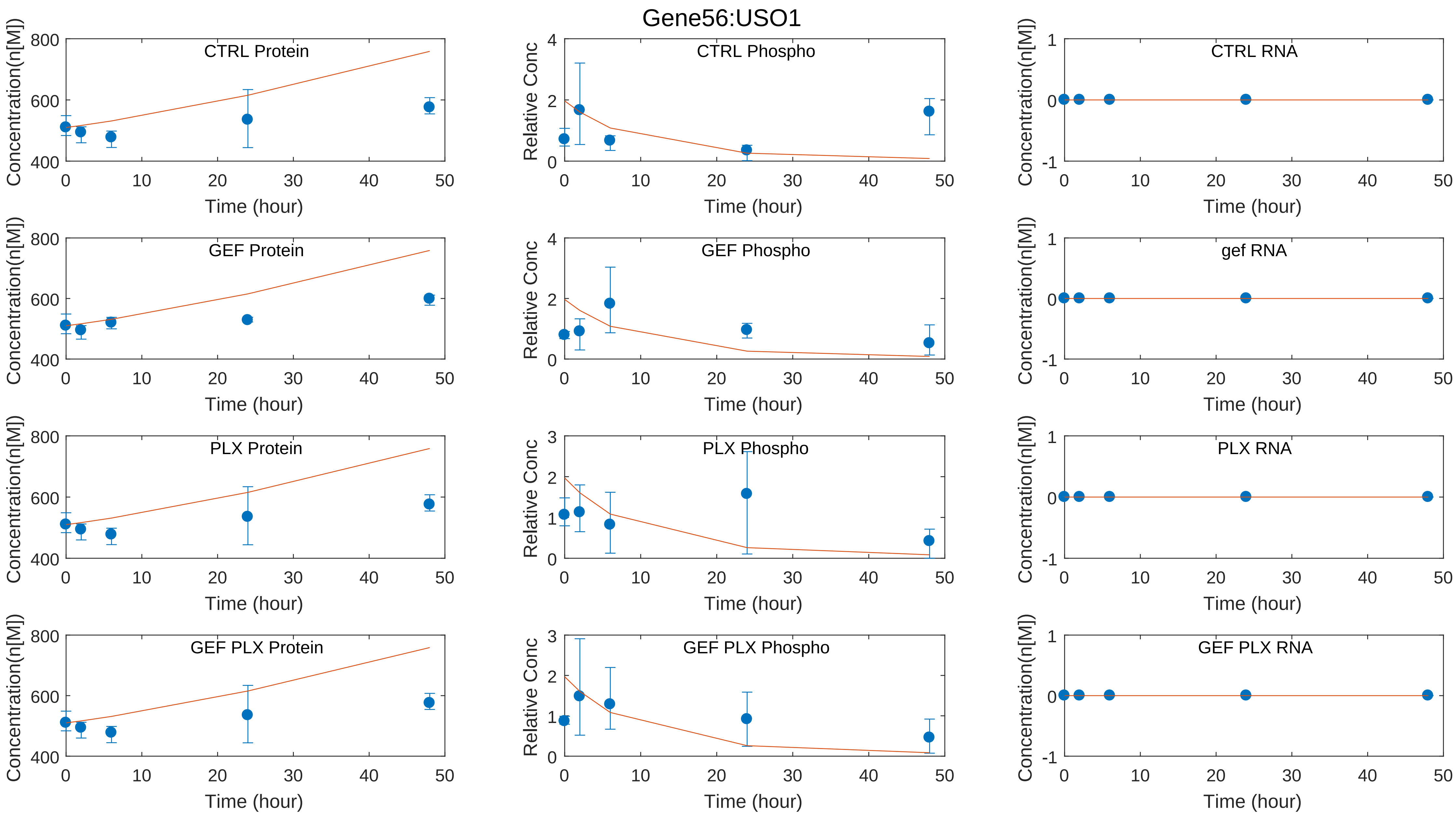

Gene56:USO1
CTRL Protein
GEF Protein
PLX Protein
GEF PLX Protein
CTRL Phospho
GEF Phospho
PLX Phospho
GEF PLX Phospho
CTRL RNA
gef RNA
PLX RNA
GEF PLX RNA
Concentration(n[M])
Relative Conc
Time (hour)

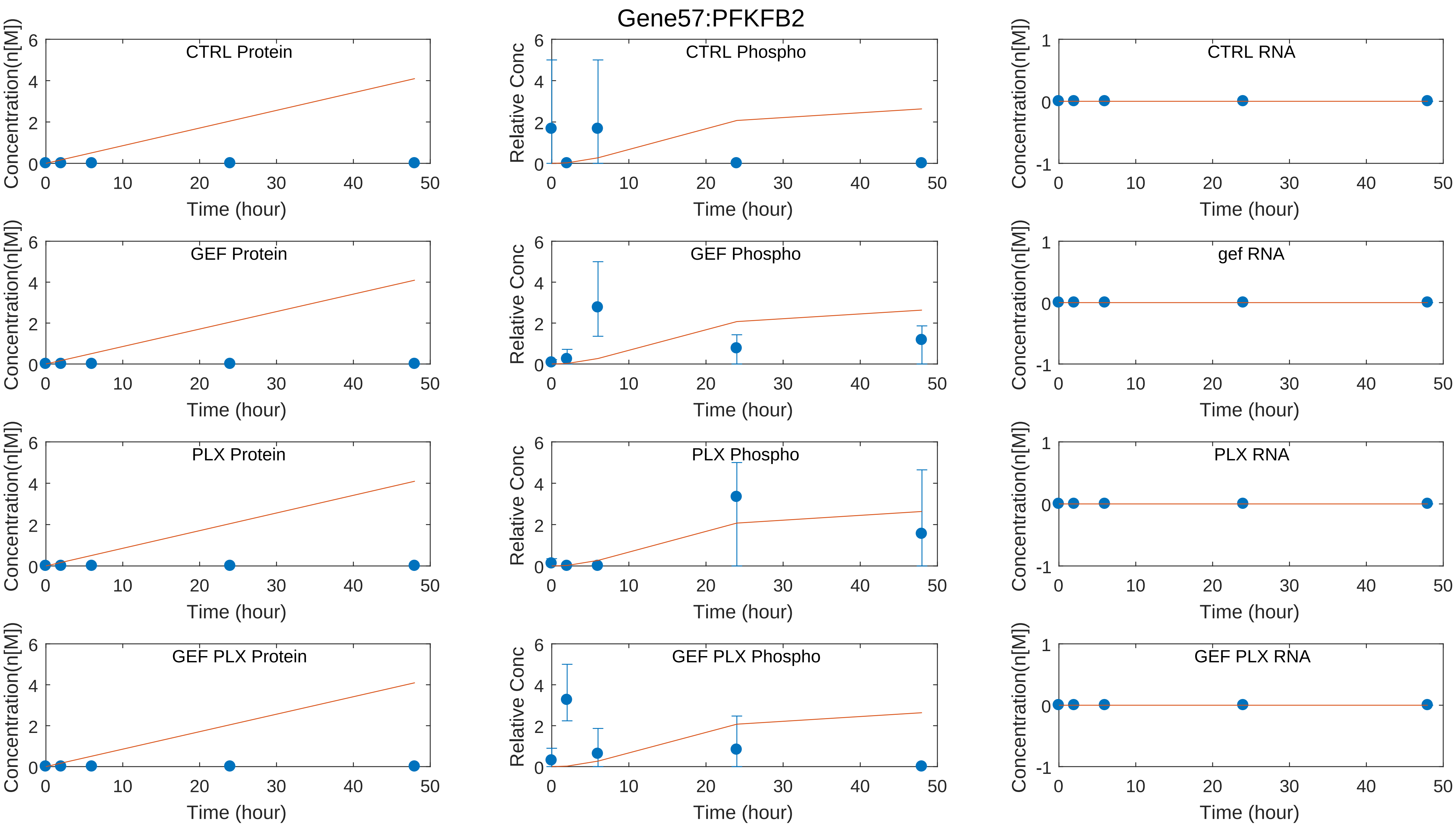

Gene57:PFKFB2
CTRL Protein
CTRL Phospho
CTRL RNA
GEF Protein
GEF Phospho
gef RNA
PLX Protein
PLX Phospho
PLX RNA
GEF PLX Protein
GEF PLX Phospho
GEF PLX RNA
Concentration(n[M])
Relative Conc
Time (hour)

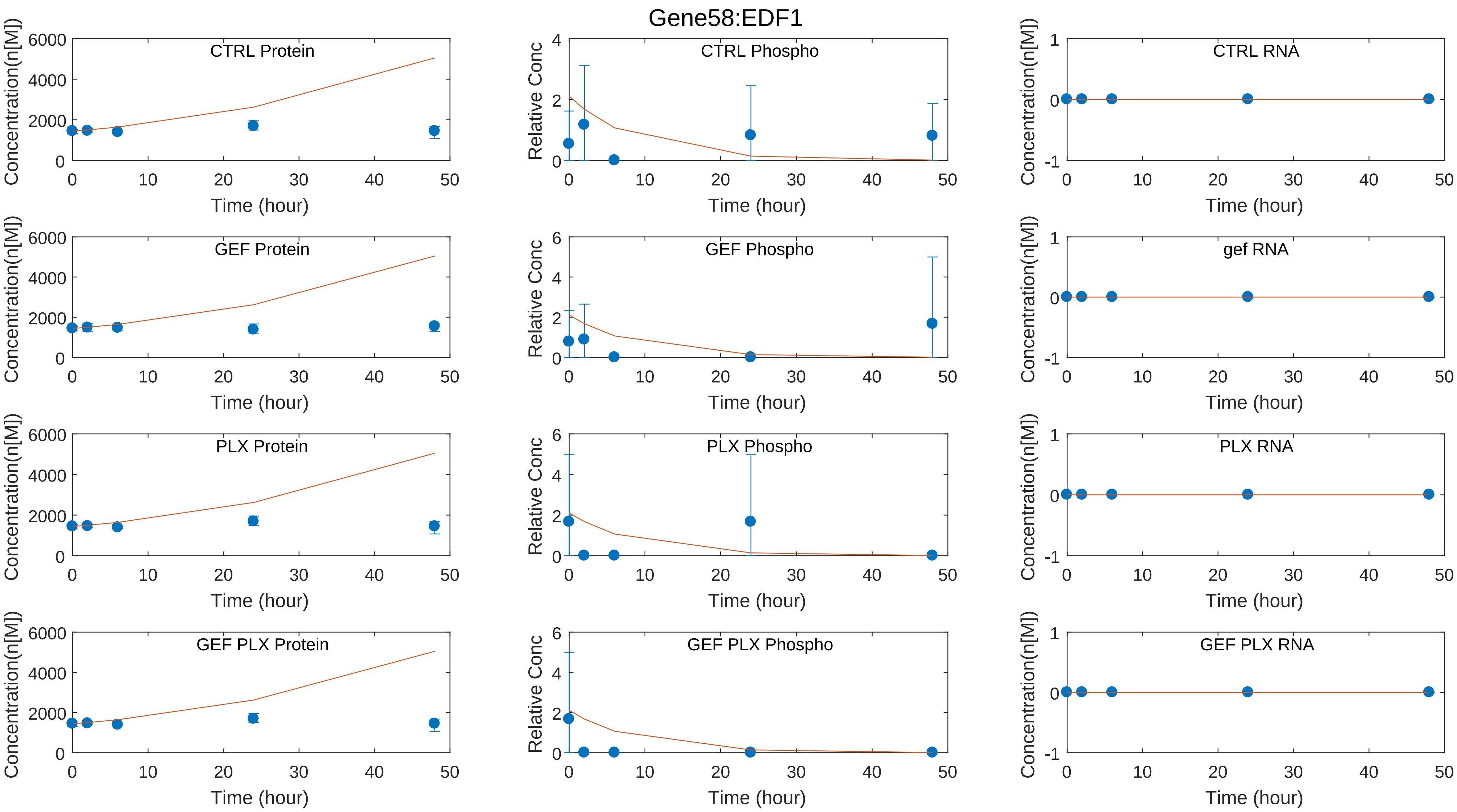
Gene58:EDF1
CTRL Protein
GEF Protein
PLX Protein
GEF PLX Protein
CTRL Phospho
GEF Phospho
PLX Phospho
GEF PLX Phospho
CTRL RNA
gef RNA
PLX RNA
GEF PLX RNA
Concentration(n[M])
Relative Conc
Time (hour)

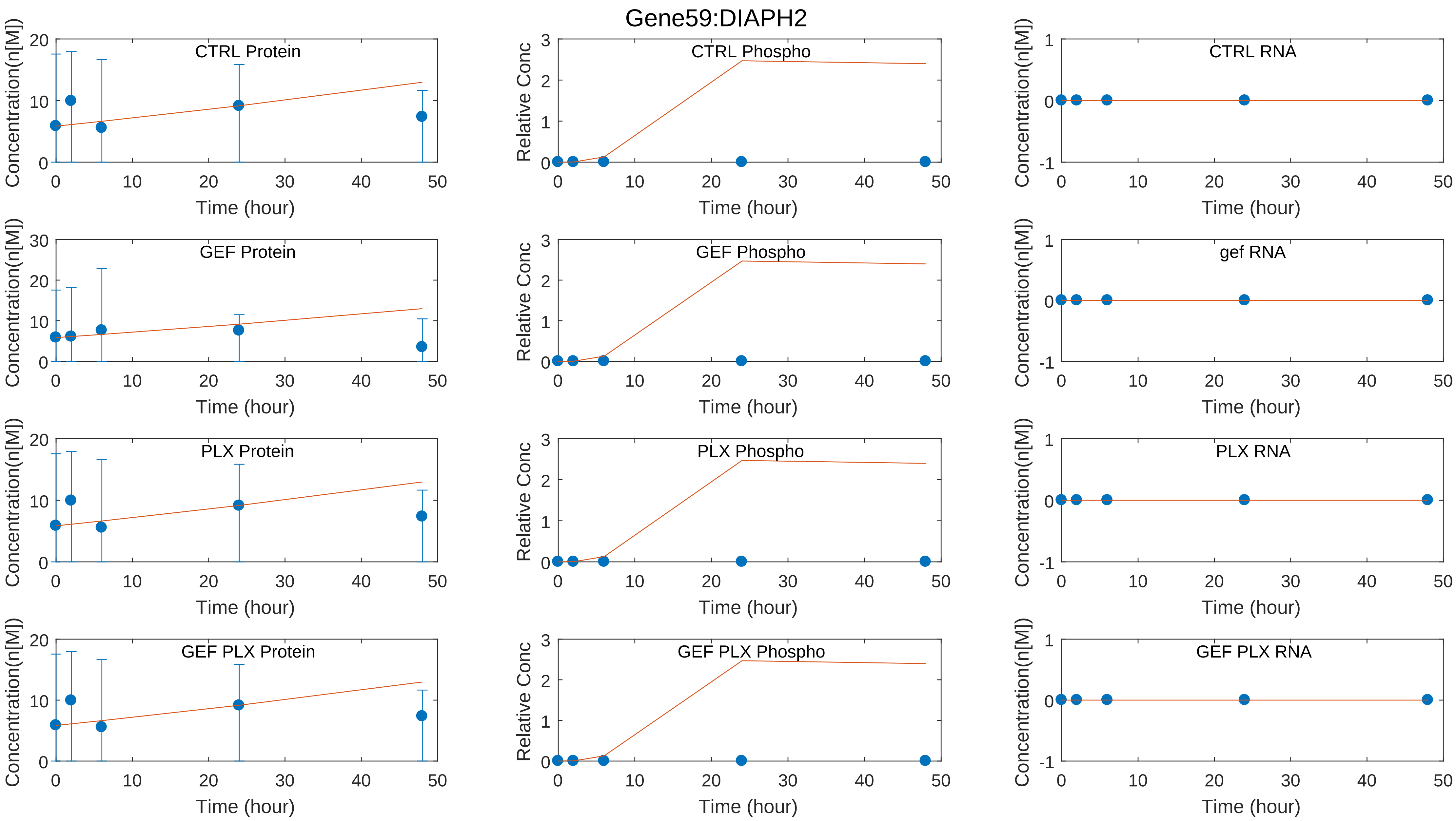

Gene59:DIAPH2
CTRL Protein
GEF Protein
PLX Protein
GEF PLX Protein
CTRL Phospho
GEF Phospho
PLX Phospho
GEF PLX Phospho
CTRL RNA
gef RNA
PLX RNA
GEF PLX RNA
Concentration(n[M])
Relative Conc
Time (hour)

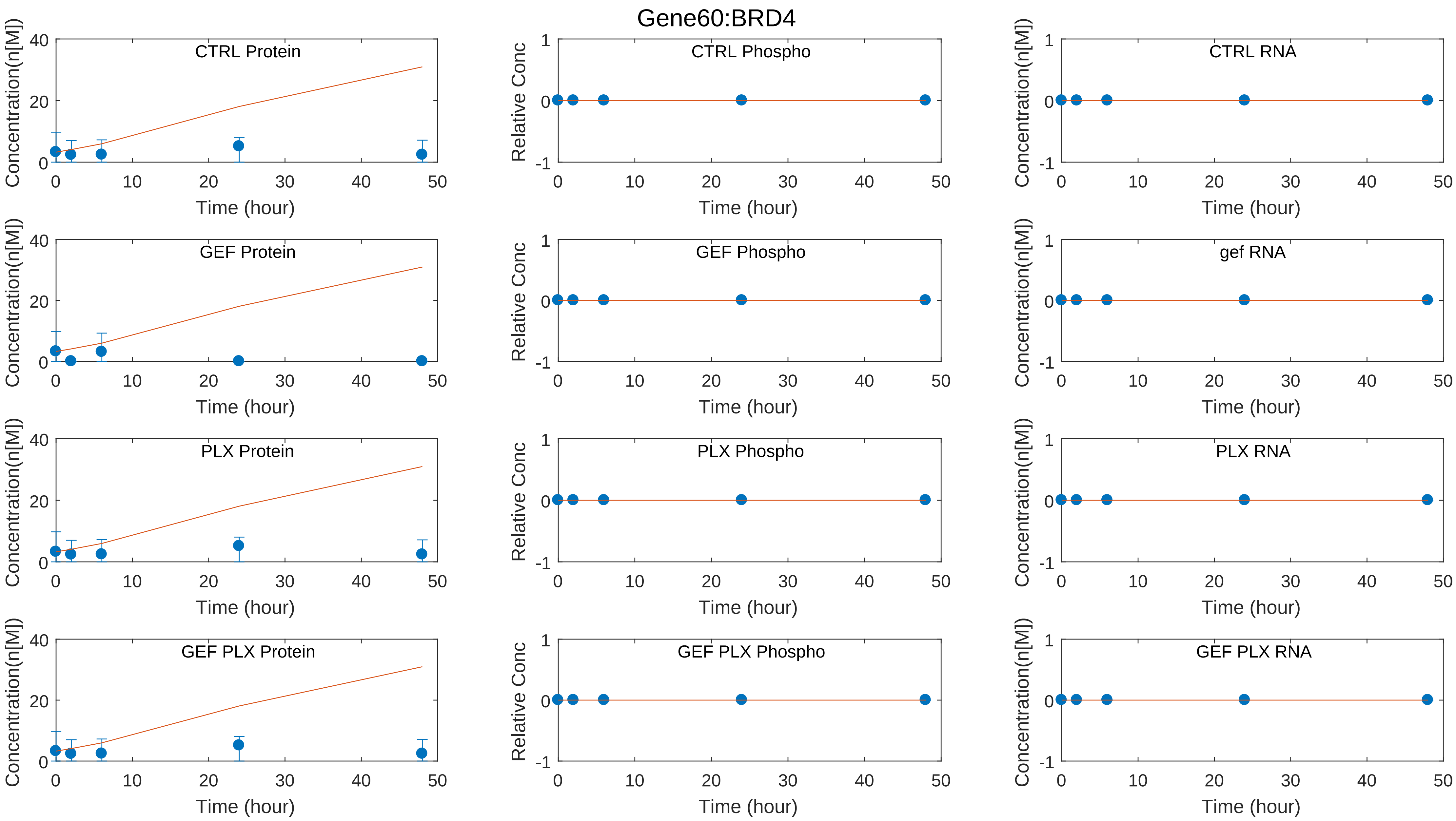

Gene60:BRD4
CTRL Protein
GEF Protein
PLX Protein
GEF PLX Protein
CTRL Phospho
GEF Phospho
PLX Phospho
GEF PLX Phospho
CTRL RNA
gef RNA
PLX RNA
GEF PLX RNA
Concentration(n[M])
Relative Conc
Time (hour)

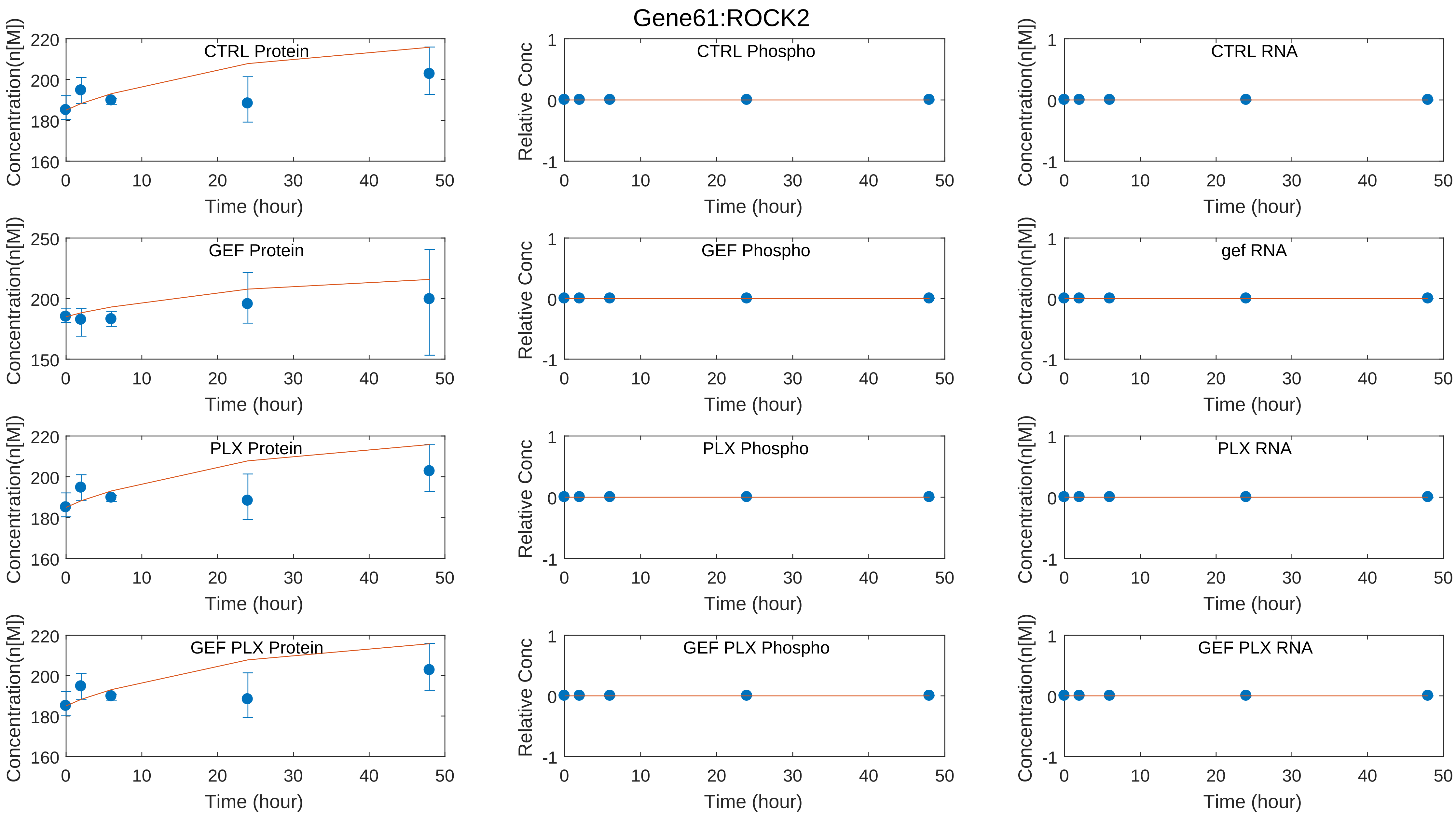
Gene61:ROCK2
CTRL Protein
CTRL Phospho
CTRL RNA
GEF Protein
GEF Phospho
gef RNA
PLX Protein
PLX Phospho
PLX RNA
GEF PLX Protein
GEF PLX Phospho
GEF PLX RNA
Concentration(n[M])
Relative Conc
Time (hour)

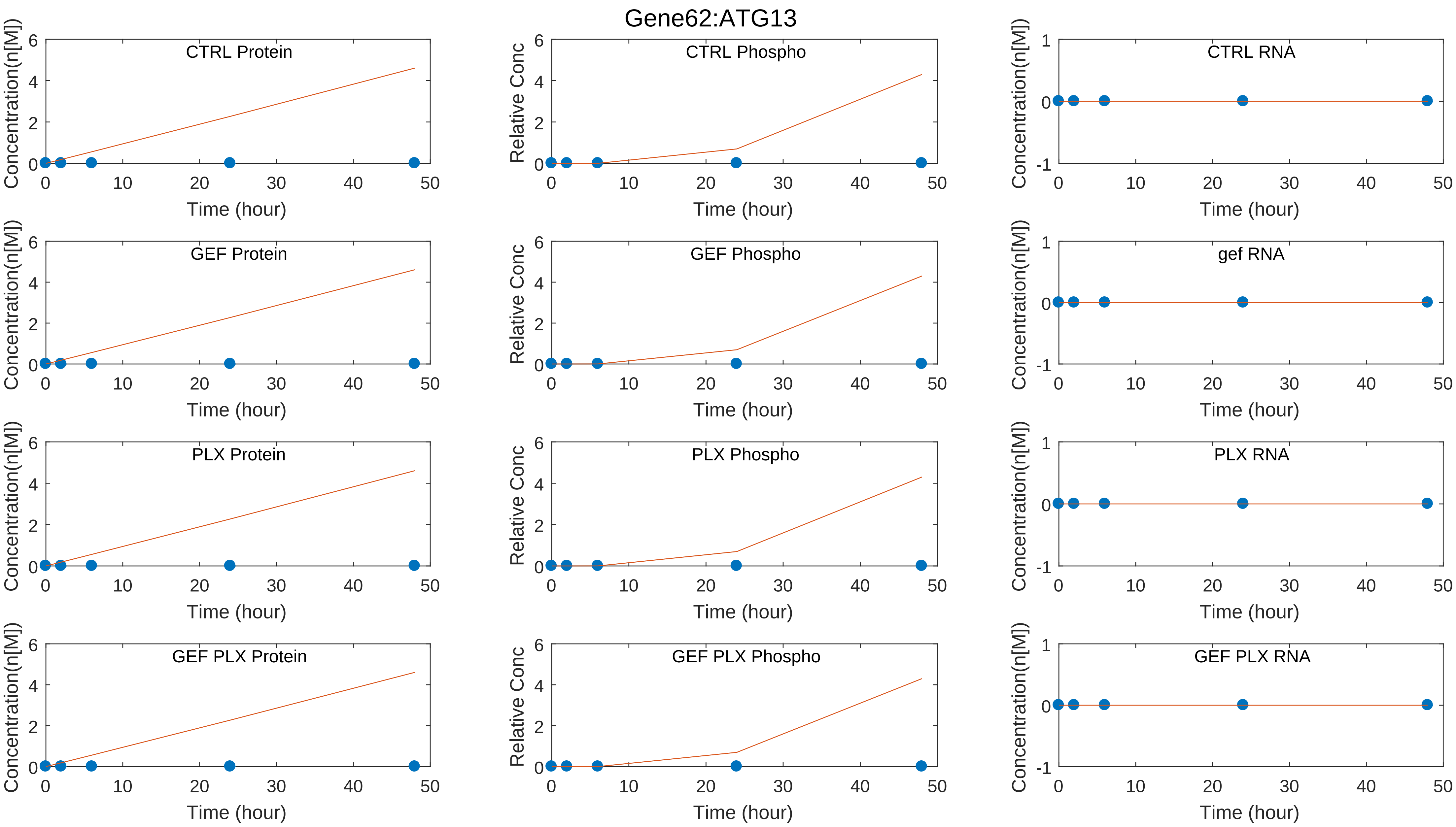
Gene62:ATG13
CTRL Protein
CTRL Phospho
CTRL RNA
GEF Protein
GEF Phospho
gef RNA
PLX Protein
PLX Phospho
PLX RNA
GEF PLX Protein
GEF PLX Phospho
GEF PLX RNA
Concentration(n[M])
Relative Conc
Time (hour)

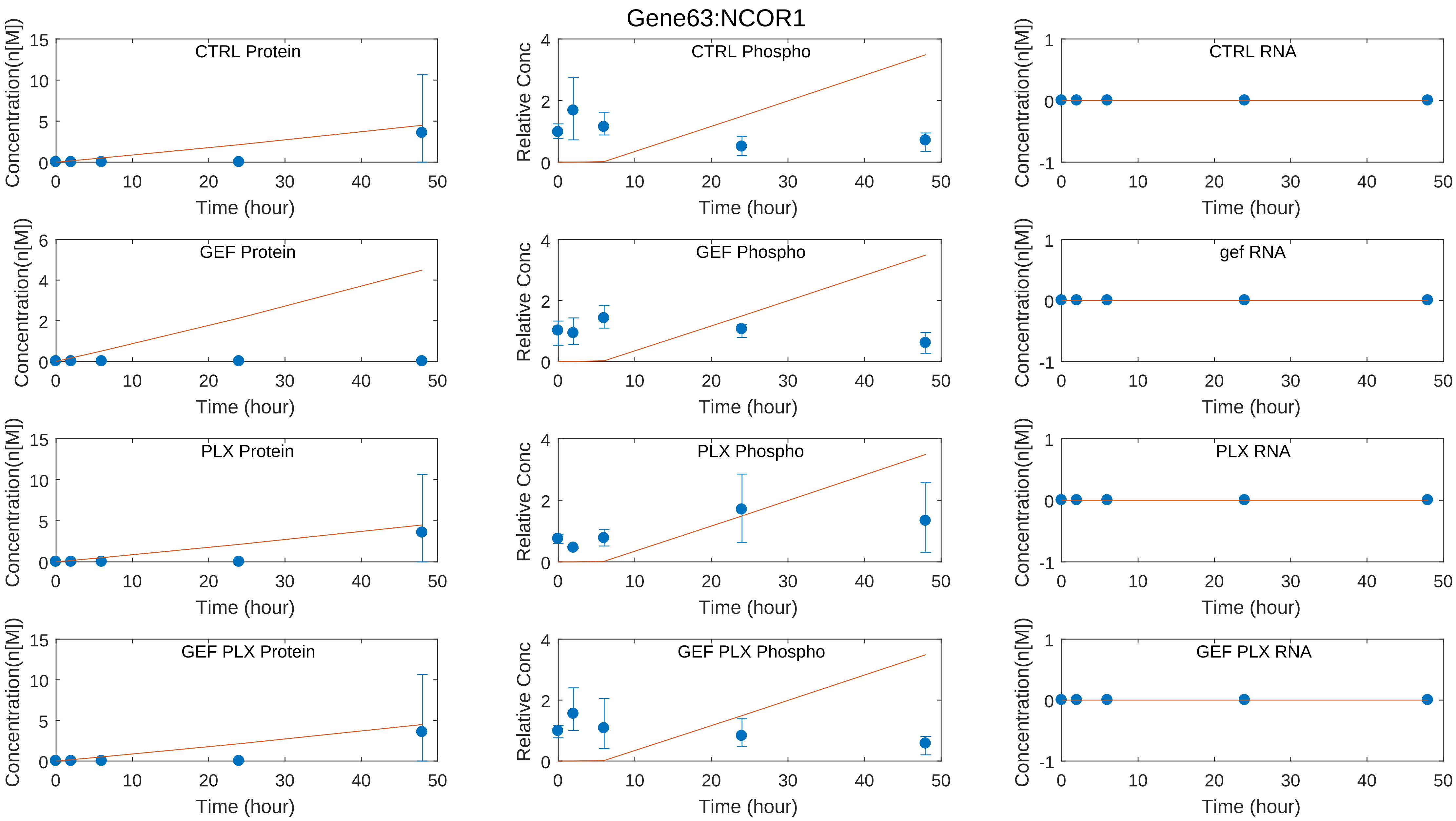

Gene63:NCOR1
CTRL Protein
CTRL Phospho
CTRL RNA
GEF Protein
GEF Phospho
gef RNA
PLX Protein
PLX Phospho
PLX RNA
GEF PLX Protein
GEF PLX Phospho
GEF PLX RNA
Concentration(n[M])
Relative Conc
Time (hour)

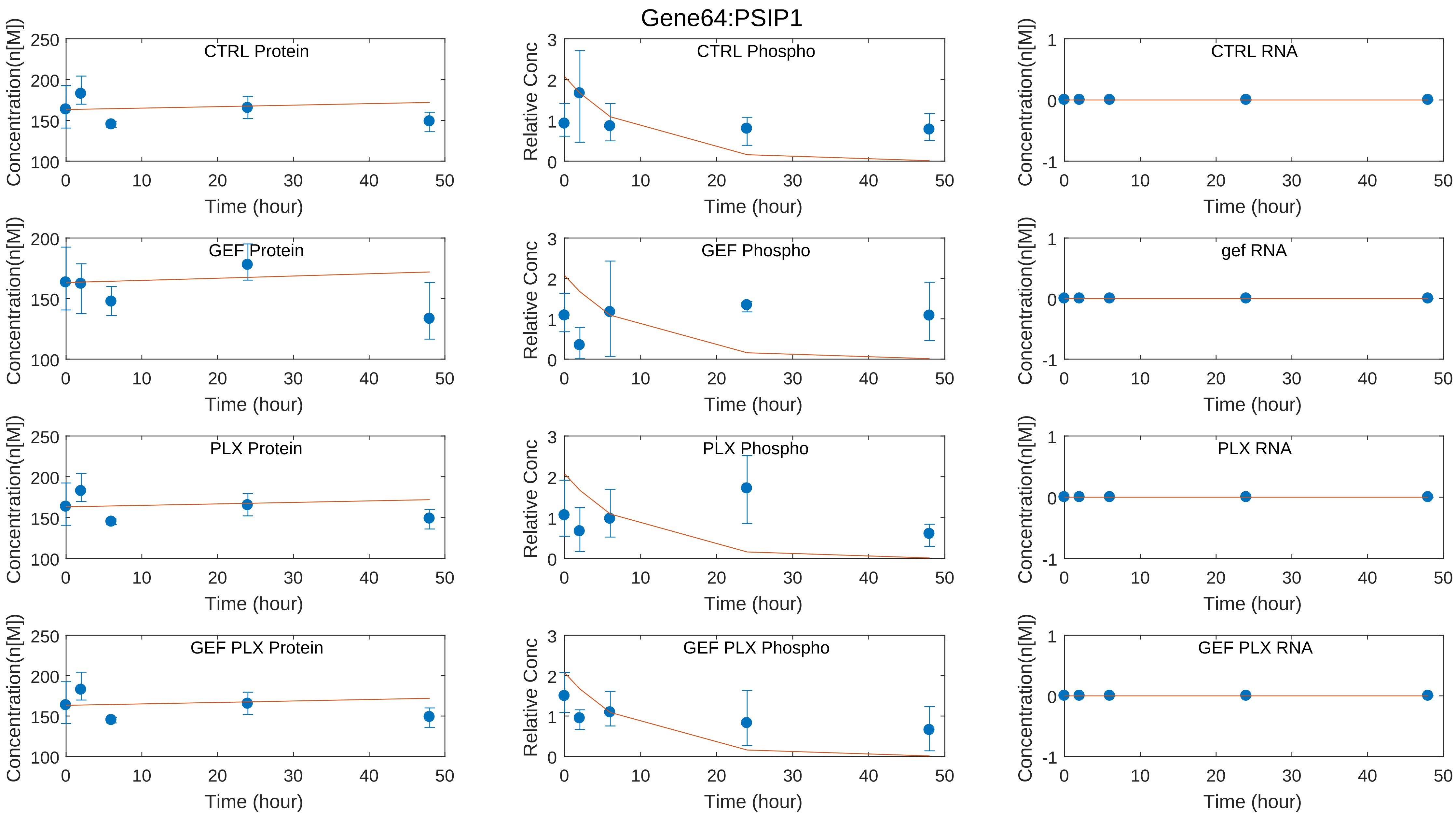

Gene64:PSIP1
CTRL Protein
CTRL Phospho
CTRL RNA
GEF Protein
GEF Phospho
gef RNA
PLX Protein
PLX Phospho
PLX RNA
GEF PLX Protein
GEF PLX Phospho
GEF PLX RNA
Concentration(n[M])
Relative Conc
Time (hour)

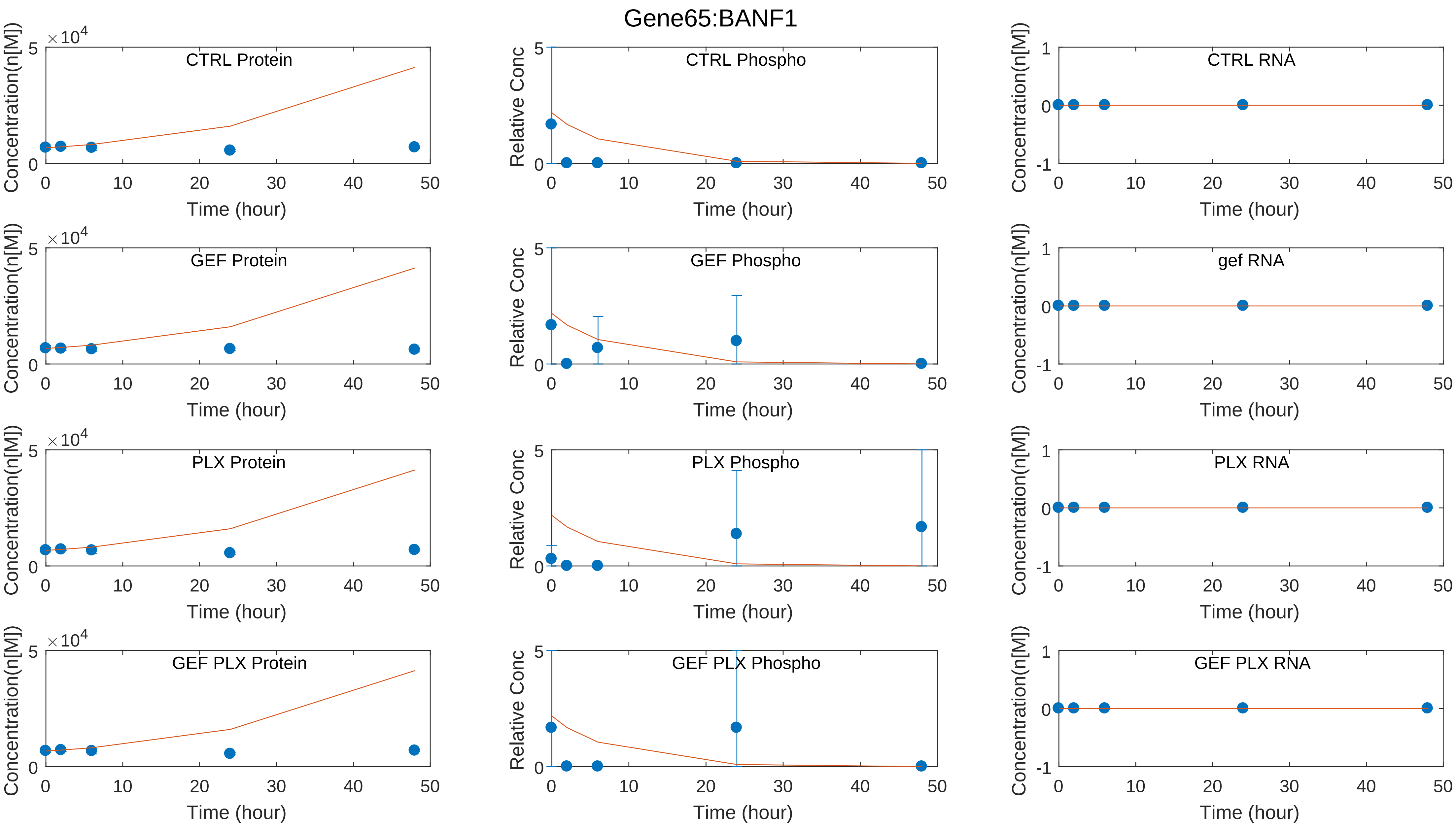

Gene65:BANF1
CTRL Protein
GEF Protein
PLX Protein
GEF PLX Protein
CTRL Phospho
GEF Phospho
PLX Phospho
GEF PLX Phospho
CTRL RNA
gef RNA
PLX RNA
GEF PLX RNA
Concentration(n[M])
Relative Conc
Time (hour)
×10^4

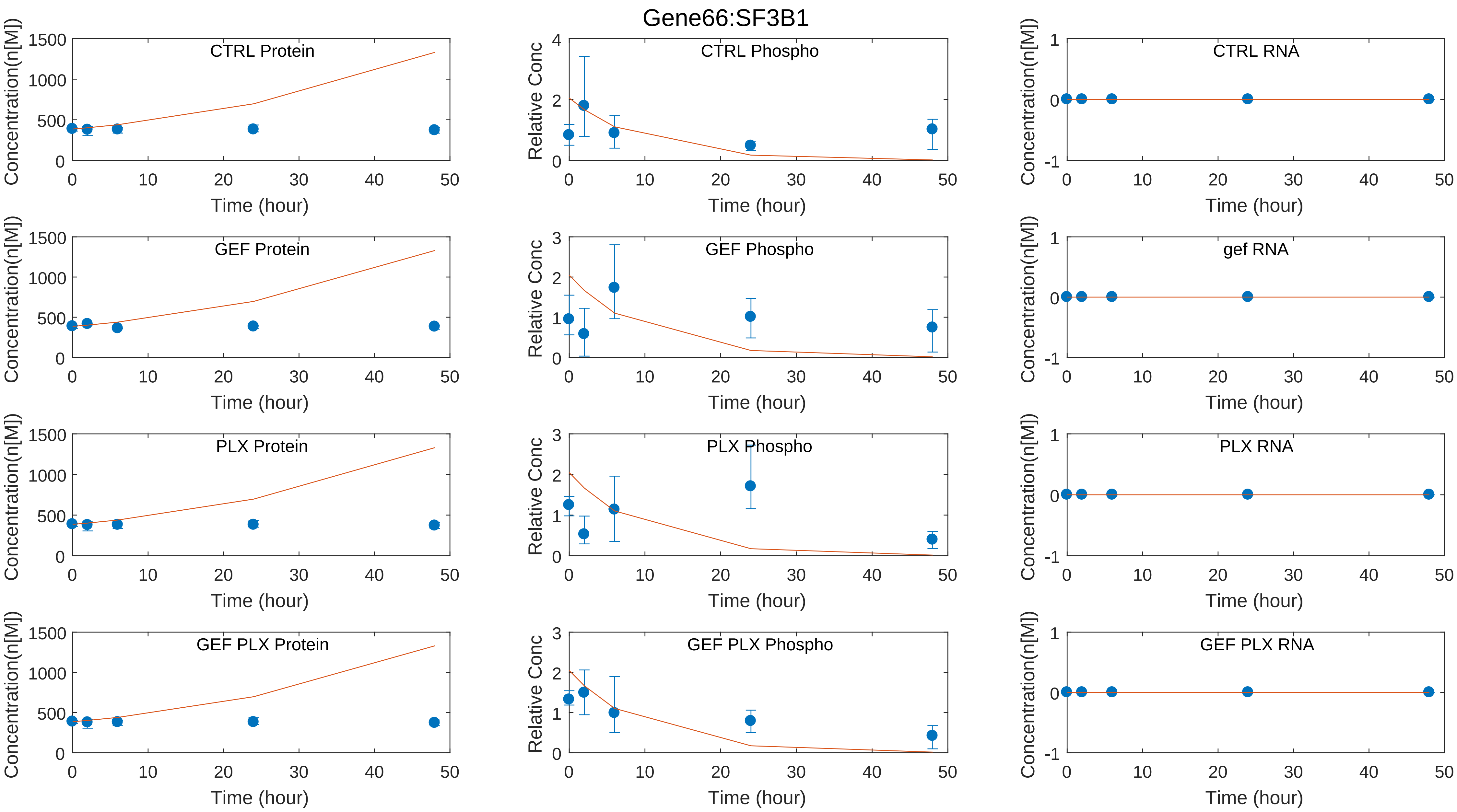

Gene66:SF3B1
CTRL Protein
GEF Protein
PLX Protein
GEF PLX Protein
CTRL Phospho
GEF Phospho
PLX Phospho
GEF PLX Phospho
CTRL RNA
gef RNA
PLX RNA
GEF PLX RNA
Concentration(n[M])
Relative Conc
Time (hour)

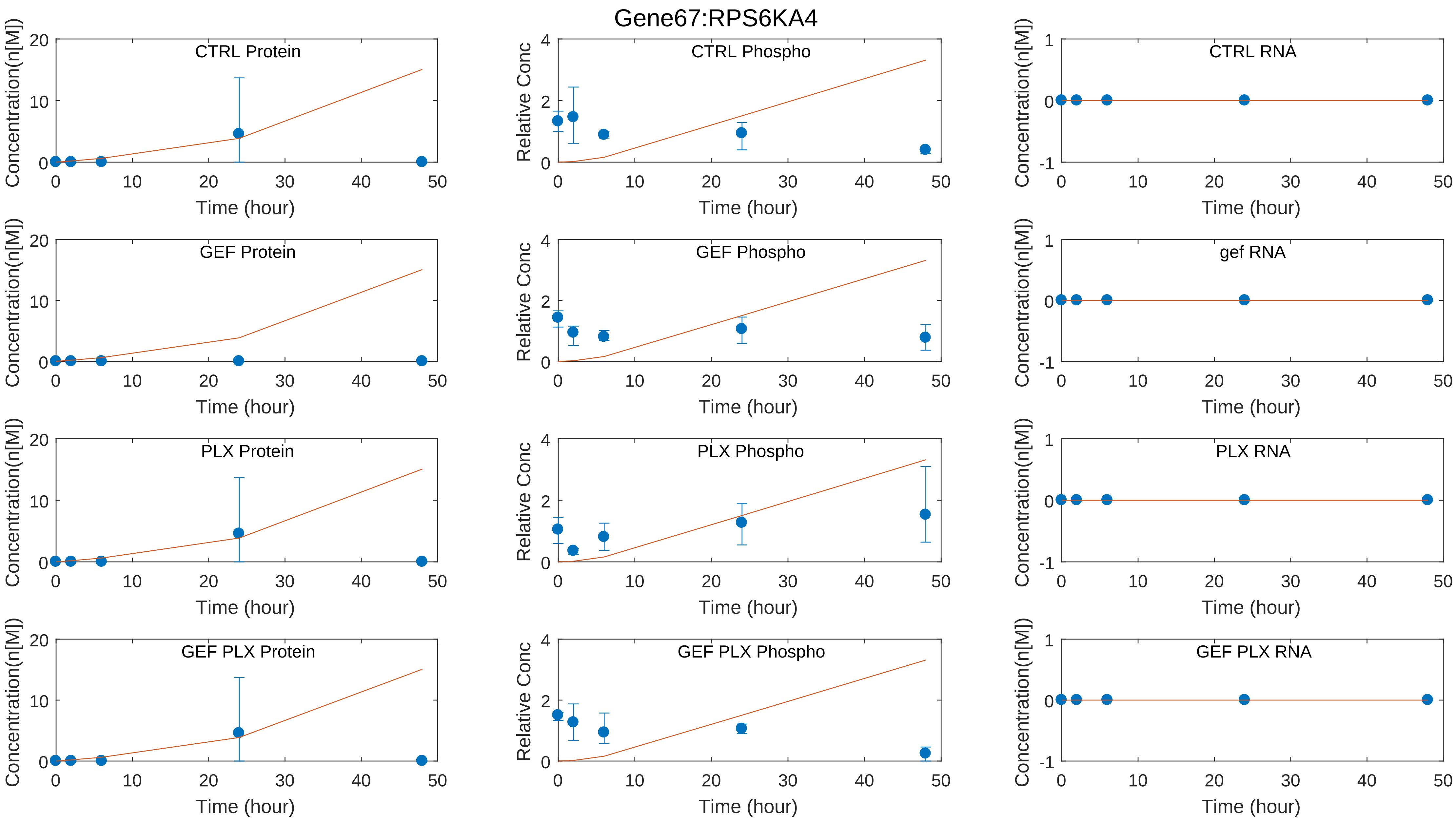
Gene67:RPS6KA4
CTRL Protein
CTRL Phospho
CTRL RNA
GEF Protein
GEF Phospho
gef RNA
PLX Protein
PLX Phospho
PLX RNA
GEF PLX Protein
GEF PLX Phospho
GEF PLX RNA
Concentration(n[M])
Relative Conc
Time (hour)

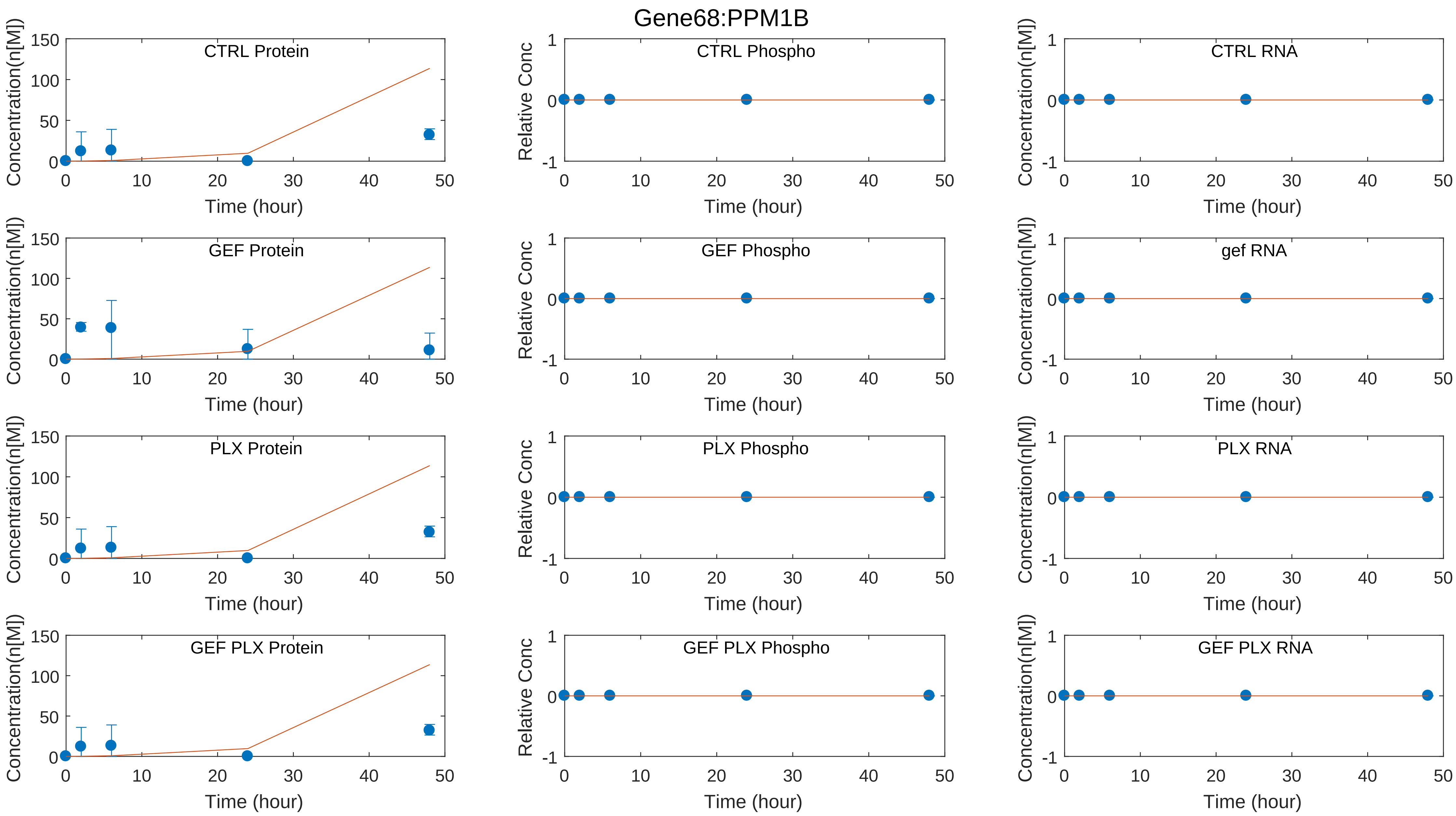
Gene68:PPM1B
CTRL Protein
CTRL Phospho
CTRL RNA
GEF Protein
GEF Phospho
gef RNA
PLX Protein
PLX Phospho
PLX RNA
GEF PLX Protein
GEF PLX Phospho
GEF PLX RNA
Concentration(n[M])
Relative Conc
Time (hour)
0
10
20
30
40
50
150
100
1
-1

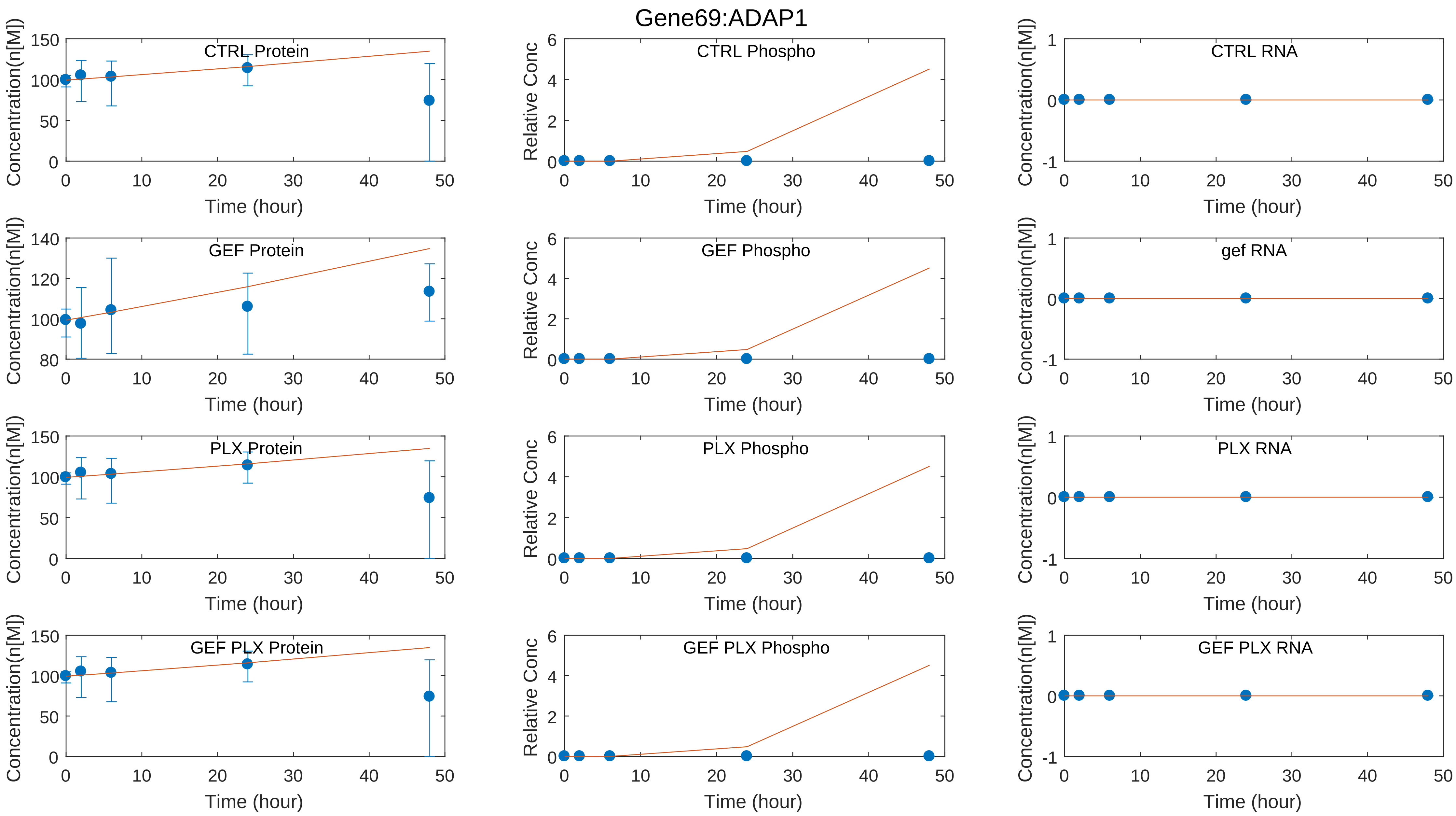

Gene69:ADAP1
CTRL Protein
GEF Protein
PLX Protein
GEF PLX Protein
CTRL Phospho
GEF Phospho
PLX Phospho
GEF PLX Phospho
CTRL RNA
gef RNA
PLX RNA
GEF PLX RNA
Concentration(n[M])
Relative Conc
Time (hour)

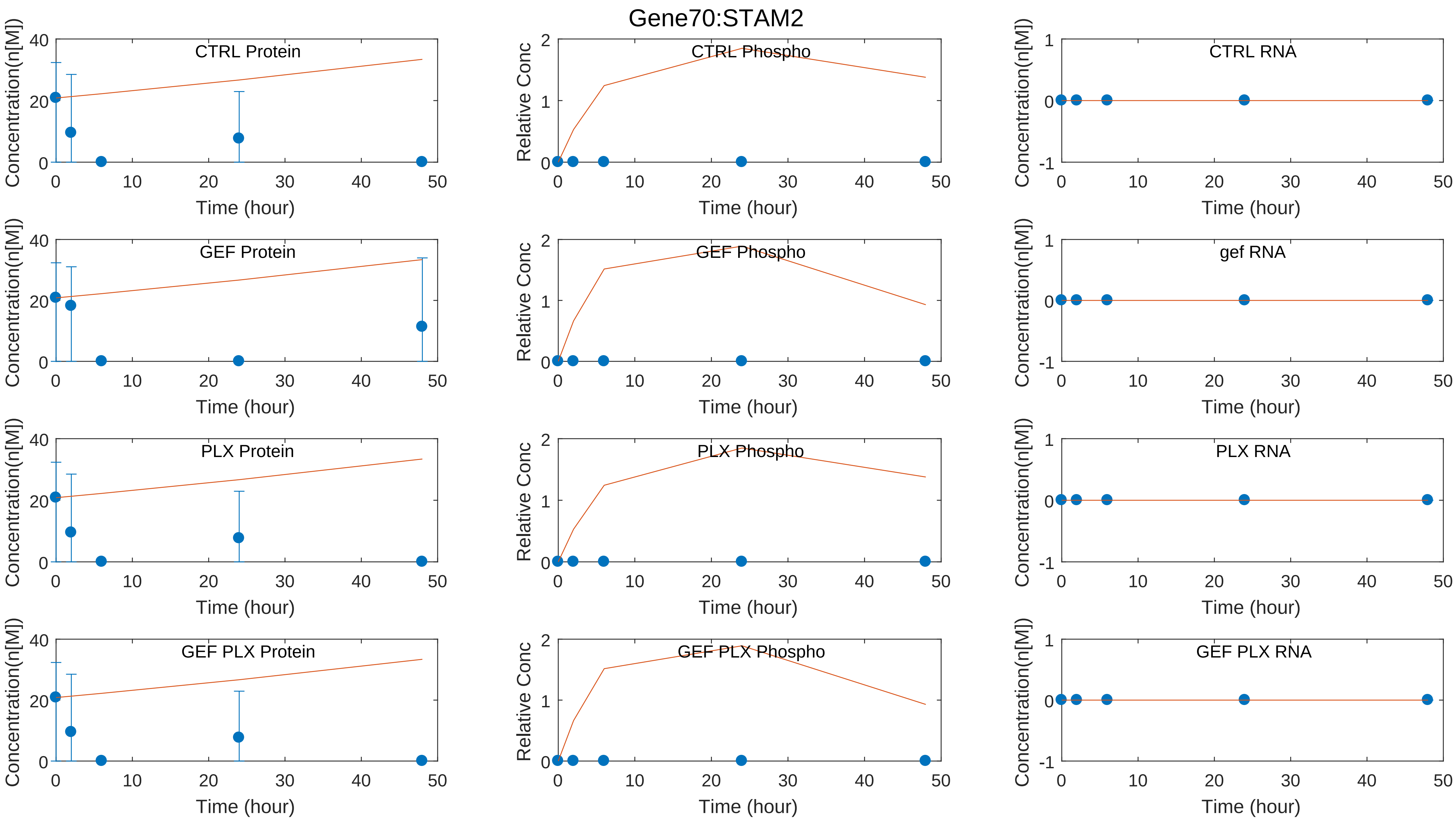

Gene70:STAM2
CTRL Protein
CTRL Phospho
CTRL RNA
GEF Protein
GEF Phospho
gef RNA
PLX Protein
PLX Phospho
PLX RNA
GEF PLX Protein
GEF PLX Phospho
GEF PLX RNA
Concentration(n[M])
Relative Conc
Time (hour)
0
10
20
30
40
50
1
2
-1

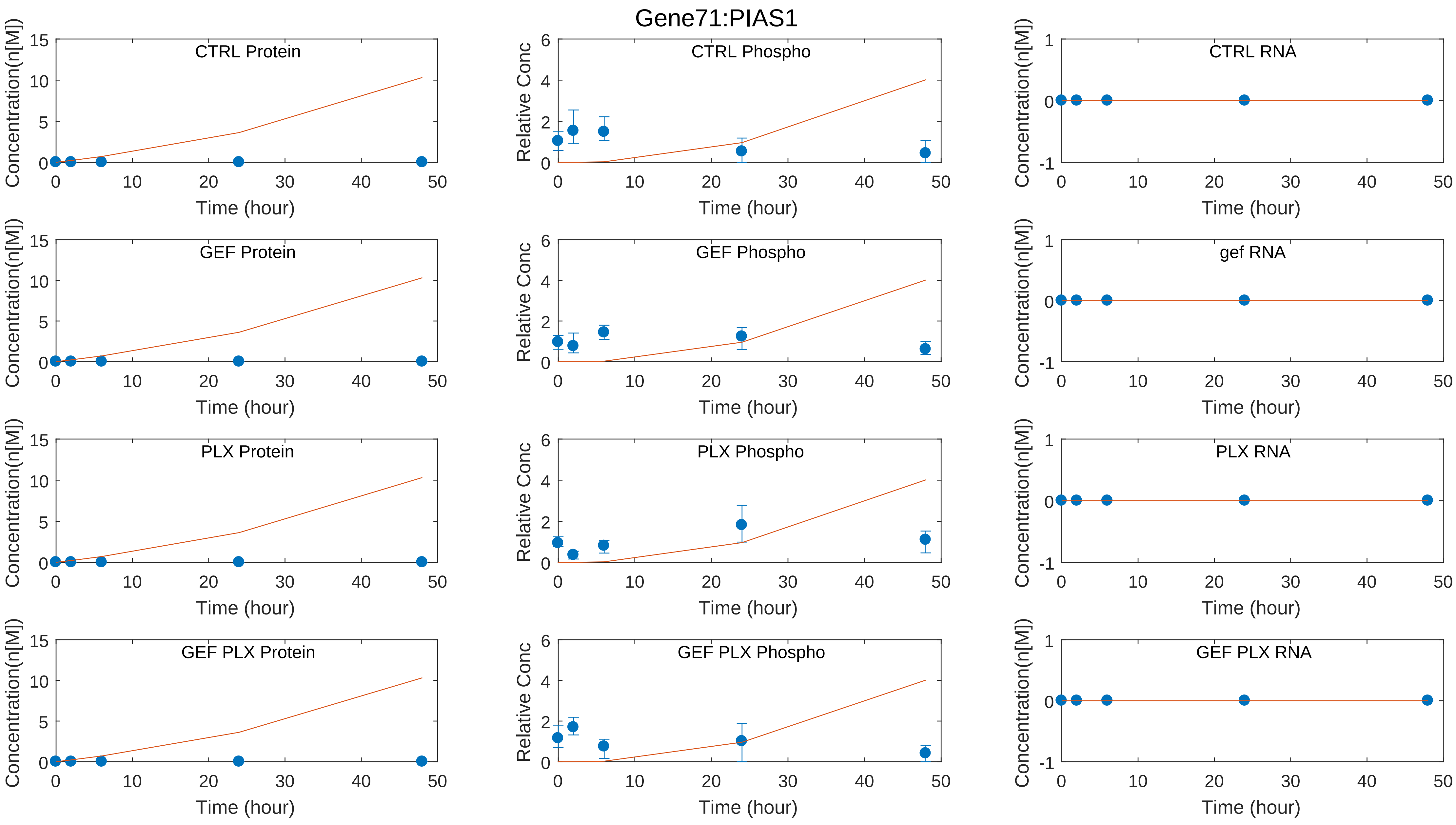

Gene71:PIAS1
CTRL Protein
GEF Protein
PLX Protein
GEF PLX Protein
CTRL Phospho
GEF Phospho
PLX Phospho
GEF PLX Phospho
CTRL RNA
gef RNA
PLX RNA
GEF PLX RNA
Concentration(n[M])
Relative Conc
Time (hour)

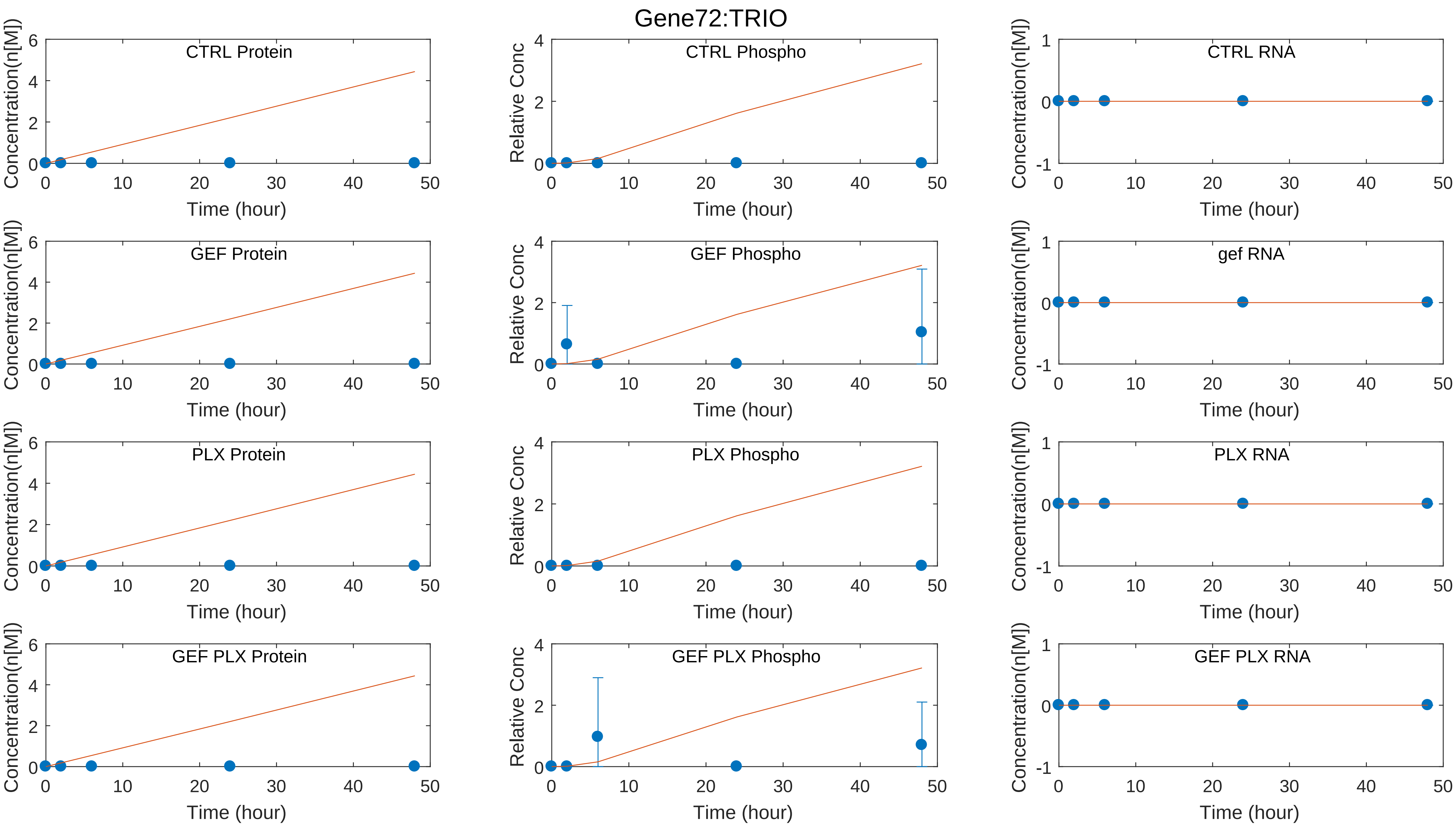
Gene72:TRIO
CTRL Protein
CTRL Phospho
CTRL RNA
GEF Protein
GEF Phospho
gef RNA
PLX Protein
PLX Phospho
PLX RNA
GEF PLX Protein
GEF PLX Phospho
GEF PLX RNA
Concentration(n[M])
Relative Conc
Time (hour)

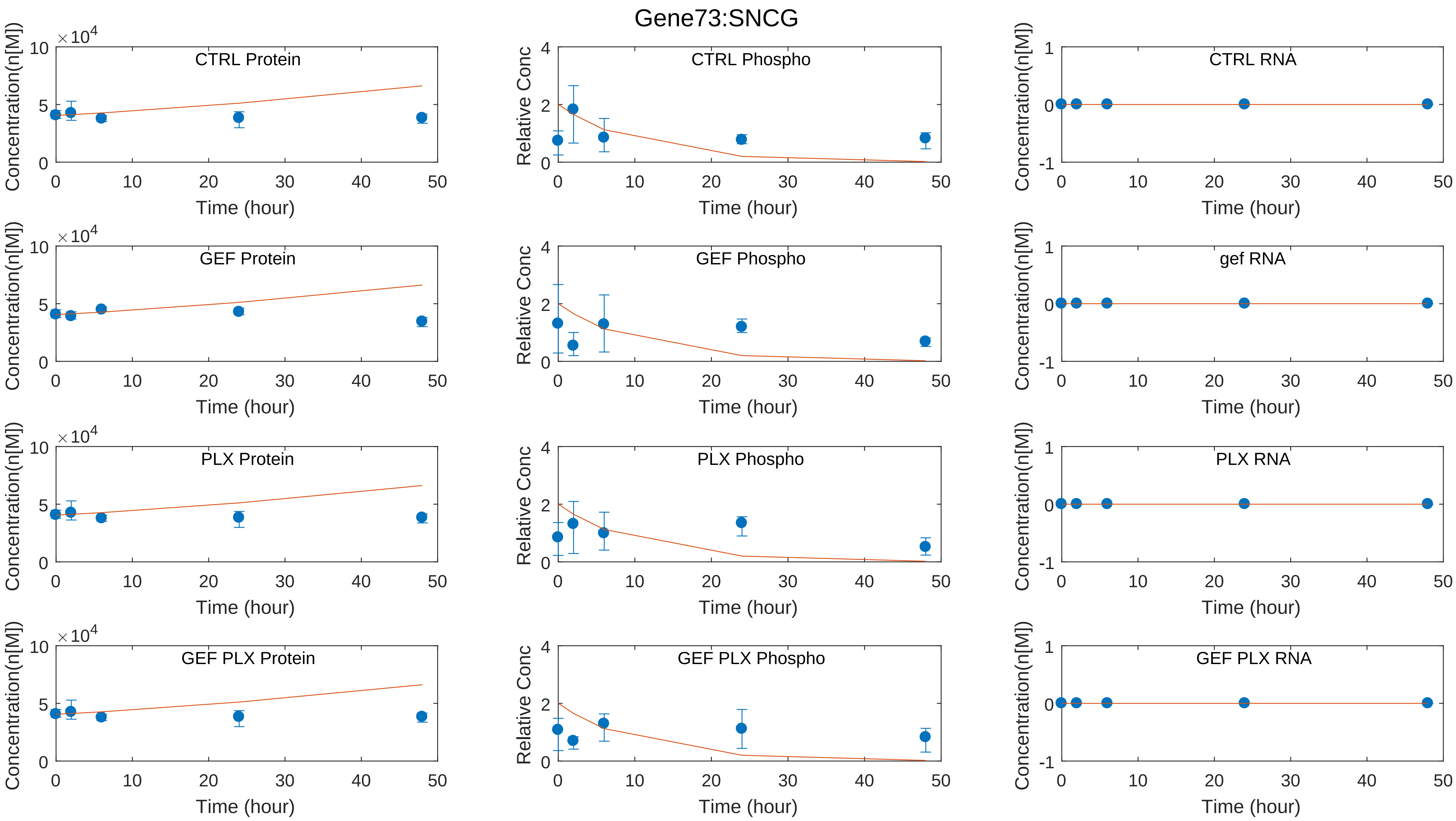

Gene73:SNCG
CTRL Protein
CTRL Phospho
CTRL RNA
GEF Protein
GEF Phospho
gef RNA
PLX Protein
PLX Phospho
PLX RNA
GEF PLX Protein
GEF PLX Phospho
GEF PLX RNA
Concentration(n[M])
Relative Conc
Time (hour)
×10^4

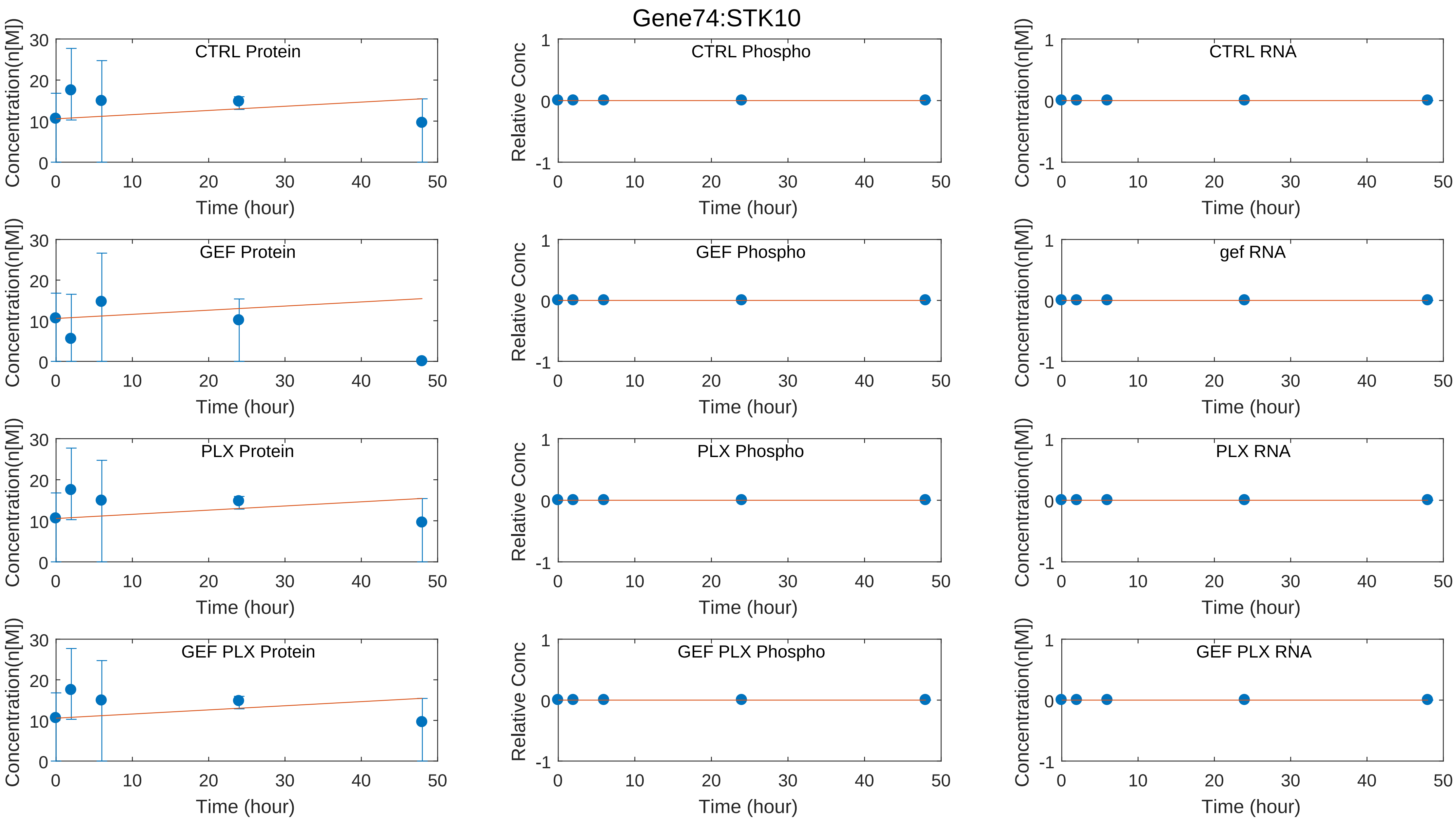

Gene74:STK10
CTRL Protein
CTRL Phospho
CTRL RNA
GEF Protein
GEF Phospho
gef RNA
PLX Protein
PLX Phospho
PLX RNA
GEF PLX Protein
GEF PLX Phospho
GEF PLX RNA
Concentration(n[M])
Relative Conc
Time (hour)

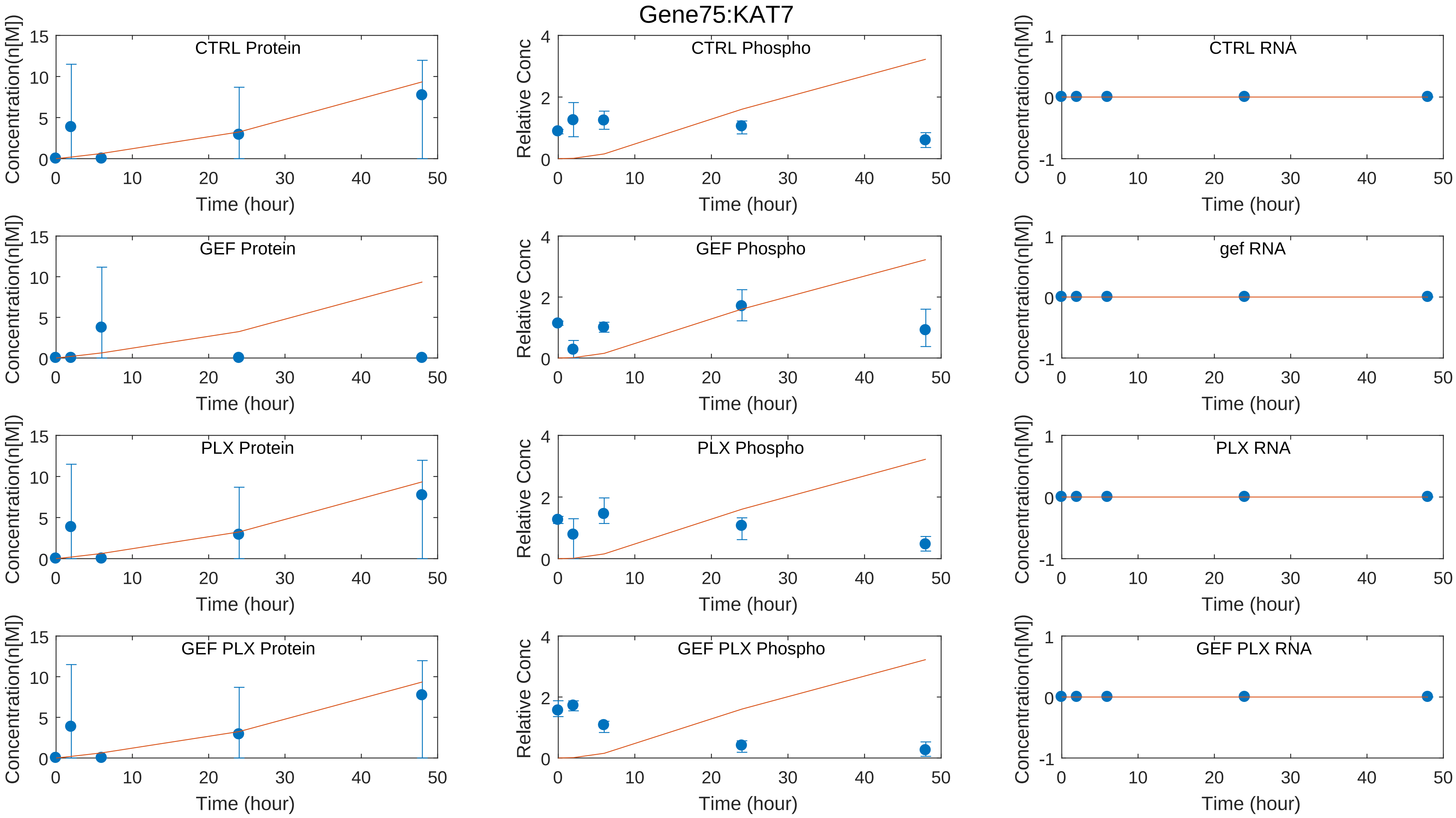
Gene75:KAT7
CTRL Protein
CTRL Phospho
CTRL RNA
GEF Protein
GEF Phospho
gef RNA
PLX Protein
PLX Phospho
PLX RNA
GEF PLX Protein
GEF PLX Phospho
GEF PLX RNA
Concentration(n[M])
Relative Conc
Time (hour)

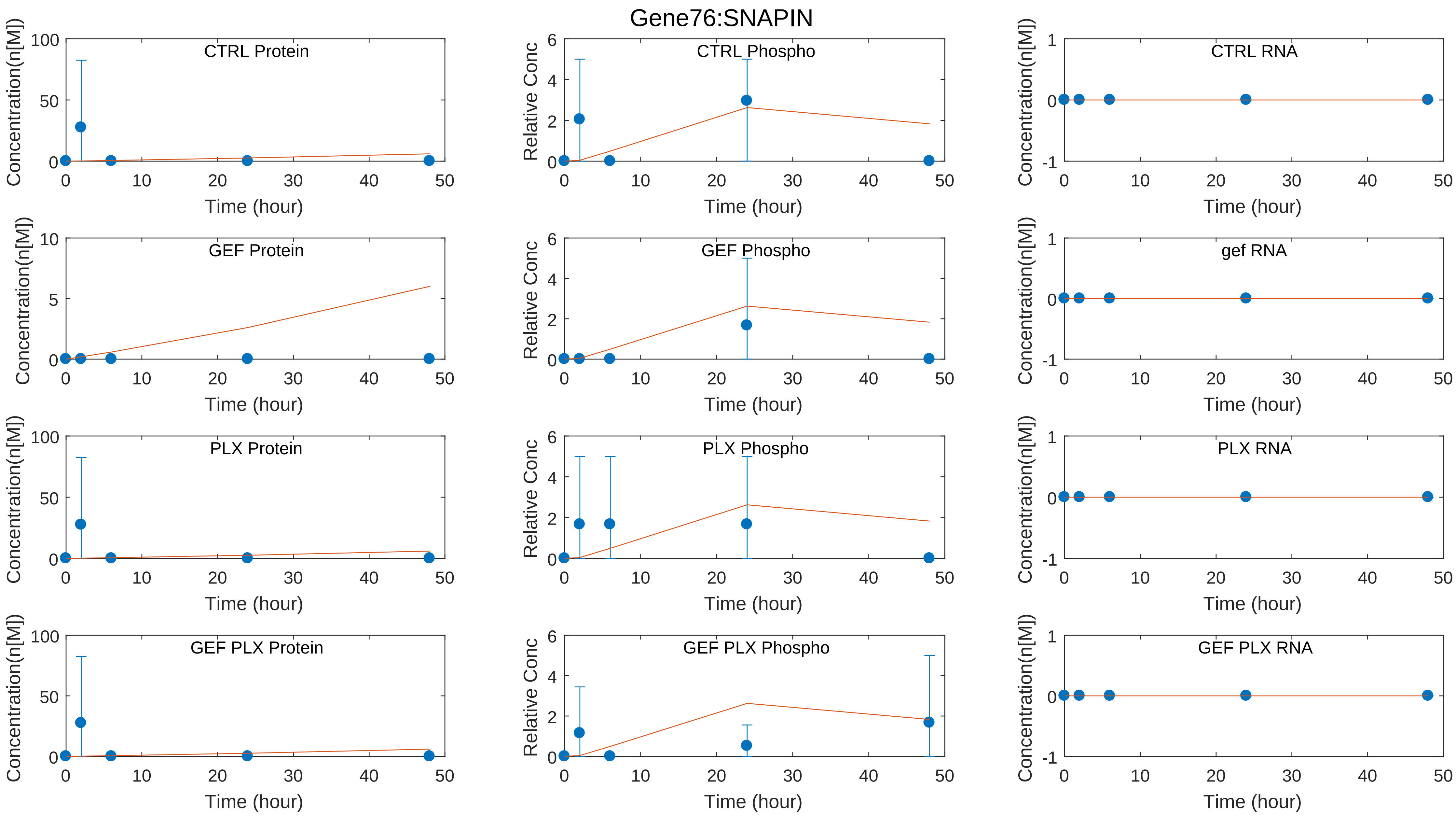

Gene76:SNAPIN
CTRL Protein
CTRL Phospho
CTRL RNA
GEF Protein
GEF Phospho
gef RNA
PLX Protein
PLX Phospho
PLX RNA
GEF PLX Protein
GEF PLX Phospho
GEF PLX RNA
Concentration(n[M])
Relative Conc
Time (hour)

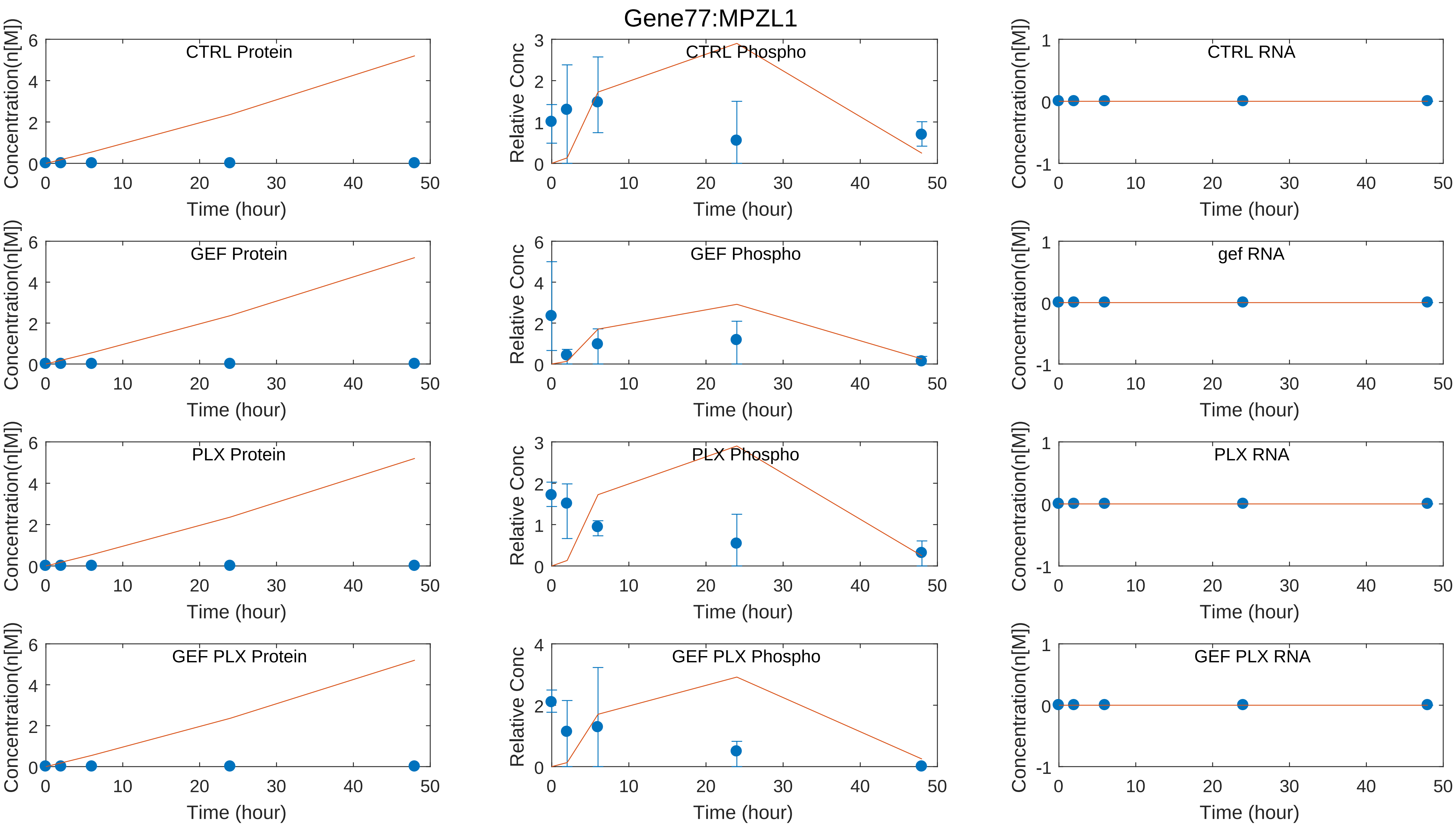
Gene77:MPZL1
CTRL Protein
CTRL Phospho
CTRL RNA
GEF Protein
GEF Phospho
gef RNA
PLX Protein
PLX Phospho
PLX RNA
GEF PLX Protein
GEF PLX Phospho
GEF PLX RNA
Concentration(n[M])
Relative Conc
Time (hour)

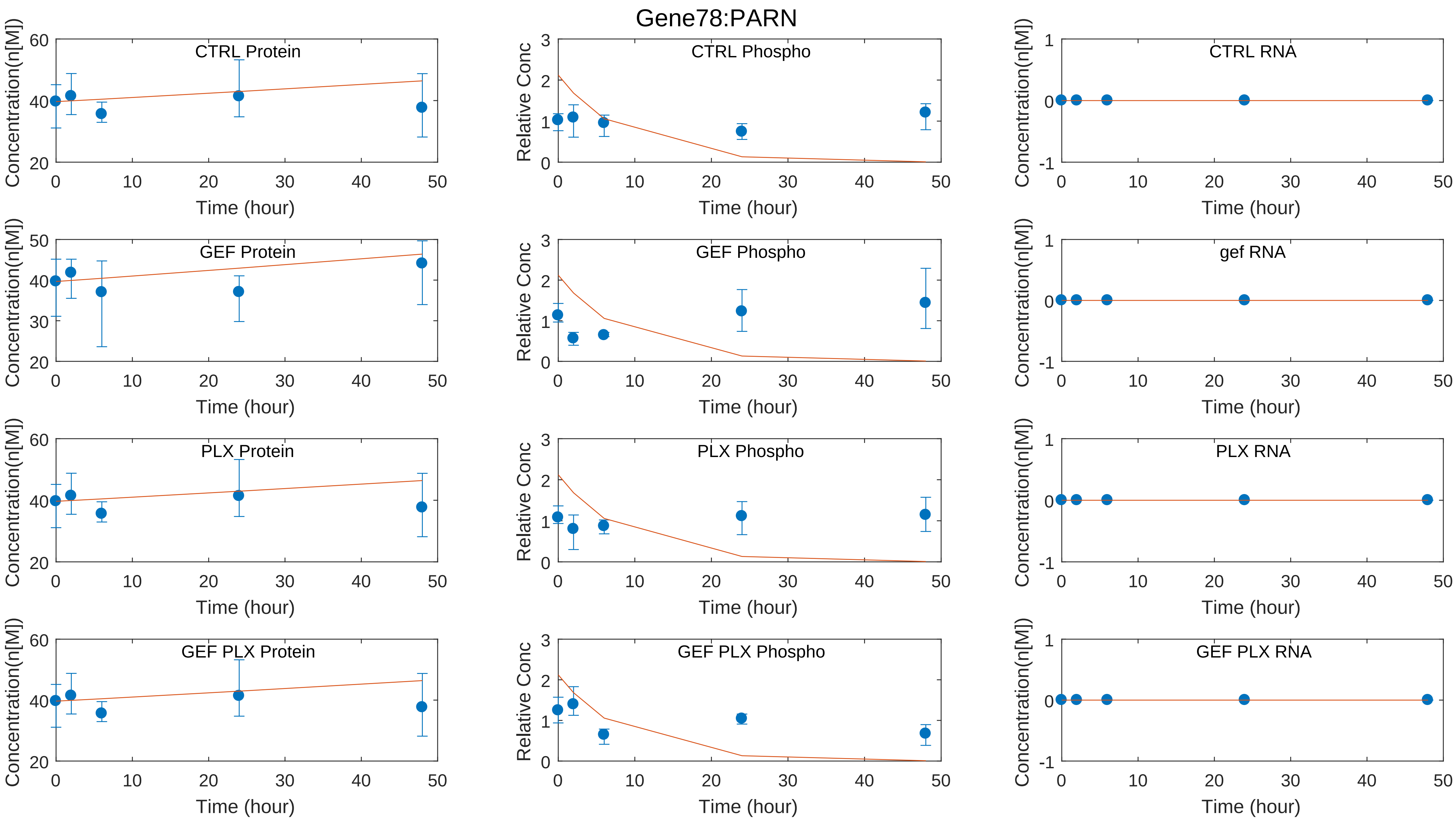
Gene78:PARN
CTRL Protein
CTRL Phospho
CTRL RNA
GEF Protein
GEF Phospho
gef RNA
PLX Protein
PLX Phospho
PLX RNA
GEF PLX Protein
GEF PLX Phospho
GEF PLX RNA
Concentration(n[M])
Relative Conc
Time (hour)

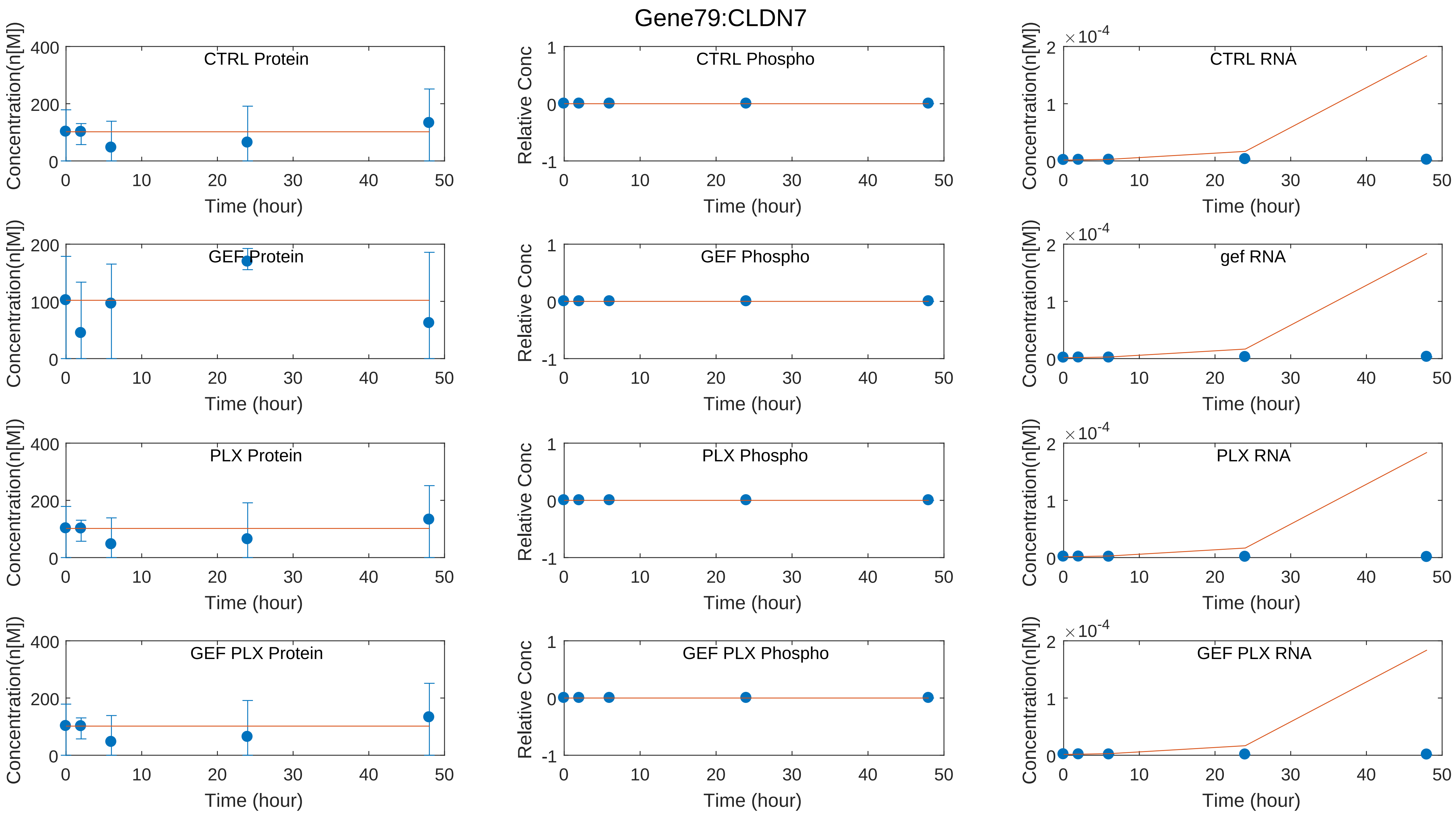

Gene79:CLDN7
CTRL Protein
CTRL Phospho
CTRL RNA
GEF Protein
GEF Phospho
gef RNA
PLX Protein
PLX Phospho
PLX RNA
GEF PLX Protein
GEF PLX Phospho
GEF PLX RNA
Concentration(n[M])
Relative Conc
Time (hour)
×10-4

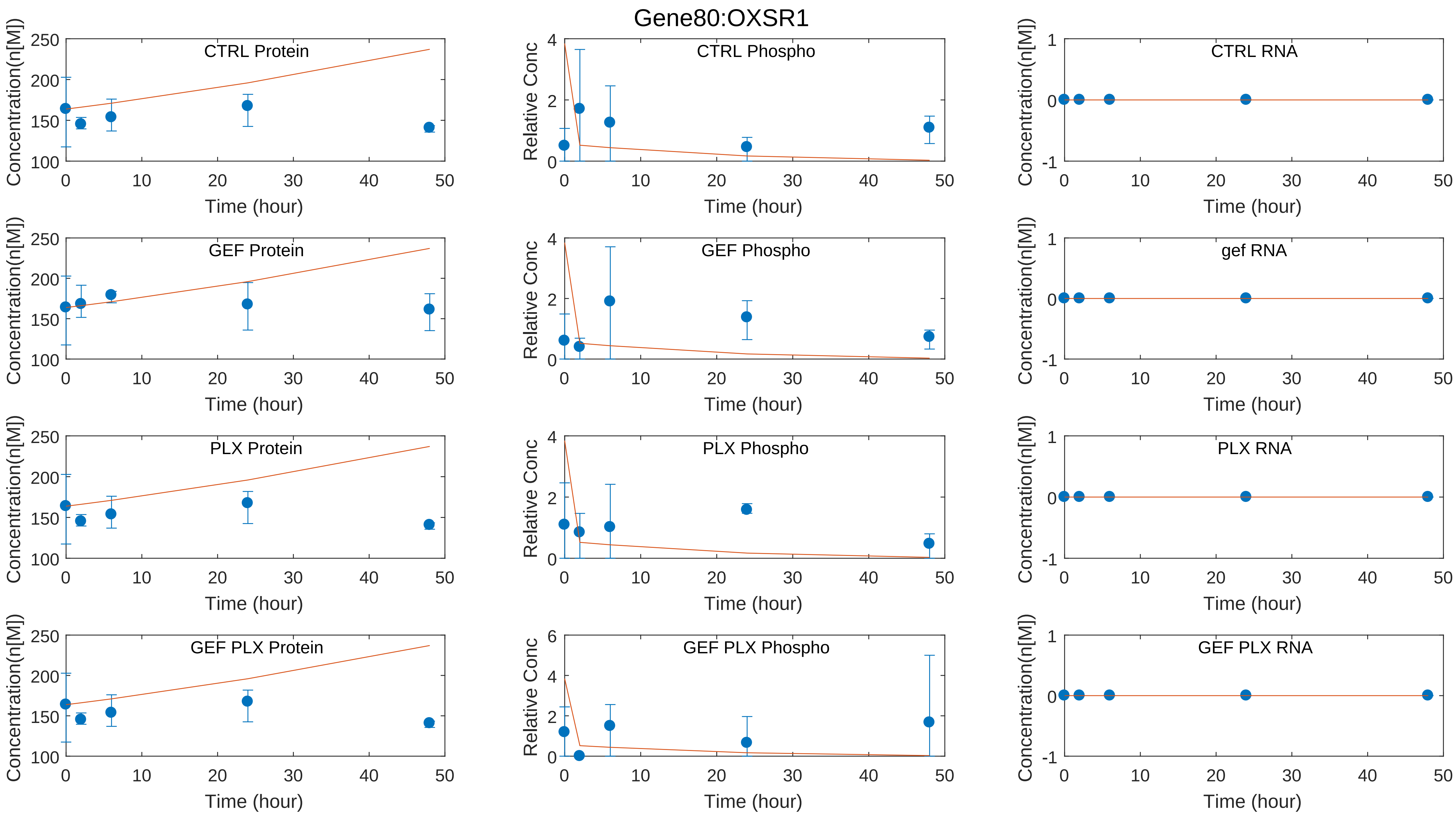

Gene80:OXSR1
CTRL Protein
GEF Protein
PLX Protein
GEF PLX Protein
CTRL Phospho
GEF Phospho
PLX Phospho
GEF PLX Phospho
CTRL RNA
gef RNA
PLX RNA
GEF PLX RNA
Concentration(n[M])
Relative Conc
Time (hour)

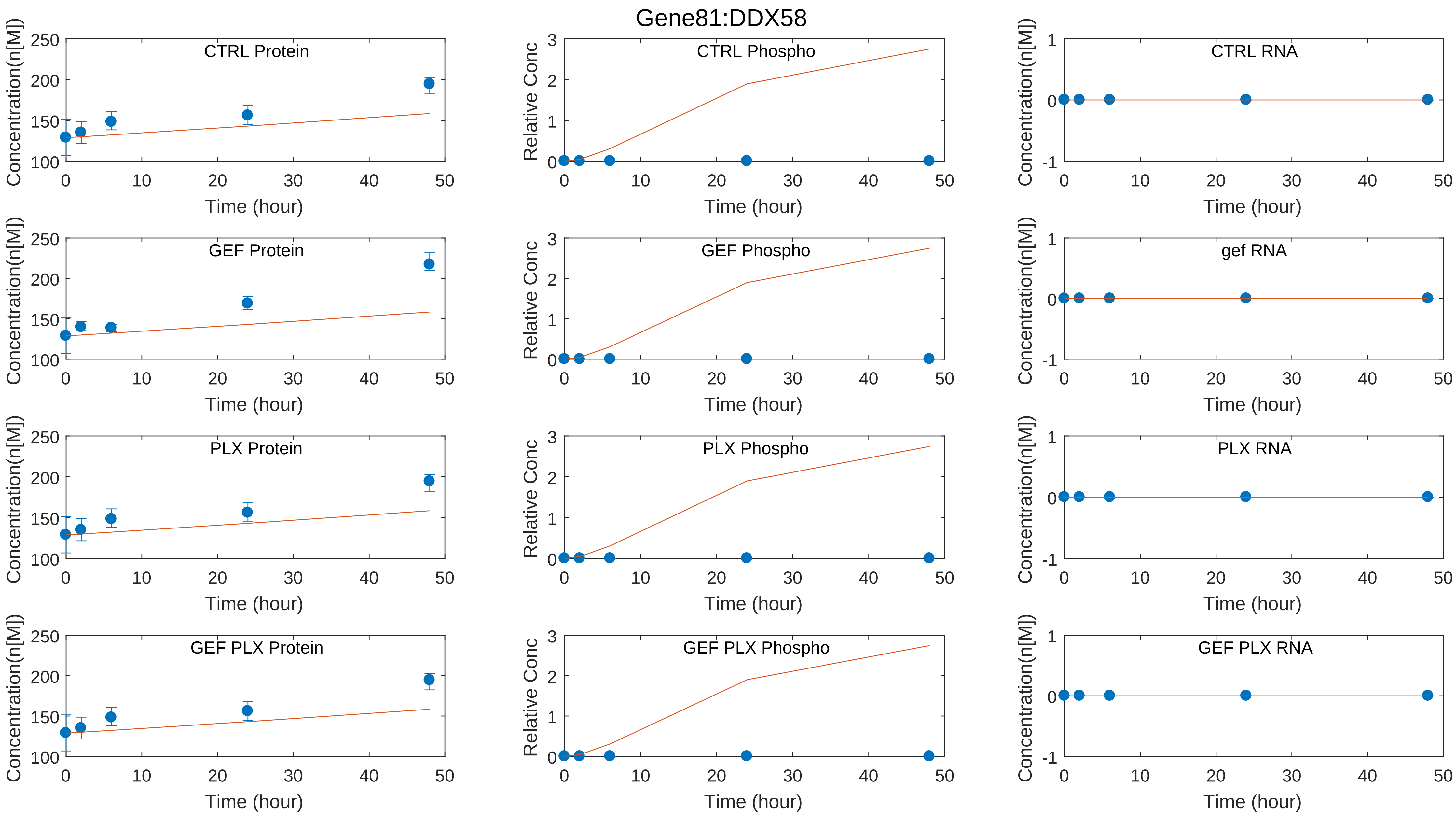

Gene81:DDX58
CTRL Protein
CTRL Phospho
CTRL RNA
GEF Protein
GEF Phospho
gef RNA
PLX Protein
PLX Phospho
PLX RNA
GEF PLX Protein
GEF PLX Phospho
GEF PLX RNA
Concentration(n[M])
Relative Conc
Time (hour)

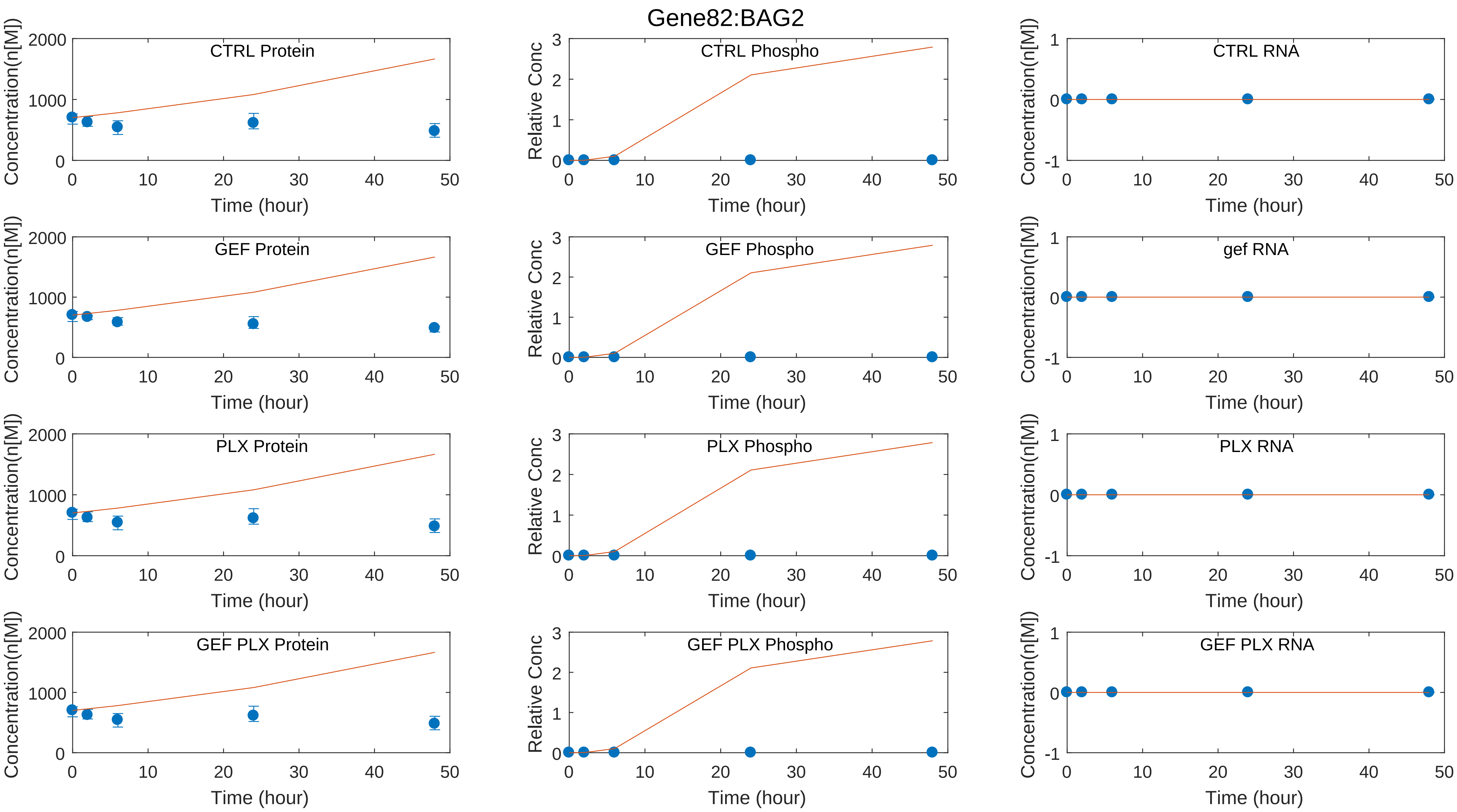
Gene82:BAG2
CTRL Protein
GEF Protein
PLX Protein
GEF PLX Protein
CTRL Phospho
GEF Phospho
PLX Phospho
GEF PLX Phospho
CTRL RNA
gef RNA
PLX RNA
GEF PLX RNA
Concentration(n[M])
Relative Conc
Time (hour)
0
10
20
30
40
50
1000
2000
1
2
3
-1

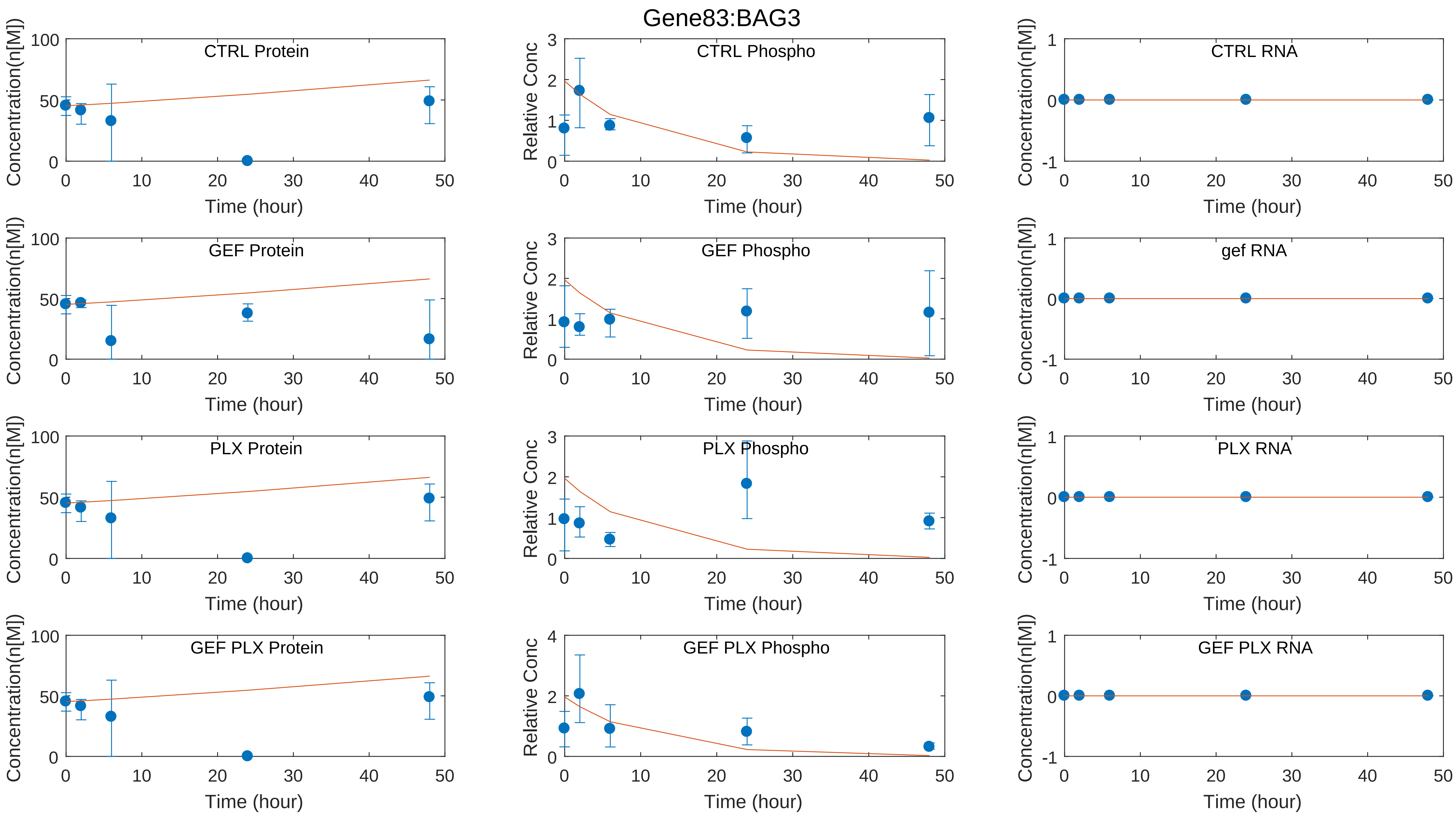

Gene83:BAG3
CTRL Protein
CTRL Phospho
CTRL RNA
GEF Protein
GEF Phospho
gef RNA
PLX Protein
PLX Phospho
PLX RNA
GEF PLX Protein
GEF PLX Phospho
GEF PLX RNA
Concentration(n[M])
Relative Conc
Time (hour)

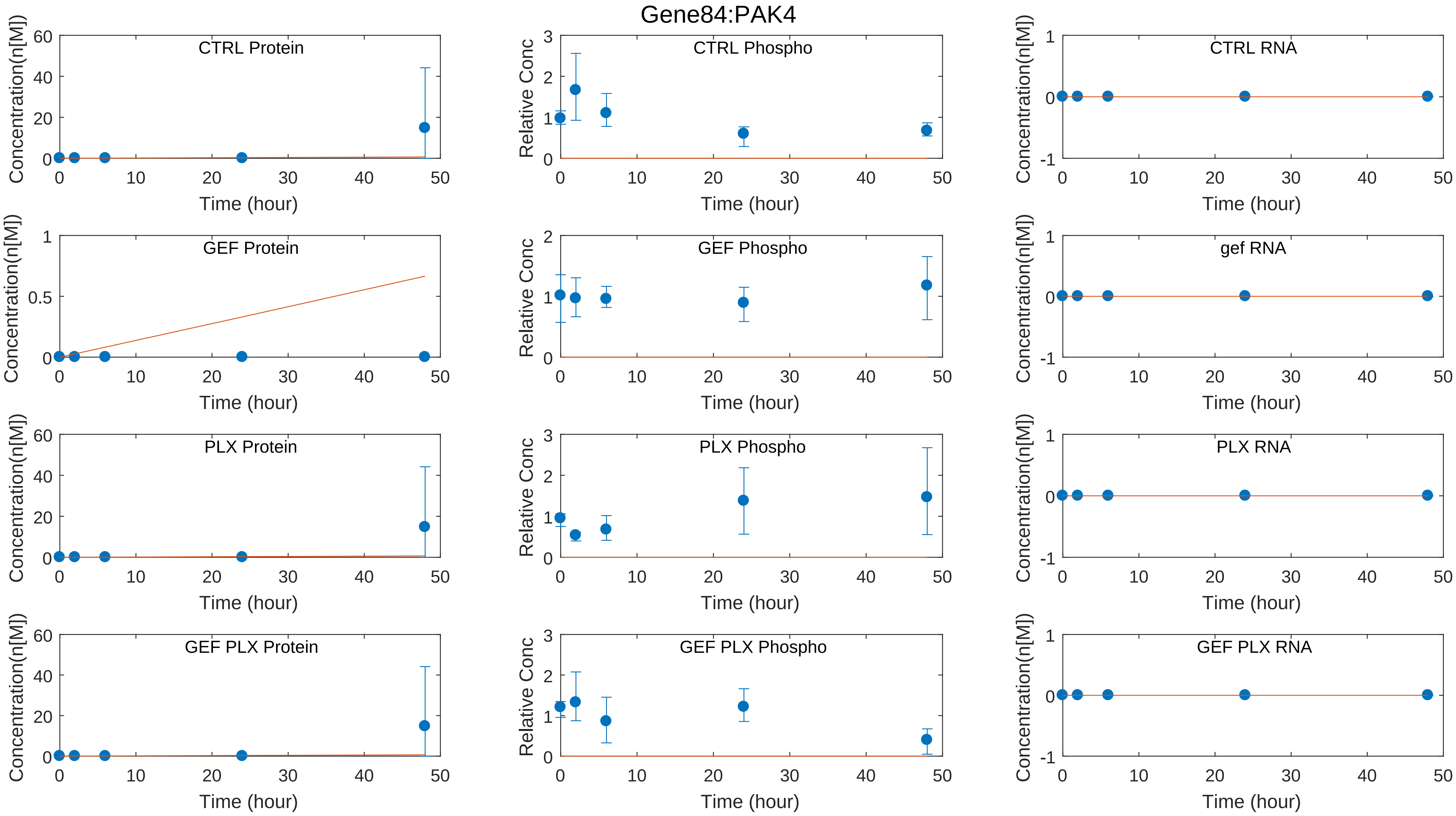
Gene84:PAK4
CTRL Protein
CTRL Phospho
CTRL RNA
GEF Protein
GEF Phospho
gef RNA
PLX Protein
PLX Phospho
PLX RNA
GEF PLX Protein
GEF PLX Phospho
GEF PLX RNA
Concentration(n[M])
Relative Conc
Time (hour)

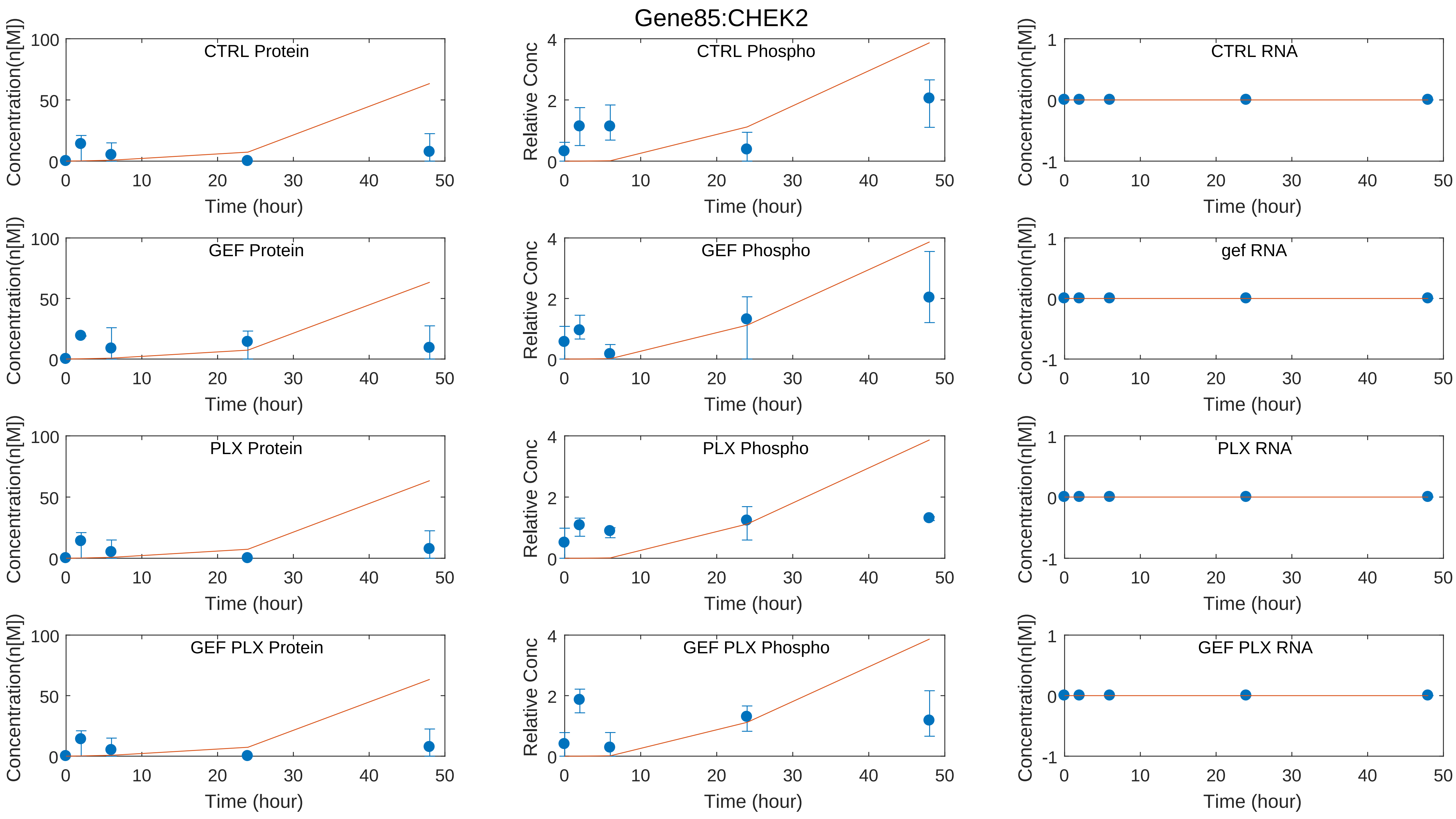
Gene85:CHEK2
CTRL Protein
CTRL Phospho
CTRL RNA
GEF Protein
GEF Phospho
gef RNA
PLX Protein
PLX Phospho
PLX RNA
GEF PLX Protein
GEF PLX Phospho
GEF PLX RNA
Concentration(n[M])
Relative Conc
Time (hour)
0
10
20
30
40
50
100
4
2
1
-1

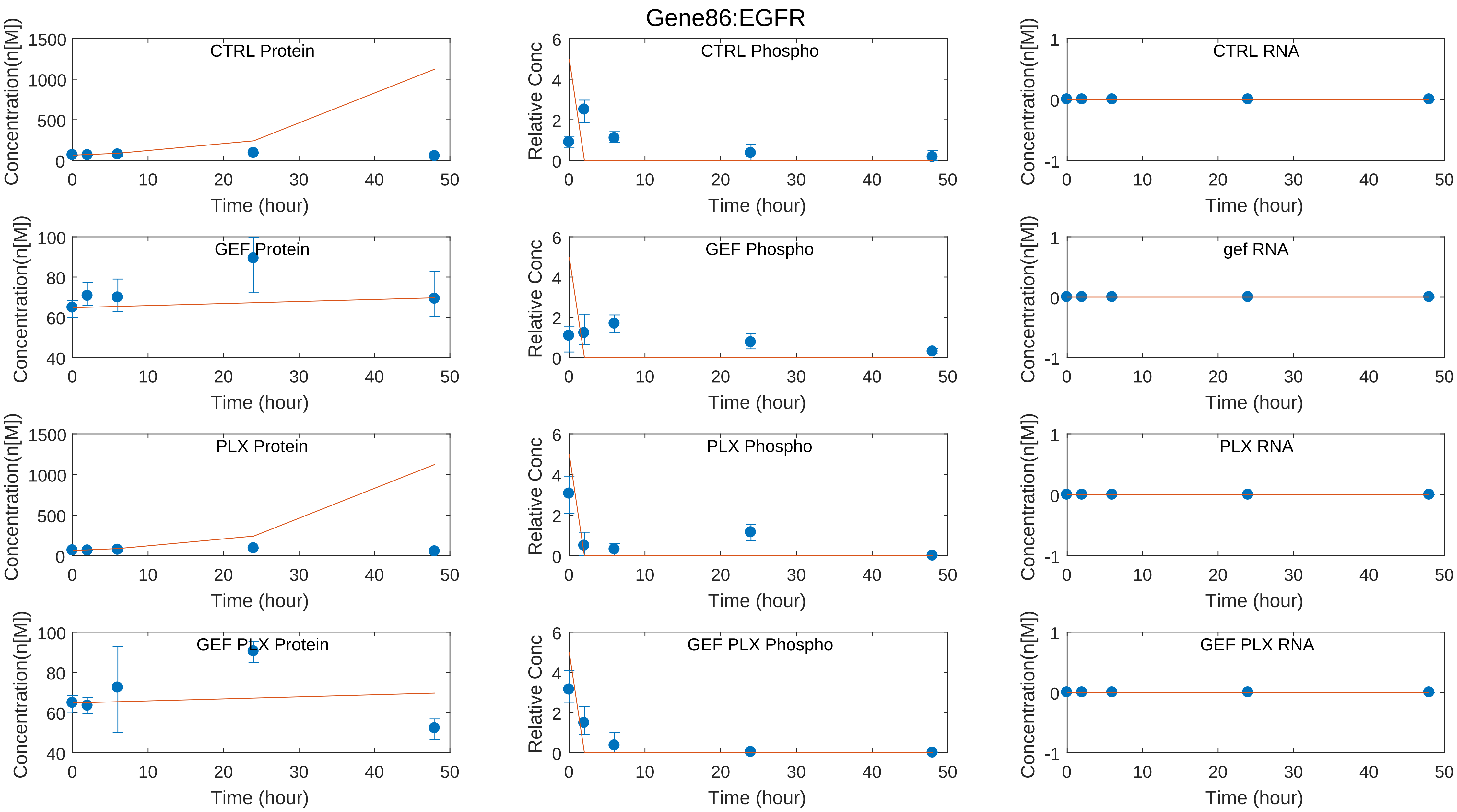
Gene86:EGFR
CTRL Protein
CTRL Phospho
CTRL RNA
GEF Protein
GEF Phospho
gef RNA
PLX Protein
PLX Phospho
PLX RNA
GEF PLX Protein
GEF PLX Phospho
GEF PLX RNA
Concentration(n[M])
Relative Conc
Time (hour)

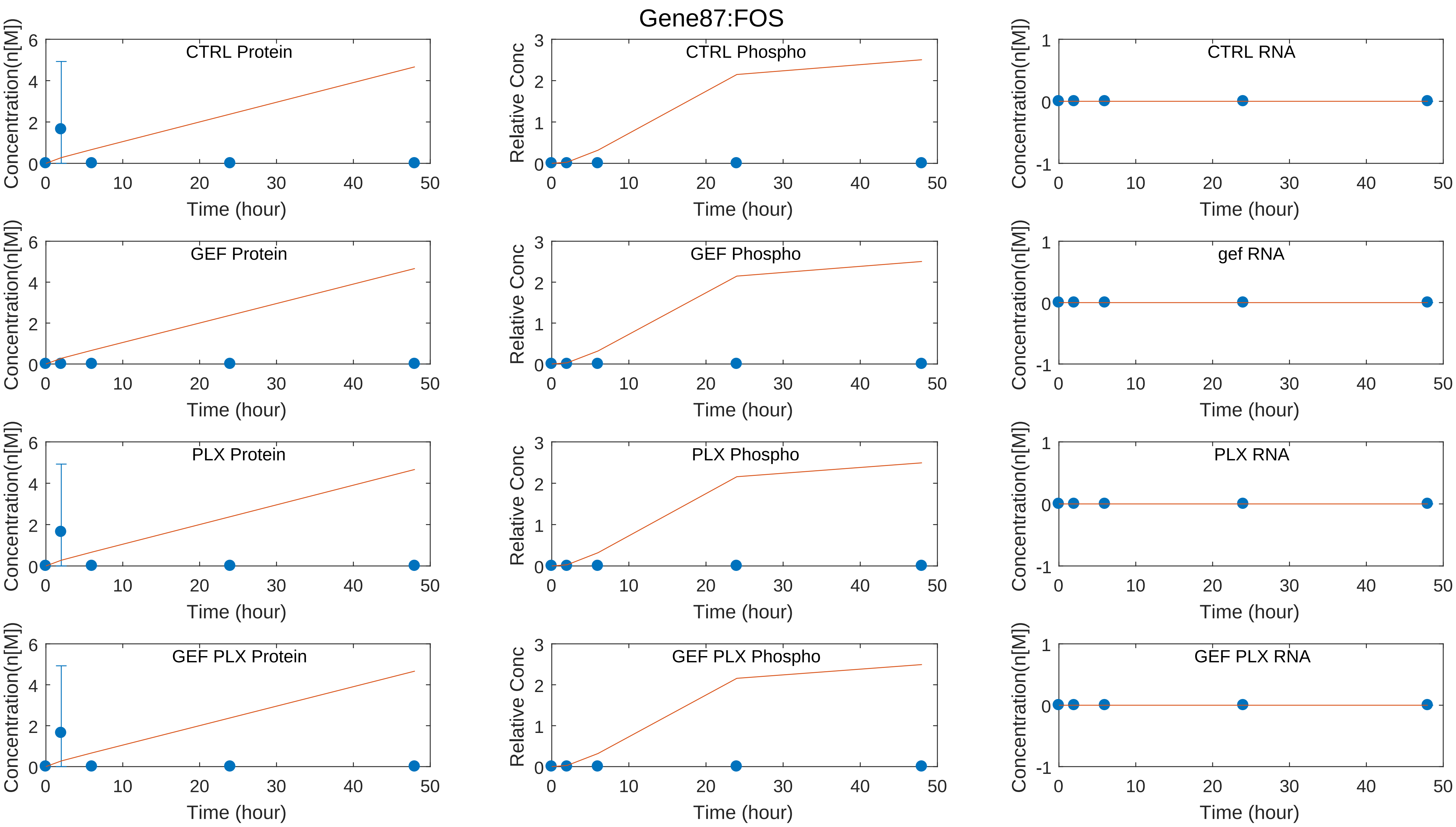
Gene87:FOS
CTRL Protein
CTRL Phospho
CTRL RNA
GEF Protein
GEF Phospho
gef RNA
PLX Protein
PLX Phospho
PLX RNA
GEF PLX Protein
GEF PLX Phospho
GEF PLX RNA
Concentration(n[M])
Relative Conc
Time (hour)
0
10
20
30
40
50
0
2
4
6
1
3
-1

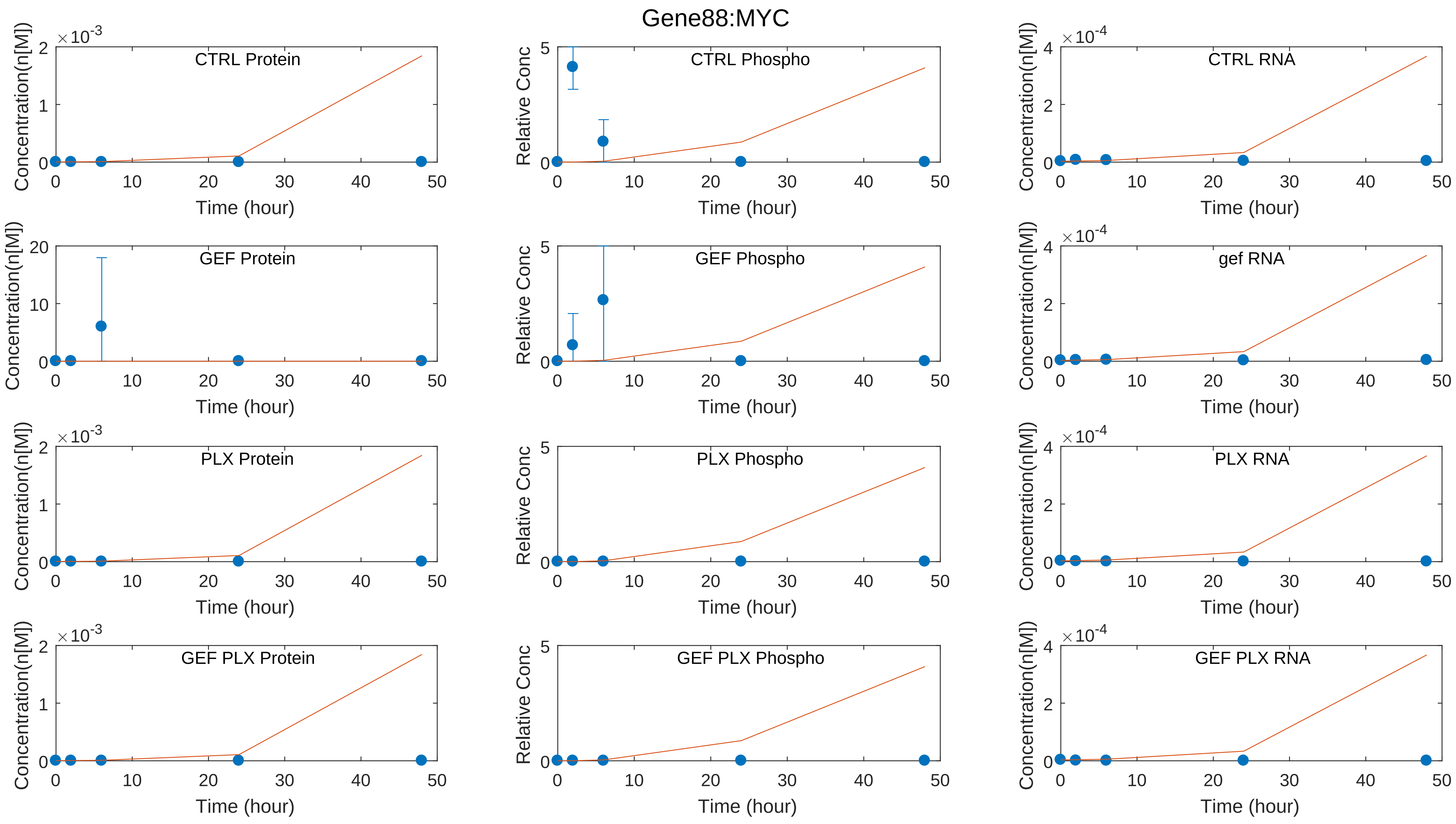

Gene88:MYC
CTRL Protein
CTRL Phospho
CTRL RNA
GEF Protein
GEF Phospho
gef RNA
PLX Protein
PLX Phospho
PLX RNA
GEF PLX Protein
GEF PLX Phospho
GEF PLX RNA
Concentration(n[M])
Relative Conc
Time (hour)
×10-3
×10-4

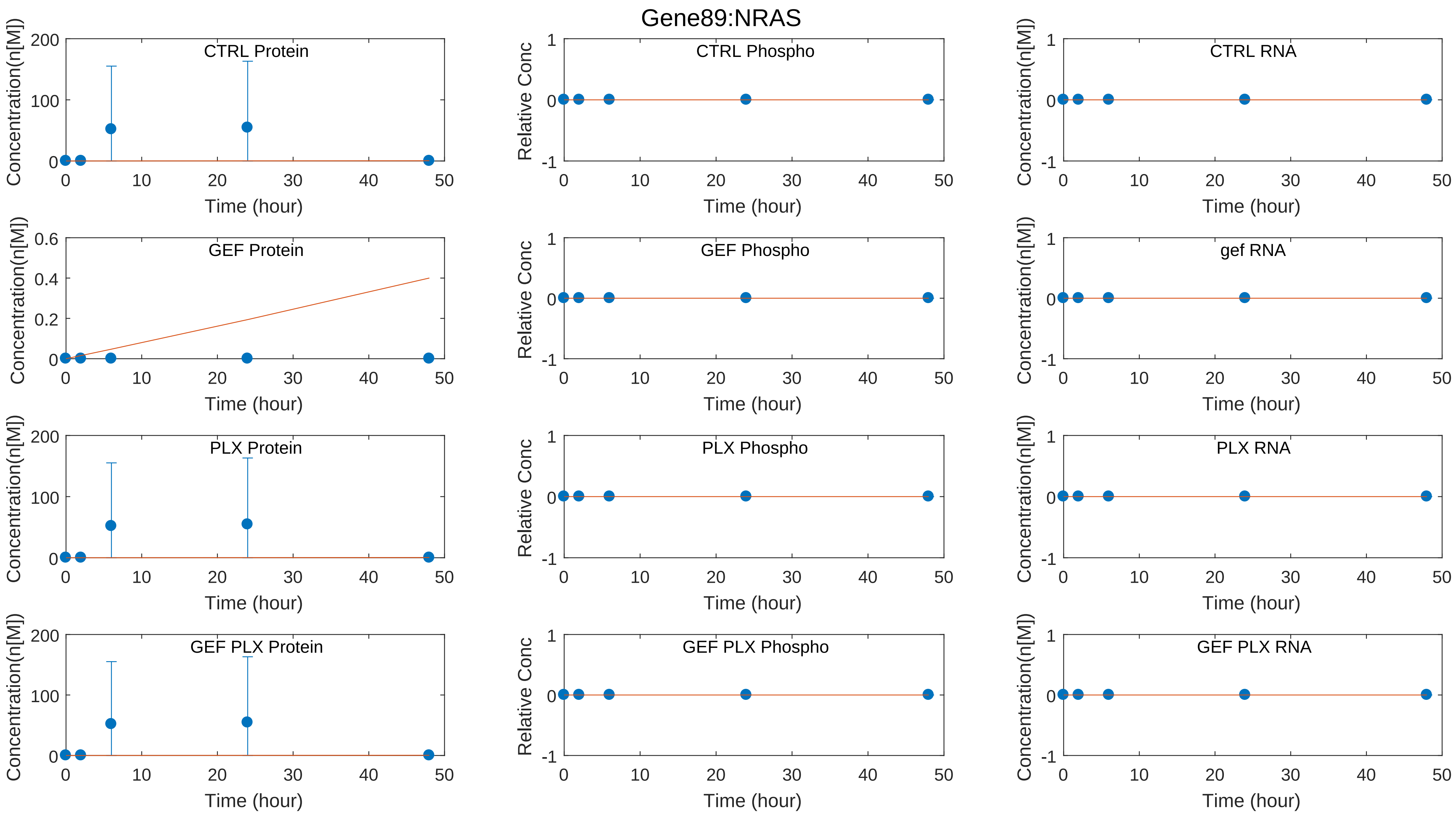
Gene89:NRAS
CTRL Protein
CTRL Phospho
CTRL RNA
GEF Protein
GEF Phospho
gef RNA
PLX Protein
PLX Phospho
PLX RNA
GEF PLX Protein
GEF PLX Phospho
GEF PLX RNA
Concentration(n[M])
Relative Conc
Time (hour)

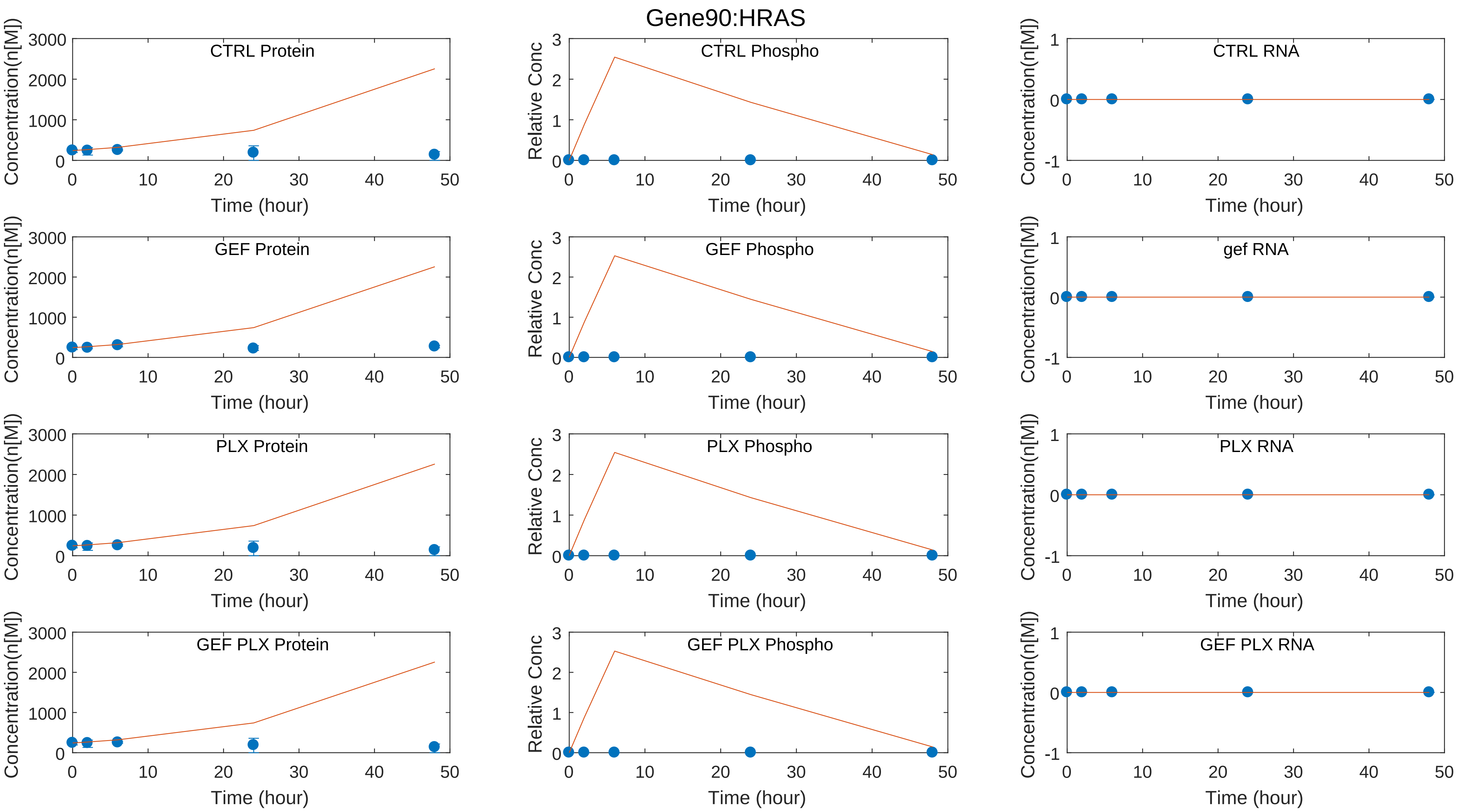

Gene90:HRAS
CTRL Protein
CTRL Phospho
CTRL RNA
GEF Protein
GEF Phospho
gef RNA
PLX Protein
PLX Phospho
PLX RNA
GEF PLX Protein
GEF PLX Phospho
GEF PLX RNA
Concentration(n[M])
Relative Conc
Time (hour)

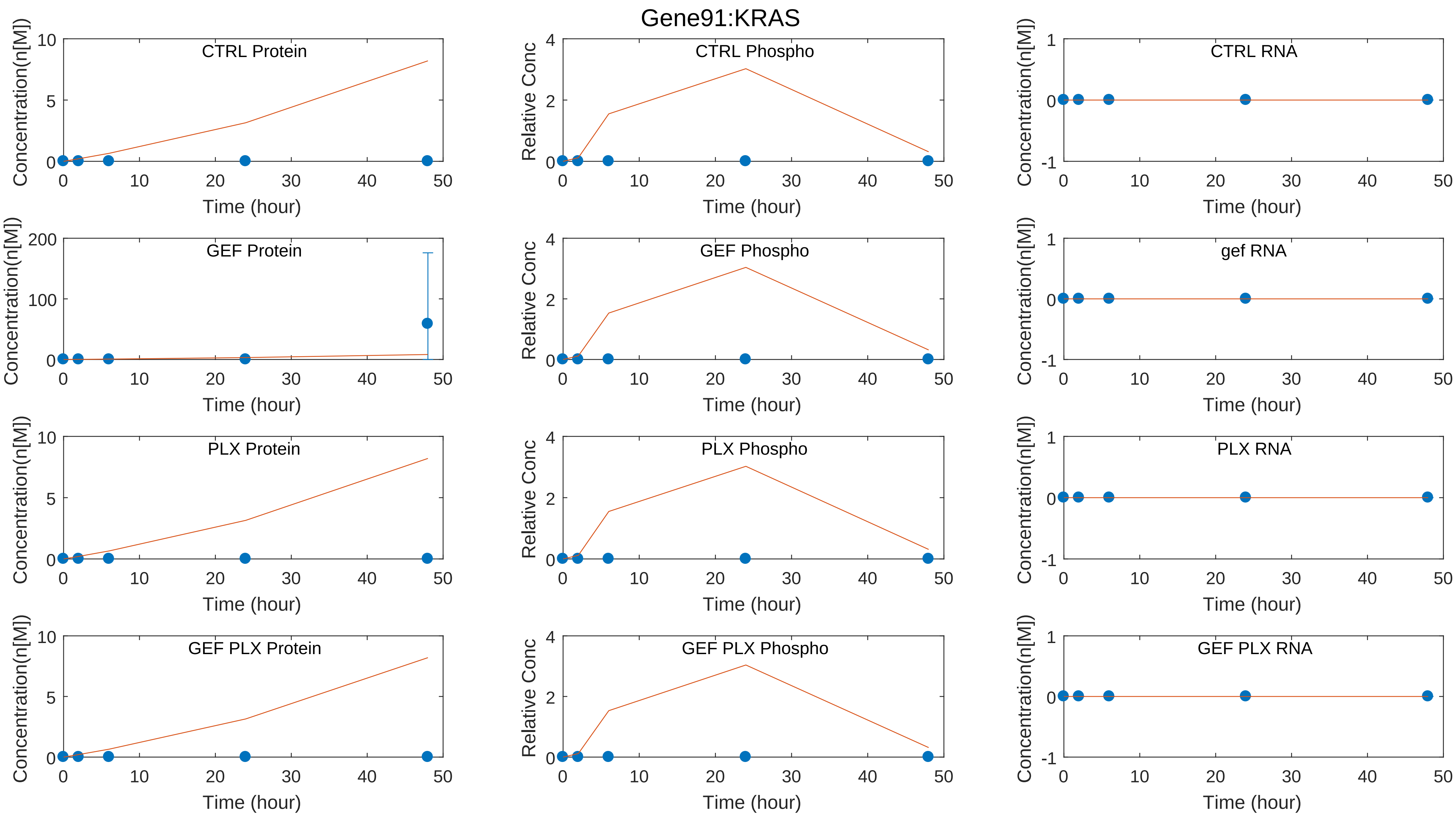

Gene91:KRAS
CTRL Protein
CTRL Phospho
CTRL RNA
GEF Protein
GEF Phospho
gef RNA
PLX Protein
PLX Phospho
PLX RNA
GEF PLX Protein
GEF PLX Phospho
GEF PLX RNA
Concentration(n[M])
Relative Conc
Time (hour)

Gene92:TGFB1

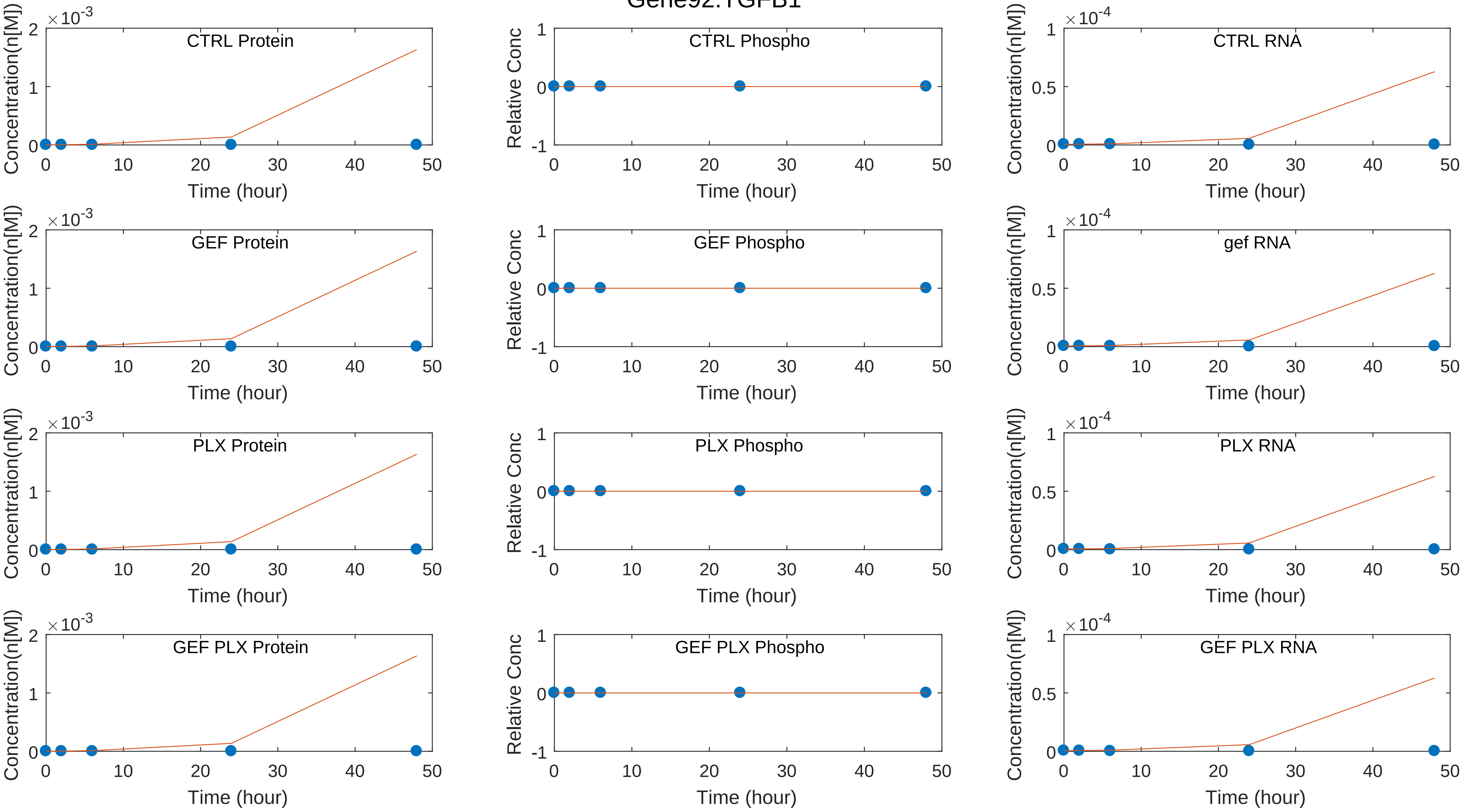

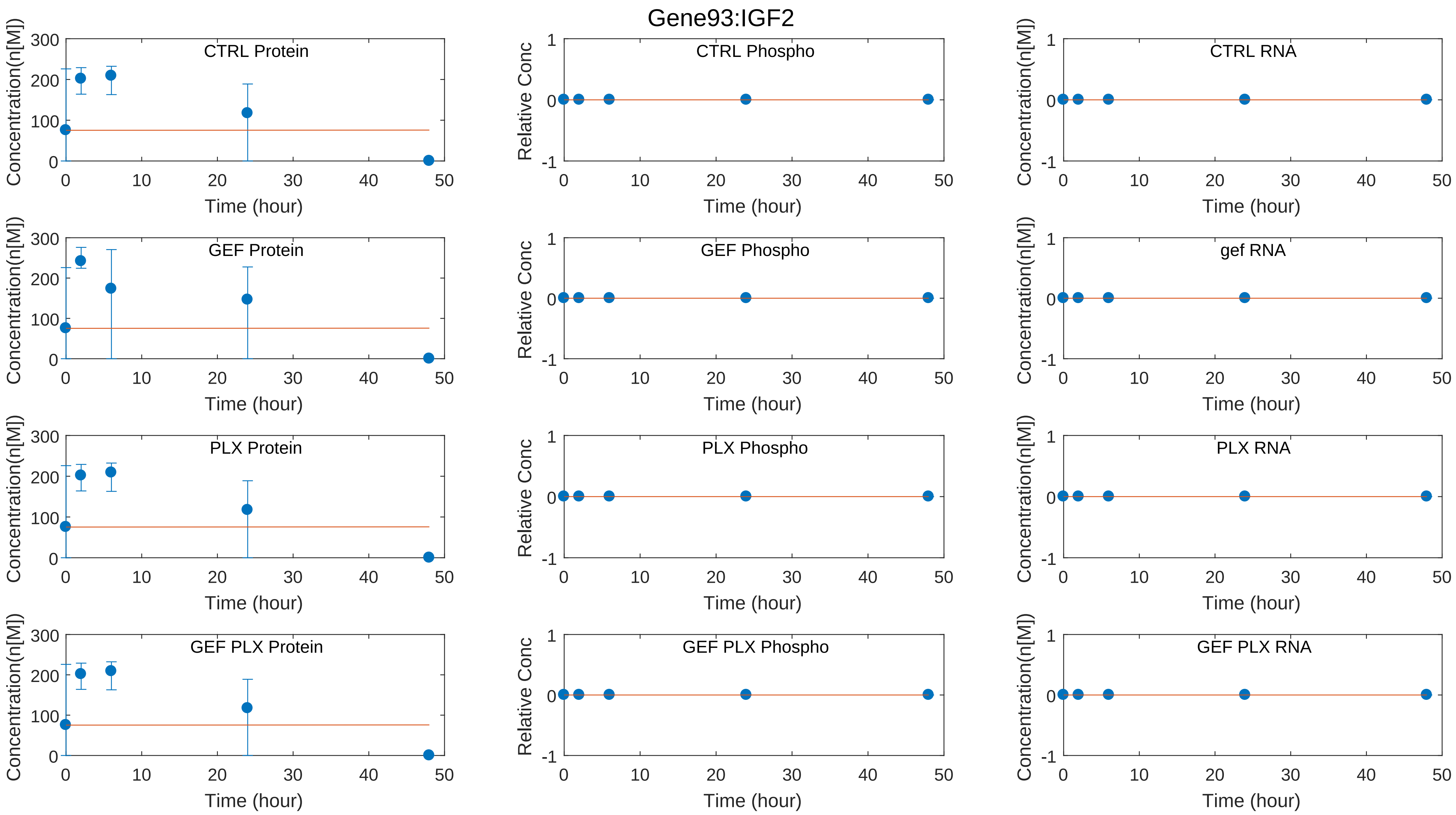
Gene93:IGF2
CTRL Protein
CTRL Phospho
CTRL RNA
GEF Protein
GEF Phospho
gef RNA
PLX Protein
PLX Phospho
PLX RNA
GEF PLX Protein
GEF PLX Phospho
GEF PLX RNA
Concentration(n[M])
Relative Conc
Time (hour)

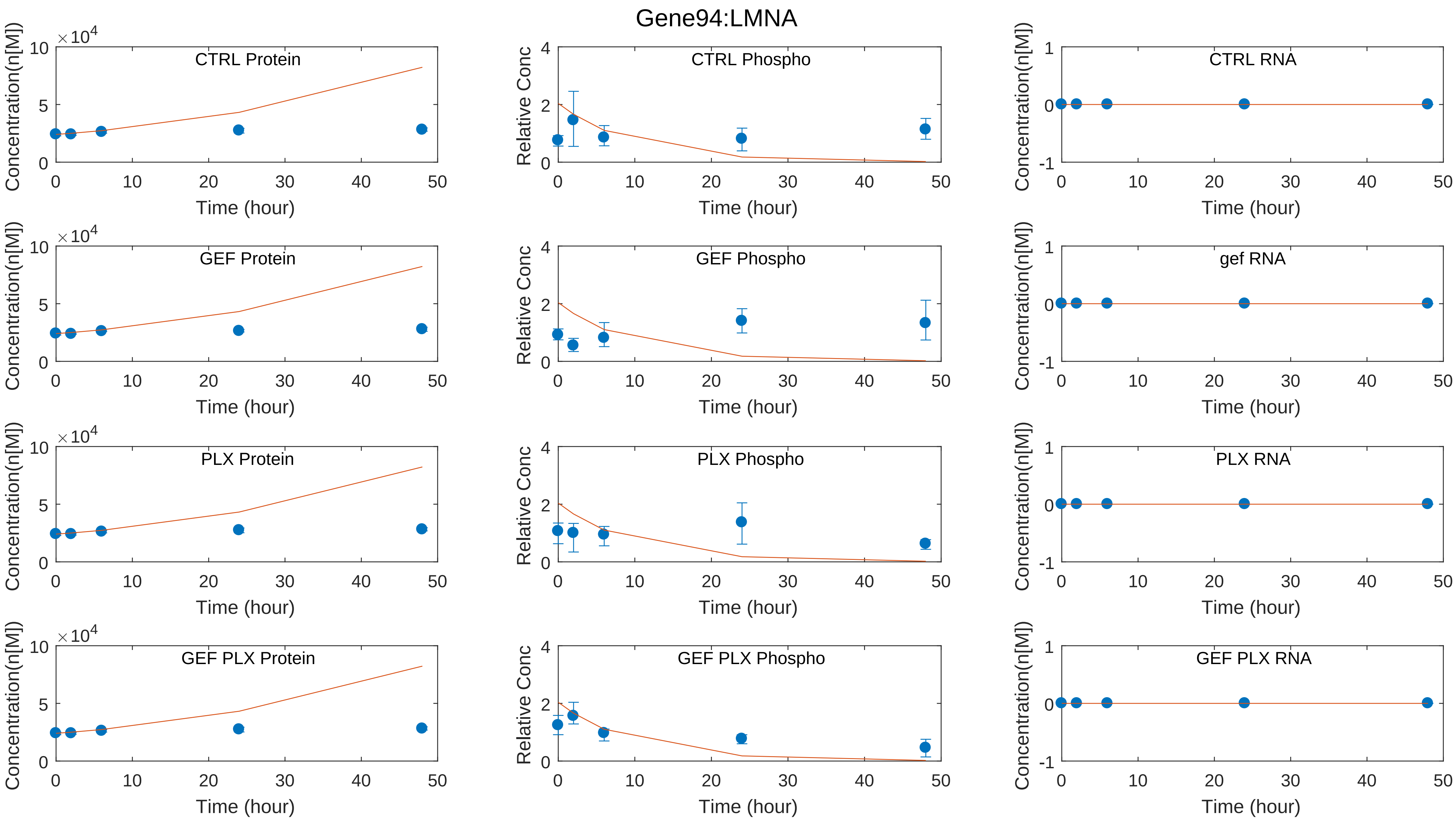

Gene94:LMNA
CTRL Protein
GEF Protein
PLX Protein
GEF PLX Protein
CTRL Phospho
GEF Phospho
PLX Phospho
GEF PLX Phospho
CTRL RNA
gef RNA
PLX RNA
GEF PLX RNA
Concentration(n[M])
Relative Conc
Time (hour)
$\times 10^4$

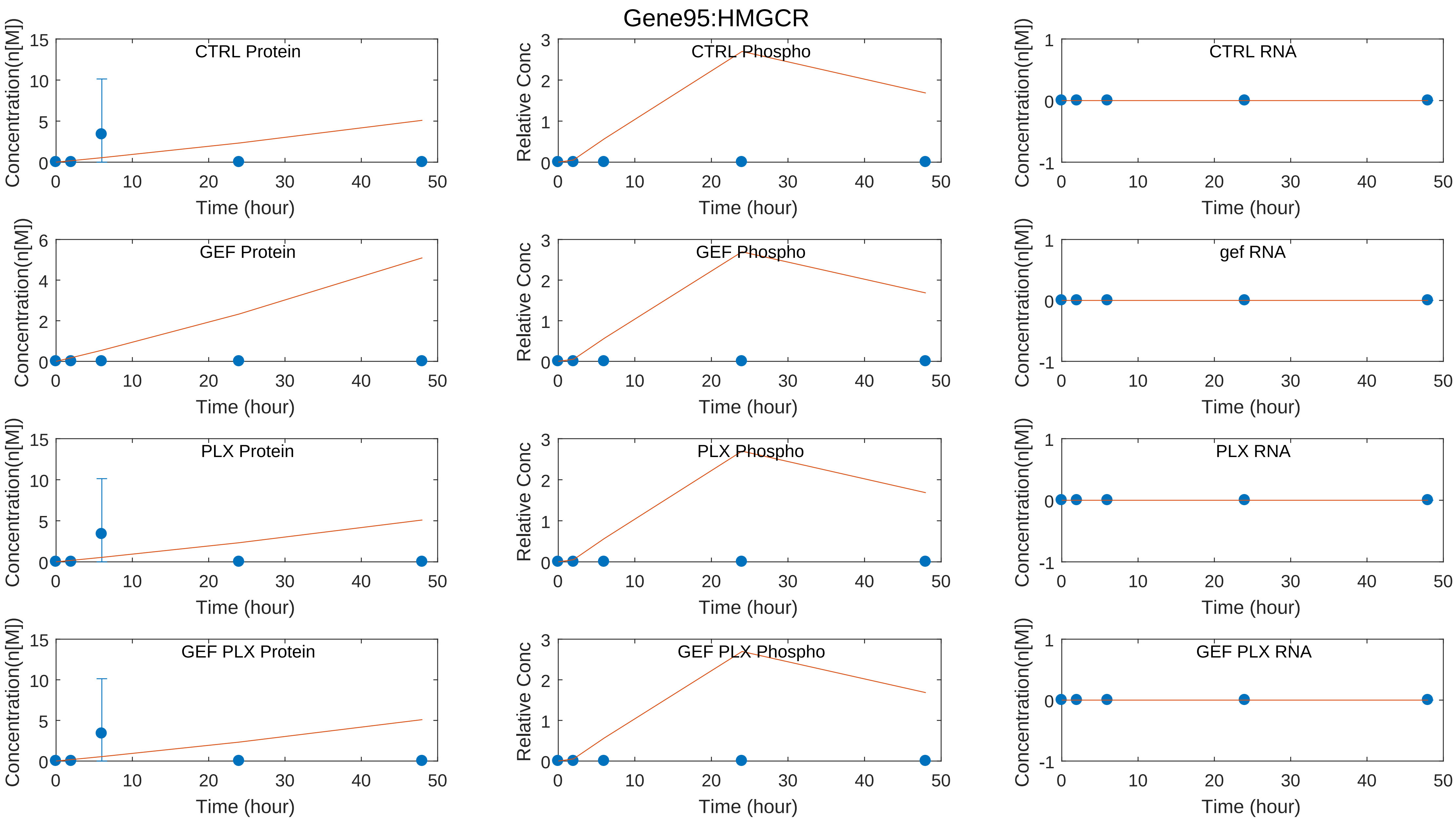

Gene95:HMGCR
CTRL Protein
CTRL Phospho
CTRL RNA
GEF Protein
GEF Phospho
gef RNA
PLX Protein
PLX Phospho
PLX RNA
GEF PLX Protein
GEF PLX Phospho
GEF PLX RNA
Concentration(n[M])
Relative Conc
Time (hour)

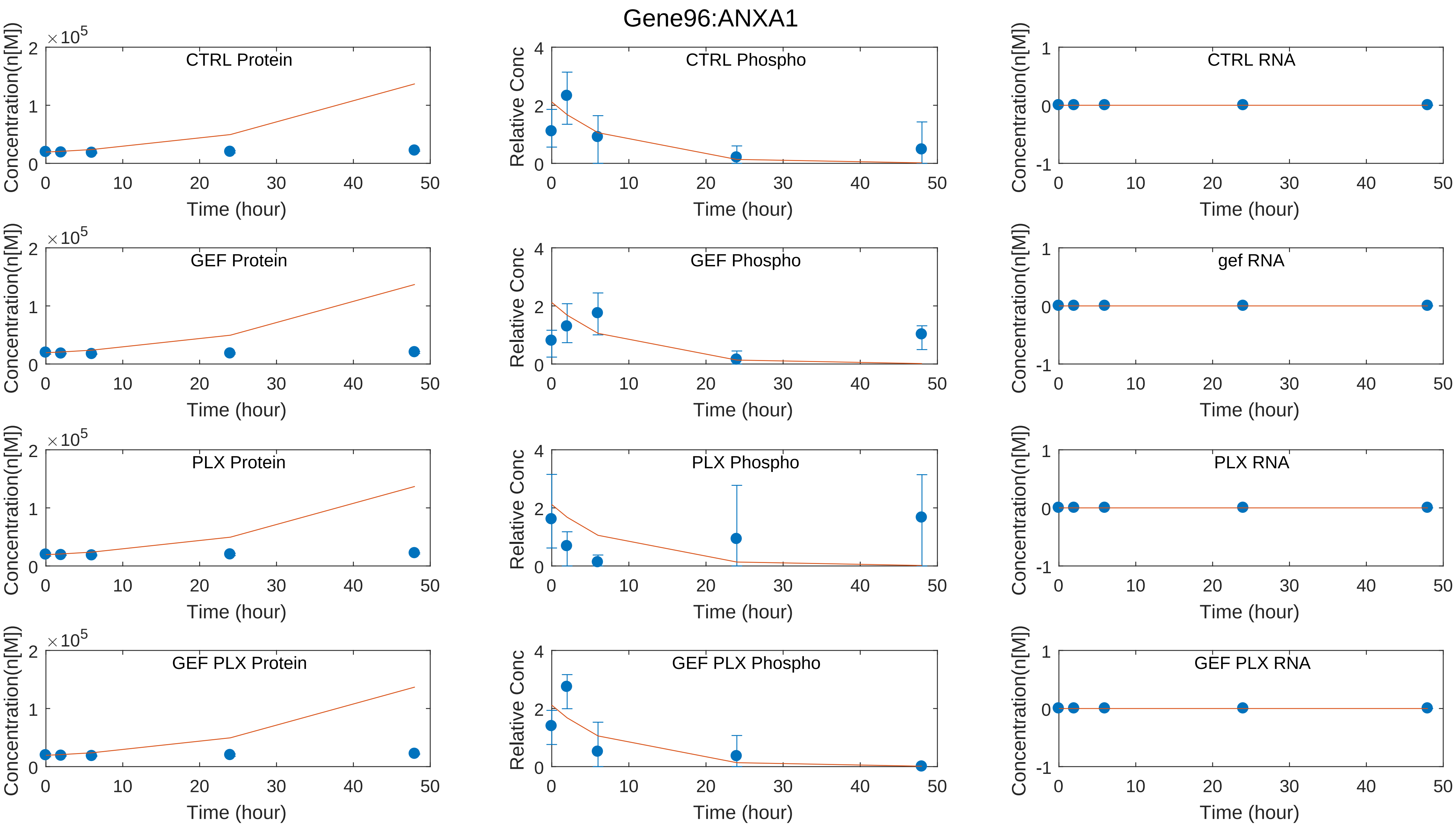
Gene96:ANXA1
CTRL Protein
GEF Protein
PLX Protein
GEF PLX Protein
CTRL Phospho
GEF Phospho
PLX Phospho
GEF PLX Phospho
CTRL RNA
gef RNA
PLX RNA
GEF PLX RNA
Concentration(n[M])
Relative Conc
Time (hour)
×10^5

Gene97:TK1

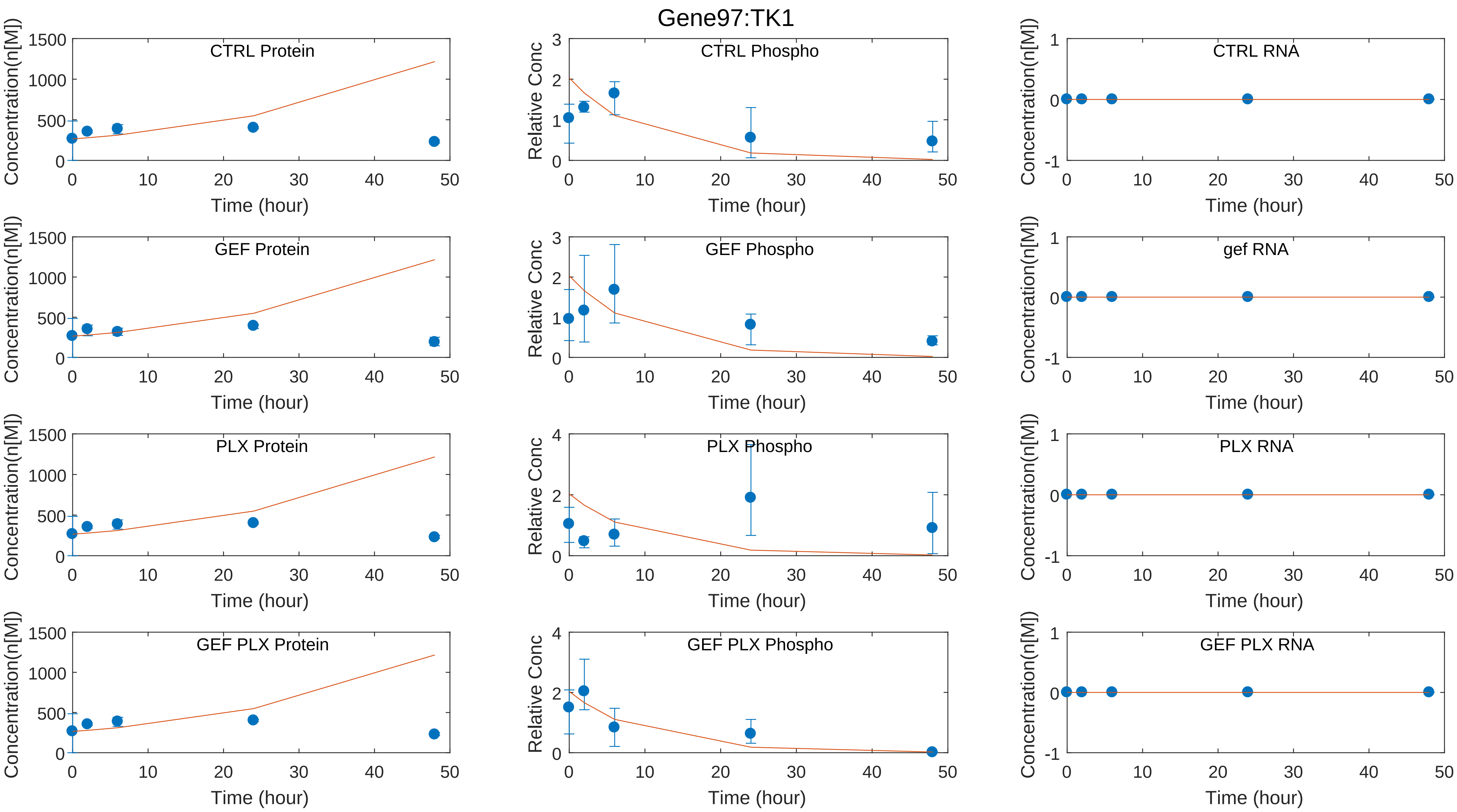

Gene97:TK1
CTRL Protein
CTRL Phospho
CTRL RNA
GEF Protein
GEF Phospho
gef RNA
PLX Protein
PLX Phospho
PLX RNA
GEF PLX Protein
GEF PLX Phospho
GEF PLX RNA
Concentration(n[M])
Relative Conc
Time (hour)

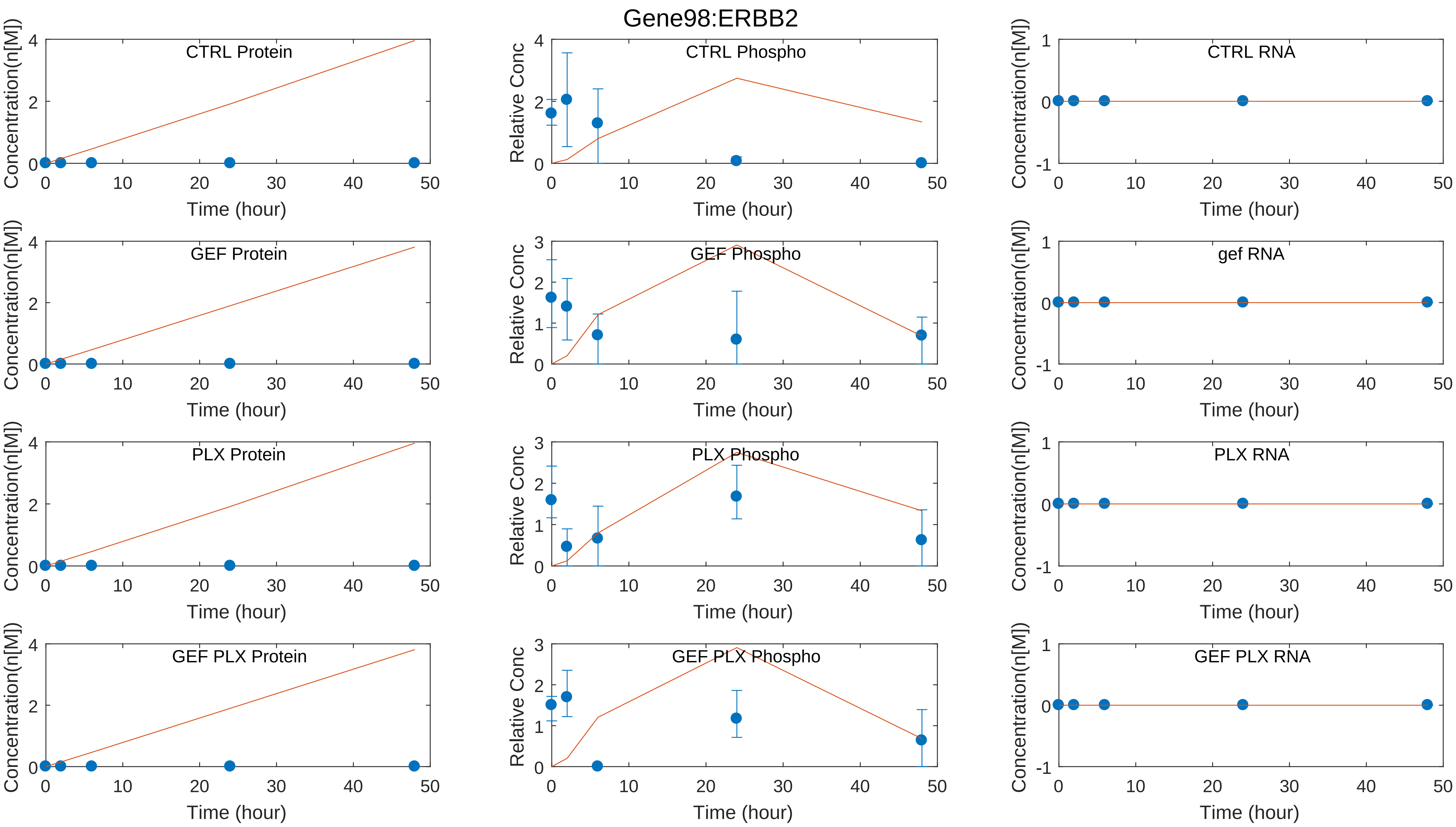

Gene98:ERBB2
CTRL Protein
CTRL Phospho
CTRL RNA
GEF Protein
GEF Phospho
gef RNA
PLX Protein
PLX Phospho
PLX RNA
GEF PLX Protein
GEF PLX Phospho
GEF PLX RNA
Concentration(n[M])
Relative Conc
Time (hour)

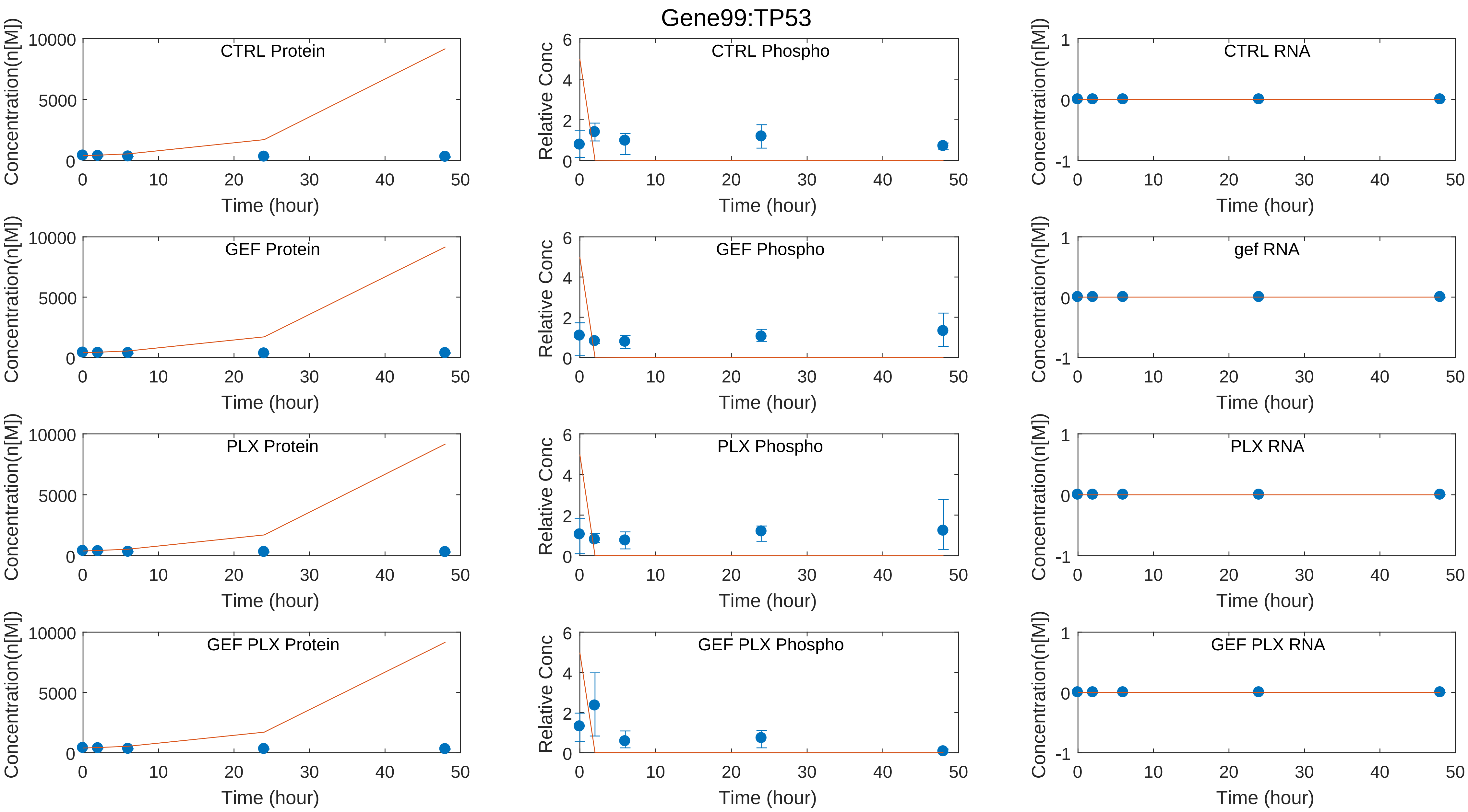
Gene99:TP53
CTRL Protein
GEF Protein
PLX Protein
GEF PLX Protein
CTRL Phospho
GEF Phospho
PLX Phospho
GEF PLX Phospho
CTRL RNA
gef RNA
PLX RNA
GEF PLX RNA
Concentration(n[M])
Relative Conc
Time (hour)
0
10
20
30
40
50
5000
10000
2
4
6
1
-1

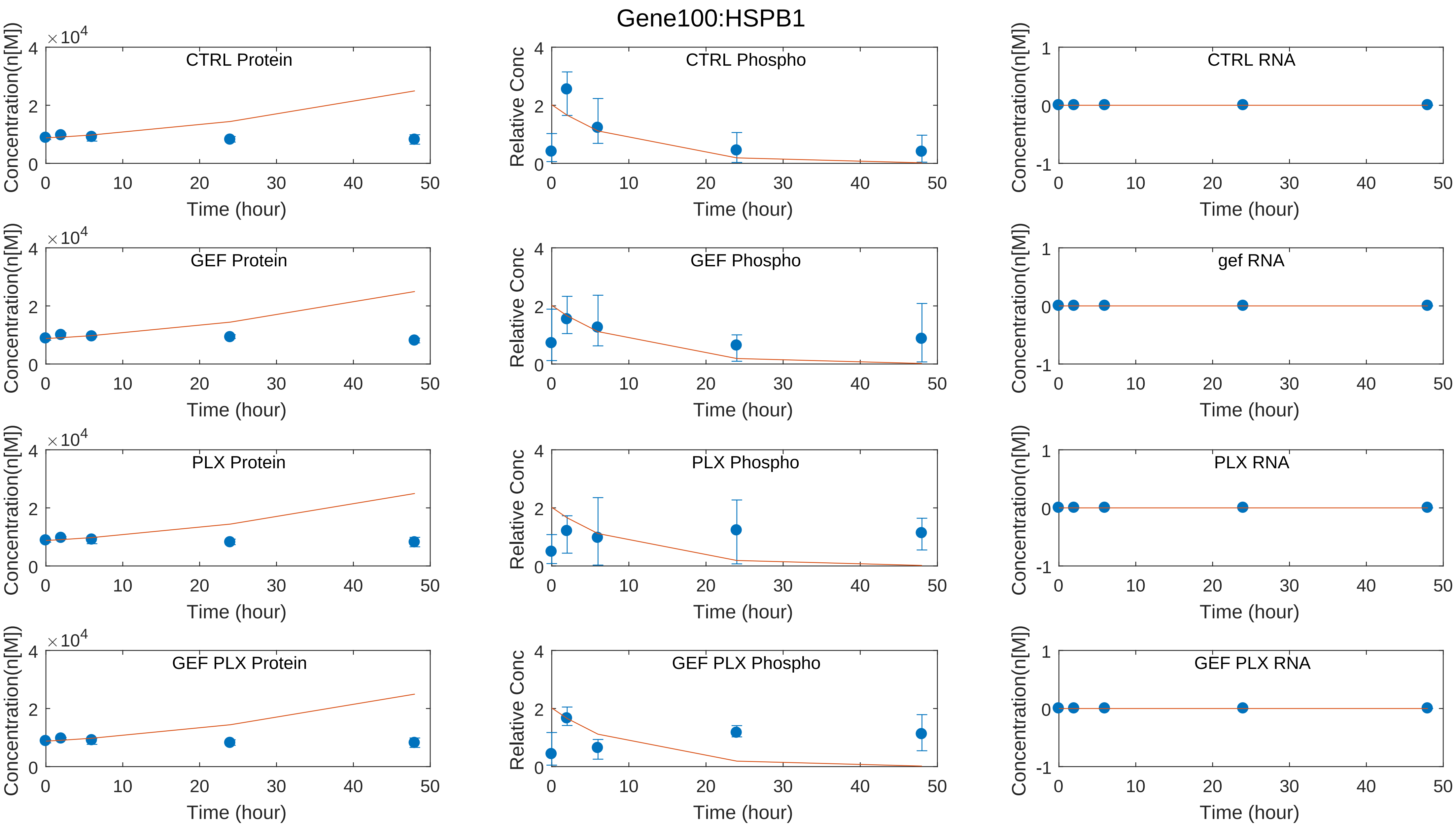
Gene100:HSPB1
CTRL Protein
GEF Protein
PLX Protein
GEF PLX Protein
CTRL Phospho
GEF Phospho
PLX Phospho
GEF PLX Phospho
CTRL RNA
gef RNA
PLX RNA
GEF PLX RNA
Concentration(n[M])
Relative Conc
Time (hour)
×10^4

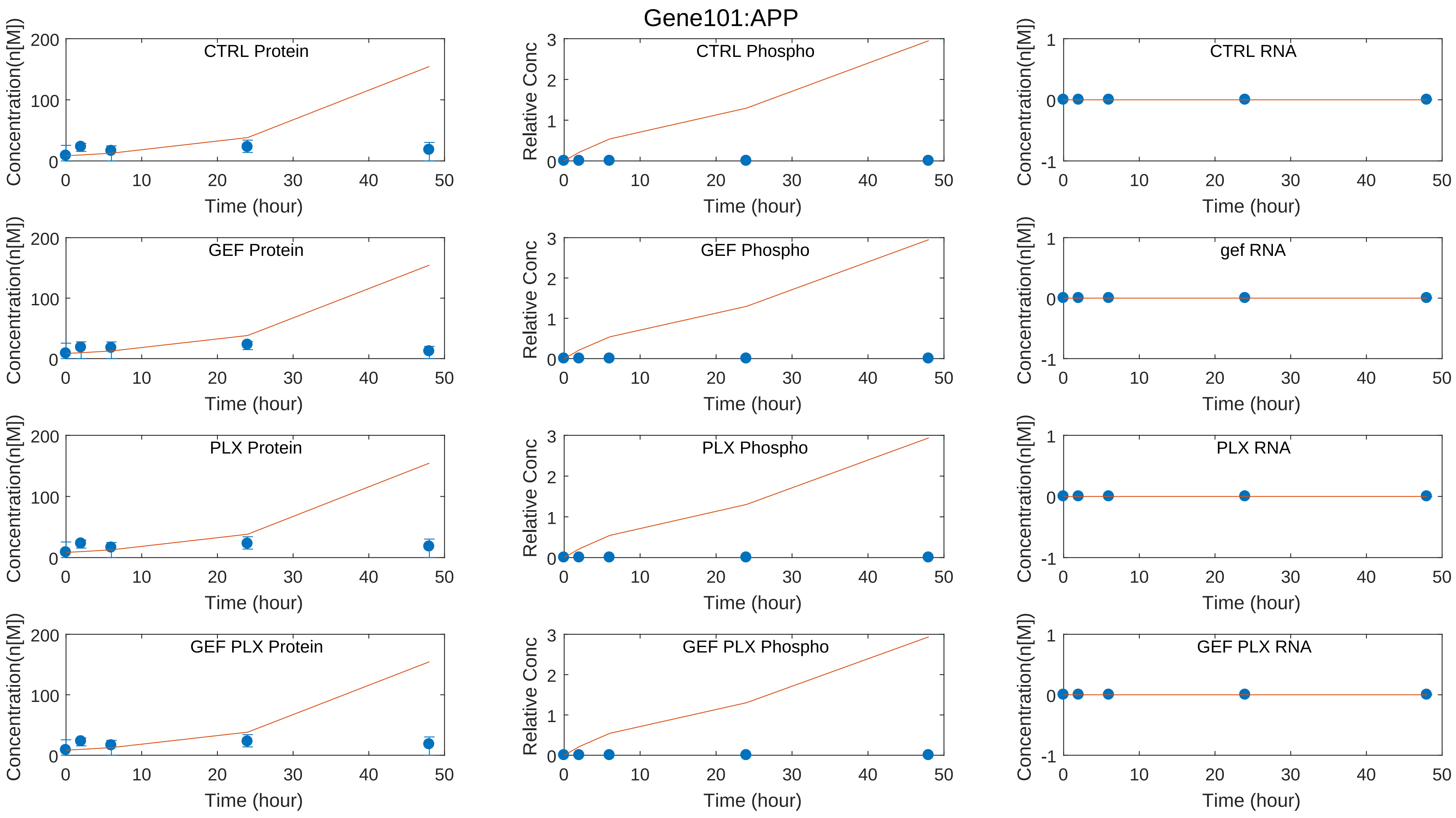

Gene101:APP
CTRL Protein
CTRL Phospho
CTRL RNA
GEF Protein
GEF Phospho
gef RNA
PLX Protein
PLX Phospho
PLX RNA
GEF PLX Protein
GEF PLX Phospho
GEF PLX RNA
Concentration(n[M])
Relative Conc
Time (hour)

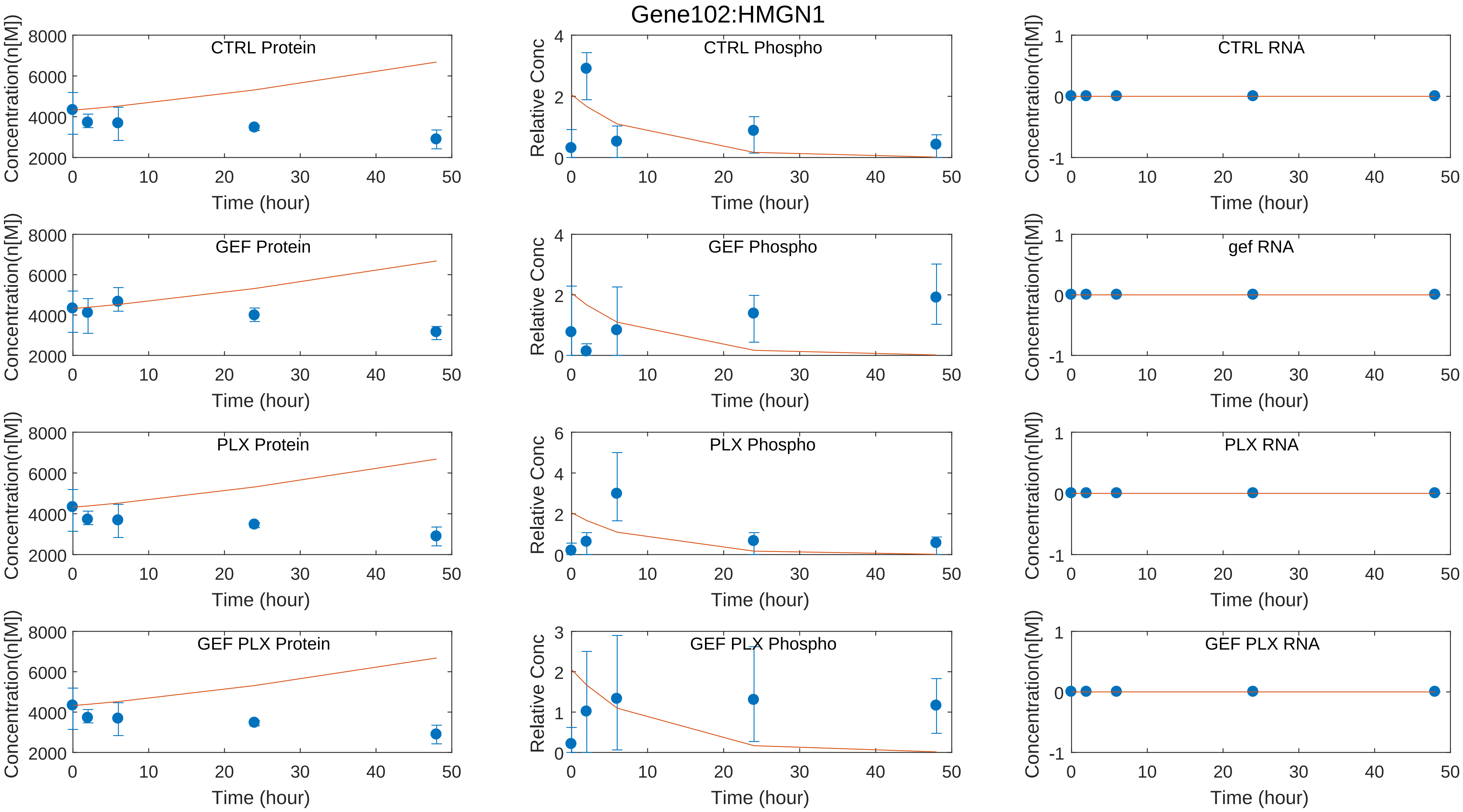
Gene102:HMGN1
CTRL Protein
CTRL Phospho
CTRL RNA
GEF Protein
GEF Phospho
gef RNA
PLX Protein
PLX Phospho
PLX RNA
GEF PLX Protein
GEF PLX Phospho
GEF PLX RNA
Concentration(n[M])
Relative Conc
Time (hour)
8000
6000
4000
2000
0
10
20
30
40
50
4
2
6
3
1
-1

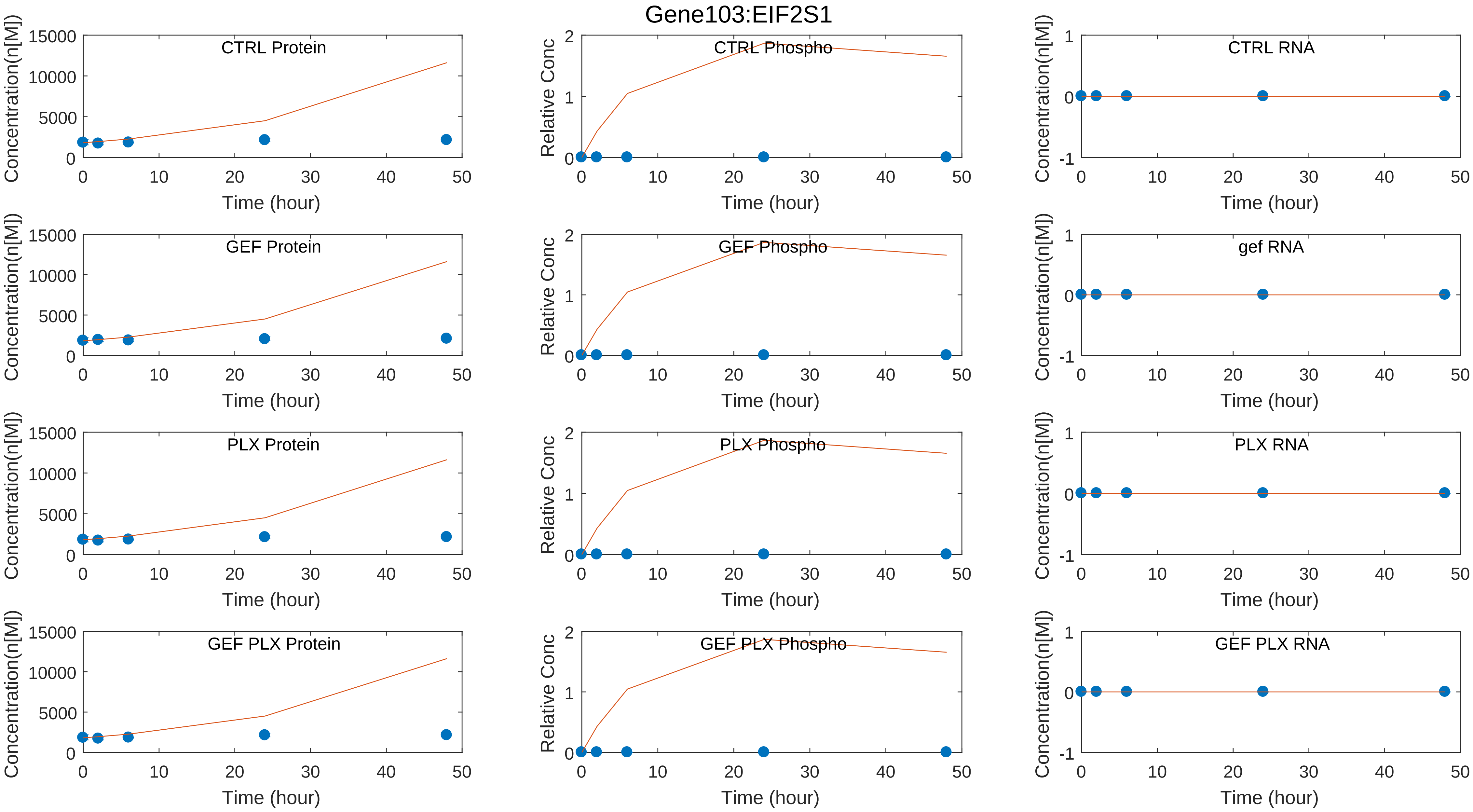
Gene103:EIF2S1
CTRL Protein
CTRL Phospho
CTRL RNA
GEF Protein
GEF Phospho
gef RNA
PLX Protein
PLX Phospho
PLX RNA
GEF PLX Protein
GEF PLX Phospho
GEF PLX RNA
Concentration(n[M])
Relative Conc
Time (hour)

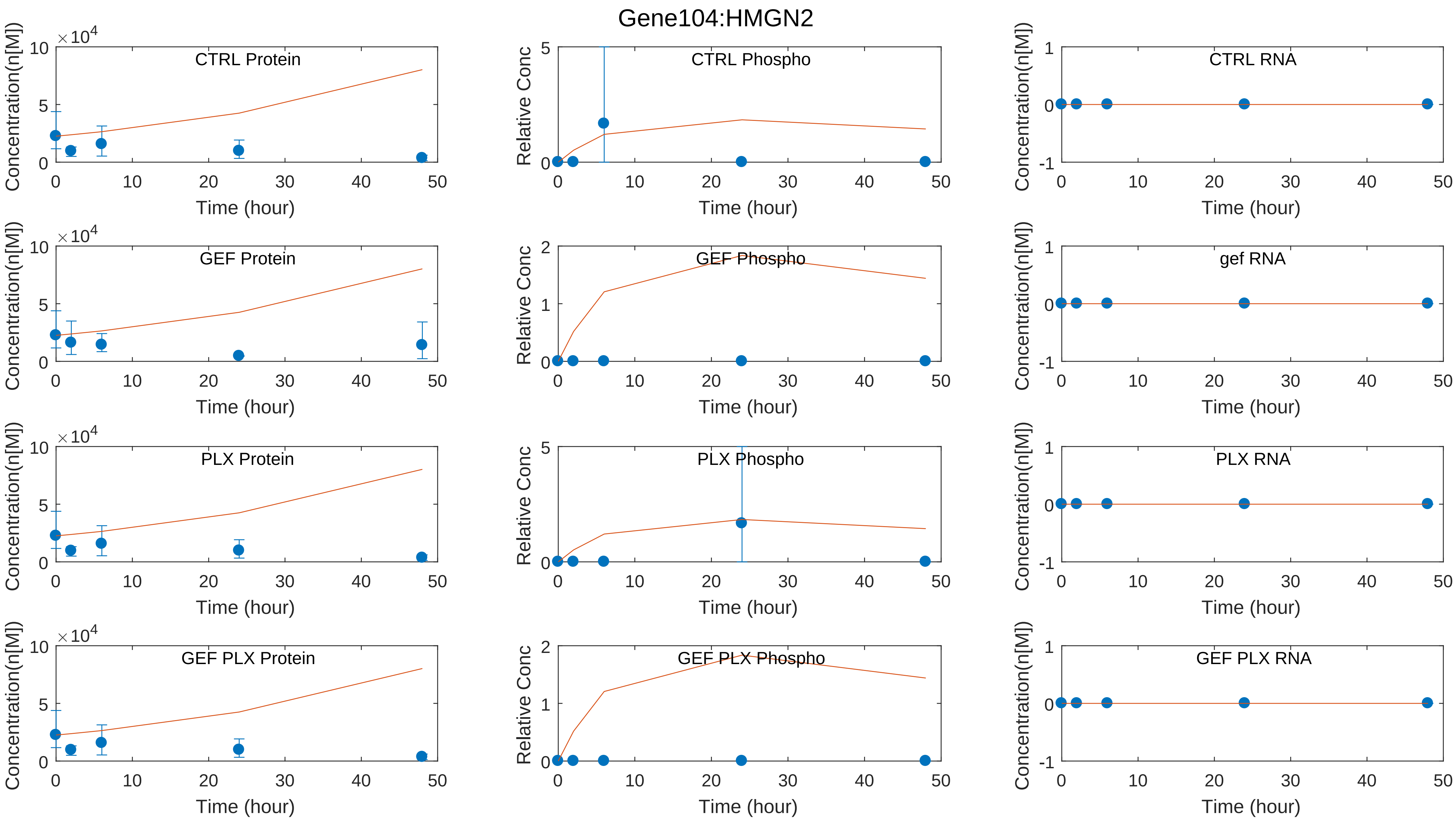

Gene104:HMGN2
CTRL Protein
GEF Protein
PLX Protein
GEF PLX Protein
CTRL Phospho
GEF Phospho
PLX Phospho
GEF PLX Phospho
CTRL RNA
gef RNA
PLX RNA
GEF PLX RNA
Concentration(n[M])
Relative Conc
Time (hour)
×10^4

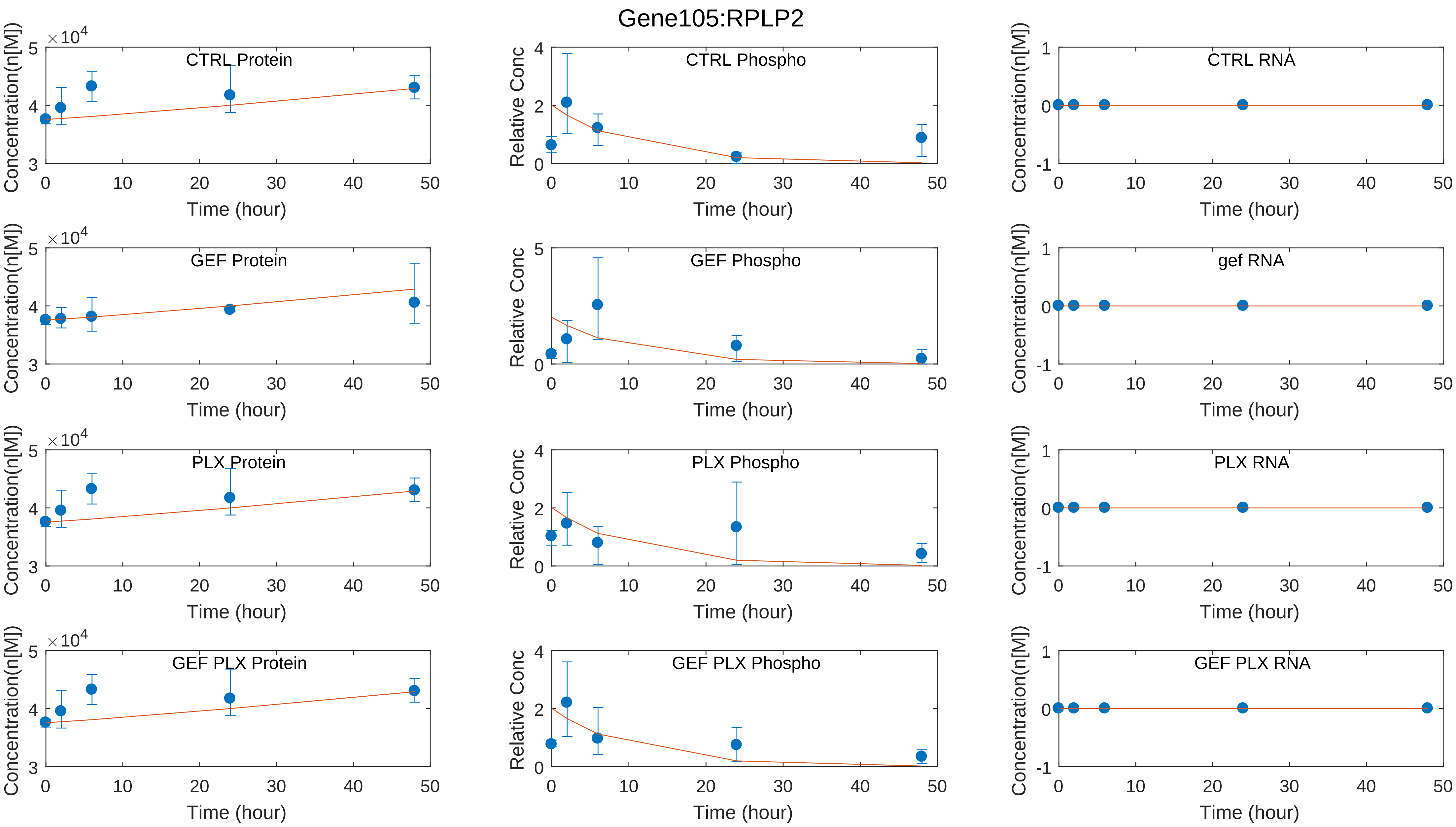

Gene105:RPLP2
CTRL Protein
CTRL Phospho
CTRL RNA
GEF Protein
GEF Phospho
gef RNA
PLX Protein
PLX Phospho
PLX RNA
GEF PLX Protein
GEF PLX Phospho
GEF PLX RNA
Concentration(n[M])
Relative Conc
Time (hour)
×10^4

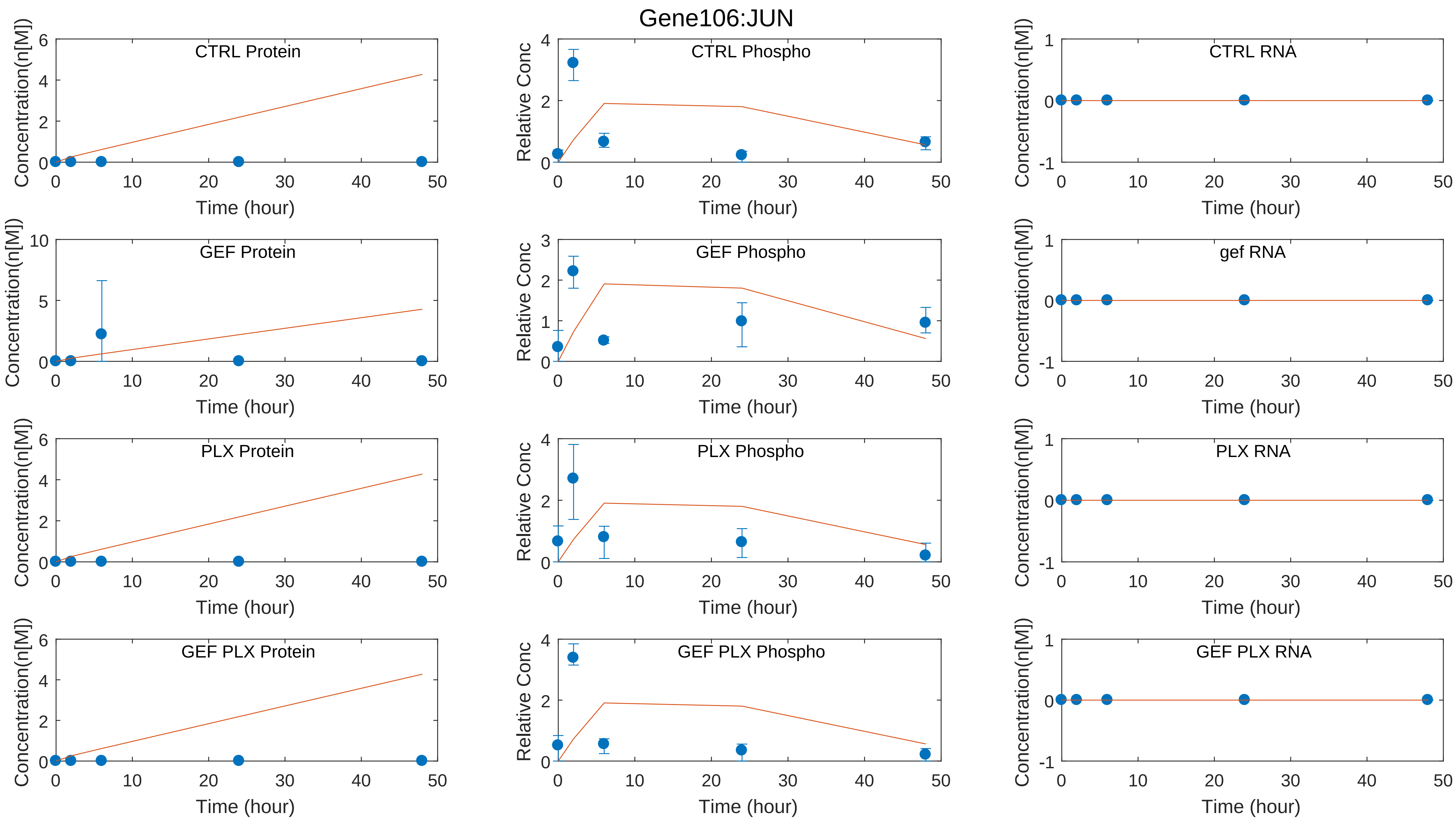
Gene106:JUN
CTRL Protein
CTRL Phospho
CTRL RNA
GEF Protein
GEF Phospho
gef RNA
PLX Protein
PLX Phospho
PLX RNA
GEF PLX Protein
GEF PLX Phospho
GEF PLX RNA
Concentration(n[M])
Relative Conc
Time (hour)
0
10
20
30
40
50
6
4
2
5
3
1
-1

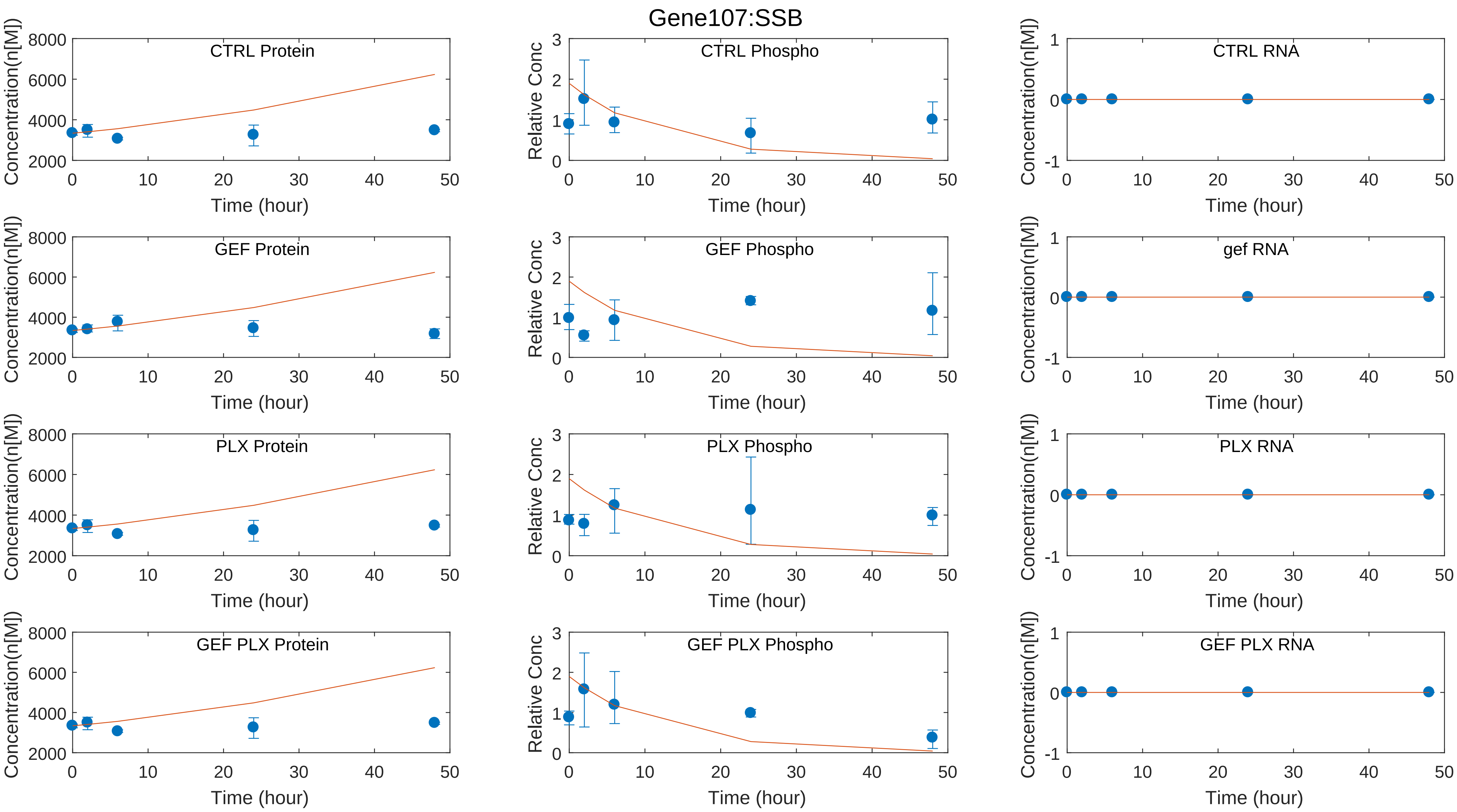

Gene107:SSB
CTRL Protein
CTRL Phospho
CTRL RNA
GEF Protein
GEF Phospho
gef RNA
PLX Protein
PLX Phospho
PLX RNA
GEF PLX Protein
GEF PLX Phospho
GEF PLX RNA
Concentration(n[M])
Relative Conc
Time (hour)

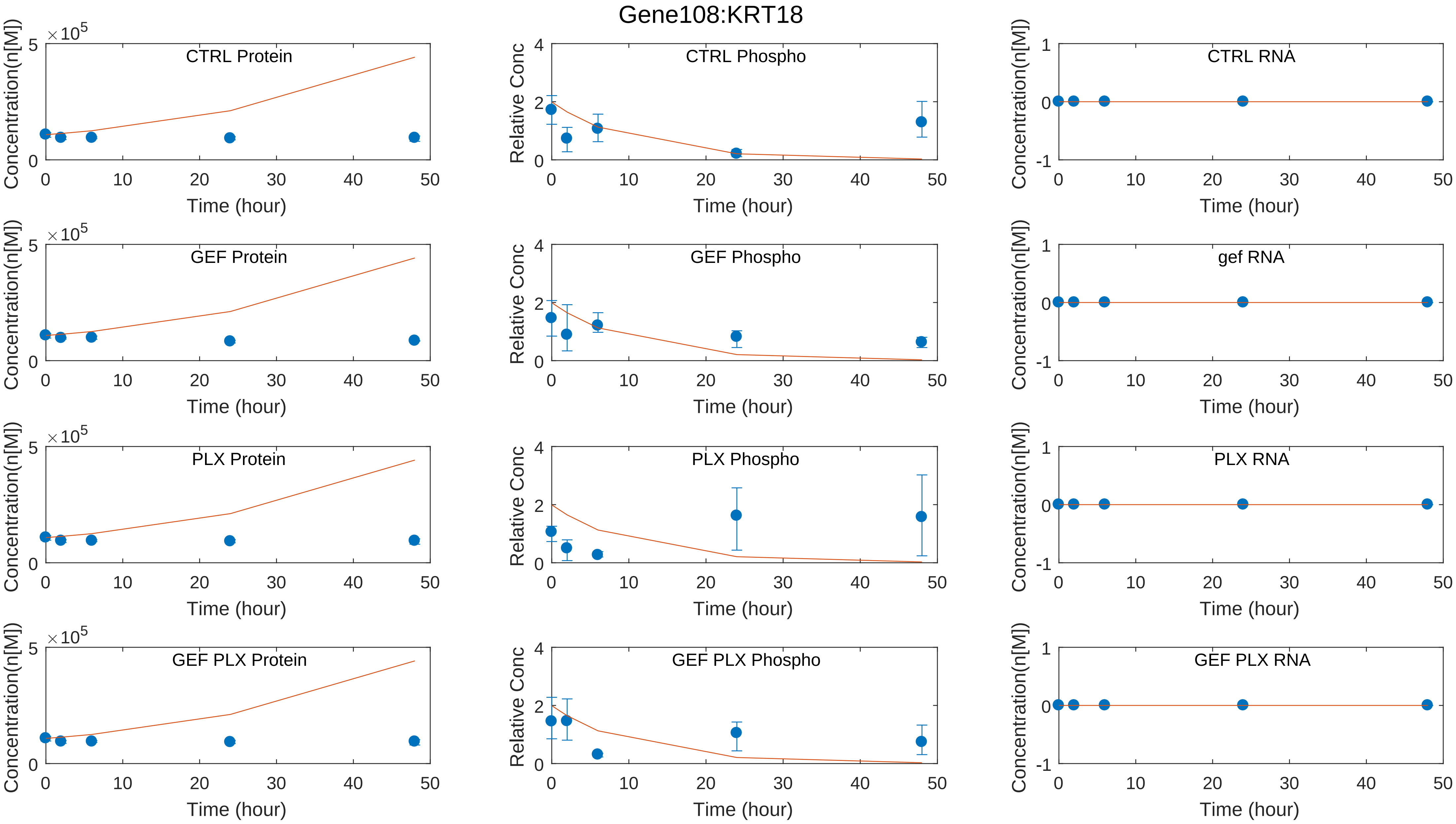
Gene108:KRT18
CTRL Protein
GEF Protein
PLX Protein
GEF PLX Protein
CTRL Phospho
GEF Phospho
PLX Phospho
GEF PLX Phospho
CTRL RNA
gef RNA
PLX RNA
GEF PLX RNA
Concentration(n[M])
Relative Conc
Time (hour)
$\times 10^5$

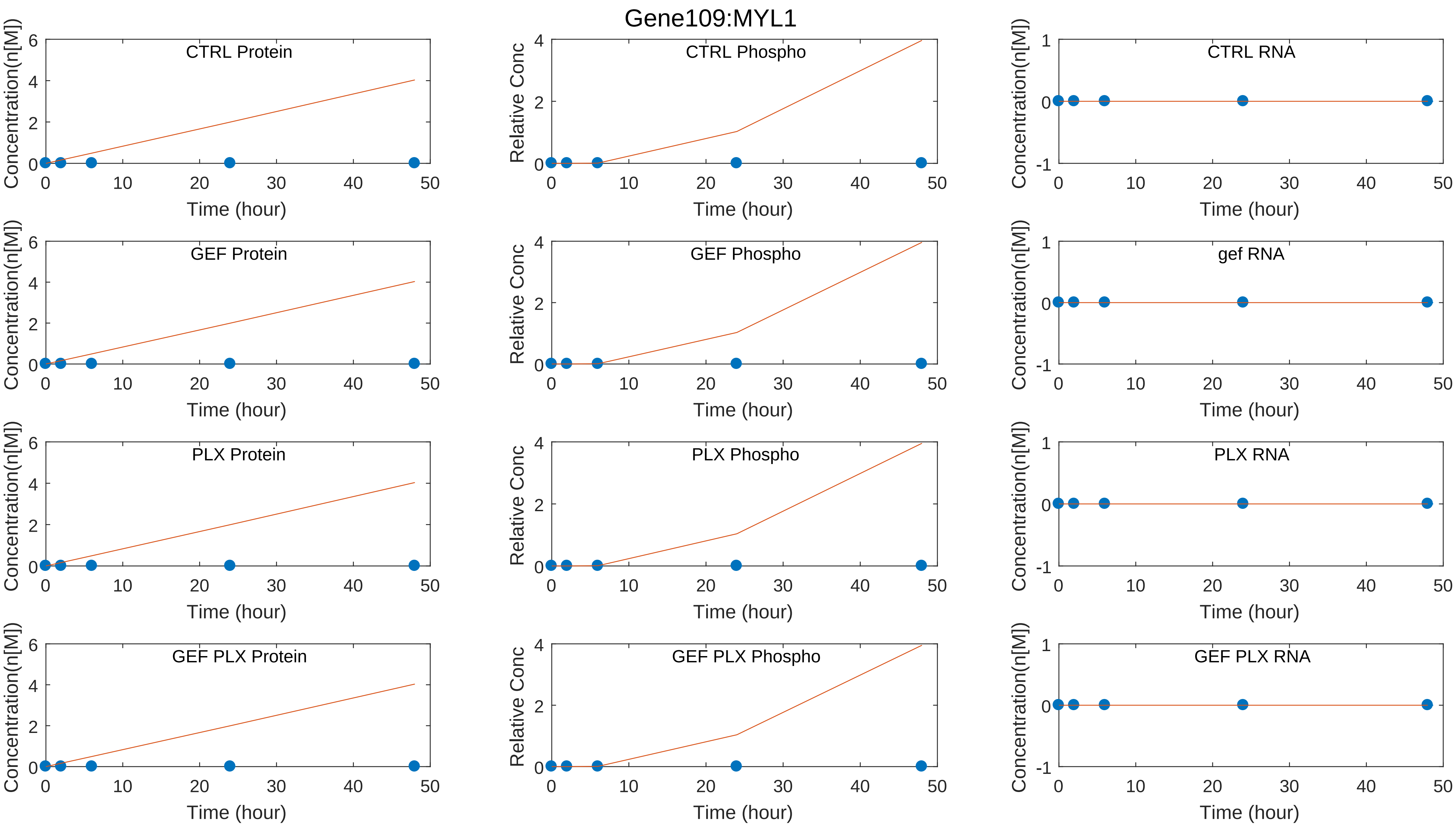

Gene109:MYL1
CTRL Protein
CTRL Phospho
CTRL RNA
GEF Protein
GEF Phospho
gef RNA
PLX Protein
PLX Phospho
PLX RNA
GEF PLX Protein
GEF PLX Phospho
GEF PLX RNA
Concentration(n[M])
Relative Conc
Time (hour)

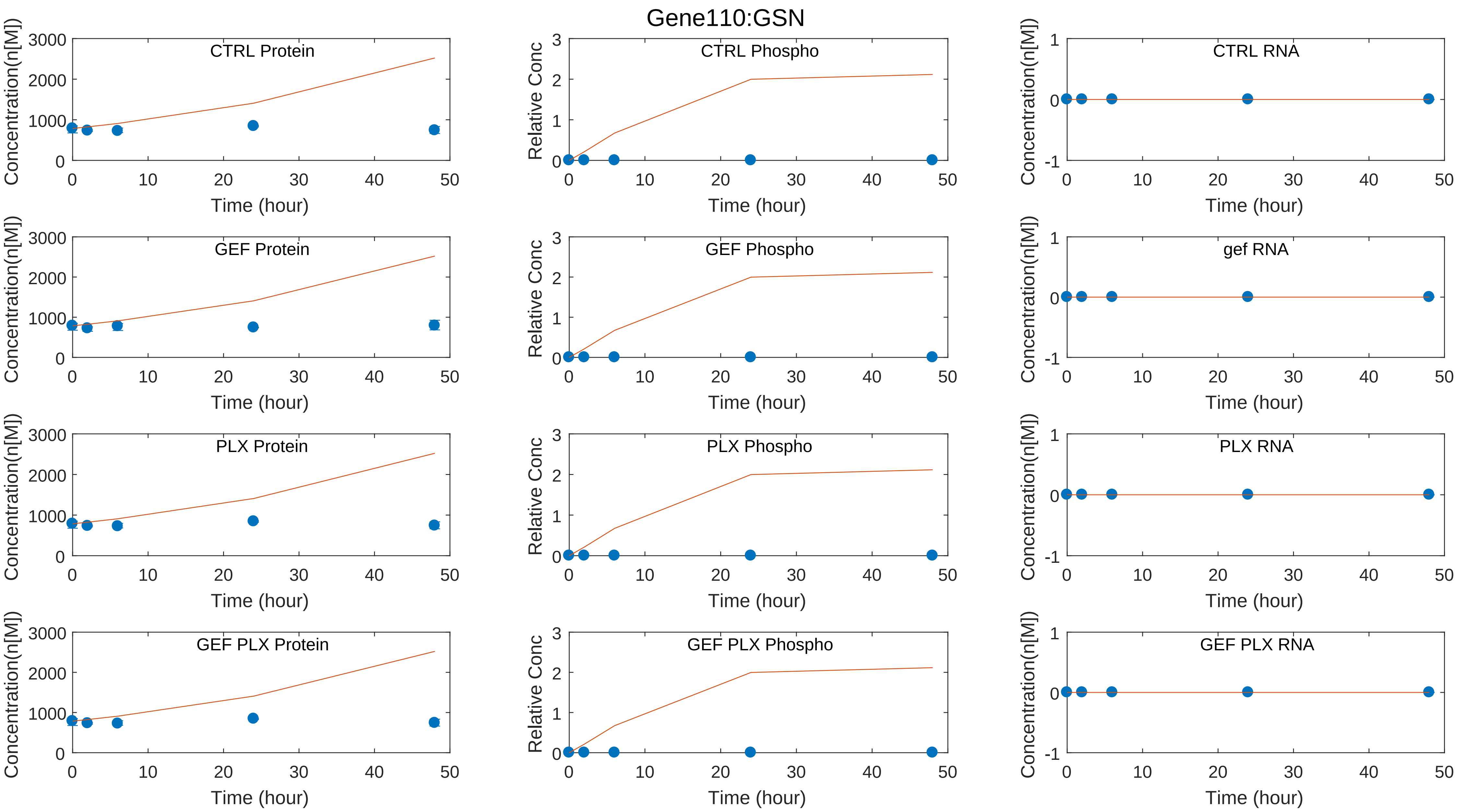
Gene110:GSN
CTRL Protein
GEF Protein
PLX Protein
GEF PLX Protein
CTRL Phospho
GEF Phospho
PLX Phospho
GEF PLX Phospho
CTRL RNA
gef RNA
PLX RNA
GEF PLX RNA
Concentration(n[M])
Relative Conc
Time (hour)

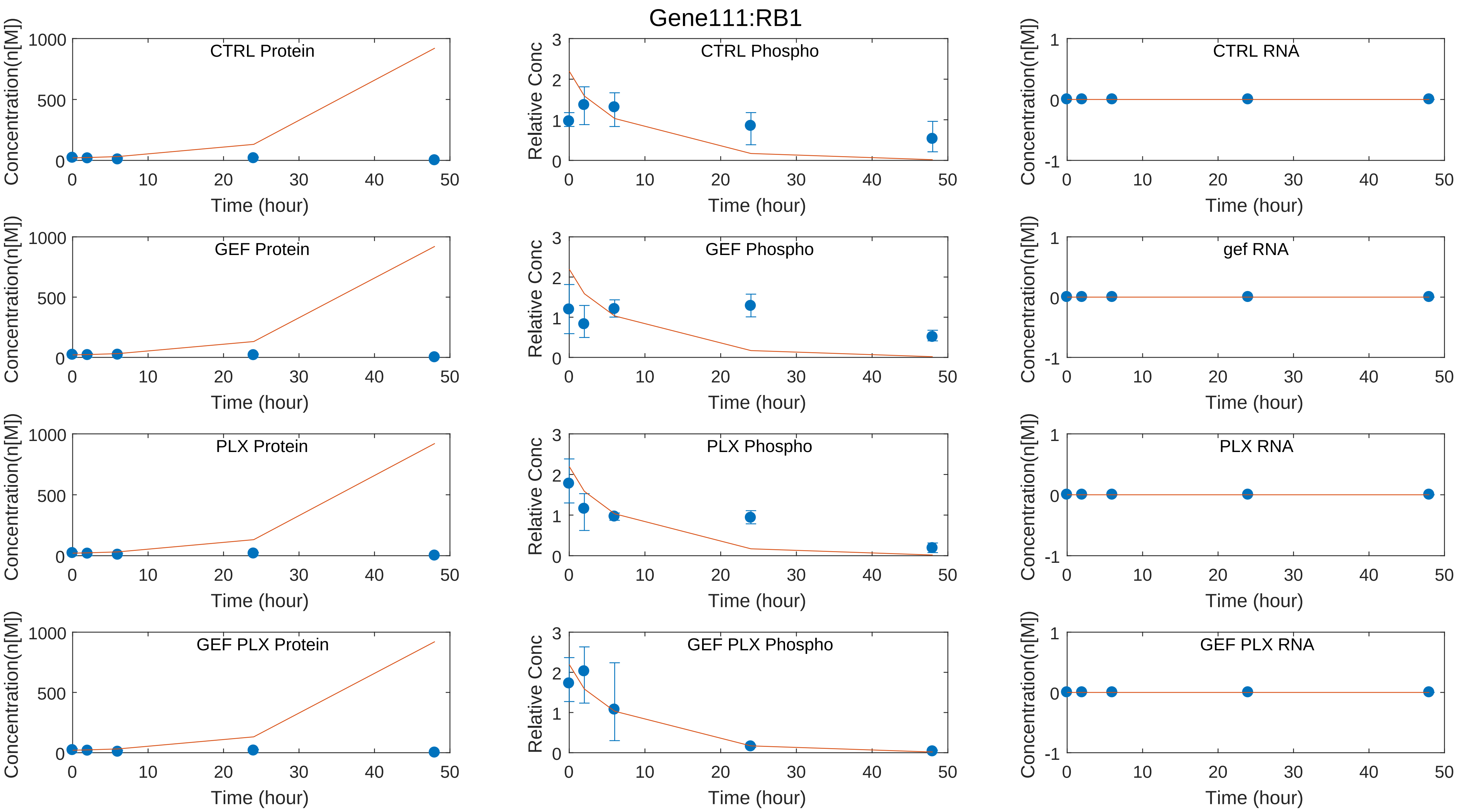

Gene111:RB1
CTRL Protein
CTRL Phospho
CTRL RNA
GEF Protein
GEF Phospho
gef RNA
PLX Protein
PLX Phospho
PLX RNA
GEF PLX Protein
GEF PLX Phospho
GEF PLX RNA
Concentration(n[M])
Relative Conc
Time (hour)

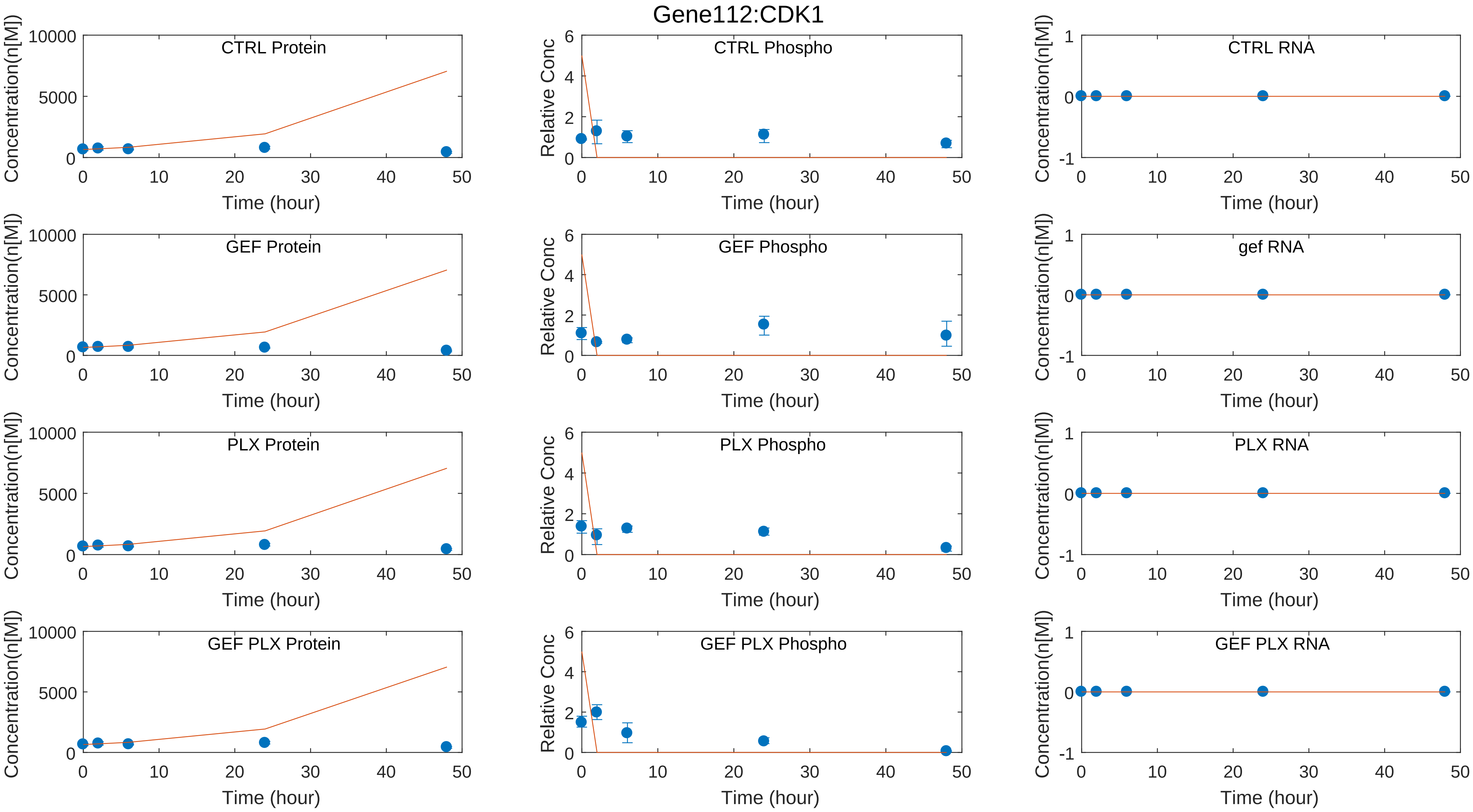

Gene112:CDK1
CTRL Protein
GEF Protein
PLX Protein
GEF PLX Protein
CTRL Phospho
GEF Phospho
PLX Phospho
GEF PLX Phospho
CTRL RNA
gef RNA
PLX RNA
GEF PLX RNA
Concentration(n[M])
Relative Conc
Time (hour)

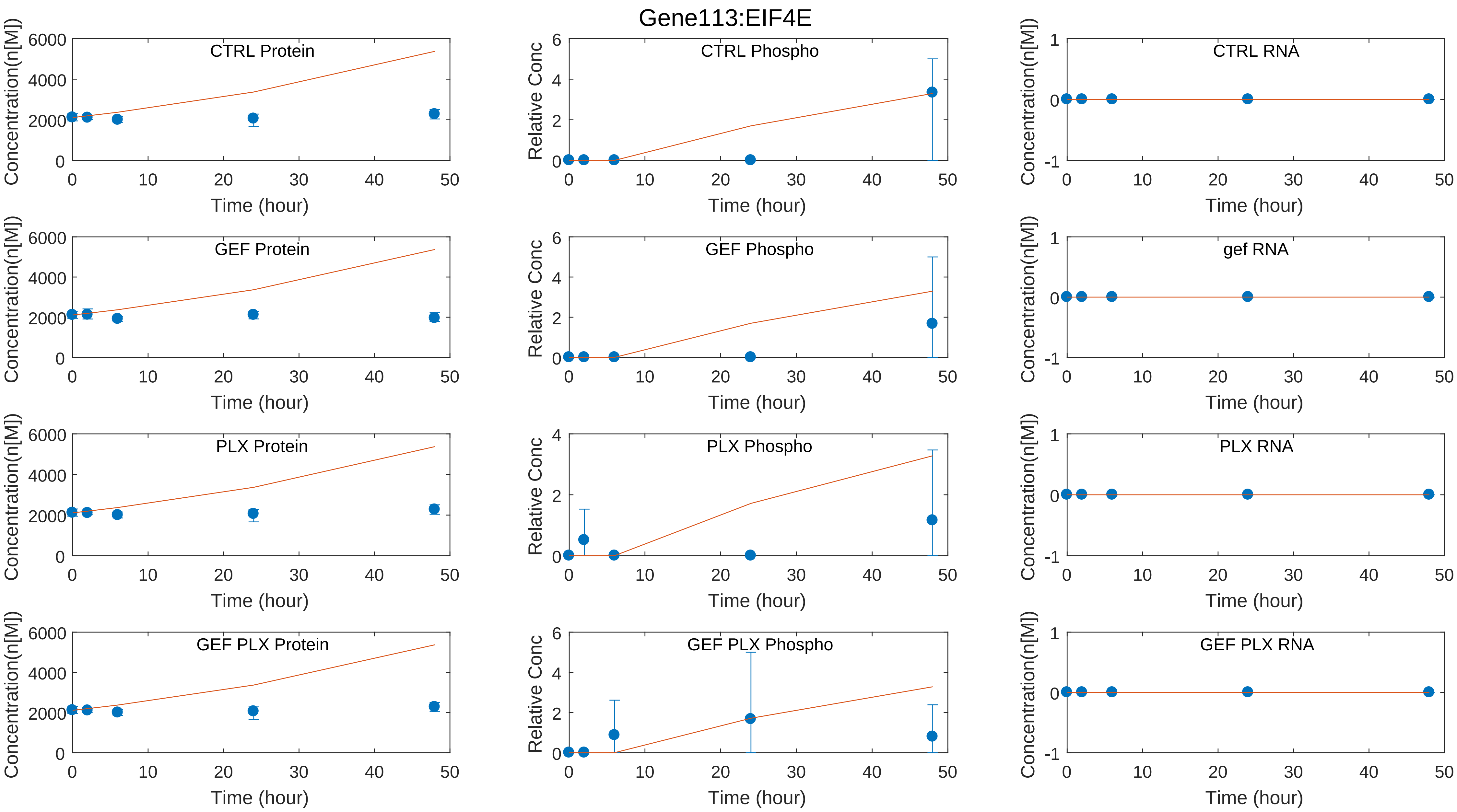
Gene113:EIF4E
CTRL Protein
CTRL Phospho
CTRL RNA
GEF Protein
GEF Phospho
gef RNA
PLX Protein
PLX Phospho
PLX RNA
GEF PLX Protein
GEF PLX Phospho
GEF PLX RNA
Concentration(n[M])
Relative Conc
Time (hour)

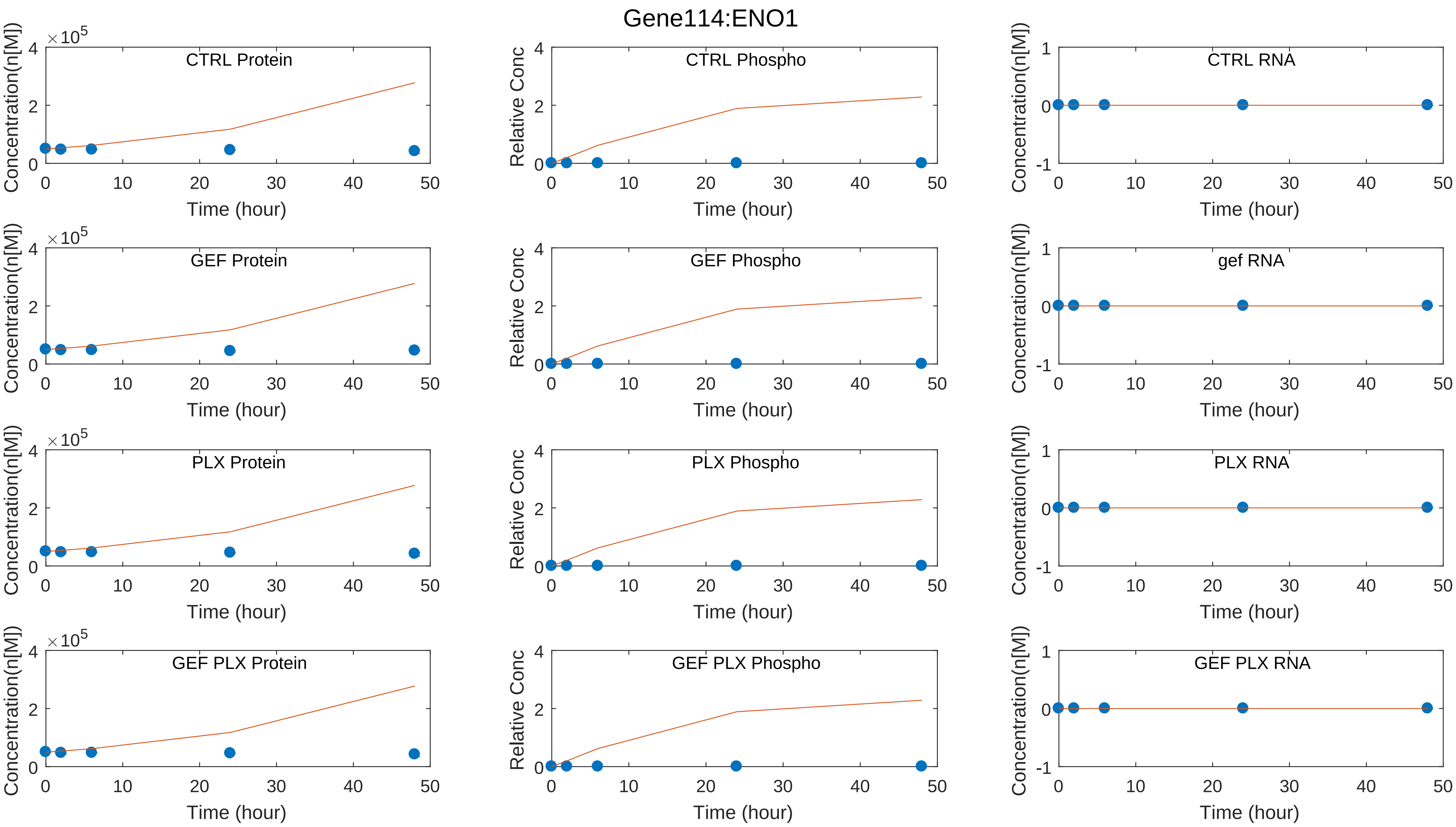
Gene114:ENO1
CTRL Protein
CTRL Phospho
CTRL RNA
GEF Protein
GEF Phospho
gef RNA
PLX Protein
PLX Phospho
PLX RNA
GEF PLX Protein
GEF PLX Phospho
GEF PLX RNA
Concentration(n[M])
Relative Conc
Time (hour)
×10^5

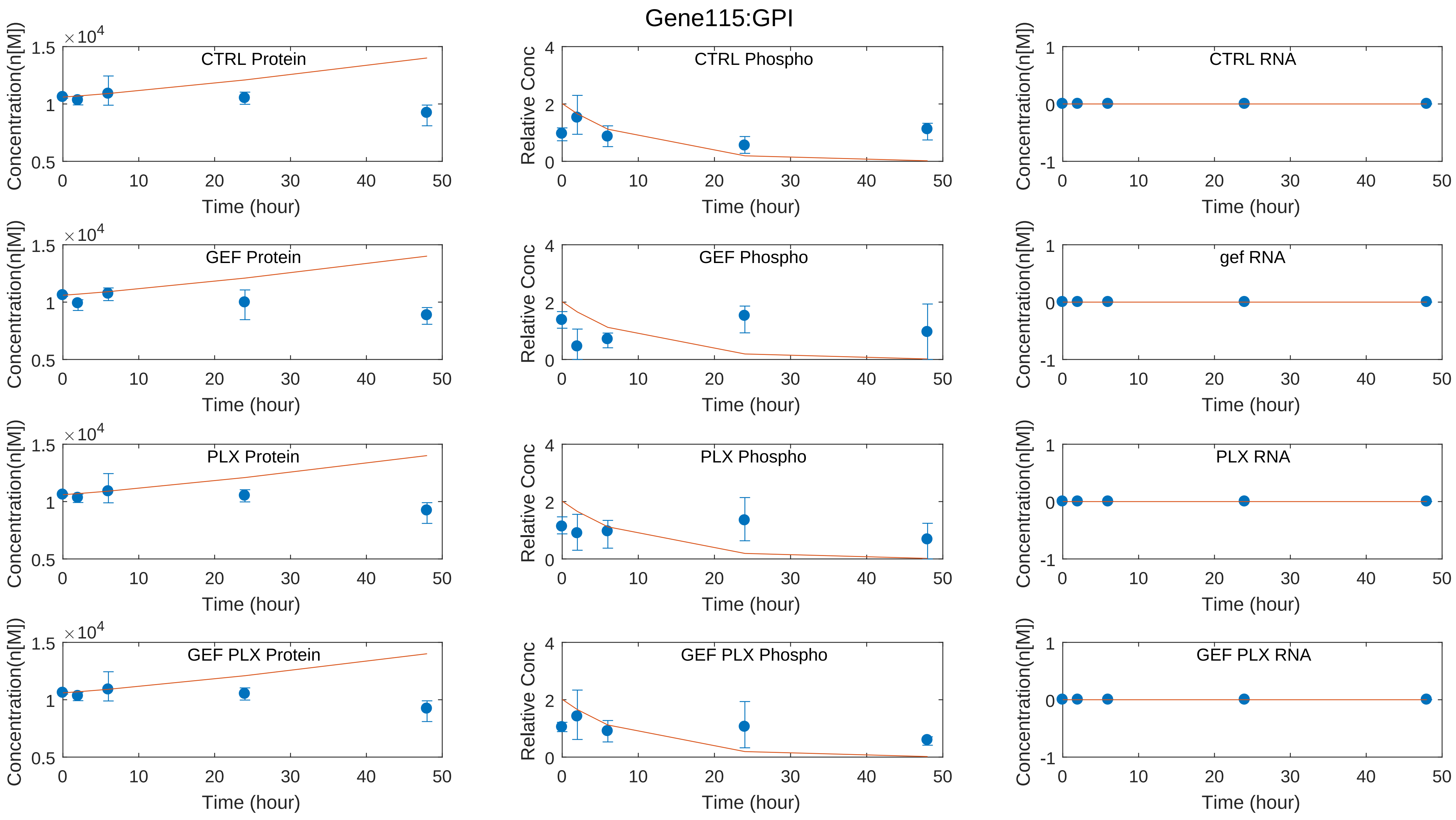
Gene115:GPI
CTRL Protein
GEF Protein
PLX Protein
GEF PLX Protein
CTRL Phospho
GEF Phospho
PLX Phospho
GEF PLX Phospho
CTRL RNA
gef RNA
PLX RNA
GEF PLX RNA
Concentration(n[M])
Relative Conc
Time (hour)
×10^4

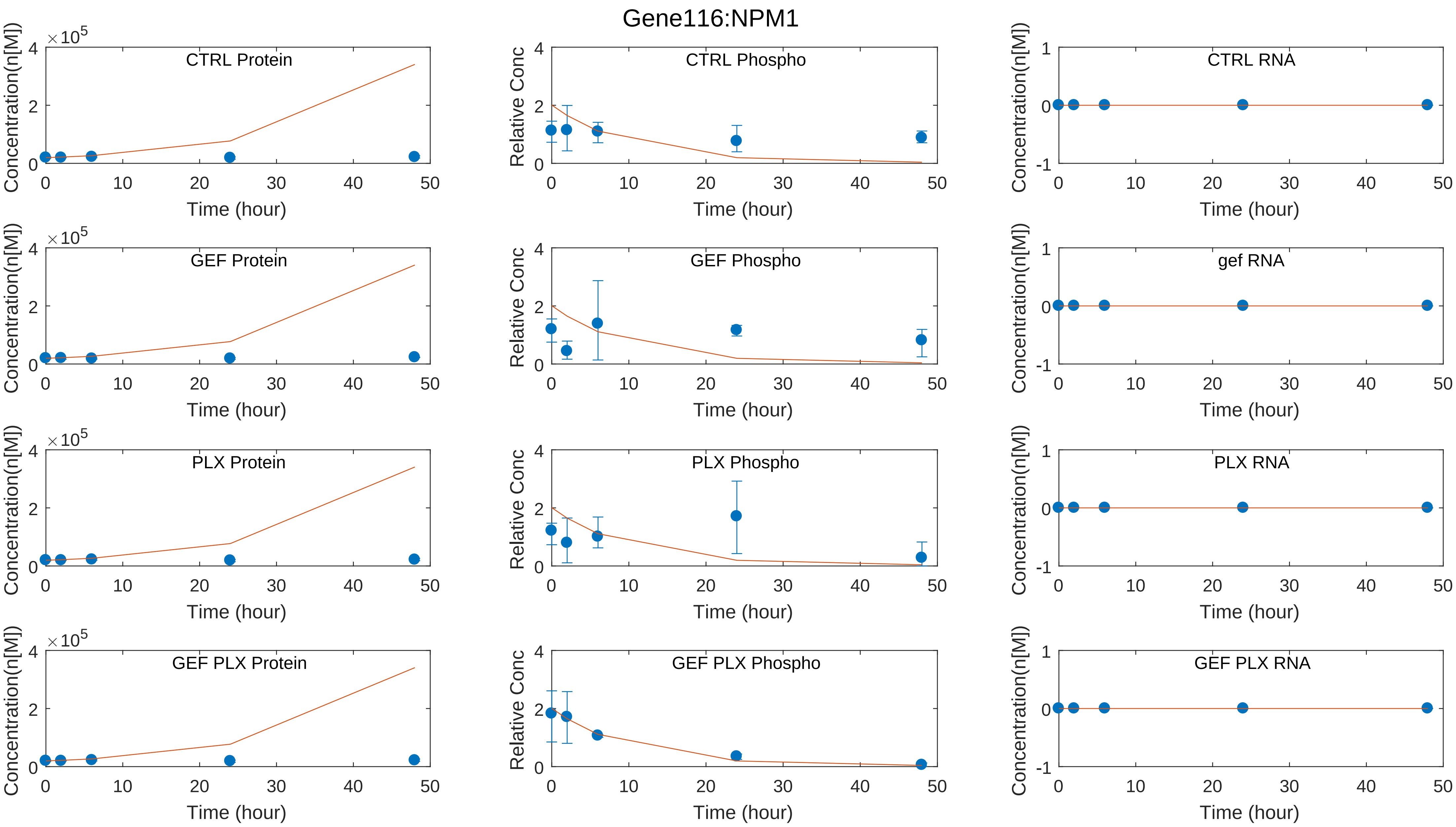
Gene116:NPM1
CTRL Protein
GEF Protein
PLX Protein
GEF PLX Protein
CTRL Phospho
GEF Phospho
PLX Phospho
GEF PLX Phospho
CTRL RNA
gef RNA
PLX RNA
GEF PLX RNA
Concentration(n[M])
Relative Conc
Time (hour)
×10^5

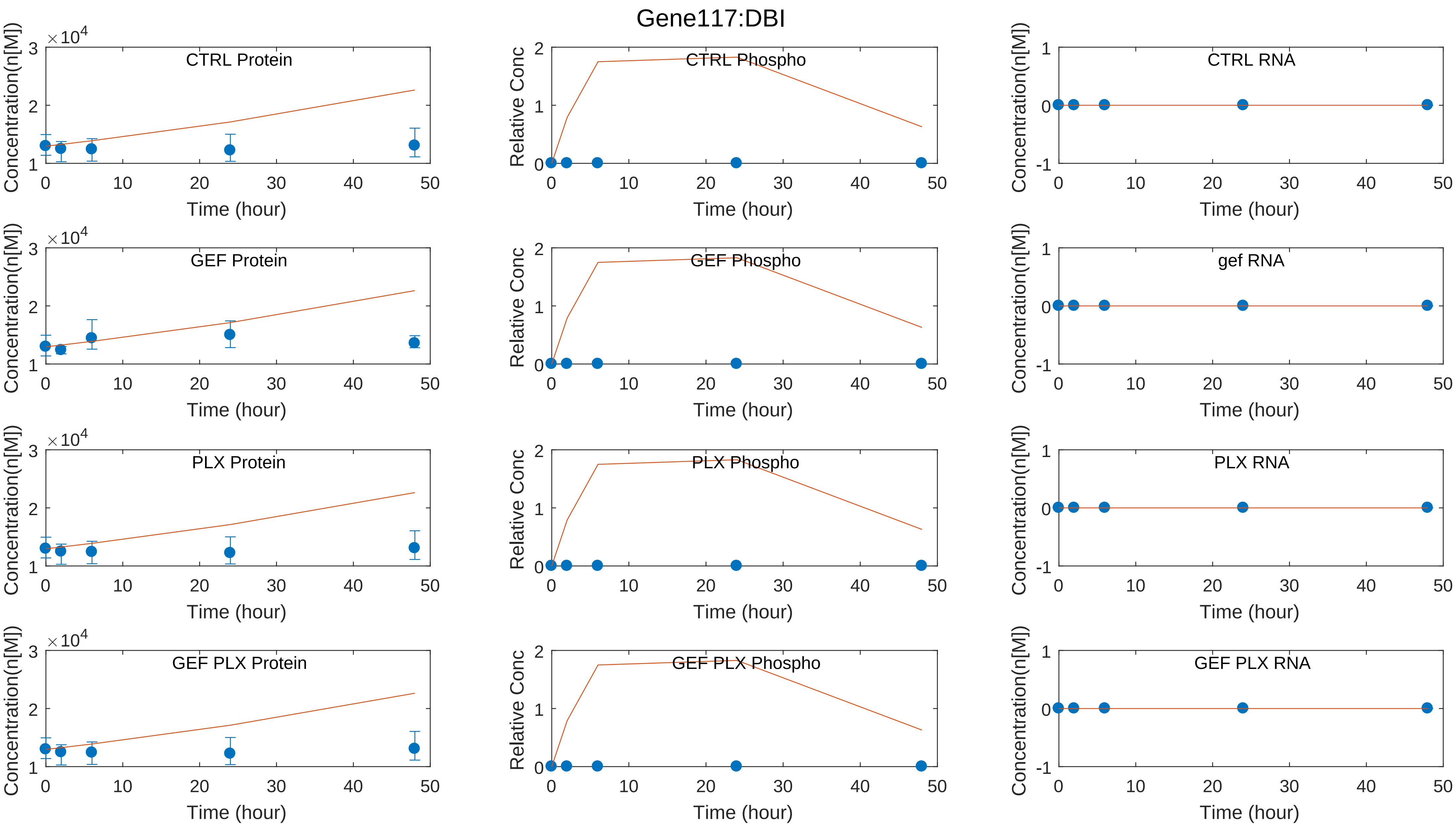
Gene117:DBI
CTRL Protein
GEF Protein
PLX Protein
GEF PLX Protein
CTRL Phospho
GEF Phospho
PLX Phospho
GEF PLX Phospho
CTRL RNA
gef RNA
PLX RNA
GEF PLX RNA
Concentration(n[M])
Relative Conc
Time (hour)
×10^4
0
10
20
30
40
50
1
2
3
-1

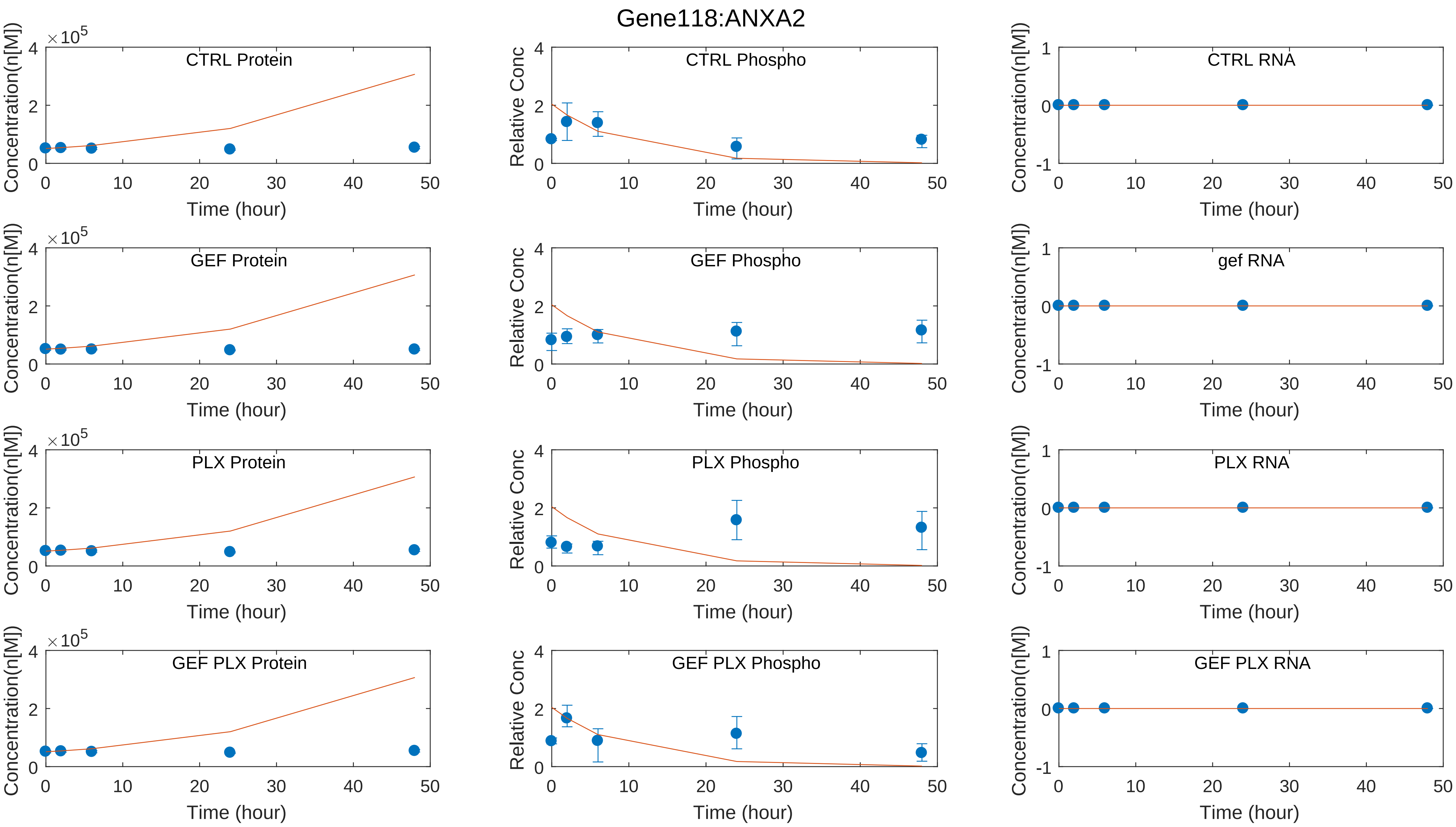

Gene118:ANXA2
CTRL Protein
GEF Protein
PLX Protein
GEF PLX Protein
CTRL Phospho
GEF Phospho
PLX Phospho
GEF PLX Phospho
CTRL RNA
gef RNA
PLX RNA
GEF PLX RNA
Concentration(n[M])
Relative Conc
Time (hour)
$\times 10^5$

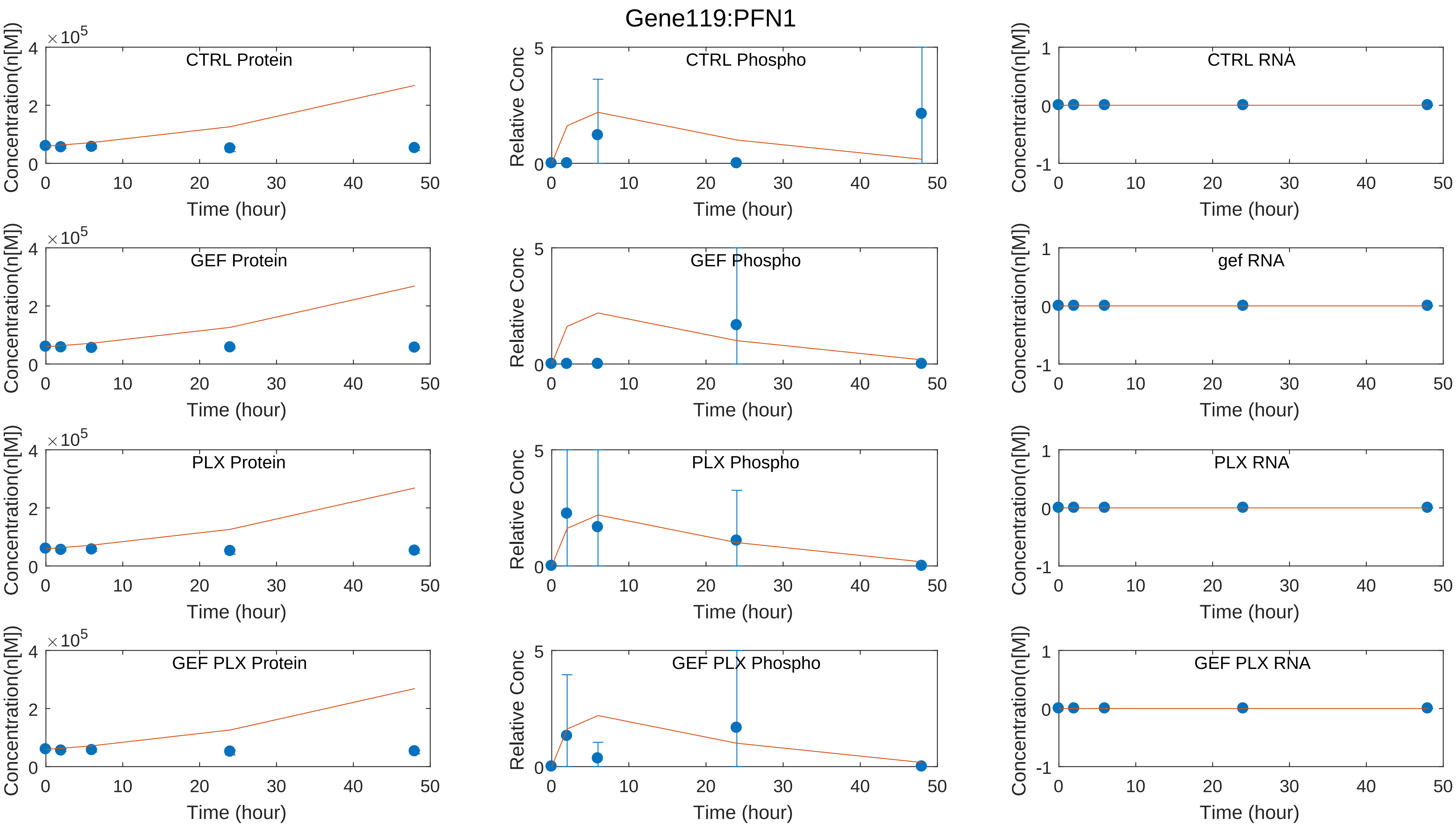
Gene119:PFN1
CTRL Protein
CTRL Phospho
CTRL RNA
GEF Protein
GEF Phospho
gef RNA
PLX Protein
PLX Phospho
PLX RNA
GEF PLX Protein
GEF PLX Phospho
GEF PLX RNA
Concentration(n[M])
Relative Conc
Time (hour)
×10^5

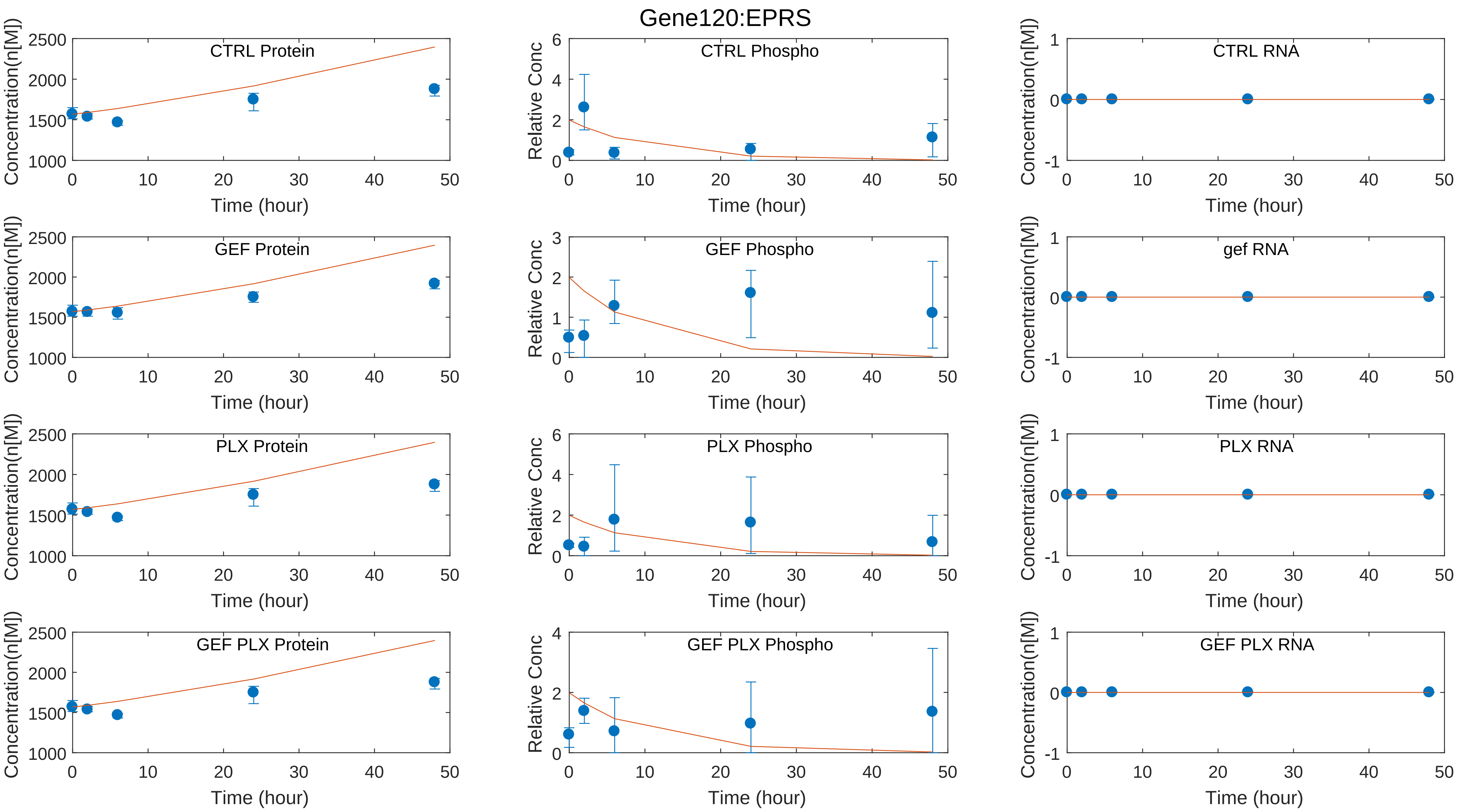

Gene120:EPRS
CTRL Protein
GEF Protein
PLX Protein
GEF PLX Protein
CTRL Phospho
GEF Phospho
PLX Phospho
GEF PLX Phospho
CTRL RNA
gef RNA
PLX RNA
GEF PLX RNA
Concentration(n[M])
Relative Conc
Time (hour)

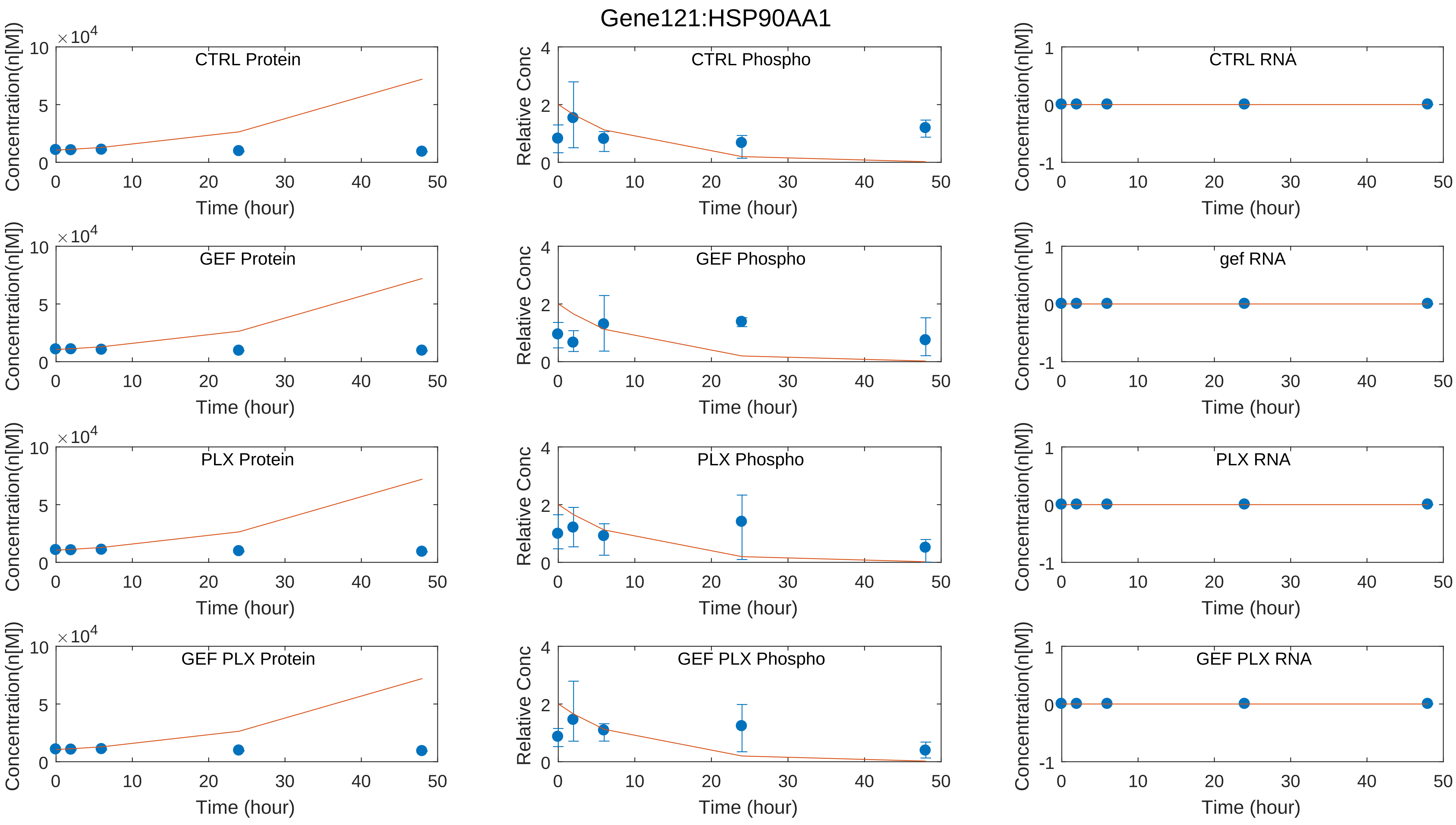
Gene121:HSP90AA1
CTRL Protein
GEF Protein
PLX Protein
GEF PLX Protein
CTRL Phospho
GEF Phospho
PLX Phospho
GEF PLX Phospho
CTRL RNA
gef RNA
PLX RNA
GEF PLX RNA
Concentration(n[M])
Relative Conc
Time (hour)
$\times 10^4$

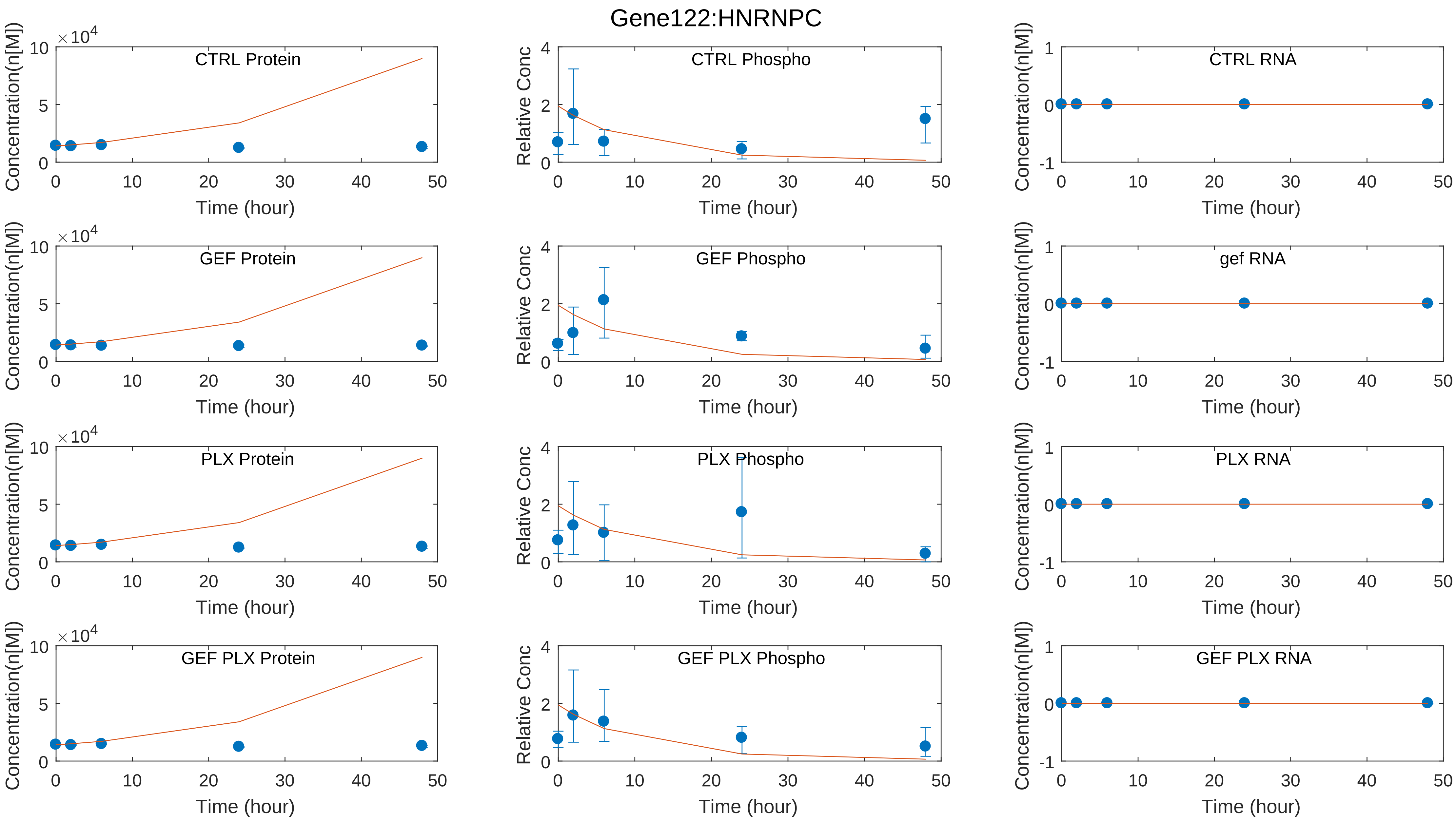
Gene122:HNRNPC
CTRL Protein
GEF Protein
PLX Protein
GEF PLX Protein
CTRL Phospho
GEF Phospho
PLX Phospho
GEF PLX Phospho
CTRL RNA
gef RNA
PLX RNA
GEF PLX RNA
Concentration(n[M])
Relative Conc
Time (hour)
×10^4

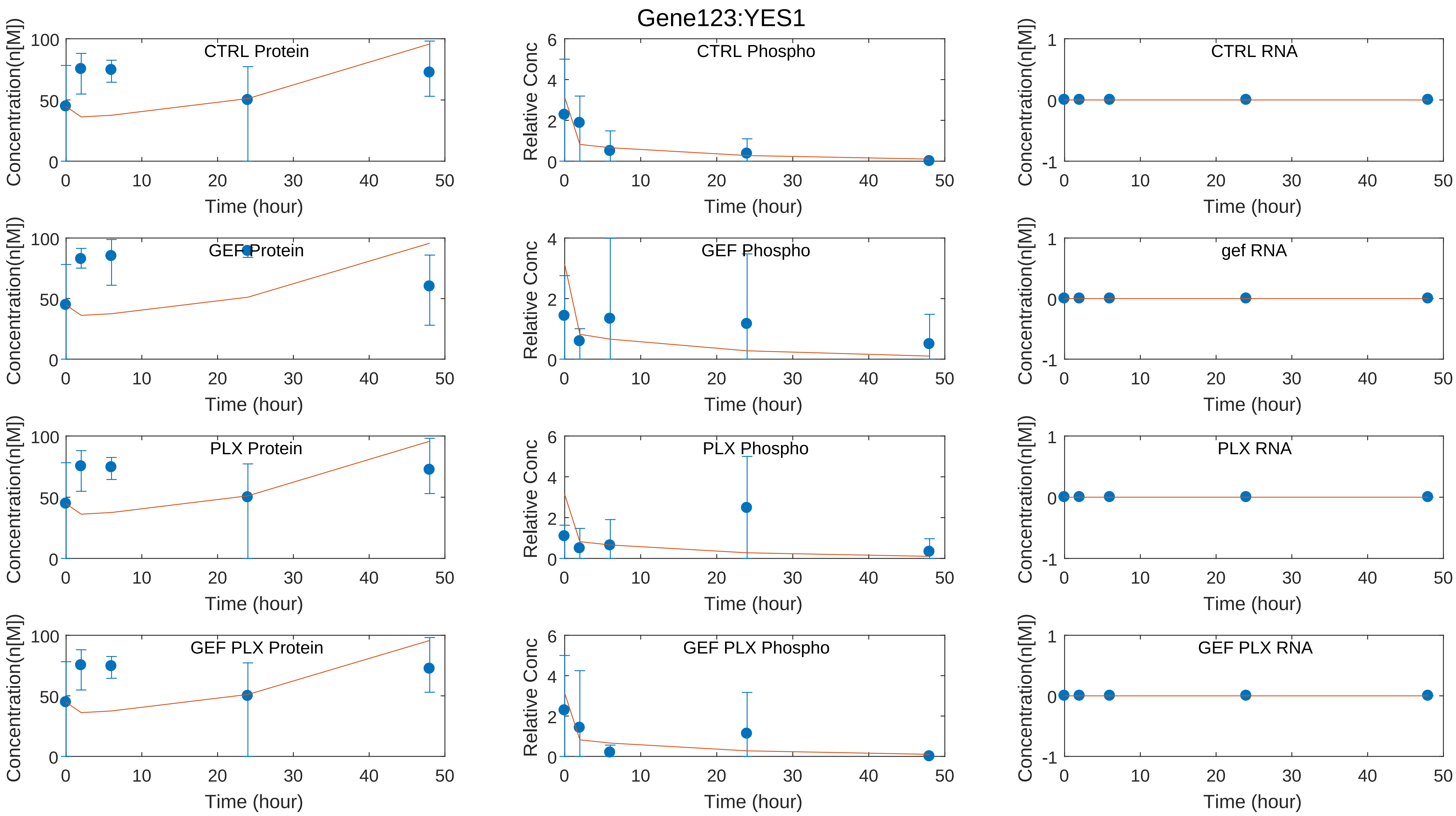

Gene123:YES1
CTRL Protein
CTRL Phospho
CTRL RNA
GEF Protein
GEF Phospho
gef RNA
PLX Protein
PLX Phospho
PLX RNA
GEF PLX Protein
GEF PLX Phospho
GEF PLX RNA
Concentration(n[M])
Relative Conc
Time (hour)

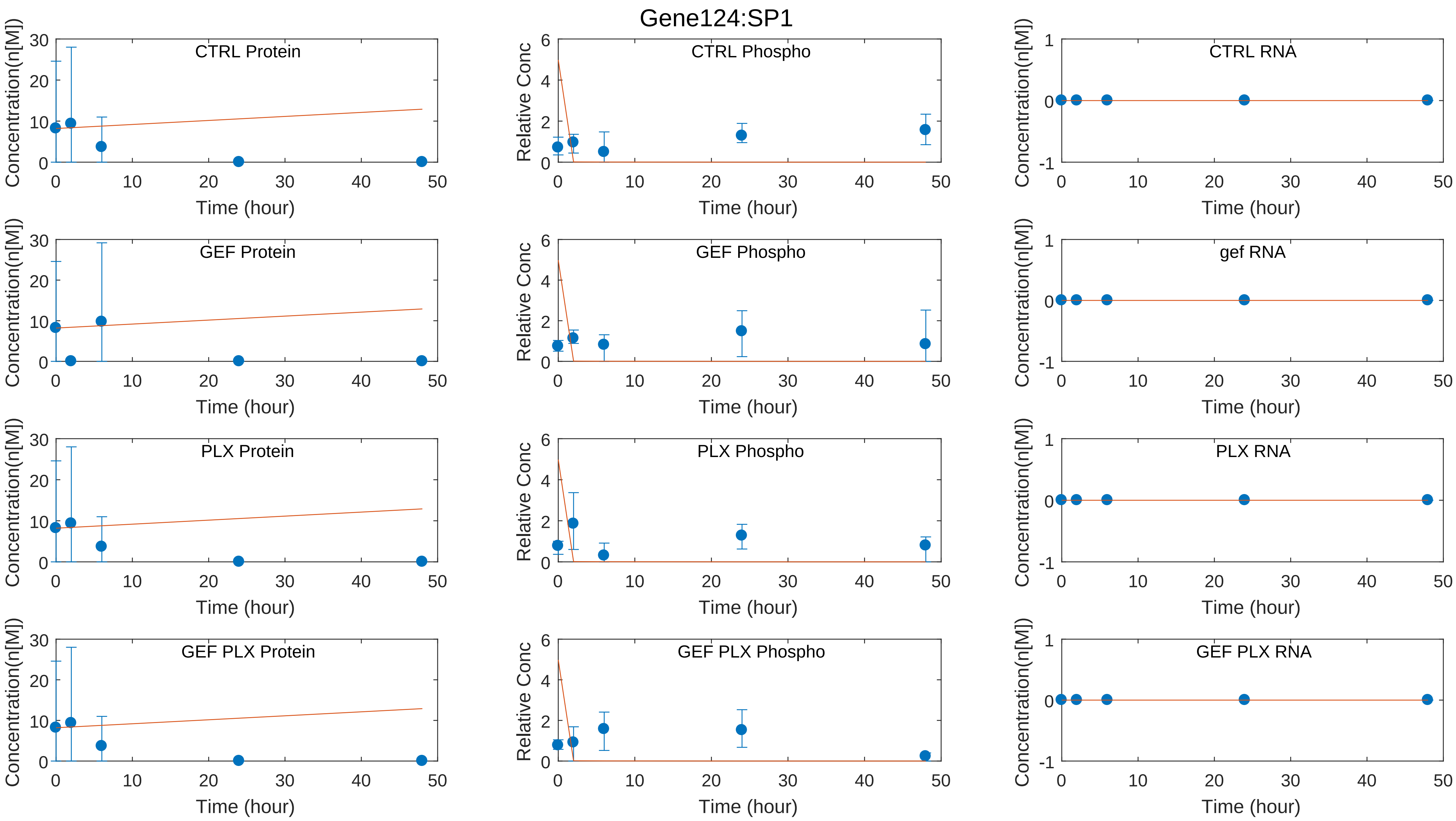

Gene124:SP1
CTRL Protein
CTRL Phospho
CTRL RNA
GEF Protein
GEF Phospho
gef RNA
PLX Protein
PLX Phospho
PLX RNA
GEF PLX Protein
GEF PLX Phospho
GEF PLX RNA
Concentration(n[M])
Relative Conc
Time (hour)

Gene125:IGF1R

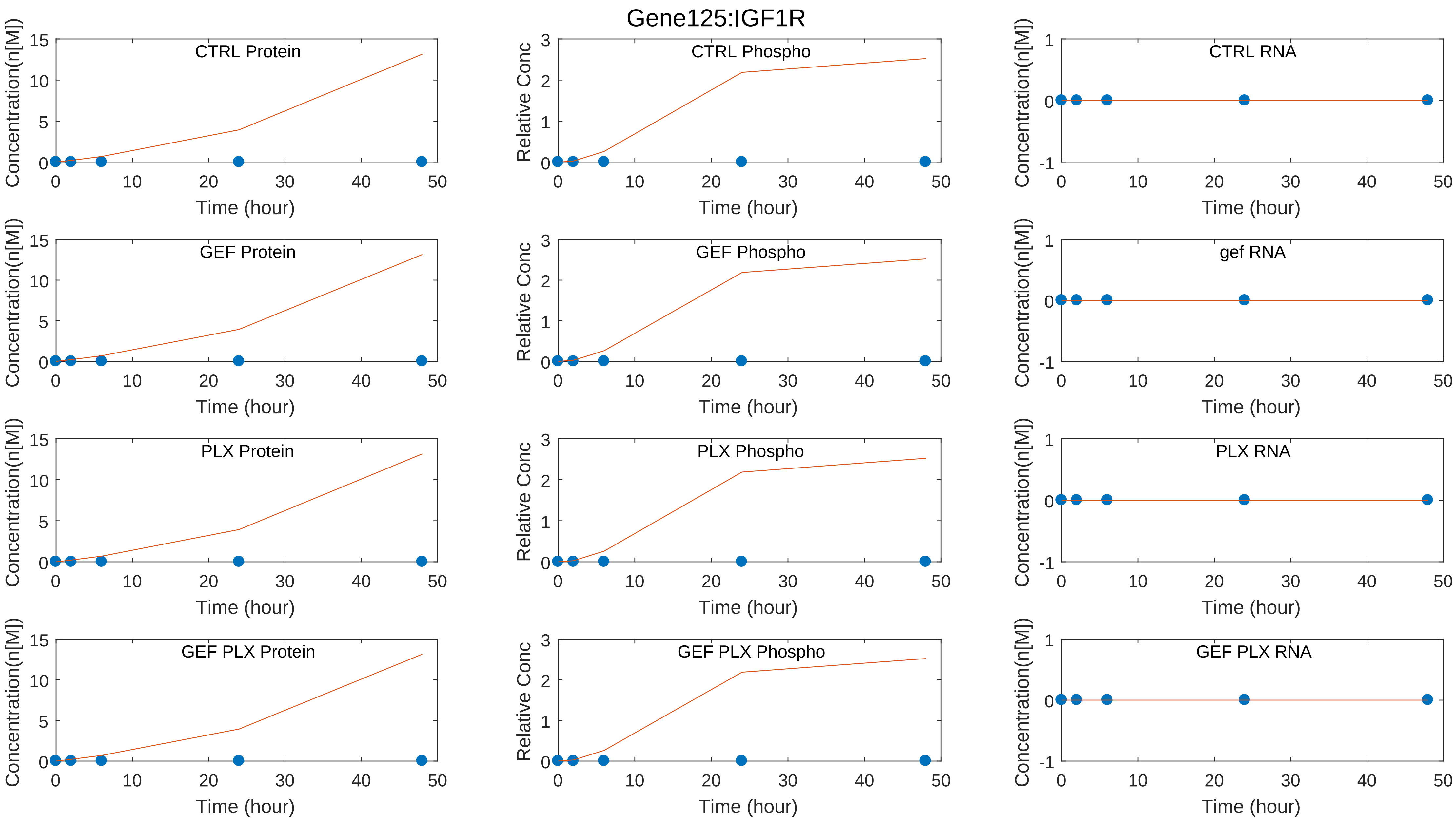

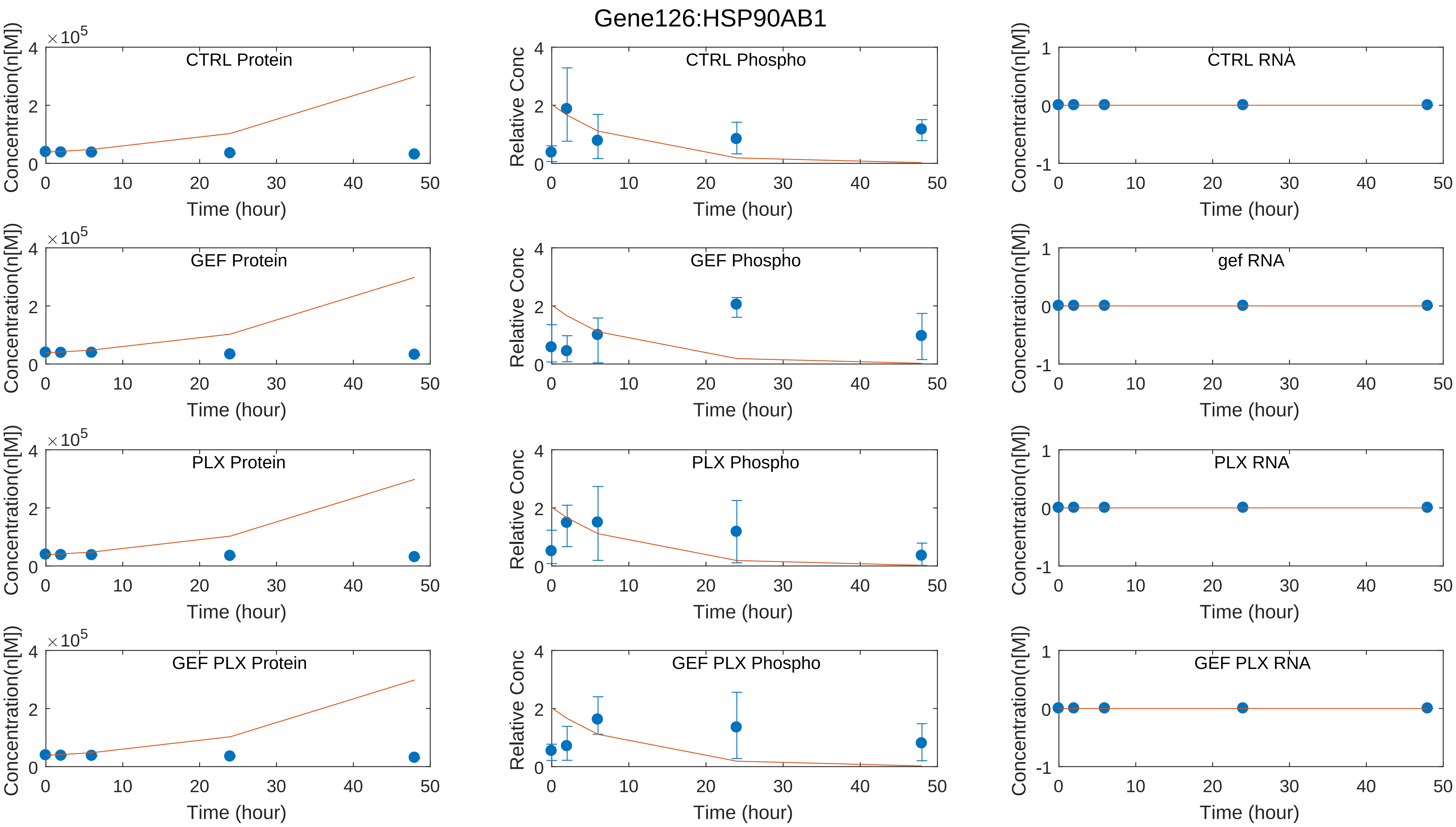

Gene126:HSP90AB1
CTRL Protein
CTRL Phospho
CTRL RNA
GEF Protein
GEF Phospho
gef RNA
PLX Protein
PLX Phospho
PLX RNA
GEF PLX Protein
GEF PLX Phospho
GEF PLX RNA
Concentration(n[M])
Relative Conc
Time (hour)
×10^5

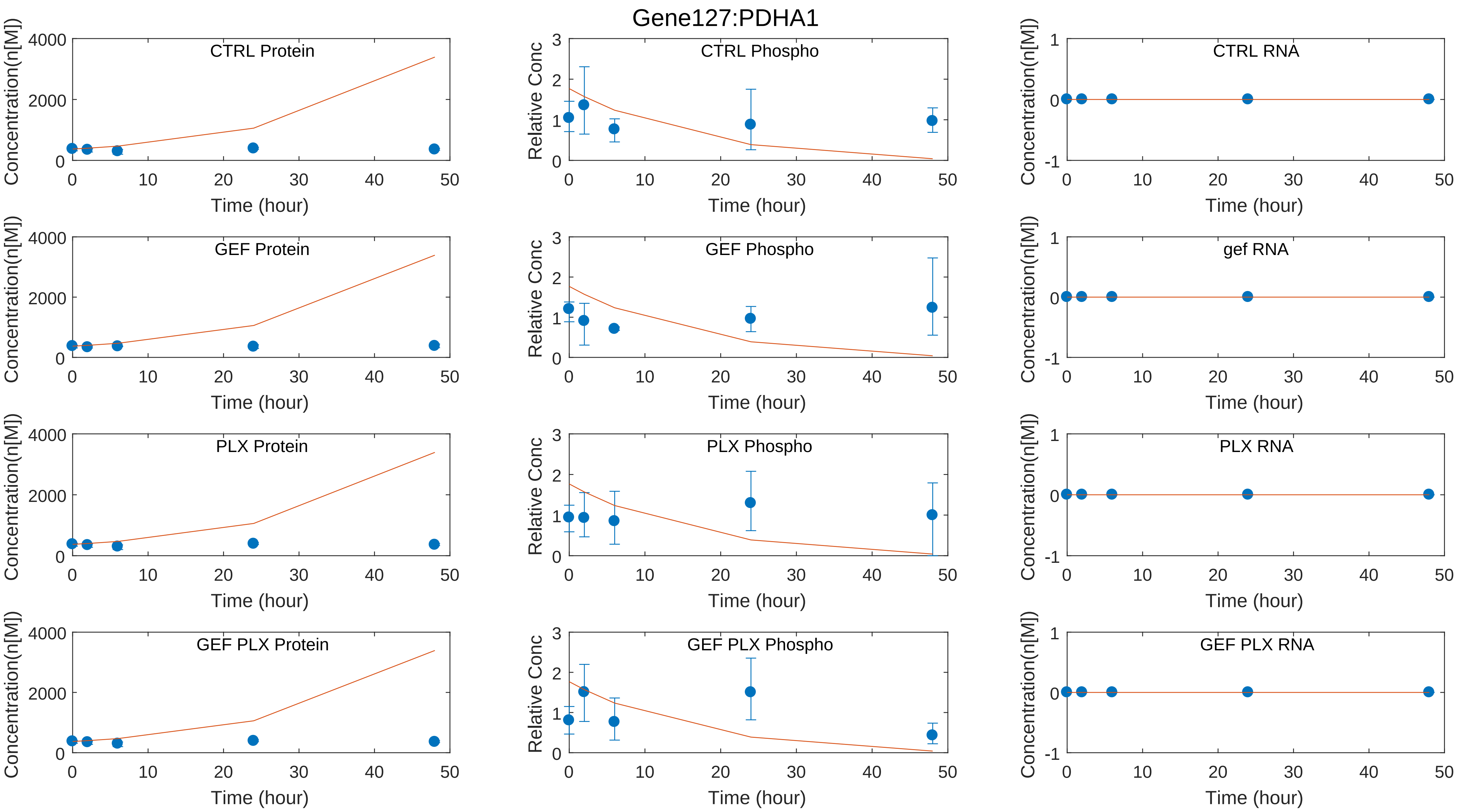
Gene127:PDHA1
CTRL Protein
CTRL Phospho
CTRL RNA
GEF Protein
GEF Phospho
gef RNA
PLX Protein
PLX Phospho
PLX RNA
GEF PLX Protein
GEF PLX Phospho
GEF PLX RNA
Concentration(n[M])
Relative Conc
Time (hour)

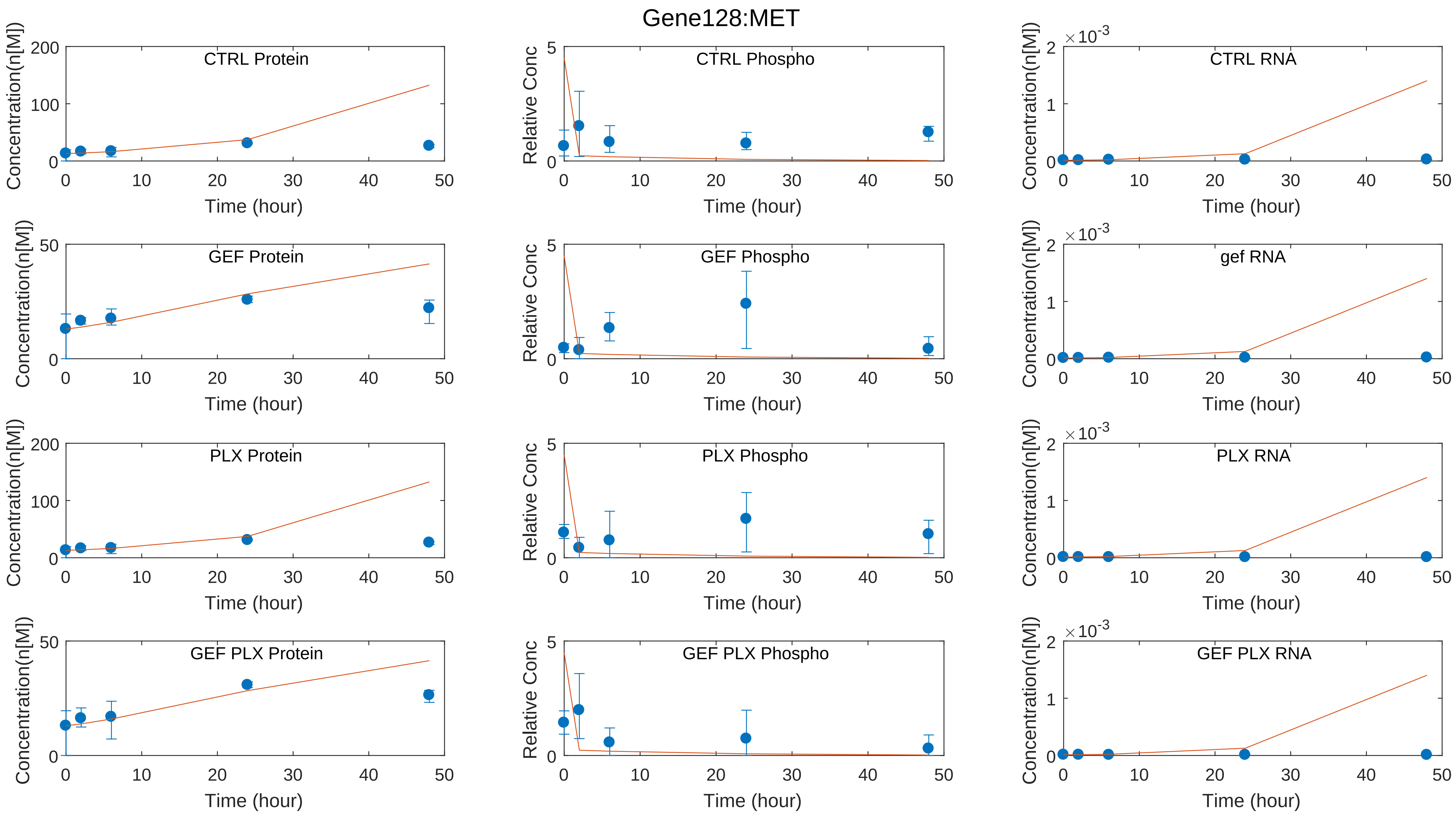
Gene128:MET
CTRL Protein
GEF Protein
PLX Protein
GEF PLX Protein
CTRL Phospho
GEF Phospho
PLX Phospho
GEF PLX Phospho
CTRL RNA
gef RNA
PLX RNA
GEF PLX RNA
Concentration(n[M])
Relative Conc
Time (hour)
×10-3

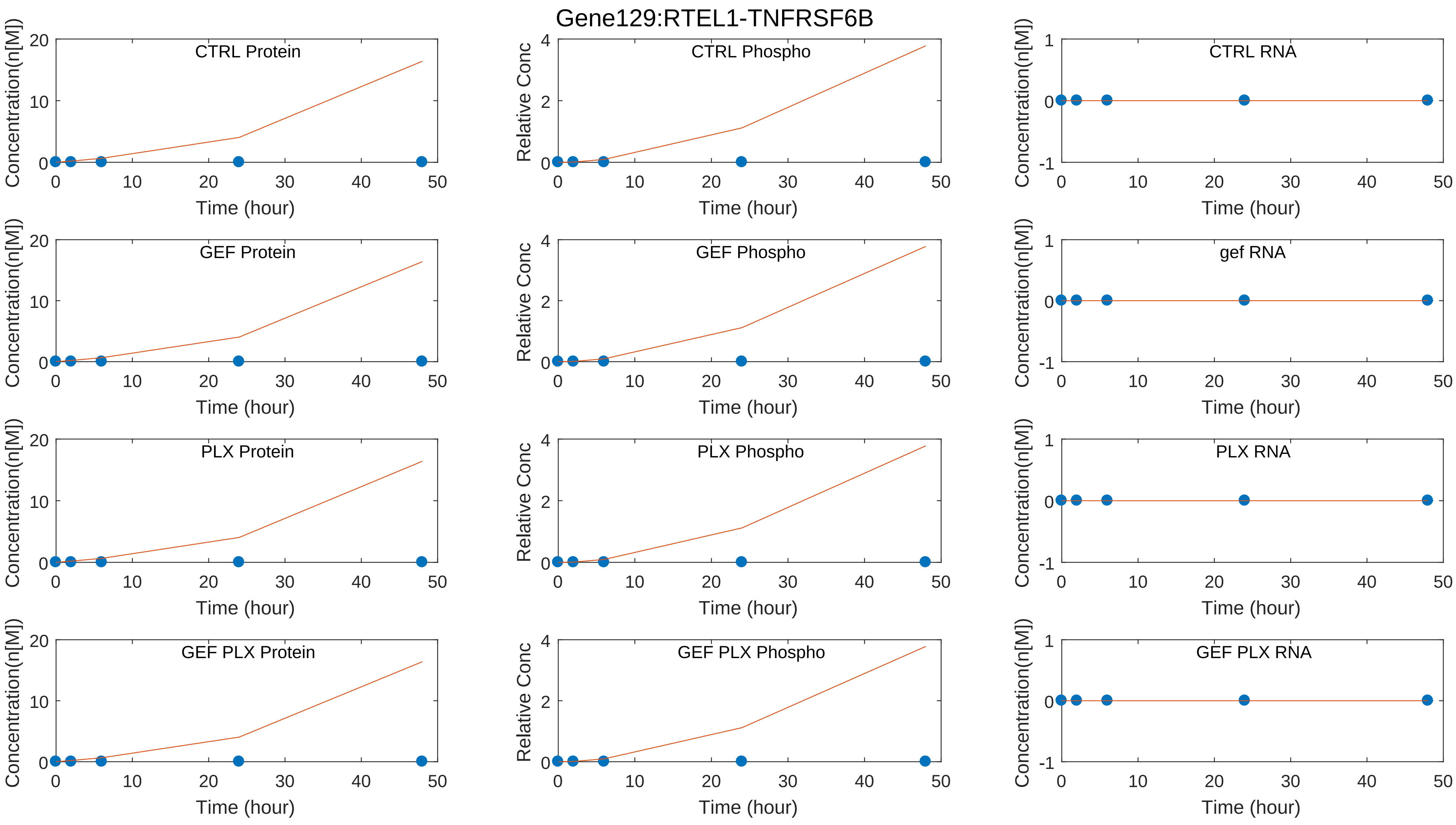
Gene129:RTEL1-TNFRSF6B
CTRL Protein
CTRL Phospho
CTRL RNA
GEF Protein
GEF Phospho
gef RNA
PLX Protein
PLX Phospho
PLX RNA
GEF PLX Protein
GEF PLX Phospho
GEF PLX RNA
Concentration(n[M])
Relative Conc
Time (hour)
0
10
20
30
40
50
2
4
1
-1

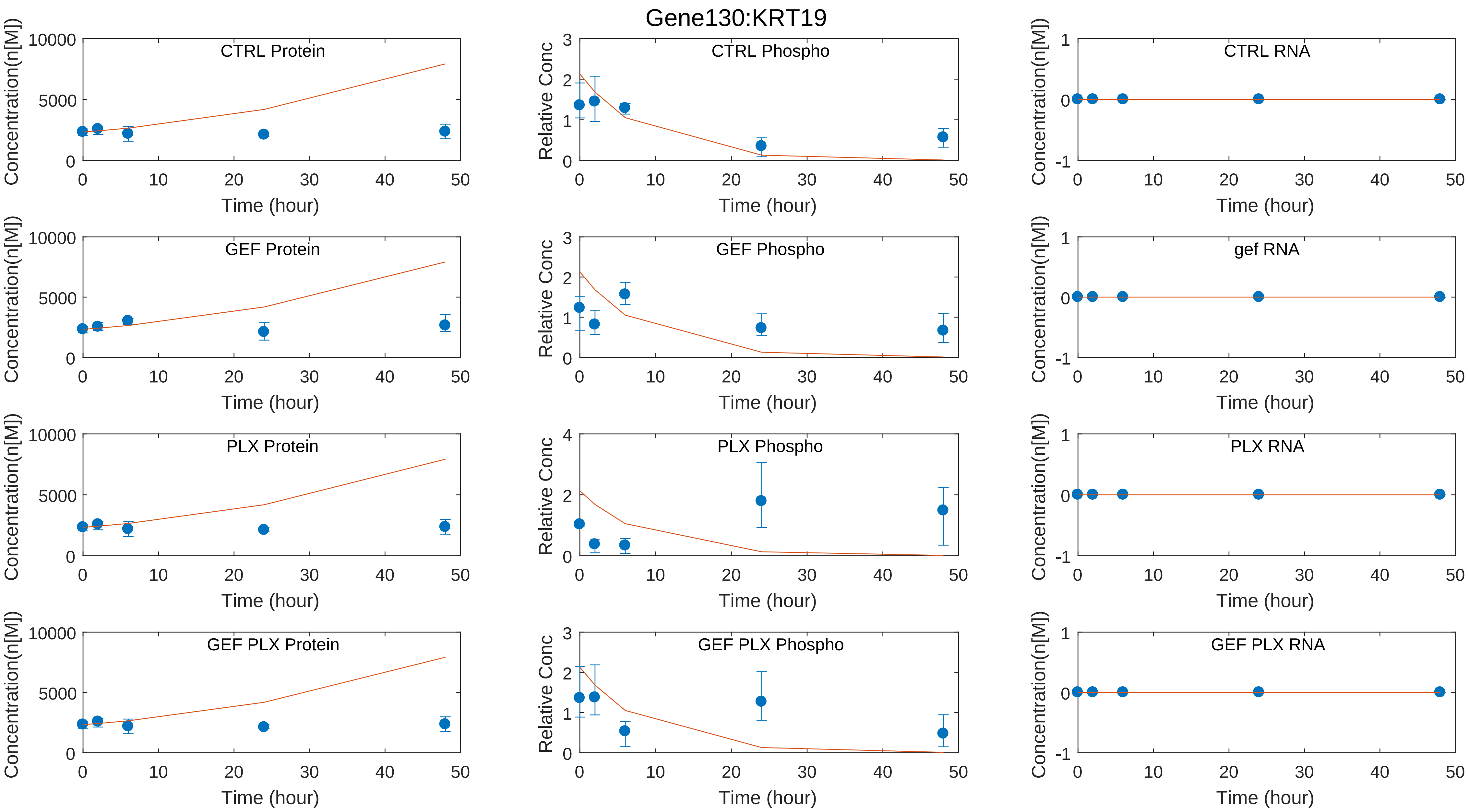

Gene130:KRT19
CTRL Protein
CTRL Phospho
CTRL RNA
GEF Protein
GEF Phospho
gef RNA
PLX Protein
PLX Phospho
PLX RNA
GEF PLX Protein
GEF PLX Phospho
GEF PLX RNA
Concentration(n[M])
Relative Conc
Time (hour)

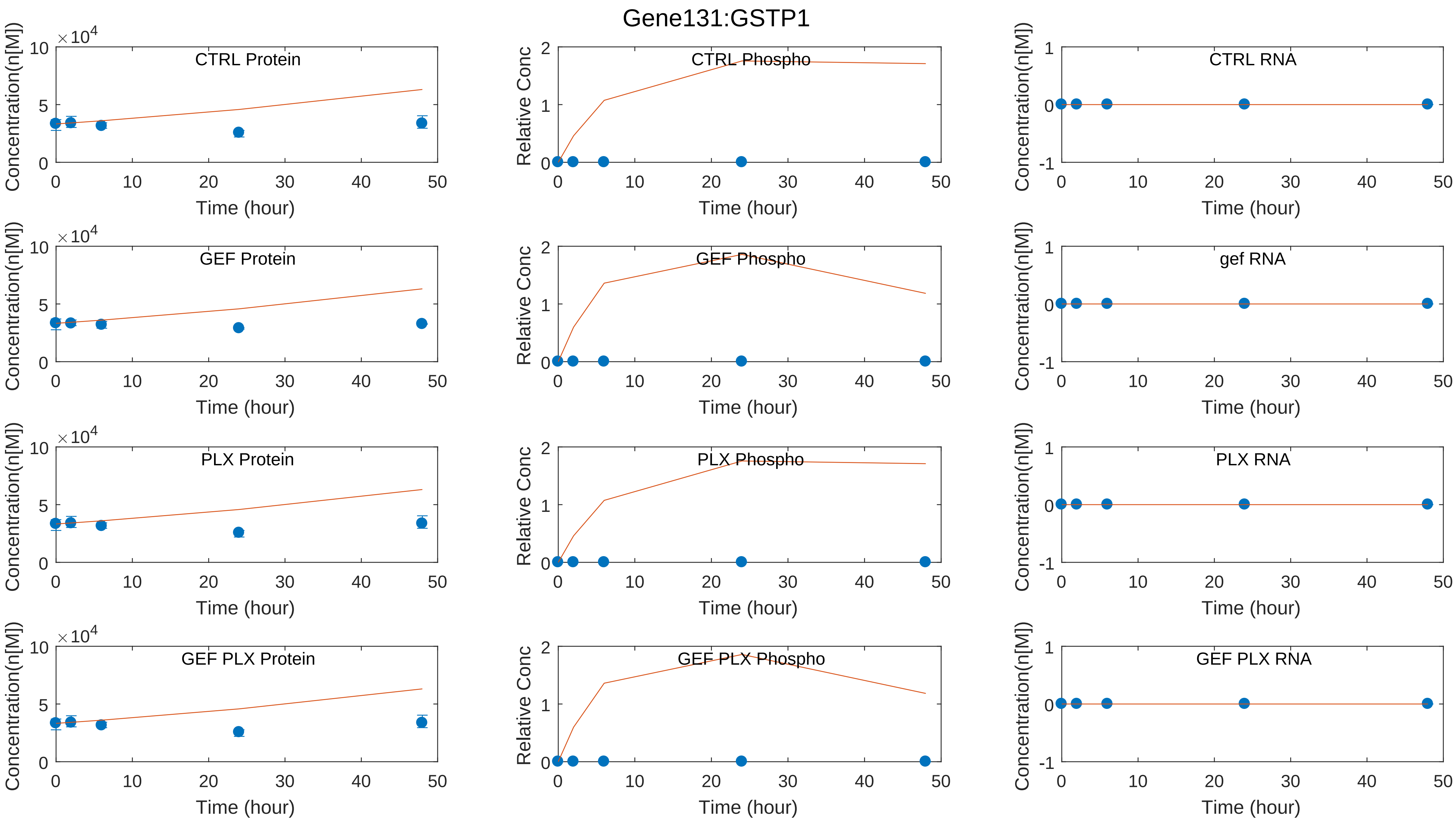

Gene131:GSTP1
CTRL Protein
GEF Protein
PLX Protein
GEF PLX Protein
CTRL Phospho
GEF Phospho
PLX Phospho
GEF PLX Phospho
CTRL RNA
gef RNA
PLX RNA
GEF PLX RNA
Concentration(n[M])
Relative Conc
Time (hour)
$\times 10^4$

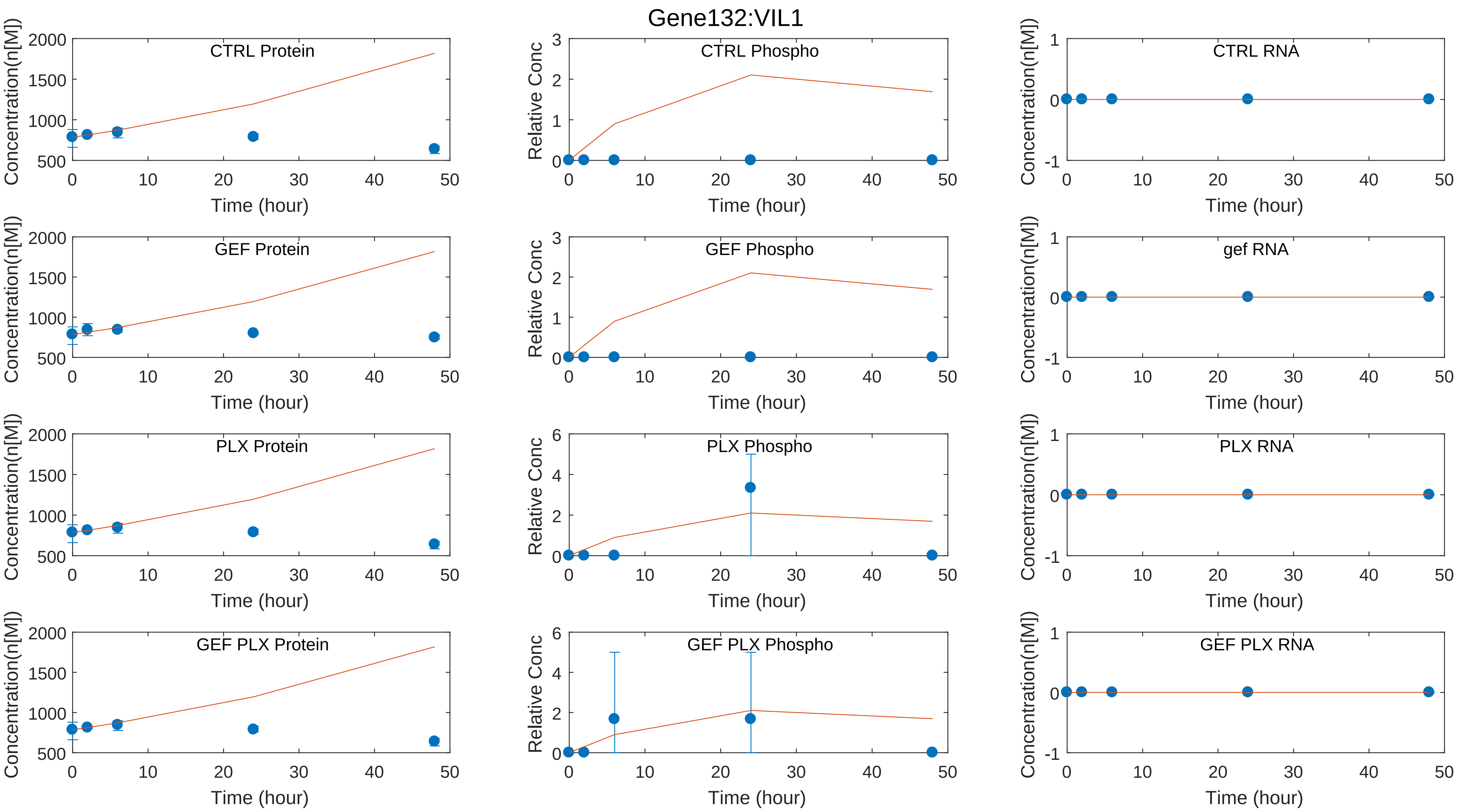
Gene132:VIL1
CTRL Protein
CTRL Phospho
CTRL RNA
GEF Protein
GEF Phospho
gef RNA
PLX Protein
PLX Phospho
PLX RNA
GEF PLX Protein
GEF PLX Phospho
GEF PLX RNA
Concentration(n[M])
Relative Conc
Time (hour)

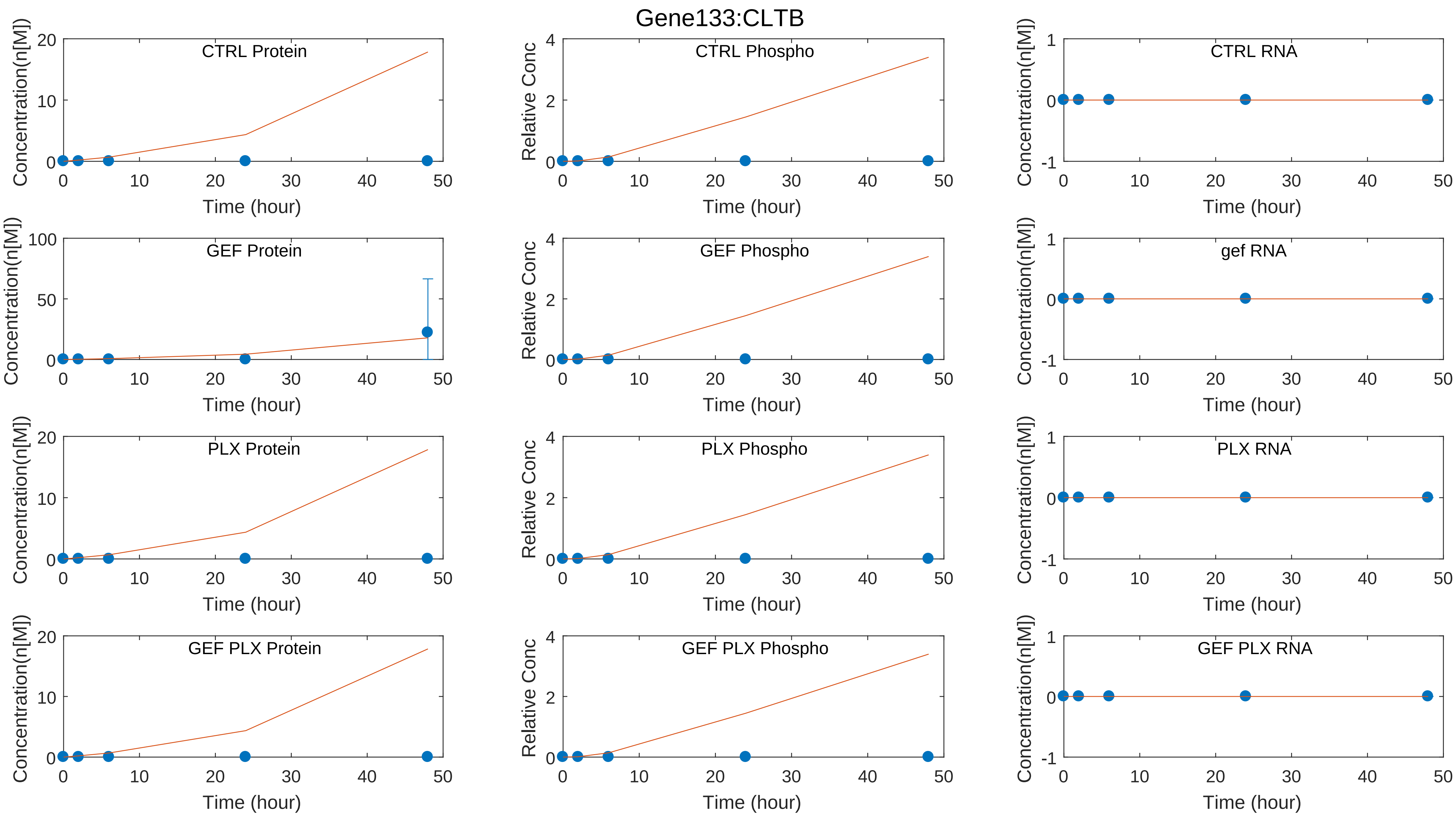
Gene133:CLTB
CTRL Protein
CTRL Phospho
CTRL RNA
GEF Protein
GEF Phospho
gef RNA
PLX Protein
PLX Phospho
PLX RNA
GEF PLX Protein
GEF PLX Phospho
GEF PLX RNA
Concentration(n[M])
Relative Conc
Time (hour)
0
10
20
30
40
50
100
2
4
1
-1

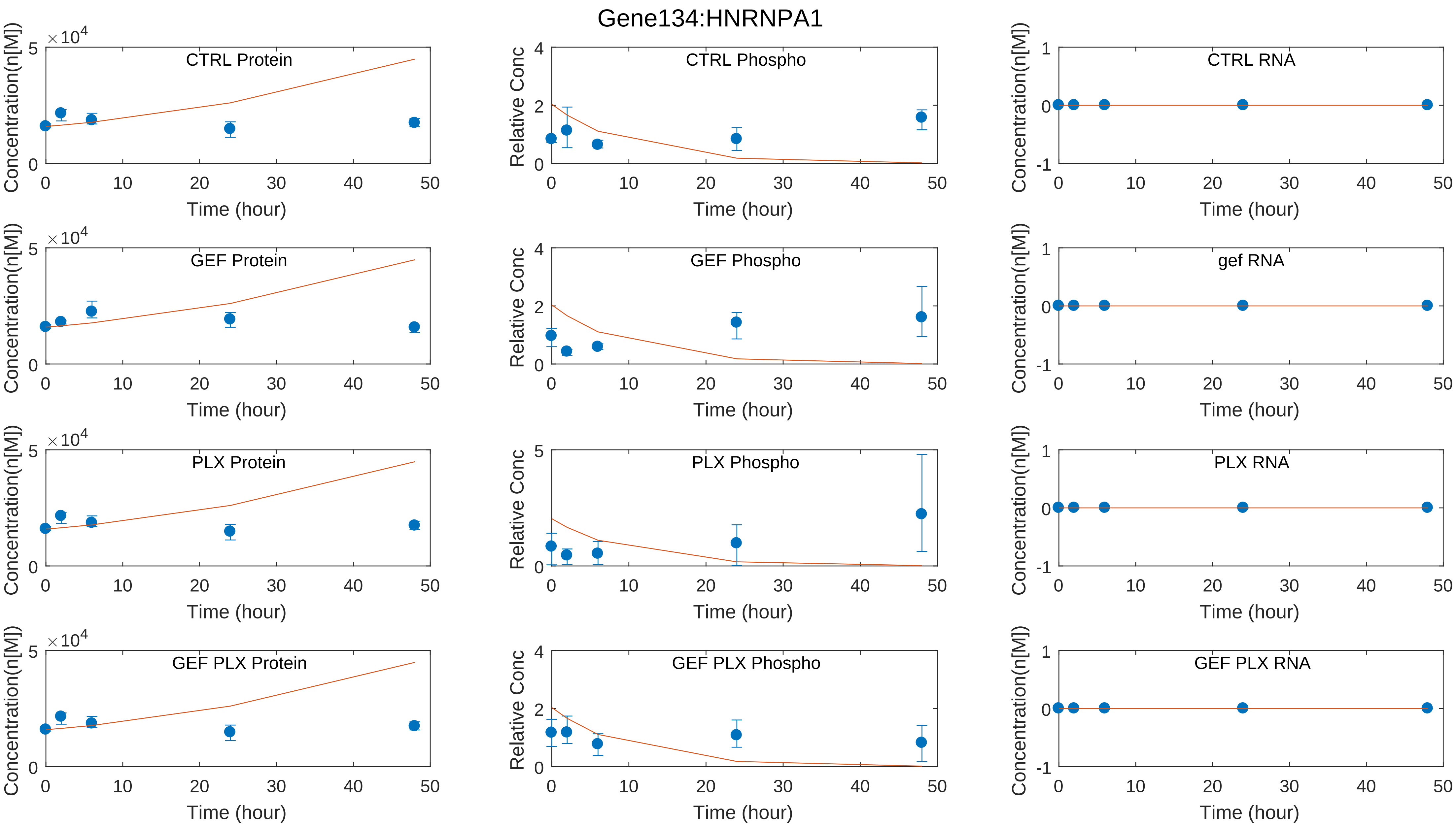
Gene134:HNRNPA1
CTRL Protein
CTRL Phospho
CTRL RNA
GEF Protein
GEF Phospho
gef RNA
PLX Protein
PLX Phospho
PLX RNA
GEF PLX Protein
GEF PLX Phospho
GEF PLX RNA
Concentration(n[M])
Relative Conc
Time (hour)
×10^4

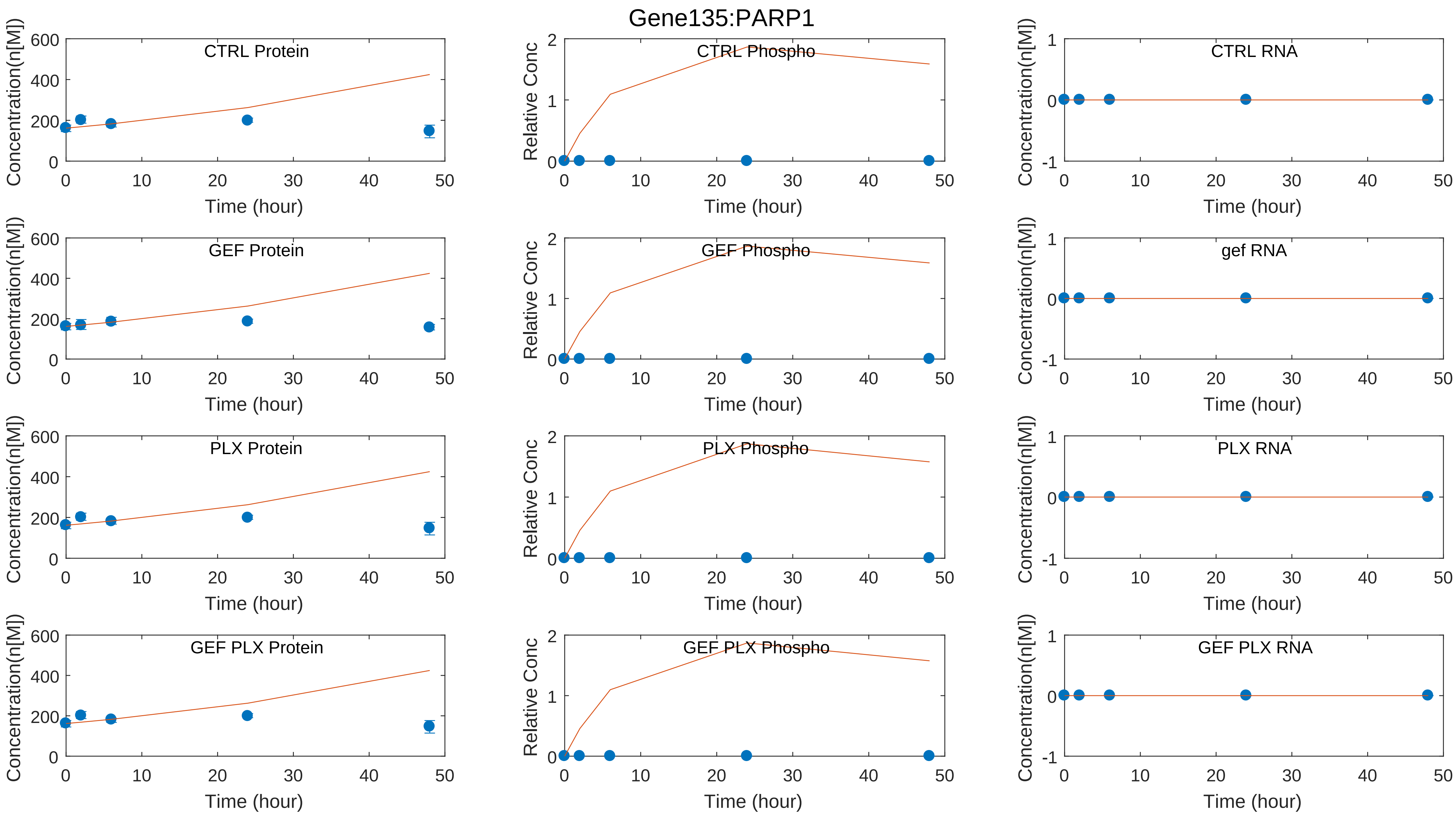

Gene135:PARP1
CTRL Protein
GEF Protein
PLX Protein
GEF PLX Protein
CTRL Phospho
GEF Phospho
PLX Phospho
GEF PLX Phospho
CTRL RNA
gef RNA
PLX RNA
GEF PLX RNA
Concentration(n[M])
Relative Conc
Time (hour)

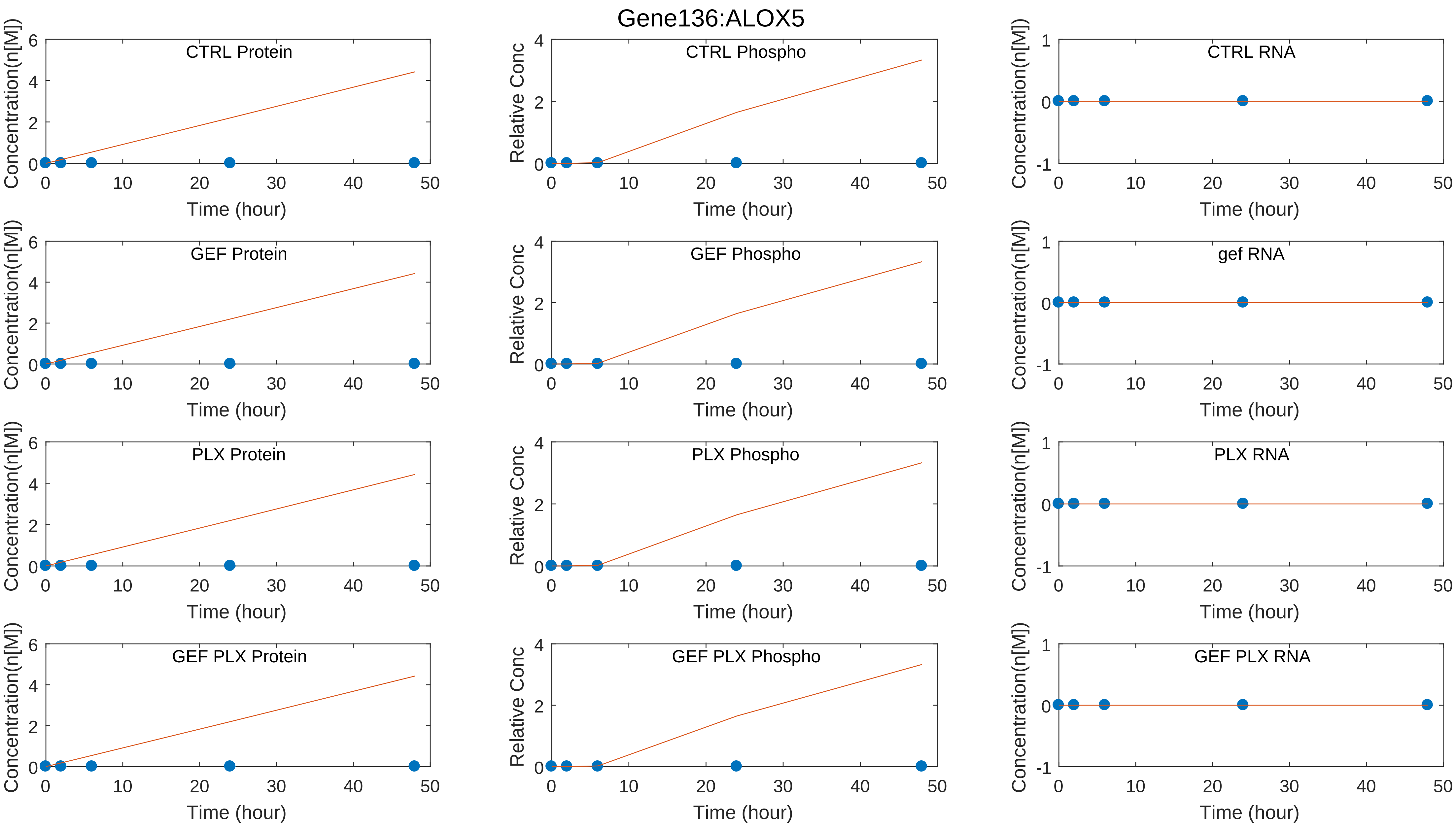
Gene136:ALOX5
CTRL Protein
CTRL Phospho
CTRL RNA
GEF Protein
GEF Phospho
gef RNA
PLX Protein
PLX Phospho
PLX RNA
GEF PLX Protein
GEF PLX Phospho
GEF PLX RNA
Concentration(n[M])
Relative Conc
Time (hour)

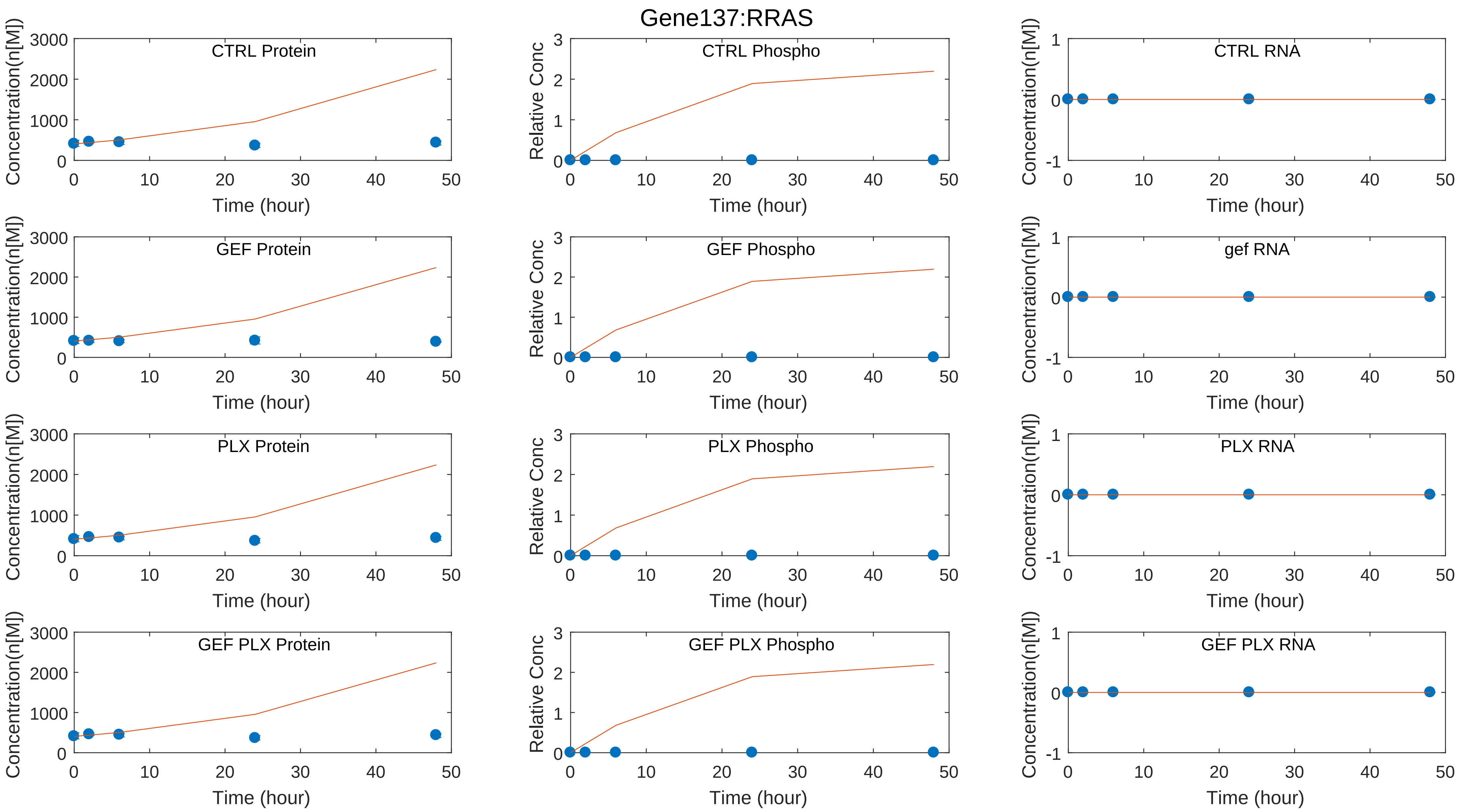
Gene137:RRAS
CTRL Protein
CTRL Phospho
CTRL RNA
GEF Protein
GEF Phospho
gef RNA
PLX Protein
PLX Phospho
PLX RNA
GEF PLX Protein
GEF PLX Phospho
GEF PLX RNA
Concentration(n[M])
Relative Conc
Time (hour)

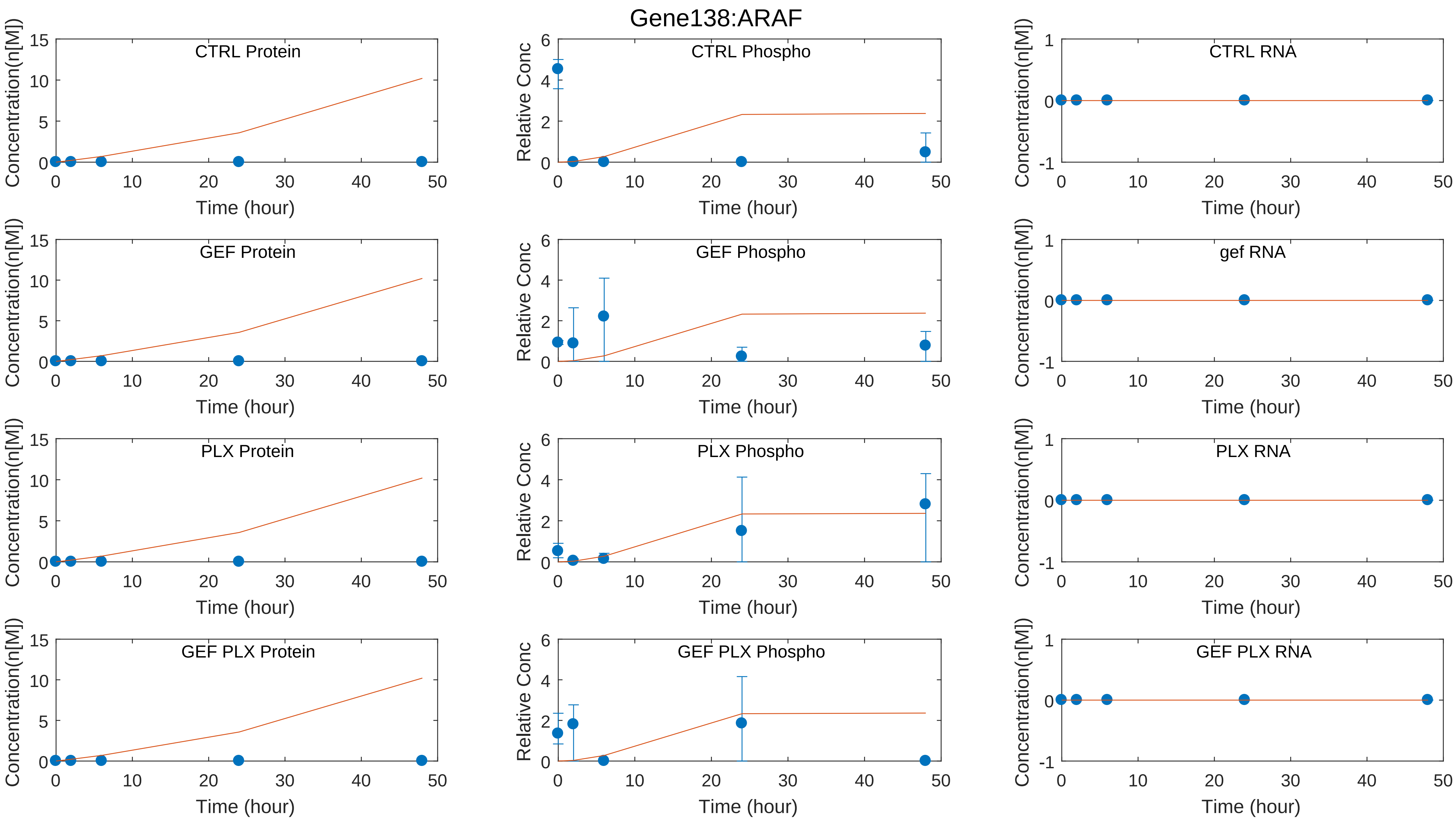
Gene138:ARAF
CTRL Protein
CTRL Phospho
CTRL RNA
GEF Protein
GEF Phospho
gef RNA
PLX Protein
PLX Phospho
PLX RNA
GEF PLX Protein
GEF PLX Phospho
GEF PLX RNA
Concentration(n[M])
Relative Conc
Time (hour)

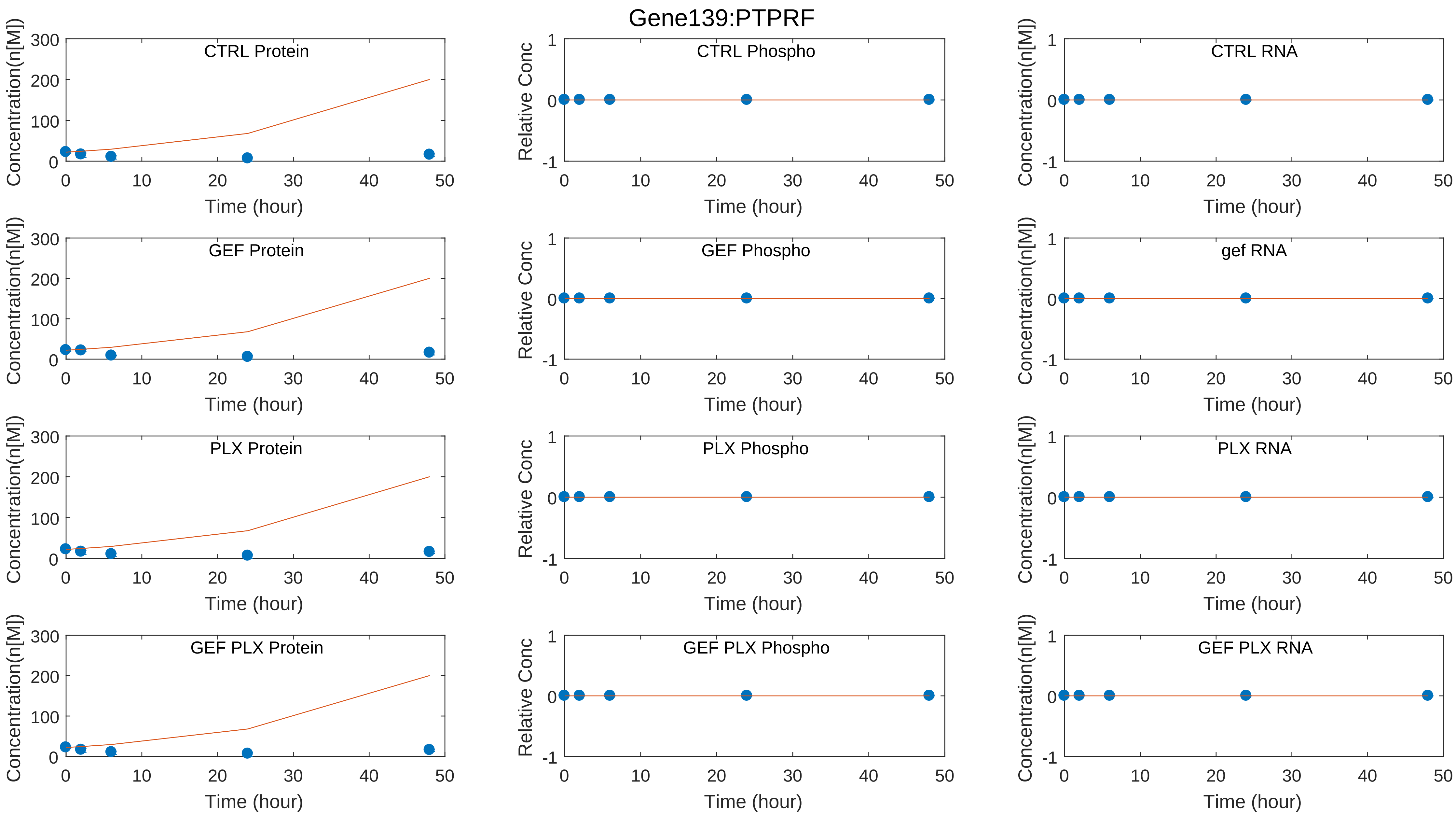
Gene139:PTPRF
CTRL Protein
CTRL Phospho
CTRL RNA
GEF Protein
GEF Phospho
gef RNA
PLX Protein
PLX Phospho
PLX RNA
GEF PLX Protein
GEF PLX Phospho
GEF PLX RNA
Concentration(n[M])
Relative Conc
Time (hour)
0
10
20
30
40
50
0
100
200
300
-1
1

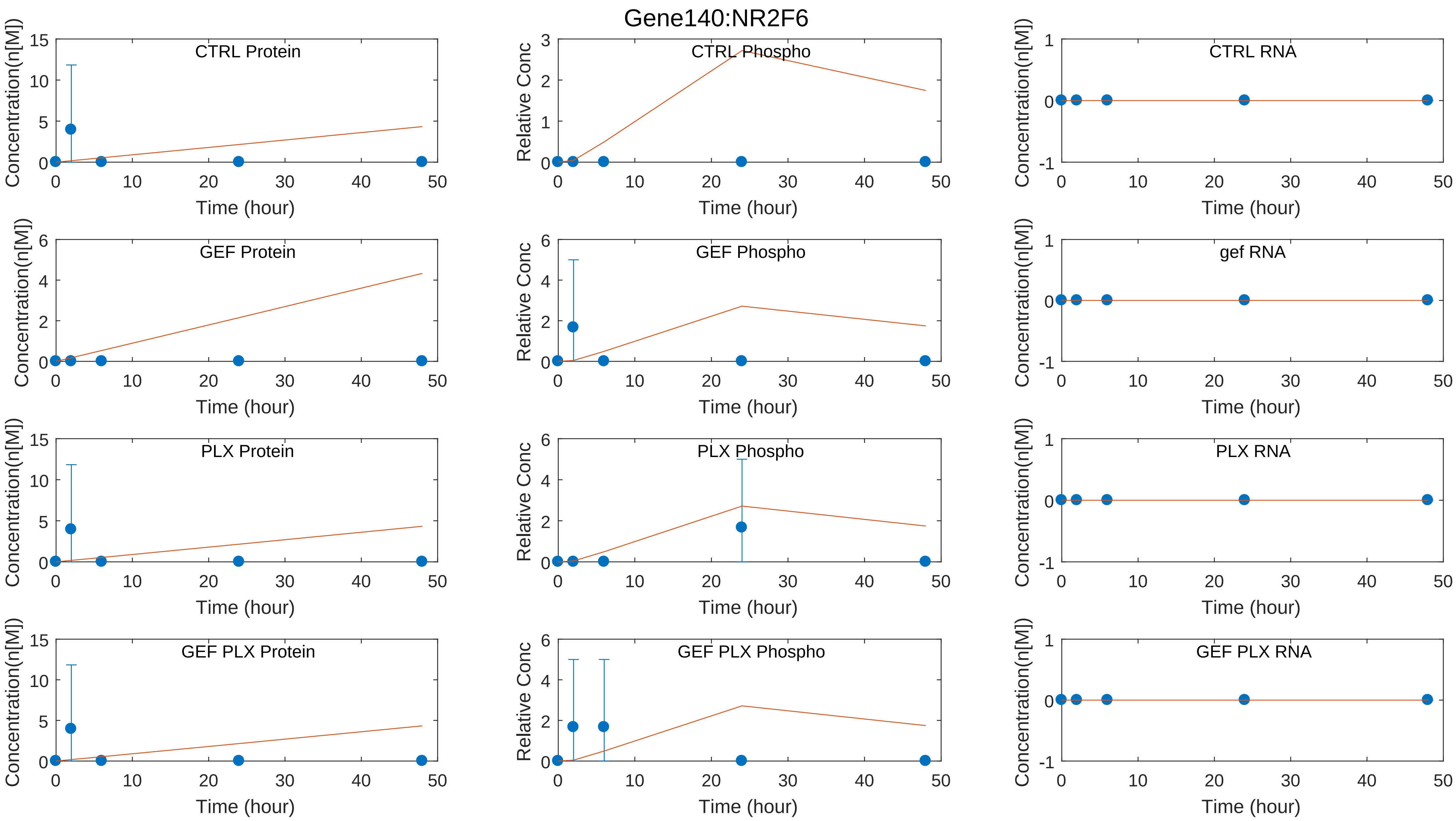

Gene140:NR2F6
CTRL Protein
CTRL Phospho
CTRL RNA
GEF Protein
GEF Phospho
gef RNA
PLX Protein
PLX Phospho
PLX RNA
GEF PLX Protein
GEF PLX Phospho
GEF PLX RNA
Concentration(n[M])
Relative Conc
Time (hour)

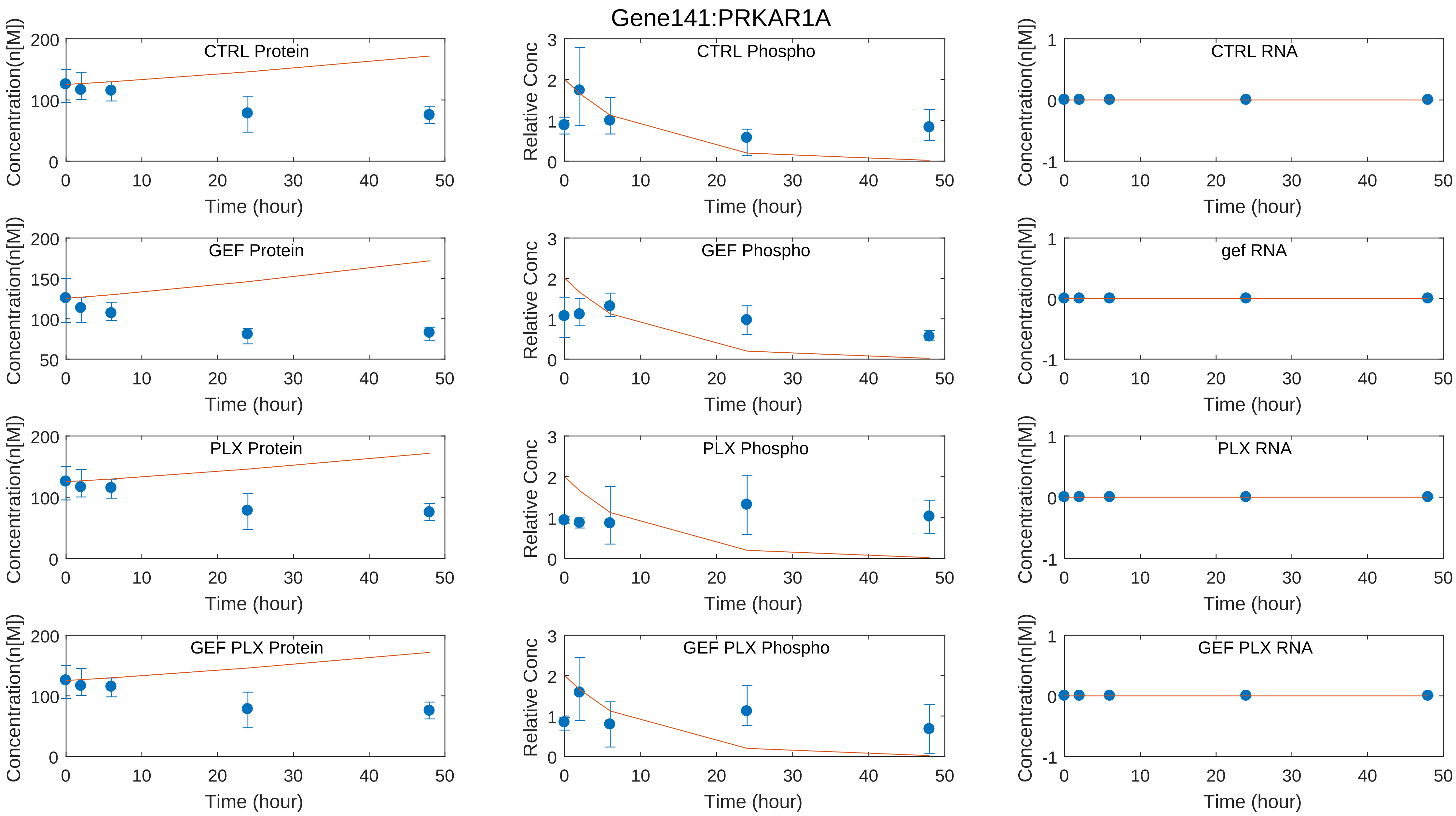
Gene141:PRKAR1A
CTRL Protein
CTRL Phospho
CTRL RNA
GEF Protein
GEF Phospho
gef RNA
PLX Protein
PLX Phospho
PLX RNA
GEF PLX Protein
GEF PLX Phospho
GEF PLX RNA
Concentration(n[M])
Relative Conc
Time (hour)

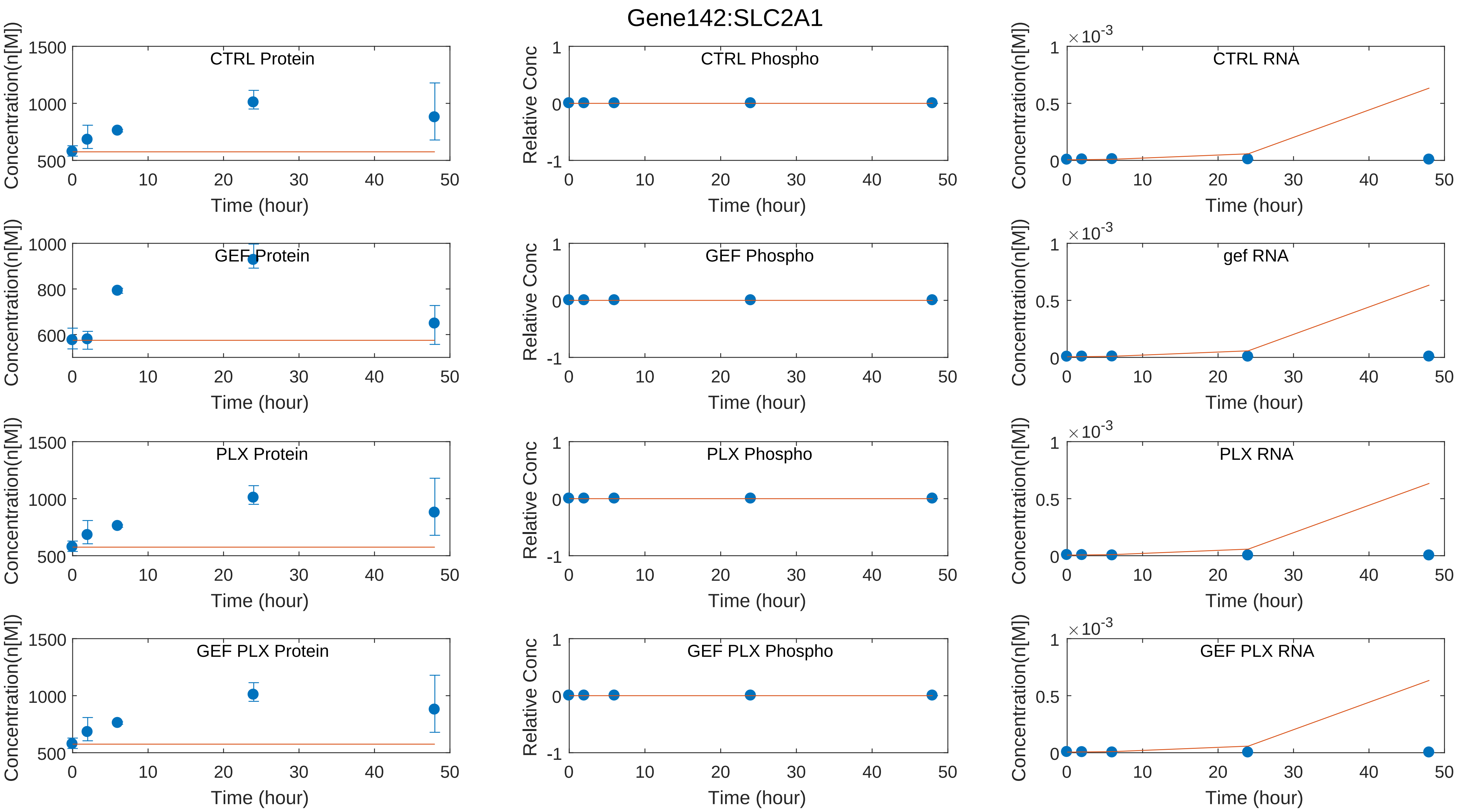
Gene142:SLC2A1
CTRL Protein
CTRL Phospho
CTRL RNA
GEF Protein
GEF Phospho
gef RNA
PLX Protein
PLX Phospho
PLX RNA
GEF PLX Protein
GEF PLX Phospho
GEF PLX RNA
Concentration(n[M])
Relative Conc
Time (hour)
×10^-3

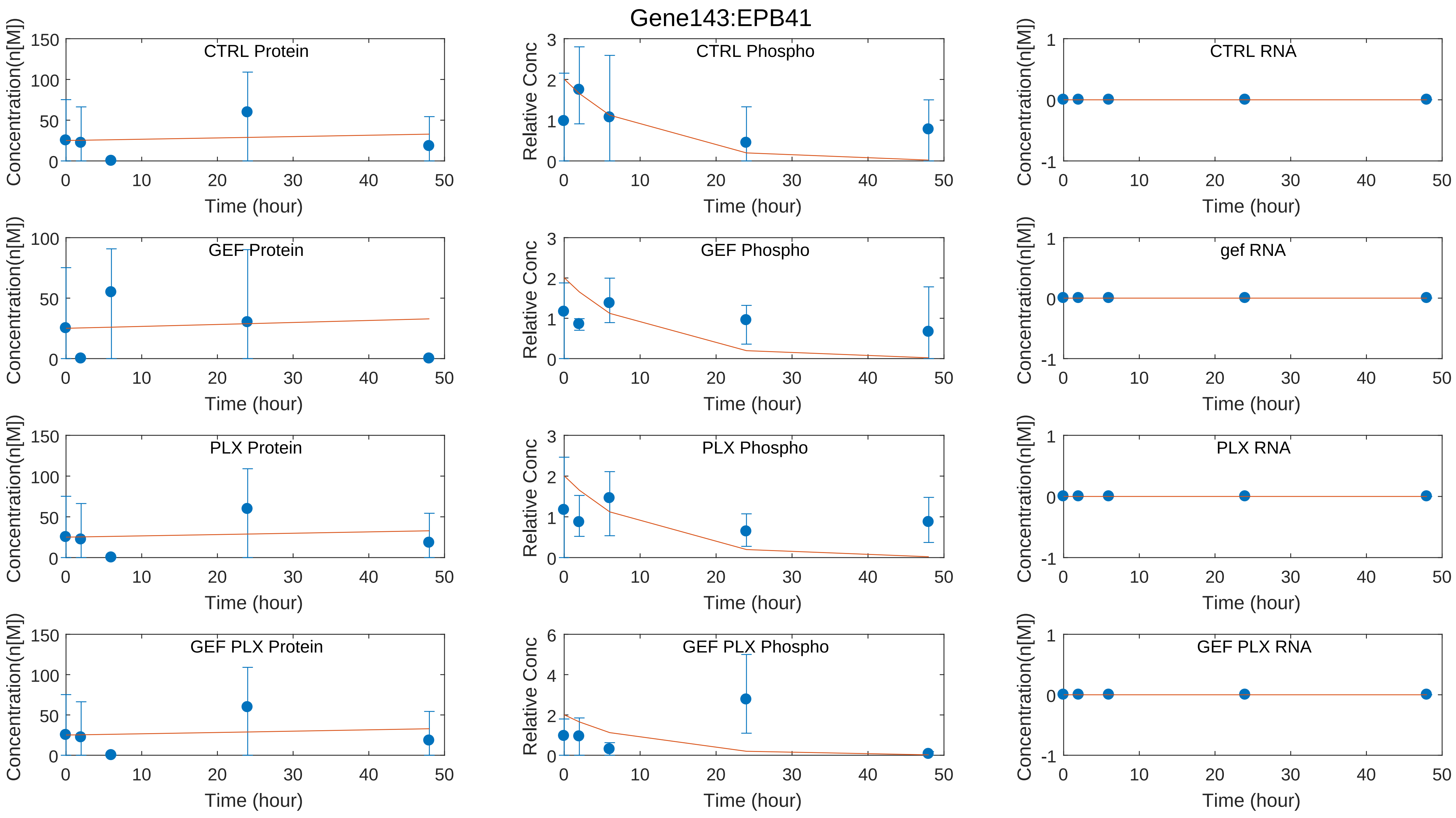

Gene143:EPB41
CTRL Protein
CTRL Phospho
CTRL RNA
GEF Protein
GEF Phospho
gef RNA
PLX Protein
PLX Phospho
PLX RNA
GEF PLX Protein
GEF PLX Phospho
GEF PLX RNA
Concentration(n[M])
Relative Conc
Time (hour)

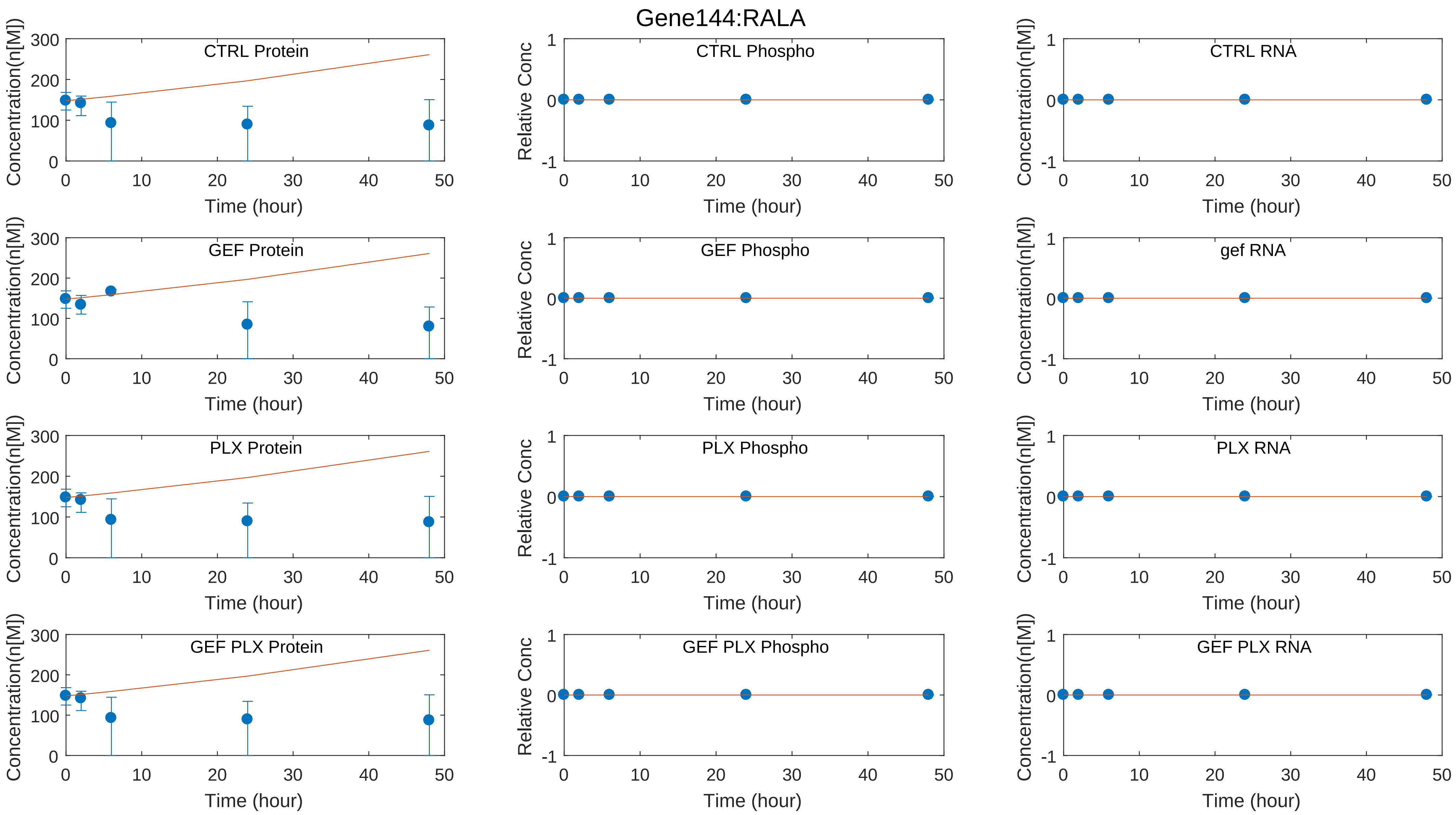

Gene144:RALA
CTRL Protein
CTRL Phospho
CTRL RNA
GEF Protein
GEF Phospho
gef RNA
PLX Protein
PLX Phospho
PLX RNA
GEF PLX Protein
GEF PLX Phospho
GEF PLX RNA
Concentration(n[M])
Relative Conc
Time (hour)
0
10
20
30
40
50
100
200
300
1
-1

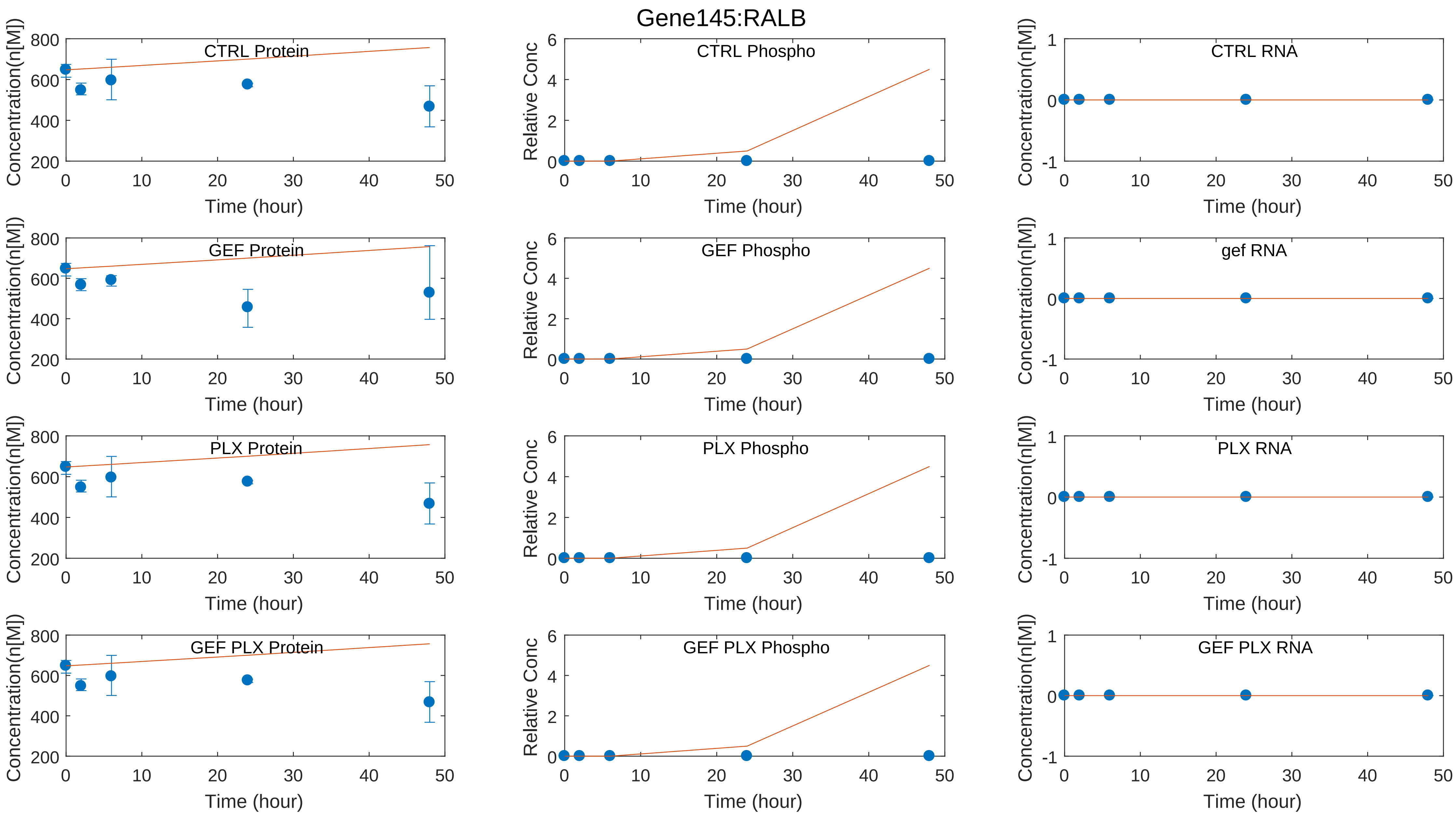
Gene145:RALB
CTRL Protein
CTRL Phospho
CTRL RNA
GEF Protein
GEF Phospho
gef RNA
PLX Protein
PLX Phospho
PLX RNA
GEF PLX Protein
GEF PLX Phospho
GEF PLX RNA
Concentration(n[M])
Relative Conc
Time (hour)
0
10
20
30
40
50
200
400
600
800
0
2
4
6
-1
0
1

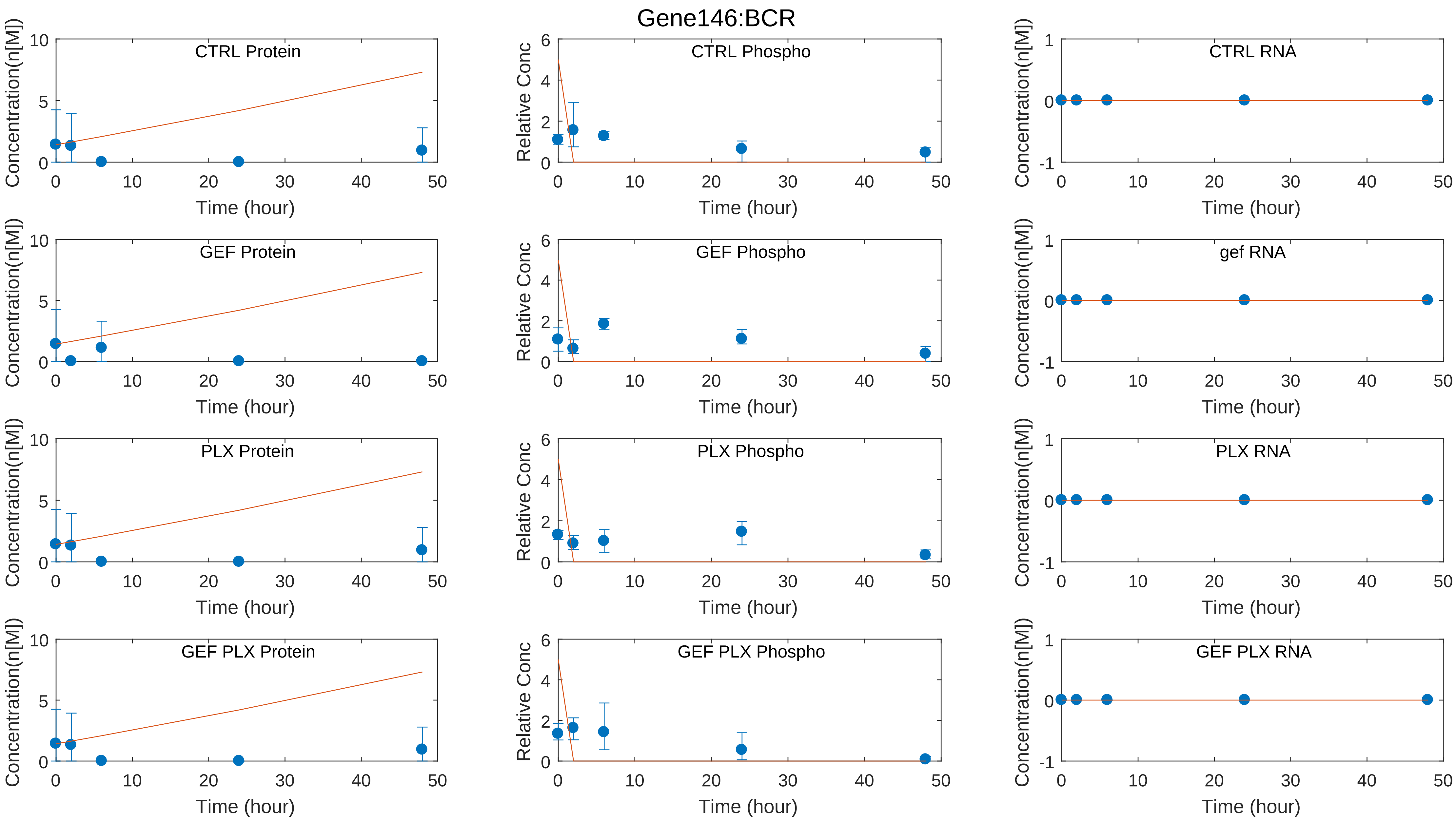

Gene146:BCR
CTRL Protein
GEF Protein
PLX Protein
GEF PLX Protein
CTRL Phospho
GEF Phospho
PLX Phospho
GEF PLX Phospho
CTRL RNA
gef RNA
PLX RNA
GEF PLX RNA
Concentration(n[M])
Relative Conc
Time (hour)
0
10
20
30
40
50

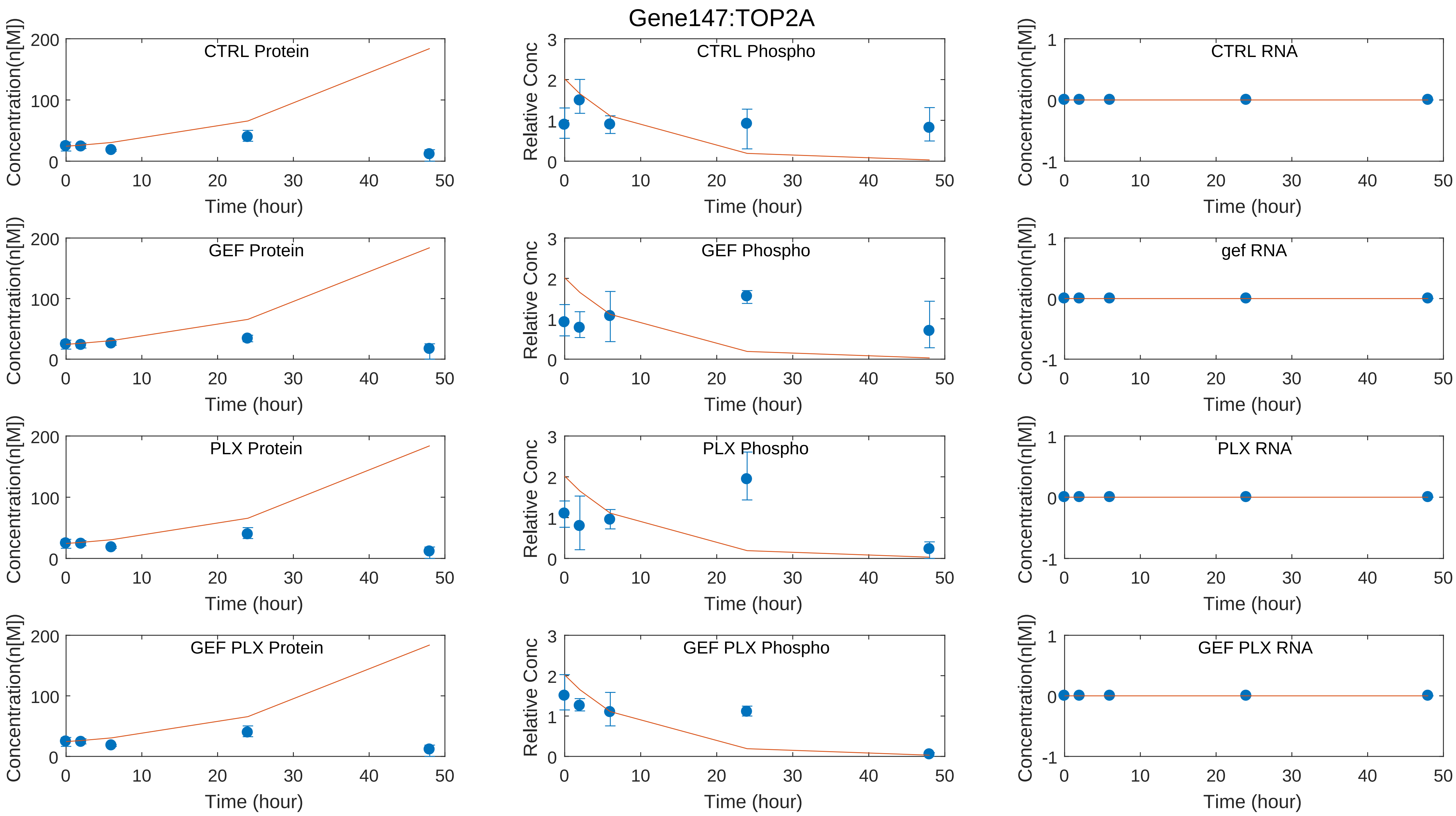
Gene147:TOP2A
CTRL Protein
CTRL Phospho
CTRL RNA
GEF Protein
GEF Phospho
gef RNA
PLX Protein
PLX Phospho
PLX RNA
GEF PLX Protein
GEF PLX Phospho
GEF PLX RNA
Concentration(n[M])
Relative Conc
Time (hour)

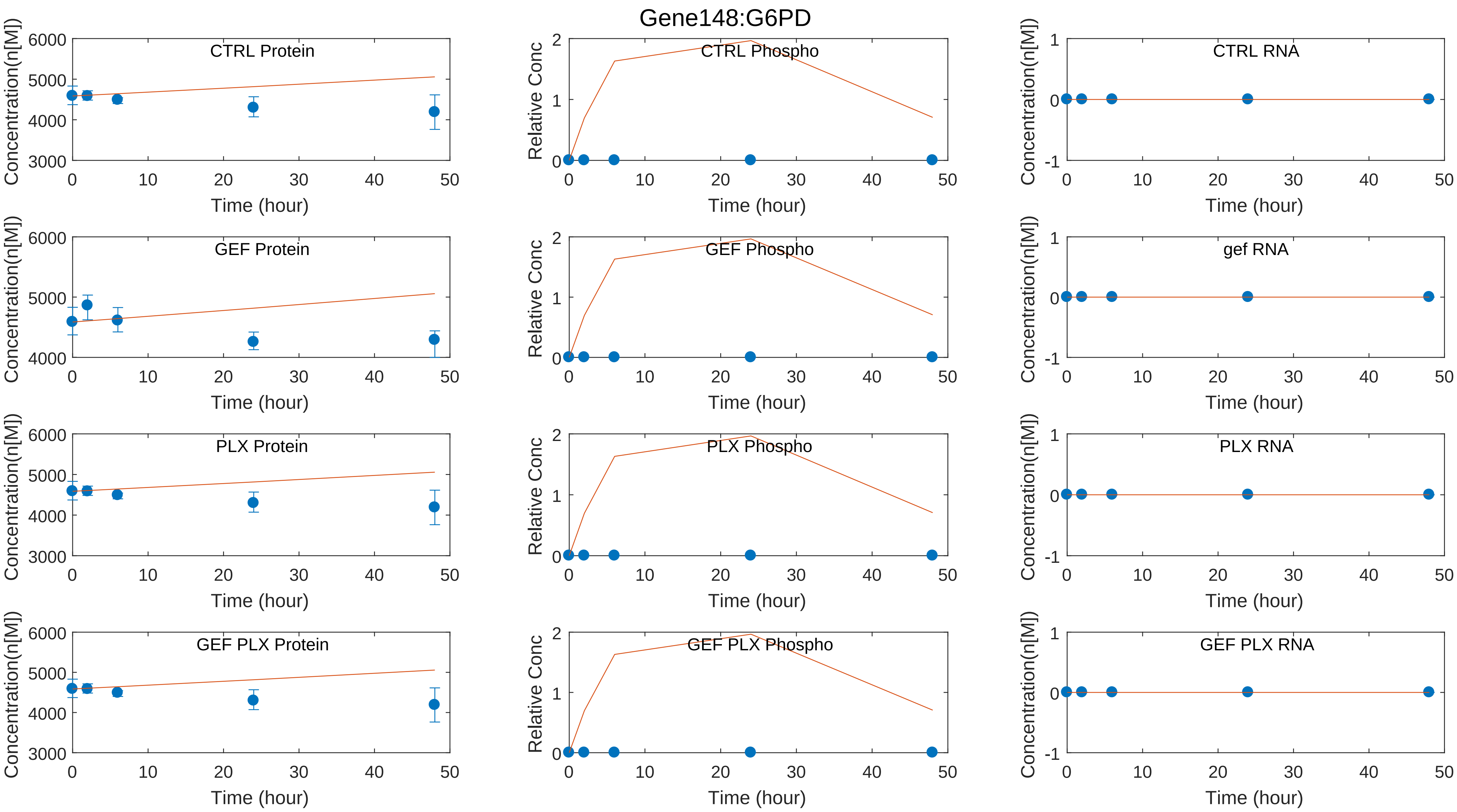
Gene148:G6PD
CTRL Protein
CTRL Phospho
CTRL RNA
GEF Protein
GEF Phospho
gef RNA
PLX Protein
PLX Phospho
PLX RNA
GEF PLX Protein
GEF PLX Phospho
GEF PLX RNA
Concentration(n[M])
Relative Conc
Time (hour)

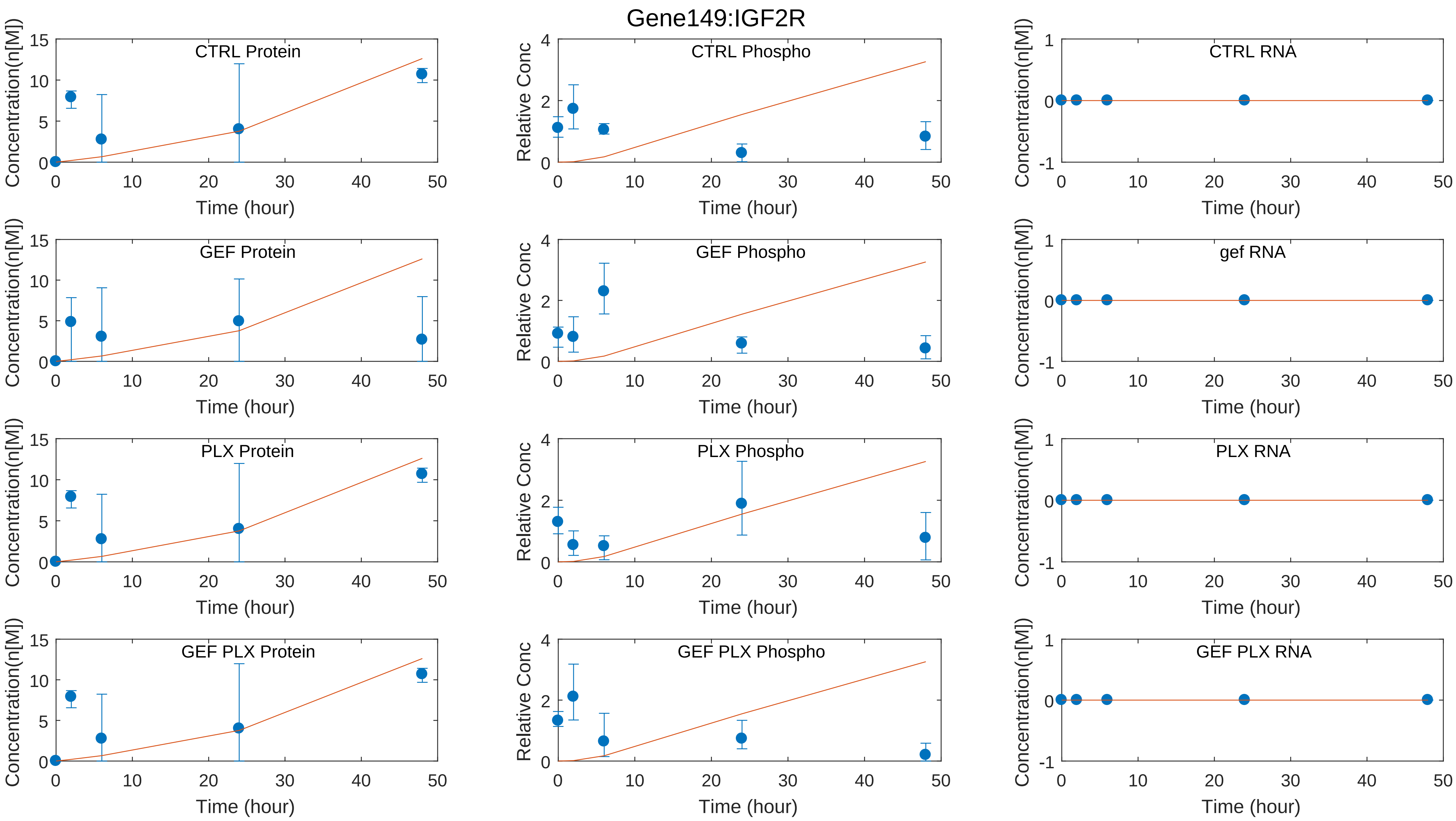

Gene149:IGF2R
CTRL Protein
CTRL Phospho
CTRL RNA
GEF Protein
GEF Phospho
gef RNA
PLX Protein
PLX Phospho
PLX RNA
GEF PLX Protein
GEF PLX Phospho
GEF PLX RNA
Concentration(n[M])
Relative Conc
Time (hour)
0
10
20
30
40
50
15
5
4
2
1
-1

Gene150:CDK4

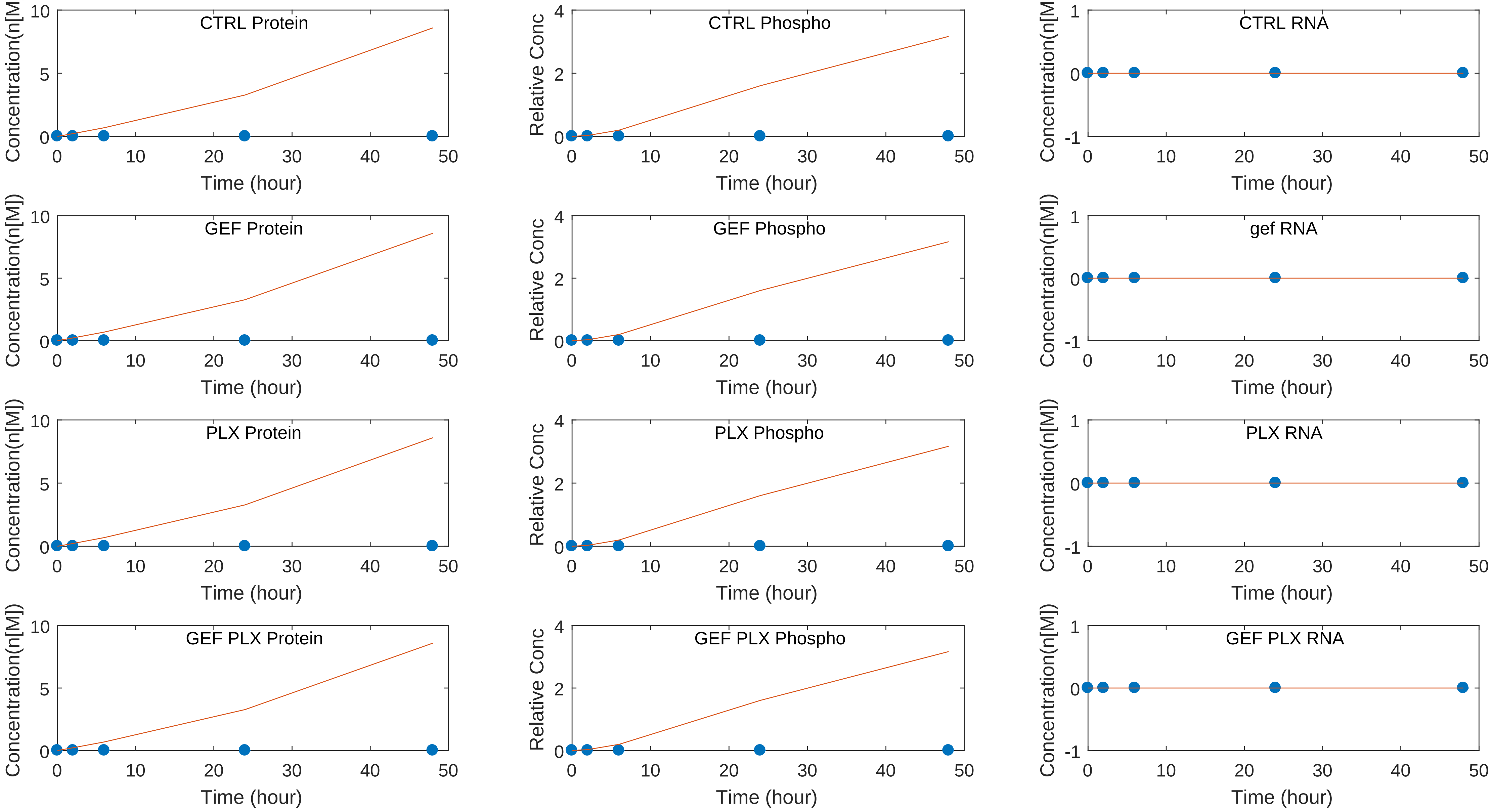

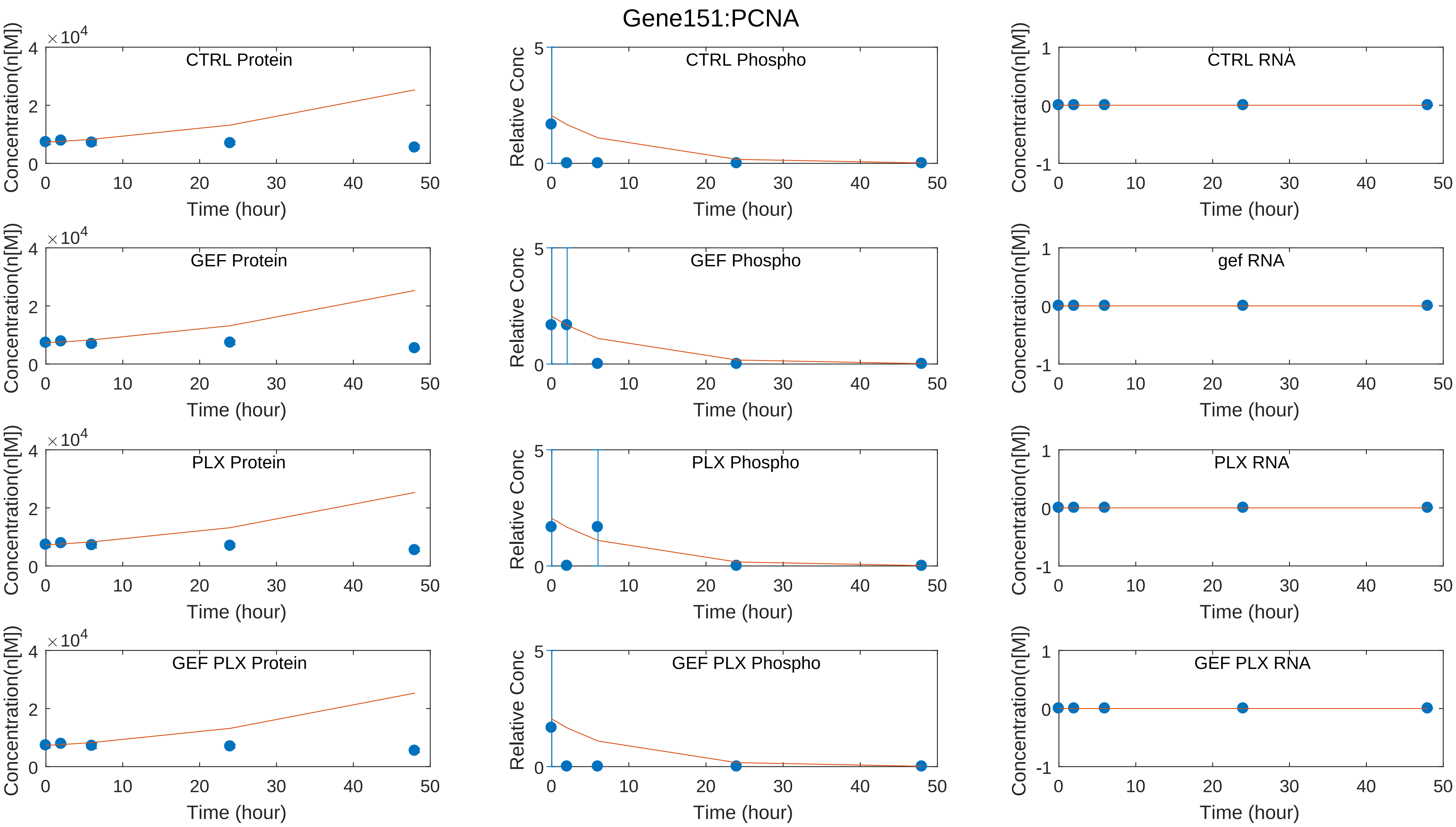
Gene151:PCNA
CTRL Protein
GEF Protein
PLX Protein
GEF PLX Protein
CTRL Phospho
GEF Phospho
PLX Phospho
GEF PLX Phospho
CTRL RNA
gef RNA
PLX RNA
GEF PLX RNA
Concentration(n[M])
Relative Conc
Time (hour)
×10^4

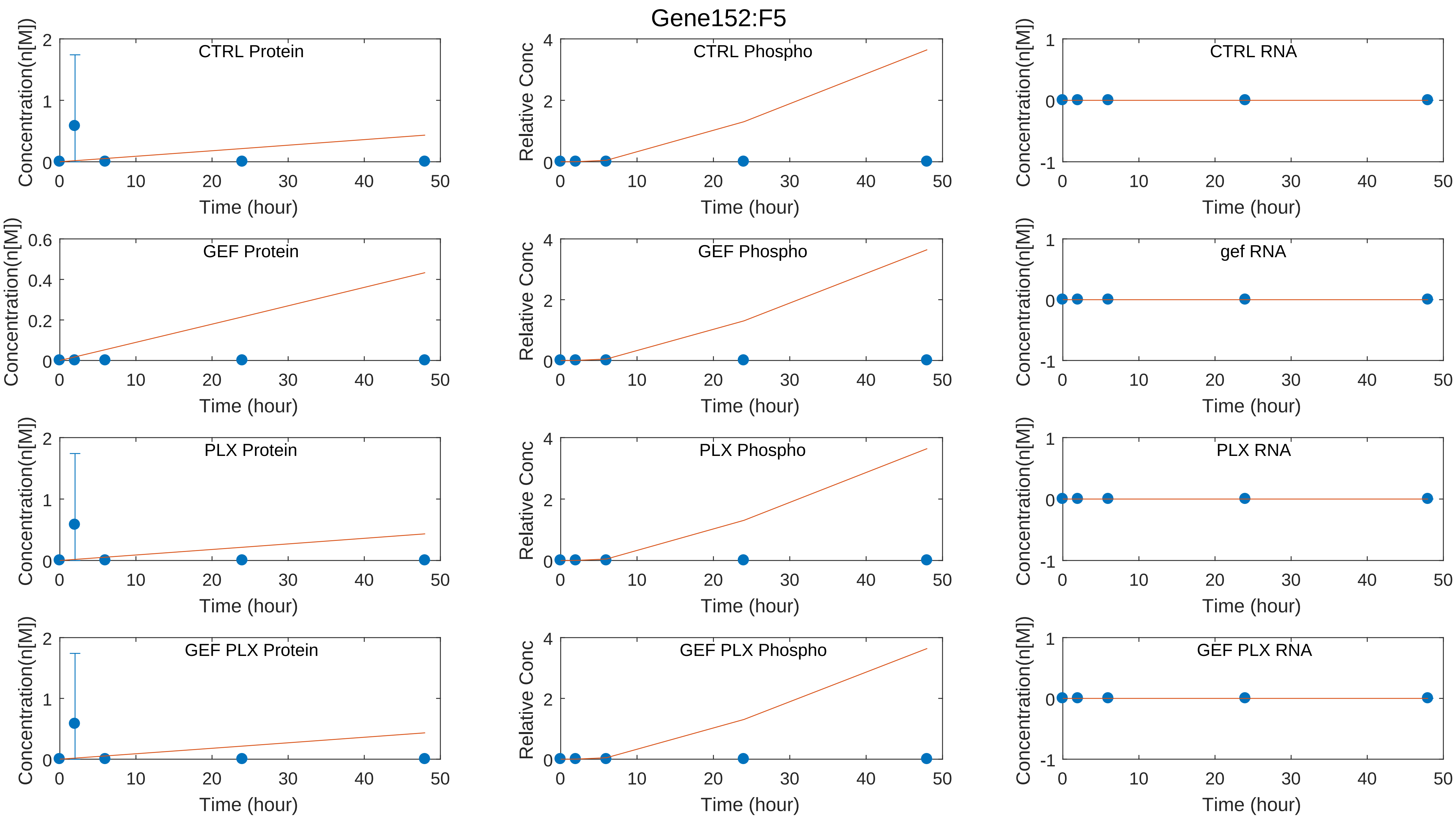
Gene152:F5
CTRL Protein
CTRL Phospho
CTRL RNA
GEF Protein
GEF Phospho
gef RNA
PLX Protein
PLX Phospho
PLX RNA
GEF PLX Protein
GEF PLX Phospho
GEF PLX RNA
Concentration(n[M])
Relative Conc
Time (hour)

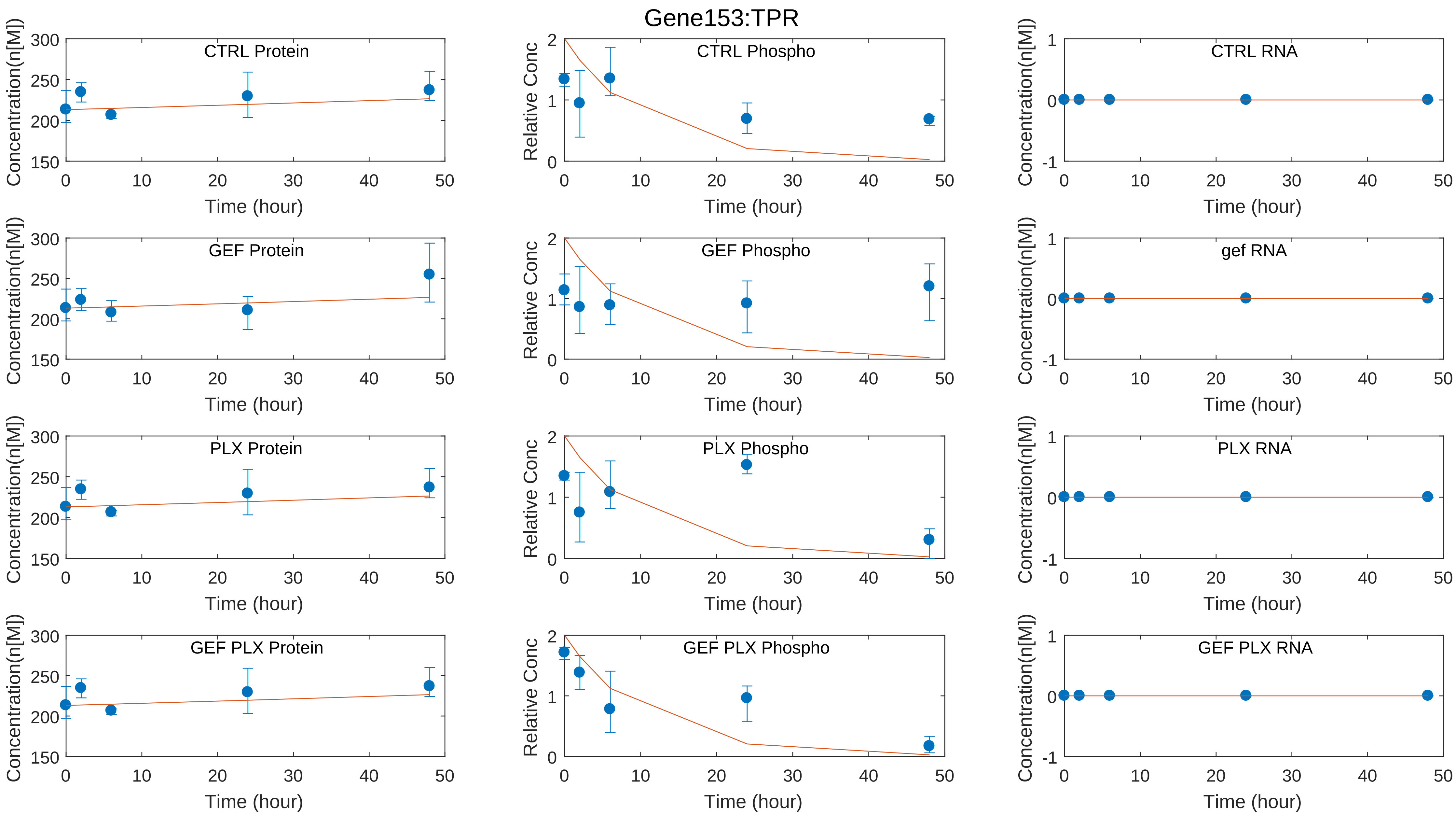
Gene153:TPR
CTRL Protein
GEF Protein
PLX Protein
GEF PLX Protein
CTRL Phospho
GEF Phospho
PLX Phospho
GEF PLX Phospho
CTRL RNA
gef RNA
PLX RNA
GEF PLX RNA
Concentration(n[M])
Relative Conc
Time (hour)

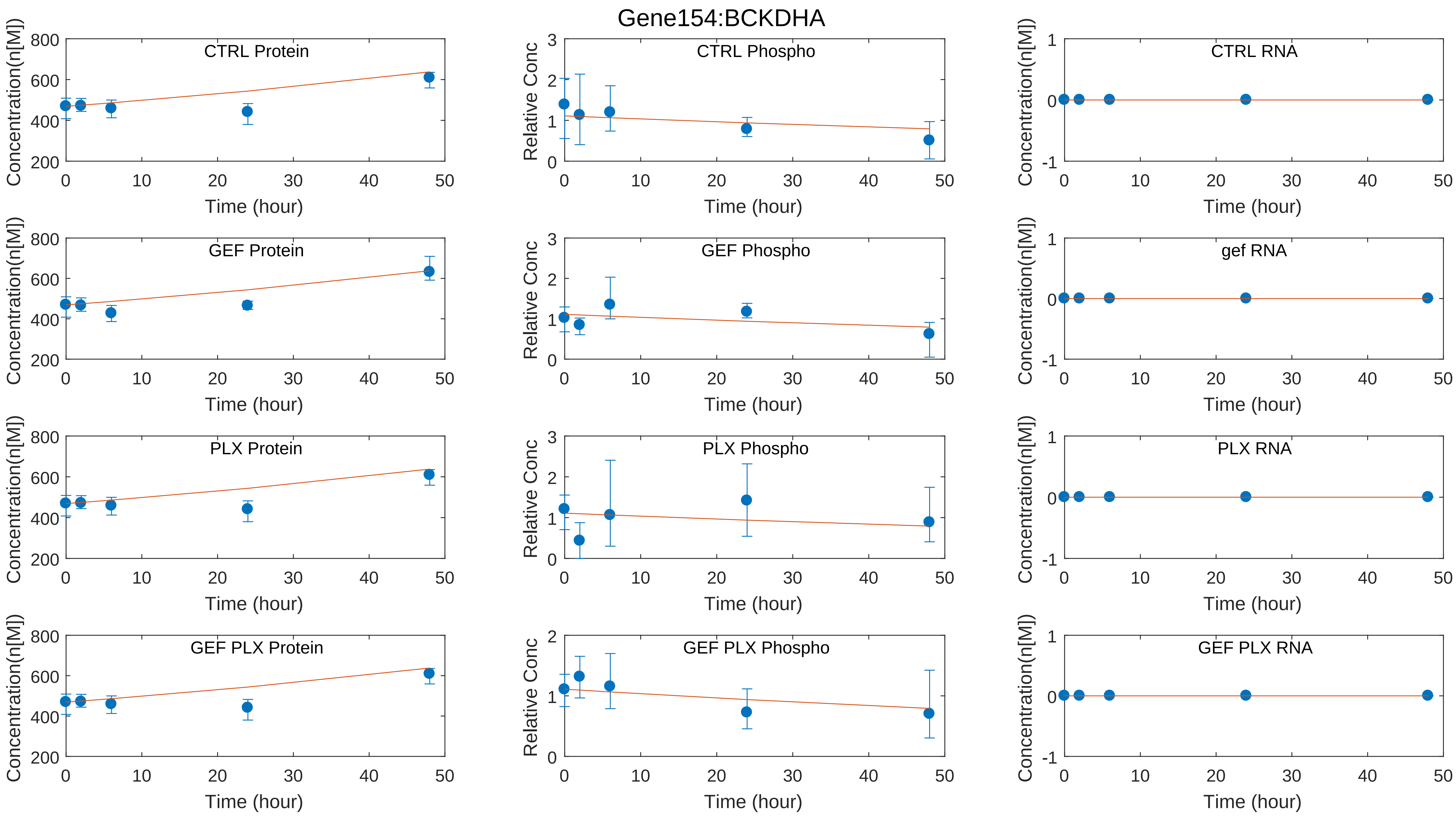
Gene154:BCKDHA
CTRL Protein
CTRL Phospho
CTRL RNA
GEF Protein
GEF Phospho
gef RNA
PLX Protein
PLX Phospho
PLX RNA
GEF PLX Protein
GEF PLX Phospho
GEF PLX RNA
Concentration(n[M])
Relative Conc
Time (hour)

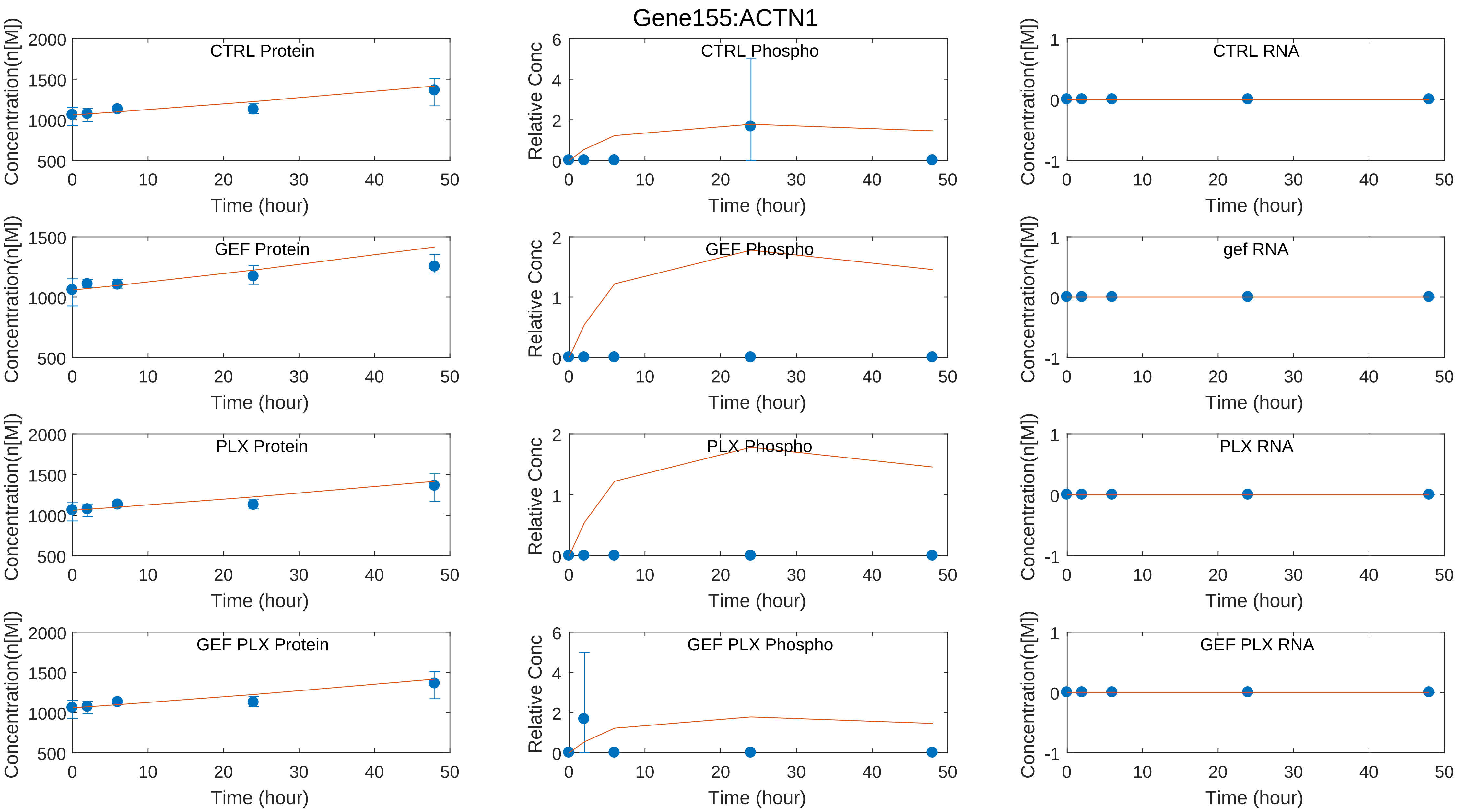

Gene155:ACTN1
CTRL Protein
CTRL Phospho
CTRL RNA
GEF Protein
GEF Phospho
gef RNA
PLX Protein
PLX Phospho
PLX RNA
GEF PLX Protein
GEF PLX Phospho
GEF PLX RNA
Concentration(n[M])
Relative Conc
Time (hour)

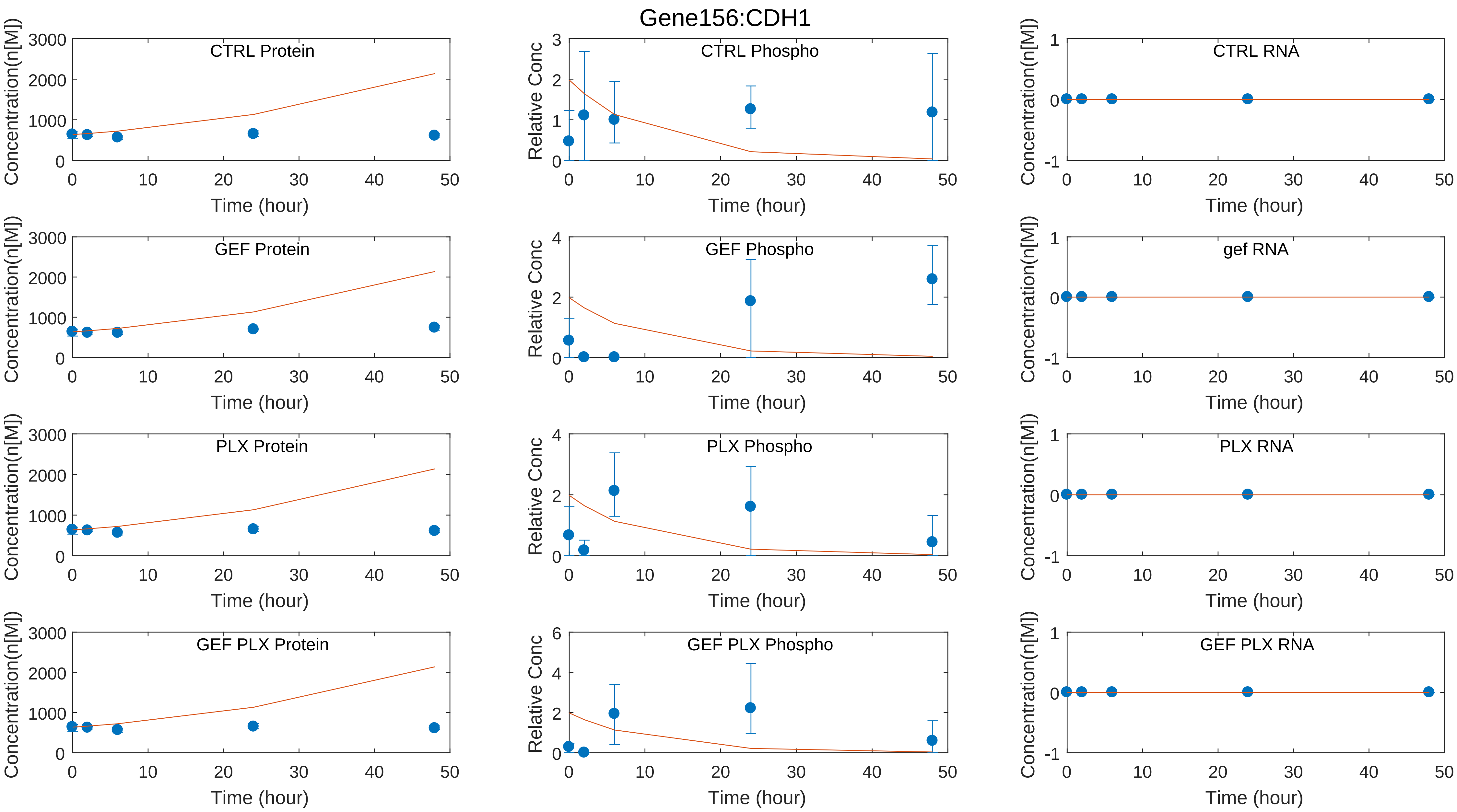

Gene156:CDH1
CTRL Protein
GEF Protein
PLX Protein
GEF PLX Protein
CTRL Phospho
GEF Phospho
PLX Phospho
GEF PLX Phospho
CTRL RNA
gef RNA
PLX RNA
GEF PLX RNA
Concentration(n[M])
Relative Conc
Time (hour)

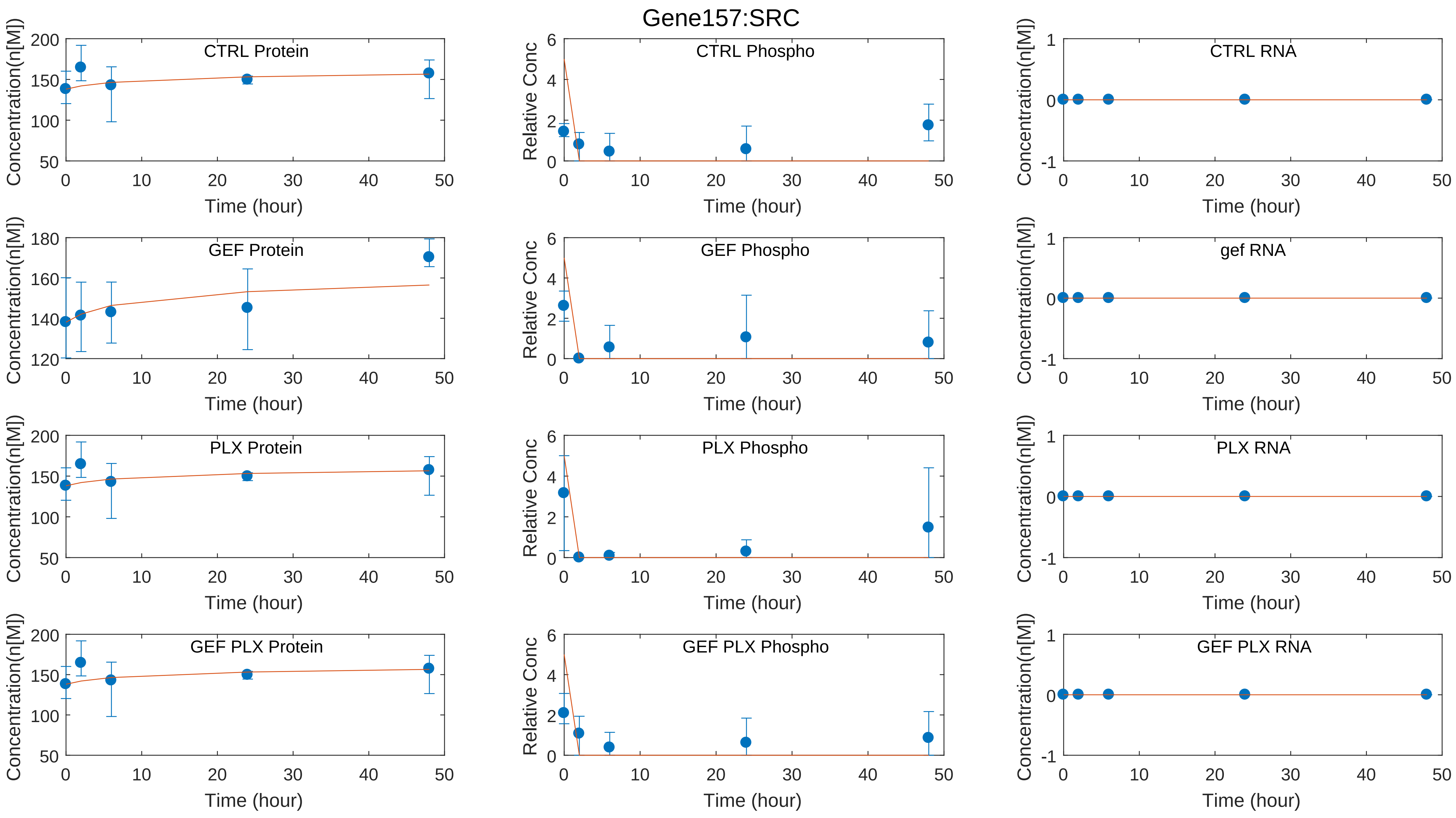
Gene157:SRC
CTRL Protein
CTRL Phospho
CTRL RNA
GEF Protein
GEF Phospho
gef RNA
PLX Protein
PLX Phospho
PLX RNA
GEF PLX Protein
GEF PLX Phospho
GEF PLX RNA
Concentration(n[M])
Relative Conc
Time (hour)

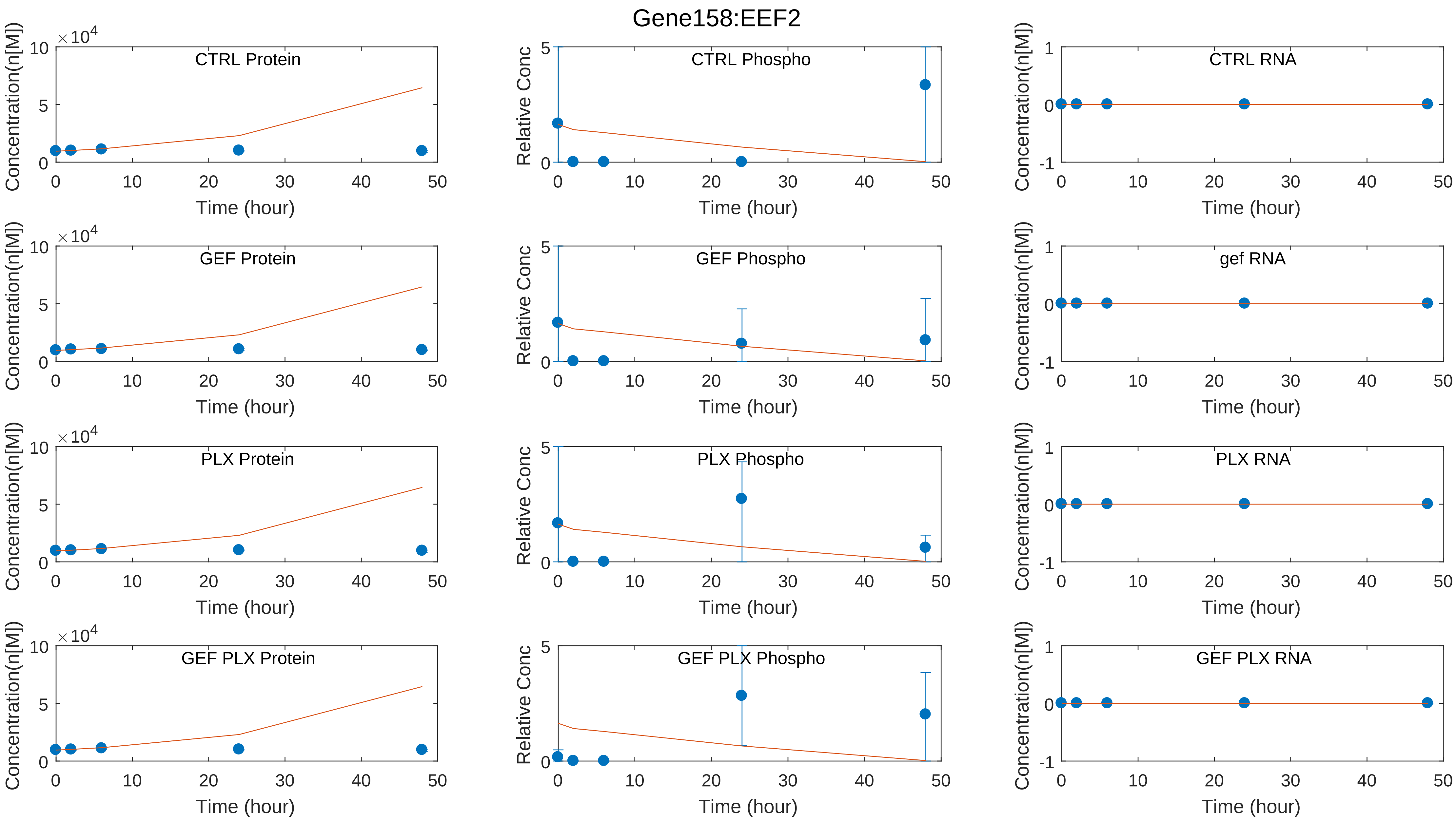

Gene158:EEF2
CTRL Protein
CTRL Phospho
CTRL RNA
GEF Protein
GEF Phospho
gef RNA
PLX Protein
PLX Phospho
PLX RNA
GEF PLX Protein
GEF PLX Phospho
GEF PLX RNA
Concentration(n[M])
Relative Conc
Time (hour)
$\times 10^4$

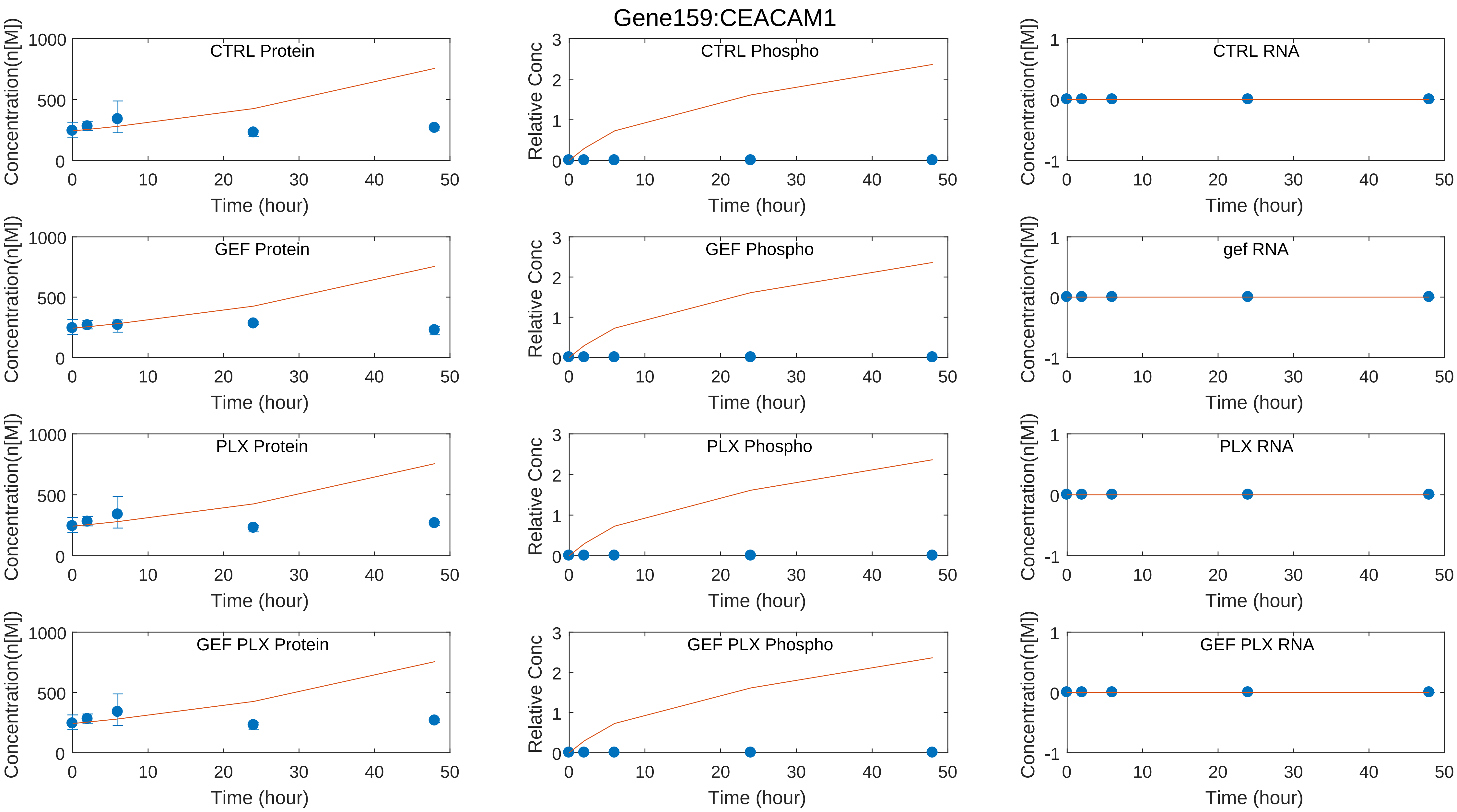
Gene159:CEACAM1
CTRL Protein
CTRL Phospho
CTRL RNA
GEF Protein
GEF Phospho
gef RNA
PLX Protein
PLX Phospho
PLX RNA
GEF PLX Protein
GEF PLX Phospho
GEF PLX RNA
Concentration(n[M])
Relative Conc
Time (hour)

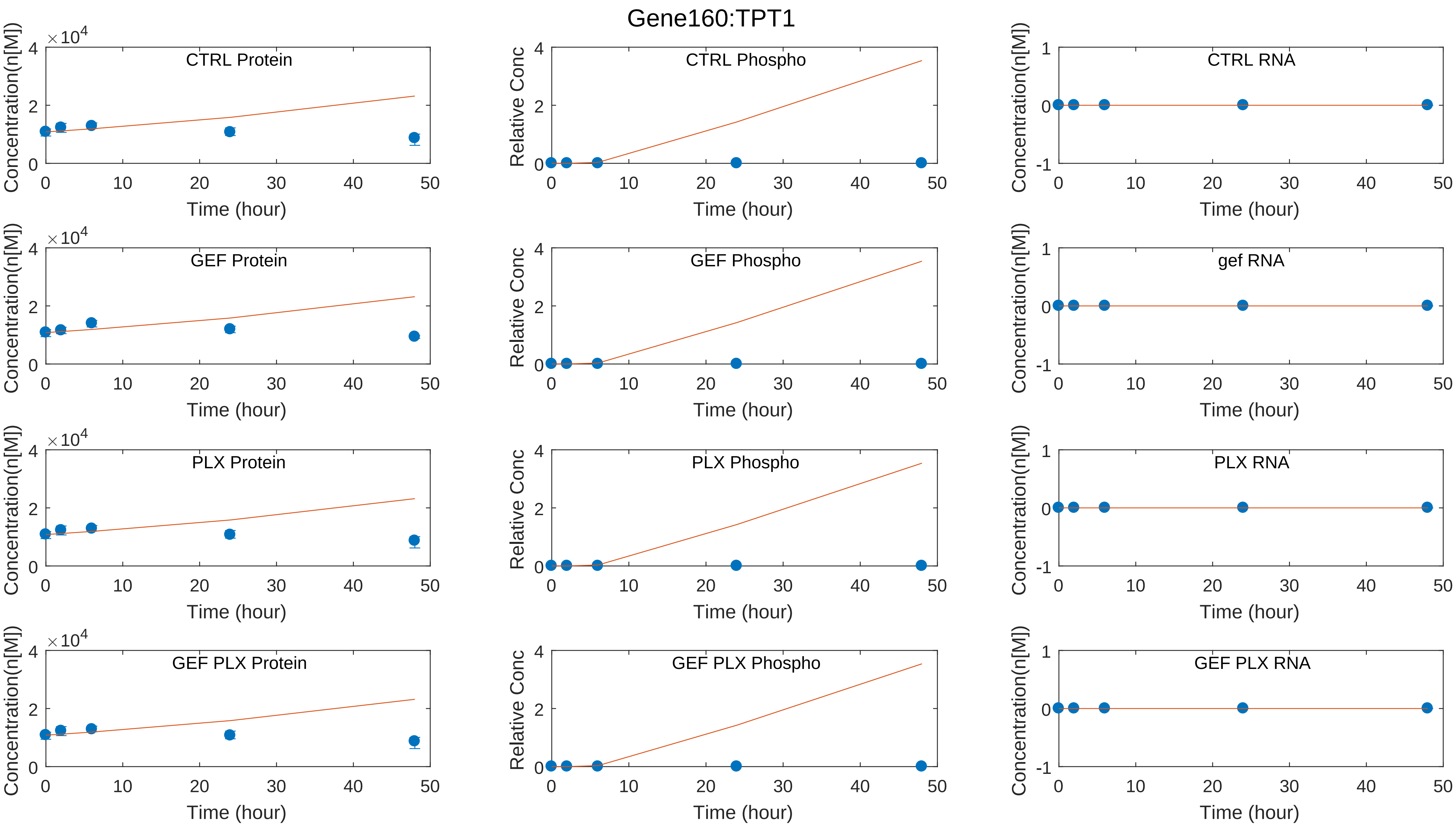

Gene160:TPT1
CTRL Protein
CTRL Phospho
CTRL RNA
GEF Protein
GEF Phospho
gef RNA
PLX Protein
PLX Phospho
PLX RNA
GEF PLX Protein
GEF PLX Phospho
GEF PLX RNA
Concentration(n[M])
Relative Conc
Time (hour)
×10^4

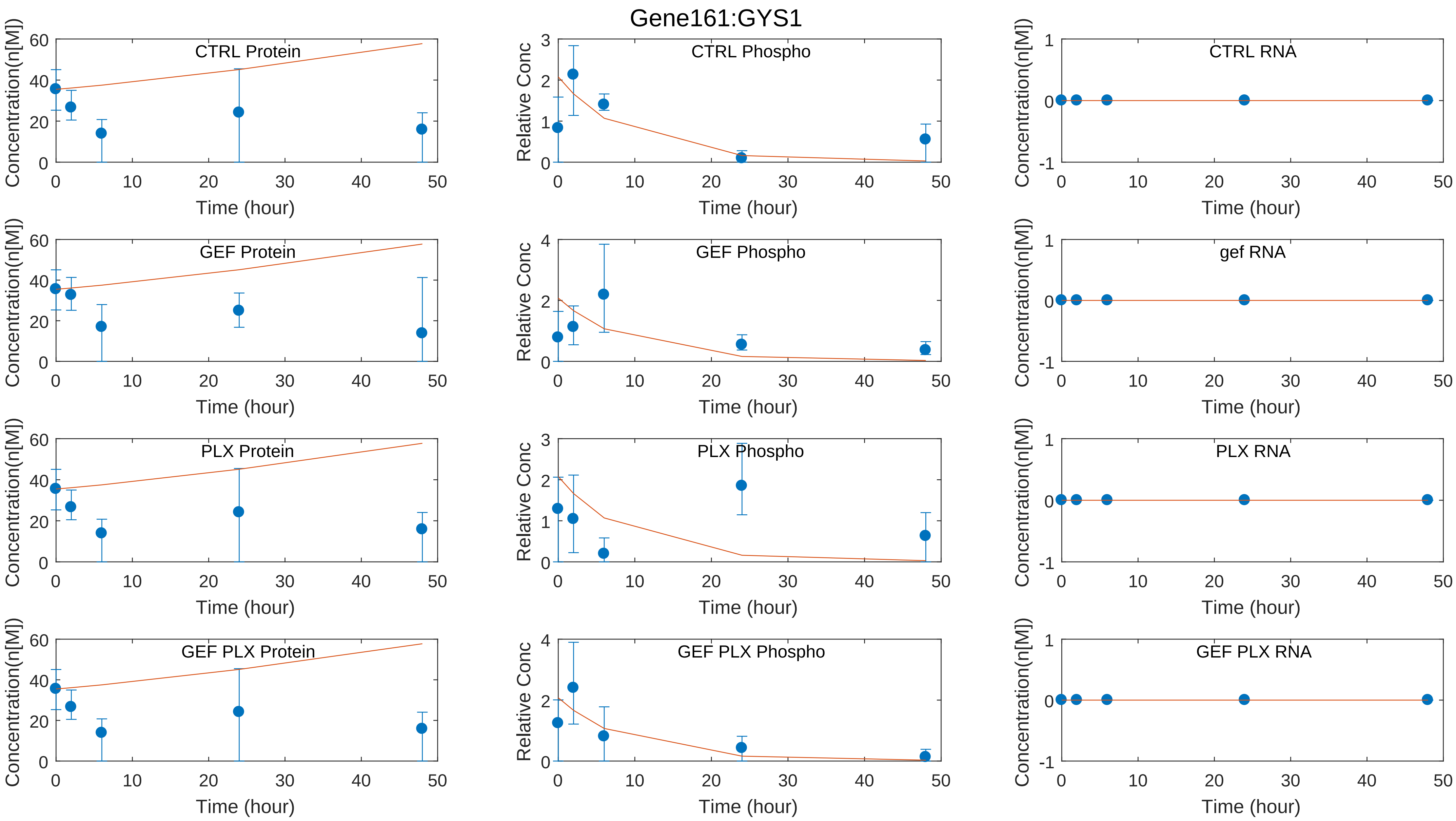

Gene161:GYS1
CTRL Protein
CTRL Phospho
CTRL RNA
GEF Protein
GEF Phospho
gef RNA
PLX Protein
PLX Phospho
PLX RNA
GEF PLX Protein
GEF PLX Phospho
GEF PLX RNA
Concentration(n[M])
Relative Conc
Time (hour)

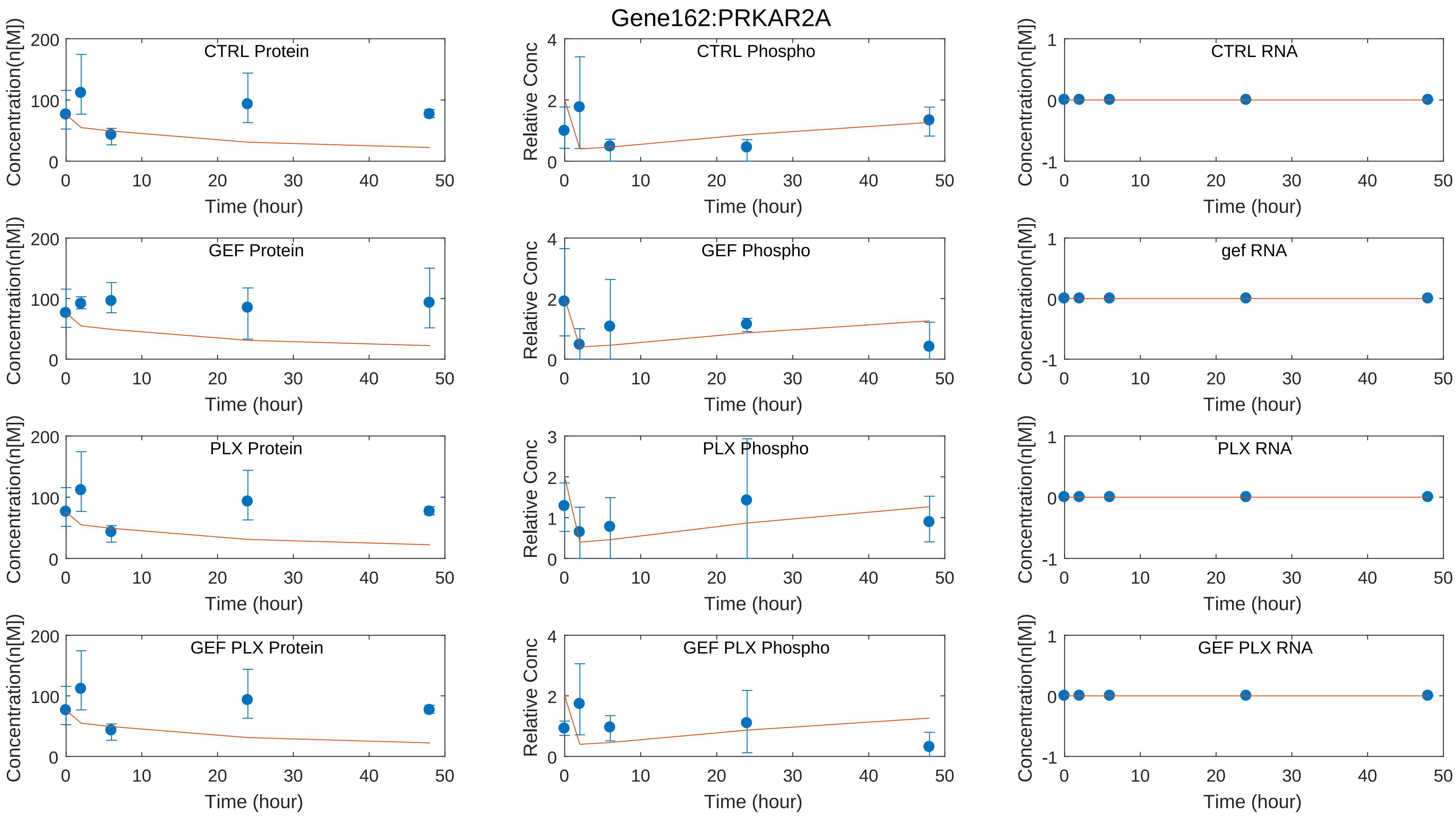

Gene162:PRKAR2A
CTRL Protein
CTRL Phospho
CTRL RNA
GEF Protein
GEF Phospho
gef RNA
PLX Protein
PLX Phospho
PLX RNA
GEF PLX Protein
GEF PLX Phospho
GEF PLX RNA
Concentration(n[M])
Relative Conc
Time (hour)

Gene163:PKM

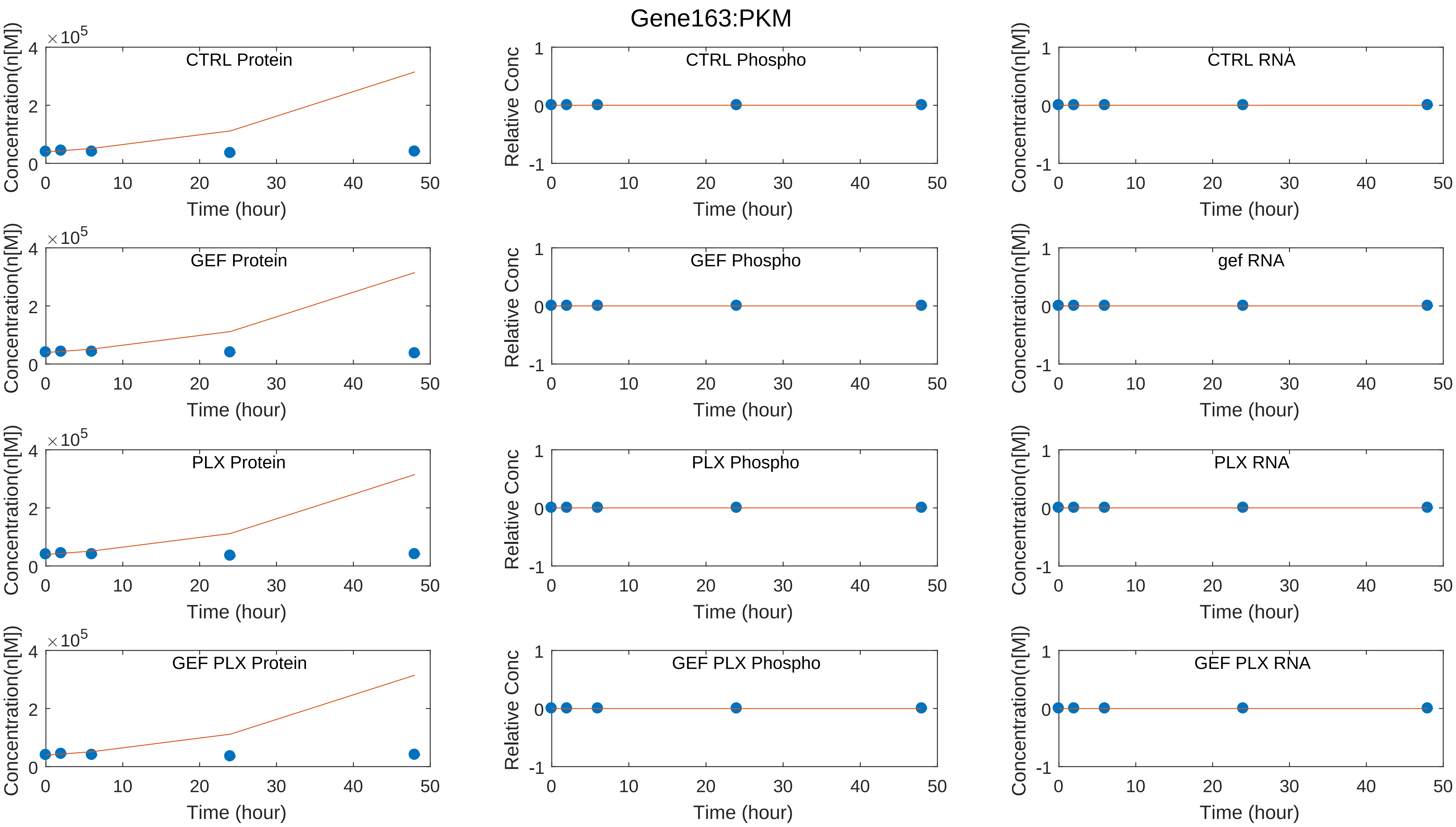

Gene163:PKM
CTRL Protein
GEF Protein
PLX Protein
GEF PLX Protein
CTRL Phospho
GEF Phospho
PLX Phospho
GEF PLX Phospho
CTRL RNA
gef RNA
PLX RNA
GEF PLX RNA
Concentration(n[M])
Relative Conc
Time (hour)
×10^5

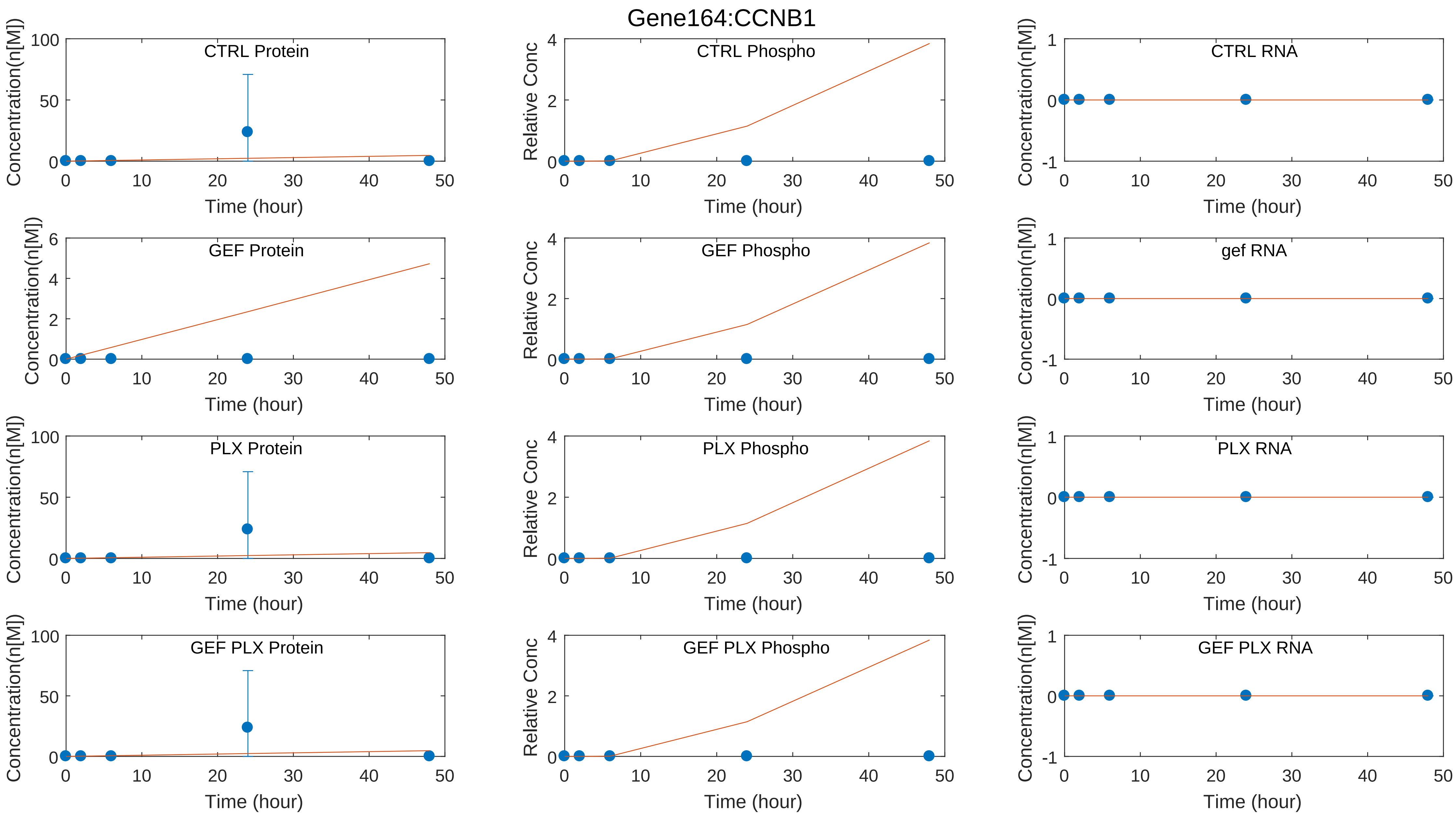
Gene164:CCNB1
CTRL Protein
CTRL Phospho
CTRL RNA
GEF Protein
GEF Phospho
gef RNA
PLX Protein
PLX Phospho
PLX RNA
GEF PLX Protein
GEF PLX Phospho
GEF PLX RNA
Concentration(n[M])
Relative Conc
Time (hour)

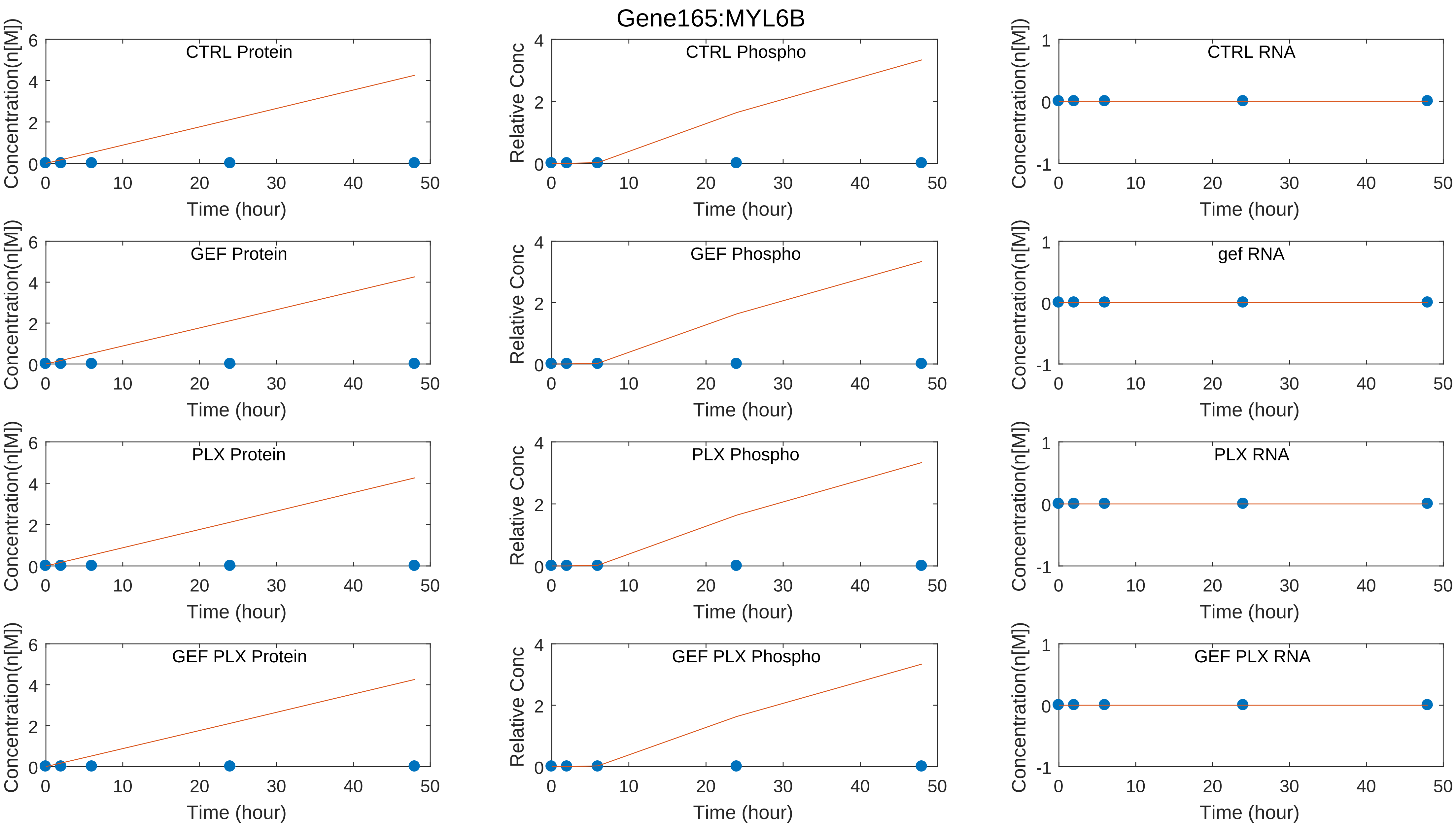
Gene165:MYL6B
CTRL Protein
CTRL Phospho
CTRL RNA
GEF Protein
GEF Phospho
gef RNA
PLX Protein
PLX Phospho
PLX RNA
GEF PLX Protein
GEF PLX Phospho
GEF PLX RNA
Concentration(n[M])
Relative Conc
Time (hour)

Gene166:POU2F1

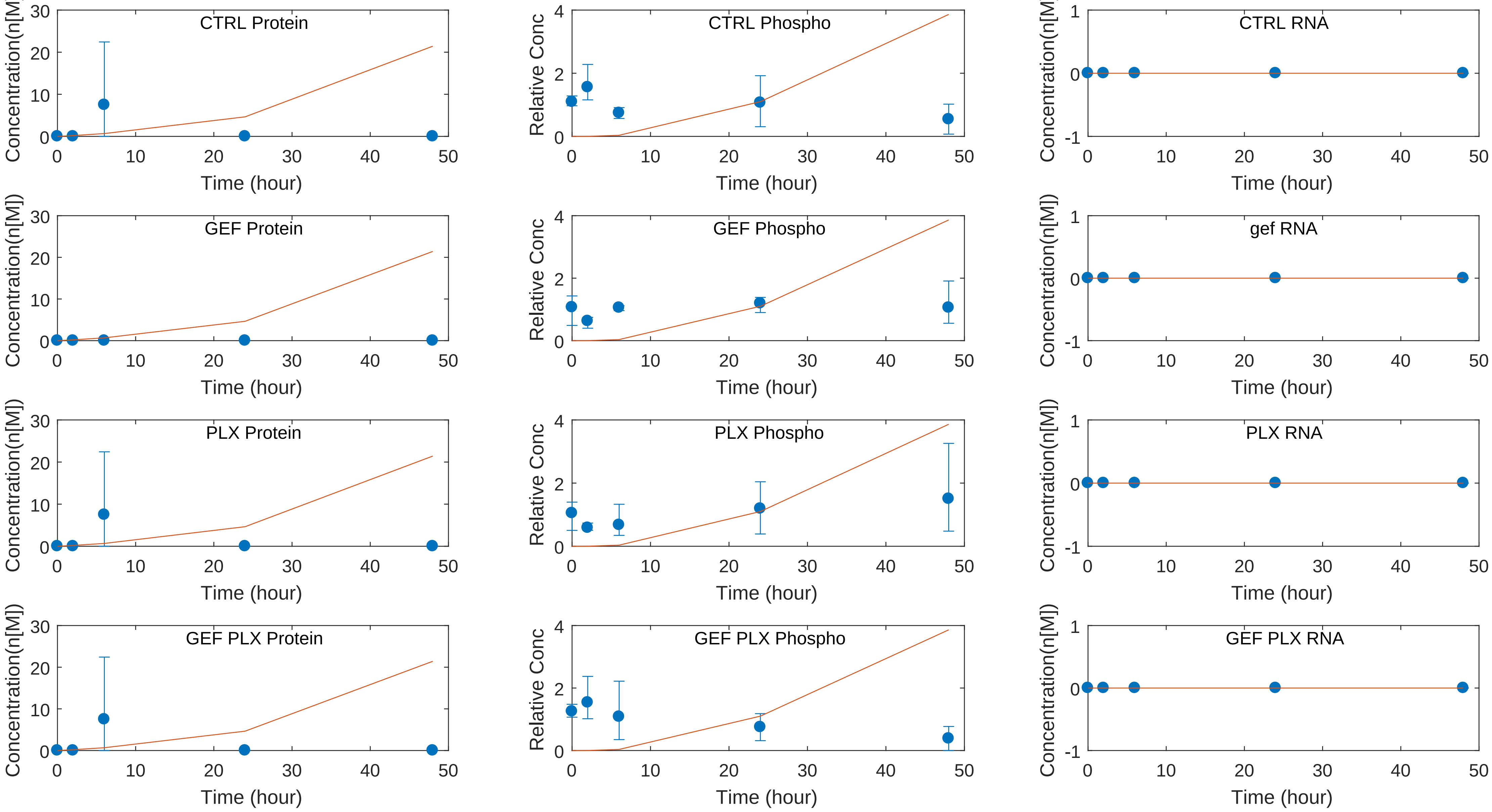

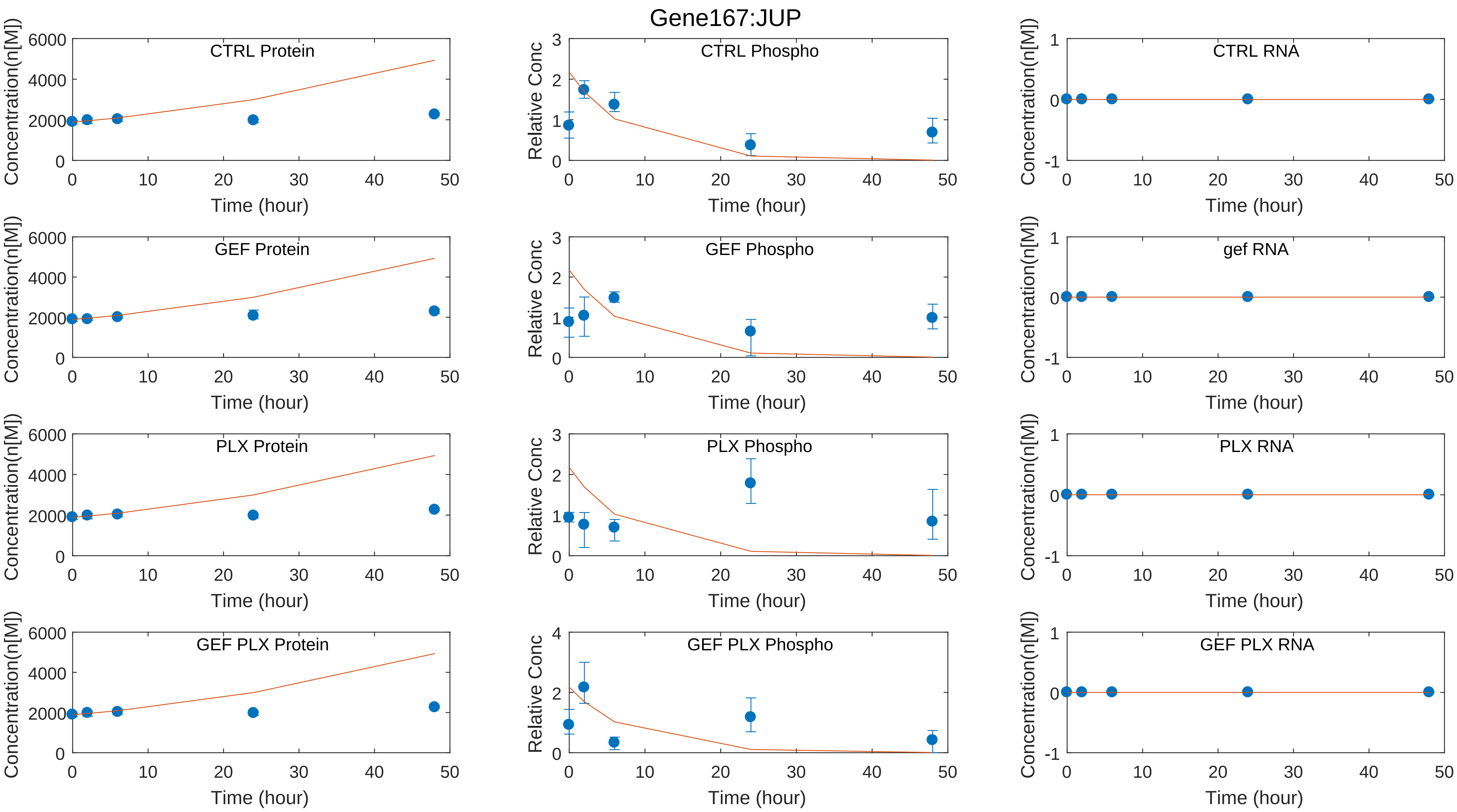

Gene167:JUP
CTRL Protein
GEF Protein
PLX Protein
GEF PLX Protein
CTRL Phospho
GEF Phospho
PLX Phospho
GEF PLX Phospho
CTRL RNA
gef RNA
PLX RNA
GEF PLX RNA
Concentration(n[M])
Relative Conc
Time (hour)

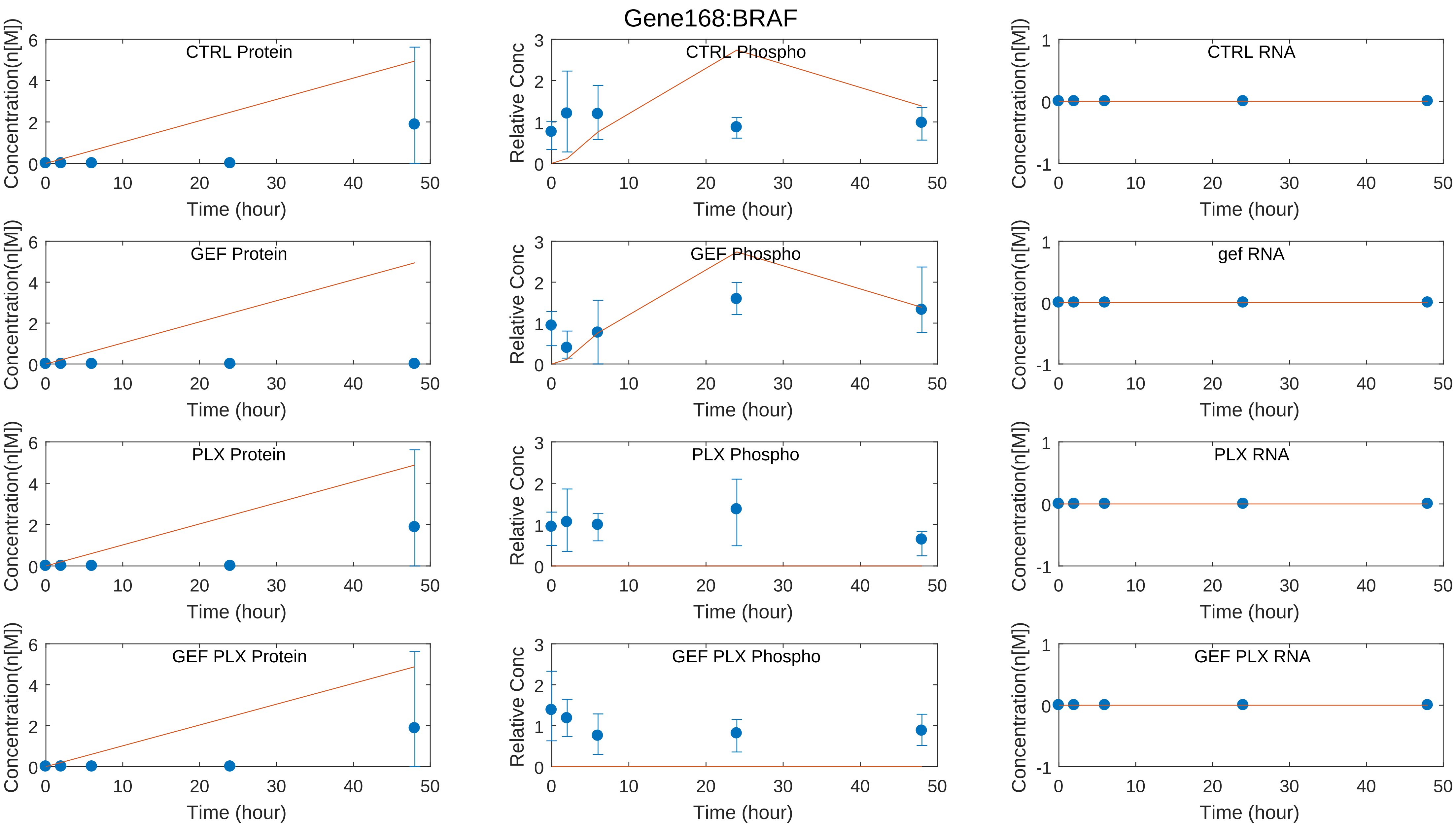

Gene168:BRAF
CTRL Protein
CTRL Phospho
CTRL RNA
GEF Protein
GEF Phospho
gef RNA
PLX Protein
PLX Phospho
PLX RNA
GEF PLX Protein
GEF PLX Phospho
GEF PLX RNA
Concentration(n[M])
Relative Conc
Time (hour)

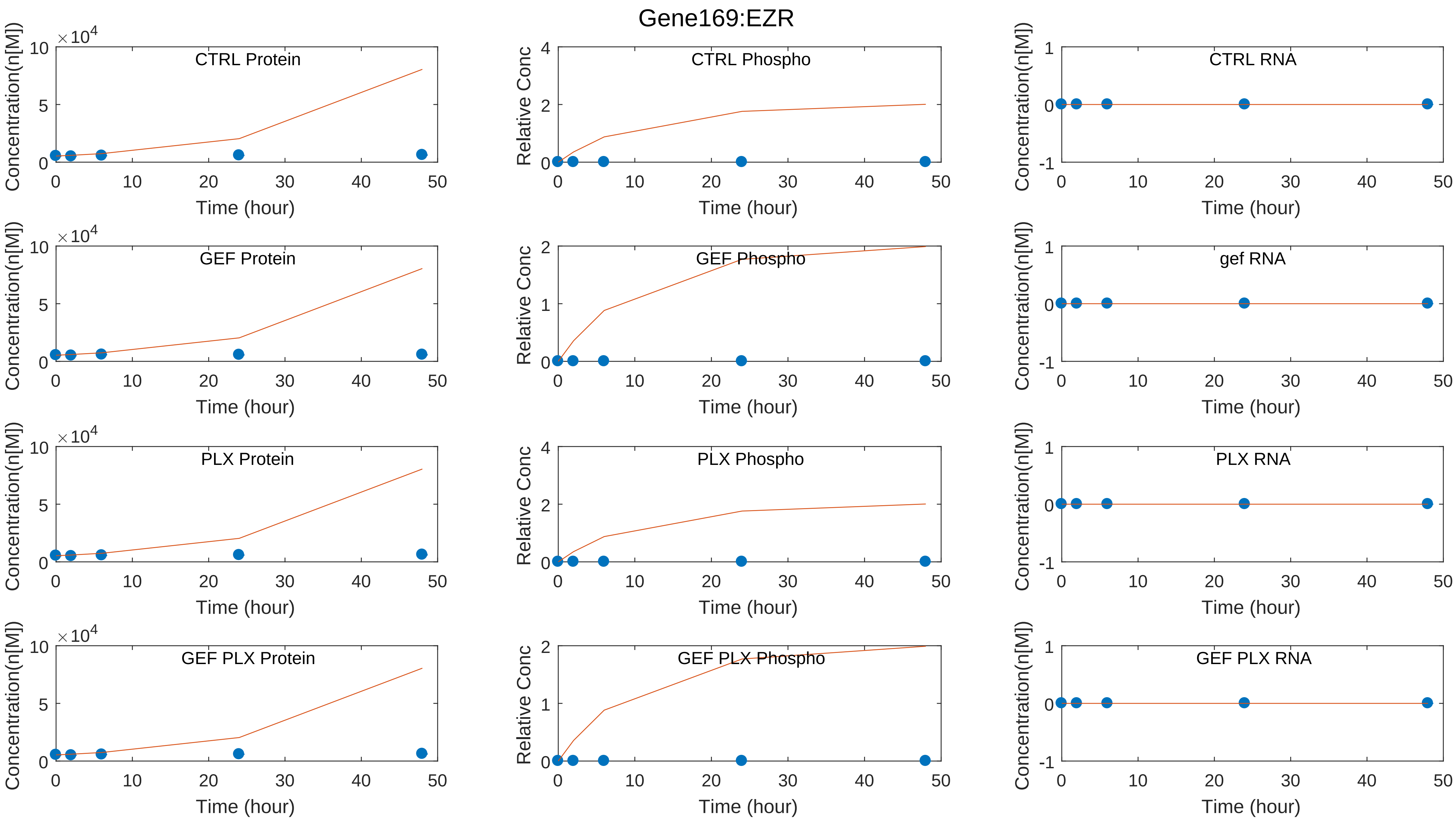
Gene169:EZR
CTRL Protein
CTRL Phospho
CTRL RNA
GEF Protein
GEF Phospho
gef RNA
PLX Protein
PLX Phospho
PLX RNA
GEF PLX Protein
GEF PLX Phospho
GEF PLX RNA
Concentration(n[M])
Relative Conc
Time (hour)
×10^4

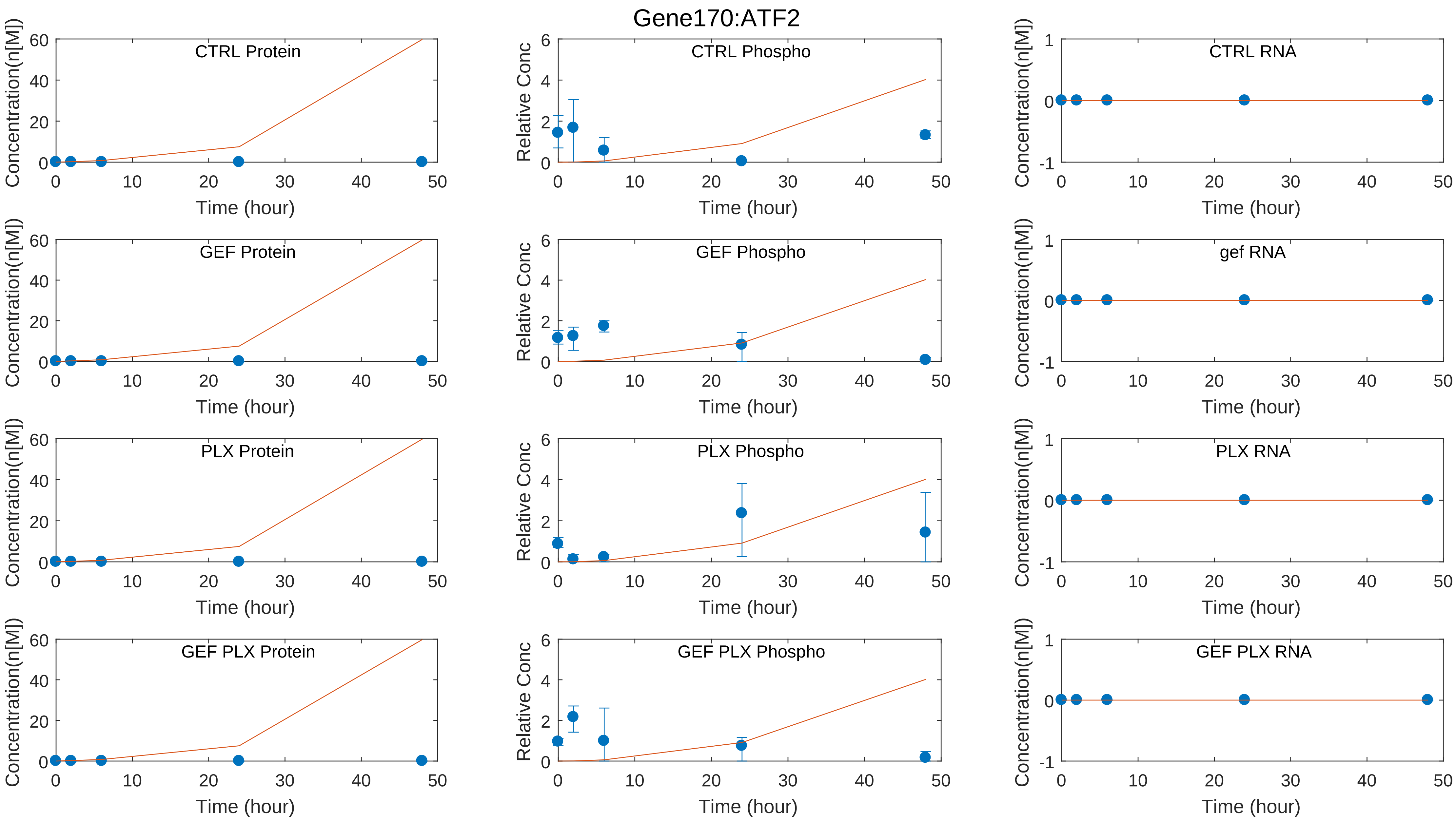
Gene170:ATF2
CTRL Protein
CTRL Phospho
CTRL RNA
GEF Protein
GEF Phospho
gef RNA
PLX Protein
PLX Phospho
PLX RNA
GEF PLX Protein
GEF PLX Phospho
GEF PLX RNA
Concentration(n[M])
Relative Conc
Time (hour)

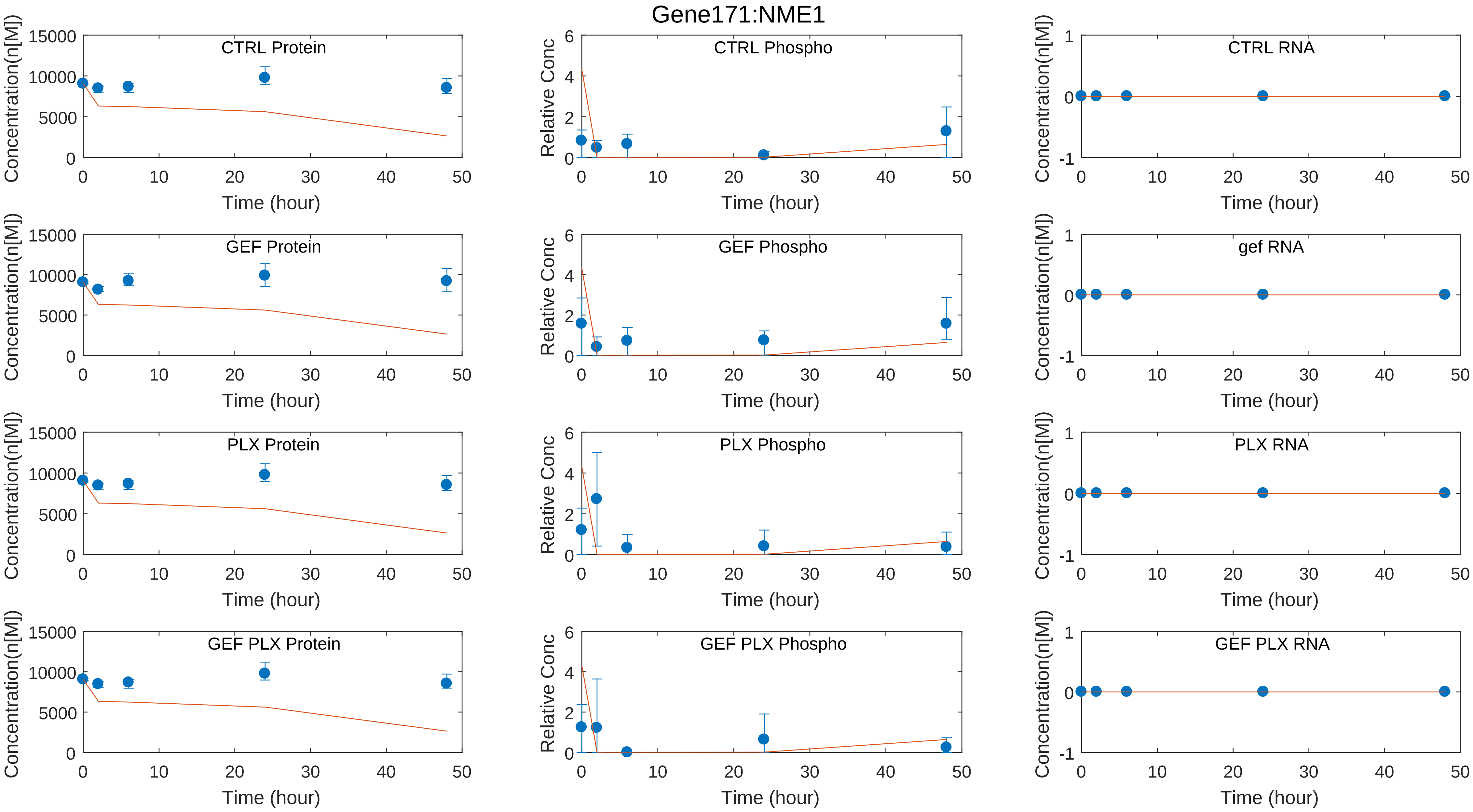
Gene171:NME1
CTRL Protein
CTRL Phospho
CTRL RNA
GEF Protein
GEF Phospho
gef RNA
PLX Protein
PLX Phospho
PLX RNA
GEF PLX Protein
GEF PLX Phospho
GEF PLX RNA
Concentration(n[M])
Relative Conc
Time (hour)

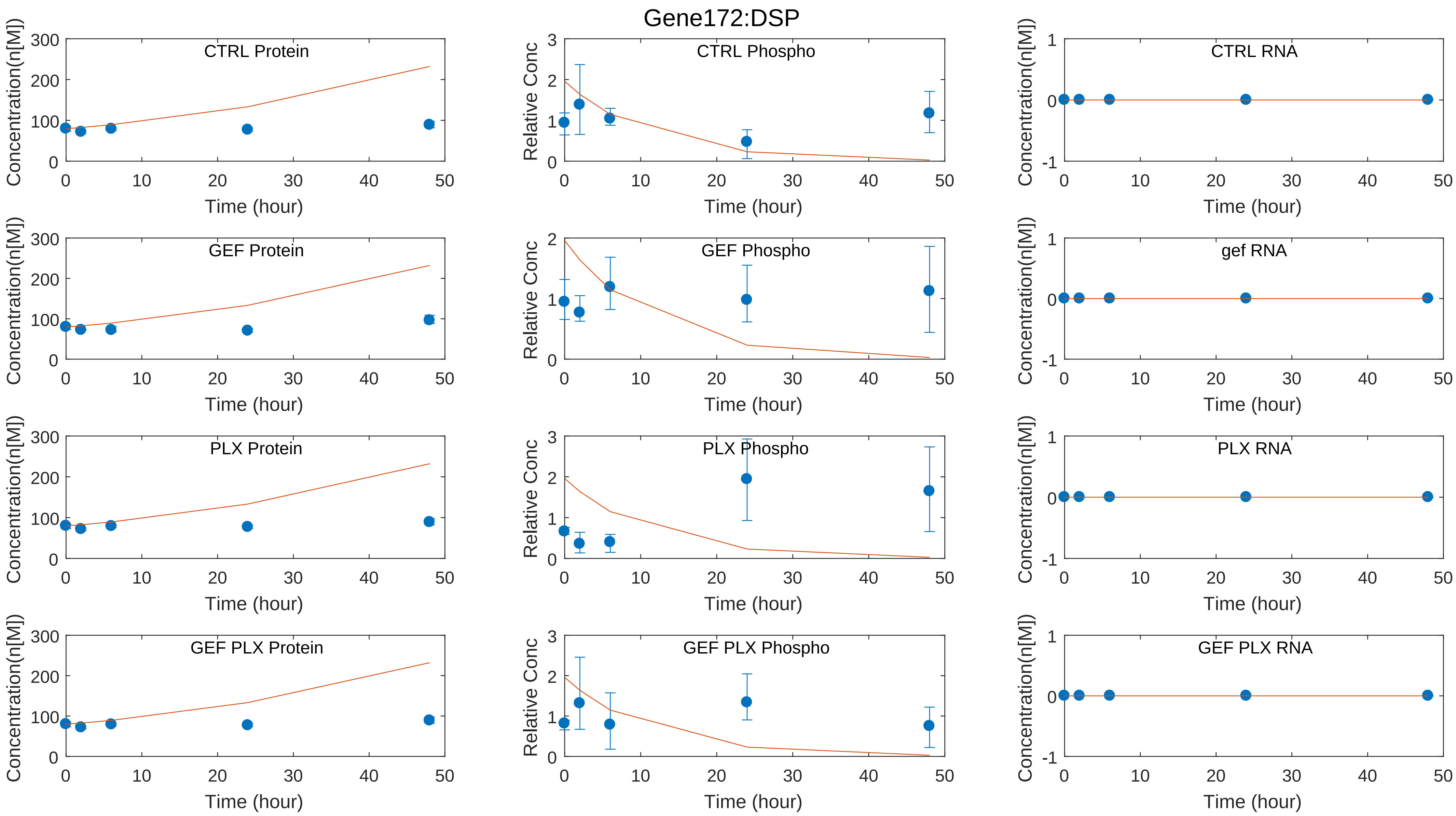

Gene172:DSP
CTRL Protein
CTRL Phospho
CTRL RNA
GEF Protein
GEF Phospho
gef RNA
PLX Protein
PLX Phospho
PLX RNA
GEF PLX Protein
GEF PLX Phospho
GEF PLX RNA
Concentration(n[M])
Relative Conc
Time (hour)

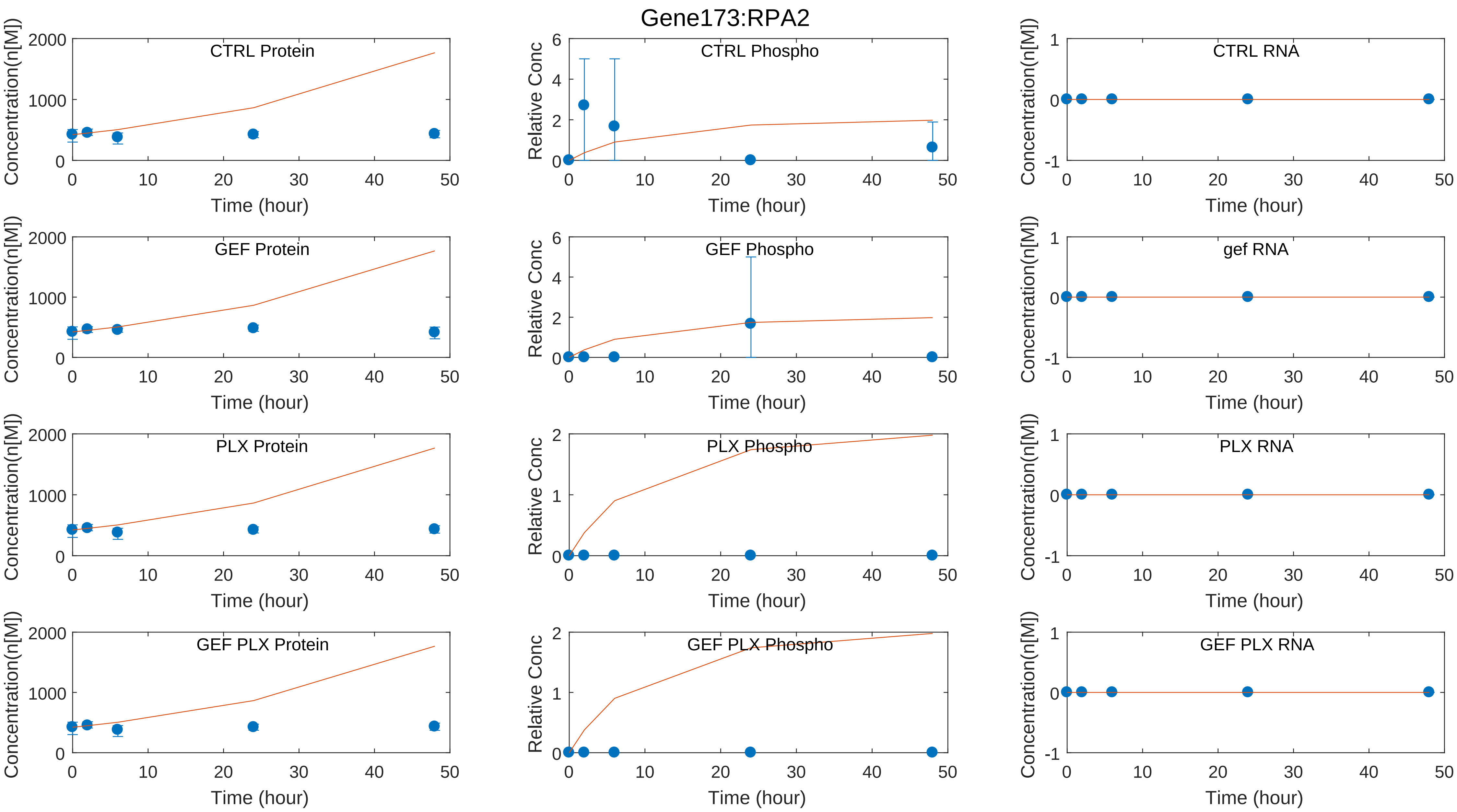
Gene173:RPA2
CTRL Protein
CTRL Phospho
CTRL RNA
GEF Protein
GEF Phospho
gef RNA
PLX Protein
PLX Phospho
PLX RNA
GEF PLX Protein
GEF PLX Phospho
GEF PLX RNA
Concentration(n[M])
Relative Conc
Time (hour)

Gene174:CD44

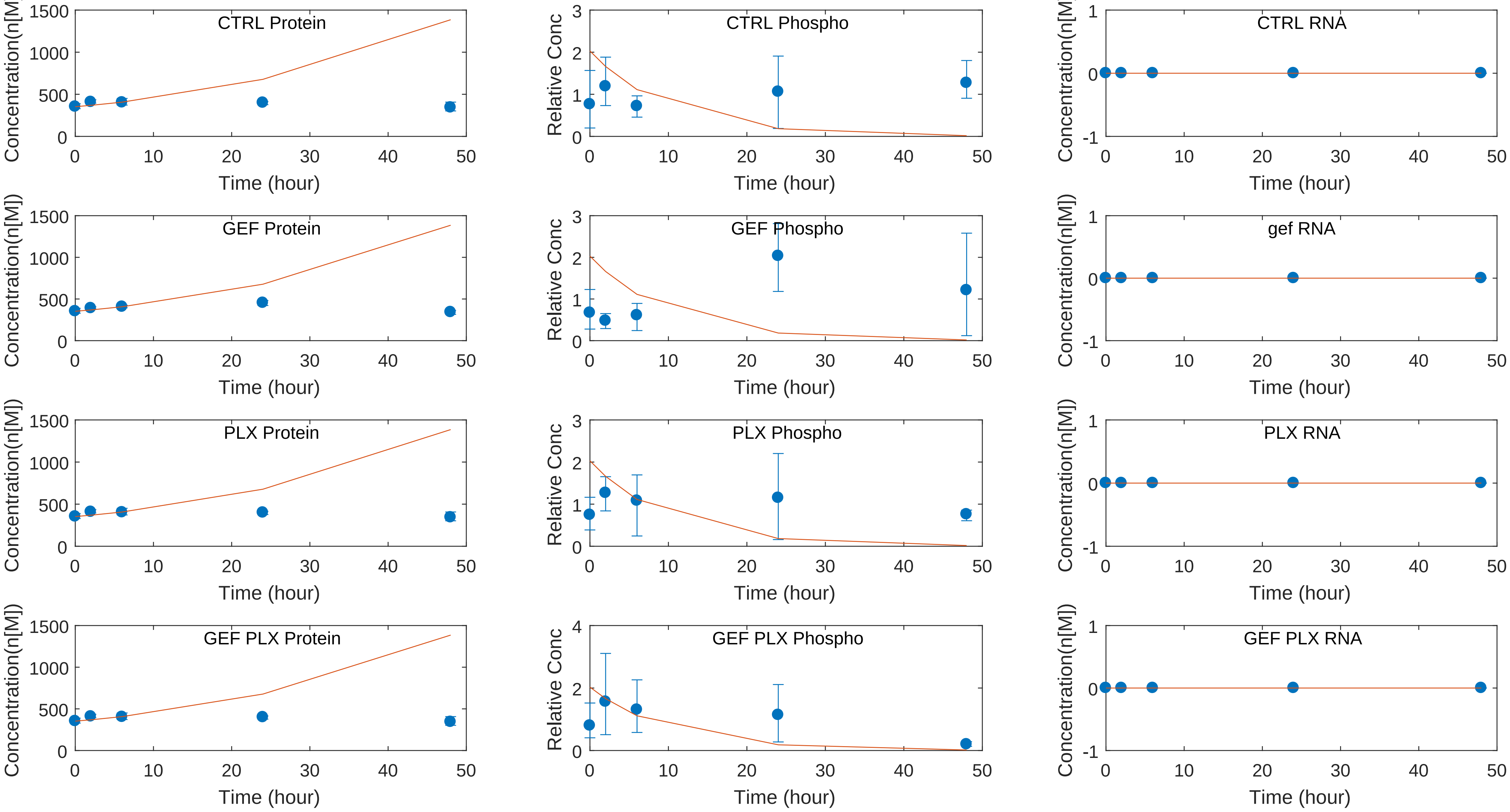

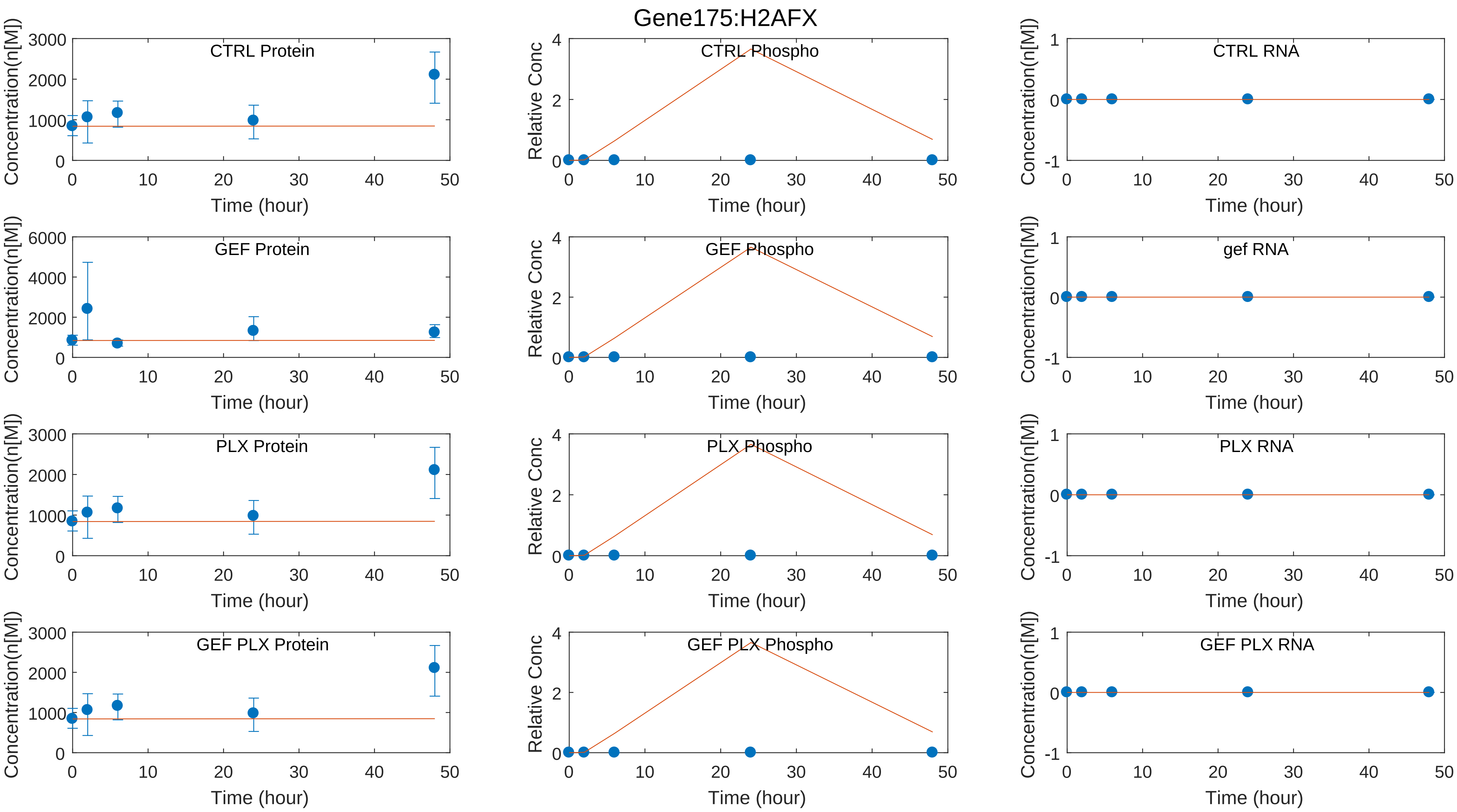
Gene175:H2AFX
CTRL Protein
CTRL Phospho
CTRL RNA
GEF Protein
GEF Phospho
gef RNA
PLX Protein
PLX Phospho
PLX RNA
GEF PLX Protein
GEF PLX Phospho
GEF PLX RNA
Concentration(n[M])
Relative Conc
Time (hour)

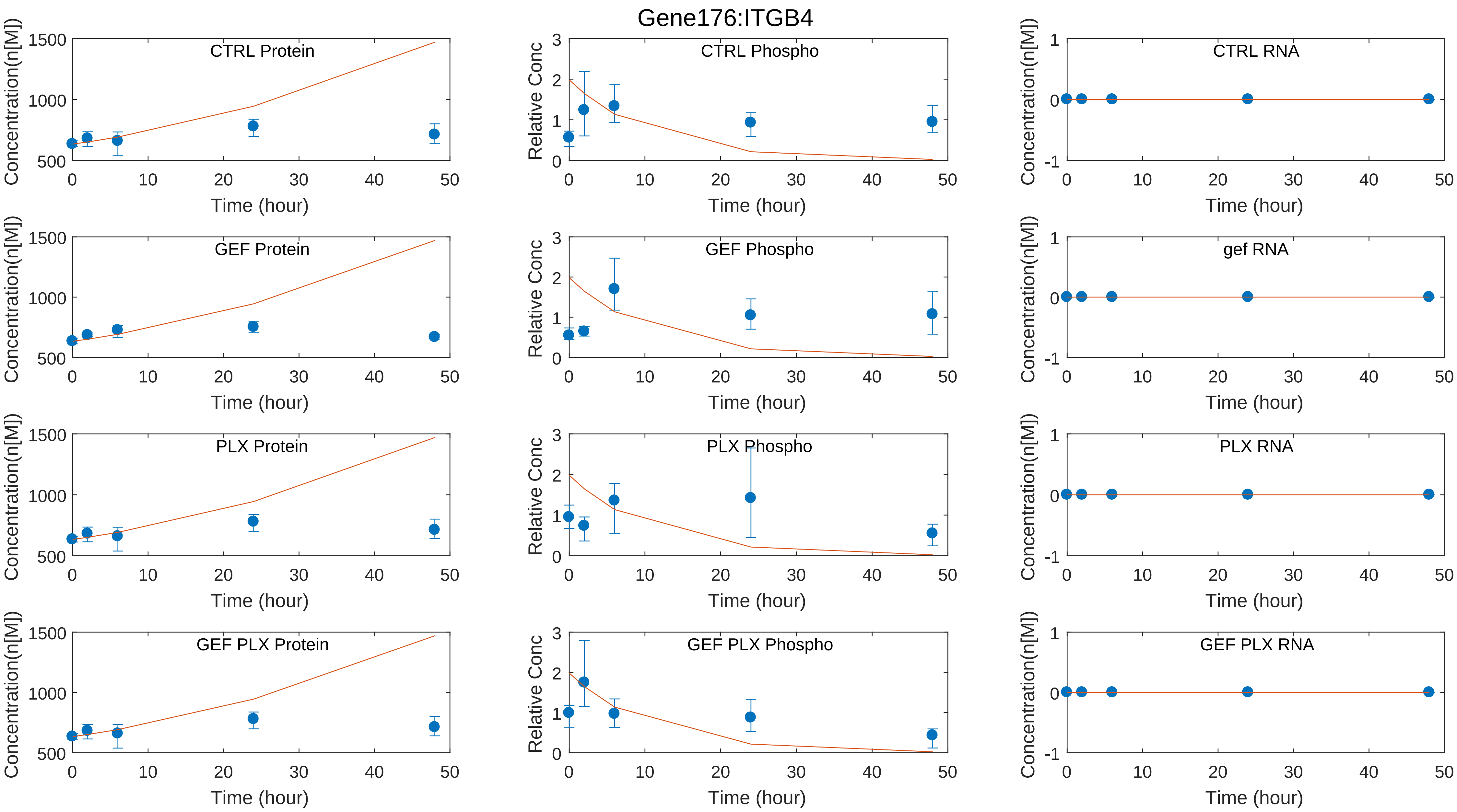
Gene176:ITGB4
CTRL Protein
GEF Protein
PLX Protein
GEF PLX Protein
CTRL Phospho
GEF Phospho
PLX Phospho
GEF PLX Phospho
CTRL RNA
gef RNA
PLX RNA
GEF PLX RNA
Concentration(n[M])
Relative Conc
Time (hour)
1500
1000
500
3
2
1
0
-1
10
20
30
40
50

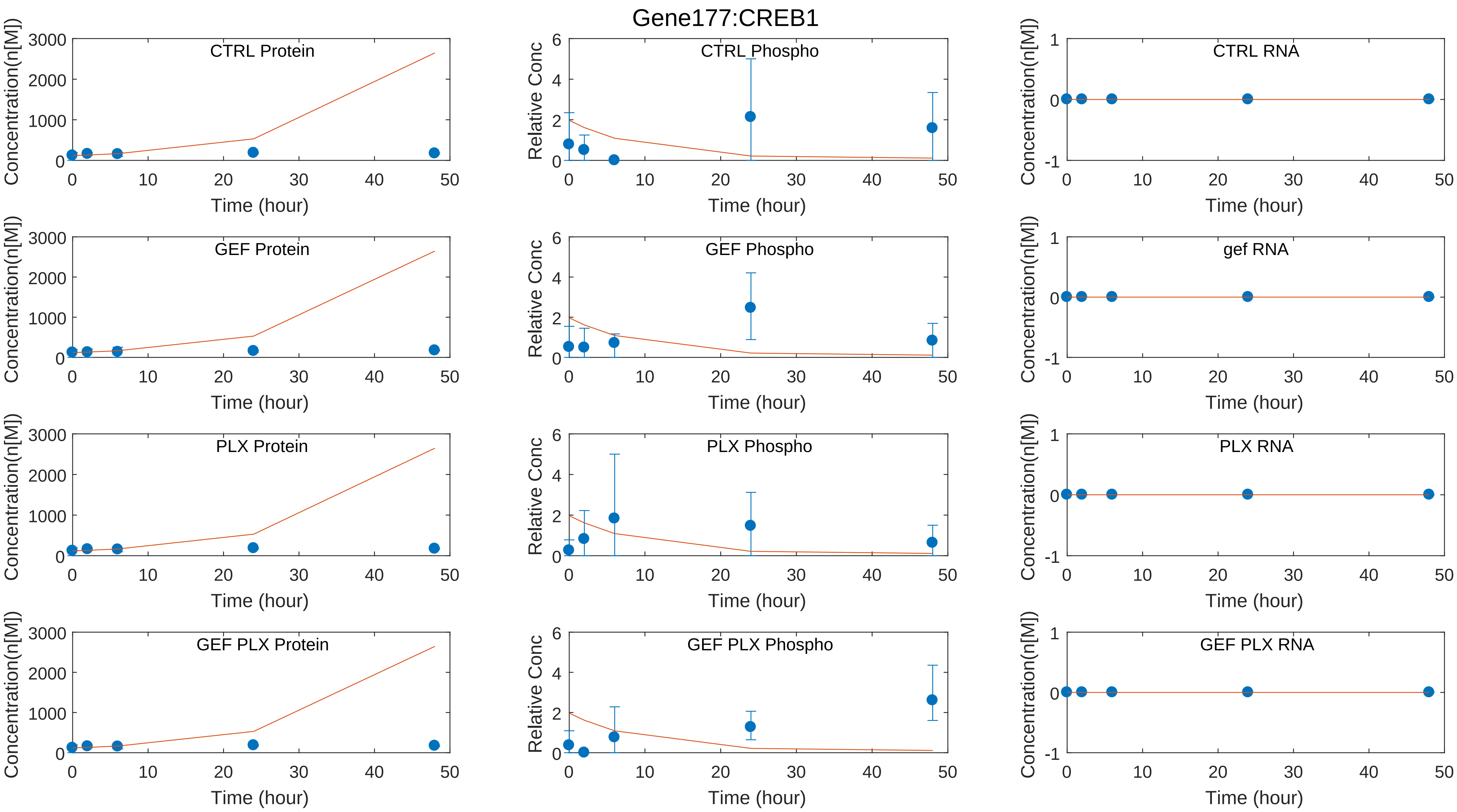

Gene177:CREB1
CTRL Protein
GEF Protein
PLX Protein
GEF PLX Protein
CTRL Phospho
GEF Phospho
PLX Phospho
GEF PLX Phospho
CTRL RNA
gef RNA
PLX RNA
GEF PLX RNA
Concentration(n[M])
Relative Conc
Time (hour)

Gene178:PPP3CB

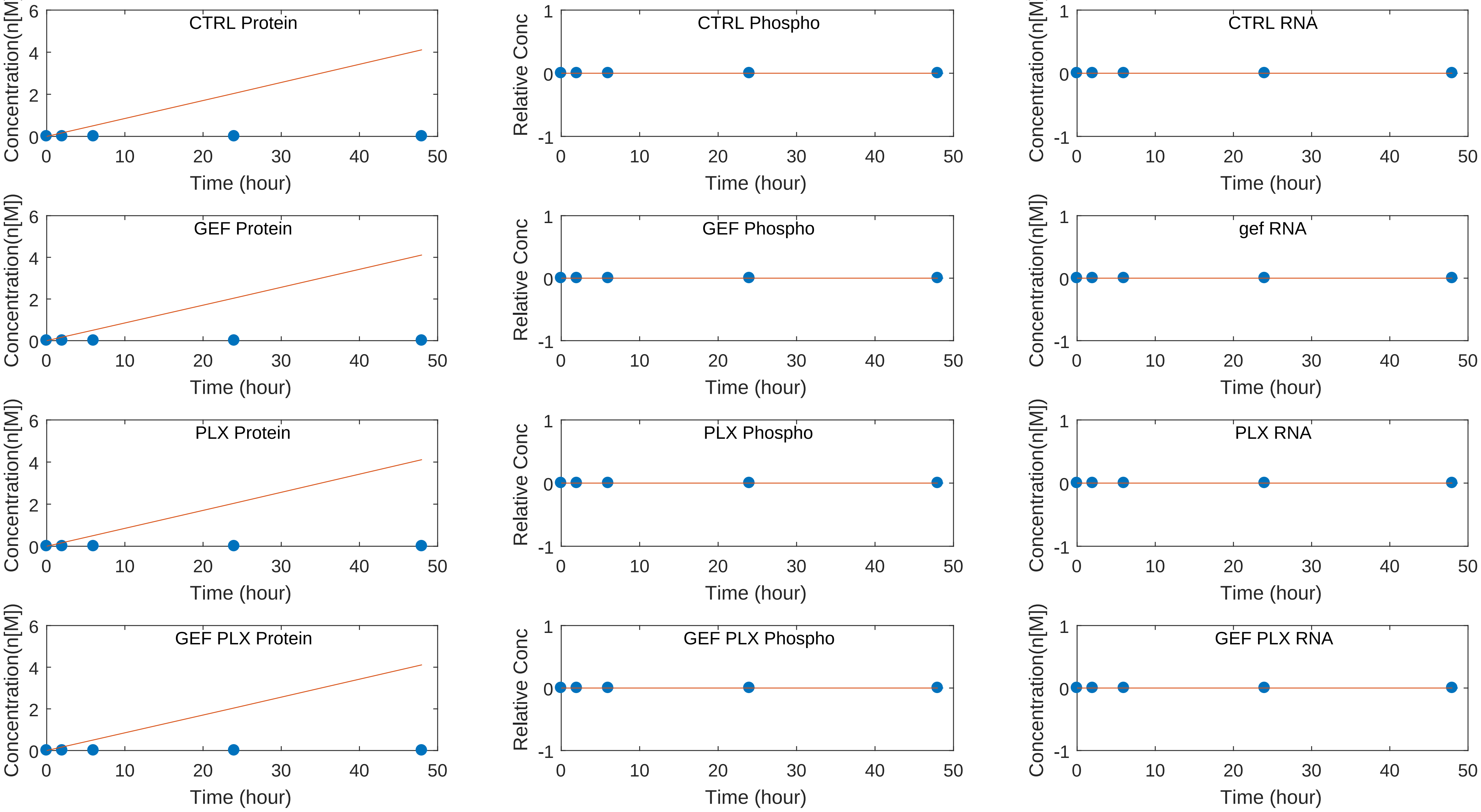

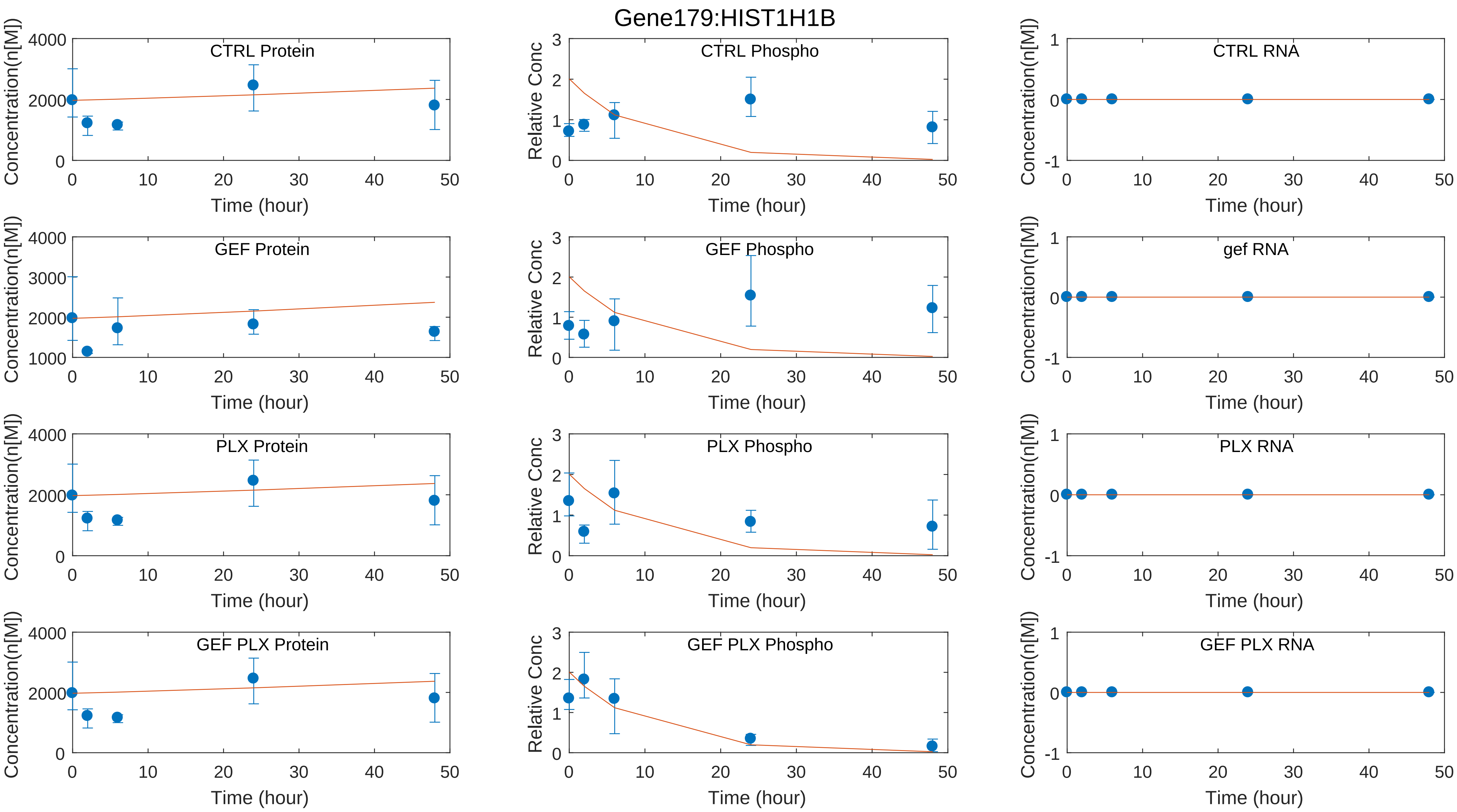

Gene179:HIST1H1B
CTRL Protein
CTRL Phospho
CTRL RNA
GEF Protein
GEF Phospho
gef RNA
PLX Protein
PLX Phospho
PLX RNA
GEF PLX Protein
GEF PLX Phospho
GEF PLX RNA
Concentration(n[M])
Relative Conc
Time (hour)

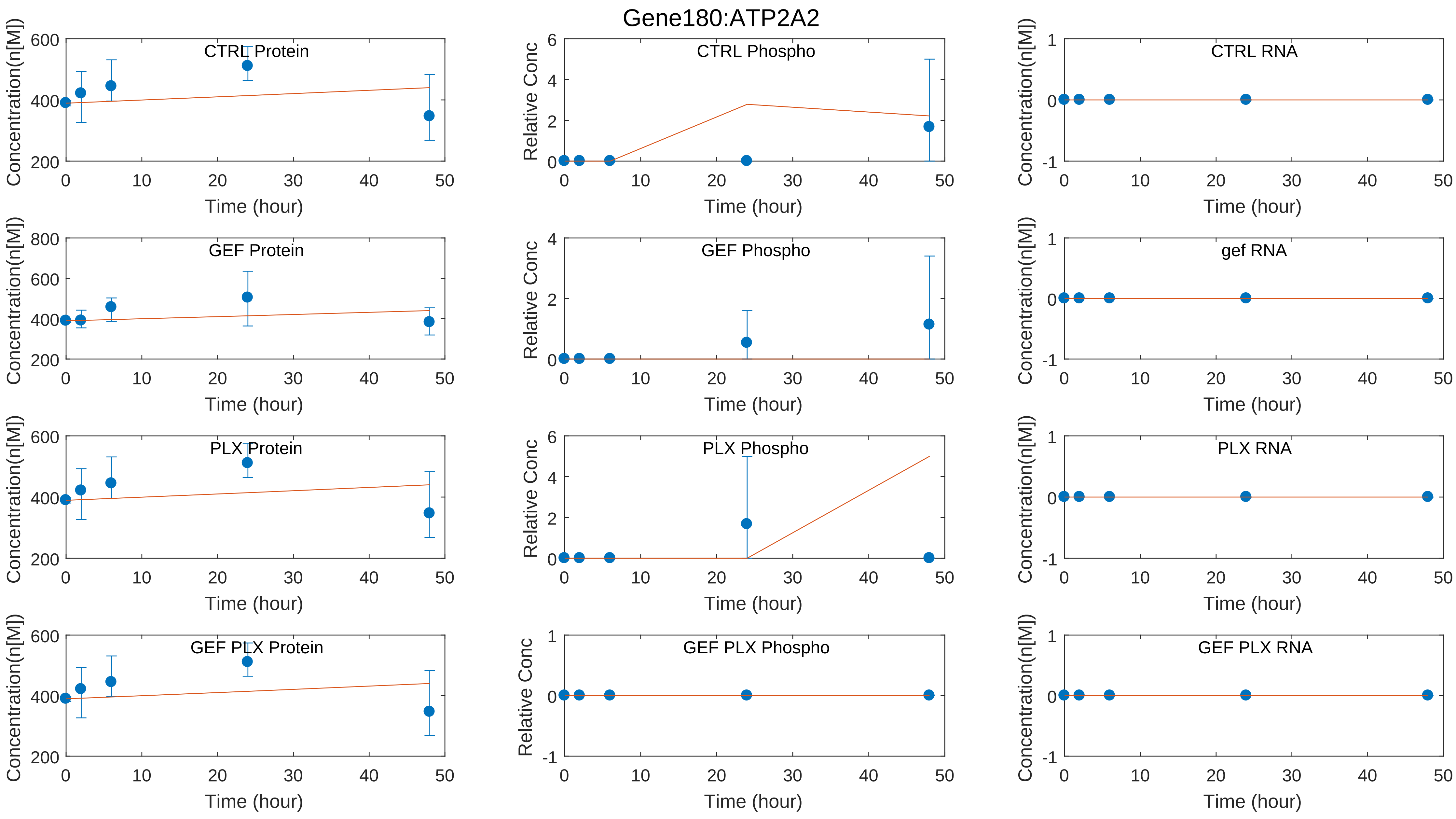
Gene180:ATP2A2
CTRL Protein
CTRL Phospho
CTRL RNA
GEF Protein
GEF Phospho
gef RNA
PLX Protein
PLX Phospho
PLX RNA
GEF PLX Protein
GEF PLX Phospho
GEF PLX RNA
Concentration(n[M])
Relative Conc
Time (hour)
0
10
20
30
40
50
200
400
600
800
-1
1
2
4
6

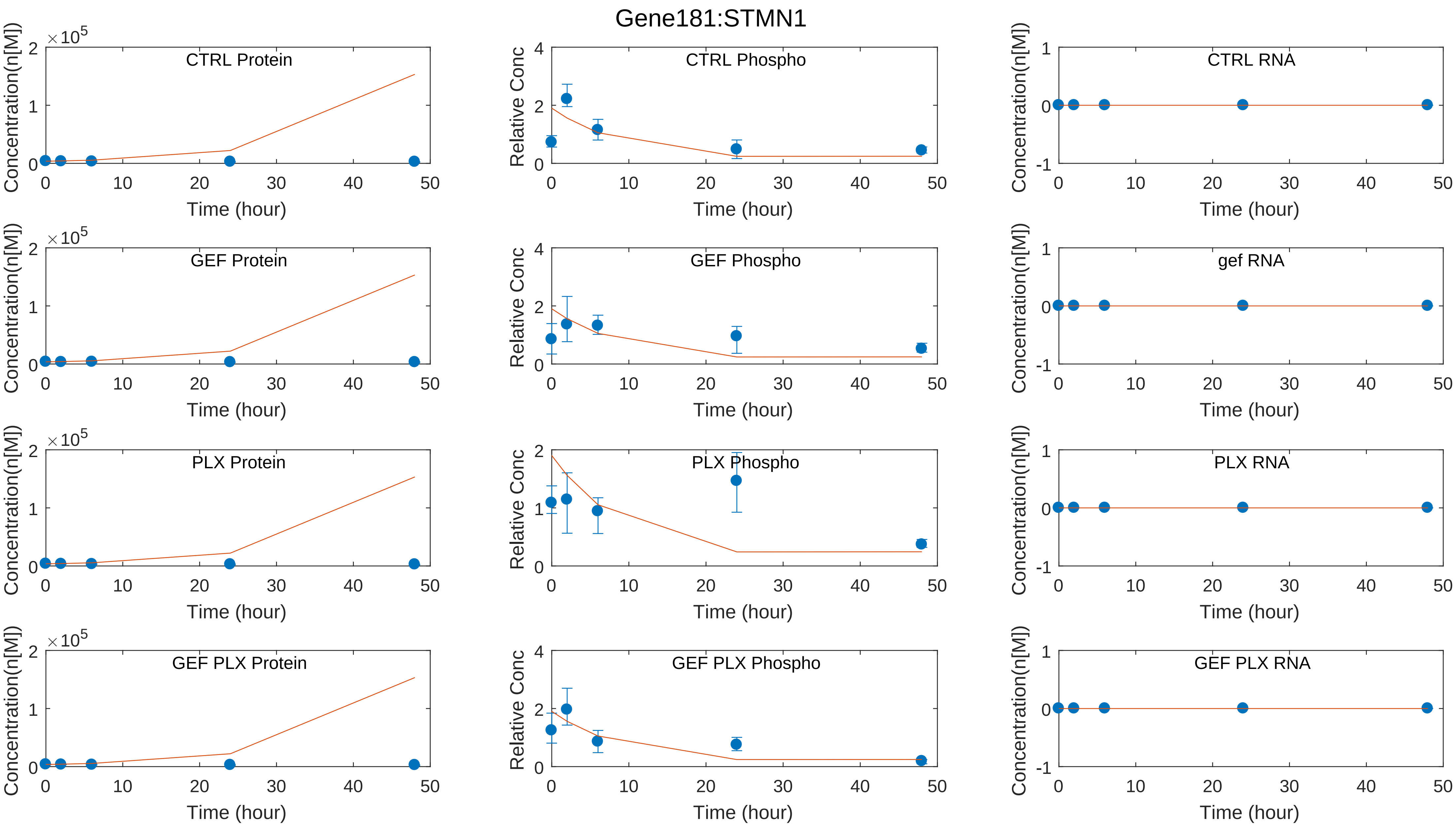
Gene181:STMN1
CTRL Protein
CTRL Phospho
CTRL RNA
GEF Protein
GEF Phospho
gef RNA
PLX Protein
PLX Phospho
PLX RNA
GEF PLX Protein
GEF PLX Phospho
GEF PLX RNA
Concentration(n[M])
Relative Conc
Time (hour)
$\times 10^5$

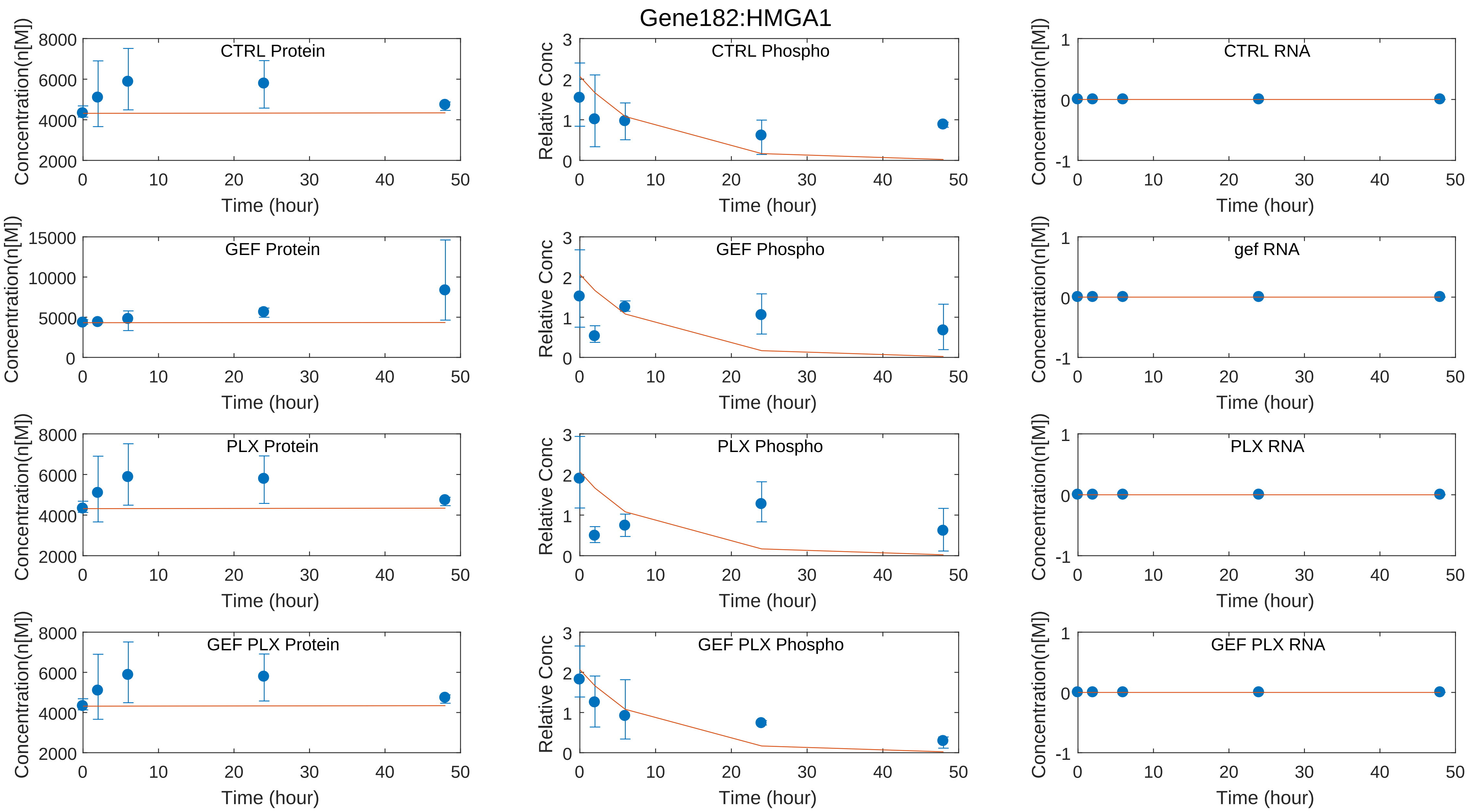
Gene182:HMGA1
CTRL Protein
CTRL Phospho
CTRL RNA
GEF Protein
GEF Phospho
gef RNA
PLX Protein
PLX Phospho
PLX RNA
GEF PLX Protein
GEF PLX Phospho
GEF PLX RNA
Concentration(n[M])
Relative Conc
Time (hour)

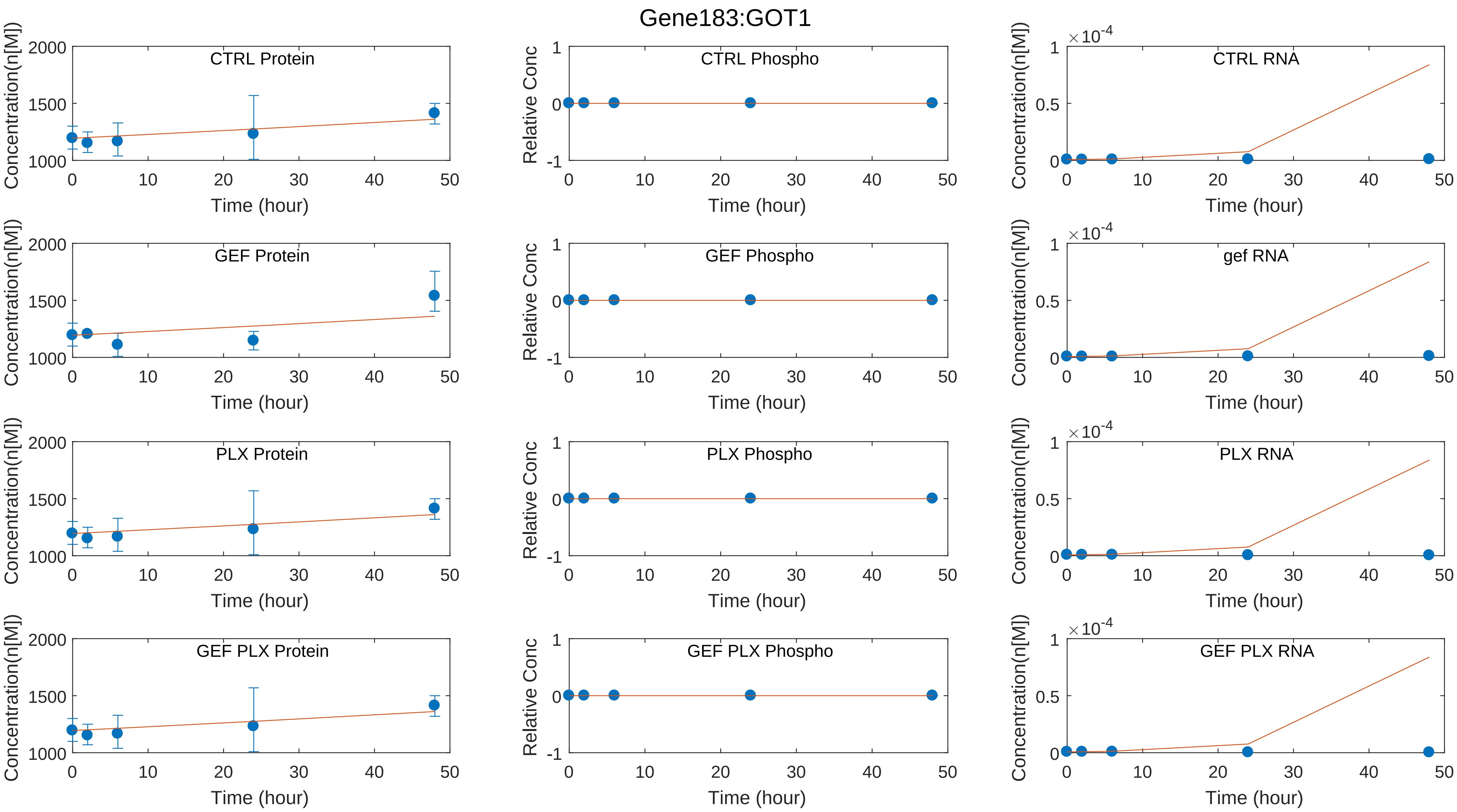
Gene183:GOT1
CTRL Protein
CTRL Phospho
CTRL RNA
GEF Protein
GEF Phospho
gef RNA
PLX Protein
PLX Phospho
PLX RNA
GEF PLX Protein
GEF PLX Phospho
GEF PLX RNA
Concentration(n[M])
Relative Conc
Time (hour)
×10-4

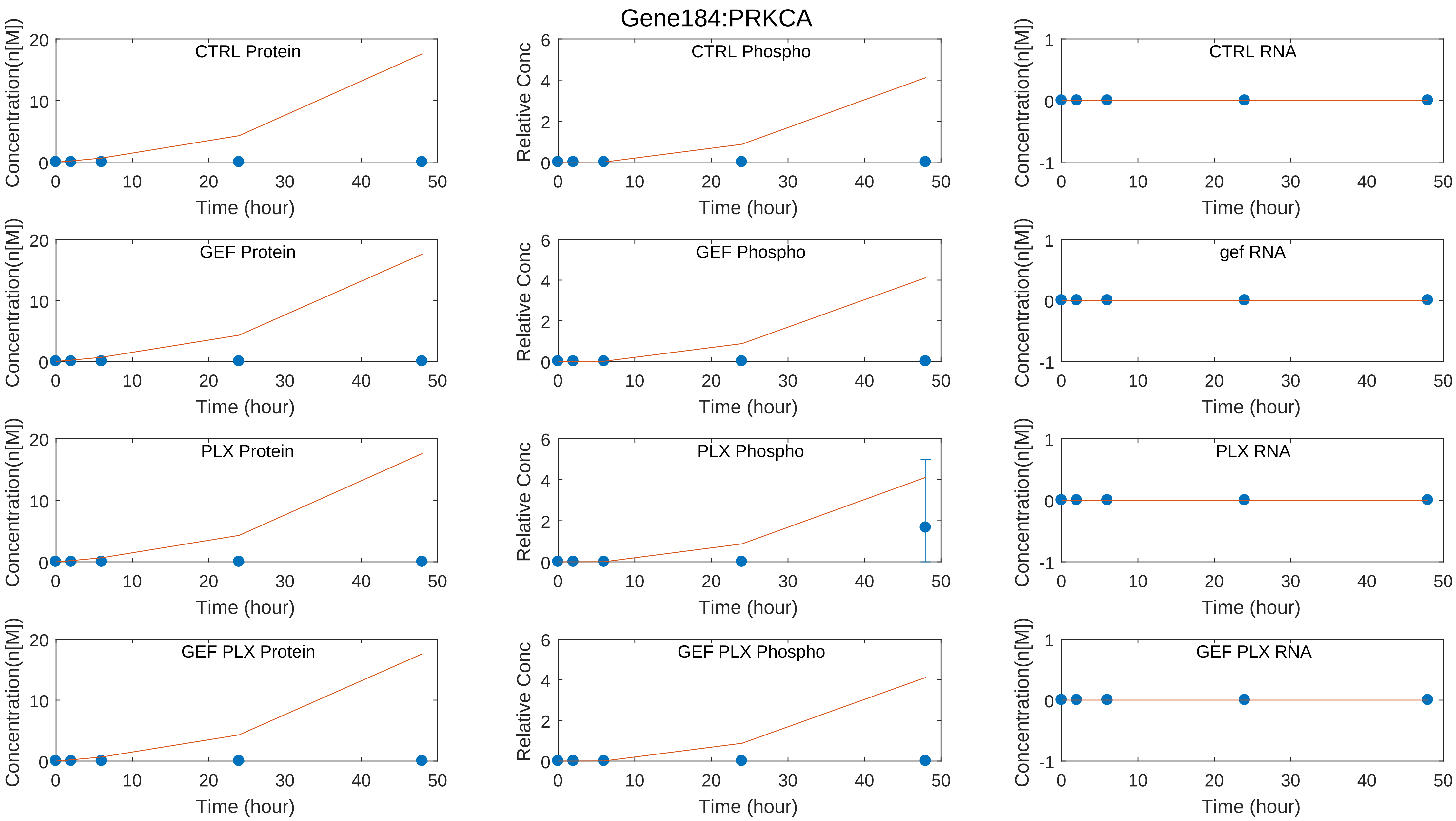
Gene184:PRKCA
CTRL Protein
CTRL Phospho
CTRL RNA
GEF Protein
GEF Phospho
gef RNA
PLX Protein
PLX Phospho
PLX RNA
GEF PLX Protein
GEF PLX Phospho
GEF PLX RNA
Concentration(n[M])
Relative Conc
Time (hour)

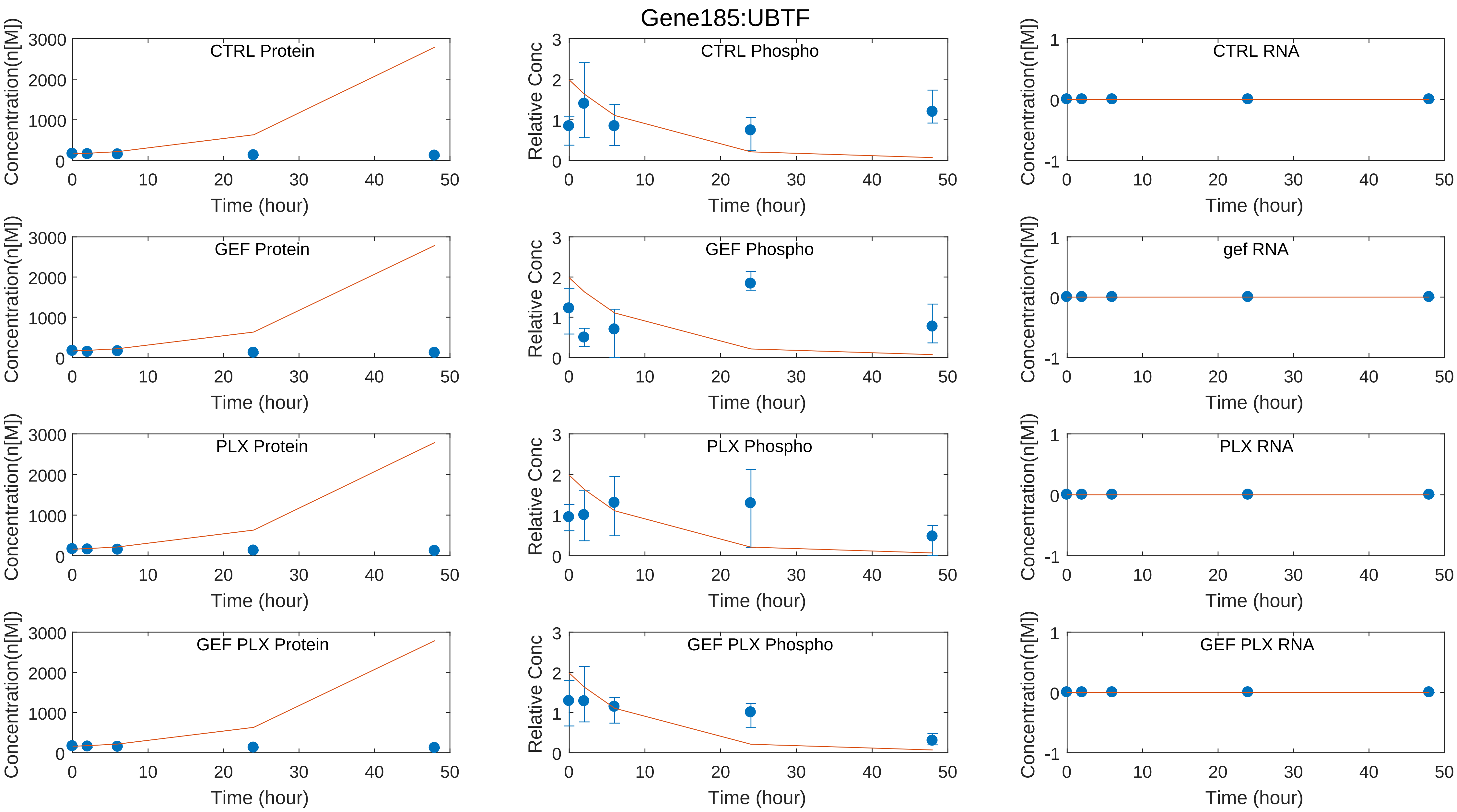
Gene185:UBTF
CTRL Protein
GEF Protein
PLX Protein
GEF PLX Protein
CTRL Phospho
GEF Phospho
PLX Phospho
GEF PLX Phospho
CTRL RNA
gef RNA
PLX RNA
GEF PLX RNA
Concentration(n[M])
Relative Conc
Time (hour)

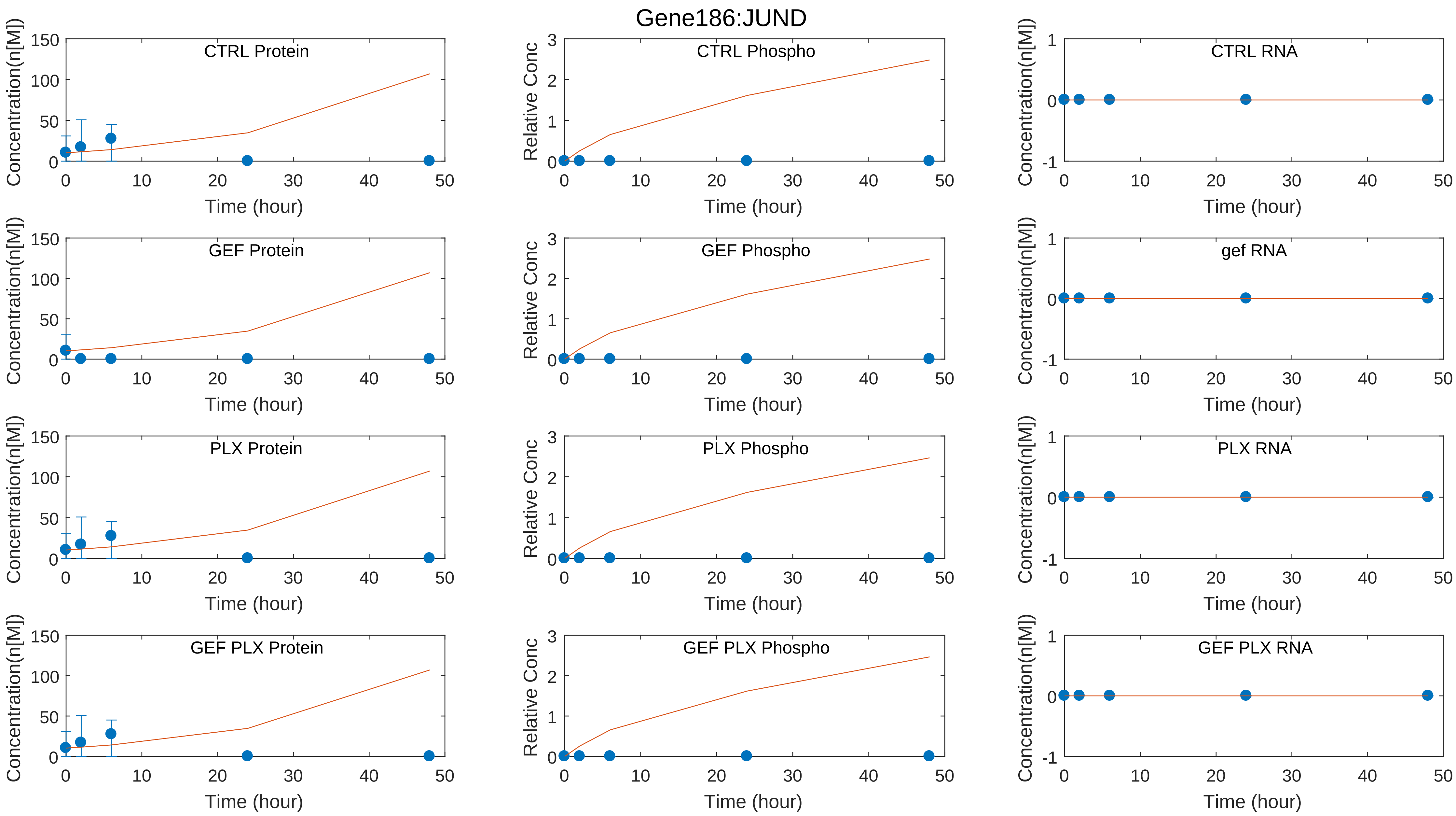

Gene186:JUND
CTRL Protein
GEF Protein
PLX Protein
GEF PLX Protein
CTRL Phospho
GEF Phospho
PLX Phospho
GEF PLX Phospho
CTRL RNA
gef RNA
PLX RNA
GEF PLX RNA
Concentration(n[M])
Relative Conc
Time (hour)

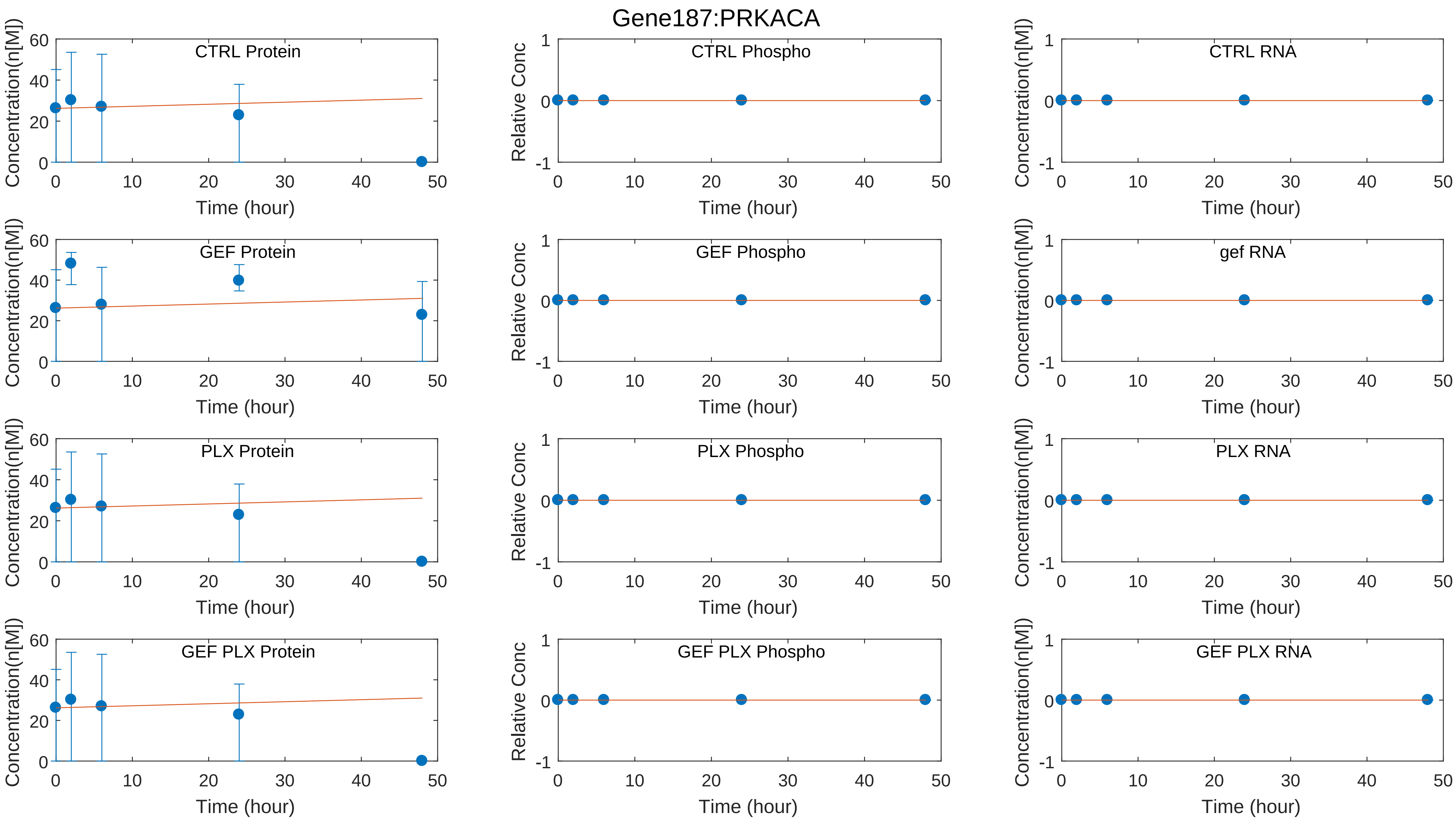
Gene187:PRKACA
CTRL Protein
CTRL Phospho
CTRL RNA
GEF Protein
GEF Phospho
gef RNA
PLX Protein
PLX Phospho
PLX RNA
GEF PLX Protein
GEF PLX Phospho
GEF PLX RNA
Concentration(n[M])
Relative Conc
Time (hour)

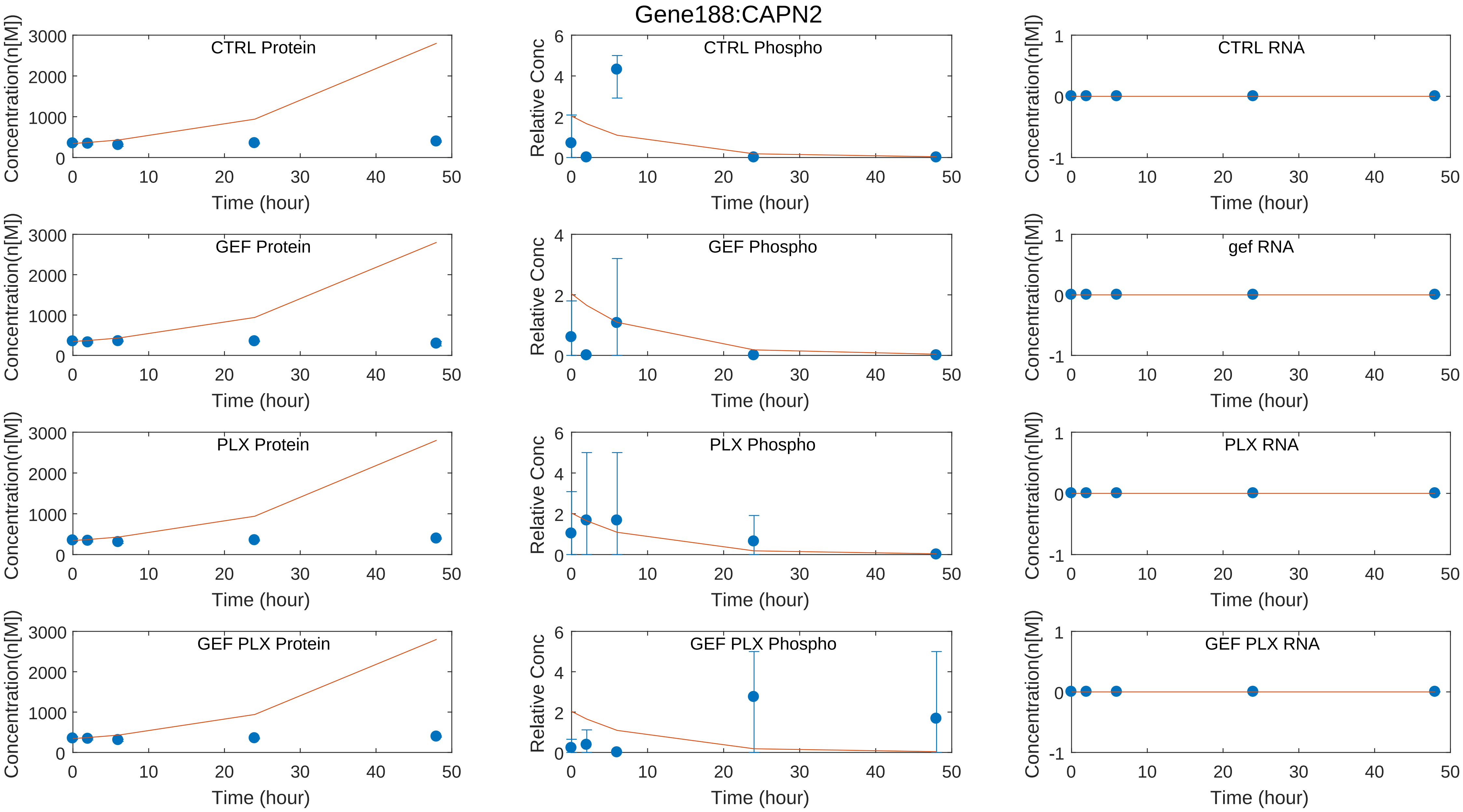

Gene188:CAPN2
CTRL Protein
CTRL Phospho
CTRL RNA
GEF Protein
GEF Phospho
gef RNA
PLX Protein
PLX Phospho
PLX RNA
GEF PLX Protein
GEF PLX Phospho
GEF PLX RNA
Concentration(n[M])
Relative Conc
Time (hour)

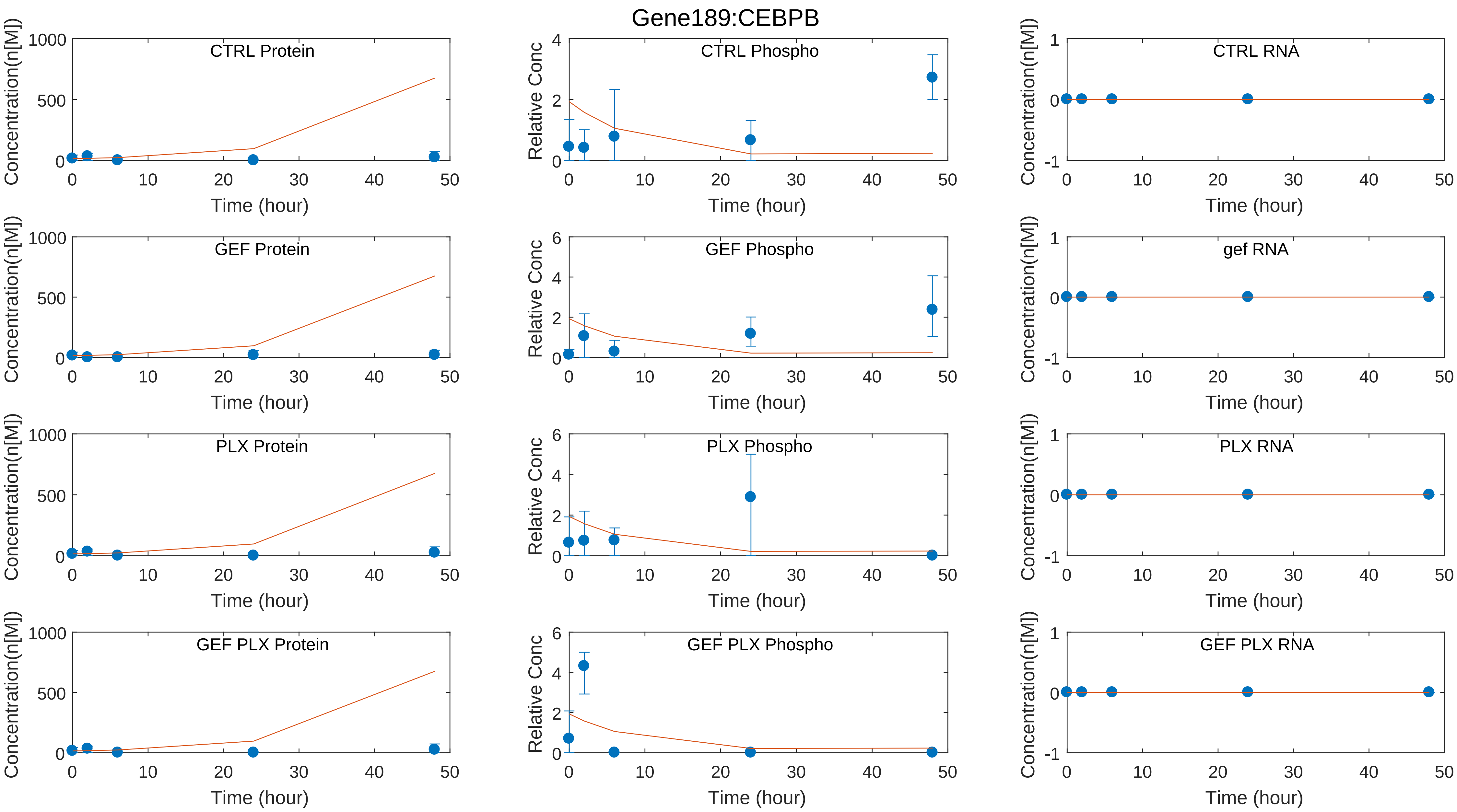

Gene189:CEBPB
CTRL Protein
CTRL Phospho
CTRL RNA
GEF Protein
GEF Phospho
gef RNA
PLX Protein
PLX Phospho
PLX RNA
GEF PLX Protein
GEF PLX Phospho
GEF PLX RNA
Concentration(n[M])
Relative Conc
Time (hour)

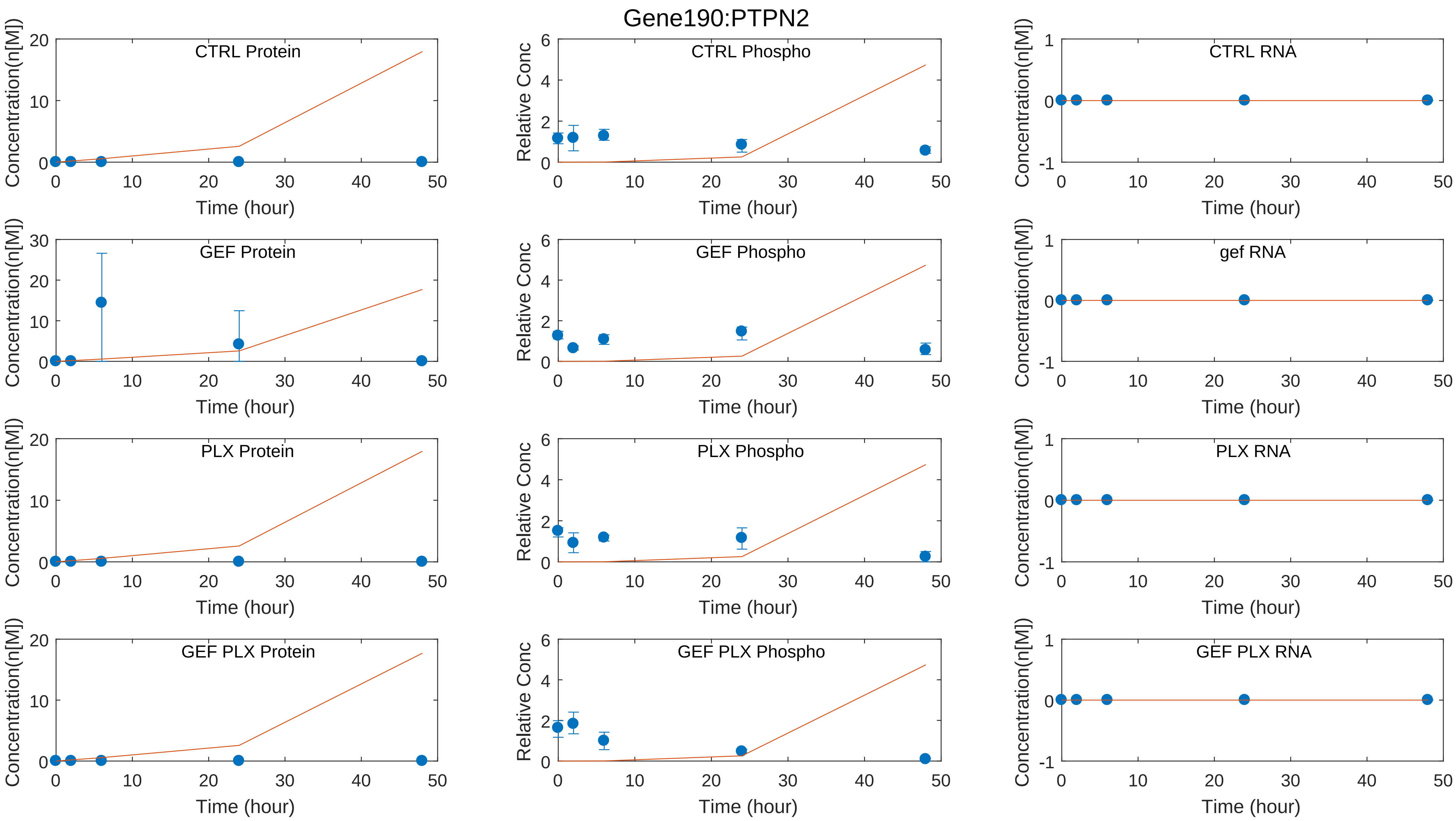
Gene190:PTPN2
CTRL Protein
CTRL Phospho
CTRL RNA
GEF Protein
GEF Phospho
gef RNA
PLX Protein
PLX Phospho
PLX RNA
GEF PLX Protein
GEF PLX Phospho
GEF PLX RNA
Concentration(n[M])
Relative Conc
Time (hour)

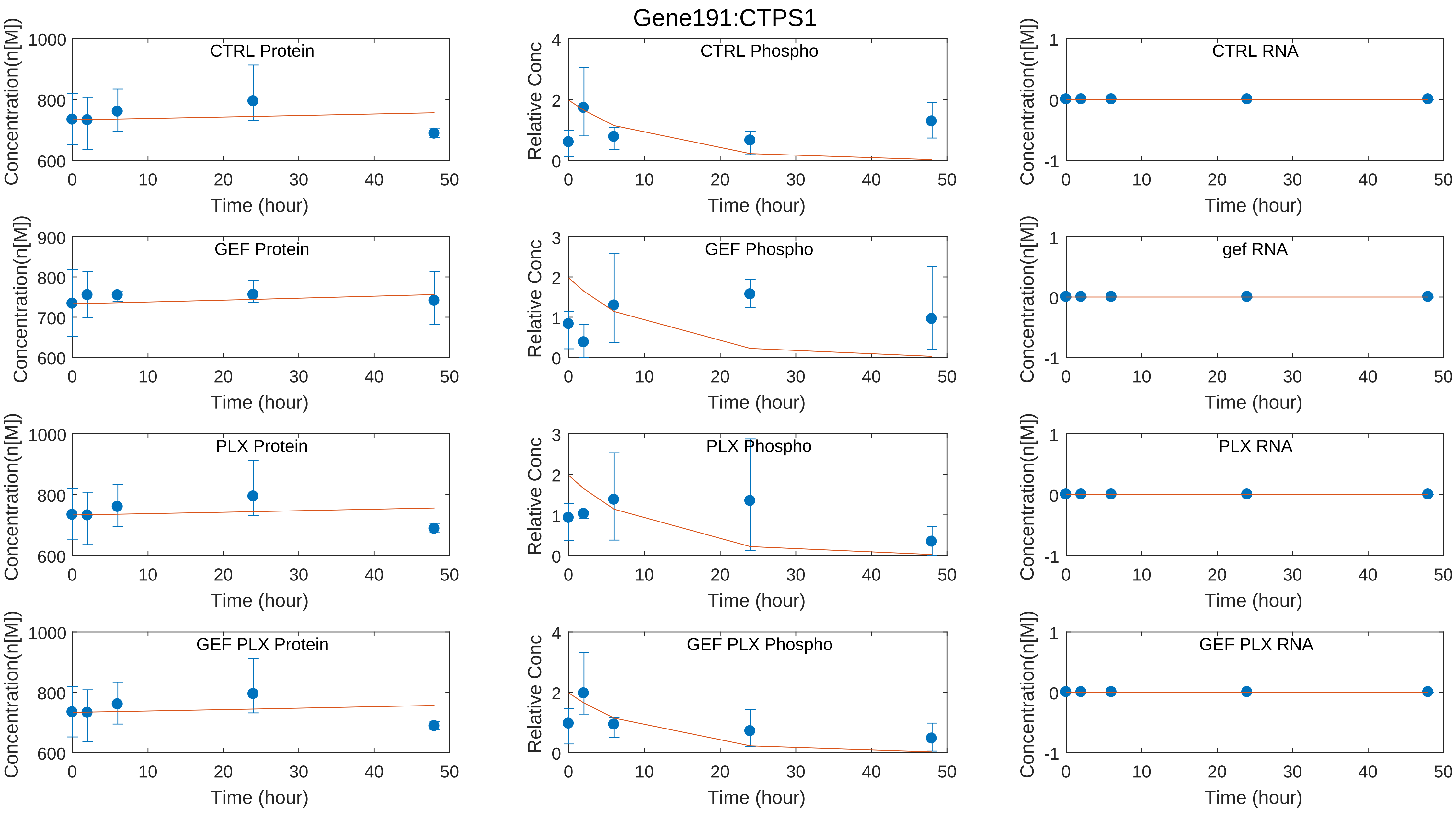

Gene191:CTPS1
CTRL Protein
CTRL Phospho
CTRL RNA
GEF Protein
GEF Phospho
gef RNA
PLX Protein
PLX Phospho
PLX RNA
GEF PLX Protein
GEF PLX Phospho
GEF PLX RNA
Concentration(n[M])
Relative Conc
Time (hour)

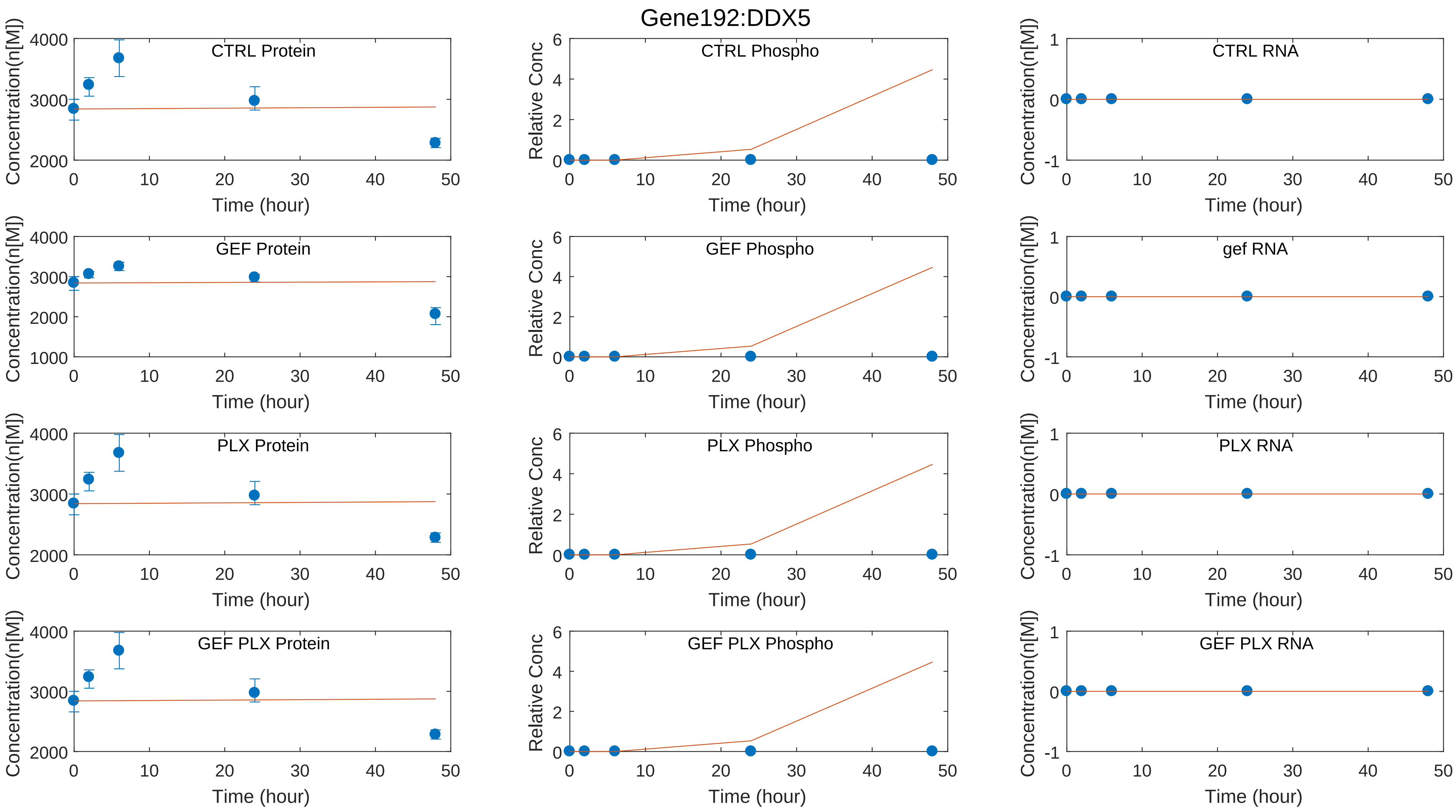

Gene192:DDX5
CTRL Protein
GEF Protein
PLX Protein
GEF PLX Protein
CTRL Phospho
GEF Phospho
PLX Phospho
GEF PLX Phospho
CTRL RNA
gef RNA
PLX RNA
GEF PLX RNA
Concentration(n[M])
Relative Conc
Time (hour)

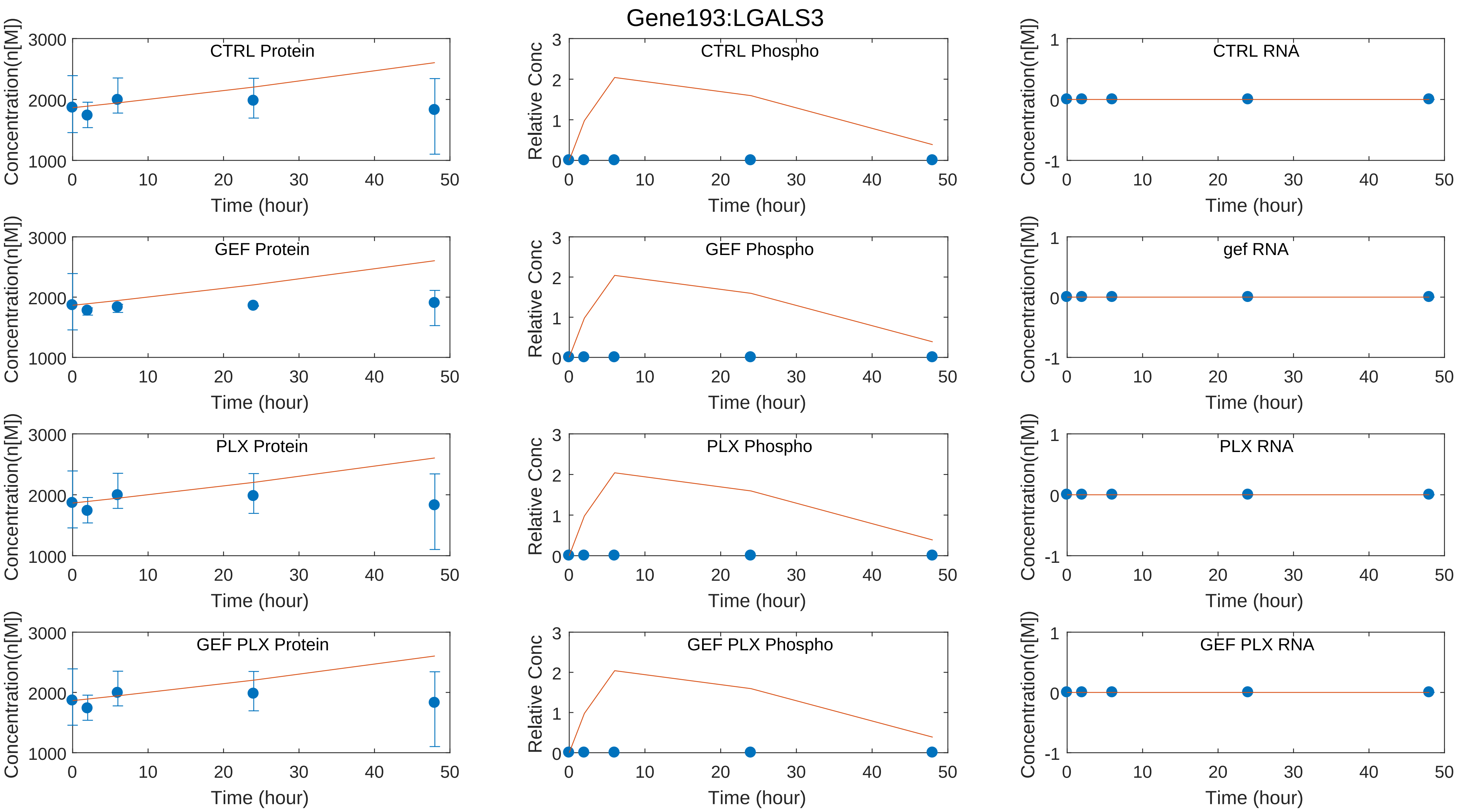
Gene193:LGALS3
CTRL Protein
CTRL Phospho
CTRL RNA
GEF Protein
GEF Phospho
gef RNA
PLX Protein
PLX Phospho
PLX RNA
GEF PLX Protein
GEF PLX Phospho
GEF PLX RNA
Concentration(n[M])
Relative Conc
Time (hour)

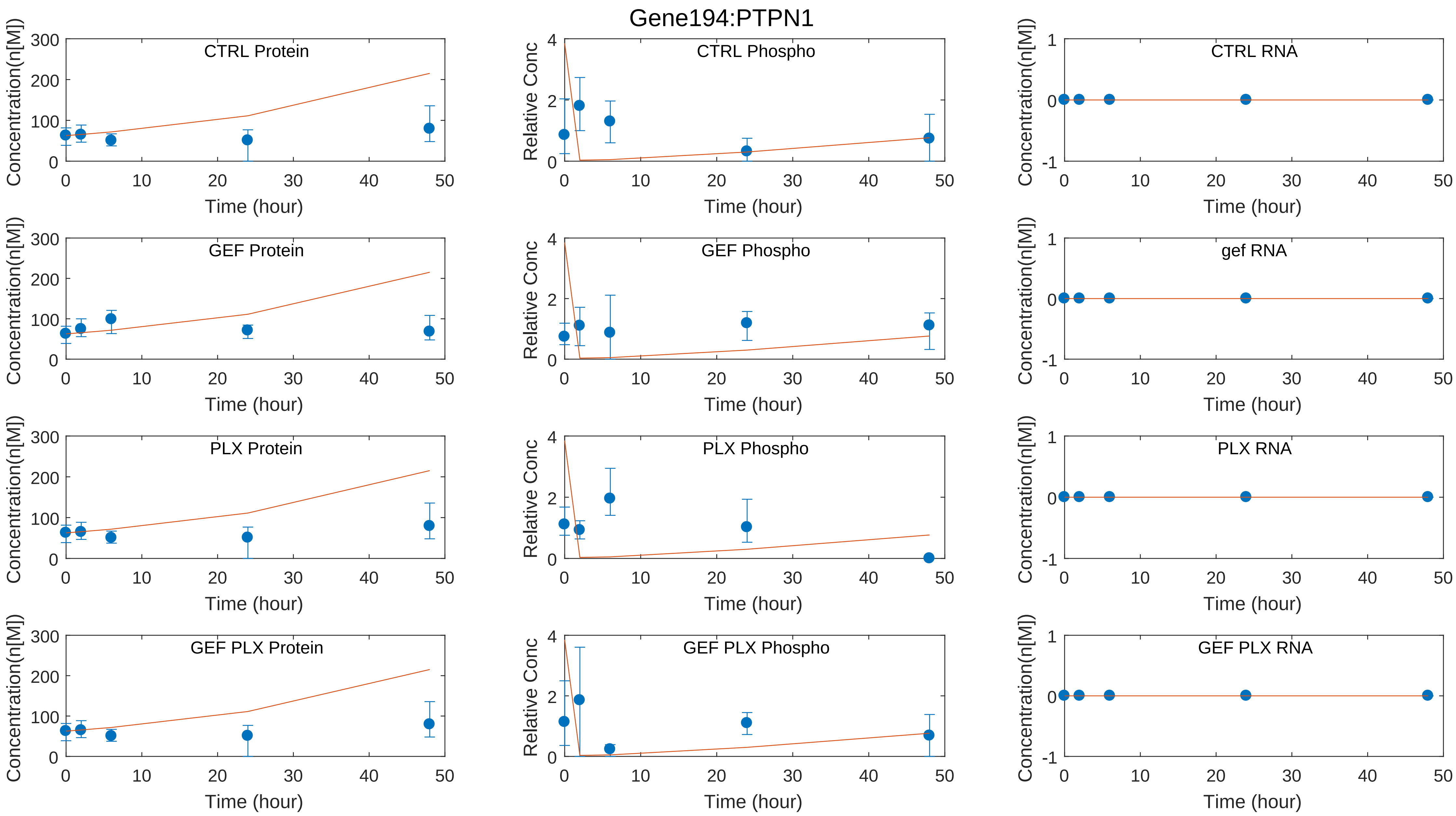
Gene194:PTPN1
CTRL Protein
CTRL Phospho
CTRL RNA
GEF Protein
GEF Phospho
gef RNA
PLX Protein
PLX Phospho
PLX RNA
GEF PLX Protein
GEF PLX Phospho
GEF PLX RNA
Concentration(n[M])
Relative Conc
Time (hour)

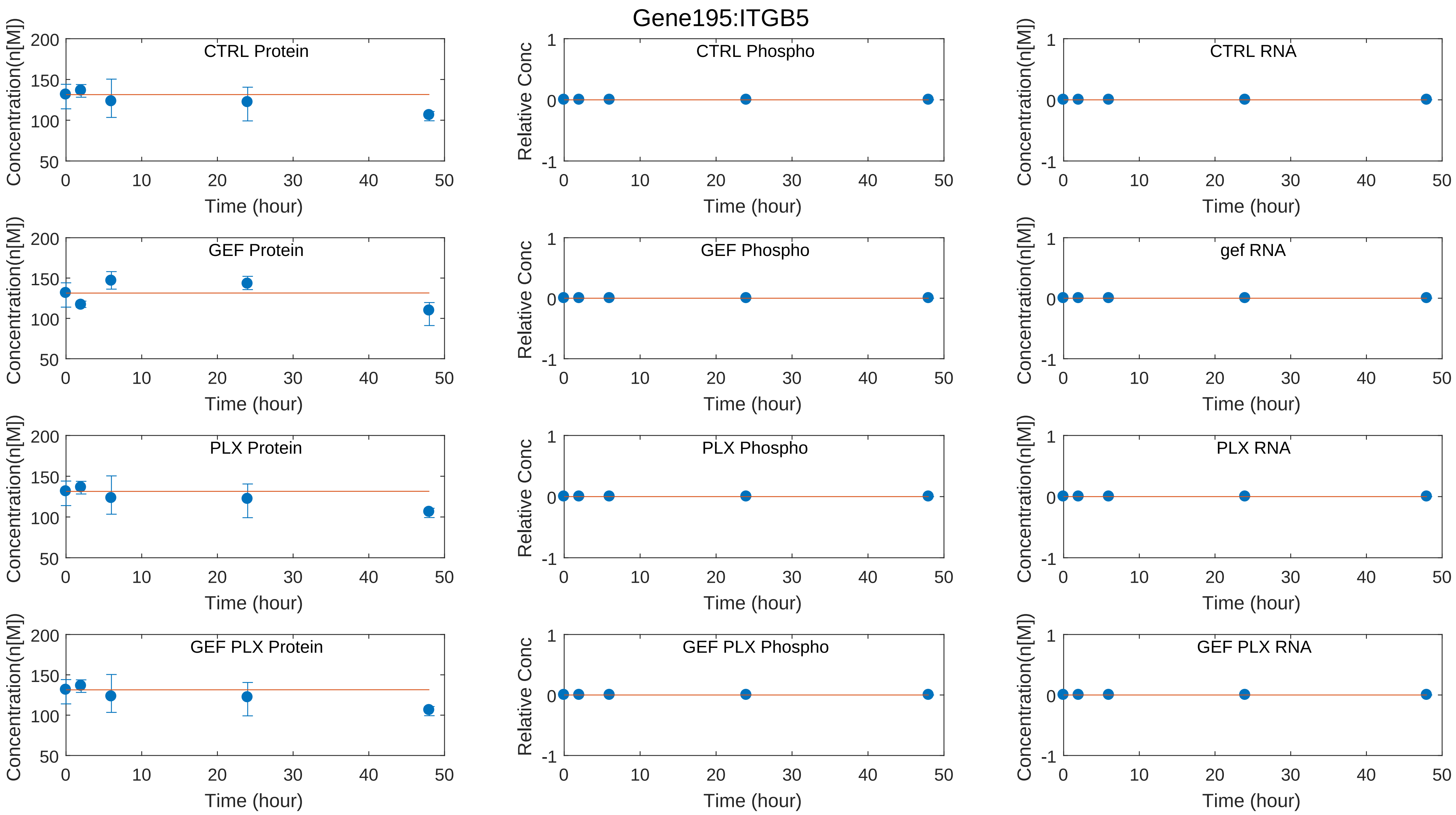

Gene195:ITGB5
CTRL Protein
CTRL Phospho
CTRL RNA
GEF Protein
GEF Phospho
gef RNA
PLX Protein
PLX Phospho
PLX RNA
GEF PLX Protein
GEF PLX Phospho
GEF PLX RNA
Concentration(n[M])
Relative Conc
Time (hour)

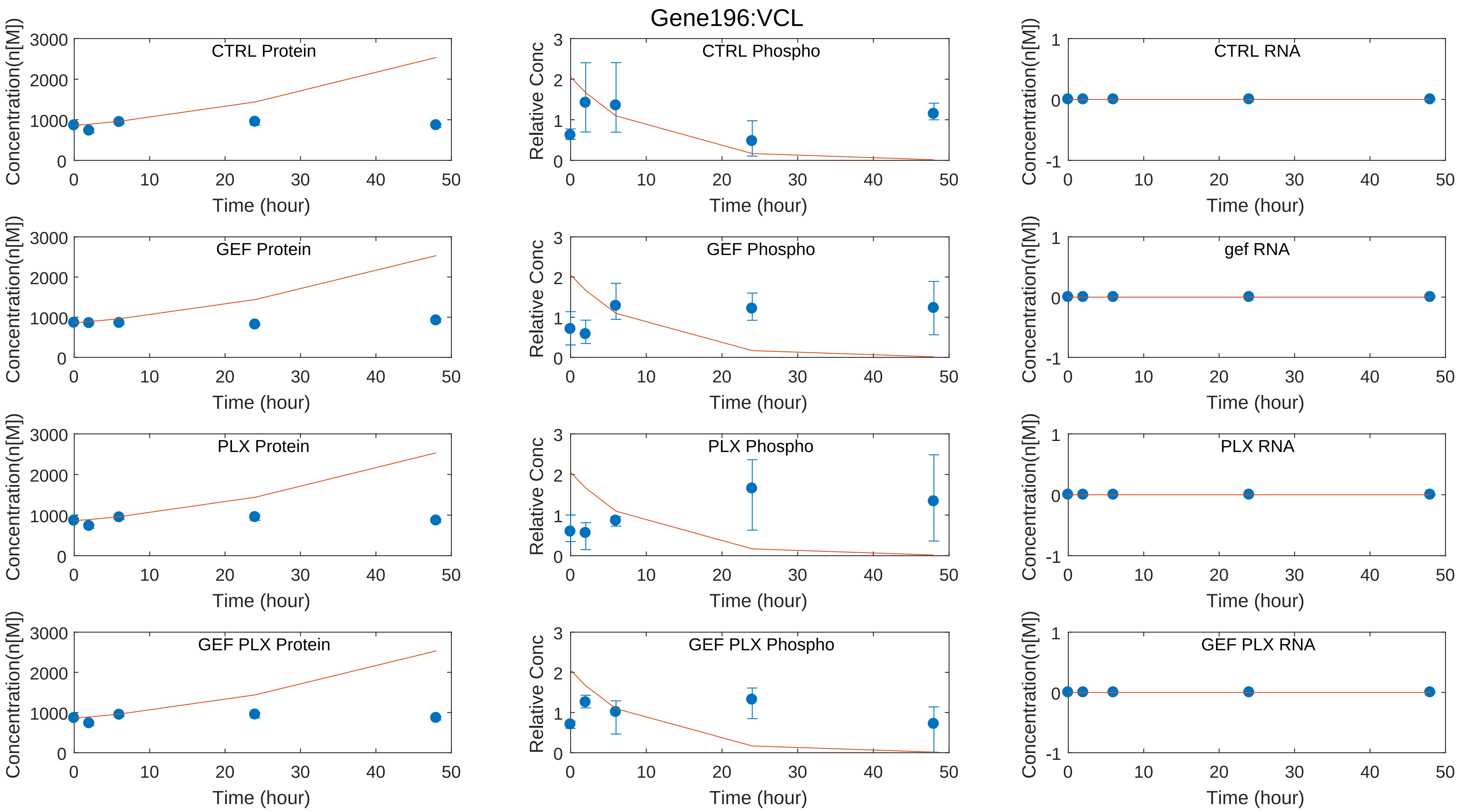
Gene196:VCL
CTRL Protein
GEF Protein
PLX Protein
GEF PLX Protein
CTRL Phospho
GEF Phospho
PLX Phospho
GEF PLX Phospho
CTRL RNA
gef RNA
PLX RNA
GEF PLX RNA
Concentration(n[M])
Relative Conc
Time (hour)

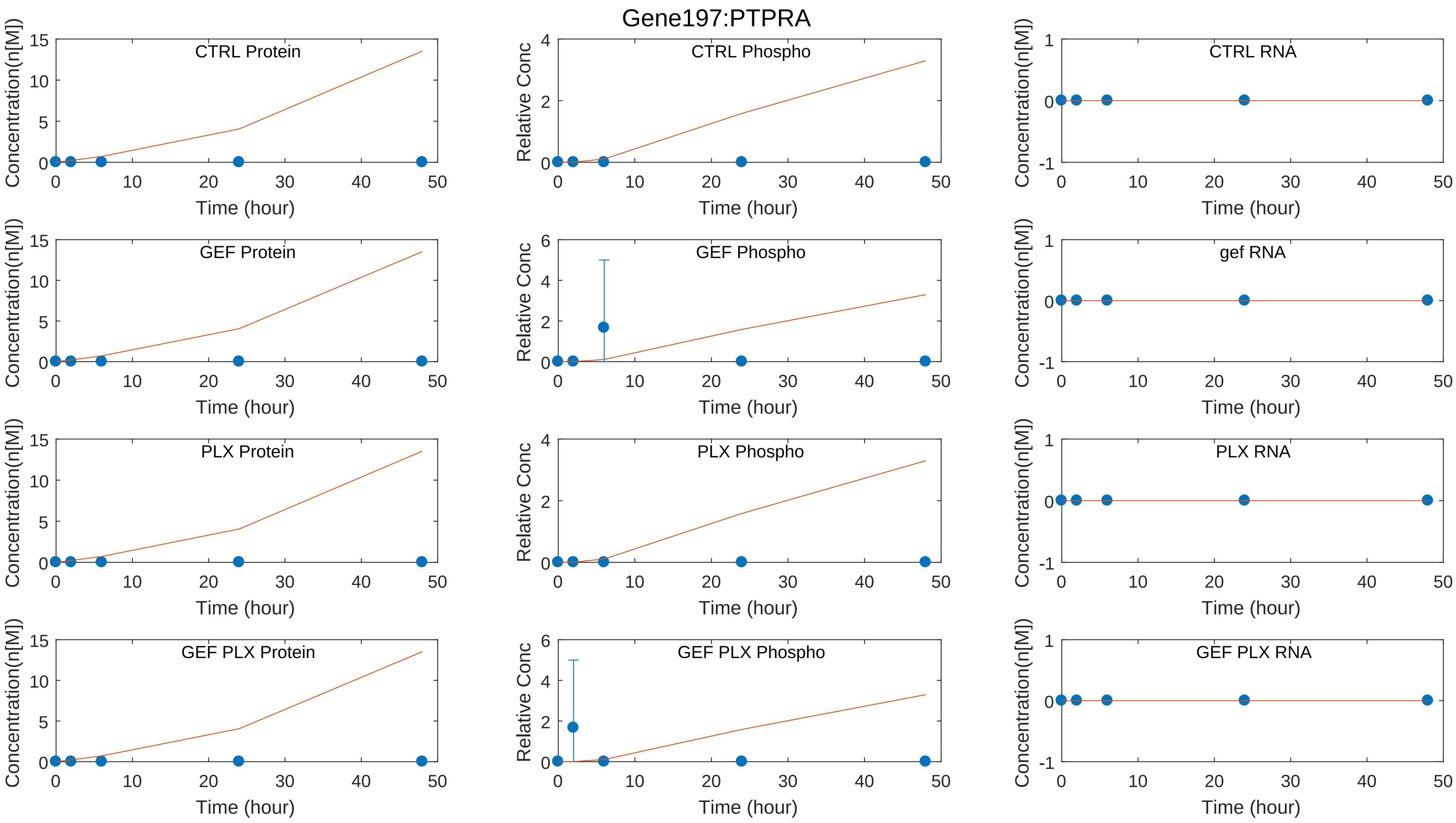

Gene197:PTPRA
CTRL Protein
CTRL Phospho
CTRL RNA
GEF Protein
GEF Phospho
gef RNA
PLX Protein
PLX Phospho
PLX RNA
GEF PLX Protein
GEF PLX Phospho
GEF PLX RNA
Concentration(n[M])
Relative Conc
Time (hour)

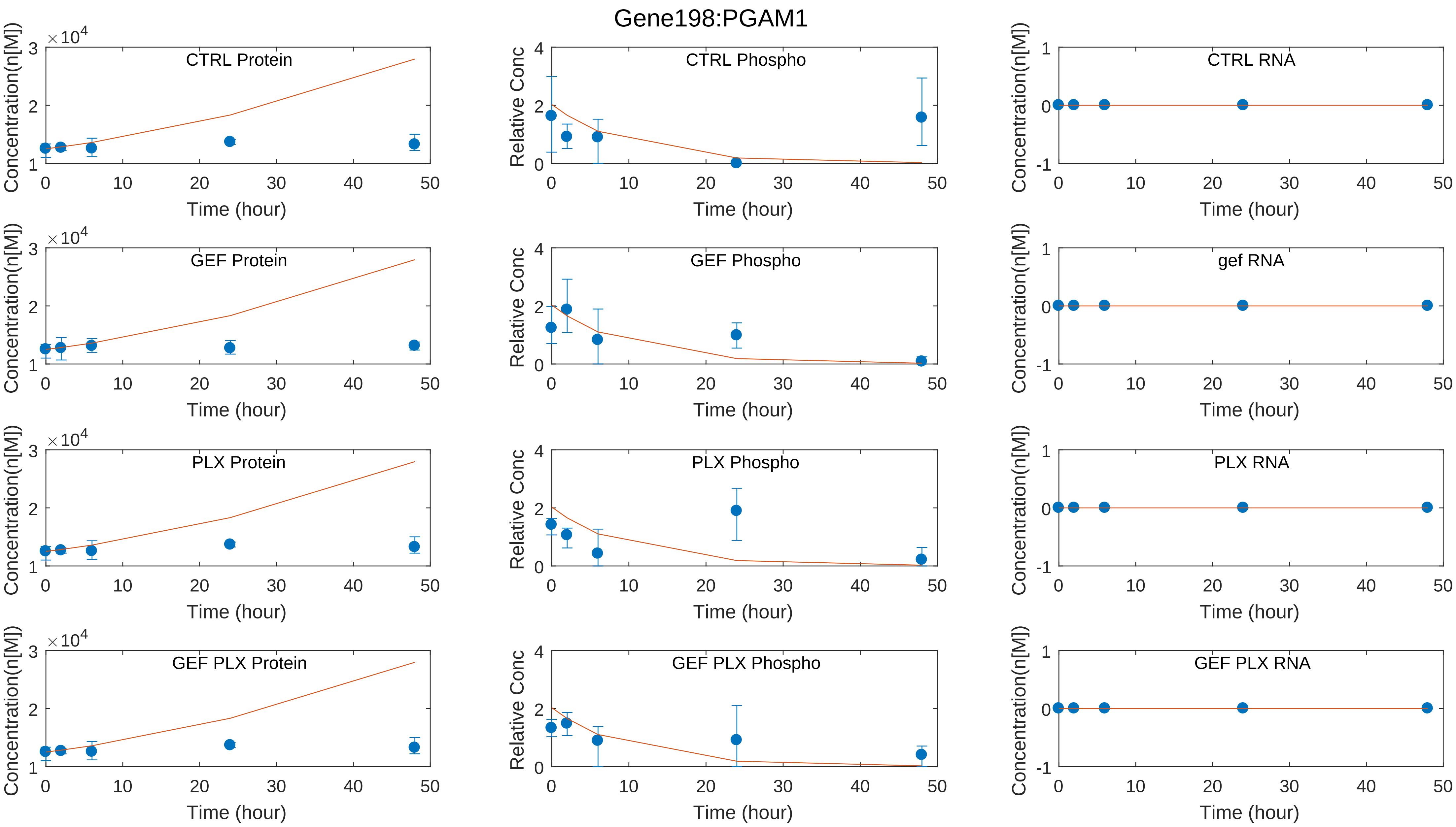
Gene198:PGAM1
CTRL Protein
GEF Protein
PLX Protein
GEF PLX Protein
CTRL Phospho
GEF Phospho
PLX Phospho
GEF PLX Phospho
CTRL RNA
gef RNA
PLX RNA
GEF PLX RNA
Concentration(n[M])
Relative Conc
Time (hour)
×10^4

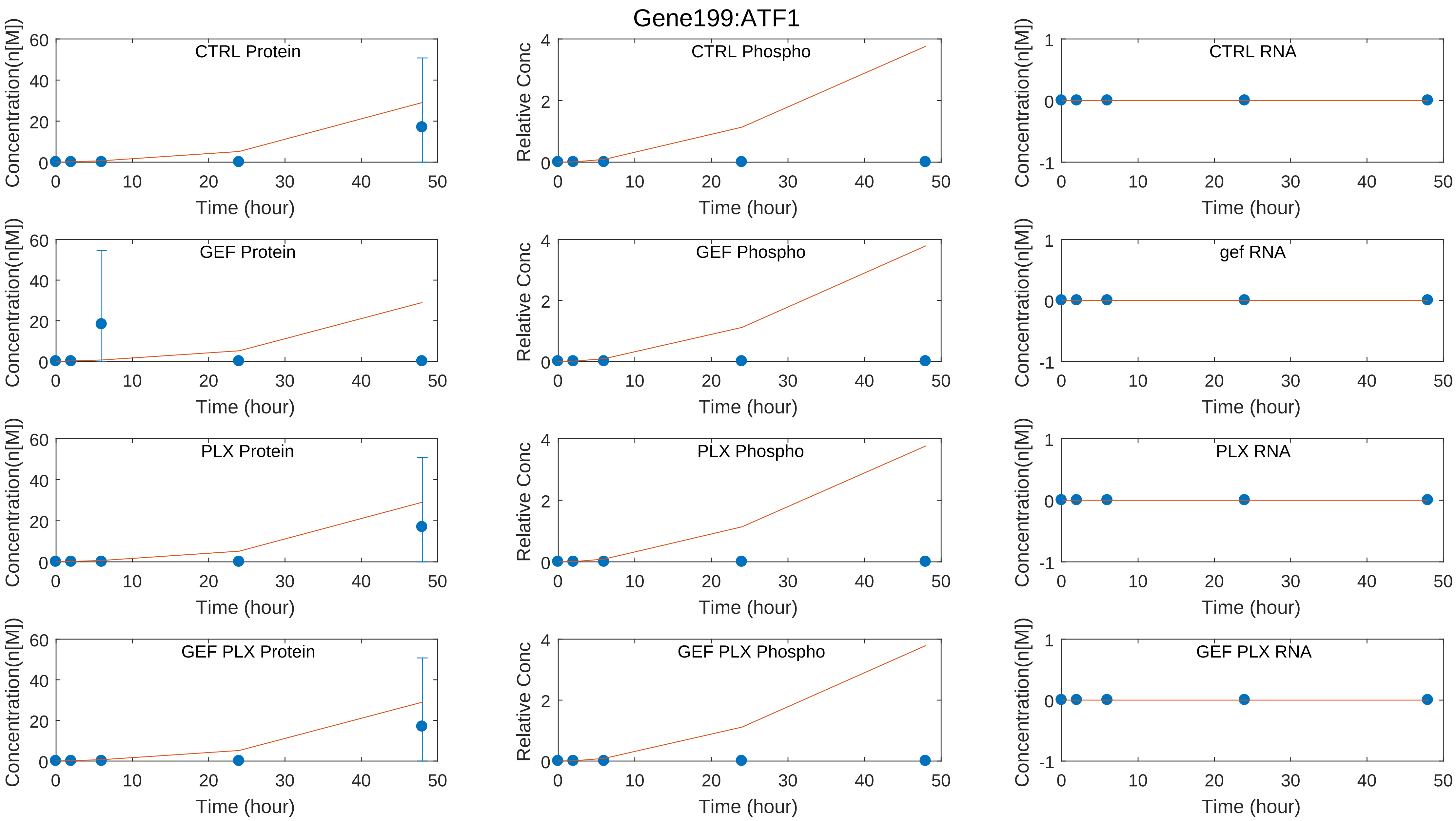

Gene199:ATF1
CTRL Protein
CTRL Phospho
CTRL RNA
GEF Protein
GEF Phospho
gef RNA
PLX Protein
PLX Phospho
PLX RNA
GEF PLX Protein
GEF PLX Phospho
GEF PLX RNA
Concentration(n[M])
Relative Conc
Time (hour)

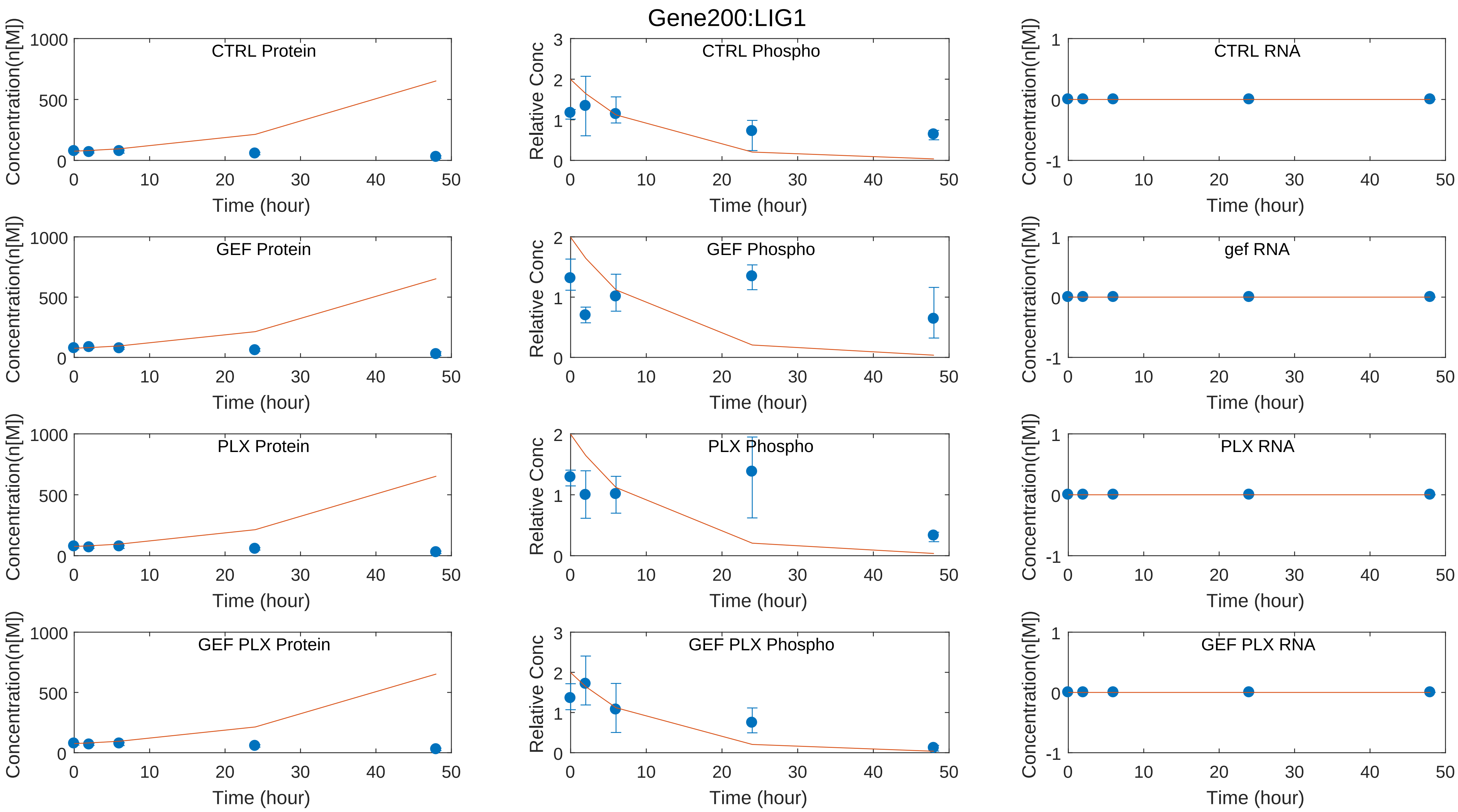

Gene200:LIG1
CTRL Protein
GEF Protein
PLX Protein
GEF PLX Protein
CTRL Phospho
GEF Phospho
PLX Phospho
GEF PLX Phospho
CTRL RNA
gef RNA
PLX RNA
GEF PLX RNA
Concentration(n[M])
Relative Conc
Time (hour)

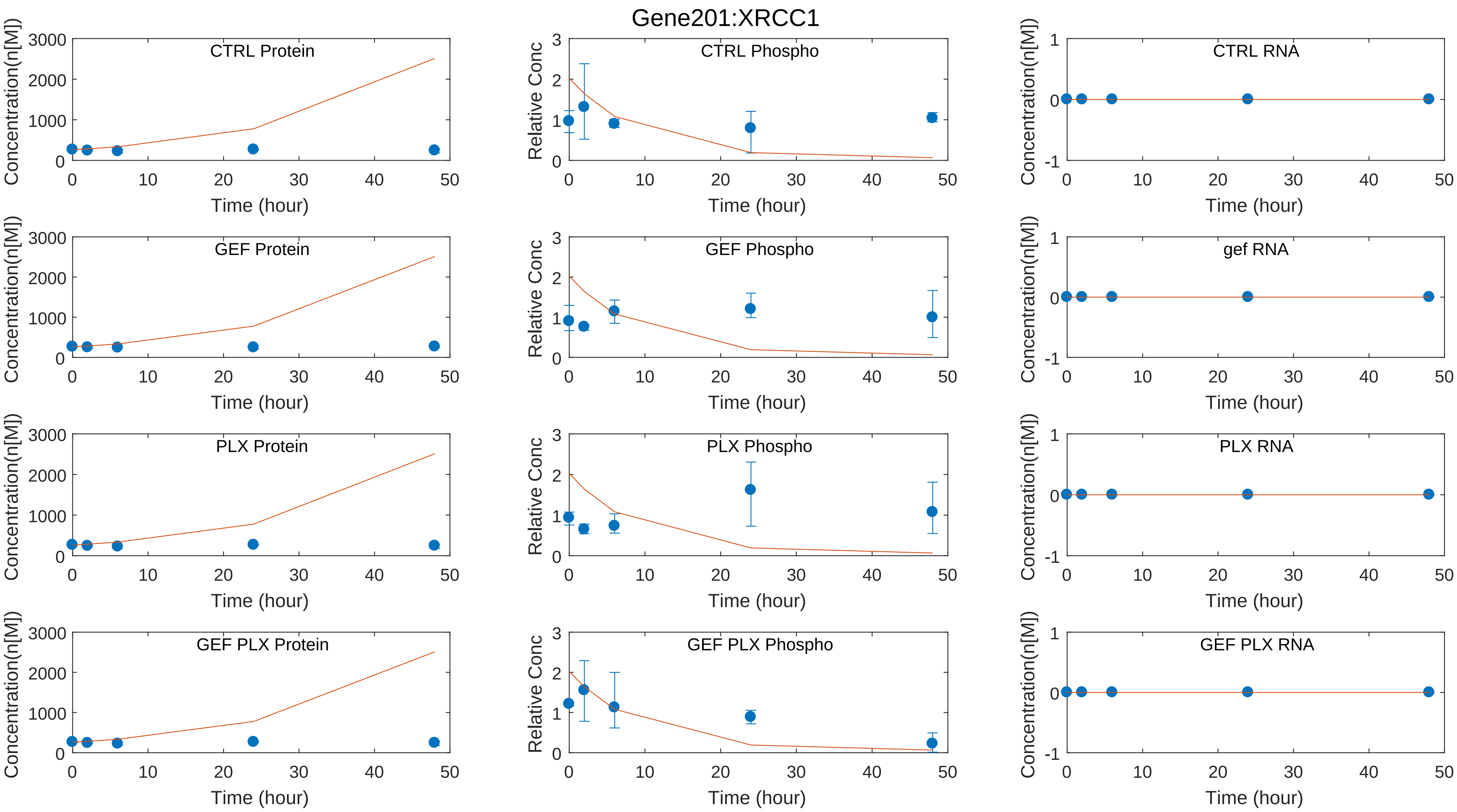

Gene201:XRCC1
CTRL Protein
CTRL Phospho
CTRL RNA
GEF Protein
GEF Phospho
gef RNA
PLX Protein
PLX Phospho
PLX RNA
GEF PLX Protein
GEF PLX Phospho
GEF PLX RNA
Concentration(n[M])
Relative Conc
Time (hour)

Gene202:TRIM21

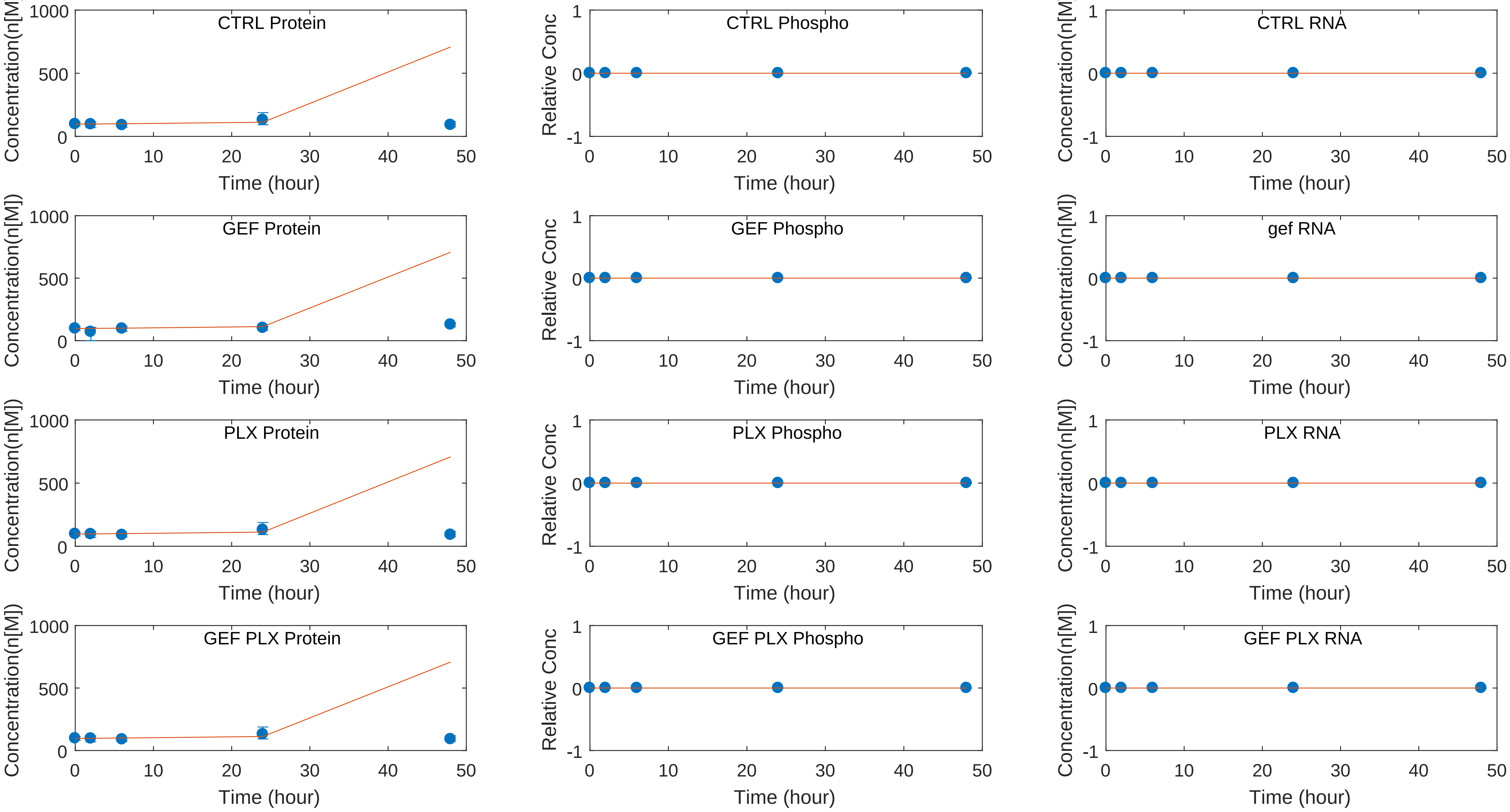

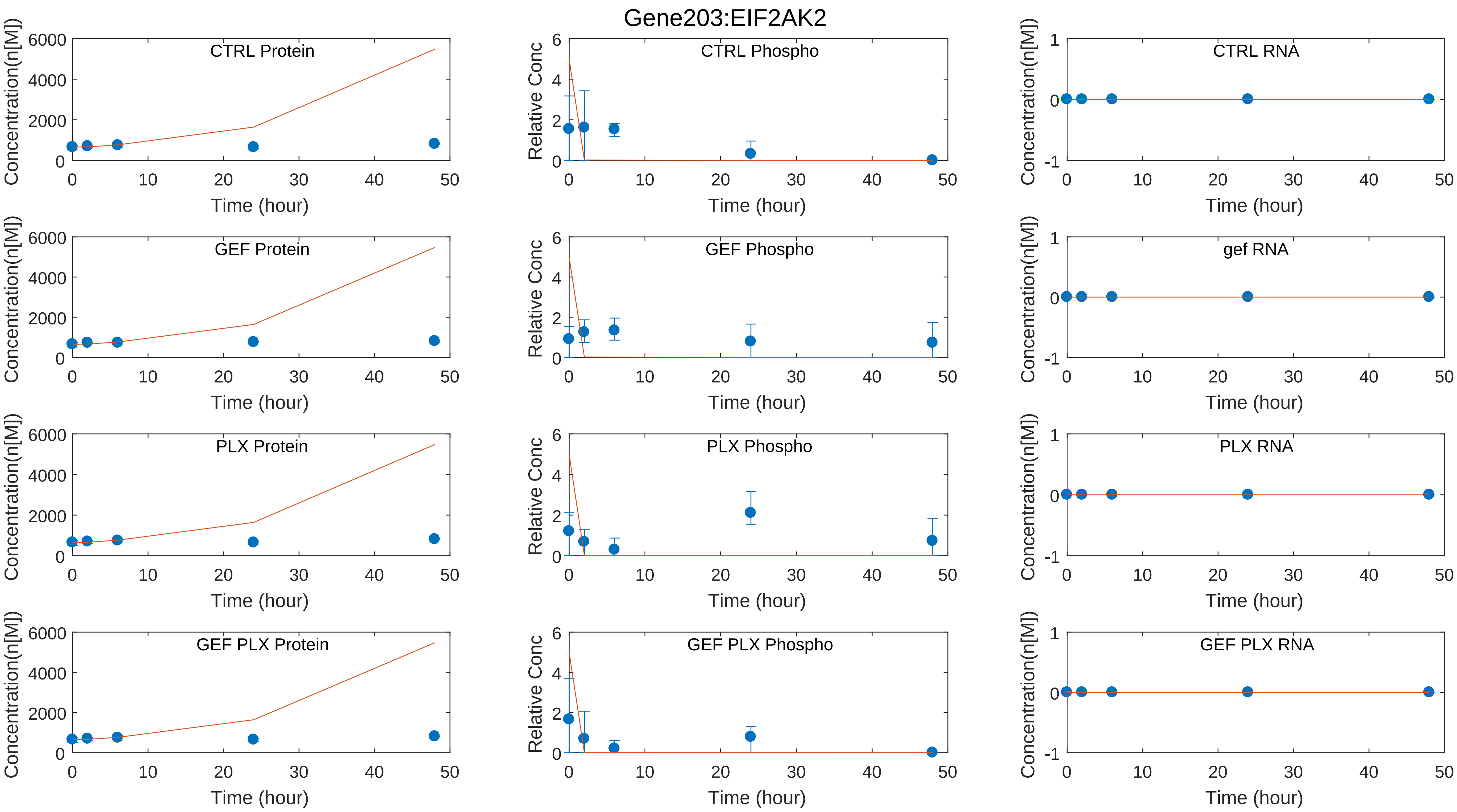

Gene203:EIF2AK2
CTRL Protein
CTRL Phospho
CTRL RNA
GEF Protein
GEF Phospho
gef RNA
PLX Protein
PLX Phospho
PLX RNA
GEF PLX Protein
GEF PLX Phospho
GEF PLX RNA
Concentration(n[M])
Relative Conc
Time (hour)

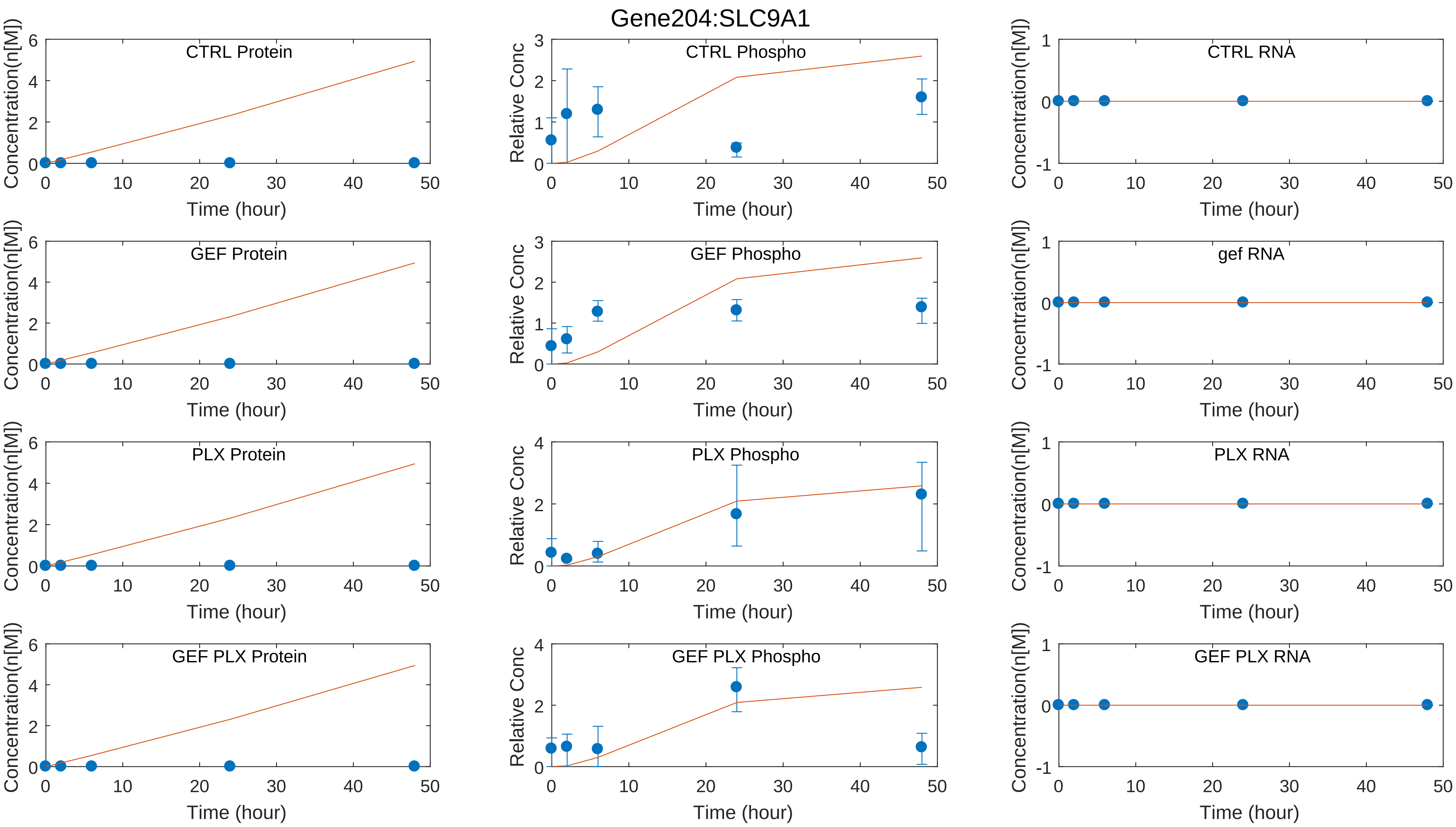
Gene204:SLC9A1
CTRL Protein
CTRL Phospho
CTRL RNA
GEF Protein
GEF Phospho
gef RNA
PLX Protein
PLX Phospho
PLX RNA
GEF PLX Protein
GEF PLX Phospho
GEF PLX RNA
Concentration(n[M])
Relative Conc
Time (hour)

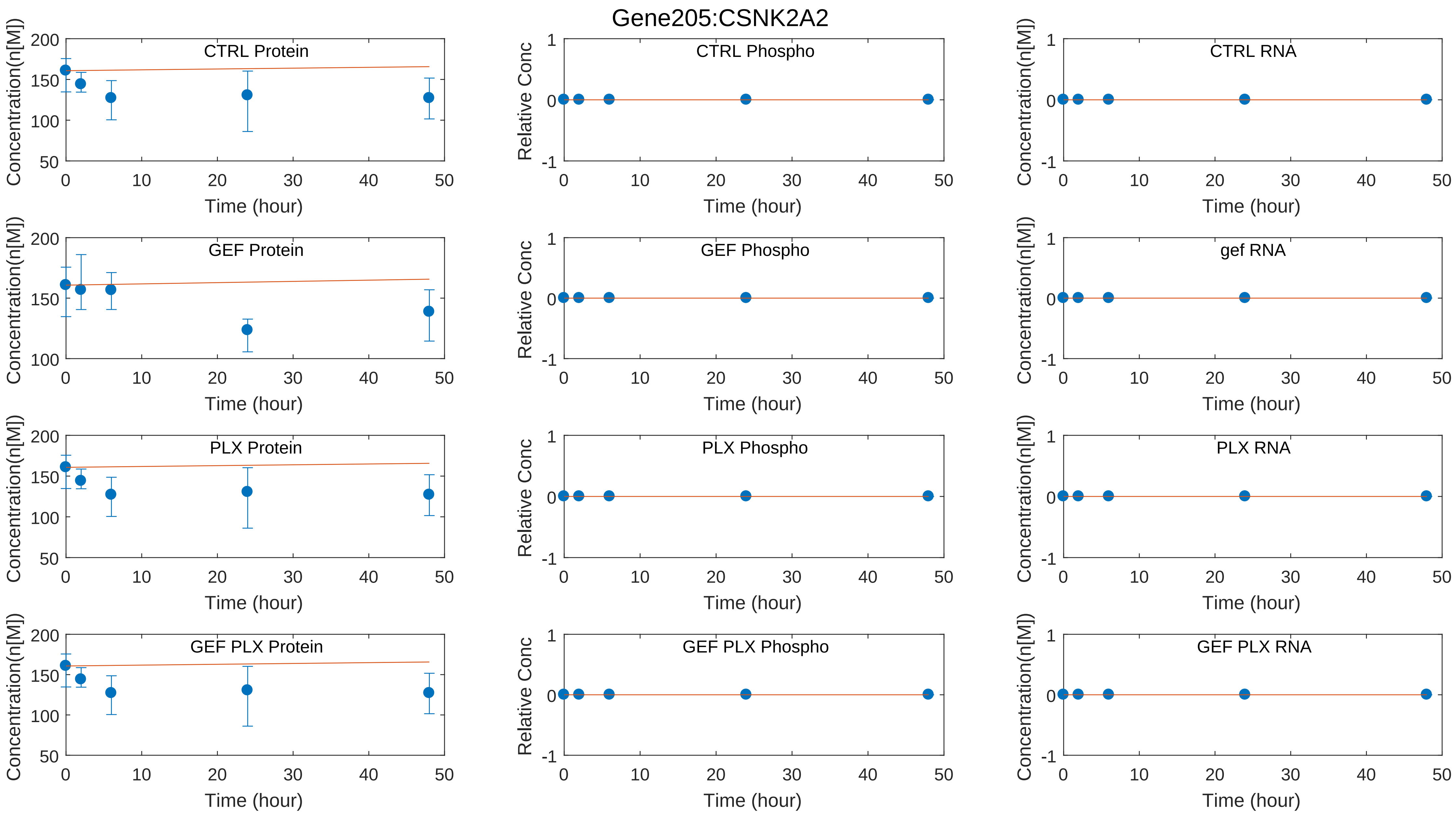
Gene205:CSNK2A2
CTRL Protein
CTRL Phospho
CTRL RNA
GEF Protein
GEF Phospho
gef RNA
PLX Protein
PLX Phospho
PLX RNA
GEF PLX Protein
GEF PLX Phospho
GEF PLX RNA
Concentration(n[M])
Relative Conc
Time (hour)

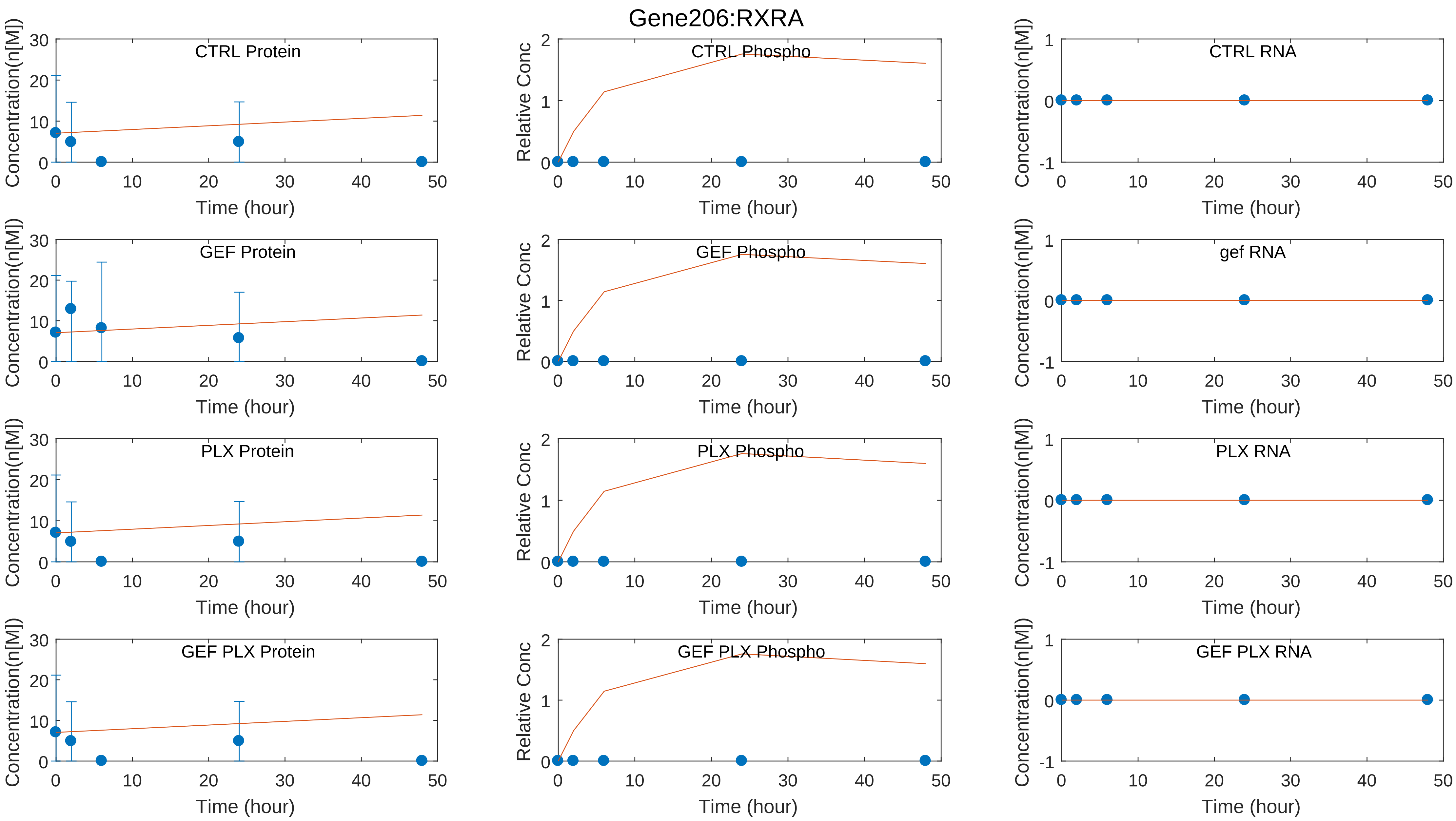

Gene206:RXRA
CTRL Protein
CTRL Phospho
CTRL RNA
GEF Protein
GEF Phospho
gef RNA
PLX Protein
PLX Phospho
PLX RNA
GEF PLX Protein
GEF PLX Phospho
GEF PLX RNA
Concentration(n[M])
Relative Conc
Time (hour)

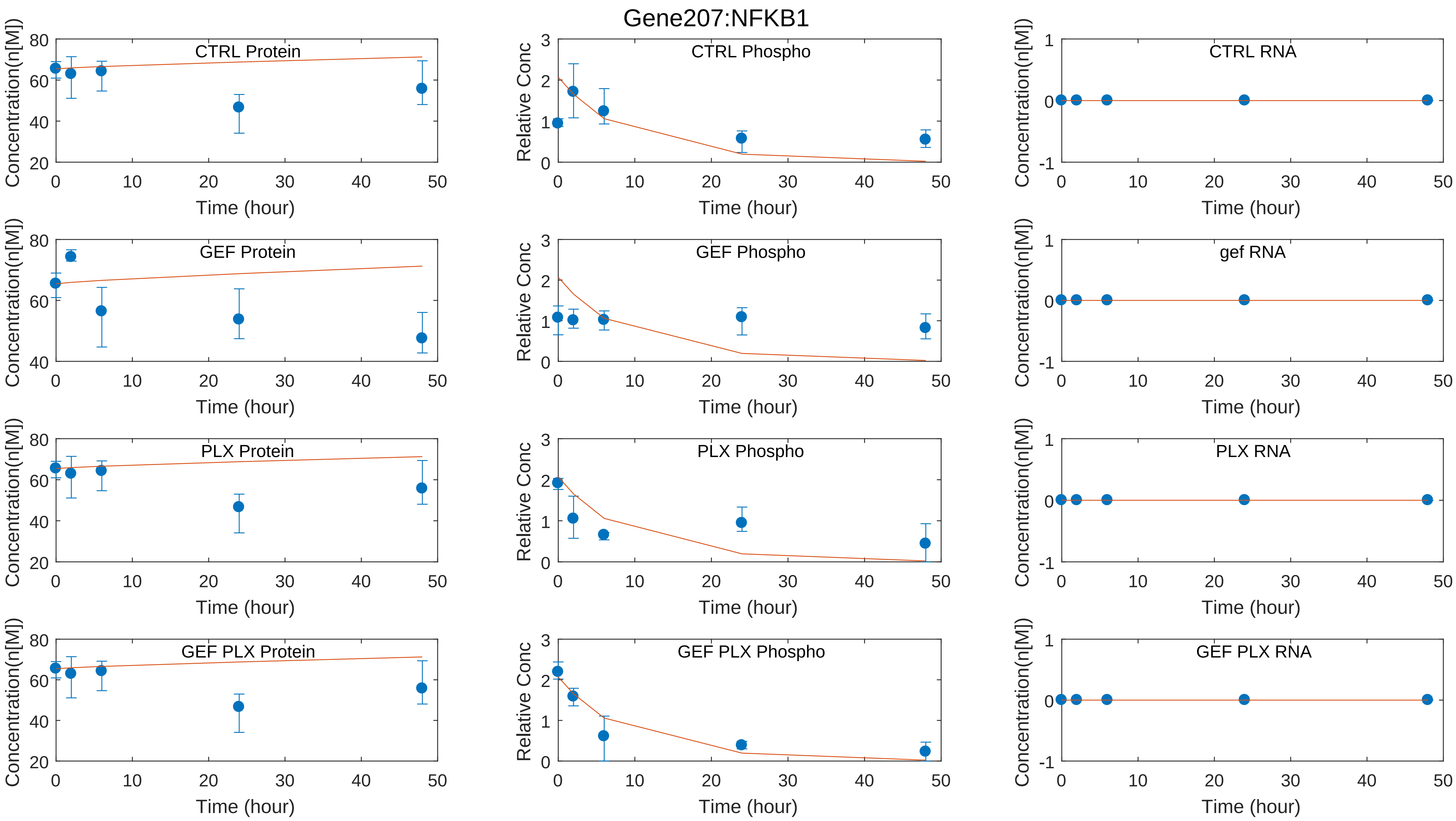
Gene207:NFKB1
CTRL Protein
CTRL Phospho
CTRL RNA
GEF Protein
GEF Phospho
gef RNA
PLX Protein
PLX Phospho
PLX RNA
GEF PLX Protein
GEF PLX Phospho
GEF PLX RNA
Concentration(n[M])
Relative Conc
Time (hour)

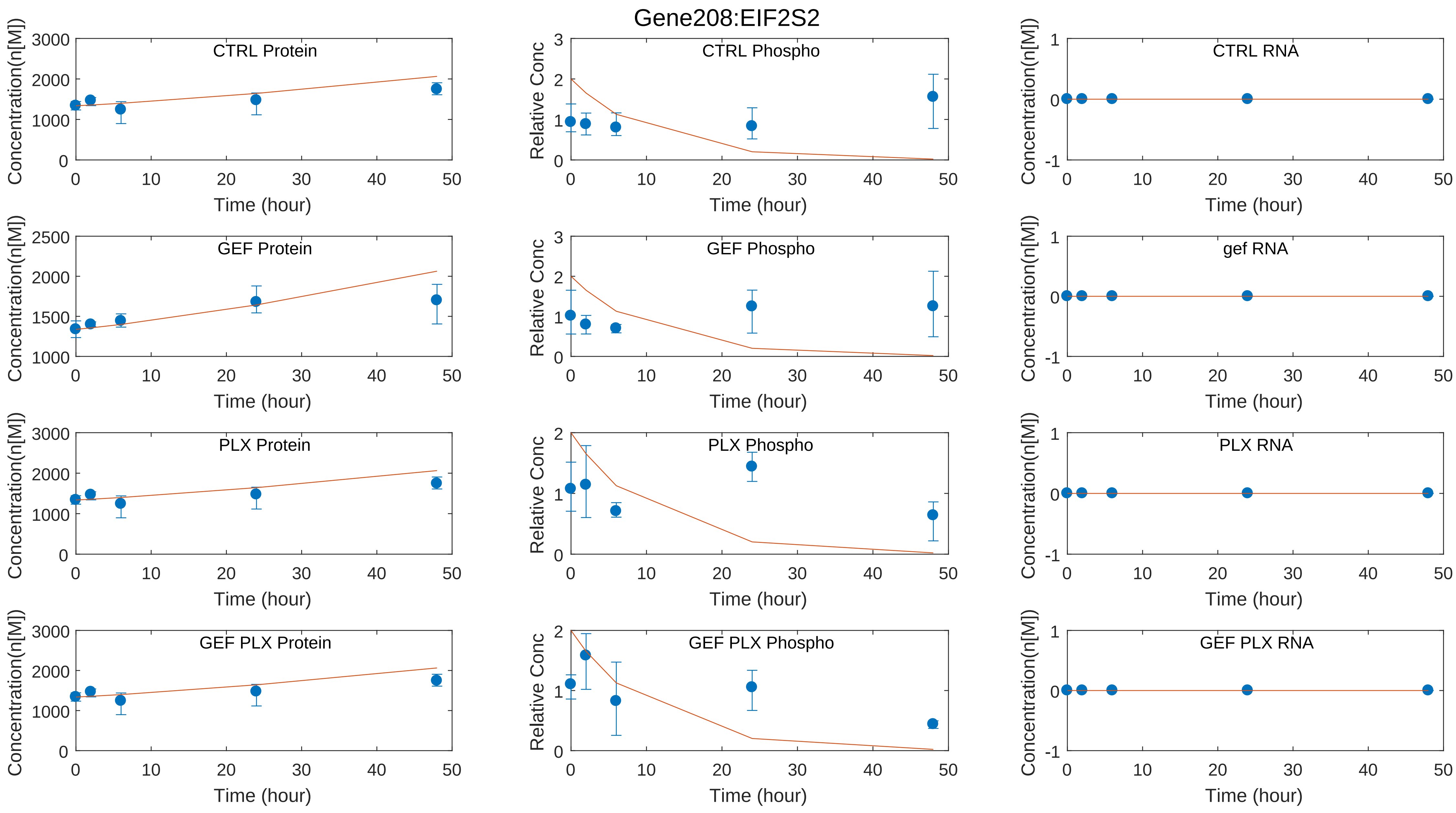
Gene208:EIF2S2
CTRL Protein
CTRL Phospho
CTRL RNA
GEF Protein
GEF Phospho
gef RNA
PLX Protein
PLX Phospho
PLX RNA
GEF PLX Protein
GEF PLX Phospho
GEF PLX RNA
Concentration(n[M])
Relative Conc
Time (hour)

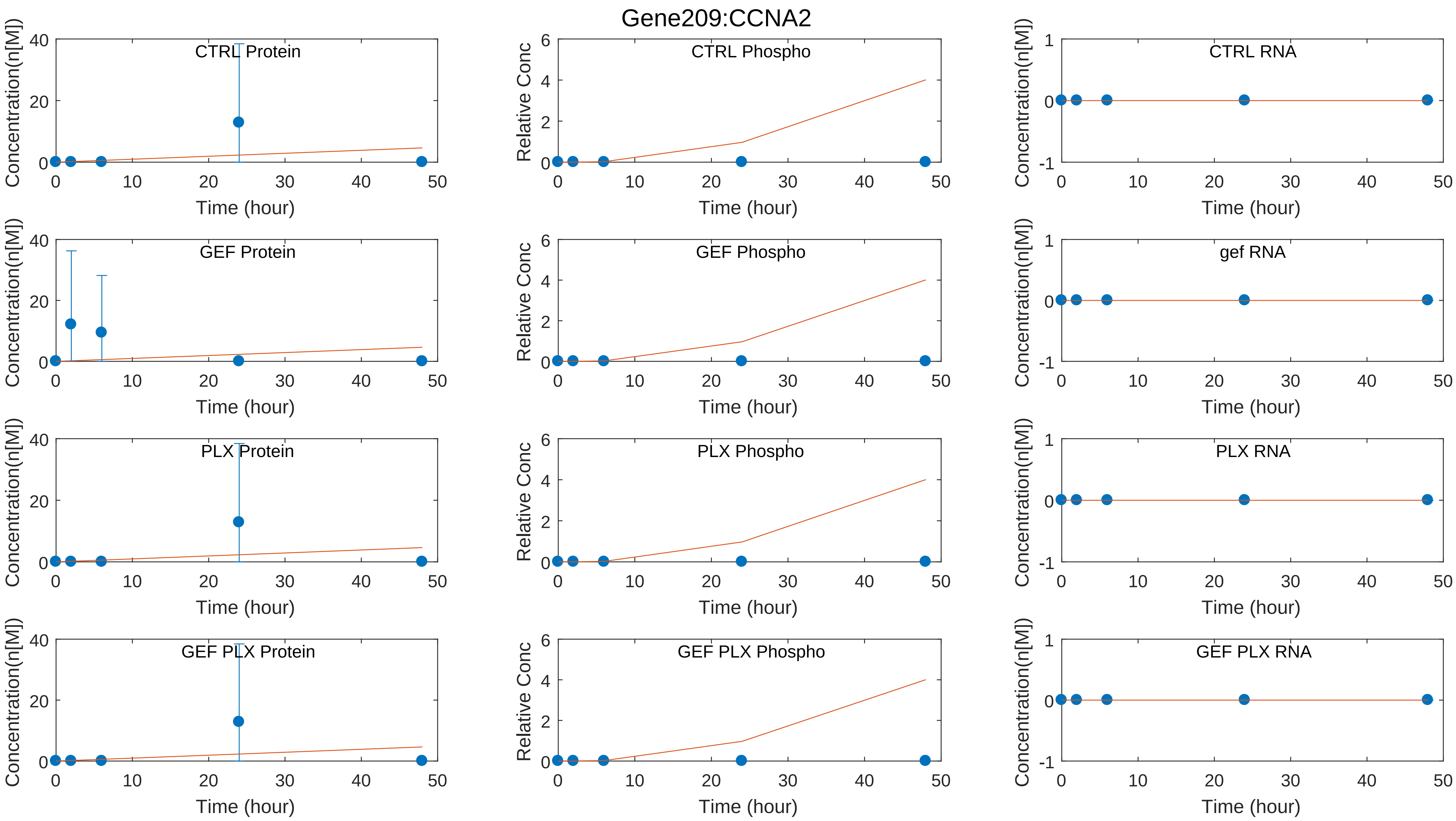
Gene209:CCNA2
CTRL Protein
CTRL Phospho
CTRL RNA
GEF Protein
GEF Phospho
gef RNA
PLX Protein
PLX Phospho
PLX RNA
GEF PLX Protein
GEF PLX Phospho
GEF PLX RNA
Concentration(n[M])
Relative Conc
Time (hour)

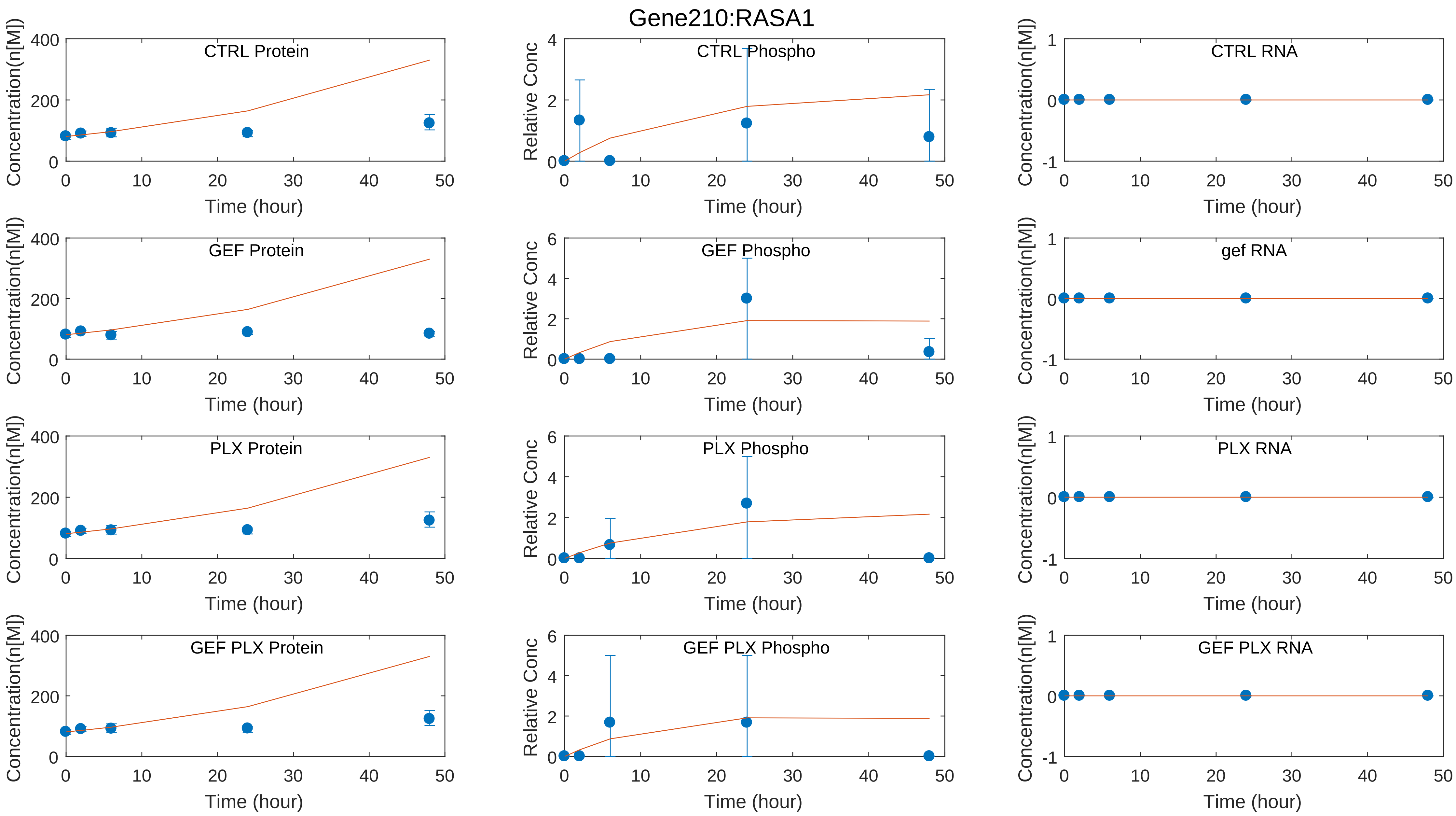

Gene210:RASA1
CTRL Protein
CTRL Phospho
CTRL RNA
GEF Protein
GEF Phospho
gef RNA
PLX Protein
PLX Phospho
PLX RNA
GEF PLX Protein
GEF PLX Phospho
GEF PLX RNA
Concentration(n[M])
Relative Conc
Time (hour)

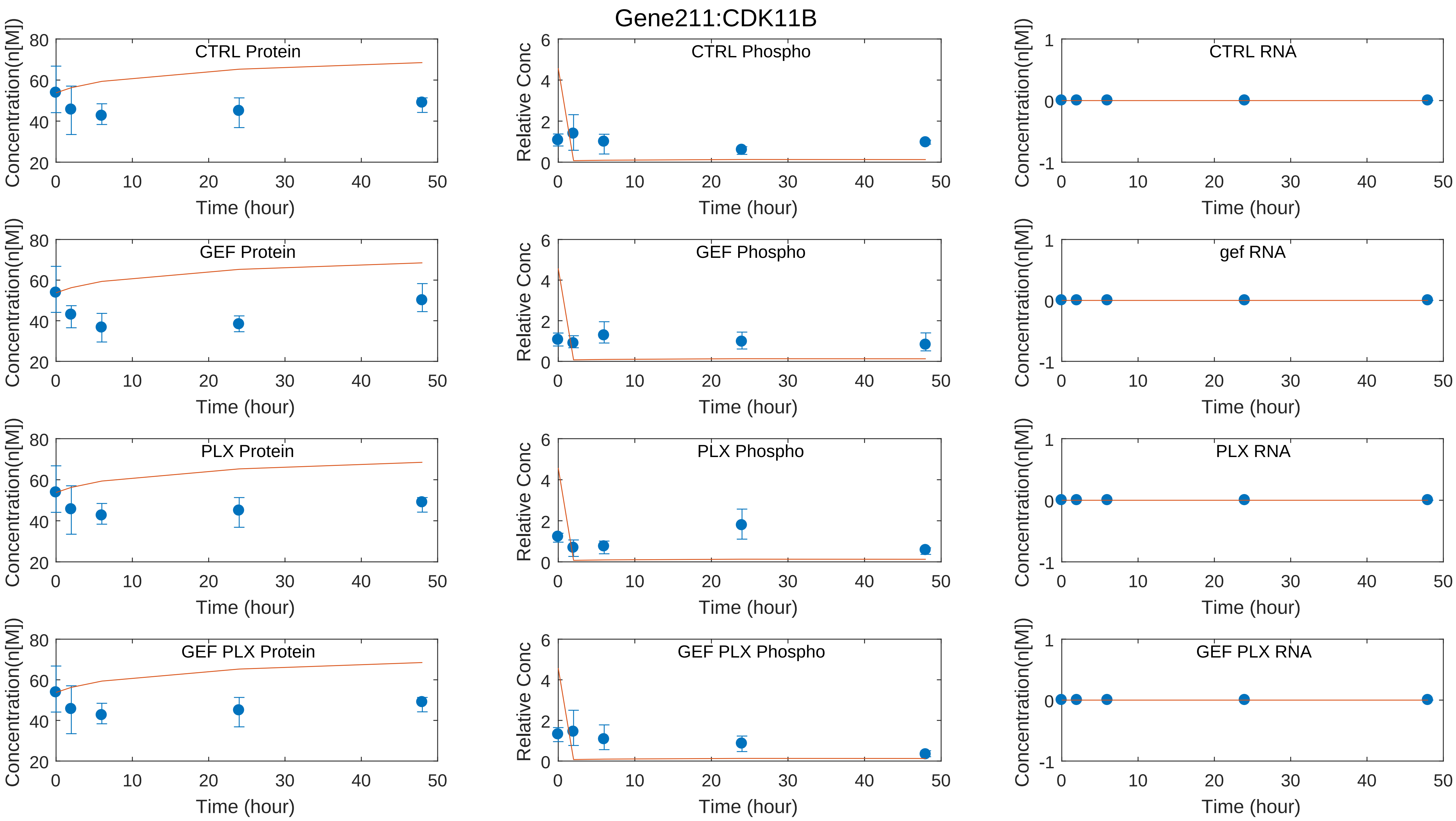

Gene211:CDK11B
CTRL Protein
CTRL Phospho
CTRL RNA
GEF Protein
GEF Phospho
gef RNA
PLX Protein
PLX Phospho
PLX RNA
GEF PLX Protein
GEF PLX Phospho
GEF PLX RNA
Concentration(n[M])
Relative Conc
Time (hour)

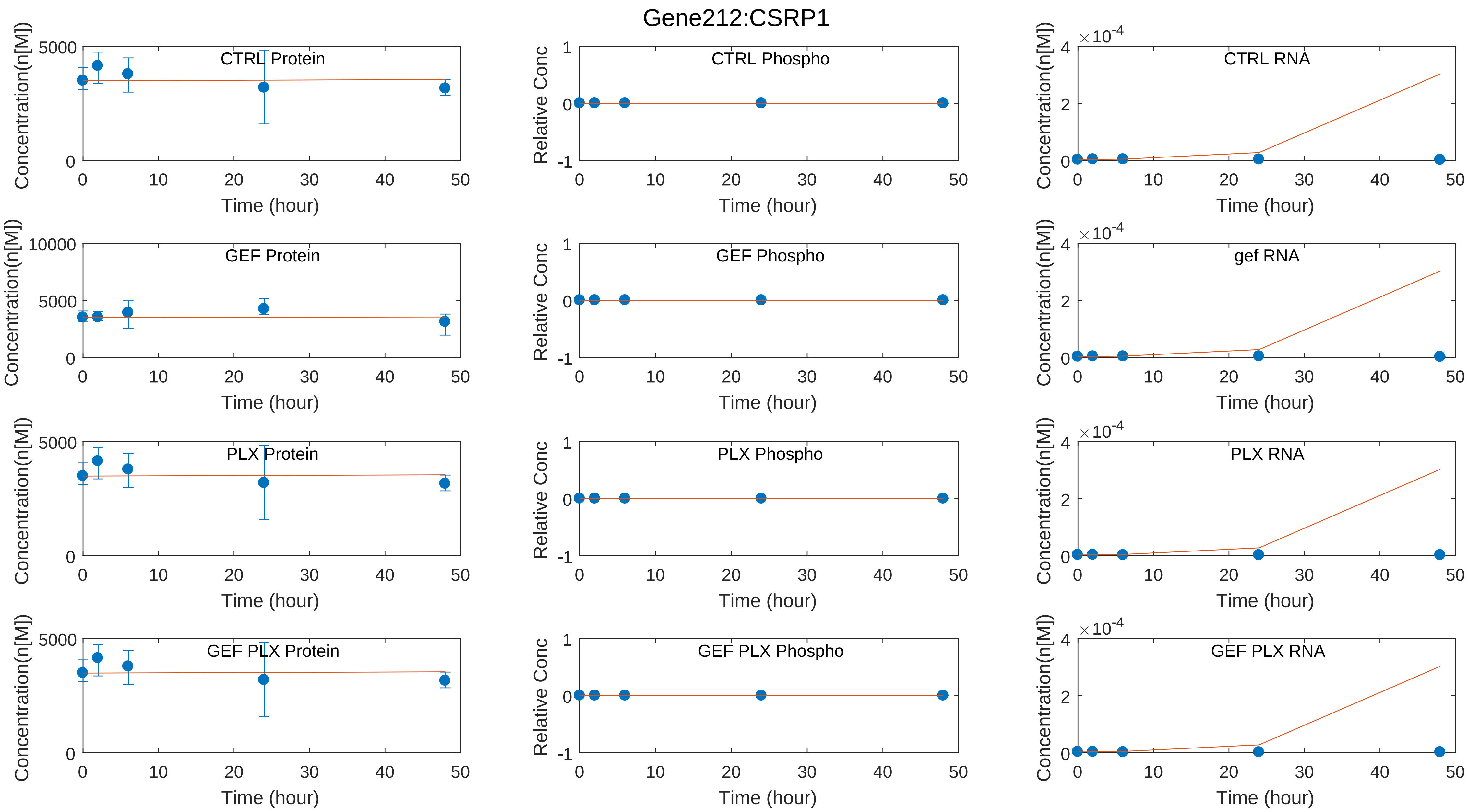

Gene212:CSRP1
CTRL Protein
CTRL Phospho
CTRL RNA
GEF Protein
GEF Phospho
gef RNA
PLX Protein
PLX Phospho
PLX RNA
GEF PLX Protein
GEF PLX Phospho
GEF PLX RNA
Concentration(n[M])
Relative Conc
Time (hour)
×10-4

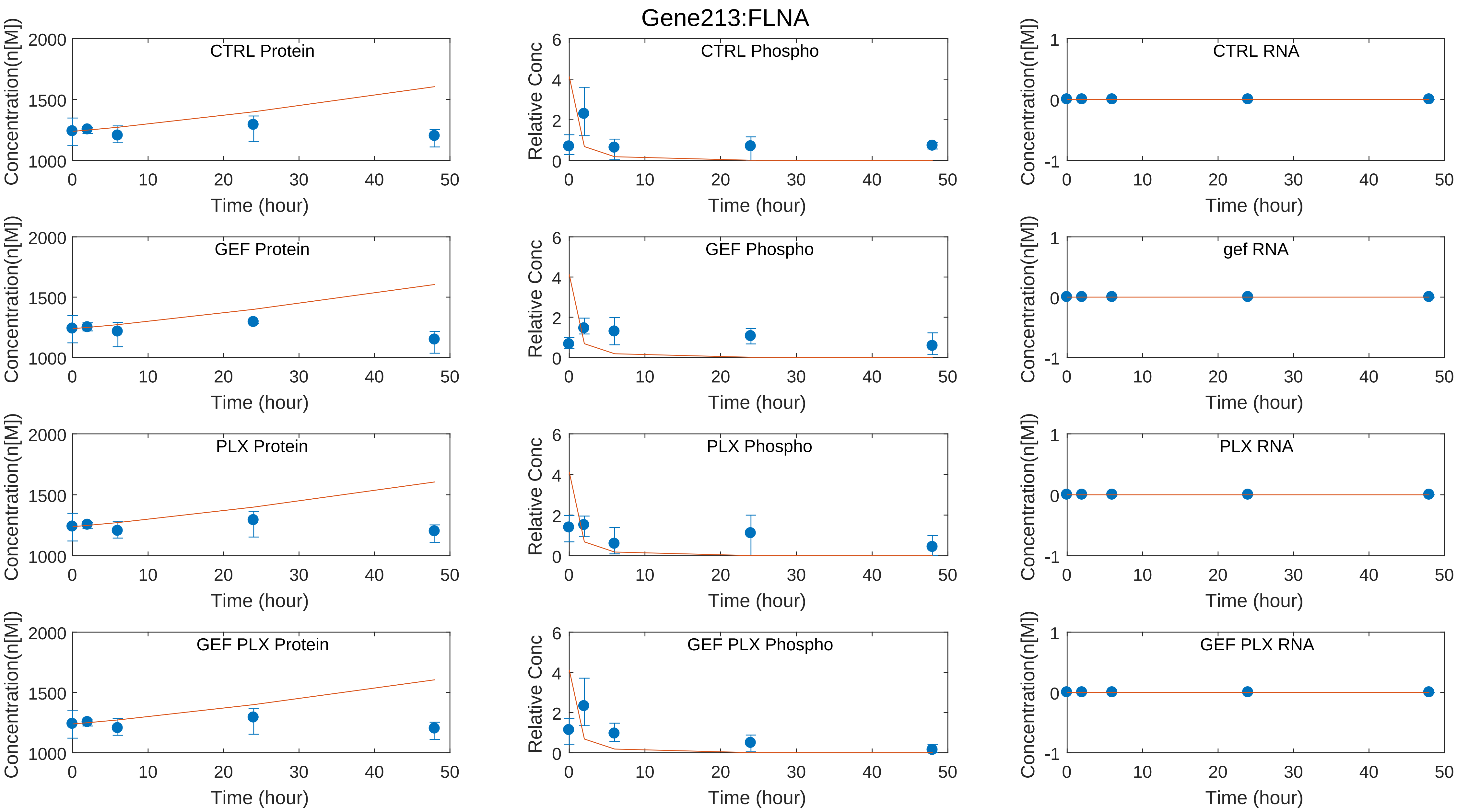
Gene213:FLNA
CTRL Protein
GEF Protein
PLX Protein
GEF PLX Protein
CTRL Phospho
GEF Phospho
PLX Phospho
GEF PLX Phospho
CTRL RNA
gef RNA
PLX RNA
GEF PLX RNA
Concentration(n[M])
Relative Conc
Time (hour)

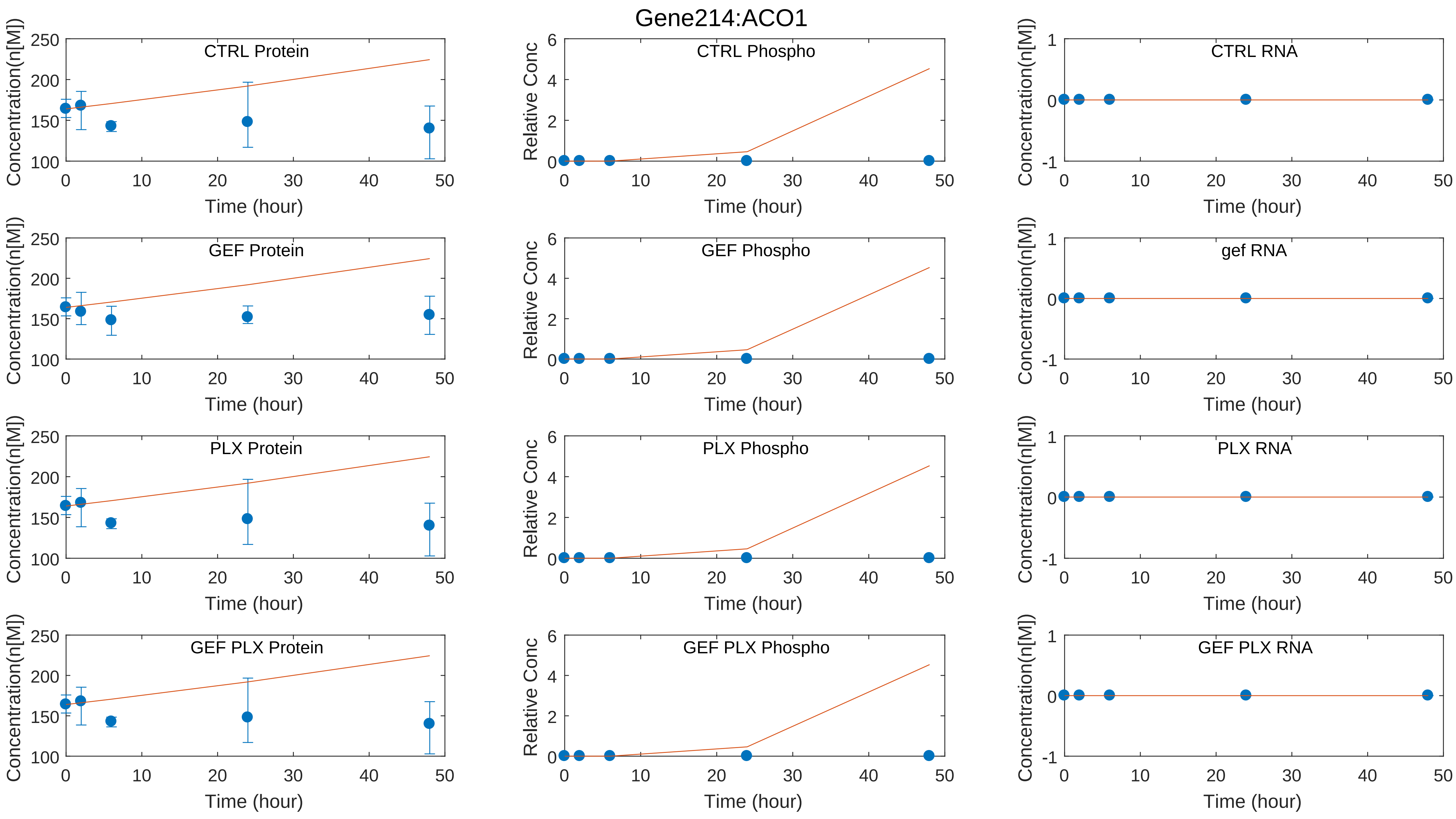

Gene214:ACO1
CTRL Protein
GEF Protein
PLX Protein
GEF PLX Protein
CTRL Phospho
GEF Phospho
PLX Phospho
GEF PLX Phospho
CTRL RNA
gef RNA
PLX RNA
GEF PLX RNA
Concentration(n[M])
Relative Conc
Time (hour)

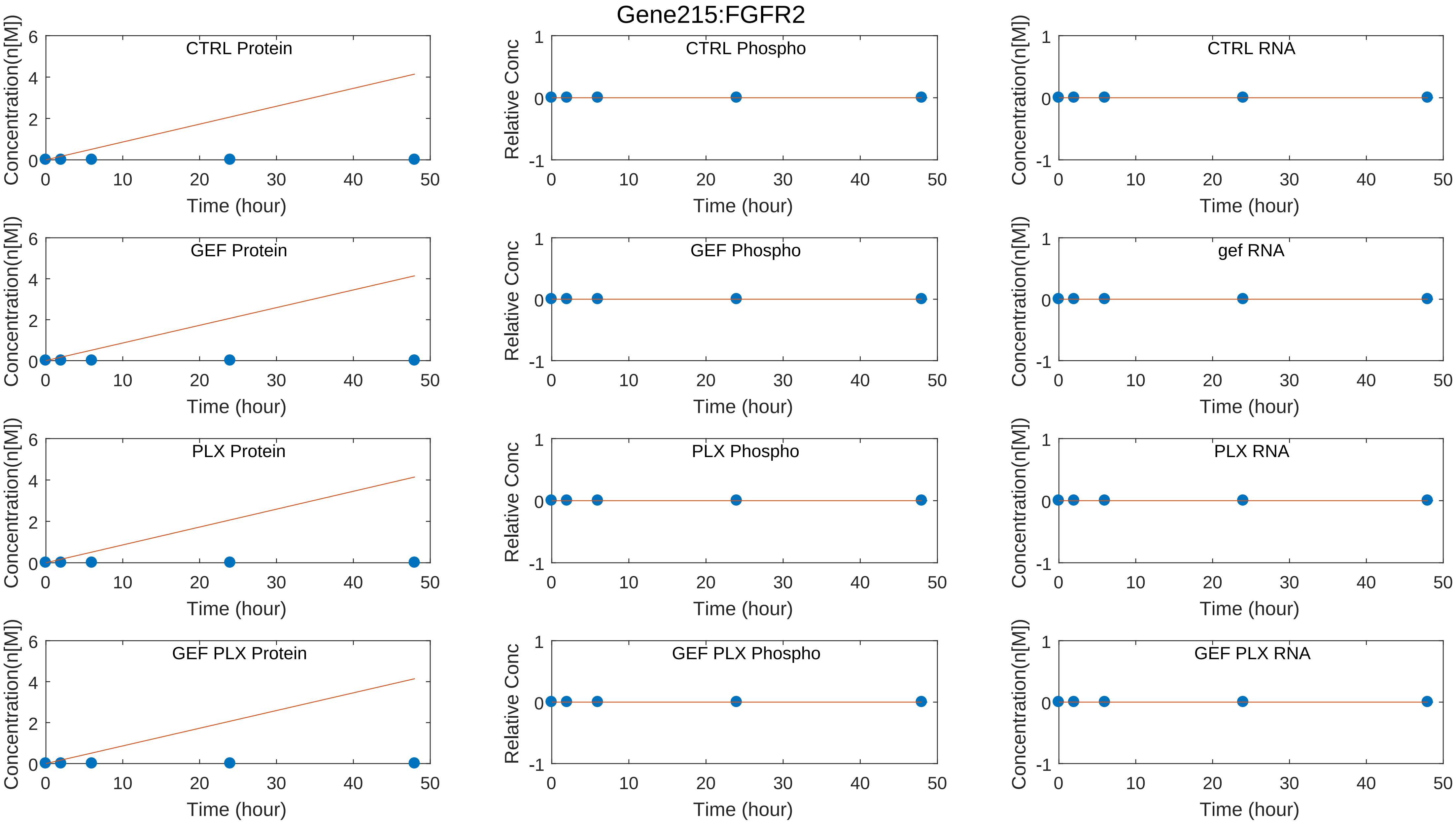

Gene215:FGFR2
CTRL Protein
CTRL Phospho
CTRL RNA
GEF Protein
GEF Phospho
gef RNA
PLX Protein
PLX Phospho
PLX RNA
GEF PLX Protein
GEF PLX Phospho
GEF PLX RNA
Concentration(n[M])
Relative Conc
Time (hour)

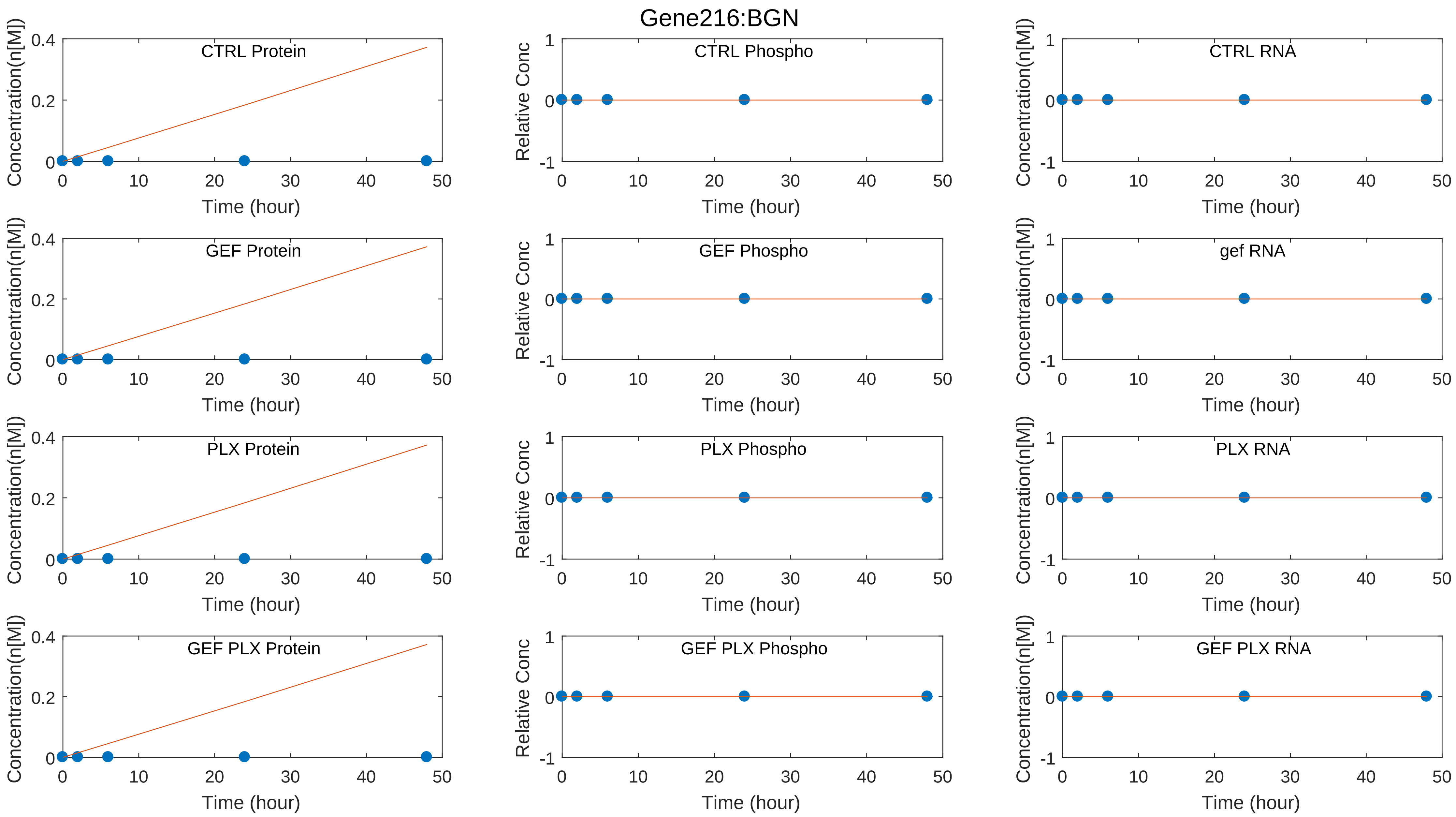

Gene216:BGN
CTRL Protein
CTRL Phospho
CTRL RNA
GEF Protein
GEF Phospho
gef RNA
PLX Protein
PLX Phospho
PLX RNA
GEF PLX Protein
GEF PLX Phospho
GEF PLX RNA
Concentration(n[M])
Relative Conc
Time (hour)
0.4
0.2
0
1
-1
10
20
30
40
50

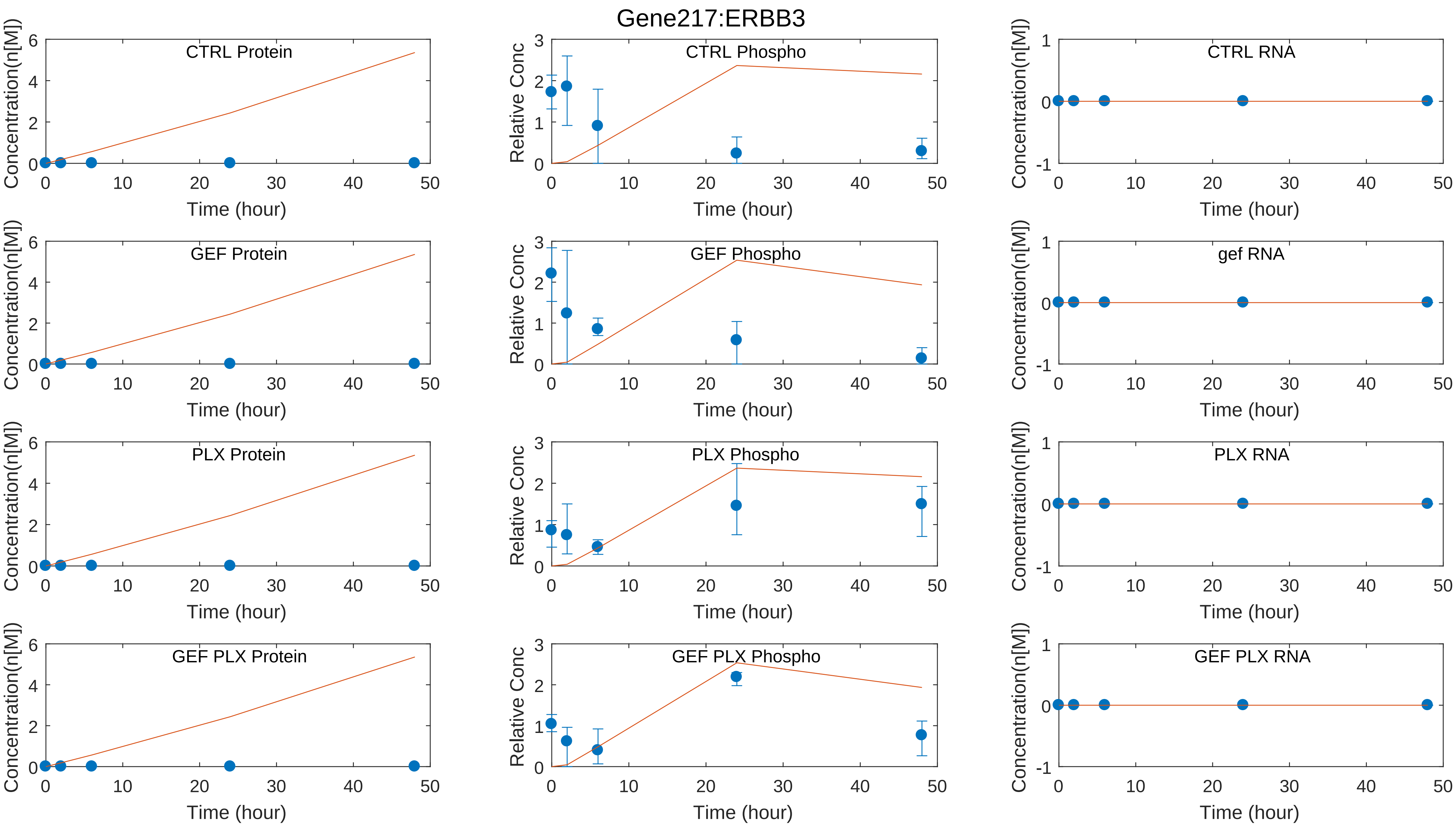
Gene217:ERBB3
CTRL Protein
CTRL Phospho
CTRL RNA
GEF Protein
GEF Phospho
gef RNA
PLX Protein
PLX Phospho
PLX RNA
GEF PLX Protein
GEF PLX Phospho
GEF PLX RNA
Concentration(n[M])
Relative Conc
Time (hour)

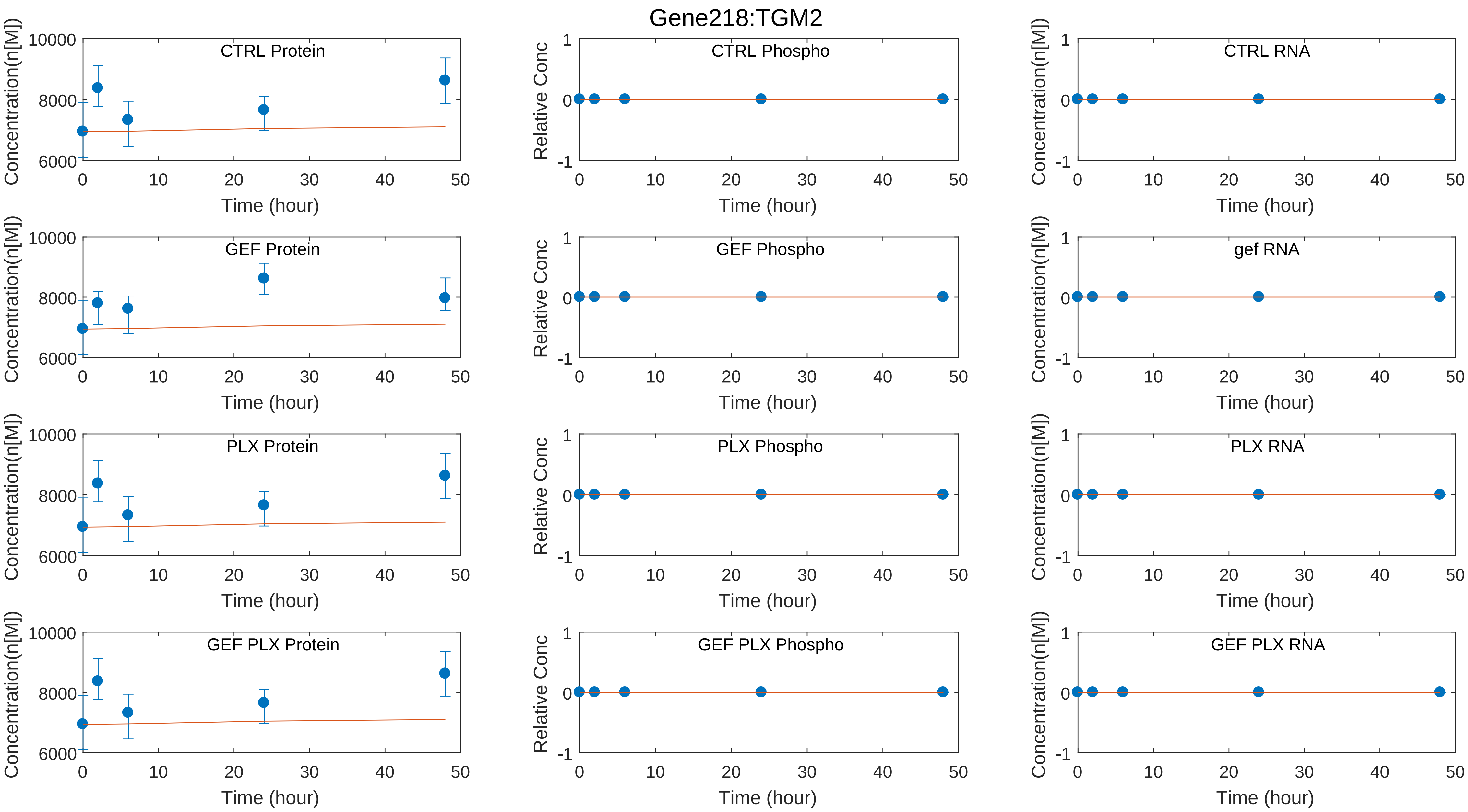

Gene218:TGM2
CTRL Protein
CTRL Phospho
CTRL RNA
GEF Protein
GEF Phospho
gef RNA
PLX Protein
PLX Phospho
PLX RNA
GEF PLX Protein
GEF PLX Phospho
GEF PLX RNA
Concentration(n[M])
Relative Conc
Time (hour)
10000
8000
6000
1
0
-1
0
10
20
30
40
50

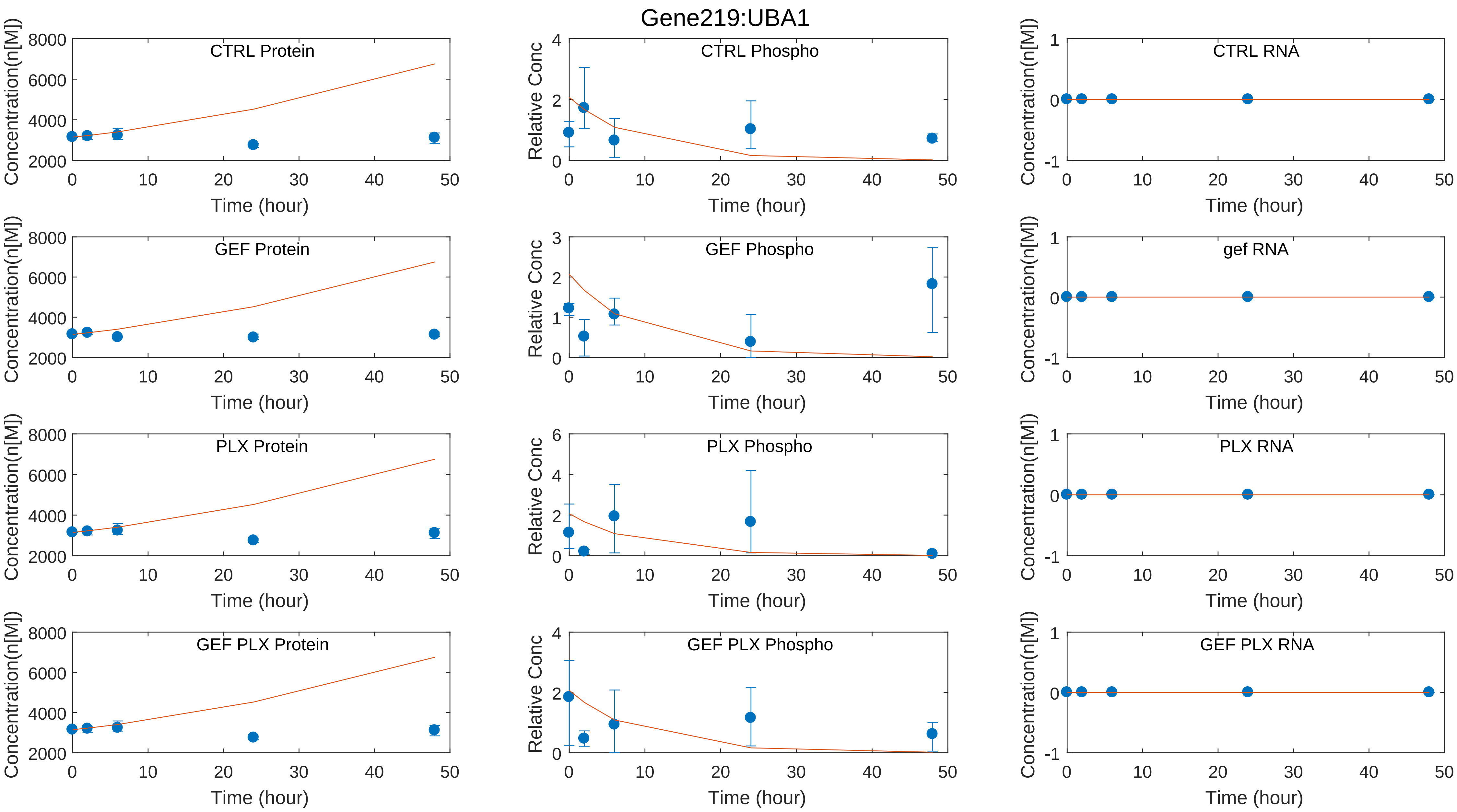
Gene219:UBA1
CTRL Protein
CTRL Phospho
CTRL RNA
GEF Protein
GEF Phospho
gef RNA
PLX Protein
PLX Phospho
PLX RNA
GEF PLX Protein
GEF PLX Phospho
GEF PLX RNA
Concentration(n[M])
Relative Conc
Time (hour)

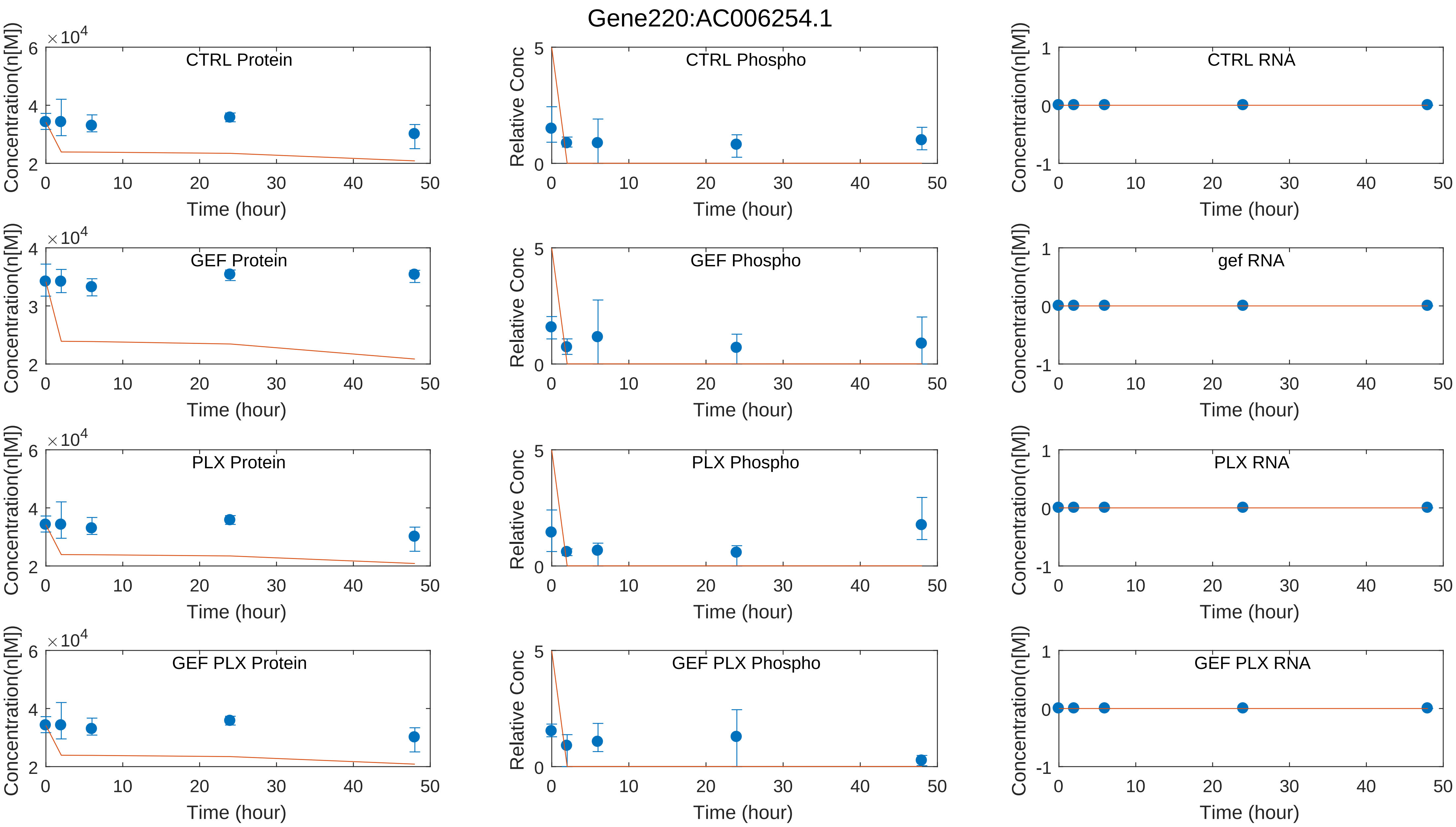
Gene220:AC006254.1
CTRL Protein
GEF Protein
PLX Protein
GEF PLX Protein
CTRL Phospho
GEF Phospho
PLX Phospho
GEF PLX Phospho
CTRL RNA
gef RNA
PLX RNA
GEF PLX RNA
Concentration(n[M])
Relative Conc
Time (hour)
×10^4

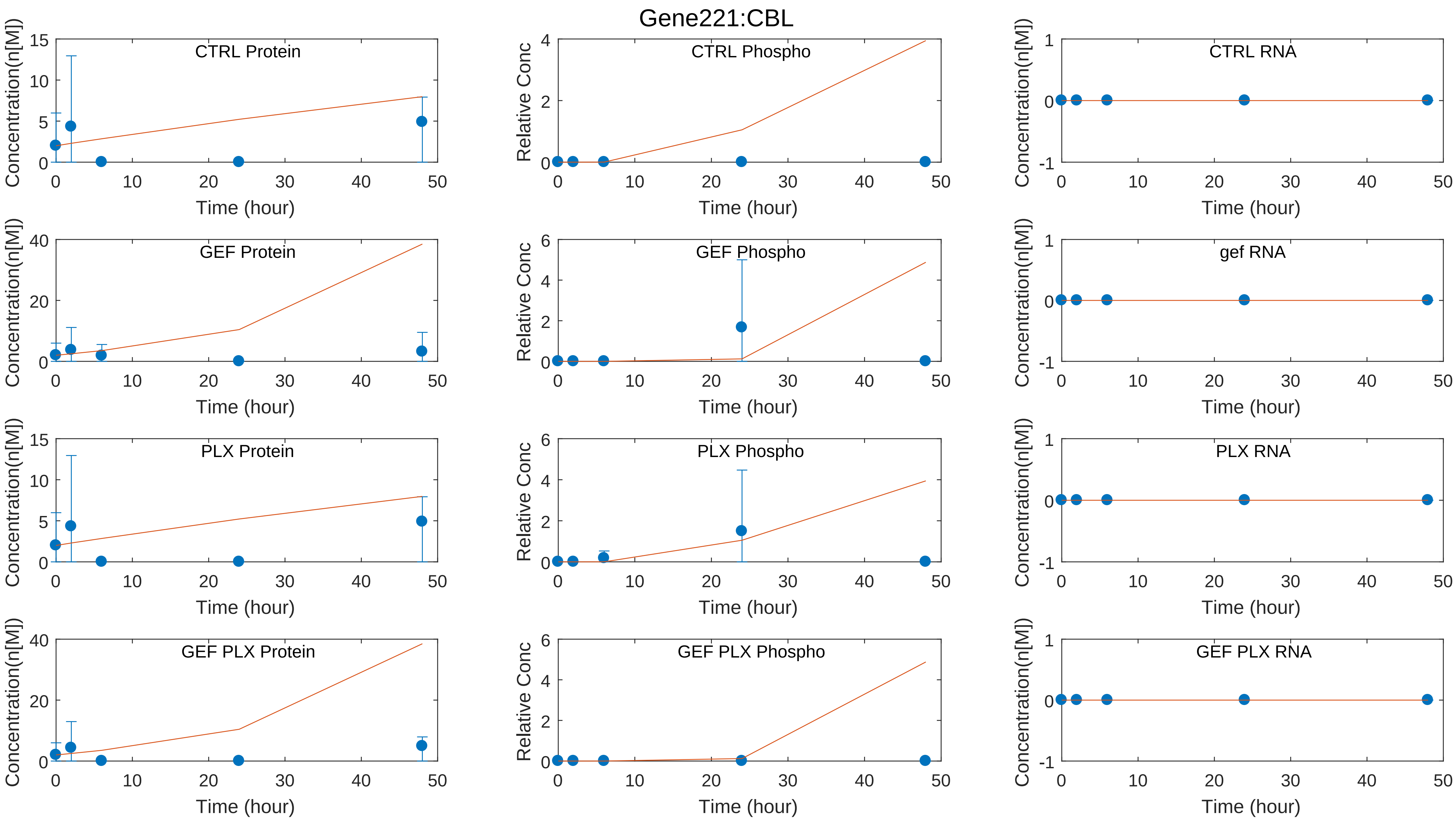
Gene221:CBL
CTRL Protein
CTRL Phospho
CTRL RNA
GEF Protein
GEF Phospho
gef RNA
PLX Protein
PLX Phospho
PLX RNA
GEF PLX Protein
GEF PLX Phospho
GEF PLX RNA
Concentration(n[M])
Relative Conc
Time (hour)

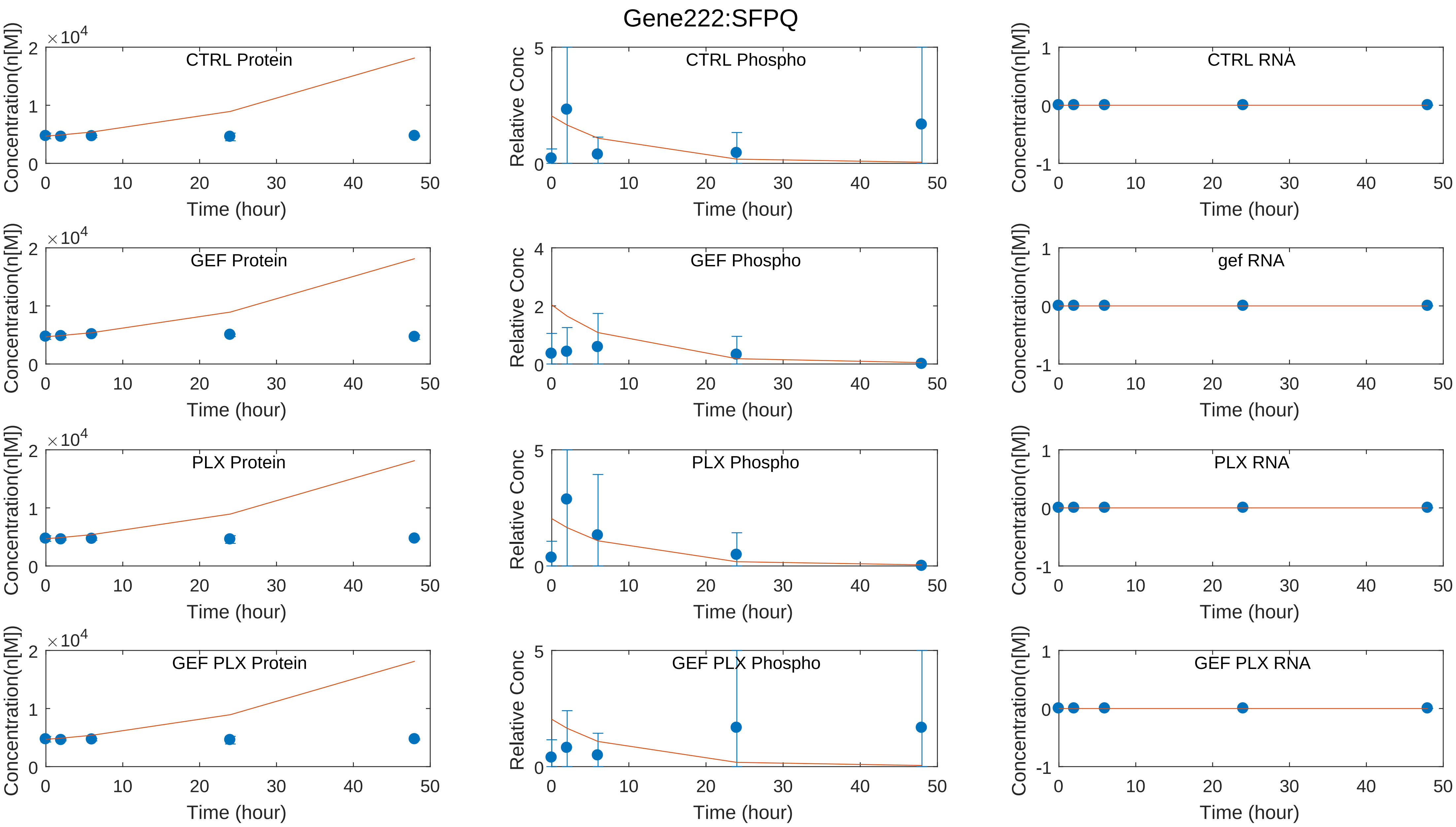
Gene222:SFPQ
CTRL Protein
GEF Protein
PLX Protein
GEF PLX Protein
CTRL Phospho
GEF Phospho
PLX Phospho
GEF PLX Phospho
CTRL RNA
gef RNA
PLX RNA
GEF PLX RNA
Concentration(n[M])
Relative Conc
Time (hour)
×10^4

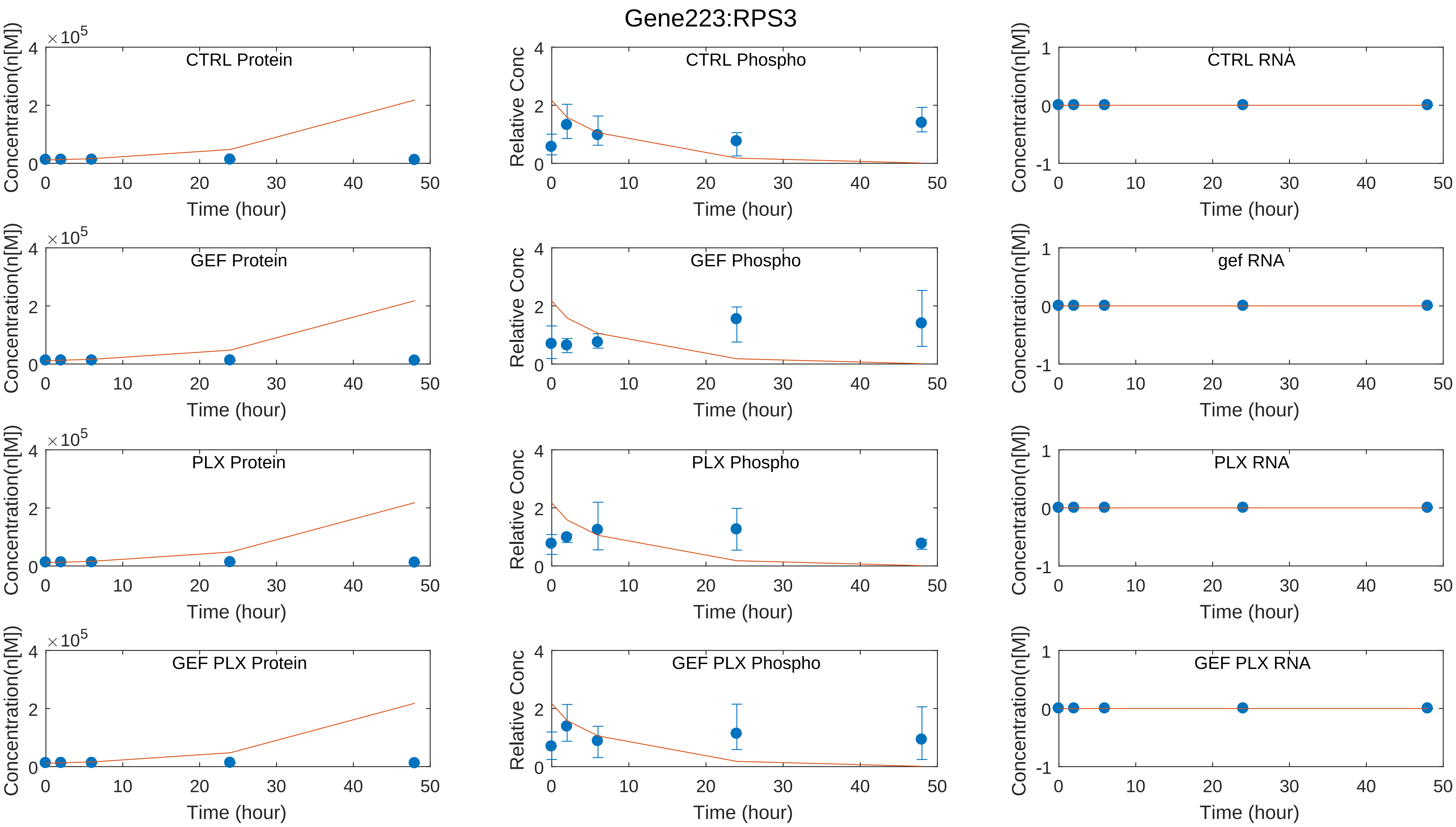

Gene223:RPS3
CTRL Protein
GEF Protein
PLX Protein
GEF PLX Protein
CTRL Phospho
GEF Phospho
PLX Phospho
GEF PLX Phospho
CTRL RNA
gef RNA
PLX RNA
GEF PLX RNA
Concentration(n[M])
Relative Conc
Time (hour)
×10^5

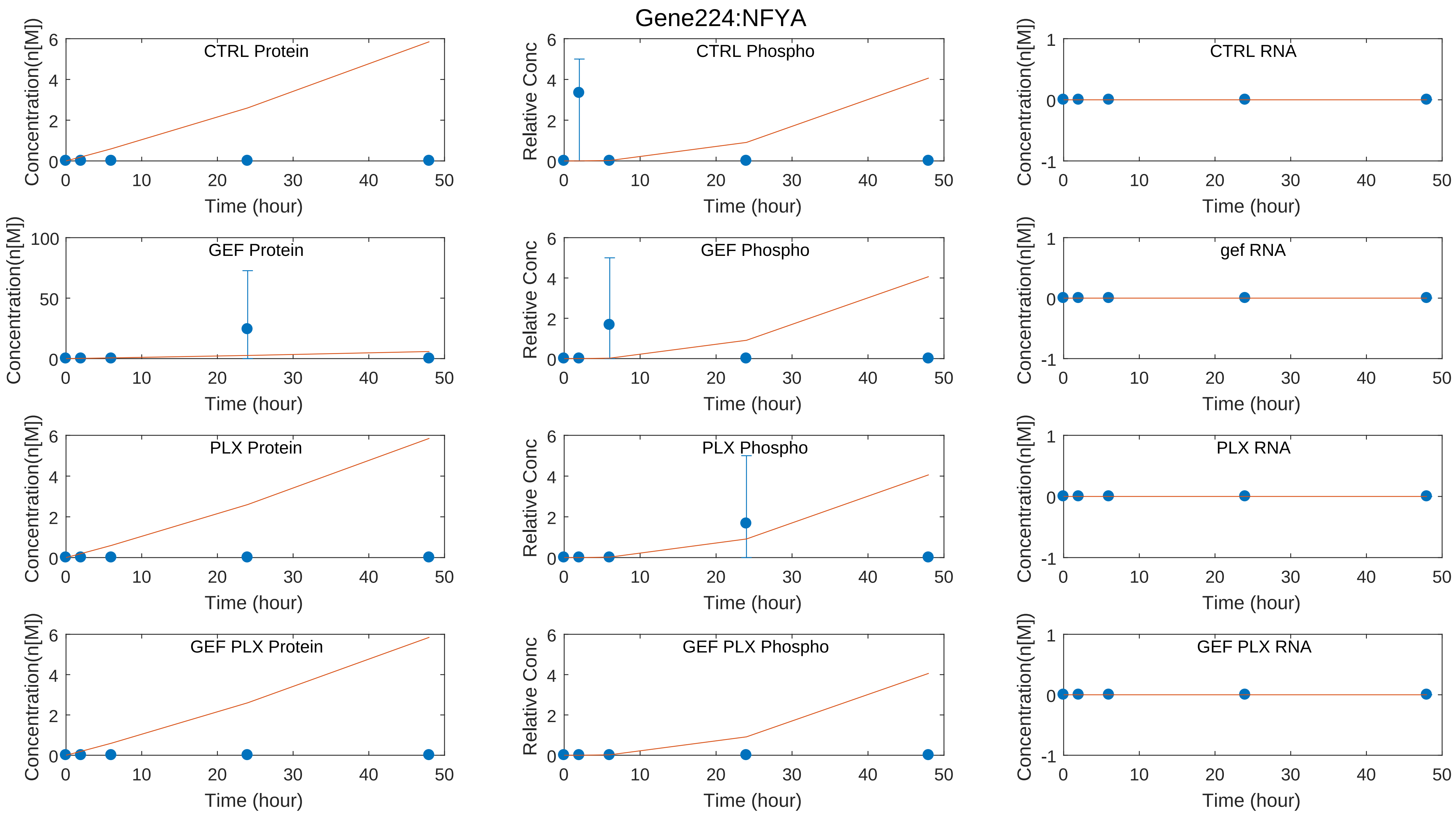
Gene224:NFYA
CTRL Protein
CTRL Phospho
CTRL RNA
GEF Protein
GEF Phospho
gef RNA
PLX Protein
PLX Phospho
PLX RNA
GEF PLX Protein
GEF PLX Phospho
GEF PLX RNA
Concentration(n[M])
Relative Conc
Time (hour)

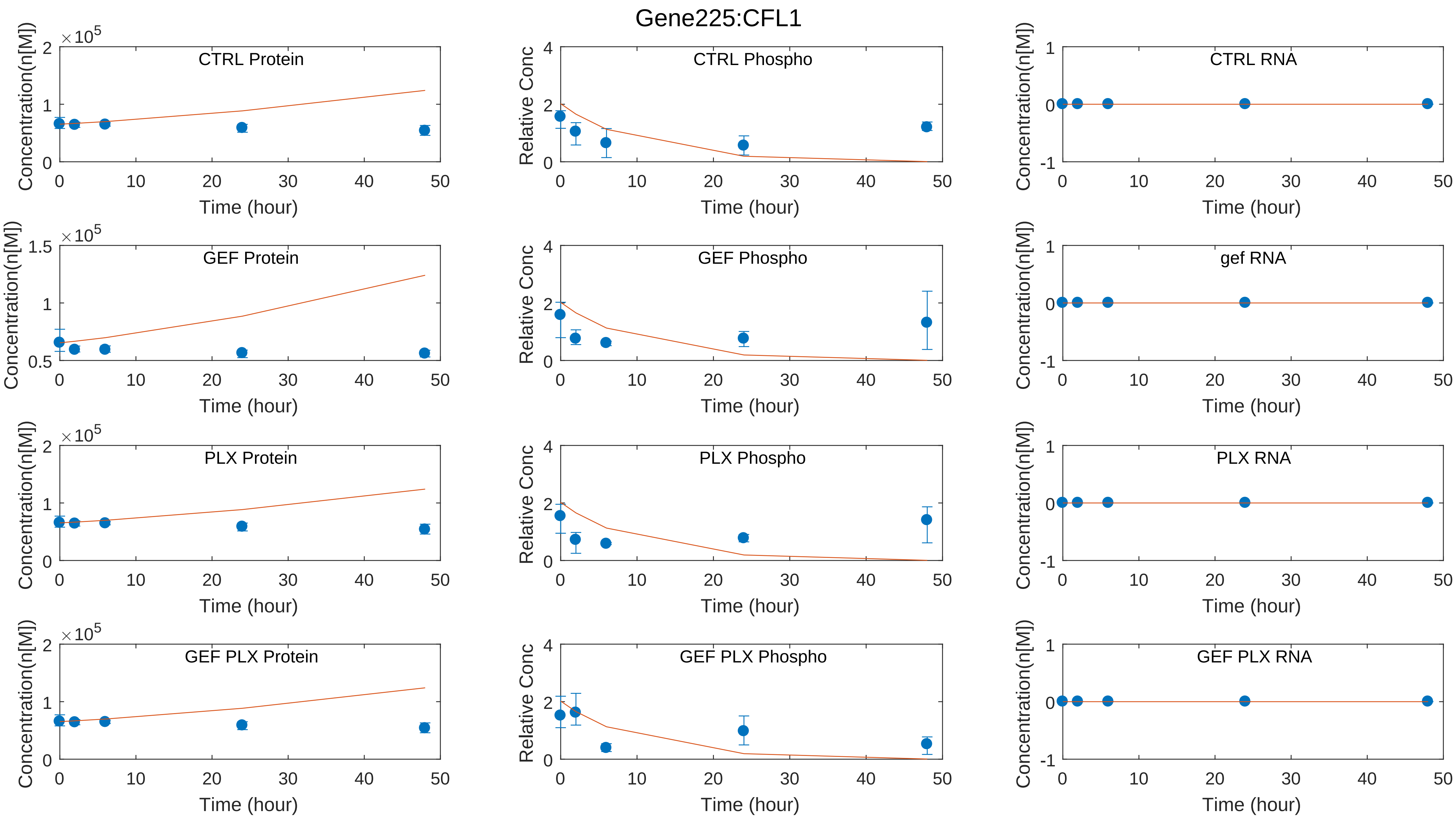

Gene225:CFL1
CTRL Protein
CTRL Phospho
CTRL RNA
GEF Protein
GEF Phospho
gef RNA
PLX Protein
PLX Phospho
PLX RNA
GEF PLX Protein
GEF PLX Phospho
GEF PLX RNA
Concentration(n[M])
Relative Conc
Time (hour)
$\times 10^5$

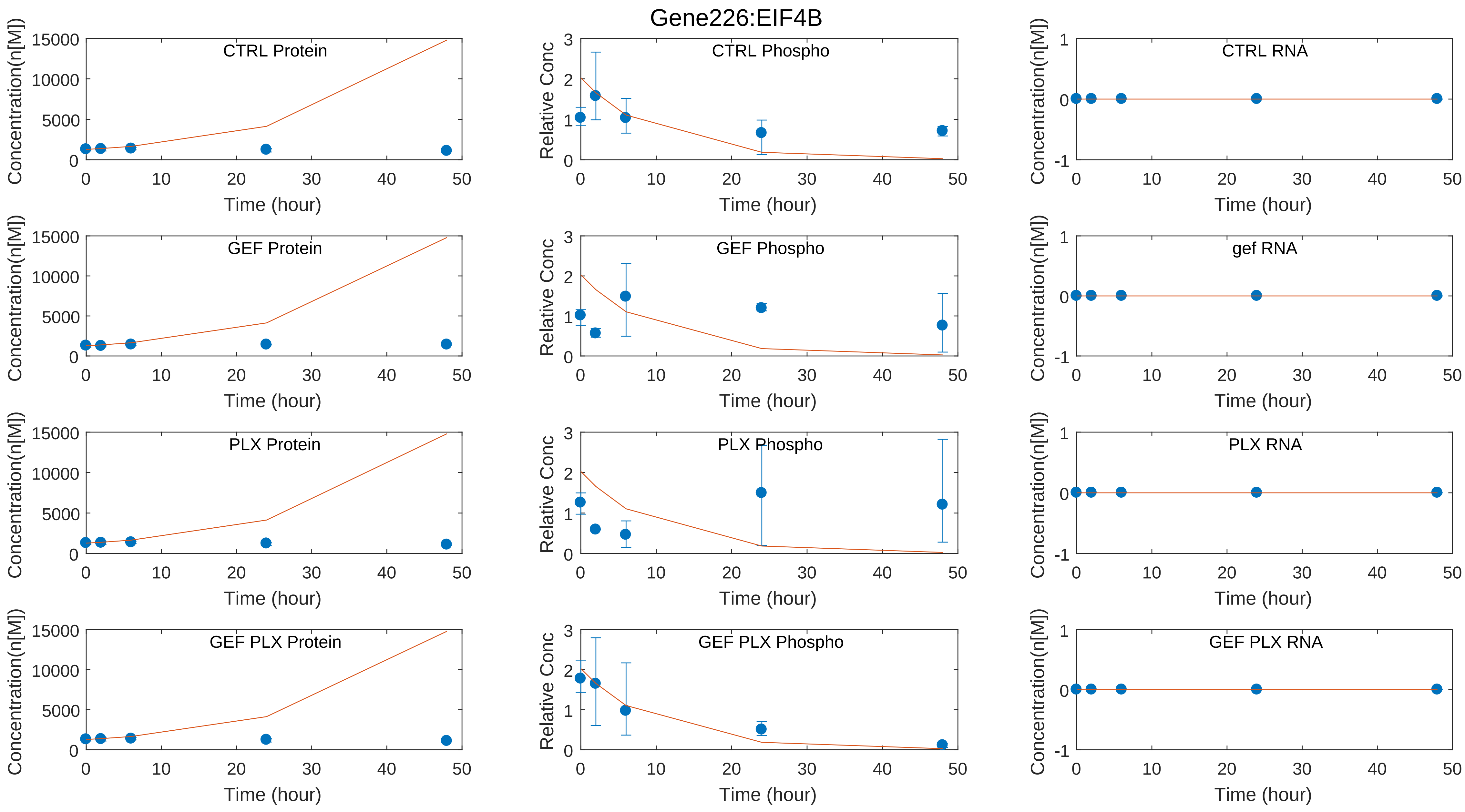
Gene226:EIF4B
CTRL Protein
GEF Protein
PLX Protein
GEF PLX Protein
CTRL Phospho
GEF Phospho
PLX Phospho
GEF PLX Phospho
CTRL RNA
gef RNA
PLX RNA
GEF PLX RNA
Concentration(n[M])
Relative Conc
Time (hour)

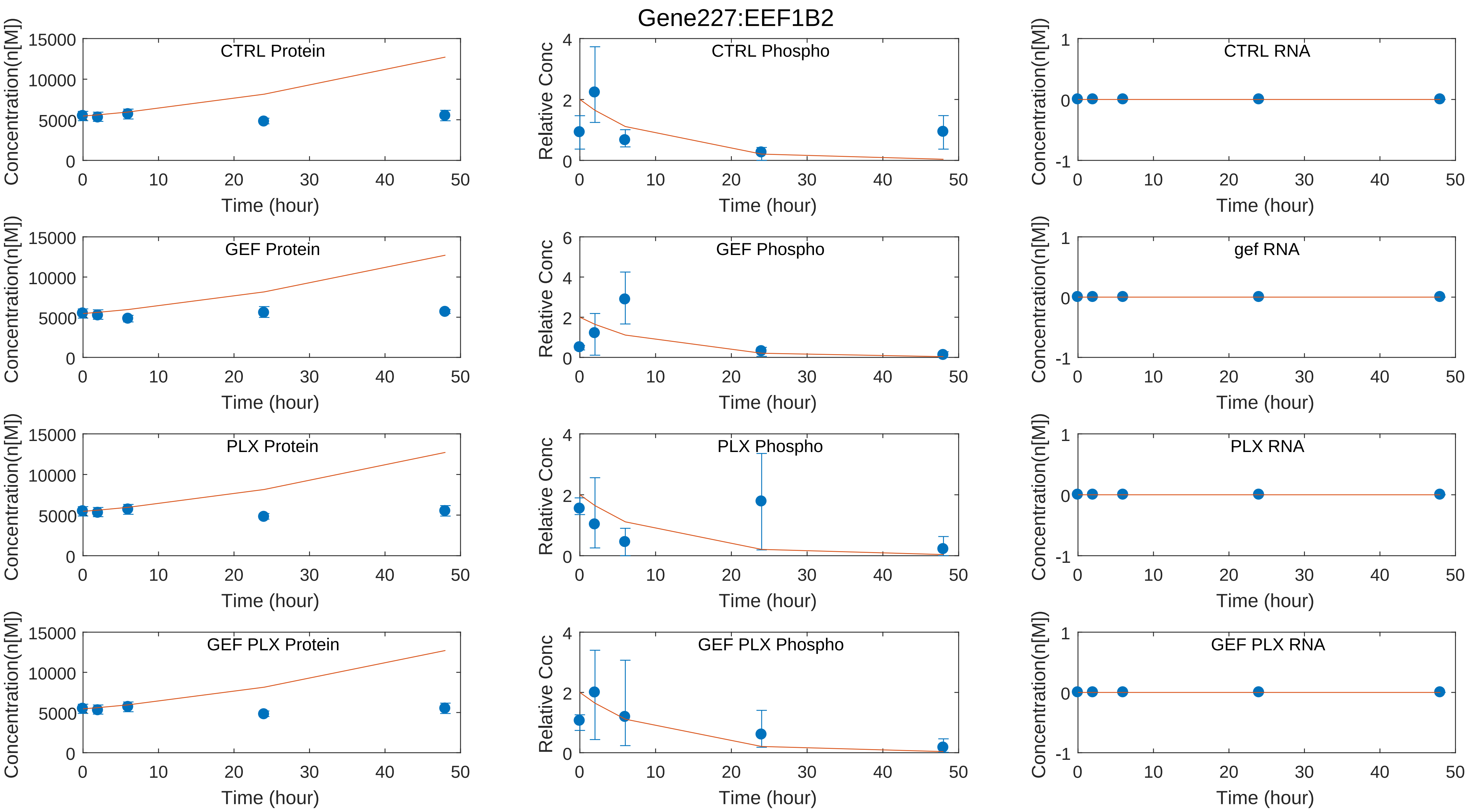
Gene227:EEF1B2
CTRL Protein
GEF Protein
PLX Protein
GEF PLX Protein
CTRL Phospho
GEF Phospho
PLX Phospho
GEF PLX Phospho
CTRL RNA
gef RNA
PLX RNA
GEF PLX RNA
Concentration(n[M])
Relative Conc
Time (hour)

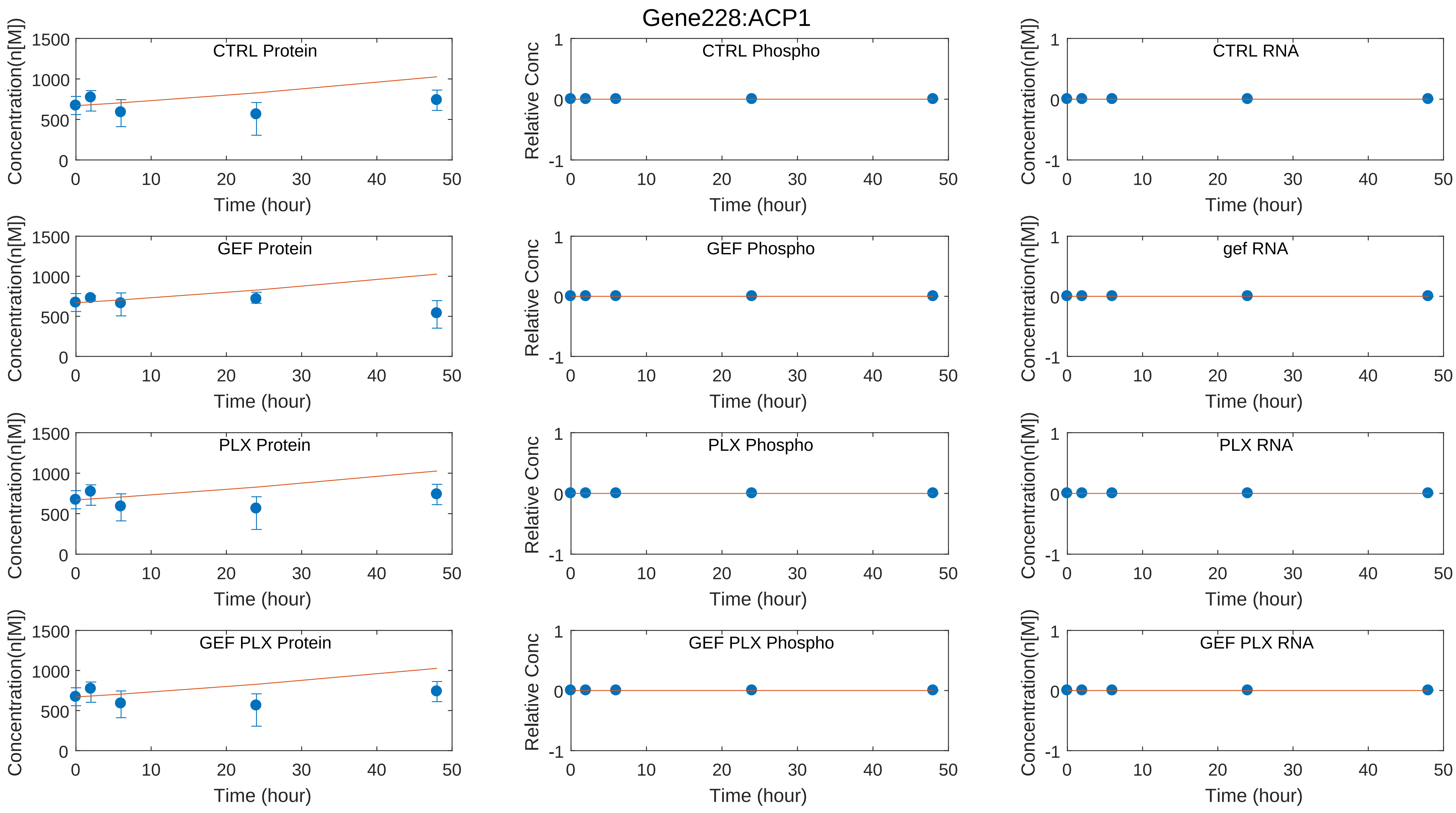
Gene228:ACP1
CTRL Protein
CTRL Phospho
CTRL RNA
GEF Protein
GEF Phospho
gef RNA
PLX Protein
PLX Phospho
PLX RNA
GEF PLX Protein
GEF PLX Phospho
GEF PLX RNA
Concentration(n[M])
Relative Conc
Time (hour)
0
10
20
30
40
50
1500
1000
500
1
-1

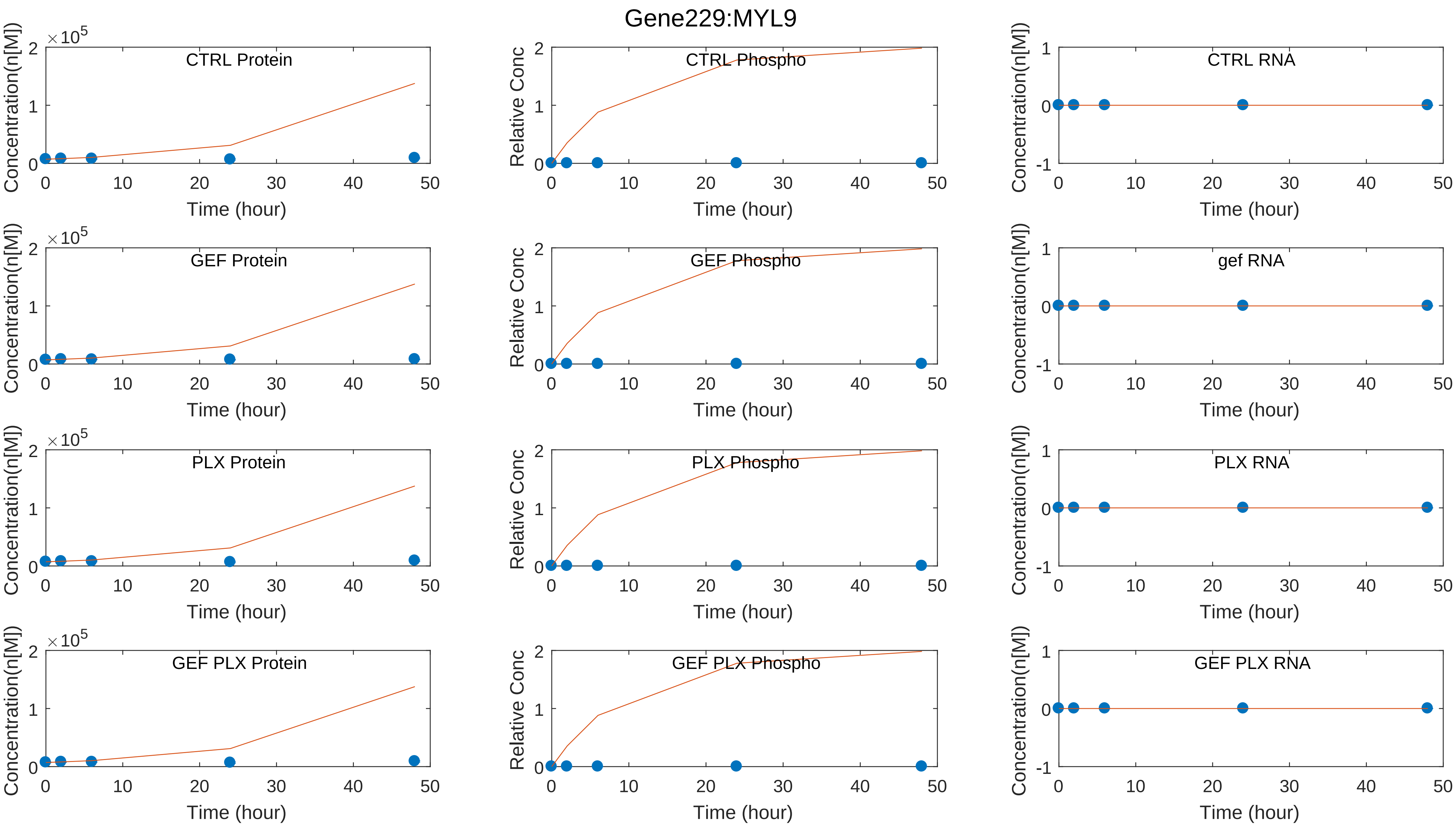

Gene229:MYL9
CTRL Protein
GEF Protein
PLX Protein
GEF PLX Protein
CTRL Phospho
GEF Phospho
PLX Phospho
GEF PLX Phospho
CTRL RNA
gef RNA
PLX RNA
GEF PLX RNA
Concentration(n[M])
Relative Conc
Time (hour)
×10^5

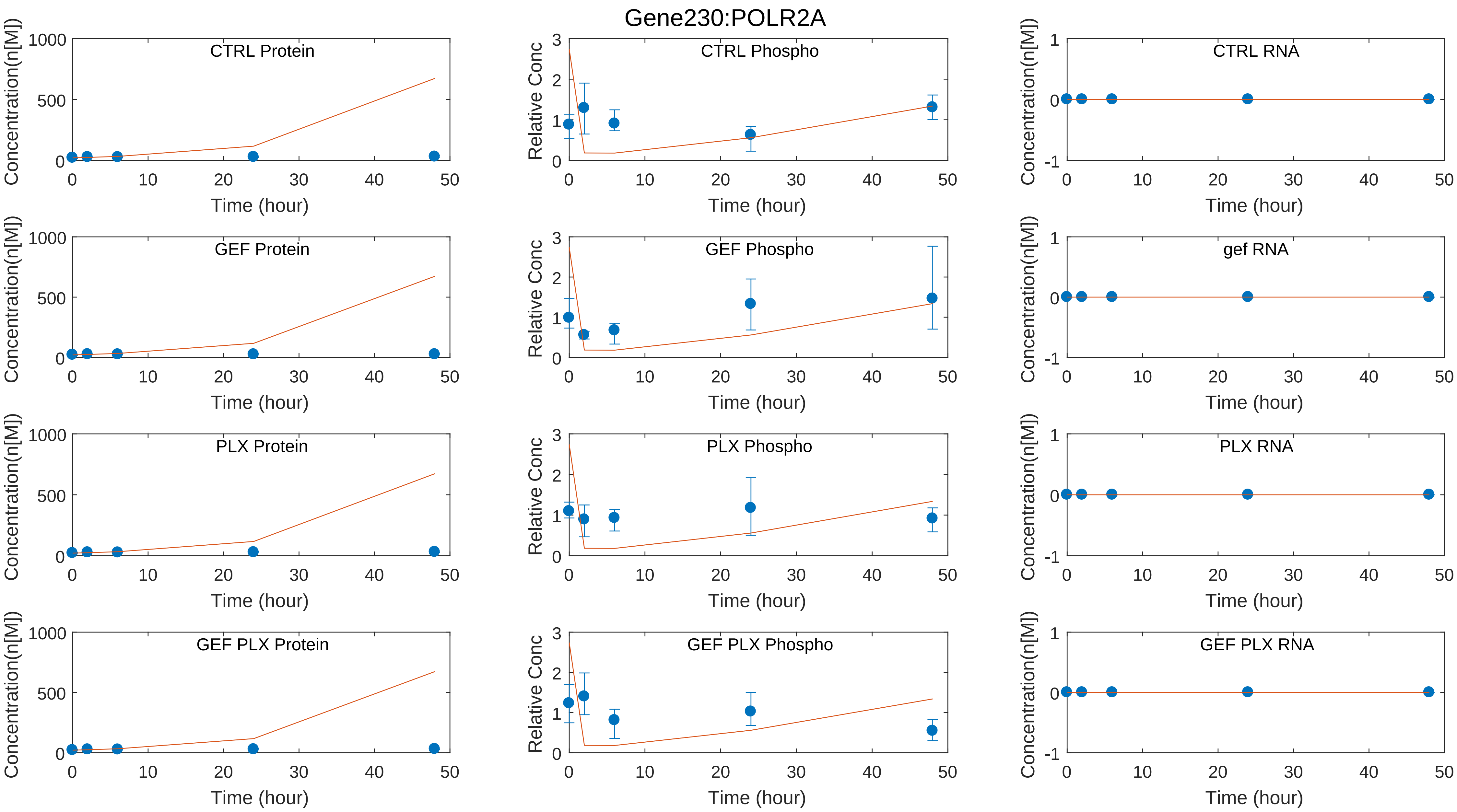

Gene230:POLR2A
CTRL Protein
CTRL Phospho
CTRL RNA
GEF Protein
GEF Phospho
gef RNA
PLX Protein
PLX Phospho
PLX RNA
GEF PLX Protein
GEF PLX Phospho
GEF PLX RNA
Concentration(n[M])
Relative Conc
Time (hour)

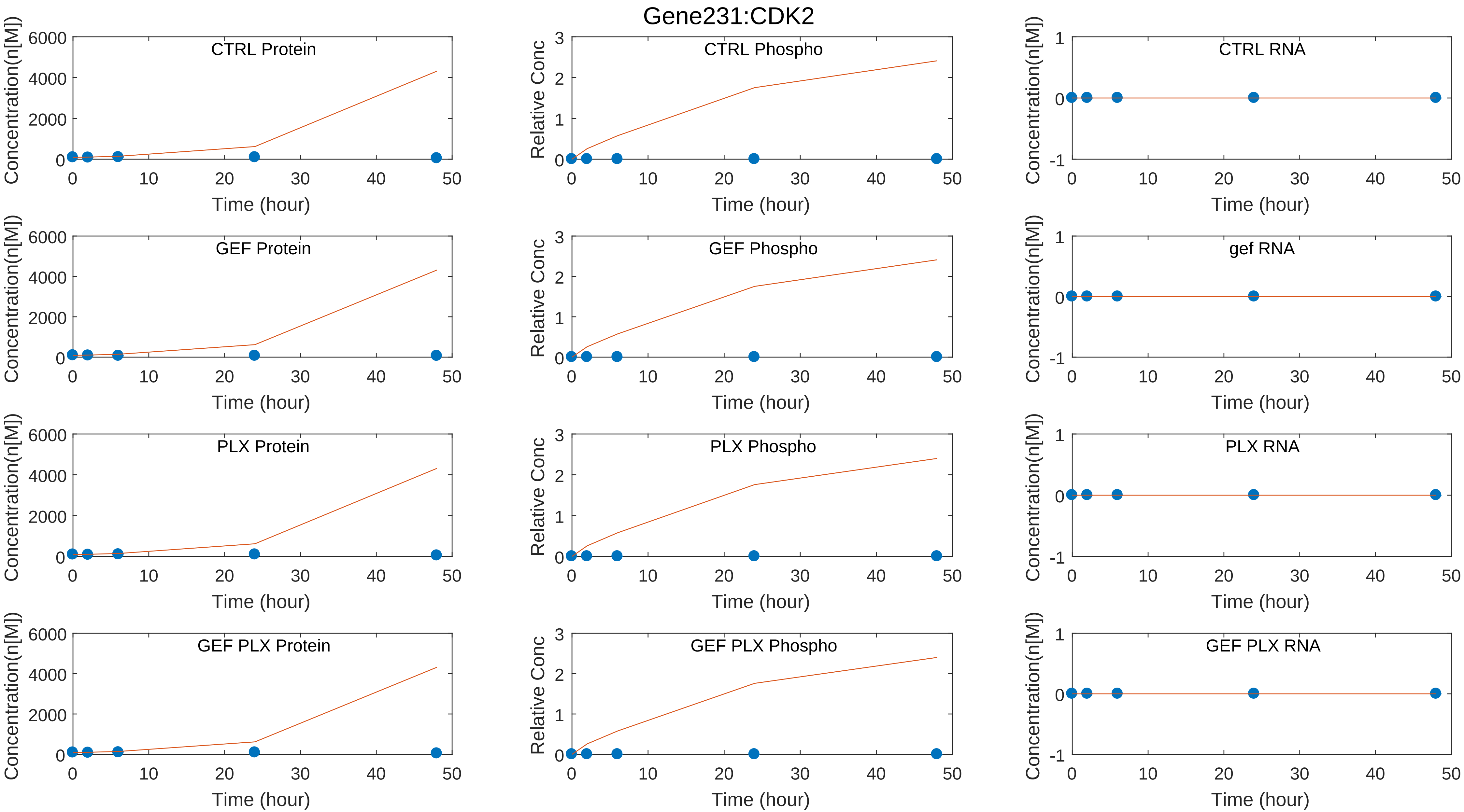
Gene231:CDK2
CTRL Protein
CTRL Phospho
CTRL RNA
GEF Protein
GEF Phospho
gef RNA
PLX Protein
PLX Phospho
PLX RNA
GEF PLX Protein
GEF PLX Phospho
GEF PLX RNA
Concentration(n[M])
Relative Conc
Time (hour)

Gene232:GRK2

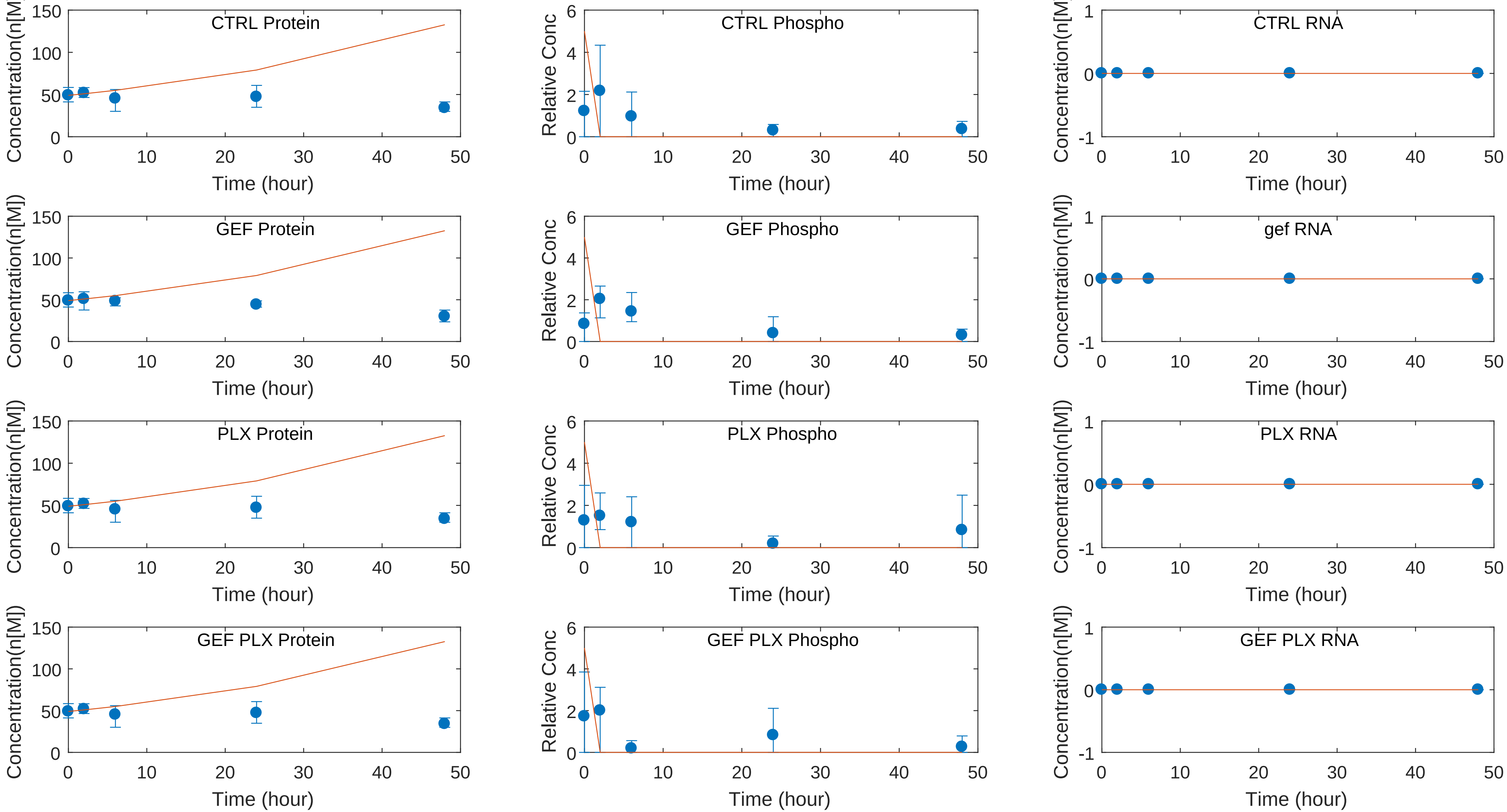

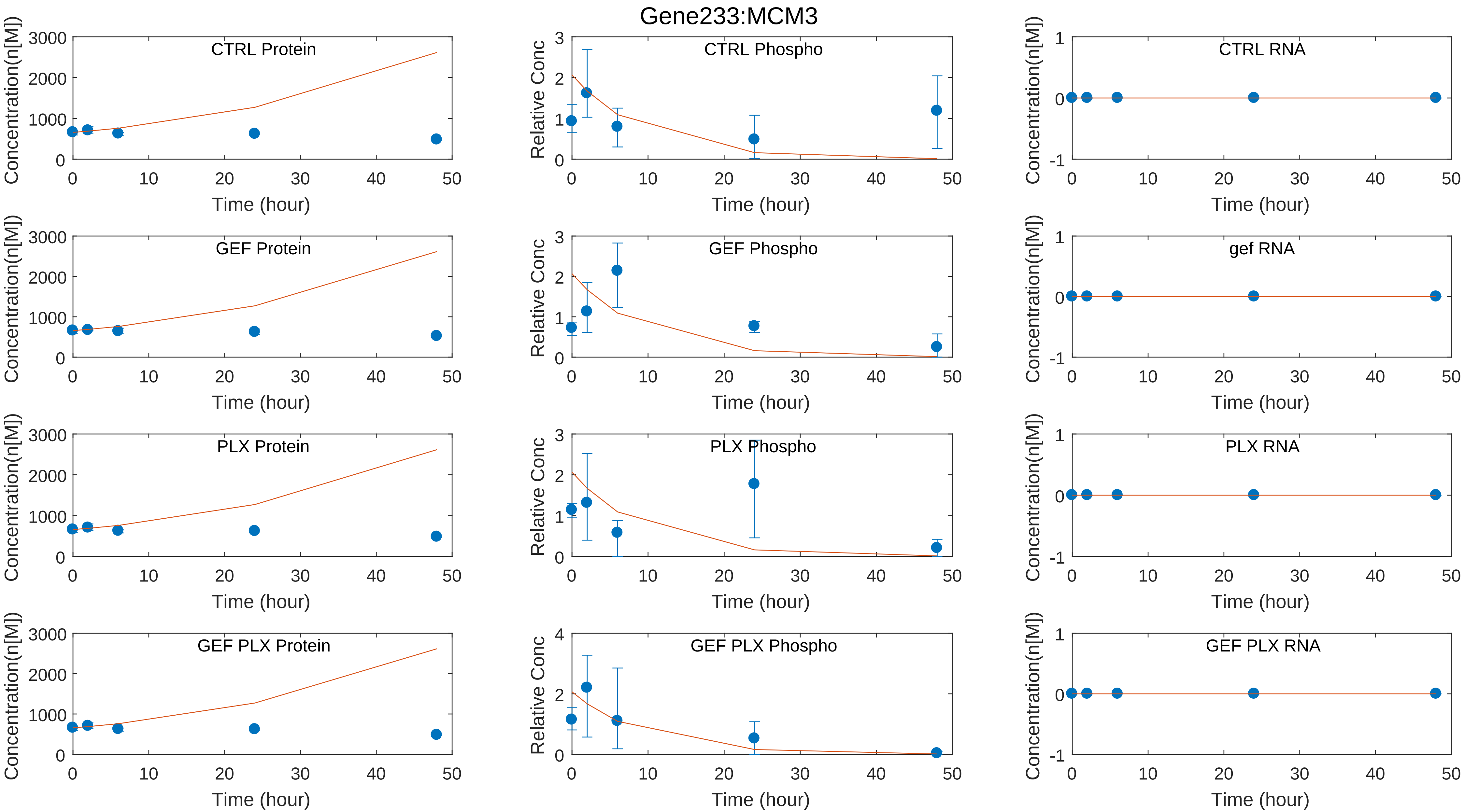
Gene233:MCM3
CTRL Protein
CTRL Phospho
CTRL RNA
GEF Protein
GEF Phospho
gef RNA
PLX Protein
PLX Phospho
PLX RNA
GEF PLX Protein
GEF PLX Phospho
GEF PLX RNA
Concentration(n[M])
Relative Conc
Time (hour)

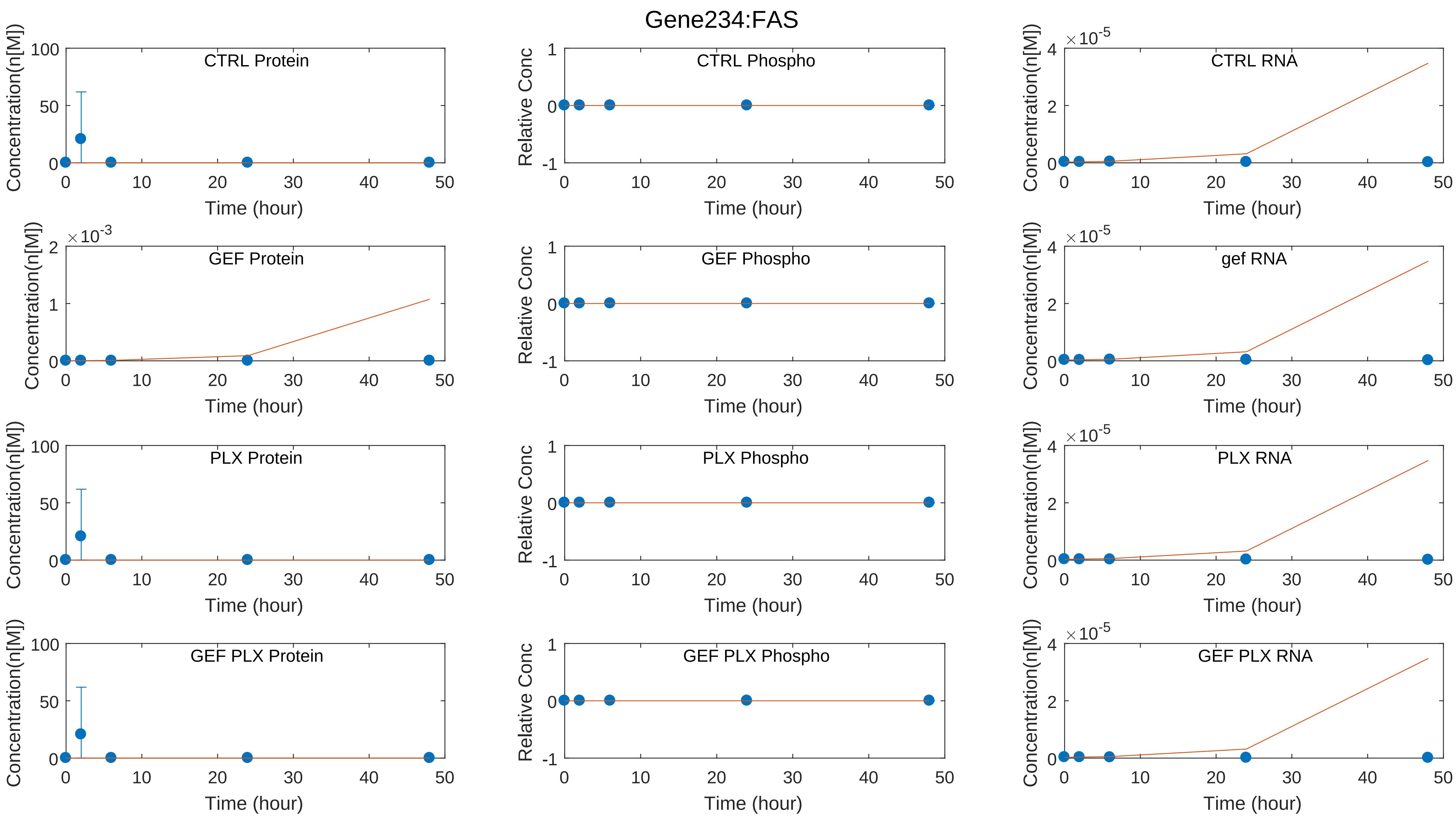

Gene234:FAS
CTRL Protein
CTRL Phospho
CTRL RNA
GEF Protein
GEF Phospho
gef RNA
PLX Protein
PLX Phospho
PLX RNA
GEF PLX Protein
GEF PLX Phospho
GEF PLX RNA
Concentration(n[M])
Relative Conc
Time (hour)
×10-5
×10-3

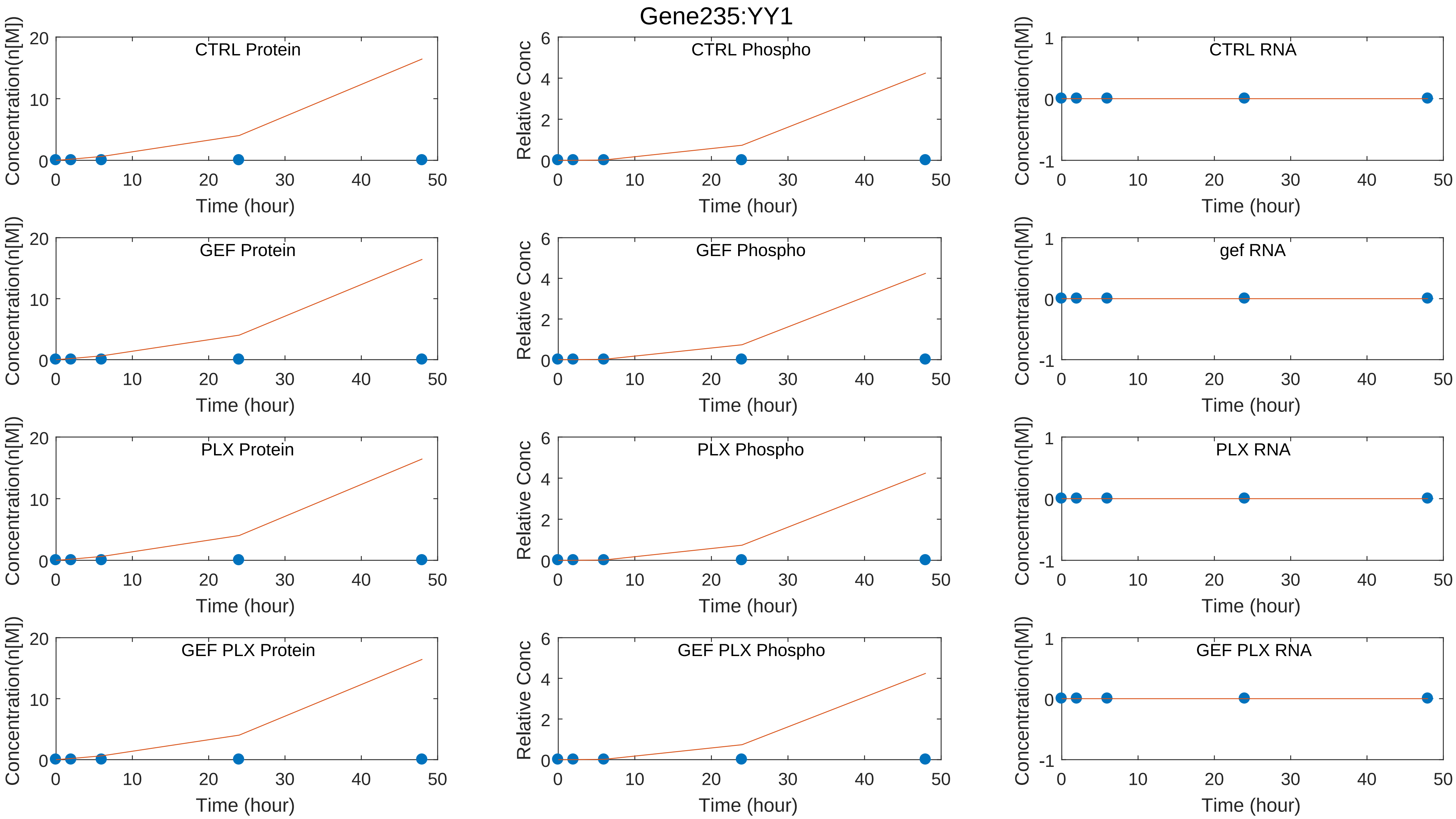
Gene235:YY1
CTRL Protein
CTRL Phospho
CTRL RNA
GEF Protein
GEF Phospho
gef RNA
PLX Protein
PLX Phospho
PLX RNA
GEF PLX Protein
GEF PLX Phospho
GEF PLX RNA
Concentration(n[M])
Relative Conc
Time (hour)

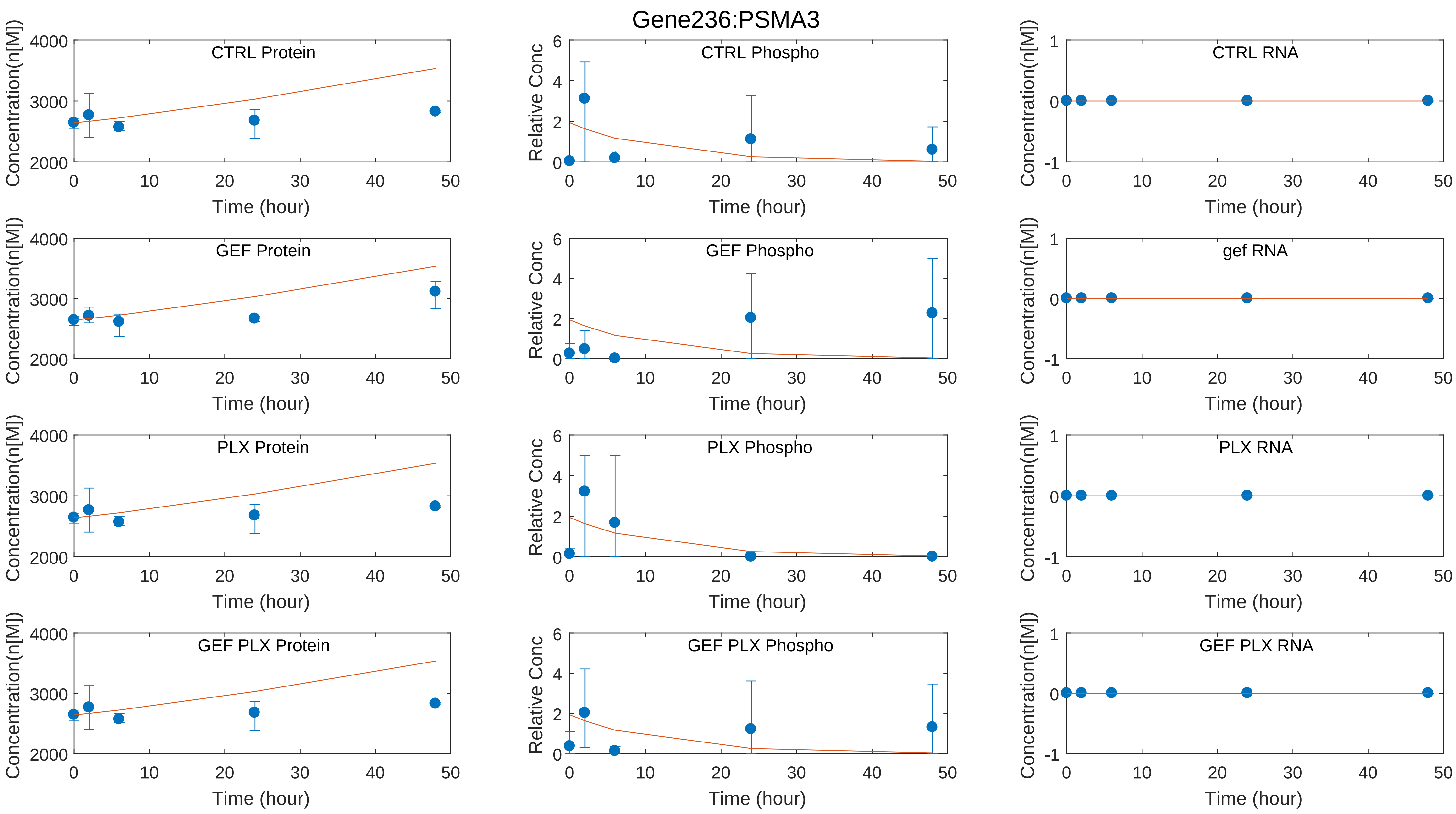

Gene236:PSMA3
CTRL Protein
CTRL Phospho
CTRL RNA
GEF Protein
GEF Phospho
gef RNA
PLX Protein
PLX Phospho
PLX RNA
GEF PLX Protein
GEF PLX Phospho
GEF PLX RNA
Concentration(n[M])
Relative Conc
Time (hour)

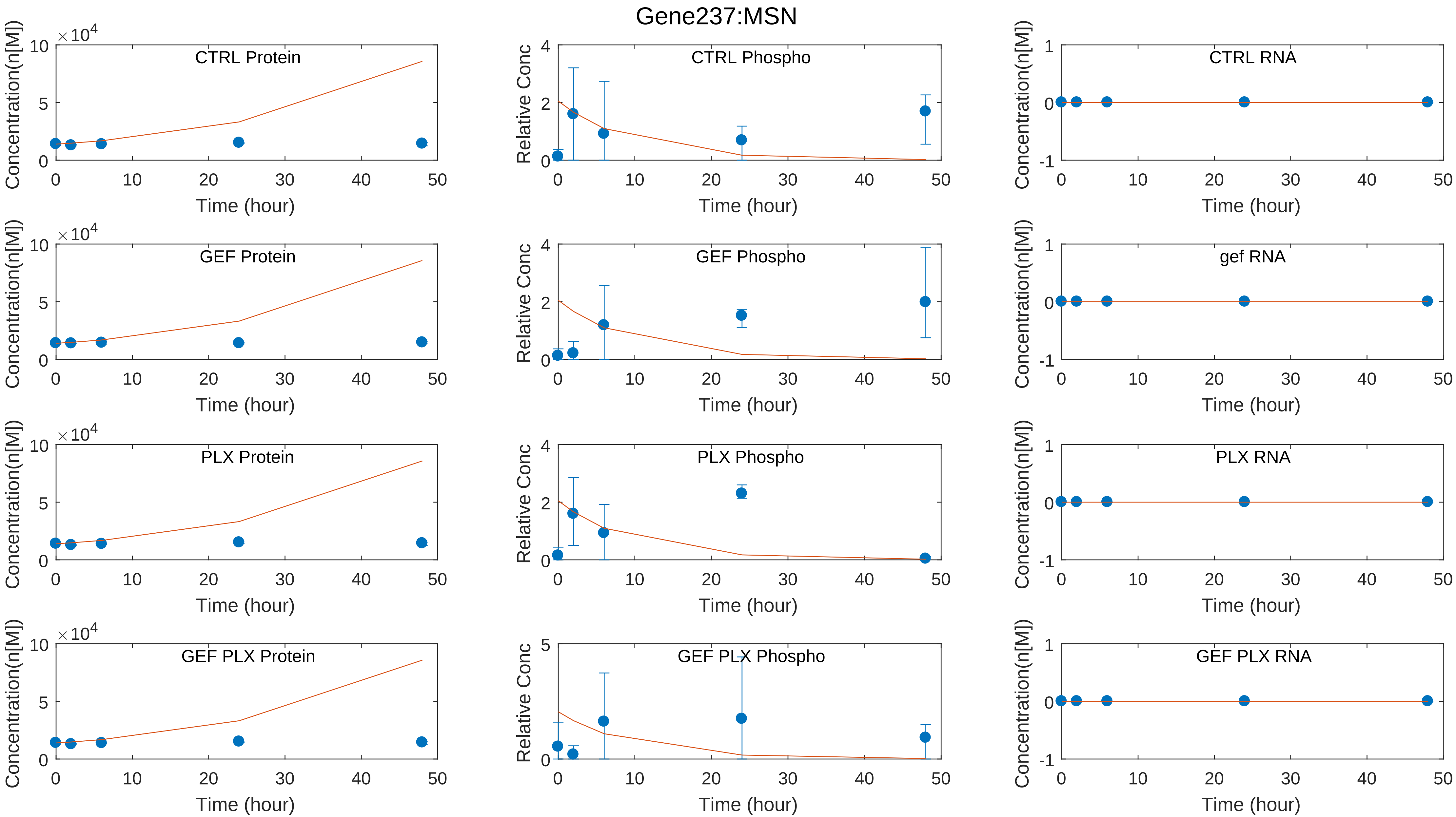
Gene237:MSN
CTRL Protein
GEF Protein
PLX Protein
GEF PLX Protein
CTRL Phospho
GEF Phospho
PLX Phospho
GEF PLX Phospho
CTRL RNA
gef RNA
PLX RNA
GEF PLX RNA
Concentration(n[M])
Relative Conc
Time (hour)
×10^4

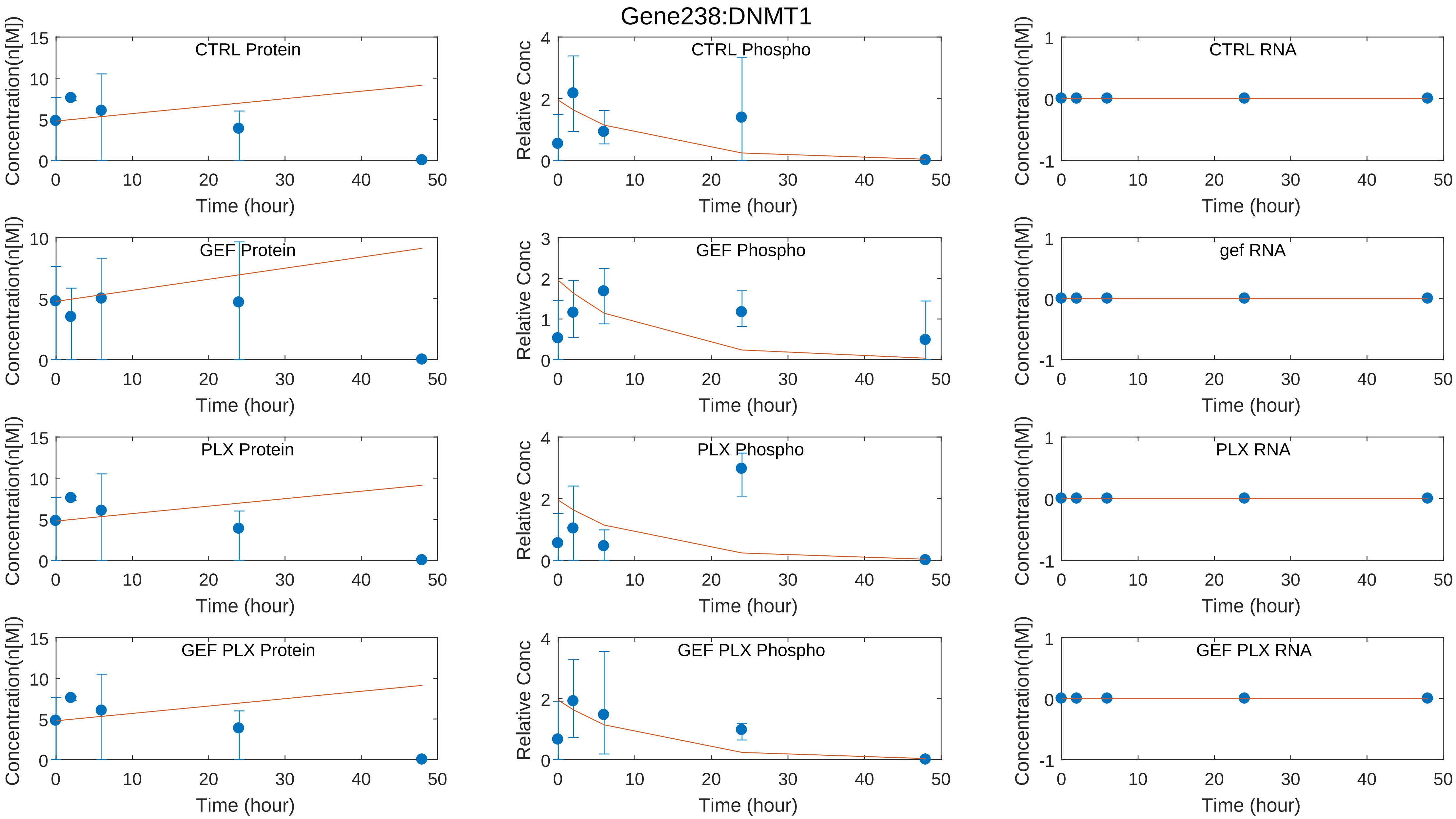

Gene238:DNMT1
CTRL Protein
CTRL Phospho
CTRL RNA
GEF Protein
GEF Phospho
gef RNA
PLX Protein
PLX Phospho
PLX RNA
GEF PLX Protein
GEF PLX Phospho
GEF PLX RNA
Concentration(n[M])
Relative Conc
Time (hour)

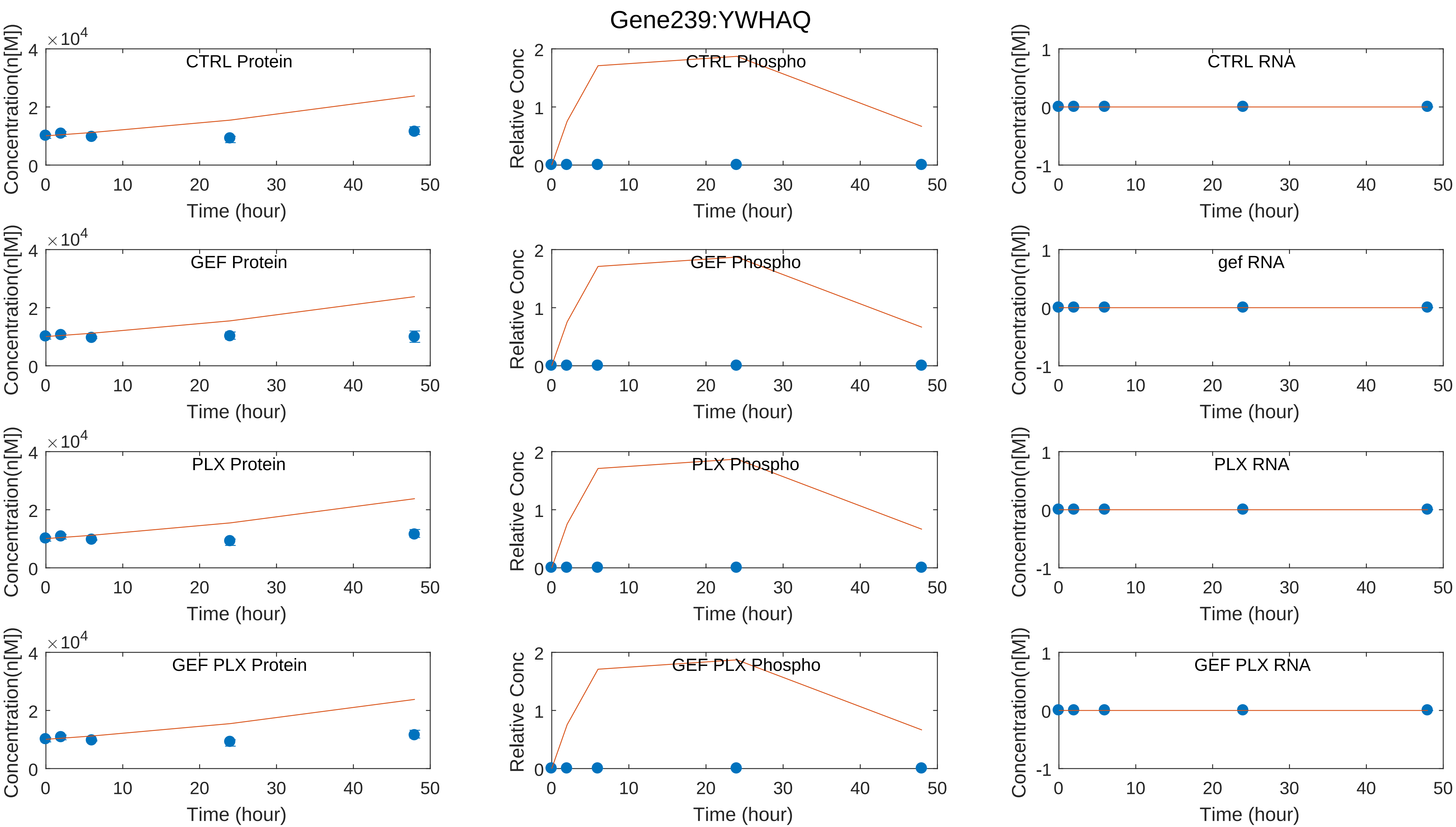

Gene239:YWHAQ
CTRL Protein
CTRL Phospho
CTRL RNA
GEF Protein
GEF Phospho
gef RNA
PLX Protein
PLX Phospho
PLX RNA
GEF PLX Protein
GEF PLX Phospho
GEF PLX RNA
Concentration(n[M])
Relative Conc
Time (hour)
$\times 10^4$

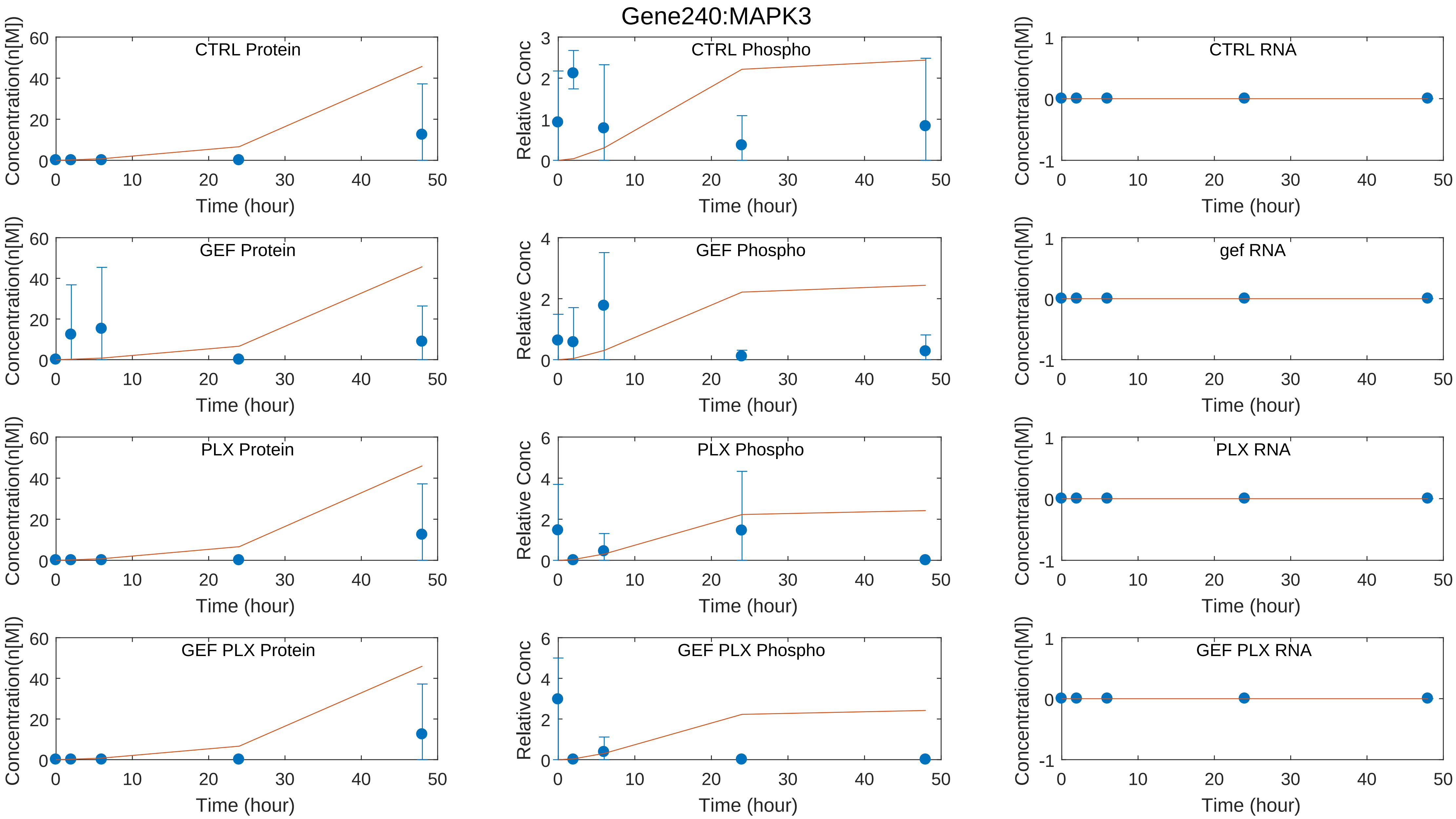
Gene240:MAPK3
CTRL Protein
CTRL Phospho
CTRL RNA
GEF Protein
GEF Phospho
gef RNA
PLX Protein
PLX Phospho
PLX RNA
GEF PLX Protein
GEF PLX Phospho
GEF PLX RNA
Concentration(n[M])
Relative Conc
Time (hour)

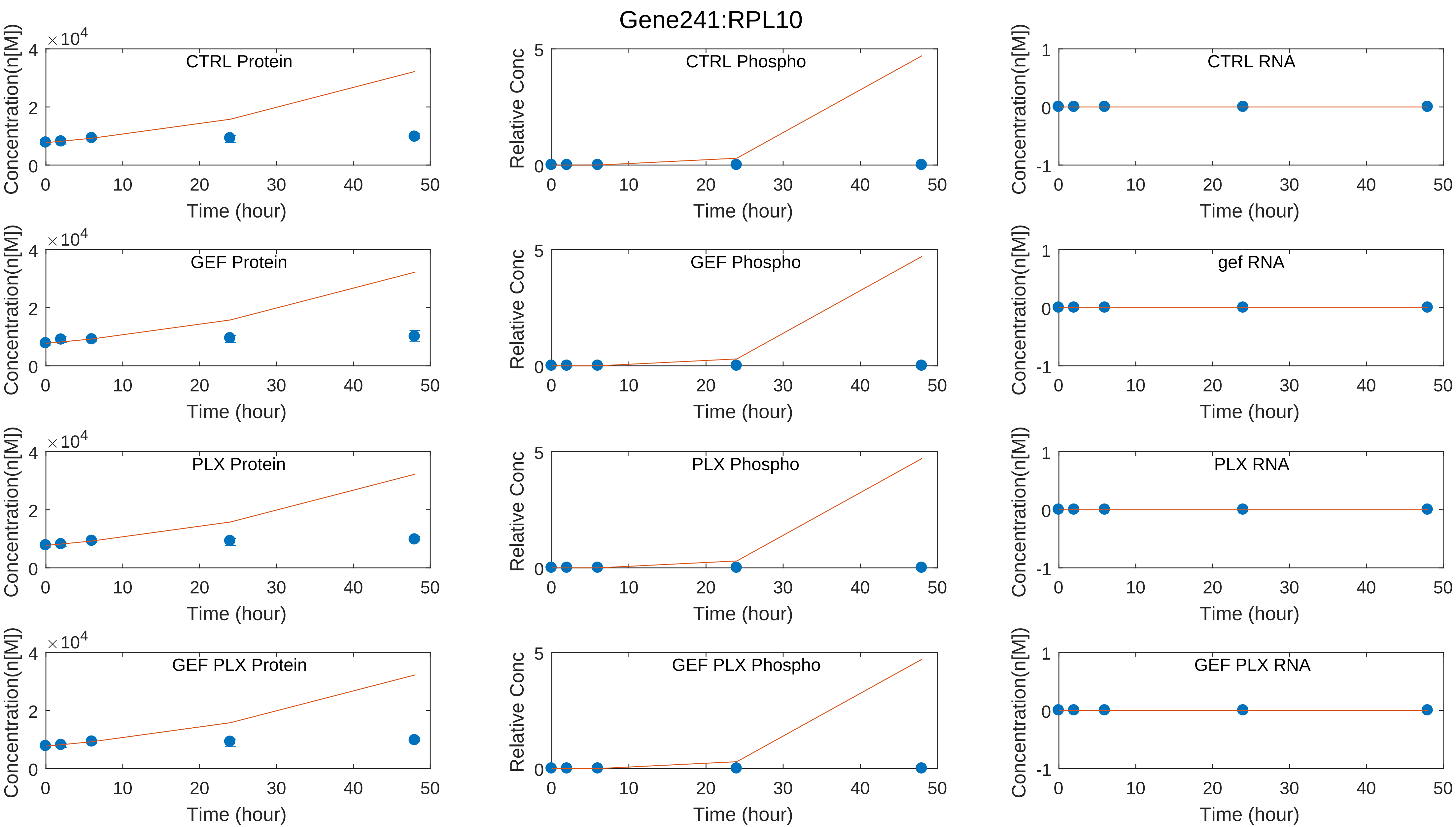
Gene241:RPL10
CTRL Protein
GEF Protein
PLX Protein
GEF PLX Protein
CTRL Phospho
GEF Phospho
PLX Phospho
GEF PLX Phospho
CTRL RNA
gef RNA
PLX RNA
GEF PLX RNA
Concentration(n[M])
Relative Conc
Time (hour)
$\times 10^4$

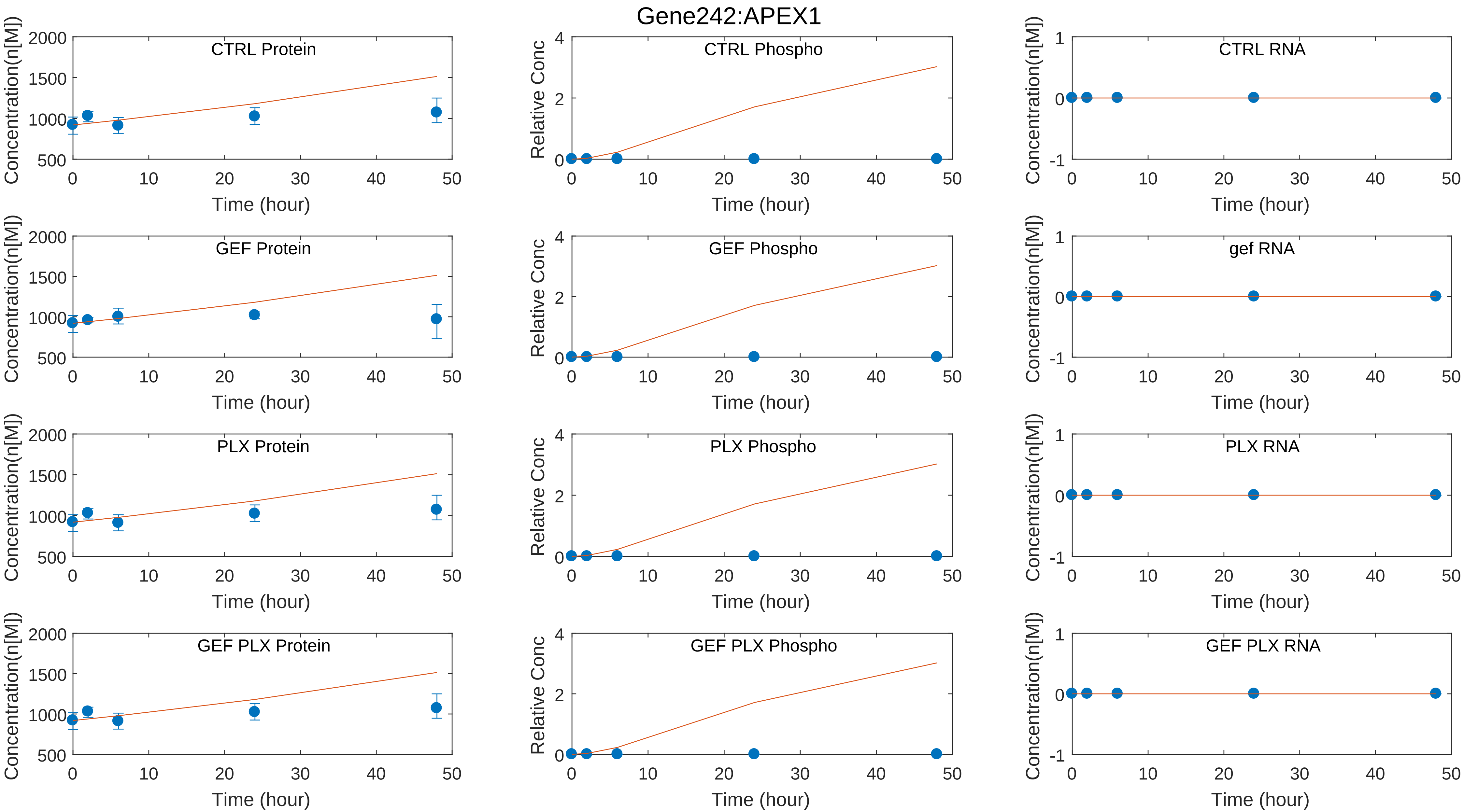
Gene242:APEX1
CTRL Protein
CTRL Phospho
CTRL RNA
GEF Protein
GEF Phospho
gef RNA
PLX Protein
PLX Phospho
PLX RNA
GEF PLX Protein
GEF PLX Phospho
GEF PLX RNA
Concentration(n[M])
Relative Conc
Time (hour)

Gene243:CAD

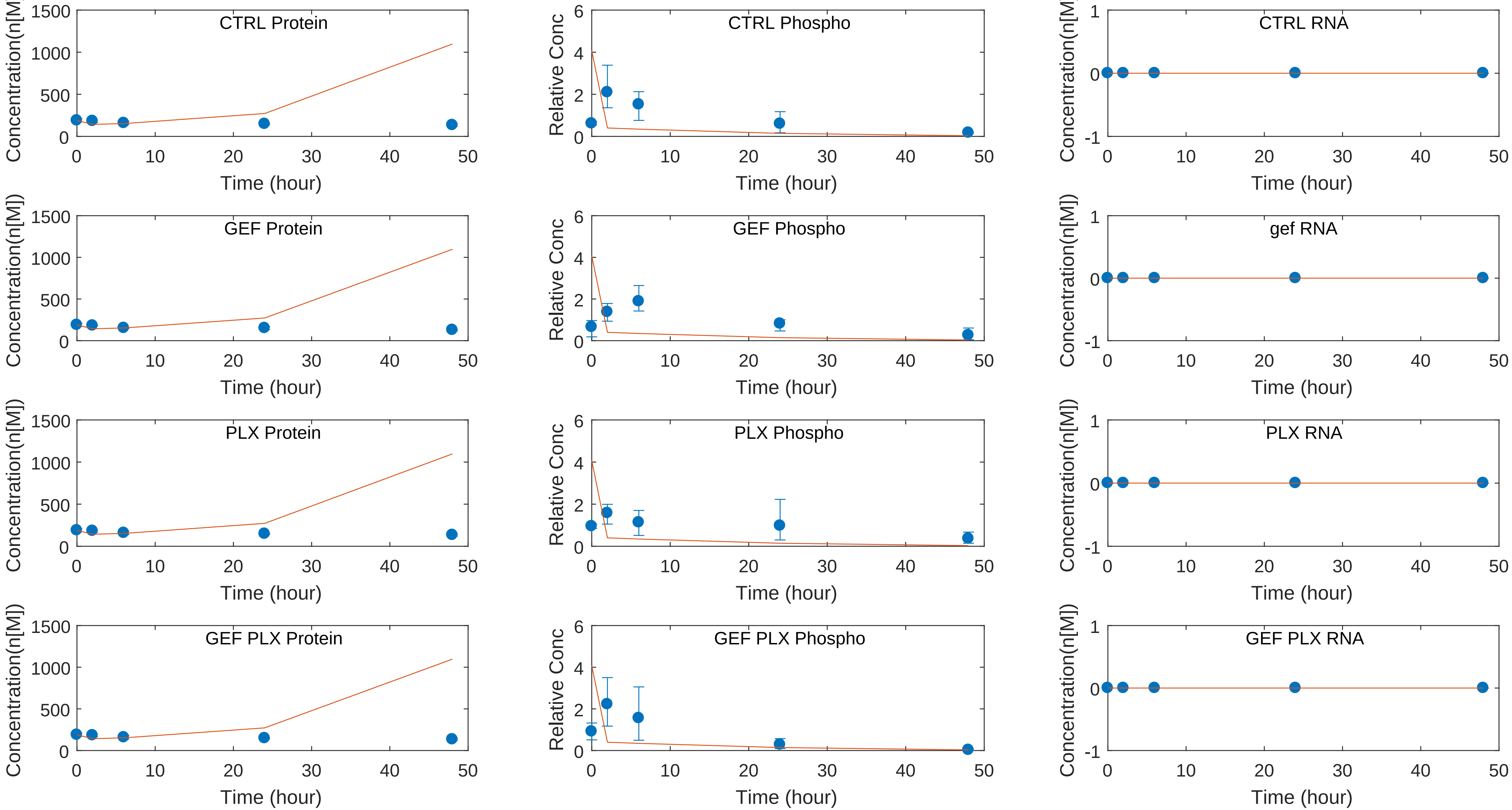

CTRL Protein
CTRL Phospho
CTRL RNA
GEF Protein
GEF Phospho
gef RNA
PLX Protein
PLX Phospho
PLX RNA
GEF PLX Protein
GEF PLX Phospho
GEF PLX RNA
Concentration(n[M])
Relative Conc
Time (hour)

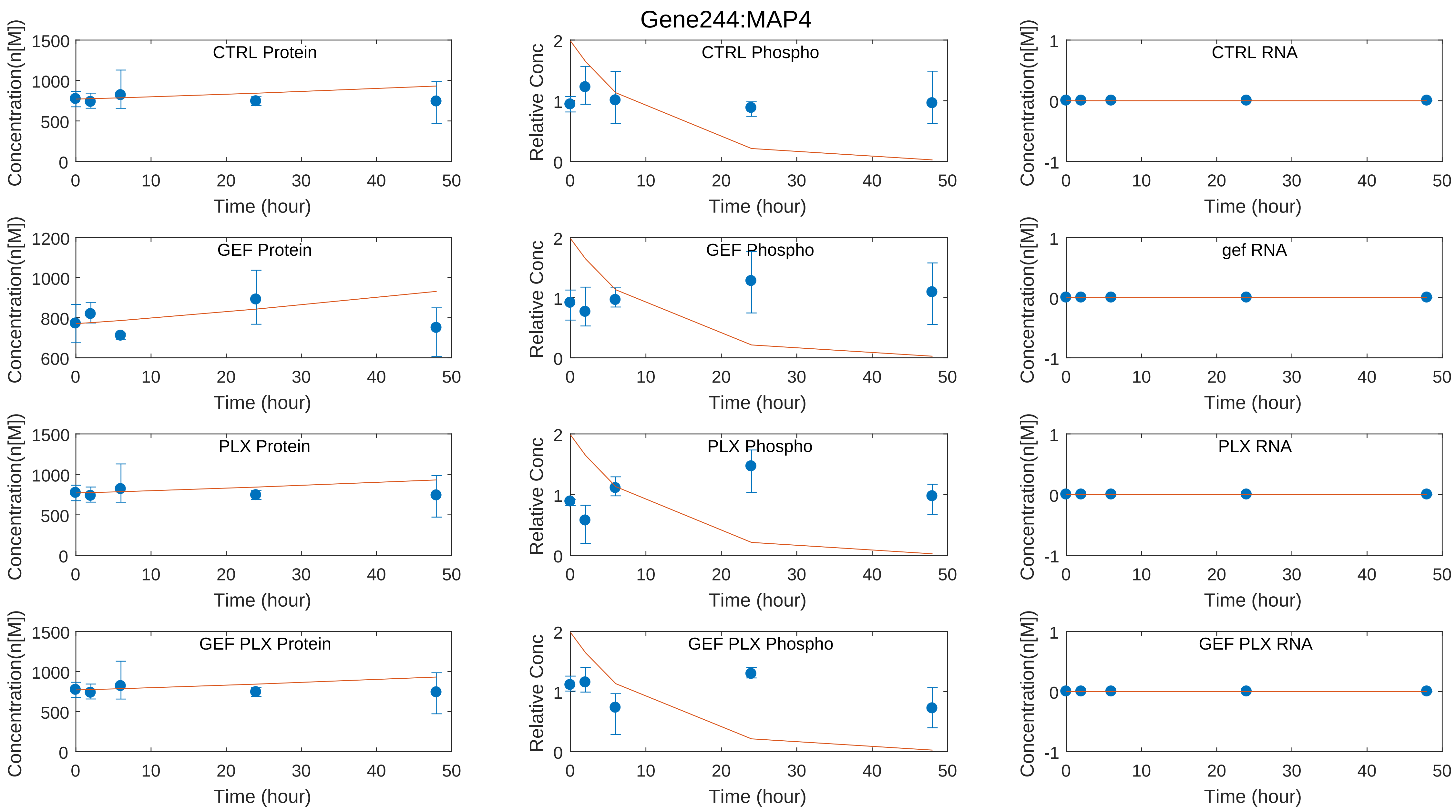
Gene244:MAP4
CTRL Protein
CTRL Phospho
CTRL RNA
GEF Protein
GEF Phospho
gef RNA
PLX Protein
PLX Phospho
PLX RNA
GEF PLX Protein
GEF PLX Phospho
GEF PLX RNA
Concentration(n[M])
Relative Conc
Time (hour)

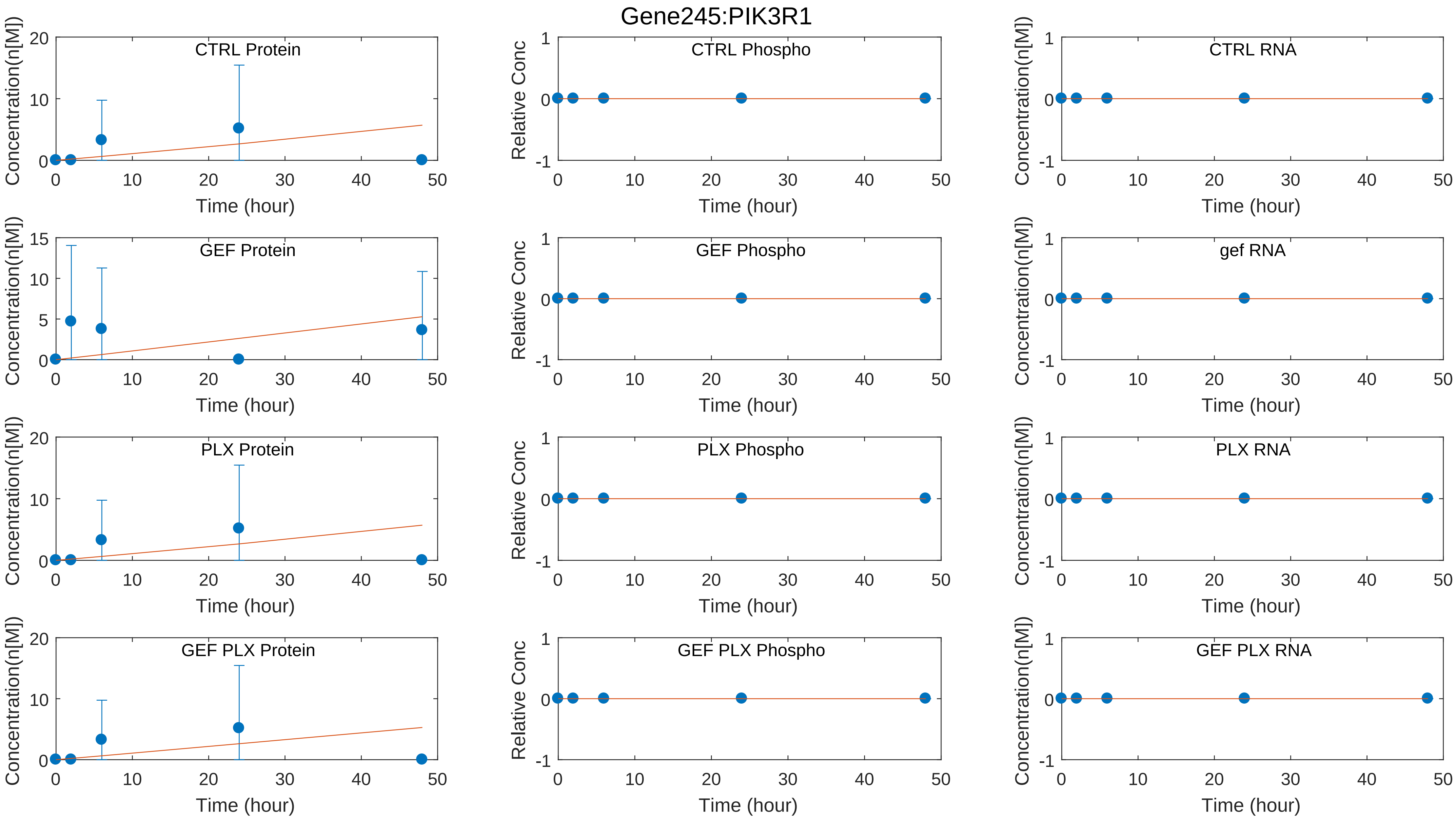

Gene245:PIK3R1
CTRL Protein
CTRL Phospho
CTRL RNA
GEF Protein
GEF Phospho
gef RNA
PLX Protein
PLX Phospho
PLX RNA
GEF PLX Protein
GEF PLX Phospho
GEF PLX RNA
Concentration(n[M])
Relative Conc
Time (hour)

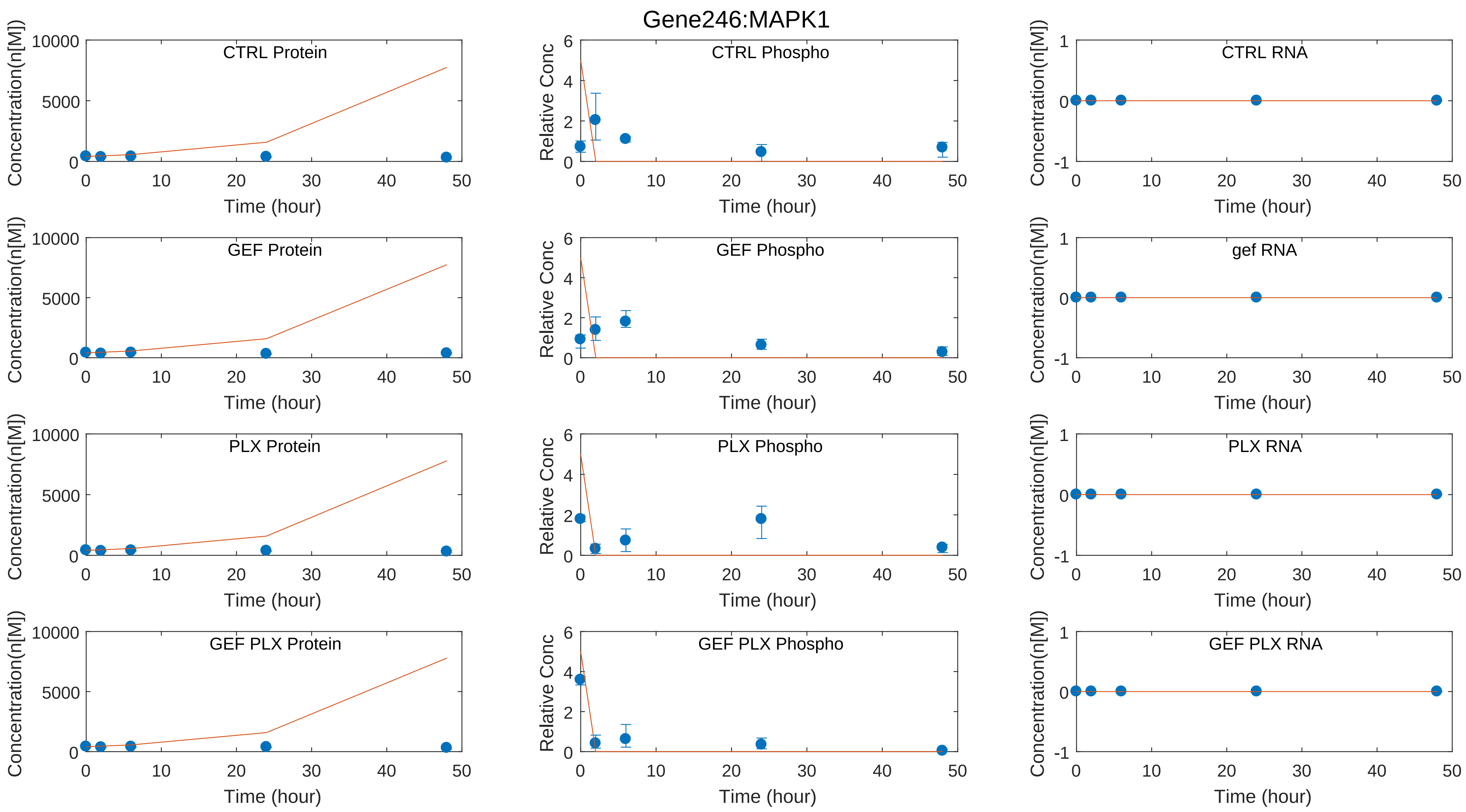

Gene246:MAPK1
CTRL Protein
GEF Protein
PLX Protein
GEF PLX Protein
CTRL Phospho
GEF Phospho
PLX Phospho
GEF PLX Phospho
CTRL RNA
gef RNA
PLX RNA
GEF PLX RNA
Concentration(n[M])
Relative Conc
Time (hour)

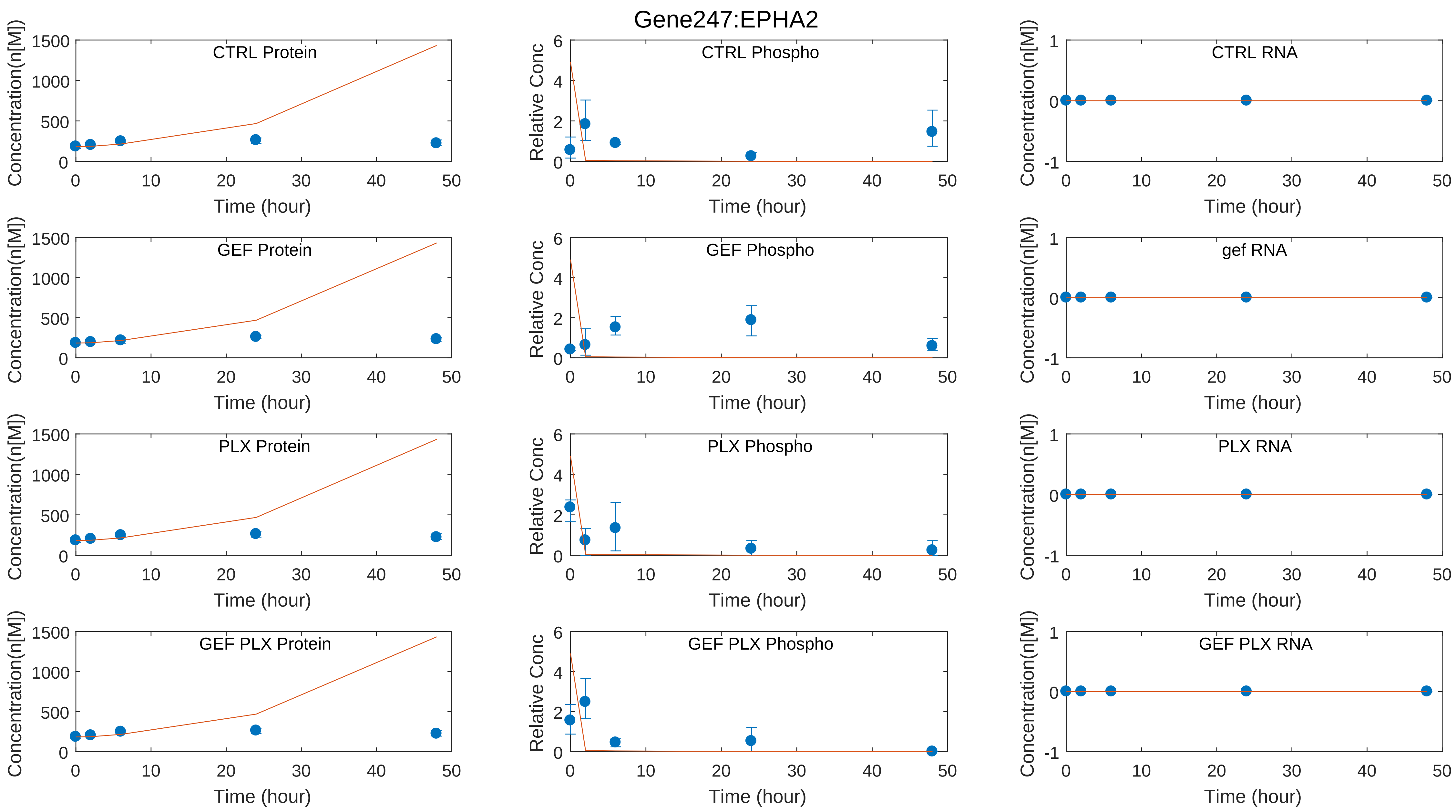

Gene247:EPHA2
CTRL Protein
CTRL Phospho
CTRL RNA
GEF Protein
GEF Phospho
gef RNA
PLX Protein
PLX Phospho
PLX RNA
GEF PLX Protein
GEF PLX Phospho
GEF PLX RNA
Concentration(n[M])
Relative Conc
Time (hour)

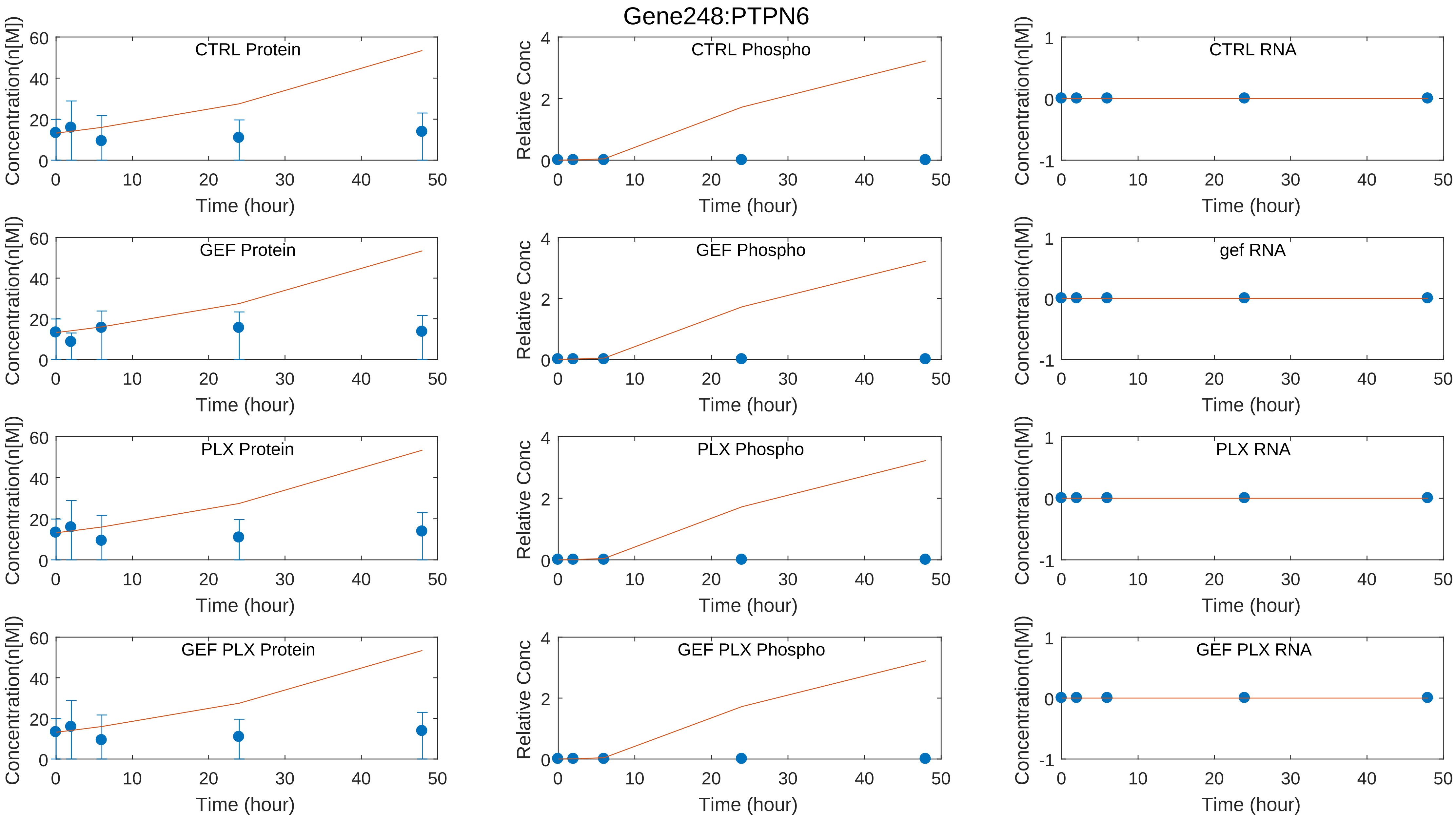
Gene248:PTPN6
CTRL Protein
CTRL Phospho
CTRL RNA
GEF Protein
GEF Phospho
gef RNA
PLX Protein
PLX Phospho
PLX RNA
GEF PLX Protein
GEF PLX Phospho
GEF PLX RNA
Concentration(n[M])
Relative Conc
Time (hour)

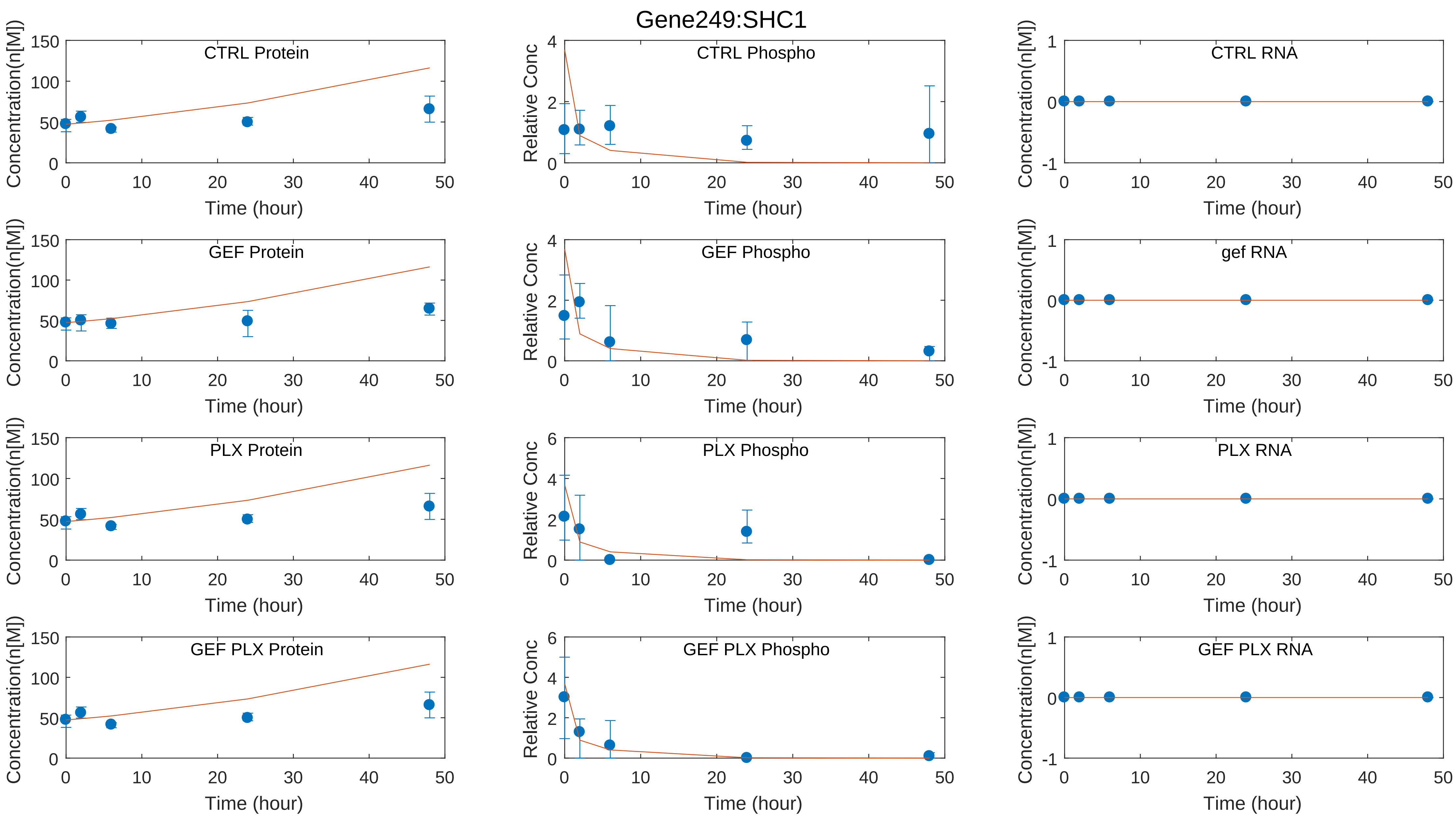

Gene249:SHC1
CTRL Protein
CTRL Phospho
CTRL RNA
GEF Protein
GEF Phospho
gef RNA
PLX Protein
PLX Phospho
PLX RNA
GEF PLX Protein
GEF PLX Phospho
GEF PLX RNA
Concentration(n[M])
Relative Conc
Time (hour)

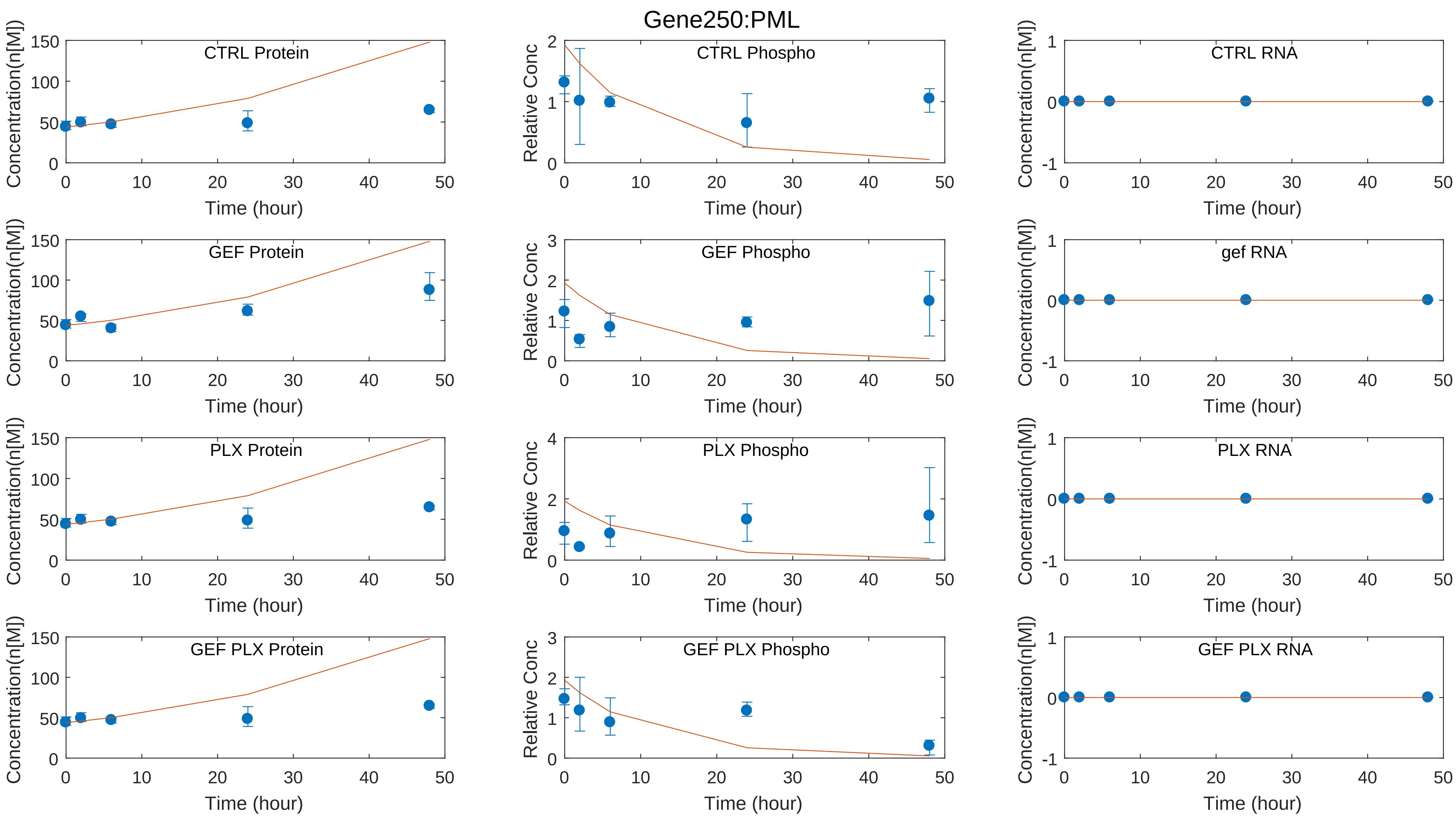

Gene250:PML
CTRL Protein
CTRL Phospho
CTRL RNA
GEF Protein
GEF Phospho
gef RNA
PLX Protein
PLX Phospho
PLX RNA
GEF PLX Protein
GEF PLX Phospho
GEF PLX RNA
Concentration(n[M])
Relative Conc
Time (hour)

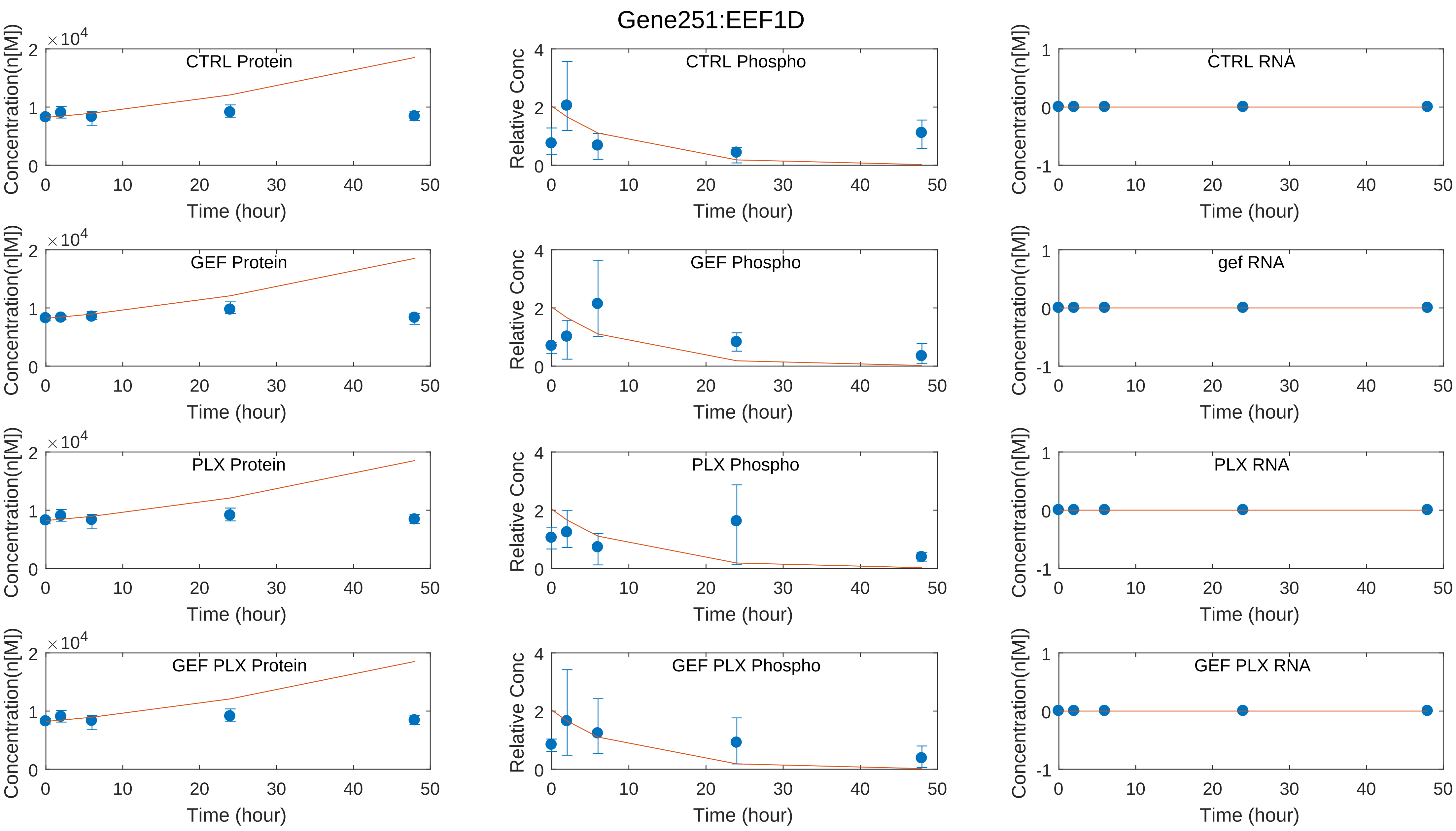
Gene251:EEF1D
CTRL Protein
GEF Protein
PLX Protein
GEF PLX Protein
CTRL Phospho
GEF Phospho
PLX Phospho
GEF PLX Phospho
CTRL RNA
gef RNA
PLX RNA
GEF PLX RNA
Concentration(n[M])
Relative Conc
Time (hour)
×10^4

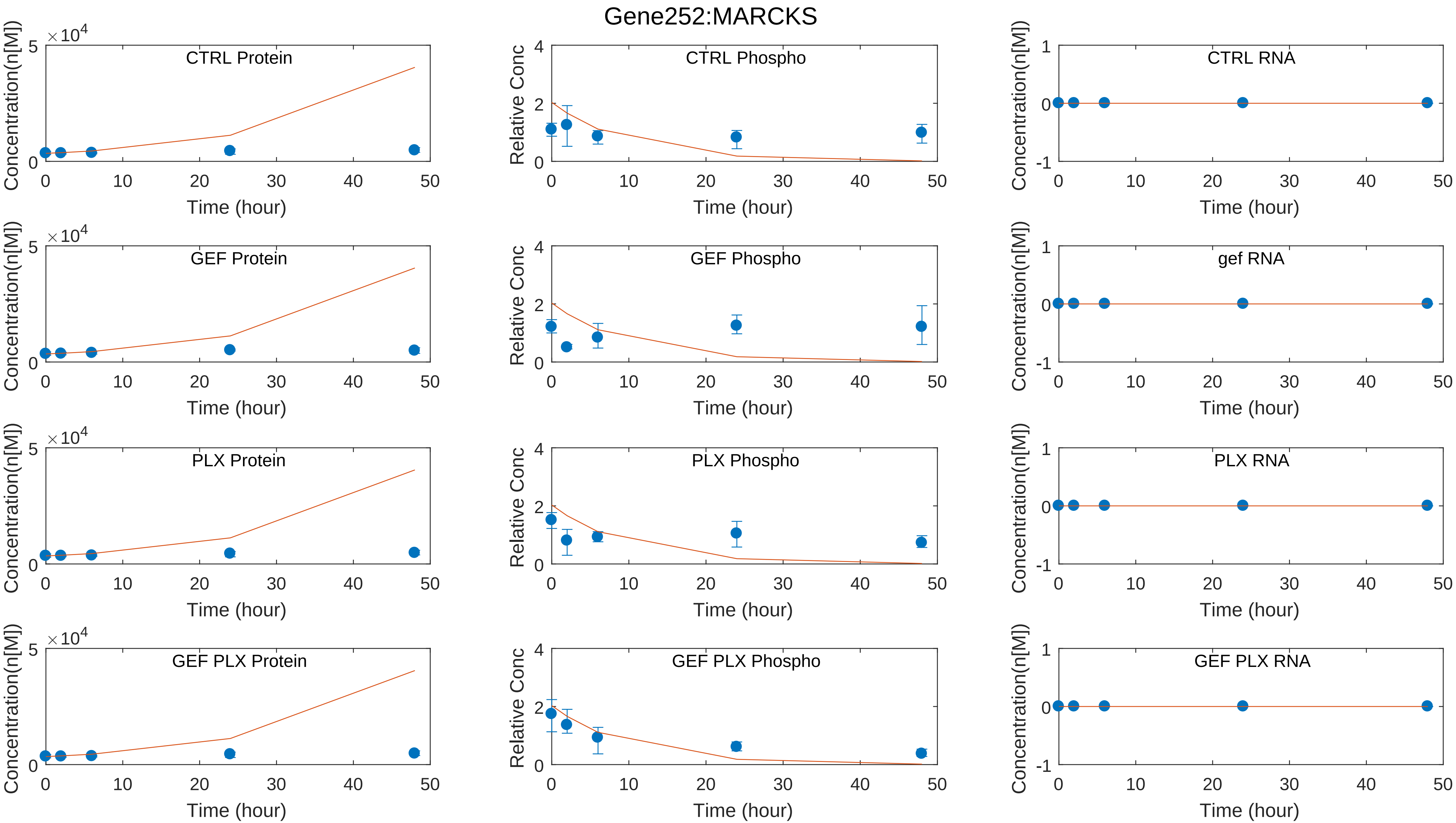

Gene252:MARCKS
CTRL Protein
GEF Protein
PLX Protein
GEF PLX Protein
CTRL Phospho
GEF Phospho
PLX Phospho
GEF PLX Phospho
CTRL RNA
gef RNA
PLX RNA
GEF PLX RNA
Concentration(n[M])
Relative Conc
Time (hour)
×10^4

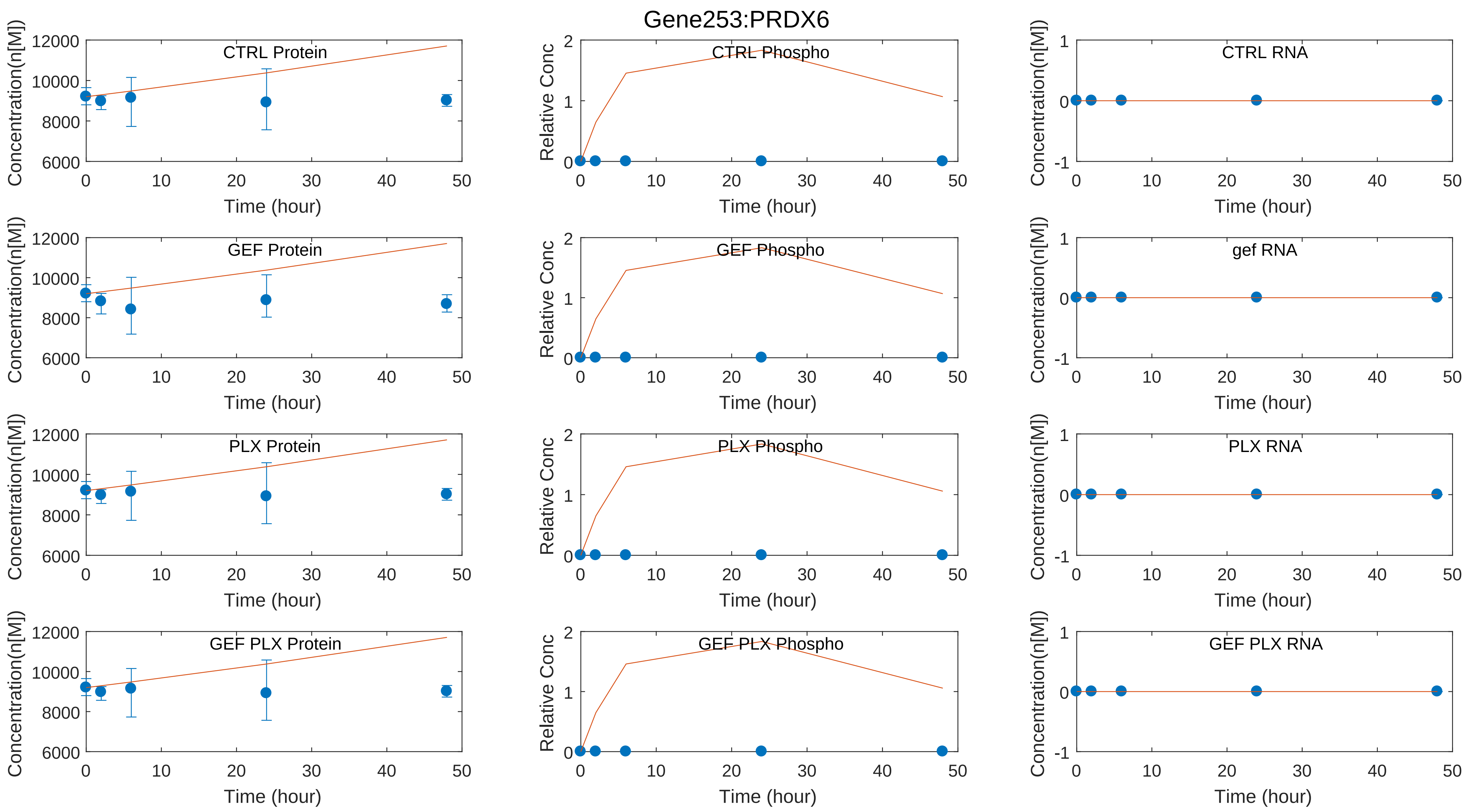

Gene253:PRDX6
CTRL Protein
CTRL Phospho
CTRL RNA
GEF Protein
GEF Phospho
gef RNA
PLX Protein
PLX Phospho
PLX RNA
GEF PLX Protein
GEF PLX Phospho
GEF PLX RNA
Concentration(n[M])
Relative Conc
Time (hour)

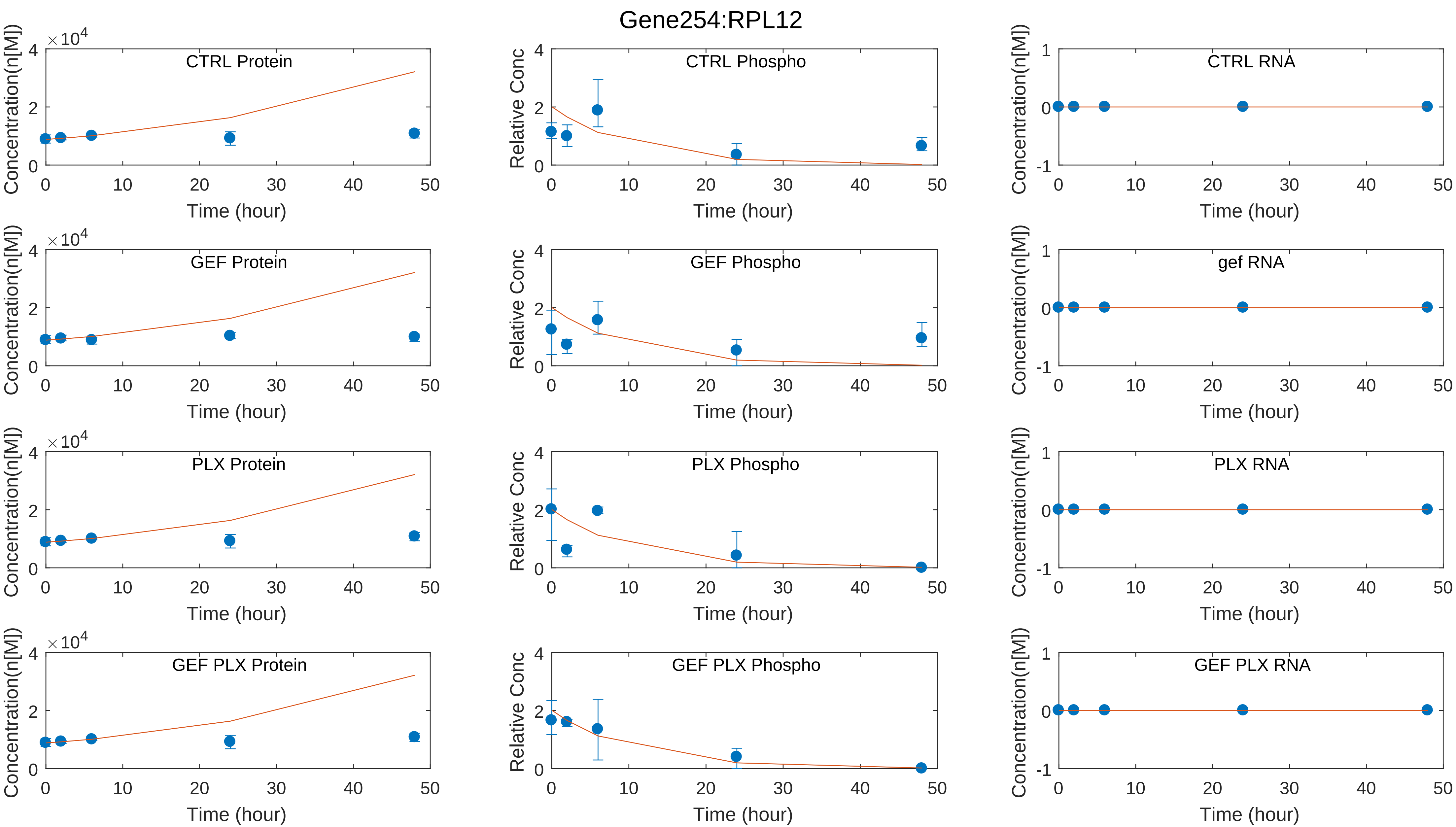

Gene254:RPL12
CTRL Protein
CTRL Phospho
CTRL RNA
GEF Protein
GEF Phospho
gef RNA
PLX Protein
PLX Phospho
PLX RNA
GEF PLX Protein
GEF PLX Phospho
GEF PLX RNA
Concentration(n[M])
Relative Conc
Time (hour)
$\times 10^4$

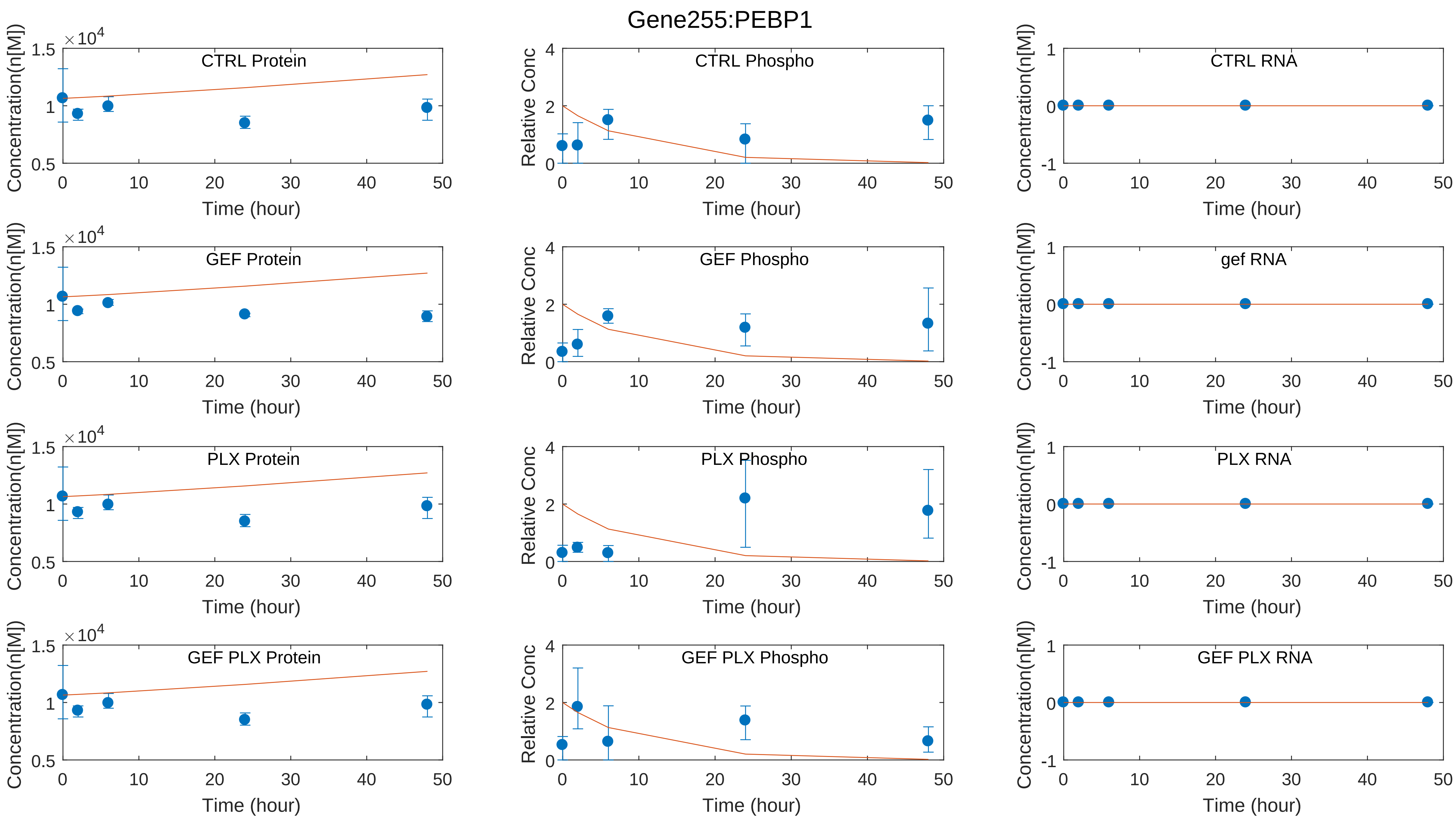

Gene255:PEBP1
CTRL Protein
CTRL Phospho
CTRL RNA
GEF Protein
GEF Phospho
gef RNA
PLX Protein
PLX Phospho
PLX RNA
GEF PLX Protein
GEF PLX Phospho
GEF PLX RNA
Concentration(n[M])
Relative Conc
Time (hour)
$\times 10^4$

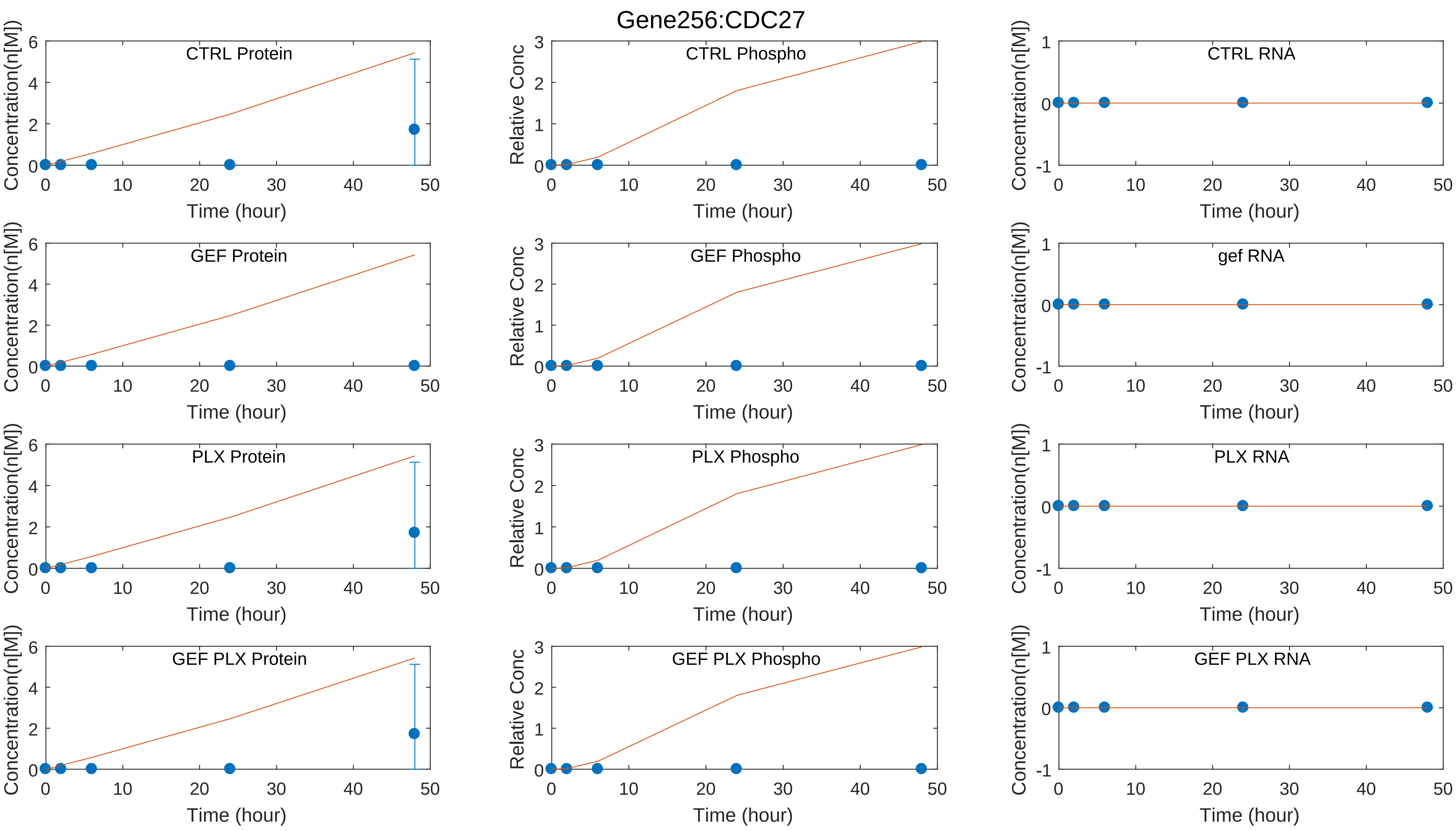

Gene256:CDC27
CTRL Protein
CTRL Phospho
CTRL RNA
GEF Protein
GEF Phospho
gef RNA
PLX Protein
PLX Phospho
PLX RNA
GEF PLX Protein
GEF PLX Phospho
GEF PLX RNA
Concentration(n[M])
Relative Conc
Time (hour)

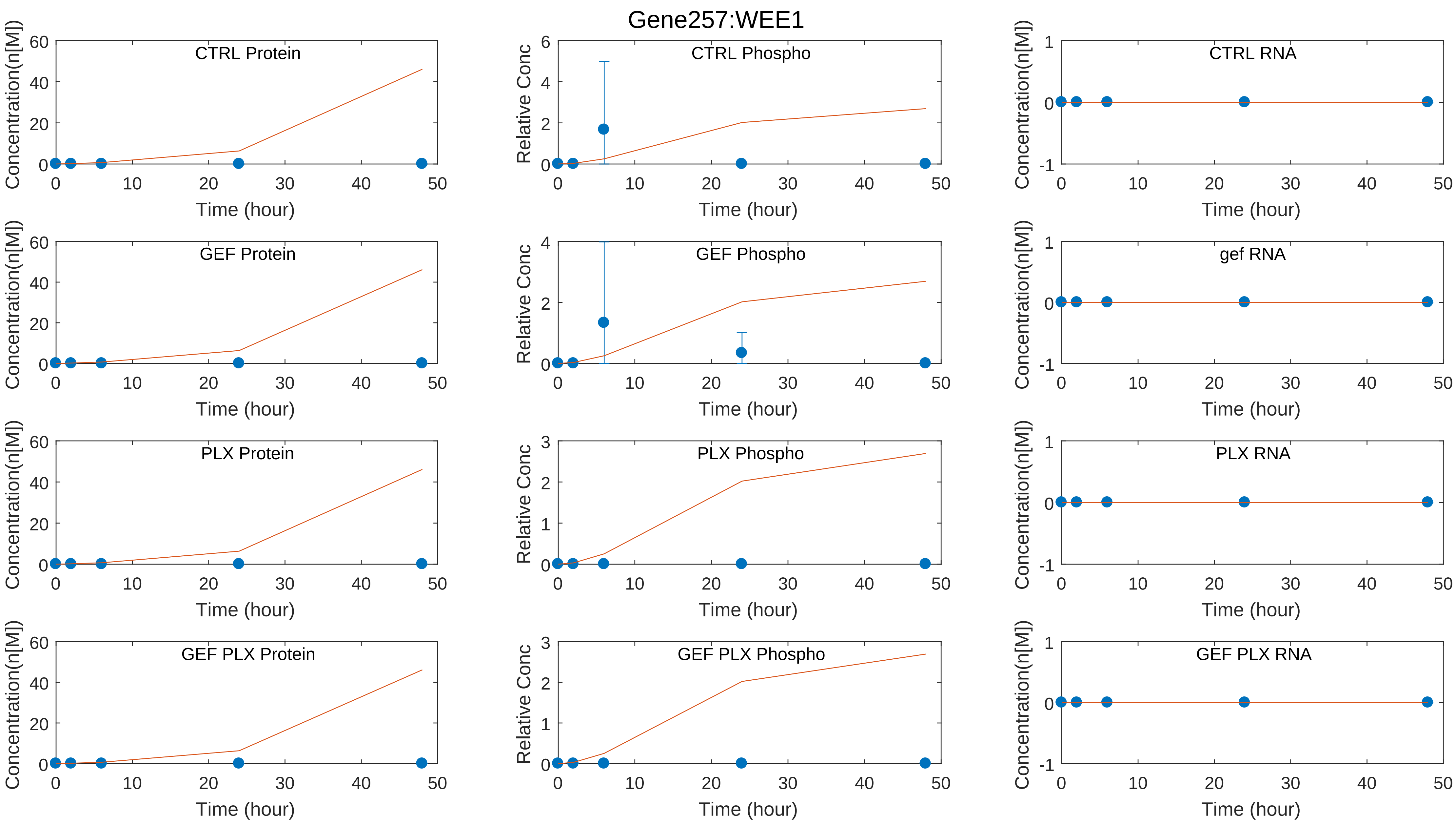

Gene257:WEE1
CTRL Protein
CTRL Phospho
CTRL RNA
GEF Protein
GEF Phospho
gef RNA
PLX Protein
PLX Phospho
PLX RNA
GEF PLX Protein
GEF PLX Phospho
GEF PLX RNA
Concentration(n[M])
Relative Conc
Time (hour)

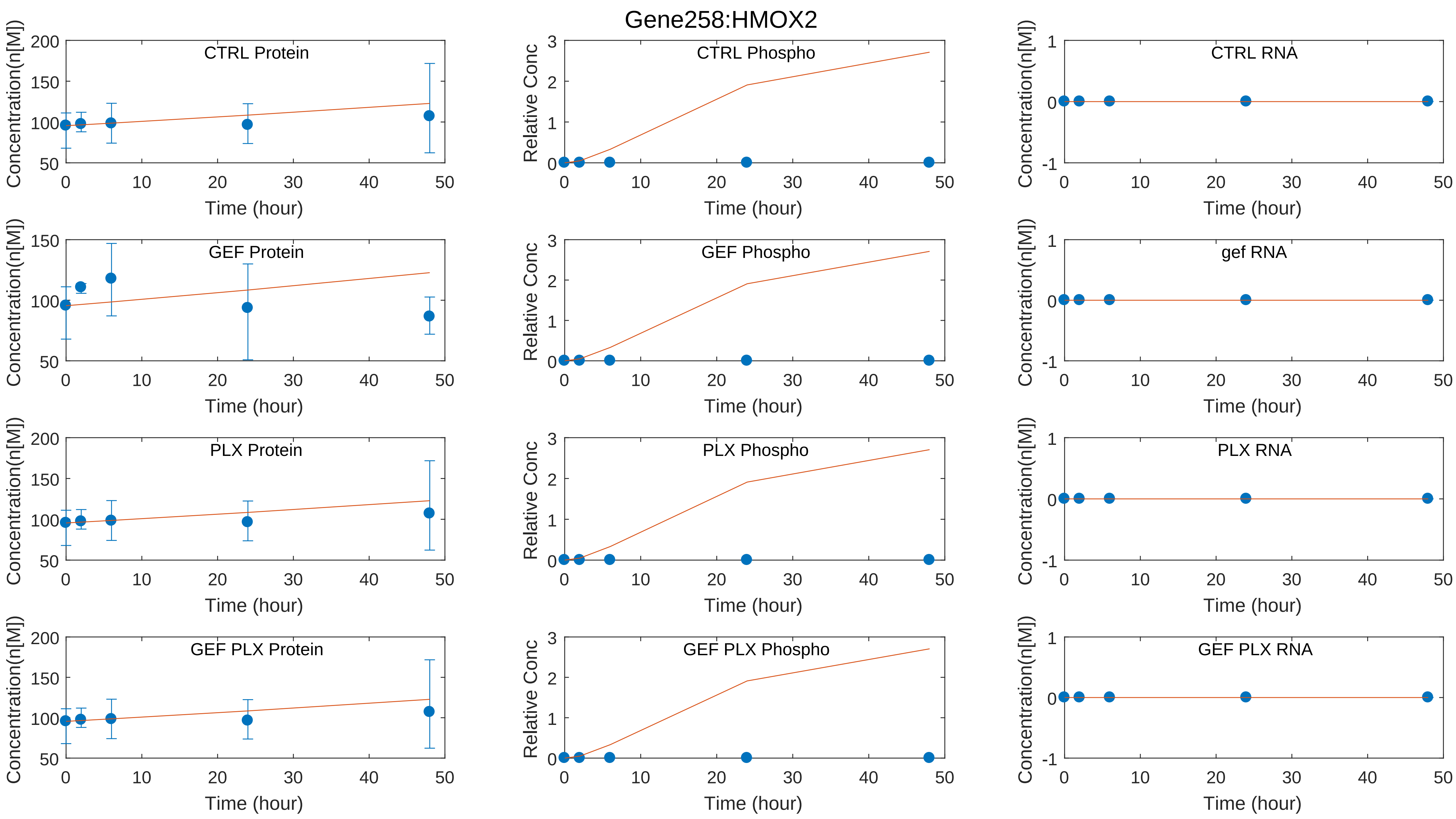
Gene258:HMOX2
CTRL Protein
GEF Protein
PLX Protein
GEF PLX Protein
CTRL Phospho
GEF Phospho
PLX Phospho
GEF PLX Phospho
CTRL RNA
gef RNA
PLX RNA
GEF PLX RNA
Concentration(n[M])
Relative Conc
Time (hour)

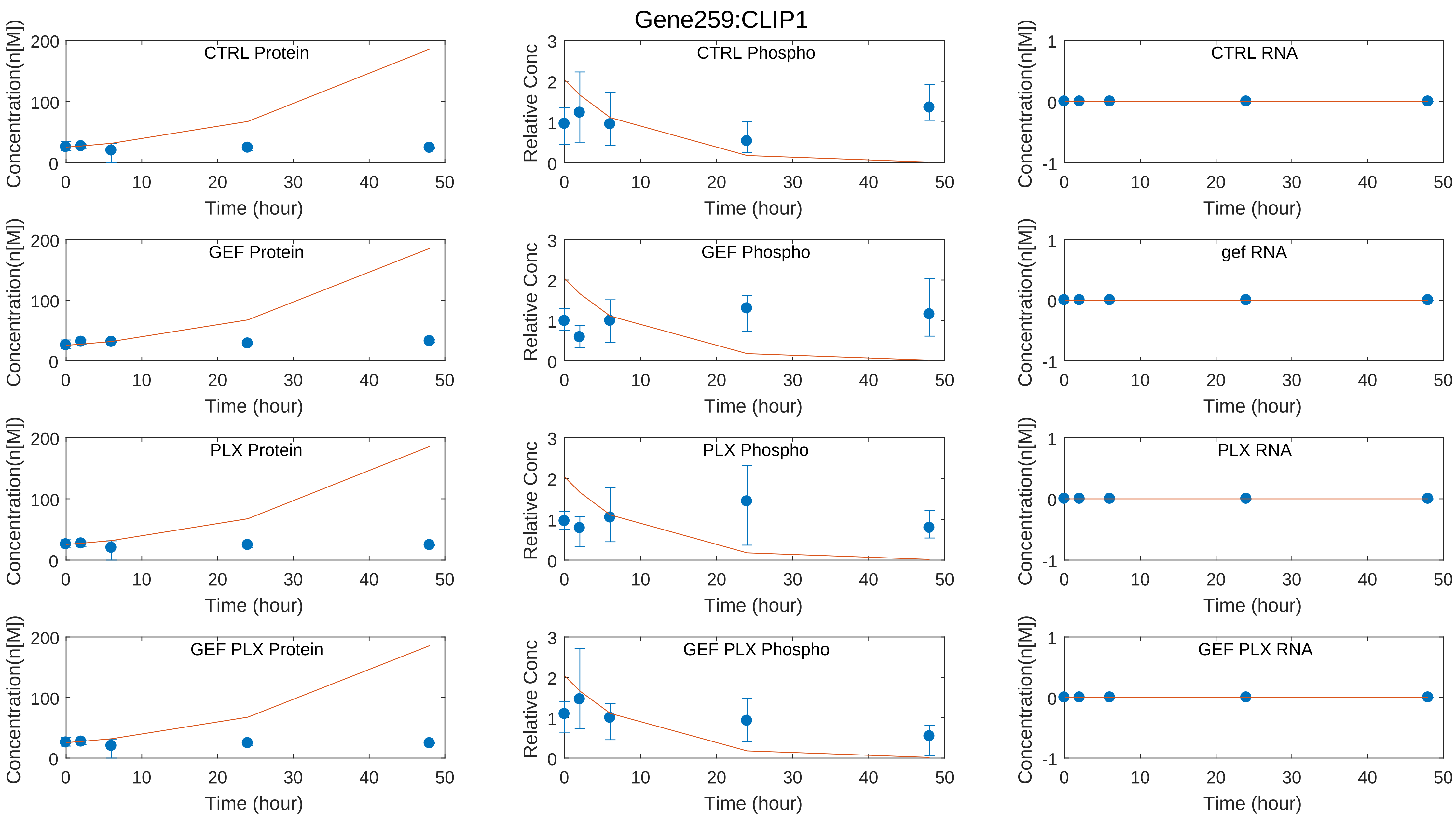
Gene259:CLIP1
CTRL Protein
GEF Protein
PLX Protein
GEF PLX Protein
CTRL Phospho
GEF Phospho
PLX Phospho
GEF PLX Phospho
CTRL RNA
gef RNA
PLX RNA
GEF PLX RNA
Concentration(n[M])
Relative Conc
Time (hour)

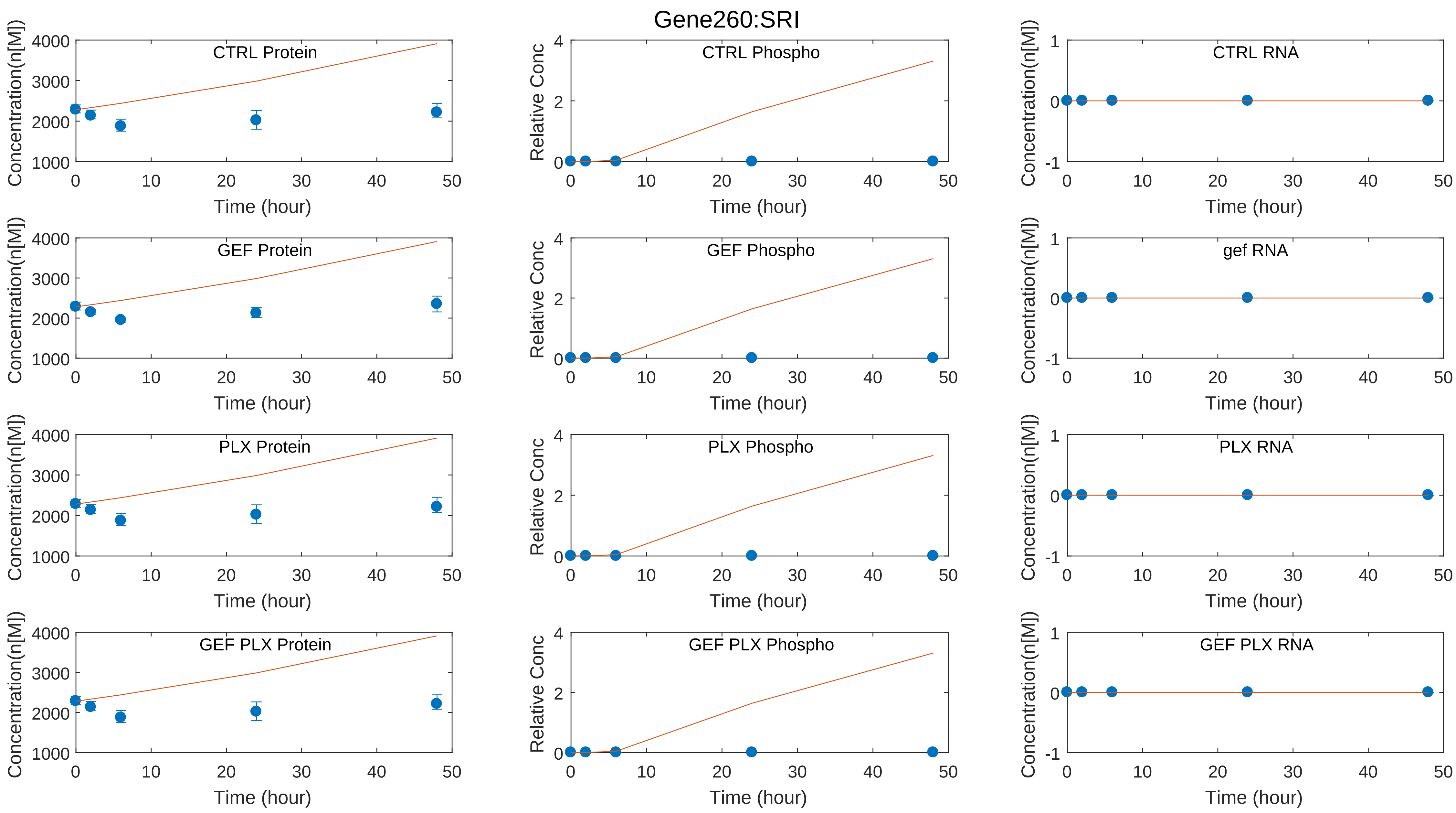

Gene260:SRI
CTRL Protein
CTRL Phospho
CTRL RNA
GEF Protein
GEF Phospho
gef RNA
PLX Protein
PLX Phospho
PLX RNA
GEF PLX Protein
GEF PLX Phospho
GEF PLX RNA
Concentration(n[M])
Relative Conc
Time (hour)

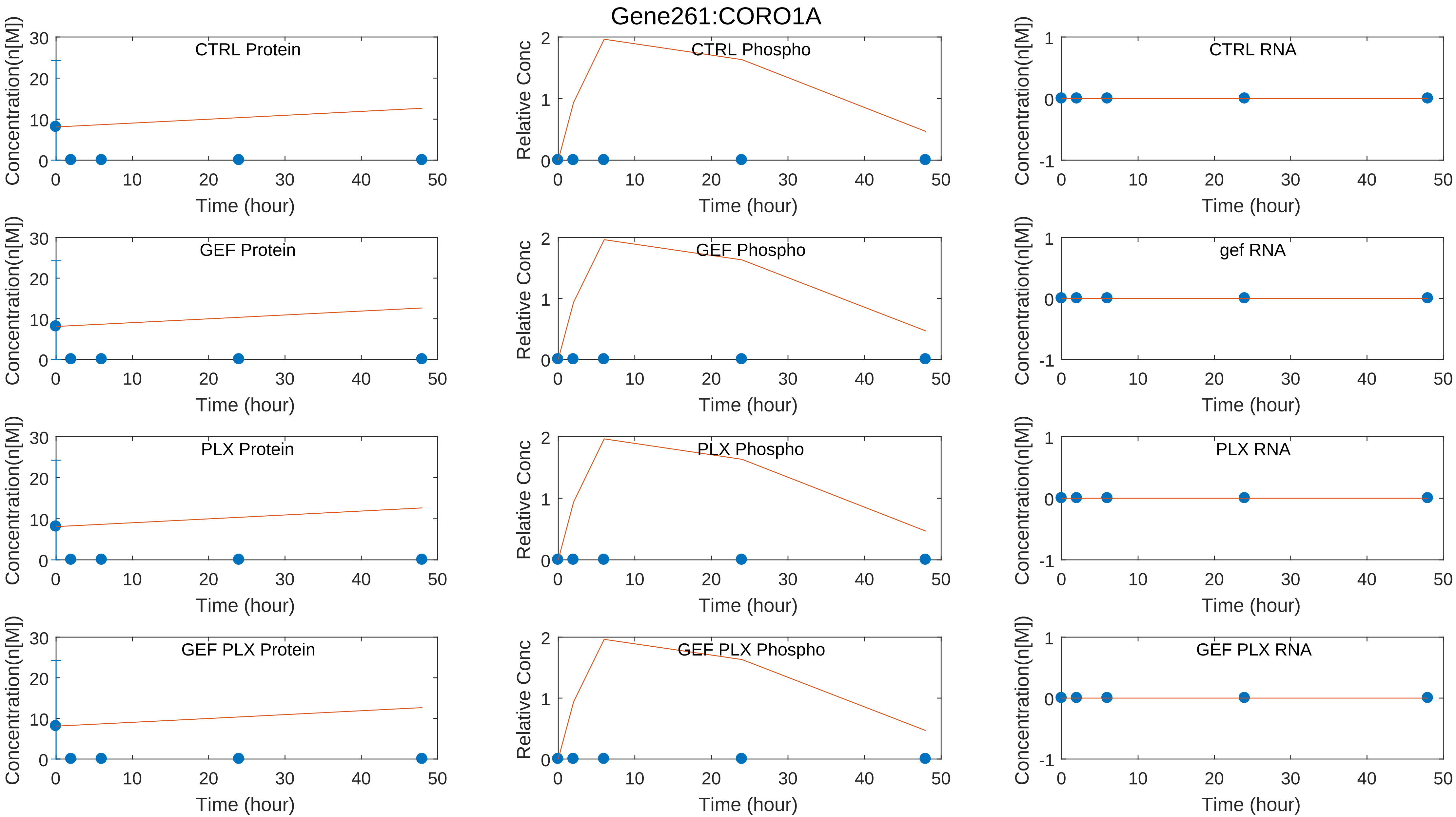

Gene261:CORO1A
CTRL Protein
CTRL Phospho
CTRL RNA
GEF Protein
GEF Phospho
gef RNA
PLX Protein
PLX Phospho
PLX RNA
GEF PLX Protein
GEF PLX Phospho
GEF PLX RNA
Concentration(n[M])
Relative Conc
Time (hour)

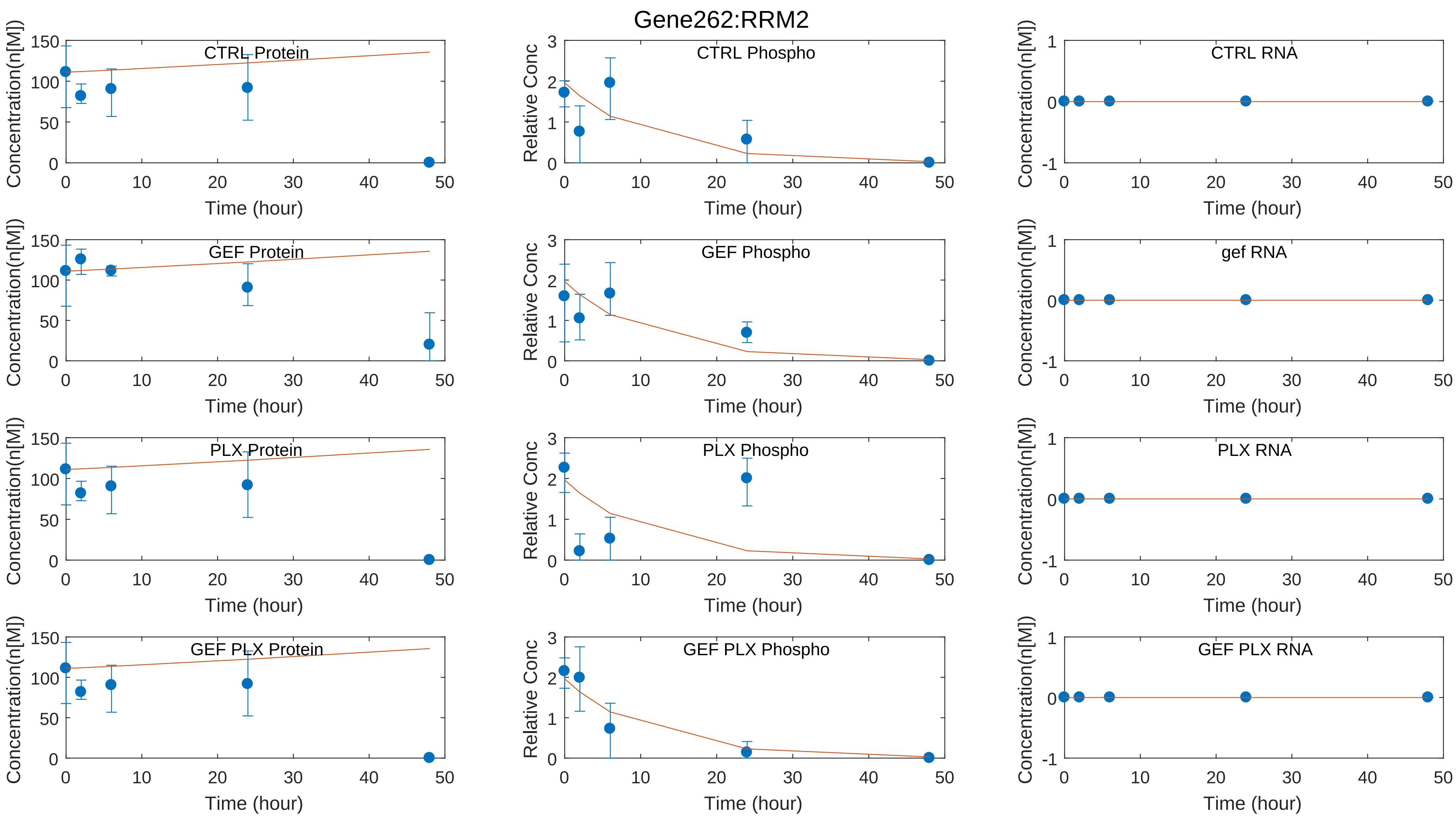
Gene262:RRM2
CTRL Protein
CTRL Phospho
CTRL RNA
GEF Protein
GEF Phospho
gef RNA
PLX Protein
PLX Phospho
PLX RNA
GEF PLX Protein
GEF PLX Phospho
GEF PLX RNA
Concentration(n[M])
Relative Conc
Time (hour)

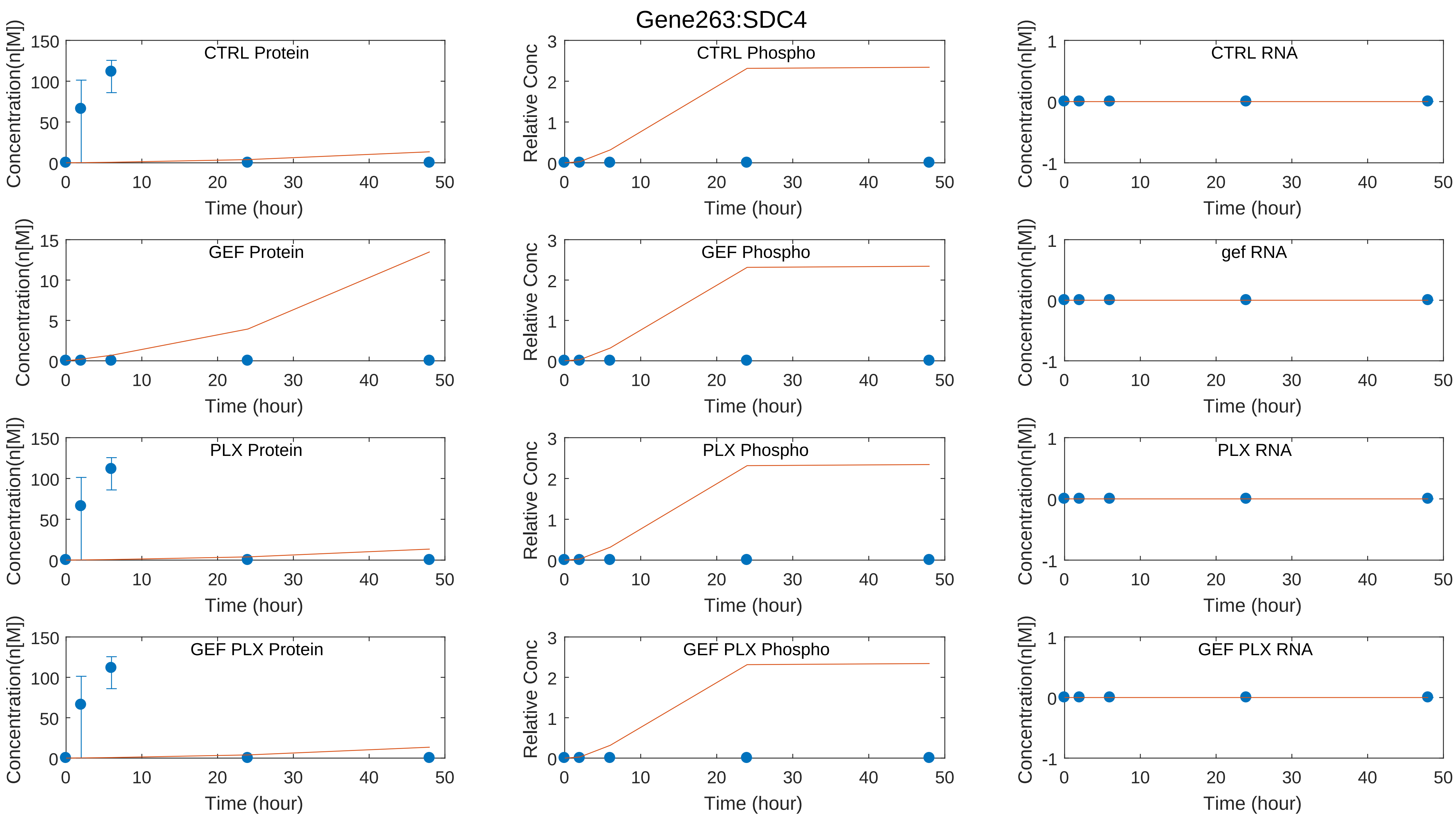
Gene263:SDC4
CTRL Protein
CTRL Phospho
CTRL RNA
GEF Protein
GEF Phospho
gef RNA
PLX Protein
PLX Phospho
PLX RNA
GEF PLX Protein
GEF PLX Phospho
GEF PLX RNA
Concentration(n[M])
Relative Conc
Time (hour)

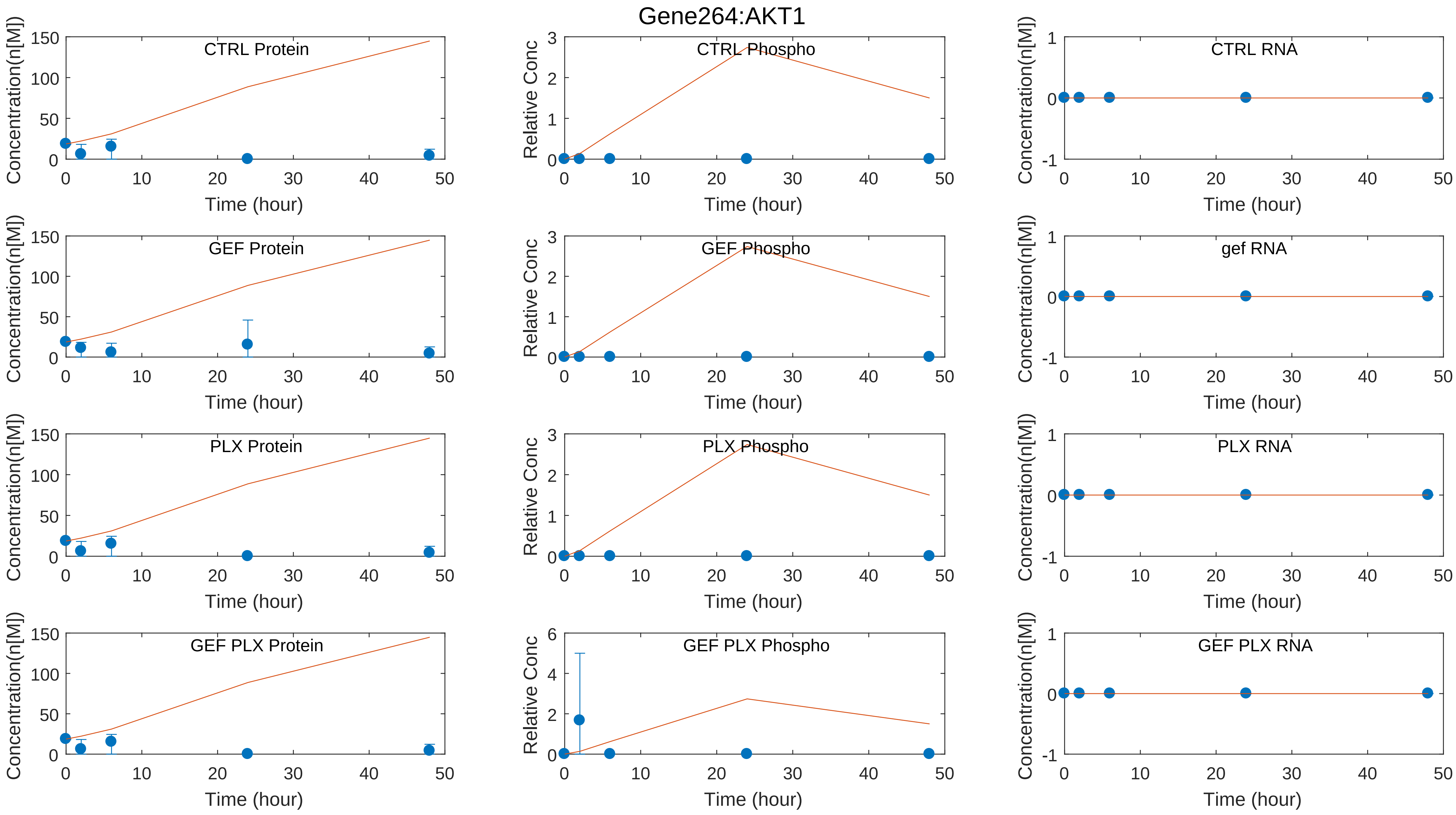
Gene264:AKT1
CTRL Protein
CTRL Phospho
CTRL RNA
GEF Protein
GEF Phospho
gef RNA
PLX Protein
PLX Phospho
PLX RNA
GEF PLX Protein
GEF PLX Phospho
GEF PLX RNA
Concentration(n[M])
Relative Conc
Time (hour)

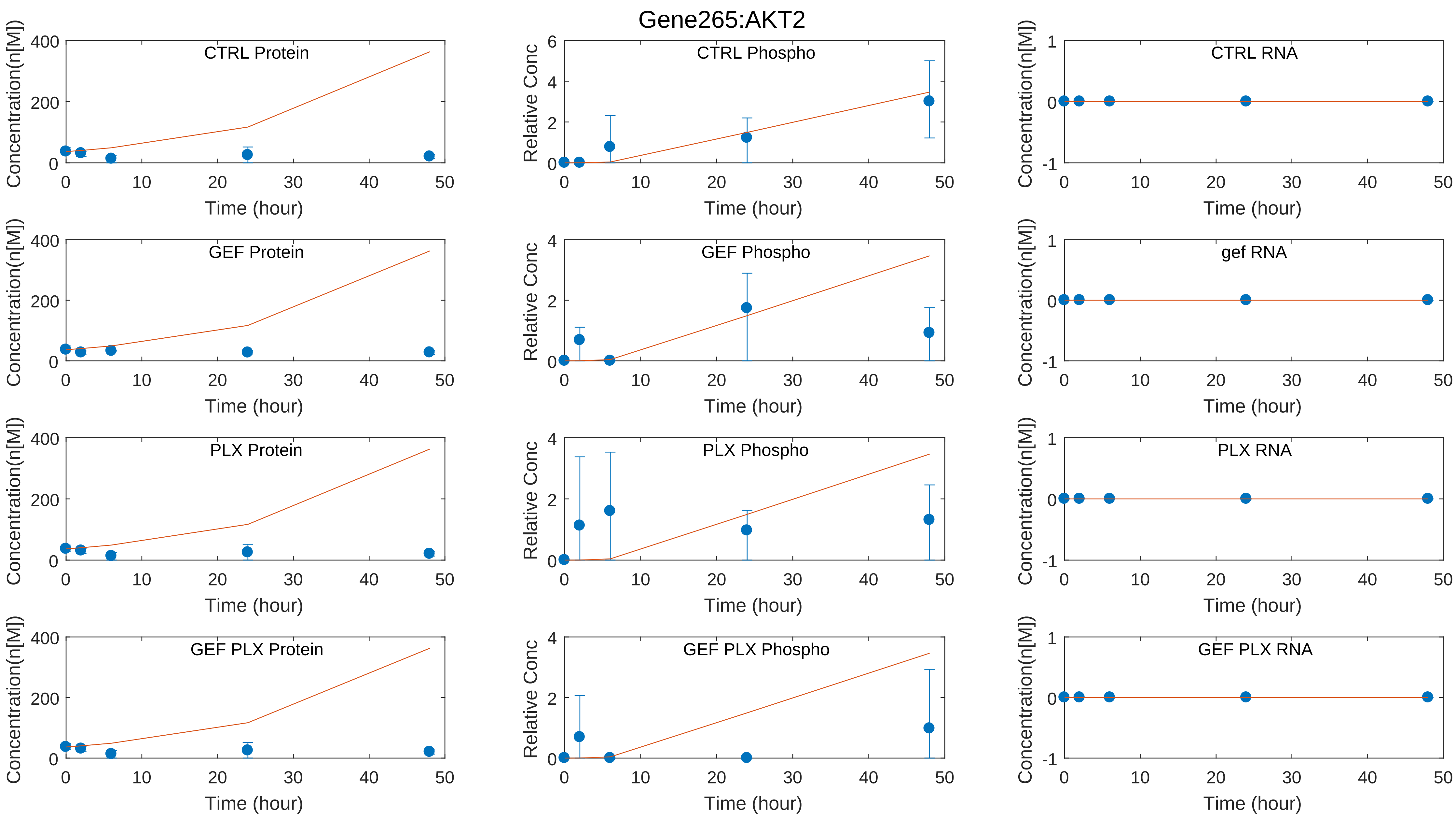
Gene265:AKT2
CTRL Protein
CTRL Phospho
CTRL RNA
GEF Protein
GEF Phospho
gef RNA
PLX Protein
PLX Phospho
PLX RNA
GEF PLX Protein
GEF PLX Phospho
GEF PLX RNA
Concentration(n[M])
Relative Conc
Time (hour)
0
10
20
30
40
50
200
400
2
4
6
1
-1

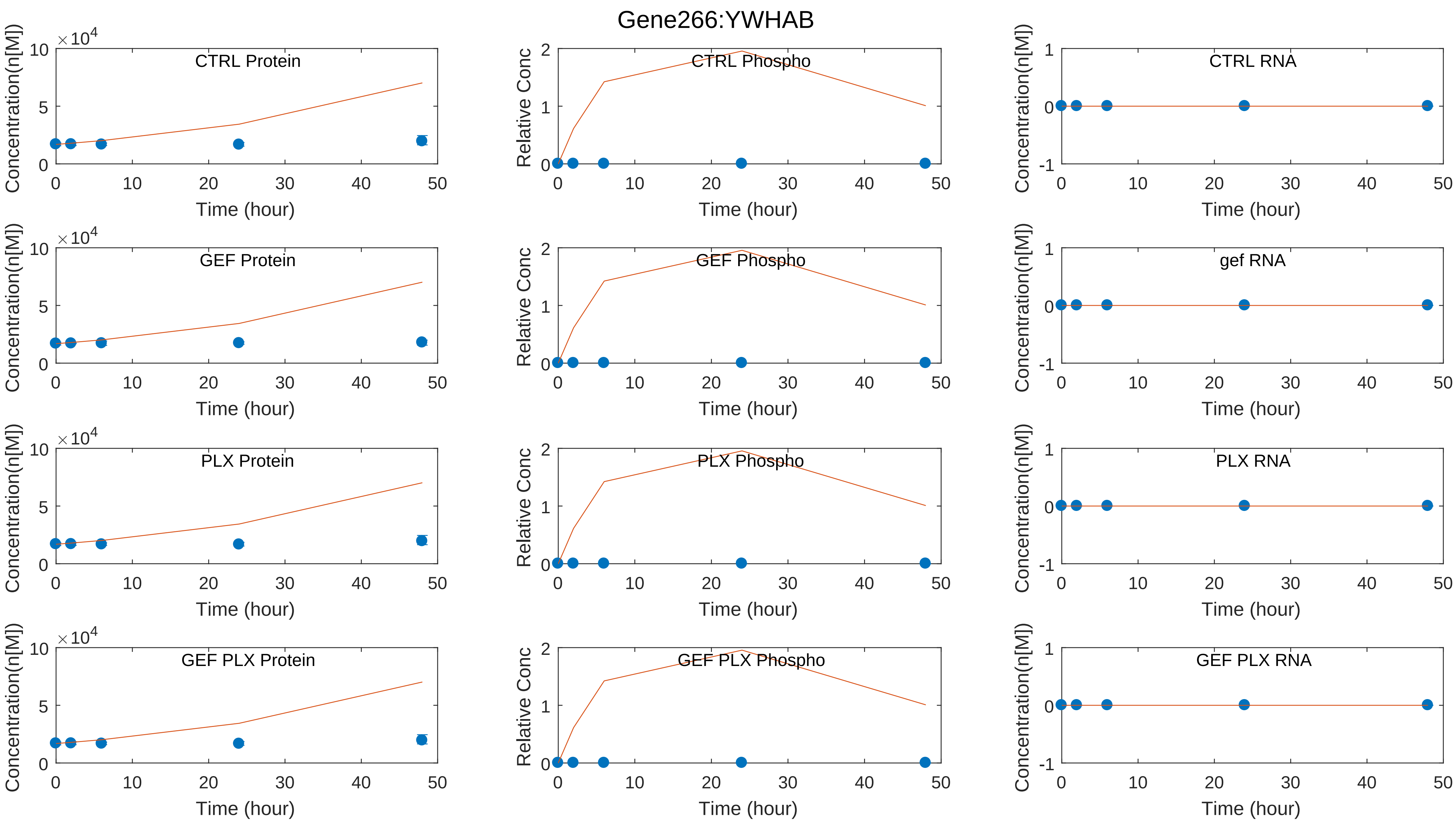
Gene266:YWHAB
CTRL Protein
GEF Protein
PLX Protein
GEF PLX Protein
CTRL Phospho
GEF Phospho
PLX Phospho
GEF PLX Phospho
CTRL RNA
gef RNA
PLX RNA
GEF PLX RNA
Concentration(n[M])
Relative Conc
Time (hour)
×10^4

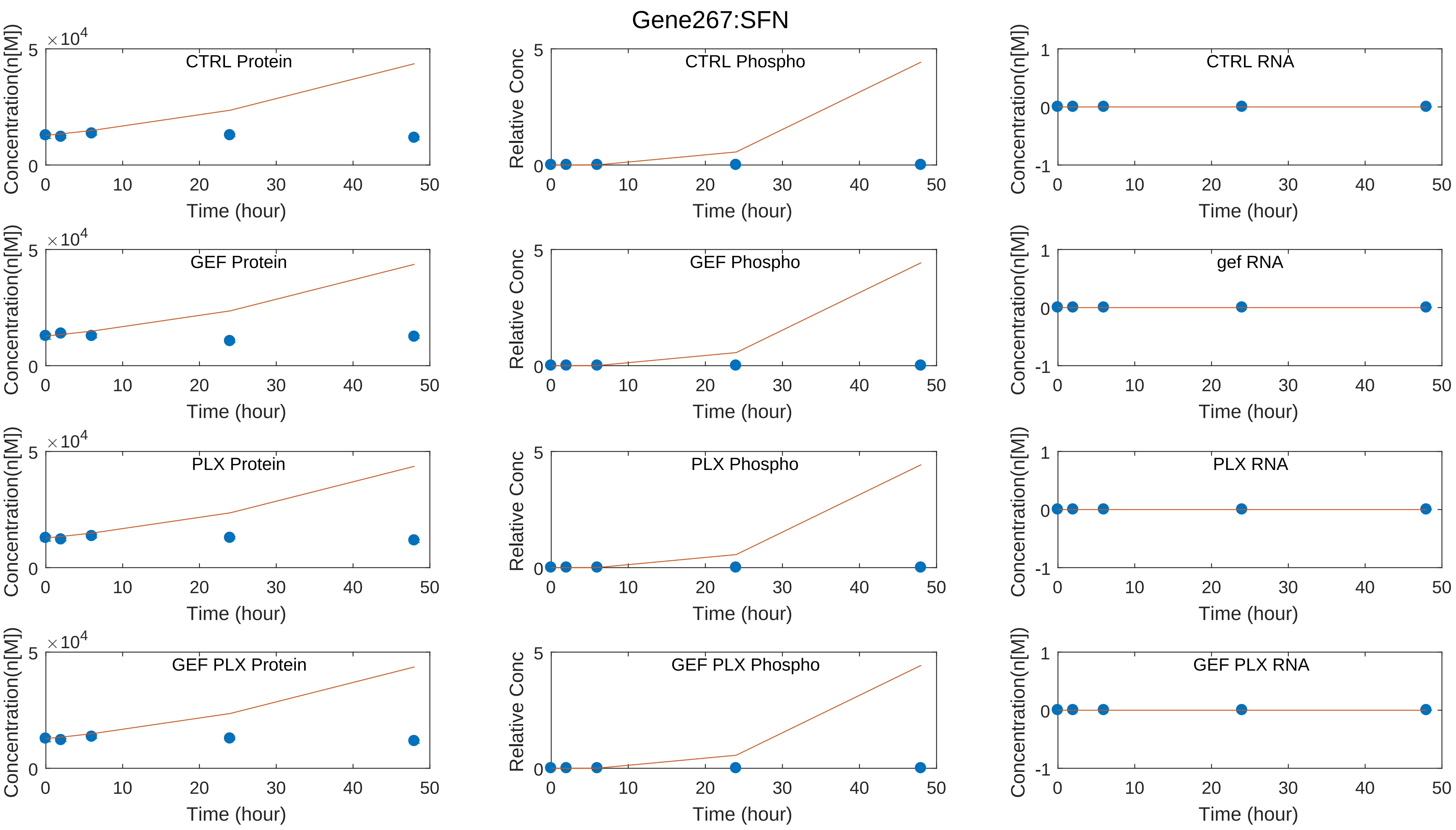
Gene267:SFN
CTRL Protein
GEF Protein
PLX Protein
GEF PLX Protein
CTRL Phospho
GEF Phospho
PLX Phospho
GEF PLX Phospho
CTRL RNA
gef RNA
PLX RNA
GEF PLX RNA
Concentration(n[M])
Relative Conc
Time (hour)
×10^4

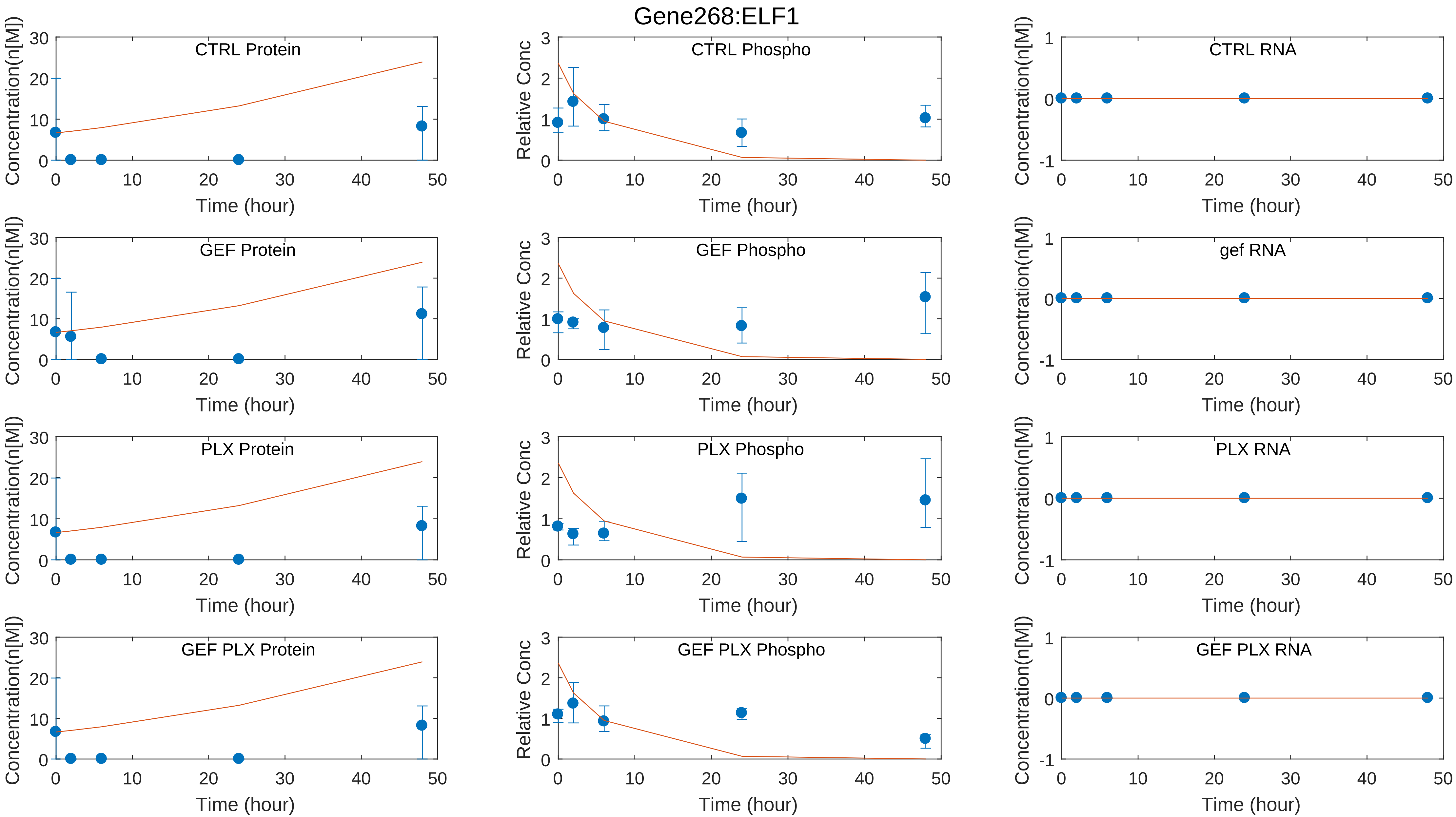

Gene268:ELF1
CTRL Protein
GEF Protein
PLX Protein
GEF PLX Protein
CTRL Phospho
GEF Phospho
PLX Phospho
GEF PLX Phospho
CTRL RNA
gef RNA
PLX RNA
GEF PLX RNA
Concentration(n[M])
Relative Conc
Time (hour)

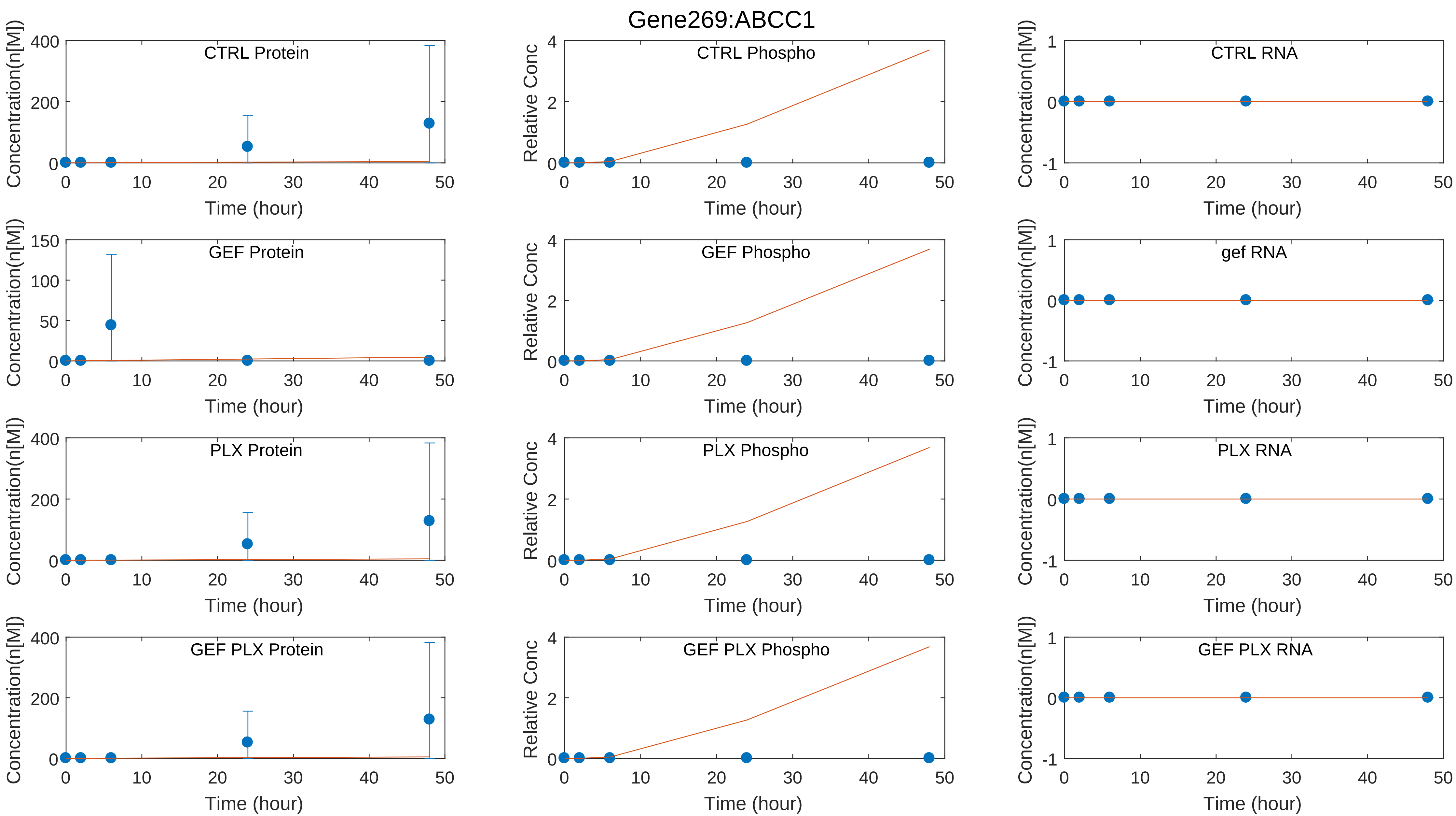

Gene269:ABCC1
CTRL Protein
GEF Protein
PLX Protein
GEF PLX Protein
CTRL Phospho
GEF Phospho
PLX Phospho
GEF PLX Phospho
CTRL RNA
gef RNA
PLX RNA
GEF PLX RNA
Concentration(n[M])
Relative Conc
Time (hour)

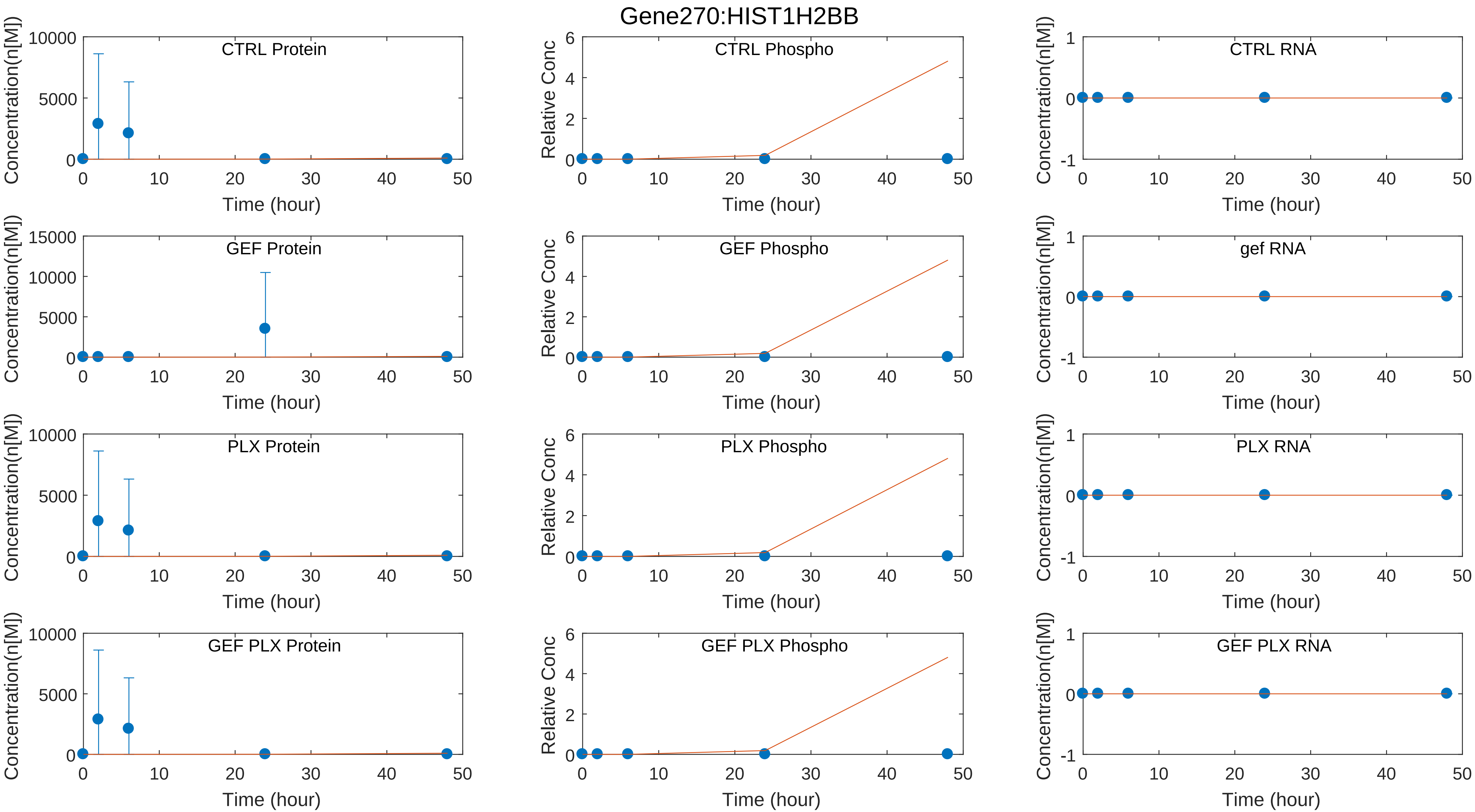
Gene270:HIST1H2BB
CTRL Protein
GEF Protein
PLX Protein
GEF PLX Protein
CTRL Phospho
GEF Phospho
PLX Phospho
GEF PLX Phospho
CTRL RNA
gef RNA
PLX RNA
GEF PLX RNA
Concentration(n[M])
Relative Conc
Time (hour)

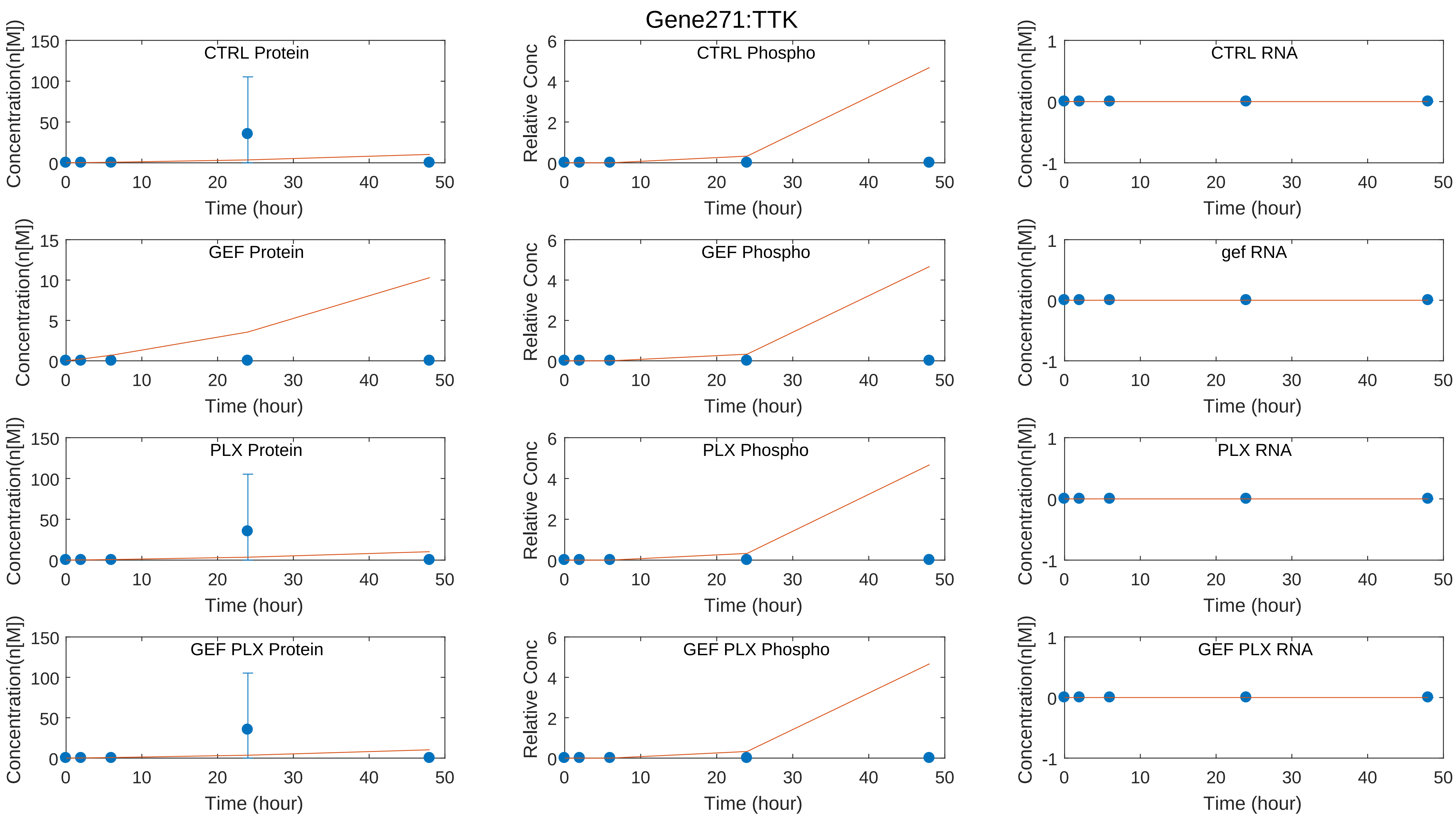

Gene271:TTK
CTRL Protein
CTRL Phospho
CTRL RNA
GEF Protein
GEF Phospho
gef RNA
PLX Protein
PLX Phospho
PLX RNA
GEF PLX Protein
GEF PLX Phospho
GEF PLX RNA
Concentration(n[M])
Relative Conc
Time (hour)

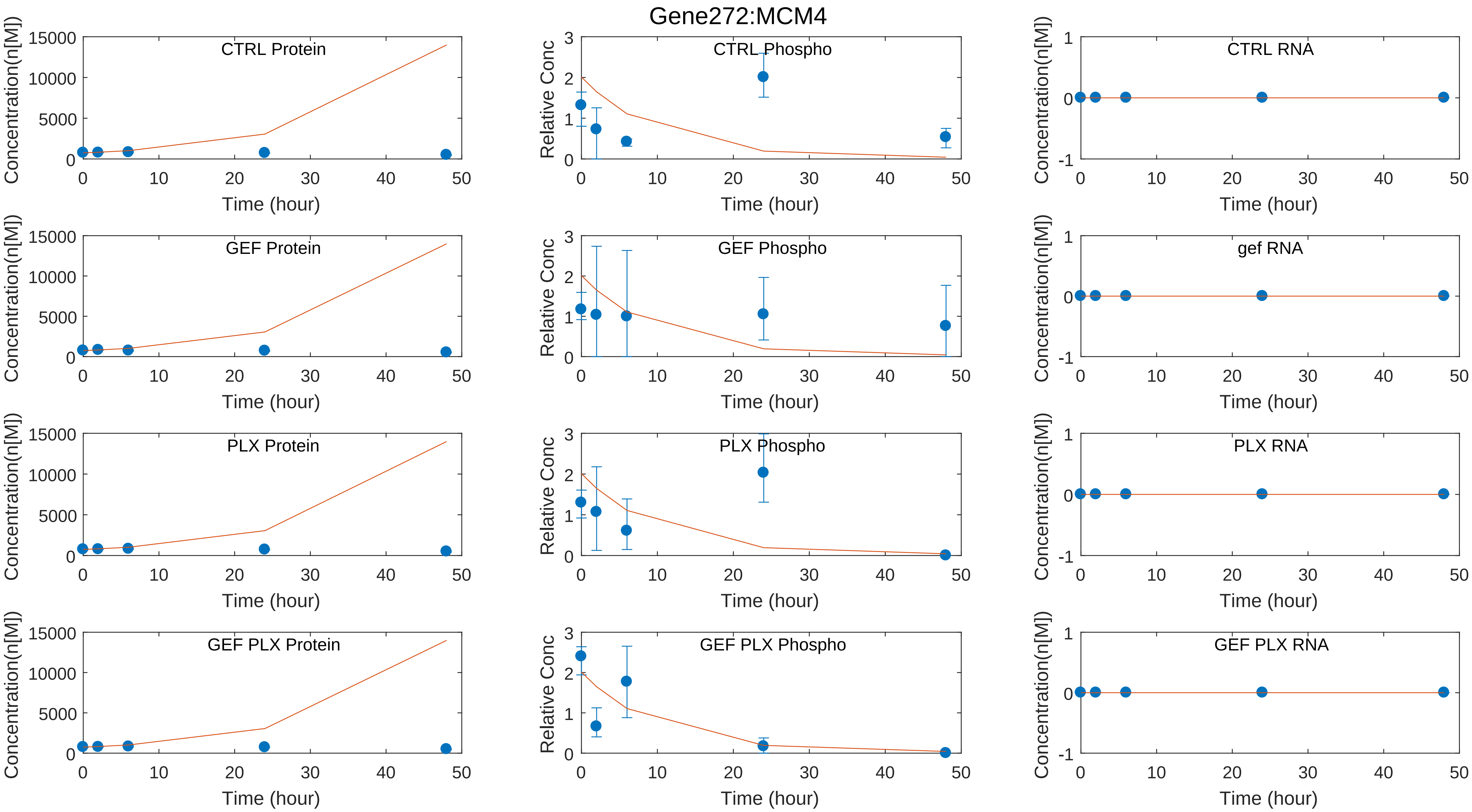

Gene272:MCM4
CTRL Protein
CTRL Phospho
CTRL RNA
GEF Protein
GEF Phospho
gef RNA
PLX Protein
PLX Phospho
PLX RNA
GEF PLX Protein
GEF PLX Phospho
GEF PLX RNA
Concentration(n[M])
Relative Conc
Time (hour)

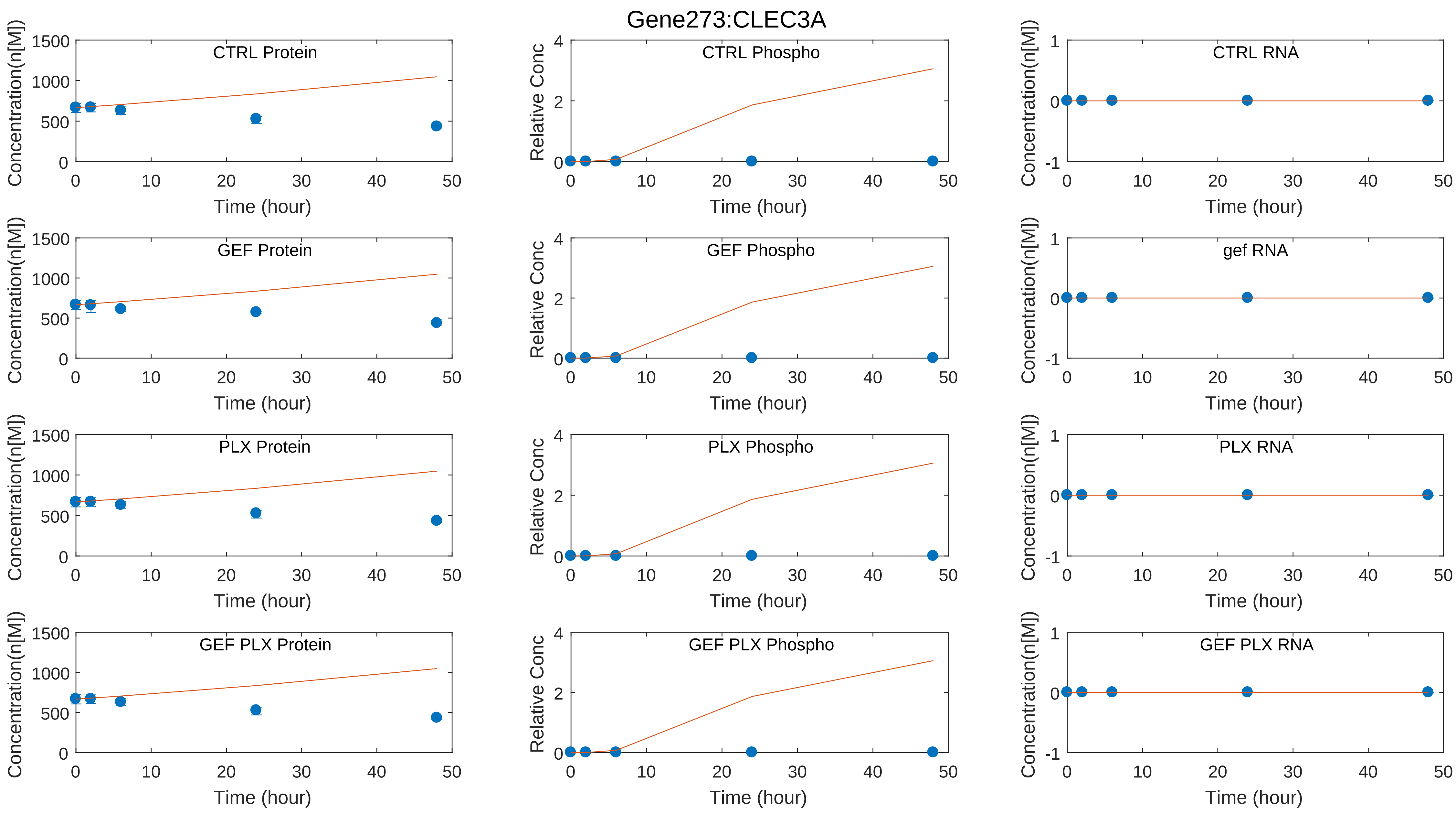

Gene273:CLEC3A
CTRL Protein
CTRL Phospho
CTRL RNA
GEF Protein
GEF Phospho
gef RNA
PLX Protein
PLX Phospho
PLX RNA
GEF PLX Protein
GEF PLX Phospho
GEF PLX RNA
Concentration(n[M])
Relative Conc
Time (hour)

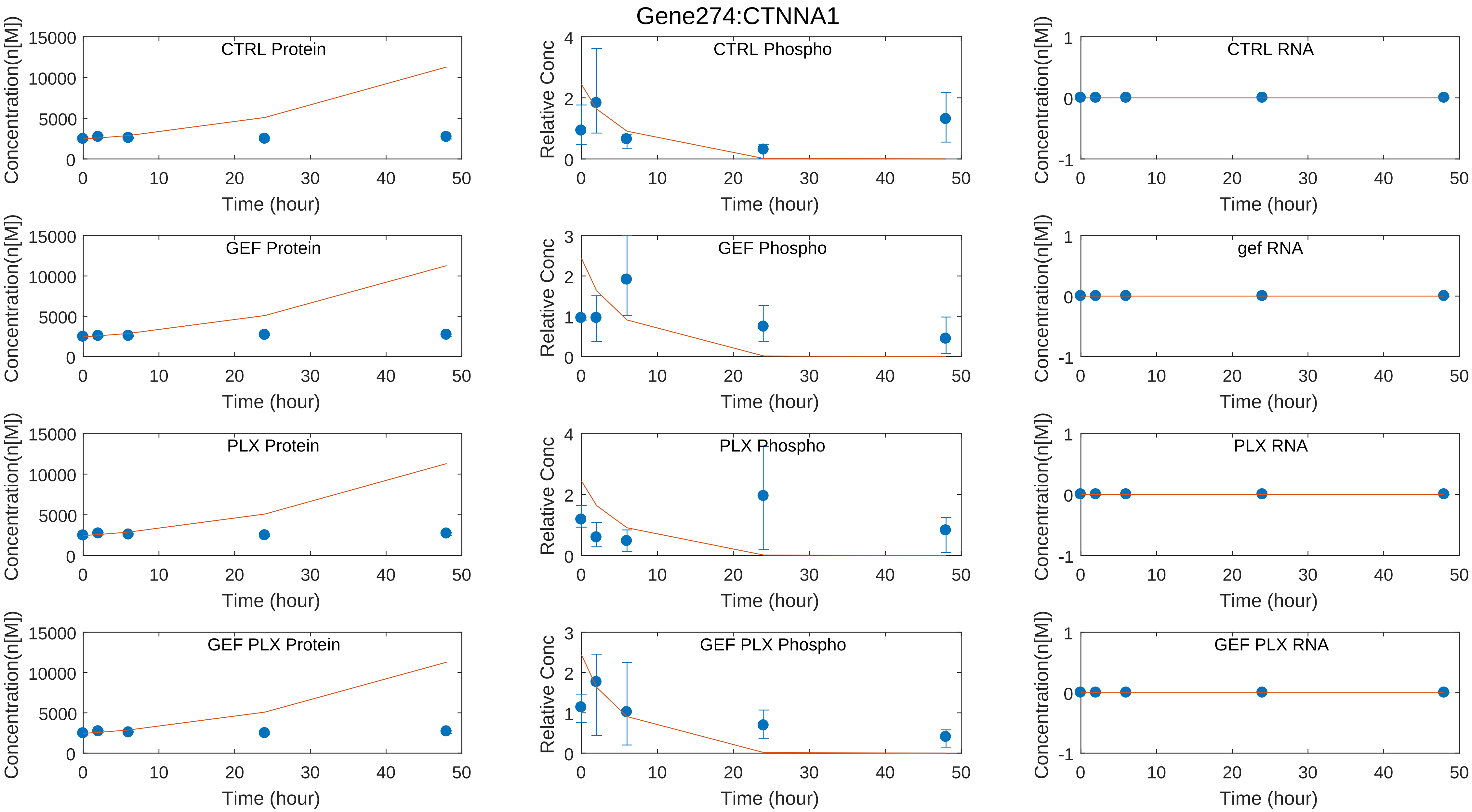
Gene274:CTNNA1
CTRL Protein
CTRL Phospho
CTRL RNA
GEF Protein
GEF Phospho
gef RNA
PLX Protein
PLX Phospho
PLX RNA
GEF PLX Protein
GEF PLX Phospho
GEF PLX RNA
Concentration(n[M])
Relative Conc
Time (hour)

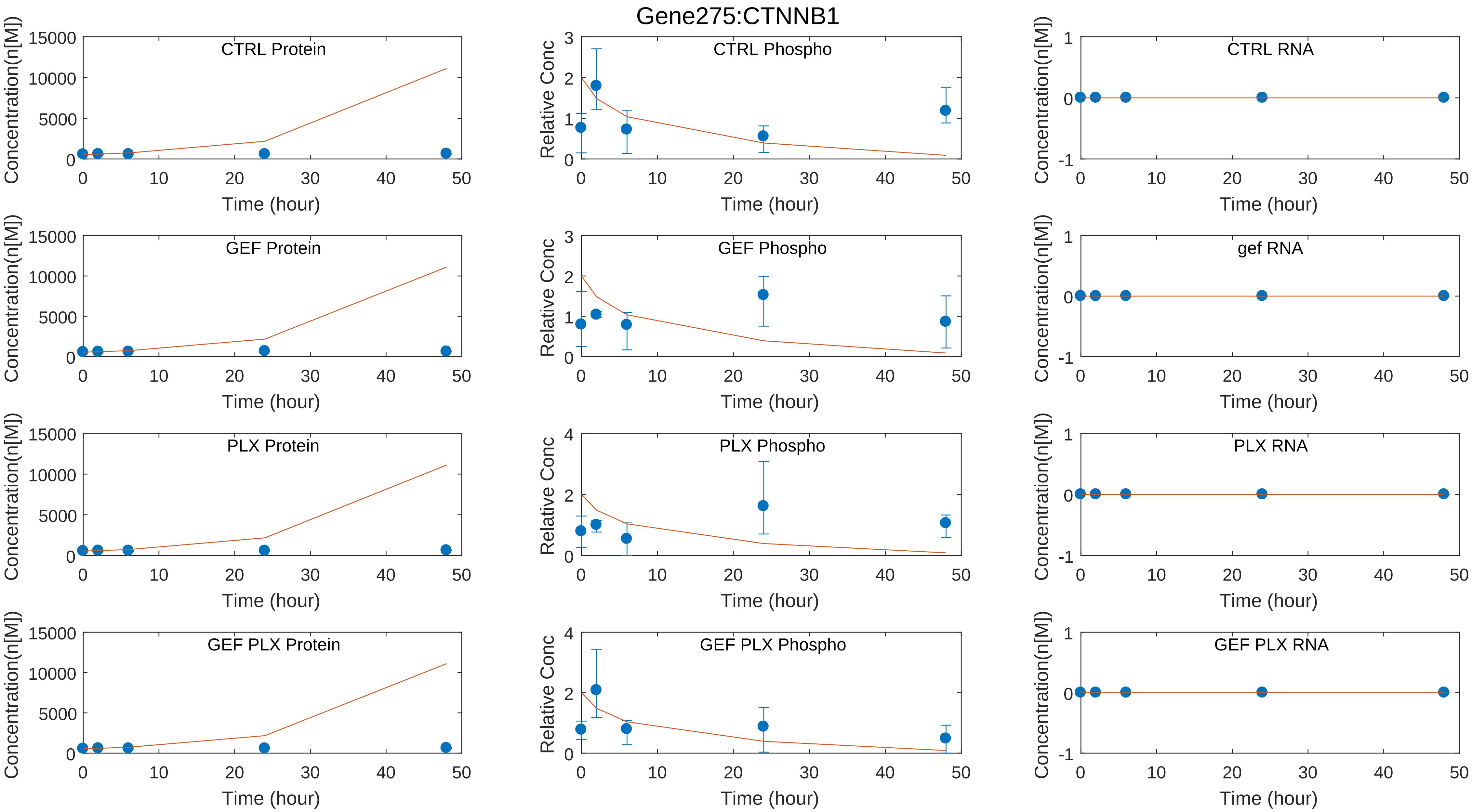
Gene275:CTNNB1
CTRL Protein
CTRL Phospho
CTRL RNA
GEF Protein
GEF Phospho
gef RNA
PLX Protein
PLX Phospho
PLX RNA
GEF PLX Protein
GEF PLX Phospho
GEF PLX RNA
Concentration(n[M])
Relative Conc
Time (hour)

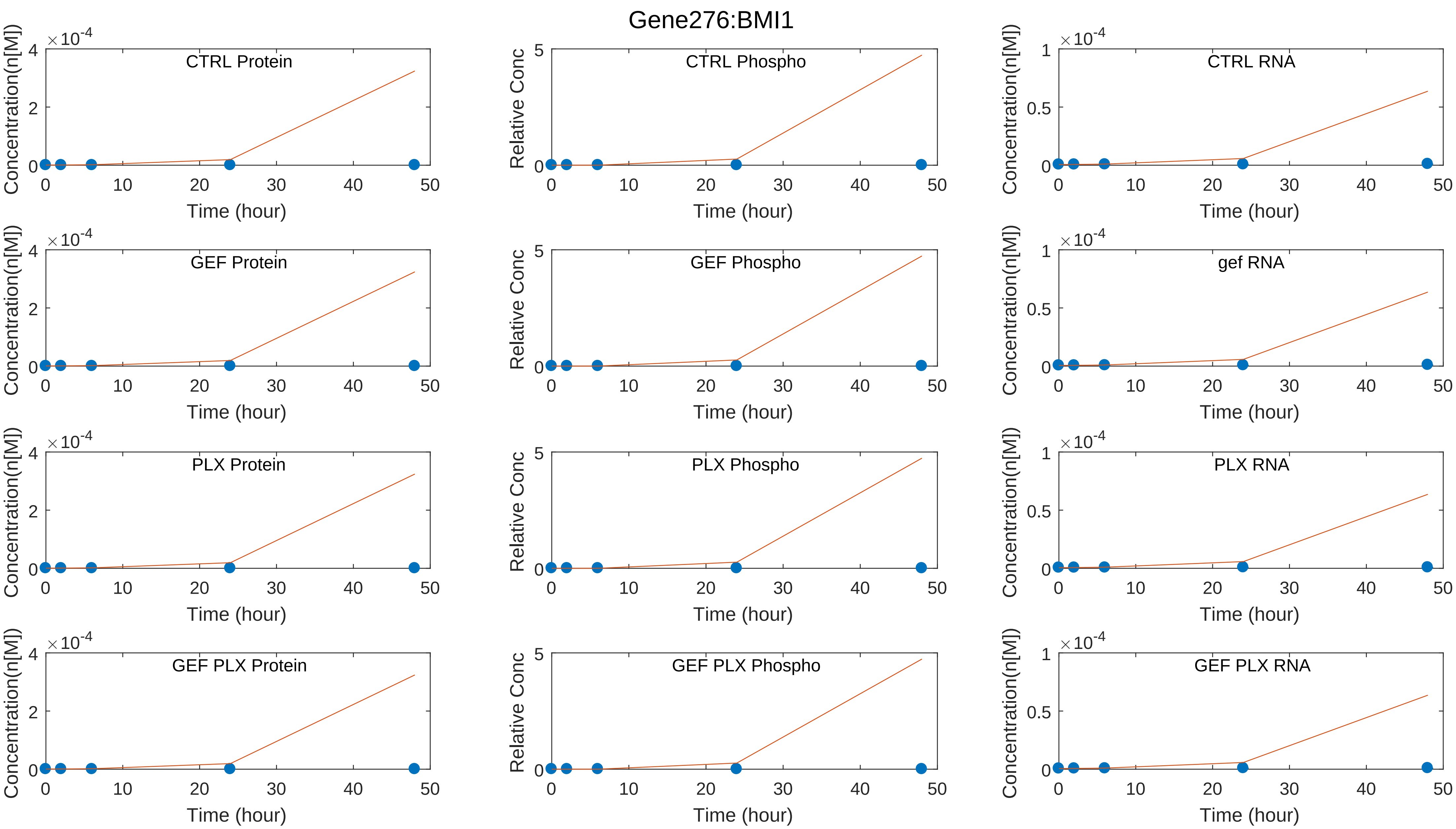
Gene276:BMI1
CTRL Protein
CTRL Phospho
CTRL RNA
GEF Protein
GEF Phospho
gef RNA
PLX Protein
PLX Phospho
PLX RNA
GEF PLX Protein
GEF PLX Phospho
GEF PLX RNA
Concentration(n[M])
Relative Conc
Time (hour)
×10-4

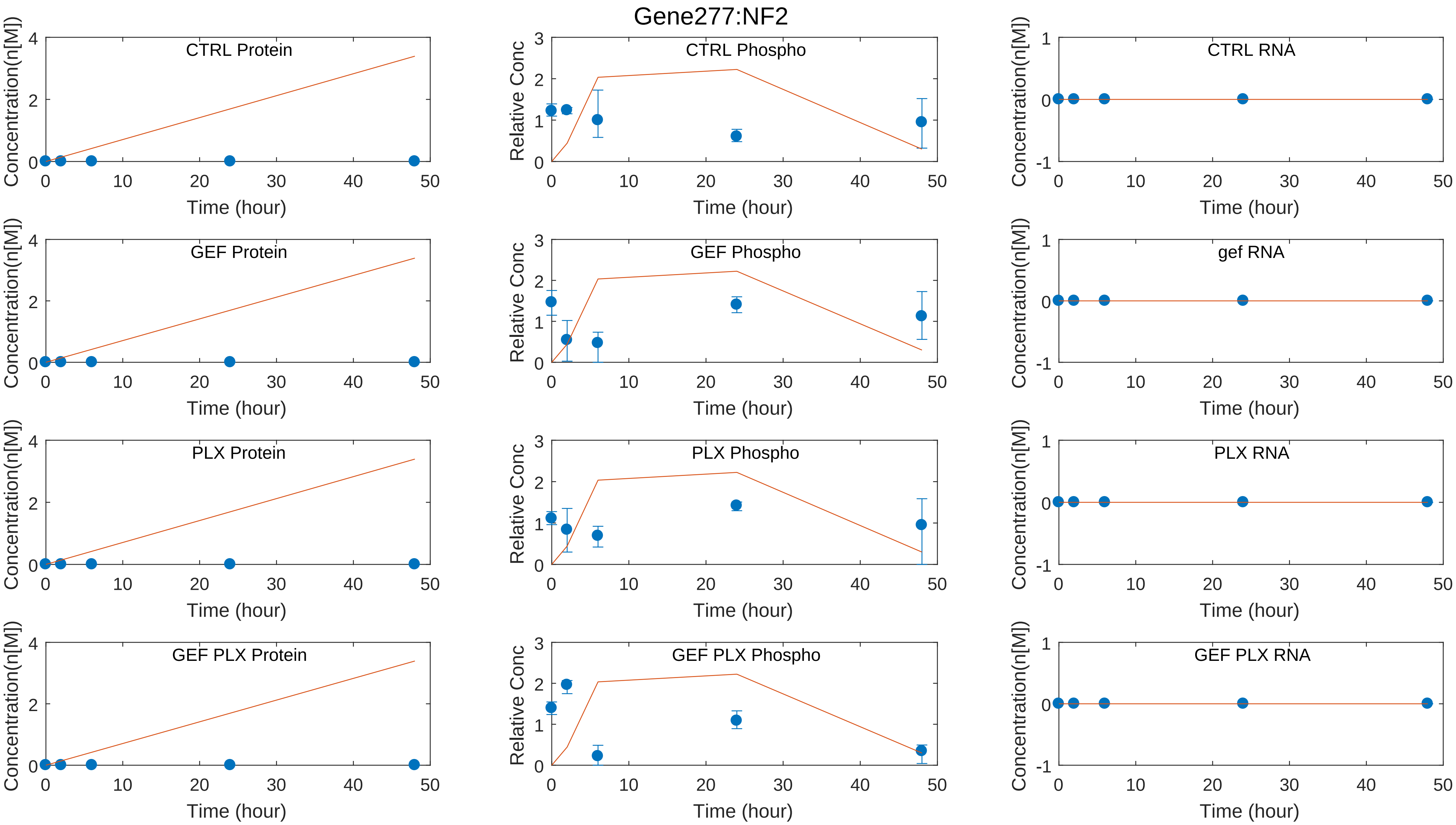

Gene277:NF2
CTRL Protein
GEF Protein
PLX Protein
GEF PLX Protein
CTRL Phospho
GEF Phospho
PLX Phospho
GEF PLX Phospho
CTRL RNA
gef RNA
PLX RNA
GEF PLX RNA
Concentration(n[M])
Relative Conc
Time (hour)
0
10
20
30
40
50

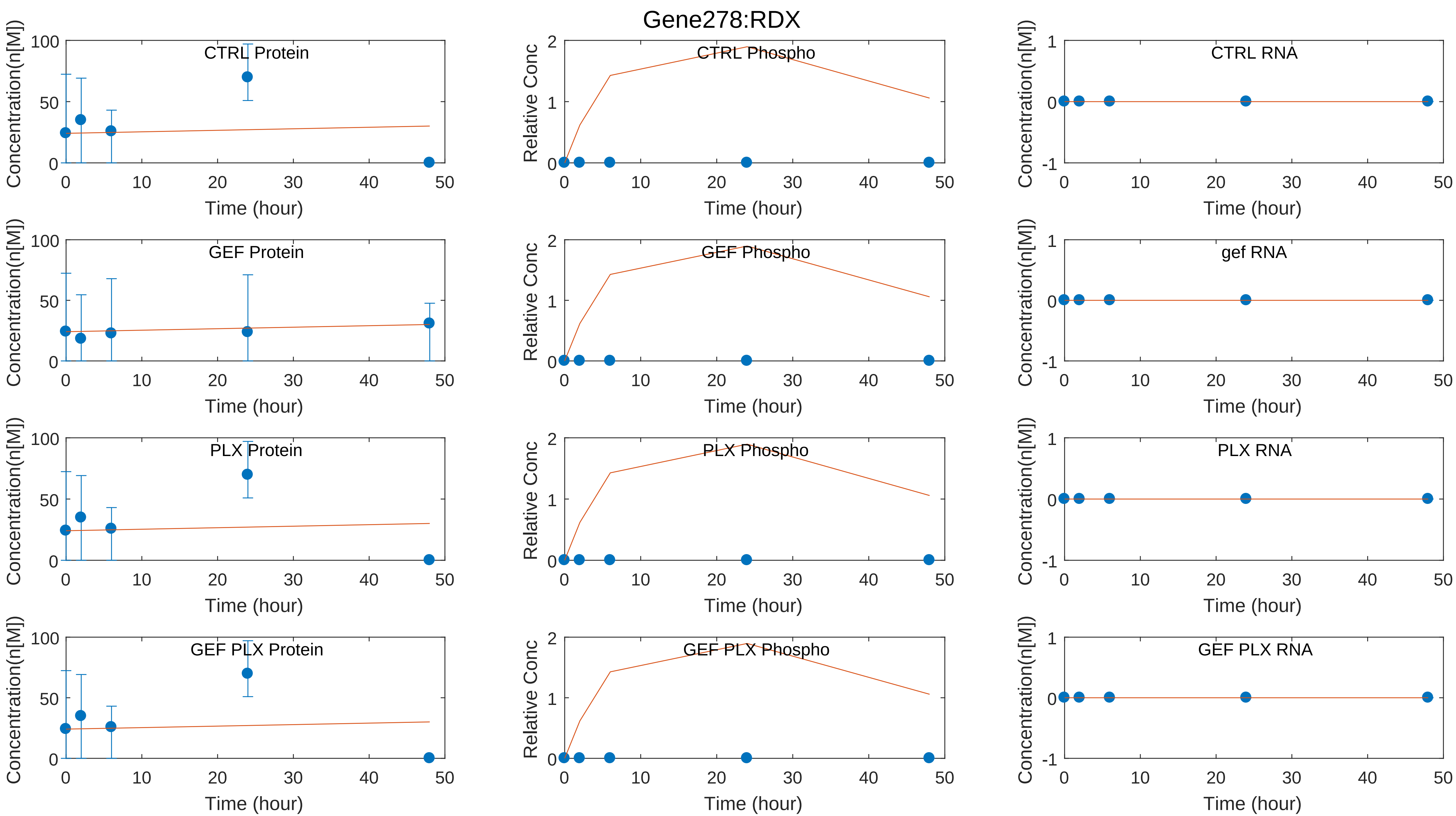

Gene278:RDX
CTRL Protein
CTRL Phospho
CTRL RNA
GEF Protein
GEF Phospho
gef RNA
PLX Protein
PLX Phospho
PLX RNA
GEF PLX Protein
GEF PLX Phospho
GEF PLX RNA
Concentration(n[M])
Relative Conc
Time (hour)

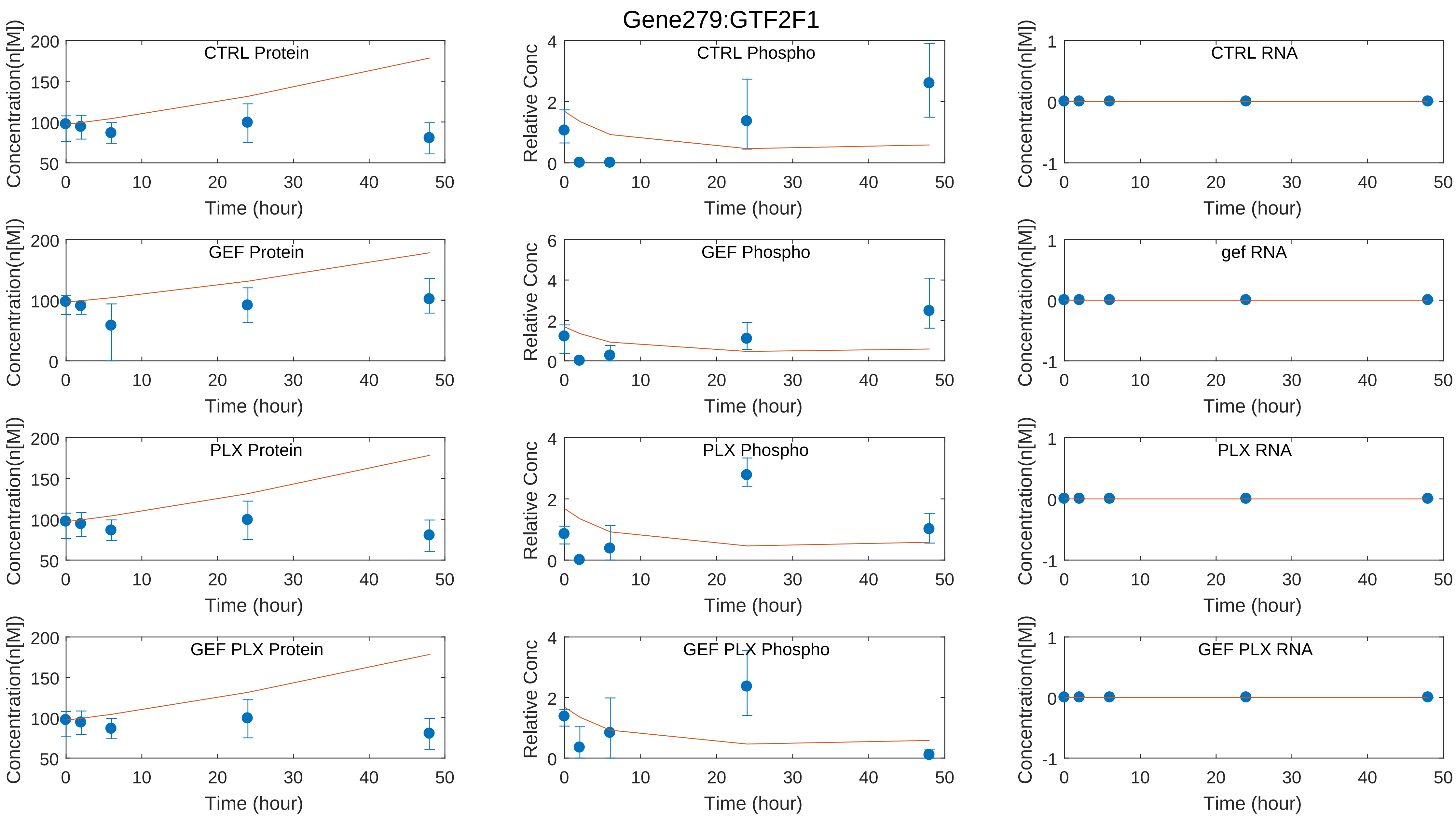

Gene279:GTF2F1
CTRL Protein
CTRL Phospho
CTRL RNA
GEF Protein
GEF Phospho
gef RNA
PLX Protein
PLX Phospho
PLX RNA
GEF PLX Protein
GEF PLX Phospho
GEF PLX RNA
Concentration(n[M])
Relative Conc
Time (hour)

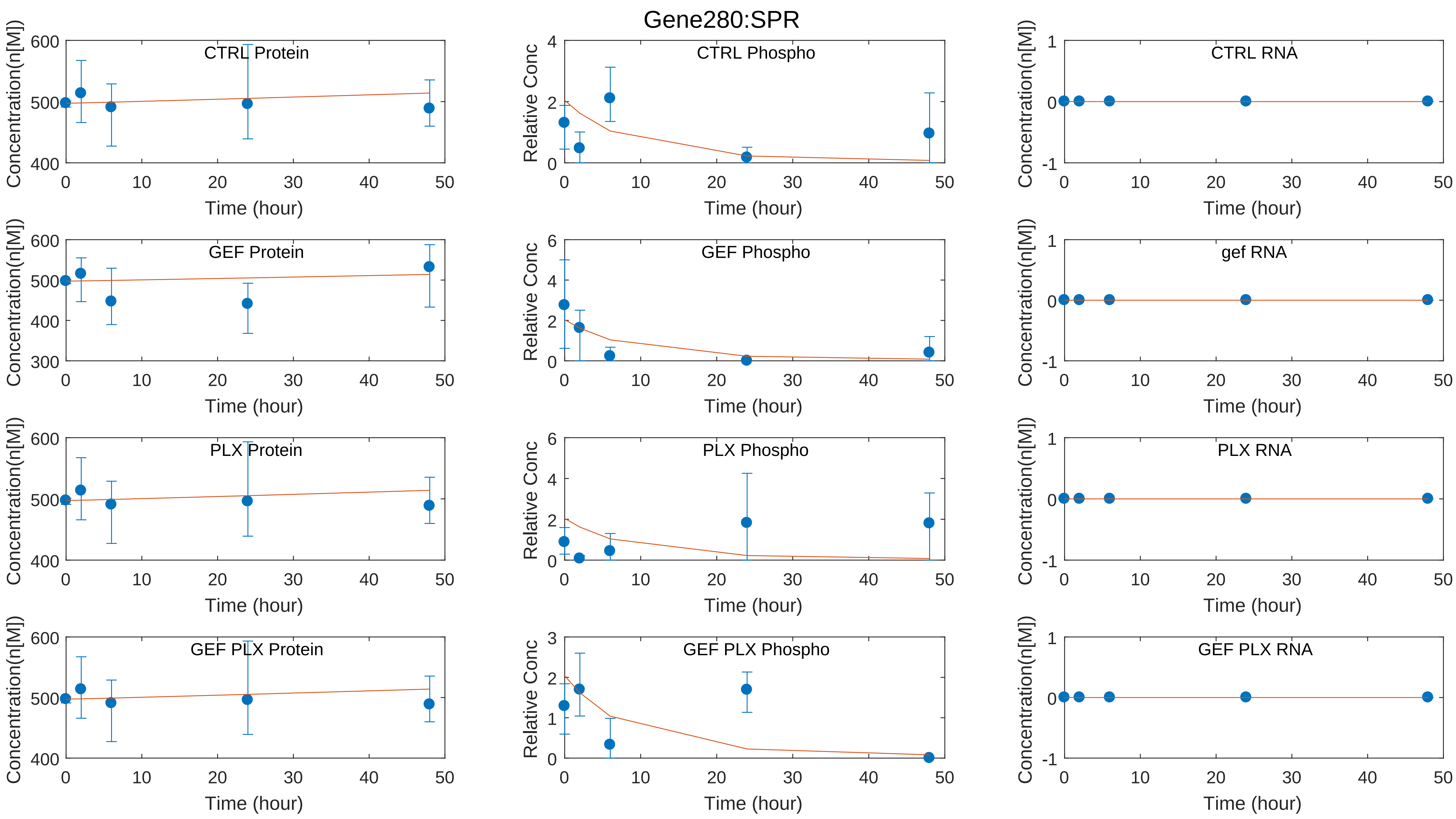

Gene280:SPR
CTRL Protein
CTRL Phospho
CTRL RNA
GEF Protein
GEF Phospho
gef RNA
PLX Protein
PLX Phospho
PLX RNA
GEF PLX Protein
GEF PLX Phospho
GEF PLX RNA
Concentration(n[M])
Relative Conc
Time (hour)

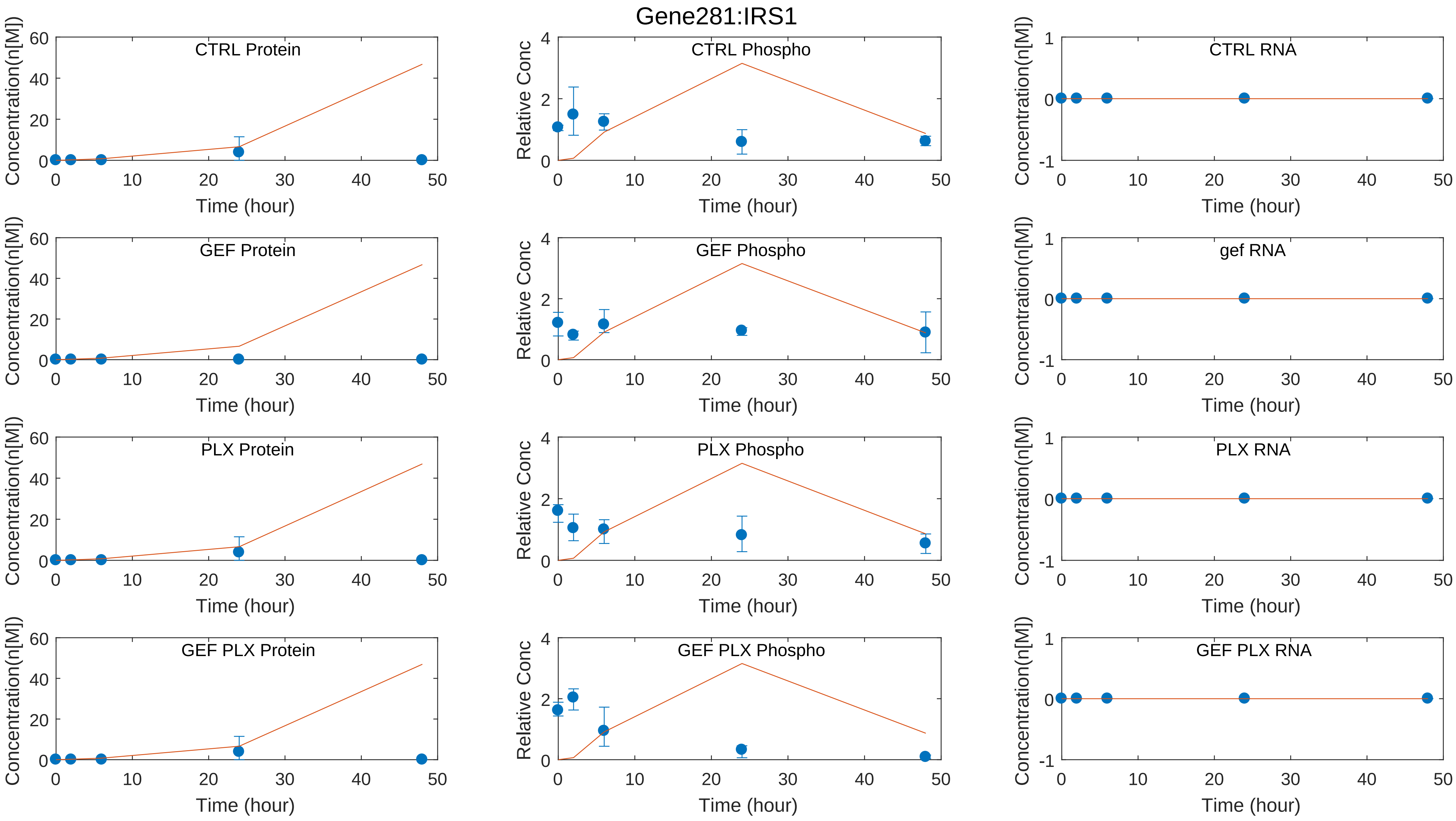

Gene281:IRS1
CTRL Protein
CTRL Phospho
CTRL RNA
GEF Protein
GEF Phospho
gef RNA
PLX Protein
PLX Phospho
PLX RNA
GEF PLX Protein
GEF PLX Phospho
GEF PLX RNA
Concentration(n[M])
Relative Conc
Time (hour)

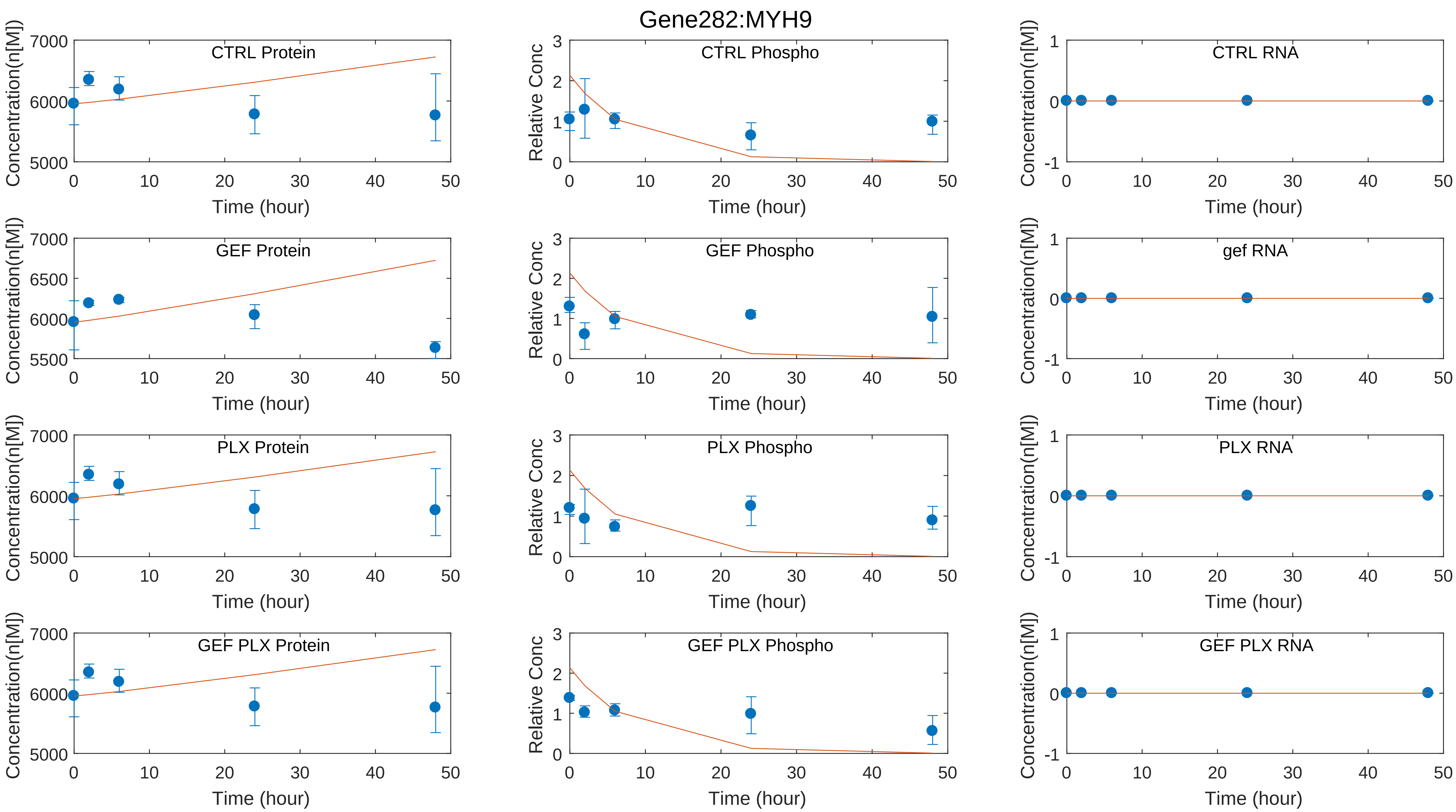

Gene282:MYH9
CTRL Protein
CTRL Phospho
CTRL RNA
GEF Protein
GEF Phospho
gef RNA
PLX Protein
PLX Phospho
PLX RNA
GEF PLX Protein
GEF PLX Phospho
GEF PLX RNA
Concentration(n[M])
Relative Conc
Time (hour)

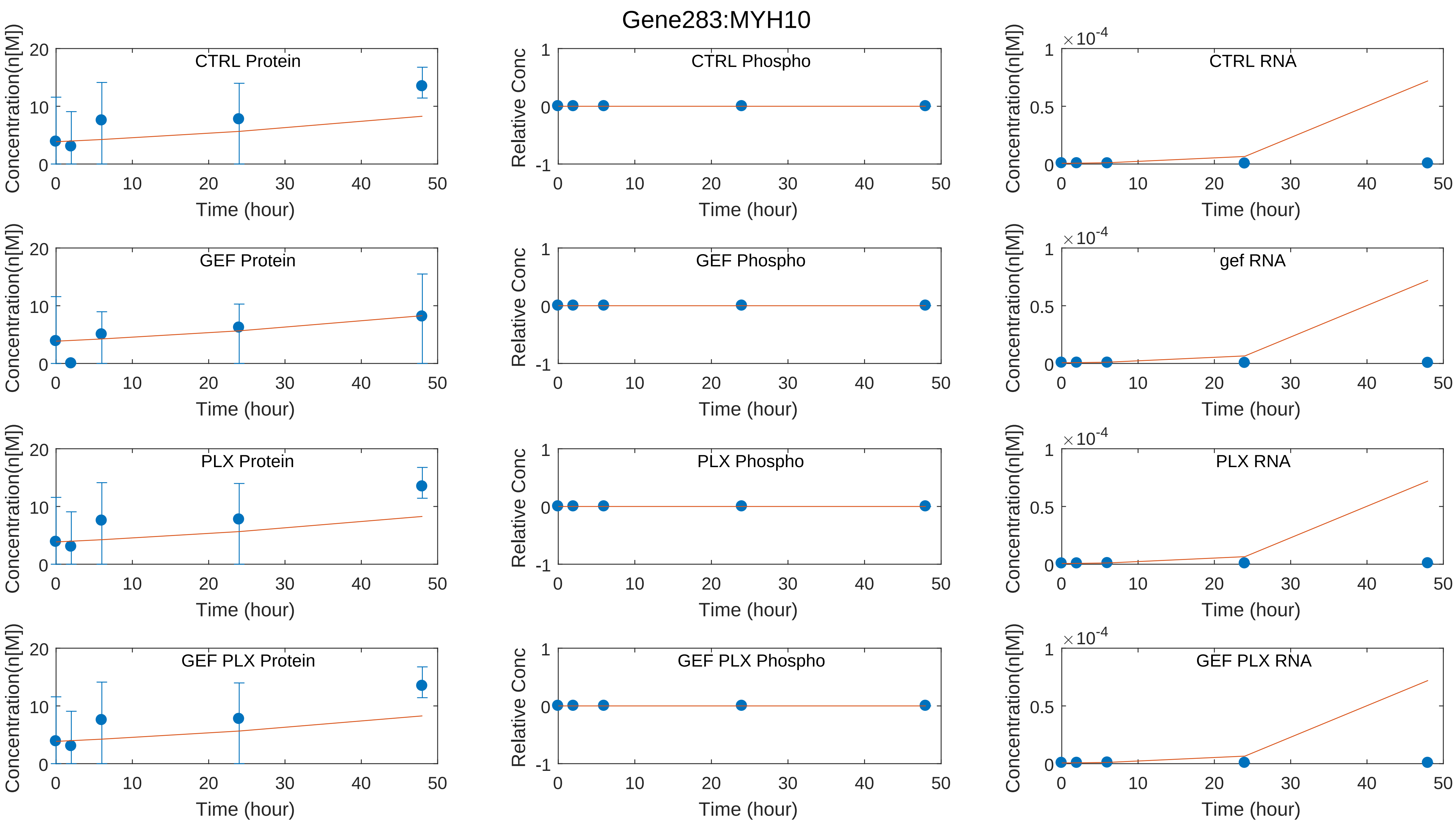

Gene283:MYH10
CTRL Protein
CTRL Phospho
CTRL RNA
GEF Protein
GEF Phospho
gef RNA
PLX Protein
PLX Phospho
PLX RNA
GEF PLX Protein
GEF PLX Phospho
GEF PLX RNA
Concentration(n[M])
Relative Conc
Time (hour)
×10^-4

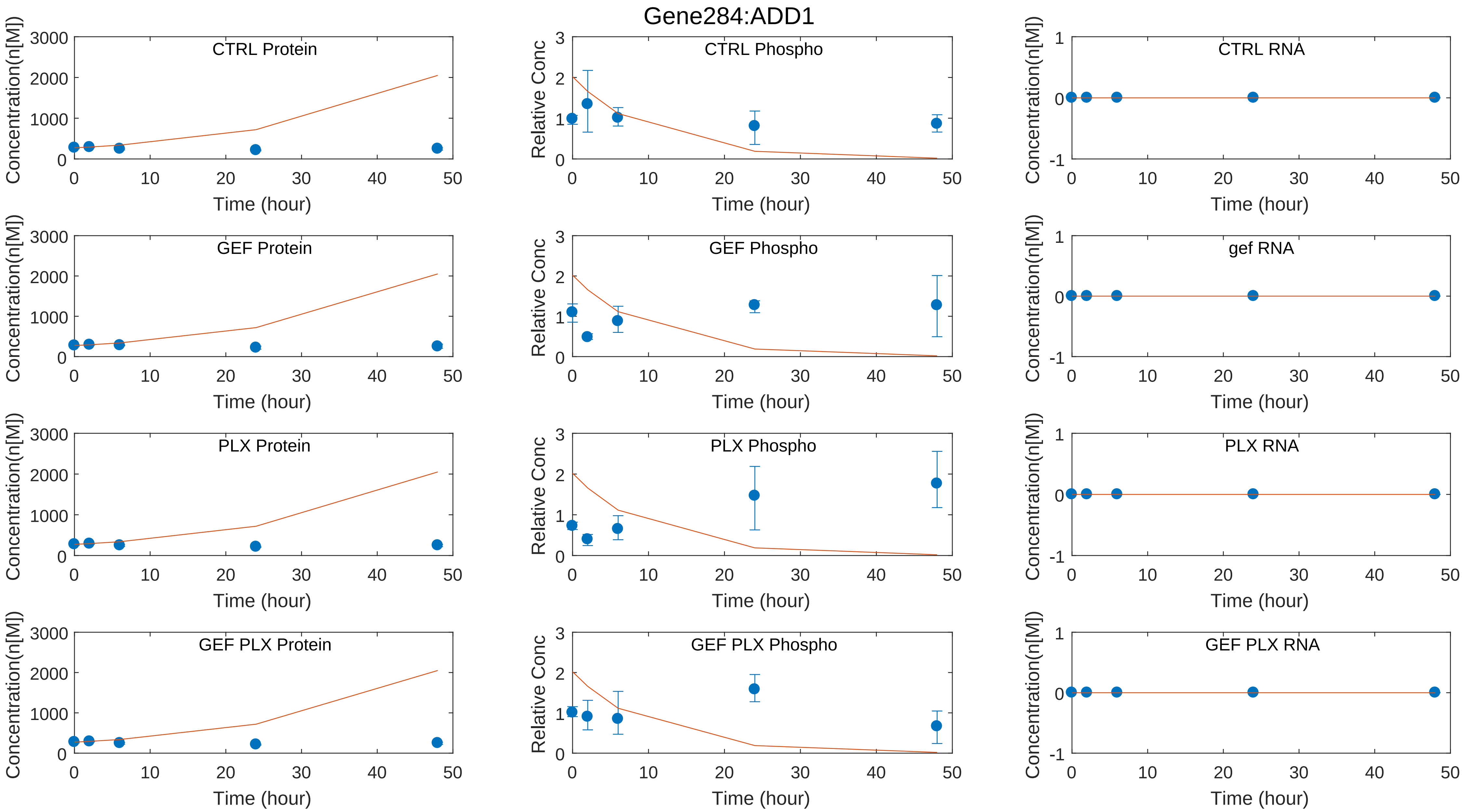

Gene284:ADD1
CTRL Protein
CTRL Phospho
CTRL RNA
GEF Protein
GEF Phospho
gef RNA
PLX Protein
PLX Phospho
PLX RNA
GEF PLX Protein
GEF PLX Phospho
GEF PLX RNA
Concentration(n[M])
Relative Conc
Time (hour)

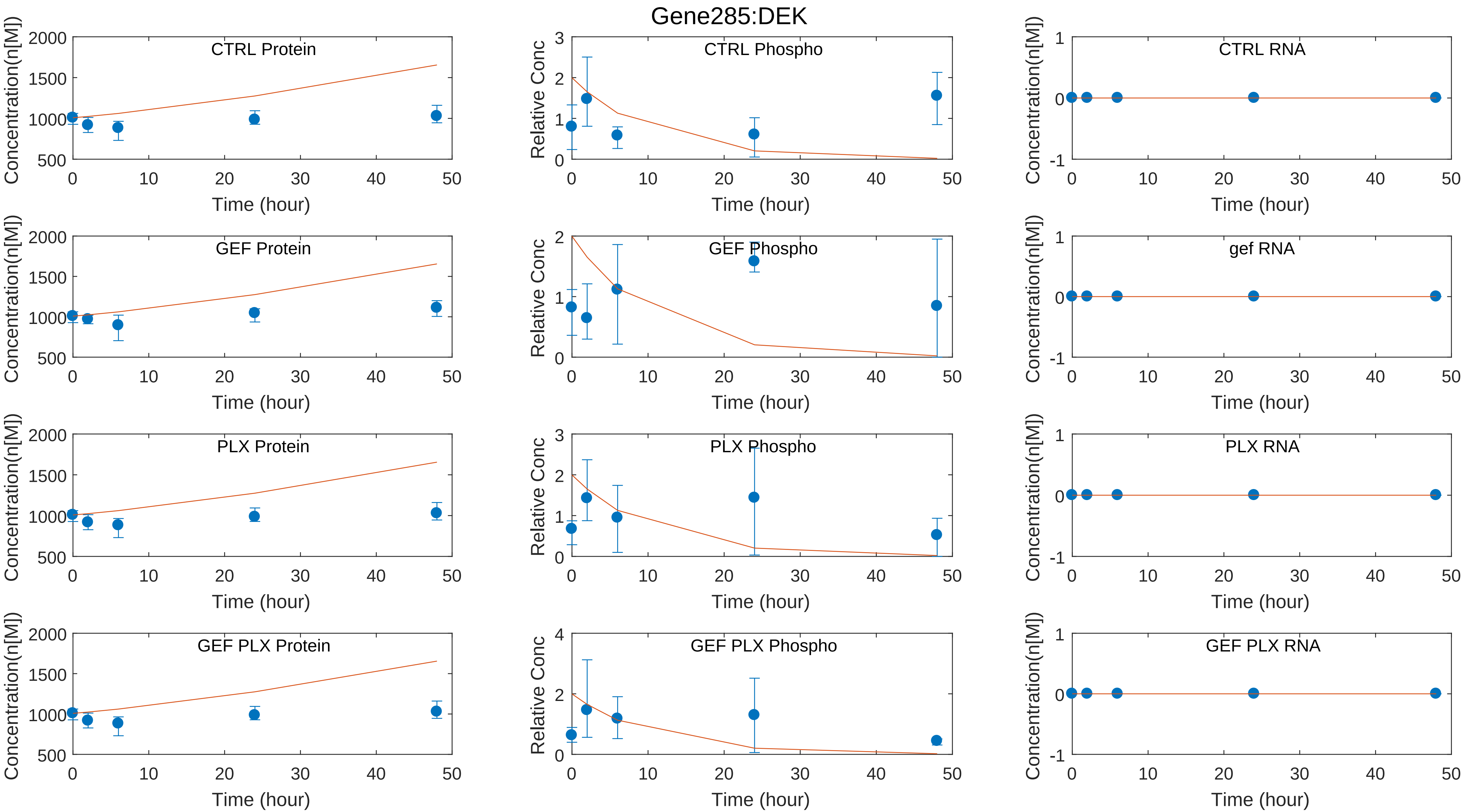

Gene285:DEK
CTRL Protein
GEF Protein
PLX Protein
GEF PLX Protein
CTRL Phospho
GEF Phospho
PLX Phospho
GEF PLX Phospho
CTRL RNA
gef RNA
PLX RNA
GEF PLX RNA
Concentration(n[M])
Relative Conc
Time (hour)

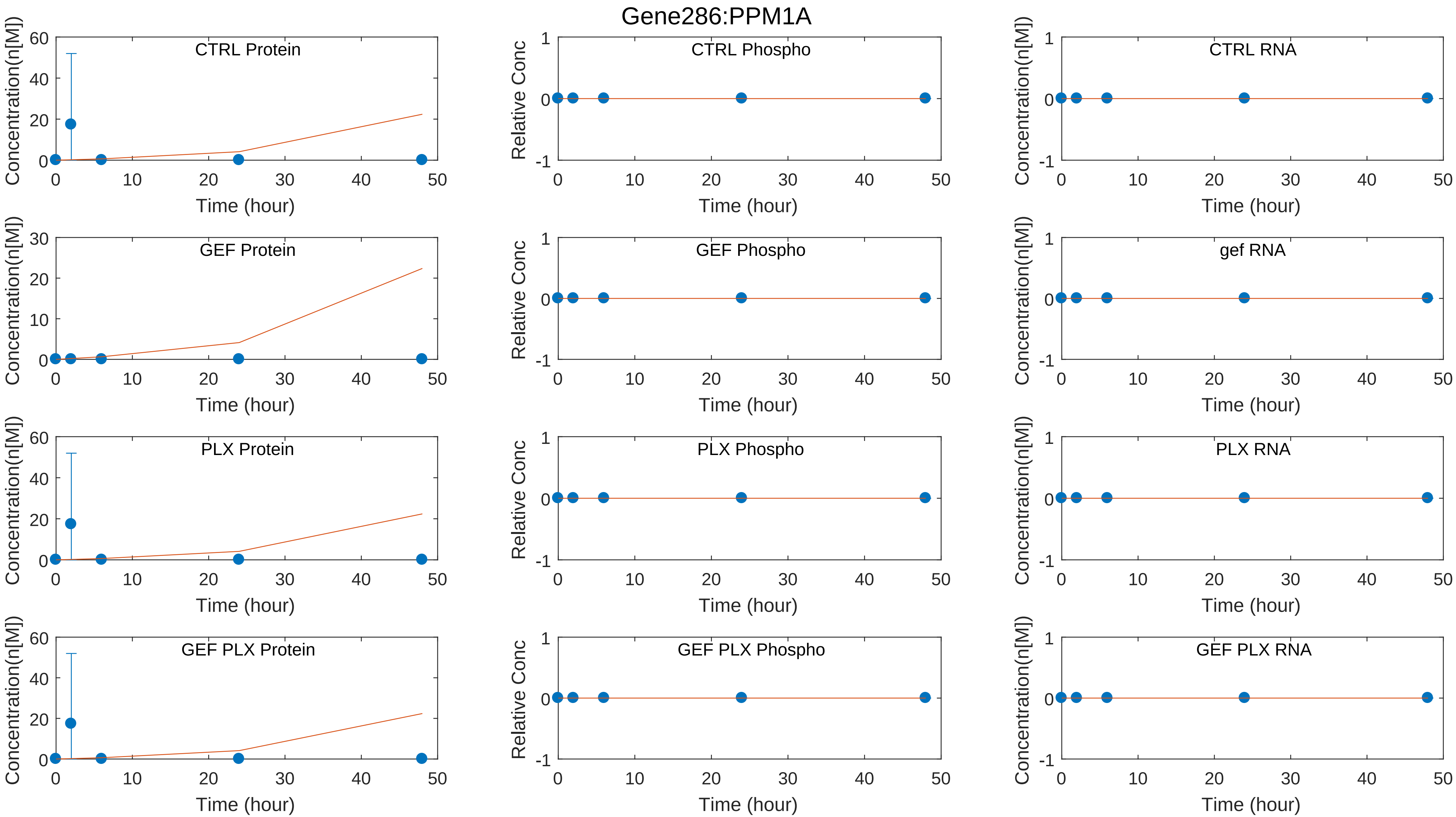
Gene286:PPM1A
CTRL Protein
CTRL Phospho
CTRL RNA
GEF Protein
GEF Phospho
gef RNA
PLX Protein
PLX Phospho
PLX RNA
GEF PLX Protein
GEF PLX Phospho
GEF PLX RNA
Concentration(n[M])
Relative Conc
Time (hour)

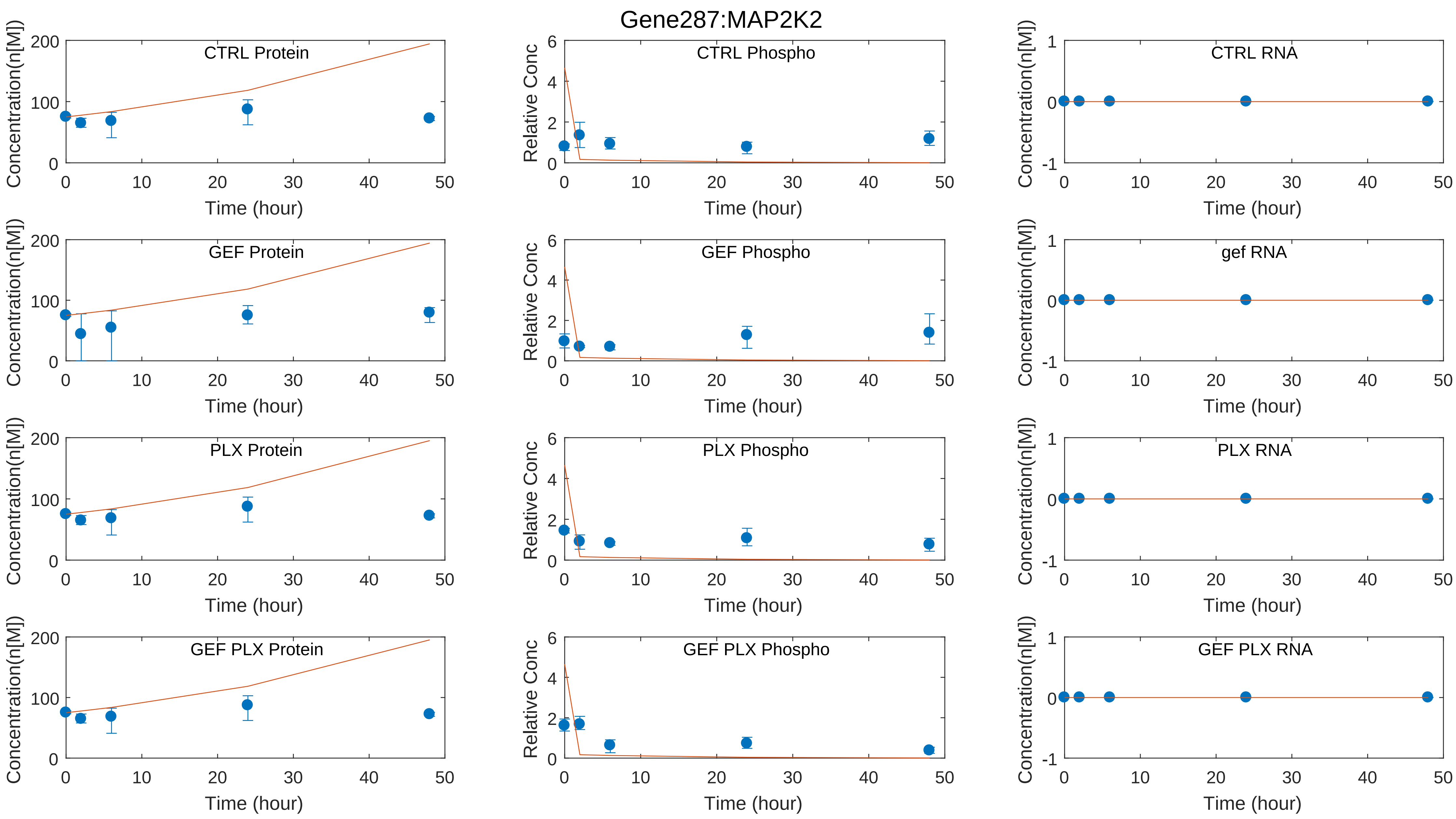

Gene287:MAP2K2
CTRL Protein
CTRL Phospho
CTRL RNA
GEF Protein
GEF Phospho
gef RNA
PLX Protein
PLX Phospho
PLX RNA
GEF PLX Protein
GEF PLX Phospho
GEF PLX RNA
Concentration(n[M])
Relative Conc
Time (hour)

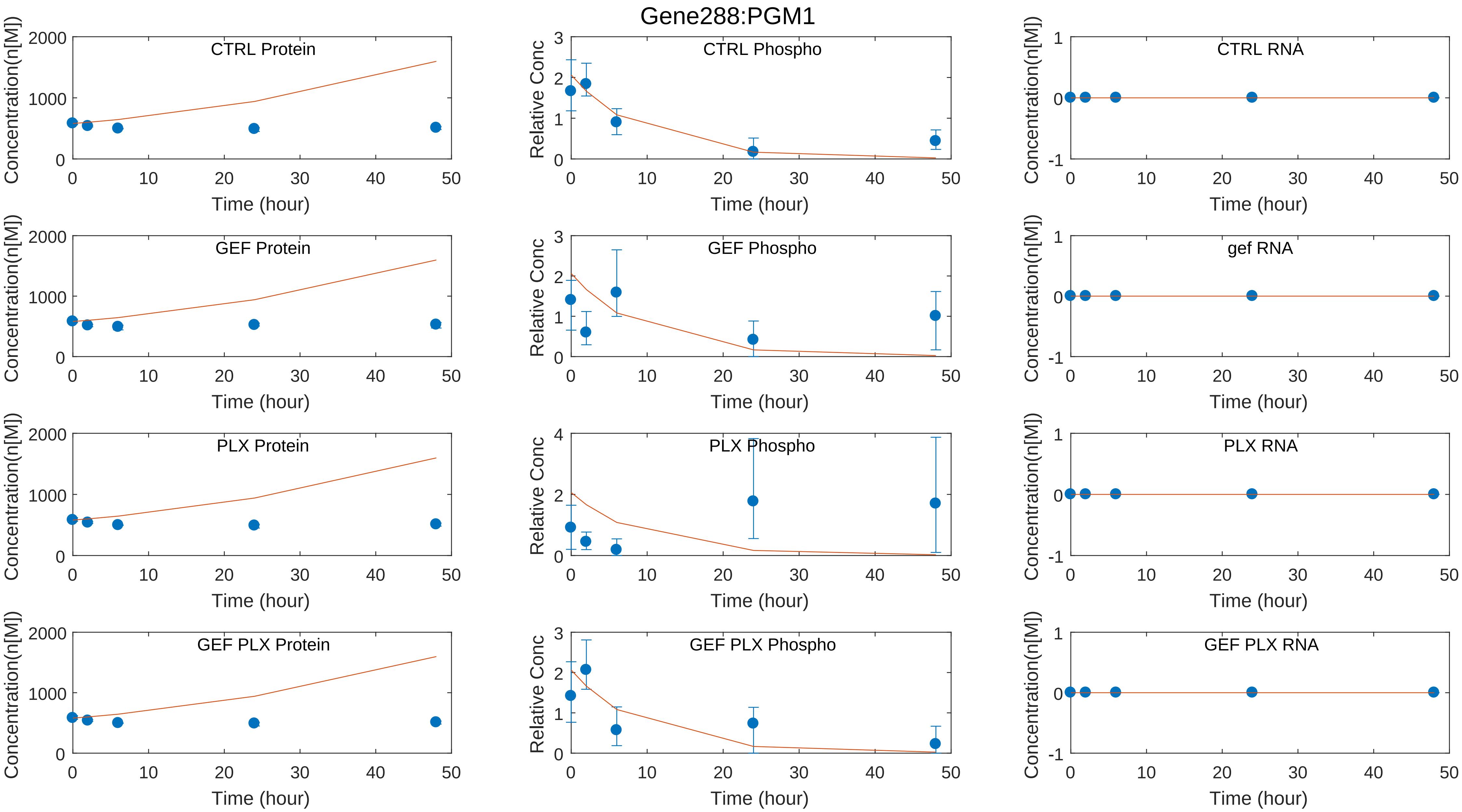

Gene288:PGM1
CTRL Protein
CTRL Phospho
CTRL RNA
GEF Protein
GEF Phospho
gef RNA
PLX Protein
PLX Phospho
PLX RNA
GEF PLX Protein
GEF PLX Phospho
GEF PLX RNA
Concentration(n[M])
Relative Conc
Time (hour)

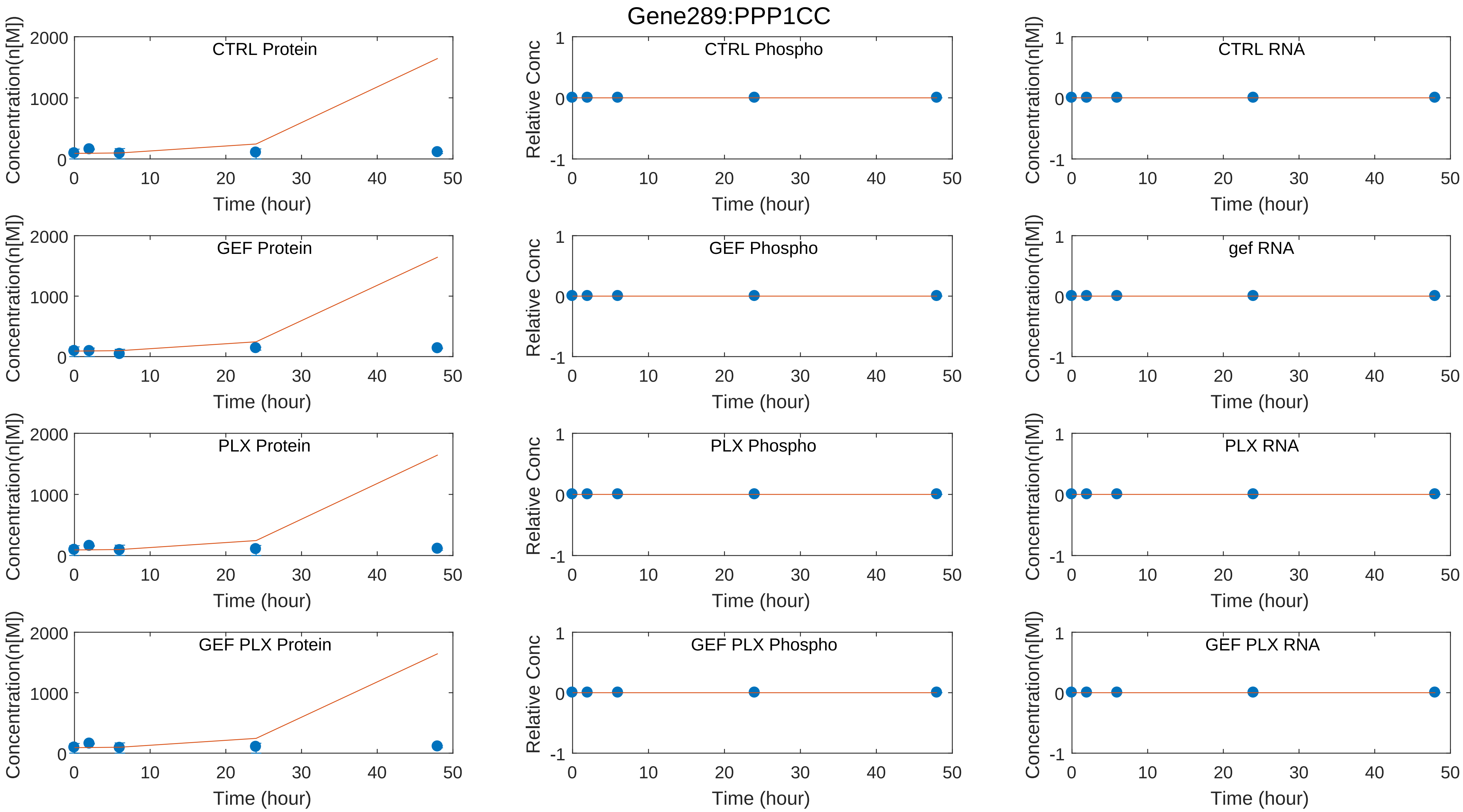

Gene289:PPP1CC
CTRL Protein
CTRL Phospho
CTRL RNA
GEF Protein
GEF Phospho
gef RNA
PLX Protein
PLX Phospho
PLX RNA
GEF PLX Protein
GEF PLX Phospho
GEF PLX RNA
Concentration(n[M])
Relative Conc
Time (hour)

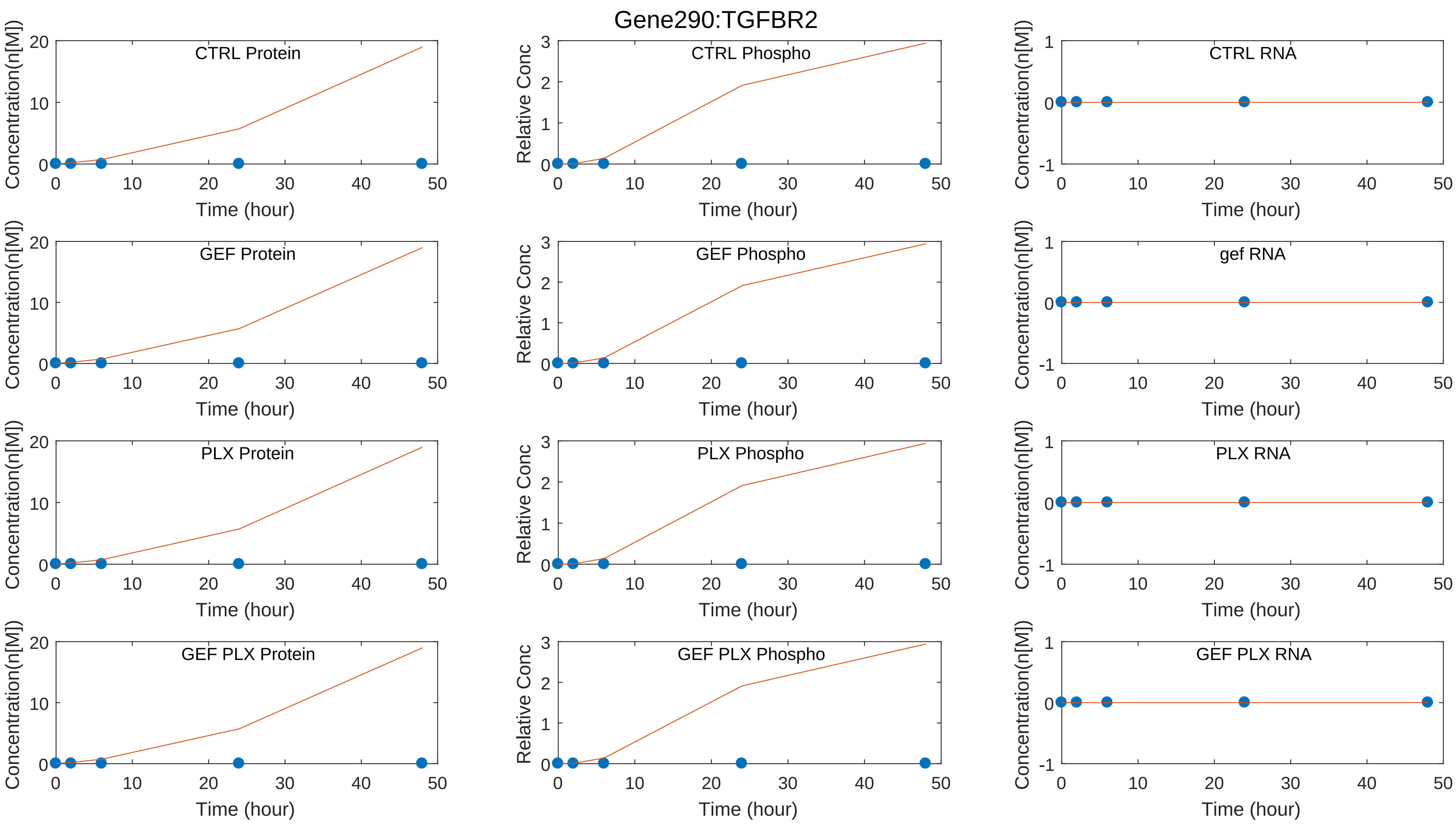

Gene290:TGFBR2
CTRL Protein
CTRL Phospho
CTRL RNA
GEF Protein
GEF Phospho
gef RNA
PLX Protein
PLX Phospho
PLX RNA
GEF PLX Protein
GEF PLX Phospho
GEF PLX RNA
Concentration(n[M])
Relative Conc
Time (hour)

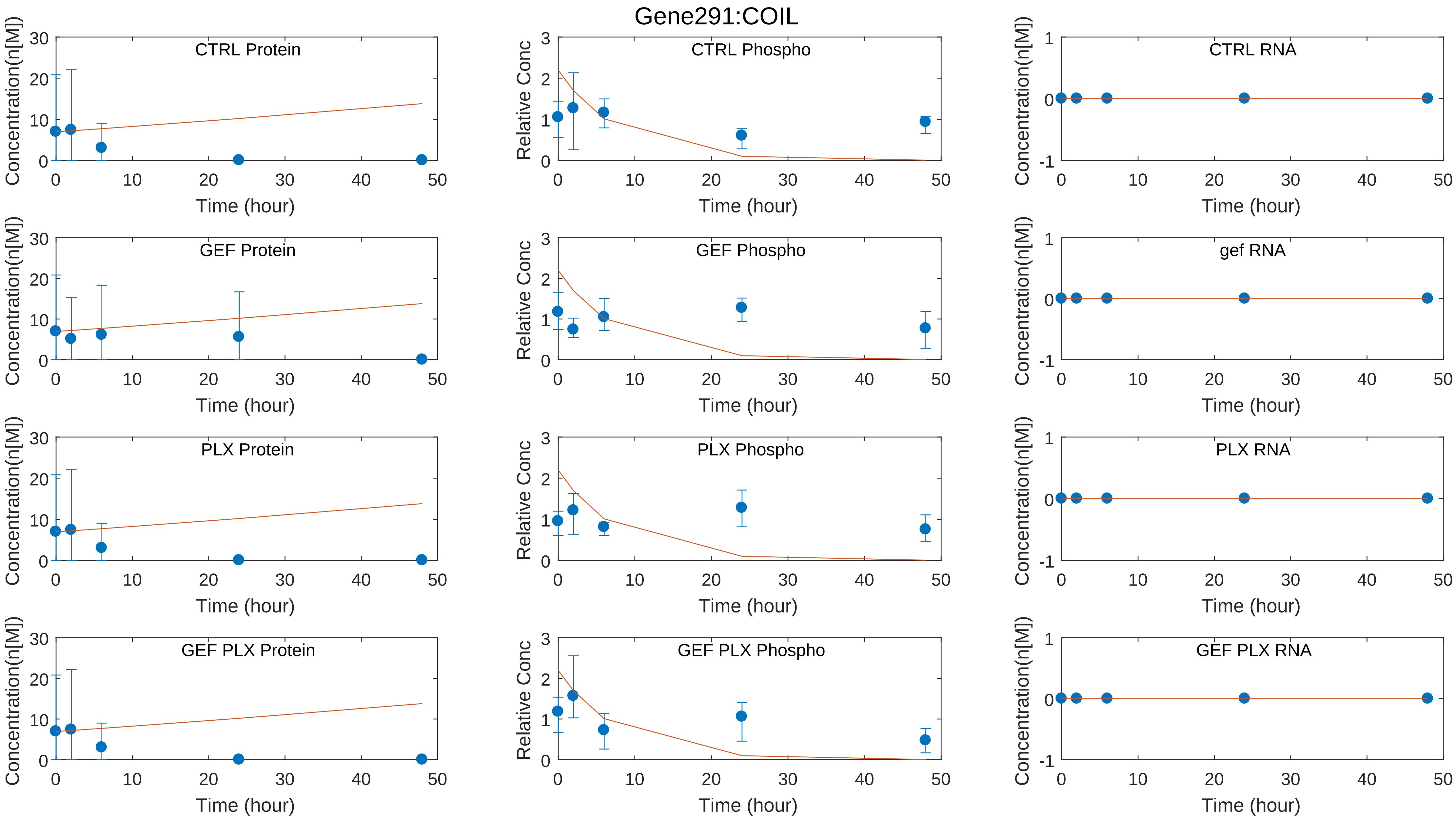

Gene291:COIL
CTRL Protein
CTRL Phospho
CTRL RNA
GEF Protein
GEF Phospho
gef RNA
PLX Protein
PLX Phospho
PLX RNA
GEF PLX Protein
GEF PLX Phospho
GEF PLX RNA
Concentration(n[M])
Relative Conc
Time (hour)

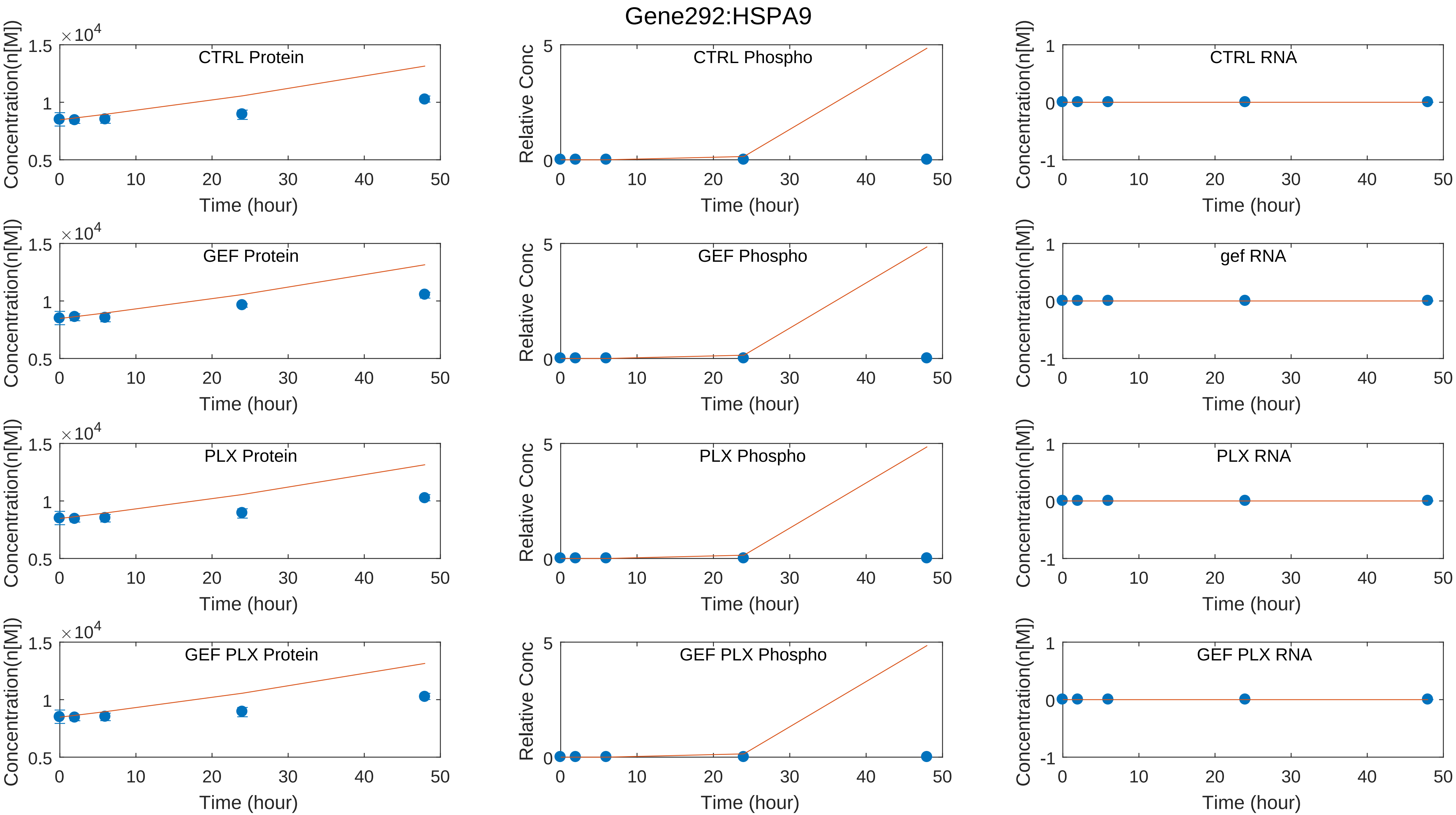

Gene292:HSPA9
CTRL Protein
GEF Protein
PLX Protein
GEF PLX Protein
CTRL Phospho
GEF Phospho
PLX Phospho
GEF PLX Phospho
CTRL RNA
gef RNA
PLX RNA
GEF PLX RNA
Concentration(n[M])
Relative Conc
Time (hour)
×10^4

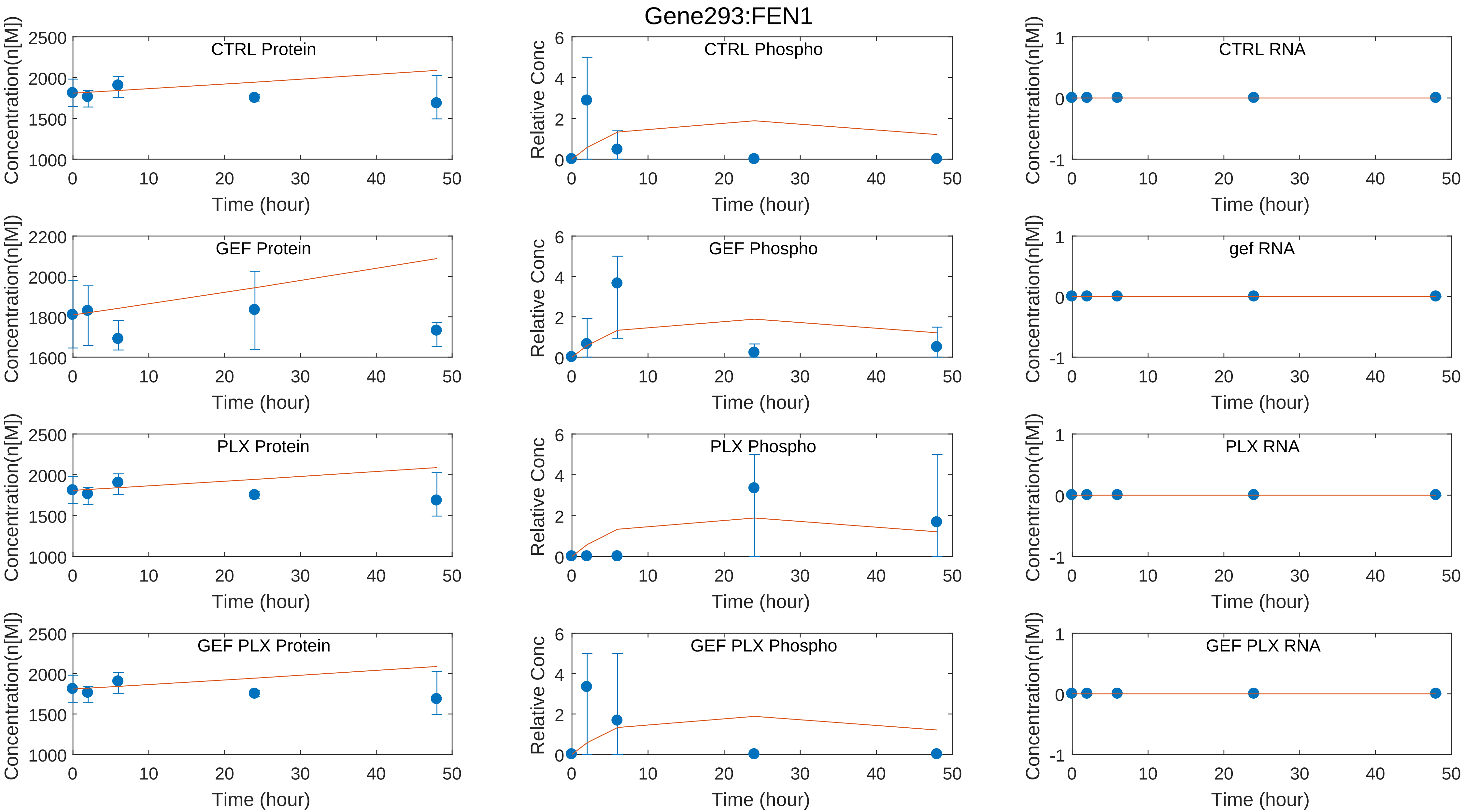

Gene293:FEN1
CTRL Protein
CTRL Phospho
CTRL RNA
GEF Protein
GEF Phospho
gef RNA
PLX Protein
PLX Phospho
PLX RNA
GEF PLX Protein
GEF PLX Phospho
GEF PLX RNA
Concentration(n[M])
Relative Conc
Time (hour)

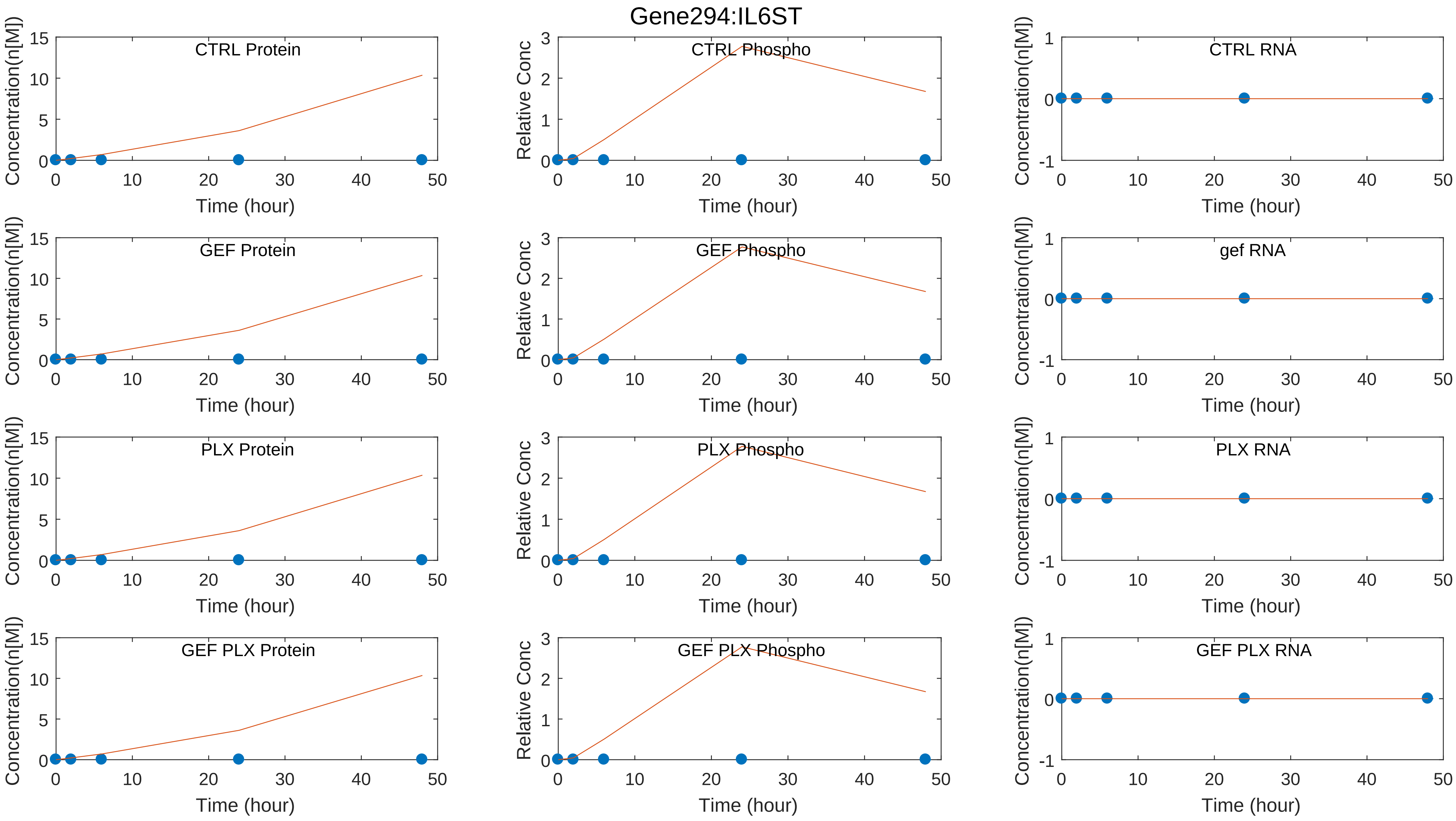
Gene294:IL6ST
CTRL Protein
CTRL Phospho
CTRL RNA
GEF Protein
GEF Phospho
gef RNA
PLX Protein
PLX Phospho
PLX RNA
GEF PLX Protein
GEF PLX Phospho
GEF PLX RNA
Concentration(n[M])
Relative Conc
Time (hour)

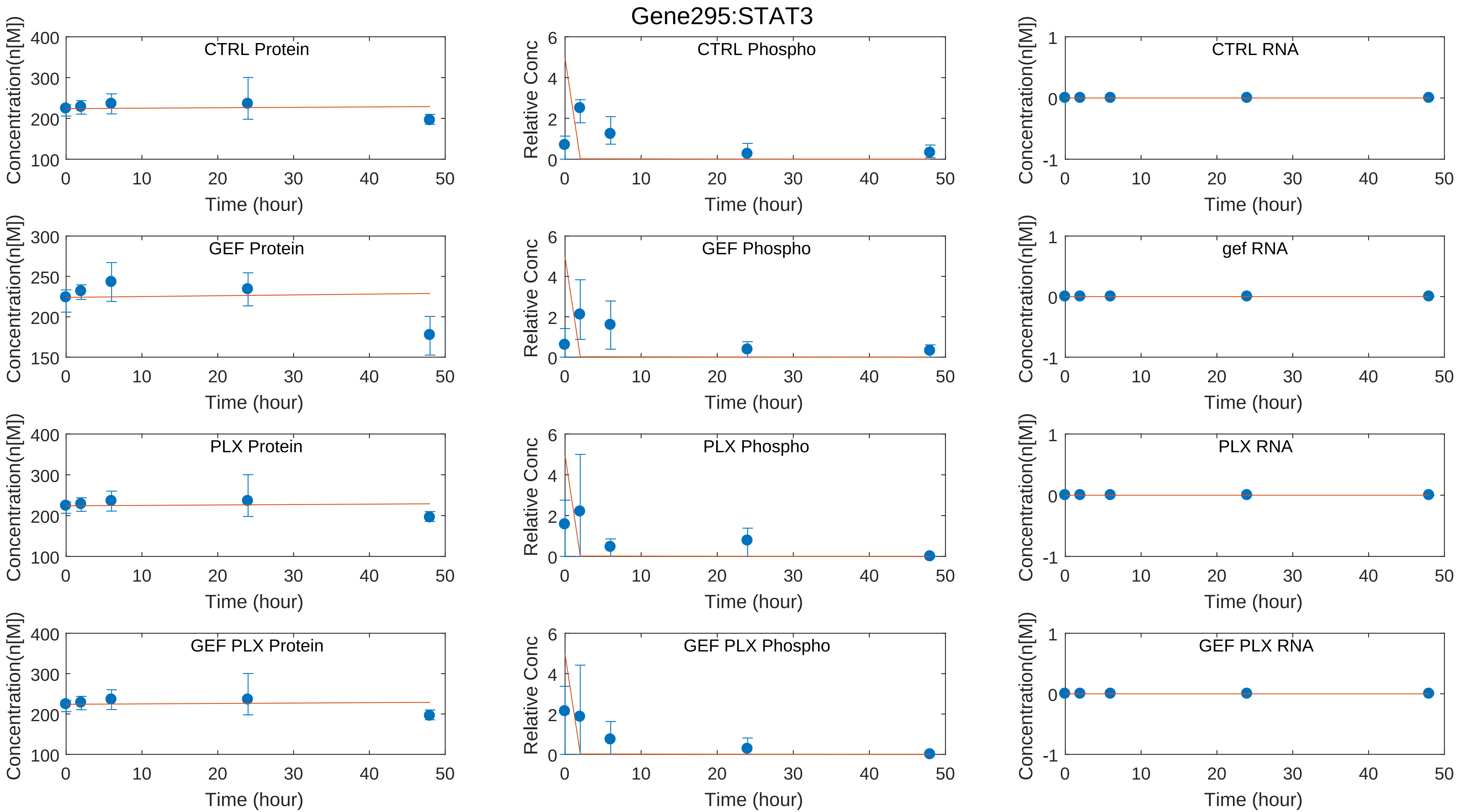

Gene295:STAT3
CTRL Protein
CTRL Phospho
CTRL RNA
GEF Protein
GEF Phospho
gef RNA
PLX Protein
PLX Phospho
PLX RNA
GEF PLX Protein
GEF PLX Phospho
GEF PLX RNA
Concentration(n[M])
Relative Conc
Time (hour)

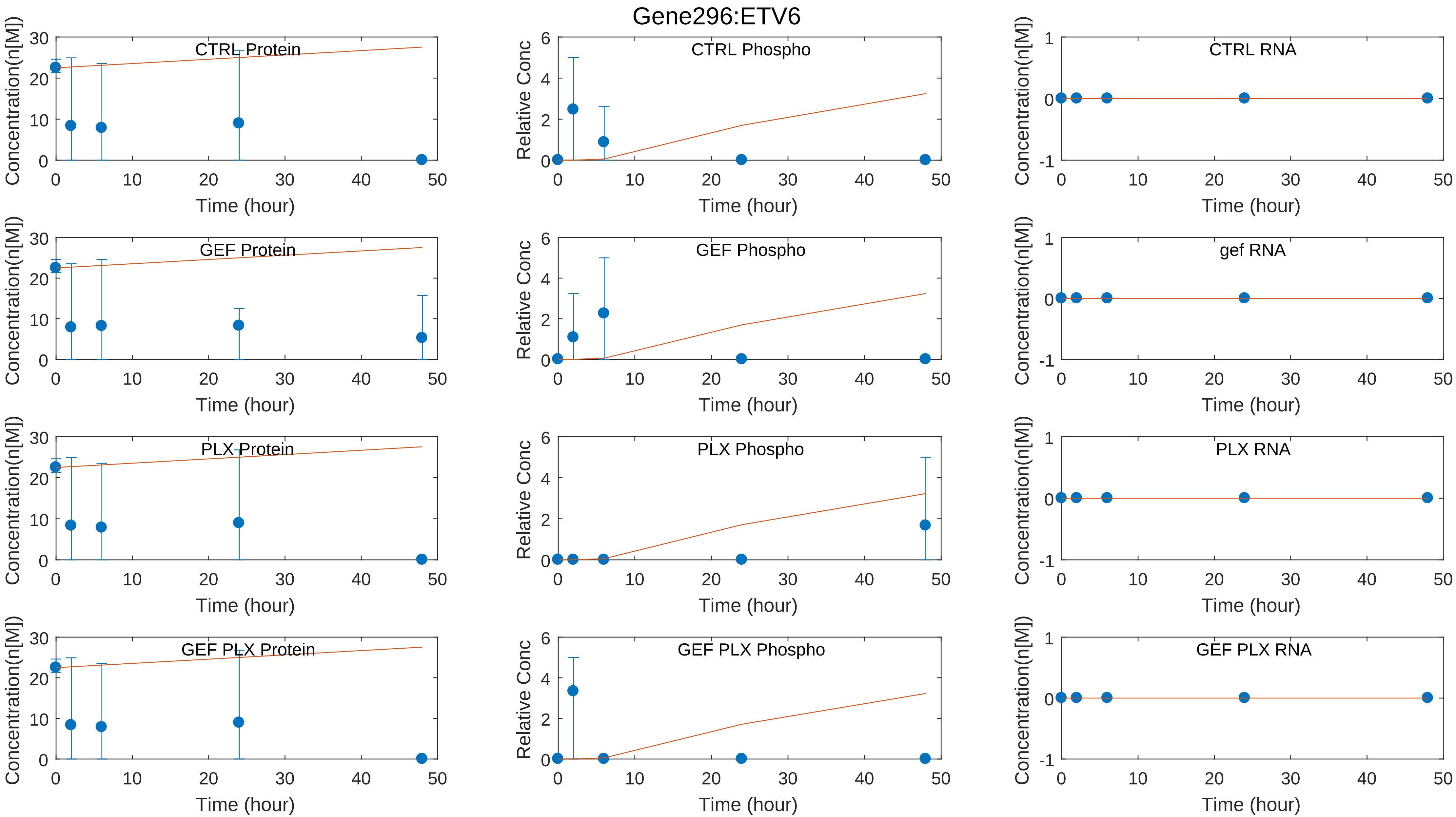
Gene296:ETV6
CTRL Protein
CTRL Phospho
CTRL RNA
GEF Protein
GEF Phospho
gef RNA
PLX Protein
PLX Phospho
PLX RNA
GEF PLX Protein
GEF PLX Phospho
GEF PLX RNA
Concentration(n[M])
Relative Conc
Time (hour)

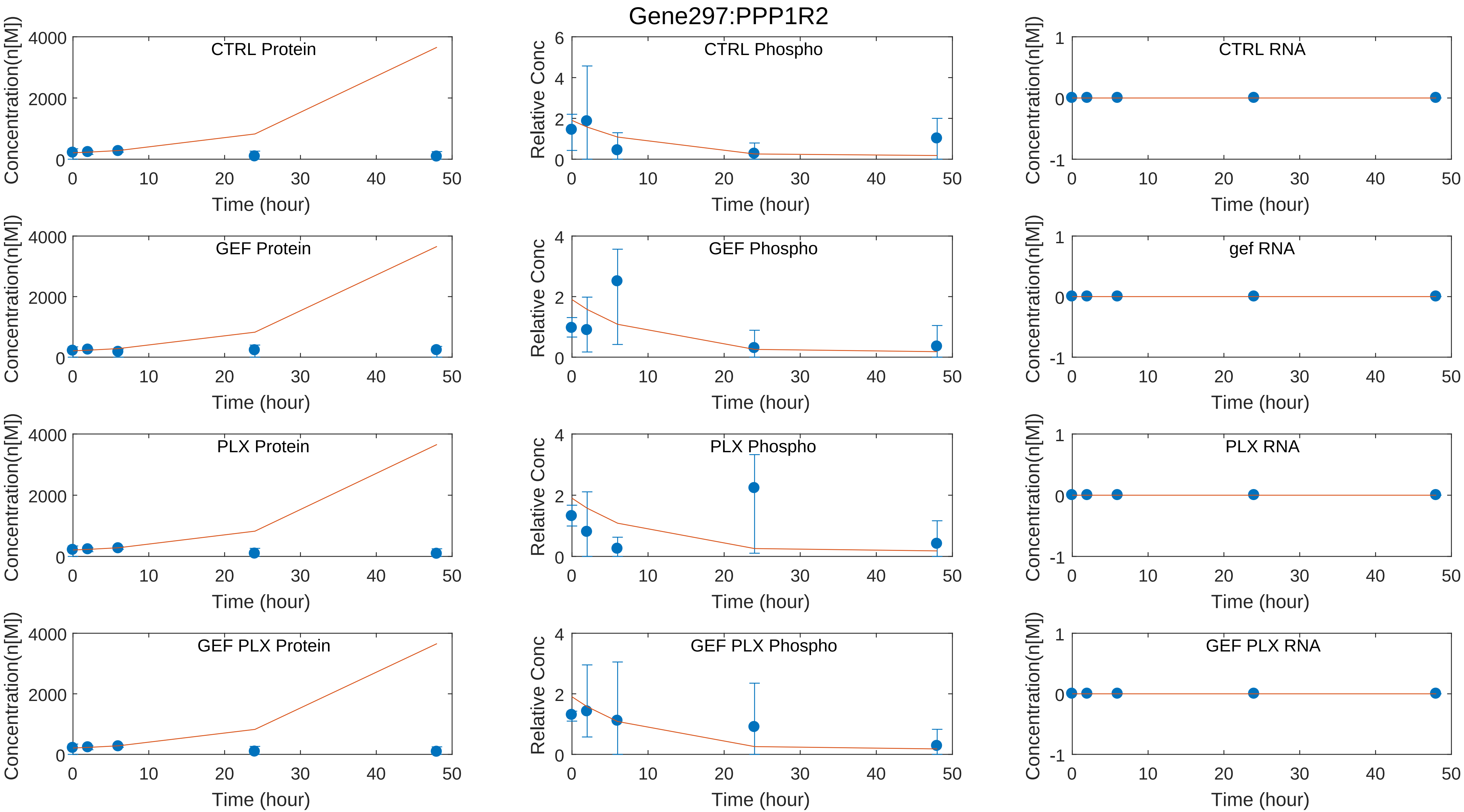

Gene297:PPP1R2
CTRL Protein
CTRL Phospho
CTRL RNA
GEF Protein
GEF Phospho
gef RNA
PLX Protein
PLX Phospho
PLX RNA
GEF PLX Protein
GEF PLX Phospho
GEF PLX RNA
Concentration(n[M])
Relative Conc
Time (hour)

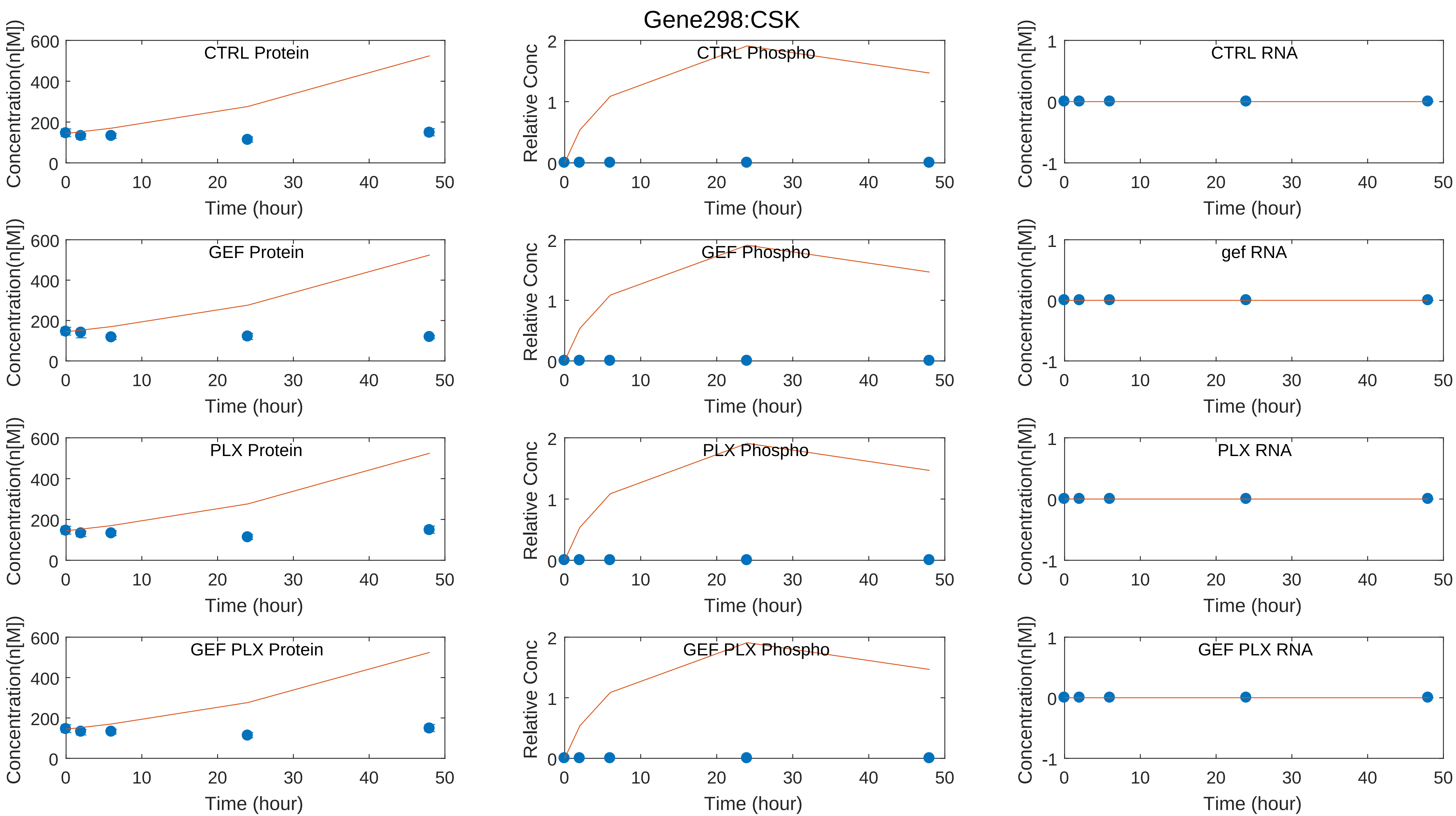

Gene298:CSK
CTRL Protein
CTRL Phospho
CTRL RNA
GEF Protein
GEF Phospho
gef RNA
PLX Protein
PLX Phospho
PLX RNA
GEF PLX Protein
GEF PLX Phospho
GEF PLX RNA
Concentration(n[M])
Relative Conc
Time (hour)

Gene299:MAP3K8

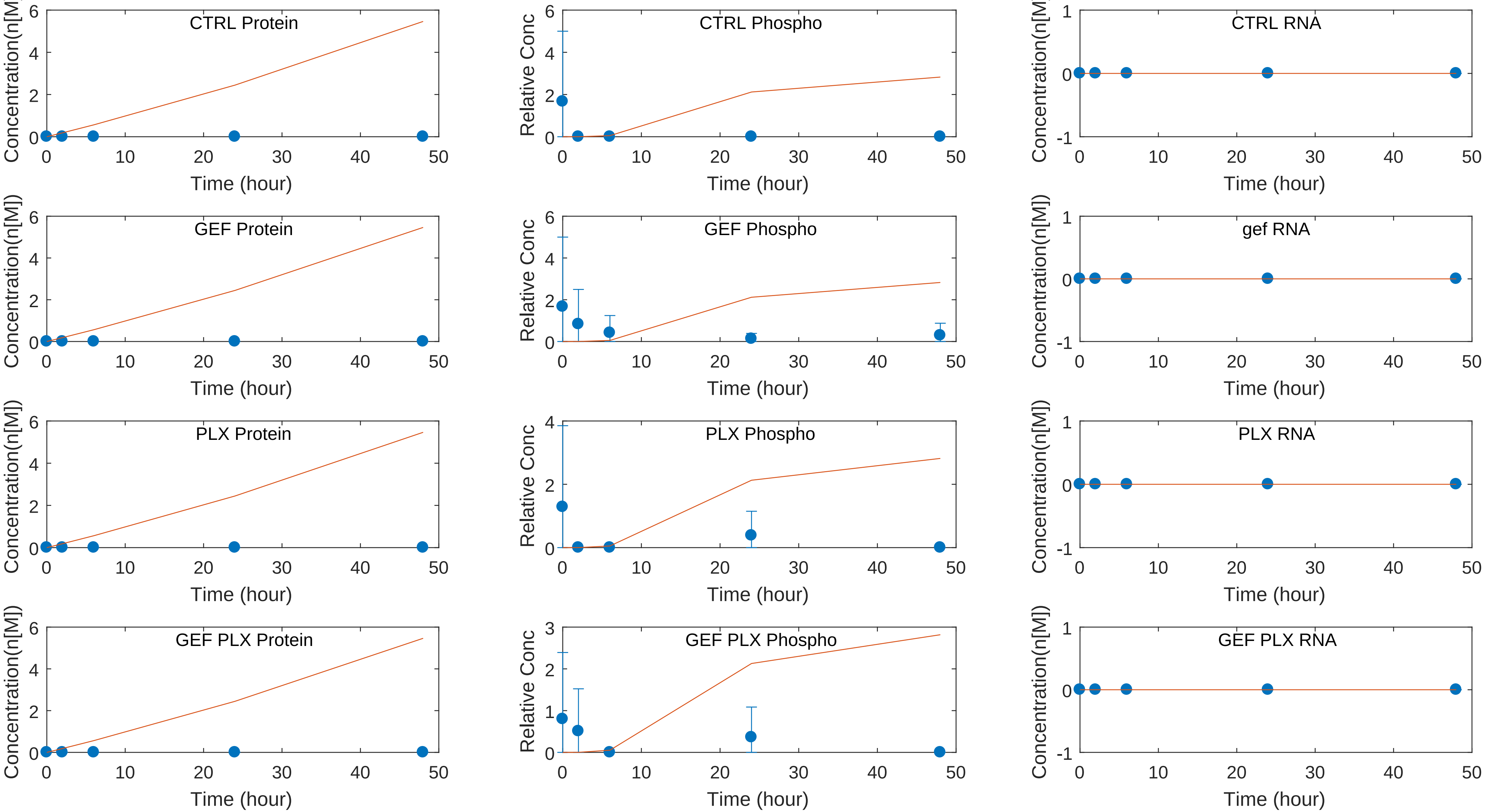

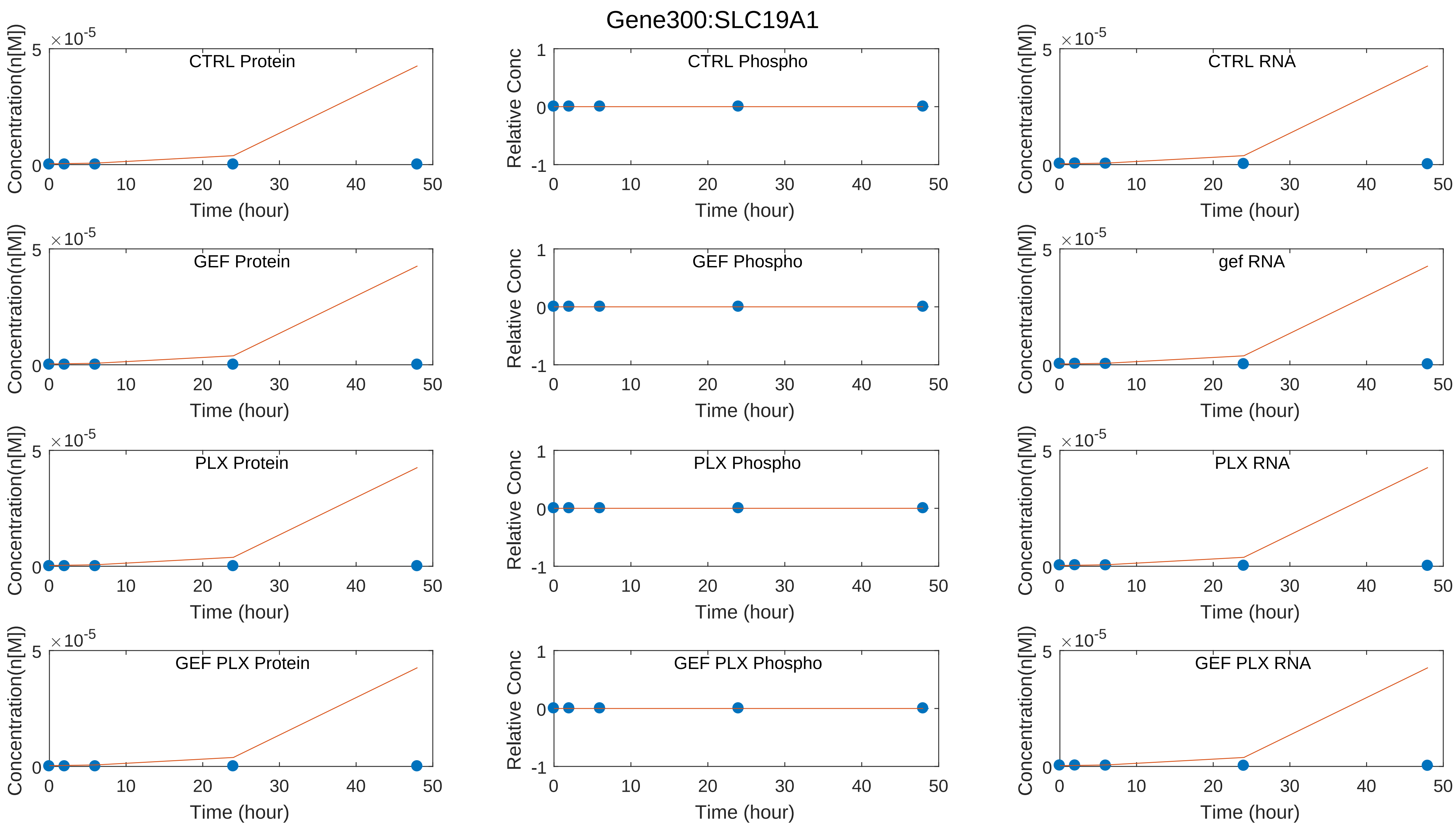
Gene300:SLC19A1
CTRL Protein
CTRL Phospho
CTRL RNA
GEF Protein
GEF Phospho
gef RNA
PLX Protein
PLX Phospho
PLX RNA
GEF PLX Protein
GEF PLX Phospho
GEF PLX RNA
Concentration(n[M])
Relative Conc
Time (hour)
×10-5

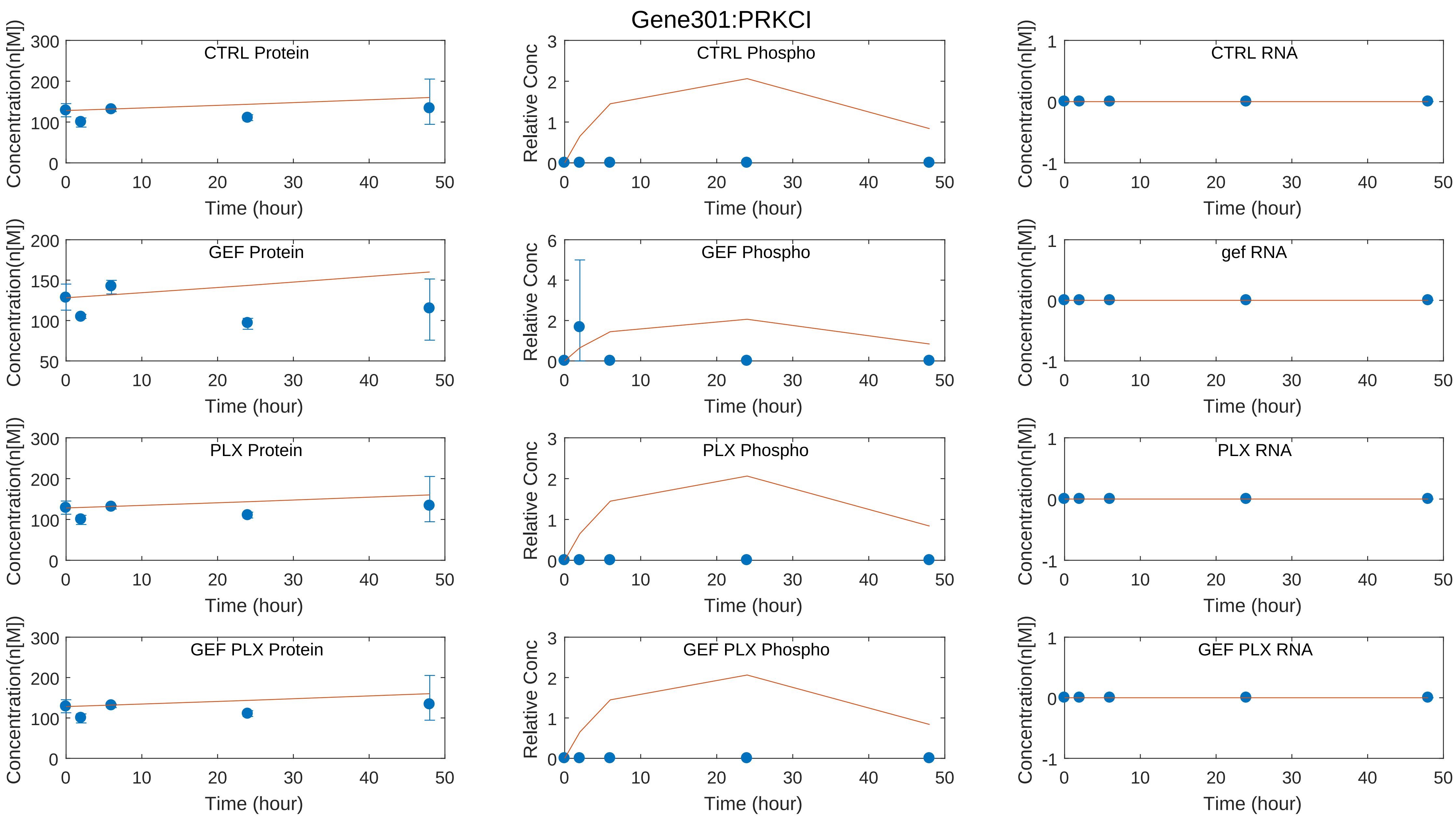

Gene301:PRKCI
CTRL Protein
CTRL Phospho
CTRL RNA
GEF Protein
GEF Phospho
gef RNA
PLX Protein
PLX Phospho
PLX RNA
GEF PLX Protein
GEF PLX Phospho
GEF PLX RNA
Concentration(n[M])
Relative Conc
Time (hour)

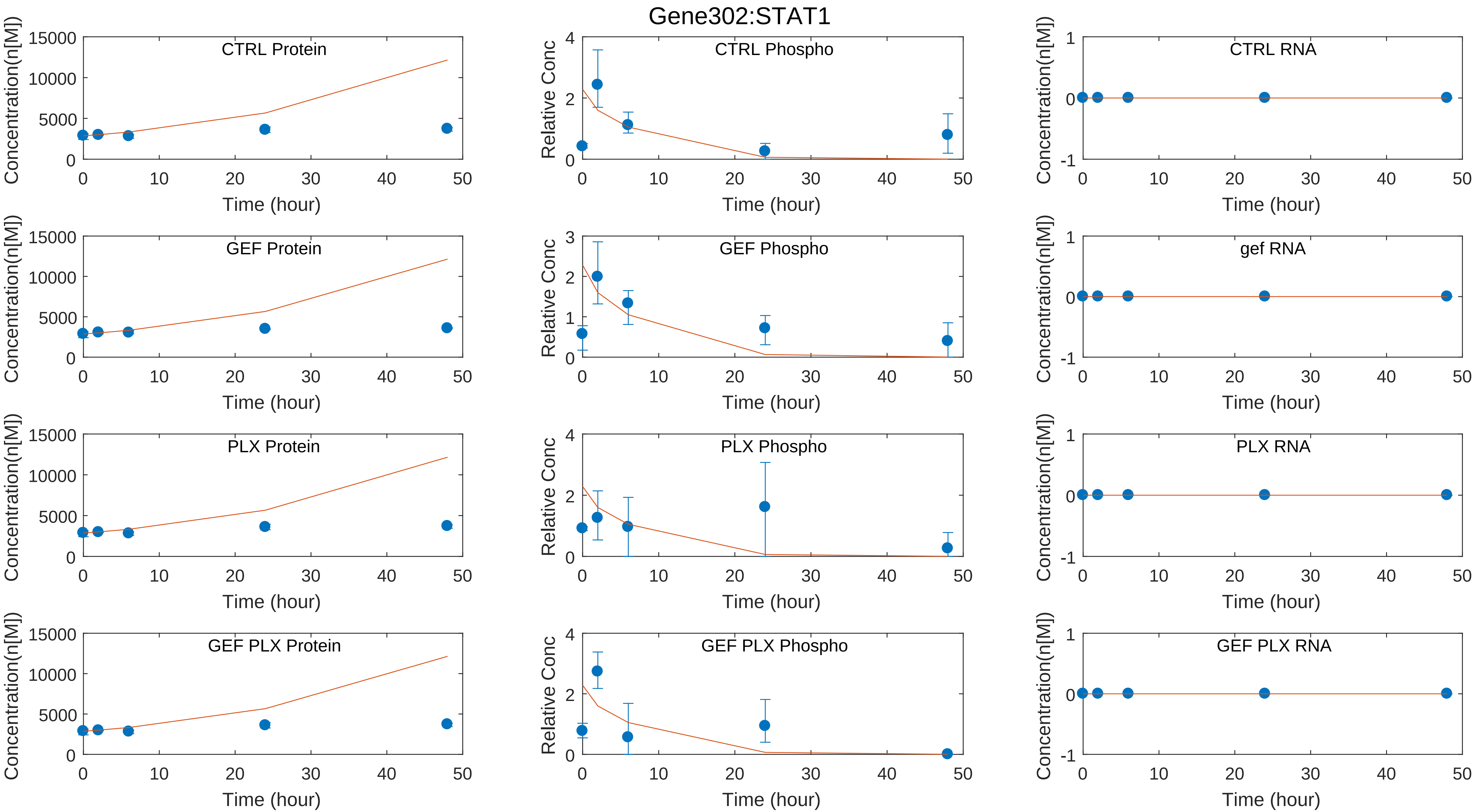

Gene302:STAT1
CTRL Protein
GEF Protein
PLX Protein
GEF PLX Protein
CTRL Phospho
GEF Phospho
PLX Phospho
GEF PLX Phospho
CTRL RNA
gef RNA
PLX RNA
GEF PLX RNA
Concentration(n[M])
Relative Conc
Time (hour)

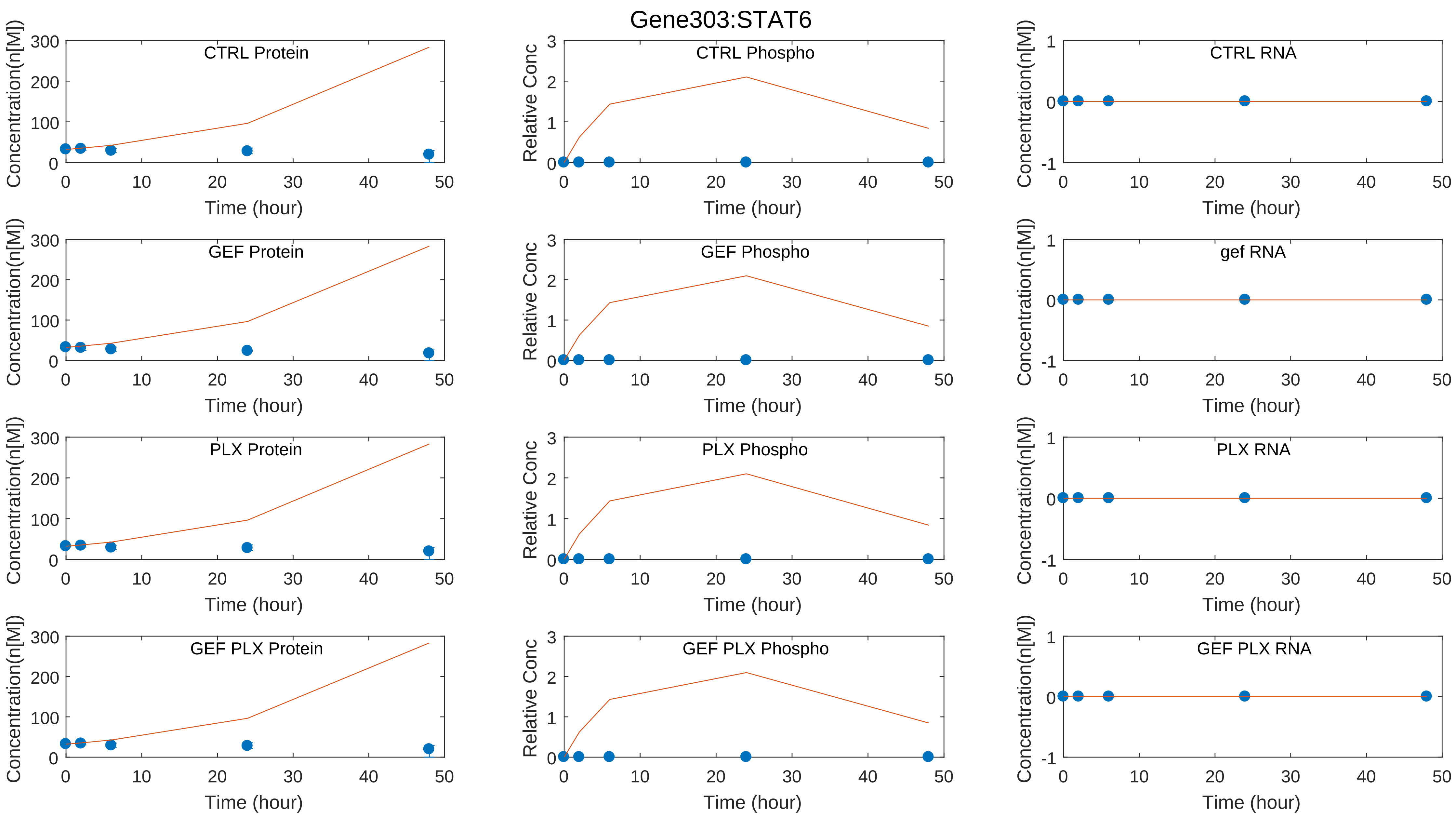

Gene303:STAT6
CTRL Protein
CTRL Phospho
CTRL RNA
GEF Protein
GEF Phospho
gef RNA
PLX Protein
PLX Phospho
PLX RNA
GEF PLX Protein
GEF PLX Phospho
GEF PLX RNA
Concentration(n[M])
Relative Conc
Time (hour)
0
10
20
30
40
50
100
200
300
1
2
3
-1

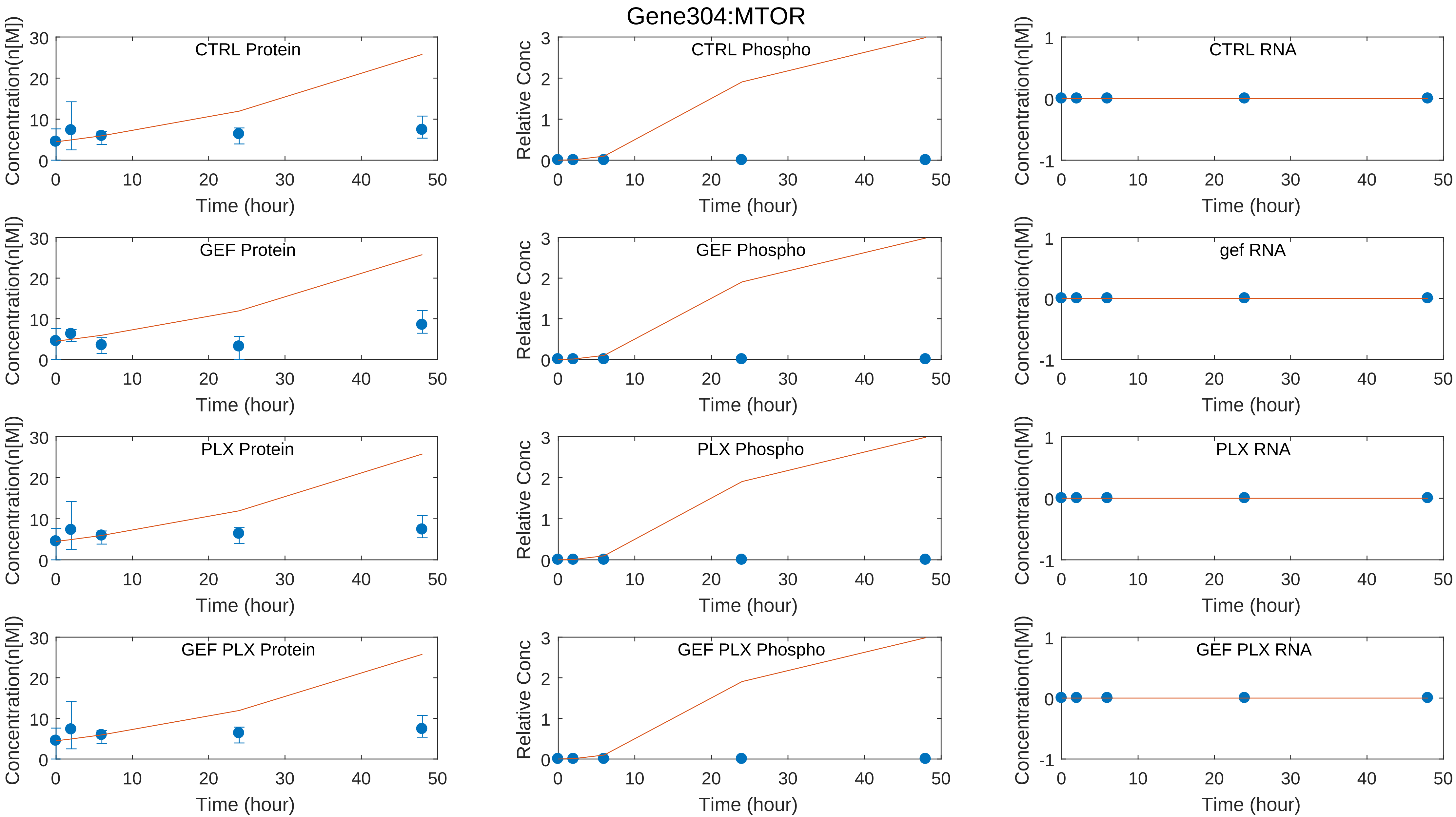

Gene304:MTOR
CTRL Protein
GEF Protein
PLX Protein
GEF PLX Protein
CTRL Phospho
GEF Phospho
PLX Phospho
GEF PLX Phospho
CTRL RNA
gef RNA
PLX RNA
GEF PLX RNA
Concentration(n[M])
Relative Conc
Time (hour)

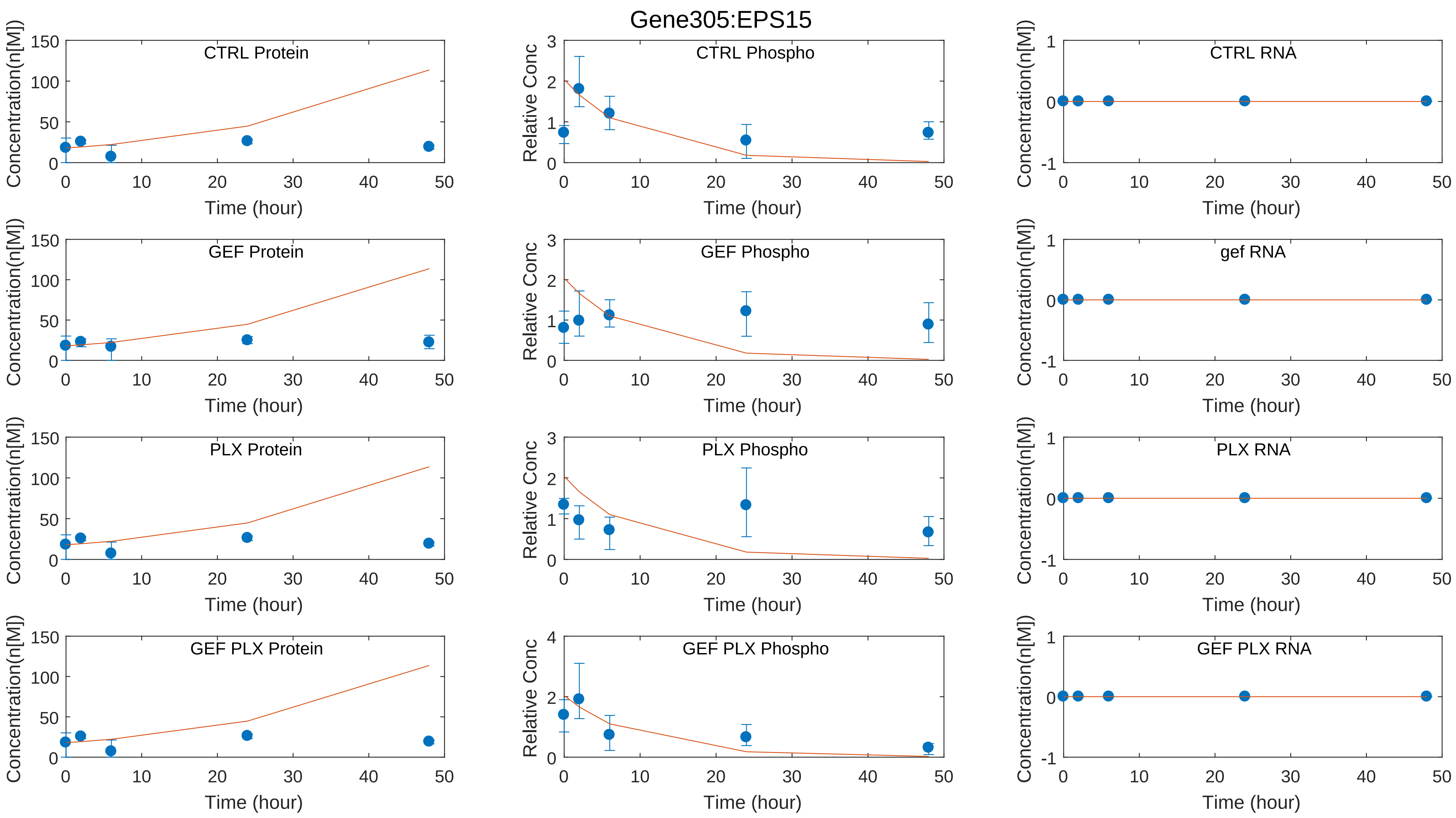
Gene305:EPS15
CTRL Protein
CTRL Phospho
CTRL RNA
GEF Protein
GEF Phospho
gef RNA
PLX Protein
PLX Phospho
PLX RNA
GEF PLX Protein
GEF PLX Phospho
GEF PLX RNA
Concentration(n[M])
Relative Conc
Time (hour)

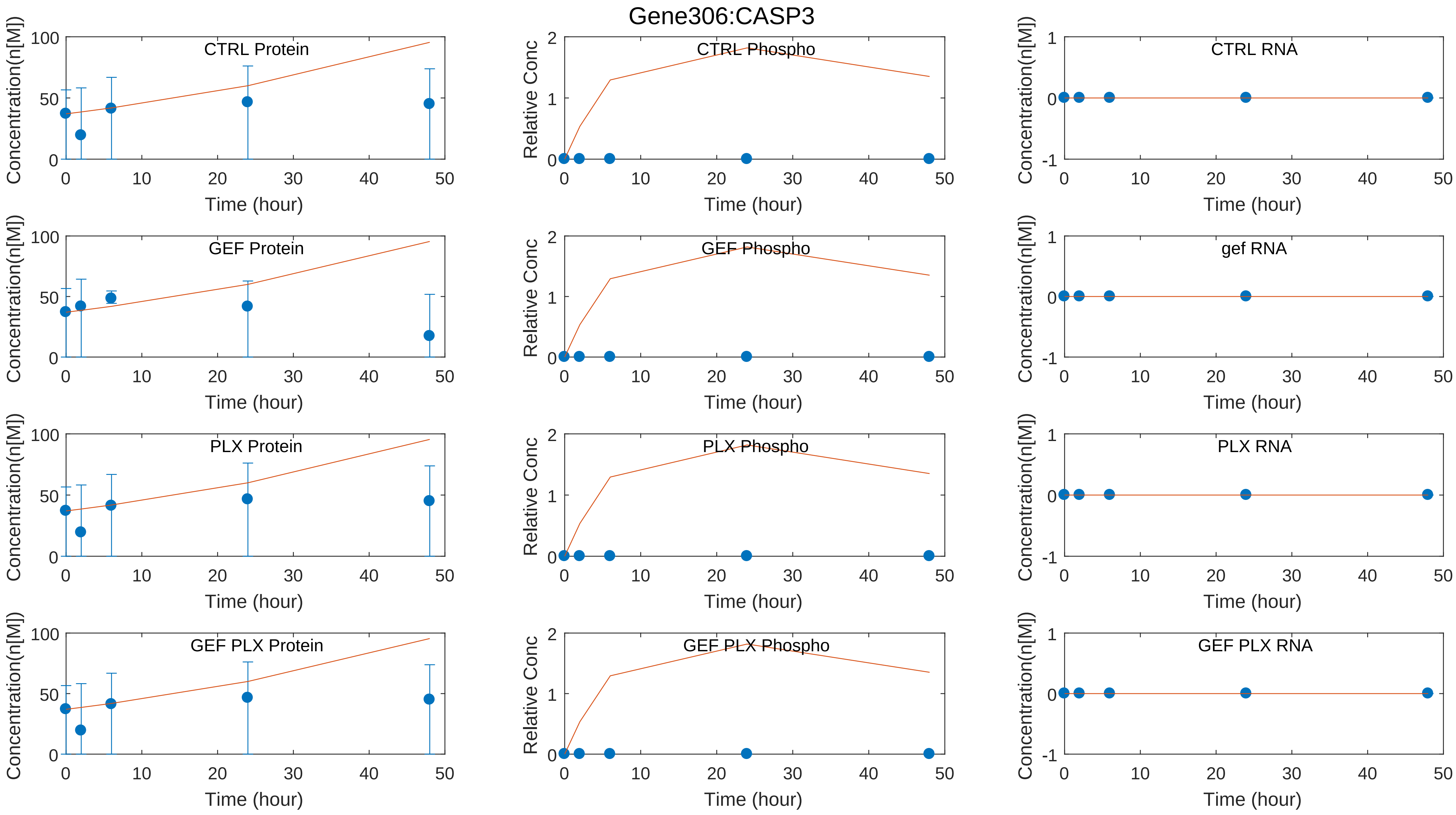
Gene306:CASP3
CTRL Protein
GEF Protein
PLX Protein
GEF PLX Protein
CTRL Phospho
GEF Phospho
PLX Phospho
GEF PLX Phospho
CTRL RNA
gef RNA
PLX RNA
GEF PLX RNA
Concentration(n[M])
Relative Conc
Time (hour)

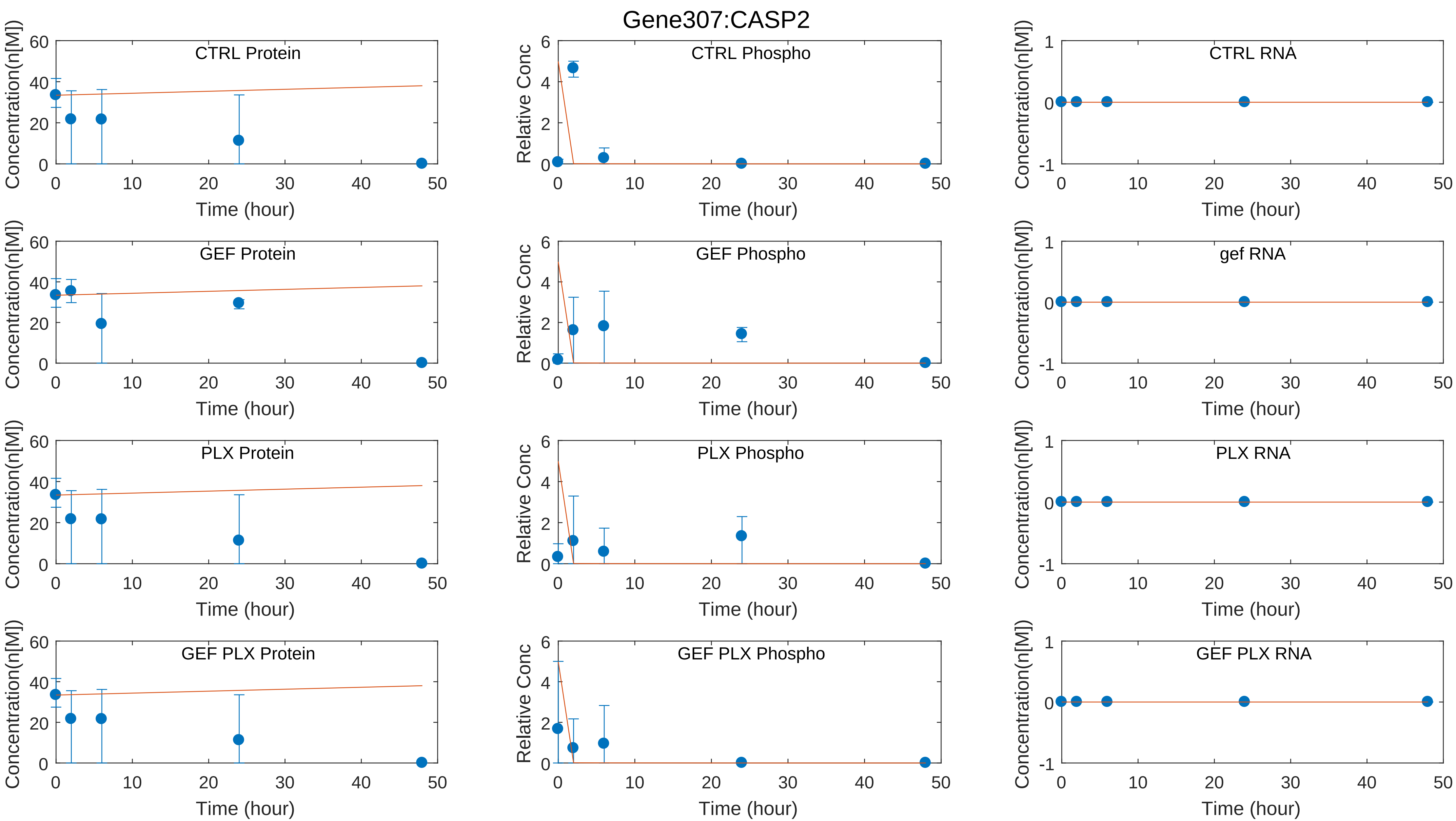

Gene307:CASP2
CTRL Protein
CTRL Phospho
CTRL RNA
GEF Protein
GEF Phospho
gef RNA
PLX Protein
PLX Phospho
PLX RNA
GEF PLX Protein
GEF PLX Phospho
GEF PLX RNA
Concentration(n[M])
Relative Conc
Time (hour)

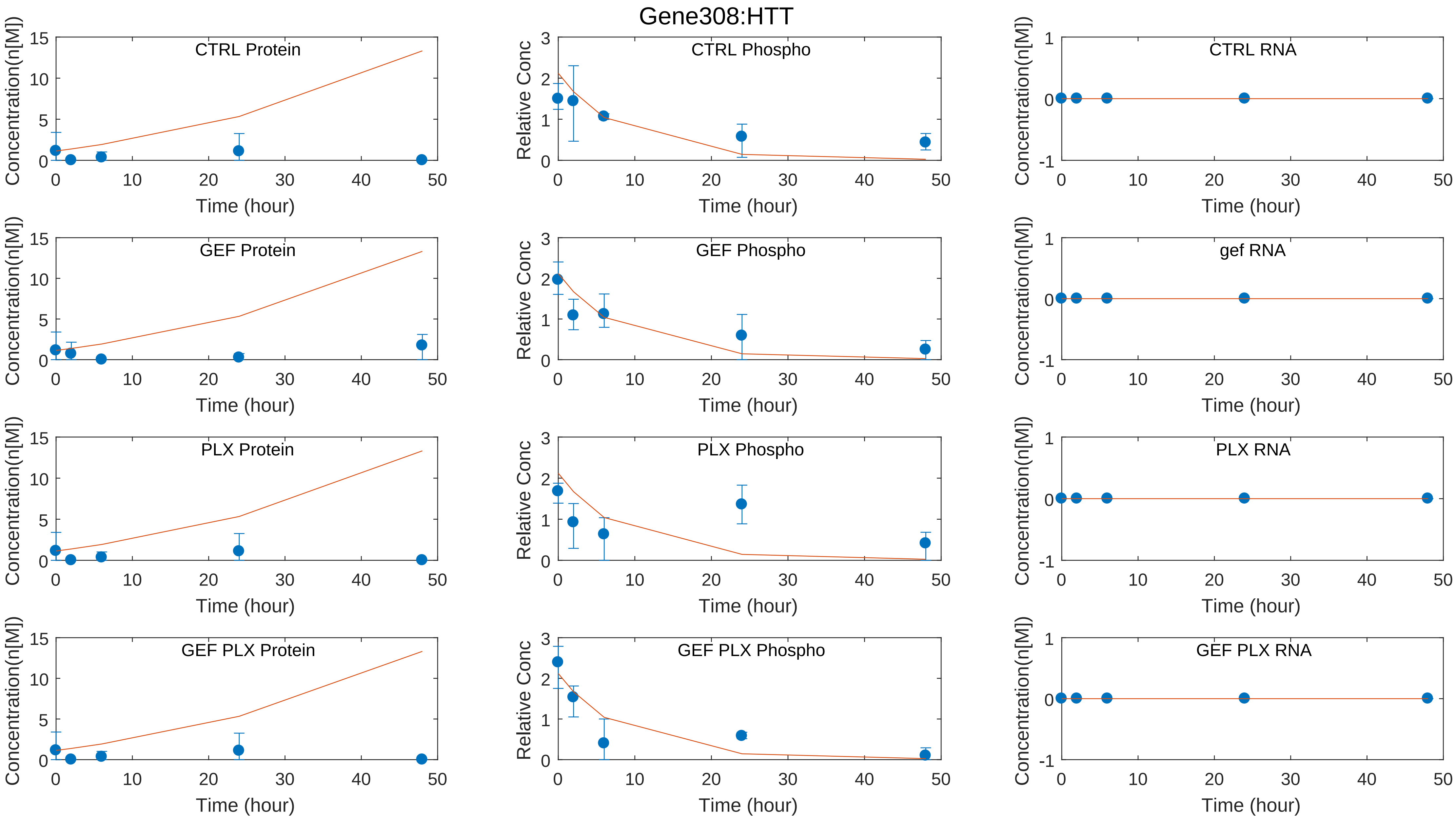
Gene308:HTT
CTRL Protein
CTRL Phospho
CTRL RNA
GEF Protein
GEF Phospho
gef RNA
PLX Protein
PLX Phospho
PLX RNA
GEF PLX Protein
GEF PLX Phospho
GEF PLX RNA
Concentration(n[M])
Relative Conc
Time (hour)

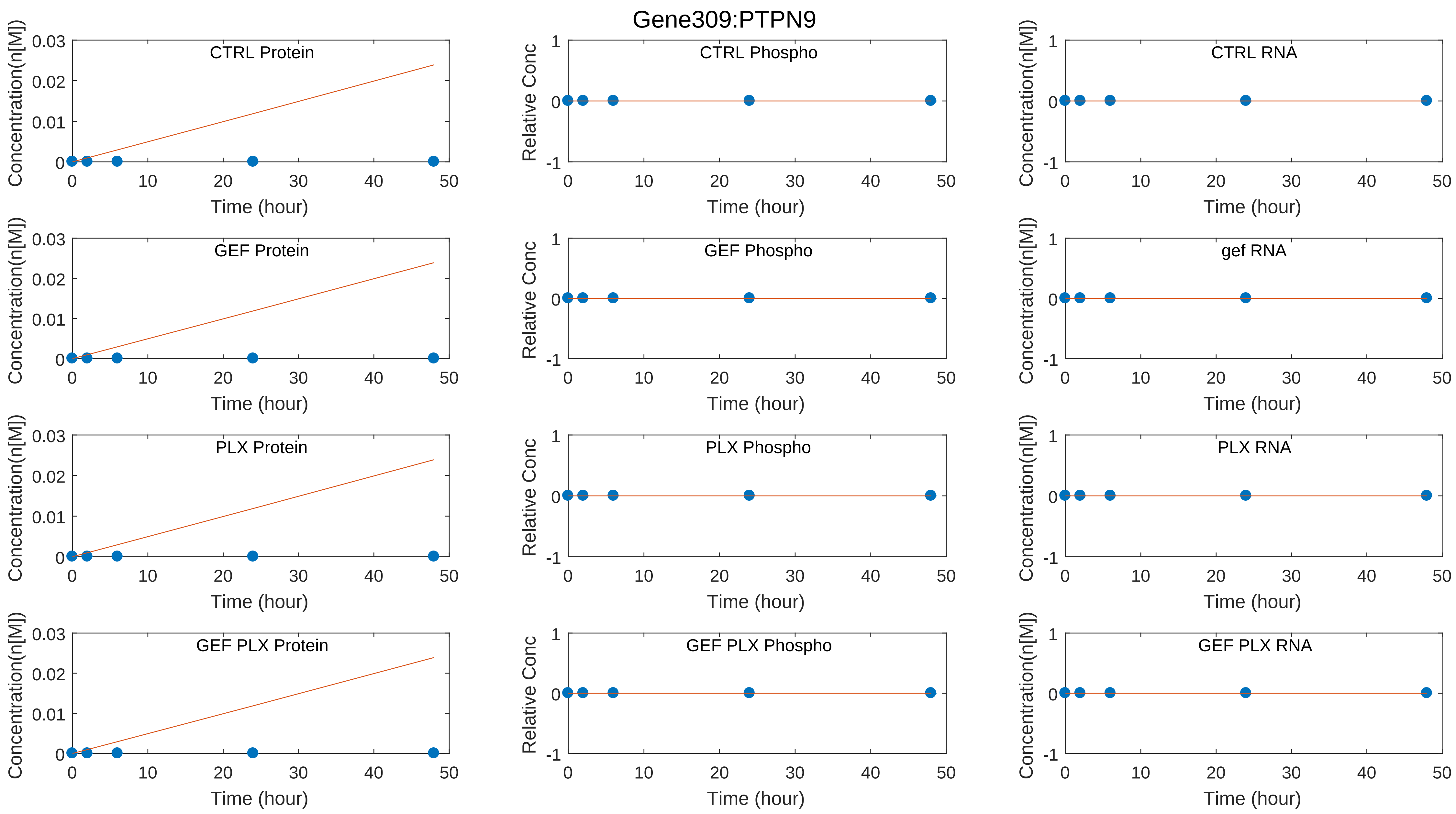

Gene309:PTPN9
CTRL Protein
CTRL Phospho
CTRL RNA
GEF Protein
GEF Phospho
gef RNA
PLX Protein
PLX Phospho
PLX RNA
GEF PLX Protein
GEF PLX Phospho
GEF PLX RNA
Concentration(n[M])
Relative Conc
Time (hour)

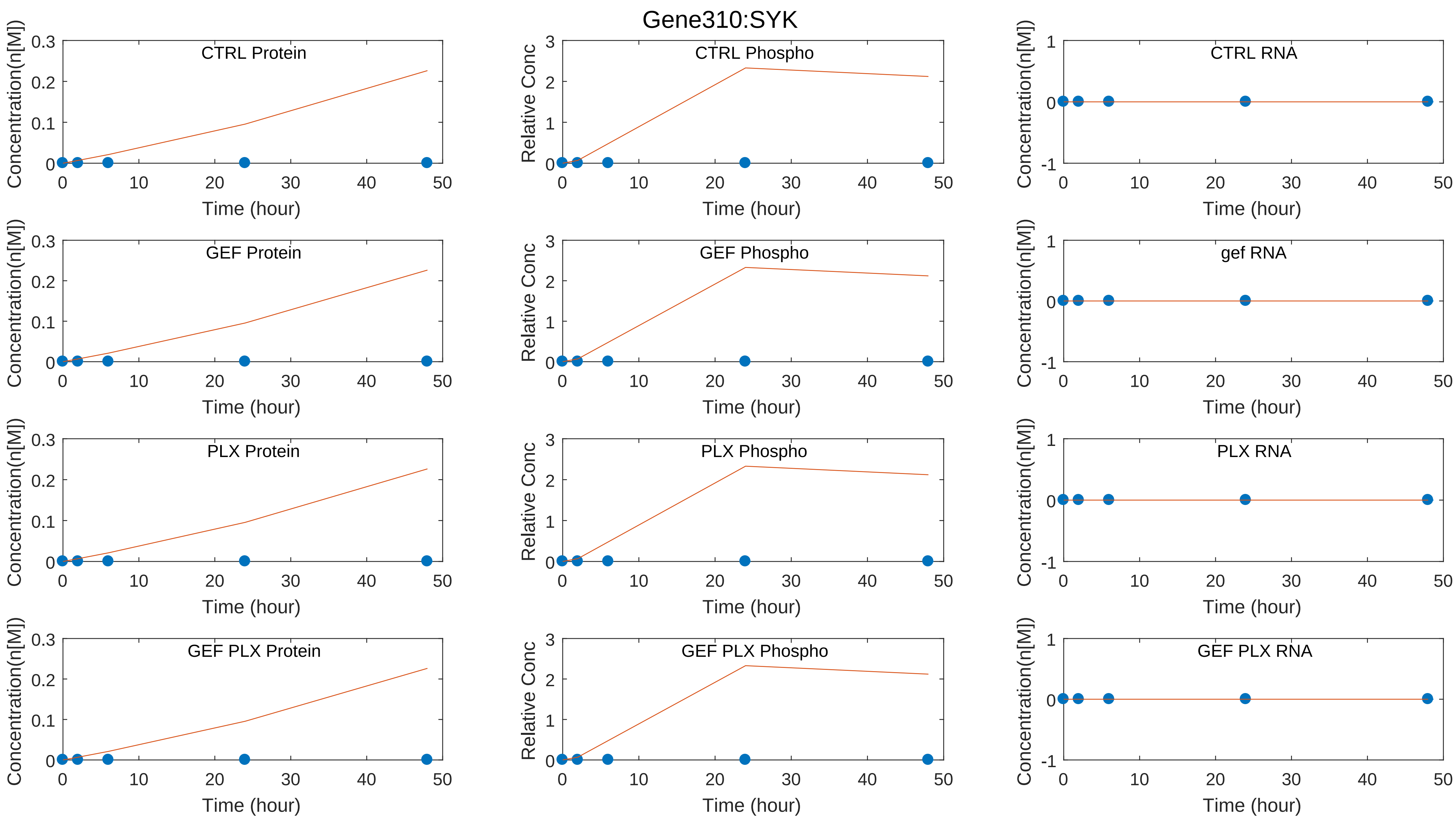
Gene310:SYK
CTRL Protein
CTRL Phospho
CTRL RNA
GEF Protein
GEF Phospho
gef RNA
PLX Protein
PLX Phospho
PLX RNA
GEF PLX Protein
GEF PLX Phospho
GEF PLX RNA
Concentration(n[M])
Relative Conc
Time (hour)
0.3
0.2
0.1
0
10
20
30
40
50
3
2
1
-1

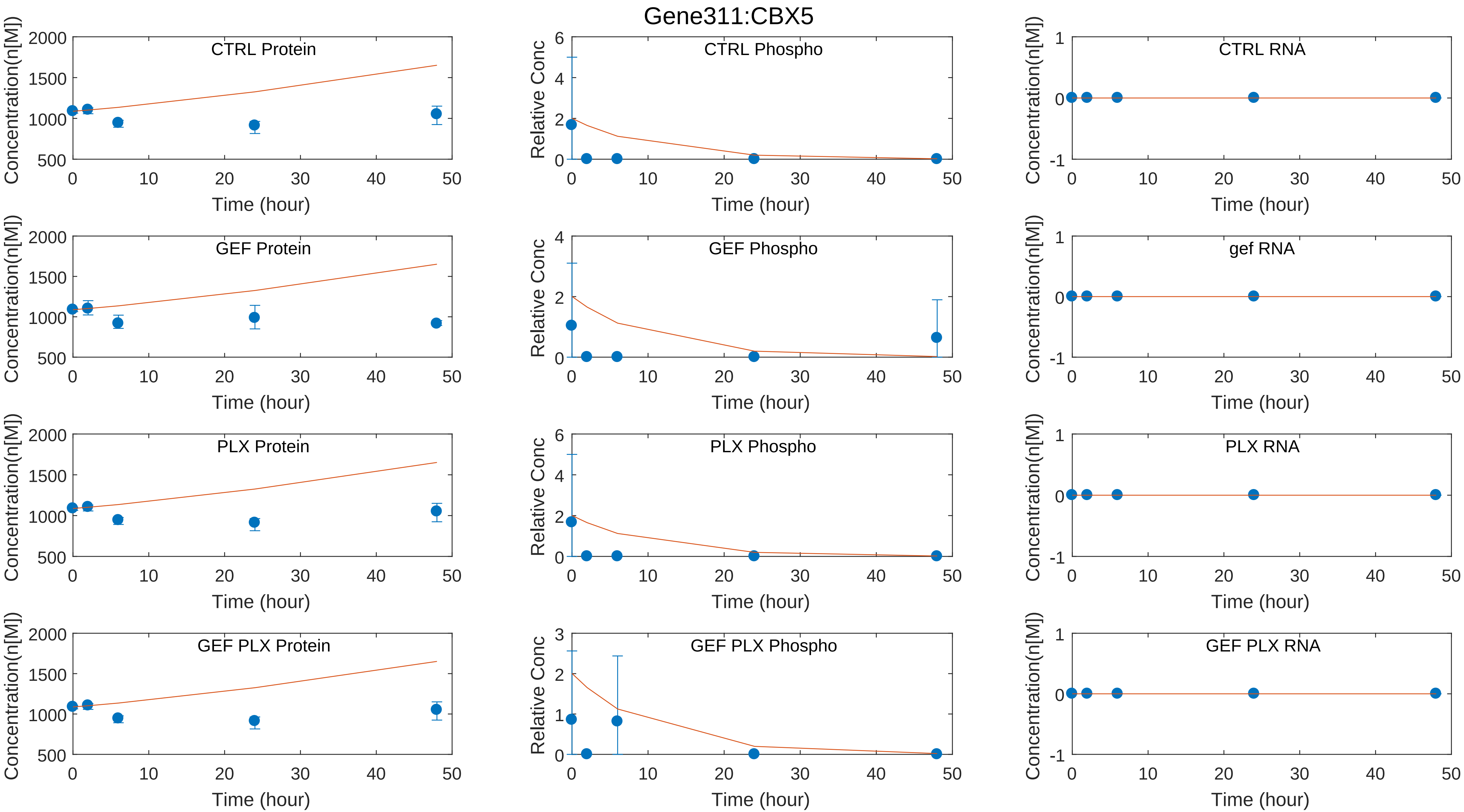

Gene311:CBX5
CTRL Protein
GEF Protein
PLX Protein
GEF PLX Protein
CTRL Phospho
GEF Phospho
PLX Phospho
GEF PLX Phospho
CTRL RNA
gef RNA
PLX RNA
GEF PLX RNA
Concentration(n[M])
Relative Conc
Time (hour)

Gene312:MAPK8

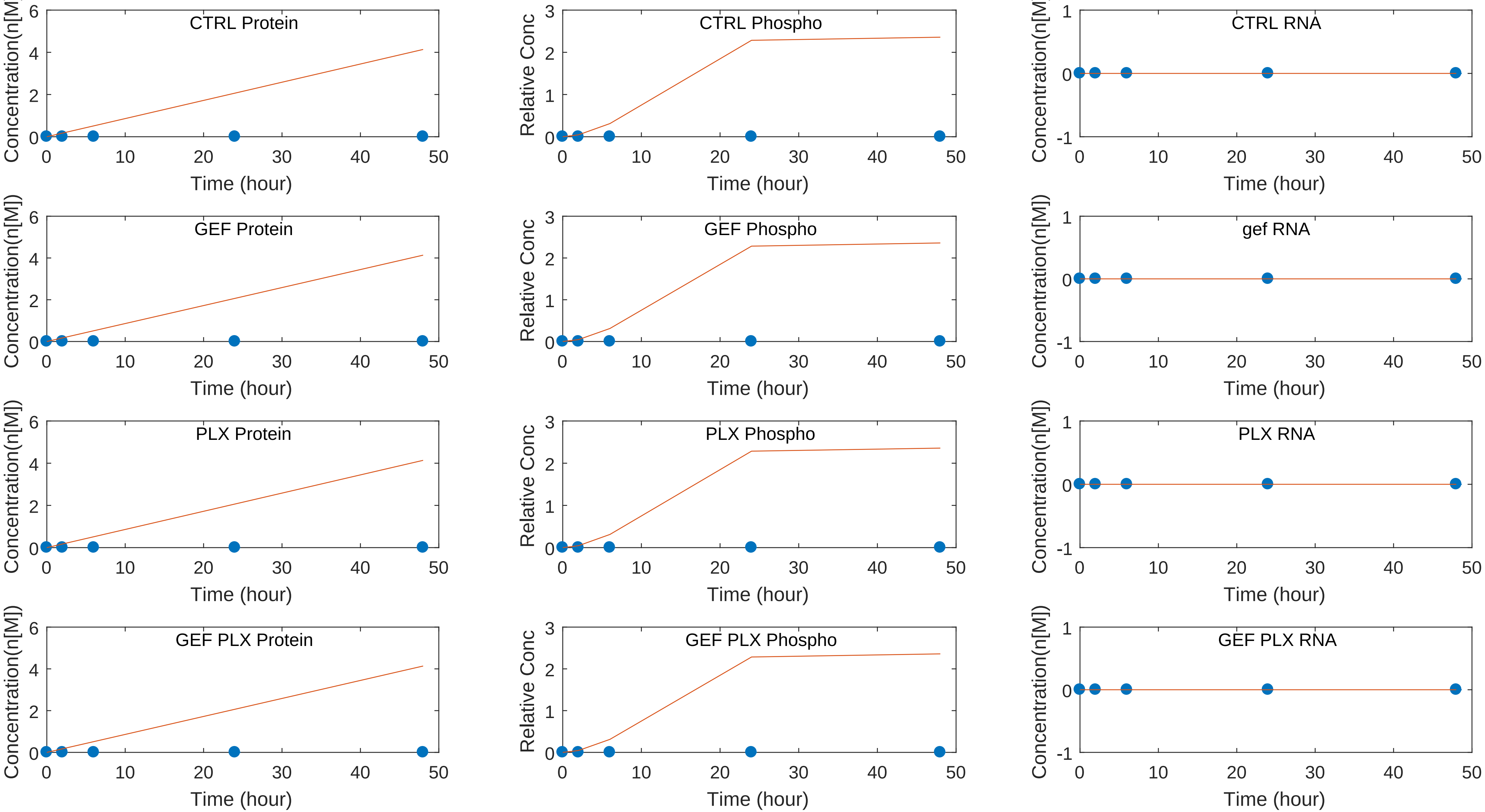

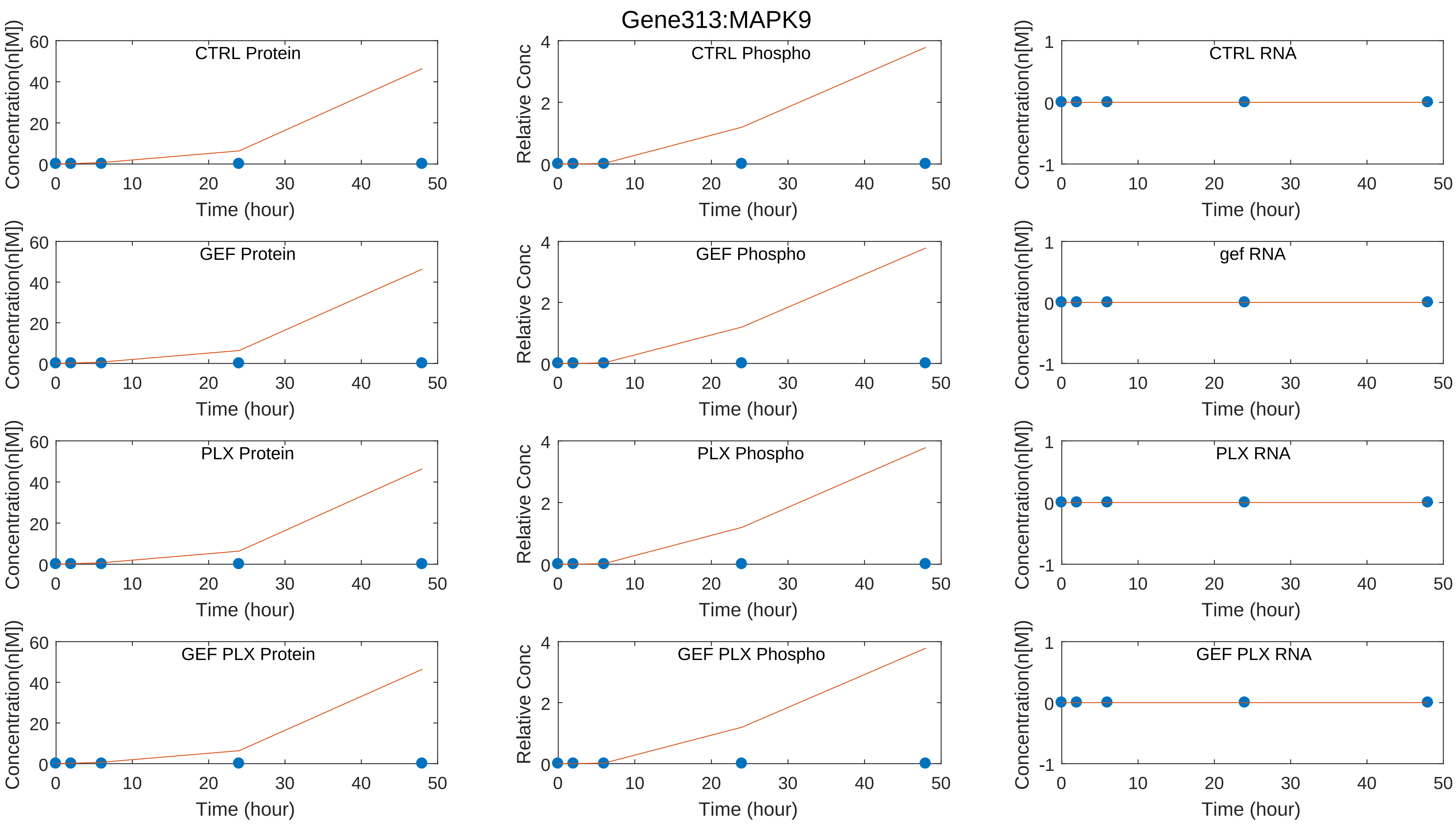

Gene313:MAPK9
CTRL Protein
CTRL Phospho
CTRL RNA
GEF Protein
GEF Phospho
gef RNA
PLX Protein
PLX Phospho
PLX RNA
GEF PLX Protein
GEF PLX Phospho
GEF PLX RNA
Concentration(n[M])
Relative Conc
Time (hour)

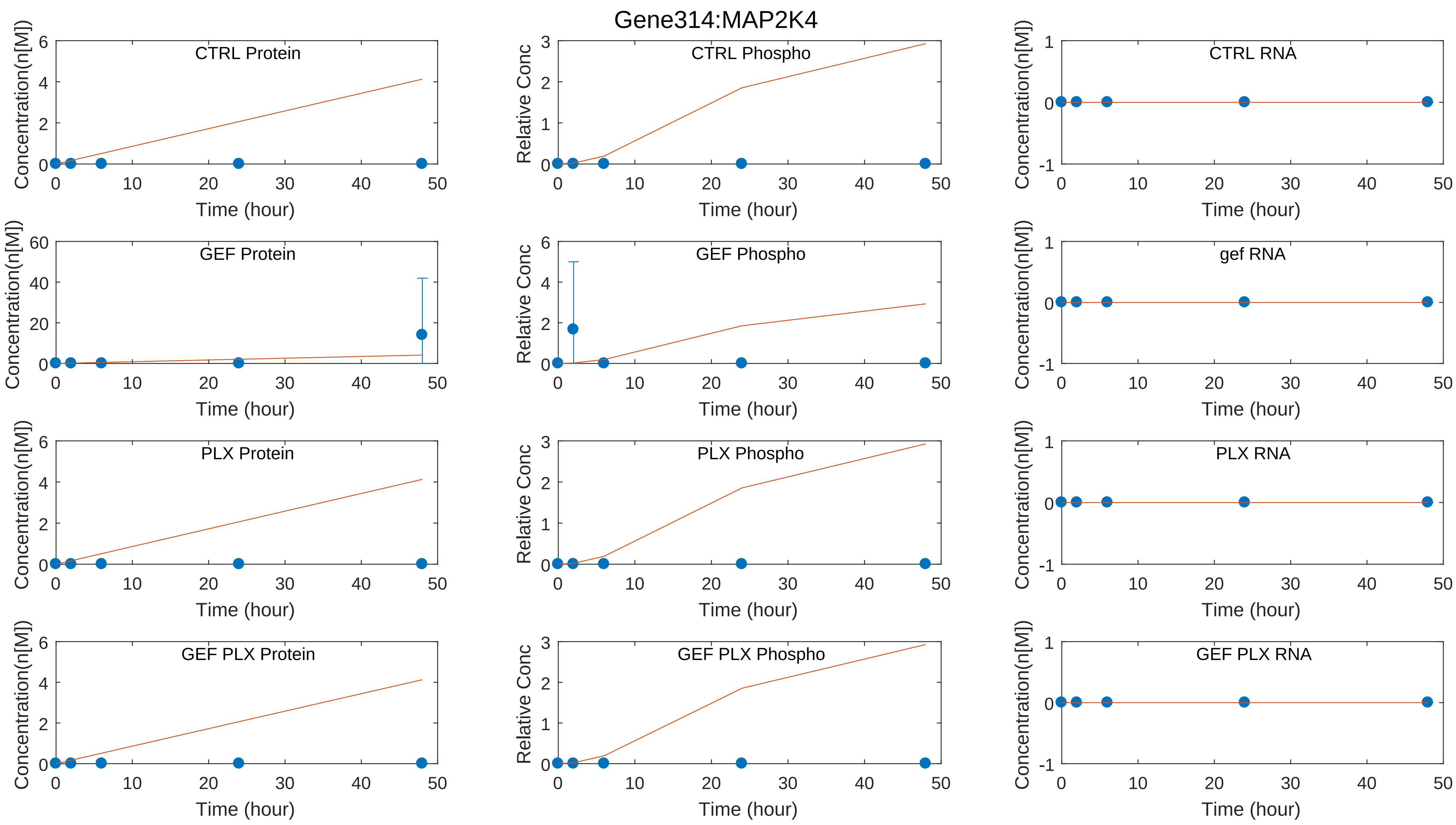

Gene314:MAP2K4
CTRL Protein
CTRL Phospho
CTRL RNA
GEF Protein
GEF Phospho
gef RNA
PLX Protein
PLX Phospho
PLX RNA
GEF PLX Protein
GEF PLX Phospho
GEF PLX RNA
Concentration(n[M])
Relative Conc
Time (hour)

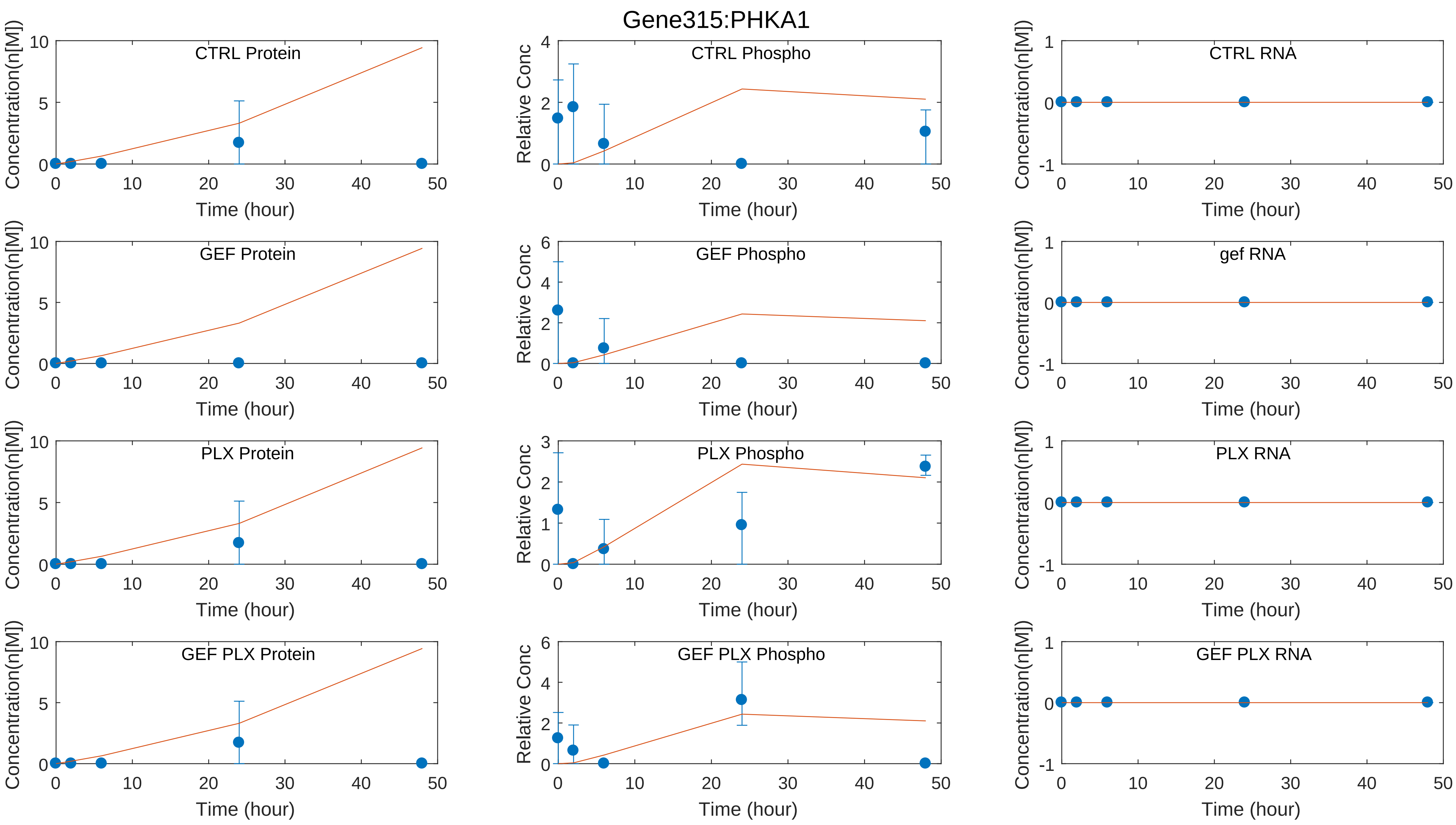

Gene315:PHKA1
CTRL Protein
CTRL Phospho
CTRL RNA
GEF Protein
GEF Phospho
gef RNA
PLX Protein
PLX Phospho
PLX RNA
GEF PLX Protein
GEF PLX Phospho
GEF PLX RNA
Concentration(n[M])
Relative Conc
Time (hour)

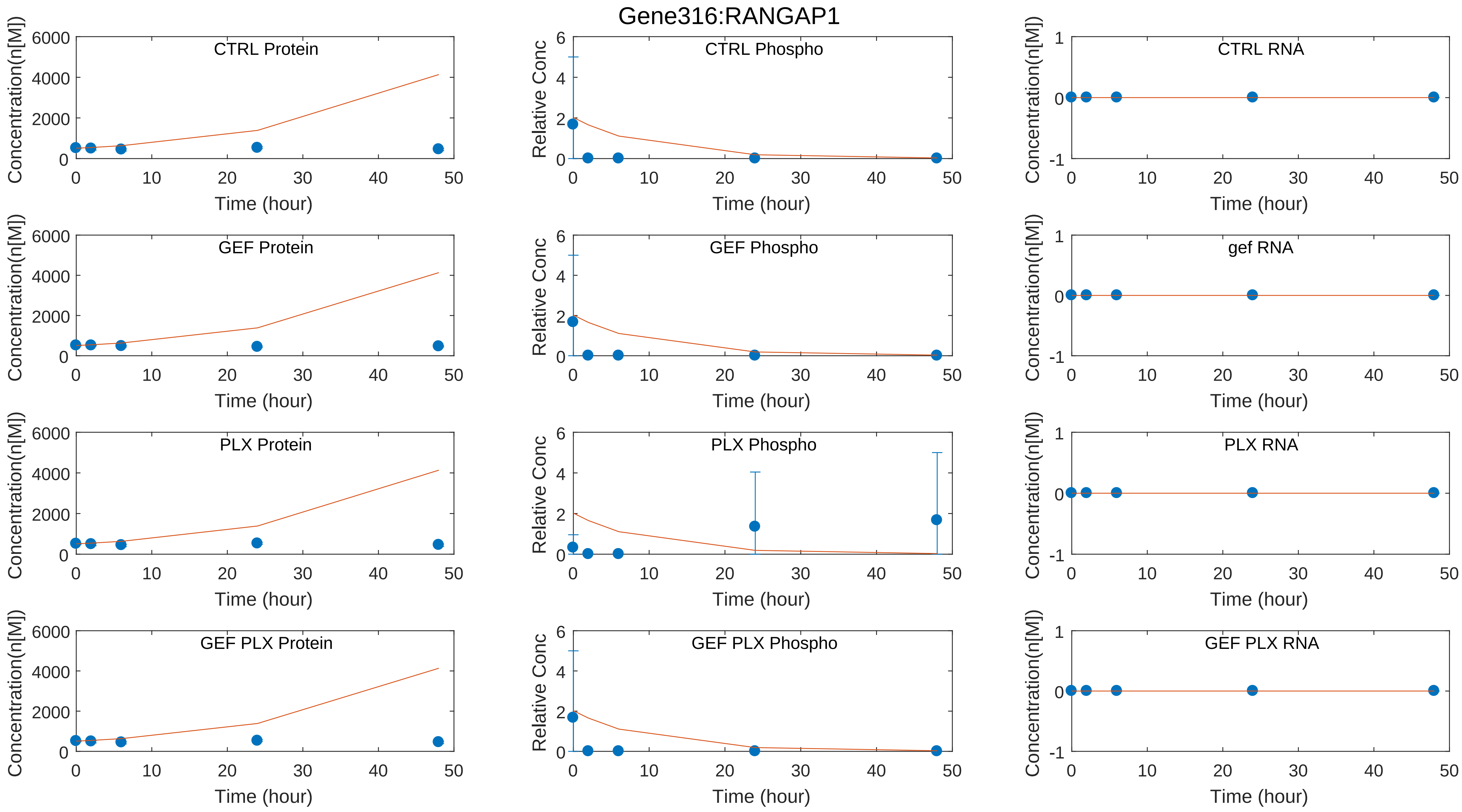
Gene316:RANGAP1
CTRL Protein
GEF Protein
PLX Protein
GEF PLX Protein
CTRL Phospho
GEF Phospho
PLX Phospho
GEF PLX Phospho
CTRL RNA
gef RNA
PLX RNA
GEF PLX RNA
Concentration(n[M])
Relative Conc
Time (hour)

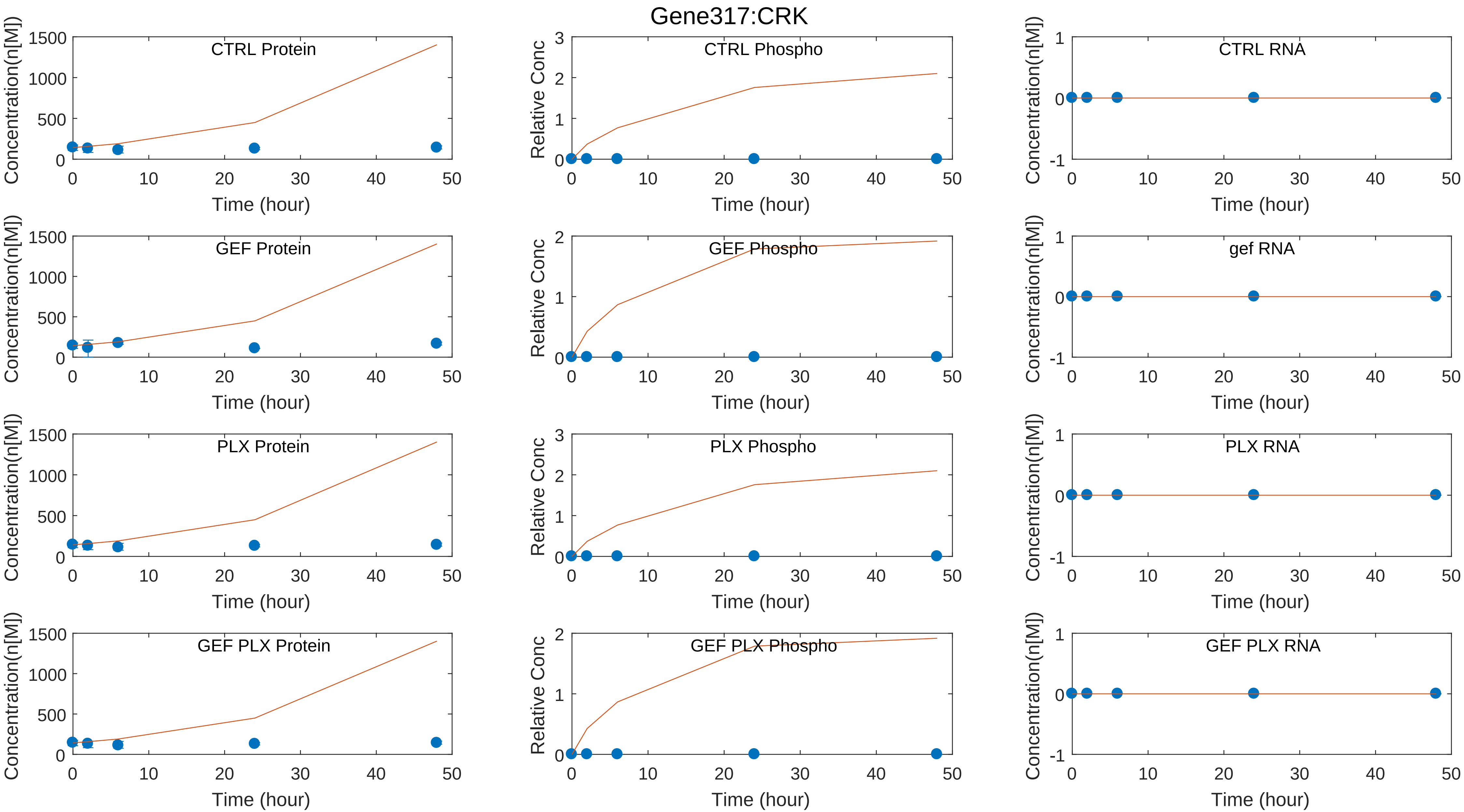

Gene317:CRK
CTRL Protein
GEF Protein
PLX Protein
GEF PLX Protein
CTRL Phospho
GEF Phospho
PLX Phospho
GEF PLX Phospho
CTRL RNA
gef RNA
PLX RNA
GEF PLX RNA
Concentration(n[M])
Relative Conc
Time (hour)

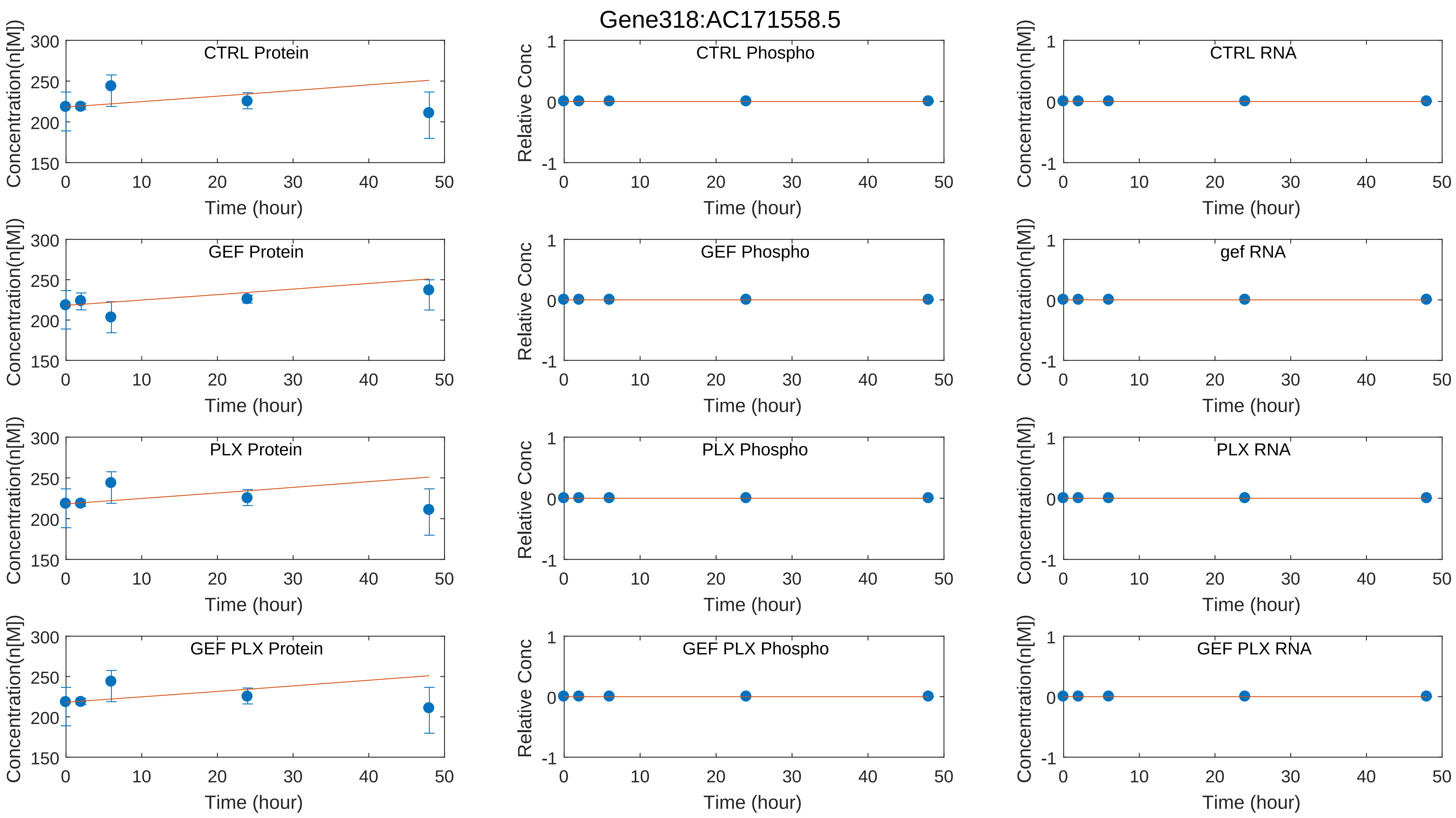
Gene318:AC171558.5
CTRL Protein
CTRL Phospho
CTRL RNA
GEF Protein
GEF Phospho
gef RNA
PLX Protein
PLX Phospho
PLX RNA
GEF PLX Protein
GEF PLX Phospho
GEF PLX RNA
Concentration(n[M])
Relative Conc
Time (hour)

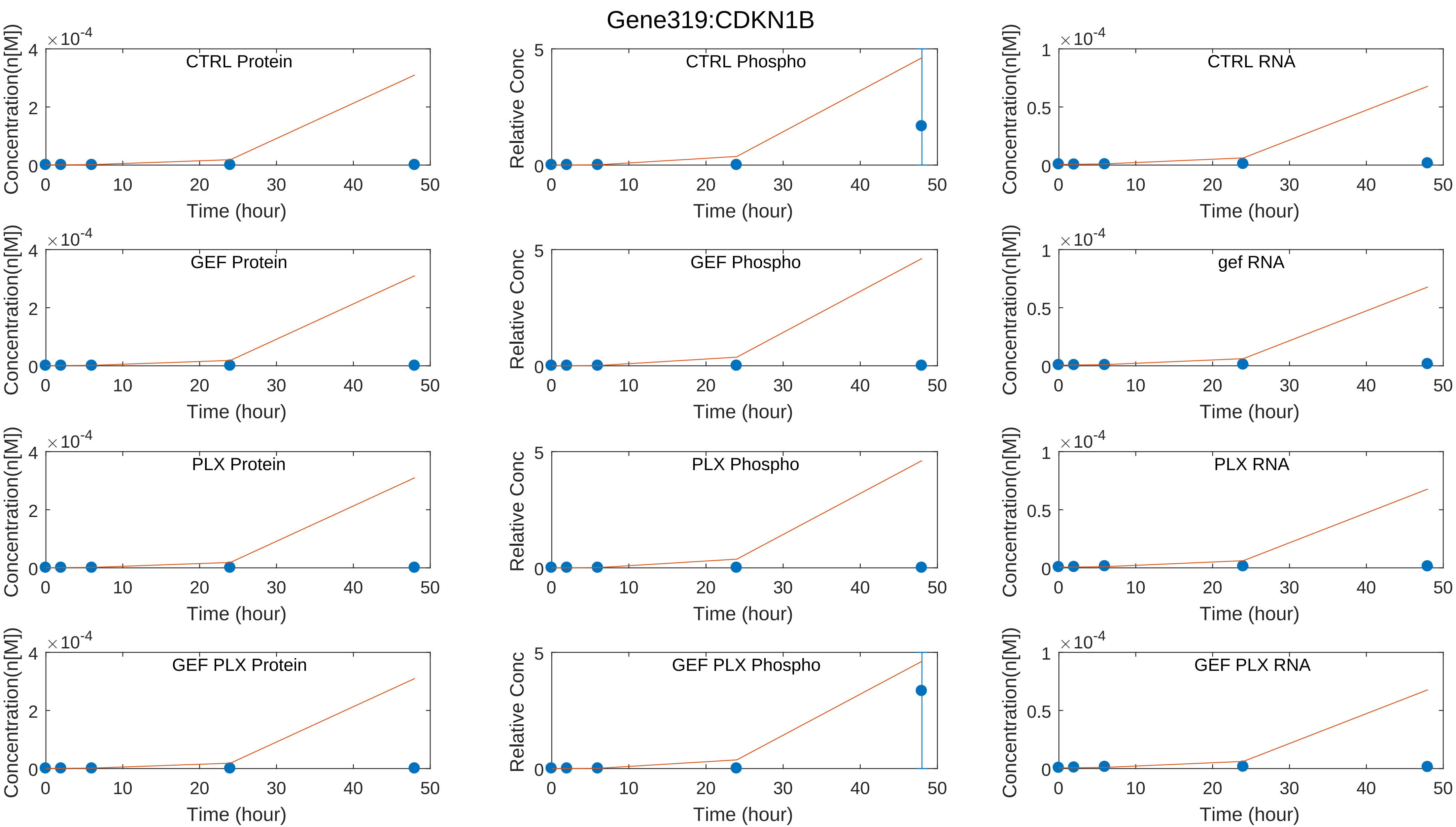

Gene319:CDKN1B
CTRL Protein
CTRL Phospho
CTRL RNA
GEF Protein
GEF Phospho
gef RNA
PLX Protein
PLX Phospho
PLX RNA
GEF PLX Protein
GEF PLX Phospho
GEF PLX RNA
Concentration(n[M])
Relative Conc
Time (hour)
×10-4

Gene320:MAP2K3

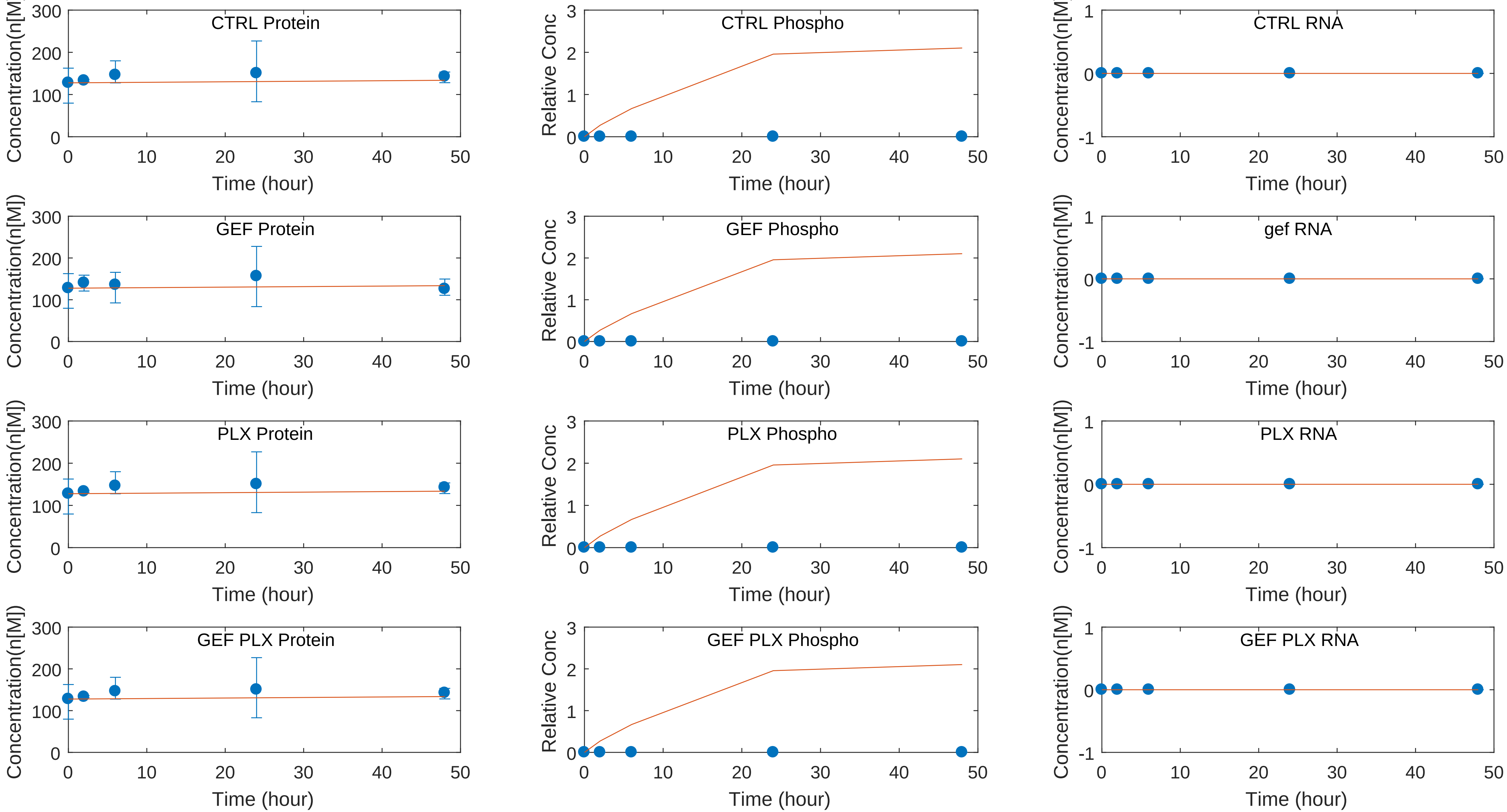

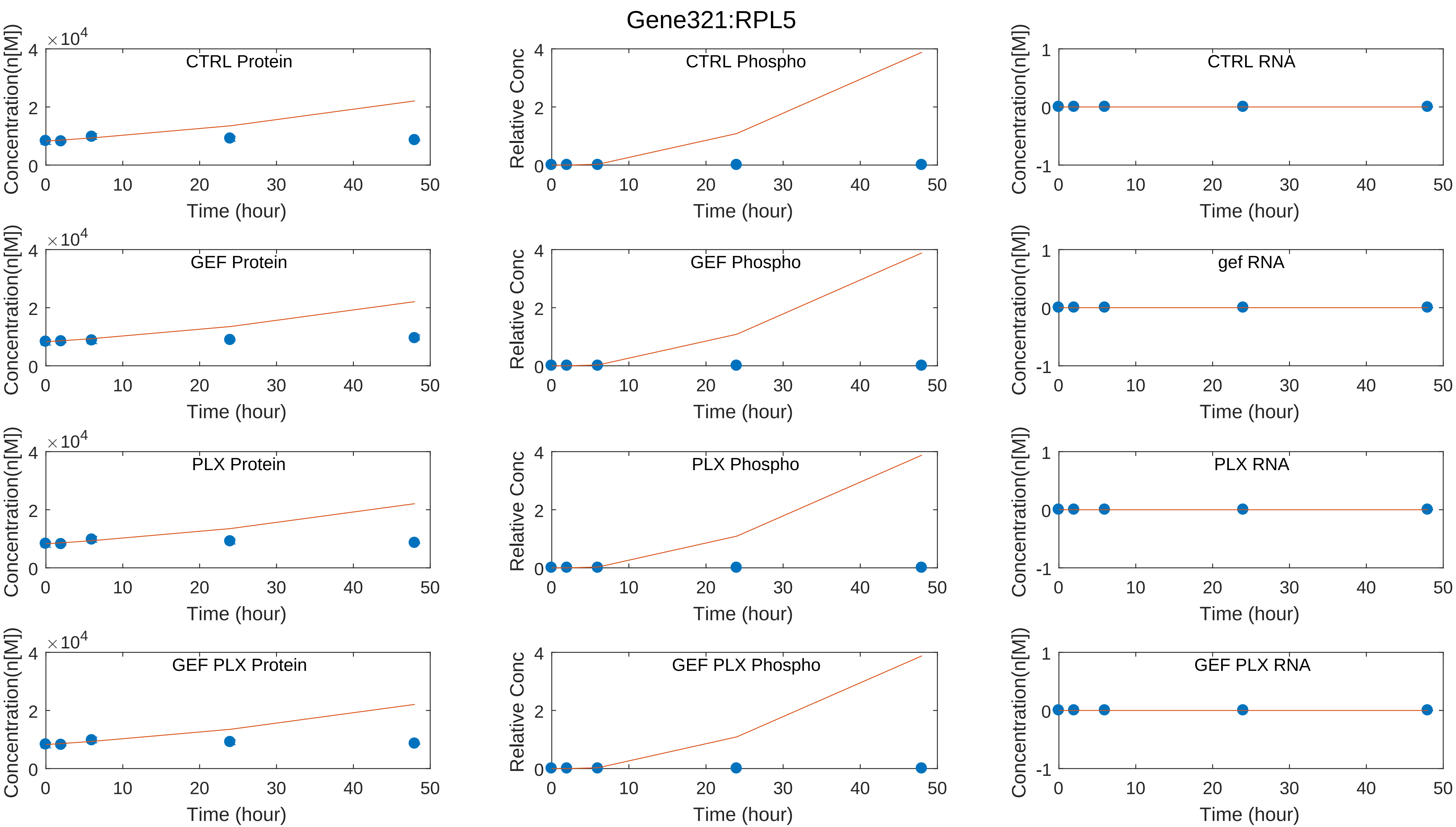
Gene321:RPL5
CTRL Protein
GEF Protein
PLX Protein
GEF PLX Protein
CTRL Phospho
GEF Phospho
PLX Phospho
GEF PLX Phospho
CTRL RNA
gef RNA
PLX RNA
GEF PLX RNA
Concentration(n[M])
Relative Conc
Time (hour)
×10^4

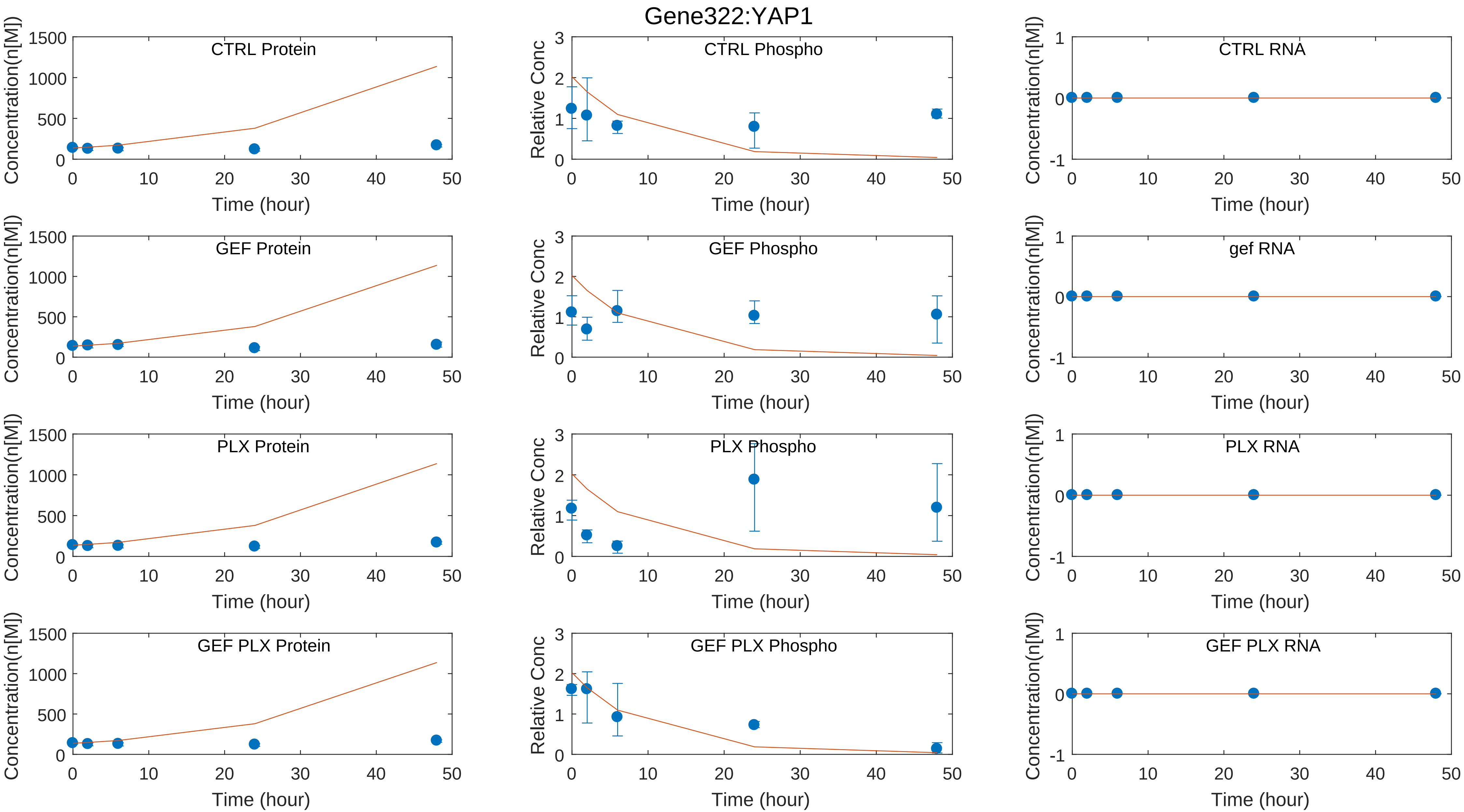

Gene322:YAP1
CTRL Protein
CTRL Phospho
CTRL RNA
GEF Protein
GEF Phospho
gef RNA
PLX Protein
PLX Phospho
PLX RNA
GEF PLX Protein
GEF PLX Phospho
GEF PLX RNA
Concentration(n[M])
Relative Conc
Time (hour)

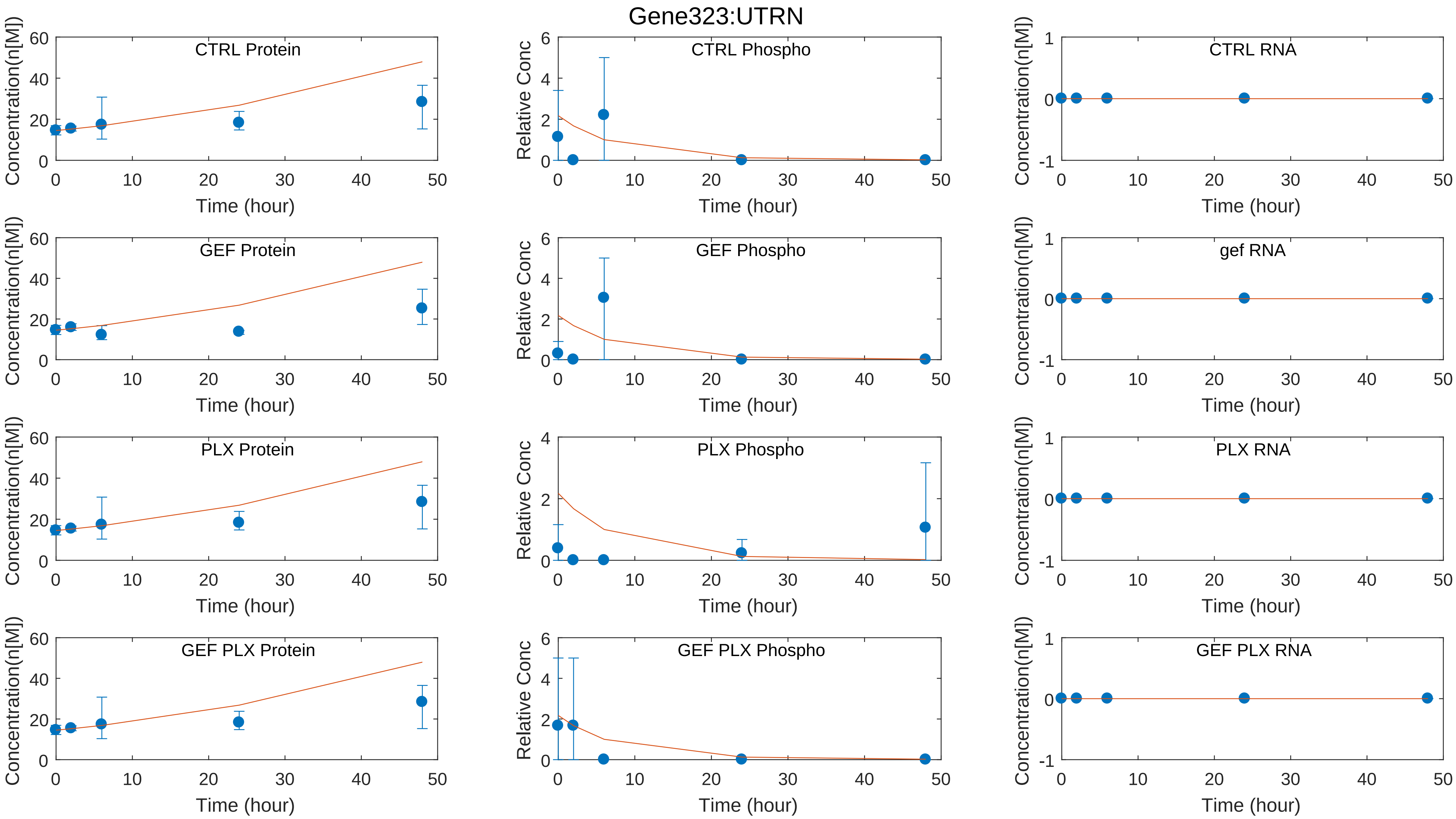
Gene323:UTRN
CTRL Protein
CTRL Phospho
CTRL RNA
GEF Protein
GEF Phospho
gef RNA
PLX Protein
PLX Phospho
PLX RNA
GEF PLX Protein
GEF PLX Phospho
GEF PLX RNA
Concentration(n[M])
Relative Conc
Time (hour)

Gene324:IQGAP1

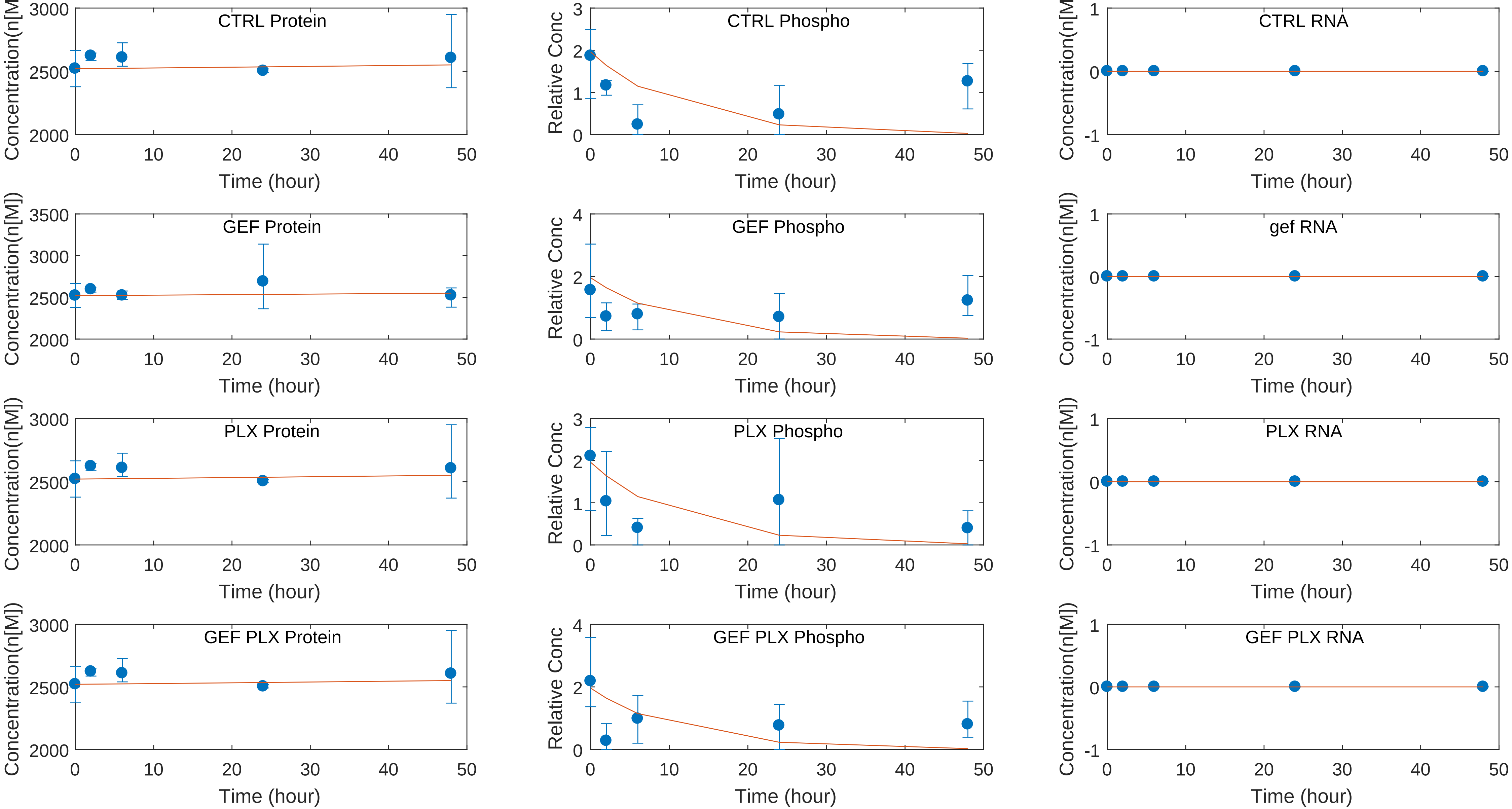

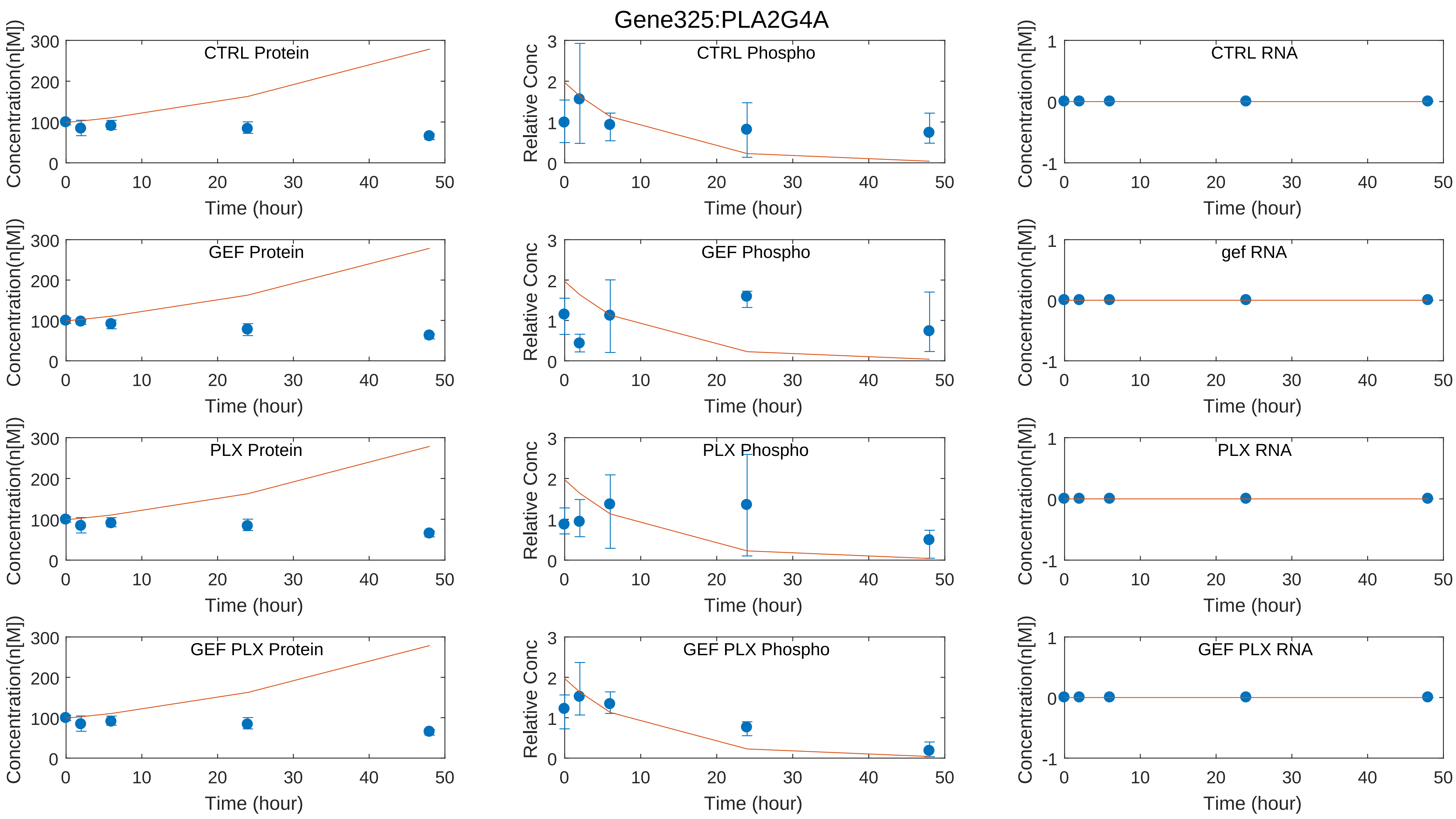
Gene325:PLA2G4A
CTRL Protein
GEF Protein
PLX Protein
GEF PLX Protein
CTRL Phospho
GEF Phospho
PLX Phospho
GEF PLX Phospho
CTRL RNA
gef RNA
PLX RNA
GEF PLX RNA
Concentration(n[M])
Relative Conc
Time (hour)

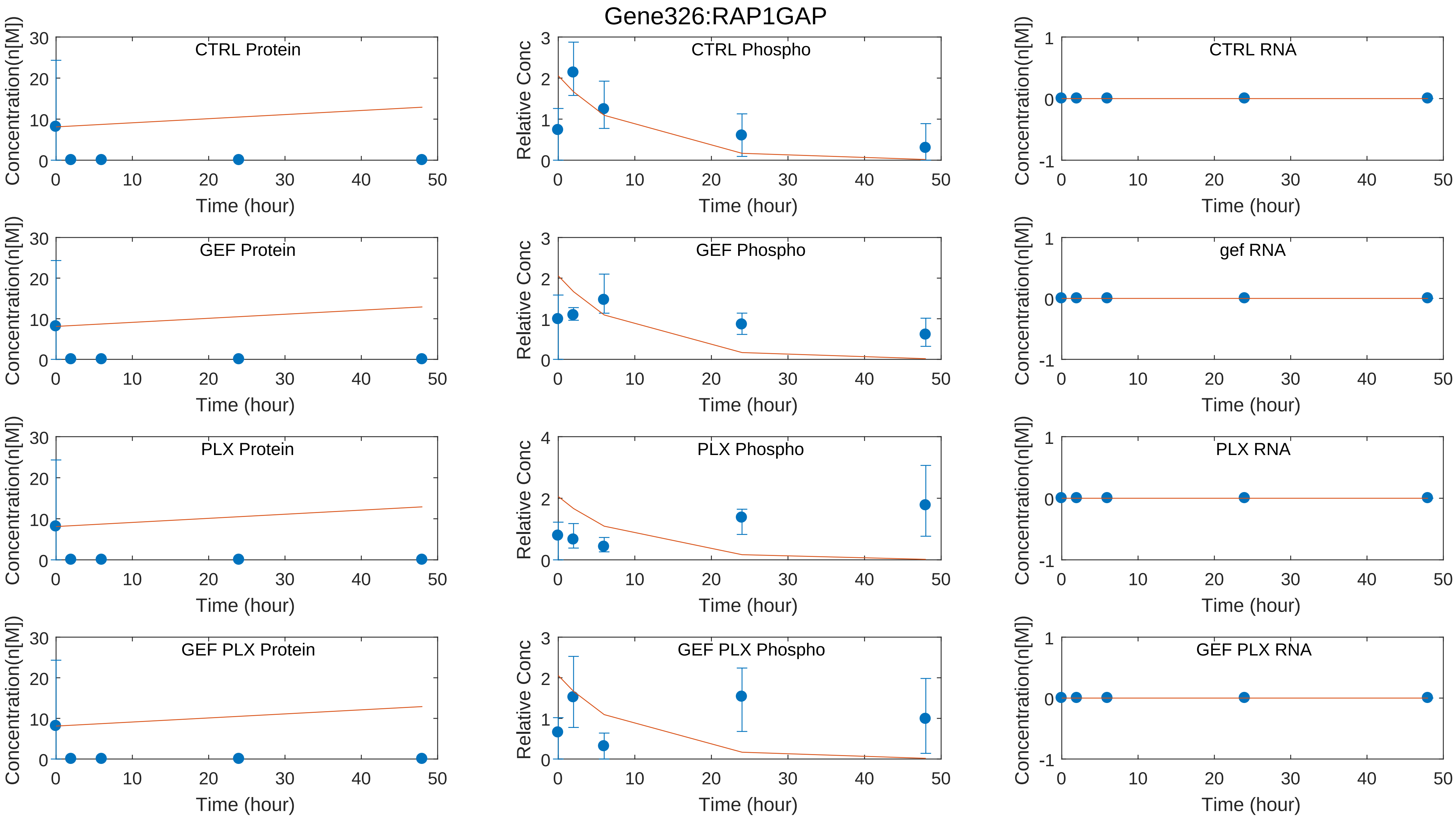
Gene326:RAP1GAP
CTRL Protein
CTRL Phospho
CTRL RNA
GEF Protein
GEF Phospho
gef RNA
PLX Protein
PLX Phospho
PLX RNA
GEF PLX Protein
GEF PLX Phospho
GEF PLX RNA
Concentration(n[M])
Relative Conc
Time (hour)

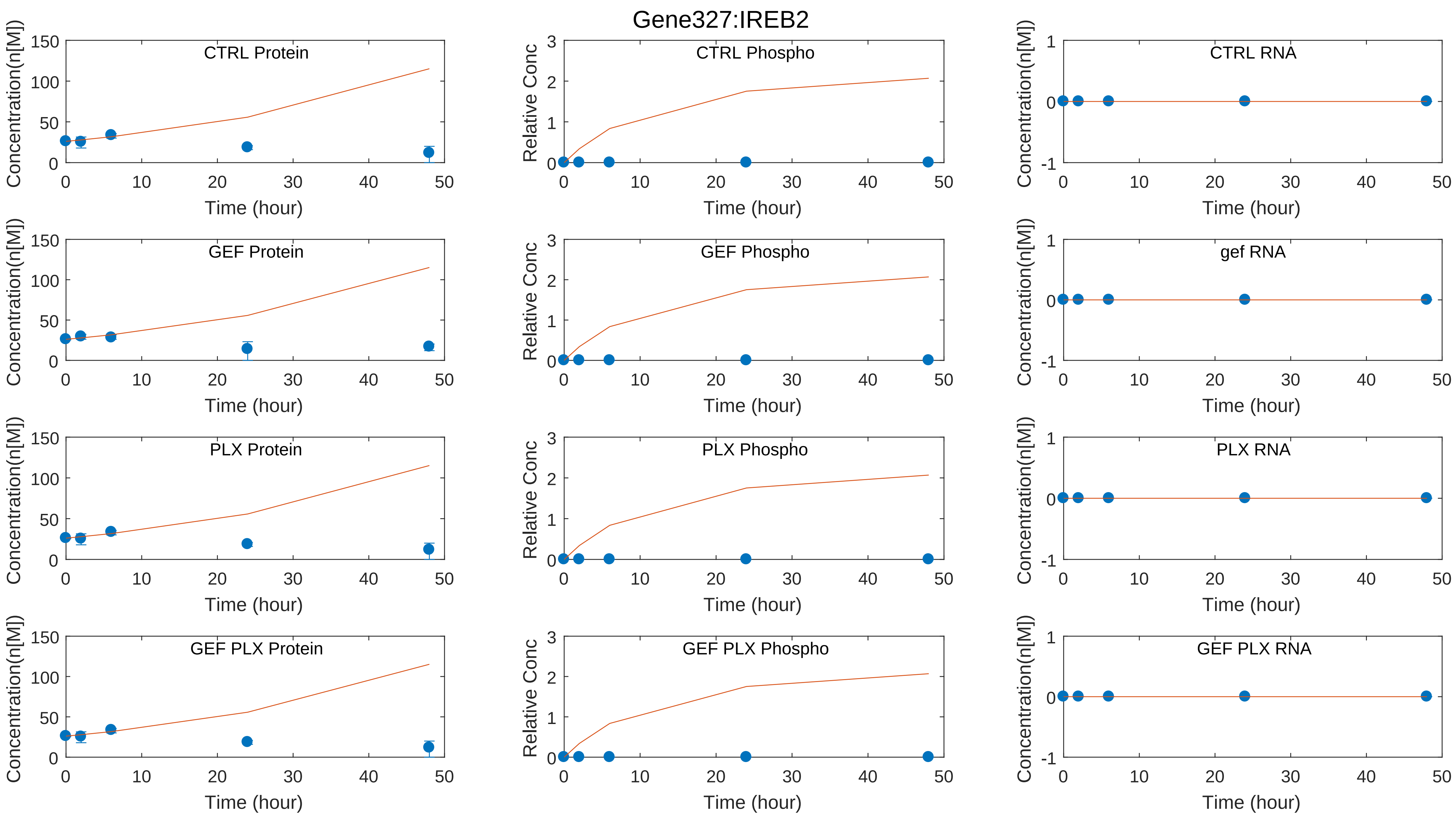

Gene327:IREB2
CTRL Protein
CTRL Phospho
CTRL RNA
GEF Protein
GEF Phospho
gef RNA
PLX Protein
PLX Phospho
PLX RNA
GEF PLX Protein
GEF PLX Phospho
GEF PLX RNA
Concentration(n[M])
Relative Conc
Time (hour)

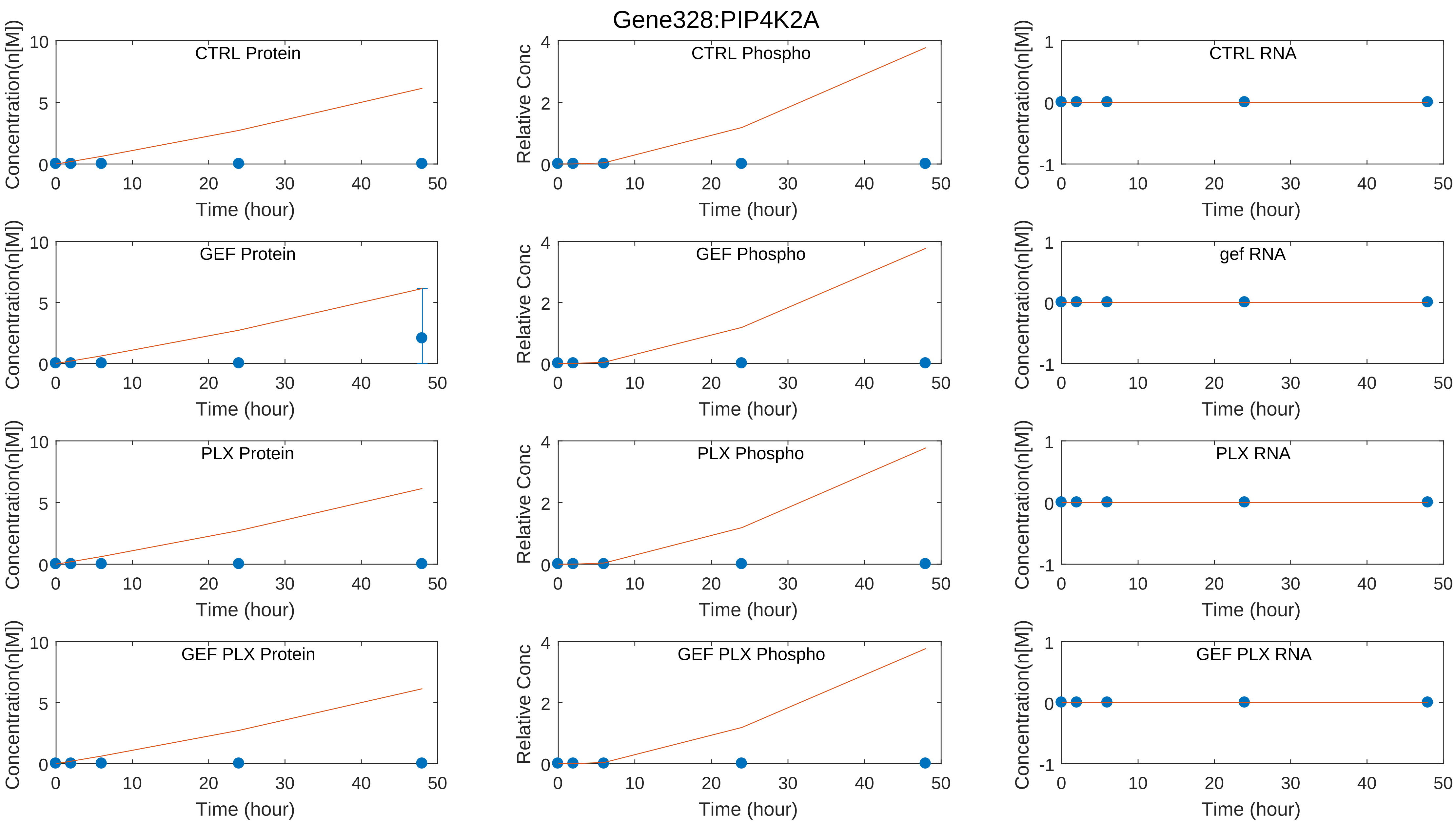
Gene328:PIP4K2A
CTRL Protein
CTRL Phospho
CTRL RNA
GEF Protein
GEF Phospho
gef RNA
PLX Protein
PLX Phospho
PLX RNA
GEF PLX Protein
GEF PLX Phospho
GEF PLX RNA
Concentration(n[M])
Relative Conc
Time (hour)

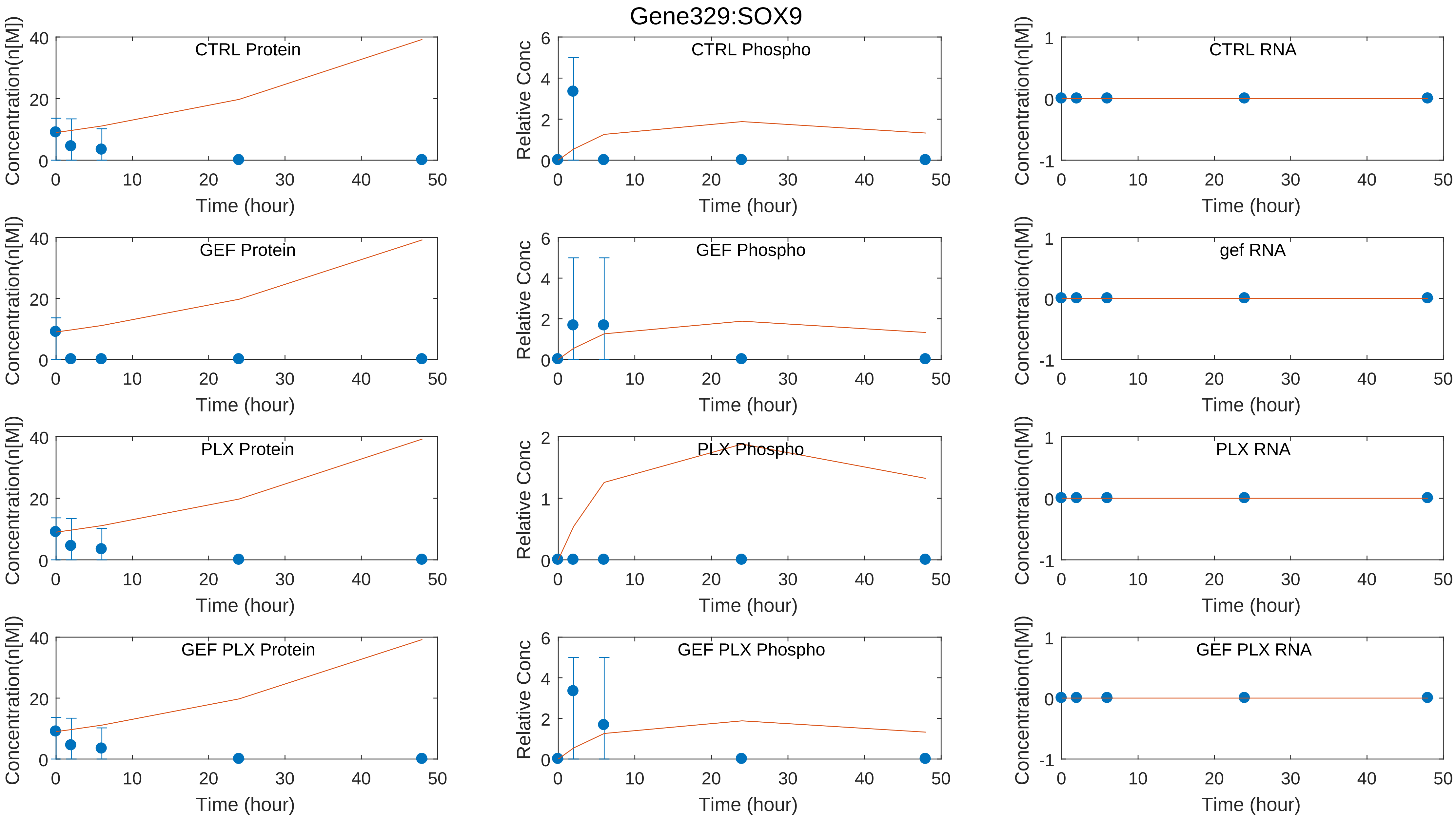

Gene329:SOX9
CTRL Protein
CTRL Phospho
CTRL RNA
GEF Protein
GEF Phospho
gef RNA
PLX Protein
PLX Phospho
PLX RNA
GEF PLX Protein
GEF PLX Phospho
GEF PLX RNA
Concentration(n[M])
Relative Conc
Time (hour)

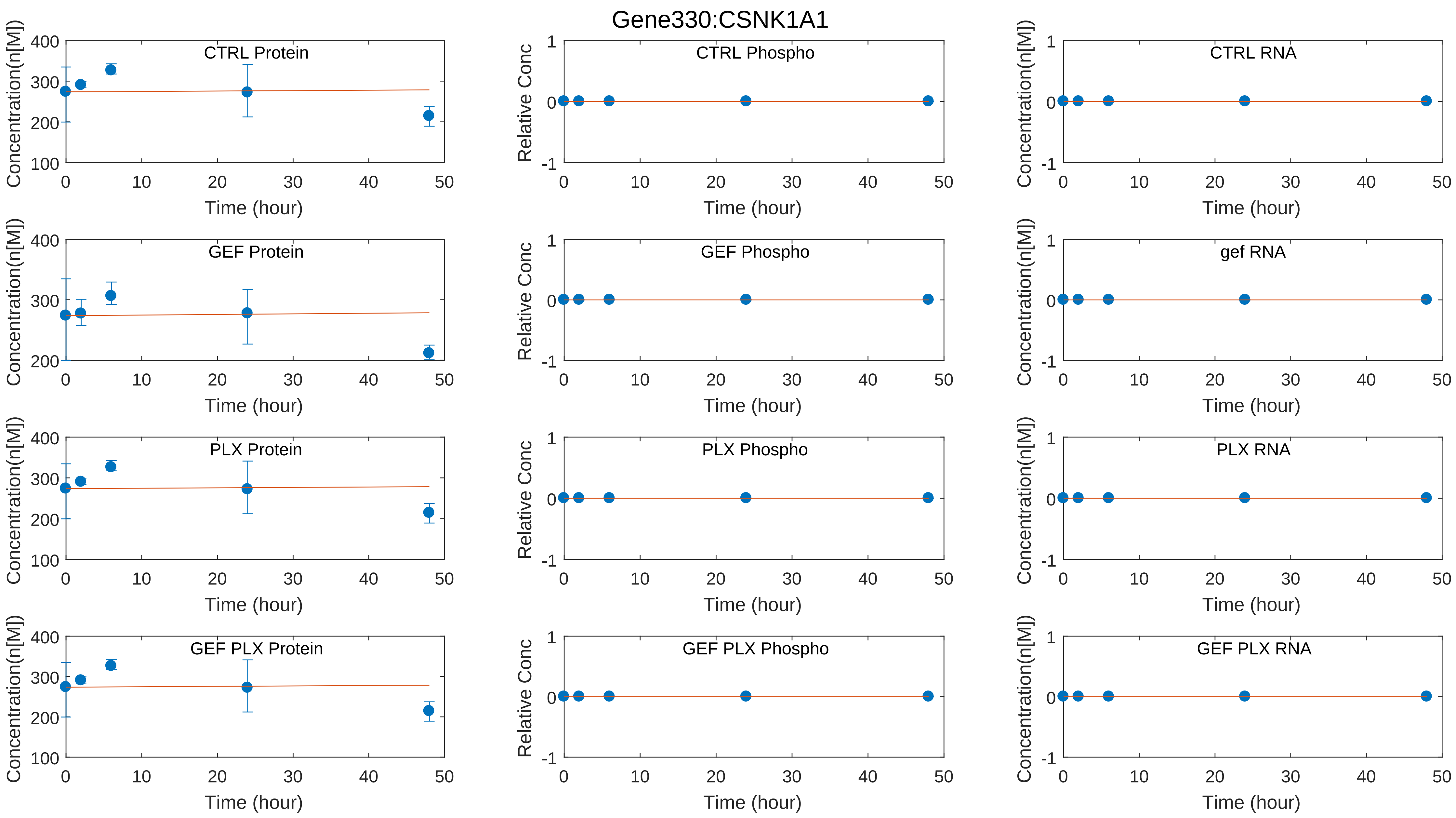

Gene330:CSNK1A1
CTRL Protein
CTRL Phospho
CTRL RNA
GEF Protein
GEF Phospho
gef RNA
PLX Protein
PLX Phospho
PLX RNA
GEF PLX Protein
GEF PLX Phospho
GEF PLX RNA
Concentration(n[M])
Relative Conc
Time (hour)

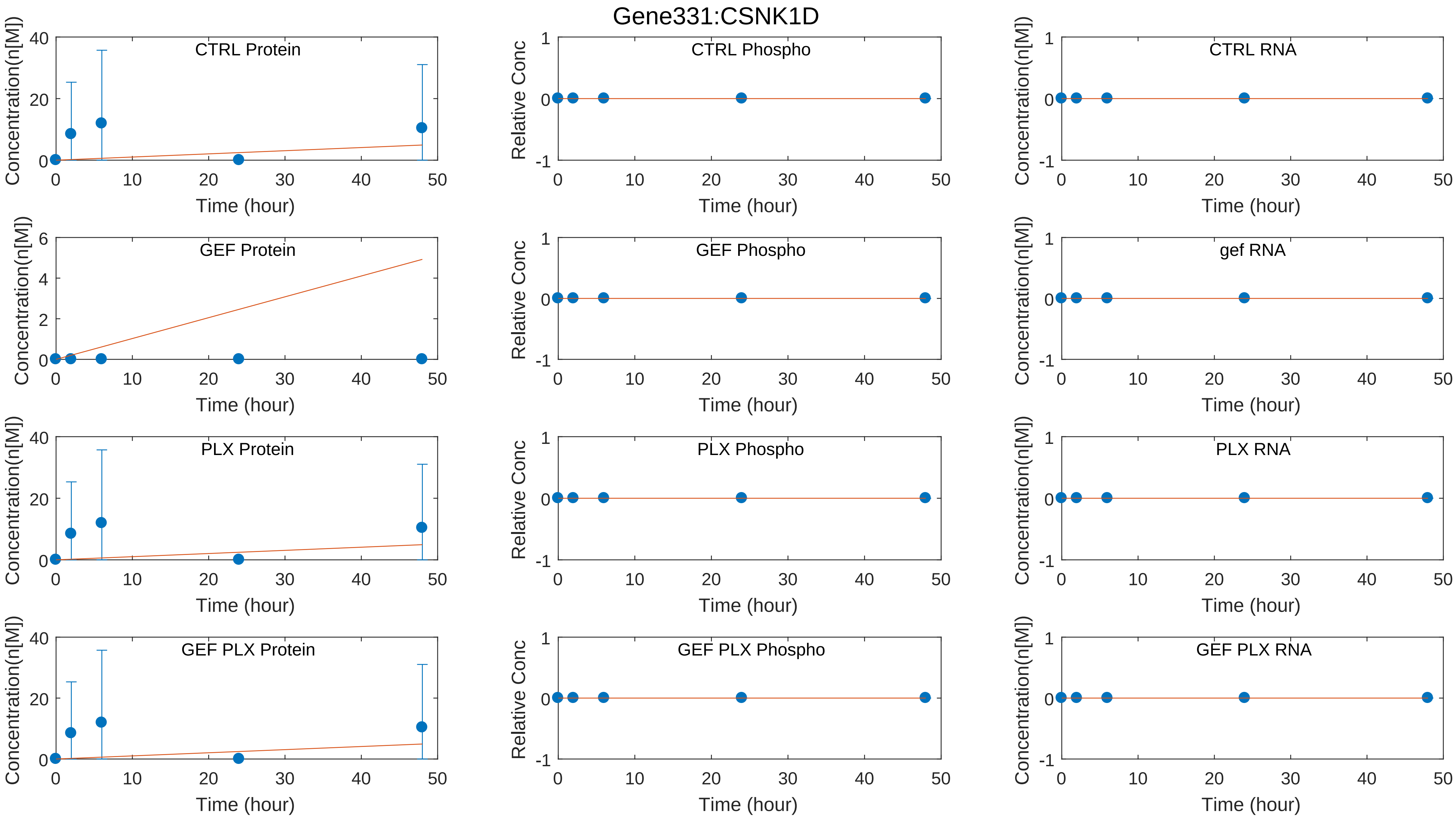
Gene331:CSNK1D
CTRL Protein
CTRL Phospho
CTRL RNA
GEF Protein
GEF Phospho
gef RNA
PLX Protein
PLX Phospho
PLX RNA
GEF PLX Protein
GEF PLX Phospho
GEF PLX RNA
Concentration(n[M])
Relative Conc
Time (hour)

Gene332:PXN

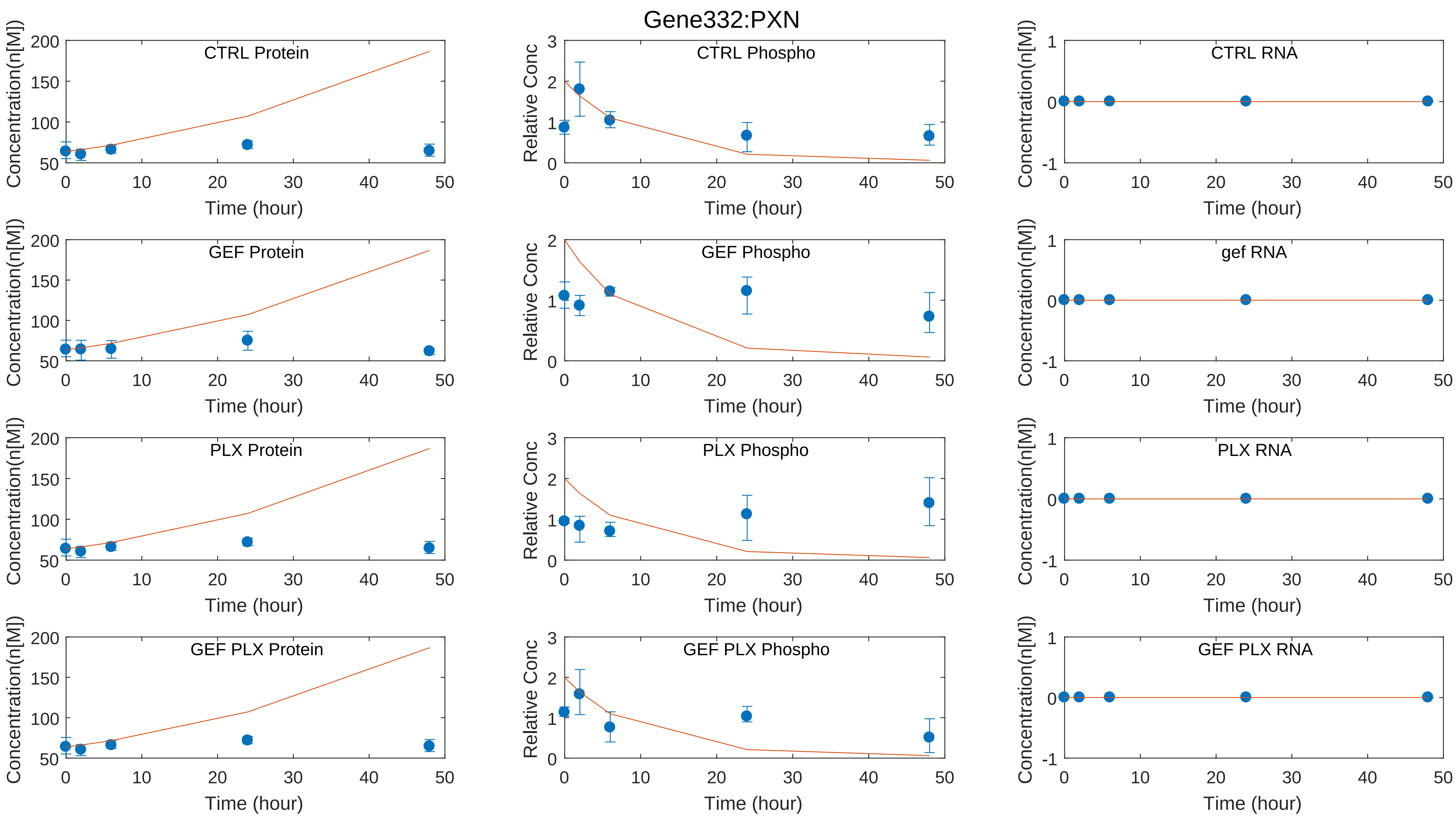

Gene332:PXN
CTRL Protein
CTRL Phospho
CTRL RNA
GEF Protein
GEF Phospho
gef RNA
PLX Protein
PLX Phospho
PLX RNA
GEF PLX Protein
GEF PLX Phospho
GEF PLX RNA
Concentration(n[M])
Relative Conc
Time (hour)

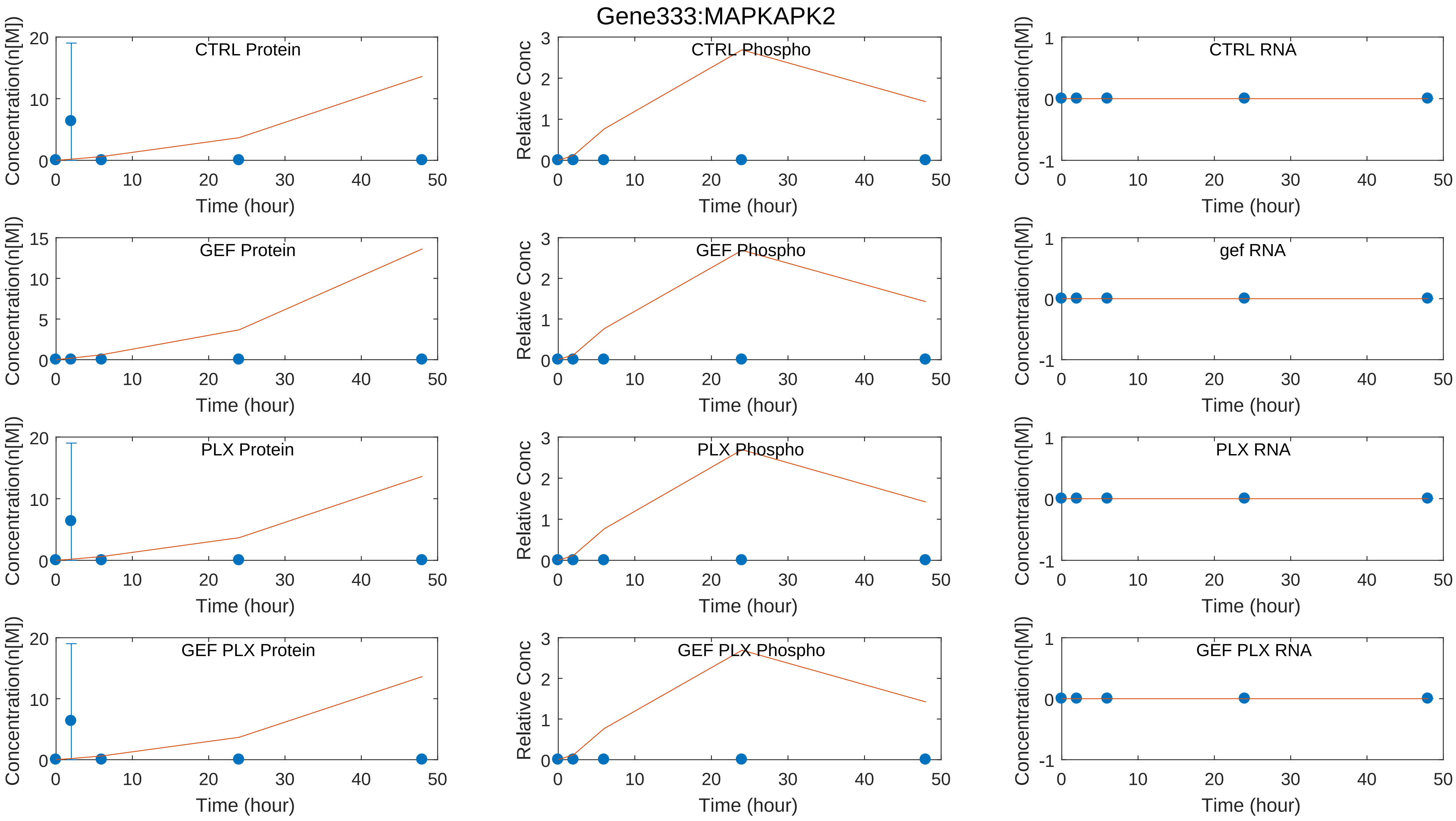

Gene333:MAPKAPK2
CTRL Protein
CTRL Phospho
CTRL RNA
GEF Protein
GEF Phospho
gef RNA
PLX Protein
PLX Phospho
PLX RNA
GEF PLX Protein
GEF PLX Phospho
GEF PLX RNA
Concentration(n[M])
Relative Conc
Time (hour)

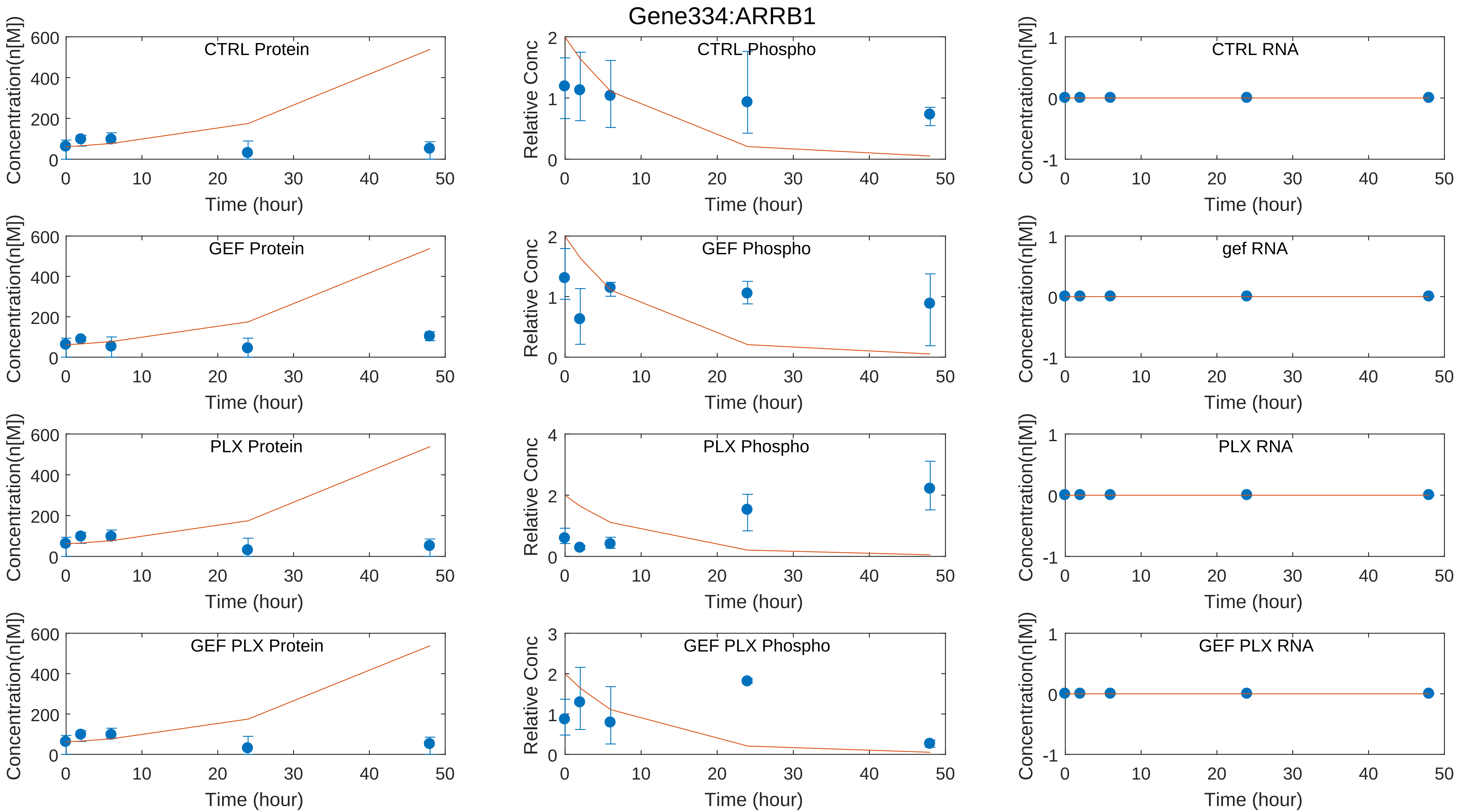
Gene334:ARRB1
CTRL Protein
CTRL Phospho
CTRL RNA
GEF Protein
GEF Phospho
gef RNA
PLX Protein
PLX Phospho
PLX RNA
GEF PLX Protein
GEF PLX Phospho
GEF PLX RNA
Concentration(n[M])
Relative Conc
Time (hour)

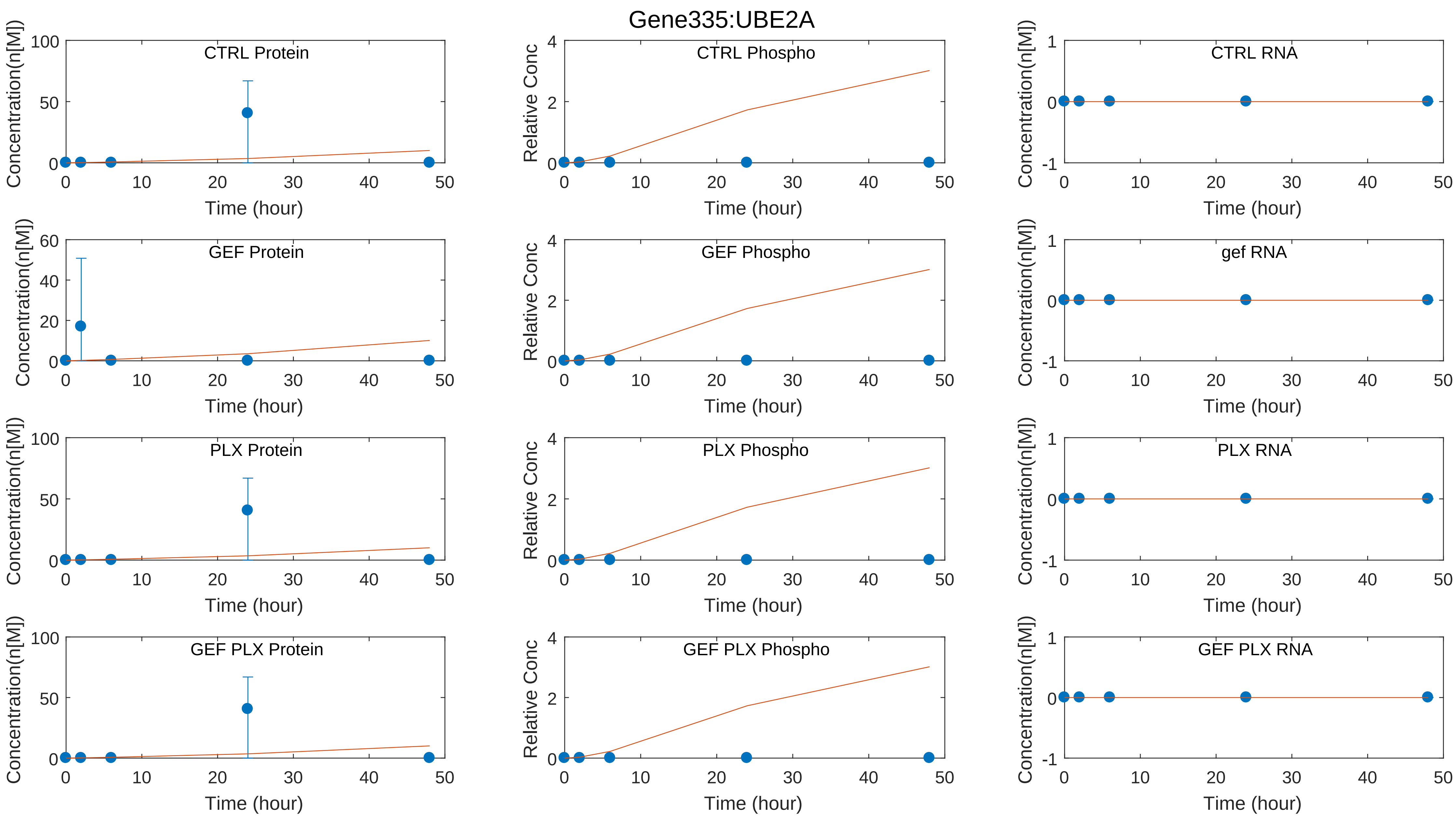

Gene335:UBE2A
CTRL Protein
CTRL Phospho
CTRL RNA
GEF Protein
GEF Phospho
gef RNA
PLX Protein
PLX Phospho
PLX RNA
GEF PLX Protein
GEF PLX Phospho
GEF PLX RNA
Concentration(n[M])
Relative Conc
Time (hour)

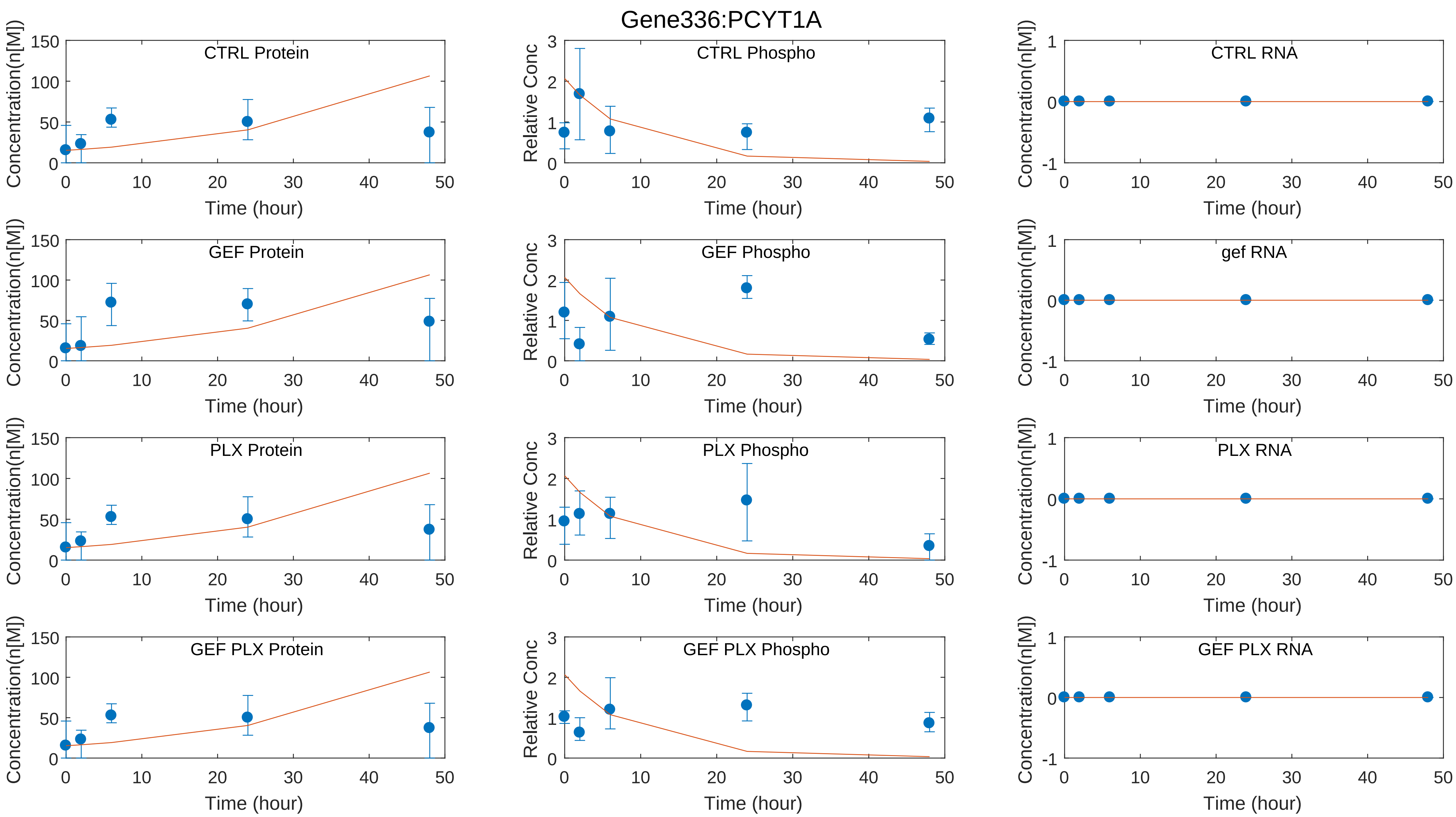

Gene336:PCYT1A
CTRL Protein
CTRL Phospho
CTRL RNA
GEF Protein
GEF Phospho
gef RNA
PLX Protein
PLX Phospho
PLX RNA
GEF PLX Protein
GEF PLX Phospho
GEF PLX RNA
Concentration(n[M])
Relative Conc
Time (hour)

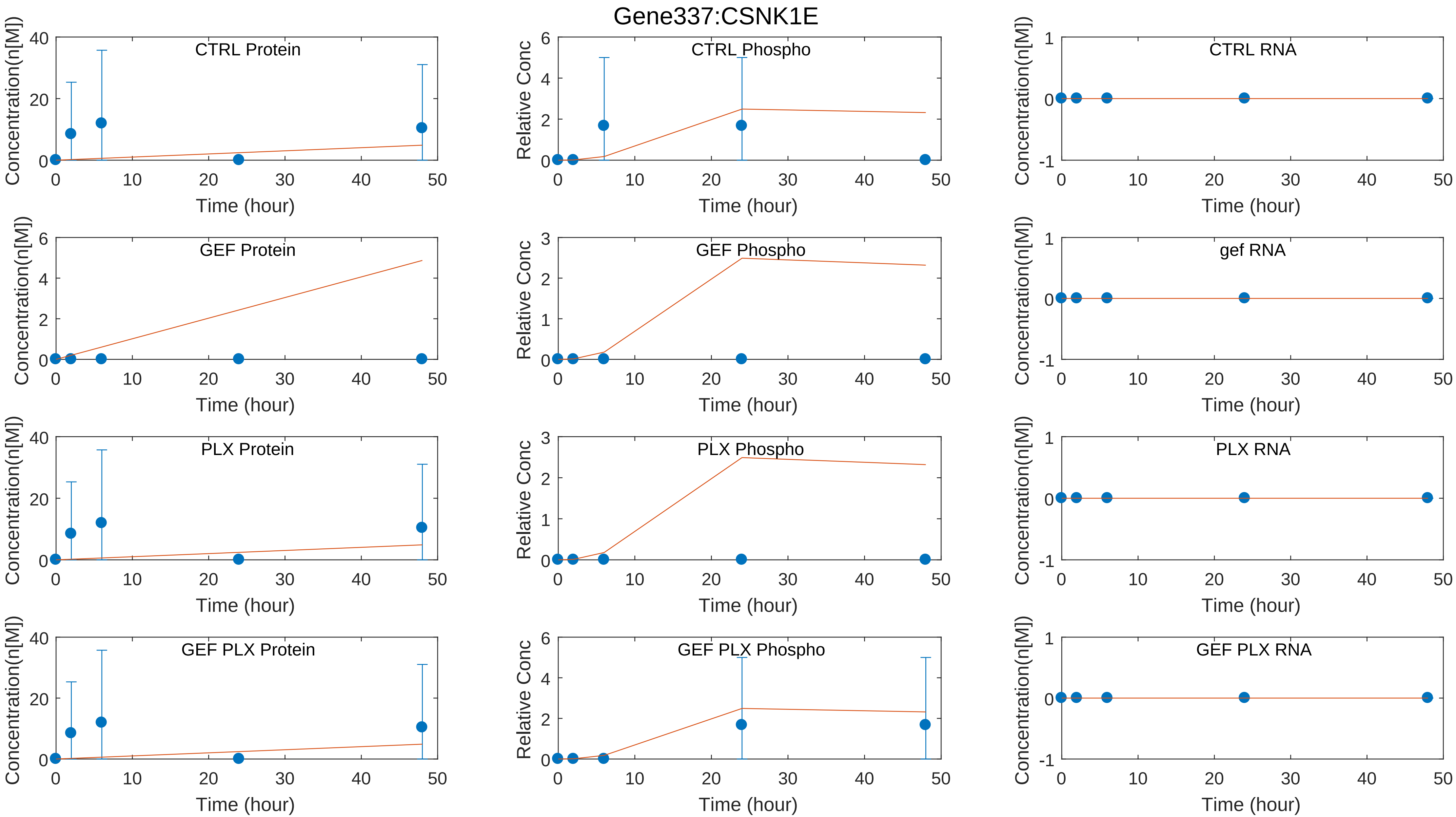

Gene337:CSNK1E
CTRL Protein
CTRL Phospho
CTRL RNA
GEF Protein
GEF Phospho
gef RNA
PLX Protein
PLX Phospho
PLX RNA
GEF PLX Protein
GEF PLX Phospho
GEF PLX RNA
Concentration(n[M])
Relative Conc
Time (hour)

Gene338:MCM2

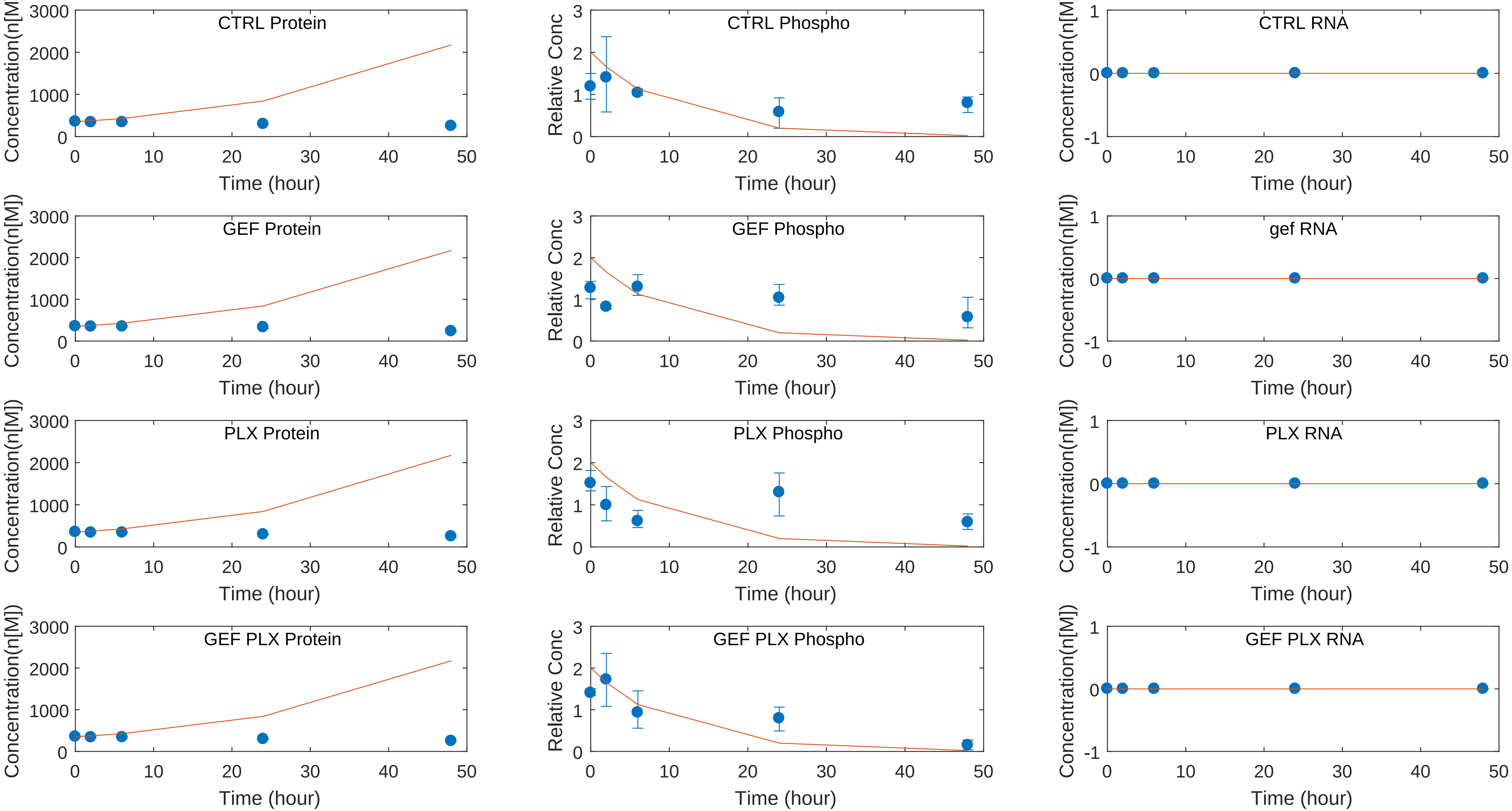

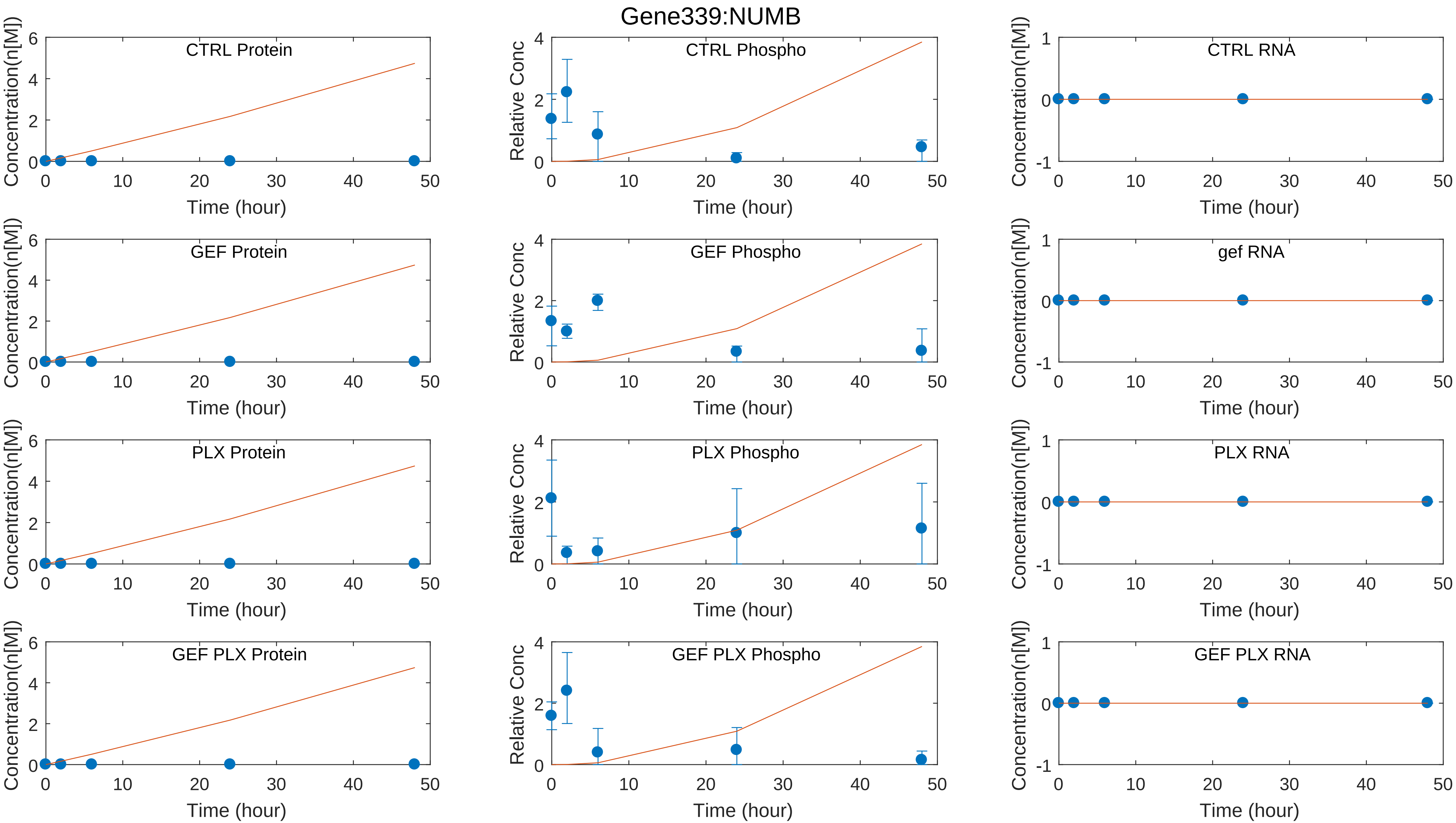
Gene339:NUMB
CTRL Protein
GEF Protein
PLX Protein
GEF PLX Protein
CTRL Phospho
GEF Phospho
PLX Phospho
GEF PLX Phospho
CTRL RNA
gef RNA
PLX RNA
GEF PLX RNA
Concentration(n[M])
Relative Conc
Time (hour)

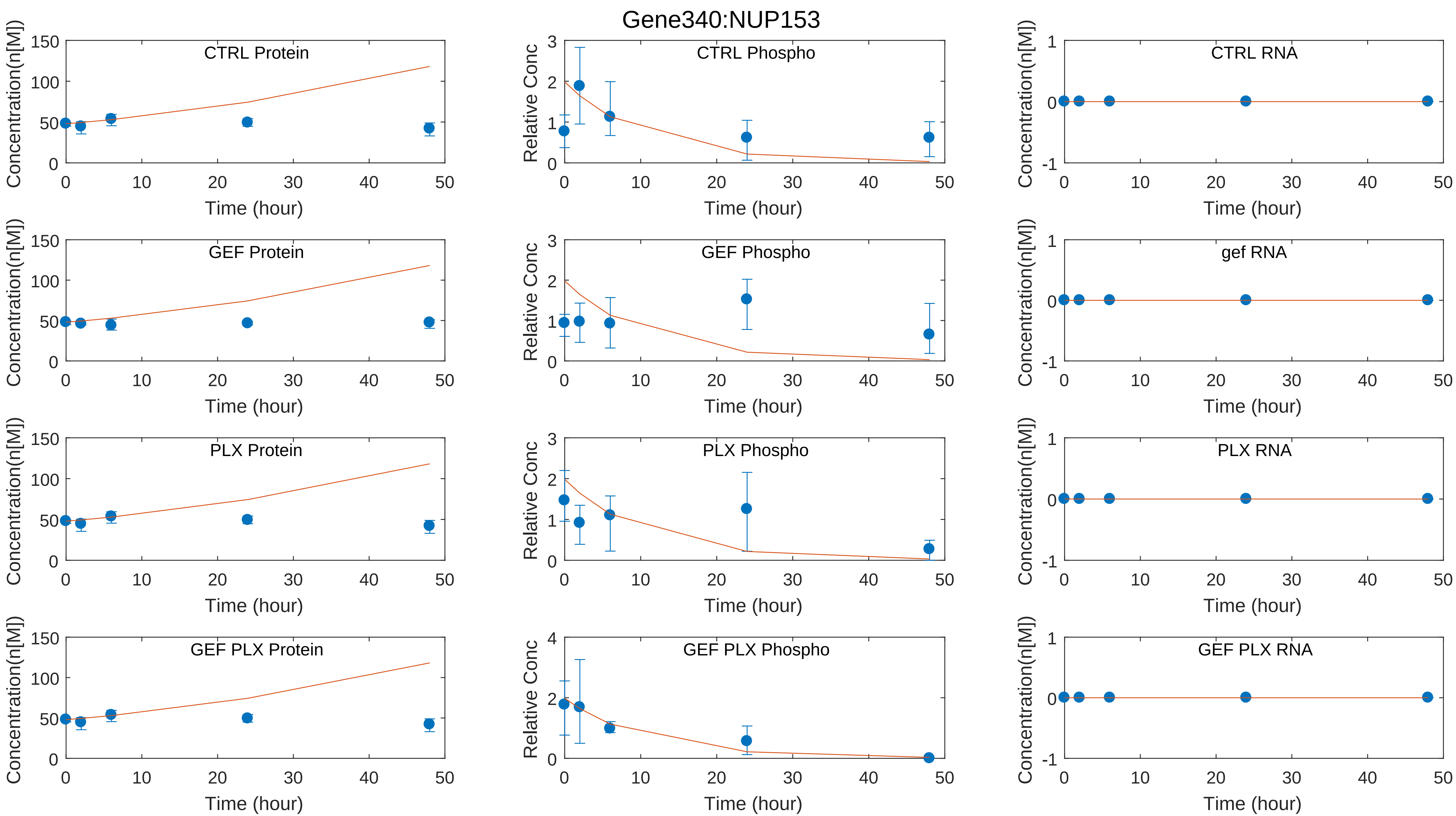

Gene340:NUP153
CTRL Protein
CTRL Phospho
CTRL RNA
GEF Protein
GEF Phospho
gef RNA
PLX Protein
PLX Phospho
PLX RNA
GEF PLX Protein
GEF PLX Phospho
GEF PLX RNA
Concentration(n[M])
Relative Conc
Time (hour)

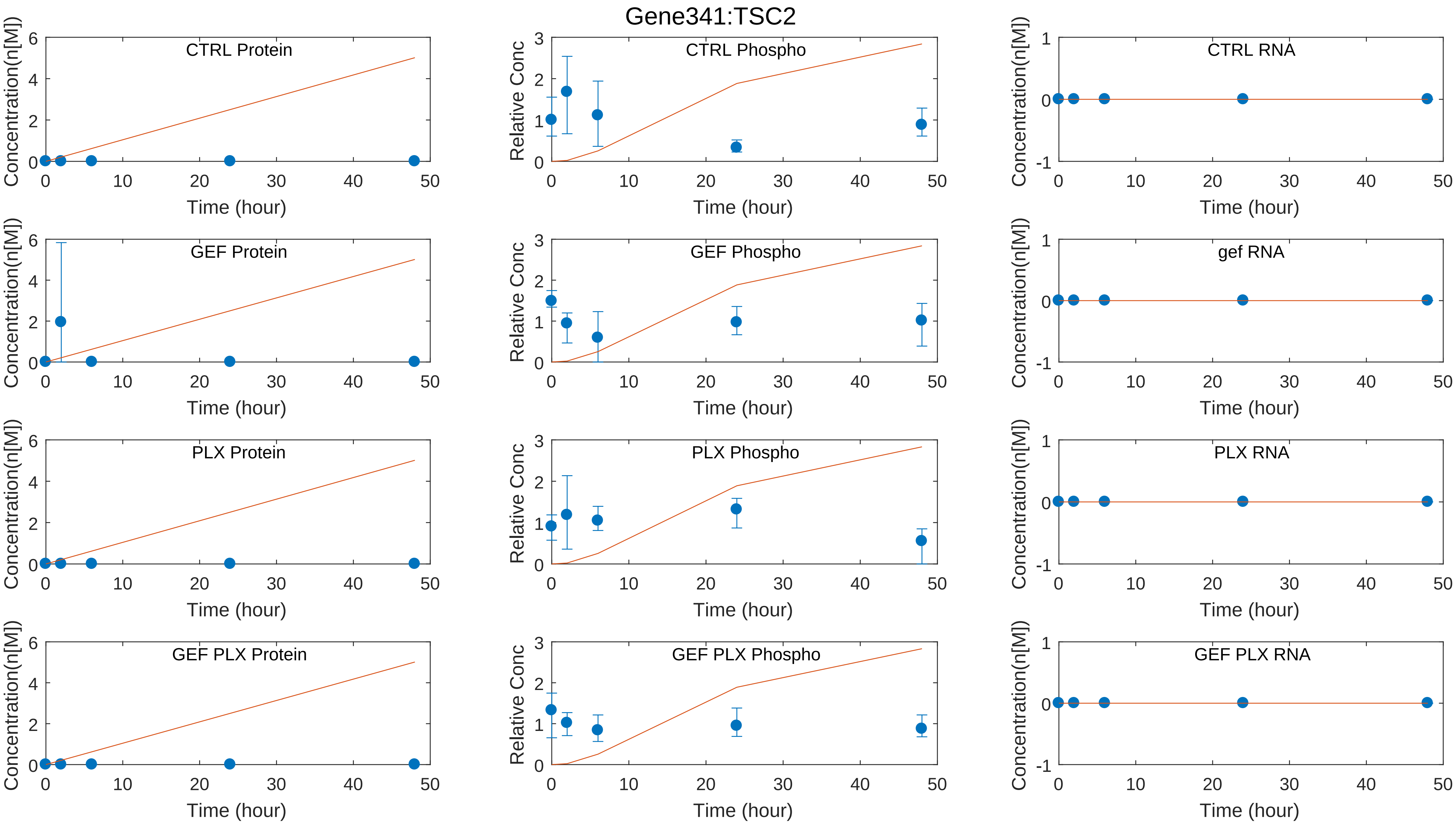

Gene341:TSC2
CTRL Protein
GEF Protein
PLX Protein
GEF PLX Protein
CTRL Phospho
GEF Phospho
PLX Phospho
GEF PLX Phospho
CTRL RNA
gef RNA
PLX RNA
GEF PLX RNA
Concentration(n[M])
Relative Conc
Time (hour)

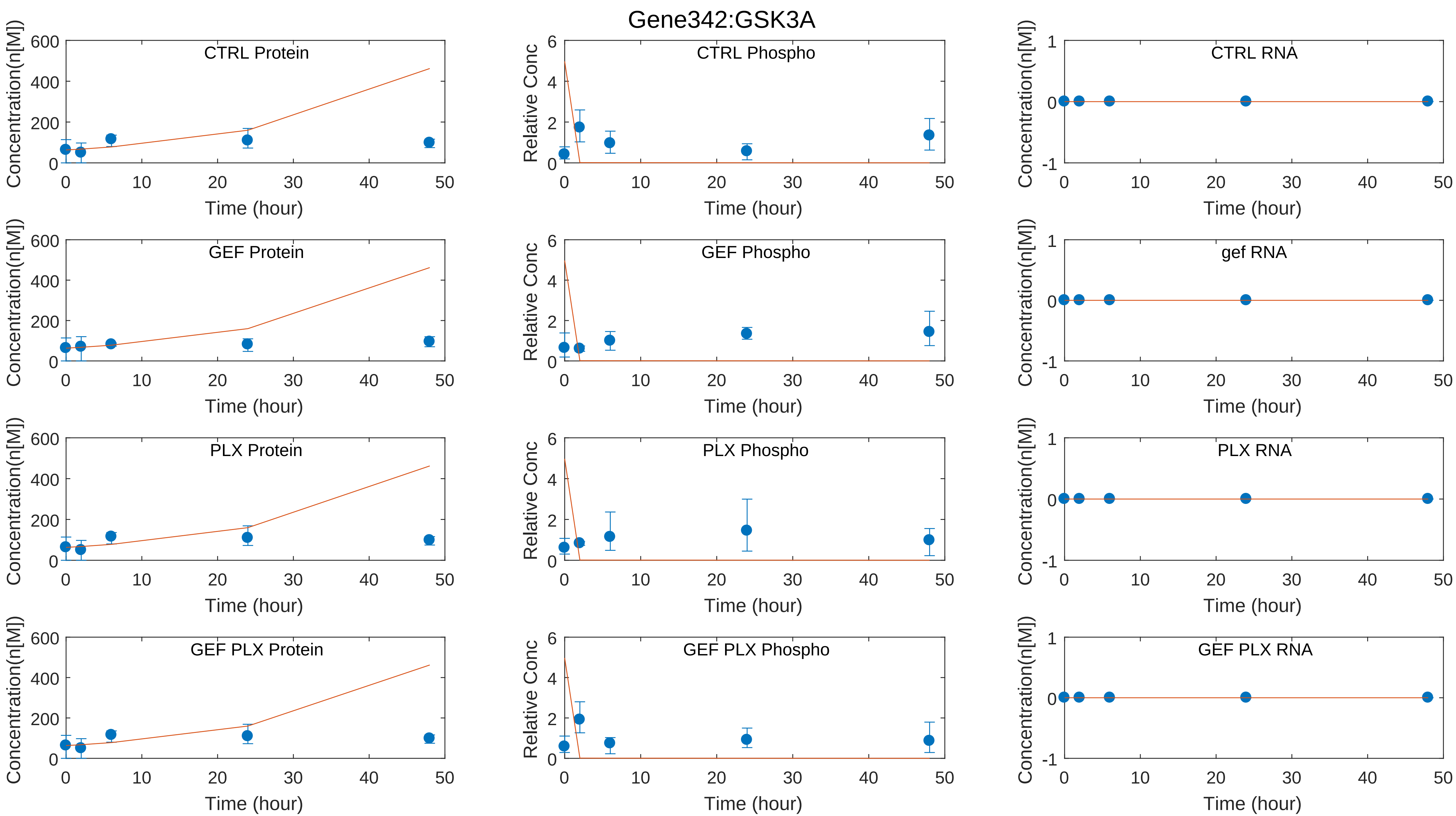
Gene342:GSK3A
CTRL Protein
CTRL Phospho
CTRL RNA
GEF Protein
GEF Phospho
gef RNA
PLX Protein
PLX Phospho
PLX RNA
GEF PLX Protein
GEF PLX Phospho
GEF PLX RNA
Concentration(n[M])
Relative Conc
Time (hour)

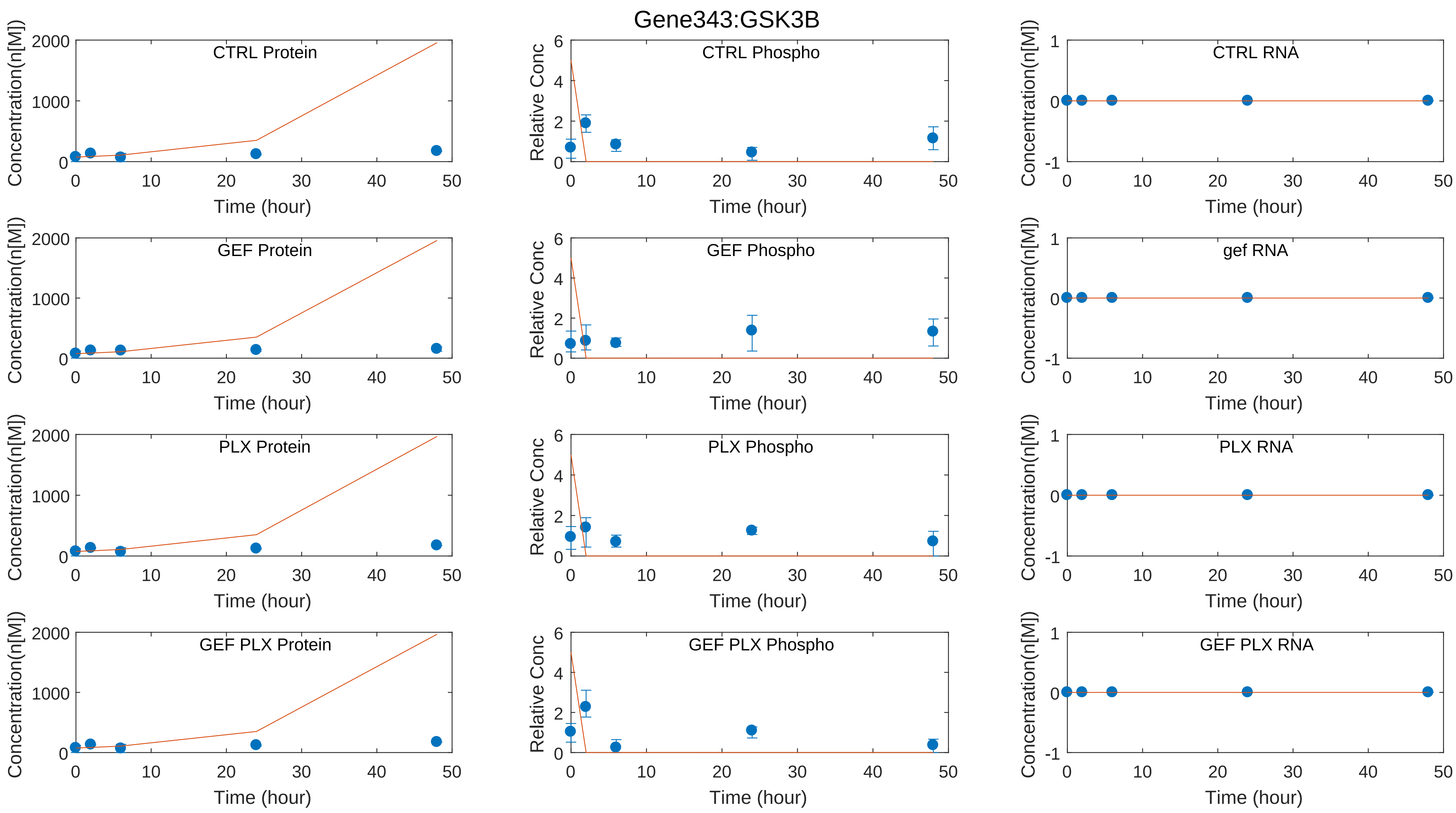

Gene343:GSK3B
CTRL Protein
GEF Protein
PLX Protein
GEF PLX Protein
CTRL Phospho
GEF Phospho
PLX Phospho
GEF PLX Phospho
CTRL RNA
gef RNA
PLX RNA
GEF PLX RNA
Concentration(n[M])
Relative Conc
Time (hour)

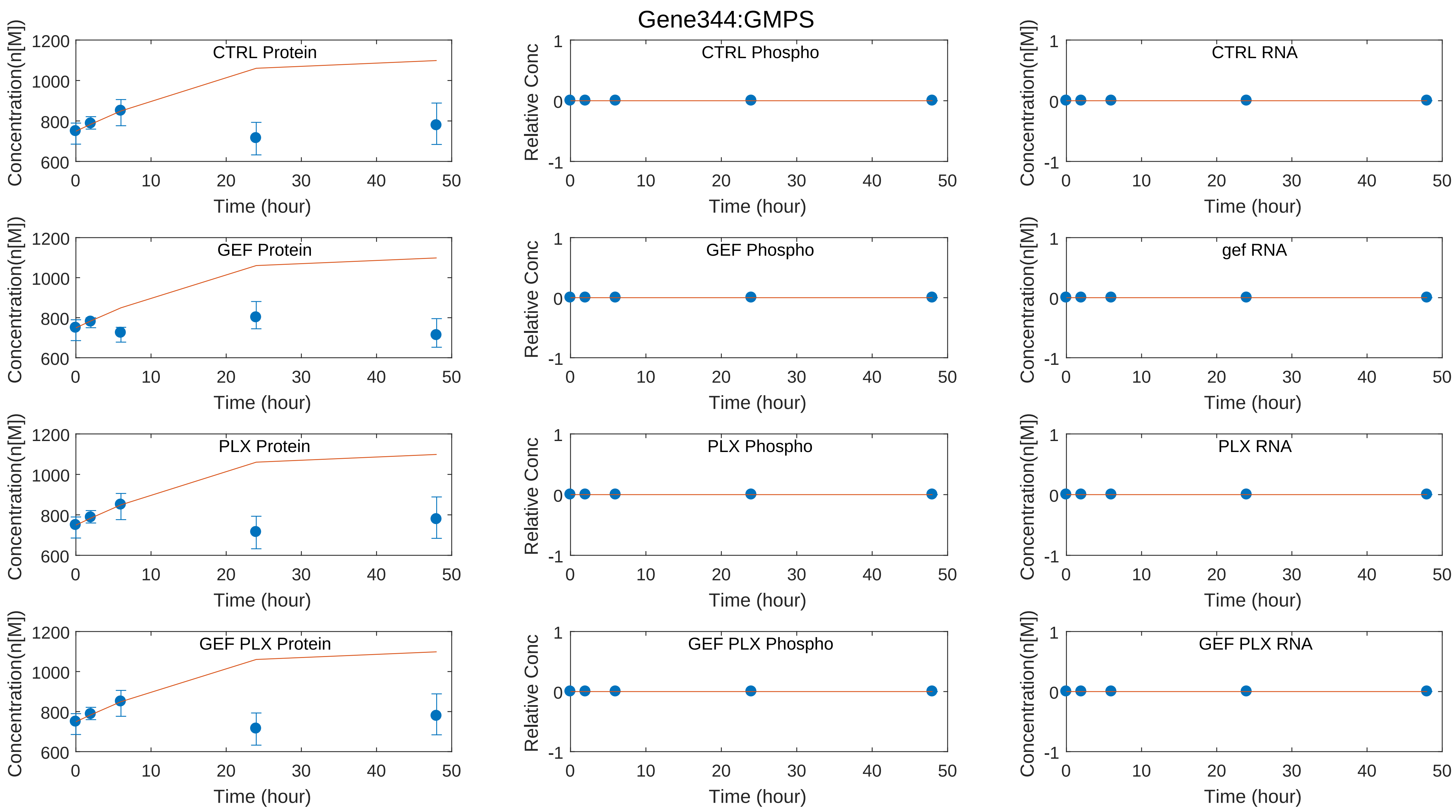

Gene344:GMPS
CTRL Protein
CTRL Phospho
CTRL RNA
GEF Protein
GEF Phospho
gef RNA
PLX Protein
PLX Phospho
PLX RNA
GEF PLX Protein
GEF PLX Phospho
GEF PLX RNA
Concentration(n[M])
Relative Conc
Time (hour)

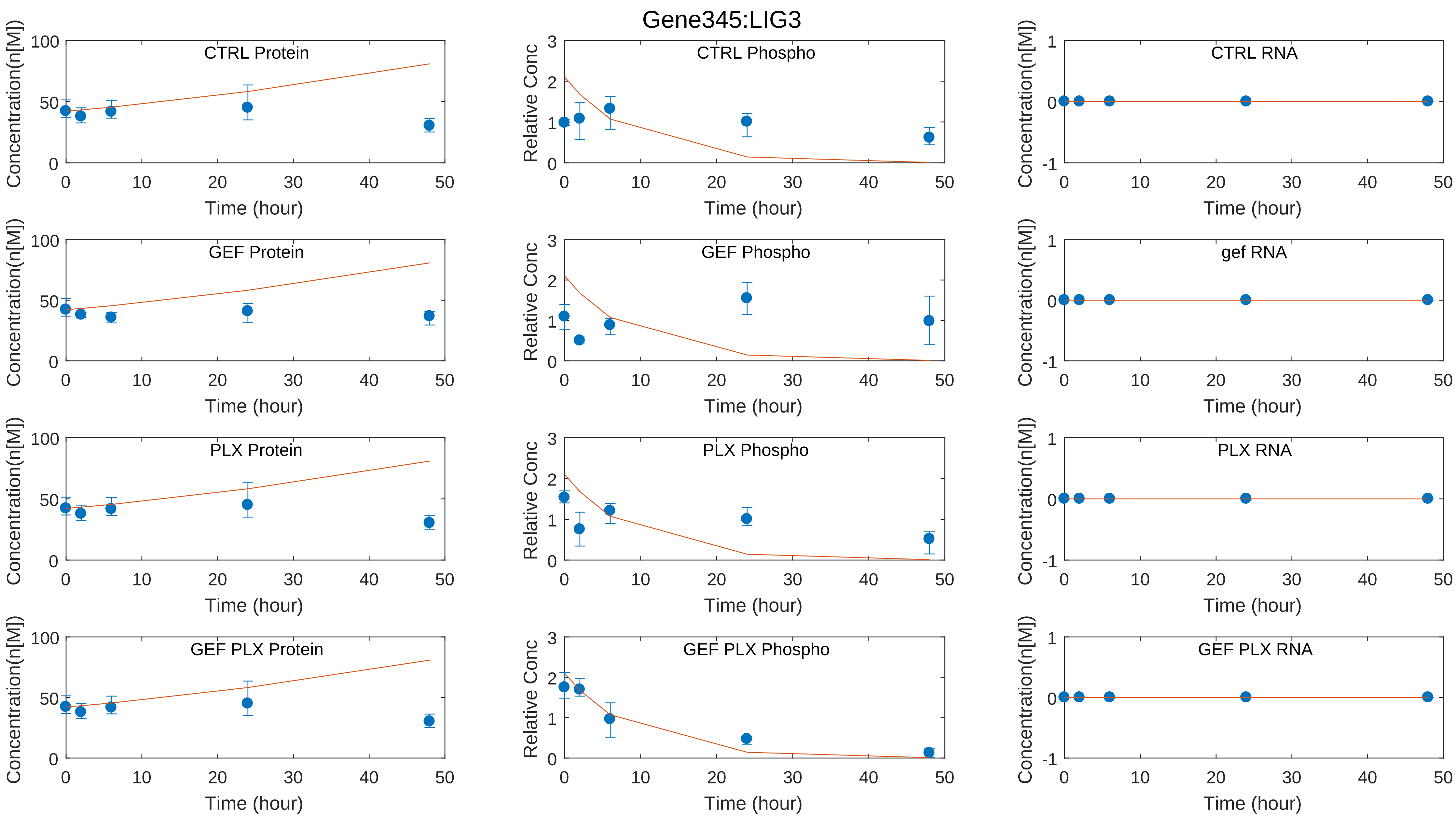
Gene345:LIG3
CTRL Protein
GEF Protein
PLX Protein
GEF PLX Protein
CTRL Phospho
GEF Phospho
PLX Phospho
GEF PLX Phospho
CTRL RNA
gef RNA
PLX RNA
GEF PLX RNA
Concentration(n[M])
Relative Conc
Time (hour)

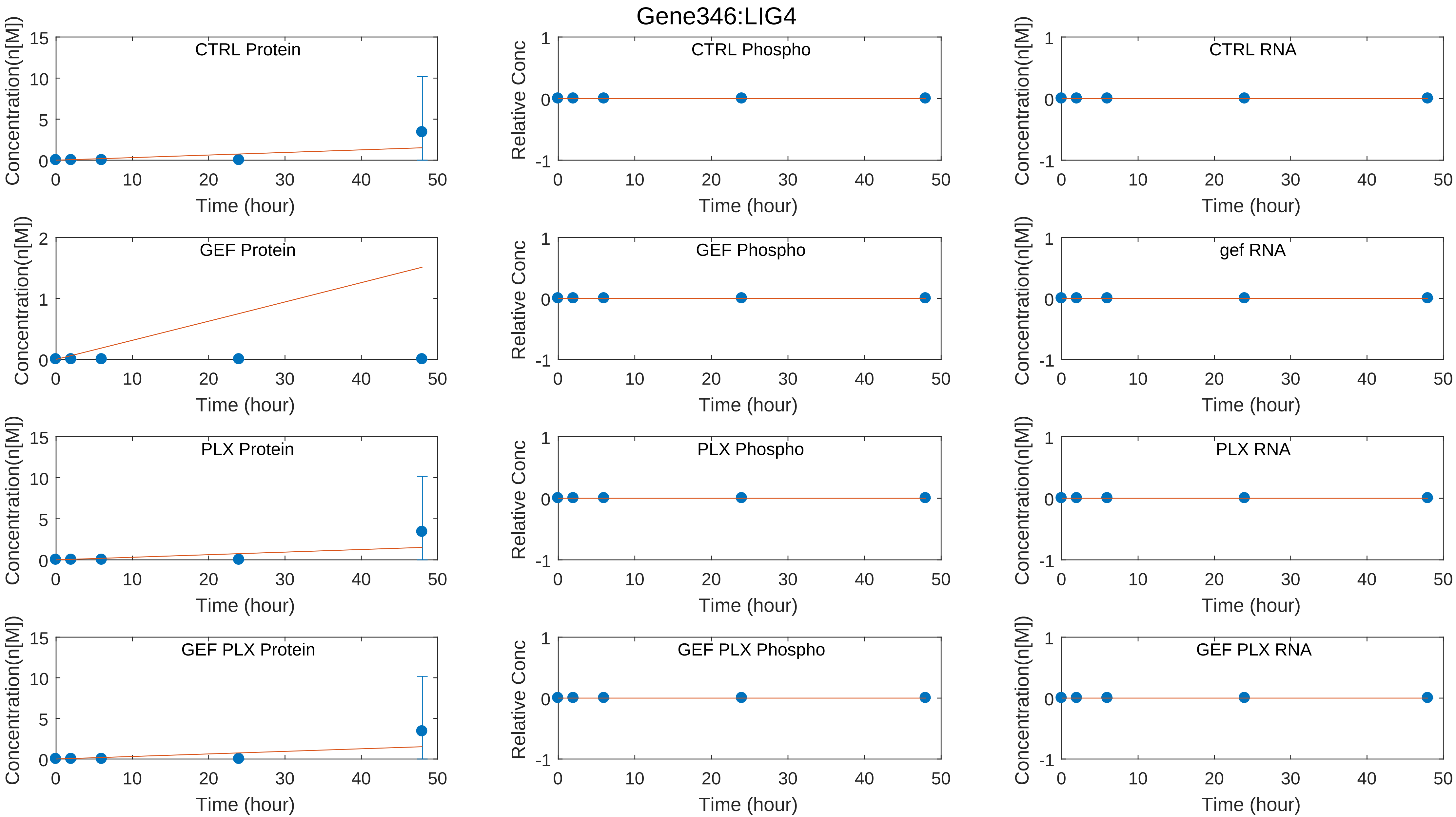

Gene346:LIG4
CTRL Protein
CTRL Phospho
CTRL RNA
GEF Protein
GEF Phospho
gef RNA
PLX Protein
PLX Phospho
PLX RNA
GEF PLX Protein
GEF PLX Phospho
GEF PLX RNA
Concentration(n[M])
Relative Conc
Time (hour)

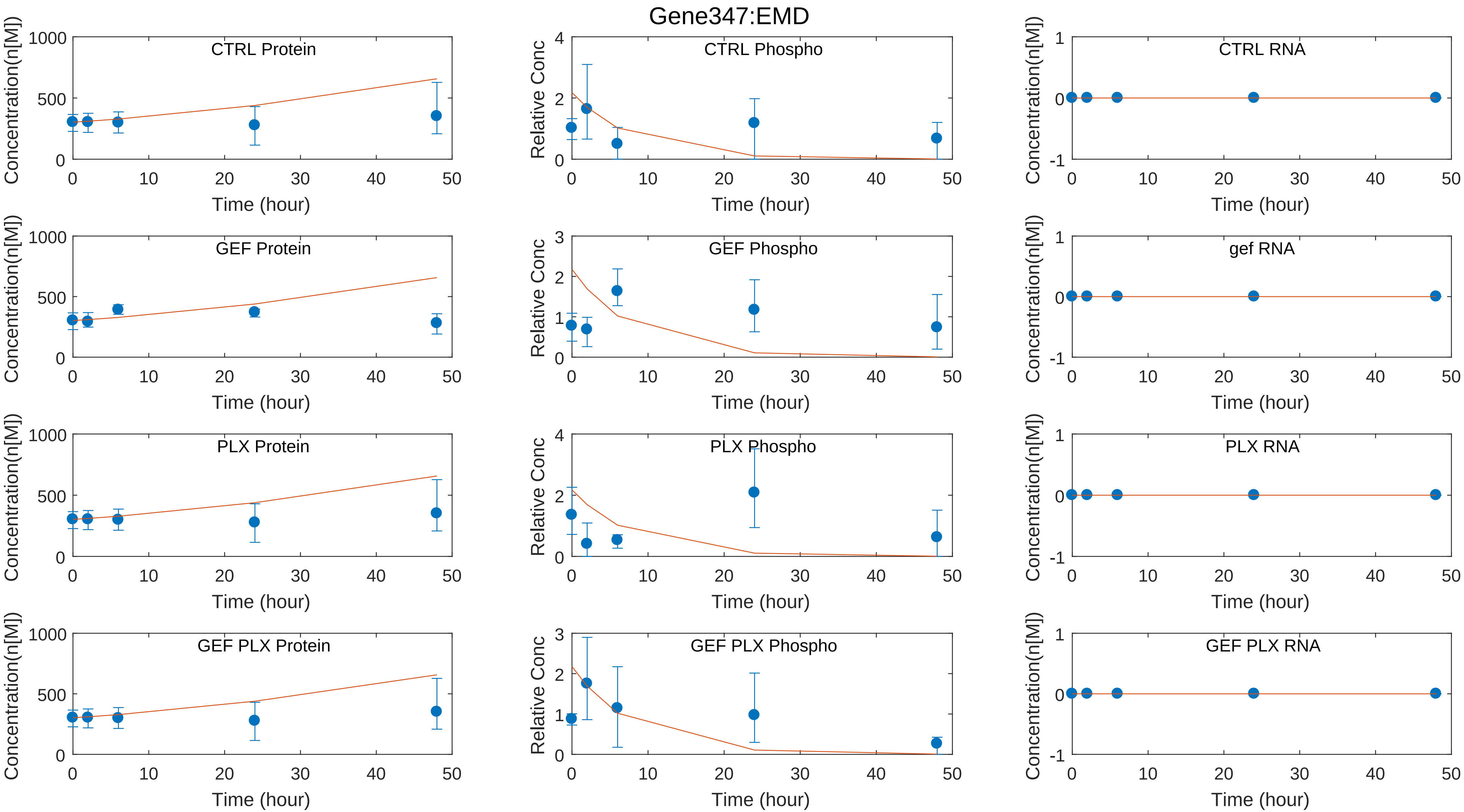

Gene347:EMD
CTRL Protein
CTRL Phospho
CTRL RNA
GEF Protein
GEF Phospho
gef RNA
PLX Protein
PLX Phospho
PLX RNA
GEF PLX Protein
GEF PLX Phospho
GEF PLX RNA
Concentration(n[M])
Relative Conc
Time (hour)

Gene348:PEX5

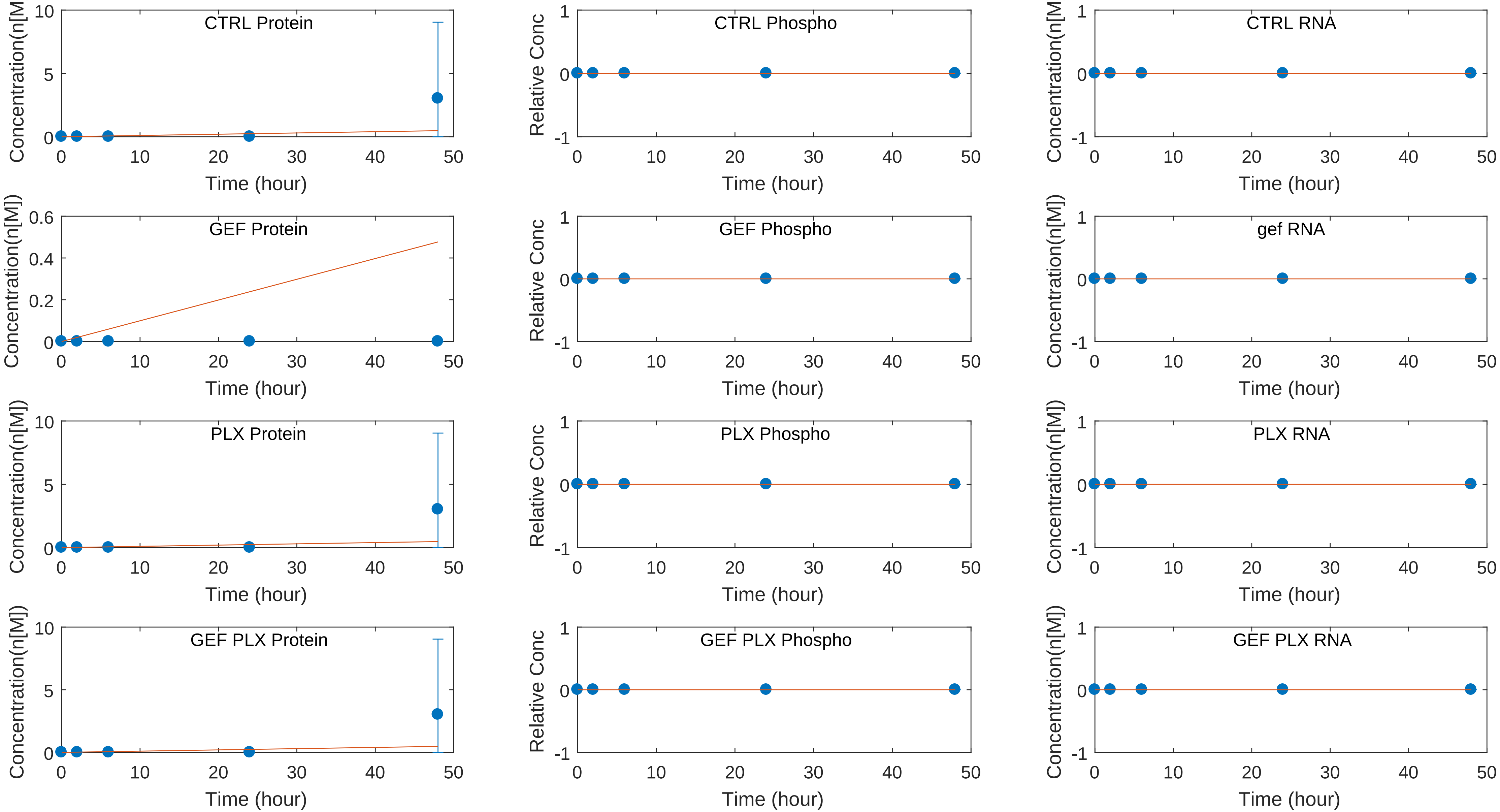

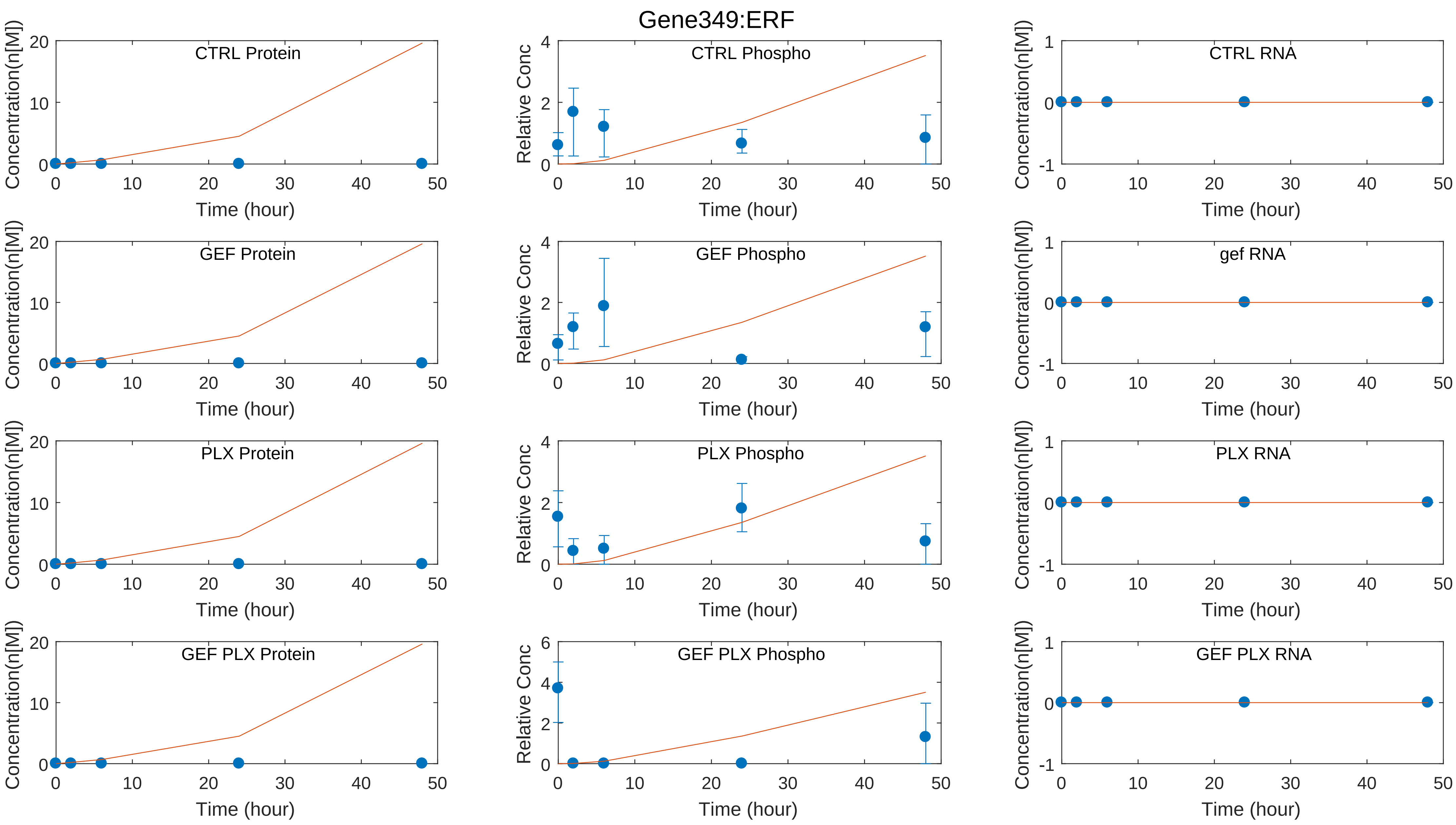

Gene349:ERF
CTRL Protein
CTRL Phospho
CTRL RNA
GEF Protein
GEF Phospho
gef RNA
PLX Protein
PLX Phospho
PLX RNA
GEF PLX Protein
GEF PLX Phospho
GEF PLX RNA
Concentration(n[M])
Relative Conc
Time (hour)

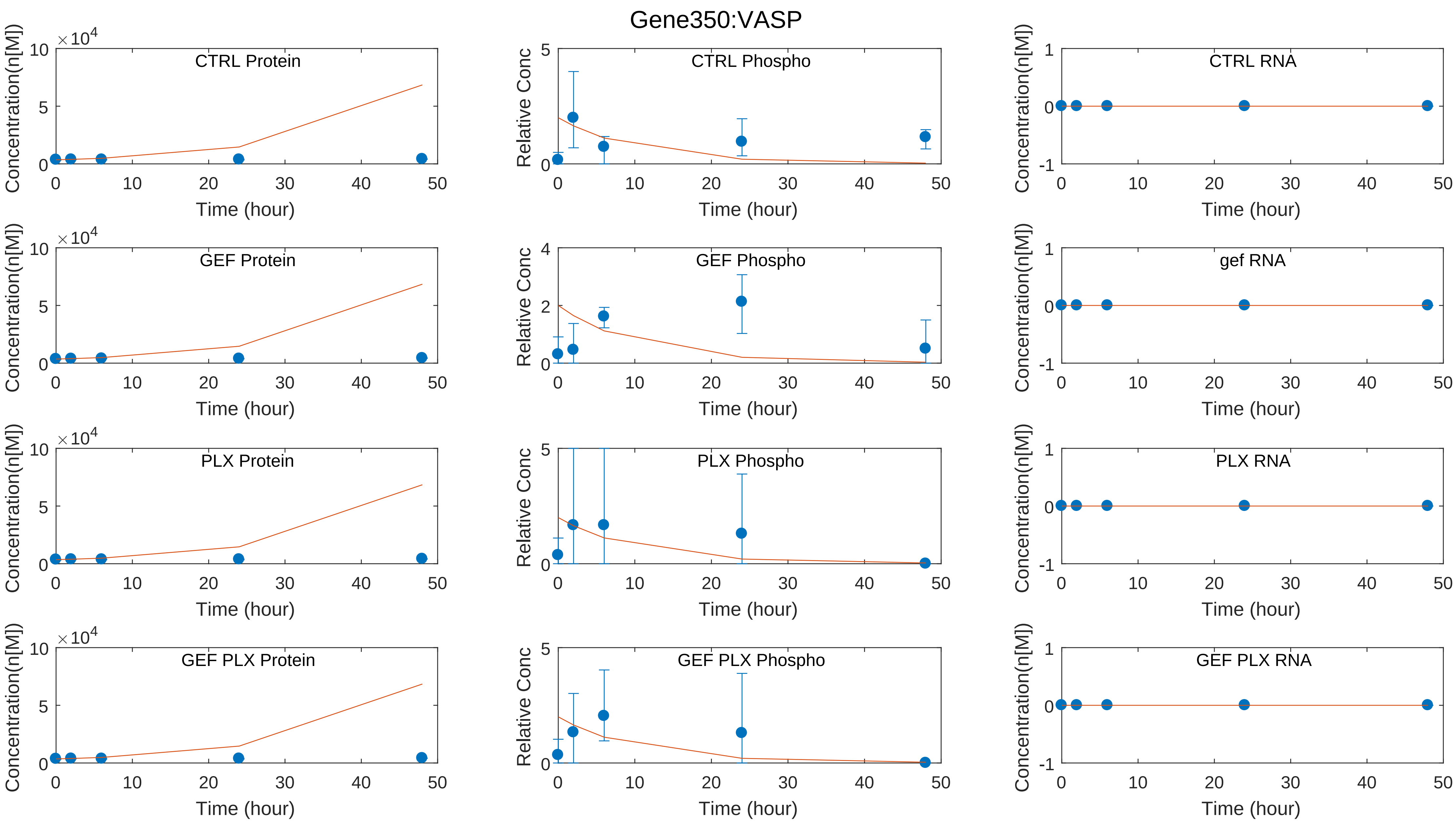

Gene350:VASP
CTRL Protein
GEF Protein
PLX Protein
GEF PLX Protein
CTRL Phospho
GEF Phospho
PLX Phospho
GEF PLX Phospho
CTRL RNA
gef RNA
PLX RNA
GEF PLX RNA
Concentration(n[M])
Relative Conc
Time (hour)
×10^4

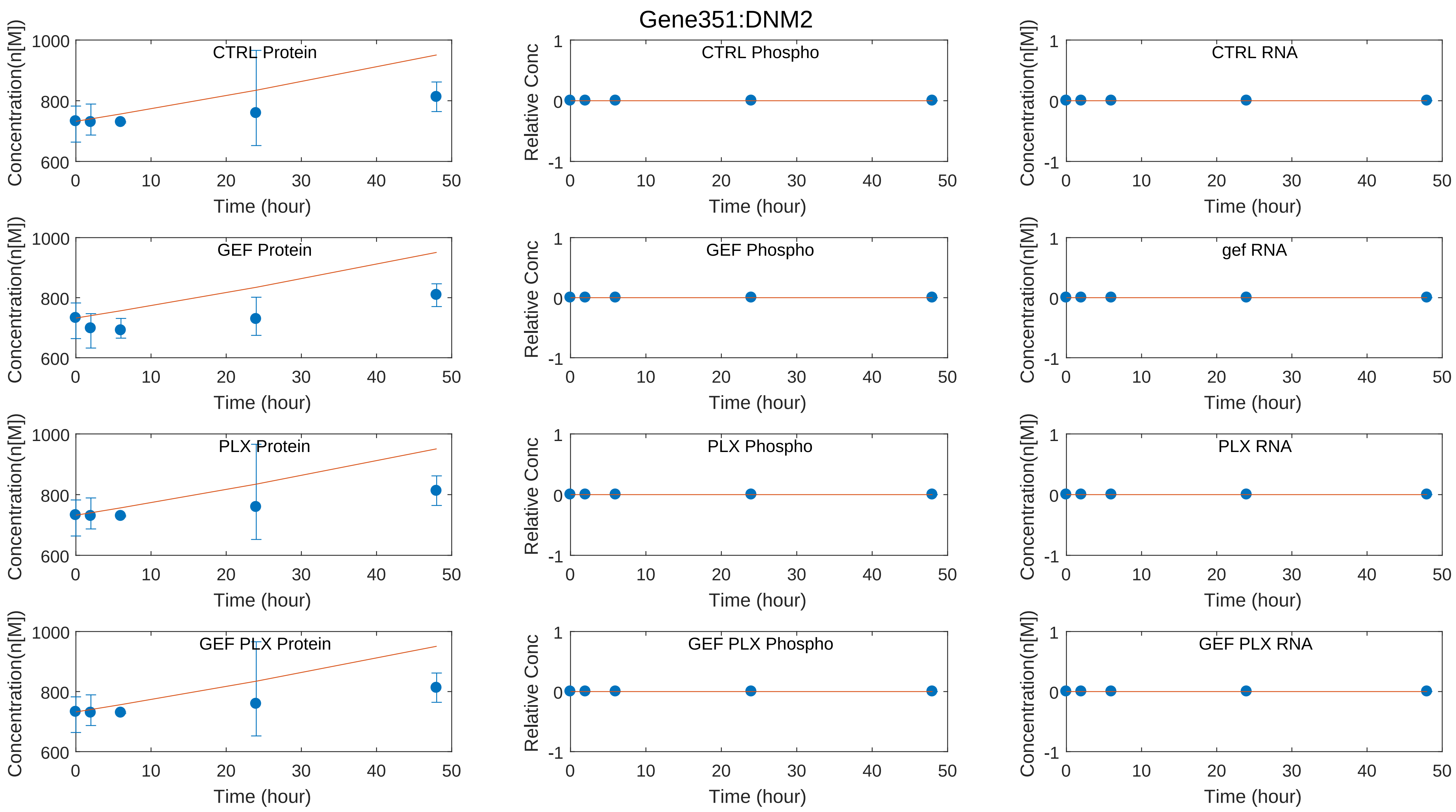

Gene351:DNM2
CTRL Protein
CTRL Phospho
CTRL RNA
GEF Protein
GEF Phospho
gef RNA
PLX Protein
PLX Phospho
PLX RNA
GEF PLX Protein
GEF PLX Phospho
GEF PLX RNA
Concentration(n[M])
Relative Conc
Time (hour)
1000
800
600
1
0
-1
0
10
20
30
40
50

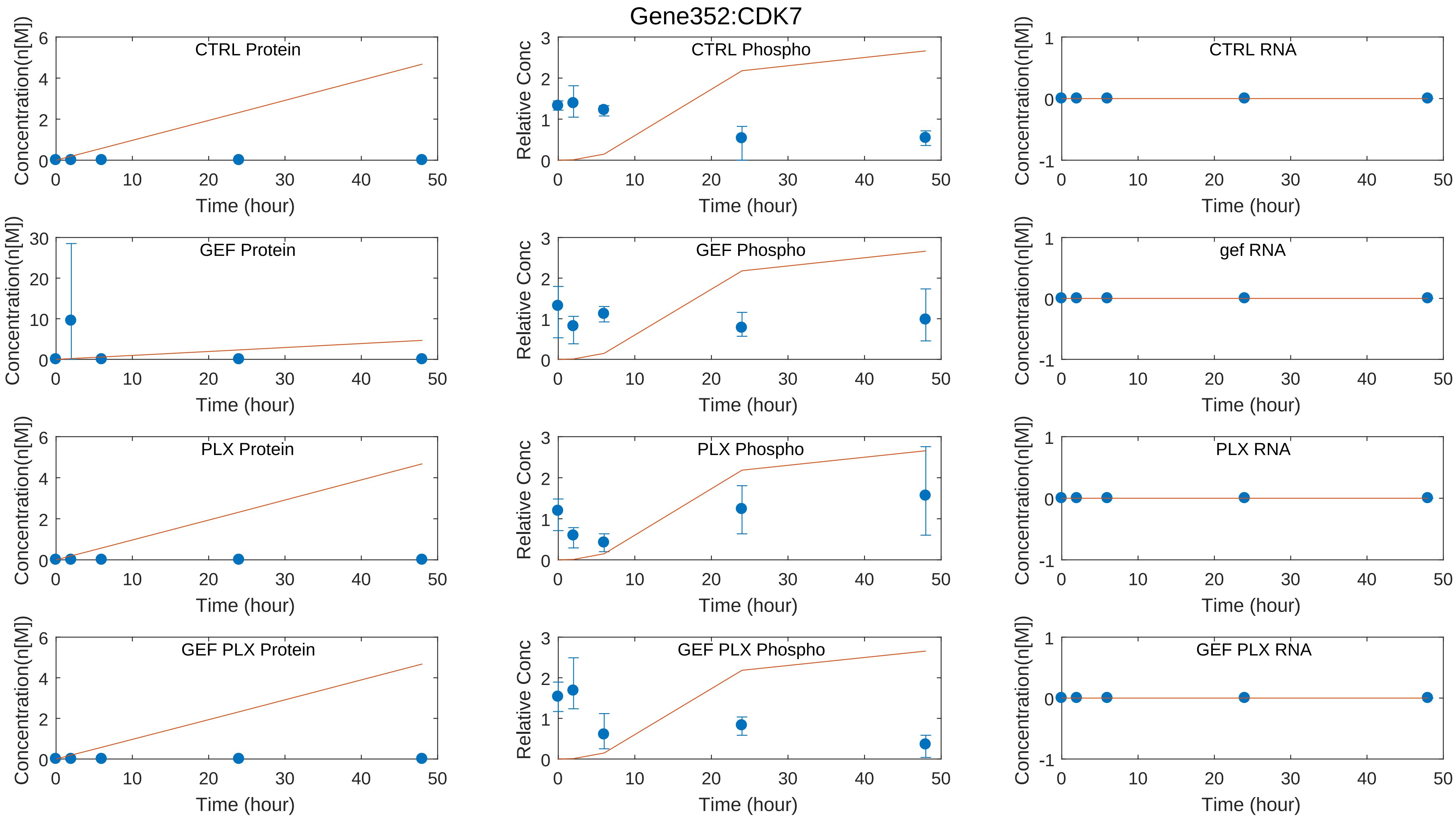

Gene352:CDK7
CTRL Protein
CTRL Phospho
CTRL RNA
GEF Protein
GEF Phospho
gef RNA
PLX Protein
PLX Phospho
PLX RNA
GEF PLX Protein
GEF PLX Phospho
GEF PLX RNA
Concentration(n[M])
Relative Conc
Time (hour)

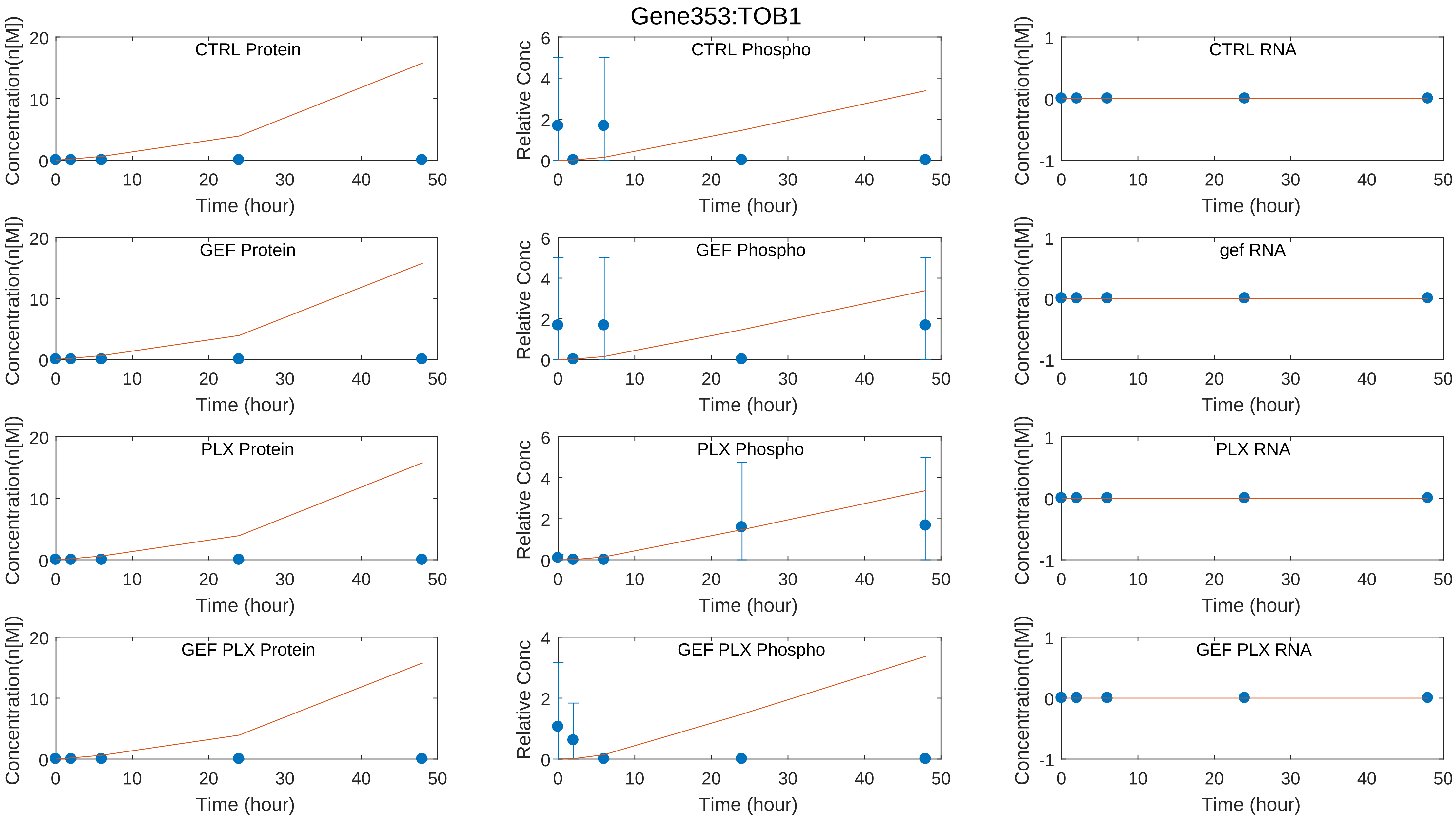

Gene353:TOB1
CTRL Protein
CTRL Phospho
CTRL RNA
GEF Protein
GEF Phospho
gef RNA
PLX Protein
PLX Phospho
PLX RNA
GEF PLX Protein
GEF PLX Phospho
GEF PLX RNA
Concentration(n[M])
Relative Conc
Time (hour)

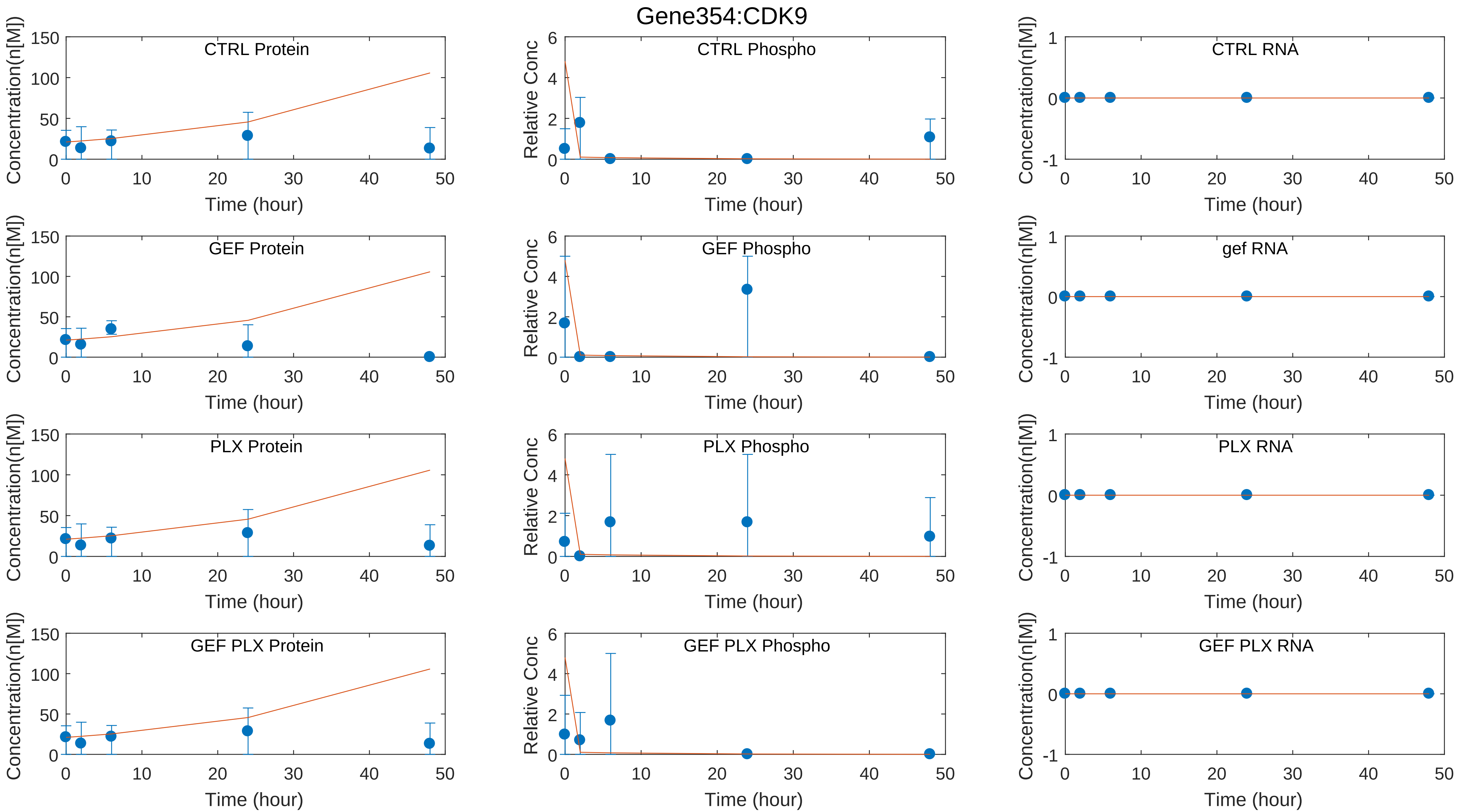

Gene354:CDK9
CTRL Protein
CTRL Phospho
CTRL RNA
GEF Protein
GEF Phospho
gef RNA
PLX Protein
PLX Phospho
PLX RNA
GEF PLX Protein
GEF PLX Phospho
GEF PLX RNA
Concentration(n[M])
Relative Conc
Time (hour)

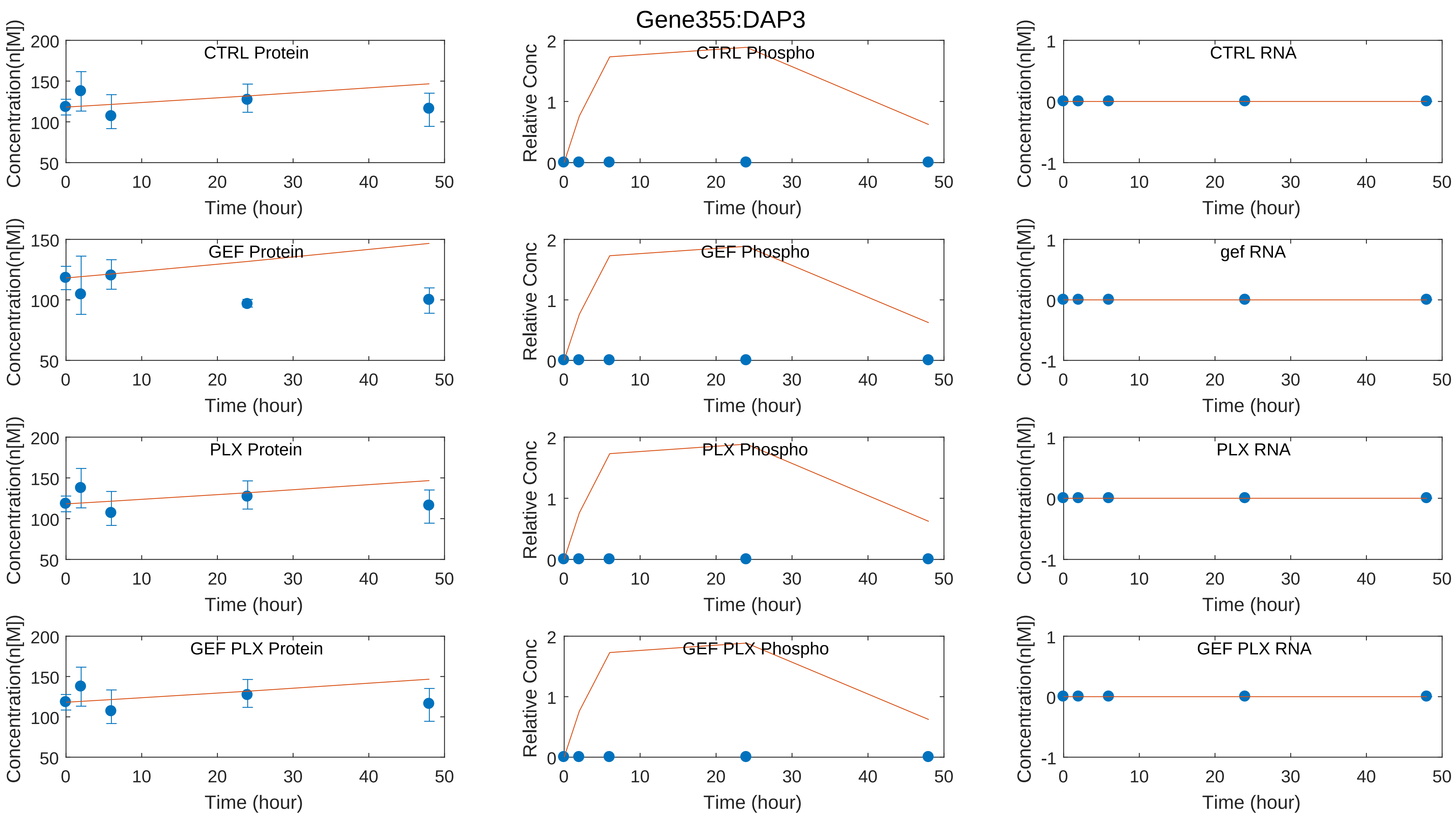

Gene355:DAP3
CTRL Protein
CTRL Phospho
CTRL RNA
GEF Protein
GEF Phospho
gef RNA
PLX Protein
PLX Phospho
PLX RNA
GEF PLX Protein
GEF PLX Phospho
GEF PLX RNA
Concentration(n[M])
Relative Conc
Time (hour)

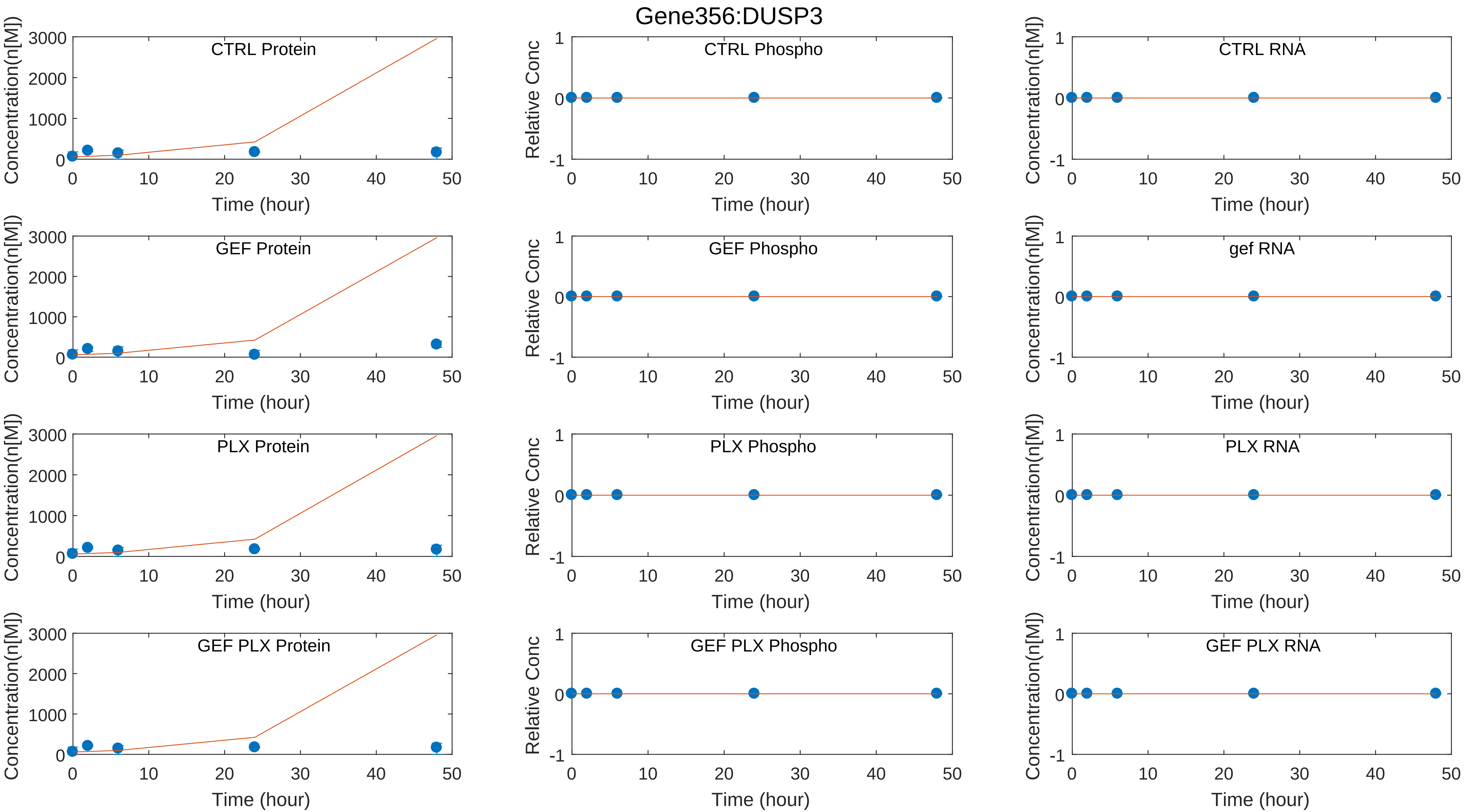
Gene356:DUSP3
CTRL Protein
GEF Protein
PLX Protein
GEF PLX Protein
CTRL Phospho
GEF Phospho
PLX Phospho
GEF PLX Phospho
CTRL RNA
gef RNA
PLX RNA
GEF PLX RNA
Concentration(n[M])
Relative Conc
Time (hour)

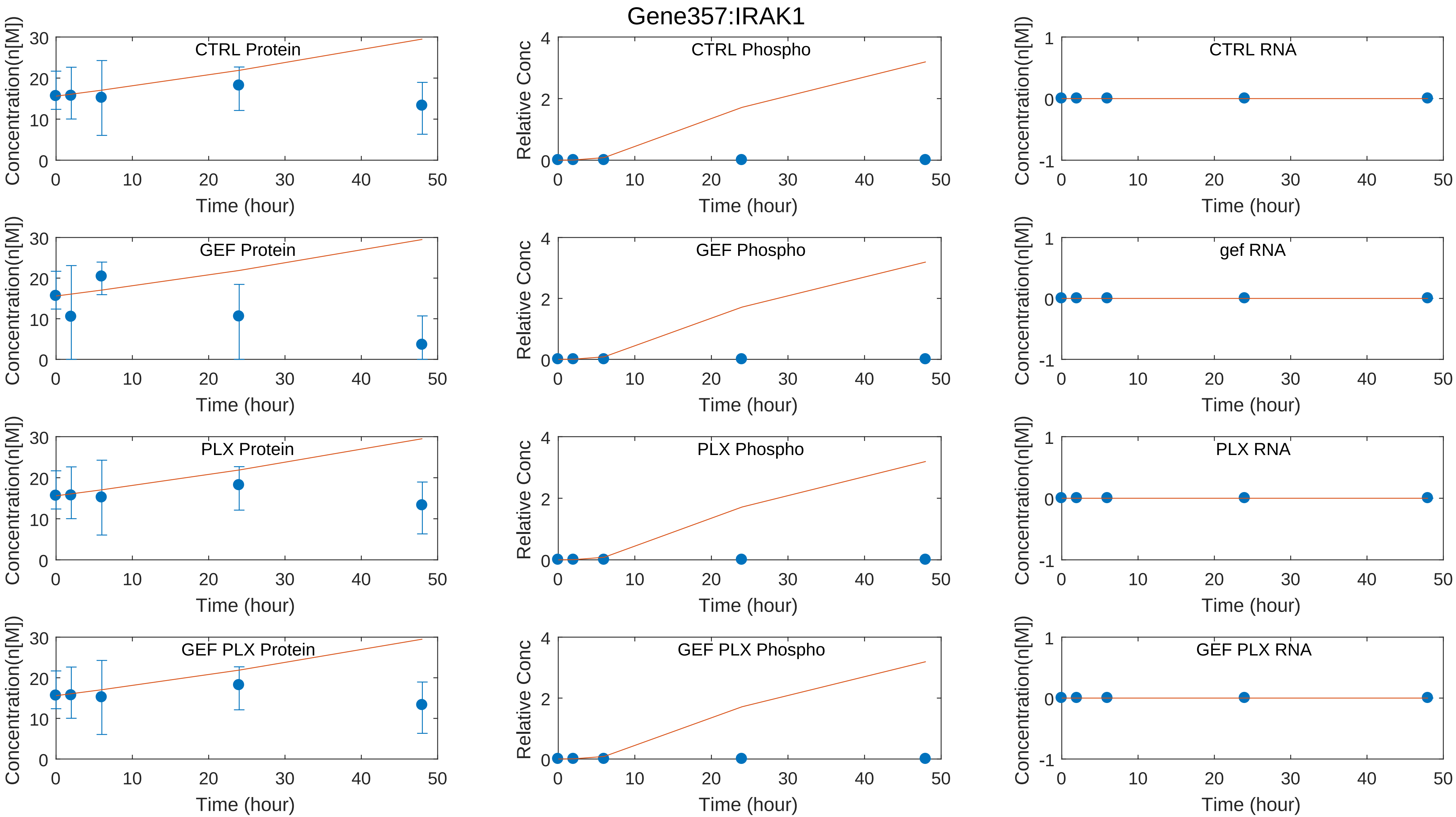

Gene357:IRAK1
CTRL Protein
GEF Protein
PLX Protein
GEF PLX Protein
CTRL Phospho
GEF Phospho
PLX Phospho
GEF PLX Phospho
CTRL RNA
gef RNA
PLX RNA
GEF PLX RNA
Concentration(n[M])
Relative Conc
Time (hour)

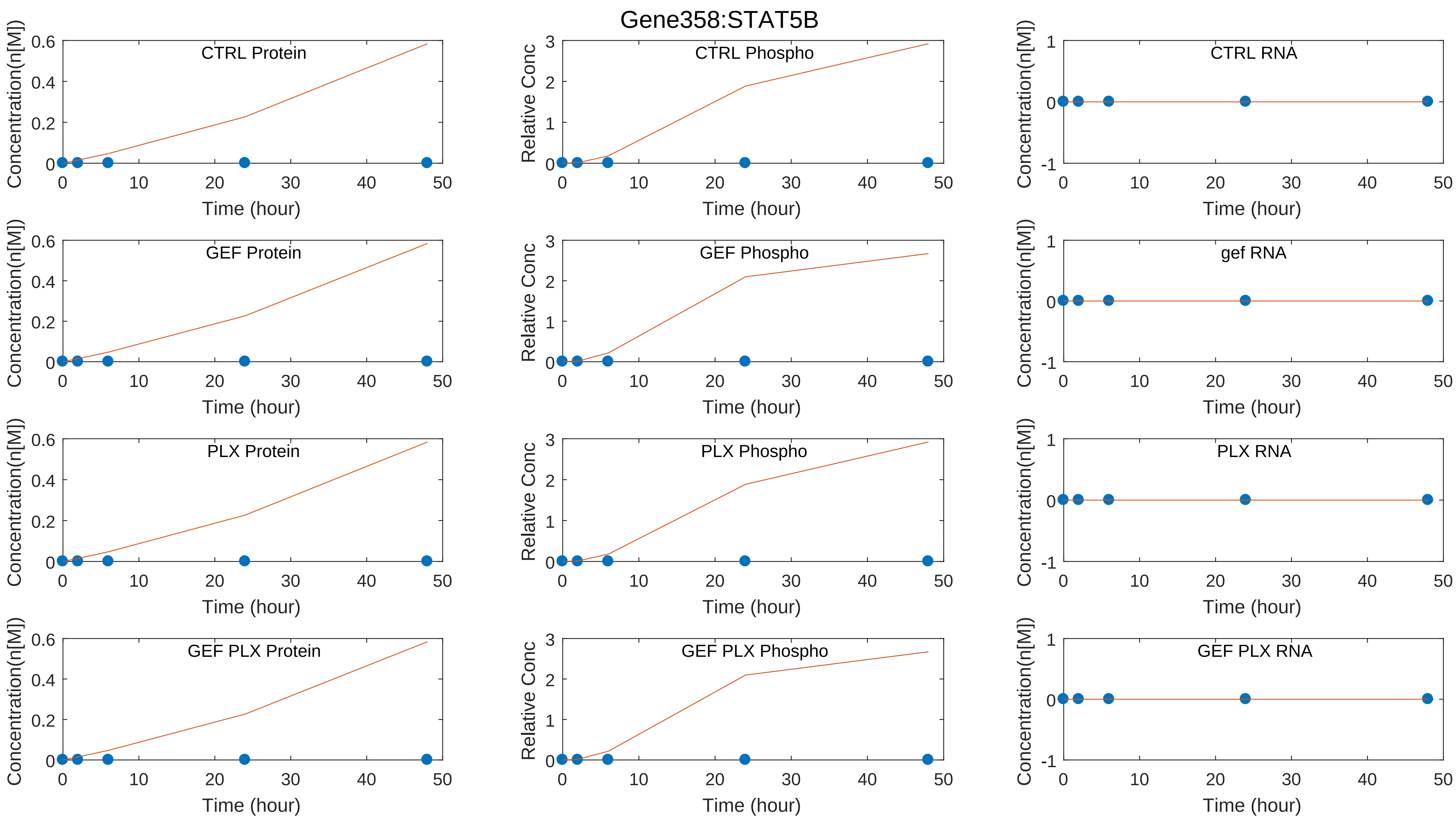

Gene358:STAT5B
CTRL Protein
CTRL Phospho
CTRL RNA
GEF Protein
GEF Phospho
gef RNA
PLX Protein
PLX Phospho
PLX RNA
GEF PLX Protein
GEF PLX Phospho
GEF PLX RNA
Concentration(n[M])
Relative Conc
Time (hour)

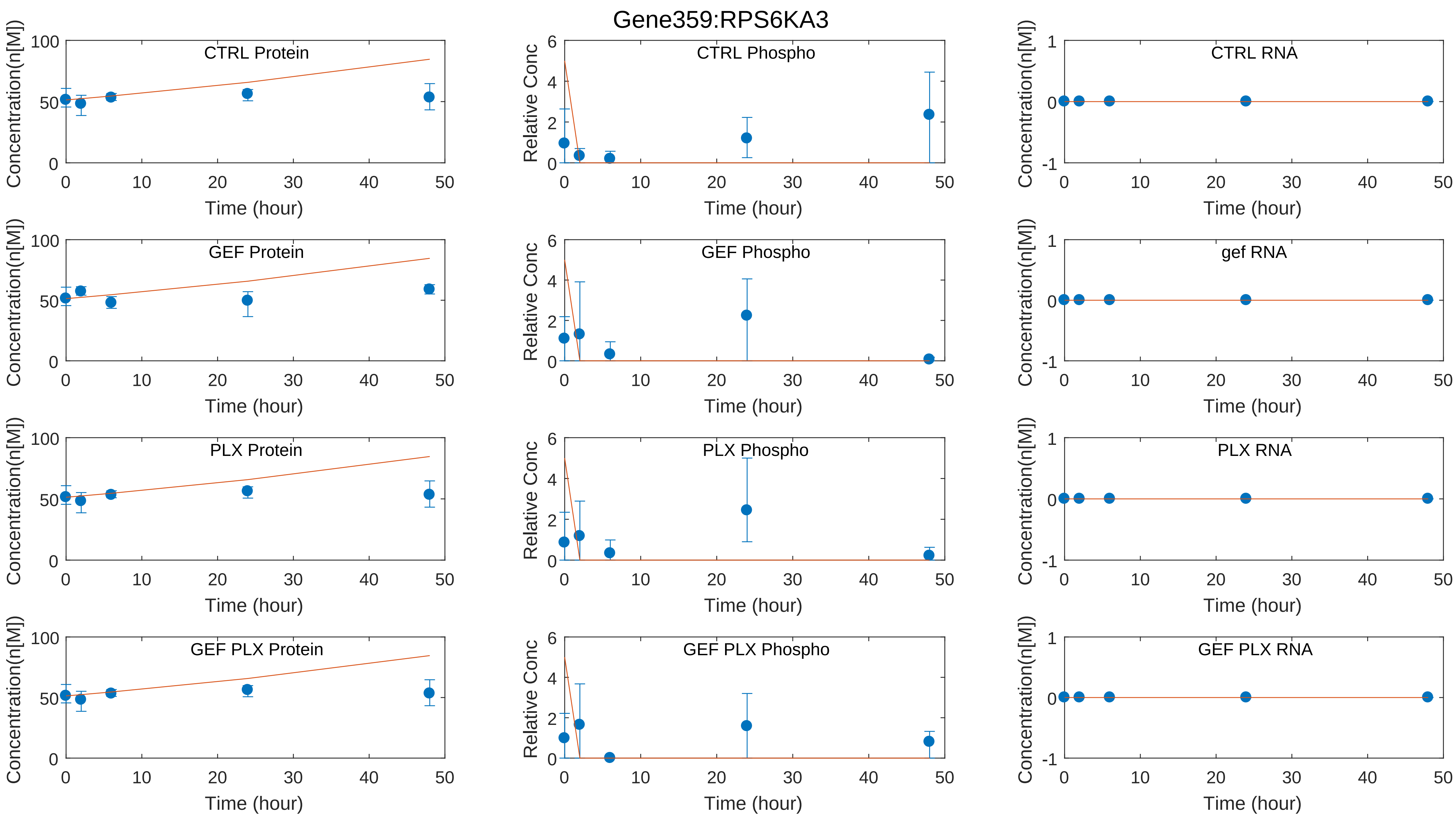
Gene359:RPS6KA3
CTRL Protein
CTRL Phospho
CTRL RNA
GEF Protein
GEF Phospho
gef RNA
PLX Protein
PLX Phospho
PLX RNA
GEF PLX Protein
GEF PLX Phospho
GEF PLX RNA
Concentration(n[M])
Relative Conc
Time (hour)

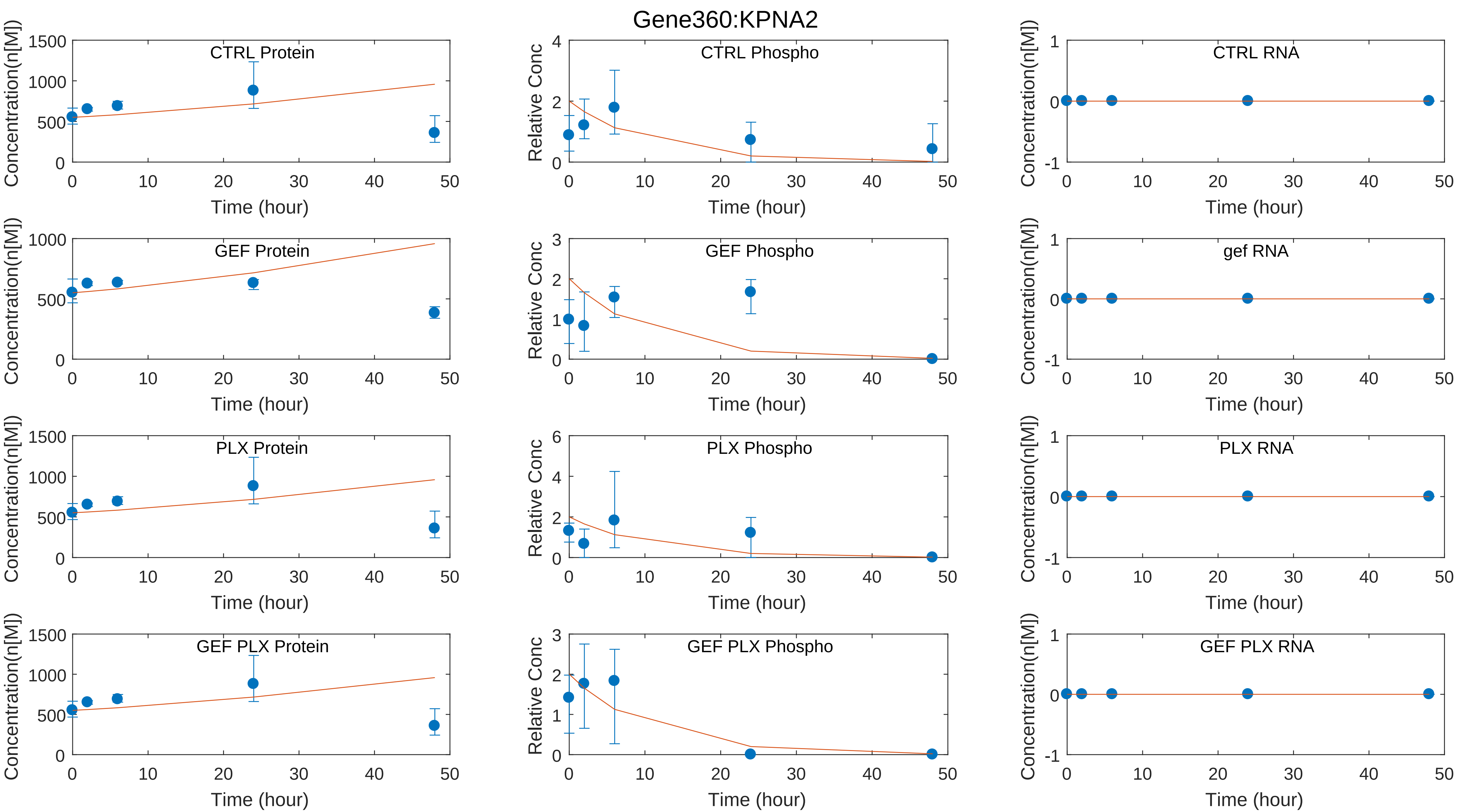
Gene360:KPNA2
CTRL Protein
CTRL Phospho
CTRL RNA
GEF Protein
GEF Phospho
gef RNA
PLX Protein
PLX Phospho
PLX RNA
GEF PLX Protein
GEF PLX Phospho
GEF PLX RNA
Concentration(n[M])
Relative Conc
Time (hour)

Gene361:MAP2K6

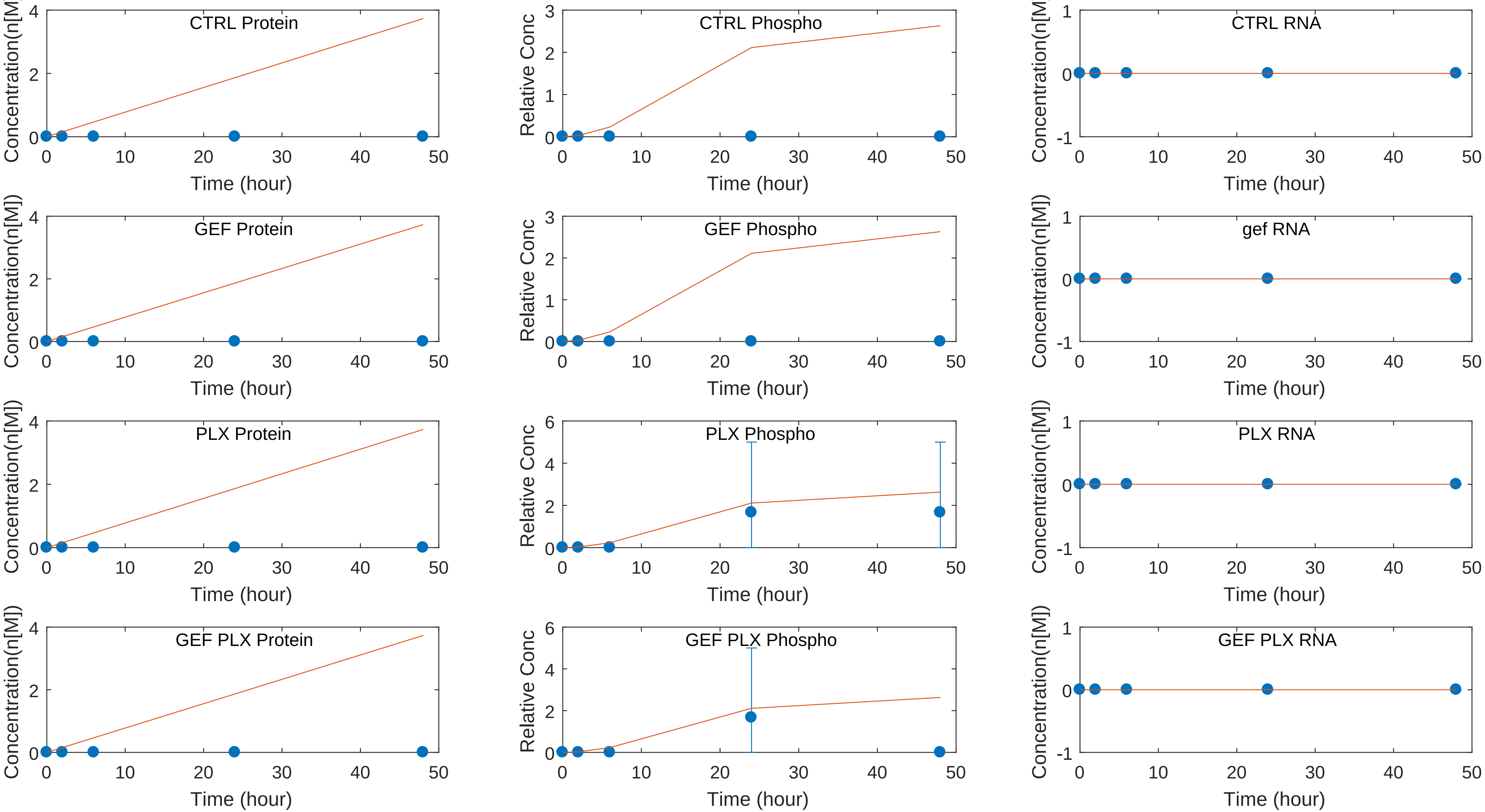

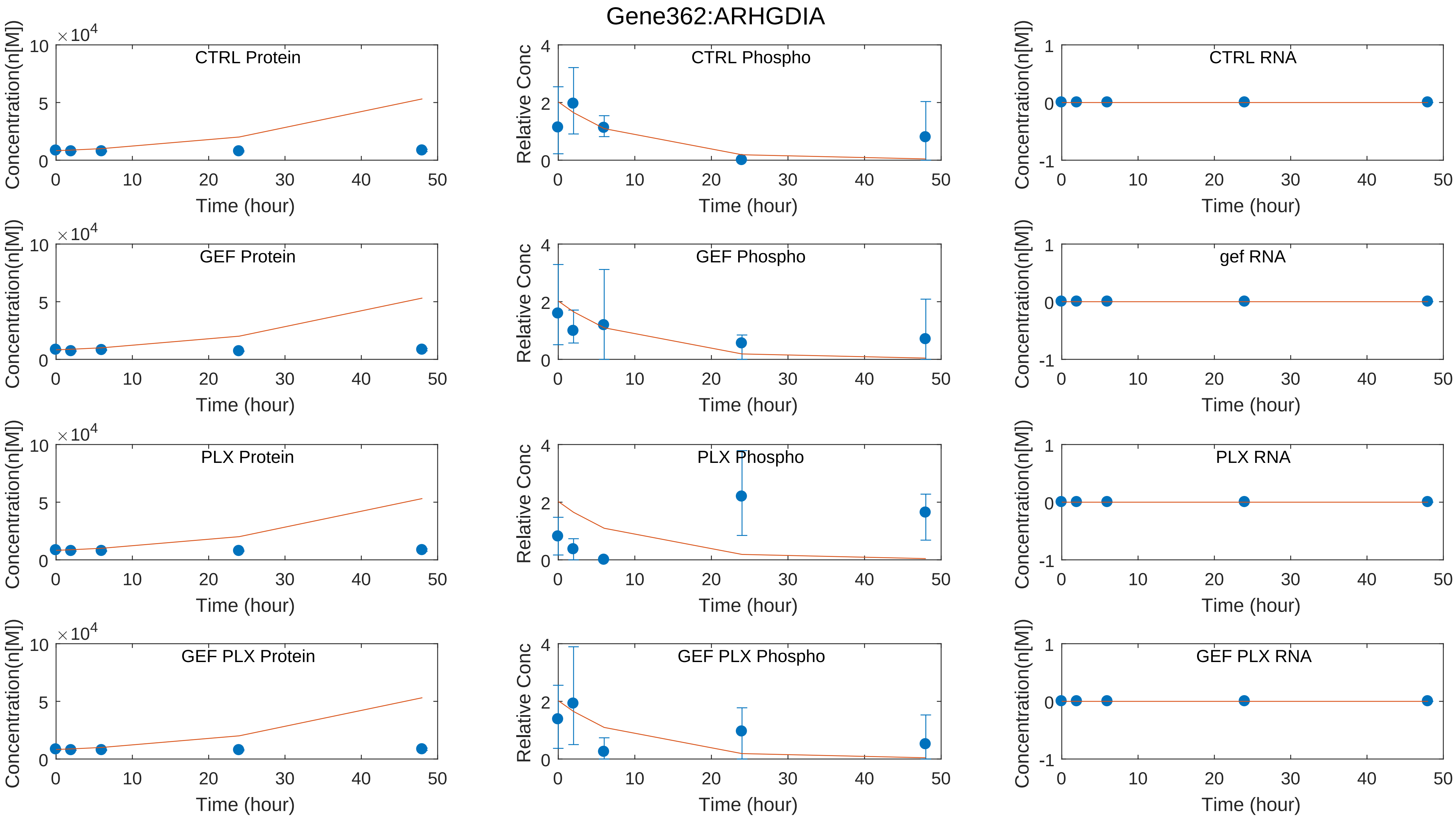

Gene362:ARHGDIA
CTRL Protein
CTRL Phospho
CTRL RNA
GEF Protein
GEF Phospho
gef RNA
PLX Protein
PLX Phospho
PLX RNA
GEF PLX Protein
GEF PLX Phospho
GEF PLX RNA
Concentration(n[M])
Relative Conc
Time (hour)
×10^4

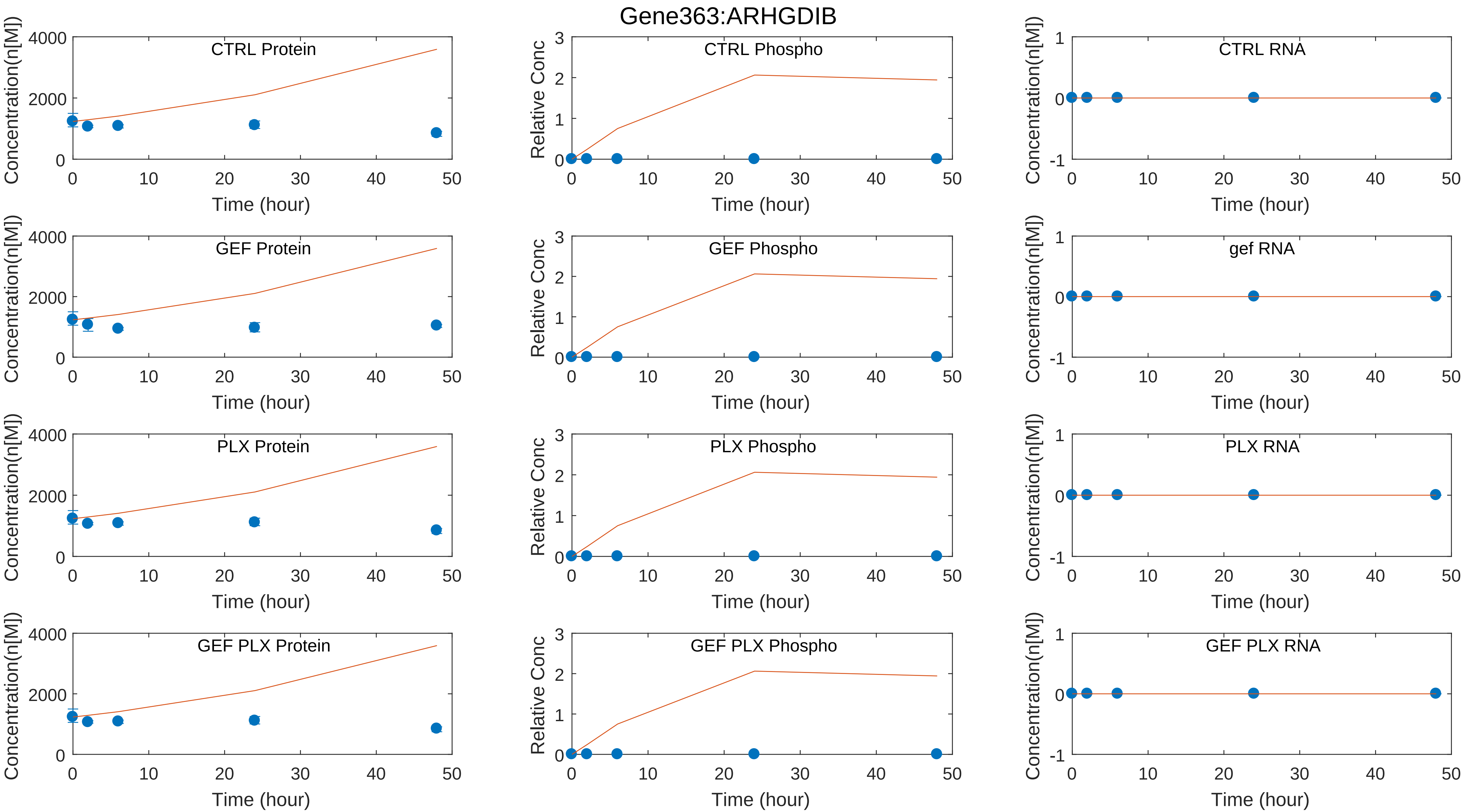

Gene363:ARHGDIB
CTRL Protein
GEF Protein
PLX Protein
GEF PLX Protein
CTRL Phospho
GEF Phospho
PLX Phospho
GEF PLX Phospho
CTRL RNA
gef RNA
PLX RNA
GEF PLX RNA
Concentration(n[M])
Relative Conc
Time (hour)

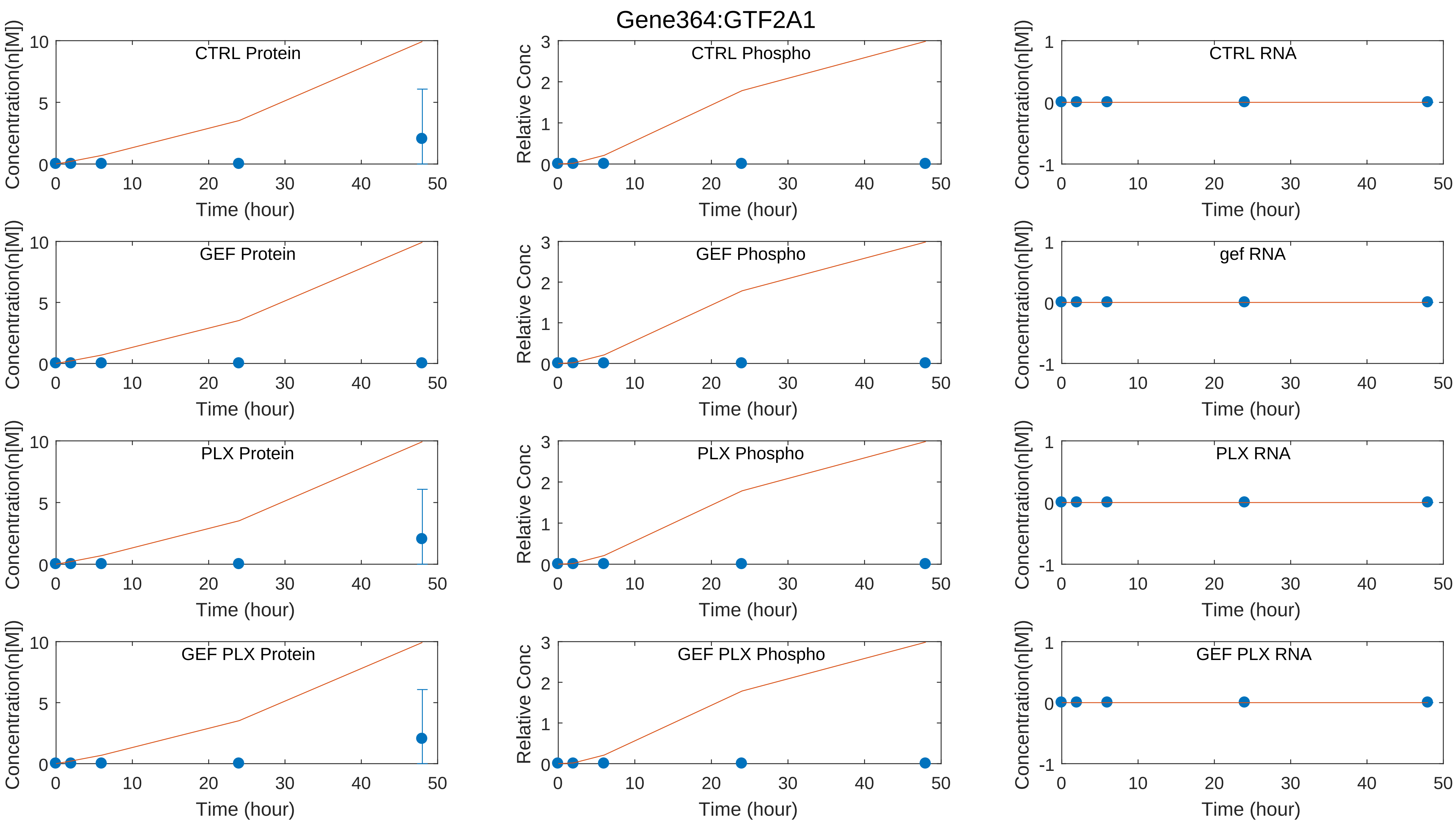

Gene364:GTF2A1
CTRL Protein
CTRL Phospho
CTRL RNA
GEF Protein
GEF Phospho
gef RNA
PLX Protein
PLX Phospho
PLX RNA
GEF PLX Protein
GEF PLX Phospho
GEF PLX RNA
Concentration(n[M])
Relative Conc
Time (hour)

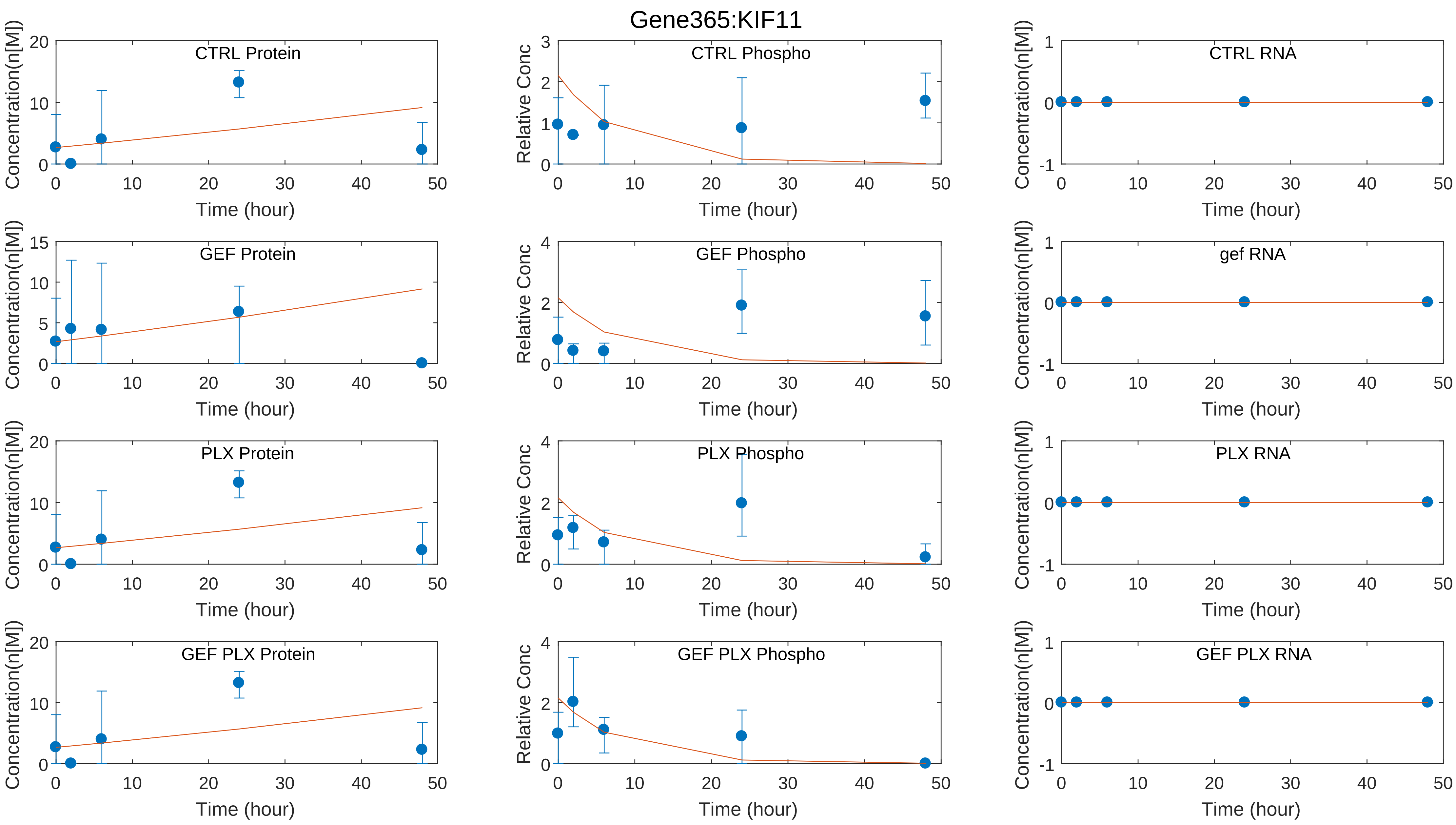

Gene365:KIF11
CTRL Protein
CTRL Phospho
CTRL RNA
GEF Protein
GEF Phospho
gef RNA
PLX Protein
PLX Phospho
PLX RNA
GEF PLX Protein
GEF PLX Phospho
GEF PLX RNA
Concentration(n[M])
Relative Conc
Time (hour)

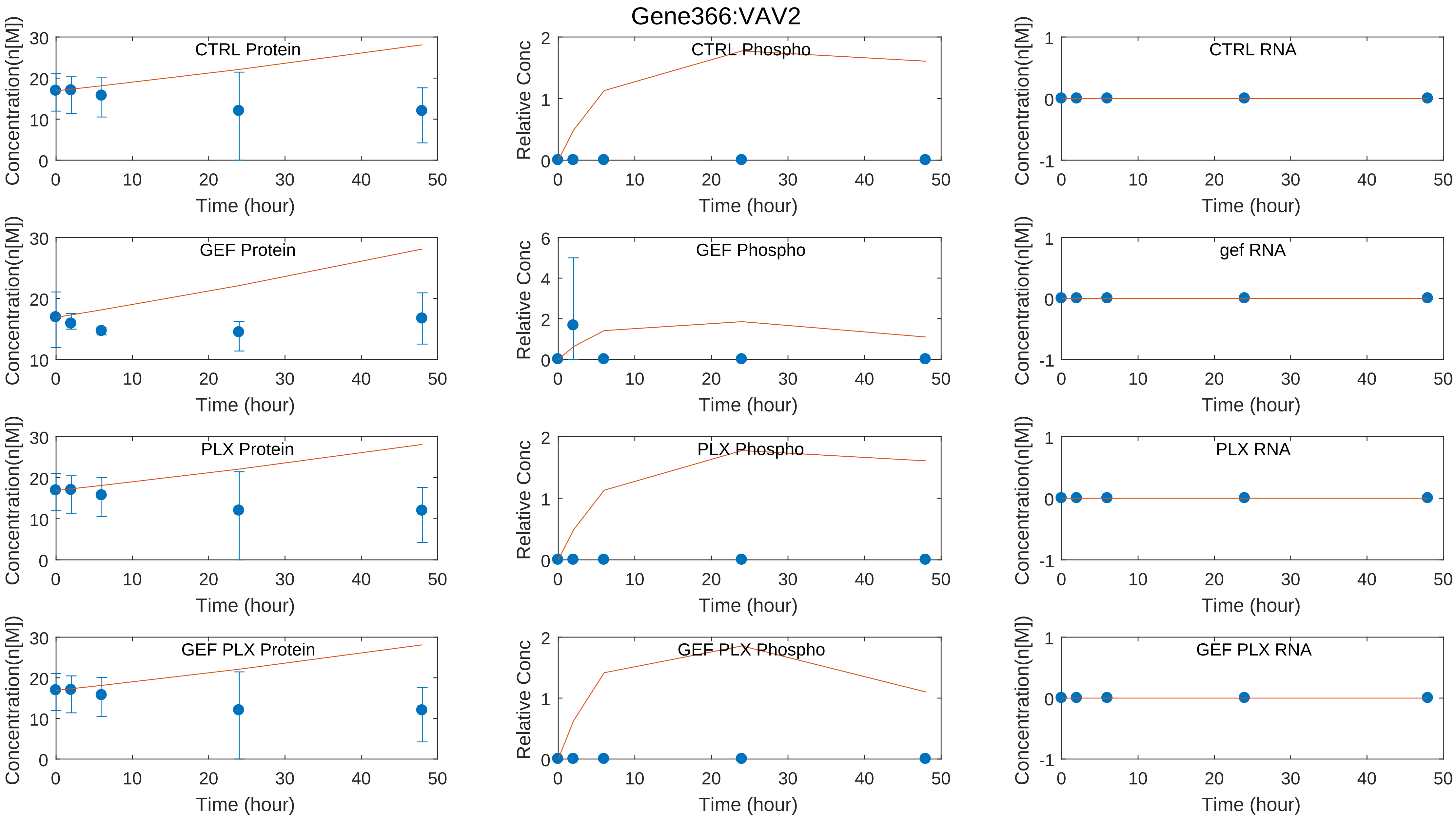

Gene366:VAV2
CTRL Protein
CTRL Phospho
CTRL RNA
GEF Protein
GEF Phospho
gef RNA
PLX Protein
PLX Phospho
PLX RNA
GEF PLX Protein
GEF PLX Phospho
GEF PLX RNA
Concentration(n[M])
Relative Conc
Time (hour)

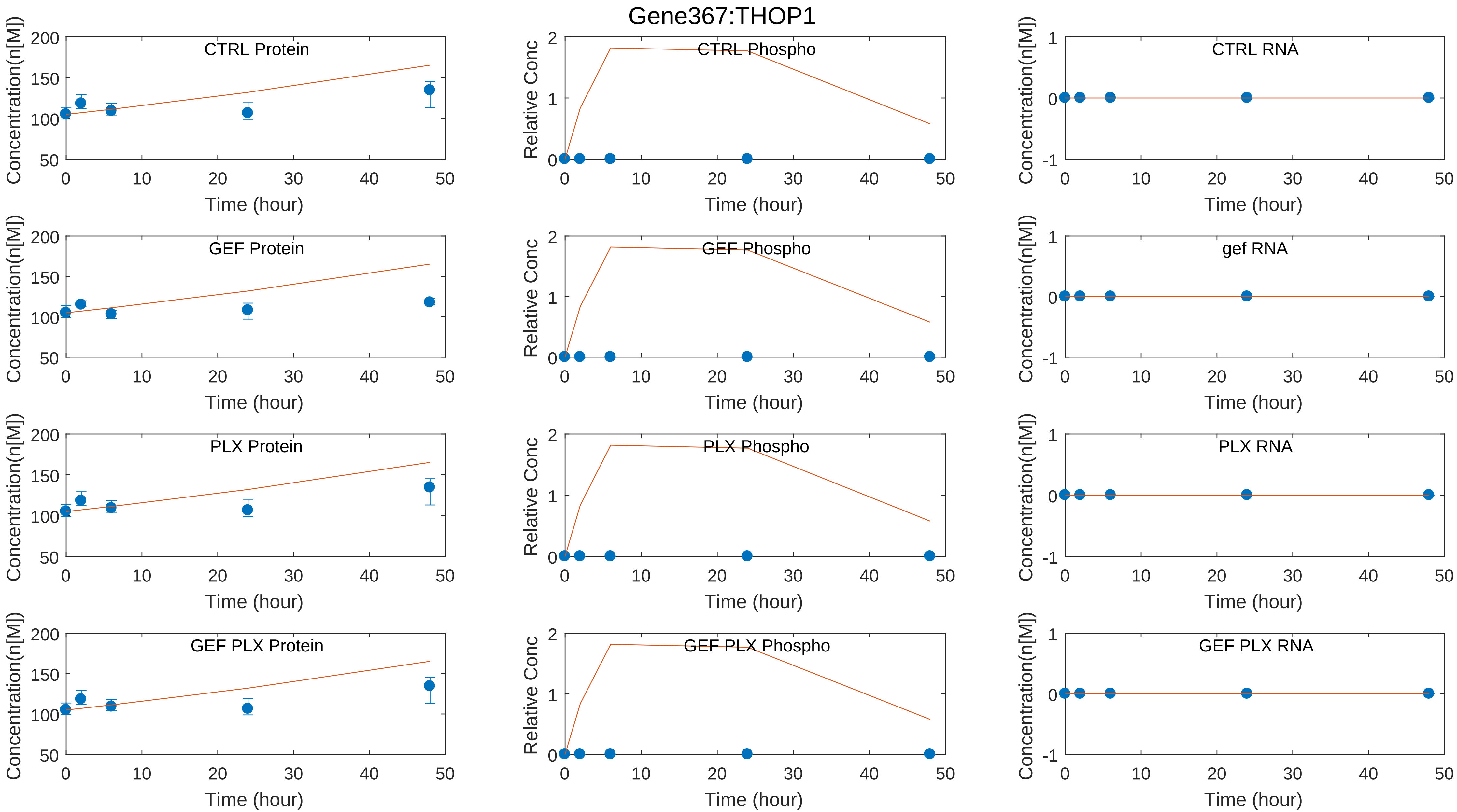

Gene367:THOP1
CTRL Protein
CTRL Phospho
CTRL RNA
GEF Protein
GEF Phospho
gef RNA
PLX Protein
PLX Phospho
PLX RNA
GEF PLX Protein
GEF PLX Phospho
GEF PLX RNA
Concentration(n[M])
Relative Conc
Time (hour)

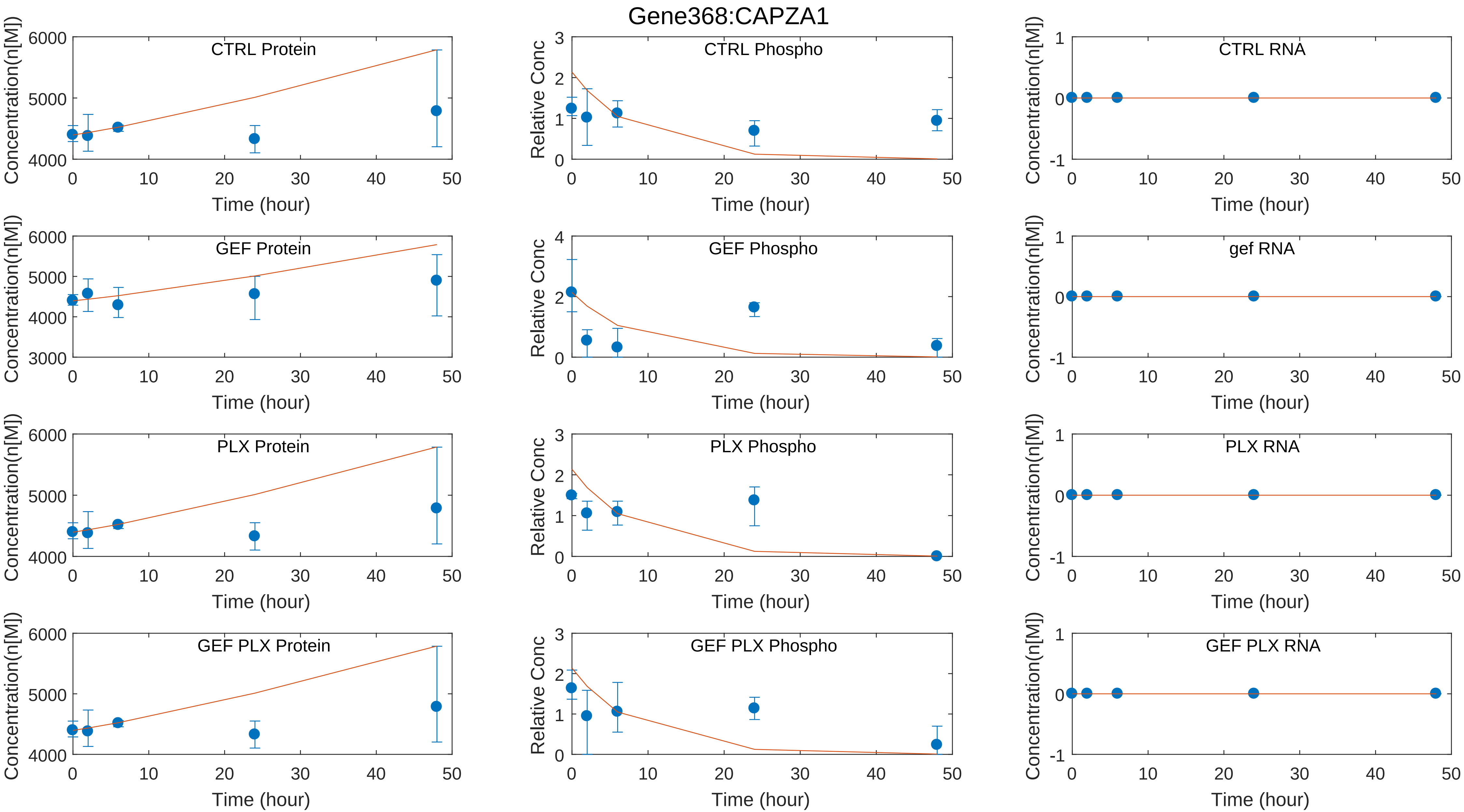

Gene368:CAPZA1
CTRL Protein
CTRL Phospho
CTRL RNA
GEF Protein
GEF Phospho
gef RNA
PLX Protein
PLX Phospho
PLX RNA
GEF PLX Protein
GEF PLX Phospho
GEF PLX RNA
Concentration(n[M])
Relative Conc
Time (hour)

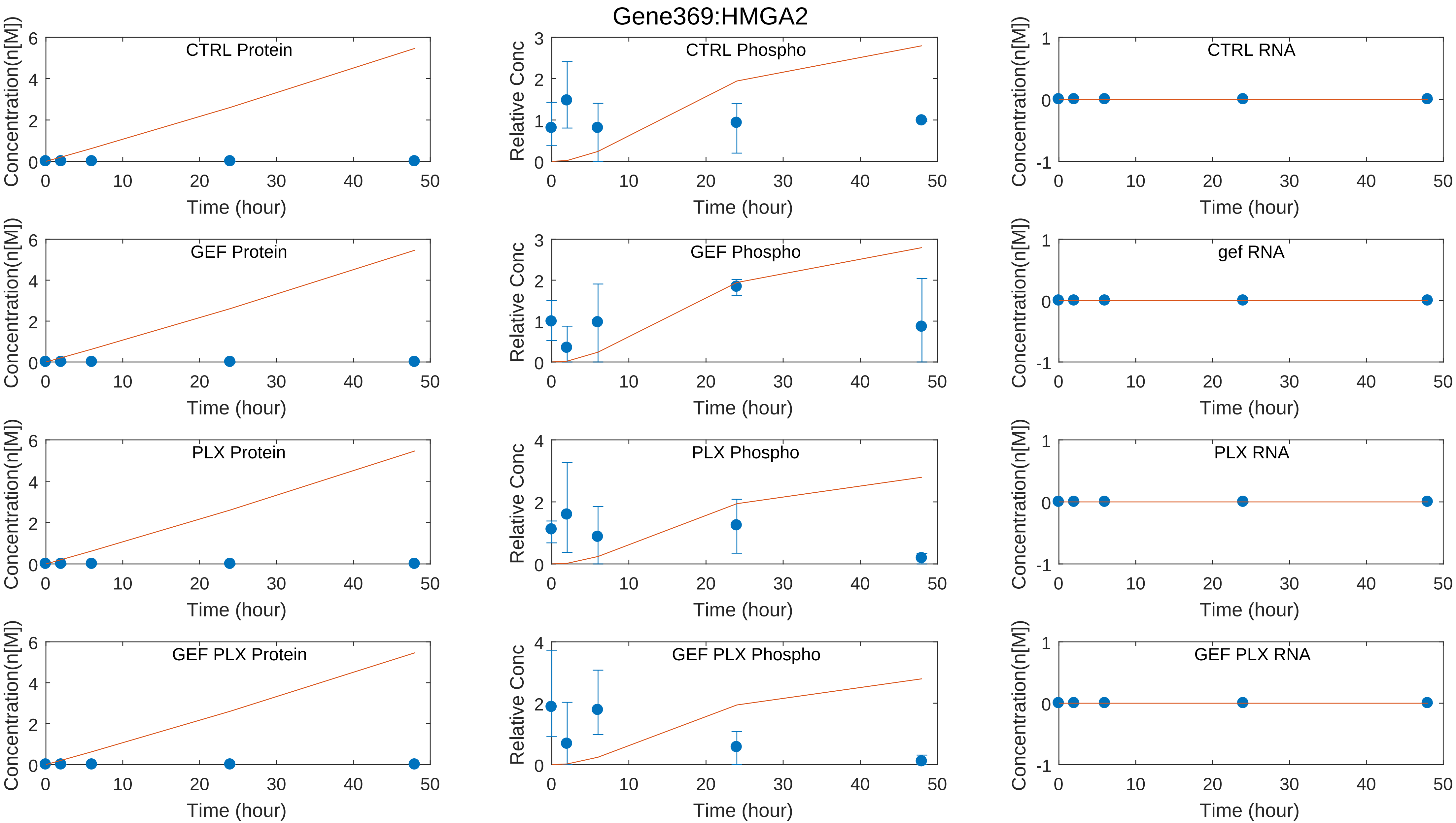
Gene369:HMGA2
CTRL Protein
CTRL Phospho
CTRL RNA
GEF Protein
GEF Phospho
gef RNA
PLX Protein
PLX Phospho
PLX RNA
GEF PLX Protein
GEF PLX Phospho
GEF PLX RNA
Concentration(n[M])
Relative Conc
Time (hour)

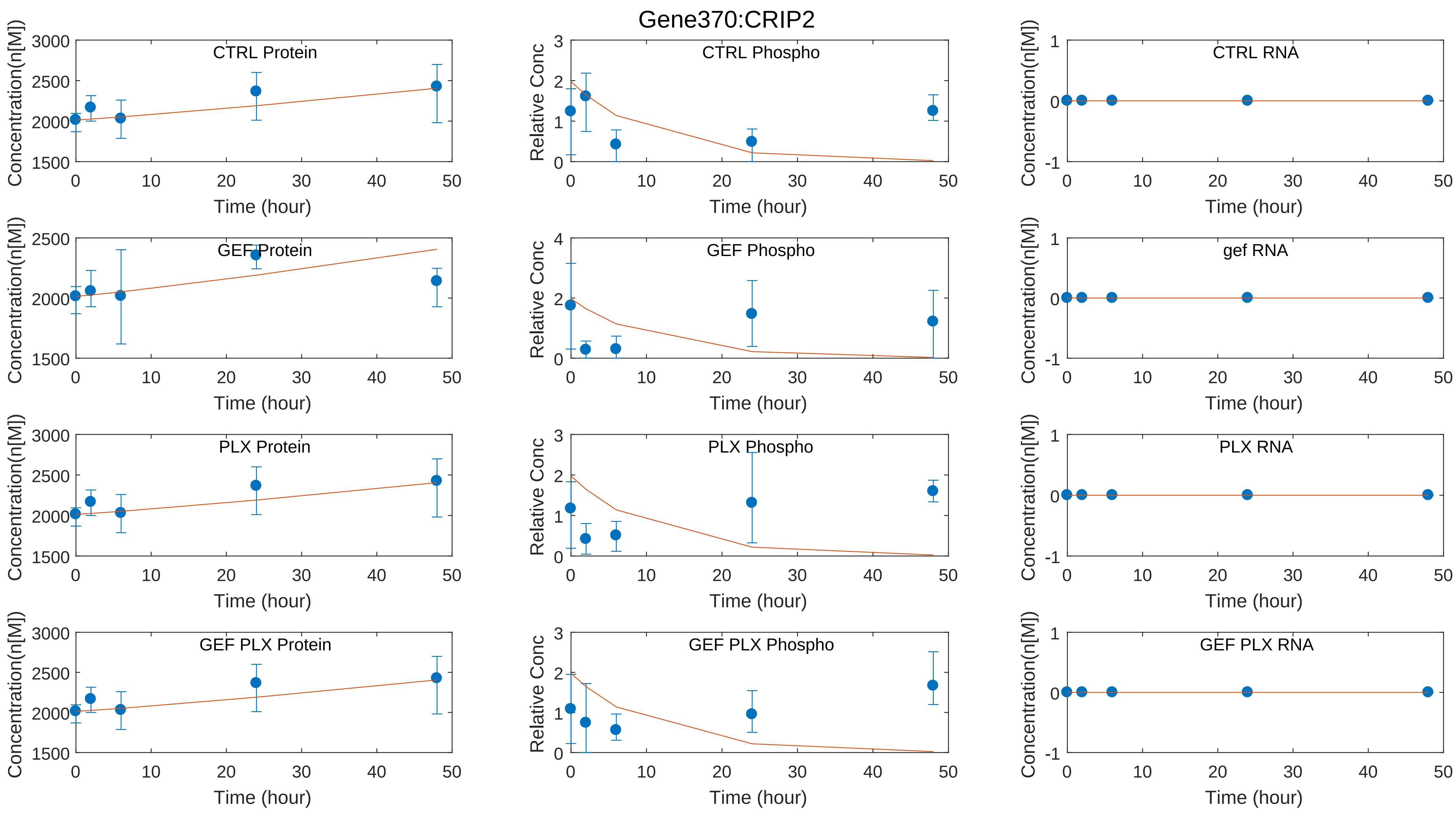

Gene370:CRIP2
CTRL Protein
CTRL Phospho
CTRL RNA
GEF Protein
GEF Phospho
gef RNA
PLX Protein
PLX Phospho
PLX RNA
GEF PLX Protein
GEF PLX Phospho
GEF PLX RNA
Concentration(n[M])
Relative Conc
Time (hour)

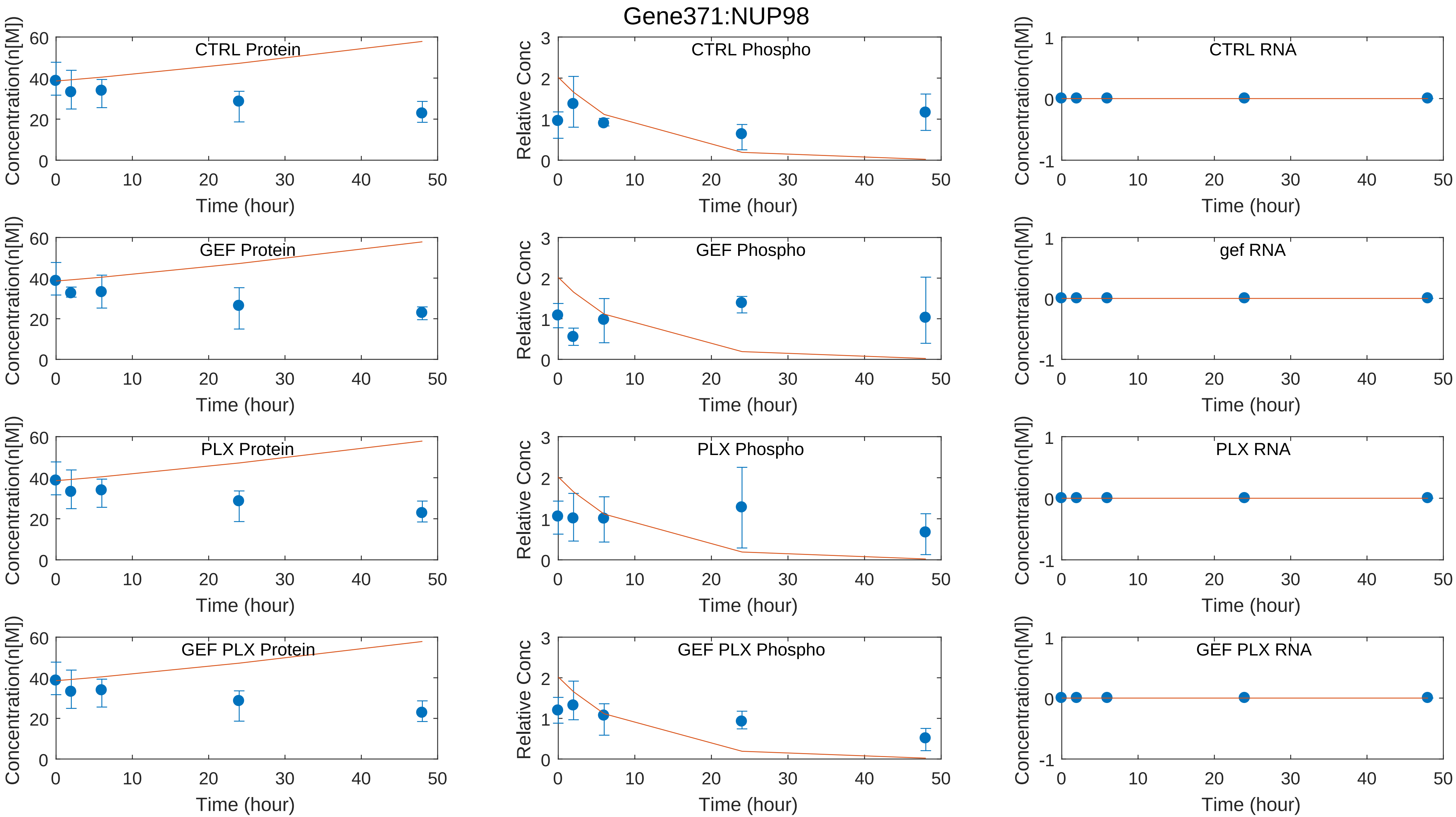

Gene371:NUP98
CTRL Protein
CTRL Phospho
CTRL RNA
GEF Protein
GEF Phospho
gef RNA
PLX Protein
PLX Phospho
PLX RNA
GEF PLX Protein
GEF PLX Phospho
GEF PLX RNA
Concentration(n[M])
Relative Conc
Time (hour)

Gene372:PPP5C

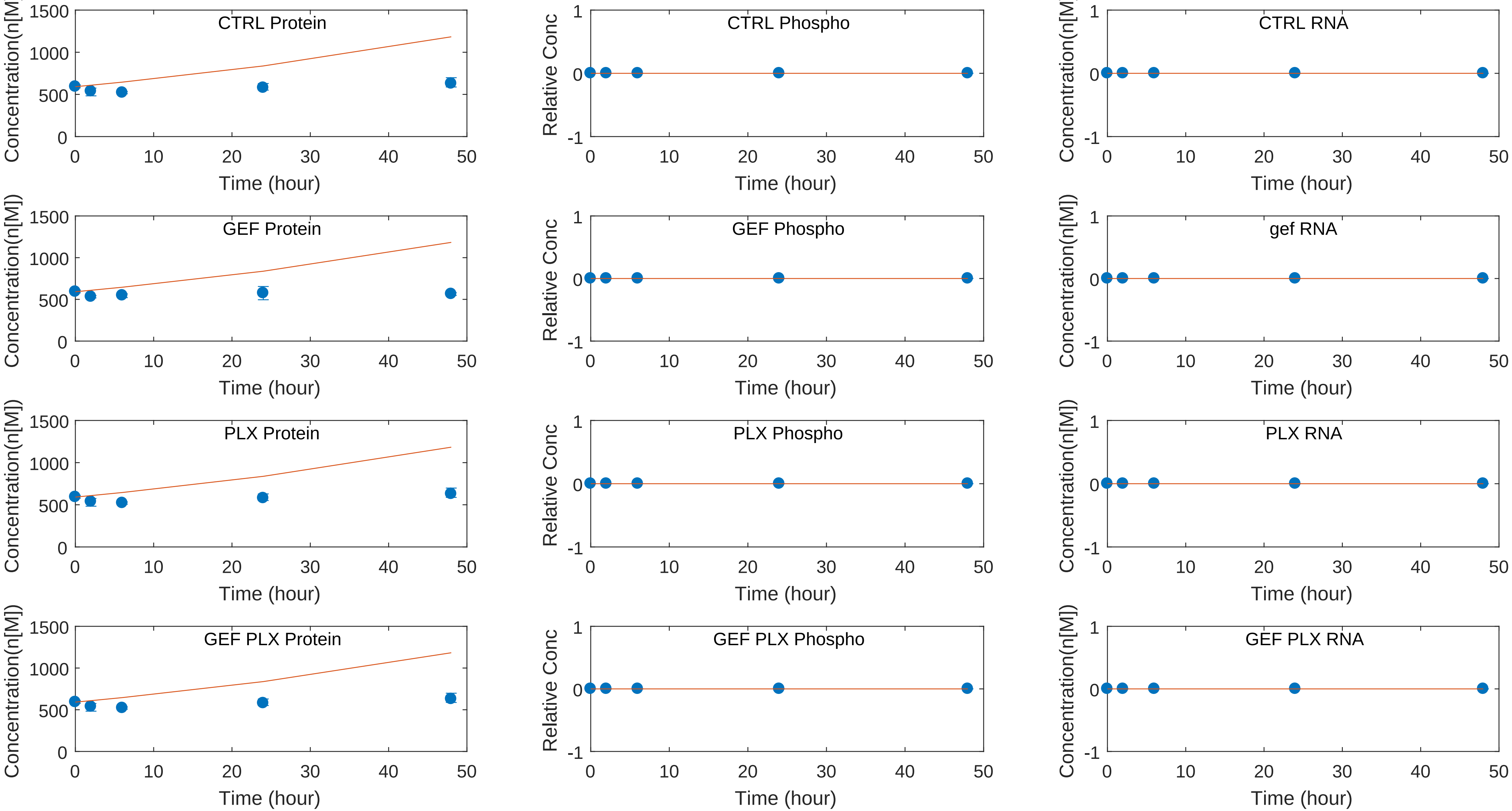

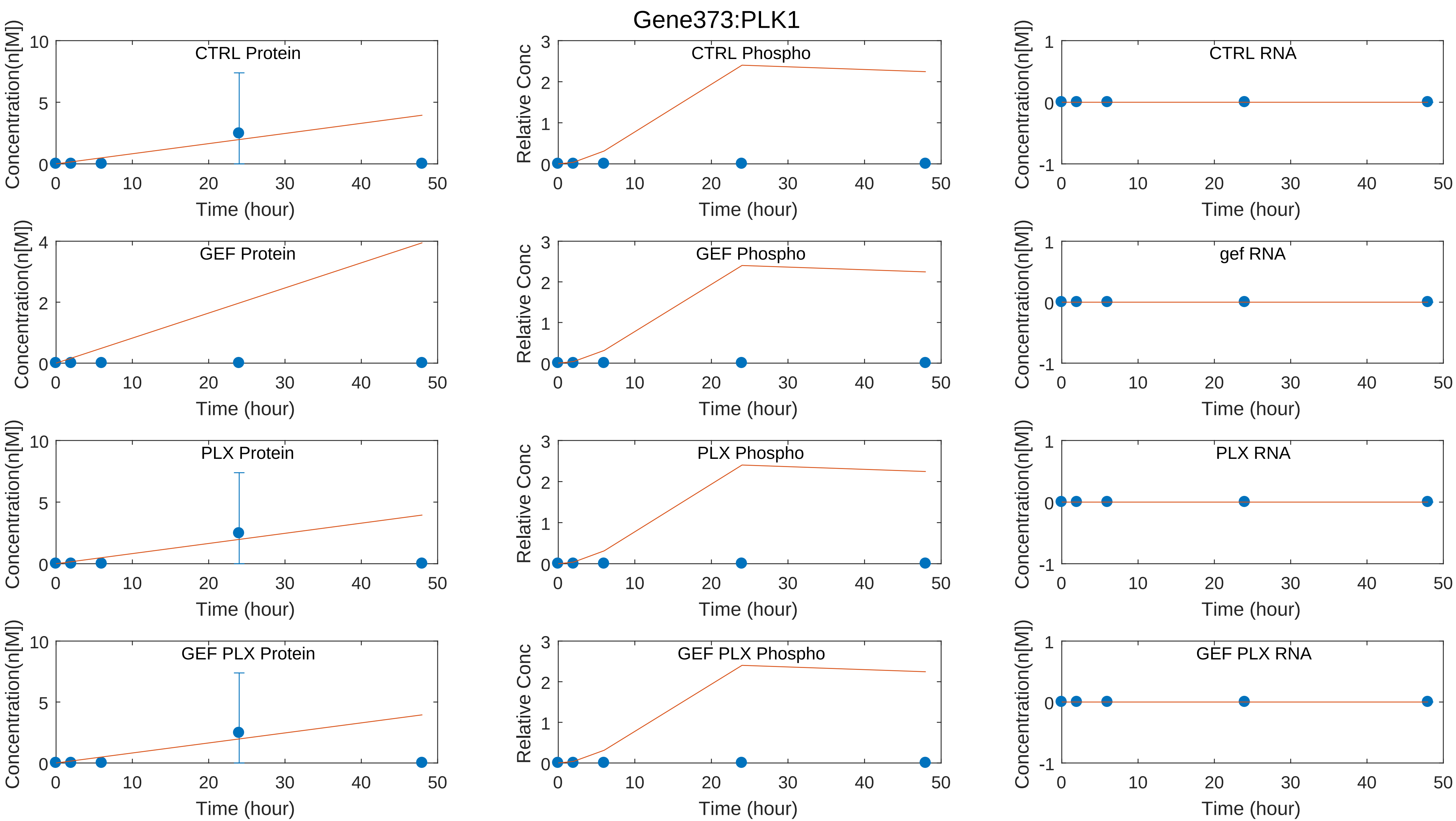
Gene373:PLK1
CTRL Protein
CTRL Phospho
CTRL RNA
GEF Protein
GEF Phospho
gef RNA
PLX Protein
PLX Phospho
PLX RNA
GEF PLX Protein
GEF PLX Phospho
GEF PLX RNA
Concentration(n[M])
Relative Conc
Time (hour)

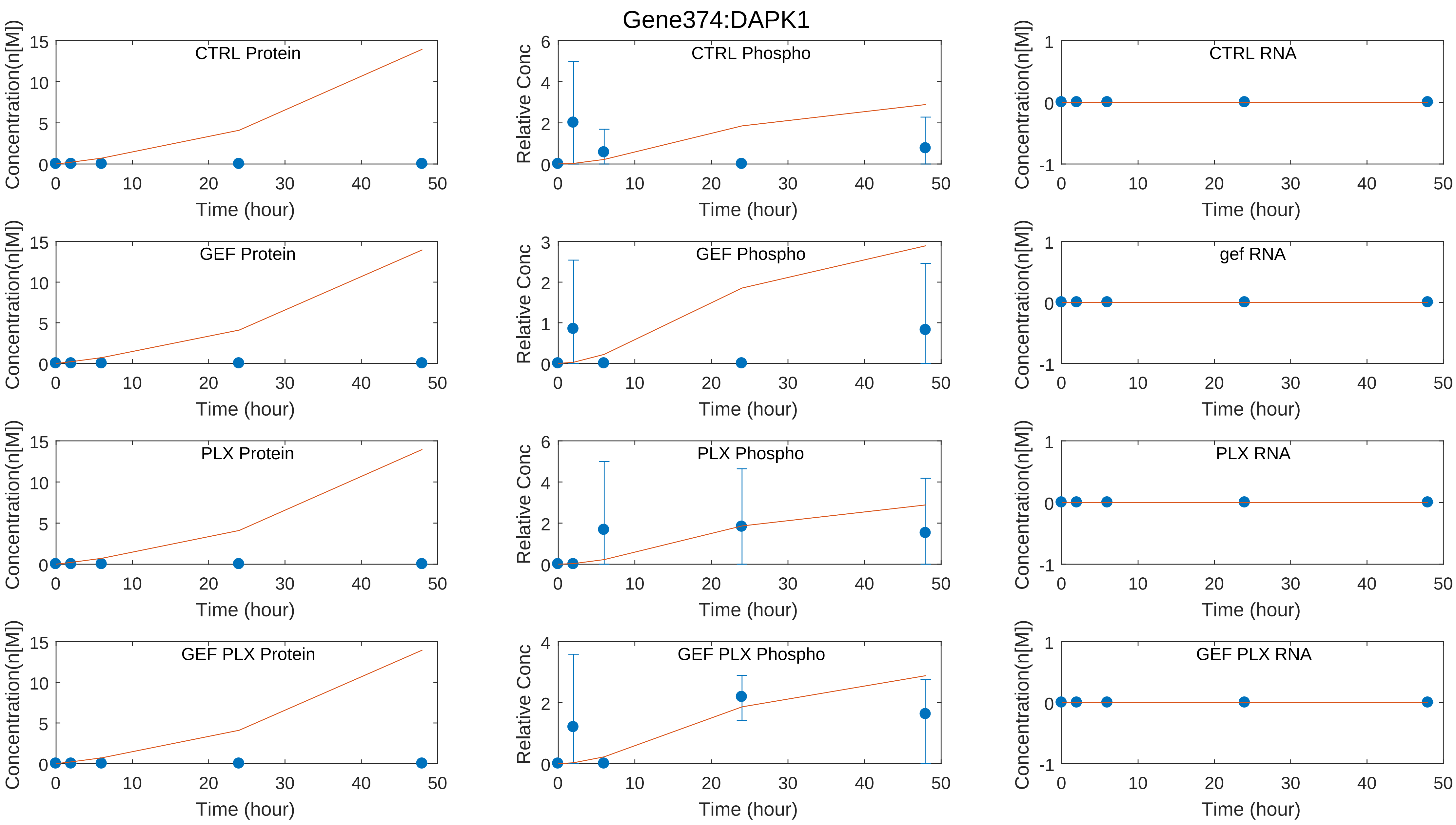
Gene374:DAPK1
CTRL Protein
CTRL Phospho
CTRL RNA
GEF Protein
GEF Phospho
gef RNA
PLX Protein
PLX Phospho
PLX RNA
GEF PLX Protein
GEF PLX Phospho
GEF PLX RNA
Concentration(n[M])
Relative Conc
Time (hour)

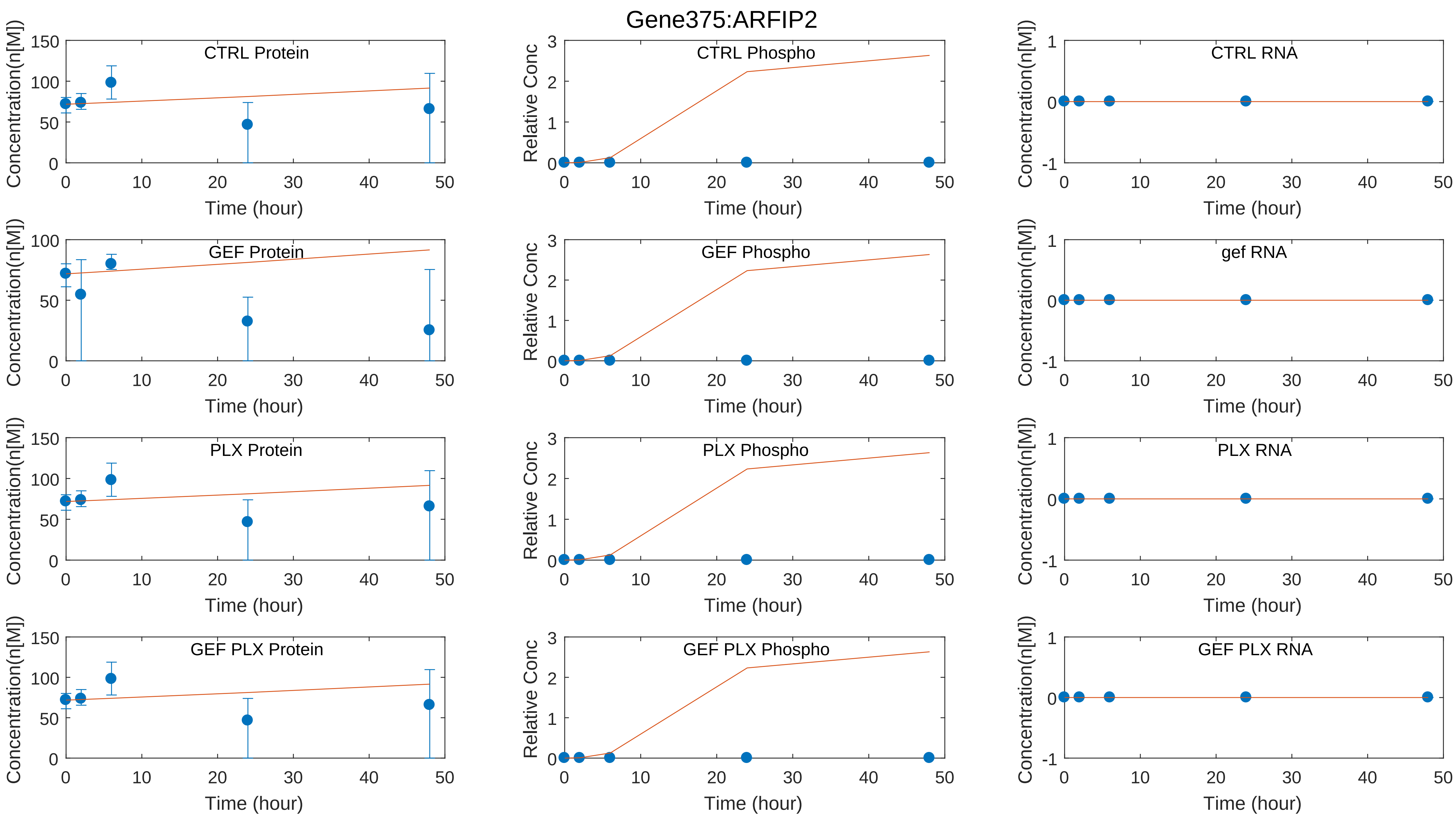
Gene375:ARFIP2
CTRL Protein
CTRL Phospho
CTRL RNA
GEF Protein
GEF Phospho
gef RNA
PLX Protein
PLX Phospho
PLX RNA
GEF PLX Protein
GEF PLX Phospho
GEF PLX RNA
Concentration(n[M])
Relative Conc
Time (hour)

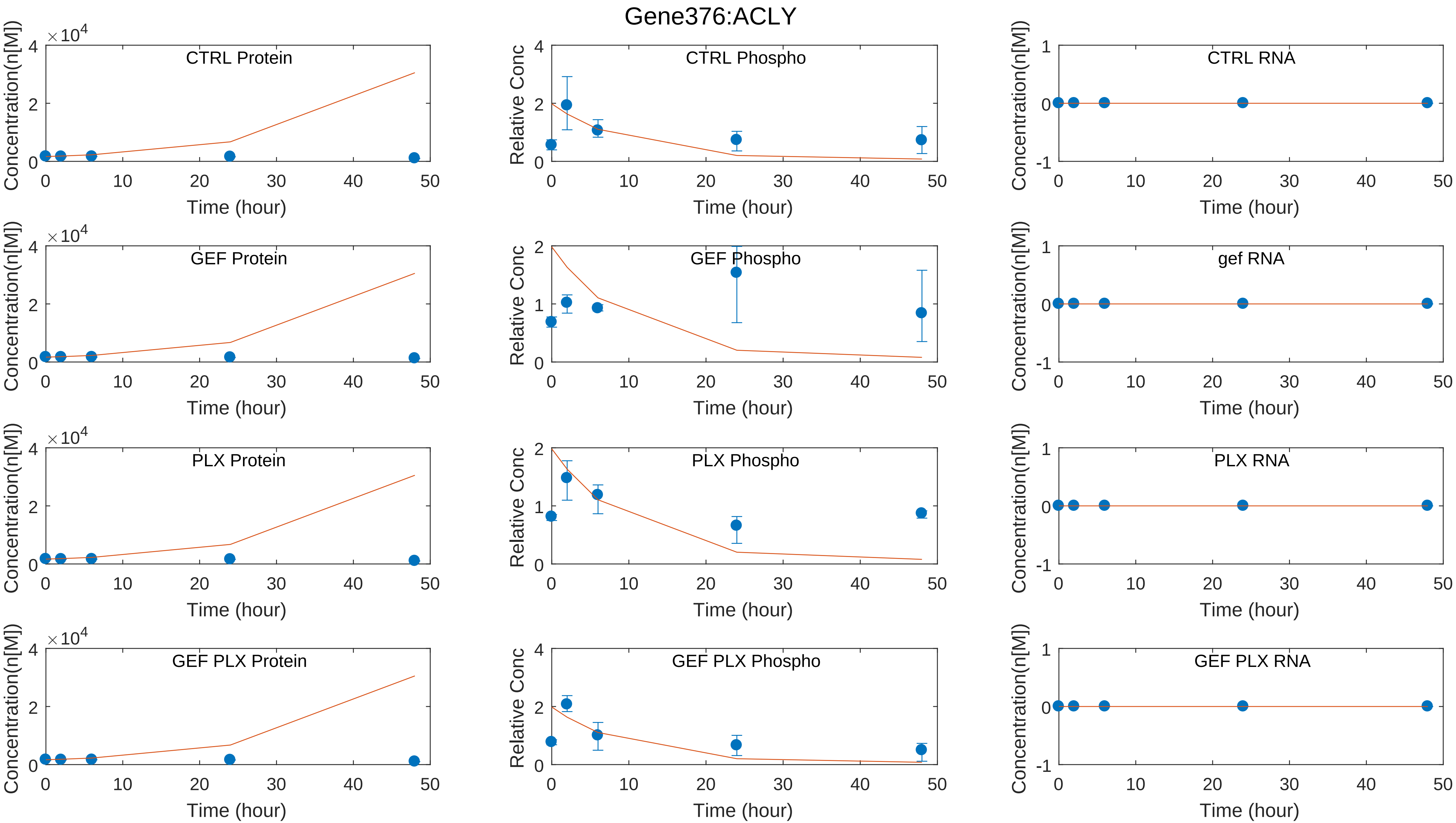
Gene376:ACLY
CTRL Protein
CTRL Phospho
CTRL RNA
GEF Protein
GEF Phospho
gef RNA
PLX Protein
PLX Phospho
PLX RNA
GEF PLX Protein
GEF PLX Phospho
GEF PLX RNA
Concentration(n[M])
Relative Conc
Time (hour)
×10^4

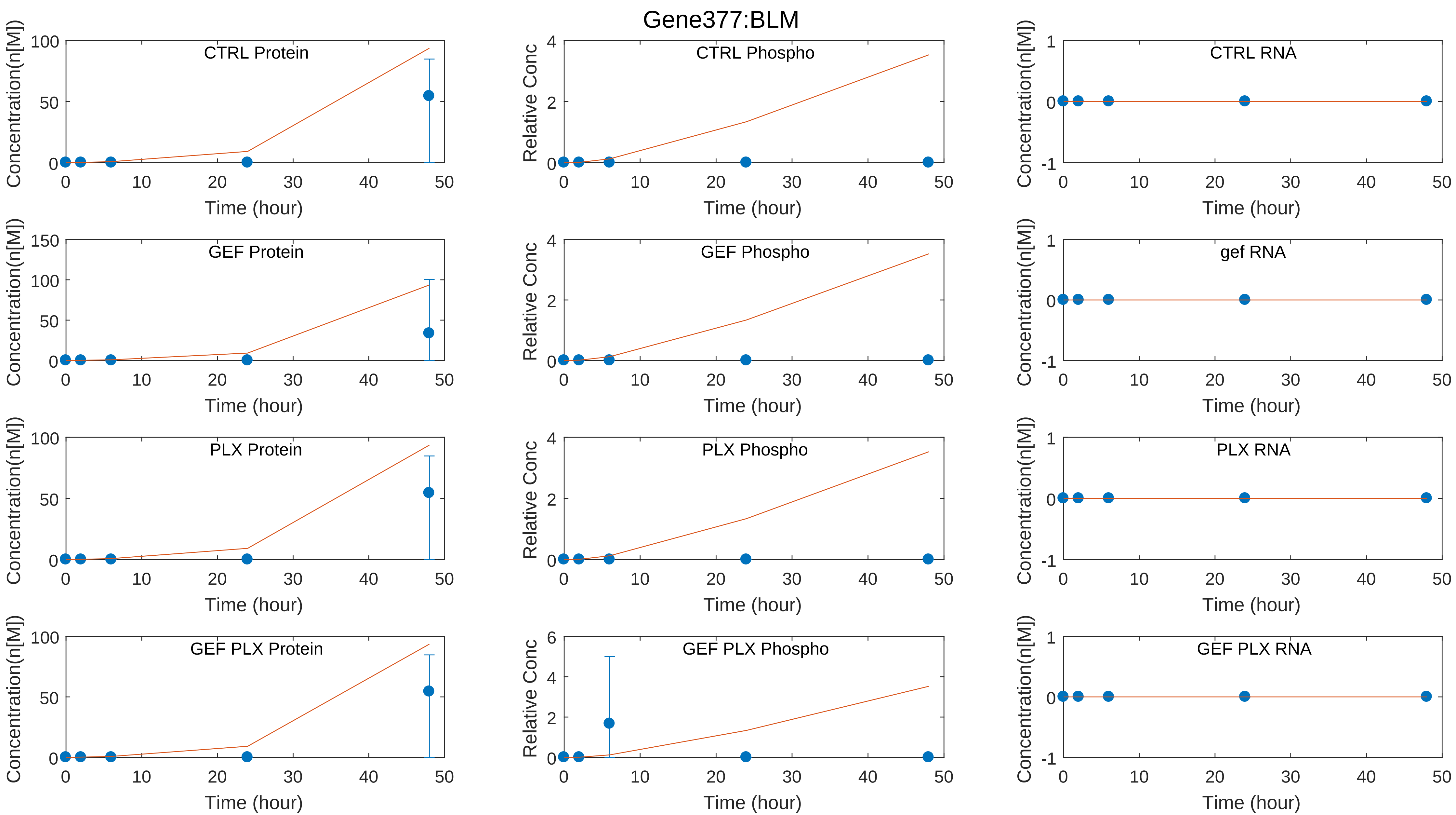
Gene377:BLM
CTRL Protein
CTRL Phospho
CTRL RNA
GEF Protein
GEF Phospho
gef RNA
PLX Protein
PLX Phospho
PLX RNA
GEF PLX Protein
GEF PLX Phospho
GEF PLX RNA
Concentration(n[M])
Relative Conc
Time (hour)

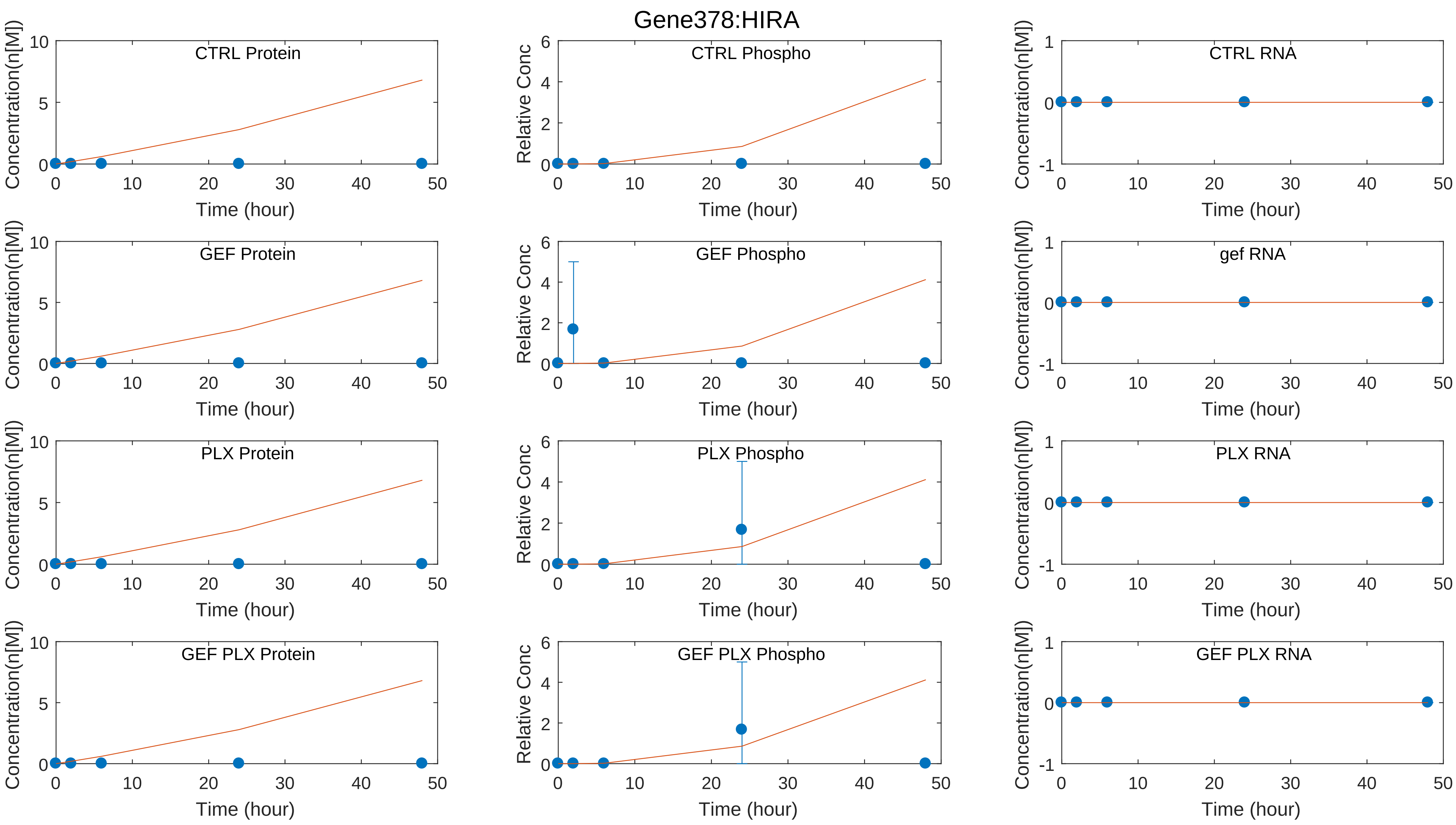

Gene378:HIRA
CTRL Protein
CTRL Phospho
CTRL RNA
GEF Protein
GEF Phospho
gef RNA
PLX Protein
PLX Phospho
PLX RNA
GEF PLX Protein
GEF PLX Phospho
GEF PLX RNA
Concentration(n[M])
Relative Conc
Time (hour)

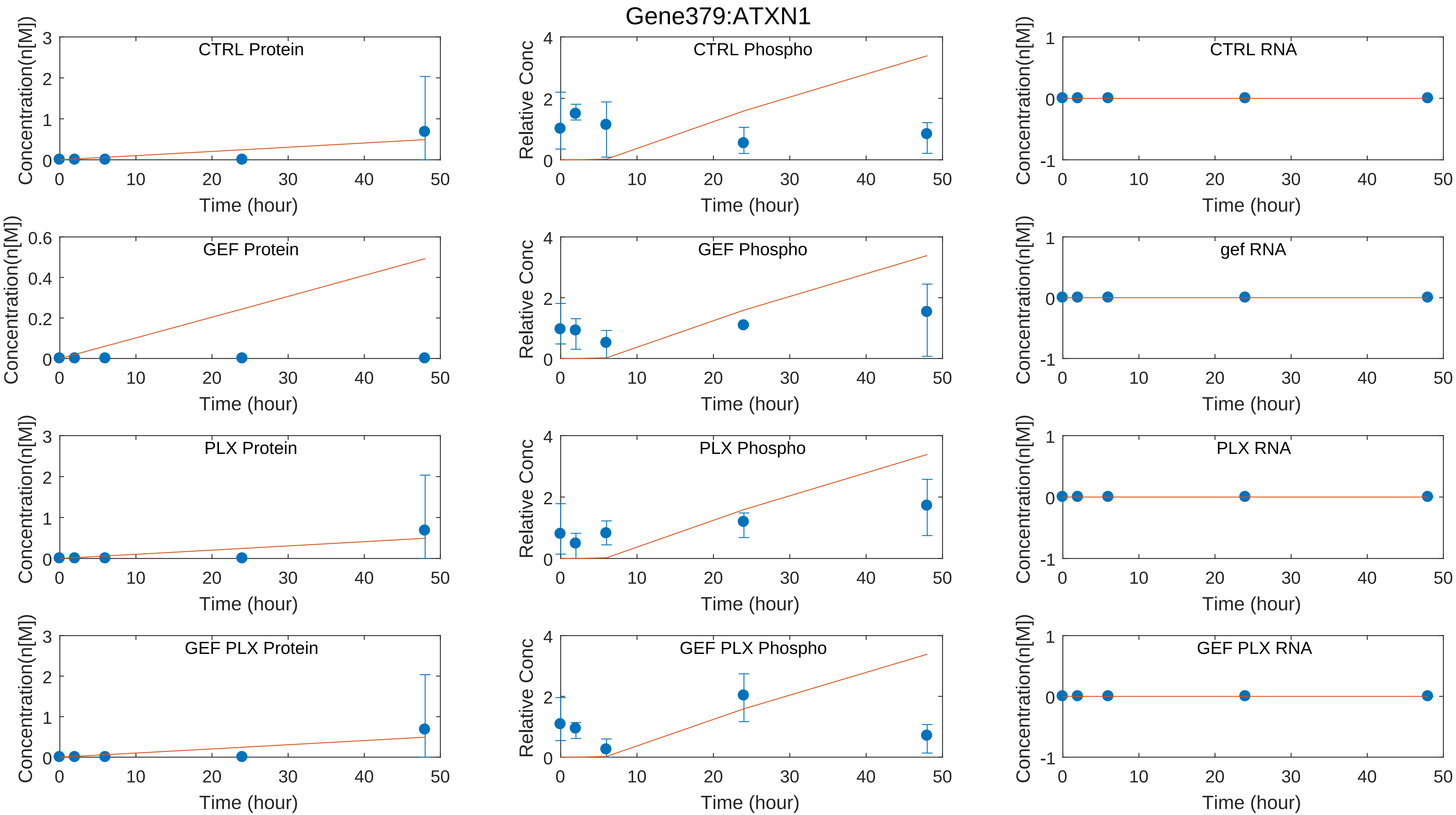

Gene379:ATXN1
CTRL Protein
CTRL Phospho
CTRL RNA
GEF Protein
GEF Phospho
gef RNA
PLX Protein
PLX Phospho
PLX RNA
GEF PLX Protein
GEF PLX Phospho
GEF PLX RNA
Concentration(n[M])
Relative Conc
Time (hour)

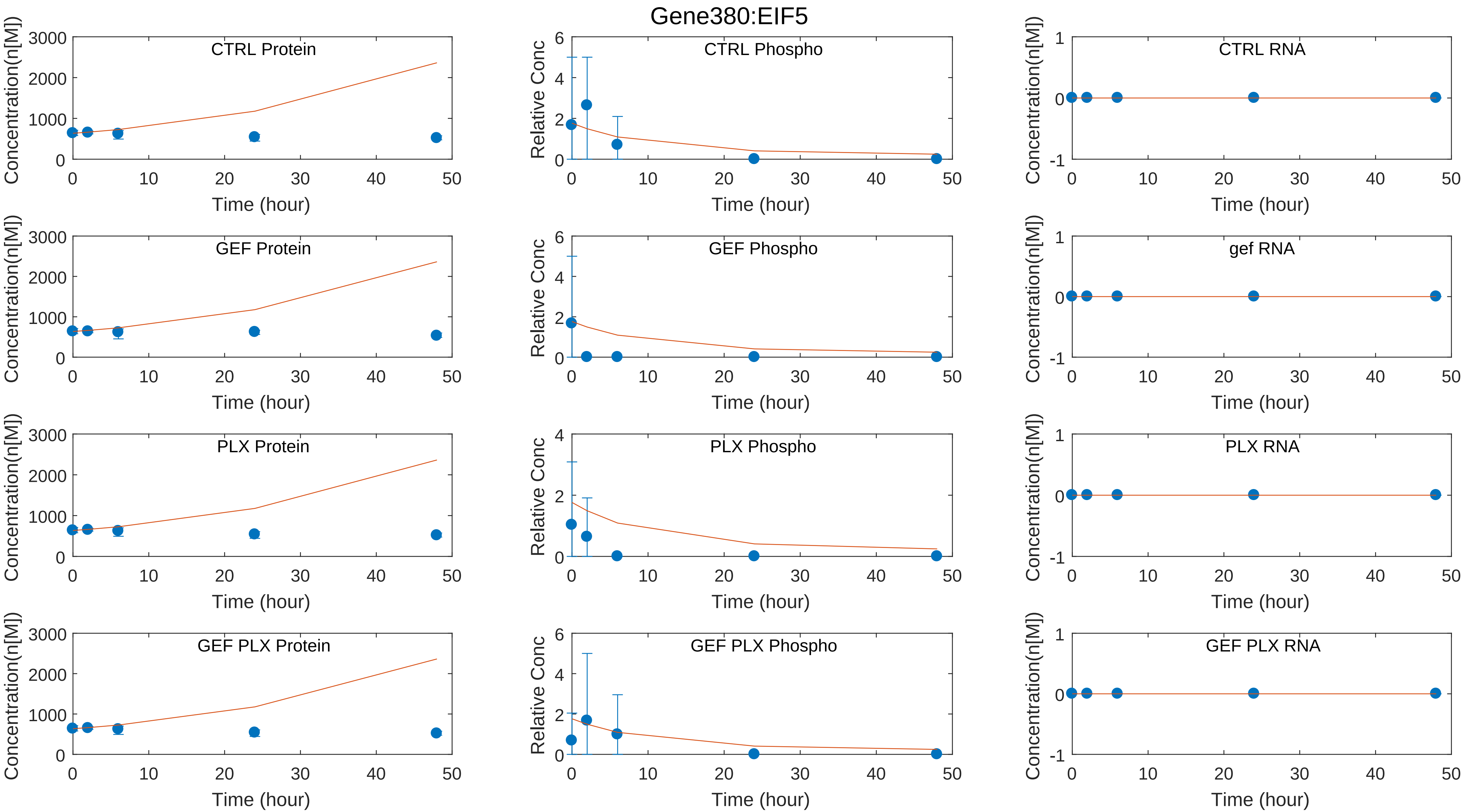
Gene380:EIF5
CTRL Protein
CTRL Phospho
CTRL RNA
GEF Protein
GEF Phospho
gef RNA
PLX Protein
PLX Phospho
PLX RNA
GEF PLX Protein
GEF PLX Phospho
GEF PLX RNA
Concentration(n[M])
Relative Conc
Time (hour)

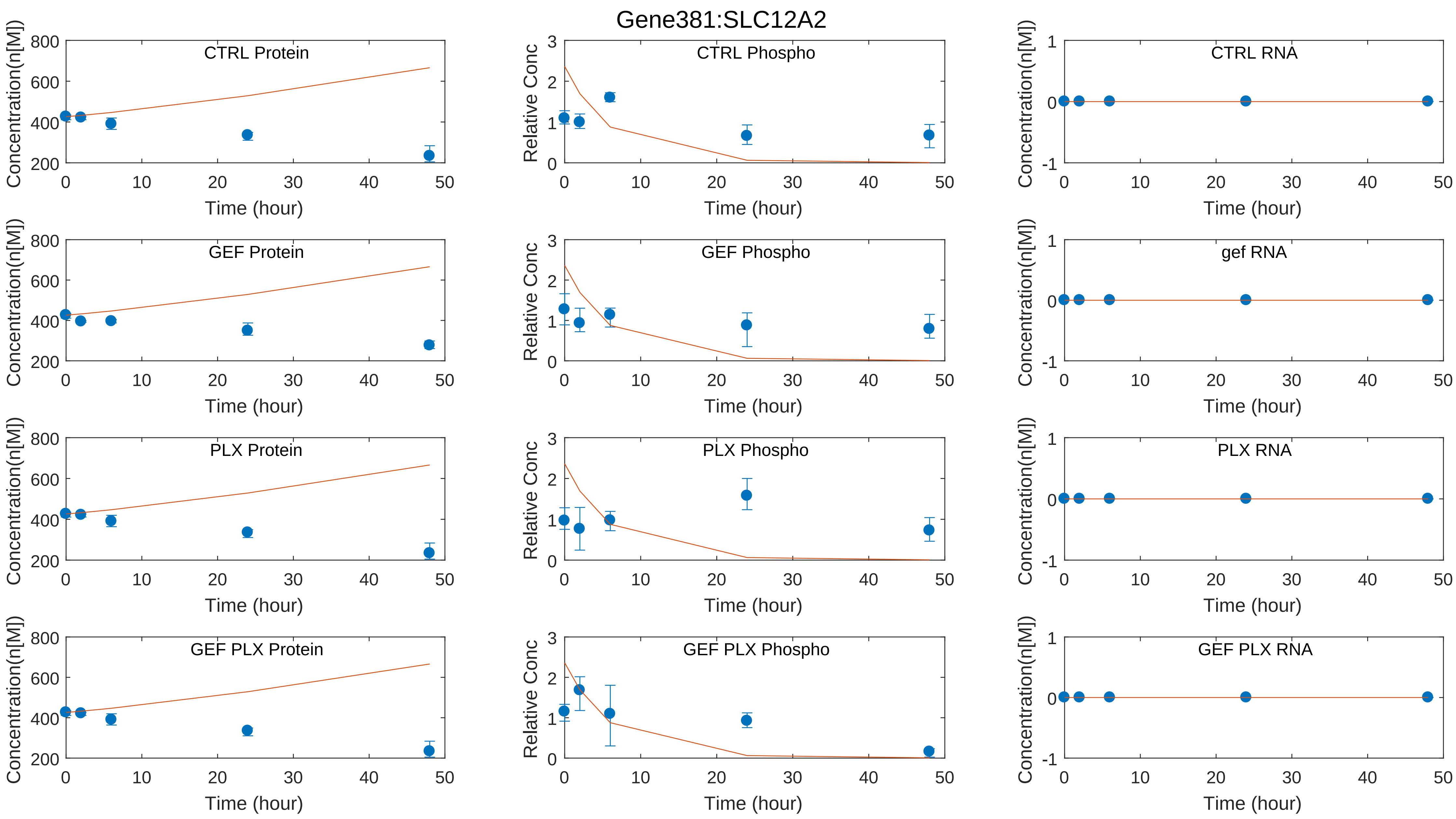
Gene381:SLC12A2
CTRL Protein
CTRL Phospho
CTRL RNA
GEF Protein
GEF Phospho
gef RNA
PLX Protein
PLX Phospho
PLX RNA
GEF PLX Protein
GEF PLX Phospho
GEF PLX RNA
Concentration(n[M])
Relative Conc
Time (hour)

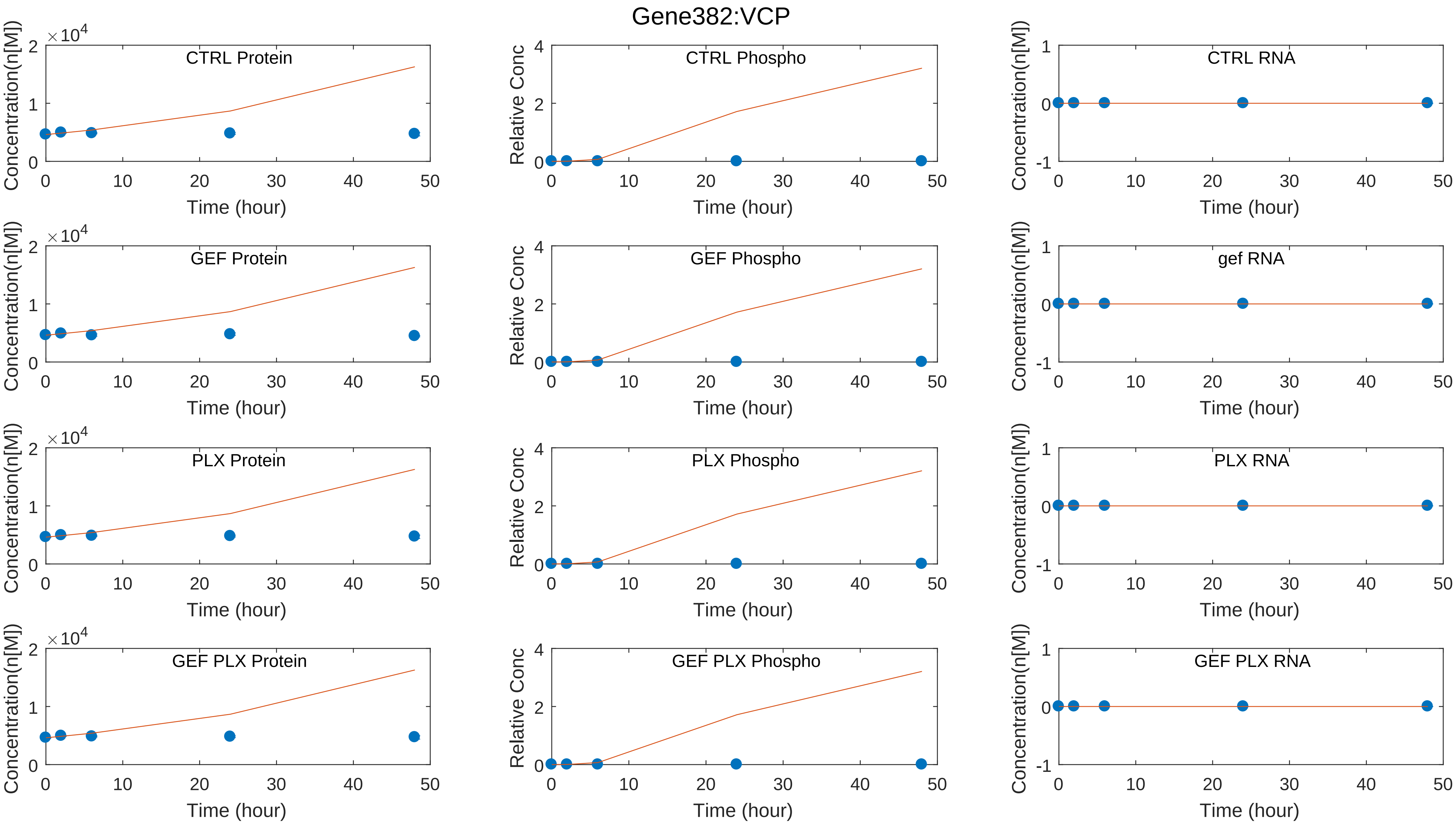

Gene382:VCP
CTRL Protein
GEF Protein
PLX Protein
GEF PLX Protein
CTRL Phospho
GEF Phospho
PLX Phospho
GEF PLX Phospho
CTRL RNA
gef RNA
PLX RNA
GEF PLX RNA
Concentration(n[M])
Relative Conc
Time (hour)
$\times 10^4$

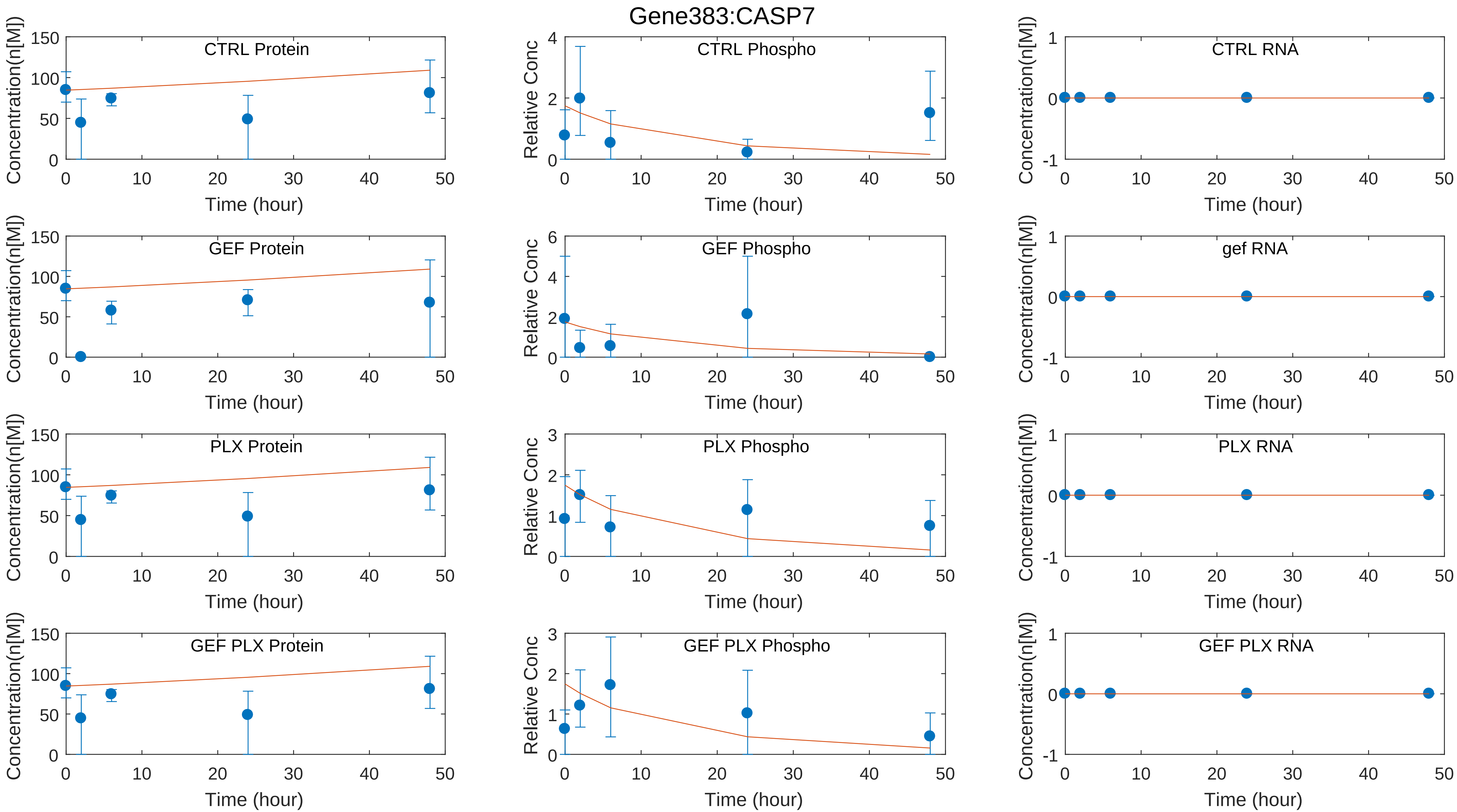

Gene383:CASP7
CTRL Protein
CTRL Phospho
CTRL RNA
GEF Protein
GEF Phospho
gef RNA
PLX Protein
PLX Phospho
PLX RNA
GEF PLX Protein
GEF PLX Phospho
GEF PLX RNA
Concentration(n[M])
Relative Conc
Time (hour)

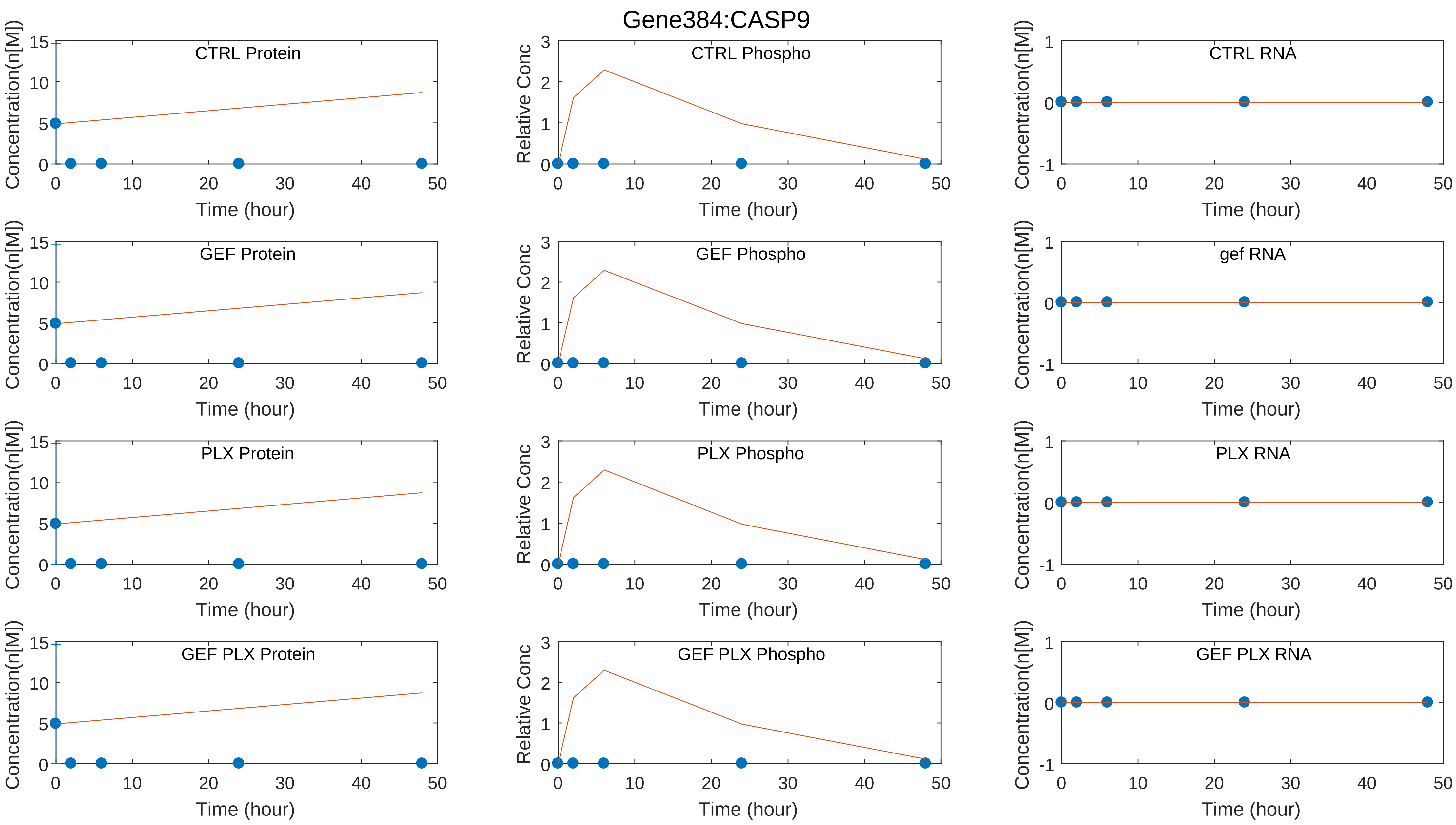

Gene384:CASP9
CTRL Protein
CTRL Phospho
CTRL RNA
GEF Protein
GEF Phospho
gef RNA
PLX Protein
PLX Phospho
PLX RNA
GEF PLX Protein
GEF PLX Phospho
GEF PLX RNA
Concentration(n[M])
Relative Conc
Time (hour)

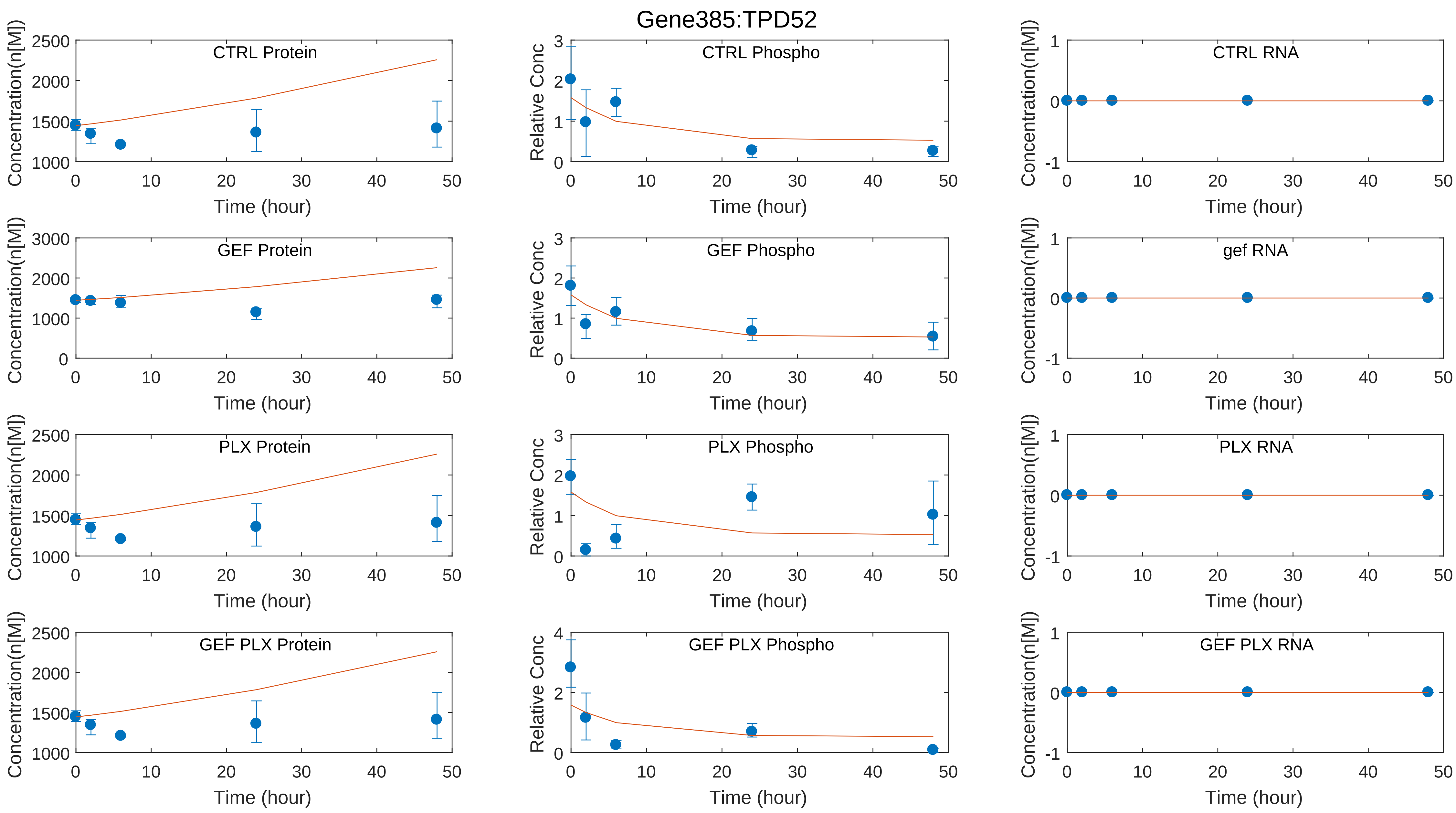
Gene385:TPD52
CTRL Protein
CTRL Phospho
CTRL RNA
GEF Protein
GEF Phospho
gef RNA
PLX Protein
PLX Phospho
PLX RNA
GEF PLX Protein
GEF PLX Phospho
GEF PLX RNA
Concentration(n[M])
Relative Conc
Time (hour)

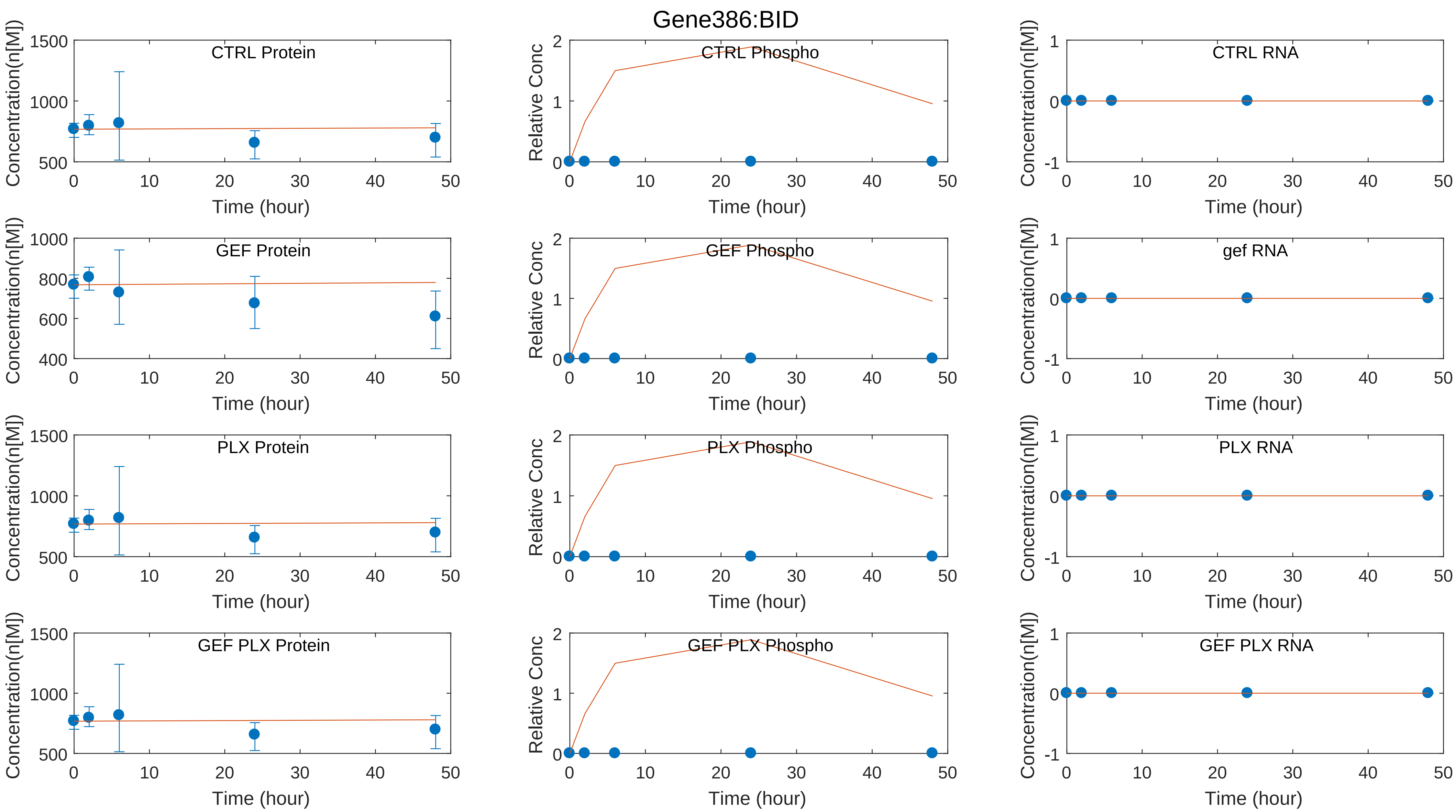

Gene386:BID
CTRL Protein
CTRL Phospho
CTRL RNA
GEF Protein
GEF Phospho
gef RNA
PLX Protein
PLX Phospho
PLX RNA
GEF PLX Protein
GEF PLX Phospho
GEF PLX RNA
Concentration(n[M])
Relative Conc
Time (hour)

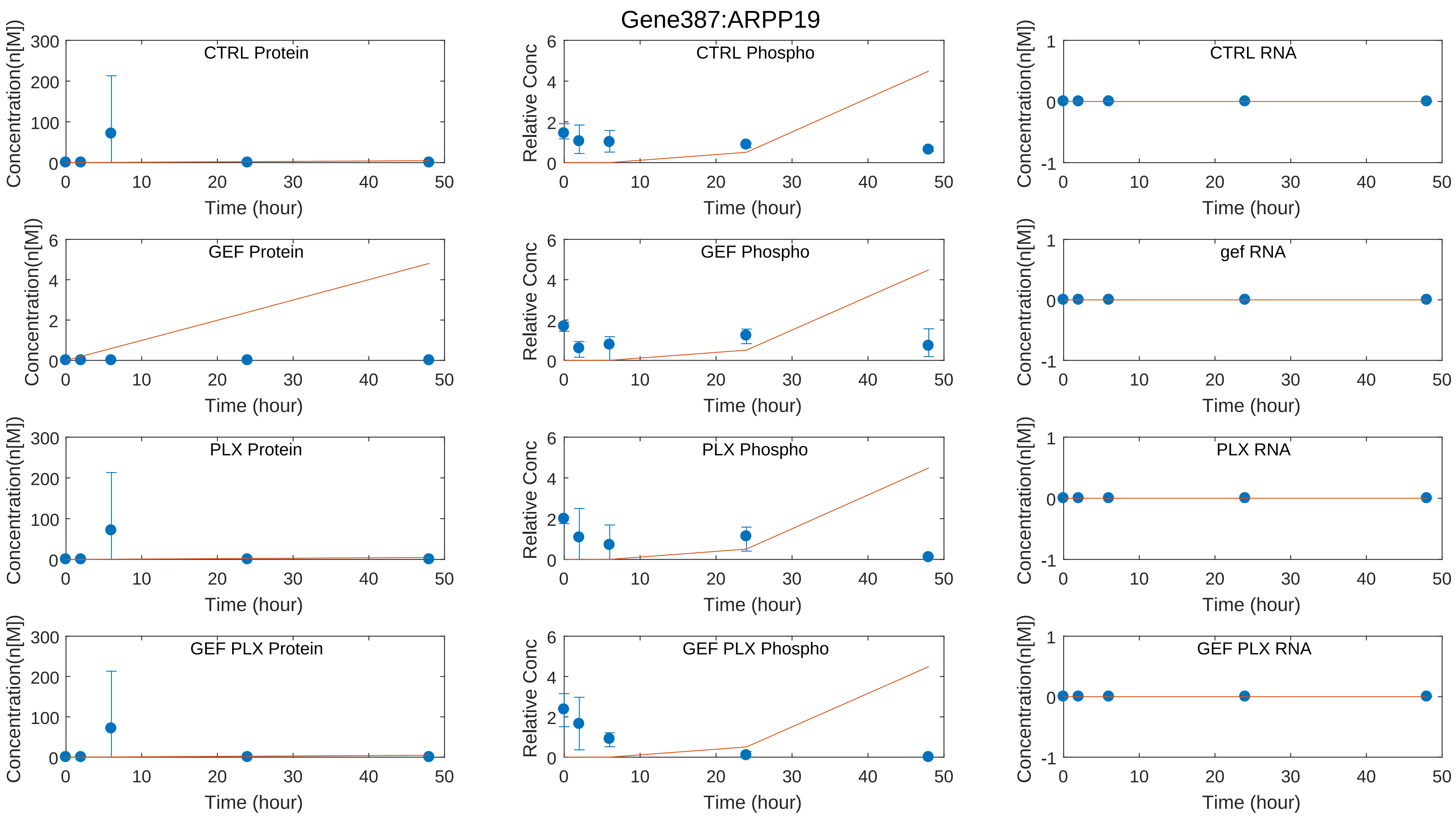
Gene387:ARPP19
CTRL Protein
CTRL Phospho
CTRL RNA
GEF Protein
GEF Phospho
gef RNA
PLX Protein
PLX Phospho
PLX RNA
GEF PLX Protein
GEF PLX Phospho
GEF PLX RNA
Concentration(n[M])
Relative Conc
Time (hour)

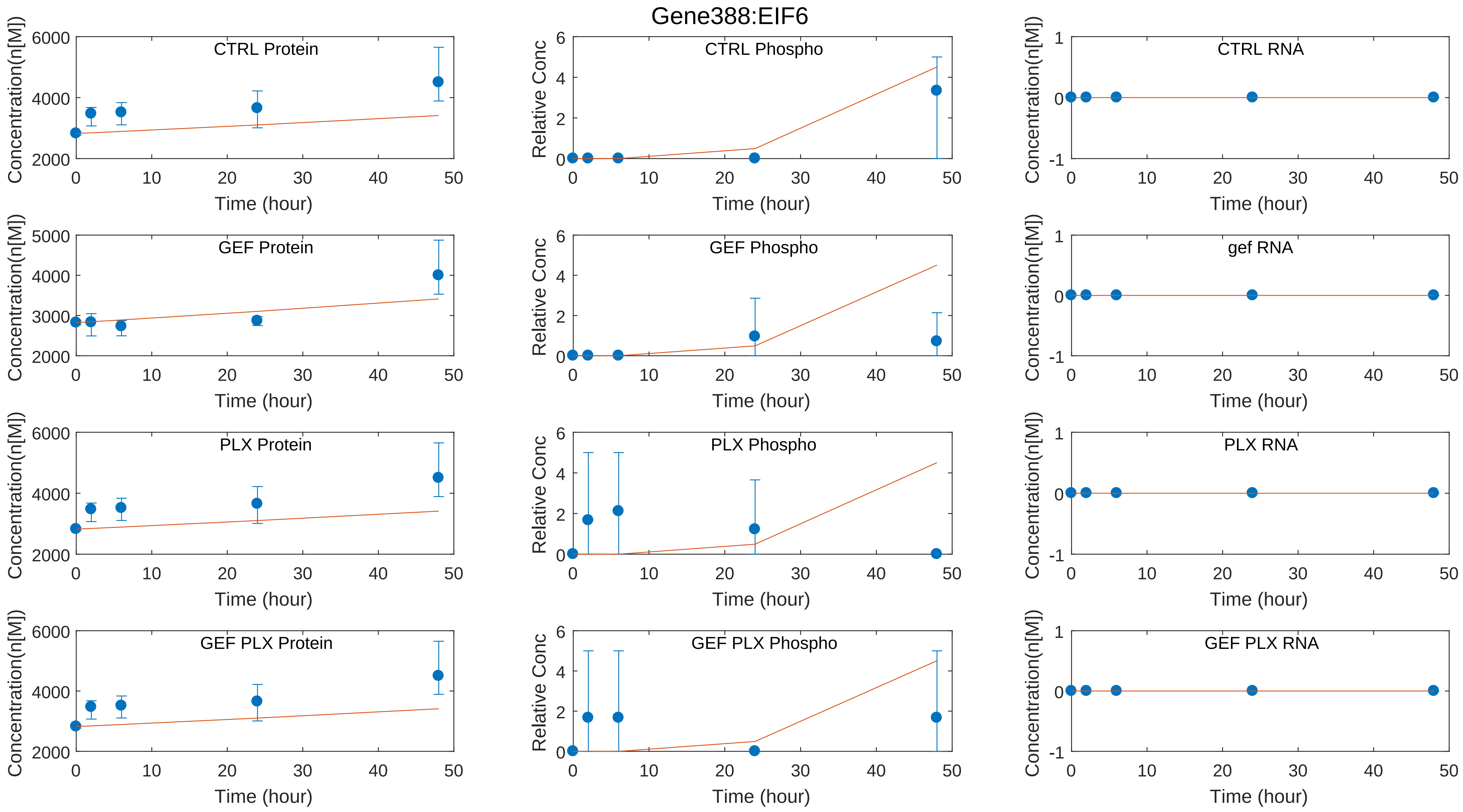

Gene388:EIF6
CTRL Protein
CTRL Phospho
CTRL RNA
GEF Protein
GEF Phospho
gef RNA
PLX Protein
PLX Phospho
PLX RNA
GEF PLX Protein
GEF PLX Phospho
GEF PLX RNA
Concentration(n[M])
Relative Conc
Time (hour)

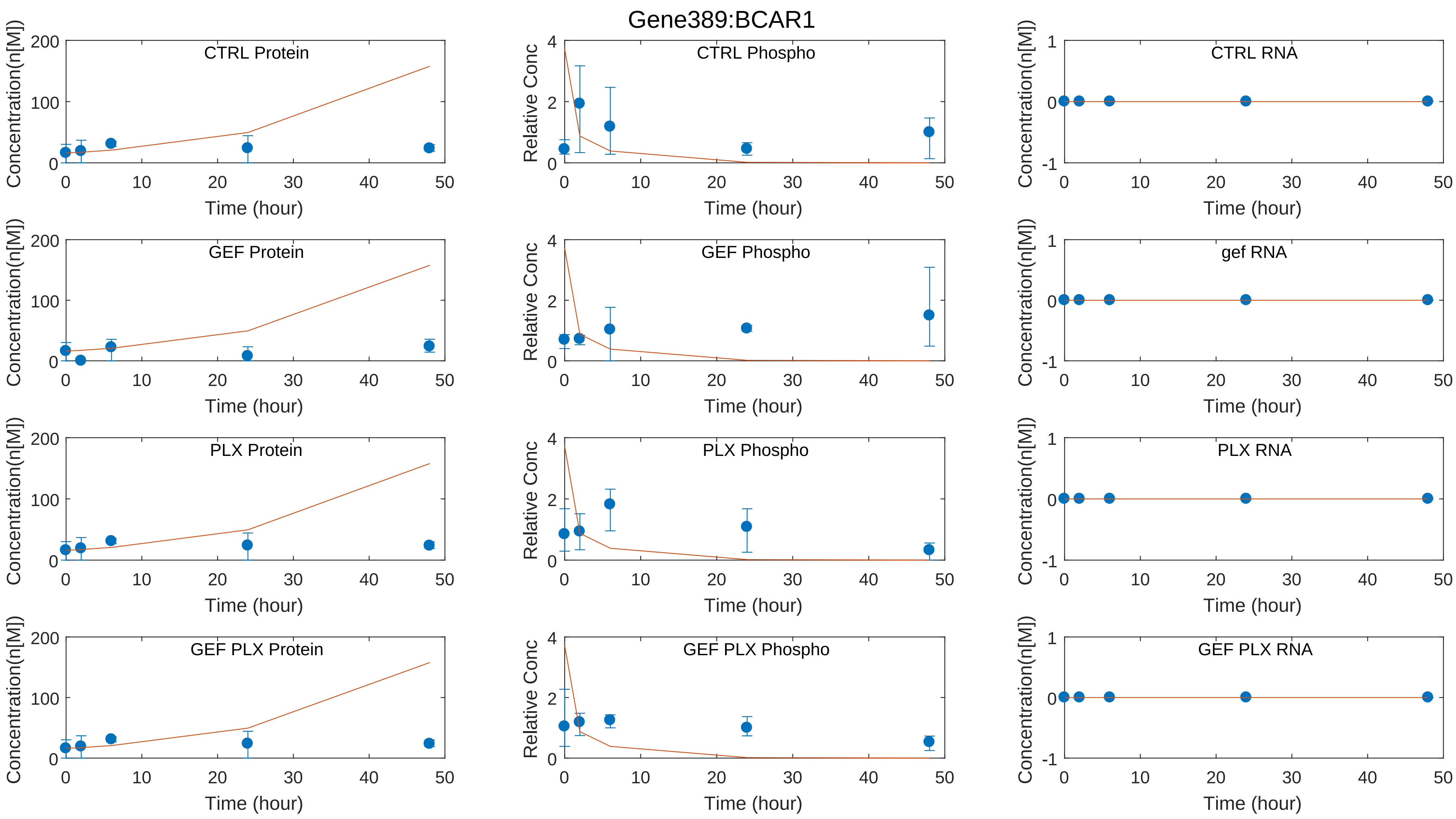

Gene389:BCAR1
CTRL Protein
CTRL Phospho
CTRL RNA
GEF Protein
GEF Phospho
gef RNA
PLX Protein
PLX Phospho
PLX RNA
GEF PLX Protein
GEF PLX Phospho
GEF PLX RNA
Concentration(n[M])
Relative Conc
Time (hour)

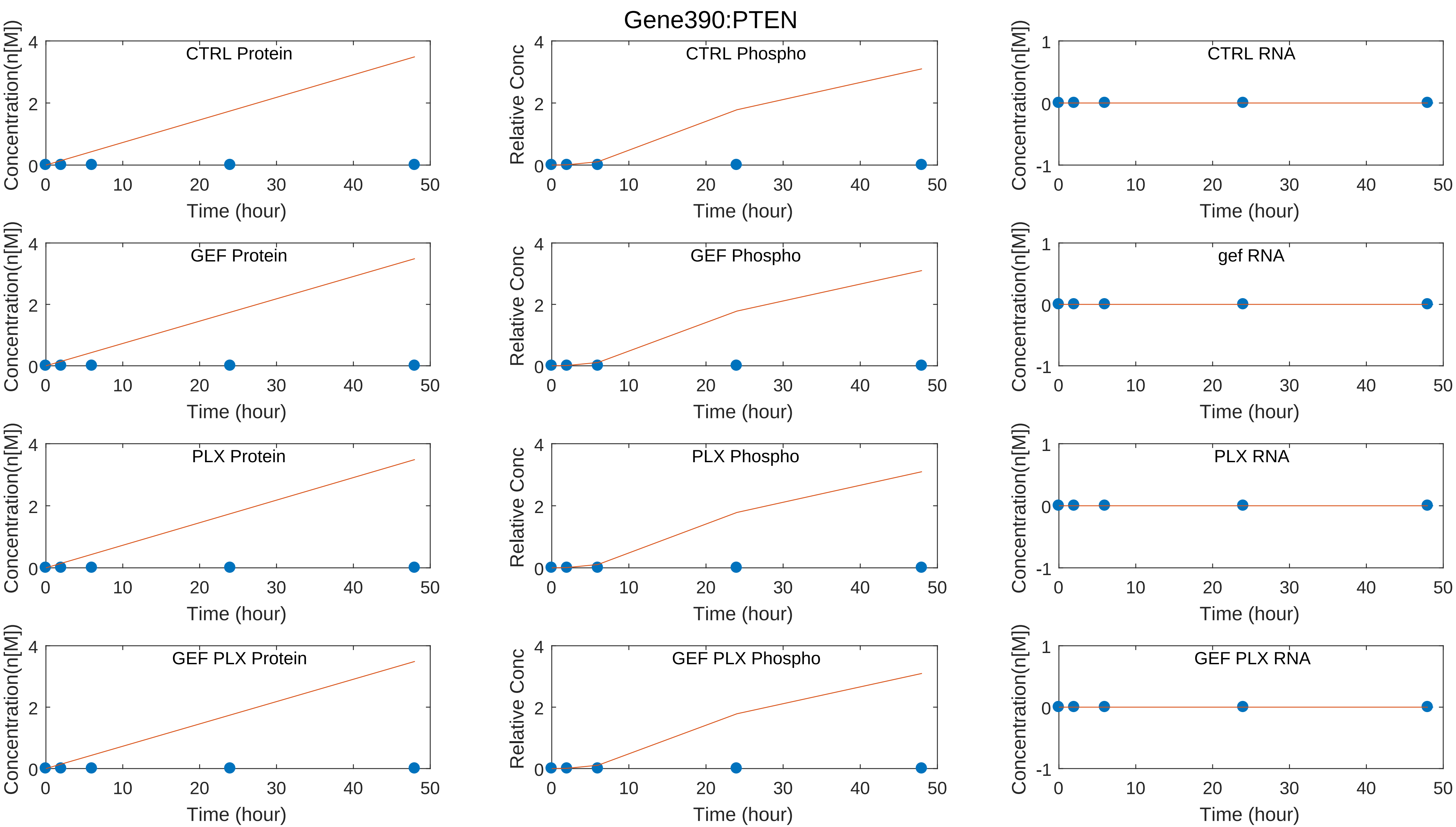
Gene390:PTEN
CTRL Protein
CTRL Phospho
CTRL RNA
GEF Protein
GEF Phospho
gef RNA
PLX Protein
PLX Phospho
PLX RNA
GEF PLX Protein
GEF PLX Phospho
GEF PLX RNA
Concentration(n[M])
Relative Conc
Time (hour)

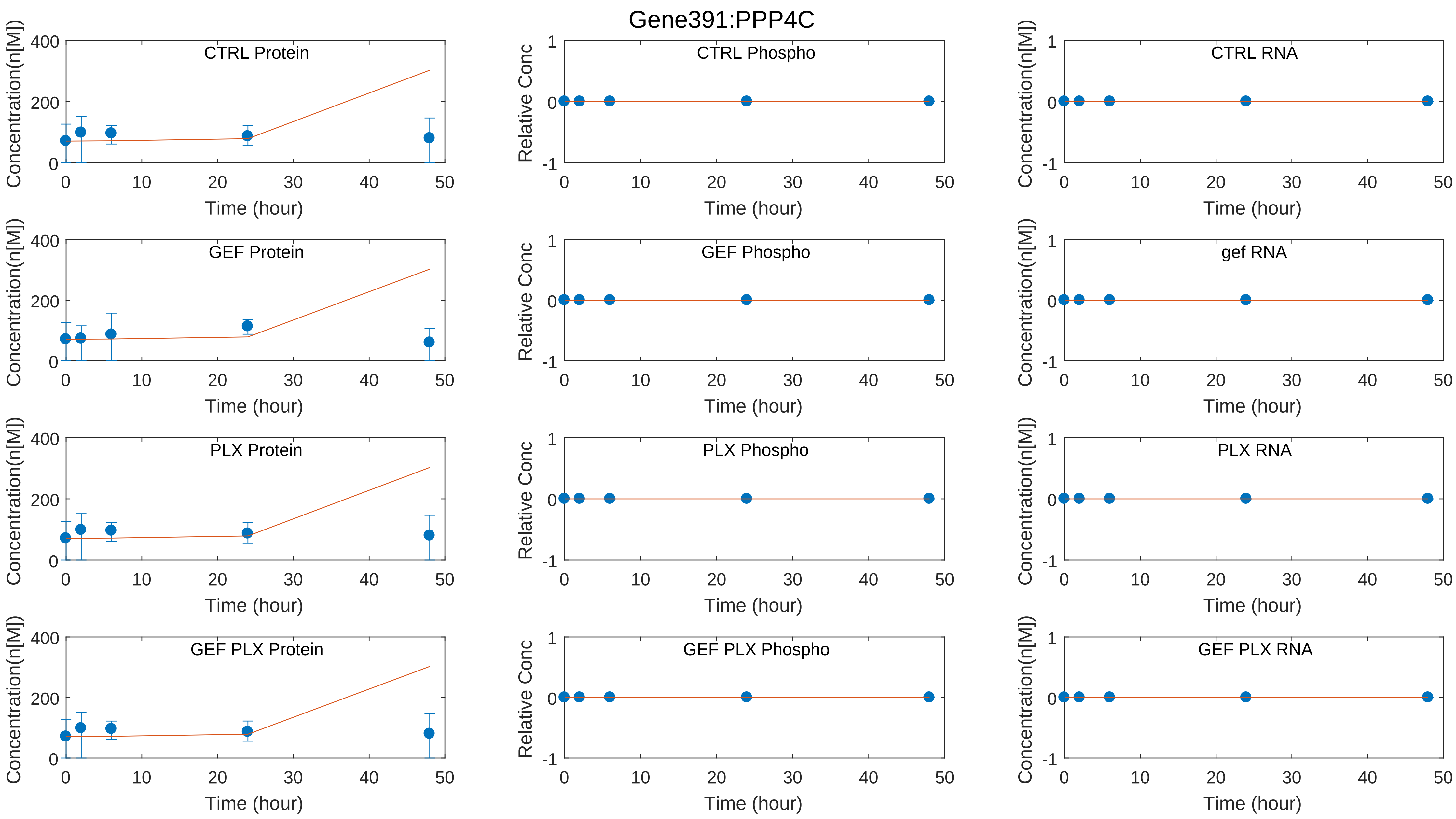
Gene391:PPP4C
CTRL Protein
CTRL Phospho
CTRL RNA
GEF Protein
GEF Phospho
gef RNA
PLX Protein
PLX Phospho
PLX RNA
GEF PLX Protein
GEF PLX Phospho
GEF PLX RNA
Concentration(n[M])
Relative Conc
Time (hour)
0
10
20
30
40
50
400
200
1
-1

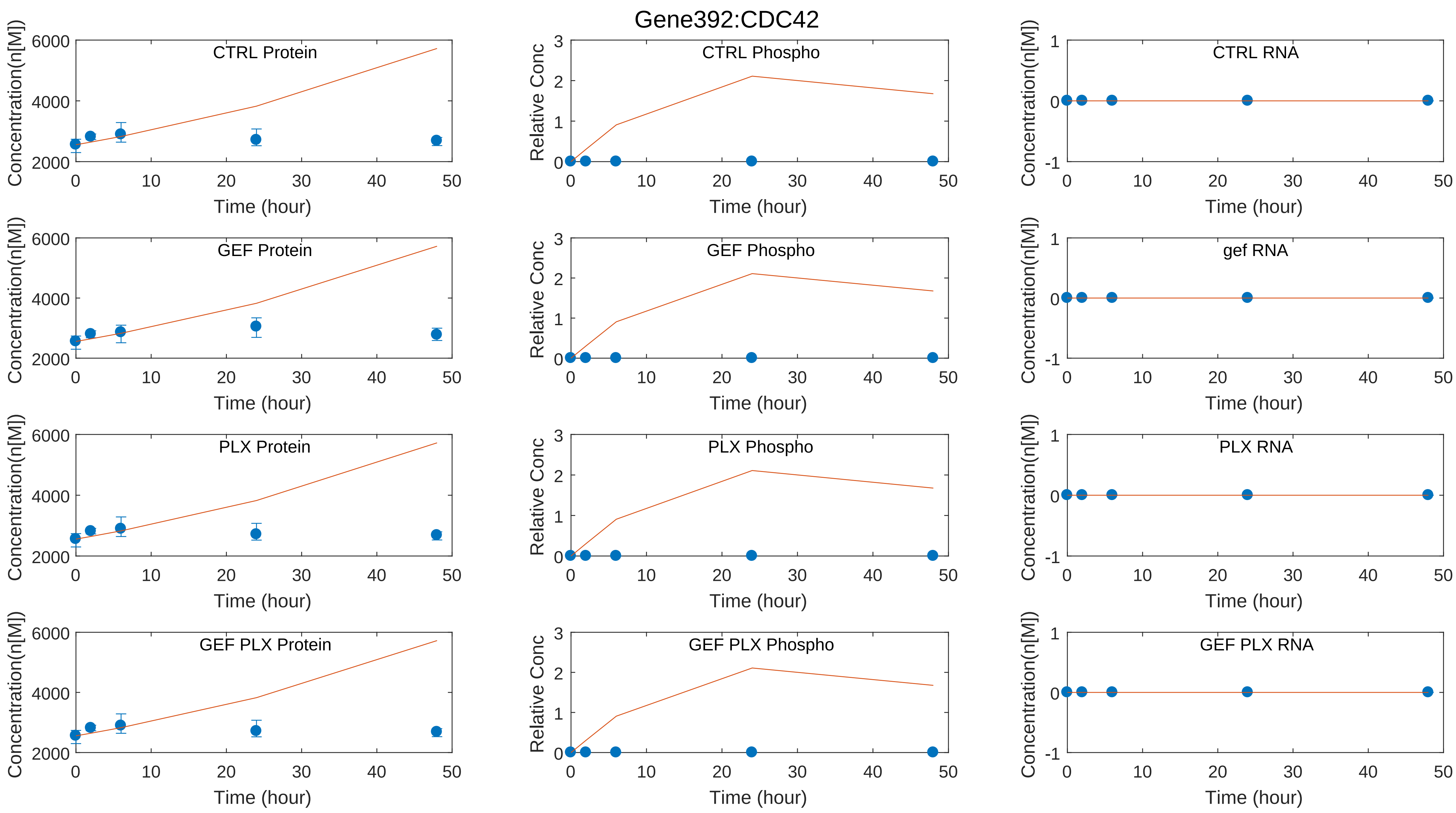
Gene392:CDC42
CTRL Protein
GEF Protein
PLX Protein
GEF PLX Protein
CTRL Phospho
GEF Phospho
PLX Phospho
GEF PLX Phospho
CTRL RNA
gef RNA
PLX RNA
GEF PLX RNA
Concentration(n[M])
Relative Conc
Time (hour)

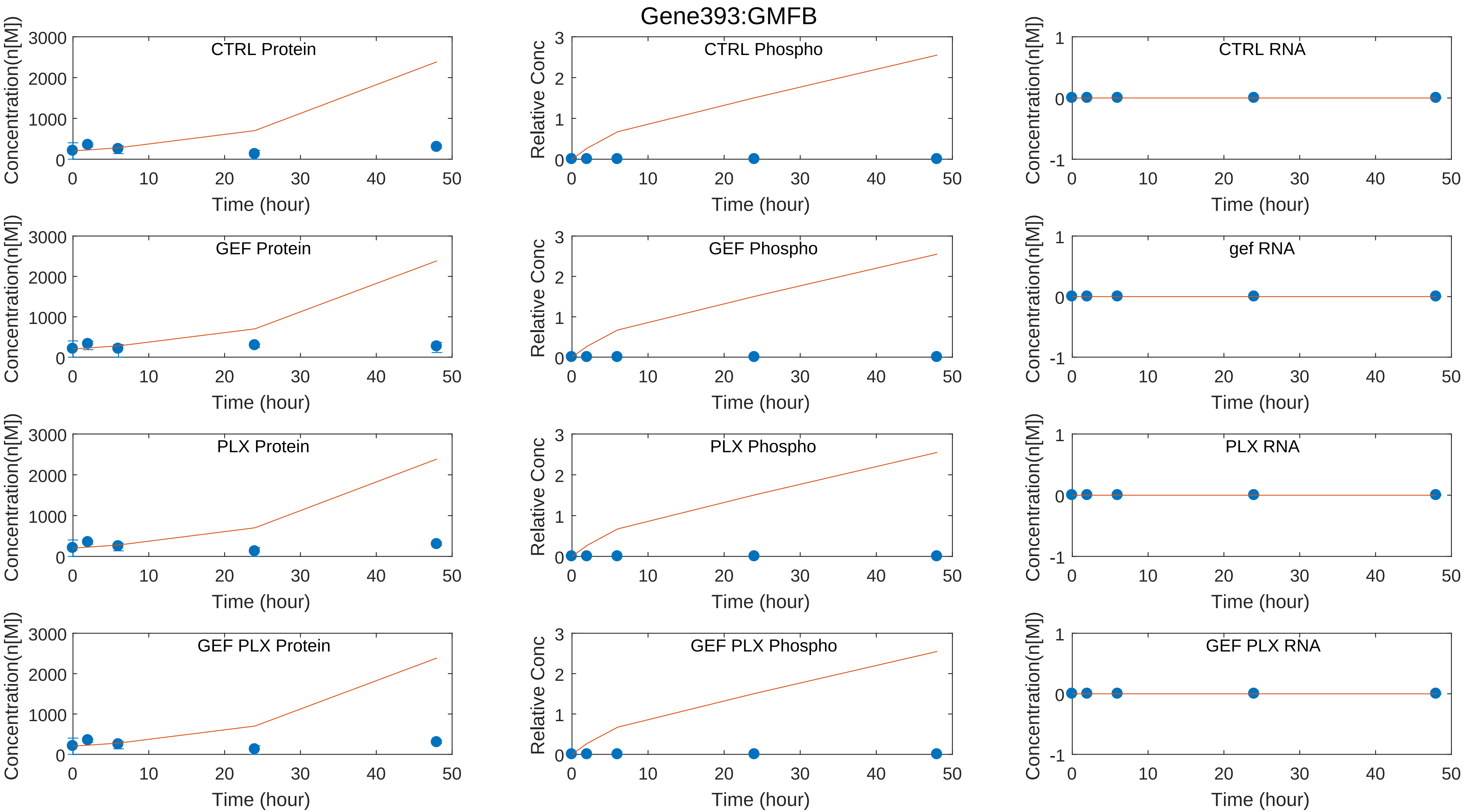

Gene393:GMFB
CTRL Protein
CTRL Phospho
CTRL RNA
GEF Protein
GEF Phospho
gef RNA
PLX Protein
PLX Phospho
PLX RNA
GEF PLX Protein
GEF PLX Phospho
GEF PLX RNA
Concentration(n[M])
Relative Conc
Time (hour)

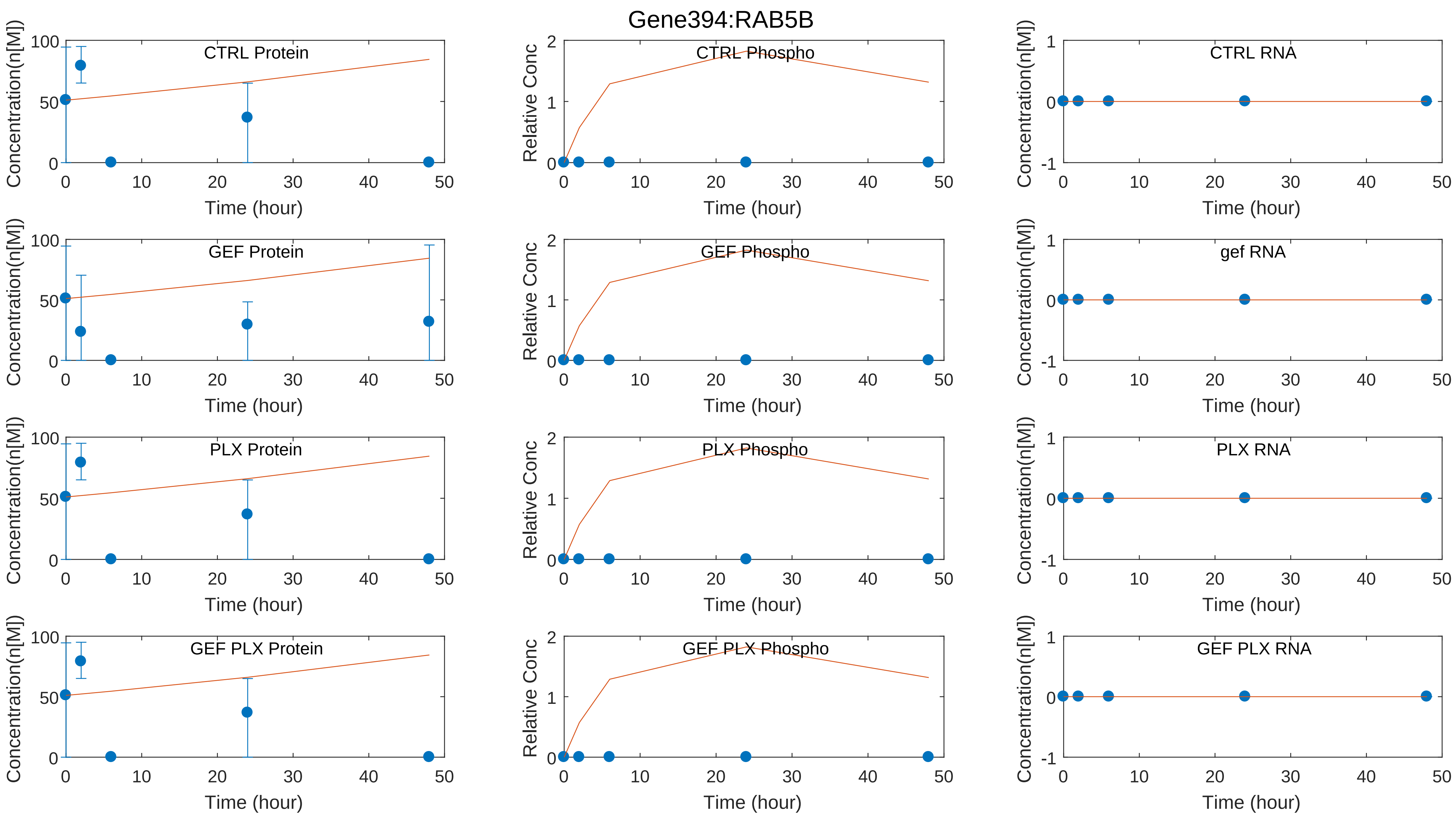

Gene394:RAB5B
CTRL Protein
CTRL Phospho
CTRL RNA
GEF Protein
GEF Phospho
gef RNA
PLX Protein
PLX Phospho
PLX RNA
GEF PLX Protein
GEF PLX Phospho
GEF PLX RNA
Concentration(n[M])
Relative Conc
Time (hour)

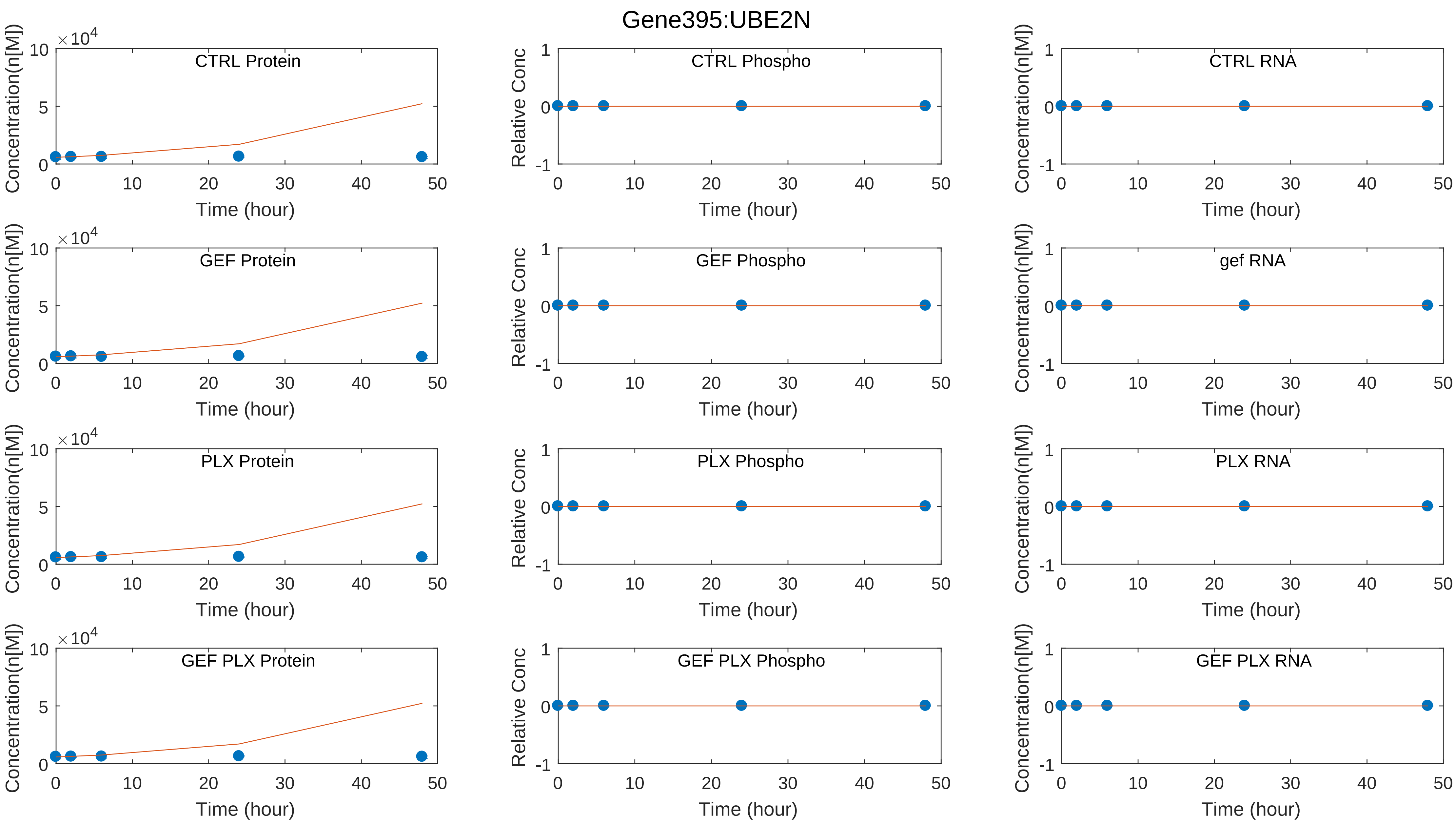

Gene395:UBE2N
CTRL Protein
CTRL Phospho
CTRL RNA
GEF Protein
GEF Phospho
gef RNA
PLX Protein
PLX Phospho
PLX RNA
GEF PLX Protein
GEF PLX Phospho
GEF PLX RNA
Concentration(n[M])
Relative Conc
Time (hour)
$\times 10^4$

Gene396:RAP1B

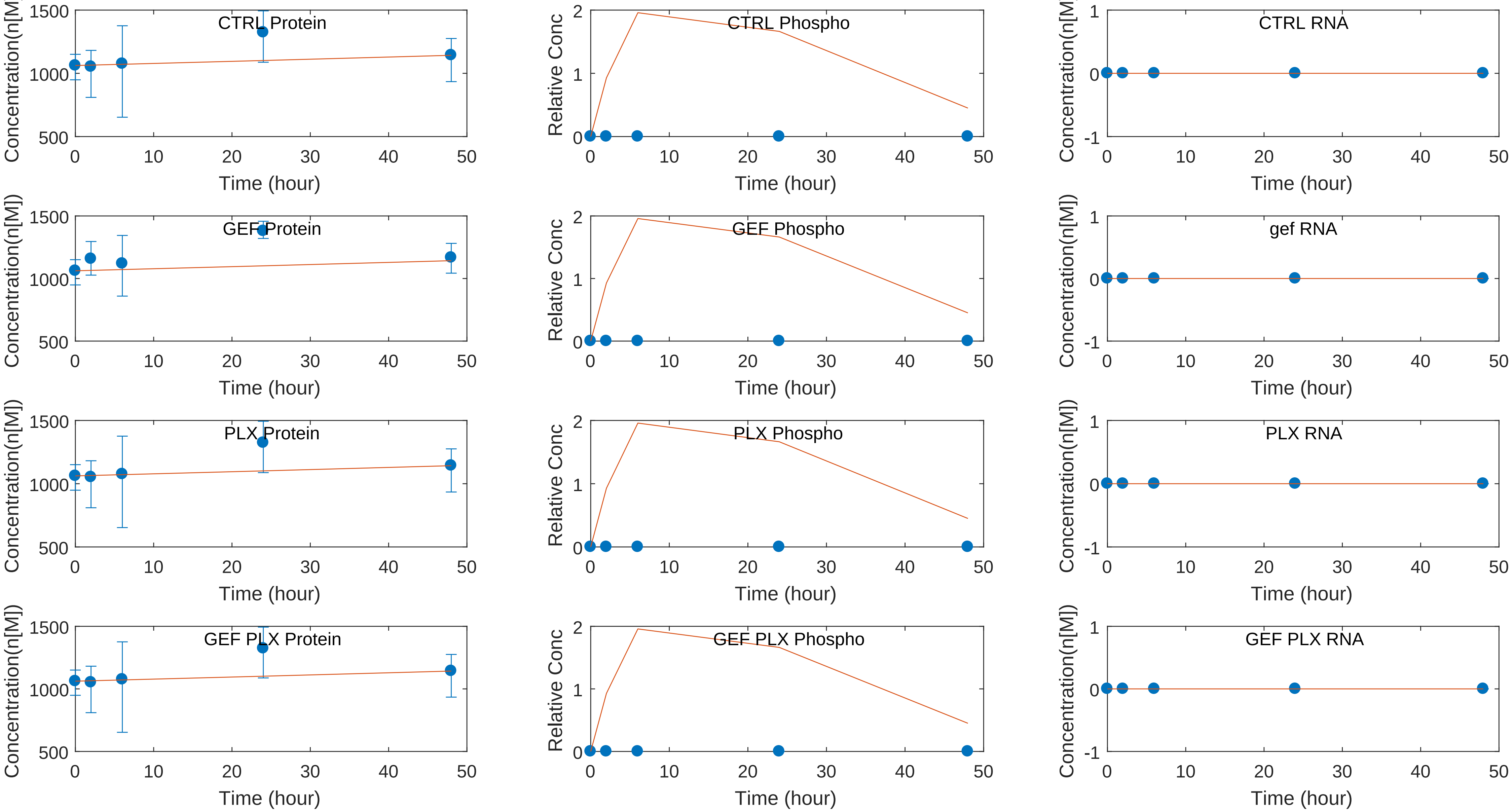

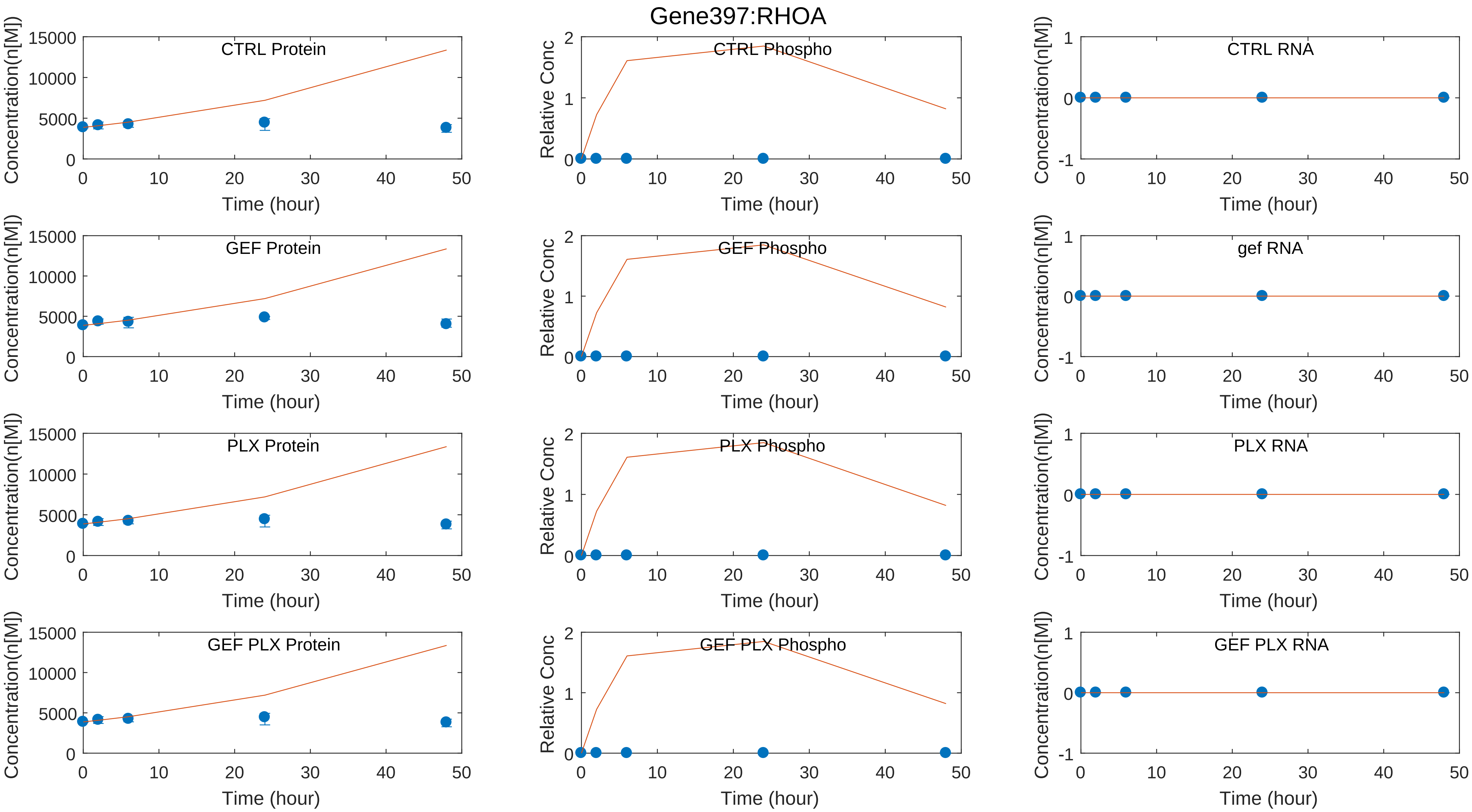

Gene397:RHOA
CTRL Protein
CTRL Phospho
CTRL RNA
GEF Protein
GEF Phospho
gef RNA
PLX Protein
PLX Phospho
PLX RNA
GEF PLX Protein
GEF PLX Phospho
GEF PLX RNA
Concentration(n[M])
Relative Conc
Time (hour)

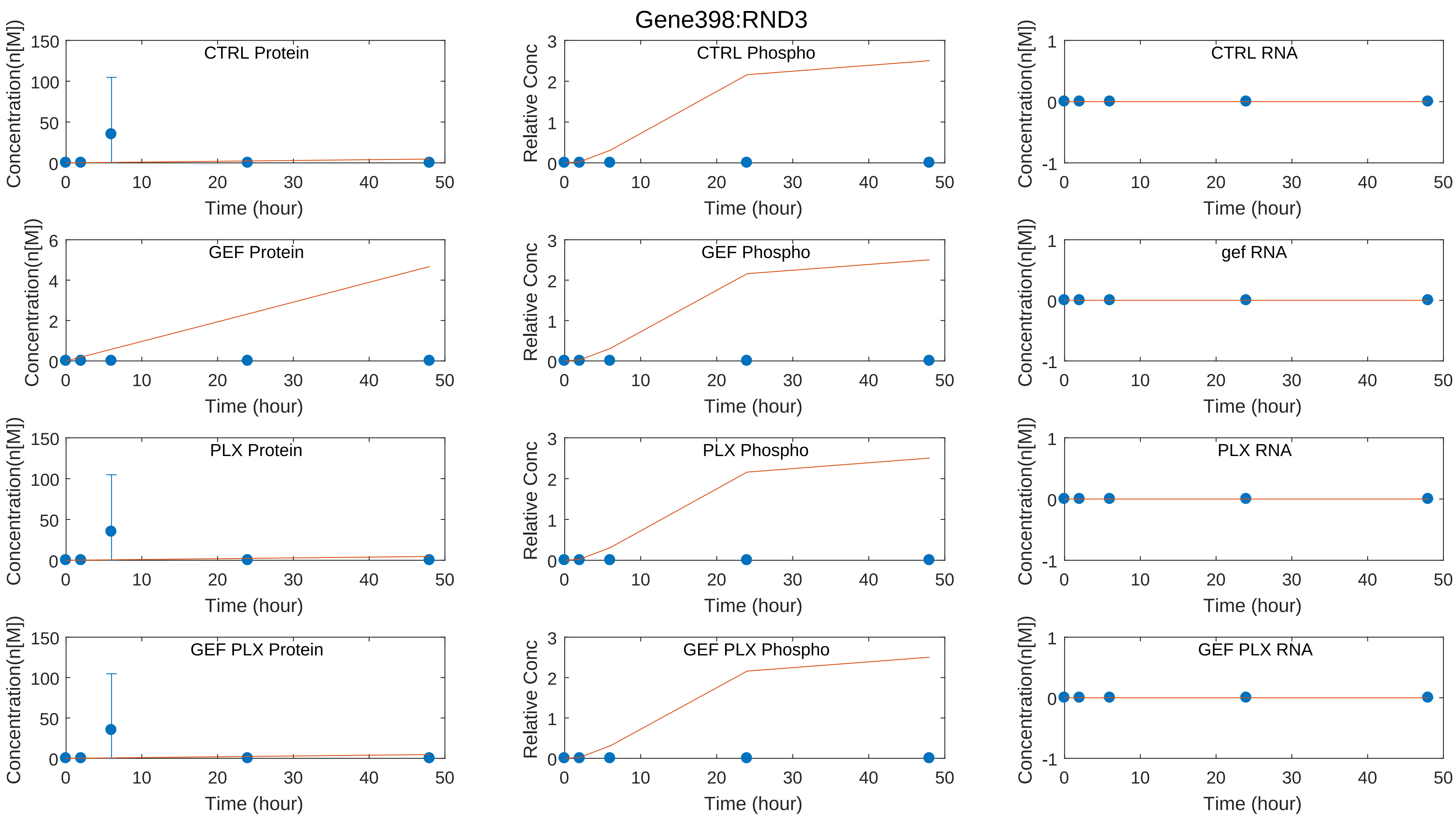

Gene398:RND3
CTRL Protein
CTRL Phospho
CTRL RNA
GEF Protein
GEF Phospho
gef RNA
PLX Protein
PLX Phospho
PLX RNA
GEF PLX Protein
GEF PLX Phospho
GEF PLX RNA
Concentration(n[M])
Relative Conc
Time (hour)

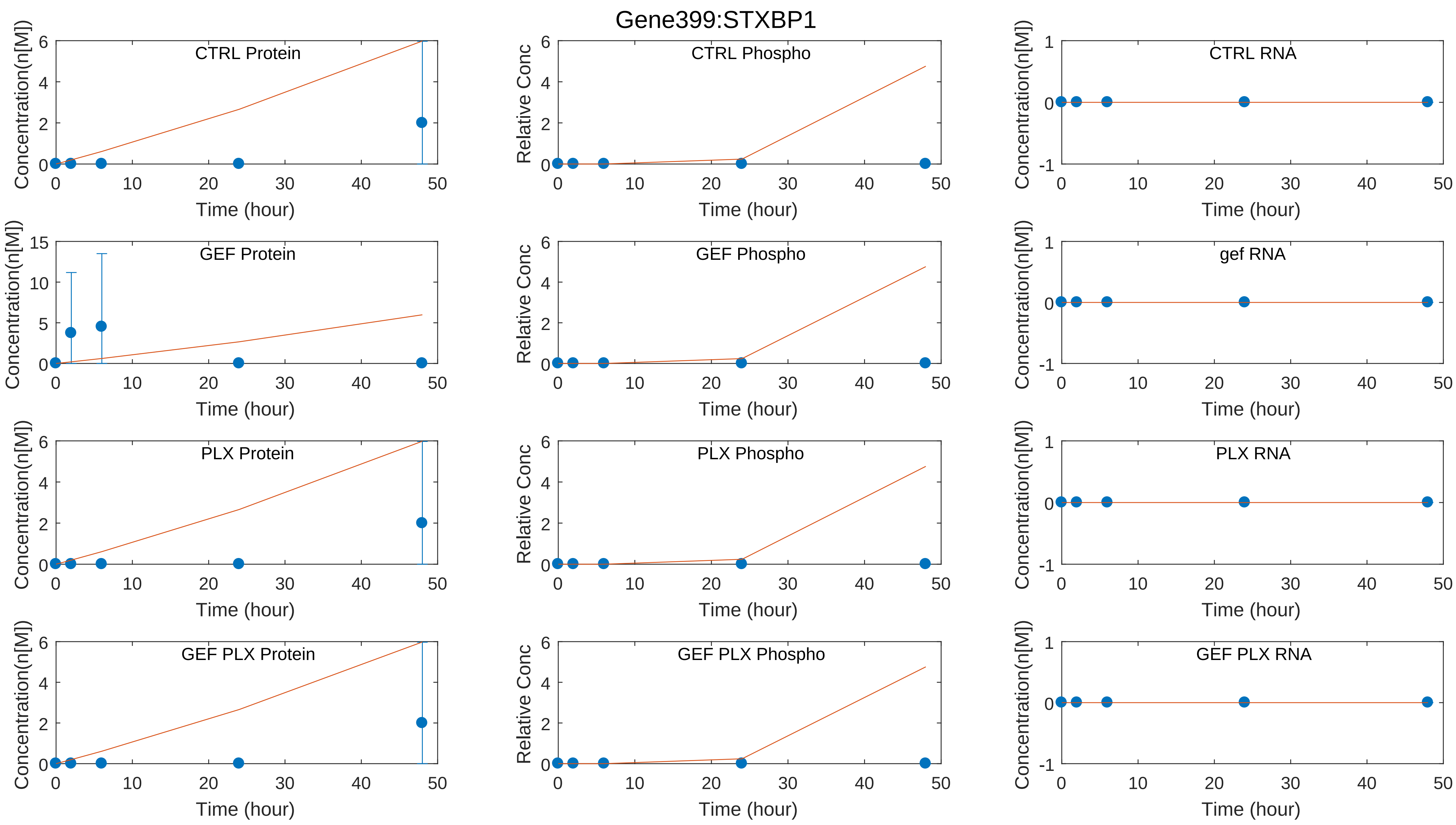

Gene399:STXBP1
CTRL Protein
CTRL Phospho
CTRL RNA
GEF Protein
GEF Phospho
gef RNA
PLX Protein
PLX Phospho
PLX RNA
GEF PLX Protein
GEF PLX Phospho
GEF PLX RNA
Concentration(n[M])
Relative Conc
Time (hour)

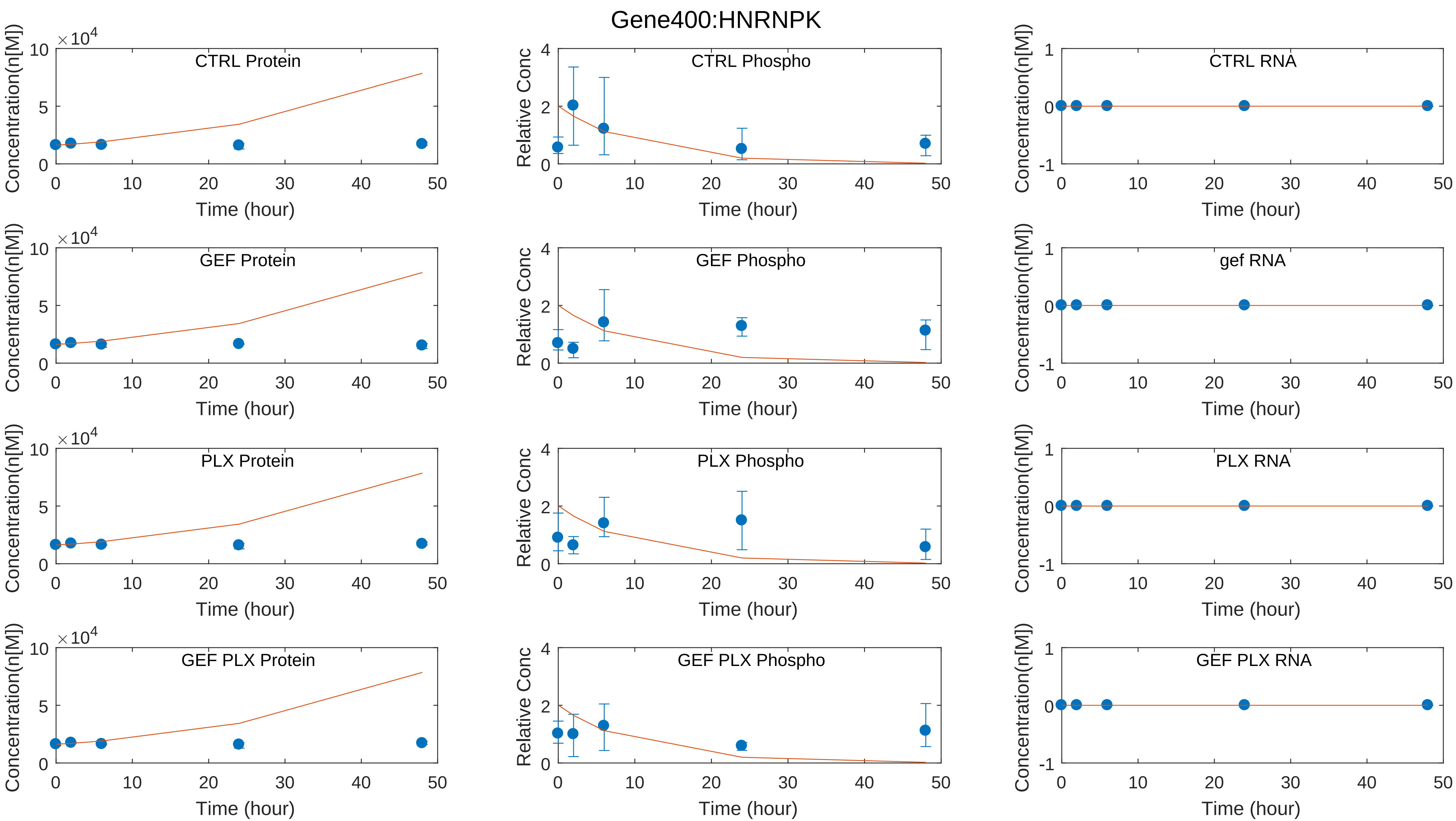

Gene400:HNRNPK
CTRL Protein
CTRL Phospho
CTRL RNA
GEF Protein
GEF Phospho
gef RNA
PLX Protein
PLX Phospho
PLX RNA
GEF PLX Protein
GEF PLX Phospho
GEF PLX RNA
Concentration(n[M])
Relative Conc
Time (hour)
×10^4

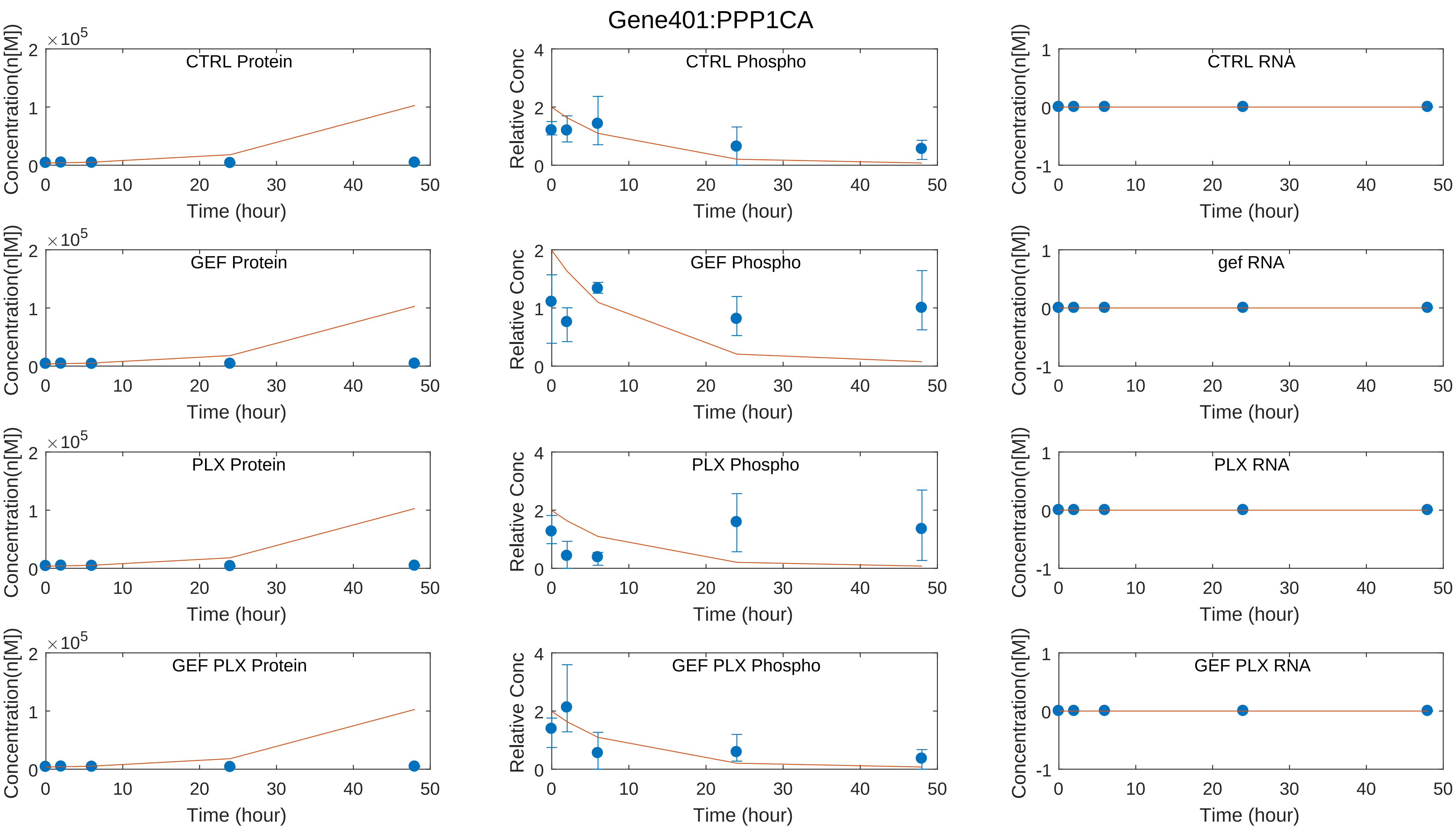

Gene401:PPP1CA
CTRL Protein
CTRL Phospho
CTRL RNA
GEF Protein
GEF Phospho
gef RNA
PLX Protein
PLX Phospho
PLX RNA
GEF PLX Protein
GEF PLX Phospho
GEF PLX RNA
Concentration(n[M])
Relative Conc
Time (hour)
×10^5

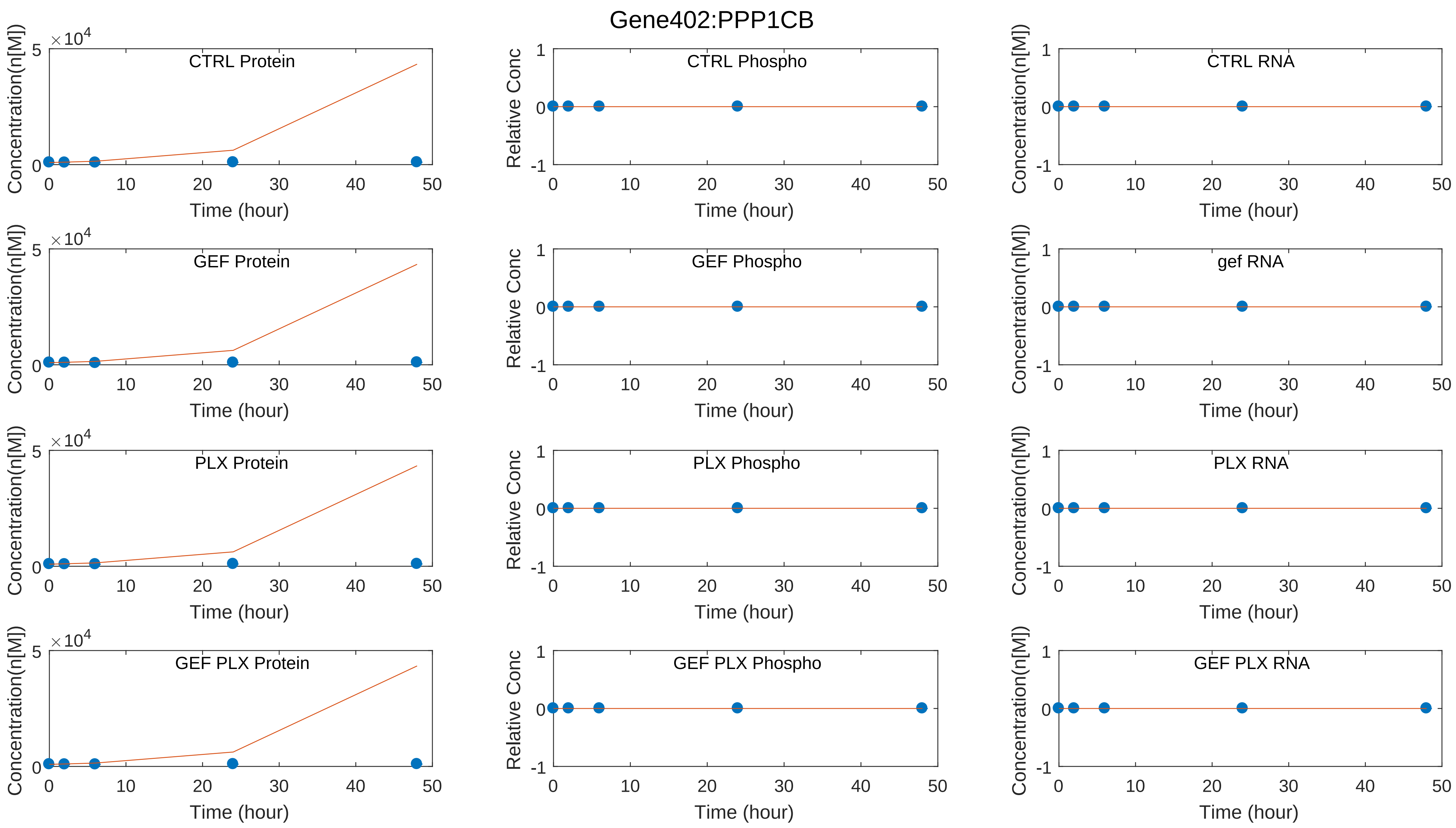

Gene402:PPP1CB
CTRL Protein
GEF Protein
PLX Protein
GEF PLX Protein
CTRL Phospho
GEF Phospho
PLX Phospho
GEF PLX Phospho
CTRL RNA
gef RNA
PLX RNA
GEF PLX RNA
Concentration(n[M])
Relative Conc
Time (hour)
×10^4

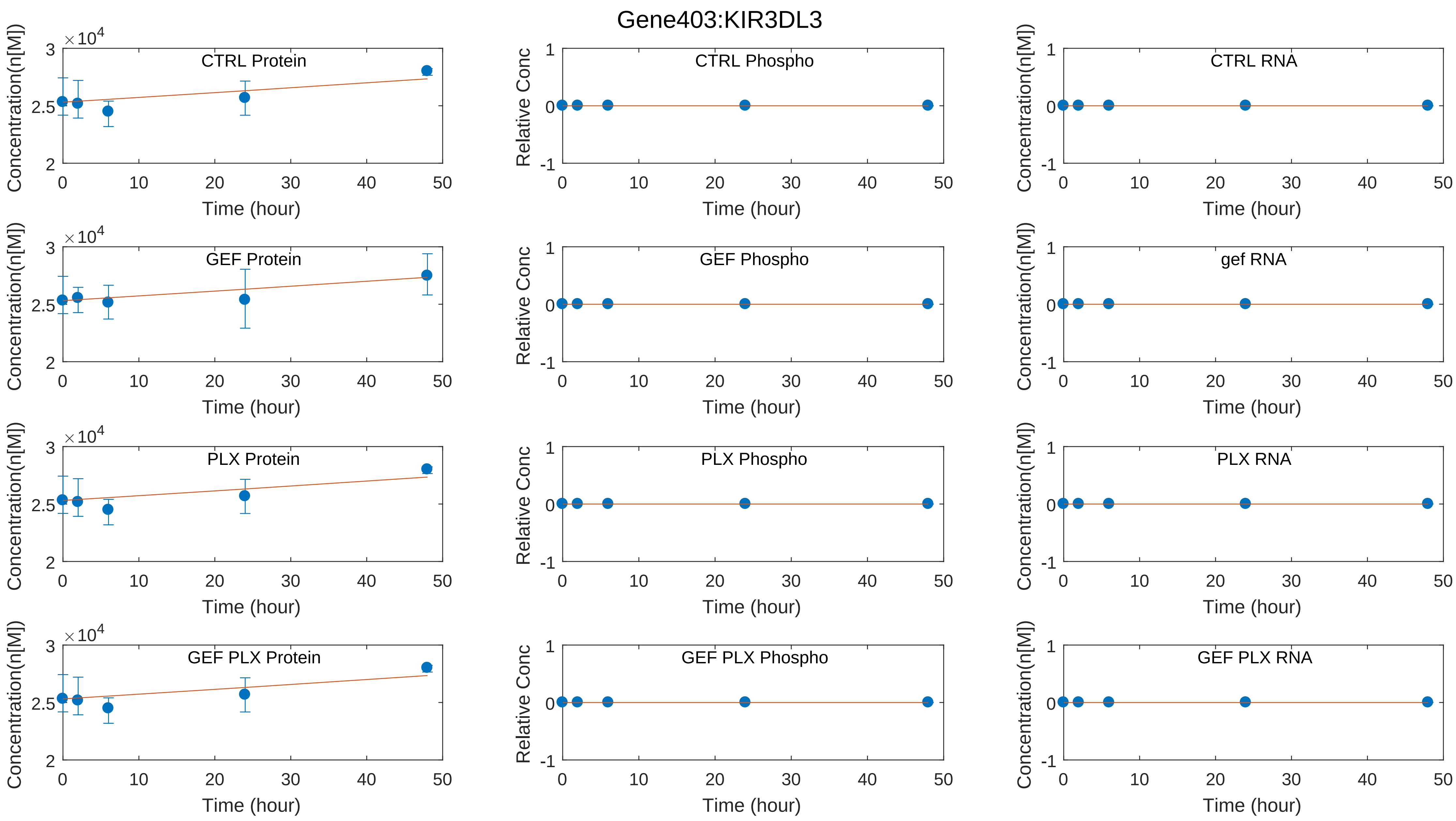
Gene403:KIR3DL3
CTRL Protein
GEF Protein
PLX Protein
GEF PLX Protein
CTRL Phospho
GEF Phospho
PLX Phospho
GEF PLX Phospho
CTRL RNA
gef RNA
PLX RNA
GEF PLX RNA
Concentration(n[M])
Relative Conc
Time (hour)
×10^4

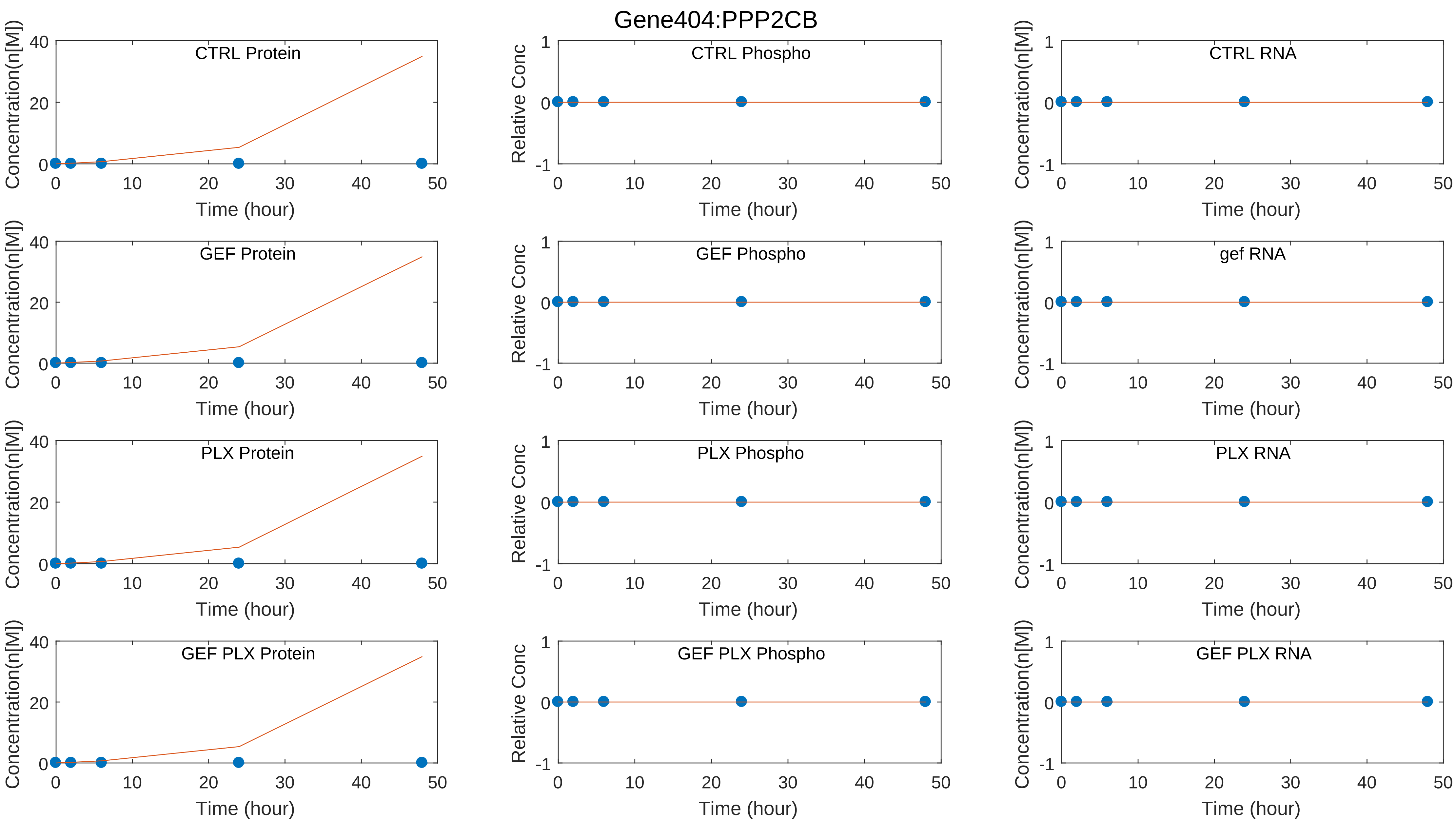
Gene404:PPP2CB
CTRL Protein
CTRL Phospho
CTRL RNA
GEF Protein
GEF Phospho
gef RNA
PLX Protein
PLX Phospho
PLX RNA
GEF PLX Protein
GEF PLX Phospho
GEF PLX RNA
Concentration(n[M])
Relative Conc
Time (hour)

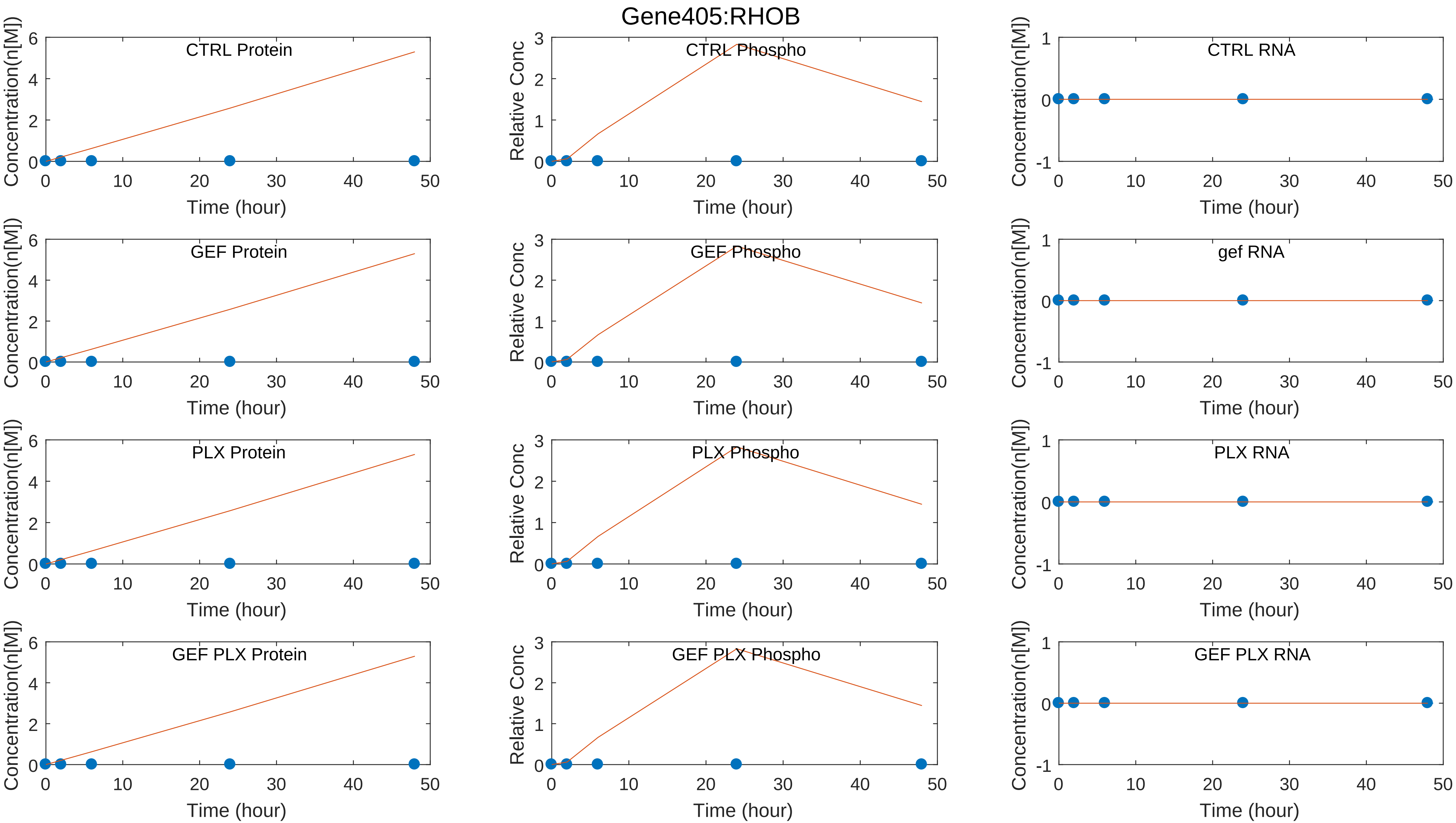

Gene405:RHOB
CTRL Protein
CTRL Phospho
CTRL RNA
GEF Protein
GEF Phospho
gef RNA
PLX Protein
PLX Phospho
PLX RNA
GEF PLX Protein
GEF PLX Phospho
GEF PLX RNA
Concentration(n[M])
Relative Conc
Time (hour)

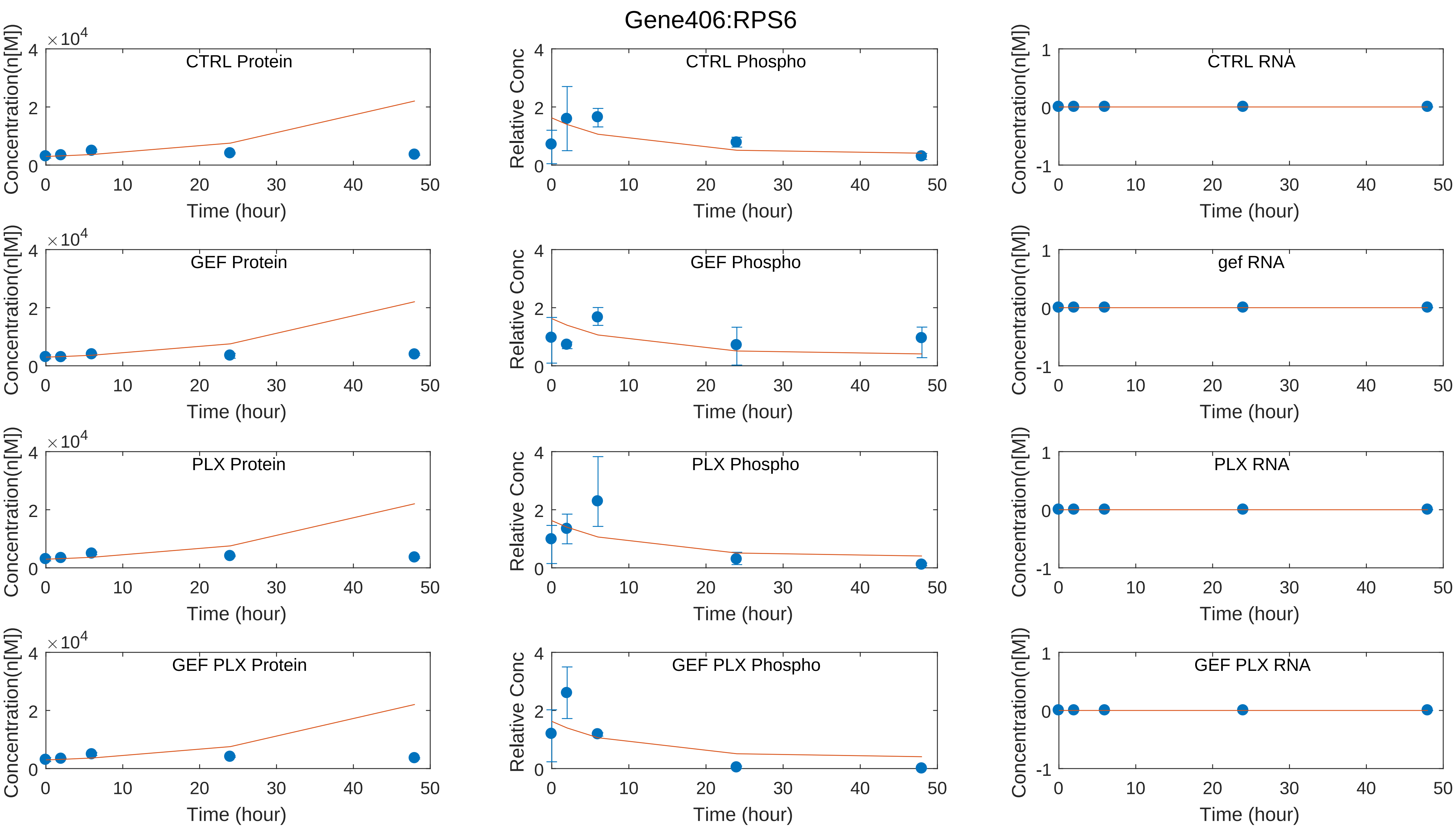
Gene406:RPS6
CTRL Protein
CTRL Phospho
CTRL RNA
GEF Protein
GEF Phospho
gef RNA
PLX Protein
PLX Phospho
PLX RNA
GEF PLX Protein
GEF PLX Phospho
GEF PLX RNA
Concentration(n[M])
Relative Conc
Time (hour)
×10^4

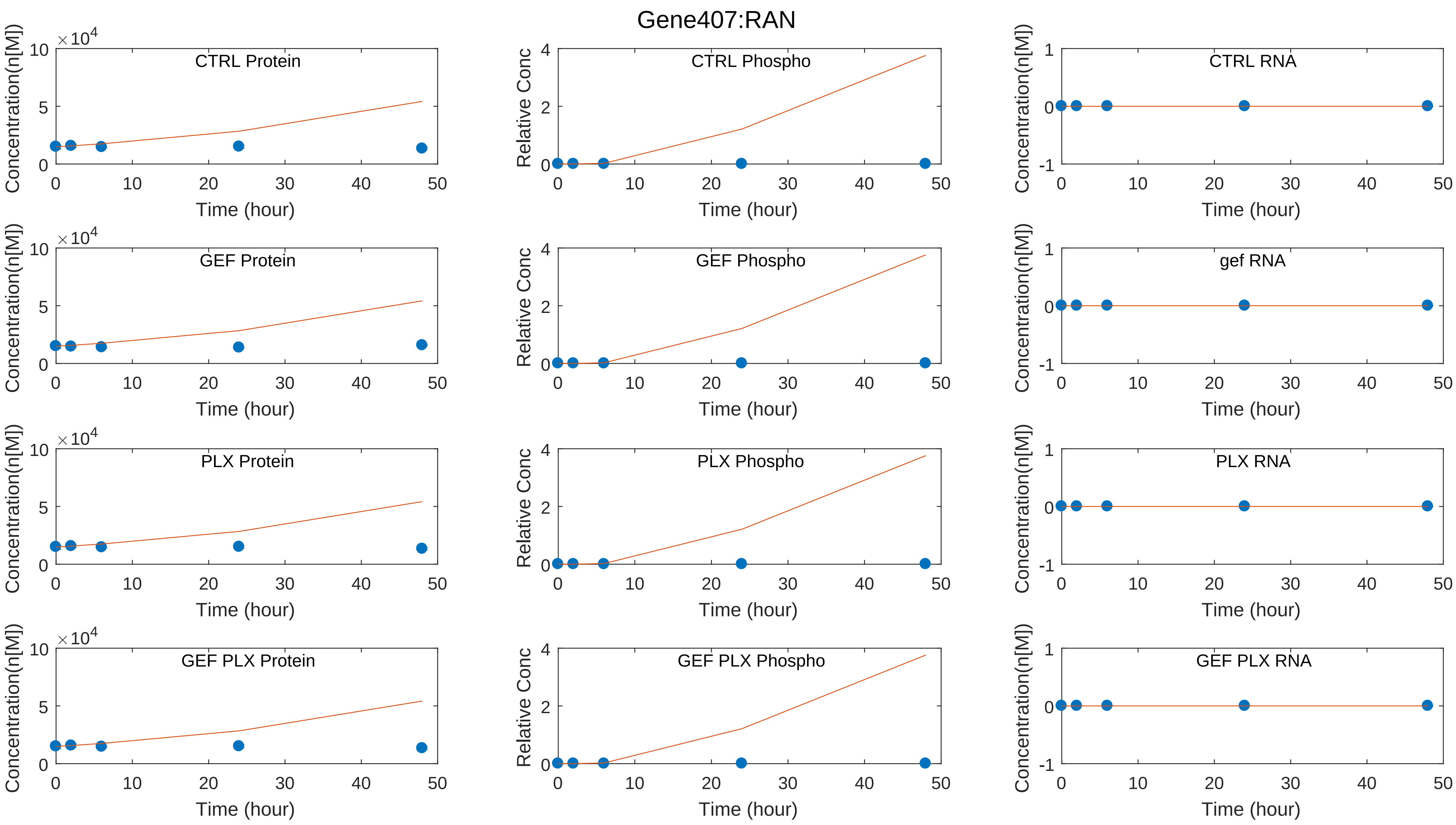
Gene407:RAN
CTRL Protein
GEF Protein
PLX Protein
GEF PLX Protein
CTRL Phospho
GEF Phospho
PLX Phospho
GEF PLX Phospho
CTRL RNA
gef RNA
PLX RNA
GEF PLX RNA
Concentration(n[M])
Relative Conc
Time (hour)
×10^4

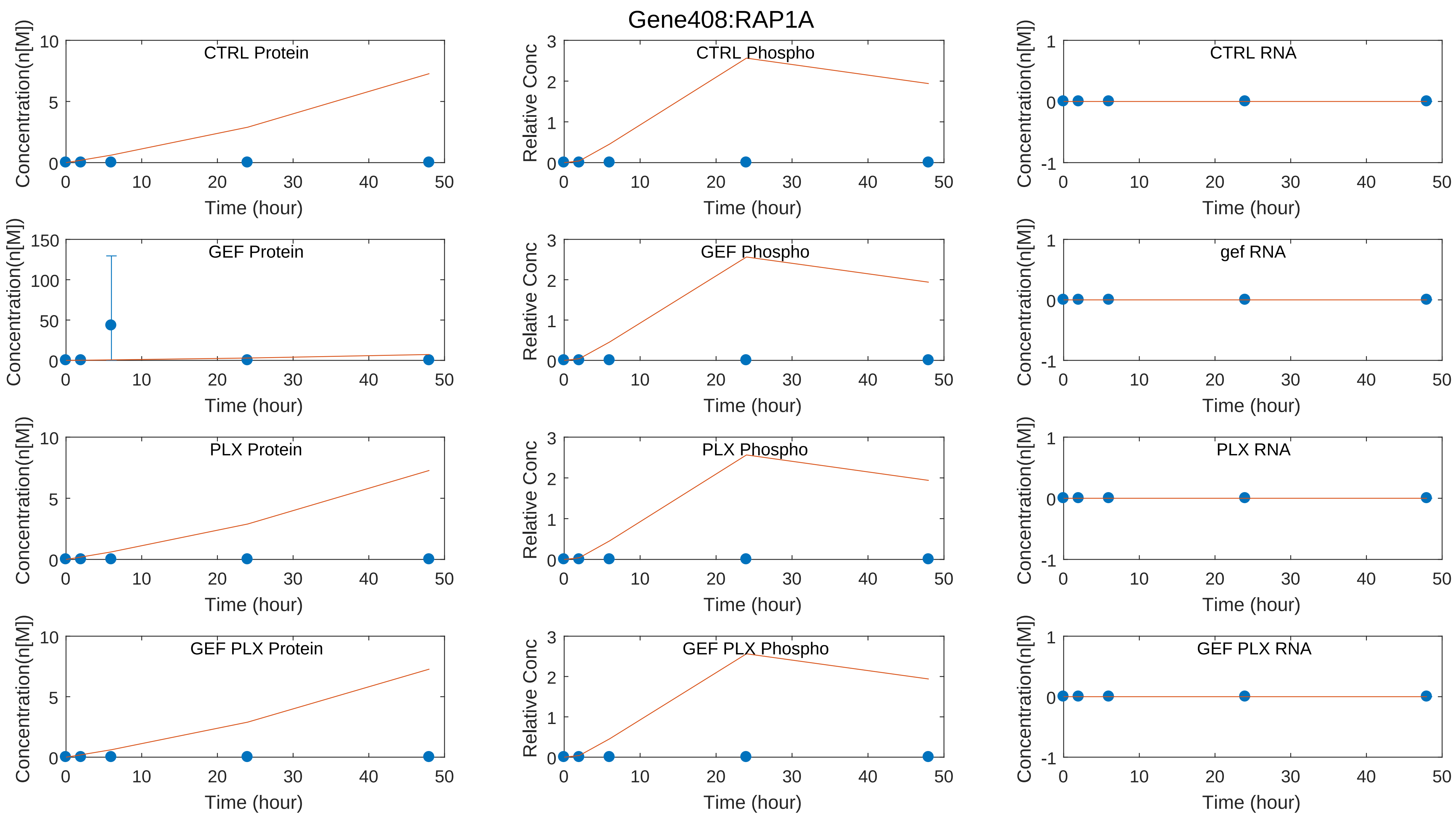

Gene408:RAP1A
CTRL Protein
CTRL Phospho
CTRL RNA
GEF Protein
GEF Phospho
gef RNA
PLX Protein
PLX Phospho
PLX RNA
GEF PLX Protein
GEF PLX Phospho
GEF PLX RNA
Concentration(n[M])
Relative Conc
Time (hour)

Gene409:UBE2D2

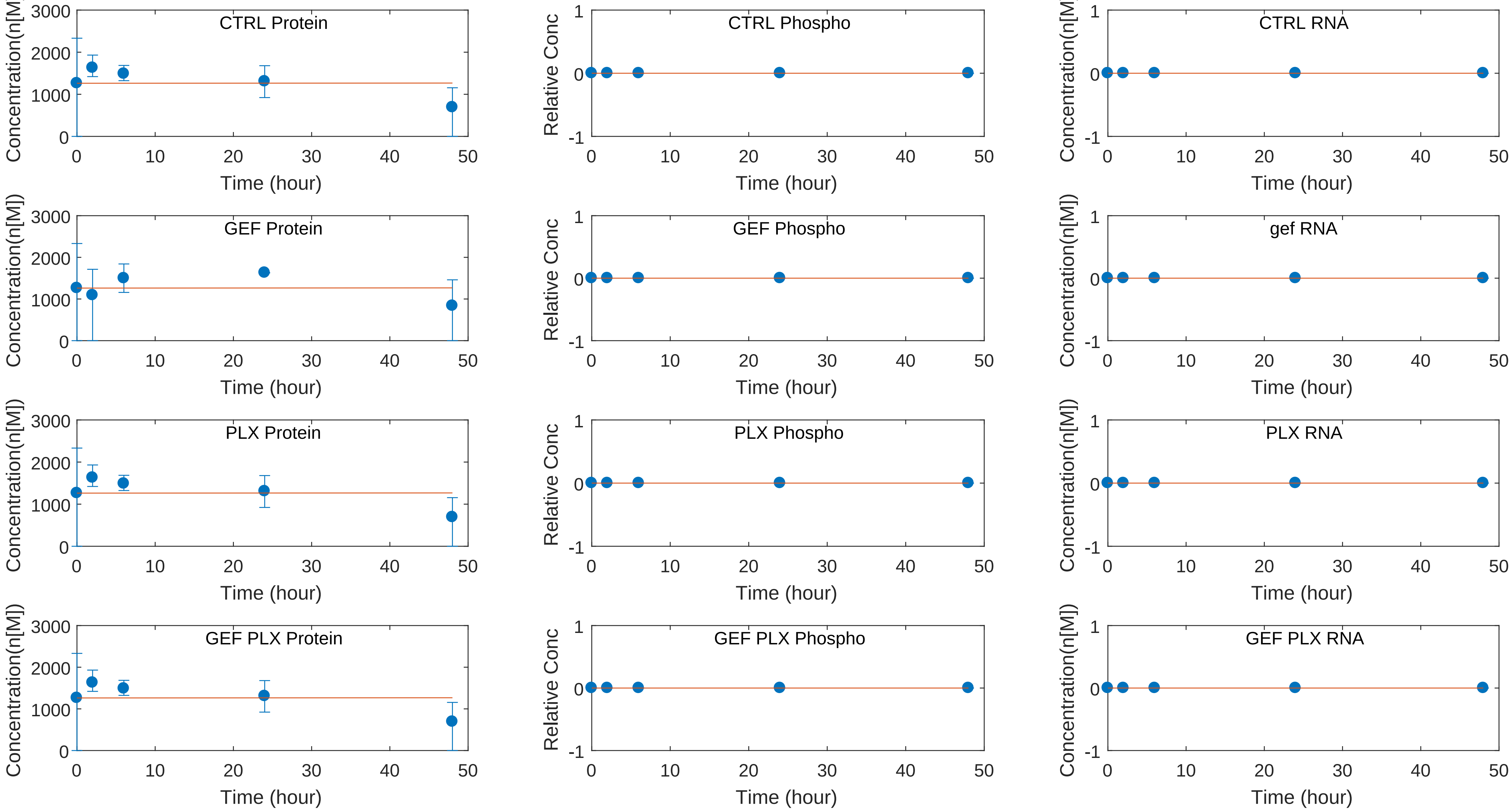

Gene410:RBX1

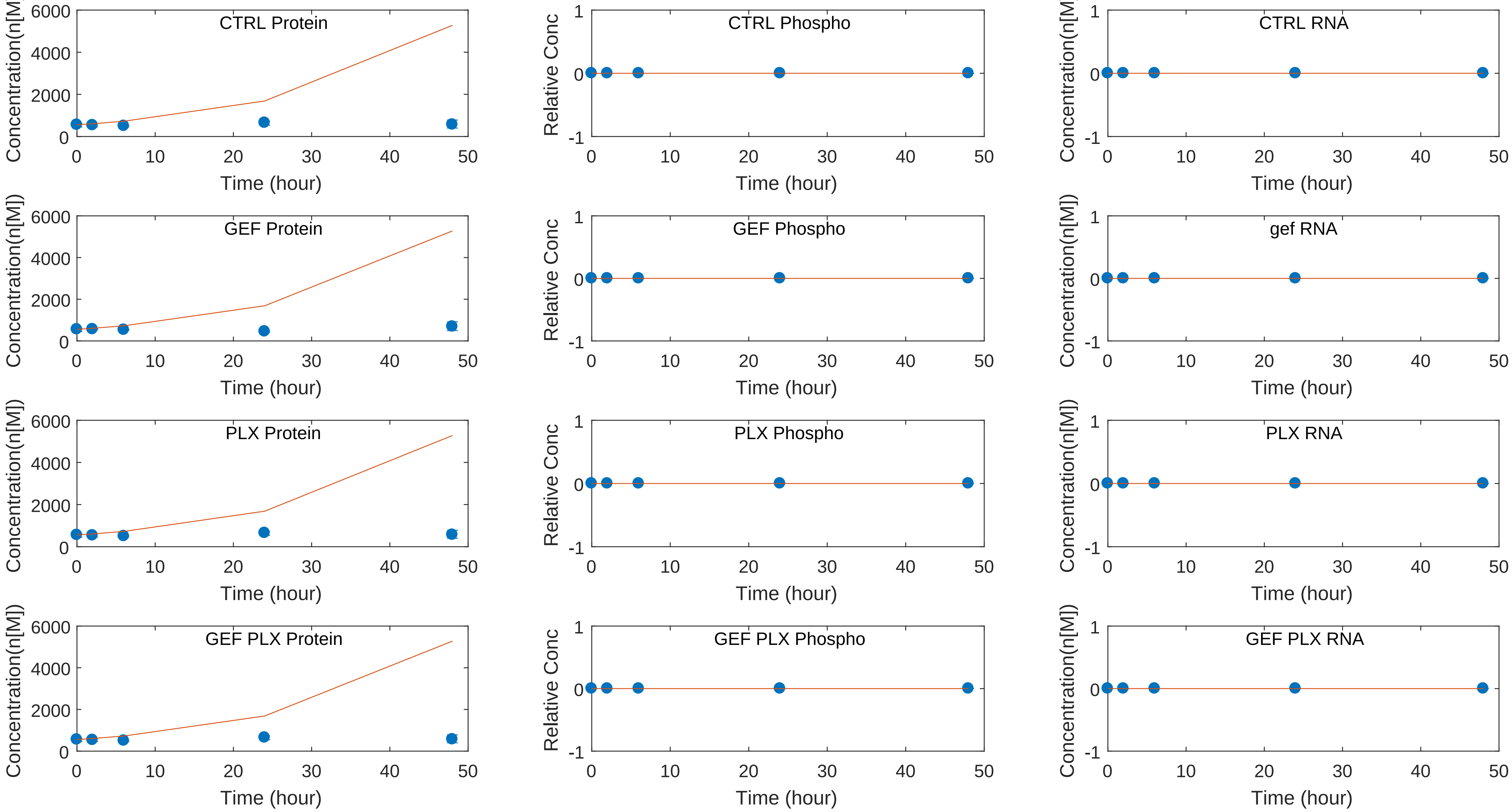

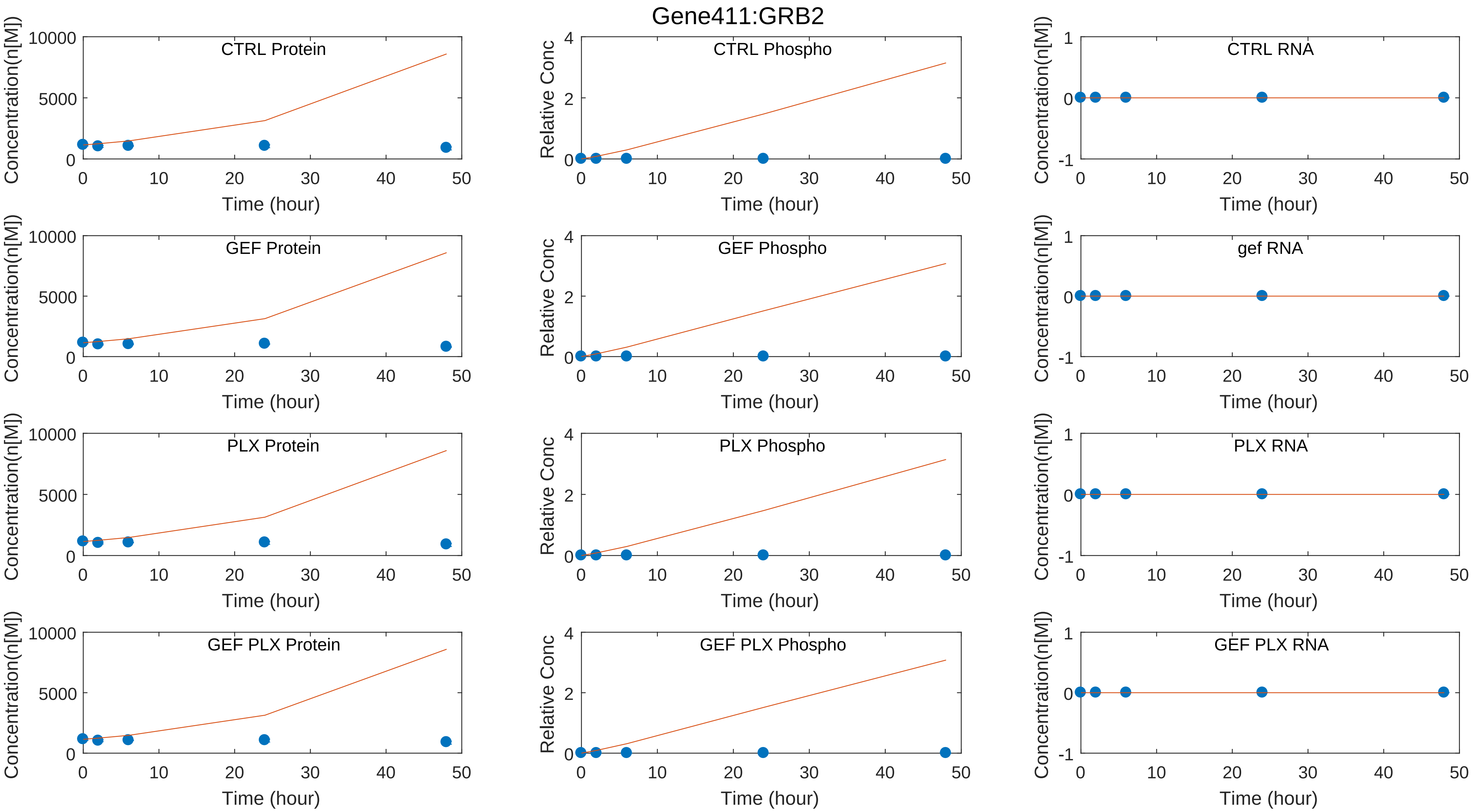

Gene411:GRB2
CTRL Protein
CTRL Phospho
CTRL RNA
GEF Protein
GEF Phospho
gef RNA
PLX Protein
PLX Phospho
PLX RNA
GEF PLX Protein
GEF PLX Phospho
GEF PLX RNA
Concentration(n[M])
Relative Conc
Time (hour)

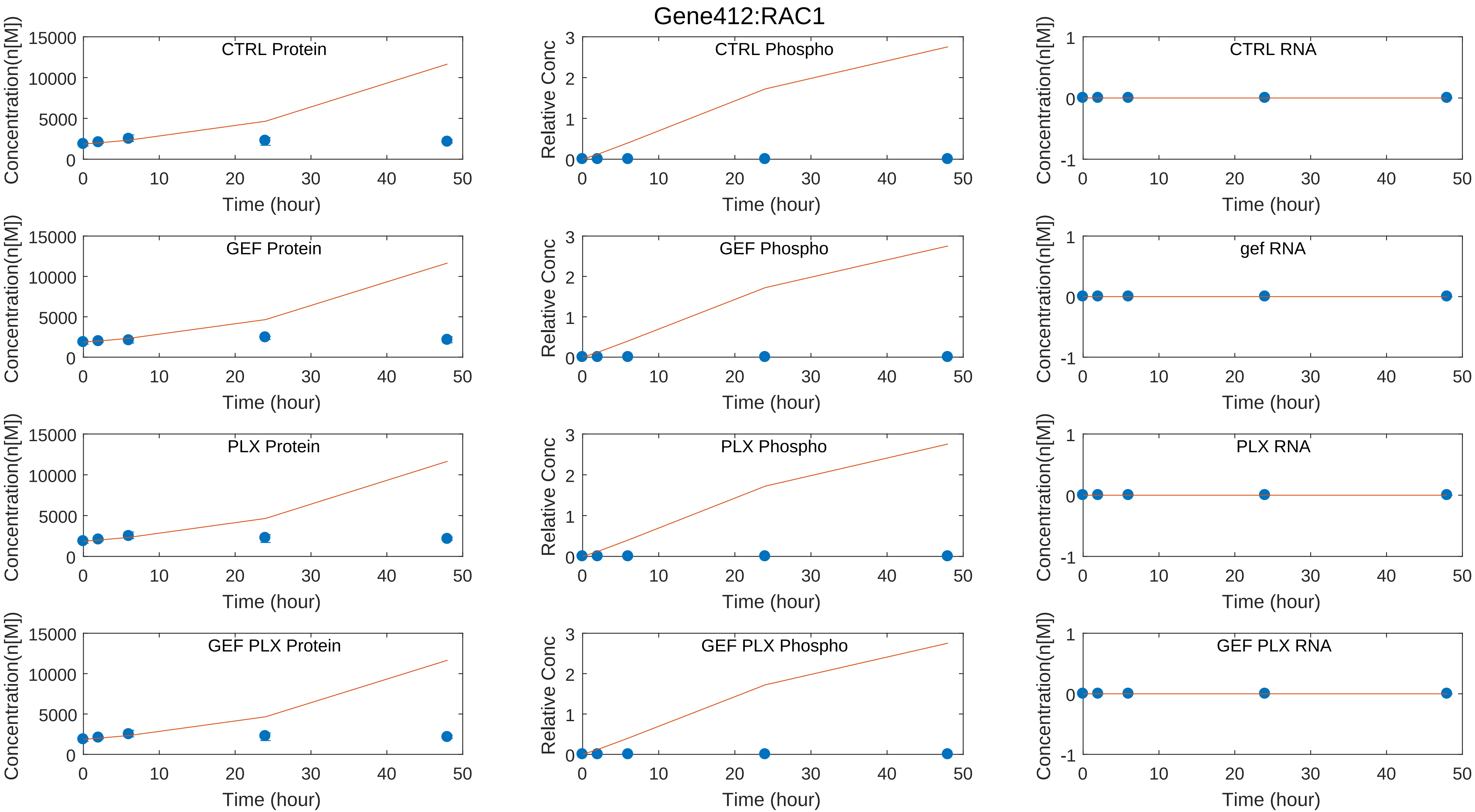

Gene412:RAC1
CTRL Protein
GEF Protein
PLX Protein
GEF PLX Protein
CTRL Phospho
GEF Phospho
PLX Phospho
GEF PLX Phospho
CTRL RNA
gef RNA
PLX RNA
GEF PLX RNA
Concentration(n[M])
Relative Conc
Time (hour)

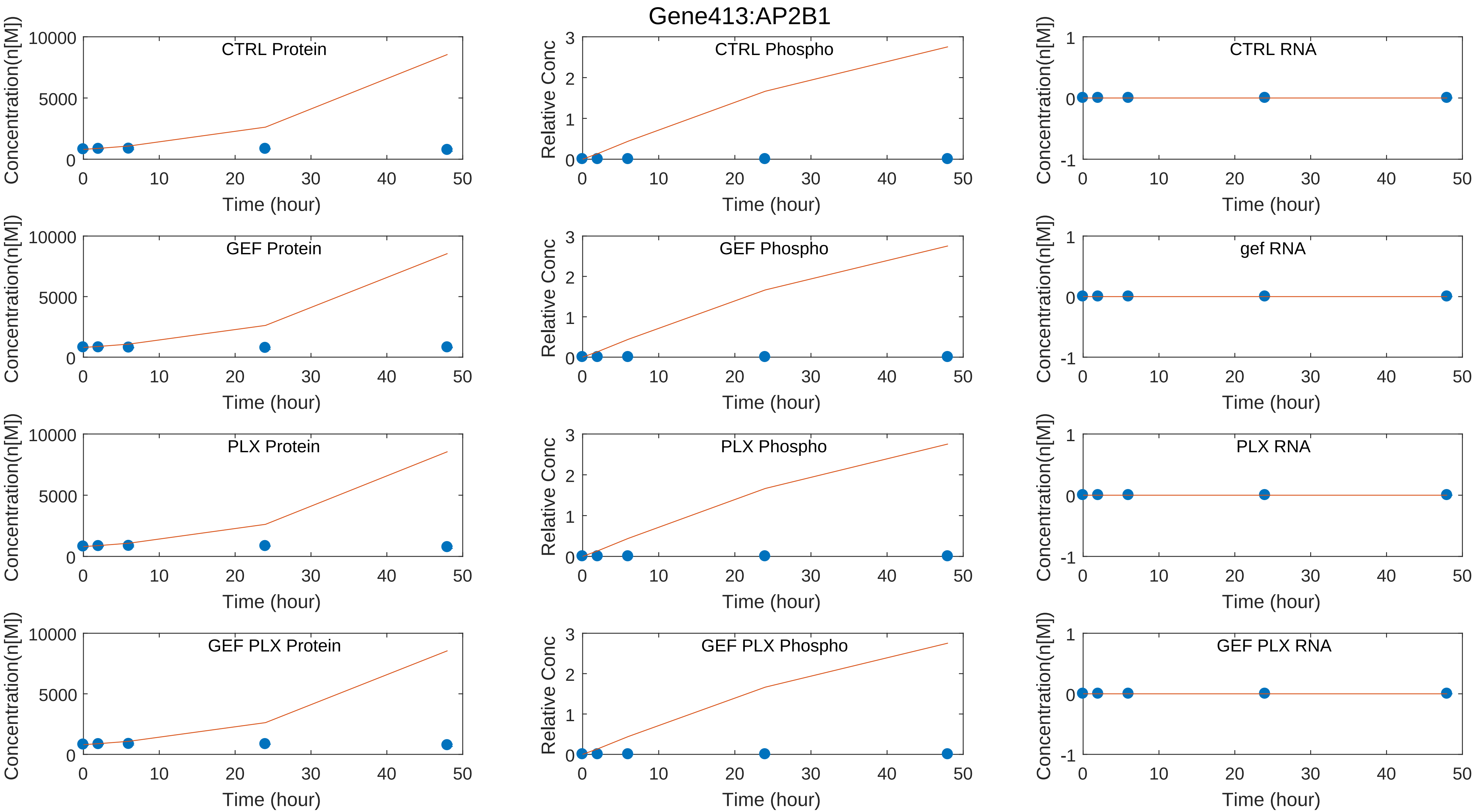

Gene413:AP2B1
CTRL Protein
CTRL Phospho
CTRL RNA
GEF Protein
GEF Phospho
gef RNA
PLX Protein
PLX Phospho
PLX RNA
GEF PLX Protein
GEF PLX Phospho
GEF PLX RNA
Concentration(n[M])
Relative Conc
Time (hour)
0
10
20
30
40
50
5000
10000
1
2
3
-1

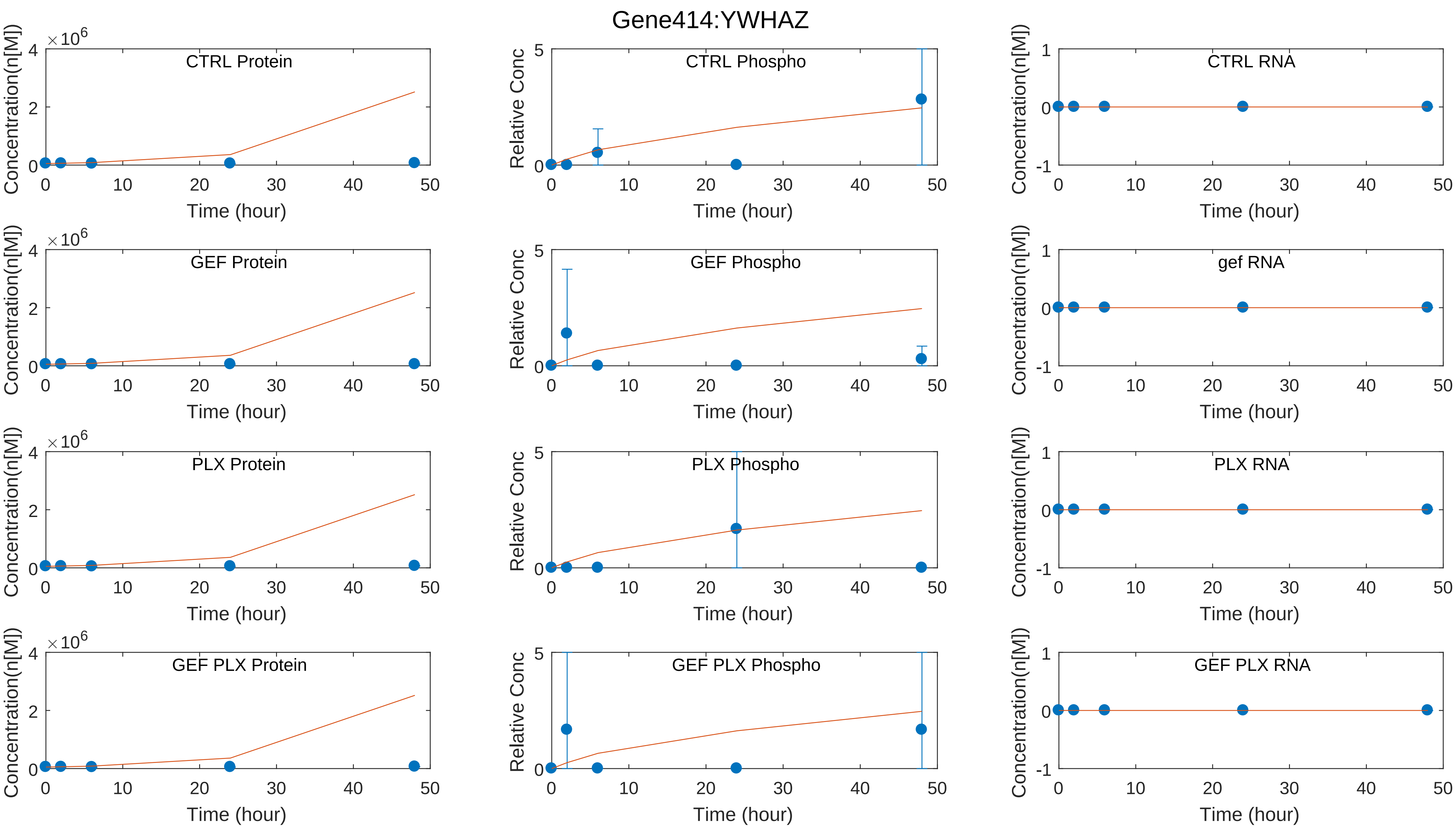

Gene414:YWHAZ
CTRL Protein
CTRL Phospho
CTRL RNA
GEF Protein
GEF Phospho
gef RNA
PLX Protein
PLX Phospho
PLX RNA
GEF PLX Protein
GEF PLX Phospho
GEF PLX RNA
Concentration(n[M])
Relative Conc
Time (hour)
×10^6

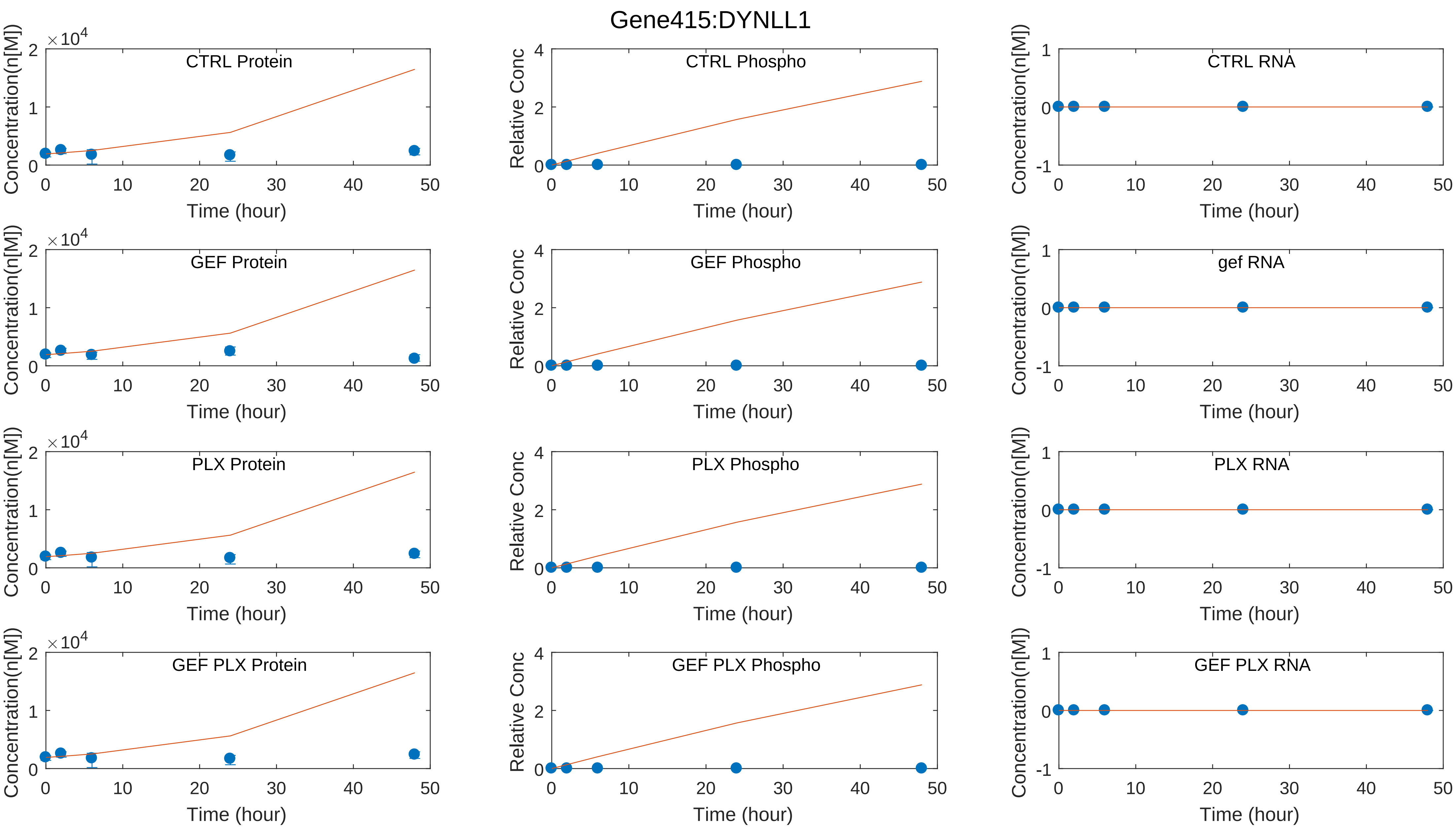
Gene415:DYNLL1
CTRL Protein
GEF Protein
PLX Protein
GEF PLX Protein
CTRL Phospho
GEF Phospho
PLX Phospho
GEF PLX Phospho
CTRL RNA
gef RNA
PLX RNA
GEF PLX RNA
Concentration(n[M])
Relative Conc
Time (hour)
×10^4

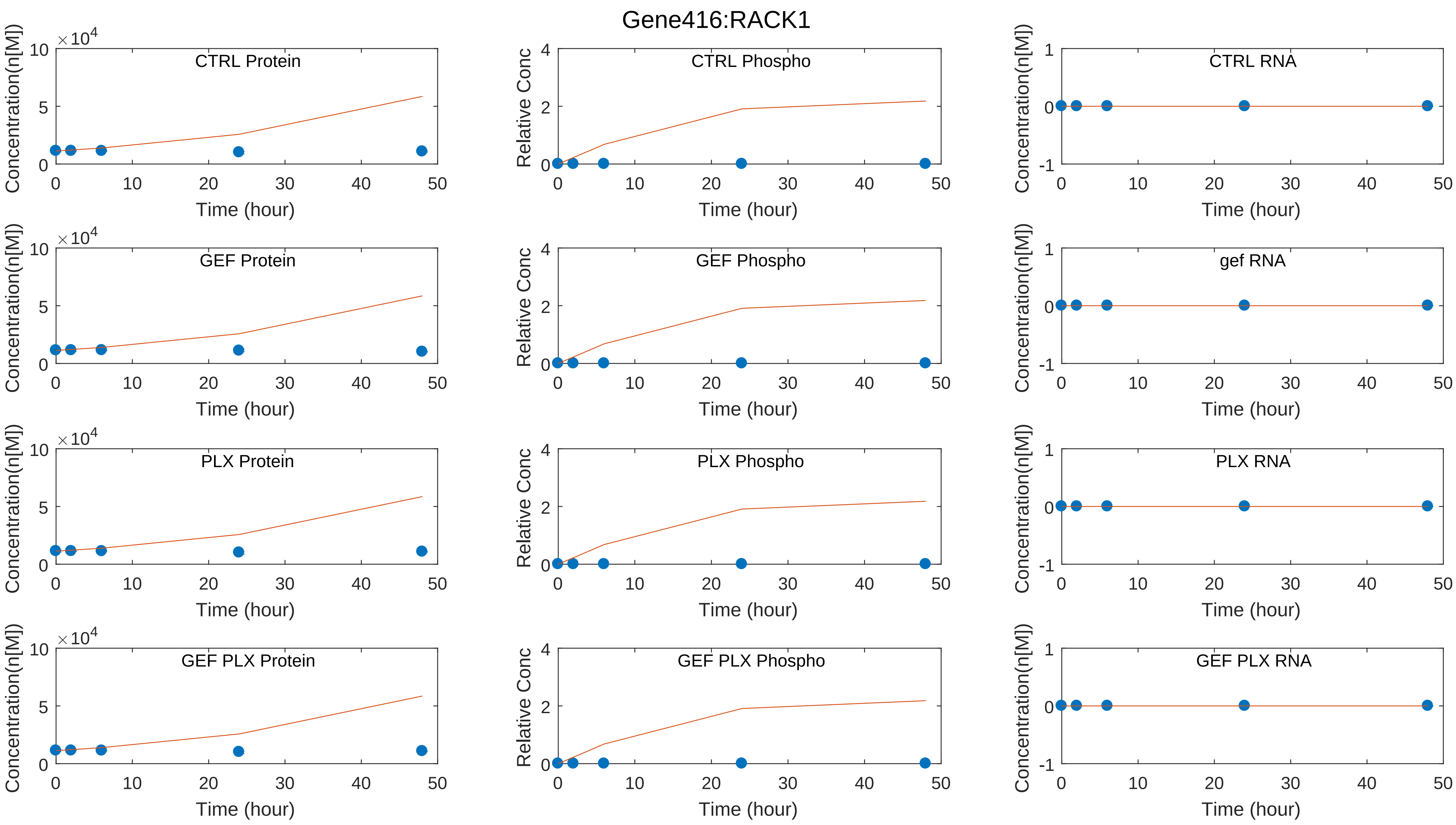

Gene416:RACK1
CTRL Protein
GEF Protein
PLX Protein
GEF PLX Protein
CTRL Phospho
GEF Phospho
PLX Phospho
GEF PLX Phospho
CTRL RNA
gef RNA
PLX RNA
GEF PLX RNA
Concentration(n[M])
Relative Conc
Time (hour)
×10^4

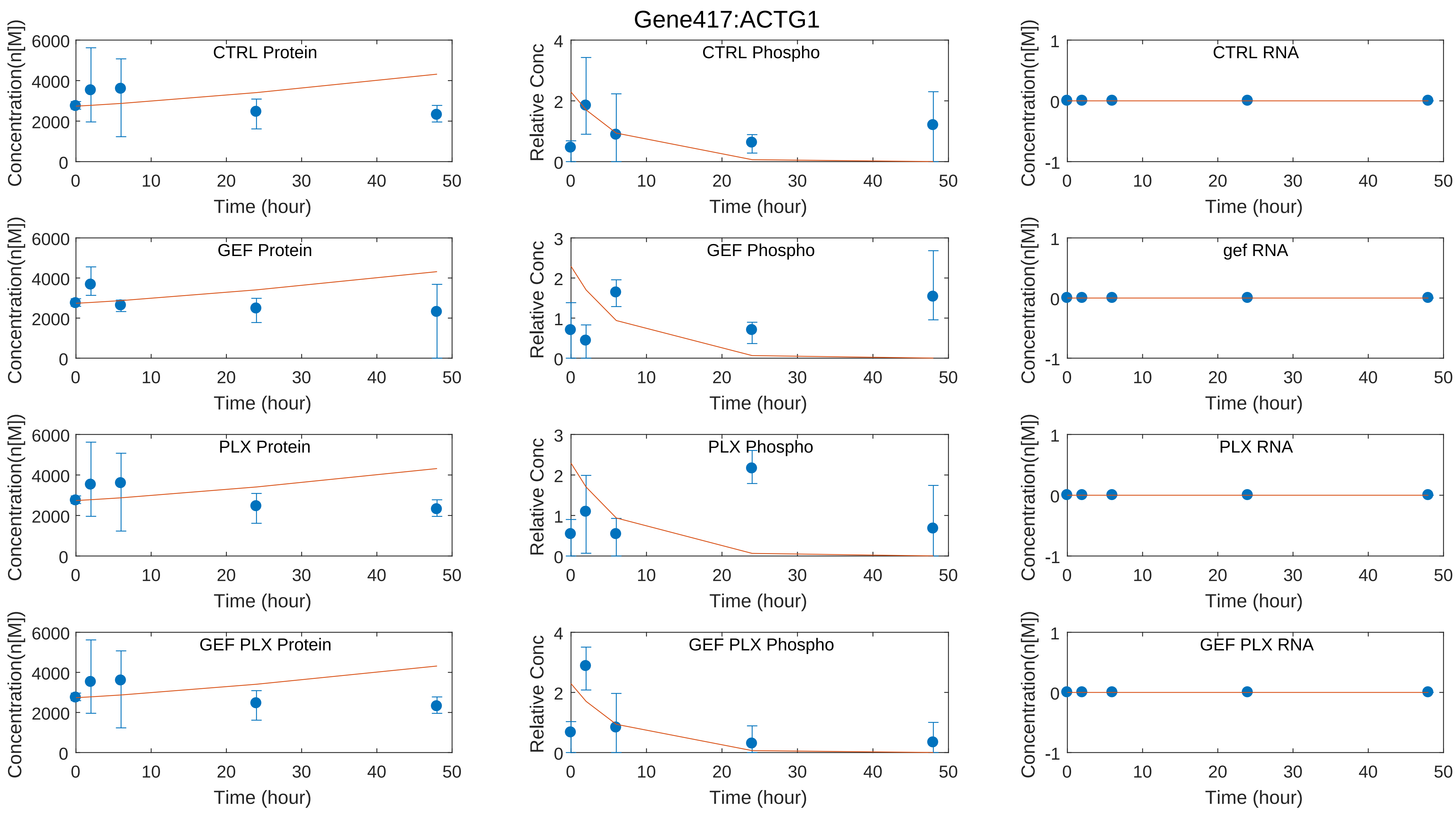
Gene417:ACTG1
CTRL Protein
CTRL Phospho
CTRL RNA
GEF Protein
GEF Phospho
gef RNA
PLX Protein
PLX Phospho
PLX RNA
GEF PLX Protein
GEF PLX Phospho
GEF PLX RNA
Concentration(n[M])
Relative Conc
Time (hour)

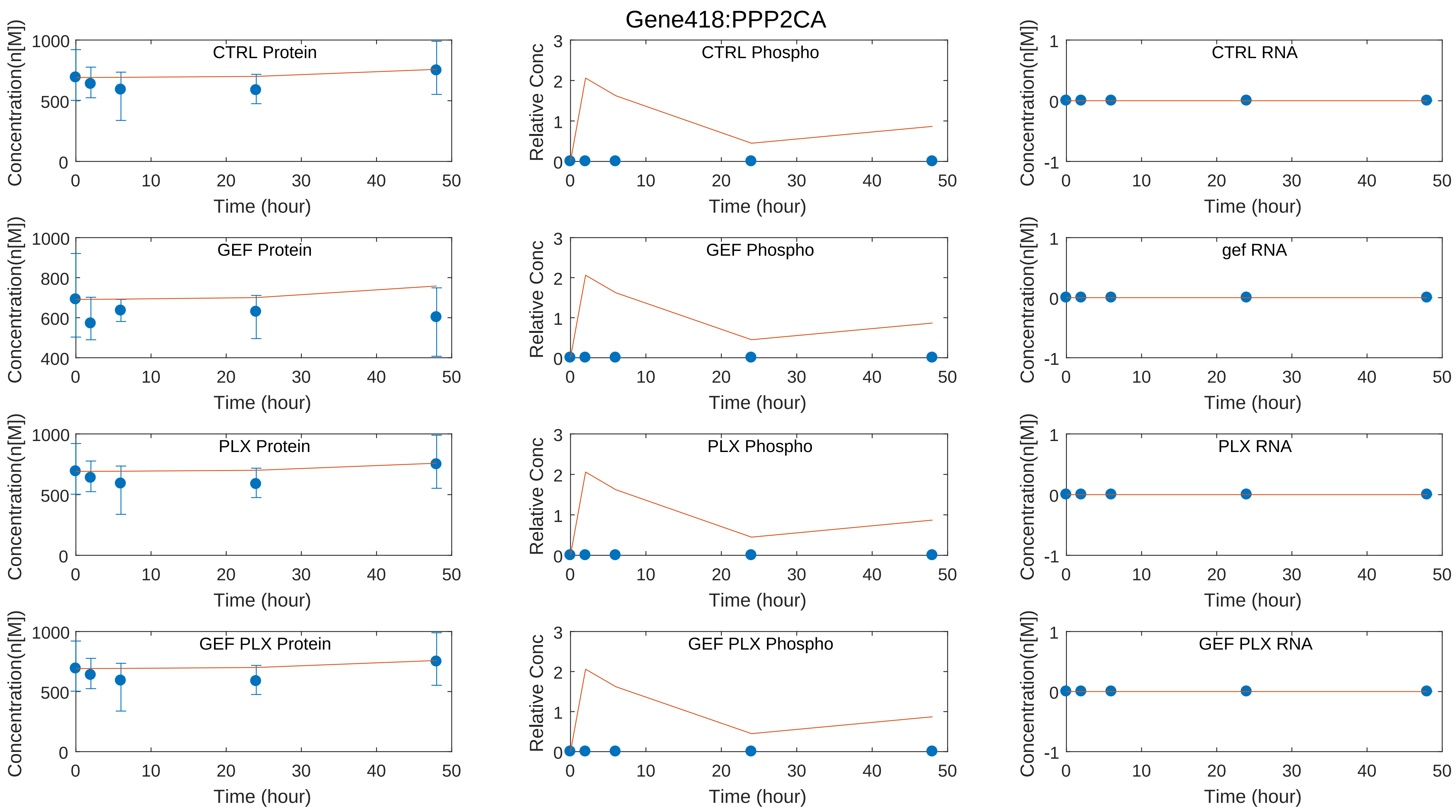
Gene418:PPP2CA
CTRL Protein
CTRL Phospho
CTRL RNA
GEF Protein
GEF Phospho
gef RNA
PLX Protein
PLX Phospho
PLX RNA
GEF PLX Protein
GEF PLX Phospho
GEF PLX RNA
Concentration(n[M])
Relative Conc
Time (hour)

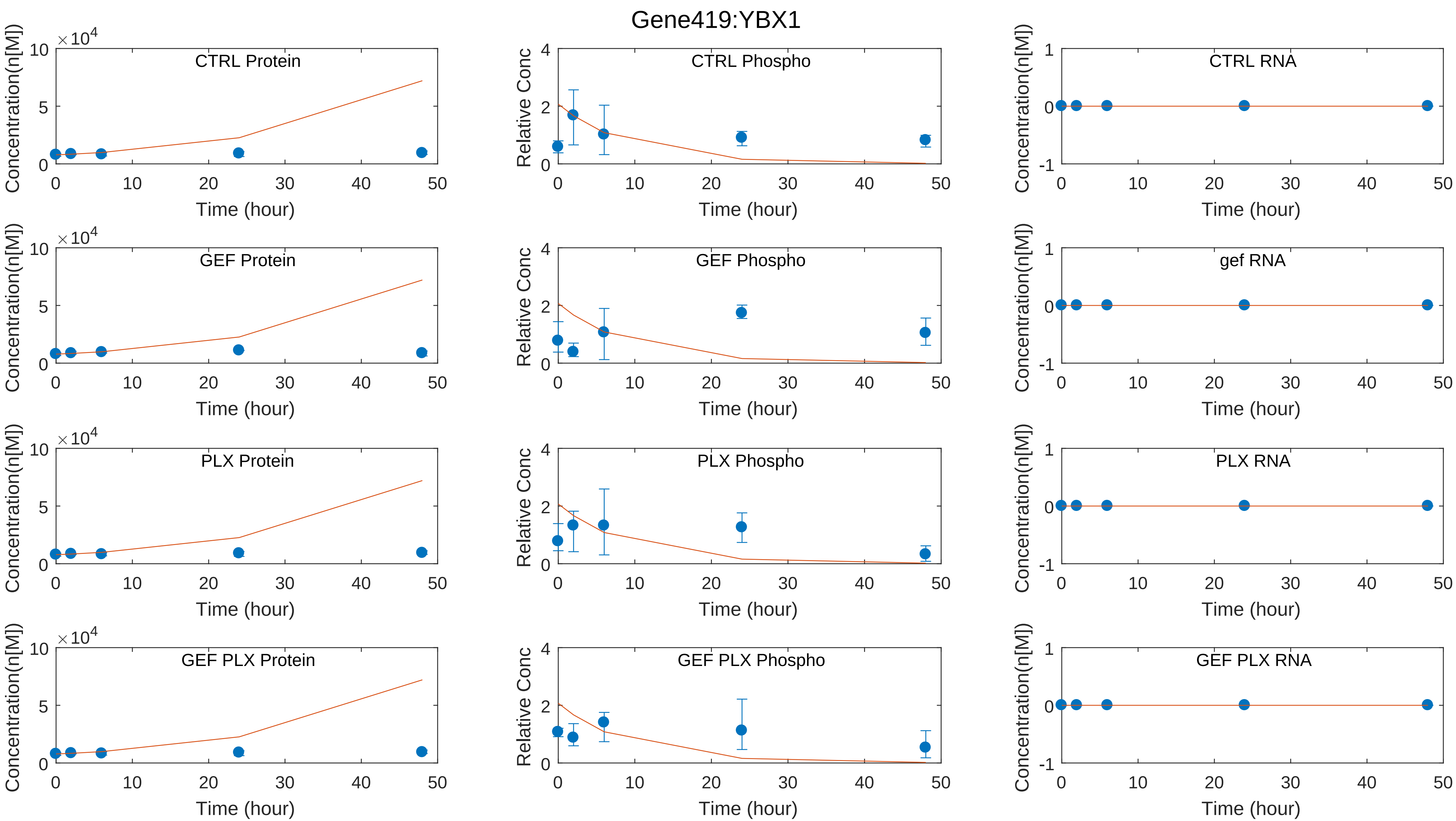

Gene419:YBX1
CTRL Protein
CTRL Phospho
CTRL RNA
GEF Protein
GEF Phospho
gef RNA
PLX Protein
PLX Phospho
PLX RNA
GEF PLX Protein
GEF PLX Phospho
GEF PLX RNA
Concentration(n[M])
Relative Conc
Time (hour)
$\times 10^4$

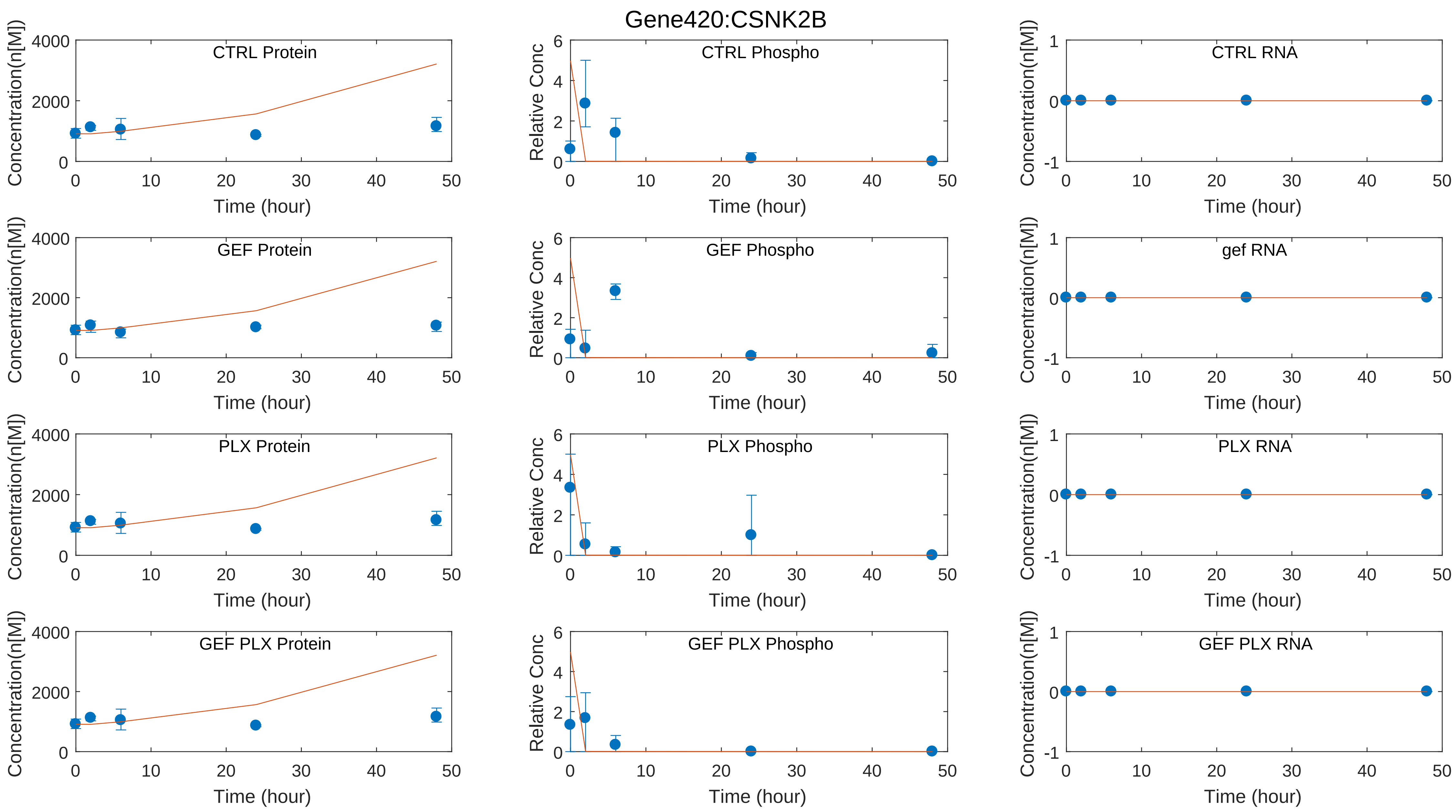

Gene420:CSNK2B
CTRL Protein
CTRL Phospho
CTRL RNA
GEF Protein
GEF Phospho
gef RNA
PLX Protein
PLX Phospho
PLX RNA
GEF PLX Protein
GEF PLX Phospho
GEF PLX RNA
Concentration(n[M])
Relative Conc
Time (hour)

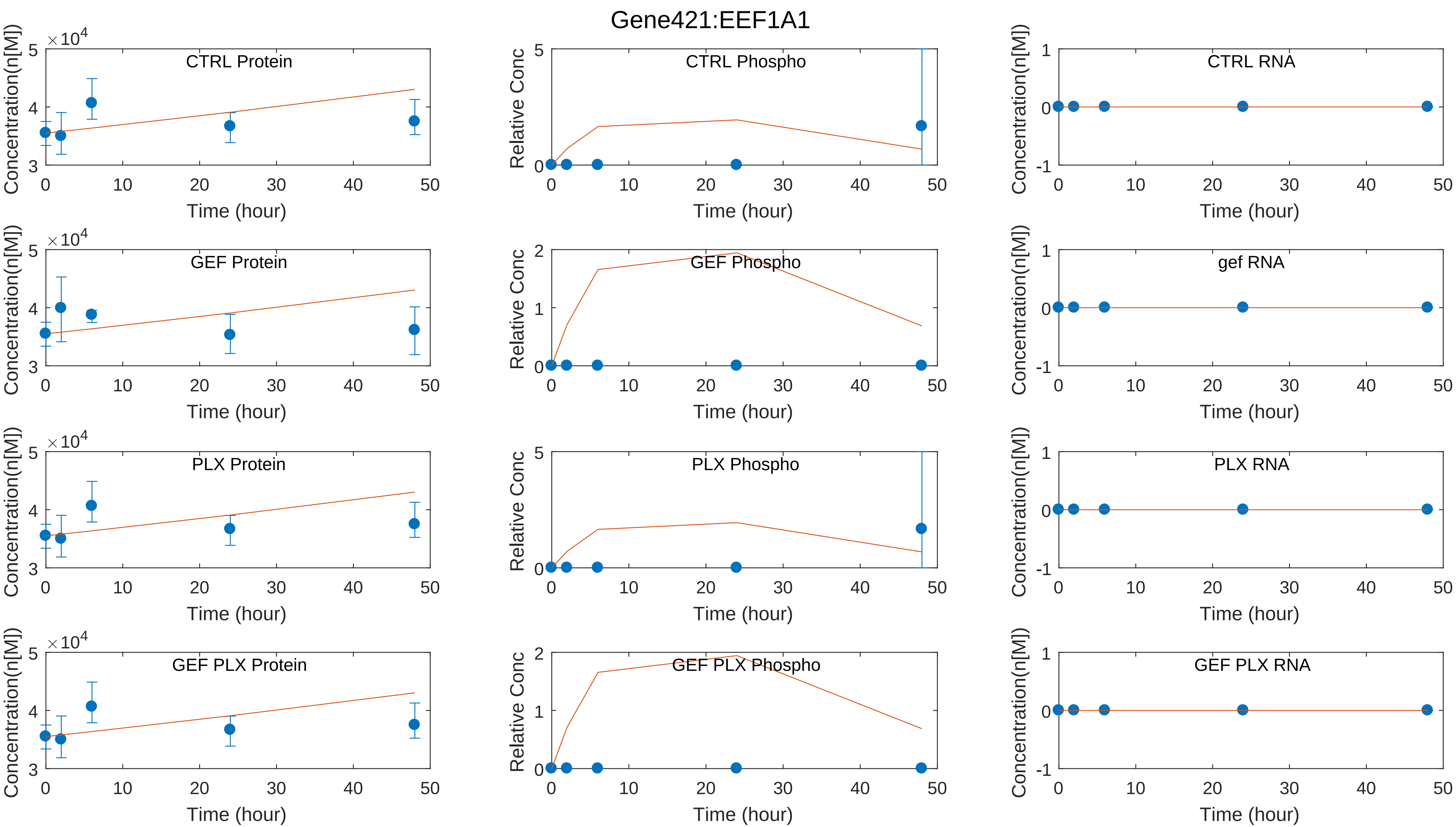
Gene421:EEF1A1
CTRL Protein
GEF Protein
PLX Protein
GEF PLX Protein
CTRL Phospho
GEF Phospho
PLX Phospho
GEF PLX Phospho
CTRL RNA
gef RNA
PLX RNA
GEF PLX RNA
Concentration(n[M])
Relative Conc
Time (hour)
×10^4

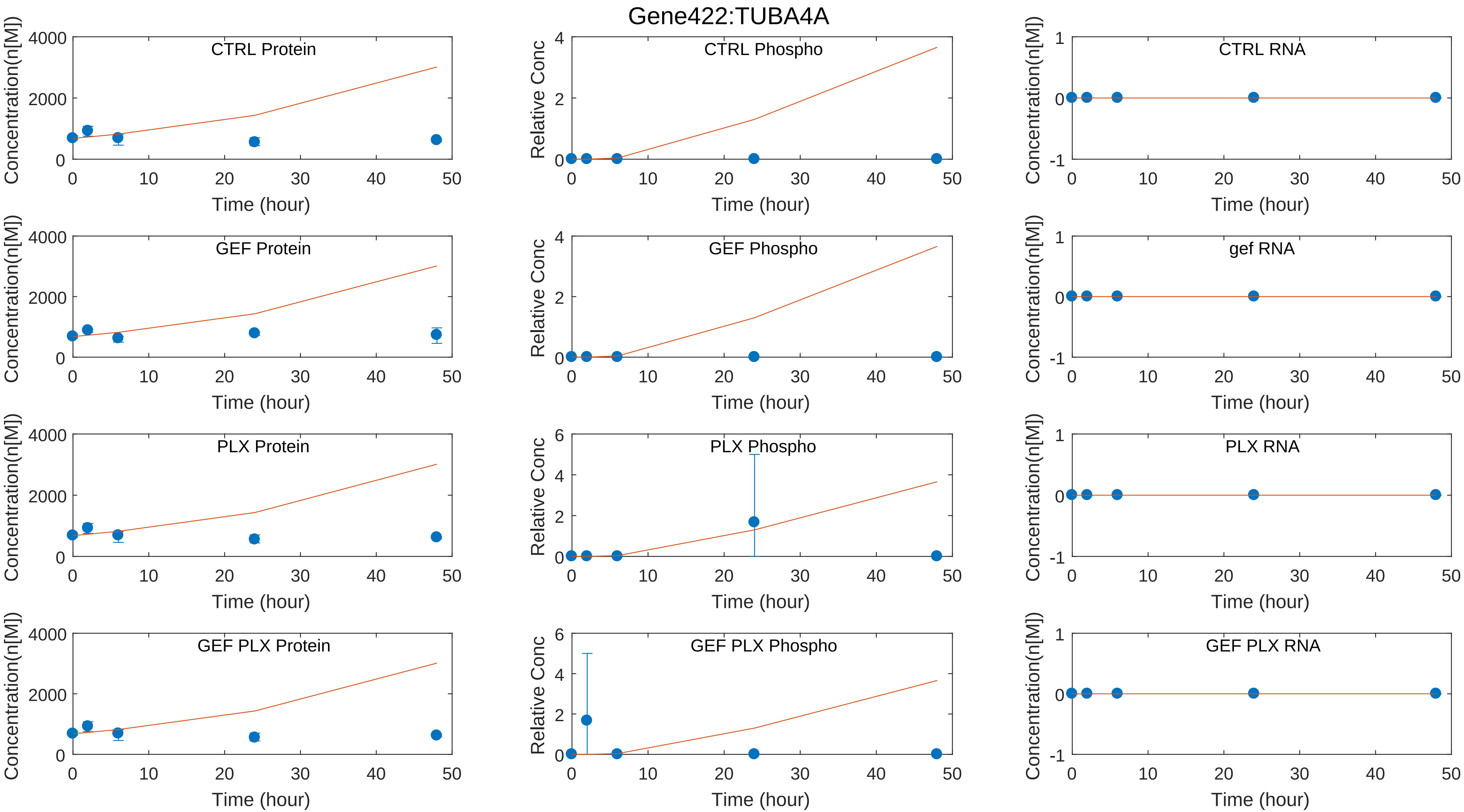

Gene422:TUBA4A
CTRL Protein
CTRL Phospho
CTRL RNA
GEF Protein
GEF Phospho
gef RNA
PLX Protein
PLX Phospho
PLX RNA
GEF PLX Protein
GEF PLX Phospho
GEF PLX RNA
Concentration(n[M])
Relative Conc
Time (hour)

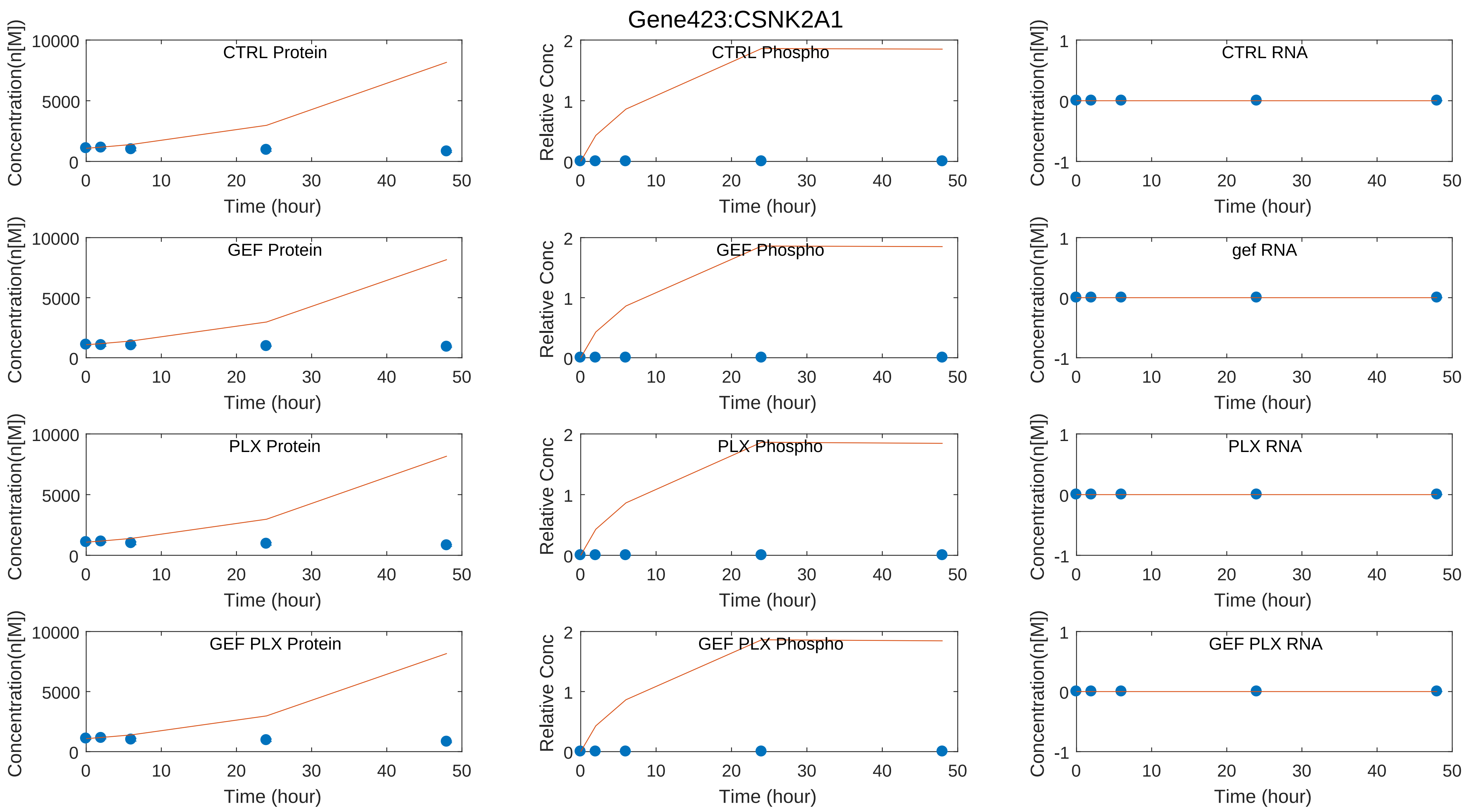
Gene423:CSNK2A1
CTRL Protein
GEF Protein
PLX Protein
GEF PLX Protein
CTRL Phospho
GEF Phospho
PLX Phospho
GEF PLX Phospho
CTRL RNA
gef RNA
PLX RNA
GEF PLX RNA
Concentration(n[M])
Relative Conc
Time (hour)

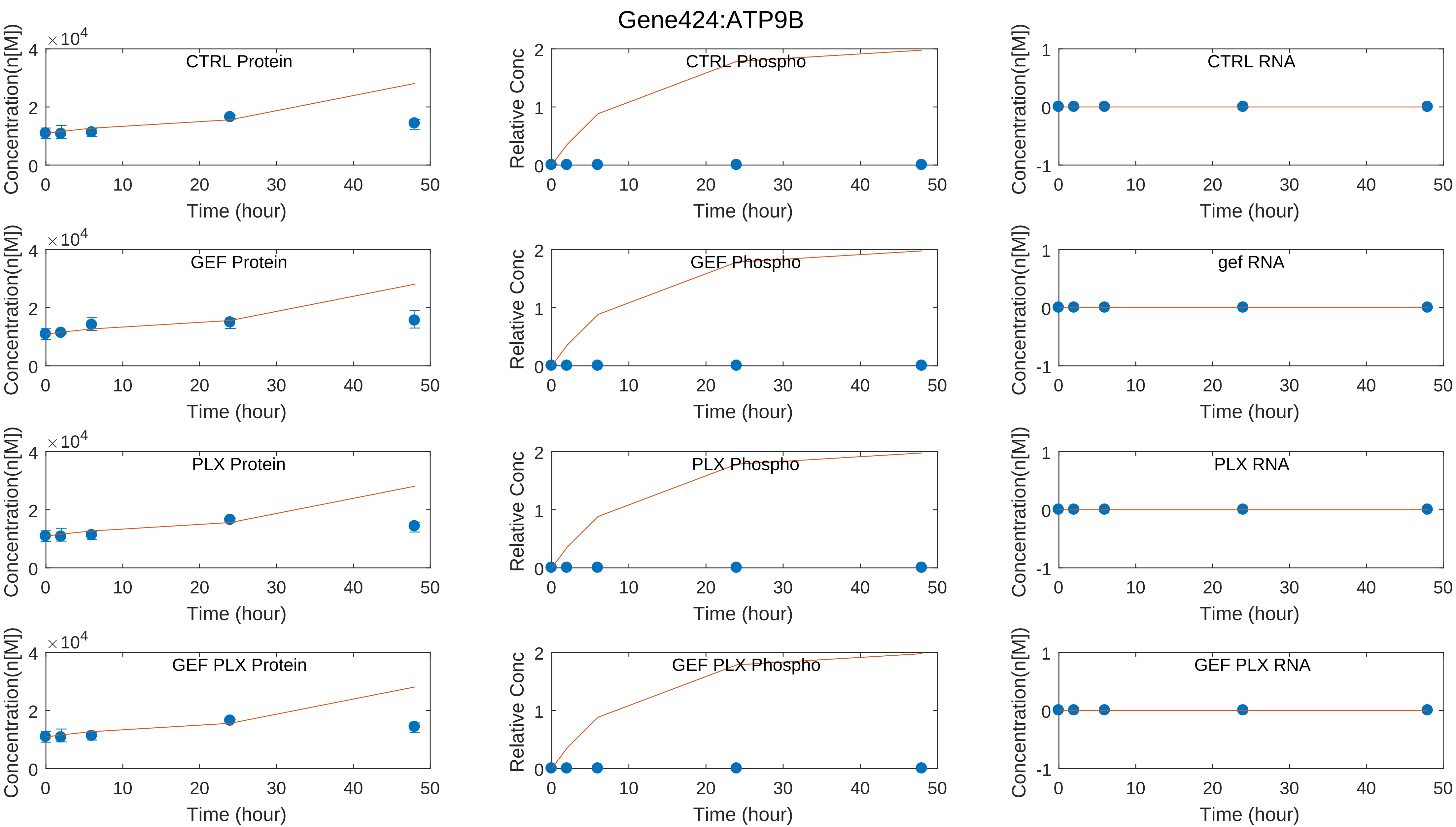
Gene424:ATP9B
CTRL Protein
CTRL Phospho
CTRL RNA
GEF Protein
GEF Phospho
gef RNA
PLX Protein
PLX Phospho
PLX RNA
GEF PLX Protein
GEF PLX Phospho
GEF PLX RNA
Concentration(n[M])
Relative Conc
Time (hour)
$\times 10^4$

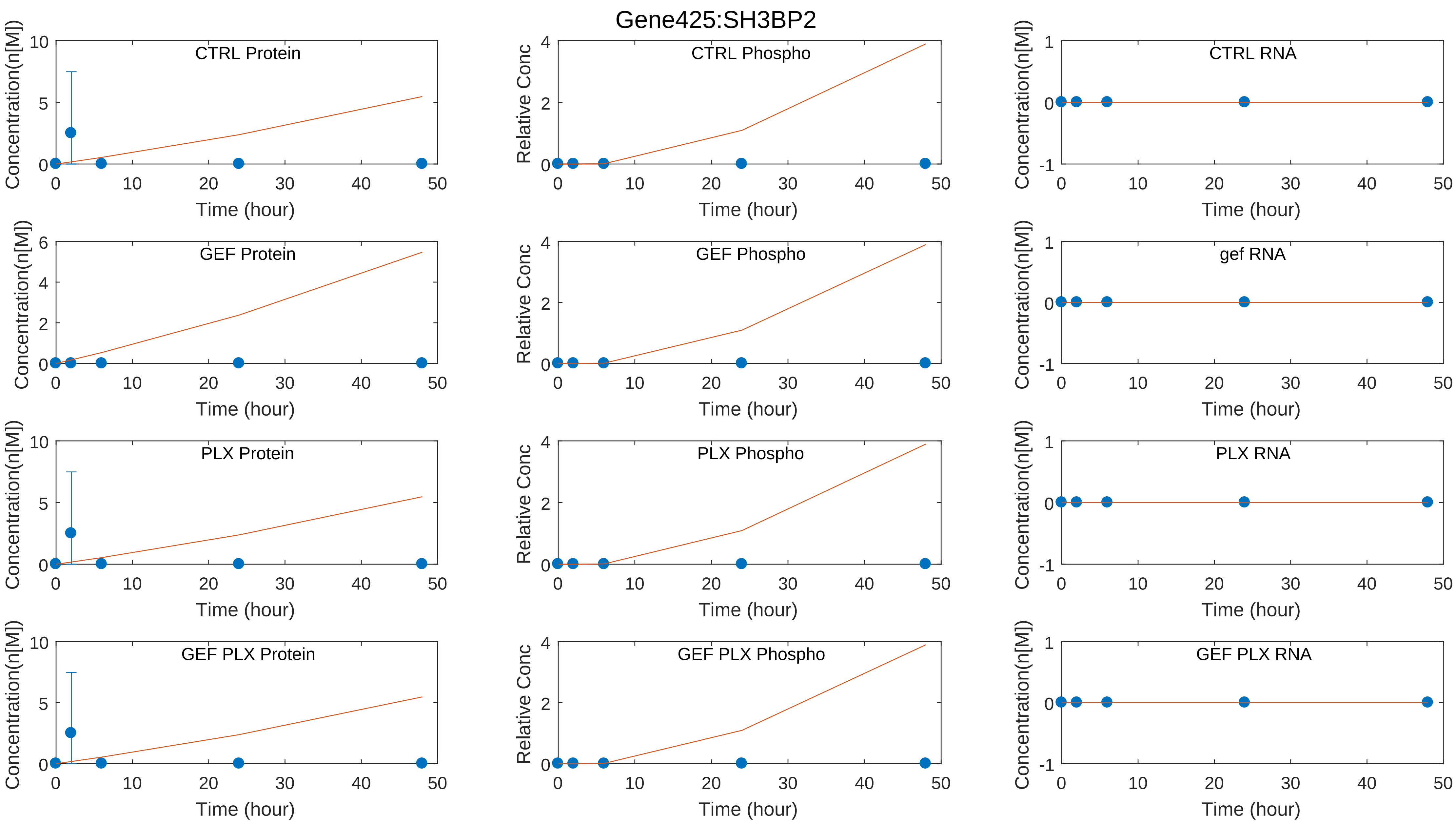

Gene425:SH3BP2
CTRL Protein
CTRL Phospho
CTRL RNA
GEF Protein
GEF Phospho
gef RNA
PLX Protein
PLX Phospho
PLX RNA
GEF PLX Protein
GEF PLX Phospho
GEF PLX RNA
Concentration(n[M])
Relative Conc
Time (hour)

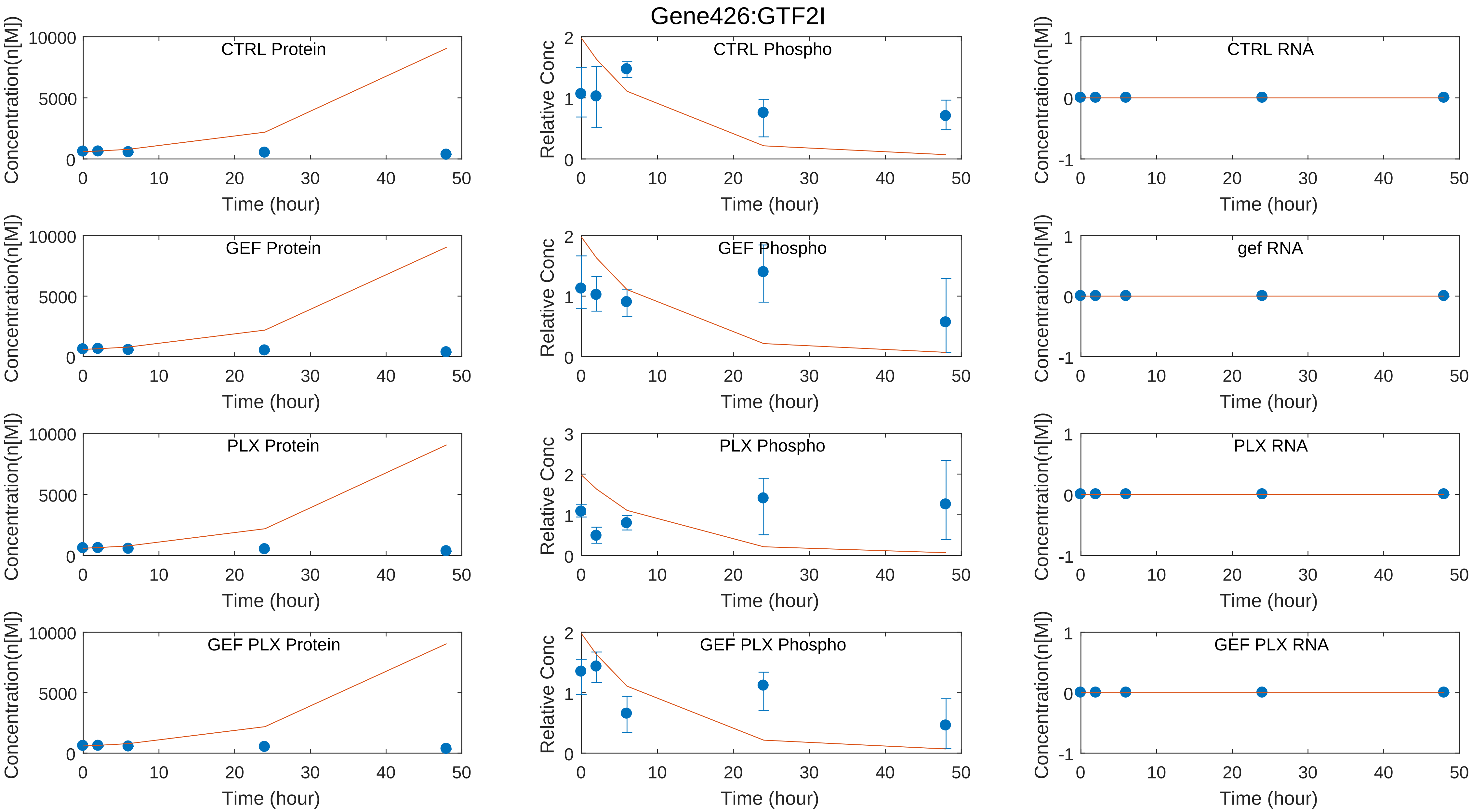
Gene426:GTF2I
CTRL Protein
CTRL Phospho
CTRL RNA
GEF Protein
GEF Phospho
gef RNA
PLX Protein
PLX Phospho
PLX RNA
GEF PLX Protein
GEF PLX Phospho
GEF PLX RNA
Concentration(n[M])
Relative Conc
Time (hour)

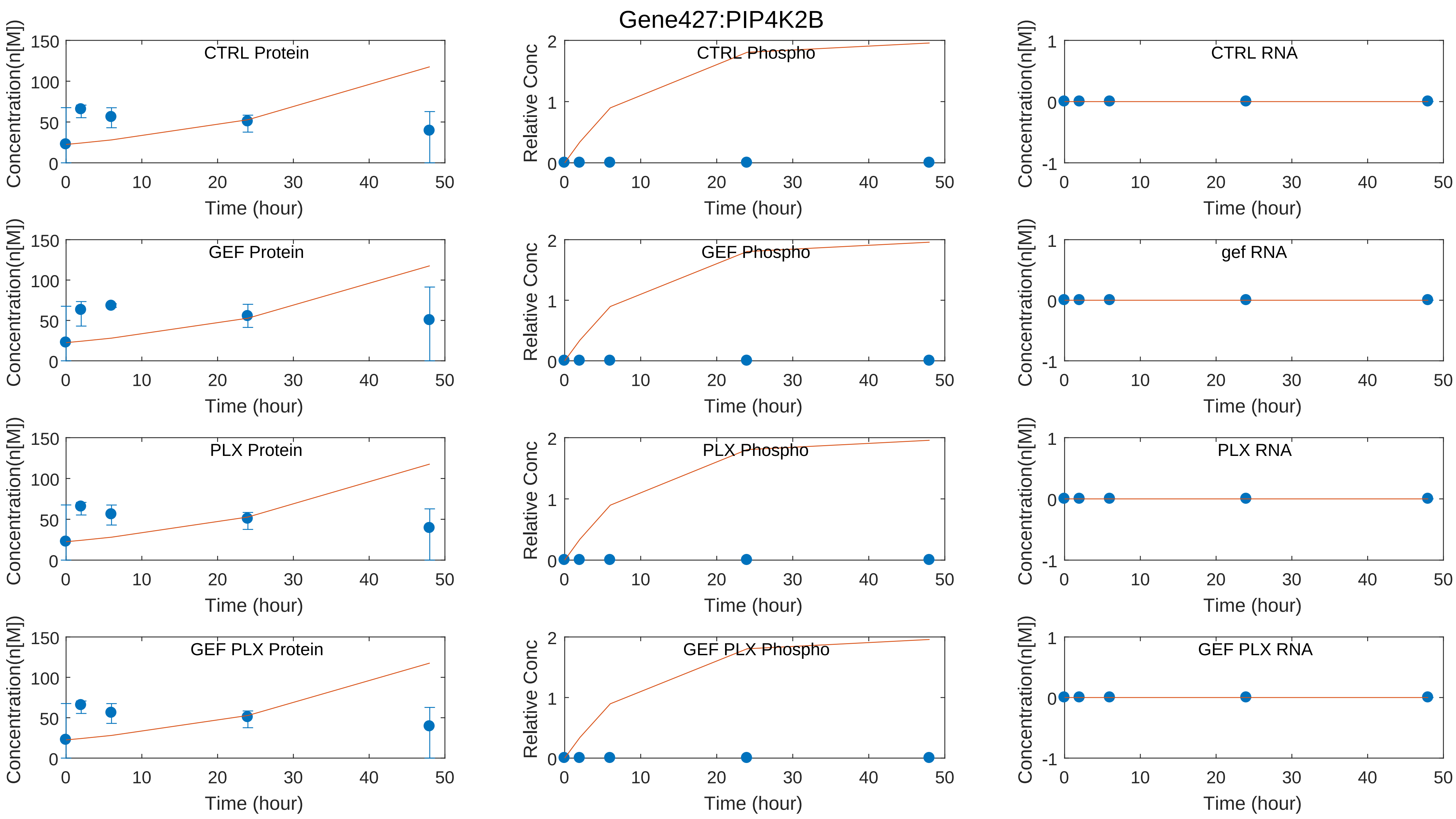

Gene427:PIP4K2B
CTRL Protein
CTRL Phospho
CTRL RNA
GEF Protein
GEF Phospho
gef RNA
PLX Protein
PLX Phospho
PLX RNA
GEF PLX Protein
GEF PLX Phospho
GEF PLX RNA
Concentration(n[M])
Relative Conc
Time (hour)

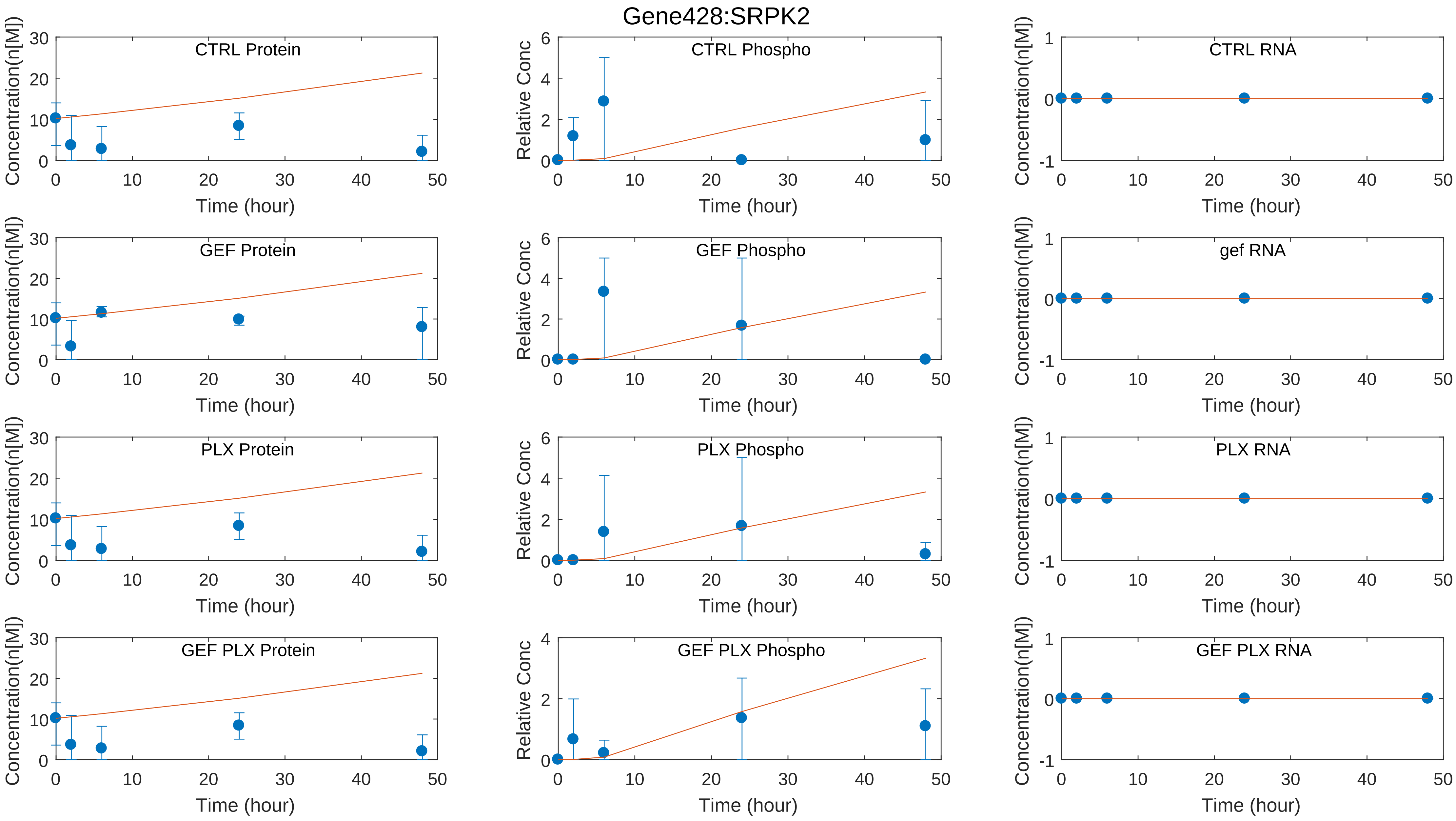
Gene428:SRPK2
CTRL Protein
CTRL Phospho
CTRL RNA
GEF Protein
GEF Phospho
gef RNA
PLX Protein
PLX Phospho
PLX RNA
GEF PLX Protein
GEF PLX Phospho
GEF PLX RNA
Concentration(n[M])
Relative Conc
Time (hour)

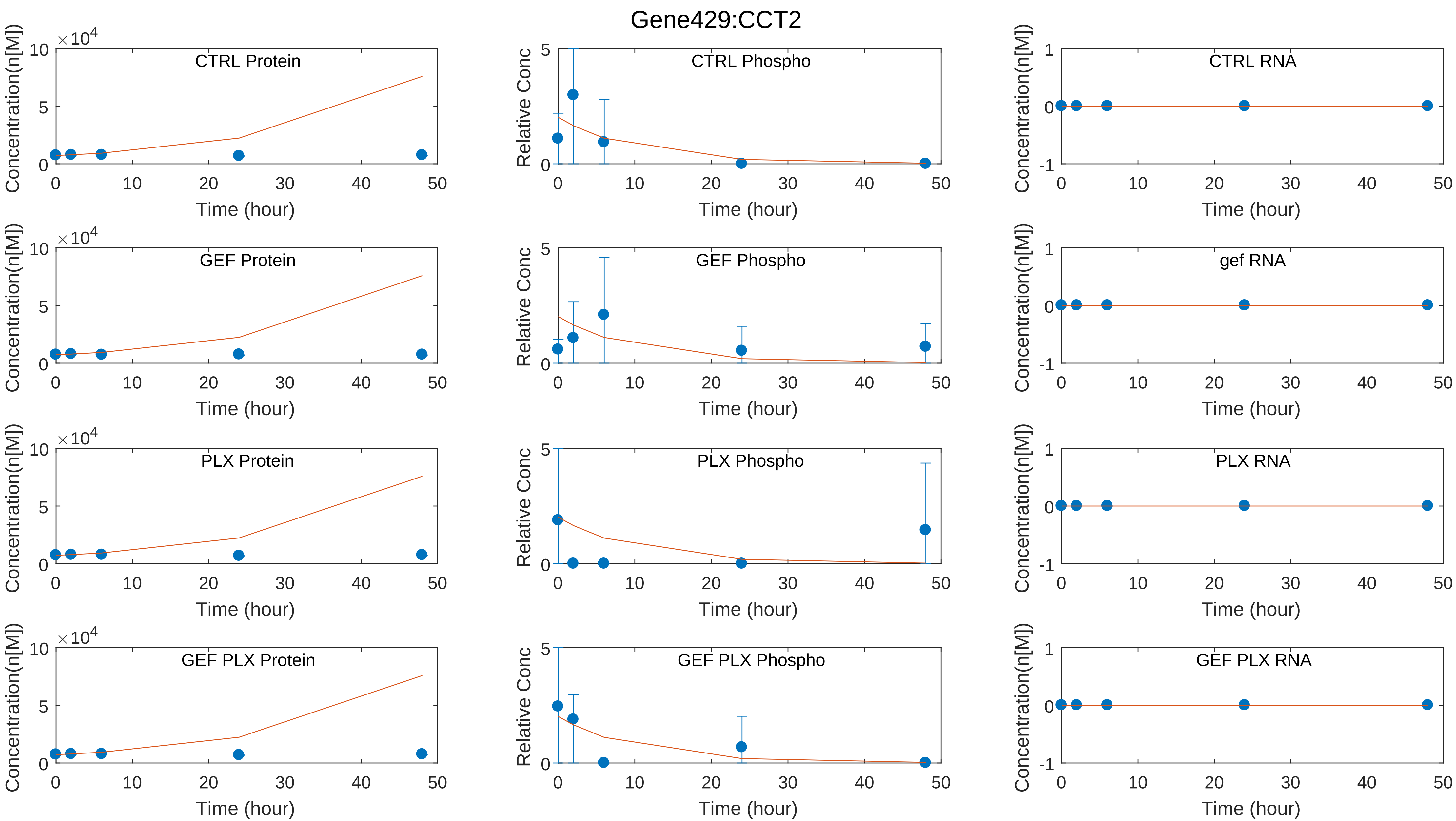

Gene429:CCT2
CTRL Protein
CTRL Phospho
CTRL RNA
GEF Protein
GEF Phospho
gef RNA
PLX Protein
PLX Phospho
PLX RNA
GEF PLX Protein
GEF PLX Phospho
GEF PLX RNA
Concentration(n[M])
Relative Conc
Time (hour)
×10^4

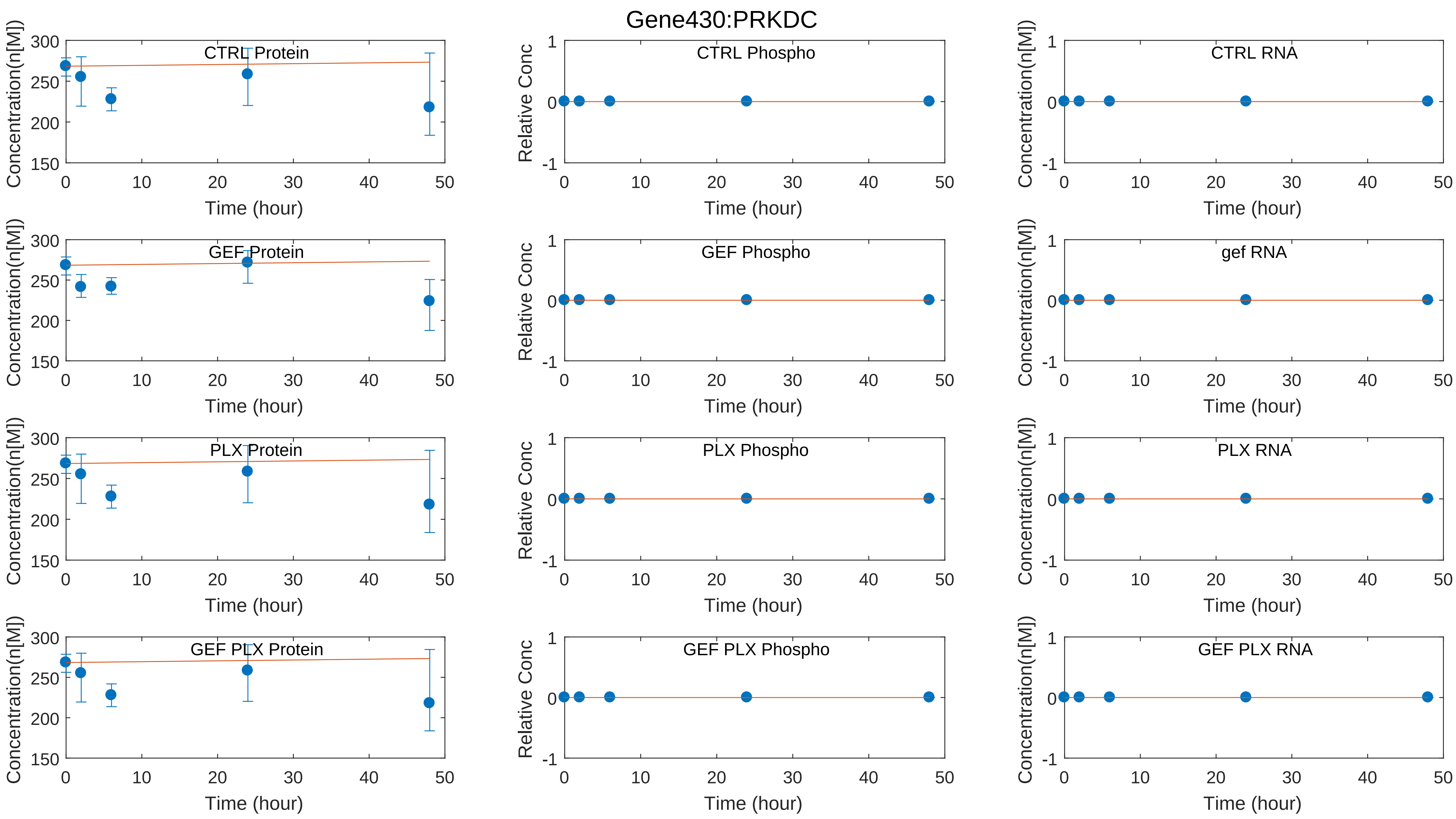
Gene430:PRKDC
CTRL Protein
CTRL Phospho
CTRL RNA
GEF Protein
GEF Phospho
gef RNA
PLX Protein
PLX Phospho
PLX RNA
GEF PLX Protein
GEF PLX Phospho
GEF PLX RNA
Concentration(n[M])
Relative Conc
Time (hour)

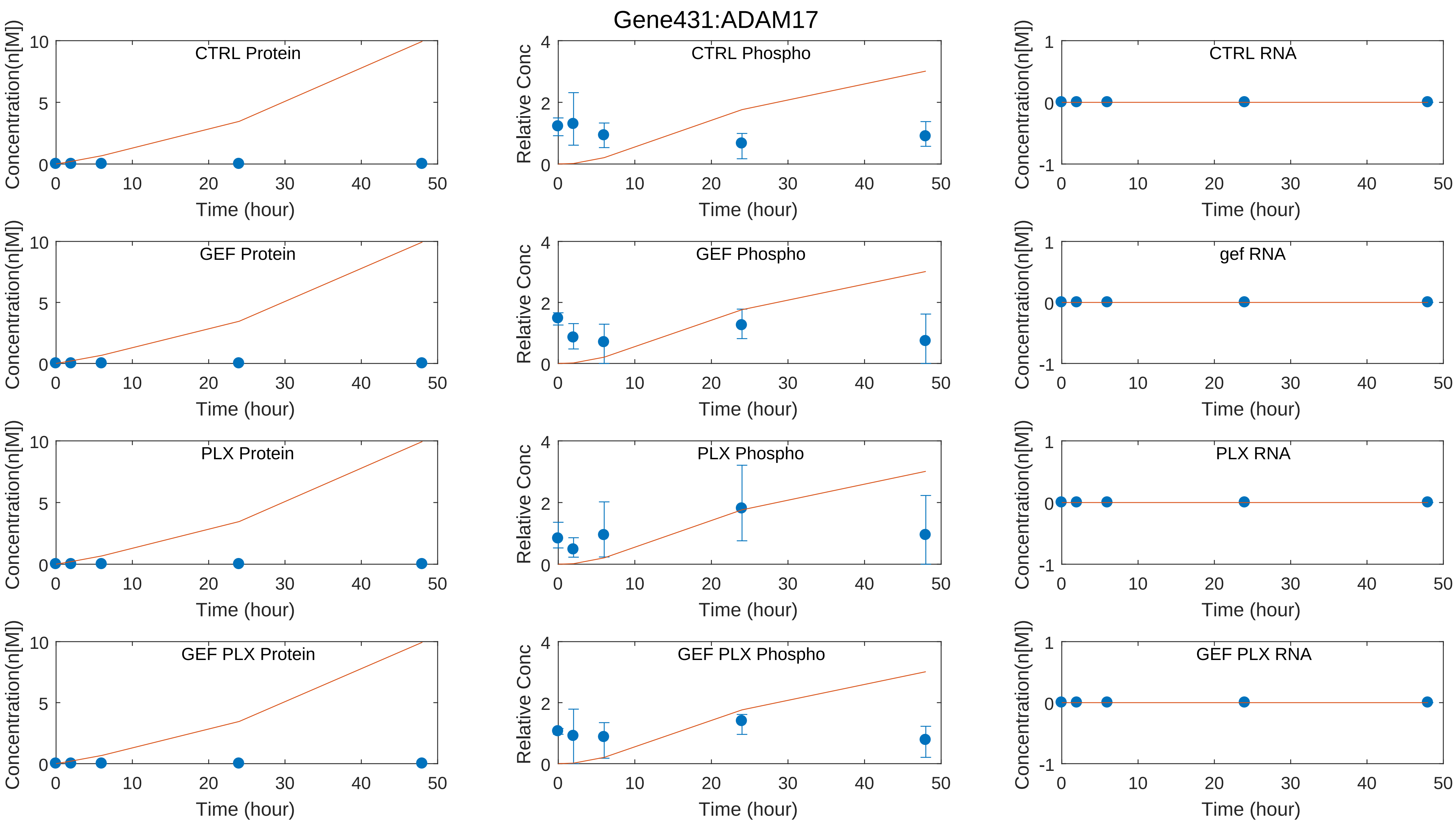
Gene431:ADAM17
CTRL Protein
CTRL Phospho
CTRL RNA
GEF Protein
GEF Phospho
gef RNA
PLX Protein
PLX Phospho
PLX RNA
GEF PLX Protein
GEF PLX Phospho
GEF PLX RNA
Concentration(n[M])
Relative Conc
Time (hour)
0
10
20
30
40
50
5
2
4
1
-1

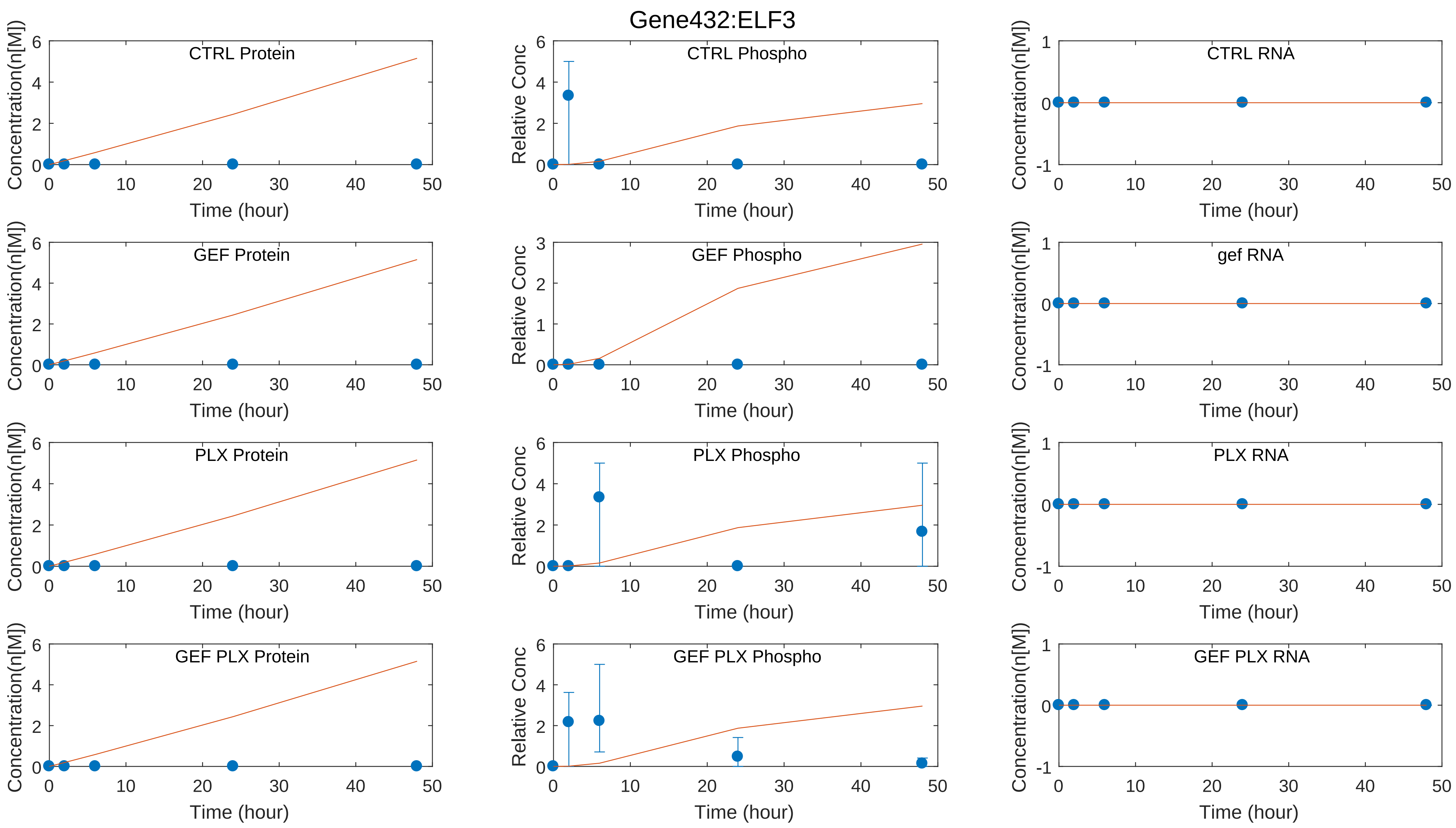

Gene432:ELF3
CTRL Protein
CTRL Phospho
CTRL RNA
GEF Protein
GEF Phospho
gef RNA
PLX Protein
PLX Phospho
PLX RNA
GEF PLX Protein
GEF PLX Phospho
GEF PLX RNA
Concentration(n[M])
Relative Conc
Time (hour)

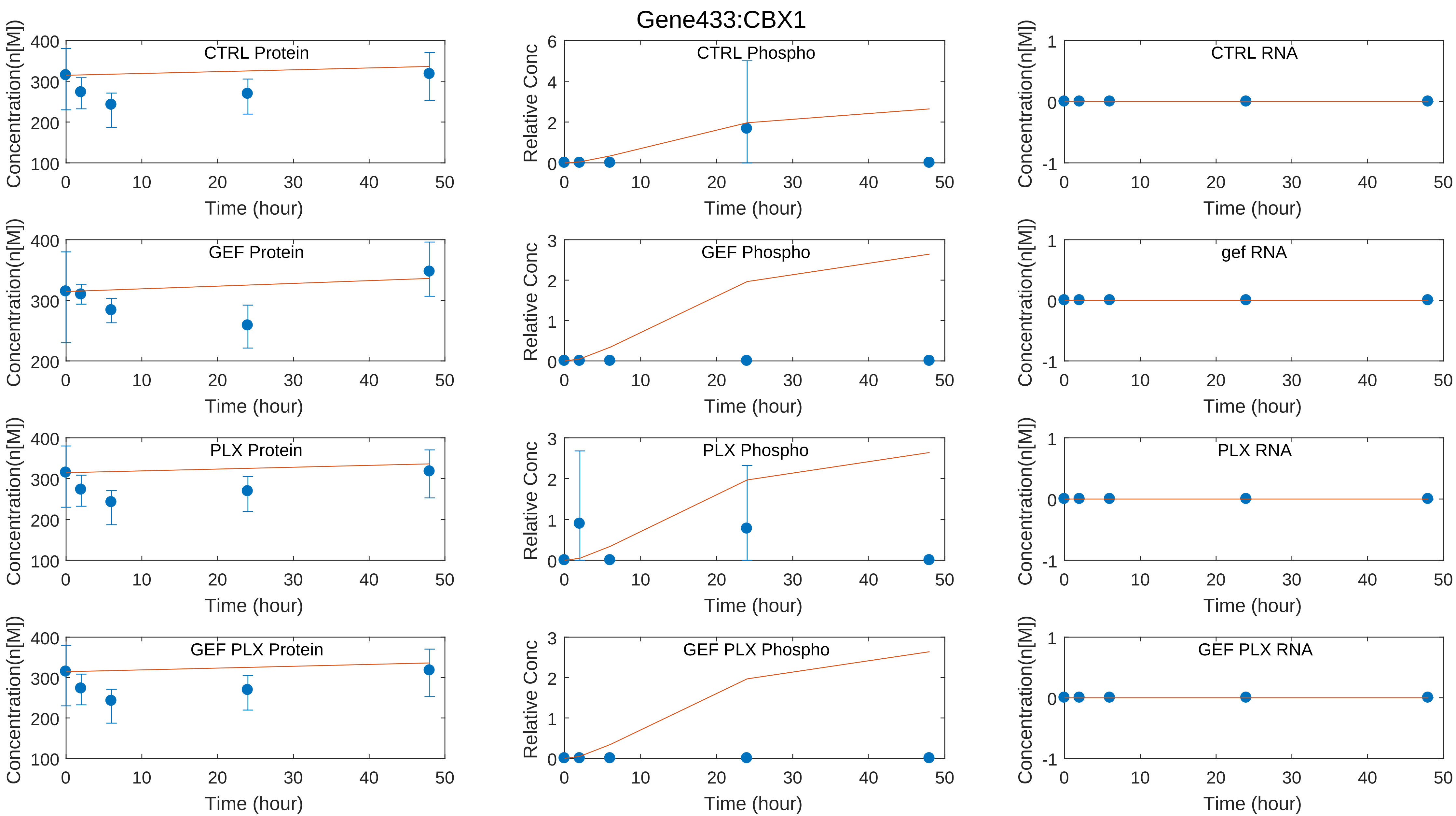

Gene433:CBX1
CTRL Protein
CTRL Phospho
CTRL RNA
GEF Protein
GEF Phospho
gef RNA
PLX Protein
PLX Phospho
PLX RNA
GEF PLX Protein
GEF PLX Phospho
GEF PLX RNA
Concentration(n[M])
Relative Conc
Time (hour)

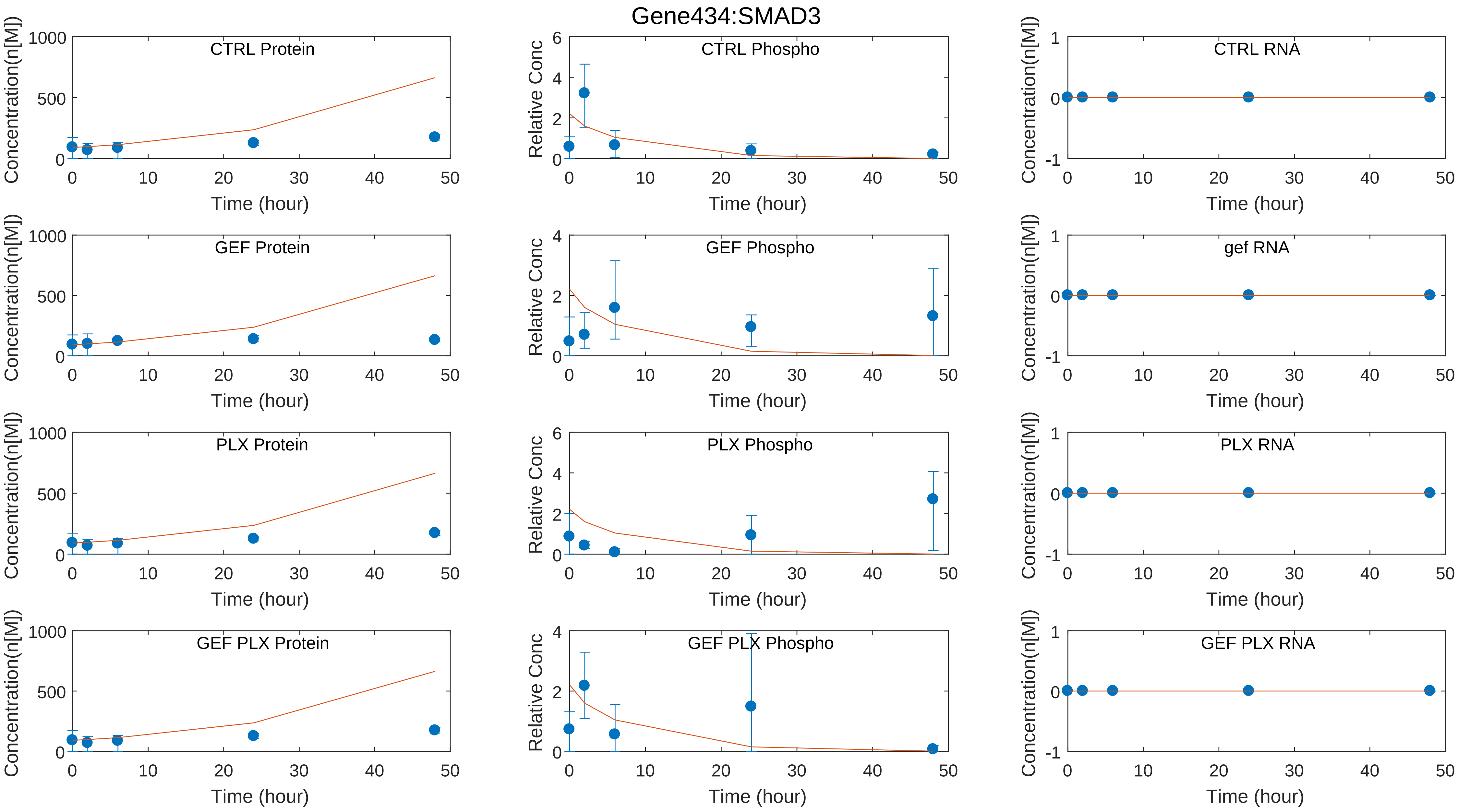
Gene434:SMAD3
CTRL Protein
CTRL Phospho
CTRL RNA
GEF Protein
GEF Phospho
gef RNA
PLX Protein
PLX Phospho
PLX RNA
GEF PLX Protein
GEF PLX Phospho
GEF PLX RNA
Concentration(n[M])
Relative Conc
Time (hour)

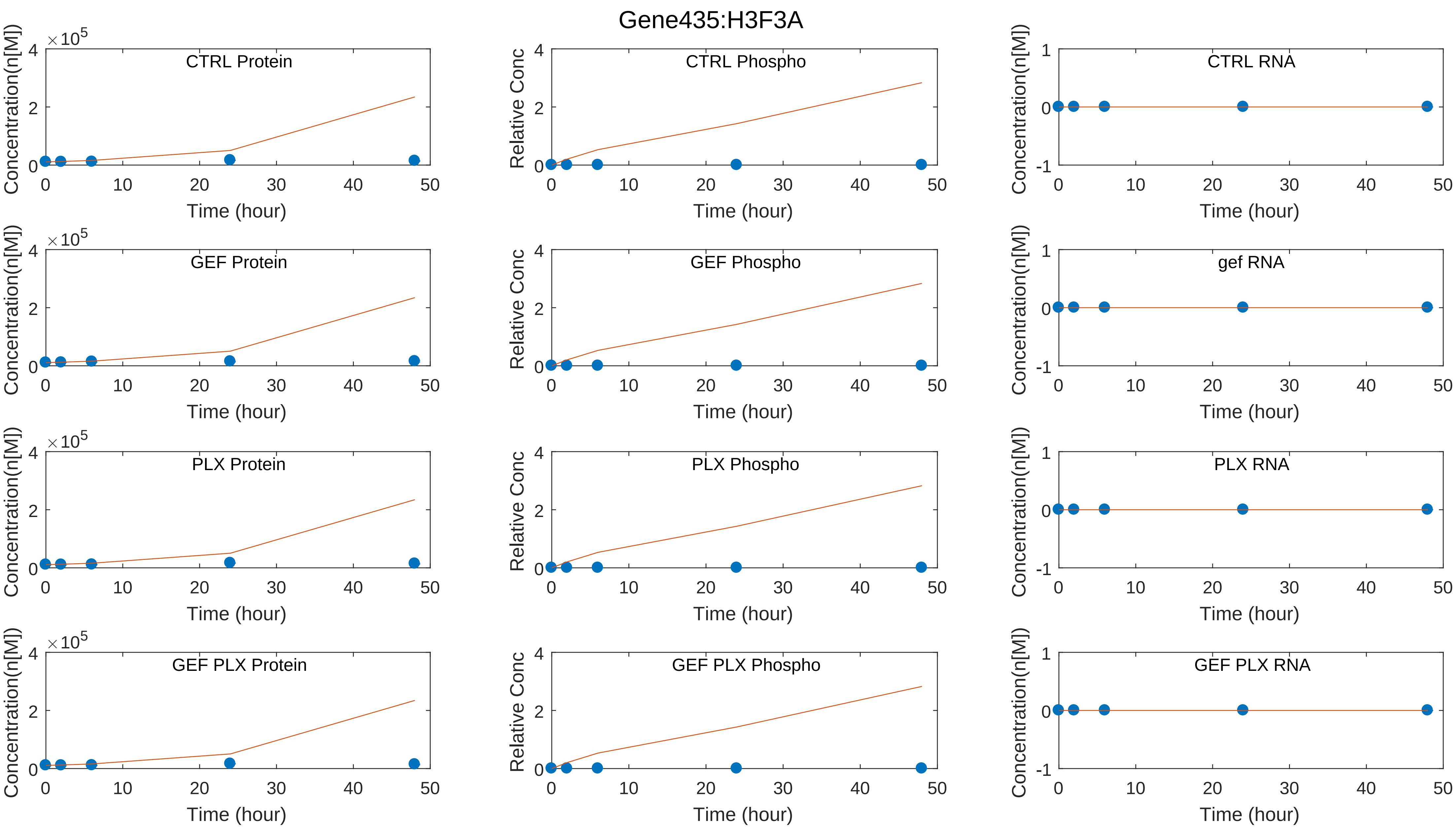

Gene435:H3F3A
CTRL Protein
CTRL Phospho
CTRL RNA
GEF Protein
GEF Phospho
gef RNA
PLX Protein
PLX Phospho
PLX RNA
GEF PLX Protein
GEF PLX Phospho
GEF PLX RNA
Concentration(n[M])
Relative Conc
Time (hour)
×10^5

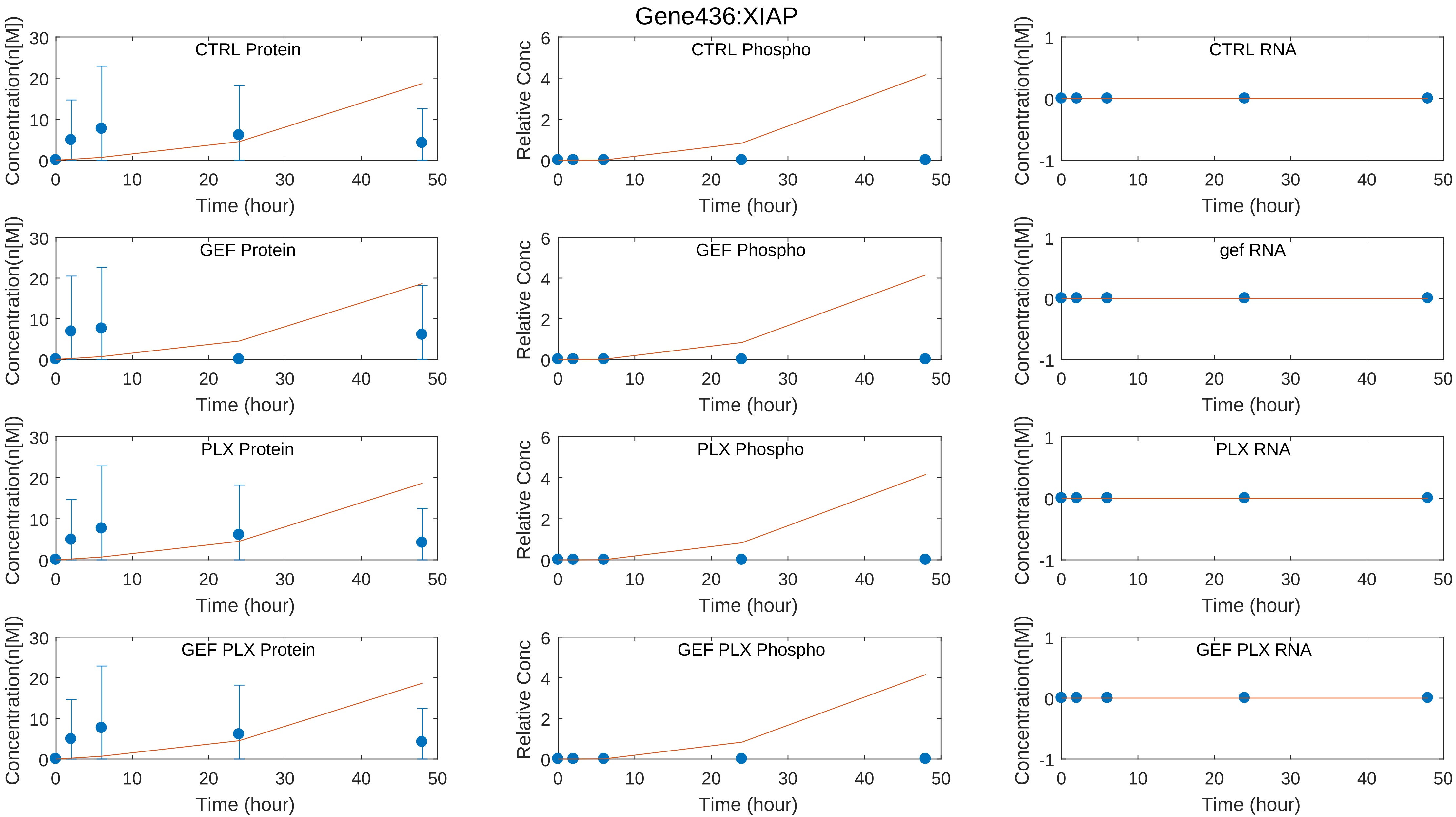

Gene436:XIAP
CTRL Protein
CTRL Phospho
CTRL RNA
GEF Protein
GEF Phospho
gef RNA
PLX Protein
PLX Phospho
PLX RNA
GEF PLX Protein
GEF PLX Phospho
GEF PLX RNA
Concentration(n[M])
Relative Conc
Time (hour)

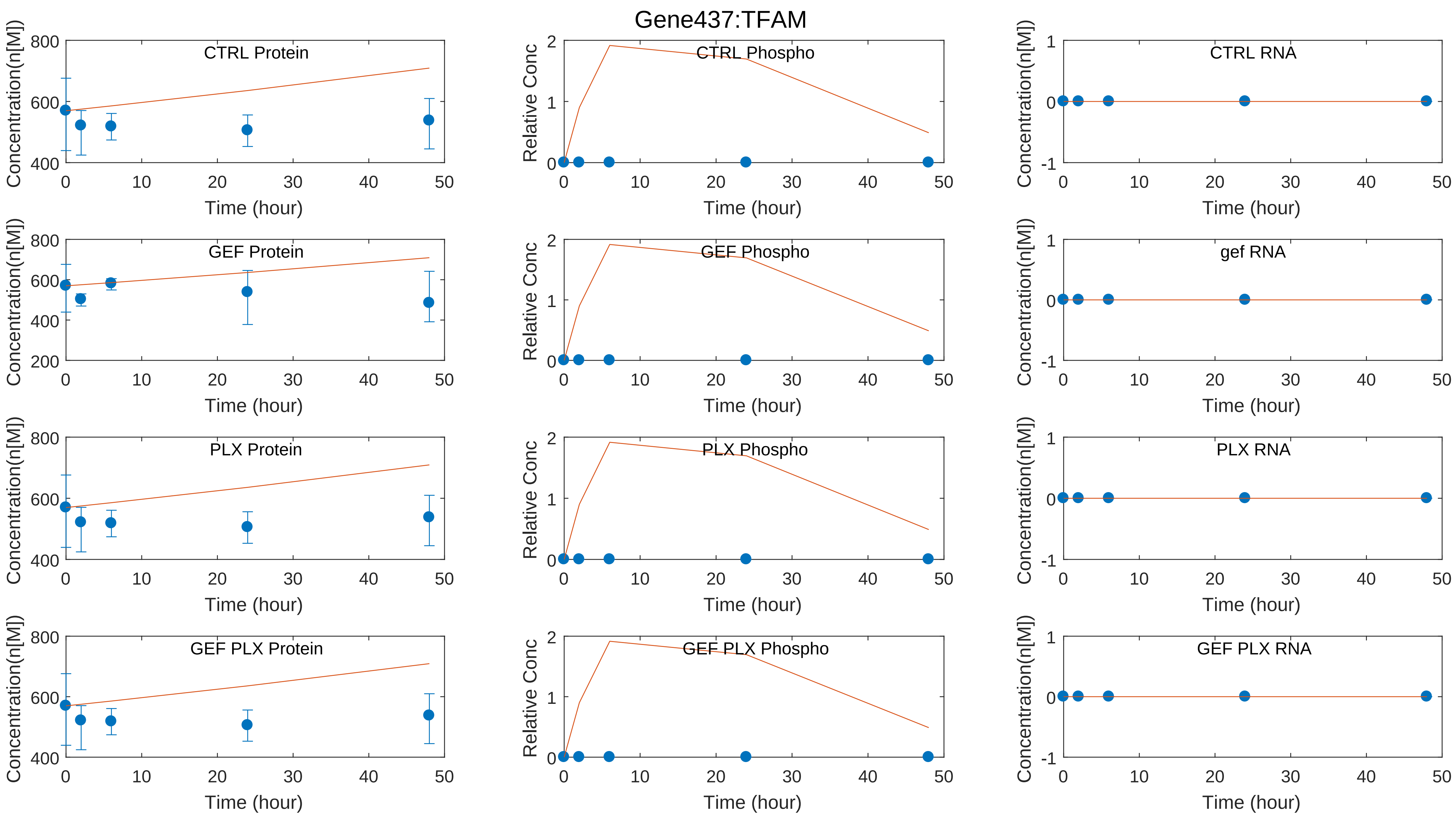
Gene437:TFAM
CTRL Protein
GEF Protein
PLX Protein
GEF PLX Protein
CTRL Phospho
GEF Phospho
PLX Phospho
GEF PLX Phospho
CTRL RNA
gef RNA
PLX RNA
GEF PLX RNA
Concentration(n[M])
Relative Conc
Time (hour)

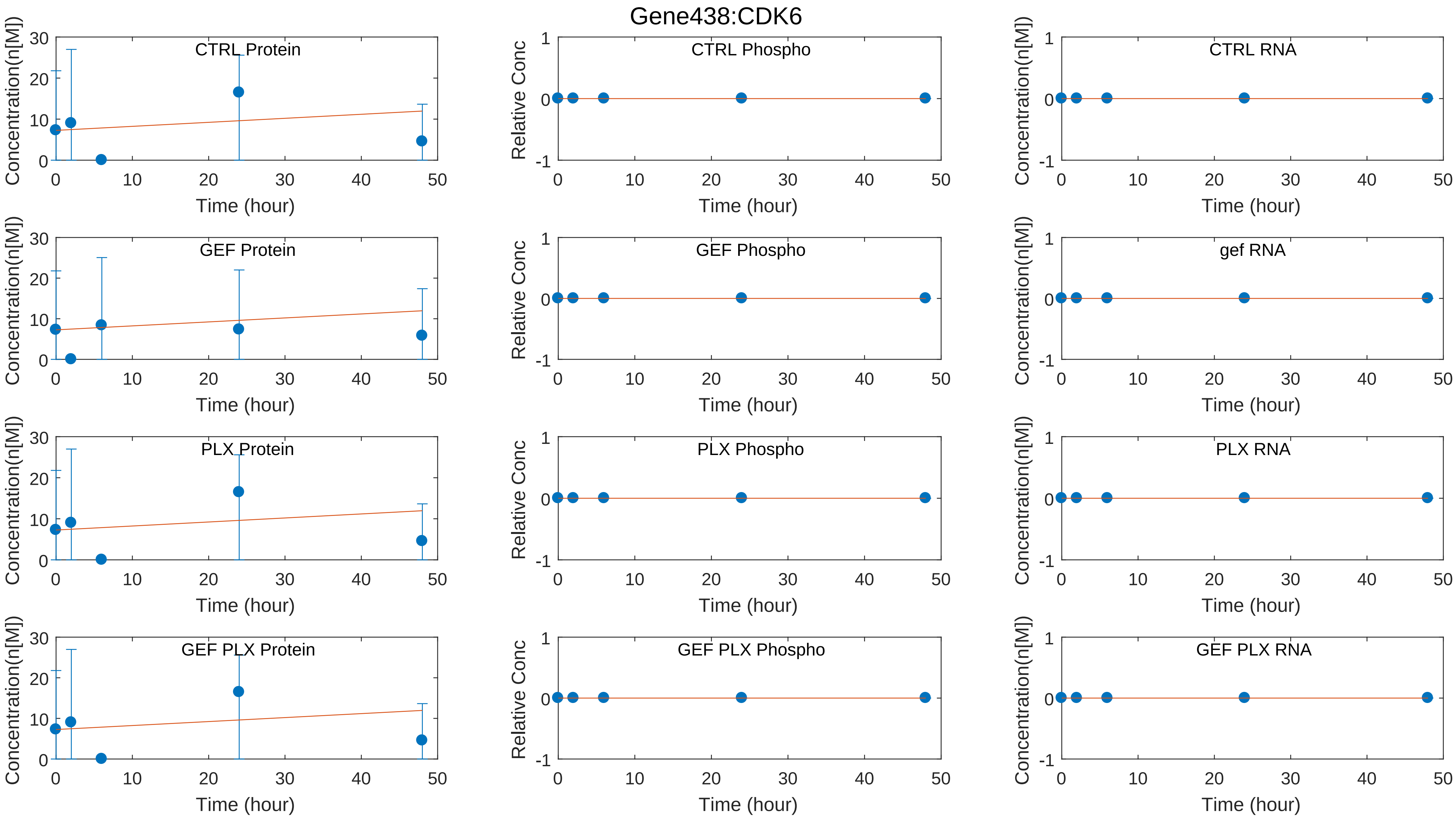
Gene438:CDK6
CTRL Protein
CTRL Phospho
CTRL RNA
GEF Protein
GEF Phospho
gef RNA
PLX Protein
PLX Phospho
PLX RNA
GEF PLX Protein
GEF PLX Phospho
GEF PLX RNA
Concentration(n[M])
Relative Conc
Time (hour)

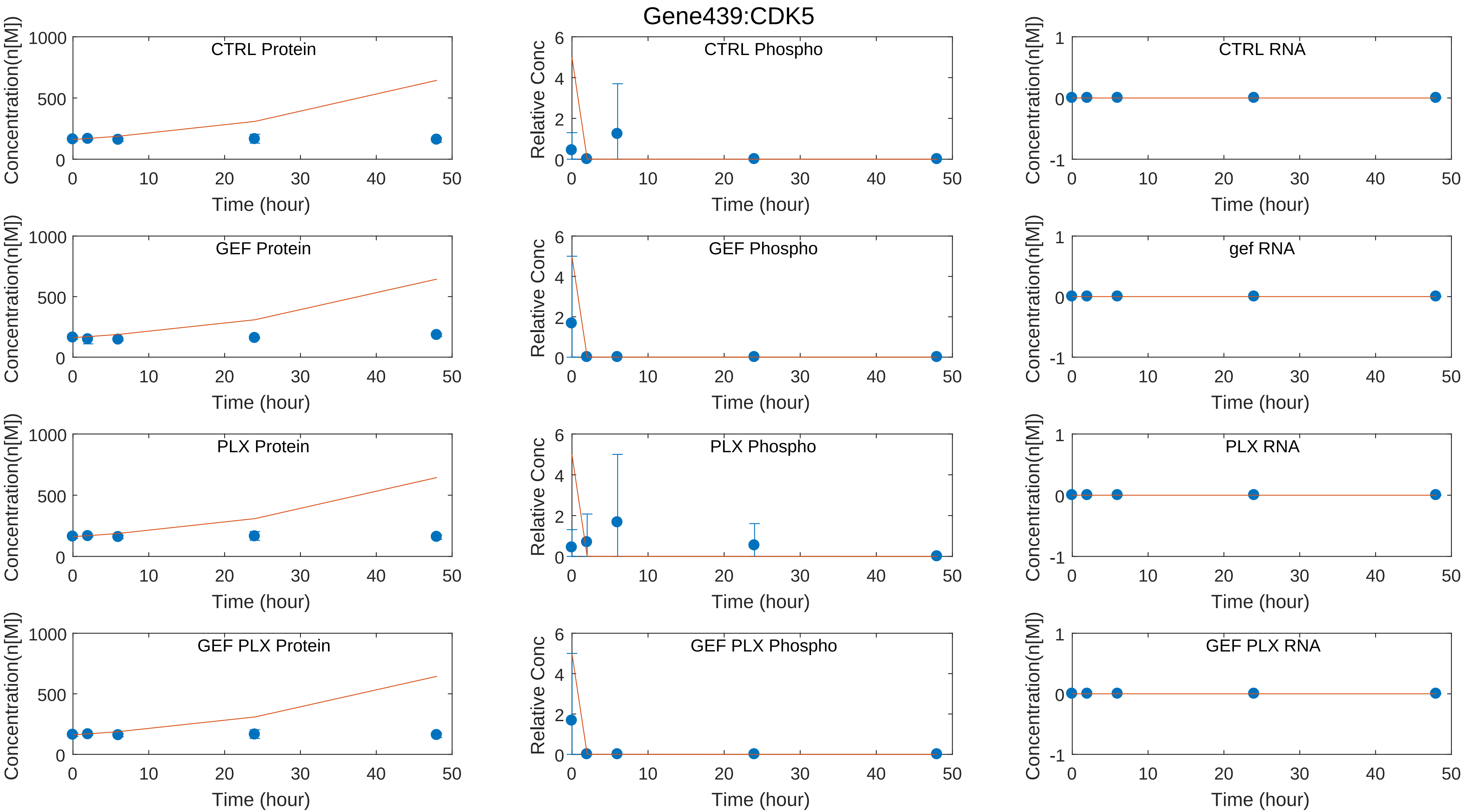

Gene439:CDK5
CTRL Protein
GEF Protein
PLX Protein
GEF PLX Protein
CTRL Phospho
GEF Phospho
PLX Phospho
GEF PLX Phospho
CTRL RNA
gef RNA
PLX RNA
GEF PLX RNA
Concentration(n[M])
Relative Conc
Time (hour)
1000
500
0
6
4
2
1
-1
10
20
30
40
50

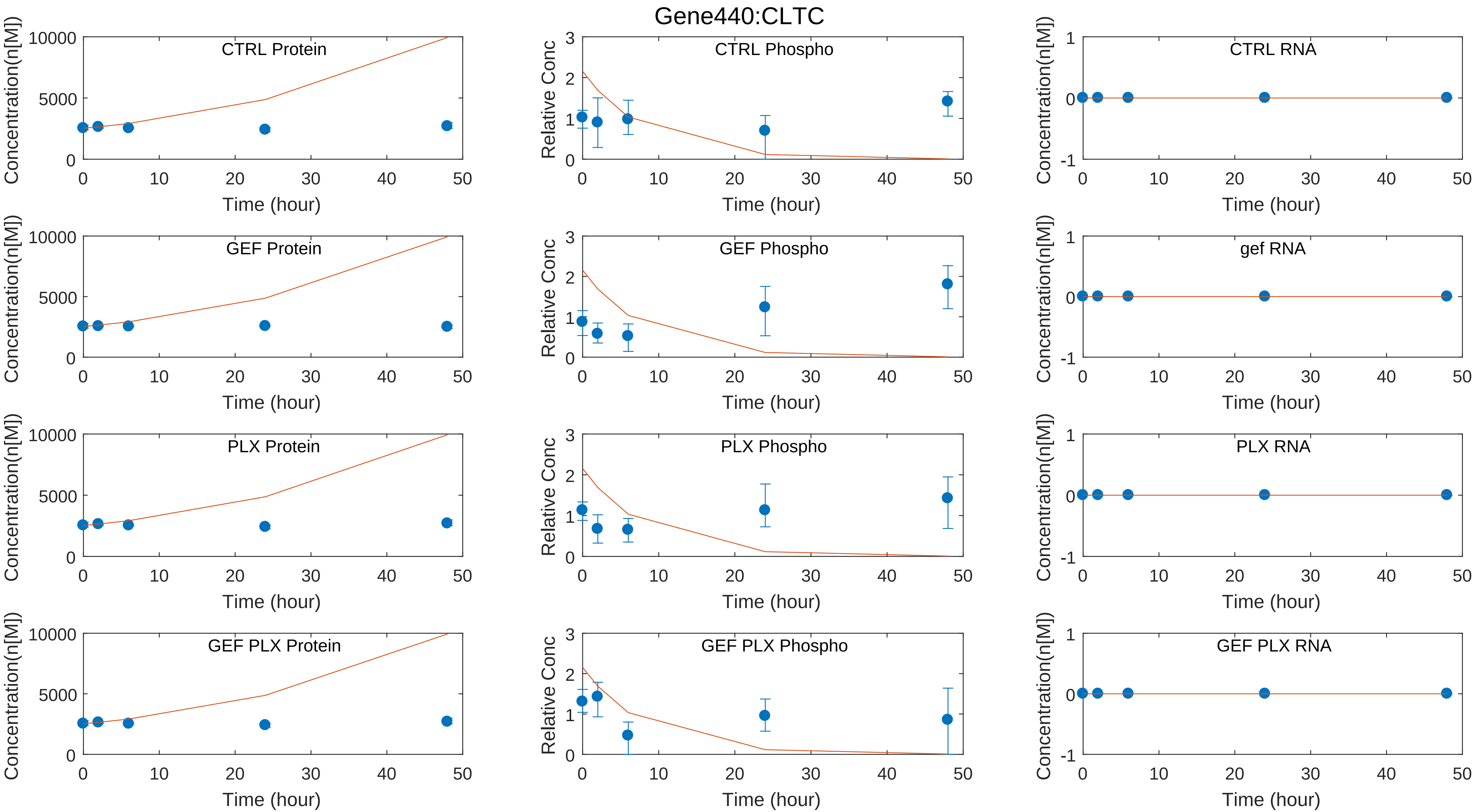
Gene440:CLTC
CTRL Protein
GEF Protein
PLX Protein
GEF PLX Protein
CTRL Phospho
GEF Phospho
PLX Phospho
GEF PLX Phospho
CTRL RNA
gef RNA
PLX RNA
GEF PLX RNA
Concentration(n[M])
Relative Conc
Time (hour)

Gene441:HSF1

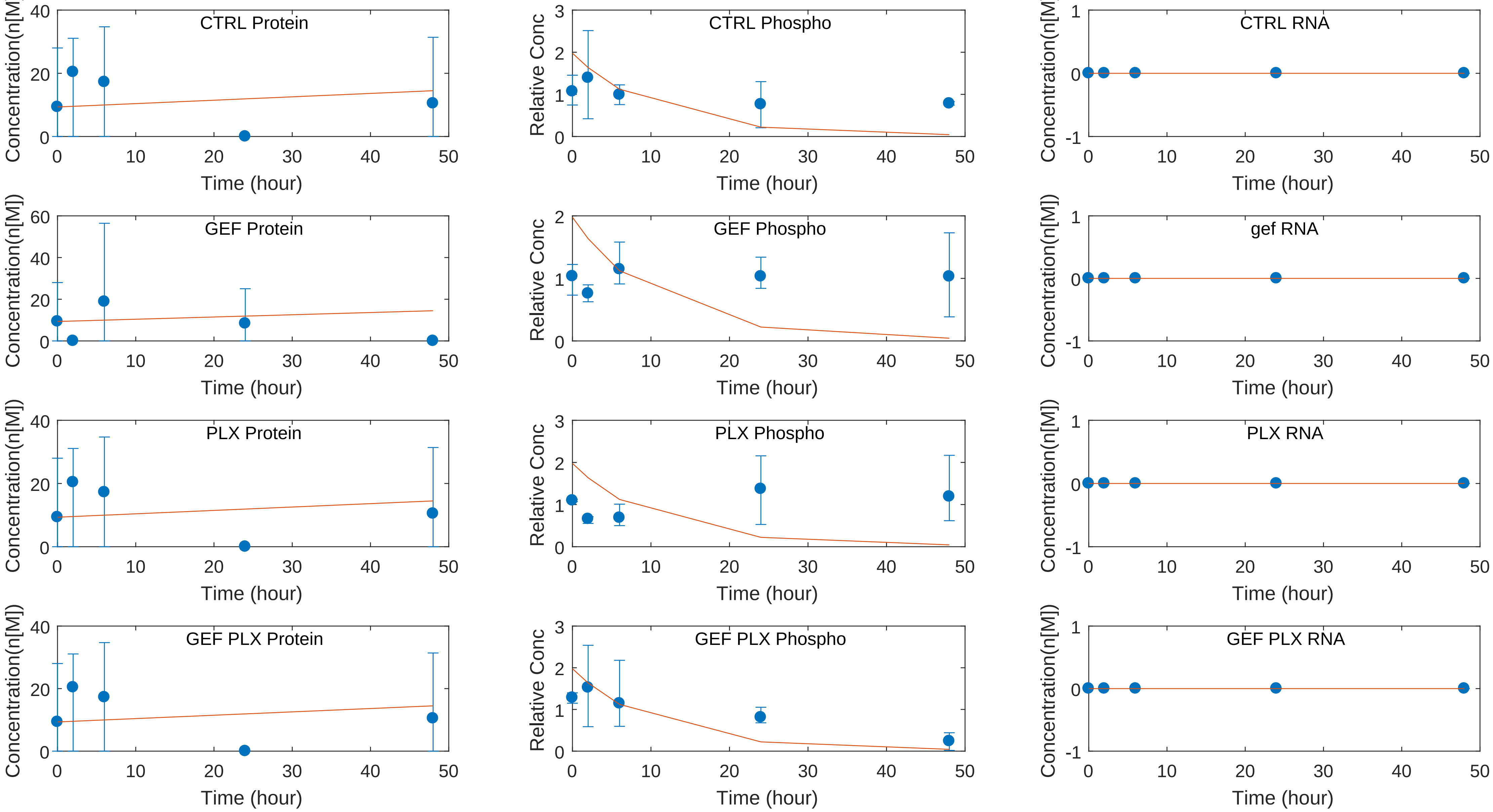

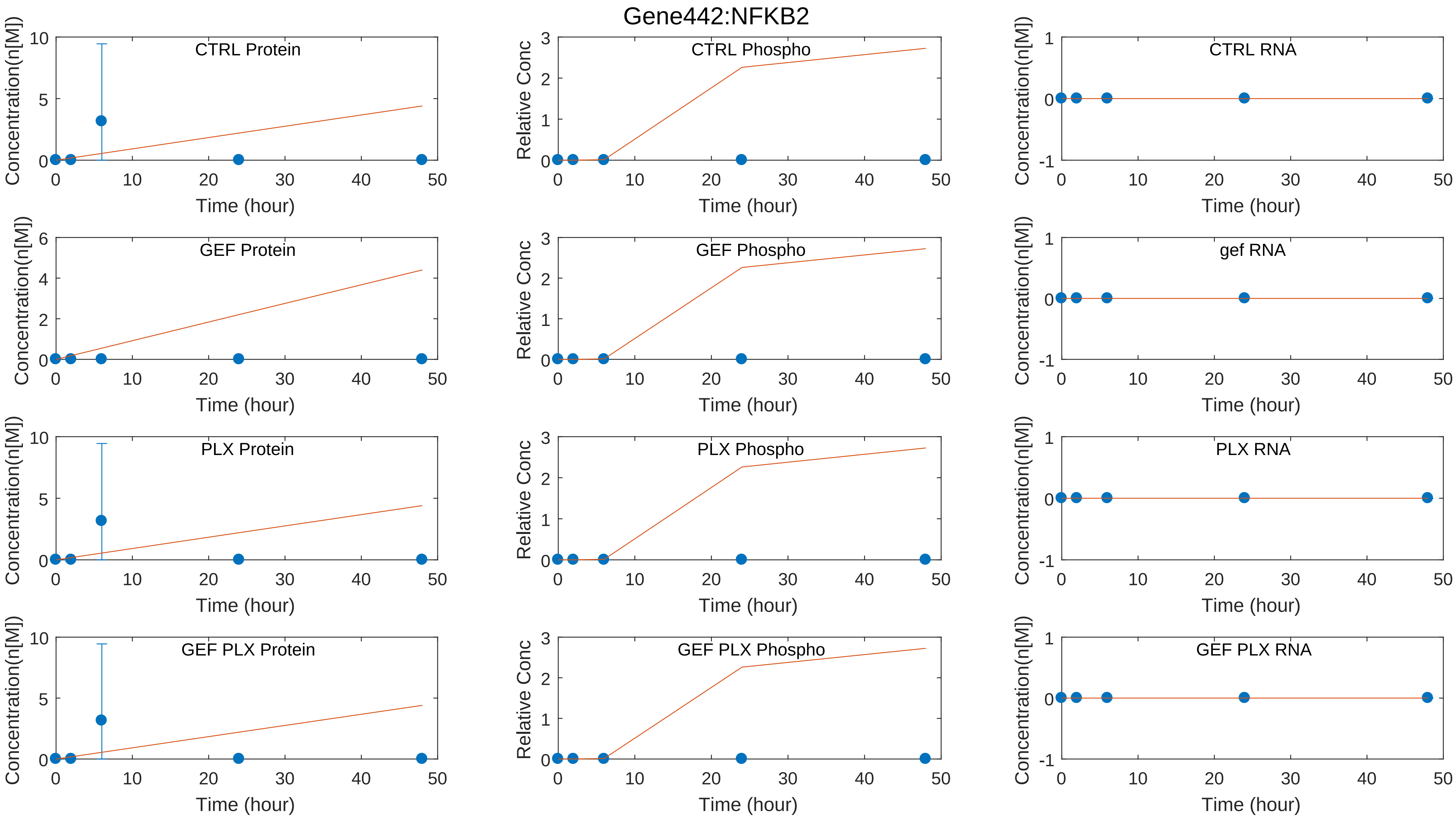
Gene442:NFKB2
CTRL Protein
CTRL Phospho
CTRL RNA
GEF Protein
GEF Phospho
gef RNA
PLX Protein
PLX Phospho
PLX RNA
GEF PLX Protein
GEF PLX Phospho
GEF PLX RNA
Concentration(n[M])
Relative Conc
Time (hour)

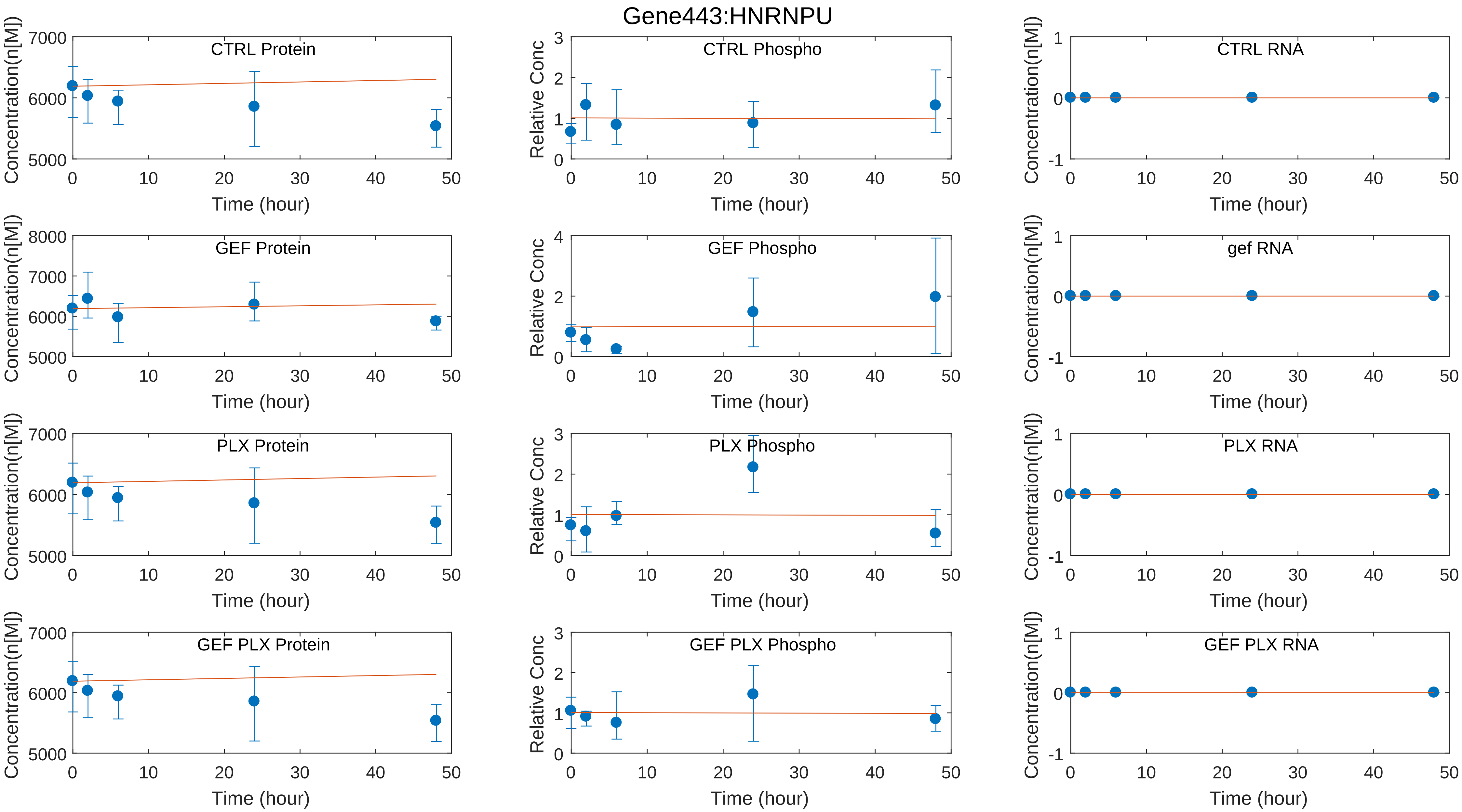
Gene443:HNRNPU
CTRL Protein
CTRL Phospho
CTRL RNA
GEF Protein
GEF Phospho
gef RNA
PLX Protein
PLX Phospho
PLX RNA
GEF PLX Protein
GEF PLX Phospho
GEF PLX RNA
Concentration(n[M])
Relative Conc
Time (hour)

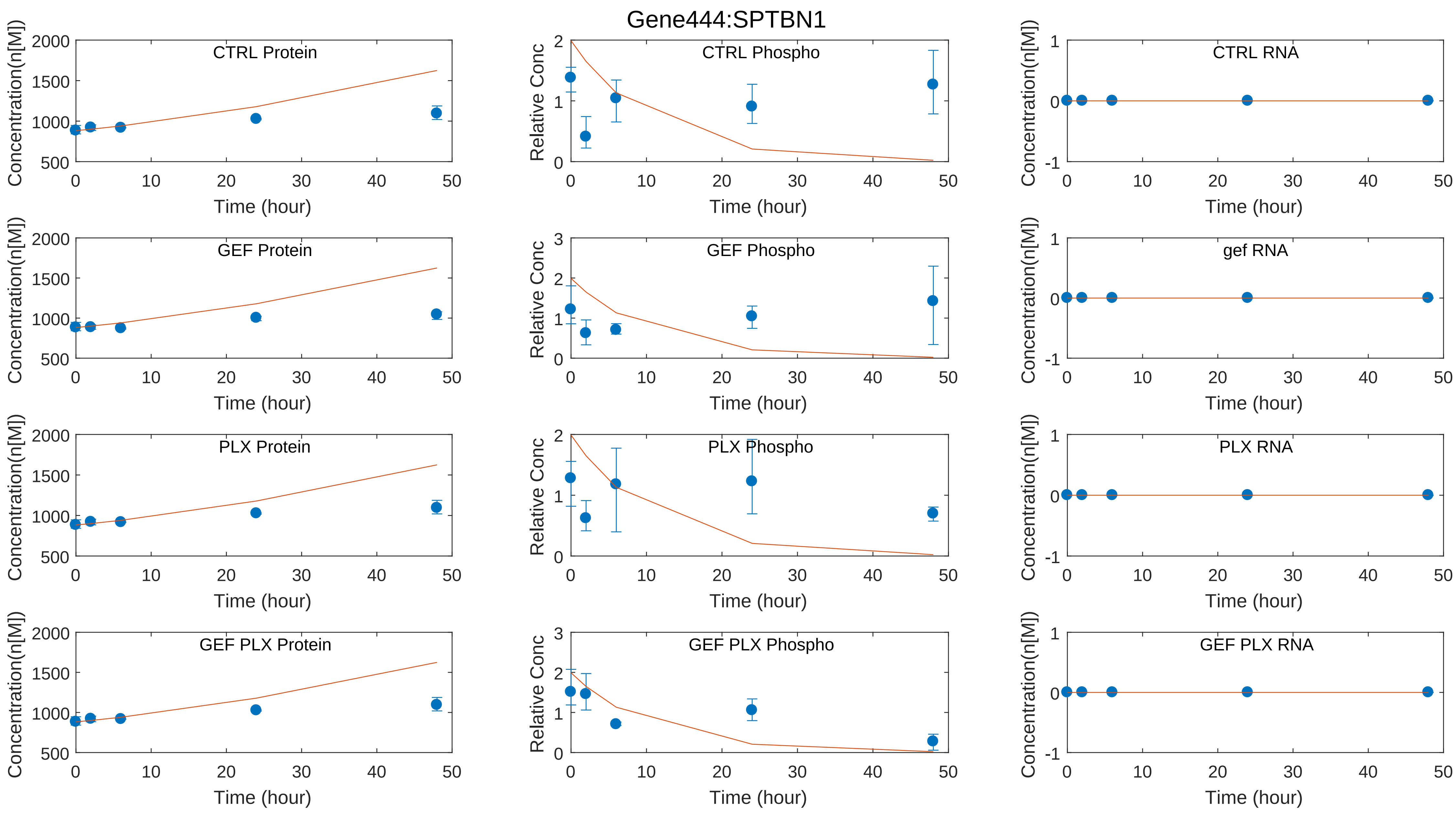
Gene444:SPTBN1
CTRL Protein
GEF Protein
PLX Protein
GEF PLX Protein
CTRL Phospho
GEF Phospho
PLX Phospho
GEF PLX Phospho
CTRL RNA
gef RNA
PLX RNA
GEF PLX RNA
Concentration(n[M])
Relative Conc
Time (hour)

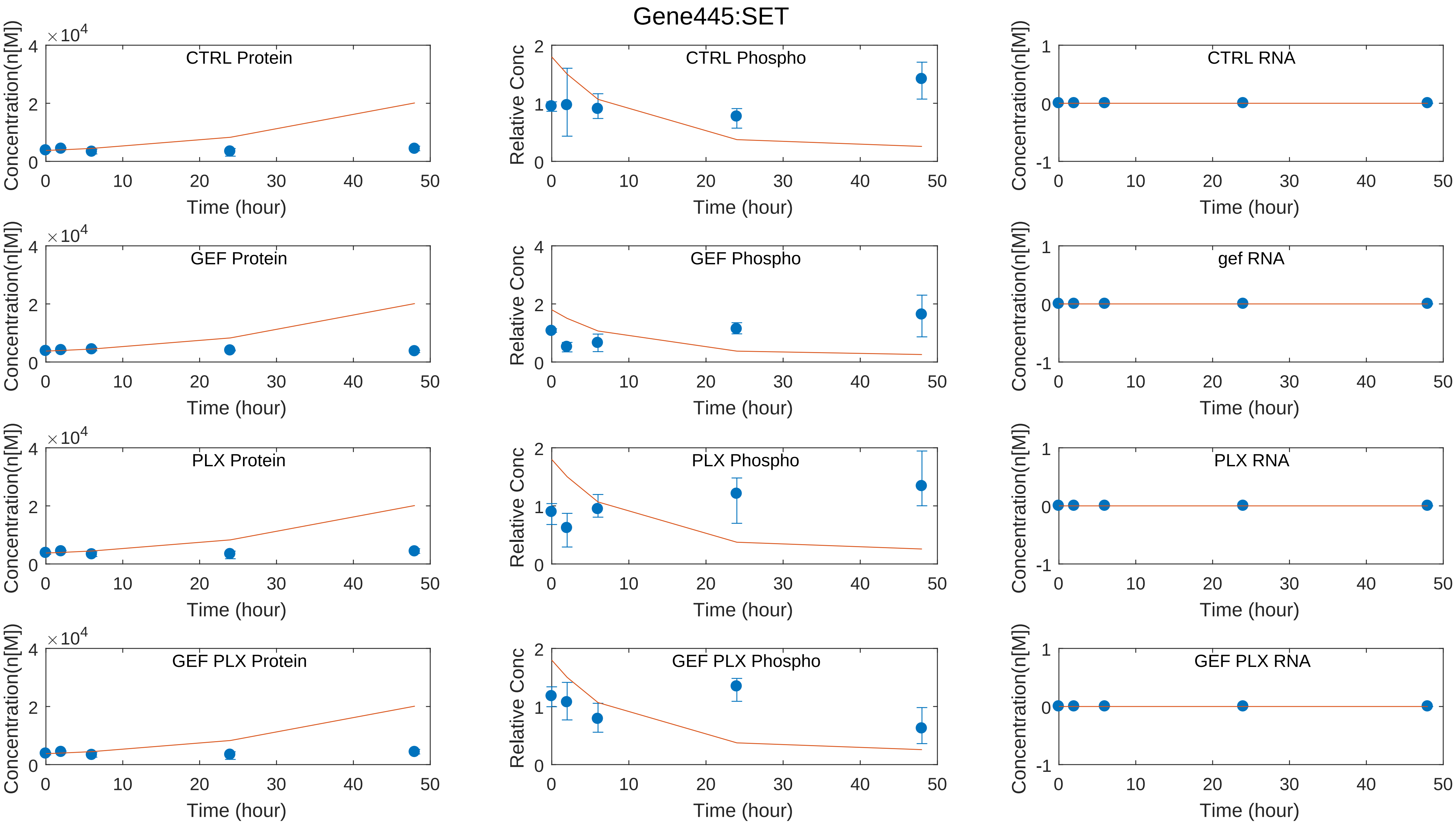

Gene445:SET
CTRL Protein
CTRL Phospho
CTRL RNA
GEF Protein
GEF Phospho
gef RNA
PLX Protein
PLX Phospho
PLX RNA
GEF PLX Protein
GEF PLX Phospho
GEF PLX RNA
Concentration(n[M])
Relative Conc
Time (hour)
×10^4

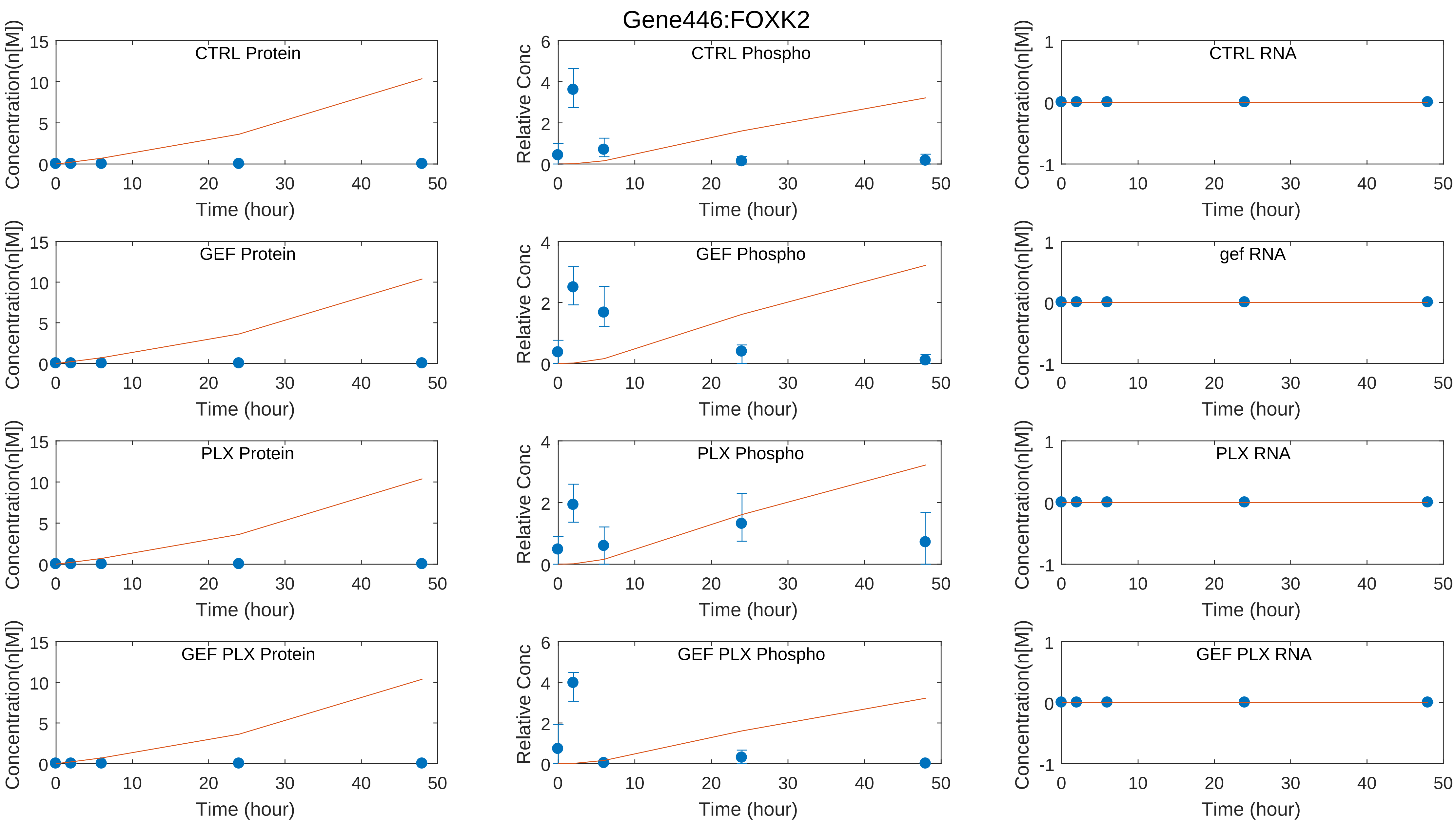

Gene446:FOXK2
CTRL Protein
CTRL Phospho
CTRL RNA
GEF Protein
GEF Phospho
gef RNA
PLX Protein
PLX Phospho
PLX RNA
GEF PLX Protein
GEF PLX Phospho
GEF PLX RNA
Concentration(n[M])
Relative Conc
Time (hour)

Gene447:EWSR1

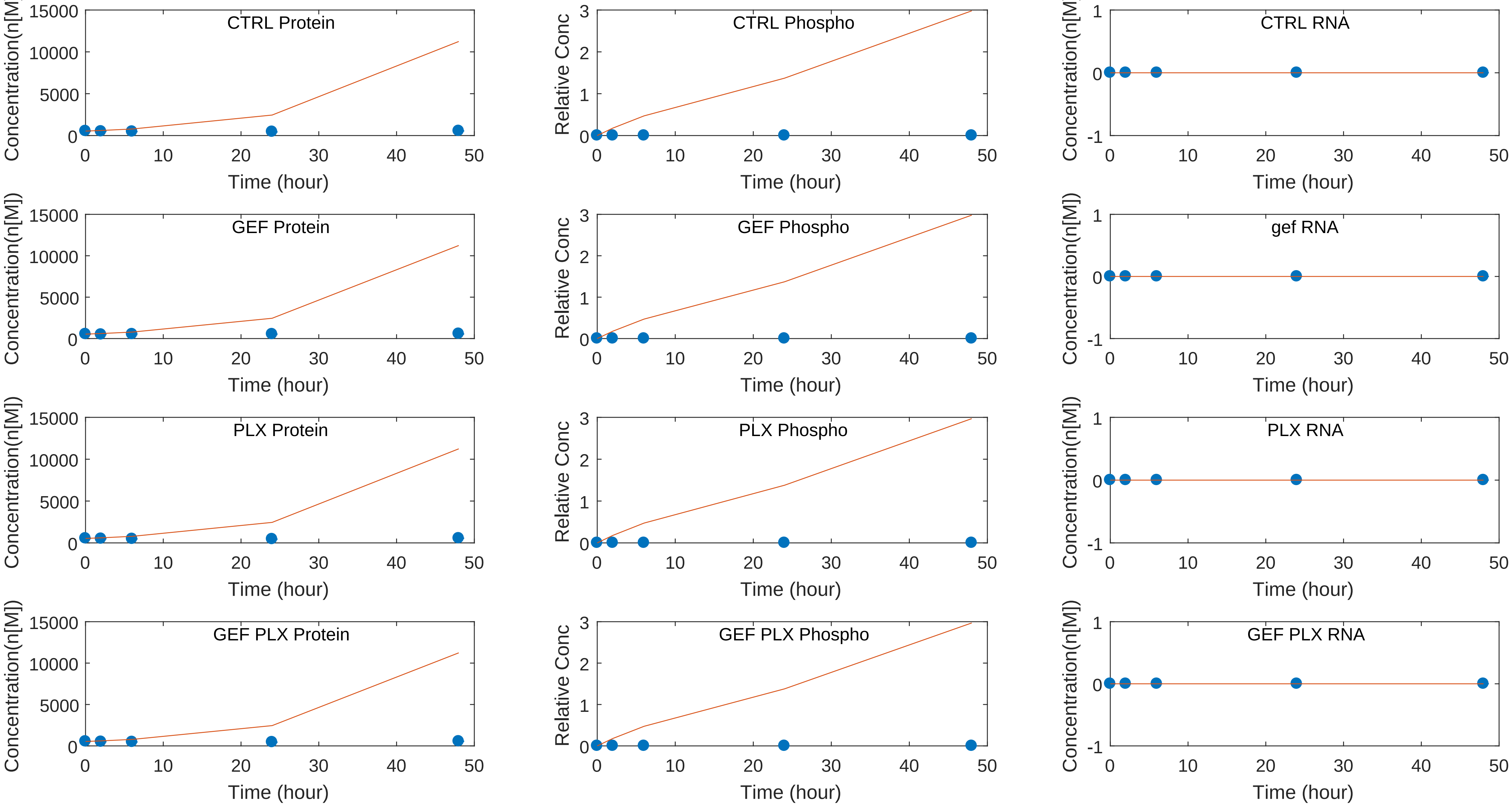

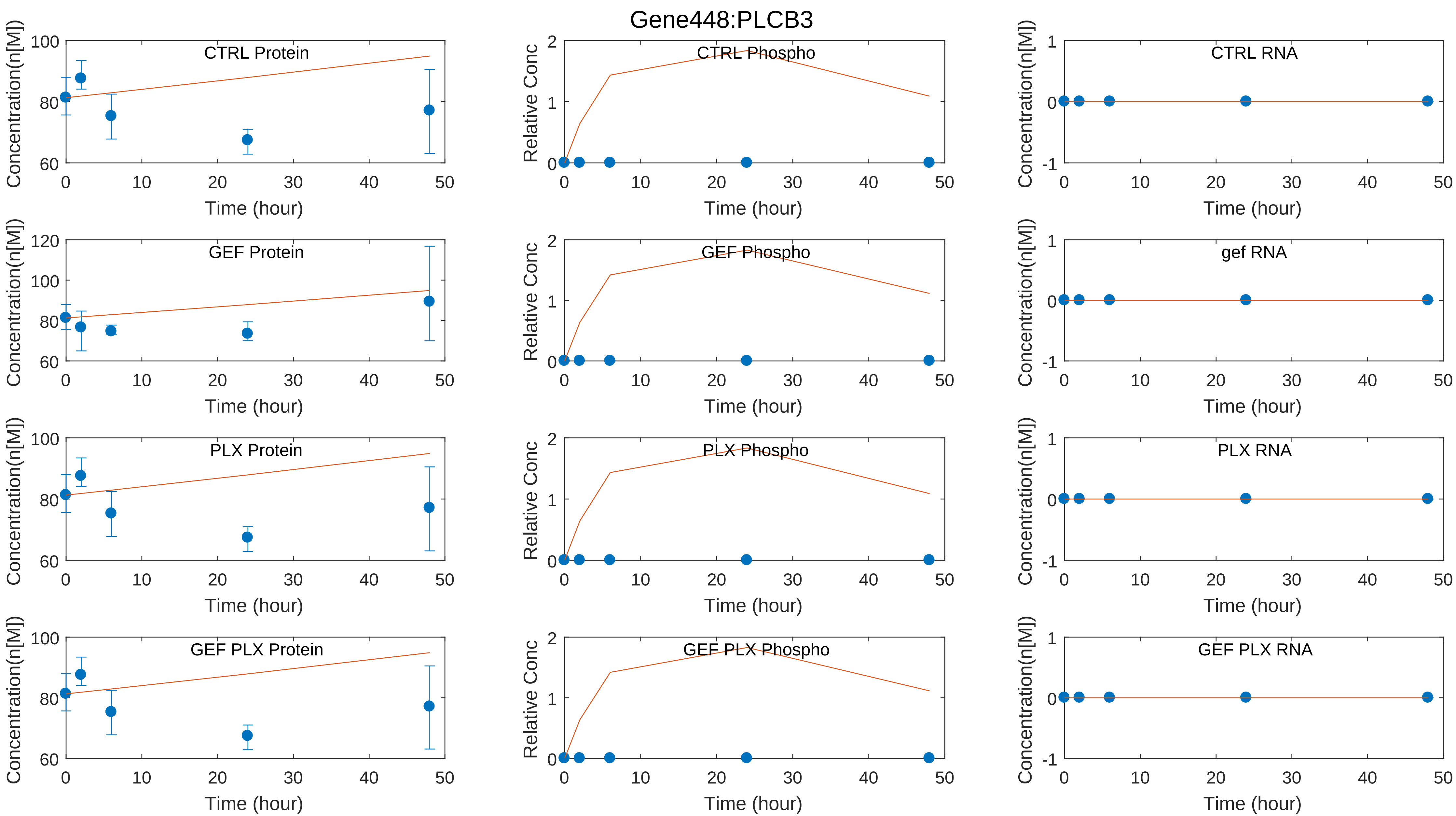

Gene448:PLCB3
CTRL Protein
CTRL Phospho
CTRL RNA
GEF Protein
GEF Phospho
gef RNA
PLX Protein
PLX Phospho
PLX RNA
GEF PLX Protein
GEF PLX Phospho
GEF PLX RNA
Concentration(n[M])
Relative Conc
Time (hour)

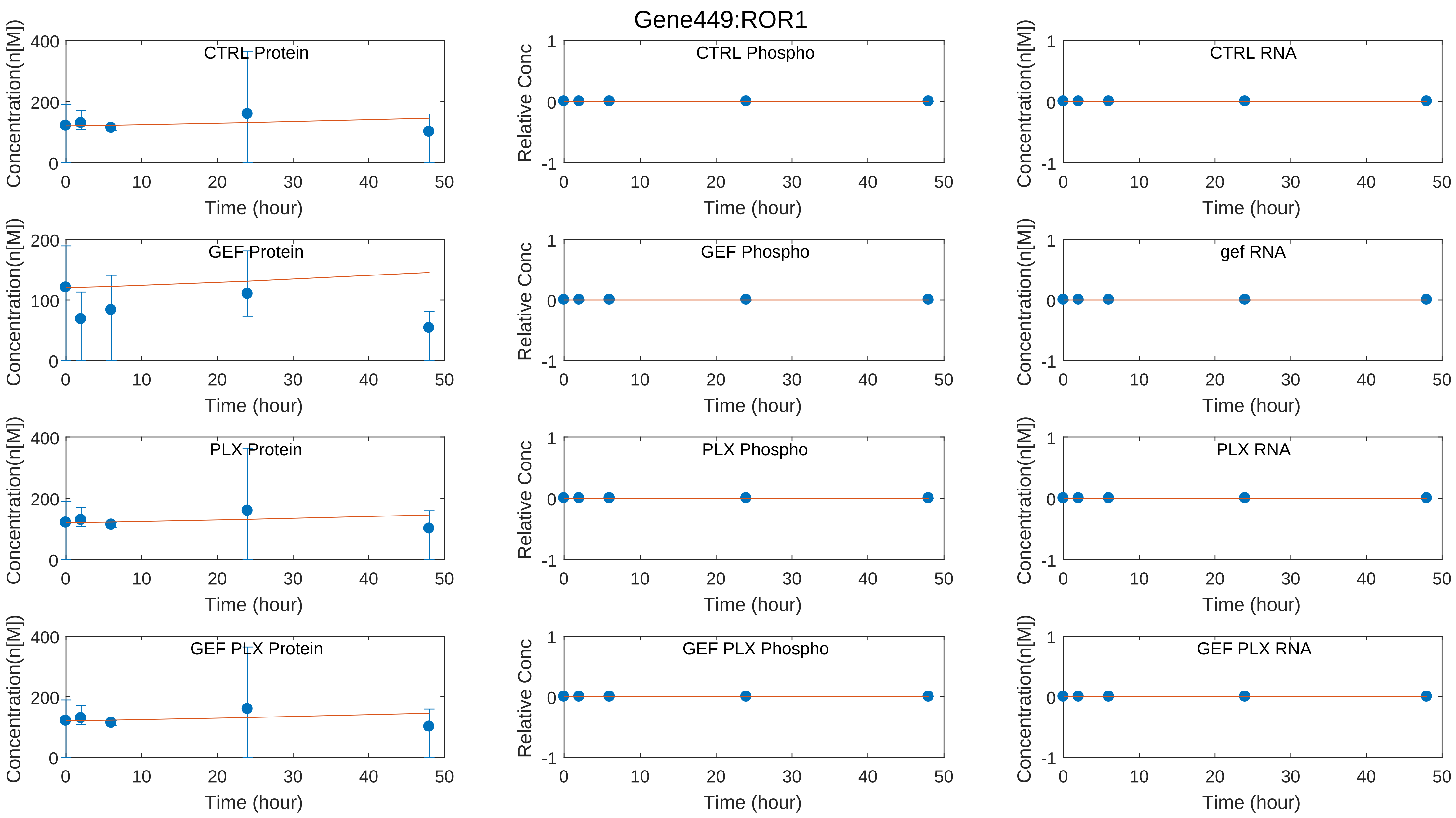

Gene449:ROR1
CTRL Protein
CTRL Phospho
CTRL RNA
GEF Protein
GEF Phospho
gef RNA
PLX Protein
PLX Phospho
PLX RNA
GEF PLX Protein
GEF PLX Phospho
GEF PLX RNA
Concentration(n[M])
Relative Conc
Time (hour)

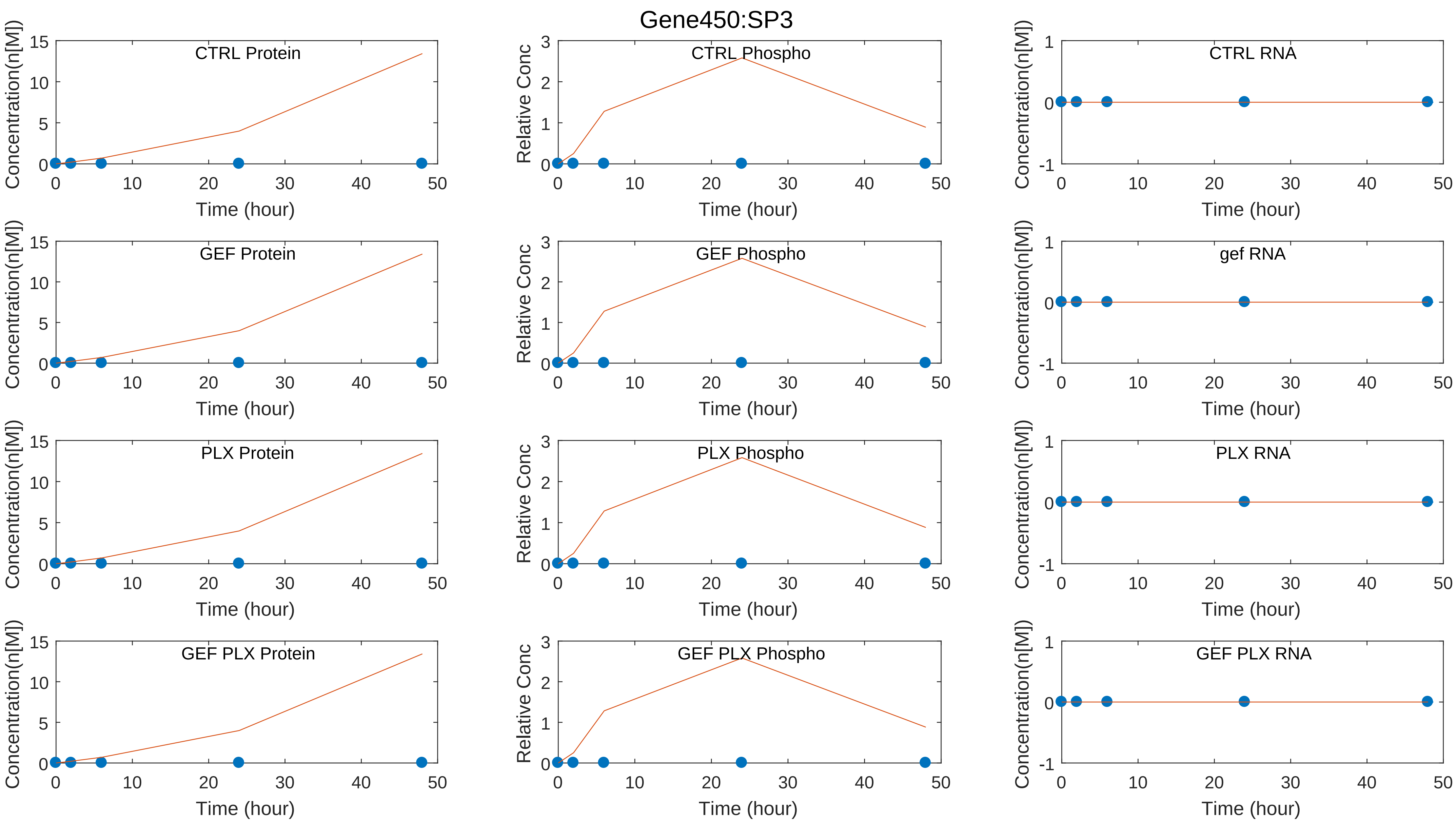
Gene450:SP3
CTRL Protein
CTRL Phospho
CTRL RNA
GEF Protein
GEF Phospho
gef RNA
PLX Protein
PLX Phospho
PLX RNA
GEF PLX Protein
GEF PLX Phospho
GEF PLX RNA
Concentration(n[M])
Relative Conc
Time (hour)

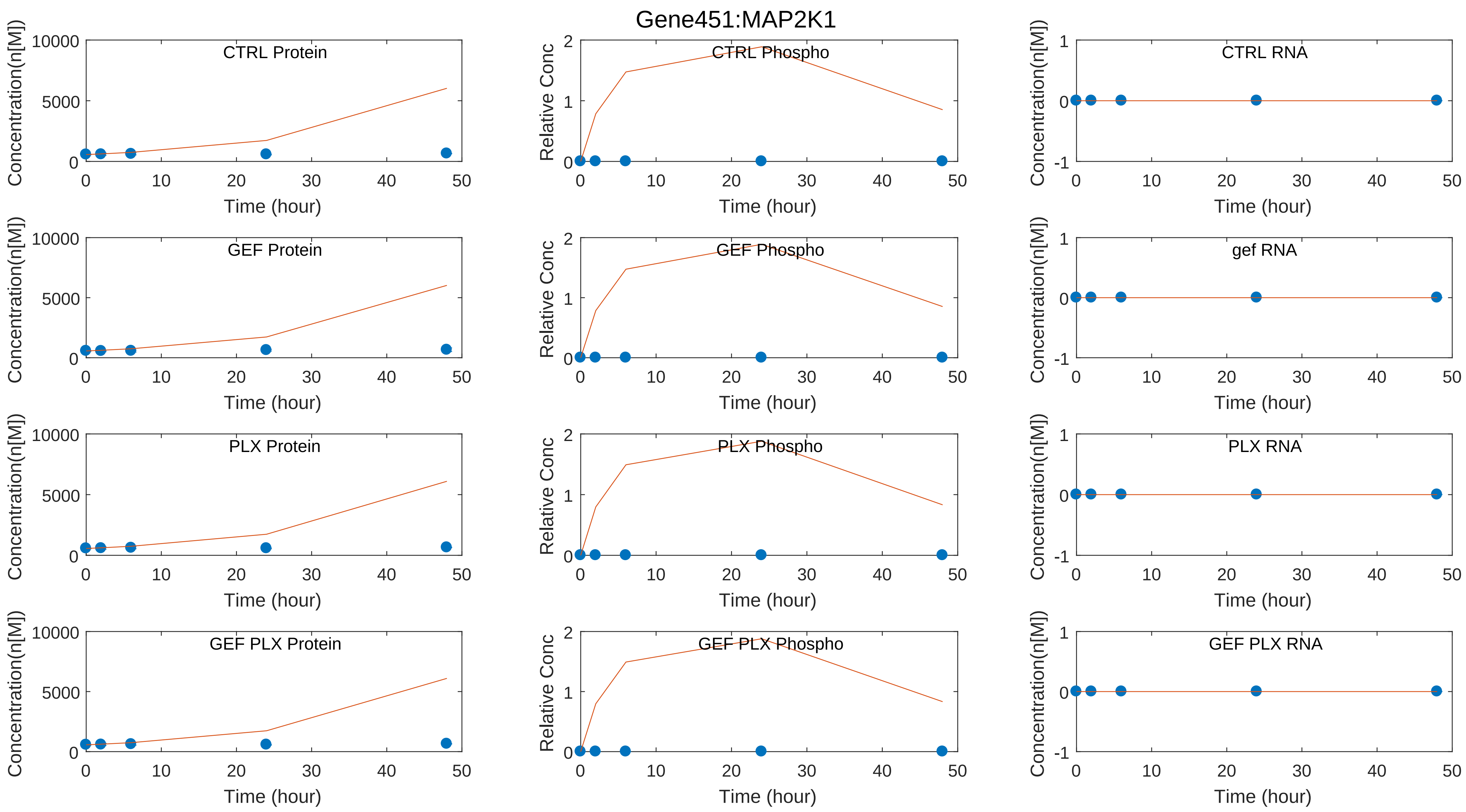

Gene451:MAP2K1
CTRL Protein
CTRL Phospho
CTRL RNA
GEF Protein
GEF Phospho
gef RNA
PLX Protein
PLX Phospho
PLX RNA
GEF PLX Protein
GEF PLX Phospho
GEF PLX RNA
Concentration(n[M])
Relative Conc
Time (hour)

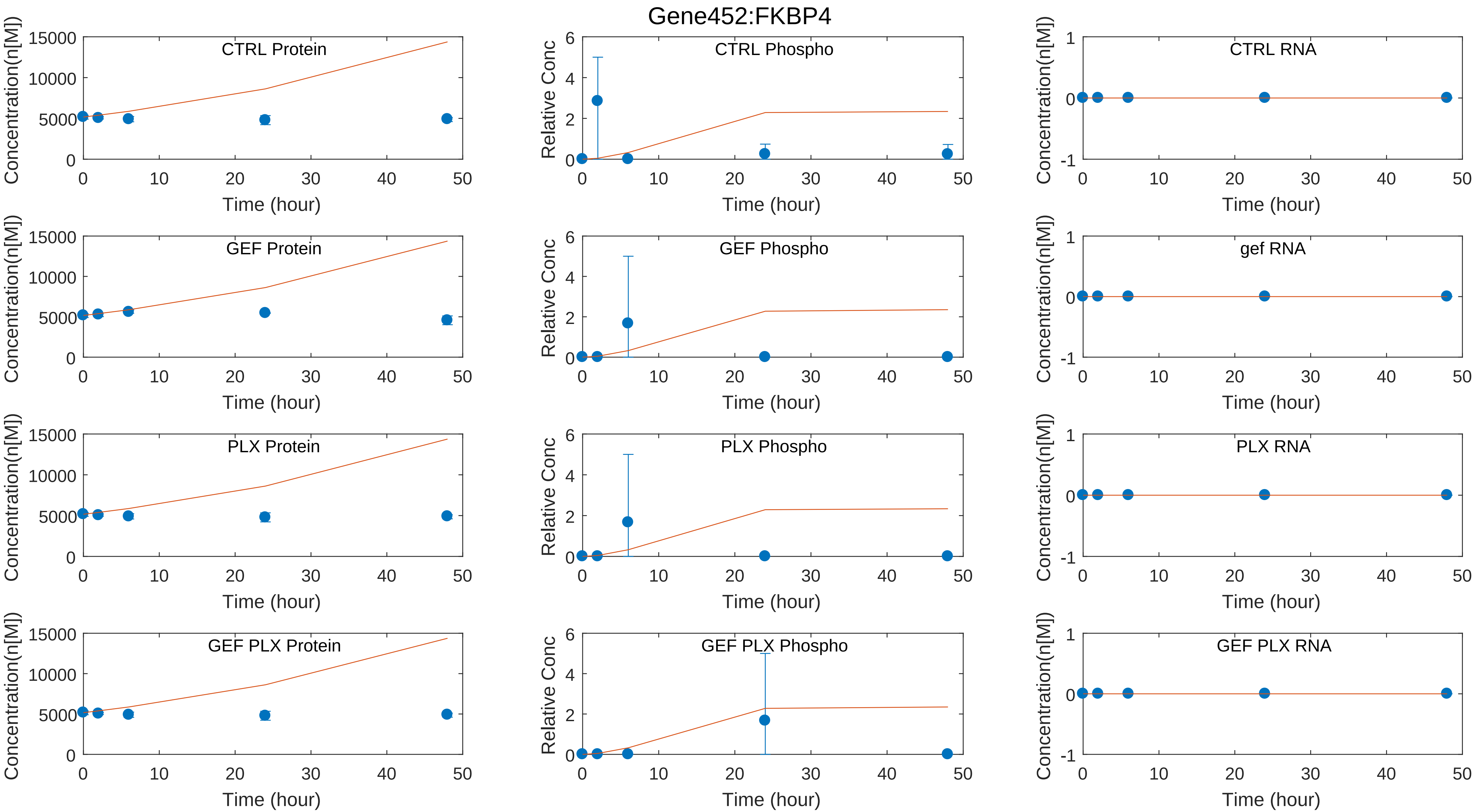

Gene452:FKBP4
CTRL Protein
GEF Protein
PLX Protein
GEF PLX Protein
CTRL Phospho
GEF Phospho
PLX Phospho
GEF PLX Phospho
CTRL RNA
gef RNA
PLX RNA
GEF PLX RNA
Concentration(n[M])
Relative Conc
Time (hour)

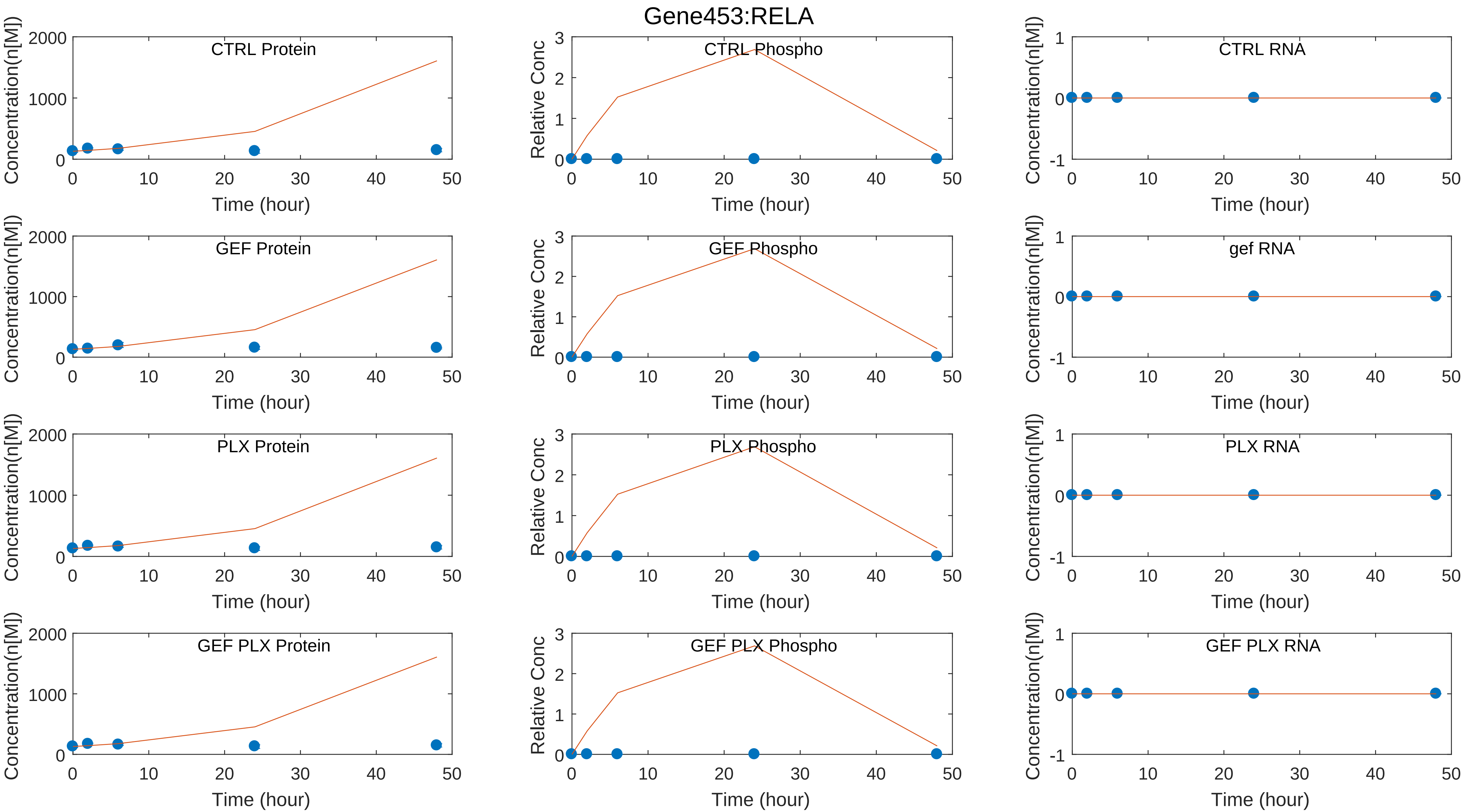

Gene453:RELA
CTRL Protein
CTRL Phospho
CTRL RNA
GEF Protein
GEF Phospho
gef RNA
PLX Protein
PLX Phospho
PLX RNA
GEF PLX Protein
GEF PLX Phospho
GEF PLX RNA
Concentration(n[M])
Relative Conc
Time (hour)

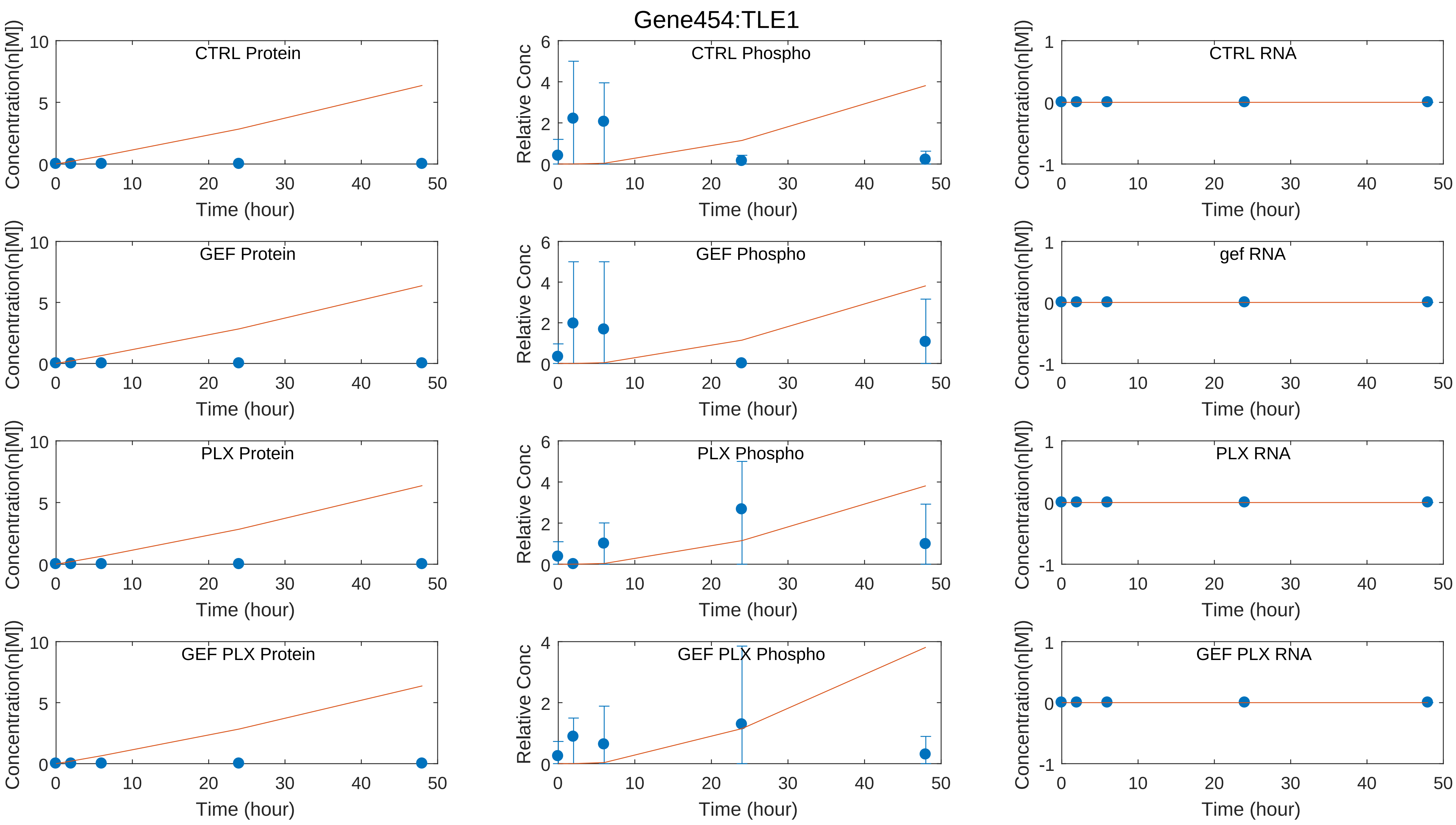

Gene454:TLE1
CTRL Protein
CTRL Phospho
CTRL RNA
GEF Protein
GEF Phospho
gef RNA
PLX Protein
PLX Phospho
PLX RNA
GEF PLX Protein
GEF PLX Phospho
GEF PLX RNA
Concentration(n[M])
Relative Conc
Time (hour)

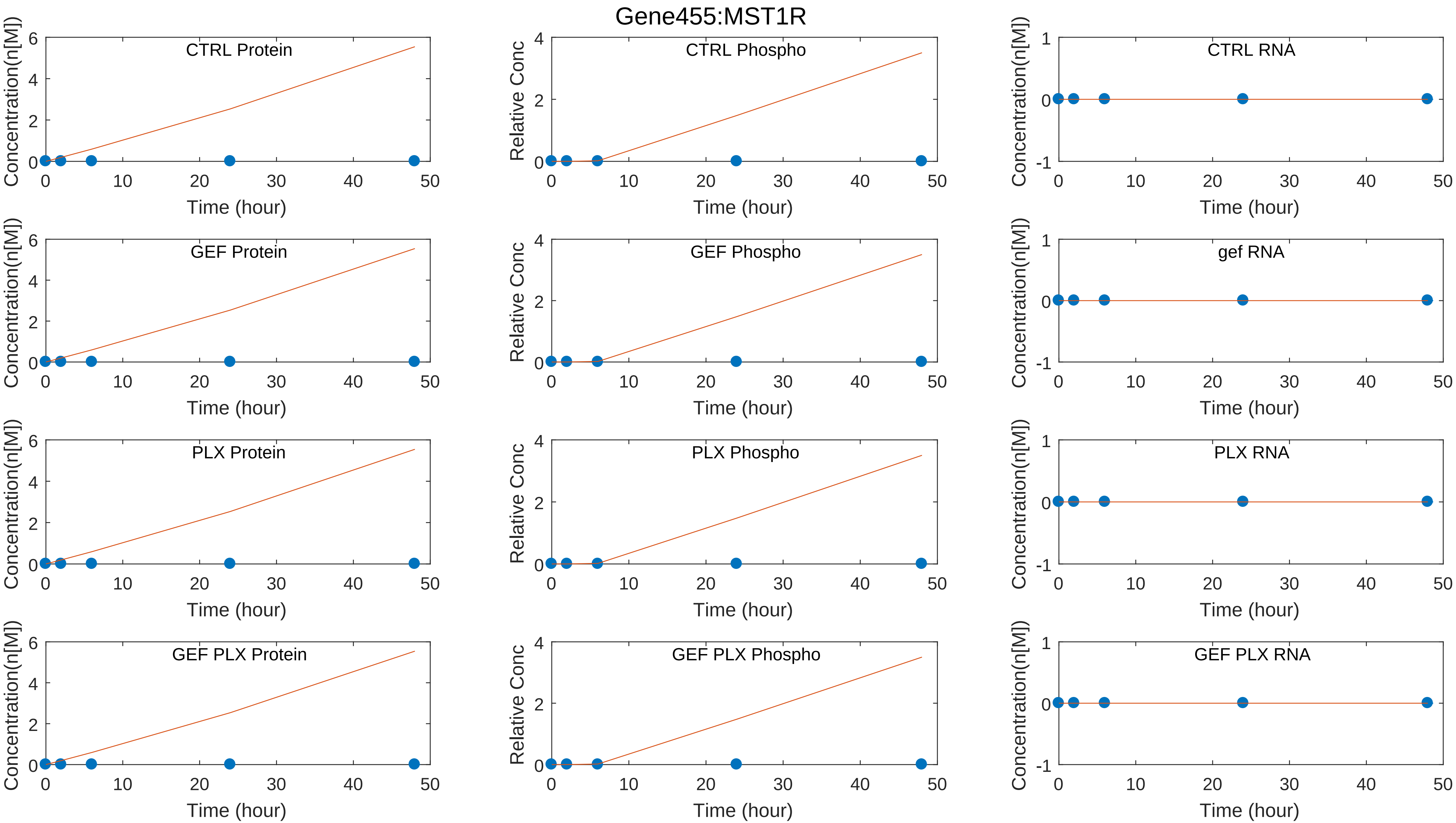

Gene455:MST1R
CTRL Protein
CTRL Phospho
CTRL RNA
GEF Protein
GEF Phospho
gef RNA
PLX Protein
PLX Phospho
PLX RNA
GEF PLX Protein
GEF PLX Phospho
GEF PLX RNA
Concentration(n[M])
Relative Conc
Time (hour)

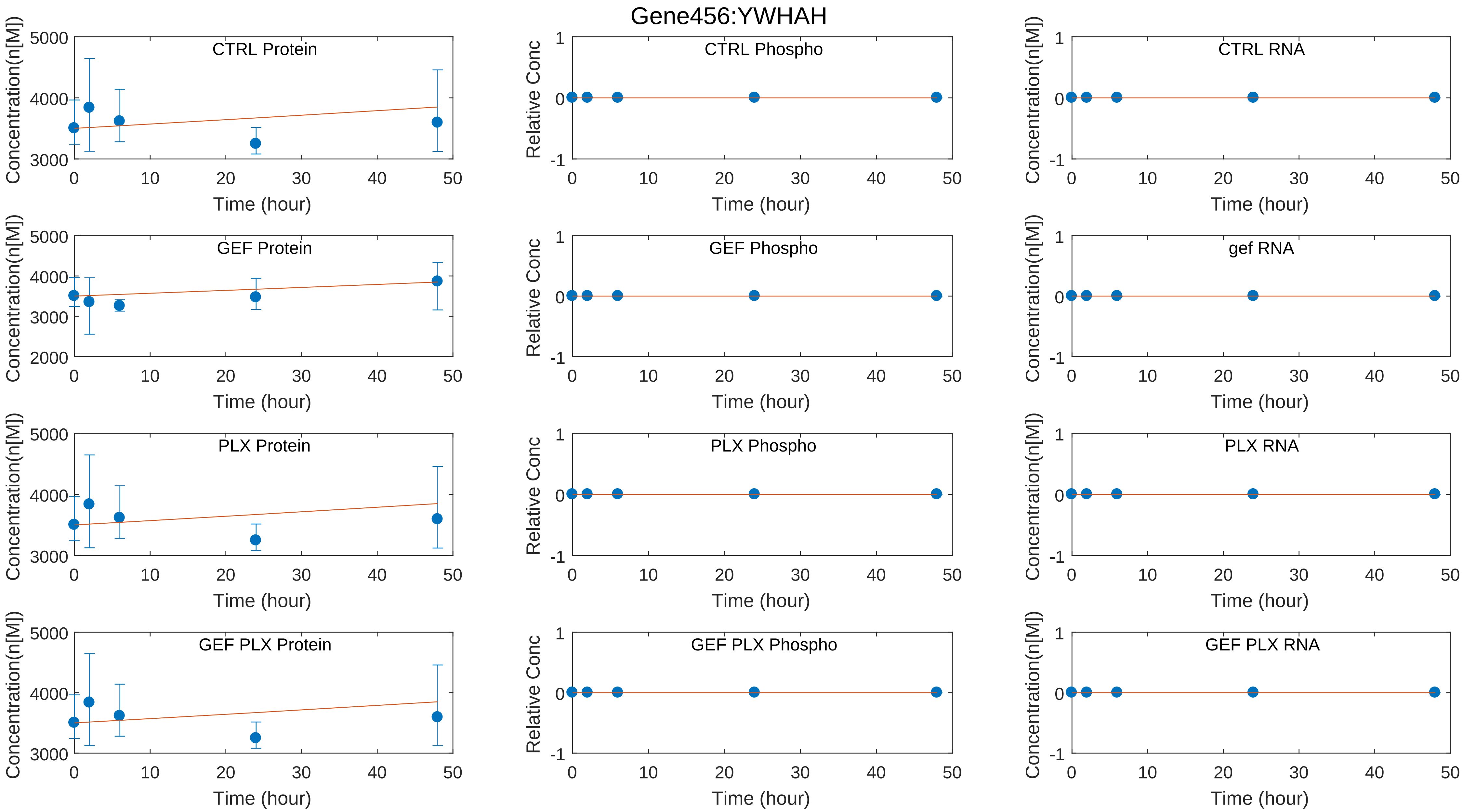

Gene456:YWHAH
CTRL Protein
CTRL Phospho
CTRL RNA
GEF Protein
GEF Phospho
gef RNA
PLX Protein
PLX Phospho
PLX RNA
GEF PLX Protein
GEF PLX Phospho
GEF PLX RNA
Concentration(n[M])
Relative Conc
Time (hour)

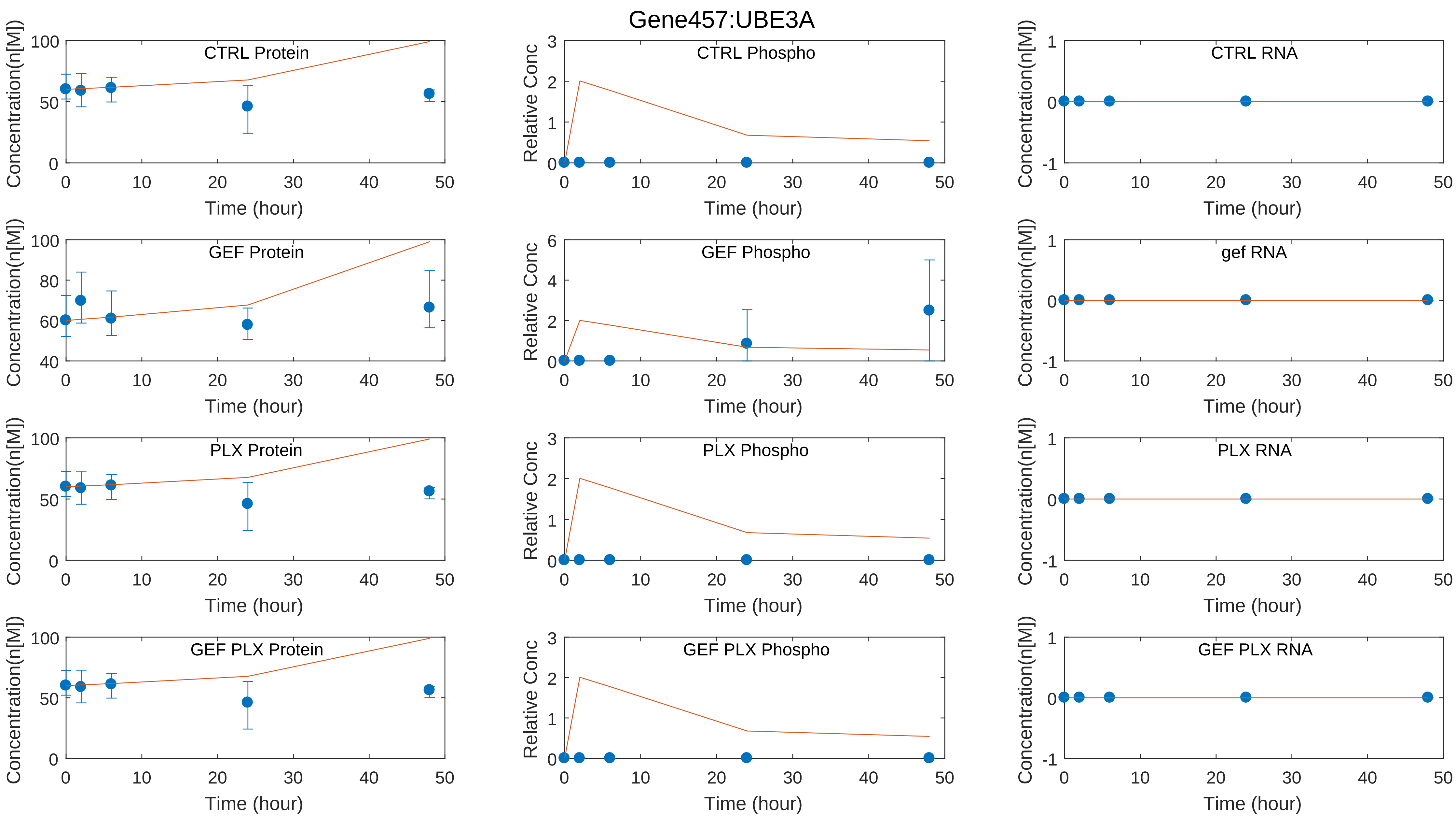
Gene457:UBE3A
CTRL Protein
CTRL Phospho
CTRL RNA
GEF Protein
GEF Phospho
gef RNA
PLX Protein
PLX Phospho
PLX RNA
GEF PLX Protein
GEF PLX Phospho
GEF PLX RNA
Concentration(n[M])
Relative Conc
Time (hour)

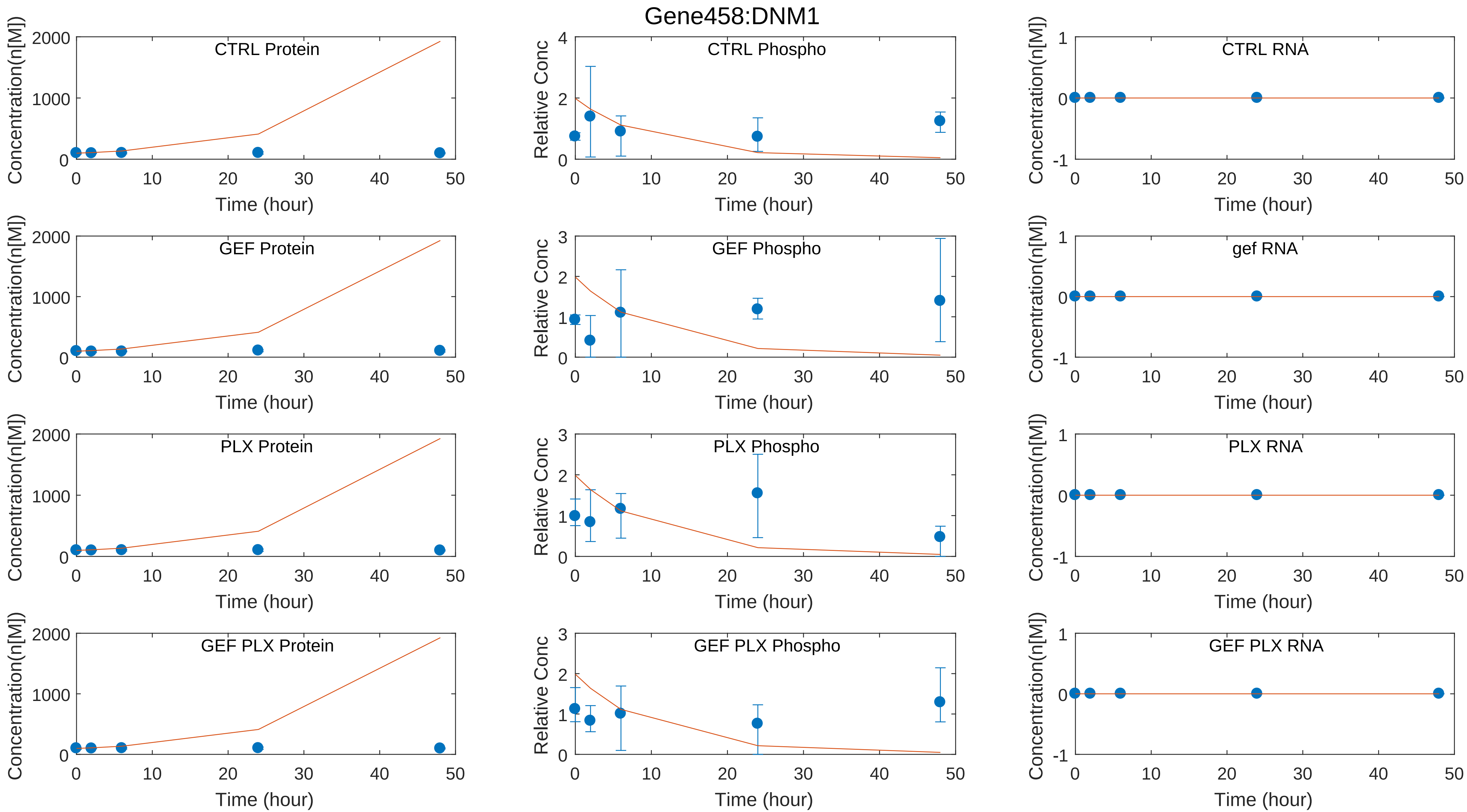
Gene458:DNM1
CTRL Protein
CTRL Phospho
CTRL RNA
GEF Protein
GEF Phospho
gef RNA
PLX Protein
PLX Phospho
PLX RNA
GEF PLX Protein
GEF PLX Phospho
GEF PLX RNA
Concentration(n[M])
Relative Conc
Time (hour)

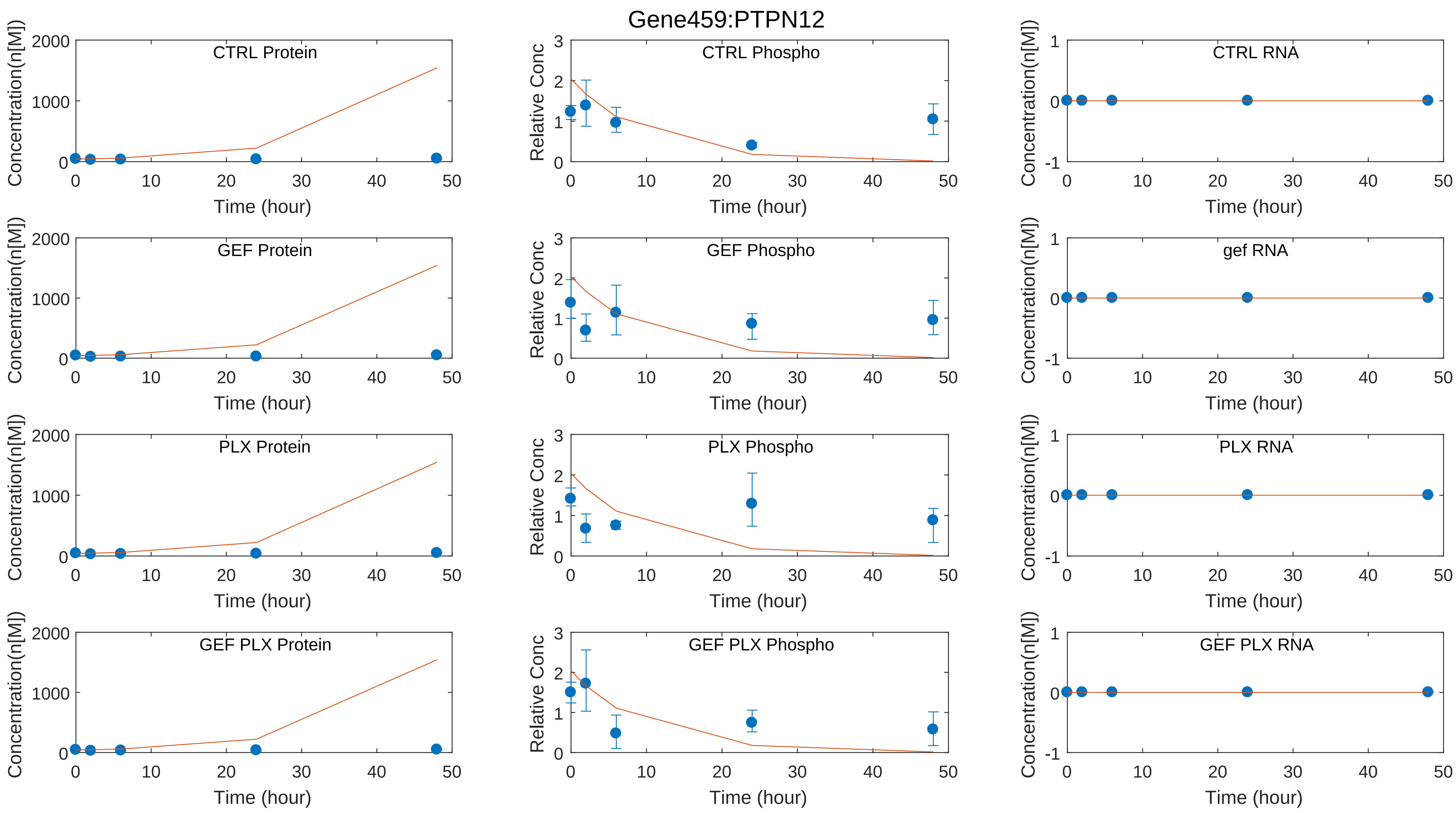
Gene459:PTPN12
CTRL Protein
GEF Protein
PLX Protein
GEF PLX Protein
CTRL Phospho
GEF Phospho
PLX Phospho
GEF PLX Phospho
CTRL RNA
gef RNA
PLX RNA
GEF PLX RNA
Concentration(n[M])
Relative Conc
Time (hour)

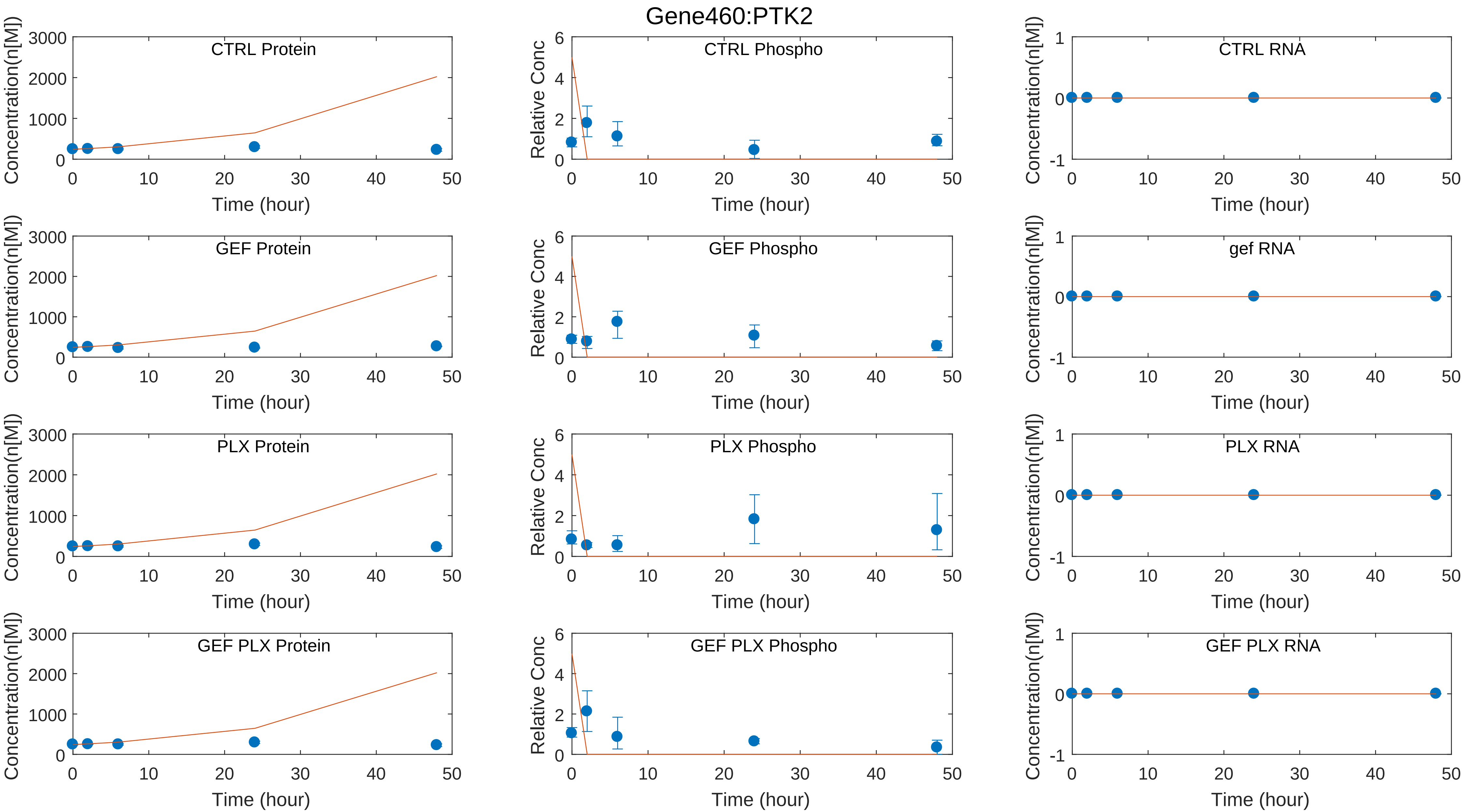

Gene460:PTK2
CTRL Protein
GEF Protein
PLX Protein
GEF PLX Protein
CTRL Phospho
GEF Phospho
PLX Phospho
GEF PLX Phospho
CTRL RNA
gef RNA
PLX RNA
GEF PLX RNA
Concentration(n[M])
Relative Conc
Time (hour)

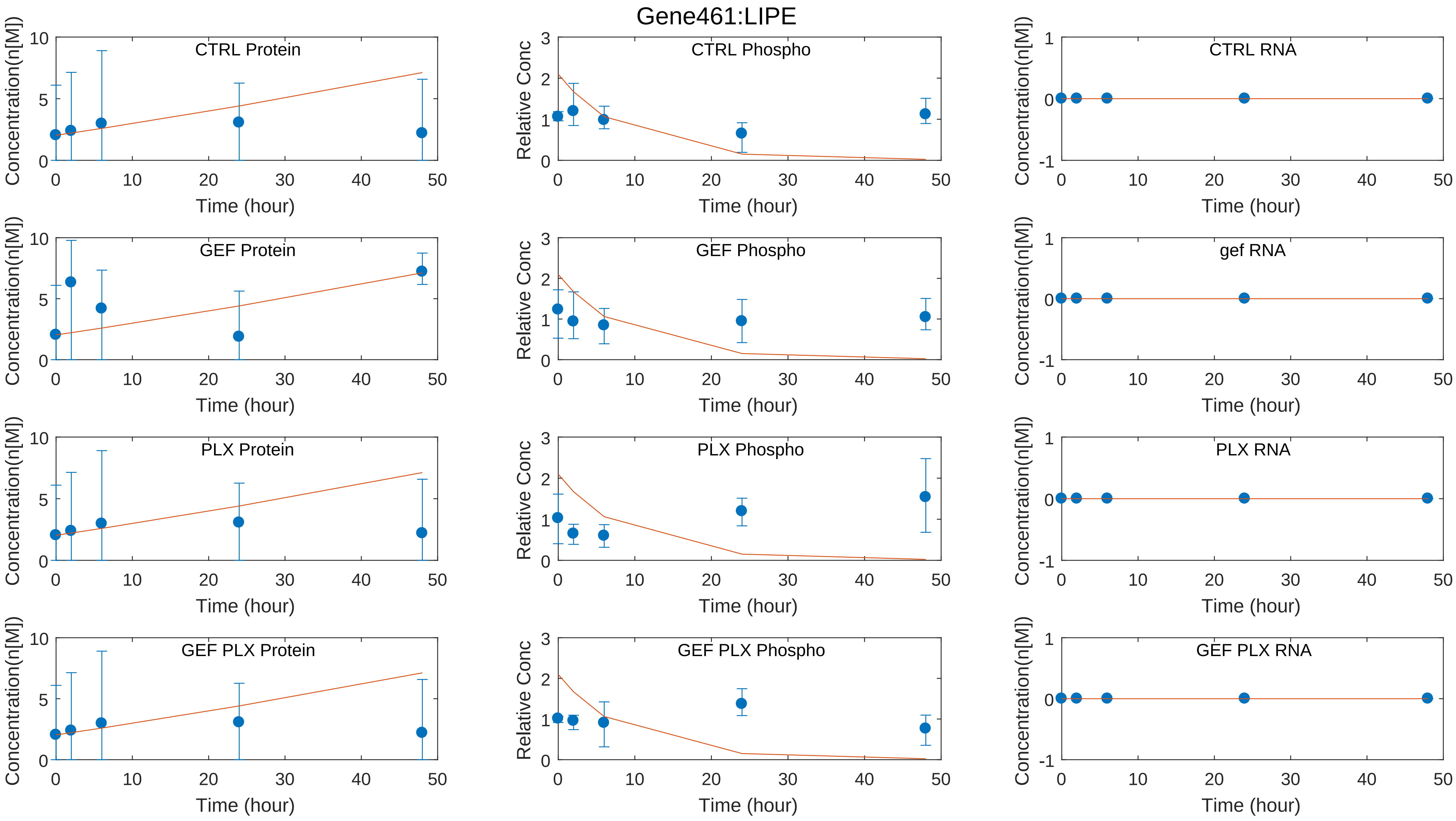

Gene461:LIPE
CTRL Protein
CTRL Phospho
CTRL RNA
GEF Protein
GEF Phospho
gef RNA
PLX Protein
PLX Phospho
PLX RNA
GEF PLX Protein
GEF PLX Phospho
GEF PLX RNA
Concentration(n[M])
Relative Conc
Time (hour)

Gene462:PRKCD

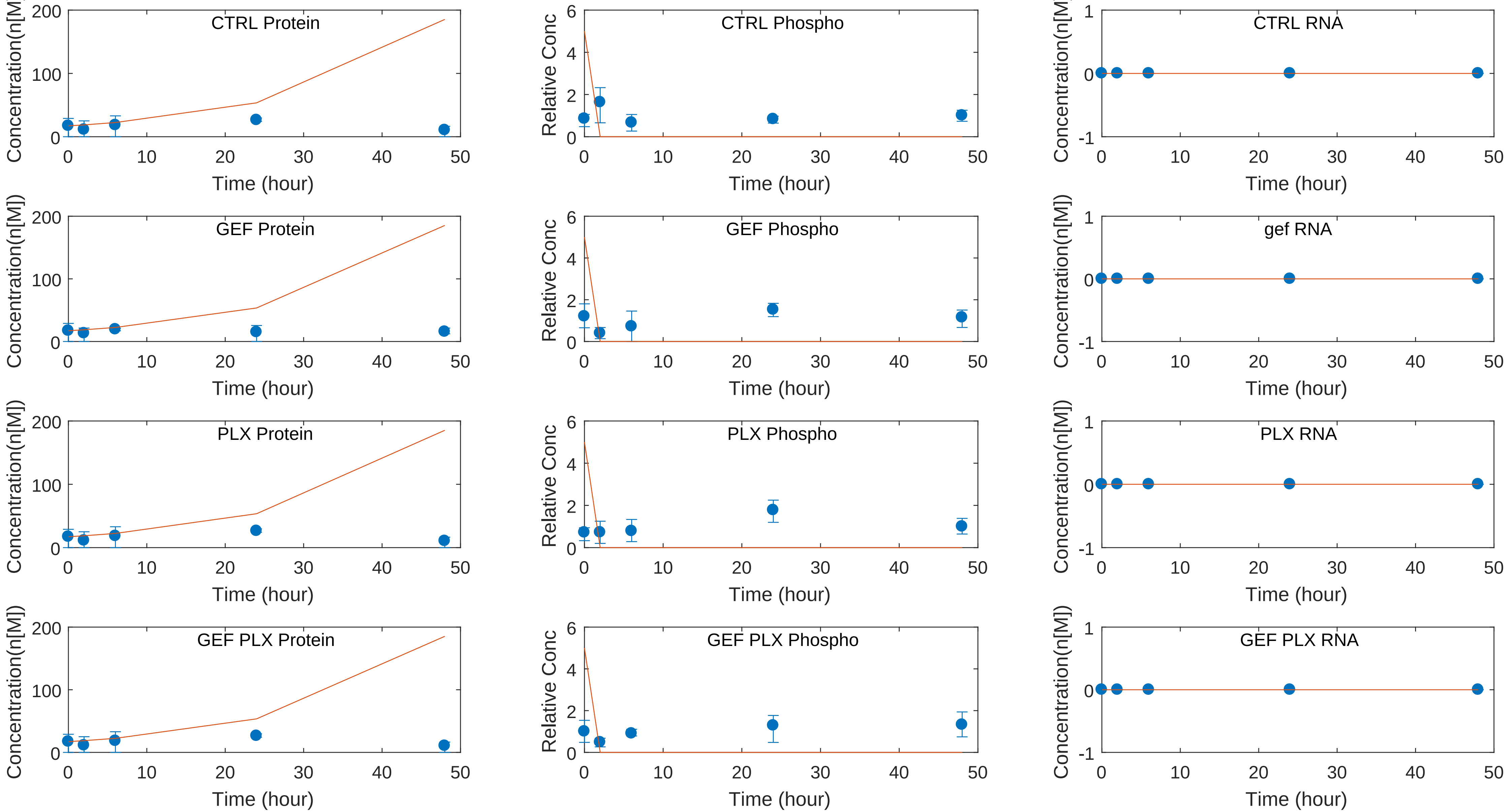

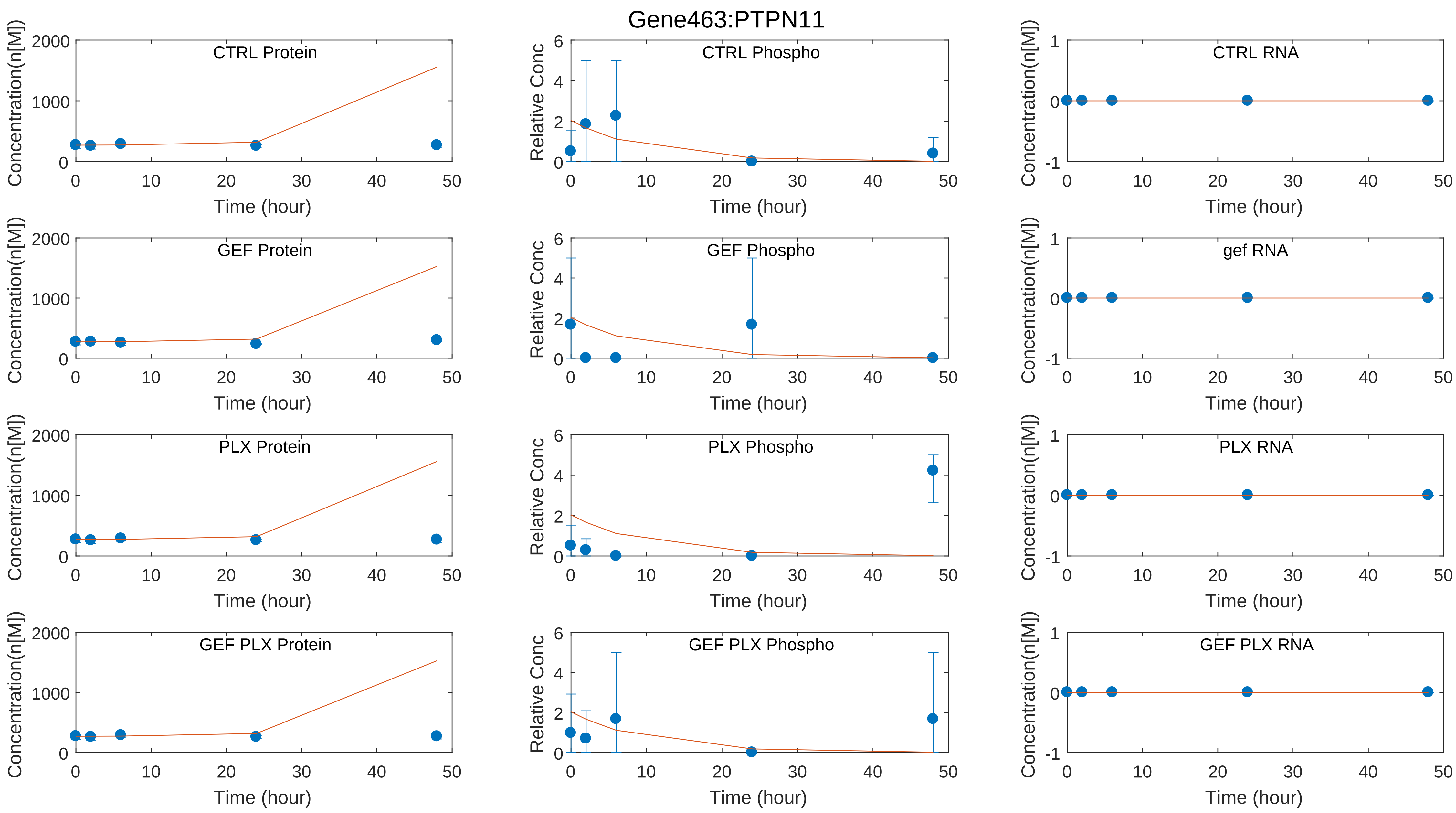
Gene463:PTPN11
CTRL Protein
GEF Protein
PLX Protein
GEF PLX Protein
CTRL Phospho
GEF Phospho
PLX Phospho
GEF PLX Phospho
CTRL RNA
gef RNA
PLX RNA
GEF PLX RNA
Concentration(n[M])
Relative Conc
Time (hour)

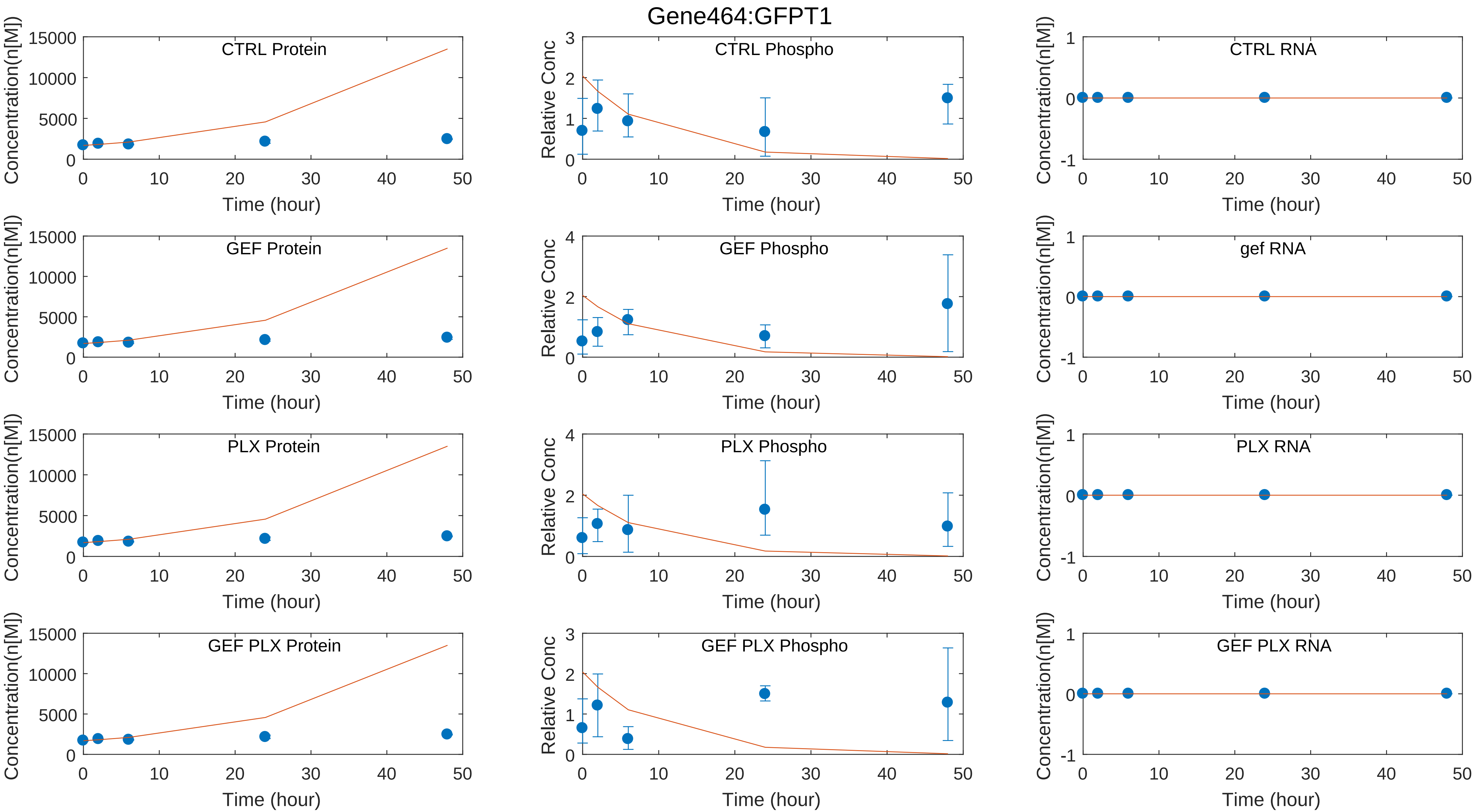
Gene464:GFPT1
CTRL Protein
GEF Protein
PLX Protein
GEF PLX Protein
CTRL Phospho
GEF Phospho
PLX Phospho
GEF PLX Phospho
CTRL RNA
gef RNA
PLX RNA
GEF PLX RNA
Concentration(n[M])
Relative Conc
Time (hour)

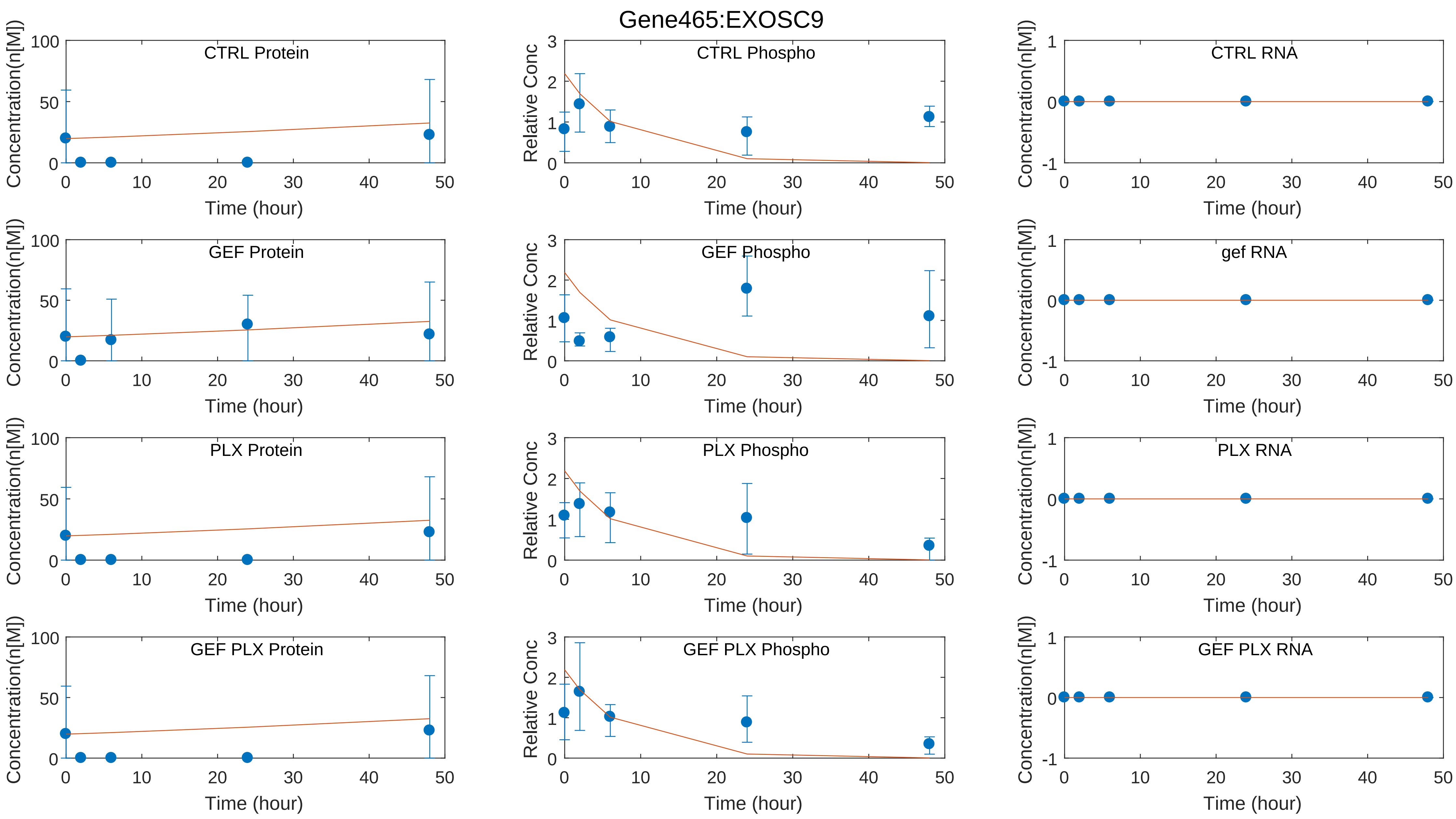

Gene465:EXOSC9
CTRL Protein
CTRL Phospho
CTRL RNA
GEF Protein
GEF Phospho
gef RNA
PLX Protein
PLX Phospho
PLX RNA
GEF PLX Protein
GEF PLX Phospho
GEF PLX RNA
Concentration(n[M])
Relative Conc
Time (hour)

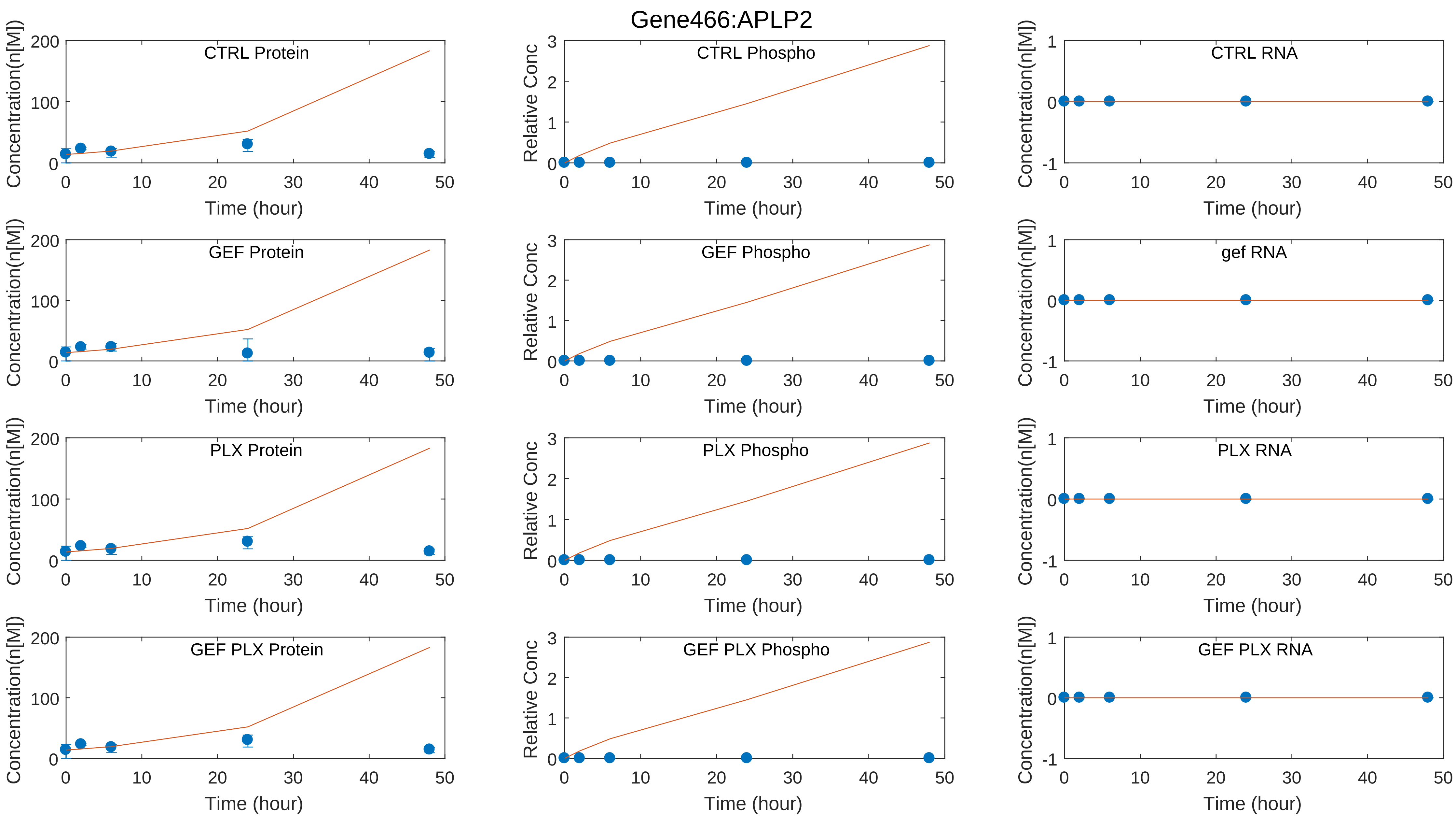

Gene466:APLP2
CTRL Protein
CTRL Phospho
CTRL RNA
GEF Protein
GEF Phospho
gef RNA
PLX Protein
PLX Phospho
PLX RNA
GEF PLX Protein
GEF PLX Phospho
GEF PLX RNA
Concentration(n[M])
Relative Conc
Time (hour)

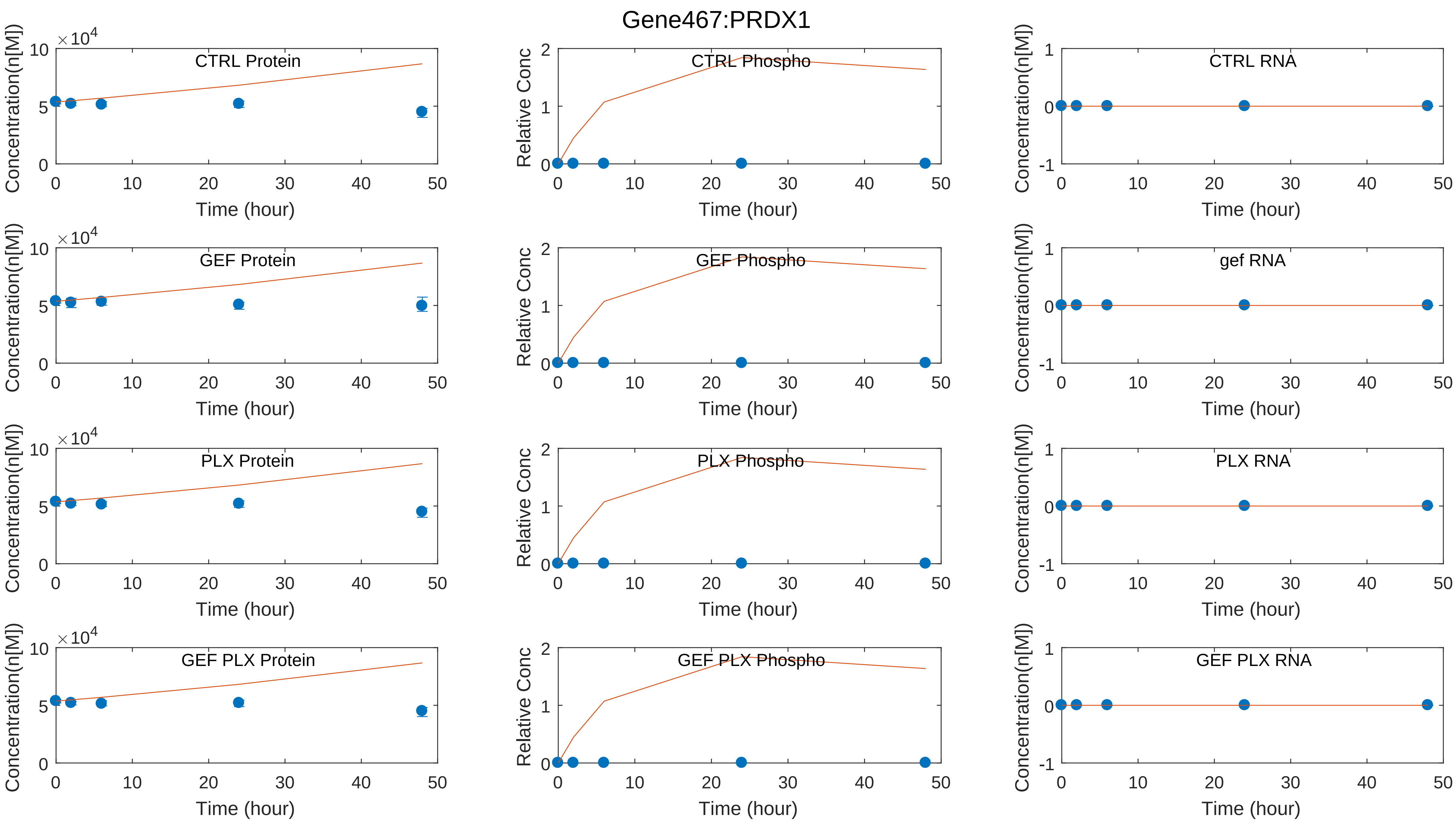

Gene467:PRDX1
CTRL Protein
GEF Protein
PLX Protein
GEF PLX Protein
CTRL Phospho
GEF Phospho
PLX Phospho
GEF PLX Phospho
CTRL RNA
gef RNA
PLX RNA
GEF PLX RNA
Concentration(n[M])
Relative Conc
Time (hour)
×10^4

Gene468:ZFP36L1

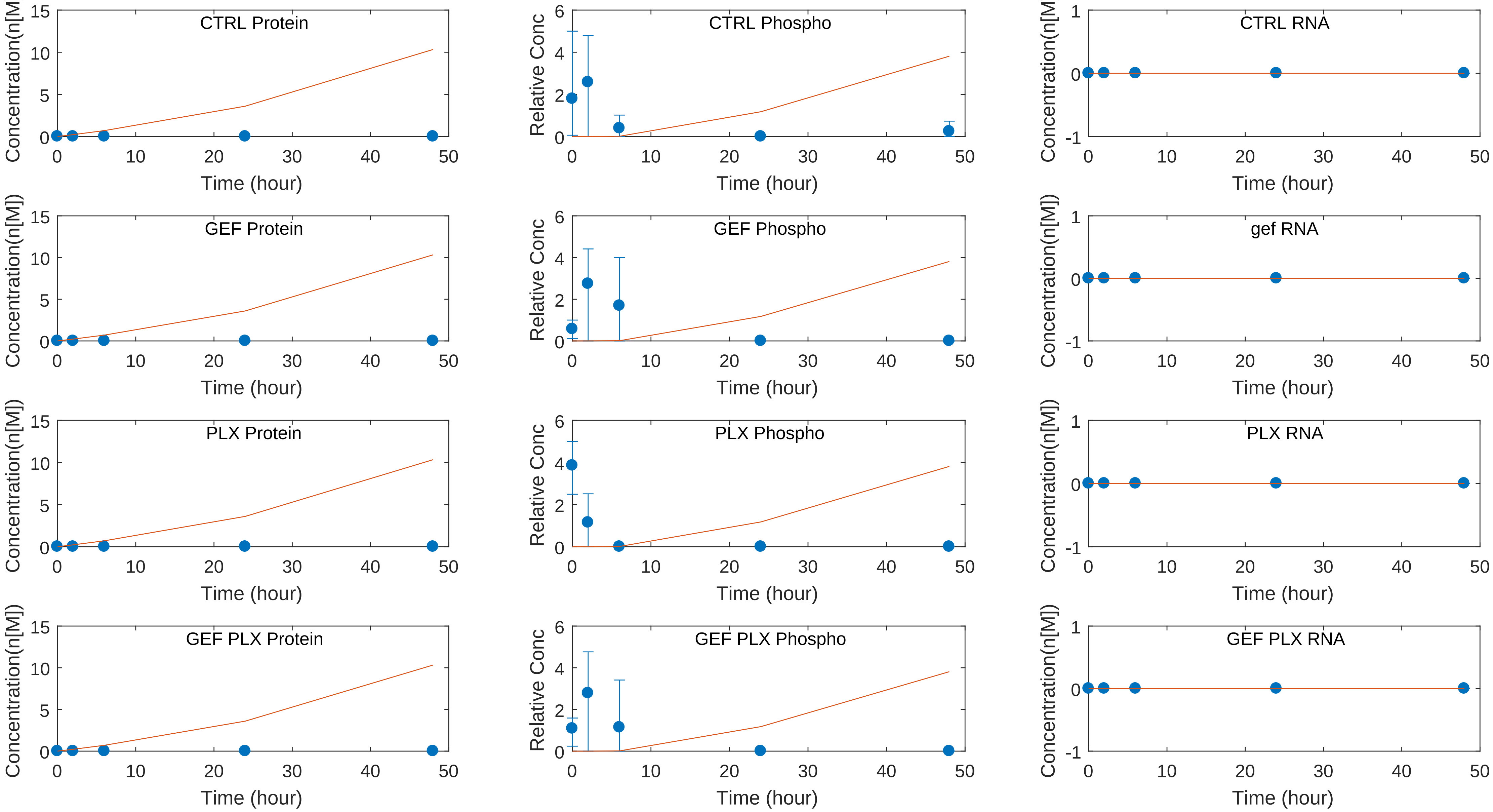

Gene469:SPAG1

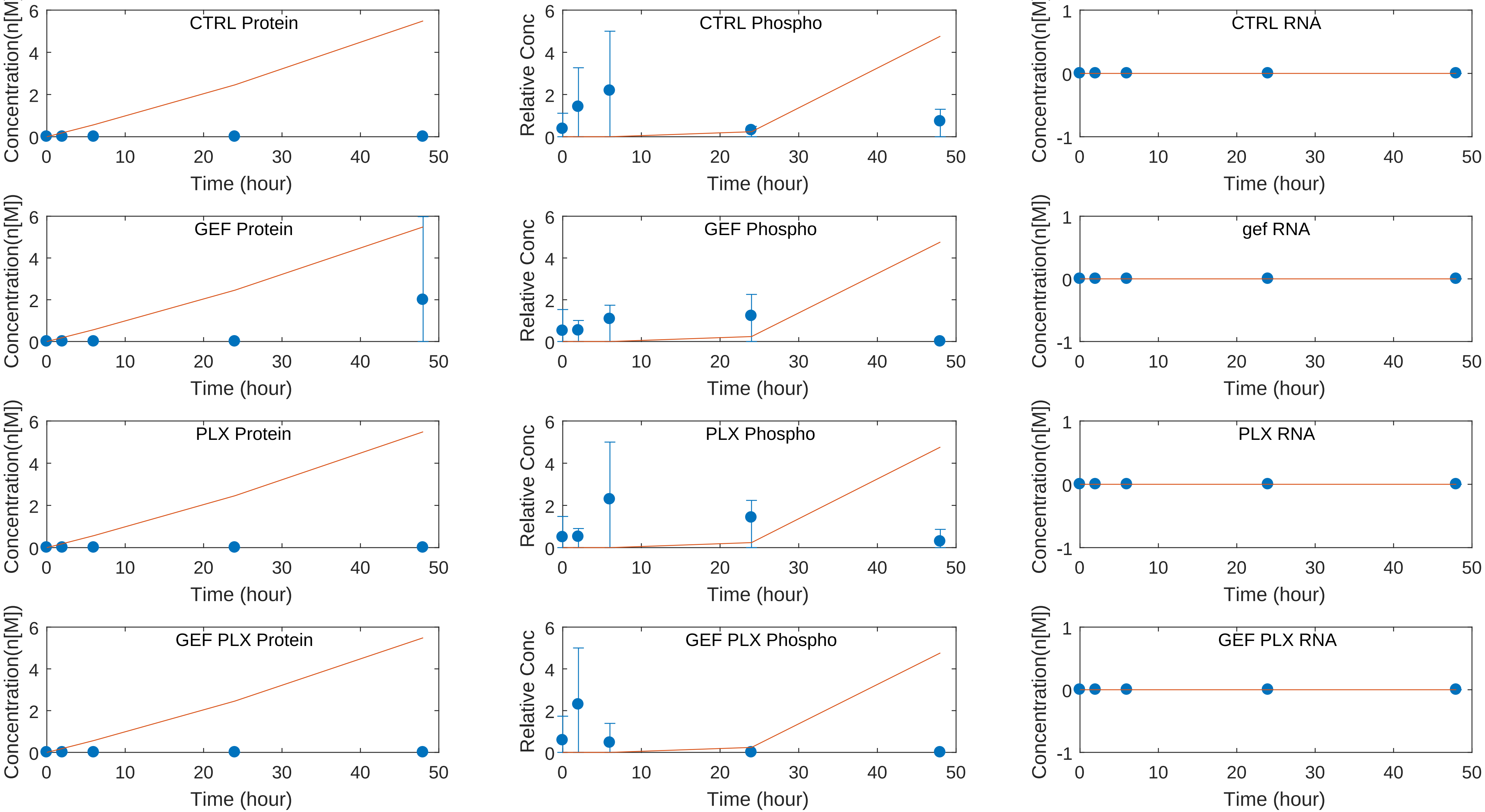

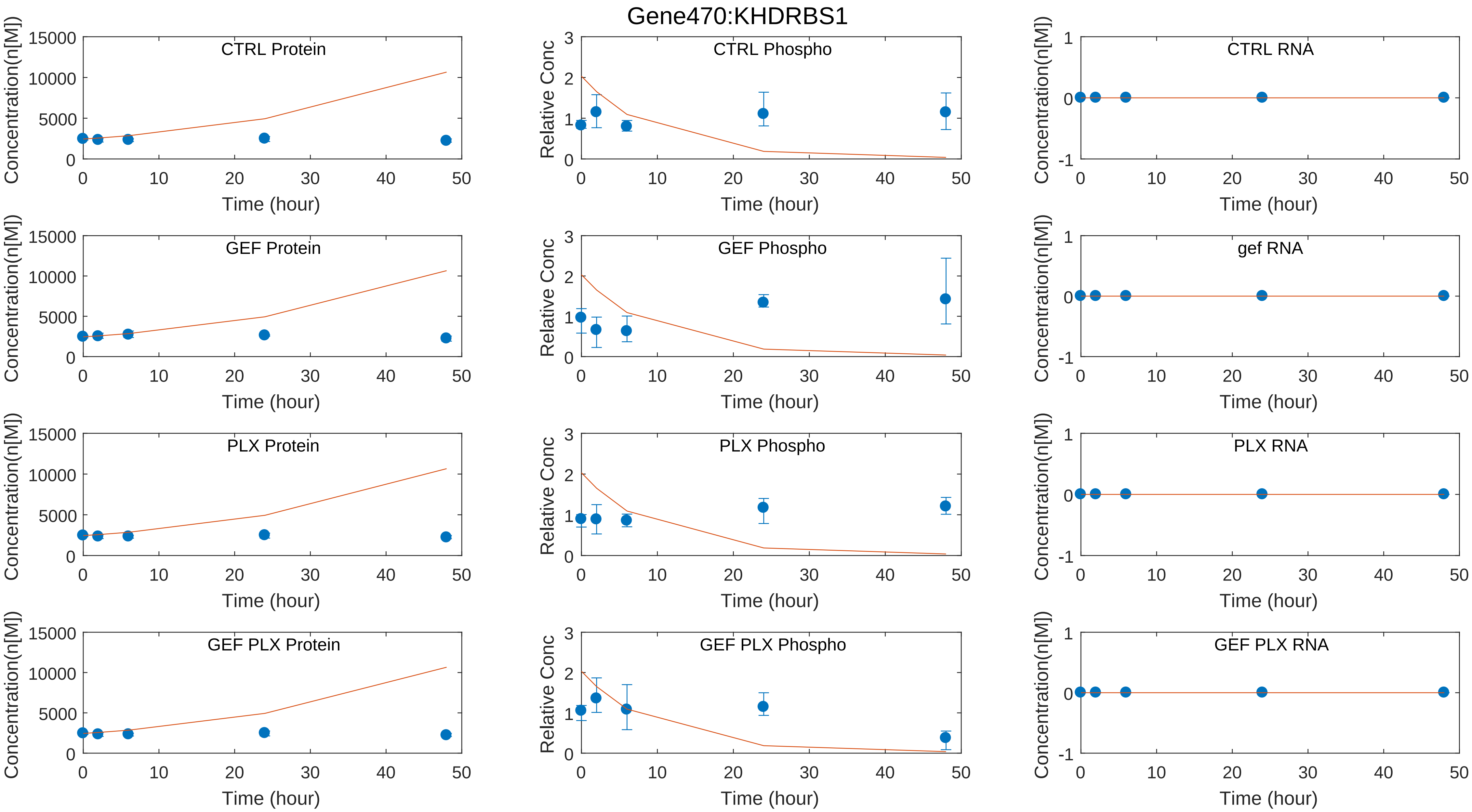
Gene470:KHDRBS1
CTRL Protein
GEF Protein
PLX Protein
GEF PLX Protein
CTRL Phospho
GEF Phospho
PLX Phospho
GEF PLX Phospho
CTRL RNA
gef RNA
PLX RNA
GEF PLX RNA
Concentration(n[M])
Relative Conc
Time (hour)

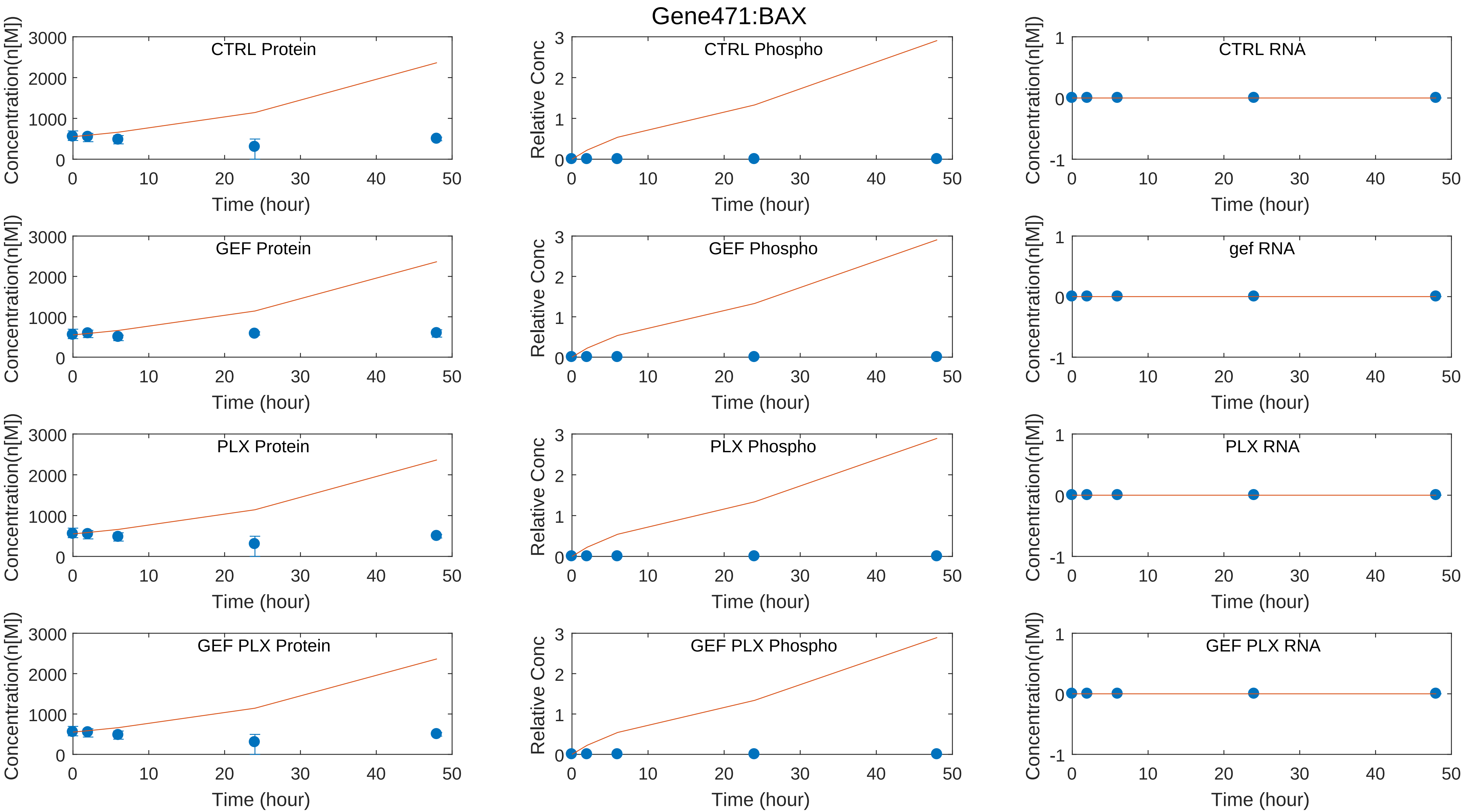
Gene471:BAX
CTRL Protein
CTRL Phospho
CTRL RNA
GEF Protein
GEF Phospho
gef RNA
PLX Protein
PLX Phospho
PLX RNA
GEF PLX Protein
GEF PLX Phospho
GEF PLX RNA
Concentration(n[M])
Relative Conc
Time (hour)

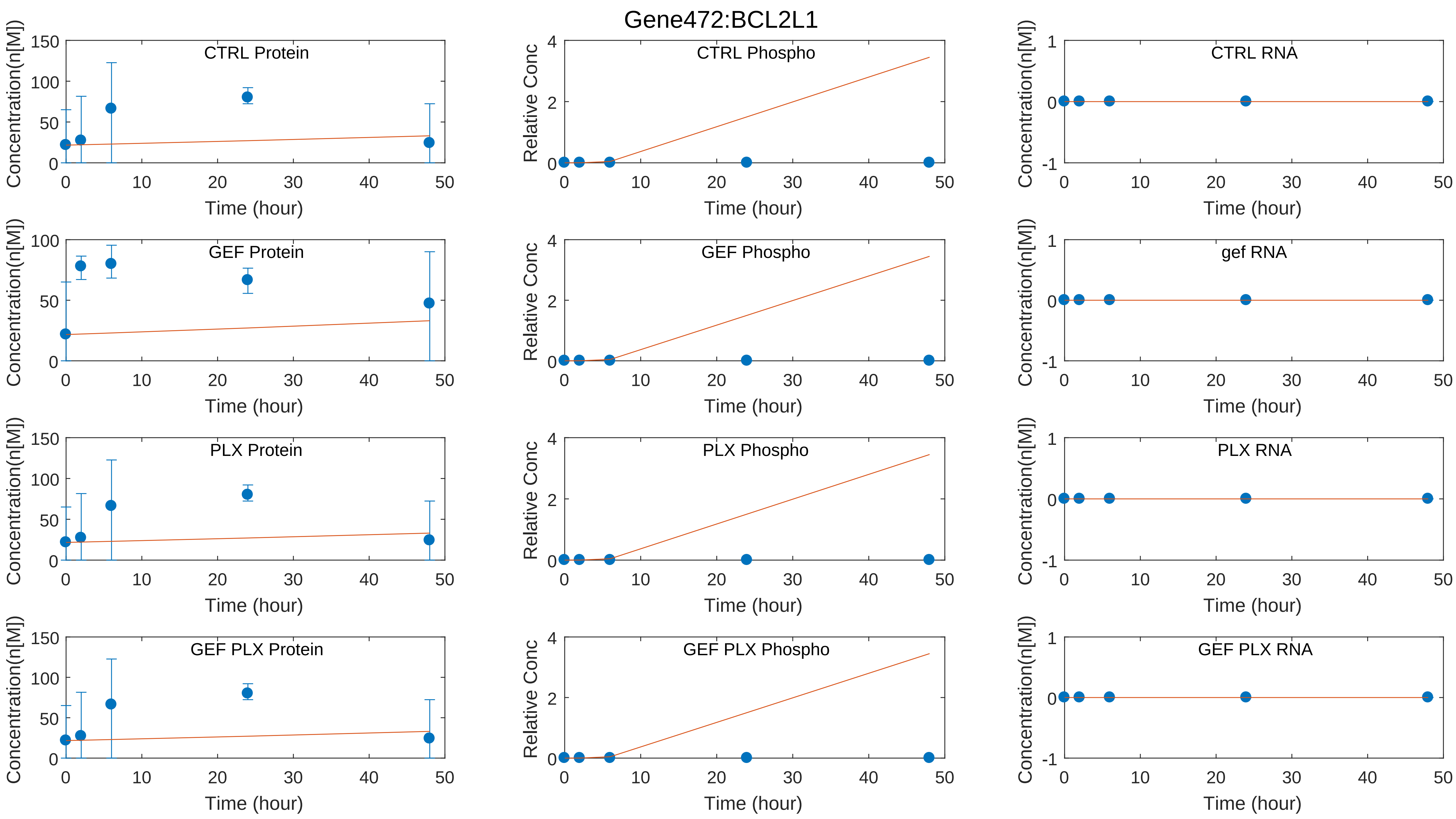

Gene472:BCL2L1
CTRL Protein
CTRL Phospho
CTRL RNA
GEF Protein
GEF Phospho
gef RNA
PLX Protein
PLX Phospho
PLX RNA
GEF PLX Protein
GEF PLX Phospho
GEF PLX RNA
Concentration(n[M])
Relative Conc
Time (hour)

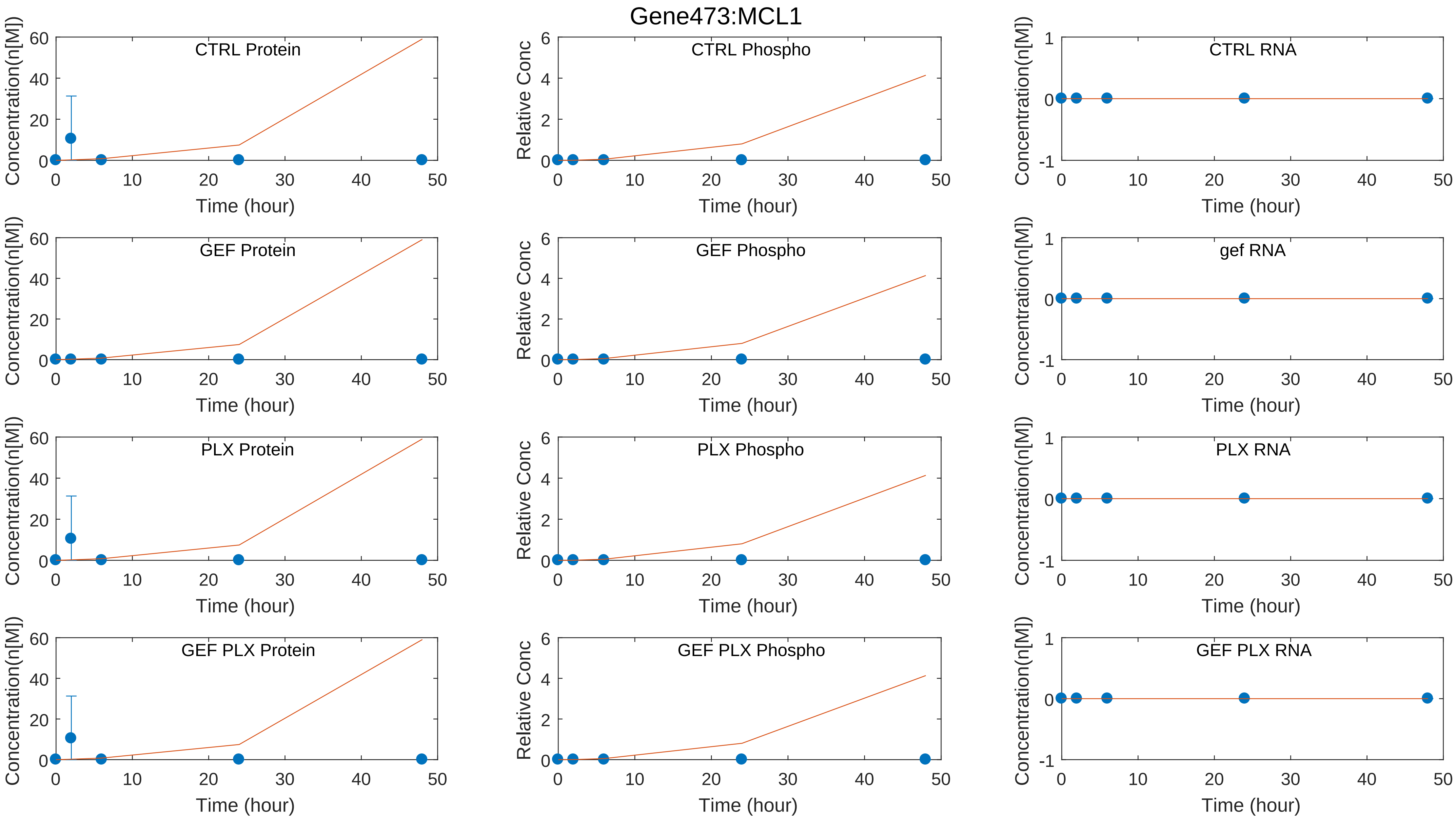

Gene473:MCL1
CTRL Protein
CTRL Phospho
CTRL RNA
GEF Protein
GEF Phospho
gef RNA
PLX Protein
PLX Phospho
PLX RNA
GEF PLX Protein
GEF PLX Phospho
GEF PLX RNA
Concentration(n[M])
Relative Conc
Time (hour)

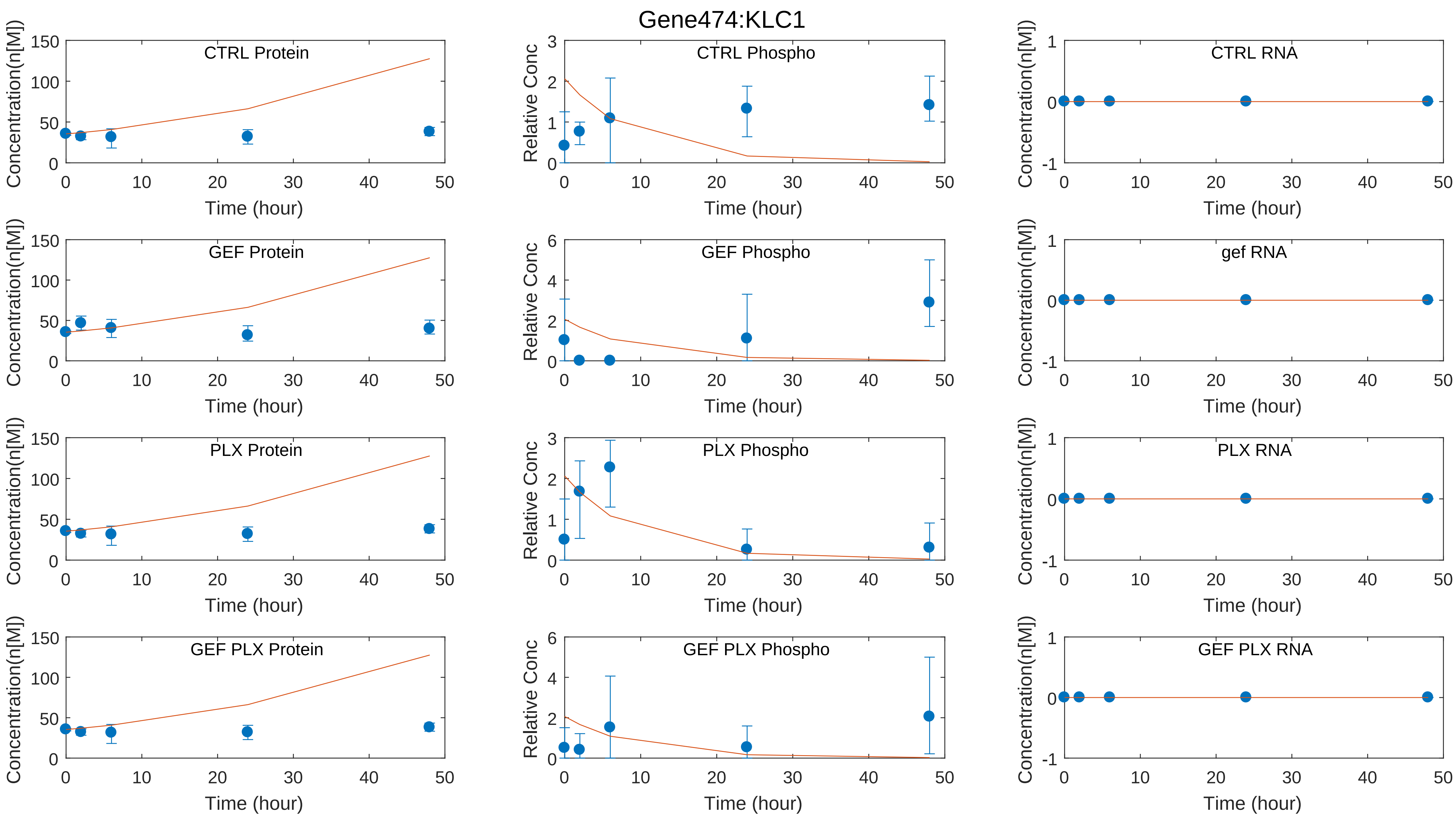

Gene474:KLC1
CTRL Protein
CTRL Phospho
CTRL RNA
GEF Protein
GEF Phospho
gef RNA
PLX Protein
PLX Phospho
PLX RNA
GEF PLX Protein
GEF PLX Phospho
GEF PLX RNA
Concentration(n[M])
Relative Conc
Time (hour)

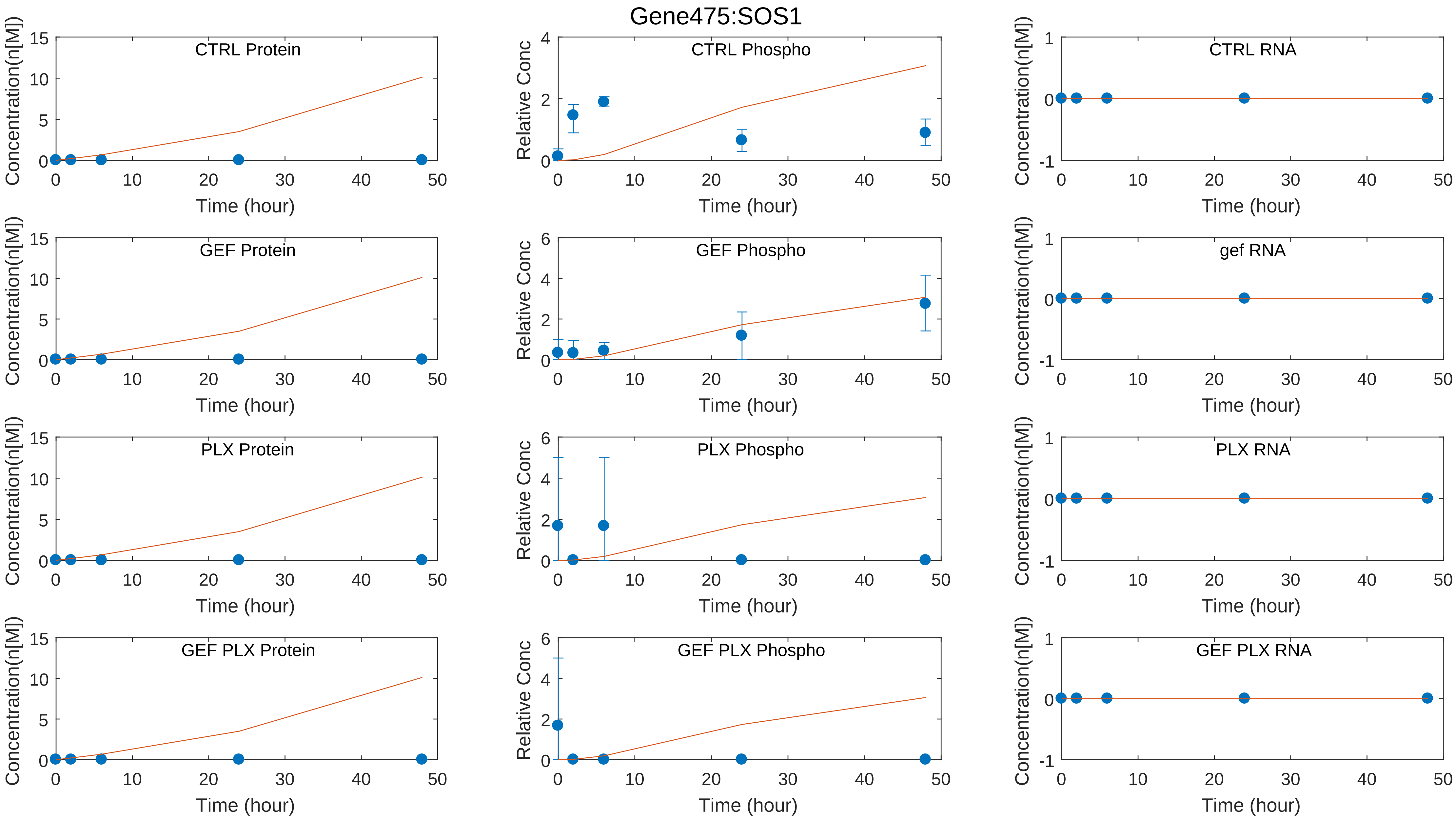

Gene475:SOS1
CTRL Protein
CTRL Phospho
CTRL RNA
GEF Protein
GEF Phospho
gef RNA
PLX Protein
PLX Phospho
PLX RNA
GEF PLX Protein
GEF PLX Phospho
GEF PLX RNA
Concentration(n[M])
Relative Conc
Time (hour)

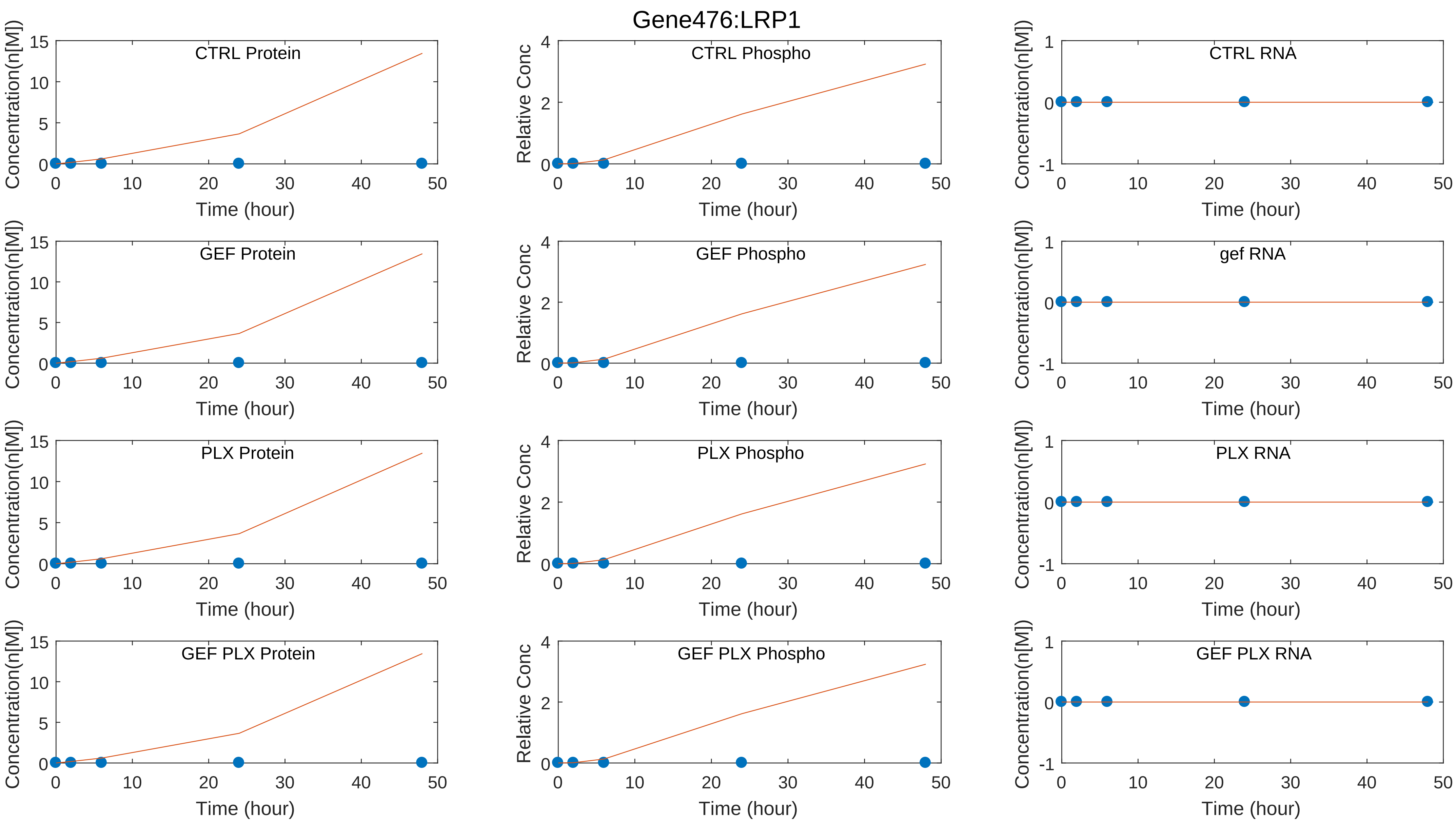

Gene476:LRP1
CTRL Protein
CTRL Phospho
CTRL RNA
GEF Protein
GEF Phospho
gef RNA
PLX Protein
PLX Phospho
PLX RNA
GEF PLX Protein
GEF PLX Phospho
GEF PLX RNA
Concentration(n[M])
Relative Conc
Time (hour)

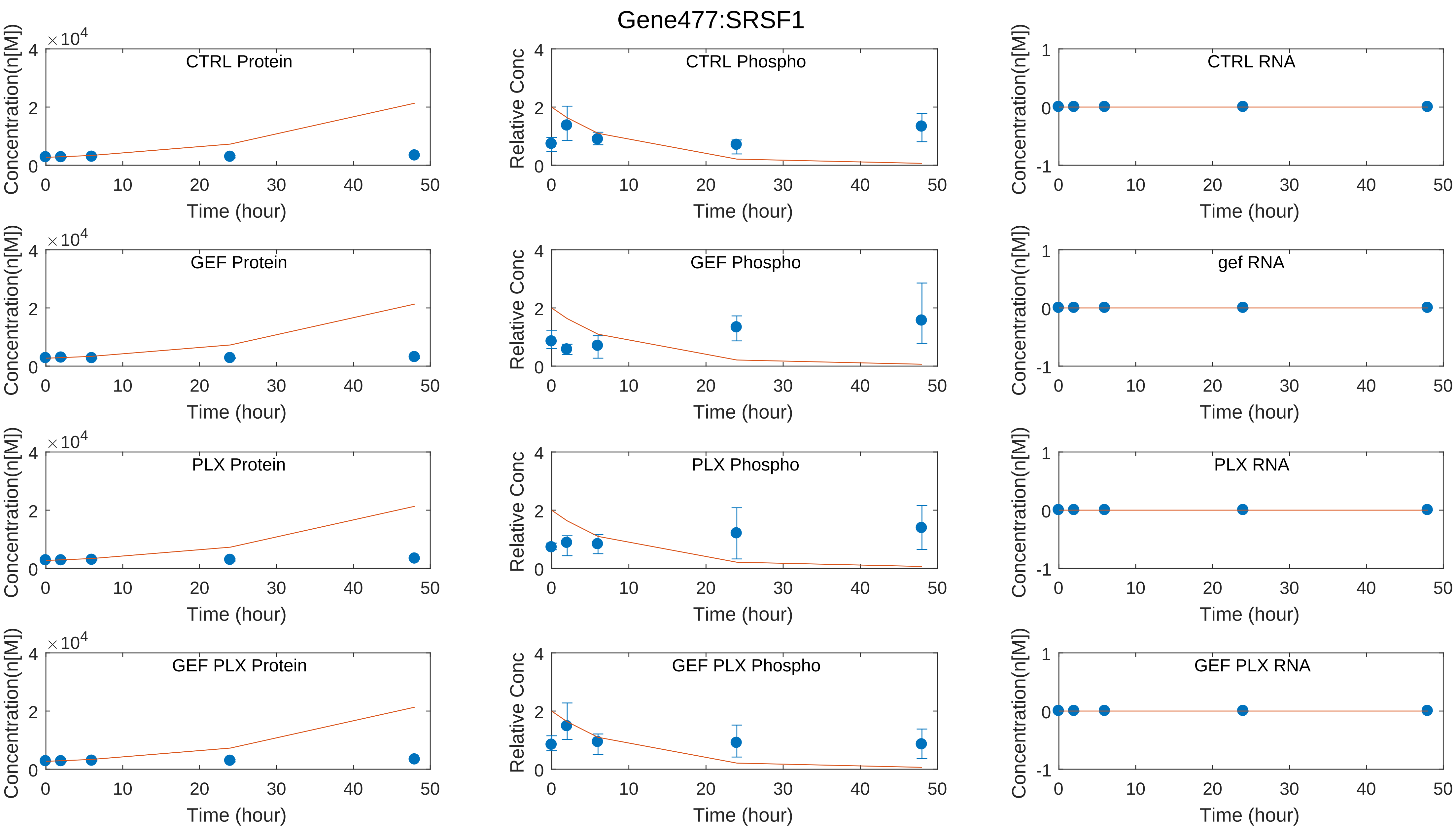

Gene477:SRSF1
CTRL Protein
GEF Protein
PLX Protein
GEF PLX Protein
CTRL Phospho
GEF Phospho
PLX Phospho
GEF PLX Phospho
CTRL RNA
gef RNA
PLX RNA
GEF PLX RNA
Concentration(n[M])
Relative Conc
Time (hour)
×10^4

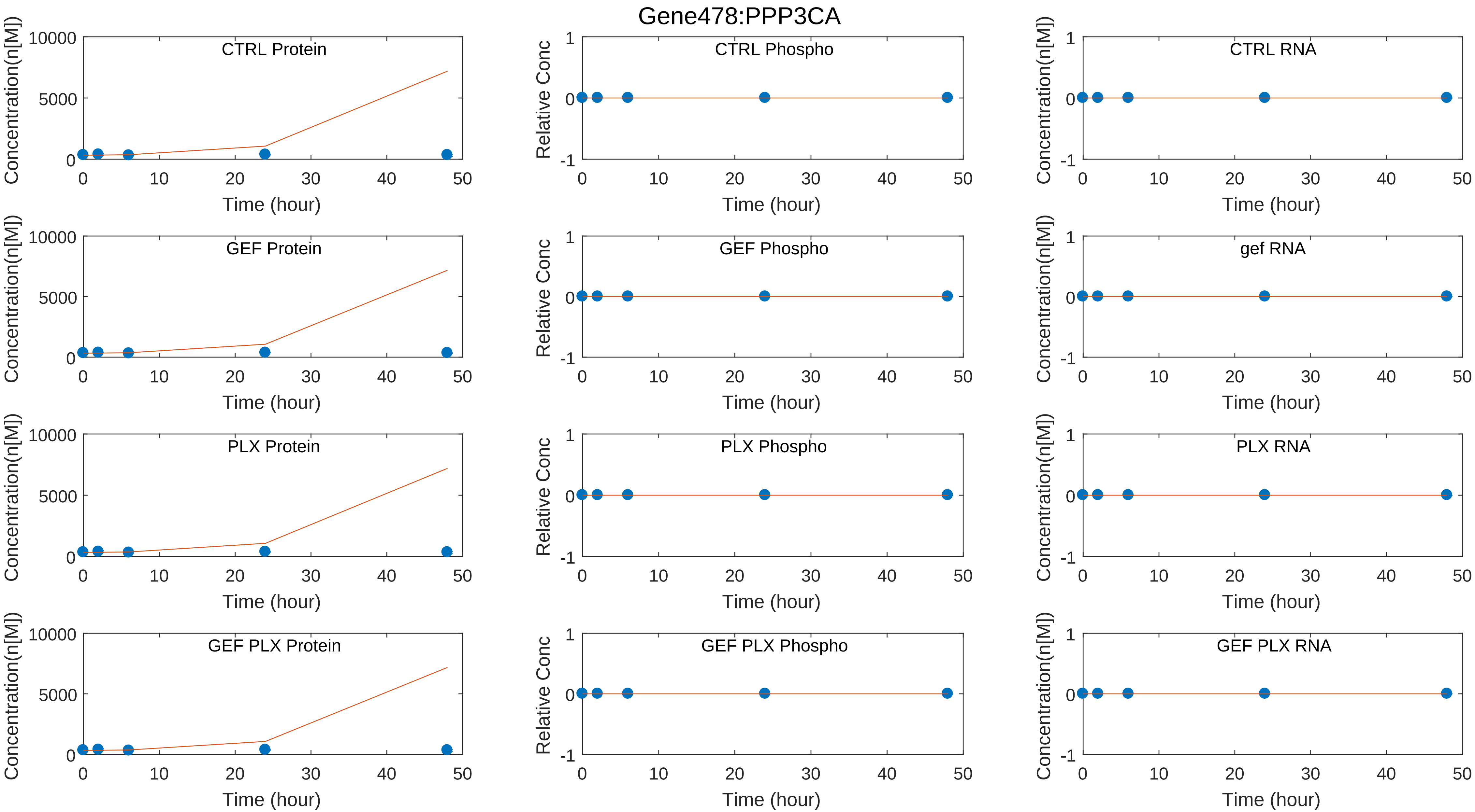

Gene478:PPP3CA
CTRL Protein
CTRL Phospho
CTRL RNA
GEF Protein
GEF Phospho
gef RNA
PLX Protein
PLX Phospho
PLX RNA
GEF PLX Protein
GEF PLX Phospho
GEF PLX RNA
Concentration(n[M])
Relative Conc
Time (hour)
10000
5000
0
1
-1
10
20
30
40
50

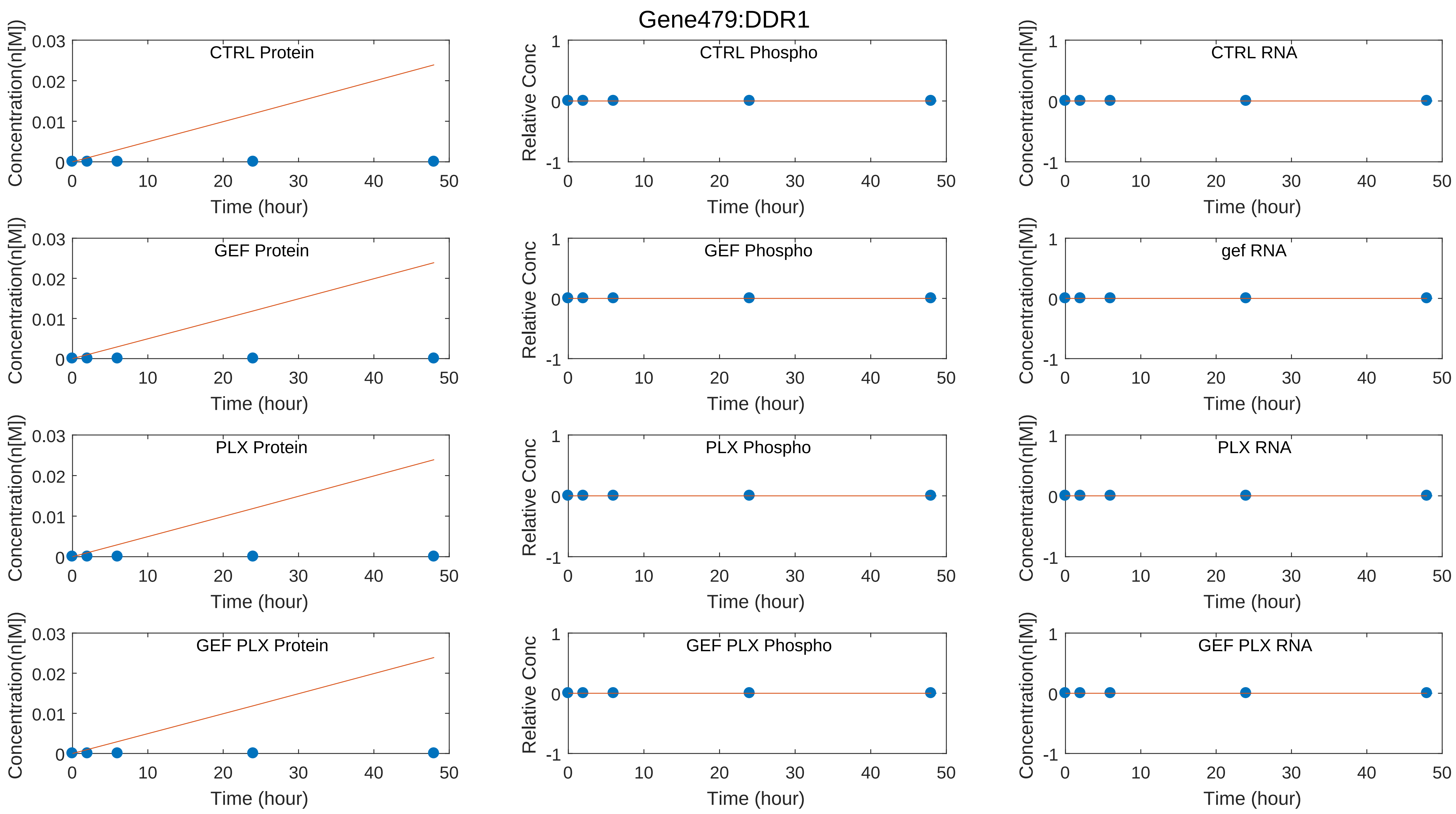
Gene479:DDR1
CTRL Protein
CTRL Phospho
CTRL RNA
GEF Protein
GEF Phospho
gef RNA
PLX Protein
PLX Phospho
PLX RNA
GEF PLX Protein
GEF PLX Phospho
GEF PLX RNA
Concentration(n[M])
Relative Conc
Time (hour)
0 10 20 30 40 50
0 0.01 0.02 0.03
-1 0 1

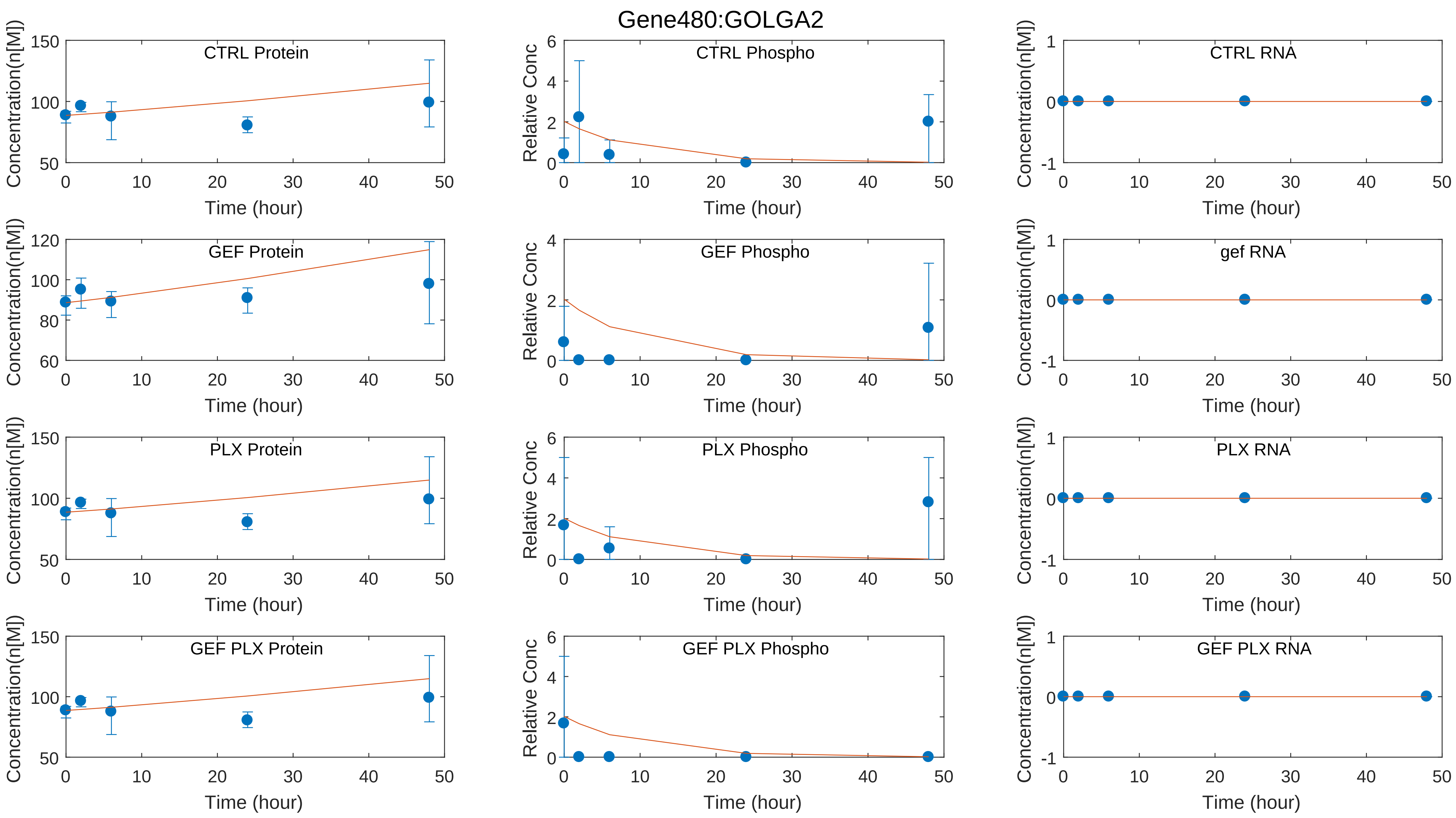
Gene480:GOLGA2
CTRL Protein
CTRL Phospho
CTRL RNA
GEF Protein
GEF Phospho
gef RNA
PLX Protein
PLX Phospho
PLX RNA
GEF PLX Protein
GEF PLX Phospho
GEF PLX RNA
Concentration(n[M])
Relative Conc
Time (hour)

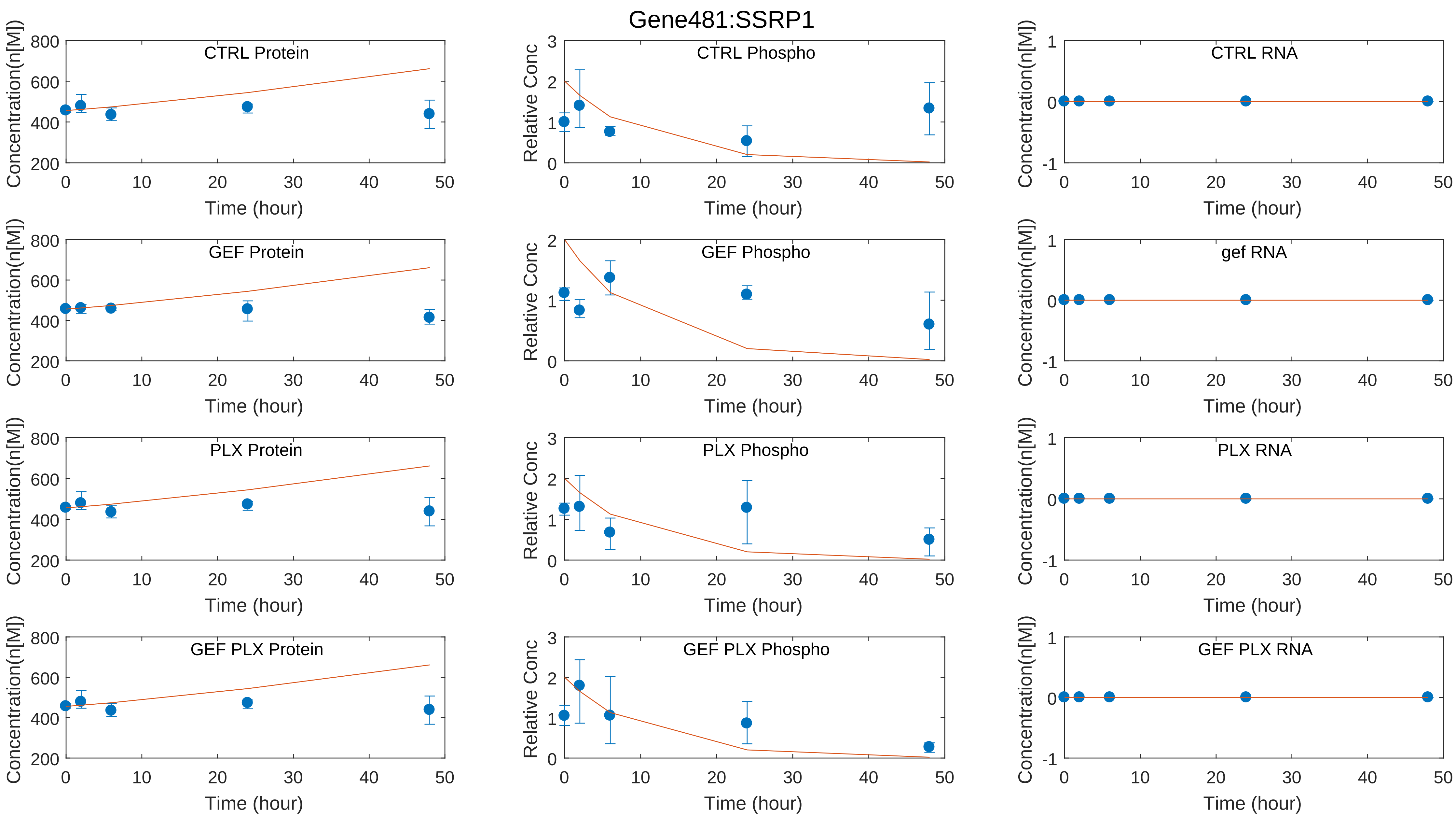

Gene481:SSRP1
CTRL Protein
CTRL Phospho
CTRL RNA
GEF Protein
GEF Phospho
gef RNA
PLX Protein
PLX Phospho
PLX RNA
GEF PLX Protein
GEF PLX Phospho
GEF PLX RNA
Concentration(n[M])
Relative Conc
Time (hour)

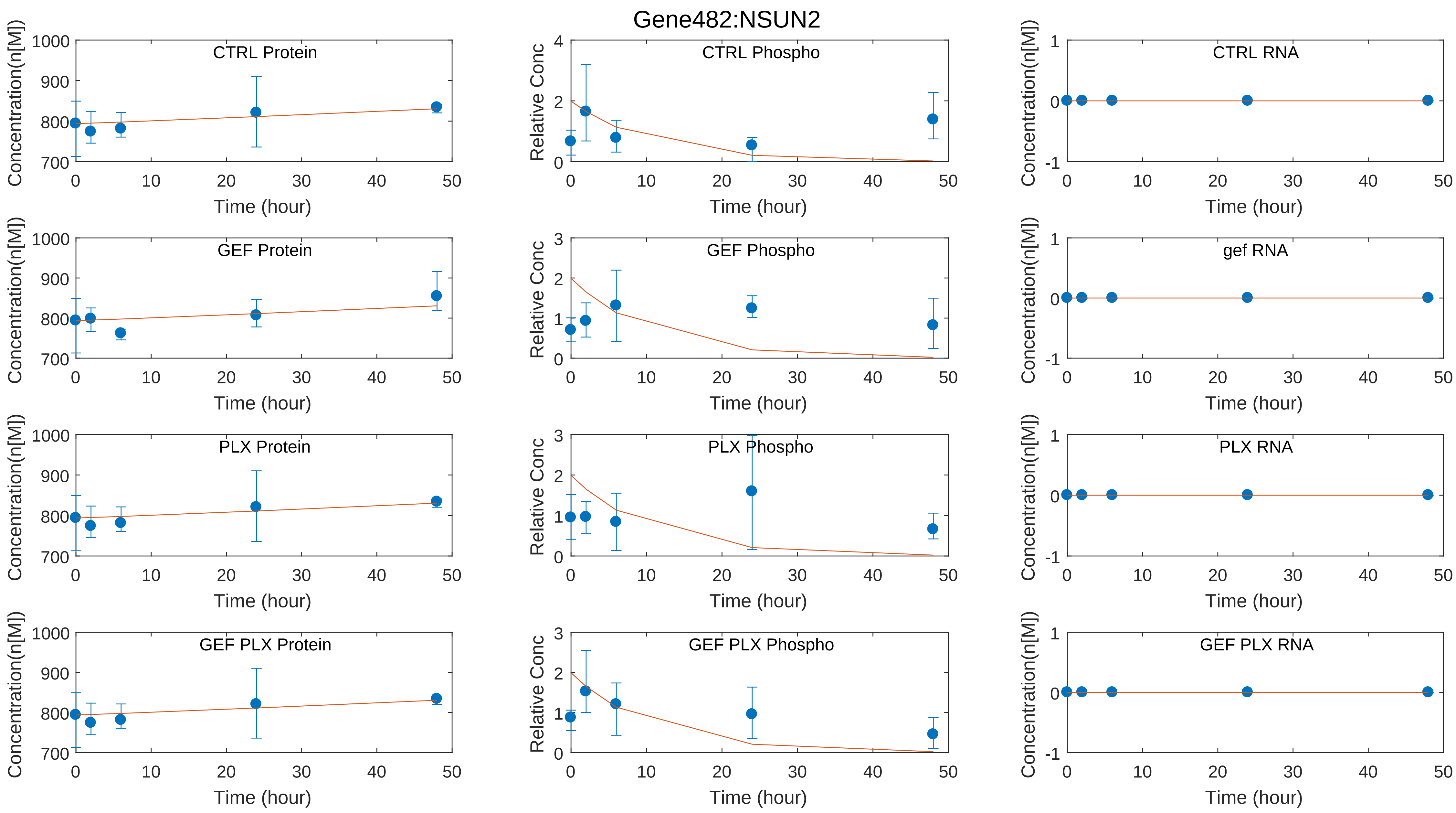

Gene482:NSUN2
CTRL Protein
GEF Protein
PLX Protein
GEF PLX Protein
CTRL Phospho
GEF Phospho
PLX Phospho
GEF PLX Phospho
CTRL RNA
gef RNA
PLX RNA
GEF PLX RNA
Concentration(n[M])
Relative Conc
Time (hour)

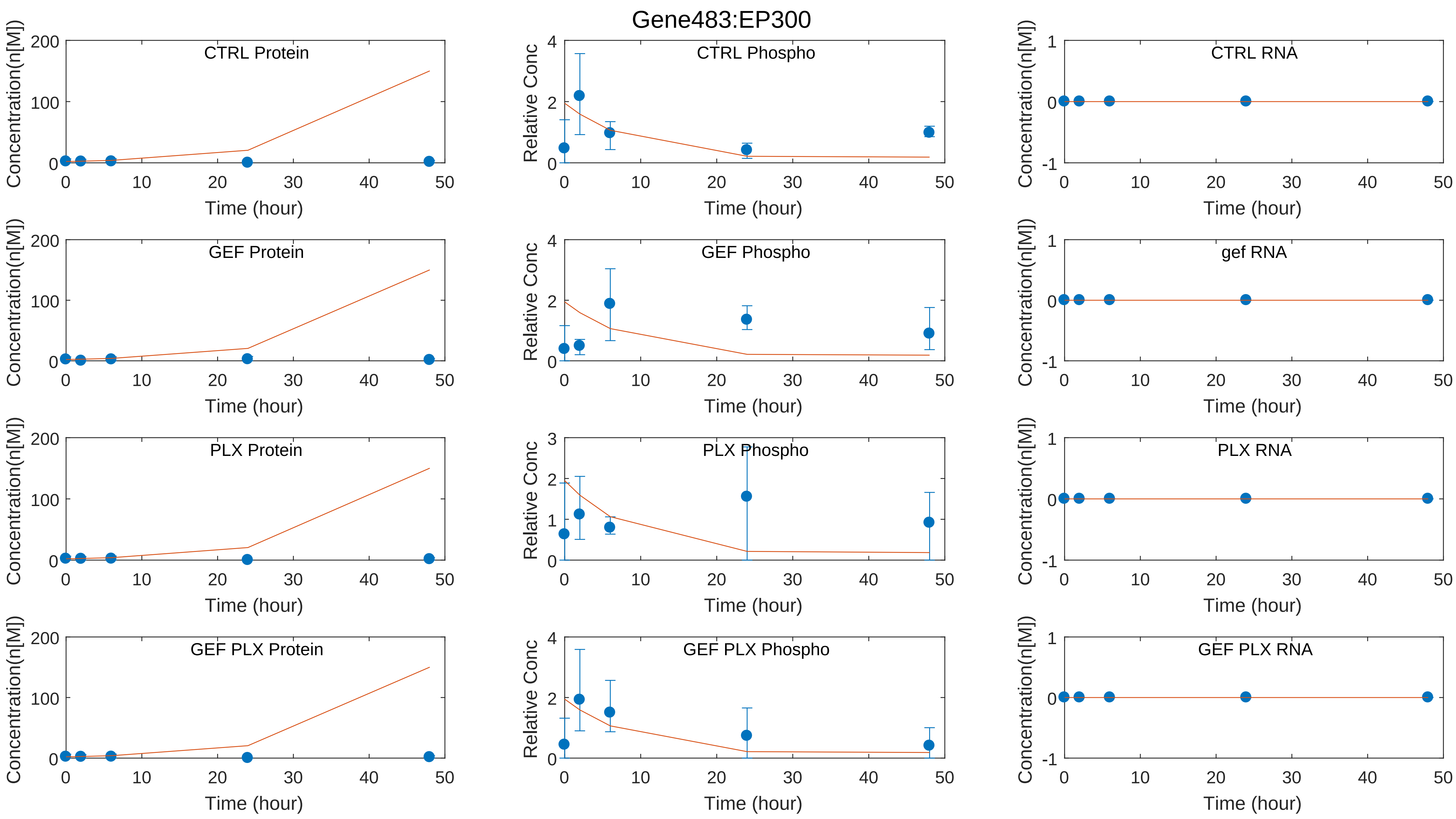

Gene483:EP300
CTRL Protein
CTRL Phospho
CTRL RNA
GEF Protein
GEF Phospho
gef RNA
PLX Protein
PLX Phospho
PLX RNA
GEF PLX Protein
GEF PLX Phospho
GEF PLX RNA
Concentration(n[M])
Relative Conc
Time (hour)

Gene484:FOXO1

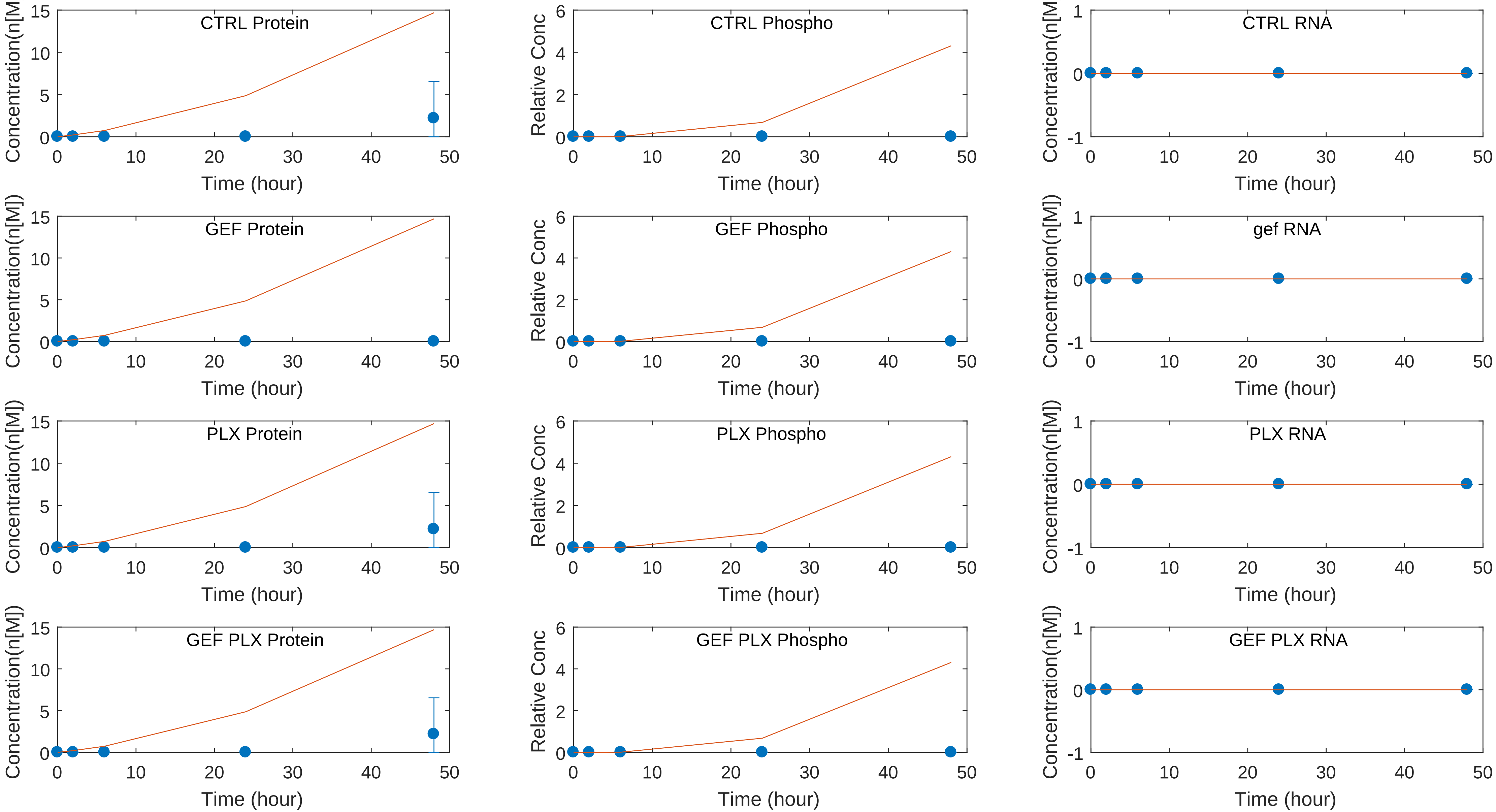

Gene485:TFCP2

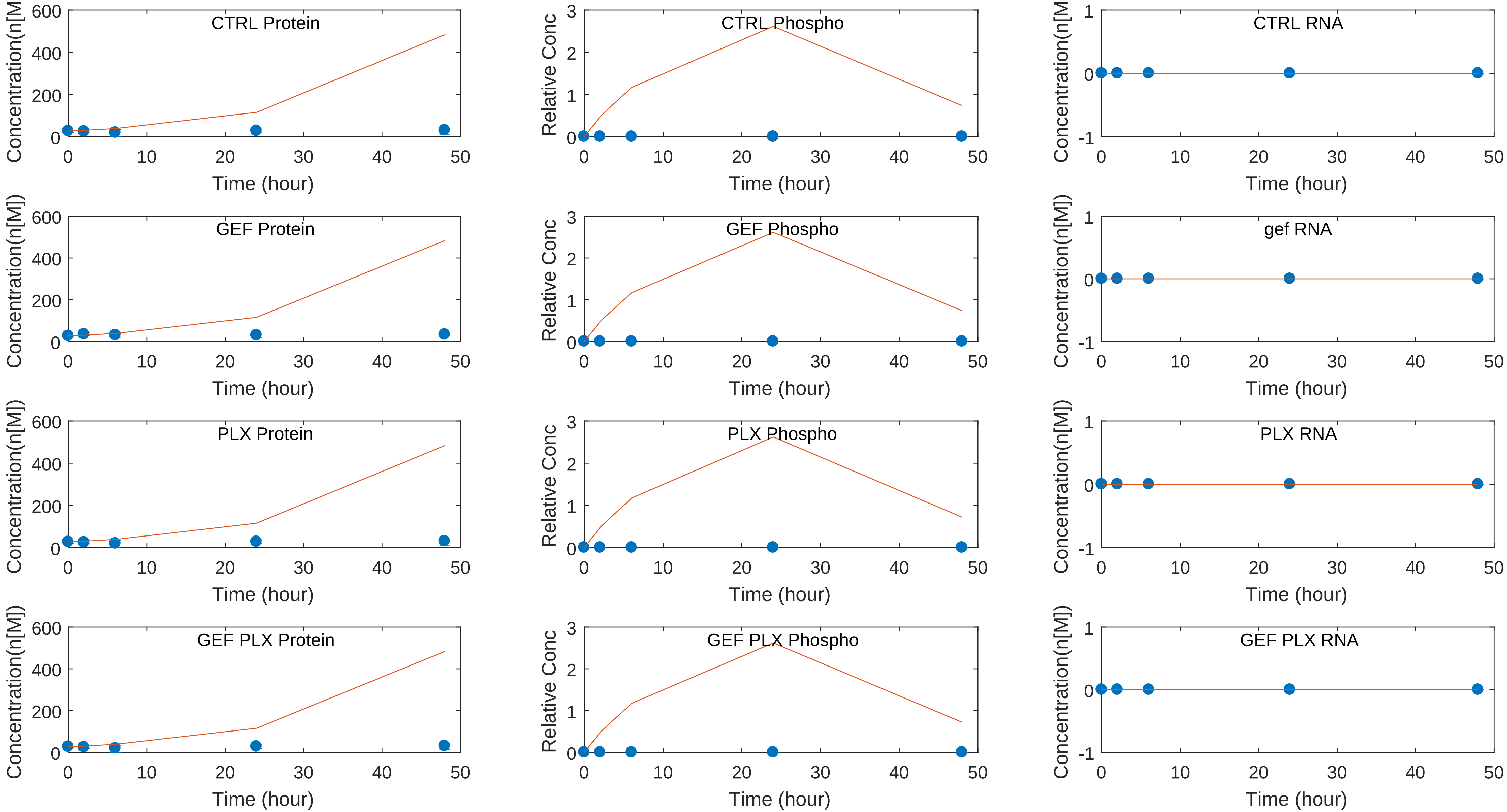

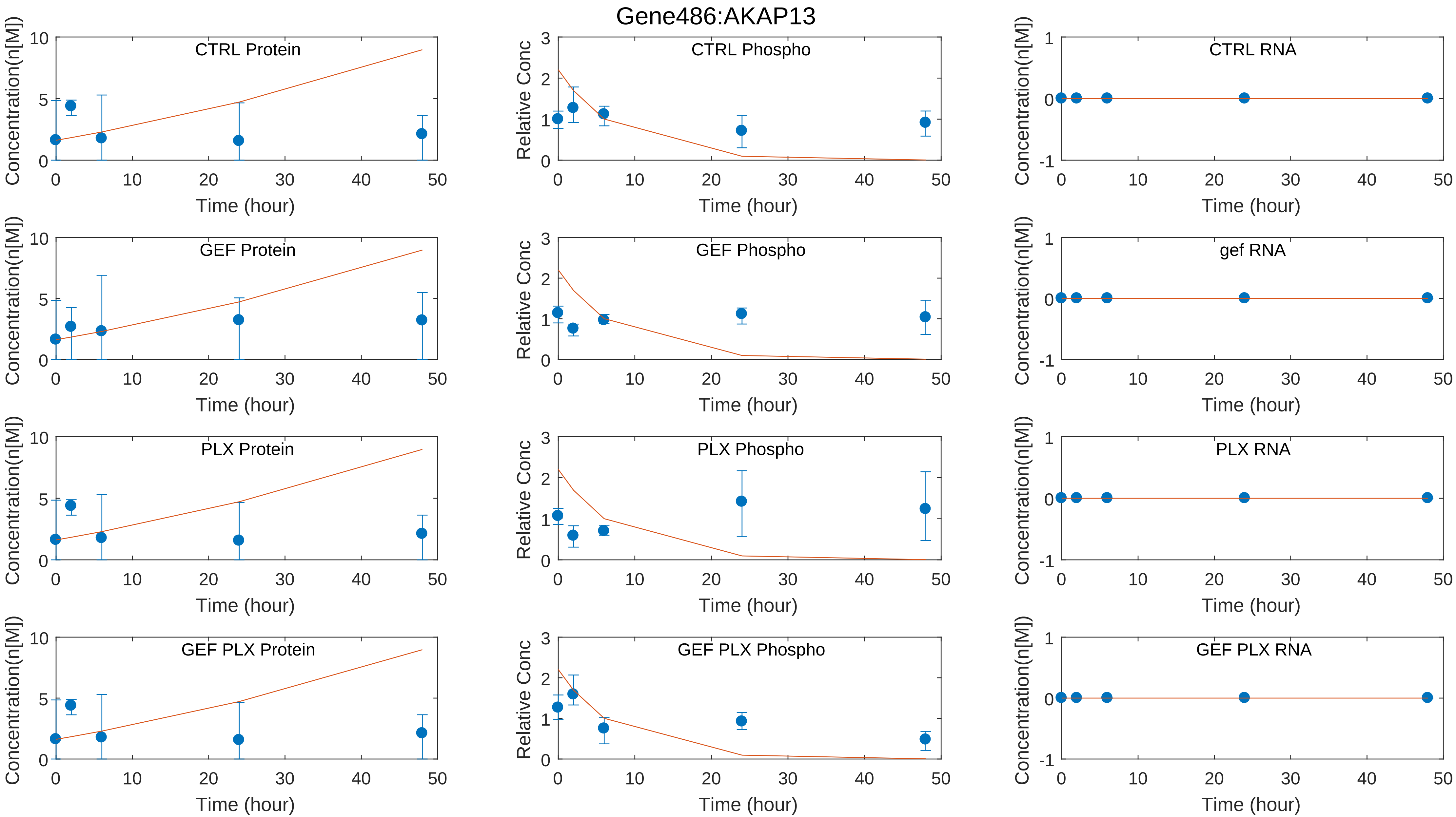

Gene486:AKAP13
CTRL Protein
GEF Protein
PLX Protein
GEF PLX Protein
CTRL Phospho
GEF Phospho
PLX Phospho
GEF PLX Phospho
CTRL RNA
gef RNA
PLX RNA
GEF PLX RNA
Concentration(n[M])
Relative Conc
Time (hour)

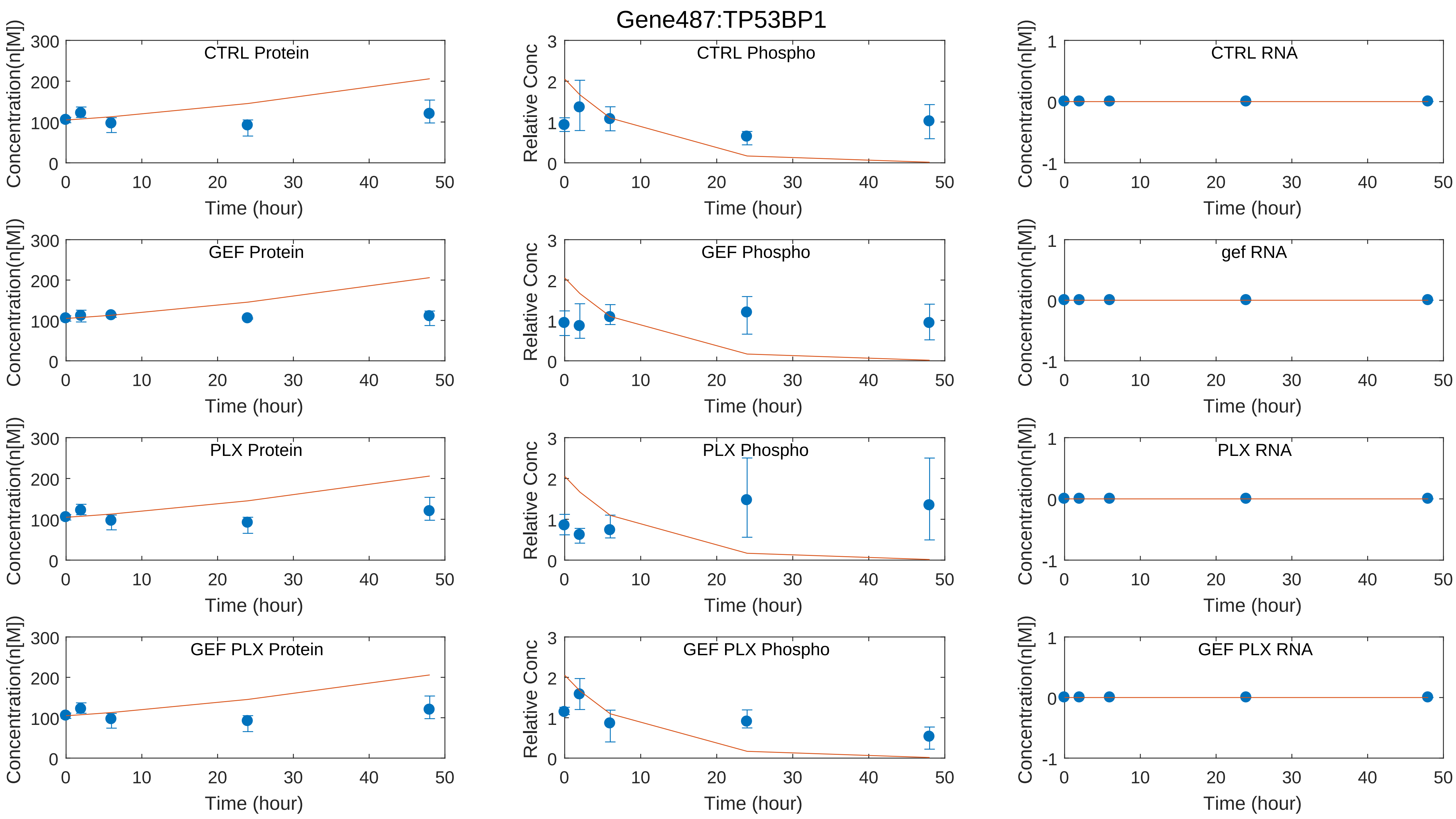

Gene487:TP53BP1
CTRL Protein
CTRL Phospho
CTRL RNA
GEF Protein
GEF Phospho
gef RNA
PLX Protein
PLX Phospho
PLX RNA
GEF PLX Protein
GEF PLX Phospho
GEF PLX RNA
Concentration(n[M])
Relative Conc
Time (hour)

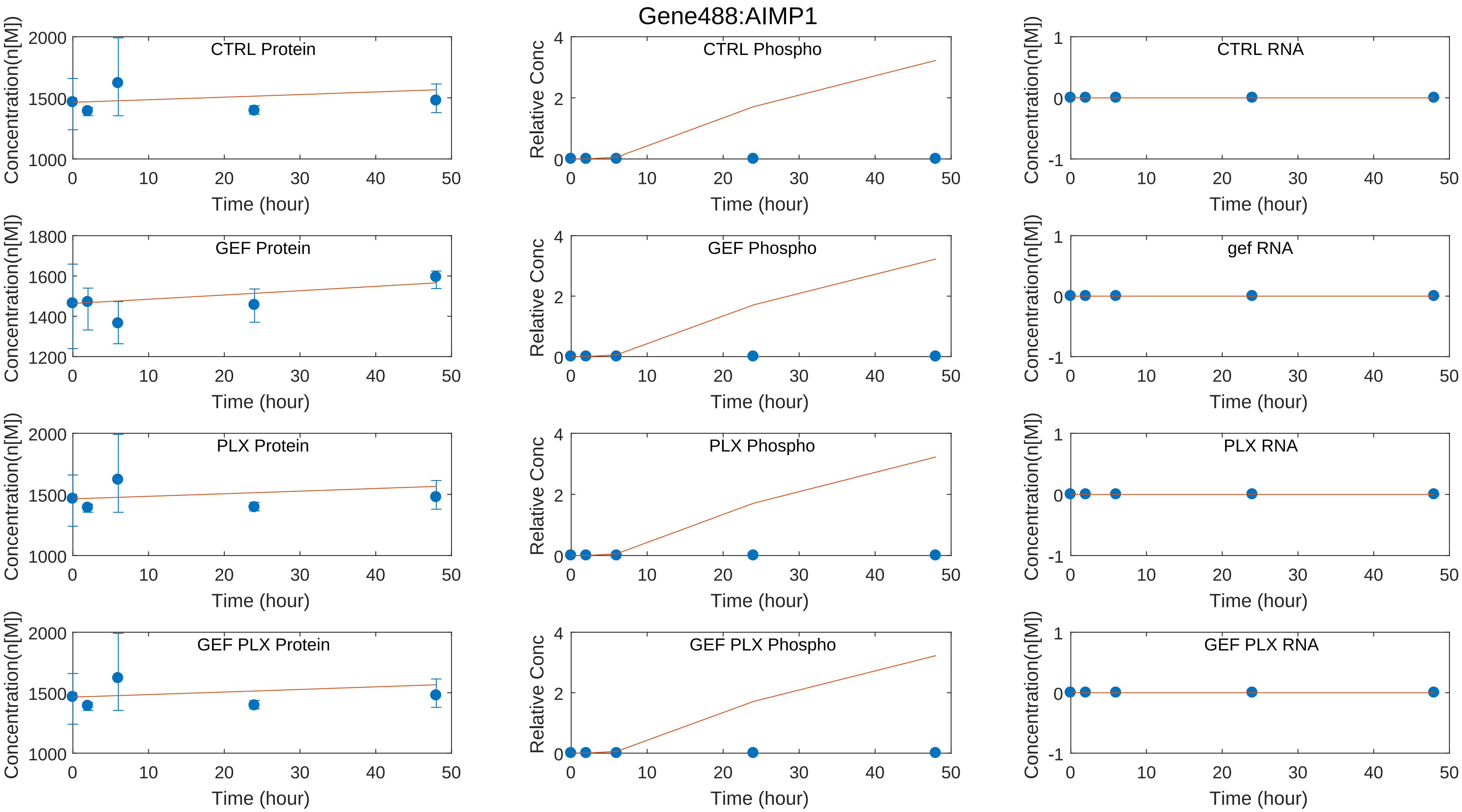
Gene488:AIMP1
CTRL Protein
CTRL Phospho
CTRL RNA
GEF Protein
GEF Phospho
gef RNA
PLX Protein
PLX Phospho
PLX RNA
GEF PLX Protein
GEF PLX Phospho
GEF PLX RNA
Concentration(n[M])
Relative Conc
Time (hour)

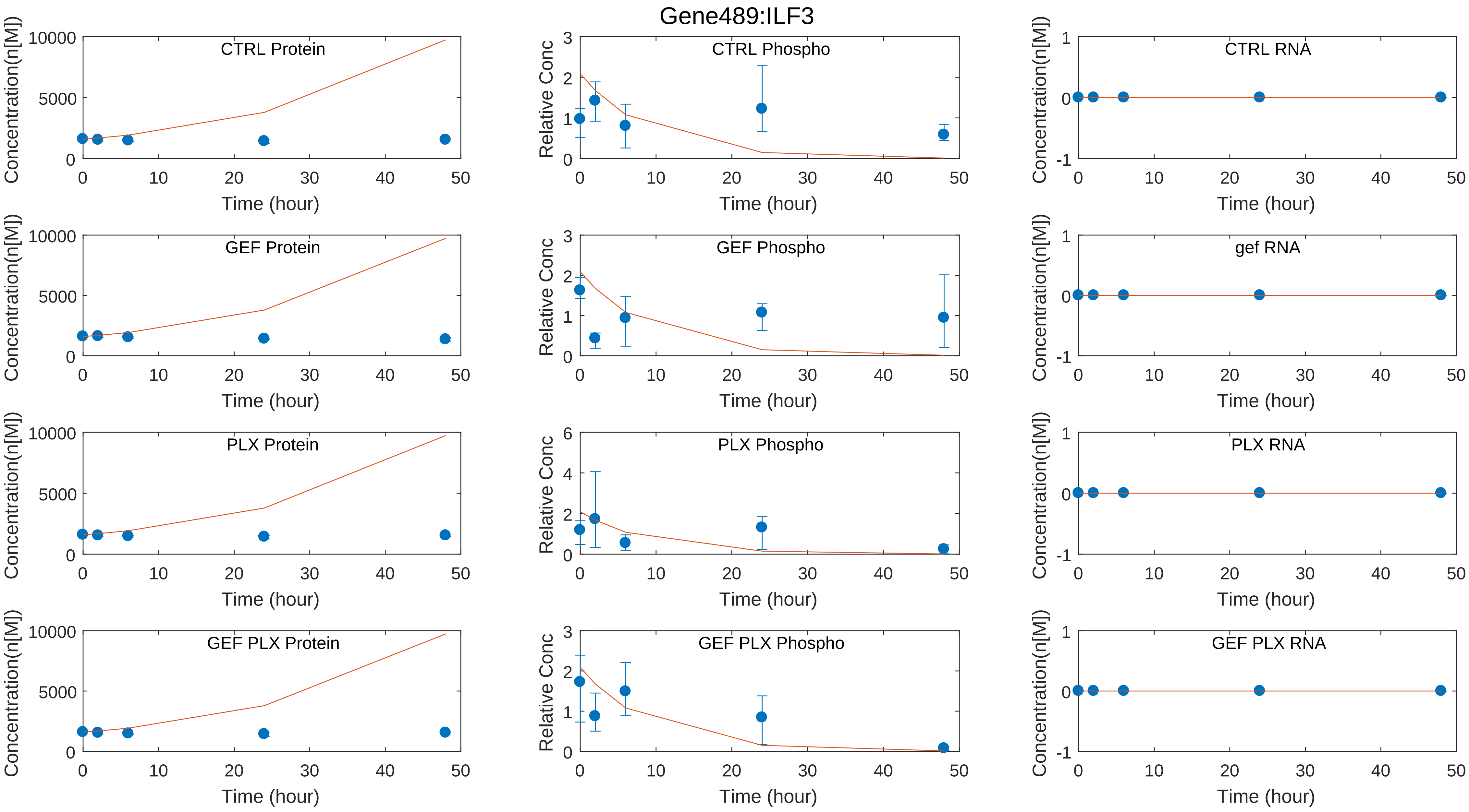

Gene489:ILF3
CTRL Protein
CTRL Phospho
CTRL RNA
GEF Protein
GEF Phospho
gef RNA
PLX Protein
PLX Phospho
PLX RNA
GEF PLX Protein
GEF PLX Phospho
GEF PLX RNA
Concentration(n[M])
Relative Conc
Time (hour)

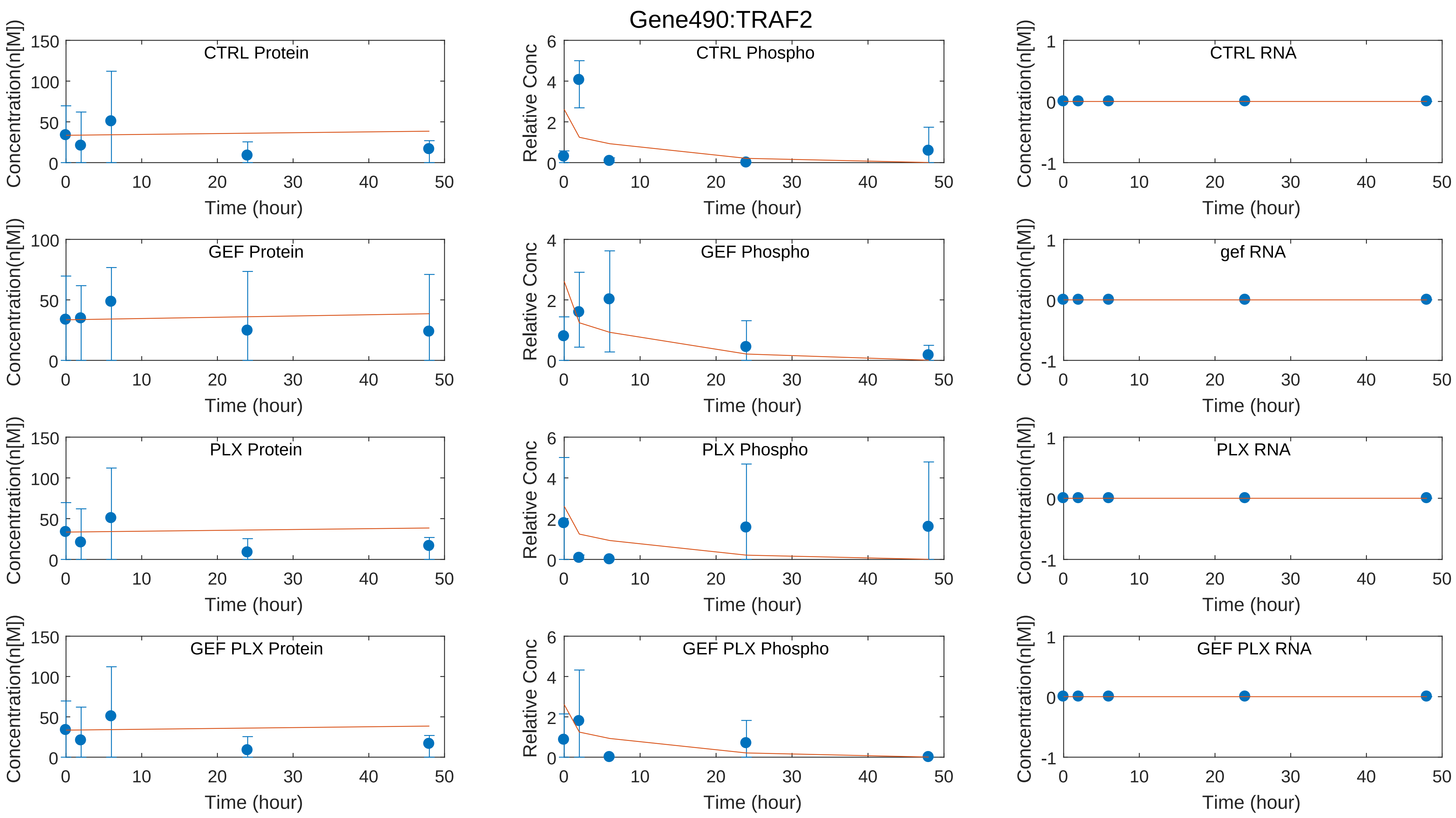

Gene490:TRAF2
CTRL Protein
CTRL Phospho
CTRL RNA
GEF Protein
GEF Phospho
gef RNA
PLX Protein
PLX Phospho
PLX RNA
GEF PLX Protein
GEF PLX Phospho
GEF PLX RNA
Concentration(n[M])
Relative Conc
Time (hour)

Gene491:DLG1

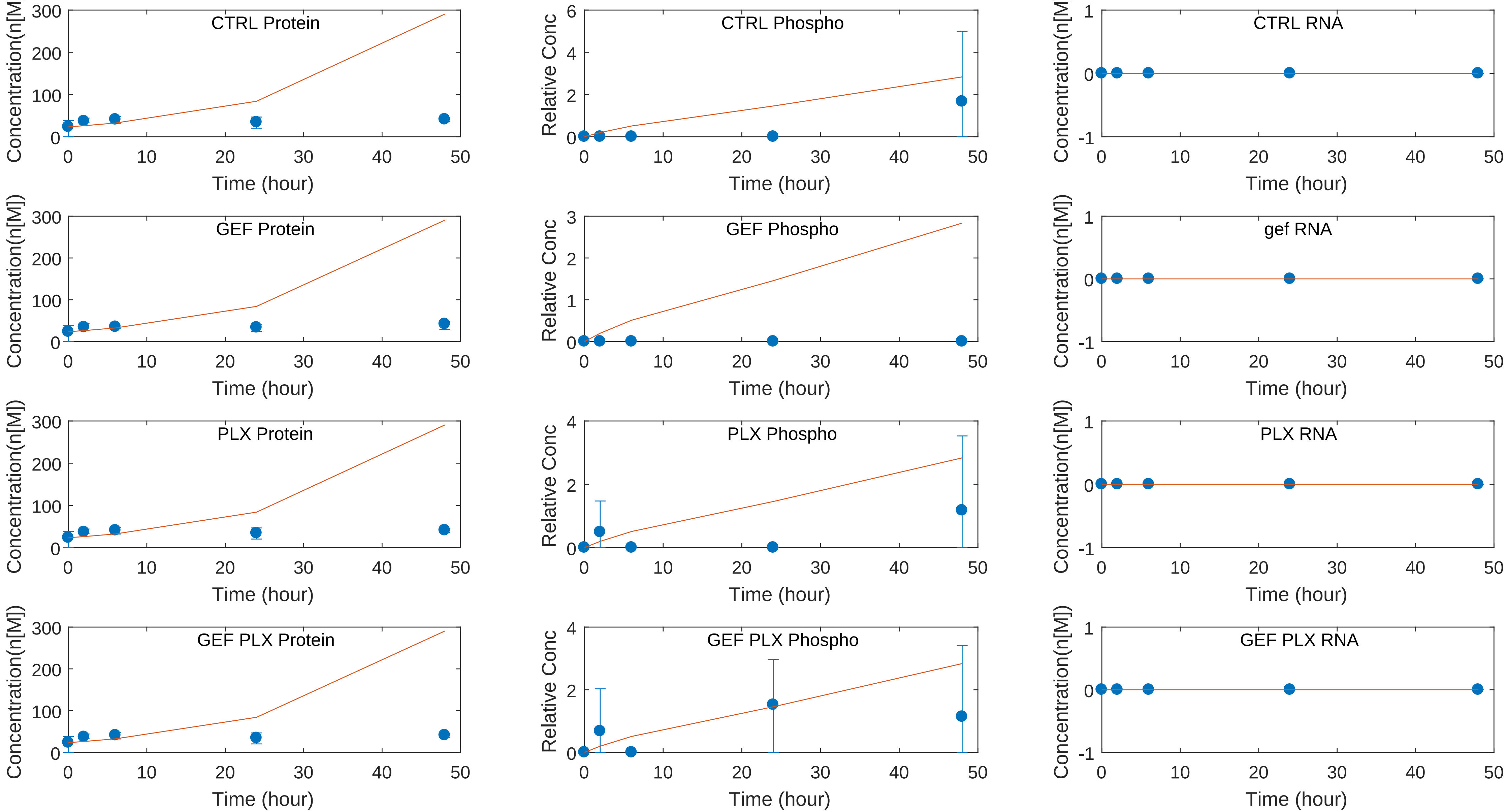

Gene492:PPP1R8

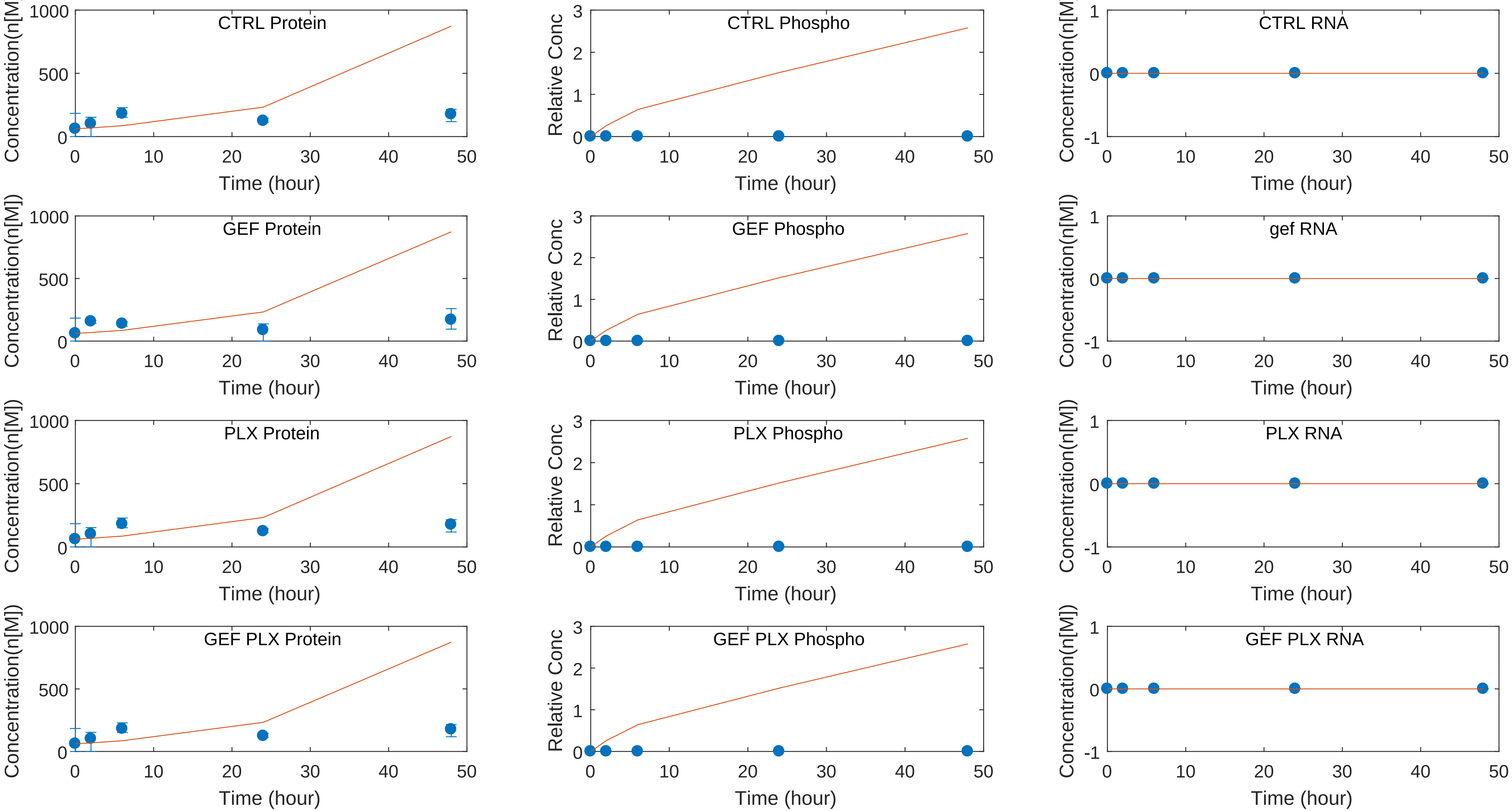

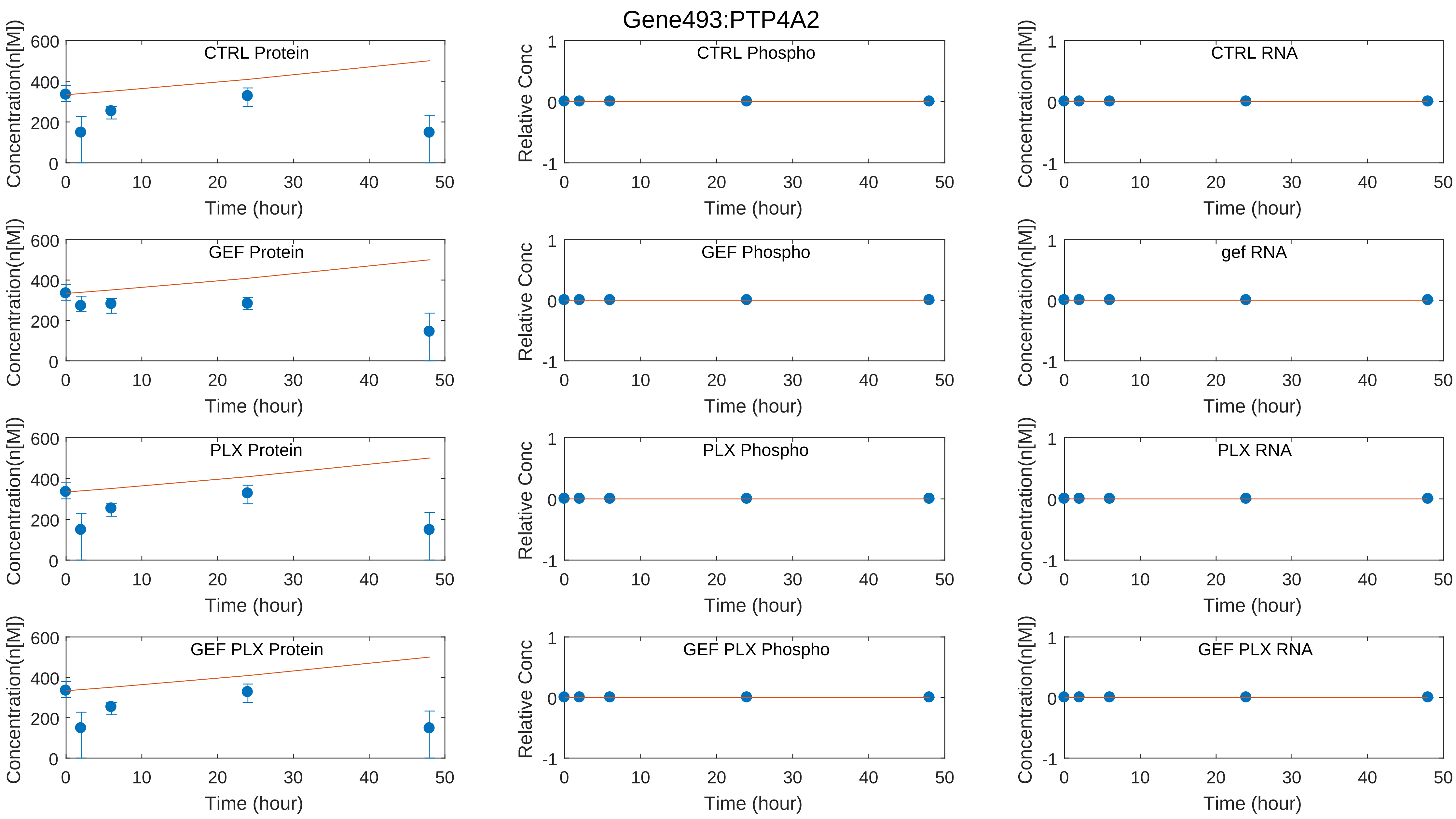
Gene493:PTP4A2
CTRL Protein
CTRL Phospho
CTRL RNA
GEF Protein
GEF Phospho
gef RNA
PLX Protein
PLX Phospho
PLX RNA
GEF PLX Protein
GEF PLX Phospho
GEF PLX RNA
Concentration(n[M])
Relative Conc
Time (hour)

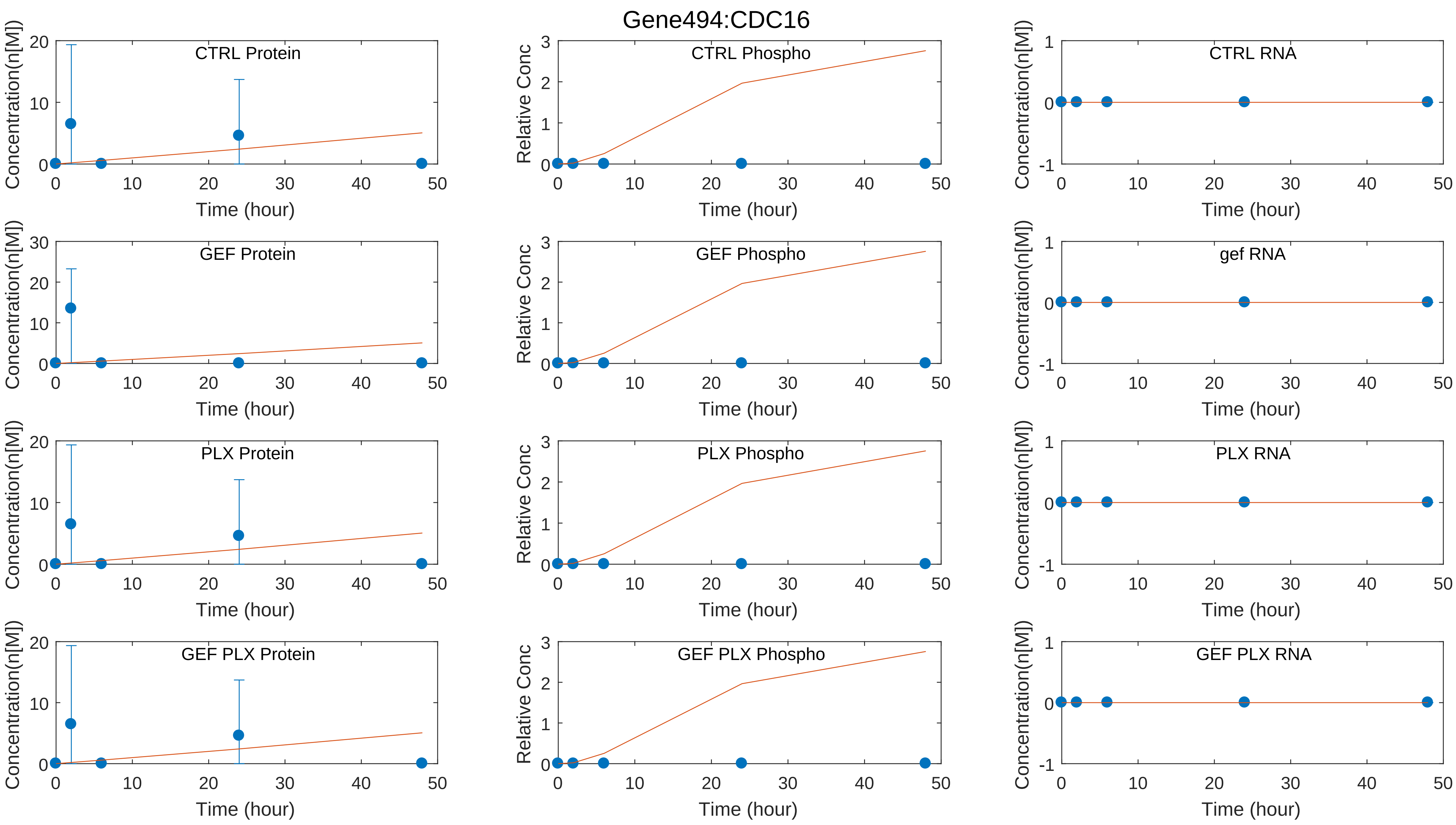

Gene494:CDC16
CTRL Protein
CTRL Phospho
CTRL RNA
GEF Protein
GEF Phospho
gef RNA
PLX Protein
PLX Phospho
PLX RNA
GEF PLX Protein
GEF PLX Phospho
GEF PLX RNA
Concentration(n[M])
Relative Conc
Time (hour)

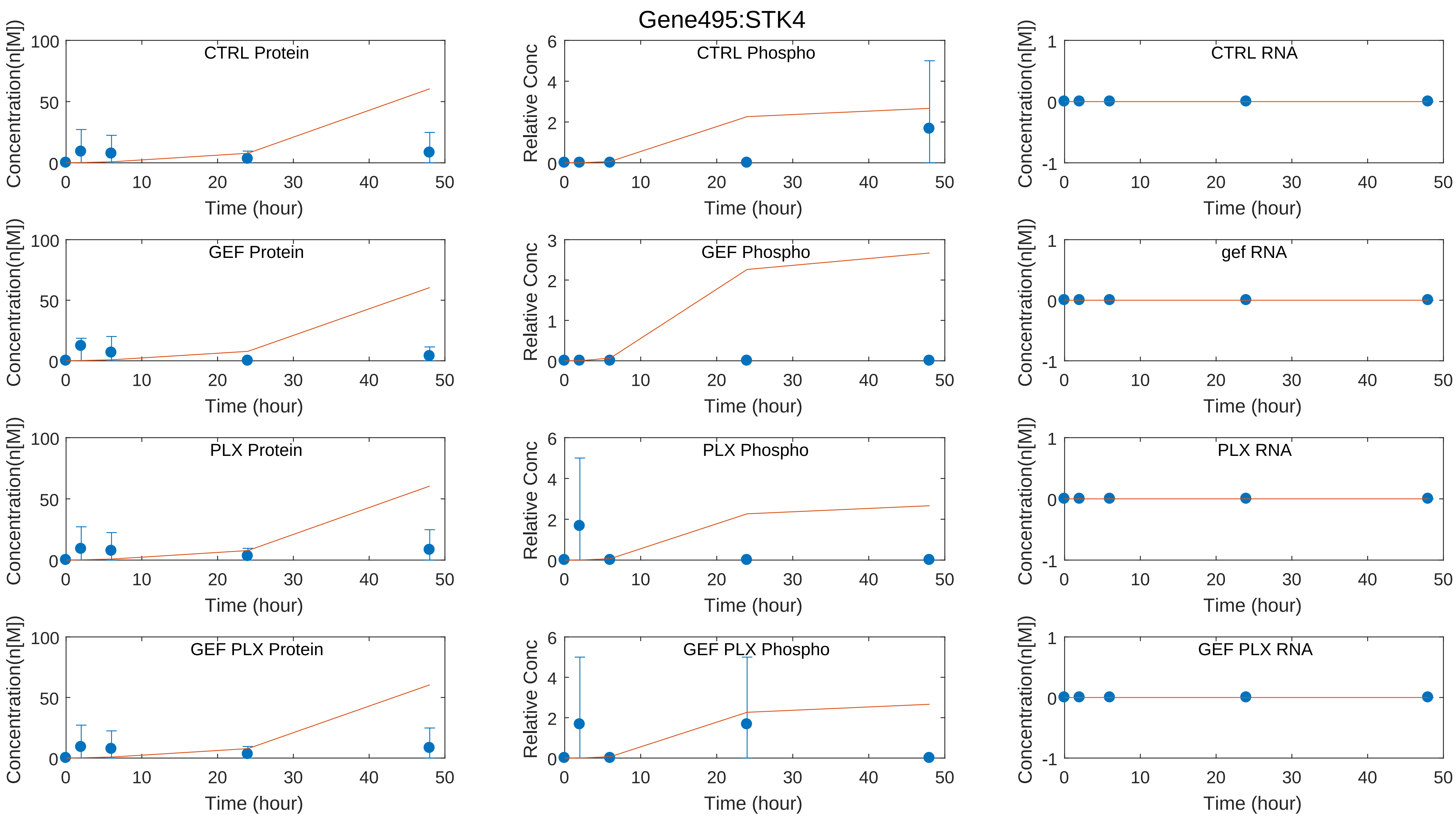

Gene495:STK4
CTRL Protein
CTRL Phospho
CTRL RNA
GEF Protein
GEF Phospho
gef RNA
PLX Protein
PLX Phospho
PLX RNA
GEF PLX Protein
GEF PLX Phospho
GEF PLX RNA
Concentration(n[M])
Relative Conc
Time (hour)

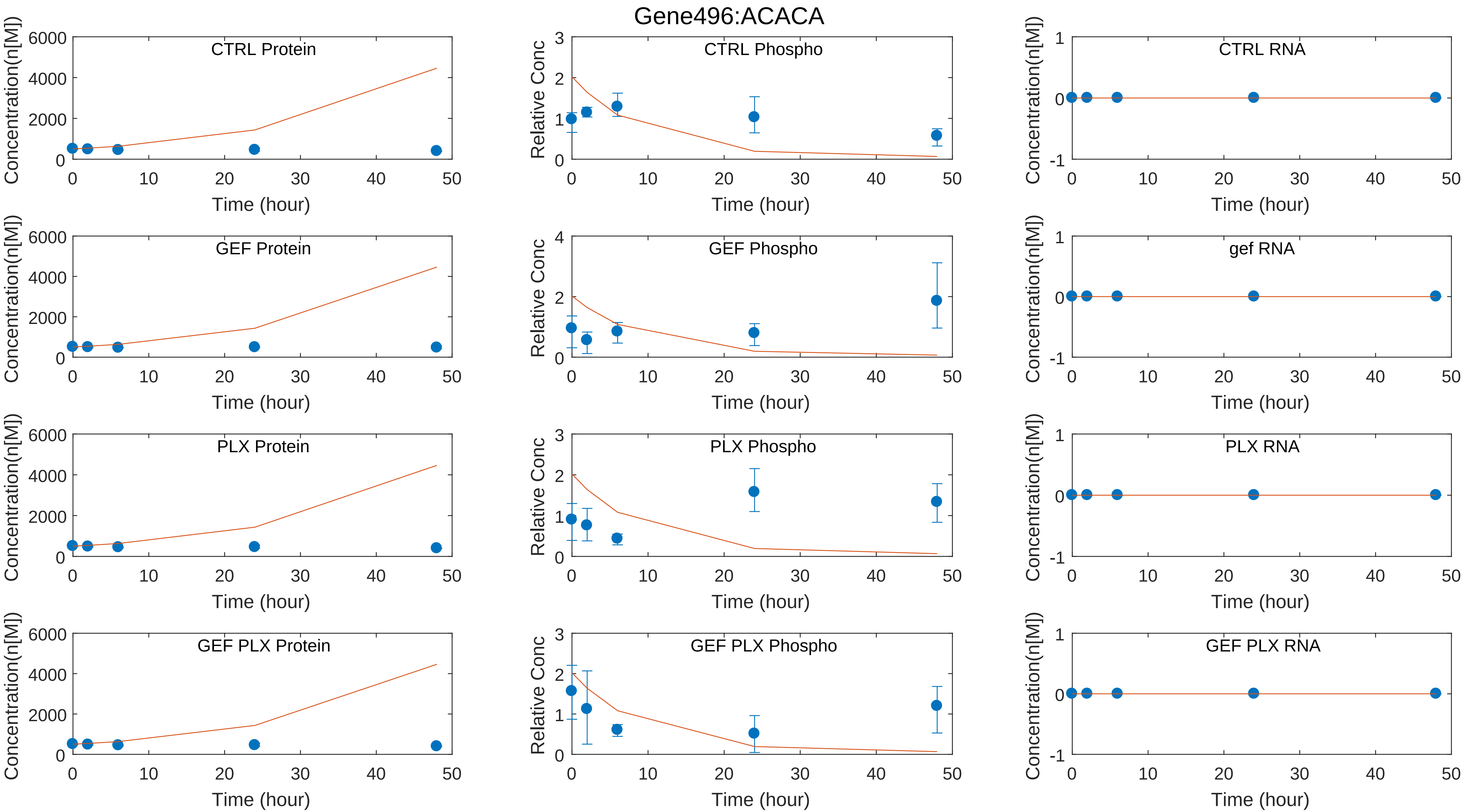
Gene496:ACACA
CTRL Protein
GEF Protein
PLX Protein
GEF PLX Protein
CTRL Phospho
GEF Phospho
PLX Phospho
GEF PLX Phospho
CTRL RNA
gef RNA
PLX RNA
GEF PLX RNA
Concentration(n[M])
Relative Conc
Time (hour)

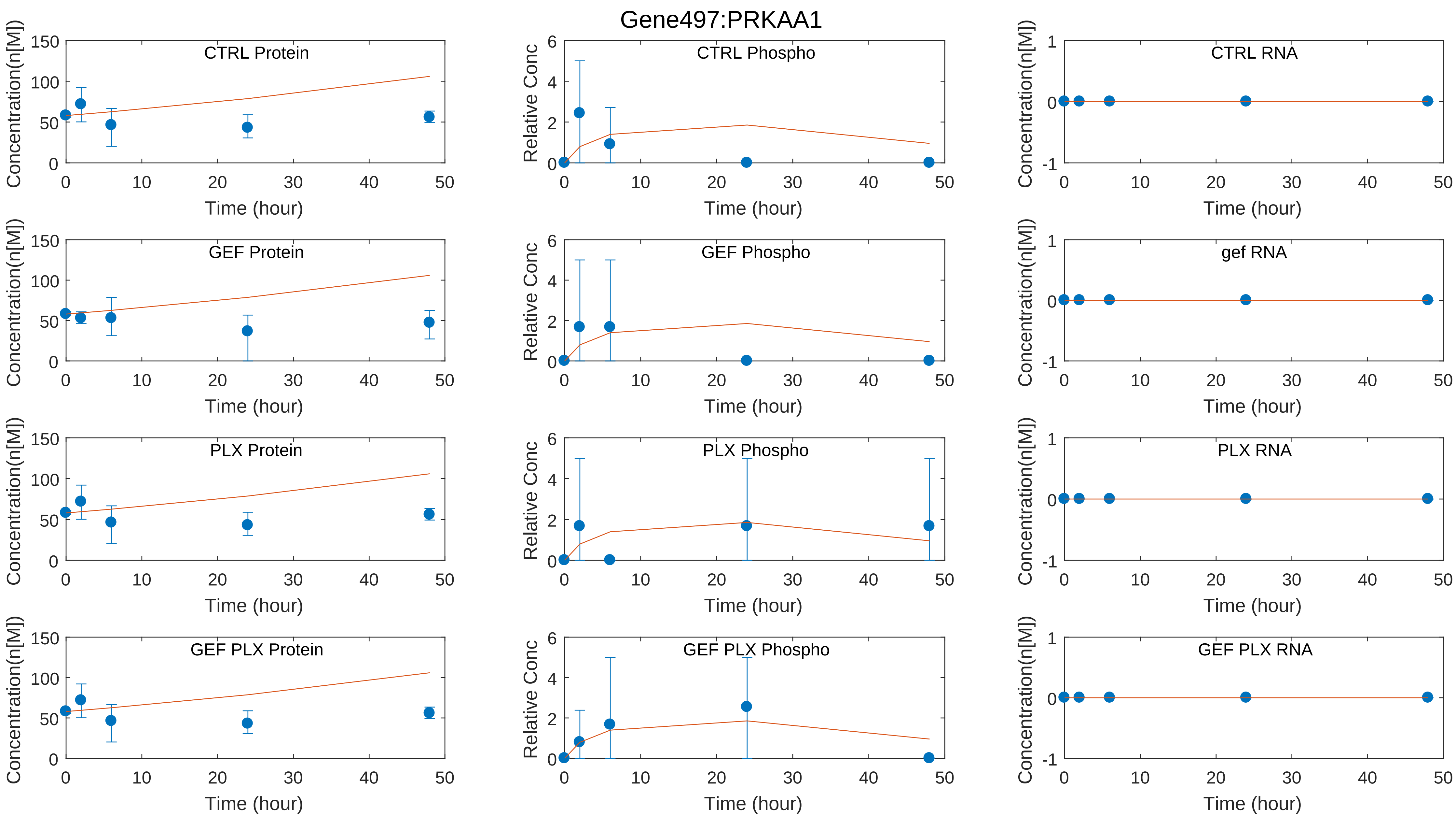

Gene497:PRKAA1
CTRL Protein
CTRL Phospho
CTRL RNA
GEF Protein
GEF Phospho
gef RNA
PLX Protein
PLX Phospho
PLX RNA
GEF PLX Protein
GEF PLX Phospho
GEF PLX RNA
Concentration(n[M])
Relative Conc
Time (hour)

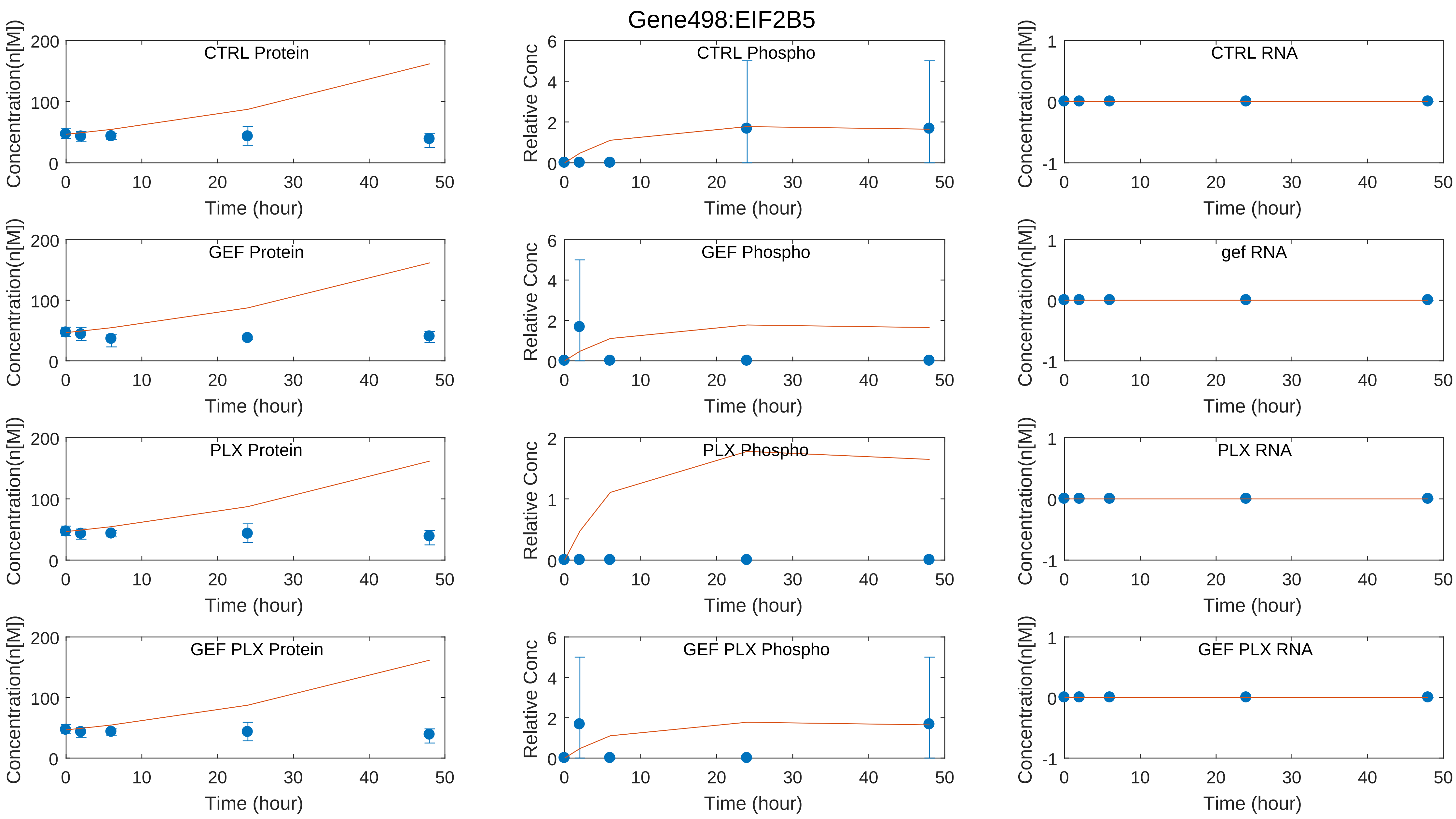
Gene498:EIF2B5
CTRL Protein
CTRL Phospho
CTRL RNA
GEF Protein
GEF Phospho
gef RNA
PLX Protein
PLX Phospho
PLX RNA
GEF PLX Protein
GEF PLX Phospho
GEF PLX RNA
Concentration(n[M])
Relative Conc
Time (hour)

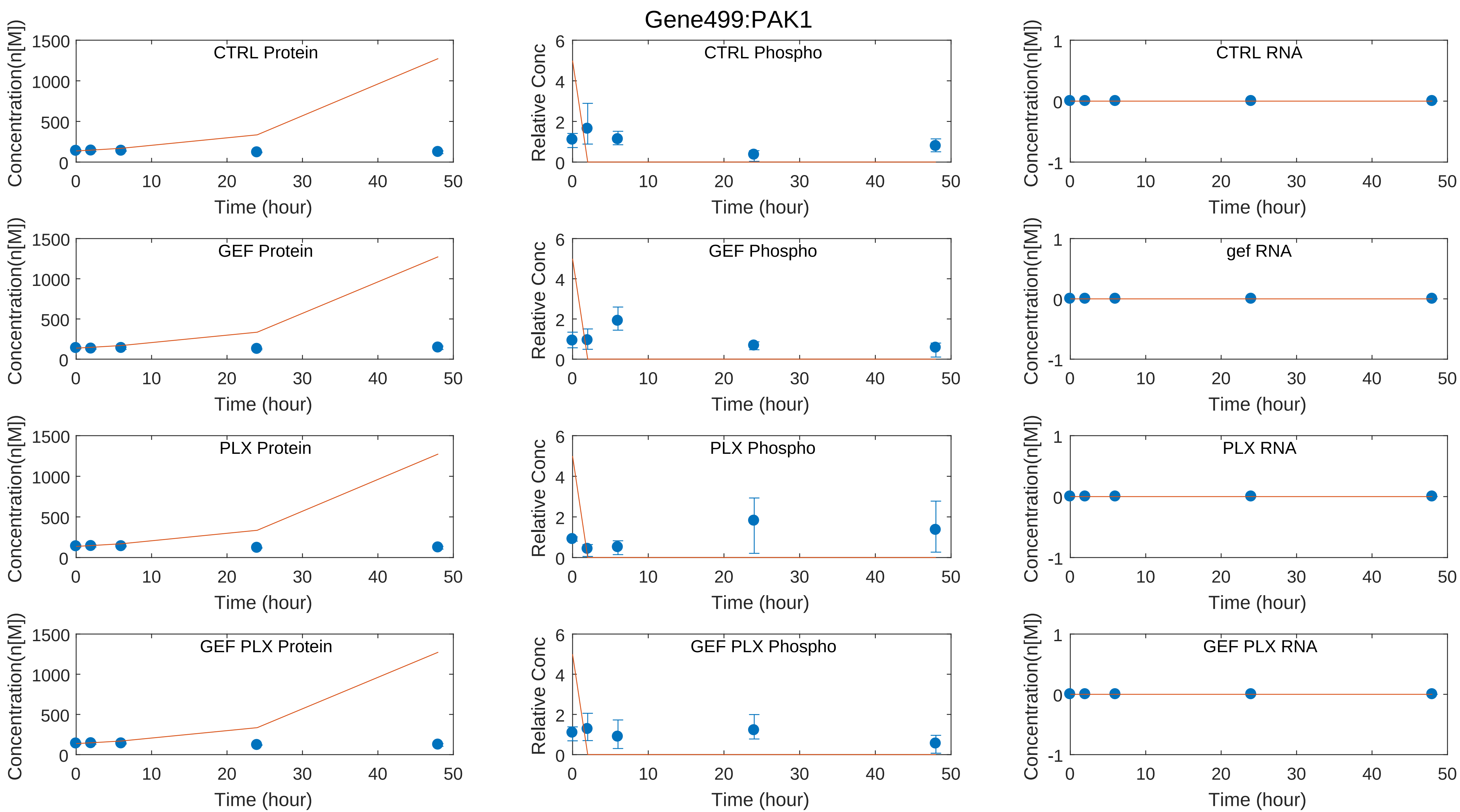
Gene499:PAK1
CTRL Protein
GEF Protein
PLX Protein
GEF PLX Protein
CTRL Phospho
GEF Phospho
PLX Phospho
GEF PLX Phospho
CTRL RNA
gef RNA
PLX RNA
GEF PLX RNA
Concentration(n[M])
Relative Conc
Time (hour)

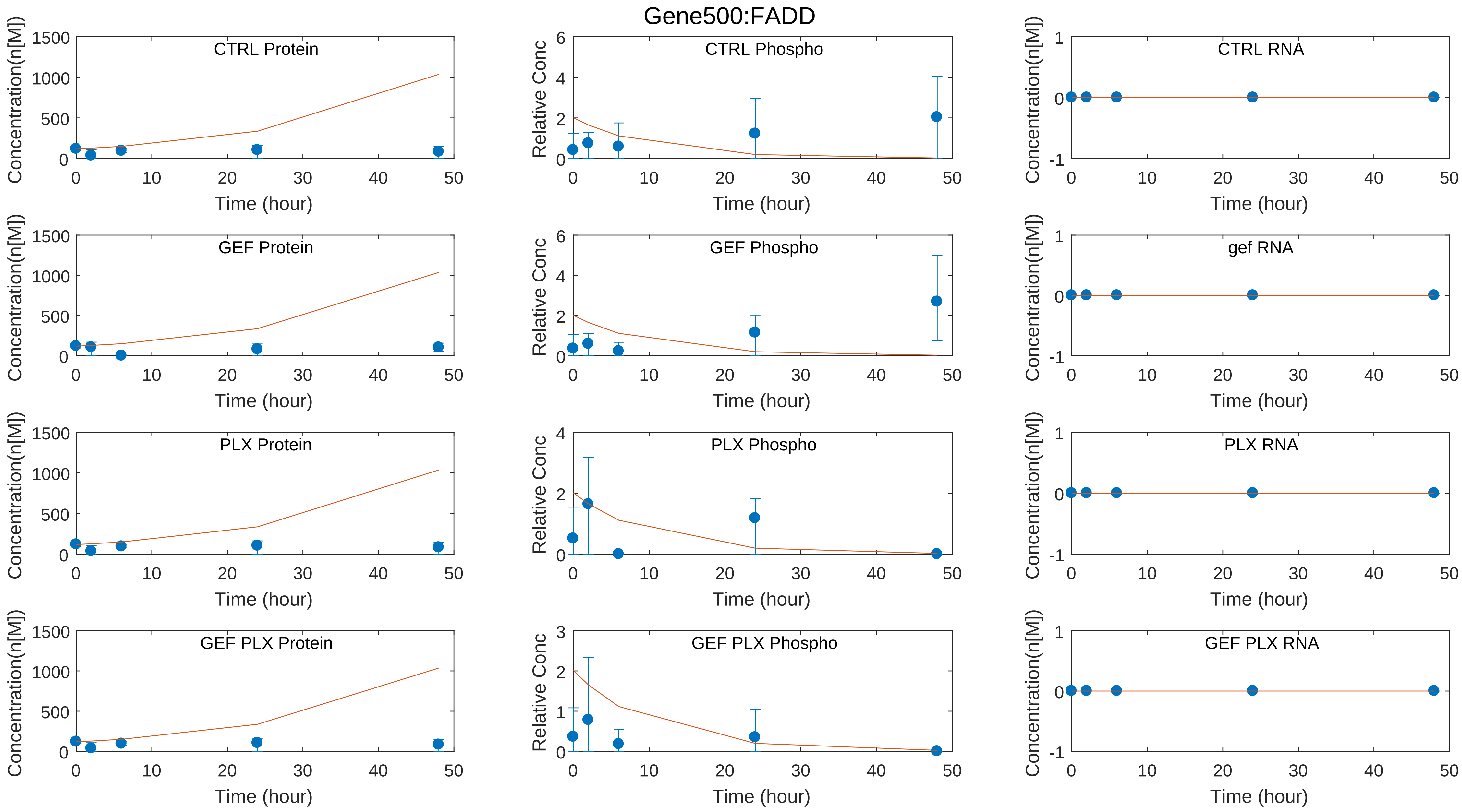

Gene500:FADD
CTRL Protein
CTRL Phospho
CTRL RNA
GEF Protein
GEF Phospho
gef RNA
PLX Protein
PLX Phospho
PLX RNA
GEF PLX Protein
GEF PLX Phospho
GEF PLX RNA
Concentration(n[M])
Relative Conc
Time (hour)

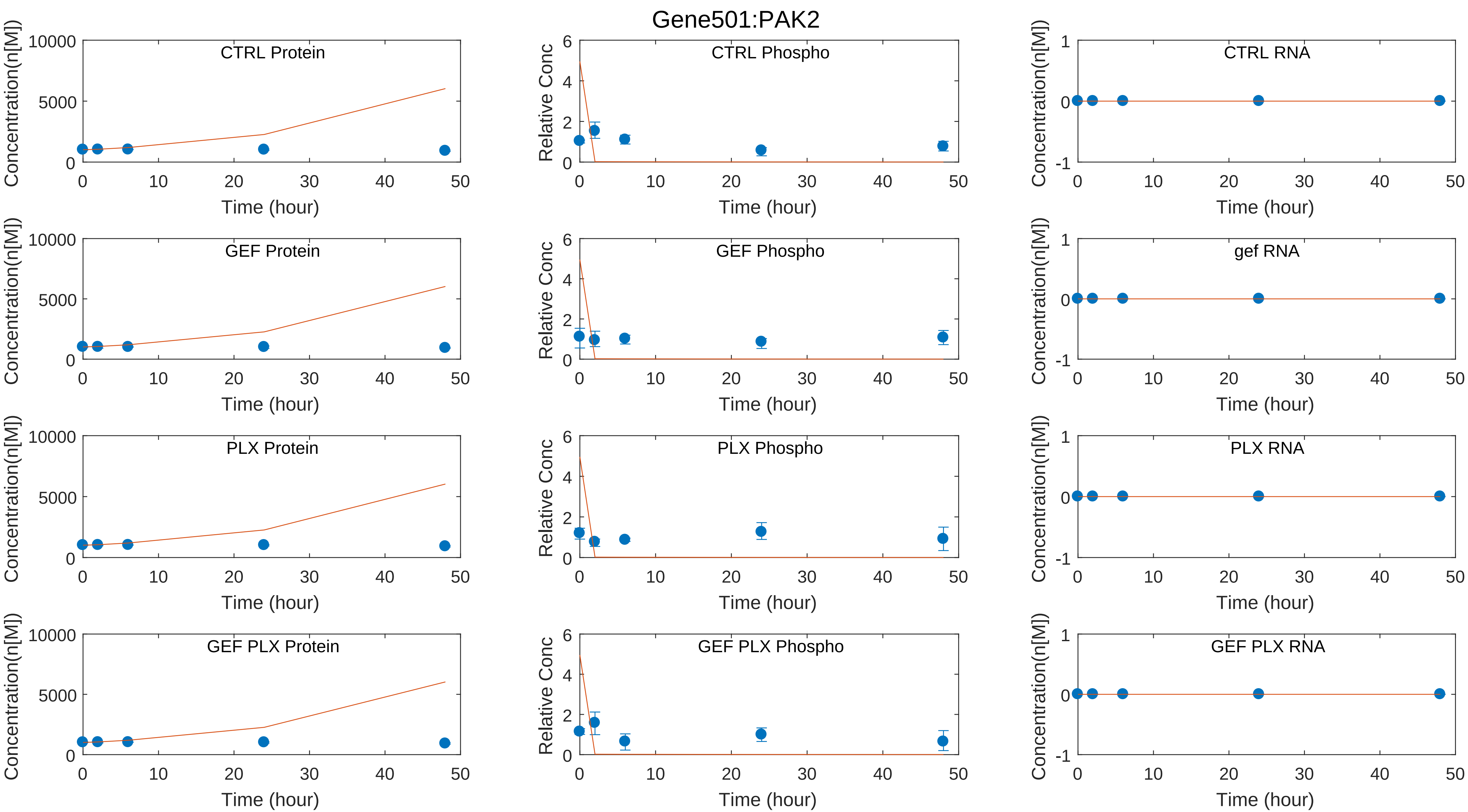
Gene501:PAK2
CTRL Protein
CTRL Phospho
CTRL RNA
GEF Protein
GEF Phospho
gef RNA
PLX Protein
PLX Phospho
PLX RNA
GEF PLX Protein
GEF PLX Phospho
GEF PLX RNA
Concentration(n[M])
Relative Conc
Time (hour)

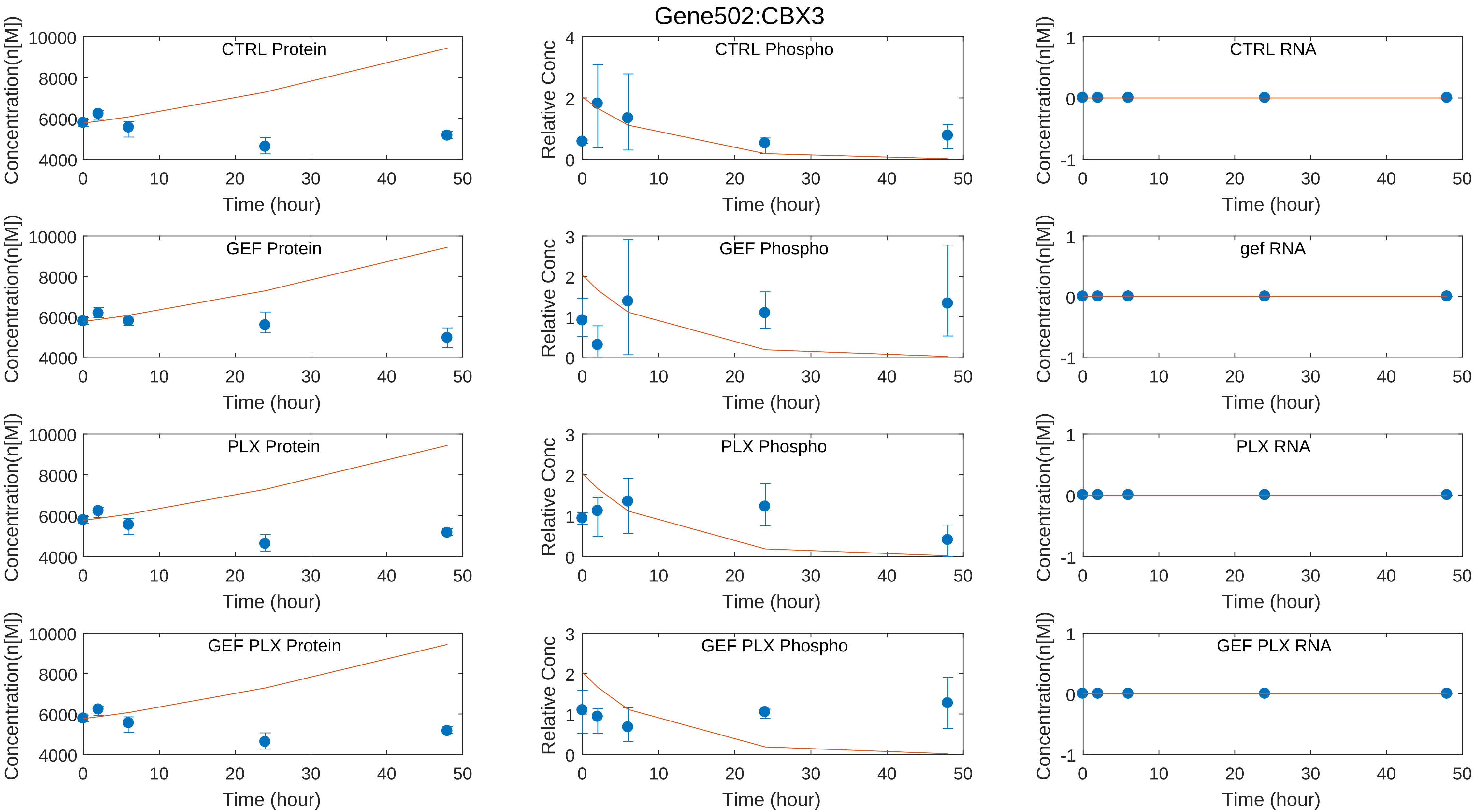
Gene502:CBX3
CTRL Protein
GEF Protein
PLX Protein
GEF PLX Protein
CTRL Phospho
GEF Phospho
PLX Phospho
GEF PLX Phospho
CTRL RNA
gef RNA
PLX RNA
GEF PLX RNA
Concentration(n[M])
Relative Conc
Time (hour)

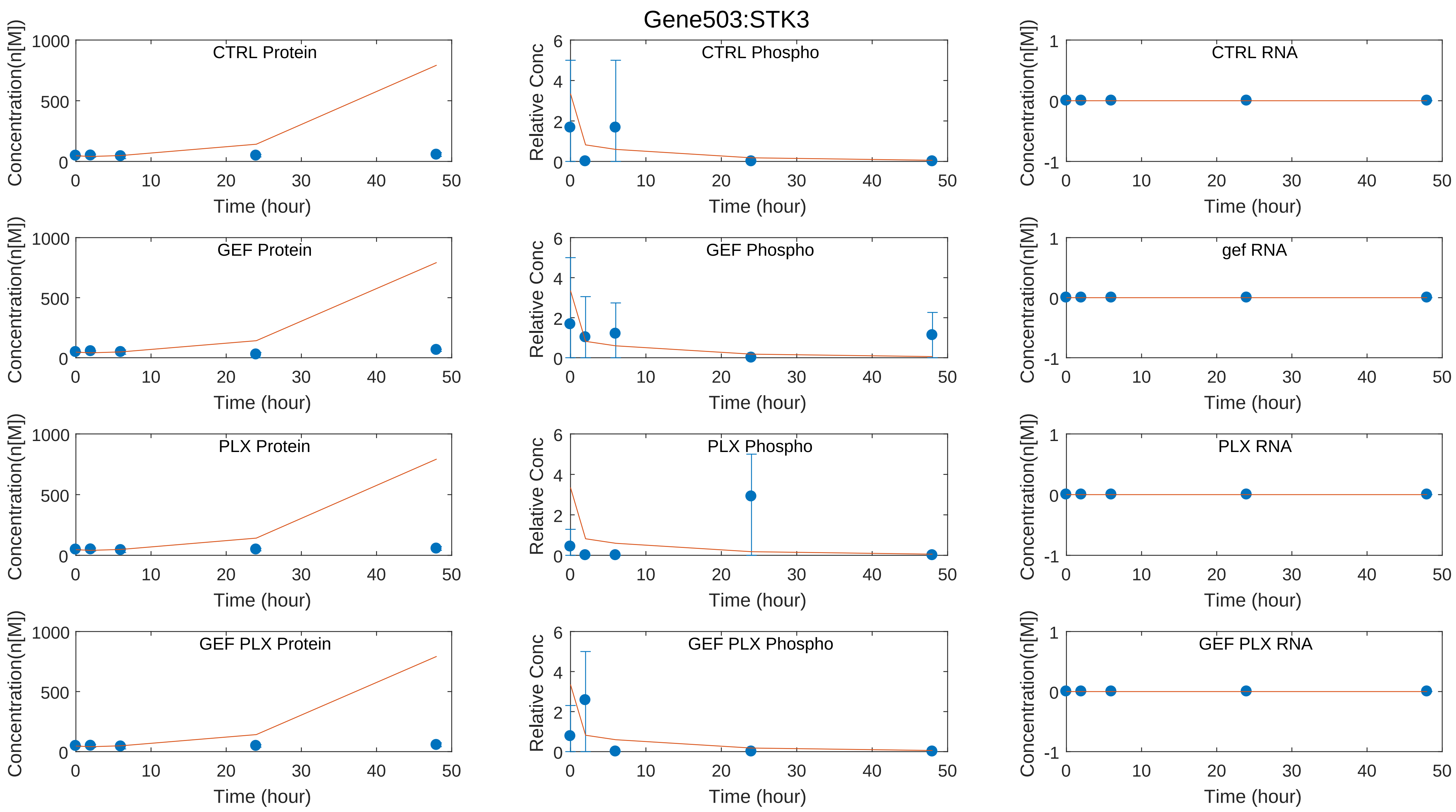

Gene503:STK3
CTRL Protein
CTRL Phospho
CTRL RNA
GEF Protein
GEF Phospho
gef RNA
PLX Protein
PLX Phospho
PLX RNA
GEF PLX Protein
GEF PLX Phospho
GEF PLX RNA
Concentration(n[M])
Relative Conc
Time (hour)

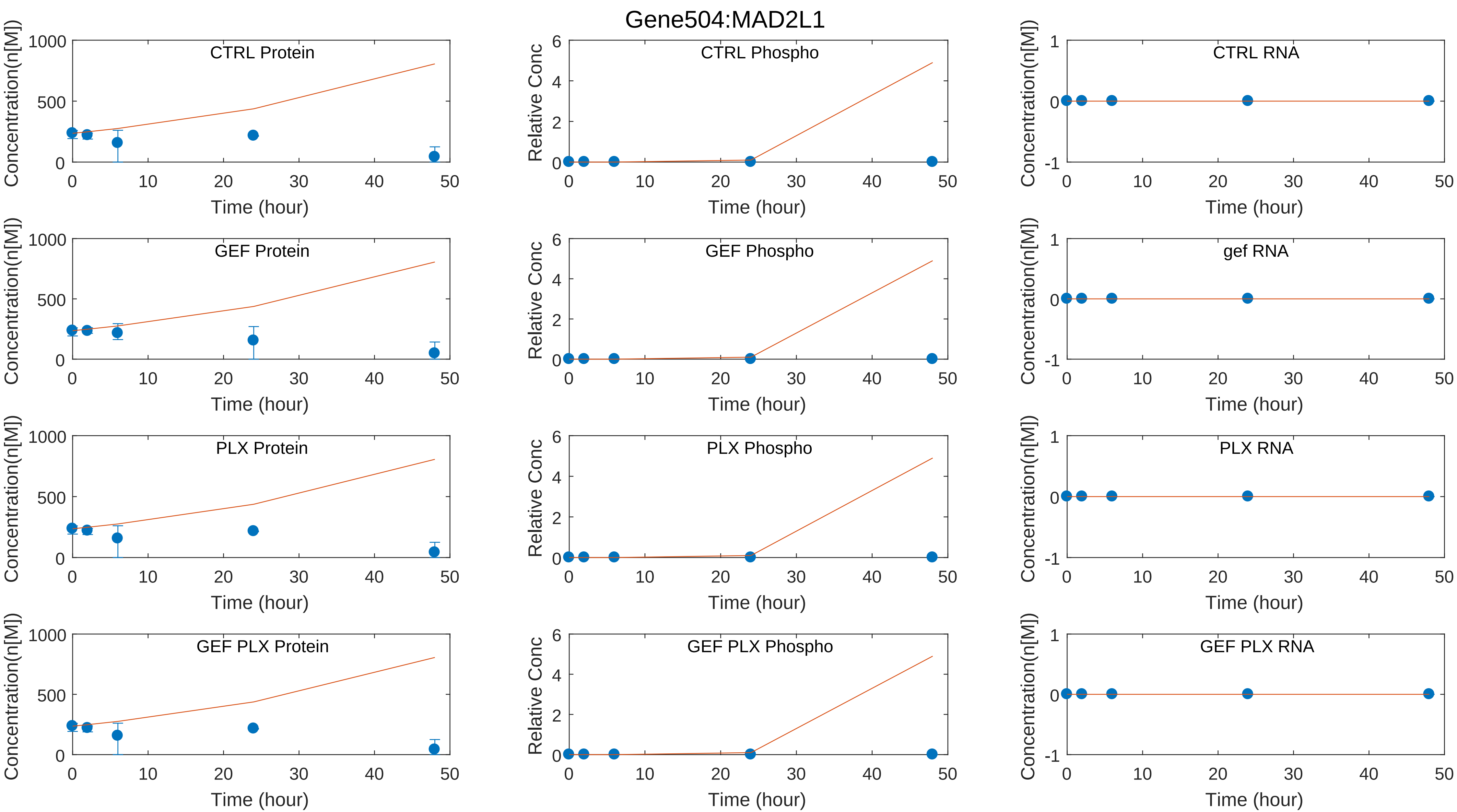
Gene504:MAD2L1
CTRL Protein
CTRL Phospho
CTRL RNA
GEF Protein
GEF Phospho
gef RNA
PLX Protein
PLX Phospho
PLX RNA
GEF PLX Protein
GEF PLX Phospho
GEF PLX RNA
Concentration(n[M])
Relative Conc
Time (hour)

Gene505:TRIM28

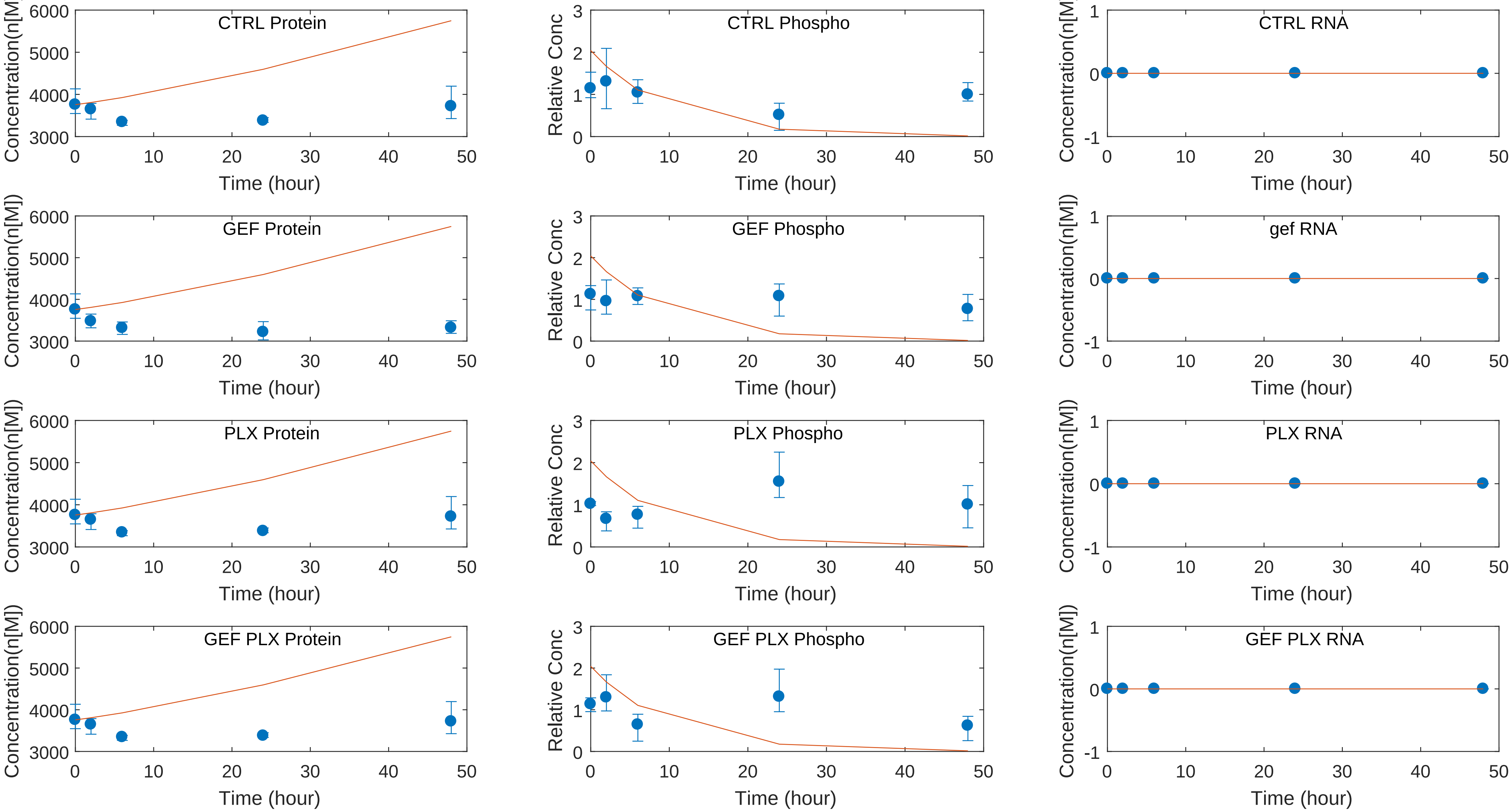

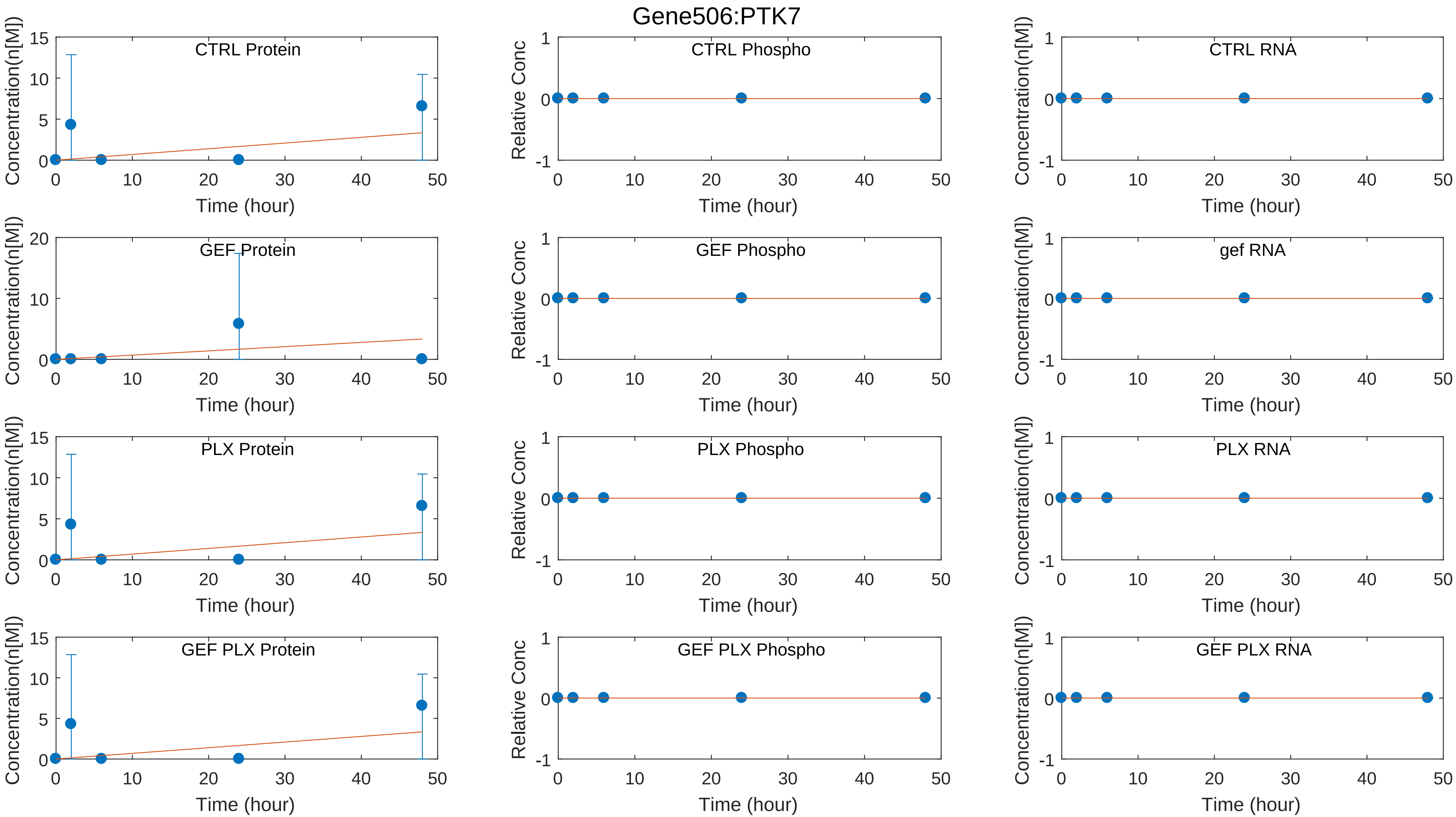

Gene506:PTK7
CTRL Protein
CTRL Phospho
CTRL RNA
GEF Protein
GEF Phospho
gef RNA
PLX Protein
PLX Phospho
PLX RNA
GEF PLX Protein
GEF PLX Phospho
GEF PLX RNA
Concentration(n[M])
Relative Conc
Time (hour)

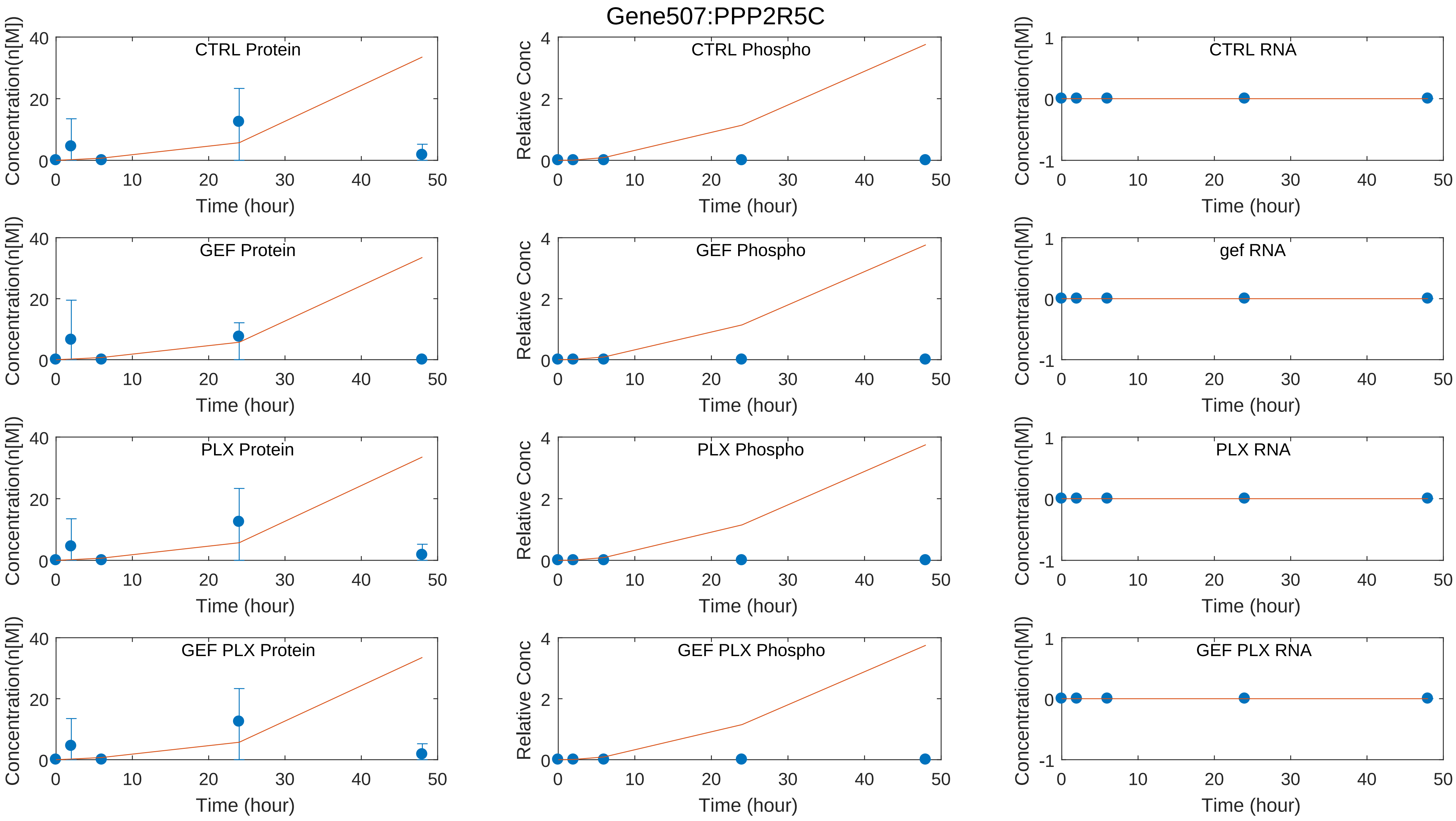

Gene507:PPP2R5C
CTRL Protein
CTRL Phospho
CTRL RNA
GEF Protein
GEF Phospho
gef RNA
PLX Protein
PLX Phospho
PLX RNA
GEF PLX Protein
GEF PLX Phospho
GEF PLX RNA
Concentration(n[M])
Relative Conc
Time (hour)

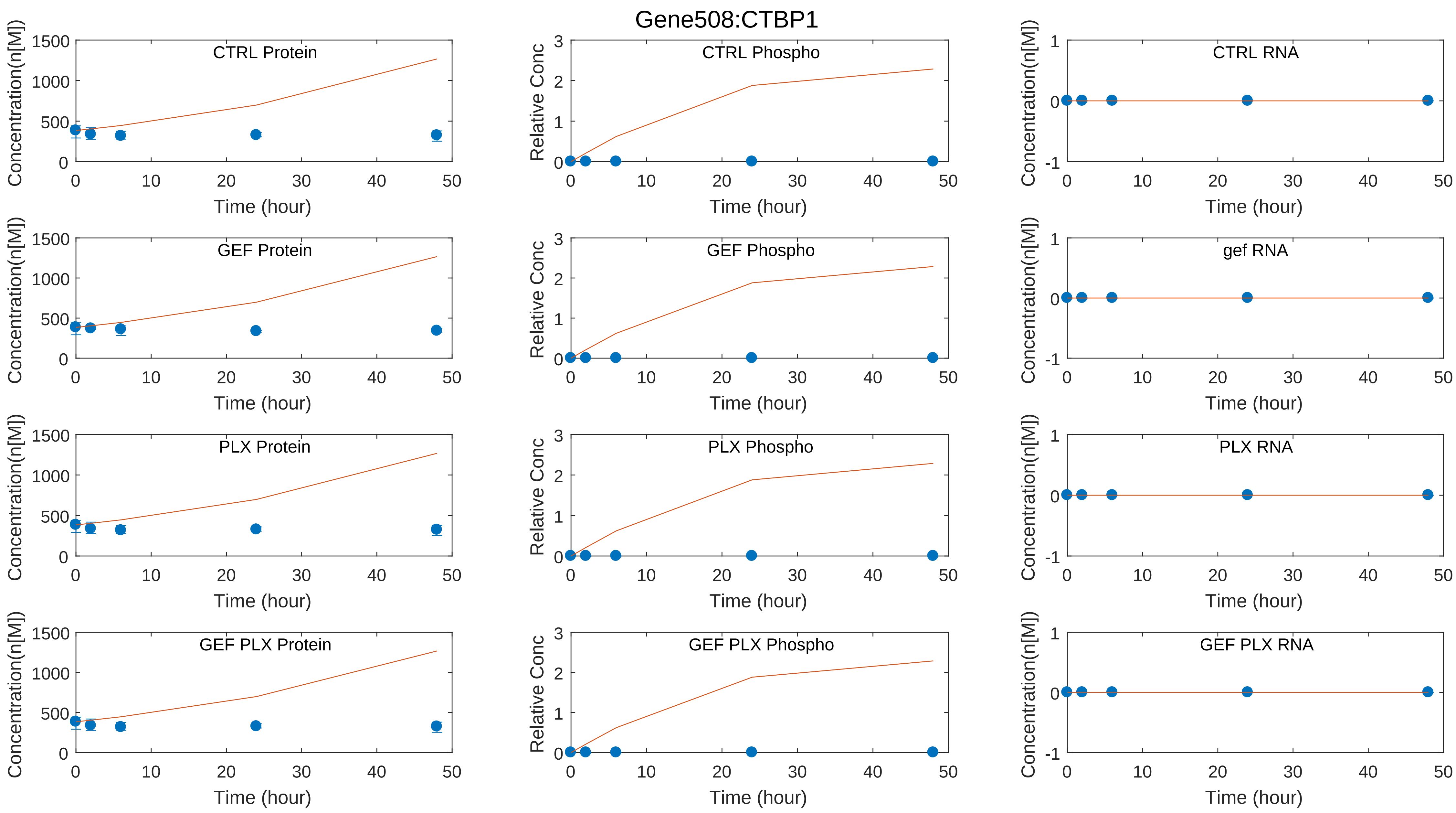

Gene508:CTBP1
CTRL Protein
CTRL Phospho
CTRL RNA
GEF Protein
GEF Phospho
gef RNA
PLX Protein
PLX Phospho
PLX RNA
GEF PLX Protein
GEF PLX Phospho
GEF PLX RNA
Concentration(n[M])
Relative Conc
Time (hour)

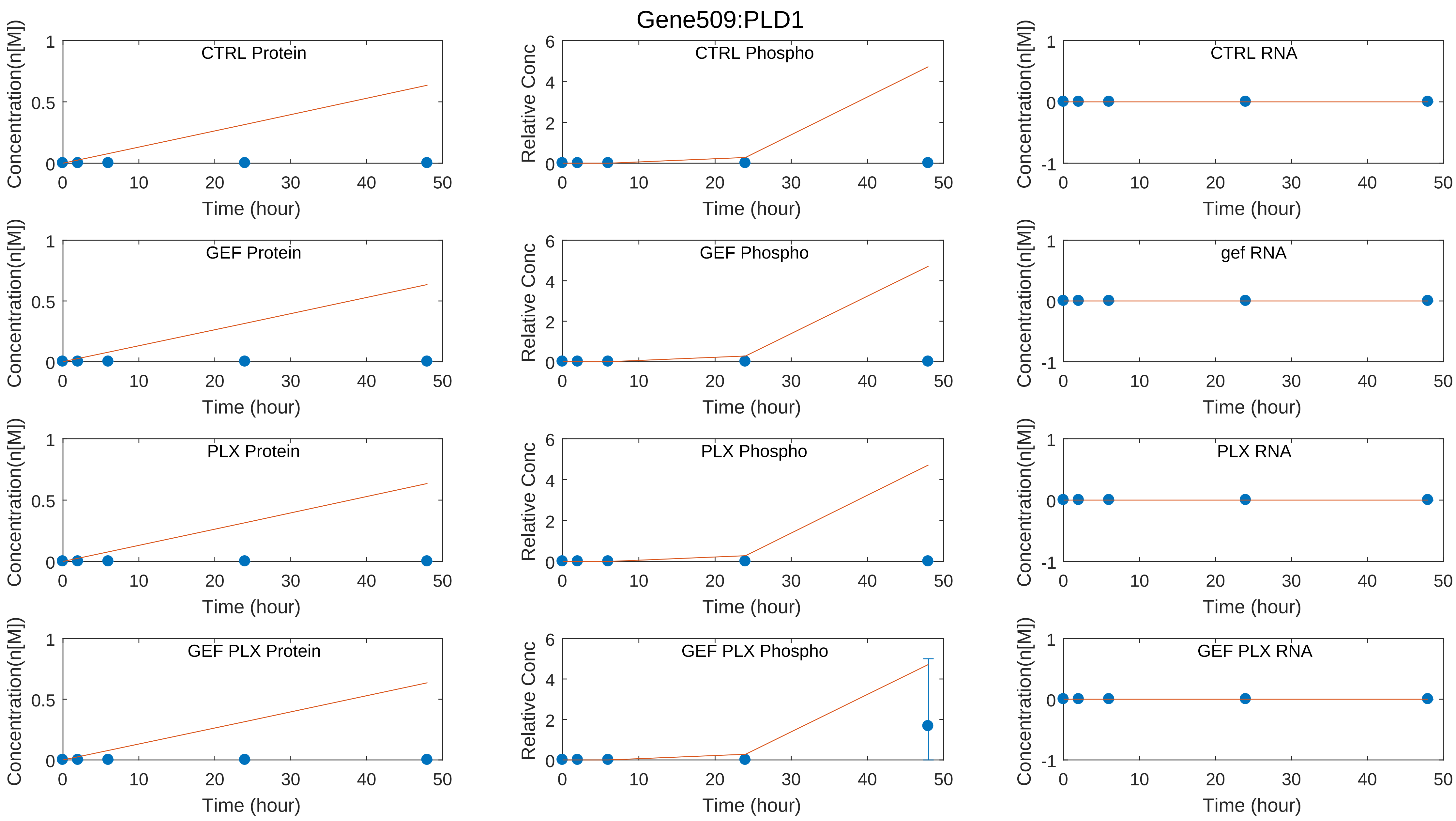
Gene509:PLD1
CTRL Protein
CTRL Phospho
CTRL RNA
GEF Protein
GEF Phospho
gef RNA
PLX Protein
PLX Phospho
PLX RNA
GEF PLX Protein
GEF PLX Phospho
GEF PLX RNA
Concentration(n[M])
Relative Conc
Time (hour)

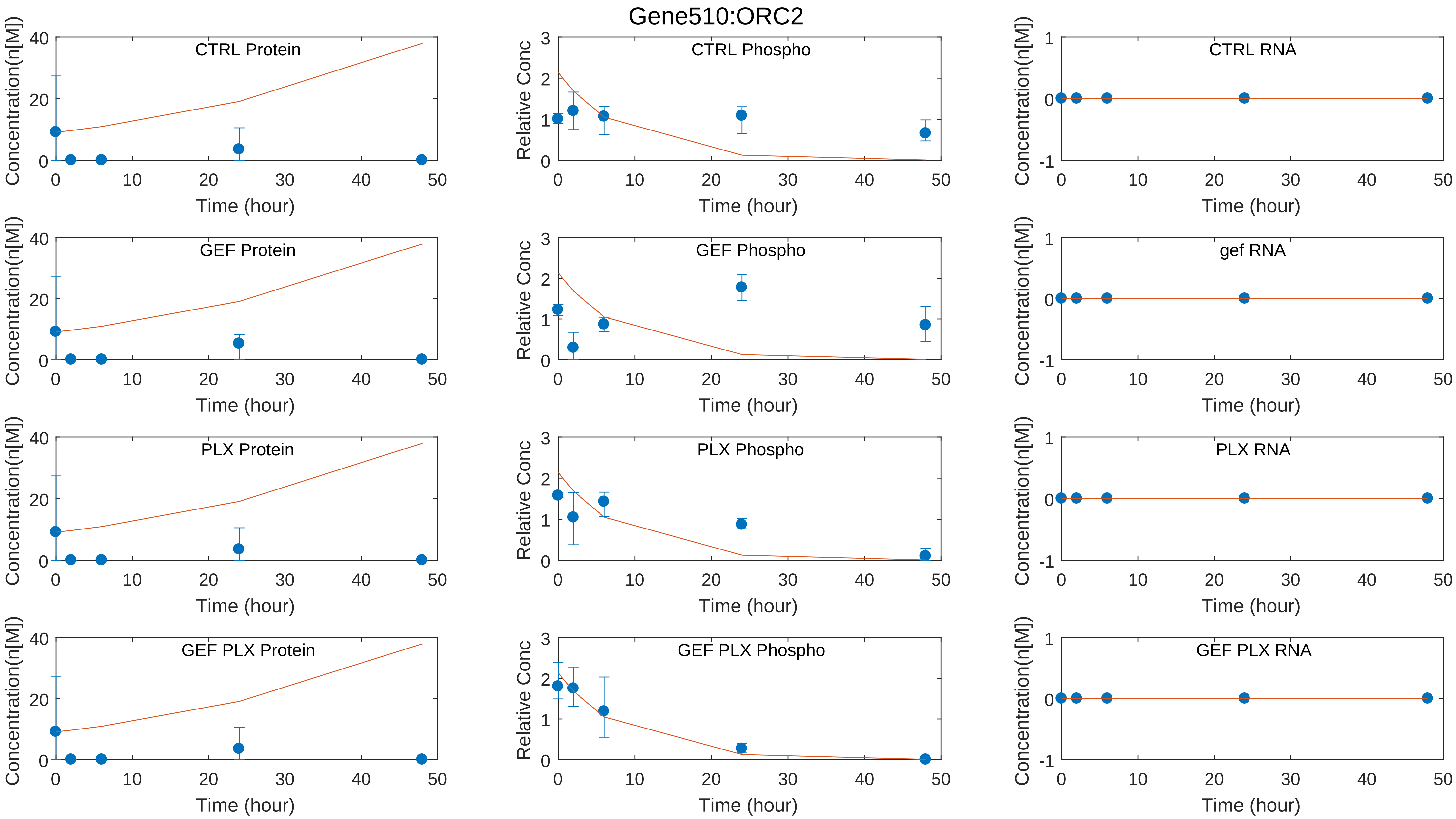
Gene510:ORC2
CTRL Protein
CTRL Phospho
CTRL RNA
GEF Protein
GEF Phospho
gef RNA
PLX Protein
PLX Phospho
PLX RNA
GEF PLX Protein
GEF PLX Phospho
GEF PLX RNA
Concentration(n[M])
Relative Conc
Time (hour)
0
10
20
30
40
50
1
2
3
-1

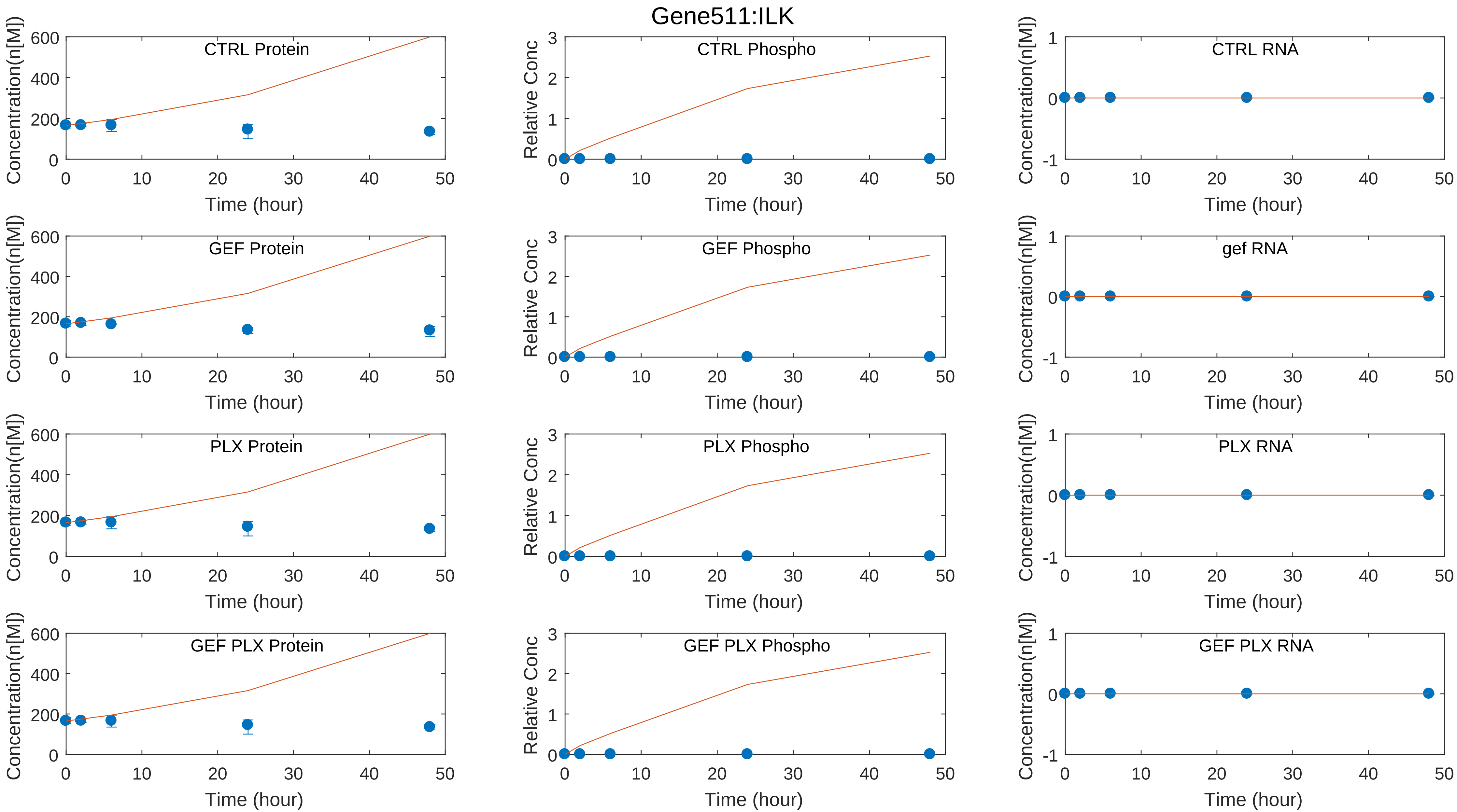
Gene511:ILK
CTRL Protein
CTRL Phospho
CTRL RNA
GEF Protein
GEF Phospho
gef RNA
PLX Protein
PLX Phospho
PLX RNA
GEF PLX Protein
GEF PLX Phospho
GEF PLX RNA
Concentration(n[M])
Relative Conc
Time (hour)

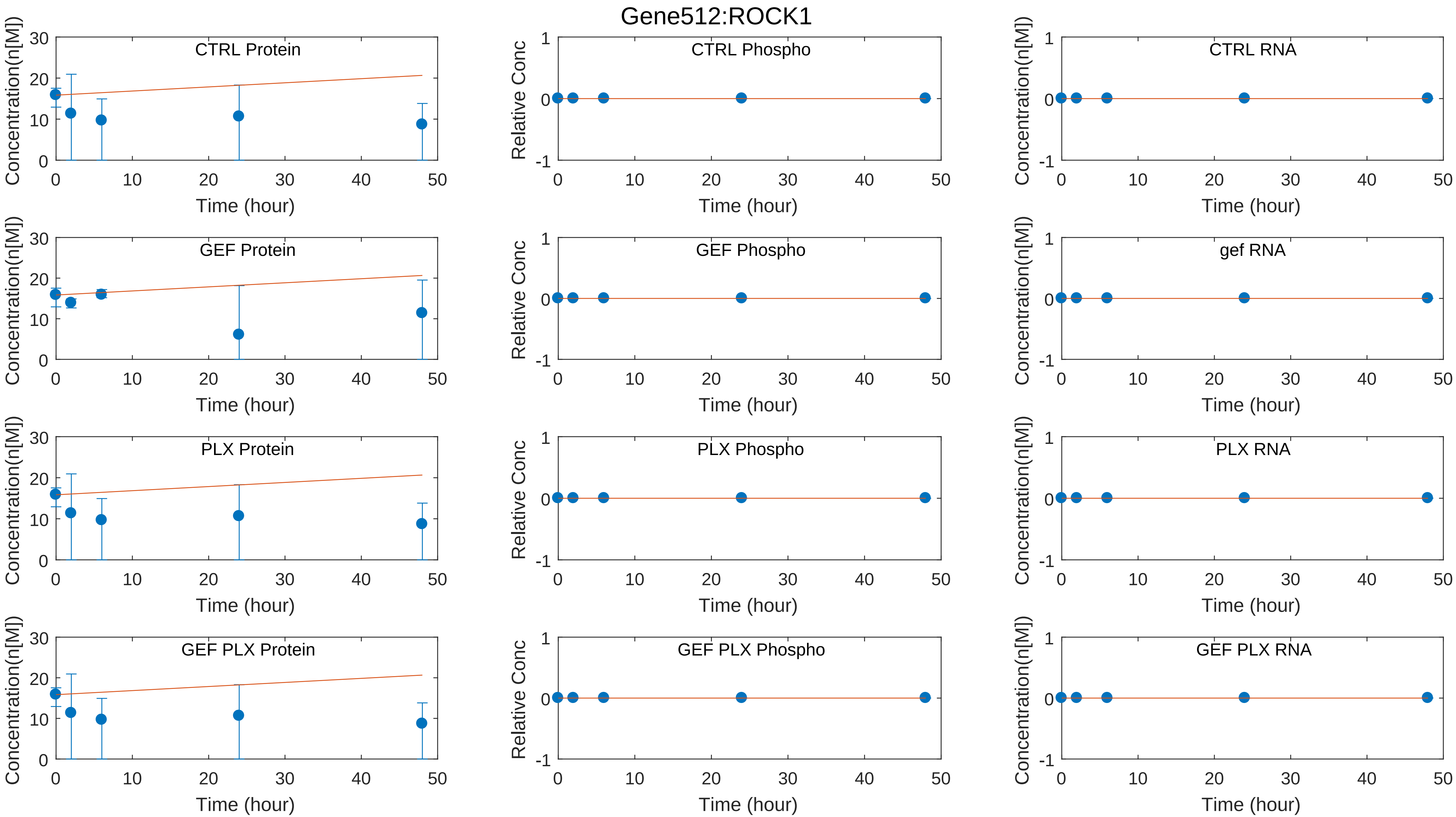

Gene512:ROCK1
CTRL Protein
CTRL Phospho
CTRL RNA
GEF Protein
GEF Phospho
gef RNA
PLX Protein
PLX Phospho
PLX RNA
GEF PLX Protein
GEF PLX Phospho
GEF PLX RNA
Concentration(n[M])
Relative Conc
Time (hour)

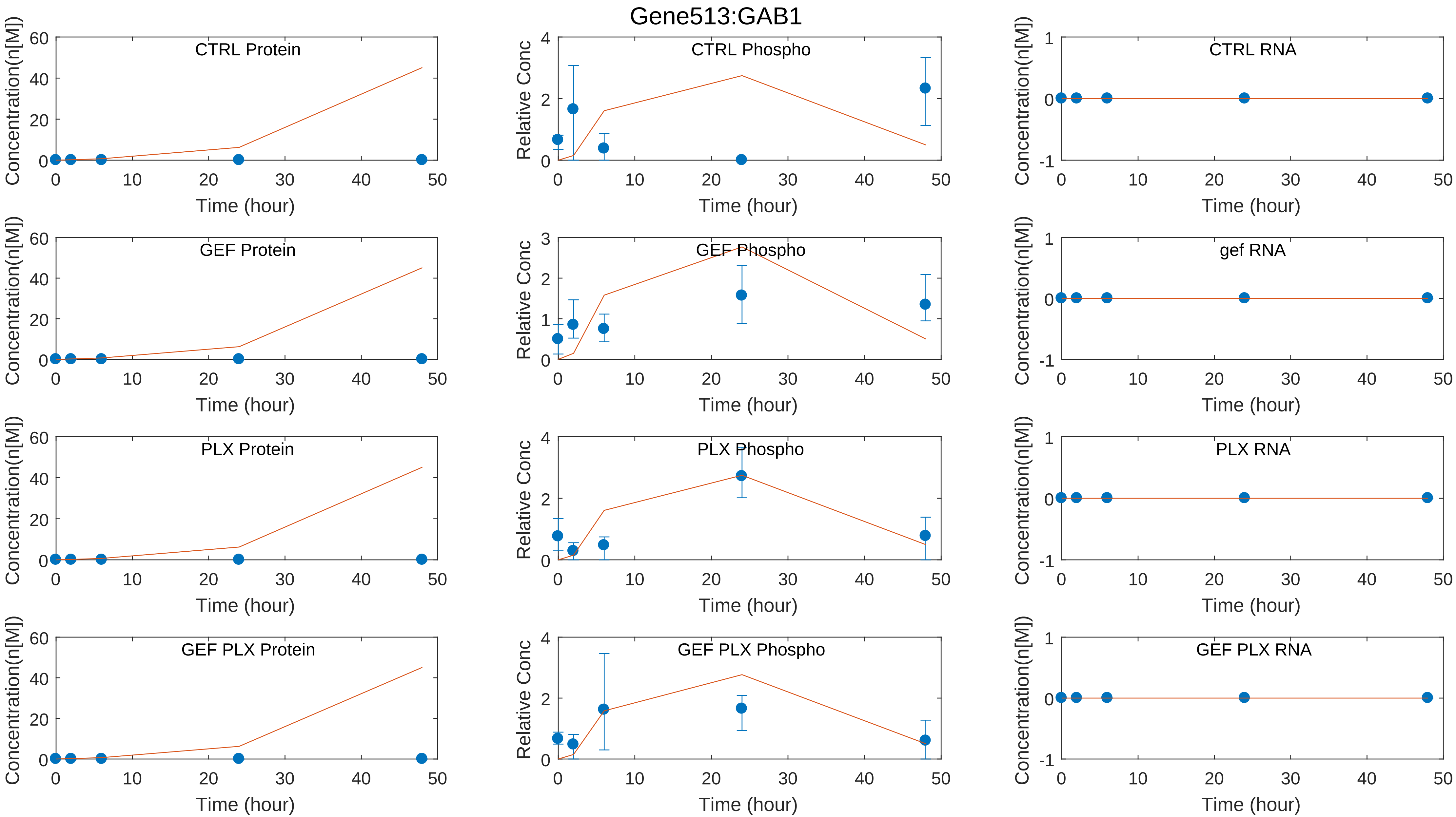

Gene513:GAB1
CTRL Protein
GEF Protein
PLX Protein
GEF PLX Protein
CTRL Phospho
GEF Phospho
PLX Phospho
GEF PLX Phospho
CTRL RNA
gef RNA
PLX RNA
GEF PLX RNA
Concentration(n[M])
Relative Conc
Time (hour)

Gene514:SQSTM1

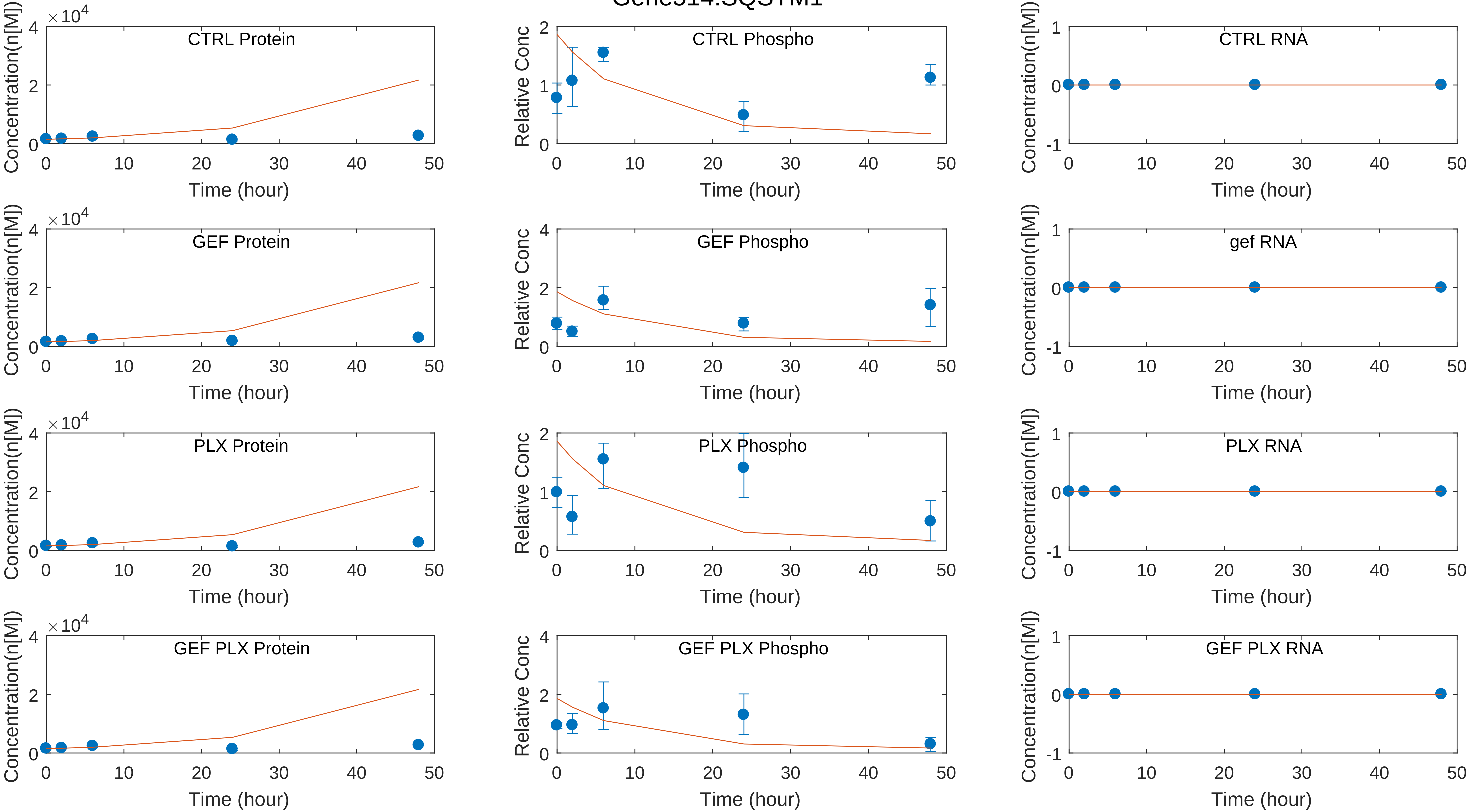

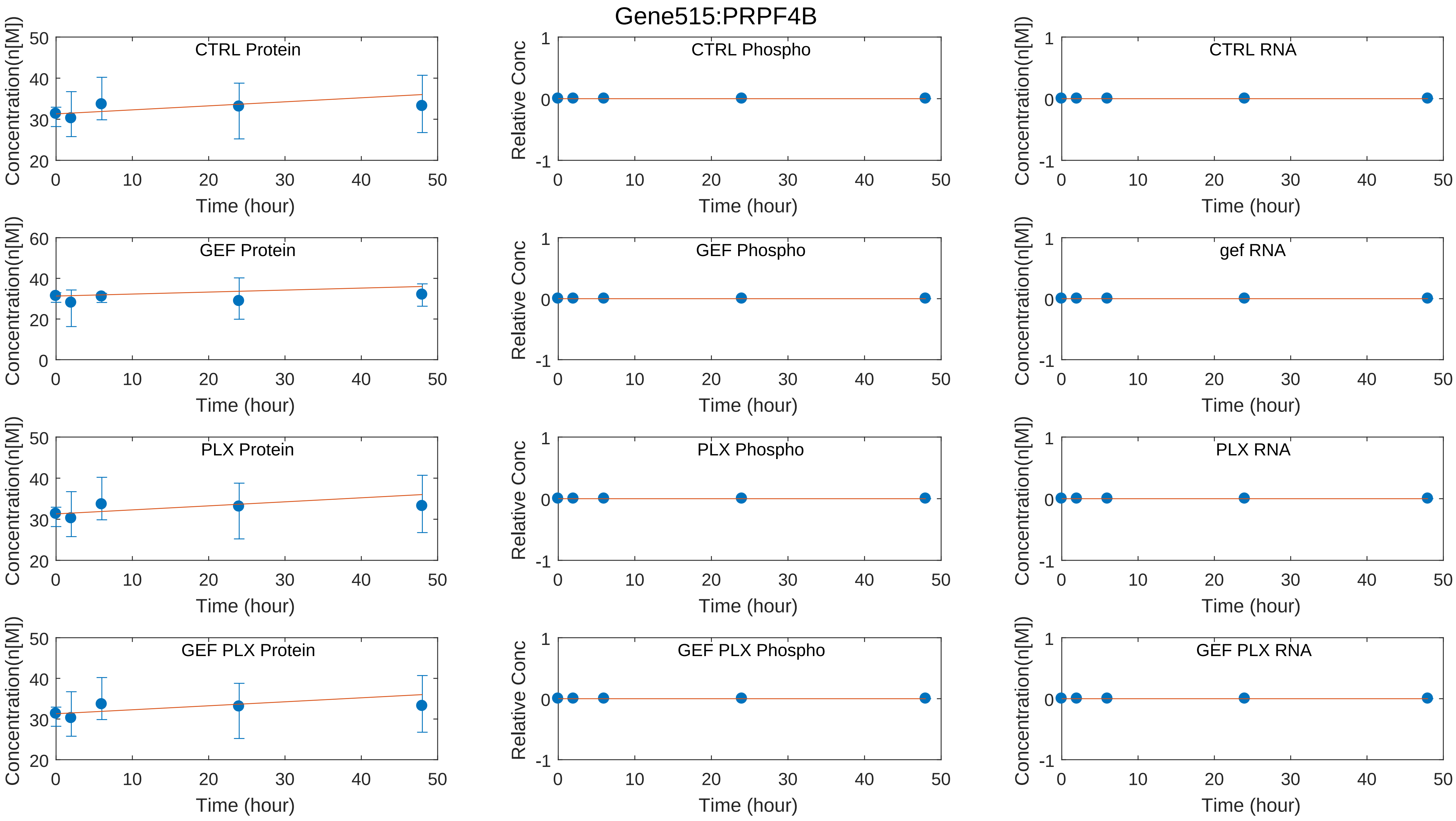

Gene515:PRPF4B
CTRL Protein
CTRL Phospho
CTRL RNA
GEF Protein
GEF Phospho
gef RNA
PLX Protein
PLX Phospho
PLX RNA
GEF PLX Protein
GEF PLX Phospho
GEF PLX RNA
Concentration(n[M])
Relative Conc
Time (hour)

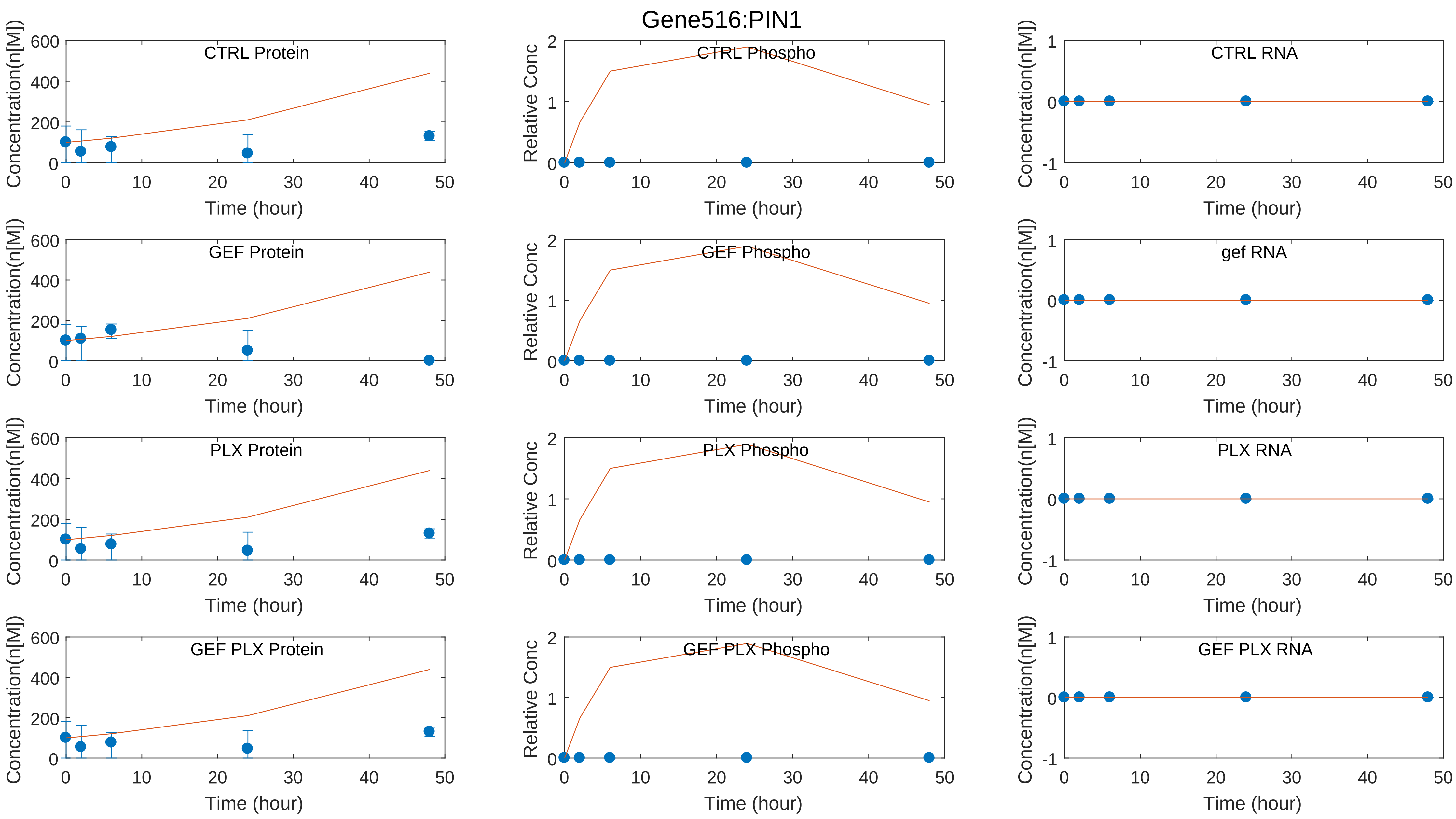
Gene516:PIN1
CTRL Protein
CTRL Phospho
CTRL RNA
GEF Protein
GEF Phospho
gef RNA
PLX Protein
PLX Phospho
PLX RNA
GEF PLX Protein
GEF PLX Phospho
GEF PLX RNA
Concentration(n[M])
Relative Conc
Time (hour)

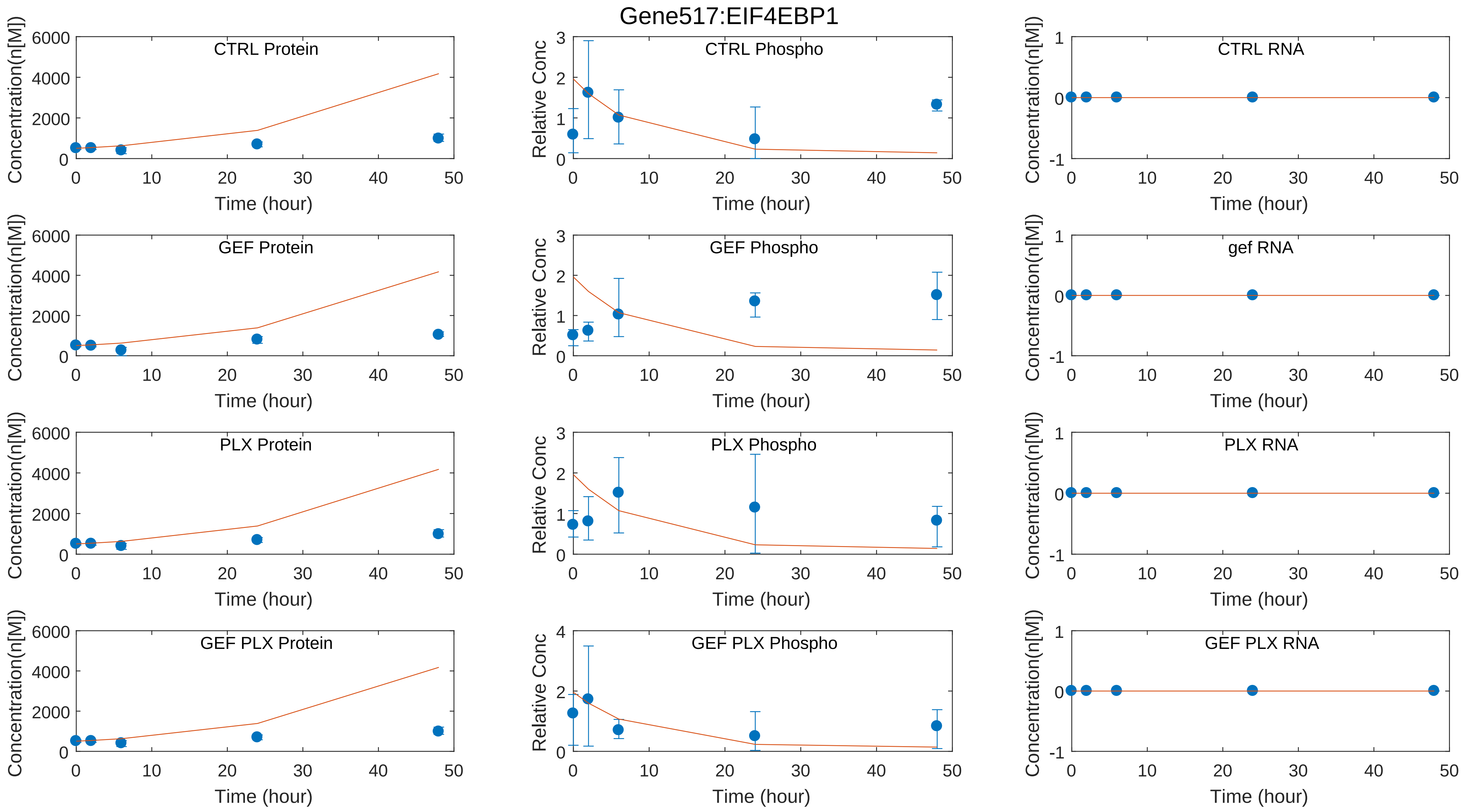
Gene517:EIF4EBP1
CTRL Protein
CTRL Phospho
CTRL RNA
GEF Protein
GEF Phospho
gef RNA
PLX Protein
PLX Phospho
PLX RNA
GEF PLX Protein
GEF PLX Phospho
GEF PLX RNA
Concentration(n[M])
Relative Conc
Time (hour)

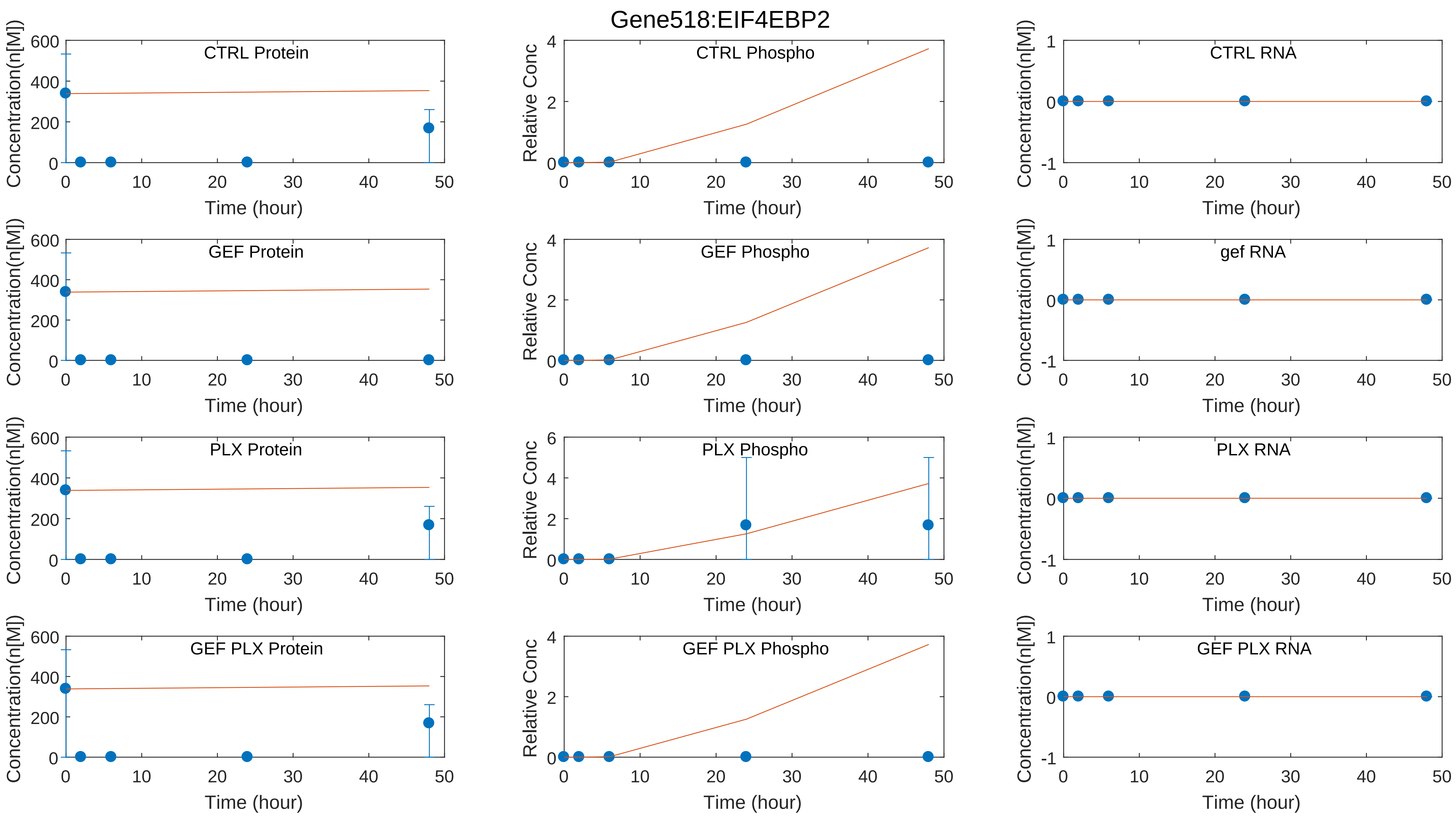

Gene518:EIF4EBP2
CTRL Protein
GEF Protein
PLX Protein
GEF PLX Protein
CTRL Phospho
GEF Phospho
PLX Phospho
GEF PLX Phospho
CTRL RNA
gef RNA
PLX RNA
GEF PLX RNA
Concentration(n[M])
Relative Conc
Time (hour)

Gene519:RIPK1

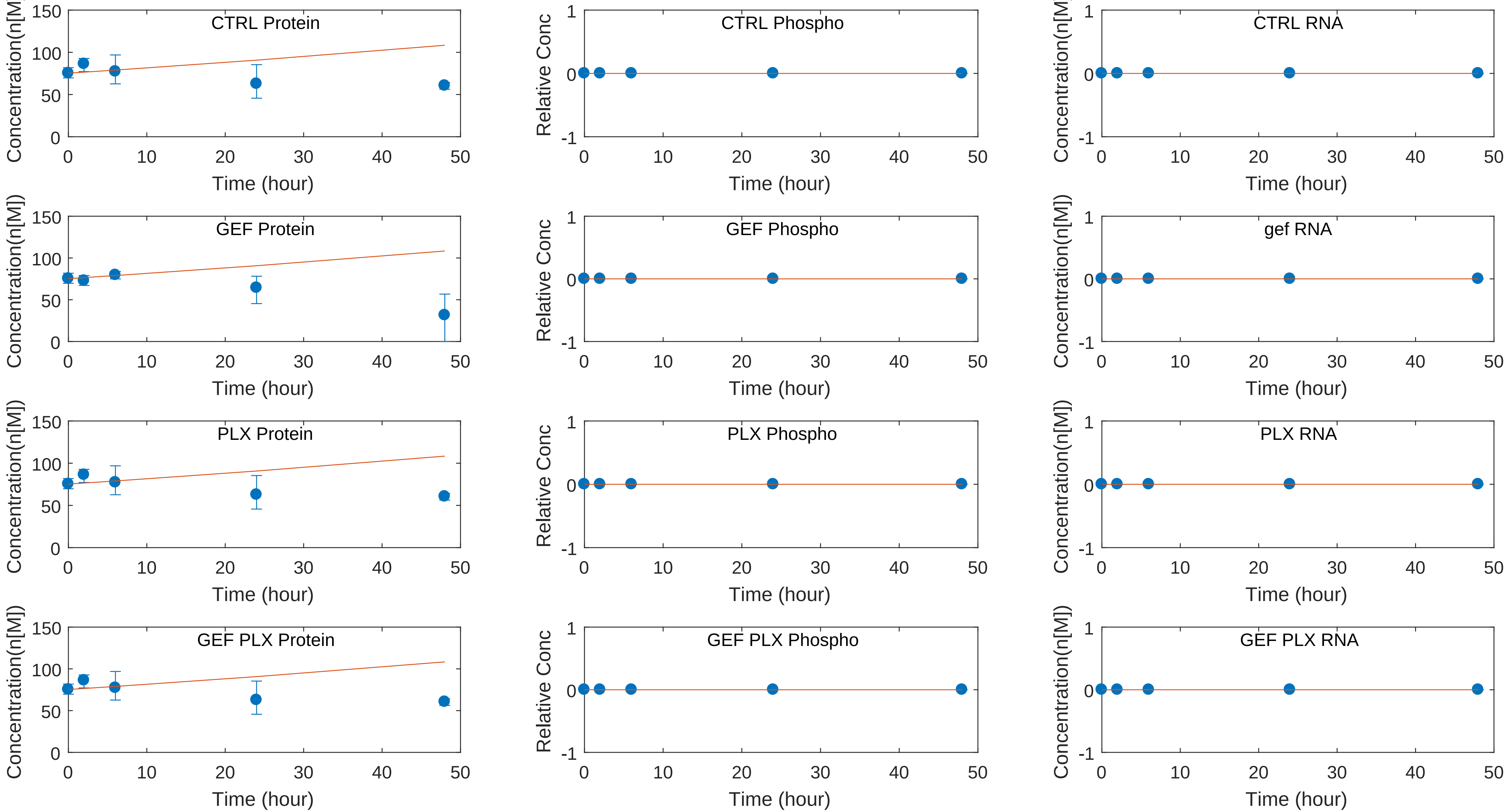

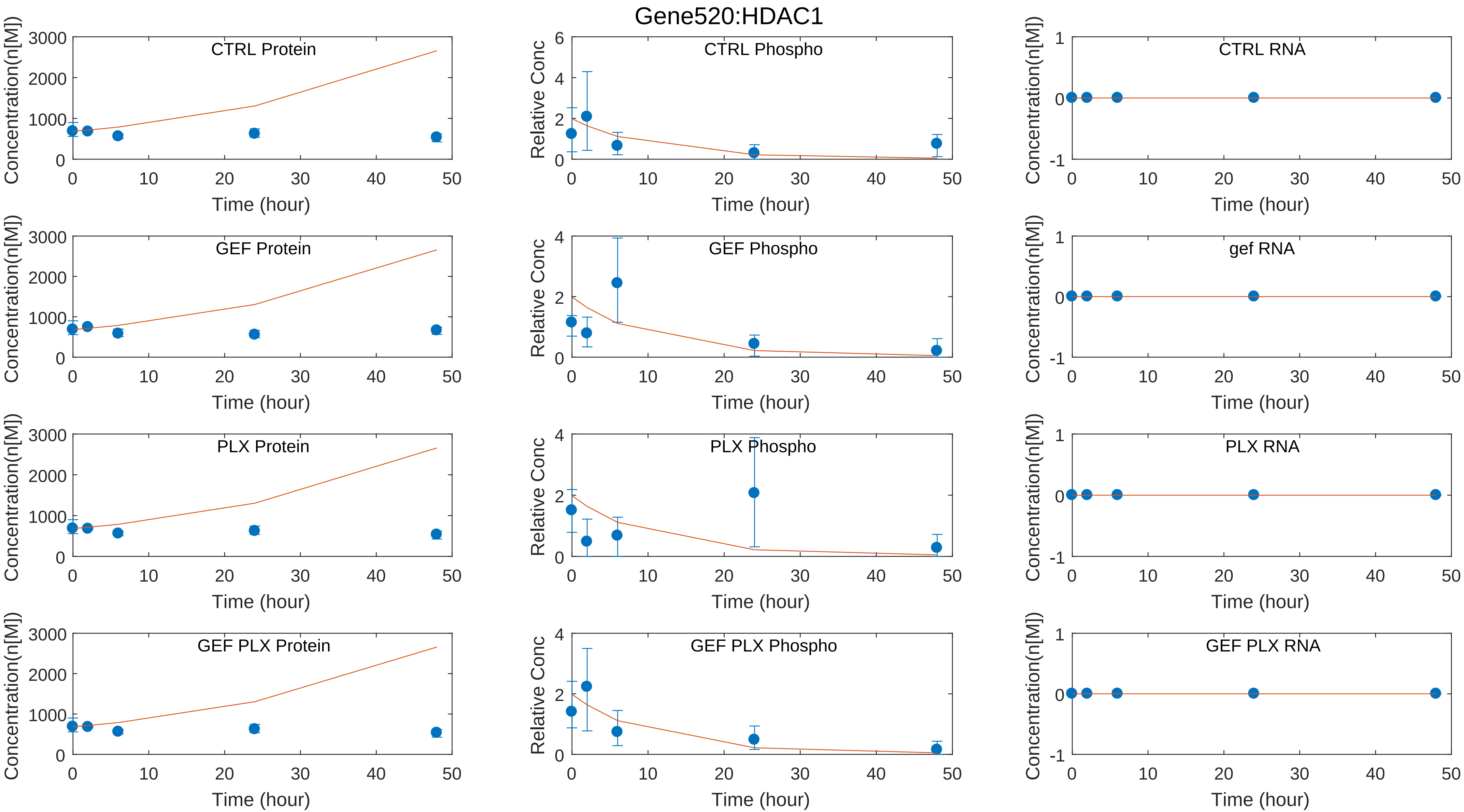
Gene520:HDAC1
CTRL Protein
GEF Protein
PLX Protein
GEF PLX Protein
CTRL Phospho
GEF Phospho
PLX Phospho
GEF PLX Phospho
CTRL RNA
gef RNA
PLX RNA
GEF PLX RNA
Concentration(n[M])
Relative Conc
Time (hour)

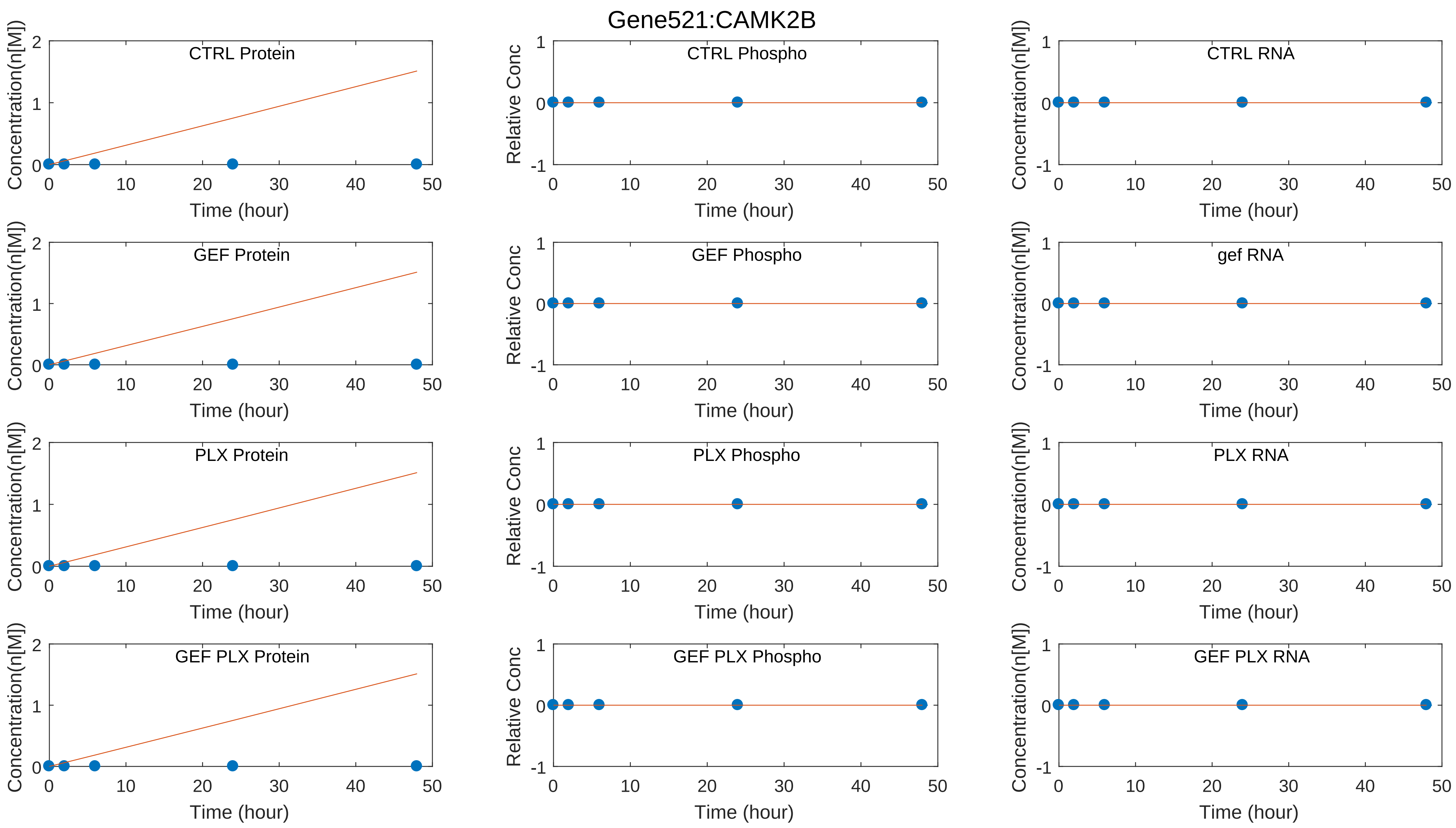
Gene521:CAMK2B
CTRL Protein
CTRL Phospho
CTRL RNA
GEF Protein
GEF Phospho
gef RNA
PLX Protein
PLX Phospho
PLX RNA
GEF PLX Protein
GEF PLX Phospho
GEF PLX RNA
Concentration(n[M])
Relative Conc
Time (hour)
0
10
20
30
40
50
-1
1
2

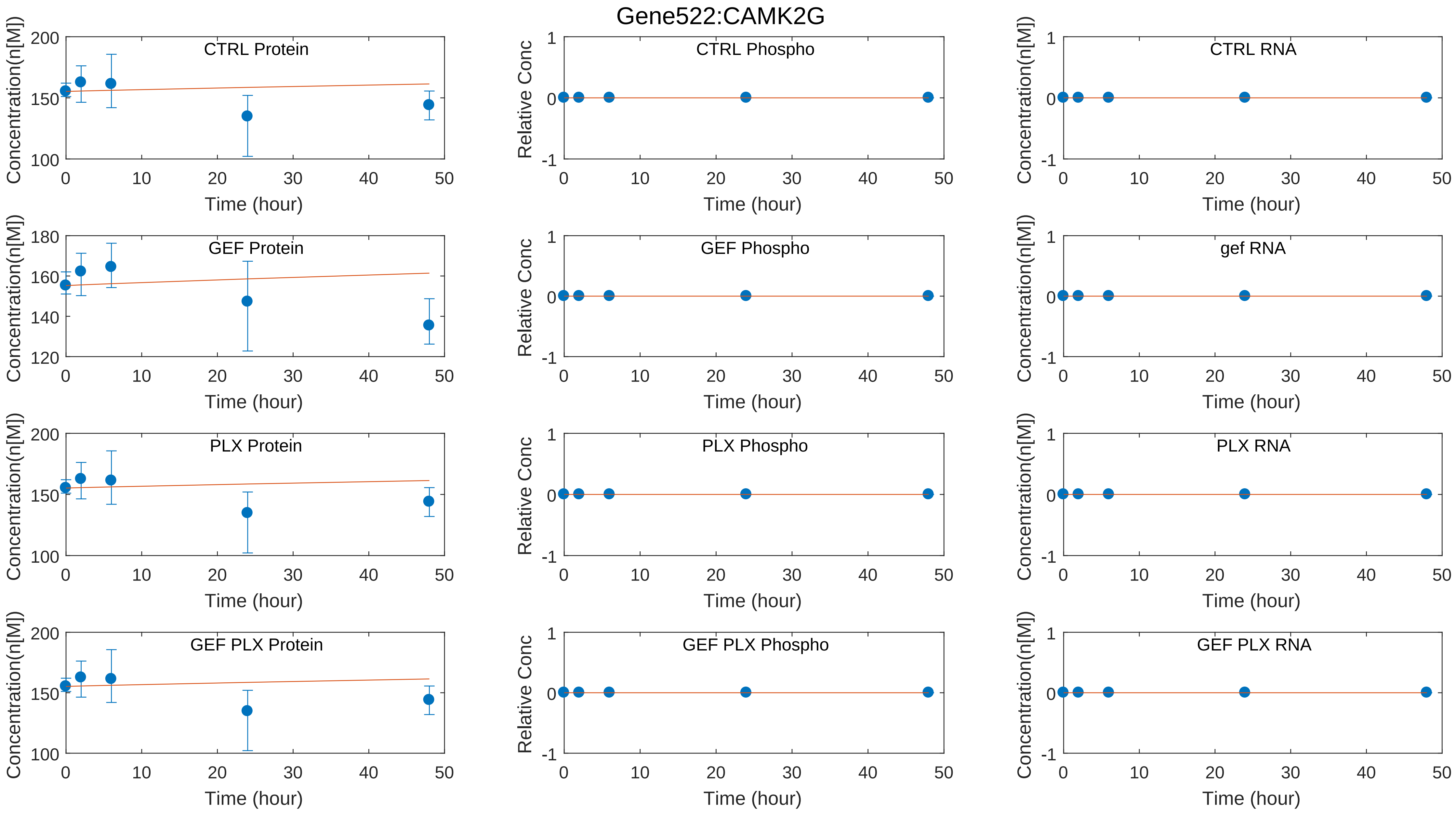

Gene522:CAMK2G
CTRL Protein
CTRL Phospho
CTRL RNA
GEF Protein
GEF Phospho
gef RNA
PLX Protein
PLX Phospho
PLX RNA
GEF PLX Protein
GEF PLX Phospho
GEF PLX RNA
Concentration(n[M])
Relative Conc
Time (hour)

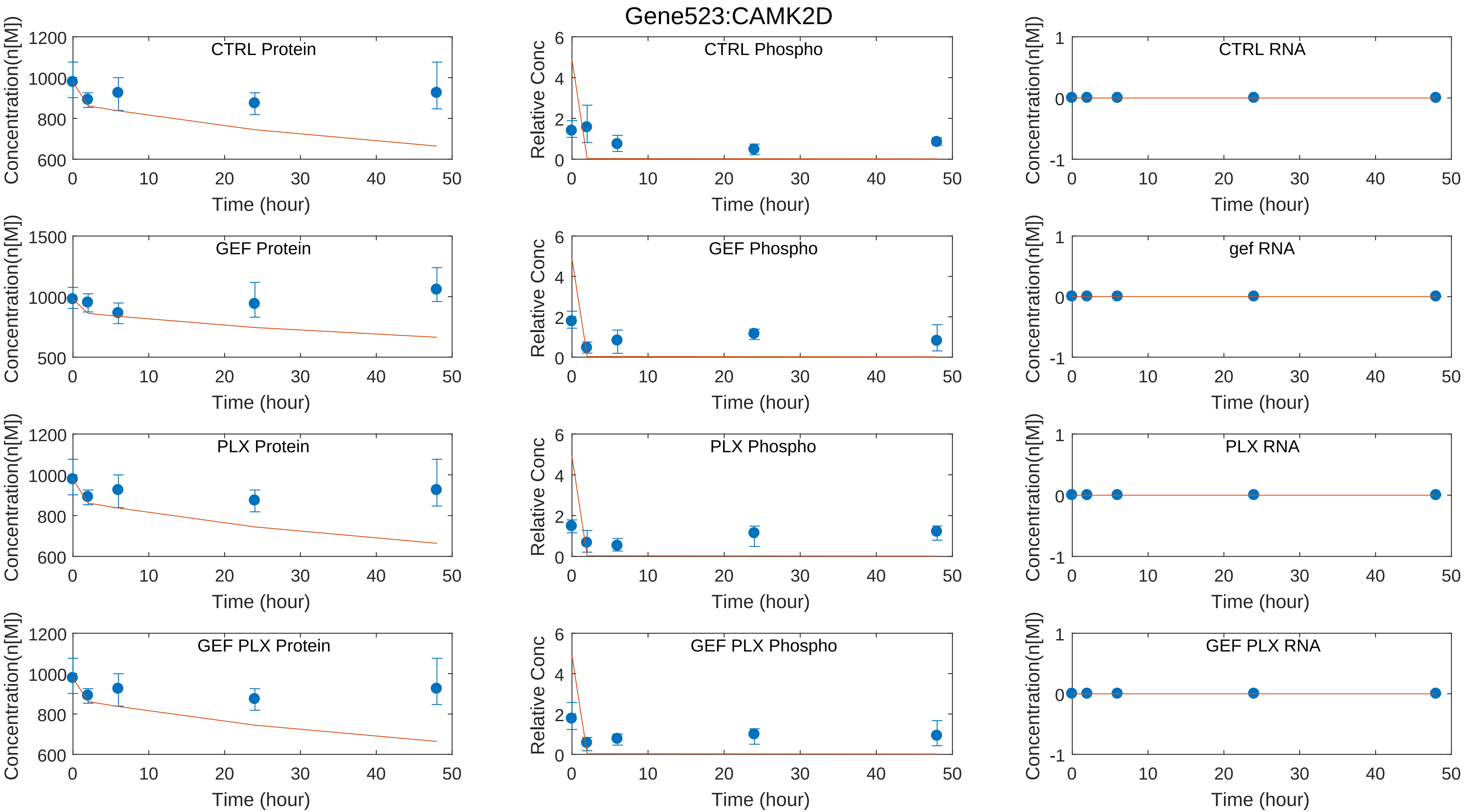

Gene523:CAMK2D
CTRL Protein
GEF Protein
PLX Protein
GEF PLX Protein
CTRL Phospho
GEF Phospho
PLX Phospho
GEF PLX Phospho
CTRL RNA
gef RNA
PLX RNA
GEF PLX RNA
Concentration(n[M])
Relative Conc
Time (hour)

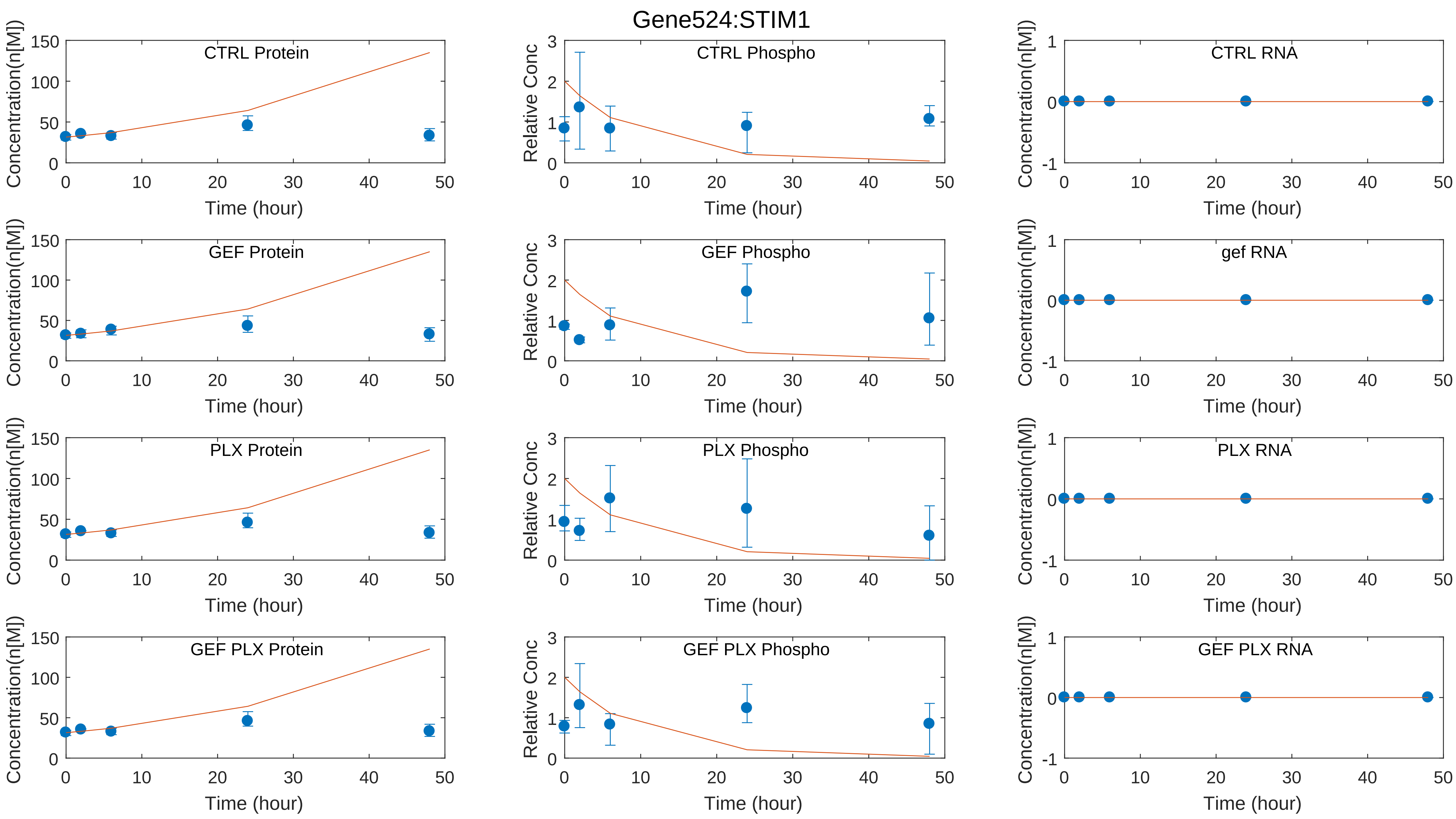

Gene524:STIM1
CTRL Protein
CTRL Phospho
CTRL RNA
GEF Protein
GEF Phospho
gef RNA
PLX Protein
PLX Phospho
PLX RNA
GEF PLX Protein
GEF PLX Phospho
GEF PLX RNA
Concentration(n[M])
Relative Conc
Time (hour)

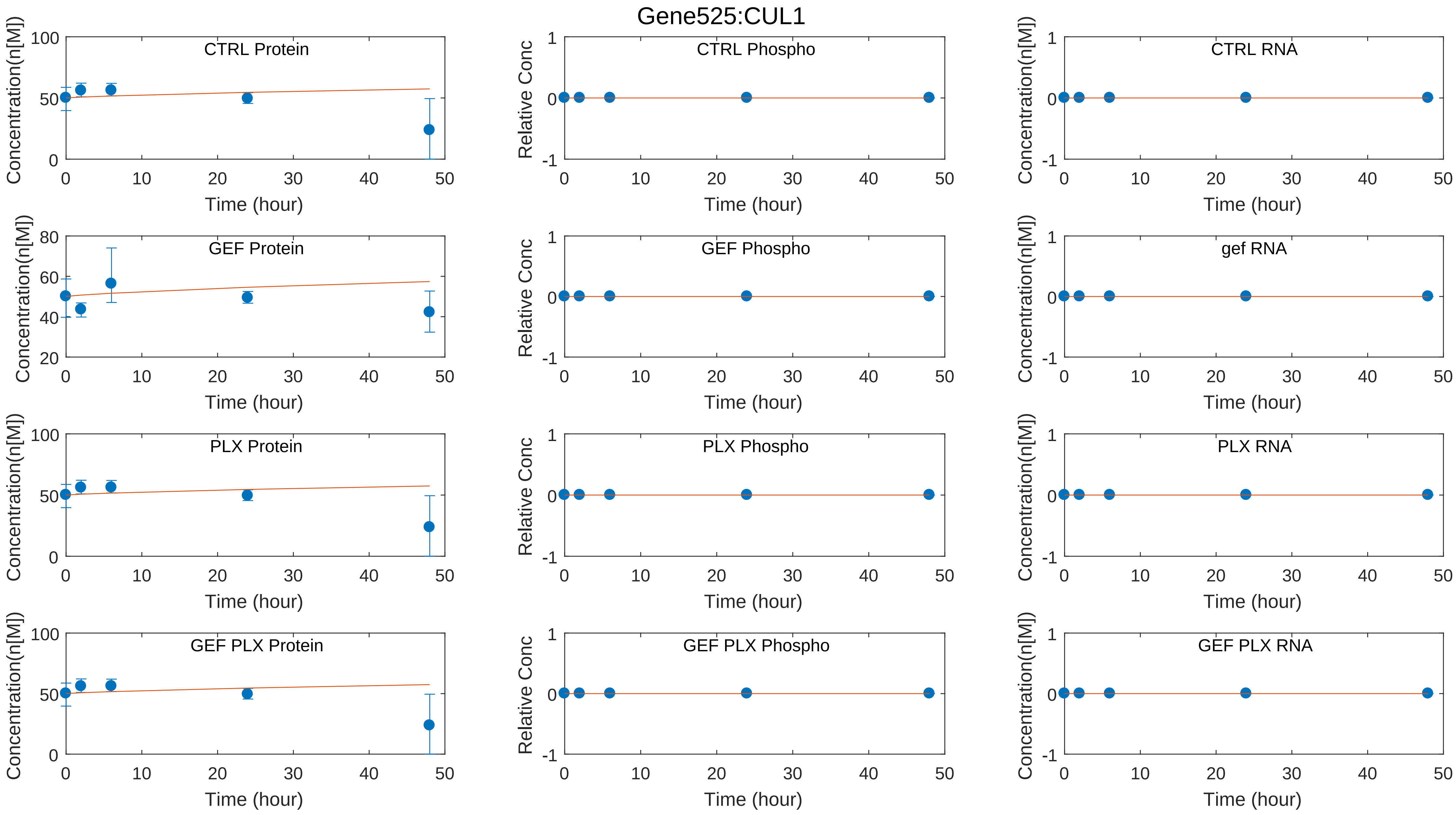

Gene525:CUL1
CTRL Protein
CTRL Phospho
CTRL RNA
GEF Protein
GEF Phospho
gef RNA
PLX Protein
PLX Phospho
PLX RNA
GEF PLX Protein
GEF PLX Phospho
GEF PLX RNA
Concentration(n[M])
Relative Conc
Time (hour)

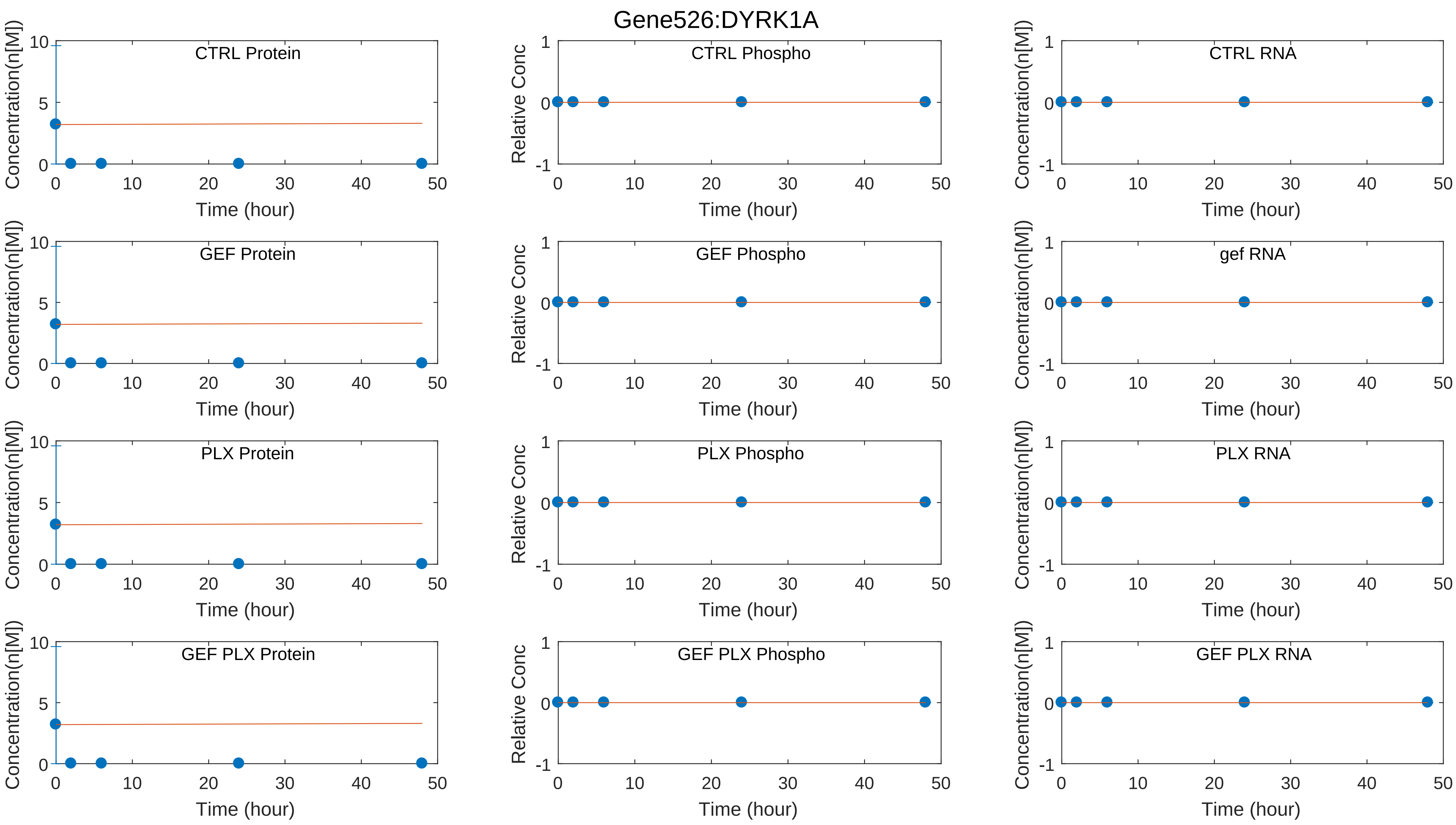

Gene526:DYRK1A
CTRL Protein
CTRL Phospho
CTRL RNA
GEF Protein
GEF Phospho
gef RNA
PLX Protein
PLX Phospho
PLX RNA
GEF PLX Protein
GEF PLX Phospho
GEF PLX RNA
Concentration(n[M])
Relative Conc
Time (hour)

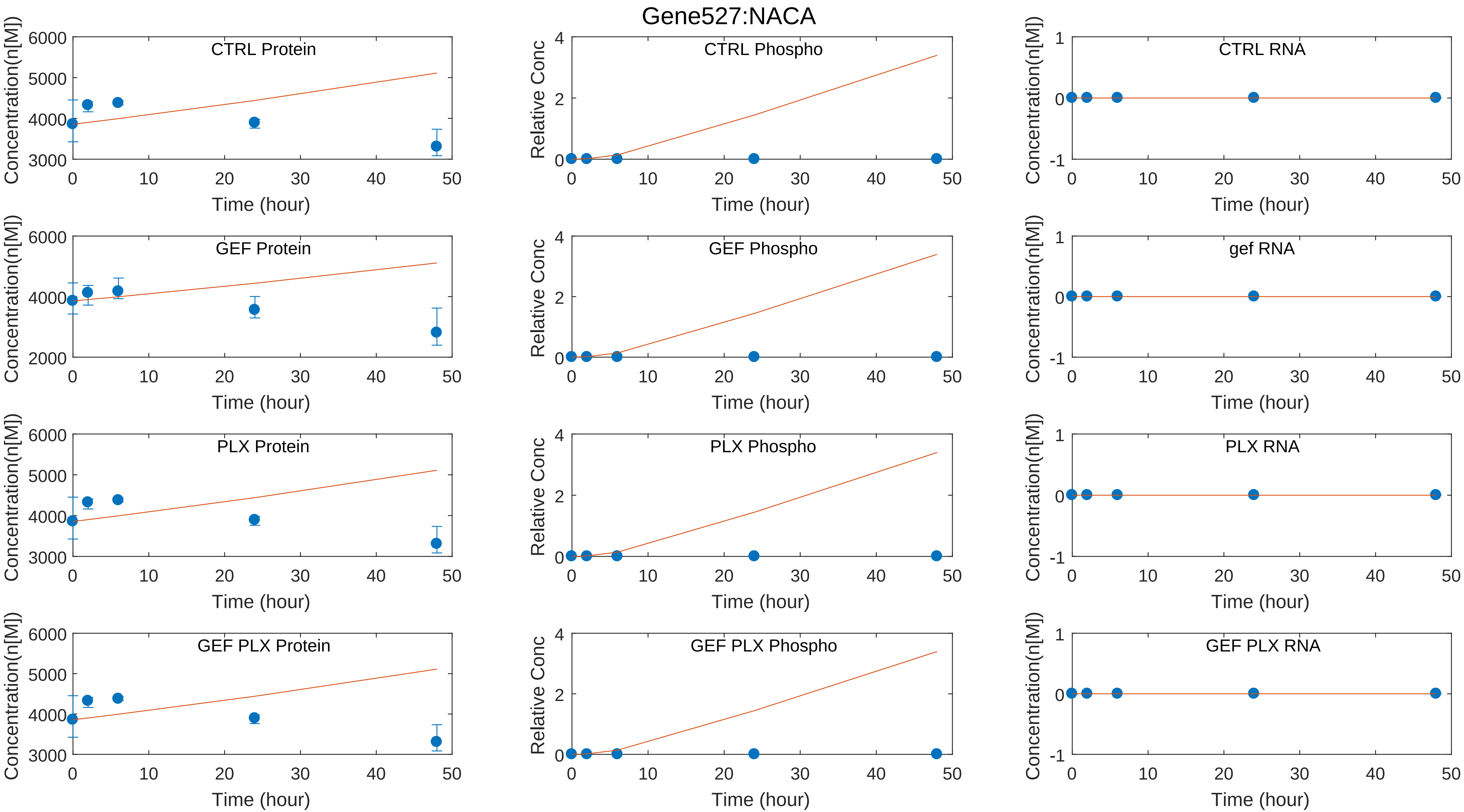

Gene527:NACA
CTRL Protein
GEF Protein
PLX Protein
GEF PLX Protein
CTRL Phospho
GEF Phospho
PLX Phospho
GEF PLX Phospho
CTRL RNA
gef RNA
PLX RNA
GEF PLX RNA
Concentration(n[M])
Relative Conc
Time (hour)

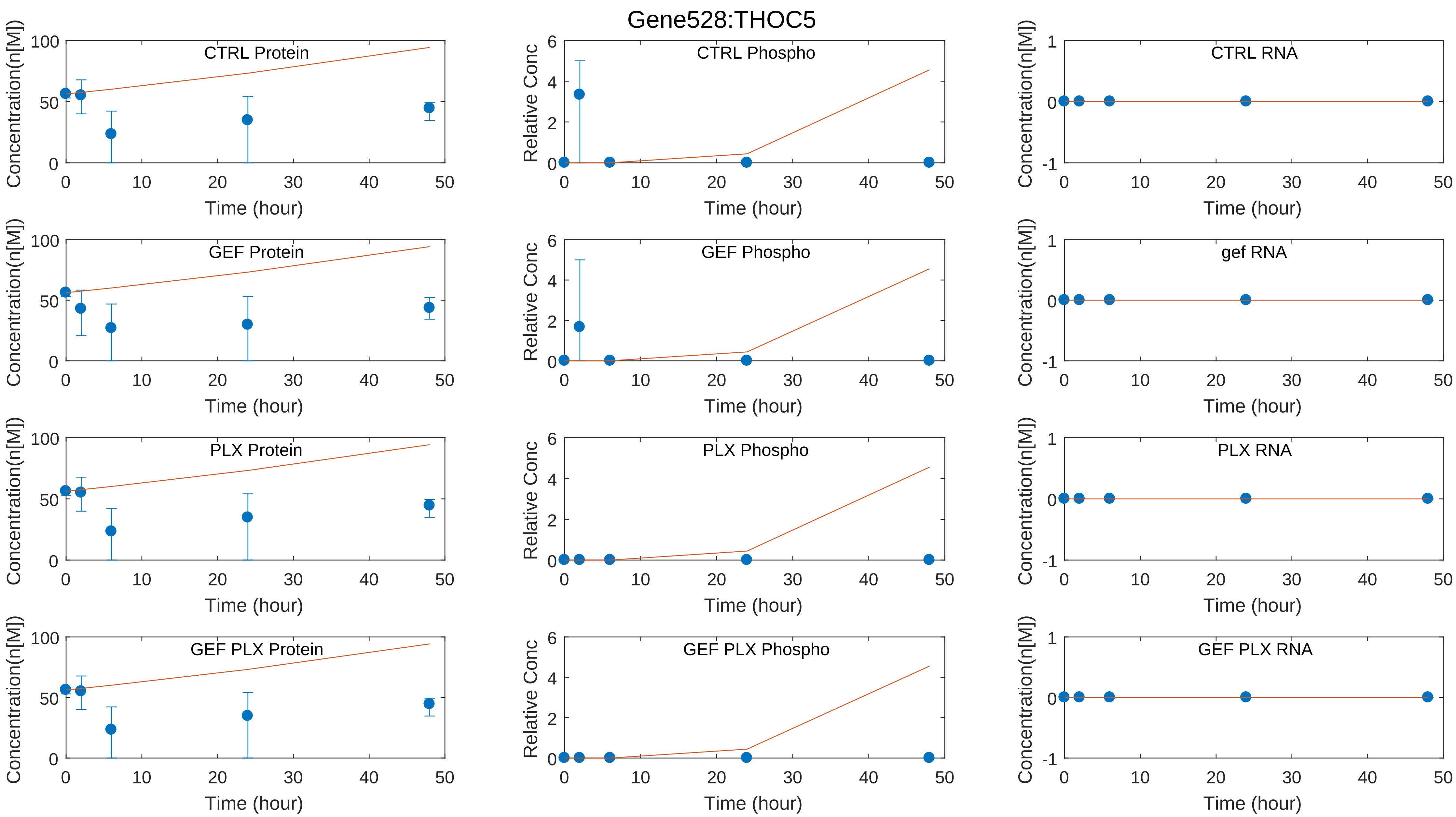

Gene528:THOC5
CTRL Protein
CTRL Phospho
CTRL RNA
GEF Protein
GEF Phospho
gef RNA
PLX Protein
PLX Phospho
PLX RNA
GEF PLX Protein
GEF PLX Phospho
GEF PLX RNA
Concentration(n[M])
Relative Conc
Time (hour)

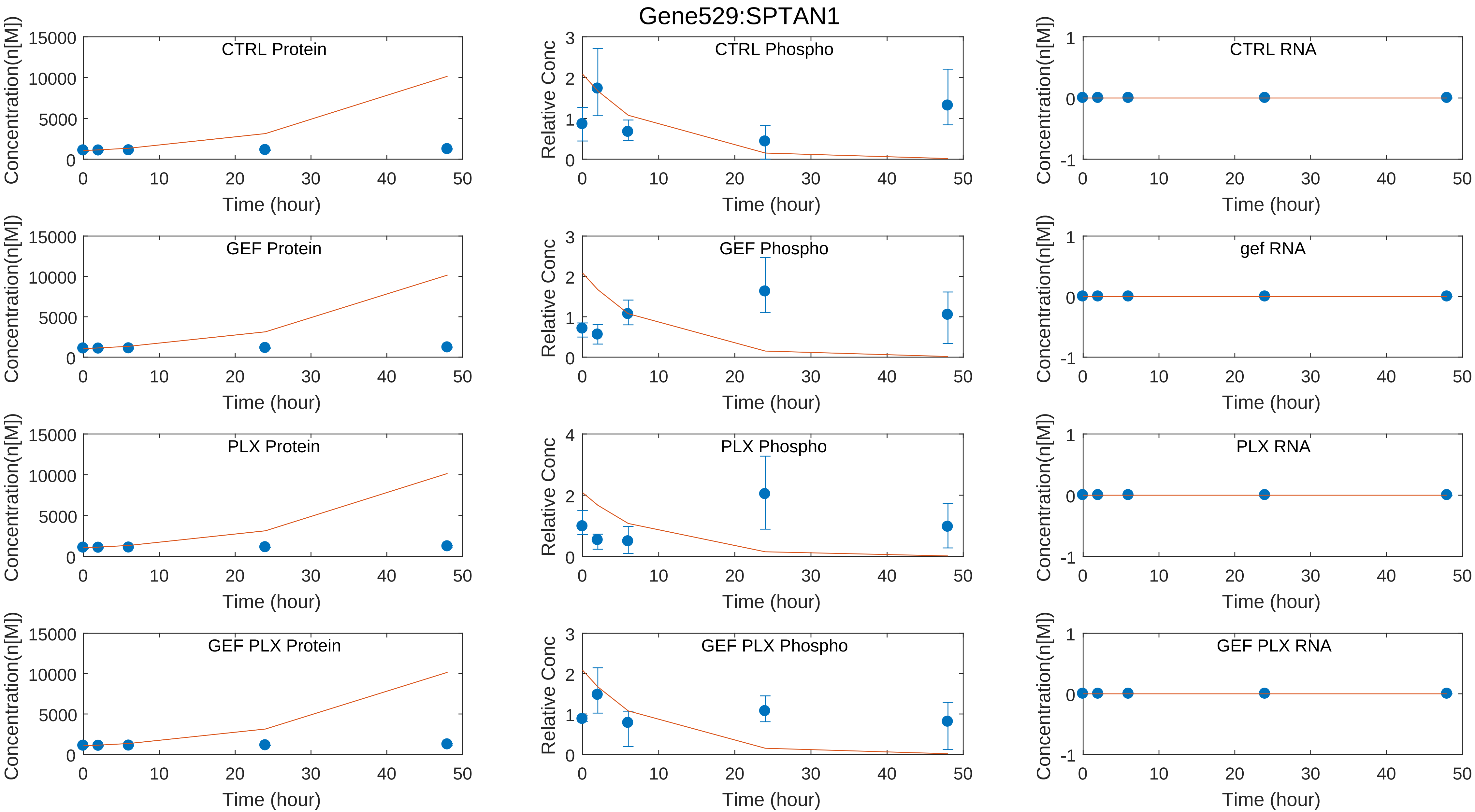

Gene529:SPTAN1
CTRL Protein
GEF Protein
PLX Protein
GEF PLX Protein
CTRL Phospho
GEF Phospho
PLX Phospho
GEF PLX Phospho
CTRL RNA
gef RNA
PLX RNA
GEF PLX RNA
Concentration(n[M])
Relative Conc
Time (hour)

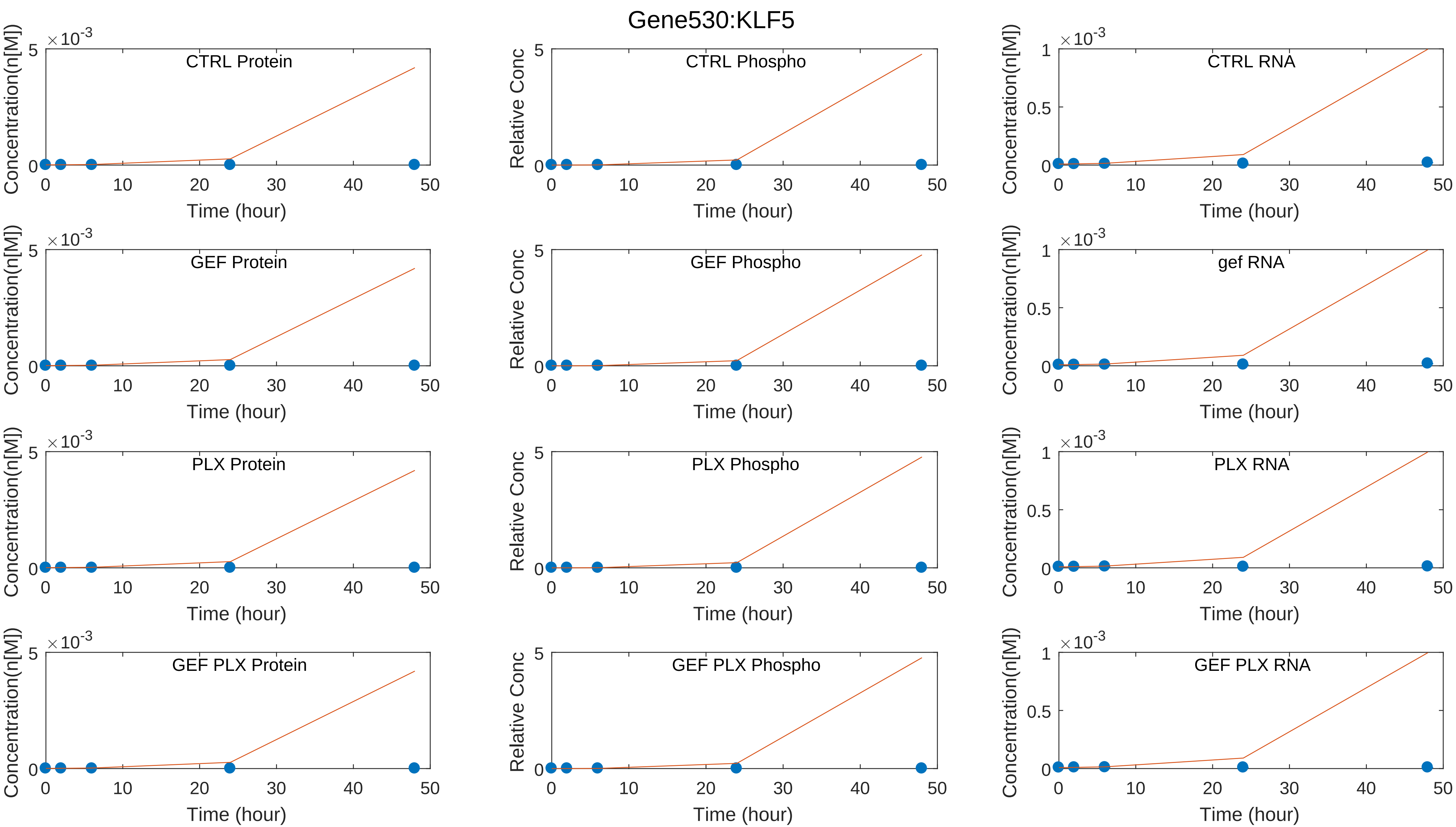

Gene530:KLF5
CTRL Protein
CTRL Phospho
CTRL RNA
GEF Protein
GEF Phospho
gef RNA
PLX Protein
PLX Phospho
PLX RNA
GEF PLX Protein
GEF PLX Phospho
GEF PLX RNA
Concentration(n[M])
Relative Conc
Time (hour)
$\times 10^{-3}$

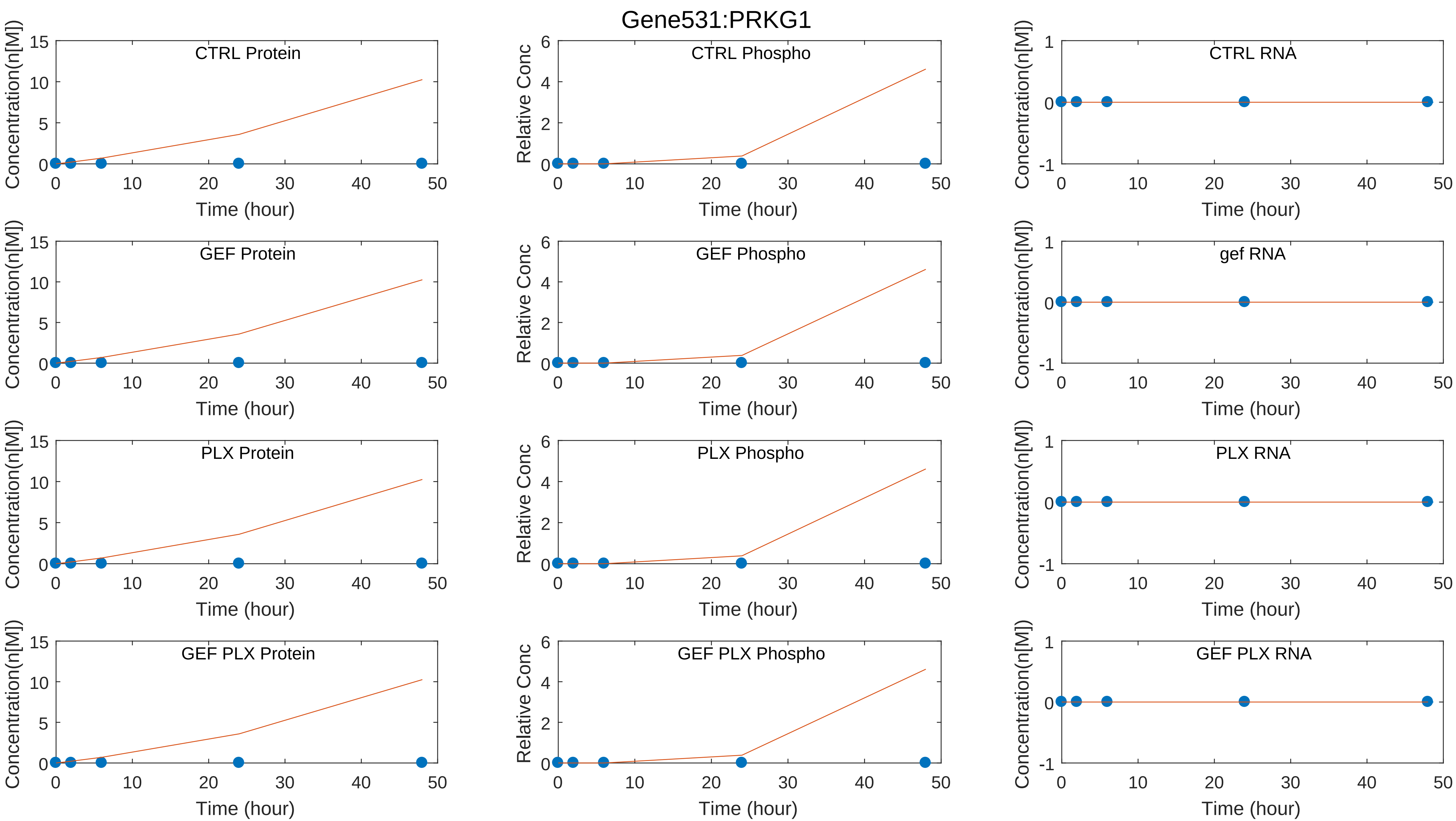

Gene531:PRKG1
CTRL Protein
CTRL Phospho
CTRL RNA
GEF Protein
GEF Phospho
gef RNA
PLX Protein
PLX Phospho
PLX RNA
GEF PLX Protein
GEF PLX Phospho
GEF PLX RNA
Concentration(n[M])
Relative Conc
Time (hour)

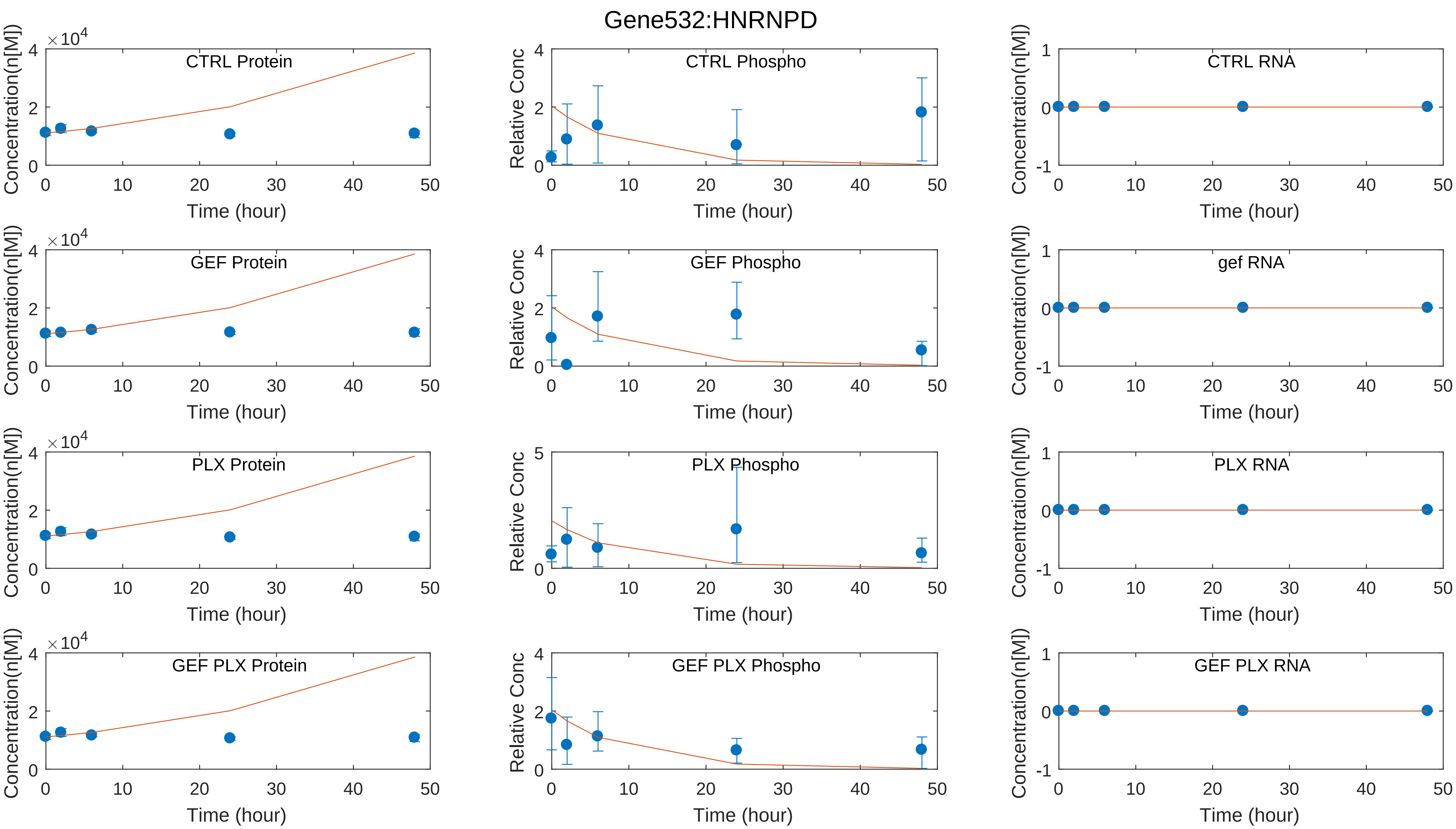
Gene532:HNRNPD
CTRL Protein
GEF Protein
PLX Protein
GEF PLX Protein
CTRL Phospho
GEF Phospho
PLX Phospho
GEF PLX Phospho
CTRL RNA
gef RNA
PLX RNA
GEF PLX RNA
Concentration(n[M])
Relative Conc
Time (hour)
$\times 10^4$

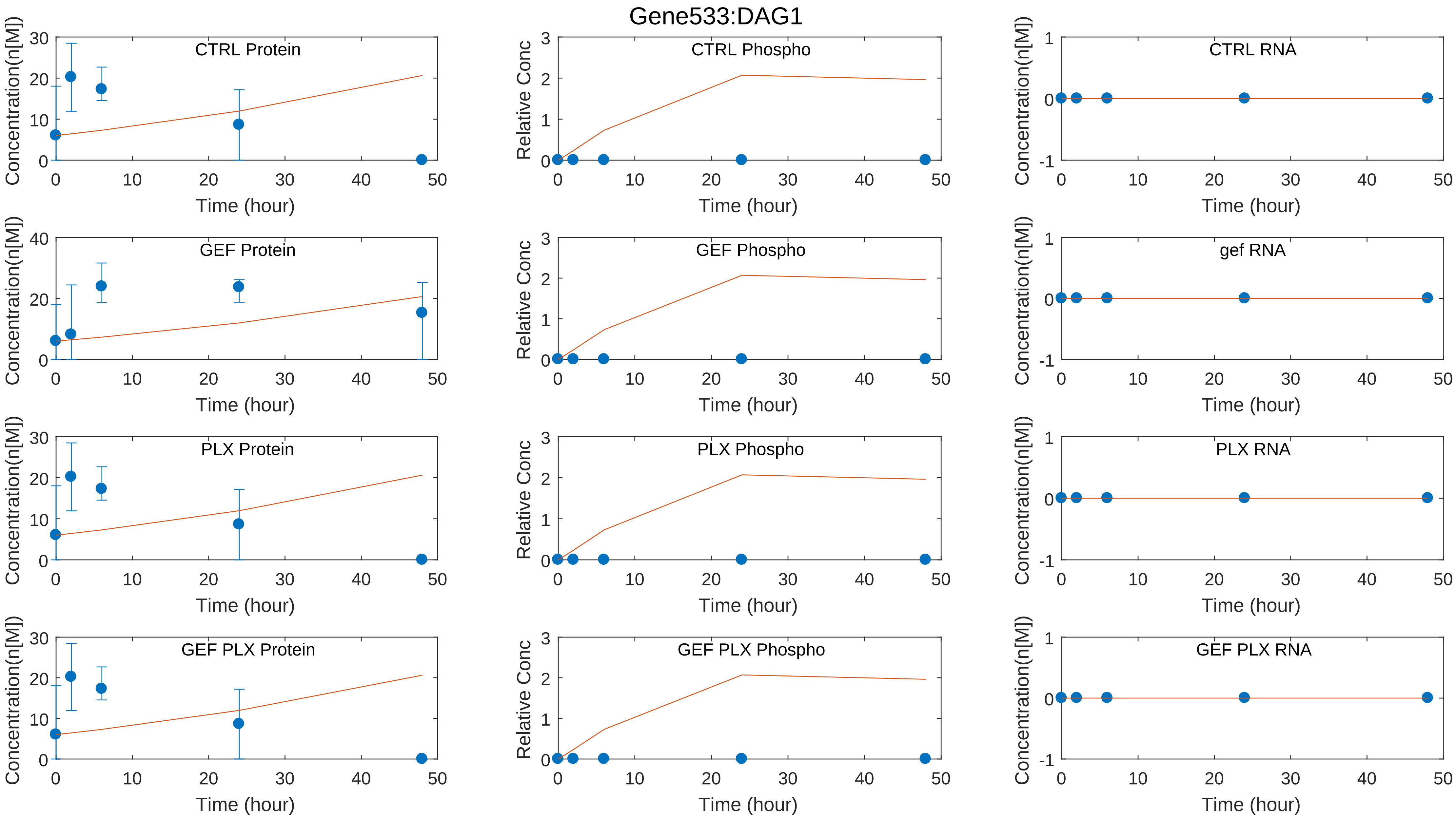

Gene533:DAG1
CTRL Protein
CTRL Phospho
CTRL RNA
GEF Protein
GEF Phospho
gef RNA
PLX Protein
PLX Phospho
PLX RNA
GEF PLX Protein
GEF PLX Phospho
GEF PLX RNA
Concentration(n[M])
Relative Conc
Time (hour)

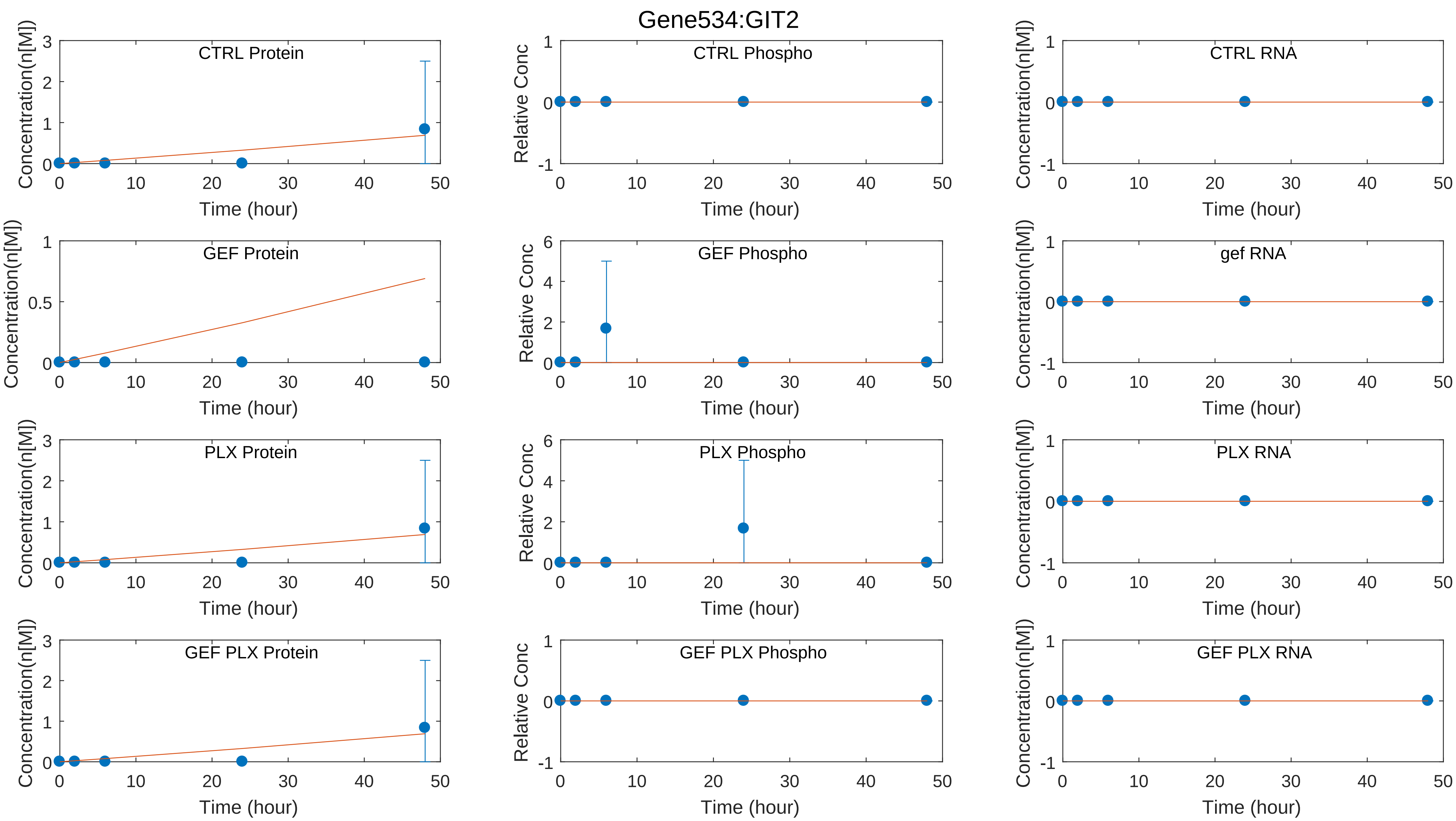

Gene534:GIT2
CTRL Protein
CTRL Phospho
CTRL RNA
GEF Protein
GEF Phospho
gef RNA
PLX Protein
PLX Phospho
PLX RNA
GEF PLX Protein
GEF PLX Phospho
GEF PLX RNA
Concentration(n[M])
Relative Conc
Time (hour)

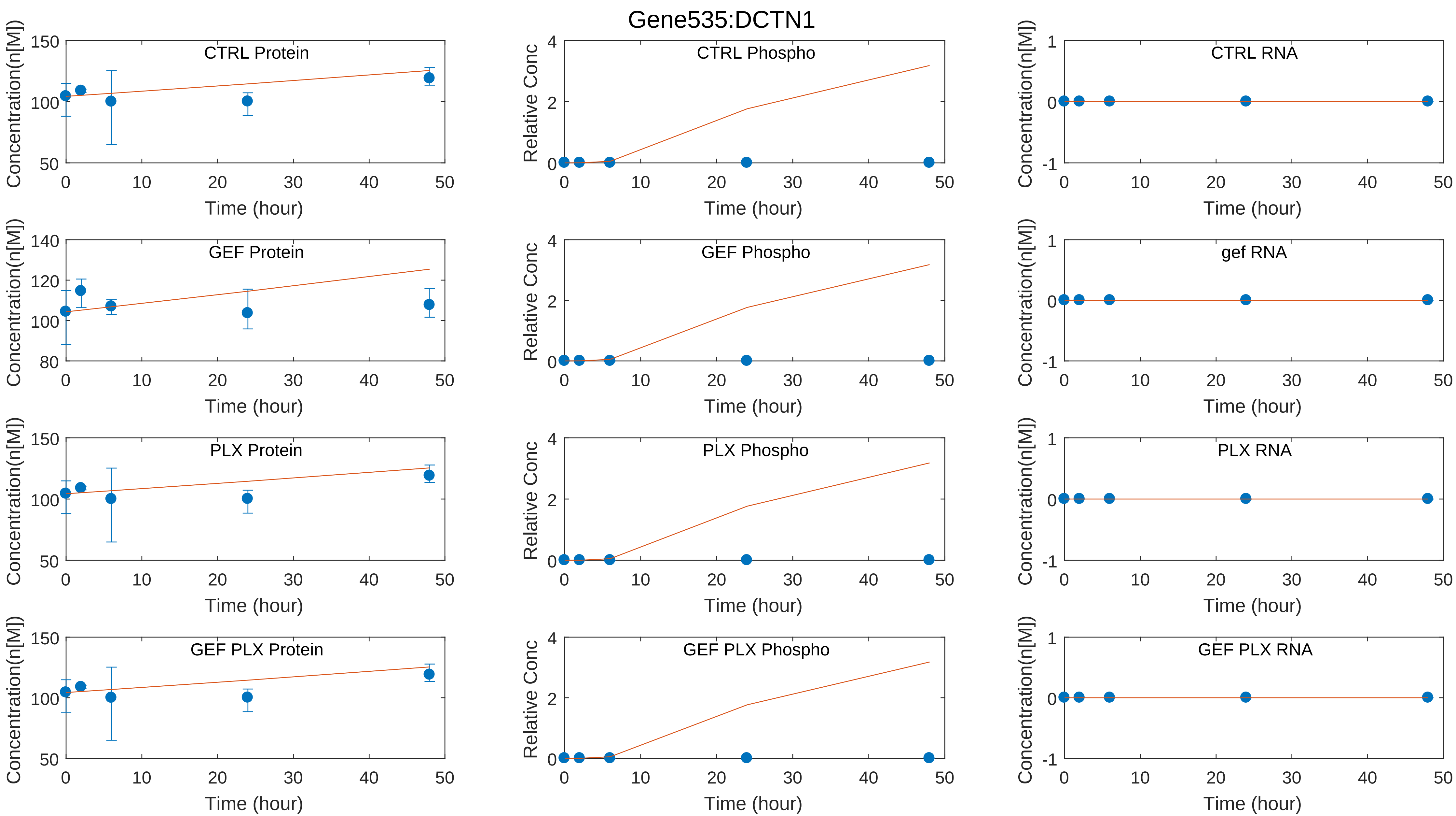

Gene535:DCTN1
CTRL Protein
GEF Protein
PLX Protein
GEF PLX Protein
CTRL Phospho
GEF Phospho
PLX Phospho
GEF PLX Phospho
CTRL RNA
gef RNA
PLX RNA
GEF PLX RNA
Concentration(n[M])
Relative Conc
Time (hour)

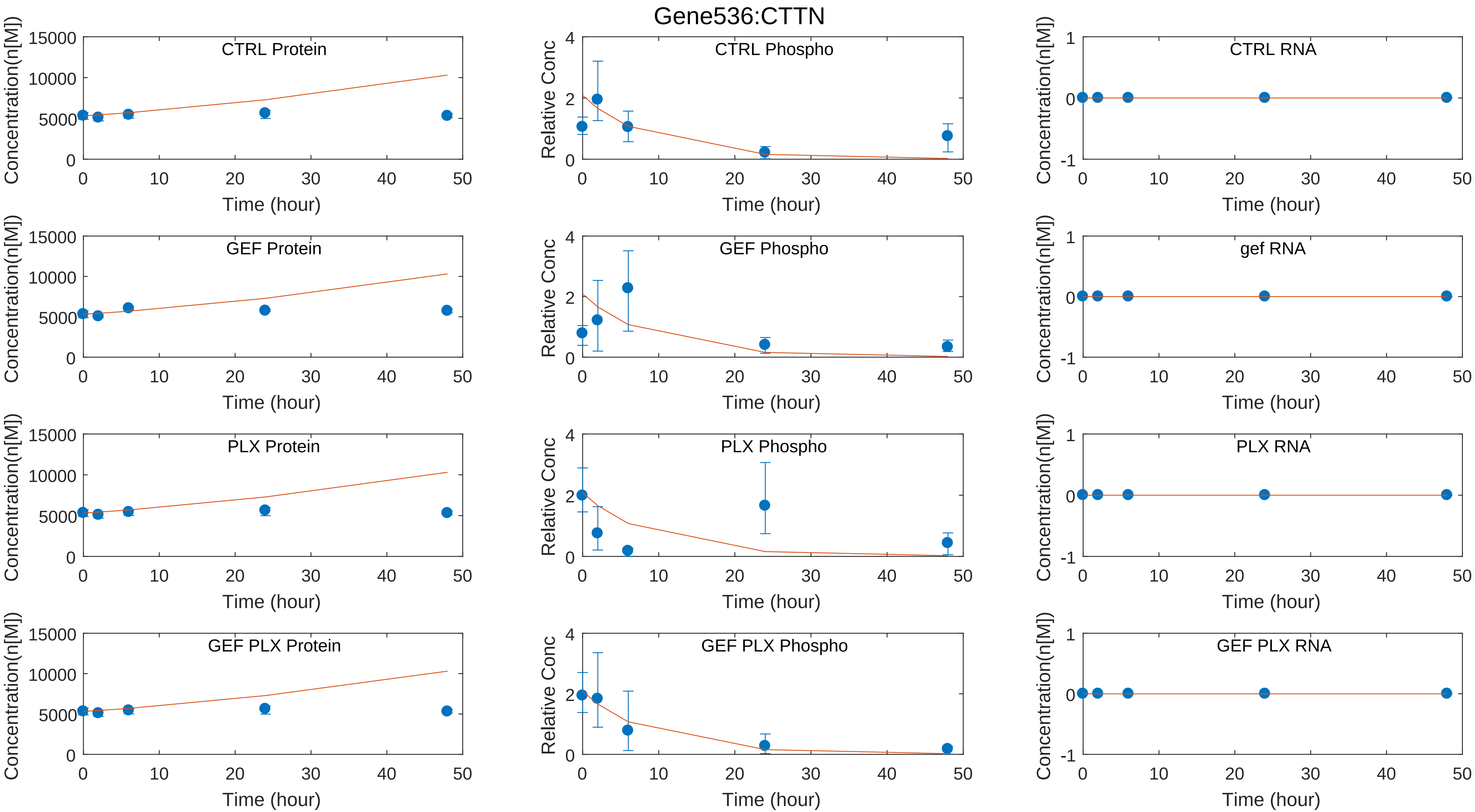

Gene536:CTTN
CTRL Protein
CTRL Phospho
CTRL RNA
GEF Protein
GEF Phospho
gef RNA
PLX Protein
PLX Phospho
PLX RNA
GEF PLX Protein
GEF PLX Phospho
GEF PLX RNA
Concentration(n[M])
Relative Conc
Time (hour)

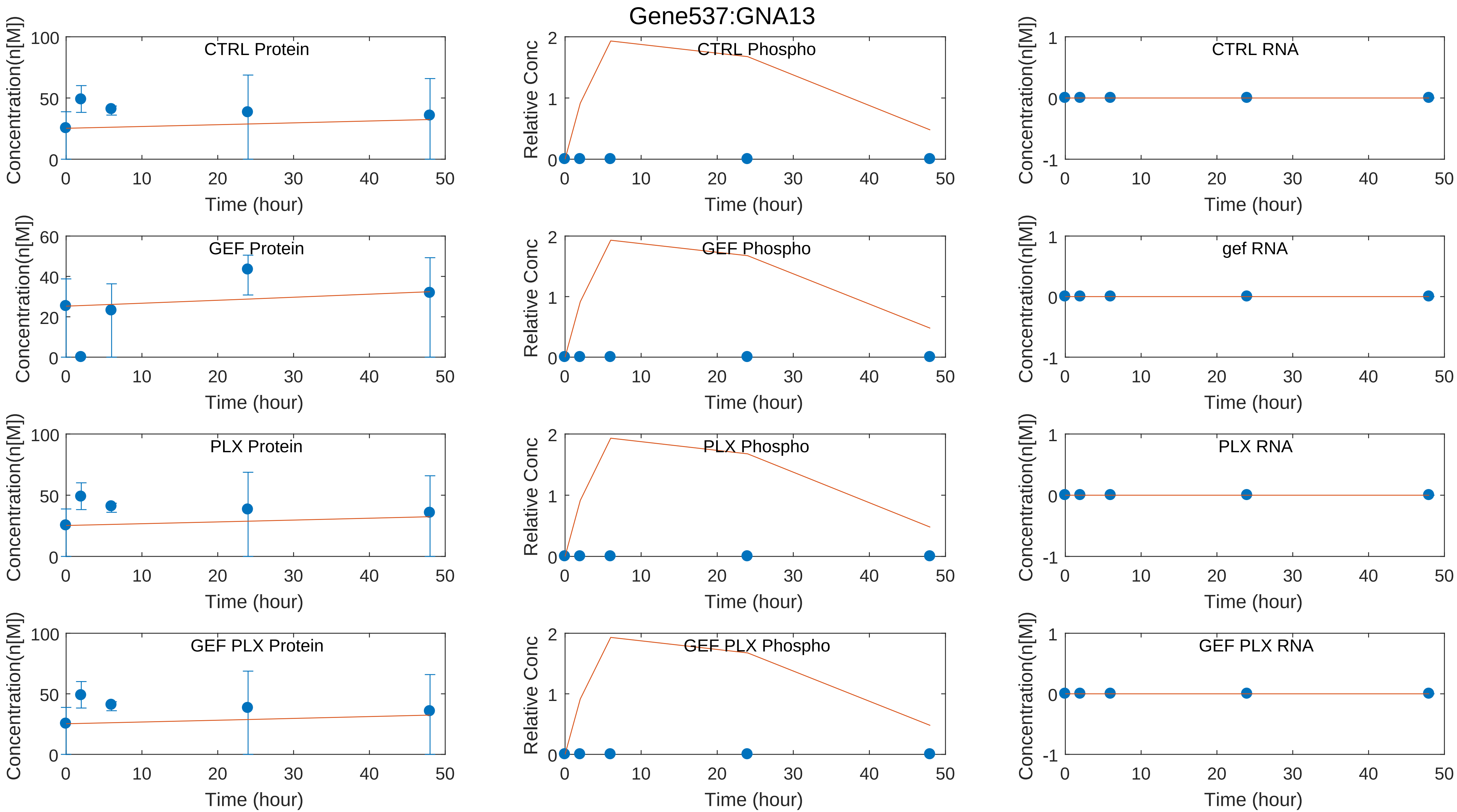

Gene537:GNA13
CTRL Protein
CTRL Phospho
CTRL RNA
GEF Protein
GEF Phospho
gef RNA
PLX Protein
PLX Phospho
PLX RNA
GEF PLX Protein
GEF PLX Phospho
GEF PLX RNA
Concentration(n[M])
Relative Conc
Time (hour)

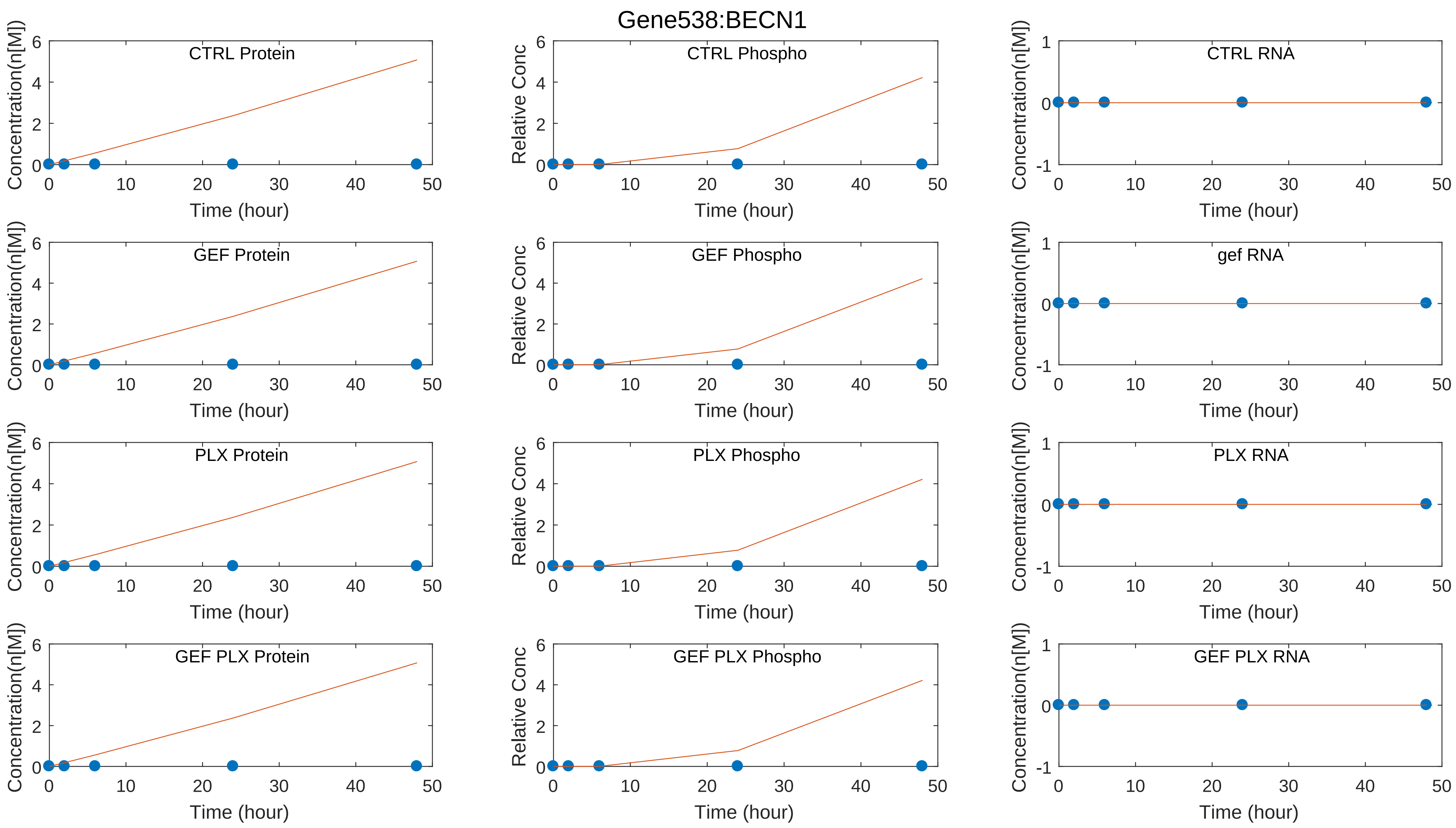

Gene538:BECN1
CTRL Protein
CTRL Phospho
CTRL RNA
GEF Protein
GEF Phospho
gef RNA
PLX Protein
PLX Phospho
PLX RNA
GEF PLX Protein
GEF PLX Phospho
GEF PLX RNA
Concentration(n[M])
Relative Conc
Time (hour)

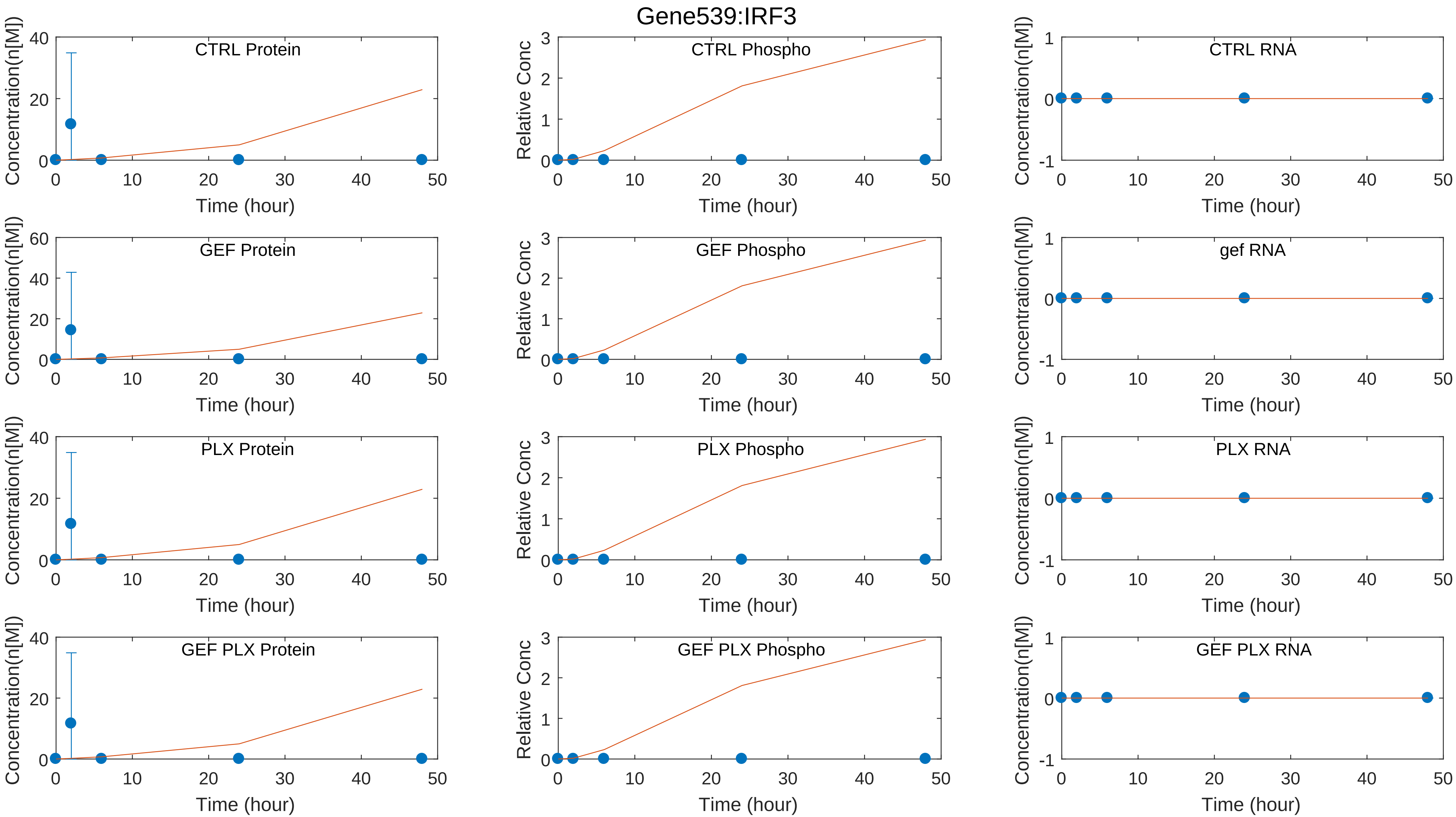

Gene539:IRF3
CTRL Protein
CTRL Phospho
CTRL RNA
GEF Protein
GEF Phospho
gef RNA
PLX Protein
PLX Phospho
PLX RNA
GEF PLX Protein
GEF PLX Phospho
GEF PLX RNA
Concentration(n[M])
Relative Conc
Time (hour)

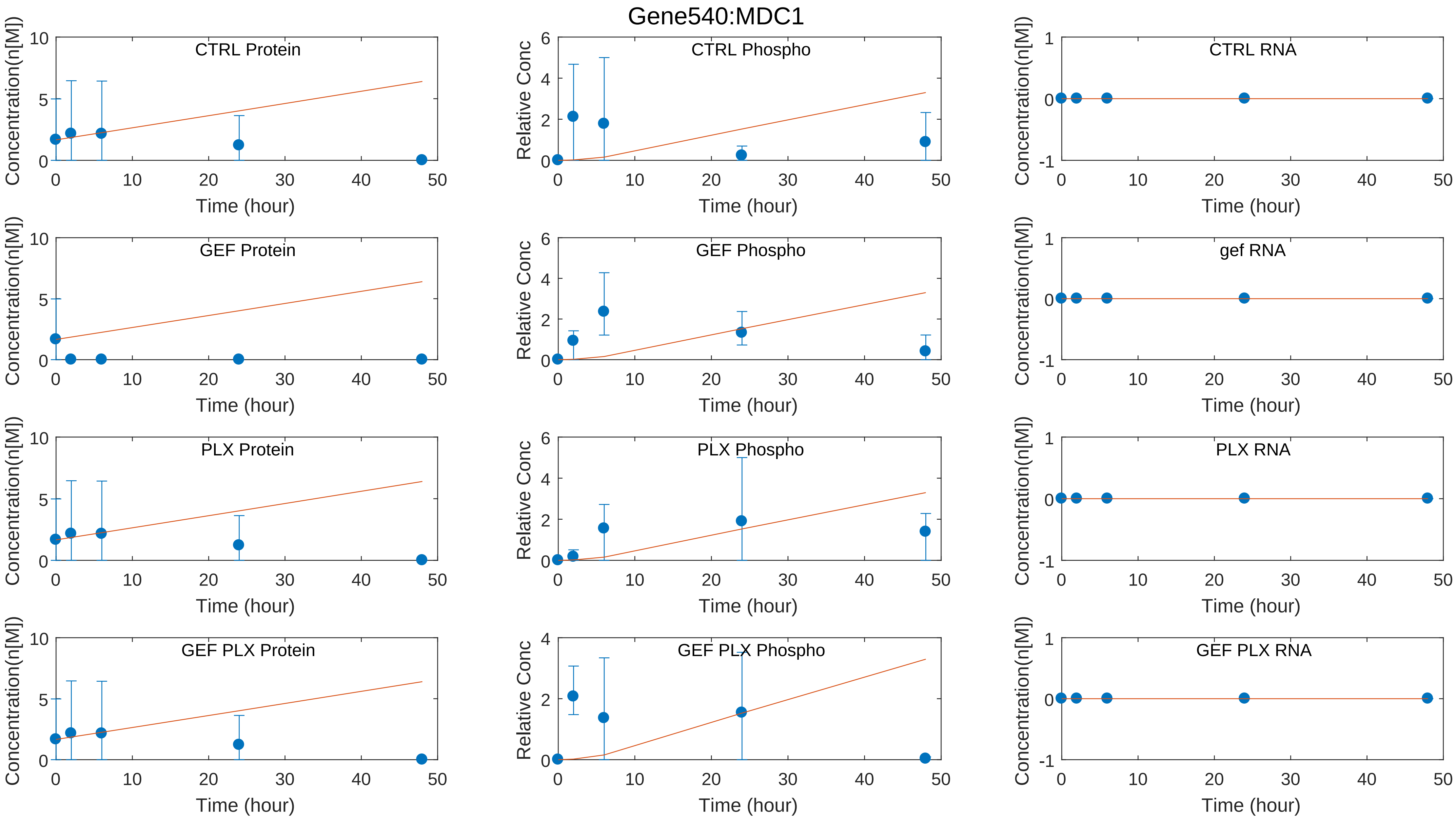

Gene540:MDC1
CTRL Protein
CTRL Phospho
CTRL RNA
GEF Protein
GEF Phospho
gef RNA
PLX Protein
PLX Phospho
PLX RNA
GEF PLX Protein
GEF PLX Phospho
GEF PLX RNA
Concentration(n[M])
Relative Conc
Time (hour)

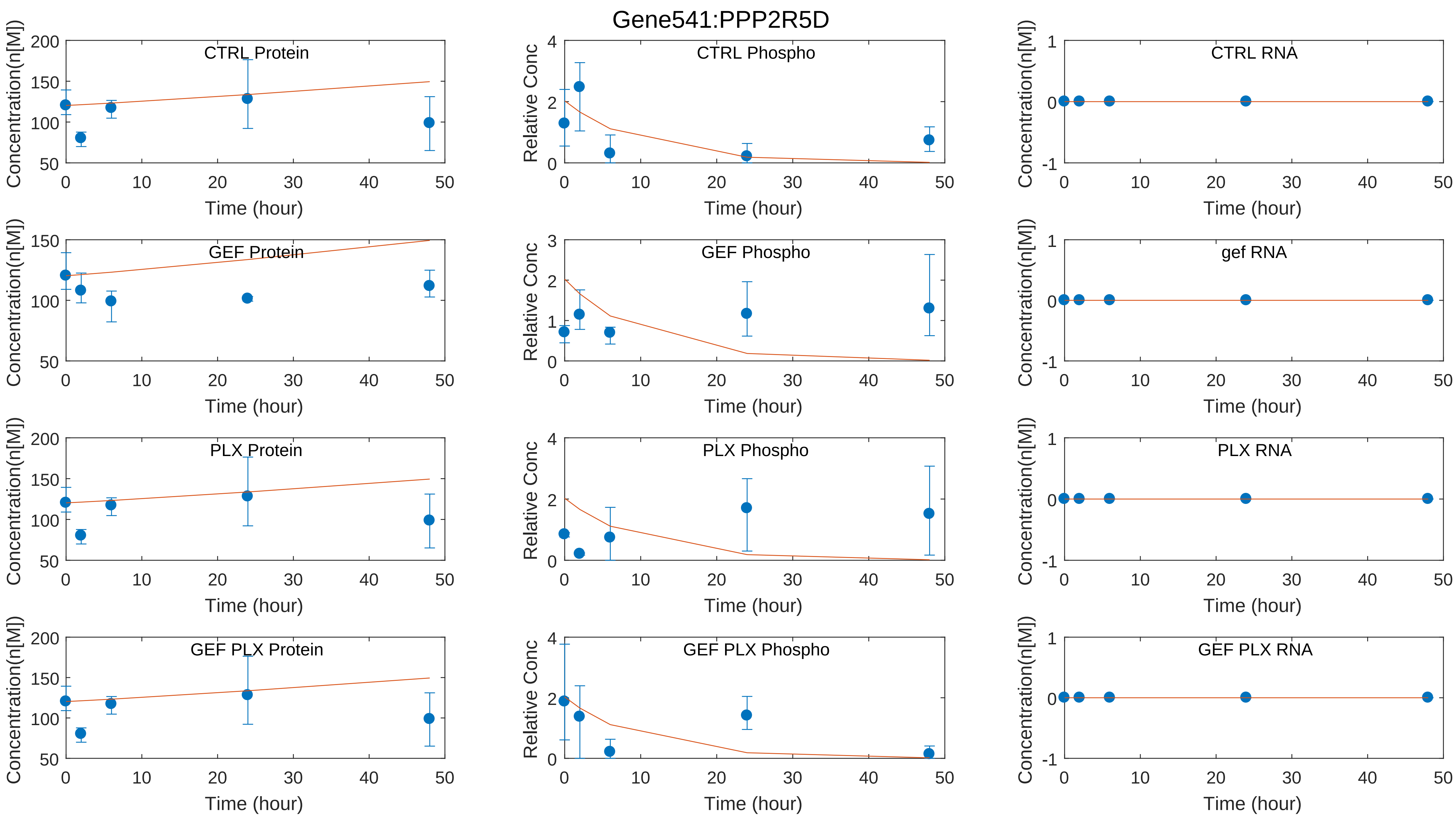

Gene541:PPP2R5D
CTRL Protein
CTRL Phospho
CTRL RNA
GEF Protein
GEF Phospho
gef RNA
PLX Protein
PLX Phospho
PLX RNA
GEF PLX Protein
GEF PLX Phospho
GEF PLX RNA
Concentration(n[M])
Relative Conc
Time (hour)

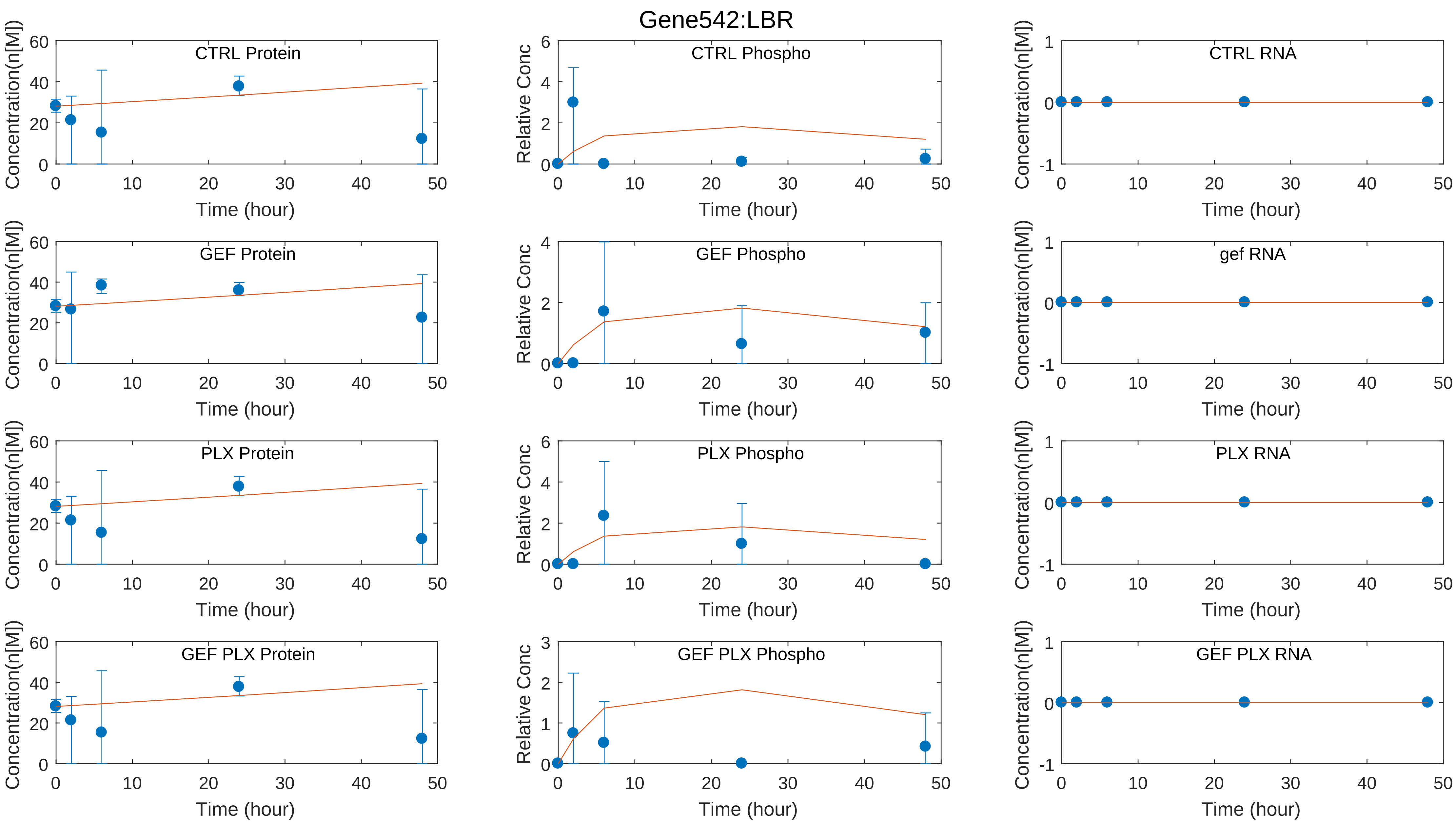

Gene542:LBR
CTRL Protein
CTRL Phospho
CTRL RNA
GEF Protein
GEF Phospho
gef RNA
PLX Protein
PLX Phospho
PLX RNA
GEF PLX Protein
GEF PLX Phospho
GEF PLX RNA
Concentration(n[M])
Relative Conc
Time (hour)

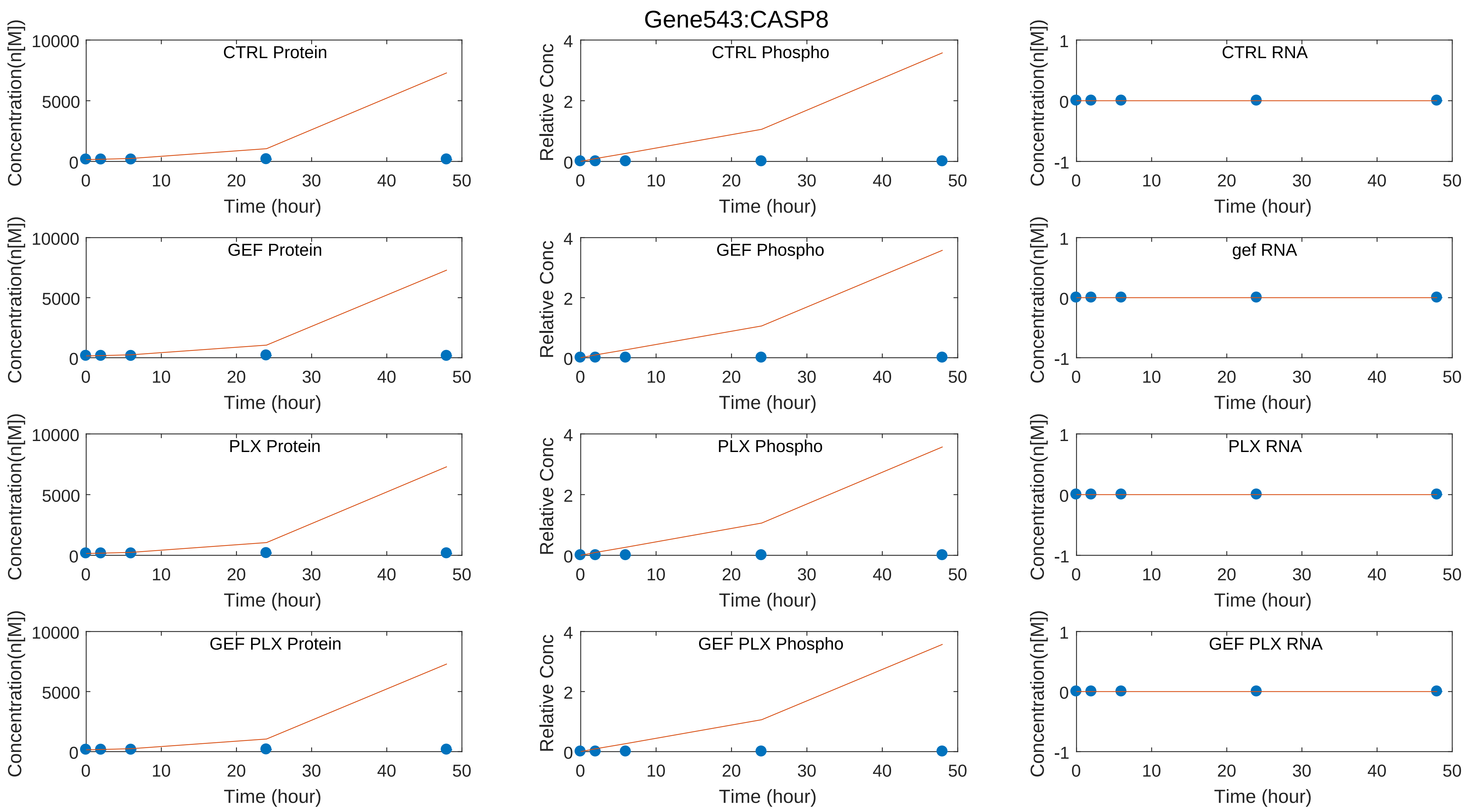

Gene543:CASP8
CTRL Protein
CTRL Phospho
CTRL RNA
GEF Protein
GEF Phospho
gef RNA
PLX Protein
PLX Phospho
PLX RNA
GEF PLX Protein
GEF PLX Phospho
GEF PLX RNA
Concentration(n[M])
Relative Conc
Time (hour)

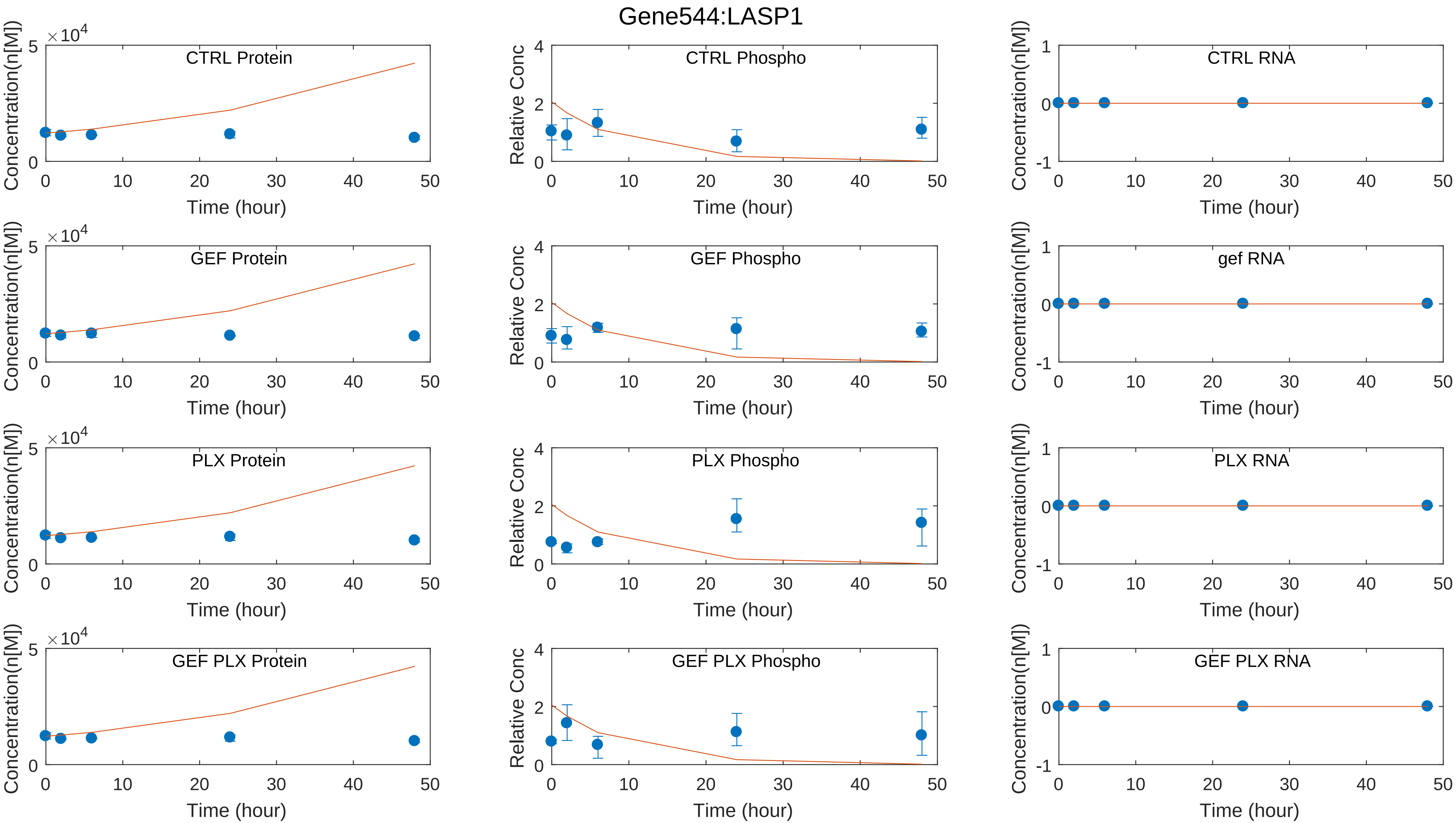

Gene544:LASP1
CTRL Protein
CTRL Phospho
CTRL RNA
GEF Protein
GEF Phospho
gef RNA
PLX Protein
PLX Phospho
PLX RNA
GEF PLX Protein
GEF PLX Phospho
GEF PLX RNA
Concentration(n[M])
Relative Conc
Time (hour)
$\times 10^4$

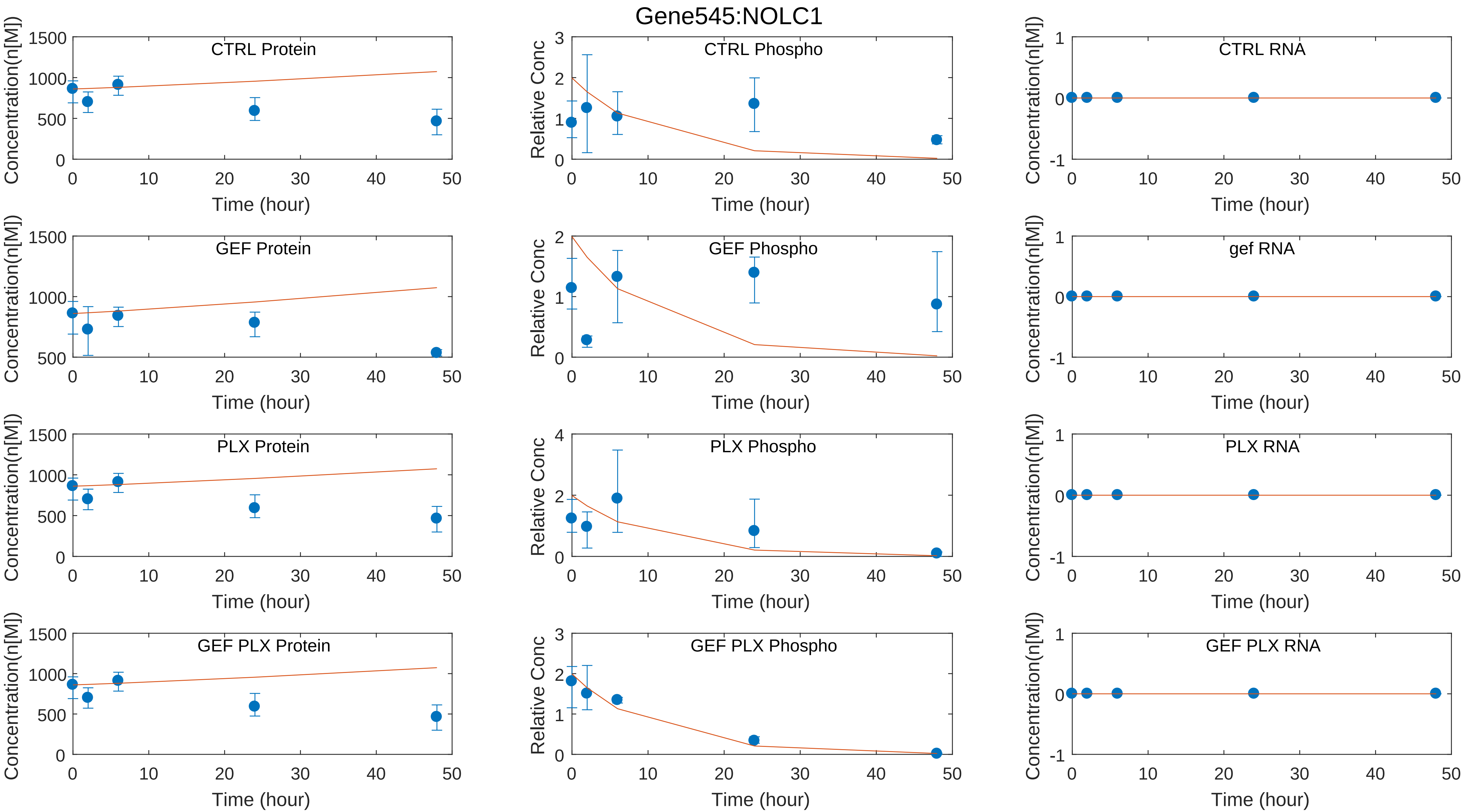
Gene545:NOLC1
CTRL Protein
CTRL Phospho
CTRL RNA
GEF Protein
GEF Phospho
gef RNA
PLX Protein
PLX Phospho
PLX RNA
GEF PLX Protein
GEF PLX Phospho
GEF PLX RNA
Concentration(n[M])
Relative Conc
Time (hour)

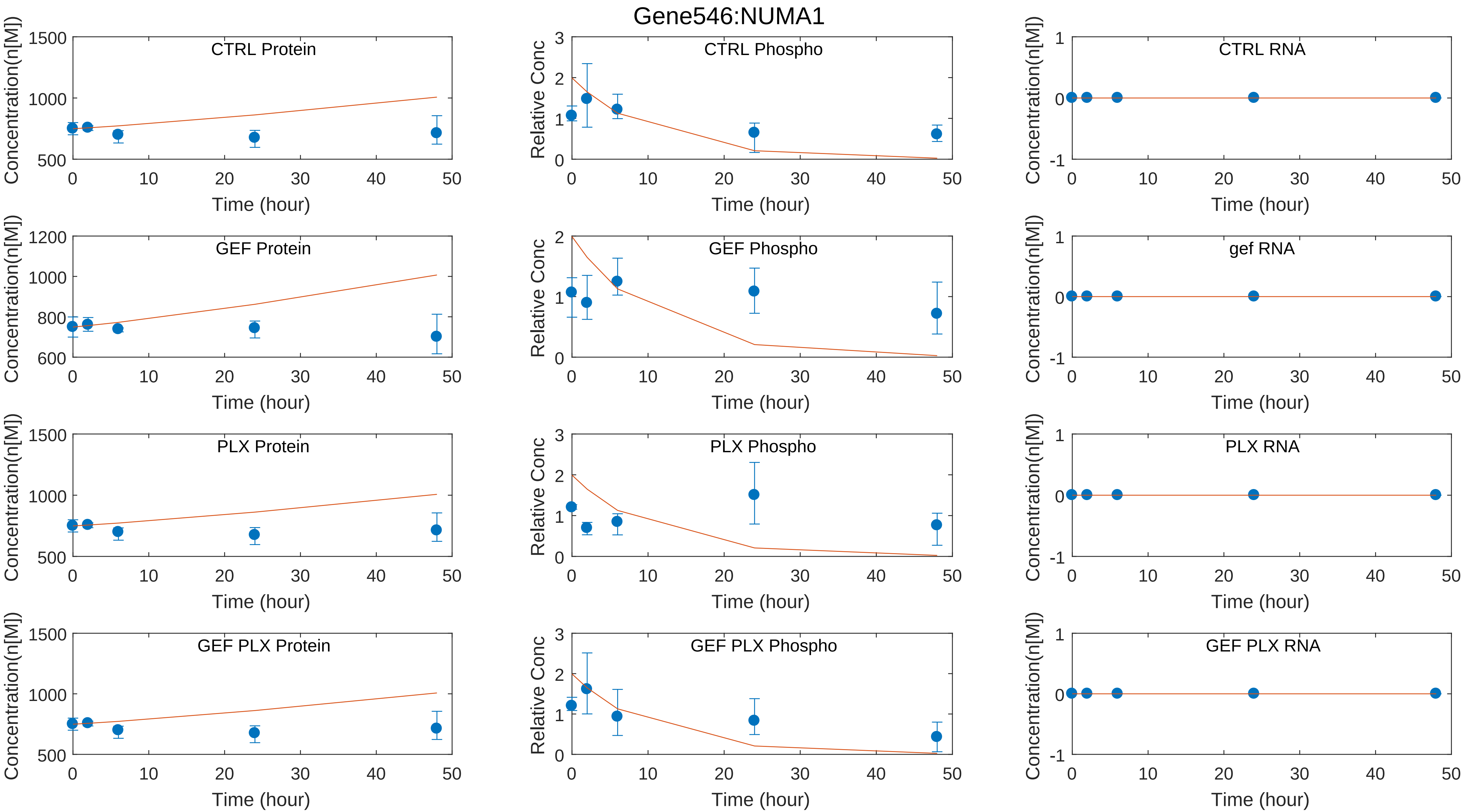

Gene546:NUMA1
CTRL Protein
CTRL Phospho
CTRL RNA
GEF Protein
GEF Phospho
gef RNA
PLX Protein
PLX Phospho
PLX RNA
GEF PLX Protein
GEF PLX Phospho
GEF PLX RNA
Concentration(n[M])
Relative Conc
Time (hour)

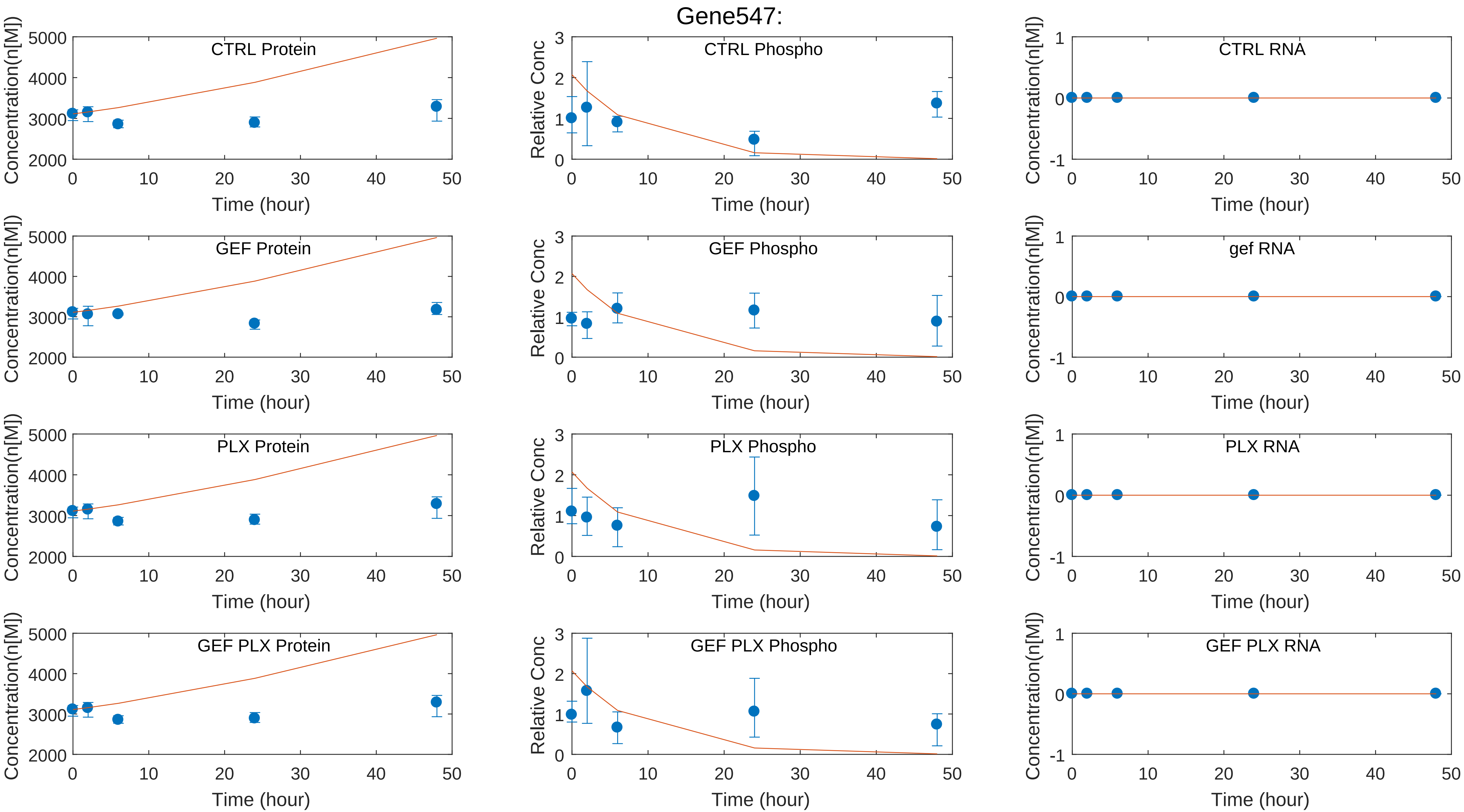

Gene547:
CTRL Protein
GEF Protein
PLX Protein
GEF PLX Protein
CTRL Phospho
GEF Phospho
PLX Phospho
GEF PLX Phospho
CTRL RNA
gef RNA
PLX RNA
GEF PLX RNA
Concentration(n[M])
Relative Conc
Time (hour)

Gene548:ACAP1

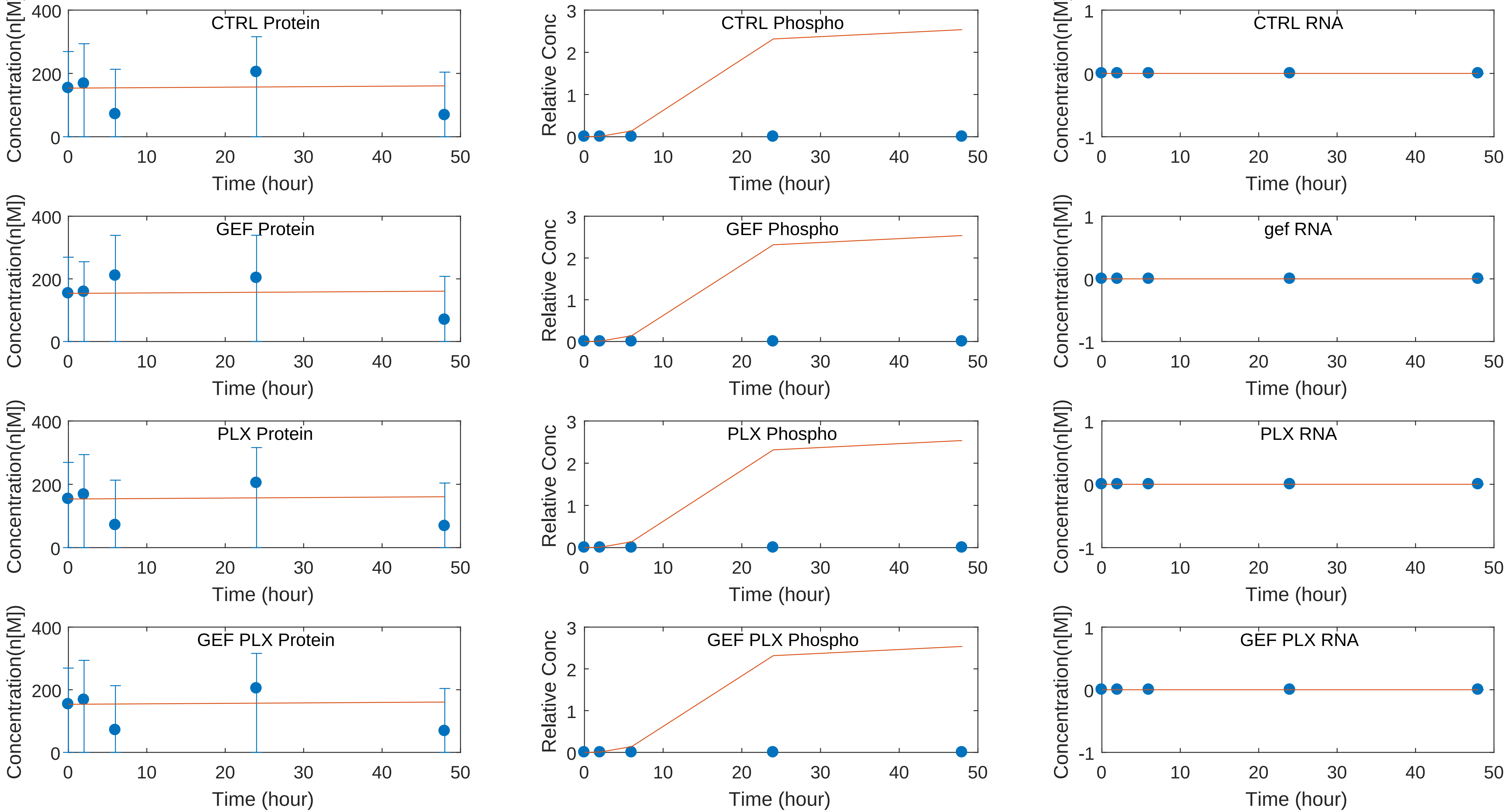

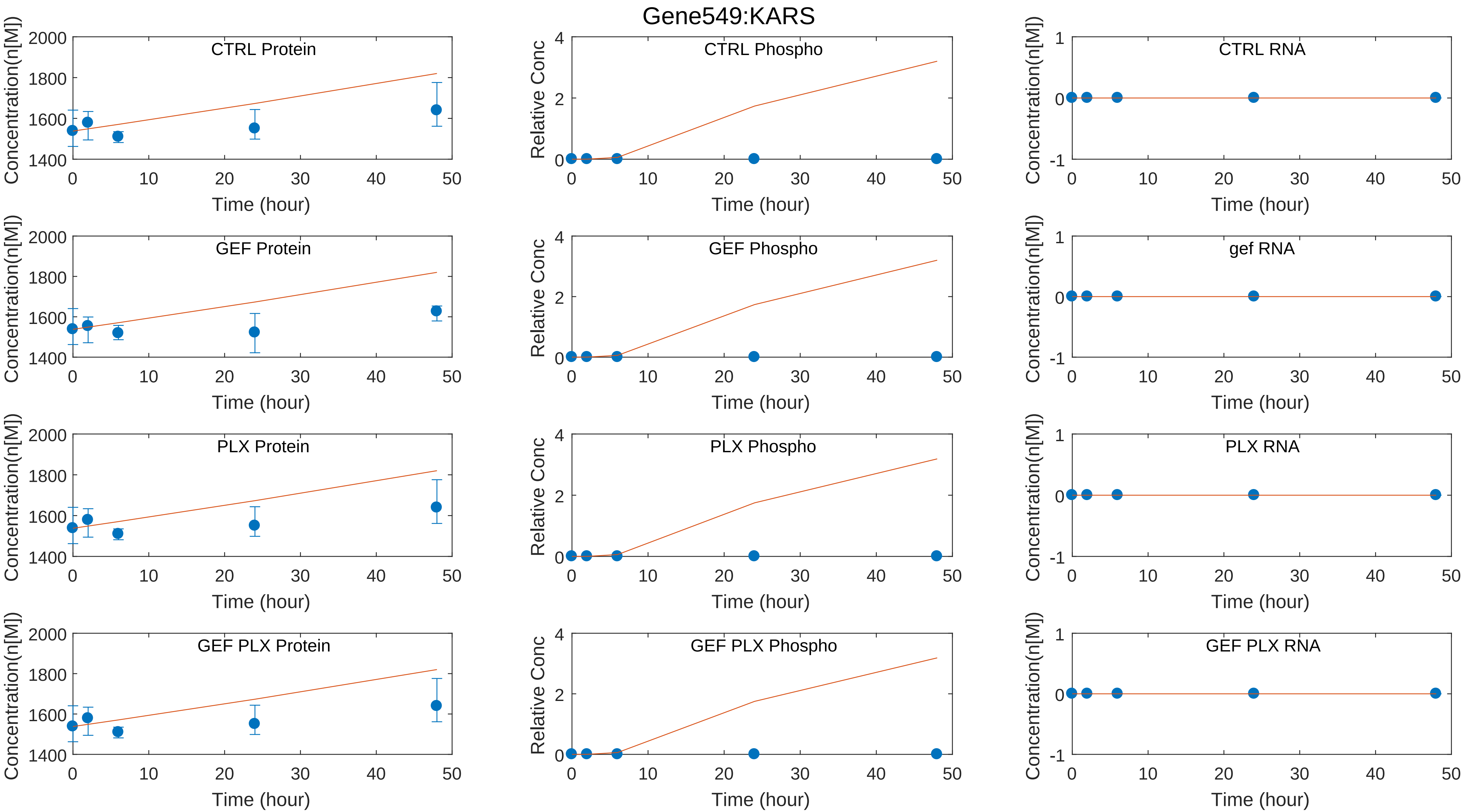

Gene549:KARS
CTRL Protein
GEF Protein
PLX Protein
GEF PLX Protein
CTRL Phospho
GEF Phospho
PLX Phospho
GEF PLX Phospho
CTRL RNA
gef RNA
PLX RNA
GEF PLX RNA
Concentration(n[M])
Relative Conc
Time (hour)

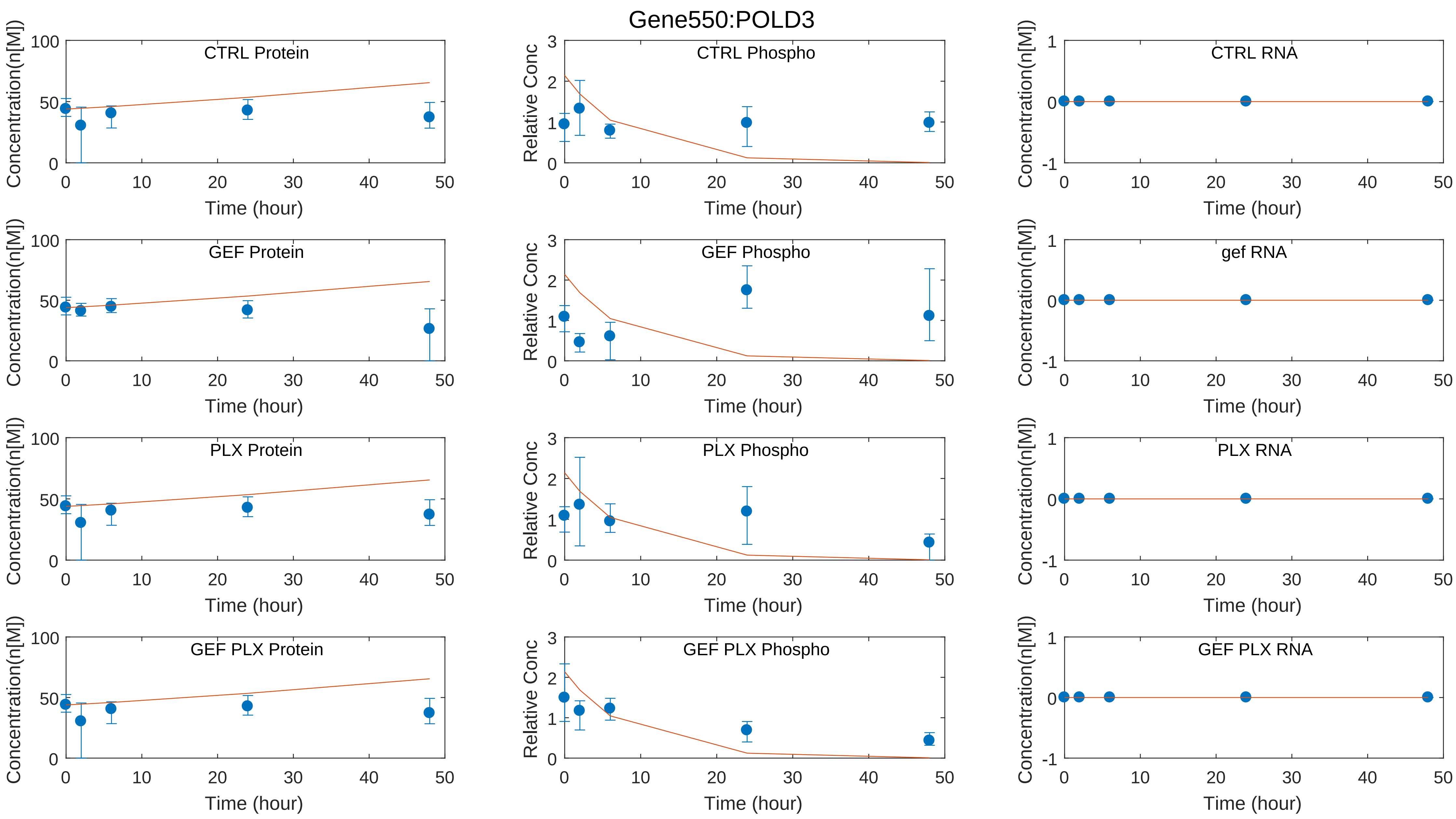
Gene550:POLD3
CTRL Protein
CTRL Phospho
CTRL RNA
GEF Protein
GEF Phospho
gef RNA
PLX Protein
PLX Phospho
PLX RNA
GEF PLX Protein
GEF PLX Phospho
GEF PLX RNA
Concentration(n[M])
Relative Conc
Time (hour)

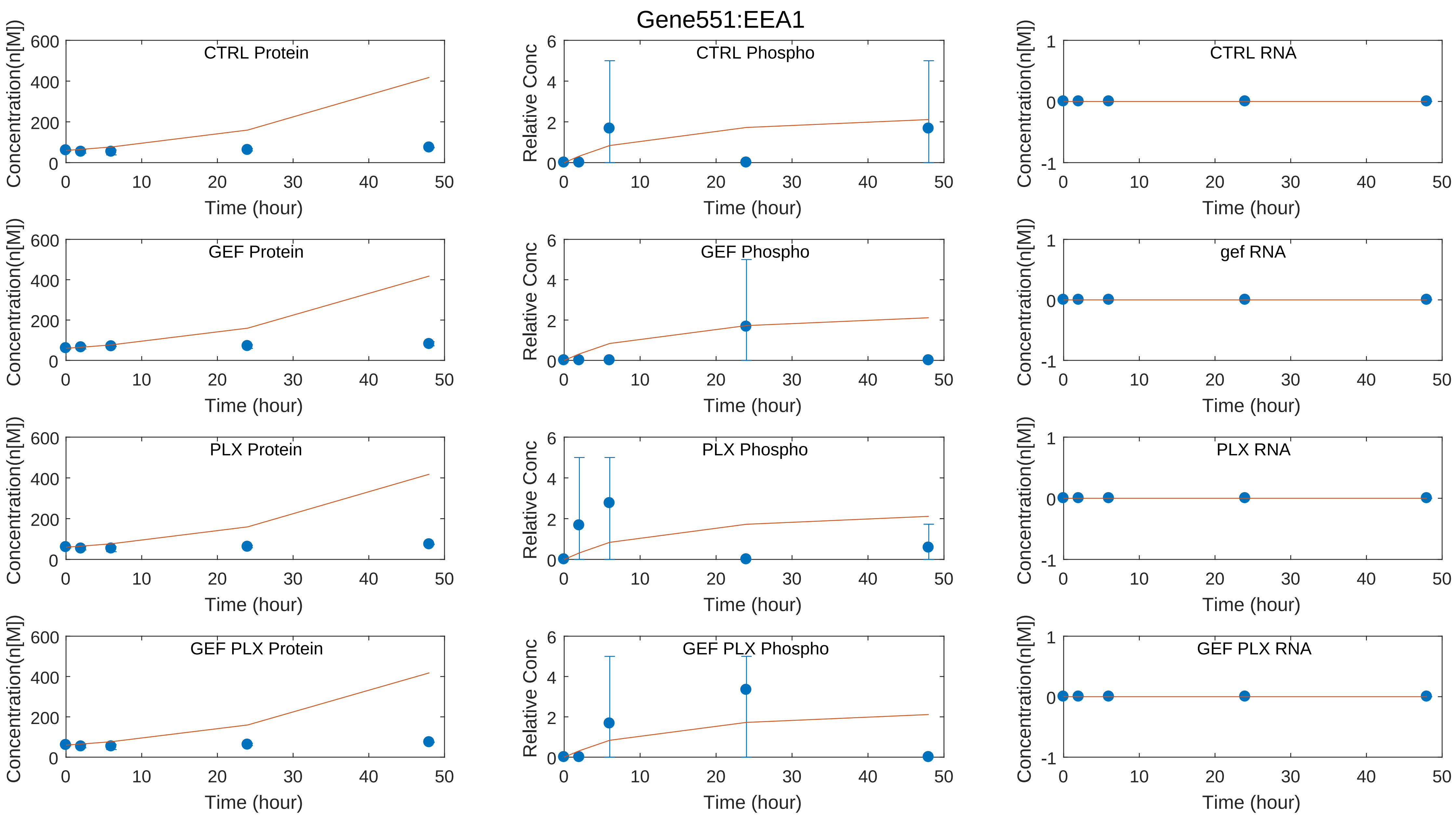
Gene551:EEA1
CTRL Protein
CTRL Phospho
CTRL RNA
GEF Protein
GEF Phospho
gef RNA
PLX Protein
PLX Phospho
PLX RNA
GEF PLX Protein
GEF PLX Phospho
GEF PLX RNA
Concentration(n[M])
Relative Conc
Time (hour)

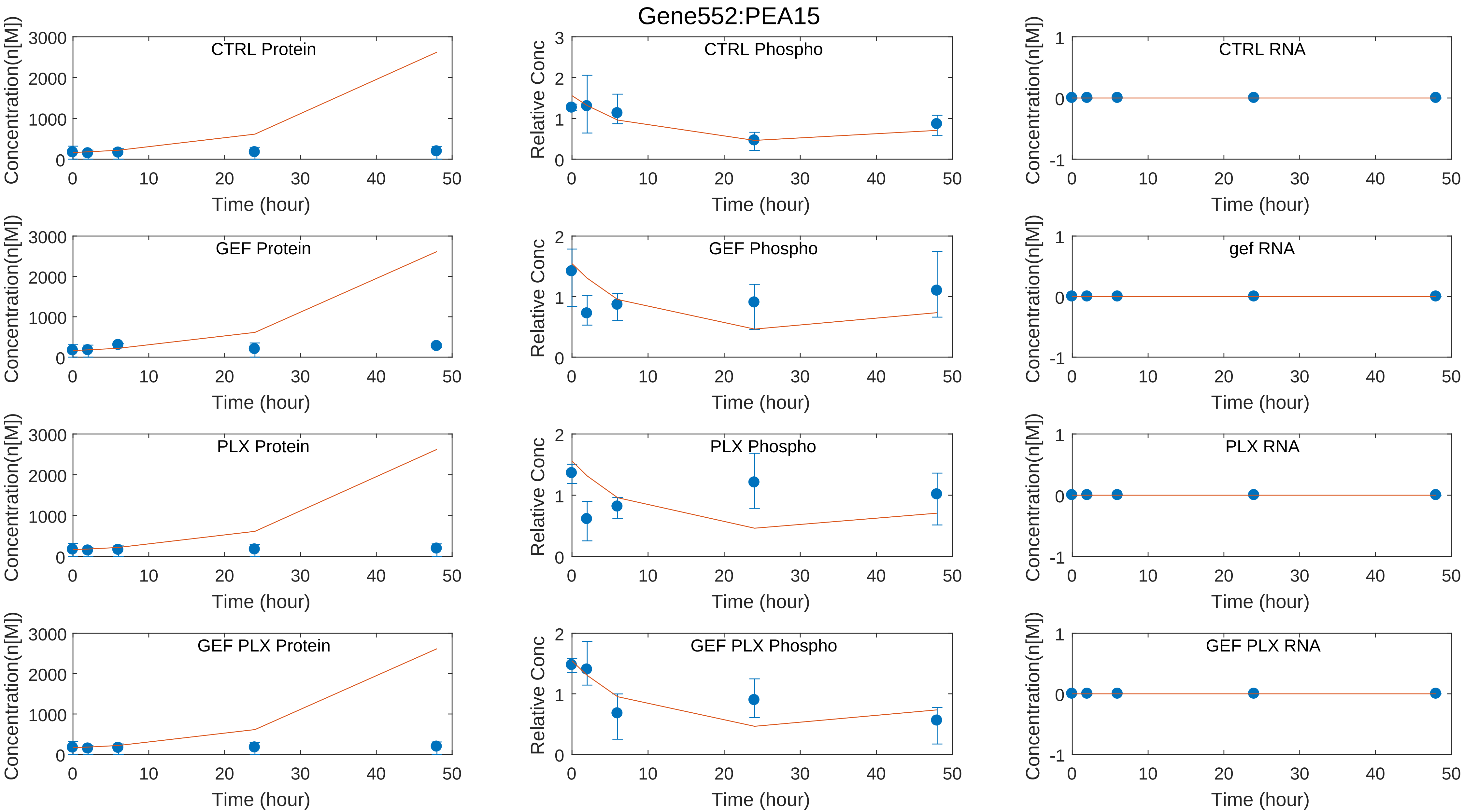

Gene552:PEA15
CTRL Protein
GEF Protein
PLX Protein
GEF PLX Protein
CTRL Phospho
GEF Phospho
PLX Phospho
GEF PLX Phospho
CTRL RNA
gef RNA
PLX RNA
GEF PLX RNA
Concentration(n[M])
Relative Conc
Time (hour)

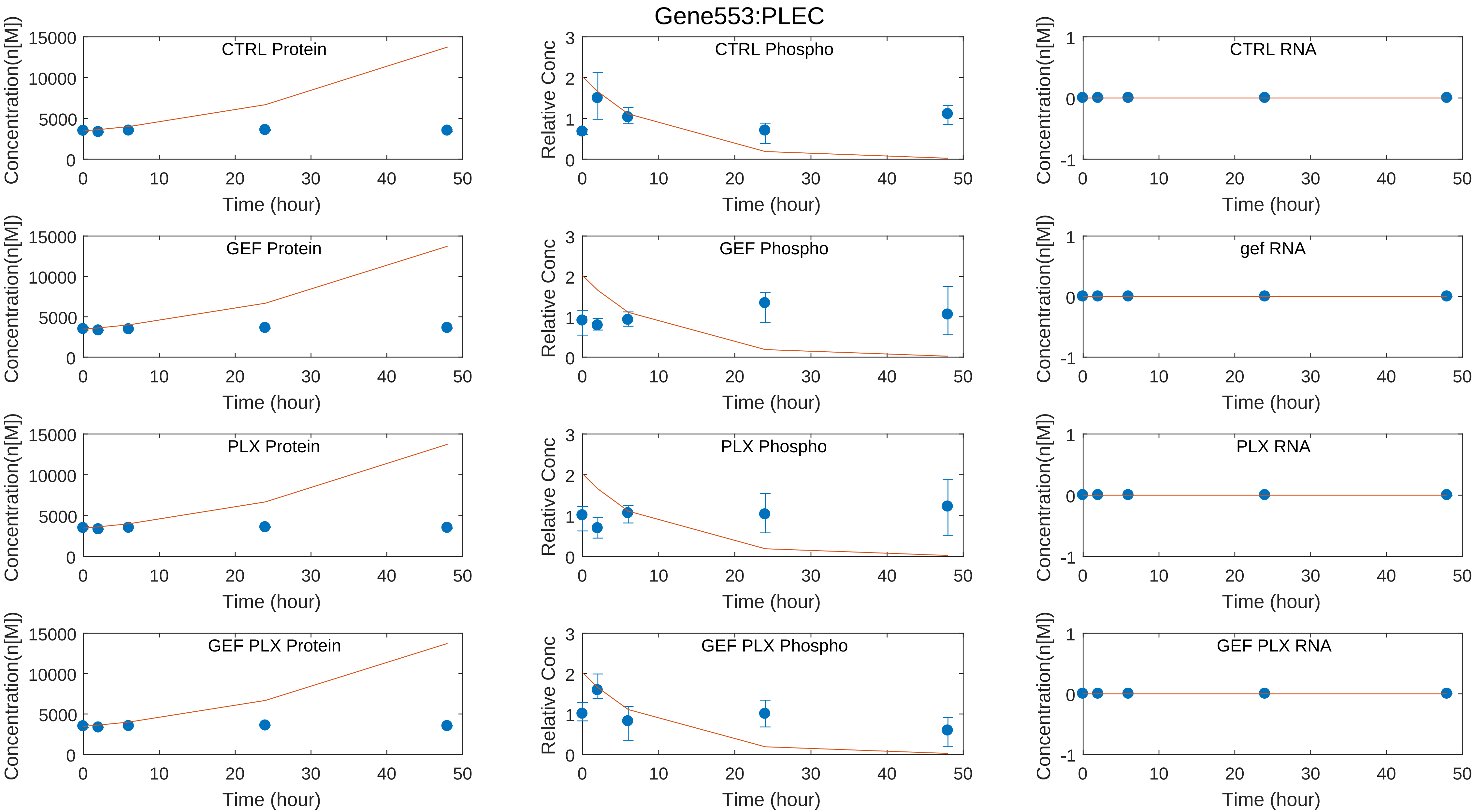

Gene553:PLEC
CTRL Protein
GEF Protein
PLX Protein
GEF PLX Protein
CTRL Phospho
GEF Phospho
PLX Phospho
GEF PLX Phospho
CTRL RNA
gef RNA
PLX RNA
GEF PLX RNA
Concentration(n[M])
Relative Conc
Time (hour)

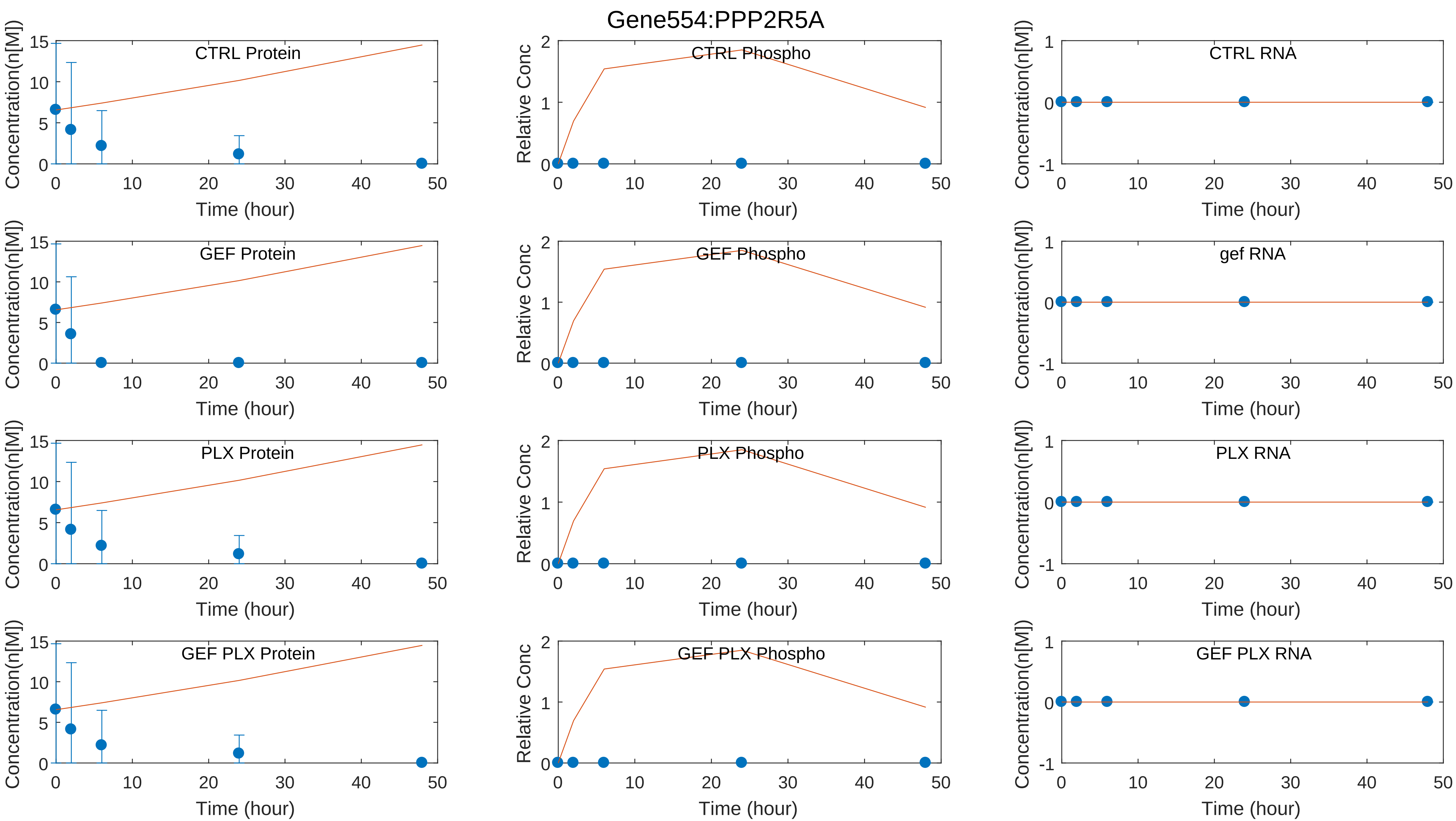
Gene554:PPP2R5A
CTRL Protein
CTRL Phospho
CTRL RNA
GEF Protein
GEF Phospho
gef RNA
PLX Protein
PLX Phospho
PLX RNA
GEF PLX Protein
GEF PLX Phospho
GEF PLX RNA
Concentration(n[M])
Relative Conc
Time (hour)

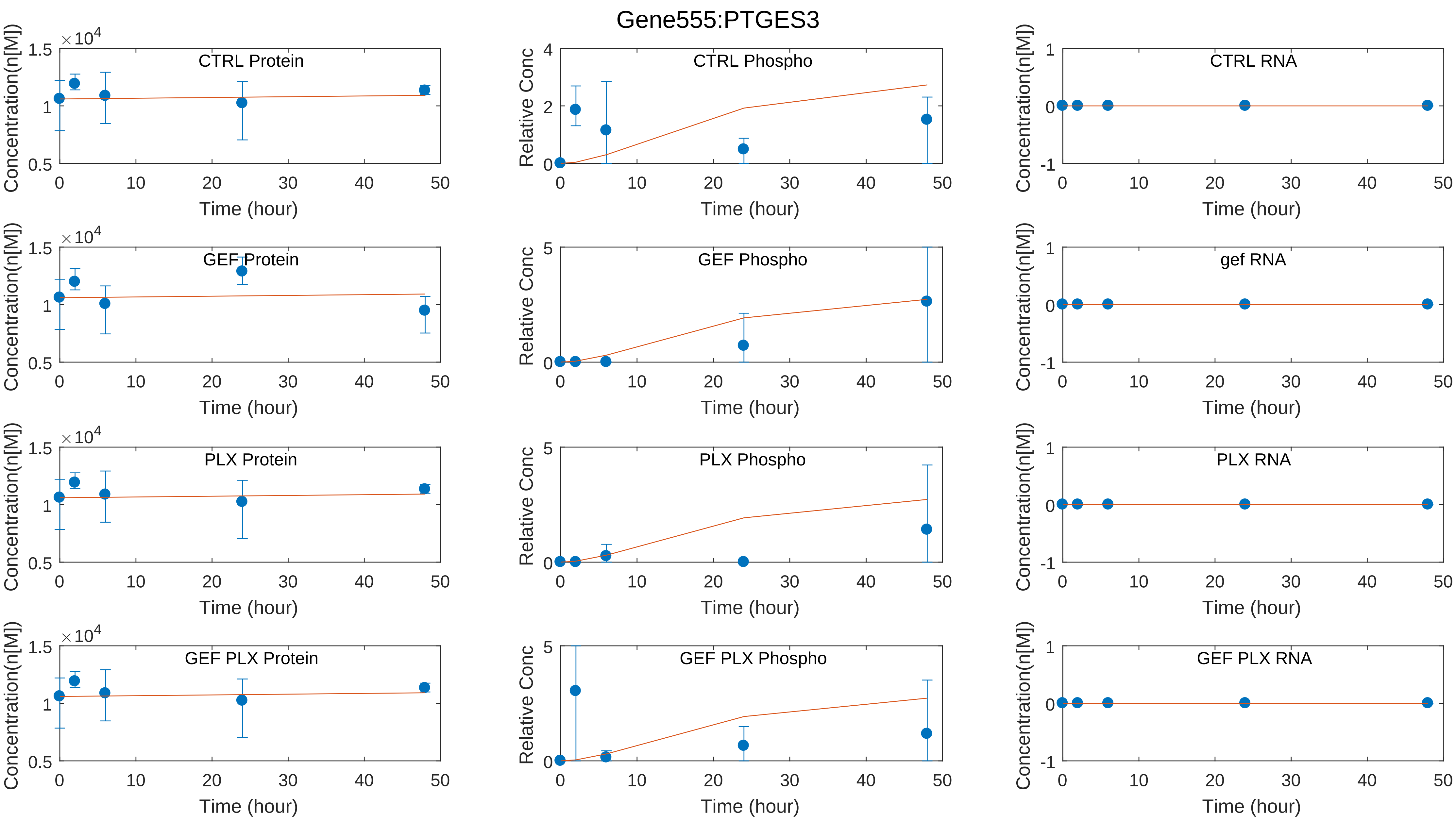

Gene555:PTGES3
CTRL Protein
CTRL Phospho
CTRL RNA
GEF Protein
GEF Phospho
gef RNA
PLX Protein
PLX Phospho
PLX RNA
GEF PLX Protein
GEF PLX Phospho
GEF PLX RNA
Concentration(n[M])
Relative Conc
Time (hour)
$\times 10^4$

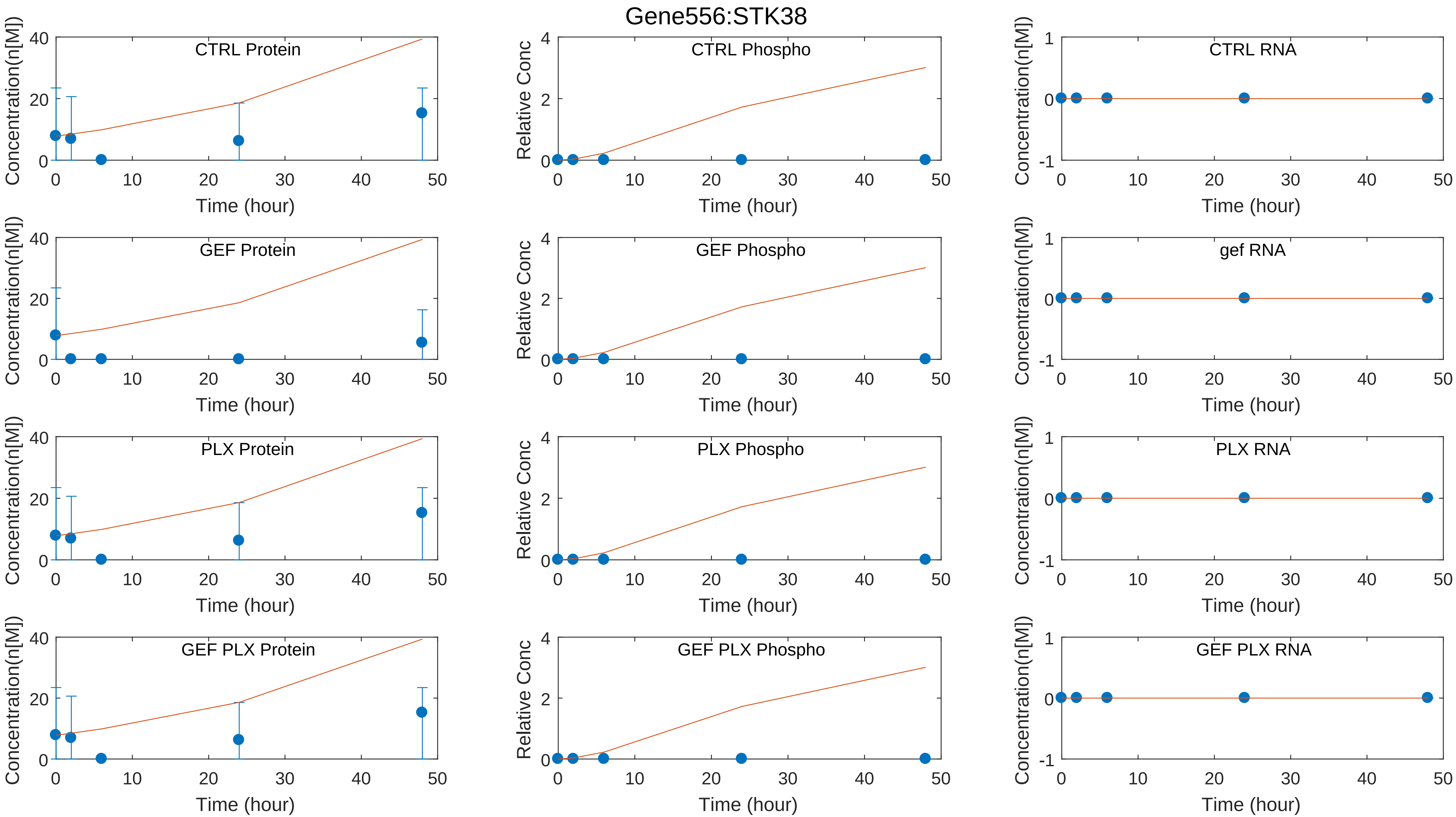
Gene556:STK38
CTRL Protein
CTRL Phospho
CTRL RNA
GEF Protein
GEF Phospho
gef RNA
PLX Protein
PLX Phospho
PLX RNA
GEF PLX Protein
GEF PLX Phospho
GEF PLX RNA
Concentration(n[M])
Relative Conc
Time (hour)

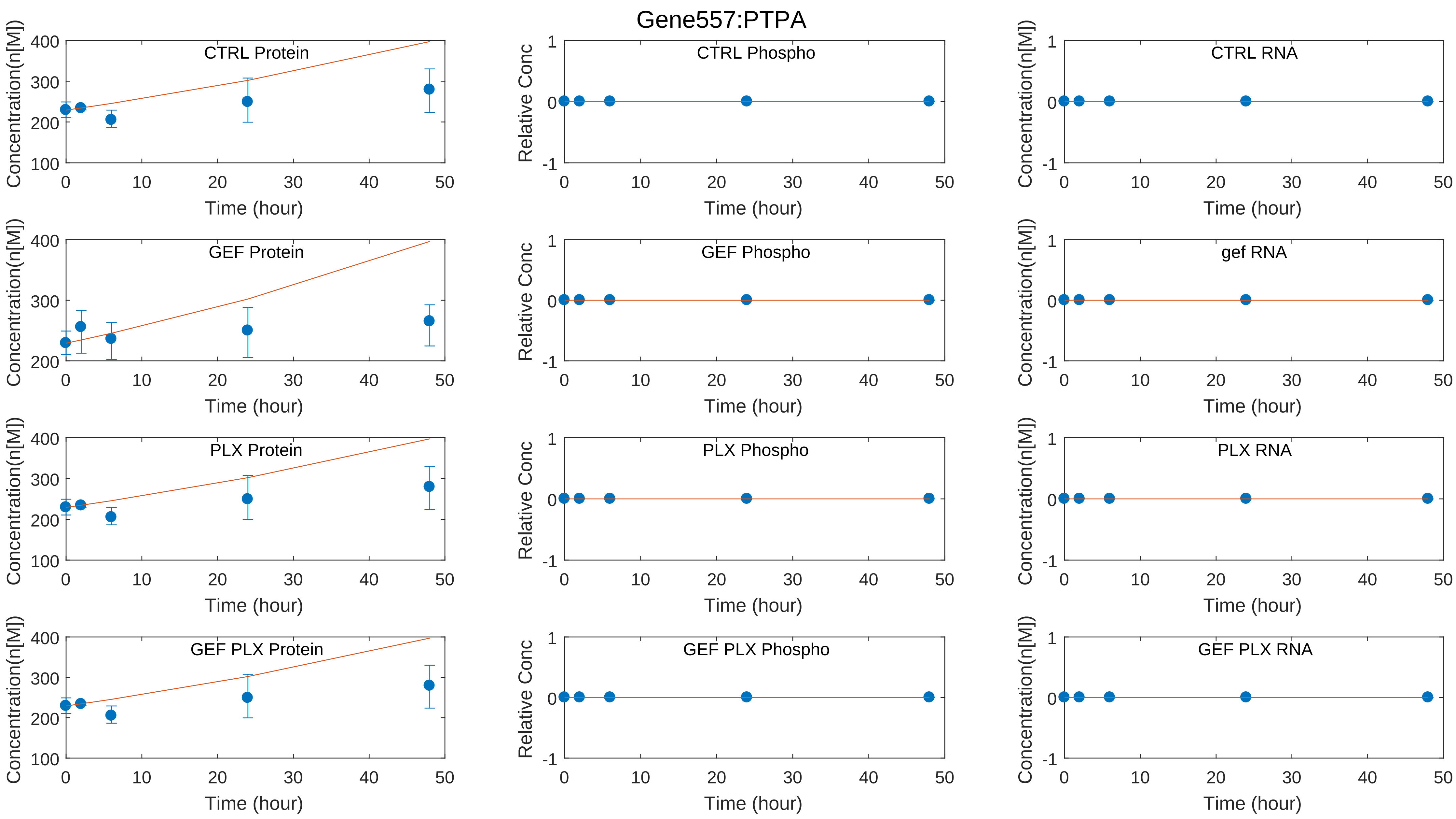

Gene557:PTPA
CTRL Protein
CTRL Phospho
CTRL RNA
GEF Protein
GEF Phospho
gef RNA
PLX Protein
PLX Phospho
PLX RNA
GEF PLX Protein
GEF PLX Phospho
GEF PLX RNA
Concentration(n[M])
Relative Conc
Time (hour)

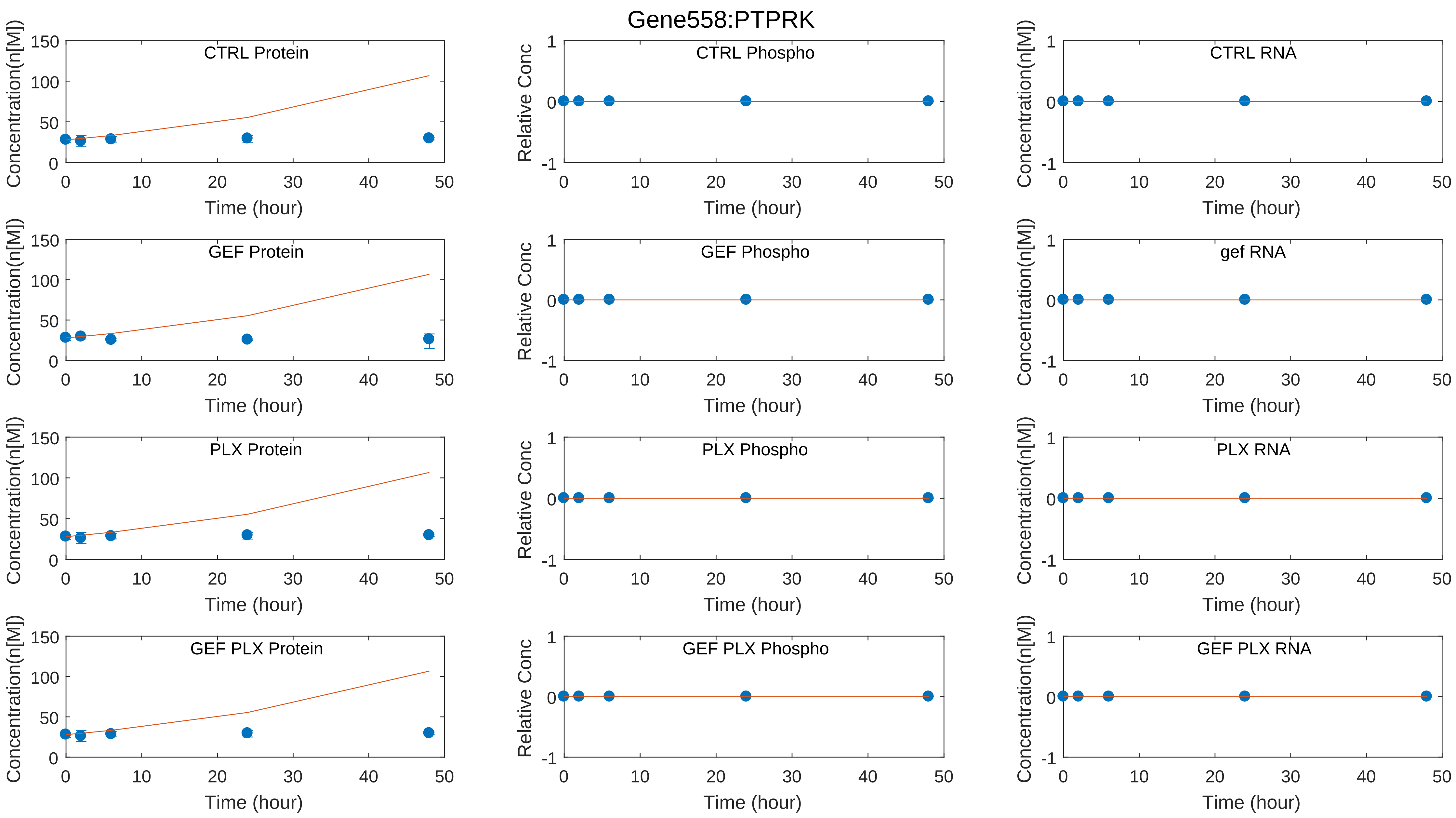

Gene558:PTPRK
CTRL Protein
CTRL Phospho
CTRL RNA
GEF Protein
GEF Phospho
gef RNA
PLX Protein
PLX Phospho
PLX RNA
GEF PLX Protein
GEF PLX Phospho
GEF PLX RNA
Concentration(n[M])
Relative Conc
Time (hour)

Gene559:ABO

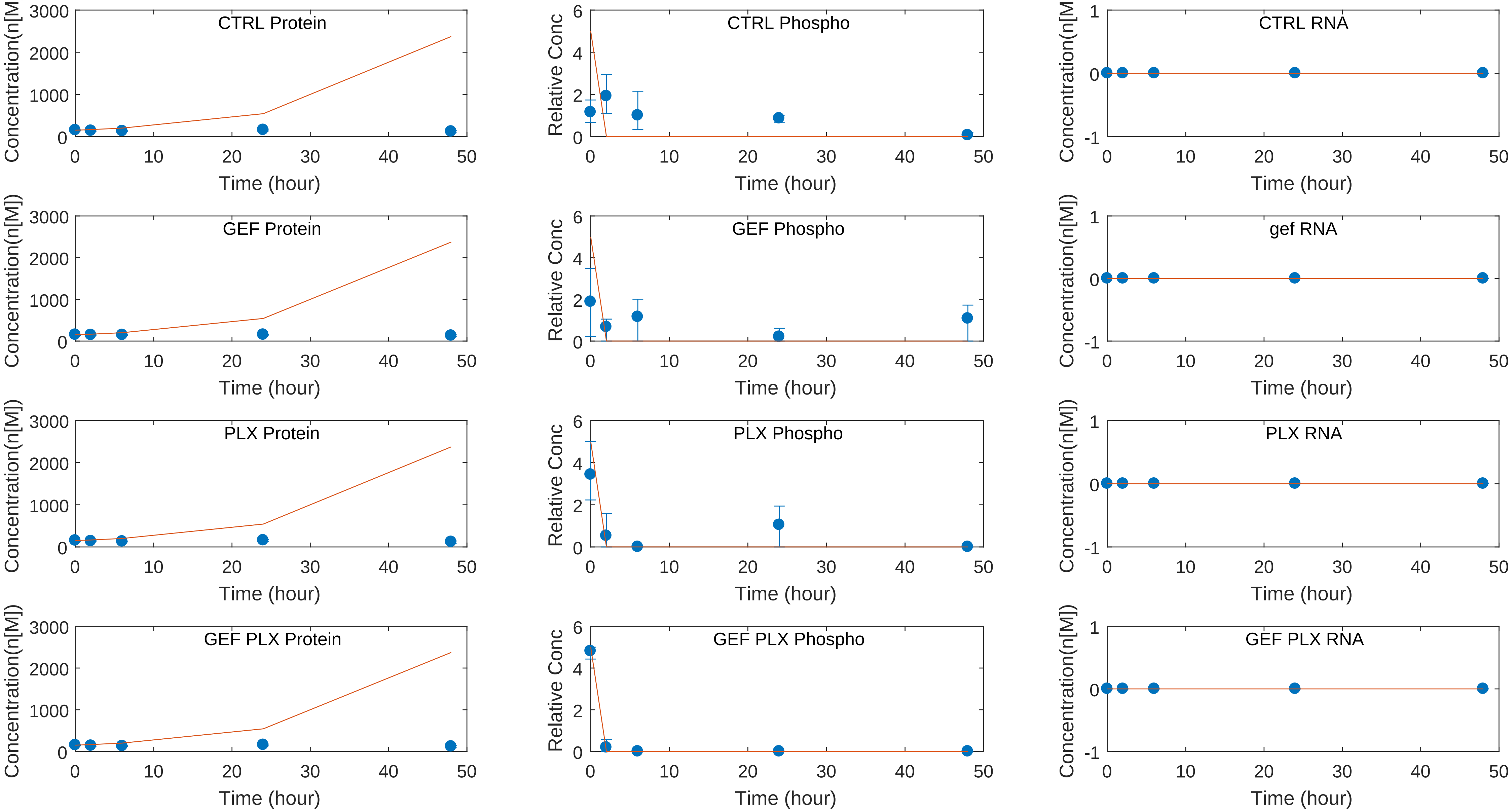

CTRL Protein
GEF Protein
PLX Protein
GEF PLX Protein
CTRL Phospho
GEF Phospho
PLX Phospho
GEF PLX Phospho
CTRL RNA
gef RNA
PLX RNA
GEF PLX RNA
Concentration(n[M])
Relative Conc
Time (hour)

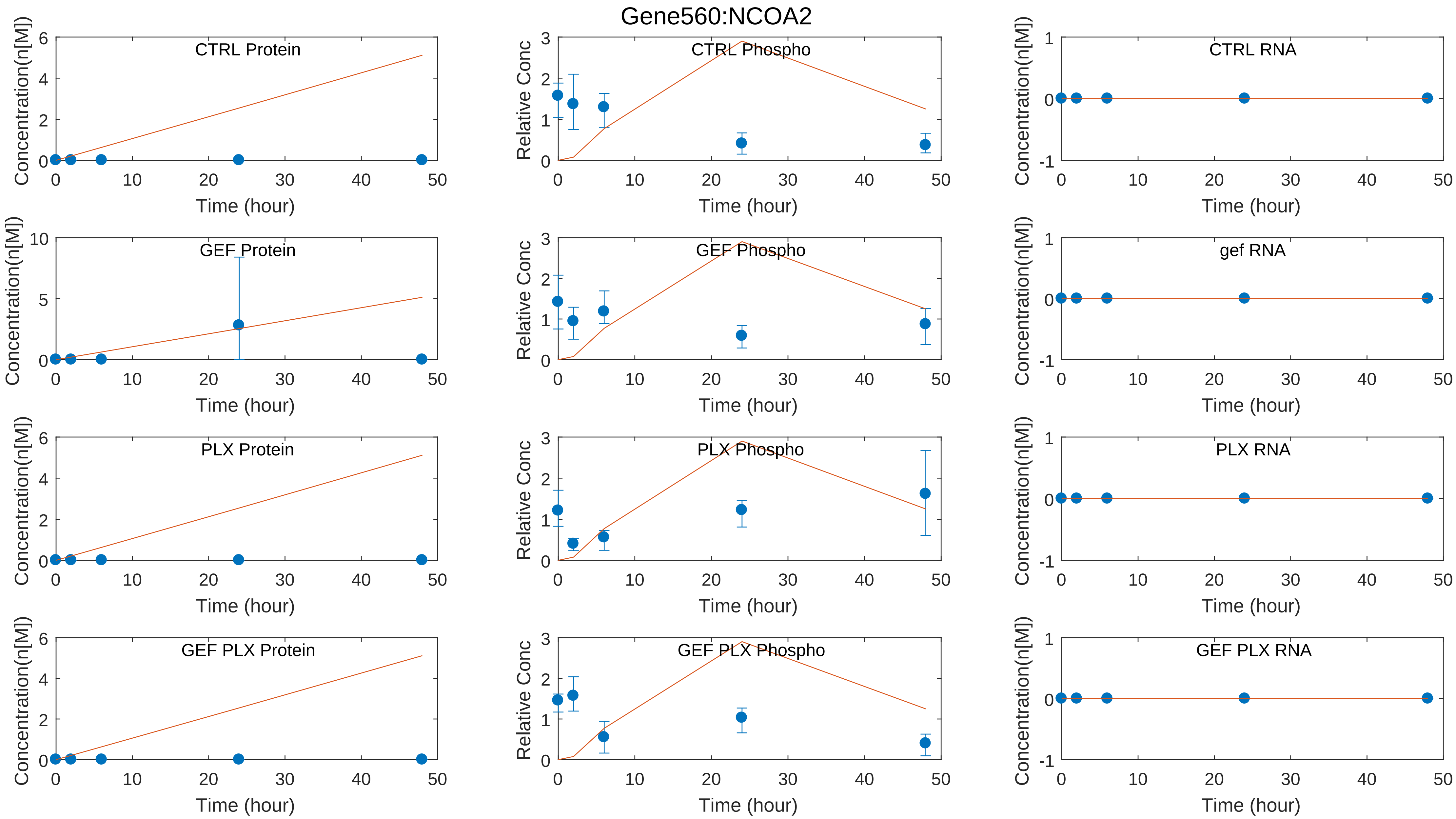

Gene560:NCOA2
CTRL Protein
CTRL Phospho
CTRL RNA
GEF Protein
GEF Phospho
gef RNA
PLX Protein
PLX Phospho
PLX RNA
GEF PLX Protein
GEF PLX Phospho
GEF PLX RNA
Concentration(n[M])
Relative Conc
Time (hour)

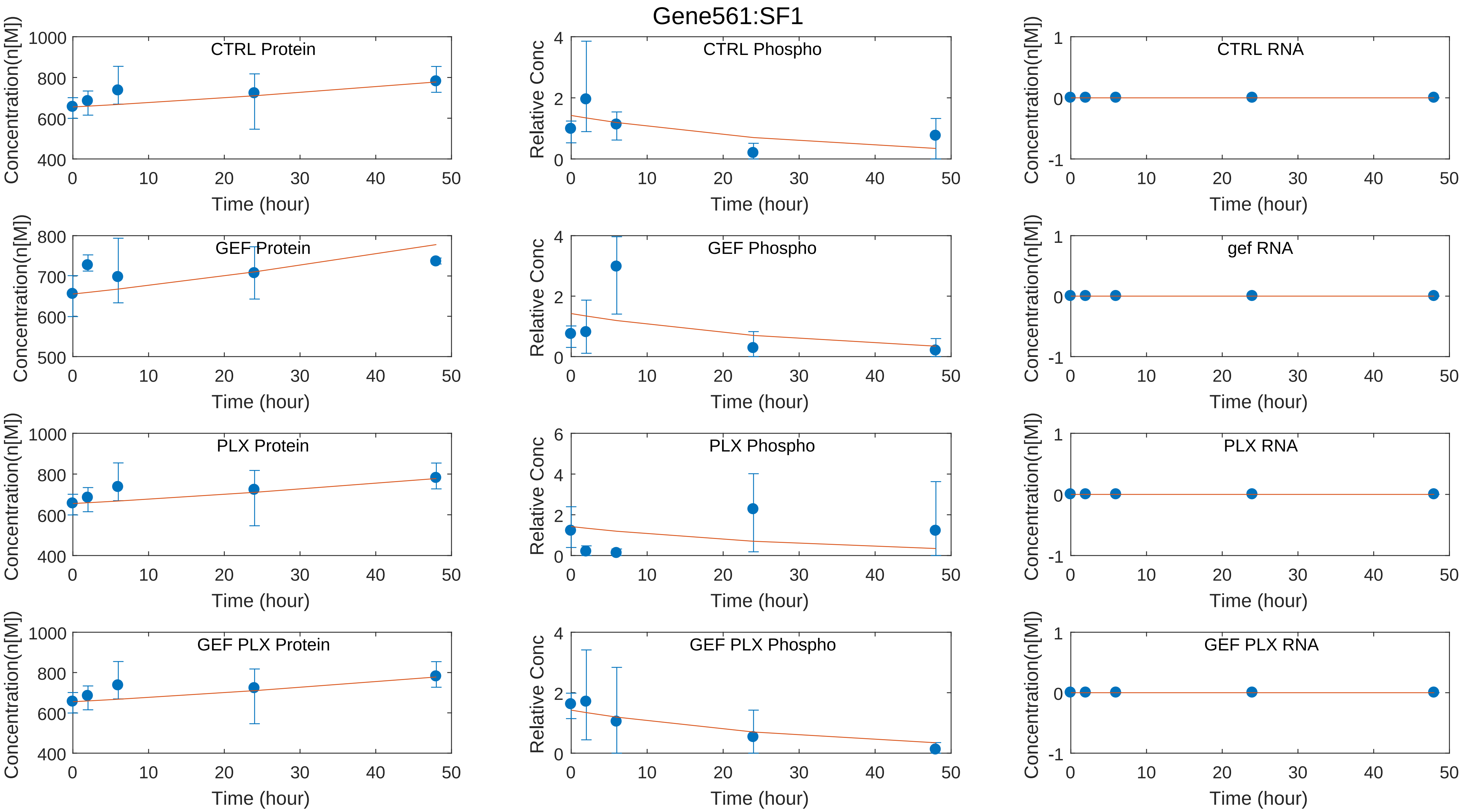

Gene561:SF1
CTRL Protein
CTRL Phospho
CTRL RNA
GEF Protein
GEF Phospho
gef RNA
PLX Protein
PLX Phospho
PLX RNA
GEF PLX Protein
GEF PLX Phospho
GEF PLX RNA
Concentration(n[M])
Relative Conc
Time (hour)

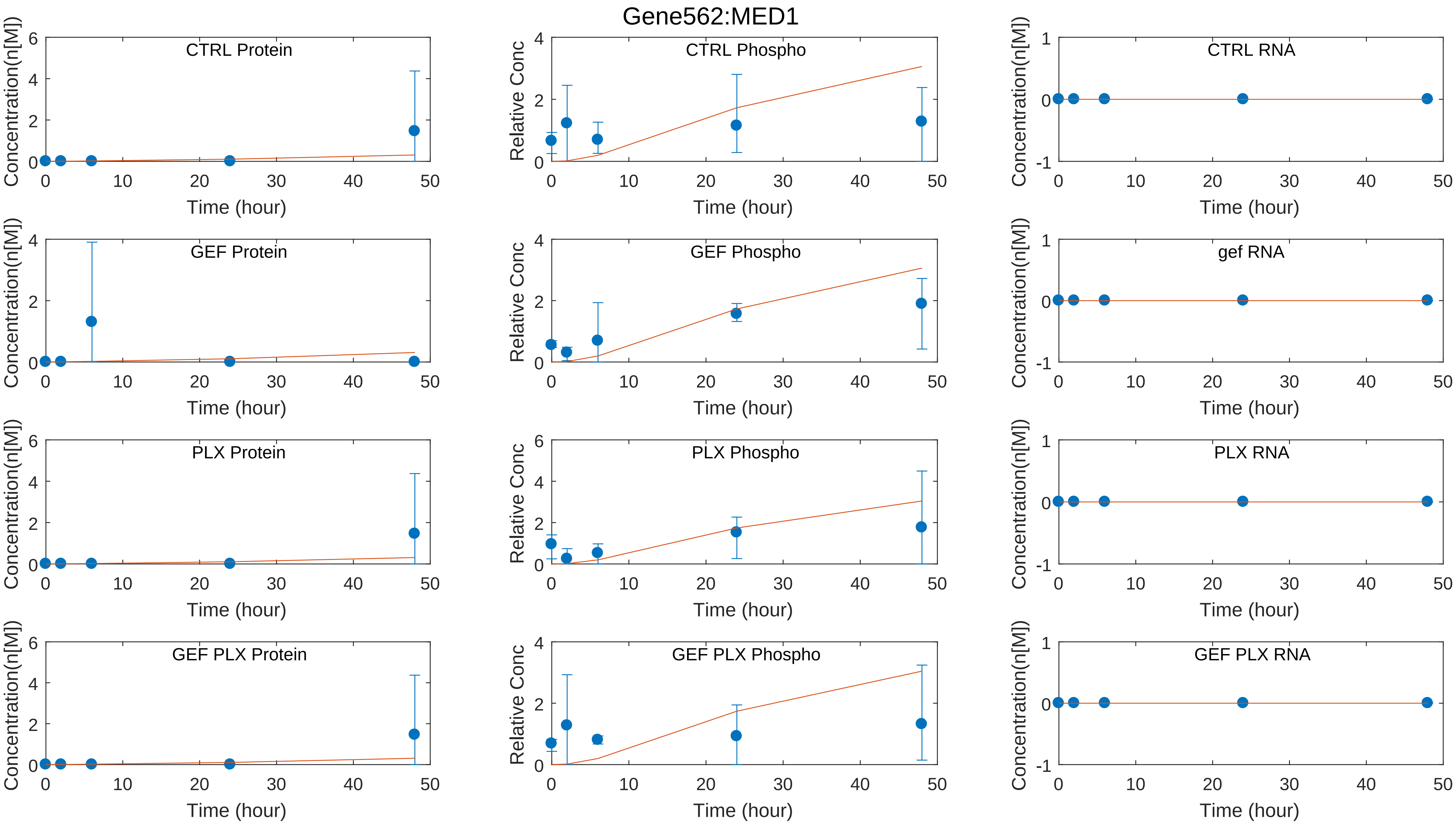

Gene562:MED1
CTRL Protein
CTRL Phospho
CTRL RNA
GEF Protein
GEF Phospho
gef RNA
PLX Protein
PLX Phospho
PLX RNA
GEF PLX Protein
GEF PLX Phospho
GEF PLX RNA
Concentration(n[M])
Relative Conc
Time (hour)

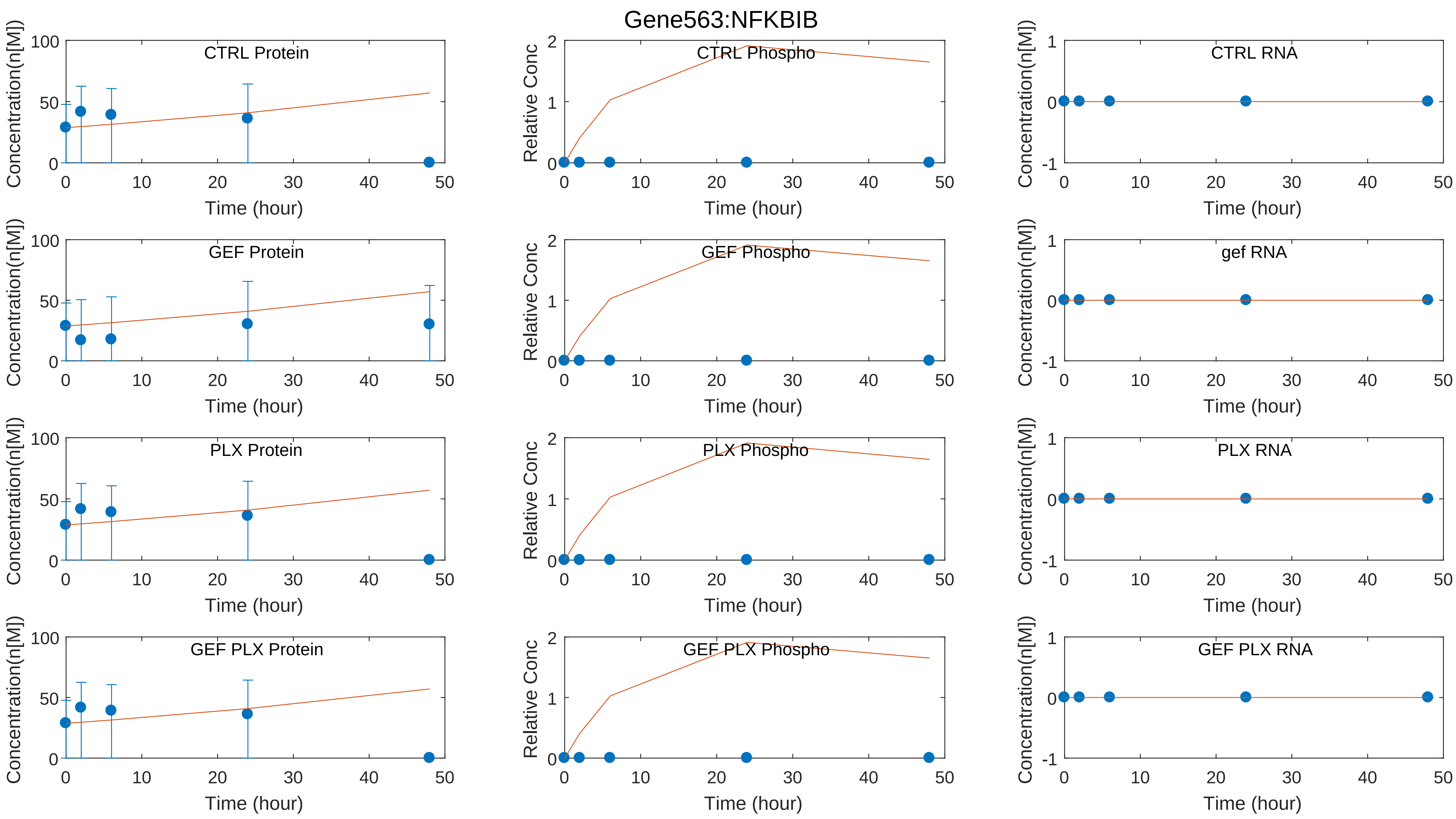

Gene563:NFKBIB
CTRL Protein
CTRL Phospho
CTRL RNA
GEF Protein
GEF Phospho
gef RNA
PLX Protein
PLX Phospho
PLX RNA
GEF PLX Protein
GEF PLX Phospho
GEF PLX RNA
Concentration(n[M])
Relative Conc
Time (hour)

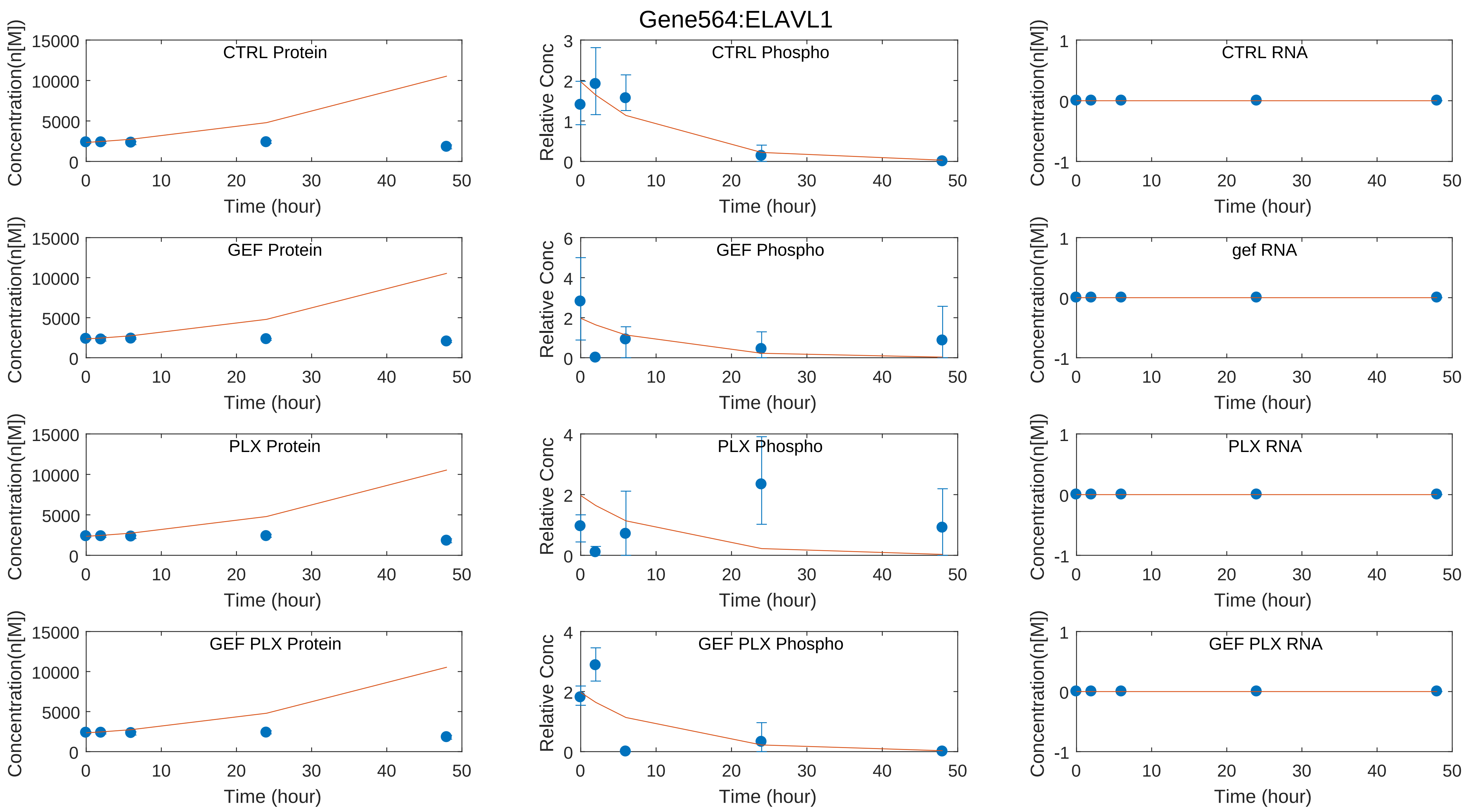
Gene564:ELAVL1
CTRL Protein
CTRL Phospho
CTRL RNA
GEF Protein
GEF Phospho
gef RNA
PLX Protein
PLX Phospho
PLX RNA
GEF PLX Protein
GEF PLX Phospho
GEF PLX RNA
Concentration(n[M])
Relative Conc
Time (hour)

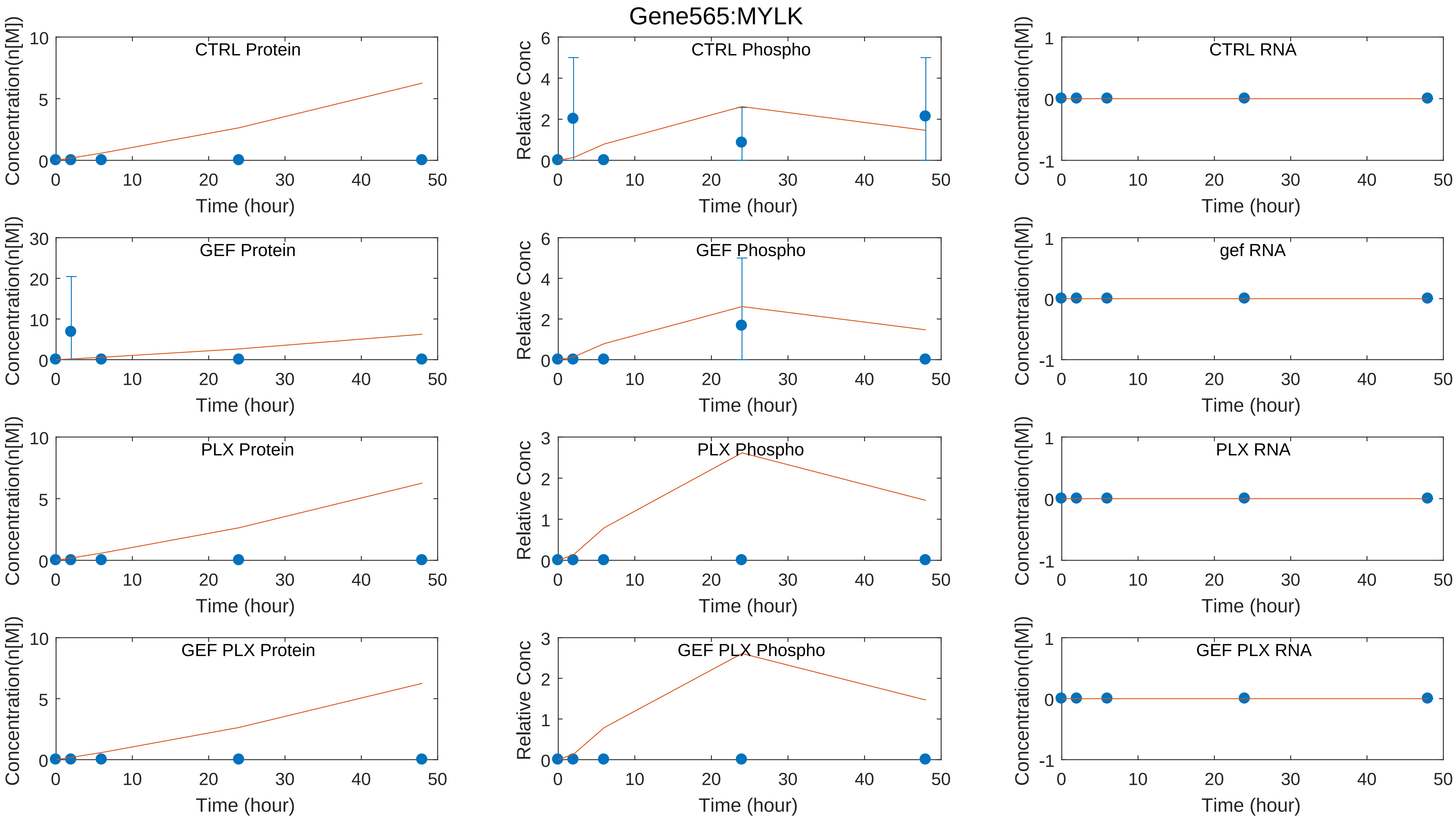

Gene565:MYLK
CTRL Protein
CTRL Phospho
CTRL RNA
GEF Protein
GEF Phospho
gef RNA
PLX Protein
PLX Phospho
PLX RNA
GEF PLX Protein
GEF PLX Phospho
GEF PLX RNA
Concentration(n[M])
Relative Conc
Time (hour)

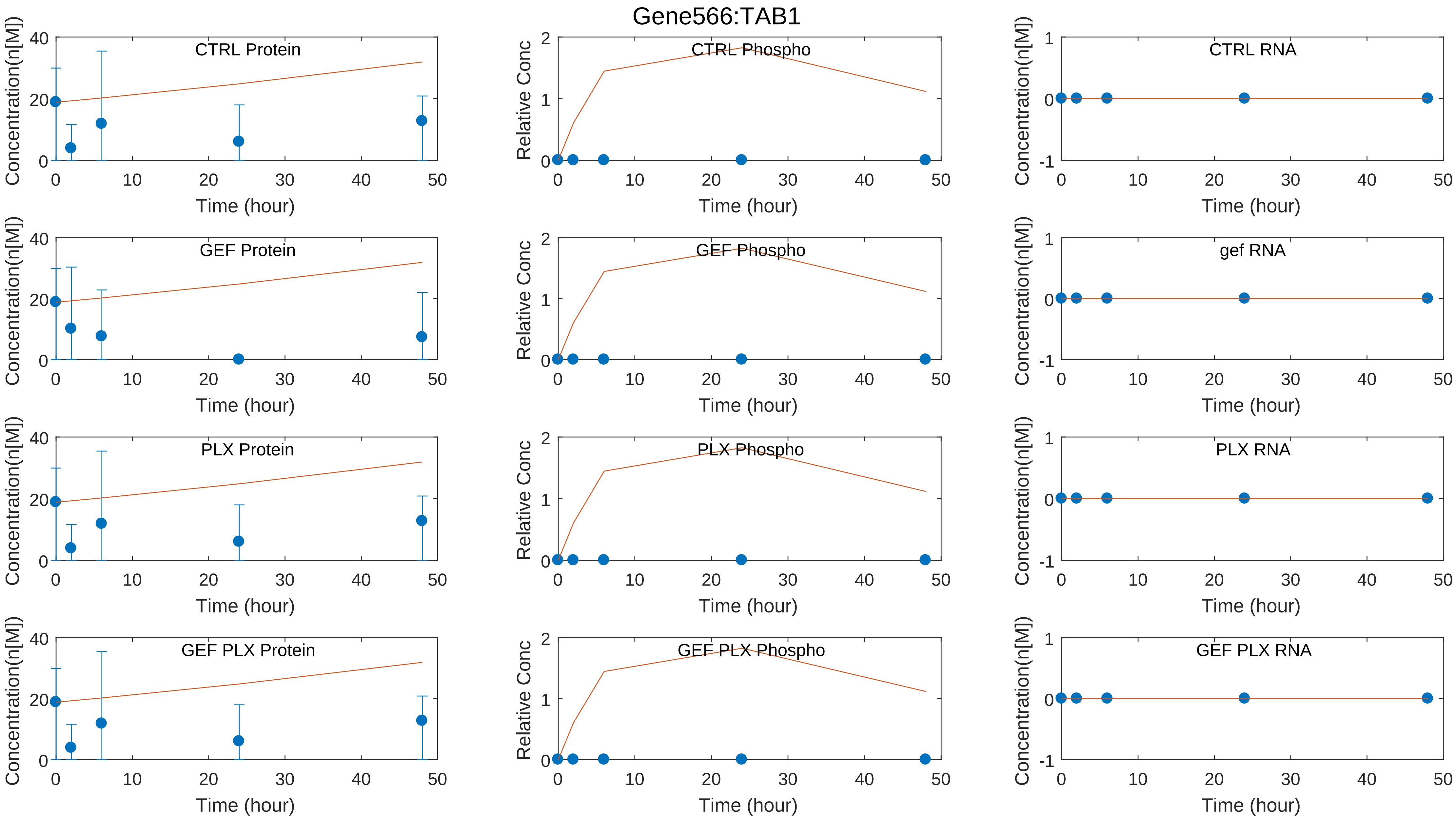

Gene566:TAB1
CTRL Protein
CTRL Phospho
CTRL RNA
GEF Protein
GEF Phospho
gef RNA
PLX Protein
PLX Phospho
PLX RNA
GEF PLX Protein
GEF PLX Phospho
GEF PLX RNA
Concentration(n[M])
Relative Conc
Time (hour)

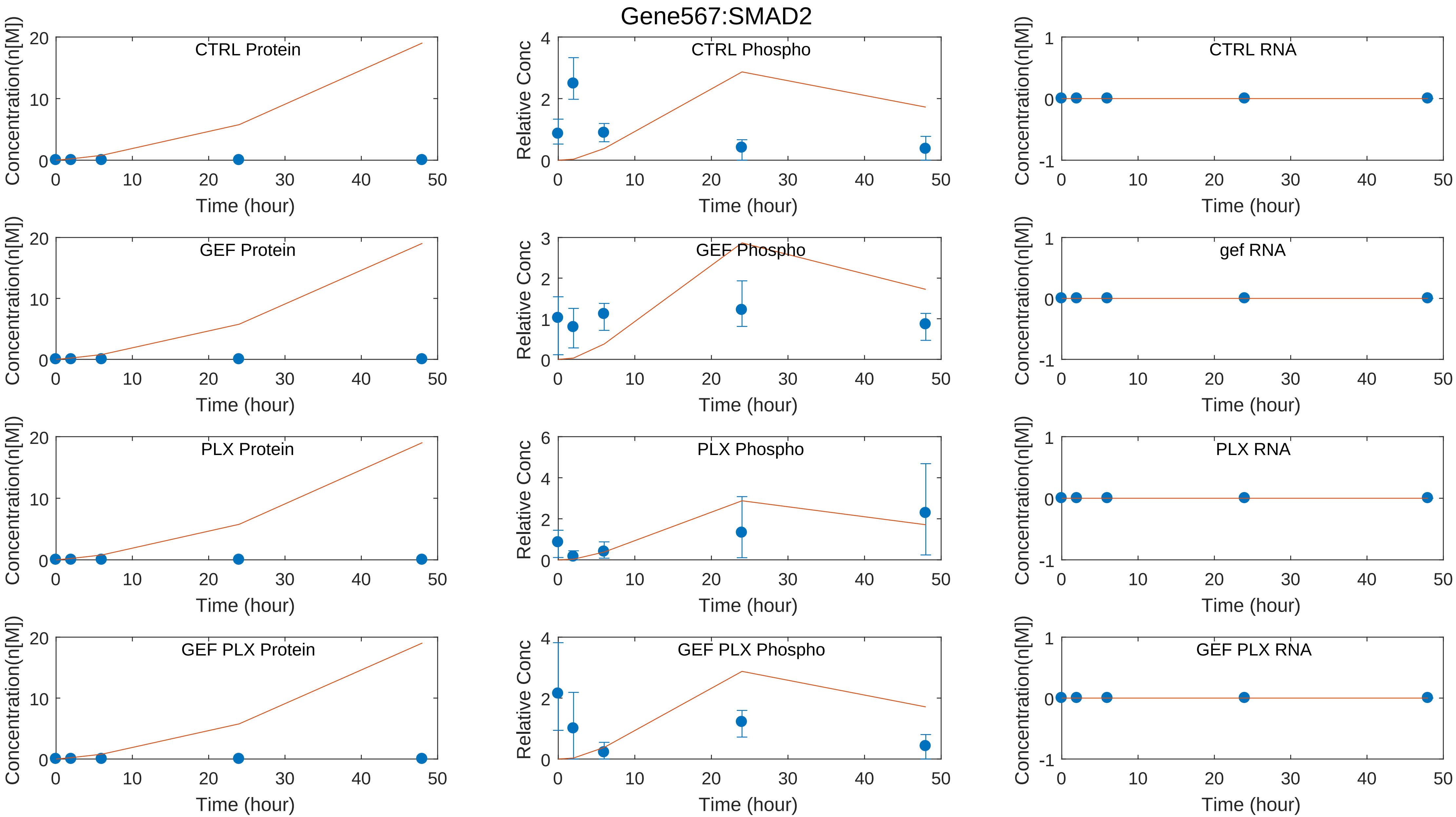

Gene567:SMAD2
CTRL Protein
CTRL Phospho
CTRL RNA
GEF Protein
GEF Phospho
gef RNA
PLX Protein
PLX Phospho
PLX RNA
GEF PLX Protein
GEF PLX Phospho
GEF PLX RNA
Concentration(n[M])
Relative Conc
Time (hour)

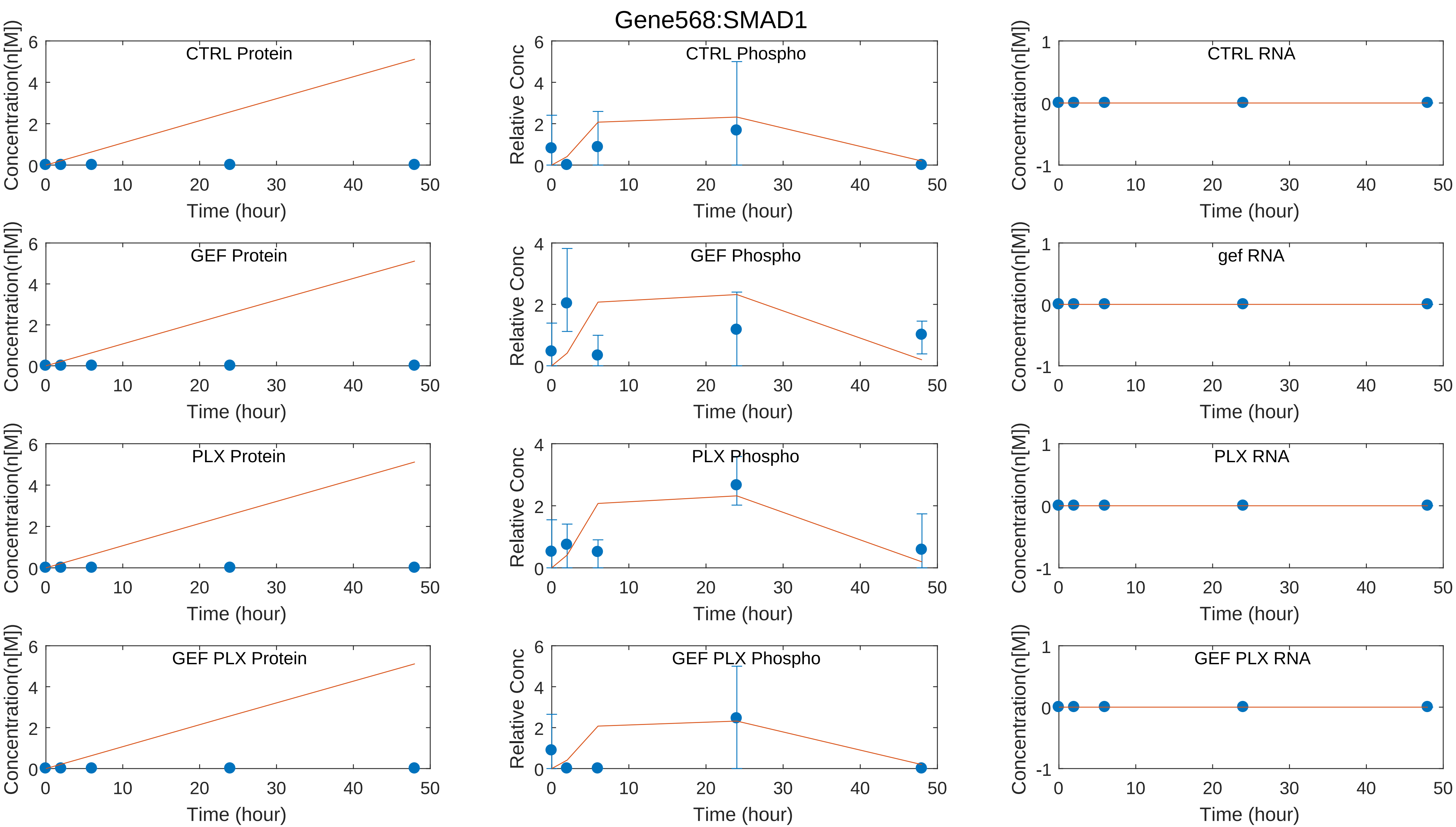
Gene568:SMAD1
CTRL Protein
CTRL Phospho
CTRL RNA
GEF Protein
GEF Phospho
gef RNA
PLX Protein
PLX Phospho
PLX RNA
GEF PLX Protein
GEF PLX Phospho
GEF PLX RNA
Concentration(n[M])
Relative Conc
Time (hour)

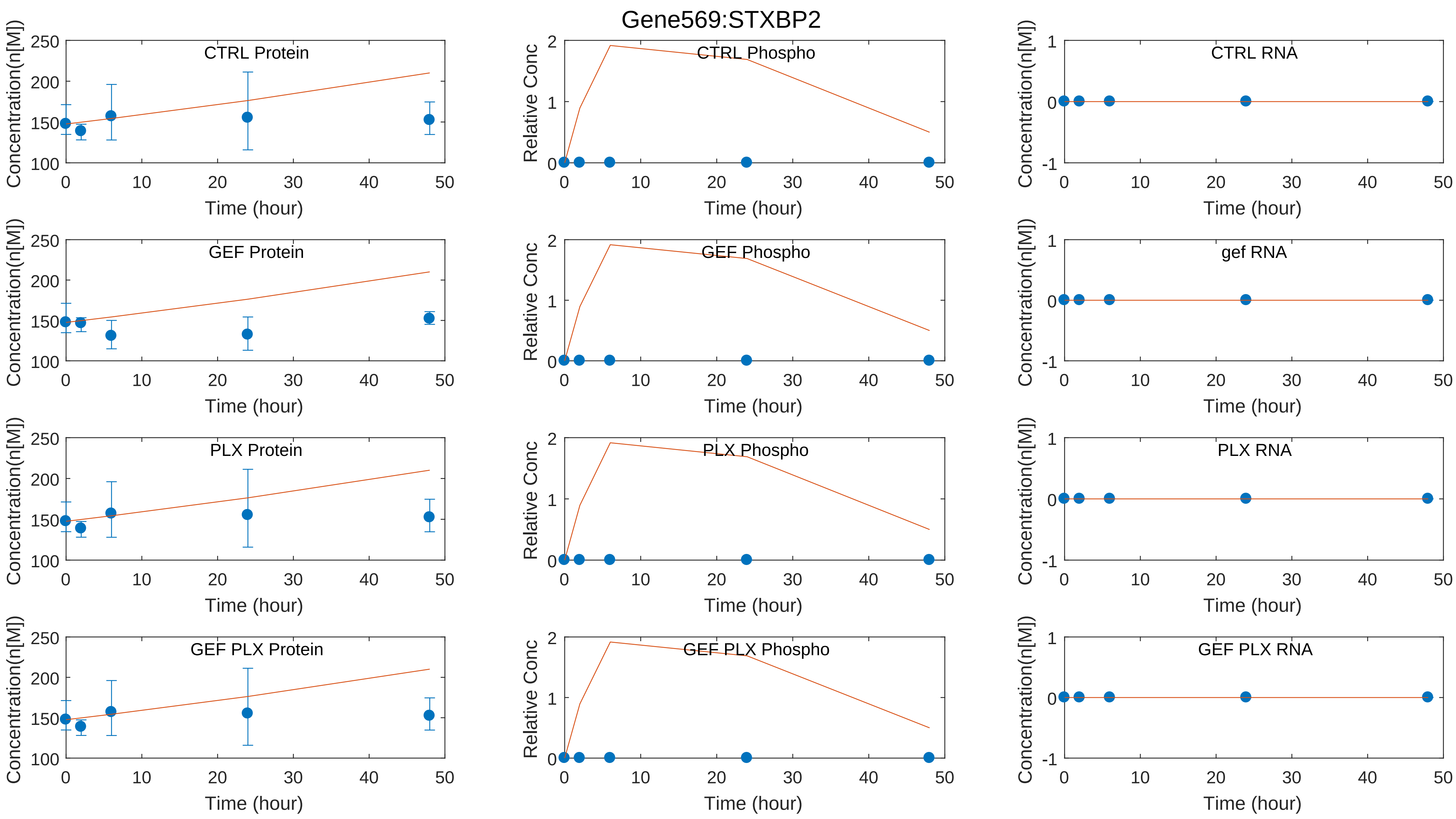

Gene569:STXBP2
CTRL Protein
GEF Protein
PLX Protein
GEF PLX Protein
CTRL Phospho
GEF Phospho
PLX Phospho
GEF PLX Phospho
CTRL RNA
gef RNA
PLX RNA
GEF PLX RNA
Concentration(n[M])
Relative Conc
Time (hour)

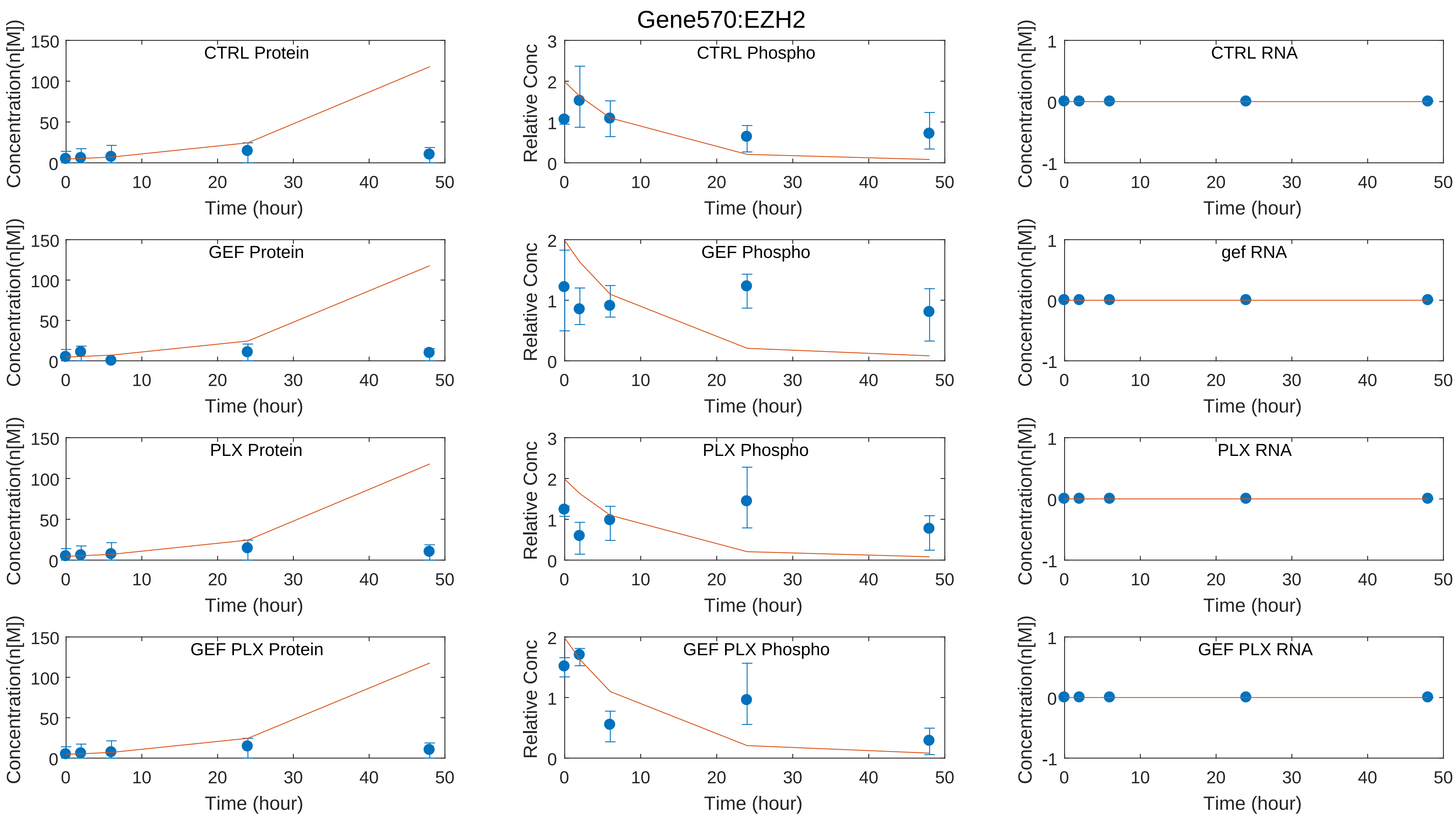
Gene570:EZH2
CTRL Protein
CTRL Phospho
CTRL RNA
GEF Protein
GEF Phospho
gef RNA
PLX Protein
PLX Phospho
PLX RNA
GEF PLX Protein
GEF PLX Phospho
GEF PLX RNA
Concentration(n[M])
Relative Conc
Time (hour)

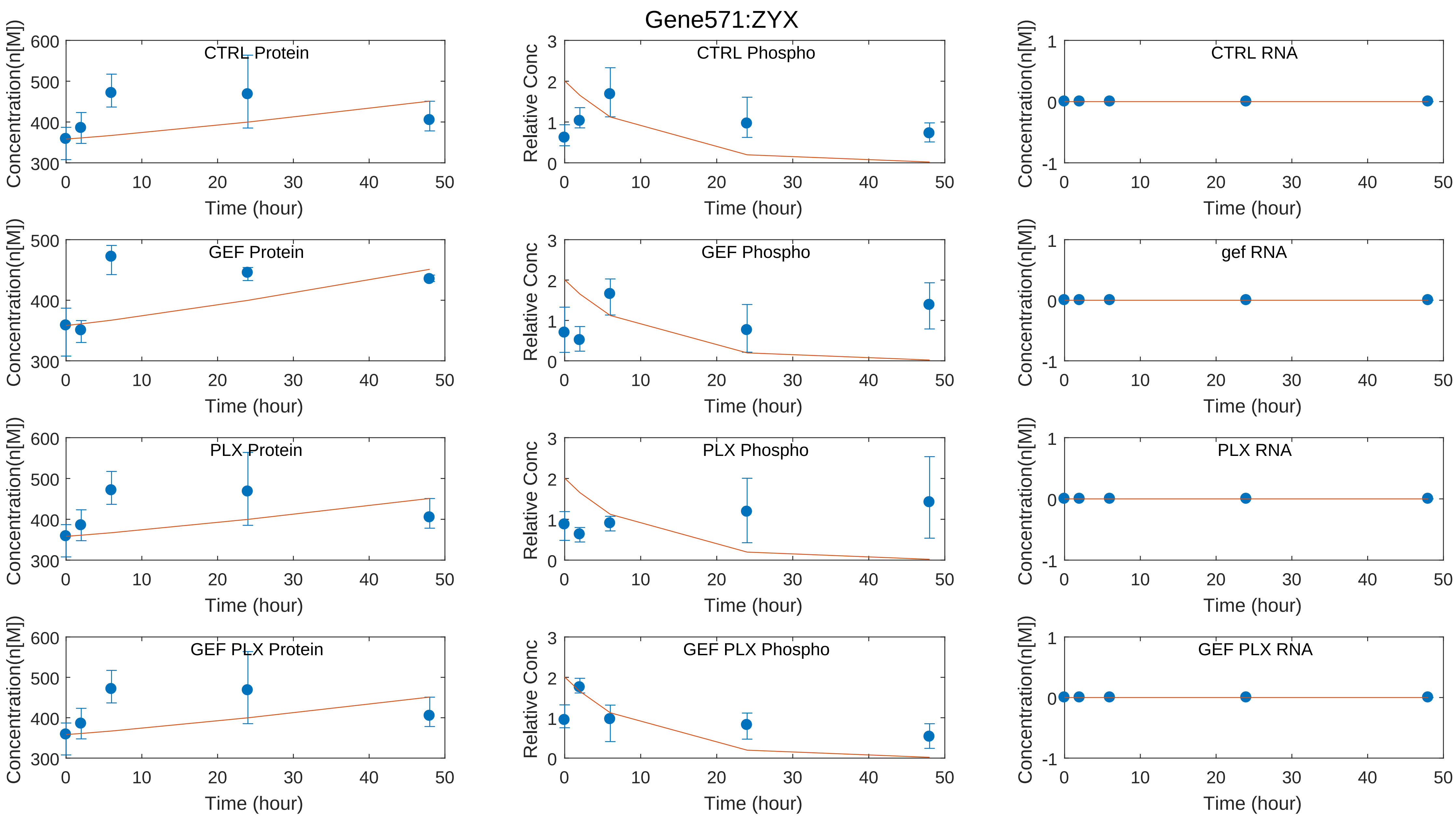

Gene571:ZYX
CTRL Protein
CTRL Phospho
CTRL RNA
GEF Protein
GEF Phospho
gef RNA
PLX Protein
PLX Phospho
PLX RNA
GEF PLX Protein
GEF PLX Phospho
GEF PLX RNA
Concentration(n[M])
Relative Conc
Time (hour)

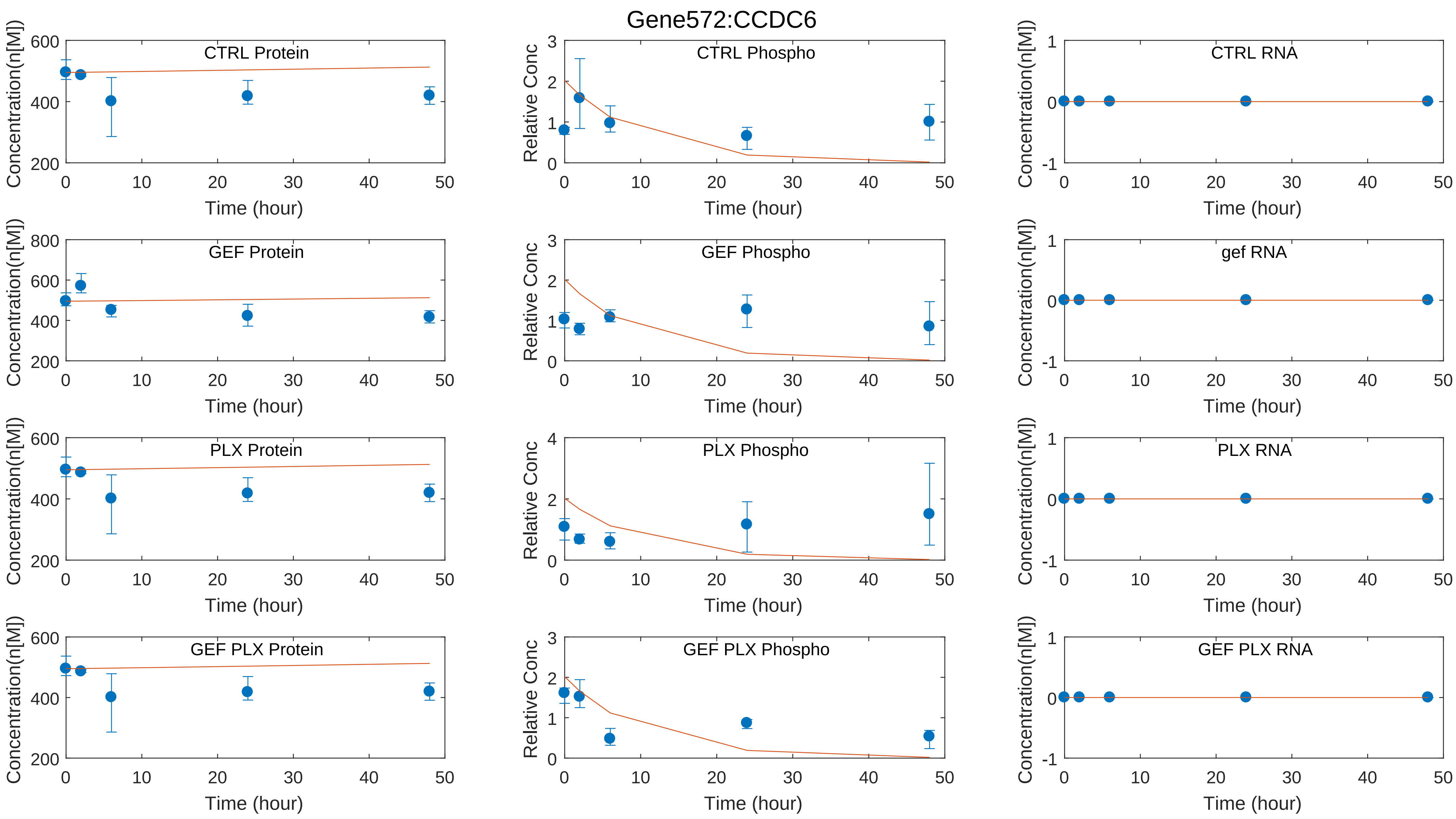

Gene572:CCDC6
CTRL Protein
CTRL Phospho
CTRL RNA
GEF Protein
GEF Phospho
gef RNA
PLX Protein
PLX Phospho
PLX RNA
GEF PLX Protein
GEF PLX Phospho
GEF PLX RNA
Concentration(n[M])
Relative Conc
Time (hour)

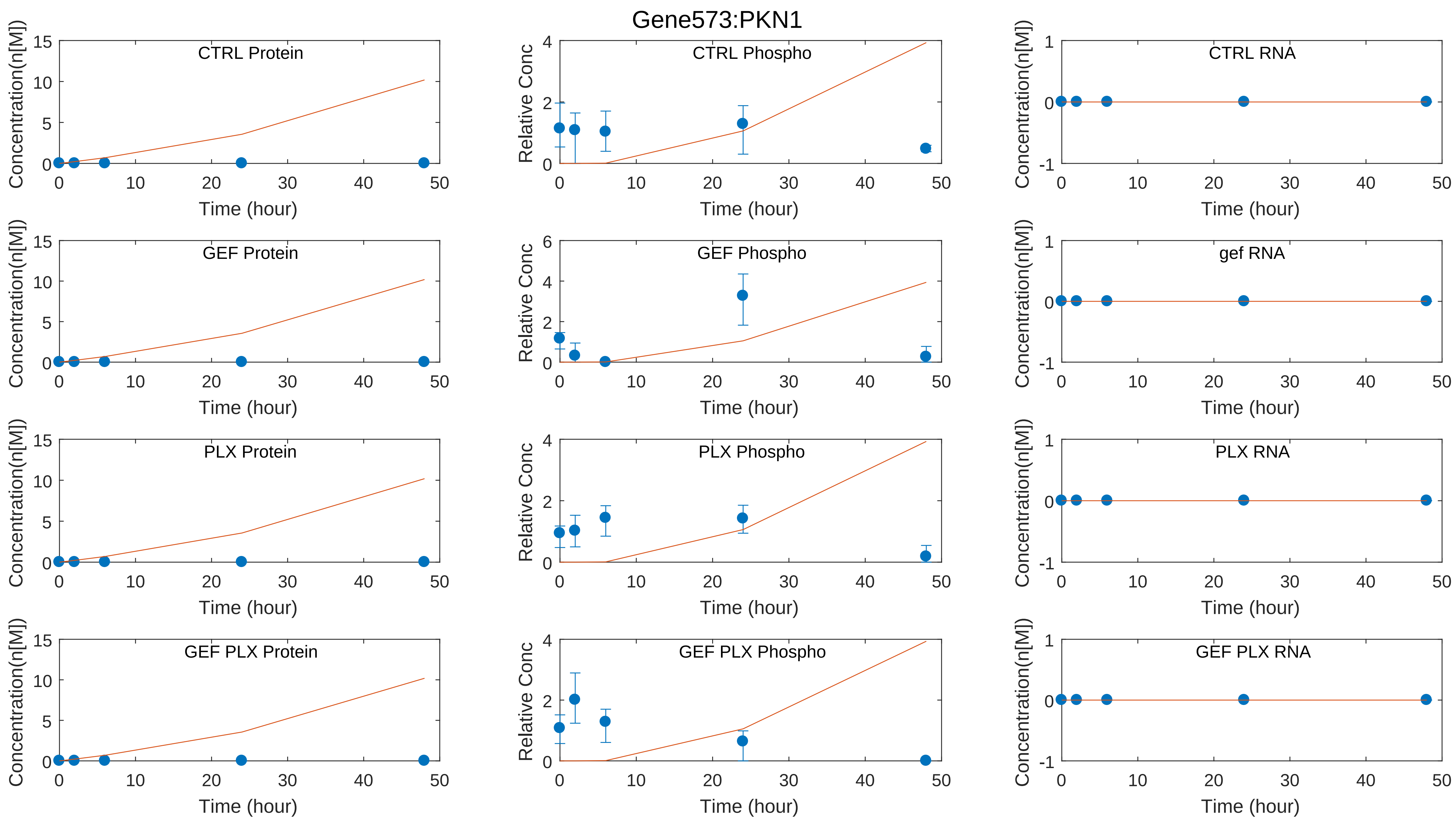
Gene573:PKN1
CTRL Protein
CTRL Phospho
CTRL RNA
GEF Protein
GEF Phospho
gef RNA
PLX Protein
PLX Phospho
PLX RNA
GEF PLX Protein
GEF PLX Phospho
GEF PLX RNA
Concentration(n[M])
Relative Conc
Time (hour)

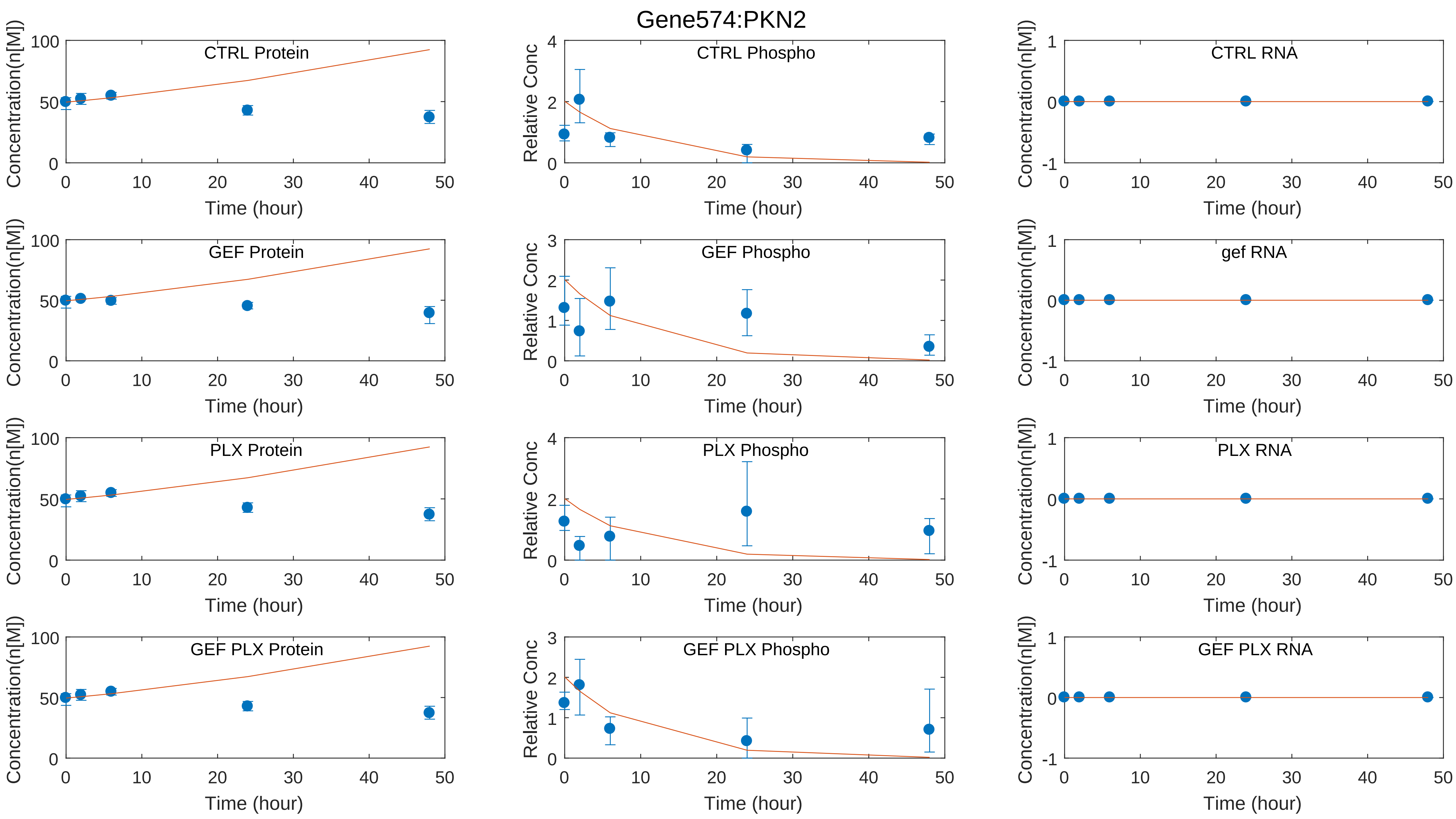

Gene574:PKN2
CTRL Protein
CTRL Phospho
CTRL RNA
GEF Protein
GEF Phospho
gef RNA
PLX Protein
PLX Phospho
PLX RNA
GEF PLX Protein
GEF PLX Phospho
GEF PLX RNA
Concentration(n[M])
Relative Conc
Time (hour)

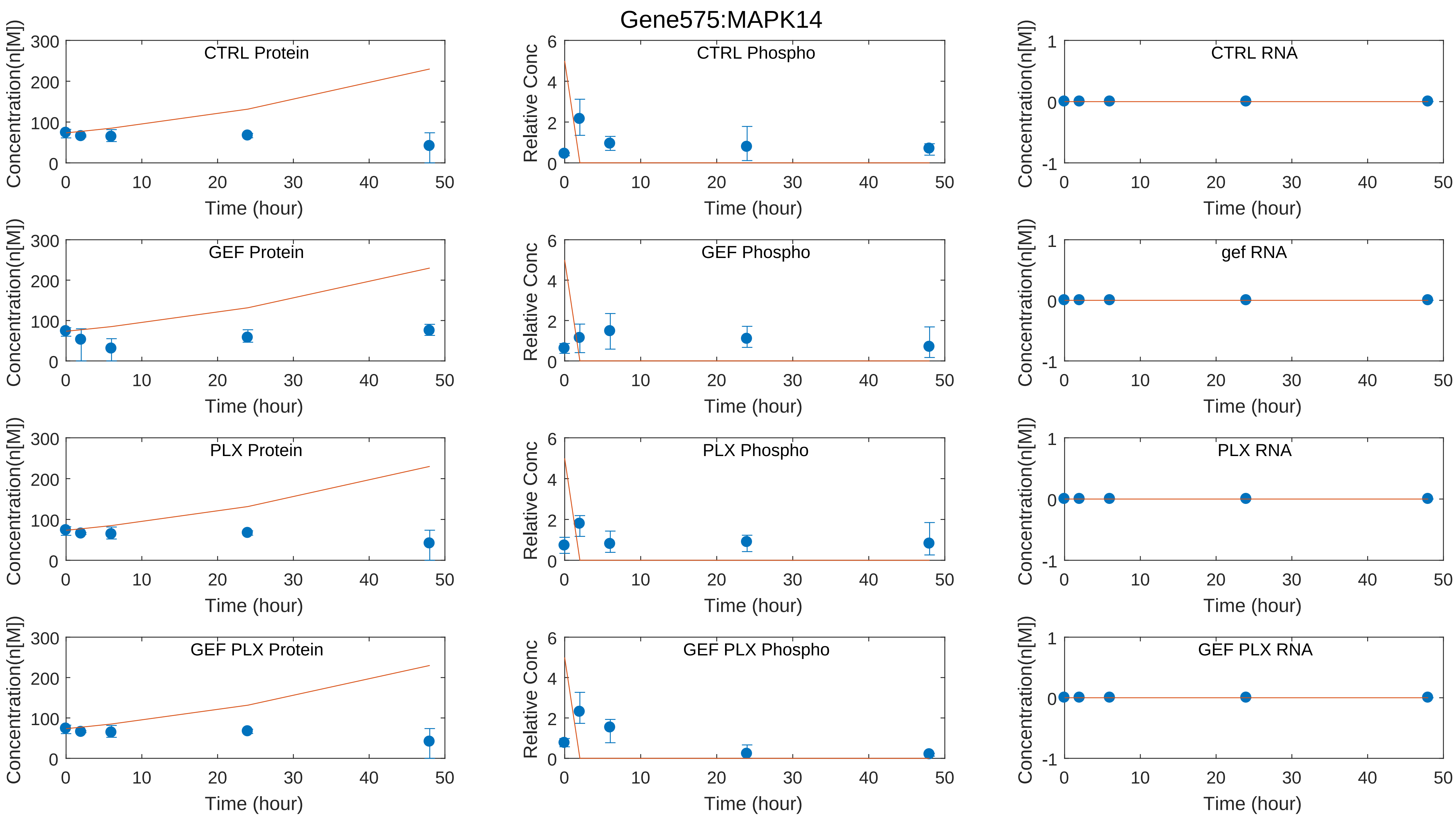

Gene575:MAPK14
CTRL Protein
CTRL Phospho
CTRL RNA
GEF Protein
GEF Phospho
gef RNA
PLX Protein
PLX Phospho
PLX RNA
GEF PLX Protein
GEF PLX Phospho
GEF PLX RNA
Concentration(n[M])
Relative Conc
Time (hour)

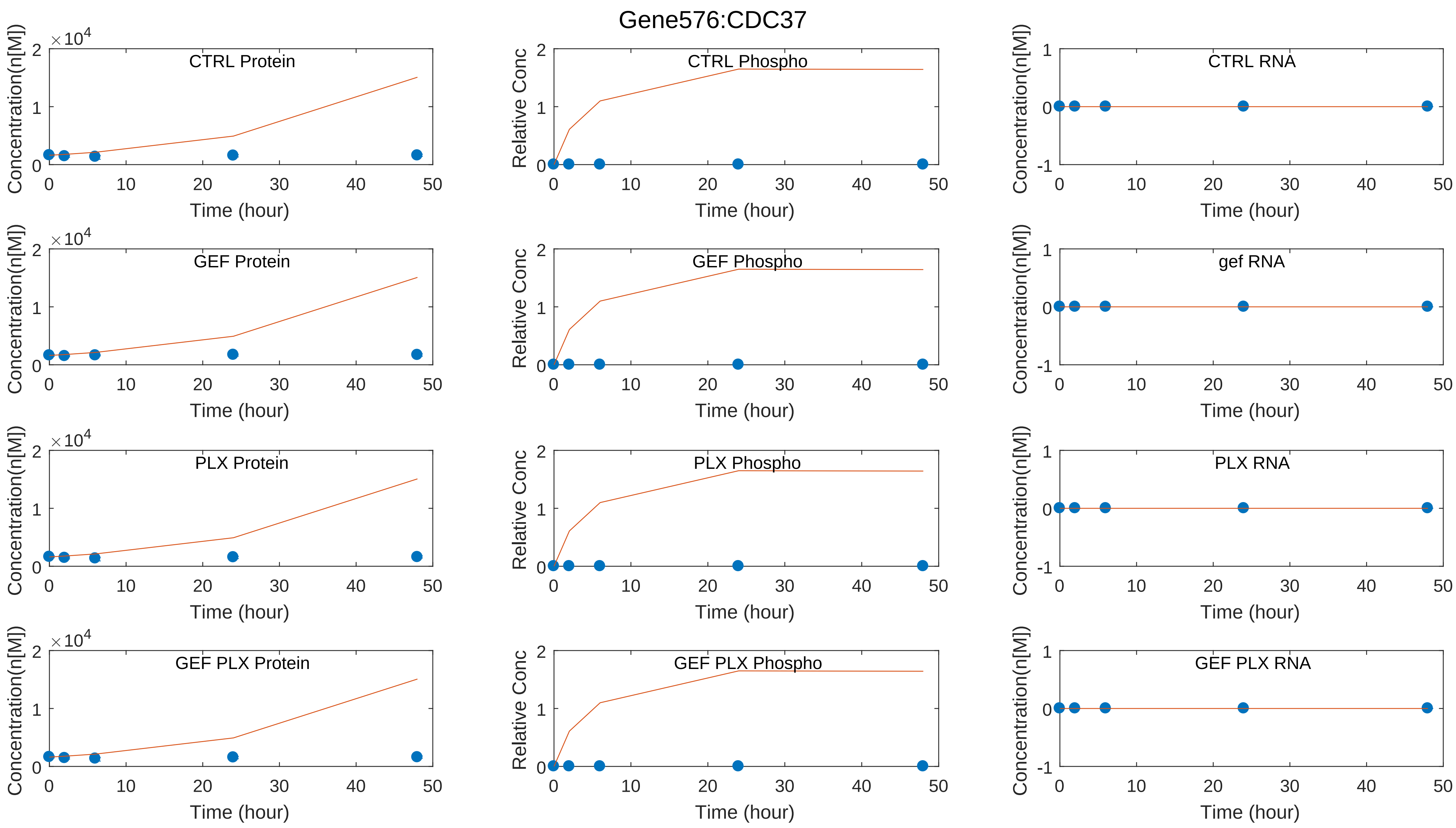

Gene576:CDC37
CTRL Protein
GEF Protein
PLX Protein
GEF PLX Protein
CTRL Phospho
GEF Phospho
PLX Phospho
GEF PLX Phospho
CTRL RNA
gef RNA
PLX RNA
GEF PLX RNA
Concentration(n[M])
Relative Conc
Time (hour)
×10^4

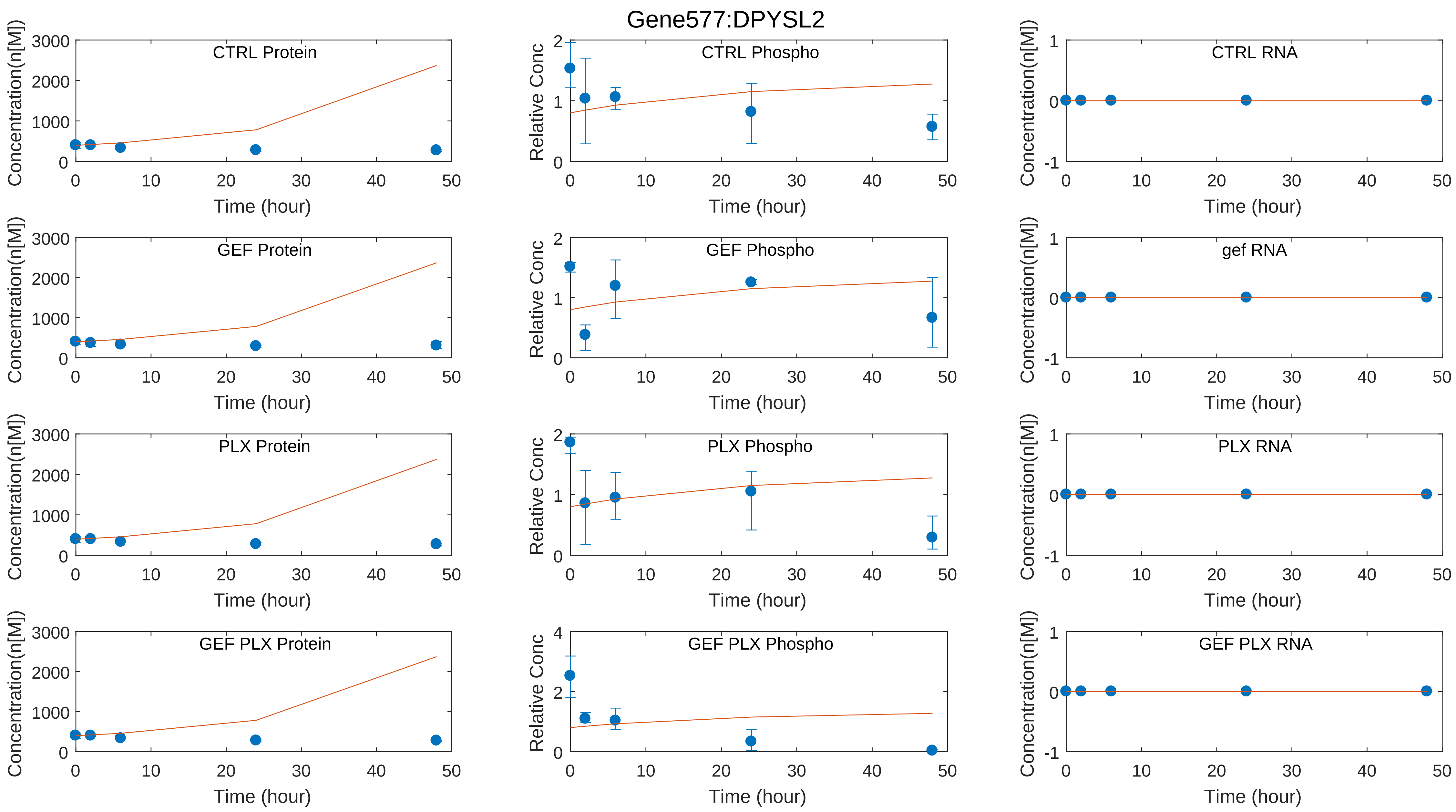
Gene577:DPYSL2
CTRL Protein
CTRL Phospho
CTRL RNA
GEF Protein
GEF Phospho
gef RNA
PLX Protein
PLX Phospho
PLX RNA
GEF PLX Protein
GEF PLX Phospho
GEF PLX RNA
Concentration(n[M])
Relative Conc
Time (hour)

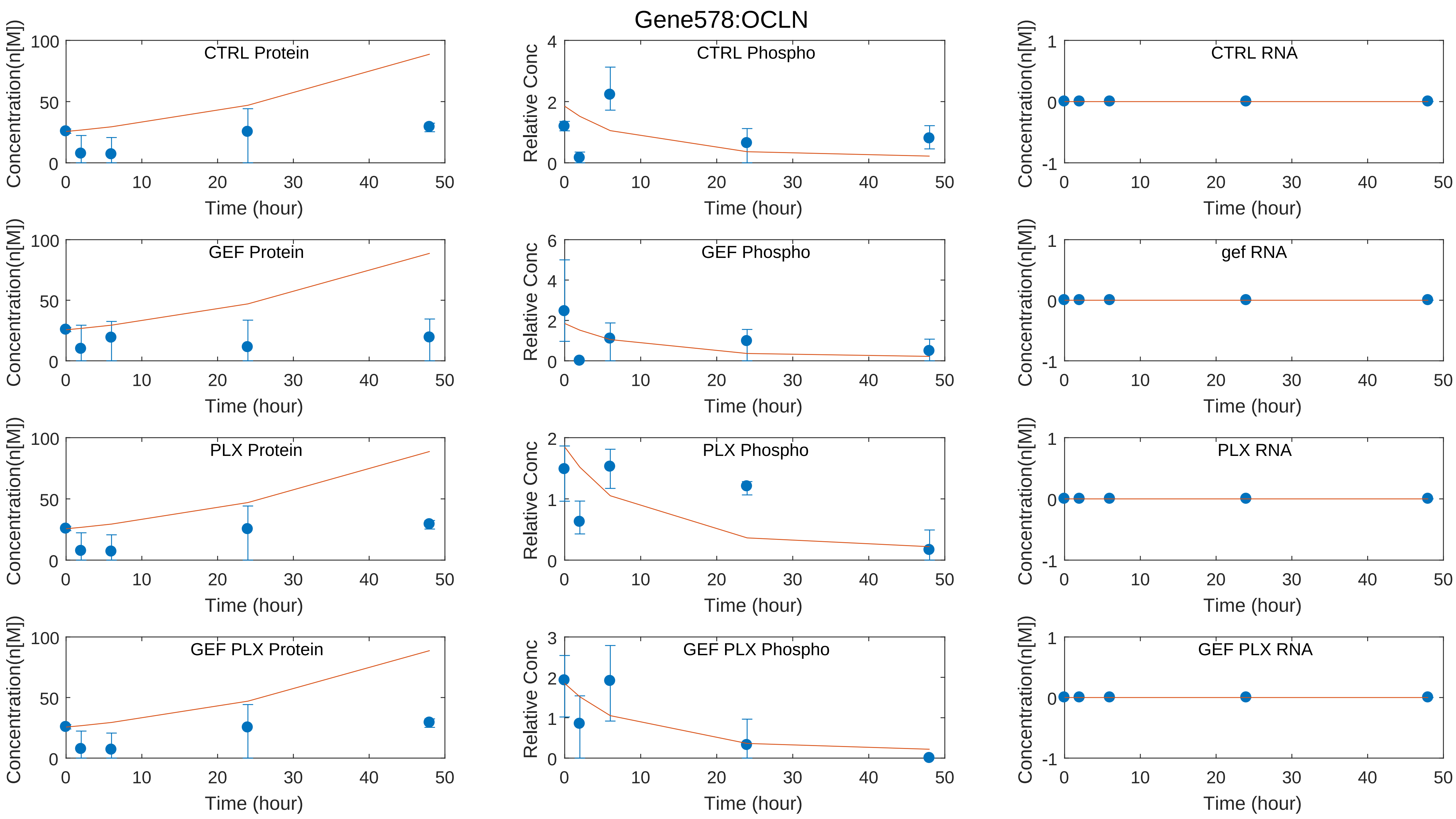
Gene578:OCLN
CTRL Protein
CTRL Phospho
CTRL RNA
GEF Protein
GEF Phospho
gef RNA
PLX Protein
PLX Phospho
PLX RNA
GEF PLX Protein
GEF PLX Phospho
GEF PLX RNA
Concentration(n[M])
Relative Conc
Time (hour)

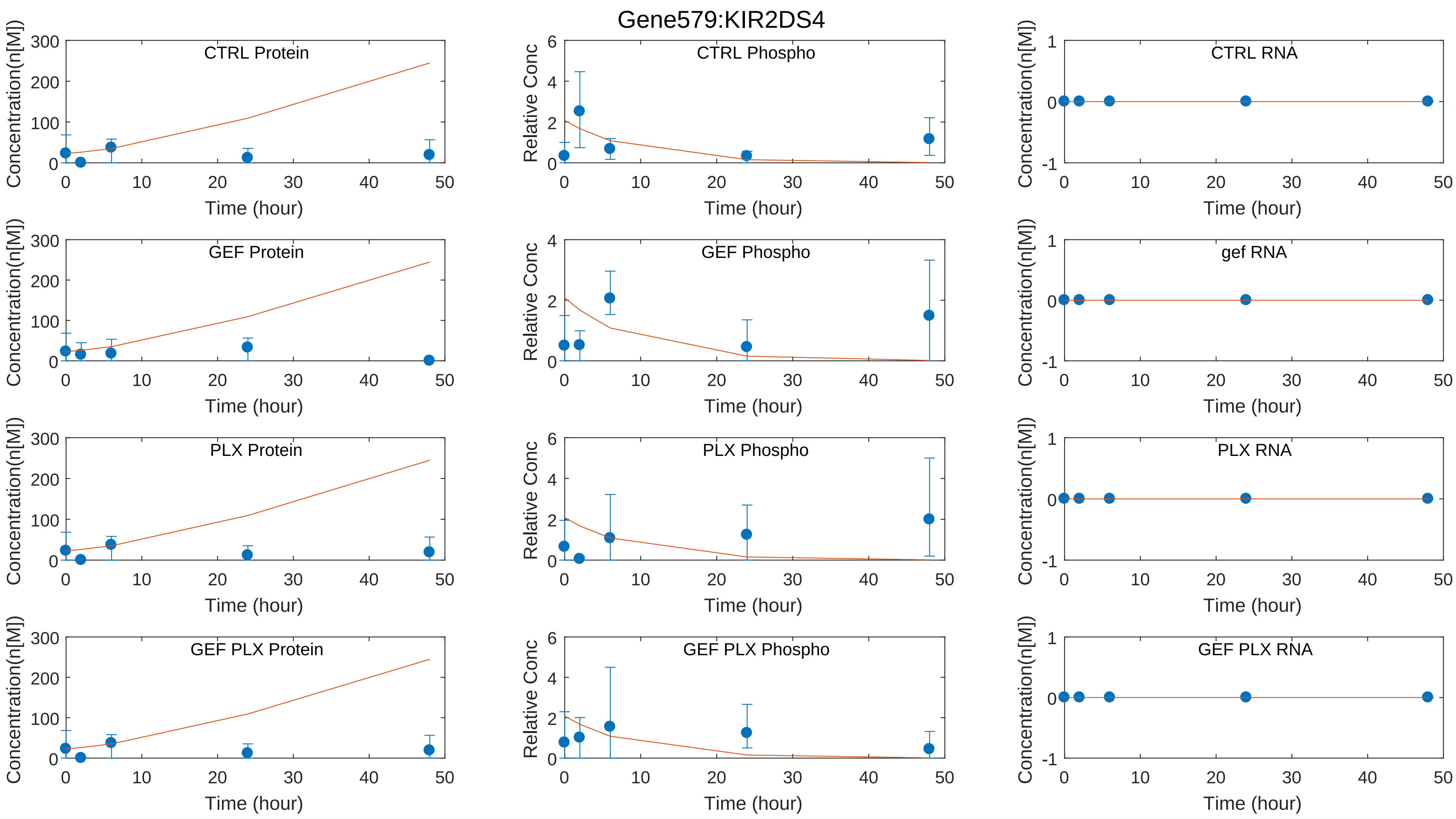

Gene579:KIR2DS4
CTRL Protein
CTRL Phospho
CTRL RNA
GEF Protein
GEF Phospho
gef RNA
PLX Protein
PLX Phospho
PLX RNA
GEF PLX Protein
GEF PLX Phospho
GEF PLX RNA
Concentration(n[M])
Relative Conc
Time (hour)

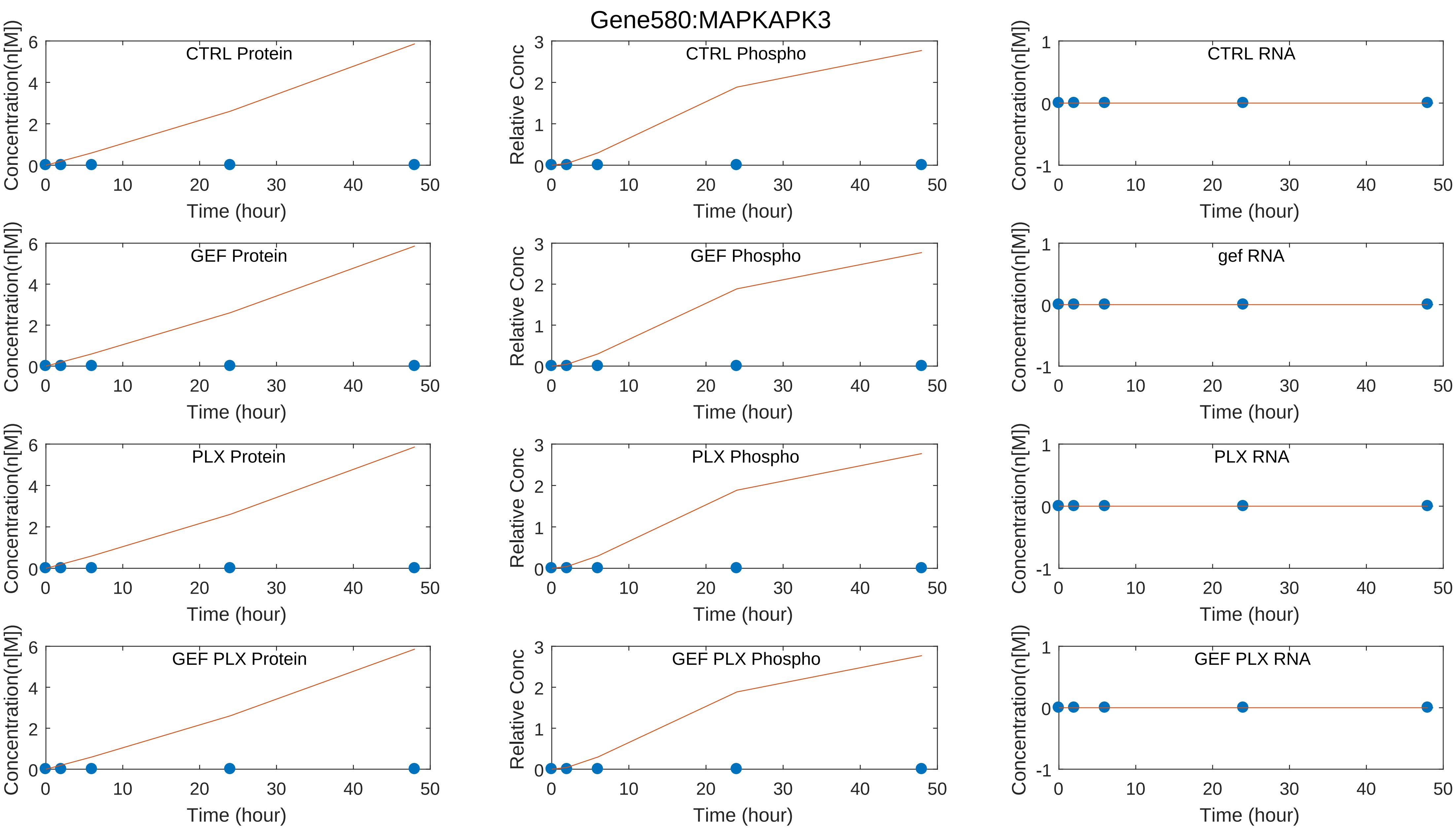

Gene580:MAPKAPK3
CTRL Protein
CTRL Phospho
CTRL RNA
GEF Protein
GEF Phospho
gef RNA
PLX Protein
PLX Phospho
PLX RNA
GEF PLX Protein
GEF PLX Phospho
GEF PLX RNA
Concentration(n[M])
Relative Conc
Time (hour)

Gene581:IFI16

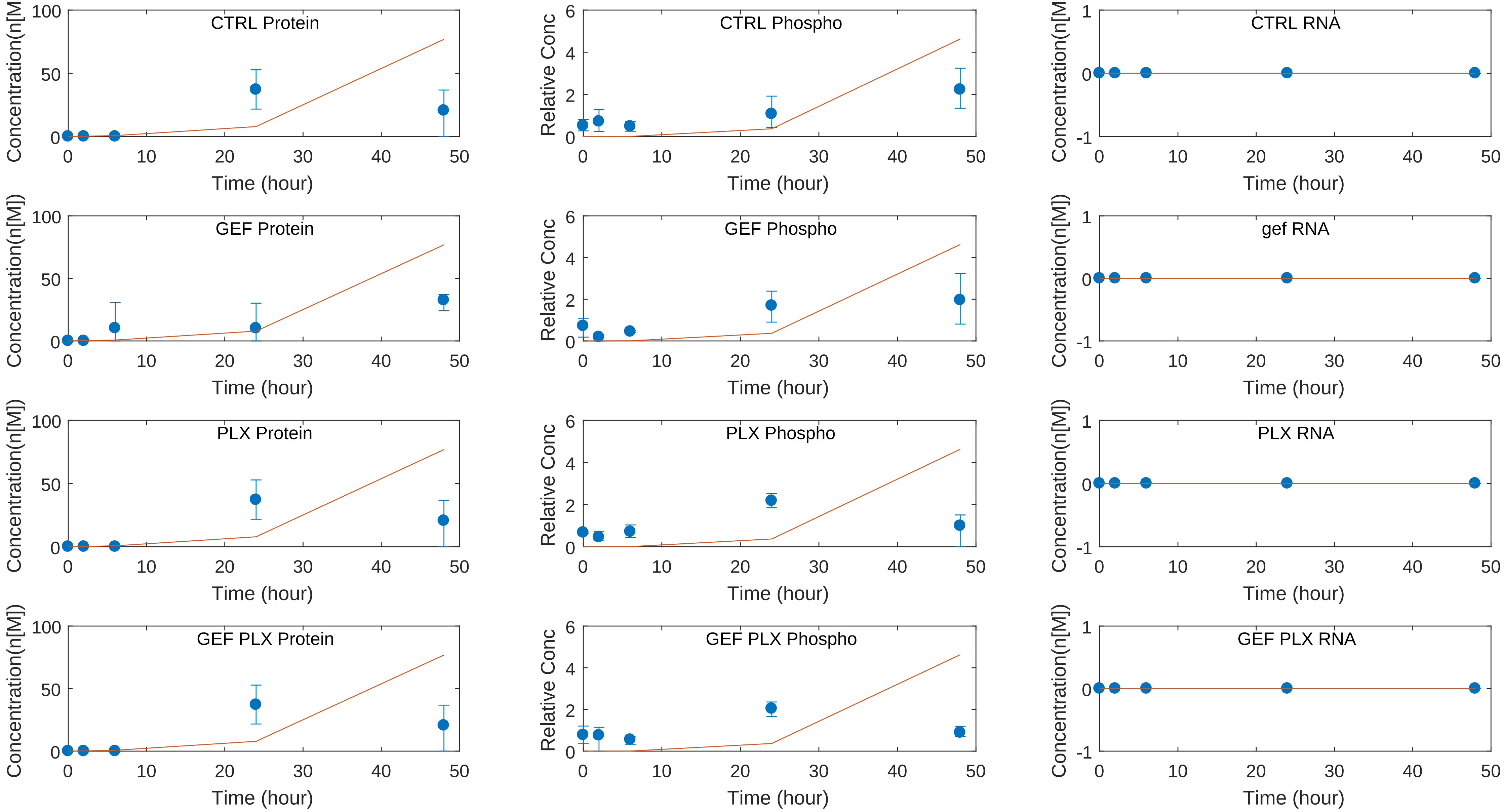

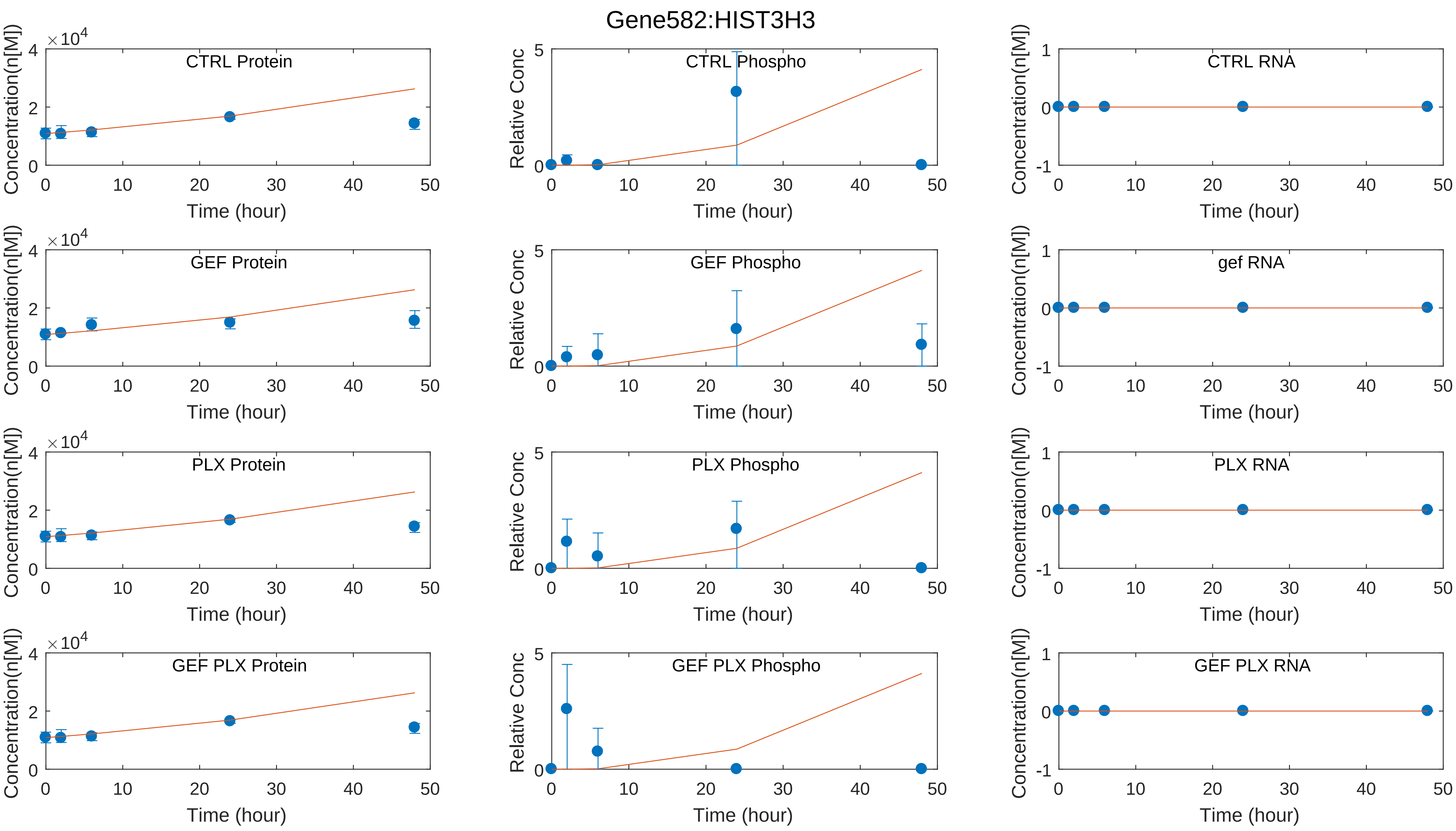

Gene582:HIST3H3
CTRL Protein
GEF Protein
PLX Protein
GEF PLX Protein
CTRL Phospho
GEF Phospho
PLX Phospho
GEF PLX Phospho
CTRL RNA
gef RNA
PLX RNA
GEF PLX RNA
Concentration(n[M])
Relative Conc
Time (hour)
×10^4

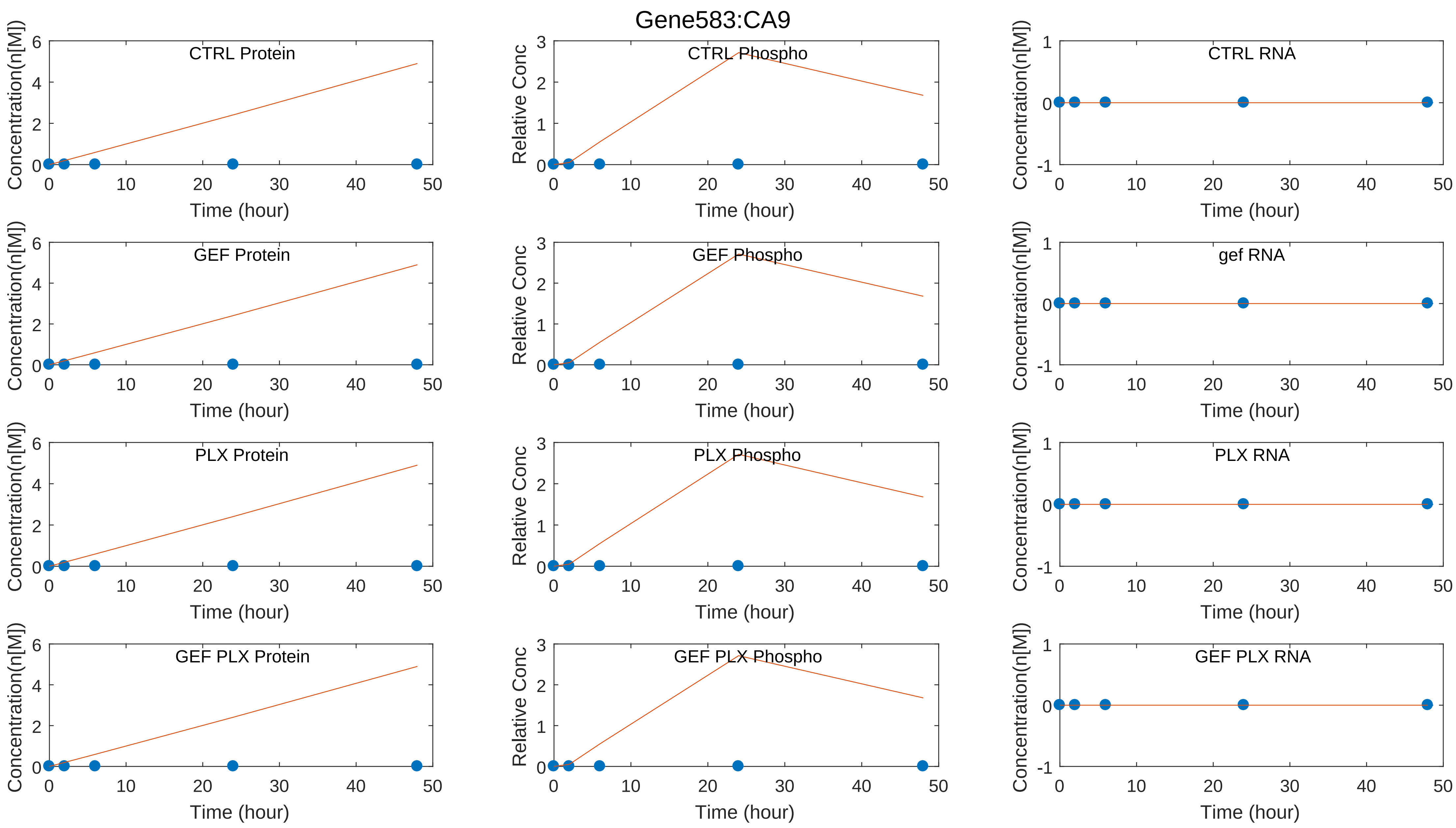
Gene583:CA9
CTRL Protein
CTRL Phospho
CTRL RNA
GEF Protein
GEF Phospho
gef RNA
PLX Protein
PLX Phospho
PLX RNA
GEF PLX Protein
GEF PLX Phospho
GEF PLX RNA
Concentration(n[M])
Relative Conc
Time (hour)

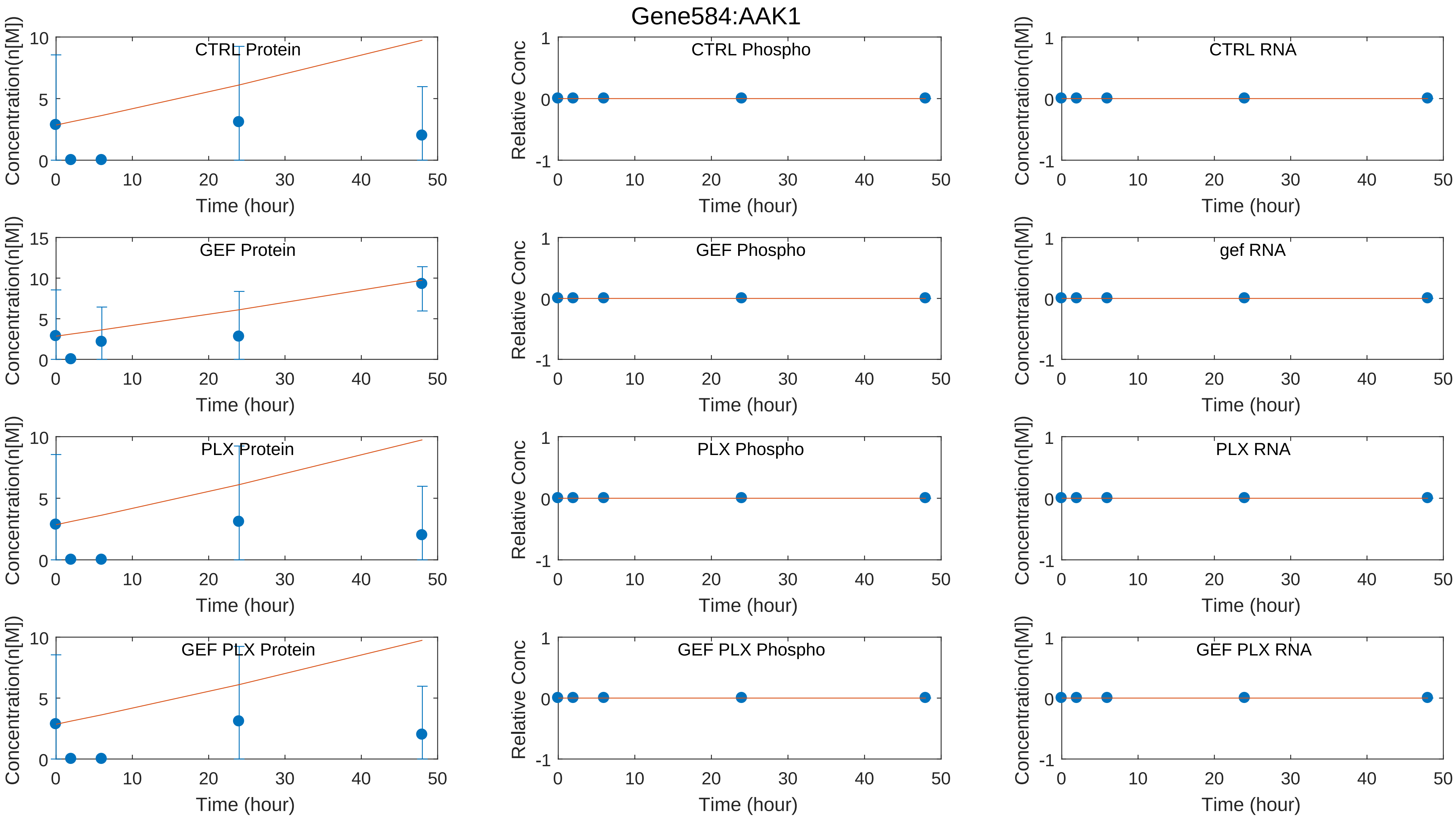

Gene584:AAK1
CTRL Protein
CTRL Phospho
CTRL RNA
GEF Protein
GEF Phospho
gef RNA
PLX Protein
PLX Phospho
PLX RNA
GEF PLX Protein
GEF PLX Phospho
GEF PLX RNA
Concentration(n[M])
Relative Conc
Time (hour)

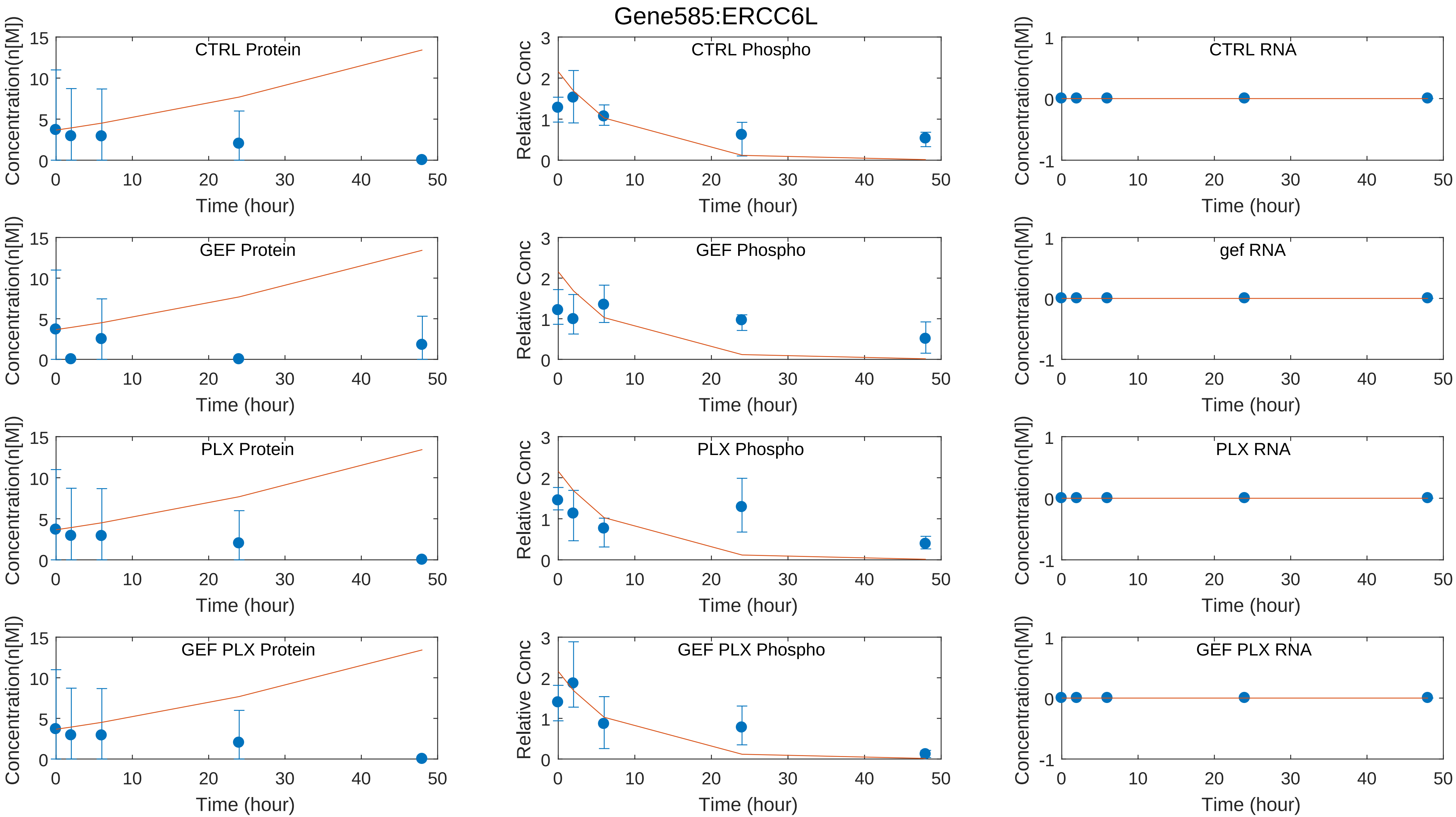

Gene585:ERCC6L
CTRL Protein
CTRL Phospho
CTRL RNA
GEF Protein
GEF Phospho
gef RNA
PLX Protein
PLX Phospho
PLX RNA
GEF PLX Protein
GEF PLX Phospho
GEF PLX RNA
Concentration(n[M])
Relative Conc
Time (hour)

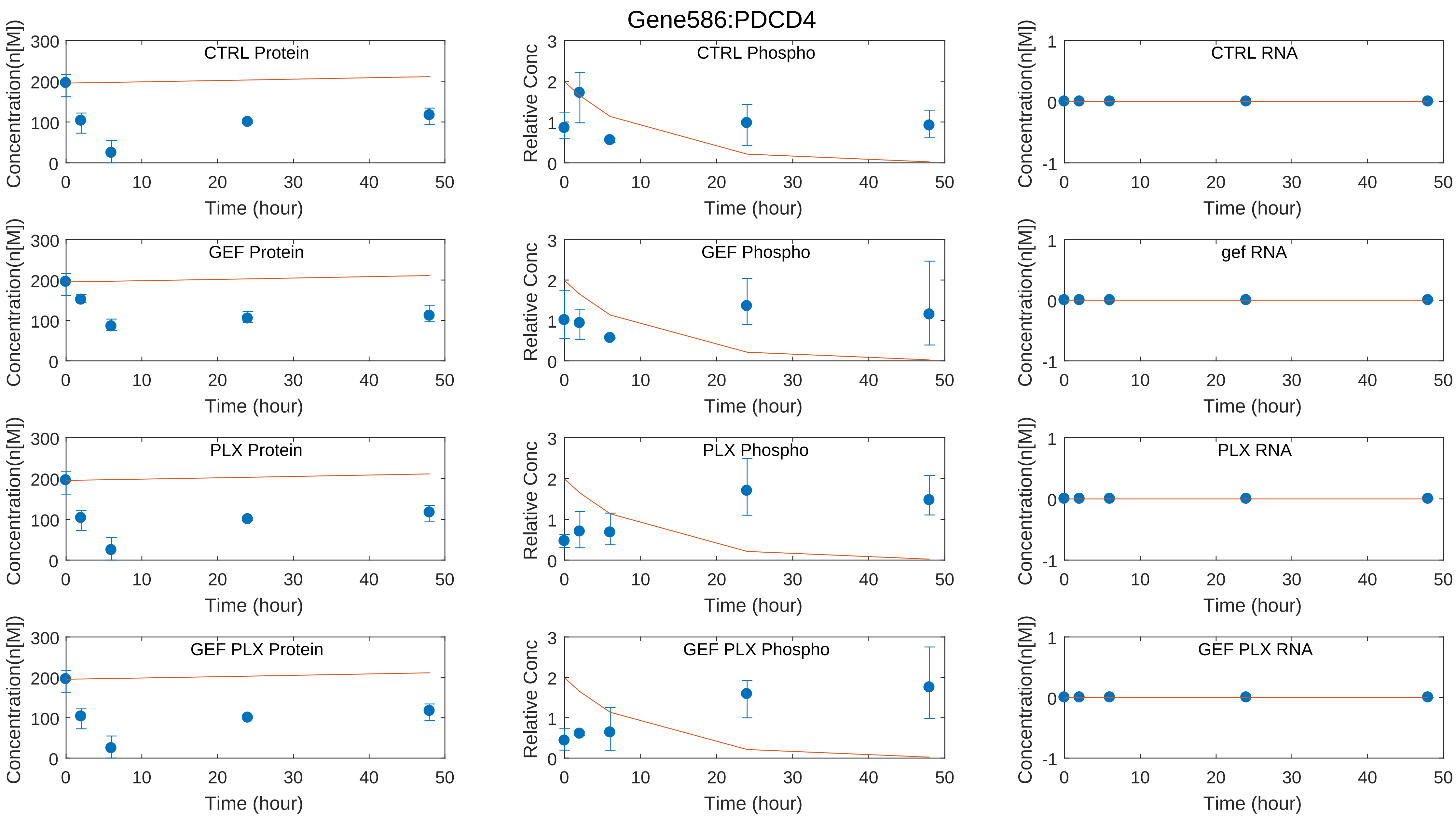

Gene586:PDCD4
CTRL Protein
CTRL Phospho
CTRL RNA
GEF Protein
GEF Phospho
gef RNA
PLX Protein
PLX Phospho
PLX RNA
GEF PLX Protein
GEF PLX Phospho
GEF PLX RNA
Concentration(n[M])
Relative Conc
Time (hour)

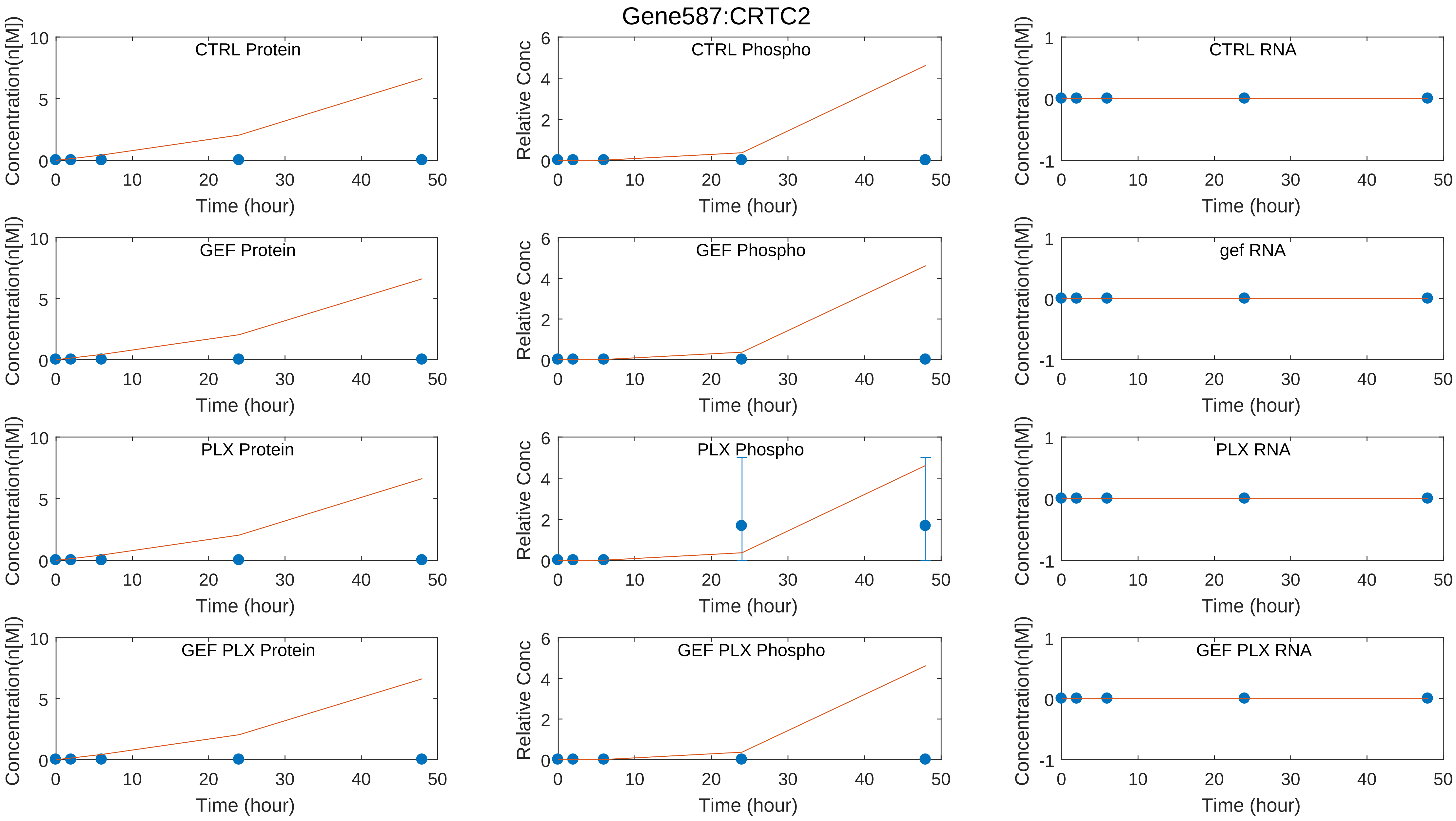

Gene587:CRTC2
CTRL Protein
CTRL Phospho
CTRL RNA
GEF Protein
GEF Phospho
gef RNA
PLX Protein
PLX Phospho
PLX RNA
GEF PLX Protein
GEF PLX Phospho
GEF PLX RNA
Concentration(n[M])
Relative Conc
Time (hour)

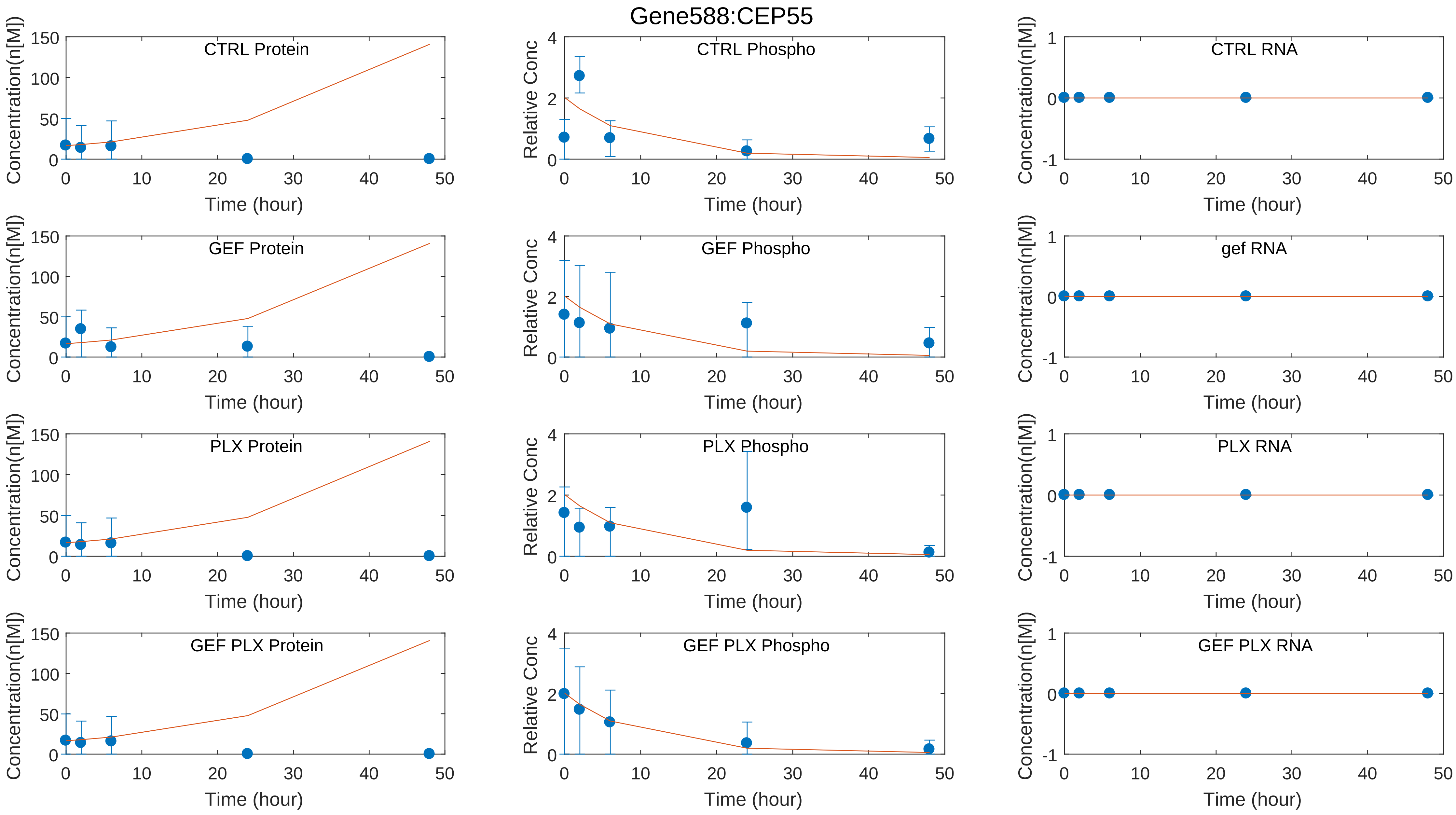

Gene588:CEP55
CTRL Protein
CTRL Phospho
CTRL RNA
GEF Protein
GEF Phospho
gef RNA
PLX Protein
PLX Phospho
PLX RNA
GEF PLX Protein
GEF PLX Phospho
GEF PLX RNA
Concentration(n[M])
Relative Conc
Time (hour)

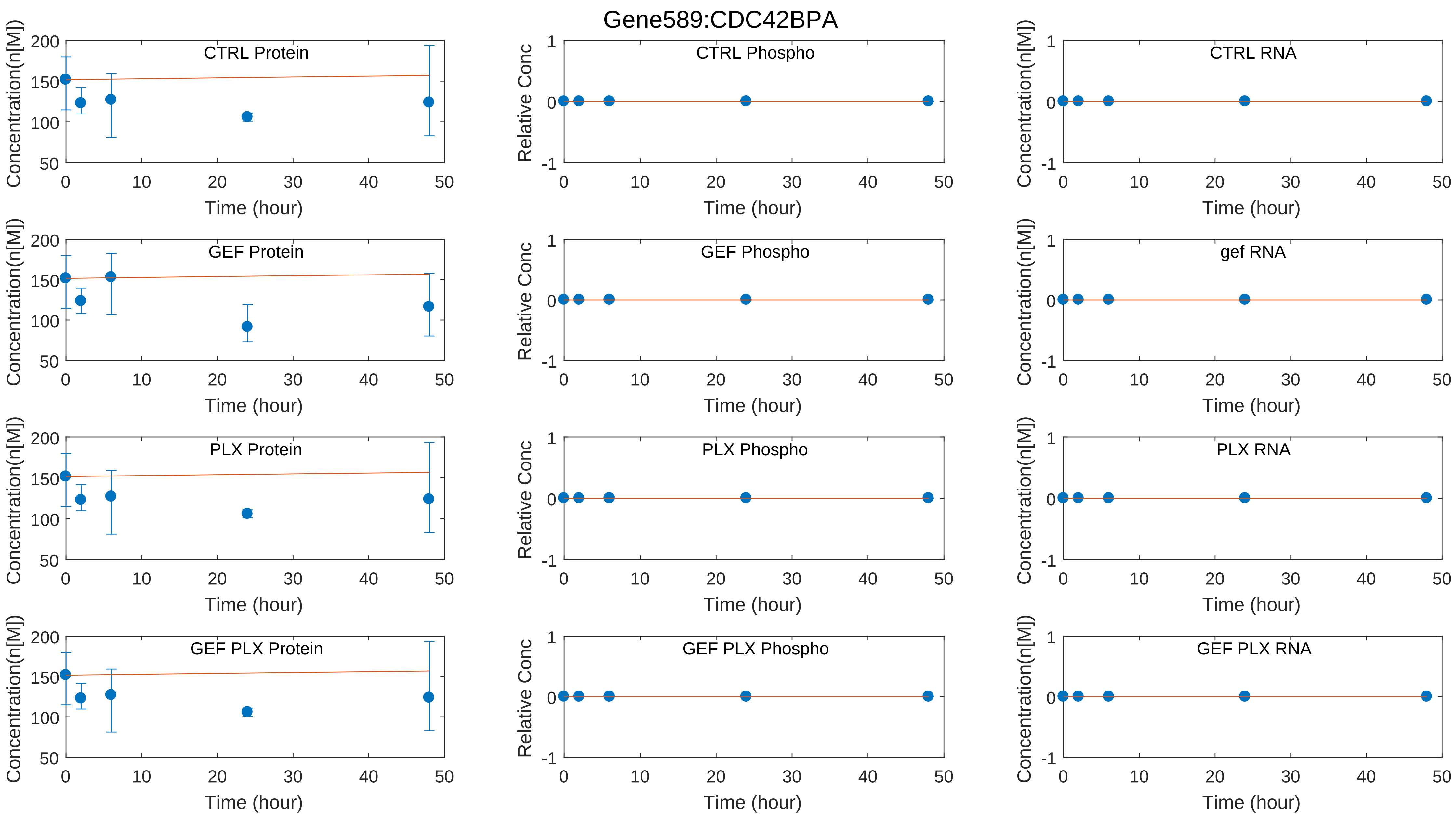

Gene589:CDC42BPA
CTRL Protein
CTRL Phospho
CTRL RNA
GEF Protein
GEF Phospho
gef RNA
PLX Protein
PLX Phospho
PLX RNA
GEF PLX Protein
GEF PLX Phospho
GEF PLX RNA
Concentration(n[M])
Relative Conc
Time (hour)

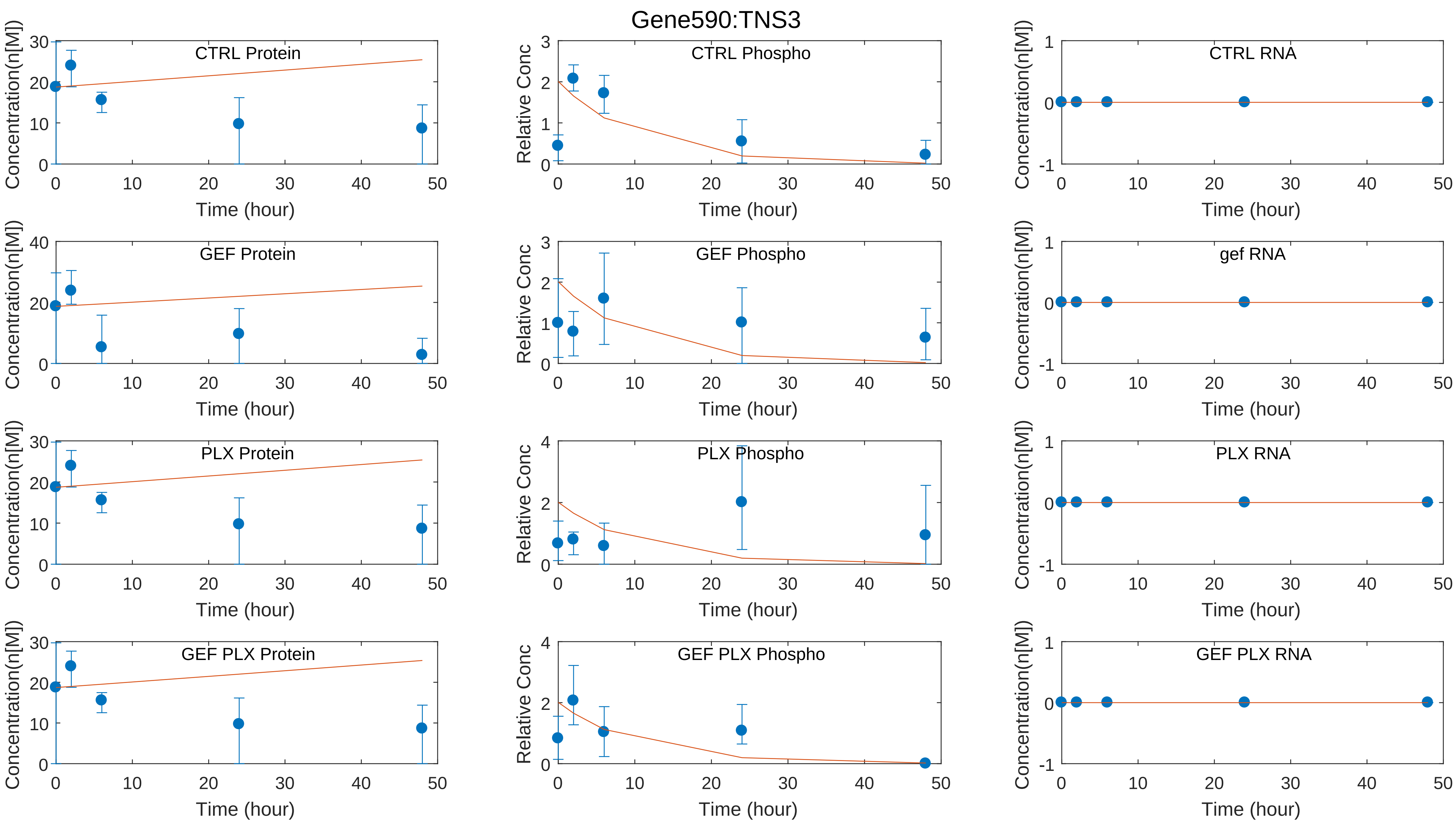

Gene590:TNS3
CTRL Protein
CTRL Phospho
CTRL RNA
GEF Protein
GEF Phospho
gef RNA
PLX Protein
PLX Phospho
PLX RNA
GEF PLX Protein
GEF PLX Phospho
GEF PLX RNA
Concentration(n[M])
Relative Conc
Time (hour)

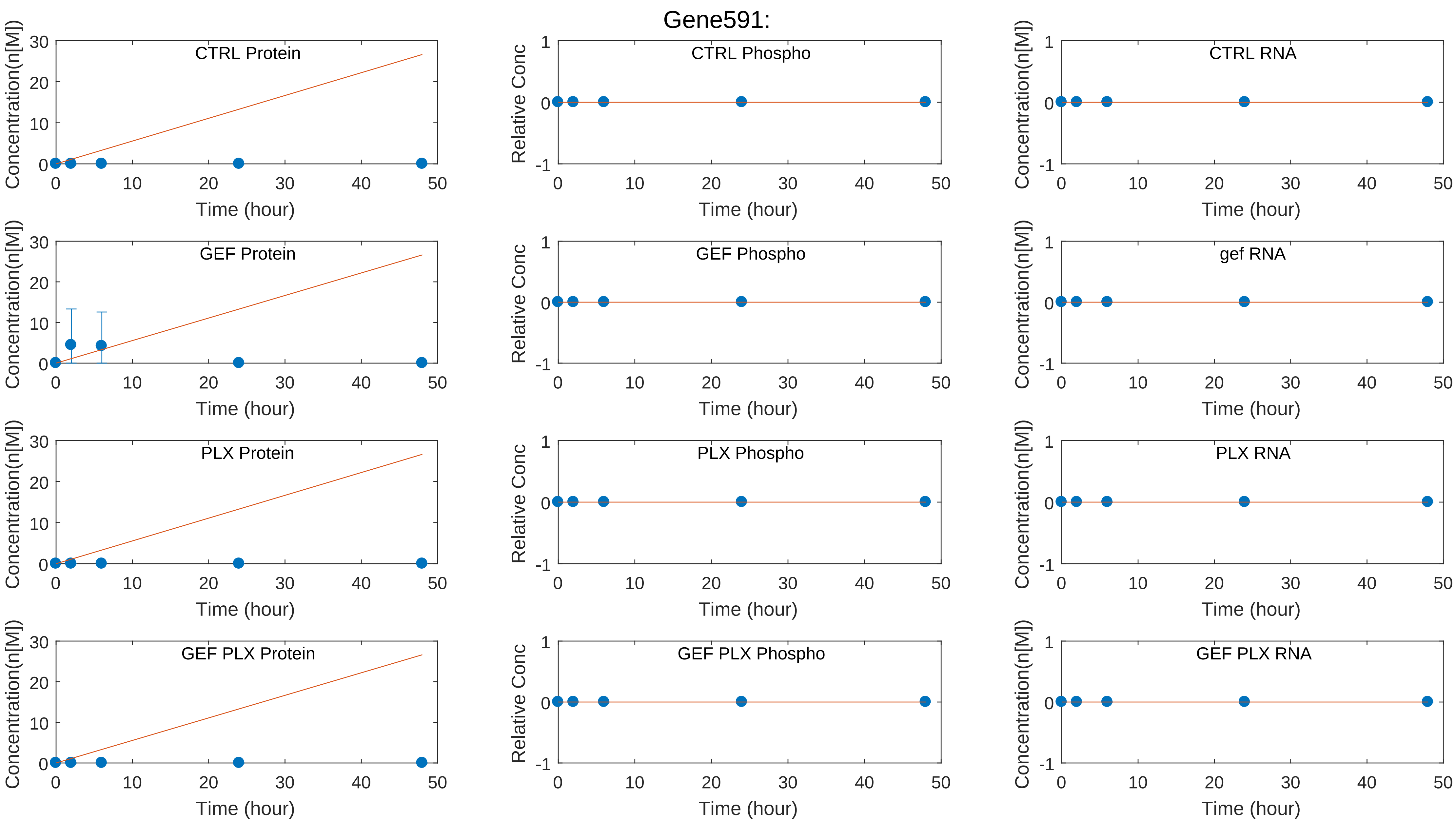

Gene591:
CTRL Protein
CTRL Phospho
CTRL RNA
GEF Protein
GEF Phospho
gef RNA
PLX Protein
PLX Phospho
PLX RNA
GEF PLX Protein
GEF PLX Phospho
GEF PLX RNA
Concentration(n[M])
Relative Conc
Time (hour)

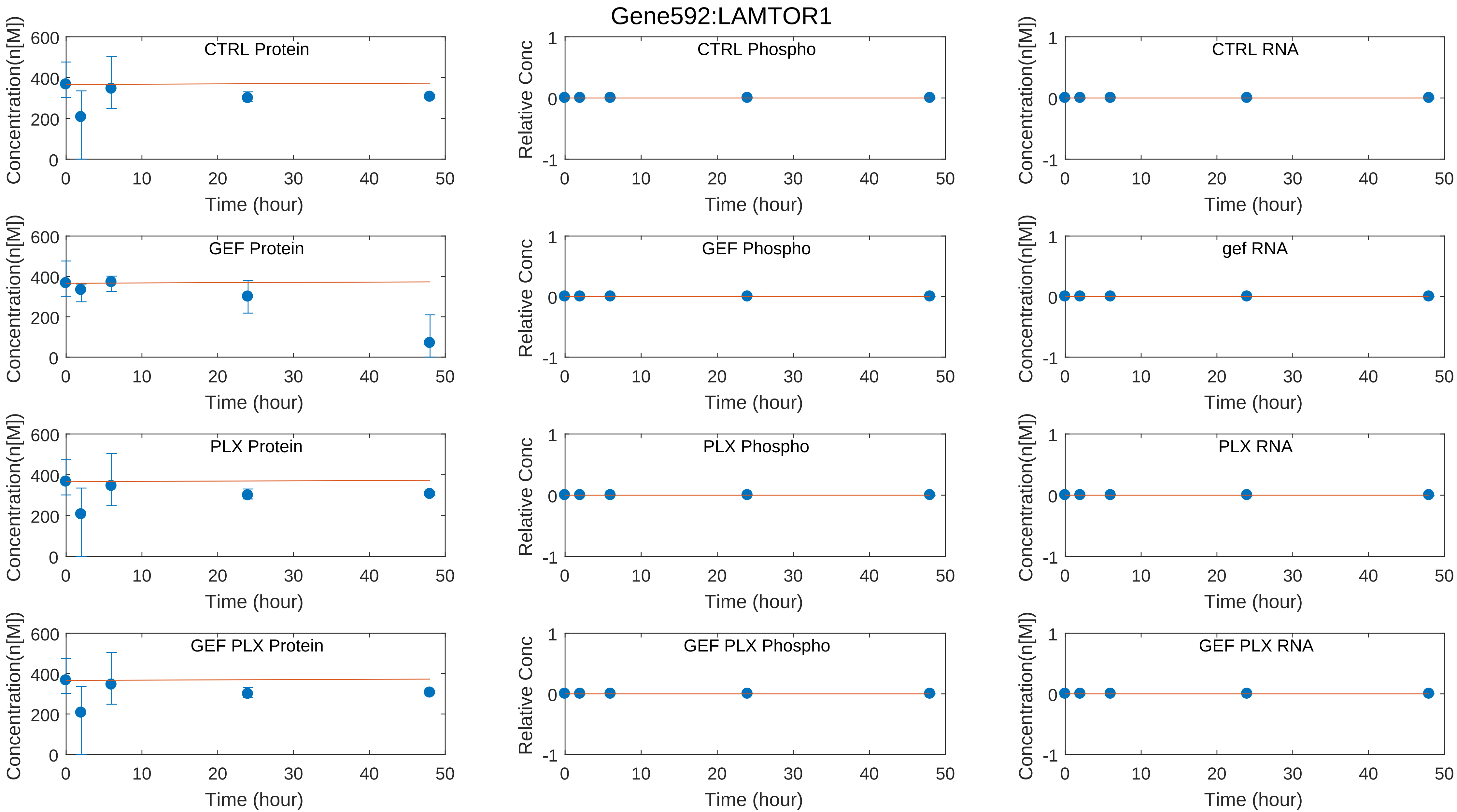

Gene592:LAMTOR1
CTRL Protein
CTRL Phospho
CTRL RNA
GEF Protein
GEF Phospho
gef RNA
PLX Protein
PLX Phospho
PLX RNA
GEF PLX Protein
GEF PLX Phospho
GEF PLX RNA
Concentration(n[M])
Relative Conc
Time (hour)
0
10
20
30
40
50
600
400
200
1
-1

Gene593:PACS1

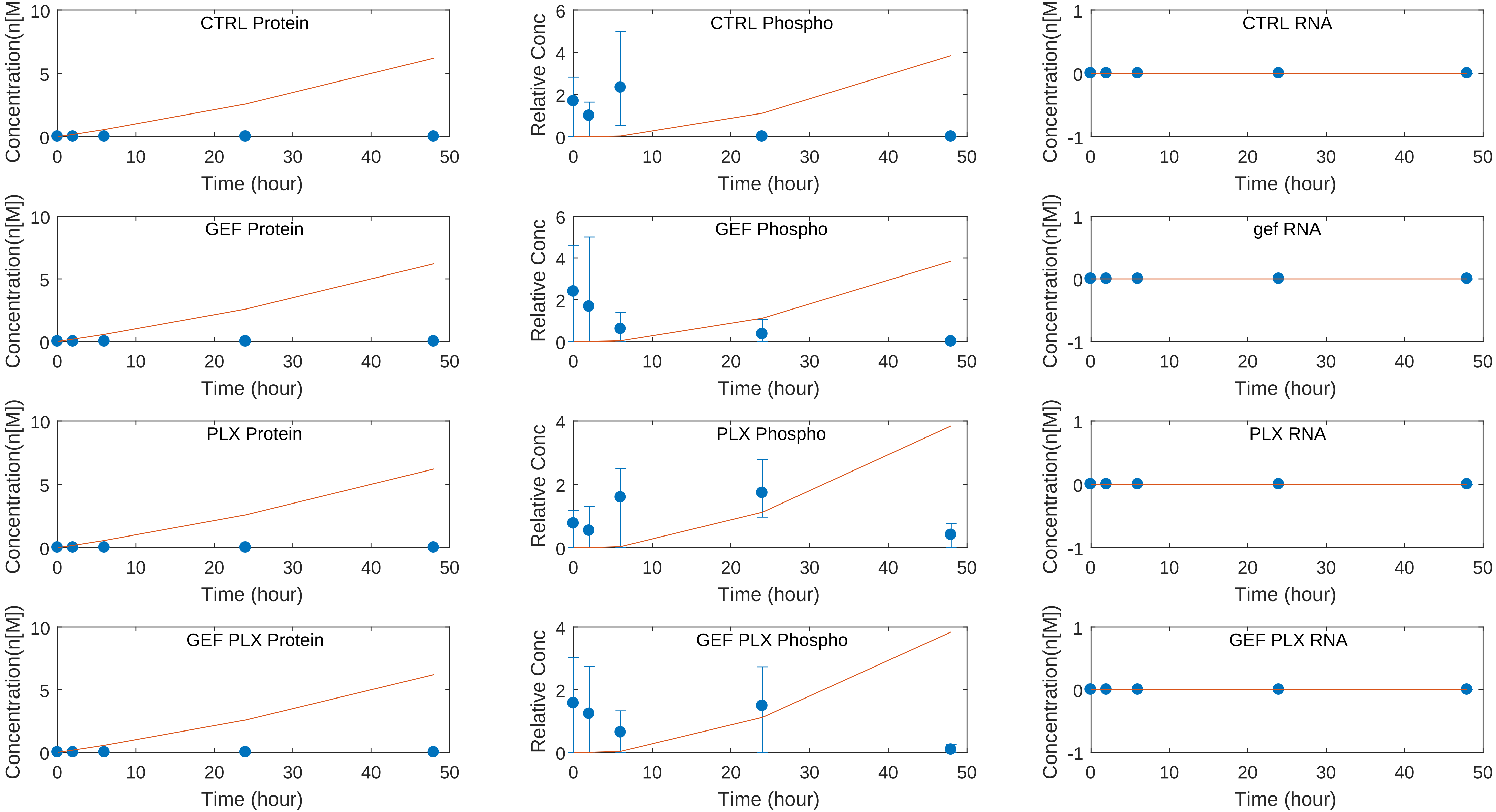

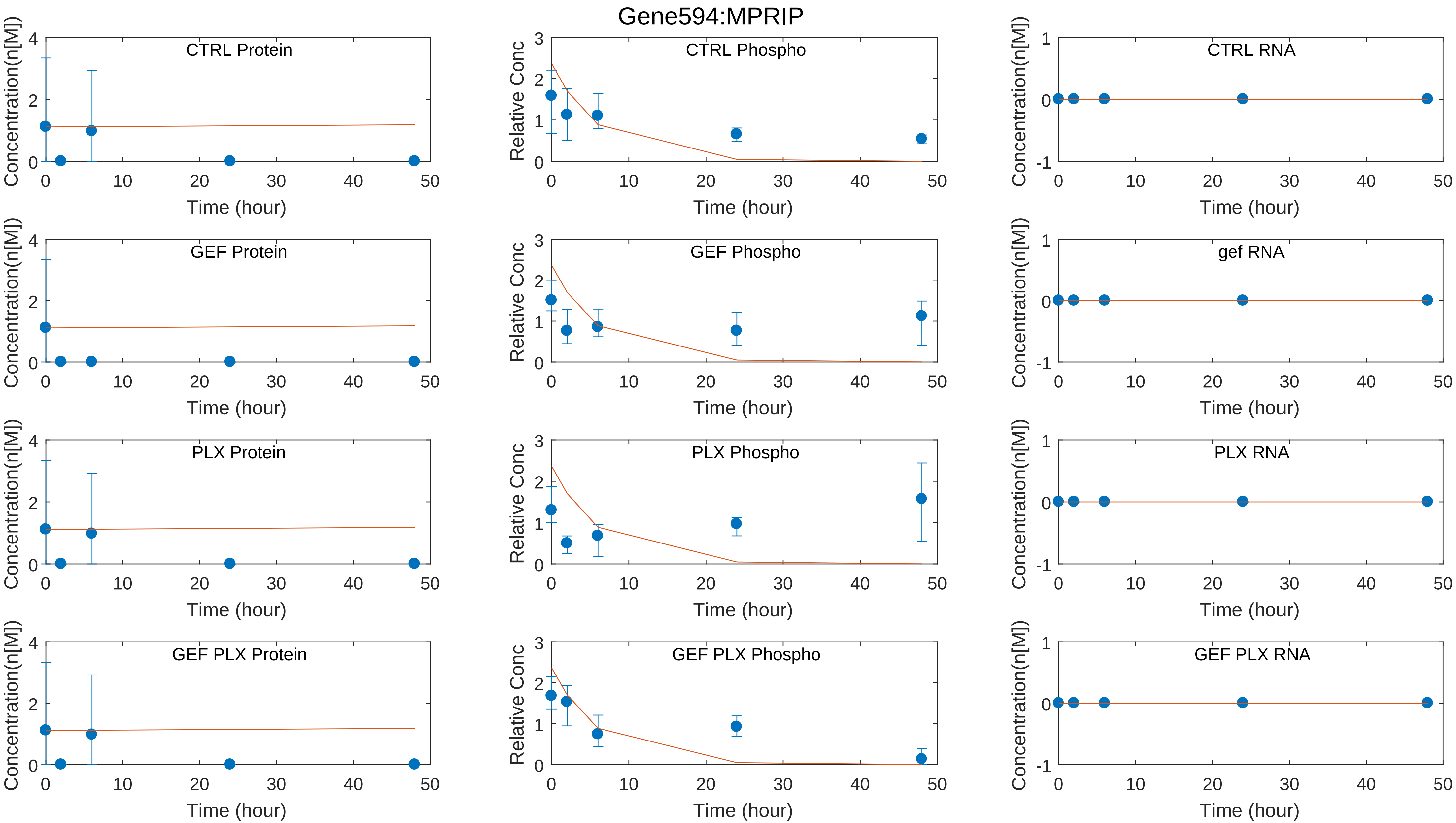

Gene594:MPRIP
CTRL Protein
CTRL Phospho
CTRL RNA
GEF Protein
GEF Phospho
gef RNA
PLX Protein
PLX Phospho
PLX RNA
GEF PLX Protein
GEF PLX Phospho
GEF PLX RNA
Concentration(n[M])
Relative Conc
Time (hour)

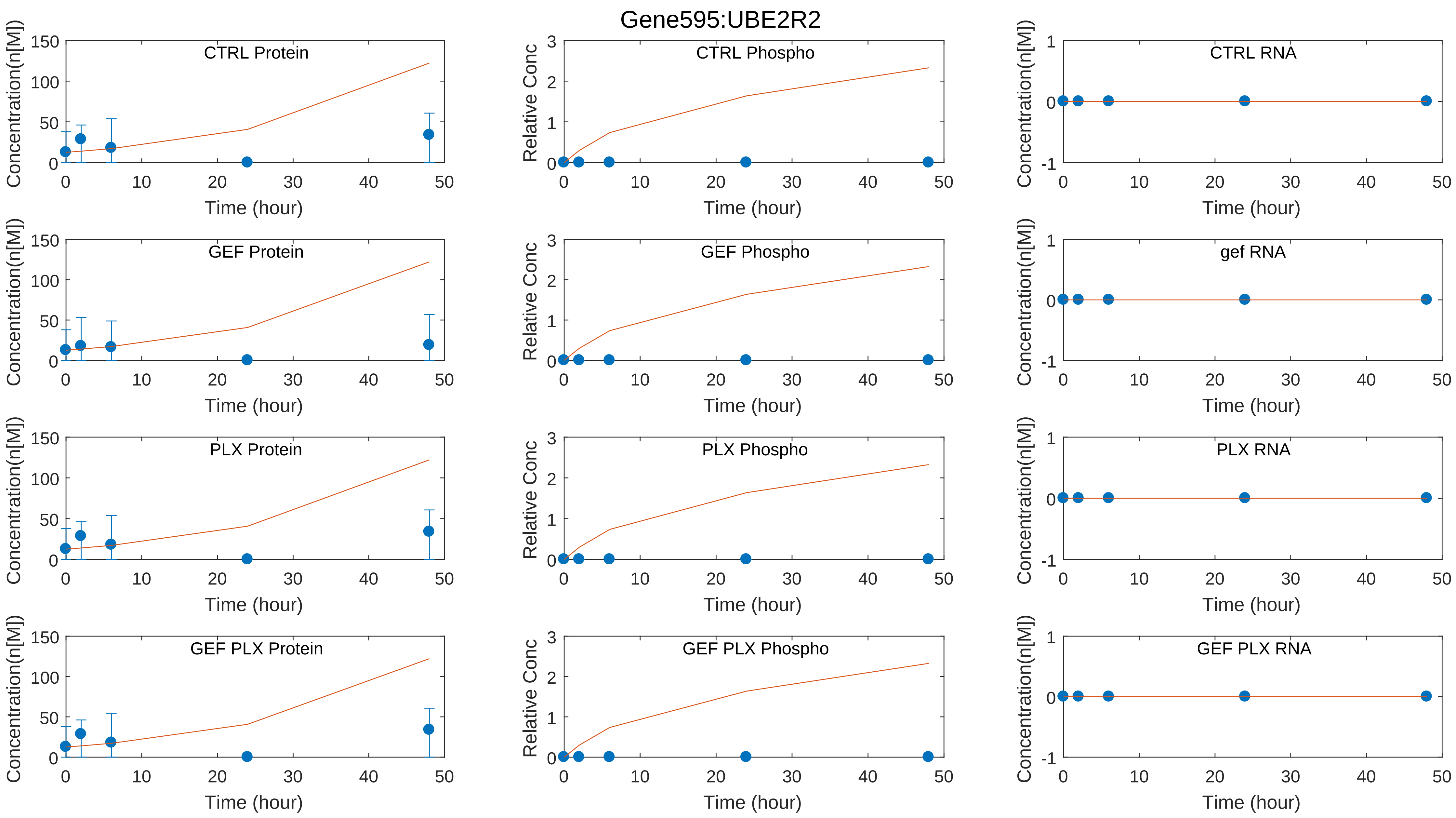

Gene595:UBE2R2
CTRL Protein
CTRL Phospho
CTRL RNA
GEF Protein
GEF Phospho
gef RNA
PLX Protein
PLX Phospho
PLX RNA
GEF PLX Protein
GEF PLX Phospho
GEF PLX RNA
Concentration(n[M])
Relative Conc
Time (hour)

Gene596:MARK2

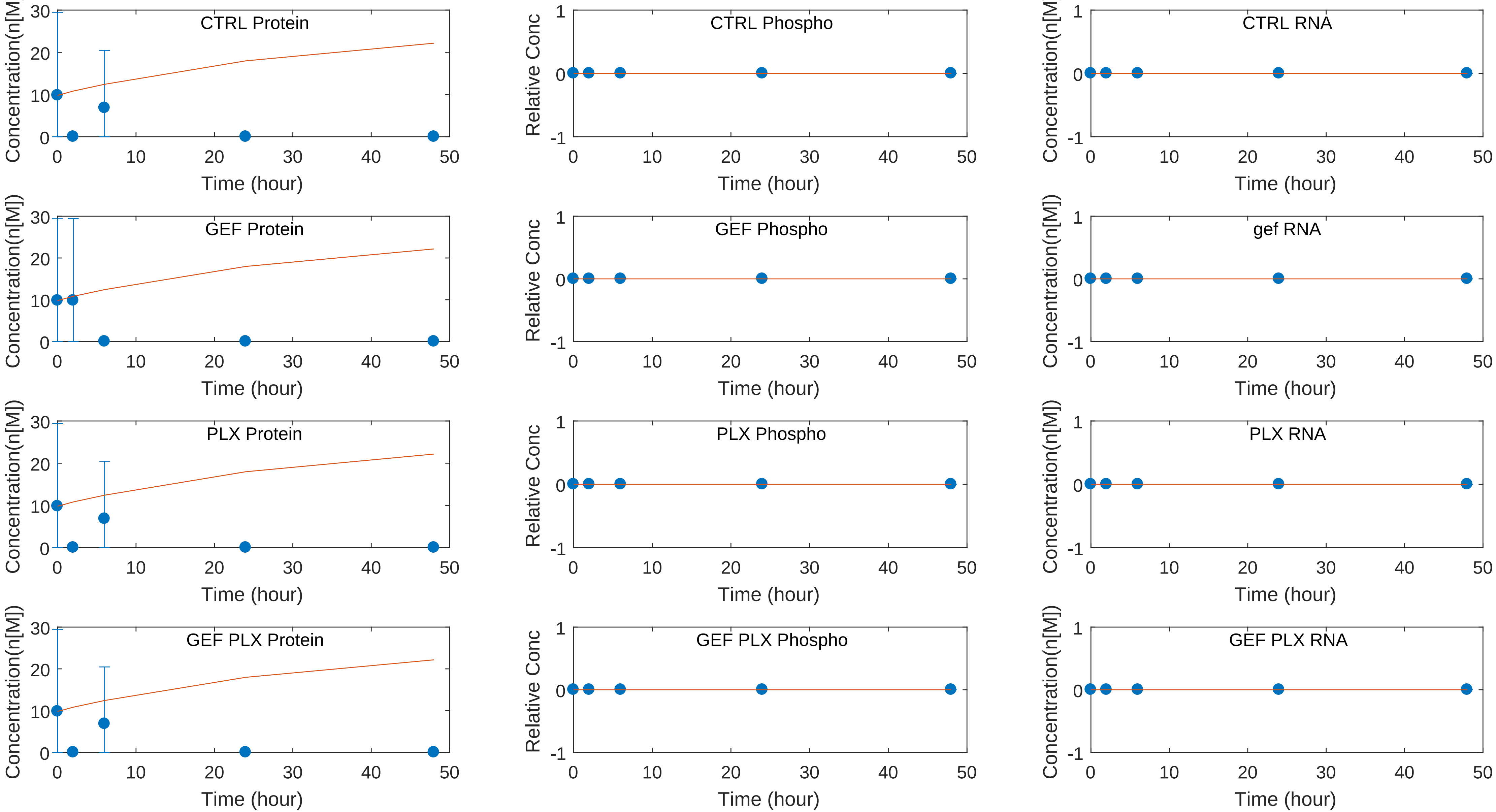

Gene597:COPS6

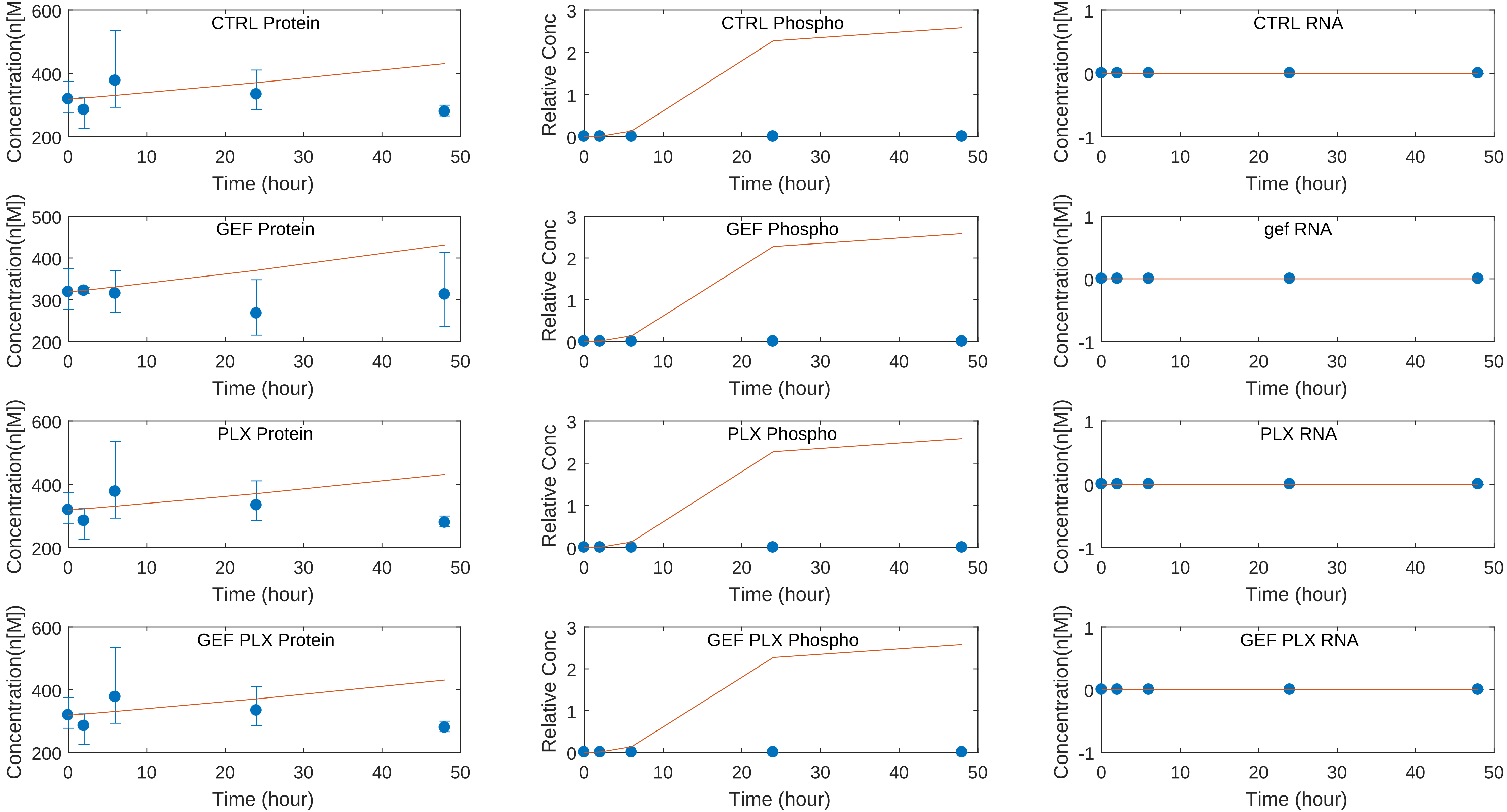

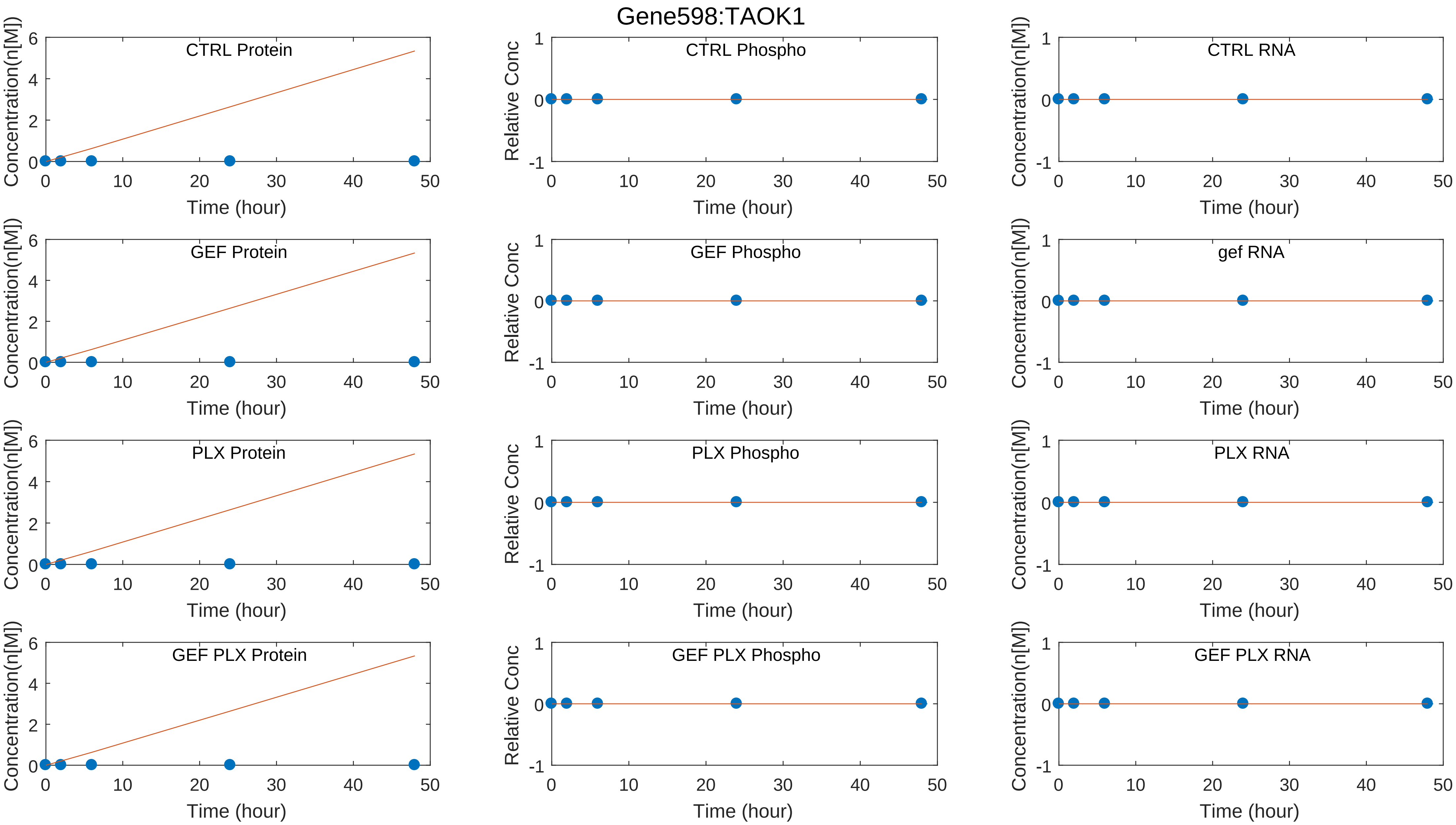

Gene598:TAOK1
CTRL Protein
CTRL Phospho
CTRL RNA
GEF Protein
GEF Phospho
gef RNA
PLX Protein
PLX Phospho
PLX RNA
GEF PLX Protein
GEF PLX Phospho
GEF PLX RNA
Concentration(n[M])
Relative Conc
Time (hour)

Gene599:TBC1D1

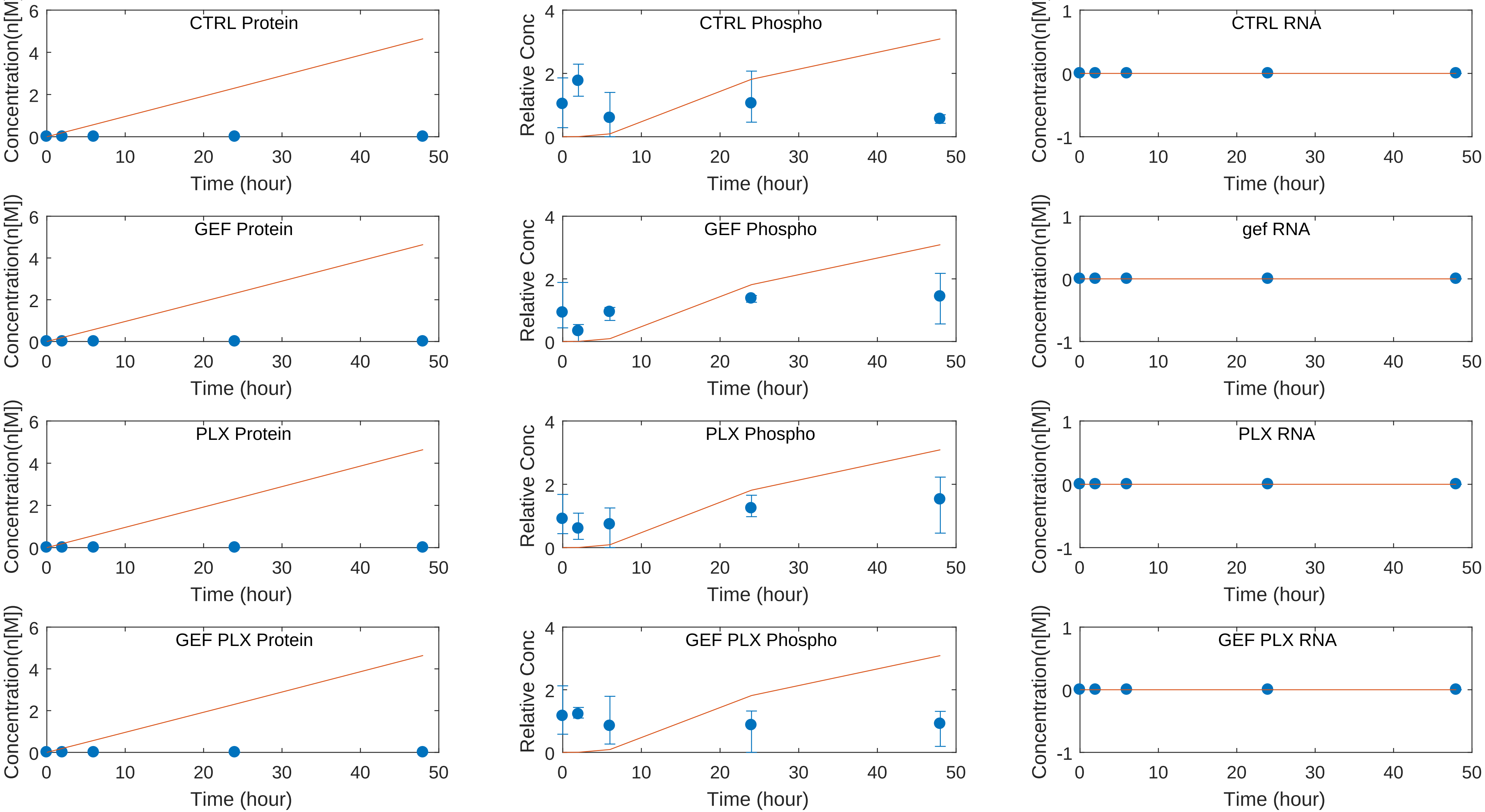

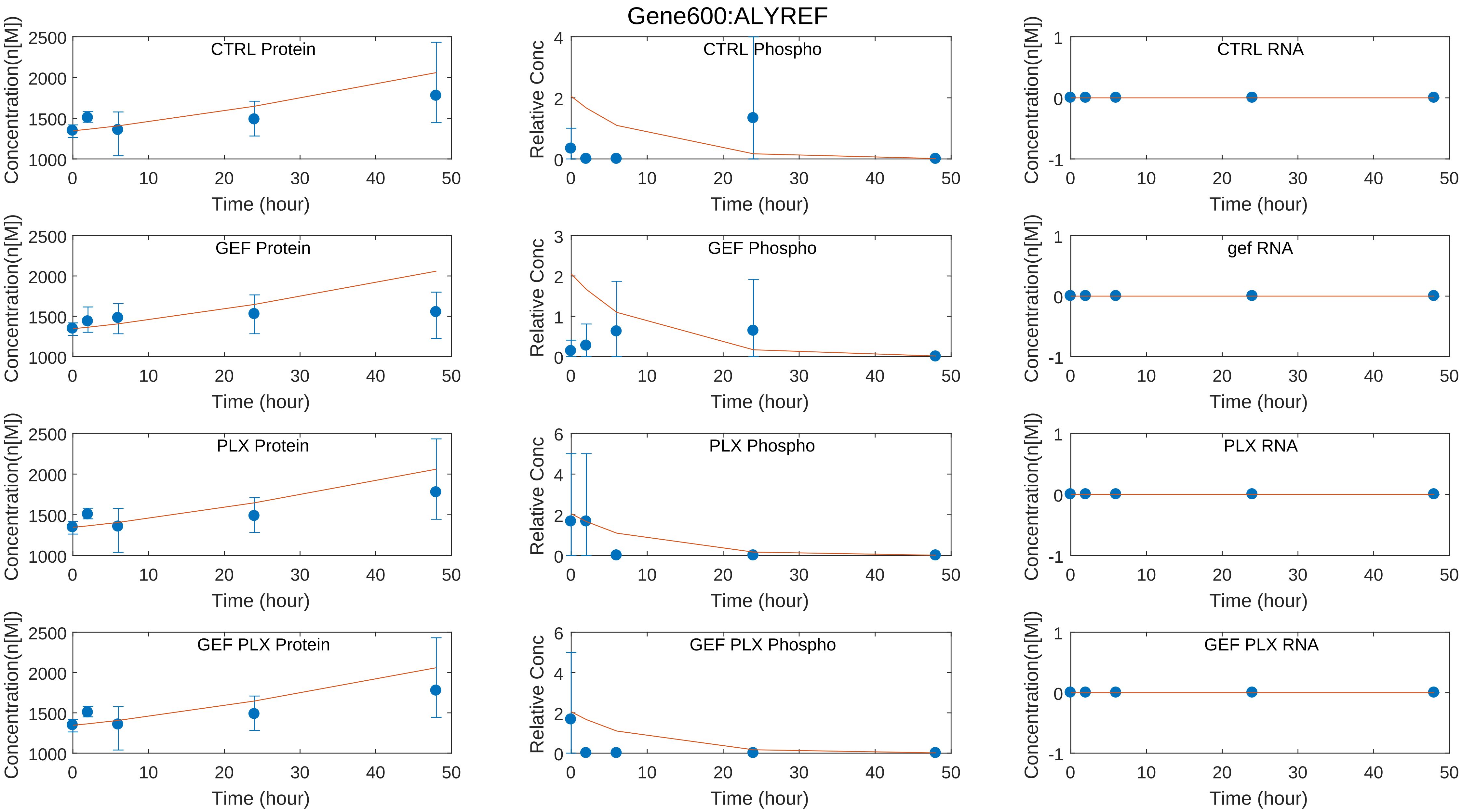

Gene600:ALYREF
CTRL Protein
CTRL Phospho
CTRL RNA
GEF Protein
GEF Phospho
gef RNA
PLX Protein
PLX Phospho
PLX RNA
GEF PLX Protein
GEF PLX Phospho
GEF PLX RNA
Concentration(n[M])
Relative Conc
Time (hour)

Gene601:TAX1BP1

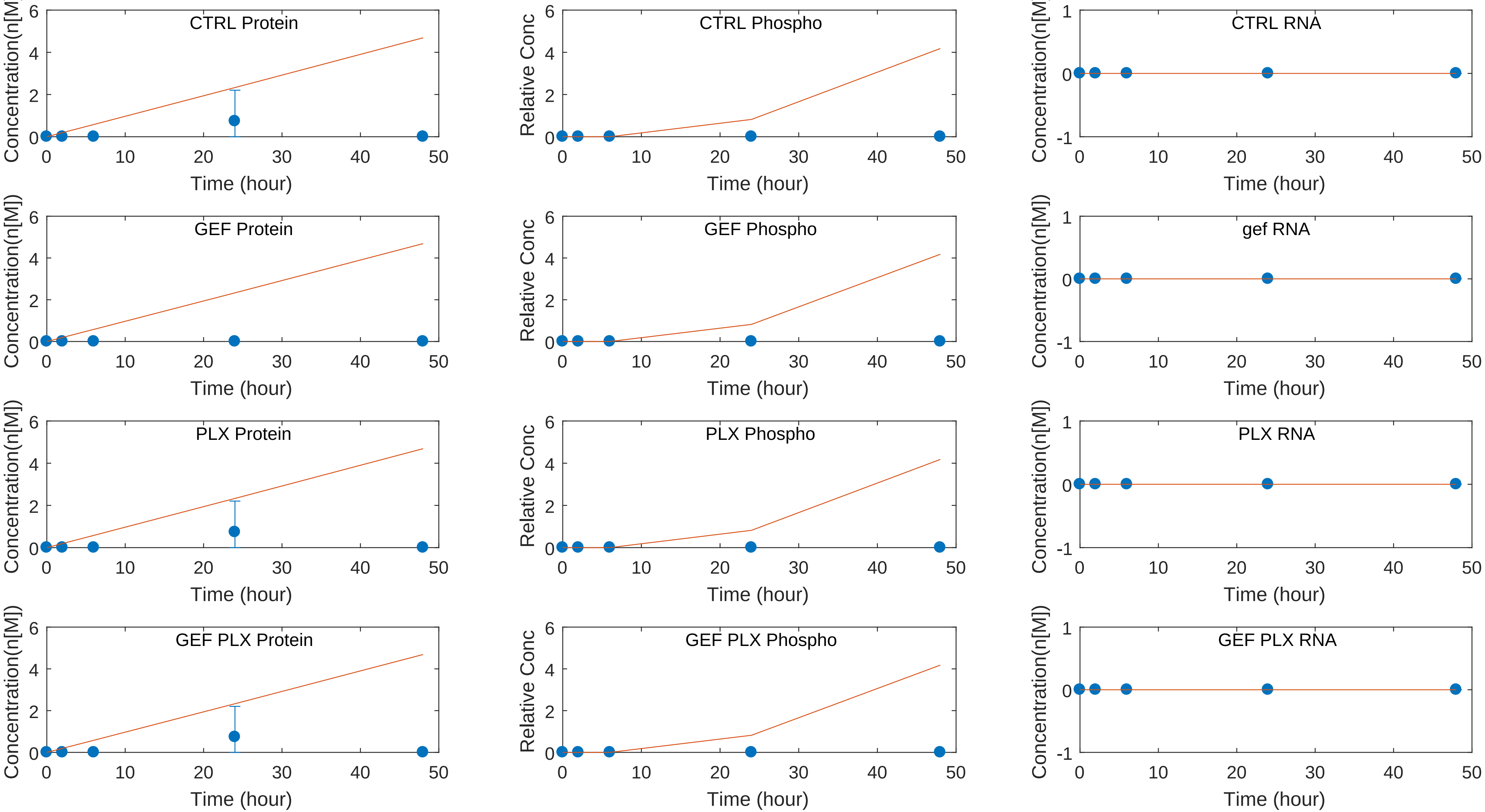

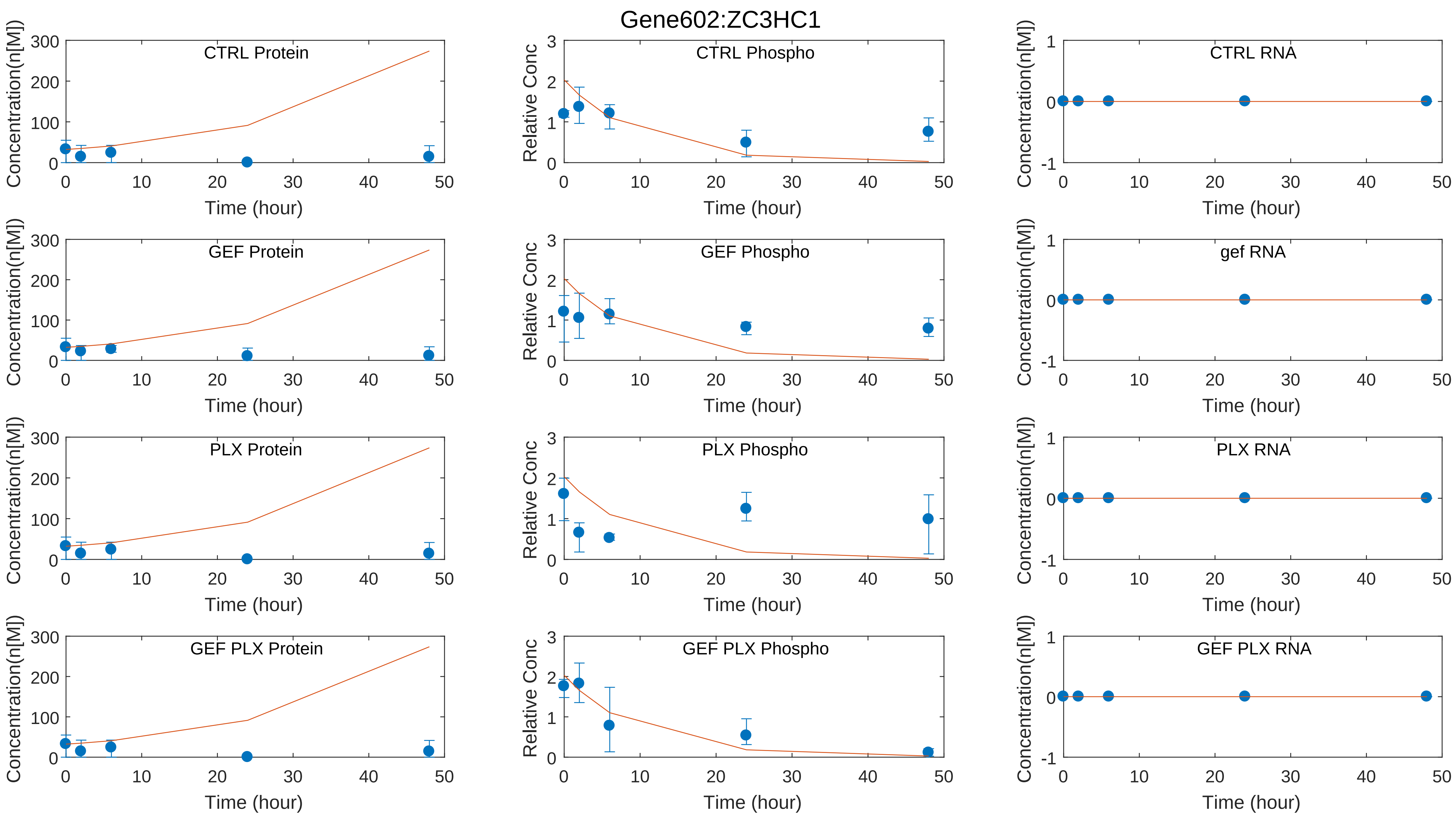

Gene602:ZC3HC1
CTRL Protein
CTRL Phospho
CTRL RNA
GEF Protein
GEF Phospho
gef RNA
PLX Protein
PLX Phospho
PLX RNA
GEF PLX Protein
GEF PLX Phospho
GEF PLX RNA
Concentration(n[M])
Relative Conc
Time (hour)

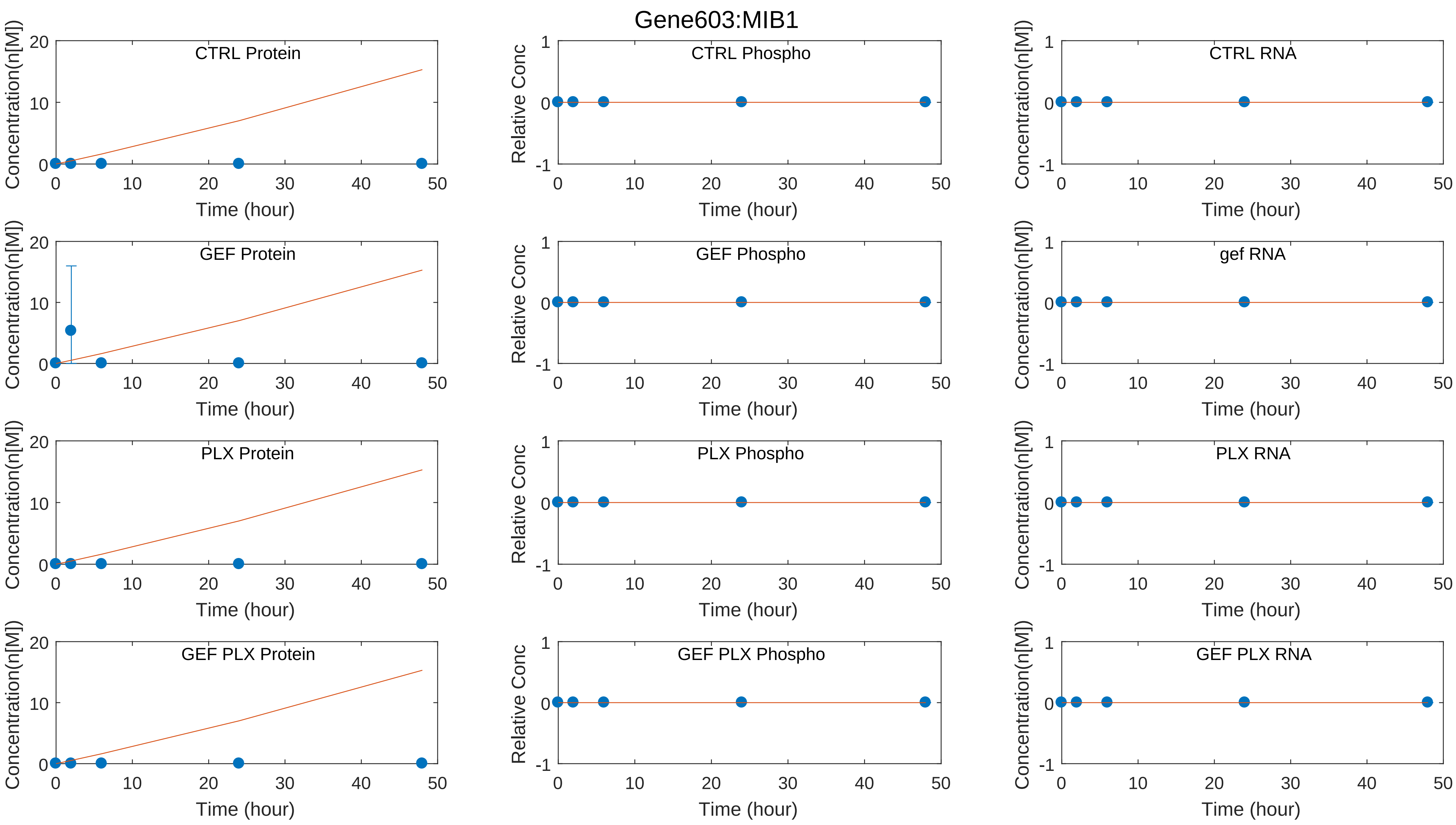

Gene603:MIB1
CTRL Protein
CTRL Phospho
CTRL RNA
GEF Protein
GEF Phospho
gef RNA
PLX Protein
PLX Phospho
PLX RNA
GEF PLX Protein
GEF PLX Phospho
GEF PLX RNA
Concentration(n[M])
Relative Conc
Time (hour)

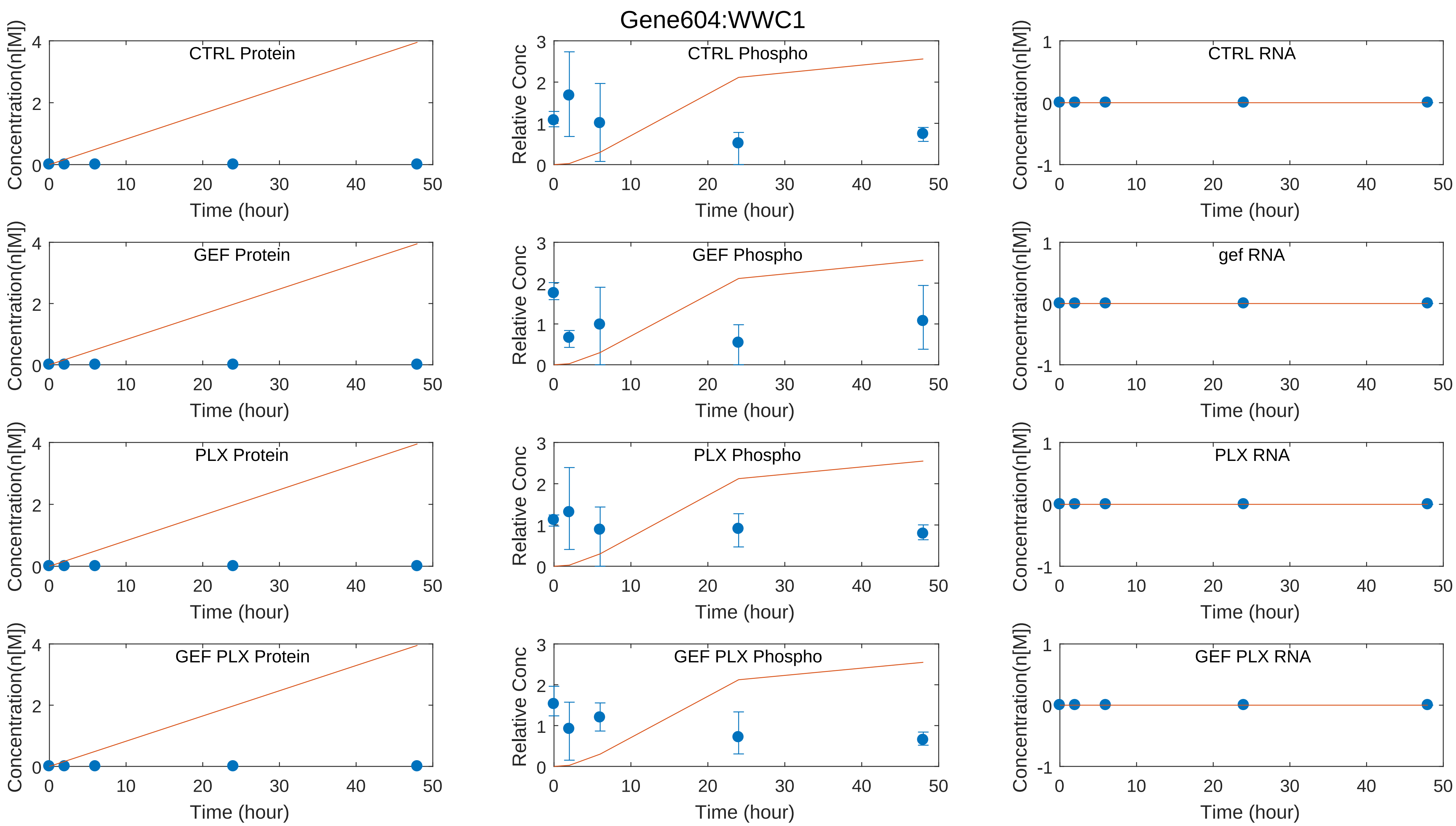

Gene604:WWC1
CTRL Protein
CTRL Phospho
CTRL RNA
GEF Protein
GEF Phospho
gef RNA
PLX Protein
PLX Phospho
PLX RNA
GEF PLX Protein
GEF PLX Phospho
GEF PLX RNA
Concentration(n[M])
Relative Conc
Time (hour)

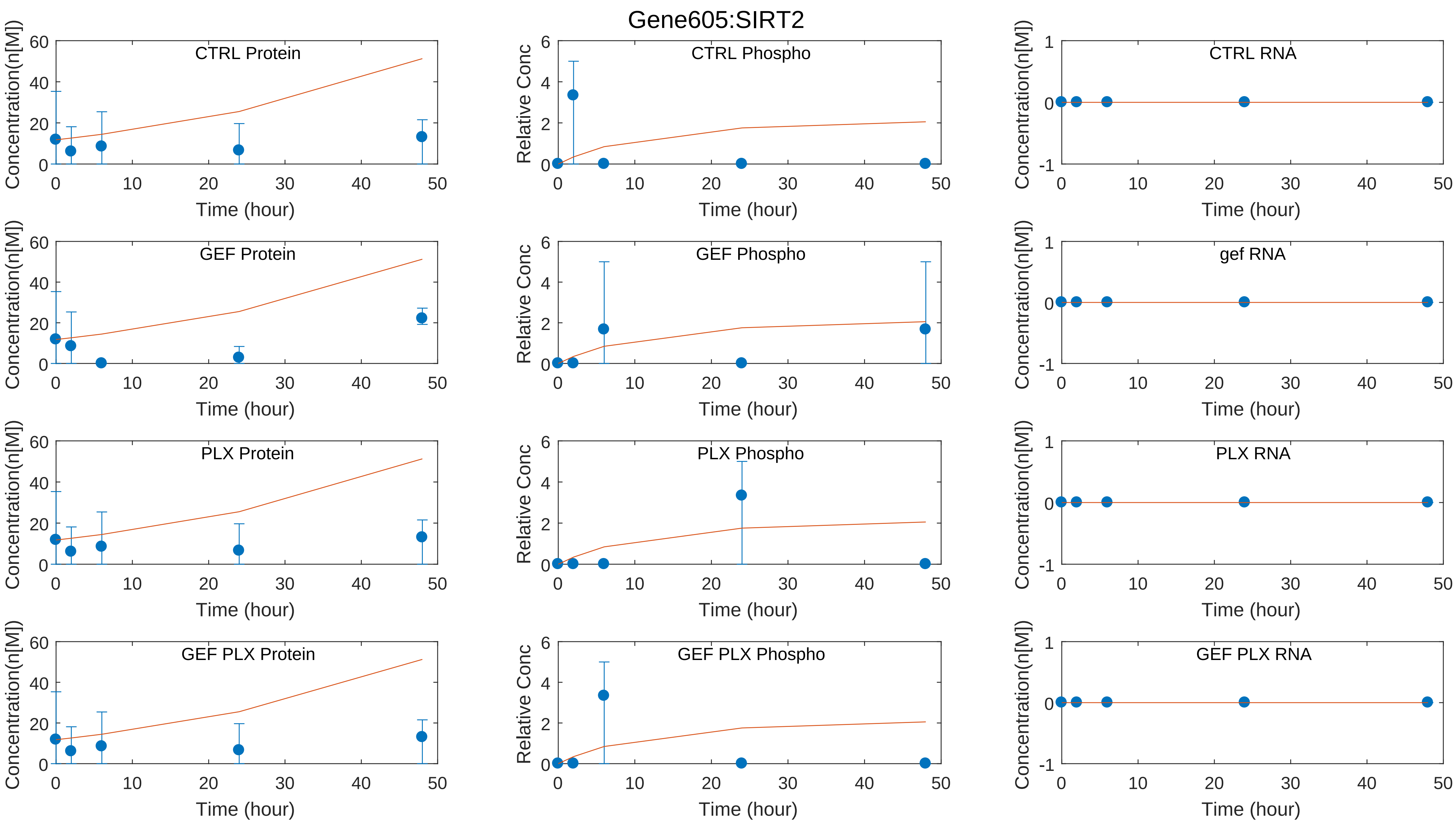

Gene605:SIRT2
CTRL Protein
CTRL Phospho
CTRL RNA
GEF Protein
GEF Phospho
gef RNA
PLX Protein
PLX Phospho
PLX RNA
GEF PLX Protein
GEF PLX Phospho
GEF PLX RNA
Concentration(n[M])
Relative Conc
Time (hour)

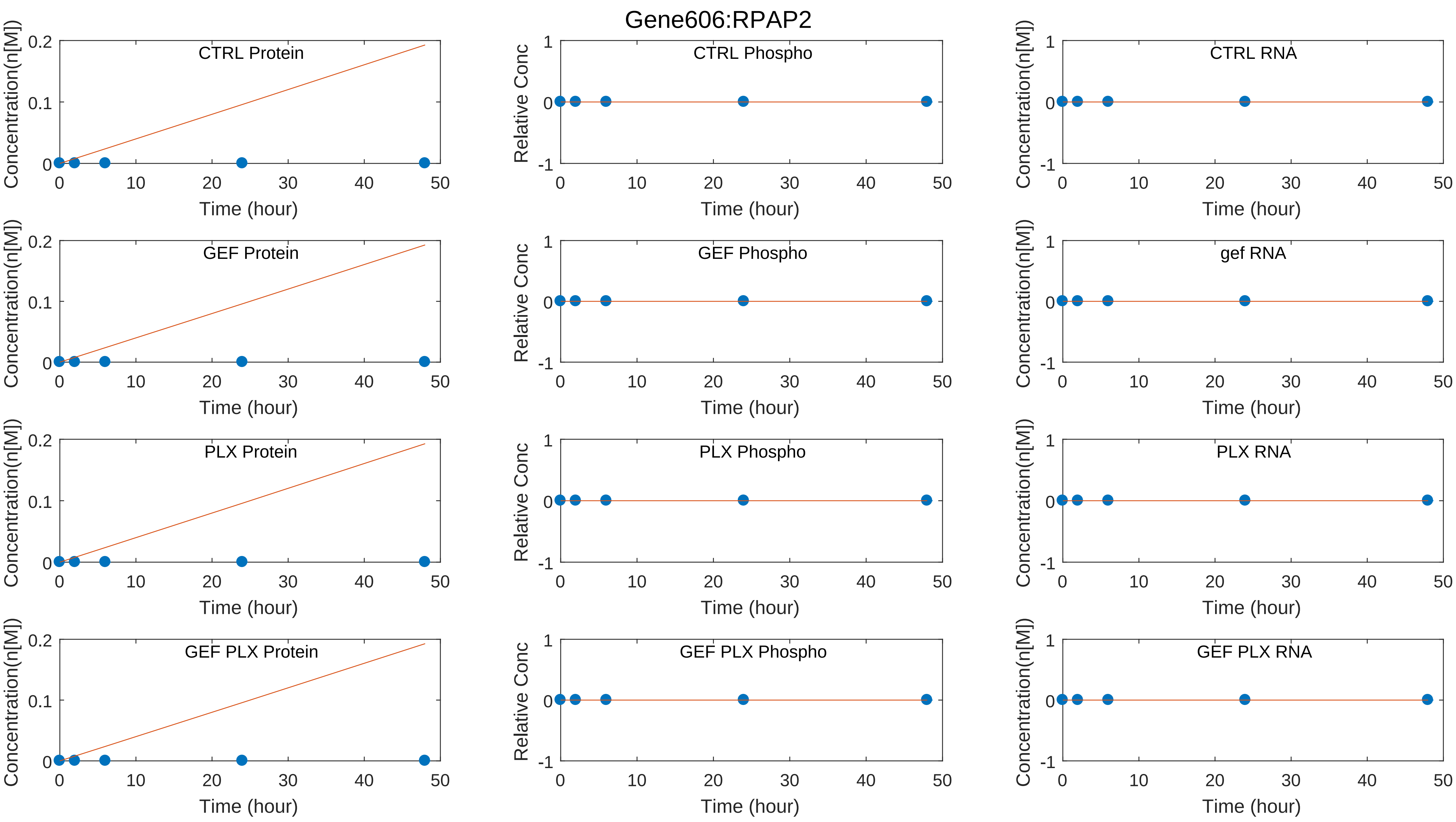
Gene606:RPAP2
CTRL Protein
CTRL Phospho
CTRL RNA
GEF Protein
GEF Phospho
gef RNA
PLX Protein
PLX Phospho
PLX RNA
GEF PLX Protein
GEF PLX Phospho
GEF PLX RNA
Concentration(n[M])
Relative Conc
Time (hour)

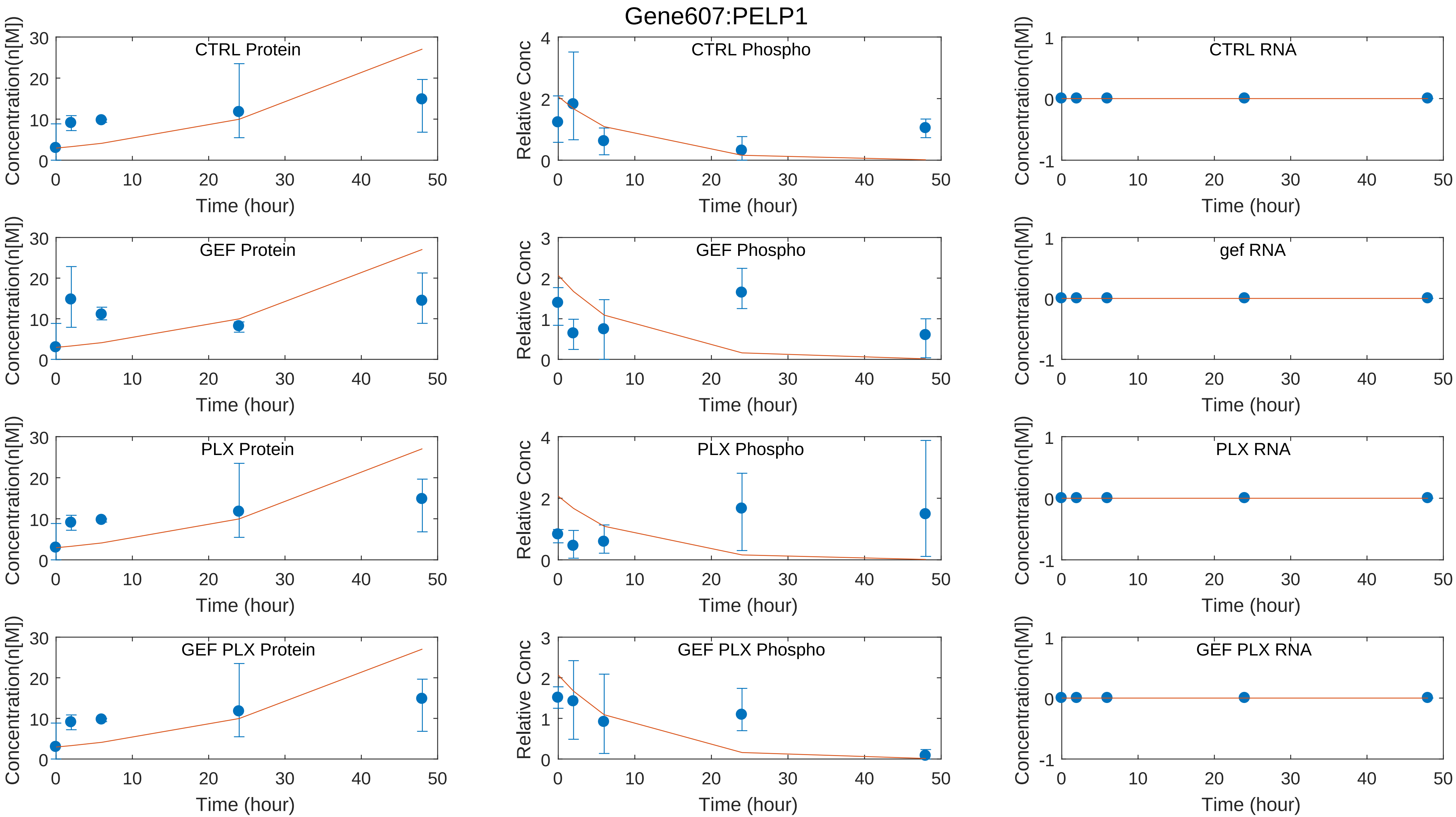

Gene607:PELP1
CTRL Protein
CTRL Phospho
CTRL RNA
GEF Protein
GEF Phospho
gef RNA
PLX Protein
PLX Phospho
PLX RNA
GEF PLX Protein
GEF PLX Phospho
GEF PLX RNA
Concentration(n[M])
Relative Conc
Time (hour)

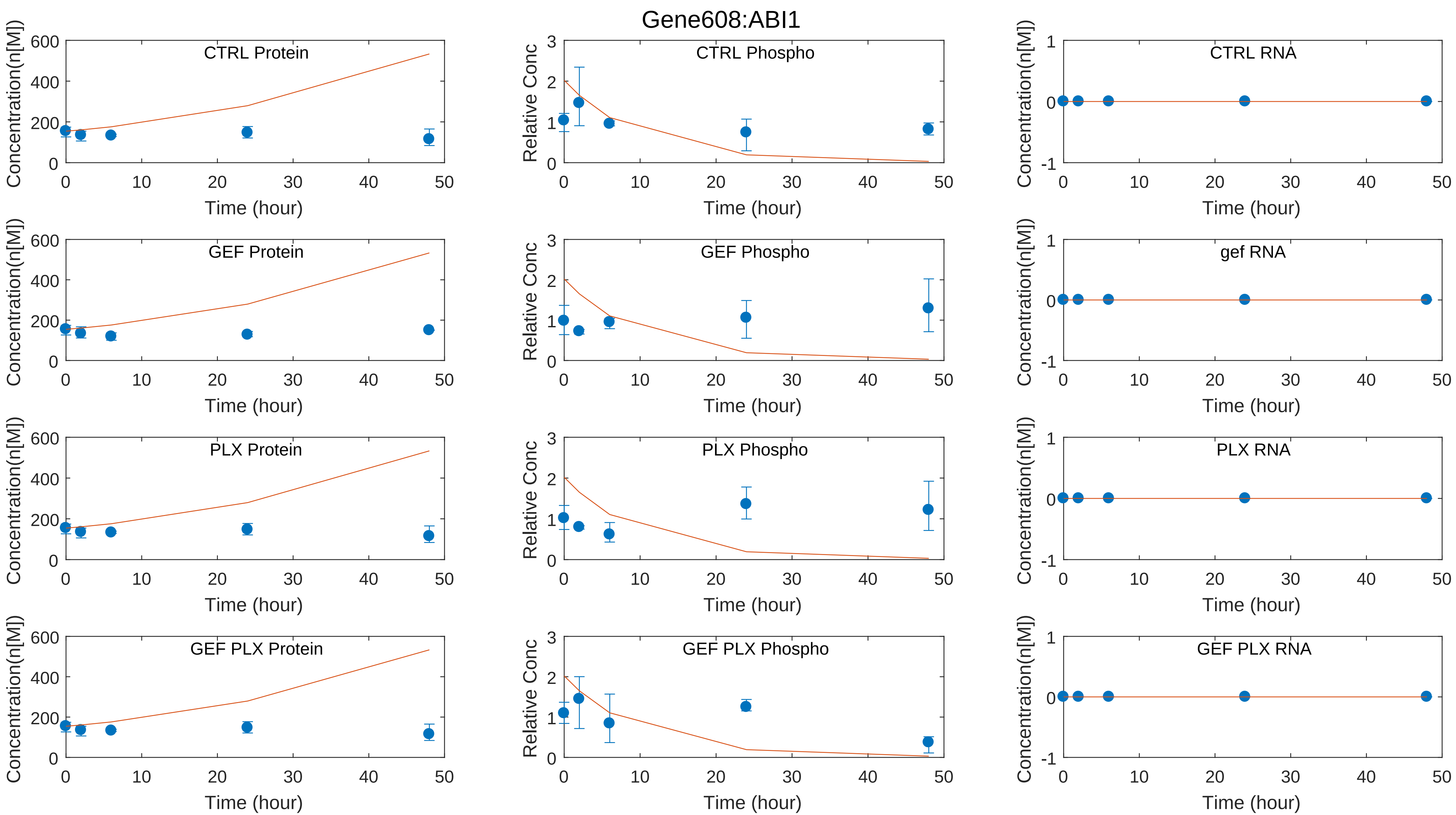

Gene608:ABI1
CTRL Protein
GEF Protein
PLX Protein
GEF PLX Protein
CTRL Phospho
GEF Phospho
PLX Phospho
GEF PLX Phospho
CTRL RNA
gef RNA
PLX RNA
GEF PLX RNA
Concentration(n[M])
Relative Conc
Time (hour)

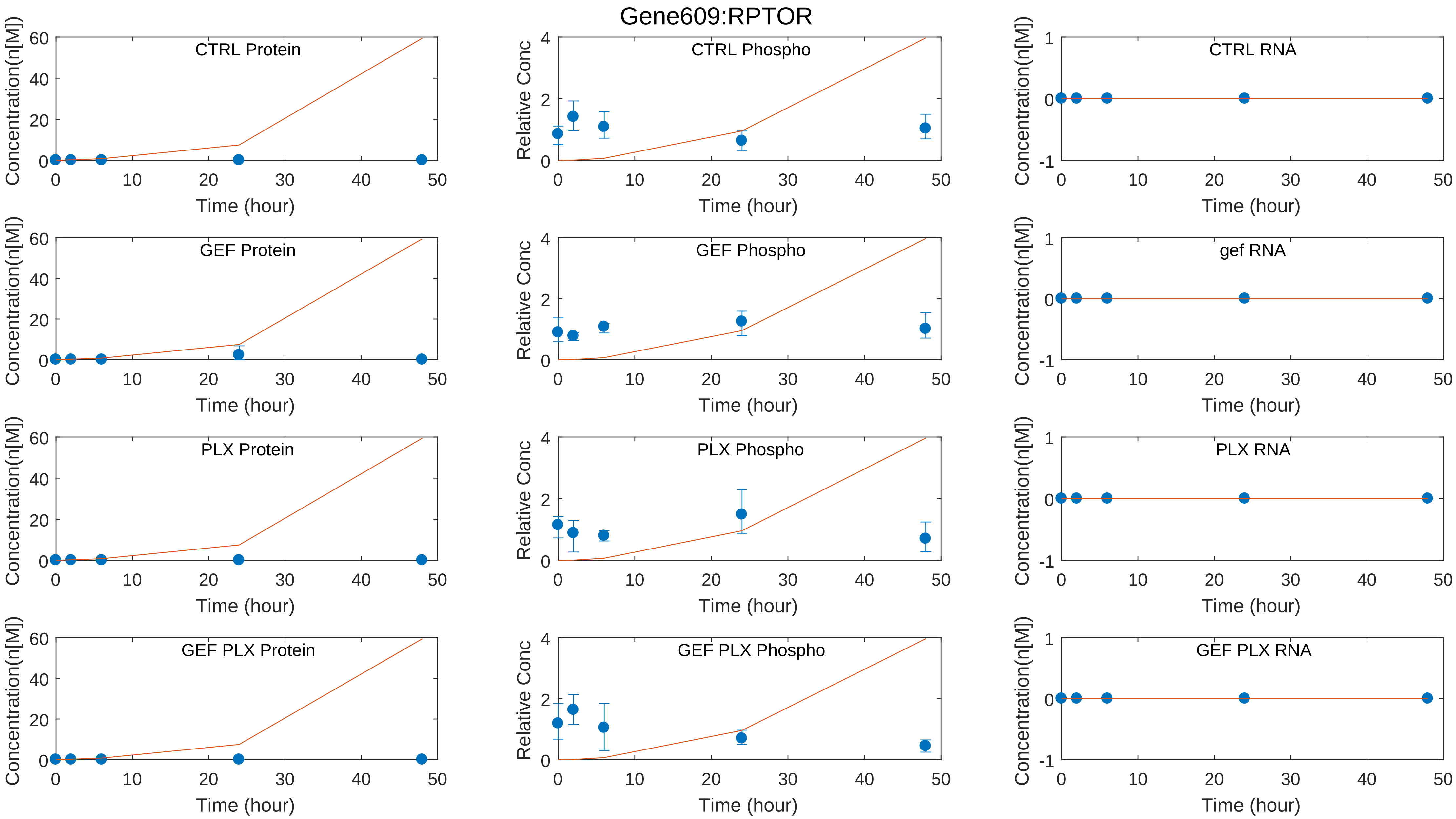

Gene609:RPTOR
CTRL Protein
CTRL Phospho
CTRL RNA
GEF Protein
GEF Phospho
gef RNA
PLX Protein
PLX Phospho
PLX RNA
GEF PLX Protein
GEF PLX Phospho
GEF PLX RNA
Concentration(n[M])
Relative Conc
Time (hour)

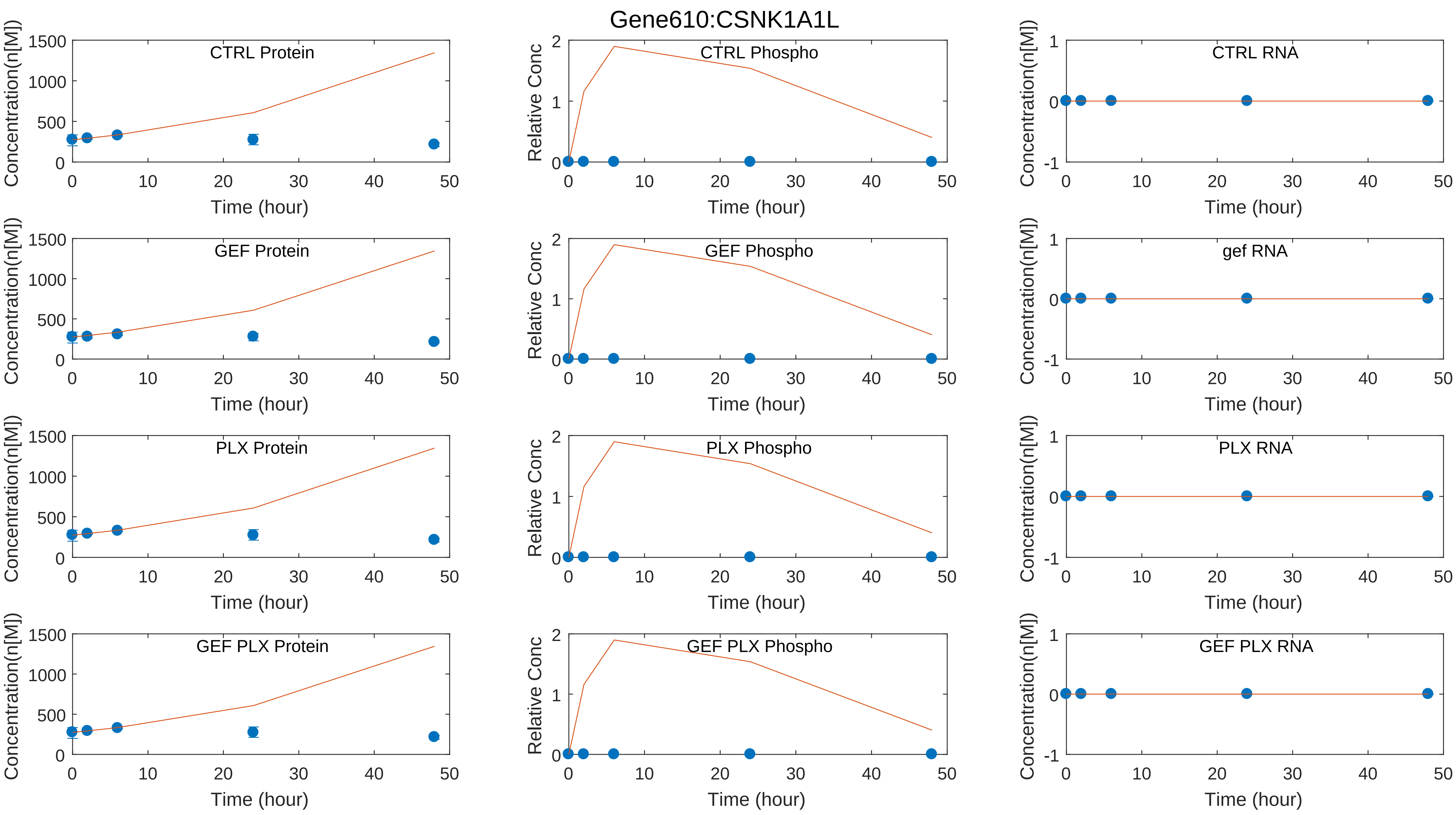

Gene610:CSNK1A1L
CTRL Protein
GEF Protein
PLX Protein
GEF PLX Protein
CTRL Phospho
GEF Phospho
PLX Phospho
GEF PLX Phospho
CTRL RNA
gef RNA
PLX RNA
GEF PLX RNA
Concentration(n[M])
Relative Conc
Time (hour)
0
500
1000
1500
1
2
-1
10
20
30
40
50

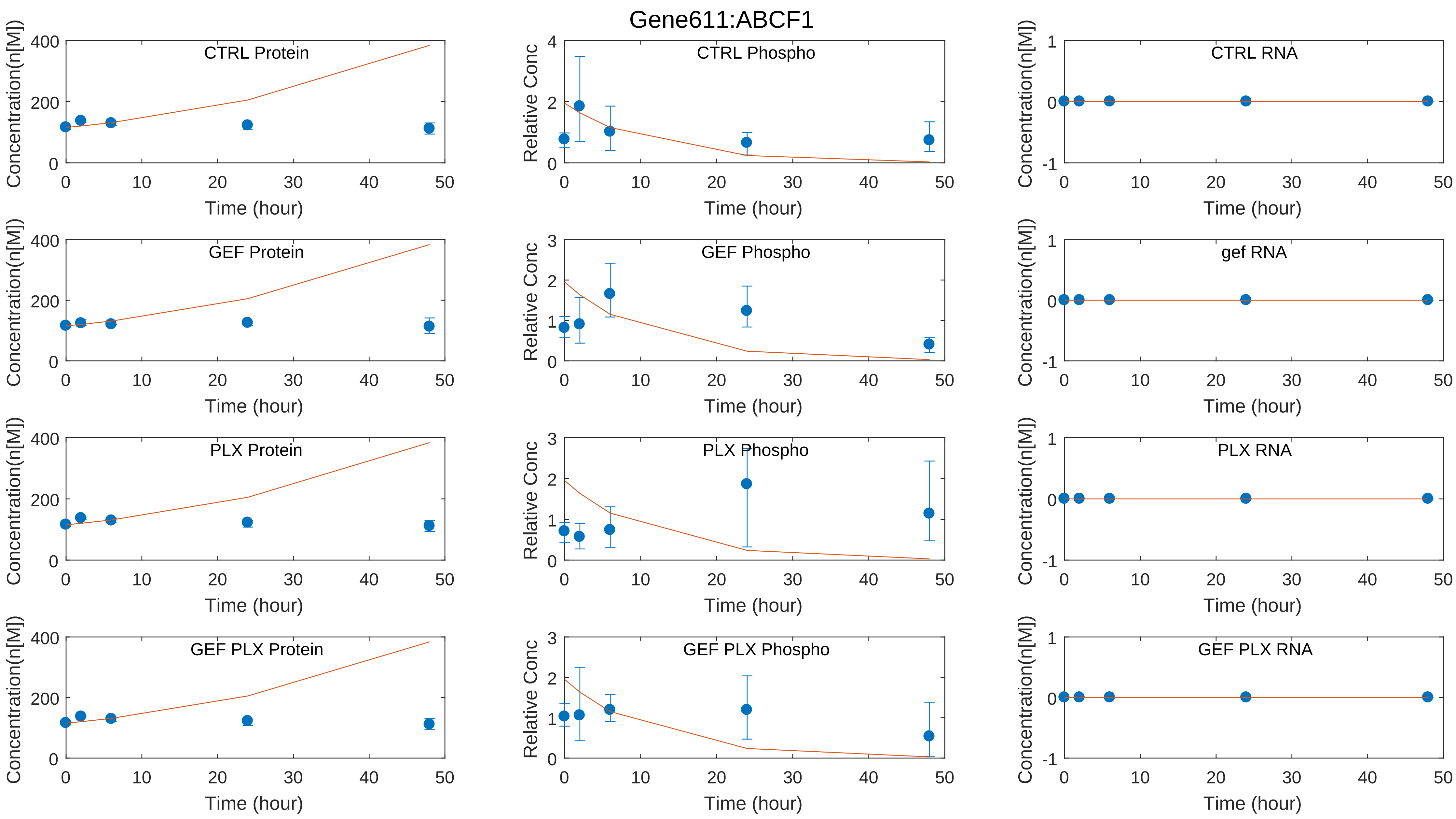
Gene611:ABCF1
CTRL Protein
CTRL Phospho
CTRL RNA
GEF Protein
GEF Phospho
gef RNA
PLX Protein
PLX Phospho
PLX RNA
GEF PLX Protein
GEF PLX Phospho
GEF PLX RNA
Concentration(n[M])
Relative Conc
Time (hour)

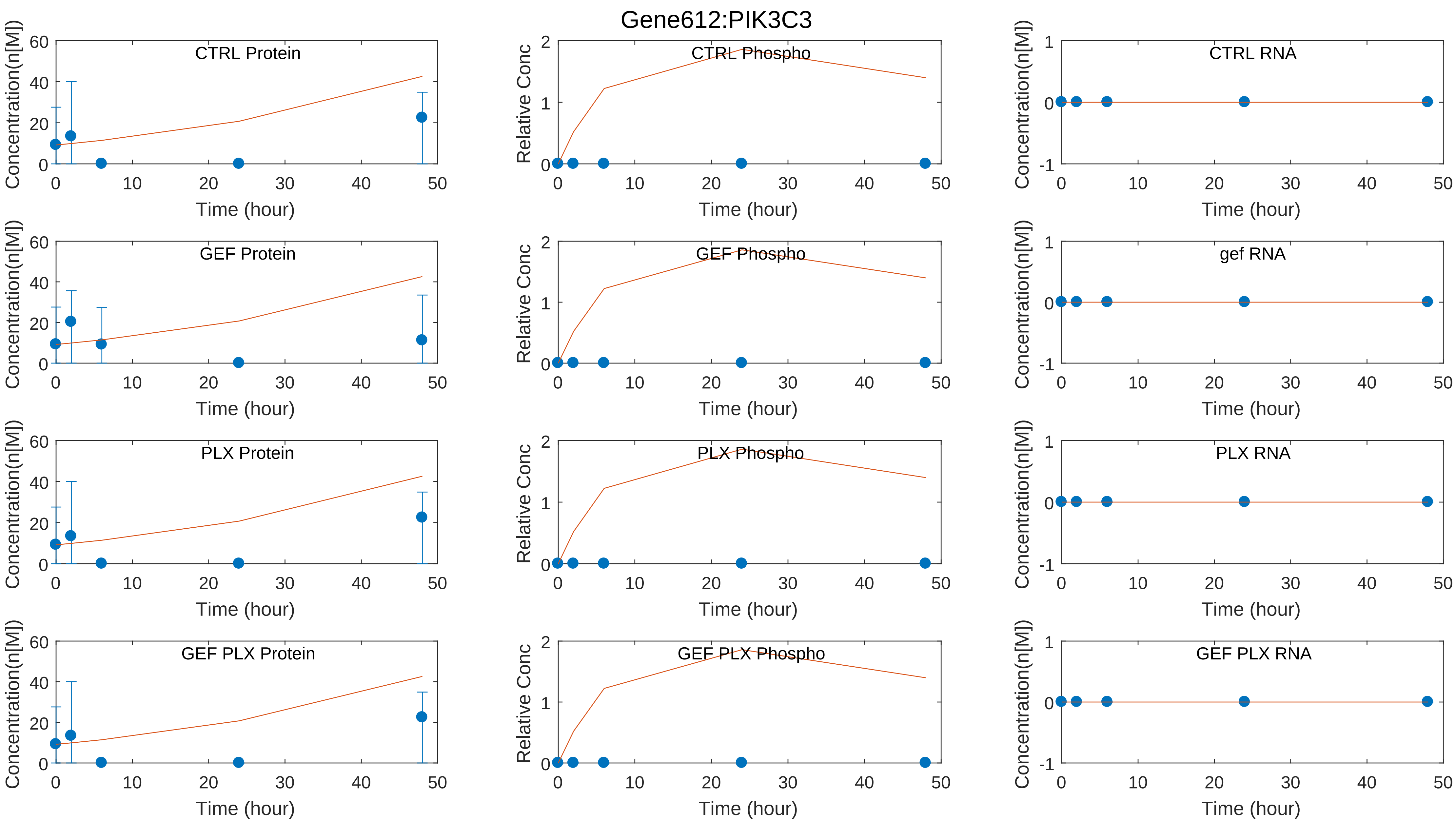
Gene612:PIK3C3
CTRL Protein
CTRL Phospho
CTRL RNA
GEF Protein
GEF Phospho
gef RNA
PLX Protein
PLX Phospho
PLX RNA
GEF PLX Protein
GEF PLX Phospho
GEF PLX RNA
Concentration(n[M])
Relative Conc
Time (hour)

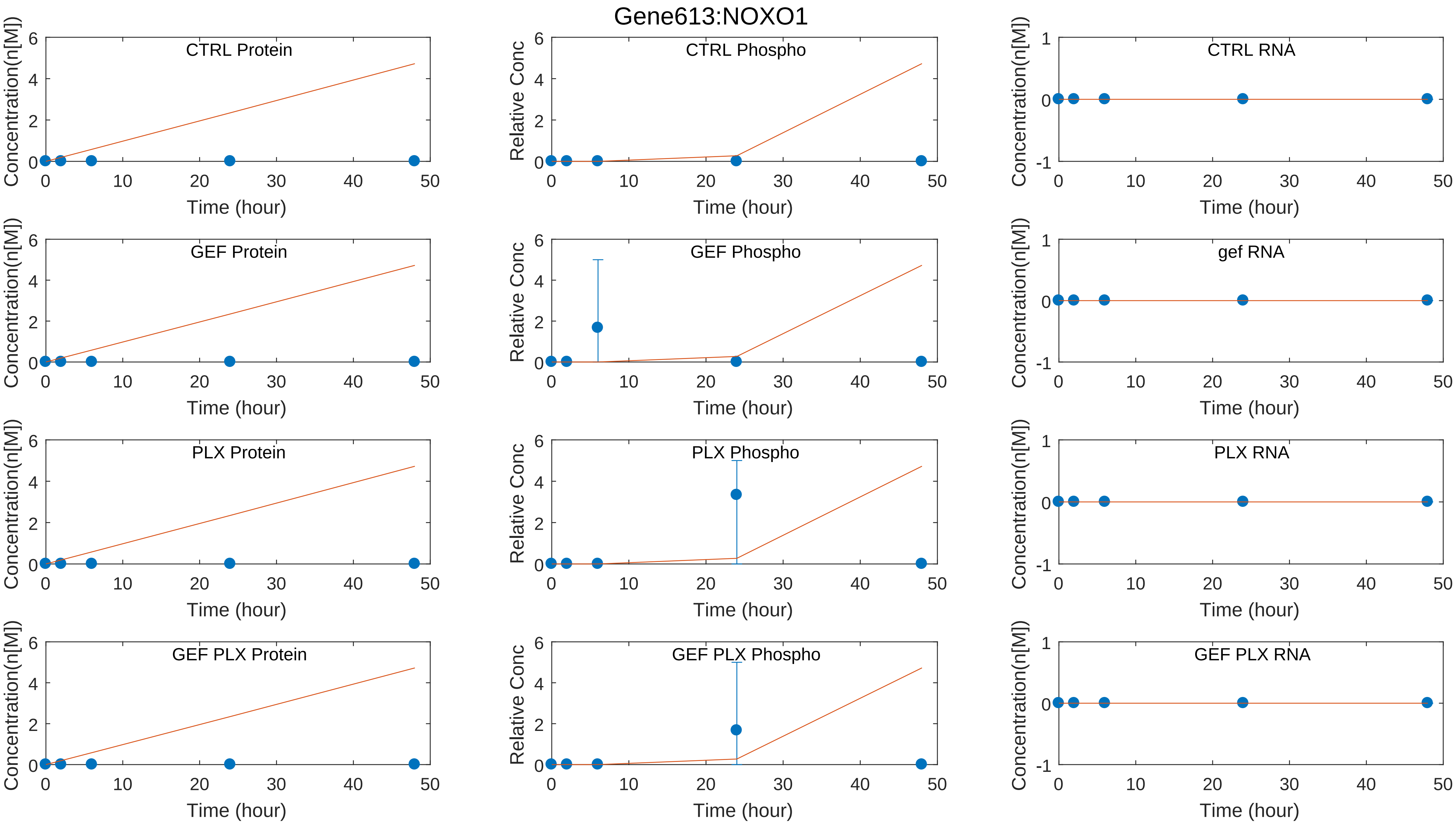

Gene613:NOXO1
CTRL Protein
CTRL Phospho
CTRL RNA
GEF Protein
GEF Phospho
gef RNA
PLX Protein
PLX Phospho
PLX RNA
GEF PLX Protein
GEF PLX Phospho
GEF PLX RNA
Concentration(n[M])
Relative Conc
Time (hour)

Gene614:MPLKIP

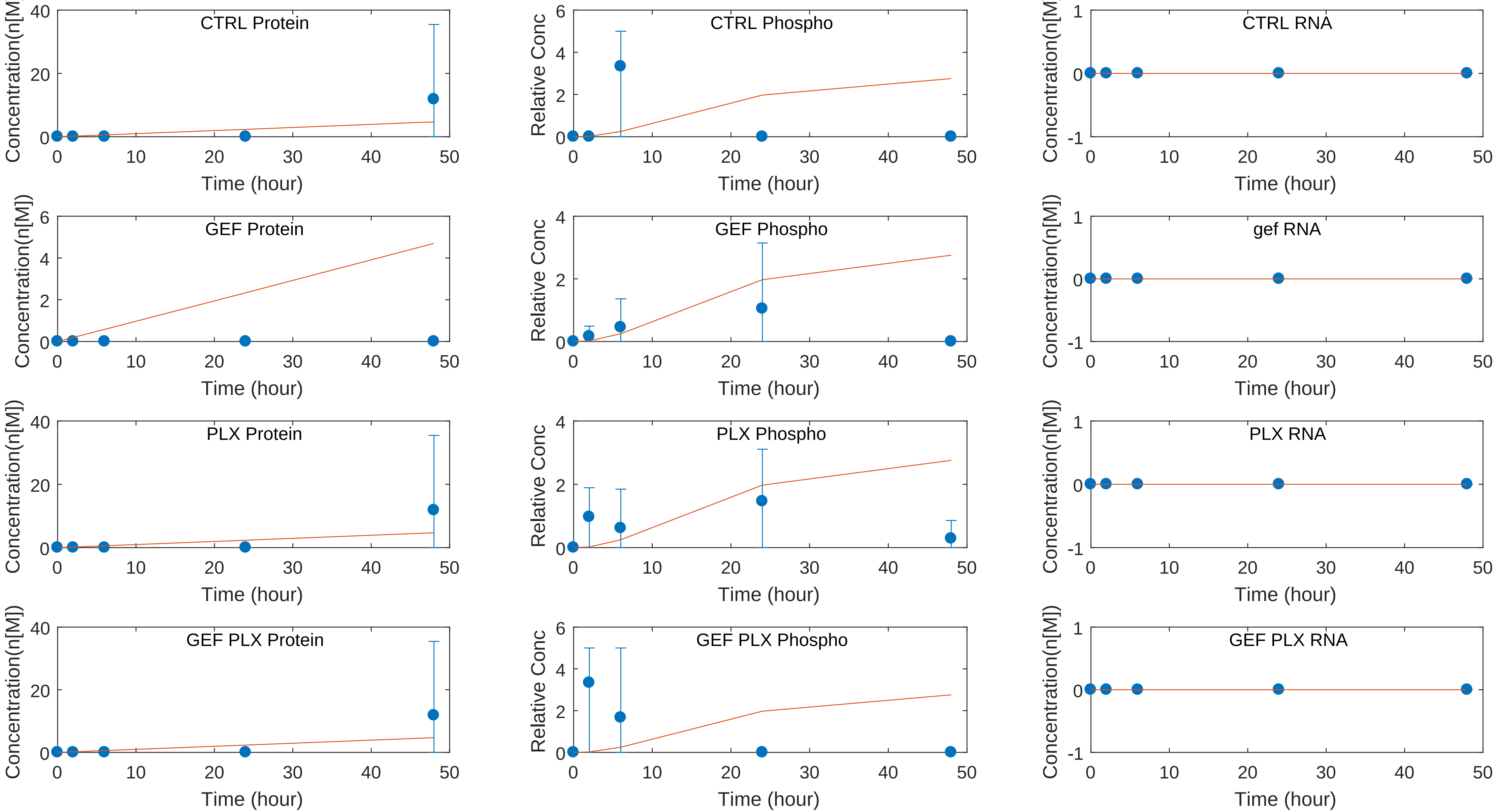

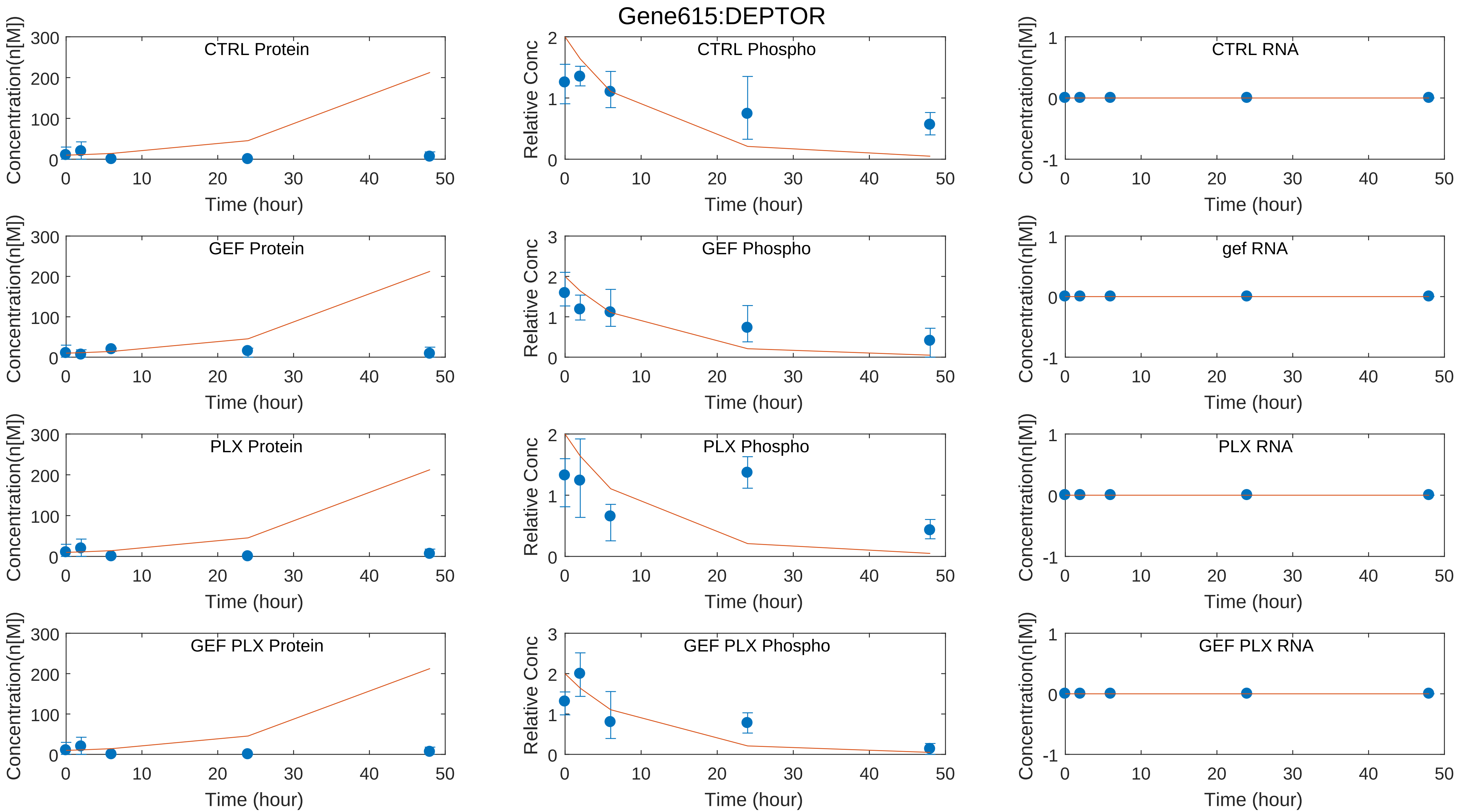

Gene615:DEPTOR
CTRL Protein
CTRL Phospho
CTRL RNA
GEF Protein
GEF Phospho
gef RNA
PLX Protein
PLX Phospho
PLX RNA
GEF PLX Protein
GEF PLX Phospho
GEF PLX RNA
Concentration(n[M])
Relative Conc
Time (hour)

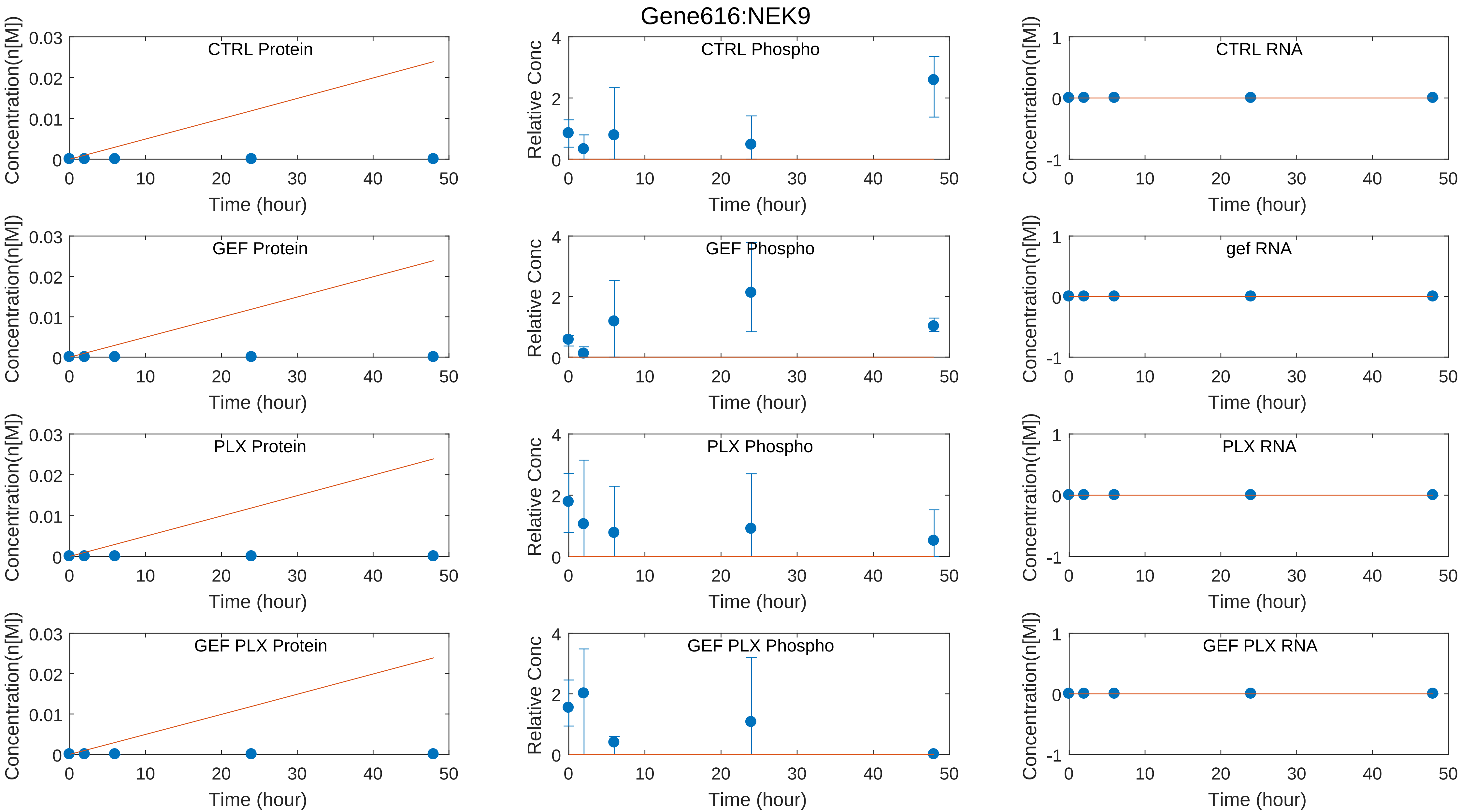

Gene616:NEK9
CTRL Protein
GEF Protein
PLX Protein
GEF PLX Protein
CTRL Phospho
GEF Phospho
PLX Phospho
GEF PLX Phospho
CTRL RNA
gef RNA
PLX RNA
GEF PLX RNA
Concentration(n[M])
Relative Conc
Time (hour)

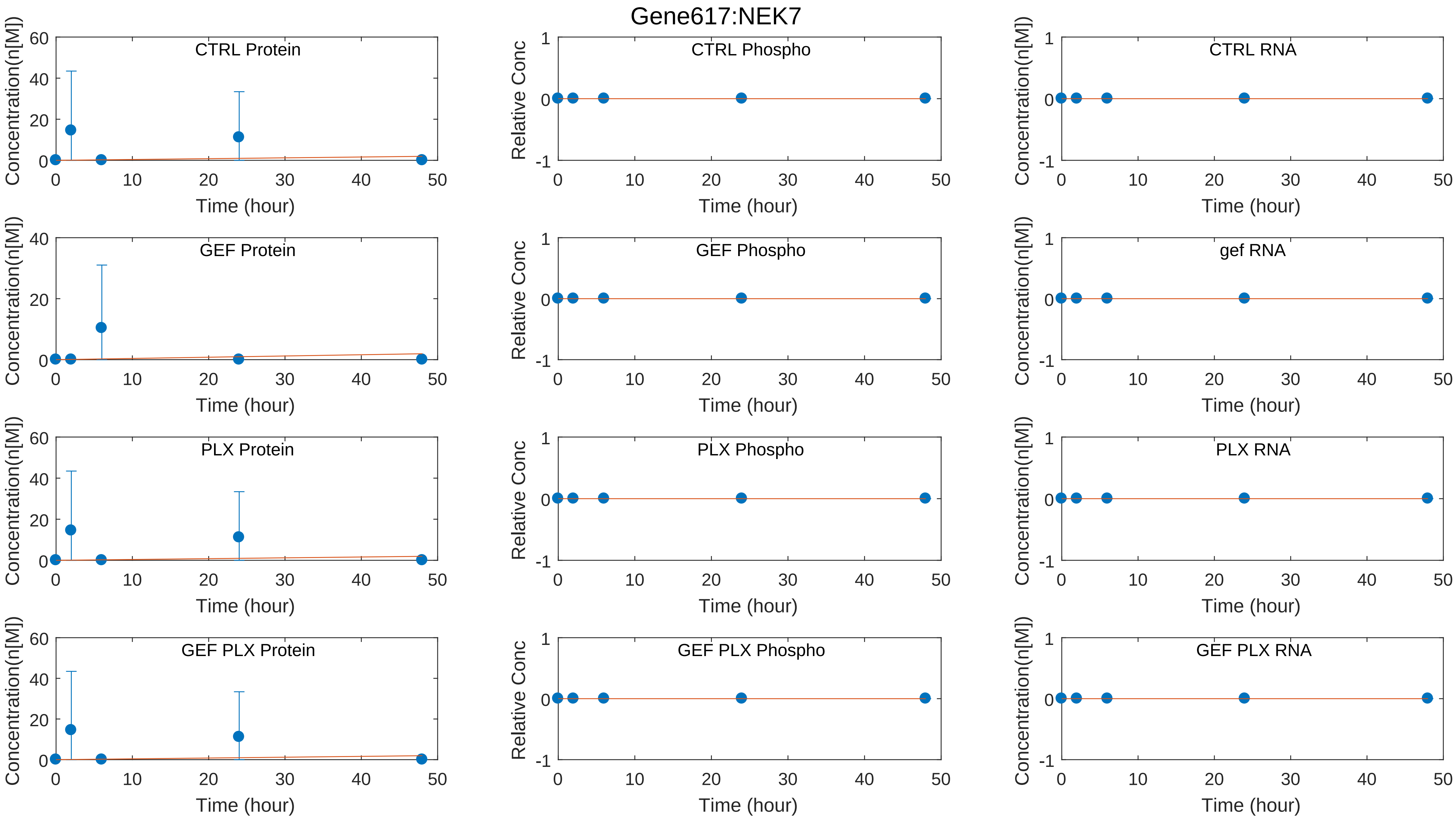

Gene617:NEK7
CTRL Protein
CTRL Phospho
CTRL RNA
GEF Protein
GEF Phospho
gef RNA
PLX Protein
PLX Phospho
PLX RNA
GEF PLX Protein
GEF PLX Phospho
GEF PLX RNA
Concentration(n[M])
Relative Conc
Time (hour)

Gene618:SSH3

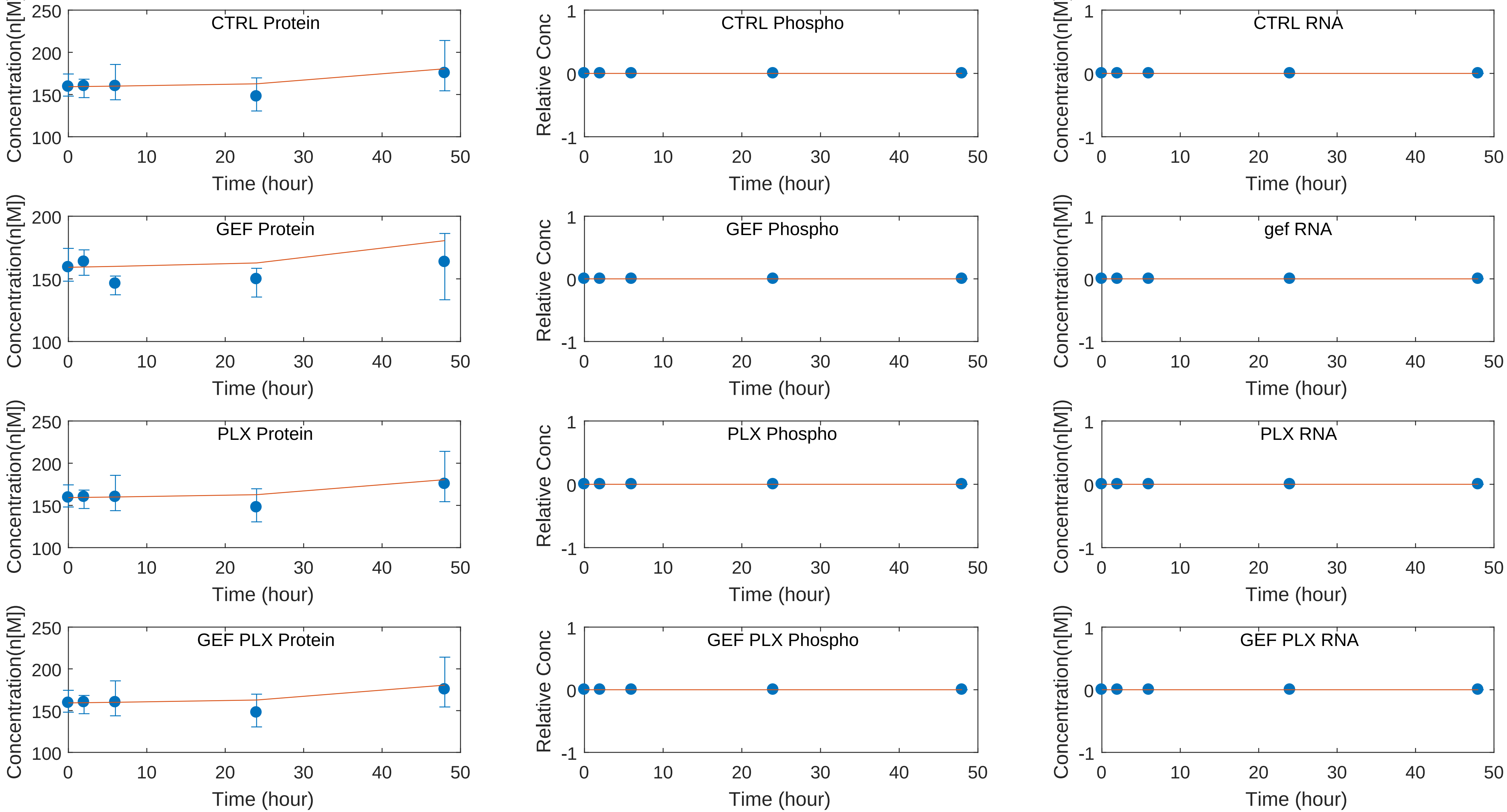

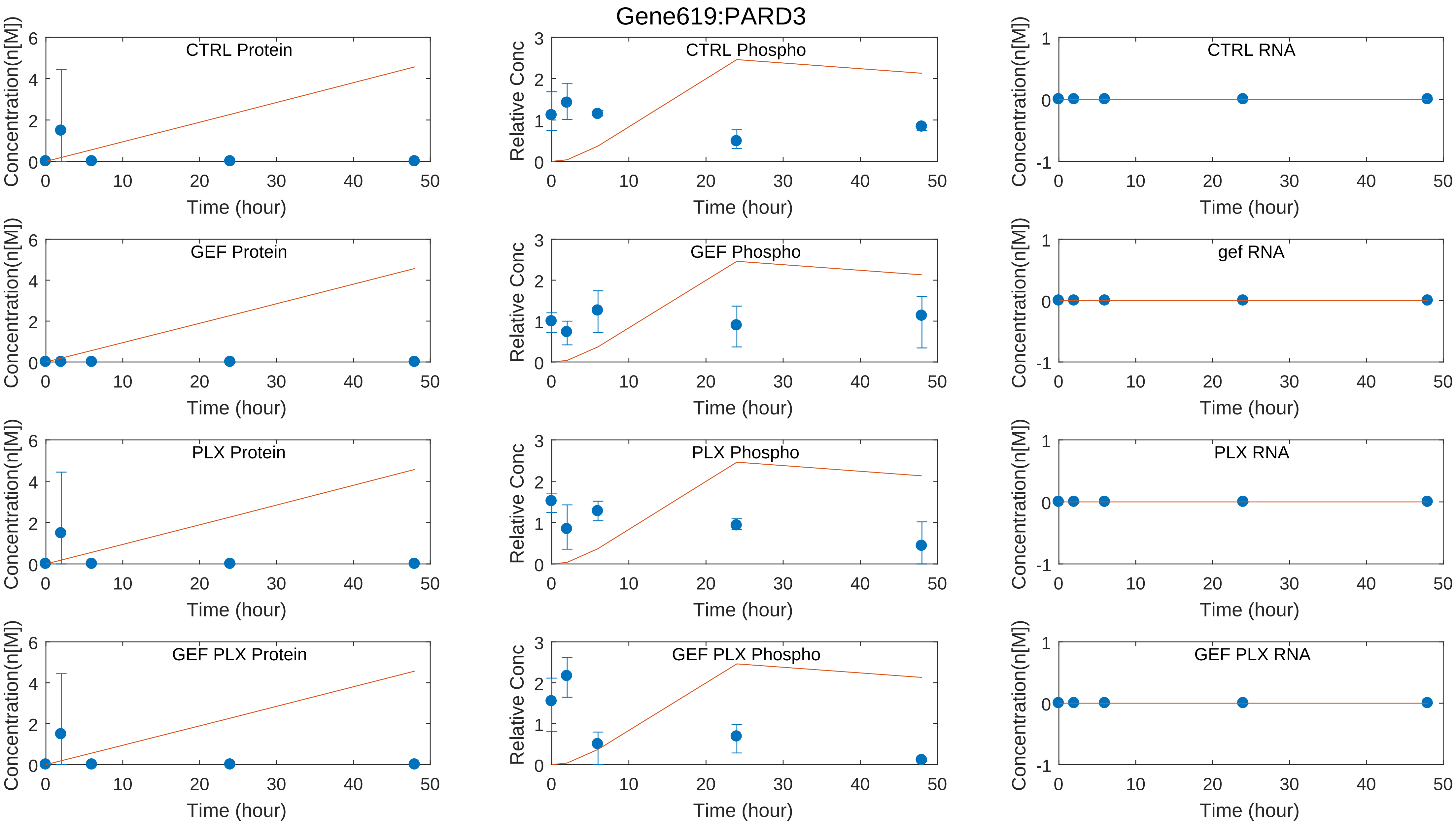
Gene619:PARD3
CTRL Protein
CTRL Phospho
CTRL RNA
GEF Protein
GEF Phospho
gef RNA
PLX Protein
PLX Phospho
PLX RNA
GEF PLX Protein
GEF PLX Phospho
GEF PLX RNA
Concentration(n[M])
Relative Conc
Time (hour)

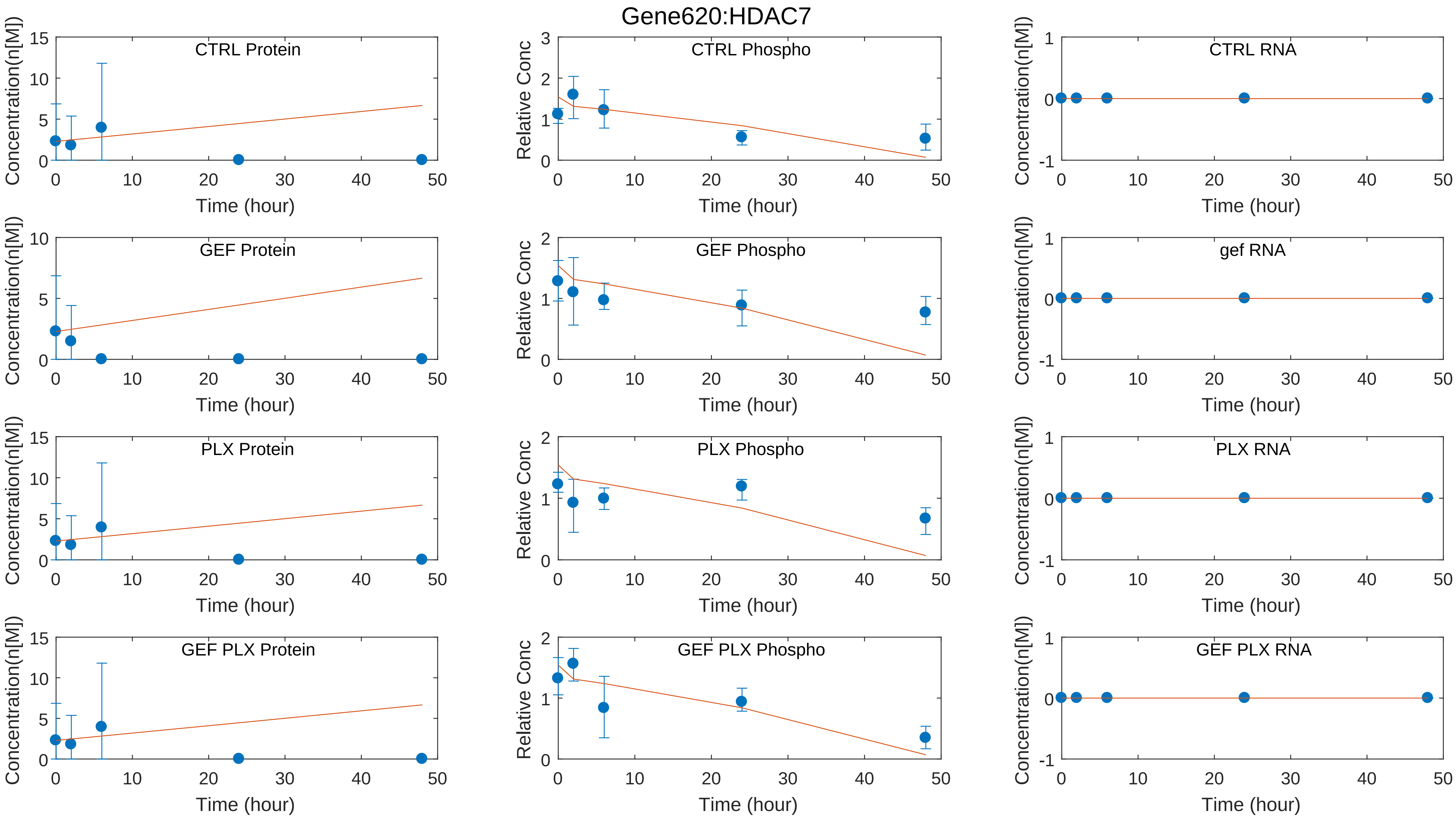

Gene620:HDAC7
CTRL Protein
CTRL Phospho
CTRL RNA
GEF Protein
GEF Phospho
gef RNA
PLX Protein
PLX Phospho
PLX RNA
GEF PLX Protein
GEF PLX Phospho
GEF PLX RNA
Concentration(n[M])
Relative Conc
Time (hour)

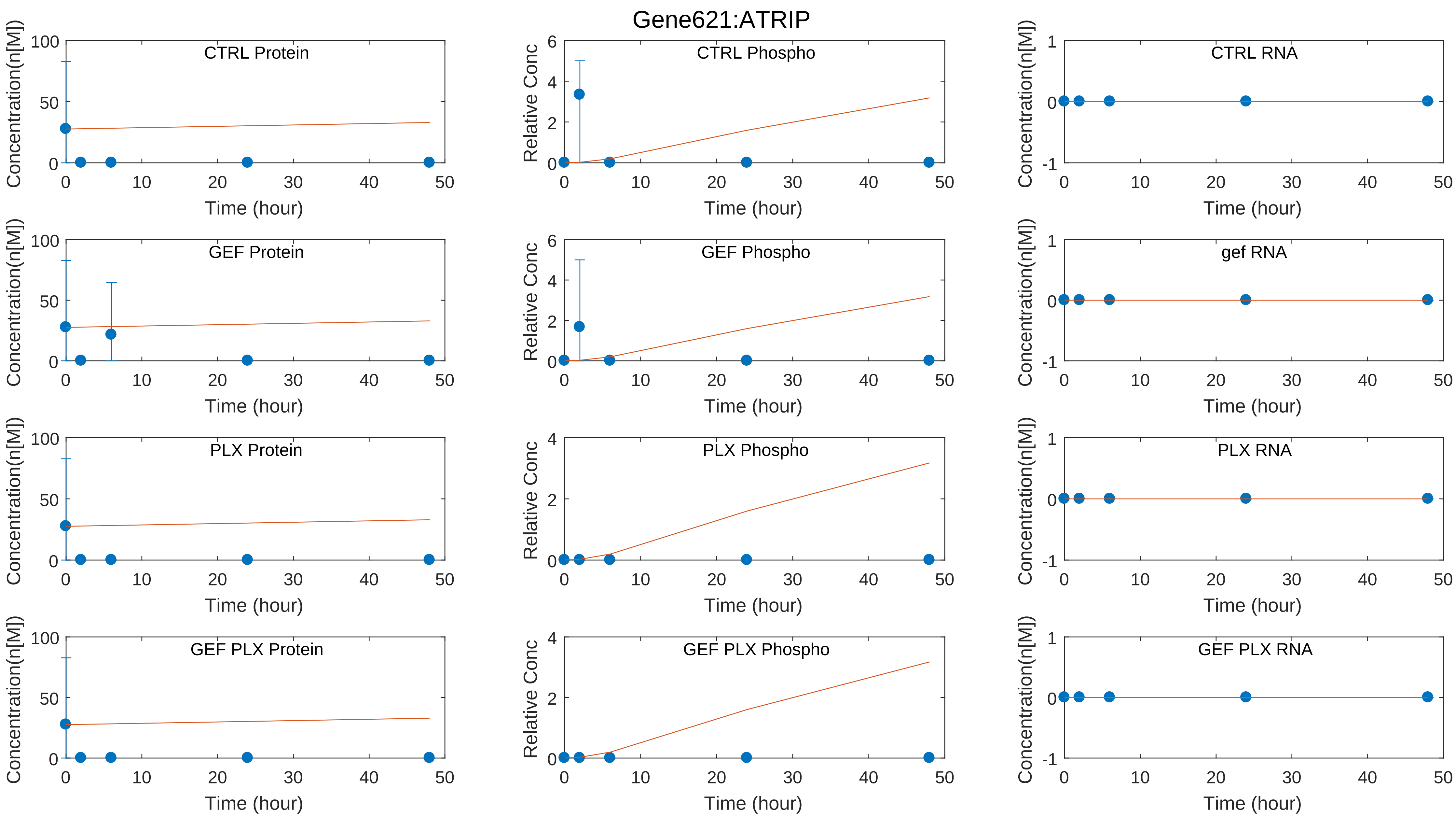
Gene621:ATRIP
CTRL Protein
CTRL Phospho
CTRL RNA
GEF Protein
GEF Phospho
gef RNA
PLX Protein
PLX Phospho
PLX RNA
GEF PLX Protein
GEF PLX Phospho
GEF PLX RNA
Concentration(n[M])
Relative Conc
Time (hour)

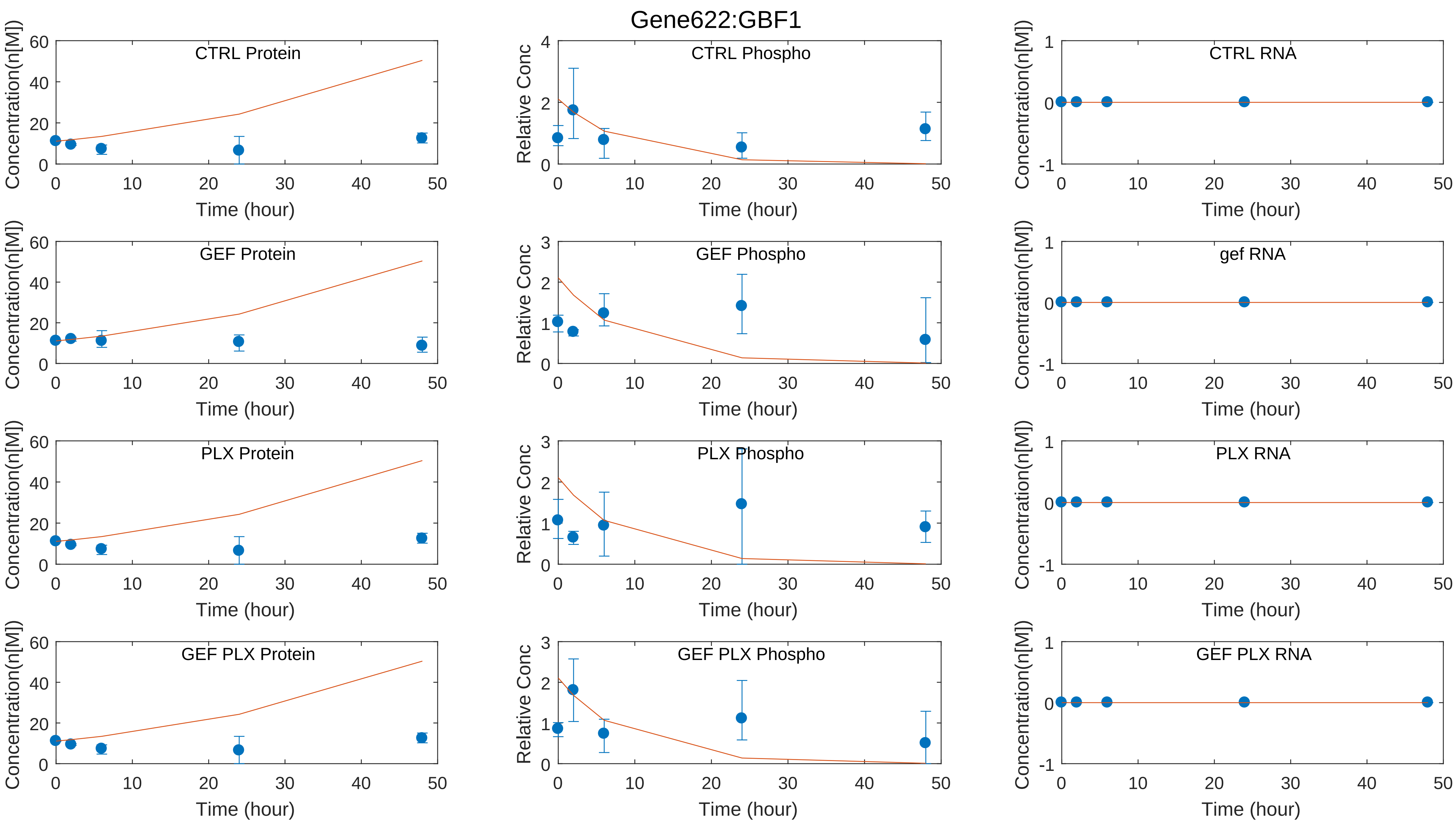
Gene622:GBF1
CTRL Protein
CTRL Phospho
CTRL RNA
GEF Protein
GEF Phospho
gef RNA
PLX Protein
PLX Phospho
PLX RNA
GEF PLX Protein
GEF PLX Phospho
GEF PLX RNA
Concentration(n[M])
Relative Conc
Time (hour)

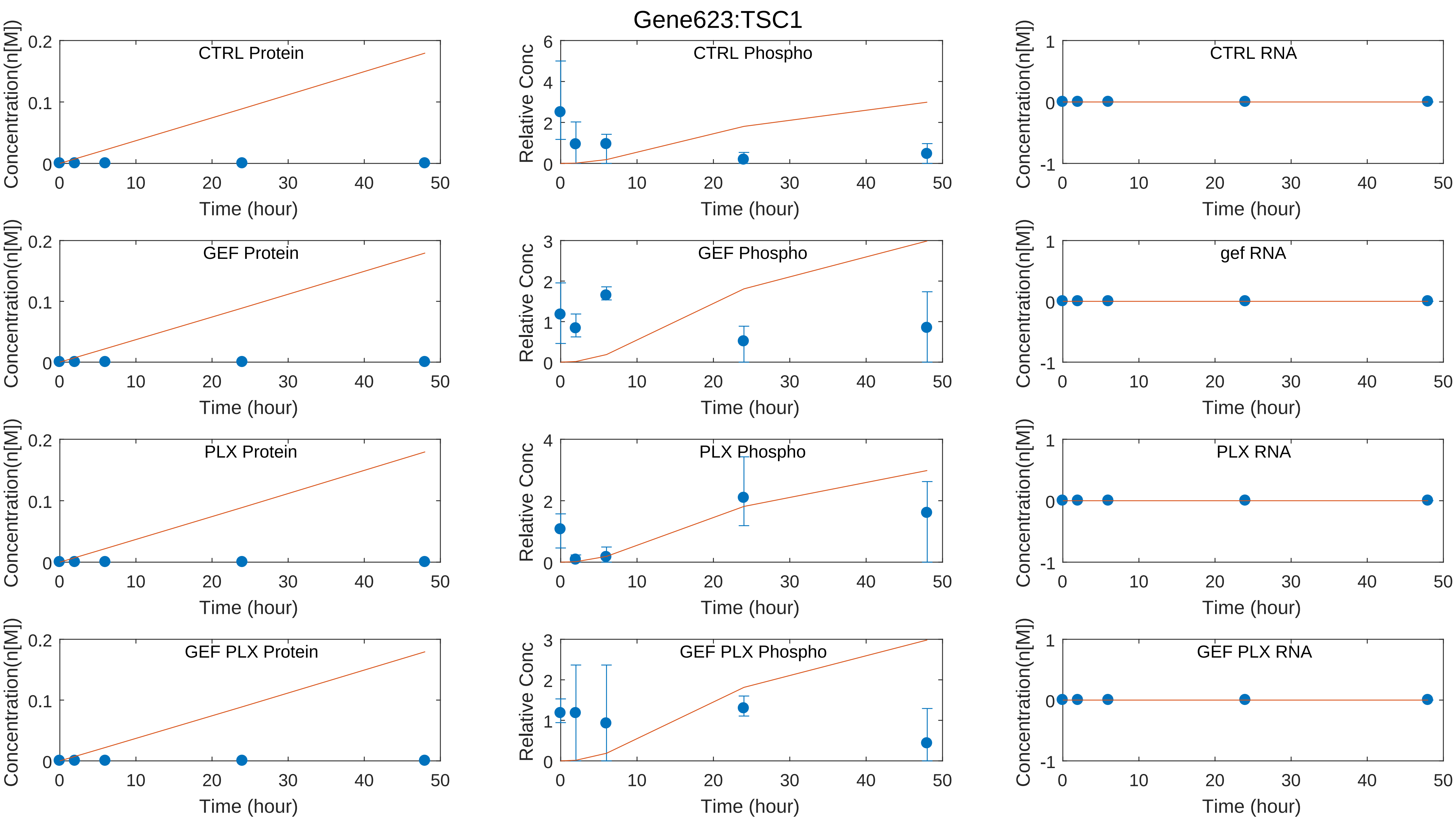

Gene623:TSC1
CTRL Protein
CTRL Phospho
CTRL RNA
GEF Protein
GEF Phospho
gef RNA
PLX Protein
PLX Phospho
PLX RNA
GEF PLX Protein
GEF PLX Phospho
GEF PLX RNA
Concentration(n[M])
Relative Conc
Time (hour)

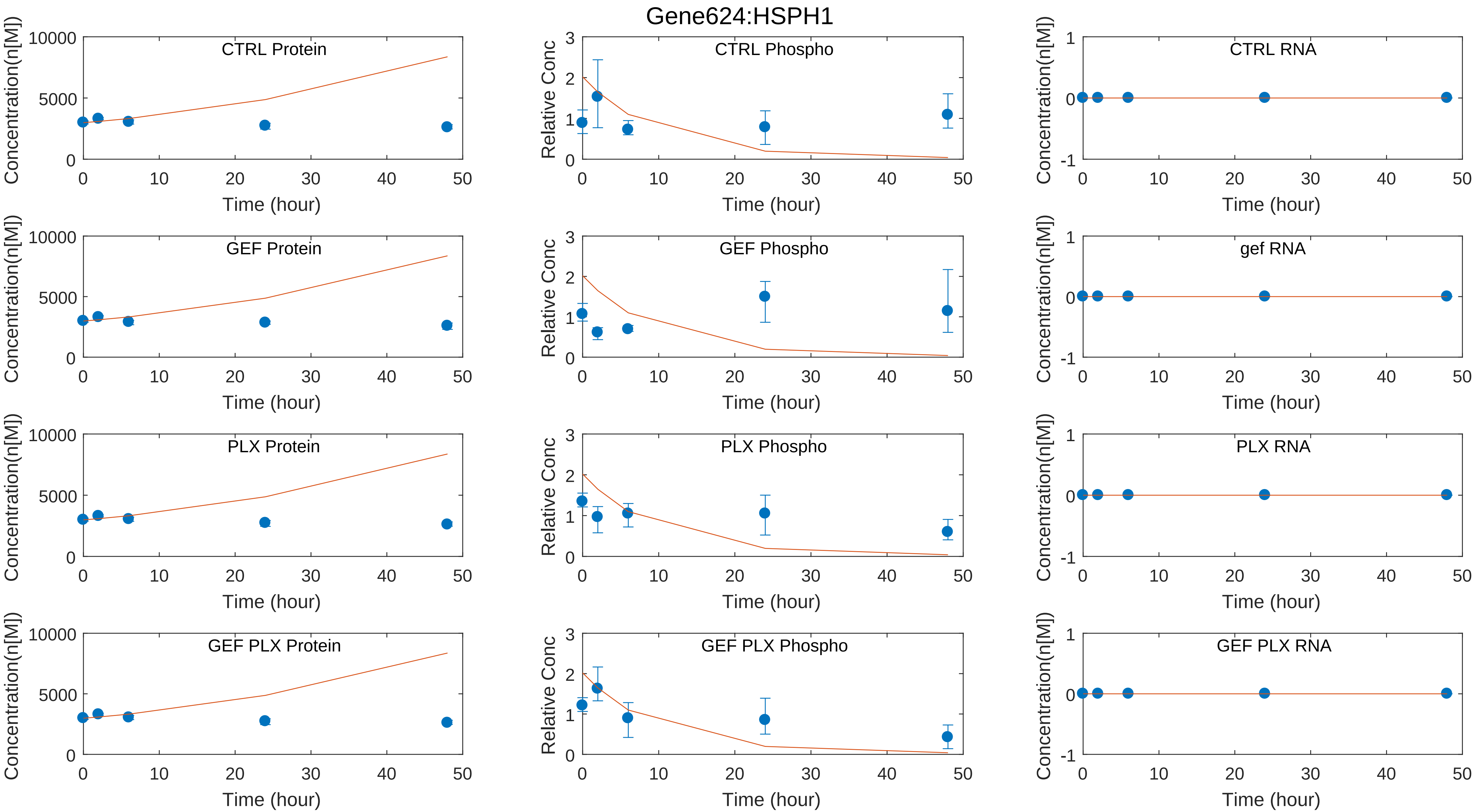

Gene624:HSPH1
CTRL Protein
CTRL Phospho
CTRL RNA
GEF Protein
GEF Phospho
gef RNA
PLX Protein
PLX Phospho
PLX RNA
GEF PLX Protein
GEF PLX Phospho
GEF PLX RNA
Concentration(n[M])
Relative Conc
Time (hour)

Gene625:NRGN

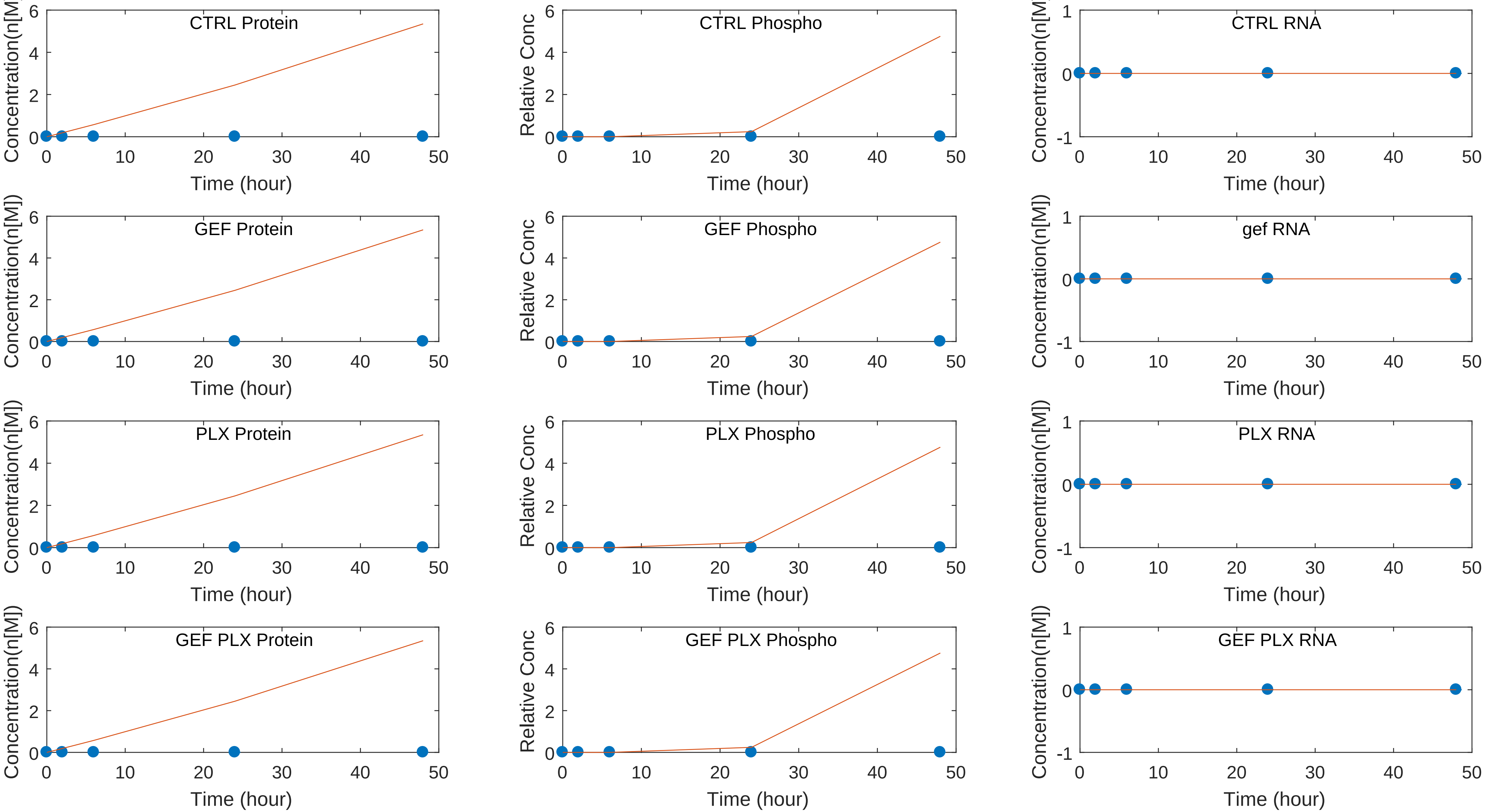

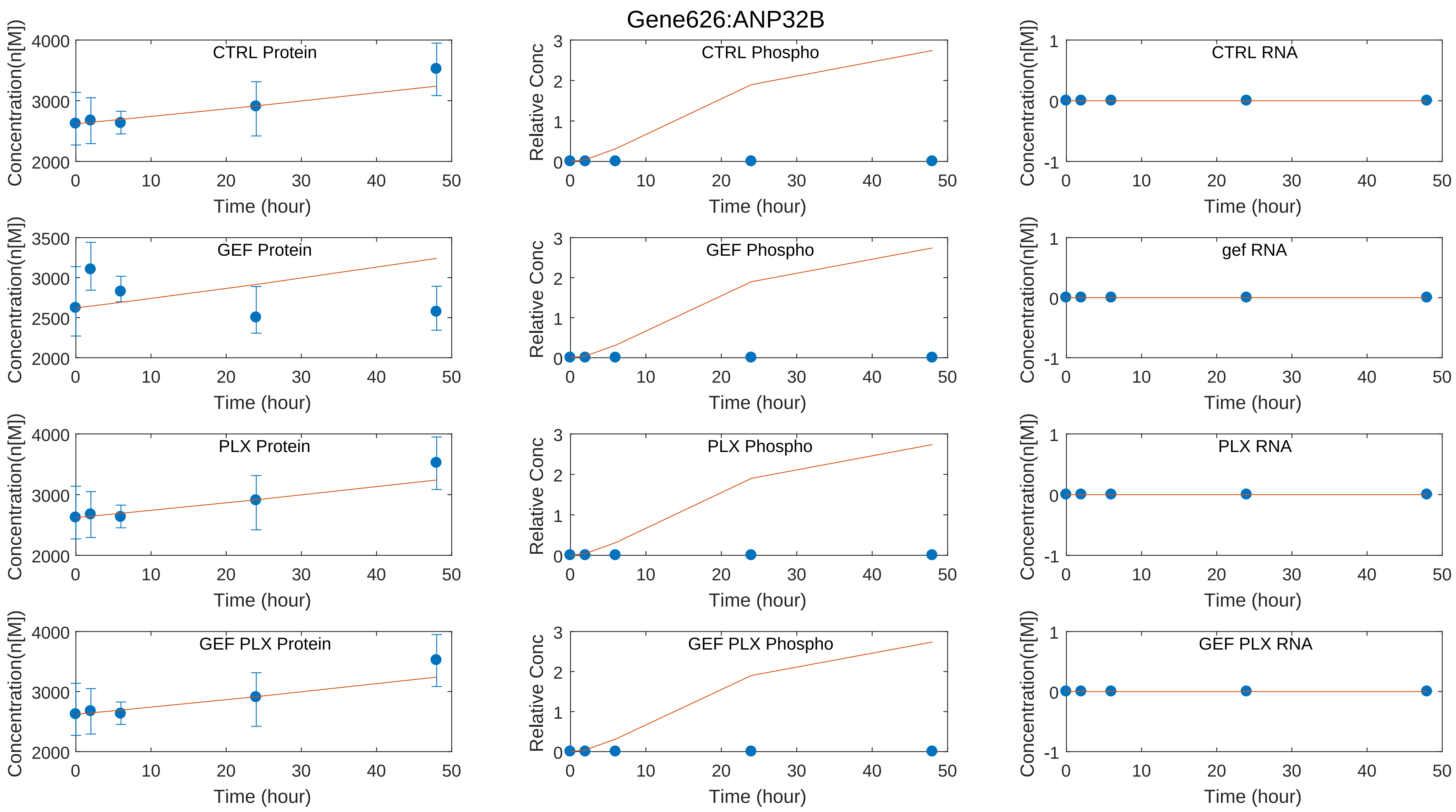
Gene626:ANP32B
CTRL Protein
Concentration(n[M])
Time (hour)
CTRL Phospho
Relative Conc
CTRL RNA
GEF Protein
GEF Phospho
gef RNA
PLX Protein
PLX Phospho
PLX RNA
GEF PLX Protein
GEF PLX Phospho
GEF PLX RNA

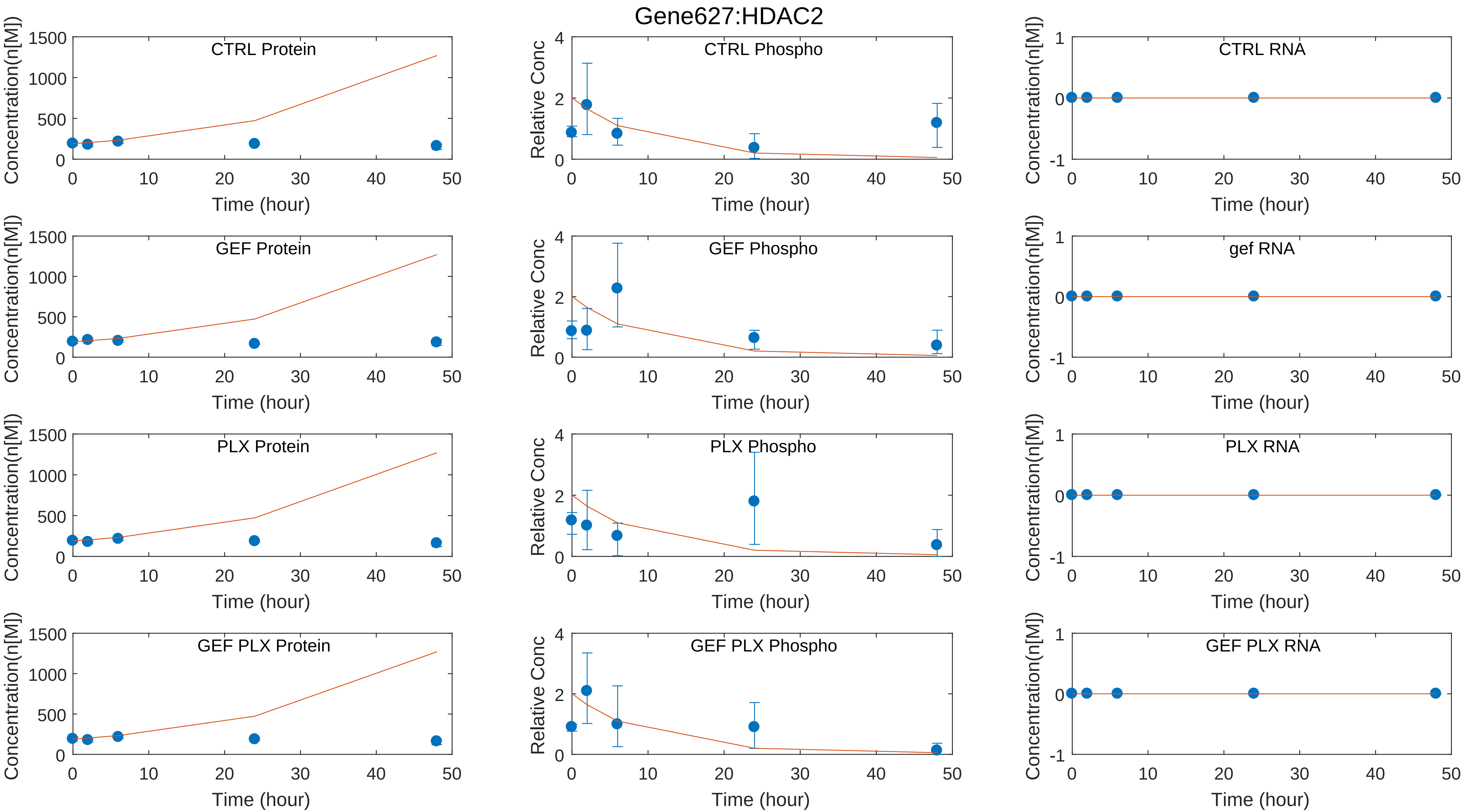

Gene627:HDAC2
CTRL Protein
GEF Protein
PLX Protein
GEF PLX Protein
CTRL Phospho
GEF Phospho
PLX Phospho
GEF PLX Phospho
CTRL RNA
gef RNA
PLX RNA
GEF PLX RNA
Concentration(n[M])
Relative Conc
Time (hour)

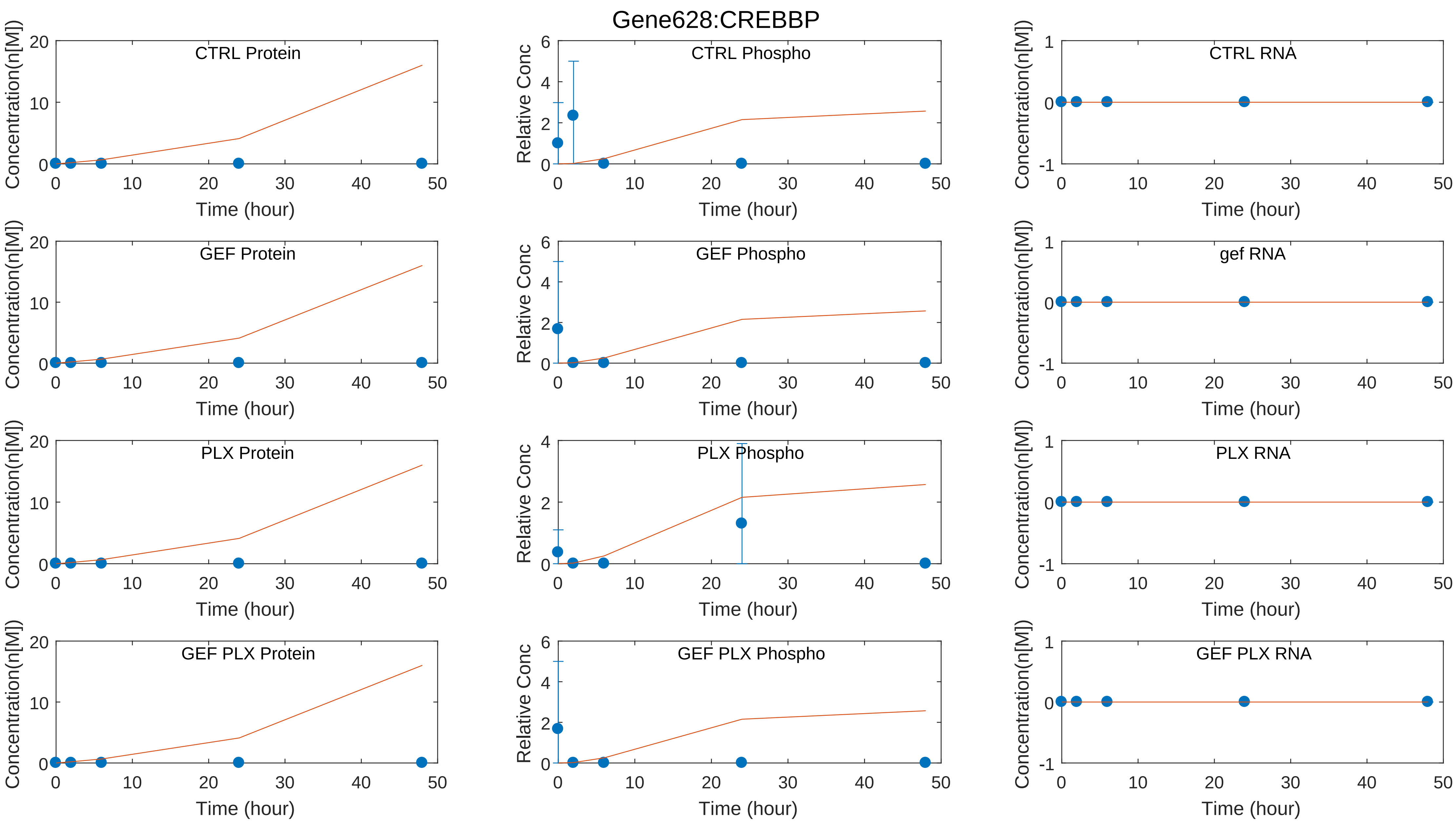
Gene628:CREBBP
CTRL Protein
CTRL Phospho
CTRL RNA
GEF Protein
GEF Phospho
gef RNA
PLX Protein
PLX Phospho
PLX RNA
GEF PLX Protein
GEF PLX Phospho
GEF PLX RNA
Concentration(n[M])
Relative Conc
Time (hour)

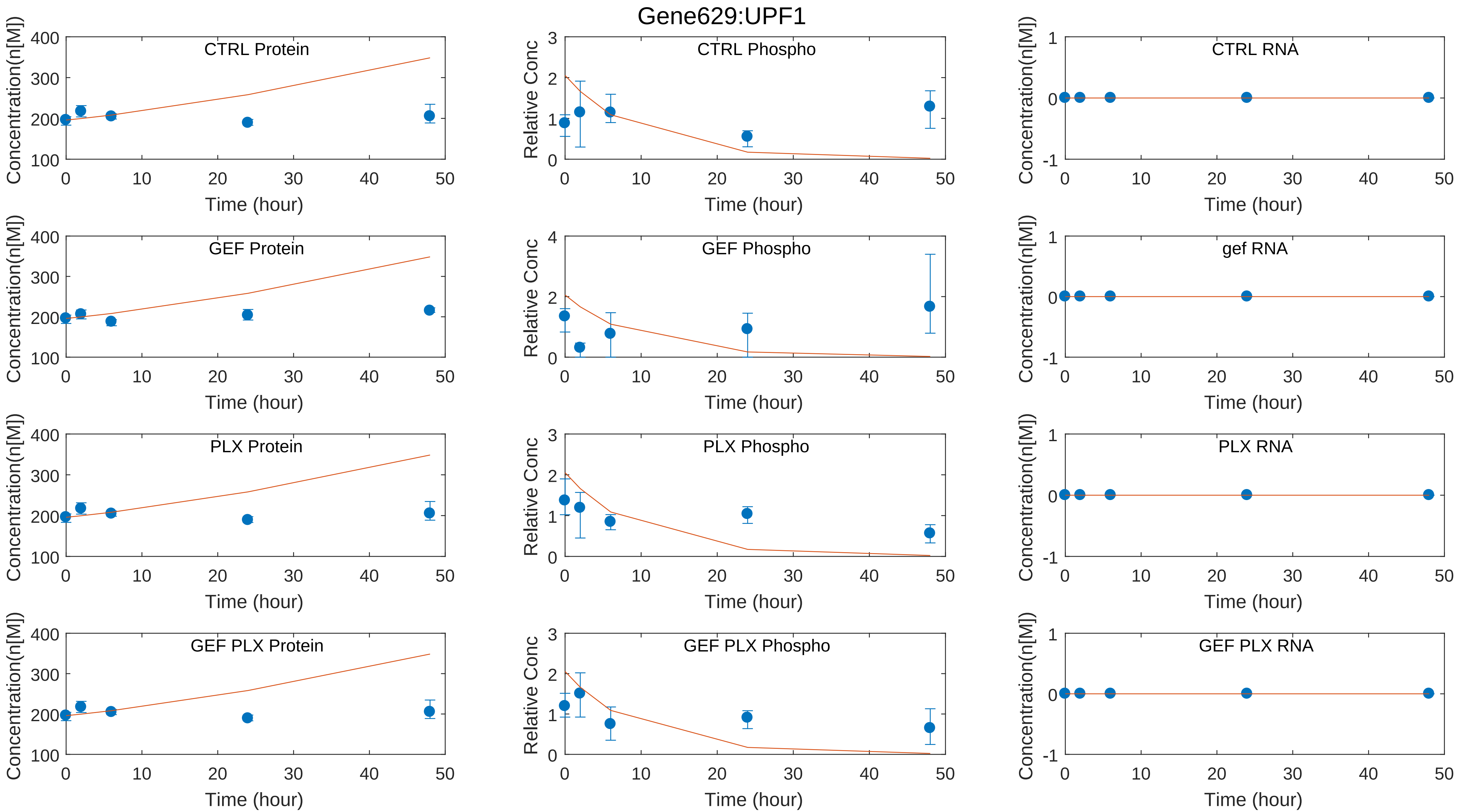

Gene629:UPF1
CTRL Protein
CTRL Phospho
CTRL RNA
GEF Protein
GEF Phospho
gef RNA
PLX Protein
PLX Phospho
PLX RNA
GEF PLX Protein
GEF PLX Phospho
GEF PLX RNA
Concentration(n[M])
Relative Conc
Time (hour)

Gene630:BAD

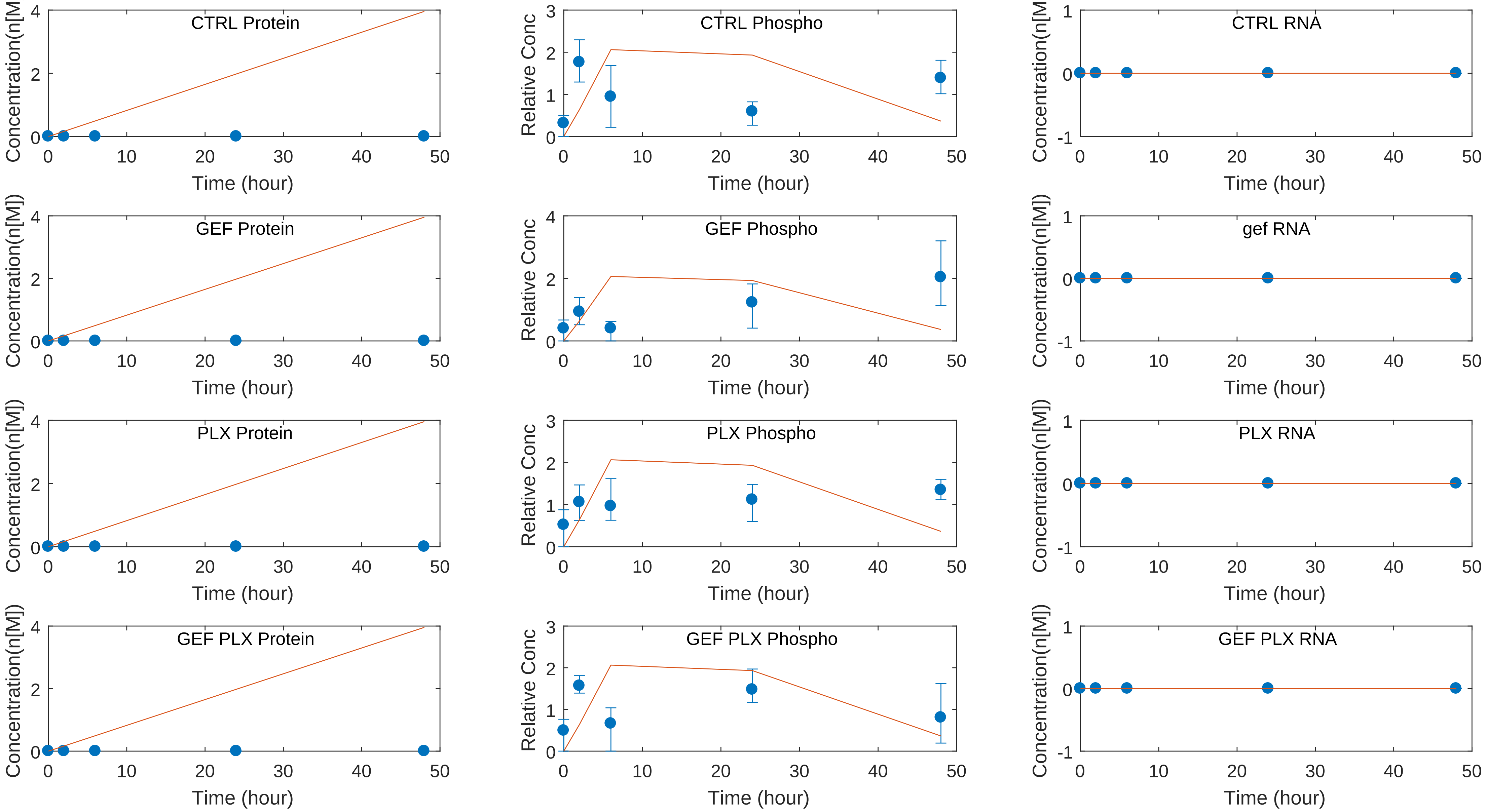
CTRL Protein
CTRL Phospho
CTRL RNA
GEF Protein
GEF Phospho
gef RNA
PLX Protein
PLX Phospho
PLX RNA
GEF PLX Protein
GEF PLX Phospho
GEF PLX RNA
Concentration(n[M])
Relative Conc
Time (hour)

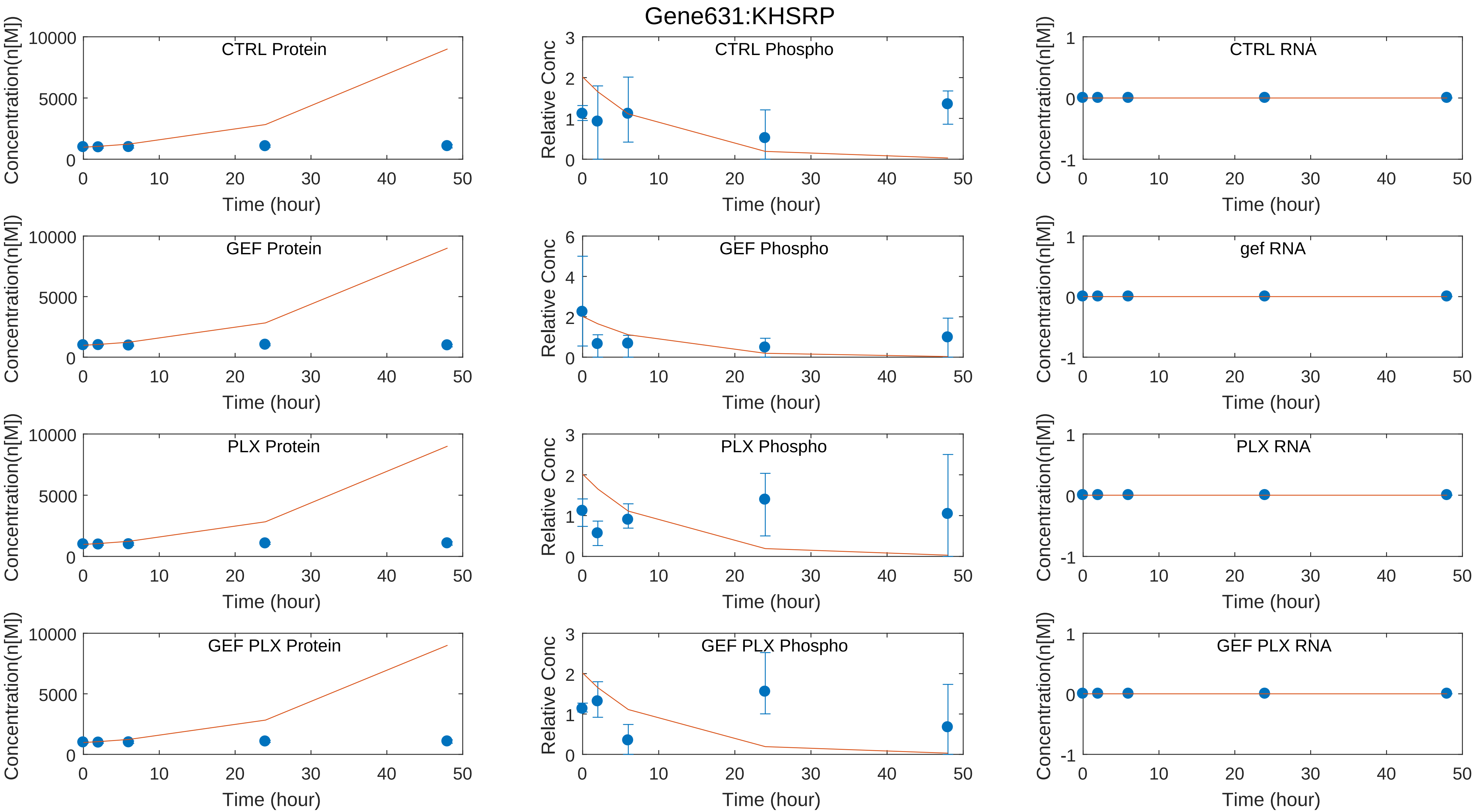

Gene631:KHSRP
CTRL Protein
CTRL Phospho
CTRL RNA
GEF Protein
GEF Phospho
gef RNA
PLX Protein
PLX Phospho
PLX RNA
GEF PLX Protein
GEF PLX Phospho
GEF PLX RNA
Concentration(n[M])
Relative Conc
Time (hour)

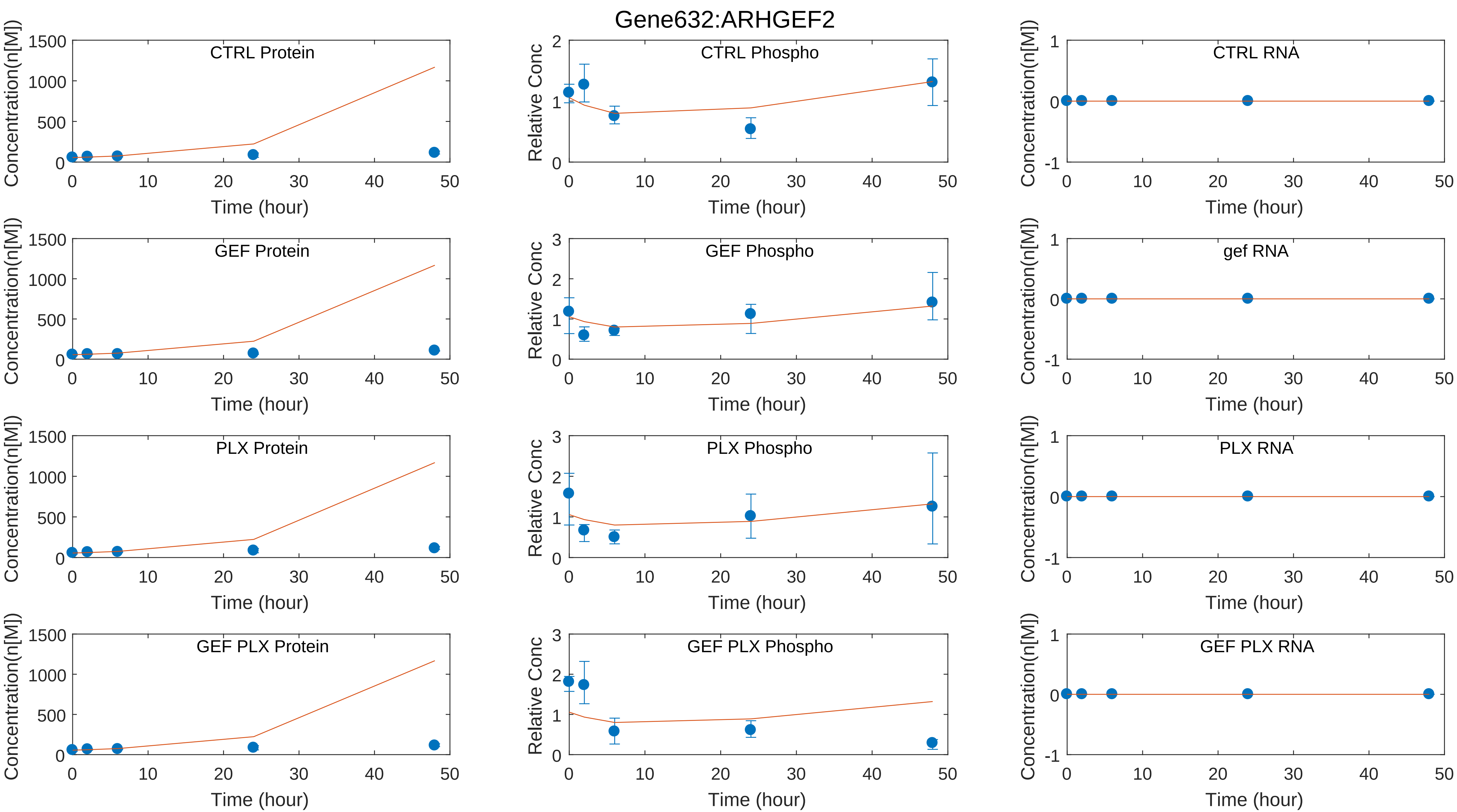

Gene632:ARHGEF2
CTRL Protein
CTRL Phospho
CTRL RNA
GEF Protein
GEF Phospho
gef RNA
PLX Protein
PLX Phospho
PLX RNA
GEF PLX Protein
GEF PLX Phospho
GEF PLX RNA
Concentration(n[M])
Relative Conc
Time (hour)

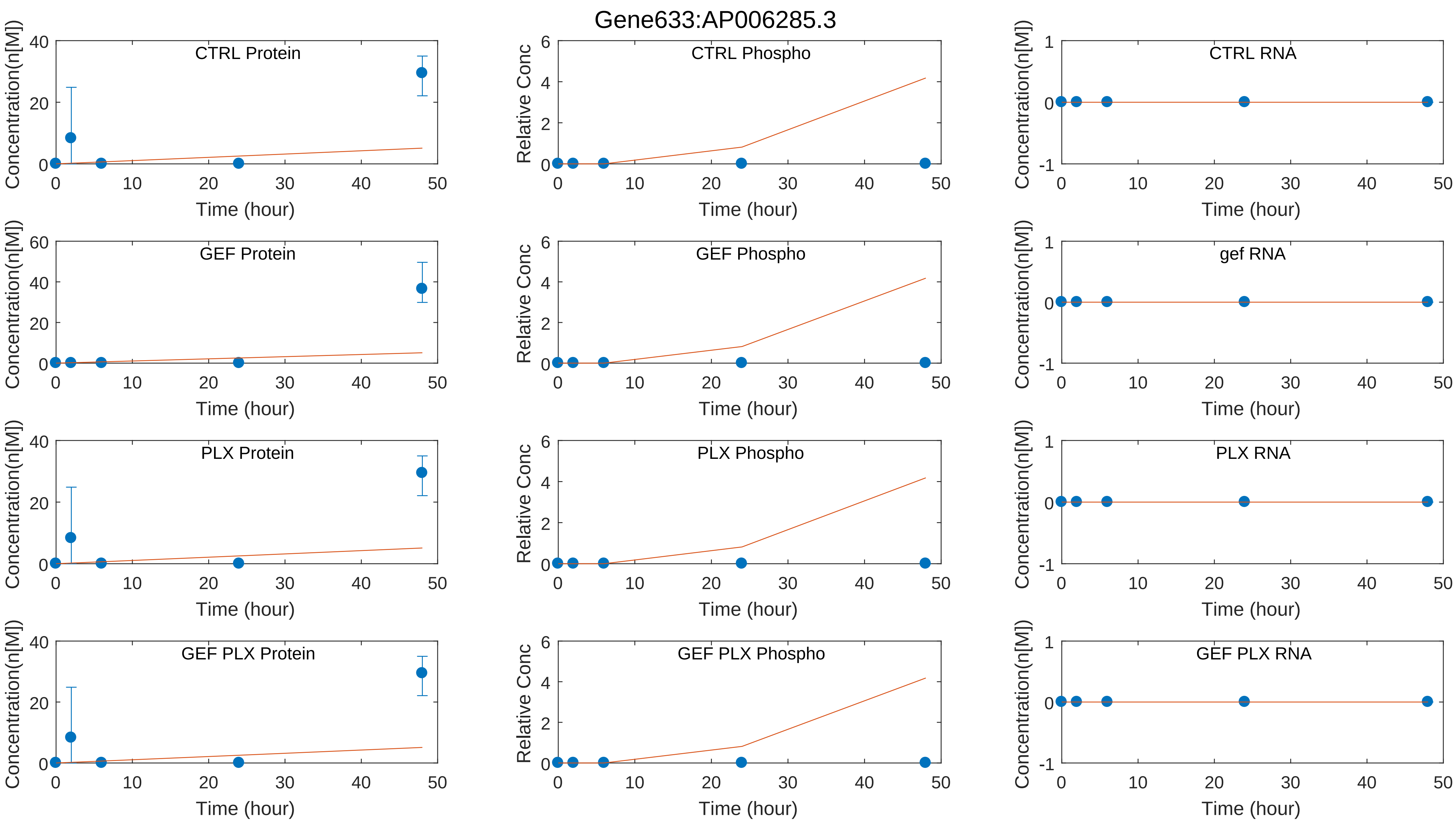
Gene633:AP006285.3
CTRL Protein
CTRL Phospho
CTRL RNA
GEF Protein
GEF Phospho
gef RNA
PLX Protein
PLX Phospho
PLX RNA
GEF PLX Protein
GEF PLX Phospho
GEF PLX RNA
Concentration(n[M])
Relative Conc
Time (hour)

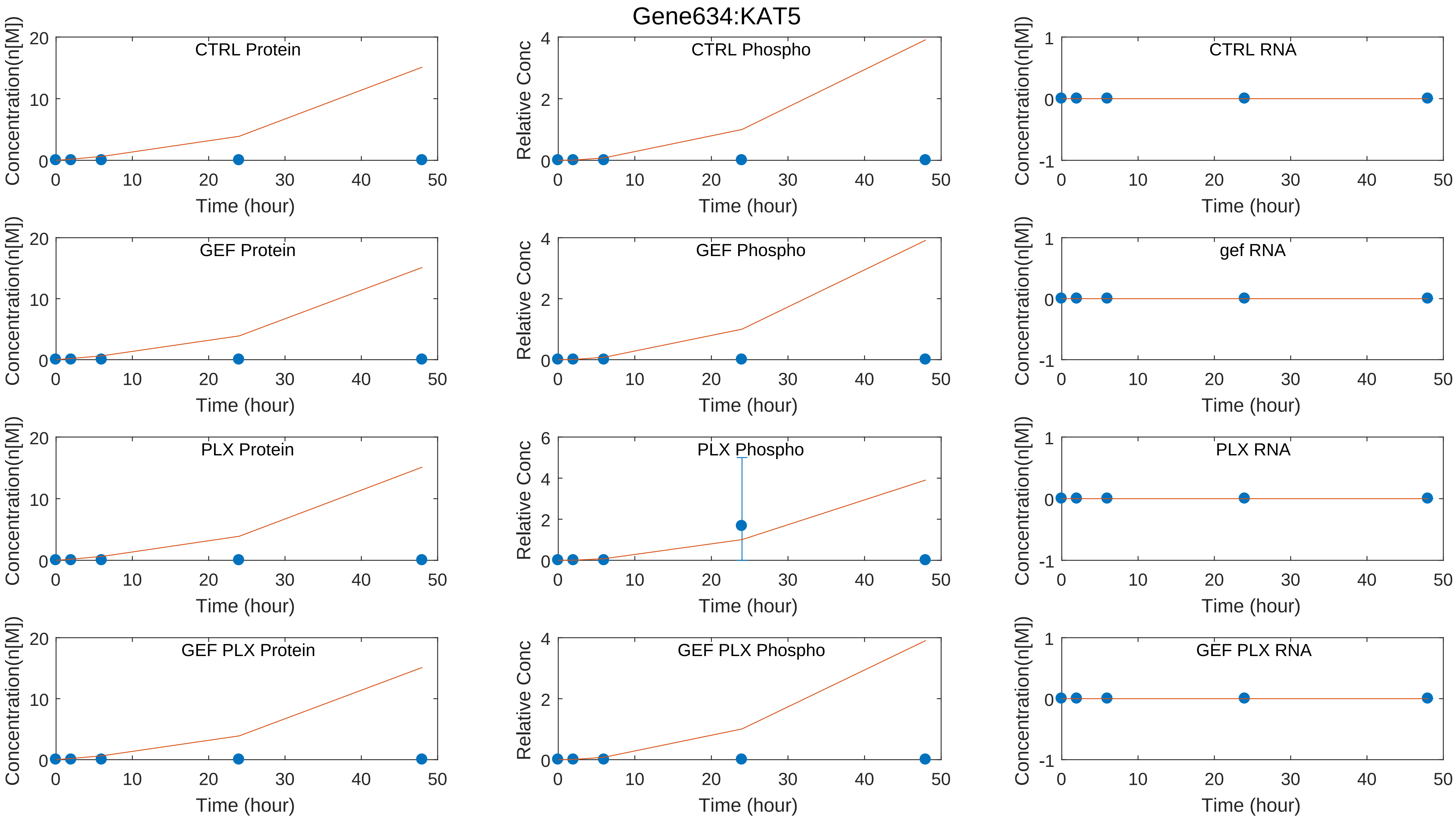

Gene634:KAT5
CTRL Protein
CTRL Phospho
CTRL RNA
GEF Protein
GEF Phospho
gef RNA
PLX Protein
PLX Phospho
PLX RNA
GEF PLX Protein
GEF PLX Phospho
GEF PLX RNA
Concentration(n[M])
Relative Conc
Time (hour)

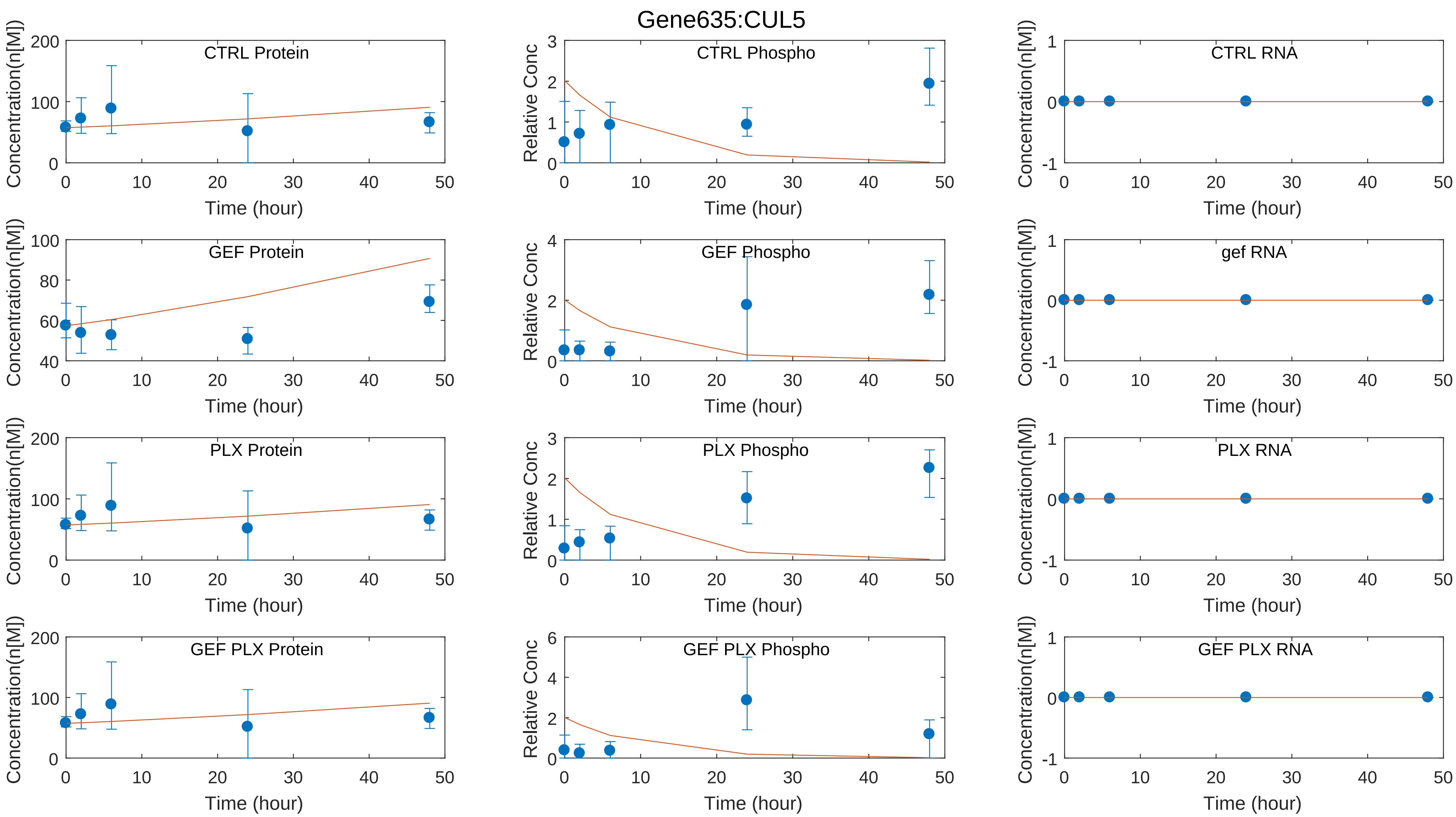

Gene635:CUL5
CTRL Protein
CTRL Phospho
CTRL RNA
GEF Protein
GEF Phospho
gef RNA
PLX Protein
PLX Phospho
PLX RNA
GEF PLX Protein
GEF PLX Phospho
GEF PLX RNA
Concentration(n[M])
Relative Conc
Time (hour)

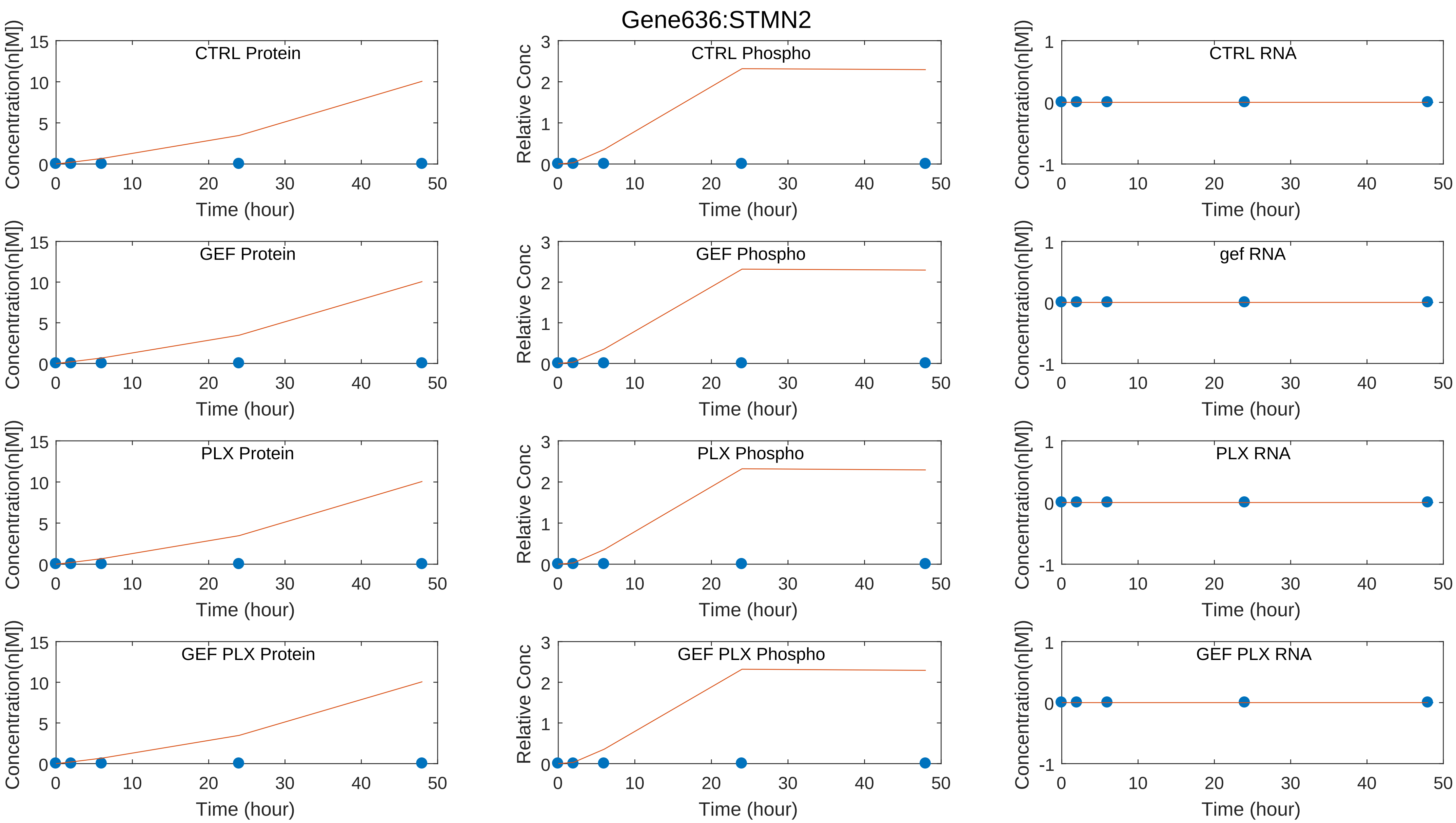

Gene636:STMN2
CTRL Protein
CTRL Phospho
CTRL RNA
GEF Protein
GEF Phospho
gef RNA
PLX Protein
PLX Phospho
PLX RNA
GEF PLX Protein
GEF PLX Phospho
GEF PLX RNA
Concentration(n[M])
Relative Conc
Time (hour)

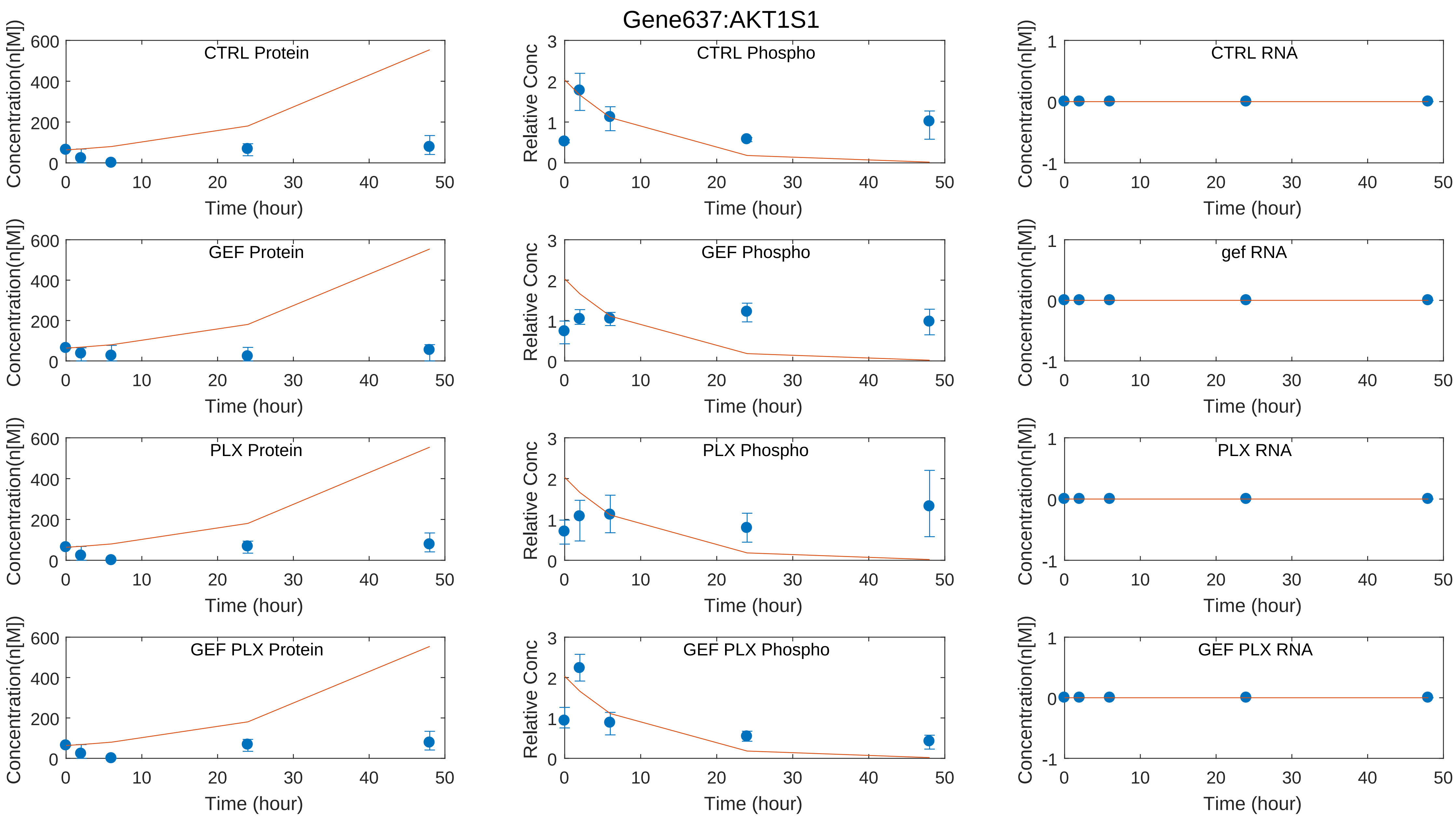

Gene637:AKT1S1
CTRL Protein
CTRL Phospho
CTRL RNA
GEF Protein
GEF Phospho
gef RNA
PLX Protein
PLX Phospho
PLX RNA
GEF PLX Protein
GEF PLX Phospho
GEF PLX RNA
Concentration(n[M])
Relative Conc
Time (hour)

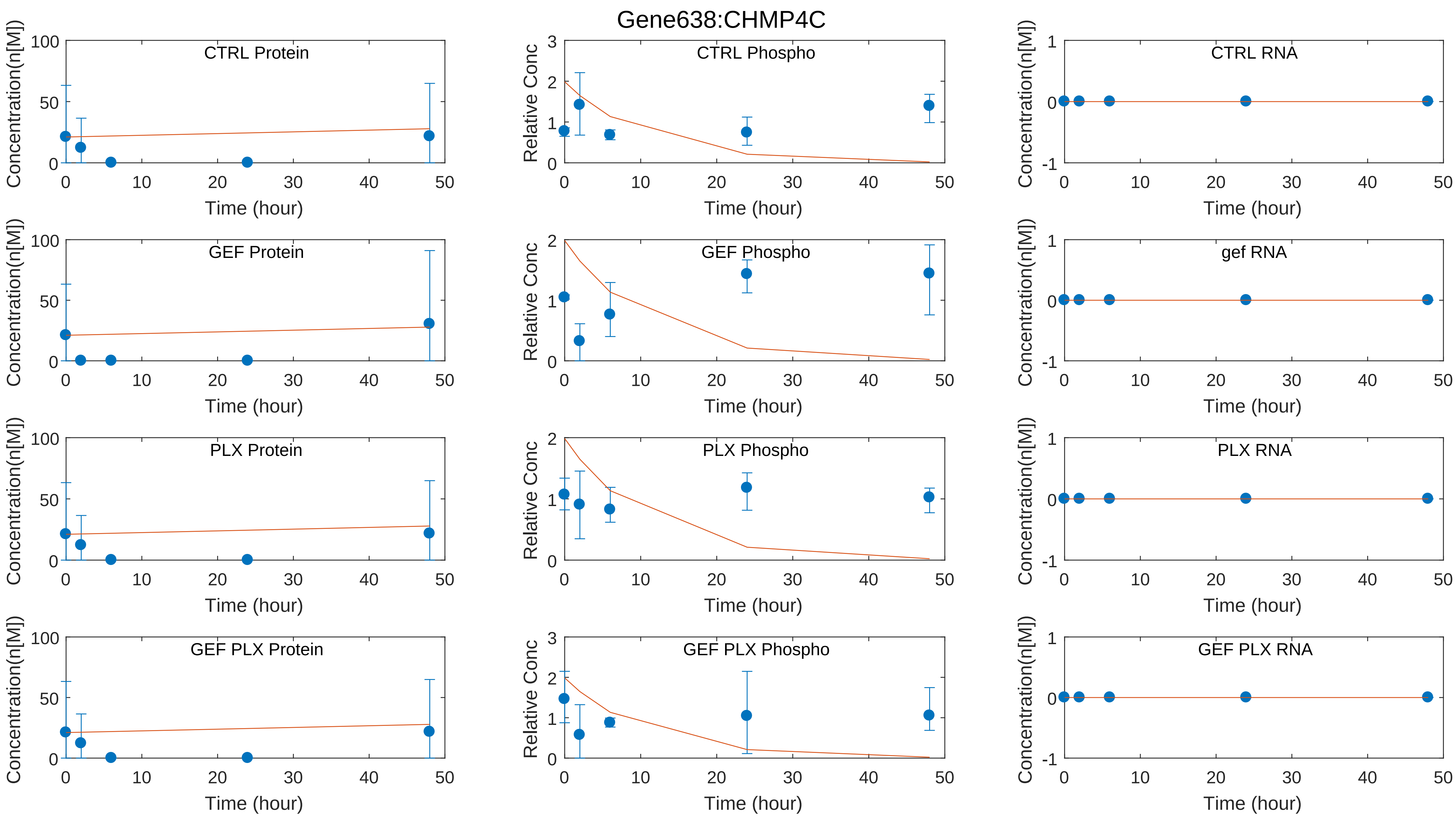

Gene638:CHMP4C
CTRL Protein
CTRL Phospho
CTRL RNA
GEF Protein
GEF Phospho
gef RNA
PLX Protein
PLX Phospho
PLX RNA
GEF PLX Protein
GEF PLX Phospho
GEF PLX RNA
Concentration(n[M])
Relative Conc
Time (hour)

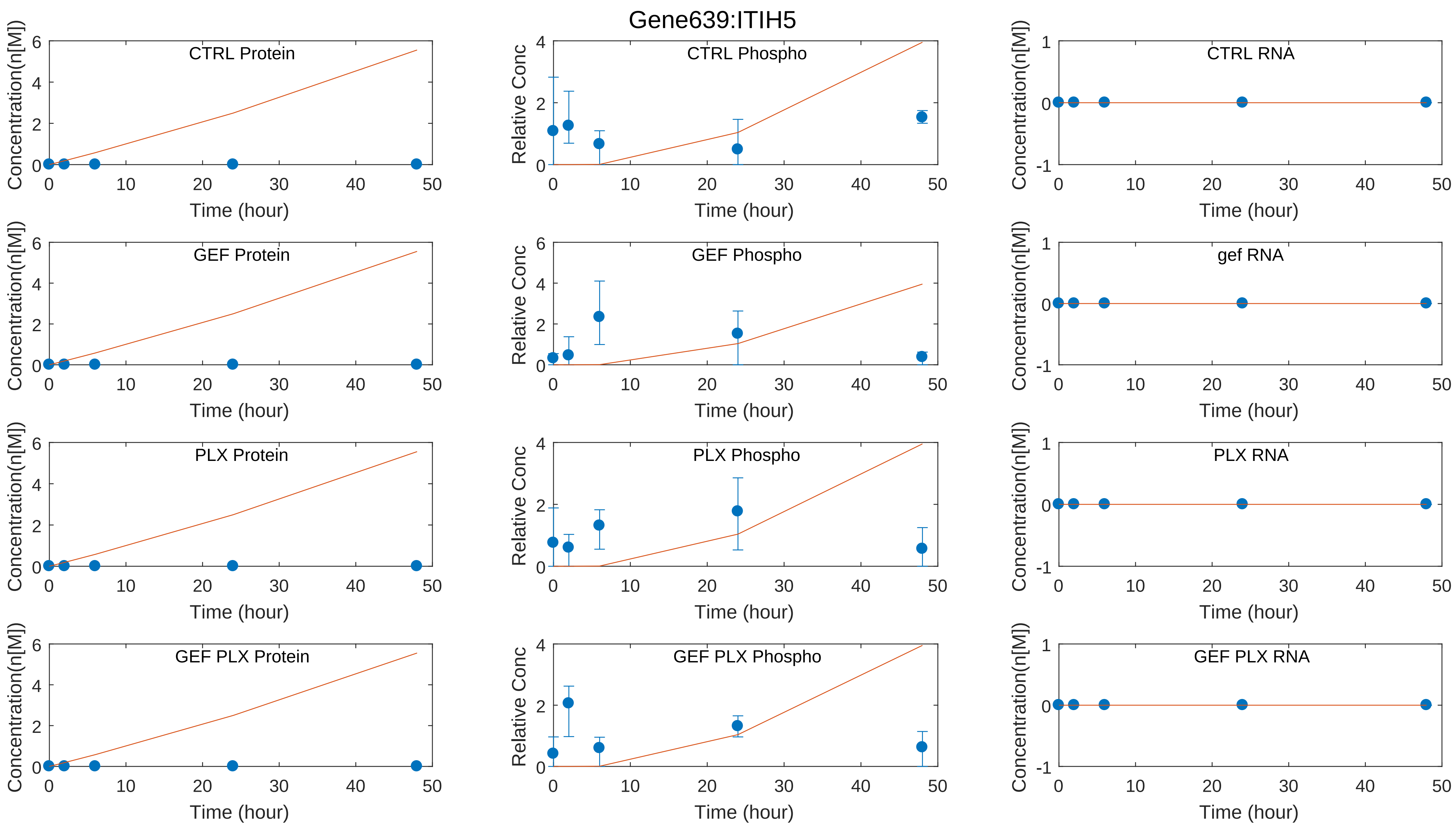

Gene639:ITIH5
CTRL Protein
CTRL Phospho
CTRL RNA
GEF Protein
GEF Phospho
gef RNA
PLX Protein
PLX Phospho
PLX RNA
GEF PLX Protein
GEF PLX Phospho
GEF PLX RNA
Concentration(n[M])
Relative Conc
Time (hour)

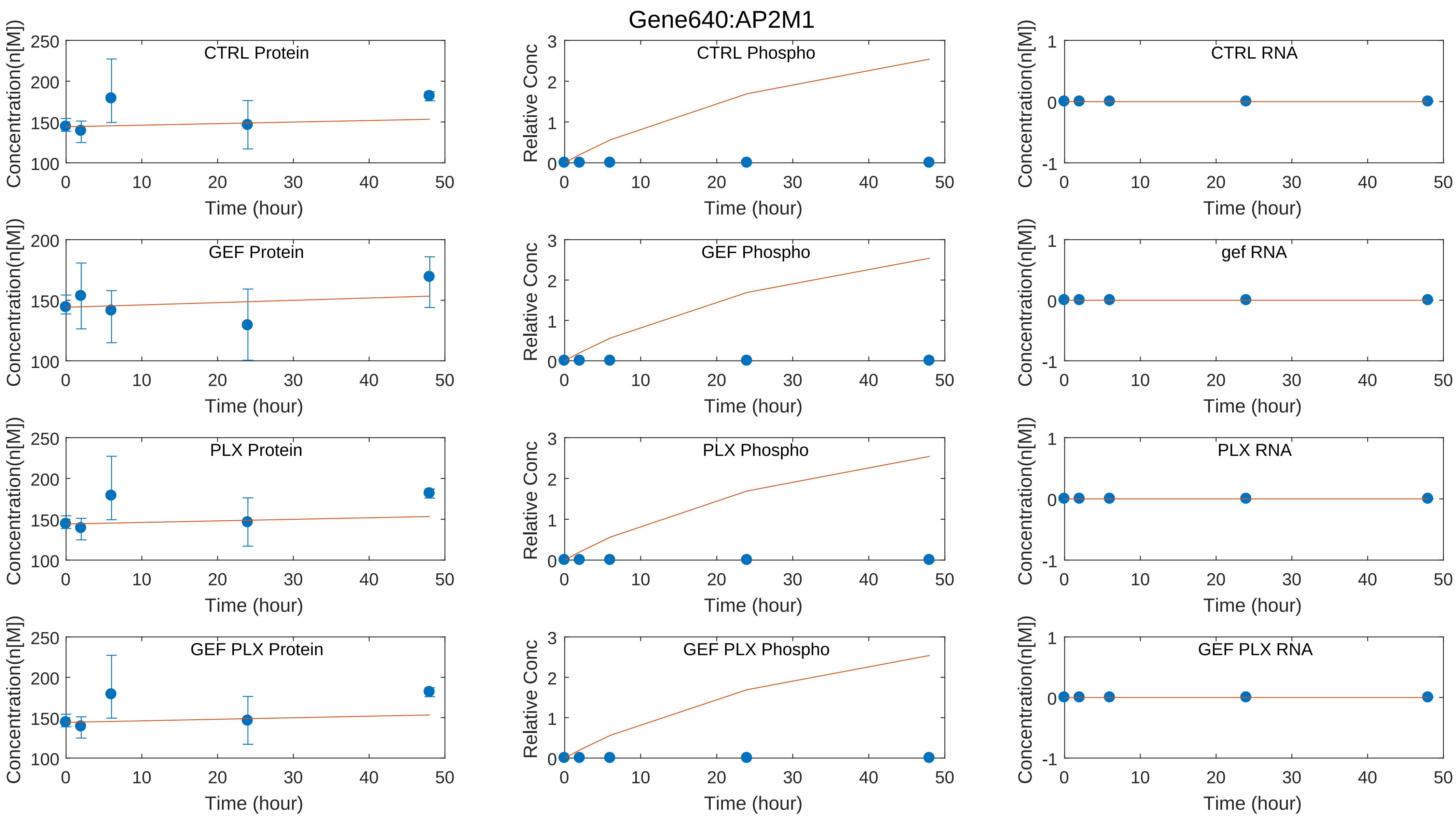
Gene640:AP2M1
CTRL Protein
CTRL Phospho
CTRL RNA
GEF Protein
GEF Phospho
gef RNA
PLX Protein
PLX Phospho
PLX RNA
GEF PLX Protein
GEF PLX Phospho
GEF PLX RNA
Concentration(n[M])
Relative Conc
Time (hour)

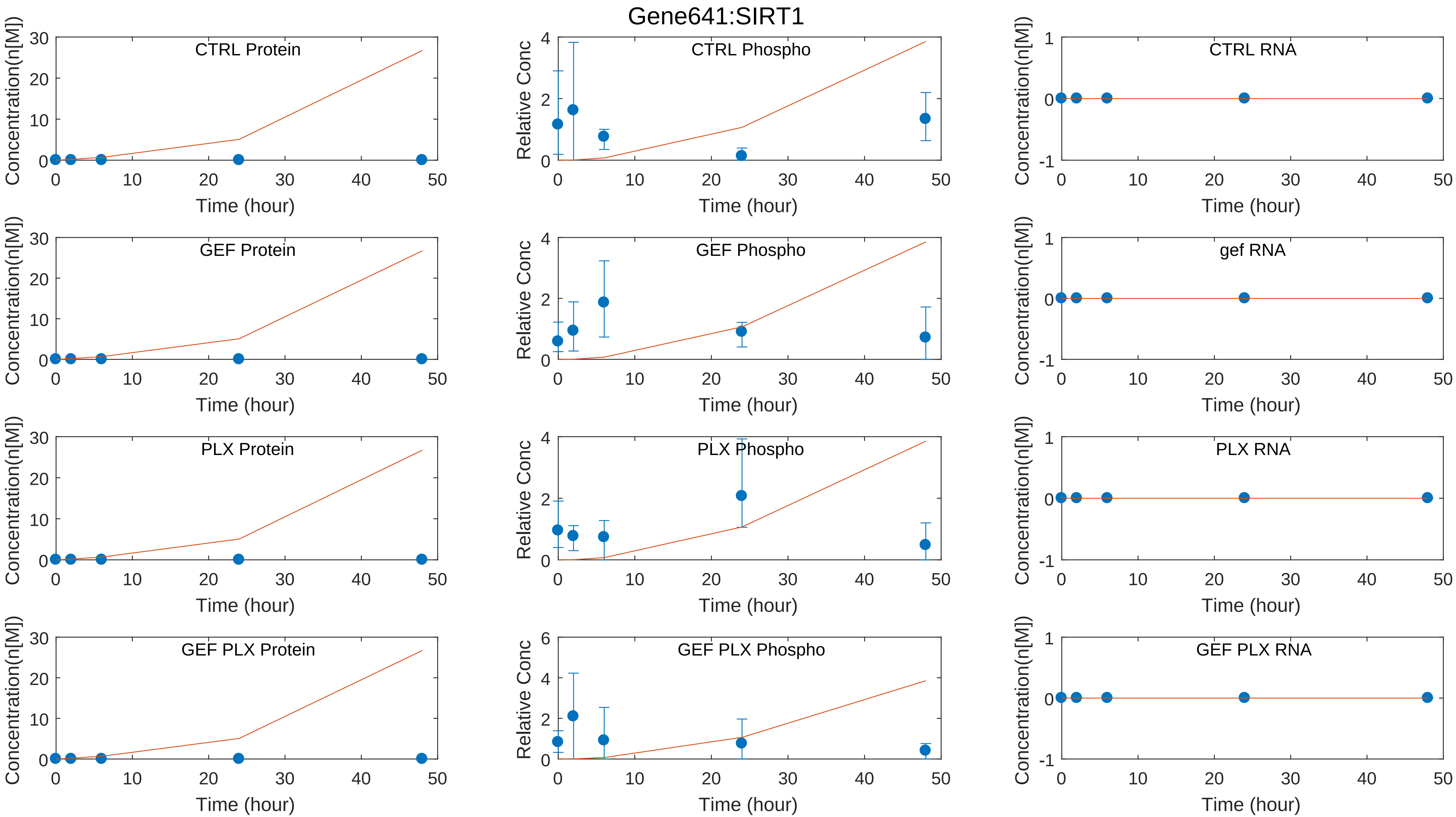
Gene641:SIRT1
CTRL Protein
GEF Protein
PLX Protein
GEF PLX Protein
CTRL Phospho
GEF Phospho
PLX Phospho
GEF PLX Phospho
CTRL RNA
gef RNA
PLX RNA
GEF PLX RNA
Concentration(n[M])
Relative Conc
Time (hour)

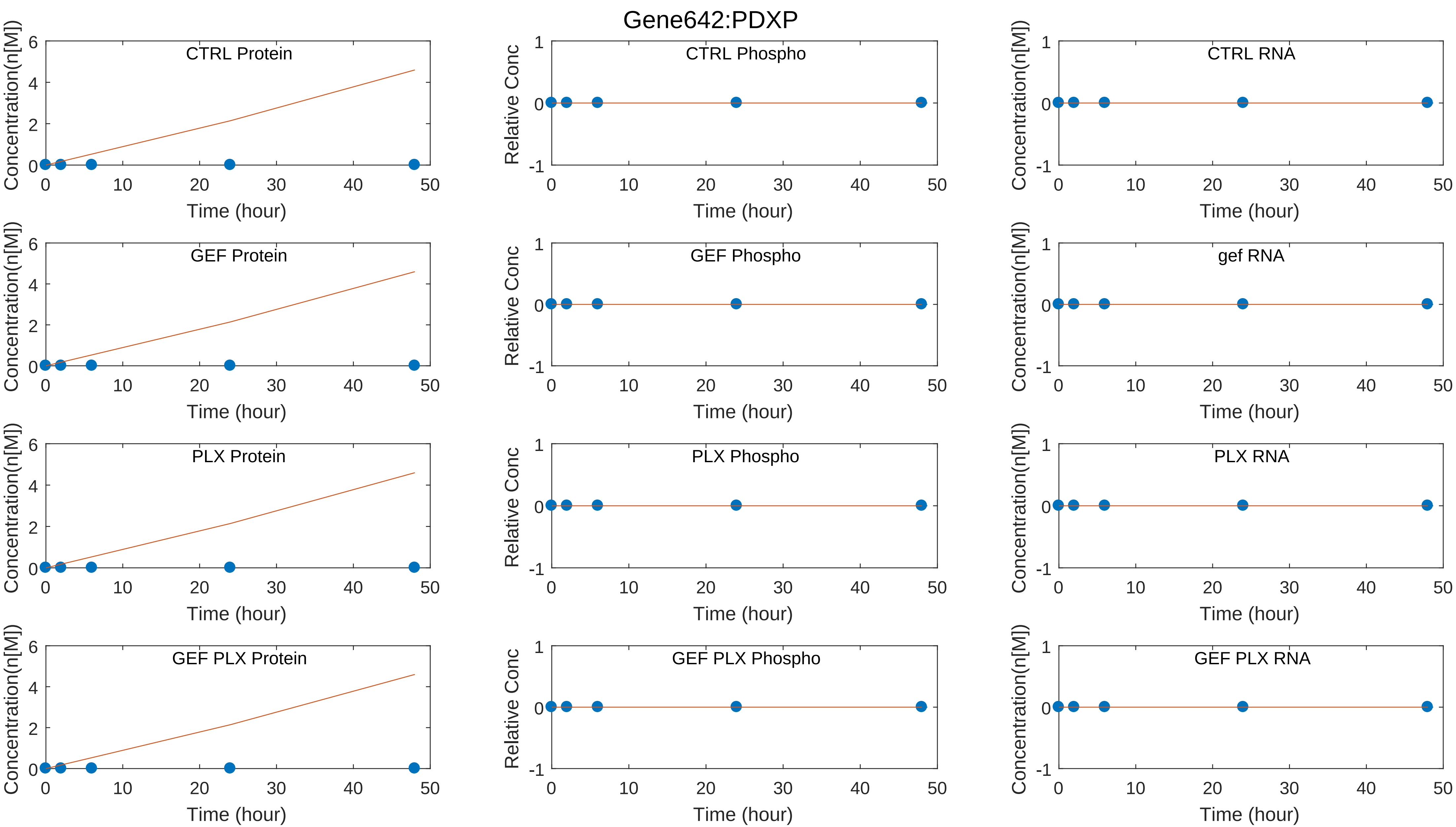

Gene642:PDXP
CTRL Protein
CTRL Phospho
CTRL RNA
GEF Protein
GEF Phospho
gef RNA
PLX Protein
PLX Phospho
PLX RNA
GEF PLX Protein
GEF PLX Phospho
GEF PLX RNA
Concentration(n[M])
Relative Conc
Time (hour)

Gene643:AURKB

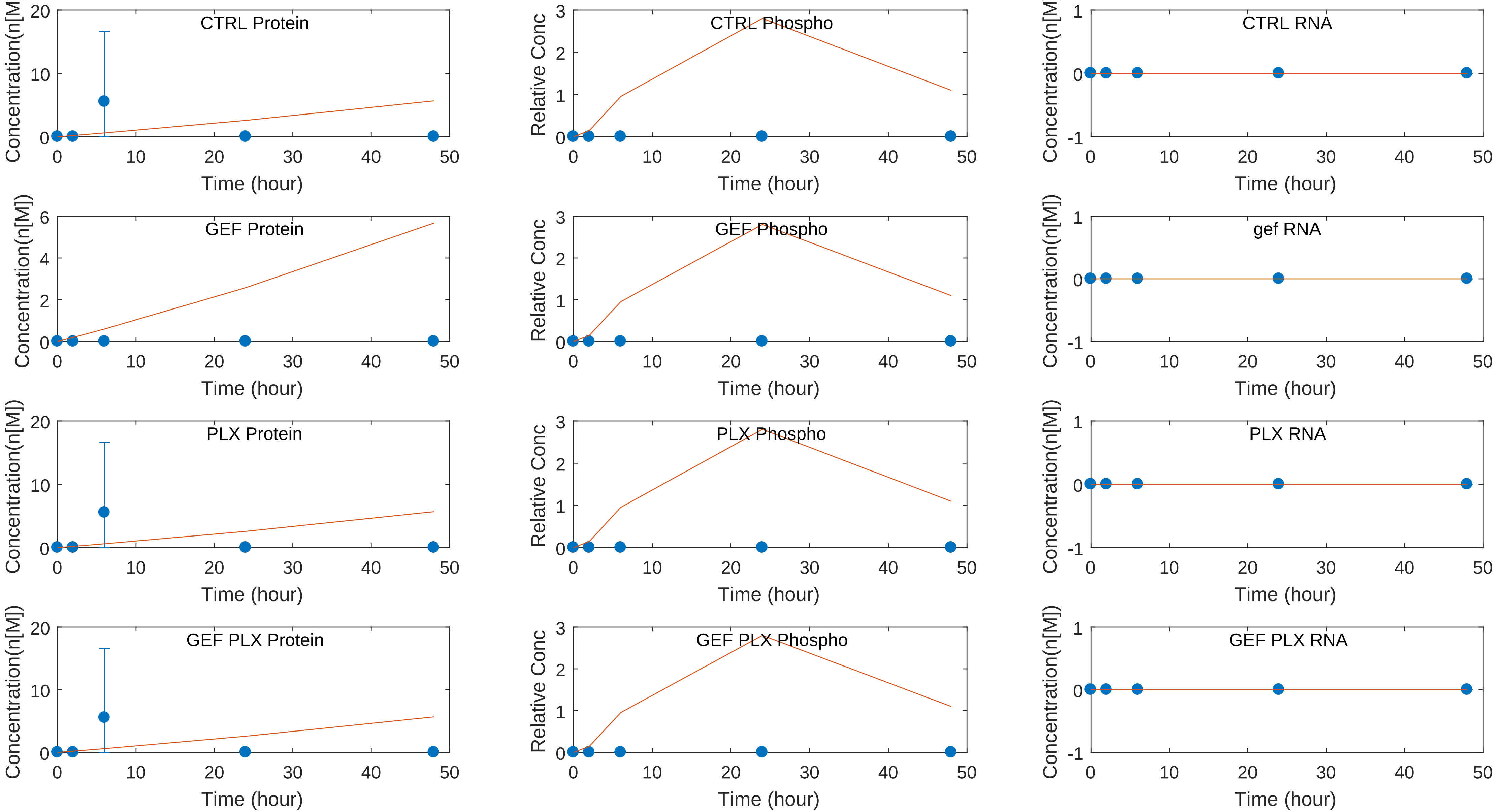

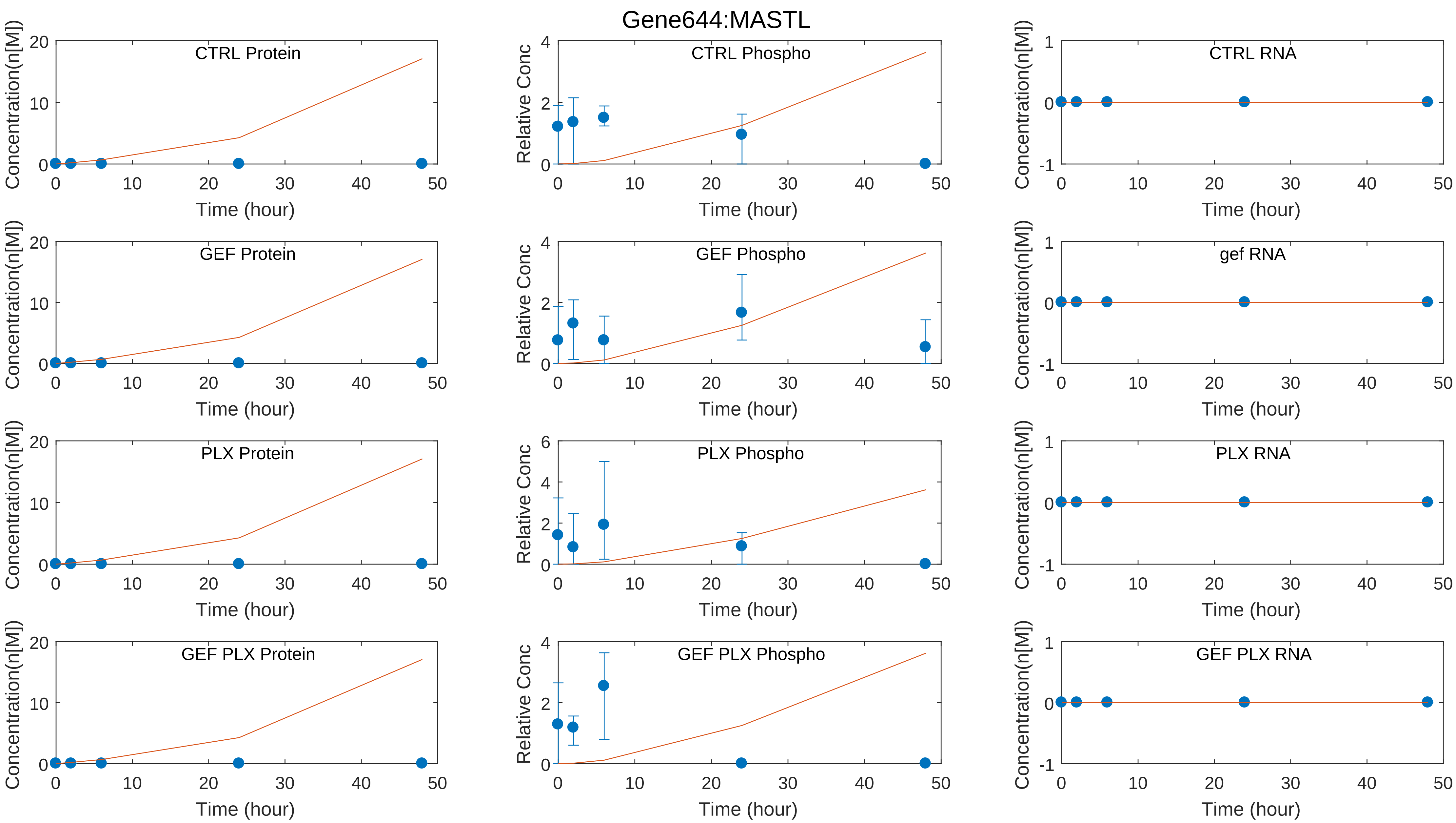

Gene644:MASTL
CTRL Protein
CTRL Phospho
CTRL RNA
GEF Protein
GEF Phospho
gef RNA
PLX Protein
PLX Phospho
PLX RNA
GEF PLX Protein
GEF PLX Phospho
GEF PLX RNA
Concentration(n[M])
Relative Conc
Time (hour)

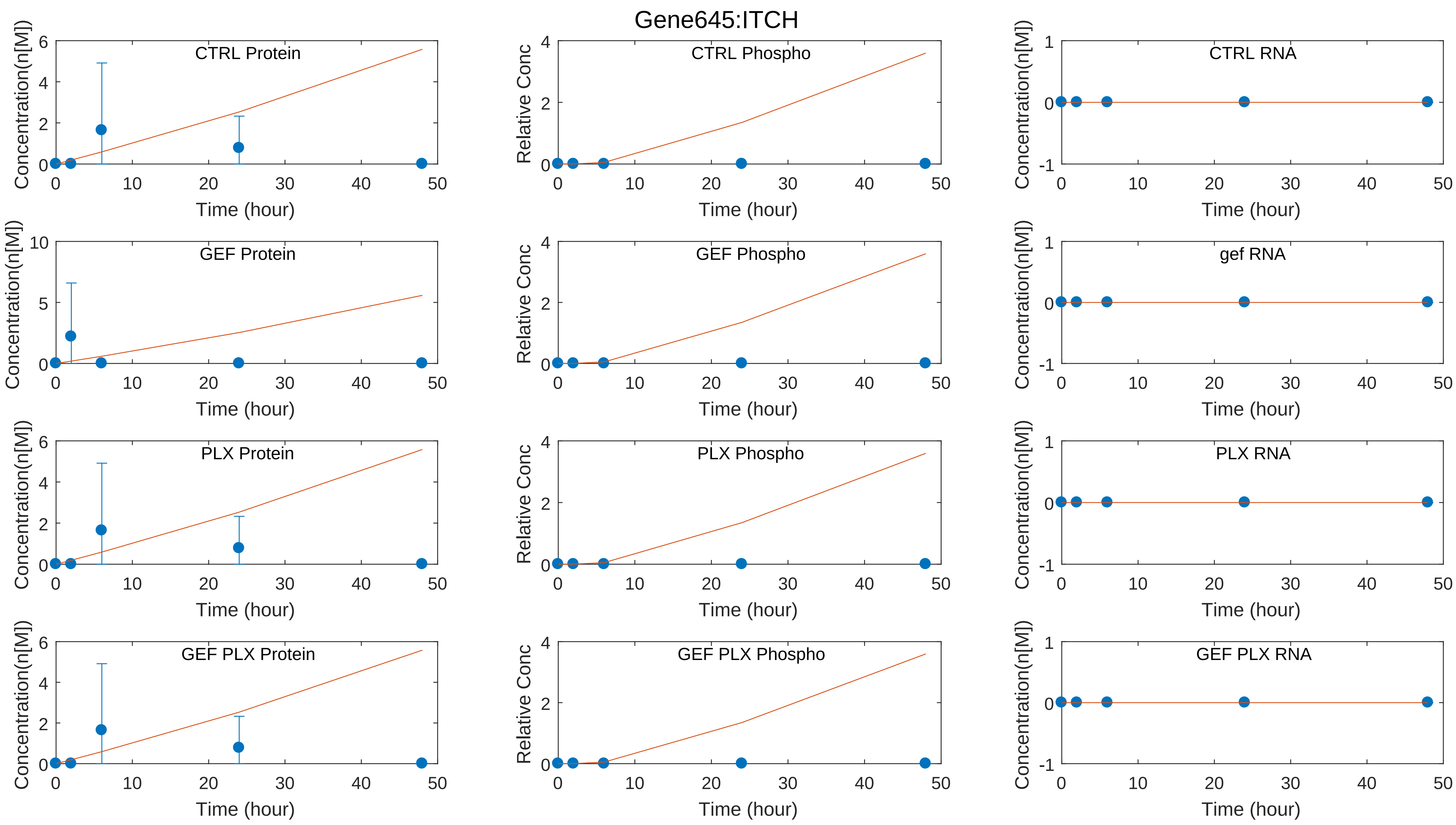

Gene645:ITCH
CTRL Protein
CTRL Phospho
CTRL RNA
GEF Protein
GEF Phospho
gef RNA
PLX Protein
PLX Phospho
PLX RNA
GEF PLX Protein
GEF PLX Phospho
GEF PLX RNA
Concentration(n[M])
Relative Conc
Time (hour)

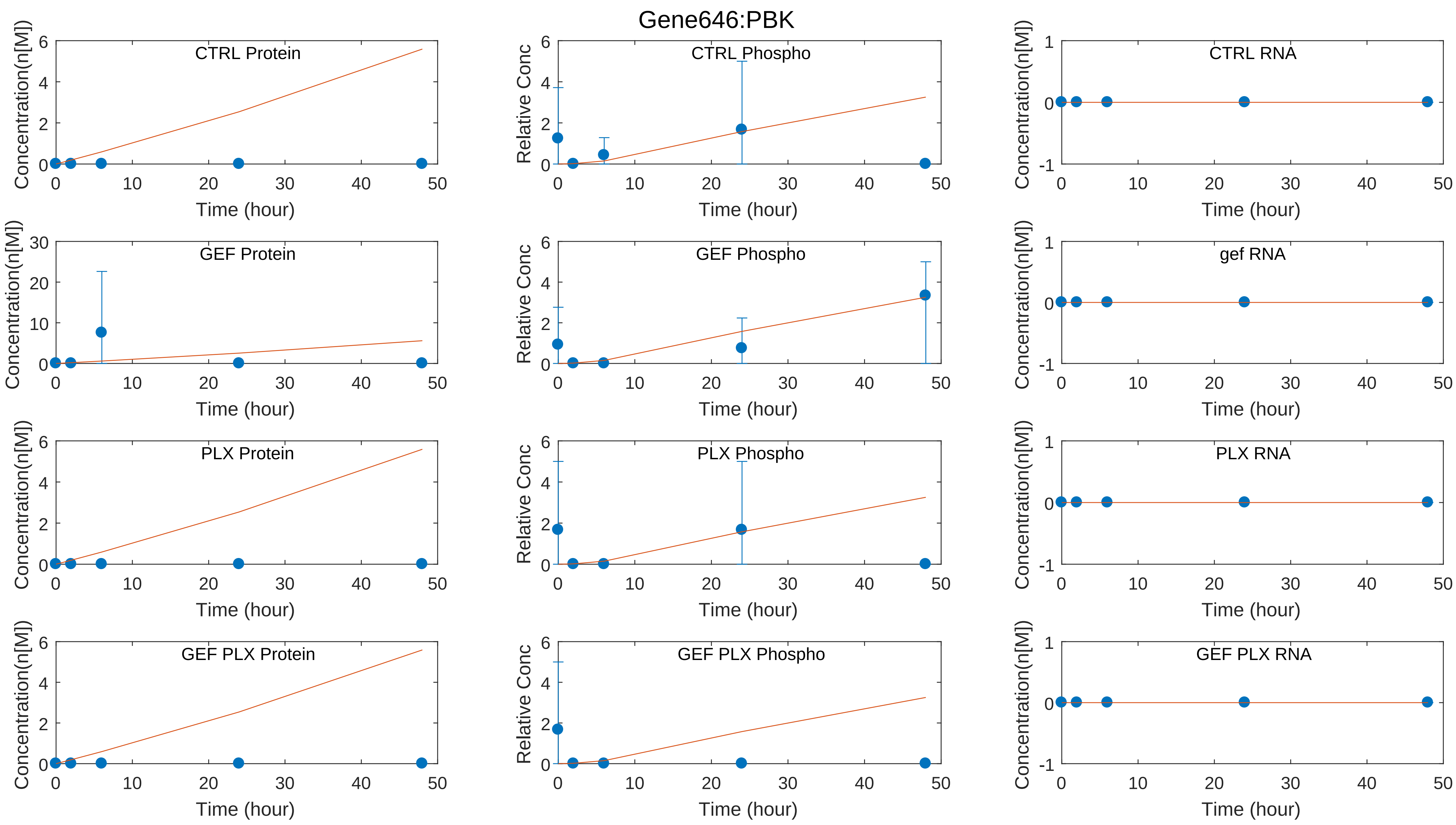
Gene646:PBK
CTRL Protein
CTRL Phospho
CTRL RNA
GEF Protein
GEF Phospho
gef RNA
PLX Protein
PLX Phospho
PLX RNA
GEF PLX Protein
GEF PLX Phospho
GEF PLX RNA
Concentration(n[M])
Relative Conc
Time (hour)

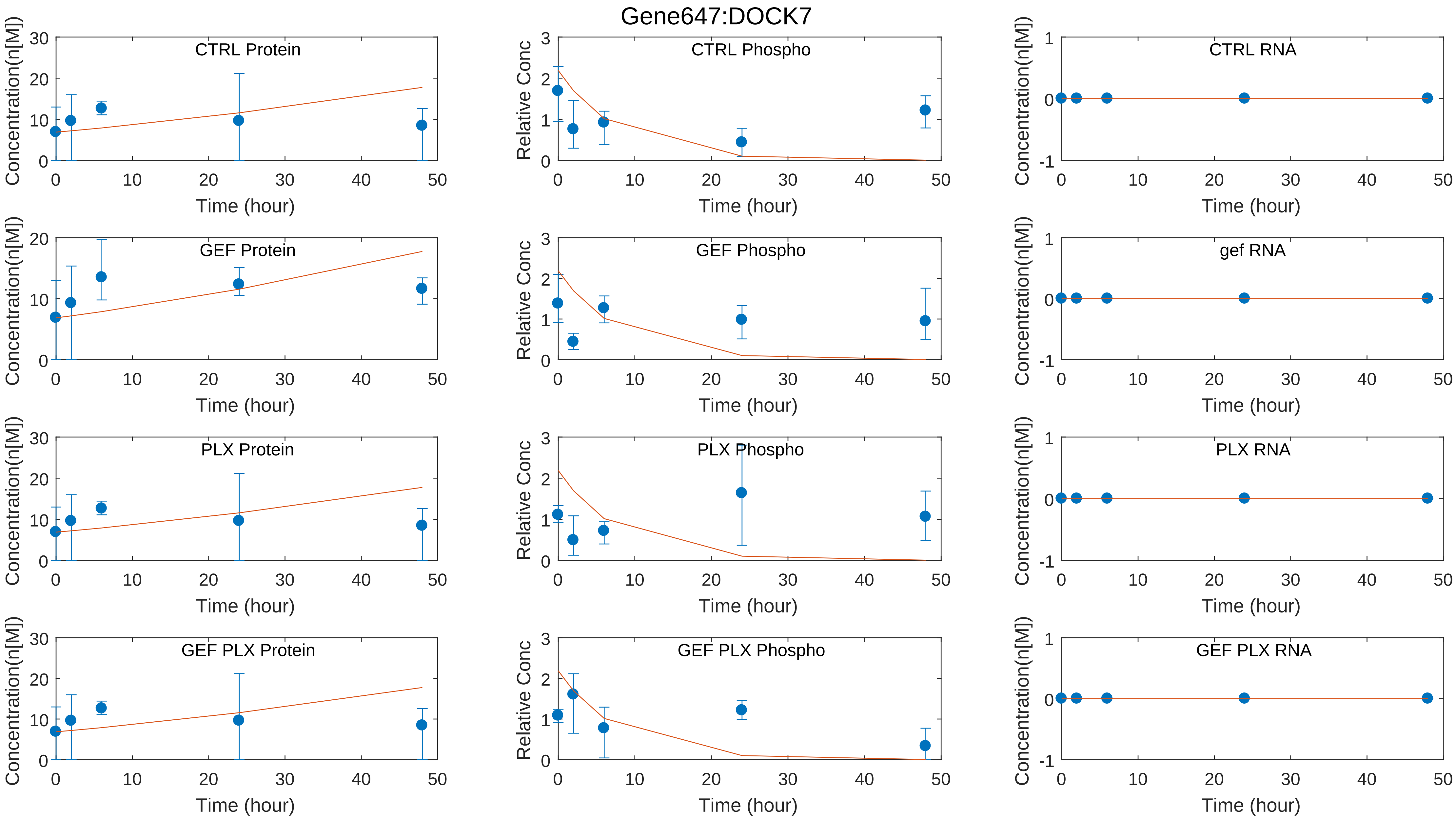
Gene647:DOCK7
CTRL Protein
CTRL Phospho
CTRL RNA
GEF Protein
GEF Phospho
gef RNA
PLX Protein
PLX Phospho
PLX RNA
GEF PLX Protein
GEF PLX Phospho
GEF PLX RNA
Concentration(n[M])
Relative Conc
Time (hour)

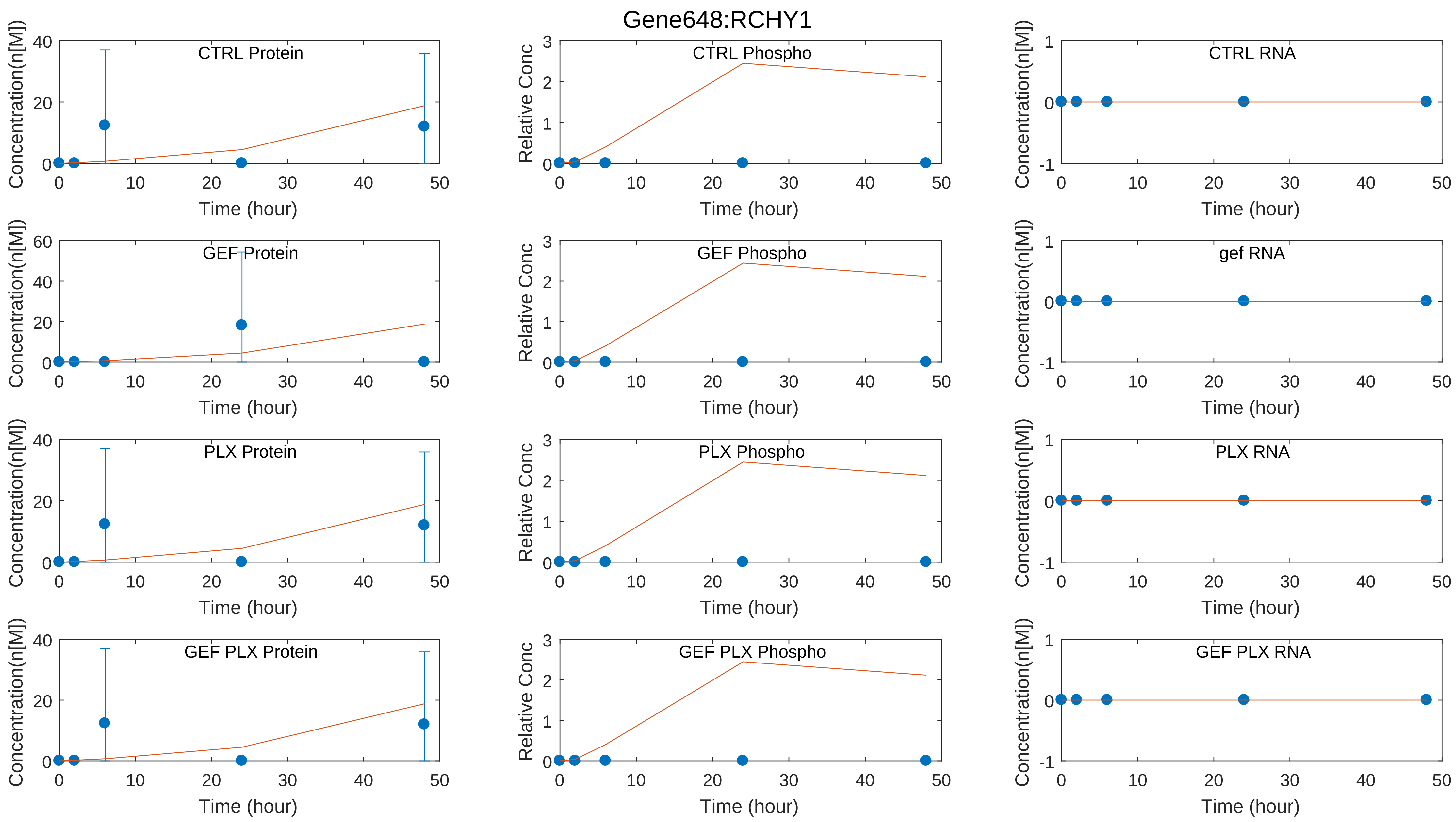
Gene648:RCHY1
CTRL Protein
CTRL Phospho
CTRL RNA
GEF Protein
GEF Phospho
gef RNA
PLX Protein
PLX Phospho
PLX RNA
GEF PLX Protein
GEF PLX Phospho
GEF PLX RNA
Concentration(n[M])
Relative Conc
Time (hour)

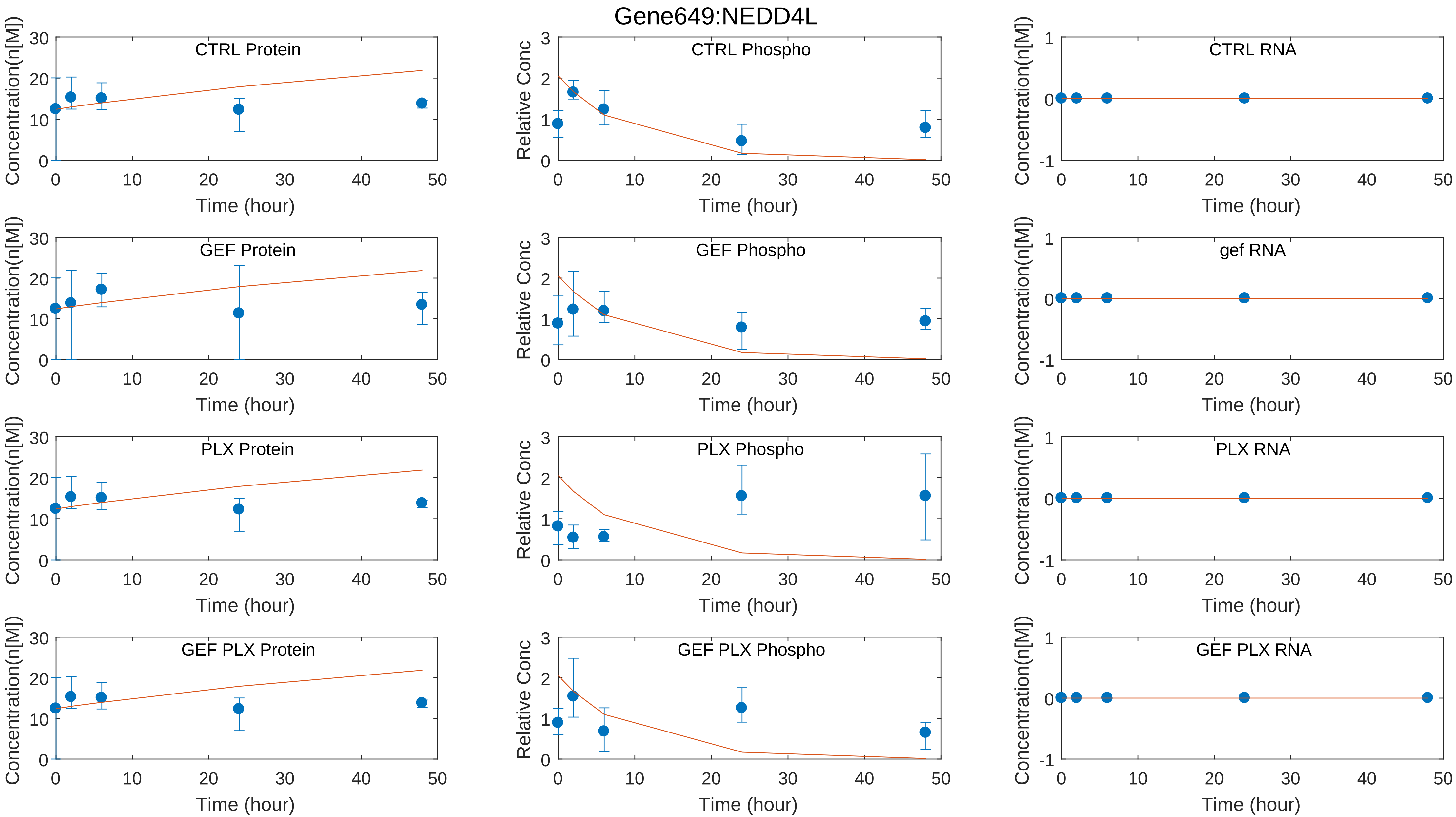

Gene649:NEDD4L
CTRL Protein
CTRL Phospho
CTRL RNA
GEF Protein
GEF Phospho
gef RNA
PLX Protein
PLX Phospho
PLX RNA
GEF PLX Protein
GEF PLX Phospho
GEF PLX RNA
Concentration(n[M])
Relative Conc
Time (hour)

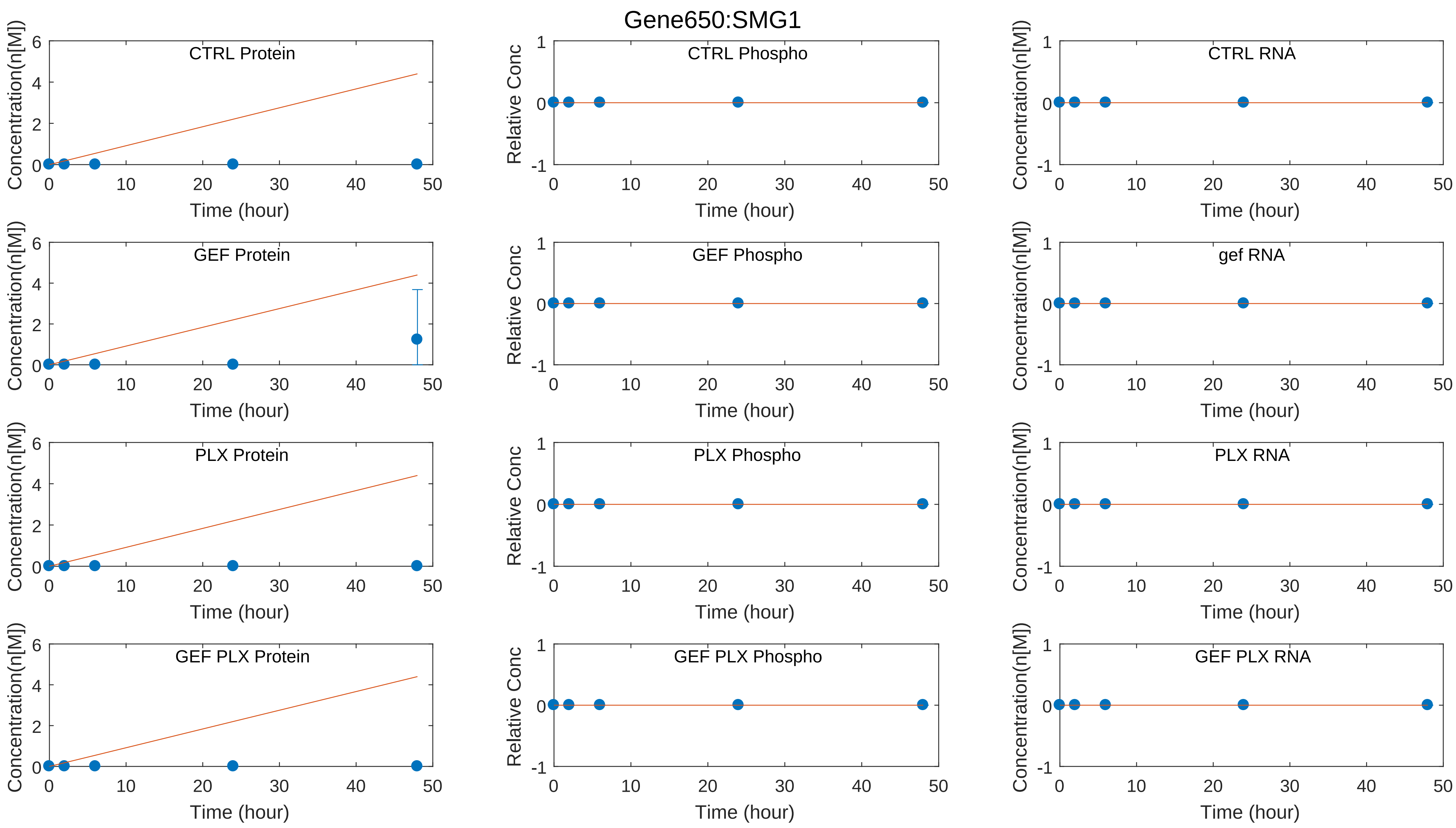

Gene650:SMG1
CTRL Protein
CTRL Phospho
CTRL RNA
GEF Protein
GEF Phospho
gef RNA
PLX Protein
PLX Phospho
PLX RNA
GEF PLX Protein
GEF PLX Phospho
GEF PLX RNA
Concentration(n[M])
Relative Conc
Time (hour)

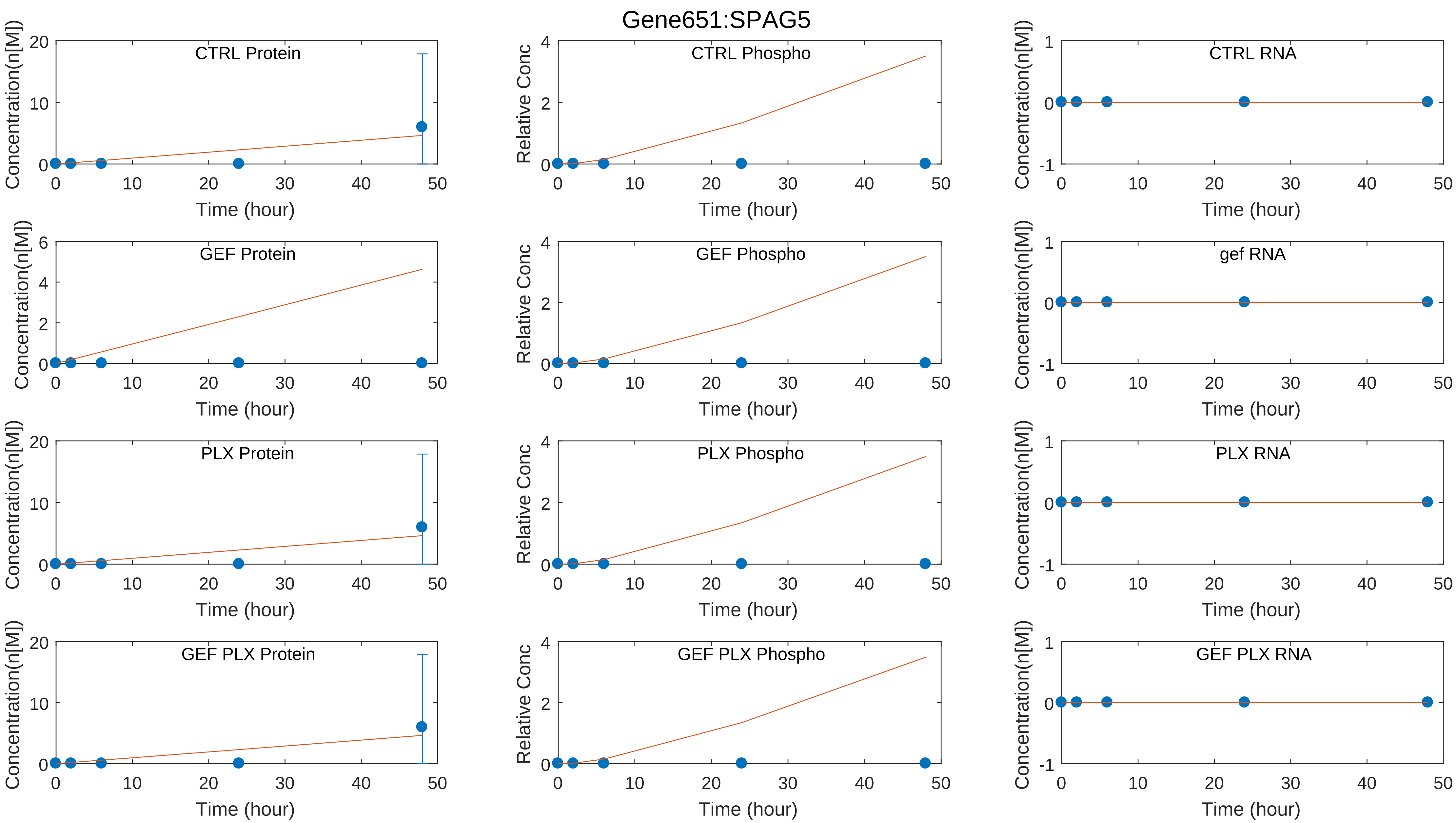

Gene651:SPAG5
CTRL Protein
CTRL Phospho
CTRL RNA
GEF Protein
GEF Phospho
gef RNA
PLX Protein
PLX Phospho
PLX RNA
GEF PLX Protein
GEF PLX Phospho
GEF PLX RNA
Concentration(n[M])
Relative Conc
Time (hour)

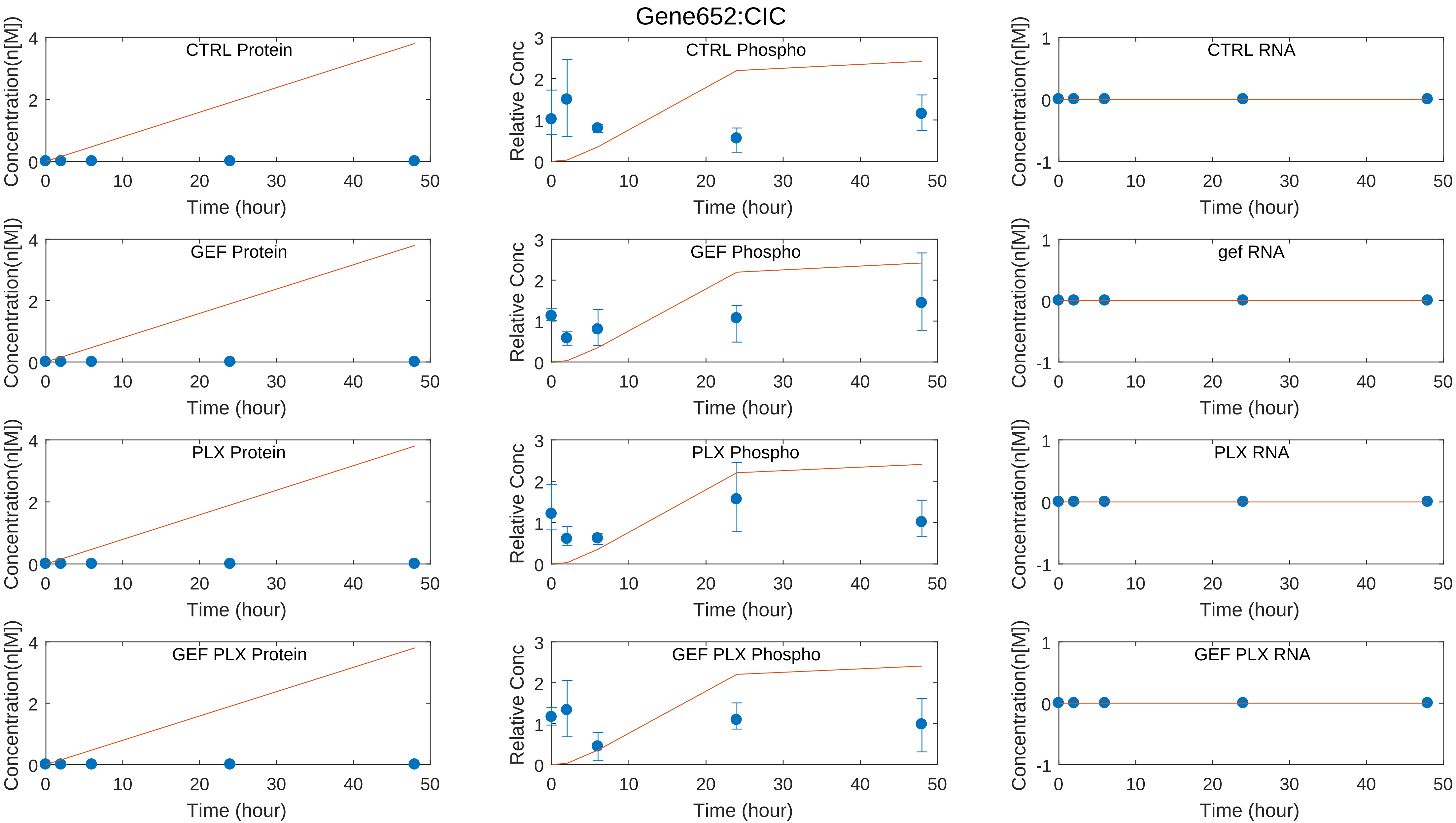

Gene652:CIC
CTRL Protein
CTRL Phospho
CTRL RNA
GEF Protein
GEF Phospho
gef RNA
PLX Protein
PLX Phospho
PLX RNA
GEF PLX Protein
GEF PLX Phospho
GEF PLX RNA
Concentration(n[M])
Relative Conc
Time (hour)

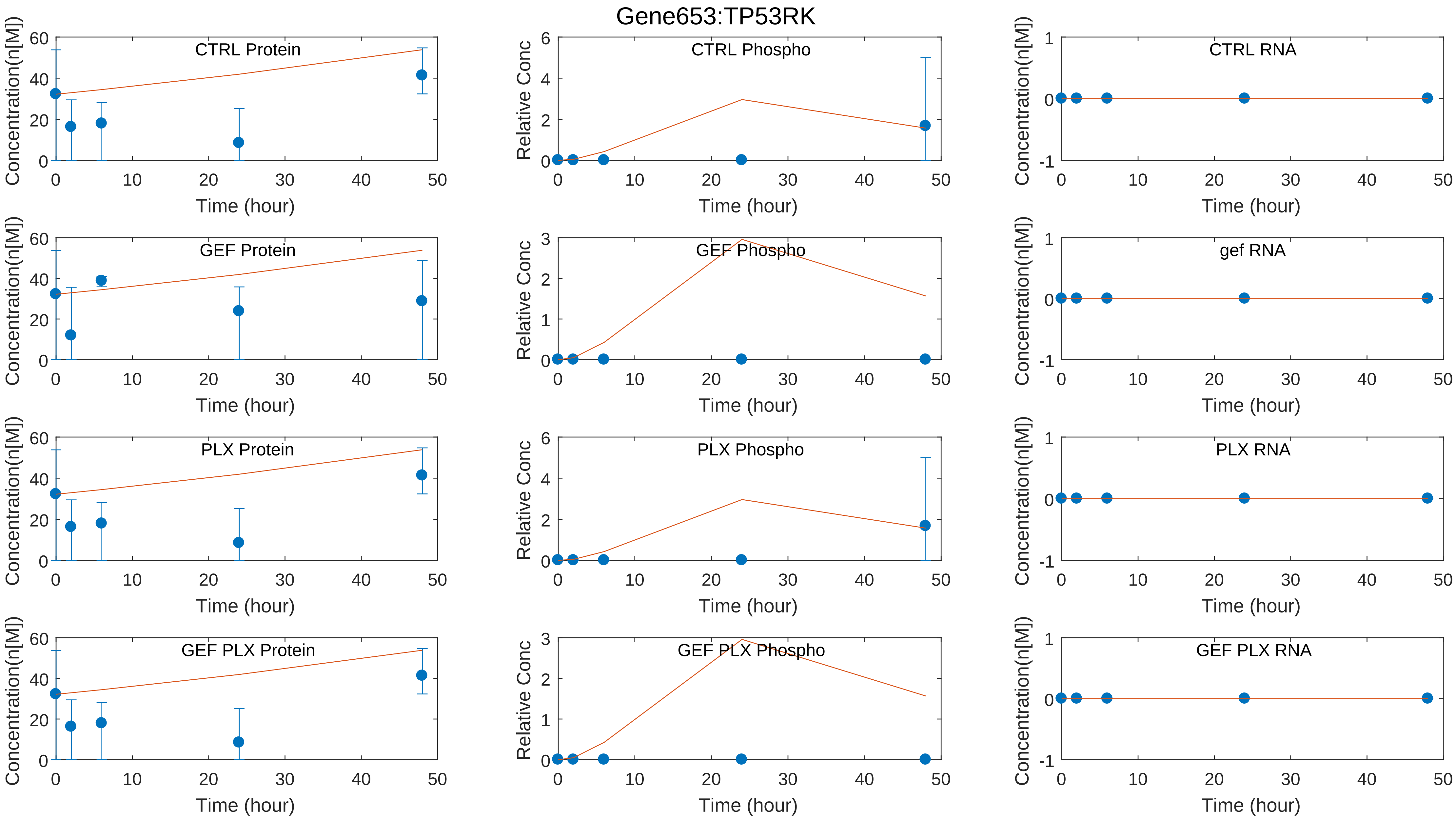

Gene653:TP53RK
CTRL Protein
CTRL Phospho
CTRL RNA
GEF Protein
GEF Phospho
gef RNA
PLX Protein
PLX Phospho
PLX RNA
GEF PLX Protein
GEF PLX Phospho
GEF PLX RNA
Concentration(n[M])
Relative Conc
Time (hour)

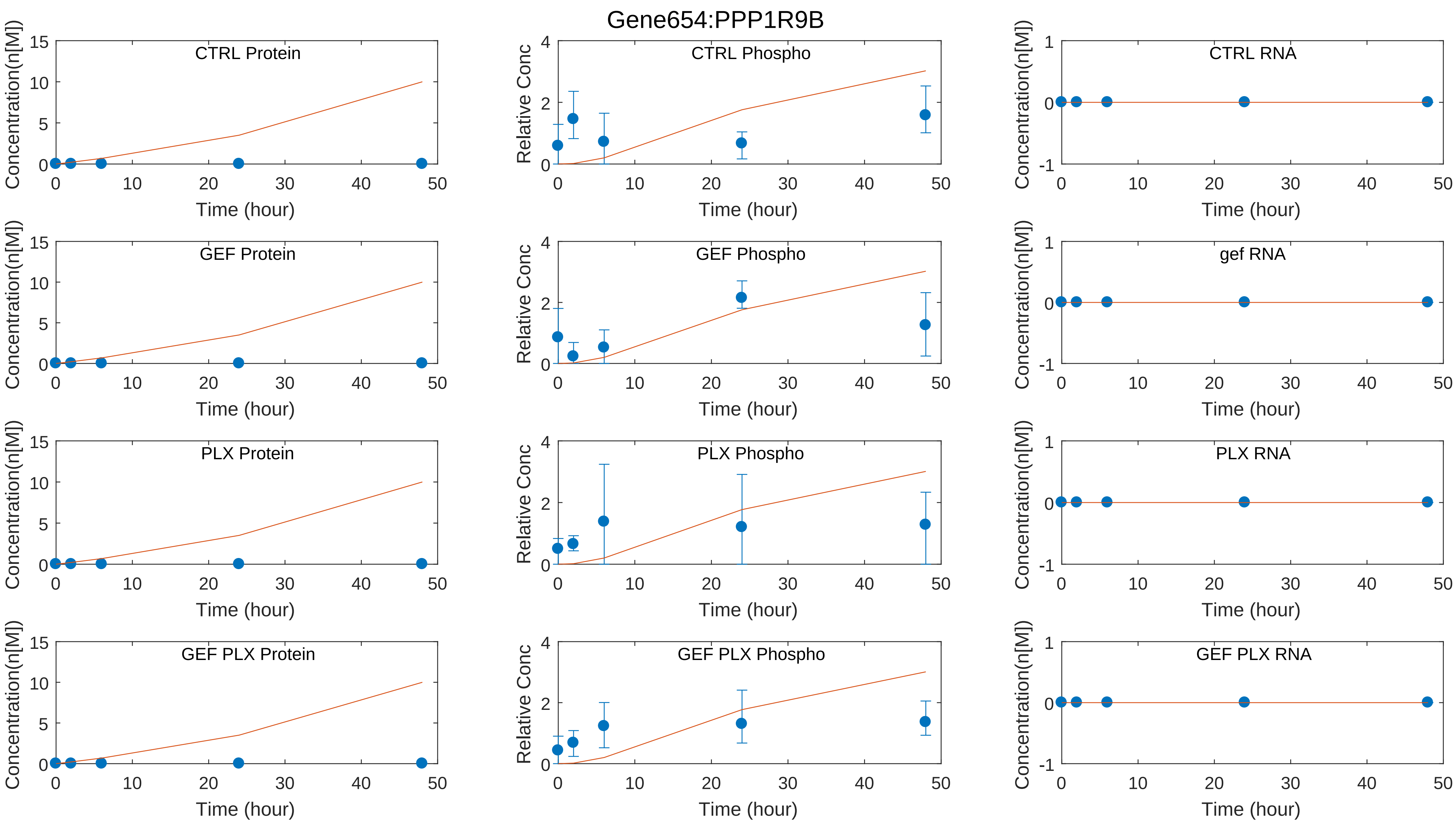

Gene654:PPP1R9B
CTRL Protein
CTRL Phospho
CTRL RNA
GEF Protein
GEF Phospho
gef RNA
PLX Protein
PLX Phospho
PLX RNA
GEF PLX Protein
GEF PLX Phospho
GEF PLX RNA
Concentration(n[M])
Relative Conc
Time (hour)

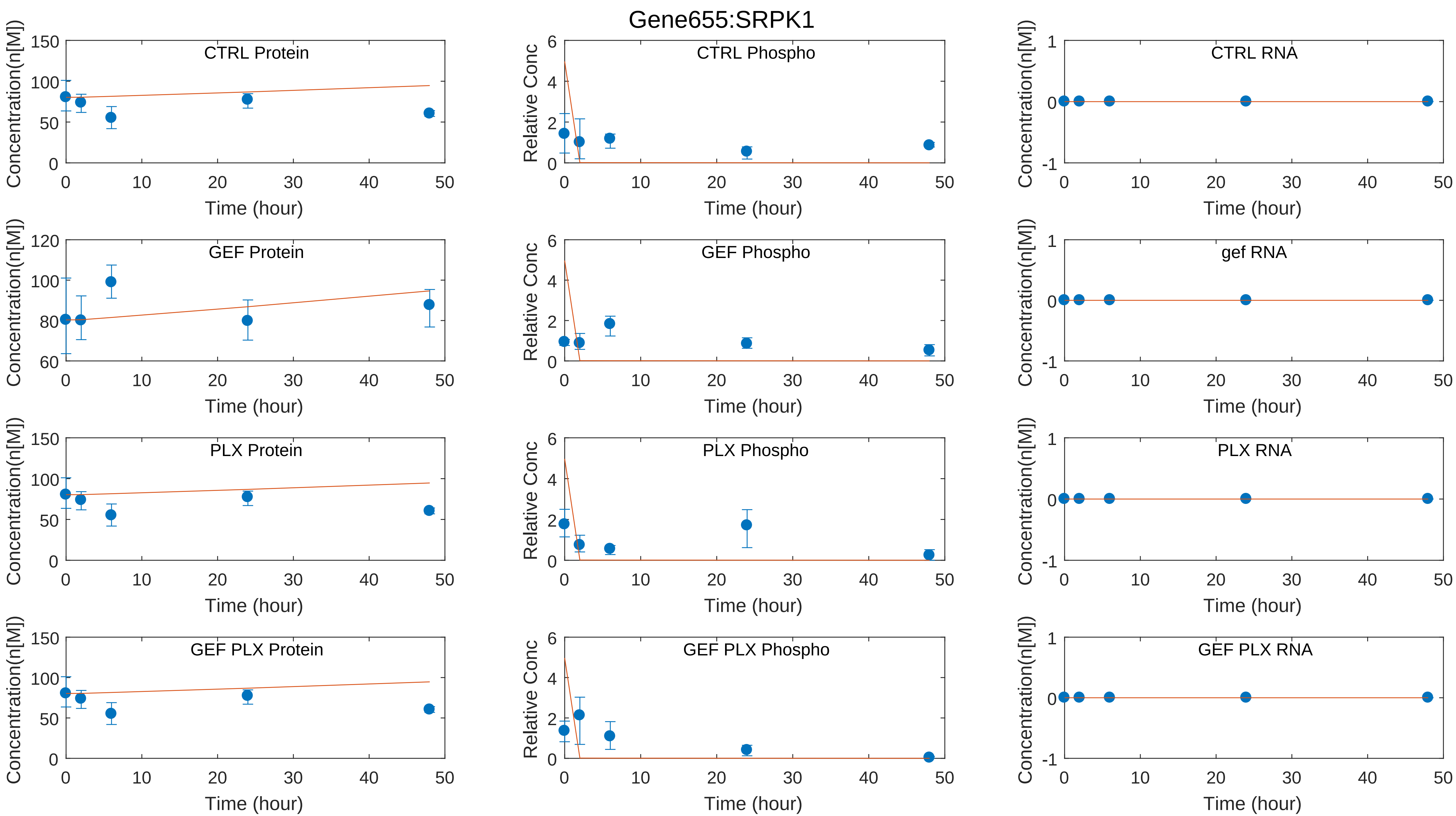
Gene655:SRPK1
CTRL Protein
CTRL Phospho
CTRL RNA
GEF Protein
GEF Phospho
gef RNA
PLX Protein
PLX Phospho
PLX RNA
GEF PLX Protein
GEF PLX Phospho
GEF PLX RNA
Concentration(n[M])
Relative Conc
Time (hour)

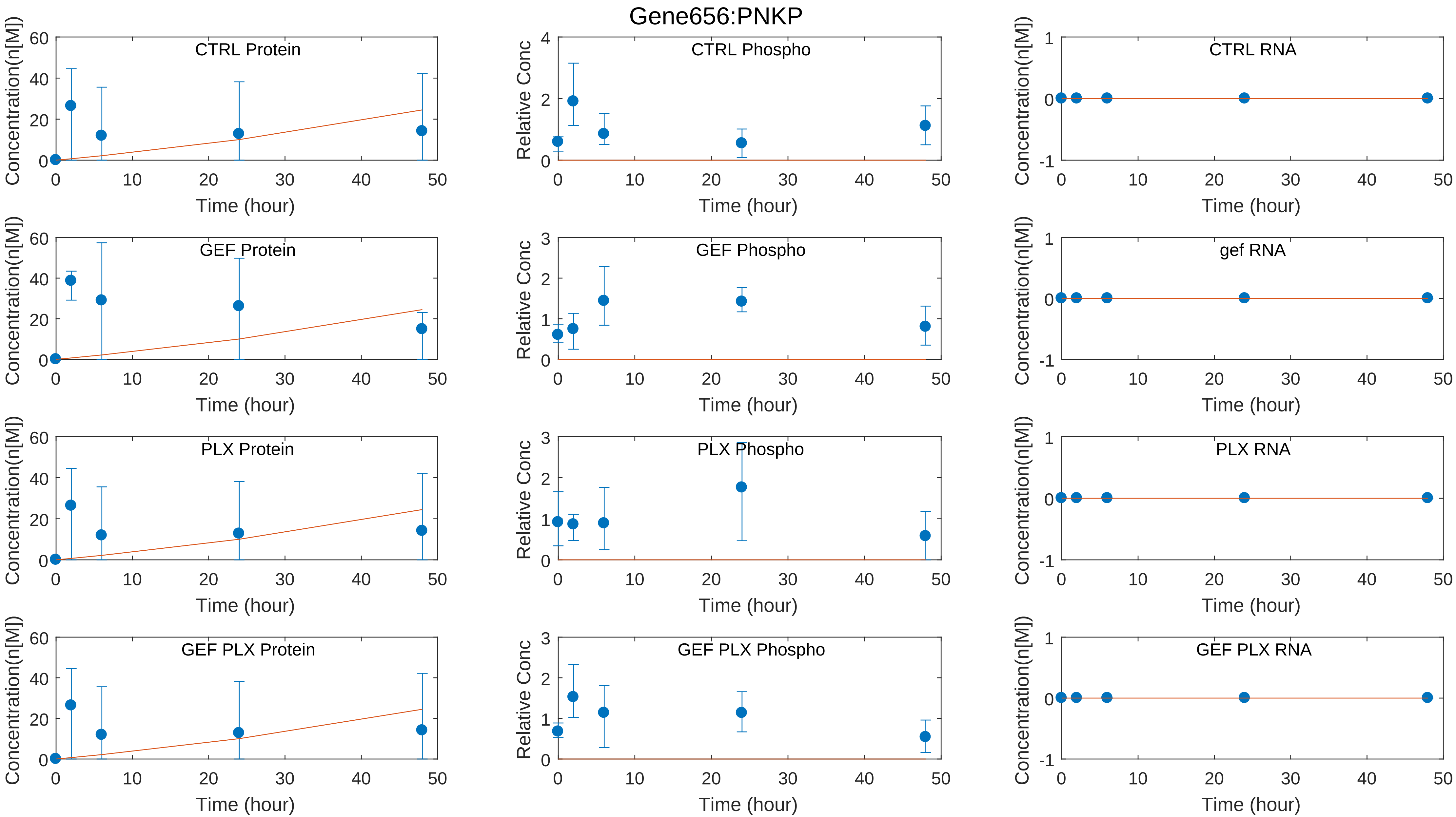
Gene656:PNKP
CTRL Protein
GEF Protein
PLX Protein
GEF PLX Protein
CTRL Phospho
GEF Phospho
PLX Phospho
GEF PLX Phospho
CTRL RNA
gef RNA
PLX RNA
GEF PLX RNA
Concentration(n[M])
Relative Conc
Time (hour)

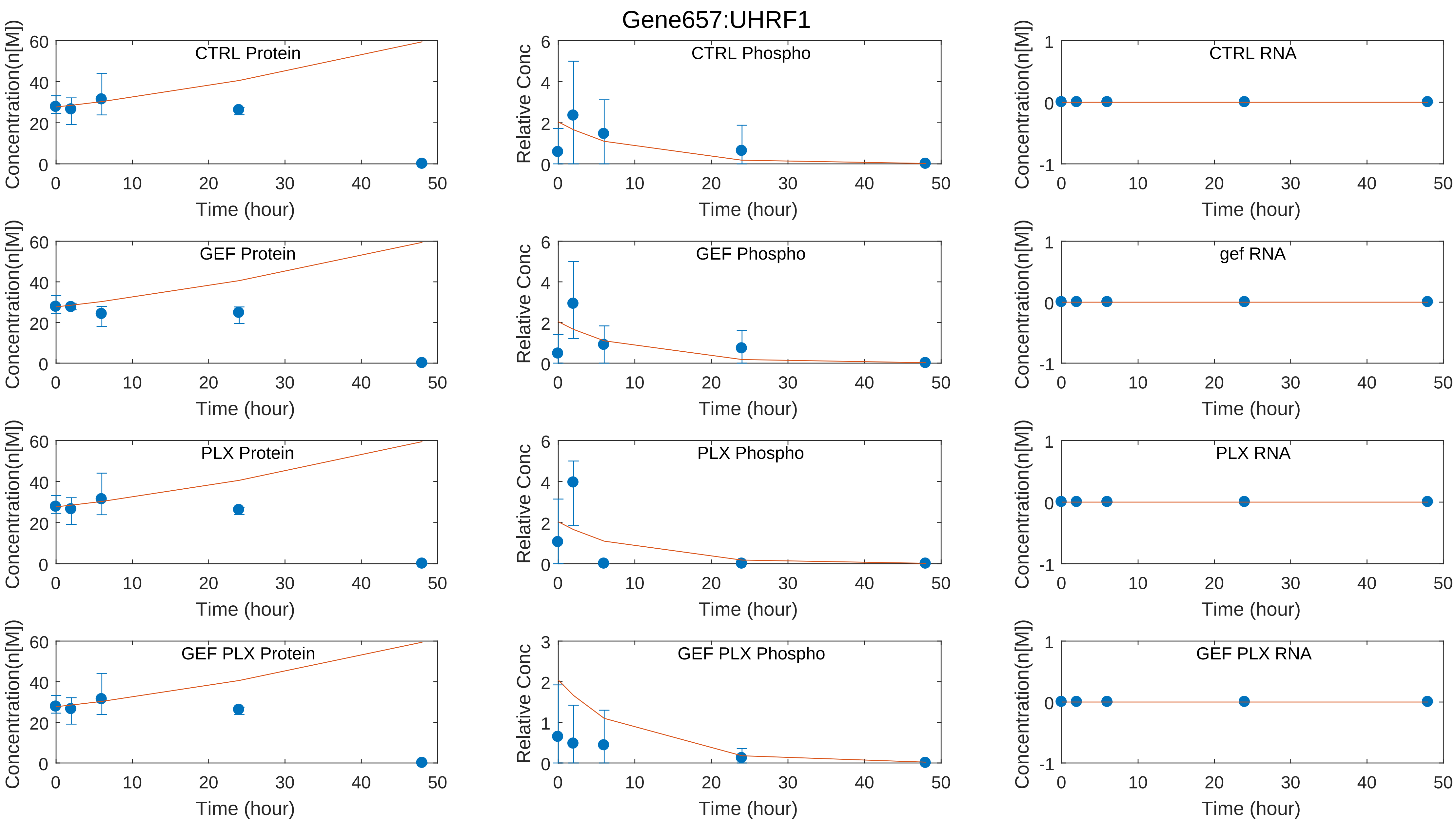
Gene657:UHRF1
CTRL Protein
CTRL Phospho
CTRL RNA
GEF Protein
GEF Phospho
gef RNA
PLX Protein
PLX Phospho
PLX RNA
GEF PLX Protein
GEF PLX Phospho
GEF PLX RNA
Concentration(n[M])
Relative Conc
Time (hour)

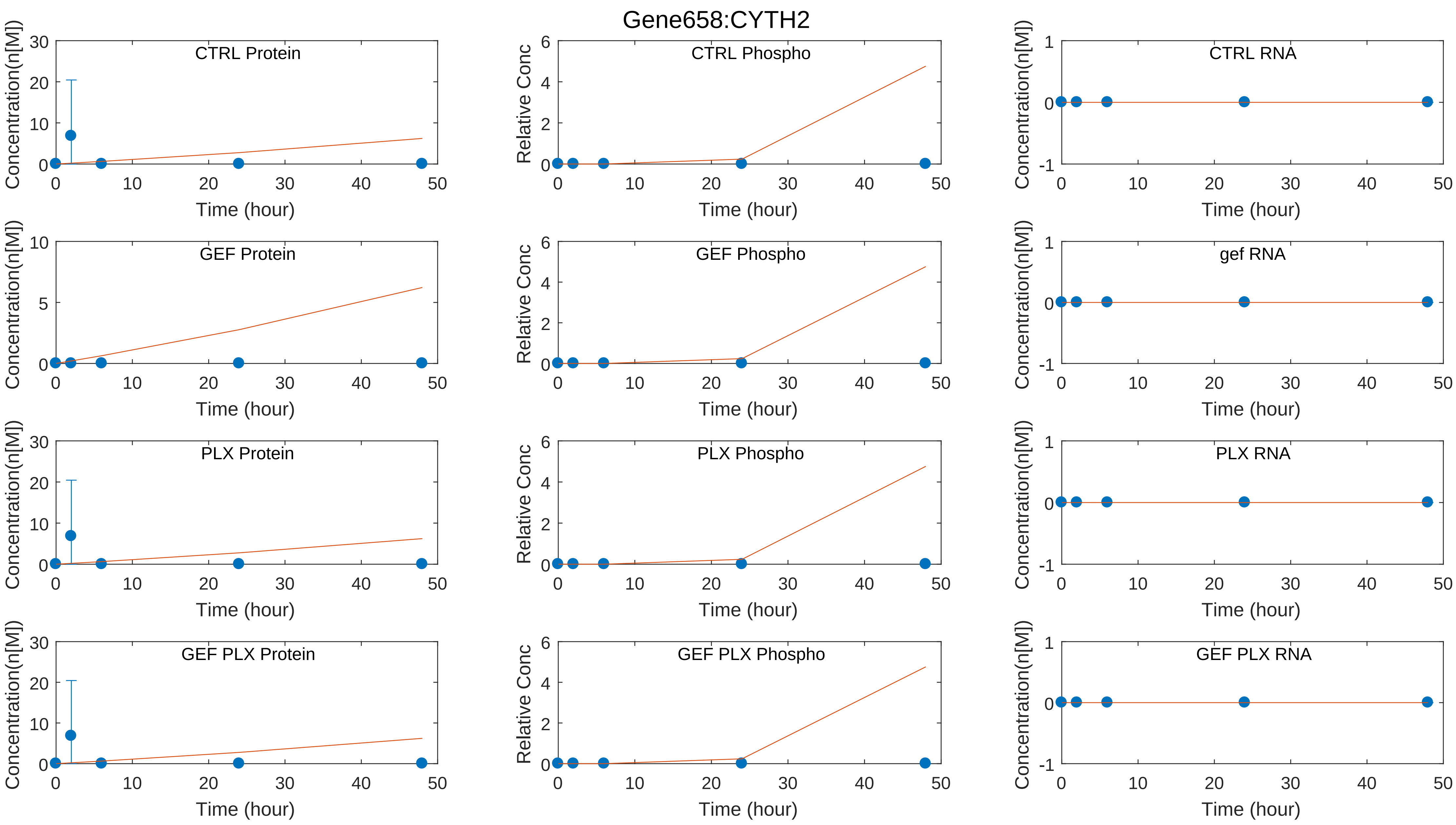
Gene658:CYTH2
CTRL Protein
CTRL Phospho
CTRL RNA
GEF Protein
GEF Phospho
gef RNA
PLX Protein
PLX Phospho
PLX RNA
GEF PLX Protein
GEF PLX Phospho
GEF PLX RNA
Concentration(n[M])
Relative Conc
Time (hour)

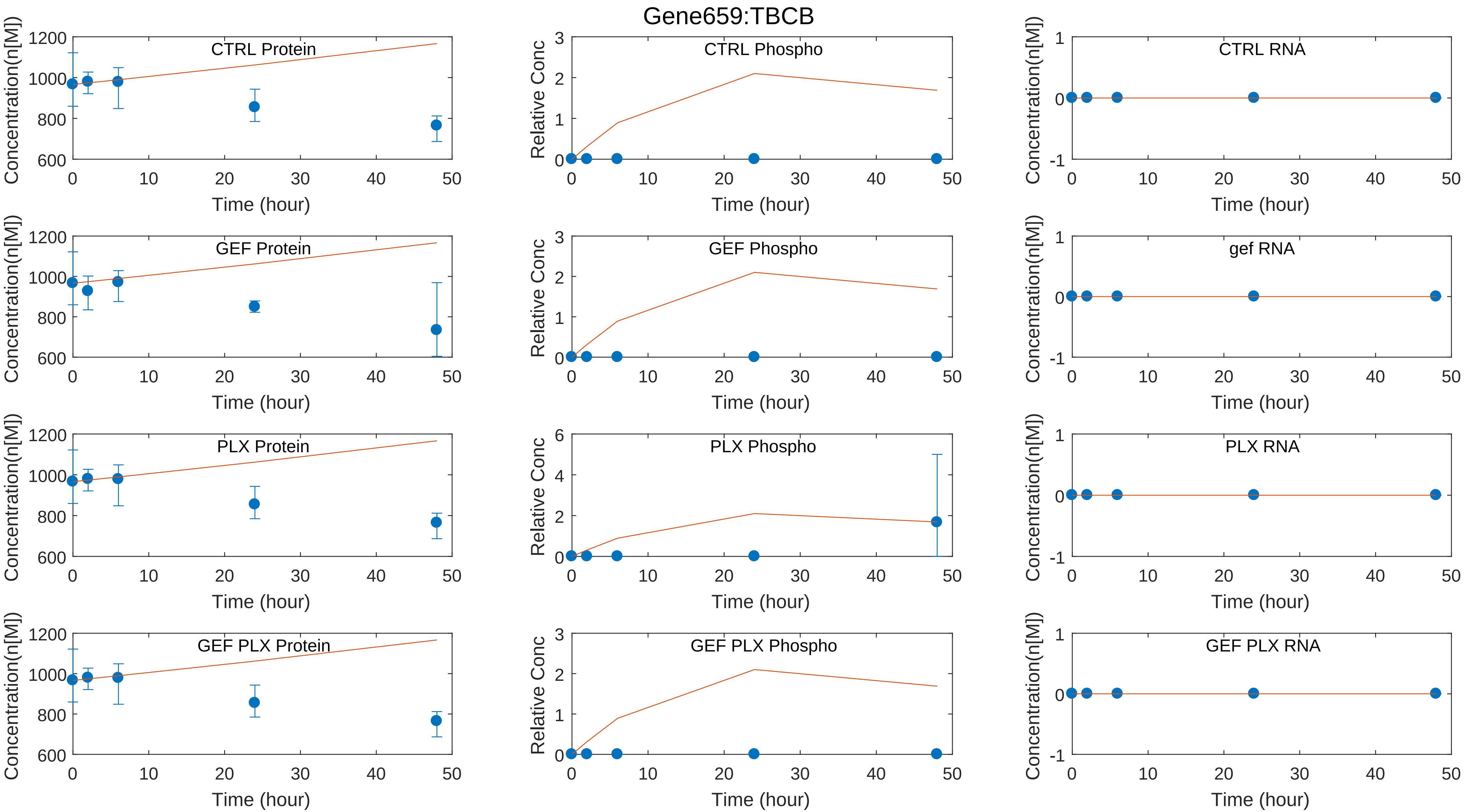

Gene659:TBCB
CTRL Protein
CTRL Phospho
CTRL RNA
GEF Protein
GEF Phospho
gef RNA
PLX Protein
PLX Phospho
PLX RNA
GEF PLX Protein
GEF PLX Phospho
GEF PLX RNA
Concentration(n[M])
Relative Conc
Time (hour)

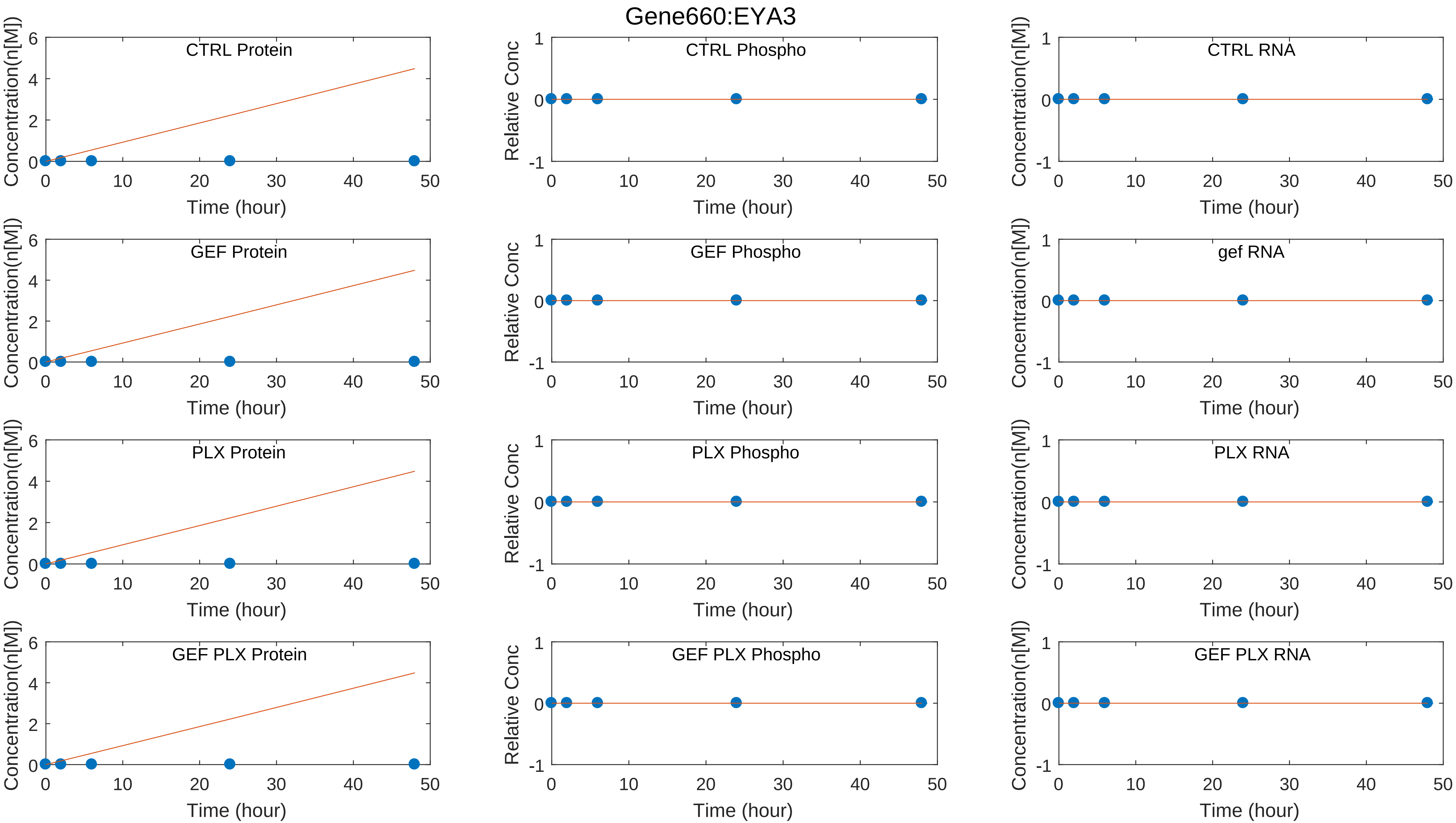

Gene660:EYA3
CTRL Protein
CTRL Phospho
CTRL RNA
GEF Protein
GEF Phospho
gef RNA
PLX Protein
PLX Phospho
PLX RNA
GEF PLX Protein
GEF PLX Phospho
GEF PLX RNA
Concentration(n[M])
Relative Conc
Time (hour)

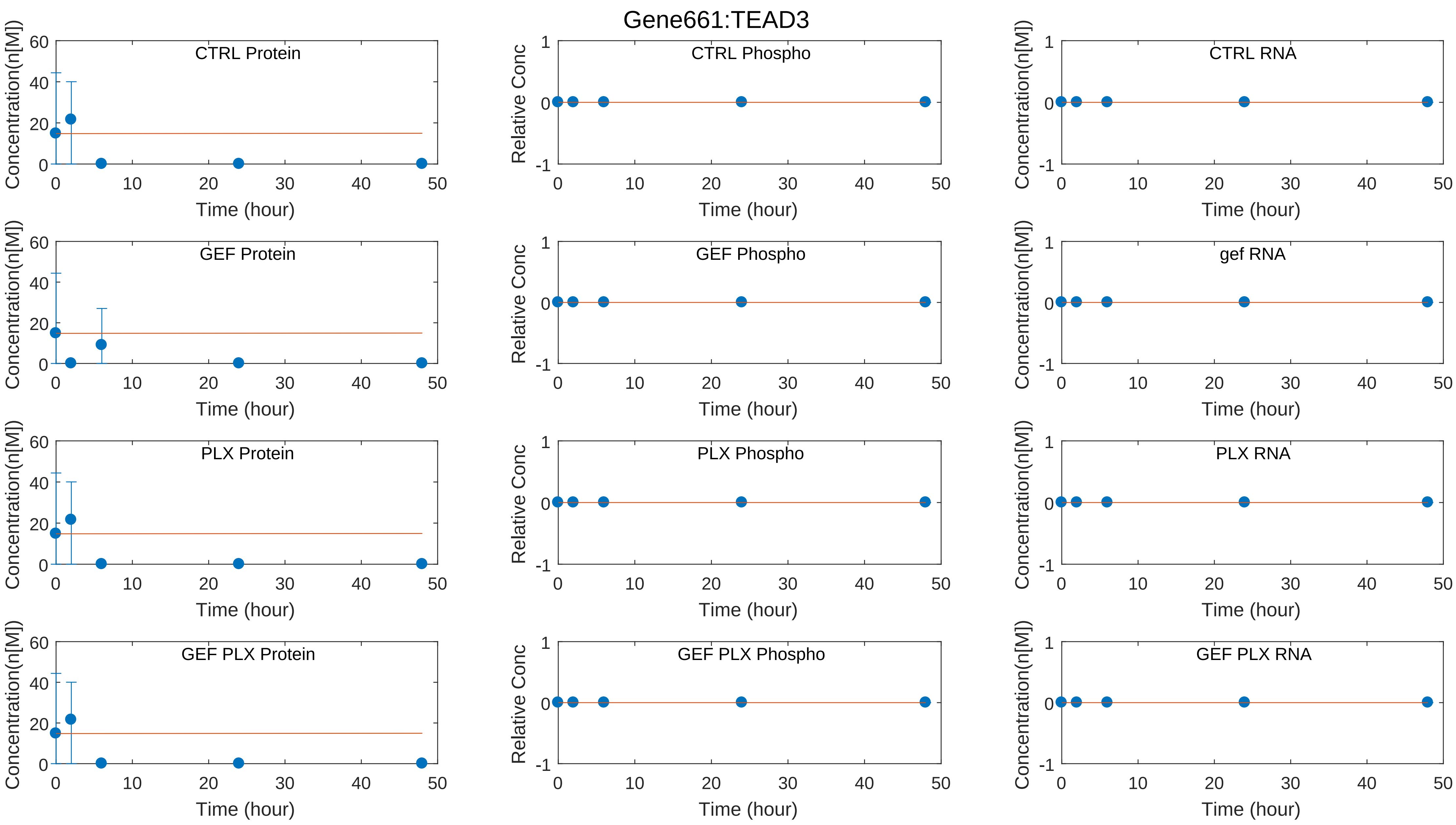

Gene661:TEAD3
CTRL Protein
CTRL Phospho
CTRL RNA
GEF Protein
GEF Phospho
gef RNA
PLX Protein
PLX Phospho
PLX RNA
GEF PLX Protein
GEF PLX Phospho
GEF PLX RNA
Concentration(n[M])
Relative Conc
Time (hour)

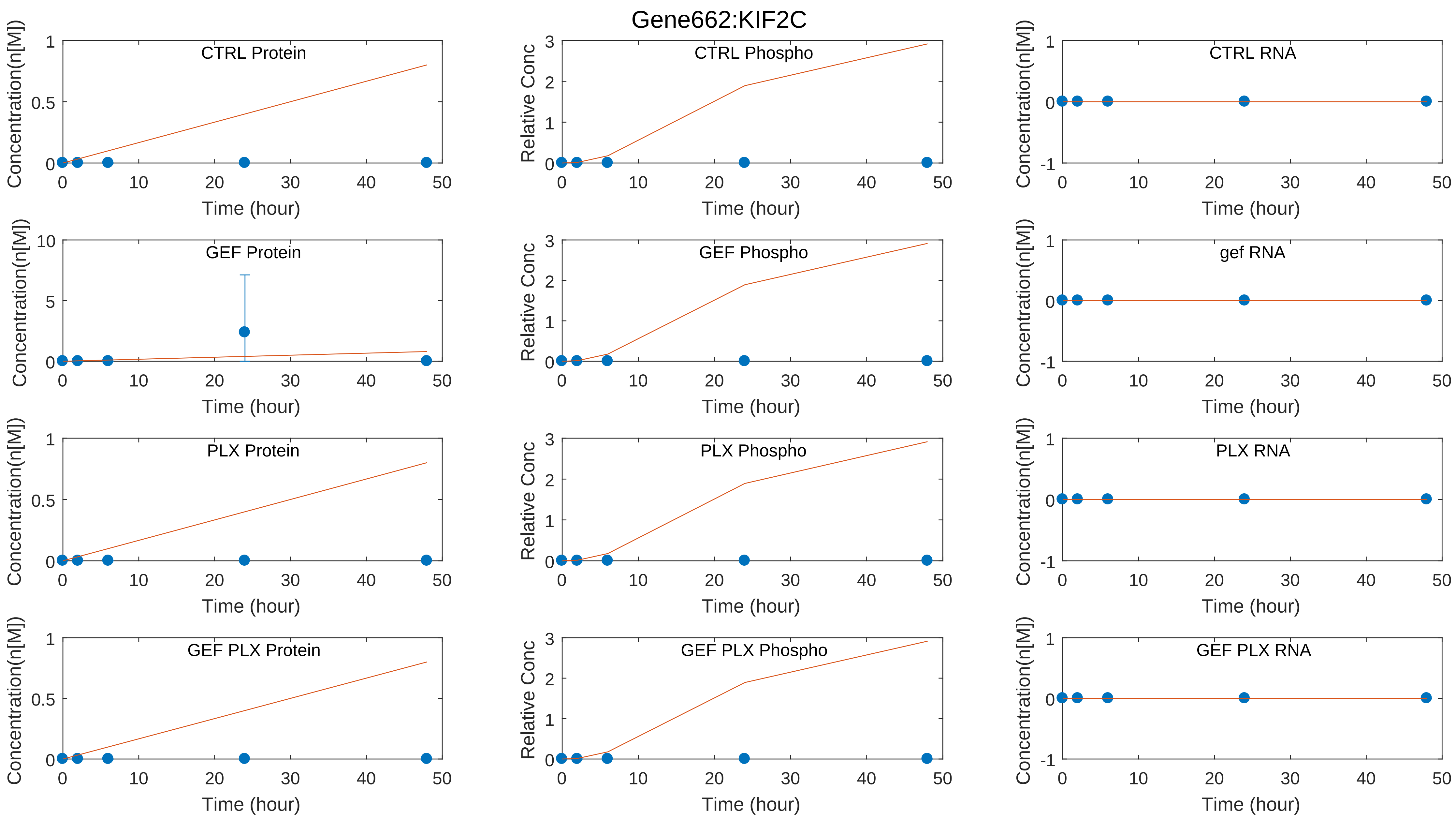

Gene662:KIF2C
CTRL Protein
CTRL Phospho
CTRL RNA
GEF Protein
GEF Phospho
gef RNA
PLX Protein
PLX Phospho
PLX RNA
GEF PLX Protein
GEF PLX Phospho
GEF PLX RNA
Concentration(n[M])
Relative Conc
Time (hour)

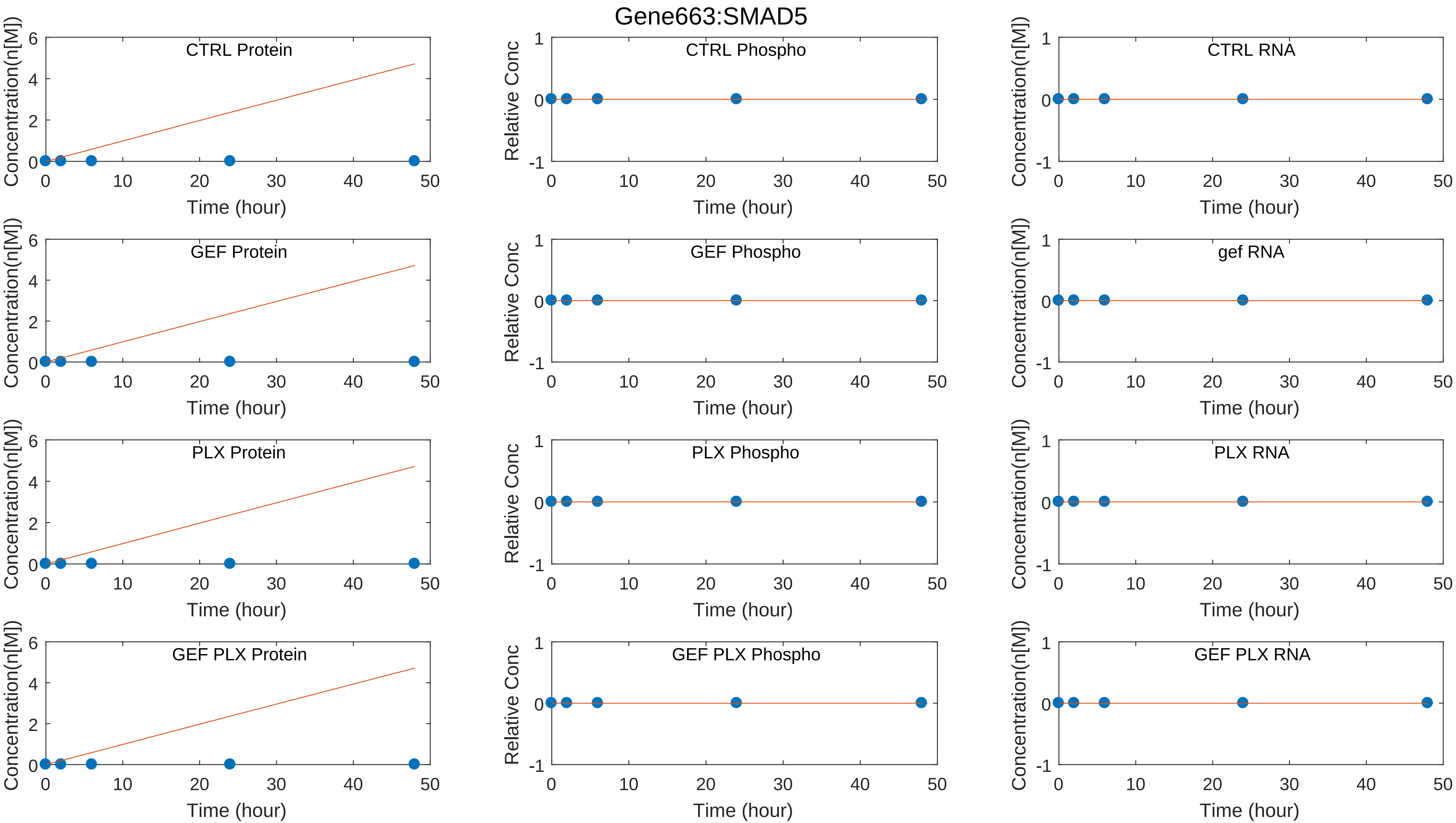

Gene663:SMAD5
CTRL Protein
CTRL Phospho
CTRL RNA
GEF Protein
GEF Phospho
gef RNA
PLX Protein
PLX Phospho
PLX RNA
GEF PLX Protein
GEF PLX Phospho
GEF PLX RNA
Concentration(n[M])
Relative Conc
Time (hour)

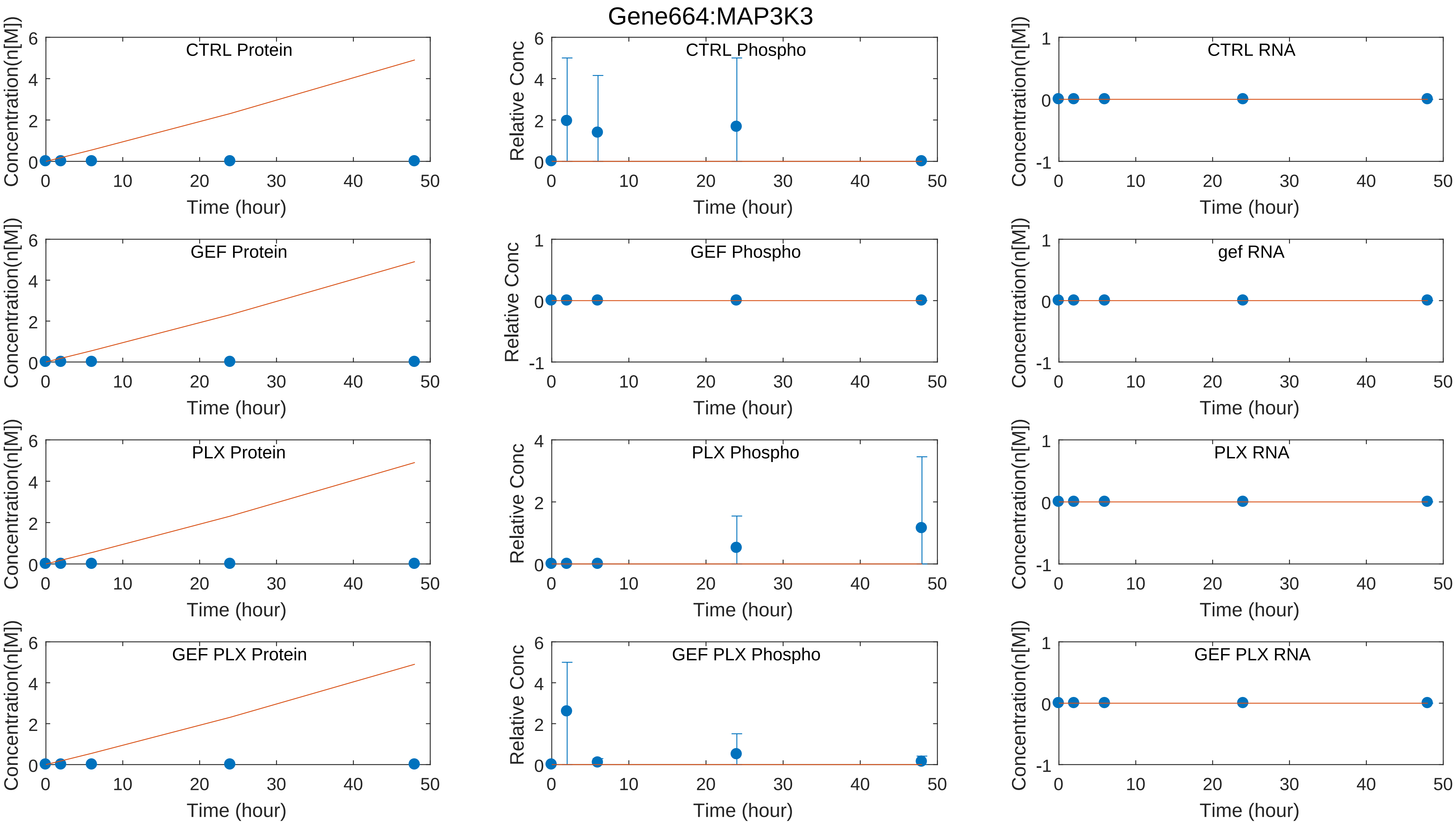
Gene664:MAP3K3
CTRL Protein
CTRL Phospho
CTRL RNA
GEF Protein
GEF Phospho
gef RNA
PLX Protein
PLX Phospho
PLX RNA
GEF PLX Protein
GEF PLX Phospho
GEF PLX RNA
Concentration(n[M])
Relative Conc
Time (hour)

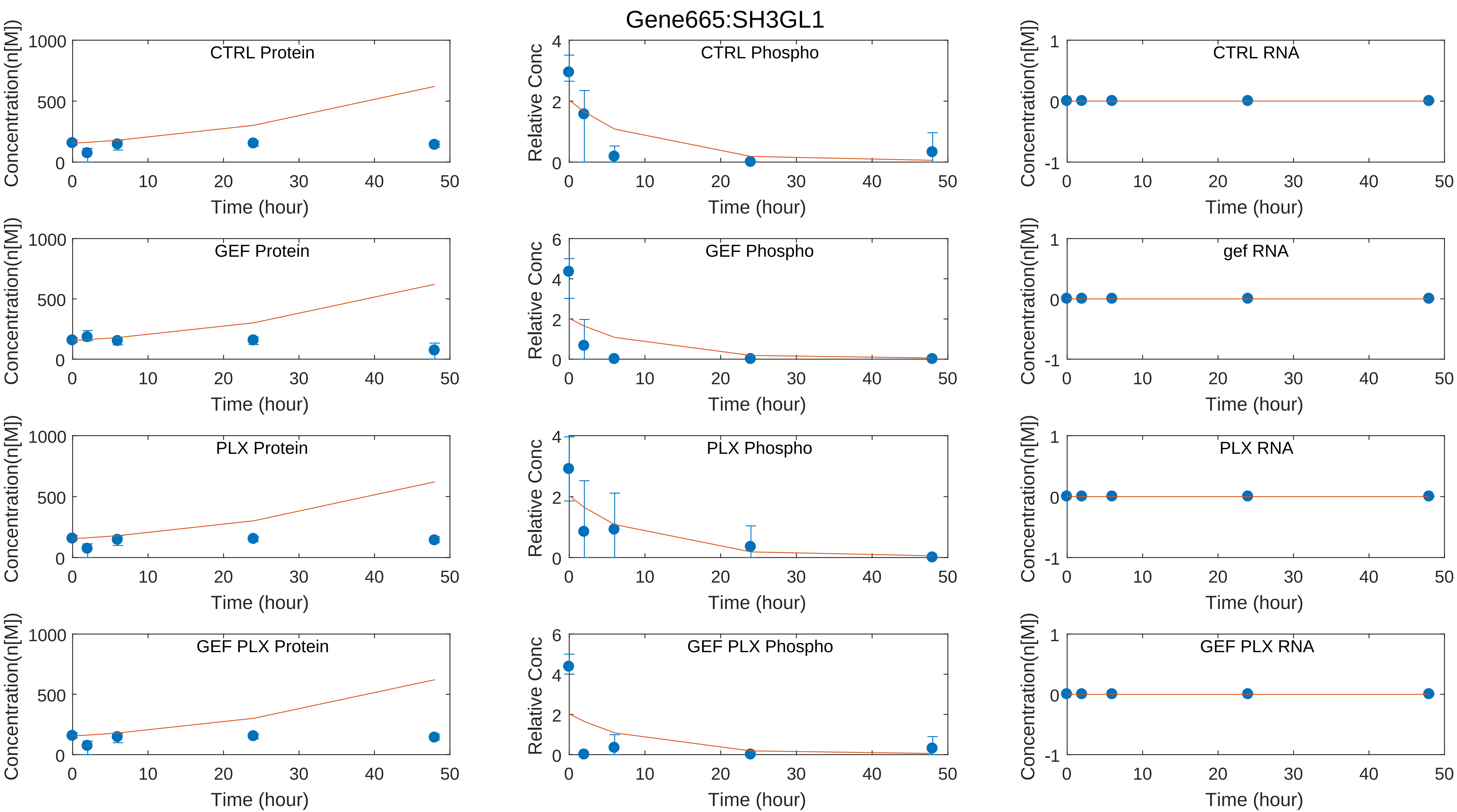
Gene665:SH3GL1
CTRL Protein
CTRL Phospho
CTRL RNA
GEF Protein
GEF Phospho
gef RNA
PLX Protein
PLX Phospho
PLX RNA
GEF PLX Protein
GEF PLX Phospho
GEF PLX RNA
Concentration(n[M])
Relative Conc
Time (hour)

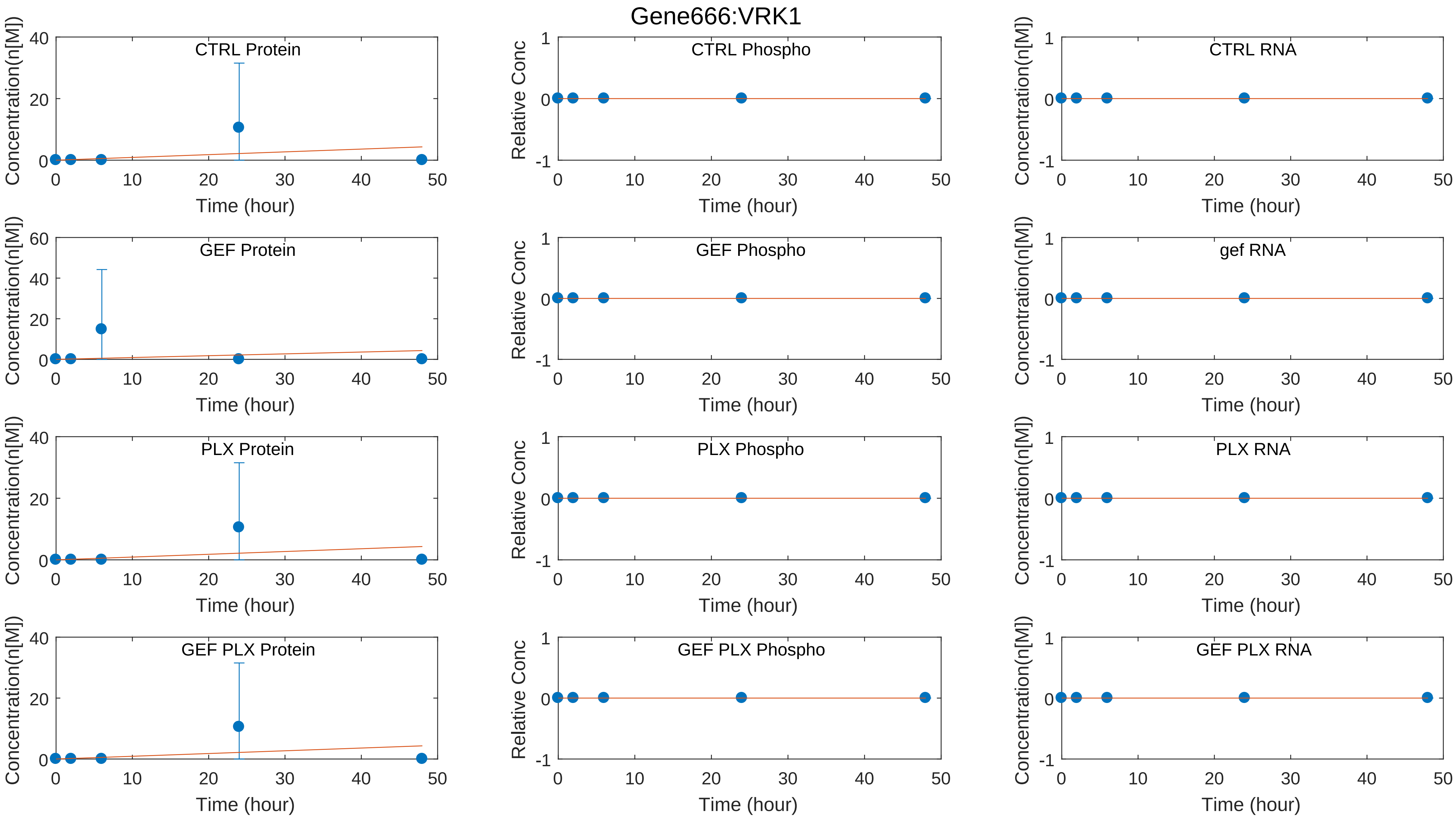
Gene666:VRK1
CTRL Protein
CTRL Phospho
CTRL RNA
GEF Protein
GEF Phospho
gef RNA
PLX Protein
PLX Phospho
PLX RNA
GEF PLX Protein
GEF PLX Phospho
GEF PLX RNA
Concentration(n[M])
Relative Conc
Time (hour)

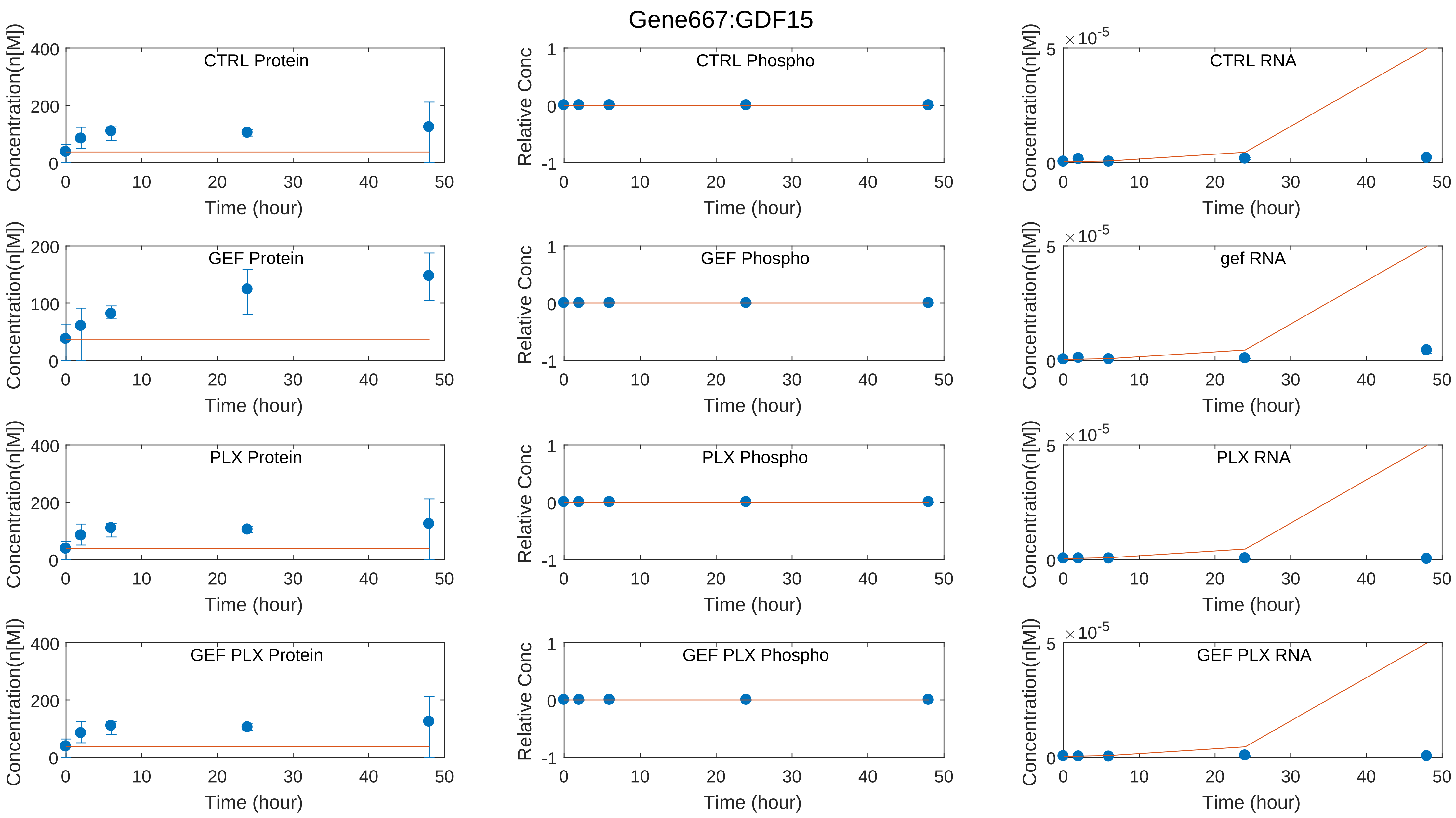
Gene667:GDF15
CTRL Protein
CTRL Phospho
CTRL RNA
GEF Protein
GEF Phospho
gef RNA
PLX Protein
PLX Phospho
PLX RNA
GEF PLX Protein
GEF PLX Phospho
GEF PLX RNA
Concentration(n[M])
Relative Conc
Time (hour)
$\times 10^{-5}$

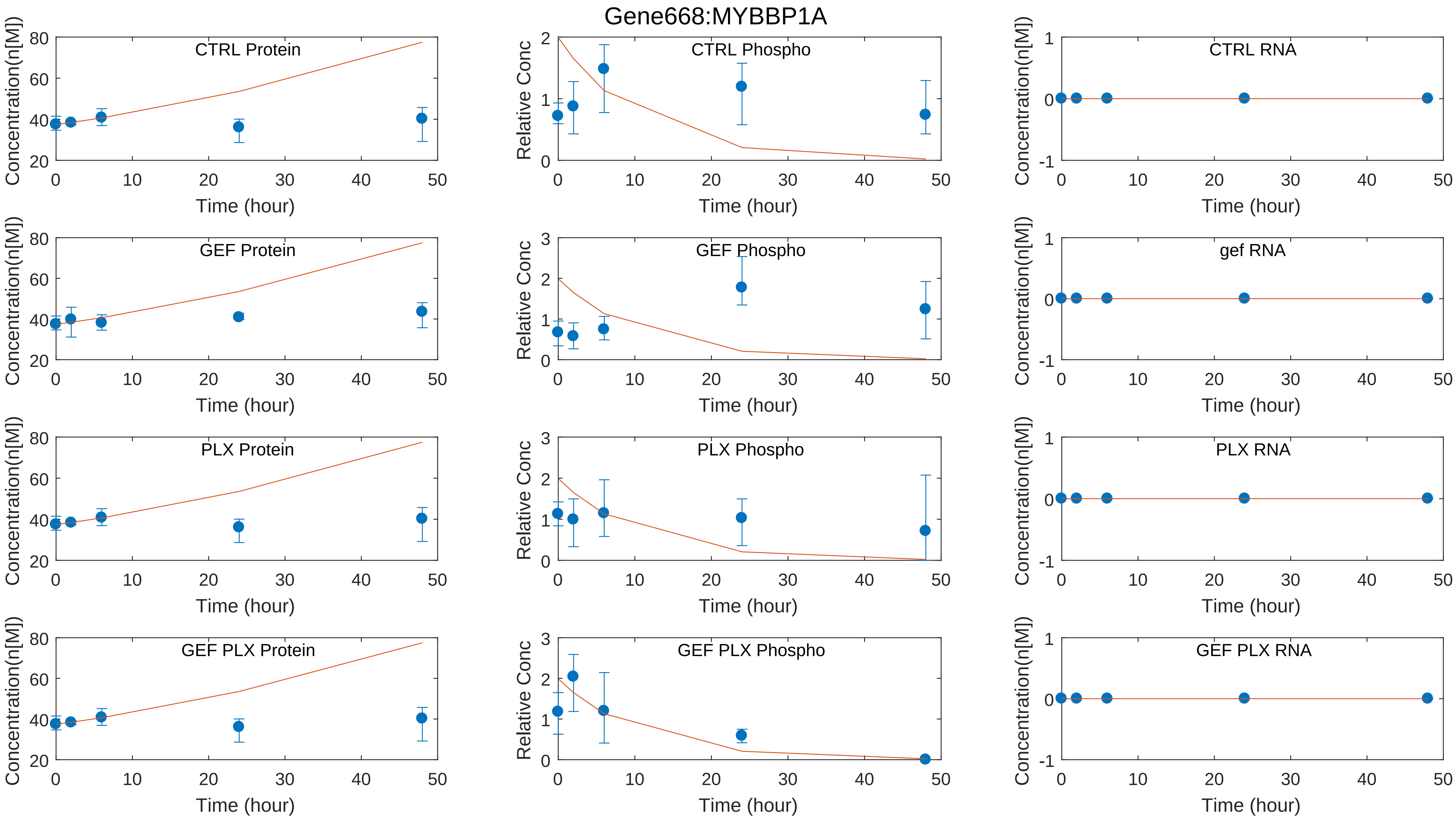

Gene668:MYBBP1A
CTRL Protein
GEF Protein
PLX Protein
GEF PLX Protein
CTRL Phospho
GEF Phospho
PLX Phospho
GEF PLX Phospho
CTRL RNA
gef RNA
PLX RNA
GEF PLX RNA
Concentration(n[M])
Relative Conc
Time (hour)

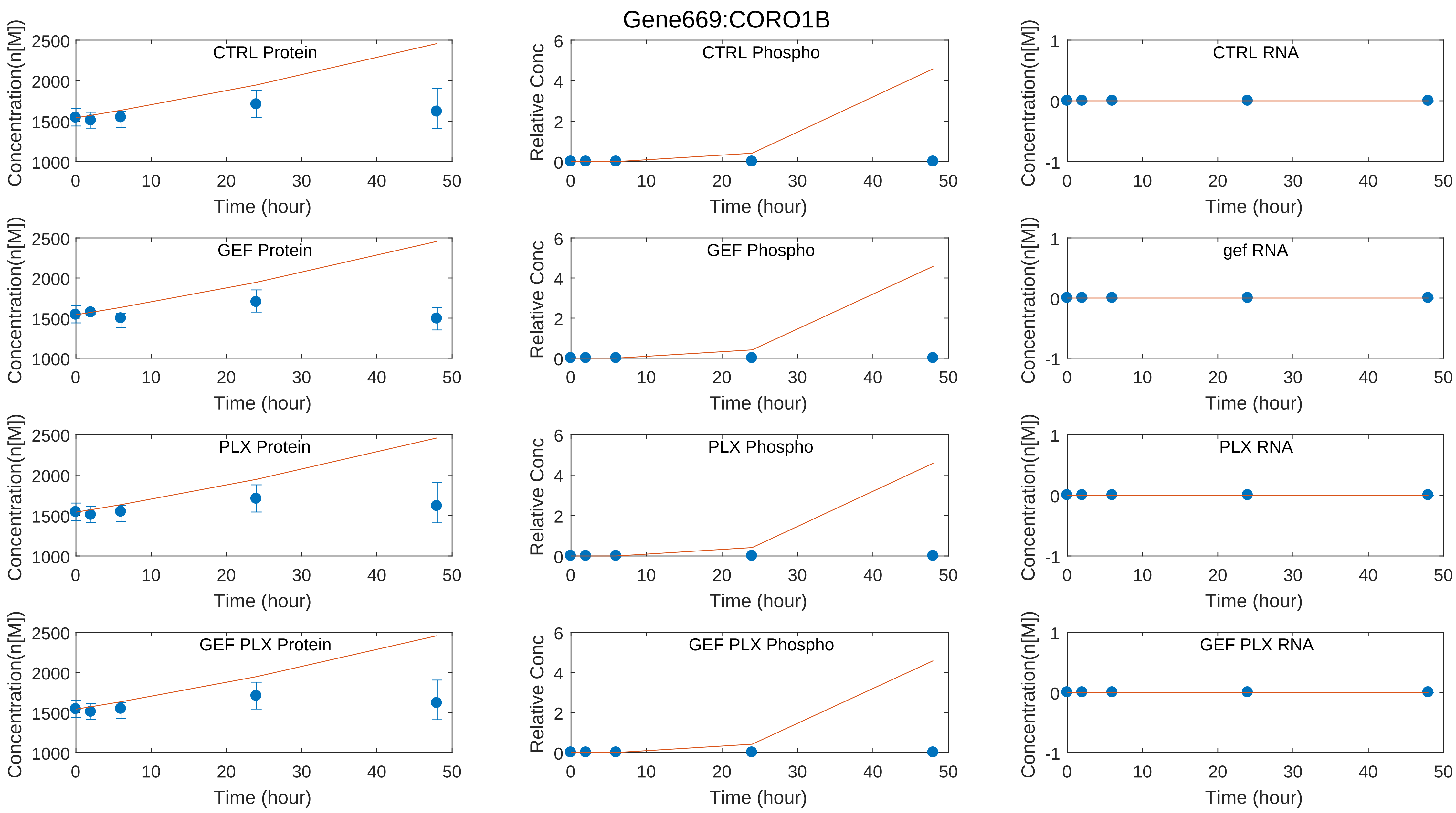
Gene669:CORO1B
CTRL Protein
CTRL Phospho
CTRL RNA
GEF Protein
GEF Phospho
gef RNA
PLX Protein
PLX Phospho
PLX RNA
GEF PLX Protein
GEF PLX Phospho
GEF PLX RNA
Concentration(n[M])
Relative Conc
Time (hour)

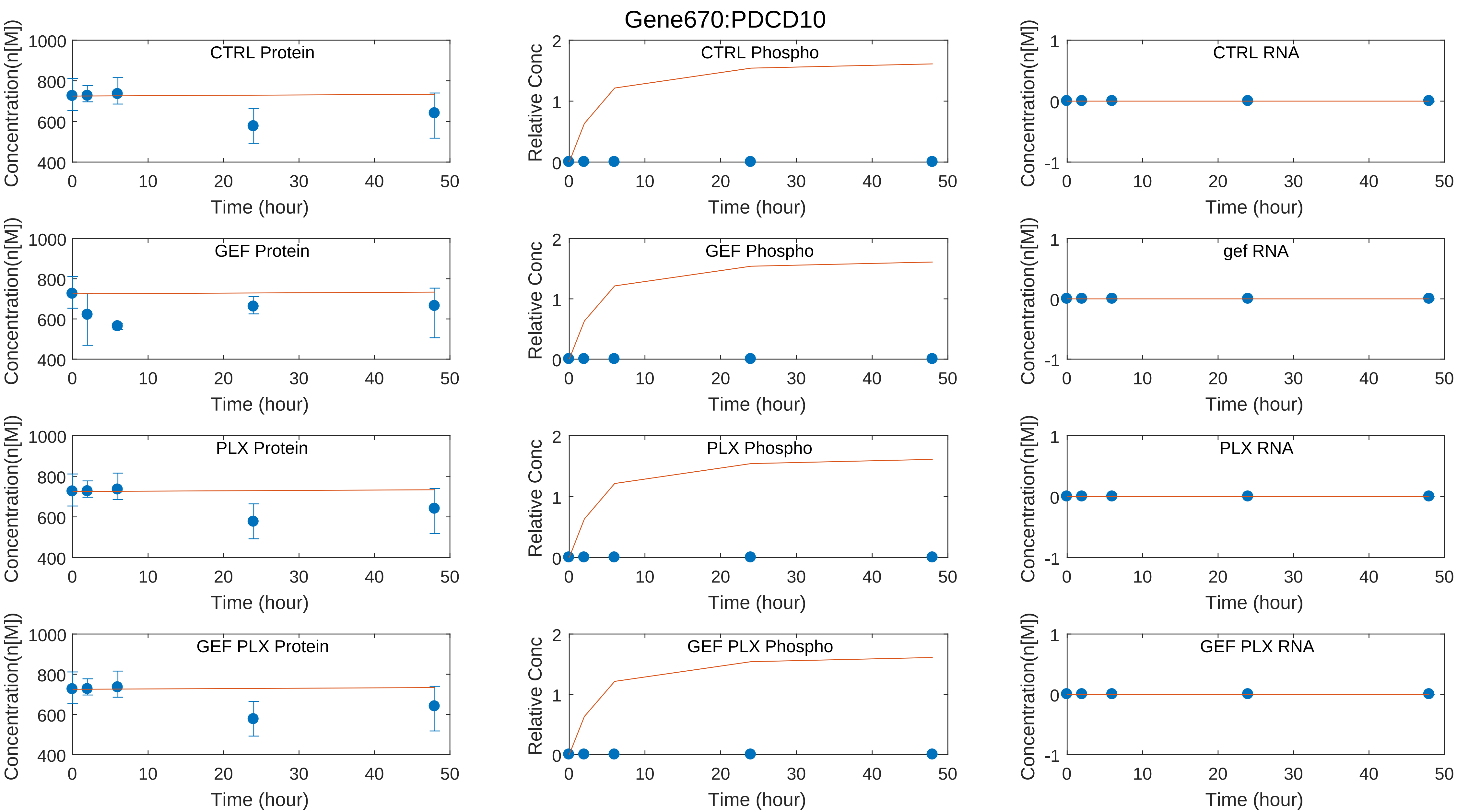

Gene670:PDCD10
CTRL Protein
CTRL Phospho
CTRL RNA
GEF Protein
GEF Phospho
gef RNA
PLX Protein
PLX Phospho
PLX RNA
GEF PLX Protein
GEF PLX Phospho
GEF PLX RNA
Concentration(n[M])
Relative Conc
Time (hour)

Gene671:AP1M1

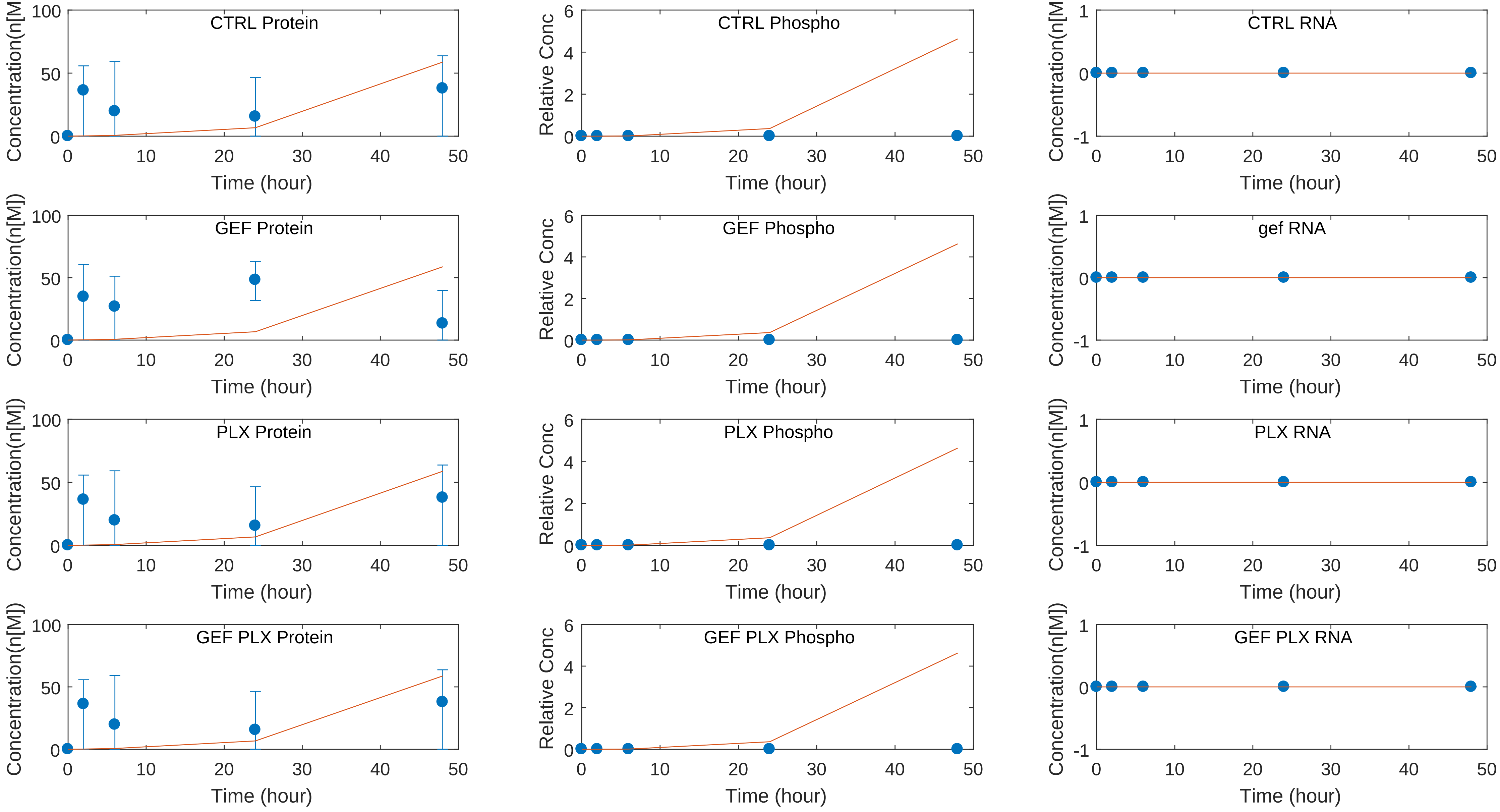

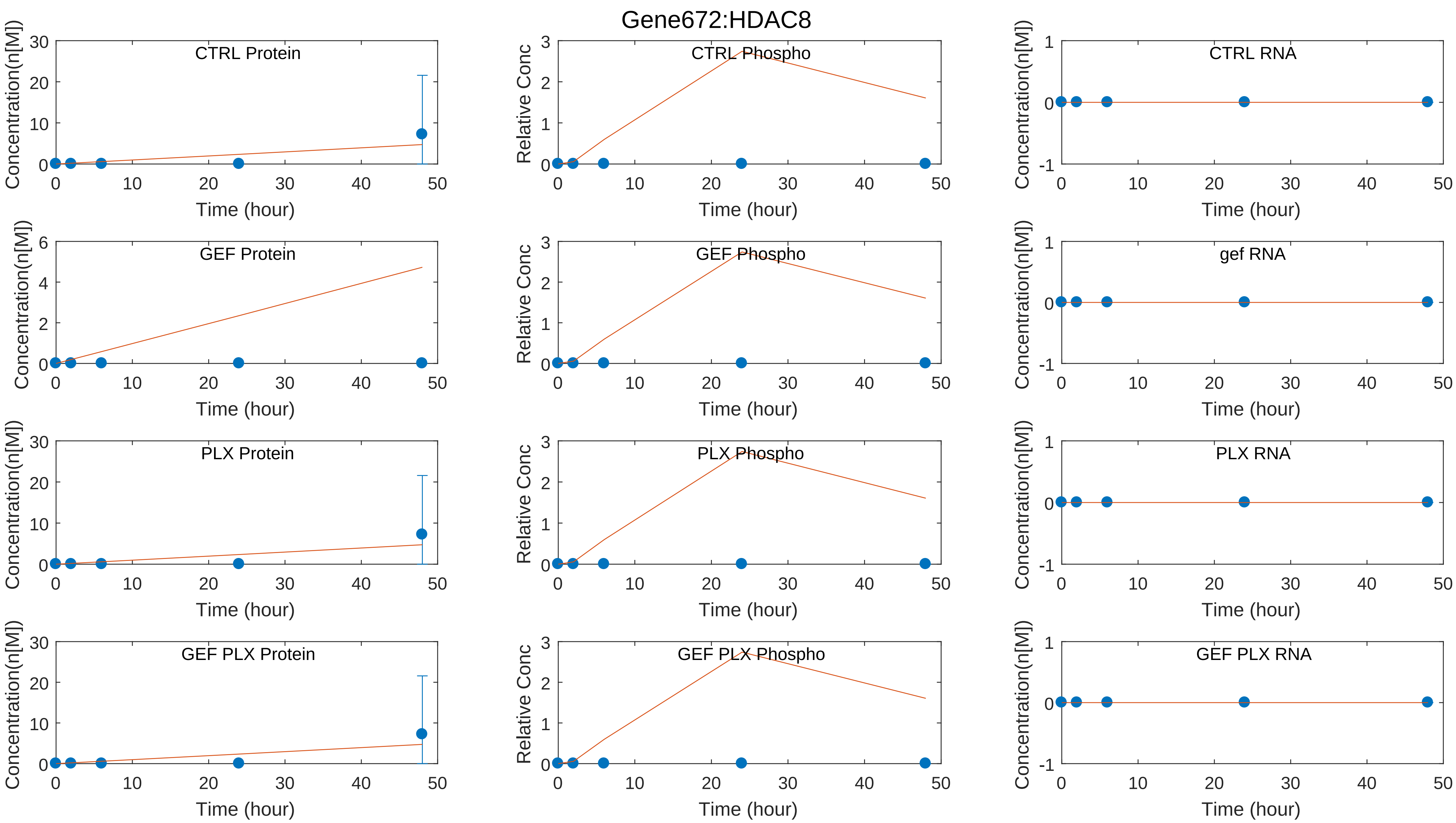

Gene672:HDAC8
CTRL Protein
CTRL Phospho
CTRL RNA
GEF Protein
GEF Phospho
gef RNA
PLX Protein
PLX Phospho
PLX RNA
GEF PLX Protein
GEF PLX Phospho
GEF PLX RNA
Concentration(n[M])
Relative Conc
Time (hour)

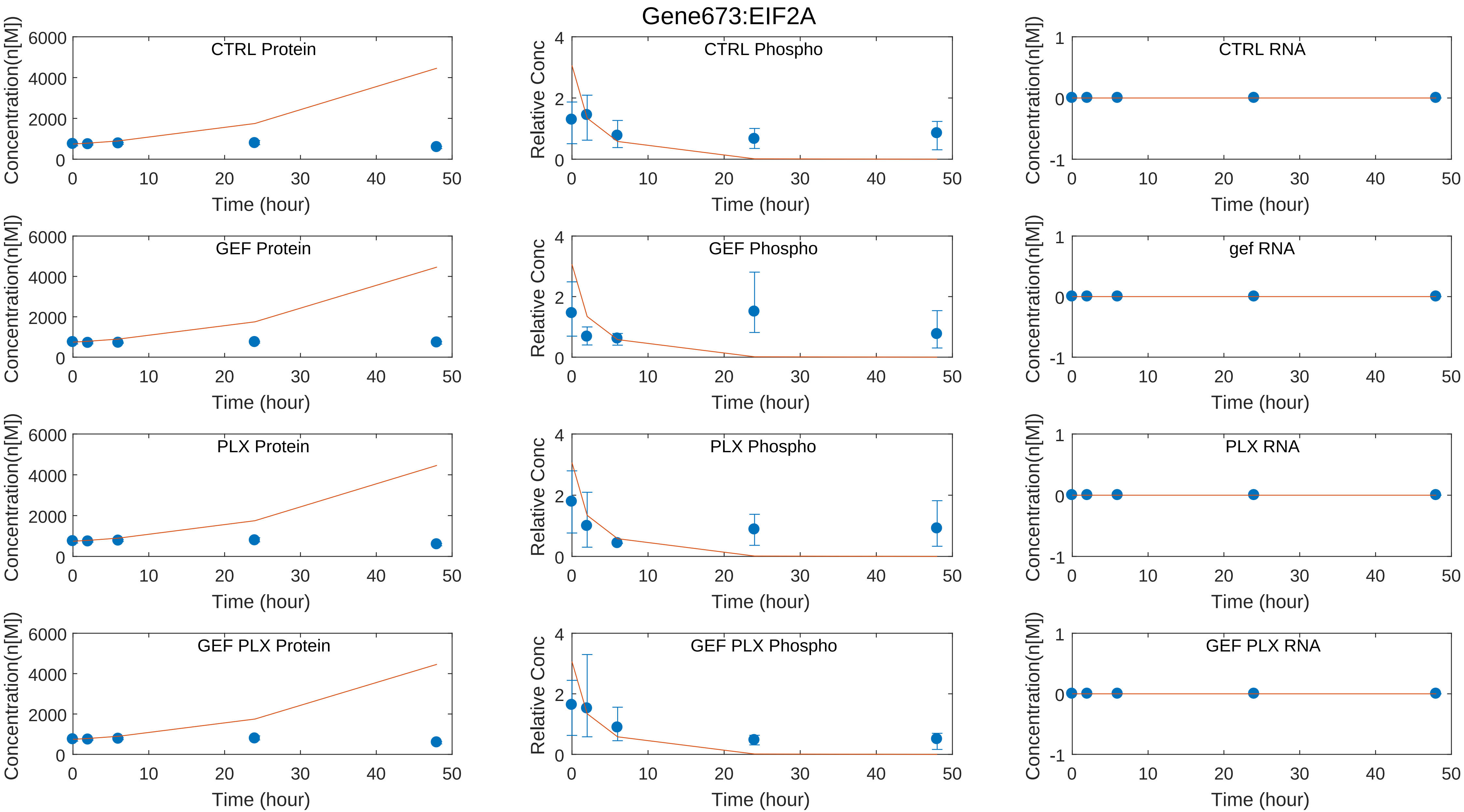

Gene673:EIF2A
CTRL Protein
CTRL Phospho
CTRL RNA
GEF Protein
GEF Phospho
gef RNA
PLX Protein
PLX Phospho
PLX RNA
GEF PLX Protein
GEF PLX Phospho
GEF PLX RNA
Concentration(n[M])
Relative Conc
Time (hour)

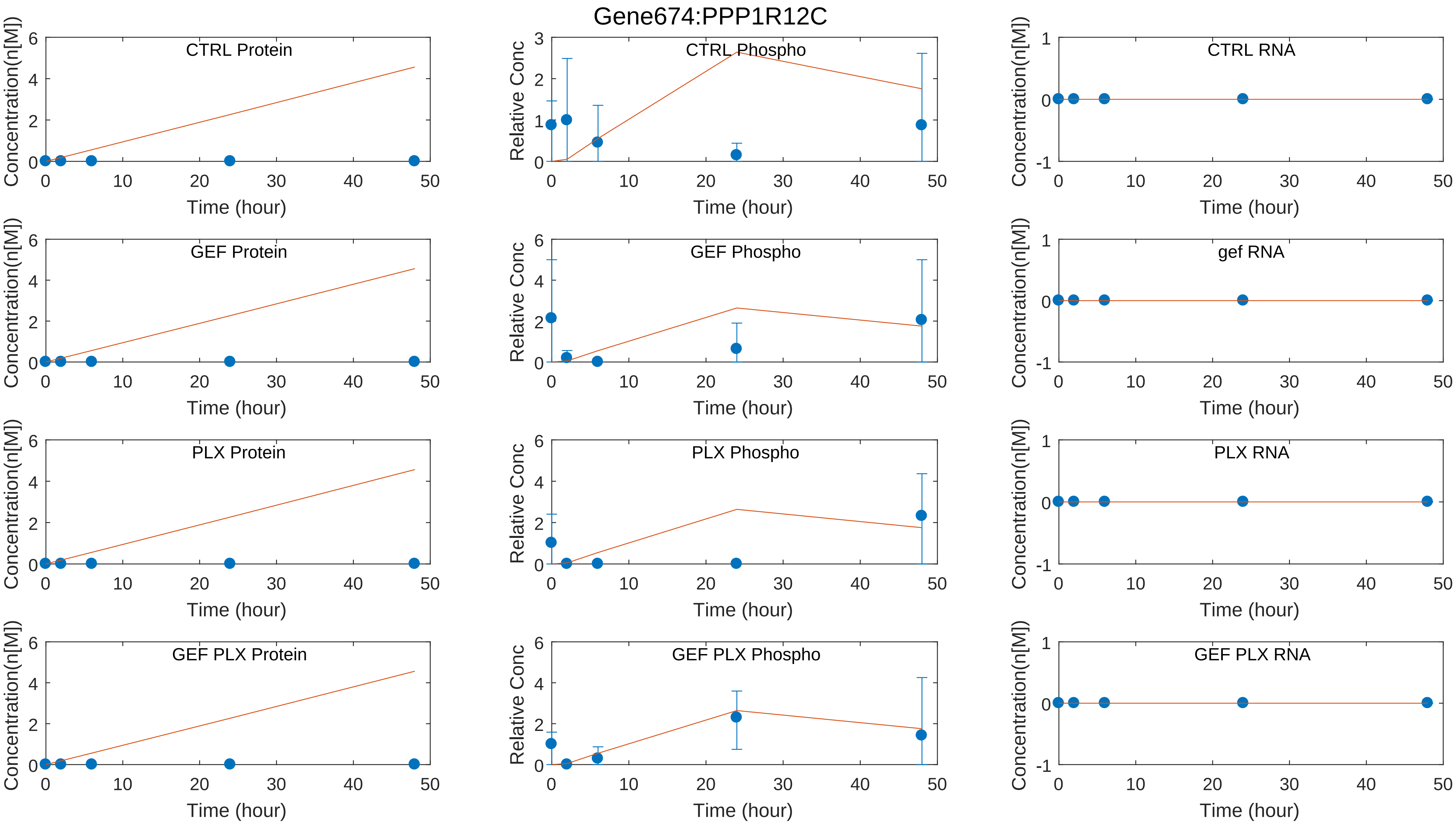

Gene674:PPP1R12C
CTRL Protein
CTRL Phospho
CTRL RNA
GEF Protein
GEF Phospho
gef RNA
PLX Protein
PLX Phospho
PLX RNA
GEF PLX Protein
GEF PLX Phospho
GEF PLX RNA
Concentration(n[M])
Relative Conc
Time (hour)

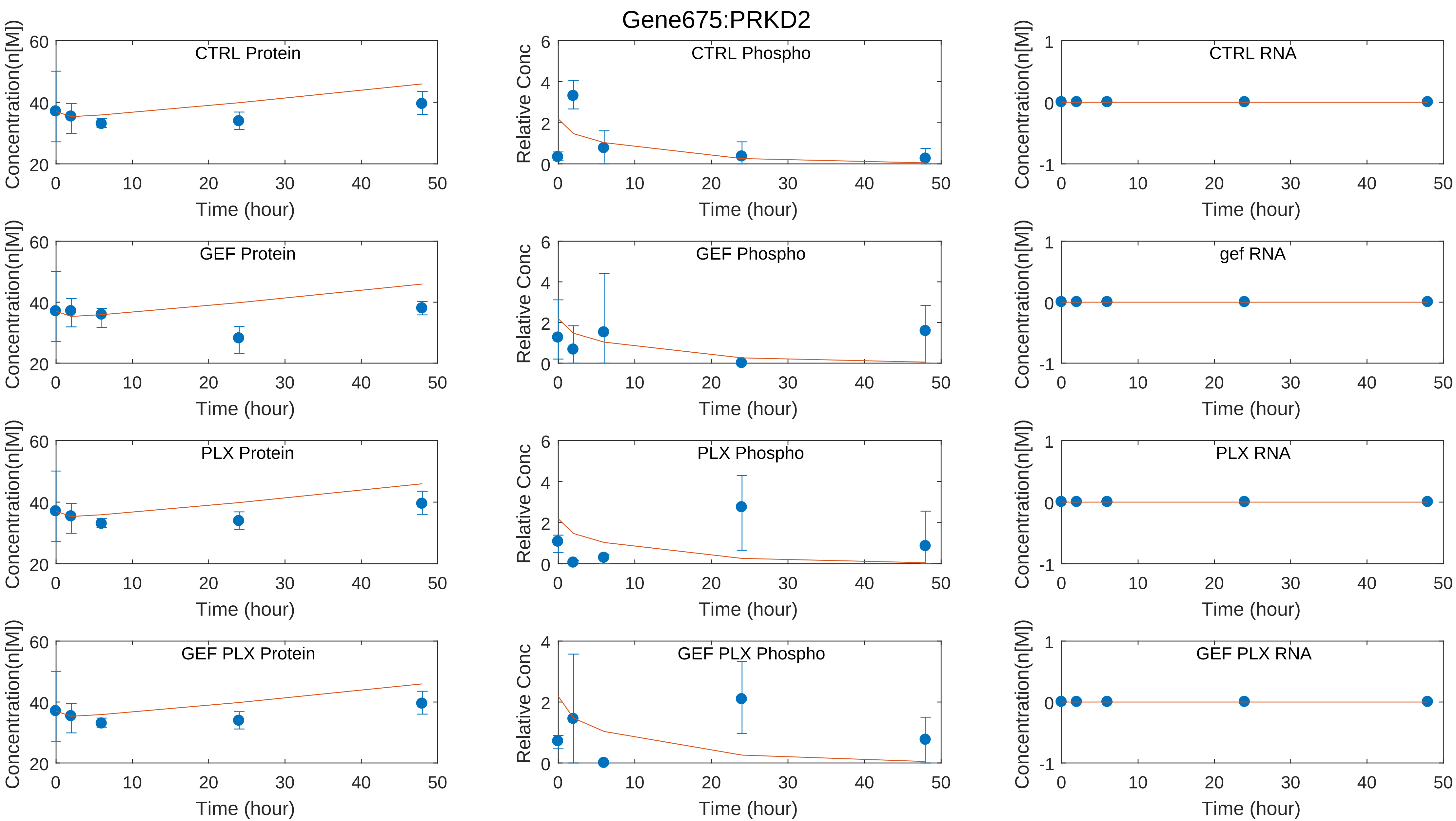
Gene675:PRKD2
CTRL Protein
CTRL Phospho
CTRL RNA
GEF Protein
GEF Phospho
gef RNA
PLX Protein
PLX Phospho
PLX RNA
GEF PLX Protein
GEF PLX Phospho
GEF PLX RNA
Concentration(n[M])
Relative Conc
Time (hour)

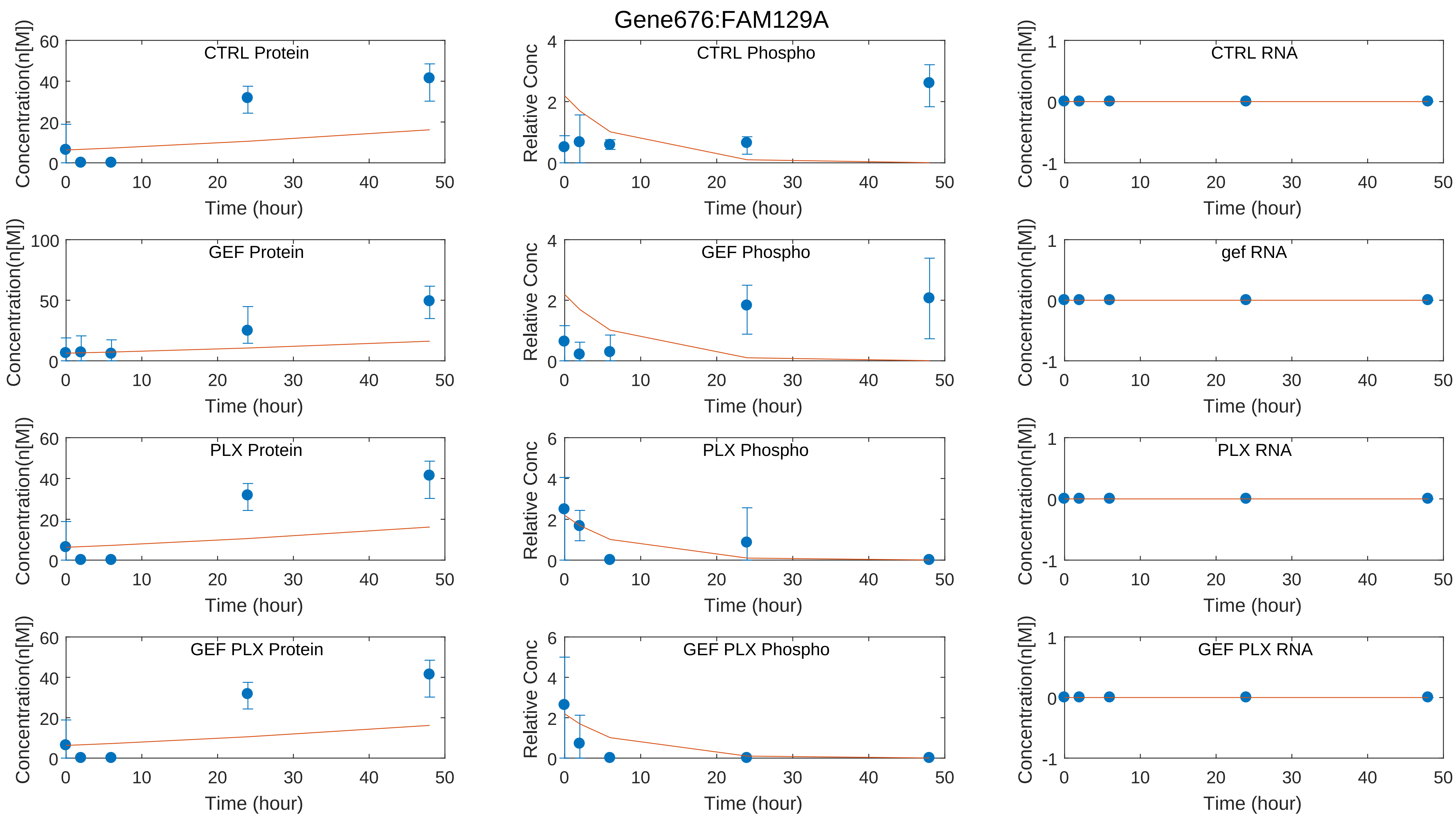
Gene676:FAM129A
CTRL Protein
CTRL Phospho
CTRL RNA
GEF Protein
GEF Phospho
gef RNA
PLX Protein
PLX Phospho
PLX RNA
GEF PLX Protein
GEF PLX Phospho
GEF PLX RNA
Concentration(n[M])
Relative Conc
Time (hour)

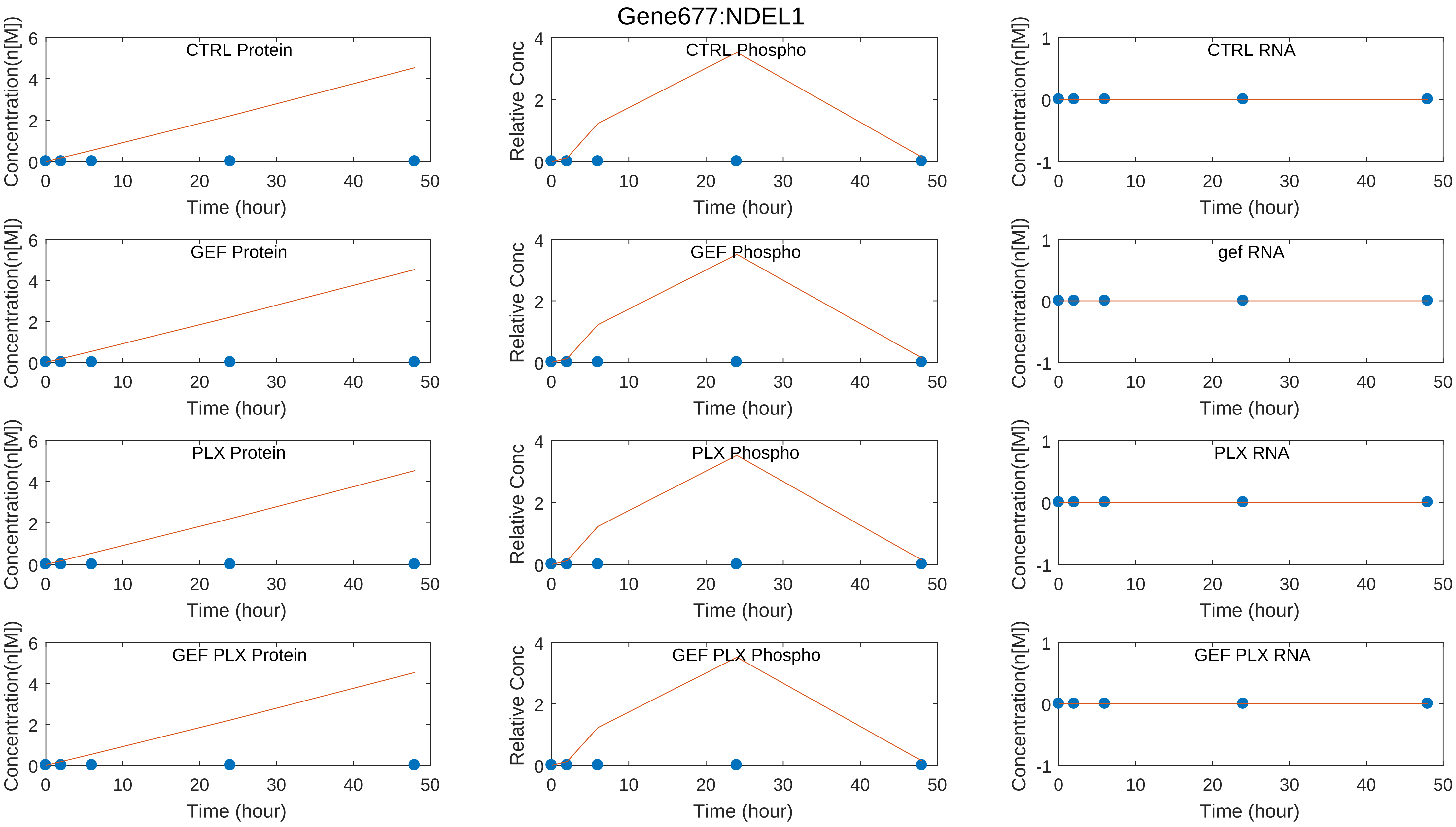
Gene677:NDEL1
CTRL Protein
CTRL Phospho
CTRL RNA
GEF Protein
GEF Phospho
gef RNA
PLX Protein
PLX Phospho
PLX RNA
GEF PLX Protein
GEF PLX Phospho
GEF PLX RNA
Concentration(n[M])
Relative Conc
Time (hour)

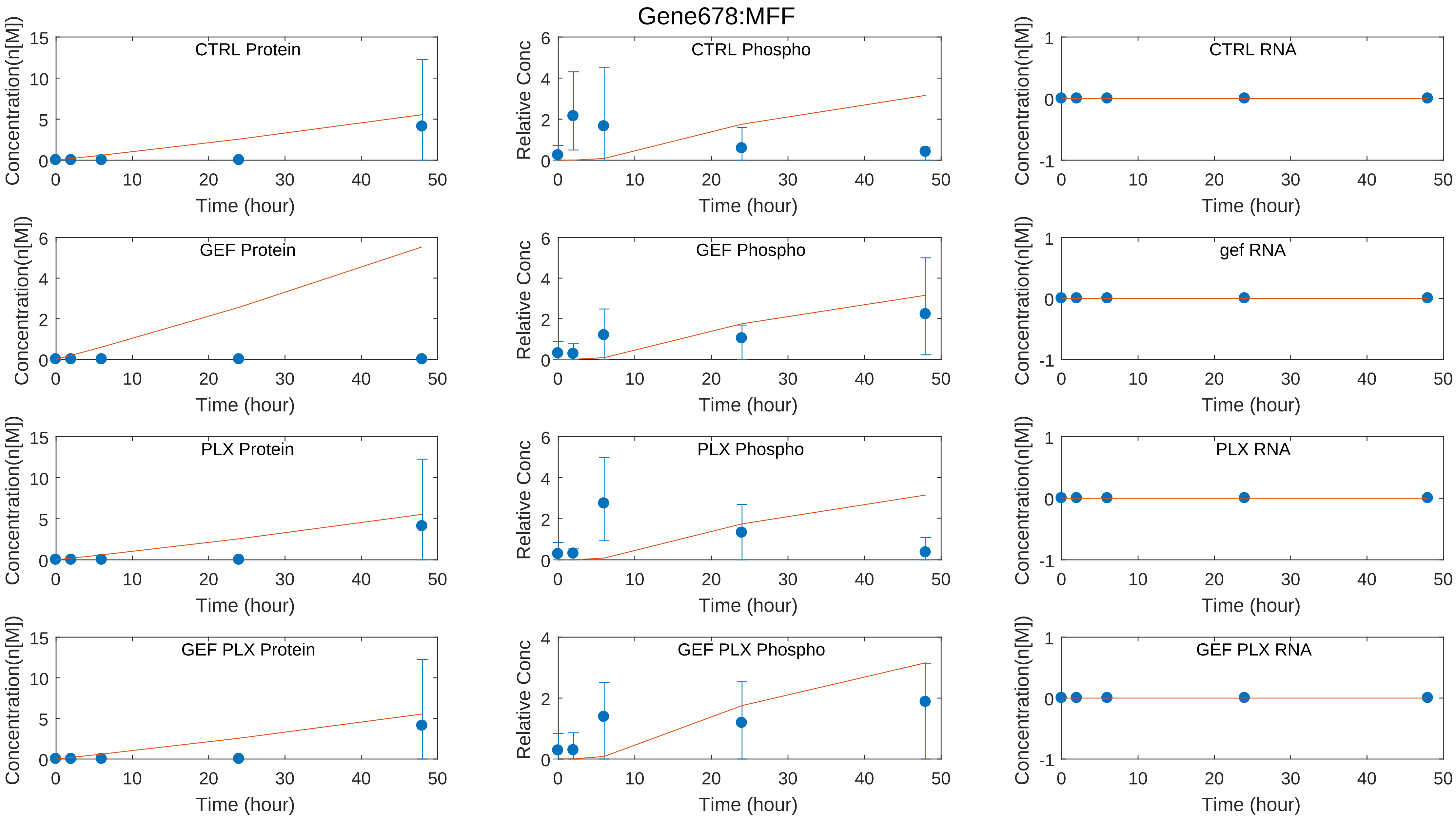

Gene678:MFF
CTRL Protein
CTRL Phospho
CTRL RNA
GEF Protein
GEF Phospho
gef RNA
PLX Protein
PLX Phospho
PLX RNA
GEF PLX Protein
GEF PLX Phospho
GEF PLX RNA
Concentration(n[M])
Relative Conc
Time (hour)

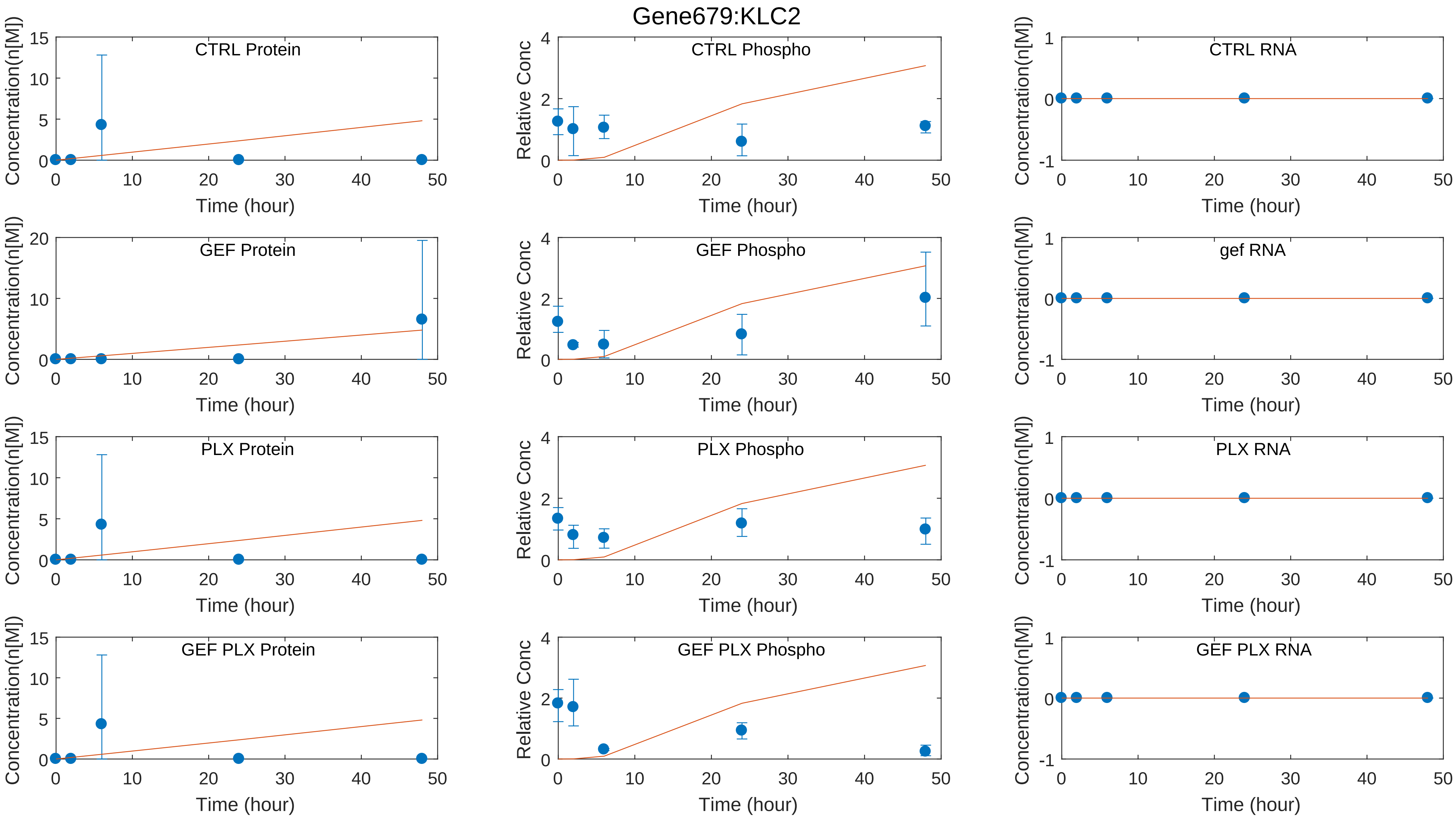

Gene679:KLC2
CTRL Protein
CTRL Phospho
CTRL RNA
GEF Protein
GEF Phospho
gef RNA
PLX Protein
PLX Phospho
PLX RNA
GEF PLX Protein
GEF PLX Phospho
GEF PLX RNA
Concentration(n[M])
Relative Conc
Time (hour)

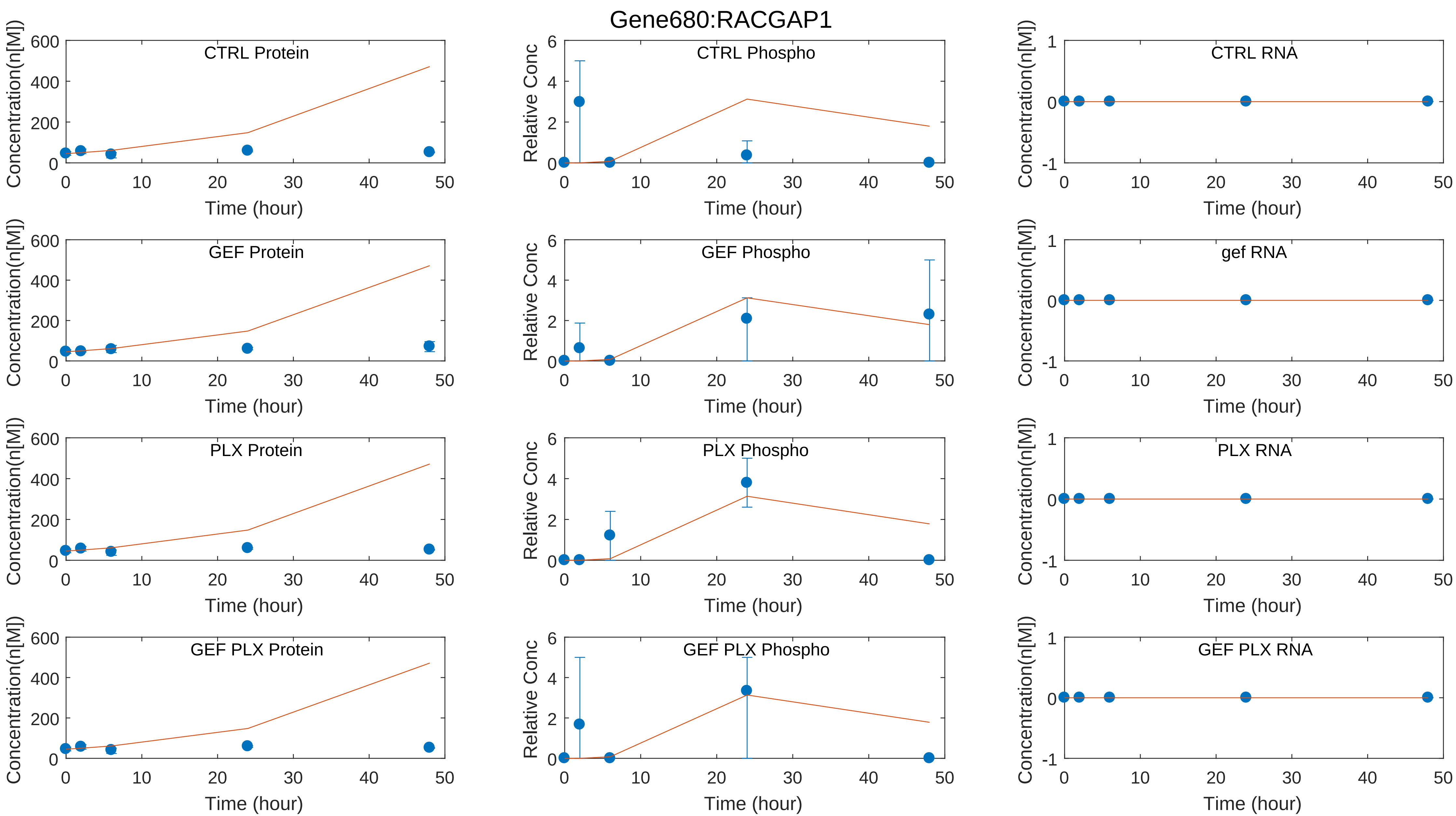

Gene680:RACGAP1
CTRL Protein
CTRL Phospho
CTRL RNA
GEF Protein
GEF Phospho
gef RNA
PLX Protein
PLX Phospho
PLX RNA
GEF PLX Protein
GEF PLX Phospho
GEF PLX RNA
Concentration(n[M])
Relative Conc
Time (hour)

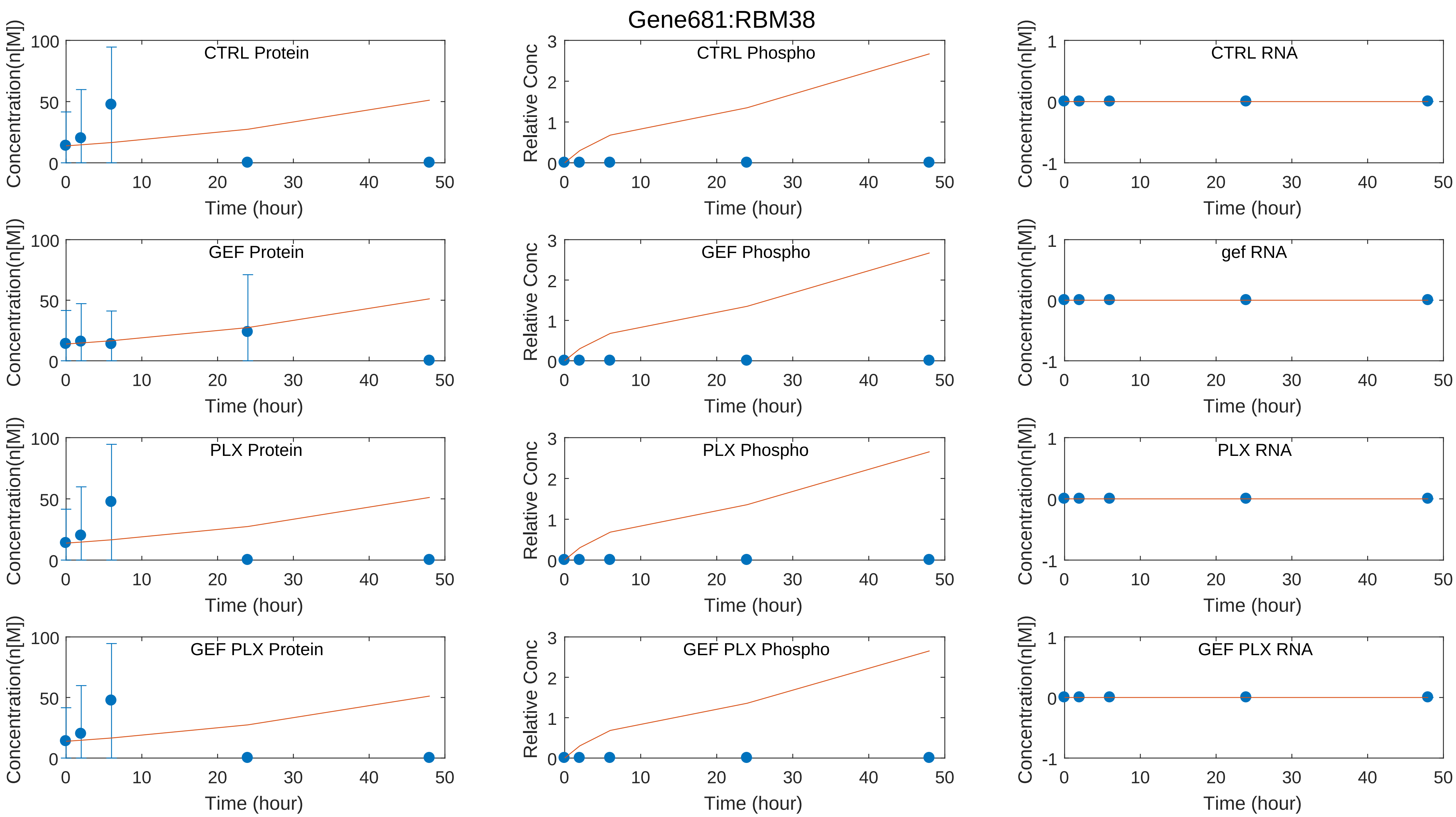

Gene681:RBM38
CTRL Protein
CTRL Phospho
CTRL RNA
GEF Protein
GEF Phospho
gef RNA
PLX Protein
PLX Phospho
PLX RNA
GEF PLX Protein
GEF PLX Phospho
GEF PLX RNA
Concentration(n[M])
Relative Conc
Time (hour)

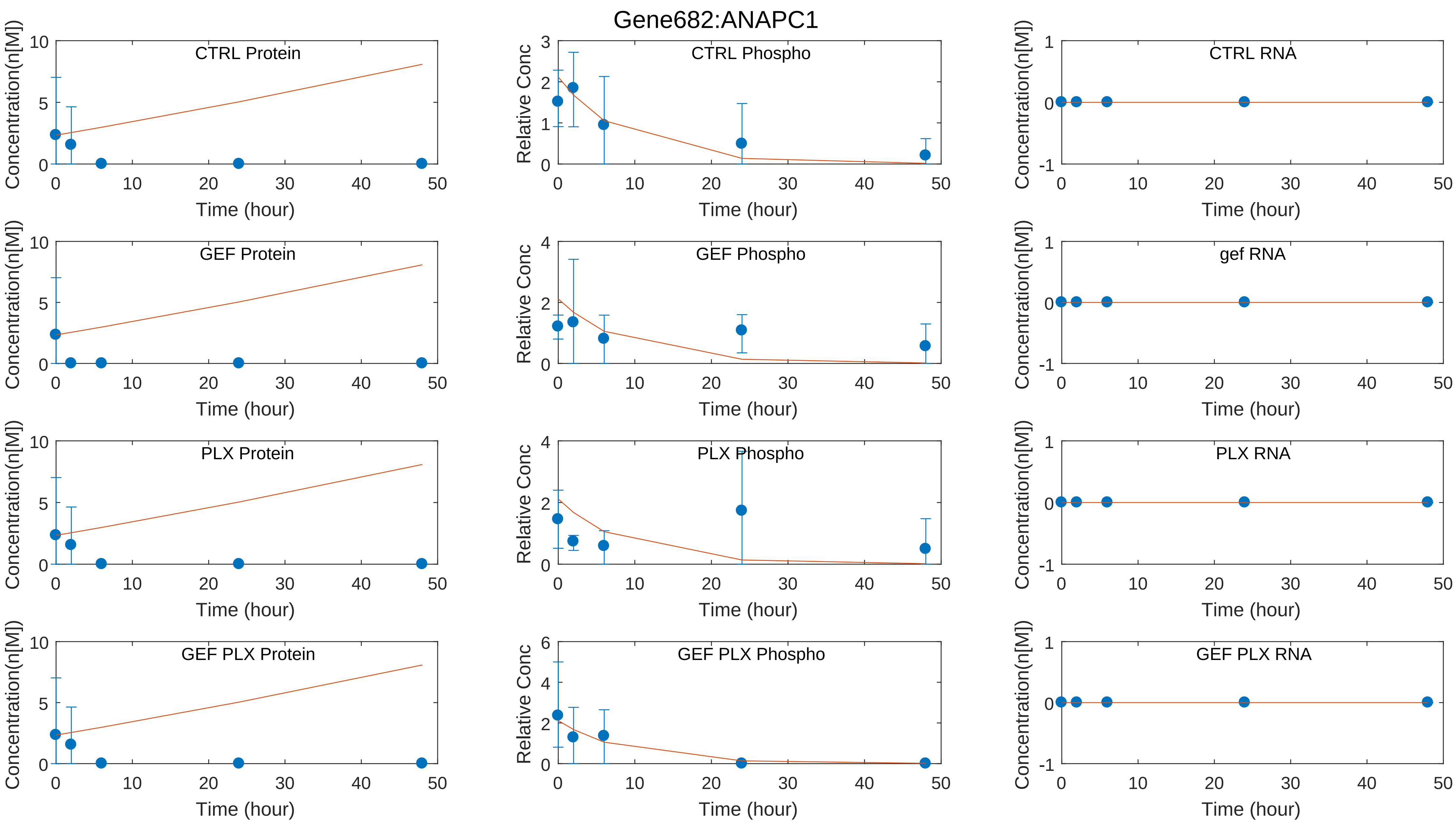

Gene682:ANAPC1
CTRL Protein
CTRL Phospho
CTRL RNA
GEF Protein
GEF Phospho
gef RNA
PLX Protein
PLX Phospho
PLX RNA
GEF PLX Protein
GEF PLX Phospho
GEF PLX RNA
Concentration(n[M])
Relative Conc
Time (hour)

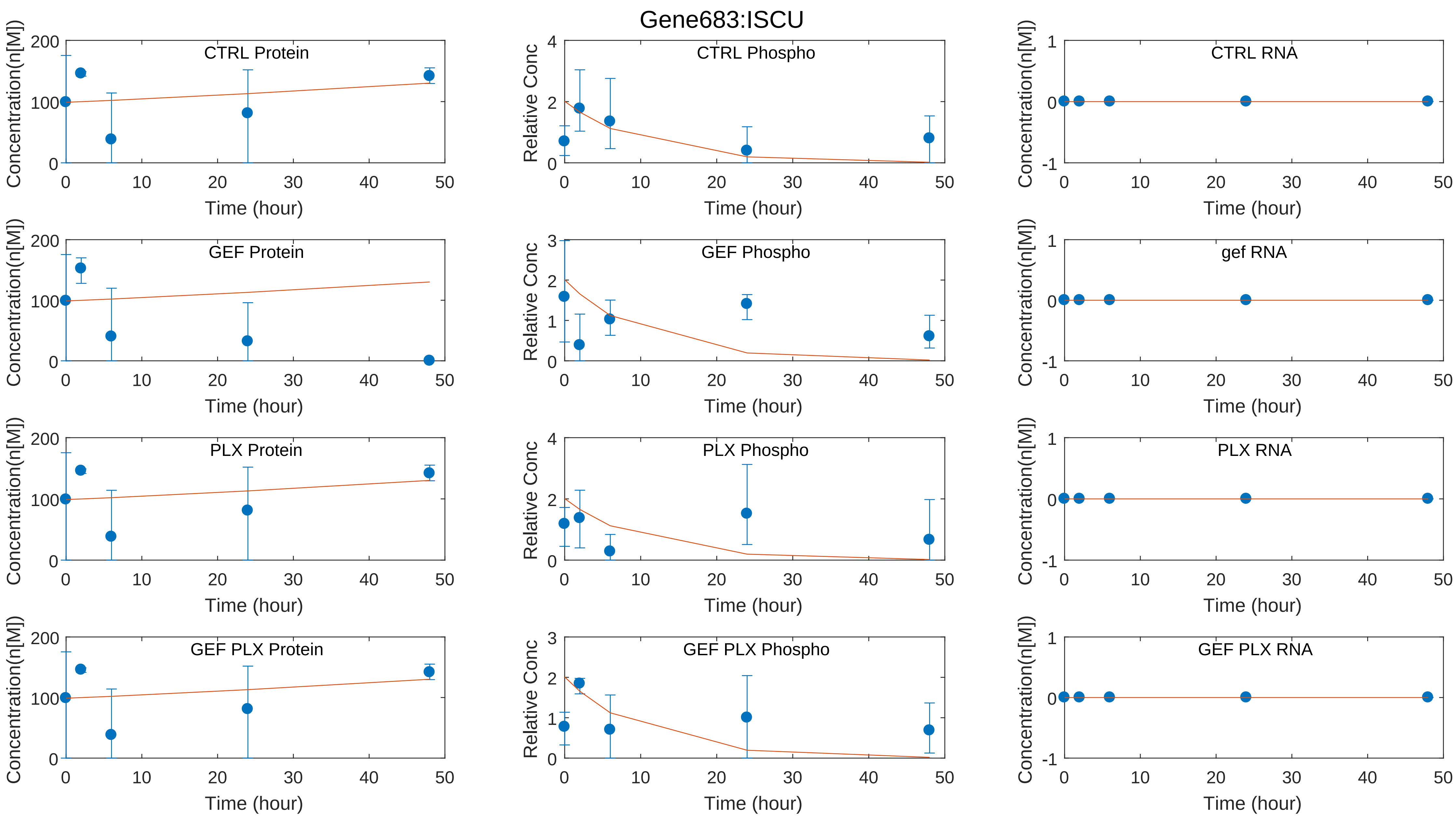

Gene683:ISCU
CTRL Protein
CTRL Phospho
CTRL RNA
GEF Protein
GEF Phospho
gef RNA
PLX Protein
PLX Phospho
PLX RNA
GEF PLX Protein
GEF PLX Phospho
GEF PLX RNA
Concentration(n[M])
Relative Conc
Time (hour)

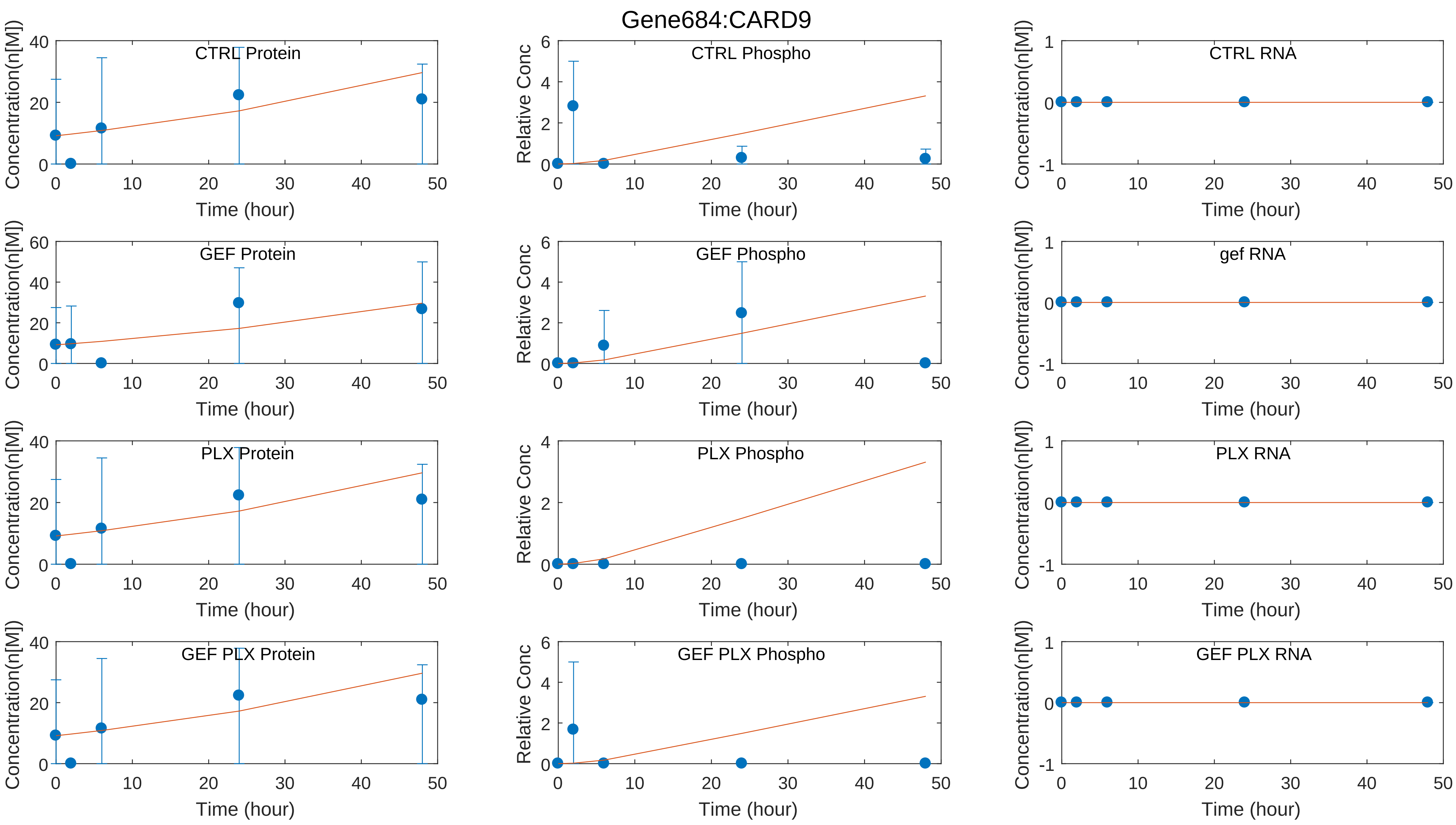

Gene684:CARD9
CTRL Protein
CTRL Phospho
CTRL RNA
GEF Protein
GEF Phospho
gef RNA
PLX Protein
PLX Phospho
PLX RNA
GEF PLX Protein
GEF PLX Phospho
GEF PLX RNA
Concentration(n[M])
Relative Conc
Time (hour)

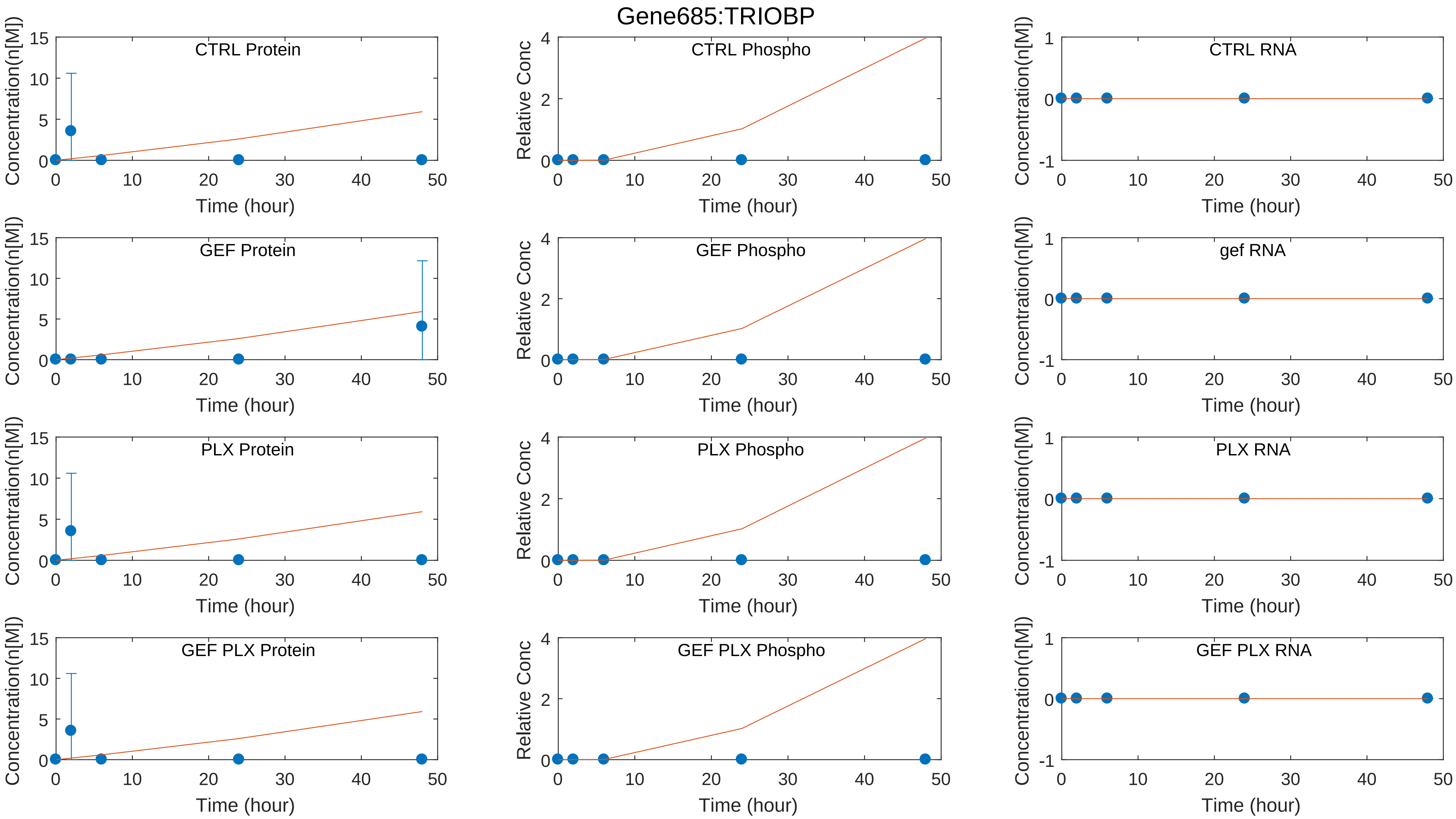

Gene685:TRIOBP
CTRL Protein
CTRL Phospho
CTRL RNA
GEF Protein
GEF Phospho
gef RNA
PLX Protein
PLX Phospho
PLX RNA
GEF PLX Protein
GEF PLX Phospho
GEF PLX RNA
Concentration(n[M])
Relative Conc
Time (hour)

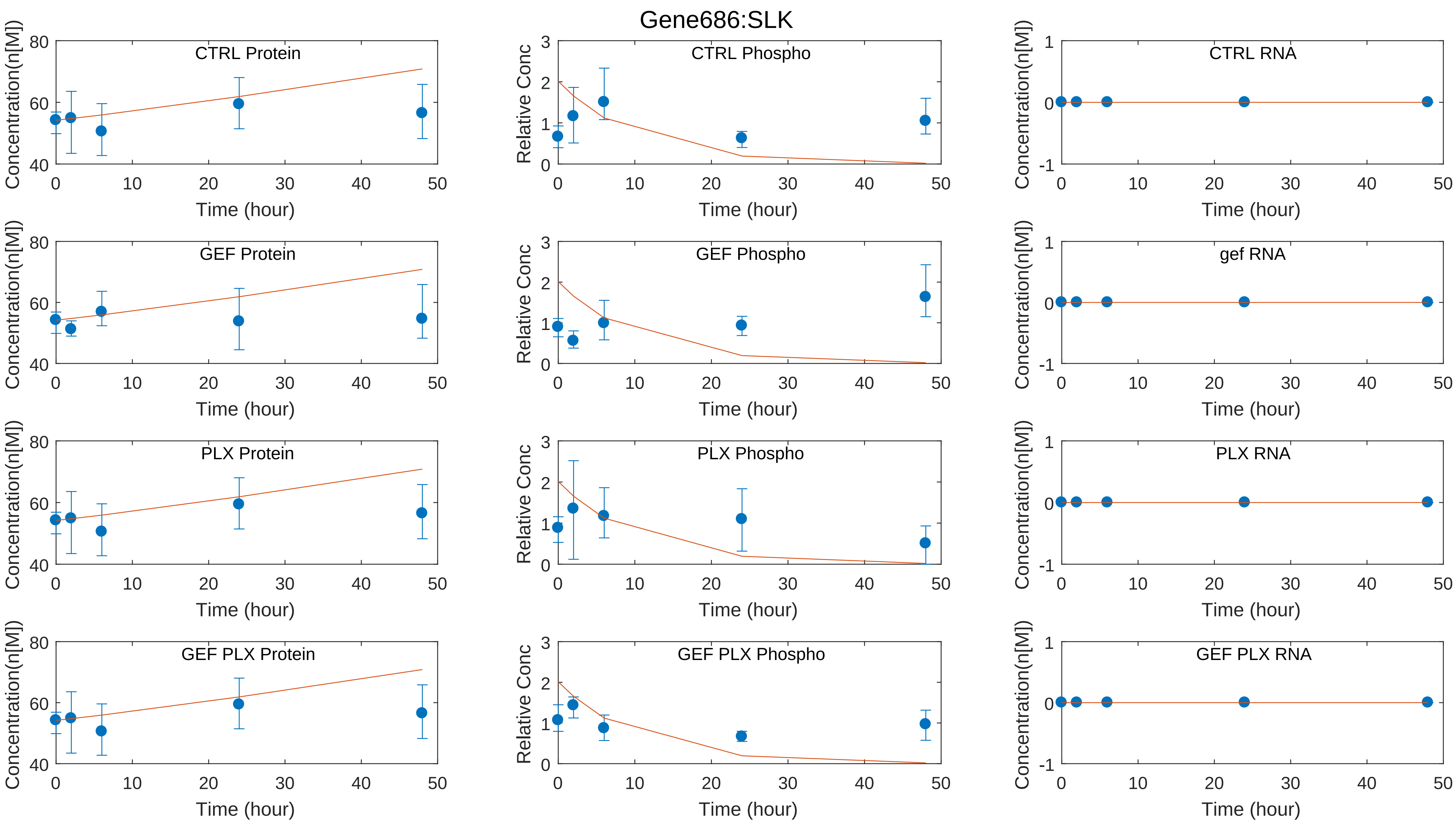

Gene686:SLK
CTRL Protein
CTRL Phospho
CTRL RNA
GEF Protein
GEF Phospho
gef RNA
PLX Protein
PLX Phospho
PLX RNA
GEF PLX Protein
GEF PLX Phospho
GEF PLX RNA
Concentration(n[M])
Relative Conc
Time (hour)

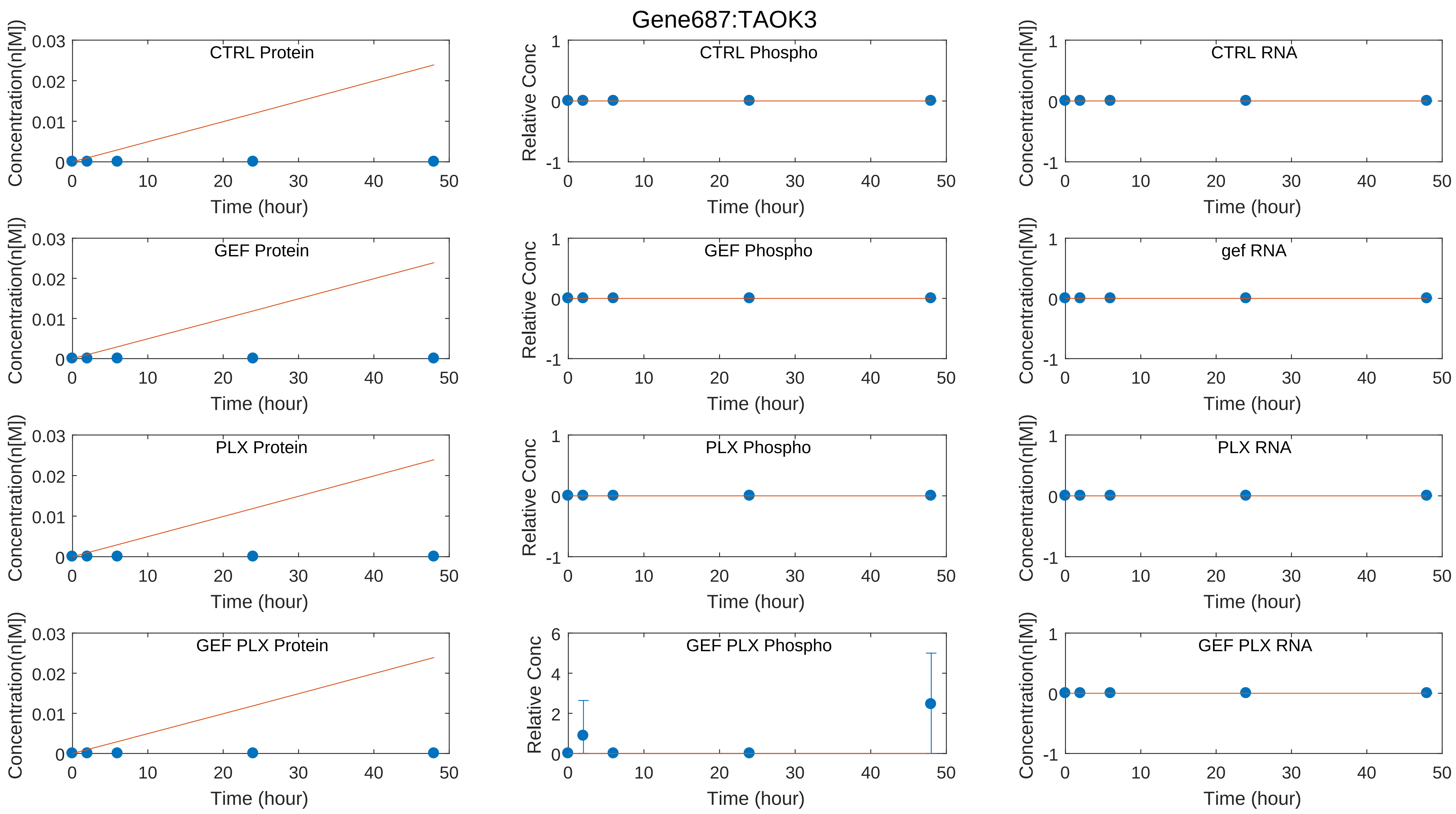

Gene687:TAOK3
CTRL Protein
CTRL Phospho
CTRL RNA
GEF Protein
GEF Phospho
gef RNA
PLX Protein
PLX Phospho
PLX RNA
GEF PLX Protein
GEF PLX Phospho
GEF PLX RNA
Concentration(n[M])
Relative Conc
Time (hour)

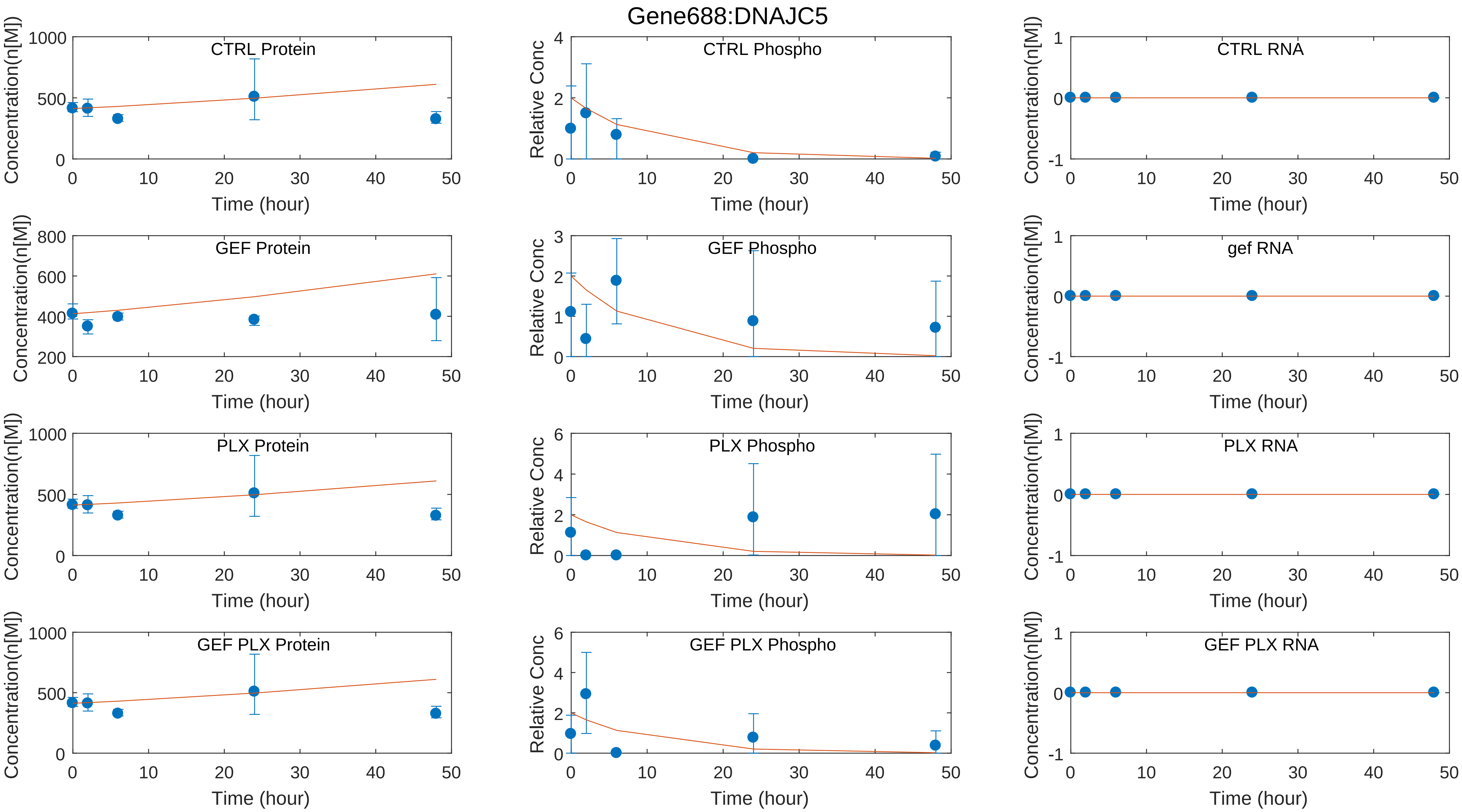

Gene688:DNAJC5
CTRL Protein
CTRL Phospho
CTRL RNA
GEF Protein
GEF Phospho
gef RNA
PLX Protein
PLX Phospho
PLX RNA
GEF PLX Protein
GEF PLX Phospho
GEF PLX RNA
Concentration(n[M])
Relative Conc
Time (hour)

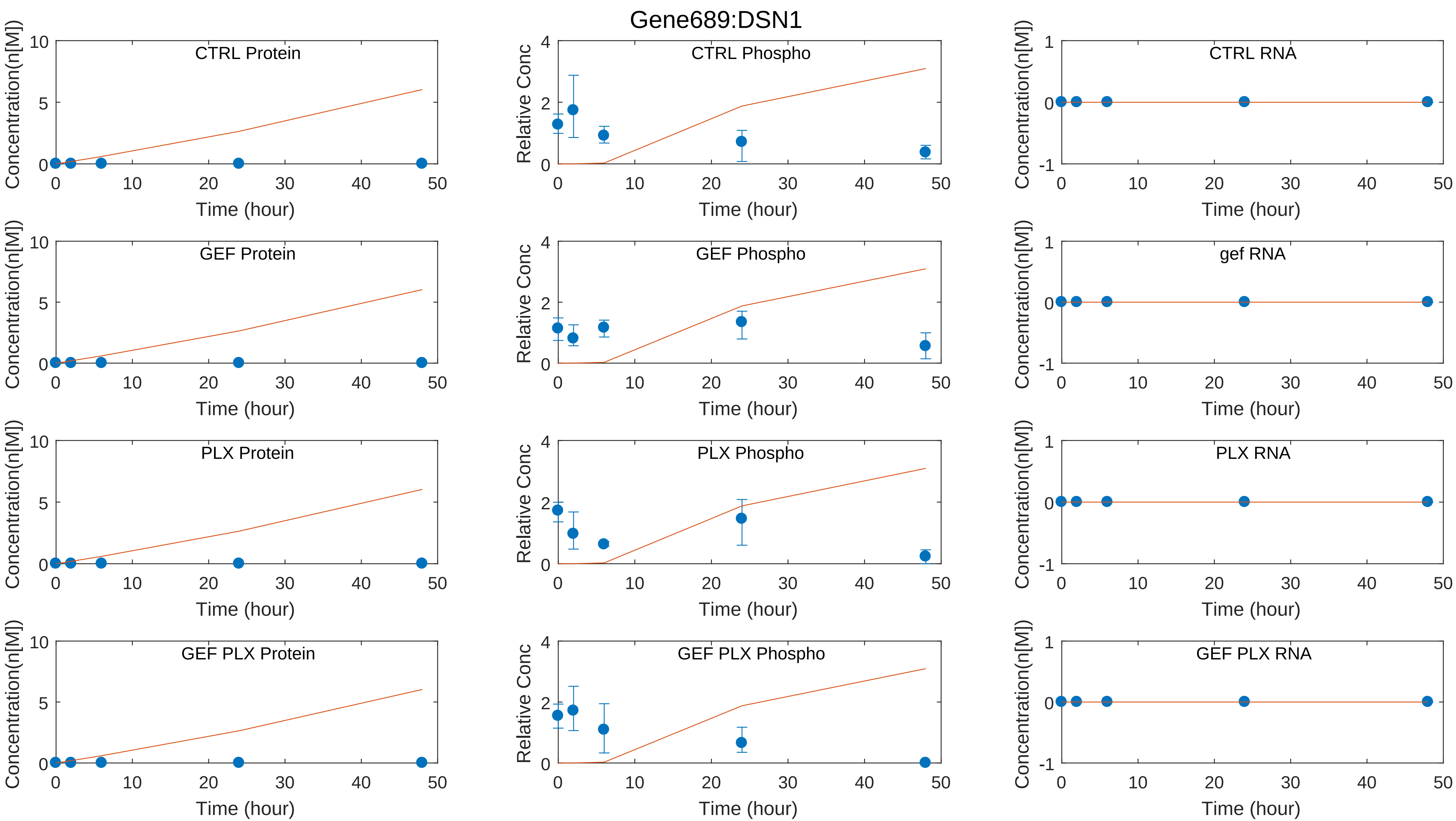

Gene689:DSN1
CTRL Protein
CTRL Phospho
CTRL RNA
GEF Protein
GEF Phospho
gef RNA
PLX Protein
PLX Phospho
PLX RNA
GEF PLX Protein
GEF PLX Phospho
GEF PLX RNA
Concentration(n[M])
Relative Conc
Time (hour)

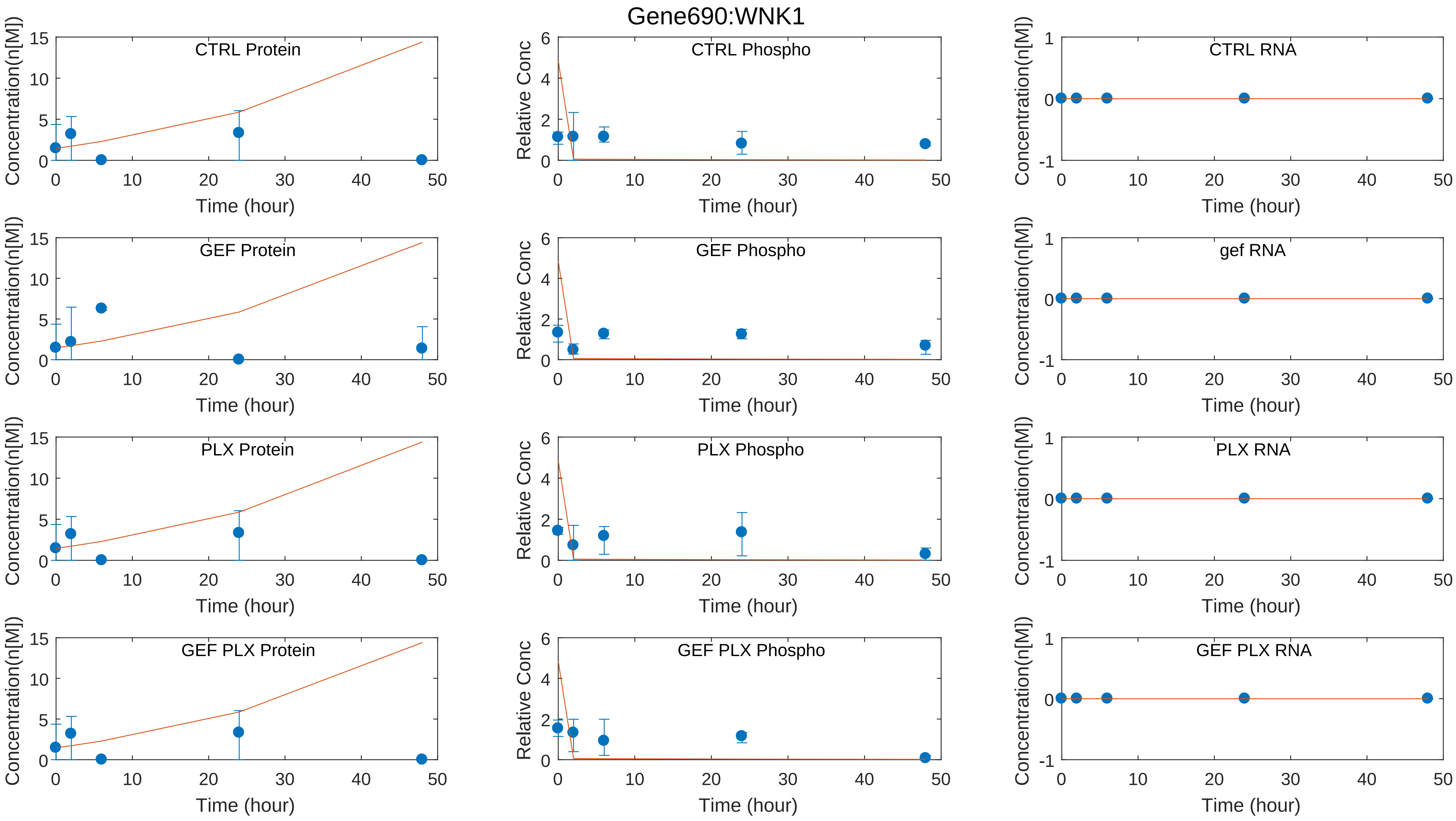

Gene690:WNK1
CTRL Protein
GEF Protein
PLX Protein
GEF PLX Protein
CTRL Phospho
GEF Phospho
PLX Phospho
GEF PLX Phospho
CTRL RNA
gef RNA
PLX RNA
GEF PLX RNA
Concentration(n[M])
Relative Conc
Time (hour)

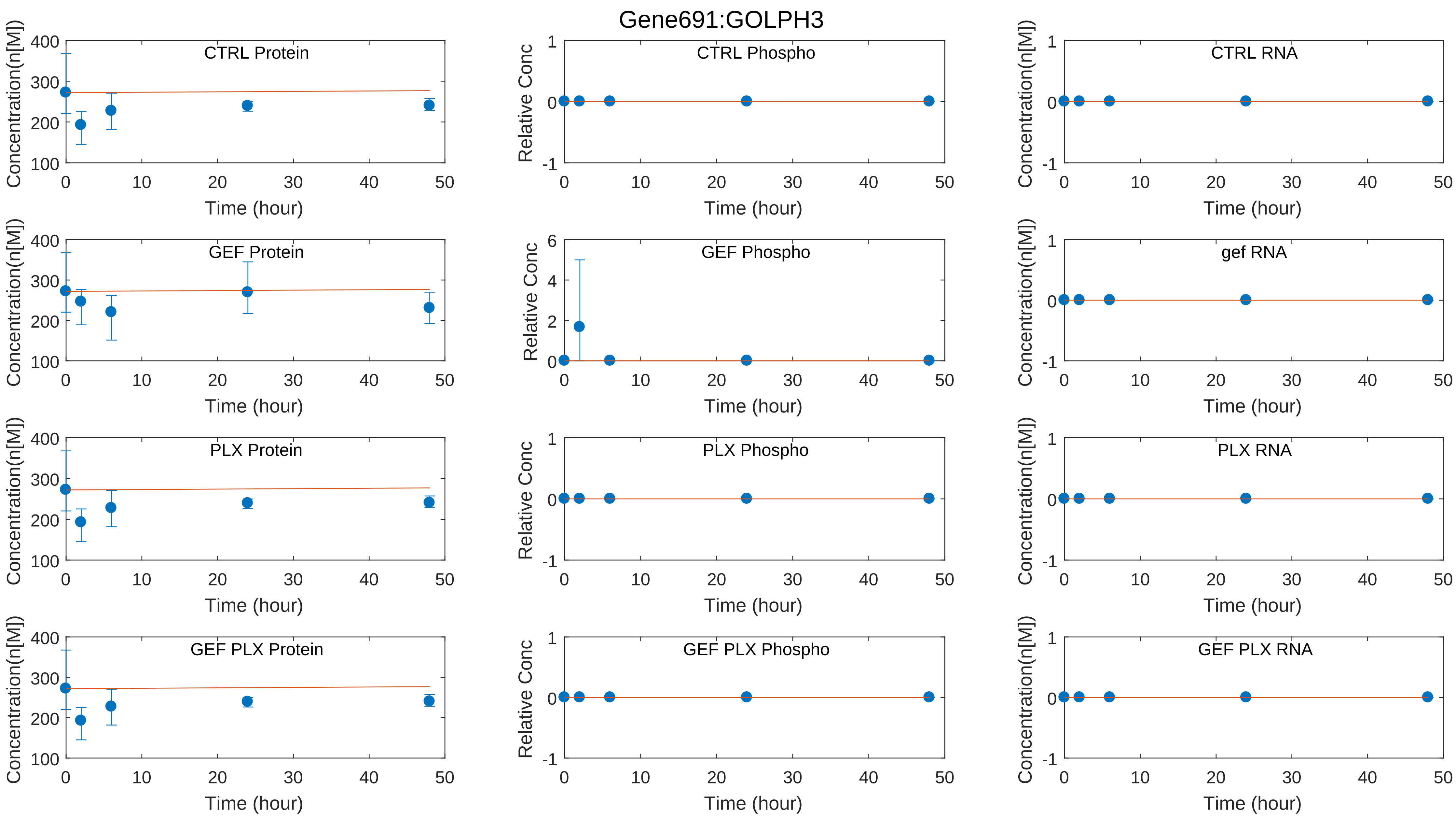

Gene691:GOLPH3
CTRL Protein
CTRL Phospho
CTRL RNA
GEF Protein
GEF Phospho
gef RNA
PLX Protein
PLX Phospho
PLX RNA
GEF PLX Protein
GEF PLX Phospho
GEF PLX RNA
Concentration(n[M])
Relative Conc
Time (hour)

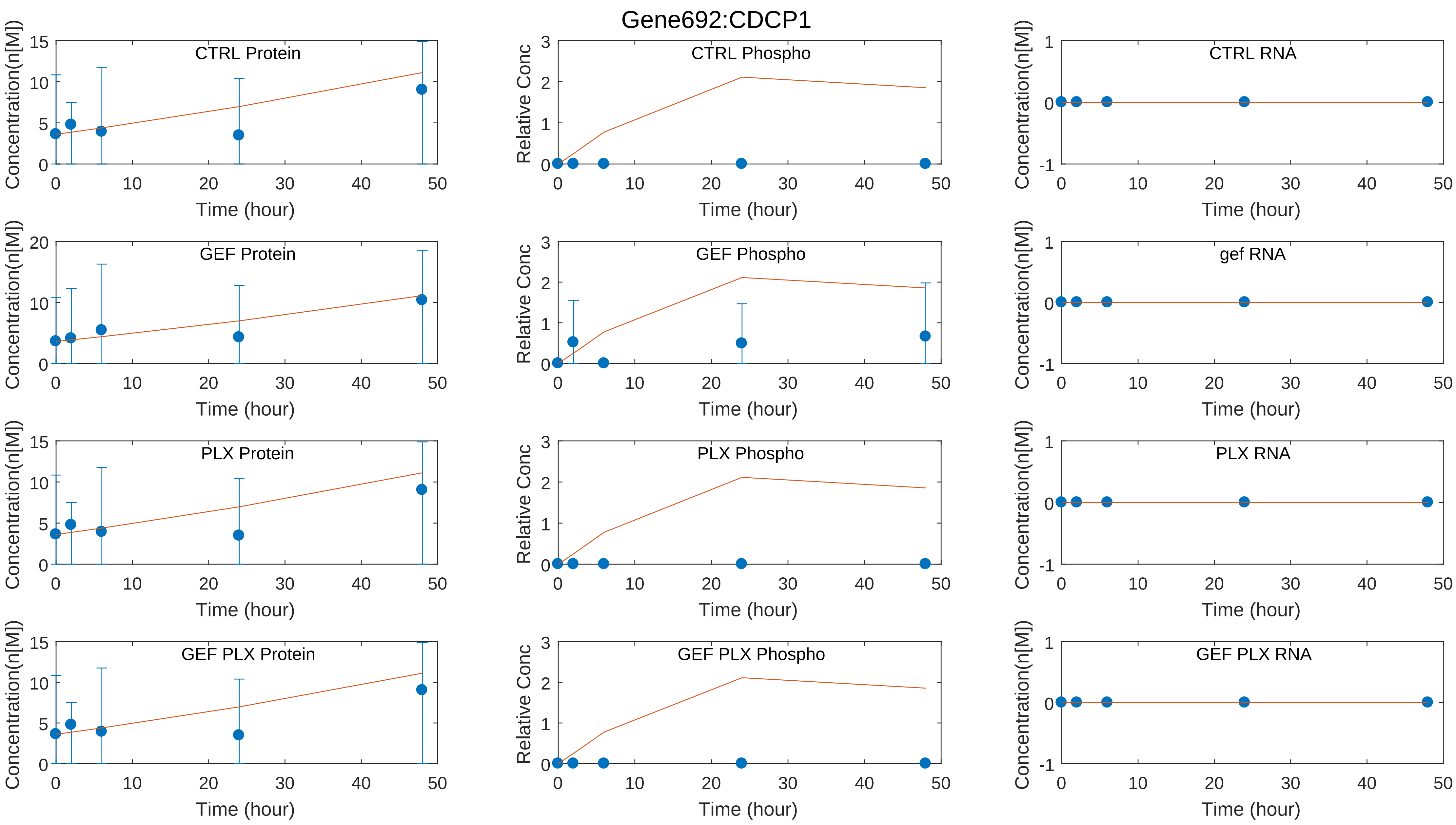
Gene692:CDCP1
CTRL Protein
CTRL Phospho
CTRL RNA
GEF Protein
GEF Phospho
gef RNA
PLX Protein
PLX Phospho
PLX RNA
GEF PLX Protein
GEF PLX Phospho
GEF PLX RNA
Concentration(n[M])
Relative Conc
Time (hour)

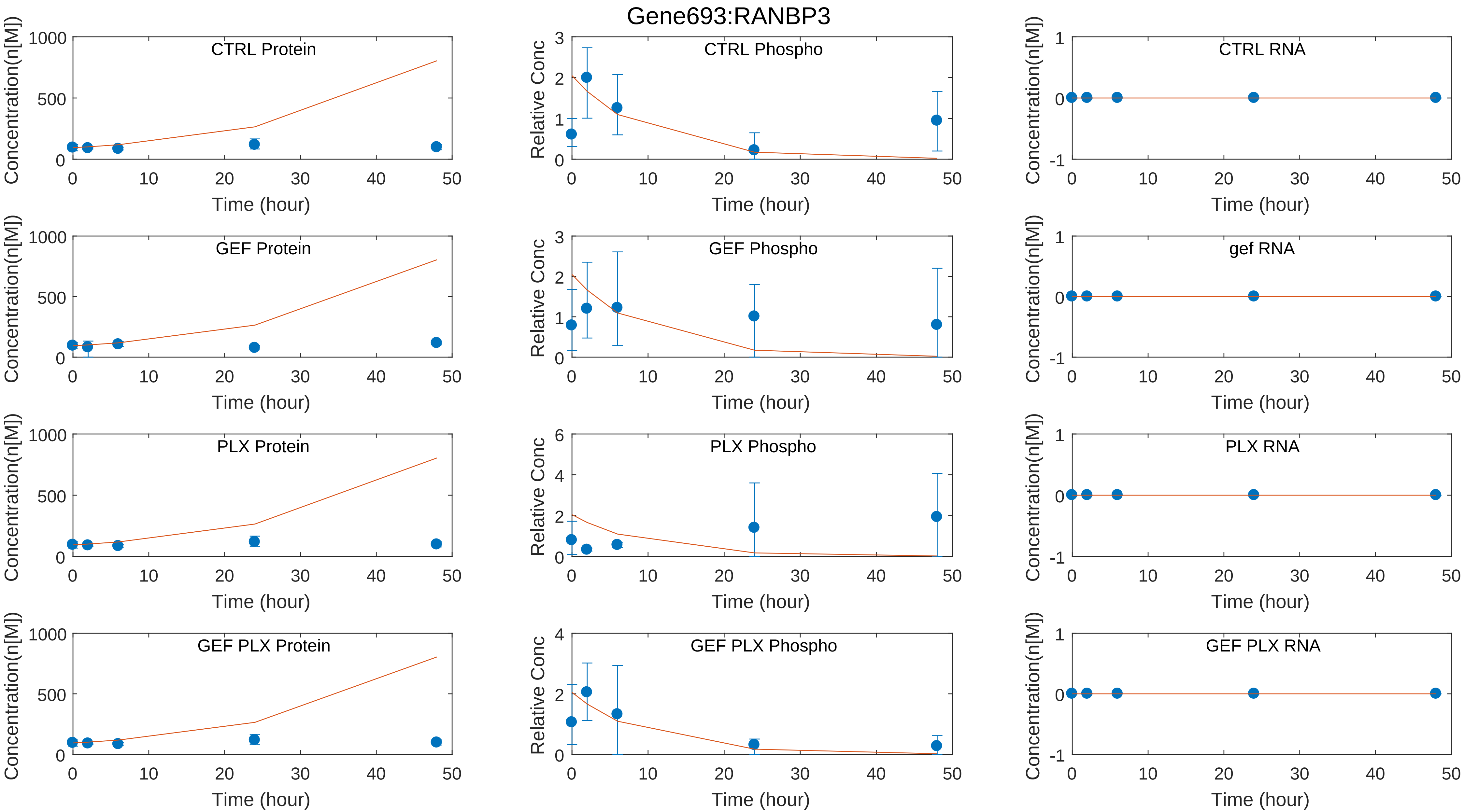

Gene693:RANBP3
CTRL Protein
CTRL Phospho
CTRL RNA
GEF Protein
GEF Phospho
gef RNA
PLX Protein
PLX Phospho
PLX RNA
GEF PLX Protein
GEF PLX Phospho
GEF PLX RNA
Concentration(n[M])
Relative Conc
Time (hour)

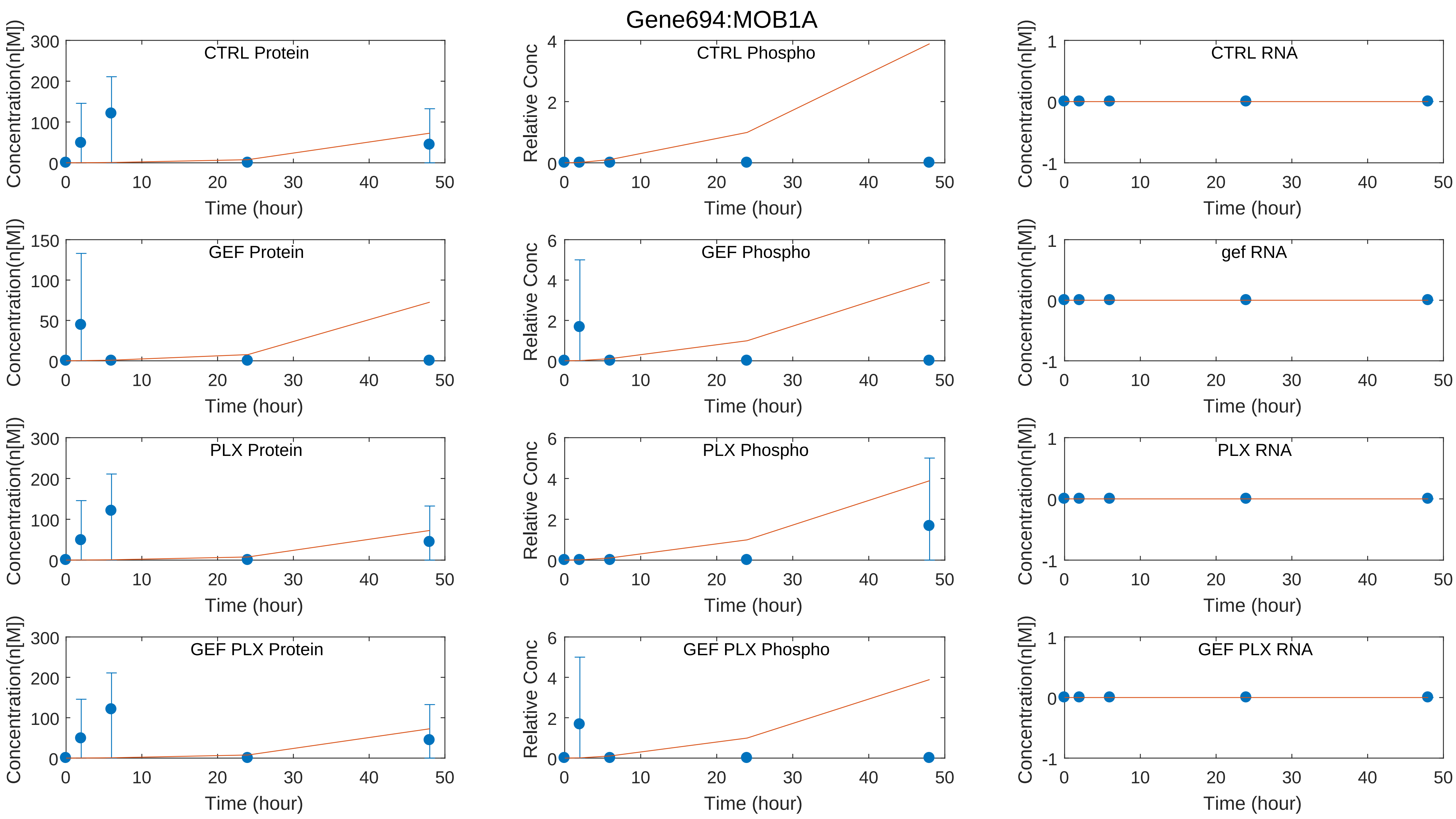
Gene694:MOB1A
CTRL Protein
CTRL Phospho
CTRL RNA
GEF Protein
GEF Phospho
gef RNA
PLX Protein
PLX Phospho
PLX RNA
GEF PLX Protein
GEF PLX Phospho
GEF PLX RNA
Concentration(n[M])
Relative Conc
Time (hour)

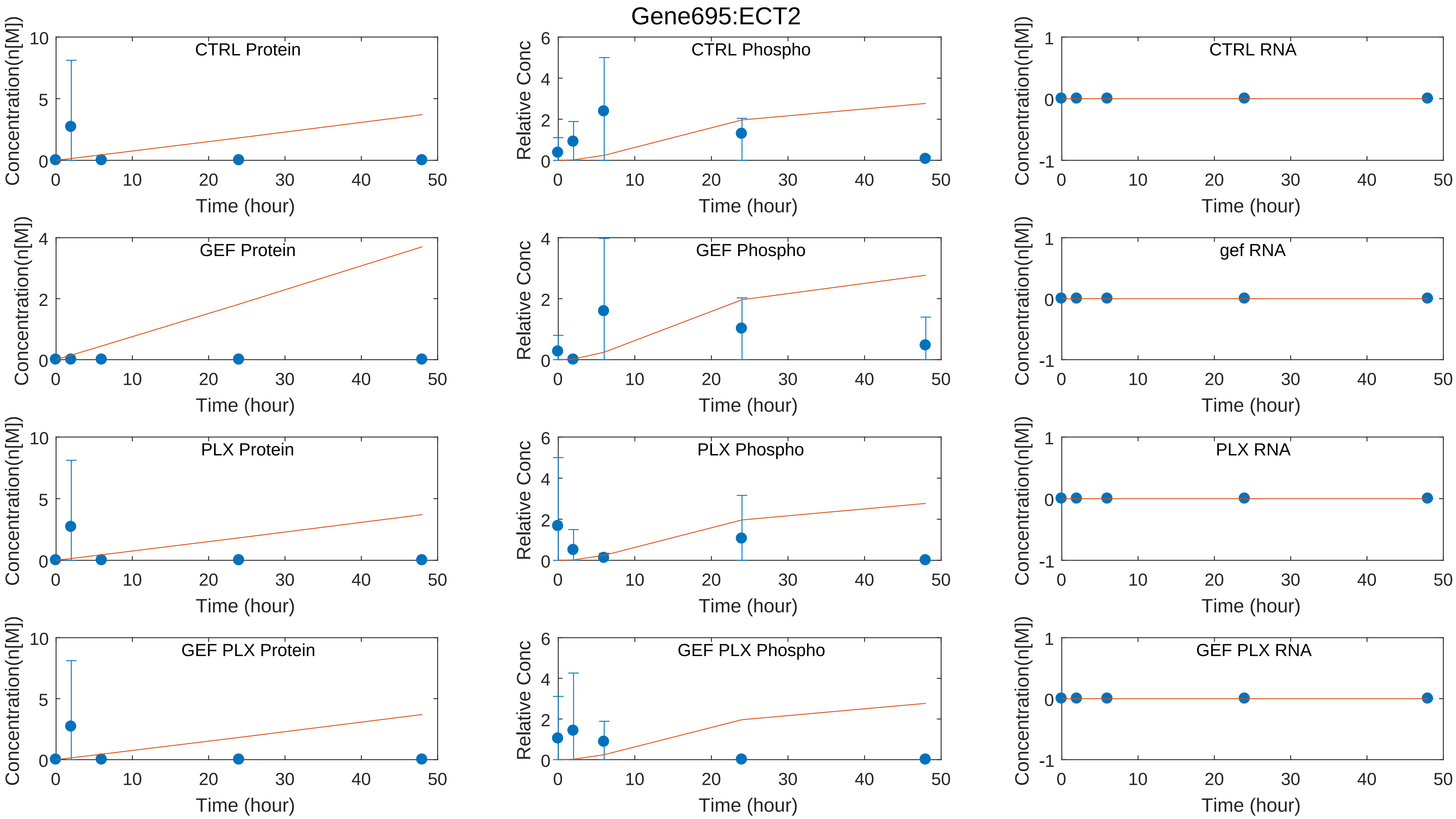

Gene695:ECT2
CTRL Protein
CTRL Phospho
CTRL RNA
GEF Protein
GEF Phospho
gef RNA
PLX Protein
PLX Phospho
PLX RNA
GEF PLX Protein
GEF PLX Phospho
GEF PLX RNA
Concentration(n[M])
Relative Conc
Time (hour)

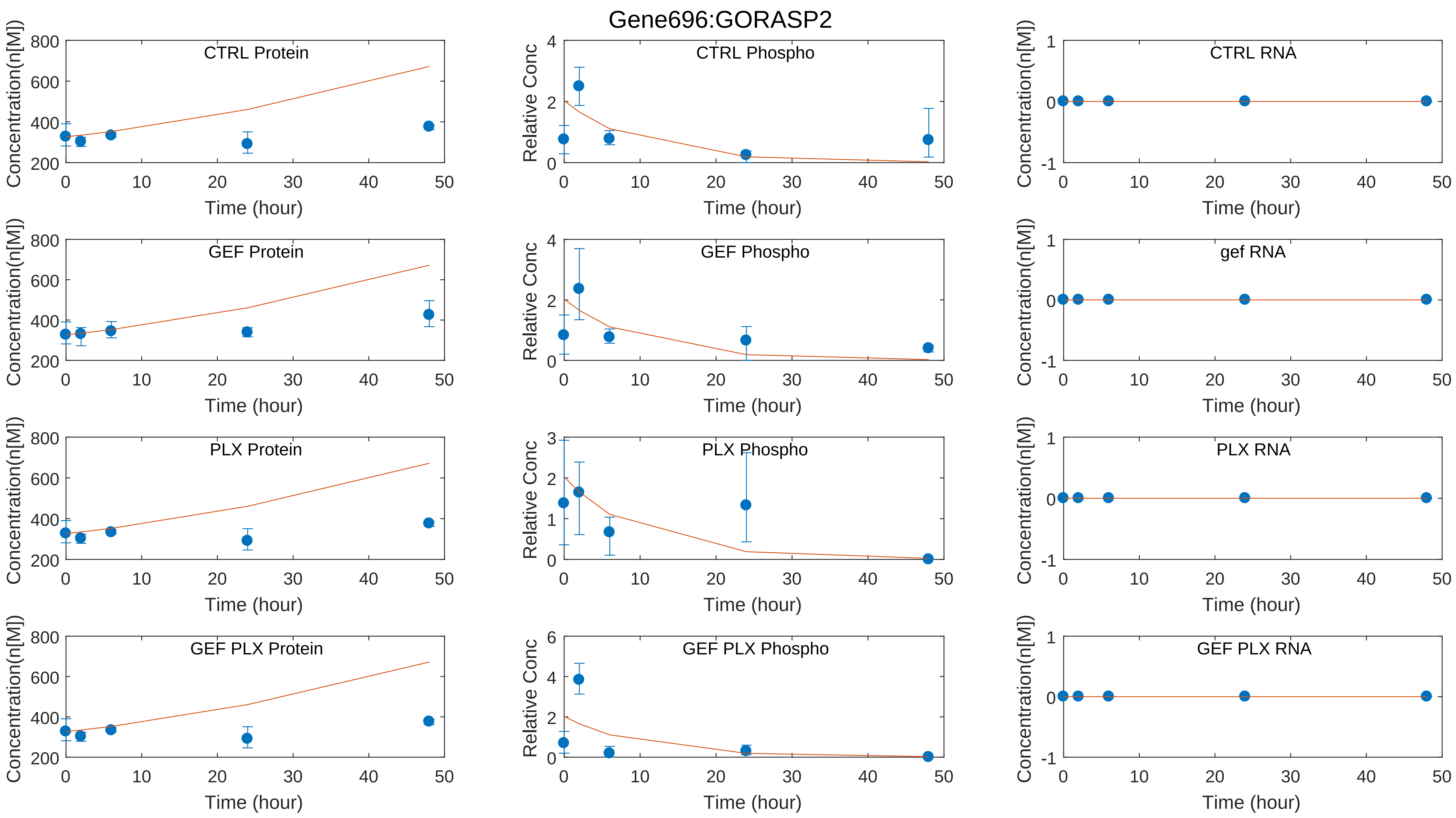

Gene696:GORASP2
CTRL Protein
CTRL Phospho
CTRL RNA
GEF Protein
GEF Phospho
gef RNA
PLX Protein
PLX Phospho
PLX RNA
GEF PLX Protein
GEF PLX Phospho
GEF PLX RNA
Concentration(n[M])
Relative Conc
Time (hour)

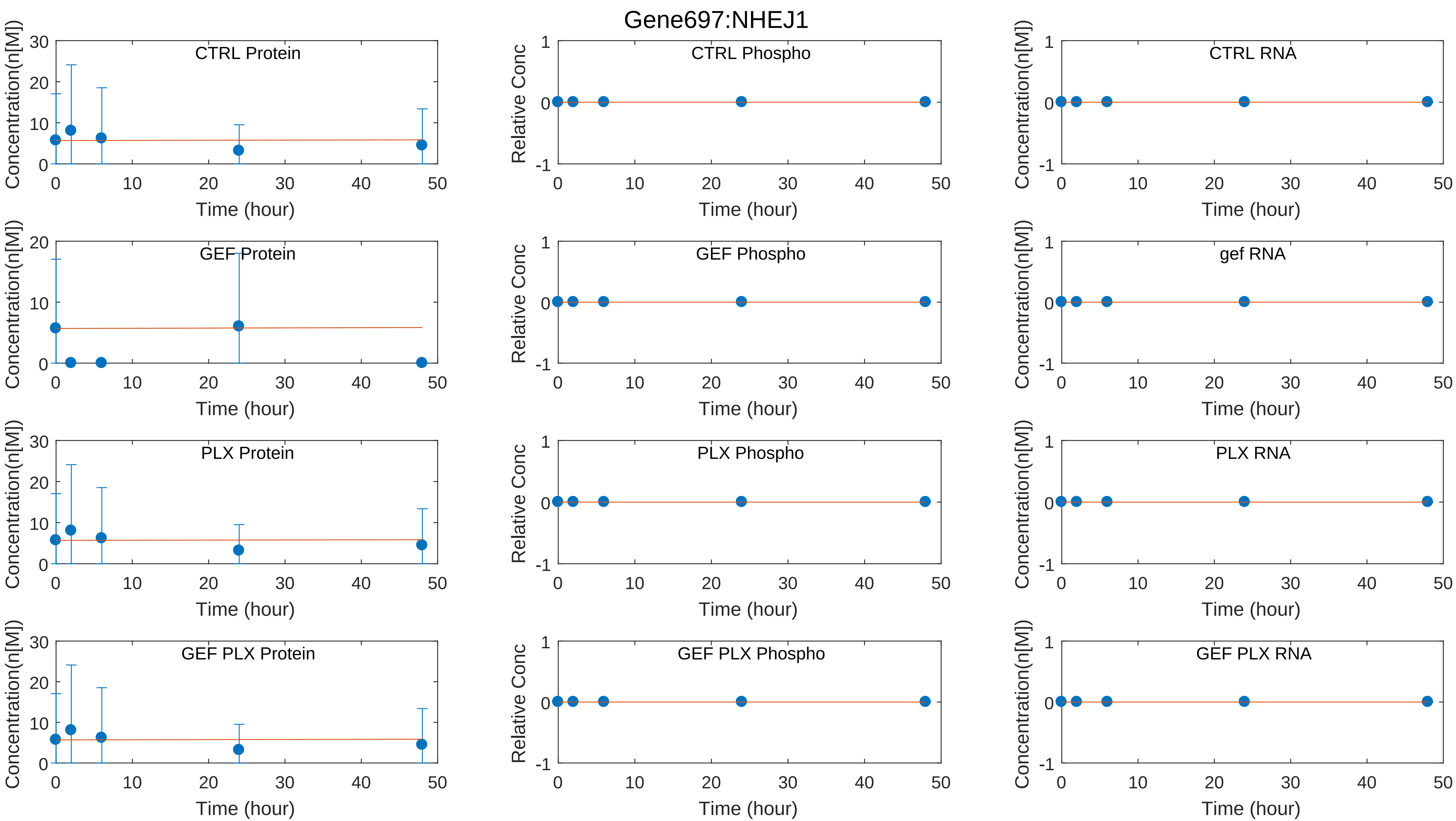

Gene697:NHEJ1
CTRL Protein
CTRL Phospho
CTRL RNA
GEF Protein
GEF Phospho
gef RNA
PLX Protein
PLX Phospho
PLX RNA
GEF PLX Protein
GEF PLX Phospho
GEF PLX RNA
Concentration(n[M])
Relative Conc
Time (hour)

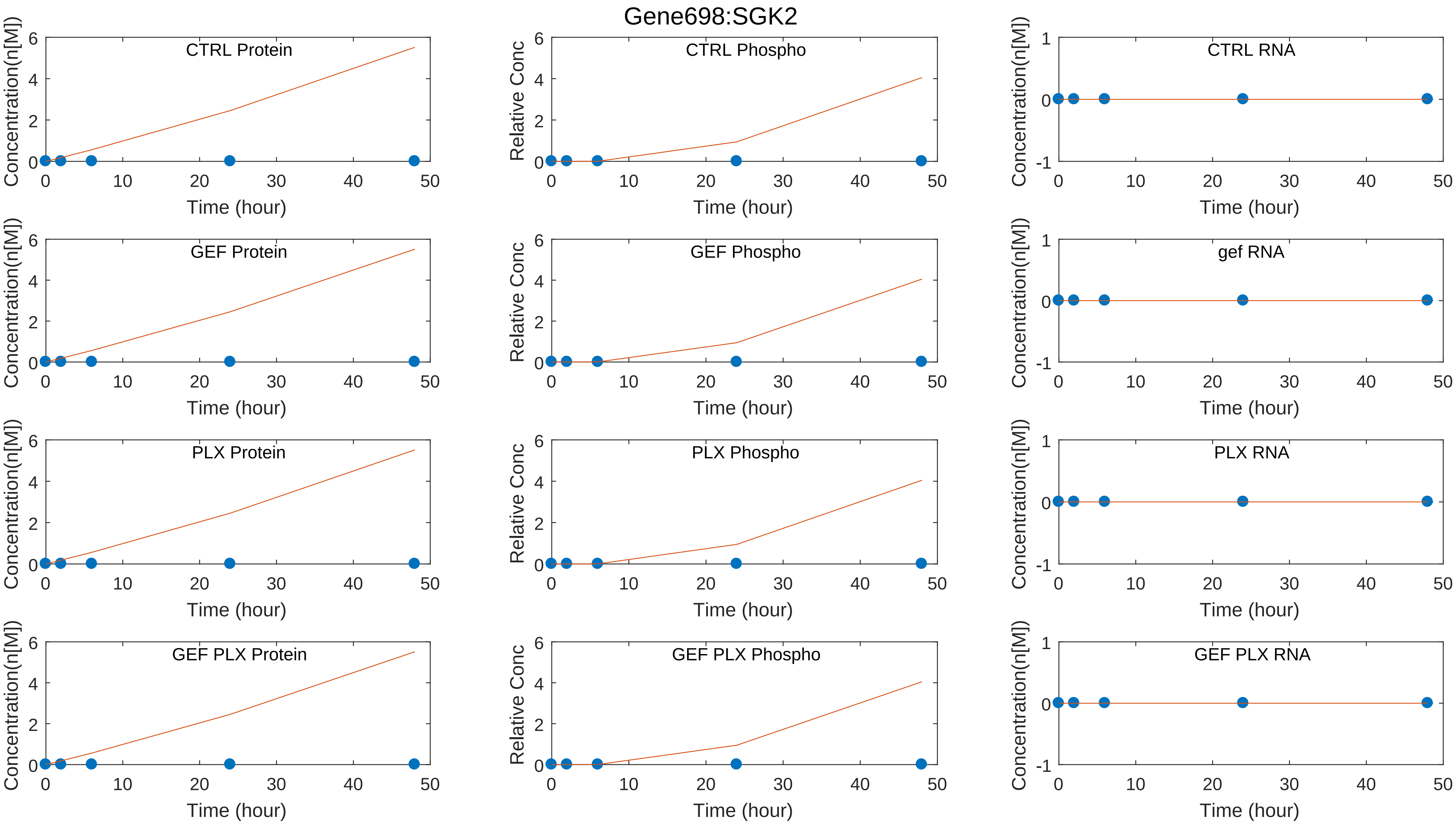
Gene698:SGK2
CTRL Protein
CTRL Phospho
CTRL RNA
GEF Protein
GEF Phospho
gef RNA
PLX Protein
PLX Phospho
PLX RNA
GEF PLX Protein
GEF PLX Phospho
GEF PLX RNA
Concentration(n[M])
Relative Conc
Time (hour)

Gene699:KIR2DL3

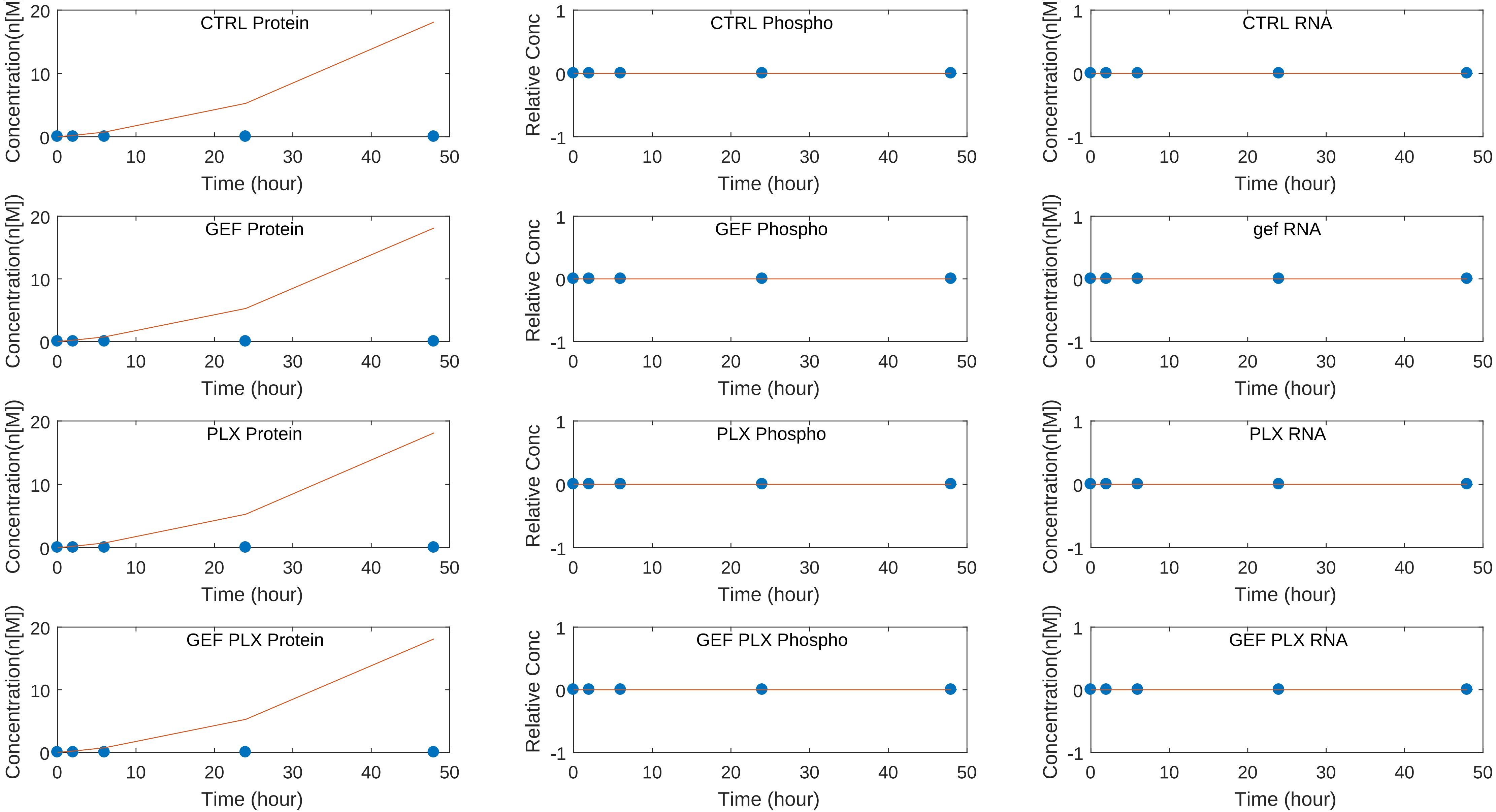

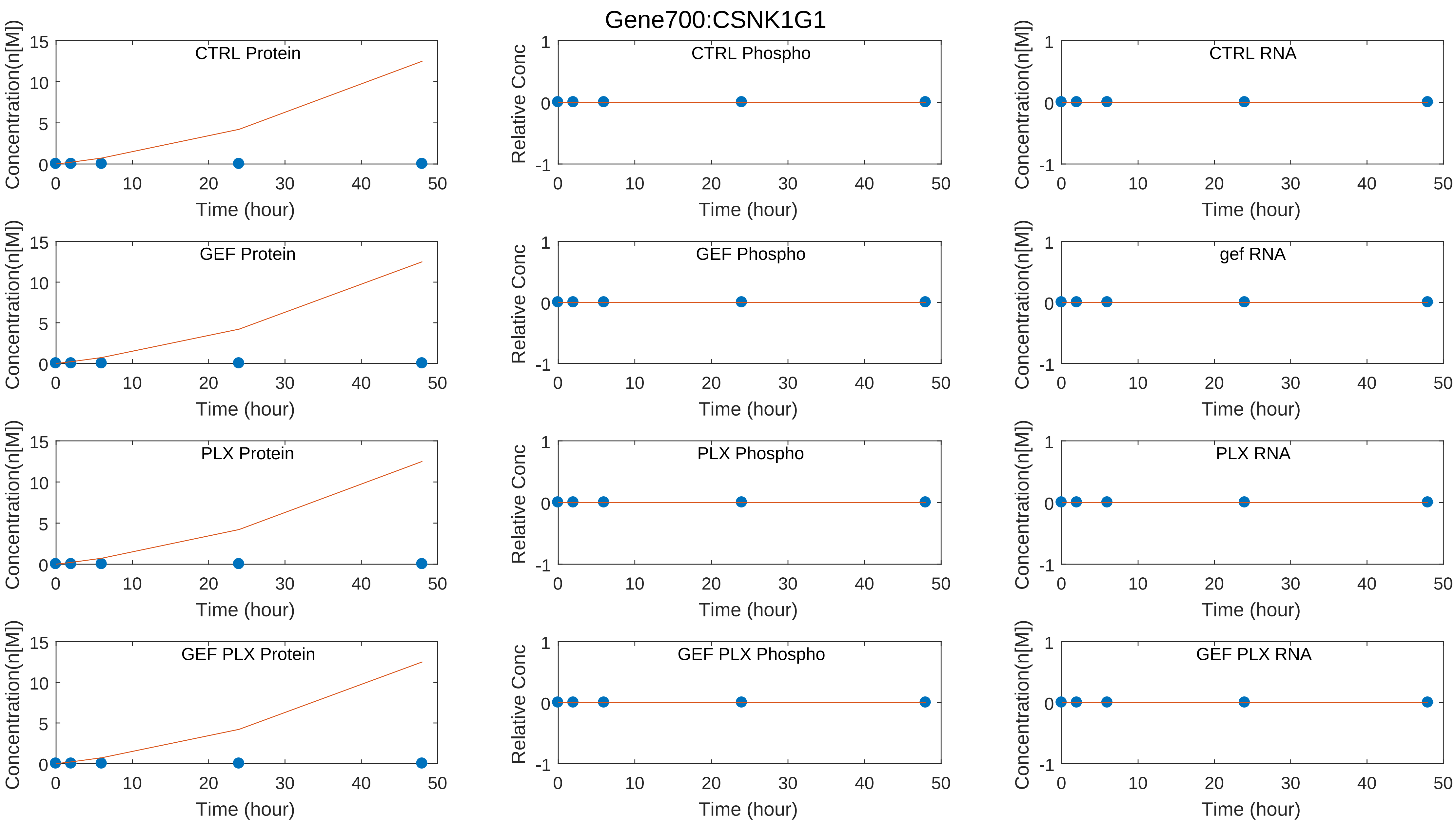
Gene700:CSNK1G1
CTRL Protein
GEF Protein
PLX Protein
GEF PLX Protein
CTRL Phospho
GEF Phospho
PLX Phospho
GEF PLX Phospho
CTRL RNA
gef RNA
PLX RNA
GEF PLX RNA
Concentration(n[M])
Relative Conc
Time (hour)

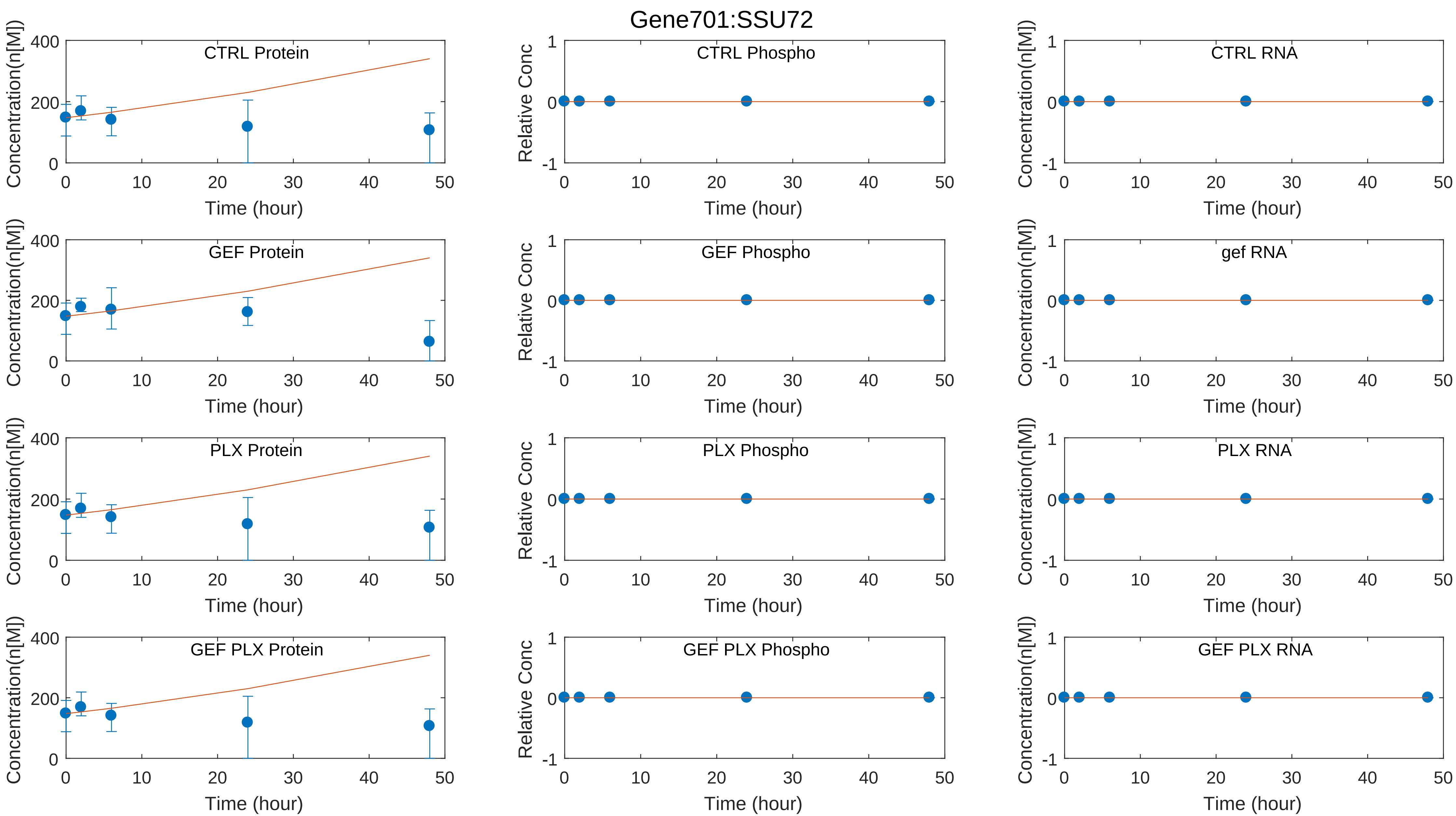

Gene701:SSU72
CTRL Protein
CTRL Phospho
CTRL RNA
GEF Protein
GEF Phospho
gef RNA
PLX Protein
PLX Phospho
PLX RNA
GEF PLX Protein
GEF PLX Phospho
GEF PLX RNA
Concentration(n[M])
Relative Conc
Time (hour)

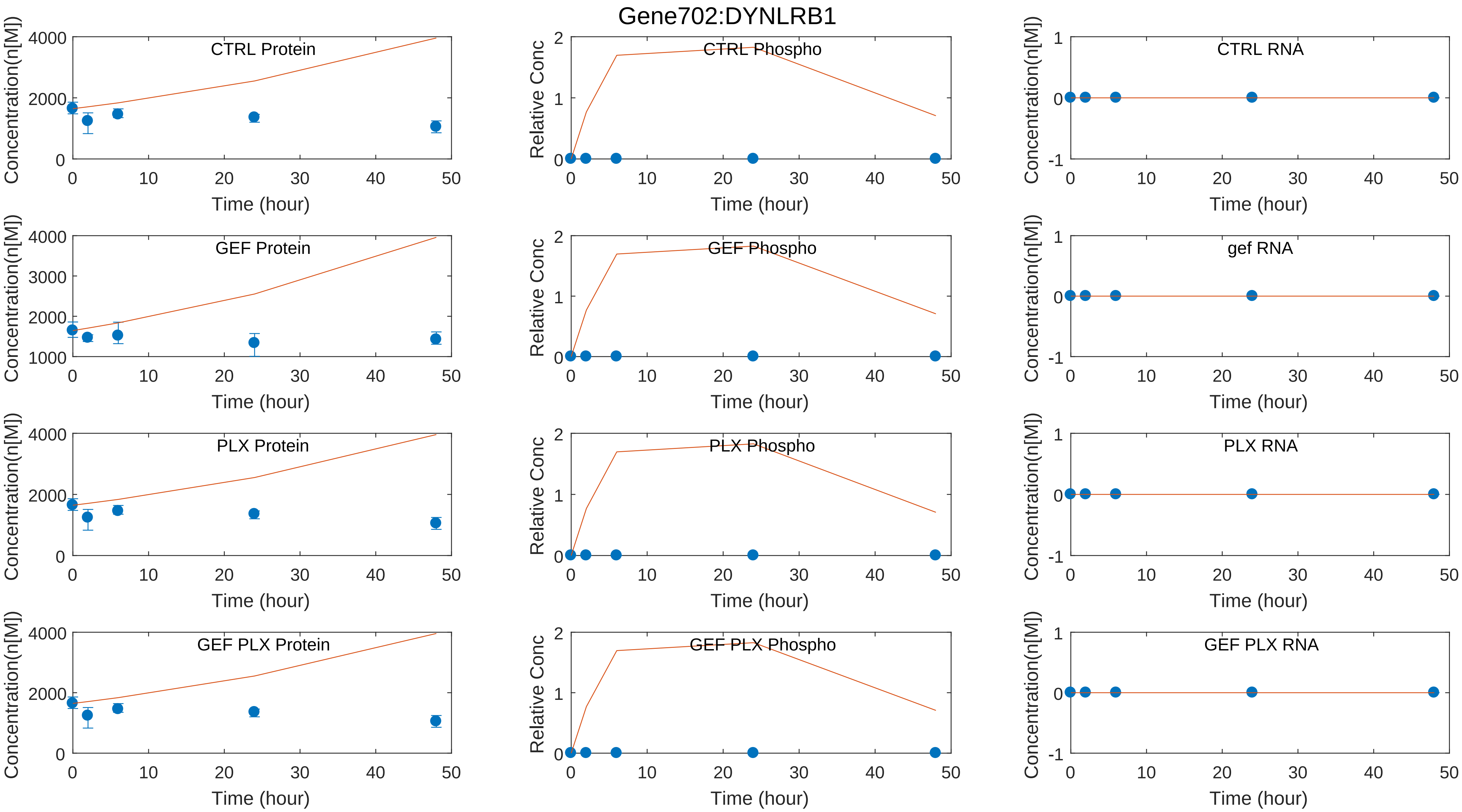
Gene702:DYNLRB1
CTRL Protein
CTRL Phospho
CTRL RNA
GEF Protein
GEF Phospho
gef RNA
PLX Protein
PLX Phospho
PLX RNA
GEF PLX Protein
GEF PLX Phospho
GEF PLX RNA
Concentration(n[M])
Relative Conc
Time (hour)

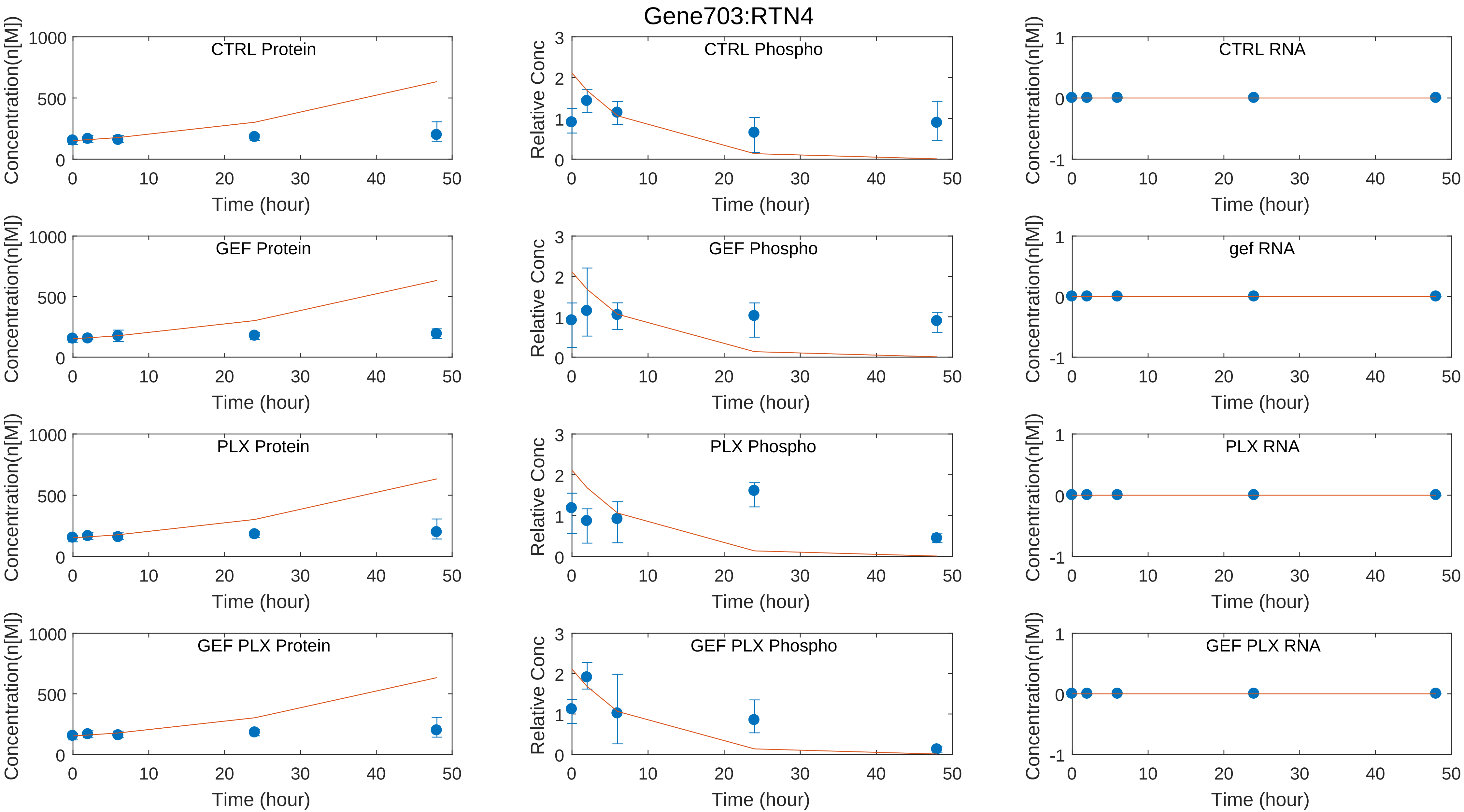

Gene703:RTN4
CTRL Protein
CTRL Phospho
CTRL RNA
GEF Protein
GEF Phospho
gef RNA
PLX Protein
PLX Phospho
PLX RNA
GEF PLX Protein
GEF PLX Phospho
GEF PLX RNA
Concentration(n[M])
Relative Conc
Time (hour)

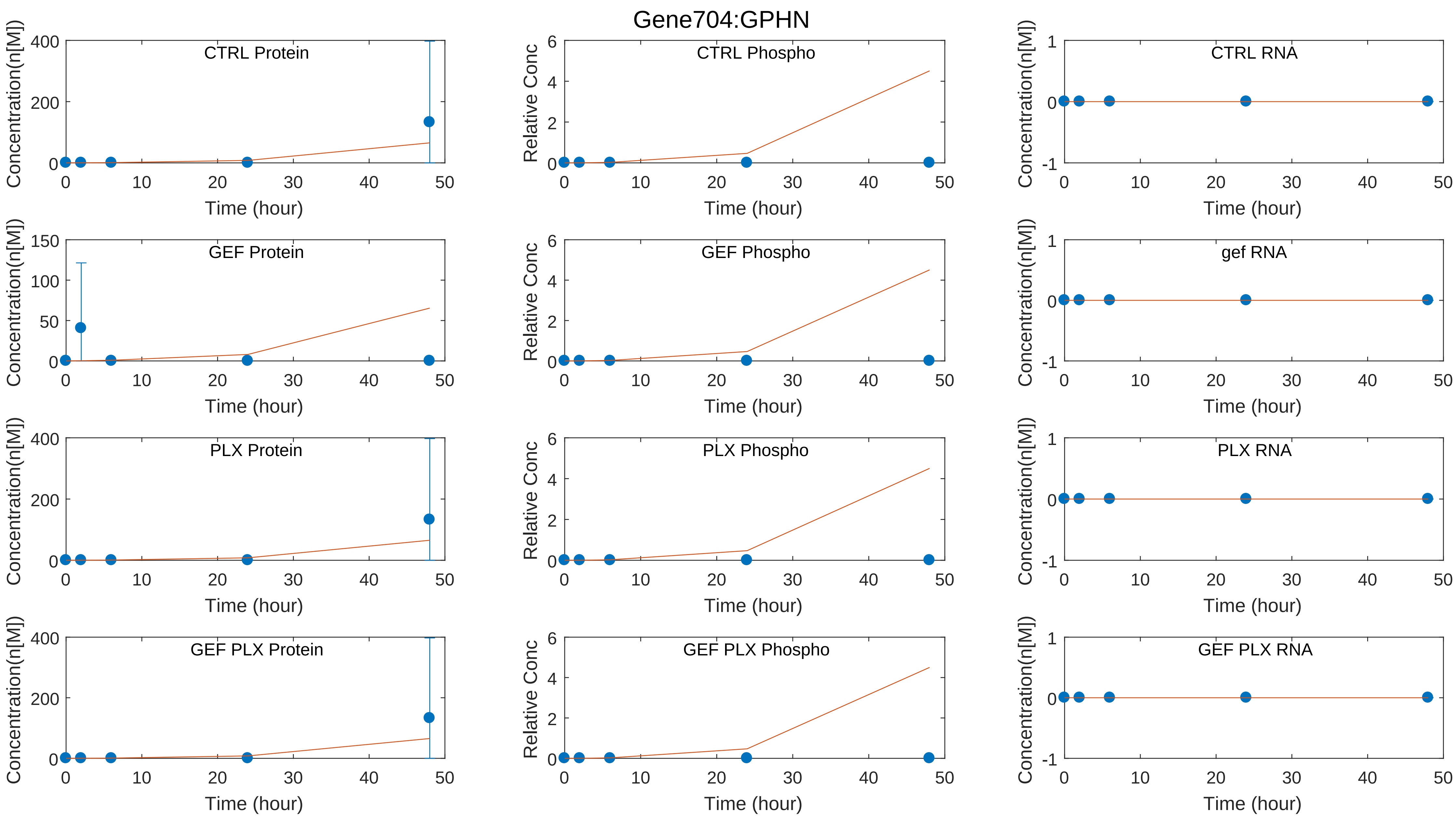

Gene704:GPHN
CTRL Protein
CTRL Phospho
CTRL RNA
GEF Protein
GEF Phospho
gef RNA
PLX Protein
PLX Phospho
PLX RNA
GEF PLX Protein
GEF PLX Phospho
GEF PLX RNA
Concentration(n[M])
Relative Conc
Time (hour)

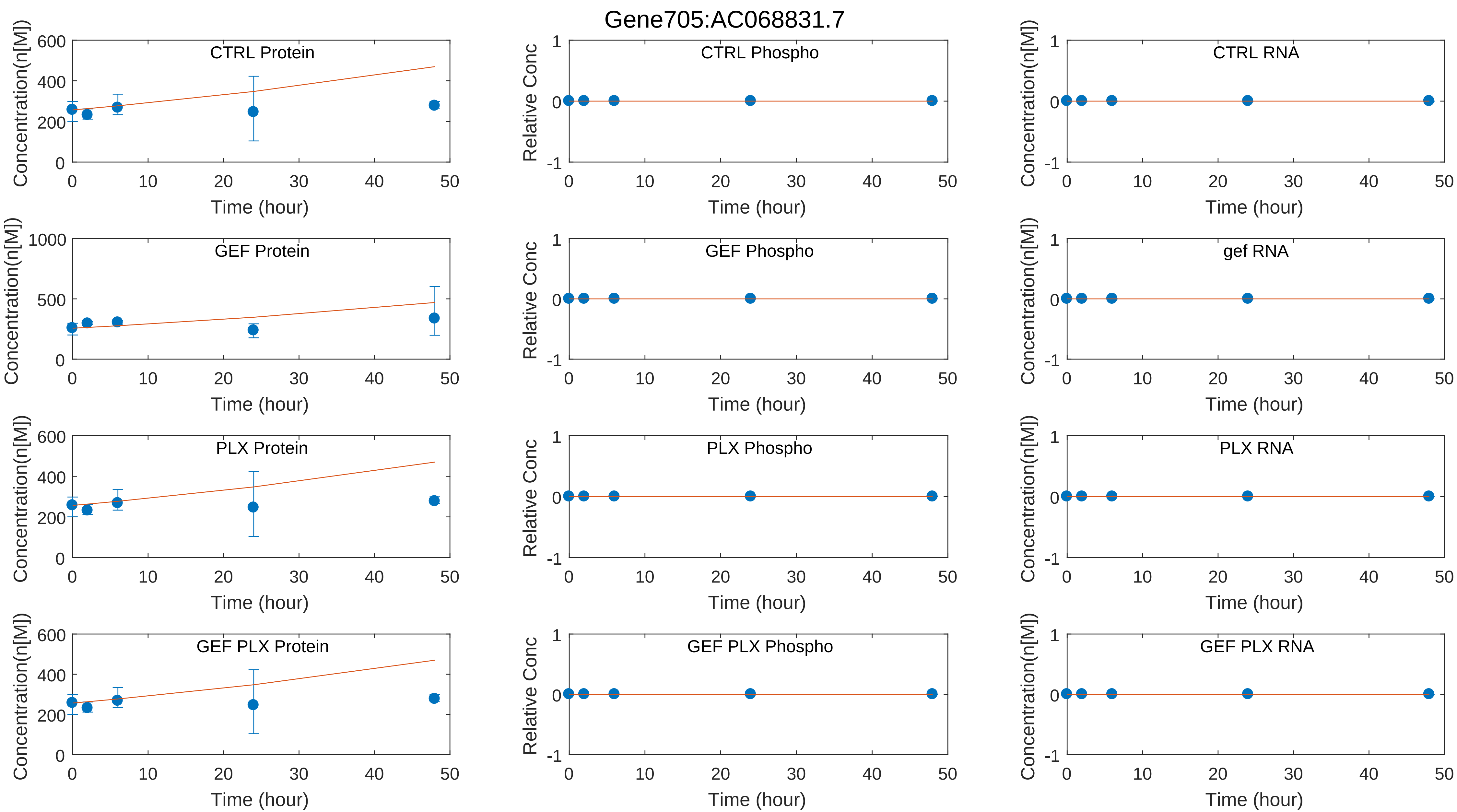
Gene705:AC068831.7
CTRL Protein
CTRL Phospho
CTRL RNA
GEF Protein
GEF Phospho
gef RNA
PLX Protein
PLX Phospho
PLX RNA
GEF PLX Protein
GEF PLX Phospho
GEF PLX RNA
Concentration(n[M])
Relative Conc
Time (hour)

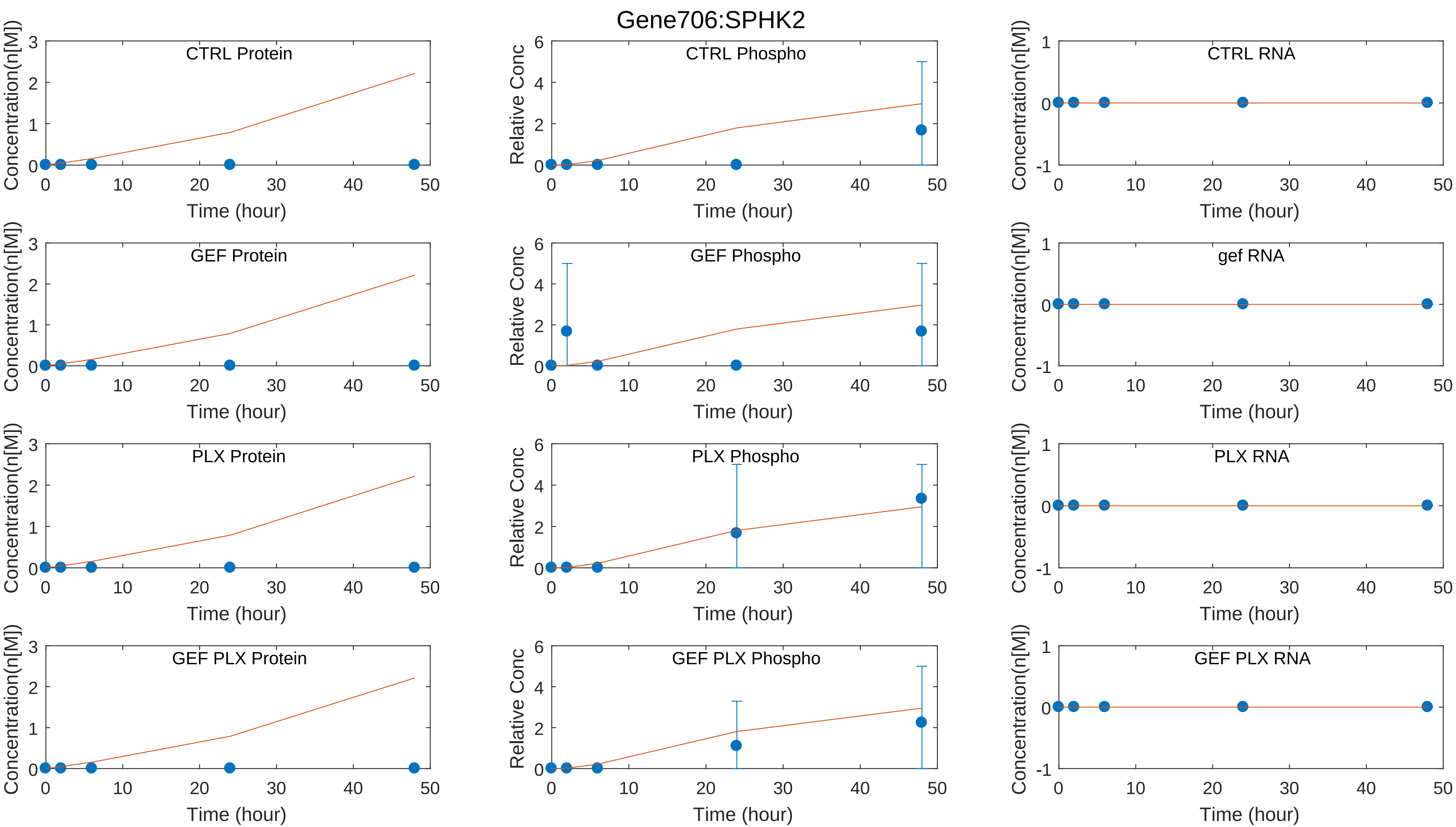

Gene706:SPHK2
CTRL Protein
CTRL Phospho
CTRL RNA
GEF Protein
GEF Phospho
gef RNA
PLX Protein
PLX Phospho
PLX RNA
GEF PLX Protein
GEF PLX Phospho
GEF PLX RNA
Concentration(n[M])
Relative Conc
Time (hour)

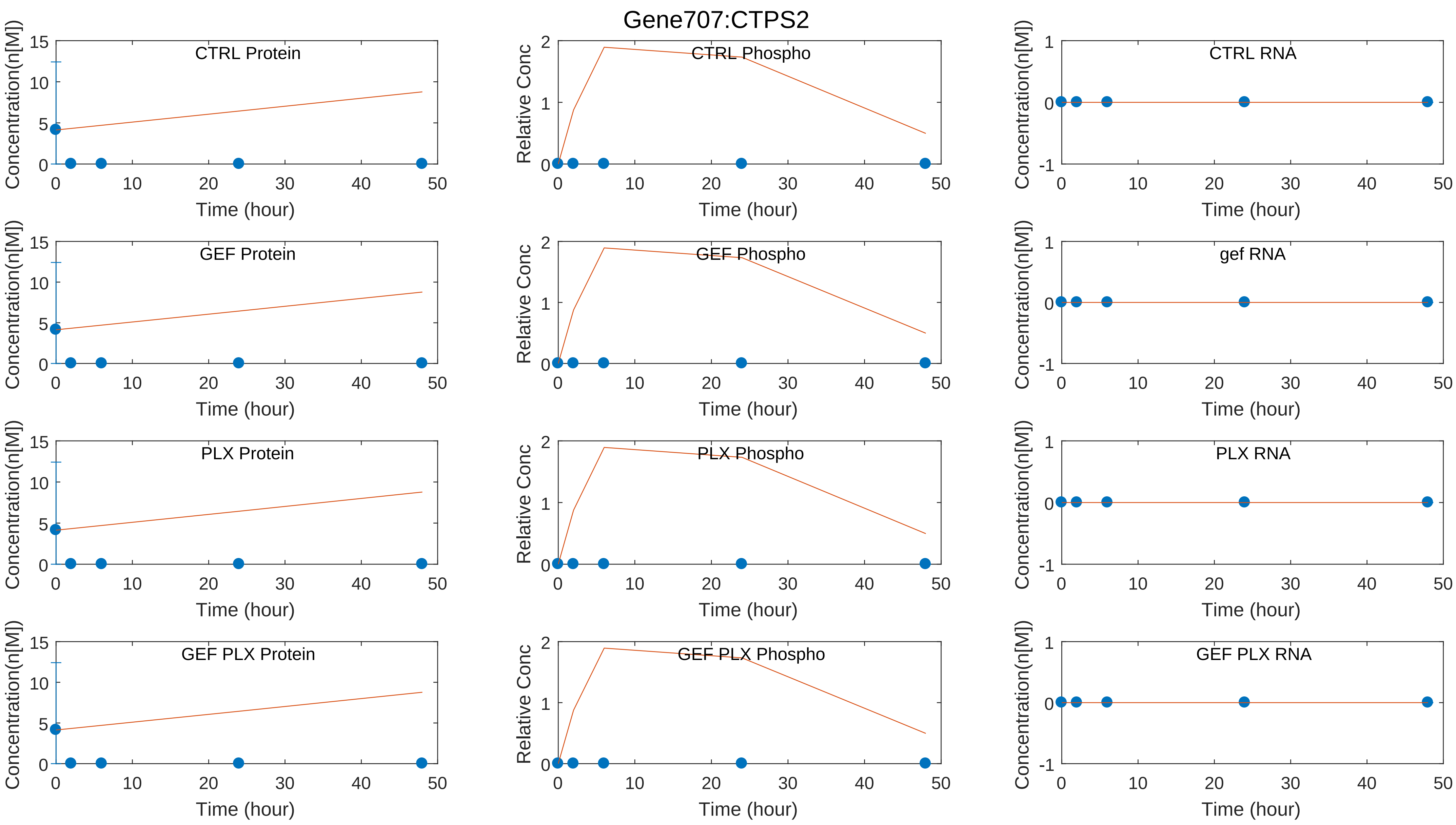

Gene707:CTPS2
CTRL Protein
CTRL Phospho
CTRL RNA
GEF Protein
GEF Phospho
gef RNA
PLX Protein
PLX Phospho
PLX RNA
GEF PLX Protein
GEF PLX Phospho
GEF PLX RNA
Concentration(n[M])
Relative Conc
Time (hour)

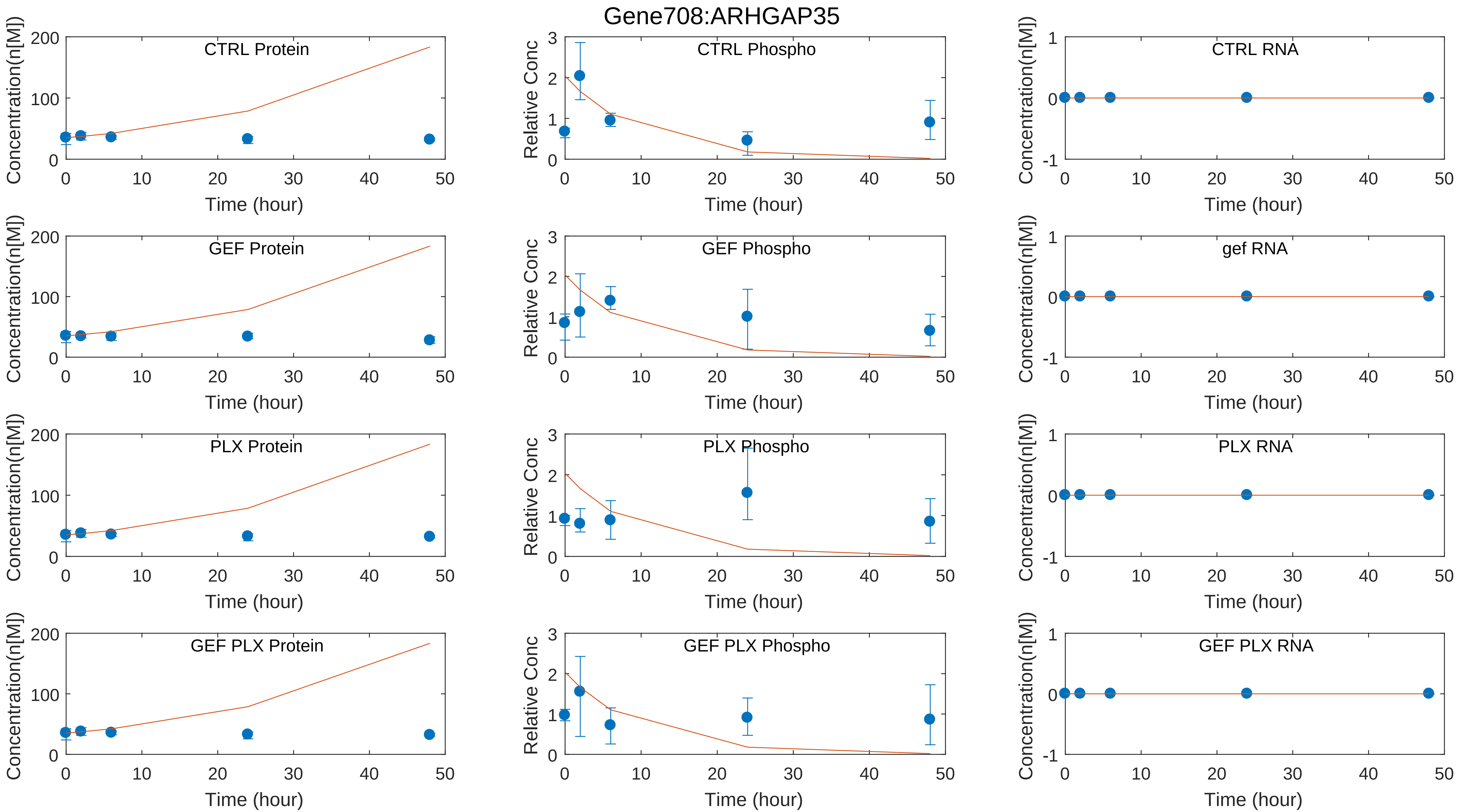

Gene708:ARHGAP35
CTRL Protein
GEF Protein
PLX Protein
GEF PLX Protein
CTRL Phospho
GEF Phospho
PLX Phospho
GEF PLX Phospho
CTRL RNA
gef RNA
PLX RNA
GEF PLX RNA
Concentration(n[M])
Relative Conc
Time (hour)

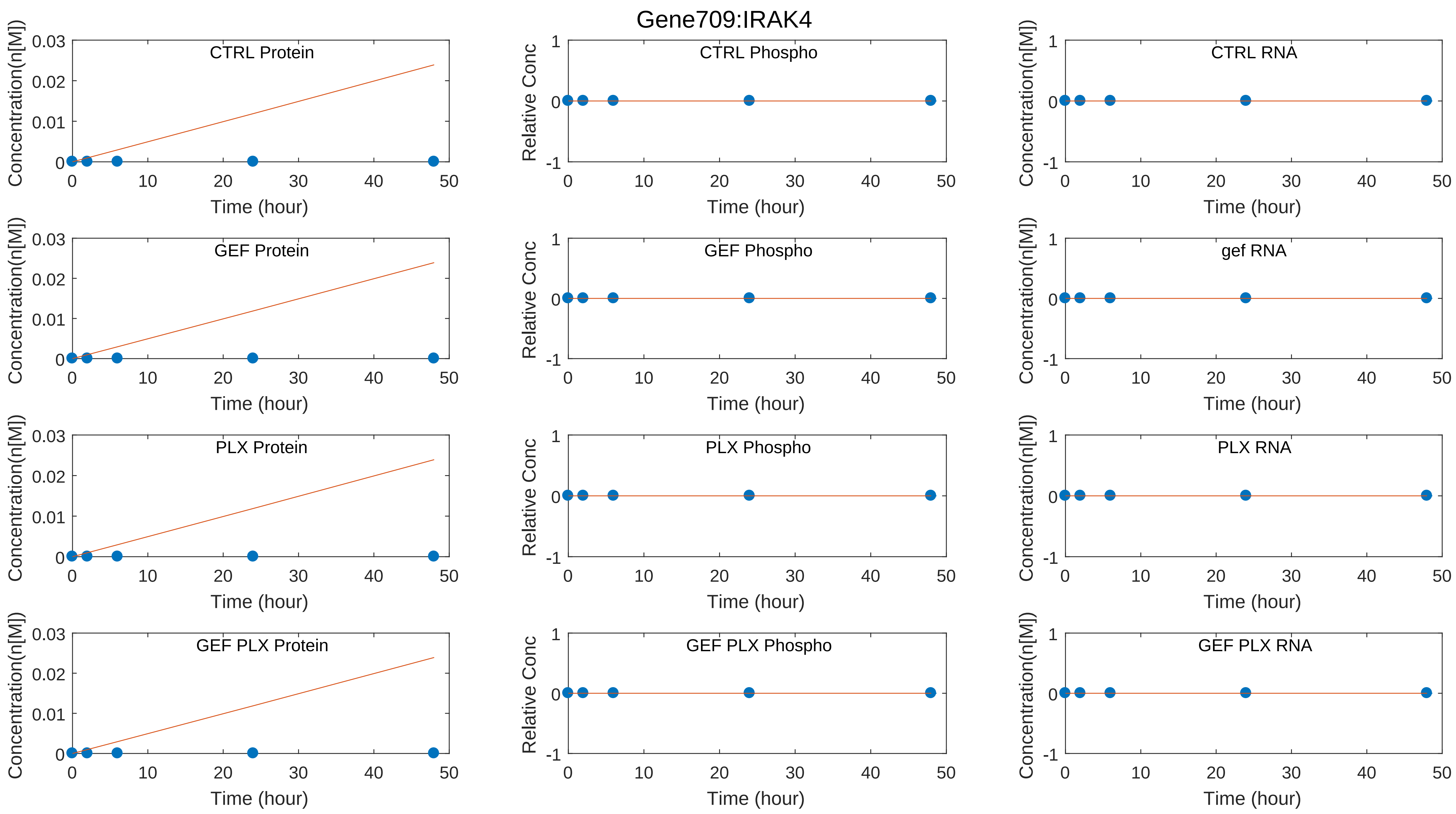

Gene709:IRAK4
CTRL Protein
CTRL Phospho
CTRL RNA
GEF Protein
GEF Phospho
gef RNA
PLX Protein
PLX Phospho
PLX RNA
GEF PLX Protein
GEF PLX Phospho
GEF PLX RNA
Concentration(n[M])
Relative Conc
Time (hour)

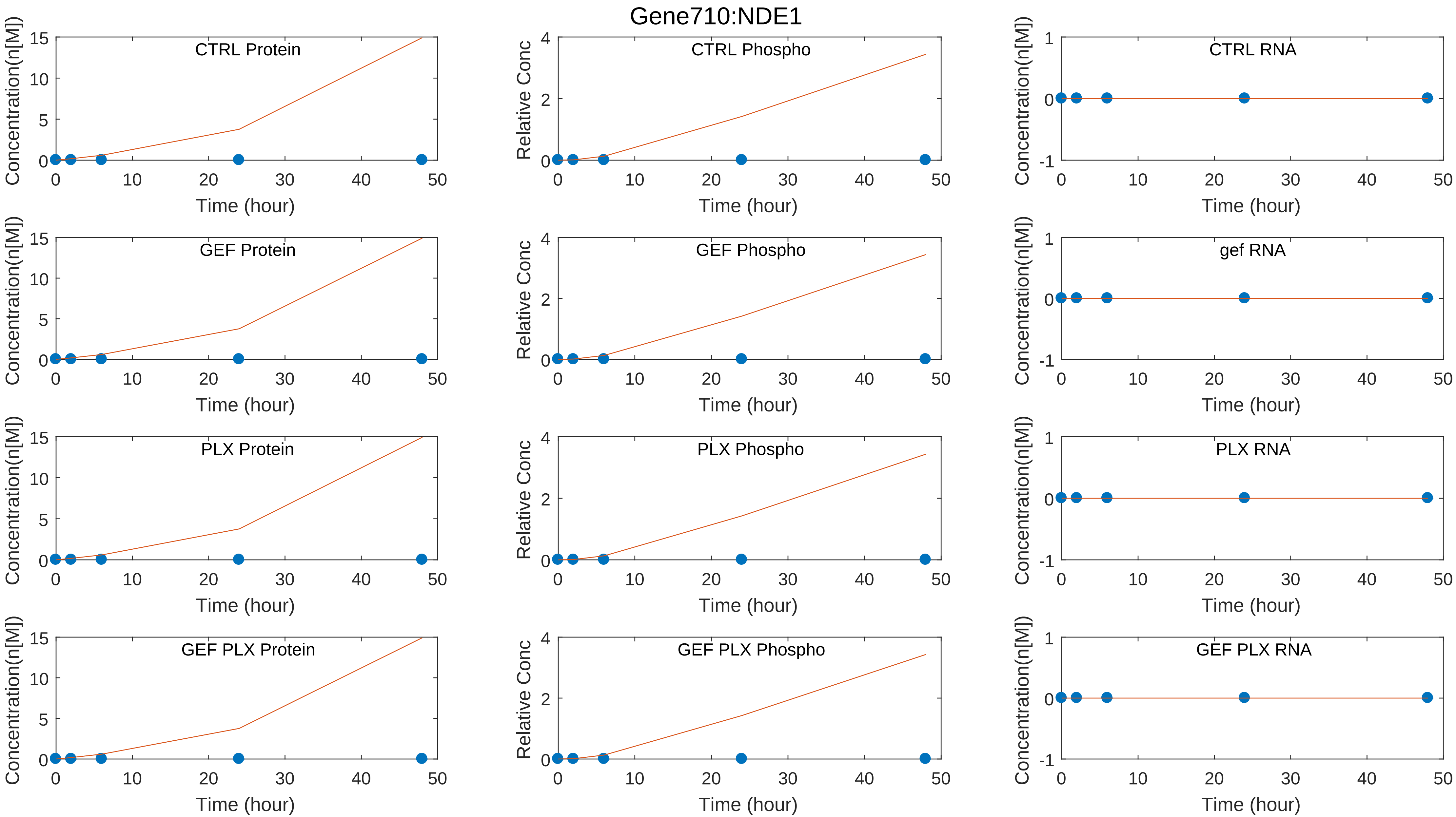

Gene710:NDE1
CTRL Protein
CTRL Phospho
CTRL RNA
GEF Protein
GEF Phospho
gef RNA
PLX Protein
PLX Phospho
PLX RNA
GEF PLX Protein
GEF PLX Phospho
GEF PLX RNA
Concentration(n[M])
Relative Conc
Time (hour)

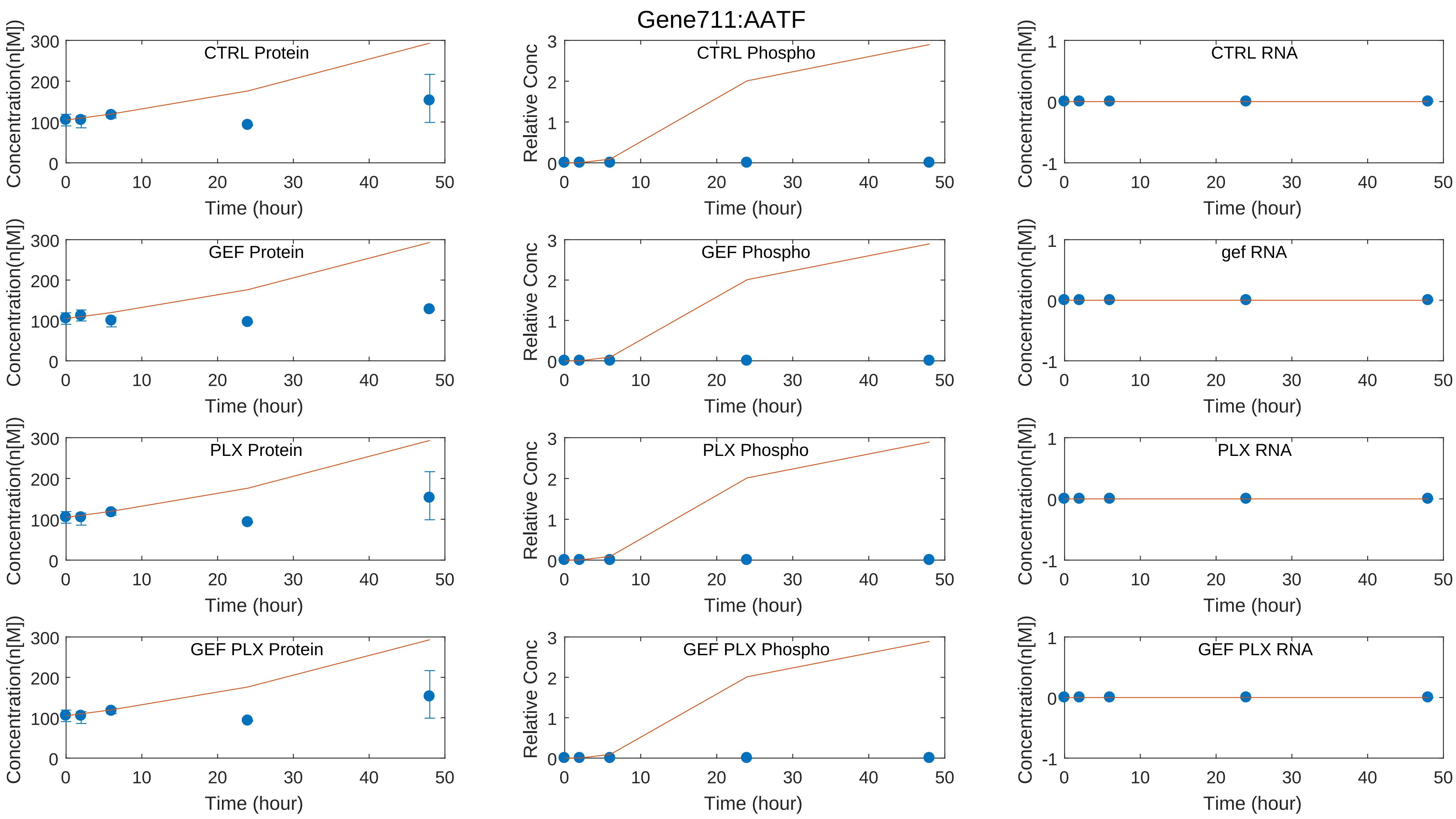

Gene711:AATF
CTRL Protein
GEF Protein
PLX Protein
GEF PLX Protein
CTRL Phospho
GEF Phospho
PLX Phospho
GEF PLX Phospho
CTRL RNA
gef RNA
PLX RNA
GEF PLX RNA
Concentration(n[M])
Relative Conc
Time (hour)

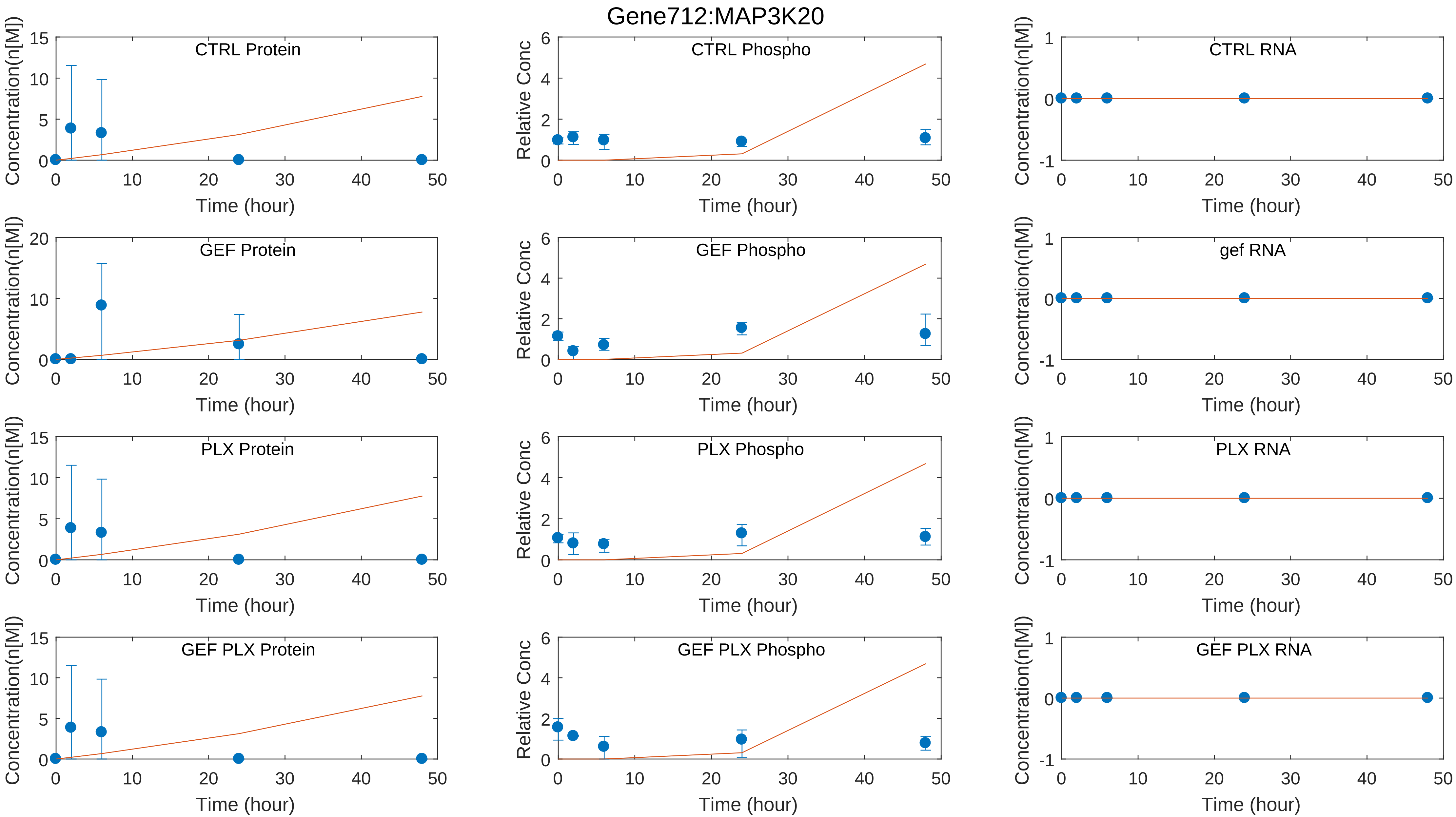

Gene712:MAP3K20
CTRL Protein
GEF Protein
PLX Protein
GEF PLX Protein
CTRL Phospho
GEF Phospho
PLX Phospho
GEF PLX Phospho
CTRL RNA
gef RNA
PLX RNA
GEF PLX RNA
Concentration(n[M])
Relative Conc
Time (hour)

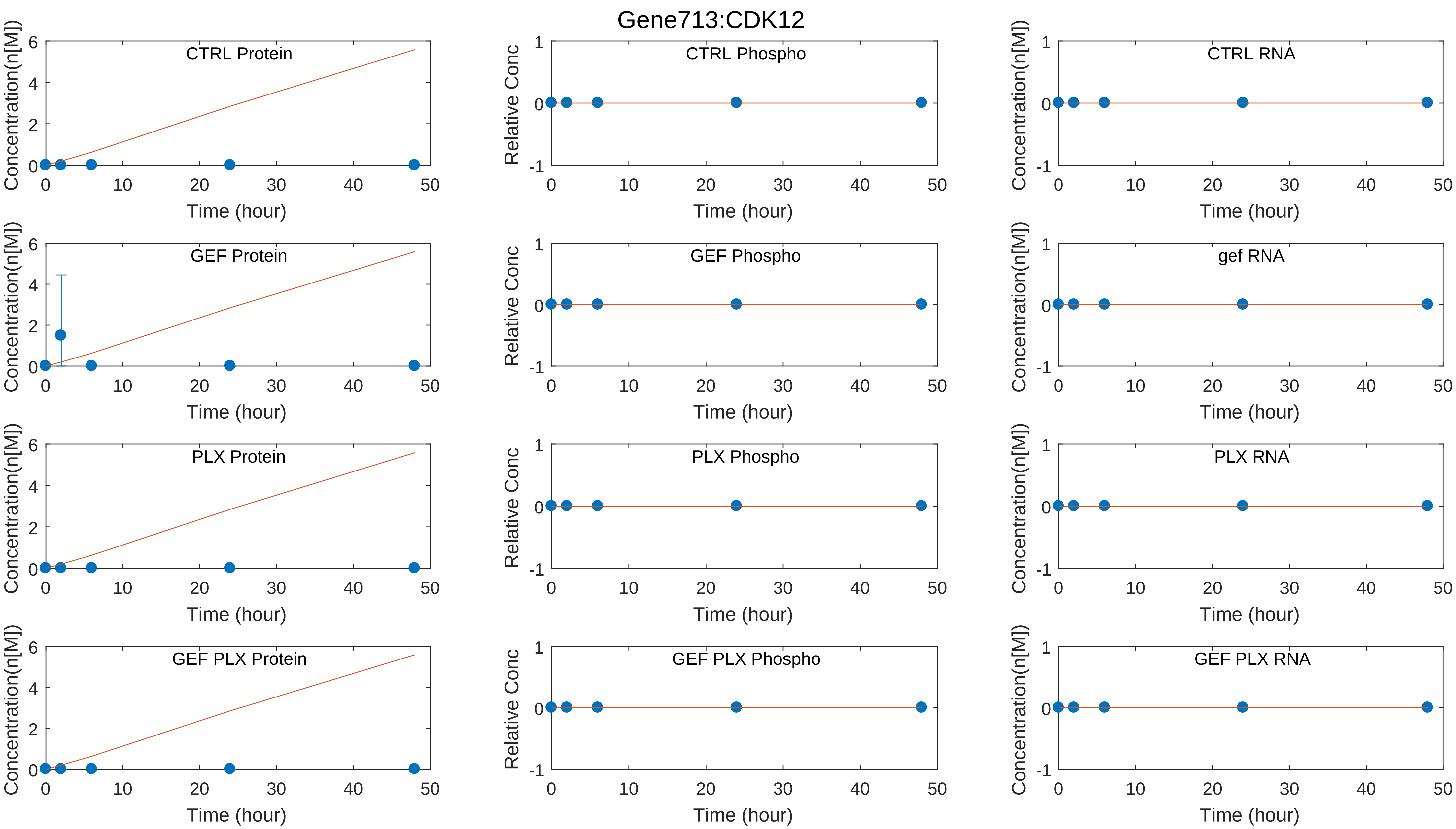

Gene713:CDK12
CTRL Protein
CTRL Phospho
CTRL RNA
GEF Protein
GEF Phospho
gef RNA
PLX Protein
PLX Phospho
PLX RNA
GEF PLX Protein
GEF PLX Phospho
GEF PLX RNA
Concentration(n[M])
Relative Conc
Time (hour)

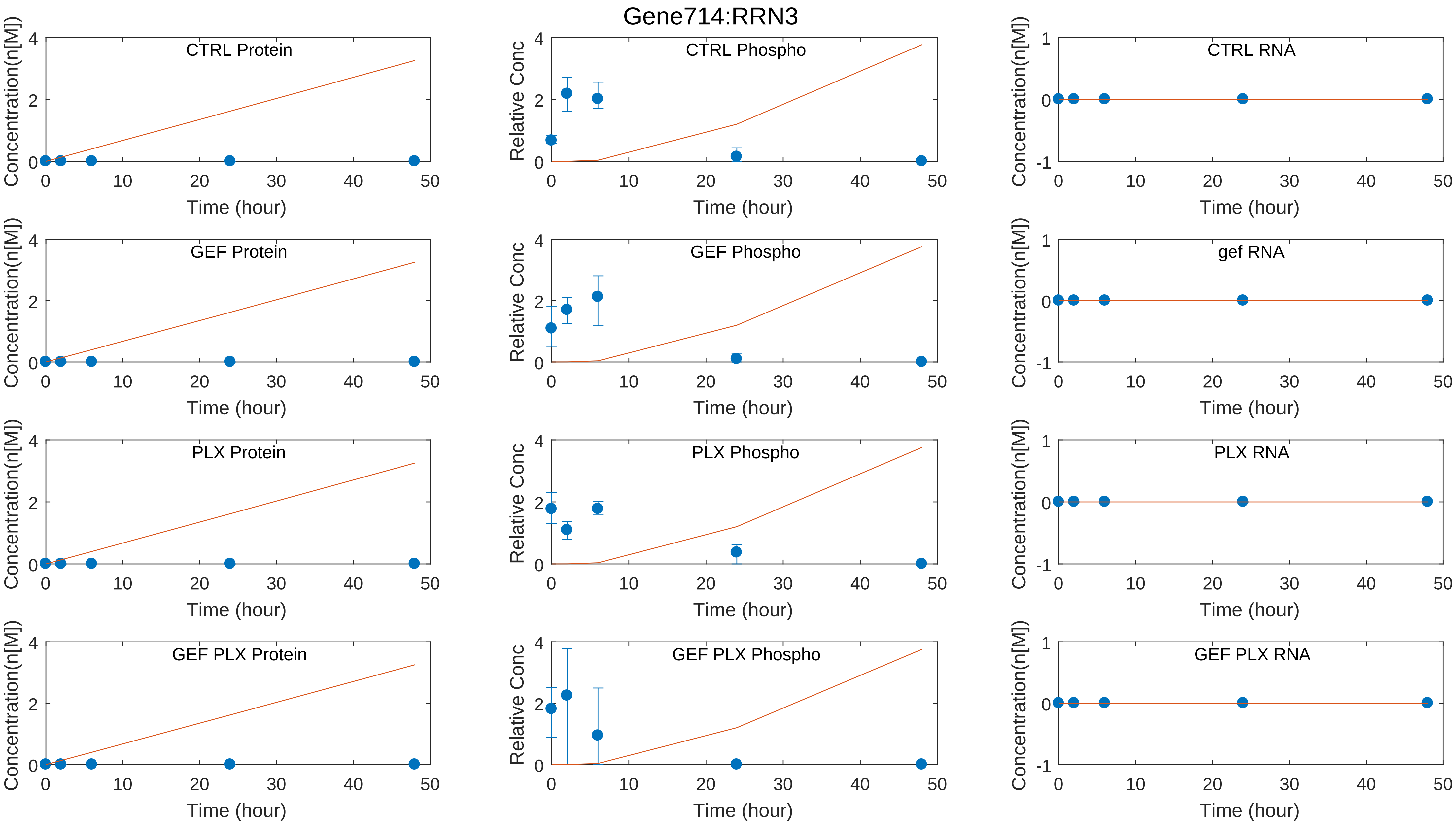

Gene714:RRN3
CTRL Protein
GEF Protein
PLX Protein
GEF PLX Protein
CTRL Phospho
GEF Phospho
PLX Phospho
GEF PLX Phospho
CTRL RNA
gef RNA
PLX RNA
GEF PLX RNA
Concentration(n[M])
Relative Conc
Time (hour)

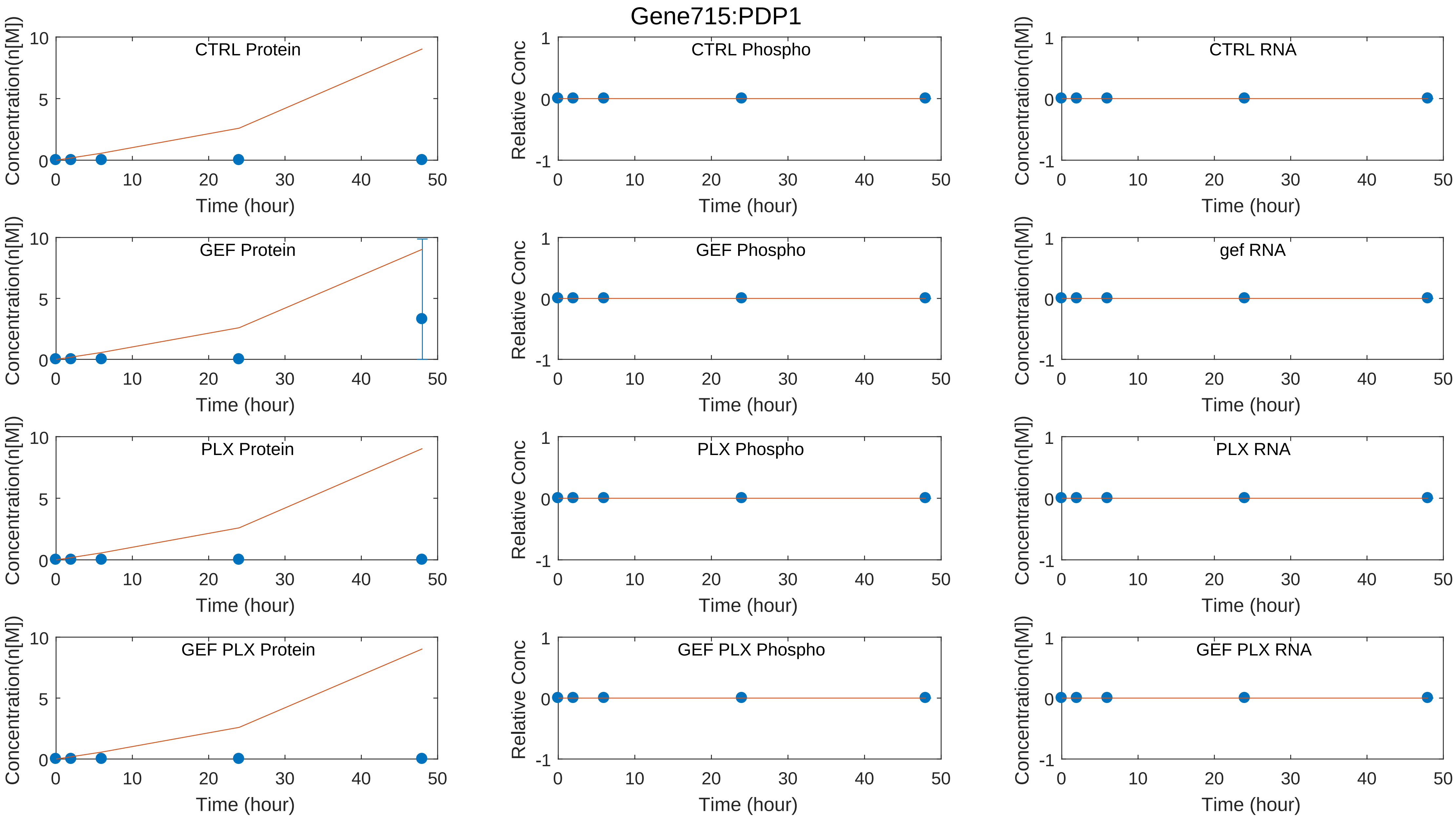

Gene715:PDP1
CTRL Protein
CTRL Phospho
CTRL RNA
GEF Protein
GEF Phospho
gef RNA
PLX Protein
PLX Phospho
PLX RNA
GEF PLX Protein
GEF PLX Phospho
GEF PLX RNA
Concentration(n[M])
Relative Conc
Time (hour)

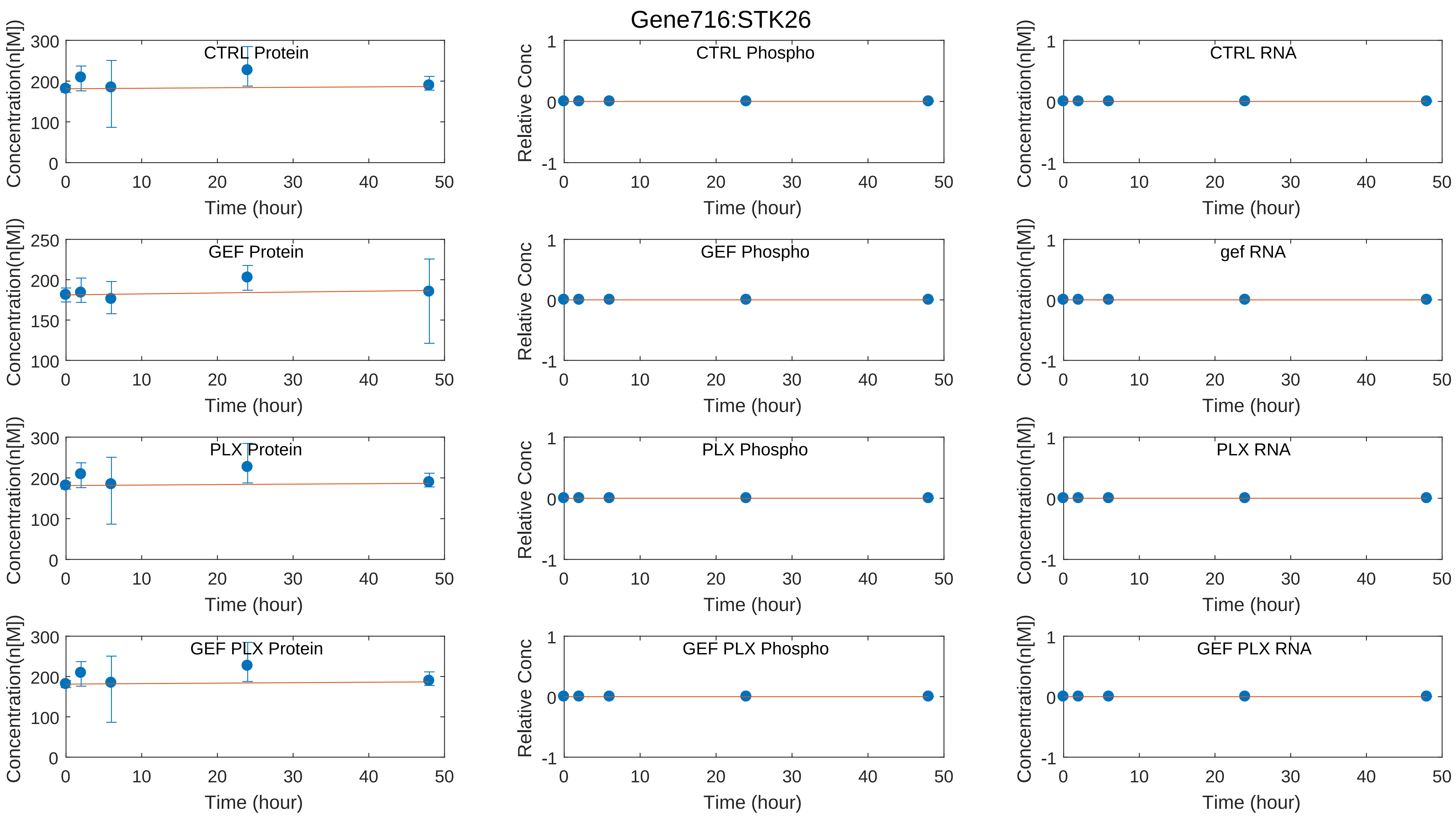

Gene716:STK26
CTRL Protein
CTRL Phospho
CTRL RNA
GEF Protein
GEF Phospho
gef RNA
PLX Protein
PLX Phospho
PLX RNA
GEF PLX Protein
GEF PLX Phospho
GEF PLX RNA
Concentration(n[M])
Relative Conc
Time (hour)

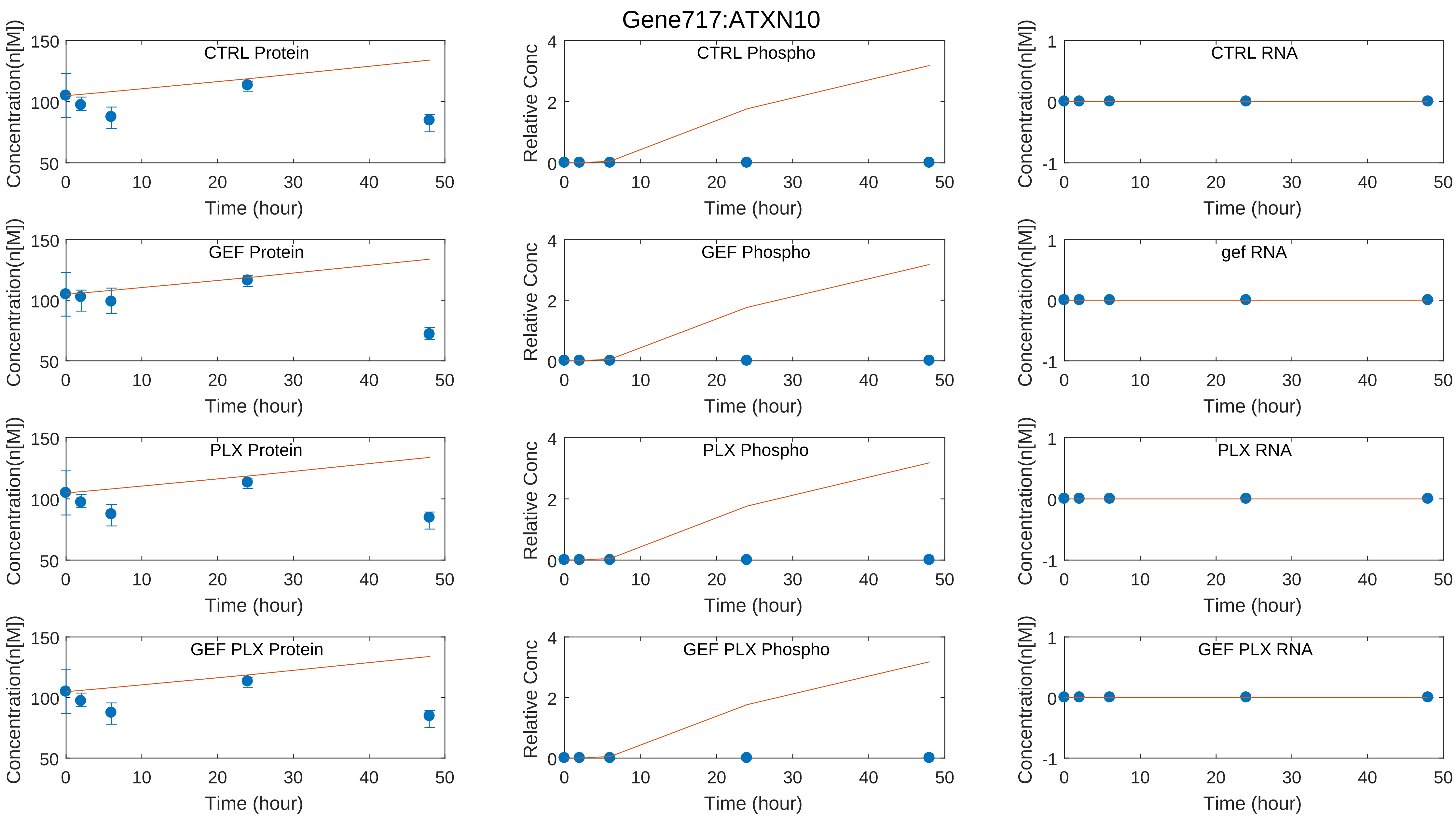

Gene717:ATXN10
CTRL Protein
CTRL Phospho
CTRL RNA
GEF Protein
GEF Phospho
gef RNA
PLX Protein
PLX Phospho
PLX RNA
GEF PLX Protein
GEF PLX Phospho
GEF PLX RNA
Concentration(n[M])
Relative Conc
Time (hour)

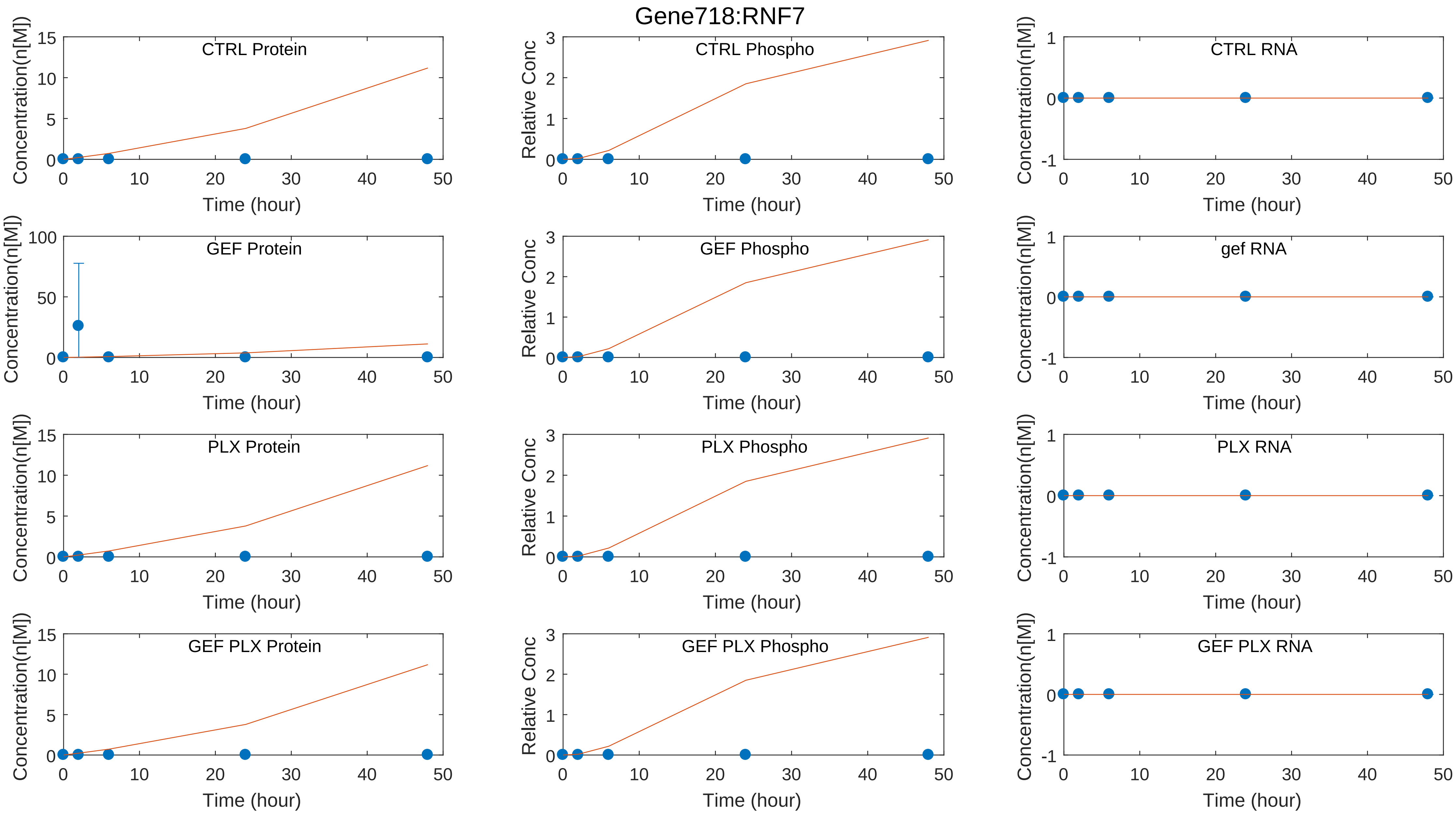
Gene718:RNF7
CTRL Protein
CTRL Phospho
CTRL RNA
GEF Protein
GEF Phospho
gef RNA
PLX Protein
PLX Phospho
PLX RNA
GEF PLX Protein
GEF PLX Phospho
GEF PLX RNA
Concentration(n[M])
Relative Conc
Time (hour)
0
10
20
30
40
50
5
15
100
1
2
3
-1

Gene719:PI4KB

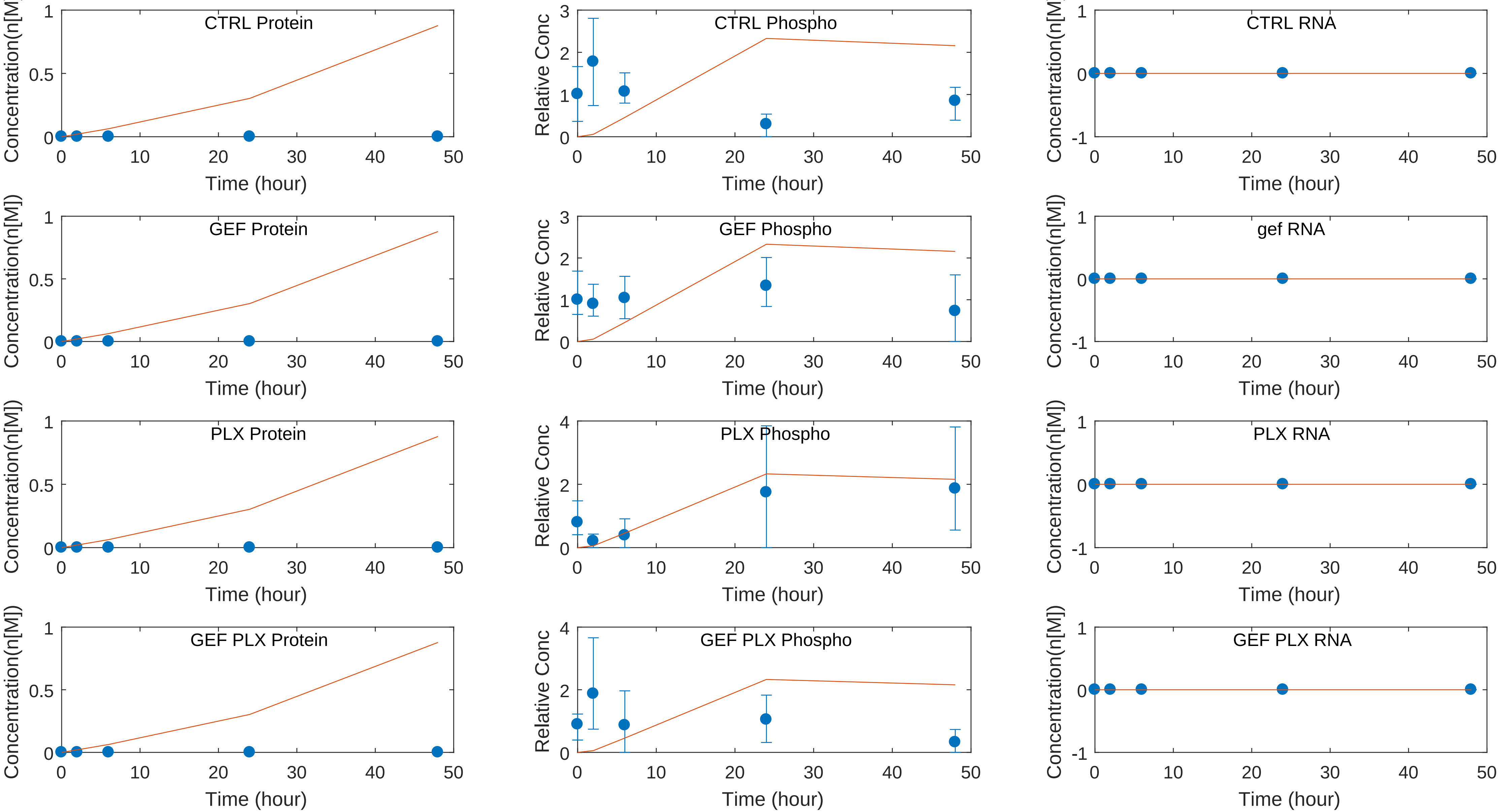

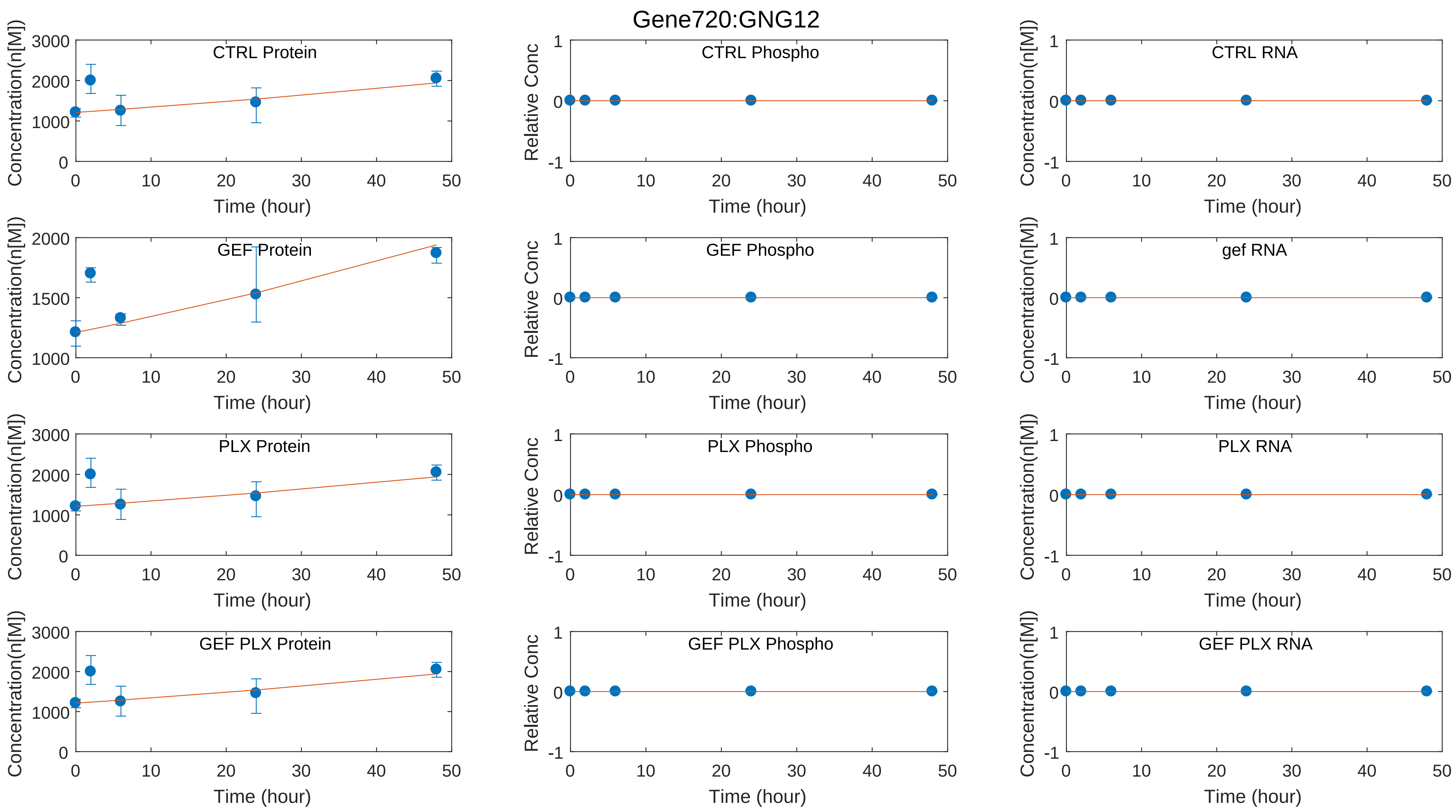
Gene720:GNG12
CTRL Protein
CTRL Phospho
CTRL RNA
GEF Protein
GEF Phospho
gef RNA
PLX Protein
PLX Phospho
PLX RNA
GEF PLX Protein
GEF PLX Phospho
GEF PLX RNA
Concentration(n[M])
Relative Conc
Time (hour)

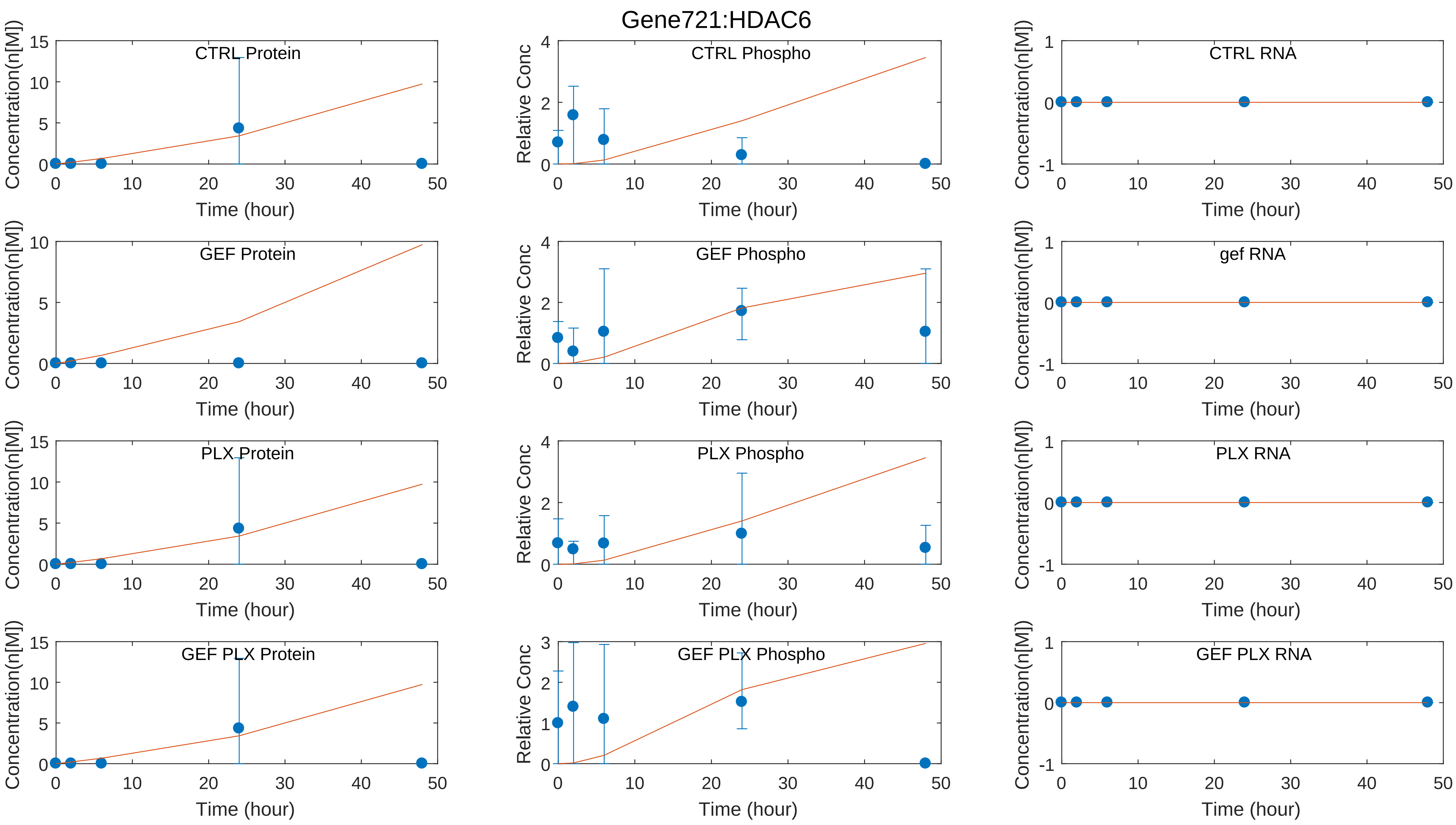

Gene721:HDAC6
CTRL Protein
CTRL Phospho
CTRL RNA
GEF Protein
GEF Phospho
gef RNA
PLX Protein
PLX Phospho
PLX RNA
GEF PLX Protein
GEF PLX Phospho
GEF PLX RNA
Concentration(n[M])
Relative Conc
Time (hour)

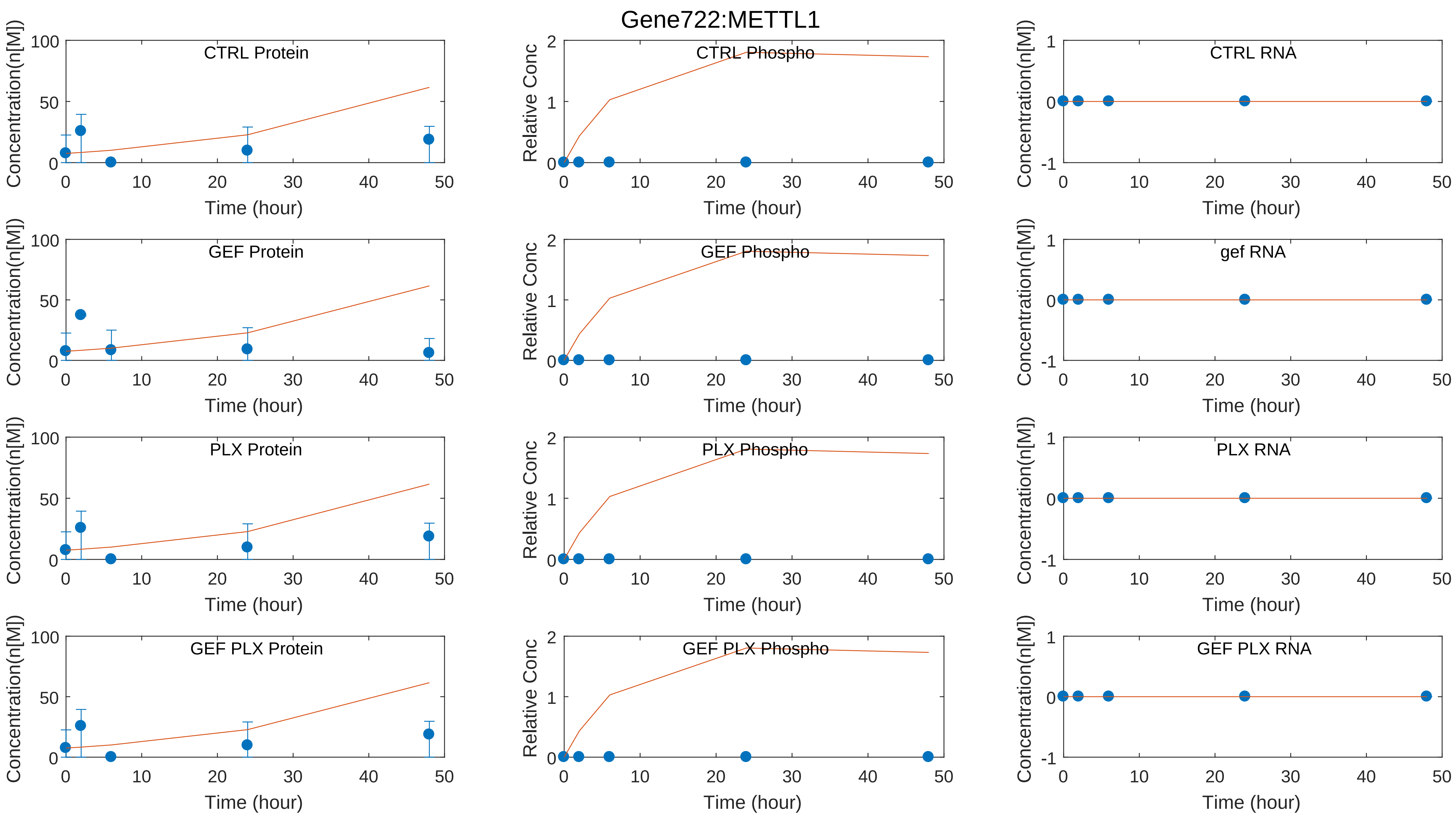
Gene722:METTL1
CTRL Protein
CTRL Phospho
CTRL RNA
GEF Protein
GEF Phospho
gef RNA
PLX Protein
PLX Phospho
PLX RNA
GEF PLX Protein
GEF PLX Phospho
GEF PLX RNA
Concentration(n[M])
Relative Conc
Time (hour)

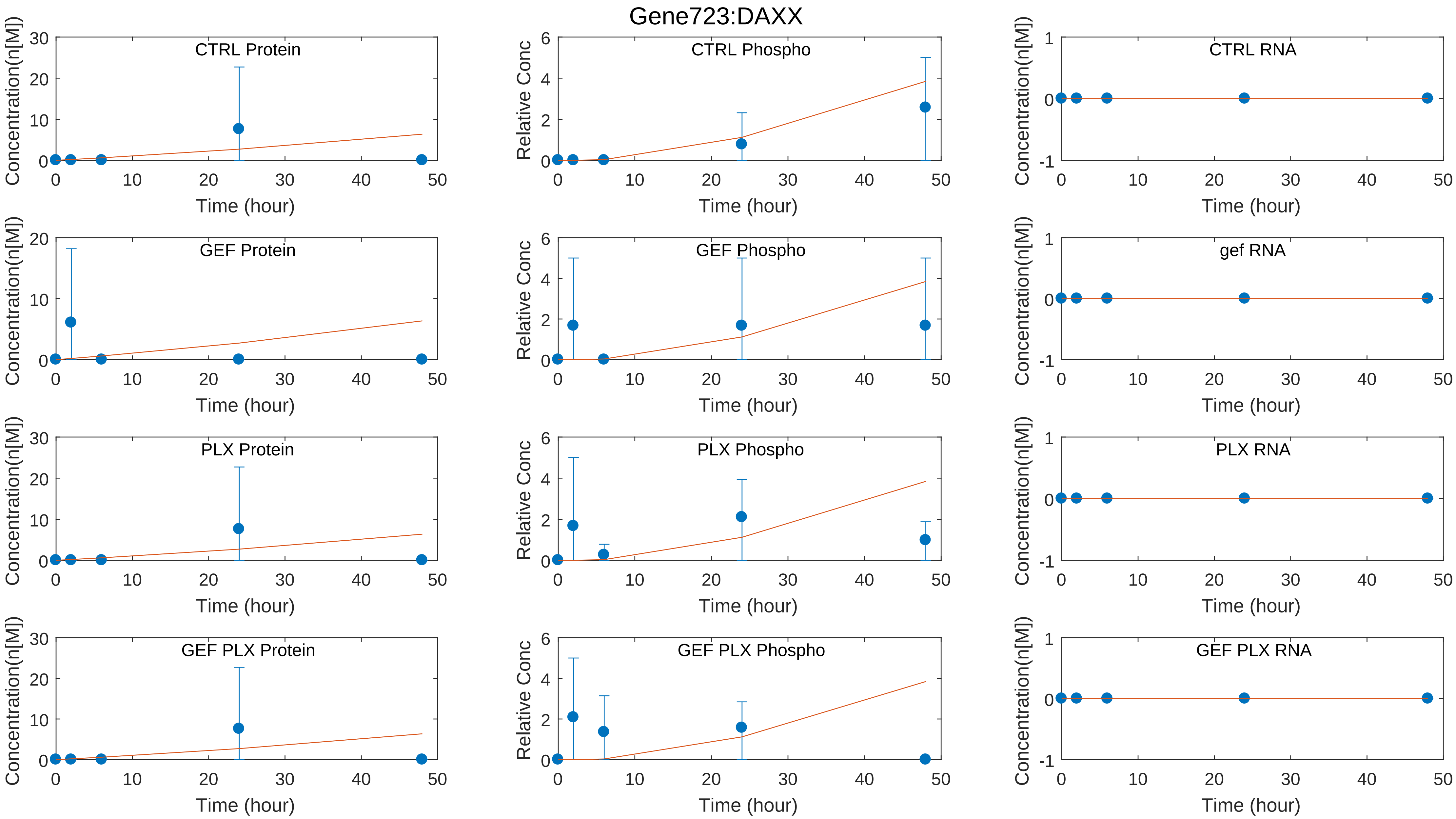
Gene723:DAXX
CTRL Protein
CTRL Phospho
CTRL RNA
GEF Protein
GEF Phospho
gef RNA
PLX Protein
PLX Phospho
PLX RNA
GEF PLX Protein
GEF PLX Phospho
GEF PLX RNA
Concentration(n[M])
Relative Conc
Time (hour)

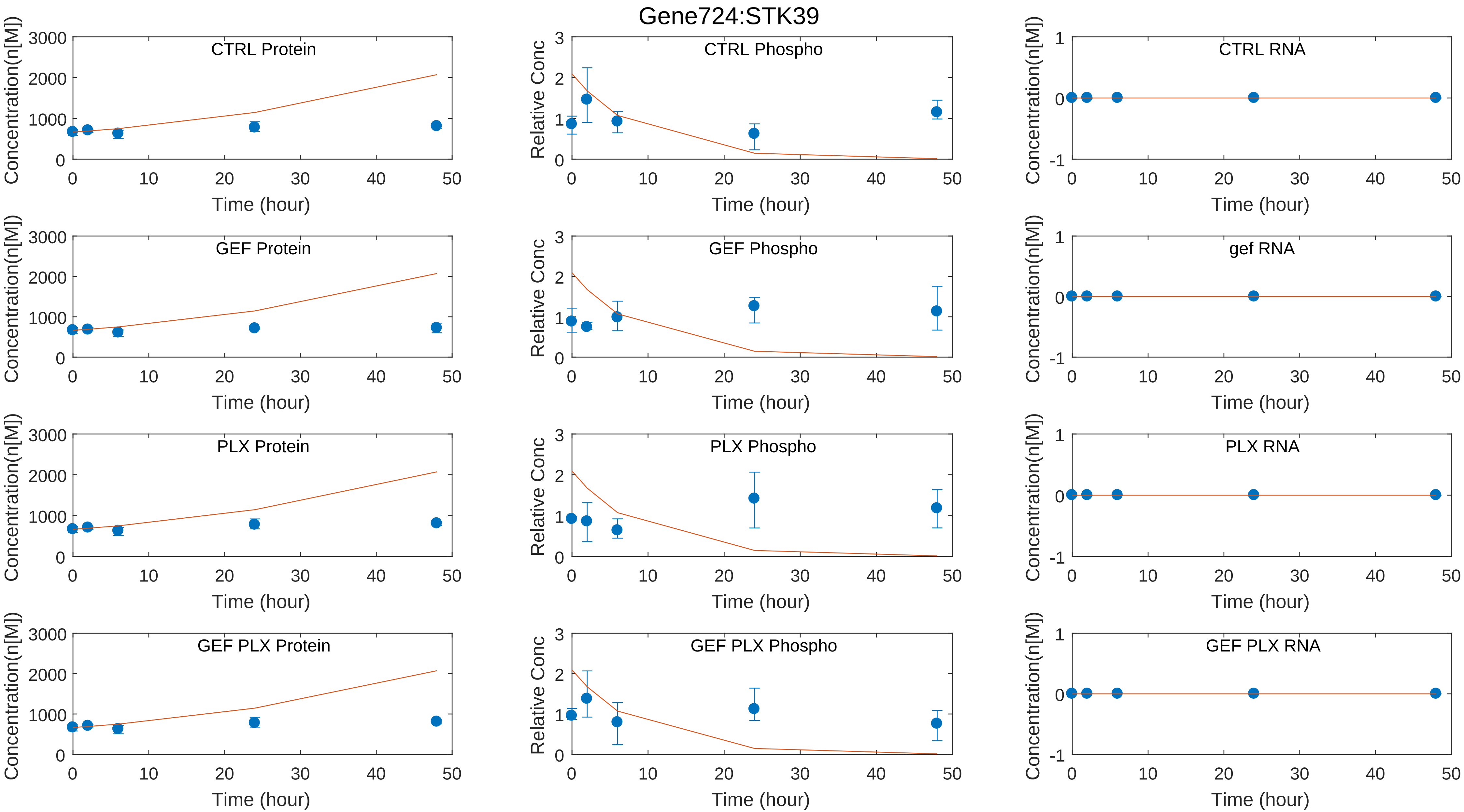
Gene724:STK39
CTRL Protein
CTRL Phospho
CTRL RNA
GEF Protein
GEF Phospho
gef RNA
PLX Protein
PLX Phospho
PLX RNA
GEF PLX Protein
GEF PLX Phospho
GEF PLX RNA
Concentration(n[M])
Relative Conc
Time (hour)

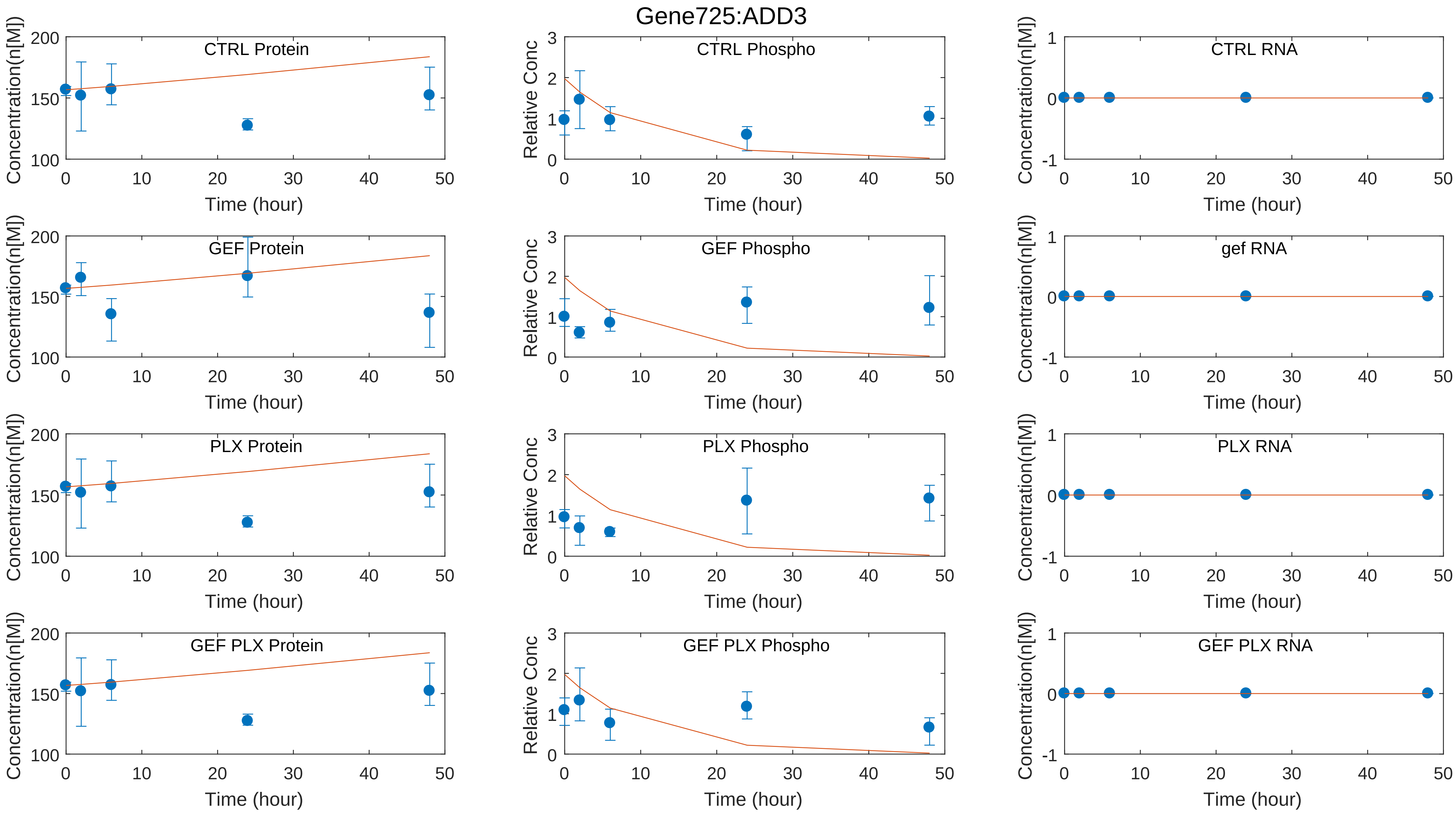

Gene725:ADD3
CTRL Protein
CTRL Phospho
CTRL RNA
GEF Protein
GEF Phospho
gef RNA
PLX Protein
PLX Phospho
PLX RNA
GEF PLX Protein
GEF PLX Phospho
GEF PLX RNA
Concentration(n[M])
Relative Conc
Time (hour)

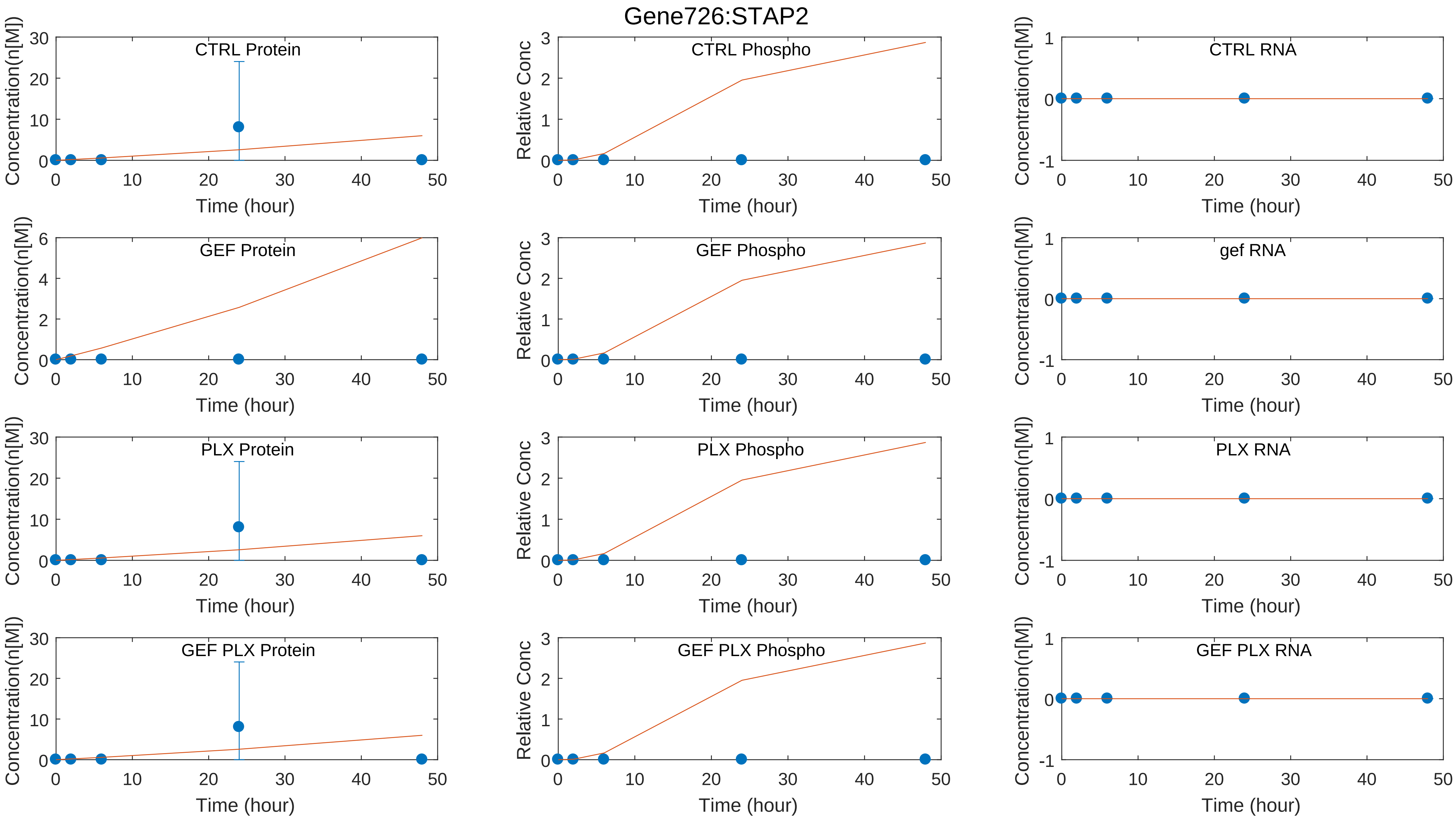
Gene726:STAP2
CTRL Protein
CTRL Phospho
CTRL RNA
GEF Protein
GEF Phospho
gef RNA
PLX Protein
PLX Phospho
PLX RNA
GEF PLX Protein
GEF PLX Phospho
GEF PLX RNA
Concentration(n[M])
Relative Conc
Time (hour)

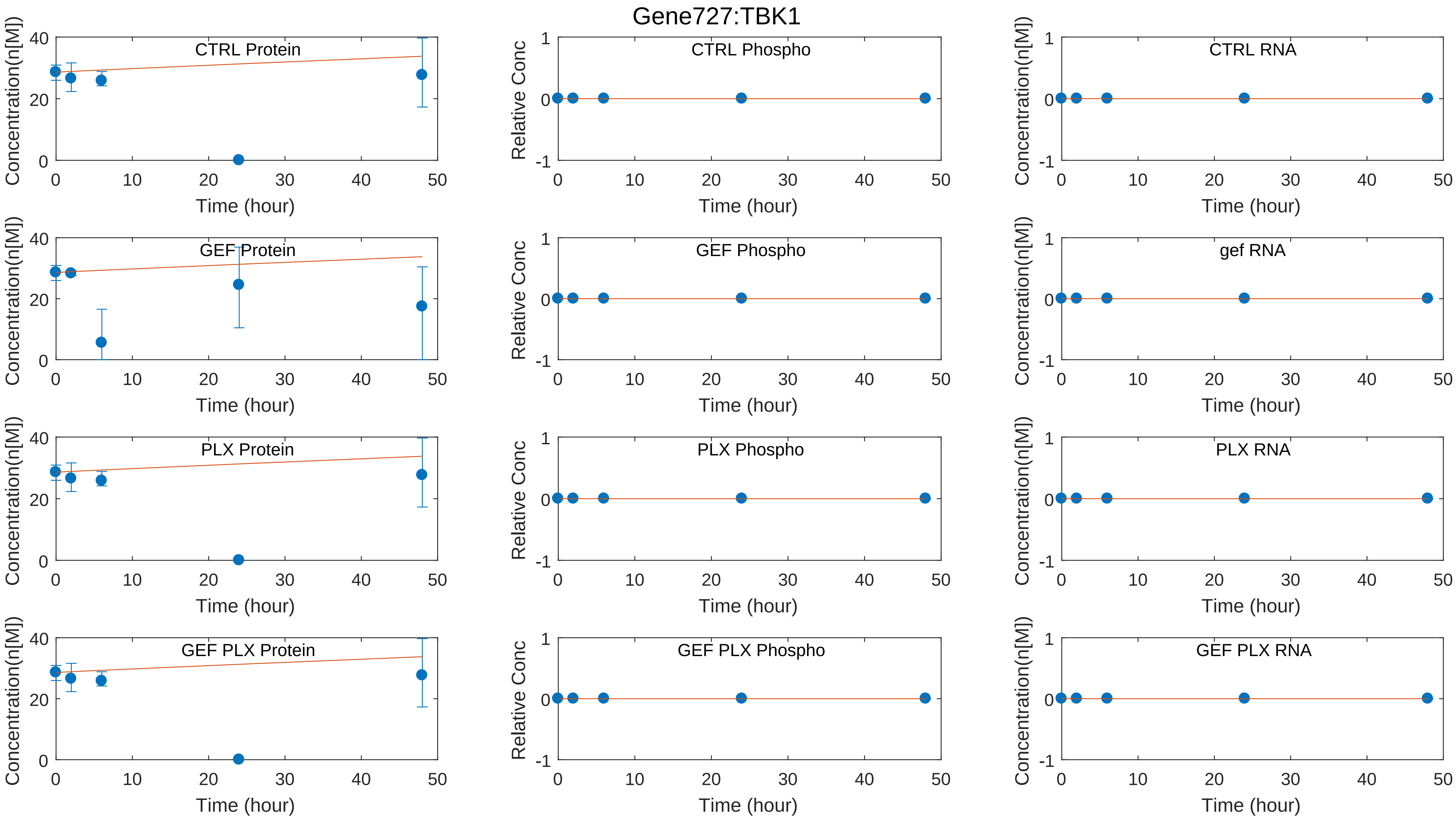
Gene727:TBK1
CTRL Protein
CTRL Phospho
CTRL RNA
GEF Protein
GEF Phospho
gef RNA
PLX Protein
PLX Phospho
PLX RNA
GEF PLX Protein
GEF PLX Phospho
GEF PLX RNA
Concentration(n[M])
Relative Conc
Time (hour)

Gene728:BAZ1B

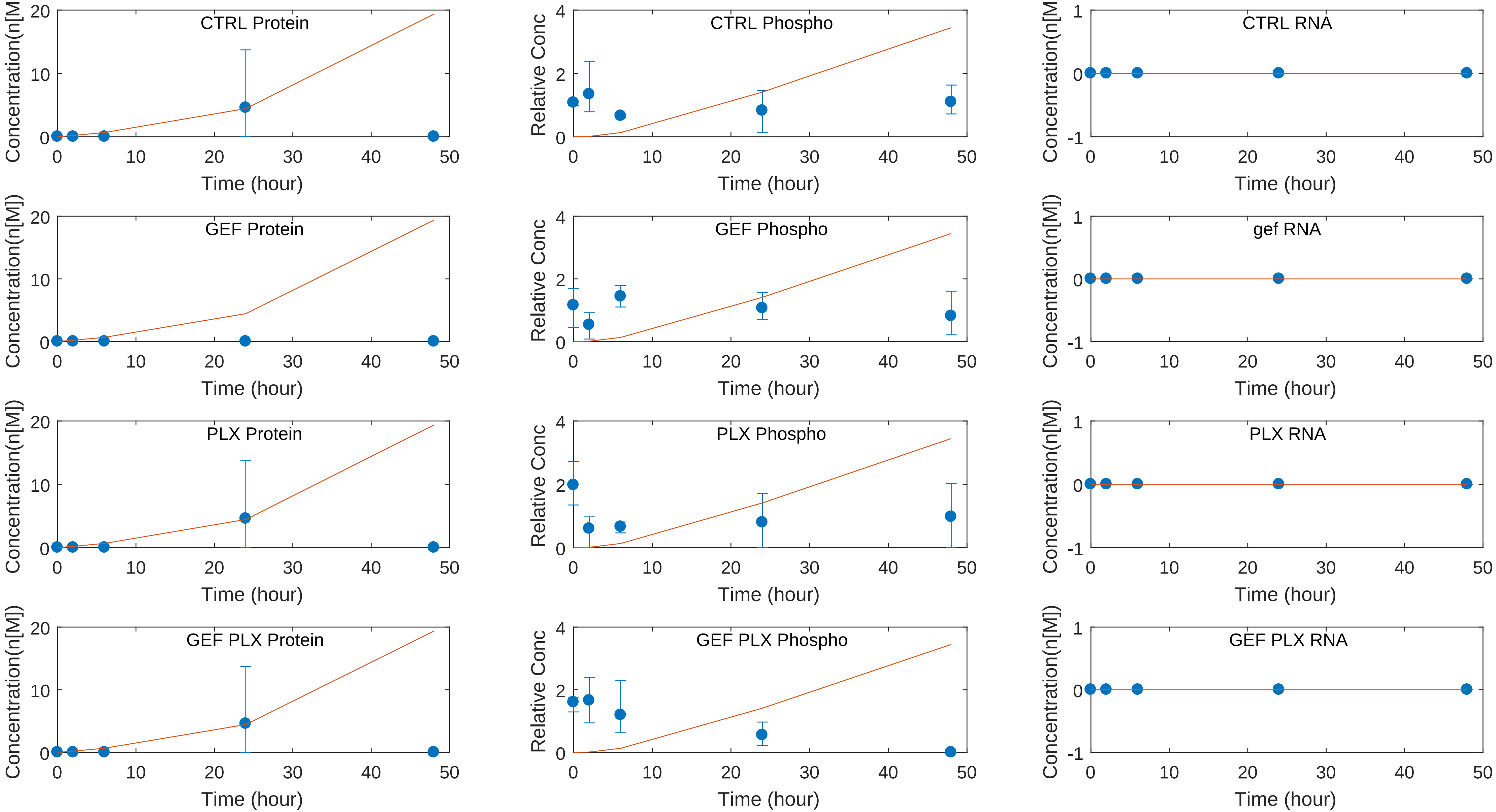

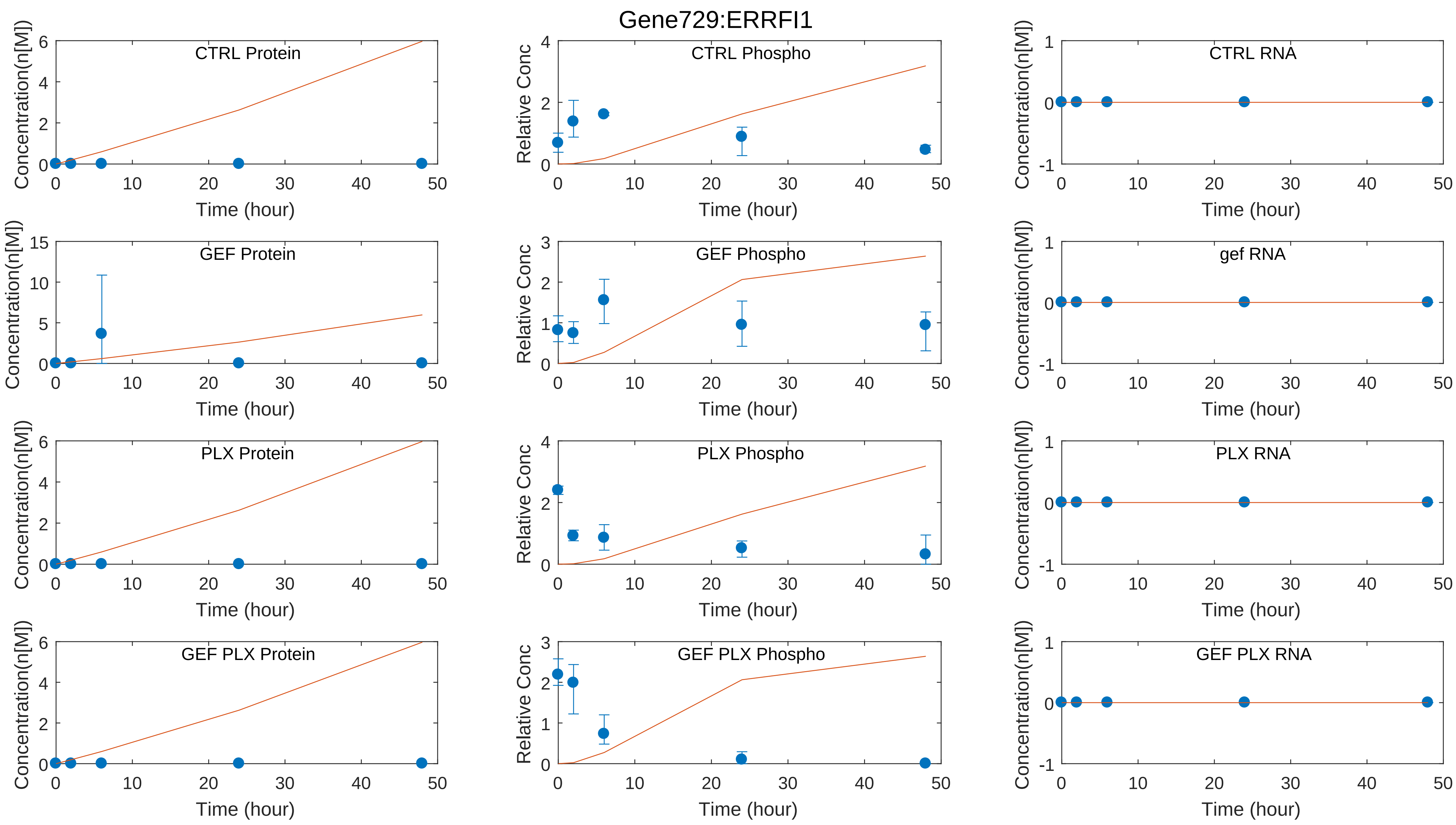

Gene729:ERRFI1
CTRL Protein
CTRL Phospho
CTRL RNA
GEF Protein
GEF Phospho
gef RNA
PLX Protein
PLX Phospho
PLX RNA
GEF PLX Protein
GEF PLX Phospho
GEF PLX RNA
Concentration(n[M])
Relative Conc
Time (hour)

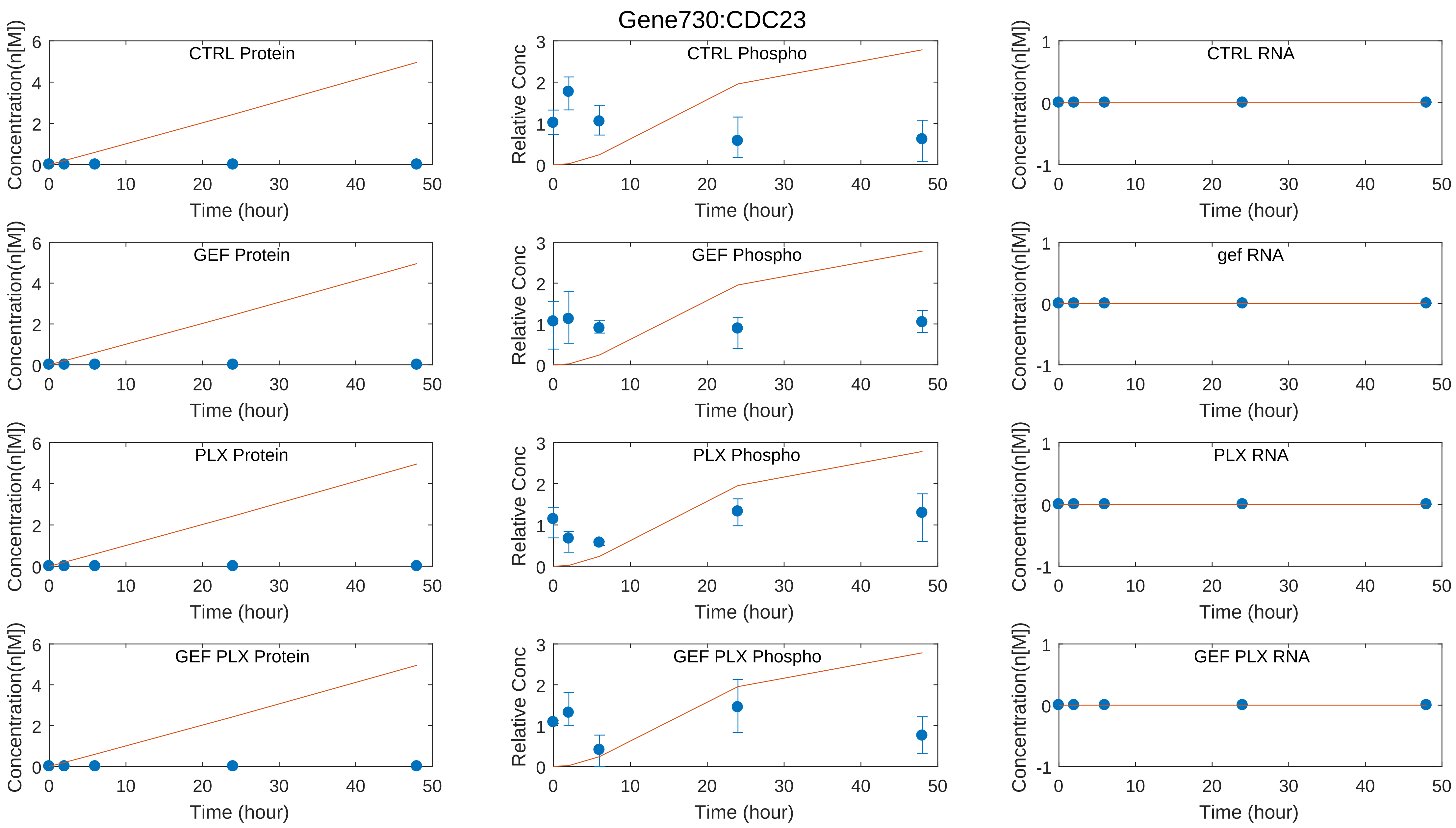

Gene730:CDC23
CTRL Protein
CTRL Phospho
CTRL RNA
GEF Protein
GEF Phospho
gef RNA
PLX Protein
PLX Phospho
PLX RNA
GEF PLX Protein
GEF PLX Phospho
GEF PLX RNA
Concentration(n[M])
Relative Conc
Time (hour)

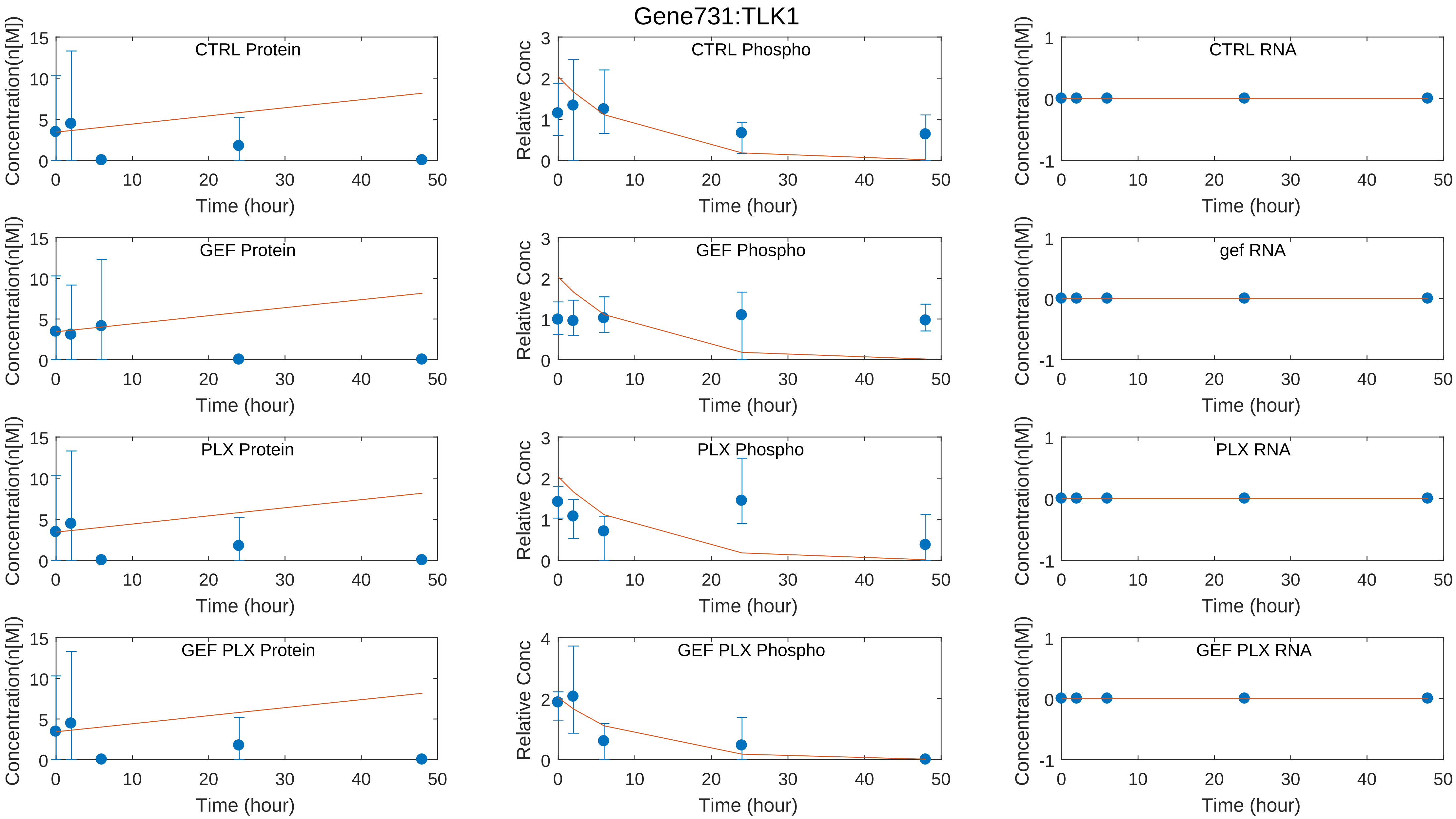

Gene731:TLK1
CTRL Protein
CTRL Phospho
CTRL RNA
GEF Protein
GEF Phospho
gef RNA
PLX Protein
PLX Phospho
PLX RNA
GEF PLX Protein
GEF PLX Phospho
GEF PLX RNA
Concentration(n[M])
Relative Conc
Time (hour)

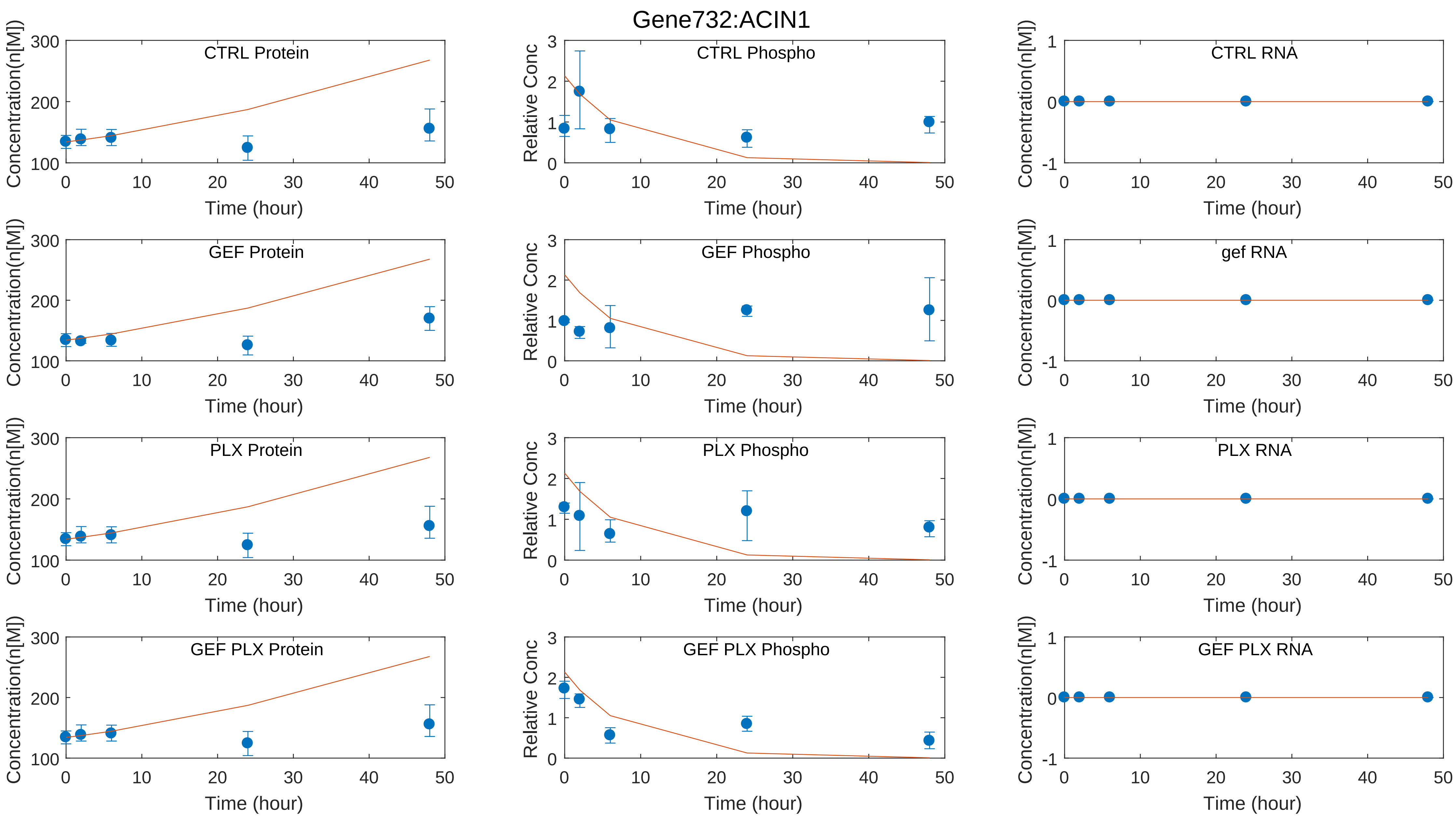

Gene732:ACIN1
CTRL Protein
CTRL Phospho
CTRL RNA
GEF Protein
GEF Phospho
gef RNA
PLX Protein
PLX Phospho
PLX RNA
GEF PLX Protein
GEF PLX Phospho
GEF PLX RNA
Concentration(n[M])
Relative Conc
Time (hour)

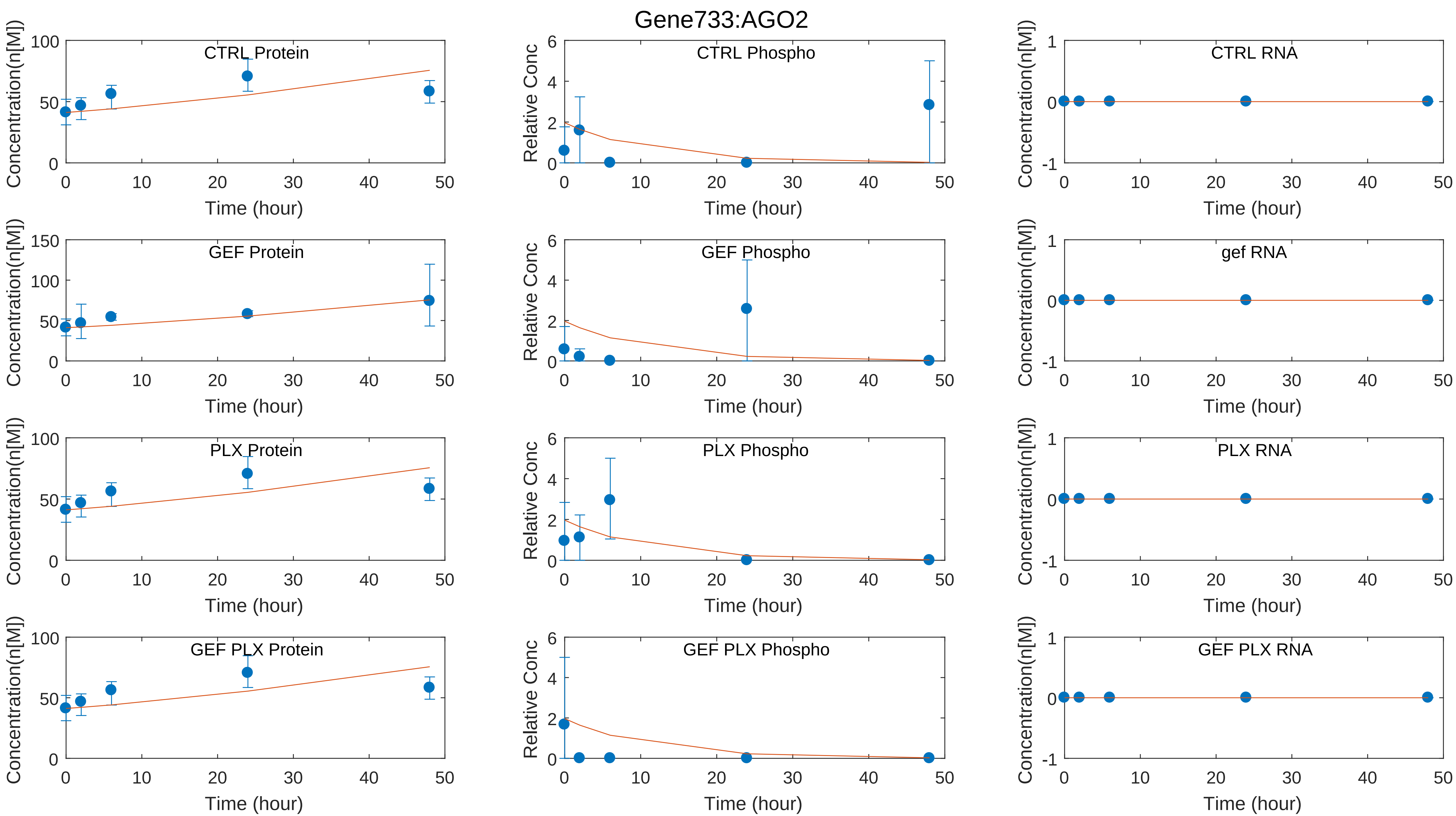

Gene733:AGO2
CTRL Protein
CTRL Phospho
CTRL RNA
GEF Protein
GEF Phospho
gef RNA
PLX Protein
PLX Phospho
PLX RNA
GEF PLX Protein
GEF PLX Phospho
GEF PLX RNA
Concentration(n[M])
Relative Conc
Time (hour)

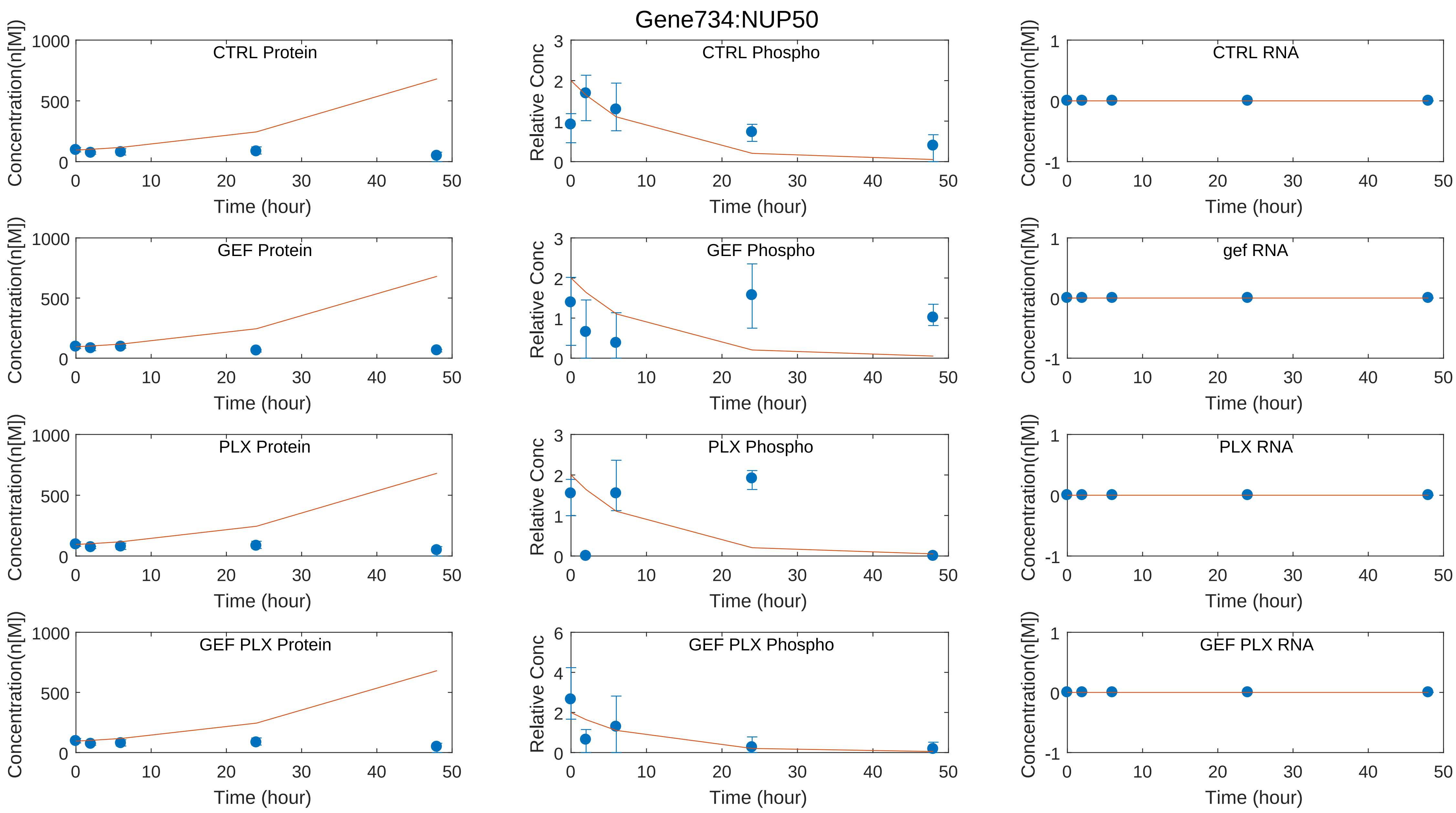

Gene734:NUP50
CTRL Protein
CTRL Phospho
CTRL RNA
GEF Protein
GEF Phospho
gef RNA
PLX Protein
PLX Phospho
PLX RNA
GEF PLX Protein
GEF PLX Phospho
GEF PLX RNA
Concentration(n[M])
Relative Conc
Time (hour)

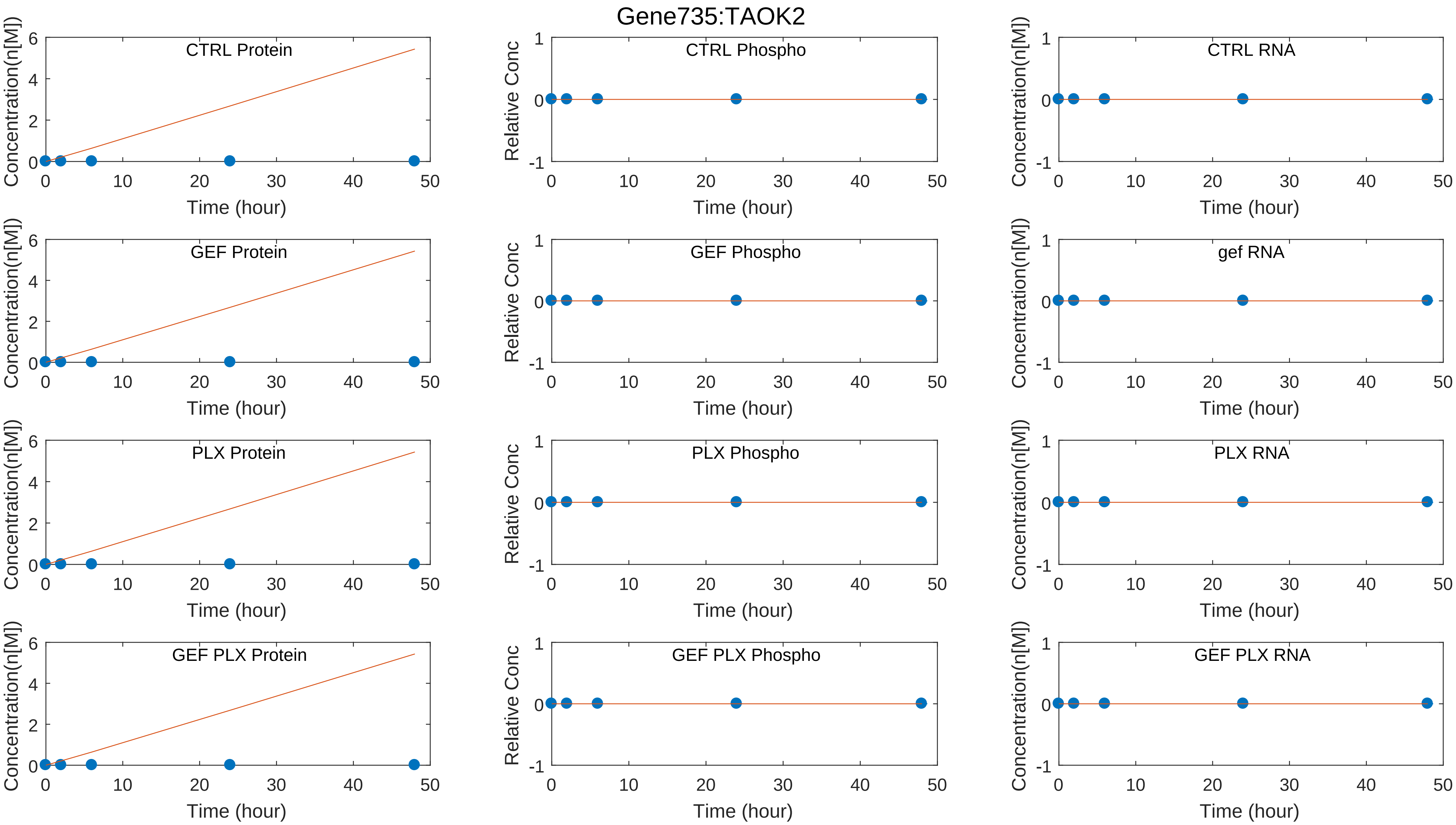

Gene735:TAOK2
CTRL Protein
CTRL Phospho
CTRL RNA
GEF Protein
GEF Phospho
gef RNA
PLX Protein
PLX Phospho
PLX RNA
GEF PLX Protein
GEF PLX Phospho
GEF PLX RNA
Concentration(n[M])
Relative Conc
Time (hour)

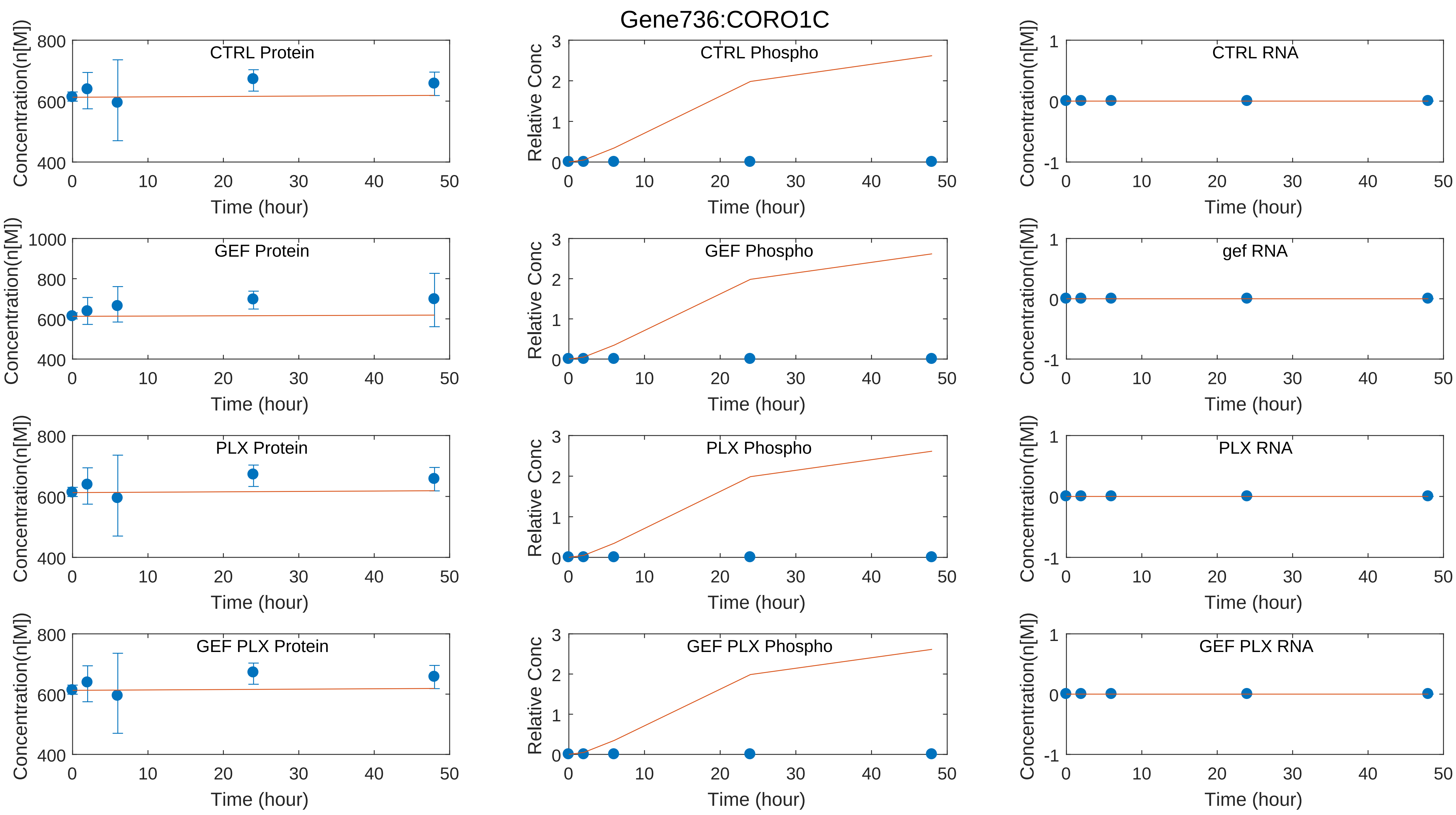

Gene736:CORO1C
CTRL Protein
CTRL Phospho
CTRL RNA
GEF Protein
GEF Phospho
gef RNA
PLX Protein
PLX Phospho
PLX RNA
GEF PLX Protein
GEF PLX Phospho
GEF PLX RNA
Concentration(n[M])
Relative Conc
Time (hour)

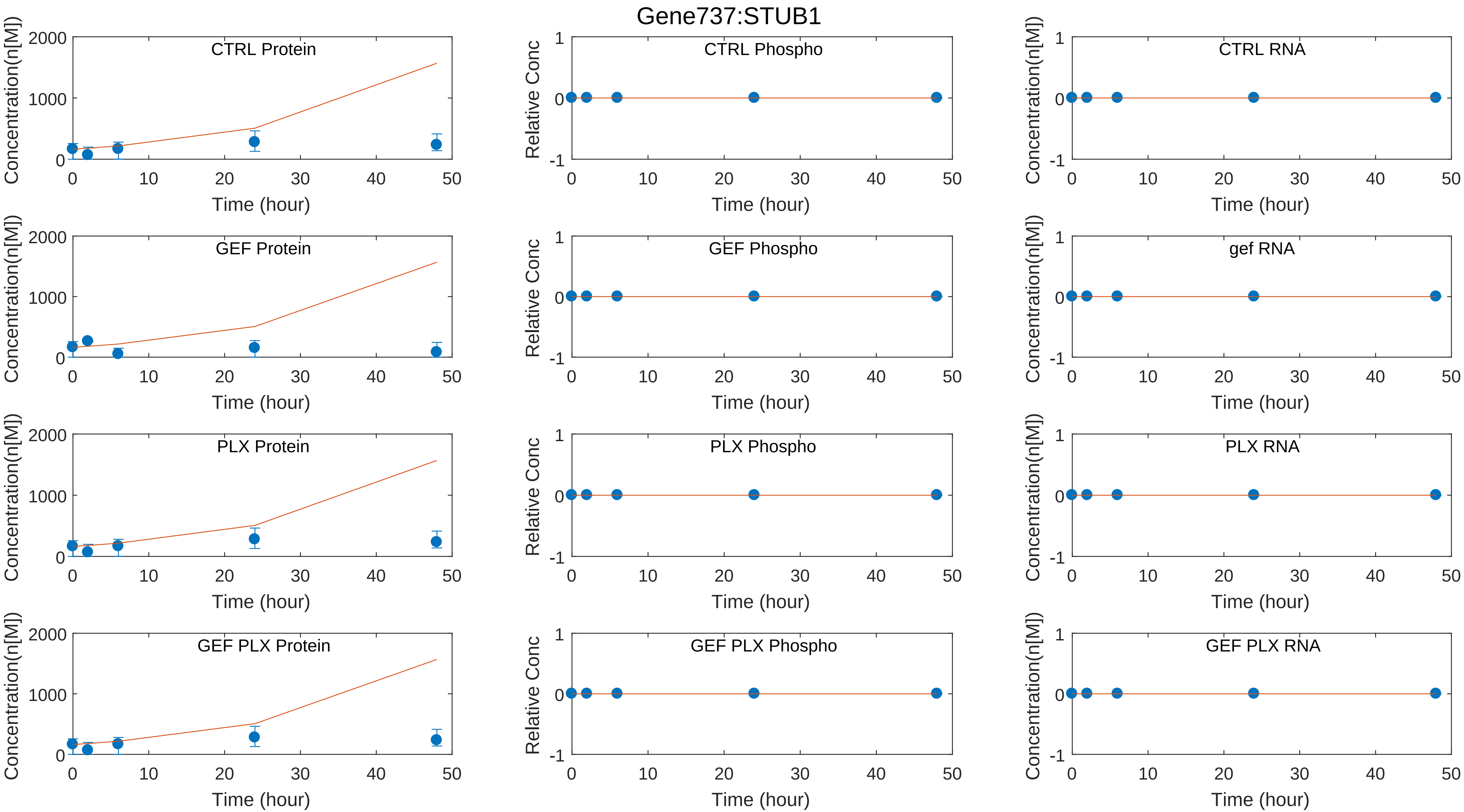
Gene737:STUB1
CTRL Protein
CTRL Phospho
CTRL RNA
GEF Protein
GEF Phospho
gef RNA
PLX Protein
PLX Phospho
PLX RNA
GEF PLX Protein
GEF PLX Phospho
GEF PLX RNA
Concentration(n[M])
Relative Conc
Time (hour)

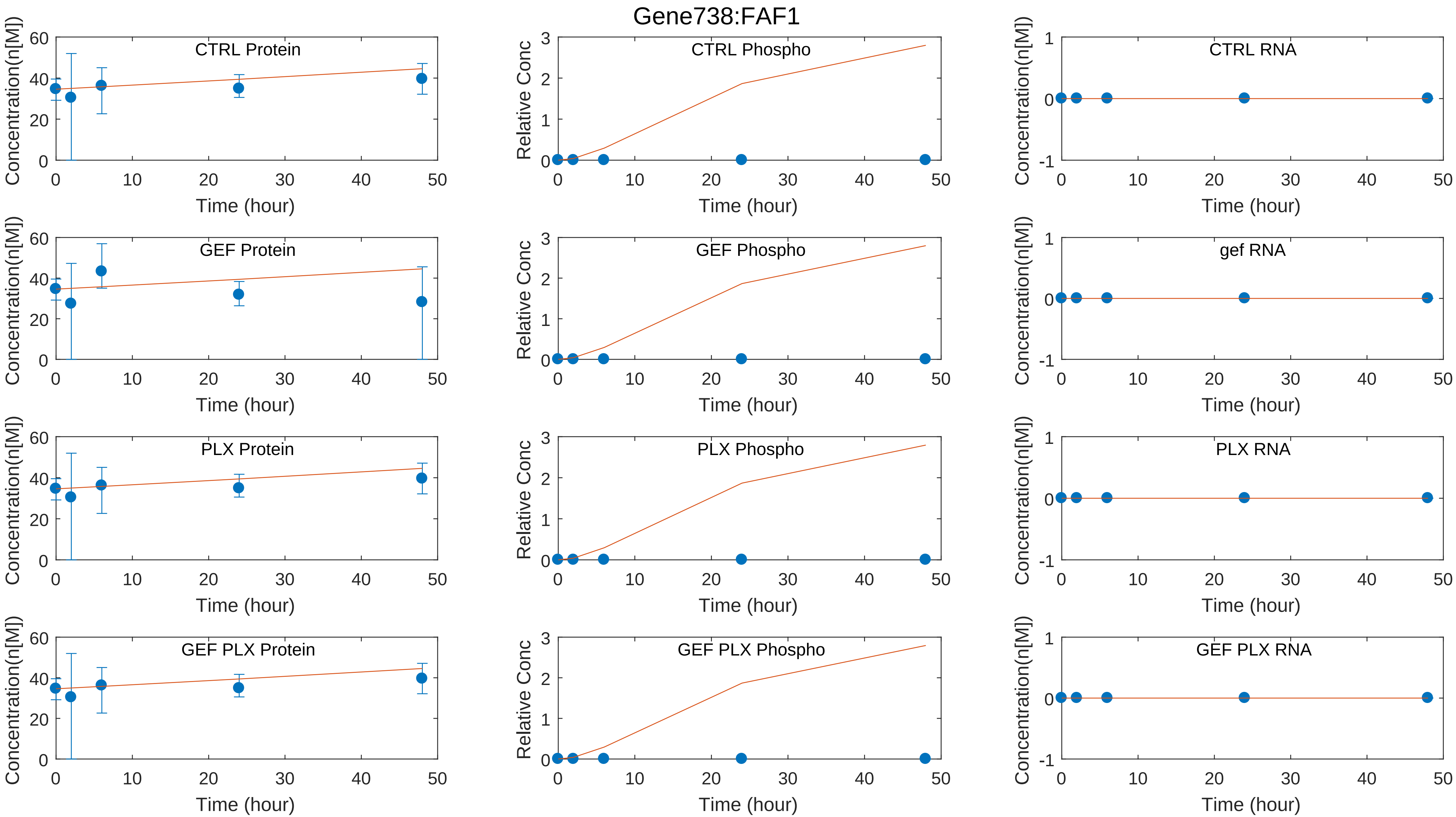
Gene738:FAF1
CTRL Protein
GEF Protein
PLX Protein
GEF PLX Protein
CTRL Phospho
GEF Phospho
PLX Phospho
GEF PLX Phospho
CTRL RNA
gef RNA
PLX RNA
GEF PLX RNA
Concentration(n[M])
Relative Conc
Time (hour)

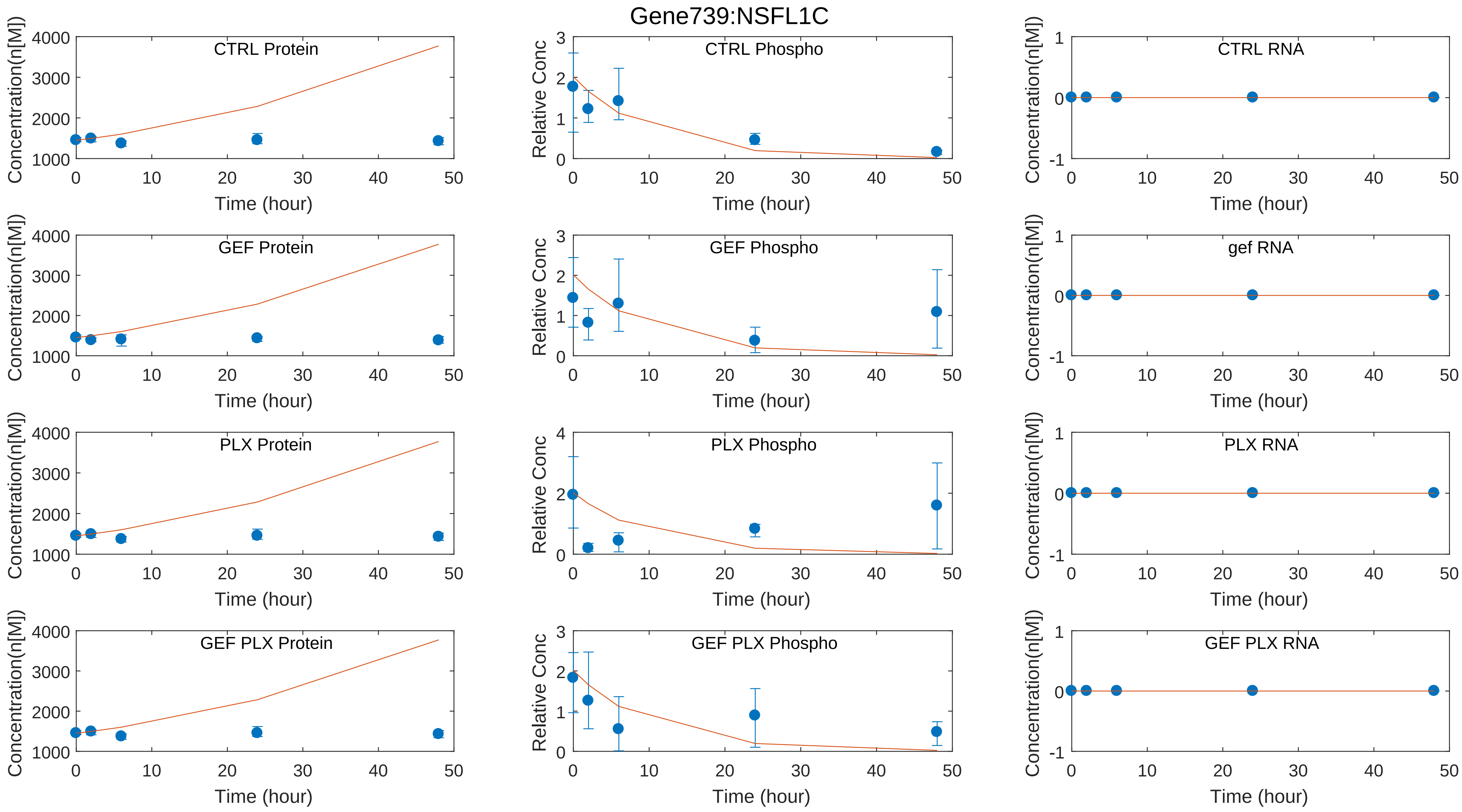

Gene739:NSFL1C
CTRL Protein
GEF Protein
PLX Protein
GEF PLX Protein
CTRL Phospho
GEF Phospho
PLX Phospho
GEF PLX Phospho
CTRL RNA
gef RNA
PLX RNA
GEF PLX RNA
Concentration(n[M])
Relative Conc
Time (hour)

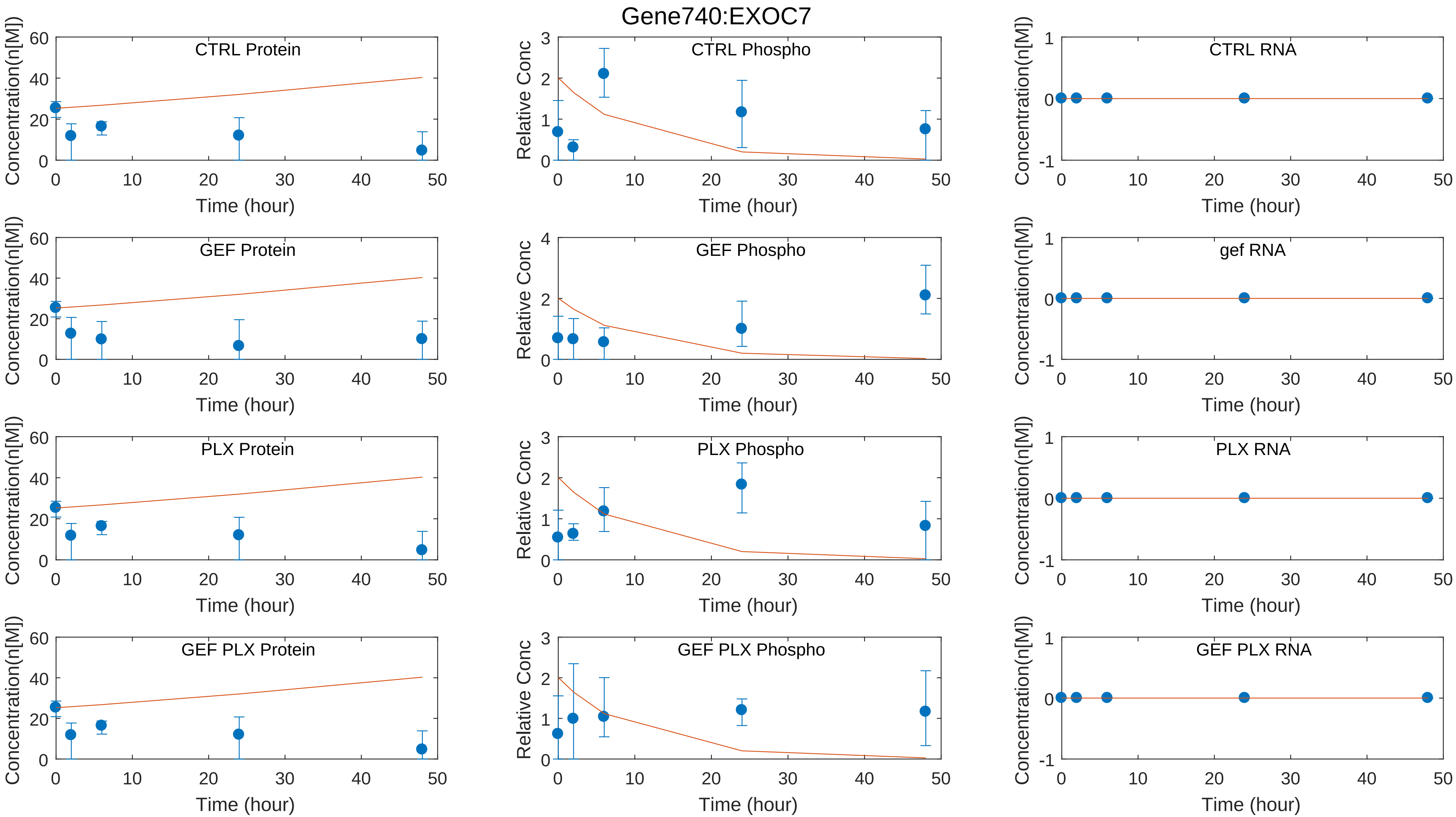

Gene740:EXOC7
CTRL Protein
CTRL Phospho
CTRL RNA
GEF Protein
GEF Phospho
gef RNA
PLX Protein
PLX Phospho
PLX RNA
GEF PLX Protein
GEF PLX Phospho
GEF PLX RNA
Concentration(n[M])
Relative Conc
Time (hour)

Gene741:MAPRE3

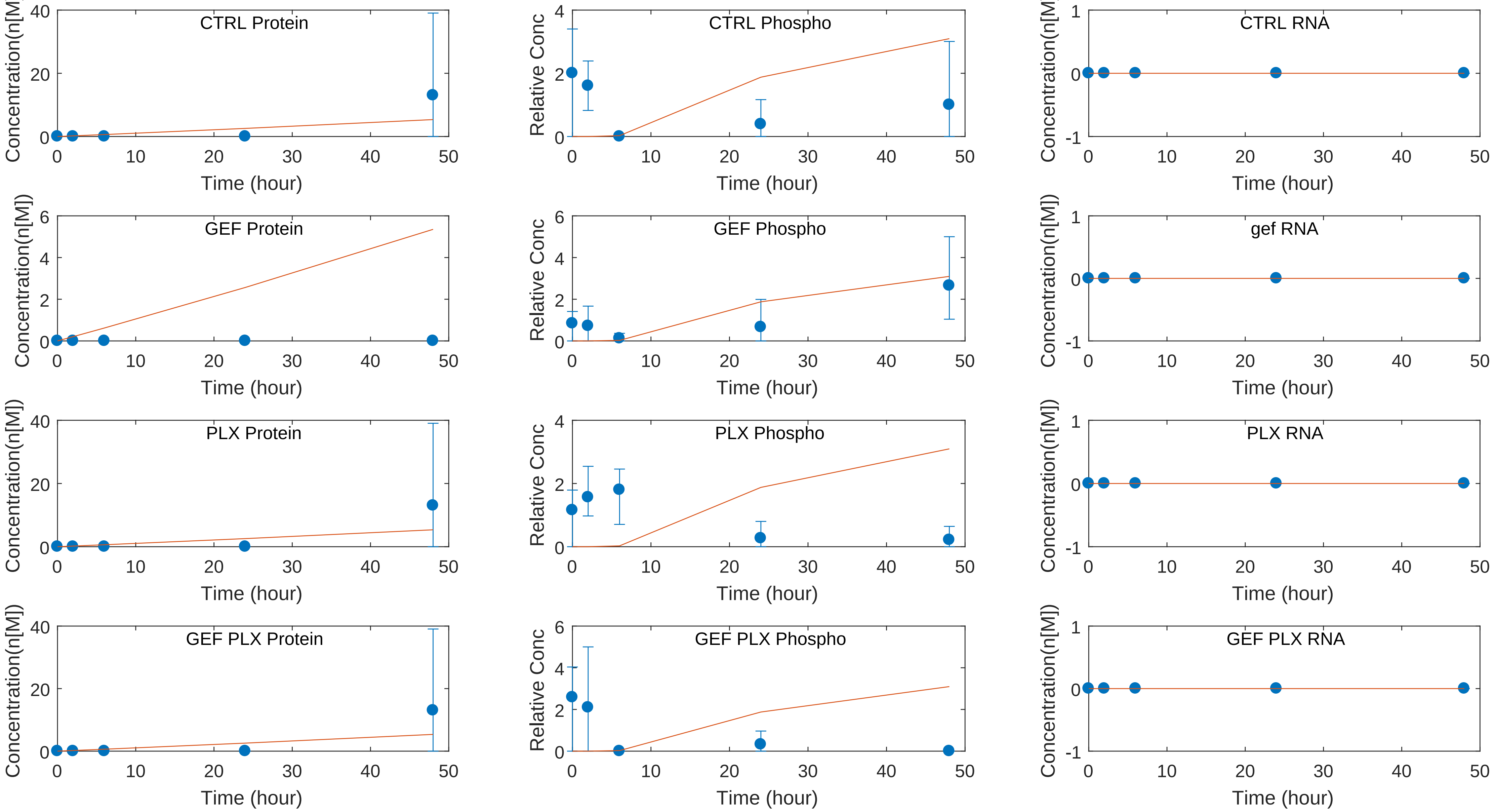

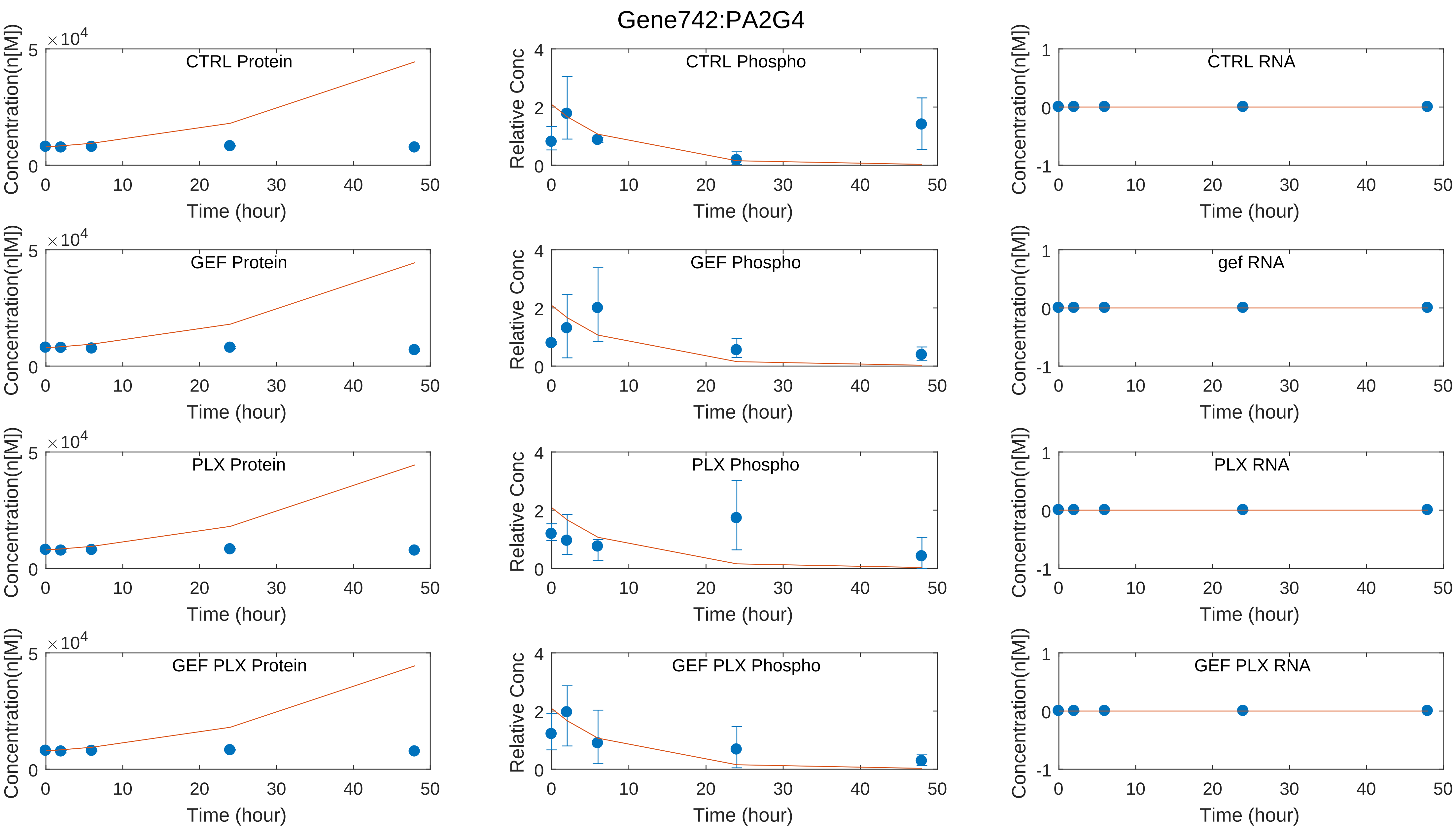
Gene742:PA2G4
CTRL Protein
GEF Protein
PLX Protein
GEF PLX Protein
CTRL Phospho
GEF Phospho
PLX Phospho
GEF PLX Phospho
CTRL RNA
gef RNA
PLX RNA
GEF PLX RNA
Concentration(n[M])
Relative Conc
Time (hour)
×10^4

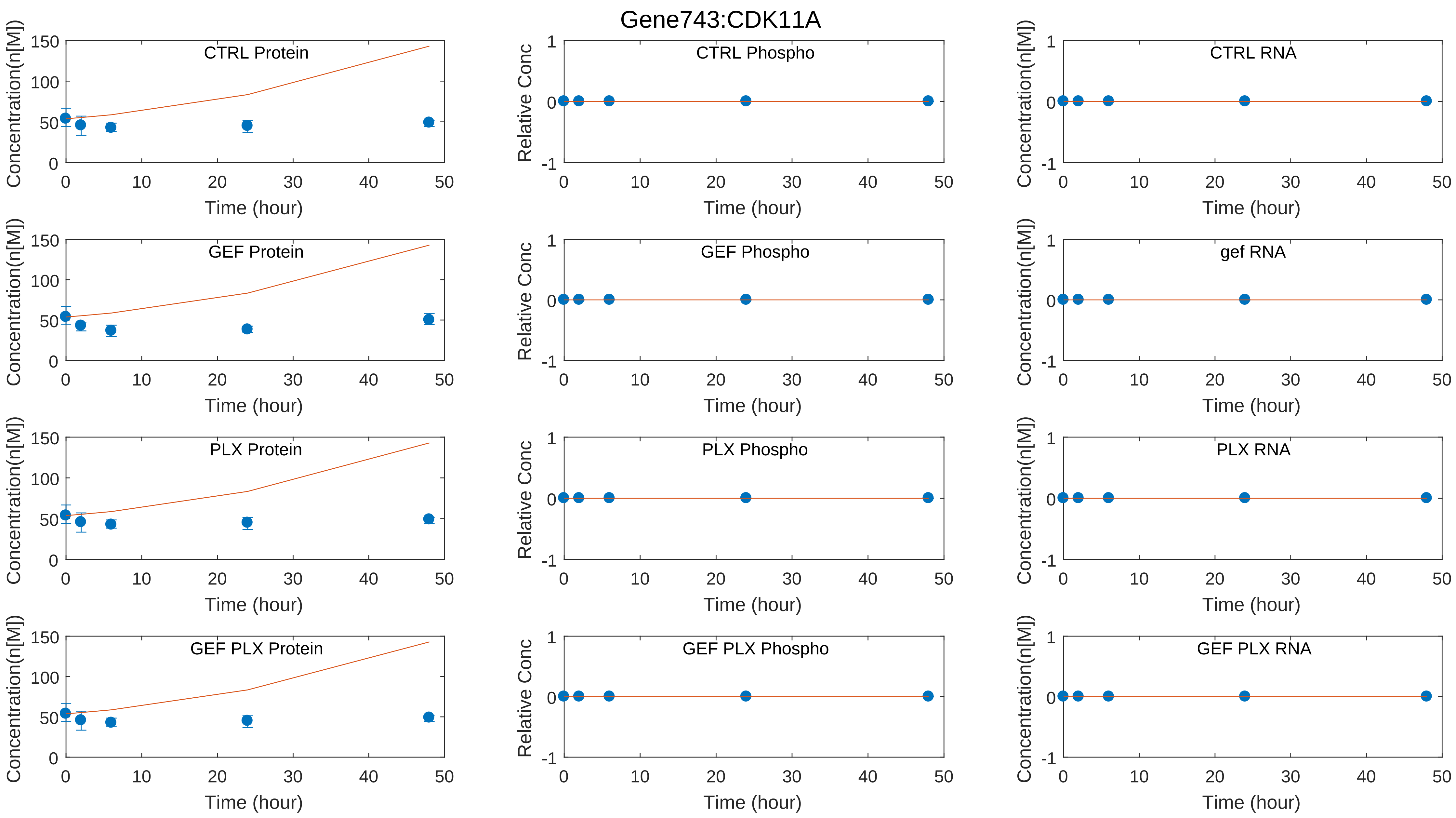

Gene743:CDK11A
CTRL Protein
CTRL Phospho
CTRL RNA
GEF Protein
GEF Phospho
gef RNA
PLX Protein
PLX Phospho
PLX RNA
GEF PLX Protein
GEF PLX Phospho
GEF PLX RNA
Concentration(n[M])
Relative Conc
Time (hour)

Gene744:SMC3

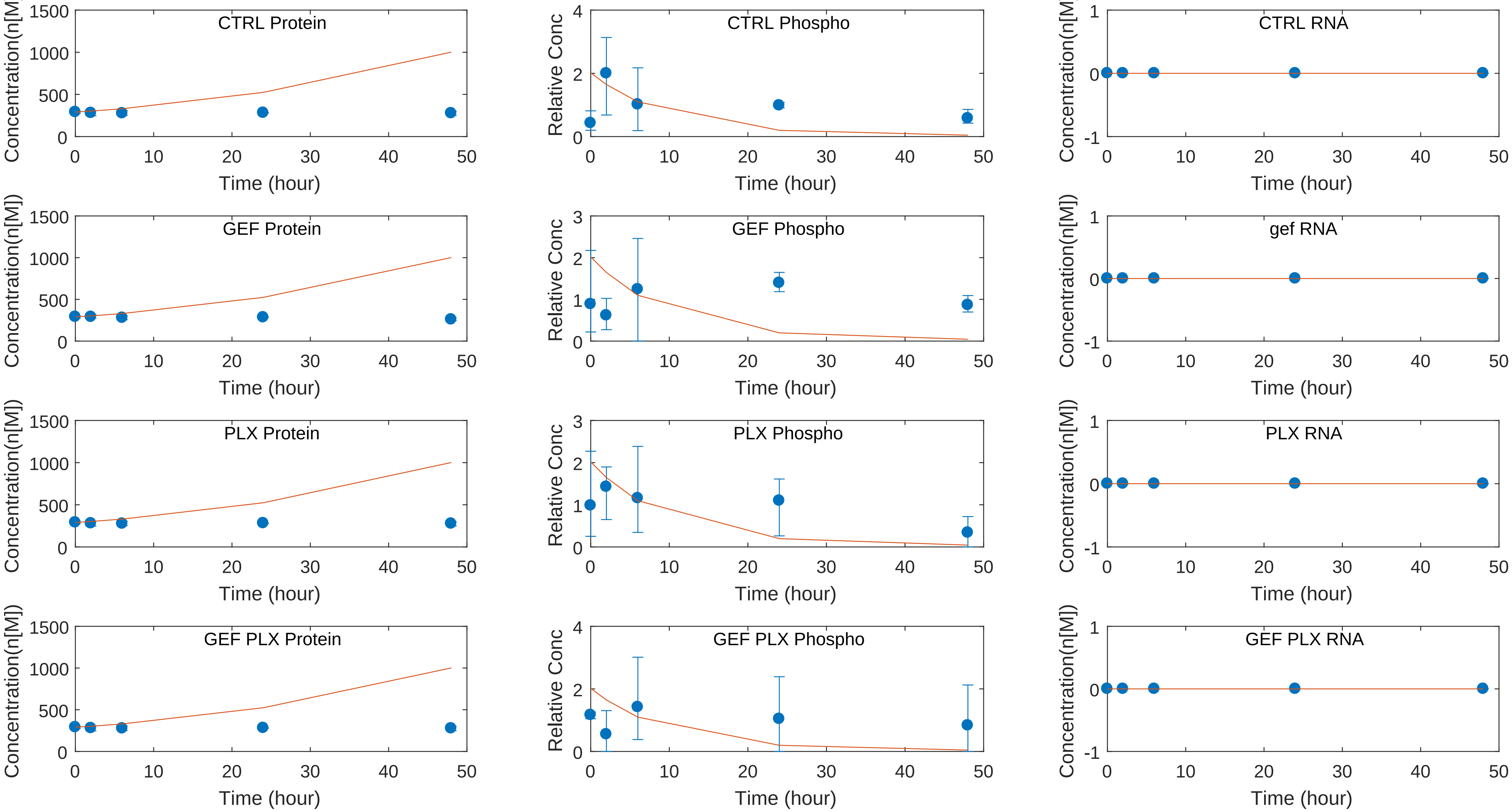

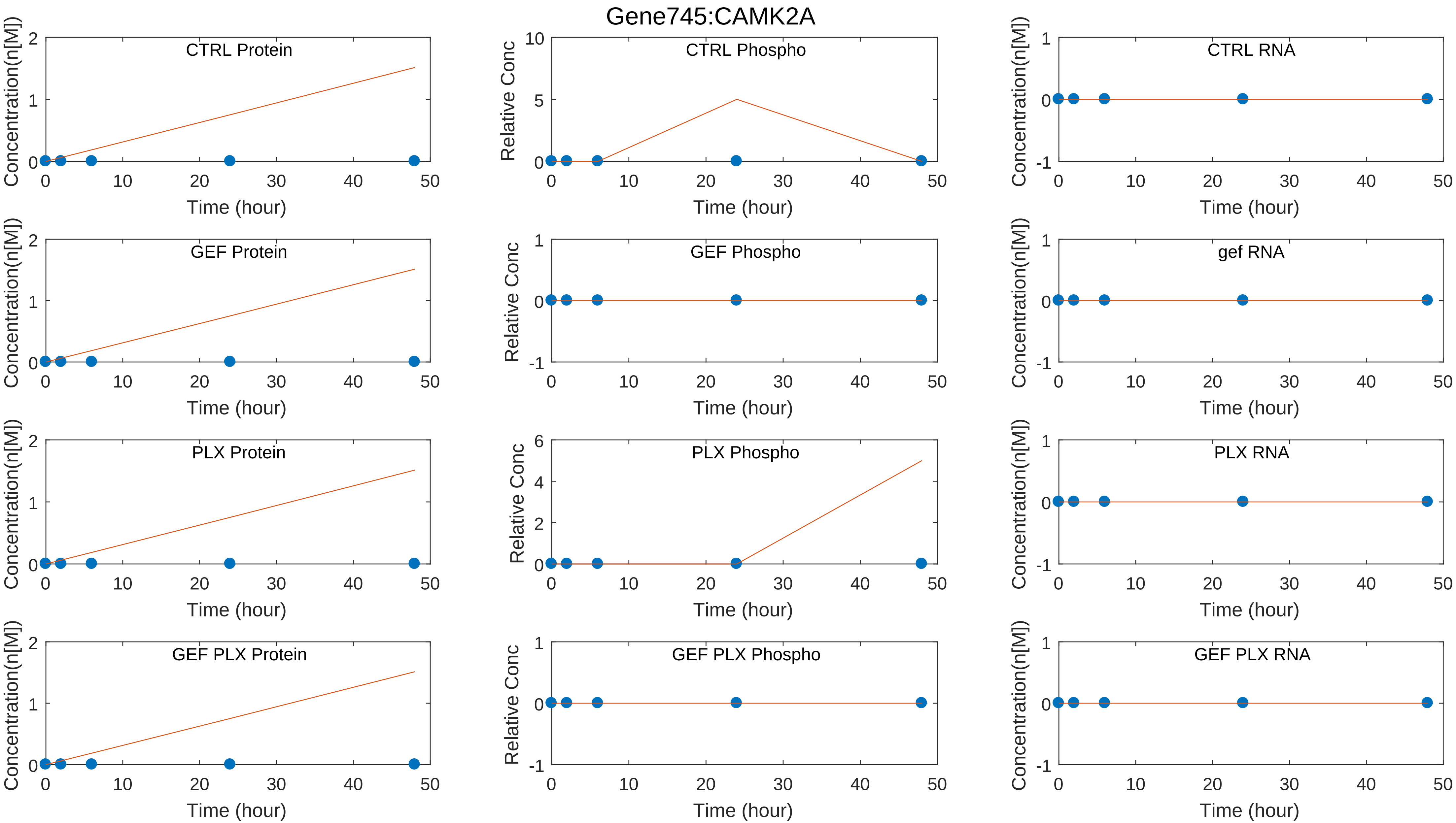

Gene745:CAMK2A
CTRL Protein
CTRL Phospho
CTRL RNA
GEF Protein
GEF Phospho
gef RNA
PLX Protein
PLX Phospho
PLX RNA
GEF PLX Protein
GEF PLX Phospho
GEF PLX RNA
Concentration(n[M])
Relative Conc
Time (hour)

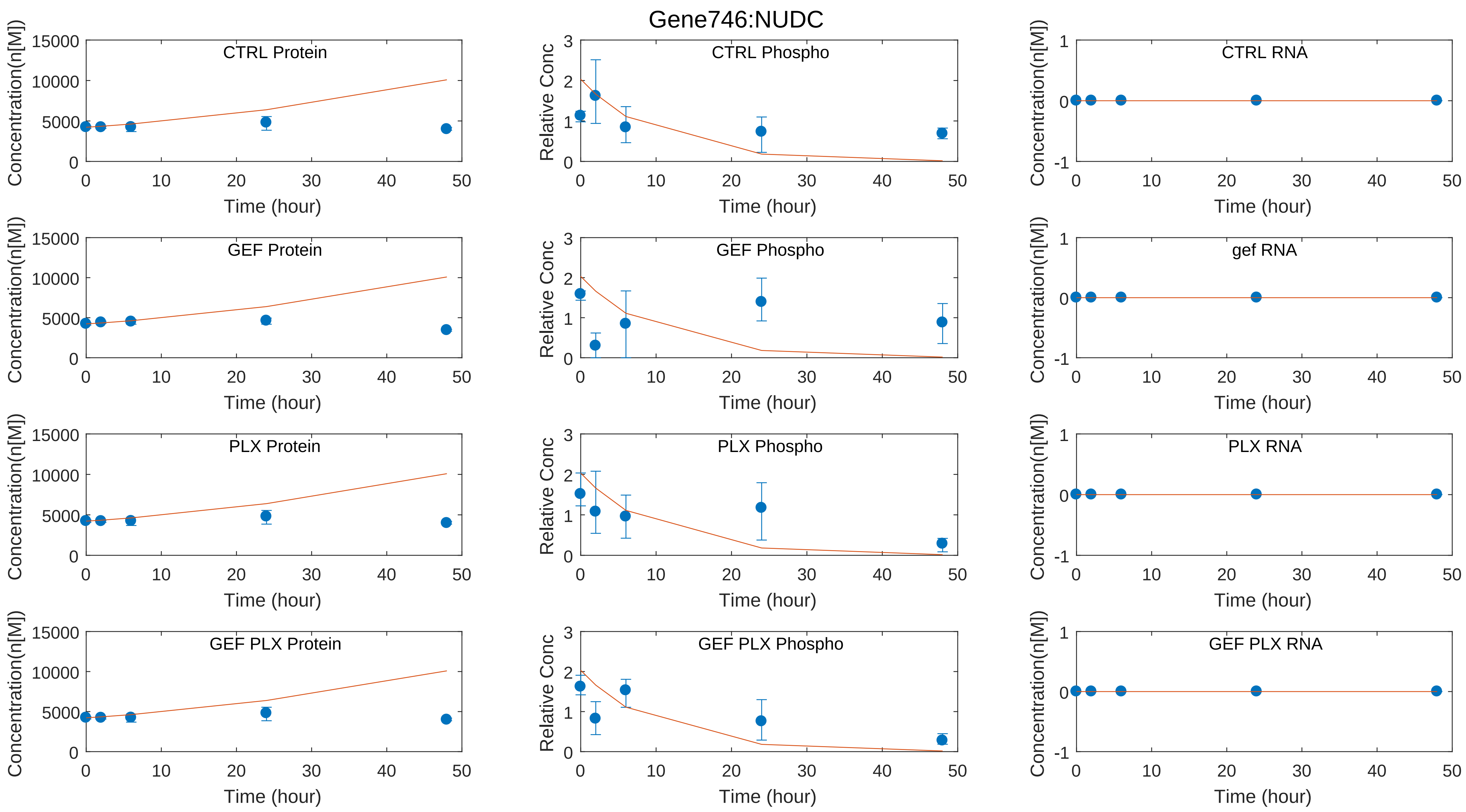

Gene746:NUDC
CTRL Protein
GEF Protein
PLX Protein
GEF PLX Protein
CTRL Phospho
GEF Phospho
PLX Phospho
GEF PLX Phospho
CTRL RNA
gef RNA
PLX RNA
GEF PLX RNA
Concentration(n[M])
Relative Conc
Time (hour)

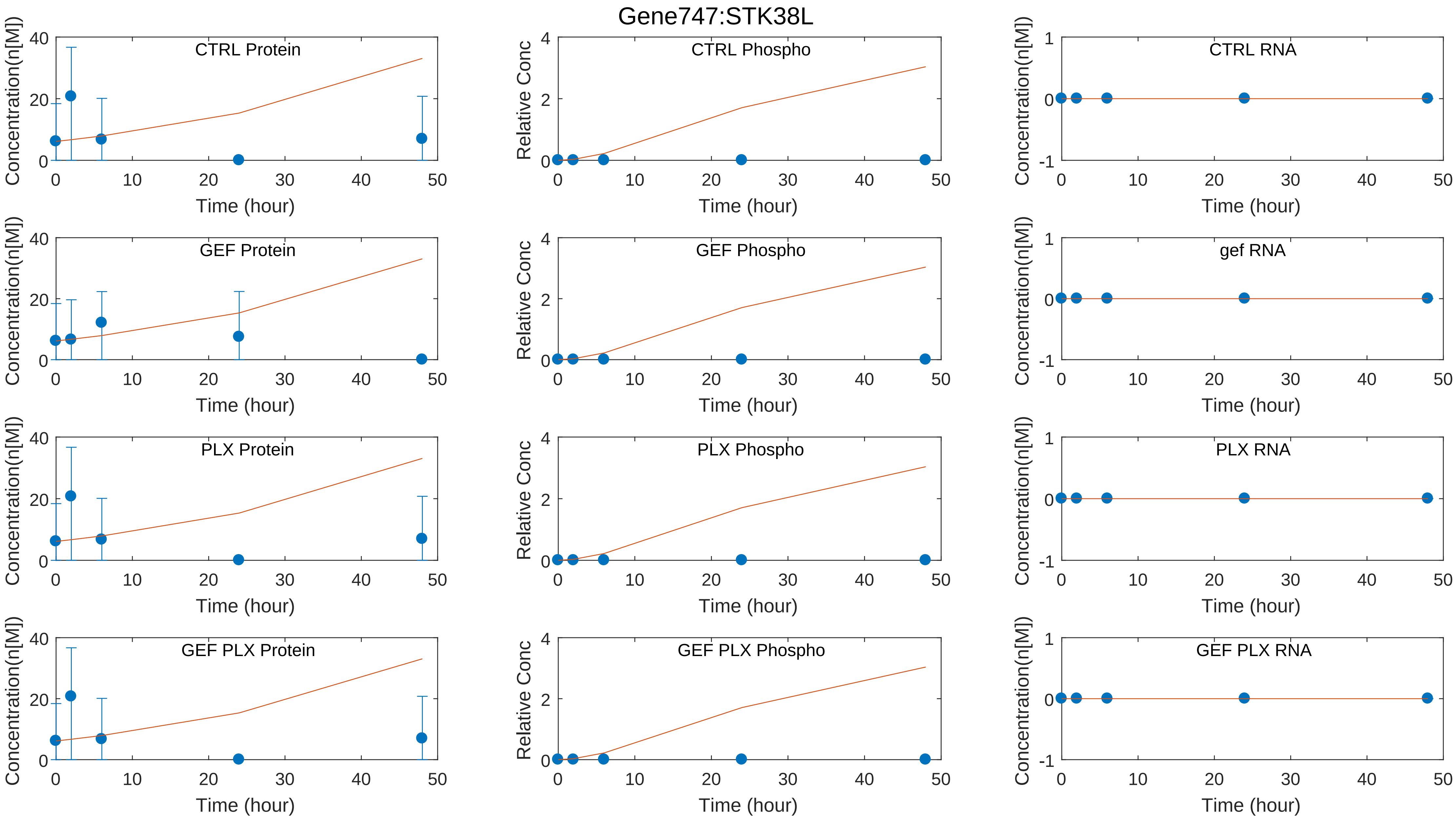

Gene747:STK38L
CTRL Protein
CTRL Phospho
CTRL RNA
GEF Protein
GEF Phospho
gef RNA
PLX Protein
PLX Phospho
PLX RNA
GEF PLX Protein
GEF PLX Phospho
GEF PLX RNA
Concentration(n[M])
Relative Conc
Time (hour)

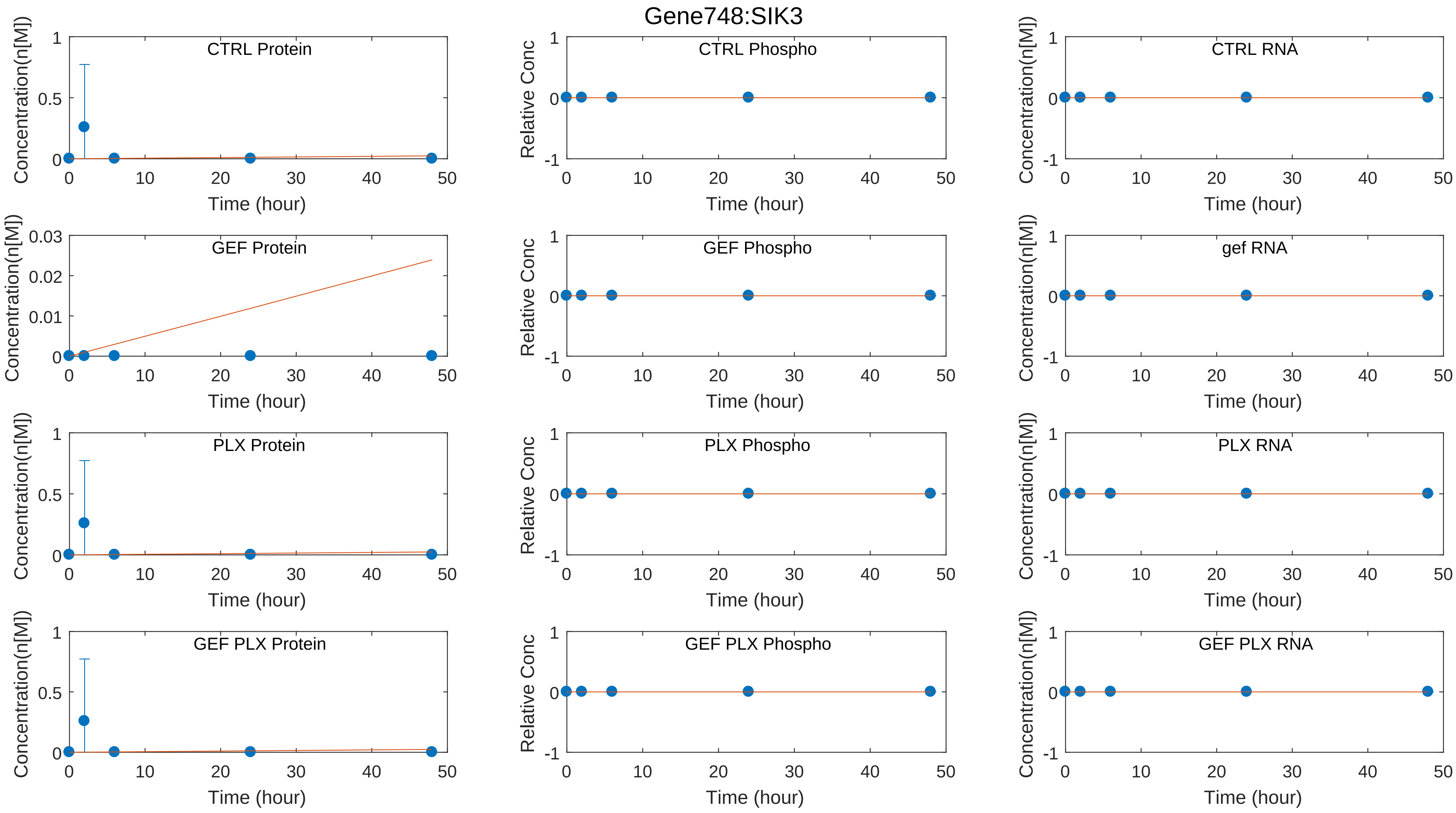
Gene748:SIK3
CTRL Protein
CTRL Phospho
CTRL RNA
GEF Protein
GEF Phospho
gef RNA
PLX Protein
PLX Phospho
PLX RNA
GEF PLX Protein
GEF PLX Phospho
GEF PLX RNA
Concentration(n[M])
Relative Conc
Time (hour)

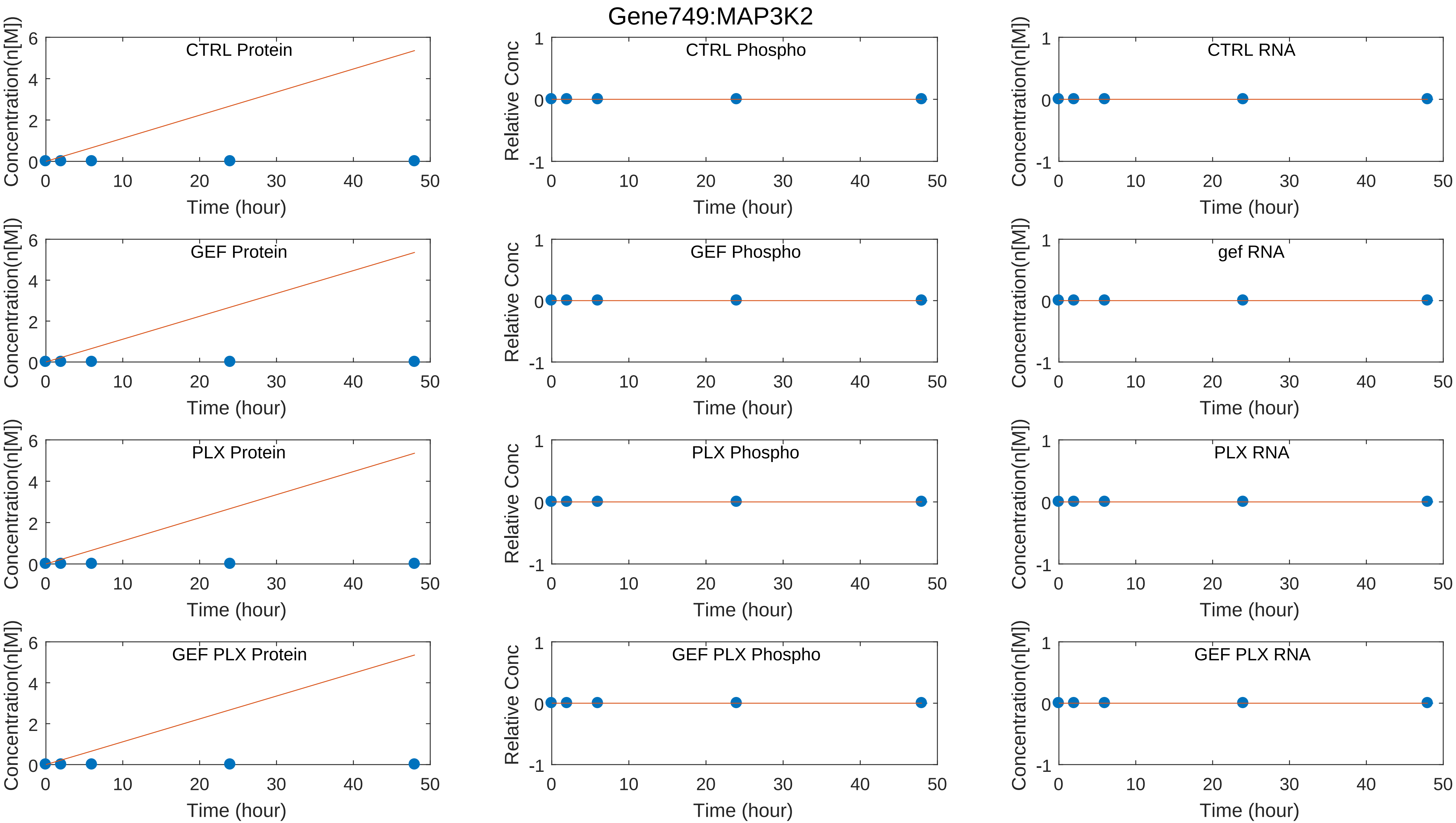

Gene749:MAP3K2
CTRL Protein
CTRL Phospho
CTRL RNA
GEF Protein
GEF Phospho
gef RNA
PLX Protein
PLX Phospho
PLX RNA
GEF PLX Protein
GEF PLX Phospho
GEF PLX RNA
Concentration(n[M])
Relative Conc
Time (hour)

Gene750:CARHSP1

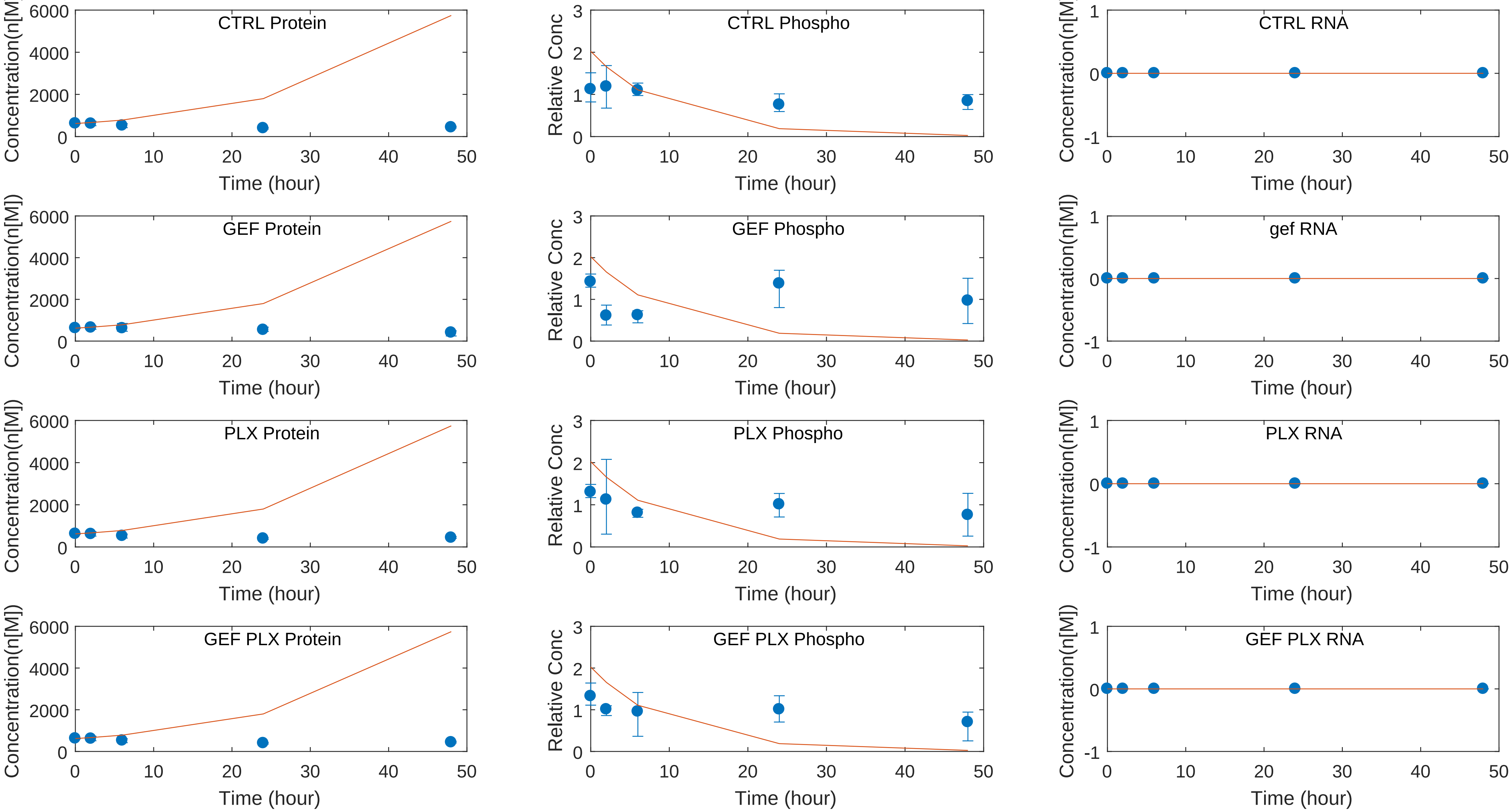

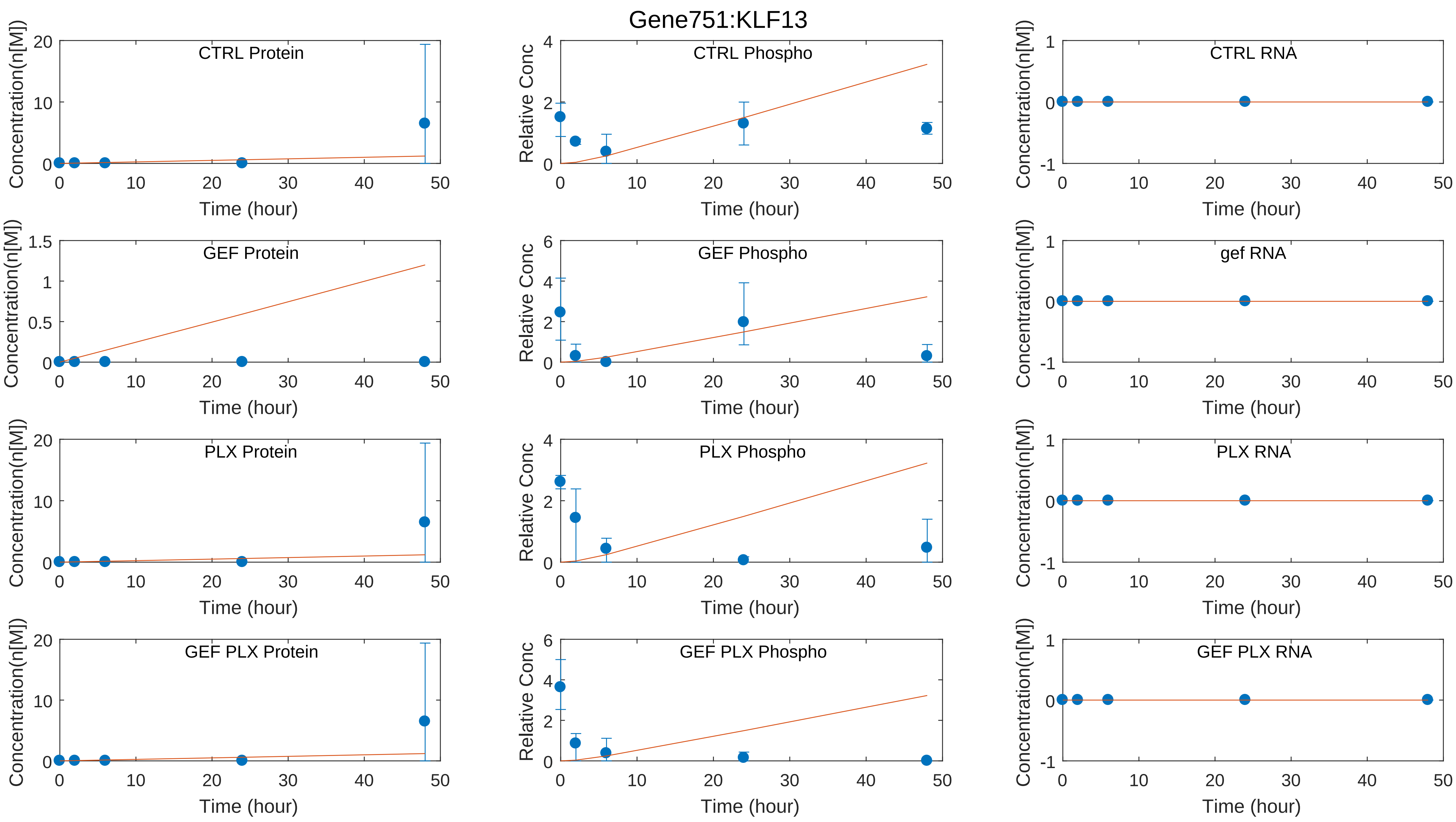
Gene751:KLF13
CTRL Protein
CTRL Phospho
CTRL RNA
GEF Protein
GEF Phospho
gef RNA
PLX Protein
PLX Phospho
PLX RNA
GEF PLX Protein
GEF PLX Phospho
GEF PLX RNA
Concentration(n[M])
Relative Conc
Time (hour)

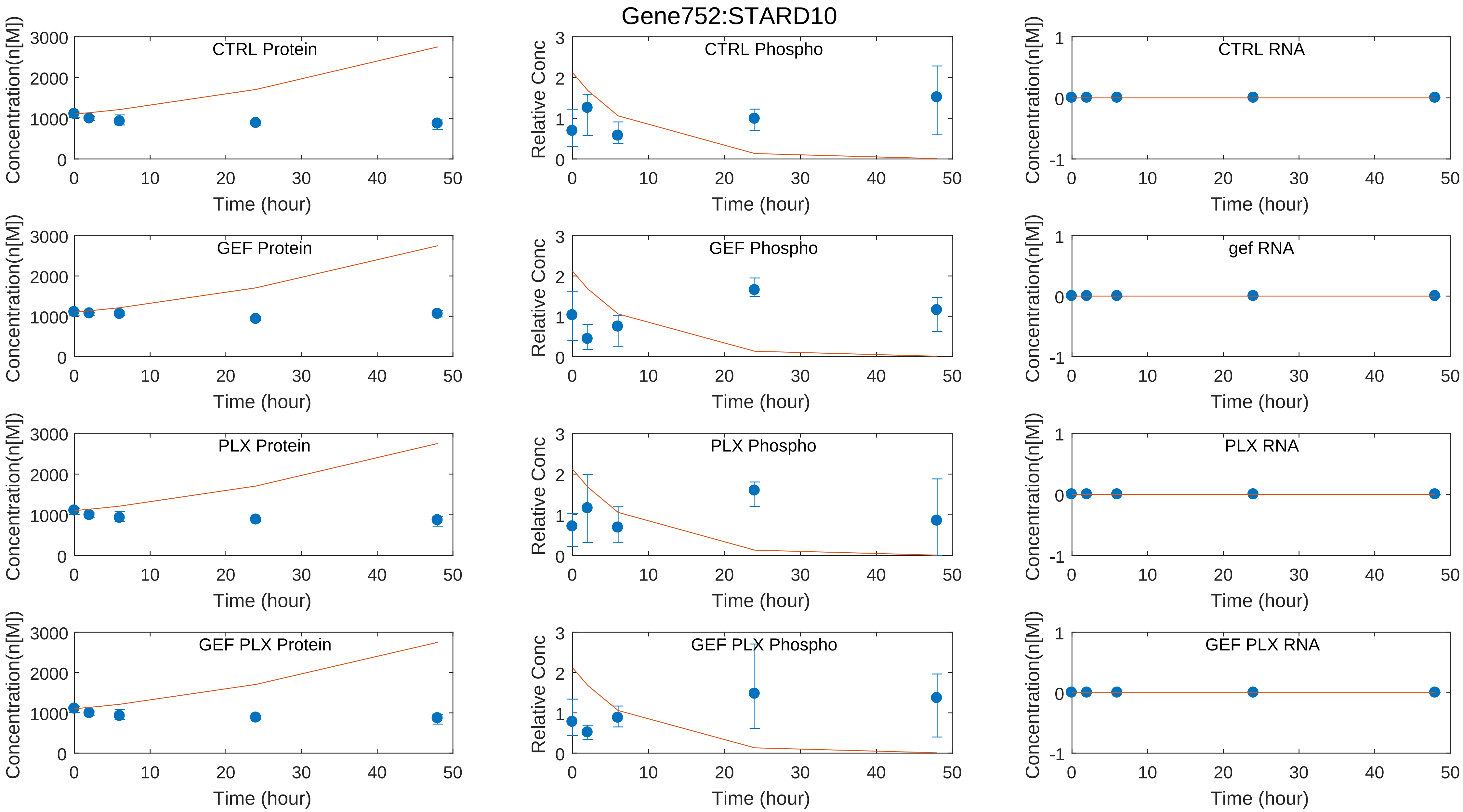

Gene752:STARD10
CTRL Protein
CTRL Phospho
CTRL RNA
GEF Protein
GEF Phospho
gef RNA
PLX Protein
PLX Phospho
PLX RNA
GEF PLX Protein
GEF PLX Phospho
GEF PLX RNA
Concentration(n[M])
Relative Conc
Time (hour)

Gene753:SAMHD1

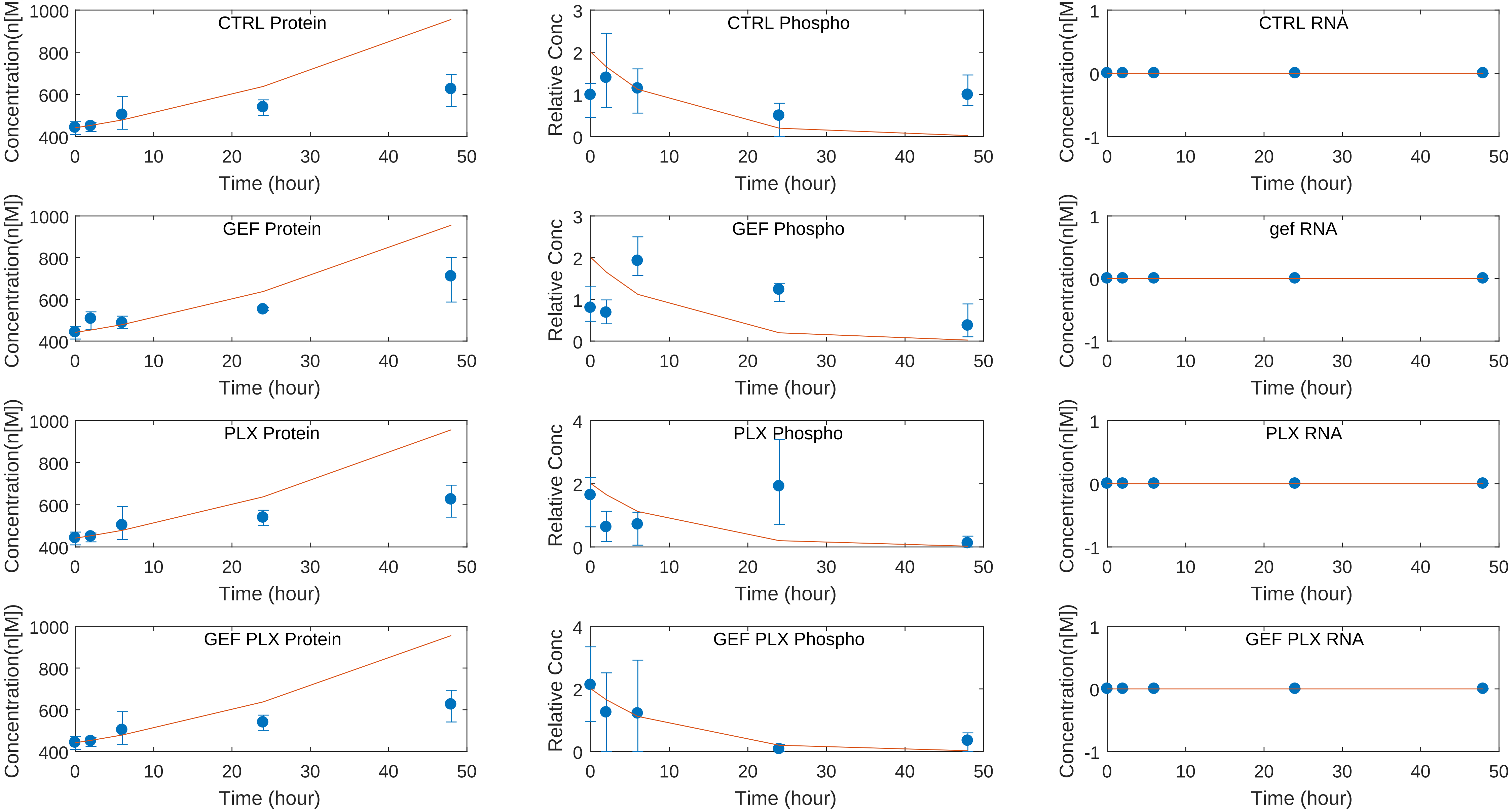

Gene754:PRKAB1

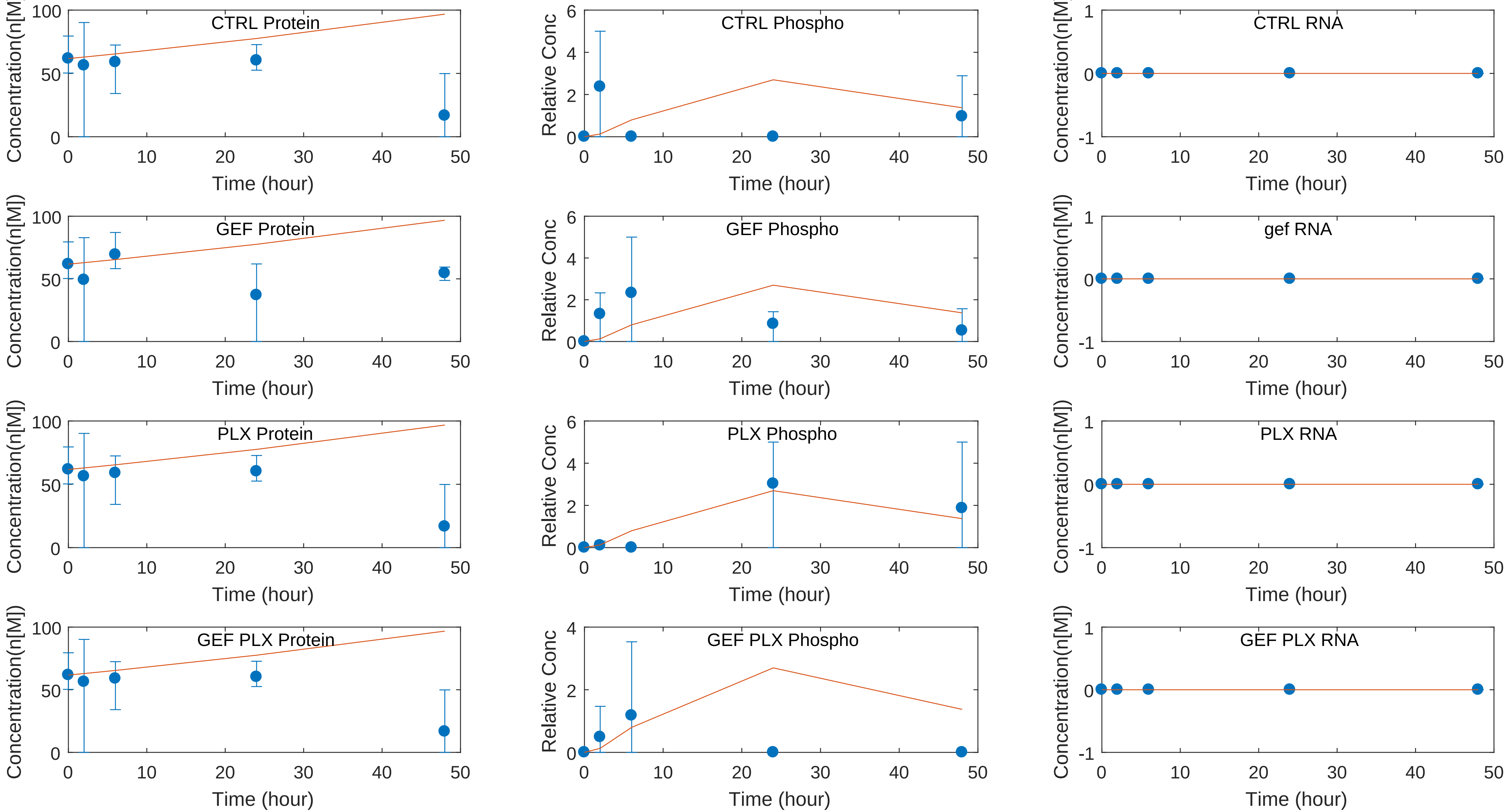

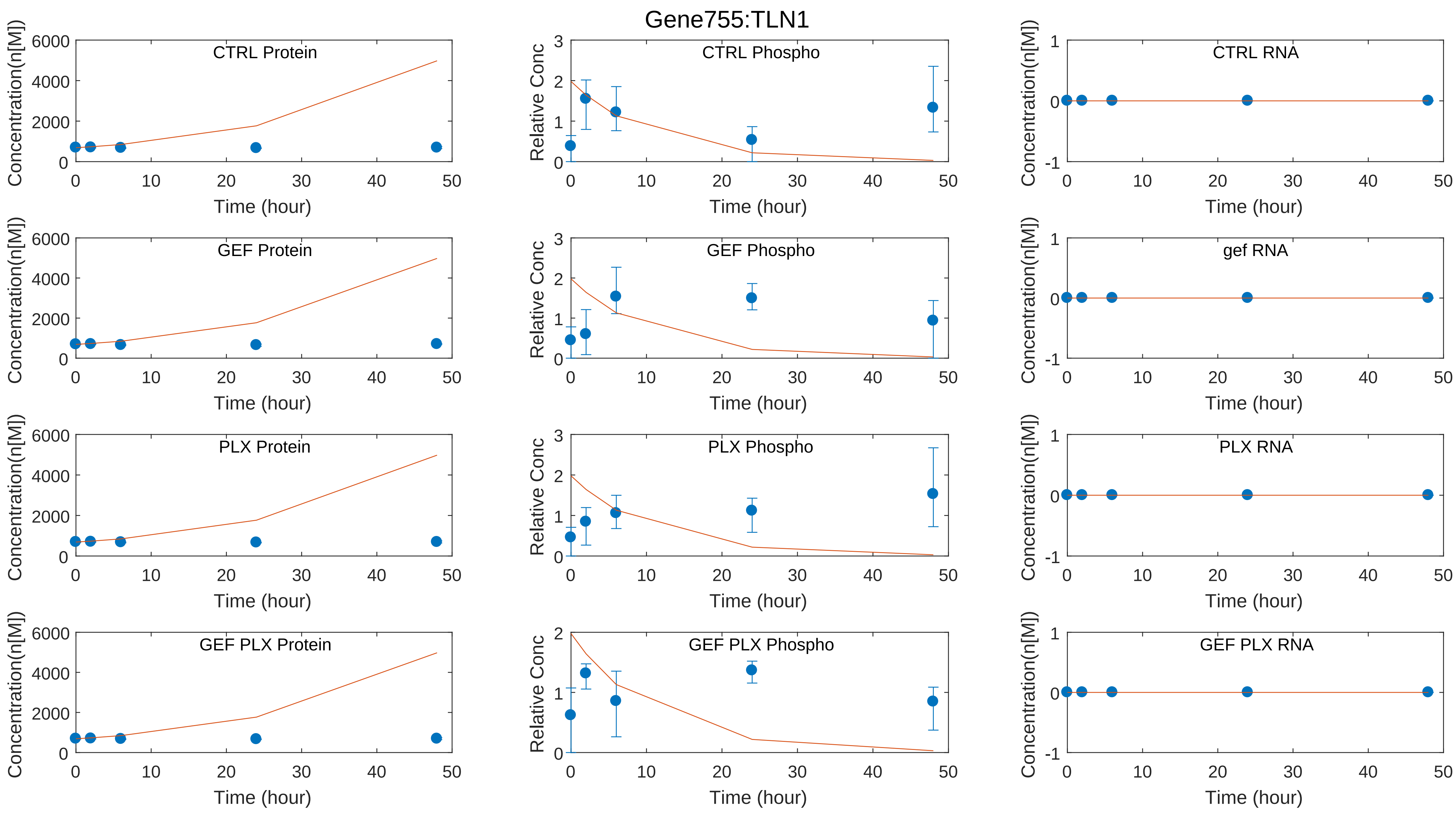
Gene755:TLN1
CTRL Protein
CTRL Phospho
CTRL RNA
GEF Protein
GEF Phospho
gef RNA
PLX Protein
PLX Phospho
PLX RNA
GEF PLX Protein
GEF PLX Phospho
GEF PLX RNA
Concentration(n[M])
Relative Conc
Time (hour)

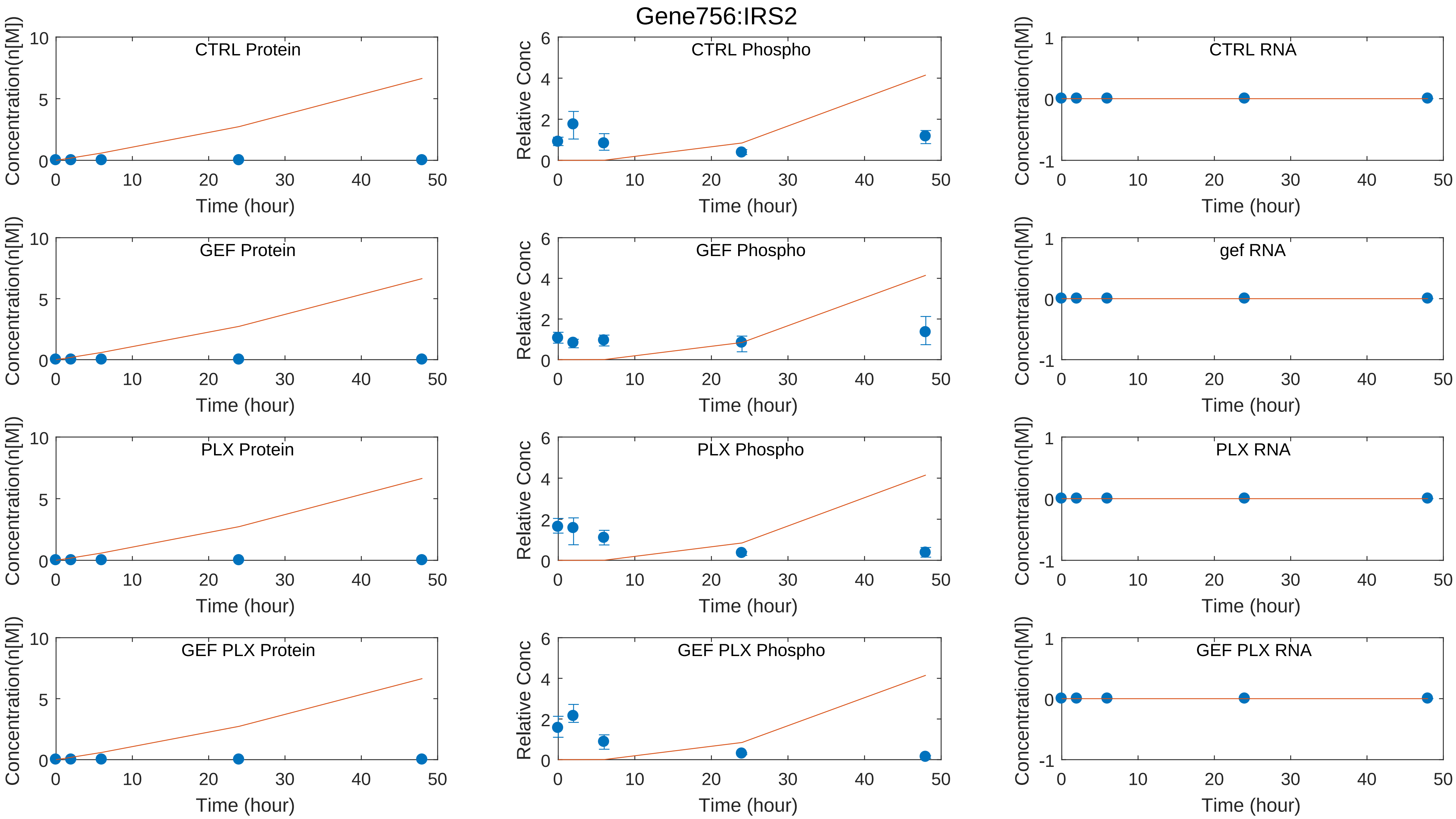

Gene756:IRS2
CTRL Protein
CTRL Phospho
CTRL RNA
GEF Protein
GEF Phospho
gef RNA
PLX Protein
PLX Phospho
PLX RNA
GEF PLX Protein
GEF PLX Phospho
GEF PLX RNA
Concentration(n[M])
Relative Conc
Time (hour)

Gene757:TRAF6

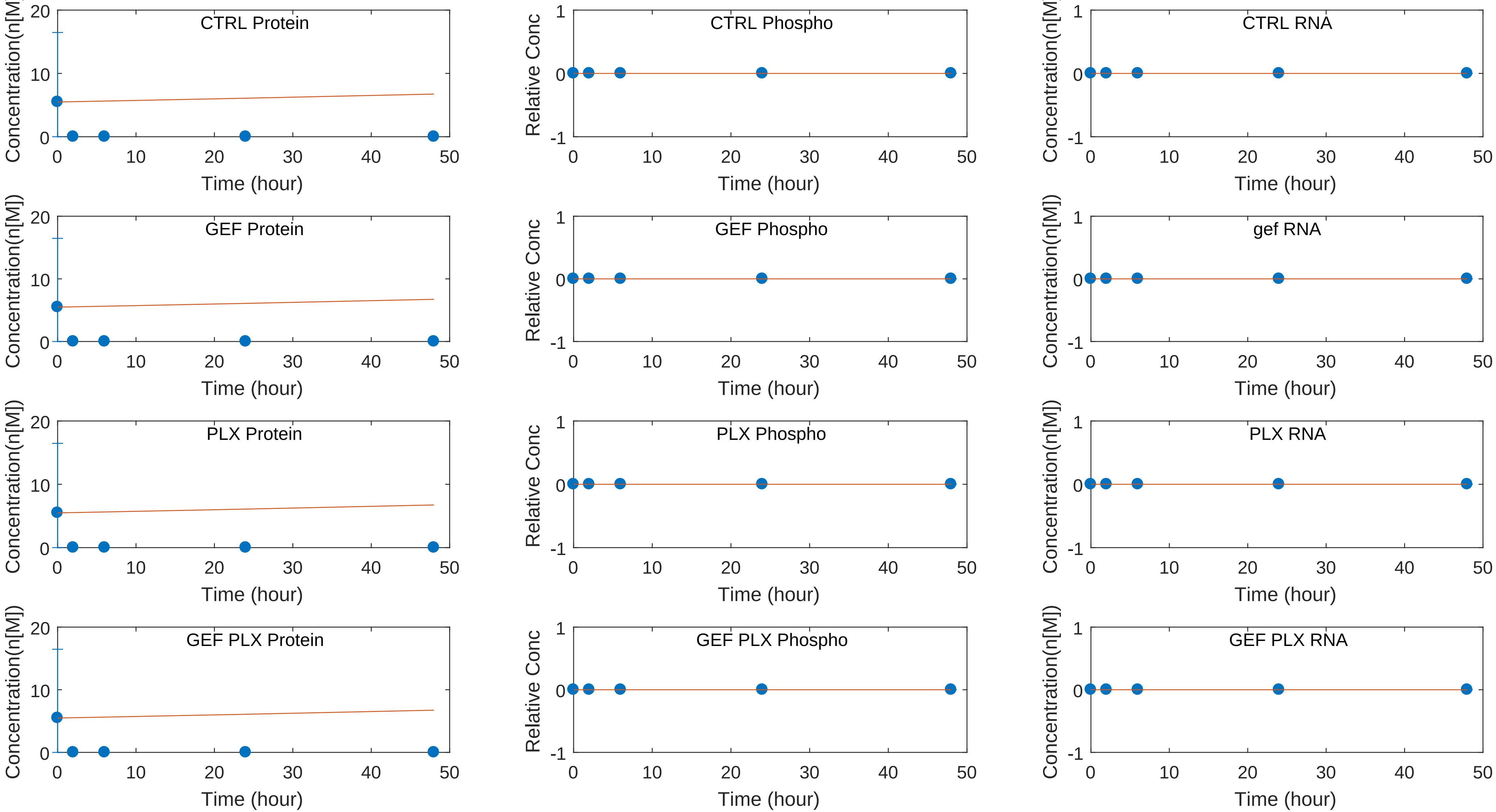

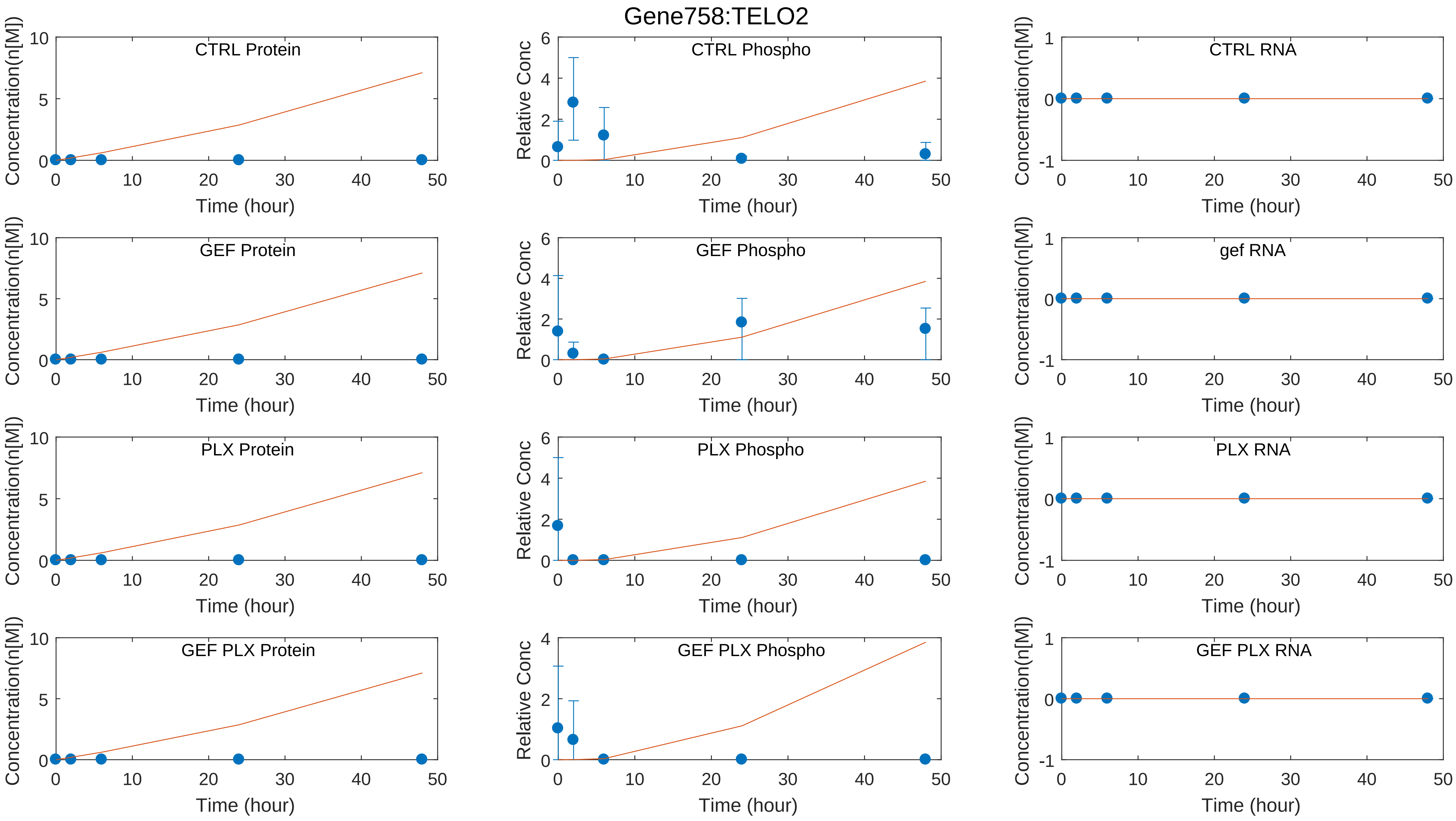

Gene758:TELO2
CTRL Protein
GEF Protein
PLX Protein
GEF PLX Protein
CTRL Phospho
GEF Phospho
PLX Phospho
GEF PLX Phospho
CTRL RNA
gef RNA
PLX RNA
GEF PLX RNA
Concentration(n[M])
Relative Conc
Time (hour)

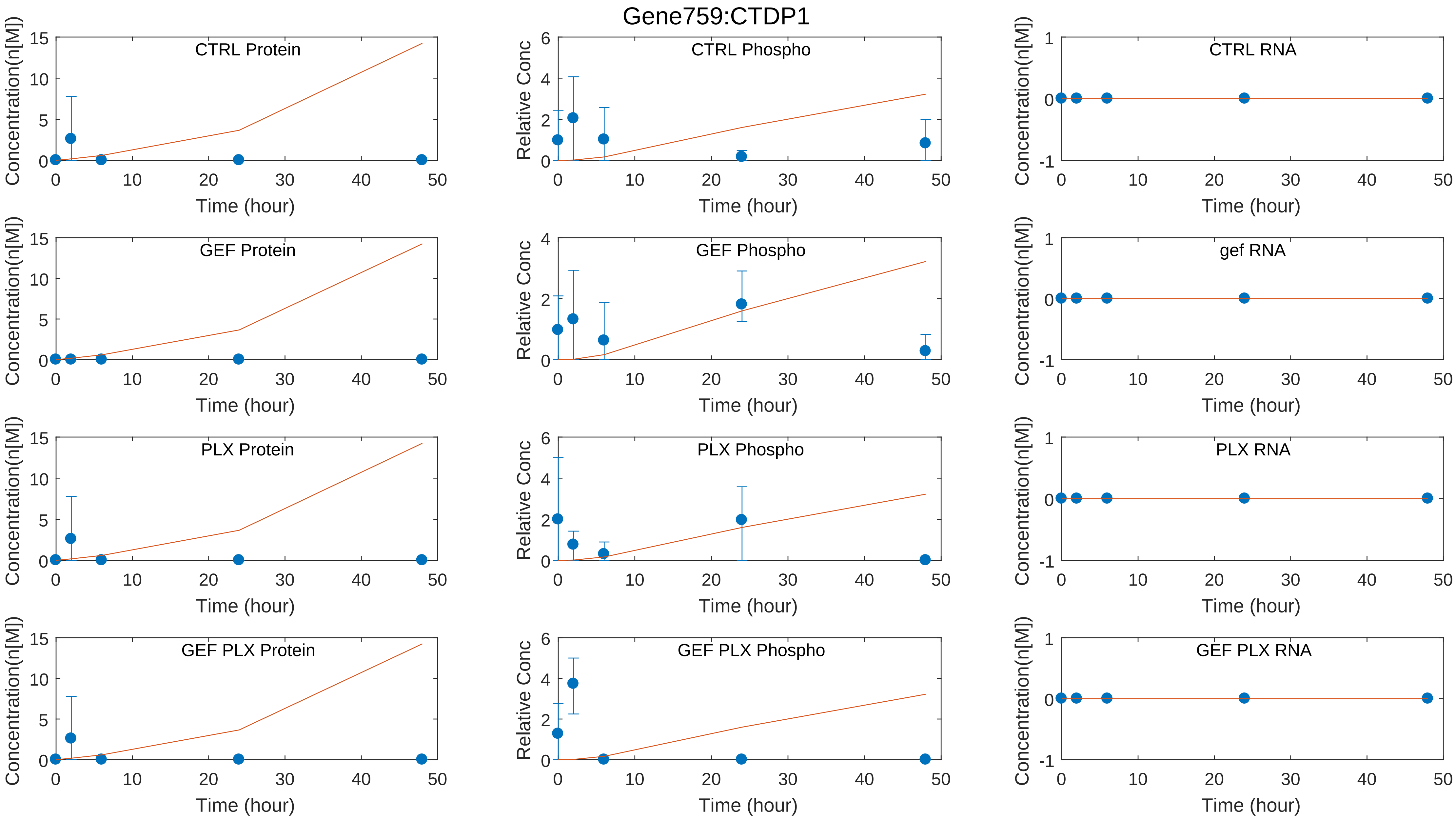

Gene759:CTDP1
CTRL Protein
CTRL Phospho
CTRL RNA
GEF Protein
GEF Phospho
gef RNA
PLX Protein
PLX Phospho
PLX RNA
GEF PLX Protein
GEF PLX Phospho
GEF PLX RNA
Concentration(n[M])
Relative Conc
Time (hour)

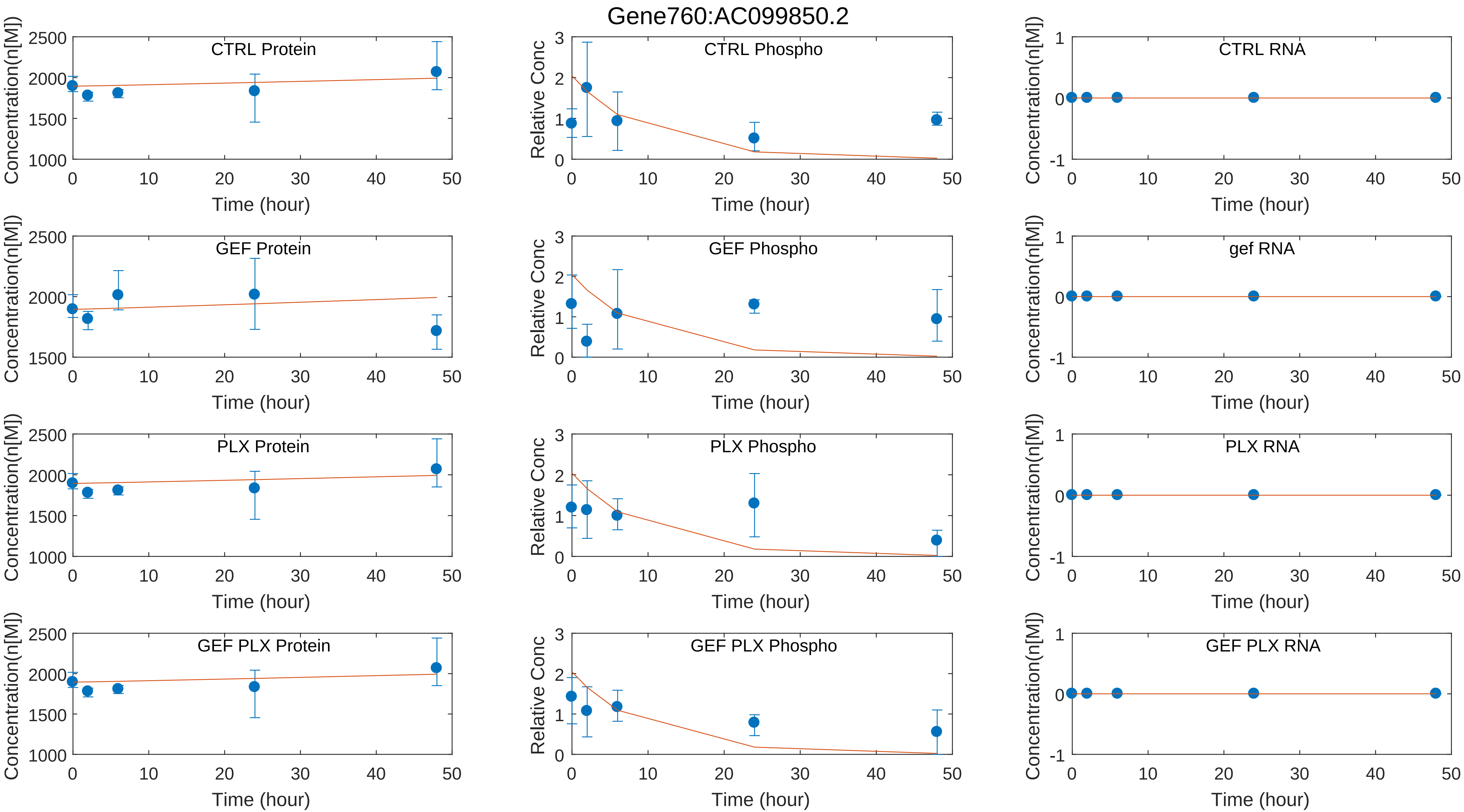
Gene760:AC099850.2
CTRL Protein
CTRL Phospho
CTRL RNA
GEF Protein
GEF Phospho
gef RNA
PLX Protein
PLX Phospho
PLX RNA
GEF PLX Protein
GEF PLX Phospho
GEF PLX RNA
Concentration(n[M])
Relative Conc
Time (hour)

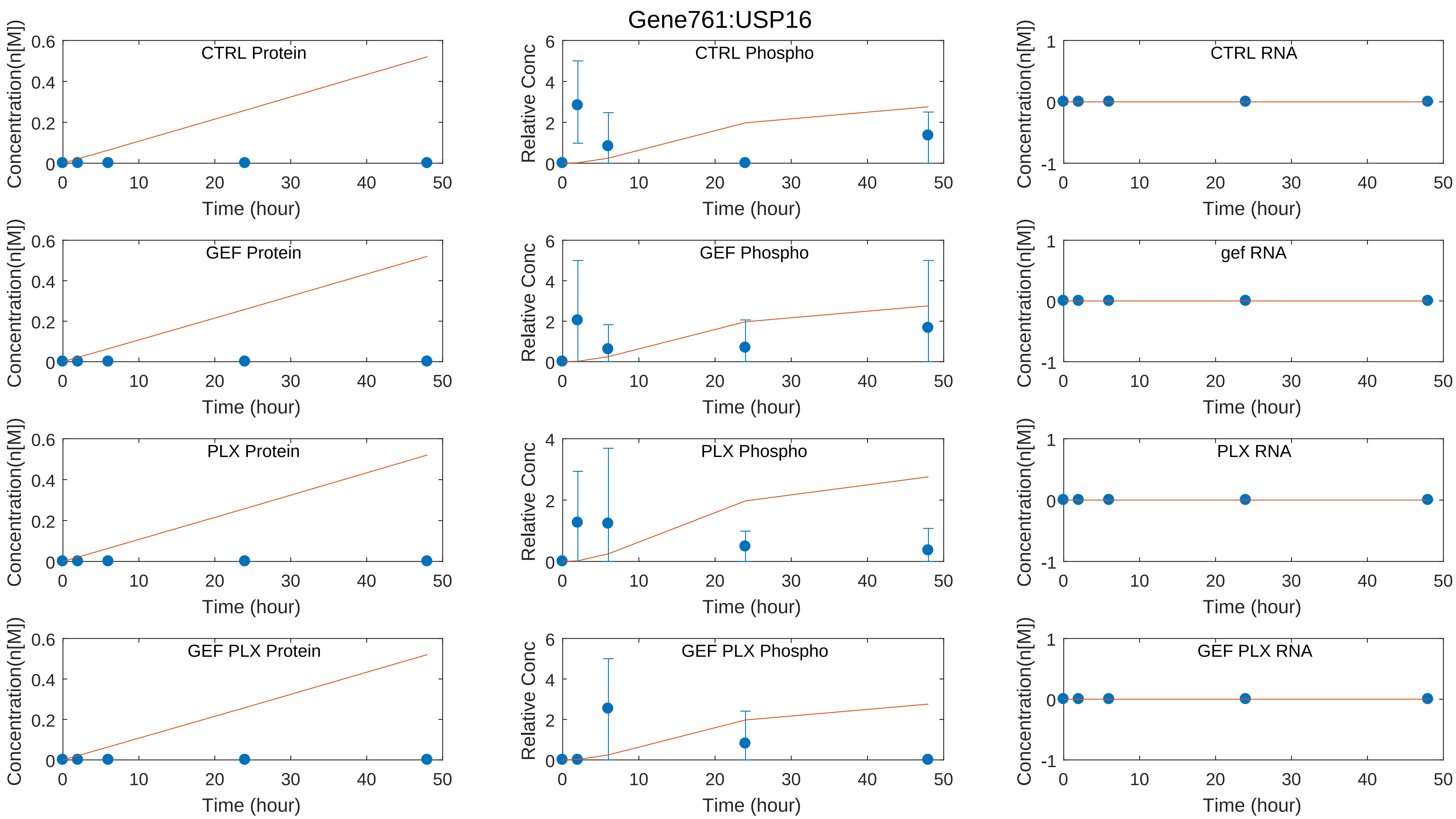

Gene761:USP16
CTRL Protein
CTRL Phospho
CTRL RNA
GEF Protein
GEF Phospho
gef RNA
PLX Protein
PLX Phospho
PLX RNA
GEF PLX Protein
GEF PLX Phospho
GEF PLX RNA
Concentration(n[M])
Relative Conc
Time (hour)

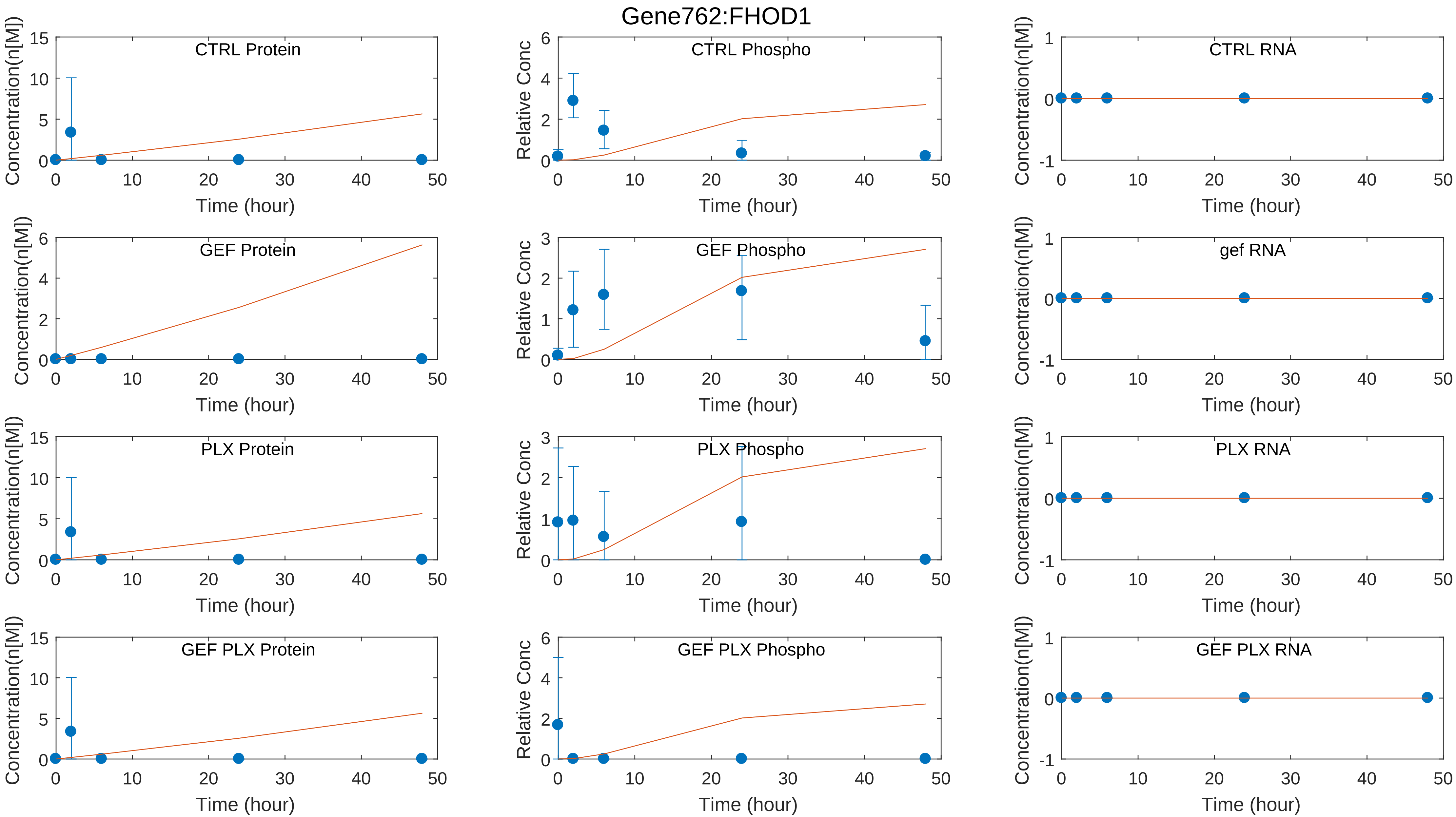

Gene762:FHOD1
CTRL Protein
CTRL Phospho
CTRL RNA
GEF Protein
GEF Phospho
gef RNA
PLX Protein
PLX Phospho
PLX RNA
GEF PLX Protein
GEF PLX Phospho
GEF PLX RNA
Concentration(n[M])
Relative Conc
Time (hour)

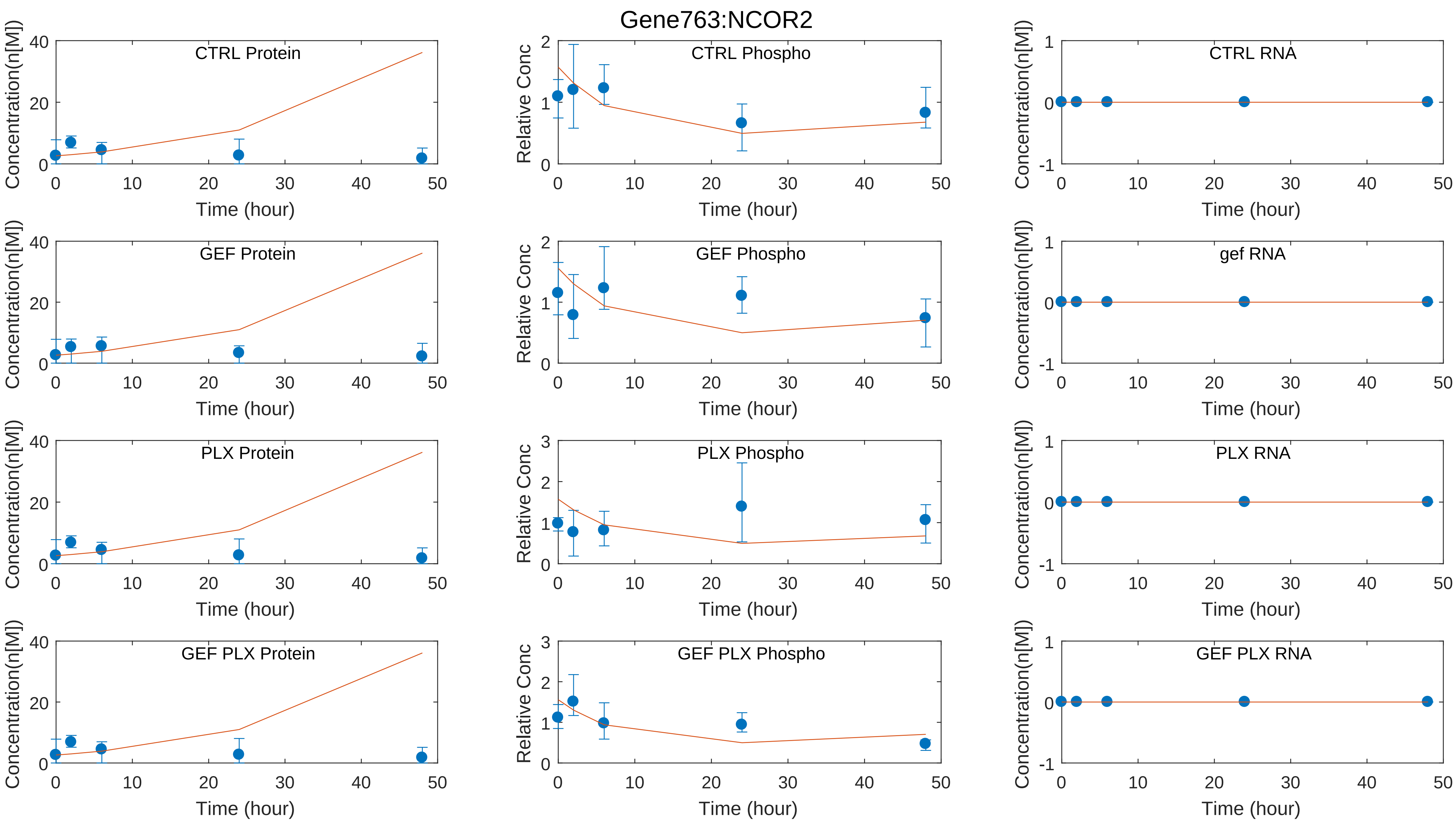
Gene763:NCOR2
CTRL Protein
CTRL Phospho
CTRL RNA
GEF Protein
GEF Phospho
gef RNA
PLX Protein
PLX Phospho
PLX RNA
GEF PLX Protein
GEF PLX Phospho
GEF PLX RNA
Concentration(n[M])
Relative Conc
Time (hour)
0
10
20
30
40
50
1
2
3
-1

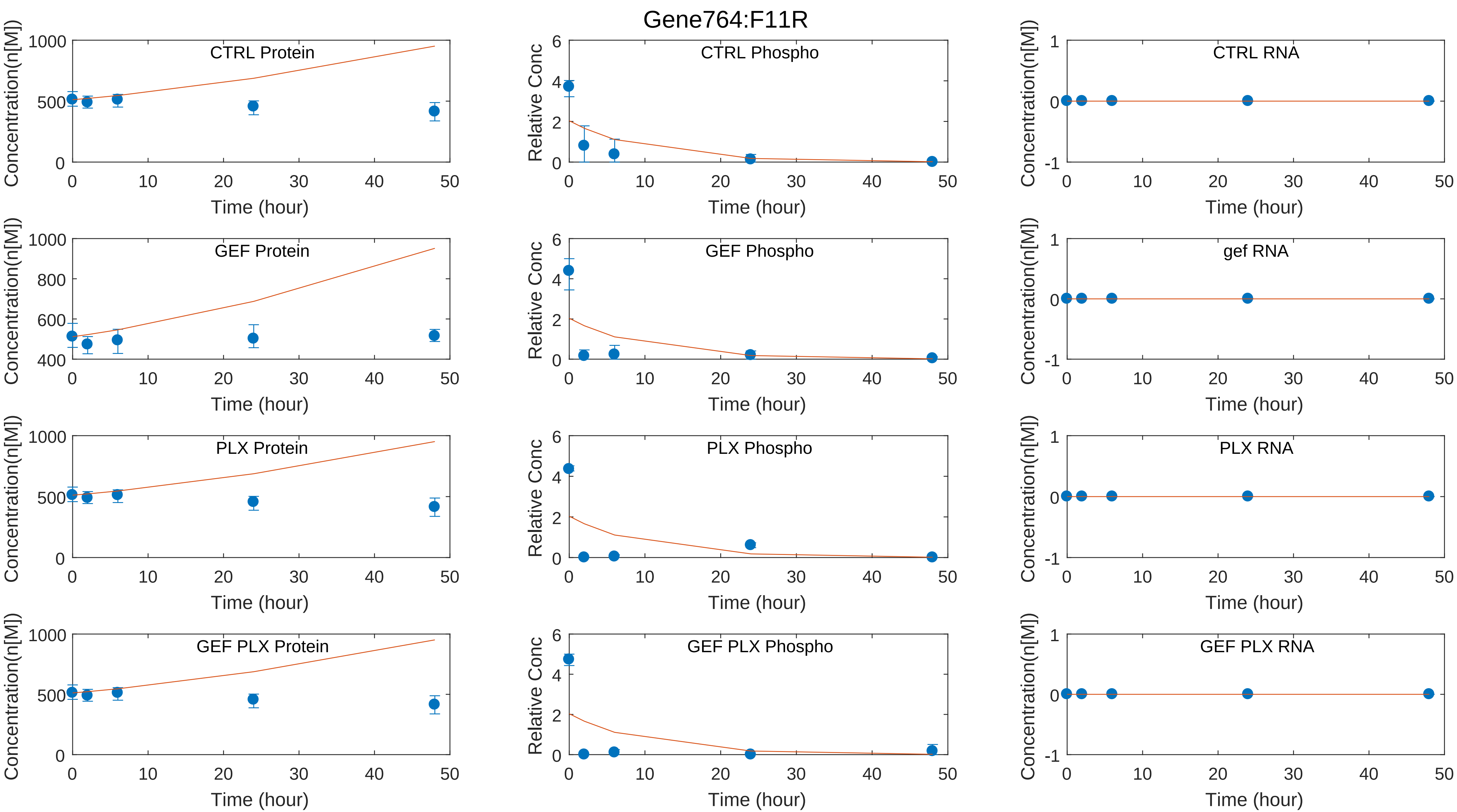

Gene764:F11R
CTRL Protein
CTRL Phospho
CTRL RNA
GEF Protein
GEF Phospho
gef RNA
PLX Protein
PLX Phospho
PLX RNA
GEF PLX Protein
GEF PLX Phospho
GEF PLX RNA
Concentration(n[M])
Relative Conc
Time (hour)

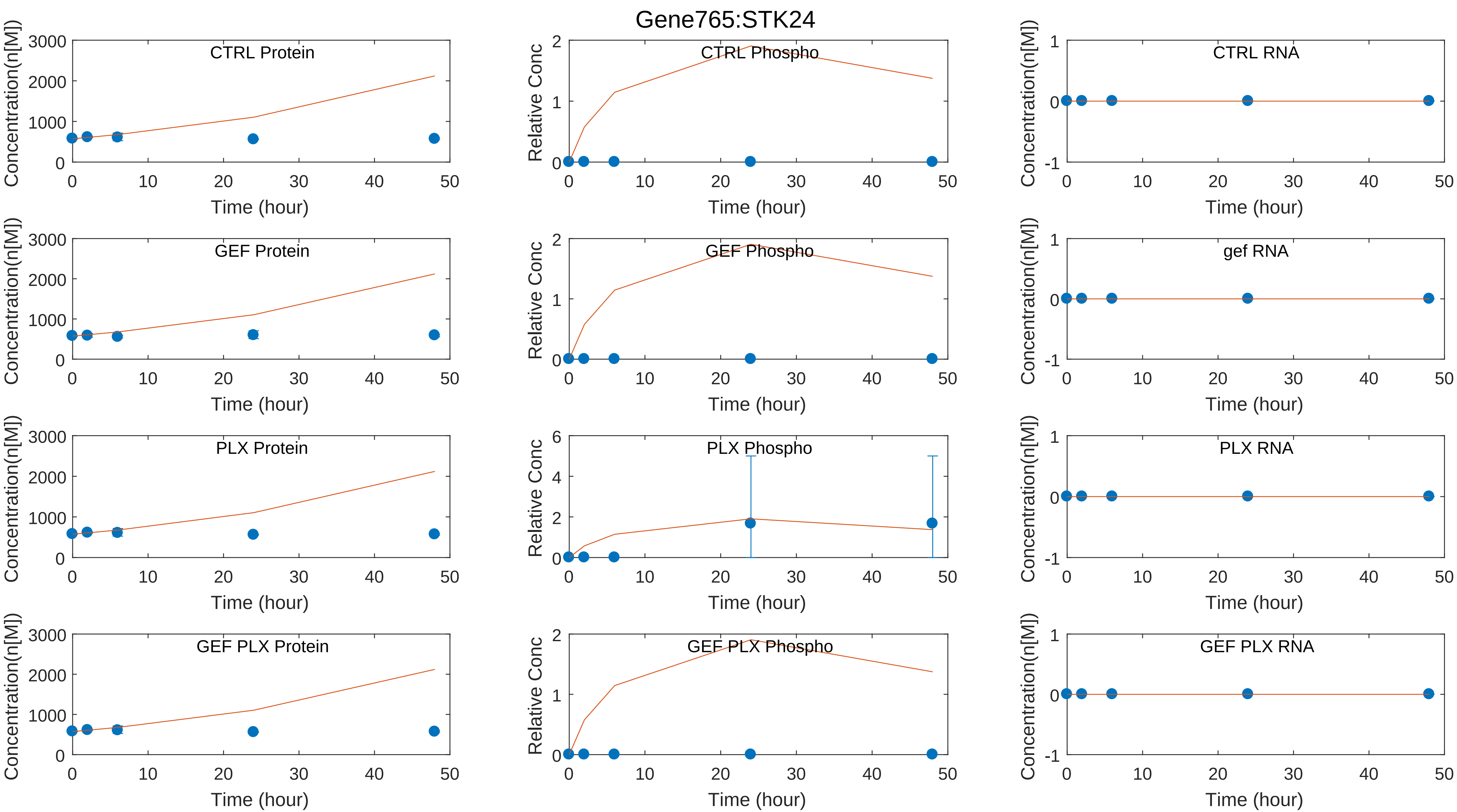

Gene765:STK24
CTRL Protein
CTRL Phospho
CTRL RNA
GEF Protein
GEF Phospho
gef RNA
PLX Protein
PLX Phospho
PLX RNA
GEF PLX Protein
GEF PLX Phospho
GEF PLX RNA
Concentration(n[M])
Relative Conc
Time (hour)

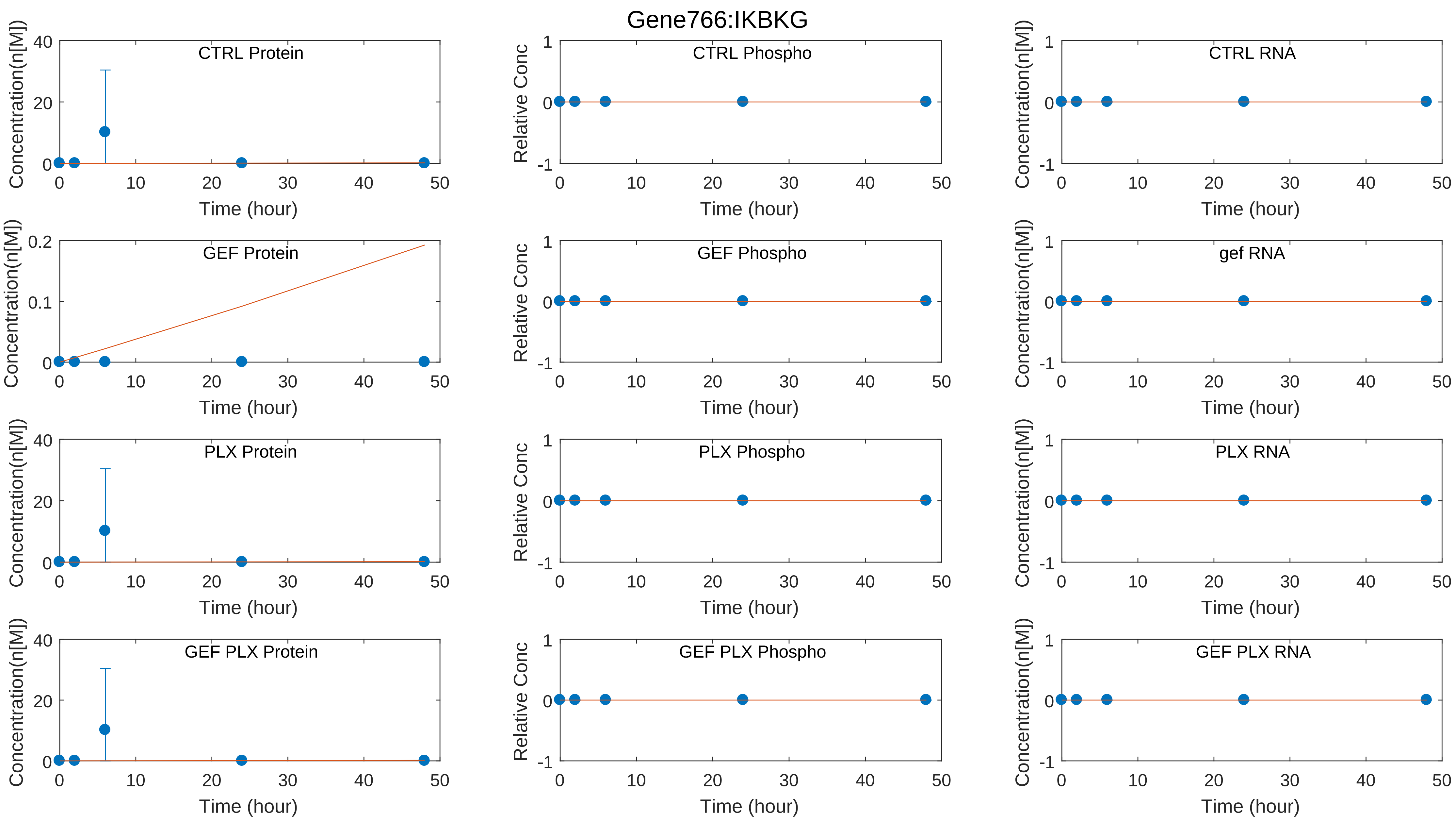
Gene766:IKBKG
CTRL Protein
CTRL Phospho
CTRL RNA
GEF Protein
GEF Phospho
gef RNA
PLX Protein
PLX Phospho
PLX RNA
GEF PLX Protein
GEF PLX Phospho
GEF PLX RNA
Concentration(n[M])
Relative Conc
Time (hour)

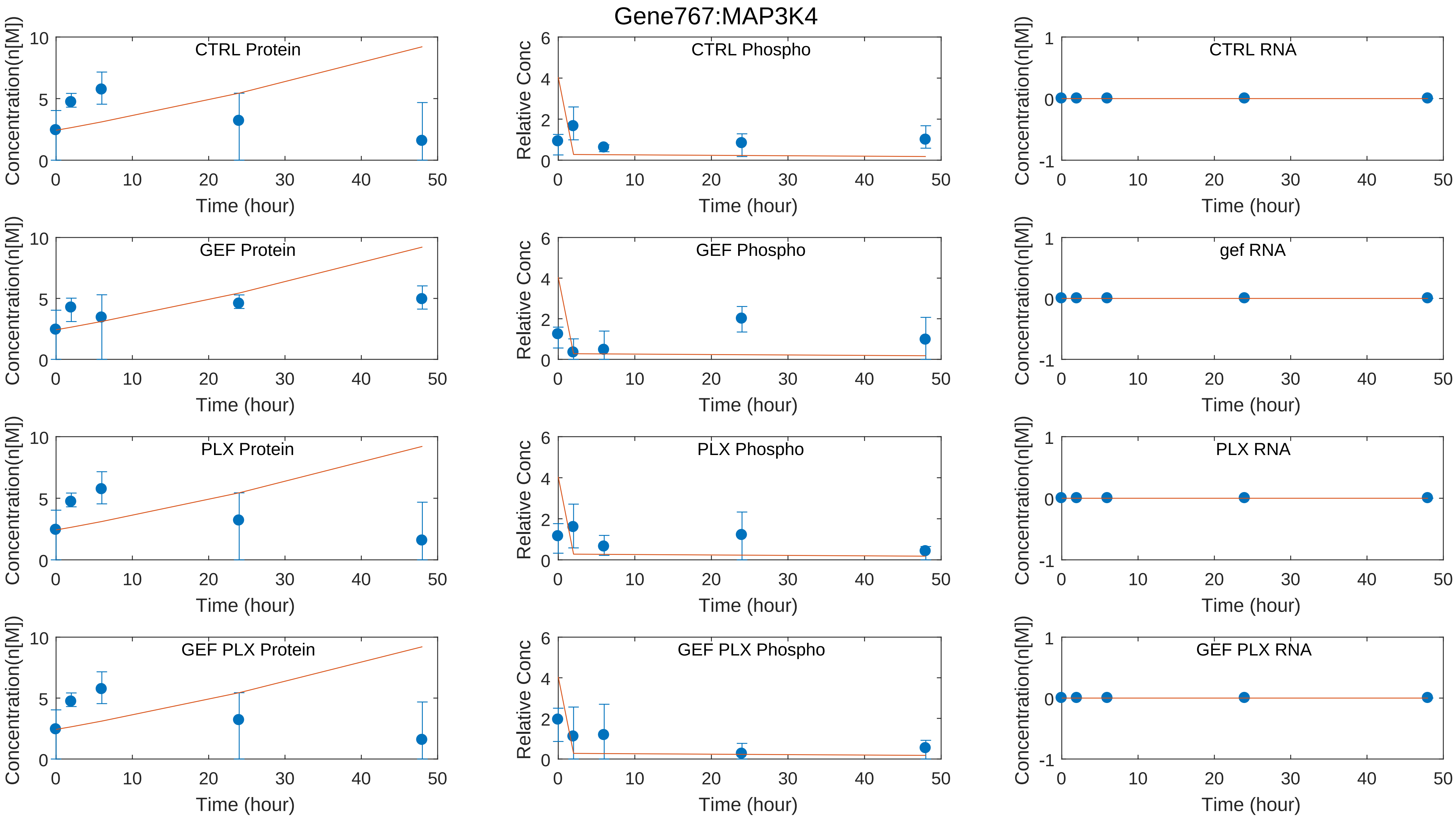
Gene767:MAP3K4
CTRL Protein
CTRL Phospho
CTRL RNA
GEF Protein
GEF Phospho
gef RNA
PLX Protein
PLX Phospho
PLX RNA
GEF PLX Protein
GEF PLX Phospho
GEF PLX RNA
Concentration(n[M])
Relative Conc
Time (hour)

Gene768:WASF2

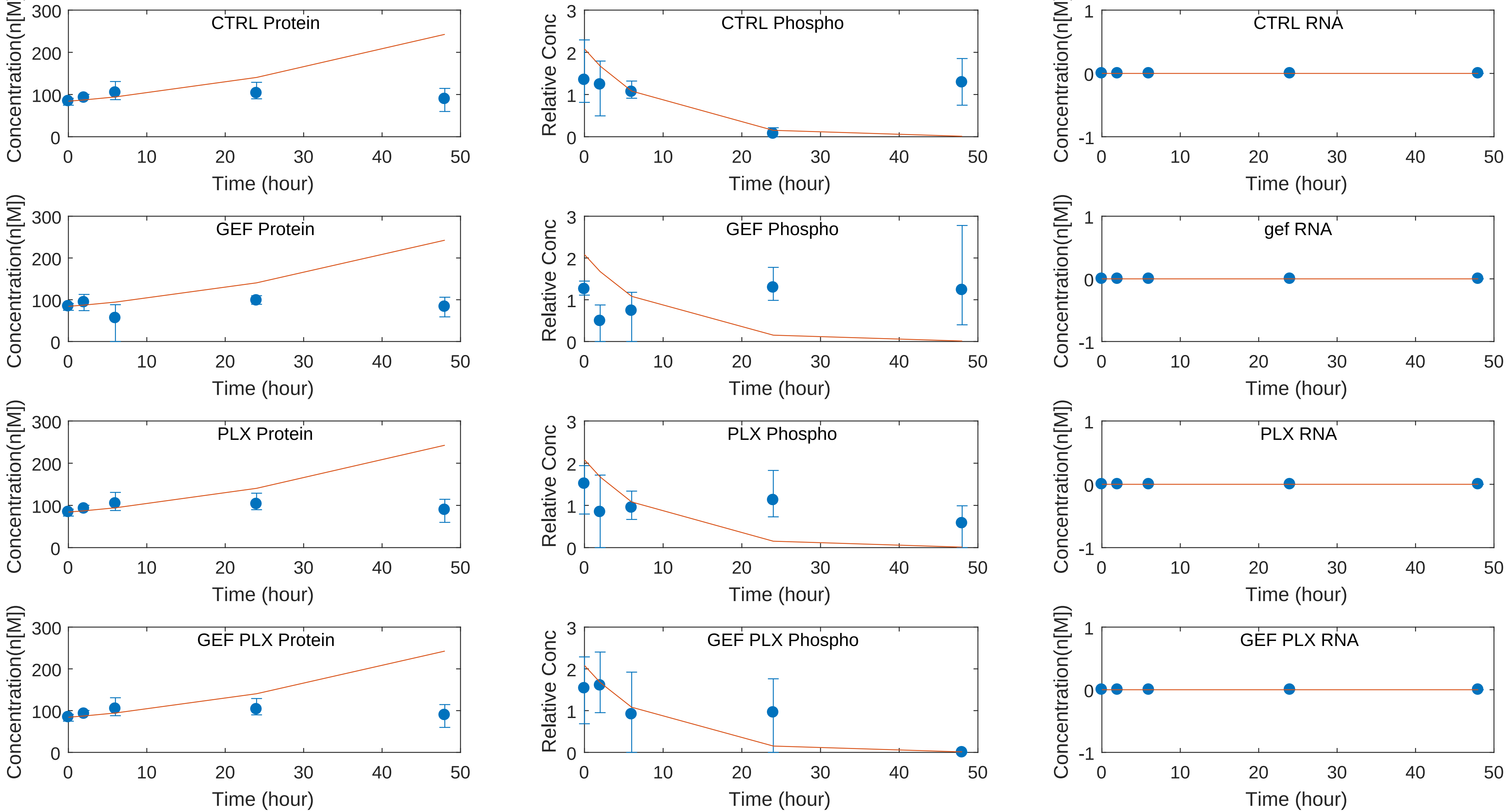

Supplement: btad079_Supplementary_Data [file btad079_supplementary_data.pdf]
